# P21 Ablation Unveils Strain-Specific Transcriptional Reprogramming in *Trypanosoma cruzi* Amastigotes

**DOI:** 10.1155/ijm/9919200

**Published:** 2025-07-04

**Authors:** Anna Clara Azevedo Silveira, Iara Dantas de Souza, João Vitor Ferreira Cavalcante, Thaise Lara Teixeira, Cristina Mary Orikaza, Rodrigo Juliani Siqueira Dalmolin, José Franco da Silveira, Claudio Vieira da Silva

**Affiliations:** ^1^Universidade Federal de Uberlândia, Uberlândia, Brazil; ^2^Universidade Federal do Rio Grande do Norte, Natal, Brazil; ^3^Universidade Federal de São Paulo, São Paulo, Brazil

**Keywords:** DTUs, P21 protein, RNA-seq, strain-specific responses, transcription, *Trypanosoma cruzi*

## Abstract

*Trypanosoma cruzi* is the causative agent of Chagas disease and is capable of invading any nucleated cell in the vertebrate host. The parasite utilizes various virulence factors during cell invasion, including the P21 protein. P21 is encoded by a single-copy, nonconserved gene expressed across all *T. cruzi* life cycle stages. Its sequence codes for a protein implicated in cell invasion and parasite multiplication. Given the significant differences in biological behavior between distinct strains of *T. cruzi*, we ablated the P21-coding gene in two phylogenetically distant strains (G and Y strains) and assessed its impact on the transcriptome profile of intracellular amastigotes. Our findings revealed that P21 depletion affected the transcription of different genes in the G and Y strains, with each strain exhibiting enrichment for distinct biological processes. Notably, protein translation was the major biological process impacted by P21 depletion, showing upregulation in the G strain and downregulation in the Y strain. In conclusion, our findings demonstrate that P21 gene ablation induces strain-specific transcriptional reprogramming in *T. cruzi* amastigotes, revealing divergent roles for P21 in modulating fundamental cellular processes like protein translation and potentially influencing host–parasite interactions, contingent upon the parasite's genetic background.

## 1. Introduction

*Trypanosoma cruzi* (*T. cruzi*), the etiological agent of Chagas disease, is an intracellular protozoan parasite capable of invading a wide range of nucleated cells within the vertebrate host. The parasite employs a variety of membrane-anchored and secreted proteins to facilitate cellular invasion [1, 2]. Among these, the P21 protein has been identified as a key player in the invasion process [3]. P21 is encoded by a single-copy gene in the *T. cruzi* genome and is expressed throughout the parasite's life cycle [3]. Previous studies have demonstrated that the ablation of the P21-coding gene leads to reduced cellular invasion and increased multiplication in the Y strain of *T. cruzi* [4, 5]. These observations suggest that P21 may function to maintain intracellular persistence of the parasite, potentially by shielding it from host immune responses and modulating the cell cycle of intracellular amastigotes, thus contributing to the establishment of chronic infection.

*T. cruzi* exhibits remarkable genetic diversity and is currently classified into six discrete typing units (DTUs), TcI–TcVI. These DTUs exhibit significant variations in biological characteristics, including virulence and the expression of proteins involved in host cell invasion [2, 6]. This inherent diversity underscores the importance of strain-specific studies to fully understand *T. cruzi* biology and pathogenesis. For instance, highly virulent strains (DTU II and VI) express and shed larger quantities of active trans-sialidase, leading to severe damage to the thymus and spleen, whereas low-virulent DTU I strains cause milder effects [6]. Furthermore, proteomic analysis has revealed that trypanolytic factors in the hemolymph and salivary glands of *Rhodnius prolixus* can lyse epimastigotes and trypomastigotes of the Y strain (TcII) but not of the Dm28c strain (TcI) [7]. Transcriptomic studies have also highlighted differences between TcI and TcII strains in their response to temperature stress, with TcII being less affected and showing increased expression of the surface metalloprotease GP63 [8]. Given this established strain diversity and the known involvement of P21 in *T. cruzi* virulence, we hypothesized that P21's functional role and regulatory impact might also diverge across different strains. For instance, strain-specific differences in tissue tropism, disease severity, and drug susceptibility have been well-documented in Chagas disease. Understanding the molecular basis of such strain variation is critical for developing broadly effective diagnostic and therapeutic strategies. Therefore, in the present work, we sought to investigate the impact of P21 gene deletion on the transcriptome of intracellular amastigotes from two phylogenetically distant *T. cruzi* strains, G and Y. The amastigote stage was specifically chosen for this study as it is the intracellular replicative form of the parasite, and our previous data indicated that P21 impact on multiplication varies between strains. While we acknowledge the importance of examining gene expression in the trypomastigote stage, which is responsible for host cell invasion, our primary focus in this study was to explore the strain-specific effects of P21 on the critical intracellular multiplication phase. By comparing the transcriptomic profiles of P21 knockout and Cas9 amastigotes from both strains, we aimed to identify genes and pathways differentially regulated in response to P21 ablation. This approach allowed us to gain insights into the diverse roles of P21 in different *T. cruzi* strains and its potential contribution to the distinct virulence phenotypes observed.

## 2. Material and Methods

### 2.1. Parasite and Cell Cultures

*T. cruzi* epimastigotes of both the Cas9 (parental) and TcP21-/- (P21 knockout) lines, belonging to the G (DTU I) and Y (DTU II) strains, were cultured at 28°C in liver infusion tryptose (LIT) medium (composition: 0.05% liver infusion, 0.5% tryptose, 0.004% hemin, 0.4% NaCl, 0.042% KCl, 0.8% Na_2_HPO_4_, 0.2% glucose, pH 7.3) supplemented with 20% fetal bovine serum (FBS; Invitrogen). Metacyclogenesis was induced by maintaining epimastigotes in LIT for 14 days, utilizing nutritional stress to promote differentiation, followed by purification of metacyclic trypomastigotes as previously described [9]. P21 knockout parasites from the G strain were generated upon early-log phase epimastigotes transfection with Cas9/pTREX-n (Addgene Plasmid #68708) [10]. Selection was performed with G418 (250 *μ*g/mL) 24 h post-transfection, and GFP-positive parasites were sorted 15 days post-transfection using BD FACSARIA II. sgRNA sequences were designed with EuPatGDT [11]. DNA templates for sgRNA in vitro transcription were generated by PCR. sgRNAs were transcribed in vitro using the MEGAShortscript T7 kit (Thermo Fisher Scientific). Donor DNA for homologous recombination was produced by PCR using 100 bp ultramer primers. For transfection, 1 × 10^7^ early-log phase Cas9-GFP expressing epimastigotes were electroporated with sgRNAs and donor DNA. CRISPR mutant cell lines were maintained under selection with G418, blasticidin, and hygromycin. Genomic DNA was extracted from wild-type (WT), Cas9-GFP (Cas9), and knockout lineages (TcP21-/-) and analyzed by PCR. Total RNA from WT, Cas9, and TcP21-/- parasites was extracted and treated with DNAse I. First-strand cDNA was synthesized from total RNA using the Superscript III First-Strand Synthesis System. PCR was performed to amplify P21 and the endogenous controls TcHGPRT and TcMVK [12]. WT and TcP21-/- parasites were fixed, washed, and incubated with anti-P21 antibodies. Subsequently, they were incubated with anti-mouse conjugated to Alexa Fluor 568 and DAPI. Images were acquired using confocal microscopy and analyzed with Imaris software (Supporting Information 1: Figure S1).

Vero cells (obtained from Instituto Adolfo Lutz) were cultured in Dulbecco's Modified Eagle Medium (DMEM) (Sigma Chemical Co.) supplemented with 10% FBS (Cultilab), 10 *μ*g/mL streptomycin, 100 U/mL penicillin, and 40 *μ*g/mL gentamicin. The Vero cell line was obtained from Instituto Adolfo Lutz, and cell line identity was confirmed by method of authentication, for example, STR profiling. These cells were maintained at 37°C in a humidified atmosphere containing 5% CO_2_. To generate tissue culture–derived trypomastigotes (TCTs), Vero cells were infected with purified metacyclic trypomastigotes from both the parental Cas9 and TcP21-/- lines of the G and Y strains. Vero cells (0.7 × 10^6^) were seeded in 75 cm^2^ culture flasks and incubated at 37°C with 5% CO_2_. Infection was carried out using TCTs present in the supernatant of pre-established cultures of knockout and control parasites. To ensure that parasites were collected at a similar stage, the time of infection was adjusted based on the known growth rates of the different strains and their respective P21 deletion mutants. After overnight infection, cells were washed with PBS to remove extracellular parasites and cultured for an additional 6 days to allow intracellular amastigote development. Infected cells were then harvested by washing with ice-cold Krebs–Henseleit buffer (KHB) containing 0.5 mM glucose, 118 mM NaCl, 4.7 mM KCl, 1.2 mM MgSO_4_, 1.25 mM CaCl_2_, 1.2 mM KH_2_PO_4_, and 25 mM NaHCO_3_, followed by detachment using a cell scraper and 1 mL of KHB. The detached cells were collected in a final volume of 10 mL of KHB +0.5 mM glucose, centrifuged, and then the supernatant was discarded. The cell pellet was resuspended in 1 mL of KHB +0.5 mM glucose, transferred to an Eppendorf tube, vortexed for 45 s, and passed 20 times through a 1 mL syringe with a 27G needle to release the amastigotes. Infected cells and debris were pelleted by centrifugation at 100 g for 5 min, and the supernatant containing amastigotes was further centrifuged at 1000 g for 10 min at room temperature to pellet the amastigotes. Amastigotes were counted and stored in RNAlater solution until RNA extraction.

### 2.2. RNA Extraction and RNA-Seq

Total RNA was extracted from the amastigotes using the Qiagen RNeasy Mini Kit. Amastigotes were resuspended in 350 *μ*L of RLT buffer with 0.1% *β*-mercaptoethanol, homogenized, and vortexed. The samples were centrifuged for 2 min at maximum speed at 4°C. The supernatant was collected and mixed with 350 *μ*L of 70% ethanol, and 700 *μ*L of this mixture was added to the column. RNA extraction was then performed according to the manufacturer's instructions. RNA quality was assessed using a NanoDrop 2000 spectrophotometer and an Agilent Bioanalyzer, confirming RNA Integrity Numbers (RIN) > 8.0 for all samples, indicating high RNA quality suitable for RNA-seq. The extracted RNA was quantified using a NanoDrop 2000 spectrophotometer (Thermo Scientific) and stored at −80°C. RNA samples were obtained from both Cas9 and TcP21-/- groups for each strain (Y and G), resulting in a total of four groups. Each group had three replicates, yielding a total of 12 samples for the experimental design. mRNA sequencing was performed by the Laboratório Central de Tecnologias de Alto Desempenho em Ciências da Vida (LaCTAD) using an Illumina HiSeq2500 sequencer. Three independent replicates of each condition were sequenced, generating paired-end 2 × 100 bp reads (30 million reads per sample).

### 2.3. Data Processing

Fastq files were processed using the fastp program to remove low-quality reads and Illumina adapters [13]. Kraken2 was used for taxonomic classification and to filter out reads derived from Vero cells [14]. The Kraken2 database was built using the EuPathDB database [15]. The mean percentage of classified reads was 35.8% and 42.3% for G and Y strains, respectively. Of these, the mean percentage annotated to the *T. cruzi* genome was 97.6% and 96.4% for G and Y strains, respectively. The classified reads were aligned to the *T. cruzi* G strain genome using STAR, using the genome-guided alignment approach with default parameters and gene annotation from TriTrypDB (Version 64). The G strain genomic sequence (Version 64) and annotation files were obtained from TriTrypDB [16, 17]. Gene expression quantification using featureCounts was performed at the gene level, counting reads that uniquely mapped to exons according to the gene annotation file. The featureCounts program [18] was used to quantify reads at the gene level and generate gene expression count tables. The preprocessing was implemented as a Nextflow pipeline (available at https://github.com/iaradsouza1/gene_exp_tcruzi/tree/add-fastp). PCA of gene expression was performed using the “prcomp” function in R. Differential expression analysis was performed using DESeq2 in R/Bioconductor, employing a design formula of “~strain + condition” to model gene expression, where “strain” accounts for strain-specific baseline differences and “condition” represents the comparison between TcP21-/- and Cas9 within each strain [19]. Raw counts of 13,504 protein-coding genes were tested. Genes with FDR < 0.001 were considered differentially expressed. Functional enrichment analysis was performed using the GOstats package in R, using the Gene Ontology database as a reference. GO term enrichment was assessed using Fisher's exact test, with *p* values adjusted for multiple testing using the Benjamini–Hochberg FDR method. Terms with at least three genes in each ontology were considered. *p* values were adjusted, and enriched terms with FDR < 0.05 were identified. Raw sequencing data is available at the National Library of Medicine under accession number PRJNA1156032. Moreover, Supporting Information 8: Table S7 and Supporting Information 9: Table S8 listing all differentially expressed genes between G (Cas9 vs. P21−/−) and Y (Cas9 vs. P21−/−), respectively, including fold changes, are provided.

## 3. Results

**Figure 1:**
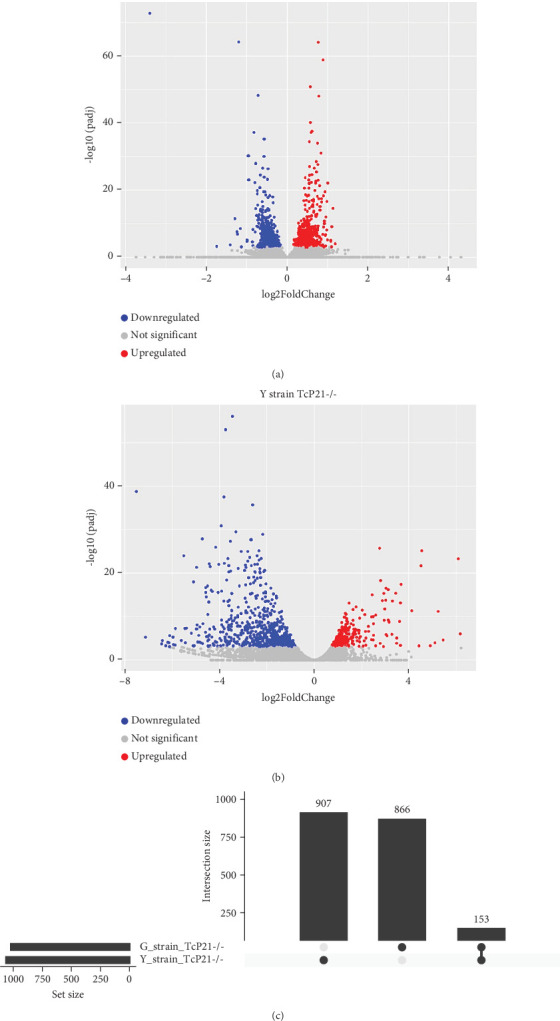
(a, b) Volcano plots depicting differentially expressed genes (DEGs) in TcP21-/- intracellular amastigotes compared to Cas9 controls for G and Y strains, respectively. Upregulated genes are shown in red, and downregulated genes are shown in blue. The *x*-axis represents the log_2_ fold change in expression, and the *y*-axis represents the −log_10_ adjusted *p* value. (c) Venn diagram illustrating the overlap of DEGs between TcP21-/- amastigotes of G and Y strains compared to their respective Cas9 controls. Numbers within each section represent the number of genes uniquely differentially expressed in each strain or shared between them.

**Figure 2:**
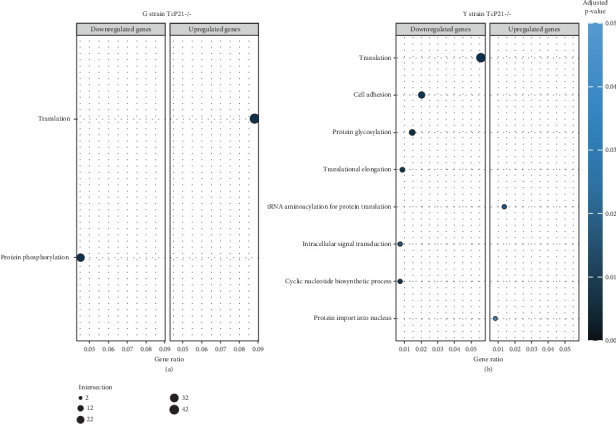
Biological processes enriched in TcP21-/- intracellular amastigotes of G and Y strains. (a, b) Bar plots showing the enrichment of differentially expressed genes associated with various biological processes in G and Y strain TcP21-/- amastigotes, respectively, compared to Cas9 controls. The *x*-axis represents the biological processes, and the *y*-axis represents the −log_10_ of the adjusted *p* value for enrichment. The color intensity of the bars corresponds to the level of enrichment, with darker colors indicating higher significance.

**Figure 3:**
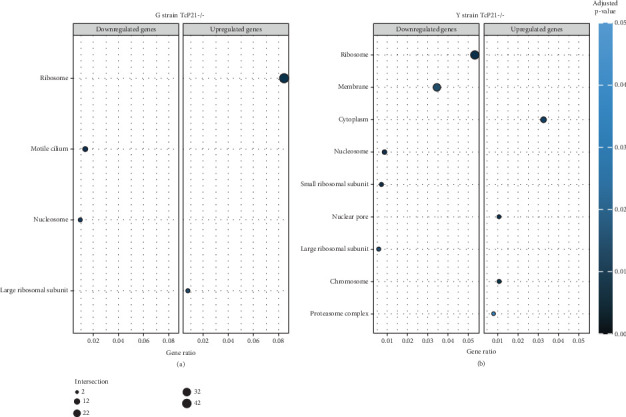
Cellular components enriched in TcP21-/- intracellular amastigotes of G and Y strains. (a, b) Bar plots showing the enrichment of differentially expressed genes associated with various cellular components in G and Y strain TcP21-/- amastigotes, respectively, compared to Cas9 controls. The *x*-axis represents the cellular components, and the *y*-axis represents the −log_10_ of the adjusted *p* value for enrichment. The color intensity of the bars corresponds to the level of enrichment, with darker colors indicating higher significance.

**Figure 4:**
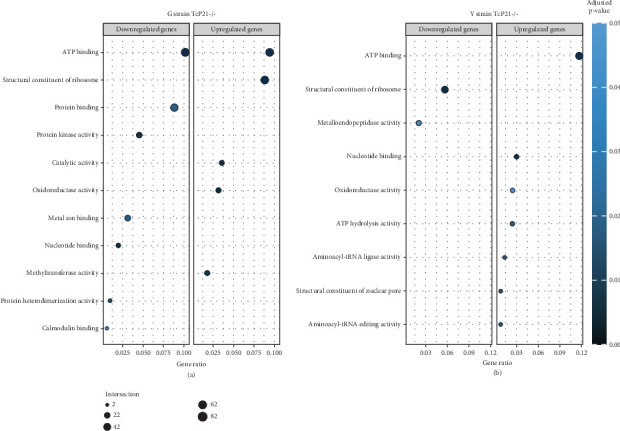
Molecular functions enriched in TcP21-/- intracellular amastigotes of G and Y strains. (a, b) Bar plots showing the enrichment of differentially expressed genes associated with various molecular functions in G and Y strain TcP21-/- amastigotes, respectively, compared to Cas9 controls. The *x*-axis represents the molecular functions, and the *y*-axis represents the −log_10_ of the adjusted *p* value for enrichment. The color intensity of the bars corresponds to the level of enrichment, with darker colors indicating higher significance.

**Figure 5:**
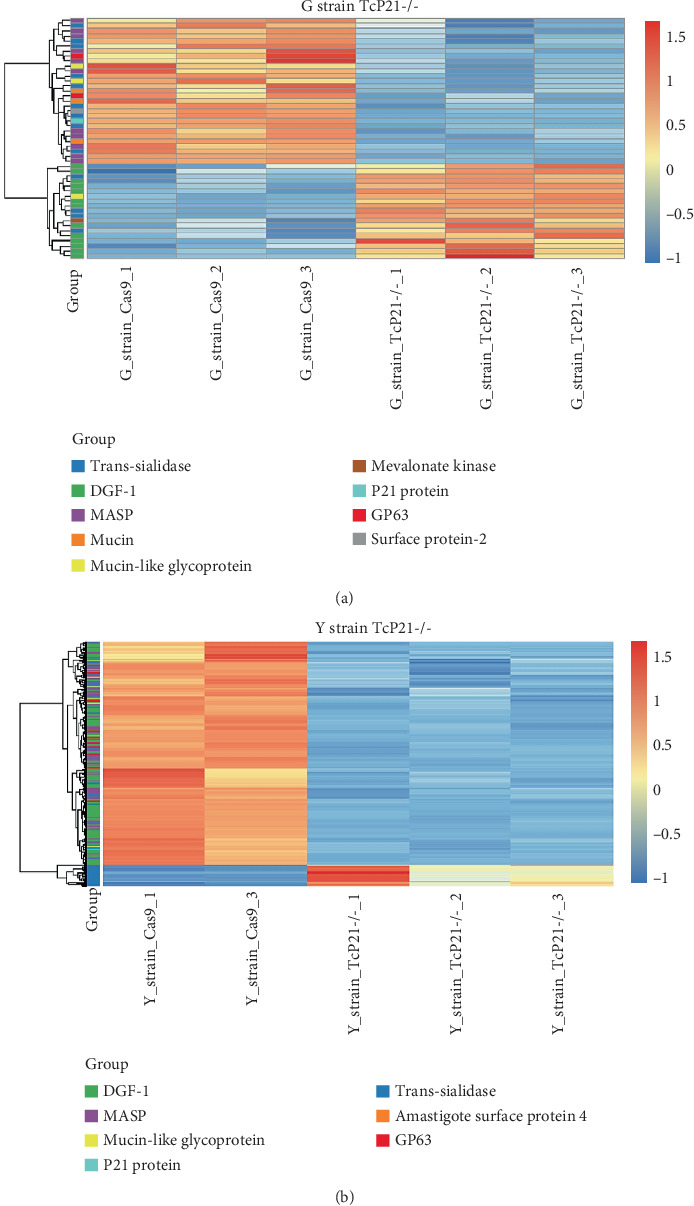
Heatmap of differentially expressed genes encoding membrane and secreted proteins in TcP21-/- amastigotes. (a, b) Heatmap visualization of the expression levels of genes coding for membrane and secreted proteins in TcP21-/- intracellular amastigotes compared to Cas9 controls for G and Y strains, respectively. Red indicates upregulation, and blue indicates downregulation. The color intensity corresponds to the magnitude of the log_2_ fold change.

RNA sequencing was performed on samples from Cas9 (parental) and P21 knockout (TcP21-/-) intracellular amastigotes of both G and Y strains. A total of 1019 and 1060 transcripts were differentially expressed in TcP21-/- amastigotes of G and Y strains, respectively, compared to their Cas9 counterparts. Of these, 866 transcripts were specifically differentially expressed in TcP21-/- parasites of the G strain, while 907 transcripts were uniquely differentially expressed in TcP21-/- parasites of the Y strain. One hundred fifty-three transcripts were differentially expressed in both strains (Figures 1a, 1b, and 1c). A group of 442 genes from TcP21-/- amastigotes of the G strain and 350 genes from the TcP21-/- amastigotes of the Y strain were associated with enriched biological processes. Functional enrichment analysis of the differentially expressed genes in amastigotes of both strains revealed the biological terms potentially altered by P21 knockout. Two biological processes were enriched in TcP21-/- samples of the G strain, while eight were enriched in TcP21-/- samples of the Y strain. Translation was upregulated, and protein phosphorylation was downregulated in TcP21-/- parasites of the G strain. In contrast, translation, cell adhesion, protein glycosylation, translational elongation, intracellular signal transduction, and cyclic nucleotide biosynthetic processes were downregulated in TcP21-/- parasites of the Y strain, while tRNA aminoacylation for protein translation and protein import into the nucleus were upregulated. Notably, translation, a fundamental process for cellular growth and metabolism, exhibited strikingly divergent regulation: it was upregulated in the G strain TcP21-/- parasites while being downregulated in the Y strain TcP21-/- parasites, suggesting distinct impacts of P21 ablation on fundamental cellular processes in these strains. Forty-four transcripts related to translation were upregulated in TcP21-/- parasites of the G strain. The modulation of 40 of these 44 transcripts was exclusive to the G strain. Fifteen downregulated transcripts were related to protein phosphorylation. In contrast, 39 transcripts related to translation were downregulated in TcP21-/- parasites of the Y strain. Ten transcripts were related to protein glycosylation, 14 to cell adhesion, six to translational elongation, five to cyclic nucleotide biosynthetic process and intracellular signal transduction, and five to tRNA aminoacylation for protein translation. Three transcripts were related to protein import into the nucleus (Figure 2a,b). The modulation of 36 of these 39 transcripts was exclusive to TcP21-/- amastigotes of the Y strain. The genes associated with translation encode ribosomal proteins such as 60S ribosomal protein L11 (TcG_07781), 60S ribosomal subunit protein L31 (TcG_00575), 60S ribosomal protein L17 (TcG_00791), 40S ribosomal protein S14 (TcG_00946), and 40S ribosomal protein S15a (TcG_01906). The consistent upregulation of multiple ribosomal protein transcripts in the G strain TcP21-/- parasites indicates a potential increase in ribosome biogenesis or translational capacity in response to P21 ablation in this strain. Genes encoding for protein phosphorylation are represented by putative mitogen-activated protein kinase (TcG_00063), putative serine/threonine protein kinase (TcG_00884), and casein kinase II, alpha chain (TcG_03076). Regarding cell adhesion, several copies of GP63 and putative GP63 genes were downregulated in TcP21-/- parasites of the Y strain, such as surface protease GP63 (TcG_07731) and GP63 Group II protein (TcG_08787). The downregulation of GP63, a well-established *T. cruzi* virulence factor involved in host cell invasion and immune modulation, in the Y strain TcP21-/- parasites, suggests that P21 may indirectly influence the expression of key surface proteins relevant to virulence in a strain-specific manner. Some representative transcripts of protein glycosylation are UDP-Gal or UDP-GlcNAc-dependent glycosyltransferase (TcG_12364) and alpha-(1,3)-fucosyltransferase, family GT10 (TcG_11677). Representative transcripts of translational elongation are 60S acidic ribosomal protein P2 (TcG_00916) and elongation factor-1 alpha (TcG_02589). For the cyclic nucleotide biosynthetic process and intracellular signal transduction, differentially expressed genes are represented by receptor-type adenylate cyclase (TcG_01431) and adenylyl cyclase (TcG_01432). Valyl-tRNA synthetase (TcG_00996) and isoleucine-tRNA ligase (TcG_04183) are the representative transcripts of tRNA aminoacylation for protein translation. Karyopherin beta (TcG_06807) is an example of protein import into the nucleus. For the complete list of differentially expressed transcripts in each strain, see Supporting Information 2: Table S1 and Supporting Information 3: Table S2. Four cellular components in the G strain and nine in the Y strain were significantly affected by the absence of P21 gene expression (Figure 3a,b). TcP21-/- parasites of both G and Y strains displayed increased differential gene expression of ribosome, motile cilium, nucleosome components, and large ribosomal subunit cellular components compared to Cas9 parasites. Additionally, TcP21-/- parasites of the Y strain also showed significant differential expression of membrane, cytoplasm, small ribosomal subunits, nuclear pore, chromosome, and proteasome complex cellular components. Ribosomal transcripts were upregulated in TcP21-/- parasites of the G strain but downregulated in those of the Y strain, consistent with the biological process analysis. The same strain-specific transcripts described previously were also observed in this analysis. Regarding nucleosomes, transcripts such as histone H2A (TcG_03830) were downregulated in TcP21-/- parasites of both G and Y strains. In relation to motile cilium enriched in the G strain, the following transcripts were downregulated: intraflagellar transport 172-like protein (TcG_07868), paraflagellar rod component (TcG_05176), and paraflagellar rod protein 2C (TcG_02132). Transcripts related to the small ribosomal subunit, for example, 40S ribosomal protein S15 (TcG_06395) and 40S ribosomal protein AS (TcG_09354), were downregulated in Y strain TcP21-/- parasites. Transcripts related to the nuclear pore, for example, putative nuclear pore complex protein (NUP155) (TcG_04832) and putative ATP-dependent RNA helicase (TcG_05048), were upregulated in Y strain TcP21-/- parasites. Large ribosomal subunit transcripts, such as 60S ribosomal protein L17 (TcG_01077) and 60S ribosomal protein L26 (TcG_02092), were upregulated in the G strain and downregulated in the Y strain TcP21-/- parasites. Chromosome-related transcripts, for example, putative structural maintenance of chromosomes (SMC) family protein (TcG_00603), were upregulated in Y strain TcP21-/- parasites. The downregulated transcripts related to the membrane are those encoding for GP63 and UDP-Gal or UDP-GlcNAc-dependent glycosyltransferase (cell adhesion and glycosylation biological processes). Some upregulated transcripts related to the cytoplasm are valyl-tRNA synthetase (TcG_00996) and karyopherin beta (TcG_06807). Proteasome complex transcripts were upregulated in Y strain TcP21-/- parasites. Putative proteasome regulatory non-ATPase subunit (TcG_01492) is an example of a gene transcript of this cellular component. For the complete list of transcripts, see Supporting Information 4: Table S3 and Supporting Information 5: Table S4. Next, we analyzed the differential gene expression associated with molecular function. The results revealed 11 molecular functions differently expressed in the G strain and nine in the Y strain TcP21-/- parasites. ATP-binding transcripts were both upregulated and downregulated in the G strain and upregulated in the Y strain TcP21-/-, exemplified by ATP-dependent DEAD/H RNA helicase (TcG_00892) and ATP-binding cassette protein subfamily B, Member 1 (TcG_02742). Protein binding was downregulated in the G strain TcP21-/-. Representative transcripts include leucine-rich repeat protein (TcG_00090), putative protein transport protein Sec31 (TcG_01901), and putative eukaryotic translation initiation factor 4 gamma (TcG_02191). Transcripts related to structural constituents of ribosomes were upregulated in the G strain and downregulated in the Y strain TcP21-/-, as previously noted in the translation biological process and ribosome cellular component sections. Protein kinase activity was downregulated in the G strain TcP21-/-, as mentioned in the protein phosphorylation section. Nucleotide binding transcripts were downregulated in the G strain and upregulated in the Y strain TcP21-/-, including succinyl-CoA synthetase alpha subunit (TcG_06609) and isoleucine--tRNA ligase (TcG_04183). Metal ion binding was downregulated in the G strain TcP21-/-, such as the putative zinc finger protein (TcG00146). ATP hydrolysis activity transcripts were upregulated in the Y strain TcP21-/-, such as ATP-binding cassette protein subfamily A, Member 10 (TcG_01541). Catalytic activity was upregulated in G strain TcP21-/- parasites with the transcription of triosephosphate isomerase (TcG_00710) and 2-amino-3-ketobutyrate coenzyme A ligase (TcG_07370). Oxidoreductase activity transcripts were upregulated in both strains, for example, tryparedoxin peroxidase (TcG_08583 in G strain; TcG_08077 in Y strain). Methyltransferase activity was upregulated in the G strain TcP21-/- with the transcription of putative FtsJ cell division protein (TcG_01406) and tRNA guanosine-2-O-methyltransferase TRM13 (TcG_04372). G strain TcP21-/- data were also enriched in protein heterodimerization activity and calmodulin binding. The transcripts for these molecular functions were histones and paraflagellar rod proteins, respectively. The Y strain TcP21-/- showed the downregulation of metalloendopeptidase activity with transcripts that encode for GP63. In addition, the Y strain TcP21-/- showed the upregulation of aminoacyl-tRNA ligase activity, aminoacyl-tRNA editing activity, and structural constituent of the nuclear pore. The transcripts for aminoacyl-tRNA ligase and editing activities were those related to valyl-tRNA synthetase, isoleucine--tRNA ligase, and others. Regarding the constituent of the nuclear pore, putative nuclear pore complex protein (NUP155) (TcG_04832) is an example of a transcript (Figure 4a,b). The complete list of transcripts can be found in Supporting Information 6: Table S5 and Supporting Information 7: Table S6. Transcripts associated with plasma membrane and secreted proteins, likely involved in parasite virulence, were also analyzed. Transcripts of trans-sialidase were both upregulated and downregulated in the G and Y strains of TcP21-/- parasites. Dispersed gene protein family-1 (DGF-1) transcripts were upregulated in parasites from the G strain and downregulated in parasites from the Y strain. Mucin-associated surface protein (MASP) and mucin transcripts were downregulated in both strains. Mucin-like glycoprotein and GP63 transcripts were both upregulated and downregulated in the G strain but only downregulated in the Y strain. Mevalonate kinase transcripts were upregulated in the G strain. Surface protein-2 transcripts were downregulated in the G strain, and amastigote surface protein-4 transcripts were downregulated in the Y strain (Figure 5a,b). Supporting Information 8: Table S7 and Supporting Information 9: Table S8 provide all differentially expressed genes between G (Cas9 vs. P21−/−) and Y (Cas9 vs. P21−/−), respectively, including fold changes.

## 4. Discussion

The dynamic interplay between *T. cruzi* and its host cell involves intricate molecular mechanisms that govern parasite invasion, intracellular survival, and ultimately, the establishment of chronic infection. To address the relevance of strain-specific characteristics, it is important to note that while the G and Y strains exhibit well-documented differences in virulence and infectivity, there is no conclusive published evidence for distinct tissue tropisms. Therefore, the divergent outcomes observed in this study are more likely attributable to intrinsic functional differences in virulence factors between the strains rather than a predetermined preference for different host cell environments. Previous research by Li et al. [20] has shed light on the extensive transcriptome remodeling that occurs in both the parasite and the host cell during infection, highlighting the complex network of host–parasite interactions. By using a deconvolution technique, authors identified six distinct subpopulations of intracellular amastigotes, confirming their heterogeneity [21]. Our study builds upon this foundation by focusing on the role of the P21 protein, a secreted factor implicated in parasite virulence. We specifically investigated the transcriptomic consequences of P21 deletion in intracellular amastigotes collected between 5 and 8 days postinfection. This timeframe corresponds to the period of active amastigote replication allowing us to capture the transcriptional changes associated with this crucial process. The timing of collection was carefully adjusted based on the growth rates of each strain and its P21 knockout counterpart to ensure that parasites were at a comparable stage of intracellular development, minimizing potential confounding effects due to differences in the parasite's life cycle progression. Highlighting amastigote heterogeneity is relevant as it provides crucial context for interpreting our bulk RNA-seq data. Our approach captures the net effect of P21 deletion across the entire intracellular population, thereby establishing a foundational overview that complements future single-cell resolution studies.

In the G strain, P21 deletion led to the upregulation of translation-related processes, suggesting a role for P21 in controlling protein synthesis in this low-virulence strain. This might contribute to the slower replication rate observed in G strain TcP21-/- parasites. In contrast, the Y strain showed the downregulation of translation-related genes, indicating a different regulatory mechanism for P21 in this more virulent strain. The opposing regulation of translation in the G and Y strains TcP21-/- parasites may offer insights into the strain-specific effects of P21 on parasite multiplication. In the low-virulence G strain, the observed upregulation of translation, while seemingly paradoxical given the reported slower replication rate in the knockout, could represent an inefficient or unbalanced compensatory response. This increased translational activity might be metabolically costly or lead to the production of proteins that are not optimally required for replication, ultimately hindering parasite growth. Conversely, the downregulation of translation in the more virulent Y strain TcP21-/- parasites, which show increased multiplication in previous studies, might indicate a more efficient or targeted shift in protein synthesis, prioritizing the production of specific proteins required for rapid intracellular replication at the expense of overall translational output.

Protein kinase activity was downregulated in G strain TcP21-/- parasites, aligning with the results obtained for the protein phosphorylation biological process. The downregulation of signaling events dependent on protein phosphorylation by protein kinases may disrupt the parasites' ability to invade host cells and multiply intracellularly. Serine–threonine kinases are crucial enzymes in cellular proliferation and differentiation. Protein phosphorylation plays a significant role in cell signaling, gene expression, differentiation, and global control of DNA/RNA-mediated processes [22].

Oxidoreductase activity was upregulated in TcP21-/- parasites of both strains. Among the transcripts, a common one is tryparedoxin peroxidase. *T. cruzi* cytosolic tryparedoxin peroxidase is a 2-Cys peroxiredoxin with a vital role in detoxifying host cell; its overexpression enhances parasite infectivity and resistance to exogenous oxidation. Tryparedoxin peroxidase induces the recruitment of IL-12/23p40-producing innate antigen-presenting cells and promotes a strong specific Th1 immune response, also inducing proliferation and high levels of IFN-*γ* secretion in PBMCs from chronic patients without clear cardiac manifestations [23]. Studies have shown that the cytosolic tryparedoxin peroxidase of *T. cruzi* is secreted by epimastigotes and trypomastigotes associated with extracellular vesicles and as a vesicle-free protein. Transcriptomic analysis revealed that cytosolic tryparedoxin peroxidase induces endoplasmic reticulum stress and interleukin-8 expression in epithelial cells. Moreover, the enzyme exhibited a mitogenic, proliferative, and proinflammatory effect on these cells in a dose-dependent manner and acts as a paracrine virulence factor, increasing the susceptibility to infection in pretreated epithelial cells [24].

Methyltransferase activity was an upregulated molecular function in G strain TcP21-/- parasites. Methyltransferases catalyze the transfer of a methyl group from S-adenosyl-l-methionine to their substrates. It has been shown that trimethylation of histone H3K76 by Dot1B enhances cell cycle progression after mitosis in *T. cruzi* [25]. These strain-specific differences suggest that P21 function extends beyond simply controlling replication and may involve modulating diverse cellular processes critical for parasite survival and virulence (Figure 4a,b). The divergent effects of P21 deletion in G and Y strains can be interpreted through several nonexclusive mechanisms. Firstly, the P21 protein sequences themselves are known to differ between these strains. This genetic divergence likely translates into structural variations that could alter the protein's stability, enzymatic activity, or its affinity for binding partners within the parasite, thus leading to distinct downstream regulatory consequences. Secondly, as P21 is a secreted factor, its influence may not be entirely cell-autonomous. P21 could modulate the host cell environment, for instance, by altering host signaling pathways or metabolic states. This altered host milieu would then exert a secondary, indirect effect on the intracellular amastigotes, contributing to the observed strain-specific transcriptomic shifts. This possibility underscores the complexity of the three-way interaction between the parasite genotype, the virulence factor's function, and the host cell response. The observed upregulation of translation-related genes in the G strain TcP21-/- parasites is intriguing. One possible mechanism could involve P21 acting as a repressor of translation in the G strain under normal conditions. P21 deletion might thus relieve this repression, leading to a compensatory upregulation of translation. Alternatively, P21 could be involved in signaling pathways that indirectly influence translation initiation or ribosome biogenesis. Future studies could investigate whether P21 interacts with known translational regulators or affects the activity of key signaling kinases or phosphatases involved in translational control.

The downregulation of GP63 transcripts in the Y strain TcP21-/- amastigotes raises questions about the role of P21 in regulating surface protein expression. GP63 is crucial for host cell invasion, parasite survival within macrophages, and modulation of the host immune response. Reduced GP63 levels in the knockout parasites could potentially impact their virulence, although the precise consequences require further investigation. It is possible that P21, in the Y strain, is involved in pathways that positively regulate GP63 expression, or that its absence indirectly affects GP63 transcription through broader changes in cellular signaling or regulatory networks.

Finally, we addressed the differential expression of transcripts coding for parasite membrane and secreted proteins. Surface proteins play diverse roles in parasite life cycle progression, host-cell interplay, immune system evasion, and parasite persistence [26]. An interesting observation was the downregulation of mucin and MASP transcripts in TcP21-/- parasites of both G and Y strains. Generally, P21 ablation resulted in the differential regulation of genes and biological processes in both strains. When the same biological process was regulated in both strains, this regulation was antagonistic. The consistency of similar transcriptional regulation of mucin-type proteins in both strains suggests that P21 directly controls the transcription of these genes. Mevalonate kinase is a glycosomal and secreted enzyme involved in parasite host cell invasion [12]. Transcripts coding for this enzyme were upregulated in G strain TcP21-/- intracellular amastigotes. The mevalonate pathway is highly conserved and mediates the production of metabolites essential for cellular metabolism, growth, and differentiation, such as isoprenoids, which feed into biosynthetic pathways for sterols, dolichol, ubiquinone, heme, isopentenyl adenine, and prenylated proteins [27] (Figure 5a,b).

## 5. Conclusion

Our findings provide novel insights into the complex and strain-specific functions of the *T. cruzi* P21 protein. This knowledge enhances our understanding of parasite adaptability and its strategies for establishing chronic infections. Future research should prioritize investigating the proteomic consequences of P21 ablation in both G and Y strains to validate the observed transcriptional changes and assess post-transcriptional regulation. Functional studies are necessary to dissect the molecular mechanisms by which P21 differentially regulates translation in these strains, potentially through interactions with translational machinery or signaling pathways. Furthermore, in vivo studies are warranted to determine how these strain-specific transcriptional responses and P21 function ultimately impact parasite virulence, chronic infection establishment, and interaction with the host immune system in the context of different *T. cruzi* strains. In conclusion, this study emphasizes that dissecting the strain-specific nuances of *T. cruzi* virulence factors like P21 is not merely an academic exercise but a fundamental imperative for advancing our understanding of Chagas disease pathogenesis and for the development of truly effective and broadly applicable therapeutic strategies in the face of *T. cruzi*'s remarkable genetic and phenotypic diversity.

## Data Availability

The data that support the findings of this study are available from the corresponding author upon reasonable request.

## Nomenclature

DTU: discrete typing unit
LIT: liver infusion tryptose
TCT: tissue culture–derived trypomastigote
DGF-1: dispersed gene protein family-1
MASP: mucin-associated surface protein

## Supporting Information

Supporting Information 1 Figure S1: Strategy and confirmation of P21 knockout clones. (A) Schematic representation of the cloning strategy. (B) sgRNA transcripts obtained by in vitro transcription of DNA_sgRNA using the MEGAShortscript T7 kit. pTRI-RNA (control); sgRNA_36 and sgRNA_424 target Cas9 for cleavage at nucleotides 36 and 424 of the P21 gene, respectively. (C) Donor DNAs obtained by PCR using ultramer primers (Table S1) and pGEM-Bsd or TOPO_hygro vectors as templates. (D) PCR of gDNA isolated from knockout clones and controls (WT and Cas9) using specific primers for P21 and the UTR region of the P21 gene. (E) PCR of cDNA from knockout clones and controls (WT and Cas9) analyzing the expression of P21 and endogenous TcMVK and TcHGPRT. (F) Epimastigotes were incubated with polyclonal anti-P21 antibodies (mouse) and anti-mouse antibodies conjugated to Alexa Fluor 568 (red). Nuclei were labeled with DAPI (blue). DIC, differential interference contrast. Single-plane images were acquired by confocal microscopy. Scale bars represent 3 *μ*m.

Supporting Information 2 Table S1: Biological process transcripts enriched in G strain TcP21-/intracellular amastigotes.

Supporting Information 3 Table S2: Biological process transcripts enriched in Y strain TcP21-/intracellular amastigotes.

Supporting Information 4 Table S3: Cellular component transcripts enriched in G strain TcP21-/- intracellular amastigotes.

Supporting Information 5 Table S4: Cellular component transcripts enriched in Y strain TcP21-/- intracellular amastigotes.

Supporting Information 6 Table S5: Molecular function transcripts enriched in G strain TcP21-/- intracellular amastigotes.

Supporting Information 7 Table S6: Molecular function transcripts enriched in Y strain TcP21-/- intracellular amastigotes.

Supporting Information 8 Table S7: Comprehensive table listing all differentially expressed genes by G strain (Cas9 vs. P21−/−) amastigotes along with the fold change values.

Supporting Information 9 Table S8: Comprehensive table listing all differentially expressed genes by Y strain (Cas9 vs. P21−/−) amastigotes along with the fold change values.
