# Supplementary material for: P21 Ablation Unveils Strain-Specific Transcriptional Reprogramming in Trypanosoma cruzi Amastigotes
Source: Int J Microbiol. 2025 Jul 4;2025:9919200. doi: 10.1155/ijm/9919200 (PMC12253989; doi:10.1155/ijm/9919200)
Supplement: Supporting Information 1 — Figure S1: Strategy and confirmation of P21 knockout clones. (A) Schematic representation of the cloning strategy. (B) sgRNA transcripts obtained by in vitro transcription of DNA_sgRNA using the MEGAShortscript T7 kit. pTRI-RNA (control); sgRNA_36 and sgRNA_424 target Cas9 for cleavage at nucleotides 36 and 424 of the P21 gene, respectively. (C) Donor DNAs obtained by PCR using ultramer primers (Table S1) and pGEM-Bsd or TOPO_hygro vectors as templates. (D) PCR of gDNA isolated from knockout clones and controls (WT and Cas9) using specific primers for P21 and the UTR region of the P21 gene. (E) PCR of cDNA from knockout clones and controls (WT and Cas9) analyzing the expression of P21 and endogenous TcMVK and TcHGPRT. (F) Epimastigotes were incubated with polyclonal anti-P21 antibodies (mouse) and anti-mouse antibodies conjugated to Alexa Fluor 568 (red). Nuclei were labeled with DAPI (blue). DIC, differential interference contrast. Single-plane images were acquired by confocal microscopy. Scale bars represent 3 μm. [file 9919200.f1.pdf]

Supplementary Figure 1: G strain P21 *gene* knockout strategy

T. cruzi G parasites were transfected with Cas9/pTREX-n (Lander et al., 2015) to generate a lineage expressing Cas9 protein fused to eGFP. Following selection and enrichment for eGFP-positive cells, these Cas9-eGFP expressing parasites were transfected with two single guide RNAs (sgRNAs) targeting P21 (at nucleotides 36 and 424) (Fig. 1A and B) along with donor DNA harboring the Bsd resistance gene (Fig 1C) and selected with 50 µg/mL blasticidin and 250 µg/mL G418.

The selected parasites were cloned by limiting dilution, and genomic DNA from grown clones was extracted and analyzed by PCR. We identified P21+/- clones, containing one allele with the intact P21 gene and the other allele harboring the Bsd gene. To generate homozygous knockout (P21-/-) clones, the P21+/- parasites were electroporated with sgRNAs and donor DNA containing the hygromycin resistance gene, selected using 50 µg/mL blasticidin and 300 µg/mL hygromycin B, and cloned again by limiting dilution. Genomic DNA from these clones was extracted and analyzed by PCR with primers specific for P21 and the UTR regions (Fig. 1D). Our results indicated the absence of P21 gene amplification in these clones (Fig. 1D - gel 1). Conversely, the Bsd and Hygro resistance genes were amplified at the P21 genomic locus (Fig. 1D – gel 2), confirming the successful generation of two P21-/- clones. To verify the absence of P21 transcripts in knockout clones, cDNA synthesized by RT-PCR from total RNA was analyzed using primers for the P21 coding sequence. Two housekeeping genes, *T. cruzi* mevalonate kinase (TcMVK) (Ferreira et al., 2016) and hypoxanthine-guanine phosphoribosyltransferase (TcHGPRT) (Murta et al., 2006), served as internal controls. The results demonstrated the absence of P21 transcripts in knockout clones, while both clones expressed TcMVK and HGPRT transcripts (Fig. 1E).

The presence of P21 protein in epimastigotes was further assessed by immunofluorescence assay using confocal microscopy. In WT epimastigotes, P21 protein exhibited a clustered distribution at multiple cytoplasmic locations. However, no P21 protein labeling was observed in knockout parasites (Fig. 1F). Collectively, these data confirm the successful knockout of P21 protein using the employed strategy. We opted not to generate add-back parasites to maintain a clear experimental system and avoid potential confounding factors from gene re-introduction, focusing on a thorough analysis of the knockout phenotype within our resource constraints.

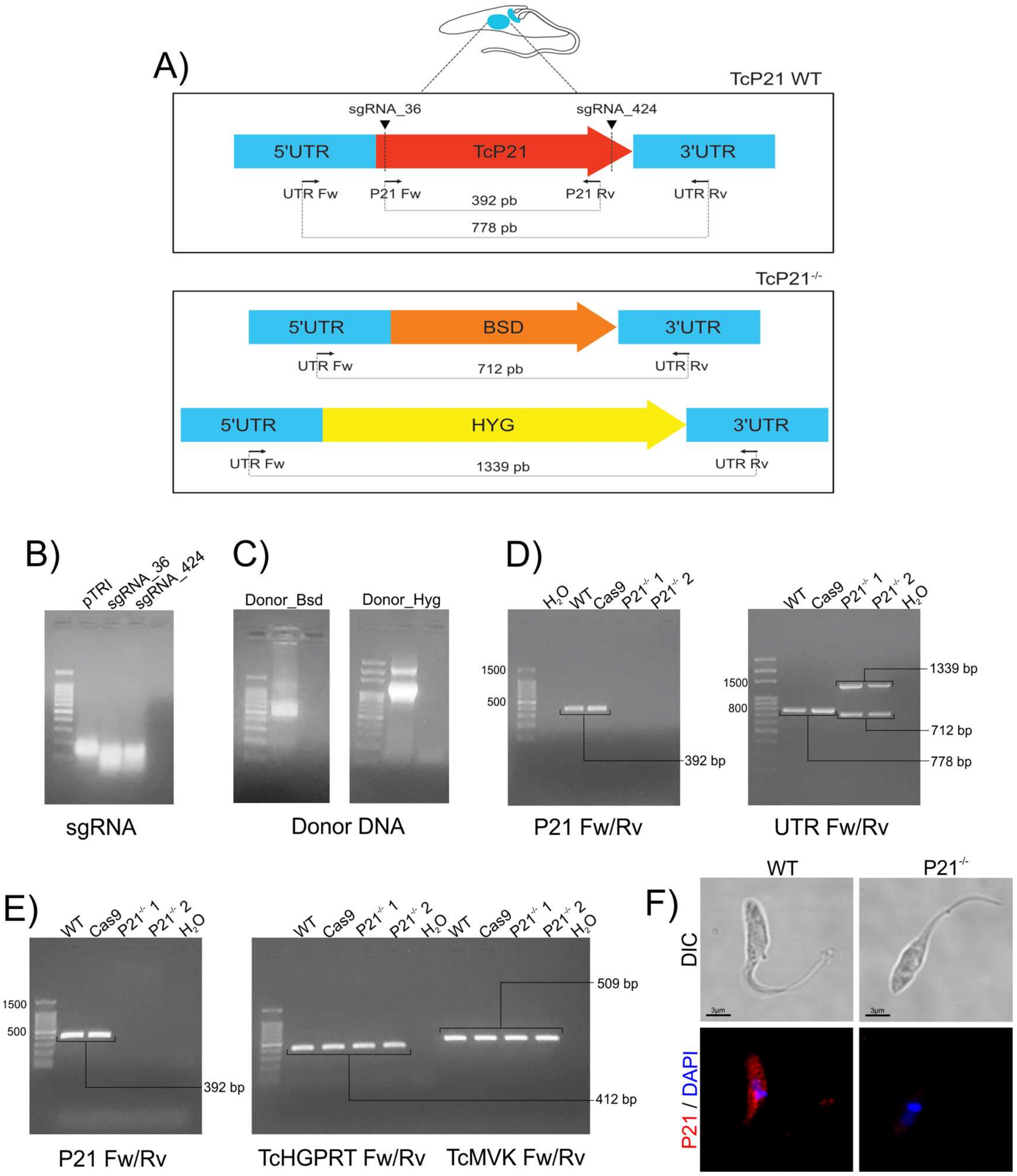
A)
TcP21 WT
sgRNA_36
sgRNA_424
5'UTR
TcP21
3'UTR
UTR Fw
P21 Fw
392 pb
P21 Rv
UTR Rv
778 pb
TcP21-/-
5'UTR
BSD
3'UTR
UTR Fw
712 pb
UTR Rv
5'UTR
HYG
3'UTR
UTR Fw
1339 pb
UTR Rv
B)
pTRI
sgRNA_36
sgRNA_424
sgRNA
C)
Donor_Bsd
Donor_Hyg
Donor DNA
D)
H2O
WT
Cas9
P21-/- 1
P21-/- 2
1500
500
392 bp
P21 Fw/Rv
WT
Cas9
P21-/- 1
P21-/- 2
H2O
1500
800
1339 bp
712 bp
778 bp
UTR Fw/Rv
E)
WT
Cas9
P21-/- 1
P21-/- 2
H2O
1500
500
392 bp
P21 Fw/Rv
509 bp
412 bp
TcHGPRT Fw/Rv
TcMVK Fw/Rv
F)
WT
P21-/-
DIC
3μm
P21 / DAPI
