# Supplementary material for: P21 Ablation Unveils Strain-Specific Transcriptional Reprogramming in Trypanosoma cruzi Amastigotes
Source: Int J Microbiol. 2025 Jul 4;2025:9919200. doi: 10.1155/ijm/9919200 (PMC12253989; doi:10.1155/ijm/9919200)
Supplement: Supporting Information 2 — Table S1: Biological process transcripts enriched in G strain TcP21-/intracellular amastigotes. [file 9919200.f2.pdf]

Supplementary Table 1: Biological processes transcripts enriched in G strain TcP21$^{-/-}$ intracellular amastigotes

ID	*DESCRIPTION*
UPREGULATED TRANSLATION	
TCG_00575	60S ribosomal subunit protein L31
TCG_00764	methyltransferase
TCG_00791	60S ribosomal protein L17
TCG_00940	ribosomal protein L15
TCG_00946	40S ribosomal protein S14
TCG_00970	putative 60S ribosomal protein L9
TCG_01380	ribosomal protein L21E (60S)
TCG_01628	putative 60S ribosomal protein L23a
TCG_01770	ribosomal protein S29
TCG_01906	40S ribosomal protein S15a
TCG_02057	60S ribosomal protein L12
TCG_02092	60S ribosomal protein L26
TCG_02510	60S ribosomal protein L13a
TCG_02639	ribosomal protein S19
TCG_02649	putative ribosomal protein L3
TCG_02796	ribosomal protein L35A
TCG_02966	40S ribosomal protein S24E
TCG_03508	putative ribosomal protein S7
TCG_03549	hypothetical protein
TCG_03847	40S ribosomal protein S3A
TCG_04538	60S acidic ribosomal protein P2
TCG_04979	ribosomal protein S26
TCG_05410	40S ribosomal protein S6
TCG_05529	60S ribosomal protein L26
TCG_05966	60S ribosomal protein L14
TCG_06224	ribosomal proteins L36
TCG_06314	40S ribosomal protein S15a
TCG_06395	40S ribosomal protein S15
TCG_06732	40S ribosomal protein L14
TCG_07213	60S ribosomal protein L35
TCG_07214	60S ribosomal protein L35
TCG_07369	60S ribosomal protein L12
TCG_07781	60S ribosomal protein L11
TCG_08072	60S ribosomal protein L6
TCG_08129	40S ribosomal protein S14
TCG_08135	ribosomal protein S20
TCG_08281	40S ribosomal protein S12
TCG_08913	60S ribosomal protein L44
TCG_08967	60S ribosomal protein L2
TCG_09183	60S ribosomal protein L6

TCG_09273	40S ribosomal protein S33
TCG_10488	40S ribosomal protein S13
TCG_11208	60S ribosomal protein L34
TCG_13465	40S ribosomal protein S8
TCG_13471	putative 40S ribosomal protein S23
PROTEIN PHOSPHORYLATION	
DOWNREGULATED	
TCG_00063	putative mitogen-activated protein kinase, putative,kinase
TCG_00242	transferase
TCG_00884	putative serine/threonine protein kinase, putative,protein kinase
TCG_01098	putative protein kinase
TCG_01099	putative protein kinase
TCG_01102	putative protein kinase
TCG_01148	putative protein kinase, putative,serine/threonine protein kinase
TCG_01179	putative protein kinase
TCG_02217	putative protein kinase
TCG_02514	putative serine/threonine protein kinase, putative,protein kinase
TCG_03076	casein kinase II, alpha chain
TCG_03170	putative protein kinase
TCG_04057	putative protein kinase
TCG_04220	putative protein kinase, putative,serine/threonine-protein kinase Nek1
TCG_04236	putative protein kinase, putative,serine/threonine protein kinase
TCG_05071	putative serine/threonine protein kinase
TCG_05295	putative protein kinase
TCG_06077	putative serine/threonine protein kinase, putative,protein kinase
TCG_06667	putative mitogen-activated protein kinase
TCG_07418	putative protein kinase
TCG_08019	putative mitogen-activated protein kinase 3
TCG_08097	putative protein kinase
TCG_09911	putative protein kinase
