# Supplementary material for: P21 Ablation Unveils Strain-Specific Transcriptional Reprogramming in Trypanosoma cruzi Amastigotes
Source: Int J Microbiol. 2025 Jul 4;2025:9919200. doi: 10.1155/ijm/9919200 (PMC12253989; doi:10.1155/ijm/9919200)
Supplement: Supporting Information 3 — Table S2: Biological process transcripts enriched in Y strain TcP21-/intracellular amastigotes. [file 9919200.f3.pdf]

Supplementary Table 2: Biological processes transcripts enriched in Y strain TcP21-/- intracellular amastigotes

ID	*DESCRIPTION*
UPREGULATED	
tRNA aminoacylation for protein translation	
TCG_00996	valyl-tRNA synthetase
TCG_01485	putative tryptophanyl-tRNA synthetase
TCG_04183	isoleucine--tRNA ligase
TCG_06042	uncharacterized protein
TCG_06182	putative arginyl-tRNA synthetase
UPREGULATED	
Protein import into nucleus	
TCG_00380	putative importin alpha
TCG_04944	putative importin beta-1 subunit
TCG_06807	karyopherin beta
DOWNREGULATED	
Translation	
TCG_00575	60S ribosomal subunit protein L31
TCG_00916	60S acidic ribosomal protein P2
TCG_00931	60S acidic ribosomal protein P2 beta (H6.4)
TCG_01077	60S ribosomal protein L17
TCG_01080	putative 40S ribosomal protein S2
TCG_01091	putative 40S ribosomal protein S2
TCG_01258	small subunit ribosomal protein S9e
TCG_01290	40S ribosomal protein S18
TCG_01628	putative 60S ribosomal protein L23a
TCG_01758	40S ribosomal protein S17
TCG_01858	40S ribosomal protein S21
TCG_02092	60S ribosomal protein L26
TCG_02464	ubiquitin/ribosomal protein S27a
TCG_02870	putative 60S ribosomal protein L4
TCG_03508	putative ribosomal protein S7
TCG_03960	60S ribosomal protein
TCG_04156	60S ribosomal protein L2
TCG_04512	ubiquitin/ribosomal protein S27a
TCG_04538	60S acidic ribosomal protein P2
TCG_04928	60S ribosomal protein L32
TCG_04979	ribosomal protein S26
TCG_05410	40S ribosomal protein S6
TCG_05510	60S ribosomal protein L13a
TCG_05529	60S ribosomal protein L26
TCG_06155	polyubiquitin
TCG_06224	ribosomal proteins L36

TCG_06395	40S ribosomal protein S15
TCG_06732	40S ribosomal protein L14
TCG_07214	60S ribosomal protein L35
TCG_07781	60S ribosomal protein L11
TCG_08004	putative 60S ribosomal protein L2
TCG_08072	60S ribosomal protein L6
TCG_08443	60S ribosomal protein L34
TCG_08967	60S ribosomal protein L2
TCG_09354	40S ribosomal protein SA
TCG_11208	60S ribosomal protein L34
TCG_12209	putative ribosomal protein L11
TCG_13465	40S ribosomal protein S8
TCG_13471	putative 40S ribosomal protein S23
DOWNREGULATED	
Cell adhesion	
TCG_07731	surface protease GP63
TCG_07894	putative surface protease GP63
TCG_08211	surface protease GP63
TCG_08787	GP63 group II protein
TCG_08789	surface protease GP63
TCG_08836	surface protease GP63
TCG_08837	surface protease GP63
TCG_09033	putative surface protease GP63
TCG_09600	surface protease GP63
TCG_10132	putative surface protease GP63
TCG_11623	putative surface protease GP63
TCG_11823	putative surface protease GP63
TCG_12560	surface protease GP63
TCG_12563	surface protease GP63
DOWNREGULATED	
Protein glycosylation	
TCG_07267	putative UDP-Gal or UDP-GlcNAc-dependent glycosyltransferase
TCG_07540	putative UDP-Gal or UDP-GlcNAc-dependent glycosyltransferase
TCG_10088	UDP-Gal or UDP-GlcNAc-dependent glycosyltransferase
TCG_10095	UDP-Gal or UDP-GlcNAc-dependent glycosyltransferase
TCG_10794	putative UDP-Gal or UDP-GlcNAc-dependent glycosyltransferase
TCG_11677	Alpha-(1,3)-fucosyltransferase, family GT10
TCG_11727	putative UDP-Gal or UDP-GlcNAc-dependent glycosyltransferase
TCG_12364	UDP-Gal or UDP-GlcNAc-dependent glycosyltransferase

TCG_12471	putative UDP-Gal or UDP-GlcNAc-dependent glycosyltransferase
TCG_13295	UDP-Gal or UDP-GlcNAc-dependent glycosyltransferase
DOWNREGULATED	
Translational elongation	
TCG_00916	60S acidic ribosomal protein P2
TCG_00931	60S acidic ribosomal protein P2 beta (H6.4)
TCG_02589	elongation factor-1 alpha
TCG_04538	60S acidic ribosomal protein P2
TCG_09233	putative elongation factor 1-gamma (EF-1-gamma)
TCG_11789	elongation factor 1-gamma (EF-1-gamma)
DOWNREGULATED	
Intracellular signal transduction	
TCG_01431	receptor-type adenylate cyclase
TCG_01432	adenylyl cyclase
TCG_06813	adenylate cyclase
TCG_10649	receptor-type adenylate cyclase
TCG_13155	receptor-type adenylate cyclase
DOWNREGULATED	
Cyclic nucleotide biosynthetic process	
TCG_01431	receptor-type adenylate cyclase
TCG_01432	adenylyl cyclase
TCG_06813	adenylate cyclase
TCG_10649	receptor-type adenylate cyclase
TCG_13155	receptor-type adenylate cyclase
