# Supplementary material for: P21 Ablation Unveils Strain-Specific Transcriptional Reprogramming in Trypanosoma cruzi Amastigotes
Source: Int J Microbiol. 2025 Jul 4;2025:9919200. doi: 10.1155/ijm/9919200 (PMC12253989; doi:10.1155/ijm/9919200)
Supplement: Supporting Information 6 — Table S5: Molecular function transcripts enriched in G strain TcP21-/- intracellular amastigotes. [file 9919200.f6.pdf]

Suplementary Table 5: Molecular functions transcripts enriched in G strain TcP21-/- intracellular amastigotes

ID	*DESCRIPTION*
UPREGULATED	
ATP binding	
TCG_00218	putative DEAD/DEAH box helicase-like protein
TCG_00682	putative pre-mRNA splicing factor
TCG_00686	putative helicase-like protein
TCG_00696	putative replication factor C, subunit 1
TCG_00704	putative minchromosome maintenance (MCM) complex subunit
TCG_00719	putative DNA repair and recombination protein RAD54
TCG_00730	putative protein kinase
TCG_00739	putative DNA helicase
TCG_00772	ATP-dependent RNA helicase
TCG_01002	putative homoserine kinase
TCG_01407	transferase
TCG_01450	ATP-binding cassette protein subfamily C, member 1
TCG_01454	rac serine-threonine kinase
TCG_01467	putative protein kinase
TCG_01886	pyruvate phosphate dikinase 1
TCG_02051	protein kinase
TCG_02955	DNA dependent protein kinase catalytic subunit
TCG_03414	putative DNA repair protein
TCG_03493	REL1 protein
TCG_03525	putative RNA helicase
TCG_03546	putative T-complex protein 1, gamma subunit
TCG_04349	putative ATP-dependent RNA helicase
TCG_04374	glycosomal ABC transporter member 1
TCG_04376	putative mevalonate kinase
TCG_04462	acetyl-CoA carboxylase
TCG_04472	putative protein kinase

TCG_04606	putative glycosomal phosphoenolpyruvate carboxykinase
TCG_05140	putative ATP-dependent RNA helicase
TCG_05447	transferase
TCG_05452	mismatch repair protein MLH1
TCG_05453	putative kinesin
TCG_05466	putative ATP-dependent DEAD/H DNA helicase recQ
TCG_05659	putative kinesin
TCG_06906	putative mismatch repair protein MSH4
TCG_07101	putative protein kinase
TCG_07108	putative cell division cycle protein
TCG_07307	putative serine/threonine protein kinase
TCG_07368	hypothetical protein
TCG_07416	Hsp90
TCG_07567	101 kDa heat shock protein
TCG_08163	chaperonin HSP60, mitochondrial precursor
TCG_08332	myosin heavy chain kinase A
TCG_08951	putative protein kinase
TCG_09372	putative phosphatidylinositol-4-phosphate 5-kinase-like protein
TCG_09682	hypothetical protein
TCG_09684	putative tryptophanyl-tRNA synthetase
TCG_09692	ATP-binding cassette protein subfamily C, member 2
TCG_12127	ATP-binding cassette protein subfamily C, member 1
UPREGULATED	
Structural constituent of ribosome	
TCG_00575	60S ribosomal subunit protein L31
TCG_00791	60S ribosomal protein L17
TCG_00940	ribosomal protein L15
TCG_00946	40S ribosomal protein S14

TCG_00970	putative 60S ribosomal protein L9
TCG_01380	ribosomal protein L21E (60S)
TCG_01468	hypothetical protein
TCG_01628	putative 60S ribosomal protein L23a
TCG_01770	ribosomal protein S29
TCG_01906	40S ribosomal protein S15a
TCG_02057	60S ribosomal protein L12
TCG_02092	60S ribosomal protein L26
TCG_02510	60S ribosomal protein L13a
TCG_02639	ribosomal protein S19
TCG_02649	putative ribosomal protein L3
TCG_02796	ribosomal protein L35A
TCG_02966	40S ribosomal protein S24E
TCG_03508	putative ribosomal protein S7
TCG_03549	hypothetical protein
TCG_03847	40S ribosomal protein S3A
TCG_04538	60S acidic ribosomal protein P2
TCG_04979	ribosomal protein S26
TCG_05410	40S ribosomal protein S6
TCG_05529	60S ribosomal protein L26
TCG_05966	60S ribosomal protein L14
TCG_06224	ribosomal proteins L36
TCG_06314	40S ribosomal protein S15a
TCG_06395	40S ribosomal protein S15
TCG_06732	40S ribosomal protein L14
TCG_07213	60S ribosomal protein L35
TCG_07214	60S ribosomal protein L35
TCG_07369	60S ribosomal protein L12
TCG_07781	60S ribosomal protein L11
TCG_08072	60S ribosomal protein L6
TCG_08129	40S ribosomal protein S14

TCG_08135	ribosomal protein S20
TCG_08281	40S ribosomal protein S12
TCG_08913	60S ribosomal protein L44
TCG_08967	60S ribosomal protein L2
TCG_09183	60S ribosomal protein L6
TCG_09273	40S ribosomal protein S33
TCG_10488	40S ribosomal protein S13
TCG_11208	60S ribosomal protein L34
TCG_13465	40S ribosomal protein S8
TCG_13471	putative 40S ribosomal protein S23
UPREGULATED	
Catalytic activity	
TCG_00400	putative dTDP-glucose 4,6-dehydratase
TCG_00710	triosephosphate isomerase
TCG_00760	putative aspartate aminotransferase, mitochondrial
TCG_00765	putative fumarate hydratase
TCG_01886	pyruvate phosphate dikinase 1
TCG_02805	protein G6
TCG_03529	acetyltransferase-like protein
TCG_04681	prostaglandin F2alpha synthase
TCG_04847	putative histidine ammonia-lyase
TCG_05528	transketolase 1
TCG_05968	putative cytosolic malate dehydrogenase
TCG_06686	putative aldose 1-epimerase-like protein
TCG_07370	2-amino-3-ketobutyrate coenzyme A ligase
TCG_07373	putative 2-amino-3-ketobutyrate coenzyme A ligase
TCG_07854	actin interacting protein-like protein
TCG_08739	putative 3,2-trans-enoyl-CoA isomerase, mitochondrial precursor
TCG_09067	putative dihydrouridine synthase (Dus)

TCG_12830	tyrosine aminotransferase
UPREGULATED	
Oxidoreductase activity	
TCG_01462	hypothetical protein
TCG_03535	putative lathosterol oxidase
TCG_04378	putative C-5 sterol desaturase
TCG_04681	prostaglandin F2alpha synthase
TCG_05330	putative glycerol-3-phosphate dehydrogenase (FAD-dependent)
TCG_05968	putative cytosolic malate dehydrogenase
TCG_06704	putative oxidoreductase
TCG_06818	putative delta-4 fatty acid desaturase
TCG_07244	hypothetical protein
TCG_07854	actin interacting protein-like protein
TCG_08449	NADH-cytochrome b5 reductase
TCG_08583	tryparedoxin peroxidase
TCG_09163	putative dihydrolipoamide dehydrogenase
TCG_09691	putative NAD(P)-dependent oxidoreductase
TCG_09726	putative NADP-dependent alcohol hydrogenase
TCG_09949	hypothetical protein
UPREGULATED	
Methyltransferase activity	
TCG_00371	putative DREV methyltransferase
TCG_00764	methyltransferase
TCG_01406	putative FtsJ cell division protein
TCG_01473	putative diphthine synthase
TCG_03547	putative S-adenosyl-methyltransferase mraW-like protein
TCG_04372	tRNA guanosine-2-O-methyltransferase TRM13
TCG_06697	putative ribosomal RNA methyltransferase

TCG_06839	putative sterol 24-c-methyltransferase
TCG_08984	putative nucleolar protein
DOWNREGULATED	
ATP binding	
TCG_00059	putative chaperonin alpha subunit
TCG_00063	putative mitogen-activated protein kinase, putative,kinase
TCG_00156	ATP-binding cassette protein subfamily F, member 2
TCG_00242	transferase
TCG_00316	putative OSM3-like kinesin
TCG_00882	putative 6-phosphofructo-2-kinase/fructose-2,6-biphosphatase
TCG_00884	putative serine/threonine protein kinase, putative,protein kinase
TCG_00907	putative 26S protease regulatory subunit
TCG_00953	S-adenosylmethionine synthetase
TCG_01073	putative ATP-dependent RNA helicase
TCG_01098	putative protein kinase
TCG_01099	putative protein kinase
TCG_01102	putative protein kinase
TCG_01110	C-terminal kinesin KIFC1
TCG_01148	putative protein kinase, putative,serine/threonine protein kinase
TCG_01158	putative kinesin
TCG_01179	putative protein kinase
TCG_01541	ATP-binding cassette protein subfamily A, member 10
TCG_01559	putative glucose regulated protein 94
TCG_01717	putative vesicular-fusion ATPase-like protein
TCG_01741	T-complex protein 1 subunit beta
TCG_02127	putative kinesin
TCG_02217	putative protein kinase
TCG_02514	putative serine/threonine protein kinase, putative,protein kinase

TCG_02924	mitochondrial ATP-dependent zinc metallopeptidase
TCG_03076	casein kinase II, alpha chain
TCG_03140	dynein, axonemal, heavy polypeptide 1
TCG_03170	putative protein kinase
TCG_03307	putative MCAK-like kinesin
TCG_03399	putative seryl-tRNA synthetase
TCG_03580	ATPase beta subunit
TCG_03750	putative cation transporting ATPase
TCG_04057	putative protein kinase
TCG_04183	isoleucine--tRNA ligase
TCG_04220	putative protein kinase, putative,serine/threonine-protein kinase Nek1
TCG_04236	putative protein kinase, putative,serine/threonine protein kinase
TCG_04588	hypothetical protein
TCG_05071	putative serine/threonine protein kinase
TCG_05295	putative protein kinase
TCG_05802	putative phospholipid-translocating P-type ATPase (flippase)
TCG_06042	uncharacterized protein
TCG_06077	putative serine/threonine protein kinase, putative,protein kinase
TCG_06214	putative methionyl-tRNA synthetase
TCG_06667	putative mitogen-activated protein kinase
TCG_07418	putative protein kinase
TCG_08019	putative mitogen-activated protein kinase 3
TCG_08097	putative protein kinase
TCG_08705	pyruvate kinase
TCG_09911	putative protein kinase
TCG_10163	metallo-peptidase, Clan MA(E), Family M41
TCG_10652	putative proteasome regulatory ATPase subunit 2
TCG_11092	ubiquitin-conjugating enzyme E2

DOWNREGULATED	
Protein binding	
TCG_00073	hypothetical protein
TCG_00090	leucine-rich repeat protein
TCG_00267	putative dynein
TCG_00476	putative paraflagellar rod component
TCG_00882	putative 6-phosphofructo-2-kinase/fructose-2,6-biphosphatase
TCG_01084	hypothetical protein
TCG_01092	putative peroxin 13
TCG_01901	putative protein transport protein Sec31
TCG_02191	putative eukaryotic translation initiation factor 4 gamma
TCG_02210	hypothetical protein
TCG_02218	hypothetical protein
TCG_02223	WD domain-containing protein
TCG_02343	hypothetical protein
TCG_02520	ribonucleoprotein p18
TCG_02905	hypothetical protein
TCG_03044	hypothetical protein
TCG_03180	flagellar associated protein
TCG_03277	hypothetical protein
TCG_03307	putative MCAK-like kinesin
TCG_03649	putative intraflagellar transport protein IFT88
TCG_03736	hypothetical protein
TCG_04215	Intraflagellar Transport Protein 140
TCG_04216	putative leucine-rich repeat protein (LRRP)
TCG_04234	vacuolar protein 8
TCG_04411	hypothetical protein
TCG_04957	putative eukaryotic translation initiation factor 5
TCG_05035	putative immunodominant antigen, putative,tc40 antigen-like
TCG_05904	pf20-like protein
TCG_05911	Protein XRP2
TCG_06028	putative vacuolar protein sorting complex subunit
TCG_06068	hypothetical protein
TCG_06486	nuclear protein Tc22

TCG_06664	putative leucine-rich repeat protein
TCG_07254	flagellar associated protein
TCG_07389	flagellar inner dynein arm I1 intermediate chain IC140
TCG_07390	programmed cell death 6-interacting protein
TCG_07555	hypothetical protein
TCG_07773	tetratricopeptide repeat protein 21B isoform a
TCG_07868	intraflagellar transport 172-like protein
TCG_07891	hypothetical protein
TCG_08168	putative vacuolar protein sorting-associated protein 41
TCG_08342	putative calreticulin
TCG_08642	WDdomain 60
TCG_08698	hypothetical protein
TCG_09148	hypothetical protein
DOWNREGULATED	
Protein kinase activity	
TCG_00063	putative mitogen-activated protein kinase, putative,kinase
TCG_00242	transferase
TCG_00884	putative serine/threonine protein kinase, putative,protein kinase
TCG_01098	putative protein kinase
TCG_01099	putative protein kinase
TCG_01102	putative protein kinase
TCG_01148	putative protein kinase, putative,serine/threonine protein kinase
TCG_01179	putative protein kinase
TCG_02217	putative protein kinase
TCG_02514	putative serine/threonine protein kinase, putative,protein kinase
TCG_03076	casein kinase II, alpha chain
TCG_03170	putative protein kinase
TCG_04057	putative protein kinase
TCG_04220	putative protein kinase, putative,serine/threonine-protein kinase Nek1

TCG_04236	putative protein kinase, putative,serine/threonine protein kinase
TCG_05071	putative serine/threonine protein kinase
TCG_05295	putative protein kinase
TCG_06077	putative serine/threonine protein kinase, putative,protein kinase
TCG_06667	putative mitogen-activated protein kinase
TCG_07418	putative protein kinase
TCG_08019	putative mitogen-activated protein kinase 3
TCG_08097	putative protein kinase
TCG_09911	putative protein kinase
DOWNREGULATED	
Metal ion binding	
TCG_00146	putative zinc finger protein
TCG_00953	S-adenosylmethionine synthetase
TCG_01116	putative RNA-binding protein
TCG_02076	putative reiske iron-sulfur protein precursor
TCG_02236	hypothetical protein
TCG_02241	endonuclease G
TCG_02474	hypothetical protein
TCG_02486	putative zinc finger protein family member
TCG_02504	hypothetical protein
TCG_03111	zinc finger protein
TCG_03694	putative fructose-1,6-bisphosphatase, cytosolic
TCG_03750	putative cation transporting ATPase
TCG_05909	cytosolic aconitase
TCG_06183	hypothetical protein
TCG_08438	zinc finger protein family memeber
TCG_10161	putative FYVE, RhoGEF and PH domain-containing protein 2
DOWNREGULATED	
Nucleotide binding	
TCG_03399	putative seryl-tRNA synthetase
TCG_03700	P-ATPase family transporter: proton

TCG_03750	putative cation transporting ATPase
TCG_04024	sarcoplasmic/endoplasmic reticulum calcium ATPase 3
TCG_04183	isoleucine--tRNA ligase
TCG_05802	putative phospholipid-translocating P-type ATPase (flippase)
TCG_06042	uncharacterized protein
TCG_06214	putative methionyl-tRNA synthetase
TCG_06609	succinyl-CoA synthetase alpha subunit
TCG_13098	putative P-type H+-ATPase
DOWNREGULATED	
Protein heterodimerization activity	
TCG_01152	histone H3 variant
TCG_03830	histone H2A
TCG_03832	histone H2A
TCG_05567	histone H2A
TCG_08085	histone H2B
DOWNREGULATED	
Calmodulin binding	
TCG_02132	paraflagellar rod protein 2C
TCG_05176	paraflagellar rod component
TCG_06311	putative paraflagellar rod protein 1D
