# Supplementary material for: P21 Ablation Unveils Strain-Specific Transcriptional Reprogramming in Trypanosoma cruzi Amastigotes
Source: Int J Microbiol. 2025 Jul 4;2025:9919200. doi: 10.1155/ijm/9919200 (PMC12253989; doi:10.1155/ijm/9919200)
Supplement: Supporting Information 7 — Table S6: Molecular function transcripts enriched in Y strain TcP21-/- intracellular amastigotes. [file 9919200.f7.pdf]

Supplementary Table 6: Molecular functions transcripts enriched in Y strain TcP21-/- intracellular amastigotes

ID	*DESCRIPTION*
UPREGULATED	
ATP binding	
TCG_00254	putative ABC transporter
TCG_00273	T-complex protein 1 subunit epsilon
TCG_00603	putative structural maintenance of chromosome (SMC) family protein
TCG_00825	putative protein kinase
TCG_00892	ATP-dependent DEAD/H RNA helicase
TCG_00907	putative 26S protease regulatory subunit
TCG_00947	putative protein kinase
TCG_00996	valyl-tRNA synthetase
TCG_01005	putative kinesin
TCG_01320	cytoplasmic dynein 2 heavy chain 1 isoform X1
TCG_01485	putative tryptophanyl-tRNA synthetase
TCG_01541	ATP-binding cassette protein subfamily A, member 10
TCG_01717	putative vesicular-fusion ATPase-like protein
TCG_02689	putative RNA editing associated helicase 2,putative
TCG_02715	ruvB-like 1
TCG_02742	ATP-binding cassette protein subfamily B, member 1
TCG_02833	putative protein kinase
TCG_03307	putative MCAK-like kinesin
TCG_03474	putative eukaryotic initiation factor 4a
TCG_03750	putative cation transporting ATPase
TCG_03916	putative nucleotide-binding protein
TCG_04057	putative protein kinase
TCG_04091	putative heat shock protein
TCG_04183	isoleucine--tRNA ligase
TCG_04349	putative ATP-dependent RNA helicase
TCG_04462	acetyl-CoA carboxylase
TCG_04548	topoisomerase
TCG_04791	cell division control protein 48-like protein E
TCG_04899	putative RNA helicase

TCG_05481	mitochondrial ATP-dependent zinc metallopeptidase
TCG_05829	putative DNA ligase
TCG_05830	DNA ligase
TCG_06042	uncharacterized protein
TCG_06182	putative arginyl-tRNA synthetase
TCG_07033	putative T-complex protein 1, delta subunit
TCG_07402	putative phenylalanyl-tRNA synthetase
TCG_07411	putative mismatch repair protein MSH2
TCG_07417	ATP-binding cassette protein subfamily F, member 3
TCG_07445	ATP-binding cassette protein subfamily F, member 1
TCG_07604	uncharacterized protein
TCG_07874	putative kinesin
TCG_07879	putative structural maintenance of chromosome protein 4
TCG_08098	putative structural maintenance of chromosome (SMC)
UPREGULATED	
Nucleotide binding	
TCG_00996	valyl-tRNA synthetase
TCG_01485	putative tryptophanyl-tRNA synthetase
TCG_03750	putative cation transporting ATPase
TCG_04183	isoleucine--tRNA ligase
TCG_04663	DNA polymerase epsilon catalytic subunit A
TCG_05256	putative calcium-transporting ATPase
TCG_05402	putative calcium motive p-type ATPase
TCG_06042	uncharacterized protein
TCG_06182	putative arginyl-tRNA synthetase
TCG_07200	glutamate dehydrogenase
TCG_07402	putative phenylalanyl-tRNA synthetase
UPREGULATED	
Oxidoreductase activity	
TCG_01027	putative aldehyde dehydrogenase
TCG_03914	oxidoreductase
TCG_03941	hypothetical protein

TCG_04256	alkyldihydroxyacetonephosphate synthase
TCG_06131	putative ribonucleoside-diphosphate reductase small chain
TCG_07200	glutamate dehydrogenase
TCG_08073	glutamate dehydrogenase
TCG_08077	tryparedoxin peroxidase
TCG_11294	trifunctional enzyme alpha subunit, mitochondrial precursor-like protein
UPREGULATED	
ATP hydrolysis activity	
TCG_00907	putative 26S protease regulatory subunit
TCG_01541	ATP-binding cassette protein subfamily A, member 10
TCG_01717	putative vesicular-fusion ATPase-like protein
TCG_02742	ATP-binding cassette protein subfamily B, member 1
TCG_03750	putative cation transporting ATPase
TCG_04791	cell division control protein 48-like protein E
TCG_05481	mitochondrial ATP-dependent zinc metallopeptidase
TCG_07417	ATP-binding cassette protein subfamily F, member 3
TCG_07445	ATP-binding cassette protein subfamily F, member 1
UPREGULATED	
Aminoacyl-trna ligase activity	
TCG_00996	valyl-tRNA synthetase
TCG_01485	putative tryptophanyl-tRNA synthetase
TCG_04183	isoleucine--tRNA ligase
TCG_06042	uncharacterized protein
TCG_06182	putative arginyl-tRNA synthetase
UPREGULATED	
Aminoacyl-trna editing activity	
TCG_00996	valyl-tRNA synthetase
TCG_04183	isoleucine--tRNA ligase
TCG_06042	uncharacterized protein
UPREGULATED	
Structural constituent of nuclear pore	

TCG_04832	putative nuclear pore complex protein (NUP155)
TCG_05048	putative ATP-dependent RNA helicase
TCG_06764	hypothetical protein
DOWNREGULATED	
Structural constituent of ribosome	
TCG_00575	60S ribosomal subunit protein L31
TCG_00916	60S acidic ribosomal protein P2
TCG_00931	60S acidic ribosomal protein P2 beta (H6.4)
TCG_01077	60S ribosomal protein L17
TCG_01080	putative 40S ribosomal protein S2
TCG_01091	putative 40S ribosomal protein S2
TCG_01258	small subunit ribosomal protein S9e
TCG_01290	40S ribosomal protein S18
TCG_01628	putative 60S ribosomal protein L23a
TCG_01758	40S ribosomal protein S17
TCG_01858	40S ribosomal protein S21
TCG_02092	60S ribosomal protein L26
TCG_02464	ubiquitin/ribosomal protein S27a
TCG_02870	putative 60S ribosomal protein L4
TCG_03508	putative ribosomal protein S7
TCG_03960	60S ribosomal protein
TCG_04156	60S ribosomal protein L2
TCG_04512	ubiquitin/ribosomal protein S27a
TCG_04538	60S acidic ribosomal protein P2
TCG_04928	60S ribosomal protein L32
TCG_04979	ribosomal protein S26
TCG_05410	40S ribosomal protein S6
TCG_05510	60S ribosomal protein L13a
TCG_05529	60S ribosomal protein L26
TCG_06155	polyubiquitin
TCG_06224	ribosomal proteins L36
TCG_06395	40S ribosomal protein S15
TCG_06732	40S ribosomal protein L14
TCG_07214	60S ribosomal protein L35
TCG_07781	60S ribosomal protein L11
TCG_08004	putative 60S ribosomal protein L2
TCG_08072	60S ribosomal protein L6
TCG_08443	60S ribosomal protein L34

TCG_08967	60S ribosomal protein L2
TCG_09354	40S ribosomal protein SA
TCG_11208	60S ribosomal protein L34
TCG_12209	putative ribosomal protein L11
TCG_13465	40S ribosomal protein S8
TCG_13471	putative 40S ribosomal protein S23
DOWNREGULATED	
Metalloendopeptidase activity	
TCG_07731	surface protease GP63
TCG_07894	putative surface protease GP63
TCG_08211	surface protease GP63
TCG_08787	GP63 group II protein
TCG_08789	surface protease GP63
TCG_08836	surface protease GP63
TCG_08837	surface protease GP63
TCG_09033	putative surface protease GP63
TCG_09600	surface protease GP63
TCG_10132	putative surface protease GP63
TCG_11623	putative surface protease GP63
TCG_11823	putative surface protease GP63
TCG_12560	surface protease GP63
TCG_12563	surface protease GP63
