# Supplementary material for: P21 Ablation Unveils Strain-Specific Transcriptional Reprogramming in Trypanosoma cruzi Amastigotes
Source: Int J Microbiol. 2025 Jul 4;2025:9919200. doi: 10.1155/ijm/9919200 (PMC12253989; doi:10.1155/ijm/9919200)
Supplement: Supporting Information 8 — Table S7: Comprehensive table listing all differentially expressed genes by G strain (Cas9 vs. P21−/−) amastigotes along with the fold change values. [file 9919200.f8.pdf]

G
(Cas9 vs.
P21−/−)

gene_id	baseMean	log2FoldChange	lfcSE	stat	pvalue	padj	type	description
TcG_00002	67,32149148	0,016750716	0,22912437	0,07310753	0,94172055	0,97296008	protein_codir	rhoptry protein
TcG_00003	509,6049534	-0,136734814	0,08601816	-1,58960397	0,1119241	0,28971061	protein_codir	hypothetical protein
TcG_00004	267,4062127	-0,160914656	0,11126245	-1,44626207	0,14810371	0,3469074	protein_codir	hypothetical protein
TcG_00005	285,2653049	-0,243088835	0,11389719	-2,13428293	0,03281963	0,12234498	protein_codir	hypothetical protein
TcG_00006	167,9781029	-0,34758614	0,13755762	-2,52684039	0,01150938	0,05519357	protein_codir	hypothetical protein
TcG_00007	211,4145772	-0,012539236	0,12375817	-0,10132047	0,91929607	0,96120058	protein_codir	COMPASS component SWD2
TcG_00008	285,6265811	-0,045589697	0,10612752	-0,4295747	0,66750504	0,82581306	protein_codir	protein SHQ1
TcG_00009	250,169546	-0,224223152	0,11439764	-1,96003304	0,04999193	0,16548757	protein_codir	hypothetical protein
TcG_00010	17,13858077	-1,12654426	0,43339726	-2,59933403	0,00934048	0,04678722	protein_codir	kinetoplast DNA-associated protein
TcG_00011	381,5212046	-0,784635582	0,09267413	-8,46660848	2,5264E-17	3,4889E-15	protein_codir	kinetoplast DNA-associated protein 3
TcG_00012	219,7292782	-0,360192885	0,12086354	-2,98016167	0,00288096	0,01844135	protein_codir	putative enolase
TcG_00013	121,7052966	-0,028946324	0,17071923	-0,16955514	0,86536	0,93509242	protein_codir	tRNA wybutosine-synthesizing protein 3
TcG_00014	116,6092249	0,08502413	0,17061452	0,49834053	0,61824405	0,793034	protein_codir	hypothetical protein
TcG_00015	185,2586073	-0,043732979	0,13656348	-0,32023921	0,748787	0,87322056	protein_codir	hypothetical protein
TcG_00016	212,3144838	0,052715133	0,12179379	0,43282284	0,66514348	0,82500292	protein_codir	hypothetical protein
TcG_00017	389,628074	-0,304488684	0,10053969	-3,02854201	0,00245737	0,01622284	protein_codir	hypothetical protein
TcG_00018	550,6400845	-0,326620783	0,08145015	-4,01006969	6,0701E-05	0,00072839	protein_codir	hypothetical protein
TcG_00019	424,6790982	-0,227861692	0,09591457	-2,37567348	0,01751696	0,0759406	protein_codir	putative enoyl-CoA hydratase/isomerase family protein
TcG_00020	323,3864505	-0,131615677	0,10496002	-1,25396012	0,2098565	0,43247908	protein_codir	putative enoyl-CoA hydratase/isomerase family protein
TcG_00021	209,0430728	-0,198711355	0,12489535	-1,5910229	0,11160442	0,28927267	protein_codir	transducin family protein / WD-40 repeat family protein
TcG_00022	199,5705799	-0,119552106	0,12541429	-0,95325742	0,34045966	0,57467448	protein_codir	hypothetical protein
TcG_00023	716,4718685	0,043837232	0,07121874	0,61552945	0,53820512	0,73759694	protein_codir	hypothetical protein
TcG_00024	636,9325052	-0,223414946	0,07397263	-3,02023791	0,00252576	0,01656111	protein_codir	hypothetical protein
TcG_00025	118,5878322	0,175497811	0,16342587	1,073868	0,28288185	0,51614876	protein_codir	hypothetical protein
TcG_00026	155,6971989	-0,255311919	0,14093271	-1,8115874	0,07004997	0,21201645	protein_codir	hypothetical protein
TcG_00027	559,5453515	-0,153161969	0,07977741	-1,91986639	0,05487478	0,17679942	protein_codir	hypothetical protein
TcG_00028	77,84822553	0,040440973	0,20019311	0,20200981	0,83990905	0,9222125	protein_codir	TBC1 domain family member 14
TcG_00029	104,0511625	0,362756097	0,1802533	2,01247964	0,0441694	0,15176355	protein_codir	hypothetical protein
TcG_00030	64,62576017	-0,648959906	0,21545032	-3,01210924	0,00259439	0,0169249	protein_codir	hypothetical protein
TcG_00031	503,8819187	-0,097116455	0,08628755	-1,12549785	0,26037813	0,49269002	protein_codir	putative CMP-sialic acid transporter
TcG_00032	280,3619162	-0,016608951	0,11092791	-0,14972744	0,88097966	0,94206932	protein_codir	putative ATP-dependent DEAD/H RNA helicase
TcG_00033	314,6297706	-0,251245915	0,10344742	-2,42873067	0,01515178	0,06816091	protein_codir	mediator of RNA polymerase II transcription subunit 24
TcG_00034	369,7774674	0,09246622	0,0997188	0,92726971	0,35378652	0,58808501	protein_codir	putative RNA-binding protein
TcG_00035	259,4989666	0,140377723	0,11833243	1,18629963	0,23550399	0,46332981	protein_codir	exosome-associated protein 2
TcG_00036	493,9625354	-0,224823747	0,09008594	-2,49565852	0,01257236	0,05911039	protein_codir	hypothetical protein
TcG_00037	215,3064973	-0,015120931	0,12372363	-0,12221538	0,90272844	0,95297279	protein_codir	hypothetical protein
TcG_00038	415,6655508	-0,132446978	0,09063341	-1,46134825	0,14391989	0,34057514	protein_codir	hypothetical protein
TcG_00039	161,434705	-0,000776225	0,13922772	-0,00557522	0,99555164	0,99830402	protein_codir	hypothetical protein
TcG_00040	241,5815004	-0,276914899	0,11485062	-2,41108749	0,01590503	0,07073923	protein_codir	hypothetical protein
TcG_00041	159,2213754	-0,11763716	0,14910044	-0,78897927	0,43012412	0,65502341	protein_codir	hypothetical protein
TcG_00042	251,7106092	-0,222358387	0,11855489	-1,87557332	0,06071389	0,19138343	protein_codir	hypothetical protein
TcG_00043	148,0234246	-0,109739434	0,14805982	-0,74118309	0,45858244	0,67743881	protein_codir	hypothetical protein
TcG_00044	793,3015992	0,019014994	0,07223796	0,26322716	0,7923755	0,89624823	protein_codir	hypothetical protein
TcG_00045	93,79136634	-0,224419035	0,18173451	-1,23487299	0,21687778	0,44089387	protein_codir	hypothetical protein
TcG_00046	2485,403916	-0,267736895	0,04646456	-5,76217487	8,3037E-09	2,6873E-07	protein_codir	hypothetical protein
TcG_00047	421,6799283	-0,198413881	0,09501995	-2,08812865	0,03678623	0,13335584	protein_codir	hypothetical protein
TcG_00048	300,2903321	0,147291364	0,1039012	1,41760985	0,1563047	0,35769079	protein_codir	tRNA pseudouridine13 synthase
TcG_00049	261,4273843	-0,220784122	0,11179703	-1,97486564	0,0482834	0,16153955	protein_codir	putative chloride channel protein

TcG_00050	385,3621804	-0,277344502	0,09983803	-2,77794448	0,0054704	0,03071502	protein_codir	putative lipase
TcG_00051	328,4962183	-0,238856009	0,10805149	-2,21057582	0,02706522	0,10590263	protein_codir	hypothetical protein
TcG_00052	483,1698054	-0,130464442	0,08356282	-1,56127387	0,11845915	0,30062612	protein_codir	hypothetical protein
TcG_00053	173,5816604	-0,410791994	0,13935167	-2,94787991	0,00319961	0,02010383	protein_codir	hypothetical protein
TcG_00054	355,5802612	-0,375502851	0,10304902	-3,64392461	0,00026851	0,00254373	protein_codir	putative CYC2-like cyclin, putative,cyclin 6
TcG_00055	995,4364559	-0,240701117	0,06530919	-3,68556297	0,0002282	0,00222926	protein_codir	putative dihydrolipoamide dehydrogenase
TcG_00056	461,0359266	-0,307972918	0,1005855	-3,06180236	0,00220009	0,01485443	protein_codir	putative chaperone DNAJ protein
TcG_00057	136,9346284	-0,153582994	0,1538727	-0,99811726	0,3182225	0,55334323	protein_codir	hypothetical protein
TcG_00058	114,2515281	-0,062596072	0,16400709	-0,38166686	0,70270849	0,84634944	protein_codir	hypothetical protein
TcG_00059	1097,639708	-0,403511726	0,06045731	-6,67432441	2,4837E-11	1,4317E-09	protein_codir	putative chaperonin alpha subunit
TcG_00060	143,2736187	-0,153025824	0,14577773	-1,04972018	0,29384678	0,52685064	protein_codir	hypothetical protein
TcG_00061	1057,605709	-0,118523093	0,0617791	-1,9184982	0,05504787	0,17711984	protein_codir	putative glucosamine-6-phosphate isomerase, putative,glucosamine-6-phosphate deaminase
TcG_00062	195,9015663	-0,466090652	0,13042851	-3,57353351	0,0003522	0,00320798		
TcG_00063	502,433043	-0,454551979	0,08206416	-5,53898288	3,0423E-08	8,792E-07	protein_codir	putative mitogen-activated protein kinase, putative,kinase
TcG_00064	325,3679422	-0,029433792	0,10372061	-0,28377958	0,7765793	0,88831084	protein_codir	hypothetical protein
TcG_00065	360,7541536	-0,313236924	0,10862865	-2,88355726	0,00393211	0,0237402	protein_codir	putative dynein light intermediate chain
TcG_00066	5,38257034	1,286492518	0,80371633	1,60067982	0,10944785	1	protein_codir	hypothetical protein
TcG_00067	218,0378946	-0,235339427	0,12601206	-1,86759442	0,06181862	0,19394275	protein_codir	Rab family, other
TcG_00068	359,8524031	-0,135339045	0,09460551	-1,43056197	0,15255579	0,35281603	protein_codir	hypothetical protein
TcG_00069	438,5080417	-0,119276175	0,08635665	-1,38120436	0,16721613	0,37314448	protein_codir	putative conserved oligomeric Golgi complex subunit 5-like
TcG_00070	375,6503268	0,039544339	0,09433104	0,41920811	0,67506404	0,82984531	protein_codir	putative mitochondrial RNA binding protein
TcG_00071	337,9397658	-0,001181101	0,09801975	-0,01204962	0,99038603	0,99603651	protein_codir	putative NADH dehydrogenase subunit NI8M
TcG_00072	452,7995244	-0,073063527	0,08712848	-0,83857228	0,40170937	0,63220648	protein_codir	hypothetical protein
TcG_00073	1696,382468	-0,236278556	0,05504096	-4,29277716	1,7645E-05	0,00024841	protein_codir	hypothetical protein
TcG_00074	713,9666735	-0,19925636	0,07174394	-2,77732678	0,0054808	0,03075058	protein_codir	delta-1-pyrroline-5-carboxylate synthetase
TcG_00075	351,5835263	-0,071796267	0,10360348	-0,69299088	0,48831527	0,69976756	protein_codir	hypothetical protein
TcG_00076	223,4634915	-0,029827549	0,12601375	-0,23670075	0,81288896	0,90759432	protein_codir	hypothetical protein
TcG_00077	280,6662301	-0,14150613	0,11376329	-1,24386458	0,21354941	0,43698047	protein_codir	hypothetical protein
TcG_00078	448,6111135	-0,047354476	0,08769618	-0,53998337	0,5892085	0,77564856	protein_codir	putative ATP-binding cassette protein subfamily B, member 3
TcG_00079	445,7349032	-0,360214769	0,09137396	-3,94220377	8,0736E-05	0,00092615	protein_codir	hypothetical protein
TcG_00080	189,9670902	-0,048173719	0,12945577	-0,37212492	0,70979984	0,85035063	protein_codir	hypothetical protein
TcG_00081	387,0587369	-0,28331541	0,09798383	-2,89145056	0,00383468	0,02328543	protein_codir	hypothetical protein
TcG_00082	644,0197328	-0,131159429	0,07566733	-1,7333693	0,08303005	0,23681114	protein_codir	hypothetical protein
TcG_00083	47,70027137	-0,163374593	0,25337195	-0,64480141	0,51905589	0,7237563	protein_codir	hypothetical protein
TcG_00084	53,69766374	-0,018669679	0,23587412	-0,07915103	0,9369125	0,97067587	protein_codir	hypothetical protein
TcG_00085	67,47093987	-0,427842864	0,21966697	-1,94768869	0,05145222	0,1686864	protein_codir	hypothetical protein
TcG_00086	60,07118313	-0,079599661	0,22979675	-0,34639159	0,72904844	0,86056342	protein_codir	hypothetical protein
TcG_00087	352,5601867	-0,256860662	0,09703122	-2,64719614	0,00811623	0,04194228	protein_codir	putative protein phosphatase 2A catalytic subunit
TcG_00088	255,0195716	-0,23083238	0,11187643	-2,06327975	0,03908606	0,13912475	protein_codir	putative chaperone DNAJ protein
TcG_00089	315,985289	-0,094578373	0,10685108	-0,88514198	0,37608006	0,60660777	protein_codir	GTP-binding protein
TcG_00090	2415,132875	-0,494459058	0,04637987	-10,6610696	1,5481E-26	5,7858E-24	protein_codir	leucine-rich repeat protein
TcG_00091	463,856426	-0,304386448	0,08494995	-3,58312666	0,00033951	0,00311196	protein_codir	CBS domain-containing protein
TcG_00092	224,0229706	-0,304468377	0,12340005	-2,46732774	0,01361257	0,0631366	protein_codir	hypothetical protein
TcG_00093	163,7547416	-0,287436621	0,14043117	-2,04681501	0,04067625	0,14281062	protein_codir	Rab5-interacting family protein
TcG_00094	76,14741636	-0,249677903	0,19946865	-1,25171499	0,21067374	0,43316165	protein_codir	cytochrome-c oxidase
TcG_00095	119,1453952	-0,222511311	0,16503934	-1,34823191	0,17758381	0,38791214	protein_codir	hypothetical protein
TcG_00096	217,3550353	-0,242640804	0,12785079	-1,89784365	0,05771668	0,18325718	protein_codir	hypothetical protein
TcG_00097	179,8146801	-0,338841506	0,14502171	-2,3364882	0,01946581	0,08231055	protein_codir	hypothetical protein
TcG_00098	32,40286867	-0,093735528	0,31978199	-0,29312323	0,76942796	0,88436428	protein_codir	hypothetical protein
TcG_00099	110,4894224	0,773930063	0,17353175	4,45987578	8,2007E-06	0,00012927	protein_codir	hypothetical protein
TcG_00100	71,30189977	0,487338875	0,22358457	2,17966237	0,0292825	0,11222859	protein_codir	trans-sialidase
TcG_00101	41,06241506	-0,257649362	0,27023061	-0,95344256	0,34036589	0,57459991	protein_codir	hypothetical protein

TcG_00102	179,3247205	-0,301026311	0,13356403	-2,25379773	0,0242089	0,09752582	protein_codir	putative class 3 lipase
TcG_00103	229,2272901	-0,098906039	0,12022276	-0,82268982	0,4106844	0,6373998	protein_codir	TATA binding protein
TcG_00104	336,4429842	-0,236801631	0,10051756	-2,35582341	0,0184817	0,07898524	protein_codir	putative cation transporter
TcG_00105	230,4317945	-0,288712314	0,11920059	-2,42207114	0,01543233	0,06919465	protein_codir	hypothetical protein
TcG_00106	120,2883349	-0,19549	0,16055859	-1,21756175	0,22339056	0,44980935	protein_codir	hypothetical protein
TcG_00107	381,903406	-0,050125992	0,09353612	-0,53589984	0,59202777	0,7775146	protein_codir	putative phosphatidic acid phosphatase protein
TcG_00108	269,0780953	-0,314781202	0,10906463	-2,88618962	0,00389937	0,02360618	protein_codir	putative GUP1
TcG_00109	96,25456123	-0,304164871	0,17834365	-1,70549879	0,08810144	0,24655634	protein_codir	glycerol uptake protein
TcG_00110	160,3132682	0,052984211	0,14934943	0,35476673	0,72276435	0,85733557	protein_codir	hypothetical protein
TcG_00111	36,95259042	0,238571395	0,28932176	0,8245885	0,40960525	0,63686685	protein_codir	putative glycerol uptake protein
TcG_00112	129,5727643	-0,162460667	0,15814881	-1,02726455	0,30429591	0,53710731	protein_codir	putative glycerol uptake protein
TcG_00113	263,9427432	-0,159244317	0,1133666	-1,40468464	0,16011508	0,36306159	protein_codir	hypothetical protein
TcG_00114	216,0938671	0,00192035	0,12924119	0,01485866	0,98814494	0,9951884	protein_codir	putative protein kinase
TcG_00115	171,5943205	-0,242183672	0,13832432	-1,75083942	0,07997358	0,23112343	protein_codir	putative RNA-binding protein
TcG_00116	176,2032225	-0,102267568	0,13114115	-0,77982823	0,43549199	0,65939964	protein_codir	hypothetical protein
TcG_00117	263,6632248	-0,254133731	0,10997158	-2,31090377	0,02083817	0,08690823	protein_codir	putative peroxisome assembly protein
TcG_00118	344,0459717	-0,170502532	0,09756061	-1,74765745	0,08052336	0,23213328	protein_codir	hypothetical protein
TcG_00119	1,525078208	0,329011134	1,39775154	0,23538599	0,81390916	1		
TcG_00120	0,611155623	-0,424209629	2,24295747	-0,18912959	0,84999125	1		
TcG_00121	0					1		
TcG_00122	11,13185034	-0,700882823	0,52021406	-1,34729696	0,17788461	1	protein_codir	putative RNA-binding protein 4
TcG_00123	171,5659236	-0,108460539	0,14488765	-0,74858372	0,45410815	0,67417952	protein_codir	hypothetical protein
TcG_00124	663,8937395	-0,200367505	0,07894439	-2,53808403	0,01114612	0,05383033	protein_codir	kinesin-14
TcG_00125	118,1901889	-0,056604069	0,16086648	-0,35186987	0,72493585	0,85817259	protein_codir	hypothetical protein
TcG_00126	159,4182693	-0,2866039	0,14369301	-1,99455701	0,04609122	0,15609846	protein_codir	U3 small nucleolar RNA-associated protein 24
TcG_00127	249,7387186	-0,360024725	0,11654937	-3,08903203	0,0020081	0,01378307	protein_codir	putative actin-related protein 2
TcG_00128	165,8986728	-0,426189179	0,14515961	-2,93600384	0,0033247	0,02069713	protein_codir	Cytochrome c oxidase assembly protein COX19
TcG_00129	407,7502408	-0,382248206	0,09388137	-4,07160863	4,669E-05	0,00058354	protein_codir	hypothetical protein
TcG_00130	341,6351049	-0,254468869	0,1039324	-2,44840744	0,01434893	0,06573614	protein_codir	hypothetical protein
TcG_00131	251,1908048	-0,196246971	0,11239484	-1,74604958	0,08080234	0,23270591	protein_codir	hypothetical protein
TcG_00132	1223,144238	-0,405705634	0,06378374	-6,36064334	2,0091E-10	9,2006E-09	protein_codir	hypothetical protein
TcG_00133	1465,793522	-0,484264165	0,05822799	-8,31669078	9,0453E-17	1,1516E-14	protein_codir	hypothetical protein
TcG_00134	226,4090568	-0,326111504	0,11783075	-2,76762651	0,00564661	0,03149815	protein_codir	cleavage stimulation factor subunit 1
TcG_00135	1349,58184	-0,042887872	0,06220961	-0,6894091	0,49056586	0,70169087	protein_codir	hypothetical protein
TcG_00136	421,3516826	-0,370832539	0,09021687	-4,11045652	3,9488E-05	0,00050001	protein_codir	proteasome regulatory non-ATPase subunit
TcG_00137	159,8698725	-0,385074046	0,13979275	-2,75460679	0,00587627	0,0324976	protein_codir	putative mitochondrial carrier protein
TcG_00138	492,2277161	-0,613589422	0,09435755	-6,50281227	7,8832E-11	3,9539E-09	protein_codir	hypothetical protein
TcG_00139	155,8390725	-0,314735944	0,14033867	-2,2426886	0,0249169	0,09947872	protein_codir	anaphase promoting complex subunit 10
TcG_00140	536,4304544	-0,112974161	0,0835807	-1,35167765	0,17647846	0,38656115	protein_codir	hypothetical protein
TcG_00141	269,7251449	-0,057446827	0,12945472	-0,44375999	0,65721611	0,81920451	protein_codir	hypothetical protein
TcG_00142	298,7924131	-0,108190927	0,10410354	-1,03926275	0,29868256	0,5322319	protein_codir	putative sphingosine kinase A, B
TcG_00143	1255,838737	-0,381107985	0,06114891	-6,23245729	4,5917E-10	2E-08	protein_codir	hypothetical protein
TcG_00144	331,0376348	-0,236116275	0,10117405	-2,33376329	0,01960812	0,08273113	protein_codir	putative tRNA pseudouridine synthase A-like protein
TcG_00145	615,0623991	-0,296915774	0,07772849	-3,81990926	0,0001335	0,00141773	protein_codir	WD repeat-containing protein wat1
TcG_00146	272,1063719	-0,471081417	0,11026943	-4,27209431	1,9365E-05	0,00026934	protein_codir	putative zinc finger protein
TcG_00147	132,3742507	0,177648167	0,1561043	1,13800948	0,25511653	0,48638803	protein_codir	putative FKBP-type peptidyl-prolyl cis-trans isomerase
TcG_00148	400,4341877	-0,460410577	0,0984995	-4,67424269	2,9504E-06	5,2997E-05	protein_codir	macrophage infectivity potentiator, precursor
TcG_00149	82,95832345	-0,409605483	0,2047396	-2,00061683	0,0454337	0,15454927	protein_codir	dynein light chain
TcG_00150	337,4879073	-0,511329911	0,10568846	-4,83808651	1,311E-06	2,57E-05	protein_codir	transcription factor IIA alpha-beta subunit
TcG_00151	249,0818305	-0,277615943	0,11164141	-2,48667547	0,0128943	0,06036094	protein_codir	hypothetical protein
TcG_00152	112,1628379	0,03142457	0,16982051	0,18504578	0,85319313	0,92887573	protein_codir	putative ADP-ribosylation factor family
TcG_00153	675,3000987	-0,223338159	0,07884545	-2,83260683	0,00461701	0,02704384	protein_codir	hypothetical protein

TcG_00154	531,3106499	-0,279286352	0,08411989	-3,32009893	0,00089986	0,00715561	protein_codir	vps53-like domain protein
TcG_00155	108,2963988	-0,238913193	0,1670266	-1,43039008	0,15260509	0,35284027	protein_codir	hypothetical protein
TcG_00156	618,9603478	-0,373922554	0,07906332	-4,72940622	2,2518E-06	4,1477E-05	protein_codir	ATP-binding cassette protein subfamily F, member 2
TcG_00157	607,3674027	-0,137201233	0,07902674	-1,73613678	0,08253965	0,23618286	protein_codir	putative signal recognition particle protein
TcG_00158	86,04288517	-0,257207218	0,19069855	-1,34876334	0,17741299	0,38761209	protein_codir	hypothetical protein
TcG_00159	135,5100919	-0,144137167	0,15375402	-0,93745302	0,34852558	0,58310373	protein_codir	putative GDP-mannose 4,6 dehydratase
TcG_00160	184,6914314	0,12192153	0,13084924	0,93177101	0,35145488	0,5859773	protein_codir	hypothetical protein
TcG_00161	520,0826113	-0,098604359	0,08578449	-1,14944274	0,25037346	0,48003094	protein_codir	putative transmembrane protein
TcG_00162	410,1316241	-0,017500285	0,09074902	-0,19284269	0,84708217	0,92596415	protein_codir	hypothetical protein
TcG_00163	129,0937241	0,070205106	0,15283719	0,4593457	0,64598593	0,81201944	protein_codir	hypothetical protein
TcG_00164	289,2797514	-0,068170324	0,10697744	-0,63724019	0,5239684	0,72776714	protein_codir	hypothetical protein
TcG_00165	288,1552045	0,308784889	0,10731612	2,87733929	0,00401044	0,0241126	protein_codir	hypothetical protein
TcG_00166	224,7216449	0,10296281	0,12465051	0,82601193	0,40879732	0,63660535	protein_codir	hypothetical protein
TcG_00167	403,5487922	-0,015716303	0,09711836	-0,16182627	0,87144267	0,93807811	protein_codir	putative chaperonin HSP60, mitochondrial precursor
TcG_00168	170,4471292	-0,229133563	0,14353068	-1,59640826	0,11039767	0,28717275	protein_codir	putative rab11B GTPase
TcG_00169	37,94769869	-0,58572458	0,29332256	-1,99686168	0,04584021	0,15547561	protein_codir	putative iron superoxide dismutase
TcG_00170	507,2577705	-0,206352126	0,0861559	-2,3951015	0,01661577	0,07311552	protein_codir	putative protein kinase
TcG_00171	182,9293073	-0,103455881	0,13446022	-0,76941625	0,44164625	0,66418918	protein_codir	hypothetical protein
TcG_00172	138,7592286	-0,133944061	0,15139831	-0,88471303	0,37631143	0,60681199	protein_codir	hypothetical protein
TcG_00173	302,4960867	-0,117175389	0,10448798	-1,12142461	0,26210716	0,49428247	protein_codir	hypothetical protein
TcG_00174	260,4833169	-0,013923615	0,11863333	-0,11736681	0,90656939	0,95471088	protein_codir	hypothetical protein
TcG_00175	433,9316528	-0,081785825	0,08937829	-0,91505244	0,3601641	0,59282019	protein_codir	hypothetical protein
TcG_00176	390,1327477	-0,159417503	0,0981743	-1,62382106	0,104414	0,27733624	protein_codir	hypothetical protein
TcG_00177	752,0259309	0,137645868	0,06991255	1,96882906	0,04897272	0,16318166	protein_codir	putative pumilio/PUF RNA binding protein 7
TcG_00178	305,0119979	-0,214645829	0,11003762	-1,95065866	0,05109766	0,16809129	protein_codir	CCCH zinc-finger 2
TcG_00179	247,8625687	0,073152116	0,11942424	0,61253993	0,54018059	0,73911316	protein_codir	methyltransferase
TcG_00180	663,2070347	-0,067218451	0,07691276	-0,87395712	0,38214161	0,61215602	protein_codir	hypothetical protein
TcG_00181	845,4875699	0,065400998	0,07870908	0,83092066	0,40601845	0,63466403	protein_codir	hypothetical protein
TcG_00182	343,396954	-0,082956046	0,10965873	-0,75649284	0,44935376	0,67029904	protein_codir	hypothetical protein
TcG_00183	268,0443621	-0,293260196	0,10854826	-2,70165717	0,00689949	0,03690556	protein_codir	putative protein phosphatase 2C
TcG_00184	234,5823053	0,246175862	0,12568211	1,9587184	0,05014577	0,16573473	protein_codir	putative mitochondrial carrier protein
TcG_00185	293,1845698	-0,030859682	0,11238246	-0,27459518	0,78362727	0,89273408	protein_codir	putative coatomer epsilon subunit
TcG_00186	330,9582944	-0,109987957	0,09948845	-1,10553497	0,26892785	0,50149655	protein_codir	DNA polymerase alpha subunit B
TcG_00187	492,2623549	-0,287504193	0,08725344	-3,29504704	0,00098405	0,00767755	protein_codir	hypothetical protein
TcG_00188	255,7512955	-0,122831367	0,11162238	-1,10041886	0,27114966	0,50434099	protein_codir	putative NAD synthase
TcG_00189	328,2795477	-0,044750545	0,10093017	-0,44338126	0,65748999	0,81928146	protein_codir	hypothetical protein
TcG_00190	5,352566293	0,405108401	0,7867859	0,51489026	0,6066297	1		
TcG_00191	307,1436927	-0,128155764	0,10266816	-1,24825223	0,2119387	0,43491353	protein_codir	hypothetical protein
TcG_00192	1062,404178	-0,064355532	0,0621882	-1,03485112	0,30073845	0,53419983	protein_codir	putative chromosomal passenger protein
TcG_00193	216,4036187	-0,004690917	0,12103359	-0,03875715	0,96908401	0,98671301	protein_codir	hypothetical protein
TcG_00194	326,9329721	-0,087086257	0,10224157	-0,85176959	0,39434199	0,62424461	protein_codir	hypothetical protein
TcG_00195	194,8295786	0,041616012	0,12643813	0,3291413	0,74204889	0,86877308	protein_codir	hypothetical protein
TcG_00196	295,0804906	-0,107101677	0,11117372	-0,96337229	0,33536072	0,56988697	protein_codir	hypothetical protein
TcG_00197	259,2197562	0,308493243	0,11168892	2,76207572	0,00574352	0,03188518	protein_codir	putative PIF1 helicase-like protein
TcG_00198	518,7122629	0,13724806	0,08136392	1,68684184	0,09163377	0,25289872	protein_codir	putative phosphomannose isomerase
TcG_00199	301,4909214	0,090846917	0,10791047	0,84187305	0,39985902	0,62979426	protein_codir	stromal membrane-associated protein
TcG_00200	137,6298484	0,07764834	0,14985937	0,51814137	0,60435964	0,7861526	protein_codir	hypothetical protein
TcG_00201	271,3021916	-0,248817886	0,11046816	-2,25239449	0,02429735	0,09778018	protein_codir	hypothetical protein
TcG_00202	318,024922	-0,332222327	0,10109365	-3,28628285	0,00101519	0,00787282	protein_codir	putative cell cycle sequence binding phosphoprotein (RBP33)
TcG_00203	299,943365	0,146013329	0,10728163	1,36102828	0,17350475	0,38209961	protein_codir	hypothetical protein
TcG_00204	209,222193	-0,2004311	0,12325095	-1,62620329	0,10390642	0,27630474	protein_codir	oligoribonuclease
TcG_00205	171,0413114	0,128477385	0,13818096	0,92977634	0,3524869	0,58676914	protein_codir	hypothetical protein

TcG_00206	481,7373385	-0,24842096	0,0858835	-2,89253402	0,00382148	0,02322961	protein_codir putative ATP-dependent zinc metallopeptidase
TcG_00207	220,0499113	0,278562358	0,12263977	2,27138675	0,02312358	0,09417789	protein_codir hypothetical protein
TcG_00208	284,9768816	0,071335835	0,11972812	0,59581521	0,55129868	0,74682127	protein_codir hypothetical protein
TcG_00209	355,8716871	0,075170969	0,1029191	0,73038888	0,46515252	0,6821385	protein_codir hypothetical protein
TcG_00210	434,4490554	0,026507883	0,08848497	0,29957498	0,76450137	0,88129227	protein_codir putative TRAF3-interacting protein 1-like
TcG_00211	283,4652461	0,343373028	0,12629932	2,71872435	0,00655342	0,03548034	protein_codir putative phosphatidylinositol 3-related kinase
TcG_00212	929,6831412	0,298644513	0,07314031	4,0831726	4,4425E-05	0,00055825	protein_codir putative phosphatidylinositol 3-related kinase
TcG_00213	139,9872196	0,194620048	0,15119996	1,28716993	0,19803507	0,4166396	protein_codir hypothetical protein
TcG_00214	284,6818873	0,196068551	0,10563884	1,8560271	0,0634497	0,19781709	protein_codir hypothetical protein
TcG_00215	740,6295641	-0,025968265	0,07029323	-0,36942766	0,71180899	0,85187676	protein_codir hypothetical protein
TcG_00216	298,0428711	0,095762752	0,10447716	0,91659034	0,35935735	0,5926302	protein_codir hypothetical protein
TcG_00217	212,9120131	0,016194975	0,13003795	0,12454037	0,90088744	0,95174891	protein_codir hypothetical protein
TcG_00218	437,1449208	0,567260811	0,09323344	6,08430628	1,17E-09	4,6106E-08	protein_codir putative DEAD/DEAH box helicase-like protein
TcG_00219	253,8087021	0,047504055	0,11352531	0,41844464	0,67562206	0,83017833	protein_codir hypothetical protein
TcG_00220	112,3622879	-0,065518332	0,16398015	-0,39955038	0,68948771	0,8386805	protein_codir hypothetical protein
TcG_00221	135,4297045	0,184578974	0,1541898	1,19708936	0,23127173	0,45851772	protein_codir hypothetical protein
TcG_00222	897,1733842	-0,782750953	0,06707492	-11,6698005	1,8185E-31	1,1089E-28	protein_codir trypomastigote small surface antigen
TcG_00223	47,85485859	0,057806059	0,25297399	0,22850594	0,81925294	0,91087139	protein_codir hypothetical protein
TcG_00224	1,867924756	-0,764781126	1,29606574	-0,59007896	0,55513771	1	
TcG_00225	37,42259348	-0,119853022	0,29673938	-0,40389995	0,68628629	0,83720952	protein_codir hypothetical protein
TcG_00226	53,72495219	-0,00931021	0,24752111	-0,0376138	0,9699956	0,98703399	protein_codir hypothetical protein
TcG_00227	0					1	protein_codir hypothetical protein
TcG_00228	3,046413349	0,812560764	0,99782833	0,81432922	0,41545638	1	protein_codir hypothetical protein
TcG_00229	16,78029875	0,05995677	0,43188892	0,13882452	0,88958882	0,94653573	protein_codir hypothetical protein
TcG_00230	33,79256316	-0,253204731	0,31838613	-0,79527562	0,42645319	0,65178385	protein_codir hypothetical protein
TcG_00231	43,67388121	-0,173650327	0,26414905	-0,65739523	0,51092682	0,71750523	protein_codir hypothetical protein
TcG_00232	58,41115106	-0,179578698	0,22976176	-0,78158653	0,4344576	0,65873534	
TcG_00233	194,0877733	0,528133391	0,14662358	3,60196759	0,00031582	0,0029296	protein_codir hypothetical protein
TcG_00234	660,0055877	-0,348901004	0,07304978	-4,77620913	1,7863E-06	3,3674E-05	protein_codir hypothetical protein
TcG_00235	590,9543704	-0,238947258	0,08132021	-2,93835006	0,00329964	0,02058678	protein_codir RNA guanylyltransferase
TcG_00236	296,8273296	0,263561991	0,103578	2,54457493	0,01094108	0,05297258	protein_codir hypothetical protein
TcG_00237	1238,274487	0,030830429	0,06132236	0,50276	0,61513302	0,79205726	protein_codir cleavage and polyadenylation specificity factor-like protein
TcG_00238	298,7945539	0,087891226	0,10931245	0,80403673	0,42137576	0,647488	protein_codir hypothetical protein
TcG_00239	359,4049928	-0,167676605	0,09511996	-1,7627909	0,07793576	0,22659065	protein_codir hypothetical protein
TcG_00240	367,1607598	-0,095372709	0,09366348	-1,01824861	0,30855982	0,54149866	protein_codir hypothetical protein
TcG_00241	230,612419	0,238602437	0,12456934	1,91541872	0,05543913	0,17802597	protein_codir putative polypeptide deformylase-like protein
TcG_00242	664,0803014	-0,307844132	0,07340583	-4,19372877	2,7441E-05	0,00036585	protein_codir transferase
TcG_00243	190,5007087	0,052358829	0,13653289	0,38348877	0,70135739	0,84532228	protein_codir RNA polymerase-like protein
TcG_00244	331,454585	-0,041428567	0,1049439	-0,39476871	0,69301359	0,8410627	protein_codir hypothetical protein
TcG_00245	224,6712527	-0,030294907	0,12194121	-0,24843862	0,80379505	0,90318531	protein_codir hypothetical protein
TcG_00246	260,0629772	0,187644517	0,11701566	1,6035847	0,10880562	0,28482195	protein_codir ubiquitin hydrolase
TcG_00247	398,4256081	0,002823208	0,10014802	0,02819036	0,97751033	0,99022484	protein_codir Bardet-Biedl syndrome 9
TcG_00248	632,7704389	-0,047711842	0,07858273	-0,60715428	0,54374853	0,74093036	protein_codir putative ADP-ribosylation factor GTPase activating protein 1
TcG_00249	576,9484864	-0,202493053	0,07834058	-2,58477868	0,00974415	0,04845311	protein_codir hypothetical protein
TcG_00250	485,8214867	-0,121074024	0,0829989	-1,4587426	0,14463597	0,34150241	protein_codir hypothetical protein
TcG_00251	316,8707991	-0,208776172	0,10193955	-2,04803908	0,04055617	0,14256183	protein_codir proteasome regulatory non-ATPase subunit
TcG_00252	197,0101555	0,259740574	0,13068191	1,98757863	0,04685831	0,15809563	protein_codir hypothetical protein
TcG_00253	262,1343684	0,007877274	0,11140751	0,07070685	0,94363107	0,97337158	protein_codir LmrCD-specific DARPin
TcG_00254	1547,625722	-0,006557018	0,05679321	-0,11545425	0,90808508	0,95541898	protein_codir putative ABC transporter
TcG_00255	231,6073581	0,053018436	0,1313174	0,40374265	0,68640197	0,83720952	protein_codir hypothetical protein
TcG_00256	382,5180784	0,034750407	0,09892635	0,35127556	0,72538163	0,85817259	protein_codir putative phosphatidylinositol-4-phosphate 5-kinase
TcG_00257	218,6803104	-0,079269423	0,12608727	-0,62868697	0,52955401	0,73154832	

TcG_00258	155,3032501	0,078386149	0,15388577	0,50937882	0,61048672	0,78905613	protein_codir	hypothetical protein
TcG_00259	498,4985171	-0,214487892	0,08511957	-2,51984241	0,01174074	0,05604788	protein_codir	polyadenylation/uridylation factor 2
TcG_00260	803,4692183	-0,196265938	0,06930125	-2,83206938	0,00462478	0,02705714	protein_codir	hypothetical protein
TcG_00261	254,2757745	0,182692473	0,11185056	1,63336223	0,10239284	0,27334981	protein_codir	putative mitochondrial carrier protein
TcG_00262	205,3152598	0,143227713	0,12478322	1,14781229	0,25104606	0,48084306	protein_codir	putative mitochondrial carrier protein
TcG_00263	886,8265237	-0,117603841	0,0677724	-1,73527637	0,08269187	0,23644323	protein_codir	putative exportin 1
TcG_00264	881,5334106	-0,184922119	0,07140162	-2,58988688	0,00960075	0,04782212	protein_codir	E3 ubiquitin-protein ligase TRIP12
TcG_00265	363,5123437	-0,064506305	0,09427831	-0,68421152	0,49384161	0,70424661	protein_codir	hypothetical protein
TcG_00266	349,7809271	-0,1849449	0,098753	-1,87280293	0,0610956	0,19193429	protein_codir	hypothetical protein
TcG_00267	741,8820286	-0,395441884	0,07206952	-5,48695058	4,0893E-08	1,15E-06	protein_codir	putative dynein
TcG_00268	178,3997654	-0,036169175	0,14593939	-0,24783696	0,80426056	0,90318531	protein_codir	vacuolar sorting protein
TcG_00269	384,8135038	0,022020462	0,09642905	0,2283592	0,81936701	0,91087139	protein_codir	hypothetical protein
TcG_00270	266,7221123	-0,02610419	0,11011391	-0,23706533	0,81260611	0,90759432	protein_codir	septum formation protein
TcG_00271	417,1956261	0,076367636	0,09124712	0,83693206	0,40263076	0,6328518	protein_codir	putative protein kinase
TcG_00272	471,6713959	0,103597932	0,08922194	1,16112622	0,24559057	0,47419697	protein_codir	hypothetical protein
TcG_00273	1337,93585	-0,078995985	0,05652744	-1,39748038	0,16226916	0,36605345	protein_codir	T-complex protein 1 subunit epsilon
TcG_00274	880,0040259	-0,034384664	0,06824815	-0,50381825	0,6143891	0,79153921	protein_codir	putative jouberin-like
TcG_00275	460,8007885	-0,058823874	0,0856228	-0,68701178	0,49207531	0,70263551	protein_codir	hypothetical protein
TcG_00276	11,64973494	-0,152345018	0,50055673	-0,30435116	0,76086038	1	protein_codir	hypothetical protein
TcG_00277	11,82541525	-0,034134906	0,49694921	-0,06868892	0,94523724	1	protein_codir	hypothetical protein
TcG_00278	711,5908686	-0,134988228	0,07437624	-1,81493746	0,06953352	0,21100455	protein_codir	calpain-like cysteine peptidase
TcG_00279	634,293495	-0,226847744	0,07952797	-2,85242705	0,00433868	0,02573882	protein_codir	putative ubiquitin-conjugating enzyme protein
TcG_00280	1395,91648	-0,194468307	0,05495317	-3,53880032	0,00040195	0,00359311	protein_codir	4SNc-Tudor domain protein
TcG_00281	423,7929785	0,001120512	0,09360005	0,01197128	0,99044853	0,99603651	protein_codir	putative CLIP-associating protein 1-like
TcG_00282	255,5827523	0,103046199	0,11311593	0,91097867	0,36230661	0,59490992	protein_codir	hypothetical protein
TcG_00283	226,135009	-0,163230396	0,11937854	-1,36733448	0,17152049	0,37866547	protein_codir	hypothetical protein
TcG_00284	515,5561183	-0,073966672	0,08688089	-0,85135725	0,39457093	0,62431497	protein_codir	putative vacuolar proton-ATPase-like protein
TcG_00285	333,9147555	-0,1243735	0,10669493	-1,16569268	0,24373872	0,47183907	protein_codir	protein phosphatase methylesterase 1
TcG_00286	186,830462	-0,062085644	0,1377177	-0,45081818	0,6521206	0,8159254	protein_codir	hypothetical protein
TcG_00287	459,3763346	-0,095446604	0,08721002	-1,09444541	0,27375968	0,50691699	protein_codir	putative ethanolamine-phosphate cytidylyltransferase
TcG_00288	229,3233181	0,053444269	0,11939255	0,44763487	0,65441673	0,81720977	protein_codir	60S ribosomal protein L18a
TcG_00289	352,9163912	0,238017148	0,10246246	2,32296935	0,0201808	0,0846848	protein_codir	60S ribosomal protein L18a
TcG_00290	732,597013	-0,075957703	0,07037308	-1,07935741	0,28042843	0,51360161	protein_codir	putative phenylalanyl-tRNA synthetase alpha chain
TcG_00291	655,3716556	0,030508508	0,08447266	0,36116429	0,71797663	0,85508792	protein_codir	hypothetical protein
TcG_00292	604,6548037	0,17848357	0,0779251	2,29045028	0,02199523	0,09043176	protein_codir	transcription factor subunit 2
TcG_00293	1042,434278	-0,05236696	0,06388355	-0,81972531	0,41237272	0,63907843	protein_codir	polypyrimidine binding protein
TcG_00294	74,68398931	-0,088284318	0,20975574	-0,42089107	0,67383463	0,82902391		
TcG_00295	724,1058445	-0,120096875	0,08437067	-1,42344349	0,15460763	0,35576644	protein_codir	hypothetical protein
TcG_00296	178,4684818	0,062722786	0,13370552	0,46911141	0,63899	0,80783774	protein_codir	CYC2-like cyclin 6
TcG_00297	254,2266166	-0,031234012	0,11998388	-0,2603184	0,79461818	0,89753819	protein_codir	putative serine/threonine protein kinase
TcG_00298	258,0279649	0,117051089	0,11603572	1,00875048	0,31309432	0,5472184	protein_codir	putative serine/threonine protein kinase
TcG_00299	97,8375089	0,178806702	0,18103179	0,98770888	0,32329524	0,55757366	protein_codir	serine/threonine protein kinase
TcG_00300	233,8961511	0,298145096	0,12335156	2,4170355	0,01564749	0,06978131	protein_codir	hypothetical protein
TcG_00301	480,5829494	-0,138533991	0,08921772	-1,55276315	0,12047973	0,3039301	protein_codir	putative MAP protein kinase
TcG_00302	160,0177867	0,061561361	0,14443379	0,42622547	0,66994356	0,82688463	protein_codir	putative ubiquitin-protein ligase
TcG_00303	234,3347235	0,104925453	0,12621676	0,83131156	0,40579764	0,63464372	protein_codir	glycosyl transferase family 2
TcG_00304	425,84453	0,509028072	0,09527299	5,34283732	9,1503E-08	2,3985E-06	protein_codir	hypothetical protein
TcG_00305	142,898068	0,135244438	0,14607489	0,92585687	0,35452037	0,58843087	protein_codir	cyclin 9
TcG_00306	1753,373114	0,210995076	0,05599754	3,76793443	0,0001646	0,00169822	protein_codir	putative RNA-binding protein
TcG_00307	193,628478	-0,133036732	0,13831791	-0,96181858	0,33614075	0,57078138	protein_codir	holo-[acyl-carrier-protein] synthase
TcG_00308	1010,192256	0,131766845	0,06515539	2,02234745	0,04314047	0,14915711	protein_codir	RNA binding protein-like protein
TcG_00309	249,0532403	0,334042249	0,12150209	2,74927161	0,00597279	0,03287002	protein_codir	hypothetical protein

TcG_00310	362,7694791	0,053258599	0,09606176	0,5544204	0,57929117	0,76889305	protein_codir	putative GDP-L-fucose synthetase
TcG_00311	449,5828898	-0,111912538	0,09068847	-1,23403274	0,2171907	0,44123645	protein_codir	Leucine-rich repeat-containing protein ODA7
TcG_00312	324,0167645	0,023913722	0,10708604	0,22331316	0,82329179	0,9127903	protein_codir	Pab1p-dependent poly(A) ribonuclease subunit
TcG_00313	150,5052851	0,209004627	0,14743459	1,41760915	0,15630491	0,35769079	protein_codir	U6 snRNA-associated Sm-like protein LSm4
TcG_00314	630,942921	0,057889704	0,07639138	0,75780419	0,44856822	0,6693858	protein_codir	putative ubiquitin carrier protein 4
TcG_00315	208,4842182	0,24544391	0,12607139	1,94686455	0,05155098	0,1687679	protein_codir	putative U3 small nucleolar ribonucleoprotein IMP3
TcG_00316	946,3506735	-0,258214294	0,06502038	-3,97128247	7,1487E-05	0,00084257	protein_codir	putative OSM3-like kinesin
TcG_00317	201,039642	0,02016594	0,12761724	0,15801893	0,87444188	0,93957209	protein_codir	hypothetical protein
TcG_00318	188,5372567	-0,034301611	0,13013445	-0,26358594	0,792099	0,89604169	protein_codir	centrin
TcG_00319	202,4824479	-0,062353231	0,12667703	-0,49222207	0,62256236	0,79666529	protein_codir	A kinase (PRKA) anchor protein 7
TcG_00320	349,063662	0,050482898	0,10131947	0,49825465	0,61830456	0,793034	protein_codir	ammecr1
TcG_00321	183,7757615	0,116942125	0,13698864	0,85366292	0,39329178	0,62300774	protein_codir	putative Mg transporter
TcG_00322	213,5599175	-0,150118867	0,12832386	-1,16984377	0,24206384	0,47087839	protein_codir	hypothetical protein
TcG_00323	200,5833126	0,126718008	0,13199754	0,96000277	0,33705382	0,57159039	protein_codir	nucleoporin SEH1
TcG_00324	397,4134718	-0,284444581	0,09659363	-2,94475499	0,00323211	0,02026362	protein_codir	hypothetical protein
TcG_00325	325,5594173	-0,000982613	0,099006	-0,00992478	0,9920813	0,99678187	protein_codir	hypothetical protein
TcG_00326	236,6489185	-0,247793813	0,11667168	-2,12385576	0,03368221	0,12471782	protein_codir	hypothetical protein
TcG_00327	427,525864	-0,147353657	0,09093559	-1,62041783	0,10514255	0,27863257	protein_codir	putative ATP-dependent RNA helicase-like protein
TcG_00328	86,1902272	0,594677417	0,19990317	2,97482738	0,00293153	0,01868249	protein_codir	hypothetical protein
TcG_00329	95,6580229	0,135012281	0,18666869	0,72327222	0,46951265	0,68614703	protein_codir	hypothetical protein
TcG_00330	1025,950502	-0,061841036	0,06229734	-0,99267536	0,32086819	0,5560344	protein_codir	co-chaperone GrpE
TcG_00331	900,8107665	-0,059459536	0,06861917	-0,86651489	0,38620786	0,61657291	protein_codir	kinesin
TcG_00332	115,122161	0,350239532	0,16438971	2,13054415	0,03312672	0,12310099	protein_codir	hypothetical protein
TcG_00333	154,9105821	-0,204316176	0,14126787	-1,44630322	0,14809217	0,3469074	protein_codir	hypothetical protein
TcG_00334	129,9315872	-0,045657666	0,17181435	-0,26573838	0,79044071	0,89521466	protein_codir	hypothetical protein
TcG_00335	1127,896549	0,237085544	0,06025457	3,93473101	8,329E-05	0,00095074	protein_codir	putative 4-methyl-5(beta-hydroxyethyl)-thiazole monophosphate synthesis protein
TcG_00336	173,1832162	0,080661293	0,13516686	0,59675345	0,550672	0,74654606	protein_codir	putative metallo-beta-lactamase superfamily
TcG_00337	560,6311869	0,058696132	0,07860268	0,74674462	0,45521773	0,67513474	protein_codir	hypothetical protein
TcG_00338	161,9098926	-0,228095859	0,13821239	-1,65032856	0,09887575	0,26647464	protein_codir	antigen 2
TcG_00339	185,437212	0,033386469	0,13066193	0,25551796	0,79832307	0,89939432	protein_codir	mitochondrial binding protein TBRGG1
TcG_00340	802,9913681	-0,119256194	0,06691542	-1,782193	0,07471776	0,22066725	protein_codir	mitochondrial oligo-U binding protein TBRGG1
TcG_00341	191,671989	0,081403977	0,12899979	0,63103959	0,52801464	0,73036982	protein_codir	hypothetical protein
TcG_00342	313,1937677	-0,146144242	0,10438938	-1,39999146	0,16151588	0,36513618	protein_codir	rac serine-threonine kinase-like protein
TcG_00343	176,478676	-0,037280989	0,13432961	-0,27753367	0,78137035	0,89130225	protein_codir	hypothetical protein
TcG_00344	145,233977	0,184136692	0,28675762	0,64213356	0,52078647	0,72530737	protein_codir	hypothetical protein
TcG_00345	354,8763498	-0,016397753	0,09924618	-0,16522301	0,86876845	0,93674123	protein_codir	SAM-dependent methyltransferase
TcG_00346	720,3415432	-0,103484654	0,07531488	-1,37402673	0,16943338	0,37606421	protein_codir	hypothetical protein
TcG_00347	350,7082939	-0,175930393	0,10420383	-1,68832946	0,091348	0,25241068	protein_codir	hypothetical protein
TcG_00348	392,2323568	0,0529218	0,09646778	0,5485956	0,583283	0,77198045	protein_codir	hypothetical protein
TcG_00349	43,08768326	0,119462789	0,27058836	0,44149271	0,65885634	0,820631	protein_codir	amastin
TcG_00350	130,1234718	0,391181779	0,17710482	2,20875853	0,02719144	0,10636058	protein_codir	amastin
TcG_00351	557,6798163	-0,040836319	0,07977299	-0,51190661	0,60871637	0,78817478	protein_codir	putative dTDP-glucose 4,6-dehydratase
TcG_00352	702,0430139	-0,1781456	0,07216362	-2,46863431	0,01356297	0,06295698	protein_codir	putative adenosine kinase
TcG_00353	391,4460282	-0,059920925	0,09510862	-0,63002624	0,52867742	0,73102477	protein_codir	hypothetical protein
TcG_00354	634,2589178	-0,083012959	0,07448559	-1,11448343	0,26507185	0,4975089	protein_codir	putative ubiquitin-protein ligase
TcG_00355	486,1510105	0,083639968	0,08613435	0,97104079	0,33152797	0,56578038	protein_codir	hypothetical protein
TcG_00356	671,7039103	0,475799334	0,07496984	6,34654351	2,2021E-10	1,0045E-08	protein_codir	Aspartate ammonia-lyase
TcG_00357	322,6120202	0,111190114	0,10017679	1,10993889	0,26702536	0,49939561	protein_codir	p22 protein precursor
TcG_00358	258,4432896	0,026308977	0,11152458	0,23590296	0,81350798	0,90788142		
TcG_00359	158,3318617	0,10762684	0,14072081	0,76482534	0,44437556	0,66642694	protein_codir	hypothetical protein
TcG_00360	683,597611	0,009492265	0,07381454	0,12859613	0,89767723	0,95018119	protein_codir	p22 protein precursor
TcG_00361	284,6740477	-0,031319217	0,11112064	-0,28184878	0,77805947	0,88925638	protein_codir	hypothetical protein

TcG_00362	292,2484585	0,155207433	0,10497607	1,47850296	0,13927321	0,33366819	protein_codir hypothetical protein
TcG_00363	376,3626721	0,077357343	0,09382614	0,82447542	0,40966947	0,63686685	protein_codir putative protein kinase
TcG_00364	895,2703224	-0,135255073	0,06807547	-1,9868402	0,04694011	0,15827942	protein_codir putative ubiquitin conjugation factor E4 B
TcG_00365	235,5360402	-0,101744879	0,12258945	-0,82996442	0,4065589	0,63483385	protein_codir hypothetical protein
TcG_00366	266,177088	0,092488891	0,11694145	0,79089914	0,42900285	0,65398767	protein_codir putative DnaJ chaperone protein
TcG_00367	432,969857	-0,170645648	0,09535179	-1,78964277	0,07351136	0,21849733	protein_codir succinate dehydrogenase subunit
TcG_00368	99,5040323	0,153993988	0,17579888	0,87596685	0,38104806	0,61104814	protein_codir ribosome-associated protein
TcG_00369	221,2122631	0,124237213	0,12014998	1,03401775	0,30112787	0,53435138	protein_codir hypothetical protein
TcG_00370	62,52936991	0,059278282	0,24647079	0,24050834	0,8099362	0,90627702	protein_codir hypothetical protein
TcG_00371	426,5170215	0,404795518	0,09443805	4,28636019	1,8162E-05	0,00025438	protein_codir putative DREV methyltransferase
TcG_00372	825,0749668	0,081684861	0,06880604	1,18717565	0,23515834	0,46296424	protein_codir putative RNA-binding protein
TcG_00373	503,1522597	0,080279223	0,08503877	0,94403089	0,34515387	0,57929591	protein_codir hypothetical protein
TcG_00374	252,9749784	0,180571801	0,11450903	1,57692197	0,11481348	0,29482025	protein_codir hypothetical protein
TcG_00375	359,07912	0,088427007	0,10578866	0,83588363	0,40322037	0,63298674	protein_codir hypothetical protein
TcG_00376	198,8268462	0,089551974	0,12720159	0,70401616	0,48142271	0,69556847	protein_codir hypothetical protein
TcG_00377	337,2405266	-0,067974234	0,09760447	-0,6964254	0,48616246	0,69827461	protein_codir ER membrane DUF1077 domain-containing protein
TcG_00378	91,22577804	0,013898794	0,18920019	0,07346079	0,94143945	0,97292993	protein_codir hypothetical protein
TcG_00379	212,6941863	0,129946385	0,13054299	0,99542983	0,31952725	0,55453007	protein_codir hypothetical protein
TcG_00380	903,4109958	-0,224345337	0,06499158	-3,45191384	0,00055663	0,00475276	protein_codir putative importin alpha
TcG_00381	386,5907465	-0,053400789	0,0924904	-0,57736573	0,56369242	0,75677498	protein_codir putative nucleolar MIF4G domain-containing protein 1-like
TcG_00382	126,4825092	0,012111606	0,16946478	0,07146975	0,9430239	0,97319113	protein_codir putative nucleolar MIF4G domain-containing protein 1-like
TcG_00383	413,7899103	-0,093030129	0,08913377	-1,04371357	0,29661793	0,52976959	protein_codir hypothetical protein
TcG_00384	190,3642152	-0,27651723	0,13254932	-2,08614595	0,0369654	0,13358739	protein_codir NUDIX hydrolase
TcG_00385	100,8087523	0,022466466	0,17478166	0,12854018	0,8977215	0,95018119	protein_codir hypothetical protein
TcG_00386	419,2196112	-0,429090538	0,09129027	-4,70028799	2,5979E-06	4,7178E-05	protein_codir putative ubiquitin hydrolase
TcG_00387	125,0107467	-0,668561071	0,16475905	-4,05781081	4,9535E-05	0,00061119	protein_codir putative ubiquitin hydrolase, putative,cysteine peptidase, Clan CA, family C19
TcG_00388	196,6257214	0,16051633	0,13082655	1,22693998	0,21984515	0,44499055	
TcG_00389	509,3042268	-0,164004404	0,09701907	-1,69043479	0,0909448	0,25165668	protein_codir hypothetical protein
TcG_00390	502,3451344	-0,070280917	0,08389732	-0,83770154	0,40219835	0,63244708	protein_codir zinc finger, c2h2 type domain containing protein
TcG_00391	178,3968818	0,011062508	0,13194519	0,08384169	0,9331823	0,96819648	protein_codir putative calcium-binding protein
TcG_00392	592,4997479	0,11887404	0,07993656	1,48710477	0,13698714	0,33007767	protein_codir hypothetical protein
TcG_00393	243,670428	0,389505307	0,11450082	3,40176877	0,00066951	0,00552885	protein_codir Pyridoxal kinase
TcG_00394	120,7268668	-0,098955501	0,16825542	-0,58812668	0,55644727	0,75122326	protein_codir transferase
TcG_00395	433,2488966	-0,014993335	0,08874927	-0,16894039	0,86584353	0,93535321	protein_codir hypothetical protein
TcG_00396	413,5785906	-0,012519399	0,089731	-0,13952144	0,88903811	0,9464367	protein_codir hypothetical protein
TcG_00397	354,3787825	-0,093534964	0,09648738	-0,96940098	0,33234516	0,5663408	protein_codir putative vacuolar assembly protein vps41
TcG_00398	1,235553853	2,181924197	1,74632122	1,24944035	0,21150406	1	protein_codir vacuolar assembly protein vps41
TcG_00399	2053,153326	0,279003115	0,05204543	5,36076077	8,2872E-08	2,1871E-06	protein_codir U3 small nuclear ribonucleoprotein (snRNP)
TcG_00400	2025,465091	0,5454744	0,05272305	10,3460335	4,3618E-25	1,2326E-22	protein_codir putative dTDP-glucose 4,6-dehydratase
TcG_00401	904,7831264	0,076278474	0,07148843	1,06700452	0,28596979	0,51918774	protein_codir hypothetical protein
TcG_00402	0					1	protein_codir vacuolar assembly protein vps41
TcG_00403	244,9223501	0,259507496	0,11461222	2,26422186	0,02356047	0,09564528	protein_codir hypothetical protein
TcG_00404	75,32328115	0,05777992	0,20665066	0,27960191	0,77978294	0,89039	protein_codir hypothetical protein
TcG_00405	248,6501451	0,081958819	0,11624019	0,70508163	0,48075944	0,69504353	protein_codir putative ABC transporter
TcG_00406	185,9598785	-0,274804036	0,12950896	-2,121892	0,03384681	0,12520725	protein_codir hypothetical protein
TcG_00407	249,2809483	-0,071623749	0,12310939	-0,58178951	0,56070847	0,75442638	protein_codir putative GTPase activating protein
TcG_00408	346,558723	-0,060270437	0,09721375	-0,61997854	0,53527192	0,73545133	protein_codir RNA helicase
TcG_00409	379,107597	0,003766926	0,09490016	0,03969357	0,96833743	0,98638627	protein_codir hypothetical protein
TcG_00410	94,86442783	0,255695538	0,1937531	1,31969781	0,18693593	0,40093293	protein_codir putative heat shock protein HslVU, ATPase subunit HslU
TcG_00411	240,1271737	0,027368023	0,1227193	0,2230132	0,82352523	0,91296176	protein_codir pleiotropic regulator 1
TcG_00412	266,8182985	0,089670963	0,1185246	0,75655996	0,44931354	0,67029904	protein_codir hypothetical protein
TcG_00413	296,2791926	0,235556601	0,10485814	2,24643127	0,0246764	0,09875673	protein_codir 200 kDa antigen p200

TcG_00414	390,7953492	-0,453449703	0,09371098	-4,83881055	1,3062E-06	2,565E-05	protein_codir	putative radial spoke protein 3
TcG_00415	325,1651706	0,043001651	0,09924705	0,43327887	0,66481219	0,82476879	protein_codir	hypothetical protein
TcG_00416	369,1349541	0,131326861	0,09952251	1,31956943	0,18697881	0,40095068	protein_codir	hypothetical protein
TcG_00417	91,14090045	0,131972882	0,18359691	0,71881864	0,47225267	0,68832802	protein_codir	phosphatidylinositol glycan, class P
TcG_00418	546,7847368	0,020468827	0,08242523	0,24833207	0,80387749	0,90318531	protein_codir	hypothetical protein
TcG_00419	252,0900292	0,019121934	0,12512783	0,15281919	0,87854087	0,94148439	protein_codir	hypothetical protein
TcG_00420	382,3264798	-0,233997978	0,09941606	-2,35372417	0,0185864	0,07922811	protein_codir	hypothetical protein
TcG_00421	134,5159758	-0,260498583	0,15467748	-1,68414038	0,09215454	0,2537919	protein_codir	hypothetical protein
TcG_00422	314,1422237	0,115792823	0,1020613	1,1345419	0,25656731	0,48858318	protein_codir	WD domain containing protein
TcG_00423	271,6799051	-0,132111109	0,10797004	-1,2235905	0,22110675	0,4463718	protein_codir	hypothetical protein
TcG_00424	181,7673258	-0,166791735	0,13275586	-1,2563795	0,20897841	0,43156443	protein_codir	hypothetical protein
TcG_00425	300,1804419	0,053012084	0,10502981	0,50473368	0,6137459	0,79123846	protein_codir	hypothetical protein
TcG_00426	335,5728487	0,133612705	0,10121318	1,32011171	0,18679772	0,40078489	protein_codir	putative MCAK-like kinesin
TcG_00427	305,2872613	0,144530456	0,10217433	1,41454763	0,15720117	0,35895404	protein_codir	60S acidic ribosomal protein P0
TcG_00428	189,6775266	0,194479777	0,13605738	1,42939527	0,15289066	0,35328902	protein_codir	putative CDC16
TcG_00429	95,80858277	0,302341787	0,1821521	1,65983147	0,09694836	0,26341002	protein_codir	Inosine triphosphate pyrophosphatase family protein isoform 1
TcG_00430	224,650959	0,088405934	0,12303475	0,71854443	0,47242166	0,68836848	protein_codir	putative serine/threonine protein kinase, putative,protein kinase
TcG_00431	623,3312878	-0,234989238	0,07656304	-3,06922559	0,00214614	0,01456663	protein_codir	hypothetical protein
TcG_00432	484,5164035	0,06576163	0,08408244	0,78210898	0,43415053	0,65855826	protein_codir	putative myosin heavy chain
TcG_00433	233,3821416	0,207497488	0,12143709	1,70868291	0,0875097	0,24531512	protein_codir	hypothetical protein
TcG_00434	469,4085707	0,00447972	0,08890375	0,05038843	0,95981286	0,98149972	protein_codir	syntaxin 1B/2/3
TcG_00435	121,9330616	0,115642072	0,16521227	0,69996056	0,48395194	0,69730968	protein_codir	hypothetical protein
TcG_00436	281,787217	-0,106725206	0,1169733	-0,91238945	0,36156374	0,59419539	protein_codir	hypothetical protein
TcG_00437	470,6427867	0,096132354	0,08571798	1,12149575	0,2620769	0,49428247	protein_codir	hypothetical protein
TcG_00438	324,5595424	-0,156144803	0,11438577	-1,36507193	0,17223044	0,37987091	protein_codir	putative cell cycle sequence binding phosphoprotein (RBP45)
TcG_00439	493,3603627	0,00158049	0,08425944	0,01875742	0,98503462	0,99412989	protein_codir	putative ABC1 protein
TcG_00440	498,1140277	0,025233393	0,08605845	0,29321226	0,76935991	0,88436428	protein_codir	putative phosphoribosylpyrophosphate synthetase
TcG_00441	2622,570741	0,114351239	0,04780077	2,39224673	0,01674558	0,07346244	protein_codir	60S ribosomal protein L18
TcG_00442	329,9572697	-0,30310525	0,11022088	-2,74998042	0,00595988	0,03281901	protein_codir	hypothetical protein
TcG_00443	309,7436675	-0,089572769	0,10556323	-0,84852246	0,39614707	0,62590481	protein_codir	methyltransferase
TcG_00444	248,0932471	-0,03167488	0,11845215	-0,26740653	0,78915617	0,89494301	protein_codir	putative amino acid permease-like protein
TcG_00445	376,9574927	0,106138703	0,09557043	1,11058102	0,26674874	0,49939561	protein_codir	putative chaperone DNAJ protein
TcG_00446	436,9086101	-0,199965886	0,0924977	-2,16184716	0,03062996	0,11604928	protein_codir	hypothetical protein
TcG_00447	724,2439691	-0,106309007	0,07205315	-1,47542492	0,14009834	0,3345817	protein_codir	metallo-peptidase, Clan MA(E), Family M3
TcG_00448	256,6640547	-0,092701199	0,11365371	-0,81564607	0,41470259	0,64165922	protein_codir	hypothetical protein
TcG_00449	728,1342939	-0,076098416	0,06976025	-1,09085643	0,27533606	0,50773647	protein_codir	hypothetical protein
TcG_00450	103,3055865	0,284102977	0,18296917	1,55273685	0,12048602	0,3039301	protein_codir	putative cytoplasmic l-asparaginase i-like protein
TcG_00451	778,4934171	0,216599488	0,07127312	3,0390068	0,00237359	0,01576862	protein_codir	60S ribosomal protein L22
TcG_00452	214,612156	0,001765164	0,12318337	0,01432957	0,98856705	0,99544045	protein_codir	hypothetical protein
TcG_00453	363,2102732	0,041389843	0,09684907	0,42736439	0,66911394	0,8263077	protein_codir	hypothetical protein
TcG_00454	670,9706459	-0,100027572	0,07375308	-1,3562495	0,1750198	0,38412188	protein_codir	putative ATP-dependent RNA helicase
TcG_00455	286,6652114	-0,094066044	0,10762917	-0,87398283	0,38212761	0,61215602	protein_codir	ADP-ribosylation factor-like protein
TcG_00456	330,7915822	0,151927787	0,10880778	1,39629528	0,16262559	0,36657201	protein_codir	putative gamma-tubulin complex subunit
TcG_00457	134,7736211	0,306662068	0,15629184	1,96211179	0,04974947	0,16499381	protein_codir	putative GTPase activating protein of Rab-like GTPase
TcG_00458	1311,881198	0,100876533	0,0700932	1,43917721	0,15010032	0,3500528	protein_codir	ubiquitin-protein ligase
TcG_00459	340,8795539	-0,193084554	0,09782782	-1,97371827	0,04841379	0,16166191	protein_codir	proteasome regulatory ATPase subunit
TcG_00460	256,1439909	-0,079974148	0,11265762	-0,70988674	0,47777438	0,69260885	protein_codir	putative kinesin
TcG_00461	276,2588011	0,152398021	0,11077176	1,37578409	0,16888849	0,37528616	protein_codir	hypothetical protein
TcG_00462	327,3683425	-0,378028523	0,09844071	-3,84016468	0,00012295	0,00132884	protein_codir	hypothetical protein
TcG_00463	409,6787409	-0,317082931	0,10197755	-3,10934068	0,00187505	0,01305279	protein_codir	putative C2 domain protein
TcG_00464	577,165096	0,080337724	0,08453204	0,95038189	0,34191824	0,5764646	protein_codir	hypothetical protein
TcG_00465	203,8636324	0,243490358	0,13375952	1,82035913	0,06870433	0,2093657	protein_codir	hypothetical protein

TcG_00466	567,7739074	0,004472819	0,0786392	0,05687772	0,9546426	0,97888932	protein_codir	putative trypanothione synthetase
TcG_00467	909,6857869	0,124477841	0,06554589	1,89909441	0,05755206	0,18280385	protein_codir	putative ADP-ribosylation factor-like 2, arl2
TcG_00468	391,5655172	-0,190064285	0,09159018	-2,07516018	0,03797169	0,13614561	protein_codir	crooked neck
TcG_00469	723,8946175	-0,257759577	0,07149695	-3,60518269	0,00031193	0,0028982	protein_codir	putative dispersed gene family protein 1 (DGF-1)
TcG_00470	250,3631421	-0,211566994	0,11201701	-1,88870416	0,05893148	0,18655195	protein_codir	putative immunodominant antigen, putative,tc40 antigen-like
TcG_00471	6,230421776	-0,417581614	0,72140214	-0,57884721	0,56269228	1		
TcG_00472	5,440191641	-0,980253655	0,77512352	-1,26464187	0,20599974	1	protein_codir	putative protein kinase
TcG_00473	440,7044174	0,096650229	0,08859272	1,09094995	0,27529491	0,50773647	protein_codir	hypothetical protein
TcG_00474	164,896286	-0,025216649	0,13563136	-0,18592048	0,85250712	0,92865245	protein_codir	hypothetical protein
TcG_00475	133,5930857	0,045935087	0,16040064	0,28637721	0,77458922	0,88749909	protein_codir	hypothetical protein
TcG_00476	573,5128393	-0,654732846	0,0792556	-8,26102917	1,4442E-16	1,7613E-14	protein_codir	putative paraflagellar rod component
TcG_00477	74,14989203	0,229982196	0,20827227	1,1042382	0,26948982	0,50230198	protein_codir	putative oxidoreductase
TcG_00478	3,181517306	-0,63410607	0,96582008	-0,65654678	0,51147238	1	protein_codir	hypothetical protein
TcG_00479	0,389577086	1,883588912	2,66752822	0,70611771	0,48011494	1	protein_codir	target of rapamycin (TOR) kinase 1
TcG_00480	116,6789372	0,003098897	0,16364321	0,01893691	0,98489144	0,99412989	protein_codir	trans-sialidase
TcG_00481	94,70979656	0,189886591	0,18336364	1,03557386	0,30040099	0,53413841	protein_codir	helicase-like protein
TcG_00482	474,9215284	-0,0273957	0,09208059	-0,29751874	0,7660705	0,88245107	protein_codir	putative tyrosine specific protein phosphatase
TcG_00483	652,8162588	0,168096475	0,07512955	2,23742162	0,0252588	0,1004974	protein_codir	putative NADH dehydrogenase
TcG_00484	802,9396812	-0,066528636	0,06781166	-0,98107961	0,32655348	0,56100959	protein_codir	putative phosphoribosylpyrophosphate synthetase
TcG_00485	410,1606046	0,054214531	0,09241266	0,58665695	0,55743412	0,75211736	protein_codir	putative ATP-dependent chaperone
TcG_00486	394,6308443	0,044182776	0,09663653	0,45720573	0,64752318	0,81260383	protein_codir	putative selenophosphate synthetase
TcG_00487	260,969041	0,015533543	0,11624834	0,13362378	0,89370008	0,94872724	protein_codir	branch point binding protein
TcG_00488	202,2459113	0,196038428	0,13040754	1,50327529	0,13276807	0,323683	protein_codir	macrocin-O-methyltransferase domain-containing protein
TcG_00489	397,8847737	-0,170389814	0,0954347	-1,78540737	0,07419526	0,2199658	protein_codir	hypothetical protein
TcG_00490	363,5268889	0,052206721	0,1041371	0,50132684	0,61614112	0,79256256	protein_codir	hypothetical protein
TcG_00491	513,7696577	-0,956771236	0,09007426	-10,622027	2,3539E-26	8,5227E-24	protein_codir	putative trans-sialidase
TcG_00492	9,442952925	-0,146892782	0,58187746	-0,25244625	0,80069616	1	protein_codir	hypothetical protein
TcG_00493	66,25419693	0,533438056	0,22565297	2,36397533	0,01808002	0,0777273	protein_codir	hypothetical protein
TcG_00494	74,57245657	-0,070988331	0,20495419	-0,34636193	0,72907073	0,86056342	protein_codir	hypothetical protein
TcG_00495	82,01856895	-0,062959116	0,19752108	-0,31874631	0,74991889	0,87392479	protein_codir	hypothetical protein
TcG_00496	200,048079	-0,098741795	0,12869631	-0,76724649	0,44293497	0,66499591	protein_codir	hypothetical protein
TcG_00497	139,5457995	-0,055686805	0,14896444	-0,37382616	0,70853366	0,84953647	protein_codir	hypothetical protein
TcG_00498	374,845694	-0,303781815	0,10848542	-2,80020867	0,00510696	0,02916176	protein_codir	DHHC-type zinc finger family protein
TcG_00499	203,2136739	-0,026796266	0,13364071	-0,20050975	0,84108193	0,92246657	protein_codir	nuclear lim interactor-interacting factor
TcG_00500	276,2035581	-0,013260777	0,11530854	-0,11500256	0,90844309	0,95550022	protein_codir	hypothetical protein
TcG_00501	249,6504171	-0,049445461	0,11654685	-0,42425395	0,67138062	0,82777091	protein_codir	hypothetical protein
TcG_00502	144,711337	-0,098999032	0,15033499	-0,65852289	0,51020219	0,71703088	protein_codir	hypothetical protein
TcG_00503	317,322234	-0,137493796	0,10394249	-1,32278716	0,18590617	0,39987989	protein_codir	hypothetical protein
TcG_00504	163,1544683	-0,017833898	0,14264142	-0,12502608	0,90050291	0,95167624	protein_codir	ankyrin repeat protein
TcG_00505	354,4923627	-0,279563621	0,10012257	-2,79221375	0,00523488	0,02968736	protein_codir	hypothetical protein
TcG_00506	370,8743952	-0,154406763	0,1007692	-1,53228132	0,12545303	0,31237885	protein_codir	hypothetical protein
TcG_00507	295,1156624	0,140667447	0,10991431	1,27979188	0,20061834	0,41986345	protein_codir	hypothetical protein
TcG_00508	184,7487604	0,016453634	0,13787282	0,11933922	0,90500661	0,95390315	protein_codir	syntaxin
TcG_00509	385,3455341	-0,105154851	0,09630778	-1,09186247	0,27489356	0,50755645	protein_codir	putative acyl-CoA dehydrogenase
TcG_00510	229,919335	-0,221435443	0,12346223	-1,7935481	0,07288533	0,21724965	protein_codir	putative transporter
TcG_00511	0,467697622	2,091063019	2,51804996	0,83042952	0,40629598	1		
TcG_00512	246,900817	0,288161302	0,11546997	2,49555192	0,01257614	0,05911039	protein_codir	putative galactokinase-like protein
TcG_00513	213,0362435	0,007422728	0,12444997	0,05964427	0,95243896	0,97749648	protein_codir	hypothetical protein
TcG_00514	156,9883893	-0,051345015	0,14118362	-0,36367543	0,71610039	0,85427594	protein_codir	hypothetical protein
TcG_00515	328,3833022	0,276211746	0,10056905	2,74648869	0,0060237	0,03310747	protein_codir	hypothetical protein
TcG_00516	478,4584848	-0,070548967	0,08612478	-0,81914833	0,41270179	0,6393939	protein_codir	hypothetical protein
TcG_00517	273,162215	-0,000514351	0,10994169	-0,0046784	0,99626719	0,99876713	protein_codir	hypothetical protein

TcG_00518	30,26646853	0,424379993	0,32906538	1,2896525	0,19717134	0,41560128	
TcG_00519	259,6291175	-0,102689984	0,11520595	-0,89136011	0,37273601	0,60390427	protein_codir putative transporter
TcG_00520	194,8630578	0,158741855	0,13472738	1,17824496	0,23869896	0,46668346	protein_codir putative transporter
TcG_00521	199,5922592	0,139041604	0,12457227	1,11615213	0,26435702	0,49664999	protein_codir hypothetical protein
TcG_00522	273,1079006	0,221125909	0,12091094	1,82883294	0,06742464	0,20673673	protein_codir putative cysteine desulfurase
TcG_00523	452,7177509	0,031158996	0,08934277	0,3487579	0,72727107	0,85937406	protein_codir hypothetical protein
TcG_00524	154,0988764	0,159076522	0,14233281	1,11763777	0,26372173	0,49602159	protein_codir hypothetical protein
TcG_00525	356,4832808	0,107226584	0,0961954	1,11467472	0,26498984	0,4975089	protein_codir putative protein kinase-like protein
TcG_00526	127,409156	0,174482684	0,15433057	1,13057757	0,25823293	0,49064584	protein_codir trafficking protein particle complex subunit 1
TcG_00527	222,7355618	0,151835577	0,12600804	1,20496736	0,2282159	0,4553314	protein_codir tRNA/rRNA methyltransferase YsgA
TcG_00528	248,5488222	0,304255035	0,11362446	2,67772473	0,00741241	0,03901872	protein_codir hypothetical protein
TcG_00529	97,84863207	-0,159241795	0,18900848	-0,8425114	0,39950176	0,6294027	protein_codir putative dynein light chain
TcG_00530	178,1406228	0,235267771	0,13870268	1,69620201	0,08984763	0,24975399	protein_codir poly(ADP-ribose) glycohydrolase
TcG_00531	428,6999539	-0,220669049	0,09058873	-2,43594379	0,01485299	0,06732284	protein_codir hypothetical protein
TcG_00532	429,5212799	-0,024801213	0,09075459	-0,27327778	0,78463969	0,8934482	protein_codir putative dolichol kinase
TcG_00533	849,9984793	0,22559866	0,07453146	3,02689182	0,00247082	0,01628382	protein_codir tRNA wybutosine-synthesizing protein 4
TcG_00534	740,2015622	0,039791726	0,07032747	0,56580633	0,57152545	0,76269221	protein_codir hypothetical protein
TcG_00535	189,6008465	0,110339638	0,12880276	0,85665588	0,39163509	0,62139829	protein_codir Suppression of tumorigenicity 5
TcG_00536	329,3137231	-0,228730096	0,10820997	-2,11376168	0,03453563	0,12718684	protein_codir hypothetical protein
TcG_00537	253,2567201	0,160366929	0,11386004	1,40845669	0,15899588	0,36134294	protein_codir ATP/GTP nucleotide-binding protein
TcG_00538	603,7963529	0,248873331	0,07633364	3,26033626	0,0011128	0,00850457	protein_codir putative chaperone DNAJ protein
TcG_00539	194,7318616	0,002954763	0,13075043	0,0225985	0,98197054	0,99328993	protein_codir zinc finger protein, predicted
TcG_00540	164,4827867	0,395951571	0,14323427	2,76436335	0,0057034	0,03169285	protein_codir hypothetical protein
TcG_00541	128,3317429	0,233842218	0,15607007	1,49831556	0,13405128	0,3256013	protein_codir lactoylglutathione lyase-like protein
TcG_00542	449,7639016	-0,192476335	0,08534152	-2,2553656	0,02411039	0,09723041	protein_codir putative phospholipase A1
TcG_00543	175,0857094	0,056549475	0,13576823	0,41651479	0,67703335	0,83085567	protein_codir putative phospholipase A2-like protein
TcG_00544	160,9998627	-0,23352696	0,1430245	-1,63277592	0,10251613	0,2735495	protein_codir putative prenyl protein specific carboxyl methyltransferase
TcG_00545	310,1889415	-0,105709123	0,10702612	-0,98769458	0,32330225	0,55757366	protein_codir NADH:ubiquinone reductase (H(+)-translocating)
TcG_00546	322,6311299	0,092286608	0,09985961	0,92416349	0,3554012	0,58930918	protein_codir putative nucleic acid binding protein
TcG_00547	1994,225053	-0,061549948	0,05220625	-1,17897669	0,23840746	0,46642837	protein_codir putative ubiquitin-activating enzyme e1
TcG_00548	350,1495545	0,026935522	0,10028803	0,26858164	0,78825164	0,89483476	protein_codir hypothetical protein
TcG_00549	347,7752357	-0,223142682	0,09716185	-2,296608	0,02164114	0,08947769	protein_codir putative glycerol kinase, glycosomal
TcG_00550	166,6322581	-0,028139062	0,13951439	-0,20169289	0,84015682	0,92224543	protein_codir hypothetical protein
TcG_00551	387,3538915	-0,348884897	0,09364588	-3,72557658	0,00019487	0,00195252	
TcG_00552	1775,9602	-0,167425796	0,06090071	-2,74916009	0,00597482	0,03287002	protein_codir putative ATP-dependent RNA helicase
TcG_00553	154,0866478	0,063979425	0,15211015	0,42061247	0,67403808	0,82902391	protein_codir MFS transporter, OPA family, solute carrier family 37 (glycerol-3-phosphate transporter), member 1/2
TcG_00554	346,2429957	-0,066461666	0,09991267	-0,6651976	0,50592412	0,71267956	protein_codir hypothetical protein
TcG_00555	376,9031292	0,140255747	0,09695139	1,44666047	0,14799204	0,3468885	protein_codir hypothetical protein
TcG_00556	172,3388018	0,002054034	0,1356602	0,01514102	0,98791968	0,99514516	protein_codir dynein light chain-like protein
TcG_00557	69,27891504	0,102821082	0,20850198	0,49314201	0,62191225	0,79636111	protein_codir hypothetical protein
TcG_00558	315,887352	0,223076279	0,11110015	2,00788456	0,04465556	0,15261927	protein_codir inositol-trisphosphate 3-kinase
TcG_00559	273,1923137	-0,044709444	0,108769	-0,41104952	0,68103623	0,83364878	protein_codir hypothetical protein
TcG_00560	147,7153688	-0,114085984	0,14429814	-0,79062686	0,42916177	0,65398767	protein_codir hypothetical protein
TcG_00561	254,6599734	0,400409145	0,11743264	3,40969196	0,00065036	0,00540151	protein_codir riboflavin kinase
TcG_00562	6,144213236	-0,241898584	0,70297792	-0,34410552	0,73076693	1	
TcG_00563	69,55647919	0,235845538	0,22065282	1,06885352	0,28513568	0,51877427	protein_codir putative protein kinase
TcG_00564	358,8224245	-0,099369358	0,096051	-1,03454785	0,30088012	0,53419983	protein_codir hypothetical protein
TcG_00565	419,6993613	-0,222553465	0,09202407	-2,41842658	0,01558779	0,06964219	protein_codir hypothetical protein
TcG_00566	602,8982091	-0,176622683	0,08136214	-2,1708215	0,02994467	0,11431266	protein_codir putative RNA-binding protein
TcG_00567	456,7649629	-0,133685849	0,08875182	-1,5062886	0,1319931	0,32282246	protein_codir protein phosphatase 2 (formerly 2A), regulatory subunit B
TcG_00568	267,2093022	0,038780388	0,1164705	0,33296317	0,73916208	0,86708727	protein_codir hypothetical protein
TcG_00569	1206,507559	0,093495262	0,0580736	1,60994423	0,10741003	0,28225281	protein_codir putative cystathione gamma lyase

TcG_00570	487,0791448	-0,12217782	0,08778514	-1,39178248	0,16398828	0,36892586	protein_codir	hypothetical protein
TcG_00571	266,566184	-0,2017744	0,11094391	-1,81870644	0,06895623	0,20980222	protein_codir	putative replication factor C, subunit 3
TcG_00572	441,283475	-0,1070042	0,08624661	-1,24067715	0,21472504	0,43861148	protein_codir	peroxin 19
TcG_00573	120,0392542	-0,020506188	0,17023067	-0,12046118	0,90411783	0,95353444	protein_codir	hypothetical protein
TcG_00574	133,2814614	-0,232911021	0,15785868	-1,47544007	0,14009427	0,3345817	protein_codir	60S ribosomal subunit protein L31
TcG_00575	967,5883271	0,392230433	0,06465934	6,06610662	1,3105E-09	5,1122E-08	protein_codir	60S ribosomal subunit protein L31
TcG_00576	0,27291574	1,35431792	3,0630933	0,4421406	0,65838746	1	protein_codir	large subunit ribosomal protein L31e
TcG_00577	344,2988804	0,06120435	0,0998593	0,61290588	0,53993858	0,73897829	protein_codir	leucine rich repeat protein
TcG_00578	339,0873158	0,03407457	0,09886525	0,34465668	0,73035249	0,86117076	protein_codir	hypothetical protein
TcG_00579	236,3784725	0,032582213	0,11570149	0,28160582	0,77824578	0,88925638	protein_codir	putative transport protein particle (TRAPP) subunit
TcG_00580	143,451427	0,055537041	0,15151357	0,36654831	0,71395597	0,85321236	protein_codir	putative mitochondrial carrier protein
TcG_00581	0					1	protein_codir	putative mitochondrial carrier protein
TcG_00582	437,8794559	0,141608679	0,09028814	1,5684085	0,11678583	0,2980592	protein_codir	putative 6-phosphogluconate dehydrogenase,decarboxylating
TcG_00583	267,5471691	0,015040539	0,1094081	0,13747189	0,89065781	0,94671205	protein_codir	16S rRNA (cytidine1402-2-O)-methyltransferase
TcG_00584	920,5005762	0,136891692	0,07015247	1,95134539	0,05101597	0,16801338	protein_codir	hypothetical protein
TcG_00585	182,3585884	0,130878325	0,13302498	0,98386277	0,32518299	0,55965169	protein_codir	hypothetical protein
TcG_00586	1031,362773	-0,002744378	0,06366325	-0,04310773	0,96561566	0,98482597	protein_codir	ATP-dependent RNA helicase
TcG_00587	265,150503	0,061423884	0,11608304	0,52913743	0,59671012	0,78083165	protein_codir	hypothetical protein
TcG_00588	294,3160924	-0,129442565	0,11084856	-1,16774244	0,24291067	0,47118082	protein_codir	dihydroxyacetone kinase 1-like protein
TcG_00589	124,3623656	0,238339634	0,16454217	1,44850186	0,14747675	0,34622763	protein_codir	hypothetical protein
TcG_00590	499,7186449	-0,02056639	0,08504472	-0,24183031	0,80891165	0,90533717	protein_codir	hypothetical protein
TcG_00591	339,3789483	0,086861244	0,10479707	0,82885185	0,40718824	0,63546377	protein_codir	hypothetical protein
TcG_00592	283,6589153	-0,173050293	0,10648081	-1,62517824	0,10412459	0,27669437	protein_codir	hypothetical protein
TcG_00593	291,3101457	-0,121224983	0,10768964	-1,1256884	0,26029744	0,49261779	protein_codir	putative replication factor A protein 3
TcG_00594	552,7702473	0,243013797	0,0795774	3,05380408	0,0022596	0,01518543	protein_codir	putative DNA-repair protein
TcG_00595	260,7430342	0,281576585	0,11292289	2,49352979	0,012648	0,05937589	protein_codir	putative ascorbate-dependent peroxidase
TcG_00596	262,4580512	0,253749549	0,12306277	2,06195212	0,03921229	0,13943541	protein_codir	acyl transferase-like protein
TcG_00597	136,2399666	-0,049155709	0,16015144	-0,30693267	0,75889462	0,87811376	protein_codir	hypothetical protein
TcG_00598	292,3464651	-0,038477233	0,11243393	-0,34222082	0,73218472	0,86246393	protein_codir	hypothetical protein
TcG_00599	433,5835357	0,088131248	0,09155675	0,96258607	0,33575529	0,57030653	protein_codir	hypothetical protein
TcG_00600	135,7249444	-0,117407601	0,15429228	-0,7609428	0,44669123	0,66822009	protein_codir	zinc finger protein
TcG_00601	55,24940191	0,086553098	0,23972535	0,36105108	0,71806126	0,85508792	protein_codir	hypothetical protein
TcG_00602	335,4539486	0,092088266	0,10205656	0,9023258	0,36688382	0,59923712	protein_codir	hypothetical protein
TcG_00603	1532,649324	-0,116902888	0,05236689	-2,23238177	0,02558974	0,10159363	protein_codir	putative structural maintenance of chromosome (SMC) family protein
TcG_00604	257,109681	0,031340036	0,11092173	0,2825419	0,77752803	0,88900067	protein_codir	RNA-binding protein PNO1
TcG_00605	340,5753824	0,088744244	0,10330054	0,85908788	0,39029204	0,62040037	protein_codir	hypothetical protein
TcG_00606	289,8527839	0,077908467	0,10551252	0,73838126	0,4602828	0,67839985	protein_codir	cellular retinaldehyde-binding protein/triple function domain-containing protein
TcG_00607	171,8615835	0,144689162	0,1356713	1,06646846	0,28621193	0,51943161	protein_codir	putative membrane transporter protein
TcG_00608	337,574317	0,216491653	0,10402782	2,08109372	0,03742533	0,13473772	protein_codir	putative membrane transporter protein
TcG_00609	681,2186083	0,045082674	0,07565044	0,59593404	0,55121929	0,74682127	protein_codir	hypothetical protein
TcG_00610	465,4553053	0,035349053	0,08973724	0,3939173	0,6936421	0,84125796	protein_codir	putative DNA repair protein RAD2
TcG_00611	223,8258412	0,30251194	0,12844433	2,35519889	0,01851279	0,07908895	protein_codir	methyltransferase like 6
TcG_00612	183,4311707	0,251812838	0,13361502	1,88461469	0,05948188	0,18793483	protein_codir	peptidyl-prolyl cis-trans isomerase (cyclophilin)
TcG_00613	303,2206882	-0,26428281	0,10849497	-2,4358992	0,01485482	0,06732284	protein_codir	iron-sulfur cluster assembly protein
TcG_00614	153,8817578	0,40840422	0,14638758	2,78988298	0,00527271	0,02984348	protein_codir	hypothetical protein
TcG_00615	649,6793865	0,096865245	0,07753677	1,24928141	0,21156217	0,43452566	protein_codir	hypothetical protein
TcG_00616	533,1292062	0,204920475	0,0832452	2,46164927	0,01382998	0,06379917	protein_codir	hypothetical protein
TcG_00617	209,5603106	0,008868512	0,13029062	0,06806716	0,94573217	0,97469438	protein_codir	NADH dehydrogenase
TcG_00618	135,9122692	0,475079053	0,15209535	3,12356067	0,00178677	0,01257686	protein_codir	putative galactokinase
TcG_00619	130,3128496	0,273866737	0,15941333	1,71796631	0,08580276	0,24217071	protein_codir	ribulose-phosphate 3-epimerase
TcG_00620	119,67721	0,137904611	0,1600024	0,86189088	0,38874757	0,61911056	protein_codir	Gim5A protein
TcG_00621	109,3733519	0,058383898	0,16634942	0,35097146	0,72560976	0,85819872	protein_codir	hypothetical protein

TcG_00622	1343,630384	0,08946588	0,05759182	1,55344763	0,12031623	0,30370407	protein_codir	Gim5A protein
TcG_00623	511,0919682	0,079709691	0,08608395	0,92595302	0,3544704	0,58843087	protein_codir	hypothetical protein
TcG_00624	37,42188149	0,104226972	0,29330187	0,35535734	0,72232191	0,85728556	protein_codir	hypothetical protein
TcG_00625	1202,219899	0,013822643	0,05959942	0,23192579	0,81659566	0,90954406	protein_codir	hypothetical protein
TcG_00626	260,6697995	-0,002429619	0,1136689	-0,02137453	0,98294689	0,99369442	protein_codir	hypothetical protein
TcG_00627	213,3707795	-0,015106149	0,12452974	-0,12130556	0,90344902	0,95322469	protein_codir	hypothetical protein
TcG_00628	607,7414655	0,258625906	0,07923027	3,26423116	0,00109762	0,00843247	protein_codir	anaphase-promoting complex subunit 2
TcG_00629	21,63738457	0,274810197	0,39140994	0,70210326	0,48261477	0,69628628	protein_codir	60S ribosomal protein L27A/L29
TcG_00630	6,669653127	-0,183873557	0,66311987	-0,27728555	0,78156085	1	protein_codir	60S ribosomal protein L27A/L29
TcG_00631	34,86608889	0,287609074	0,3028344	0,94972392	0,34225255	0,57657632	protein_codir	60S ribosomal protein L27A/L29
TcG_00632	269,3310784	0,281995508	0,11666969	2,41704179	0,01564722	0,06978131	protein_codir	60S ribosomal protein L23
TcG_00633	558,4358239	0,307023242	0,08474107	3,62307482	0,00029112	0,00273112	protein_codir	60S ribosomal protein L23
TcG_00634	182,939512	-0,118713149	0,13641018	-0,87026604	0,38415503	0,61449954	protein_codir	hypothetical protein
TcG_00635	162,399856	-0,033873735	0,13812374	-0,24524195	0,8062691	0,90430143	protein_codir	hypothetical protein
TcG_00636	110,9506603	-0,197762446	0,17076598	-1,15809039	0,24682715	0,47559278	protein_codir	hypothetical protein
TcG_00637	299,3767095	0,225157715	0,107068	2,10294133	0,0354709	0,12985405	protein_codir	protein DENND6A
TcG_00638	514,8047402	-0,1581605	0,08436447	-1,87472878	0,06083004	0,19150771	protein_codir	putative proteasome beta 2 subunit
TcG_00639	191,7952936	-0,258255996	0,13416012	-1,92498337	0,05423144	0,17526512	protein_codir	putative 30S Ribosomal protein S17
TcG_00640	1057,882019	-0,052717631	0,06350223	-0,8301697	0,40644284	0,63483385	protein_codir	T-complex protein 1 subunit eta
TcG_00641	188,5486933	-0,405113248	0,12877418	-3,14591983	0,00165565	0,01180456	protein_codir	putative nucleoside diphosphate kinase
TcG_00642	298,7537395	-0,165313813	0,10380204	-1,59258738	0,11125279	0,28842578	protein_codir	Sof1-like protein
TcG_00643	576,7023788	-0,24418001	0,07785857	-3,13619967	0,00171153	0,01212828	protein_codir	calmodulin
TcG_00644	238,0491957	-0,150236048	0,11560121	-1,29960622	0,19373597	0,41080251	protein_codir	hypothetical protein
TcG_00645	362,3561396	-0,0381148	0,10181436	-0,37435582	0,70813961	0,84943749	protein_codir	hypothetical protein
TcG_00646	377,6525238	-0,143709265	0,09741352	-1,47524967	0,14014543	0,3345817	protein_codir	hypothetical protein
TcG_00647	130,1505245	-0,121620895	0,15495817	-0,78486277	0,43253403	0,65696635	protein_codir	unc-50 related protein
TcG_00648	278,4075935	-0,081105292	0,11075661	-0,73228401	0,46399524	0,68152242	protein_codir	hypothetical protein
TcG_00649	50,54448476	-0,234061183	0,24969032	-0,93740593	0,34854979	0,58310373	protein_codir	hypothetical protein
TcG_00650	64,06199472	0,306712868	0,23190409	1,32258501	0,18597342	0,39987989	protein_codir	hypothetical protein
TcG_00651	862,0305516	0,083687949	0,08053111	1,03920021	0,29871163	0,5322319	protein_codir	pre-mRNA-processing-splicing factor 8-like
TcG_00652	482,9740528	0,043797326	0,08401219	0,52132107	0,60214313	0,78475506	protein_codir	U5 snRNA-associated splicing factor
TcG_00653	40,04176581	-0,289657056	0,28582198	-1,01341772	0,31086068	0,54446303	protein_codir	protein kinase A catalytic subunit isoform 1
TcG_00654	170,0682499	-0,138286425	0,13510033	-1,02358314	0,30603221	0,53910433	protein_codir	protein kinase-A catalytic subunit
TcG_00655	344,2270673	-0,220856197	0,10467243	-2,10997496	0,03486051	0,12819561	protein_codir	zinc finger protein, predicted
TcG_00656	804,7547948	-0,107577165	0,0699662	-1,53755902	0,12415647	0,31021181	protein_codir	putative 4E-interacting protein
TcG_00657	128,2296385	0,272858967	0,15965047	1,70910214	0,08743203	0,24520181	protein_codir	putative mitochondrial carrier protein
TcG_00658	364,3227907	0,313026124	0,09693248	3,22932144	0,00124084	0,00929309	protein_codir	putative DNAJ-domain protein
TcG_00659	346,9269847	0,284775993	0,09872675	2,88448662	0,00392052	0,02368258	protein_codir	WASH complex subunit 7-like
TcG_00660	250,6620215	0,180320632	0,11455257	1,57413001	0,1154574	0,29583886	protein_codir	hypothetical protein
TcG_00661	82,88026241	0,305913924	0,20413249	1,49860475	0,1339762	0,32557556	protein_codir	putative UPF0598 protein C8orf82
TcG_00662	128,1297825	0,133183473	0,15450328	0,86201064	0,38868166	0,6190907	protein_codir	hypothetical protein
TcG_00663	94,0683513	0,288124832	0,18402694	1,56566655	0,1174267	0,29881523	protein_codir	transferase
TcG_00664	183,753479	0,22410988	0,13236677	1,69309771	0,09043687	0,25095367	protein_codir	putative glycogenin glucosyltransferase
TcG_00665	97,49431155	-0,161107478	0,1771805	-0,90928446	0,36319999	0,59526856	protein_codir	protein kinase
TcG_00666	890,7966841	0,184005054	0,0713075	2,58044455	0,00986732	0,04889768	protein_codir	hypothetical protein
TcG_00667	279,1855763	0,282482173	0,10931656	2,58407488	0,00976406	0,04853127	protein_codir	translation factor SUI1
TcG_00668	186,7413969	0,589647485	0,14082756	4,18701755	2,8264E-05	0,00037425	protein_codir	hypothetical protein
TcG_00669	346,3898213	0,505637039	0,10217291	4,94883681	7,4658E-07	1,5642E-05	protein_codir	hypothetical protein
TcG_00670	162,1359893	0,17045463	0,14271759	1,19434913	0,23234143	0,45960522	protein_codir	secreted protein
TcG_00671	122,0528288	0,758528913	0,16072973	4,71928204	2,3668E-06	4,3252E-05	protein_codir	putative UDP-Gal or UDP-GlcNAc-dependent glycosyltransferase
TcG_00672	578,1718276	0,457645472	0,07876134	5,81053415	6,2274E-09	2,053E-07	protein_codir	hypothetical protein
TcG_00673	84,24572135	0,159421542	0,19208752	0,82994222	0,40657145	0,63483385	protein_codir	hypothetical protein

TcG_00674	478,0042141	0,336275865	0,09501241	3,53928364	0,00040121	0,00359066	protein_codir	hypothetical protein
TcG_00675	946,152465	0,413139184	0,06875046	6,00925659	1,8638E-09	6,9657E-08	protein_codir	putative mitochondrial DNA-directed RNA polymerase
TcG_00676	1704,657715	0,341418887	0,05650349	6,04243879	1,518E-09	5,7665E-08	protein_codir	hypothetical protein
TcG_00677	234,6610825	0,477771472	0,11868496	4,02554367	5,6844E-05	0,00068818	protein_codir	hypothetical protein
TcG_00678	319,4585327	0,449044982	0,10231724	4,38875196	1,14E-05	0,00017013	protein_codir	hypothetical protein
TcG_00679	404,1524132	0,486876506	0,09795082	4,97062197	6,6738E-07	1,411E-05	protein_codir	hypothetical protein
TcG_00680	238,9437324	0,317758895	0,11797106	2,69353252	0,00706992	0,03757438	protein_codir	putative formin
TcG_00681	341,7006764	0,42408233	0,10003255	4,23944324	2,2407E-05	0,00030615	protein_codir	putative formin
TcG_00682	482,7977311	0,362138666	0,08796792	4,11671279	3,8431E-05	0,00048877	protein_codir	putative pre-mRNA splicing factor
TcG_00683	355,821934	0,211567194	0,09673143	2,18716071	0,02873079	0,11061934	protein_codir	hypothetical protein
TcG_00684	192,7889437	0,176090872	0,13968237	1,2606521	0,20743422	0,42962689	protein_codir	putative DnaJ chaperone protein
TcG_00685	638,045209	0,277947981	0,07928051	3,50588053	0,0004551	0,00398849	protein_codir	hypothetical protein
TcG_00686	605,0149734	0,449972097	0,07974284	5,64279009	1,6732E-08	5,0747E-07	protein_codir	putative helicase-like protein
TcG_00687	299,1247647	0,42786925	0,11472232	3,72960771	0,00019178	0,00193061	protein_codir	hypothetical protein
TcG_00688	163,7650193	0,033019944	0,13853389	0,23835282	0,81160746	0,90721505	protein_codir	dynein light chain
TcG_00689	0					1	protein_codir	dynein light chain
TcG_00690	1392,095	0,195451607	0,05500164	3,55355932	0,00038006	0,00342405	protein_codir	putative nucleoside transporter 1
TcG_00691	156,9007795	0,372184426	0,1491206	2,49586199	0,01256515	0,0591067	protein_codir	hypothetical protein
TcG_00692	781,6100424	0,492684712	0,07274102	6,77313449	1,2602E-11	7,8924E-10	protein_codir	hypothetical protein
TcG_00693	18,50975724	1,42342162	0,42995558	3,31062484	0,00093088	0,00736692	protein_codir	dynein light chain
TcG_00694	243,307555	0,278945214	0,11647182	2,39495888	0,01662223	0,07311552	protein_codir	hypothetical protein
TcG_00695	272,8872834	0,352731443	0,11200129	3,14935152	0,00163633	0,01169851	protein_codir	putative DNA mismatch repair protein MSH2
TcG_00696	435,0827006	0,500093798	0,09043779	5,52969955	3,2078E-08	9,1994E-07	protein_codir	putative replication factor C, subunit 1
TcG_00697	845,7734264	0,006422931	0,07266432	0,08839181	0,92956527	0,96574096	protein_codir	hypothetical protein
TcG_00698	211,2667044	0,466741181	0,13232317	3,52728244	0,00041985	0,00372463	protein_codir	putative pre-mRNA splicing factor ATP-dependent RNA helicase
TcG_00699	342,3454958	0,324428207	0,10396504	3,12055106	0,00180513	0,01266604	protein_codir	hypothetical protein
TcG_00700	242,5651579	0,670931403	0,11562358	5,80272133	6,5247E-09	2,1355E-07	protein_codir	putative GTPase activating protein
TcG_00701	305,6620928	0,485524663	0,10378142	4,6783391	2,8921E-06	5,2111E-05	protein_codir	hypothetical protein
TcG_00702	282,4784095	0,34811117	0,11102185	3,13551936	0,0017155	0,01214902	protein_codir	putative cell division cycle protein
TcG_00703	162,4788995	0,7973018	0,14374931	5,54647382	2,9149E-08	8,4641E-07	protein_codir	putative protein transport protein Sec31
TcG_00704	458,7272289	0,39238208	0,08599995	4,56258497	5,0528E-06	8,4597E-05	protein_codir	putative minchromosome maintenance (MCM) complex subunit
TcG_00705	423,646738	0,341046089	0,08868701	3,84550198	0,00012031	0,0013039	protein_codir	hypothetical protein
TcG_00706	415,3431876	0,413534217	0,09818543	4,21176757	2,5338E-05	0,00034175	protein_codir	putative mitochondrial DNA polymerase I protein A
TcG_00707	205,4489682	0,486871884	0,12391893	3,92895497	8,5316E-05	0,0009729	protein_codir	putative transmembrane protein
TcG_00708	109,9206617	0,809760629	0,17039037	4,75238483	2,0103E-06	3,7446E-05	protein_codir	hypothetical protein
TcG_00709	224,6134993	0,566779789	0,12011153	4,71877901	2,3726E-06	4,329E-05	protein_codir	hypothetical protein
TcG_00710	978,8736931	0,494139499	0,06416948	7,70053735	1,355E-14	1,3418E-12	protein_codir	triosephosphate isomerase
TcG_00711	274,3589485	0,092882404	0,1073917	0,8648937	0,38709713	0,61733142	protein_codir	putative 33 kDa inner dynein arm light chain, axonemal
TcG_00712	522,9049527	0,33853633	0,0811421	4,17214137	3,0175E-05	0,00039728	protein_codir	hypothetical protein
TcG_00713	668,421863	0,351643523	0,08456428	4,15829876	3,2063E-05	0,00041739	protein_codir	putative synaptojanin (N-terminal domain), putative,inositol/phosphatidylinositol phosphatase
TcG_00714	338,6896628	0,367328077	0,1029917	3,56657925	0,00036167	0,00328138	protein_codir	phosphotransferase
TcG_00715	231,7090826	0,326364752	0,12076344	2,70251292	0,00688175	0,03684791	protein_codir	Trypanosoma vivax
TcG_00716	371,1891199	0,812395037	0,09738564	8,34204118	7,3019E-17	9,4E-15	protein_codir	hypothetical protein
TcG_00717	498,0573521	0,308137175	0,08534685	3,61041044	0,00030571	0,00284732	protein_codir	malic enzyme
TcG_00718	1666,482447	0,335188295	0,05324511	6,29519434	3,0701E-10	1,3734E-08	protein_codir	malic enzyme
TcG_00719	1037,960186	0,449997901	0,06109099	7,36602734	1,7579E-13	1,4652E-11	protein_codir	putative DNA repair and recombination protein RAD54
TcG_00720	63,75265484	0,860236976	0,22328744	3,85259906	0,00011687	0,00127621	protein_codir	hypothetical protein
TcG_00721	446,8904844	0,347991812	0,09272353	3,75300429	0,00017473	0,00178676	protein_codir	hypothetical protein
TcG_00722	492,1775957	0,491532323	0,08397399	5,85338763	4,8166E-09	1,6365E-07	protein_codir	hypothetical protein
TcG_00723	590,7933568	0,334772646	0,08685531	3,85437174	0,00011603	0,0012694	protein_codir	putative signal recognition particle
TcG_00724	113,5754798	0,510304837	0,16482106	3,09611423	0,00196075	0,01353828	protein_codir	hypothetical protein
TcG_00725	175,8725151	0,314902387	0,13576005	2,3195512	0,02036517	0,08529856	protein_codir	Trypanosoma vivax

TcG_00726	43,19584862	0,095833114	0,27253025	0,35164212	0,72510667	0,85817259	protein_codir	hypothetical protein
TcG_00727	126,4739457	0,278038582	0,1676306	1,65863862	0,09718864	0,26370668	protein_codir	putative aldo/keto reductase
TcG_00728	218,922597	0,485650545	0,12095492	4,0151366	5,9411E-05	0,00071553	protein_codir	kelch repeat protein
TcG_00729	553,9338389	0,454504388	0,0811617	5,59998583	2,1437E-08	6,4012E-07	protein_codir	putative sugar transporter
TcG_00730	405,3064669	0,378323486	0,09311713	4,06287728	4,8472E-05	0,00059935	protein_codir	putative protein kinase
TcG_00731	403,8938786	0,446328313	0,09031104	4,94212372	7,7276E-07	1,6085E-05	protein_codir	hypothetical protein
TcG_00732	263,7897986	0,272652371	0,11707271	2,32891487	0,01986358	0,08350486	protein_codir	hypothetical protein
TcG_00733	384,3151448	0,452300506	0,0996953	4,5368287	5,7106E-06	9,4116E-05	protein_codir	hypothetical protein
TcG_00734	478,3043336	0,088428214	0,08607251	1,0273688	0,30424683	0,53710252	protein_codir	putative MCAK-like kinesin
TcG_00735	300,54188	0,392689143	0,10665051	3,68201844	0,00023139	0,00225478	protein_codir	putative mitochondrial carrier protein
TcG_00736	405,3556135	0,196296378	0,09360143	2,0971515	0,03598017	0,13129644	protein_codir	ATPase subunit 9
TcG_00737	966,3153331	0,238093994	0,06401492	3,71935135	0,00019974	0,00198979	protein_codir	putative ubiquitin hydrolase
TcG_00738	356,2106587	0,529641477	0,09959651	5,31787189	1,0499E-07	2,7091E-06	protein_codir	transferase
TcG_00739	360,588993	0,429888345	0,10051143	4,27700946	1,8942E-05	0,00026441	protein_codir	putative DNA helicase
TcG_00740	788,2128699	0,503557416	0,07338846	6,8615337	6,8125E-12	4,5103E-10	protein_codir	hypothetical protein
TcG_00741	165,251312	0,392934919	0,14007274	2,80522042	0,00502822	0,02888296	protein_codir	hexosyltransferase
TcG_00742	161,0786723	0,484877025	0,14016448	3,45934309	0,00054149	0,00464723	protein_codir	tRNA guanosine-2-O-methyltransferase TRM13
TcG_00743	50,01883718	0,465326142	0,25734347	1,80819098	0,07057678	0,21310988	protein_codir	tRNA guanosine-2-O-methyltransferase TRM13
TcG_00744	286,1694946	0,373735483	0,10750356	3,4764941	0,00050802	0,004399	protein_codir	hypothetical protein
TcG_00745	587,6284368	0,384434958	0,08381822	4,58653231	4,5067E-06	7,6561E-05	protein_codir	putative chaperone DNAJ protein
TcG_00746	202,2984799	0,588233697	0,12730418	4,62069418	3,8246E-06	6,6236E-05	protein_codir	hypothetical protein
TcG_00747	138,1952969	0,250168027	0,1511115	1,65551948	0,09781917	0,26473555	protein_codir	hypothetical protein
TcG_00748	707,6967019	0,237367766	0,07479857	3,1734266	0,00150651	0,01090901	protein_codir	hypothetical protein
TcG_00749	243,1602959	0,153609647	0,11506161	1,33502081	0,18186954	0,39371085	protein_codir	hypothetical protein
TcG_00750	166,0096969	0,546577224	0,14853568	3,67977056	0,00023344	0,00227093	protein_codir	hypothetical protein
TcG_00751	201,1932613	0,459865707	0,1301008	3,53468785	0,00040826	0,00363939	protein_codir	putative ubiquitin-activating enzyme
TcG_00752	416,2880015	0,315009132	0,09096921	3,46281057	0,00053456	0,00459797	protein_codir	hypothetical protein
TcG_00753	212,0509419	0,228315084	0,12319279	1,85331525	0,06383719	0,19850178	protein_codir	putative protein kinase
TcG_00754	328,369709	0,488944204	0,1950845	2,50632016	0,01219951	0,05783287	protein_codir	putative ubiquitin carboxyl-terminal hydrolase, putative,cysteine peptidase, Clan CA, family C12
TcG_00755	345,7789821	0,476089032	0,09958667	4,7806504	1,7473E-06	3,3037E-05	protein_codir	hypothetical protein
TcG_00756	307,973813	0,683753028	0,10454152	6,54049276	6,1316E-11	3,2145E-09	protein_codir	putative actin
TcG_00757	421,989663	0,934827331	0,09555651	9,78297939	1,3323E-22	3,1502E-20	protein_codir	hypothetical protein
TcG_00758	409,0504952	0,615034556	0,09124293	6,74062706	1,577E-11	9,5165E-10	protein_codir	hypothetical protein
TcG_00759	405,6066779	0,350650724	0,09298752	3,77094411	0,00016263	0,00168236	protein_codir	hypothetical protein
TcG_00760	302,4758906	0,615229348	0,10892403	5,64824239	1,621E-08	4,9293E-07	protein_codir	putative aspartate aminotransferase, mitochondrial
TcG_00761	20,16109446	1,005587945	0,40810325	2,46405275	0,01373759	0,06346579		
TcG_00762	148,3528343	0,877381599	0,1505192	5,82903432	5,5749E-09	1,8776E-07	protein_codir	hypothetical protein
TcG_00763	442,7438087	0,331278133	0,08742947	3,78909023	0,0001512	0,00158391	protein_codir	putative ethanolamine phosphotransferase
TcG_00764	758,7600499	0,631572105	0,07171428	8,80678278	1,2879E-18	2,0164E-16	protein_codir	methyltransferase
TcG_00765	752,6092909	0,651736106	0,07087459	9,19562424	3,7282E-20	6,8564E-18	protein_codir	putative fumarate hydratase
TcG_00766	293,8093148	0,429563784	0,10965376	3,91745619	8,9488E-05	0,00101648	protein_codir	putative leucine-rich repeat protein (LRRP)
TcG_00767	460,3223474	0,370087641	0,08962866	4,12912156	3,6415E-05	0,00046698	protein_codir	hypothetical protein
TcG_00768	591,6732422	0,523531224	0,07956276	6,58010398	4,7012E-11	2,5452E-09	protein_codir	hypothetical protein
TcG_00769	386,3424746	0,131808151	0,09306669	1,41627639	0,1566946	0,35829162	protein_codir	putative TFIIF-stimulated CTD phosphatase
TcG_00770	427,8612951	0,179247635	0,09300701	1,92724871	0,05394864	0,1744972	protein_codir	dynein intermediate-chain-like protein
TcG_00771	206,1447731	0,558266351	0,13434522	4,15546115	3,2463E-05	0,00042213	protein_codir	putative protein phosphatase
TcG_00772	493,4279595	0,606427746	0,09219745	6,57748905	4,7846E-11	2,5783E-09	protein_codir	ATP-dependent RNA helicase
TcG_00773	295,6992598	0,472333319	0,11082435	4,26199934	2,0261E-05	0,00028045	protein_codir	putative WD40 repeat protein
TcG_00774	102,7151573	0,529812766	0,18907389	2,80214669	0,00507638	0,02903007	protein_codir	hypothetical protein
TcG_00775	180,2768149	0,523654396	0,13593592	3,85221497	0,00011705	0,00127701	protein_codir	putative transcription elongation factor
TcG_00776	128,1859645	0,624799759	0,1607844	3,88594765	0,00010193	0,00113665	protein_codir	putative transcription elongation factor
TcG_00777	459,5197738	0,090892036	0,09117047	0,99694599	0,31879073	0,55374953	protein_codir	serine/threonine-protein phosphatase 4 regulatory subunit 4

TcG_00778	150,4141547	0,300757585	0,1514695	1,98559836	0,04707794	0,15847633	protein_codir	ATPase domain protein
TcG_00779	289,982436	0,473895196	0,10746088	4,40993225	1,034E-05	0,0001564	protein_codir	hypothetical protein
TcG_00780	334,1124533	0,022555262	0,10544702	0,21390138	0,83062397	0,91760178	protein_codir	hypothetical protein
TcG_00781	96,17192585	0,225527172	0,18737118	1,20363854	0,22872931	0,45574613	protein_codir	putative glycosomal membrane protein
TcG_00782	747,781634	0,2915477	0,0716034	4,07170205	4,6671E-05	0,00058354	protein_codir	putative ankyrin repeat family protein
TcG_00783	417,4386912	0,164425311	0,09151354	1,79673212	0,07237816	0,21618286	protein_codir	guanine nucleotide-binding protein beta subunit-like protein
TcG_00784	315,6180348	0,39338593	0,10468256	3,75789367	0,00017135	0,00175531	protein_codir	hypothetical protein
TcG_00785	318,0467486	-0,056302389	0,10028766	-0,56140895	0,57451878	0,76562855	protein_codir	enkurin
TcG_00786	254,1707454	0,472829697	0,11368738	4,15903428	3,196E-05	0,00041652	protein_codir	hypothetical protein
TcG_00787	174,370533	0,332032257	0,13867171	2,39437635	0,01664866	0,07320354	protein_codir	excreted/secreted protein 66
TcG_00788	304,3666875	0,150235036	0,10962136	1,3704905	0,17053384	0,37713401	protein_codir	hypothetical protein
TcG_00789	370,4271674	0,314032374	0,0977561	3,21240678	0,00131628	0,0097759	protein_codir	hypothetical protein
TcG_00790	183,0003769	0,191935373	0,13051471	1,47060339	0,14139841	0,3366698	protein_codir	hypothetical protein
TcG_00791	1182,362046	0,7177622	0,0608717	11,7913933	4,3233E-32	2,7828E-29	protein_codir	60S ribosomal protein L17
TcG_00792	206,965825	0,264460805	0,12436039	2,12656783	0,03345601	0,12399914	protein_codir	hypothetical protein
TcG_00793	227,8567029	0,122966499	0,12641883	0,97269132	0,33070674	0,56523583	protein_codir	putative copper homeostasis protein
TcG_00794	622,5876827	0,19584104	0,07600055	2,57683728	0,00997089	0,04927909	protein_codir	putative clathrin coat assembly protein
TcG_00795	252,0204116	0,134222827	0,11208639	1,19749442	0,2311139	0,45850782	protein_codir	hypothetical protein
TcG_00796	291,3824236	0,04292635	0,10582703	0,40562747	0,68501634	0,83655708	protein_codir	putative pyruvate dehydrogenase (lipoamide) kinase
TcG_00797	11,77652167	0,655911311	0,51301972	1,2785304	0,20106247	1	protein_codir	putative trans-sialidase
TcG_00798	6,447853186	-0,360461708	0,69320228	-0,51999498	0,60306707	1	protein_codir	trans-sialidase
TcG_00799	16,5382623	-0,077095658	0,4452736	-0,17314222	0,86253964	1		
TcG_00800	7,544788332	0,644062923	0,64744384	0,99477805	0,31984423	1	protein_codir	dispersed gene family protein 1 (DGF-1)
TcG_00801	0,27264935	1,353296468	3,06414982	0,4416548	0,65873902	1	protein_codir	dispersed gene family protein 1 (DGF-1)
TcG_00802	5,181714975	-0,536891778	0,80938543	-0,66333265	0,50711753	1	protein_codir	dispersed gene family protein 1 (DGF-1)
TcG_00803	12,69893477	0,075243285	0,49531819	0,15190899	0,87925872	1	protein_codir	dispersed gene family protein 1 (DGF-1)
TcG_00804	11,87530708	-0,081548256	0,53104832	-0,1535609	0,87795597	1	protein_codir	nuclear lim interactor-interacting factor
TcG_00805	212,5950541	-0,369461437	0,12604585	-2,93116697	0,00337691	0,02094481	protein_codir	hypothetical protein
TcG_00806	227,3190627	-0,047972262	0,11926531	-0,40223146	0,68751369	0,83768363	protein_codir	putative acyltransferase
TcG_00807	346,5925491	-0,236306986	0,09988528	-2,36578385	0,01799195	0,07749245	protein_codir	putative acyltransferase
TcG_00808	328,634868	-0,37203637	0,10701076	-3,47662595	0,00050777	0,004399	protein_codir	hypothetical protein
TcG_00809	137,5451437	0,142641207	0,14921924	0,95591699	0,33911417	0,57332217	protein_codir	hypothetical protein
TcG_00810	94,70911282	-0,132695768	0,18904352	-0,70193239	0,48272133	0,69628628	protein_codir	cAMP-phosphodiesterase D
TcG_00811	427,0950532	-0,070914333	0,09119415	-0,77761931	0,43679347	0,66040574	protein_codir	heat shock protein 20
TcG_00812	1339,268592	0,102117414	0,05798502	1,76110004	0,07822147	0,22719328	protein_codir	60S ribosomal protein L13
TcG_00813	31,54437081	0,149063698	0,30790845	0,48411694	0,62830288	0,80073888	protein_codir	60S ribosomal protein L13
TcG_00814	567,4948805	-0,452272049	0,0789545	-5,72826189	1,0146E-08	3,2207E-07	protein_codir	hypothetical protein
TcG_00815	1058,290018	-0,373109069	0,06111916	-6,10461728	1,0305E-09	4,0887E-08	protein_codir	hypothetical protein
TcG_00816	222,5847564	-0,244037352	0,12292025	-1,98533069	0,04710769	0,15852156	protein_codir	putative protein kinase
TcG_00817	587,059353	-0,214371651	0,07999042	-2,67996665	0,00736295	0,03884918	protein_codir	vacuolar protein sorting-associated protein 4
TcG_00818	581,687456	-0,090137986	0,07995737	-1,12732553	0,25960487	0,4921081	protein_codir	ATP-dependent phosphofructokinase
TcG_00819	129,7919995	-0,231326596	0,16374885	-1,41269145	0,15774647	0,35984457	protein_codir	hypothetical protein
TcG_00820	151,8253903	-0,117907601	0,1426188	-0,82673253	0,40838868	0,63660535	protein_codir	hypothetical protein
TcG_00821	357,1917372	0,069178053	0,10380114	0,66644792	0,50512484	0,71227448	protein_codir	tRNA (cytosine34-C5)-methyltransferase
TcG_00822	544,5661394	-0,122136866	0,07935996	-1,53902371	0,12379851	0,30965663	protein_codir	RNA polymerase-associated protein CTR9
TcG_00823	536,0700226	-0,211757971	0,08200178	-2,58235811	0,00981277	0,04868983	protein_codir	hypothetical protein
TcG_00824	114,35706	-0,189500027	0,16918166	-1,12009793	0,26267203	0,49484848		
TcG_00825	645,4804833	-0,284932156	0,074439	-3,82772696	0,00012933	0,00138488	protein_codir	putative protein kinase
TcG_00826	243,7259344	-0,146207093	0,1182994	-1,2359073	0,21649302	0,44059164	protein_codir	hypothetical protein
TcG_00827	91,23891082	0,052797777	0,19941615	0,26476179	0,79119297	0,8956289	protein_codir	hypothetical protein
TcG_00828	195,9973337	-0,305105197	0,12780542	-2,38726331	0,01697433	0,0742691	protein_codir	hypothetical protein
TcG_00829	182,6537911	-0,485132385	0,13504302	-3,59242833	0,00032761	0,00301007	protein_codir	hypothetical protein

TcG_00830	104,3829992	0,171655499	0,17176347	0,9993714	0,31761481	0,55273912	protein_codir	poly(A) polymerase
TcG_00831	292,5358236	0,338747015	0,10729591	3,15712887	0,00159331	0,01145166	protein_codir	putative poly(A) polymerase, putative,polynucleotide adenylyltransferase
TcG_00832	430,5562151	-0,078832435	0,08869204	-0,88883324	0,37409271	0,60449626	protein_codir	hypothetical protein
TcG_00833	337,2331585	-0,051910879	0,10264551	-0,50572967	0,61304644	0,79081972	protein_codir	hypothetical protein
TcG_00834	1820,662883	-0,129541748	0,05666264	-2,28619326	0,02224296	0,0912883	protein_codir	hypothetical protein
TcG_00835	210,5033366	-0,094830564	0,12266635	-0,77307726	0,43947666	0,66264661	protein_codir	hypothetical protein
TcG_00836	737,1647764	-0,217964095	0,07361546	-2,9608468	0,00306795	0,01941596	protein_codir	condensin complex subunit 3
TcG_00837	112,3075739	0,195306458	0,17152758	1,13863004	0,25485749	0,48614229	protein_codir	peptidyl-prolyl cis-trans isomerase
TcG_00838	502,4527091	-0,144578679	0,08362143	-1,72896681	0,08381504	0,23833704	protein_codir	putative helicase
TcG_00839	256,4512667	-0,158063898	0,11539974	-1,36970754	0,17077821	0,37747622	protein_codir	putative serine/threonine-protein kinase Nek1, putative,protein kinase
TcG_00840	232,2351139	0,093806991	0,11813883	0,79404027	0,42717199	0,65241427	protein_codir	putative cAMP phosphodiesterase A
TcG_00841	489,8738963	-0,223326804	0,09585721	-2,3297862	0,01981745	0,08336787	protein_codir	hypothetical protein
TcG_00842	460,1546825	-0,097834023	0,08779746	-1,11431495	0,2651441	0,4975639	protein_codir	hypothetical protein
TcG_00843	155,8501075	-0,481434689	0,14553905	-3,30794164	0,00093984	0,00741255	protein_codir	parallel beta-helix repeat-containing protein
TcG_00844	213,1193239	-0,058821038	0,12430054	-0,47321627	0,63605889	0,80601316	protein_codir	protein kinase Wee570
TcG_00845	454,9591591	-0,194579689	0,08904254	-2,18524425	0,02887094	0,11105536	protein_codir	hypothetical protein
TcG_00846	126,4472827	-0,088226257	0,16288003	-0,54166406	0,58804996	0,77564856	protein_codir	putative actin-like protein
TcG_00847	395,1413366	-0,351181432	0,09146636	-3,83946007	0,00012331	0,00133142	protein_codir	tetratricopeptide repeat protein 26 isoform X1
TcG_00848	94,0108025	0,109294685	0,18558654	0,58891494	0,55591834	0,75077163	protein_codir	putative trypsin-like cysteine/serine peptidase
TcG_00849	154,564106	0,028233261	0,14147736	0,19956027	0,8418245	0,92300356	protein_codir	putative mitochondrial carrier protein
TcG_00850	148,1262073	-0,109233993	0,14907716	-0,73273459	0,46372032	0,68129135	protein_codir	hypothetical protein
TcG_00851	518,8359054	-0,276758744	0,08117833	-3,40926896	0,00065137	0,00540602	protein_codir	putative inosine-adenosine-guanosine-nucleoside hydrolase
TcG_00852	410,4281104	-0,244795853	0,09003909	-2,71877317	0,00655245	0,03548034	protein_codir	ribonuclease inhibitor-like protein
TcG_00853	80,00619353	0,27960274	0,20191834	1,38473176	0,1661345	0,3716614	protein_codir	hypothetical protein
TcG_00854	22,28046457	-0,899926808	0,37720414	-2,38578189	0,01704286	0,07444098	protein_codir	hypothetical protein
TcG_00855	33,14855609	-0,222458995	0,33124557	-0,67158331	0,50184901	0,71072272	protein_codir	RNA-binding protein 6
TcG_00856	5,247198576	-0,304628128	0,85676168	-0,3555576	0,7221719	1		
TcG_00857	152,4343352	0,140647091	0,14712785	0,95595154	0,33909672	0,57332217	protein_codir	hypothetical protein
TcG_00858	205,7630275	-0,191148293	0,13168666	-1,45153877	0,1466299	0,34466504	protein_codir	hypothetical protein
TcG_00859	114,7444675	-0,221220392	0,16832835	-1,31421948	0,18877236	0,40322945	protein_codir	hypothetical protein
TcG_00860	879,2478088	-0,343870275	0,06630913	-5,18586621	2,1501E-07	5,1791E-06	protein_codir	eukaryotic initiation factor 2 alpha subunit
TcG_00861	286,1183807	-0,10206455	0,10787179	-0,94616531	0,34406428	0,5783155	protein_codir	putative protein kinase
TcG_00862	272,3339569	-0,591249387	0,10990984	-5,37940371	7,4733E-08	1,9951E-06	protein_codir	hypothetical protein
TcG_00863	483,0077796	-0,330016641	0,08628897	-3,82455173	0,00013101	0,0013964	protein_codir	hypothetical protein
TcG_00864	397,1611431	-0,088824013	0,09060459	-0,98034788	0,32691443	0,56146317	protein_codir	hypothetical protein
TcG_00865	575,4220923	-0,187359128	0,08054319	-2,32619443	0,02000818	0,08405177	protein_codir	hypothetical protein
TcG_00866	99,81770943	-0,151773236	0,18735757	-0,8100726	0,41789845	0,64453826	protein_codir	hypothetical protein
TcG_00867	161,971995	-0,172878474	0,14250263	-1,21315986	0,22506871	0,45161864	protein_codir	hypothetical protein
TcG_00868	144,0029497	-0,29541345	0,1456619	-2,02807635	0,04255246	0,1476132	protein_codir	hypothetical protein
TcG_00869	253,2758493	0,045960269	0,12253625	0,37507488	0,70760478	0,84921369	protein_codir	putative inorganic pyrophosphatase
TcG_00870	260,9088035	-0,233000739	0,1181068	-1,9727969	0,04851871	0,16190603	protein_codir	putative peter pan protein
TcG_00871	357,9043109	-0,253175367	0,0961701	-2,63257894	0,00847393	0,04340362	protein_codir	putative CTD nuclear envelope phosphatase 1A-like
TcG_00872	899,7691845	-0,233060866	0,07143003	-3,26278546	0,00110323	0,00844813	protein_codir	hypothetical protein
TcG_00873	65,4493322	-0,112850646	0,21490143	-0,52512747	0,59949458	0,78310281	protein_codir	hypothetical protein
TcG_00874	332,3315382	-0,117859819	0,09862335	-1,19504988	0,23206754	0,45922596	protein_codir	hypothetical protein
TcG_00875	358,2198474	-0,351519095	0,09689668	-3,62777217	0,00028588	0,00269064	protein_codir	hypothetical protein
TcG_00876	308,630301	-0,128496737	0,10645266	-1,20707868	0,22740185	0,45433312	protein_codir	hypothetical protein
TcG_00877	802,4323401	-0,138183	0,06962866	-1,98457075	0,04719225	0,15875794	protein_codir	hypothetical protein
TcG_00878	633,8811214	-0,06460841	0,07833926	-0,8247258	0,40952728	0,63686685	protein_codir	hypothetical protein
TcG_00879	184,5636196	-0,083927555	0,14470727	-0,57998159	0,56192703	0,7553645	protein_codir	hypothetical protein
TcG_00880	599,8139765	-0,283192169	0,07622959	-3,71498989	0,00020321	0,00201749	protein_codir	aminoalcohol phosphotransferase
TcG_00881	902,1099037	-5,00389E-05	0,06843868	-0,00073115	0,99941663	0,99981271	protein_codir	hypothetical protein

TcG_00882	805,4031867	-0,491880329	0,07600941	-6,47130812	9,7158E-11	4,7901E-09	protein_codir	putative 6-phosphofructo-2-kinase/fructose-2,6-biphosphatase
TcG_00883	425,9023384	-0,21116695	0,08767998	-2,40838286	0,01602337	0,07112902	protein_codir	hypothetical protein
TcG_00884	338,1338468	-0,442492146	0,09983671	-4,43215865	9,3294E-06	0,00014412	protein_codir	putative serine/threonine protein kinase, putative,protein kinase
TcG_00885	645,8270132	0,141779263	0,07890381	1,79686204	0,07235752	0,21618229	protein_codir	tubulin-specific chaperone E
TcG_00886	270,639504	0,019575532	0,11202272	0,17474608	0,86127916	0,93373073	protein_codir	hypothetical protein
TcG_00887	144,8184714	-0,132323324	0,145618	-0,90870168	0,36350762	0,59544737	protein_codir	hypothetical protein
TcG_00888	337,1020447	-0,300952485	0,10752423	-2,7989272	0,00512727	0,02923452	protein_codir	hypothetical protein
TcG_00889	189,6978766	0,037762751	0,13975851	0,27020002	0,78700638	0,8939307	protein_codir	EbsC protein
TcG_00890	864,9404681	-0,378859092	0,07050897	-5,37320408	7,735E-08	2,0602E-06	protein_codir	hypothetical protein
TcG_00891	454,0992394	-0,229031206	0,08801491	-2,60218634	0,00926315	0,04644001	protein_codir	GPI inositol deacylase 2
TcG_00892	1040,857572	-0,182804517	0,06611437	-2,76497406	0,00569273	0,03166394	protein_codir	ATP-dependent DEAD/H RNA helicase
TcG_00893	706,0157225	-0,220677256	0,07416053	-2,97566979	0,00292349	0,0186415	protein_codir	ATP-dependent DEAD/H RNA helicase
TcG_00894	67,45764874	-0,23351861	0,21315913	-1,09551304	0,27329193	0,50653661	protein_codir	hypothetical protein
TcG_00895	528,3964242	-0,237821949	0,08861485	-2,68377072	0,0072797	0,03849503	protein_codir	hypothetical protein
TcG_00896	381,6482071	-0,220164424	0,09173303	-2,40005616	0,01639256	0,07232614	protein_codir	hypothetical protein
TcG_00897	278,6266782	-0,186632668	0,10767865	-1,73323738	0,08305349	0,23681114	protein_codir	hypothetical protein
TcG_00898	245,5697523	-0,063534414	0,12214415	-0,52015927	0,60295257	0,78518697	protein_codir	hypothetical protein
TcG_00899	573,6117019	-0,099377247	0,07944614	-1,25087574	0,21097982	0,43356016	protein_codir	RNA editing complex protein MP99
TcG_00900	441,0152327	-0,092156162	0,09121287	-1,01034167	0,31233162	0,54629743	protein_codir	putative arginine N-methyltransferase
TcG_00901	597,2081334	0,015609236	0,07554477	0,20662232	0,83630483	0,92052325	protein_codir	putative DNA repair helicase
TcG_00902	167,9402852	-0,117130064	0,14228334	-0,82321701	0,4103846	0,63727596	protein_codir	hypothetical protein
TcG_00903	230,2001985	-0,075514676	0,11657063	-0,6478019	0,51711309	0,72262597	protein_codir	hypothetical protein
TcG_00904	176,5210858	-0,049710757	0,13514429	-0,36783467	0,71299651	0,85259341	protein_codir	hypothetical protein
TcG_00905	213,8586036	-0,060980311	0,12703077	-0,48004364	0,63119636	0,80248448	protein_codir	putative quinone oxidoreductase
TcG_00906	610,0757941	-0,380636668	0,07713149	-4,9349059	8,0189E-07	1,665E-05	protein_codir	hypothetical protein
TcG_00907	538,8231633	-0,330050358	0,08313547	-3,97003037	7,1863E-05	0,00084529	protein_codir	putative 26S protease regulatory subunit
TcG_00908	983,07977	-0,04301556	0,07211972	-0,59644657	0,55087694	0,74654917	protein_codir	hypothetical protein
TcG_00909	694,4225457	-0,121533801	0,07333	-1,65735446	0,09744783	0,26410072	protein_codir	U2AF65-like splicing factor
TcG_00910	1138,144508	-0,227208817	0,05893058	-3,8555334	0,00011548	0,00126565	protein_codir	protein-tyrosine phosphatase
TcG_00911	535,8439953	-0,348910812	0,09028488	-3,86455426	0,00011129	0,00122105	protein_codir	hypothetical protein
TcG_00912	438,614951	-0,166290105	0,08878106	-1,87303583	0,06106344	0,19193429	protein_codir	putative 6-phosphofructo-2-kinase/fructose-2,6-biphosphatase
TcG_00913	368,710397	-0,172730416	0,09700775	-1,78058371	0,07498048	0,22110559	protein_codir	hypothetical protein
TcG_00914	4,061597169	-1,640345947	0,98787336	-1,66048201	0,09681752	1	protein_codir	hypothetical protein
TcG_00915	589,7001521	-0,058303747	0,07687596	-0,7584133	0,4482036	0,66910023	protein_codir	conserved TLD domain protein
TcG_00916	914,203089	0,160442782	0,06646002	2,41412479	0,01577306	0,07028718	protein_codir	60S acidic ribosomal protein P2
TcG_00917	304,9046471	-0,195489798	0,10484929	-1,8644838	0,06225379	0,19488583	protein_codir	putative PTP1-interacting protein, 39 kDa
TcG_00918	467,8814482	-0,022705803	0,09350932	-0,24281862	0,80814591	0,90509218	protein_codir	putative MFS transporter
TcG_00919	287,0304797	-0,120711567	0,10823366	-1,11528679	0,26472754	0,49718484	protein_codir	putative calcium channel protein
TcG_00920	48,35702861	-0,164314196	0,25620313	-0,64134343	0,52129958	0,72540161	protein_codir	hypothetical protein
TcG_00921	365,4011672	-0,122447842	0,09446137	-1,29627428	0,19488101	0,41224965	protein_codir	protein kinase, putative,serine/threonine protein kinase
TcG_00922	61,40573229	0,21874196	0,22186774	0,98591151	0,32417654	0,5584165	protein_codir	hypothetical protein
TcG_00923	239,2188102	-0,018226018	0,11893572	-0,15324259	0,87820697	0,94142358	protein_codir	putative retrotransposon hot spot (RHS) protein
TcG_00924	27,31240633	0,316438085	0,34255078	0,92376985	0,35560616	0,58942102	protein_codir	retrotransposon hot spot (RHS) protein
TcG_00925	36,61193044	0,134268621	0,29330116	0,45778414	0,64710753	0,81242026	protein_codir	hypothetical protein
TcG_00926	57,98136785	-0,148927051	0,22917969	-0,64982656	0,51580426	0,72175219	protein_codir	hypothetical protein
TcG_00927	18,68822965	-0,045960226	0,40717512	-0,11287582	0,91012901	0,95641431		
TcG_00928	148,6401618	-0,01313758	0,14303211	-0,09185057	0,92681677	0,96430637	protein_codir	putative trans-sialidase
TcG_00929	860,3954681	0,04675294	0,07112627	0,65732305	0,51097322	0,71750523	protein_codir	putative AGP2beta-2
TcG_00930	243,2817219	0,151633749	0,12108401	1,25230202	0,21045983	0,43302467	protein_codir	putative ribosomal P protein AGP2beta-1
TcG_00931	1537,13346	0,17118722	0,06388079	2,67979203	0,00736679	0,03884918	protein_codir	60S acidic ribosomal protein P2 beta (H6.4)
TcG_00932	597,3208956	-0,044194761	0,07670971	-0,57612997	0,56452733	0,75736611	protein_codir	putative damage-specific DNA binding protein
TcG_00933	176,9148203	-0,048233041	0,14228682	-0,33898461	0,73462133	0,86407606	protein_codir	putative damage-specific DNA binding protein

TcG_00934	998,8964689	-0,076276096	0,0657001	-1,16097386	0,24565253	0,47419697	protein_codir	putative protein kinase, putative,polo-like protein kinase
TcG_00935	706,4929827	-0,274754573	0,07287942	-3,76998865	0,00016325	0,00168731	protein_codir	hypothetical protein
TcG_00936	811,8457784	-0,142574895	0,07357087	-1,93792585	0,05263227	0,17128186	protein_codir	tetratricopeptide repeat domain 39B
TcG_00937	839,9997955	-0,381894124	0,06774602	-5,63714477	1,7289E-08	5,2301E-07	protein_codir	hypothetical protein
TcG_00938	151,3418114	0,311082742	0,16299672	1,90852152	0,05632384	0,17991951	protein_codir	hypothetical protein
TcG_00939	197,2005665	-0,079544327	0,12965549	-0,61350528	0,53954229	0,73873044	protein_codir	putative vacuolar ATP synthase
TcG_00940	358,5122096	0,394996584	0,09701071	4,07168037	4,6675E-05	0,00058354	protein_codir	ribosomal protein L15
TcG_00941	408,0549863	0,041910238	0,08993366	0,46601281	0,64120633	0,80952561	protein_codir	putative leucine-rich repeat protein
TcG_00942	124,8072208	0,728069507	0,1607711	4,52860938	5,9373E-06	9,7436E-05	protein_codir	cyclin
TcG_00943	696,7366152	-0,111386444	0,07073231	-1,5747605	0,11531174	0,29577193	protein_codir	hypothetical protein
TcG_00944	453,8545881	-0,032826373	0,09482235	-0,34618814	0,72920132	0,86056342	protein_codir	putative dynein heavy chain
TcG_00945	565,1720577	-0,015253537	0,08953661	-0,1703609	0,86472632	0,93498506	protein_codir	ATP synthase, epsilon chain
TcG_00946	1221,519745	0,253757108	0,05965373	4,25383462	2,1014E-05	0,00028976	protein_codir	40S ribosomal protein S14
TcG_00947	1075,146734	-0,151901761	0,0634971	-2,39226309	0,01674483	0,07346244	protein_codir	putative protein kinase
TcG_00948	513,3010578	-0,02448159	0,08328439	-0,29395172	0,7687948	0,88431385	protein_codir	zinc finger-domain protein
TcG_00949	447,503342	0,111426118	0,08805156	1,26546437	0,20570491	0,42719074	protein_codir	mago nashi-like protein
TcG_00950	270,2467402	0,297043846	0,10875356	2,73134834	0,00630758	0,03434191	protein_codir	cytochrome P450-like protein
TcG_00951	375,9449275	-0,021656177	0,09511983	-0,22767258	0,81990079	0,91102214	protein_codir	putative peptide chain release factor 1
TcG_00952	171,2162625	0,033458038	0,14185117	0,23586719	0,81353573	0,90788142	protein_codir	S-adenosylmethionine synthetase
TcG_00953	1819,402845	-0,285916476	0,05288226	-5,4066611	6,421E-08	1,7335E-06	protein_codir	S-adenosylmethionine synthetase
TcG_00954	235,4979252	0,301107162	0,123177	2,44450797	0,01450499	0,06621675	protein_codir	hypothetical protein
TcG_00955	423,9197037	-0,05599307	0,09155361	-0,61158776	0,54081054	0,73959288	protein_codir	lorien protein
TcG_00956	422,969251	-0,04036211	0,08964221	-0,45025788	0,65252451	0,81625448	protein_codir	putative chromosomal passenger protein
TcG_00957	364,2200719	0,088391607	0,10840551	0,8153793	0,41485523	0,64180968	protein_codir	putative casein kinase 1 isoform 2
TcG_00958	178,9023987	0,067813799	0,13221349	0,51291134	0,60801334	0,78802211	protein_codir	zinc-binding protein (Yippee)
TcG_00959	460,5667261	0,139948219	0,08884873	1,57512914	0,11522664	0,2956191	protein_codir	tetratricopeptide repeat domain 5
TcG_00960	106,7861703	0,254136477	0,16894565	1,50424988	0,13251704	0,32357058	protein_codir	putative zinc-binding protein (Yippee)
TcG_00961	547,0367749	0,039919815	0,0791164	0,50457064	0,61386044	0,79129807	protein_codir	putative DNA ligase I
TcG_00962	982,0601948	-0,275238227	0,06270001	-4,38976391	1,1347E-05	0,00016964	protein_codir	viral life cyclerelated protein
TcG_00963	816,6324049	-0,145419867	0,06668486	-2,18070299	0,02920539	0,11204426	protein_codir	hypothetical protein
TcG_00964	826,6296832	-0,148697236	0,07073387	-2,10220688	0,03553516	0,13003108	protein_codir	hypothetical protein
TcG_00965	663,0003431	-0,229032679	0,07331052	-3,12414484	0,00178323	0,01255955	protein_codir	putative CAS/CSE/importin domain protein
TcG_00966	708,5570629	-0,102139625	0,07393282	-1,38151945	0,1671193	0,37307211	protein_codir	putative PAS-domain containing phosphoglycerate kinase
TcG_00967	388,8363728	-0,287312567	0,09530211	-3,01475557	0,00257186	0,01679685	protein_codir	putative calmodulin
TcG_00968	368,2613144	-0,116796819	0,09645731	-1,21086538	0,225947	0,45243566	protein_codir	hypothetical protein
TcG_00969	303,6068966	0,239680509	0,10443303	2,2950643	0,02172944	0,08965715	protein_codir	hypothetical protein
TcG_00970	2349,963462	0,233854789	0,04574143	5,1125383	3,1786E-07	7,2494E-06	protein_codir	putative 60S ribosomal protein L9
TcG_00971	183,9452987	-0,29435053	0,14460658	-2,03552653	0,04179792	0,14577686	protein_codir	hypothetical protein
TcG_00972	684,0077247	-0,178446854	0,07349126	-2,428137	0,01517661	0,06821665	protein_codir	hypothetical protein
TcG_00973	751,4078604	-0,23774024	0,07169903	-3,31580813	0,00091379	0,0072614	protein_codir	flagellar component
TcG_00974	140,6158884	-0,16415965	0,15529576	-1,05707744	0,29047625	0,52345679	protein_codir	putative cationic amino acid transporter
TcG_00975	522,746723	-0,147374589	0,0847022	-1,73991447	0,08187404	0,23491644	protein_codir	putative UV excision repair RAD23-like protein
TcG_00976	424,3301547	-0,151207469	0,09973792	-1,51604801	0,12950721	0,31918115	protein_codir	hypothetical protein
TcG_00977	277,4821826	-0,143200049	0,10933521	-1,30973401	0,19028583	0,40552085	protein_codir	hypothetical protein
TcG_00978	313,7571258	0,003479211	0,10371357	0,03354634	0,97323891	0,98816458	protein_codir	putative serine/threonine protein phosphatase
TcG_00979	242,9018721	-0,055746034	0,11504361	-0,48456438	0,62798539	0,80068655	protein_codir	hypothetical protein
TcG_00980	266,9231859	-0,208491639	0,10905378	-1,91182397	0,05589878	0,17890697	protein_codir	ankyrin repeat protein
TcG_00981	505,0562282	-0,192141121	0,08331927	-2,3060826	0,02110602	0,0878356	protein_codir	putative ATP-dependent RNA helicase
TcG_00982	99,50621788	-0,119029094	0,17570633	-0,67743202	0,49813189	0,70742498	protein_codir	ATP-dependent RNA helicase
TcG_00983	750,303631	-0,250889559	0,07120869	-3,52329977	0,00042621	0,00377527	protein_codir	putative glutamyl-tRNA synthetase
TcG_00984	93,19531082	-0,04696959	0,18152441	-0,25875082	0,7958275	0,89816468	protein_codir	hypothetical protein
TcG_00985	105,1421144	-0,193906037	0,17727108	-1,09383911	0,27402555	0,50708513	protein_codir	hypothetical protein

TcG_00986	273,1488731	-0,34982968	0,11377156	-3,07484307	0,00210613	0,01437083	protein_codir	hypothetical protein
TcG_00987	420,5524759	-0,362874885	0,09031054	-4,01807899	5,8675E-05	0,00070887	protein_codir	hypothetical protein
TcG_00988	676,8698433	-0,040011521	0,0747657	-0,5351588	0,59254005	0,7778347	protein_codir	hydroxymethylglutaryl CoA reductase
TcG_00989	170,1096908	-0,020212496	0,13384712	-0,1510118	0,8799664	0,94165427	protein_codir	putative RNA-binding protein
TcG_00990	286,2244522	0,003749436	0,11049878	0,03393192	0,97293144	0,98811217	protein_codir	putative amino acid permease
TcG_00991	308,2823354	-0,11875359	0,10798328	-1,09974054	0,27144519	0,50457365	protein_codir	hypothetical protein
TcG_00992	335,9665818	-0,641235853	0,09864914	-6,50016675	8,0231E-11	3,9895E-09	protein_codir	meiosis-specific nuclear structural 1
TcG_00993	231,2218837	-0,153234732	0,12073081	-1,26922645	0,20436031	0,42546605	protein_codir	hypothetical protein
TcG_00994	675,8092987	-0,379320661	0,07309963	-5,18909131	2,1132E-07	5,1212E-06	protein_codir	translocon-associated protein subunit beta
TcG_00995	351,6963268	-0,098919306	0,10148511	-0,97471748	0,32970042	0,56434119	protein_codir	hypothetical protein
TcG_00996	1591,657445	-0,098390503	0,05371914	-1,83157272	0,0670151	0,20589684	protein_codir	valyl-tRNA synthetase
TcG_00997	249,1645512	-0,121077624	0,116728	-1,0372629	0,29961335	0,53340814	protein_codir	putative S-adenosylmethionine decarboxylase proenzyme
TcG_00998	138,7663252	-0,282107594	0,16185437	-1,74297178	0,08133854	0,23384327		
TcG_00999	627,6182396	-0,062841747	0,07851354	-0,80039375	0,4234827	0,64943356	protein_codir	S-adenosylmethionine decarboxylase
TcG_01000	356,8599886	-0,05095193	0,09726212	-0,52386202	0,60037452	0,78350294	protein_codir	hypothetical protein
TcG_01001	3185,775487	0,110332693	0,04375492	2,52160655	0,01168203	0,0558136	protein_codir	putative RNA-binding protein
TcG_01002	606,1697128	0,339221998	0,07634399	4,44333588	8,8575E-06	0,00013793	protein_codir	putative homoserine kinase
TcG_01003	361,2953364	-0,220873133	0,09696721	-2,27781268	0,02273774	0,09305527	protein_codir	hypothetical protein
TcG_01004	243,6511902	0,198907818	0,12505049	1,59062003	0,11169511	0,28931357	protein_codir	tRNA (guanine-N(1)-)-methyltransferase TRM10
TcG_01005	964,3855796	-0,058367249	0,06413297	-0,91009734	0,36277118	0,59508238	protein_codir	putative kinesin
TcG_01006	1026,457495	0,066017275	0,06952408	0,9495598	0,34233597	0,57657632	protein_codir	protein kinase-like protein
TcG_01007	353,9920636	-0,347257814	0,0980813	-3,54050976	0,00039935	0,00358121	protein_codir	protein fam63a
TcG_01008	55,57717523	-0,146882427	0,23650821	-0,6210458	0,53456949	0,73491866		
TcG_01009	1125,881026	-0,123613171	0,05997516	-2,06107292	0,03929608	0,13957217	protein_codir	putative eukaryotic translation initiation factor 3 subunit 7-like protein
TcG_01010	365,7612549	0,141096546	0,09421192	1,49765073	0,13422402	0,32570991	protein_codir	hypothetical protein
TcG_01011	215,4741845	0,075523284	0,1236161	0,61095024	0,54123252	0,73989536	protein_codir	hypothetical protein
TcG_01012	315,4323226	0,152164552	0,10548567	1,44251391	0,14915745	0,34834473	protein_codir	ribosomal RNA assembly protein
TcG_01013	149,8870475	0,395916011	0,14568631	2,71759235	0,00657588	0,03558531	protein_codir	U6 snRNA-associated Sm-like protein LSm5p
TcG_01014	141,1274478	0,434388258	0,15159039	2,86553953	0,00416299	0,02493921	protein_codir	hypothetical protein
TcG_01015	597,9461717	0,063714623	0,07966738	0,79975797	0,42385104	0,64974043	protein_codir	hypothetical protein
TcG_01016	185,2537988	0,318513734	0,13921341	2,287953	0,02214026	0,09096349	protein_codir	hypothetical protein
TcG_01017	590,7905772	-0,030230289	0,07623779	-0,39652632	0,6917168	0,84050813	protein_codir	hypothetical protein
TcG_01018	390,4079473	0,210064219	0,09232406	2,27529214	0,02288841	0,09357597	protein_codir	putative DNA topoisomerase III
TcG_01019	230,3477133	-0,189721787	0,12606922	-1,50490178	0,13234933	0,32329735	protein_codir	putative calpain-like cysteine peptidase
TcG_01020	5760,473614	-0,21735872	0,03925068	-5,53770613	3,0646E-08	8,8324E-07	protein_codir	glycosomal glyceraldehyde-3-phosphate dehydrogenase
TcG_01021	312,0027794	0,118008916	0,10250625	1,15123633	0,24963502	0,47885288	protein_codir	hypothetical protein
TcG_01022	373,9804884	-0,070846924	0,10459617	-0,67733762	0,49819177	0,70742498	protein_codir	hypothetical protein
TcG_01023	198,2618078	0,149874978	0,13496345	1,11048568	0,2667898	0,49939561		
TcG_01024	146,5513476	-0,030452846	0,15779649	-0,19298811	0,84696828	0,92596191	protein_codir	hypothetical protein
TcG_01025	297,4616977	0,003547561	0,10544733	0,03364297	0,97316186	0,98816458	protein_codir	hypothetical protein
TcG_01026	302,0036529	-0,146553709	0,10317921	-1,42038021	0,15549702	0,35689154	protein_codir	putative mitogen-activated protein kinase 5, putative,protein kinase
TcG_01027	800,6528916	0,150313423	0,07348482	2,04550312	0,04080528	0,14322023	protein_codir	putative aldehyde dehydrogenase
TcG_01028	375,5639309	-0,049467608	0,09438414	-0,52410932	0,60020251	0,78350294	protein_codir	hypothetical protein
TcG_01029	338,3954292	0,178560762	0,10007164	1,78432936	0,07437016	0,22015933	protein_codir	putative mitochondrial protein
TcG_01030	206,2694174	-0,440239179	0,12458525	-3,53363811	0,00040988	0,00364738	protein_codir	hypothetical protein
TcG_01031	443,8819764	0,103167326	0,08954284	1,15215605	0,24925695	0,47852379	protein_codir	hypothetical protein
TcG_01032	268,1295784	-0,088399981	0,11507508	-0,76819395	0,44237197	0,66466715	protein_codir	hypothetical protein
TcG_01033	176,606953	0,004636162	0,13277903	0,03491637	0,97214643	0,98777616	protein_codir	hypothetical protein
TcG_01034	744,8112685	0,039296403	0,07091686	0,55411936	0,57949716	0,76901886	protein_codir	hypothetical protein
TcG_01035	470,3823953	-0,696917886	0,09258614	-7,52723767	5,1825E-14	4,7654E-12	protein_codir	hypothetical protein
TcG_01036	377,5063157	-0,155519651	0,1082385	-1,43682383	0,15076806	0,35076279	protein_codir	succinate dehydrogenase cytochrome B subunit
TcG_01037	263,2004972	-0,02083389	0,113989	-0,18277106	0,85497766	0,92959564	protein_codir	putative GTPase activating protein of Rab-like GTPase

TcG_01038	627,1756866	-0,158390205	0,07878259	-2,01047229	0,04438123	0,15222051	protein_codir trichohyalin
TcG_01039	352,501682	-0,084992458	0,0969941	-0,87626419	0,38088643	0,61104814	protein_codir putative chaperonin HSP60/CNP60
TcG_01040	207,9168299	-0,192419779	0,12775173	-1,50620091	0,13201561	0,32282246	protein_codir hypothetical protein
TcG_01041	371,7941009	-0,067746929	0,09457364	-0,71634053	0,47378111	0,68951487	protein_codir hypothetical protein
TcG_01042	135,7229299	0,176424932	0,15385722	1,14667955	0,25151409	0,48158027	protein_codir hypothetical protein
TcG_01043	260,115918	0,36123197	0,12220569	2,95593408	0,00311724	0,01966747	protein_codir hypothetical protein
TcG_01044	322,337083	0,085323795	0,10830957	0,78777704	0,43082713	0,65564072	protein_codir 39S ribosomal protein L28
TcG_01045	399,0598486	0,168969947	0,10210204	1,65491248	0,09794225	0,26500676	protein_codir putative superoxide dismutase
TcG_01046	320,9447575	-0,128858833	0,10540525	-1,22250871	0,22151531	0,44696559	protein_codir hypothetical protein
TcG_01047	1457,923313	0,022394488	0,05439862	0,41167384	0,6805785	0,83339466	protein_codir putative small glutamine-rich tetratricopeptide repeat protein
TcG_01048	90,17064247	0,090525703	0,18410048	0,49171899	0,62291801	0,79676839	protein_codir hypothetical protein
TcG_01049	365,2448974	-0,000353778	0,09471578	-0,00373515	0,99701979	0,99900296	protein_codir hypothetical protein
TcG_01050	460,0685405	-0,007279383	0,09051538	-0,08042151	0,93590202	0,96997592	protein_codir GRAM domain containing protein
TcG_01051	476,7389194	-0,272604298	0,09378244	-2,90677343	0,00365178	0,0223623	protein_codir hypothetical protein
TcG_01052	234,7921736	-0,194126523	0,11694843	-1,65993266	0,096928	0,26341002	protein_codir hypothetical protein
TcG_01053	465,7505029	0,133746105	0,08516599	1,5704168	0,11631818	0,29730033	protein_codir SH3 domain protein
TcG_01054	291,2361925	4,78738E-05	0,10778355	0,00044417	0,99964561	0,99981271	protein_codir hypothetical protein
TcG_01055	205,8890609	0,213251115	0,13233544	1,61144379	0,10708303	0,28208285	protein_codir hypothetical protein
TcG_01056	623,9866632	-0,049912032	0,07542757	-0,66172132	0,50814984	0,71484022	protein_codir hypothetical protein
TcG_01057	392,3228228	-0,026246919	0,09280258	-0,28282531	0,77731075	0,88888065	protein_codir hypothetical protein
TcG_01058	432,0314302	-0,132408023	0,09895336	-1,33808518	0,18086867	0,39227742	protein_codir hypothetical protein
TcG_01059	144,3821154	-0,15574886	0,1471383	-1,05852019	0,28981835	0,52327695	protein_codir hypothetical protein
TcG_01060	198,8559585	0,502616396	0,12885272	3,90070469	9,5913E-05	0,00107679	protein_codir hypothetical protein
TcG_01061	415,9289093	-0,070013287	0,09153686	-0,76486438	0,4443523	0,66642694	protein_codir hypothetical protein
TcG_01062	374,0356152	-0,281490416	0,09776559	-2,87923823	0,00398637	0,02400473	protein_codir putative replication factor C, subunit 2
TcG_01063	321,597778	-0,039344539	0,10130796	-0,38836571	0,69774541	0,843778	protein_codir putative RNA-binding protein
TcG_01064	428,5581728	0,034112585	0,08981551	0,37980727	0,70408848	0,84742878	protein_codir C-1-tetrahydrofolate synthase, cytoplasmic
TcG_01065	293,7955953	0,054519054	0,10781629	0,50566619	0,61309101	0,79081972	protein_codir hypothetical protein
TcG_01066	277,7778943	0,096989113	0,11180094	0,86751607	0,38565931	0,61622518	protein_codir putative chaperone DNAJ protein
TcG_01067	1023,114411	-0,161445374	0,06130864	-2,6333219	0,00845542	0,04334711	protein_codir putative reticulon domain protein
TcG_01068	367,7774577	-0,045868776	0,10118089	-0,4533344	0,65030797	0,81444904	protein_codir hypothetical protein
TcG_01069	494,6804655	-0,214011316	0,08580468	-2,49416844	0,01262526	0,05929318	protein_codir hypothetical protein
TcG_01070	303,8523507	-0,059541734	0,10720483	-0,55540162	0,57861999	0,76835429	protein_codir hypothetical protein
TcG_01071	16,52643405	-0,127500882	0,43333987	-0,29422837	0,7685834	1	
TcG_01072	0,155988004	0,503022807	4,08047286	0,12327562	0,90188885	1	protein_codir mitochondrial heat shock
TcG_01073	454,0753689	-0,376764144	0,09524103	-3,95590179	7,6246E-05	0,00088428	protein_codir putative ATP-dependent RNA helicase
TcG_01074	606,1625714	-0,219588266	0,07807607	-2,81249126	0,00491594	0,02843536	protein_codir hypothetical protein
TcG_01075	400,5861207	-0,111947697	0,09425462	-1,1877158	0,2349454	0,46285961	protein_codir hypothetical protein
TcG_01076	316,9865451	-0,361095447	0,10260921	-3,51913282	0,00043296	0,00383214	protein_codir putative kinesin
TcG_01077	1159,954989	-0,012479735	0,06379913	-0,19560979	0,8449156	0,92481739	protein_codir 60S ribosomal protein L17
TcG_01078	347,6422443	-0,224121196	0,09755442	-2,29739671	0,02159615	0,08937536	protein_codir hypothetical protein
TcG_01079	316,0067289	0,365535944	0,10488125	3,48523635	0,0004917	0,00427693	protein_codir putative 40S ribosomal protein S2
TcG_01080	110,8427583	-0,087838322	0,1677892	-0,52350403	0,60062355	0,7835886	protein_codir putative 40S ribosomal protein S2
TcG_01081	278,663622	-0,172122489	0,10947326	-1,57227877	0,11588591	0,2964571	protein_codir hypothetical protein
TcG_01082	587,4306175	0,162186773	0,07829094	2,07159046	0,03830365	0,13697102	protein_codir prostaglandin F synthase
TcG_01083	51,88880068	0,487721807	0,26926659	1,81129718	0,07009486	0,21209691	protein_codir aldo-keto reductase
TcG_01084	690,1769802	-0,51172311	0,07264883	-7,04378999	1,8708E-12	1,3718E-10	protein_codir hypothetical protein
TcG_01085	194,0874269	-0,233021396	0,14014268	-1,66274396	0,0963637	0,26258838	protein_codir putative replication termination factor
TcG_01086	127,6611412	0,18260693	0,15845372	1,15243071	0,24914413	0,47849319	protein_codir hypothetical protein
TcG_01087	214,6925741	-0,401309334	0,12435092	-3,2272325	0,00124994	0,00935516	protein_codir hypothetical protein
TcG_01088	482,149549	-0,189076471	0,08657186	-2,18404064	0,02895926	0,11135812	protein_codir putative ribosome biogenesis protein
TcG_01089	383,5478505	-0,283658062	0,1001282	-2,83294884	0,00461208	0,02702859	protein_codir putative syntaxin

TcG_01090	1480,429433	-0,371422686	0,05351256	-6,94085006	3,8975E-12	2,672E-10	protein_codir	nucleic acid binding protein Tc38
TcG_01091	248,5400671	-0,002228353	0,11710849	-0,01902811	0,98481868	0,99412989	protein_codir	putative 40S ribosomal protein S2
TcG_01092	338,0694952	-0,536650205	0,09851202	-5,4475609	5,1065E-08	1,402E-06	protein_codir	putative peroxin 13
TcG_01093	690,2084612	-0,613145469	0,07320221	-8,37605128	5,4733E-17	7,2061E-15	protein_codir	putative chaperone protein DNAj
TcG_01094	483,6745686	-0,52333173	0,08398744	-6,23107119	4,6326E-10	2,0102E-08	protein_codir	hypothetical protein
TcG_01095	396,2683389	-0,281737238	0,0949116	-2,96841724	0,00299338	0,01900343	protein_codir	putative fibrillarin
TcG_01096	748,3940211	-0,199457986	0,07452563	-2,6763676	0,0074425	0,03914152	protein_codir	hypothetical protein
TcG_01097	195,0782898	-0,368229086	0,13834558	-2,66166129	0,00777561	0,040562	protein_codir	syntaxin 7
TcG_01098	275,0235984	-0,571120225	0,10969954	-5,20622262	1,9272E-07	4,7408E-06	protein_codir	putative protein kinase
TcG_01099	603,0416714	-0,586855639	0,07597445	-7,72438176	1,124E-14	1,1423E-12	protein_codir	putative protein kinase
TcG_01100	1153,264663	-0,364379318	0,05948708	-6,12535187	9,0483E-10	3,6275E-08	protein_codir	putative aminopeptidase, putative,metallo-peptidase, Clan MG, Family M24
TcG_01101	422,5760594	-0,154587511	0,09131255	-1,69294928	0,09046512	0,25095367	protein_codir	hypothetical protein
TcG_01102	373,1716004	-0,652588187	0,09813083	-6,65018511	2,9272E-11	1,6544E-09	protein_codir	putative protein kinase
TcG_01103	391,8490578	-0,468902381	0,09192189	-5,10109621	3,3769E-07	7,6716E-06	protein_codir	transcription factor
TcG_01104	1896,987977	-0,477767446	0,05106196	-9,35662212	8,2327E-21	1,5897E-18	protein_codir	putative ADP,ATP carrier protein 1, mitochondrial precursor, putative,ADP/ATP translocase 1
TcG_01105	444,6855294	-0,175016078	0,09122634	-1,9184819	0,05504994	0,17711984	protein_codir	putative polyprenyl synthase
TcG_01106	600,6170817	-0,272683257	0,07542637	-3,61522457	0,00030009	0,00280614	protein_codir	hypothetical protein
TcG_01107	162,7260661	-0,500060129	0,14888152	-3,35877894	0,00078288	0,00634294	protein_codir	hypothetical protein
TcG_01108	571,2647831	-0,294994689	0,07967591	-3,70243241	0,00021354	0,00210562	protein_codir	hypothetical protein
TcG_01109	219,3100651	-0,595476922	0,1261416	-4,72070207	2,3503E-06	4,3019E-05	protein_codir	hypothetical protein
TcG_01110	525,3858721	-0,505236657	0,08171397	-6,18299033	6,2899E-10	2,6214E-08	protein_codir	C-terminal kinesin KIFC1
TcG_01111	57,64586566	-0,315244769	0,23537366	-1,3393375	0,18046083	0,39185364		
TcG_01112	421,3302459	-0,1676408	0,09925426	-1,68900357	0,09121875	0,25224962	protein_codir	hypothetical protein
TcG_01113	443,4758736	-0,216578616	0,08616259	-2,51360389	0,01195046	0,05676835	protein_codir	putative sarcoplasmic reticulum glycoprotein, putative,sarcalumenin precursor
TcG_01114	241,7260871	0,103620403	0,11903541	0,87050064	0,38402687	0,614473	protein_codir	hypothetical protein
TcG_01115	502,6812417	-0,314858093	0,08896301	-3,53920241	0,00040134	0,00359066	protein_codir	RNA-binding protein
TcG_01116	884,5943424	-0,36963478	0,06691117	-5,52426115	3,3088E-08	9,4422E-07	protein_codir	putative RNA-binding protein
TcG_01117	161,0862939	-0,371585625	0,15159953	-2,45110004	0,01424204	0,0653498	protein_codir	membrane protein
TcG_01118	407,0657215	-0,399678709	0,09107279	-4,38856358	1,141E-05	0,00017013	protein_codir	putative intraflagellar transport protein component
TcG_01119	487,4572102	-0,306925826	0,08417704	-3,64619422	0,00026615	0,00253173	protein_codir	intraflagellar transport protein 80
TcG_01120	165,3153008	-0,609053677	0,13964505	-4,36144115	1,2921E-05	0,00018974	protein_codir	calcyphosin
TcG_01121	230,1879807	-0,21974242	0,11852007	-1,85405234	0,06373167	0,19833338	protein_codir	putative lipoic acid synthetase, mitochondrial precursor
TcG_01122	442,9712431	-0,154634431	0,09481576	-1,63089379	0,10291273	0,27429191	protein_codir	putative protein kinase
TcG_01123	532,3941143	-0,224855574	0,08826759	-2,5474308	0,01085194	0,05266933	protein_codir	hypothetical protein
TcG_01124	110,5351704	-1,29284286	0,17073418	-7,57225577	3,668E-14	3,4551E-12	protein_codir	mucin-associated surface protein (MASP)
TcG_01125	160,9150114	-0,306569802	0,14291697	-2,1450902	0,03194564	0,11989703	protein_codir	hypothetical protein
TcG_01126	562,915463	-0,20170336	0,08275421	-2,43737875	0,01479417	0,06721777	protein_codir	hypothetical protein
TcG_01127	1093,843398	-0,464765676	0,06030652	-7,70672402	1,2909E-14	1,2893E-12	protein_codir	hypothetical protein
TcG_01128	876,6160545	-0,076709723	0,06579329	-1,16592019	0,24364671	0,47181875	protein_codir	40S ribosomal protein S13
TcG_01129	234,3269824	-0,088380627	0,12011386	-0,73580708	0,46184811	0,67953581	protein_codir	synaptobrevin-type transport protein
TcG_01130	299,4138476	-0,129539809	0,11235553	-1,15294555	0,24893273	0,47837695	protein_codir	hypothetical protein
TcG_01131	170,0629955	-0,084017307	0,13865614	-0,60594004	0,54455457	0,74164914	protein_codir	RNA polymerase III C11 subunit
TcG_01132	326,1379482	-0,145804225	0,10051064	-1,45063472	0,1468816	0,34518666	protein_codir	hypothetical protein
TcG_01133	598,773181	-0,189877339	0,07659652	-2,47892901	0,01317775	0,06146435	protein_codir	hypothetical protein
TcG_01134	667,6836	-0,21171755	0,07407517	-2,85814463	0,00426126	0,0253967	protein_codir	putative nucleosome assembly protein
TcG_01135	394,0932476	-0,254149012	0,09418307	-2,69845763	0,00696616	0,03719002	protein_codir	hypothetical protein
TcG_01136	95,32477783	-0,226410223	0,18267006	-1,239449	0,21517926	0,4388705	protein_codir	coiled-coil domain-containing protein
TcG_01137	161,1718842	-0,20417033	0,14209149	-1,43689349	0,15074826	0,35076279	protein_codir	nuclear cap binding complex subunit CBP30
TcG_01138	191,5681468	-0,298729925	0,12691075	-2,35385841	0,01857969	0,07922811	protein_codir	hypothetical protein
TcG_01139	288,4661519	-0,336405277	0,10935118	-3,0763753	0,00209534	0,01431403	protein_codir	hypothetical protein
TcG_01140	867,7276173	-0,06696028	0,06714404	-0,99726326	0,31863674	0,55357619	protein_codir	hypothetical protein
TcG_01141	227,8011366	0,018595517	0,12002032	0,1549364	0,87687146	0,94102958	protein_codir	putative arginase

TcG_01142	559,1908655	-0,658344039	0,07824443	-8,41394075	3,9646E-17	5,404E-15	protein_codir	hypothetical protein
TcG_01143	404,8576068	-0,715879598	0,09304949	-7,69353632	1,4312E-14	1,4053E-12	protein_codir	putative leucine-rich repeat protein (LRRP)
TcG_01144	609,8940238	-0,203119671	0,07863101	-2,58320051	0,00978884	0,04861137	protein_codir	putative methionine aminopeptidase, putative,metallo-peptidase, Clan MG, Family M24
TcG_01145	418,7572666	-0,352545733	0,09336652	-3,77593311	0,00015941	0,00165941	protein_codir	spermatogenesis-associated protein 4
TcG_01146	126,6662869	-0,138412951	0,16124542	-0,85839925	0,39067204	0,62055474	protein_codir	hypothetical protein
TcG_01147	575,2216383	-0,454821349	0,08211335	-5,53894547	3,043E-08	8,792E-07	protein_codir	transferase
TcG_01148	858,4544088	-0,483825232	0,06602591	-7,32780912	2,3395E-13	1,9223E-11	protein_codir	putative protein kinase, putative,serine/threonine protein kinase
TcG_01149	57,40220348	0,011537601	0,23442754	0,04921606	0,96074711	0,98198472	protein_codir	hypothetical protein
TcG_01150	198,1350943	-0,280539943	0,12771685	-2,1965774	0,02805064	0,10873026	protein_codir	hypothetical protein
TcG_01151	762,0273637	-0,482585319	0,07469045	-6,46113836	1,0392E-10	5,0802E-09	protein_codir	hypothetical protein
TcG_01152	809,3259526	-0,510899644	0,06927238	-7,37522826	1,6406E-13	1,408E-11	protein_codir	histone H3 variant
TcG_01153	445,2371478	-0,186485682	0,08835263	-2,1106976	0,03479831	0,12803215	protein_codir	putative extracellular receptor
TcG_01154	1076,082773	-0,238697434	0,06358915	-3,7537447	0,00017421	0,00178306	protein_codir	putative RNA helicase
TcG_01155	373,0296635	-0,022223038	0,09660641	-0,23003689	0,8180631	0,91031113	protein_codir	putative DNA-directed RNA polymerase, alpha subunit
TcG_01156	301,2619816	-0,412414368	0,10758356	-3,8334331	0,00012637	0,00135942	protein_codir	U3 snoRNA-associated protein UTP11
TcG_01157	441,9439478	-0,375804397	0,09896037	-3,79752434	0,00014615	0,00153934	protein_codir	putative kinesin
TcG_01158	3388,493187	-0,47795558	0,04217411	-11,3329132	9,0155E-30	4,3522E-27	protein_codir	putative kinesin
TcG_01159	1774,610662	-0,112794919	0,05414565	-2,08317586	0,0372352	0,13422744	protein_codir	putative malate dehydrogenase
TcG_01160	224,5553372	-0,134761383	0,12281413	-1,09727914	0,27251939	0,50550907	protein_codir	S-adenosyl-L-methionine-dependent methyltransferase
TcG_01161	233,0950042	-0,294921337	0,11973874	-2,46304031	0,01377644	0,06361653	protein_codir	hypothetical protein
TcG_01162	179,4919873	-0,314226142	0,13086991	-2,40105723	0,01634778	0,07223699	protein_codir	hypothetical protein
TcG_01163	252,5646703	-0,629280336	0,11942269	-5,26935333	1,3691E-07	3,4709E-06	protein_codir	hypothetical protein
TcG_01164	188,4769478	-0,422972876	0,12958854	-3,26396815	0,00109864	0,00843247	protein_codir	hypothetical protein
TcG_01165	249,2904191	-0,224769637	0,11708583	-1,91969978	0,05489583	0,17679942	protein_codir	ESCRT-II complex subunit VPS36
TcG_01166	209,6289259	-0,027417433	0,13057929	-0,20996769	0,83369289	0,91924811	protein_codir	hypothetical protein
TcG_01167	434,6273439	-0,192093825	0,08766183	-2,19130529	0,02842971	0,10986878	protein_codir	putative transporter
TcG_01168	175,285427	-0,114237931	0,13339536	-0,85638611	0,39178425	0,62139829	protein_codir	putative transporter
TcG_01169	261,547647	0,578071572	0,11409796	5,06644973	4,053E-07	8,9787E-06	protein_codir	hypothetical protein
TcG_01170	27,52628479	0,654750465	0,33257707	1,96871799	0,04898548	0,16318166	protein_codir	hypothetical protein
TcG_01171	727,622286	-0,128756006	0,07054219	-1,82523401	0,06796572	0,20781967	protein_codir	putative cyclophilin
TcG_01172	86,89725654	-0,156611674	0,19214191	-0,81508337	0,41502459	0,64198597	protein_codir	p53 and DNA damage-regulated protein 1
TcG_01173	153,2509122	-0,058652482	0,15053616	-0,38962388	0,69681469	0,84298788	protein_codir	hypothetical protein
TcG_01174	161,7989498	-0,435519162	0,14226467	-3,06133039	0,00220356	0,01486369	protein_codir	hypothetical protein
TcG_01175	198,7985087	-0,156217902	0,12954534	-1,20589368	0,22785849	0,4546966	protein_codir	UDP-GlcNAc:PI a1-6 GlcNAc-transferase
TcG_01176	56,75447041	0,245720463	0,23826858	1,03127516	0,3024118	0,5353728	protein_codir	hypothetical protein
TcG_01177	794,3246001	-0,014994469	0,07526338	-0,19922662	0,84208548	0,9231054	protein_codir	DNA polymerase delta catalytic subunit
TcG_01178	1189,875223	-0,032507682	0,05872557	-0,55355244	0,57988518	0,76932895	protein_codir	putative DNA-dependent ATPase
TcG_01179	505,1719365	-0,455476199	0,08829732	-5,15843746	2,4902E-07	5,876E-06	protein_codir	putative protein kinase
TcG_01180	284,0681678	-0,03953736	0,10803273	-0,36597575	0,71438317	0,85349203	protein_codir	TPR Domain containing protein
TcG_01181	563,9275687	-0,208272896	0,08242245	-2,52689517	0,01150758	0,05519357	protein_codir	hypothetical protein
TcG_01182	306,0817574	-0,121744513	0,11148165	-1,09205873	0,27480729	0,50755645	protein_codir	autophagocytosis associated protein
TcG_01183	1843,808561	0,126732063	0,05036105	2,5164698	0,01185371	0,05645812	protein_codir	putative peptidase M20/M25/M40
TcG_01184	768,7550783	0,115539636	0,07017126	1,64653781	0,09965308	0,26802034	protein_codir	hypothetical protein
TcG_01185	1081,185435	-0,312922749	0,05922289	-5,28381386	1,2652E-07	3,2217E-06	protein_codir	putative short-chain dehydrogenase
TcG_01186	197,4460353	-0,327348481	0,13475781	-2,42916149	0,01513379	0,06816091	protein_codir	hypothetical protein
TcG_01187	206,8054975	-0,182617622	0,1236067	-1,47740876	0,1395661	0,33409356	protein_codir	hypothetical protein
TcG_01188	304,4382602	0,102315751	0,10495337	0,97486862	0,32962544	0,56434119	protein_codir	putative ribulose-5-phosphate 3-epimerase
TcG_01189	191,5283414	0,442599016	0,12886585	3,43457173	0,00059349	0,00500086	protein_codir	hypothetical protein
TcG_01190	295,582635	-0,20531864	0,1060977	-1,93518467	0,05296764	0,17194818	protein_codir	RAB-interacting protein
TcG_01191	1870,664138	-0,245771544	0,05056639	-4,86037369	1,1716E-06	2,3284E-05	protein_codir	hypothetical protein
TcG_01192	248,6088359	0,00199215	0,11196938	0,01779192	0,98580485	0,99438751	protein_codir	putative RAB-interacting protein
TcG_01193	195,5553713	-0,129349789	0,13593097	-0,95158442	0,34130779	0,57568671	protein_codir	hypothetical protein

TcG_01194	240,6644126	0,226252229	0,11728593	1,92906541	0,05372274	0,17391218	protein_codir	hypothetical protein
TcG_01195	483,1394493	-0,063868228	0,09772134	-0,65357504	0,51338563	0,7194975	protein_codir	transmembrane protein NRF-6
TcG_01196	690,2551336	0,015916151	0,0758513	0,2098336	0,83379755	0,91924811	protein_codir	hypothetical protein
TcG_01197	1428,215131	-0,090633215	0,06064663	-1,4944476	0,13505867	0,3270198	protein_codir	hypothetical protein
TcG_01198	269,5571983	-0,17349424	0,11826518	-1,46699344	0,14237784	0,33832855	protein_codir	hypothetical protein
TcG_01199	23,88757249	-0,181566942	0,35423475	-0,51256107	0,60825839	0,78802211	protein_codir	RNA-binding protein
TcG_01200	43,86118275	0,032622067	0,26892206	0,12130678	0,90344805	0,95322469	protein_codir	RNA-binding protein
TcG_01201	4,553959778	0,357144171	0,84740907	0,42145427	0,6734234	1		
TcG_01202	342,9525477	-0,074818735	0,09713772	-0,77023359	0,44116134	0,66354606	protein_codir	RNA-binding protein
TcG_01203	150,1224793	0,38780628	0,14418483	2,68964687	0,00715277	0,03792767	protein_codir	hypothetical protein
TcG_01204	970,3888988	-0,053386789	0,06303119	-0,84699002	0,39700068	0,62669278	protein_codir	hypothetical protein
TcG_01205	445,3310973	-0,085846731	0,09377497	-0,91545461	0,35995302	0,5926302	protein_codir	putative amino acid permease-like protein
TcG_01206	334,4307179	0,08384739	0,10249759	0,81804253	0,41333291	0,64005409	protein_codir	putative protein kinase
TcG_01207	352,0023591	-0,09911296	0,10053979	-0,98580835	0,32422717	0,55842069	protein_codir	hypothetical protein
TcG_01208	574,2073735	-0,319398488	0,08303782	-3,84642175	0,00011986	0,00130139	protein_codir	putative mitogen activated protein kinase
TcG_01209	434,1531551	-0,120542653	0,08769374	-1,37458678	0,16925959	0,3758945	protein_codir	hypothetical protein
TcG_01210	202,8878809	0,088899722	0,12783979	0,69539948	0,48680498	0,6985537	protein_codir	putative DNA repair protein
TcG_01211	223,6946656	0,329146389	0,12248525	2,68723292	0,00720467	0,03817008	protein_codir	hypothetical protein
TcG_01212	264,4405743	-0,085337703	0,10987307	-0,77669351	0,43733961	0,66071414	protein_codir	hypothetical protein
TcG_01213	389,5574155	0,032659147	0,09129618	0,35772741	0,72054732	0,85670516	protein_codir	RNA-binding protein
TcG_01214	342,2062523	-0,129708695	0,09846186	-1,31734957	0,18772148	0,4020967	protein_codir	putative vesicle-associated membrane protein, putative,syntaxin-like protein
TcG_01215	261,6223488	-0,151179354	0,12528274	-1,20670537	0,22754563	0,45444535	protein_codir	hypothetical protein
TcG_01216	357,5874094	0,157074482	0,09748601	1,61125152	0,10712491	0,28208285	protein_codir	hypothetical protein
TcG_01217	288,1510902	-0,004133784	0,10722024	-0,03855414	0,96924587	0,9867567	protein_codir	hypothetical protein
TcG_01218	232,2186231	0,128119247	0,11987306	1,06879095	0,28516388	0,51877427	protein_codir	MFS transporter, PPP family, 3-phenylpropionic acid transporter
TcG_01219	207,9316172	0,033187475	0,12550347	0,26443471	0,79144497	0,89576419	protein_codir	hypothetical protein
TcG_01220	590,078334	0,101241216	0,076952	1,31564112	0,18829453	0,40265419	protein_codir	putative ABC transporter
TcG_01221	350,7671331	-0,175936593	0,09718341	-1,81035632	0,07024055	0,21231594	protein_codir	putative NAD(P)-dependent oxidoreductase
TcG_01222	103,361738	-0,086811762	0,17956399	-0,48345863	0,62877013	0,80107001	protein_codir	hypothetical protein
TcG_01223	214,3127614	-0,087744307	0,12337902	-0,71117689	0,47697463	0,69213601	protein_codir	hypothetical protein
TcG_01224	229,1256787	0,07993469	0,11924887	0,6703182	0,50265497	0,71125555	protein_codir	hypothetical protein
TcG_01225	275,2054734	-0,065568254	0,10809699	-0,60656872	0,54413717	0,74125493	protein_codir	hypothetical protein
TcG_01226	628,0509545	-0,121940282	0,07887171	-1,54605861	0,12209041	0,306775	protein_codir	hypothetical protein
TcG_01227	394,3339704	0,08220694	0,09269794	0,88682592	0,37517263	0,6057344	protein_codir	putative amidinotransferase
TcG_01228	131,4354162	0,18205605	0,16503255	1,10315236	0,26996101	0,5027758	protein_codir	hypothetical protein
TcG_01229	202,5505805	-0,097470079	0,22807663	-0,42735672	0,66911953	0,8263077	protein_codir	hypothetical protein
TcG_01230	160,8827365	-0,067266234	0,14053763	-0,47863503	0,63219829	0,80290602	protein_codir	hypothetical protein
TcG_01231	55,78582746	0,175304861	0,24125085	0,72664972	0,46744056	0,684068	protein_codir	hypothetical protein
TcG_01232	664,7936723	0,048969605	0,07361891	0,66517701	0,50593729	0,71267956	protein_codir	hypothetical protein
TcG_01233	375,4139412	0,026343418	0,09355851	0,28157159	0,77827203	0,88925638	protein_codir	hypothetical protein
TcG_01234	335,3143148	-0,006803916	0,10388679	-0,06549357	0,94778103	0,97539448	protein_codir	hypothetical protein
TcG_01235	283,9965044	-0,005712201	0,1097554	-0,05204483	0,95849297	0,98075595	protein_codir	putative 33 kDa inner dynein arm light chain, axonemal
TcG_01236	815,2696185	-0,313700147	0,06934663	-4,52365348	6,0781E-06	9,9372E-05	protein_codir	hypothetical protein
TcG_01237	346,0383357	-0,115430051	0,10576901	-1,09134096	0,27512288	0,50763484	protein_codir	hypothetical protein
TcG_01238	755,0358862	-0,320071238	0,07639583	-4,18964277	2,7939E-05	0,00037122	protein_codir	putative pumilio protein 6
TcG_01239	296,6209366	-0,075790987	0,10546398	-0,71864337	0,47236068	0,68836848		
TcG_01240	270,3553279	0,461724742	0,12345761	3,7399455	0,00018406	0,00186551		
TcG_01241	1027,615494	0,13616798	0,06349031	2,14470477	0,03197646	0,11991016	protein_codir	putative trans-sialidase
TcG_01242	337,1593657	0,275499561	0,11031385	2,49741598	0,01251021	0,058944	protein_codir	putative trans-sialidase
TcG_01243	284,2734317	0,041541902	0,10704842	0,38806645	0,69796686	0,84388746	protein_codir	hypothetical protein
TcG_01244	224,9169119	0,173043536	0,1277149	1,35492051	0,17544289	0,3847589	protein_codir	hypothetical protein
TcG_01245	169,2011116	0,177929033	0,13772939	1,29187413	0,19640074	0,41463173	protein_codir	hypothetical protein

TcG_01246	271,0965199	0,186306089	0,12237101	1,52246911	0,12789158	0,31668131	protein_codir	hypothetical protein
TcG_01247	138,0721765	0,310429013	0,16188673	1,91756921	0,05516566	0,17739365	protein_codir	hypothetical protein
TcG_01248	198,4892607	0,25411364	0,13691973	1,85593146	0,06346333	0,19781709	protein_codir	trans-sialidase
TcG_01249	230,4322566	0,166427167	0,12934254	1,28671643	0,19819315	0,4168208	protein_codir	amino acid transporter
TcG_01250	301,4229769	-0,141092085	0,10669293	-1,32241264	0,18603078	0,39987989	protein_codir	putative N-acetyltransferase subunit Nat1
TcG_01251	973,3324905	-0,069501995	0,0619887	-1,12120423	0,26220094	0,49428247	protein_codir	putative N-acetyltransferase subunit Nat1
TcG_01252	422,8991115	0,041624297	0,09089859	0,45792015	0,64700981	0,81242026	protein_codir	heat shock protein
TcG_01253	395,4681955	0,065432918	0,09107205	0,71847418	0,47246497	0,68836848	protein_codir	hypothetical protein
TcG_01254	352,5612918	-0,117043958	0,10047178	-1,16494356	0,24404184	0,47218917	protein_codir	calmodulin-like protein containing EF hand domain
TcG_01255	415,8541685	-0,082290039	0,08972404	-0,91714591	0,35906619	0,5926302	protein_codir	hypothetical protein
TcG_01256	208,3626016	-0,232791493	0,13077476	-1,780095	0,07506041	0,22117242	protein_codir	hypothetical protein
TcG_01257	2421,204616	-0,104654257	0,04534442	-2,30798554	0,02099994	0,08755139	protein_codir	fructose-bisphosphate aldolase (ald)
TcG_01258	2557,729853	0,128090024	0,04672826	2,74116804	0,00612212	0,03355292	protein_codir	small subunit ribosomal protein S9e
TcG_01259	282,7893144	0,166165718	0,10803191	1,53811707	0,12401999	0,31011278	protein_codir	hypothetical protein
TcG_01260	381,0625419	0,003567848	0,09526093	0,03745342	0,97012348	0,98704108	protein_codir	putative protein phosphatase 2C
TcG_01261	295,6582803	0,156217637	0,10997867	1,4204358	0,15548085	0,35689154	protein_codir	hypothetical protein
TcG_01262	205,2311444	0,018482382	0,12627732	0,14636343	0,8836345	0,94348809	protein_codir	hypothetical protein
TcG_01263	673,4262252	-0,145401824	0,07381601	-1,96978699	0,04886279	0,16291346	protein_codir	GPI alpha-mannosyltransferase III
TcG_01264	403,1816197	-0,034018817	0,09296322	-0,36593847	0,71441099	0,85349203	protein_codir	hypothetical protein
TcG_01265	402,1314284	-0,267049436	0,09508154	-2,8086361	0,00497518	0,02867786	protein_codir	hypothetical protein
TcG_01266	46,3740635	0,316454238	0,25624324	1,23497593	0,21683946	0,44089387		
TcG_01267	965,3378087	0,334832983	0,06314404	5,30268559	1,1411E-07	2,9121E-06	protein_codir	hypothetical protein
TcG_01268	453,6507961	-0,077751131	0,08741146	-0,88948445	0,37374278	0,60449626	protein_codir	protein MEMO1
TcG_01269	295,2959651	-0,086860149	0,10697546	-0,81196329	0,4168127	0,6436309	protein_codir	putative endonuclease/exonuclease/phosphatase
TcG_01270	379,8184522	-0,095804329	0,09628444	-0,99501366	0,31972962	0,55479817	protein_codir	hypothetical protein
TcG_01271	412,9360557	0,408846373	0,08985946	4,54984237	5,3686E-06	8,9113E-05	protein_codir	HORMA domain containing protein
TcG_01272	112,9820374	0,18301658	0,17134307	1,06812944	0,28546213	0,51904649	protein_codir	ribosomal protein L24
TcG_01273	199,5841836	0,080565407	0,13004293	0,61952933	0,53556771	0,73555572	protein_codir	hypothetical protein
TcG_01274	0,300145286	-1,420535481	3,2277253	-0,44010421	0,65986164	1		
TcG_01275	91,1344933	0,536157873	0,1933177	2,77345462	0,00554646	0,0310291	protein_codir	ribosomal protein L24
TcG_01276	950,0894148	-0,002027962	0,06406887	-0,03165284	0,97474891	0,98897419	protein_codir	putative nucleoplasmin-like protein (NLP)
TcG_01277	469,1922628	-0,147473222	0,08478872	-1,73930228	0,08198161	0,2349617	protein_codir	putative RNA editing complex protein MP61
TcG_01278	295,3074561	-0,196590949	0,10599371	-1,85474159	0,06363314	0,19813317	protein_codir	flagellar associated protein
TcG_01279	686,4419559	0,081118552	0,07452297	1,08850407	0,27637264	0,50882781	protein_codir	hypothetical protein
TcG_01280	515,2600327	-0,295347421	0,08397707	-3,51700063	0,00043645	0,00385716	protein_codir	putative 3-transmembrane protein A13
TcG_01281	601,9597947	0,067958422	0,07623436	0,8914409	0,37269269	0,60390427	protein_codir	hypothetical protein
TcG_01282	641,5830918	-0,242143575	0,07815164	-3,09838113	0,00194581	0,01345117	protein_codir	intraflagellar transport 122-like protein
TcG_01283	134,8367807	0,280016503	0,15291525	1,83118759	0,06707255	0,2060187	protein_codir	40S ribosomal protein S10
TcG_01284	351,3168237	0,218395885	0,09967092	2,19116948	0,02843953	0,10986956	protein_codir	40S ribosomal protein S10
TcG_01285	60,8578588	0,293393546	0,23417359	1,2528891	0,21024607	0,43289563	protein_codir	40S ribosomal protein S10
TcG_01286	208,8566834	-0,013122914	0,12389673	-0,10591817	0,91564728	0,95893423	protein_codir	hypothetical protein
TcG_01287	318,9452104	-0,03596168	0,10664117	-0,33722136	0,73595004	0,86494473	protein_codir	hypothetical protein
TcG_01288	2156,613681	-0,184444627	0,10201855	-1,80795194	0,07061398	0,21316665	protein_codir	putative dynein heavy chain
TcG_01289	241,2014629	0,368451265	0,11777149	3,12852686	0,00175685	0,01240394	protein_codir	40S ribosomal protein S18
TcG_01290	295,205036	0,02142017	0,11412427	0,18769163	0,85111839	0,92805389	protein_codir	40S ribosomal protein S18
TcG_01291	394,7245668	0,131013856	0,09166571	1,42925704	0,15293037	0,35331032	protein_codir	MP67 protein
TcG_01292	292,4401583	-0,227197408	0,10396142	-2,18540115	0,02885945	0,11104801	protein_codir	cyclic nucleotide-binding protein
TcG_01293	230,1459297	-0,152453727	0,11586582	-1,31577822	0,18824849	0,40265419	protein_codir	hypothetical protein
TcG_01294	278,6864428	-0,143689509	0,10803396	-1,33004025	0,18350501	0,39591975	protein_codir	1,2-Dihydroxy-3-keto-5-methylthiopentene dioxygenase
TcG_01295	150,4992597	0,077459358	0,15191425	0,50988868	0,61012945	0,7890164	protein_codir	hypothetical protein
TcG_01296	157,762352	-0,247887556	0,15887556	-1,56026238	0,11869789	0,30069066	protein_codir	hypothetical protein
TcG_01297	67,19062458	0,156180458	0,21622575	0,72230277	0,47010835	0,68645032	protein_codir	hypothetical protein

TcG_01298	528,684321	-0,112209341	0,08118159	-1,38220179	0,16690975	0,37291003	protein_codir putative RNA-binding protein
TcG_01299	222,2038266	-0,213100701	0,12166122	-1,75159107	0,07984415	0,23080696	protein_codir putative cell differentiation protein
TcG_01300	869,7207831	-0,044076462	0,07553863	-0,58349563	0,5595597	0,75401472	protein_codir phosphatidylinositol 3-kinase tor2
TcG_01301	300,7090575	-0,019598416	0,10865008	-0,18038106	0,85685343	0,9308407	protein_codir putative proteasome beta 7 subunit
TcG_01302	245,2704369	0,219861492	0,12047168	1,82500557	0,06800019	0,20782121	protein_codir putative quinonoid dihydropteridine reductase
TcG_01303	175,6301974	0,15446065	0,13603269	1,13546715	0,25617964	0,48809362	protein_codir hypothetical protein
TcG_01304	1065,708009	-0,025217647	0,06523727	-0,38655276	0,69908734	0,84414248	protein_codir coatomer protein
TcG_01305	280,7150829	0,153681709	0,10807443	1,42199881	0,1550266	0,35638871	protein_codir alkylated DNA repair protein
TcG_01306	111,9802151	0,226549255	0,1664762	1,3608507	0,17356088	0,38215057	protein_codir hypothetical protein
TcG_01307	195,9600536	0,034695043	0,12678006	0,27366325	0,78434342	0,89328643	protein_codir snoRNP protein GAR1
TcG_01308	0,467697622	2,091063019	2,51804996	0,83042952	0,40629598	1	protein_codir D-alanyl-glycyl endopeptidase-like protein
TcG_01309	141,2503519	-0,132223094	0,14697615	-0,89962282	0,36832101	0,60036117	protein_codir DCN1-like protein 2
TcG_01310	709,1163748	-0,054992222	0,07331842	-0,75004652	0,45322669	0,67382066	protein_codir putative Unc104-like kinesin
TcG_01311	279,0360104	-0,041360524	0,11345868	-0,36454262	0,71545285	0,85396046	protein_codir hypothetical protein
TcG_01312	173,3669324	0,225877281	0,13653122	1,65440023	0,09804622	0,26514068	protein_codir hypothetical protein
TcG_01313	130,0374457	0,254684314	0,15366838	1,65736321	0,09744606	0,26410072	protein_codir hypothetical protein
TcG_01314	202,9069152	0,348064975	0,12517077	2,78072095	0,00542383	0,03050656	protein_codir hypothetical protein
TcG_01315	278,8270912	0,255290817	0,10894103	2,34338543	0,01910963	0,08104106	protein_codir hypothetical protein
TcG_01316	206,0685224	0,303719103	0,1284441	2,36460151	0,01804948	0,07764255	protein_codir putative serine/threonine protein phosphatase
TcG_01317	489,4327696	0,243861366	0,08701345	2,80257106	0,0050697	0,02900622	protein_codir hypothetical protein
TcG_01318	249,2507175	0,303069612	0,11926708	2,54110035	0,01105042	0,05339039	protein_codir hypothetical protein
TcG_01319	467,9027129	0,174361459	0,08478275	2,05656767	0,03972783	0,14050264	protein_codir cytoplasmic dynein 2 heavy chain 1 isoform X1
TcG_01320	894,5769921	0,279178469	0,15322134	1,82205995	0,06844589	0,20874285	protein_codir cytoplasmic dynein 2 heavy chain 1 isoform X1
TcG_01321	189,7180835	0,222223163	0,13036219	1,70465965	0,08825792	0,246875	protein_codir hypothetical protein
TcG_01322	198,255202	0,006574213	0,12504979	0,05257276	0,95807232	0,98064539	protein_codir hypothetical protein
TcG_01323	918,0943976	-0,0783659	0,0640559	-1,22339857	0,22117919	0,4463718	protein_codir hypothetical protein
TcG_01324	176,9675631	0,125710946	0,13753281	0,91404331	0,36069409	0,59335535	protein_codir hypothetical protein
TcG_01325	463,7048272	0,012988555	0,08545879	0,15198617	0,87919784	0,94152752	protein_codir hypothetical protein
TcG_01326	937,7053408	-0,034410911	0,07322645	-0,46992461	0,63840888	0,80773199	protein_codir hypothetical protein
TcG_01327	132,9942146	-0,086229142	0,15215781	-0,5667086	0,57091217	0,7624407	protein_codir hypothetical protein
TcG_01328	179,5400848	-0,15500001	0,14311875	-1,08301679	0,27880097	0,51175349	protein_codir hypothetical protein
TcG_01329	218,1546774	0,101592418	0,12292276	0,82647365	0,40853546	0,63660535	protein_codir chaperone protein DNAJ
TcG_01330	392,1794756	-0,249484591	0,0923438	-2,70169291	0,00689874	0,03690556	protein_codir putative chaperone protein DNAj
TcG_01331	16,80514785	0,356313241	0,42672605	0,83499294	0,40372169	0,63312391	
TcG_01332	467,9305823	-0,133346719	0,0888293	-1,50115696	0,13331497	0,32445352	protein_codir ubiquitin hydrolase
TcG_01333	264,2507808	0,024762042	0,10935137	0,22644472	0,82085555	0,91123655	protein_codir hypothetical protein
TcG_01334	180,7812267	0,136687941	0,13421118	1,01845418	0,30846217	0,54140928	protein_codir hypothetical protein
TcG_01335	167,4440129	-0,170212716	0,13810059	-1,23252707	0,21775226	0,44195661	protein_codir putative geranylgeranyl transferase type II beta subunit
TcG_01336	174,1144949	-0,275168149	0,13614271	-2,02117425	0,04326173	0,14935352	protein_codir hypothetical protein
TcG_01337	473,5131304	0,255417324	0,08398779	3,04112436	0,00235696	0,01569413	protein_codir dihydroflavonol-4-reductase
TcG_01338	364,2505502	-0,085630662	0,09577742	-0,89405902	0,37129031	0,60280376	protein_codir hypothetical protein
TcG_01339	302,4008427	0,072943914	0,10472215	0,69654715	0,48608624	0,69827461	protein_codir hypothetical protein
TcG_01340	194,7280745	0,113843709	0,1299391	0,87613128	0,38095867	0,61104814	protein_codir hypothetical protein
TcG_01341	364,9866531	0,14222807	0,09730987	1,46159964	0,14385095	0,34048154	protein_codir 50S ribosomal protein L7Ae
TcG_01342	519,4003644	-0,128069221	0,09090425	-1,40883652	0,15888351	0,36122928	protein_codir putative adaptor gamma-1 chain
TcG_01343	205,2580717	0,154568824	0,129455	1,19399657	0,23247931	0,45964254	protein_codir putative ubiquitin-protein ligase
TcG_01344	689,2146368	0,081461094	0,07771187	1,04824524	0,29452563	0,52749637	protein_codir putative ubiquitin-protein ligase
TcG_01345	158,9200617	0,05275222	0,14103191	0,37404458	0,70837115	0,84943749	protein_codir hypothetical protein
TcG_01346	1187,610949	0,005731663	0,07491738	0,07650645	0,93901618	0,97168077	protein_codir phosphatidylinositol 3-kinase-like protein
TcG_01347	345,0356707	0,504411509	0,10034366	5,02683991	4,9863E-07	1,088E-05	protein_codir ESAG-like protein
TcG_01348	220,5554552	0,043528095	0,1225736	0,35511803	0,72250117	0,85733557	protein_codir hypothetical protein
TcG_01349	139,6563727	-0,255513411	0,14822471	-1,72382462	0,08473951	0,23992961	protein_codir hypothetical protein

TcG_01350	348,4431807	-0,105769755	0,09935858	-1,06452565	0,28709064	0,52029285	protein_codir	transferase
TcG_01351	166,6026137	-0,491115003	0,14024802	-3,50176057	0,00046219	0,00404088	protein_codir	hypothetical protein
TcG_01352	148,7831549	0,094296199	0,14280237	0,66032655	0,50904429	0,71575087	protein_codir	hypothetical protein
TcG_01353	1591,872223	-0,177806456	0,06364781	-2,79359903	0,00521251	0,02961849	protein_codir	dynein-1-alpha heavy chain, flagellar inner arm I1 complex protein
TcG_01354	371,3450423	0,139758998	0,09611287	1,45411326	0,14591491	0,34340242	protein_codir	putative TPR-repeat-containing chaperone protein DNAJ,putative
TcG_01355	146,7806843	-0,058679811	0,14564428	-0,40289816	0,68702314	0,83752632	protein_codir	small nuclear ribonucleoprotein SmD3
TcG_01356	182,0657957	0,288492749	0,14144905	2,03955245	0,04139492	0,14476353	protein_codir	Aquaporin 2
TcG_01357	129,1013343	-0,054773201	0,16275266	-0,33654259	0,73646174	0,86530636	protein_codir	hypothetical protein
TcG_01358	202,5655707	0,112916996	0,13109295	0,86135064	0,38904495	0,61928495	protein_codir	hypothetical protein
TcG_01359	261,9244845	-0,268987262	0,11735085	-2,29216281	0,02189625	0,09019841	protein_codir	TPR protein
TcG_01360	135,7670888	0,362044092	0,15165602	2,38727149	0,01697395	0,0742691	protein_codir	ribosomal protein L14
TcG_01361	110,1621189	-0,082013436	0,16901672	-0,4852386	0,6275071	0,80025287	protein_codir	hypothetical protein
TcG_01362	164,6364441	0,133420889	0,13925983	0,95807161	0,33802665	0,57268153	protein_codir	hypothetical protein
TcG_01363	435,4813865	0,263145545	0,08843106	2,97571394	0,00292307	0,0186415	protein_codir	hypothetical protein
TcG_01364	402,4983759	0,351206149	0,09255981	3,79436992	0,00014802	0,0015548	protein_codir	putative phosphomannomutase-like protein
TcG_01365	109,1991449	0,205788449	0,17371324	1,18464462	0,23615799	0,46360093	protein_codir	hypothetical protein
TcG_01366	123,1760587	0,148804007	0,1649973	0,90185724	0,36713271	0,59939061	protein_codir	putative actin
TcG_01367	345,1787685	-0,242243644	0,10329944	-2,34506241	0,01902389	0,08073657	protein_codir	hypothetical protein
TcG_01368	119,0967518	0,144495051	0,16288214	0,88711417	0,37501744	0,60565264	protein_codir	hypothetical protein
TcG_01369	476,049188	0,21774801	0,08828586	2,46639734	0,01364798	0,06325022	protein_codir	putative serine-palmitoyl-CoA transferase
TcG_01370	185,8649863	0,116963942	0,13146908	0,88966879	0,37364376	0,60449626	protein_codir	hypothetical protein
TcG_01371	131,2171445	0,272056223	0,15964459	1,70413676	0,08835554	0,24704966	protein_codir	hypothetical protein
TcG_01372	111,9975438	-0,24326103	0,17154784	-1,41803608	0,15618023	0,35769079	protein_codir	hypothetical protein
TcG_01373	151,6910636	0,27657431	0,14325594	1,93063062	0,05352875	0,17342955	protein_codir	inositol-pentakisphosphate 2-kinase
TcG_01374	414,4846583	0,339257513	0,09881096	3,43339965	0,00059606	0,00501888	protein_codir	hypothetical protein
TcG_01375	121,5315365	0,161313817	0,16416376	0,98263962	0,32578484	0,5602746	protein_codir	ribosomal protein L13
TcG_01376	557,3028604	0,027623761	0,07959537	0,34705234	0,728552	0,86018582	protein_codir	vacuolar H+-ATPase
TcG_01377	382,5365593	-0,472420579	0,09841778	-4,80015468	1,5854E-06	3,0395E-05	protein_codir	endosomal integral membrane protein
TcG_01378	17,23875482	-0,529481555	0,41338202	-1,2808529	0,20024534	0,41933119	protein_codir	endosomal integral membrane protein
TcG_01379	844,5236009	-0,265961006	0,06702018	-3,9683718	7,2365E-05	0,00084861	protein_codir	endomembrane protein
TcG_01380	4689,435406	0,587023589	0,04384118	13,389775	6,9391E-41	7,3087E-38	protein_codir	ribosomal protein L21E (60S)
TcG_01381	2961,373568	-0,074261318	0,04313083	-1,72176865	0,08511144	0,2405856	protein_codir	beta-fructofuranosidase-like protein
TcG_01382	68,11160931	0,179057166	0,21528508	0,8317212	0,40556633	0,6344709	protein_codir	endomembrane protein
TcG_01383	284,047078	0,114198284	0,10696303	1,06764255	0,28568178	0,51912745	protein_codir	beta-fructofuranosidase-like protein
TcG_01384	468,0791802	-0,056986104	0,08735685	-0,65233695	0,51418383	0,72009354	protein_codir	hypothetical protein
TcG_01385	243,1542608	0,050715288	0,11770791	0,43085708	0,66657229	0,82543809	protein_codir	patatin family phospholipase
TcG_01386	150,2770156	-0,026196762	0,14441168	-0,18140334	0,856051	0,93026807	protein_codir	LYR motif containing protein 1
TcG_01387	135,8262299	-0,002764078	0,16306289	-0,01695099	0,98647571	0,99463124	protein_codir	putative adenosine kinase
TcG_01388	321,0446156	-0,170565148	0,10275302	-1,65995268	0,09692397	0,26341002	protein_codir	hypothetical protein
TcG_01389	206,3726066	-0,213276659	0,12568054	-1,69697438	0,0897015	0,2496079	protein_codir	putative phosphatidylinositol 4-kinase
TcG_01390	210,5017949	-0,144879701	0,1223304	-1,18433111	0,23628202	0,46360093	protein_codir	hypothetical protein
TcG_01391	247,7102526	0,069271341	0,11602392	0,59704363	0,55047825	0,74654606	protein_codir	hypothetical protein
TcG_01392	122,8847438	-0,055544692	0,17109398	-0,32464434	0,74545027	0,87134653	protein_codir	hypothetical protein
TcG_01393	297,2945405	-0,051797303	0,10641731	-0,48673756	0,62644432	0,79939806	protein_codir	putative ankyrin
TcG_01394	116,0075673	-0,076181934	0,16447229	-0,46319009	0,64322812	0,8106874	protein_codir	pre-rRNA-processing protein TSR4
TcG_01395	204,7117745	0,350137633	0,13065168	2,6799321	0,00736371	0,03884918	protein_codir	hypothetical protein
TcG_01396	387,7617796	0,13647737	0,09524834	1,43285829	0,15189834	0,35209328	protein_codir	hypothetical protein
TcG_01397	141,5397532	-0,098367251	0,1542581	-0,63767965	0,52368223	0,72767838	protein_codir	hypothetical protein
TcG_01398	0					1		
TcG_01399	0					1		
TcG_01400	0					1		
TcG_01401	2,021113092	0,529151953	1,20895379	0,43769411	0,66160804	1	protein_codir	hypothetical protein

TcG_01402	0					1		
TcG_01403	453,5967159	0,558878797	0,09224641	6,05854251	1,3736E-09	5,3226E-08	protein_codir	hypothetical protein
TcG_01404	1199,446017	0,200534627	0,05949979	3,37034149	0,00075075	0,00612549	protein_codir	putative mitochondrial RNA binding complex 1 subunit
TcG_01405	813,4406208	0,237941029	0,06799829	3,49922067	0,00046662	0,00407098	protein_codir	putative mitochondrial RNA binding protein
TcG_01406	951,9071845	0,70292774	0,06763757	10,3925639	2,6805E-25	7,9631E-23	protein_codir	putative FtsJ cell division protein
TcG_01407	872,0777588	0,26989323	0,0663998	4,06466914	4,8101E-05	0,00059667	protein_codir	transferase
TcG_01408	636,2912319	0,229188943	0,08030326	2,8540429	0,00431667	0,02564518	protein_codir	hypothetical protein
TcG_01409	259,5838458	0,573412505	0,11356869	5,04903674	4,4404E-07	9,7437E-06	protein_codir	c2 domain protein
TcG_01410	10,1258996	0,922886997	0,56695048	1,62780883	0,10356543	1	protein_codir	hypothetical protein
TcG_01411	88,10770259	0,995798392	0,19534949	5,09752231	3,4413E-07	7,8025E-06	protein_codir	hypothetical protein
TcG_01412	605,3422458	0,150897485	0,07879749	1,91500362	0,05549204	0,17810882	protein_codir	putative ATP-dependent zinc metallopeptidase
TcG_01413	492,0633511	0,239135529	0,08645311	2,76607192	0,0056736	0,03160252	protein_codir	putative small GTP-binding rab protein
TcG_01414	201,7853323	0,371938749	0,22664863	1,64103681	0,10078977	0,27018749	protein_codir	hypothetical protein
TcG_01415	386,5147732	0,107313389	0,09468682	1,13335091	0,25706692	0,48902898	protein_codir	hypothetical protein
TcG_01416	685,2439143	0,138819824	0,08496302	1,63388523	0,10228295	0,27324194	protein_codir	putative RNA-binding protein
TcG_01417	42,64649042	-0,021861343	0,29086598	-0,0751595	0,9400878	0,97222684		
TcG_01418	219,9683656	0,087503346	0,12464621	0,70201372	0,48267061	0,69628628	protein_codir	hypothetical protein
TcG_01419	343,8953255	0,294229742	0,09881591	2,97755443	0,00290558	0,01856815	protein_codir	hypothetical protein
TcG_01420	621,0979501	0,01667083	0,07583065	0,2198429	0,82599351	0,91511289	protein_codir	hypothetical protein
TcG_01421	120,0199056	0,349166529	0,16115772	2,16661367	0,03026432	0,11518024	protein_codir	divalent cation tolerance protein
TcG_01422	180,693099	0,231192906	0,13401582	1,72511653	0,08450647	0,23954982	protein_codir	serine/threonine protein kinase
TcG_01423	285,3818932	0,365368051	0,1127339	3,24097772	0,0011912	0,00899108	protein_codir	hypothetical protein
TcG_01424	510,0521995	0,087243179	0,0907236	0,96163714	0,33623191	0,57078138	protein_codir	endosomal P24B protein
TcG_01425	24,14893906	-0,022153635	0,36476608	-0,06073381	0,9515712	0,97721184	protein_codir	trans-sialidase
TcG_01426	648,0309207	0,215189276	0,07641423	2,81608921	0,00486122	0,02817553	protein_codir	putative monoglyceride lipase
TcG_01427	898,9819425	0,176983709	0,0644345	2,7467226	0,0060194	0,03309957	protein_codir	putative leucine-rich repeat protein (LRRP)
TcG_01428	891,1250665	0,505117337	0,06990369	7,22590355	4,9778E-13	4,0331E-11	protein_codir	hypothetical protein
TcG_01429	313,9096673	0,421016022	0,10872085	3,87244976	0,00010775	0,00118891	protein_codir	hypothetical protein
TcG_01430	124,6464164	0,082297071	0,16052481	0,51267508	0,60817863	0,78802211	protein_codir	receptor-type adenylate cyclase
TcG_01431	64,09504024	0,033052267	0,21954364	0,15054987	0,88033081	0,94195722	protein_codir	receptor-type adenylate cyclase
TcG_01432	71,37692433	0,423786167	0,20644448	2,05278517	0,04009342	0,14153636	protein_codir	adenylyl cyclase
TcG_01433	282,4756218	0,305806939	0,10908575	2,80336285	0,00505727	0,02897803	protein_codir	adenylyl cyclase
TcG_01434	43,7169251	0,375351466	0,26567758	1,41280823	0,15771212	0,35983706	protein_codir	receptor-type adenylate cyclase
TcG_01435	82,35031438	0,484729583	0,20196663	2,40004789	0,01639293	0,07232614	protein_codir	hypothetical protein
TcG_01436	18,46729698	0,526714504	0,41306355	1,27514158	0,20225913	0,42238277	protein_codir	adenylyl cyclase
TcG_01437	2761,995502	0,365293051	0,10988204	3,32441078	0,00088606	0,00706527	protein_codir	hypothetical protein
TcG_01438	1317,692861	0,27675012	0,06129555	4,51501191	6,3313E-06	0,00010245	protein_codir	vacuolar H+-PPase
TcG_01439	115,0093039	0,218063041	0,16658546	1,30901608	0,1905289	0,40593377	protein_codir	hypothetical protein
TcG_01440	190,6026145	-0,01942467	0,13337415	-0,14564045	0,88420524	0,94348884	protein_codir	hypothetical protein
TcG_01441	165,7558189	0,098660724	0,13856296	0,71202814	0,47644735	0,69175791	protein_codir	hypothetical protein
TcG_01442	51,1233289	0,297318445	0,24179041	1,22965359	0,21882685	0,44362693	protein_codir	hypothetical protein
TcG_01443	334,6168731	0,205756339	0,09993287	2,05894566	0,03949944	0,14000054	protein_codir	putative kinesin-like protein
TcG_01444	933,783354	0,084667925	0,06828669	1,23988922	0,21501637	0,4388705	protein_codir	hypothetical protein
TcG_01445	59,28844042	0,338806744	0,24752725	1,36876544	0,17107261	0,37787336	protein_codir	hypothetical protein
TcG_01446	424,290319	0,140208267	0,08952849	1,56607424	0,11733124	0,29871451	protein_codir	hypothetical protein
TcG_01447	533,4157262	0,199735101	0,08471523	2,35772354	0,01838738	0,07861114	protein_codir	hypothetical protein
TcG_01448	918,0194137	0,154344749	0,06517018	2,36833388	0,0178684	0,07704558	protein_codir	hypothetical protein
TcG_01449	850,1234475	0,248116733	0,06828878	3,63334531	0,00027977	0,00263959	protein_codir	dynein heavy chain, cytosolic
TcG_01450	1068,452427	0,370749367	0,06264908	5,91787387	3,2613E-09	1,1626E-07	protein_codir	ATP-binding cassette protein subfamily C, member 1
TcG_01451	246,5024237	0,508661353	0,12017684	4,23260723	2,31E-05	0,00031412	protein_codir	hypothetical protein
TcG_01452	671,9326053	0,148942753	0,07387137	2,01624453	0,04377441	0,15058502	protein_codir	hypothetical protein
TcG_01453	1476,959337	0,33703275	0,0618247	5,45142523	4,9968E-08	1,3782E-06	protein_codir	putative protein phosphatase 2C

TcG_01454	488,0638353	0,435021045	0,08663385	5,02137523	5,1303E-07	1,1152E-05	protein_codir rac serine-threonine kinase
TcG_01455	9,519045281	-0,211347506	0,61806648	-0,34194947	0,73238892	1	
TcG_01456	26,18579618	0,280048863	0,34669294	0,80777203	0,41922183	0,64580563	protein_codir hypothetical protein
TcG_01457	316,8239979	0,678726588	0,10295565	6,592417	4,3272E-11	2,3538E-09	protein_codir hypothetical protein
TcG_01458	678,5286125	0,474536294	0,07375634	6,43383738	1,2442E-10	6,0065E-09	protein_codir hypothetical protein
TcG_01459	524,7665771	0,227911806	0,08449039	2,69748783	0,00698648	0,03725053	protein_codir zinc-finger protein Lsd1
TcG_01460	967,7252933	0,392290972	0,06892476	5,69158248	1,2587E-08	3,8785E-07	protein_codir hypothetical protein
TcG_01461	1284,888689	0,721966788	0,06479049	11,1430979	7,738E-29	3,5861E-26	protein_codir hypothetical protein
TcG_01462	201,9665892	0,728648679	0,1303065	5,59180629	2,2472E-08	6,6588E-07	protein_codir hypothetical protein
TcG_01463	705,8346	0,488382552	0,07243573	6,74228841	1,5591E-11	9,4575E-10	protein_codir hypothetical protein
TcG_01464	677,1158929	0,374787276	0,07396309	5,06722033	4,0367E-07	8,9733E-06	protein_codir hypothetical protein
TcG_01465	119,0843777	0,30479744	0,16009496	1,90385403	0,0569292	0,18135323	
TcG_01466	729,5024435	0,182722276	0,07152434	2,5546868	0,01062834	0,05180613	protein_codir hypothetical protein
TcG_01467	457,8096507	0,386209531	0,08841343	4,36822232	1,2526E-05	0,00018464	protein_codir putative protein kinase
TcG_01468	296,6018402	0,429328353	0,10510156	4,0848904	4,4098E-05	0,00055474	protein_codir hypothetical protein
TcG_01469	212,6524669	0,337228028	0,1252981	2,69140568	0,00711516	0,03774554	protein_codir hypothetical protein
TcG_01470	1909,248188	0,176277874	0,05024336	3,5084809	0,00045067	0,00396169	protein_codir cytochrome c oxidase VIII (COX VIII)
TcG_01471	94,93351265	0,45647935	0,19411429	2,3516009	0,01869282	0,0795939	protein_codir hypothetical protein
TcG_01472	250,0731418	0,341204767	0,12167484	2,80423444	0,00504362	0,02894158	protein_codir hypothetical protein
TcG_01473	257,9805189	0,537966715	0,11253184	4,78057335	1,748E-06	3,3037E-05	protein_codir putative diphthine synthase
TcG_01474	205,2891574	0,31679668	0,12990383	2,43870154	0,01474014	0,06699852	protein_codir hypothetical protein
TcG_01475	899,5417653	0,4910212	0,06664121	7,36813163	1,7304E-13	1,4527E-11	protein_codir thiolase protein-like protein
TcG_01476	30,39098055	-0,095328023	0,34675993	-0,27491072	0,78338483	0,89263342	protein_codir dispersed protein family protein 1
TcG_01477	49,16402018	-0,109701504	0,25094817	-0,43714805	0,66200398	0,82295903	protein_codir hypothetical protein
TcG_01478	464,5444974	-0,156452958	0,08987454	-1,74079289	0,08171989	0,23453224	protein_codir putative protein kinase
TcG_01479	321,4198657	-0,072590585	0,10302002	-0,70462601	0,48104301	0,69536181	protein_codir putative nuclear transcription factor
TcG_01480	312,2054704	0,111130285	0,10299195	1,07901911	0,28057921	0,51365478	protein_codir putative 3,2-trans-enoyl-CoA isomerase, mitochondrial precursor
TcG_01481	1268,106749	-0,230103293	0,06083313	-3,78253246	0,00015524	0,00162038	protein_codir transcription factor-like protein
TcG_01482	372,6801718	-0,26317211	0,09666587	-2,72249263	0,00647915	0,03519335	protein_codir ribosomal protein L3-like protein
TcG_01483	177,8733785	-0,187827047	0,13541219	-1,38707635	0,16541848	0,3709156	protein_codir hypothetical protein
TcG_01484	224,1671597	-0,103343898	0,12329281	-0,83819887	0,40191902	0,63220648	protein_codir hypothetical protein
TcG_01485	1095,545245	-0,203428544	0,0620952	-3,27607528	0,0010526	0,0081509	protein_codir putative tryptophanyl-tRNA synthetase
TcG_01486	216,6911067	-0,335710789	0,12855339	-2,61145021	0,00901591	0,04555532	protein_codir putative syntaxin
TcG_01487	69,11886014	0,138209962	0,2234478	0,61853357	0,53622368	0,73627489	protein_codir hypothetical protein
TcG_01488	249,5599316	-0,198315987	0,12039327	-1,64723479	0,0995098	0,26780964	protein_codir small GTP-binding protein
TcG_01489	164,1902521	0,103237225	0,15165288	0,6807469	0,49603166	0,70584903	protein_codir WD repeat domain 34
TcG_01490	936,252735	-0,380742109	0,06435449	-5,91632574	3,2921E-09	1,1664E-07	protein_codir putative membrane associated protein
TcG_01491	112,5303299	-0,36463822	0,18031962	-2,02217715	0,04315805	0,14917339	protein_codir putative membrane associated protein
TcG_01492	835,671827	-0,400977677	0,06730502	-5,95761893	2,5594E-09	9,3543E-08	protein_codir putative proteasome regulatory non-ATPase subunit
TcG_01493	404,8574102	-0,205213878	0,09469866	-2,16701995	0,03023333	0,11517668	protein_codir IQ calmodulin-binding protein motif family protein
TcG_01494	303,0835303	-0,180548107	0,10716599	-1,68475198	0,09203643	0,25370786	protein_codir putative DNA-directed RNA polymerase II subunit 3
TcG_01495	563,8785542	-0,283371023	0,08177141	-3,46540446	0,00052943	0,00455723	protein_codir tetratricopeptide repeat protein 30A
TcG_01496	113,5198051	0,07298331	0,16807092	0,43424114	0,66411334	0,82434295	protein_codir transmembrane protein 180
TcG_01497	62,1549931	0,23477186	0,23012242	1,02020422	0,30763162	0,54090194	protein_codir hypothetical protein
TcG_01498	243,6687317	-0,087360041	0,1194316	-0,73146507	0,46449513	0,68173811	protein_codir putative protein phosphatase inhibitor
TcG_01499	602,4646342	-0,026387735	0,08225899	-0,32078844	0,74837072	0,87311203	protein_codir hypothetical protein
TcG_01500	650,410562	0,006816518	0,08036454	0,08481997	0,93240452	0,96764948	protein_codir hypothetical protein
TcG_01501	948,9081815	-0,075395244	0,06387374	-1,18037938	0,23784936	0,46565101	protein_codir SNF2 DNA repair protein
TcG_01502	263,958483	-0,078374877	0,11319745	-0,69237317	0,48870301	0,69997689	protein_codir hypothetical protein
TcG_01503	774,5767991	-0,371194328	0,0731288	-5,07589797	3,8567E-07	8,6933E-06	protein_codir putative protein transport protein Sec24A
TcG_01504	158,8893823	0,218917148	0,14332456	1,52742241	0,12665601	0,31469794	protein_codir hypothetical protein
TcG_01505	436,4796059	-0,272226532	0,08835242	-3,08114408	0,00206207	0,01411171	protein_codir RIO kinase 1

TcG_01506	160,2890105	-0,175150619	0,14080728	-1,24390311	0,21353522	0,43698047	protein_codir	putative thymine-7-hydroxylase
TcG_01507	193,985143	0,106839098	0,13818611	0,7731537	0,43943143	0,66264661	protein_codir	putative tubulin-tyrosine ligase
TcG_01508	860,6701561	-0,455015174	0,06646694	-6,84573713	7,6083E-12	4,9522E-10	protein_codir	host cell surface-exposed lipoprotein
TcG_01509	121,4886101	-0,006539967	0,17490574	-0,03739138	0,97017295	0,98704108	protein_codir	Smad nuclear-interacting protein 1
TcG_01510	211,591471	-0,455008143	0,12133519	-3,75000979	0,00017683	0,00180346	protein_codir	hypothetical protein
TcG_01511	1111,00185	-0,08998796	0,06041404	-1,48952069	0,13635031	0,32916375	protein_codir	Hsc70-interacting protein
TcG_01512	326,3010608	-0,216732015	0,10085793	-2,14888426	0,03164358	0,11891744	protein_codir	ADP-ribosylation factor GTPase activating protein
TcG_01513	503,8580768	-0,245191538	0,0880249	-2,78547925	0,00534487	0,03017818	protein_codir	UBX domain protein 7
TcG_01514	532,8126528	-0,214666629	0,08267656	-2,59646295	0,00941891	0,04705798	protein_codir	hypothetical protein
TcG_01515	173,2342563	0,168876231	0,13585916	1,24302429	0,21385888	0,43745922	protein_codir	hypothetical protein
TcG_01516	451,3598892	-0,251767461	0,09883115	-2,54745039	0,01085133	0,05266933	protein_codir	hypothetical protein
TcG_01517	351,9784152	0,001318739	0,09841075	0,01340036	0,98930838	0,99554162	protein_codir	putative RNA-binding protein
TcG_01518	160,5120757	0,033673984	0,14799671	0,22753197	0,82001011	0,91102214	protein_codir	DNA-directed RNA polymerase
TcG_01519	242,2656253	-0,17804227	0,12370752	-1,43921949	0,15008835	0,3500528	protein_codir	hypothetical protein
TcG_01520	317,4928174	-0,408303787	0,10753882	-3,7968035	0,00014657	0,00154242	protein_codir	hypothetical protein
TcG_01521	548,2098878	-0,097526582	0,07855735	-1,24146988	0,21443222	0,43816785	protein_codir	hypothetical protein
TcG_01522	301,5319198	-0,142551816	0,11697467	-1,21865538	0,22297502	0,44921697	protein_codir	putative serine/threonine protein phosphatase
TcG_01523	194,0542014	0,038624114	0,12800831	0,3017313	0,76285691	0,88014938	protein_codir	putative DNA repair protein
TcG_01524	70,11205917	-0,166275237	0,21174597	-0,78525808	0,43230227	0,65678653	protein_codir	hypothetical protein
TcG_01525	73,34905704	0,181459261	0,20626983	0,87971792	0,37901214	0,61023272	protein_codir	hypothetical protein
TcG_01526	206,7026915	-0,497632236	0,12558941	-3,96237405	7,4208E-05	0,00086846	protein_codir	hypothetical protein
TcG_01527	275,4876597	-0,112974988	0,11032674	-1,02400367	0,30583354	0,53883629	protein_codir	uncharacterized protein
TcG_01528	143,4385672	-0,410221529	0,15561559	-2,63612102	0,00838598	0,04308647	protein_codir	hypothetical protein
TcG_01529	721,2850054	-0,448828957	0,07423004	-6,04646015	1,4806E-09	5,6429E-08	protein_codir	cofilin/actin depolymerizing factor
TcG_01530	210,949941	0,148003762	0,12161954	1,21694064	0,2236268	0,45017916	protein_codir	hypothetical protein
TcG_01531	526,3147952	-0,194941717	0,08235078	-2,36721165	0,01792268	0,07722282	protein_codir	megakaryocyte stimulating factor
TcG_01532	397,3100007	0,098758111	0,09055637	1,0905706	0,27546187	0,50773647	protein_codir	exonuclease
TcG_01533	290,9908633	-0,369111742	0,10427054	-3,53994277	0,00040021	0,00358614	protein_codir	hypothetical protein
TcG_01534	41,13183494	-0,356830027	0,27090664	-1,31716975	0,18778173	0,40215141	protein_codir	hypothetical protein
TcG_01535	286,9435498	-0,130990512	0,11046986	-1,18575792	0,23571791	0,46344584	protein_codir	hypothetical protein
TcG_01536	259,548361	-0,207538567	0,11241432	-1,8461934	0,06486413	0,200779	protein_codir	hypothetical protein
TcG_01537	204,269867	-0,505591192	0,12517869	-4,03895587	5,369E-05	0,00065765	protein_codir	hypothetical protein
TcG_01538	553,5370726	-0,050812658	0,08514194	-0,5967994	0,55064132	0,74654606	protein_codir	putative DNA repair helicase
TcG_01539	276,6914972	-0,443428999	0,11035724	-4,01812328	5,8664E-05	0,00070887	protein_codir	hypothetical protein
TcG_01540	948,9616153	-0,284832274	0,06996392	-4,07113094	4,6785E-05	0,00058411	protein_codir	zinc finger protein
TcG_01541	2980,184544	-0,450709758	0,05388821	-8,36379235	6,0734E-17	7,9063E-15	protein_codir	ATP-binding cassette protein subfamily A, member 10
TcG_01542	79,93586361	0,070979939	0,20040419	0,35418391	0,72320106	0,85736289	protein_codir	Golgi vesicular membrane trafficking protein
TcG_01543	171,589966	-0,071396977	0,14554187	-0,4905597	0,62373789	0,79706621	protein_codir	aprataxin
TcG_01544	147,4965085	-0,253959317	0,14981905	-1,69510701	0,09005512	0,25021071	protein_codir	putative phosphate transporter
TcG_01545	118,4864421	-0,328911342	0,16351671	-2,01148459	0,0442743	0,15194372	protein_codir	putative phosphate transporter
TcG_01546	536,9900846	-0,420929046	0,08129236	-5,17796552	2,2432E-07	5,3587E-06	protein_codir	putative flagellar radial spoke protein-like
TcG_01547	120,4539852	-0,172031976	0,16687315	-1,03091463	0,30258085	0,5354669	protein_codir	hypothetical protein
TcG_01548	396,9895345	-0,061267506	0,09101201	-0,67318048	0,50083248	0,70971686	protein_codir	nuclear cap binding protein
TcG_01549	221,3041248	-0,298847119	0,12261938	-2,43719325	0,01480177	0,0672259	protein_codir	hypothetical protein
TcG_01550	226,3318621	-0,174306204	0,12328645	-1,41383099	0,15741153	0,35929275	protein_codir	hypothetical protein
TcG_01551	406,0181793	-0,136870024	0,09479427	-1,4438639	0,14877726	0,34787757	protein_codir	putative intergrin alpha chain protein
TcG_01552	51,04865711	0,025762946	0,2418529	0,10652321	0,91516724	0,95875395	protein_codir	hypothetical protein
TcG_01553	240,7591021	0,16530804	0,11624693	1,4220422	0,155014	0,35638871	protein_codir	putative elongation factor ts
TcG_01554	250,6484421	-0,145263468	0,12065826	-1,20392473	0,22861867	0,45574259	protein_codir	NAD-dependent epimerase/dehydratase family protein
TcG_01555	181,6919567	-0,390155756	0,13274257	-2,93919095	0,0032907	0,02054207	protein_codir	hypothetical protein
TcG_01556	322,1217936	-0,170586824	0,10696828	-1,59474211	0,11076992	0,28762444	protein_codir	putative peroxisomal targeting signal type 2 receptor
TcG_01557	97,26409785	-0,073637954	0,19506655	-0,37750169	0,70580081	0,84845489	protein_codir	hypothetical protein

TcG_01558	631,2828225	-0,244846891	0,07686049	-3,18560145	0,00144453	0,01052602	protein_codir	putative U3 small nucleolar ribonucleoprotein MPP10
TcG_01559	2618,987143	-0,435369193	0,04585067	-9,49537152	2,1943E-21	4,6223E-19	protein_codir	putative glucose regulated protein 94
TcG_01560	109,5044029	0,118462623	0,18469011	0,64141292	0,52125444	0,72540161	protein_codir	hypothetical protein
TcG_01561	551,7785928	-0,353672275	0,08203721	-4,31112007	1,6243E-05	0,00023145	protein_codir	putative U-box domain protein
TcG_01562	356,196017	-0,106061539	0,09719112	-1,09126775	0,27515509	0,50763484	protein_codir	hypersensitive-induced reaction protein 1
TcG_01563	917,87366	0,039533237	0,06454367	0,61250366	0,54020457	0,73911316	protein_codir	putative dolicholphosphate-mannose synthase
TcG_01564	255,412579	-0,191914459	0,11347268	-1,69128344	0,09078268	0,25143492	protein_codir	putative nucleoporin (NUP54/57)
TcG_01565	0					1	protein_codir	mucin-associated surface protein (MASP)
TcG_01566	5,415518995	0,303046299	0,77315724	0,39195947	0,69508816	1	protein_codir	hypothetical protein
TcG_01567	343,7799738	-0,140693793	0,1021593	-1,37719999	0,16845042	0,37494155	protein_codir	L1Tc protein
TcG_01568	61,31899467	-0,119759189	0,22448647	-0,53348065	0,5937009	0,77886311	protein_codir	mucin-associated surface protein (MASP)
TcG_01569	50,3000527	0,38310184	0,24554158	1,56023205	0,11870506	0,30069066	protein_codir	hypothetical protein
TcG_01570	31,90289371	0,051228752	0,3426275	0,14951734	0,88114543	0,94206932	protein_codir	receptor-type adenylate cyclase
TcG_01571	12,40320674	0,515944371	0,54057957	0,95442818	0,33986695	1	protein_codir	putative receptor-type adenylate cyclase
TcG_01572	133,9324625	0,212711177	0,1508281	1,4102888	0,15845442	0,36060753	protein_codir	putative receptor-type adenylate cyclase
TcG_01573	67,66689055	0,358909875	0,21428593	1,6749111	0,09395168	0,25751694	protein_codir	receptor-type adenylate cyclase
TcG_01574	456,7370635	-0,446657516	0,09140487	-4,88658345	1,026E-06	2,0674E-05	protein_codir	hypothetical protein
TcG_01575	754,5509593	-0,095973106	0,07598841	-1,26299664	0,20659039	0,42864546	protein_codir	hypothetical protein
TcG_01576	46,18339428	0,133892367	0,2629888	0,50911813	0,61066942	0,78920423	protein_codir	receptor-type adenylate cyclase
TcG_01577	28,56783279	-0,045947916	0,3190973	-0,14399344	0,88550565	0,94437554	protein_codir	surface protease GP63
TcG_01578	157,3454787	0,211989982	0,14750355	1,4371856	0,15066526	0,35066447	protein_codir	hypothetical protein
TcG_01579	482,9915439	-0,266184162	0,09176417	-2,90074181	0,0037228	0,02266548	protein_codir	hypothetical protein
TcG_01580	766,1587391	-0,038155749	0,0690551	-0,55254062	0,58057801	0,77015993	protein_codir	hypothetical protein
TcG_01581	727,3525017	0,127947641	0,07229929	1,76969422	0,07677809	0,22458873	protein_codir	hypothetical protein
TcG_01582	272,3046121	-0,162347895	0,11208004	-1,44849958	0,14747738	0,34622763	protein_codir	putative cell cycle associated protein MOB1
TcG_01583	429,2473962	-0,139267145	0,09014051	-1,54500071	0,12234609	0,30728416	protein_codir	putative glucosamine-fructose-6-phosphate aminotransferase
TcG_01584	200,710035	-0,218649698	0,12394367	-1,76410543	0,07771423	0,22617359	protein_codir	hypothetical protein
TcG_01585	384,8664803	0,017957303	0,09981542	0,17990509	0,85722708	0,93107816	protein_codir	2,4-dienoyl-CoA reductase-like protein
TcG_01586	339,6709388	0,039840448	0,09871732	0,40358114	0,68652075	0,83726626	protein_codir	hypothetical protein
TcG_01587	283,3627566	-0,132030982	0,12139333	-1,08762959	0,27675866	0,50904889	protein_codir	peptidyl-tRNA hydrolase, PTH2 family
TcG_01588	567,3102875	0,04275762	0,07918654	0,5399607	0,58922413	0,77564856	protein_codir	putative protein phosphatase
TcG_01589	154,6156345	-0,040272714	0,14355547	-0,28053764	0,77906505	0,88989921	protein_codir	hypothetical protein
TcG_01590	630,1020885	-0,062397657	0,07548332	-0,82664167	0,40844019	0,63660535	protein_codir	hypothetical protein
TcG_01591	176,2189222	0,075777731	0,1393083	0,54395703	0,58647104	0,77504888	protein_codir	hypothetical protein
TcG_01592	408,2801651	-0,015984961	0,09733845	-0,16422041	0,86955763	0,93709374	protein_codir	putative acyl-CoA dehydrogenase
TcG_01593	318,7795621	0,013069967	0,10472944	0,12479744	0,90068391	0,95174891	protein_codir	hypothetical protein
TcG_01594	549,3136881	0,057918971	0,07924582	0,73087728	0,46485411	0,68209217	protein_codir	putative arginine N-methyltransferase, type III
TcG_01595	1017,137443	0,002015508	0,06249973	0,03224826	0,97427407	0,98878235	protein_codir	dihydrofolate reductase-thymidylate synthetase
TcG_01596	225,1624823	0,07516002	0,13100424	0,57372204	0,56615591	0,75832167	protein_codir	hypothetical protein
TcG_01597	231,6517404	0,226627013	0,1217606	1,86125082	0,06270876	0,19593952	protein_codir	exosome-associated protein 3
TcG_01598	463,7938851	-0,188185245	0,08899053	-2,11466598	0,03445843	0,12698325	protein_codir	putative lipin
TcG_01599	277,4451208	0,086134219	0,1122427	0,7673926	0,44284813	0,66499591	protein_codir	putative Qb-SNARE protein
TcG_01600	887,0314858	0,2042302	0,06936181	2,94441876	0,00323562	0,02026372	protein_codir	hypothetical protein
TcG_01601	548,2974691	-0,143134806	0,08261027	-1,73265143	0,08315764	0,23695633	protein_codir	hypothetical protein
TcG_01602	88,46359772	-0,066894381	0,18363254	-0,36428391	0,71564601	0,85396046	protein_codir	hypothetical protein
TcG_01603	558,1536432	0,124291474	0,08351204	1,48830595	0,13667023	0,32947591	protein_codir	hypothetical protein
TcG_01604	10,07190723	0,319932617	0,55330284	0,57822334	0,56311334	1	protein_codir	putative RNA-binding protein
TcG_01605	480,3304475	-0,138047848	0,08463465	-1,63110317	0,10286855	0,27423724	protein_codir	hypothetical protein
TcG_01606	225,2001932	0,143358229	0,12068819	1,1878397	0,23489657	0,46284212	protein_codir	adiponectin receptor
TcG_01607	612,5970195	-0,029597865	0,07738181	-0,38249123	0,70209703	0,84610945	protein_codir	hypothetical protein
TcG_01608	55,26915194	0,04873064	0,24762974	0,19678832	0,8439932	0,92439431	protein_codir	sodium stibogluconate resistance protein
TcG_01609	456,7035661	0,017484752	0,08703965	0,20088261	0,84079037	0,92246657	protein_codir	hypothetical protein

TcG_01610	541,8517817	-0,008101949	0,08415215	-0,09627739	0,92330027	0,96264894	protein_codir	hypothetical protein
TcG_01611	247,9804899	-0,187909051	0,11495694	-1,63460382	0,10213213	0,27291888	protein_codir	putative serine-threonine dehydratase
TcG_01612	355,1707564	0,020158763	0,09602717	0,2099277	0,8337241	0,91924811	protein_codir	putative protein AF-9
TcG_01613	888,4935516	-0,031431734	0,06783612	-0,46334803	0,64311493	0,8106874	protein_codir	hypothetical protein
TcG_01614	721,188832	0,018742714	0,07039032	0,26626833	0,79003256	0,89502471	protein_codir	hypothetical protein
TcG_01615	1272,302701	0,045338953	0,06865034	0,66043302	0,50897599	0,71575087	protein_codir	hypothetical protein
TcG_01616	270,562308	0,104939089	0,11333369	0,92593025	0,35448223	0,58843087	protein_codir	hypothetical protein
TcG_01617	247,1452527	-0,032619039	0,12010362	-0,27159081	0,78593667	0,89351999	protein_codir	hypothetical protein
TcG_01618	623,0687172	-0,025974567	0,08266439	-0,31421715	0,75335612	0,87552748	protein_codir	hypothetical protein
TcG_01619	290,3471493	-0,464379905	0,10632612	-4,36750552	1,2567E-05	0,00018501	protein_codir	hypothetical protein
TcG_01620	946,5909078	0,003319393	0,06421076	0,05169528	0,9587715	0,98091703	protein_codir	lanosterol synthase
TcG_01621	1307,696698	-0,09214085	0,06463669	-1,42551936	0,15400713	0,3550448	protein_codir	putative protein kinase
TcG_01622	1144,662551	-0,1981825	0,06753401	-2,93455856	0,00334023	0,02076172	protein_codir	hypothetical protein
TcG_01623	1790,743958	0,105116896	0,05019597	2,09413017	0,03624839	0,13205252	protein_codir	hypothetical protein
TcG_01624	455,6335972	0,050783941	0,08561152	0,59319053	0,55305365	0,74829844	protein_codir	Phosphoethanolamine/phosphocholine phosphatase
TcG_01625	223,8044069	0,185530689	0,12484327	1,48610889	0,13725032	0,33025591	protein_codir	hypothetical protein
TcG_01626	176,4185444	0,298999074	0,13289598	2,24987303	0,02445701	0,09818395	protein_codir	hypothetical protein
TcG_01627	835,2631226	-0,200499411	0,07125229	-2,81393633	0,00489389	0,02832199	protein_codir	hypothetical protein
TcG_01628	2682,838916	0,196150838	0,04770729	4,11154833	3,9301E-05	0,00049819	protein_codir	putative 60S ribosomal protein L23a
TcG_01629	434,2896889	0,100064519	0,08918292	1,12201433	0,26185635	0,49419574	protein_codir	deoxyuridine triphosphatase
TcG_01630	538,6695748	0,010679911	0,08155197	0,13095834	0,89580826	0,94939943	protein_codir	hypothetical protein
TcG_01631	601,7989487	0,051885706	0,07960355	0,65180142	0,51452928	0,7202291	protein_codir	Basic immunoglobulin-like variable motif-containing protein
TcG_01632	128,6496151	0,154445616	0,16274629	0,94899622	0,34262253	0,57679606	protein_codir	hypothetical protein
TcG_01633	383,5271549	0,199416413	0,09341429	2,13475267	0,03278122	0,12228049	protein_codir	hypothetical protein
TcG_01634	199,8271523	0,269591043	0,13344915	2,02017802	0,04336493	0,14953156	protein_codir	23S rRNA (guanosine2251-2-O)-methyltransferase
TcG_01635	421,5229081	0,099398217	0,09107045	1,09144318	0,27507792	0,50763484	protein_codir	hypothetical protein
TcG_01636	379,0864793	-0,12721786	0,09523469	-1,33583522	0,18160314	0,39328112	protein_codir	hypothetical protein
TcG_01637	663,8283602	-0,199605664	0,07356612	-2,7132824	0,00666203	0,0359006	protein_codir	chromosome-associated protein H
TcG_01638	630,8733826	-0,165254689	0,07906102	-2,09021708	0,0365983	0,13300751	protein_codir	putative inorganic polyphosphate/ATP-NAD kinase, putative,poly(p)/ATP NAD kinase
TcG_01639	116,4426733	-0,32738348	0,16088621	-2,03487595	0,04186336	0,14582948		
TcG_01640	228,9815237	-0,160033583	0,1229871	-1,30122253	0,1931823	0,41000368	protein_codir	hypothetical protein
TcG_01641	687,3222074	-0,243086176	0,07312071	-3,32445042	0,00088593	0,00706527	protein_codir	hypothetical protein
TcG_01642	442,9072808	-0,126828217	0,086245	-1,47055741	0,14141085	0,3366698	protein_codir	hypothetical protein
TcG_01643	195,3646031	-0,10802841	0,13065461	-0,82682432	0,40833665	0,63660535	protein_codir	hypothetical protein
TcG_01644	296,1685326	0,291590078	0,10601679	2,75041415	0,005952	0,03279118	protein_codir	60S ribosomal protein L19
TcG_01645	320,5986926	-0,076071341	0,10154925	-0,74910789	0,45379218	0,67403555	protein_codir	hypothetical protein
TcG_01646	785,6688387	0,157366636	0,06854978	2,29565473	0,02169563	0,08958146	protein_codir	60S ribosomal protein L19
TcG_01647	377,761181	-0,125016999	0,09660414	-1,29411638	0,19562522	0,41344653	protein_codir	hypothetical protein
TcG_01648	232,4633366	-0,012989216	0,11649091	-0,11150412	0,9112166	0,95684497	protein_codir	hypothetical protein
TcG_01649	75,06379363	-0,118421494	0,20028875	-0,59125383	0,55435036	0,74917803	protein_codir	calpain-like cysteine peptidase
TcG_01650	52,79764198	-0,442125123	0,24166944	-1,8294623	0,06733038	0,20653688	protein_codir	putative calpain-like cysteine peptidase, putative,cysteine peptidase, Clan CA, family C2
TcG_01651	418,3310793	-0,620695112	0,09137856	-6,79256803	1,1015E-11	6,9741E-10	protein_codir	putative radial spoke protein 3
TcG_01652	335,3391593	-0,216990419	0,09752326	-2,22501193	0,02608044	0,1029533	protein_codir	hypothetical protein
TcG_01653	278,5668617	0,070619602	0,1096994	0,64375561	0,51973393	0,72427679	protein_codir	hypothetical protein
TcG_01654	40,64156929	-0,591722114	0,27419539	-2,15803085	0,03092543	0,11690116		
TcG_01655	16,91146704	-0,666546261	0,43647333	-1,52711796	0,12673168	0,31481845	protein_codir	hypothetical protein
TcG_01656	77,91056886	-0,619975637	0,19741687	-3,14043892	0,00168695	0,01199079	protein_codir	mucin TcMUCI
TcG_01657	38,60477568	-0,115250174	0,28936643	-0,39828454	0,69042045	0,83922987	protein_codir	hypothetical protein
TcG_01658	23,7597744	-0,056524242	0,38028984	-0,14863464	0,88184194	0,94218407	protein_codir	hypothetical protein
TcG_01659	41,03286646	0,119364885	0,28281683	0,42205722	0,67298325	0,82890021	protein_codir	RNaseH
TcG_01660	53,8243127	0,118986562	0,23831129	0,49929049	0,61757475	0,793034	protein_codir	hypothetical protein
TcG_01661	34,71579219	0,160709884	0,30868706	0,52062398	0,60262874	0,785033	protein_codir	hypothetical protein

TcG_01662	90,06258073	-0,334293441	0,18563789	-1,80078235	0,07173719	0,21511728		
TcG_01663	254,314423	-0,084780005	0,11480616	-0,73846214	0,46023366	0,67839985	protein_codir	hypothetical protein
TcG_01664	271,4608469	-0,198787288	0,11039747	-1,80065077	0,07175794	0,21511728	protein_codir	PHD and RING finger domain-containing protein 1-like
TcG_01665	278,2612799	-0,114038665	0,10817715	-1,05418436	0,29179852	0,52445086	protein_codir	WD40 repeat-containing protein SMU1
TcG_01666	370,7138959	-0,171600737	0,0987308	-1,73806689	0,08219903	0,23549899	protein_codir	putative dynein assembly factor 3, axonemal
TcG_01667	518,7282097	-0,147890446	0,08313646	-1,77888797	0,07525813	0,22152965	protein_codir	hypothetical protein
TcG_01668	238,9131534	-0,240348295	0,11849342	-2,02836822	0,04252268	0,1475937	protein_codir	glycoprotein 96-92
TcG_01669	243,8556463	-0,092848496	0,11429729	-0,81234209	0,41659537	0,64347073	protein_codir	hypothetical protein
TcG_01670	452,4718157	-0,168045721	0,08647033	-1,94339165	0,05196886	0,16970438	protein_codir	hypothetical protein
TcG_01671	283,0214816	-0,194039831	0,11019818	-1,76082616	0,07826783	0,22727095	protein_codir	copper-transporting ATPase-like protein
TcG_01672	79,47137026	-0,099607407	0,19946631	-0,49936958	0,61751904	0,793034	protein_codir	hypothetical protein
TcG_01673	166,4473847	0,28764955	0,13886874	2,07137724	0,03832356	0,13699991	protein_codir	hypothetical protein
TcG_01674	2394,130286	-0,000144631	0,04885999	-0,0029601	0,99763818	0,99910416	protein_codir	putative copper-transporting ATPase-like protein
TcG_01675	63,63644697	0,111458243	0,22254231	0,50084068	0,61648326	0,79265743	protein_codir	hypothetical protein
TcG_01676	324,4478082	-0,401092314	0,10867308	-3,69081573	0,00022354	0,00218622	protein_codir	hypothetical protein
TcG_01677	208,6860573	-0,096887587	0,12179121	-0,79552201	0,42630991	0,65178385	protein_codir	hypothetical protein
TcG_01678	295,594545	-0,26248218	0,10739013	-2,44419278	0,01451767	0,06622252	protein_codir	ubiquilin
TcG_01679	231,1927703	0,132085511	0,11826932	1,11681975	0,2640714	0,49619385	protein_codir	putative NADH-cytochrome b5 reductase
TcG_01680	264,7321979	-0,098375503	0,11421699	-0,86130357	0,38907087	0,61928495	protein_codir	NADH-ubiquinone oxidoreductase 20 kDa subunit
TcG_01681	258,4345564	-0,013464125	0,11917568	-0,11297712	0,9100487	0,95641431	protein_codir	diagnostic antigen
TcG_01682	72,21250733	0,142519774	0,2113915	0,67419824	0,50018529	0,70914669	protein_codir	hypothetical protein
TcG_01683	57,03164153	-0,136971845	0,23081142	-0,59343618	0,55288928	0,74825081	protein_codir	hypothetical protein
TcG_01684	137,8880919	-0,078640524	0,15011501	-0,52386851	0,60037	0,78350294	protein_codir	putative calcium uniporter protein, mitochondrial
TcG_01685	420,5698346	-0,064737212	0,09278902	-0,6976818	0,48537621	0,69814634	protein_codir	multidrug resistance protein, MATE family
TcG_01686	257,4910959	-0,218247605	0,11308563	-1,92993224	0,05361523	0,1736612	protein_codir	hypothetical protein
TcG_01687	619,7832304	-0,097171223	0,08091363	-1,20092529	0,22978018	0,45688956	protein_codir	hypothetical protein
TcG_01688	330,6416767	-0,004389178	0,10572853	-0,04151366	0,9668864	0,98551473	protein_codir	hypothetical protein
TcG_01689	440,5748104	-0,354757403	0,09223573	-3,84620372	0,00011996	0,00130139	protein_codir	hypothetical protein
TcG_01690	218,8537258	-0,112281948	0,12409324	-0,90481924	0,36556116	0,59769234	protein_codir	hypothetical protein
TcG_01691	582,7168728	-0,169540137	0,07729588	-2,19339168	0,02827917	0,10943302	protein_codir	hypothetical protein
TcG_01692	574,3284132	-0,539752136	0,07985761	-6,75893174	1,3901E-11	8,6129E-10	protein_codir	hypothetical protein
TcG_01693	225,2606362	-0,154563011	0,12424193	-1,24404872	0,21348163	0,43698047	protein_codir	hypothetical protein
TcG_01694	897,0623094	-0,334142434	0,06454483	-5,17690455	2,256E-07	5,3781E-06	protein_codir	oxoglutarate dehydrogenase (succinyl-transferring)
TcG_01695	841,0696781	-0,226824991	0,06750213	-3,36026415	0,00077868	0,0063222	protein_codir	putative phosphatidylinositol (3,5) kinase
TcG_01696	3,670854085	0,429013326	0,94519856	0,45388699	0,64991017	1		
TcG_01697	24,78740552	0,335700244	0,34597424	0,97030415	0,33189491	0,56623979		
TcG_01698	877,6515974	0,114477014	0,06456783	1,77297288	0,0762332	0,22337832	protein_codir	putative retrotransposon hot spot (RHS) protein
TcG_01699	142,9583379	0,237354105	0,14673992	1,61751559	0,10576703	0,27983943	protein_codir	putative retrotransposon hot spot (RHS) protein
TcG_01700	50,66736359	0,061236294	0,24951572	0,24542059	0,80613079	0,90423384		
TcG_01701	292,4968083	-0,13357137	0,1045182	-1,27797237	0,20125917	0,4207486	protein_codir	isovaleryl-CoA dehydrogenase
TcG_01702	353,7505586	-0,087914927	0,09536209	-0,92190643	0,35657739	0,59060838	protein_codir	hypothetical protein
TcG_01703	801,9521241	0,441381873	0,07281965	6,06130176	1,3502E-09	5,2496E-08	protein_codir	putative 1,2-Dihydroxy-3-keto-5-methylthiopentene dioxygenase
TcG_01704	411,9842456	0,034736392	0,09149311	0,37966128	0,70419687	0,84742878	protein_codir	hypothetical protein
TcG_01705	240,1314556	0,11564989	0,11489271	1,00659028	0,31413171	0,54836974	protein_codir	hypothetical protein
TcG_01706	120,8130306	0,018076637	0,15984029	0,11309186	0,90995773	0,95641431	protein_codir	hypothetical protein
TcG_01707	173,3987982	-0,259839465	0,13877308	-1,87240546	0,06115053	0,19200272	protein_codir	hypothetical protein
TcG_01708	206,5596321	0,054071111	0,12367782	0,43719329	0,66197118	0,82295903	protein_codir	TatD related deoxyribonuclease
TcG_01709	439,2119278	-0,311485965	0,09471971	-3,28850227	0,00100722	0,00782673	protein_codir	hypothetical protein
TcG_01710	302,5121064	0,040333953	0,1031223	0,39112738	0,69570308	0,84234674	protein_codir	hypothetical protein
TcG_01711	434,5706762	-0,042806061	0,08884398	-0,48181161	0,62993978	0,80168887	protein_codir	hypothetical protein
TcG_01712	190,8129466	-0,056706839	0,13480396	-0,42066151	0,67400227	0,82902391	protein_codir	hypothetical protein
TcG_01713	384,8170239	-0,08658525	0,10172539	-0,85116658	0,39467682	0,62431497	protein_codir	hypothetical protein

TcG_01714	74,95051894	-0,21217304	0,20093551	-1,05592604	0,29100201	0,52365178	protein_codir	hypothetical protein
TcG_01715	415,2480836	-0,082008012	0,0898342	-0,91288182	0,3613047	0,59395184	protein_codir	putative vesicular transport protein
TcG_01716	239,7461449	0,016558263	0,12126928	0,13654129	0,89139339	0,94697266	protein_codir	putative cysteine desulfurase
TcG_01717	425,4285824	-0,376155792	0,0891074	-4,22137535	2,4282E-05	0,00032827	protein_codir	putative vesicular-fusion ATPase-like protein
TcG_01718	285,3365674	-0,293063758	0,10711765	-2,73590542	0,00622089	0,03396713	protein_codir	cholestenol Delta-isomerase
TcG_01719	562,7731374	-0,341884458	0,0793266	-4,30983371	1,6338E-05	0,00023226	protein_codir	hypothetical protein
TcG_01720	491,1646174	-0,272766685	0,08951098	-3,04729863	0,00230908	0,01545524	protein_codir	putative mitochondrial RNA binding protein 1
TcG_01721	200,0977923	0,039649322	0,12661814	0,31314092	0,7541736	0,87597548	protein_codir	pre-rRNA-processing protein TSR2
TcG_01722	60,52017255	-0,07539993	0,22752609	-0,33139027	0,74034972	0,86763449	protein_codir	hypothetical protein
TcG_01723	501,9505562	-0,317603109	0,08589371	-3,69762939	0,00021762	0,00213857	protein_codir	putative intraflagellar transport protein IFT88
TcG_01724	312,8546885	-0,05781259	0,10738385	-0,53837321	0,59031942	0,77623888	protein_codir	hypothetical protein
TcG_01725	131,6418348	-0,34086098	0,15710682	-2,16961284	0,03003619	0,11458652	protein_codir	putative transporter
TcG_01726	151,5964407	0,09116258	0,14171761	0,64326925	0,5200494	0,72462927	protein_codir	hypothetical protein
TcG_01727	440,2668894	-0,179264495	0,08955567	-2,0017102	0,04531591	0,15432984	protein_codir	hypothetical protein
TcG_01728	206,1957095	-0,055242525	0,12643083	-0,43693872	0,66215579	0,82305943	protein_codir	hypothetical protein
TcG_01729	129,166712	0,036653667	0,15324786	0,23917899	0,8109668	0,90685309	protein_codir	protein phosphatase 2C
TcG_01730	415,0905397	-0,151984271	0,09146312	-1,66169997	0,09657295	0,26284825	protein_codir	histone h1
TcG_01731	136,3347986	-0,072744696	0,15268499	-0,47643644	0,63376348	0,80419712	protein_codir	putative E3 ubiquitin-protein ligase
TcG_01732	329,9691701	-0,27963962	0,10004284	-2,79519864	0,00518678	0,02950126	protein_codir	putative translation initiation factor eIF2B delta subunit
TcG_01733	116,9135523	-0,265771603	0,17224671	-1,54297055	0,12283792	0,30774201	protein_codir	hypothetical protein
TcG_01734	184,5438571	-0,188490371	0,12849032	-1,4669616	0,1423865	0,33832855	protein_codir	hypothetical protein
TcG_01735	641,0109879	-0,52493071	0,07762465	-6,76242287	1,357E-11	8,453E-10	protein_codir	hypothetical protein
TcG_01736	320,2979987	0,091563916	0,10360767	0,88375619	0,37682784	0,60747563	protein_codir	putative dynein heavy chain
TcG_01737	193,2297348	0,275494841	0,12924085	2,13163903	0,03303653	0,12295575	protein_codir	glutamine-dependent carbamoyl-phosphate synthetase
TcG_01738	133,2580619	-0,300480517	0,15598833	-1,92630122	0,05406677	0,17483049	protein_codir	hypothetical protein
TcG_01739	127,0240079	-0,312460663	0,15503205	-2,01545851	0,04385663	0,15082306	protein_codir	hypothetical protein
TcG_01740	409,6266973	-0,311108781	0,09674081	-3,2159002	0,00130036	0,00967629	protein_codir	putative eukaryotic translation initiation factor 3 subunit
TcG_01741	1195,746374	-0,303036904	0,0580831	-5,21729913	1,8155E-07	4,485E-06	protein_codir	T-complex protein 1 subunit beta
TcG_01742	402,532482	-0,469292088	0,0948178	-4,94940927	7,4439E-07	1,5624E-05	protein_codir	putative cation transporter protein
TcG_01743	39,28911182	-0,012608499	0,28015828	-0,04500491	0,96410339	0,98397656	protein_codir	surface protease GP63
TcG_01744	247,2735985	0,000380114	0,1146591	0,00331516	0,99735489	0,99910416	protein_codir	hypothetical protein
TcG_01745	477,3443413	0,086595041	0,08427083	1,0275802	0,30414734	0,53709055	protein_codir	cytochrome oxidase assembly protein
TcG_01746	222,9900202	0,077007195	0,11959012	0,64392608	0,51962337	0,72427679	protein_codir	putative serine/threonine-protein phosphatase 2A, catalytic subunit
TcG_01747	263,5128552	0,188177551	0,11085671	1,69748447	0,08960511	0,24955883	protein_codir	peptide methionine sulfoxide reductase B8
TcG_01748	462,3237492	-0,057215312	0,0876515	-0,65275907	0,51391161	0,71997339	protein_codir	molybdenum cofactor biosynthesis protein
TcG_01749	140,7013923	-0,11055848	0,14717393	-0,75120966	0,45252649	0,67329805	protein_codir	hypothetical protein
TcG_01750	562,1042658	-0,353822088	0,08412658	-4,20582995	2,6013E-05	0,00034842	protein_codir	putative coatomer gamma subunit
TcG_01751	28,6259899	0,048907804	0,32899311	0,14865905	0,88182267	0,94218407	protein_codir	hypothetical protein
TcG_01752	190,8836622	0,042669072	0,13177027	0,3238141	0,74607878	0,87149696	protein_codir	hypothetical protein
TcG_01753	237,278525	-0,009156576	0,12205994	-0,07501705	0,94020115	0,97225728	protein_codir	hypothetical protein
TcG_01754	195,2958882	0,135280193	0,12844986	1,05317507	0,29226076	0,52498189	protein_codir	hypothetical protein
TcG_01755	375,5256526	0,017153132	0,09651699	0,17772137	0,8589418	0,93207022	protein_codir	putative leucine-rich repeat protein (LRRP)
TcG_01756	872,9949321	0,071649243	0,06560969	1,09205273	0,27480992	0,50755645	protein_codir	putative splicing factor 3B subunit 1
TcG_01757	163,7475033	-0,272215489	0,13657395	-1,99317277	0,04624253	0,15647371	protein_codir	hypothetical protein
TcG_01758	196,3607301	0,160108659	0,12802812	1,25057419	0,21108987	0,43370939	protein_codir	40S ribosomal protein S17
TcG_01759	372,403477	0,313782922	0,09471073	3,31306635	0,00092279	0,0073129	protein_codir	40S ribosomal protein S17
TcG_01760	393,333931	0,179258222	0,09797842	1,82956841	0,0673145	0,20653688	protein_codir	40S ribosomal protein S17
TcG_01761	452,9458763	-0,010950779	0,08898038	-0,1230696	0,90205198	0,95270504	protein_codir	serine/arginine repetitive matrix protein 1
TcG_01762	231,9570681	0,615255005	0,11858396	5,18834922	2,1217E-07	5,1212E-06	protein_codir	putative DNA repair and recombination protein,mitochondrial precursor
TcG_01763	214,3406324	0,018241032	0,12171199	0,14987046	0,88086681	0,94206932	protein_codir	hypothetical protein
TcG_01764	591,6910718	-0,101359604	0,08004759	-1,26624183	0,20542651	0,42684211	protein_codir	putative acyl-CoA dehydrogenase
TcG_01765	548,1000623	-0,150994455	0,08538529	-1,76838959	0,07699579	0,22481291	protein_codir	putative eukaryotic translation initiation factor

TcG_01766	188,2410437	0,321183518	0,13027495	2,46542812	0,01368496	0,06332028	protein_codir	hypothetical protein
TcG_01767	2,308126441	0,612868731	1,14084278	0,537207	0,59112463	1	protein_codir	putative membrane-bound acid phosphatase
TcG_01768	1891,915848	0,52887236	0,04981826	10,6160345	2,51E-26	8,8123E-24	protein_codir	putative membrane-bound acid phosphatase
TcG_01769	265,8136707	0,195900541	0,11145705	1,75763259	0,07881005	0,22861622	protein_codir	putative MRP protein
TcG_01770	738,3986218	0,503048851	0,07173153	7,01293887	2,3336E-12	1,669E-10	protein_codir	ribosomal protein S29
TcG_01771	282,8445834	0,029797772	0,10966395	0,27171895	0,78583813	0,89351472	protein_codir	putative FG-GAP repeat protein
TcG_01772	161,7015634	0,083104981	0,15066673	0,55158151	0,58123511	0,77048234	protein_codir	hypothetical protein
TcG_01773	739,9888763	-0,039837698	0,07878412	-0,50565644	0,61309785	0,79081972	protein_codir	GDP-mannose pyrophosphorylase
TcG_01774	891,4191249	-0,207330826	0,06692233	-3,09808142	0,00194778	0,01345675	protein_codir	vacuolar ATPase subunit B
TcG_01775	883,1710604	-0,165211239	0,06440564	-2,56516738	0,0103126	0,05060645	protein_codir	2-oxoglutarate dehydrogenase, E2 component, dihydrolipoamide succinyltransferase
TcG_01776	169,2254737	-0,265852399	0,1425593	-1,86485477	0,06220176	0,19481928	protein_codir	putative dolichyl-P-Man:GDP-Man5GlcNAc2-PP-dolichyl alpha-1,2-mannosyltranslocase
TcG_01777	340,6856938	0,111724097	0,09686007	1,15345877	0,24872213	0,47820907	protein_codir	putative tRNA methyltransferase complex subunit
TcG_01778	508,0139682	0,071172451	0,08247965	0,86290925	0,38818736	0,61847343	protein_codir	putative glutamic acid-rich protein precursor
TcG_01779	439,1188132	-0,243465248	0,09394627	-2,59153714	0,00955482	0,0476959	protein_codir	putative DNA replication licensing factor
TcG_01780	10,12296909	0,556834564	0,55729939	0,99916593	0,31771432	1	protein_codir	cation transporter
TcG_01781	259,6699177	0,423319371	0,11597116	3,65021236	0,00026202	0,00250272	protein_codir	putative cation transporter
TcG_01782	6,753434972	0,173159259	0,68185086	0,25395474	0,79953052	1	protein_codir	cation transporter
TcG_01783	381,8631546	-0,060378329	0,09473283	-0,6373538	0,52389441	0,72776714	protein_codir	putative ZIP Zn transporter
TcG_01784	22,19213698	-0,010194181	0,37891045	-0,02690393	0,97853636	0,99085144	protein_codir	ZIP Zn transporter
TcG_01785	2373,063181	-0,168012991	0,04995216	-3,36347792	0,00076967	0,00625343	protein_codir	putative cation transporter
TcG_01786	1795,465564	0,322521866	0,05451079	5,91666129	3,2854E-09	1,1664E-07	protein_codir	putative X-pro, dipeptidyl-peptidase,serine peptidase,Clan SC, family S15
TcG_01787	233,8708938	0,320340268	0,12188576	2,62820087	0,00858378	0,04384995	protein_codir	putative elongation factor G2-like protein
TcG_01788	340,5024343	0,387542161	0,10072575	3,84749855	0,00011933	0,00129717	protein_codir	elongation factor G2-like protein
TcG_01789	316,1613103	0,138485909	0,11040125	1,2543872	0,2097013	0,43223613	protein_codir	ribose 5-phosphate isomerase
TcG_01790	518,3815882	0,079247555	0,08146292	0,97280521	0,33065012	0,56523583	protein_codir	hypothetical protein
TcG_01791	727,0607347	-0,127977886	0,07850217	-1,63024652	0,10304941	0,27452987	protein_codir	leucine rich
TcG_01792	335,3979647	0,215191761	0,10370905	2,07495646	0,03799057	0,13614561	protein_codir	Small Surface Antigen
TcG_01793	271,8029063	0,103637443	0,10960251	0,94557545	0,34436517	0,57856944	protein_codir	hypothetical protein
TcG_01794	236,3891453	-0,274344226	0,11461387	-2,39363897	0,01668216	0,07329523	protein_codir	hypothetical protein
TcG_01795	153,2022787	0,14185479	0,14603896	0,97134895	0,33137454	0,56560186	protein_codir	putative NADH dehydrogenase subunit NB6M
TcG_01796	147,5059575	-0,032887527	0,15119407	-0,21751863	0,82780419	0,91588083	protein_codir	DPCD protein
TcG_01797	181,1843687	0,071223891	0,14067375	0,5063055	0,61264221	0,79081972	protein_codir	DNA-directed RNA polymerase I/III subunit
TcG_01798	338,1225626	0,065825273	0,10025814	0,65655788	0,51146524	0,71768359	protein_codir	putative translation factor (SUA5)
TcG_01799	599,5071481	-0,048427442	0,07570116	-0,63971859	0,52235557	0,72619266	protein_codir	mitochondrial DEAD box protein
TcG_01800	189,6885929	-0,288909143	0,13485268	-2,14240562	0,03216086	0,12046936	protein_codir	centrosomal protein 76kDa
TcG_01801	72,7457629	0,240576921	0,2220976	1,08320362	0,27871805	0,51168236	protein_codir	hypothetical protein
TcG_01802	203,5650359	-0,214316026	0,12779788	-1,67699202	0,09354404	0,2567641	protein_codir	glycerophosphoryl diester phosphodiesterase
TcG_01803	222,0524215	0,134379561	0,12503681	1,07472002	0,2825001	0,51568397	protein_codir	L1Tc protein
TcG_01804	5,254969218	-0,519660765	0,81012126	-0,64146047	0,52122355	1	protein_codir	hypothetical protein
TcG_01805	0					1	protein_codir	hypothetical protein
TcG_01806	0,888260645	-0,268975824	1,76814802	-0,15212291	0,87908999	1	protein_codir	hypothetical protein
TcG_01807	15,04434802	-0,569016982	0,45223763	-1,25822564	0,20831017	1	protein_codir	hypothetical protein
TcG_01808	46,07625854	0,193666652	0,25851695	0,7491449	0,45376988	0,67403555	protein_codir	helicase-like protein
TcG_01809	15,18230185	-0,178425067	0,43582575	-0,40939542	0,6822495	1	protein_codir	putative trans-sialidase
TcG_01810	16,106836	0,495512376	0,44255137	1,11967199	0,26285356	1	protein_codir	putative glycogen synthase kinase-3 alpha
TcG_01811	351,6854964	-0,306357013	0,09540124	-3,21124787	0,0013216	0,00979657	protein_codir	putative cullin 2
TcG_01812	301,0922514	-0,144380054	0,11117361	-1,29869003	0,19405033	0,41101775	protein_codir	putative endonuclease III
TcG_01813	136,7438485	-0,004707622	0,14905512	-0,0315831	0,97480452	0,98897419	protein_codir	type 11 methyltransferase
TcG_01814	1050,351357	0,029709254	0,06023688	0,49320701	0,62186632	0,79636111	protein_codir	hypothetical protein
TcG_01815	140,2521384	0,181612773	0,15116826	1,20139488	0,22959806	0,45682112	protein_codir	hypothetical protein
TcG_01816	141,7925483	-0,065005782	0,14884753	-0,43672731	0,66230912	0,82307341	protein_codir	hypothetical protein
TcG_01817	252,3489409	0,182023256	0,11799699	1,54260935	0,12292559	0,30780546	protein_codir	hypothetical protein

TcG_01818	185,9163249	0,185842283	0,13470266	1,37964816	0,167695	0,37370923	protein_codir	hypothetical protein
TcG_01819	393,5399068	-0,077043981	0,09680707	-0,79585075	0,42611879	0,6517508	protein_codir	mannosyl-oligosaccharide glucosidase
TcG_01820	367,5548145	-0,026843235	0,10173221	-0,26386171	0,79188648	0,89604169	protein_codir	mannosyl-oligosaccharide glucosidase
TcG_01821	308,6405746	-0,096430818	0,11731792	-0,82196155	0,41109878	0,63778662	protein_codir	trichohyalin
TcG_01822	257,5841426	-0,049425341	0,11073404	-0,4463428	0,65534964	0,81772823	protein_codir	trichohyalin
TcG_01823	337,198098	0,125963683	0,10018151	1,25735459	0,20862527	0,43120717	protein_codir	putative 2,3-bisphosphoglycerate-independent phosphoglycerate mutase
TcG_01824	146,8168421	-0,211029905	0,15370892	-1,37291909	0,1697775	0,37606704	protein_codir	hypothetical protein
TcG_01825	924,925312	-0,11407022	0,07027662	-1,62316025	0,10455515	0,27752285	protein_codir	putative cyclin dependent kinase-binding protein
TcG_01826	127,9308446	0,249739565	0,17103229	1,46018956	0,14423798	0,34084056	protein_codir	hypothetical protein
TcG_01827	1449,000782	0,16826855	0,06011142	2,79927757	0,00512171	0,02923159	protein_codir	putative glycosomal membrane protein
TcG_01828	813,127454	0,108150873	0,07242311	1,49331991	0,13535347	0,32759667	protein_codir	hypothetical protein
TcG_01829	417,8223765	0,163613758	0,091524	1,78765965	0,07383094	0,21927282	protein_codir	histone acetyltransferase
TcG_01830	1239,783796	-0,01290053	0,06126896	-0,21055573	0,83323396	0,91908998	protein_codir	DNA topoisomerase 2
TcG_01831	631,3243851	-0,053753517	0,07638437	-0,70372407	0,48160463	0,69570871	protein_codir	translation initiation factor eIF-2B subunit epsilon
TcG_01832	262,5877607	-0,154123181	0,12516398	-1,23137013	0,21818446	0,44255693	protein_codir	hypothetical protein
TcG_01833	511,481417	-0,121345811	0,08307853	-1,46061582	0,1441209	0,34078152	protein_codir	putative eukaryotic translation initiation factor
TcG_01834	321,0303868	0,105655246	0,10096095	1,04649613	0,29533203	0,52809782	protein_codir	hypothetical protein
TcG_01835	342,4221091	0,112982441	0,0980358	1,15246107	0,24913166	0,47849319	protein_codir	hypothetical protein
TcG_01836	388,9923834	-0,064445236	0,09651224	-0,66774158	0,50429857	0,71207637	protein_codir	hypothetical protein
TcG_01837	257,9897979	-0,090255719	0,12267101	-0,73575429	0,46188024	0,67953581	protein_codir	hypothetical protein
TcG_01838	137,1329395	-0,119395254	0,15000373	-0,79594857	0,42606193	0,6517508	protein_codir	surface protease GP63
TcG_01839	344,2742251	0,226078022	0,10040371	2,25168992	0,02434187	0,09782343	protein_codir	hypothetical protein
TcG_01840	1414,371152	0,037017171	0,05741841	0,64469167	0,51912702	0,7237563	protein_codir	aminopeptidase
TcG_01841	400,1596029	0,142301272	0,09235431	1,54081898	0,12336085	0,30869521	protein_codir	hypothetical protein
TcG_01842	410,2370368	0,022849249	0,10076188	0,22676481	0,82060663	0,91114306	protein_codir	hypothetical protein
TcG_01843	702,9201794	0,118731691	0,07668148	1,54837498	0,12153204	0,30590272	protein_codir	hypothetical protein
TcG_01844	6,819920763	-0,540917265	1,25530734	-0,43090425	0,66653799	1	protein_codir	hypothetical protein
TcG_01845	327,2242952	0,093858213	0,10839368	0,86590119	0,38654435	0,61678872	protein_codir	hypothetical protein
TcG_01846	1164,925627	0,226387006	0,06211013	3,6449288	0,00026747	0,00253789	protein_codir	hypothetical protein
TcG_01847	737,5814193	0,28803188	0,0763197	3,77401746	0,00016064	0,00166921	protein_codir	hypothetical protein
TcG_01848	364,5806724	0,261416531	0,09703062	2,69416533	0,00705652	0,03752032	protein_codir	putative membrane transporter
TcG_01849	157,6924497	-0,062683006	0,14254447	-0,43974351	0,66012288	0,82167853	protein_codir	ankyrin
TcG_01850	166,8959778	0,234056366	0,13903386	1,68344869	0,09228826	0,25409977	protein_codir	hypothetical protein
TcG_01851	373,5447175	-0,007875617	0,10233945	-0,07695583	0,93865869	0,97168077	protein_codir	putative anion-transporting ATPase-like
TcG_01852	436,9071821	-0,115944739	0,08660958	-1,33870578	0,18066647	0,39206215	protein_codir	hypothetical protein
TcG_01853	3455,059727	-0,174819233	0,04334003	-4,03366627	5,4913E-05	0,00066971	protein_codir	PUF nine target 1
TcG_01854	512,8200551	-0,133507421	0,08860993	-1,50668683	0,13189095	0,32280981	protein_codir	hypothetical protein
TcG_01855	398,7230431	-0,473891961	0,09254663	-5,12057495	3,0461E-07	6,9884E-06	protein_codir	hypothetical protein
TcG_01856	273,3859567	-0,1052239	0,11371333	-0,92534359	0,35478721	0,58873741	protein_codir	hypothetical protein
TcG_01857	272,8053804	-0,017860247	0,11611474	-0,1538155	0,87775521	0,94120054	protein_codir	40S ribosomal protein S21
TcG_01858	2145,412623	0,074214364	0,04970789	1,49300966	0,13543466	0,32772472	protein_codir	40S ribosomal protein S21
TcG_01859	249,197306	0,126862528	0,11890764	1,06689969	0,28601713	0,51918774	protein_codir	hypothetical protein
TcG_01860	293,8621843	0,318236188	0,11406399	2,78997942	0,00527114	0,02984348	protein_codir	putative helicase ARIP4 isoform X3
TcG_01861	136,2490785	-0,075224684	0,15872401	-0,47393386	0,63554707	0,80554079	protein_codir	hypothetical protein
TcG_01862	742,6504442	-0,175861507	0,07372529	-2,38536189	0,01706233	0,07445643	protein_codir	hypothetical protein
TcG_01863	202,5637778	0,005422656	0,12366661	0,04384899	0,96502478	0,98439664	protein_codir	putative serine incorporator
TcG_01864	475,0859209	-0,054752342	0,08476866	-0,64590312	0,5183421	0,7233813	protein_codir	putative serine incorporator
TcG_01865	1691,968305	-0,243879242	0,06048699	-4,03192865	5,5321E-05	0,00067397	protein_codir	hypothetical protein
TcG_01866	1877,879071	-0,344753592	0,05590456	-6,16682418	6,9675E-10	2,8831E-08	protein_codir	hypothetical protein
TcG_01867	485,1106587	-0,062193646	0,08479597	-0,73345048	0,46328372	0,68090894	protein_codir	hypothetical protein
TcG_01868	671,6119818	-0,194352933	0,07533436	-2,57987089	0,00988373	0,04895804	protein_codir	hypothetical protein
TcG_01869	501,5750174	-0,101653219	0,0829162	-1,22597535	0,22020795	0,44530063	protein_codir	hypothetical protein

TcG_01870	1406,652429	0,054072099	0,05655264	0,95613742	0,33900281	0,57332217	protein_codir hypothetical protein
TcG_01871	894,4450916	0,167156869	0,0664836	2,51425736	0,01192834	0,05673304	protein_codir ARM repeat-containing protein
TcG_01872	1210,556253	-0,104300882	0,05878756	-1,77419995	0,07603008	0,22306521	protein_codir putative kinesin
TcG_01873	349,4203443	-0,070062929	0,10071013	-0,69568902	0,4866236	0,69843889	protein_codir hypothetical protein
TcG_01874	375,0487556	-0,111536101	0,09525275	-1,17094887	0,24161933	0,47025056	protein_codir hypothetical protein
TcG_01875	601,8032581	-0,186182152	0,0807865	-2,30461973	0,02118788	0,08804031	protein_codir hypothetical protein
TcG_01876	473,8630828	-0,028456132	0,08552897	-0,33270754	0,73935506	0,86708727	protein_codir 60S ribosomal protein L24
TcG_01877	806,3909511	0,016960305	0,06785171	0,24996133	0,80261725	0,90238947	protein_codir putative ATPase
TcG_01878	320,2226098	-0,095716589	0,11246125	-0,85110731	0,39470975	0,62431497	protein_codir hypothetical protein
TcG_01879	477,2155671	-0,111732142	0,0839014	-1,33170776	0,18295624	0,39525097	protein_codir hypothetical protein
TcG_01880	252,5998433	0,163141392	0,11755362	1,3878041	0,16519671	0,3707155	protein_codir hypothetical protein
TcG_01881	266,5173455	-0,356509501	0,11421109	-3,12149639	0,00179934	0,01263467	protein_codir 40S ribosomal protein S5
TcG_01882	306,7263284	-0,25058553	0,10338629	-2,42377916	0,01535994	0,06893502	protein_codir 40S ribosomal protein S5
TcG_01883	146,9212713	0,198947295	0,15404438	1,29149339	0,19653265	0,41473232	protein_codir hypothetical protein
TcG_01884	129,094261	0,028112067	0,1699336	0,16542971	0,86860577	0,93674123	protein_codir beta-adaptin 1
TcG_01885	320,5832489	-0,172283299	0,10256977	-1,67966934	0,09302167	0,25569372	protein_codir putative beta-adaptin 1
TcG_01886	3847,24079	0,213773325	0,04159347	5,1395888	2,7534E-07	6,3802E-06	protein_codir pyruvate phosphate dikinase 1
TcG_01887	98,99801681	-0,011069492	0,18111476	-0,06111866	0,95126471	0,97713458	protein_codir inositol-1,4,5-trisphosphate (IP3) 5-phosphatase
TcG_01888	212,156531	-0,02675784	0,12253091	-0,21837624	0,82713598	0,9155856	protein_codir putative inositol-1,4,5-trisphosphate (IP3) 5-phosphatase
TcG_01889	424,5078097	-0,042891072	0,08904051	-0,4817029	0,63001702	0,80168887	protein_codir hypothetical protein
TcG_01890	741,0472965	-0,154569664	0,07166817	-2,15674072	0,03102587	0,11720436	protein_codir hypothetical protein
TcG_01891	49,96327857	0,255962757	0,24817735	1,03137032	0,30236719	0,5353728	protein_codir hypothetical protein
TcG_01892	8,130433007	0,440002598	0,6024188	0,7303932	0,46514987	1	
TcG_01893	1440,586908	-0,398738339	0,05535279	-7,2035811	5,8651E-13	4,5915E-11	protein_codir putative SEC61-like (pretranslocation process) protein
TcG_01894	433,4877269	-0,416306037	0,08845189	-4,70658177	2,519E-06	4,5817E-05	protein_codir putative zinc finger domain protein
TcG_01895	1357,242299	-0,180632302	0,0548669	-3,29219091	0,0009941	0,00774036	protein_codir putative lanosterol 14-alpha-demethylase
TcG_01896	76,4572348	0,25022739	0,19930172	1,2555205	0,20928987	0,4318721	protein_codir 60S ribosomal protein L28
TcG_01897	95,85883855	-0,024409809	0,1805651	-0,13518564	0,8924651	0,94793735	protein_codir hypothetical protein
TcG_01898	395,6228571	0,15012282	0,09591635	1,56514309	0,11754936	0,29897429	protein_codir 60S ribosomal protein L28
TcG_01899	32,4080152	0,075927582	0,30620366	0,24796432	0,80416201	0,90318531	protein_codir 60S ribosomal protein L28
TcG_01900	345,3032591	0,051253027	0,0978738	0,52366443	0,60051196	0,7835886	protein_codir phosphatidylinositol-4-phosphate 5-kinase
TcG_01901	1083,844032	-0,320616259	0,0645728	-4,96519089	6,8634E-07	1,4484E-05	protein_codir putative protein transport protein Sec31
TcG_01902	274,4177467	-0,032225684	0,11384821	-0,28305834	0,77713212	0,88883048	protein_codir putative eukaryotic release factor 3
TcG_01903	747,5169038	-0,014728436	0,06984271	-0,21088008	0,83298085	0,91908998	protein_codir putative eukaryotic release factor 3
TcG_01904	266,3040575	-0,158840641	0,11082514	-1,43325457	0,1517851	0,35199803	protein_codir preprotein translocase subunit YidC
TcG_01905	454,6834881	0,153387303	0,08838813	1,73538358	0,08267289	0,23644323	protein_codir methyltransferase
TcG_01906	891,6385589	0,412549012	0,06582987	6,26689735	3,6831E-10	1,6287E-08	protein_codir 40S ribosomal protein S15a
TcG_01907	318,0757165	-0,074928852	0,10003396	-0,74903412	0,45383664	0,67403555	protein_codir hypothetical protein
TcG_01908	283,7737511	-0,661390223	0,11118359	-5,94863178	2,7039E-09	9,8206E-08	protein_codir S-phase kinase-associated protein
TcG_01909	355,9388866	-0,735210207	0,09880901	-7,44072005	1,0014E-13	9,0641E-12	
TcG_01910	40,64958409	-0,901634392	0,2778147	-3,24545238	0,00117264	0,00889151	
TcG_01911	473,240423	0,086481547	0,0880993	0,98163717	0,32627863	0,56072112	protein_codir ABC1 transporter
TcG_01912	239,262866	0,018658735	0,120583	0,15473768	0,87702812	0,94102958	protein_codir heat shock protein-like protein
TcG_01913	245,0680518	0,090441724	0,11547658	0,78320404	0,4335073	0,65792712	protein_codir tatD related deoxyribonuclease
TcG_01914	196,6712088	-0,420157199	0,13394191	-3,13686133	0,00170767	0,01210835	protein_codir putative protein kinase
TcG_01915	221,9310747	-0,274820823	0,12244619	-2,24442127	0,02480531	0,09913567	protein_codir putative acyl-CoA thioesterase
TcG_01916	294,4006412	-0,15781007	0,10464639	-1,50803164	0,13154643	0,32235553	protein_codir putative protein kinase
TcG_01917	210,3600646	-0,107230988	0,12840998	-0,83506742	0,40367975	0,63312391	protein_codir hypothetical protein
TcG_01918	488,4977199	-0,258154891	0,08419665	-3,06609457	0,00216875	0,01467705	protein_codir putative retrotransposon hot spot (RHS) protein
TcG_01919	873,530498	-0,023281467	0,06834724	-0,34063509	0,73337831	0,86298204	protein_codir hypothetical protein
TcG_01920	457,2282831	0,042752862	0,08639811	0,49483565	0,62071614	0,79518103	protein_codir hypothetical protein
TcG_01921	192,5069444	-0,017627531	0,13119794	-0,13435829	0,89311926	0,94840219	protein_codir putative small GTP-binding protein RAB6

TcG_01922	176,5355876	-0,34453522	0,14372306	-2,39721596	0,01652018	0,07280443	protein_codir	hypothetical protein
TcG_01923	177,408189	-0,172528403	0,13406066	-1,2869428	0,19811422	0,41673047	protein_codir	hypothetical protein
TcG_01924	392,3919917	-0,208598553	0,09283623	-2,24695191	0,0246431	0,09868451	protein_codir	hypothetical protein
TcG_01925	257,945759	0,193936497	0,11228581	1,7271684	0,08413742	0,23892743	protein_codir	tRNA pseudouridine13 synthase
TcG_01926	427,0673579	0,074776323	0,08949988	0,83549072	0,40344147	0,63312391	protein_codir	hypothetical protein
TcG_01927	573,8341953	-0,023918667	0,07865929	-0,30407938	0,76106741	0,87939833	protein_codir	phosphatidylinositol-phospholipase C
TcG_01928	76,29399232	0,040291452	0,20516178	0,19638868	0,84430595	0,92449946	protein_codir	putative trans-sialidase
TcG_01929	0,286047678	-0,458748634	3,06551352	-0,14964822	0,88104216	1	protein_codir	hypothetical protein
TcG_01930	0					1		
TcG_01931	1,001587711	-2,491122606	3,85377393	-0,64641119	0,51801309	1		
TcG_01932	0					1		
TcG_01933	1,248952181	0,97832668	1,66860824	0,58631299	0,55766521	1		
TcG_01934	0					1		
TcG_01935	0,324841555	-0,458748506	2,94047964	-0,15601146	0,87602399	1		
TcG_01936	0,714418641	1,39408784	2,10103149	0,66352544	0,50699409	1		
TcG_01937	171,9210003	-0,02235972	0,15707099	-0,14235423	0,88680021	0,94530014	protein_codir	putative ubiquitin-conjugating enzyme e2
TcG_01938	111,9986126	-0,19619228	0,17013284	-1,15317113	0,24884015	0,47827837	protein_codir	hypothetical protein
TcG_01939	201,8037417	0,060656551	0,12654302	0,47933543	0,63170002	0,80290602	protein_codir	hypothetical protein
TcG_01940	624,8072846	0,307680177	0,0847975	3,62841082	0,00028517	0,00268705	protein_codir	putative dihydroxyacetone phosphate acyltransferase
TcG_01941	203,641785	0,14952396	0,12798789	1,16826645	0,2426993	0,47105644	protein_codir	hypothetical protein
TcG_01942	106,0409666	0,127963336	0,17620328	0,72622561	0,46770047	0,68431606	protein_codir	hypothetical protein
TcG_01943	245,9758138	-0,013875817	0,11361859	-0,12212628	0,90279901	0,95297279	protein_codir	SBDS-like protein
TcG_01944	139,774208	0,271219723	0,151506	1,79015832	0,07342846	0,21830695	protein_codir	sperm flagellar protein 1
TcG_01945	286,1939271	0,037524935	0,10652769	0,35225523	0,72464686	0,85802335	protein_codir	hypothetical protein
TcG_01946	152,7207963	0,174391961	0,14120011	1,23506956	0,21680462	0,44089387	protein_codir	putative tRNA-dihydrouridine synthase 4
TcG_01947	143,4667585	0,226491527	0,15542179	1,45727007	0,14504184	0,34190332	protein_codir	hypothetical protein
TcG_01948	128,1820884	0,021470044	0,15656889	0,13712841	0,89092929	0,94691375	protein_codir	hypothetical protein
TcG_01949	97,97276654	0,577870161	0,18178441	3,17887638	0,00147847	0,01073954	protein_codir	hypothetical protein
TcG_01950	301,0021636	-0,169405028	0,10616292	-1,59570801	0,110554	0,28745033	protein_codir	hypothetical protein
TcG_01951	351,0055135	-0,09434827	0,10065574	-0,93733621	0,34858564	0,58310373	protein_codir	hypothetical protein
TcG_01952	318,6946585	-0,200324654	0,10288706	-1,9470345	0,0515306	0,16876407	protein_codir	hypothetical protein
TcG_01953	232,2687267	0,061523634	0,11673655	0,52702976	0,59817292	0,78203921	protein_codir	hypothetical protein
TcG_01954	228,278739	0,448764783	0,12019221	3,73372595	0,00018867	0,00190576	protein_codir	hypothetical protein
TcG_01955	585,7189305	0,014826565	0,08708131	0,17026116	0,86480475	0,93498506	protein_codir	ATP-dependent RNA helicase-like protein
TcG_01956	165,8769674	0,432264728	0,14166447	3,05132776	0,00227832	0,0152935	protein_codir	hypothetical protein
TcG_01957	121,4727219	0,015354105	0,16052665	0,09564833	0,92379988	0,96277282	protein_codir	hypothetical protein
TcG_01958	257,9427046	-0,17873662	0,11556463	-1,54663771	0,12195063	0,30662327	protein_codir	inositol 5-phosphatase-like protein
TcG_01959	497,6976103	0,027649381	0,08644744	0,31984035	0,74908936	0,87339734	protein_codir	hypothetical protein
TcG_01960	219,2066235	-0,062854052	0,124311	-0,5056194	0,61312386	0,79081972	protein_codir	hypothetical protein
TcG_01961	132,7037	-0,083891517	0,15866682	-0,52872752	0,59699448	0,78093915	protein_codir	chaperone DnaJ protein
TcG_01962	114,7338676	0,22227224	0,17255596	1,28811685	0,19770529	0,41624814	protein_codir	hypothetical protein
TcG_01963	412,9220216	-0,079651716	0,08947204	-0,89024139	0,37333629	0,60436974	protein_codir	DNA polymerase I-like protein A
TcG_01964	851,6426269	0,321983857	0,07459623	4,31635572	1,5863E-05	0,00022634	protein_codir	hypothetical protein
TcG_01965	120,1440517	-0,118857352	0,17227096	-0,68994423	0,49022926	0,70130255	protein_codir	hypothetical protein
TcG_01966	137,4531395	0,262172698	0,15967442	1,64192044	0,10060649	0,26982102	protein_codir	hypothetical protein
TcG_01967	196,2789518	-0,099829827	0,12624488	-0,7907634	0,42908207	0,65398767	protein_codir	hypothetical protein
TcG_01968	320,5505811	-0,13636507	0,10349621	-1,3175852	0,18764254	0,40205074	protein_codir	hypothetical protein
TcG_01969	236,2486701	-0,260268216	0,11665007	-2,23118774	0,0256687	0,1018136	protein_codir	hypothetical protein
TcG_01970	467,9313176	0,062699478	0,08469218	0,74032193	0,45910468	0,6777761	protein_codir	hypothetical protein
TcG_01971	475,8427265	-0,070382426	0,08490503	-0,8289547	0,40713004	0,63545853	protein_codir	hypothetical protein
TcG_01972	1274,556974	-0,007691835	0,06463796	-0,11899874	0,90527636	0,95390315	protein_codir	hypothetical protein
TcG_01973	149,1157407	0,067654678	0,15255199	0,44348603	0,65741422	0,81928146	protein_codir	hypothetical protein

TcG_01974	1725,012711	0,283778141	0,12181064	2,3296663	0,01982379	0,08336787	protein_codir	hypothetical protein
TcG_01975	144,9790827	0,076008473	0,15131194	0,50232966	0,61543565	0,79219196	protein_codir	ESCRT-II complex subunit VPS22
TcG_01976	677,7321198	0,341522327	0,07207007	4,73875416	2,1504E-06	3,9863E-05	protein_codir	D-isomer specific 2-hydroxyacid dehydrogenase-protein
TcG_01977	693,7379804	0,283487938	0,07479777	3,79005893	0,00015061	0,00157917	protein_codir	D-isomer specific 2-hydroxyacid dehydrogenase-protein
TcG_01978	635,4812253	0,177567376	0,07993468	2,22140608	0,02632347	0,10366544	protein_codir	hypothetical protein
TcG_01979	372,7408063	0,101948709	0,09484133	1,07493968	0,28240173	0,51558564	protein_codir	hypothetical protein
TcG_01980	79,88499207	0,582884036	0,20238445	2,88008314	0,0039757	0,02395346	protein_codir	hypothetical protein
TcG_01981	358,8753778	0,14165169	0,10004	1,41595049	0,15679	0,35829762	protein_codir	putative tyrosine phosphatase isoform
TcG_01982	516,4771197	-0,239406438	0,0808796	-2,96003499	0,00307604	0,01944379	protein_codir	hypothetical protein
TcG_01983	358,3663192	0,211491763	0,09868734	2,14304861	0,0321092	0,12031602	protein_codir	hypothetical protein
TcG_01984	243,1627038	-0,046548344	0,11623811	-0,40045683	0,68882007	0,83844588	protein_codir	hypothetical protein
TcG_01985	162,9546796	-0,178823688	0,15148166	-1,1804973	0,23780248	0,46565101	protein_codir	hypothetical protein
TcG_01986	288,1054092	0,141523537	0,10915317	1,29655911	0,19478293	0,41217405	protein_codir	hypothetical protein
TcG_01987	12,28976675	0,547070667	0,50428187	1,08485095	0,27798767	1	protein_codir	hypothetical protein
TcG_01988	25,78774804	0,072082778	0,3553636	0,20284232	0,83925828	0,92192691	protein_codir	hypothetical protein
TcG_01989	293,6519277	0,073129495	0,10477351	0,69797698	0,48519159	0,69814081	protein_codir	tetratricopeptidedomain 39C
TcG_01990	5,618020417	0,218010379	0,72150459	0,30216077	0,76252951	1	protein_codir	hypothetical protein
TcG_01991	92,53307032	0,13788183	0,18579714	0,74210953	0,45802098	0,67721172	protein_codir	hypothetical protein
TcG_01992	515,9199067	-0,033208482	0,08413279	-0,39471508	0,69305317	0,8410627	protein_codir	hypothetical protein
TcG_01993	332,0239614	-0,09571644	0,09956708	-0,96132615	0,3363882	0,57080486		
TcG_01994	716,3312151	-0,030492871	0,07763948	-0,39274954	0,69450447	0,84195133	protein_codir	flagellar associated protein
TcG_01995	204,9188075	0,118804727	0,12689089	0,93627468	0,34913178	0,58361576	protein_codir	putative kinesin-like protein
TcG_01996	11,67291943	-0,149248029	0,50982834	-0,29274172	0,76971957	1	protein_codir	putative kinesin-like protein
TcG_01997	34,93976588	0,643093828	0,29483291	2,18121453	0,02916755	0,11201036	protein_codir	kinesin-like protein
TcG_01998	16,32472672	0,360016619	0,43675294	0,82430267	0,4097676	1	protein_codir	putative kinesin-like protein
TcG_01999	138,9995697	-0,00106232	0,14848078	-0,0071546	0,99429151	0,99773191	protein_codir	hypothetical protein
TcG_02000	437,8998151	0,009173212	0,09543039	0,09612465	0,92342158	0,96264894	protein_codir	putative ATP dependent DEAD-box helicase
TcG_02001	174,9144387	0,092049727	0,1410823	0,65245412	0,51410826	0,72008994	protein_codir	ubiquitin-conjugating enzyme E2
TcG_02002	430,0703526	-0,314197616	0,08964514	-3,504904	0,00045677	0,00400012	protein_codir	putative hydroxymethylglutaryl-CoA lyase, putative,3-hydroxy-3-methylglutarate-CoA lyase
TcG_02003	411,8532587	0,130956447	0,08967239	1,46038758	0,14418359	0,34078152	protein_codir	hypothetical protein
TcG_02004	263,1385802	0,055662732	0,11481986	0,48478313	0,62783019	0,80057677	protein_codir	putative pyroglutamyl-peptidase I (PGP), putative,cysteine peptidase, Clan CF, family C15
TcG_02005	422,3703941	-0,090514164	0,09090637	-0,99568558	0,31940294	0,55440022	protein_codir	hypothetical protein
TcG_02006	311,4463742	-0,077755141	0,1045788	-0,74350765	0,45717439	0,6767839	protein_codir	hypothetical protein
TcG_02007	554,0576736	0,004121519	0,08228366	0,05008916	0,96005134	0,98165694	protein_codir	hypothetical protein
TcG_02008	472,7141265	0,085953727	0,0864929	0,99376623	0,32033669	0,55551877	protein_codir	putative ATP-dependent RNA helicase
TcG_02009	346,8606183	-0,110753939	0,10074421	-1,09935782	0,27161202	0,50471482	protein_codir	putative glycogenin glucosyltransferase
TcG_02010	146,7601056	0,175466463	0,15252014	1,15044781	0,24995948	0,47931653	protein_codir	hypothetical protein
TcG_02011	63,11641749	-0,425366791	0,2182195	-1,94926115	0,05126425	0,16840021	protein_codir	folate/pteridine transporter
TcG_02012	531,1930143	0,225576503	0,18019676	1,25183437	0,21063022	0,43314905	protein_codir	NADPH cytochrome P450 reductase B
TcG_02013	283,737946	-0,359410899	0,10635819	-3,37924998	0,00072684	0,00594295	protein_codir	putative RNA binding protein
TcG_02014	220,559352	0,062711585	0,12145445	0,51633829	0,60561816	0,78688931	protein_codir	hypothetical protein
TcG_02015	220,7819747	0,099659469	0,12644156	0,78818601	0,43058791	0,65538512	protein_codir	Low complexity protein
TcG_02016	200,9428912	0,066263319	0,12706432	0,52149432	0,60202246	0,78475506	protein_codir	RING finger protein 32
TcG_02017	115,972855	0,329215434	0,1674059	1,96657007	0,0492328	0,16369351	protein_codir	hypothetical protein
TcG_02018	300,3226185	-0,028749801	0,10578537	-0,27177482	0,78579517	0,89351472	protein_codir	hypothetical protein
TcG_02019	517,6821625	-0,046500834	0,08330475	-0,55820149	0,5767068	0,7668885	protein_codir	hypothetical protein
TcG_02020	243,783614	0,06841937	0,12297846	0,55635248	0,57796992	0,76800198	protein_codir	hypothetical protein
TcG_02021	207,7786676	0,318170394	0,13995696	2,27334448	0,02300543	0,09385243	protein_codir	hypothetical protein
TcG_02022	366,4517177	-0,103473533	0,09725297	-1,06396274	0,28734558	0,52042924	protein_codir	E3 ubiquitin-protein ligase TRIP12
TcG_02023	180,8307569	0,049652793	0,13271939	0,37411861	0,70831607	0,84943749	protein_codir	fumarylpyruvate hydrolase
TcG_02024	369,3683626	0,118605902	0,09861974	1,20265885	0,22910836	0,45626889	protein_codir	hypothetical protein
TcG_02025	119,640635	0,180623599	0,16634188	1,08585761	0,277542	0,51000818	protein_codir	hypothetical protein

TcG_02026	579,6559161	-0,059745819	0,08162556	-0,73194992	0,46419914	0,68170486	protein_codir	putative phosphoinositide-binding protein
TcG_02027	68,8164557	-0,183224167	0,23545575	-0,77816817	0,43646988	0,66000718	protein_codir	ubiquitin hydrolase
TcG_02028	590,2804554	-0,18514881	0,07903185	-2,34271133	0,01914419	0,08115791	protein_codir	hypothetical protein
TcG_02029	190,3344696	0,276957504	0,13521283	2,04830787	0,04052984	0,14251252	protein_codir	hypothetical protein
TcG_02030	445,5757519	0,010939395	0,09451109	0,1157472	0,90785289	0,95536083	protein_codir	hypothetical protein
TcG_02031	144,9925183	0,357890139	0,1495033	2,39386107	0,01667206	0,07327865	protein_codir	hypothetical protein
TcG_02032	609,2093166	0,051634059	0,07538281	0,684958	0,49337042	0,70398758	protein_codir	metallo-peptidase, Clan MG, Family M24
TcG_02033	478,29628	0,050516833	0,08503715	0,59405602	0,55247464	0,74786438	protein_codir	putative ribonuclease
TcG_02034	345,0372301	0,230161232	0,10215149	2,25313626	0,02425056	0,09765971	protein_codir	hypothetical protein
TcG_02035	1267,562634	0,037849488	0,05904264	0,6410535	0,52148793	0,72540161	protein_codir	transportin 1
TcG_02036	115,8886609	-0,047860274	0,1635507	-0,29263265	0,76980295	0,88436428	protein_codir	hypothetical protein
TcG_02037	207,6729408	0,036693206	0,12423377	0,29535612	0,76772185	0,88350974	protein_codir	hypothetical protein
TcG_02038	296,5635341	0,076235518	0,11237997	0,67837283	0,49753533	0,70728133	protein_codir	hypothetical protein
TcG_02039	120,2764242	0,037151202	0,15810205	0,23498241	0,81422238	0,90826251	protein_codir	hypothetical protein
TcG_02040	103,8342	0,428483206	0,17377502	2,46573532	0,01367323	0,06329128	protein_codir	hypothetical protein
TcG_02041	409,6843924	0,111882944	0,09212534	1,2144644	0,22457044	0,45108253	protein_codir	hypothetical protein
TcG_02042	954,2878663	-0,178676376	0,07023257	-2,5440673	0,010957	0,05302747	protein_codir	ATP-dependent RNA helicase-like protein
TcG_02043	76,63093785	0,077068377	0,19730615	0,39060302	0,69609069	0,84259143	protein_codir	hypothetical protein
TcG_02044	177,2681989	0,047443188	0,13384752	0,35445699	0,72299643	0,85733557	protein_codir	biotin carboxylase
TcG_02045	592,966222	0,019365366	0,07973631	0,24286758	0,80810798	0,90509218	protein_codir	hypothetical protein
TcG_02046	141,6134808	0,298425637	0,15254355	1,95633079	0,0504262	0,16640215	protein_codir	hypothetical protein
TcG_02047	17,31934386	-0,4268385	0,41945795	-1,01759545	0,30887025	0,54196134	protein_codir	ADP-ribosylation factor 1
TcG_02048	0					1	protein_codir	hypothetical protein
TcG_02049	123,226668	0,102475452	0,16131633	0,63524538	0,52526839	0,72866385	protein_codir	ADP-ribosylation factor 1
TcG_02050	48,06652477	-0,11686146	0,25161905	-0,46443805	0,64233393	0,8100665	protein_codir	ADP-ribosylation factor 1
TcG_02051	298,2054017	0,446039577	0,10717476	4,16179672	3,1575E-05	0,00041197	protein_codir	protein kinase
TcG_02052	161,943145	-0,410062283	0,13711214	-2,99070742	0,00278332	0,01788724	protein_codir	hypothetical protein
TcG_02053	131,4199604	-0,18865509	0,15181877	-1,24263348	0,21400293	0,43752213	protein_codir	putative trans-sialidase
TcG_02054	1180,556015	-0,23035683	0,05887062	-3,9129335	9,1182E-05	0,00103268	protein_codir	kinetoplastid membrane protein KMP-11
TcG_02055	283,7442188	0,154580714	0,11046786	1,39932747	0,1617148	0,36537202	protein_codir	hypothetical protein
TcG_02056	706,6608038	-0,32636007	0,07604902	-4,29144367	1,7752E-05	0,0002496	protein_codir	putative RNA-binding protein
TcG_02057	1169,943075	0,271280779	0,05944538	4,56352974	5,0301E-06	8,4339E-05	protein_codir	60S ribosomal protein L12
TcG_02058	349,3972399	0,054221131	0,09681216	0,56006531	0,57543489	0,76625291	protein_codir	serine/threonine protein phosphatase 2B catalytic subunit
TcG_02059	619,0790766	0,219146713	0,07822395	2,80152978	0,00508609	0,02907128	protein_codir	hypothetical protein
TcG_02060	215,9690644	-0,085217816	0,12337871	-0,69070116	0,48975336	0,70087481	protein_codir	putative short-chain dehydrogenase
TcG_02061	343,0008998	0,083660813	0,10137889	0,82522911	0,40924153	0,63678114	protein_codir	hypothetical protein
TcG_02062	228,7513827	0,110390552	0,12009466	0,91919615	0,35799298	0,59193759	protein_codir	short-chain dehydrogenase
TcG_02063	26,76048531	0,109878755	0,34988039	0,31404662	0,75348563	0,87552748	protein_codir	serine/threonine protein phosphatase
TcG_02064	45,03871182	0,313453192	0,26732667	1,17254738	0,24097736	0,46972948	protein_codir	rab1 small GTP-binding protein
TcG_02065	23,53879409	-0,099437529	0,36578232	-0,27184892	0,78573819	0,89351472	protein_codir	putative mitotic centromere-associated kinesin (MCAK)
TcG_02066	0					1	protein_codir	hypothetical protein
TcG_02067	30,08078647	0,338313578	0,32019064	1,05660047	0,29069397	0,52354738	protein_codir	hypothetical protein
TcG_02068	426,5951044	-0,042384887	0,09054877	-0,46808904	0,63972092	0,80835495	protein_codir	protein kinase domain
TcG_02069	274,9568871	0,334573482	0,11692411	2,86145852	0,00421697	0,02517145	protein_codir	hypothetical protein
TcG_02070	395,3682484	-0,181296806	0,10104315	-1,79425138	0,07277305	0,21697082	protein_codir	putative vesicle-associated membrane protein
TcG_02071	552,8605247	0,101049094	0,08074949	1,25138986	0,21079228	0,43332848	protein_codir	hypothetical protein
TcG_02072	582,2557485	0,009809145	0,08475737	0,11573206	0,9078649	0,95536083	protein_codir	hypothetical protein
TcG_02073	389,9525271	0,078318516	0,10126732	0,77338393	0,4392952	0,66263171	protein_codir	hypothetical protein
TcG_02074	266,0537392	0,086239824	0,12290648	0,70167025	0,48288483	0,69637835	protein_codir	hypothetical protein
TcG_02075	110,7040479	0,19475611	0,16861332	1,15504582	0,24807166	0,47744549	protein_codir	hypothetical protein
TcG_02076	672,3525302	-0,305234301	0,07308906	-4,17619705	2,9642E-05	0,0003916	protein_codir	putative reiske iron-sulfur protein precursor
TcG_02077	287,7930273	-0,02001695	0,10639549	-0,1881372	0,8507691	0,92805389	protein_codir	hypothetical protein

TcG_02078	257,6258591	0,120518629	0,12609352	0,95578762	0,33917954	0,57334902	protein_codir	putative chaperone DNAJ protein
TcG_02079	326,5591396	0,455153643	0,10324379	4,40853275	1,0407E-05	0,00015707	protein_codir	hypothetical protein
TcG_02080	357,8678318	0,062297354	0,09893466	0,62968177	0,52890281	0,73107665	protein_codir	hypothetical protein
TcG_02081	355,837947	-0,009573322	0,09982754	-0,0958986	0,9236011	0,96265225	protein_codir	putative metacaspase 5
TcG_02082	427,1218092	-0,107333778	0,08821187	-1,21677251	0,22369078	0,45017916	protein_codir	hypothetical protein
TcG_02083	437,3734022	-0,028274513	0,08988625	-0,31455882	0,75309665	0,87551453	protein_codir	hypothetical protein
TcG_02084	408,7576243	0,037484424	0,08910244	0,42068906	0,67398215	0,82902391	protein_codir	hypothetical protein
TcG_02085	751,1082974	-0,04269908	0,06875892	-0,62099696	0,53460163	0,73491866	protein_codir	putative chaperone DNAJ protein
TcG_02086	346,0592135	-0,216185318	0,09611211	-2,24930367	0,02449318	0,09826993	protein_codir	calmodulin
TcG_02087	168,5634068	0,11626022	0,14728284	0,78936704	0,42989752	0,65483116	protein_codir	RNA helicase
TcG_02088	218,4015658	0,020407334	0,12356882	0,16514955	0,86882627	0,93674123	protein_codir	thiopurine S-methyltransferase
TcG_02089	179,2111678	0,007239155	0,13245501	0,05465369	0,95641436	0,97984055	protein_codir	putative antigenic protein
TcG_02090	399,5122508	-0,059806088	0,0941313	-0,63534749	0,52520181	0,72866385	protein_codir	putative kinesin
TcG_02091	434,0266958	0,008695521	0,08748843	0,09939052	0,92082821	0,96150609	protein_codir	mismatch repair protein
TcG_02092	1751,275302	0,538263625	0,05956024	9,03729813	1,6059E-19	2,6965E-17	protein_codir	60S ribosomal protein L26
TcG_02093	326,0110572	-0,00914317	0,10709342	-0,08537564	0,93196276	0,96736431	protein_codir	hypothetical protein
TcG_02094	282,2135111	0,082670399	0,10871487	0,76043321	0,44699568	0,66841661	protein_codir	putative leucine-rich repeat protein (LRRP)
TcG_02095	245,7842412	0,046945407	0,11455017	0,40982399	0,68193507	0,83422317	protein_codir	putative protein kinase
TcG_02096	284,5351376	-0,238990507	0,10722993	-2,22876685	0,02582942	0,10220618	protein_codir	putative cyclophilin
TcG_02097	30,76625916	0,505620594	0,31689128	1,59556489	0,11058597	0,28746894	protein_codir	hypothetical protein
TcG_02098	346,2987079	-0,338225995	0,09733762	-3,47477157	0,00051129	0,00441415	protein_codir	putative casein kinase II, alpha chain
TcG_02099	724,1524894	-0,146038451	0,07480534	-1,95224626	0,05090897	0,16775636	protein_codir	putative cysteine peptidase, Clan CA, family C19
TcG_02100	206,4063485	0,084503681	0,12765475	0,66197053	0,50799011	0,71478909	protein_codir	putative UDP-glucoronosyl and UDP-glucosyl transferase
TcG_02101	7,834701238	0,343570926	0,62525771	0,54948691	0,58267134	1		
TcG_02102	295,2388137	-0,017926258	0,10553741	-0,1698569	0,86512268	0,93501039	protein_codir	putative small GTP-binding protein Rab18
TcG_02103	228,05036	-0,213209653	0,12422499	-1,71631854	0,08610376	0,24272461	protein_codir	putative calpain cysteine peptidase
TcG_02104	210,0419294	-0,050869518	0,13409045	-0,37936721	0,7044152	0,84755533	protein_codir	hypothetical protein
TcG_02105	134,5591008	-0,118473652	0,15811185	-0,74930279	0,45367473	0,67403555	protein_codir	hypothetical protein
TcG_02106	91,59175362	0,23730999	0,18527913	1,28082417	0,20025543	0,41933119	protein_codir	hypothetical protein
TcG_02107	8,278525022	-0,844904795	0,64363969	-1,31269841	0,18928459	1	protein_codir	hypothetical protein
TcG_02108	141,5068906	-0,001503242	0,15251837	-0,00985614	0,99213607	0,99678187	protein_codir	hypothetical protein
TcG_02109	223,2807973	-0,036016167	0,13021645	-0,27658693	0,7820973	0,89186804	protein_codir	hypothetical protein
TcG_02110	363,5912825	0,013744597	0,09546942	0,14396858	0,88552528	0,94437554	protein_codir	putative prohibitin
TcG_02111	428,9968562	-0,100016182	0,09170285	-1,09065509	0,27542468	0,50773647	protein_codir	putative eukaryotic translation initiation factor 4 gamma
TcG_02112	152,7932565	0,052541375	0,14311502	0,36712692	0,71352435	0,85278452	protein_codir	putative U1 small nuclear ribonucleoprotein
TcG_02113	536,9051158	-0,208867641	0,07999574	-2,61098455	0,0090282	0,04559751	protein_codir	putative kinesin
TcG_02114	97,81924548	-0,178388884	0,17796644	-1,00237374	0,31616312	0,55115373	protein_codir	hypothetical protein
TcG_02115	334,0775259	-0,080194104	0,09861337	-0,81321733	0,41609346	0,6430384	protein_codir	putative protein transport protein Sec23A
TcG_02116	674,1227238	-0,253005163	0,08507496	-2,97390881	0,00294032	0,01870763	protein_codir	WDdomain 65
TcG_02117	724,3067934	-0,0836571	0,07771608	-1,07644523	0,28172818	0,51481079	protein_codir	putative DNA polymerase I alpha catalytic subunit
TcG_02118	309,2861181	0,242388912	0,10554712	2,29649963	0,02164733	0,08947769	protein_codir	putative endoplasmic reticulum oxidoreductin
TcG_02119	32,5016105	0,747506294	0,31006596	2,4107977	0,01591767	0,0707683	protein_codir	hypothetical protein
TcG_02120	391,8701608	-0,25328947	0,09712458	-2,60788223	0,00911043	0,0458928	protein_codir	hypothetical protein
TcG_02121	163,5070116	-0,349833602	0,13800556	-2,53492396	0,01124717	0,05422795	protein_codir	hypothetical protein
TcG_02122	689,4215415	0,019522658	0,07437442	0,26249158	0,79294247	0,89624823	protein_codir	putative protein kinase
TcG_02123	328,7865376	-0,06927708	0,10015887	-0,69167192	0,48914338	0,70034791	protein_codir	hypothetical protein
TcG_02124	226,4491221	-0,138073075	0,12186599	-1,13299107	0,257218	0,48902898	protein_codir	hypothetical protein
TcG_02125	346,5225993	0,064599189	0,10186801	0,63414595	0,52598558	0,72890472	protein_codir	putative mitochondrial DNA topoisomerase II
TcG_02126	114,5056144	-0,20429517	0,16384051	-1,24691489	0,21242871	0,43561044	protein_codir	putative aspartate aminotransferase, mitochondrial
TcG_02127	662,6605446	-0,327874785	0,08185787	-4,00541556	6,1909E-05	0,00074022	protein_codir	putative kinesin
TcG_02128	371,0414507	-0,421638741	0,09357031	-4,50611689	6,6025E-06	0,00010669	protein_codir	putative OSM3-like kinesin
TcG_02129	148,7793892	0,223065055	0,14906465	1,49643159	0,13454122	0,32611143	protein_codir	hypothetical protein

TcG_02130	498,8049941	0,148706931	0,08581585	1,73286082	0,08312041	0,2369085	protein_codir hypothetical protein
TcG_02131	15,27476713	-0,659949043	0,44117001	-1,4959064	0,13467805	1	protein_codir paraflagellar rod protein 3
TcG_02132	3305,326781	-0,573856848	0,04393683	-13,0609527	5,5035E-39	5,3136E-36	protein_codir paraflagellar rod protein 2C
TcG_02133	361,0348561	-0,383139338	0,09710883	-3,94546338	7,9646E-05	0,00091636	protein_codir hypothetical protein
TcG_02134	326,2530722	-0,392635642	0,11461266	-3,42576146	0,00061308	0,00512491	protein_codir hypothetical protein
TcG_02135	114,8518781	0,072988224	0,16365633	0,44598473	0,65560828	0,81772823	protein_codir trafficking protein particle complex subunit 3
TcG_02136	348,9088833	0,068676086	0,09864545	0,69619112	0,48630915	0,69827461	protein_codir essential for mitotic growth 1
TcG_02137	704,3923334	-0,078622247	0,07278542	-1,08019221	0,2800566	0,51321462	protein_codir hypothetical protein
TcG_02138	166,8808044	-0,259831675	0,14456072	-1,79738783	0,07227407	0,21618229	protein_codir putative ADP-ribosylation factor
TcG_02139	252,7935302	-0,189932196	0,1117898	-1,69901191	0,08931694	0,24893579	protein_codir hypothetical protein
TcG_02140	556,0515656	-0,190325409	0,08269068	-2,30165491	0,02135464	0,08855965	protein_codir fatty acid amide hydrolase 2
TcG_02141	1169,56193	0,027125007	0,07040956	0,38524608	0,70005511	0,84436727	protein_codir putative DNA-directed rna polymerase I largest subunit
TcG_02142	974,6286027	0,156050013	0,06628074	2,35437961	0,01855365	0,07916746	protein_codir hypothetical protein
TcG_02143	355,5630147	-0,200311415	0,09810037	-2,0419027	0,04116118	0,14420728	protein_codir hypothetical protein
TcG_02144	407,7838993	-0,426328217	0,09094204	-4,68791138	2,7601E-06	4,981E-05	protein_codir cytochrome c
TcG_02145	80,10026288	-0,239675271	0,19609828	-1,22222013	0,22162439	0,44710782	protein_codir glycosyl transferase family 2
TcG_02146	69,39193665	-0,10403093	0,21183293	-0,49109896	0,62335646	0,79702106	protein_codir glycosyl transferase family 2
TcG_02147	597,6345155	-0,521868304	0,07764652	-6,72107753	1,8039E-11	1,0609E-09	protein_codir hypothetical protein
TcG_02148	353,6319729	-0,215476892	0,10113611	-2,1305634	0,03312513	0,12310099	protein_codir putative diacylglycerol kinase
TcG_02149	140,1595437	-0,180308376	0,15064264	-1,1969279	0,23133466	0,45855318	protein_codir hypothetical protein
TcG_02150	978,5413119	-0,353795021	0,06861245	-5,15642597	2,5171E-07	5,9034E-06	protein_codir hypothetical protein
TcG_02151	253,9242166	-0,132311265	0,11355757	-1,16514702	0,24395949	0,47210867	protein_codir glycosyltransferase
TcG_02152	470,2804125	-0,309869331	0,09140581	-3,39003959	0,00069883	0,00574634	protein_codir hypothetical protein
TcG_02153	75,34368343	-0,292569066	0,21513056	-1,35996054	0,17384241	0,38244467	protein_codir putative small nuclear ribonucleoprotein
TcG_02154	1373,3351	-0,212819964	0,05720114	-3,72055447	0,00019879	0,00198204	protein_codir hypothetical protein
TcG_02155	774,7749601	-0,23466283	0,08042897	-2,91764055	0,00352691	0,02174707	protein_codir cullin
TcG_02156	1095,192207	-0,133769999	0,05943492	-2,25069701	0,02440473	0,09804204	protein_codir hypothetical protein
TcG_02157	180,0382579	-0,395087202	0,13204888	-2,99197681	0,00277177	0,01784437	protein_codir cyclophilin
TcG_02158	796,9824646	-0,05598088	0,06727631	-0,83210393	0,40535029	0,63430422	protein_codir hypothetical protein
TcG_02159	621,6120081	-0,293043901	0,07786747	-3,76336762	0,00016764	0,00172363	protein_codir putative coatomer delta subunit
TcG_02160	218,5956528	0,081233582	0,11968871	0,67870715	0,49732343	0,70725289	protein_codir hypothetical protein
TcG_02161	392,6547278	-0,030430267	0,09066965	-0,33561691	0,73715977	0,8655856	protein_codir putative NEDD4-like E3 ubiquitin-protein ligase WWP1
TcG_02162	591,8103342	-0,483441093	0,07642831	-6,32541925	2,5255E-10	1,1341E-08	protein_codir hypothetical protein
TcG_02163	531,6987706	0,012202964	0,08783707	0,13892727	0,88950762	0,94653573	protein_codir putative coenzyme Q-binding protein COQ10 B, mitochondrial-like
TcG_02164	93,73259157	0,267329606	0,18028117	1,48284819	0,13811475	0,33164715	protein_codir hypothetical protein
TcG_02165	191,4193672	-0,157417895	0,12854039	-1,22465701	0,22070448	0,44579535	protein_codir tyrosyl or methionyl-tRNA synthetase
TcG_02166	538,9387674	-0,045731588	0,08961529	-0,5103101	0,60983423	0,78882878	protein_codir hypothetical protein
TcG_02167	568,3527892	-0,088078064	0,08048633	-1,09432327	0,27381322	0,50693512	protein_codir hypothetical protein
TcG_02168	214,5810354	-0,303828863	0,12226742	-2,48495351	0,01295684	0,0606292	protein_codir hypothetical protein
TcG_02169	700,6322623	-0,052154609	0,07507026	-0,69474396	0,48721577	0,69871047	protein_codir putative surface antigen TASV, putative,mucin-like glycoprotein
TcG_02170	263,979158	0,033159233	0,11223515	0,29544426	0,76765452	0,88350974	
TcG_02171	112,5825861	-0,423334859	0,16551049	-2,55775243	0,01053511	0,05144417	protein_codir hypothetical protein
TcG_02172	2609,36697	-0,066186976	0,04809211	-1,37625443	0,16874288	0,37510648	protein_codir putative amino acid transporter
TcG_02173	95,53111063	-0,249721127	0,17866972	-1,39766898	0,16221249	0,36599687	protein_codir hypothetical protein
TcG_02174	532,3056291	-0,318582956	0,08207568	-3,88157565	0,00010378	0,00115064	protein_codir hypothetical protein
TcG_02175	374,995263	-0,441853984	0,0946932	-4,6661637	3,0688E-06	5,4868E-05	protein_codir coiled-coil domain protein
TcG_02176	542,4923766	-0,315359123	0,08150881	-3,86901901	0,00010927	0,00120347	protein_codir Importin-beta, N-terminal domain-containing protein
TcG_02177	97,60441261	-0,074089611	0,18408835	-0,40246769	0,68733986	0,83764801	protein_codir hypothetical protein
TcG_02178	660,442704	-0,744788421	0,07729576	-9,63556573	5,6579E-22	1,2854E-19	protein_codir putative flagellar protofilament ribbon protein
TcG_02179	68,0528417	-0,357869589	0,22337304	-1,60211631	0,10912989	0,28534844	protein_codir hypothetical protein
TcG_02180	155,8597015	-0,560056485	0,27921309	-2,00583895	0,04487343	0,15326528	protein_codir putative small GTP-binding protein Rab7
TcG_02181	447,1662324	-0,573686034	0,0859969	-6,67100841	2,5405E-11	1,4571E-09	protein_codir hypothetical protein

TcG_02182	121,2857789	-0,349261922	0,16311567	-2,14119173	0,03225858	0,12075861	protein_codir hypothetical protein
TcG_02183	426,4222978	-0,569948103	0,08902184	-6,40233997	1,5301E-10	7,2656E-09	protein_codir flagella associated protein
TcG_02184	292,7823994	-0,391235901	0,10622615	-3,68304702	0,00023046	0,00224759	protein_codir zinc finger family protein
TcG_02185	565,5262983	-0,234222927	0,07748707	-3,02273561	0,00250501	0,01644365	protein_codir hypothetical protein
TcG_02186	111,1242357	-0,12637194	0,16828131	-0,75095647	0,45267885	0,67343826	protein_codir hypothetical protein
TcG_02187	1174,166593	-0,670017982	0,06121651	-10,9450538	7,0176E-28	3,0113E-25	protein_codir putative ataxin-2 isoform X1
TcG_02188	124,2925853	0,104722753	0,16131426	0,64918471	0,51621899	0,72205516	protein_codir hypothetical protein
TcG_02189	413,3399523	-0,647788731	0,09326649	-6,94556803	3,7694E-12	2,6151E-10	protein_codir hypothetical protein
TcG_02190	287,3541274	-0,291747134	0,10896108	-2,67753538	0,0074166	0,03902305	protein_codir putative nucleoside diphosphate kinase
TcG_02191	995,4091758	-0,527200624	0,06351483	-8,30043311	1,0373E-16	1,2923E-14	protein_codir putative eukaryotic translation initiation factor 4 gamma
TcG_02192	581,9077035	-0,24555387	0,08348829	-2,94117744	0,00326967	0,0204328	protein_codir hypothetical protein
TcG_02193	372,2377801	-0,345493211	0,09496769	-3,63800813	0,00027475	0,00259862	protein_codir hypothetical protein
TcG_02194	163,8298786	-0,215136588	0,14197523	-1,51531077	0,12969372	0,31936907	protein_codir chaperone protein DNAJ
TcG_02195	487,9023776	-0,325061341	0,08715748	-3,72958648	0,00019179	0,00193061	protein_codir hypothetical protein
TcG_02196	623,8568488	-0,485409202	0,07490372	-6,4804422	9,1454E-11	4,5282E-09	protein_codir putative RNA-binding protein
TcG_02197	281,1693953	-0,581512319	0,11179592	-5,20155233	1,9763E-07	4,8512E-06	protein_codir putative carrier protein
TcG_02198	417,353824	0,181949238	0,09749064	1,86632515	0,06199589	0,194341	protein_codir nucleoside phosphorylase
TcG_02199	134,5715614	-0,324731326	0,15488975	-2,09653211	0,03603501	0,13145519	protein_codir hypothetical protein
TcG_02200	207,5444537	-0,227423122	0,13865741	-1,64018002	0,10096774	0,27053938	protein_codir putative ARP2/3 complex subunit
TcG_02201	121,393054	-0,288605883	0,17701794	-1,63037647	0,10302196	0,27451987	protein_codir hypothetical protein
TcG_02202	367,8554427	-0,552636511	0,09499284	-5,81766491	5,9675E-09	1,9868E-07	protein_codir hypothetical protein
TcG_02203	398,5553476	-0,508508358	0,09932689	-5,11954364	3,0628E-07	6,999E-06	protein_codir putative translation initiation factor eif-2b beta subunit, putative,eIF-2B GDP-GTP exchange factor
TcG_02204	399,7377541	-0,283612933	0,09967074	-2,84549848	0,0044342	0,02627213	
TcG_02205	752,9542167	-0,527971701	0,0711076	-7,42496834	1,1281E-13	9,977E-12	protein_codir hypothetical protein
TcG_02206	953,5732041	-0,623419111	0,06901219	-9,03346344	1,6632E-19	2,7529E-17	protein_codir hypothetical protein
TcG_02207	200,6401226	-0,464537711	0,12391189	-3,74893572	0,00017759	0,0018096	protein_codir hypothetical protein
TcG_02208	846,1663474	-0,502426685	0,06548009	-7,6729684	1,6806E-14	1,6363E-12	protein_codir hypothetical protein
TcG_02209	359,9414596	-0,60877231	0,10383636	-5,86280469	4,5511E-09	1,56E-07	protein_codir putative small GTP-binding protein Rab11, putative,Rab11 GTPase
TcG_02210	868,7761685	-0,679912892	0,06746063	-10,0786616	6,8655E-24	1,8499E-21	protein_codir hypothetical protein
TcG_02211	706,6864953	-0,744488068	0,07547319	-9,86427149	5,9465E-23	1,4659E-20	protein_codir hypothetical protein
TcG_02212	70,07475985	-0,358527561	0,20535968	-1,74585175	0,08083672	0,23274706	
TcG_02213	591,1130042	-0,245970996	0,07886811	-3,1187635	0,00181612	0,0127293	protein_codir hypothetical protein
TcG_02214	359,8995466	-0,548118033	0,10553173	-5,1938693	2,0597E-07	5,0345E-06	protein_codir hypothetical protein
TcG_02215	377,5009048	-0,611353887	0,09594632	-6,37183282	1,8678E-10	8,6562E-09	protein_codir nuclear transport factor 2
TcG_02216	361,0986432	-0,383801849	0,09591215	-4,00159765	6,2916E-05	0,00074995	protein_codir adenosine deaminase
TcG_02217	623,7413575	-0,415644129	0,08201226	-5,06807305	4,0186E-07	8,9711E-06	protein_codir putative protein kinase
TcG_02218	345,1689948	-0,484977987	0,10630206	-4,56226319	5,0605E-06	8,4605E-05	protein_codir hypothetical protein
TcG_02219	1318,701317	-0,559225597	0,05718475	-9,77927803	1,3819E-22	3,2022E-20	protein_codir hypothetical protein
TcG_02220	1732,227346	-0,168370575	0,05997206	-2,80748371	0,00499302	0,02874518	protein_codir hypothetical protein
TcG_02221	1465,045414	-0,532499925	0,05578194	-9,54609842	1,3467E-21	2,944E-19	protein_codir putative glutamic acid rich protein
TcG_02222	416,650006	-0,541056593	0,08994644	-6,01531979	1,7953E-09	6,7316E-08	protein_codir hypothetical protein
TcG_02223	418,8700046	-0,559750207	0,09039027	-6,1925938	5,9182E-10	2,4844E-08	protein_codir WD domain-containing protein
TcG_02224	61,86857636	-0,084197947	0,22566197	-0,37311536	0,70906259	0,84999474	
TcG_02225	331,7030416	-0,227091857	0,09999797	-2,27096469	0,02314912	0,09420641	protein_codir hypothetical protein
TcG_02226	299,6541951	-0,44813755	0,10312136	-4,34572961	1,3881E-05	0,00020179	protein_codir hypothetical protein
TcG_02227	1167,588803	-0,586229134	0,05989831	-9,78707314	1,2795E-22	3,0883E-20	protein_codir hypothetical protein
TcG_02228	152,0426521	-0,404186746	0,14739296	-2,74223918	0,00610219	0,03347536	protein_codir putative deaminase
TcG_02229	370,3699447	-0,262598573	0,0986339	-2,66235607	0,00775957	0,04049659	protein_codir hypothetical protein
TcG_02230	34,99578563	-0,52366266	0,28977182	-1,80715524	0,07073808	0,21343005	protein_codir hypothetical protein
TcG_02231	388,8812859	-0,474590671	0,09196486	-5,16056532	2,4621E-07	5,8215E-06	protein_codir hypothetical protein
TcG_02232	285,1479213	-0,419322198	0,10848258	-3,8653414	0,00011093	0,00121828	protein_codir putative plectin-like protein
TcG_02233	522,0726404	-0,257135558	0,08193866	-3,13814695	0,0017002	0,01207754	protein_codir hypothetical protein

TcG_02234	299,595877	-0,30186219	0,10350424	-2,91642349	0,0035407	0,02177509	protein_codir hypothetical protein
TcG_02235	212,0626118	-0,799740812	0,13034022	-6,13579452	8,4735E-10	3,4447E-08	protein_codir hypothetical protein
TcG_02236	262,258221	-0,482326036	0,11681461	-4,12898713	3,6436E-05	0,00046698	protein_codir hypothetical protein
TcG_02237	340,7905185	-0,542244119	0,10195076	-5,31868658	1,0452E-07	2,703E-06	protein_codir putative citrate transporter
TcG_02238	352,5083495	-0,711060463	0,09983863	-7,12209721	1,063E-12	7,9971E-11	protein_codir insect stage-specific antigen
TcG_02239	30,91824916	-0,8102685	0,31974687	-2,53409358	0,01127386	0,05433401	
TcG_02240	721,586816	-0,808773167	0,07755373	-10,4285523	1,8367E-25	5,7512E-23	protein_codir hypothetical protein
TcG_02241	481,4391609	-0,465832254	0,0854577	-5,45102737	5,008E-08	1,3782E-06	protein_codir endonuclease G
TcG_02242	749,9321405	0,054522885	0,06989298	0,780091	0,43533731	0,65939964	protein_codir putative conserved RIO1-domain protein
TcG_02243	263,9994847	0,02843906	0,11025881	0,25793003	0,79646091	0,89860707	protein_codir putative ESAG8-associated protein
TcG_02244	187,701615	-0,3769741	0,12833748	-2,93736563	0,00331014	0,02063003	protein_codir hypothetical protein
TcG_02245	155,2425727	0,301412358	0,14342035	2,10160105	0,03558824	0,1301532	protein_codir hypothetical protein
TcG_02246	434,7495789	-0,318512066	0,08936426	-3,56419963	0,00036497	0,00330611	protein_codir putative kinesin
TcG_02247	649,073845	-0,258918886	0,0744903	-3,47587383	0,00050919	0,00440261	protein_codir putative p1/s1 nuclease
TcG_02248	426,953786	-0,118219442	0,09036416	-1,30825586	0,19078654	0,40625857	protein_codir SAM domain-containing protein
TcG_02249	240,5338774	0,090868926	0,11947127	0,76059227	0,44690064	0,66836076	protein_codir pseudouridylate synthase
TcG_02250	268,8114283	-0,027108481	0,10927502	-0,24807574	0,8040758	0,90318531	protein_codir hypothetical protein
TcG_02251	328,6222575	-0,068191496	0,10095043	-0,67549486	0,49936141	0,70841207	protein_codir AAA+-type ATPase
TcG_02252	341,3728939	-0,125993221	0,09975683	-1,26300339	0,20658796	0,42864546	protein_codir putative conserved flavoprotein
TcG_02253	75,27816536	0,104615297	0,20460606	0,51130107	0,60914026	0,788218	protein_codir hypothetical protein
TcG_02254	765,5122817	-0,076975741	0,06827474	-1,12744104	0,25955606	0,4921081	protein_codir putative epsilon-adaptin, putative,AP-1/4 adapter complex gamma/epsilon subunit
TcG_02255	270,5717049	0,040141939	0,10848583	0,37002011	0,71136751	0,85161231	protein_codir hypothetical protein
TcG_02256	432,1198052	0,041601172	0,08840375	0,47058152	0,63793961	0,80751094	protein_codir putative pseudouridine synthase A-like protein
TcG_02257	501,8445717	0,059078165	0,09105528	0,64881645	0,51645703	0,72205516	protein_codir putative protein kinase
TcG_02258	266,7694304	0,264735482	0,11295033	2,34382217	0,01908727	0,08097587	protein_codir hypothetical protein
TcG_02259	279,5869599	-0,104237495	0,11067556	-0,94182942	0,34627998	0,58052378	protein_codir hypothetical protein
TcG_02260	217,2841513	0,476122665	0,12134471	3,92371994	8,7192E-05	0,00099235	protein_codir sec4-like phosphatidylinositol transfer-like protein
TcG_02261	159,9773471	-0,146168491	0,14123903	-1,03490157	0,30071488	0,53419983	protein_codir hypothetical protein
TcG_02262	331,0396369	-0,15767362	0,10381512	-1,5187924	0,12881476	0,31792545	protein_codir adenosinetriphosphatase
TcG_02263	270,5696243	-0,041939011	0,11065572	-0,37900446	0,70468455	0,84755533	protein_codir hypothetical protein
TcG_02264	276,0626956	0,013500784	0,11021799	0,12249165	0,90250966	0,9529091	protein_codir putative small GTP-binding protein Rab28
TcG_02265	266,8028065	0,021827412	0,11780499	0,18528428	0,85300607	0,92887573	protein_codir putative aldehyde dehydrogenase family
TcG_02266	155,4523431	0,00718359	0,14535813	0,04941994	0,96058464	0,98194227	protein_codir hypothetical protein
TcG_02267	212,4182289	-0,288628972	0,13942237	-2,07017695	0,03843578	0,13725406	protein_codir hypothetical protein
TcG_02268	160,6427608	-0,076932013	0,13782667	-0,55817944	0,57672186	0,7668885	protein_codir hypothetical protein
TcG_02269	258,3242194	0,116148555	0,11889585	0,97689327	0,328622	0,56308329	protein_codir hypothetical protein
TcG_02270	1190,318932	-0,2592362	0,06385555	-4,05972825	4,913E-05	0,00060684	protein_codir XMAP215 family protein
TcG_02271	603,3378408	0,02163243	0,07877334	0,27461612	0,78361118	0,89273408	protein_codir hypothetical protein
TcG_02272	354,7336127	-0,00292459	0,1005885	-0,0290748	0,97680494	0,99017719	protein_codir putative protein kinase, putative,cdc2
TcG_02273	234,2455019	-0,2177113	0,1173667	-1,85496646	0,06360102	0,19808639	protein_codir putative DNA-J protein
TcG_02274	588,8629485	0,111374469	0,08060547	1,3817234	0,16705665	0,37307211	protein_codir putative queuine tRNA-ribosyltransferase
TcG_02275	111,1743193	-0,369138503	0,16734779	-2,20581646	0,02739685	0,1069114	protein_codir hypothetical protein
TcG_02276	750,4528191	-0,242170419	0,2455668	-0,9861692	0,32405009	0,55828169	protein_codir hypothetical protein
TcG_02277	1386,932061	-0,288867375	0,06390042	-4,52058655	6,1669E-06	0,00010035	protein_codir hydin-like protein
TcG_02278	425,6109898	-0,231009919	0,09077668	-2,54481562	0,01093355	0,05297258	protein_codir putative splicing factor 3a
TcG_02279	312,9141266	0,088954858	0,10895546	0,81643321	0,41425241	0,6411339	protein_codir hypothetical protein
TcG_02280	331,7932563	-0,173528371	0,09968227	-1,74081472	0,08171606	0,23453224	protein_codir hypothetical protein
TcG_02281	294,6833328	0,040075464	0,11055799	0,36248364	0,71699065	0,85489901	protein_codir hypothetical protein
TcG_02282	172,217035	-0,003362407	0,1346511	-0,02497125	0,98007789	0,9919789	protein_codir hypothetical protein
TcG_02283	252,3879254	0,37660086	0,11904219	3,1635916	0,00155835	0,01122828	protein_codir putative kinase
TcG_02284	674,4709516	-0,093687092	0,07380503	-1,2693863	0,20430332	0,42546605	protein_codir hypothetical protein
TcG_02285	326,1178339	0,056996422	0,10134471	0,56240157	0,57384245	0,76499121	protein_codir putative RNA pseudouridylate synthase

TcG_02286	289,8711464	-0,080623263	0,10589695	-0,76133694	0,44645584	0,66804047	protein_codir	hypothetical protein
TcG_02287	145,1325588	-0,165971665	0,1533318	-1,08243472	0,27905941	0,51208848	protein_codir	hypothetical protein
TcG_02288	374,6022835	-0,097248891	0,09992071	-0,9732606	0,3304238	0,56507811	protein_codir	Zinc finger Transcription Factor family member (ztf-7)
TcG_02289	218,9210539	-0,09925357	0,12020202	-0,82572302	0,40896123	0,63660535	protein_codir	hypothetical protein
TcG_02290	98,491767	-0,103306498	0,17693459	-0,58386828	0,55930893	0,75385683	protein_codir	hypothetical protein
TcG_02291	670,5409427	-0,083144417	0,07994822	-1,03997832	0,29834997	0,53204291	protein_codir	cyclosome subunit-like protein
TcG_02292	162,0225351	-0,191621494	0,13881989	-1,38036051	0,16747567	0,37350782	protein_codir	intraflagellar transport (IFT) protein
TcG_02293	267,4233426	-0,007027172	0,1108036	-0,06342007	0,94943201	0,97650727	protein_codir	mannosyltransferase
TcG_02294	148,1004926	-0,009172541	0,15001503	-0,06114415	0,95124441	0,97713458	protein_codir	hypothetical protein
TcG_02295	342,322876	-0,07245896	0,10131295	-0,7151994	0,47448584	0,69021001	protein_codir	calpain-like cysteine peptidase
TcG_02296	66,97310266	0,022923928	0,21188116	0,10819239	0,91384309	0,95787309	protein_codir	putative ferric reductase transmembrane protein
TcG_02297	281,2541972	-0,205535647	0,10975863	-1,8726149	0,06112158	0,19196385	protein_codir	hypothetical protein
TcG_02298	176,1915186	-0,34638502	0,14866917	-2,32990491	0,01981118	0,08336787	protein_codir	coiled-coil domain-containing protein 25
TcG_02299	281,2339481	-0,145613867	0,10713436	-1,3591705	0,17409257	0,38259417	protein_codir	single-stranded nucleic acid binding protein R3H domain-containing protein
TcG_02300	217,5853674	-0,182209317	0,11945647	-1,5253198	0,12717935	0,31552462	protein_codir	hypothetical protein
TcG_02301	23,76260455	0,220709949	0,36301968	0,60798343	0,54319846	0,74084725	protein_codir	hypothetical protein
TcG_02302	67,59283794	0,110573477	0,22720397	0,48667053	0,62649184	0,79939806	protein_codir	structural maintenance of chromosome protein 4
TcG_02303	70,22272025	0,16453358	0,21106444	0,77954192	0,43566055	0,65939964	protein_codir	target of rapamycin (TOR) kinase 1
TcG_02304	61,67970575	0,021706924	0,22250004	0,09755919	0,92228233	0,9622299	protein_codir	hypothetical protein
TcG_02305	163,9024307	-0,098262717	0,14147286	-0,69456941	0,48732519	0,6987809	protein_codir	putative phosphomannomutase-like protein
TcG_02306	408,5833932	-0,231650816	0,08986137	-2,57786887	0,00994117	0,04915852	protein_codir	hypothetical protein
TcG_02307	213,2306713	-0,054200218	0,12614334	-0,42967166	0,6674345	0,82581306	protein_codir	hypothetical protein
TcG_02308	240,4714955	-0,180179879	0,12216901	-1,47484112	0,14025526	0,33471264	protein_codir	hypothetical protein
TcG_02309	462,9344045	-0,289928848	0,08619121	-3,36378688	0,00076881	0,00625082	protein_codir	putative protein kinase
TcG_02310	131,2604927	-0,026857875	0,15956339	-0,16832104	0,86633072	0,9355306	protein_codir	putative nucleoside diphosphate-linked moiety X motif 22-like
TcG_02311	357,6919215	-0,048761888	0,09961674	-0,4894949	0,62449136	0,79763609	protein_codir	hypothetical protein
TcG_02312	235,3979435	0,003116322	0,11558977	0,02696019	0,97849149	0,99085144	protein_codir	putative WD repeat-containing protein 27-like
TcG_02313	227,5636628	0,037213466	0,11897606	0,31278113	0,75444695	0,87620513	protein_codir	putative WD repeat-containing protein 27-like
TcG_02314	294,3830689	-0,04327208	0,10634715	-0,40689457	0,68408542	0,83596566	protein_codir	hypothetical protein
TcG_02315	150,3803599	-0,072437884	0,15060829	-0,48096877	0,6305387	0,80202553	protein_codir	hypothetical protein
TcG_02316	199,9763823	-0,074358783	0,12869639	-0,57778452	0,56340961	0,75668455	protein_codir	hypothetical protein
TcG_02317	181,5304919	0,049487486	0,13692503	0,36142031	0,71778527	0,85508792	protein_codir	putative RNA-binding protein
TcG_02318	51,18493556	0,53402902	0,26203405	2,03801387	0,04154855	0,14512555	protein_codir	hypothetical protein
TcG_02319	1953,011738	-0,178881279	0,04870242	-3,67294448	0,00023977	0,0023208	protein_codir	putative kinesin
TcG_02320	268,7140168	0,321460983	0,1116941	2,87804793	0,00400144	0,02407099	protein_codir	protein ARV1
TcG_02321	193,6553745	0,447368551	0,131019	3,41453183	0,00063892	0,00531407	protein_codir	retrotransposon hot spot (RHS) protein
TcG_02322	270,923598	0,446485598	0,11446101	3,90076588	9,5889E-05	0,00107679	protein_codir	retrotransposon hot spot (RHS) protein
TcG_02323	82,75950782	0,216449722	0,19178572	1,12860189	0,25906581	0,49163663	protein_codir	hypothetical protein
TcG_02324	24,34531674	0,155669055	0,36318135	0,42862624	0,66819525	0,8261349	protein_codir	trans-sialidase
TcG_02325	129,3656188	-0,080151555	0,16863322	-0,4753011	0,63457238	0,8046575	protein_codir	trans-sialidase-like protein
TcG_02326	20,15221542	-0,235098255	0,41454662	-0,56712139	0,57063171	0,76220187	protein_codir	trans-sialidase
TcG_02327	44,51135021	-0,046502028	0,26049439	-0,17851451	0,85831893	0,93174207	protein_codir	hypothetical protein
TcG_02328	63,20004929	0,245166231	0,23141171	1,05943742	0,28940061	0,52292506	protein_codir	exo-alpha-sialidase
TcG_02329	268,9982819	-0,034113287	0,10837338	-0,31477552	0,7529321	0,87541107	protein_codir	putative SpoU type methylase
TcG_02330	230,2995901	0,068334904	0,11933022	0,57265383	0,56687911	0,75876992	protein_codir	hypothetical protein
TcG_02331	328,3188758	-0,073551115	0,0989209	-0,74353466	0,45715805	0,6767839	protein_codir	serine peptidase
TcG_02332	342,7756001	-0,000686572	0,10303495	-0,00666348	0,99468335	0,99778366	protein_codir	CNH domain-containing protein
TcG_02333	277,9059647	0,067413282	0,10983955	0,61374322	0,53938502	0,73860703	protein_codir	hypothetical protein
TcG_02334	79,88077675	0,23525687	0,21051177	1,11754737	0,26376036	0,49602159	protein_codir	hypothetical protein
TcG_02335	81,49297782	0,268949034	0,19969768	1,34678097	0,17805079	0,38871234	protein_codir	putative ribonuclease H1/H2 small subunit
TcG_02336	258,0871109	0,024658777	0,11465599	0,21506751	0,82971469	0,91719057	protein_codir	hypothetical protein
TcG_02337	189,0862341	0,104776781	0,13174351	0,79530887	0,42643386	0,65178385	protein_codir	hypothetical protein

TcG_02338	213,7347184	-0,01231337	0,12086915	-0,10187355	0,91885704	0,96099275	protein_codir hypothetical protein
TcG_02339	466,8217594	-0,085193862	0,08777796	-0,97056099	0,33176694	0,56610483	protein_codir putative arginine N-methyltransferase
TcG_02340	195,2099965	0,165958543	0,12684755	1,30833066	0,19076118	0,40625857	protein_codir hypothetical protein
TcG_02341	335,3947827	0,164650442	0,10300828	1,59841942	0,10994967	0,28646723	protein_codir hypothetical protein
TcG_02342	51,39582729	-0,324759491	0,2453869	-1,32345898	0,18568279	0,39957667	
TcG_02343	882,282761	-0,456049225	0,06655671	-6,85204	7,2804E-12	4,7927E-10	protein_codir hypothetical protein
TcG_02344	840,8040683	0,089416078	0,0682351	1,31041172	0,19005659	0,40522555	protein_codir puromycin-sensitive aminopeptidase-like protein
TcG_02345	322,3245157	0,035797638	0,09991639	0,35827594	0,72013682	0,85644685	protein_codir metallo-peptidase, Clan MA(E), Family M1
TcG_02346	203,3162766	-0,168015728	0,12518171	-1,34217468	0,17953936	0,39071057	protein_codir SNARE associated golgi family protein
TcG_02347	337,6848832	-0,021859071	0,10095044	-0,2165327	0,82857254	0,91636516	protein_codir hypothetical protein
TcG_02348	578,9538332	0,129959306	0,07711166	1,68533923	0,09192314	0,25345586	protein_codir hypothetical protein
TcG_02349	145,5682305	-0,169545595	0,14709416	-1,15263312	0,249061	0,47849319	protein_codir hypothetical protein
TcG_02350	739,3320081	-0,063113031	0,07956677	-0,79320838	0,42765643	0,65289595	protein_codir calcium/potassium channel (CAKC)
TcG_02351	205,7114039	0,182806899	0,12738356	1,43509021	0,15126139	0,3515895	protein_codir ABC transporter
TcG_02352	200,7744934	-0,041833379	0,12453189	-0,33592504	0,7369274	0,86548817	protein_codir hypothetical protein
TcG_02353	148,4853994	0,168979579	0,14401031	1,17338526	0,24064134	0,46929315	protein_codir hypothetical protein
TcG_02354	349,9867107	-0,16901545	0,10553451	-1,60151831	0,10926217	0,28562985	protein_codir hypothetical protein
TcG_02355	318,8075023	0,039582203	0,10237529	0,38663826	0,69902403	0,84414248	protein_codir hypothetical protein
TcG_02356	213,9509676	0,046849661	0,12141832	0,38585331	0,69960531	0,84432352	protein_codir hypothetical protein
TcG_02357	205,124559	0,326780425	0,12771693	2,5586305	0,01050854	0,05135045	protein_codir hypothetical protein
TcG_02358	197,6008373	-0,230812888	0,1249491	-1,84725525	0,06471016	0,20037928	protein_codir hypothetical protein
TcG_02359	1201,318138	-0,235801941	0,06044303	-3,90122664	9,5706E-05	0,00107656	protein_codir hypothetical protein
TcG_02360	393,3230819	-0,291138039	0,09050214	-3,21691868	0,00129575	0,0096544	protein_codir putative chaperone DNAJ protein
TcG_02361	240,6324591	-0,025285688	0,12481441	-0,20258629	0,8394584	0,92197981	protein_codir hypothetical protein
TcG_02362	305,4118467	-0,1203642	0,10568639	-1,13888076	0,25475289	0,48614229	protein_codir hypothetical protein
TcG_02363	439,9161159	-0,17091918	0,08607079	-1,98579778	0,04705578	0,15847633	protein_codir hypothetical protein
TcG_02364	271,4334975	0,076845042	0,1213368	0,63332017	0,52652458	0,72926644	protein_codir hypothetical protein
TcG_02365	98,24698587	0,039217208	0,18174374	0,21578299	0,82915691	0,91666145	protein_codir membrane protein
TcG_02366	323,6965432	-0,09781549	0,10223745	-0,95674812	0,3386944	0,57327673	protein_codir hypothetical protein
TcG_02367	159,8901254	0,168302915	0,14049241	1,19795024	0,23093639	0,4583126	protein_codir hypothetical protein
TcG_02368	246,9069926	-0,025241232	0,11527582	-0,21896381	0,82667824	0,91523915	protein_codir bardet-Biedl syndrome 5 protein
TcG_02369	278,2926084	0,171209312	0,10763528	1,5906431	0,11168992	0,28931357	protein_codir putative kinase
TcG_02370	946,6119542	-0,000396174	0,06285565	-0,00630292	0,99497103	0,99789945	protein_codir putative cytochrome c oxidase subunit IV
TcG_02371	60,32774548	0,163150167	0,22338782	0,73034495	0,46517936	0,6821385	protein_codir hypothetical protein
TcG_02372	49,82915827	0,385518895	0,25519986	1,51065481	0,13087642	0,32139343	protein_codir hypothetical protein
TcG_02373	49,77667059	0,150913735	0,25798997	0,5849597	0,55857482	0,75321786	protein_codir hypothetical protein
TcG_02374	53,35939981	-0,56565956	0,24903328	-2,27142157	0,02312147	0,09417789	
TcG_02375	1502,146311	-0,321478107	0,05778859	-5,56300348	2,6517E-08	7,7779E-07	protein_codir Ser/Thr protein phosphatase
TcG_02376	268,2287926	0,0331557	0,11533486	0,28747337	0,77374989	0,88691888	protein_codir hypothetical protein
TcG_02377	856,0179048	-0,213835078	0,07068886	-3,02501791	0,00248618	0,01637574	protein_codir DNA repair protein RAD50
TcG_02378	15,26584337	0,070580287	0,45027057	0,15675083	0,87544122	1	protein_codir putative alanine aminotransferase
TcG_02379	95,85440229	0,028052538	0,18382872	0,15260149	0,87871255	0,94148439	protein_codir alanine transaminase
TcG_02380	34,37715681	0,634819988	0,30094826	2,10939907	0,03491015	0,12828068	
TcG_02381	51,00684486	0,092998891	0,26094184	0,35639701	0,72154327	0,85711748	protein_codir putative alanine aminotransferase
TcG_02382	384,7935069	0,226754717	0,09334416	2,42923298	0,01513081	0,06816091	protein_codir hypothetical protein
TcG_02383	350,7087934	0,11674831	0,10183391	1,1464581	0,25160566	0,48167599	protein_codir hypothetical protein
TcG_02384	725,2754654	0,013881071	0,07245833	0,19157317	0,84807656	0,92660555	protein_codir arylsulfatase G
TcG_02385	242,1383735	-0,061605937	0,13344968	-0,46164171	0,64433828	0,8110828	protein_codir hypothetical protein
TcG_02386	721,0759469	-0,033334351	0,07167416	-0,46508184	0,64187285	0,80983762	protein_codir hypothetical protein
TcG_02387	249,10163	-0,015868531	0,11599085	-0,13680847	0,89118219	0,94697266	protein_codir hypothetical protein
TcG_02388	1835,565324	0,162535376	0,05357257	3,03392911	0,00241391	0,01598147	protein_codir cytosolic glucose-6-phosphate isomerase
TcG_02389	141,8242844	0,028600756	0,15442697	0,18520571	0,85306769	0,92887573	protein_codir ATP synthase subunit

TcG_02390	65,40923456	0,256583609	0,22845656	1,12311769	0,26138751	0,49358779	protein_codir	hypothetical protein
TcG_02391	102,0971701	0,039552473	0,17139285	0,23077084	0,81749284	0,91010589	protein_codir	peroxiredoxin-like protein
TcG_02392	10,83530102	0,496211946	0,53518097	0,92718533	0,35383032	1	protein_codir	UDP-Gal or UDP-GlcNAc-dependent glycosyltransferase
TcG_02393	0					1		
TcG_02394	208,2801553	0,105519568	0,12822889	0,8229001	0,4105648	0,63729955	protein_codir	hypothetical protein
TcG_02395	777,8923214	0,051157768	0,07377044	0,69347243	0,48801312	0,69945302	protein_codir	putative N-acetylglucosamine-6-phosphate deacetylase-like protein
TcG_02396	510,3210801	0,15137501	0,08179517	1,85065962	0,06421854	0,19929349	protein_codir	pumilio protein 6
TcG_02397	251,2966393	-0,128874106	0,11237572	-1,14681456	0,25145827	0,48155298	protein_codir	putative L-ribulokinase
TcG_02398	221,6044809	-0,149102424	0,12245185	-1,21764126	0,22336033	0,44980935	protein_codir	hypothetical protein
TcG_02399	273,4045529	-0,111074514	0,11338172	-0,97965101	0,32725842	0,56180412	protein_codir	putative stress-inducible protein STI1-like
TcG_02400	355,1034739	-0,08141377	0,09541704	-0,85324145	0,39352542	0,62329262	protein_codir	putative leucine carboxyl methyltransferase
TcG_02401	415,9892808	-0,044414741	0,09289592	-0,47811295	0,63256982	0,80317303	protein_codir	putative trans-sialidase
TcG_02402	271,3701509	-0,013787075	0,1074556	-0,12830485	0,89790773	0,95018119	protein_codir	putative DNA damage repair protein
TcG_02403	381,5981187	-0,152136073	0,09420314	-1,61497883	0,10631528	0,28071304	protein_codir	hypothetical protein
TcG_02404	157,4051002	-0,125432786	0,14286685	-0,87796982	0,3799601	0,6107213	protein_codir	putative mRNA capping methyltransferase
TcG_02405	266,5715192	0,093441602	0,11179528	0,8358278	0,40325178	0,63298674	protein_codir	putative ubiquitin regulatory protein (ISS)
TcG_02406	66,68369849	-0,074021232	0,21378406	-0,34624298	0,72916011	0,86056342	protein_codir	hypothetical protein
TcG_02407	248,1775845	-0,074499329	0,11480715	-0,64890846	0,51639755	0,72205516	protein_codir	putative fructose-6-phosphate2-kinase/fructose-2,6-bisphos phatase,putative
TcG_02408	221,7670265	-0,181550867	0,11824396	-1,53539227	0,1246875	0,31110872	protein_codir	hypothetical protein
TcG_02409	136,5709428	-0,130050235	0,15369378	-0,84616461	0,39746092	0,62704006	protein_codir	ARP2/3 complex subunit
TcG_02410	104,8060399	-0,41877878	0,17193811	-2,43563679	0,0148656	0,06733107	protein_codir	hypothetical protein
TcG_02411	36,52443149	0,532233511	0,29075345	1,83053205	0,06717042	0,20626463	protein_codir	elongation factor 2
TcG_02412	8453,844183	-0,011664699	0,03418721	-0,34120064	0,73295254	0,86278448	protein_codir	elongation factor 2
TcG_02413	0,325107945	-0,458748506	2,93970254	-0,1560527	0,87599148	1	protein_codir	elongation factor 2
TcG_02414	220,0739435	0,067714808	0,1206893	0,5610672	0,57475173	0,76567477	protein_codir	hypothetical protein
TcG_02415	101,4893309	-0,053058133	0,19061413	-0,27835362	0,78074092	0,89087556	protein_codir	mitochondrial inner membrane signal peptidase
TcG_02416	292,8680385	-0,169047347	0,11241932	-1,50372155	0,13265308	0,32361438	protein_codir	putative SET domain protein
TcG_02417	252,6282851	0,079810698	0,11751389	0,67915967	0,4970367	0,70701868	protein_codir	dolicholphosphate-mannose synthase
TcG_02418	457,998603	0,195545136	0,09080975	2,15334949	0,03129122	0,11793757	protein_codir	peptidyl-prolyl cis-trans isomerase
TcG_02419	255,5444542	-0,009633147	0,11277746	-0,0854173	0,93192964	0,96736431	protein_codir	hypothetical protein
TcG_02420	808,9156911	0,151407163	0,06738652	2,24684657	0,02464983	0,09868451	protein_codir	putative eukaryotic translation initiation factor 3 (eIF-3) interacting protein
TcG_02421	398,0336066	-0,112781313	0,09047856	-1,24649772	0,21258173	0,43576997	protein_codir	hypothetical protein
TcG_02422	179,6053425	0,129417843	0,13157839	0,98357981	0,32532216	0,5598073	protein_codir	hypothetical protein
TcG_02423	239,9041851	0,156771093	0,11563253	1,35576985	0,17517241	0,384384	protein_codir	hypothetical protein
TcG_02424	204,0664157	0,087784148	0,12597622	0,69683109	0,48590851	0,69827461	protein_codir	MutT 8-oxo-dGTP pyrophosphohydrolase-like protein
TcG_02425	199,6121479	-0,167216195	0,12878937	-1,29836956	0,19416037	0,41117567	protein_codir	dynein light chain
TcG_02426	276,7176076	0,195632795	0,11063442	1,76828155	0,07701385	0,22481291	protein_codir	putative dolicholphosphate-mannose synthase
TcG_02427	331,6173244	-0,031445768	0,10061111	-0,31254766	0,75462435	0,87623549	protein_codir	putative proteasome beta 2 subunit
TcG_02428	155,9291507	-0,009047082	0,14529372	-0,06226754	0,95034978	0,97694613	protein_codir	hypothetical protein
TcG_02429	199,5109736	0,058287812	0,12490403	0,46666076	0,64074261	0,80929115	protein_codir	hypothetical protein
TcG_02430	284,2222059	-0,064253505	0,10708026	-0,60004997	0,54847293	0,7450589	protein_codir	putative ribonuclease HII
TcG_02431	186,2552304	0,120500103	0,12914036	0,93309407	0,3507714	0,58517458	protein_codir	uncharacterized protein
TcG_02432	272,7291915	0,147149303	0,10872798	1,35337108	0,17593711	0,38562379	protein_codir	hypothetical protein
TcG_02433	407,4224029	0,061802212	0,09160217	0,67468063	0,4998787	0,70879875	protein_codir	hypothetical protein
TcG_02434	244,8164468	0,053613996	0,1177058	0,45549154	0,64875565	0,81328019	protein_codir	putative phosphatidylinositol-4-phosphate 5-kinase
TcG_02435	356,9724231	0,673342172	0,10320275	6,52445931	6,8247E-11	3,4987E-09	protein_codir	putative GPI inositol deacylase precursor
TcG_02436	137,3467496	0,169329424	0,15024151	1,12704824	0,25972209	0,4921081	protein_codir	hypothetical protein
TcG_02437	273,2900804	0,091277283	0,11031962	0,82738936	0,40801641	0,63641332	protein_codir	hypothetical protein
TcG_02438	91,90681802	0,170664625	0,18462298	0,92439536	0,35528051	0,58921843	protein_codir	hypothetical protein
TcG_02439	137,6145542	-0,115927577	0,15408697	-0,75235159	0,45183965	0,67279452	protein_codir	hypothetical protein
TcG_02440	237,9260618	-0,189225999	0,11719609	-1,61461013	0,10639515	0,28080473	protein_codir	hypothetical protein
TcG_02441	169,107721	0,235475771	0,13892467	1,69498882	0,09007754	0,25021299	protein_codir	hypothetical protein

TcG_02442	110,594228	0,236508139	0,1716355	1,37796745	0,16821334	0,37464816	protein_codir	hypothetical protein
TcG_02443	363,794196	-0,139650192	0,09650563	-1,44706776	0,14787795	0,34675448	protein_codir	hypothetical protein
TcG_02444	4,837605351	-0,446168928	0,80462009	-0,55450881	0,57923068	1		
TcG_02445	454,194309	-0,369636267	0,08521046	-4,33792106	1,4384E-05	0,00020825	protein_codir	hypothetical protein
TcG_02446	310,1697895	-0,150262761	0,10884318	-1,38054369	0,16741931	0,37345401	protein_codir	putative vacuolar protein sorting-associated protein
TcG_02447	166,4359928	0,171904131	0,14179988	1,21230094	0,2253972	0,45182011	protein_codir	putative dihydrouridine synthase
TcG_02448	1157,790392	-0,294898641	0,06005525	-4,91045567	9,0865E-07	1,8694E-05	protein_codir	cytochrome-c oxidase
TcG_02449	104,2629686	-0,464039716	0,18788056	-2,46986553	0,01351638	0,06279103	protein_codir	hypothetical protein
TcG_02450	473,9009847	-0,222331773	0,08487879	-2,61940316	0,00880838	0,04472124	protein_codir	putative TPR-repeat-containing chaperone protein DNAJ
TcG_02451	248,1696859	0,152643024	0,11397158	1,33930782	0,18047048	0,39185364	protein_codir	putative mitochondrial carrier protein
TcG_02452	227,1077466	-0,254908914	0,1182984	-2,15479591	0,03117781	0,11758662	protein_codir	hypothetical protein
TcG_02453	172,2700993	-0,26254197	0,13621946	-1,92734555	0,05393658	0,1744972	protein_codir	protein phosphatase 2C-like
TcG_02454	164,5657098	-0,327751324	0,13857119	-2,36521982	0,01801937	0,07755291	protein_codir	serine/threonine protein kinase TbPK6
TcG_02455	119,0060707	0,223343446	0,17933584	1,24539212	0,21298765	0,43652484	protein_codir	small nuclear ribonucleoprotein
TcG_02456	49,3463326	0,065997464	0,25505752	0,25875522	0,79582411	0,89816468	protein_codir	hypothetical protein
TcG_02457	1054,898159	-0,047671238	0,07153588	-0,66639619	0,50515789	0,71227448	protein_codir	putative ubiquitin-like protein
TcG_02458	456,1334738	-0,232297353	0,08707048	-2,66792326	0,00763217	0,0399393	protein_codir	cdc2-related protein kinase 3
TcG_02459	420,1071984	-0,350849311	0,09467655	-3,70576797	0,00021075	0,00207987	protein_codir	hypothetical protein
TcG_02460	407,0123789	-0,210089357	0,08993646	-2,33597526	0,01949253	0,08234943	protein_codir	putative Mitochondrial elongation factor G
TcG_02461	276,9616745	-0,275334376	0,1070769	-2,57137043	0,01012969	0,04985667	protein_codir	putative Mitochondrial elongation factor G
TcG_02462	223,0972067	-0,018695178	0,12153295	-0,15382806	0,87774531	0,94120054	protein_codir	putative cytosolic factor SEC14, putative,phosphatidylinositol/phosphatidylcholine transfer protein
TcG_02463	382,1552248	-0,244838887	0,09501988	-2,57671213	0,0099745	0,04927909	protein_codir	hypothetical protein
TcG_02464	1124,893567	0,172408525	0,06080476	2,83544433	0,0045762	0,0268727	protein_codir	ubiquitin/ribosomal protein S27a
TcG_02465	796,6019093	-0,323560626	0,06865183	-4,71306625	2,4402E-06	4,4452E-05	protein_codir	putative DnaJ chaperone protein
TcG_02466	281,3256782	-0,253748128	0,1110875	-2,28421862	0,02235869	0,09166586	protein_codir	hypothetical protein
TcG_02467	273,2409148	0,053519817	0,1124206	0,4760677	0,63402615	0,80419712	protein_codir	putative ribonuclease H
TcG_02468	388,548557	-0,246448272	0,09237862	-2,66780648	0,00763482	0,0399393	protein_codir	putative GPI transamidase component Tta2
TcG_02469	323,8298057	-0,002113542	0,09995151	-0,02114567	0,98312945	0,99373875	protein_codir	putative ATP-dependent DEAD/H RNA helicase
TcG_02470	143,1464867	0,121490103	0,14958815	0,81216398	0,41669755	0,64354276	protein_codir	putative MP24
TcG_02471	356,7227553	-0,009988545	0,10032545	-0,09956142	0,92069252	0,96150609	protein_codir	RNA editing complex protein MP18
TcG_02472	276,8304518	-0,016757715	0,11765286	-0,14243356	0,88673756	0,94530014	protein_codir	Protein C21orf2
TcG_02473	704,5574714	-0,136839307	0,07793505	-1,75581207	0,0791205	0,22917254	protein_codir	putative protein kinase, putative,mitogen-activated protein kinase
TcG_02474	73,75682737	-0,854327053	0,20525969	-4,16217644	3,1523E-05	0,00041175	protein_codir	hypothetical protein
TcG_02475	994,8730464	-0,040226314	0,06333582	-0,63512739	0,52534533	0,72866385	protein_codir	hypothetical protein
TcG_02476	724,3444628	-0,271537603	0,0718528	-3,77908172	0,00015741	0,00164152	protein_codir	putative TPR-repeat-containing chaperone protein DNAJ
TcG_02477	19,46956173	-0,172063977	0,39095364	-0,44011351	0,6598549	0,82143321	protein_codir	hypothetical protein
TcG_02478	3,609060267	-0,405950519	0,91005349	-0,44607325	0,65554433	1		
TcG_02479	356,7990988	-0,199089641	0,0982026	-2,02733582	0,04262808	0,14773824	protein_codir	hypothetical protein
TcG_02480	325,1731356	-0,022093949	0,10177363	-0,21708914	0,82813888	0,91614791	protein_codir	zinc transporter
TcG_02481	247,4851831	0,118518324	0,11236361	1,05477494	0,29152827	0,52415371	protein_codir	hypothetical protein
TcG_02482	303,9995336	-0,124175587	0,10631808	-1,16796305	0,24282167	0,47116594	protein_codir	DNA repair and transcription factor protein
TcG_02483	413,7017384	-0,082564616	0,09772934	-0,84482934	0,39820613	0,6279592	protein_codir	hypothetical protein
TcG_02484	280,468041	0,026464743	0,11040133	0,23971399	0,81055199	0,90664755	protein_codir	putative ADP-ribosylation factor-like protein
TcG_02485	1179,972772	-0,255316733	0,06367074	-4,00995371	6,0731E-05	0,00072839	protein_codir	putative 40S ribosomal protein S9
TcG_02486	592,4783298	-0,313885258	0,07967852	-3,93939638	8,1687E-05	0,00093428	protein_codir	putative zinc finger protein family member
TcG_02487	70,48408338	0,43606043	0,21570714	2,02153912	0,04322399	0,1493122	protein_codir	hypothetical protein
TcG_02488	65,69589882	-0,048119476	0,22430508	-0,21452692	0,83013618	0,91748143	protein_codir	hypothetical protein
TcG_02489	378,8990887	0,181435565	0,09504705	1,90890272	0,05627464	0,17981191	protein_codir	surface protein-2
TcG_02490	140,1334208	0,031920346	0,15093244	0,21148764	0,83250678	0,9188743		
TcG_02491	669,7756139	-0,102167677	0,07350563	-1,38992987	0,16455017	0,36983091	protein_codir	lysine decarboxylase domain-containing protein
TcG_02492	291,2117561	-0,213262029	0,11136543	-1,91497516	0,05549567	0,17810882	protein_codir	NADH-cytochrome b5 reductase
TcG_02493	2136,91317	-0,169893493	0,05408313	-3,14133975	0,00168177	0,01196865	protein_codir	putative eukaryotic initiation factor 4a

TcG_02494	359,4354499	-0,154902145	0,09842783	-1,57376363	0,1155421	0,29583886	protein_codir kinectin
TcG_02495	558,4496472	0,222903359	0,08543101	2,60916232	0,00907642	0,04576126	protein_codir hypothetical protein
TcG_02496	201,3470983	-0,02645149	0,12440745	-0,21261982	0,83162351	0,9181618	protein_codir hypothetical protein
TcG_02497	48,72992078	-0,718421216	0,25578828	-2,80865577	0,00497488	0,02867786	
TcG_02498	411,5908138	-0,332202693	0,08920588	-3,72399986	0,00019609	0,00195854	protein_codir putative heat shock protein HslVU, ATPase subunit HslU
TcG_02499	239,3195628	-0,132373885	0,12353043	-1,07158929	0,28390454	0,51743245	protein_codir dimethylaniline monooxygenase (N-oxide forming)
TcG_02500	268,7398637	-0,381155134	0,10869763	-3,50656342	0,00045393	0,00398276	protein_codir hypothetical protein
TcG_02501	277,3562271	-0,08269738	0,1084755	-0,76235995	0,4458452	0,66755784	protein_codir putative mitochondrial carrier protein
TcG_02502	616,8680179	-0,147187132	0,08000551	-1,83971254	0,06581045	0,20305723	protein_codir putative ATP-dependent RNA helicase
TcG_02503	126,9514171	-0,325613241	0,15877532	-2,05077994	0,04028838	0,14200827	
TcG_02504	638,4701048	-0,492841922	0,08031374	-6,13645804	8,4382E-10	3,4424E-08	protein_codir hypothetical protein
TcG_02505	10,14887979	0,139101812	0,54221461	0,25654383	0,79753094	1	protein_codir hypothetical protein
TcG_02506	226,0684382	-0,058074645	0,11695851	-0,49654058	0,61951306	0,79399097	protein_codir putative Kelch repeat protein
TcG_02507	260,9702819	-0,208331074	0,10926398	-1,90667656	0,05656248	0,18033378	protein_codir hypothetical protein
TcG_02508	470,2399102	0,014650158	0,08538439	0,17157888	0,86376861	0,93486334	protein_codir putative protein phosphatase 1 catalitic subunit
TcG_02509	445,115559	-0,308651163	0,09109953	-3,38806535	0,00070387	0,00577966	protein_codir hypothetical protein
TcG_02510	2366,369205	0,19181023	0,04844952	3,9589705	7,5274E-05	0,00087474	protein_codir 60S ribosomal protein L13a
TcG_02511	256,7505615	-0,385367689	0,1101524	-3,4984955	0,00046789	0,004079	protein_codir hypothetical protein
TcG_02512	228,532884	0,213228878	0,11849176	1,79952494	0,07193568	0,21552801	protein_codir membrane-associated progesterone binding protein 2
TcG_02513	510,6926849	-0,298628967	0,08171797	-3,65438537	0,0002578	0,0024644	protein_codir hypothetical protein
TcG_02514	1132,405207	-0,270610416	0,0606855	-4,45922677	8,2256E-06	0,00012949	protein_codir putative serine/threonine protein kinase, putative,protein kinase
TcG_02515	785,5908436	-0,241108544	0,06784965	-3,55357094	0,00038004	0,00342405	protein_codir putative protein phosphatase 2C
TcG_02516	387,8831818	0,005656352	0,09745575	0,05804021	0,9537166	0,97828779	protein_codir WD40 repeat protein
TcG_02517	1122,861888	-0,106712641	0,06194955	-1,72257321	0,08496573	0,24027654	protein_codir hypothetical protein
TcG_02518	451,6888613	-0,293792281	0,09281149	-3,16547328	0,00154831	0,01117676	protein_codir hypothetical protein
TcG_02519	593,1547553	0,083615829	0,07726455	1,08220172	0,2791629	0,51214997	protein_codir putative replication Factor A 28 kDa subunit
TcG_02520	1319,622107	-0,247361423	0,05859679	-4,22141613	2,4277E-05	0,00032827	protein_codir ribonucleoprotein p18
TcG_02521	628,0014297	0,046071022	0,07780794	0,59211208	0,55377554	0,74883793	protein_codir hypothetical protein
TcG_02522	475,7381268	-0,017376117	0,0836339	-0,20776404	0,83541321	0,92024125	protein_codir hypothetical protein
TcG_02523	276,9566741	-0,125918005	0,11517951	-1,09323266	0,27429166	0,50733448	protein_codir putative ecotin
TcG_02524	496,7374595	-0,013651915	0,08284831	-0,16478206	0,86911552	0,93679156	protein_codir hypothetical protein
TcG_02525	345,9865645	-0,077126911	0,10105313	-0,76323132	0,44532545	0,66703823	protein_codir hypothetical protein
TcG_02526	546,3793209	0,057155617	0,08058644	0,70924604	0,47817181	0,69283432	protein_codir hypothetical protein
TcG_02527	1543,382148	0,171861815	0,05542404	3,10085332	0,00192964	0,01335531	protein_codir 60S acidic ribosomal protein
TcG_02528	191,2923876	0,616817213	0,13039406	4,73040888	2,2407E-06	4,1338E-05	protein_codir putative cytoplasmic l-asparaginase i-like protein
TcG_02529	580,2813001	0,104502877	0,08206897	1,27335428	0,20289235	0,42300656	protein_codir hypothetical protein
TcG_02530	621,6335111	-0,110827401	0,08091149	-1,36973626	0,17076925	0,37747622	protein_codir hypothetical protein
TcG_02531	551,6104826	-0,036356939	0,08342433	-0,4358074	0,66297647	0,82354941	protein_codir hypothetical protein
TcG_02532	115,812527	-0,002434306	0,17231235	-0,01412729	0,98872843	0,99551643	protein_codir hypothetical protein
TcG_02533	1931,100549	-0,216904027	0,05441257	-3,98628527	6,7116E-05	0,00079673	protein_codir putative lysosomal/endosomal membrane protein p67, putative,lysosomal membrane glycoprotein
TcG_02534	308,103388	-0,021215948	0,1031801	-0,20562053	0,83708734	0,92068482	protein_codir hypothetical protein
TcG_02535	45,12080401	0,36377024	0,27622537	1,31693275	0,18786117	0,40224718	protein_codir hypothetical protein
TcG_02536	0,572794636	0,398549884	2,34919466	0,16965383	0,86528238	1	protein_codir hypothetical protein
TcG_02537	85,86639932	-0,056665177	0,1937623	-0,29244687	0,76994498	0,88436428	protein_codir hypothetical protein
TcG_02538	23,54654434	-0,336187042	0,37547964	-0,89535358	0,37059811	0,60220893	protein_codir calpain-like cysteine peptidase
TcG_02539	719,5095454	-0,039826031	0,08146677	-0,48886228	0,6249392	0,79811388	protein_codir hypothetical protein
TcG_02540	470,8195349	0,028864487	0,08472561	0,34068198	0,73334301	0,86298204	protein_codir hypothetical protein
TcG_02541	361,1727138	0,012275994	0,09546501	0,12859155	0,89768085	0,95018119	protein_codir hypothetical protein
TcG_02542	848,6598704	0,137571207	0,07099245	1,93782878	0,05264411	0,17128186	protein_codir putative katanin-like protein, putative,serine peptidase, Clan SJ, family S16
TcG_02543	257,5308309	-0,130753802	0,11168956	-1,17068959	0,24172357	0,47037442	protein_codir ecotin
TcG_02544	888,5410212	-0,201156552	0,06747936	-2,98100873	0,00287301	0,01840058	protein_codir hypothetical protein
TcG_02545	350,8890692	-0,25669122	0,09923428	-2,58671929	0,00968945	0,04821315	protein_codir hypothetical protein

TcG_02546	341,5733459	-0,287591999	0,09982721	-2,88089777	0,00396544	0,02391651	protein_codir hypothetical protein
TcG_02547	726,1948085	-0,224965732	0,07205347	-3,12220552	0,00179502	0,01262209	protein_codir hypothetical protein
TcG_02548	604,9542835	0,068249732	0,076878	0,88776679	0,37466621	0,60525414	protein_codir putative coiled-coil domain-containing protein 13 isoform X1
TcG_02549	441,2267349	-0,43154232	0,09436021	-4,57335065	4,7999E-06	8,0713E-05	protein_codir peptide hydrolase
TcG_02550	310,867581	-0,726818022	0,10805818	-6,72617308	1,7418E-11	1,0349E-09	protein_codir hypothetical protein
TcG_02551	1211,400203	-0,062564809	0,06107437	-1,02440373	0,30564462	0,53874922	protein_codir hypothetical protein
TcG_02552	696,4720514	-0,050235433	0,07489963	-0,67070333	0,50240954	0,71099511	protein_codir hypothetical protein
TcG_02553	472,7857421	-0,059361255	0,0847591	-0,70035262	0,48370712	0,69704534	protein_codir hypothetical protein
TcG_02554	121,1644641	-0,218243896	0,1586277	-1,37582458	0,16887595	0,37528616	protein_codir hypothetical protein
TcG_02555	573,7158576	-0,094084233	0,07871338	-1,19527628	0,2319791	0,45922596	protein_codir hypothetical protein
TcG_02556	132,2842171	0,327602789	0,15239137	2,1497464	0,03157528	0,11869928	protein_codir hypothetical protein
TcG_02557	46,83423653	-0,123379921	0,25918012	-0,47603929	0,63404639	0,80419712	protein_codir hypothetical protein
TcG_02558	1399,299235	0,072146668	0,06849511	1,05331118	0,2921984	0,52495125	protein_codir myosin heavy chain
TcG_02559	139,6054832	-0,011821742	0,17436993	-0,06779691	0,94594731	0,97469438	protein_codir flagellar attachment zone protein
TcG_02560	462,6120061	-0,122407264	0,08681098	-1,41004362	0,1585268	0,3607014	protein_codir putative tetratricopeptide repeat protein 18-like
TcG_02561	777,7911788	-0,036330911	0,07075895	-0,51344615	0,60763927	0,78802211	protein_codir putative la RNA binding protein
TcG_02562	452,1382092	0,055431298	0,0864416	0,64125721	0,52135559	0,72540161	protein_codir dihydrolipoamide acetyltransferase
TcG_02563	229,5153439	0,075796054	0,12144198	0,62413385	0,53253968	0,73321506	protein_codir hypothetical protein
TcG_02564	320,3741529	0,052619773	0,10095529	0,52121859	0,6022145	0,78475506	protein_codir hypothetical protein
TcG_02565	322,4936345	-0,053047942	0,100172	-0,52956856	0,5964111	0,78066886	protein_codir hypothetical protein
TcG_02566	363,9958061	0,045838227	0,10166566	0,45087227	0,65208161	0,8159254	protein_codir hypothetical protein
TcG_02567	187,4177539	-0,231702372	0,13048408	-1,7757138	0,0757801	0,22250083	protein_codir thioesterase superfamily protein
TcG_02568	165,0683313	0,112283619	0,14182352	0,79171367	0,42852765	0,65390759	protein_codir thioesterase superfamily protein
TcG_02569	486,2745585	-0,168452688	0,08561064	-1,96766071	0,04910709	0,16339883	protein_codir putative chaperone DNAJ protein
TcG_02570	255,0646905	0,127029862	0,11356079	1,11860675	0,26330794	0,49548251	protein_codir hypothetical protein
TcG_02571	138,740483	0,292263033	0,15723879	1,85872091	0,06306671	0,19679259	protein_codir hypothetical protein
TcG_02572	785,9160623	-0,087343254	0,0678566	-1,28717398	0,19803366	0,4166396	protein_codir vacuolar sorting-associated-like protein
TcG_02573	387,57235	0,068213383	0,09344852	0,72995679	0,4654166	0,6821385	protein_codir hypothetical protein
TcG_02574	238,5653839	0,030396149	0,11951889	0,25432087	0,79924768	0,89990311	protein_codir hypothetical protein
TcG_02575	116,6861722	0,014704862	0,16464073	0,08931485	0,92883169	0,96542881	protein_codir hypothetical protein
TcG_02576	325,783523	-0,091429577	0,10150378	-0,90075049	0,367721	0,59997401	protein_codir putative ubiquitin-conjugating enzyme variant Kua
TcG_02577	1908,147136	-0,153177486	0,05091262	-3,00863479	0,00262424	0,017062	protein_codir hypothetical protein
TcG_02578	265,5179341	0,091151881	0,1181293	0,77162805	0,44033476	0,66333617	protein_codir ferredoxin NADP+ reductase-like protein
TcG_02579	441,7753592	0,20024894	0,09478061	2,11276283	0,03462107	0,1274205	protein_codir putative PIWI-like protein 1
TcG_02580	487,5173456	0,044513856	0,0824636	0,53980008	0,58933491	0,77564856	protein_codir putative ubiquitin hydrolase
TcG_02581	227,1412848	0,027534718	0,11746279	0,23441227	0,81466494	0,90837643	protein_codir hypothetical protein
TcG_02582	710,9339072	-0,373098544	0,07143797	-5,22269216	1,7634E-07	4,3656E-06	protein_codir Flagellar Associated Protein
TcG_02583	247,2865655	0,048853253	0,11351442	0,43037044	0,66692619	0,82553492	protein_codir hypothetical protein
TcG_02584	259,5020597	-0,100887031	0,11494365	-0,87770861	0,38010187	0,6107213	protein_codir hypothetical protein
TcG_02585	175,8044258	-0,01031217	0,1403288	-0,07348577	0,94141957	0,97292993	protein_codir hypothetical protein
TcG_02586	547,4134416	0,04071903	0,08303687	0,49037291	0,62387005	0,79706621	protein_codir mitochondrial processing peptidase alpha subunit
TcG_02587	277,7318885	0,01374974	0,11393961	0,12067568	0,90394792	0,95349059	protein_codir Vps51/Vps67 protein
TcG_02588	141,5970626	0,053023556	0,14781405	0,35871798	0,71980608	0,85619724	protein_codir elongation factor 1-alpha (ef-1-alpha)
TcG_02589	23331,22048	0,178566484	0,03622283	4,92966729	8,237E-07	1,7072E-05	protein_codir elongation factor-1 alpha
TcG_02590	46,31726177	-0,177683449	0,25961996	-0,68439826	0,49372371	0,70420817	protein_codir elongation factor 1-alpha (ef-1-alpha)
TcG_02591	292,1444738	0,281960912	0,10604294	2,6589316	0,00783889	0,04083694	protein_codir hypothetical protein
TcG_02592	173,4235701	0,14214771	0,13633248	1,04265476	0,29710822	0,53031825	protein_codir hypothetical protein
TcG_02593	619,1923565	0,219376532	0,07454269	2,94296524	0,00325085	0,02033712	protein_codir hypothetical protein
TcG_02594	177,1551047	0,237892086	0,14227342	1,67207685	0,09450917	0,2588001	protein_codir hypothetical protein
TcG_02595	100,591624	-0,100347637	0,1890633	-0,53076211	0,59558364	0,7806028	
TcG_02596	386,8474488	-0,118003838	0,09459671	-1,24744131	0,21223573	0,43544593	protein_codir putative protein kinase
TcG_02597	426,9584356	0,45381019	0,09113015	4,97980307	6,3649E-07	1,3556E-05	protein_codir pentatricopeptidecontaining protein

TcG_02598	1051,702846	-0,044620466	0,06042604	-0,73843107	0,46025254	0,67839985	protein_codir	putative hexokinase
TcG_02599	180,5982638	0,001164911	0,14037326	0,00829867	0,9933787	0,99751132	protein_codir	putative actin-like protein
TcG_02600	237,9747651	0,159540312	0,11759461	1,35669751	0,17487734	0,38395469	protein_codir	acyl-CoA dehydrogenase
TcG_02601	104,8184277	0,264698807	0,17411564	1,52024716	0,12844887	0,31738294	protein_codir	calmodulin
TcG_02602	250,5688663	0,093507126	0,11247286	0,83137504	0,40576179	0,63464372	protein_codir	hypothetical protein
TcG_02603	560,2858746	-0,098404036	0,07838542	-1,25538698	0,20933831	0,4318721	protein_codir	putative major vault protein
TcG_02604	379,5970511	0,090961387	0,09547924	0,95268238	0,34075102	0,57500425	protein_codir	hypothetical protein
TcG_02605	370,056416	-0,013127681	0,0987728	-0,13290786	0,89426625	0,94882077	protein_codir	TPR repeat-containing protein
TcG_02606	156,1423823	0,130946505	0,14904919	0,87854555	0,37964773	0,6107213	protein_codir	TPRcontaining protein
TcG_02607	131,0740964	0,337674757	0,16176189	2,08748029	0,03684474	0,13342337	protein_codir	hypothetical protein
TcG_02608	718,6288593	0,095214466	0,07691395	1,23793493	0,21574019	0,43952274	protein_codir	hypothetical protein
TcG_02609	249,6629575	0,232726963	0,1147533	2,02806338	0,04255378	0,1476132	protein_codir	putative leucine-rich repeat protein (LRRP)
TcG_02610	138,8588718	0,187780572	0,14979171	1,25361126	0,20998334	0,43266352	protein_codir	putative serine/threonine protein kinase
TcG_02611	343,590777	0,147735034	0,09827861	1,50322678	0,13278057	0,323683	protein_codir	hypothetical protein
TcG_02612	382,7004832	-0,277812693	0,09645741	-2,88015922	0,00397474	0,02395346	protein_codir	putative serine/threonine protein kinase
TcG_02613	1146,202786	-0,06910081	0,06456246	-1,07029403	0,28448698	0,51816792	protein_codir	topoisomerase IAmt
TcG_02614	854,4687061	-0,195504111	0,06907235	-2,83042513	0,00464862	0,02716031	protein_codir	putative calpain
TcG_02615	331,0348158	-0,098665736	0,10123726	-0,97459906	0,32975918	0,56434119	protein_codir	hypothetical protein
TcG_02616	148,4907364	-0,100594678	0,14742126	-0,68236209	0,49501002	0,70482808	protein_codir	hypothetical protein
TcG_02617	115,3106701	0,01139306	0,16528965	0,06892785	0,94504705	0,97448515	protein_codir	hypothetical protein
TcG_02618	41,39458524	0,329883277	0,28278284	1,16656044	0,24338793	0,4717117	protein_codir	MFS transporter, FLVCR family, disrupted in renal carcinoma protein 2
TcG_02619	263,7260146	-0,133773884	0,11016866	-1,21426447	0,22464675	0,45108253	protein_codir	hypothetical protein
TcG_02620	199,9947199	0,009238867	0,13050945	0,07079079	0,94356426	0,97337158	protein_codir	WD repeat domain 31
TcG_02621	245,9123316	0,099509709	0,11816368	0,84213451	0,39971267	0,62964935	protein_codir	hypothetical protein
TcG_02622	159,2717232	-0,172081332	0,1388558	-1,23928082	0,21524152	0,4388705	protein_codir	syntaxin
TcG_02623	1241,416743	0,100571938	0,05823586	1,72697597	0,08417198	0,23896509	protein_codir	hypothetical protein
TcG_02624	119,1444922	-0,19374006	0,1595222	-1,21450219	0,22455601	0,45108253	protein_codir	hypothetical protein
TcG_02625	565,7500603	0,048249753	0,07972763	0,60518235	0,54505784	0,74189851	protein_codir	hypothetical protein
TcG_02626	176,5833371	-0,08538408	0,1399549	-0,61008283	0,54180694	0,74008786	protein_codir	methyltransferase domain-containing protein
TcG_02627	349,7030846	0,05258556	0,10585114	0,49678784	0,61933867	0,79394311	protein_codir	helicase-like protein
TcG_02628	116,0115976	0,089899506	0,16357083	0,54960599	0,58258965	0,77144033	protein_codir	hypothetical protein
TcG_02629	61,91322609	-0,059805491	0,22213493	-0,26923047	0,78775234	0,89453088	protein_codir	retrotransposon hot spot (RHS) protein
TcG_02630	17,31415502	0,386371162	0,42931775	0,89996549	0,36813861	0,60027638		
TcG_02631	604,2745928	-0,626474292	0,07950031	-7,88014932	3,2699E-15	3,6081E-13	protein_codir	hypothetical protein
TcG_02632	47,81730482	0,305538307	0,25776065	1,18535666	0,23587646	0,46344584		
TcG_02633	139,4823325	-0,216855344	0,15314496	-1,41601357	0,15677153	0,35829762	protein_codir	putative trans-sialidase
TcG_02634	166,8783739	-0,2781146	0,14803431	-1,87871711	0,06028313	0,19031072	protein_codir	putative complement regulatory protein
TcG_02635	217,0940523	0,02732357	0,12127774	0,2252975	0,82174785	0,91160193	protein_codir	methyltransferase-like protein
TcG_02636	132,2750794	-0,238447437	0,1567994	-1,52071652	0,12833099	0,31731972	protein_codir	hypothetical protein
TcG_02637	220,9080906	0,055492349	0,12216619	0,45423657	0,64965857	0,81398769	protein_codir	hypothetical protein
TcG_02638	115,4750871	-0,034109245	0,16562388	-0,205944	0,83683466	0,92065507	protein_codir	putative serine/threonine-protein phosphatase 2A, catalytic subunit
TcG_02639	1943,30393	0,226803823	0,056024	4,04833358	5,1584E-05	0,00063377	protein_codir	ribosomal protein S19
TcG_02640	469,7279037	-0,05136871	0,08573352	-0,59916714	0,54906143	0,7455087	protein_codir	signal recognition particle subunit SRP68
TcG_02641	506,6048995	-0,237222377	0,08429784	-2,81409789	0,00489143	0,02832191	protein_codir	metallo-peptidase, Clan MC, Family M14
TcG_02642	1007,712334	0,172623782	0,06234909	2,76866579	0,00562863	0,03142812	protein_codir	putative regulatory subunit of protein kinase a-like protein
TcG_02643	94,7580979	0,456740789	0,18309655	2,49453524	0,01261222	0,05925595	protein_codir	ubiquitin-related modifier
TcG_02644	293,9426041	0,041024735	0,10634532	0,38576906	0,69966771	0,84432352	protein_codir	putative selenocysteine-tRNA-specific elongation factor
TcG_02645	276,3918593	0,067452542	0,10764406	0,62662579	0,53090456	0,73226908	protein_codir	putative serine acetyltransferase
TcG_02646	142,8550265	0,202965586	0,15405156	1,3175172	0,18766532	0,40205074	protein_codir	hypothetical protein
TcG_02647	220,4397338	0,226343916	0,13024561	1,73782382	0,08224186	0,23553177	protein_codir	hypothetical protein
TcG_02648	0					1	protein_codir	putative ribosomal protein L13
TcG_02649	2651,488186	0,302221088	0,04458784	6,77810582	1,2176E-11	7,667E-10	protein_codir	putative ribosomal protein L3

TcG_02650	4,293738421	-0,298123665	1,63033592	-0,18286027	0,85490766	1	protein_codir ribosomal protein L13
TcG_02651	199,4314642	0,025350296	0,13381184	0,18944733	0,84974223	0,9276306	protein_codir patatin-like phospholipase
TcG_02652	78,70068584	0,212313676	0,20599016	1,03069816	0,30268239	0,535498	
TcG_02653	231,0437662	0,322305293	0,12034398	2,6782003	0,00740189	0,03898107	protein_codir hypothetical protein
TcG_02654	441,2474312	0,051770756	0,08743076	0,59213436	0,55376062	0,74883793	protein_codir putative tyrosine phosphatase
TcG_02655	161,6540269	-0,131285648	0,14433898	-0,90956473	0,3630521	0,59526856	protein_codir hypothetical protein
TcG_02656	176,2458892	0,071393461	0,13768585	0,51852431	0,6040925	0,78596471	protein_codir hypothetical protein
TcG_02657	182,7891093	0,038118798	0,13326582	0,28603583	0,77485068	0,88753796	protein_codir hypothetical protein
TcG_02658	76,47460563	0,096106256	0,20823533	0,46152715	0,64442045	0,8110828	protein_codir hypothetical protein
TcG_02659	171,5368497	0,189898352	0,13712761	1,38482946	0,16610462	0,3716614	protein_codir hypothetical protein
TcG_02660	280,8479695	-0,183461302	0,10739121	-1,70834566	0,08757222	0,245431	protein_codir putative nucleoside diphosphate kinase
TcG_02661	265,8092639	-0,120243697	0,11600767	-1,03651502	0,29996194	0,53367513	protein_codir testis specific, 14
TcG_02662	307,3428451	-0,200673821	0,10861587	-1,84755533	0,0646667	0,20032844	protein_codir putative casein kinase
TcG_02663	171,2811869	-0,056564833	0,13896016	-0,40705791	0,68396545	0,83594728	protein_codir hypothetical protein
TcG_02664	364,5047538	-0,180462838	0,09444352	-1,9108016	0,05603008	0,17912929	protein_codir hypothetical protein
TcG_02665	142,9890151	-0,003991019	0,14792117	-0,02698072	0,97847512	0,99085144	protein_codir hypothetical protein
TcG_02666	248,3439573	0,139167458	0,11405859	1,22014004	0,22241179	0,44840924	protein_codir putative ADP,ATP carrier protein 1, mitochondrial precursor, putative,ADP/ATP translocase 1
TcG_02667	421,5831619	-0,27564555	0,09830133	-2,8040878	0,00504592	0,02894158	protein_codir hypothetical protein
TcG_02668	258,7600039	0,178553573	0,11499159	1,55275337	0,12048207	0,3039301	protein_codir putative GDP-mannose transporter
TcG_02669	285,5019484	0,045303914	0,11877753	0,38141823	0,70289294	0,8464523	protein_codir putative exosome subunit rrp6p-like protein
TcG_02670	89,4122913	0,246953734	0,20451974	1,20748116	0,2272469	0,45410185	protein_codir hypothetical protein
TcG_02671	42,85146127	-0,036890839	0,28025435	-0,13163342	0,89527425	0,9489813	protein_codir hypothetical protein
TcG_02672	151,0364502	0,166843522	0,15884675	1,05034269	0,29356058	0,52658197	protein_codir putative phosphatidylinositol-4-phosphate 5-kinase-like
TcG_02673	378,8767459	0,17598009	0,09590841	1,83487646	0,066524	0,20482249	protein_codir putative suppressive immunomodulating factor
TcG_02674	194,4067279	-0,207903216	0,12580302	-1,65260909	0,09841045	0,26571509	protein_codir retrotransposon hot spot (RHS) protein
TcG_02675	257,7982183	0,201923097	0,11623216	1,73723949	0,08234491	0,23574205	protein_codir putative trans-sialidase
TcG_02676	274,9117409	0,106375059	0,11562917	0,91996735	0,35758982	0,59135536	protein_codir hypothetical protein
TcG_02677	126,3462318	-0,131625067	0,16978918	-0,77522649	0,43820584	0,66150526	protein_codir hypothetical protein
TcG_02678	250,2001595	-0,023538091	0,11431732	-0,20590135	0,83686797	0,92065507	protein_codir hypothetical protein
TcG_02679	234,0087239	0,035973854	0,11669007	0,30828547	0,75786512	0,87801518	protein_codir hypothetical protein
TcG_02680	289,4677481	0,053995395	0,10837958	0,49820638	0,61833859	0,793034	protein_codir putative sugar transporter
TcG_02681	316,3796251	-0,210173361	0,10265758	-2,04732432	0,04062625	0,14271104	protein_codir dihydroflavonol-4-reductase
TcG_02682	513,5516922	0,076380825	0,08206166	0,93077363	0,35197067	0,58633102	protein_codir hypothetical protein
TcG_02683	185,8931374	0,00301	0,14557902	0,02067606	0,98350407	0,99376391	protein_codir hypothetical protein
TcG_02684	7,32144312	0,20787449	0,64424125	0,3226656	0,74694851	1	protein_codir hypothetical protein
TcG_02685	10,76027728	0,532908764	0,54405493	0,9795128	0,32732667	1	protein_codir hypothetical protein
TcG_02686	432,3609462	0,001164015	0,08936923	0,01302479	0,98960801	0,99570981	protein_codir putative phospholipid-transporting ATPase IIB
TcG_02687	4,703973838	-0,685031025	0,80792334	-0,84789112	0,39649861	1	protein_codir hypothetical protein
TcG_02688	29,95901825	0,584666797	0,31886424	1,83359165	0,06671463	0,20510851	protein_codir hypothetical protein
TcG_02689	1773,690922	0,057340294	0,06450867	0,8888773	0,37406903	0,60449626	protein_codir putative RNA editing associated helicase 2,putative
TcG_02690	208,9770355	-0,030280955	0,12476121	-0,2427113	0,80822905	0,9050978	protein_codir hypothetical protein
TcG_02691	301,163666	-0,152742213	0,10314819	-1,48080362	0,13865891	0,33254029	protein_codir DNA cross-link repair 1A protein
TcG_02692	205,577194	0,069211548	0,12786018	0,54130653	0,58829632	0,77564856	protein_codir acetyl-CoA C-acetyltransferase
TcG_02693	103,8575149	0,247907442	0,17179972	1,44300261	0,14901974	0,3482337	protein_codir hypothetical protein
TcG_02694	234,7387051	0,060793473	0,11909657	0,51045529	0,60973253	0,78878529	protein_codir BRCA1-associated protein
TcG_02695	198,6577038	0,185948644	0,12957702	1,43504334	0,15127475	0,3515895	protein_codir hypothetical protein
TcG_02696	148,5730733	0,238249864	0,14785023	1,61142712	0,10708666	0,28208285	protein_codir putative rRNA maturation factor
TcG_02697	205,1811743	-0,088518274	0,12658602	-0,69927371	0,48438099	0,69758088	protein_codir hypothetical protein
TcG_02698	93,95683217	0,181289404	0,18097628	1,00173021	0,31647391	0,55146139	protein_codir hypothetical protein
TcG_02699	284,0963365	0,233610644	0,10976327	2,12831345	0,0333111	0,12362026	protein_codir hypothetical protein
TcG_02700	263,6311918	0,392497632	0,11209093	3,50160018	0,00046247	0,00404088	protein_codir putative vacuolar transport protein 4A
TcG_02701	324,104584	0,044119466	0,10483437	0,42084925	0,67386517	0,82902391	protein_codir hypothetical protein

TcG_02702	272,0469378	0,655468651	0,11256025	5,82326927	5,7707E-09	1,9324E-07	protein_codir	hypothetical protein
TcG_02703	108,529289	0,028682627	0,16835973	0,17036512	0,864723	0,93498506	protein_codir	Chain A, Structural Analysis Of A Probable Eukaryotic D-Amino Acid Trna Deacylase
TcG_02704	122,9487189	0,147594041	0,16134732	0,91475981	0,36031774	0,59295581	protein_codir	hypothetical protein
TcG_02705	321,4769467	0,083506485	0,10191114	0,81940487	0,41255546	0,63927612	protein_codir	aldose 1-epimerase-like protein
TcG_02706	18,44305635	-0,231854788	0,39754576	-0,58321535	0,55974834	0,75405439	protein_codir	hypothetical protein
TcG_02707	200,7521301	0,034275602	0,13585958	0,25228698	0,80081925	0,9008925	protein_codir	hydroxyacylglutathione hydrolase
TcG_02708	547,5057108	0,037538631	0,08622584	0,43535245	0,66330662	0,82378288	protein_codir	putative cleavage and polyadenylation specificity factor
TcG_02709	786,5048257	-0,216420561	0,07001885	-3,09088986	0,00199558	0,01371337	protein_codir	DNA topoisomerase IB, large subunit
TcG_02710	350,3905643	-0,064365562	0,09879144	-0,65152975	0,51470458	0,72038744	protein_codir	hypothetical protein
TcG_02711	306,6802516	0,117641508	0,11391677	1,03269703	0,3017457	0,53501019	protein_codir	hypothetical protein
TcG_02712	1947,51938	-0,032061688	0,05030558	-0,63733859	0,52390432	0,72776714	protein_codir	linear amidine hydrolase
TcG_02713	143,0334129	-0,046839397	0,151917	-0,30832228	0,75783711	0,87801518	protein_codir	hypothetical protein
TcG_02714	279,1894612	0,148958306	0,10975064	1,35724321	0,17470395	0,38371942	protein_codir	hypothetical protein
TcG_02715	563,3552289	-0,089710801	0,07853648	-1,14228199	0,25333683	0,48445481	protein_codir	ruvB-like 1
TcG_02716	117,8558216	0,252180025	0,16551171	1,52363858	0,12759902	0,31636256	protein_codir	hypothetical protein
TcG_02717	507,2364504	0,05754538	0,08160066	0,70520733	0,48068122	0,69501718	protein_codir	peroxisome biosynthesis protein-like protein
TcG_02718	559,5796188	-0,387821876	0,08905557	-4,35482991	1,3317E-05	0,00019457	protein_codir	putative phosphatase 2C
TcG_02719	417,480548	-0,266058599	0,09611802	-2,76804083	0,00563944	0,03147329	protein_codir	putative DNA repair protein
TcG_02720	3256,869284	0,28318067	0,04499445	6,29367977	3,1003E-10	1,3815E-08	protein_codir	eukaryotic initiation factor 5a
TcG_02721	659,9239994	-0,482247046	0,07295842	-6,60988915	3,8461E-11	2,1219E-09	protein_codir	putative mucin-associated surface protein (MASP)
TcG_02722	237,197664	-0,305669249	0,12000292	-2,54718185	0,01085968	0,05266933	protein_codir	secretory carrier membrane protein 3
TcG_02723	153,691677	-0,187380627	0,14832254	-1,2633321	0,20646985	0,42854886	protein_codir	hypothetical protein
TcG_02724	270,8635363	-0,290009965	0,11574832	-2,50552202	0,01222708	0,05791616	protein_codir	putative ribose-phosphate pyrophosphokinase
TcG_02725	904,0217825	-0,211273484	0,06408257	-3,29689471	0,0009776	0,00763241	protein_codir	epsin
TcG_02726	88,60343877	0,229321223	0,20324135	1,1283197	0,25918493	0,49163663	protein_codir	hypothetical protein
TcG_02727	1309,667505	-0,18670684	0,06393934	-2,92006201	0,00349962	0,02163638	protein_codir	hypothetical protein
TcG_02728	905,1756421	-0,053685352	0,06369488	-0,84285194	0,39931126	0,62918812	protein_codir	putative RNA polymerase I second largest subunit
TcG_02729	595,698081	-0,076560072	0,08553957	-0,89502525	0,37077359	0,60232513	protein_codir	regulatory associated protein of mTOR
TcG_02730	207,4132258	-0,403189986	0,12323907	-3,27160855	0,00106938	0,00824337	protein_codir	hypothetical protein
TcG_02731	88,33146136	0,136696111	0,19073478	0,71668162	0,47357057	0,68929505	protein_codir	hypothetical protein
TcG_02732	537,623748	-0,131116673	0,08591096	-1,52619259	0,12696191	0,31518763		
TcG_02733	98,53383015	-0,311453578	0,17455202	-1,78430229	0,07437456	0,22015933	protein_codir	putative CYC2-like cyclin 6
TcG_02734	222,3982339	-0,280287334	0,11997868	-2,33614281	0,0194838	0,08234943	protein_codir	putative complement regulatory protein
TcG_02735	68,68194465	-0,116400843	0,2082917	-0,55883572	0,57627384	0,76670634	protein_codir	putative tyrosine phosphatase
TcG_02736	140,4180054	-0,262141722	0,14996514	-1,74801778	0,08046095	0,23202947	protein_codir	hypothetical protein
TcG_02737	221,9785165	-0,131031383	0,11970749	-1,09459634	0,27369352	0,50688937	protein_codir	hypothetical protein
TcG_02738	852,8930014	-0,484375943	0,06708978	-7,21981717	5,2058E-13	4,1311E-11	protein_codir	kinesin
TcG_02739	88,36467343	-0,615723084	0,18893222	-3,25896284	0,0011182	0,00854021		
TcG_02740	459,8746203	-0,008891758	0,09437734	-0,09421496	0,9249384	0,96326618	protein_codir	hypothetical protein
TcG_02741	223,3541799	-0,016864562	0,13888961	-0,12142422	0,90335504	0,95322469	protein_codir	guanine nucleotide releasing protein
TcG_02742	626,4127201	-0,119374256	0,07460741	-1,60003208	0,10959147	0,28616785	protein_codir	ATP-binding cassette protein subfamily B, member 1
TcG_02743	258,5868221	-0,141240709	0,11527087	-1,225294	0,22046447	0,44546587	protein_codir	RNA-binding protein
TcG_02744	921,5902247	-0,175082155	0,06807945	-2,57173295	0,01011909	0,04982992	protein_codir	hypothetical protein
TcG_02745	581,0916583	-0,338341504	0,07799996	-4,33771369	1,4397E-05	0,00020825	protein_codir	U-rich RNA-binding protein UBP-2
TcG_02746	243,258721	-0,370147188	0,12061364	-3,06886673	0,00214872	0,0145756		
TcG_02747	381,60141	-0,232761422	0,10324707	-2,2544119	0,02417027	0,09740408	protein_codir	RNA-binding protein
TcG_02748	663,5232304	-0,020666659	0,07732977	-0,26725358	0,78927392	0,89494301	protein_codir	putative myosin heavy chain
TcG_02749	425,2010106	-0,156727378	0,09389038	-1,6692592	0,09506602	0,25983367	protein_codir	putative hepatocyte growth factor-like
TcG_02750	141,0016636	0,227567392	0,15384572	1,4791922	0,13908895	0,33343361	protein_codir	hypothetical protein
TcG_02751	231,8182177	0,110126584	0,12122084	0,90847898	0,36362521	0,5955558	protein_codir	putative small GTP-binding protein
TcG_02752	279,606504	-0,020191976	0,11199822	-0,18028836	0,85692619	0,9308407	protein_codir	transcription factor
TcG_02753	861,1848927	-0,079022743	0,06839467	-1,15539324	0,24792943	0,4773991	protein_codir	U3 small nucleolar RNA-associated protein 13

TcG_02754	215,8575255	0,122458492	0,13081664	0,93610794	0,34921762	0,58367503	protein_codir	putative 60S ribosomal protein L7a
TcG_02755	542,0662784	-0,137977643	0,08360303	-1,65039037	0,09886312	0,26647464	protein_codir	hypothetical protein
TcG_02756	252,3098645	-0,470849088	0,11304639	-4,1650962	3,1122E-05	0,00040744	protein_codir	COP9 signalosome complex subunit 7, variant
TcG_02757	327,7626559	-0,033585873	0,1005333	-0,33407708	0,73832139	0,86642273	protein_codir	putative sigma-adaptin 3, putative,adaptor complex AP-3 small subunit
TcG_02758	174,174939	-0,0294234	0,14293015	-0,2058586	0,83690137	0,92065507	protein_codir	nucleolus protein required for cell viability
TcG_02759	126,5911142	0,288153042	0,15794159	1,82442788	0,06808741	0,20797805	protein_codir	hypothetical protein
TcG_02760	264,0554022	-0,128492681	0,11078058	-1,15988454	0,24609582	0,47465726	protein_codir	hypothetical protein
TcG_02761	544,2210245	-0,236860997	0,08149378	-2,90649189	0,00365506	0,02237061	protein_codir	hypothetical protein
TcG_02762	88,10150362	-0,33273724	0,19604825	-1,69722113	0,08965486	0,24957742	protein_codir	B9 protein domain 2
TcG_02763	440,1580502	-0,316993166	0,09251665	-3,42633648	0,00061178	0,00512103	protein_codir	repressor activator protein 1
TcG_02764	154,8331314	-0,310630858	0,14045311	-2,21163384	0,02699198	0,10575889	protein_codir	hypothetical protein
TcG_02765	213,5415374	-0,057170096	0,12203772	-0,46846251	0,63945387	0,80810565	protein_codir	putative ATP-binding protein
TcG_02766	390,9201313	-0,256445341	0,10077576	-2,54471254	0,01093677	0,05297258	protein_codir	hypothetical protein
TcG_02767	436,7730351	-0,083304172	0,08984731	-0,92717493	0,35383572	0,58808501	protein_codir	hypothetical protein
TcG_02768	610,8201714	-0,188731895	0,07844476	-2,40592089	0,01613176	0,07150059	protein_codir	hypothetical protein
TcG_02769	171,4037455	-0,010905573	0,14263918	-0,07645566	0,93905659	0,97168077		
TcG_02770	719,2355337	-0,229916076	0,07803366	-2,94637035	0,00321527	0,02017992	protein_codir	hypothetical protein
TcG_02771	213,8648197	-0,31600697	0,13147653	-2,40352381	0,01623791	0,07188858	protein_codir	hypothetical protein
TcG_02772	299,7227473	-0,185518329	0,10650979	-1,74179596	0,08154415	0,23426	protein_codir	dual specificity phosphatase
TcG_02773	364,1017789	-0,131432588	0,09467584	-1,38823783	0,16506464	0,37062769	protein_codir	hypothetical protein
TcG_02774	333,0203212	-0,28989568	0,10200705	-2,84191814	0,0044843	0,02645372	protein_codir	mitochondrial carrier protein
TcG_02775	754,6237272	-0,224185268	0,07208839	-3,10986651	0,00187172	0,01304013	protein_codir	WD repeat-containing protein 19 isoform X2
TcG_02776	236,6258639	-0,168554775	0,11653928	-1,44633444	0,14808342	0,3469074	protein_codir	ubiquitin carboxyl-terminal hydrolase
TcG_02777	579,4579362	-0,164740014	0,08410846	-1,95866168	0,05015242	0,16573473	protein_codir	hypothetical protein
TcG_02778	546,6565528	-0,13427287	0,07913823	-1,69668788	0,08975568	0,24967812	protein_codir	putative cleavage and polyadenylation specificity factor
TcG_02779	155,8859389	-0,258300028	0,14344178	-1,80073073	0,07174533	0,21511728	protein_codir	hypothetical protein
TcG_02780	458,9315523	-0,31054578	0,08563187	-3,62652093	0,00028727	0,00270151	protein_codir	hypothetical protein
TcG_02781	128,5363017	-0,210243132	0,15311867	-1,3730731	0,16972962	0,37606704	protein_codir	putative DNA-directed RNA polymerase subunit
TcG_02782	568,7432636	0,033309235	0,08143466	0,40903019	0,6825175	0,83475645	protein_codir	putative elongation factor
TcG_02783	418,2623285	-0,258301557	0,09497845	-2,71958067	0,00653647	0,03543828	protein_codir	electron transfer flavoprotein
TcG_02784	335,9494181	-0,00934486	0,10378166	-0,09004346	0,92825268	0,96532946	protein_codir	hypothetical protein
TcG_02785	390,6344527	-0,139337398	0,10446322	-1,33384175	0,18225573	0,39410505		
TcG_02786	148,8160584	-0,057921335	0,14863998	-0,38967534	0,69677664	0,84298788	protein_codir	putative Qc-SNARE protein
TcG_02787	654,9611881	0,01244447	0,07434279	0,1673931	0,86706076	0,93578156	protein_codir	ADP-ribosylation factor guanine nucleotide-exchange factor 1(brefeldin A-inhibited)
TcG_02788	41,91898959	0,07889482	0,26573649	0,29689117	0,76654959	0,8828274	protein_codir	putative protein phosphatase 2C
TcG_02789	70,11281438	-0,406953921	0,2103198	-1,93492923	0,05299898	0,17200173	protein_codir	protein phosphatase 2C
TcG_02790	216,5716925	-0,0610077	0,12425285	-0,49099637	0,62342901	0,79702106	protein_codir	hypothetical protein
TcG_02791	99,66684476	0,165597512	0,18389255	0,90051235	0,36784766	0,60009616	protein_codir	hypothetical protein
TcG_02792	398,0385137	-0,2957653	0,09594409	-3,08268376	0,00205143	0,01404721	protein_codir	coiled-coil protein required for normal flagellar motility
TcG_02793	69,80237091	-0,064053029	0,21411946	-0,29914623	0,76482847	0,88145854	protein_codir	hypothetical protein
TcG_02794	79,93532579	0,02299106	0,19594441	0,11733461	0,9065949	0,95471088	protein_codir	hypothetical protein
TcG_02795	126,7031782	0,324500877	0,16166202	2,00727958	0,0447199	0,1527941	protein_codir	hypothetical protein
TcG_02796	1717,76318	0,412496768	0,05470767	7,54001779	4,6991E-14	4,3555E-12	protein_codir	ribosomal protein L35A
TcG_02797	195,6329755	0,233899538	0,12812506	1,82555655	0,06791709	0,20778647	protein_codir	hypothetical protein
TcG_02798	179,9881838	0,025557718	0,13632282	0,18747937	0,85128479	0,9281063	protein_codir	putative GTP-binding protein
TcG_02799	181,2176499	-0,003832909	0,13263434	-0,02889831	0,97694569	0,99017719	protein_codir	hypothetical protein
TcG_02800	58,44522754	0,182621299	0,24050291	0,75933092	0,44765462	0,66876383	protein_codir	hypothetical protein
TcG_02801	492,7028248	0,040403289	0,08792507	0,45951956	0,64586111	0,81201944	protein_codir	hypothetical protein
TcG_02802	99,70992449	-0,074848608	0,17455075	-0,42880714	0,66806359	0,8261349	protein_codir	hypothetical protein
TcG_02803	515,4053672	-0,065593749	0,08249002	-0,7951719	0,42651351	0,65178385	protein_codir	putative TPR-repeat protein
TcG_02804	346,2740465	0,311783033	0,09807365	3,17907051	0,00147748	0,01073908	protein_codir	putative dolichyl-P-Man:GDP-Man1GlcNAc2-PP-dolichyl alpha-1,3-mannosyltransferase
TcG_02805	300,5998468	0,980671517	0,10837627	9,0487663	1,4459E-19	2,4636E-17	protein_codir	protein G6

TcG_02806	144,9695502	-0,067720221	0,14821337	-0,45691034	0,64773549	0,81260383	protein_codir	hypothetical protein
TcG_02807	255,3253869	-0,012657257	0,11257812	-0,11243089	0,91048177	0,95647184	protein_codir	centrin
TcG_02808	145,2399635	0,223473599	0,14743953	1,5156966	0,12959609	0,31926436	protein_codir	hypothetical protein
TcG_02809	163,594454	0,192146518	0,14000295	1,37244617	0,16992459	0,3762739	protein_codir	hypothetical protein
TcG_02810	470,5953704	-0,202687017	0,0892144	-2,27190908	0,023092	0,09416384	protein_codir	hypothetical protein
TcG_02811	481,6747076	-0,03369174	0,08817953	-0,38208118	0,70240115	0,84624626	protein_codir	hypothetical protein
TcG_02812	1237,06387	-0,036939896	0,05737922	-0,64378525	0,5197147	0,72427679	protein_codir	putative asparaginyl-tRNA synthetase
TcG_02813	127,0616323	0,107253488	0,16040823	0,66862833	0,5037326	0,7120387	protein_codir	clathrin coat assembly protein AP17
TcG_02814	138,4630783	-0,124450663	0,14880143	-0,83635395	0,40295581	0,63295092	protein_codir	hypothetical protein
TcG_02815	254,4614597	0,230149692	0,11154288	2,06332927	0,03908136	0,13912475	protein_codir	hypothetical protein
TcG_02816	454,1648852	-0,110908506	0,0870885	-1,27351498	0,20283536	0,42300656	protein_codir	putative G-actin binding protein, putative,twinfilin
TcG_02817	189,1585445	0,031764496	0,13014111	0,24407734	0,80717092	0,90472556	protein_codir	hypothetical protein
TcG_02818	273,8956866	0,204732645	0,11217442	1,82512778	0,06798175	0,20781967	protein_codir	hypothetical protein
TcG_02819	265,9079282	0,085694426	0,10895386	0,78652032	0,43156272	0,65596889	protein_codir	putative sarcoplasmic reticulum glycoprotein
TcG_02820	293,5807581	0,010513662	0,10617372	0,09902321	0,92111984	0,96162322	protein_codir	ATP-dependent RNA helicase-like protein
TcG_02821	757,7320406	0,25671571	0,06903416	3,71867633	0,00020027	0,0019934	protein_codir	hypothetical protein
TcG_02822	407,3636479	0,163730877	0,09627206	1,70071022	0,08899742	0,24828415	protein_codir	glycosyl hydrolase
TcG_02823	173,7462458	0,229271746	0,13759747	1,66624972	0,09566369	0,26103614	protein_codir	hypothetical protein
TcG_02824	247,7929771	0,074292432	0,12234771	0,60722374	0,54370244	0,74093036	protein_codir	2,5-phosphodiesterase
TcG_02825	240,4662693	0,24599036	0,11866909	2,07291008	0,03818065	0,1366155	protein_codir	hypothetical protein
TcG_02826	185,4437883	0,024177933	0,13192272	0,18327346	0,85458346	0,92942435	protein_codir	metal ion transporter, MIT family
TcG_02827	517,3335113	-0,026217161	0,08228709	-0,31860603	0,75002528	0,87396087	protein_codir	putative thioredoxin
TcG_02828	403,8446011	-0,017034329	0,09061913	-0,18797719	0,85089453	0,92805389	protein_codir	putative dual specificity protein phosphatase
TcG_02829	469,9062003	0,049288456	0,08427416	0,58485844	0,55864291	0,75322201	protein_codir	hypothetical protein
TcG_02830	349,9662141	0,296016314	0,09729126	3,04257863	0,00234561	0,01565407	protein_codir	putative 8-oxoguanine DNA glycosylase
TcG_02831	50,13188772	0,226326984	0,24432313	0,92634286	0,35426783	0,58832404	protein_codir	alpha-1,2-mannosyltransferase
TcG_02832	202,8278392	0,018285461	0,12385652	0,14763423	0,88263144	0,94267772	protein_codir	hypothetical protein
TcG_02833	765,535652	-0,180119015	0,07303693	-2,46613625	0,01365794	0,06327104	protein_codir	putative protein kinase
TcG_02834	348,4029043	0,06697638	0,09704629	0,6901488	0,49010061	0,70128513	protein_codir	hypothetical protein
TcG_02835	518,6289939	0,145956328	0,08213194	1,77709581	0,07555248	0,22215746	protein_codir	putative multidrug resistance-associated protein
TcG_02836	148,6454419	0,26609567	0,15340171	1,73463301	0,08280583	0,23665228	protein_codir	NAD-dependent deacetylase
TcG_02837	512,7868652	0,038220049	0,08799679	0,43433459	0,66404549	0,82434295	protein_codir	DoxX
TcG_02838	156,6574648	-0,17829885	0,14684895	-1,21416498	0,22468473	0,45108253	protein_codir	hypothetical protein
TcG_02839	342,2769979	0,092955271	0,10373999	0,89604087	0,37023093	0,60186553	protein_codir	putative cardiolipin synthetase
TcG_02840	231,4385819	0,298007717	0,12249143	2,43288618	0,01497901	0,06766691	protein_codir	hypothetical protein
TcG_02841	365,1040041	-0,029140065	0,09457558	-0,30811406	0,75799554	0,87801518	protein_codir	hypothetical protein
TcG_02842	240,3054357	0,175607939	0,1189859	1,47587182	0,13997831	0,3345817	protein_codir	hypothetical protein
TcG_02843	506,7700552	-0,151809098	0,08404401	-1,8063048	0,07087074	0,21360781	protein_codir	hypothetical protein
TcG_02844	65,36355111	-0,36421141	0,213738	-1,70400872	0,08837946	0,24704966		
TcG_02845	260,986442	-0,047525111	0,1115705	-0,42596484	0,67013347	0,82696679	protein_codir	putative protein kinase
TcG_02846	607,4179229	0,015254878	0,08335988	0,18300024	0,85479783	0,92948734	protein_codir	hypothetical protein
TcG_02847	626,2914395	0,176765851	0,07791971	2,26856397	0,02329485	0,09469971	protein_codir	permease, family protein
TcG_02848	23,55612306	0,294904079	0,38372962	0,7685205	0,44217802	0,66464381	protein_codir	amino acid transporter
TcG_02849	8,350172884	0,413321011	0,62537836	0,66091352	0,50866778	1	protein_codir	hypothetical protein
TcG_02850	1,372717874	-1,041142804	1,51727189	-0,68619396	0,49259081	1	protein_codir	RNA-binding protein
TcG_02851	0					1	protein_codir	target of rapamycin (TOR) kinase 1
TcG_02852	8,863219599	-1,472472368	0,61317296	-2,40139806	0,01633256	1	protein_codir	target of rapamycin (TOR) kinase 1
TcG_02853	143,7662261	-0,370548519	0,15324886	-2,41795292	0,0156081	0,06968609	protein_codir	putative tRNA isopentenyltransferase
TcG_02854	335,4279996	-0,486326899	0,09989965	-4,86815412	1,1265E-06	2,2463E-05	protein_codir	putative signal peptide peptidase, putative,aspartic peptidase, clan AD, family A22B
TcG_02855	305,2790755	-0,754459013	0,11072621	-6,81373472	9,5097E-12	6,0538E-10	protein_codir	leucine zipper-EF-hand containing transmembrane protein 2
TcG_02856	232,6848292	-0,187155914	0,12862354	-1,45506736	0,14565061	0,34312892	protein_codir	putative ABC transporter
TcG_02857	124,8286091	-0,177386765	0,1595297	-1,1119357	0,2661658	0,49883483	protein_codir	hypothetical protein

TcG_02858	799,7139898	-0,37137189	0,06719448	-5,52682144	3,2608E-08	9,3284E-07	protein_codir	generic methyltransferase
TcG_02859	173,3455457	-0,129867208	0,14550581	-0,8925225	0,37211294	0,60340105	protein_codir	hypothetical protein
TcG_02860	822,0668739	-0,166312457	0,06655465	-2,49888571	0,01245845	0,05880973	protein_codir	putative Unc104-like kinesin
TcG_02861	241,4443215	-0,083245954	0,11706789	-0,71109129	0,47702767	0,69213601	protein_codir	hypothetical protein
TcG_02862	497,4495615	-0,4541885	0,08933196	-5,08427774	3,6903E-07	8,3344E-06	protein_codir	hypothetical protein
TcG_02863	408,5659454	-0,527484424	0,09240162	-5,70860555	1,1391E-08	3,5764E-07	protein_codir	hypothetical protein
TcG_02864	278,8025782	-0,001706283	0,10652145	-0,01601821	0,98721986	0,99486208	protein_codir	putative oxidoreductase
TcG_02865	254,4838016	-0,099011334	0,11112843	-0,89096314	0,37294894	0,60416477	protein_codir	hypothetical protein
TcG_02866	167,185667	-0,308535562	0,1378548	-2,23811978	0,02521324	0,10038511	protein_codir	hypothetical protein
TcG_02867	626,9249555	-0,130320542	0,07749039	-1,68176387	0,09261464	0,25481671	protein_codir	hypothetical protein
TcG_02868	363,3642745	-0,129342116	0,09843357	-1,31400415	0,18884481	0,40323552	protein_codir	putative KU70 protein
TcG_02869	303,8667143	0,096599291	0,1032714	0,93539253	0,34958605	0,58410694	protein_codir	hypothetical protein
TcG_02870	4508,581108	0,11284276	0,04061574	2,77830146	0,00546439	0,0307034	protein_codir	putative 60S ribosomal protein L4
TcG_02871	332,0277194	-0,334451952	0,10046771	-3,32894978	0,00087174	0,00696551	protein_codir	ankyrin
TcG_02872	348,4413242	-0,201827501	0,09549488	-2,1134902	0,03455883	0,12723185	protein_codir	hypothetical protein
TcG_02873	62,8001801	-0,074905388	0,23843452	-0,31415497	0,75340334	0,87552748	protein_codir	hypothetical protein
TcG_02874	188,361135	-0,273805902	0,13296705	-2,05920121	0,03947497	0,13999295	protein_codir	tryparedoxin
TcG_02875	1942,92564	-0,265720033	0,04985759	-5,32958015	9,844E-08	2,5572E-06	protein_codir	tryparedoxin
TcG_02876	375,8366785	-0,311253095	0,09624372	-3,2340095	0,00122065	0,00916558	protein_codir	hypothetical protein
TcG_02877	925,1221865	-0,10132497	0,06640424	-1,52588099	0,1270395	0,31529063	protein_codir	hypothetical protein
TcG_02878	147,6799989	-0,198462238	0,14536268	-1,36529015	0,17216187	0,37984159	protein_codir	hypothetical protein
TcG_02879	0					1	protein_codir	serine/threonine protein phosphatase
TcG_02880	2,981815551	-1,161417546	1,03642785	-1,12059662	0,2624596	1	protein_codir	serine/threonine protein phosphatase
TcG_02881	223,6229292	-0,238431546	0,12076802	-1,97429373	0,04834835	0,16157024	protein_codir	putative serine/threonine protein phosphatase
TcG_02882	18,76962249	-0,258609413	0,40591698	-0,63709928	0,52406018	0,72776714	protein_codir	serine/threonine protein phosphatase
TcG_02883	16,92913009	-0,7804292	0,44860142	-1,73969401	0,08191276	0,2349617	protein_codir	serine/threonine protein phosphatase
TcG_02884	387,8211132	-0,51694903	0,09391993	-5,50414623	3,7096E-08	1,0508E-06	protein_codir	hypothetical protein
TcG_02885	420,1208444	-0,372755241	0,09022412	-4,13143675	3,605E-05	0,00046357	protein_codir	hypothetical protein
TcG_02886	241,8767673	-0,045567265	0,11413147	-0,39925243	0,68970722	0,8386805	protein_codir	hypothetical protein
TcG_02887	1251,186387	-0,073655843	0,06236013	-1,18113684	0,23754837	0,46529761	protein_codir	hypothetical protein
TcG_02888	110,178659	-0,035585752	0,16798868	-0,21183422	0,83223637	0,91866335	protein_codir	hypothetical protein
TcG_02889	468,849183	-0,10085578	0,09230968	-1,09258077	0,27457791	0,50755645	protein_codir	putative lipase domain protein
TcG_02890	730,8925839	-0,310350749	0,07365604	-4,21351366	2,5143E-05	0,00033952	protein_codir	putative lipase domain protein
TcG_02891	231,1777621	-0,186279793	0,11775986	-1,58186152	0,11368119	0,29269116	protein_codir	hypothetical protein
TcG_02892	245,9339793	0,032695498	0,11488887	0,28458368	0,77596312	0,88831084	protein_codir	hypothetical protein
TcG_02893	795,2051949	-0,321322683	0,06933495	-4,6343537	3,5805E-06	6,257E-05	protein_codir	carnitine/choline acetyltransferase
TcG_02894	193,9491001	-0,22960504	0,1337444	-1,71674503	0,08602577	0,24262282	protein_codir	DNA-directed RNA polymerase
TcG_02895	290,8735535	-0,183420142	0,10566955	-1,73578994	0,08260098	0,2363	protein_codir	putative serine/threonine protein kinase, putative,protein kinase
TcG_02896	66,9864449	-0,121831752	0,21778517	-0,55941252	0,57588022	0,76647309	protein_codir	putative RNA-binding protein
TcG_02897	4,25402074	0,06071667	0,87814208	0,06914219	0,94487644	1		
TcG_02898	503,9250538	0,009681458	0,08739541	0,11077766	0,91179266	0,95702042	protein_codir	protein LTV1
TcG_02899	13,10815951	-0,428210927	0,51664109	-0,82883638	0,407197	1	protein_codir	putative RNA-binding protein
TcG_02900	214,2536746	0,041031419	0,1267267	0,32377881	0,7461055	0,87149696	protein_codir	RNA binding protein
TcG_02901	512,9382489	-0,117397178	0,0824317	-1,42417509	0,15439579	0,35556145	protein_codir	hypothetical protein
TcG_02902	111,4013423	-0,098768048	0,17137223	-0,57633638	0,56438784	0,75726665	protein_codir	hypothetical protein
TcG_02903	296,8252916	-0,122085648	0,11582879	-1,05401816	0,2918746	0,52445086	protein_codir	putative asparagine synthase
TcG_02904	128,775387	-0,371372943	0,15473394	-2,40007417	0,01639175	0,07232614	protein_codir	hypothetical protein
TcG_02905	894,8373981	-0,296642077	0,06587756	-4,50293034	6,7023E-06	0,000108	protein_codir	hypothetical protein
TcG_02906	554,3096535	-0,257975514	0,07879152	-3,27415317	0,00105979	0,0081834	protein_codir	hypothetical protein
TcG_02907	704,1906255	-0,039614161	0,07371653	-0,53738507	0,59100166	0,77669524	protein_codir	putative phosphatidylinositol 4-kinase alpha
TcG_02908	20,98188809	-0,264960824	0,39902855	-0,6640147	0,5066809	0,71346681	protein_codir	hypothetical protein
TcG_02909	13,75797134	-0,391633114	0,47412451	-0,82601322	0,40879659	1	protein_codir	hypothetical protein

TcG_02910	22,91562571	-0,509908403	0,3808979	-1,338701	0,18066803	0,39206215	protein_codir	hypothetical protein
TcG_02911	20,62398766	-0,277287547	0,38292446	-0,72413119	0,4689852	0,68554914	protein_codir	hypothetical protein
TcG_02912	107,917004	-0,198192132	0,17581401	-1,12728291	0,25962289	0,4921081	protein_codir	hypothetical protein
TcG_02913	1556,213634	-0,0884903	0,05456658	-1,62169413	0,10486886	0,27816176	protein_codir	clathrin assembly protein AP19-like protein
TcG_02914	955,1973111	-0,00157469	0,07057976	-0,02231079	0,98220004	0,99334581	protein_codir	protein G7
TcG_02915	344,1560659	-0,096360341	0,09784409	-0,98483556	0,32470485	0,55907719	protein_codir	hypothetical protein
TcG_02916	328,531924	-0,457097544	0,09953392	-4,59237954	4,3822E-06	7,4665E-05	protein_codir	hypothetical protein
TcG_02917	45,90287678	0,065284882	0,25298251	0,25806085	0,79635994	0,89858067	protein_codir	hypothetical protein
TcG_02918	126,6562103	-0,234792809	0,16021909	-1,46544844	0,14279861	0,33915975	protein_codir	hypothetical protein
TcG_02919	80,10321514	-0,273613772	0,195764	-1,39767152	0,16221173	0,36599687		
TcG_02920	380,8194717	0,080512162	0,09747422	0,82598417	0,40881307	0,63660535	protein_codir	putative membrane-bound acid phosphatase 2
TcG_02921	390,5558626	0,08600156	0,09515373	0,90381699	0,36609245	0,59825161	protein_codir	putative short chain 3-hydroxyacyl-CoA dehydrogenase
TcG_02922	10,73271923	0,778458227	0,54778098	1,42111219	0,15528415	1	protein_codir	putative glyceraldehyde 3-phosphate dehydrogenase, cytosolic
TcG_02923	622,6287659	-0,376185023	0,0834905	-4,50572227	6,6148E-06	0,00010674	protein_codir	transportin2-like protein
TcG_02924	469,0446657	-0,383258235	0,0915034	-4,18845886	2,8086E-05	0,00037231	protein_codir	mitochondrial ATP-dependent zinc metallopeptidase
TcG_02925	812,4680027	-0,172206055	0,07443971	-2,3133628	0,0207027	0,08646773	protein_codir	hypothetical protein
TcG_02926	404,5833219	-0,141848397	0,10207904	-1,38959372	0,16465228	0,36998863	protein_codir	hypothetical protein
TcG_02927	32,60698664	0,018266818	0,30818309	0,05927262	0,95273497	0,97762708		
TcG_02928	153,5380963	0,260373958	0,14900444	1,74742415	0,08056379	0,2321481	protein_codir	hypothetical protein
TcG_02929	431,5227451	0,154795881	0,10014713	1,54568463	0,12218075	0,30693543	protein_codir	hypothetical protein
TcG_02930	959,9181358	-0,005346393	0,06352224	-0,08416568	0,93292471	0,96801591	protein_codir	putative dihydrolipoamide acetyltransferase precursor
TcG_02931	292,5714449	-0,016656853	0,11297032	-0,14744451	0,88278118	0,94275074	protein_codir	hypothetical protein
TcG_02932	168,3012364	0,372308107	0,15230359	2,44451298	0,01450479	0,06621675	protein_codir	tRNA methyltransferase
TcG_02933	324,7772968	-0,214678966	0,10284562	-2,08739045	0,03685285	0,13342337	protein_codir	COP9 signalosome complex subunit 12
TcG_02934	301,4634	0,164941016	0,10680392	1,54433483	0,12250724	0,30735575	protein_codir	putative phosphatidylinositol-4-phosphate 5-kinase
TcG_02935	296,8310169	0,184773976	0,10603988	1,74249513	0,08142184	0,23402468	protein_codir	putative protein kinase
TcG_02936	489,5234997	-0,212372812	0,08588645	-2,47271629	0,01340905	0,06241756	protein_codir	putative UDP-glucose/GDP-mannose dehydrogenase family, NAD binding domain
TcG_02937	342,7472119	0,229387349	0,09770744	2,34769579	0,01888994	0,08022822	protein_codir	oligoribonuclease
TcG_02938	196,9292261	0,105907307	0,13171349	0,80407334	0,42135462	0,647488	protein_codir	putative folylpolyglutamate synthase
TcG_02939	158,1164204	-0,20519822	0,14059608	-1,45948752	0,14443097	0,34108791	protein_codir	putative chromatin binding protein
TcG_02940	6,376970003	-1,080268983	0,71146376	-1,51837528	0,12891982	1	protein_codir	hypothetical protein
TcG_02941	1,136480117	-0,044216644	1,66454095	-0,02656387	0,97880759	1		
TcG_02942	0,483491558	-2,708304379	2,49000457	-1,08767045	0,27674062	1		
TcG_02943	810,0665279	-0,117123495	0,0743948	-1,5743505	0,11540644	0,29583886	protein_codir	putative cullin-like protein
TcG_02944	251,5648553	-0,084330363	0,11532519	-0,73123972	0,46463274	0,6818537	protein_codir	hypothetical protein
TcG_02945	61,54516533	0,208409061	0,22057581	0,94484095	0,34474008	0,57877968	protein_codir	RNA-binding protein
TcG_02946	225,6976488	-0,124826704	0,11824326	-1,05567714	0,29111574	0,52365178	protein_codir	putative clathrin coat assembly protein
TcG_02947	197,2540516	0,283832822	0,12963282	2,18951355	0,02855953	0,11004016	protein_codir	putative exosome complex exonuclease RRP41A
TcG_02948	1128,106627	-0,064325846	0,06023269	-1,06795578	0,28554046	0,51910745	protein_codir	superkiller viralicidic activity 2-like 2
TcG_02949	255,6445885	0,022321177	0,12171899	0,18338287	0,85449762	0,92942259	protein_codir	putative GTP-binding protein
TcG_02950	325,6035835	0,174131798	0,10360982	1,68064956	0,092831	0,25533309	protein_codir	bromodomain protein
TcG_02951	143,9841769	0,156201157	0,14686869	1,06354292	0,28753581	0,52052968	protein_codir	hypothetical protein
TcG_02952	66,55429458	-0,217614819	0,21843779	-0,99623247	0,31913721	0,55410215	protein_codir	hypothetical protein
TcG_02953	1809,231143	-0,080109721	0,05347596	-1,49805105	0,13411999	0,32569989	protein_codir	putative succinyl-CoA ligase beta-chain
TcG_02954	334,8976833	-0,00439408	0,1031049	-0,04261757	0,96600639	0,98495028		
TcG_02955	636,4248088	0,359882059	0,07762179	4,63635335	3,5461E-06	6,2345E-05	protein_codir	DNA dependent protein kinase catalytic subunit
TcG_02956	291,8696623	-0,026317168	0,111732	-0,23553833	0,81379093	0,90799131	protein_codir	hypothetical protein
TcG_02957	470,7259465	0,044997229	0,08650431	0,52017327	0,60294282	0,78518697	protein_codir	hypothetical protein
TcG_02958	87,09310506	0,582398359	0,19966017	2,91694809	0,00353475	0,02177509	protein_codir	PHD finger-like domain-containing protein 5A
TcG_02959	279,1608	-0,004784505	0,11398752	-0,04197394	0,96651947	0,98531409	protein_codir	hypothetical protein
TcG_02960	146,957651	0,051757609	0,14980644	0,34549655	0,7297211	0,86068906	protein_codir	hypothetical protein
TcG_02961	379,3905409	0,206010442	0,09560331	2,15484644	0,03117385	0,11758662	protein_codir	hypothetical protein

TcG_02962	239,2580975	0,265792846	0,11758851	2,260364	0,02379867	0,09630855	protein_codir	hypothetical protein
TcG_02963	227,9730886	0,054180131	0,12010298	0,45111395	0,65190742	0,81583489	protein_codir	putative ATP-binding cassette protein
TcG_02964	687,6771972	0,054596488	0,07228344	0,75531112	0,45006233	0,67075149	protein_codir	hypothetical protein
TcG_02965	283,9370367	0,196246513	0,11118929	1,76497678	0,07756767	0,2259741	protein_codir	40S ribosomal protein S24E
TcG_02966	281,2150082	0,526230744	0,11881423	4,42902139	9,4662E-06	0,00014526	protein_codir	40S ribosomal protein S24E
TcG_02967	177,5655561	-0,04460216	0,13515917	-0,32999729	0,74140201	0,86827896	protein_codir	hypothetical protein
TcG_02968	523,2207807	-0,324206536	0,0868784	-3,73172769	0,00019017	0,00191927	protein_codir	putative terminal uridylyltransferase 3
TcG_02969	53,71613451	0,216787	0,24832629	0,87299254	0,38266714	0,61262699	protein_codir	hypothetical protein
TcG_02970	803,3946886	-0,042997902	0,07442276	-0,57775207	0,56343152	0,75668455	protein_codir	putative serine carboxypeptidase S28
TcG_02971	1017,208453	-0,004388399	0,06679512	-0,0656994	0,94761715	0,97539448	protein_codir	hypothetical protein
TcG_02972	561,4259511	0,125468456	0,07975432	1,57318686	0,11567556	0,2960497	protein_codir	ATP-dependent RNA helicase DHX8
TcG_02973	326,1734651	-0,015534516	0,10426677	-0,14898818	0,88156296	0,94218407	protein_codir	hypothetical protein
TcG_02974	68,19844286	-0,239252814	0,21829345	-1,09601465	0,27307236	0,50629163	protein_codir	40S ribosomal protein S24e
TcG_02975	99,47416043	0,269801968	0,17982459	1,50036196	0,13352067	0,3248573	protein_codir	40S ribosomal protein S24e
TcG_02976	429,8564917	-0,127552745	0,08930235	-1,42832457	0,15319846	0,35371808	protein_codir	hypothetical protein
TcG_02977	995,4366501	-0,015157573	0,06595246	-0,22982574	0,81822718	0,91031113	protein_codir	nucleoside-diphosphate kinase
TcG_02978	204,8506016	0,147572149	0,12447925	1,18551606	0,23581347	0,46344584	protein_codir	putative meiotic recombination protein spo11
TcG_02979	122,1708383	0,29488564	0,16300513	1,80905739	0,07044209	0,21281409	protein_codir	hypothetical protein
TcG_02980	80,75957974	0,178491866	0,20211962	0,88310016	0,37718216	0,60795612	protein_codir	hypothetical protein
TcG_02981	520,2127623	-0,065683244	0,08581124	-0,76543868	0,44401037	0,66627433	protein_codir	putative serine/threonine protein phosphatase type 5
TcG_02982	336,52862	-0,167504018	0,10074783	-1,66260667	0,0963912	0,26258838	protein_codir	hypothetical protein
TcG_02983	383,262424	0,039912876	0,09782165	0,4080168	0,68326134	0,83535712	protein_codir	primary-amine oxidase
TcG_02984	32,21621464	0,149949701	0,31114036	0,48193587	0,6298515	0,80168887	protein_codir	hypothetical protein
TcG_02985	6,248051541	-0,556853019	0,68400925	-0,81410159	0,41558675	1	protein_codir	hypothetical protein
TcG_02986	53,74787833	-0,231611025	0,23967033	-0,96637339	0,33385737	0,56824909	protein_codir	hypothetical protein
TcG_02987	256,2076703	0,121219792	0,11086459	1,093404	0,27421646	0,50733448	protein_codir	ubiquitin-conjugating enzyme E2
TcG_02988	693,7679751	-0,095541959	0,07713405	-1,23864824	0,21547579	0,43913853	protein_codir	hypothetical protein
TcG_02989	507,6406146	0,090884888	0,10171285	0,89354383	0,37156601	0,60280866	protein_codir	hypothetical protein
TcG_02990	383,725016	0,033800058	0,09467377	0,35701608	0,72107977	0,85686464	protein_codir	phosphoacetylglucosamine mutase
TcG_02991	533,5566658	-0,126478382	0,08659794	-1,46052407	0,1441461	0,34078152	protein_codir	putative RNA-binding protein
TcG_02992	220,28933	0,128881526	0,12515331	1,02978916	0,30310899	0,53574687	protein_codir	hypothetical protein
TcG_02993	322,6500948	-0,117120428	0,10282539	-1,13902242	0,2546938	0,48614208	protein_codir	chaperone protein DNAJ
TcG_02994	375,1362137	-0,099584823	0,09590729	-1,03834472	0,2991096	0,53274155	protein_codir	putative 6-phosphofructo-2-kinase/fructose-2,6-biphosphatase
TcG_02995	11,84070708	0,334892343	0,54250247	0,61731027	0,53703009	1	protein_codir	hypothetical protein
TcG_02996	191,3206986	0,187942369	0,12699102	1,47996582	0,13888237	0,33300727	protein_codir	chaperone protein DNAJ
TcG_02997	148,6358122	-0,167169083	0,14476891	-1,15473057	0,24820078	0,47744549	protein_codir	hypothetical protein
TcG_02998	104,4286369	0,112677159	0,18298437	0,61577479	0,53804317	0,73746222	protein_codir	hypothetical protein
TcG_02999	260,8619297	0,160632484	0,11425459	1,40591708	0,15974875	0,36241414	protein_codir	hypothetical protein
TcG_03000	150,9355868	-0,005940116	0,15144313	-0,03922341	0,96871227	0,98659462	protein_codir	putative protein phosphatase inhibitor
TcG_03001	3,527675798	0,982717658	0,95662669	1,02727393	0,3042915	1	protein_codir	putative Protein kinase domain
TcG_03002	1,079552525	1,856485987	1,90000112	0,97709731	0,32852098	1		
TcG_03003	1121,844493	0,027567955	0,06293464	0,43804103	0,66135654	0,82259548	protein_codir	hypothetical protein
TcG_03004	292,4605527	0,034164179	0,10896593	0,31353082	0,75387741	0,87580704	protein_codir	putative malonyl-CoA decarboxylase, mitochondrial precursor
TcG_03005	732,2502035	0,238098131	0,07626103	3,12214667	0,00179537	0,01262209	protein_codir	vacuolar protein sorting protein 18
TcG_03006	661,3803998	0,171578728	0,0792102	2,16611914	0,03030208	0,11525934	protein_codir	hypothetical protein
TcG_03007	217,1530048	0,084246391	0,1223403	0,68862339	0,4910603	0,70187819	protein_codir	centrin
TcG_03008	561,9603505	-0,037465028	0,07744155	-0,48378461	0,62853874	0,80086328	protein_codir	putative DNA polymerase sigma
TcG_03009	996,8466729	-0,232220398	0,06823893	-3,4030484	0,00066638	0,00551087	protein_codir	putative protein kinase
TcG_03010	158,9717041	-0,102835039	0,14601702	-0,70426747	0,48126622	0,69542909	protein_codir	putative Qa-SNARE protein
TcG_03011	350,0070869	0,237166637	0,09750822	2,43227337	0,01500438	0,06774588	protein_codir	putative serine/threonine protein phosphatase
TcG_03012	227,0843504	0,254106678	0,12246009	2,07501629	0,03798503	0,13614561	protein_codir	hypothetical protein
TcG_03013	414,2542856	-0,020548151	0,09165482	-0,22419061	0,82260899	0,91212056	protein_codir	hypothetical protein

TcG_03014	502,3859099	-0,089240124	0,08196822	-1,08871614	0,27627908	0,5087364	protein_codir	putative vacuolar-type Ca2+-ATPase
TcG_03015	672,5826854	-0,026927724	0,0719924	-0,37403566	0,70837779	0,84943749	protein_codir	hypothetical protein
TcG_03016	70,11154966	0,026565098	0,20578562	0,12909113	0,89728554	0,95018119	protein_codir	hypothetical protein
TcG_03017	346,4288407	0,101989588	0,09725251	1,04870909	0,29431203	0,52725669	protein_codir	hypothetical protein
TcG_03018	142,5496079	0,243555525	0,14818522	1,64358854	0,10026122	0,26920661	protein_codir	hypothetical protein
TcG_03019	197,0053704	0,055752403	0,14124153	0,39473094	0,69304147	0,8410627	protein_codir	hypothetical protein
TcG_03020	0,300973289	-0,458748581	3,01449841	-0,15218073	0,87904439	1	protein_codir	hypothetical protein
TcG_03021	122,475066	-0,019862041	0,16587345	-0,11974214	0,90468742	0,95370277	protein_codir	putative electron transfer flavoprotein-ubiquinone oxidoreductase
TcG_03022	49,62591384	0,069622591	0,25497241	0,27305931	0,78480763	0,89346381	protein_codir	electron transfer flavoprotein-ubiquinone oxidoreductase
TcG_03023	816,3025958	-0,094241023	0,06716538	-1,40311898	0,16058136	0,36344905	protein_codir	hypothetical protein
TcG_03024	417,1882151	0,075950579	0,09249347	0,82114533	0,41156349	0,63808037	protein_codir	NUC156 family protein
TcG_03025	121,9233252	-0,022841639	0,16593596	-0,13765333	0,8905144	0,94667588	protein_codir	hypothetical protein
TcG_03026	700,8757391	0,097740974	0,07117695	1,37321106	0,16968674	0,37606704	protein_codir	hypothetical protein
TcG_03027	329,9201108	0,020357781	0,11454986	0,17771982	0,85894302	0,93207022	protein_codir	hypothetical protein
TcG_03028	227,0719797	-0,155085317	0,12514244	-1,23927039	0,21524538	0,4388705	protein_codir	hypothetical protein
TcG_03029	342,9250977	0,175385241	0,09827544	1,78462944	0,07432144	0,22015933	protein_codir	hypothetical protein
TcG_03030	217,1481427	0,052473884	0,12159229	0,43155601	0,66606414	0,82519673	protein_codir	putative mitochondrial carrier protein
TcG_03031	224,4784116	0,062788494	0,12901315	0,48668292	0,62648305	0,79939806	protein_codir	putative QA-SNARE protein
TcG_03032	5000,3046	0,202117389	0,04508886	4,48264533	7,3723E-06	0,00011733	protein_codir	putative 60S ribosomal protein L7a
TcG_03033	209,065741	0,437854499	0,13880718	3,15440809	0,00160824	0,01153037	protein_codir	putative 60S ribosomal protein L7a
TcG_03034	213,8974925	-0,160071818	0,12321999	-1,29907345	0,19391872	0,41081411	protein_codir	hypothetical protein
TcG_03035	257,6214656	0,167100873	0,11325886	1,47538892	0,14010801	0,3345817	protein_codir	hypothetical protein
TcG_03036	278,8227719	0,178785114	0,11508972	1,55344119	0,12031777	0,30370407	protein_codir	hypothetical protein
TcG_03037	676,6341058	0,227163344	0,15129379	1,50147169	0,1332336	0,32443138	protein_codir	hypothetical protein
TcG_03038	70,53583412	0,357607744	0,21321229	1,67723792	0,09349597	0,25669295	protein_codir	hypothetical protein
TcG_03039	94,5263414	-0,160283086	0,18152928	-0,88295997	0,3772579	0,60795612	protein_codir	hypothetical protein
TcG_03040	20,74614295	-0,132103072	0,3983792	-0,33160133	0,74019032	0,86756146		
TcG_03041	526,1910627	-0,045713594	0,08461443	-0,54025764	0,58901937	0,77564856	protein_codir	protein kinase, putative,NIMA/Nek Serine/threonine-protein kinase family
TcG_03042	539,7633479	-0,234143707	0,07982156	-2,9333393	0,00335337	0,0208211	protein_codir	small GTP-binding protein RAB6
TcG_03043	109,1125395	0,152402052	0,16615372	0,91723528	0,35901937	0,5926302	protein_codir	hypothetical protein
TcG_03044	528,2727386	-0,646836778	0,0869855	-7,43614518	1,0367E-13	9,3106E-12	protein_codir	hypothetical protein
TcG_03045	323,2016709	0,137421994	0,11002404	1,24901793	0,21165852	0,43456948	protein_codir	ribosomal RNA processing protein 41B
TcG_03046	107,8355274	0,445389691	0,1820169	2,44696892	0,01440633	0,06584288	protein_codir	hypothetical protein
TcG_03047	695,4869549	-0,162362253	0,072943	-2,22587842	0,02602233	0,10279396	protein_codir	putative histone deacetylase
TcG_03048	184,6857358	0,268236661	0,1318003	2,03517488	0,04183328	0,14581239	protein_codir	cytochrome b-domain protein
TcG_03049	232,9277744	0,264686723	0,11929391	2,21877815	0,02650182	0,1042615	protein_codir	hypothetical protein
TcG_03050	470,6318618	-0,121301523	0,08907508	-1,3617896	0,17326429	0,38164261	protein_codir	hypothetical protein
TcG_03051	718,4568435	0,157564514	0,06996601	2,25201527	0,02432131	0,09782343	protein_codir	hypothetical protein
TcG_03052	230,2748216	0,135308878	0,12656453	1,06909005	0,28502909	0,51877427	protein_codir	hypothetical protein
TcG_03053	144,4535652	0,386223854	0,15080262	2,56112163	0,01043348	0,05111303	protein_codir	hypothetical protein
TcG_03054	1144,222068	0,105060283	0,06844803	1,5348913	0,12481053	0,31118029	protein_codir	hypothetical protein
TcG_03055	827,651195	-0,240595608	0,07237738	-3,32418232	0,00088678	0,0070662	protein_codir	hypothetical protein
TcG_03056	1357,138272	-0,051136254	0,05558368	-0,9199869	0,35757961	0,59135536	protein_codir	exportin T (tRNA exportin)-like protein
TcG_03057	2031,563581	0,097932364	0,06082429	1,61008639	0,107379	0,28223527	protein_codir	phosphatidylinositol kinase related protein
TcG_03058	367,0119588	-0,034810059	0,0974388	-0,3572505	0,72090428	0,85674398	protein_codir	hypothetical protein
TcG_03059	7,53518199	0,612890047	0,64781006	0,9460953	0,34409998	1	protein_codir	SMUG L protein
TcG_03060	29,34535036	0,489856816	0,33303953	1,47086687	0,14132712	0,3366698		
TcG_03061	532,09376	0,439631187	0,08895835	4,94198885	7,733E-07	1,6085E-05	protein_codir	hypothetical protein
TcG_03062	100,6134214	-0,667805856	0,17471872	-3,82217683	0,00013228	0,00140804		
TcG_03063	591,5544932	-0,397218053	0,07941648	-5,00170782	5,6825E-07	1,2283E-05	protein_codir	putative surface antigen TASV, putative,mucin-like glycoprotein
TcG_03064	152,3851724	-0,054219608	0,15352848	-0,35315669	0,72397098	0,85783675	protein_codir	hypothetical protein
TcG_03065	134,7452775	-0,055772094	0,15814688	-0,3526601	0,72434328	0,85798219	protein_codir	hypothetical protein

TcG_03066	90,13801254	-0,100688922	0,18728798	-0,5376155	0,59084253	0,77668571	protein_codir	putative target of rapamycin (TOR) kinase 1
TcG_03067	172,0042021	-0,061950146	0,13776685	-0,44967381	0,65294566	0,8163406	protein_codir	trans-sialidase
TcG_03068	100,0031938	0,175074113	0,18304385	0,95645997	0,3388399	0,57327673	protein_codir	hypothetical protein
TcG_03069	1112,259138	0,029672508	0,06283025	0,47226468	0,63673788	0,8066089	protein_codir	phosphoric diester hydrolase
TcG_03070	878,9312667	-0,154150428	0,06714526	-2,29577518	0,02168874	0,08958146	protein_codir	hypothetical protein
TcG_03071	1141,761271	-0,047525502	0,0597624	-0,79524084	0,42647342	0,65178385	protein_codir	hypothetical protein
TcG_03072	971,7966046	-0,145561649	0,0649411	-2,24144094	0,02499753	0,09969754	protein_codir	hypothetical protein
TcG_03073	323,4707591	-0,045899212	0,10076607	-0,45550266	0,64874765	0,81328019	protein_codir	hypothetical protein
TcG_03074	763,2535862	0,018107437	0,07035707	0,25736486	0,79689712	0,89872949	protein_codir	UDP-GlcNAc:polypeptide N-acetylglucosaminyltransferase
TcG_03075	372,5904665	-0,044411993	0,09274314	-0,4788709	0,63203047	0,80290602	protein_codir	hypothetical protein
TcG_03076	627,2931353	-0,424908484	0,07921743	-5,36382568	8,1478E-08	2,1552E-06	protein_codir	casein kinase II, alpha chain
TcG_03077	41,37327266	-0,058514357	0,28714739	-0,20377813	0,83852689	0,92148084		
TcG_03078	896,972065	-0,027442384	0,06984731	-0,39289107	0,69439994	0,84191269	protein_codir	casein kinase II, alpha chain
TcG_03079	90,00987874	0,224565232	0,19984633	1,12368957	0,26114474	0,49358779	protein_codir	casein kinase II, alpha chain
TcG_03080	696,1311619	-0,352095804	0,07194285	-4,89410419	9,8755E-07	2,0108E-05	protein_codir	proteasome regulatory non-ATP-ase subunit 6
TcG_03081	395,3863394	-0,067230299	0,09481336	-0,70908047	0,47827454	0,69283432	protein_codir	putative ribosomal RNA methyltransferase
TcG_03082	330,255499	-0,23269188	0,10156981	-2,29095517	0,02196601	0,09040787	protein_codir	putative ubiquitin-conjugating enzyme e2
TcG_03083	802,0439234	-0,059389209	0,0690554	-0,86002263	0,38977657	0,6201653	protein_codir	putative RNA-editing complex protein MP81
TcG_03084	1025,903611	0,030608025	0,06134761	0,49892776	0,61783027	0,793034	protein_codir	hypothetical protein
TcG_03085	273,7958182	0,223718421	0,10866445	2,05880045	0,03951336	0,14000054	protein_codir	hypothetical protein
TcG_03086	390,8376981	-0,03921705	0,10343941	-0,37913065	0,70459084	0,84755533	protein_codir	serine peptidase, Clan S-, family S54
TcG_03087	3143,090859	0,207899791	0,04715947	4,40844163	1,0412E-05	0,00015707	protein_codir	putative voltage-dependent anion-selective channel
TcG_03088	832,0997972	0,181355509	0,06669344	2,71924039	0,0065432	0,03545817	protein_codir	hypothetical protein
TcG_03089	1329,497128	-0,002900213	0,06135653	-0,0472682	0,96229947	0,98256823	protein_codir	hypothetical protein
TcG_03090	1281,261903	-0,085336842	0,06075982	-1,40449472	0,16017158	0,36306159	protein_codir	hypothetical protein
TcG_03091	260,8092717	0,171237167	0,11043019	1,5506373	0,12098863	0,30498617	protein_codir	hypothetical protein
TcG_03092	237,7442779	0,256314882	0,12116706	2,11538422	0,03439721	0,12687874	protein_codir	hypothetical protein
TcG_03093	0					1	protein_codir	putative glycerate kinase
TcG_03094	2278,229578	0,780000361	0,05142578	15,1674967	5,8049E-52	9,6079E-49	protein_codir	putative glycerate kinase
TcG_03095	2,589718447	0,810428993	1,10010132	0,73668578	0,46131346	1	protein_codir	putative glycerate kinase
TcG_03096	194,7859718	-0,086479259	0,12927494	-0,66895609	0,50352349	0,7119628	protein_codir	hypothetical protein
TcG_03097	490,1977877	0,044185751	0,08823165	0,50079251	0,61651716	0,79265743	protein_codir	putative imidazolonepropionase
TcG_03098	1579,813923	-0,103942287	0,06363725	-1,63335616	0,10239411	0,27334981	protein_codir	hypothetical protein
TcG_03099	471,9487852	-0,0382714	0,17220518	-0,22224303	0,8241247	0,91345152	protein_codir	hypothetical protein
TcG_03100	45,2686816	-0,093343657	0,25673627	-0,363578	0,71617316	0,85427594	protein_codir	hypothetical protein
TcG_03101	44,27361363	-0,087481597	0,28038619	-0,31200394	0,75503753	0,87636394	protein_codir	hypothetical protein
TcG_03102	143,5036533	0,573739834	0,16594355	3,45743977	0,00054533	0,0046698	protein_codir	hypothetical protein
TcG_03103	305,3020781	-0,142836826	0,10498873	-1,36049668	0,1736728	0,38225172	protein_codir	hypothetical protein
TcG_03104	860,6156854	0,216923921	0,07174826	3,02340324	0,00249949	0,01644365	protein_codir	SMUG S protein
TcG_03105	248,504666	0,289348236	0,12428272	2,32814542	0,01990438	0,08364606	protein_codir	mucin TcSMUGS
TcG_03106	47,31307242	-0,145478179	0,28194061	-0,51598873	0,60586228	0,78692126		
TcG_03107	41,84346846	0,371481352	0,2826899	1,3140949	0,18881427	0,40323552	protein_codir	hypothetical protein
TcG_03108	50,45289687	0,281983319	0,24696797	1,14178096	0,25354508	0,48450821	protein_codir	hypothetical protein
TcG_03109	22,37037849	-0,459069216	0,38201652	-1,2016999	0,22947982	0,45668697	protein_codir	hypothetical protein
TcG_03110	22,86532153	0,339956809	0,36700059	0,92631134	0,35428421	0,58832404	protein_codir	hypothetical protein
TcG_03111	493,9464342	-0,464464308	0,08471287	-5,48280661	4,1863E-08	1,1744E-06	protein_codir	zinc finger protein
TcG_03112	142,957373	-0,003676387	0,14621076	-0,02514444	0,97993975	0,9919484	protein_codir	putative ras-related protein rab
TcG_03113	305,6319378	-0,14091214	0,10573418	-1,33270191	0,18262965	0,39470131	protein_codir	ribosome biogenesis protein ENP2
TcG_03114	1429,079784	-0,245734445	0,05538965	-4,43646865	9,1447E-06	0,00014164	protein_codir	70 kDa heat shock protein
TcG_03115	208,797826	-0,093194738	0,12803851	-0,72786493	0,46669627	0,68332403	protein_codir	hypothetical protein
TcG_03116	581,2345787	-0,039423471	0,08017256	-0,49173275	0,62290828	0,79676839	protein_codir	hypothetical protein
TcG_03117	290,974237	-0,226246715	0,10742042	-2,10617973	0,03518874	0,12905881	protein_codir	hypothetical protein

TcG_03118	356,4129271	-0,009265997	0,09816758	-0,09438958	0,92479969	0,96320831	protein_codir	putative chaperone protein DNAj
TcG_03119	337,8745695	-0,172098812	0,10400557	-1,65470767	0,09798381	0,2650573	protein_codir	hypothetical protein
TcG_03120	283,2278241	-0,254774057	0,10530656	-2,41935605	0,01554801	0,0696056	protein_codir	hypothetical protein
TcG_03121	25,78173477	-0,292944385	0,34899481	-0,83939467	0,40124788	0,63172413		
TcG_03122	167,3975718	0,057199182	0,13681472	0,41807769	0,67589031	0,83017833	protein_codir	hypothetical protein
TcG_03123	211,476981	0,07477221	0,1287385	0,58080691	0,5613706	0,75484505	protein_codir	tRNA pseudouridine synthase 9
TcG_03124	156,0003044	0,184549294	0,14631271	1,26133465	0,2071883	0,42942463	protein_codir	hypothetical protein
TcG_03125	297,8509385	-0,255744091	0,1037632	-2,46468976	0,01371319	0,06340026	protein_codir	hypothetical protein
TcG_03126	269,6297003	-0,204287399	0,10981506	-1,86028584	0,0628451	0,19625964	protein_codir	hypothetical protein
TcG_03127	203,2381332	-0,209546336	0,1288042	-1,62685943	0,10376696	0,27606061	protein_codir	dynein arm light chain, axonemal
TcG_03128	691,6816733	0,132015615	0,07734612	1,70681627	0,08785621	0,24598889	protein_codir	telomerase-associated protein
TcG_03129	270,5358094	0,174866231	0,13005502	1,3445558	0,17876873	0,38979933	protein_codir	putative telomerase-associated protein
TcG_03130	467,1666681	-0,114900578	0,08419846	-1,36463993	0,17236625	0,38004187	protein_codir	hypothetical protein
TcG_03131	172,0705987	0,094416824	0,14471402	0,65243731	0,51411911	0,72008994	protein_codir	hypothetical protein
TcG_03132	529,8697185	-0,085754053	0,08168372	-1,04983037	0,29379611	0,52684725	protein_codir	hypothetical protein
TcG_03133	203,0022764	0,000934283	0,12311721	0,00758856	0,99394526	0,99767711	protein_codir	hypothetical protein
TcG_03134	323,0906945	-0,079909812	0,1084697	-0,73670172	0,46130376	0,67929148	protein_codir	putative RNA-binding protein
TcG_03135	96,90314462	0,076818692	0,17779841	0,43205499	0,66570145	0,82504261	protein_codir	electron transfer protein
TcG_03136	408,9409049	0,032051047	0,08936451	0,35865521	0,71985304	0,85619724	protein_codir	nicalin
TcG_03137	299,7077064	-0,154760606	0,10616233	-1,45777328	0,14490305	0,34175483	protein_codir	hypothetical protein
TcG_03138	43,67851317	0,311493302	0,28058975	1,11013784	0,26693963	0,49939561	protein_codir	aldo/keto reductase
TcG_03139	131,3671101	0,093496198	0,16287637	0,57403169	0,56594636	0,7582163	protein_codir	aldo/keto reductase
TcG_03140	926,2806584	-0,404026635	0,06490816	-6,22458879	4,8282E-10	2,0795E-08	protein_codir	dynein, axonemal, heavy polypeptide 1
TcG_03141	83,94735764	-0,21172762	0,19129326	-1,10682217	0,26837082	0,50094157	protein_codir	hypothetical protein
TcG_03142	258,0500553	-0,063416004	0,11299921	-0,5612075	0,57465609	0,76564508	protein_codir	hypothetical protein
TcG_03143	613,8811149	-0,153773874	0,07684056	-2,00120704	0,04537008	0,1543782	protein_codir	putative protein kinase C substrate protein, heavy chain
TcG_03144	275,1344586	-0,253764588	0,11032716	-2,30010985	0,021442	0,08880394	protein_codir	hypothetical protein
TcG_03145	63,41343762	0,12695027	0,23016003	0,55157392	0,58124031	0,77048234	protein_codir	putative protein kinase
TcG_03146	515,1265989	-0,148665537	0,0921617	-1,61309455	0,10672397	0,28147141	protein_codir	nonsense-mediated mRNA decay protein 3
TcG_03147	178,4950533	0,114220239	0,13593839	0,84023533	0,40077645	0,63106768	protein_codir	hypothetical protein
TcG_03148	470,0942685	-0,211945981	0,08607988	-2,46220111	0,01380872	0,06374018	protein_codir	hypothetical protein
TcG_03149	827,421861	-0,16656295	0,07305339	-2,2800168	0,02260669	0,09261709	protein_codir	putative chaperone DNAJ protein
TcG_03150	439,6583395	-0,144814577	0,09329131	-1,55228367	0,12059436	0,3041372	protein_codir	hypothetical protein
TcG_03151	93,32282842	0,054868632	0,18199182	0,30148956	0,76304121	0,88027437	protein_codir	hypothetical protein
TcG_03152	103,6247979	0,002609826	0,17375526	0,01502013	0,98801612	0,99514516	protein_codir	hypothetical protein
TcG_03153	460,1178736	-0,0858466	0,08541543	-1,00504787	0,3148738	0,54958238	protein_codir	heat shock 70 protein-like protein
TcG_03154	515,0574082	0,257548007	0,09158843	2,81201464	0,00492323	0,02846332	protein_codir	putative 40S ribosomal protein S16
TcG_03155	115,8235271	0,255580664	0,17348331	1,47322908	0,14068928	0,33546531	protein_codir	40S ribosomal protein S16
TcG_03156	355,8913785	-0,132944118	0,09773539	-1,36024543	0,17375227	0,382354	protein_codir	hypothetical protein
TcG_03157	599,6801956	0,151851364	0,07652051	1,98445318	0,04720534	0,15875794	protein_codir	RNA editing complex protein MP100
TcG_03158	342,6230406	0,059597941	0,10757928	0,55399089	0,57958508	0,76901886	protein_codir	hypothetical protein
TcG_03159	315,9876353	0,159040822	0,10628047	1,49642562	0,13454278	0,32611143	protein_codir	hypothetical protein
TcG_03160	745,2888407	-0,00147981	0,0693313	-0,02134404	0,98297122	0,99369442	protein_codir	hypothetical protein
TcG_03161	1069,959393	-0,193423562	0,06331241	-3,05506576	0,00225011	0,01513047	protein_codir	putative asparagine synthetase a
TcG_03162	223,1380978	0,20614802	0,1218689	1,69155564	0,09073073	0,25136448	protein_codir	hypothetical protein
TcG_03163	278,0140039	0,265166227	0,10885926	2,4358628	0,01485632	0,06732284	protein_codir	glutathione peroxidase-like protein
TcG_03164	572,1170519	-0,197512587	0,07840822	-2,51902921	0,01176789	0,05610813	protein_codir	trypanothione/tryparedoxin dependent peroxidase 2
TcG_03165	585,6342128	-0,114103313	0,0814991	-1,40005615	0,16149651	0,36513618	protein_codir	putative eukaryotic initiation factor 4a
TcG_03166	94,01586825	-0,20633968	0,18798529	-1,09763737	0,27236287	0,50538055	protein_codir	hypothetical protein
TcG_03167	268,8809499	-0,017172782	0,11048094	-0,1554366	0,87647713	0,94087501	protein_codir	hypothetical protein
TcG_03168	423,4463002	-0,337718976	0,08815192	-3,8311016	0,00012757	0,00136855	protein_codir	hypothetical protein
TcG_03169	226,5282015	0,371711225	0,1221003	3,0443104	0,00233214	0,01558259	protein_codir	hypothetical protein

TcG_03170	611,0162406	-0,365060537	0,0797297	-4,57872685	4,6781E-06	7,901E-05	protein_codir putative protein kinase
TcG_03171	316,7121528	-0,154353195	0,10189563	-1,51481663	0,12981885	0,31940564	protein_codir putative ubiquinone biosynthesis methyltransferase
TcG_03172	28,29898157	0,215654126	0,33021589	0,65307011	0,51371108	0,71977949	
TcG_03173	254,969102	0,250016776	0,11946909	2,09273183	0,0363731	0,13235513	protein_codir hypothetical protein
TcG_03174	374,3896444	-0,100954333	0,09537972	-1,0584465	0,28985193	0,52327695	protein_codir hypothetical protein
TcG_03175	628,8118287	0,086450422	0,07916536	1,09202339	0,27482282	0,50755645	protein_codir U3 small nucleolar RNA-associated protein 21
TcG_03176	401,9196547	-0,147697105	0,10035675	-1,47172075	0,1410963	0,33629742	protein_codir hypothetical protein
TcG_03177	228,5081127	0,346365913	0,11999923	2,88640112	0,00389675	0,02360618	protein_codir GINS complex subunit 2
TcG_03178	200,419445	-0,057215357	0,1271019	-0,45015342	0,65259982	0,81626055	protein_codir putative UDP-N-acetylglucosamine-dolichyl-phosphate N-acetylglucosaminephosphotransferase
TcG_03179	166,3170491	-0,029840515	0,13970622	-0,21359475	0,83086309	0,91760178	protein_codir hypothetical protein
TcG_03180	422,1058729	-0,392551412	0,09129582	-4,29977417	1,7097E-05	0,00024199	protein_codir flagellar associated protein
TcG_03181	205,2393217	0,061830763	0,12591602	0,49104764	0,62339276	0,79702106	protein_codir DNA-directed RNA polymerase
TcG_03182	190,1280909	-0,102423404	0,1340596	-0,76401396	0,44485892	0,66677659	protein_codir hypothetical protein
TcG_03183	205,8524209	0,249657543	0,1301642	1,91802009	0,05510846	0,17725893	protein_codir hypothetical protein
TcG_03184	283,8267027	-0,176872728	0,11157007	-1,58530626	0,11289677	0,29124976	protein_codir hypothetical protein
TcG_03185	165,0159732	-0,130079045	0,13728826	-0,9474885	0,34338992	0,57760098	protein_codir hypothetical protein
TcG_03186	284,834832	0,141171423	0,10657075	1,3246733	0,18527953	0,39893118	protein_codir putative glycosyl hydrolase-like protein
TcG_03187	299,8650774	0,227923054	0,10955812	2,08038479	0,03749025	0,13489505	protein_codir putative glycosyl hydrolase-like protein
TcG_03188	239,9146198	-0,225736026	0,11377052	-1,98413469	0,04724083	0,15883117	protein_codir hypothetical protein
TcG_03189	183,9618888	-0,064556576	0,13162382	-0,49046273	0,6238065	0,79706621	protein_codir putative spermatogenesis-associated protein 17-like
TcG_03190	9,359158266	-0,059023084	0,56461761	-0,10453639	0,9167437	1	protein_codir hypothetical protein
TcG_03191	10,10679842	-0,582497728	0,55712755	-1,04553748	0,29577463	1	protein_codir hypothetical protein
TcG_03192	105,4894185	0,197068057	0,18460117	1,06753418	0,28573069	0,51912745	protein_codir putative phosphoglycerate mutase family member 5
TcG_03193	38,57688431	0,255321093	0,28442352	0,89767924	0,36935658	0,60112883	
TcG_03194	355,4355923	-0,179310572	0,10254948	-1,74852734	0,08037276	0,2318722	protein_codir hypothetical protein
TcG_03195	325,081436	0,174667269	0,10351455	1,68736921	0,09153238	0,25268603	protein_codir Cut9 interacting protein Scn1
TcG_03196	604,6659794	0,018201745	0,07696245	0,23650162	0,81304345	0,90759432	protein_codir hypothetical protein
TcG_03197	491,7399191	-0,155433238	0,08414518	-1,84720317	0,0647177	0,20037928	protein_codir putative zinc carboxypeptidase, putative,metallo-peptidase, Clan MC, Family M14
TcG_03198	156,3636159	-0,077257834	0,14262874	-0,54167087	0,58804526	0,77564856	protein_codir putative Golgi SNARE protein-like
TcG_03199	165,5620125	-0,197480848	0,13988414	-1,41174576	0,15802483	0,36012504	protein_codir hypothetical protein
TcG_03200	149,940214	0,159009708	0,14342282	1,10867789	0,26756916	0,49992998	protein_codir hypothetical protein
TcG_03201	554,9696346	0,093081976	0,07806878	1,19230736	0,23314075	0,46049525	protein_codir hslvu complex proteolytic subunit-like
TcG_03202	354,7740911	-0,038650083	0,09655271	-0,40030032	0,68893533	0,83844588	protein_codir parafibromin
TcG_03203	202,771108	-0,03857702	0,12842051	-0,30039609	0,76387504	0,88092016	protein_codir hypothetical protein
TcG_03204	386,8493104	-0,137658701	0,09577798	-1,43726886	0,15064161	0,35066447	protein_codir hypothetical protein
TcG_03205	255,758032	0,158512452	0,11657256	1,35977496	0,17390115	0,38246369	protein_codir putative glycosyl transferase-like
TcG_03206	339,8156201	-0,263876349	0,10749561	-2,45476393	0,01409771	0,06479021	protein_codir hypothetical protein
TcG_03207	389,3451103	0,073566266	0,09758507	0,75386805	0,45092845	0,67161389	protein_codir putative telomerase reverse transcriptase
TcG_03208	530,759955	-0,205644712	0,08130875	-2,52918297	0,01143284	0,05496303	protein_codir hypothetical protein
TcG_03209	166,7273316	0,176699798	0,13916055	1,26975494	0,20417194	0,42530314	protein_codir hypothetical protein
TcG_03210	527,443117	0,308430335	0,08354399	3,69183169	0,00022264	0,00218053	protein_codir 60S ribosomal protein L22
TcG_03211	970,6523101	-0,223082074	0,06279868	-3,55233707	0,00038183	0,00343732	protein_codir hypothetical protein
TcG_03212	295,8735122	0,047326476	0,11459499	0,41298905	0,67961464	0,83278849	protein_codir hypothetical protein
TcG_03213	311,9011508	0,186122849	0,10938831	1,70148763	0,08885146	0,24805614	protein_codir hypothetical protein
TcG_03214	374,9510933	-0,211354933	0,09605886	-2,20026492	0,0277881	0,10793286	protein_codir hypothetical protein
TcG_03215	249,5439841	-0,065181693	0,11506628	-0,56647085	0,57107374	0,7624407	protein_codir putative actin-like protein
TcG_03216	196,6866422	-0,342884162	0,12633826	-2,7140169	0,00664728	0,03585445	protein_codir hypothetical protein
TcG_03217	273,0306273	-0,299250168	0,10913304	-2,74206748	0,00610538	0,03347701	protein_codir flagella associated protein
TcG_03218	493,4415073	-0,090109782	0,08399834	-1,07275664	0,28338032	0,51680221	protein_codir hypothetical protein
TcG_03219	534,6139762	-0,040520426	0,09555257	-0,42406423	0,67151897	0,82777091	protein_codir hypothetical protein
TcG_03220	223,3852767	-0,213679288	0,12080132	-1,768849	0,07691908	0,22464946	protein_codir putative variant surface glycoprotein
TcG_03221	140,4916922	0,166959736	0,15691903	1,06398651	0,28733481	0,52042924	protein_codir hypothetical protein

TcG_03222	311,2735323	-0,303499938	0,10401786	-2,91776765	0,00352547	0,02174707	protein_codir	hypothetical protein
TcG_03223	499,9422113	0,105026636	0,08220175	1,27766909	0,20136613	0,42089626	protein_codir	putative biotin/lipoate protein ligase
TcG_03224	115,3456704	-0,067929058	0,17550251	-0,38705463	0,69871577	0,84414248	protein_codir	hypothetical protein
TcG_03225	108,1136421	0,173557529	0,18011078	0,96361543	0,33523877	0,56976329	protein_codir	hypothetical protein
TcG_03226	413,5553455	0,590575827	0,09484544	6,22671841	4,7631E-10	2,0591E-08		
TcG_03227	143,380101	0,46815916	0,15102033	3,09997433	0,00193537	0,01338701		
TcG_03228	455,8016309	0,001771583	0,08617847	0,02055714	0,98359893	0,99376391	protein_codir	short-chain dehydrogenase
TcG_03229	49,80351062	-0,407907894	0,26640936	-1,531132	0,12573677	0,31295086		
TcG_03230	411,1252636	0,083342714	0,08917789	0,93456701	0,35001149	0,58454758	protein_codir	putative vacuolar protein sorting-associated protein-like
TcG_03231	382,7993903	-0,164404711	0,09213853	-1,78432096	0,07437153	0,22015933	protein_codir	Sec14 cytosolic factor
TcG_03232	273,3756198	-0,043944301	0,1069052	-0,41105859	0,68102957	0,83364878	protein_codir	putative SNF7-like protein
TcG_03233	185,4181573	-0,144725055	0,13014342	-1,11204279	0,26611975	0,49882923	protein_codir	putative glycosyl transferase-like protein
TcG_03234	399,8332643	-0,088791753	0,09694004	-0,91594506	0,35969571	0,5926302	protein_codir	putative protein NipSnap 3A-like
TcG_03235	1057,974586	-0,245163558	0,06165554	-3,9763428	6,9983E-05	0,00082589	protein_codir	putative alpha-ketoglutarate dehydrogenase complex subunit Kgd1
TcG_03236	1532,190856	0,375193227	0,05329759	7,03959128	1,928E-12	1,4017E-10	protein_codir	mucin-like glycoprotein
TcG_03237	27,38463115	-0,342619656	0,32714441	-1,04730403	0,29495938	0,52793889	protein_codir	dispersed gene family protein 1 (DGF-1)
TcG_03238	4,827022198	-0,608563712	0,78935085	-0,77096732	0,4407263	1	protein_codir	dispersed gene family protein 1 (DGF-1)
TcG_03239	505,6716884	-0,404081138	0,08297256	-4,87005774	1,1157E-06	2,2286E-05	protein_codir	transferase
TcG_03240	801,357914	-0,112165279	0,07064673	-1,58769252	0,11235589	0,29018174	protein_codir	hydantoinase/oxoprolinase
TcG_03241	315,3571394	-0,170875064	0,10242279	-1,66833057	0,09525012	0,26009143	protein_codir	alkaline phosphatase
TcG_03242	23,49098882	-0,338110911	0,37350777	-0,90523126	0,36534288	0,59752437	protein_codir	putative trans-sialidase
TcG_03243	71,28664303	-0,340385785	0,20490157	-1,66121608	0,09667006	0,26300388	protein_codir	hypothetical protein
TcG_03244	345,1246169	-0,363810167	0,09752658	-3,73036933	0,0001912	0,00192797	protein_codir	hypothetical protein
TcG_03245	462,2565422	-0,125195122	0,09274795	-1,34984244	0,17706653	0,38718302	protein_codir	hypothetical protein
TcG_03246	241,943767	-0,505903332	0,11528454	-4,38830154	1,1424E-05	0,00017013	protein_codir	dynein light chain
TcG_03247	134,848484	-0,055292668	0,15356054	-0,36007081	0,71879418	0,85564054	protein_codir	putative mitochondrial carrier protein
TcG_03248	434,3296128	-0,224937459	0,08982416	-2,50419778	0,01227294	0,05803849	protein_codir	putative UTP-glucose-1-phosphate uridylyltransferase 2
TcG_03249	198,0018589	-0,300936253	0,14107224	-2,1332068	0,03290777	0,12255528	protein_codir	hypothetical protein
TcG_03250	399,5697361	-0,258108456	0,09331738	-2,76592066	0,00567623	0,03160252	protein_codir	hypothetical protein
TcG_03251	440,9915864	-0,382824527	0,08748873	-4,37570099	1,2104E-05	0,00017865	protein_codir	putative mannosyltransferase-II
TcG_03252	134,2100436	-0,177255604	0,15350039	-1,15475668	0,24819008	0,47744549	protein_codir	hypothetical protein
TcG_03253	169,0177272	-0,159507751	0,1345741	-1,18527821	0,23590746	0,46344584	protein_codir	hypothetical protein
TcG_03254	987,4724509	-0,218237703	0,06608997	-3,3021304	0,00095953	0,00753635	protein_codir	hypothetical protein
TcG_03255	425,979181	-0,071499181	0,0900612	-0,79389547	0,42725629	0,65245701	protein_codir	hypothetical protein
TcG_03256	180,1229274	-0,279548254	0,13983935	-1,99906722	0,04560108	0,15489127	protein_codir	putative ARP2/3 complex subunit
TcG_03257	150,4858111	0,013318675	0,14844957	0,08971852	0,9285109	0,96542881	protein_codir	hypothetical protein
TcG_03258	166,6591105	-0,071749433	0,14031191	-0,51135667	0,60910133	0,788218	protein_codir	hypothetical protein
TcG_03259	244,7283766	-0,022243311	0,11426028	-0,19467229	0,84564951	0,92503539	protein_codir	hypothetical protein
TcG_03260	214,2654979	-0,302511753	0,1270406	-2,38122107	0,01725535	0,07510162	protein_codir	putative dolichyl-P-Man:GDP-ManGlcNAc2-PP-dolichyl beta-1,4-mannosyltransferase
TcG_03261	194,3372759	-0,254999507	0,12697994	-2,00818736	0,04462339	0,15255431	protein_codir	putative cyclophilin type peptidyl-prolyl cis-trans isomerase
TcG_03262	112,7049811	-0,358991163	0,16568976	-2,16664666	0,03026181	0,11518024	protein_codir	putative cyclophilin type peptidyl-prolyl cis-trans isomerase
TcG_03263	388,2032459	-0,179854305	0,09219395	-1,95082556	0,0510778	0,16807367	protein_codir	hypothetical protein
TcG_03264	278,1006008	-0,356226586	0,11177365	-3,18703548	0,00143739	0,01049377	protein_codir	hypothetical protein
TcG_03265	665,0380017	-0,32342404	0,07502518	-4,31087343	1,6261E-05	0,00023145	protein_codir	hypothetical protein
TcG_03266	136,9186523	0,058178435	0,15713204	0,3702519	0,71119481	0,85158155	protein_codir	putative serine/threonine protein phosphatase
TcG_03267	464,1395617	-0,297322833	0,09375665	-3,1712186	0,00151801	0,01097856	protein_codir	mitochondrial ATP-dependent zinc metallopeptidase
TcG_03268	319,0269924	-0,341509466	0,101438	-3,36668177	0,00076078	0,00619427	protein_codir	hypothetical protein
TcG_03269	127,0684068	-0,310652834	0,16057558	-1,93462063	0,05303687	0,17207649	protein_codir	hypothetical protein
TcG_03270	480,3247549	-0,180435737	0,08446833	-2,1361348	0,03266843	0,1219642	protein_codir	periodic tryptophan protein 2-like protein
TcG_03271	1917,440873	-0,180973491	0,05755841	-3,14417122	0,00166558	0,01186802	protein_codir	hypothetical protein
TcG_03272	95,3774696	-0,270231209	0,18353807	-1,47234417	0,14092796	0,3359653	protein_codir	putative ethanolamine phosphotransferase
TcG_03273	184,9441547	-0,181564859	0,12942754	-1,40283019	0,16066748	0,36357294	protein_codir	putative ethanolamine phosphotransferase

TcG_03274	232,2528001	-0,451044151	0,12612614	-3,57613525	0,00034871	0,00318124	protein_codir	30S ribosomal protein S8
TcG_03275	272,395356	-0,10560545	0,11064931	-0,95441579	0,33987322	0,57399583	protein_codir	DNA-directed RNA polymerase II
TcG_03276	241,1673395	-0,30712251	0,12185066	-2,52048285	0,0117194	0,05596905	protein_codir	DNA-directed RNA polymerase II/III subunit
TcG_03277	988,6571866	-0,26279041	0,06633585	-3,96151425	7,4476E-05	0,00087071	protein_codir	hypothetical protein
TcG_03278	67,40860025	-0,007640493	0,21971546	-0,03477449	0,97225956	0,98777616	protein_codir	hypothetical protein
TcG_03279	595,8560531	0,034862242	0,08066768	0,4321711	0,66561706	0,82504261	protein_codir	hypothetical protein
TcG_03280	167,4362029	0,466853071	0,14080642	3,31556674	0,00091458	0,00726269	protein_codir	putative alcohol dehydrogenase
TcG_03281	769,6778117	-0,447469517	0,07157176	-6,25204023	4,0512E-10	1,7779E-08	protein_codir	putative elongation factor Tu
TcG_03282	110,0554284	-0,208426591	0,17350012	-1,20130516	0,22963285	0,45682112	protein_codir	hypothetical protein
TcG_03283	248,3740609	-0,220334104	0,12344457	-1,78488285	0,07428032	0,22015933	protein_codir	hypothetical protein
TcG_03284	1,67259677	-1,391992493	1,42237755	-0,97863784	0,32775895	1	protein_codir	hypothetical protein
TcG_03285	1,805124943	-0,580506831	1,35347099	-0,42890231	0,66799432	1	protein_codir	hypothetical protein
TcG_03286	238,0362234	0,028343852	0,11655686	0,24317617	0,80786892	0,90504442	protein_codir	hypothetical protein
TcG_03287	246,1312125	-0,371561503	0,11366855	-3,26881538	0,00107999	0,00830859	protein_codir	putative citrate synthase
TcG_03288	431,0858739	-0,379481559	0,08934677	-4,24728909	2,1637E-05	0,00029632	protein_codir	hypothetical protein
TcG_03289	374,751945	-0,4336017	0,09473271	-4,57710668	4,7145E-06	7,9508E-05	protein_codir	serine/threonine-protein phosphatase 4 regulatory subunit 1
TcG_03290	394,301875	-0,469341697	0,0914013	-5,13495628	2,8221E-07	6,5133E-06	protein_codir	hypothetical protein
TcG_03291	161,4852348	-0,30657352	0,13809144	-2,22007627	0,02641359	0,10394968	protein_codir	putative serine/threonine protein kinase
TcG_03292	12,52223296	0,182328564	0,48594582	0,37520348	0,70750914	1	protein_codir	hypothetical protein
TcG_03293	26,27173312	-0,313055229	0,33999748	-0,9207575	0,35717705	0,59092578	protein_codir	trans-sialidase
TcG_03294	27,11167573	-0,382387903	0,34946355	-1,09421397	0,27386114	0,50694284	protein_codir	trans-sialidase
TcG_03295	24,29326484	0,112932378	0,36371413	0,31049764	0,75618255	0,87716571	protein_codir	hypothetical protein
TcG_03296	19,44363962	0,134010798	0,416267	0,32193471	0,74750215	0,87251259	protein_codir	hypothetical protein
TcG_03297	71,27155603	0,08301621	0,20789836	0,39931153	0,68966367	0,8386805	protein_codir	beta galactofuranosyl glycosyltransferase
TcG_03298	32,97669814	-0,10725234	0,31270077	-0,34298713	0,73160814	0,86212489		
TcG_03299	36,47726863	0,406592842	0,28944156	1,40474935	0,16009582	0,36306159	protein_codir	protein kinase, putative,serine/threonine protein kinase
TcG_03300	52,02845242	0,023643541	0,2406519	0,09824789	0,92173546	0,96200586	protein_codir	hypothetical protein
TcG_03301	11,59118391	0,470437783	0,5233237	0,89894224	0,36868343	1	protein_codir	RNA-binding protein
TcG_03302	1391,909453	0,421714195	0,05676855	7,42865914	1,097E-13	9,7772E-12	protein_codir	hypothetical protein
TcG_03303	1387,120894	-0,130556137	0,05661213	-2,30615136	0,02110218	0,0878356	protein_codir	chaperonin TCP20
TcG_03304	3504,267816	-0,271021648	0,08841731	-3,06525542	0,00217484	0,0147097	protein_codir	dynein heavy chain 9, axonemal isoform 2
TcG_03305	1287,427909	-0,030459213	0,05745324	-0,5301566	0,59600336	0,78066886	protein_codir	putative mitochondrial DNA polymerase I protein D
TcG_03306	1706,25488	-0,124665677	0,0512048	-2,43464834	0,01490627	0,06743617	protein_codir	squalene monooxygenase-like protein
TcG_03307	567,6175476	-0,351712113	0,07786806	-4,51676983	6,279E-06	0,00010189	protein_codir	putative MCAK-like kinesin
TcG_03308	1116,845461	-0,010491715	0,0627215	-0,16727462	0,86715397	0,93578156	protein_codir	protein p166
TcG_03309	622,75027	-0,445054577	0,07655623	-5,81343407	6,1204E-09	2,026E-07	protein_codir	centromere protein J
TcG_03310	657,3936136	-0,008676324	0,07432796	-0,11673029	0,90707379	0,95496201	protein_codir	ubiquitin-conjugating enzyme-like
TcG_03311	489,8158809	-0,260699288	0,08658545	-3,0108904	0,00260483	0,01696433	protein_codir	putative phosphoprotein phosphatase
TcG_03312	395,343193	0,139511231	0,09250017	1,50822672	0,13149651	0,32230137	protein_codir	putative ras-family member, GTP-binding protein
TcG_03313	764,9128984	0,221316157	0,07160108	3,090961	0,0019951	0,01371337	protein_codir	hypothetical protein
TcG_03314	409,9344721	0,128400046	0,08946686	1,43516883	0,15123899	0,3515895	protein_codir	hypothetical protein
TcG_03315	183,1344683	0,155348931	0,13146484	1,18167664	0,23733403	0,46503502	protein_codir	phospholipid-transporting ATPase 1-like protein
TcG_03316	682,7009537	-0,085373149	0,07193643	-1,18678608	0,23531201	0,46318807	protein_codir	IQ motif containing with AAA domain 1
TcG_03317	282,2155558	0,355552018	0,10627306	3,34564575	0,00082091	0,00661871	protein_codir	putative DNA cross-link repair 1A protein
TcG_03318	323,7762624	-0,165781824	0,10323671	-1,6058418	0,10830867	0,28390595	protein_codir	Ran-binding protein 1
TcG_03319	1604,303687	0,094212232	0,06872078	1,37094238	0,17039292	0,37696627	protein_codir	putative ubiquitin-protein ligase
TcG_03320	189,2930168	-0,166200986	0,13110514	-1,26769237	0,20490783	0,42614649	protein_codir	calmodulin
TcG_03321	312,502013	-0,104456911	0,10251362	-1,01895641	0,30822366	0,5412602	protein_codir	putative mucin-associated surface protein (MASP)
TcG_03322	266,8942519	0,067666598	0,11775799	0,57462427	0,56554543	0,75798662	protein_codir	hypothetical protein
TcG_03323	473,0434682	0,100633113	0,09204787	1,09326934	0,27427556	0,50733448	protein_codir	hypothetical protein
TcG_03324	89,25716887	-0,34127397	0,19156294	-1,78152394	0,0748269	0,22070887	protein_codir	ATP-binding cassette transporter ABCA1
TcG_03325	1690,71961	-0,132638948	0,06238056	-2,12628675	0,03347939	0,12404612	protein_codir	ABC1 transporter

TcG_03326	421,9117325	-0,103859065	0,09826252	-1,05695501	0,29053212	0,52345679	protein_codir	putative leucine-rich repeat protein (LRRP)
TcG_03327	186,3698778	-0,121592698	0,13307683	-0,913703	0,36087293	0,59353695	protein_codir	hypothetical protein
TcG_03328	366,6154571	-0,022486613	0,09661075	-0,23275477	0,81595184	0,90921145	protein_codir	hypothetical protein
TcG_03329	29,74257697	-0,45418884	0,32615617	-1,39255022	0,16375585	0,36854608		
TcG_03330	517,7376281	0,136922741	0,08365162	1,63682117	0,10166784	0,2719463	protein_codir	hypothetical protein
TcG_03331	237,8580785	-0,111995666	0,12122013	-0,92390321	0,35553671	0,58939023	protein_codir	hypothetical protein
TcG_03332	383,1641438	-0,315204922	0,09309233	-3,38593869	0,00070935	0,00582051	protein_codir	putative endomembrane protein
TcG_03333	632,7872907	0,061120637	0,07579645	0,80637865	0,42002455	0,64656135	protein_codir	DNA-directed RNA polymerase
TcG_03334	1084,522795	-0,380637774	0,06228533	-6,11119459	9,8888E-10	3,9372E-08	protein_codir	hypothetical protein
TcG_03335	680,139799	-0,283045068	0,07266975	-3,89495049	9,8219E-05	0,00109842	protein_codir	protein dpy-30
TcG_03336	491,3608372	-0,224182503	0,08364727	-2,6800936	0,00736016	0,03884918	protein_codir	peptidyl-prolyl isomerase E (cyclophilin E)
TcG_03337	20,11647829	-0,115759947	0,41527617	-0,27875413	0,78043352	0,89067206	protein_codir	surface protein-2
TcG_03338	247,770521	-0,018854675	0,11550472	-0,16323727	0,87033162	0,93739833	protein_codir	hypothetical protein
TcG_03339	221,2346863	-0,057682016	0,12401179	-0,46513333	0,64183598	0,80983762	protein_codir	rabGTPase-activating protein
TcG_03340	134,1911028	-0,044694794	0,1562534	-0,28604046	0,77484713	0,88753796	protein_codir	hypothetical protein
TcG_03341	117,6076285	-0,15262024	0,16757854	-0,91073859	0,36243312	0,59502869	protein_codir	hypothetical protein
TcG_03342	49,87592454	0,252873578	0,24875625	1,01655166	0,30936676	0,54250391	protein_codir	hypothetical protein
TcG_03343	461,8933305	0,24566221	0,08806	2,78971383	0,00527546	0,0298445	protein_codir	putative zinc-finger protein ZPR1
TcG_03344	295,3760402	0,045769129	0,10606419	0,43152291	0,6660882	0,82519673	protein_codir	hypothetical protein
TcG_03345	336,3092832	-0,104463562	0,10290617	-1,01513412	0,3100419	0,54327669	protein_codir	hypothetical protein
TcG_03346	251,4652255	0,212610744	0,12038799	1,76604615	0,07738811	0,22562119	protein_codir	otubain
TcG_03347	226,5993538	-0,014602276	0,11928973	-0,12241017	0,90257418	0,9529091	protein_codir	myo-inositol-1(or 4)-monophosphatase 1
TcG_03348	285,4383172	-0,004924792	0,11043904	-0,04459286	0,96443184	0,98422507	protein_codir	hypothetical protein
TcG_03349	65,76435725	0,231263585	0,2177727	1,06194941	0,28825865	0,52128131	protein_codir	hypothetical protein
TcG_03350	541,0141605	0,128231454	0,08301164	1,5447406	0,12240902	0,30729757	protein_codir	protein SDA1
TcG_03351	88,58872818	0,394565763	0,20344582	1,93941448	0,05245089	0,17089963	protein_codir	putative L-gulonolactone oxidase
TcG_03352	94,66660444	0,444464413	0,18634046	2,38522766	0,01706855	0,07445643	protein_codir	putative L-gulonolactone oxidase
TcG_03353	65,3737962	-0,146473957	0,21386835	-0,68487906	0,49342024	0,70398758	protein_codir	hypothetical protein
TcG_03354	106,5712761	0,2029014	0,16872023	1,20259079	0,22913471	0,45626889	protein_codir	nicotinamide mononucleotide adenylyltransferase
TcG_03355	156,682806	0,037632241	0,143315	0,26258411	0,79287115	0,89624823	protein_codir	hypothetical protein
TcG_03356	346,2556997	-0,205696376	0,10032522	-2,05029578	0,04033558	0,14202291	protein_codir	hypothetical protein
TcG_03357	269,0775471	0,118079671	0,11060008	1,06762739	0,28568862	0,51912745	protein_codir	hypothetical protein
TcG_03358	362,0672948	-0,092024823	0,1028693	-0,89458004	0,37101162	0,60262732	protein_codir	hypothetical protein
TcG_03359	252,576984	0,167752341	0,11200548	1,49771544	0,1342072	0,32570991	protein_codir	hypothetical protein
TcG_03360	150,7018601	0,719691261	0,14857261	4,84403743	1,2723E-06	2,5069E-05	protein_codir	Appr-1-p processing domain-containing protein
TcG_03361	183,0283971	0,060353173	0,13611492	0,44339866	0,6574774	0,81928146	protein_codir	putative choline/carnitine O-acetyltransferase
TcG_03362	135,9187438	-0,091731877	0,15929822	-0,57584997	0,5647166	0,7574446	protein_codir	hypothetical protein
TcG_03363	1177,283028	-0,064640346	0,06067205	-1,0654057	0,28669238	0,51981501	protein_codir	putative translation initiation factor
TcG_03364	263,0744486	-0,154398946	0,11698224	-1,31984946	0,18688528	0,40089852	protein_codir	hypothetical protein
TcG_03365	254,0484096	-0,13696414	0,11800814	-1,160633	0,24579118	0,47432445	protein_codir	organic solute carrier partner 1
TcG_03366	384,0450349	-0,070830792	0,09428092	-0,75127391	0,45248783	0,67329805	protein_codir	nat10 protein
TcG_03367	203,1663921	0,039633931	0,12897322	0,30730357	0,75861232	0,87801518	protein_codir	hypothetical protein
TcG_03368	417,9522166	0,018354884	0,090746	0,20226659	0,83970832	0,92207948	protein_codir	tetratricopeptide repeat domain 27
TcG_03369	457,5485952	0,049382728	0,08833465	0,55904145	0,57613343	0,76670634	protein_codir	hypothetical protein
TcG_03370	111,676482	0,124293229	0,17594829	0,70641908	0,47992756	0,69419608	protein_codir	hypothetical protein
TcG_03371	150,0974473	0,117997822	0,15662001	0,75340196	0,45120841	0,67194095	protein_codir	hypothetical protein
TcG_03372	307,9384311	-0,091810837	0,11403538	-0,80510836	0,42075715	0,64722416	protein_codir	hypothetical protein
TcG_03373	167,498154	0,536121359	0,13831247	3,87616078	0,00010612	0,00117205	protein_codir	hypothetical protein
TcG_03374	369,8383183	-0,165308424	0,09812813	-1,68461813	0,09206227	0,25371871	protein_codir	Na/H hydrogen antiporter 1
TcG_03375	129,3757775	-0,190287645	0,15698432	-1,21214427	0,22545716	0,45182011	protein_codir	hypothetical protein
TcG_03376	213,5321046	0,063095442	0,12585367	0,5013397	0,61613207	0,79256256	protein_codir	hypothetical protein
TcG_03377	175,9605504	0,015691368	0,14088765	0,11137505	0,91131895	0,95684497	protein_codir	hypothetical protein

TcG_03378	459,3636377	0,228958846	0,08793406	2,60375622	0,00922083	0,04633075	protein_codir	UDP-sugar pyrophosphorylase
TcG_03379	335,9614257	-0,143734647	0,09927592	-1,44782987	0,14766464	0,34639451	protein_codir	putative 5'-3' exonuclease
TcG_03380	414,7582609	-0,005025708	0,08860591	-0,05671978	0,95476842	0,97888932	protein_codir	hypothetical protein
TcG_03381	192,0934386	-0,078523803	0,13006566	-0,60372432	0,54602694	0,74295574	protein_codir	membrane transporter protein
TcG_03382	308,7608451	0,036416434	0,1024751	0,35536859	0,72231347	0,85728556	protein_codir	tRNA (cytosine34-C5)-methyltransferase
TcG_03383	586,1907777	-0,28121351	0,07946994	-3,53861464	0,00040223	0,00359311	protein_codir	putative kinesin
TcG_03384	553,9367132	-0,138332904	0,08330165	-1,66062619	0,09678855	0,2631758	protein_codir	putative ubiquitin hydrolase
TcG_03385	284,7307246	-0,207353793	0,10704345	-1,93709925	0,05273321	0,17142733	protein_codir	hypothetical protein
TcG_03386	4,981502165	0,671464693	0,84610088	0,79359886	0,427429	1		
TcG_03387	90,10658218	0,098106909	0,18292211	0,53633161	0,59172939	0,77721083	protein_codir	hypothetical protein
TcG_03388	181,467238	0,055393576	0,13744697	0,4030178	0,68693513	0,83750715	protein_codir	hydrolase-like protein
TcG_03389	386,0886854	0,412633947	0,09536569	4,32685967	1,5125E-05	0,00021742	protein_codir	putative hydrolase, alpha/beta fold family
TcG_03390	111,4921574	0,183648865	0,17130235	1,0720744	0,28368662	0,5171166	protein_codir	hydrolase-like protein
TcG_03391	106,6009681	0,114193904	0,17699587	0,64517834	0,51881162	0,72368787	protein_codir	hypothetical protein
TcG_03392	478,2097372	-0,439209822	0,08464922	-5,18858647	2,119E-07	5,1212E-06	protein_codir	UDP-GlcNAc:polypeptide N-acetylglucosaminyltransferase
TcG_03393	448,7368653	0,096970146	0,08772955	1,10533048	0,26901641	0,50158097	protein_codir	putative SNF2/RAD54 related DNA helicase
TcG_03394	695,5865023	0,26219694	0,07420672	3,5333316	0,00041036	0,00364881	protein_codir	putative protein kinase
TcG_03395	354,7985708	0,076464918	0,0951889	0,80329656	0,42180334	0,64805908	protein_codir	putative MYH7B protein
TcG_03396	299,1181721	0,11776723	0,10543245	1,11699225	0,26399764	0,49619385	protein_codir	putative MYH7B protein
TcG_03397	216,6590198	0,161912202	0,12508362	1,29443174	0,19551633	0,4133332	protein_codir	hypothetical protein
TcG_03398	272,2750267	0,058709012	0,10978393	0,53476873	0,59280979	0,77801248	protein_codir	hypothetical protein
TcG_03399	828,4925896	-0,298760582	0,06716228	-4,44833883	8,6537E-06	0,00013549	protein_codir	putative seryl-tRNA synthetase
TcG_03400	300,4411329	0,196691265	0,10422363	1,8872042	0,05913287	0,18708722	protein_codir	putative protein kinase, putative,cyclin-dependent protein kinase
TcG_03401	183,749039	-0,039457034	0,1317716	-0,29943503	0,76460814	0,88129227	protein_codir	hypothetical protein
TcG_03402	175,7568738	0,029872516	0,13231393	0,22577	0,82138031	0,91145601	protein_codir	putative NGG1 interacting factor 3
TcG_03403	372,928712	0,07988368	0,09915305	0,80566031	0,42043874	0,64690614	protein_codir	putative cell cycle sequence binding phosphoprotein (RBP45)
TcG_03404	308,9852753	0,13250575	0,10363767	1,27854816	0,20105622	0,42055196	protein_codir	hypothetical protein
TcG_03405	357,5184787	0,212867081	0,09764074	2,18010513	0,02924967	0,11217698	protein_codir	exocyst complex component 6
TcG_03406	116,7138961	0,196617205	0,16297807	1,20640286	0,2276622	0,45451795	protein_codir	hypothetical protein
TcG_03407	187,5417443	0,261419554	0,13099392	1,99566172	0,04597076	0,15578157	protein_codir	cytoplasmic translation machinery associated protein
TcG_03408	251,2287549	0,228578252	0,11232487	2,0349745	0,04185344	0,14582948	protein_codir	putative DEAD-boc ATP-dependent (RNA) helicase
TcG_03409	274,7555394	0,39387623	0,11076601	3,55593058	0,00037664	0,00340389	protein_codir	hypothetical protein
TcG_03410	75,724576	0,253671714	0,20037653	1,26597518	0,20552197	0,42688733	protein_codir	hypothetical protein
TcG_03411	489,5497094	-0,307573394	0,08313019	-3,69989989	0,00021568	0,00212437	protein_codir	putative acidocalcisomal pyrophosphatase
TcG_03412	244,6026293	0,379213429	0,12009727	3,15755242	0,001591	0,01144214	protein_codir	hypothetical protein
TcG_03413	279,1412149	0,184389185	0,10866012	1,69693528	0,0897089	0,2496079	protein_codir	pterin-4-alpha-carbinolamine dehydratase
TcG_03414	152,5924621	0,60068808	0,14374112	4,17895798	2,9285E-05	0,00038732	protein_codir	putative DNA repair protein
TcG_03415	456,7510898	-0,135912001	0,08853524	-1,53511749	0,12475497	0,31110872	protein_codir	putative proteasome alpha 7 subunit
TcG_03416	39,1960669	0,401739225	0,28058429	1,43179517	0,15220245	0,35239414	protein_codir	putative protein kinase
TcG_03417	128,059694	0,184898748	0,15409237	1,19992147	0,23016984	0,45718289	protein_codir	putative protein kinase, putative,serine/threonine protein kinase
TcG_03418	601,2213658	-0,162398212	0,07956035	-2,04119539	0,04123141	0,14440965	protein_codir	carboxy-lyase
TcG_03419	239,1015418	0,17125555	0,12246857	1,39836328	0,162004	0,36574014	protein_codir	putative NADH-cytochrome b5 reductase
TcG_03420	239,6558719	-0,013737183	0,11550183	-0,11893476	0,90532704	0,95390315	protein_codir	Protein tipD
TcG_03421	266,534958	-0,054858752	0,11005348	-0,49847356	0,6181503	0,793034	protein_codir	hypothetical protein
TcG_03422	124,163665	0,113357423	0,1563894	0,72484082	0,46854969	0,6850854	protein_codir	hypothetical protein
TcG_03423	582,5448278	-0,124252782	0,07989771	-1,55514823	0,11991077	0,30300681	protein_codir	hypothetical protein
TcG_03424	196,5705955	0,123059931	0,1269393	0,96943916	0,33232612	0,5663408	protein_codir	hypothetical protein
TcG_03425	497,3911232	-0,06133014	0,08275563	-0,74109928	0,45863325	0,67743881	protein_codir	hypothetical protein
TcG_03426	473,2751193	-0,025919656	0,08538139	-0,303575	0,7614517	0,87949151	protein_codir	putative PIF1 helicase-like protein
TcG_03427	887,6216191	-0,319524878	0,06768548	-4,7207303	2,35E-06	4,3019E-05	protein_codir	putative DNA repair and recombination protein,mitochondrial precursor
TcG_03428	1396,058519	-0,287782809	0,05418767	-5,31085405	1,0911E-07	2,8031E-06	protein_codir	putative 14-3-3 protein
TcG_03429	413,2463869	0,253720211	0,09158696	2,77026579	0,00560106	0,03128922	protein_codir	hypothetical protein

TcG_03430	338,2012257	-0,190353211	0,09828047	-1,9368365	0,05276533	0,17148363	protein_codir hypothetical protein
TcG_03431	653,8466413	-0,081858742	0,07845644	-1,0433655	0,29677904	0,52997565	protein_codir hypothetical protein
TcG_03432	283,3249228	-0,24987789	0,11217031	-2,22766513	0,02590285	0,10242678	protein_codir hypothetical protein
TcG_03433	1178,077795	-0,067803391	0,06620841	-1,02409034	0,30579261	0,53883629	protein_codir putative tubulin-tyrsoine ligase-like protein
TcG_03434	412,9367633	0,139919402	0,09209074	1,51936457	0,12867075	0,31779564	protein_codir hypothetical protein
TcG_03435	818,4279101	0,034061104	0,06703946	0,50807547	0,61140042	0,78979655	protein_codir ribosome biogenesis protein ERB1
TcG_03436	126,4433189	0,147350073	0,16455352	0,8954538	0,37054455	0,60220637	protein_codir hypothetical protein
TcG_03437	177,1054858	-0,182929697	0,13425561	-1,36254791	0,17302504	0,38126057	protein_codir putative chromatin binding protein
TcG_03438	314,7909878	-0,089032688	0,10443247	-0,85253838	0,39391535	0,62373967	protein_codir hypothetical protein
TcG_03439	556,9873402	-0,146844541	0,08108082	-1,81108851	0,07012715	0,21213921	protein_codir hypothetical protein
TcG_03440	236,2765582	0,113192445	0,11649049	0,9716883	0,33120564	0,56548018	protein_codir hypothetical protein
TcG_03441	285,9511381	0,057490362	0,11143095	0,51592815	0,60590459	0,78692126	protein_codir DNA ligase
TcG_03442	309,6067926	-0,03272289	0,10151669	-0,32234	0,74719513	0,87232999	protein_codir putative pumilio-repeat, RNA-binding protein
TcG_03443	145,9397141	0,052423548	0,15300459	0,34262728	0,73187887	0,86235621	protein_codir pumilio
TcG_03444	0,262179411	-0,45874873	3,15671551	-0,1453247	0,88445451	1	
TcG_03445	0,574155419	1,027852573	2,2704697	0,45270482	0,65076131	1	
TcG_03446	801,8692991	-0,176978898	0,06773452	-2,61283162	0,00897955	0,04541122	protein_codir hypothetical protein
TcG_03447	278,4229043	-0,255523185	0,11093165	-2,30342897	0,02125472	0,08826422	protein_codir tetratricopeptide repeat (TPR) protein
TcG_03448	308,1708417	0,060025881	0,10135162	0,59225379	0,55368066	0,74883793	protein_codir putative protein kinase
TcG_03449	702,0938307	0,346061107	0,07622106	4,54022931	5,6193E-06	9,2743E-05	protein_codir putative amino acid permease/transporter
TcG_03450	611,9553036	0,120384541	0,07641832	1,57533604	0,1151789	0,29556207	protein_codir hypothetical protein
TcG_03451	653,4416059	-0,116997071	0,07494029	-1,5612039	0,11847565	0,30062612	protein_codir putative transcription modulator/accessory protein
TcG_03452	339,1040711	0,386376625	0,10042374	3,84746309	0,00011935	0,00129717	protein_codir putative nucleoside transporter-like
TcG_03453	442,7314373	0,344415864	0,10561075	3,26118201	0,00110949	0,00848484	protein_codir helicase-like protein
TcG_03454	53,74354909	0,478118073	0,23768166	2,01159008	0,04426317	0,15194372	protein_codir putative cytochrome P450
TcG_03455	394,7270064	0,259535594	0,09499927	2,7319747	0,0062956	0,03430263	protein_codir putative trans-sialidase
TcG_03456	55,74168719	0,229080366	0,24045991	0,95267594	0,34075429	0,57500425	protein_codir hypothetical protein
TcG_03457	24,15302199	0,100314948	0,36836386	0,2723257	0,78537159	0,89351472	protein_codir trans-sialidase
TcG_03458	272,4929929	-0,232232414	0,11619669	-1,99861475	0,04565005	0,15492577	protein_codir sialidase-like protein
TcG_03459	72,79332342	0,161912943	0,20197671	0,80164164	0,42276028	0,64889255	protein_codir hypothetical protein
TcG_03460	154,7627749	0,239955906	0,14166009	1,69388507	0,09028712	0,25061491	protein_codir putative retrotransposon hot spot (RHS) protein
TcG_03461	125,1717089	0,172144465	0,15867792	1,08486715	0,2779805	0,51065198	protein_codir putative retrotransposon hot spot (RHS) protein
TcG_03462	323,2324655	0,117112045	0,1028173	1,13903051	0,25469043	0,48614208	protein_codir retrotransposon hot spot (RHS) protein
TcG_03463	28,96317568	0,261821496	0,34399446	0,76112126	0,44658464	0,66814691	protein_codir retrotransposon hot spot (RHS) protein
TcG_03464	44,19625698	0,074579721	0,26254066	0,28406922	0,77635733	0,88831084	protein_codir hypothetical protein
TcG_03465	14,13223674	-0,193820672	0,45483266	-0,42613623	0,67000858	1	
TcG_03466	22,87846144	0,917366757	0,39952184	2,29616173	0,02166664	0,08952555	
TcG_03467	45,15771661	0,121942712	0,26586519	0,45866369	0,64647569	0,81201944	protein_codir trans-sialidase
TcG_03468	72,66462536	0,080806336	0,20825426	0,38801768	0,69800295	0,84388746	protein_codir hypothetical protein
TcG_03469	33,83053245	0,239283962	0,32629541	0,73333536	0,46335391	0,68091923	protein_codir trans-sialidase
TcG_03470	39,57753573	-0,002443288	0,283837	-0,00860807	0,99313184	0,99743633	protein_codir hypothetical protein
TcG_03471	612,6121374	0,171385482	0,07789357	2,20025185	0,02778903	0,10793286	protein_codir putative calcium/potassium channel (CAKC)
TcG_03472	244,5825974	0,306247017	0,11552429	2,65093193	0,008027	0,04161111	protein_codir hypothetical protein
TcG_03473	376,5444695	0,095588077	0,0932033	1,02558688	0,30508636	0,53795118	protein_codir hypothetical protein
TcG_03474	832,8221658	-0,117452029	0,07255421	-1,61881754	0,10548653	0,2792886	protein_codir putative eukaryotic initiation factor 4a
TcG_03475	133,6324614	0,540495152	0,16667312	3,24284543	0,00118342	0,00894193	protein_codir hypothetical protein
TcG_03476	529,813692	0,285917158	0,08100843	3,52947409	0,00041639	0,00369958	protein_codir putative protein kinase
TcG_03477	329,6622777	0,272409159	0,10055586	2,70903323	0,00674796	0,03632204	protein_codir hypothetical protein
TcG_03478	737,6416947	0,132007556	0,07511955	1,75729963	0,07886675	0,22872345	protein_codir hypothetical protein
TcG_03479	581,179439	0,106793157	0,07658764	1,39439155	0,1631994	0,36765083	protein_codir hypothetical protein
TcG_03480	446,8442726	0,148994499	0,0918657	1,62187301	0,10483054	0,2781238	protein_codir putative ubiquitin activating enzyme
TcG_03481	175,8961265	0,202042146	0,14232319	1,41960102	0,15572387	0,35727064	protein_codir hypothetical protein

TcG_03482	126,5761499	0,345502459	0,15670006	2,20486492	0,02746358	0,10708804	protein_codir hypothetical protein
TcG_03483	296,3540285	0,301594897	0,10786353	2,79607842	0,00517268	0,02943552	protein_codir hypothetical protein
TcG_03484	1116,046299	-0,077300671	0,05976519	-1,29340621	0,1958706	0,41381415	protein_codir hypothetical protein
TcG_03485	344,9001522	0,007726702	0,09690471	0,07973506	0,93644798	0,97043819	protein_codir hypothetical protein
TcG_03486	679,5681872	0,272806343	0,07340526	3,71644157	0,00020205	0,00200766	protein_codir hypothetical protein
TcG_03487	413,0685529	-0,065287014	0,08990468	-0,72618038	0,4677282	0,68431606	protein_codir putative mitochondrial processing peptide beta subunit
TcG_03488	473,7126685	0,090525479	0,08600327	1,0525818	0,2925327	0,52530749	protein_codir putative heat shock protein 70 (hsp70)
TcG_03489	222,3137099	0,25627219	0,12354985	2,07424117	0,03805692	0,13629906	protein_codir hypothetical protein
TcG_03490	335,8889728	-0,186014294	0,09821622	-1,89392635	0,05823479	0,1845987	protein_codir hypothetical protein
TcG_03491	415,624939	0,100550273	0,09488695	1,059685	0,28928793	0,52280298	protein_codir putative DNA-damage inducible protein DDI1-like protein
TcG_03492	512,7825072	0,117135438	0,08145957	1,43795804	0,15044596	0,35042645	protein_codir putative GTPase activating protein
TcG_03493	450,9486594	0,359297423	0,08856406	4,05692126	4,9724E-05	0,00061287	protein_codir REL1 protein
TcG_03494	870,5125643	-0,103548416	0,06982694	-1,48292941	0,13809317	0,33164715	protein_codir hypothetical protein
TcG_03495	341,6314194	0,135901462	0,09958948	1,36461669	0,17237356	0,38004187	protein_codir putative tricarboxylate carrier
TcG_03496	355,8732668	0,218622685	0,10082051	2,16843472	0,03012563	0,11485209	protein_codir U4/U6 small nuclear ribonucleoprotein PRP3
TcG_03497	555,3370722	0,195548662	0,08537388	2,29049747	0,0219925	0,09043176	protein_codir hypothetical protein
TcG_03498	400,9060607	-0,0798378	0,09176766	-0,86999928	0,3843008	0,61456285	protein_codir hypothetical protein
TcG_03499	323,5038841	-0,186839193	0,10548624	-1,77121859	0,07652436	0,22400486	protein_codir putative long-chain-fatty-acid-CoA ligase
TcG_03500	429,7691613	0,231732924	0,0948209	2,44390128	0,0145294	0,06622252	protein_codir putative fatty acyl CoA syntetase 1
TcG_03501	413,3396722	0,109438096	0,09271171	1,1804129	0,23783603	0,46565101	protein_codir putative fatty acyl CoA synthetase 2
TcG_03502	749,1344764	0,059659361	0,07142639	0,83525662	0,40357324	0,63312391	protein_codir putative fatty acyl CoA syntetase 1
TcG_03503	2466,299415	-0,021716052	0,05570311	-0,38985352	0,69664486	0,84295847	protein_codir hypothetical protein
TcG_03504	252,4768461	0,449908482	0,11550225	3,89523576	9,8103E-05	0,00109819	protein_codir alpha/beta-hydrolase
TcG_03505	78,88215711	0,470086872	0,19760708	2,37889688	0,01736453	0,07540685	
TcG_03506	192,2202889	0,156940121	0,13103177	1,1977257	0,23102382	0,45840761	protein_codir hypothetical protein
TcG_03507	142,522106	0,104291721	0,16607619	0,62797515	0,53002022	0,73198176	protein_codir BRCA1-associated protein
TcG_03508	3044,739601	0,452983955	0,04733983	9,56877036	1,0818E-21	2,4104E-19	protein_codir putative ribosomal protein S7
TcG_03509	121,3614741	0,330652322	0,16517245	2,00186117	0,04529967	0,15431988	protein_codir hypothetical protein
TcG_03510	323,8404121	0,041041901	0,10227694	0,40128207	0,68821246	0,83818245	protein_codir hypothetical protein
TcG_03511	602,0119076	0,10540366	0,07968635	1,32273172	0,18592461	0,39987989	protein_codir hypothetical protein
TcG_03512	159,3072208	0,216301908	0,14368339	1,50540646	0,13221961	0,32311673	protein_codir hypothetical protein
TcG_03513	136,6935098	0,357481109	0,1597695	2,2374803	0,02525497	0,1004974	protein_codir hypothetical protein
TcG_03514	0,467431231	2,090445105	2,51848818	0,83003967	0,40651635	1	protein_codir hypothetical protein
TcG_03515	45,32657911	0,573512753	0,26345263	2,17691034	0,02948726	0,11286401	protein_codir hypothetical protein
TcG_03516	101,1769749	0,633267026	0,1795955	3,52607411	0,00042177	0,0037388	protein_codir hypothetical protein
TcG_03517	18,93491283	0,167192886	0,40352732	0,41432854	0,67863352	0,83185018	
TcG_03518	230,3180594	0,47859935	0,11887833	4,02595973	5,6743E-05	0,00068769	protein_codir metal ion binding protein
TcG_03519	176,7460568	0,455919505	0,14044189	3,24632126	0,00116907	0,00887603	protein_codir zinc finger protein
TcG_03520	188,4085819	0,456677874	0,13846208	3,29821622	0,00097301	0,00761196	protein_codir zinc finger protein
TcG_03521	42,923806	0,557968813	0,26714707	2,08862035	0,03674191	0,13332032	protein_codir peptidyl-prolyl isomerase E (cyclophilin E)
TcG_03522	454,9134869	0,173788212	0,08848785	1,96397824	0,04953262	0,16448406	protein_codir electron transfer protein
TcG_03523	145,3954854	0,41815431	0,15779895	2,64991827	0,00805112	0,04168022	protein_codir putative phosphatidylinositol 3-kinase
TcG_03524	154,5861964	0,472732895	0,14555731	3,2477441	0,00116324	0,00883756	protein_codir hypothetical protein
TcG_03525	770,1722742	0,547002493	0,07041464	7,76830614	7,9543E-15	8,3025E-13	protein_codir putative RNA helicase
TcG_03526	681,2757763	0,140025303	0,07285382	1,92200368	0,0546053	0,17612944	protein_codir hypothetical protein
TcG_03527	209,3250573	0,336559074	0,12479171	2,69696668	0,00699743	0,03729172	protein_codir mitochondrial carrier protein
TcG_03528	235,1724257	0,571541351	0,12255695	4,66347578	3,1091E-06	5,5419E-05	protein_codir hypothetical protein
TcG_03529	358,7923116	0,438606378	0,09927054	4,41829334	9,9483E-06	0,00015146	protein_codir acetyltransferase-like protein
TcG_03530	443,0225982	0,424575129	0,09209459	4,6102071	4,0227E-06	6,9252E-05	protein_codir hypothetical protein
TcG_03531	791,2384474	0,554273035	0,074996	7,39070166	1,4606E-13	1,2628E-11	protein_codir putative DNA polymerase zeta catalytic subunit
TcG_03532	169,8659466	0,555522849	0,14652585	3,79129597	0,00014986	0,00157275	protein_codir PhnP protein
TcG_03533	72,84095192	0,525057698	0,20517965	2,55901451	0,01049694	0,0513154	protein_codir hypothetical protein

TcG_03534	2147,539288	0,240262288	0,10304591	2,33160442	0,01972151	0,08308852	protein_codir	1-beta dynein
TcG_03535	558,2017286	0,483771941	0,08494373	5,6952049	1,2322E-08	3,8071E-07	protein_codir	putative lathosterol oxidase
TcG_03536	134,65235	0,340351259	0,15774391	2,15761906	0,03095746	0,11698407	protein_codir	hypothetical protein
TcG_03537	205,1760591	0,550582462	0,12569557	4,38028545	1,1852E-05	0,00017538	protein_codir	hypothetical protein
TcG_03538	573,3644479	0,329268763	0,07945771	4,14394986	3,4137E-05	0,00044093	protein_codir	putative exonuclease
TcG_03539	139,7229952	0,313434138	0,15457438	2,0277237	0,04258846	0,14764508	protein_codir	hypothetical protein
TcG_03540	702,2466727	0,410931673	0,07911198	5,19430381	2,0549E-07	5,0334E-06	protein_codir	hypothetical protein
TcG_03541	216,4916773	0,51127193	0,12341546	4,14268936	3,4326E-05	0,00044238	protein_codir	hypothetical protein
TcG_03542	571,6234795	0,523671405	0,08034432	6,51784008	7,1327E-11	3,6199E-09	protein_codir	hypothetical protein
TcG_03543	307,5650957	0,619192205	0,10420893	5,9418345	2,8185E-09	1,0205E-07	protein_codir	hypothetical protein
TcG_03544	452,7125395	0,712357295	0,09182002	7,7581918	8,6149E-15	8,9118E-13	protein_codir	hypothetical protein
TcG_03545	213,6272185	0,340293045	0,12497455	2,7228987	0,00647119	0,03516661	protein_codir	hypothetical protein
TcG_03546	1003,127687	0,271861006	0,06293044	4,32002364	1,5601E-05	0,00022343	protein_codir	putative T-complex protein 1, gamma subunit
TcG_03547	401,1449222	0,854193009	0,09482711	9,00789885	2,1004E-19	3,4275E-17	protein_codir	putative S-adenosyl-methyltransferase mraW-like protein
TcG_03548	46,1629356	0,798890683	0,2649331	3,01544307	0,00256604	0,01677888	protein_codir	transmembrane protein 234
TcG_03549	368,6933895	0,534508135	0,09698856	5,51104304	3,5671E-08	1,0155E-06	protein_codir	hypothetical protein
TcG_03550	408,9370487	0,379024188	0,09522908	3,98013072	6,8877E-05	0,00081513	protein_codir	putative coronin
TcG_03551	103,9613337	0,289384962	0,18617683	1,55435539	0,12009967	0,30335181	protein_codir	hypothetical protein
TcG_03552	591,8191619	0,499056109	0,081217	6,14472443	8,0102E-10	3,3027E-08	protein_codir	hypothetical protein
TcG_03553	129,8255039	0,943234466	0,16459855	5,73051494	1,0013E-08	3,187E-07	protein_codir	hypothetical protein
TcG_03554	528,0322411	0,486907193	0,08445638	5,76519112	8,1565E-09	2,6471E-07	protein_codir	putative cytosolic leucyl aminopeptidase, putative,metallo-peptidase, Clan MF, Family M17
TcG_03555	500,9837713	0,433535441	0,08710391	4,97722156	6,4503E-07	1,3713E-05	protein_codir	hypothetical protein
TcG_03556	285,055025	0,44717236	0,10786642	4,14561216	3,3891E-05	0,00043823	protein_codir	hypothetical protein
TcG_03557	559,7886217	0,200833306	0,08037305	2,49876422	0,01246272	0,05880973	protein_codir	hypothetical protein
TcG_03558	171,4364506	0,195295684	0,13620148	1,43387343	0,15160839	0,35179947		
TcG_03559	521,5929615	0,349435129	0,08184991	4,26921846	1,9616E-05	0,00027218	protein_codir	hypothetical protein
TcG_03560	361,3683627	0,566479141	0,09930505	5,70443447	1,1673E-08	3,6495E-07	protein_codir	hypothetical protein
TcG_03561	72,73076724	0,606646899	0,22601862	2,68405713	0,00727347	0,03847963	protein_codir	hypothetical protein
TcG_03562	265,8557276	0,453412147	0,10943387	4,14325234	3,4241E-05	0,00044178	protein_codir	hypothetical protein
TcG_03563	118,6954198	0,571196075	0,1728032	3,30547162	0,00094817	0,00746401	protein_codir	hypothetical protein
TcG_03564	198,9106477	0,490318971	0,13562058	3,61537278	0,00029992	0,00280614	protein_codir	putative Bis(5-adenosyl)-triphosphatase
TcG_03565	308,3693173	0,352798839	0,10271463	3,43474785	0,00059311	0,00500086	protein_codir	OB fold-containing protein
TcG_03566	144,9834326	0,537315136	0,15469025	3,47349064	0,00051374	0,00443197	protein_codir	methyltransferase
TcG_03567	133,6699076	0,561989538	0,15417648	3,64510561	0,00026728	0,00253789	protein_codir	methyltransferase
TcG_03568	300,8547348	0,348317941	0,11049666	3,15229372	0,00161993	0,01160701	protein_codir	Leucine-rich, ribonuclease inhibitor subtype
TcG_03569	426,5233207	-0,039340272	0,08997208	-0,43724979	0,6619302	0,82295903	protein_codir	MGT2 magnesium transporter
TcG_03570	256,4025887	-0,056280966	0,12147511	-0,46331275	0,64314021	0,8106874	protein_codir	putative AAA ATPase
TcG_03571	318,2322831	-0,055697173	0,10819528	-0,51478376	0,60670414	0,78750732	protein_codir	putative AAA ATPase
TcG_03572	496,7665873	-0,238751727	0,08705481	-2,7425448	0,00609651	0,03346007	protein_codir	putative heat shock protein DNAJ
TcG_03573	1135,020399	0,037502171	0,06499062	0,57703976	0,5639126	0,75680428	protein_codir	hypothetical protein
TcG_03574	126,2134607	0,112212013	0,16420711	0,68335661	0,49438153	0,70453824	protein_codir	hypothetical protein
TcG_03575	173,2254105	0,281802339	0,13481204	2,09033509	0,03658771	0,13300751	protein_codir	mucin-associated surface protein (MASP)
TcG_03576	113,7744747	0,229138784	0,1776192	1,29005642	0,19703107	0,41550818	protein_codir	structural maintenance of chromosome protein 4
TcG_03577	1487,267863	-0,17104541	0,0560745	-3,05032412	0,00228595	0,01533582	protein_codir	cytochrome c oxidase VII
TcG_03578	197,9567161	0,046633097	0,129057	0,3613372	0,71784738	0,85508792	protein_codir	hypothetical protein
TcG_03579	191,1314978	-0,278818207	0,13362834	-2,08652004	0,03693154	0,13350666	protein_codir	LMBR1 domain-containing protein 1
TcG_03580	1742,845607	-0,390225474	0,05265122	-7,41151872	1,2486E-13	1,0959E-11	protein_codir	ATPase beta subunit
TcG_03581	1470,63732	0,154685811	0,05296273	2,92065396	0,00349298	0,02160684	protein_codir	ribosomal protein S25
TcG_03582	767,0913424	-0,129131303	0,06922881	-1,86528264	0,0621418	0,19469568	protein_codir	putative aminopeptidase, putative,metallo-peptidase, Clan MG, Family M24
TcG_03583	290,6185787	0,008035681	0,10484419	0,07664403	0,93890673	0,97168077	protein_codir	hypothetical protein
TcG_03584	595,6474001	0,074523752	0,07649703	0,97420448	0,32995502	0,56459295	protein_codir	hypothetical protein
TcG_03585	536,375585	0,03638045	0,08031893	0,45294988	0,65058484	0,81453165	protein_codir	hypothetical protein

TcG_03586	532,9659433	0,074585247	0,08301432	0,89846237	0,3689391	0,60086146	protein_codir	hypothetical protein
TcG_03587	351,1499267	-0,170721326	0,10309362	-1,65598348	0,09772516	0,26460476	protein_codir	hypothetical protein
TcG_03588	506,0819086	0,147515791	0,08611359	1,71303731	0,08670568	0,2440651	protein_codir	putative cullin 4B
TcG_03589	417,6798566	0,167317684	0,0919828	1,81901051	0,06890983	0,20974552	protein_codir	nuclear protein localization protein 4
TcG_03590	371,3545444	0,078136687	0,09557124	0,81757533	0,41359972	0,64029481	protein_codir	hypothetical protein
TcG_03591	151,8323292	-0,057779455	0,14272663	-0,40482602	0,6856054	0,83694281	protein_codir	hypothetical protein
TcG_03592	164,4983742	0,142099784	0,14539822	0,97731448	0,32841349	0,56286962	protein_codir	putative GCN5-like protein
TcG_03593	389,0852695	-0,117650952	0,09331216	-1,26083201	0,20736938	0,42962689	protein_codir	putative serine/threonine-protein phosphatase 2A, catalytic subunit
TcG_03594	494,5738807	-0,061037298	0,08435122	-0,72360898	0,46930583	0,68593129	protein_codir	structure-specific endonuclease subunit SLX1
TcG_03595	621,9027535	-0,113275904	0,0794947	-1,42494908	0,15417192	0,35518709	protein_codir	putative protein transport protein Sec24C
TcG_03596	441,1022838	0,118302469	0,09618732	1,22991756	0,21872798	0,44350408	protein_codir	hypothetical protein
TcG_03597	1114,249183	-0,054928816	0,06787633	-0,80924843	0,41837226	0,64501145	protein_codir	hypothetical protein
TcG_03598	220,9374005	0,04329266	0,12288235	0,35230982	0,72460592	0,85802335	protein_codir	hypothetical protein
TcG_03599	271,8253366	-0,051144036	0,11118703	-0,45998202	0,64552913	0,81191979	protein_codir	hypothetical protein
TcG_03600	190,7602199	0,435602867	0,13660439	3,18879113	0,00142869	0,01044341	protein_codir	mucin-associated surface protein (MASP)
TcG_03601	25,16726652	0,021564489	0,35953306	0,05997916	0,95217224	0,9773959	protein_codir	trans-sialidase
TcG_03602	232,785925	0,199530493	0,1255646	1,58906645	0,11204539	0,28988569	protein_codir	structural maintenance of chromosome protein 4
TcG_03603	138,264176	0,250339098	0,16164867	1,54866163	0,12146308	0,30579559	protein_codir	helicase-like protein
TcG_03604	332,3093348	0,164954391	0,10313726	1,59936752	0,10973896	0,28632613	protein_codir	hypothetical protein
TcG_03605	271,6781181	0,246523079	0,11147646	2,21143624	0,02700564	0,10577666	protein_codir	hypothetical protein
TcG_03606	543,542553	0,32154852	0,08554056	3,75901827	0,00017058	0,00174899	protein_codir	GPN-loop GTPase 3
TcG_03607	371,9478482	-0,173628184	0,09659231	-1,79753622	0,07225053	0,21618229	protein_codir	putative splicing factor 3 subunit
TcG_03608	391,7365936	0,006342407	0,09346766	0,06785671	0,9458997	0,97469438	protein_codir	putative DNA polymerase delta subunit 2
TcG_03609	1324,47081	-0,155876161	0,05871687	-2,65470813	0,00793771	0,04124846	protein_codir	GTP-binding nuclear protein rtb2
TcG_03610	403,1396546	-0,135753057	0,09276371	-1,46342843	0,14335018	0,33978217	protein_codir	clusterin-associated protein 1
TcG_03611	331,5490835	0,144799727	0,1053815	1,37405262	0,16942535	0,37606421	protein_codir	hypothetical protein
TcG_03612	245,8869684	-0,092850622	0,11541638	-0,8044839	0,42111757	0,647488	protein_codir	hypothetical protein
TcG_03613	305,0794176	-0,154786812	0,10926683	-1,41659463	0,15660148	0,35818781	protein_codir	putative C-14 sterol reductase
TcG_03614	187,7082099	-0,214002359	0,12963813	-1,65076708	0,09878614	0,26636667	protein_codir	putative peptide chain release factor 1
TcG_03615	594,511032	-0,385400781	0,07844936	-4,91273325	8,9815E-07	1,8516E-05	protein_codir	protein associated with the ribbon compartment of flagellar microtubule
TcG_03616	204,632378	-0,073293226	0,13172308	-0,55641899	0,57792446	0,76800198	protein_codir	putative cyclin
TcG_03617	0					1		
TcG_03618	28,39344468	-0,005386405	0,32195612	-0,01673025	0,98665182	0,9946703		
TcG_03619	8,936665733	0,493935596	0,58614301	0,84268785	0,39940305	1	protein_codir	hypothetical protein
TcG_03620	4,393760128	0,022594003	0,92099373	0,0245322	0,9804281	1	protein_codir	hypothetical protein
TcG_03621	1,483089552	1,66635831	1,52468631	1,09291878	0,27442946	1	protein_codir	putative mucin-associated surface protein (MASP)
TcG_03622	3,832556471	0,512187105	0,90752268	0,5643794	0,57249596	1	protein_codir	putative mucin-associated surface protein (MASP)
TcG_03623	32,61876189	0,034193721	0,3041564	0,11242151	0,9104892	0,95647184		
TcG_03624	34,35934448	-0,290316219	0,31928994	-0,90925576	0,36321514	0,59526856	protein_codir	trans-sialidase
TcG_03625	79,95923026	0,391714811	0,20088055	1,9499887	0,05117747	0,16825358	protein_codir	SH3 domain protein
TcG_03626	75,18732688	0,261627131	0,19976235	1,30969188	0,19030009	0,40552085	protein_codir	rab1 small GTP-binding protein
TcG_03627	125,2149914	0,113684385	0,16028794	0,70925101	0,47816872	0,69283432	protein_codir	protein kinase, putative,serine/threonine protein kinase
TcG_03628	390,2355917	-0,504237687	0,09212	-5,47370473	4,4072E-08	1,2245E-06	protein_codir	hypothetical protein
TcG_03629	403,9802776	-0,206801522	0,09921717	-2,0843319	0,03712999	0,13397323	protein_codir	elongation factor 2-like protein
TcG_03630	246,7562643	-0,300257109	0,11589951	-2,59066757	0,009579	0,04775485	protein_codir	hypothetical protein
TcG_03631	254,114176	-0,192314738	0,11096017	-1,73318705	0,08306243	0,23681114	protein_codir	protein phosphatase 2C
TcG_03632	338,6801273	-0,215726077	0,10332717	-2,0877962	0,03681622	0,13342282	protein_codir	putative helicase-like protein
TcG_03633	320,2058482	-0,094270263	0,10459692	-0,90127188	0,36744378	0,59969061	protein_codir	putative pseudouridylate synthase-like protein
TcG_03634	209,7915655	-0,419451415	0,12676556	-3,30887513	0,00093672	0,00739795	protein_codir	hypothetical protein
TcG_03635	256,5333251	-0,542801294	0,11240661	-4,8289089	1,3728E-06	2,6822E-05	protein_codir	nuclear lim interactor-interacting factor-like protein
TcG_03636	212,4319012	-0,432975984	0,12552078	-3,44943682	0,00056176	0,00478886	protein_codir	hypothetical protein
TcG_03637	346,6532118	-0,201314379	0,09971418	-2,01891417	0,04349614	0,14989479	protein_codir	putative aminopeptidase P1

TcG_03638	219,9555388	-0,063409458	0,12886751	-0,49205153	0,62268291	0,79673156	protein_codir	putative trans-sialidase
TcG_03639	197,0694542	-0,016689929	0,13110757	-0,1272995	0,89870335	0,95069557	protein_codir	3,2-trans-enoyl-CoA isomerase
TcG_03640	703,0180852	-0,118958485	0,08308896	-1,43170017	0,15222965	0,35239414	protein_codir	putative dynein heavy chain
TcG_03641	282,3062908	-0,313191393	0,11255769	-2,7824967	0,00539424	0,03039771	protein_codir	putative protein kinase
TcG_03642	1031,372806	-0,255355503	0,0732314	-3,48696752	0,00048853	0,00425253	protein_codir	chaperonin containing t-complex protein
TcG_03643	583,5786372	-0,161868538	0,08221398	-1,96886887	0,04896815	0,16318166	protein_codir	putative kinesin
TcG_03644	120,2799677	-0,064122671	0,16387098	-0,39129974	0,69557569	0,84234001	protein_codir	acylphosphatase
TcG_03645	447,5346193	-0,283439986	0,0882281	-3,21258191	0,00131548	0,0097759	protein_codir	putative kinesin
TcG_03646	311,0066595	0,057112643	0,10197907	0,56004279	0,57545025	0,76625291	protein_codir	hypothetical protein
TcG_03647	113,6956219	-0,043084061	0,175529	-0,24545266	0,80610596	0,90423384	protein_codir	hypothetical protein
TcG_03648	360,041104	0,558118542	0,09784269	5,70424352	1,1686E-08	3,6495E-07	protein_codir	urocanate hydratase isoform X1
TcG_03649	301,1524206	-0,438136731	0,10907281	-4,01691994	5,8964E-05	0,00071162	protein_codir	putative intraflagellar transport protein IFT88
TcG_03650	153,5714302	0,039011693	0,14189474	0,27493403	0,78336692	0,89263342	protein_codir	hypothetical protein
TcG_03651	120,9038054	-0,206353403	0,17814294	-1,15835855	0,24671775	0,47546104	protein_codir	putative mucin-like glycoprotein
TcG_03652	142,5111893	-0,140549962	0,14947312	-0,94030259	0,34706237	0,58158297	protein_codir	hypothetical protein
TcG_03653	254,3712028	0,25467687	0,11517758	2,21116713	0,02702427	0,10581384	protein_codir	carboxypeptidase
TcG_03654	200,7324873	-0,107291755	0,13461852	-0,79700591	0,4254476	0,65124005	protein_codir	hypothetical protein
TcG_03655	354,2588248	-0,234581904	0,09905355	-2,36823329	0,01787326	0,07704558	protein_codir	putative ras-related GTP-binding protein
TcG_03656	77,2750863	-0,352820202	0,19800848	-1,78184388	0,07477469	0,22066725	protein_codir	putative ras-related GTP-binding protein
TcG_03657	144,2060227	-0,245855529	0,14463816	-1,69979713	0,08916909	0,24858529	protein_codir	putative actin-like protein
TcG_03658	239,1110647	-0,392367344	0,11778881	-3,33110875	0,00086501	0,00692126	protein_codir	hypothetical protein
TcG_03659	311,9425301	-0,077887029	0,10689147	-0,72865523	0,46621258	0,6827884	protein_codir	Pre-mRNA-processing factor 17
TcG_03660	436,2005102	-0,701003038	0,08832878	-7,93629276	2,0831E-15	2,3662E-13	protein_codir	CCR4-NOT transcription complex subunit 3
TcG_03661	135,8460182	-0,549757386	0,15082297	-3,64505079	0,00026734	0,00253789	protein_codir	hypothetical protein
TcG_03662	264,2652476	-0,633077538	0,1119453	-5,65523983	1,5563E-08	4,7693E-07	protein_codir	hypothetical protein
TcG_03663	76,7421093	-0,996428093	0,20342622	-4,89822839	9,6705E-07	1,976E-05	protein_codir	hypothetical protein
TcG_03664	214,1479513	-0,319445327	0,12985481	-2,46001929	0,01389295	0,06395065	protein_codir	hypothetical protein
TcG_03665	96,10557165	-0,163609654	0,18866259	-0,86720773	0,3858282	0,61632504	protein_codir	hypothetical protein
TcG_03666	115,3921876	-0,16673912	0,16187597	-1,03004243	0,30299009	0,53561843	protein_codir	hypothetical protein
TcG_03667	16,1430399	-0,472639453	0,43474226	-1,08717162	0,27696097	1	protein_codir	putative D-alanyl-glycyl endopeptidase-like protein
TcG_03668	101,7042437	-0,194045385	0,17502644	-1,10866325	0,26757548	0,49992998	protein_codir	D-alanyl-glycyl endopeptidase-like protein
TcG_03669	80,78030869	-0,049144867	0,19272456	-0,25500054	0,79872268	0,89959947	protein_codir	putative D-alanyl-glycyl endopeptidase-like protein
TcG_03670	501,2141599	-0,168522941	0,08476941	-1,98801607	0,04680991	0,15809563	protein_codir	methyltransferase/D-alanine--D-alanine ligase
TcG_03671	185,1182741	-0,356837276	0,12964889	-2,75233577	0,00591718	0,03266149	protein_codir	putative protein kinase
TcG_03672	1825,440721	-0,220175059	0,05127485	-4,29401687	1,7547E-05	0,00024732	protein_codir	putative 3-oxo-5-alpha-steroid 4-dehydrogenase
TcG_03673	280,791873	-0,049351498	0,11359214	-0,43446224	0,66395281	0,82434295	protein_codir	apurinic/apyrimidinic endonuclease
TcG_03674	185,2109637	-0,32670692	0,13226903	-2,47001823	0,01351062	0,06279103		
TcG_03675	175,2880415	-0,404595793	0,15003093	-2,69674918	0,007002	0,03729892	protein_codir	putative mitochondrial RNA binding complex 1 subunit
TcG_03676	346,197377	-0,168631693	0,09898087	-1,70367956	0,08844097	0,24714835	protein_codir	hypothetical protein
TcG_03677	834,2916002	-0,302232551	0,06806319	-4,44047006	8,9763E-06	0,0001396	protein_codir	putative dynein heavy chain
TcG_03678	618,5017755	-0,382632756	0,07547795	-5,06946406	3,9894E-07	8,9402E-06	protein_codir	putative 2-oxoisovalerate dehydrogenase beta subunit, mitochondrial precursor
TcG_03679	120,6790384	-0,226225157	0,16475867	-1,37306984	0,16973064	0,37606704	protein_codir	U6 snRNA-associated Sm-like protein LSm8p
TcG_03680	78,74107144	0,294072905	0,19715667	1,49156961	0,13581201	0,32836351	protein_codir	L1Tc protein
TcG_03681	1,082480914	-0,385994369	1,7518142	-0,22033979	0,82560654	1	protein_codir	hypothetical protein
TcG_03682	645,8946004	0,170362666	0,07536032	2,2606414	0,02378147	0,09628871	protein_codir	putative target of rapamycin (TOR) kinase 1
TcG_03683	63,15159882	-0,437075104	0,22416901	-1,94975702	0,05120509	0,16825358	protein_codir	hypothetical protein
TcG_03684	18,04975425	-0,404594757	0,4033045	-1,00319922	0,31576475	0,55066198	protein_codir	hypothetical protein
TcG_03685	124,519583	-0,010293933	0,15675421	-0,06566926	0,94764115	0,97539448	protein_codir	type 11 methyltransferase
TcG_03686	116,1107309	-0,148134297	0,16249106	-0,91164585	0,36195518	0,59458566	protein_codir	putative leucine-rich repeat protein 1 (LRRP1)
TcG_03687	137,9935345	-0,622060555	0,15799311	-3,93726374	8,2416E-05	0,00094169		
TcG_03688	1850,987794	-0,281838031	0,04908458	-5,74188558	9,3628E-09	2,9966E-07	protein_codir	actin
TcG_03689	1146,424535	-0,410194785	0,06199625	-6,61644563	3,6794E-11	2,0495E-09	protein_codir	hypothetical protein

TcG_03690	193,8718126	-0,309974444	0,12867147	-2,40903788	0,01599464	0,07105551	protein_codir	cAMP-dependent protein kinase regulator
TcG_03691	281,680643	-0,28086551	0,11497204	-2,44290282	0,01456966	0,0663538	protein_codir	putative tRNA nucleotidyltransferase
TcG_03692	1909,65375	-0,319203977	0,05988915	-5,3299129	9,826E-08	2,5572E-06	protein_codir	hypothetical protein
TcG_03693	1279,457394	-0,329037635	0,0567011	-5,80302036	6,5131E-09	2,1355E-07	protein_codir	putative RNA-binding protein
TcG_03694	441,0226782	-0,453715524	0,09172125	-4,94667846	7,5491E-07	1,5788E-05	protein_codir	putative fructose-1,6-bisphosphatase, cytosolic
TcG_03695	89,79097722	-0,141459353	0,18436909	-0,76726177	0,44292589	0,66499591	protein_codir	hypothetical protein
TcG_03696	275,3911738	-0,258448364	0,11160426	-2,31575714	0,02057153	0,08598188	protein_codir	hypothetical protein
TcG_03697	139,5401137	0,047410002	0,15493673	0,30599588	0,75960778	0,87858798	protein_codir	cytochrome c oxidase assembly factor
TcG_03698	202,0616049	-0,034326468	0,12990502	-0,26424282	0,79159282	0,89581895	protein_codir	Rho-like GTP binding protein
TcG_03699	591,0795905	-0,249907637	0,08034561	-3,11040815	0,00186829	0,01302407	protein_codir	hypothetical protein
TcG_03700	1350,962733	-0,345600863	0,0651463	-5,30499578	1,1268E-07	2,8882E-06	protein_codir	P-ATPase family transporter: proton
TcG_03701	343,4244393	-0,325653972	0,09767335	-3,3341128	0,00085572	0,0068659	protein_codir	ankyrin
TcG_03702	764,7500955	-0,351688742	0,07040627	-4,99513419	5,8795E-07	1,2662E-05	protein_codir	hypothetical protein
TcG_03703	253,1325061	-0,12342524	0,11692612	-1,05558314	0,29115871	0,52365178	protein_codir	putative transcription factor 25-like
TcG_03704	158,5231186	-0,18357594	0,1414061	-1,29821793	0,19421246	0,41121081	protein_codir	putative transcription factor 25-like
TcG_03705	73,61212017	-0,080749988	0,20900787	-0,38634903	0,69923819	0,84415689	protein_codir	hypothetical protein
TcG_03706	134,2551617	-0,494408195	0,15525423	-3,18450705	0,00145001	0,01055926	protein_codir	hypothetical protein
TcG_03707	177,7168466	-0,13167893	0,13536843	-0,97274473	0,33068019	0,56523583	protein_codir	hypothetical protein
TcG_03708	591,630262	-0,162507674	0,08198214	-1,98223274	0,0474532	0,15931404	protein_codir	putative chloride channel protein
TcG_03709	321,9670959	-0,203941759	0,10169833	-2,00535993	0,04492458	0,15335775	protein_codir	hypothetical protein
TcG_03710	518,3819244	-0,245681342	0,08207854	-2,99324685	0,00276026	0,01779703	protein_codir	hypothetical protein
TcG_03711	416,3014673	-0,283116513	0,08874023	-3,19039656	0,00142078	0,01039869	protein_codir	hypothetical protein
TcG_03712	224,5500067	-0,215446163	0,12059723	-1,78649344	0,0740194	0,21961299	protein_codir	hypothetical protein
TcG_03713	148,8051786	-0,295240616	0,14883259	-1,98370948	0,04728824	0,15894445	protein_codir	adenylate kinase
TcG_03714	432,4804881	-0,071965879	0,0894981	-0,80410509	0,42133629	0,647488	protein_codir	60S ribosomal protein L10
TcG_03715	421,7587794	-0,104051706	0,08922359	-1,16619048	0,24353744	0,47180551	protein_codir	putative 60S ribosomal protein L10
TcG_03716	551,5159903	-0,283903604	0,08110211	-3,50057005	0,00046426	0,00405348	protein_codir	putative chaperone protein DNAj
TcG_03717	266,6778969	-0,378238741	0,11738048	-3,22233079	0,00127152	0,00948606	protein_codir	zinc finger protein, predicted
TcG_03718	116,7366155	-0,169238332	0,16699202	-1,01345159	0,31084451	0,54446303	protein_codir	hypothetical protein
TcG_03719	67,3407328	-0,834632751	0,21837291	-3,82205255	0,00013235	0,00140804	protein_codir	hypothetical protein
TcG_03720	386,639758	-0,506700656	0,0928604	-5,45658474	4,8538E-08	1,3422E-06	protein_codir	hypothetical protein
TcG_03721	156,6966265	-0,122999622	0,14872843	-0,82700816	0,40823244	0,63660535	protein_codir	uncharacterized protein
TcG_03722	404,048619	-0,131743797	0,09199737	-1,4320388	0,15213272	0,35233988	protein_codir	putative rhomboid-like protein, putative,serine peptidase, Clan S-, family S54
TcG_03723	251,8021082	-0,132215409	0,11478757	-1,1518269	0,24939221	0,47862484	protein_codir	putative tyrosine phospatase-like protein
TcG_03724	503,1947534	-0,29798094	0,08330859	-3,57683332	0,00034778	0,00317526	protein_codir	hypothetical protein
TcG_03725	324,697057	-0,157627738	0,10519284	-1,49846458	0,13401259	0,32557556	protein_codir	putative metalloprotease-like protein, putative,polypeptide deformylase
TcG_03726	1061,548192	-0,153931736	0,06515694	-2,36247655	0,01815329	0,07791213	protein_codir	pescadillo
TcG_03727	173,1547736	-0,403495369	0,14224104	-2,83670151	0,00455822	0,02680789	protein_codir	hypothetical protein
TcG_03728	421,5903242	-0,533555574	0,09094615	-5,86671978	4,445E-09	1,5282E-07	protein_codir	hypothetical protein
TcG_03729	193,1118079	-0,254726379	0,13045726	-1,95256579	0,05087107	0,16767915	protein_codir	putative heat shock protein
TcG_03730	1745,641044	-0,108005307	0,05193116	-2,07977842	0,03754586	0,13501188	protein_codir	nascent polypeptide associated complex subunit
TcG_03731	13,52677732	-0,00417161	0,48562979	-0,0085901	0,99314617	1		
TcG_03732	15,51693205	-0,065939456	0,4484952	-0,14702377	0,88311326	1	protein_codir	putative 60S ribosomal protein L10
TcG_03733	298,1273083	-0,135818541	0,11001126	-1,23458762	0,21698402	0,44089387	protein_codir	putative WAS protein family 3-like
TcG_03734	217,2984222	-0,345289737	0,12228538	-2,82363866	0,00474819	0,02763059	protein_codir	uncharacterized protein
TcG_03735	791,405576	-0,350360305	0,06800622	-5,1518862	2,5788E-07	6,0238E-06	protein_codir	hypothetical protein
TcG_03736	524,4364238	-0,362263429	0,08321637	-4,35327119	1,3412E-05	0,00019571	protein_codir	hypothetical protein
TcG_03737	214,1226796	-0,367244366	0,12350935	-2,9734135	0,00294507	0,01872757	protein_codir	putative Mg transporter
TcG_03738	65,65477153	-0,322410698	0,21651173	-1,48911422	0,1364573	0,32916375	protein_codir	hypothetical protein
TcG_03739	74,52963213	0,09384567	0,23694773	0,39606064	0,6920603	0,84065953	protein_codir	hypothetical protein
TcG_03740	247,6979017	0,449538153	0,11588954	3,8790226	0,00010488	0,00116056	protein_codir	peptide methionine sulfoxide reductase
TcG_03741	373,1570893	-0,027380248	0,09514277	-0,28778064	0,77351466	0,88691888	protein_codir	Ras like protein family, member T1

TcG_03742	542,0703896	-0,216906019	0,08403452	-2,58115374	0,00984707	0,04881822	protein_codir	putative proteasome regulatory non-ATPase subunit
TcG_03743	258,4615793	0,034982661	0,11041469	0,31682977	0,75137278	0,87482715	protein_codir	hypothetical protein
TcG_03744	353,8998658	0,045950037	0,10013806	0,45886685	0,64632978	0,81201944	protein_codir	putative carnitine O-palmitoyltransferase
TcG_03745	255,412959	0,114107492	0,11418052	0,99936041	0,31762013	0,55273912		
TcG_03746	113,6050391	0,070587776	0,16447696	0,42916514	0,66780305	0,82600258	protein_codir	hypothetical protein
TcG_03747	383,4838591	0,088192029	0,09478175	0,93047475	0,35212533	0,58641114	protein_codir	hypothetical protein
TcG_03748	269,9618509	0,182415005	0,10993657	1,65927499	0,09706039	0,26348213	protein_codir	RNA-editing complex protein MP42
TcG_03749	225,9337644	0,05792079	0,12679117	0,45682038	0,64780016	0,81260383	protein_codir	lipoate-protein ligase
TcG_03750	942,136823	-0,327106999	0,07129213	-4,58826245	4,4695E-06	7,6041E-05	protein_codir	putative cation transporting ATPase
TcG_03751	178,8542221	-0,15527701	0,13445374	-1,1548731	0,2481424	0,47744549	protein_codir	Sel1 domain-containing protein
TcG_03752	142,1535952	-0,036626068	0,1480402	-0,24740623	0,80459386	0,90347203	protein_codir	hypothetical protein
TcG_03753	174,5114967	0,145243085	0,1352541	1,07385345	0,28288837	0,51614876	protein_codir	hypothetical protein
TcG_03754	143,9817557	0,026533877	0,15465415	0,17156912	0,86377629	0,93486334	protein_codir	RNA-editing complex protein
TcG_03755	319,7247177	0,027582818	0,10431995	0,26440596	0,79146712	0,89576419	protein_codir	hypothetical protein
TcG_03756	303,7847047	-0,079978177	0,11061433	-0,7230363	0,46965758	0,6861914	protein_codir	peptidyl-prolyl cis-trans isomerase
TcG_03757	517,3207562	-0,014794507	0,08323862	-0,1777361	0,85893023	0,93207022	protein_codir	PIF1 helicase-like protein
TcG_03758	242,2534827	-0,106369809	0,11642442	-0,91363829	0,36090694	0,59353695	protein_codir	rabGTPase-activating protein
TcG_03759	1379,119919	-0,252081407	0,06855504	-3,6770658	0,00023593	0,00229129	protein_codir	putative RNA-binding protein
TcG_03760	46,66177247	-0,731840871	0,27284327	-2,68227568	0,00731232	0,03864987		
TcG_03761	122,6760936	-0,017705288	0,15886385	-0,11144944	0,91125995	0,95684497	protein_codir	MIP18 family protein
TcG_03762	1725,62259	0,128358833	0,05260767	2,43992644	0,01469025	0,06687673	protein_codir	nucleolar RNA-binding protein
TcG_03763	908,4751594	0,172496572	0,06439779	2,67861004	0,00739284	0,03895111	protein_codir	nucleolar RNA-binding protein
TcG_03764	34,96035138	0,332906568	0,29291114	1,13654459	0,25572871	0,48739478	protein_codir	nucleolar RNA-binding protein
TcG_03765	317,6456523	-0,019182874	0,10959318	-0,17503712	0,86105046	0,93365753	protein_codir	hypothetical protein
TcG_03766	560,1990656	-0,047565003	0,07895226	-0,60245273	0,54687282	0,74375731	protein_codir	vesicular transport-associated repeat protein
TcG_03767	397,229871	0,001501323	0,09121761	0,0164587	0,98686845	0,99474691	protein_codir	hypothetical protein
TcG_03768	2766,206322	-0,10131815	0,04615603	-2,19512254	0,02815481	0,10906105	protein_codir	hypothetical protein
TcG_03769	202,053469	-0,225014421	0,12978963	-1,73368569	0,08297387	0,23681114	protein_codir	radial spoke head protein 9
TcG_03770	627,499673	-0,05307158	0,0743496	-0,71381127	0,47534389	0,69103267	protein_codir	vacuole import and degradation
TcG_03771	63,87075355	0,070270575	0,21773412	0,32273571	0,7468954	0,87215583	protein_codir	hypothetical protein
TcG_03772	630,1915148	0,061179335	0,07846752	0,77967721	0,43558089	0,65939964	protein_codir	putative DNA-directed RNA polymerase III largest subunit
TcG_03773	308,6264227	-0,033236116	0,10698021	-0,31067538	0,75604742	0,87716571	protein_codir	hypothetical protein
TcG_03774	295,5870568	0,178295215	0,10955778	1,62740803	0,10365047	0,2758774	protein_codir	hypothetical protein
TcG_03775	365,4325928	-0,087250595	0,09955995	-0,87636236	0,38083307	0,61104814	protein_codir	hypothetical protein
TcG_03776	244,8078567	0,183198706	0,11462955	1,59818046	0,11000282	0,28646723	protein_codir	glycosyltransferase
TcG_03777	135,3484258	0,108717858	0,16066486	0,6766748	0,49861231	0,70769597	protein_codir	hypothetical protein
TcG_03778	53,56311149	-0,344604695	0,24337253	-1,41595561	0,1567885	0,35829762	protein_codir	hypothetical protein
TcG_03779	172,9459455	-0,199295403	0,14764705	-1,34980963	0,17707705	0,38718302	protein_codir	putative serine/threonine protein kinase, putative,protein kinase
TcG_03780	603,2941266	-0,156010478	0,07895688	-1,9758948	0,04816669	0,16124222	protein_codir	hypothetical protein
TcG_03781	331,3397869	-0,126471408	0,10360221	-1,2207405	0,22218429	0,44808132	protein_codir	putative protein kinase
TcG_03782	1043,984668	-0,128430057	0,06537268	-1,96458304	0,04946252	0,16434549	protein_codir	putative small GTP-binding protein Rab11, putative,Rab11 GTPase
TcG_03783	40,66410968	-0,163691747	0,28153066	-0,58143489	0,56094739	0,7545729		
TcG_03784	32,39535319	-0,817972783	0,30633574	-2,67018397	0,00758097	0,03977949		
TcG_03785	370,3562082	-0,171022154	0,09882458	-1,73056284	0,08352976	0,23772435	protein_codir	splicing factor TSR1
TcG_03786	53,04831721	-0,431658843	0,23757569	-1,81693188	0,06922754	0,21046191	protein_codir	hypothetical protein
TcG_03787	56,86743383	-0,466110706	0,22885731	-2,03668702	0,04168142	0,14545501	protein_codir	hypothetical protein
TcG_03788	99,0169395	-0,22161219	0,18307224	-1,21051772	0,2260803	0,45247302	protein_codir	hypothetical protein
TcG_03789	64,3872776	0,198359638	0,22234369	0,89213074	0,37232286	0,6035725	protein_codir	microtubial binding protein
TcG_03790	310,3909015	0,063050392	0,10377321	0,60757867	0,54346695	0,74086458	protein_codir	hypothetical protein
TcG_03791	197,9429266	0,304968145	0,12782238	2,38587438	0,01703857	0,07444098	protein_codir	hypothetical protein
TcG_03792	398,5868775	0,192349125	0,09798799	1,96298667	0,04964772	0,16481906	protein_codir	hypothetical protein
TcG_03793	518,6658748	-0,041841252	0,08474134	-0,49375253	0,62148096	0,79598479	protein_codir	hypothetical protein

TcG_03794	295,6714326	0,068789673	0,10479023	0,65645123	0,51153384	0,71768359	protein_codir	hypothetical protein
TcG_03795	467,8195527	0,042614486	0,09315183	0,45747341	0,64733081	0,81256498	protein_codir	putative translation initiation factor IF-2
TcG_03796	493,8112214	0,122285662	0,08444331	1,4481392	0,14757813	0,34633181	protein_codir	hypothetical protein
TcG_03797	168,2222688	0,15098275	0,1412823	1,06866009	0,28522286	0,51877427	protein_codir	hypothetical protein
TcG_03798	323,4265696	-0,032976592	0,09961017	-0,33105647	0,74060183	0,86769267	protein_codir	hypothetical protein
TcG_03799	361,2566638	0,210636523	0,09840563	2,14049255	0,03231498	0,12093068	protein_codir	cation transport protein ChaC
TcG_03800	227,2212796	0,09603346	0,11837807	0,81124364	0,41722577	0,64394142	protein_codir	putative protein kinase
TcG_03801	161,2252702	0,064297368	0,14883827	0,43199486	0,66574515	0,82504261	protein_codir	hypothetical protein
TcG_03802	243,7167797	0,30993439	0,12519438	2,47562544	0,0133003	0,06198605	protein_codir	serine/threonine protein kinase
TcG_03803	288,9626441	-0,054058772	0,10549188	-0,51244485	0,60833971	0,78802389	protein_codir	adaptor medium chain 1
TcG_03804	79,09997864	-0,179522346	0,20809552	-0,86269201	0,38830682	0,6185787	protein_codir	hypothetical protein
TcG_03805	130,7575768	-0,078728869	0,15252092	-0,51618407	0,60572585	0,78692126	protein_codir	hypothetical protein
TcG_03806	581,3818783	0,105564923	0,07699202	1,37111517	0,17033906	0,37691908	protein_codir	putative dynein heavy chain, cytosolic
TcG_03807	1038,457814	0,297735639	0,14058177	2,1178822	0,03418505	0,12621668	protein_codir	cytoplasmic dynein 1 heavy chain 1
TcG_03808	746,2322284	-0,026249915	0,07254556	-0,36184039	0,7174713	0,85508792	protein_codir	hypothetical protein
TcG_03809	296,795723	-0,245487954	0,10618259	-2,31194158	0,0207809	0,08670059	protein_codir	hypothetical protein
TcG_03810	205,035911	0,240625983	0,13320831	1,80638864	0,07085765	0,21360781	protein_codir	hypothetical protein
TcG_03811	274,8396417	0,010245969	0,11141073	0,09196573	0,92672527	0,96430637	protein_codir	hypothetical protein
TcG_03812	142,7062987	0,06179711	0,1528145	0,40439297	0,68592377	0,83706686	protein_codir	hypothetical protein
TcG_03813	257,3661599	-0,072089905	0,11117924	-0,64841155	0,5167188	0,72224827		
TcG_03814	404,858165	-0,124510416	0,09466539	-1,31526867	0,18841962	0,40277302	protein_codir	transporter protein
TcG_03815	247,6369687	0,003460566	0,11560787	0,02993365	0,97611997	0,98978872	protein_codir	trichohyalin
TcG_03816	546,5994801	-0,01368004	0,08011602	-0,17075287	0,86441809	0,93498506	protein_codir	hypothetical protein
TcG_03817	344,4423843	0,243317861	0,10351404	2,35057829	0,01874426	0,07975433	protein_codir	hypothetical protein
TcG_03818	90,09623814	0,03985333	0,19656417	0,20274972	0,83933066	0,92192691	protein_codir	hypothetical protein
TcG_03819	487,6849588	0,015878595	0,09931751	0,15987709	0,87297789	0,93911995	protein_codir	solute carrier family 35 (UDP-galactose transporter), member B1
TcG_03820	314,1220152	0,075331112	0,10132564	0,74345558	0,45720591	0,6767839	protein_codir	hypothetical protein
TcG_03821	613,0680528	0,29066872	0,07894915	3,68172058	0,00023167	0,00225552	protein_codir	hypothetical protein
TcG_03822	282,5463188	-0,170996586	0,10631155	-1,60844793	0,10773711	0,28272756	protein_codir	G-protein (beta)-like protein
TcG_03823	542,7829661	0,070055985	0,07988453	0,87696558	0,38050533	0,61079532	protein_codir	hypothetical protein
TcG_03824	181,0012568	-0,030218458	0,13449724	-0,22467717	0,82223043	0,91187534	protein_codir	hypothetical protein
TcG_03825	381,8735091	0,096068822	0,0978295	0,98200263	0,32609855	0,56056051	protein_codir	hypothetical protein
TcG_03826	828,0106532	0,114882351	0,06649357	1,72772131	0,0840382	0,23876082	protein_codir	putative kinesin
TcG_03827	66,8924551	0,306643714	0,22277282	1,37648619	0,16867116	0,37510151	protein_codir	protein kinase
TcG_03828	278,2251789	0,220865481	0,10828243	2,03971672	0,04137855	0,14474996	protein_codir	hypothetical protein
TcG_03829	302,4126061	0,239518528	0,10982261	2,18095824	0,02918651	0,11204426	protein_codir	a44l protein-like protein
TcG_03830	4357,660001	-0,220841969	0,04373935	-5,04904542	4,4402E-07	9,7437E-06	protein_codir	histone H2A
TcG_03831	1424,456047	0,019443202	0,06305988	0,30832919	0,75783186	0,87801518	protein_codir	histone H2A
TcG_03832	1606,757197	-0,34121796	0,05799112	-5,8839689	4,0054E-09	1,3936E-07	protein_codir	histone H2A
TcG_03833	533,9736327	0,088244023	0,08551216	1,03194703	0,30209693	0,53534644	protein_codir	trans-sialidase
TcG_03834	682,4906958	0,211641999	0,079699	2,65551621	0,00791871	0,04118059	protein_codir	3-hydroxyacyl-CoA dehydrogenase
TcG_03835	253,4180295	-0,117064204	0,11550243	-1,01352154	0,31081111	0,54446303	protein_codir	hypothetical protein
TcG_03836	231,1103504	-0,013629415	0,12444533	-0,1095213	0,91278903	0,95723874	protein_codir	hypothetical protein
TcG_03837	405,0363862	0,034682444	0,09420179	0,36817181	0,71274513	0,85248988	protein_codir	putative serine palmitoyltransferase
TcG_03838	231,8176021	0,088919977	0,11944007	0,74447361	0,45659	0,67639993	protein_codir	3-keto-dihydrosphingosine reductase
TcG_03839	118,7474085	0,421200404	0,16740495	2,51605712	0,01186759	0,05647811	protein_codir	hypothetical protein
TcG_03840	318,5943743	-0,13950746	0,10165483	-1,37236431	0,16995006	0,3762739	protein_codir	hypothetical protein
TcG_03841	237,8837229	0,05524656	0,1164862	0,47427558	0,6353034	0,80532004	protein_codir	enoyl-CoA hydratase/isomerase family protein
TcG_03842	608,8875255	-0,071875347	0,0766724	-0,93743444	0,34853513	0,58310373	protein_codir	putative ATP-dependent DEAD-box RNA helicase
TcG_03843	671,6619772	-0,109813689	0,07723162	-1,42187474	0,15506262	0,35638871	protein_codir	hypothetical protein
TcG_03844	90,09341337	-0,111315921	0,19921676	-0,55876785	0,57632017	0,76670634	protein_codir	hypothetical protein
TcG_03845	191,6381731	0,002010244	0,1315723	0,01527863	0,98780989	0,99514516	protein_codir	hypothetical protein

TcG_03846	126,373812	0,545761547	0,16039804	3,40254488	0,00066761	0,0055171	protein_codir	40S ribosomal protein S3a
TcG_03847	1903,095711	0,356028727	0,05559242	6,40426798	1,5109E-10	7,2337E-09	protein_codir	40S ribosomal protein S3A
TcG_03848	282,6621547	0,238780765	0,11089954	2,1531267	0,03130872	0,11795457	protein_codir	hypothetical protein
TcG_03849	93,08769259	-0,068361189	0,17898212	-0,38194424	0,70250272	0,84624626	protein_codir	hypothetical protein
TcG_03850	200,2360446	-0,07796971	0,12618441	-0,61790287	0,53663936	0,73660472	protein_codir	hypothetical protein
TcG_03851	289,5254309	0,160623804	0,10559516	1,52112842	0,12822761	0,31717445	protein_codir	hypothetical protein
TcG_03852	240,4830141	0,259957435	0,11811038	2,20097032	0,02773813	0,10791211	protein_codir	hypothetical protein
TcG_03853	207,6047963	-0,142145705	0,1237867	-1,14831158	0,25083996	0,4806072	protein_codir	putative protein kinase
TcG_03854	389,6469286	0,206921061	0,10750798	1,92470422	0,05426637	0,17531119	protein_codir	putative phosphatidylinositol-4-phosphate 5-kinase-like
TcG_03855	338,5302286	0,1227037	0,09885937	1,24119443	0,21453393	0,43829838	protein_codir	EF hand domain containing protein
TcG_03856	563,8324149	0,129734617	0,08134106	1,59494621	0,11072427	0,28760174	protein_codir	hypothetical protein
TcG_03857	330,6958341	-0,004881671	0,09978757	-0,04892064	0,96098254	0,98198472	protein_codir	putative SNF-7-like protein
TcG_03858	30,69400209	0,435005323	0,35116856	1,23873653	0,21544308	0,43913853	protein_codir	hypothetical protein
TcG_03859	77,19778406	0,231927075	0,20728567	1,11887656	0,2631928	0,49542678	protein_codir	hypothetical protein
TcG_03860	762,4482876	0,203902193	0,06942949	2,9368241	0,00331592	0,02065498	protein_codir	60S ribosomal protein L18a
TcG_03861	837,066329	-0,033154485	0,0714781	-0,46384118	0,64276154	0,81042933	protein_codir	hypothetical protein
TcG_03862	275,1031896	0,086500998	0,10911083	0,79278102	0,42790541	0,65319	protein_codir	hypothetical protein
TcG_03863	156,2395086	-0,025093588	0,14887556	-0,16855411	0,86614738	0,93550699	protein_codir	conserved RING finger protein
TcG_03864	2157,736363	0,191348816	0,05621441	3,4039106	0,00066428	0,00549989	protein_codir	hypothetical protein
TcG_03865	566,9280012	-0,143332635	0,08192887	-1,7494765	0,08020869	0,23148475	protein_codir	hypothetical protein
TcG_03866	19,48075232	-0,644191831	0,4113756	-1,56594566	0,11736134	0,29871451		
TcG_03867	203,5611119	-0,059498944	0,12259385	-0,48533386	0,62743954	0,80025287	protein_codir	TPR-repeat-containing protein
TcG_03868	253,439167	0,200580916	0,12057669	1,6635132	0,09620975	0,26234083	protein_codir	putative vacuolar ATP synthase subunit d
TcG_03869	162,9905886	0,135191325	0,14281197	0,94663862	0,34382296	0,57799373	protein_codir	hypothetical protein
TcG_03870	158,7069155	-0,000974264	0,14509018	-0,00671488	0,99464234	0,99778366	protein_codir	hypothetical protein
TcG_03871	231,0199282	-0,028624633	0,11613394	-0,24647948	0,80531109	0,90392698	protein_codir	hypothetical protein
TcG_03872	336,9087197	0,110713432	0,10359109	1,06875439	0,28518035	0,51877427	protein_codir	hypothetical protein
TcG_03873	499,2497542	0,087241839	0,09285412	0,93955811	0,34744427	0,58190301	protein_codir	putative proteasome activator protein pa26
TcG_03874	421,0185086	-0,185313487	0,08981311	-2,06332336	0,03908192	0,13912475	protein_codir	conserved CBS domain protein
TcG_03875	185,1668829	0,124083308	0,13286579	0,93389961	0,35035568	0,58488966	protein_codir	putative rRNA dimethyltransferase
TcG_03876	151,7680376	0,026456994	0,14587376	0,18136911	0,85607786	0,93026807	protein_codir	putative actin
TcG_03877	177,2929506	-0,149796355	0,13611477	-1,10051509	0,27110776	0,50434099	protein_codir	hypothetical protein
TcG_03878	277,5300783	-0,226124359	0,10897587	-2,07499472	0,03798702	0,13614561	protein_codir	hypothetical protein
TcG_03879	552,8816549	0,270980992	0,08186581	3,31006322	0,00093275	0,00737164	protein_codir	putative aspartate aminotransferase
TcG_03880	187,4772499	0,066671651	0,13074324	0,50994339	0,61009112	0,7890164	protein_codir	zinc finger protein
TcG_03881	243,3222855	-0,036017865	0,11492028	-0,31341609	0,75396456	0,87582047	protein_codir	hypothetical protein
TcG_03882	102,6566682	-0,20890347	0,17235097	-1,21208175	0,22548109	0,45182011	protein_codir	hypothetical protein
TcG_03883	143,0371608	-0,091361613	0,14716794	-0,62079832	0,53473233	0,73501112	protein_codir	hypothetical protein
TcG_03884	173,9711044	0,305479021	0,13436738	2,27346122	0,0229984	0,09385243	protein_codir	putative serine protease PepD
TcG_03885	179,664439	0,337510715	0,13784271	2,44852054	0,01434443	0,06573614	protein_codir	Adenine phosphoribosyltransferase
TcG_03886	373,7875787	0,021584985	0,09803052	0,22018638	0,82572601	0,91505132	protein_codir	Adenine phosphoribosyltransferase
TcG_03887	264,9299547	0,200478924	0,11075824	1,81005875	0,07028668	0,21239996	protein_codir	E2F target protein 1
TcG_03888	176,3624615	-0,101423839	0,14093636	-0,71964279	0,47174496	0,68776105	protein_codir	hypothetical protein
TcG_03889	100,4469833	0,037579121	0,18377285	0,2044868	0,83797311	0,92139664	protein_codir	putative 60S ribosomal protein L7
TcG_03890	26,08092813	0,172443519	0,36586393	0,47133239	0,63740339	0,80727544	protein_codir	putative 60S ribosomal protein L7
TcG_03891	117,4767632	0,069109732	0,17517034	0,39452873	0,69319072	0,8410627	protein_codir	putative 60S ribosomal protein L7
TcG_03892	78,78750373	0,141031678	0,19973032	0,7061105	0,48011942	0,69429152	protein_codir	hypothetical protein
TcG_03893	207,651913	0,038710177	0,12587128	0,3075378	0,75843406	0,87801518	protein_codir	putative HIRA-interacting protein 5
TcG_03894	259,9104386	-0,102461135	0,11201604	-0,91470055	0,36034886	0,59295581	protein_codir	eukaryotic peptide chain release factor subunit 1
TcG_03895	342,5201809	0,181201688	0,10161709	1,78318129	0,0745568	0,22045189	protein_codir	hypothetical protein
TcG_03896	276,1277957	0,04430432	0,11215213	0,3950377	0,69281507	0,8410627	protein_codir	putative silent information regulator 2
TcG_03897	188,0936304	-0,056597382	0,12997371	-0,43545254	0,66323398	0,82378097	protein_codir	hypothetical protein

TcG_03898	328,9948474	0,034786392	0,1062672	0,32734833	0,74340444	0,86954228	protein_codir	hypothetical protein
TcG_03899	353,1097117	-0,251478321	0,10103899	-2,48892352	0,01281305	0,06005341	protein_codir	hypothetical protein
TcG_03900	368,7911722	-0,081011883	0,10240057	-0,79112725	0,42886974	0,65398767	protein_codir	hypothetical protein
TcG_03901	433,9343962	0,029079403	0,09301537	0,31263008	0,75456172	0,87623549	protein_codir	hypothetical protein
TcG_03902	280,9216741	-0,222286937	0,10843425	-2,04996975	0,04036738	0,14207063	protein_codir	ras-like small GTPase
TcG_03903	181,1461543	-0,108589082	0,13502462	-0,80421689	0,42127173	0,647488	protein_codir	hypothetical protein
TcG_03904	141,9897825	-0,174525192	0,16501581	-1,05762709	0,29022549	0,52345679	protein_codir	hypothetical protein
TcG_03905	355,8286482	-0,14659866	0,09862725	-1,48639096	0,13717574	0,33021361	protein_codir	6-phosphofructo-2-kinase/fructose-2, 6-biphosphatase-1-like protein
TcG_03906	169,3402005	-0,010269121	0,13594793	-0,07553717	0,93978733	0,97208963	protein_codir	putative C-1-tetrahydrofolate synthase, cytoplasmic
TcG_03907	119,617789	-0,259404312	0,16068039	-1,61441173	0,10643815	0,28084546	protein_codir	putative mitotic cyclin
TcG_03908	182,2311124	-0,059022612	0,13323383	-0,44300018	0,65776561	0,81952831	protein_codir	pyrimidine-specific ribonucleoside hydrolase
TcG_03909	456,1185106	-0,041501408	0,09157663	-0,45318777	0,65041354	0,81446995	protein_codir	Vacuolar protein sorting protein
TcG_03910	518,6042282	0,105538748	0,0852324	1,23824685	0,21562455	0,4393644	protein_codir	hypothetical protein
TcG_03911	274,0603928	-0,131418504	0,10945095	-1,20070682	0,22986495	0,45688956	protein_codir	TPR Domain containing protein
TcG_03912	359,1750236	-0,153320894	0,09783521	-1,56713405	0,11708336	0,29853165	protein_codir	RET2, RNA editing complex MP57
TcG_03913	125,9849571	-0,035879327	0,17393303	-0,20628242	0,83657031	0,92063564	protein_codir	Thermonuclease precursor
TcG_03914	196,532864	0,166544387	0,13269359	1,25510497	0,20944066	0,43200631	protein_codir	oxidoreductase
TcG_03915	201,2420162	-0,260031783	0,12418741	-2,09386597	0,03627192	0,13206992	protein_codir	putative pseudouridylate synthase I
TcG_03916	395,4518655	-0,144297023	0,09276464	-1,55551753	0,11982286	0,30291726	protein_codir	putative nucleotide-binding protein
TcG_03917	149,5103148	-0,030486103	0,15104982	-0,20182813	0,84005109	0,92224543	protein_codir	hypothetical protein
TcG_03918	380,7677192	-0,102209393	0,09766477	-1,0465329	0,29531506	0,52809782	protein_codir	hypothetical protein
TcG_03919	545,2783415	-0,265700419	0,08365565	-3,1761205	0,00149259	0,01082852	protein_codir	ATPase subunit 9
TcG_03920	941,0807035	-0,290665244	0,06523425	-4,45571545	8,3614E-06	0,00013145	protein_codir	hypothetical protein
TcG_03921	326,5802956	-0,140237102	0,10243891	-1,36898276	0,17100466	0,37781465	protein_codir	hypothetical protein
TcG_03922	132,0852408	0,309123715	0,15956592	1,93727906	0,05271124	0,17142733	protein_codir	cytoplasmic protein
TcG_03923	203,9704316	-0,220278134	0,13193784	-1,66955997	0,09500646	0,25979344	protein_codir	putative DNA polymerase sigma-like protein
TcG_03924	314,1062966	-0,080851073	0,1093638	-0,73928548	0,45973366	0,67827253	protein_codir	putative leucine-rich repeat protein (LRRP)
TcG_03925	550,6982173	-0,359205584	0,08373108	-4,28999088	1,7868E-05	0,00025093	protein_codir	hypothetical protein
TcG_03926	408,2060794	-0,063232938	0,09355966	-0,67585689	0,49913151	0,70825936	protein_codir	hypothetical protein
TcG_03927	434,3347625	-0,136920301	0,0952247	-1,43786536	0,15047226	0,35042645	protein_codir	hypothetical protein
TcG_03928	342,6316283	0,028422325	0,09862883	0,28817461	0,77321308	0,88691888	protein_codir	hypothetical protein
TcG_03929	102,588974	-0,169501176	0,19734547	-0,85890584	0,39039247	0,62040037	protein_codir	hypothetical protein
TcG_03930	307,2717245	-0,013556279	0,10655625	-0,12722182	0,89876484	0,95069557	protein_codir	putative spliced leader RNA PSE-promoter transcription factor PPB1
TcG_03931	345,3357409	-0,20329064	0,10566589	-1,92390034	0,05436708	0,17555656	protein_codir	hypothetical protein
TcG_03932	273,4299805	0,121330318	0,10776971	1,12582951	0,26023769	0,49261779	protein_codir	hypothetical protein
TcG_03933	28,65673562	0,075056643	0,3304814	0,22711306	0,82033583	0,91114306	protein_codir	10 kDa heat shock protein
TcG_03934	2,292272937	0,530677983	1,24347501	0,42677012	0,66954677	1		
TcG_03935	228,5949943	0,0466903	0,12165395	0,38379602	0,70112964	0,84529532	protein_codir	hypothetical protein
TcG_03936	396,8431543	0,158376484	0,09836306	1,61012154	0,10737133	0,28223527	protein_codir	10 kDa heat shock protein
TcG_03937	1,486996936	0,707428819	1,47287859	0,48030355	0,63101156	1		
TcG_03938	675,3908293	-0,009840827	0,07377836	-0,13338365	0,89388997	0,94881323	protein_codir	thiolester hydrolase
TcG_03939	144,6025124	0,268639434	0,1509368	1,779814	0,07510641	0,22125167	protein_codir	10 kDa heat shock protein
TcG_03940	0,155988004	0,503022807	4,08047286	0,12327562	0,90188885	1	protein_codir	10 kDa heat shock protein
TcG_03941	693,8266382	-0,134723054	0,07154504	-1,88305239	0,05969327	0,18855131	protein_codir	hypothetical protein
TcG_03942	1141,197942	-0,350389762	0,06083962	-5,75923658	8,4495E-09	2,7269E-07	protein_codir	putative protein disulfide isomerase
TcG_03943	179,3597317	-0,202692259	0,13132207	-1,54347449	0,12271569	0,30767149	protein_codir	putative exosome complex exonuclease, putative,ribosomal RNA processing protein 4
TcG_03944	133,8644693	0,015585517	0,15547737	0,10024299	0,92015141	0,96146026	protein_codir	hypothetical protein
TcG_03945	343,5999051	-0,319666061	0,09684261	-3,3008824	0,00096381	0,0075553	protein_codir	putative phosphatidylinositol 3-kinase 2
TcG_03946	147,2419957	0,108059084	0,14988648	0,7209395	0,47094674	0,68692532	protein_codir	hypothetical protein
TcG_03947	623,7005608	0,099628997	0,08060943	1,23594711	0,21647822	0,44059164	protein_codir	putative j-binding protein
TcG_03948	100,1588654	-0,37165647	0,18336998	-2,02681194	0,04268165	0,14787966		
TcG_03949	174,1339461	0,135815432	0,13671641	0,99340986	0,32051026	0,55569903	protein_codir	hypothetical protein

TcG_03950	104,8235885	0,142080297	0,17949392	0,79156052	0,42861698	0,65390759	protein_codir	ADP-ribosylation factor-like protein 16
TcG_03951	140,1368495	0,026012164	0,15556798	0,1672077	0,86720663	0,93578156	protein_codir	putative leucine-rich repeat protein (LRRP)
TcG_03952	163,5916326	0,041042945	0,14212218	0,28878634	0,77274488	0,88661341	protein_codir	hypothetical protein
TcG_03953	191,9824305	-0,383093262	0,13776837	-2,78070553	0,00542409	0,03050656	protein_codir	hypothetical protein
TcG_03954	271,3296948	0,01557133	0,11165961	0,13945356	0,88909175	0,9464367	protein_codir	hypothetical protein
TcG_03955	206,1287541	0,004890754	0,12505057	0,03911021	0,96880252	0,98659981	protein_codir	putative chaperone protein DNAj
TcG_03956	19,00167521	0,232242668	0,39925284	0,58169322	0,56077334	0,75442638	protein_codir	hypothetical protein
TcG_03957	243,3540091	-0,049528162	0,11482508	-0,43133575	0,66622426	0,82519673	protein_codir	hypothetical protein
TcG_03958	171,1014796	-0,363028837	0,1393755	-2,60468177	0,00919596	0,04624324	protein_codir	putative inosine-guanine nucleoside hydrolase
TcG_03959	365,6561493	0,076160383	0,09519625	0,80003553	0,42369021	0,6496658	protein_codir	putative histone acetyltransferase
TcG_03960	216,6144319	0,008638498	0,12236678	0,07059512	0,94371999	0,97337664	protein_codir	60S ribosomal protein
TcG_03961	256,0341313	0,153314668	0,11139428	1,37632438	0,16872123	0,37510648	protein_codir	hypothetical protein
TcG_03962	135,7913756	0,222977412	0,1661137	1,34231801	0,17949291	0,39068285	protein_codir	hypothetical protein
TcG_03963	994,2544218	-0,065256984	0,0727119	-0,89747322	0,36946646	0,60112883	protein_codir	hypothetical protein
TcG_03964	442,0264608	0,125913255	0,08969157	1,40384715	0,16036437	0,36324176	protein_codir	hypothetical protein
TcG_03965	256,0764563	-0,037285128	0,11317712	-0,3294405	0,74182276	0,86859611	protein_codir	putative coiled-coil domain-containing protein 96
TcG_03966	529,5888589	0,200196914	0,08801619	2,27454646	0,02293315	0,09367365	protein_codir	hypothetical protein
TcG_03967	647,5954128	0,160236763	0,0750727	2,13442119	0,03280832	0,12234219	protein_codir	hypothetical protein
TcG_03968	293,2016763	0,213952225	0,10973228	1,9497656	0,05120406	0,16825358	protein_codir	adenosine 5'-monophosphoramidase
TcG_03969	448,2978637	-0,241586927	0,09016207	-2,67947392	0,00737379	0,03886842	protein_codir	translocon-associated protein subunit gamma
TcG_03970	294,2352104	-0,123592744	0,10852878	-1,13880152	0,25478595	0,48614229	protein_codir	hypothetical protein
TcG_03971	297,8812186	0,22182427	0,10411742	2,13052031	0,03312868	0,12310099	protein_codir	hypothetical protein
TcG_03972	540,3877198	-0,077063527	0,0887453	-0,8683674	0,38519323	0,61573521	protein_codir	hypothetical protein
TcG_03973	172,9184012	0,244984672	0,14421459	1,69875093	0,08936612	0,24901296	protein_codir	hypothetical protein
TcG_03974	138,2534443	0,009044943	0,15487774	0,05840054	0,95342959	0,97816658	protein_codir	hypothetical protein
TcG_03975	477,434247	-0,113118469	0,08597388	-1,3157306	0,18826448	0,40265419	protein_codir	putative proteasome alpha 3 subunit
TcG_03976	842,2175928	0,213774116	0,06693357	3,19382528	0,00140401	0,01028935	protein_codir	putative amino acid permease
TcG_03977	190,434251	-0,058800954	0,13184114	-0,44599854	0,6555983	0,81772823	protein_codir	hypothetical protein
TcG_03978	145,7099611	-0,059001602	0,17361895	-0,33983389	0,73398163	0,86342889	protein_codir	putative inositol polyphosphate kinase-like protein
TcG_03979	332,4937753	0,235892554	0,10150236	2,32401058	0,02012493	0,08448094	protein_codir	putative threonine synthase
TcG_03980	417,0294459	0,039671762	0,09044537	0,43862679	0,66093198	0,82224396	protein_codir	hypothetical protein
TcG_03981	448,9387855	-0,080928983	0,08825013	-0,91704094	0,35912119	0,5926302	protein_codir	hypothetical protein
TcG_03982	661,2310442	-0,96413818	0,0795031	-12,127051	7,5935E-34	5,4986E-31	protein_codir	hypothetical protein
TcG_03983	420,9721426	-0,400327985	0,08909093	-4,49347621	7,007E-06	0,00011229	protein_codir	hypothetical protein
TcG_03984	121,4794252	0,016821533	0,16687111	0,10080555	0,91970482	0,9613082	protein_codir	hypothetical protein
TcG_03985	544,9211443	0,020154037	0,08361587	0,24103123	0,80953092	0,90594274	protein_codir	U3 small nucleolar RNA-associated protein 25
TcG_03986	440,6670094	0,139240163	0,09434806	1,47581381	0,13999388	0,3345817	protein_codir	hypothetical protein
TcG_03987	227,7273675	-0,149485196	0,13192653	-1,13309426	0,25717467	0,48902898	protein_codir	hypothetical protein
TcG_03988	288,5265002	0,019394509	0,11132721	0,17421175	0,86169905	0,93381907	protein_codir	putative apoptosis-inducing factor A-like
TcG_03989	356,885273	0,216451166	0,09588659	2,25736643	0,02398519	0,09677174	protein_codir	hypothetical protein
TcG_03990	733,1126788	-0,186208903	0,06915236	-2,69273374	0,00708688	0,03764724	protein_codir	nuclear distribution protein C like protein
TcG_03991	235,135777	0,047860058	0,12990451	0,36842491	0,71255642	0,85248988	protein_codir	putative UDP-glucoronosyl and UDP-glucosyl transferase
TcG_03992	55,46713156	-0,023358786	0,23327895	-0,10013242	0,9202392	0,96146026	protein_codir	putative UDP-glucoronosyl and UDP-glucosyl transferase
TcG_03993	443,0317178	-0,060258853	0,08604097	-0,70035071	0,48370831	0,69704534	protein_codir	putative metallopeptidase, putative,metallo-peptidase, clan MP, family M67
TcG_03994	406,9070768	0,111967845	0,0971694	1,15229538	0,24919972	0,47849319		
TcG_03995	15,58637342	0,461007616	0,43946908	1,04901036	0,29417335	1		
TcG_03996	983,4087811	-0,075044846	0,06232147	-1,20415721	0,22852882	0,45564187	protein_codir	putative adaptor complex protein (AP) 3 delta subunit 1
TcG_03997	375,1450782	-0,10053743	0,09600017	-1,04726301	0,29497829	0,52793889	protein_codir	putative myosin heavy chain
TcG_03998	198,3555933	0,079355358	0,13543706	0,58592058	0,55792889	0,75269726	protein_codir	putative queuine tRNA-ribosyltransferase
TcG_03999	200,0640429	0,28891511	0,13039224	2,21573851	0,02670942	0,10497127	protein_codir	hypothetical protein
TcG_04000	107,7909453	-0,058494787	0,1695552	-0,34498965	0,73010216	0,86096321	protein_codir	putative amino acid permease
TcG_04001	349,3855736	-0,056448884	0,09711106	-0,5812817	0,56105061	0,75462415	protein_codir	transmembrane protein 67

TcG_04002	179,171037	-0,028089516	0,13145468	-0,21368214	0,83079494	0,91760178	protein_codir	hypothetical protein
TcG_04003	335,9135466	-0,140738621	0,10873095	-1,29437498	0,19553593	0,4133332	protein_codir	putative vesicle-associated membrane protein
TcG_04004	218,5725766	-0,046064302	0,12234971	-0,37649702	0,70654743	0,84873598	protein_codir	hypothetical protein
TcG_04005	277,4839813	0,166461018	0,10978282	1,51627565	0,12944967	0,3191072	protein_codir	phosphoglycerate mutase protein
TcG_04006	413,1783522	0,098876913	0,0890712	1,11008854	0,26696088	0,49939561	protein_codir	thyroid hormone receptor interactor 4
TcG_04007	491,0702498	-0,061623544	0,08288505	-0,74348201	0,45718991	0,6767839	protein_codir	putative ATP-dependent DEAD/H RNA helicase
TcG_04008	413,3354515	-0,093560633	0,09110771	-1,02692334	0,30445656	0,53722719	protein_codir	putative adaptor complex protein (AP) 3 delta subunit 1
TcG_04009	102,0558132	-0,132263738	0,19796551	-0,66811505	0,50406016	0,71207637	protein_codir	putative dual-specificity protein phosphatase
TcG_04010	263,3314821	-0,030246733	0,11073057	-0,27315612	0,78473321	0,89346381	protein_codir	hypothetical protein
TcG_04011	164,7616948	0,454766048	0,14885021	3,05519259	0,00224916	0,01513047	protein_codir	hypothetical protein
TcG_04012	367,0160108	0,115831834	0,09438497	1,22722758	0,21973707	0,44499055	protein_codir	hypothetical protein
TcG_04013	182,8013021	0,179307959	0,13898281	1,29014487	0,19700036	0,41550818	protein_codir	hypothetical protein
TcG_04014	433,5031844	0,320114996	0,08984485	3,5629756	0,00036667	0,00331898	protein_codir	putative DNA repair protein
TcG_04015	93,27556301	0,065206784	0,18149549	0,35927496	0,7193894	0,85590939	protein_codir	phosphatidylinositol glycan, class H
TcG_04016	121,0540192	0,043946962	0,15929012	0,27589258	0,78263057	0,89230051	protein_codir	hypothetical protein
TcG_04017	389,3156246	-0,046079852	0,09769669	-0,47166236	0,6371678	0,80706529	protein_codir	hypothetical protein
TcG_04018	244,218781	0,128009857	0,11530312	1,11020286	0,26691162	0,49939561	protein_codir	gamma carbonic dehydratase
TcG_04019	569,6135987	-0,203555601	0,08122854	-2,50596172	0,01221188	0,05786785	protein_codir	putative eukaryotic translation initiation factor 3 subunit
TcG_04020	153,315956	-0,053549403	0,14221126	-0,37654827	0,70650934	0,84873598	protein_codir	hypothetical protein
TcG_04021	473,5107387	-0,026298095	0,08588923	-0,30618616	0,75946291	0,87850811	protein_codir	ubiquitin-activating enzyme-like protein
TcG_04022	287,3113779	-0,003251311	0,10771291	-0,03018497	0,97591954	0,98975873	protein_codir	aarF domain-containing kinase
TcG_04023	550,1105596	0,002901296	0,08110871	0,03577046	0,97146539	0,98762955	protein_codir	hypothetical protein
TcG_04024	1094,115143	-0,325885254	0,06336686	-5,1428343	2,7062E-07	6,2835E-06	protein_codir	sarcoplasmic/endoplasmic reticulum calcium ATPase 3
TcG_04025	142,6350114	-0,143139333	0,15528536	-0,92178255	0,35664201	0,59063098	protein_codir	ADG1 protein
TcG_04026	361,5452112	0,048611231	0,09647015	0,50389922	0,6143322	0,79153921	protein_codir	putative ADG2
TcG_04027	43,8500313	0,14183655	0,26674591	0,53172905	0,59491367	0,78015504	protein_codir	hypothetical protein
TcG_04028	129,9146254	-0,095247135	0,1579341	-0,6030815	0,54645447	0,74336286	protein_codir	hypothetical protein
TcG_04029	325,9936309	0,054649793	0,10093595	0,54143043	0,58821094	0,77564856	protein_codir	putative ribosomal protein L2
TcG_04030	372,6573373	0,030810098	0,09397337	0,32785989	0,7430176	0,86930139	protein_codir	putative iron superoxide dismutase
TcG_04031	1353,250685	0,292580043	0,0684657	4,2733816	1,9253E-05	0,00026811	protein_codir	putative DNA repair and recombination protein RAD54
TcG_04032	692,5585843	-0,175562618	0,07215811	-2,4330267	0,0149732	0,06766691	protein_codir	protein kinase
TcG_04033	183,5429262	0,148800321	0,13186745	1,12840827	0,25914754	0,49163663	protein_codir	hypothetical protein
TcG_04034	49,0123572	0,537797732	0,24833289	2,16563236	0,03033929	0,11532514	protein_codir	putative protein kinase
TcG_04035	27,67931902	0,137426525	0,33263148	0,41314948	0,6794971	0,83273253	protein_codir	hypothetical protein
TcG_04036	793,7531165	-0,080899731	0,06907384	-1,17120653	0,24151577	0,47019585	protein_codir	hypothetical protein
TcG_04037	189,0810982	0,103387905	0,1300493	0,79499011	0,42661926	0,65182787	protein_codir	hypothetical protein
TcG_04038	165,0075691	-0,046740382	0,13683311	-0,34158678	0,73266188	0,86278448	protein_codir	hypothetical protein
TcG_04039	242,4425409	-0,08914218	0,11947992	-0,74608506	0,45561603	0,67563898	protein_codir	hypothetical protein
TcG_04040	295,5193113	0,015872154	0,10495532	0,15122773	0,87979608	0,94162382	protein_codir	hypothetical protein
TcG_04041	474,9822646	-0,333817931	0,08581234	-3,89009251	0,00010021	0,00111848	protein_codir	putative rab-GDP dissociation inhibitor
TcG_04042	62,34660214	-0,342603085	0,22647659	-1,51275276	0,13034247	0,32035382	protein_codir	putative protein kinase
TcG_04043	257,4578921	-0,09631148	0,11253302	-0,85585085	0,39208029	0,6215135	protein_codir	WD40 repeat-containing protein
TcG_04044	345,3888324	0,028171254	0,10264401	0,2744559	0,78373429	0,89276822	protein_codir	putative C-8 sterol isomerase
TcG_04045	352,0341974	-0,094147794	0,09927089	-0,94839273	0,34292956	0,57691039	protein_codir	putative leucine-rich repeat protein
TcG_04046	201,6723039	-0,136622994	0,1234313	-1,10687482	0,26834806	0,50094157	protein_codir	hypothetical protein
TcG_04047	154,9923053	0,11188574	0,14010122	0,79860645	0,42451865	0,65015164	protein_codir	hypothetical protein
TcG_04048	1541,071644	-0,0830051	0,05831166	-1,42347337	0,15459898	0,35576644	protein_codir	UDP-glucose:glycoprotein glucosyltransferase
TcG_04049	846,3509584	-0,03785556	0,06559724	-0,57709074	0,56387816	0,75680428	protein_codir	hypothetical protein
TcG_04050	197,9813038	0,247556071	0,12888493	1,92075265	0,0547629	0,17656406	protein_codir	retinol dehydrogenase 14
TcG_04051	608,143539	0,09083539	0,083533	1,08741925	0,27685157	0,50906241	protein_codir	hypothetical protein
TcG_04052	256,5329358	-0,100019875	0,11494384	-0,87016301	0,38421133	0,61450476	protein_codir	hypothetical protein
TcG_04053	414,1589611	-0,051432438	0,09265613	-0,55508942	0,5788335	0,76846166	protein_codir	importin 7

TcG_04054	101,4084915	-0,319043475	0,17400461	-1,83353457	0,06672311	0,20510851	protein_codir	hypothetical protein
TcG_04055	345,1693877	-0,337121615	0,09686782	-3,48022287	0,000501	0,00434472	protein_codir	hypothetical protein
TcG_04056	349,5385692	-0,408453624	0,09588056	-4,26002527	2,044E-05	0,0002826	protein_codir	putative UDP-GlcNAc:PI a1-6 GlcNAc-transferase
TcG_04057	815,1448164	-0,431391476	0,07208224	-5,98471278	2,1677E-09	7,9985E-08	protein_codir	putative protein kinase
TcG_04058	262,4050753	-0,100737637	0,11913802	-0,84555409	0,39780155	0,627492	protein_codir	hypothetical protein
TcG_04059	448,1398872	-0,00174157	0,09072708	-0,01919571	0,98468498	0,99412361	protein_codir	hypothetical protein
TcG_04060	177,5265844	0,094199423	0,13584137	0,69345164	0,48802616	0,69945302	protein_codir	hypothetical protein
TcG_04061	215,9490555	0,031243952	0,12785104	0,24437776	0,80693826	0,9046141	protein_codir	hypothetical protein
TcG_04062	716,2854283	-0,258162129	0,07053349	-3,66013565	0,00025208	0,00241973	protein_codir	hypothetical protein
TcG_04063	37,69869646	0,21038925	0,28428456	0,74006569	0,45926013	0,67791061	protein_codir	putative fumarate hydratase
TcG_04064	628,5188768	-0,509483031	0,07569712	-6,73054726	1,6903E-11	1,0095E-09	protein_codir	fumarate hydratase
TcG_04065	333,5713832	-0,337157416	0,10706475	-3,14909818	0,00163775	0,01169851	protein_codir	putative protein farnesyltransferase alpha subunit
TcG_04066	120,8973683	-0,341717646	0,15989181	-2,13718041	0,03258332	0,12177753	protein_codir	diacylglycerol kinase-like protein
TcG_04067	101,2463357	0,154614341	0,17895922	0,8639641	0,38760761	0,61797465	protein_codir	hypothetical protein
TcG_04068	179,6293336	-0,287332723	0,13929572	-2,06275344	0,03913606	0,13925995	protein_codir	protein phosphatase 2 (formerly 2A), regulatory subunit B
TcG_04069	198,0735531	-0,261671482	0,12686369	-2,06261916	0,03914883	0,1392626	protein_codir	hypothetical protein
TcG_04070	128,2979248	-0,001597314	0,15653956	-0,0102039	0,9918586	0,99678187	protein_codir	hypothetical protein
TcG_04071	139,5578033	-0,115474746	0,14821664	-0,77909433	0,43592415	0,6595217	protein_codir	hypothetical protein
TcG_04072	245,8136851	-0,000726872	0,11445324	-0,00635082	0,99493281	0,99789945	protein_codir	hypothetical protein
TcG_04073	250,4223615	-0,099048833	0,11328568	-0,87432792	0,3819397	0,61205441	protein_codir	hypothetical protein
TcG_04074	108,5919193	0,070204374	0,17992777	0,39018087	0,6964028	0,84275359	protein_codir	hypothetical protein
TcG_04075	206,5184088	-0,423599229	0,12271495	-3,45189582	0,00055666	0,00475276	protein_codir	hypothetical protein
TcG_04076	1,82305276	-3,517156309	1,70800169	-2,05922297	0,03947288	1		
TcG_04077	2,470151047	-0,52547107	1,17939033	-0,44554467	0,65592619	1		
TcG_04078	147,8503232	-0,05250281	0,1485704	-0,35338675	0,72379852	0,85772012	protein_codir	putative DnaJ chaperone protein
TcG_04079	1356,793533	-0,130507033	0,06037858	-2,16147902	0,03065836	0,1161189	protein_codir	R27-2 protein
TcG_04080	436,0357176	0,097717455	0,08931556	1,09406982	0,27392436	0,50697885	protein_codir	hypothetical protein
TcG_04081	348,2487849	0,324518198	0,09963587	3,25704182	0,0011258	0,0085869	protein_codir	putative protein kinase
TcG_04082	526,4117089	-0,048645167	0,08949172	-0,54357171	0,58673623	0,77522249	protein_codir	putative UDP-galactose 4-epimerase
TcG_04083	227,6440988	0,053243474	0,1184875	0,44935943	0,65317239	0,81653597	protein_codir	hypothetical protein
TcG_04084	372,3542311	-0,001872835	0,09433887	-0,01985221	0,98416126	0,99398772	protein_codir	putative cell division cycle protein 45 (CDC45)
TcG_04085	625,6231905	0,031723724	0,07518515	0,42194138	0,67306781	0,82890021	protein_codir	hypothetical protein
TcG_04086	477,1743176	0,099670691	0,08508503	1,17142448	0,2414282	0,47019585	protein_codir	hypothetical protein
TcG_04087	1403,267296	0,375752649	0,05455248	6,88791089	5,6618E-12	3,77E-10	protein_codir	putative succinyl-CoA:3-ketoacid-coenzyme A transferase
TcG_04088	416,9004706	-0,045299912	0,09414032	-0,48119566	0,63037744	0,80197135	protein_codir	hypothetical protein
TcG_04089	601,8334051	-0,18043588	0,07901488	-2,28356824	0,02239692	0,09179013	protein_codir	hypothetical protein
TcG_04090	433,1877487	0,060222341	0,09592916	0,62777931	0,53014852	0,73198176	protein_codir	Golgi reassembly stacking protein
TcG_04091	1544,527467	0,011593817	0,05682244	0,20403588	0,83832546	0,92144011	protein_codir	putative heat shock protein
TcG_04092	282,2036112	-0,06949702	0,10952588	-0,63452603	0,52573759	0,72887348	protein_codir	putative GTPase
TcG_04093	307,5520084	0,218665583	0,10398191	2,10291937	0,03547282	0,12985405	protein_codir	putative diphthamide synthesis protein
TcG_04094	1620,516598	-0,19021794	0,05126103	-3,71077083	0,00020663	0,00204092	protein_codir	putative OSM3-like kinesin
TcG_04095	594,1570892	-0,077135335	0,08365191	-0,92209887	0,35647701	0,59052653	protein_codir	hypothetical protein
TcG_04096	635,0712467	-0,070441099	0,07425604	-0,94862451	0,34281162	0,57679606	protein_codir	hypothetical protein
TcG_04097	164,8419524	0,277068869	0,13827405	2,00376614	0,04509512	0,15371346	protein_codir	putative retrotransposon hot spot (RHS) protein
TcG_04098	757,1929285	0,002825416	0,07150182	0,0395153	0,96847956	0,98644432	protein_codir	hypothetical protein
TcG_04099	258,8032577	-0,044005254	0,11727565	-0,37522924	0,70748999	0,84916389	protein_codir	putative dynein heavy chain
TcG_04100	264,528269	-0,032998535	0,11165821	-0,29553166	0,76758777	0,88350974	protein_codir	putative meiotic recombination protein DMC1
TcG_04101	112,0924895	0,390192348	0,17380003	2,24506494	0,02476396	0,09904653	protein_codir	helicase-like protein
TcG_04102	199,4599925	-0,013825851	0,12429888	-0,1112307	0,91143341	0,95685642	protein_codir	radial spoke protein Ci-RSP9
TcG_04103	485,631837	-0,048295563	0,08513588	-0,56727622	0,57052653	0,76219457	protein_codir	putative mitochondrial RNA binding complex 1 subunit
TcG_04104	259,741819	0,079667737	0,11170762	0,71318084	0,47573386	0,69116462	protein_codir	UDP-sugar pyrophosphorylase
TcG_04105	1023,863919	0,011190427	0,06471467	0,17291947	0,86271473	0,93423804	protein_codir	ribosomal rRNA-processing protein 12

TcG_04106	974,9700001	0,043148174	0,06455564	0,6683874	0,50388633	0,71207637	protein_codir	carboxypeptidase
TcG_04107	646,4466715	-0,237587655	0,07386186	-3,2166488	0,00129697	0,00965728	protein_codir	putative Unc104-like kinesin
TcG_04108	423,2963907	-0,145851708	0,09062665	-1,60936894	0,10753569	0,28245489	protein_codir	putative aminopeptidase, putative,metallo-peptidase, Clan MF, Family M17
TcG_04109	276,3211765	0,176158048	0,1111265	1,58520289	0,11292024	0,29124976	protein_codir	hypothetical protein
TcG_04110	800,6704268	0,062725556	0,07064024	0,88795779	0,37456346	0,60517253	protein_codir	FGF receptor activating protein
TcG_04111	195,856907	0,257202071	0,13031571	1,97368436	0,04841764	0,16166191	protein_codir	adenylate kinase
TcG_04112	315,2130528	-0,219430939	0,10478083	-2,09418982	0,03624307	0,13205252	protein_codir	putative tubulin binding cofactor c
TcG_04113	1889,719458	0,094964257	0,06015429	1,57867815	0,1144099	0,2939794	protein_codir	cytoplasmic dynein 2 heavy chain 1 isoform X1
TcG_04114	313,0358077	0,163220792	0,10747975	1,51861896	0,12885844	0,31792545	protein_codir	putative tubulin tyrosine ligase
TcG_04115	77,78075344	-0,081694065	0,20602519	-0,39652465	0,69171803	0,84050813	protein_codir	hypothetical protein
TcG_04116	156,2700216	0,205830542	0,14898819	1,38152252	0,16711836	0,37307211	protein_codir	GDP-mannose 4,6 dehydratase
TcG_04117	429,8712215	-0,049161431	0,09061299	-0,54254284	0,5874446	0,77564856	protein_codir	putative iron superoxide dismutase
TcG_04118	151,3179817	-0,107166876	0,14779836	-0,72508838	0,46839781	0,68500074	protein_codir	putative glutathione peroxidase
TcG_04119	173,2359449	-0,230996709	0,13486975	-1,71273919	0,08676053	0,24410091	protein_codir	component of TRAPP complex
TcG_04120	727,8626288	-0,109232867	0,07387434	-1,4786308	0,13923902	0,33365527	protein_codir	hypothetical protein
TcG_04121	378,0047855	-0,440015533	0,10002013	-4,3992696	1,0862E-05	0,00016301	protein_codir	putative amino acid transporter, putative,amino acid permease
TcG_04122	28,94069961	-0,218403661	0,31726155	-0,68840255	0,49119932	0,70199029		
TcG_04123	206,7764187	0,079086311	0,12759363	0,61982961	0,53536997	0,73545133	protein_codir	As/Sb Reductase
TcG_04124	483,7917366	-0,1752137	0,08717273	-2,0099601	0,04443541	0,1522642	protein_codir	hypothetical protein
TcG_04125	461,3862537	-0,240866206	0,0860557	-2,7989571	0,00512679	0,02923452	protein_codir	hypothetical protein
TcG_04126	517,0349929	-0,281383163	0,08360425	-3,36565603	0,00076362	0,00621298	protein_codir	hypothetical protein
TcG_04127	811,8539517	-0,145132361	0,07348334	-1,97503766	0,04826387	0,16152087	protein_codir	hypothetical protein
TcG_04128	35,04451583	-0,272115542	0,30122513	-0,90336268	0,36633344	0,59855299		
TcG_04129	192,7263393	0,075222664	0,13009062	0,57823279	0,56310697	0,75642404	protein_codir	exonuclease
TcG_04130	735,6007383	-0,225746276	0,07724289	-2,92255105	0,00347177	0,02149861	protein_codir	hypothetical protein
TcG_04131	333,9318777	-0,216760608	0,11065006	-1,95897408	0,05011582	0,16573473	protein_codir	putative RNA-binding protein
TcG_04132	436,6912118	-0,243499936	0,09272522	-2,62603773	0,00863852	0,04408319	protein_codir	proteasome regulatory non-ATP-ase subunit 7
TcG_04133	531,6344536	-0,139180834	0,08240555	-1,68897406	0,0912244	0,25224962	protein_codir	hypothetical protein
TcG_04134	151,2366918	-0,153988962	0,14564536	-1,0572871	0,29038058	0,52345679	protein_codir	hypothetical protein
TcG_04135	432,6561331	-0,319296659	0,09187615	-3,47529417	0,00051029	0,00440884	protein_codir	TPR Domain containing protein
TcG_04136	238,0744633	-0,222517567	0,11764967	-1,89135726	0,05857667	0,18558088	protein_codir	putative zinc finger protein
TcG_04137	214,1424334	-0,123395057	0,1286716	-0,95899217	0,33756269	0,57211839	protein_codir	putative phosphatidate cytidylyltransferase-like protein
TcG_04138	199,3770689	-0,111264795	0,12537726	-0,88744	0,37484206	0,60545379	protein_codir	hypothetical protein
TcG_04139	291,0525665	-0,237634599	0,10981826	-2,16388961	0,03047282	0,11560515	protein_codir	putative SNAP protein
TcG_04140	699,2560806	-0,525678594	0,07294002	-7,20699846	5,7199E-13	4,5082E-11	protein_codir	putative outer dynein arm docking complex
TcG_04141	797,1428168	-0,184130726	0,06831473	-2,69532989	0,0070319	0,03742378	protein_codir	putative ubiquitin hydrolase
TcG_04142	619,350208	-0,23128592	0,07612682	-3,03816597	0,00238023	0,01580362	protein_codir	putative intergrin alpha chain protein
TcG_04143	31,2608538	0,370298982	0,32133244	1,15238594	0,24916252	0,47849319	protein_codir	hypothetical protein
TcG_04144	503,4234177	-0,213242581	0,0843319	-2,52861118	0,01145148	0,05500699	protein_codir	hypothetical protein
TcG_04145	870,4989858	-0,355154719	0,06642016	-5,34709247	8,9378E-08	2,3482E-06	protein_codir	nucleoside diphosphate kinase
TcG_04146	490,4702987	-0,335840133	0,08743543	-3,84100731	0,00012253	0,00132553	protein_codir	putative minichromosome maintenance (MCM) complex subunit
TcG_04147	406,300225	-0,30314347	0,09168776	-3,30625887	0,00094551	0,00745214	protein_codir	hypothetical protein
TcG_04148	211,265947	-0,234488848	0,12098564	-1,93815434	0,0526044	0,17124881	protein_codir	tubulin binding cofactor A-like protein
TcG_04149	311,4692552	-0,182835157	0,10643934	-1,71774039	0,08584398	0,24222804	protein_codir	putative cyclic nucleotide-binding protein
TcG_04150	140,1514693	-0,11716063	0,15089997	-0,77641255	0,43750544	0,66087849	protein_codir	hypothetical protein
TcG_04151	617,8014876	-0,12200172	0,07561519	-1,61345509	0,10664567	0,28132896	protein_codir	hypothetical protein
TcG_04152	484,4308993	-0,305900658	0,0884458	-3,45862262	0,00054295	0,00465623	protein_codir	hypothetical protein
TcG_04153	309,4057963	0,069903326	0,10995128	0,63576637	0,52492871	0,7284494	protein_codir	hypothetical protein
TcG_04154	602,2442811	-0,086372957	0,07932131	-1,08889977	0,27619809	0,5087364	protein_codir	hypothetical protein
TcG_04155	313,6276758	-0,031871847	0,10455603	-0,30483032	0,76049539	0,87908805	protein_codir	putative DNA repair helicase
TcG_04156	1078,857942	-0,014048571	0,06066364	-0,2315814	0,81686316	0,90958077	protein_codir	60S ribosomal protein L2
TcG_04157	209,5790318	0,122044876	0,12695611	0,96131552	0,33639355	0,57080486	protein_codir	surface protease GP63

TcG_04158	499,4279992	0,070116138	0,08503054	0,82459951	0,409599	0,63686685	protein_codir L1Tc protein
TcG_04159	185,9795259	0,12365659	0,13315931	0,92863646	0,35307752	0,58749908	
TcG_04160	96,93671433	0,354096425	0,18259199	1,93927688	0,05246763	0,17089963	protein_codir hypothetical protein
TcG_04161	116,7320545	0,128632539	0,16928713	0,75984832	0,44734526	0,66854651	protein_codir hypothetical protein
TcG_04162	277,713639	0,105162215	0,10694764	0,98330564	0,32545704	0,55987308	protein_codir hypothetical protein
TcG_04163	147,2717968	0,046613992	0,14658643	0,31799665	0,75048748	0,8744115	
TcG_04164	515,4239603	0,103835494	0,08267011	1,25602227	0,2091079	0,43162731	protein_codir 2-hydroxyglutarate dehydrogenase
TcG_04165	710,2560966	-0,104530127	0,07354094	-1,42138693	0,1552043	0,35661201	protein_codir putative guanylate cyclase
TcG_04166	670,5564089	-0,193308302	0,07208589	-2,68163857	0,00732626	0,03870589	protein_codir hypothetical protein
TcG_04167	236,9951688	0,554169735	0,11591196	4,78095384	1,7447E-06	3,3037E-05	protein_codir hypothetical protein
TcG_04168	459,0715427	0,066649125	0,08715805	0,76469271	0,44445455	0,66642694	protein_codir adiponectin receptor protein 1
TcG_04169	1,860274304	0,788757284	1,35721957	0,58115673	0,56113483	1	
TcG_04170	215,8561964	0,360136405	0,12716838	2,83196507	0,00462629	0,02705714	protein_codir adiponectin receptor protein 1
TcG_04171	451,5724149	-0,135651544	0,09024164	-1,50320349	0,13278658	0,323683	protein_codir putative phosphorylated CTD-interacting factor 1-like
TcG_04172	465,7871856	-0,191234463	0,08872853	-2,15527601	0,03114024	0,11752145	protein_codir hypothetical protein
TcG_04173	675,5606962	0,06732557	0,07354748	0,91540285	0,35998018	0,5926302	protein_codir hypothetical protein
TcG_04174	206,5322561	0,085355488	0,12578271	0,67859476	0,49739466	0,70725677	protein_codir hypothetical protein
TcG_04175	638,9464634	-0,204197926	0,0760138	-2,68632715	0,00722423	0,03825409	protein_codir hypothetical protein
TcG_04176	342,5413437	0,23327308	0,09830311	2,37299794	0,01764436	0,07633592	protein_codir putative G-actin binding protein, putative,CAP/Srv2p
TcG_04177	324,2391543	-0,313124966	0,10019308	-3,12521561	0,00177675	0,01252153	protein_codir hypothetical protein
TcG_04178	476,088892	0,016951356	0,08379274	0,20230101	0,83968141	0,92207948	protein_codir putative beta-adaptin
TcG_04179	253,0744537	-0,299144319	0,11322592	-2,6420128	0,00824149	0,04247596	protein_codir hypothetical protein
TcG_04180	320,5408892	-0,137616526	0,10113867	-1,36067172	0,17361745	0,38220251	protein_codir hypothetical protein
TcG_04181	633,4463872	-0,124015723	0,07736837	-1,6029254	0,10895113	0,28500965	protein_codir hypothetical protein
TcG_04182	382,5705065	0,043234851	0,09377068	0,46107006	0,64474834	0,8113451	protein_codir hypothetical protein
TcG_04183	1340,776508	-0,278652861	0,05746546	-4,84904993	1,2405E-06	2,4527E-05	protein_codir isoleucine--tRNA ligase
TcG_04184	303,4773965	-0,207659299	0,11437344	-1,81562513	0,0694279	0,2108497	protein_codir hypothetical protein
TcG_04185	338,7075216	-0,145377715	0,0983866	-1,47761707	0,1395103	0,33409356	protein_codir hypothetical protein
TcG_04186	605,53484	0,0657522	0,07893835	0,83295633	0,40486936	0,63415119	protein_codir cyclin L1
TcG_04187	630,5621254	-0,247828236	0,07814345	-3,17145229	0,00151679	0,01097658	protein_codir hypothetical protein
TcG_04188	469,2157934	-0,043713657	0,08635451	-0,50621165	0,61270808	0,79081972	protein_codir hypothetical protein
TcG_04189	541,928164	-0,018176937	0,08012496	-0,22685735	0,82053467	0,91114306	protein_codir putative RNA helicase
TcG_04190	365,6756368	0,093229735	0,09696469	0,96148126	0,33631024	0,57080486	protein_codir hypothetical protein
TcG_04191	192,3217263	-0,053324696	0,12963035	-0,41135966	0,68080883	0,83354656	protein_codir hypothetical protein
TcG_04192	613,7884913	-0,055994139	0,07653202	-0,73164327	0,46438633	0,68173811	protein_codir hypothetical protein
TcG_04193	596,8333622	0,039536661	0,07555523	0,52328158	0,60077832	0,7835886	protein_codir hypothetical protein
TcG_04194	326,8971918	-0,197667392	0,09948415	-1,98692354	0,04693087	0,15827942	protein_codir hypothetical protein
TcG_04195	581,9335594	0,003803256	0,07784831	0,04885469	0,9610351	0,98198472	protein_codir hypothetical protein
TcG_04196	409,8963965	-0,256176417	0,09435849	-2,71492712	0,00662904	0,03577318	protein_codir hypothetical protein
TcG_04197	379,3126327	0,115606043	0,09819489	1,17731222	0,23907091	0,46725297	protein_codir tRNA pseudouridine13 synthase
TcG_04198	820,9793925	-0,03094672	0,06743491	-0,45891243	0,64629705	0,81201944	protein_codir hypothetical protein
TcG_04199	177,5612151	0,119464875	0,13618792	0,87720613	0,38037469	0,61073866	protein_codir hypothetical protein
TcG_04200	679,0811714	0,136090615	0,07774427	1,7504907	0,08003368	0,23118071	protein_codir tRNA (adenine-N(1)-)-methyltransferase non-catalytic subunit
TcG_04201	132,6235988	0,242320428	0,15449032	1,56851531	0,11676092	0,2980592	protein_codir putative leucine-rich repeat protein (LRRP)
TcG_04202	765,1091144	0,081537627	0,07424787	1,09818137	0,2721253	0,50504046	protein_codir hypothetical protein
TcG_04203	289,1761921	0,065881231	0,10914638	0,60360436	0,54610671	0,74297703	protein_codir hypothetical protein
TcG_04204	4453,562967	-0,325958197	0,04228317	-7,70893507	1,2687E-14	1,2782E-12	protein_codir hypothetical protein
TcG_04205	582,0516281	0,100761926	0,08212432	1,22694379	0,21984372	0,44499055	protein_codir kinetoplast-associated protein
TcG_04206	364,0489641	-0,284456627	0,0961913	-2,95719694	0,0031045	0,01960148	protein_codir hypothetical protein
TcG_04207	517,9844142	-0,262585806	0,08064746	-3,25597127	0,00113005	0,00861367	protein_codir proteasome regulatory non-ATPase subunit 8
TcG_04208	310,2627367	-0,059509782	0,10512033	-0,56611105	0,57131829	0,76259144	protein_codir hypothetical protein
TcG_04209	813,3791219	-0,410339505	0,06876525	-5,96725095	2,4128E-09	8,8747E-08	protein_codir hypothetical protein

TcG_04210	767,663726	-0,306764138	0,07218982	-4,24941002	2,1433E-05	0,00029458	protein_codir	hypothetical protein
TcG_04211	364,022943	-0,649423593	0,09505552	-6,83204511	8,3713E-12	5,3585E-10	protein_codir	hypothetical protein
TcG_04212	488,3526916	-0,629191917	0,0853894	-7,36850164	1,7256E-13	1,4527E-11	protein_codir	hypothetical protein
TcG_04213	745,4858556	-0,397794367	0,07126311	-5,58205188	2,377E-08	7,0075E-07	protein_codir	hypothetical protein
TcG_04214	706,9048841	-0,339519427	0,07694231	-4,41264944	1,0211E-05	0,00015465	protein_codir	hypothetical protein
TcG_04215	865,3820967	-0,348578313	0,06808529	-5,11973017	3,0597E-07	6,999E-06	protein_codir	Intraflagellar Transport Protein 140
TcG_04216	548,2342623	-0,48000604	0,07831955	-6,12881512	8,8536E-10	3,5617E-08	protein_codir	putative leucine-rich repeat protein (LRRP)
TcG_04217	277,0982478	-0,127543873	0,10917947	-1,16820378	0,24272458	0,47105644	protein_codir	hypothetical protein
TcG_04218	276,5832128	-0,254311106	0,11431006	-2,22474815	0,02609815	0,10298813	protein_codir	B9 protein domain 1
TcG_04219	1208,354337	-0,229144443	0,06643298	-3,44925717	0,00056213	0,00478886	protein_codir	hypothetical protein
TcG_04220	500,4986822	-0,388220442	0,0931176	-4,1691413	3,0575E-05	0,00040209	protein_codir	putative protein kinase, putative,serine/threonine-protein kinase Nek1
TcG_04221	1060,484126	-0,143515933	0,06371061	-2,25262228	0,02428297	0,09775627	protein_codir	putative protein kinase
TcG_04222	1011,582069	-0,225067981	0,06233899	-3,61038861	0,00030574	0,00284732	protein_codir	hypothetical protein
TcG_04223	1081,733183	-0,391652655	0,06009363	-6,51737372	7,1549E-11	3,6199E-09	protein_codir	hypothetical protein
TcG_04224	809,5792503	-0,415970885	0,06713986	-6,19558781	5,8068E-10	2,4465E-08	protein_codir	structure-specific recognition protein 1
TcG_04225	526,3291514	-0,178083282	0,08047831	-2,21281082	0,02691069	0,10551177	protein_codir	hypothetical protein
TcG_04226	573,4182588	-0,208149107	0,07850766	-2,65132226	0,00801773	0,04158166	protein_codir	hypothetical protein
TcG_04227	190,6741591	0,055316741	0,1290267	0,42872322	0,66812466	0,8261349	protein_codir	hypothetical protein
TcG_04228	353,0683704	-0,126907595	0,09798965	-1,29511223	0,19528152	0,41302148	protein_codir	putative U2 small nuclear ribonucleoprotein 40K
TcG_04229	185,5720548	0,0464099	0,13157891	0,35271534	0,72430186	0,85798219	protein_codir	hypothetical protein
TcG_04230	265,2264075	-0,359374792	0,11142859	-3,22515784	0,00125903	0,00941106	protein_codir	hypothetical protein
TcG_04231	547,4708236	-0,458527125	0,0828076	-5,53725918	3,0724E-08	8,833E-07	protein_codir	hypothetical protein
TcG_04232	837,6471163	-0,301974505	0,07207499	-4,18972676	2,7929E-05	0,00037122	protein_codir	U3 small nucleolar RNA-associated protein 14
TcG_04233	159,2572472	-0,495084533	0,14331097	-3,45461713	0,00055107	0,00471199	protein_codir	U3 small nucleolar RNA-associated protein 14
TcG_04234	775,4309	-0,413302977	0,07549687	-5,47443886	4,389E-08	1,2224E-06	protein_codir	vacuolar protein 8
TcG_04235	309,2836659	-0,345265081	0,10622162	-3,25042205	0,00115234	0,00876625	protein_codir	hypothetical protein
TcG_04236	414,9395519	-0,598181764	0,09743543	-6,13926325	8,2905E-10	3,3941E-08	protein_codir	putative protein kinase, putative,serine/threonine protein kinase
TcG_04237	294,4035254	-0,154402336	0,11195846	-1,37910377	0,16786276	0,37401114	protein_codir	putative mitochondrial carrier protein
TcG_04238	0,689722372	0,936118941	2,17568091	0,43026481	0,66700302	1		
TcG_04239	19,90112191	-0,750340246	0,38459846	-1,95097048	0,05106056	0,16807367	protein_codir	Dullard-like phosphatase domain-containing protein
TcG_04240	28,00252663	0,133396067	0,33294801	0,40065135	0,68867684	0,83839548	protein_codir	hypothetical protein
TcG_04241	30,73291059	-0,100942939	0,31418953	-0,3212804	0,74799791	0,87282745	protein_codir	hypothetical protein
TcG_04242	40,01483246	-0,605852589	0,281694	-2,15074722	0,03149616	0,11847872	protein_codir	beta galactofuranosyl glycosyltransferase
TcG_04243	59,56513408	-0,009168874	0,23064475	-0,03975323	0,96828986	0,98638627	protein_codir	beta galactofuranosyl glycosyltransferase
TcG_04244	13,24066071	0,445463712	0,47716532	0,93356264	0,35052955	1	protein_codir	beta galactofuranosyl glycosyltransferase
TcG_04245	51,97840504	0,05434531	0,245669	0,22121354	0,82492616	0,91425239	protein_codir	hypothetical protein
TcG_04246	62,69089015	0,126509144	0,22175217	0,57049787	0,56834006	0,76010481		
TcG_04247	115,5079828	-0,13552662	0,16196161	-0,83678237	0,40271491	0,6328518	protein_codir	cyclophilin
TcG_04248	439,6904859	0,085847576	0,08658446	0,9914894	0,32144667	0,55611008	protein_codir	hypothetical protein
TcG_04249	56,56017464	0,20298107	0,24620047	0,82445444	0,40968139	0,63686685	protein_codir	hypothetical protein
TcG_04250	556,2471278	-0,134529342	0,08332081	-1,61459477	0,10639848	0,28080473	protein_codir	trichohyalin
TcG_04251	481,0299616	0,142025838	0,08381443	1,69452724	0,09016514	0,25039629	protein_codir	hypothetical protein
TcG_04252	236,1153411	-0,180689436	0,11545138	-1,56506948	0,11756662	0,29897429	protein_codir	hypothetical protein
TcG_04253	553,2218631	-0,160850113	0,07810677	-2,05936186	0,03945959	0,13999295	protein_codir	hypothetical protein
TcG_04254	656,680762	-0,042163754	0,07398426	-0,56990165	0,56874441	0,76055779	protein_codir	hypothetical protein
TcG_04255	177,8827852	-0,284471671	0,13245571	-2,14767384	0,03173968	0,11916263	protein_codir	putative leucine-rich repeat protein (LRRP)
TcG_04256	875,0257495	-0,144206105	0,06922226	-2,08323298	0,03722999	0,13422744	protein_codir	alkyldihydroxyacetonephosphate synthase
TcG_04257	242,2532965	0,109245055	0,12444524	0,87785641	0,38002165	0,6107213	protein_codir	putative lysyl-tRNA synthetase
TcG_04258	300,7009725	0,117046896	0,10420498	1,12323707	0,26133682	0,49358779	protein_codir	glutamine amidotransferase class-I
TcG_04259	0,445396961	-2,390354933	2,60655946	-0,91705368	0,35911451	1		
TcG_04260	523,7175737	0,013510649	0,08379705	0,16123061	0,87191177	0,93840871	protein_codir	protein YIPF2
TcG_04261	352,2318354	-0,023553814	0,09979426	-0,23602374	0,81341425	0,90788142	protein_codir	putative leucine-rich repeat protein

TcG_04262	185,5286417	0,347281805	0,13230074	2,6249422	0,00866637	0,04415503	protein_codir	SET domain containing protein
TcG_04263	661,3594217	0,119948484	0,07702246	1,55731828	0,11939494	0,30216464	protein_codir	putative 2-hydroxy-3-oxopropionate reductase
TcG_04264	307,0882031	0,284426595	0,10434424	2,72584844	0,00641364	0,03487024	protein_codir	putative polynucleotide kinase 3'-phosphatase
TcG_04265	269,0316499	0,297585374	0,11134447	2,67265523	0,00752535	0,03955933	protein_codir	putative serine peptidase
TcG_04266	162,88433	0,285535294	0,14139243	2,01945244	0,04344022	0,14974661	protein_codir	hypothetical protein
TcG_04267	361,1158202	-0,078600506	0,10164744	-0,77326595	0,439365	0,66264661	protein_codir	putative ribosomal RNA adenine dimethylase family protein
TcG_04268	127,3806	0,058541561	0,15706124	0,3727308	0,70934881	0,85005287	protein_codir	hypothetical protein
TcG_04269	226,6368133	0,144207295	0,122213	1,17996688	0,23801339	0,46589341	protein_codir	hypothetical protein
TcG_04270	176,8046305	0,232609969	0,13791666	1,68659807	0,09168066	0,25296789	protein_codir	solute carrier family 25 (mitochondrial adenine nucleotide translocator), member 4/5/6/31
TcG_04271	174,5594795	0,418995682	0,13773388	3,04206684	0,0023496	0,01565407	protein_codir	hypothetical protein
TcG_04272	288,1367512	0,036165551	0,10759147	0,33613771	0,73676702	0,86538754	protein_codir	hypothetical protein
TcG_04273	626,1460927	-0,170582237	0,07510172	-2,27134917	0,02312585	0,09417789	protein_codir	putative cysteine peptidase, Clan CA, family C19
TcG_04274	120,2687412	0,252649413	0,16687652	1,51399018	0,13002833	0,31985313	protein_codir	hypothetical protein
TcG_04275	470,6971271	0,028459801	0,08995786	0,31636814	0,7517231	0,87492919	protein_codir	putative Zn-finger protein
TcG_04276	244,4672831	0,130551674	0,11438464	1,14133918	0,25372881	0,48477934	protein_codir	putative AUT2/APG4/ATG4 cysteine peptidase, putative,cysteine peptidase, Clan CA, family C54
TcG_04277	163,9600819	0,073928123	0,14845064	0,49799799	0,61848546	0,793034	protein_codir	hypothetical protein
TcG_04278	211,1352631	0,077457586	0,13509468	0,57335779	0,56640246	0,75838888	protein_codir	hypothetical protein
TcG_04279	64,17674149	0,094066002	0,2212464	0,42516398	0,67071714	0,82731063	protein_codir	hypothetical protein
TcG_04280	68,58532561	0,063445472	0,20858642	0,30416876	0,76099933	0,87939833	protein_codir	hypothetical protein
TcG_04281	630,2840467	0,000975365	0,07589125	0,01285214	0,98974575	0,99576192	protein_codir	hypothetical protein
TcG_04282	233,7342629	-0,146583739	0,11666938	-1,25640279	0,20896997	0,43156443	protein_codir	hypothetical protein
TcG_04283	478,4494524	0,027915403	0,08616113	0,32399069	0,74594508	0,87149696	protein_codir	hypothetical protein
TcG_04284	314,5673145	-0,044233222	0,10272266	-0,43060824	0,66675325	0,82543809	protein_codir	putative KU80 protein
TcG_04285	75,22721547	-0,155293879	0,19986291	-0,777002	0,43715759	0,66061143	protein_codir	KU80 protein
TcG_04286	420,4751496	-0,221877504	0,08826917	-2,5136467	0,01194901	0,05676835	protein_codir	putative kinesin
TcG_04287	578,6959657	-0,023506703	0,07959834	-0,29531649	0,76775212	0,88350974	protein_codir	putative endosomal integral membrane protein
TcG_04288	207,0519043	-0,220964784	0,13058468	-1,69211875	0,09062333	0,25112699	protein_codir	mitogen-activated protein kinase, putative,protein kinase
TcG_04289	179,8693245	-0,093146879	0,13603257	-0,68473954	0,4935083	0,70398758	protein_codir	hypothetical protein
TcG_04290	326,5290523	-0,300663676	0,10453755	-2,87613092	0,00402583	0,02418001	protein_codir	putative phosphatase 2C
TcG_04291	107,362373	0,141022508	0,16950152	0,83198374	0,40541812	0,6343247	protein_codir	putative rab6 GTPase activating protein
TcG_04292	100,5001714	0,042459491	0,1906019	0,22276531	0,82371817	0,91308828	protein_codir	rab6 GTPase activating protein
TcG_04293	317,3496578	-0,085320171	0,10118893	-0,8431769	0,39912951	0,62898729	protein_codir	hypothetical protein
TcG_04294	133,335917	-0,090383488	0,16005014	-0,56471983	0,57226435	0,76350239	protein_codir	PAP2 family protein
TcG_04295	256,7871835	0,080078432	0,11704584	0,68416301	0,49387224	0,70424661	protein_codir	WD-40 repeat protein
TcG_04296	127,0012827	0,214125029	0,16208054	1,32110265	0,18646714	0,40038787	protein_codir	putative diacylglycerol kinase
TcG_04297	0					1	protein_codir	hypothetical protein
TcG_04298	758,0477136	0,05199701	0,06893894	0,75424727	0,45070076	0,67144387	protein_codir	quiescin sulfhydryl oxidase
TcG_04299	48,6899285	0,111032506	0,26974058	0,41162701	0,68061283	0,83339466	protein_codir	dispersed gene family protein 1 (DGF-1)
TcG_04300	16,64561935	-0,362540707	0,43878609	-0,82623564	0,40867043	1	protein_codir	hypothetical protein
TcG_04301	28,02354903	-0,236569487	0,33328416	-0,70981317	0,47782	0,69260885	protein_codir	trans-sialidase
TcG_04302	0,145251675	-1,420530545	4,08047286	-0,3481289	0,72774338	1	protein_codir	trans-sialidase
TcG_04303	2,81099027	1,598708411	1,12080732	1,42639005	0,15375578	1	protein_codir	hypothetical protein
TcG_04304	76,77093871	0,417953095	0,21132976	1,97772947	0,04795923	0,16064056	protein_codir	target of rapamycin (TOR) kinase 1
TcG_04305	11,60028412	0,513044201	0,5128256	1,00042627	0,31710426	1	protein_codir	hypothetical protein
TcG_04306	143,8156401	0,650740098	0,16264819	4,00090594	6,31E-05	0,00075137	protein_codir	helicase-like protein
TcG_04307	0,116927736	0,503022807	4,08047286	0,12327562	0,90188885	1		
TcG_04308	0					1		
TcG_04309	240,348523	-0,213378092	0,11373776	-1,87605317	0,06064798	0,19125408	protein_codir	hypothetical protein
TcG_04310	109,2076823	-0,268210998	0,17611781	-1,52290669	0,12778205	0,31654541	protein_codir	hypothetical protein
TcG_04311	56,64482672	-0,472363921	0,23505587	-2,00958147	0,0444755	0,15231841	protein_codir	hypothetical protein
TcG_04312	32,86692864	-0,011031984	0,30416051	-0,03627027	0,97106686	0,98762678	protein_codir	hypothetical protein
TcG_04313	356,6489166	-0,356627779	0,10986308	-3,24611125	0,00116993	0,00887677	protein_codir	protein phosphatase 2 (formerly 2A), regulatory subunit B

TcG_04314	411,8901647	0,127225751	0,08964828	1,4191655	0,15585077	0,35742024	protein_codir	hypothetical protein
TcG_04315	192,955108	0,120972223	0,12799584	0,94512618	0,34459446	0,57877968	protein_codir	hypothetical protein
TcG_04316	11,35533753	0,055045444	0,50543523	0,10890702	0,91327624	1		
TcG_04317	589,044143	-0,2910739	0,07875878	-3,69576431	0,00021923	0,00215068	protein_codir	hypothetical protein
TcG_04318	711,7545696	0,00598445	0,07300757	0,08197027	0,93467036	0,96913288		
TcG_04319	220,2624261	-0,074837155	0,12282641	-0,60929204	0,54233088	0,74032141	protein_codir	putative P-type ATPase
TcG_04320	394,8951421	-0,046455731	0,09319765	-0,49846463	0,61815659	0,793034	protein_codir	hypothetical protein
TcG_04321	282,7513448	0,022156205	0,10719394	0,2066927	0,83624986	0,92052325	protein_codir	hypothetical protein
TcG_04322	111,5837738	0,264860267	0,17281322	1,53263893	0,12536484	0,3122935	protein_codir	hypothetical protein
TcG_04323	375,6712874	0,143209921	0,09791757	1,46255587	0,14358895	0,34000823	protein_codir	hypothetical protein
TcG_04324	156,2274646	0,473871627	0,14784494	3,2051935	0,00134972	0,00994772	protein_codir	helicase-like protein
TcG_04325	4,294612603	1,634505107	0,92243172	1,77195241	0,07640246	1	protein_codir	putative GAG protein
TcG_04326	29,28584483	0,321552353	0,35266432	0,91178023	0,36188442	0,59455373	protein_codir	hypothetical protein
TcG_04327	53,90186797	0,342128427	0,24305924	1,40759278	0,15925169	0,36185331	protein_codir	trans-sialidase
TcG_04328	64,49136684	0,091145155	0,21798088	0,41813372	0,67584936	0,83017833	protein_codir	rab1 small GTP-binding protein
TcG_04329	37,78378621	0,253281299	0,31197239	0,81187088	0,41686572	0,6436309	protein_codir	rab1 small GTP-binding protein
TcG_04330	70,10127661	0,418314314	0,21758531	1,92253012	0,05453909	0,17596487	protein_codir	target of rapamycin (TOR) kinase 1
TcG_04331	22,57149687	0,468169241	0,36516947	1,28206019	0,19982153	0,4186496	protein_codir	target of rapamycin (TOR) kinase 1
TcG_04332	7,812869155	0,33297405	0,65787331	0,50613704	0,61276045	1	protein_codir	RNA editing complex protein MP90
TcG_04333	299,2753288	0,12612149	0,1055205	1,19523212	0,23199635	0,45922596	protein_codir	hypothetical protein
TcG_04334	119,9975794	-0,052976371	0,16972176	-0,31213658	0,75493673	0,87636394	protein_codir	hypothetical protein
TcG_04335	697,2211921	0,043777865	0,07428606	0,58931471	0,55565018	0,75049697	protein_codir	hypothetical protein
TcG_04336	299,8849346	0,238673501	0,11110545	2,14817093	0,03170018	0,11905292	protein_codir	putative protein kinase
TcG_04337	1008,539047	0,210346903	0,07642215	2,75243356	0,00591541	0,03266149	protein_codir	hypothetical protein
TcG_04338	203,1691362	0,198083724	0,12475681	1,58775881	0,11234089	0,29018174	protein_codir	putative chaperone protein DNAj
TcG_04339	416,353659	0,125917655	0,08947388	1,40731186	0,15933493	0,36189689	protein_codir	putative NADH-dependent fumarate reductase
TcG_04340	175,8557035	0,322894362	0,13651087	2,36533817	0,01801361	0,07755291	protein_codir	fructosamine kinase
TcG_04341	707,6775244	-0,117085376	0,07321084	-1,59929013	0,10975615	0,28632613	protein_codir	hypothetical protein
TcG_04342	381,6330287	0,12839818	0,09328071	1,3764709	0,16867589	0,37510151	protein_codir	hypothetical protein
TcG_04343	331,9535659	-0,143872783	0,10033211	-1,43396548	0,15158211	0,35179947	protein_codir	hypothetical protein
TcG_04344	624,8995201	-0,028784837	0,09117224	-0,3157193	0,75221558	0,87492919	protein_codir	methyltransferase
TcG_04345	730,9168915	-0,029287976	0,07332801	-0,39941048	0,68959077	0,8386805	protein_codir	hypothetical protein
TcG_04346	66,82273441	0,446835556	0,22242932	2,00888781	0,04454903	0,15248009	protein_codir	hypothetical protein
TcG_04347	0					1	protein_codir	hypothetical protein
TcG_04348	457,6861757	0,529394343	0,08637791	6,12881622	8,8535E-10	3,5617E-08	protein_codir	lysine-specific demethylase NO66
TcG_04349	1094,154184	0,301905594	0,06648722	4,54080618	5,604E-06	9,2621E-05	protein_codir	putative ATP-dependent RNA helicase
TcG_04350	333,2857051	0,403786342	0,10330863	3,90854426	9,2854E-05	0,00104752	protein_codir	putative chaperone DNAJ protein
TcG_04351	325,1182973	0,495548053	0,10422553	4,75457434	1,9886E-06	3,7222E-05	protein_codir	hypothetical protein
TcG_04352	128,1247671	0,201361285	0,15651954	1,28649299	0,19827106	0,41690899	protein_codir	mitotic centromere-associated kinesin (MCAK)
TcG_04353	439,724992	0,195025506	0,09941818	1,96166841	0,0498011	0,1649973	protein_codir	hypothetical protein
TcG_04354	589,0947754	0,505600598	0,0787323	6,42176862	1,347E-10	6,4757E-09	protein_codir	hypothetical protein
TcG_04355	79,3079501	0,552148496	0,1983795	2,78329409	0,005381	0,03035262	protein_codir	hypothetical protein
TcG_04356	96,87321536	0,217136443	0,1939596	1,11949312	0,26292982	0,49509263	protein_codir	putative amino acid permease
TcG_04357	102,5117648	0,308348801	0,17726158	1,73951289	0,08194459	0,2349617	protein_codir	amino acid permease
TcG_04358	511,8537085	0,323890711	0,08523943	3,79977548	0,00014483	0,0015282	protein_codir	putative amino acid permease
TcG_04359	166,7552344	0,36972493	0,14616408	2,52951971	0,01142188	0,05493477	protein_codir	amino acid permease
TcG_04360	272,3087293	0,57154278	0,11061065	5,1671587	2,3768E-07	5,6545E-06	protein_codir	hypothetical protein
TcG_04361	1346,742628	0,318556015	0,0557076	5,71835784	1,0756E-08	3,3956E-07	protein_codir	hypothetical protein
TcG_04362	398,3662914	0,285018648	0,09576989	2,97607785	0,00291961	0,01863723	protein_codir	hypothetical protein
TcG_04363	778,5246149	0,510337865	0,070871	7,2009409	5,9798E-13	4,6498E-11	protein_codir	calpain-like protein
TcG_04364	690,3313269	0,47664058	0,08173143	5,83179031	5,4836E-09	1,8523E-07	protein_codir	putative calpain-like cysteine peptidase
TcG_04365	1520,718381	-0,147809546	0,05687602	-2,59880249	0,00935496	0,04681924	protein_codir	cytoskeleton-associated protein CAP5.5

TcG_04366	646,9888358	0,519602008	0,07946768	6,53853228	6,2125E-11	3,2423E-09	protein_codir	calpain-like cysteine peptidase
TcG_04367	145,6391491	0,064940829	0,14977078	0,43360146	0,66457787	0,82474285	protein_codir	hypothetical protein
TcG_04368	108,5457536	0,242813088	0,17862584	1,35933909	0,17403916	0,38259333	protein_codir	hypothetical protein
TcG_04369	323,0188689	0,443074238	0,10201097	4,34339789	1,403E-05	0,00020369	protein_codir	hypothetical protein
TcG_04370	425,2646467	0,1229182	0,0890693	1,38002885	0,16757776	0,37364402	protein_codir	chaperone protein DnaJ
TcG_04371	88,89888062	0,2195367	0,18713883	1,17312213	0,24074683	0,46941986	protein_codir	putative UDP-Gal or UDP-GlcNAc-dependent glycosyltransferase
TcG_04372	573,9402092	0,413500933	0,07964358	5,19189303	2,0817E-07	5,0562E-06	protein_codir	tRNA guanosine-2-O-methyltransferase TRM13
TcG_04373	208,3240562	-0,234555527	0,12654634	-1,85351483	0,0638086	0,19849537	protein_codir	hypothetical protein
TcG_04374	639,3270469	0,402738178	0,07593574	5,30367069	1,135E-07	2,9028E-06	protein_codir	glycosomal ABC transporter member 1
TcG_04375	603,3417072	0,073963101	0,0765269	0,96649811	0,33379499	0,56822639	protein_codir	3',5'-cyclic-nucleotide phosphodiesterase
TcG_04376	551,0161531	0,402797813	0,08236566	4,89036074	1,0065E-06	2,0387E-05	protein_codir	putative mevalonate kinase
TcG_04377	396,3915254	0,129172143	0,09292606	1,39005288	0,16451282	0,36983091	protein_codir	putative UDP-Gal or UDP-GlcNAc-dependent glycosyltransferase
TcG_04378	867,3914869	0,539352845	0,06930937	7,78181677	7,149E-15	7,599E-13	protein_codir	putative C-5 sterol desaturase
TcG_04379	25,86208702	0,53860847	0,35375692	1,52253834	0,12787425	0,31668131	protein_codir	putative C-5 sterol desaturase
TcG_04380	169,8969819	0,135126984	0,13626026	0,99168297	0,3213522	0,55605469	protein_codir	hypothetical protein
TcG_04381	340,8724082	0,308803729	0,11012554	2,80410628	0,00504563	0,02894158	protein_codir	hypothetical protein
TcG_04382	214,7056816	0,454788658	0,12426656	3,65978303	0,00025243	0,00242106	protein_codir	hypothetical protein
TcG_04383	0,625814844	-0,250879225	2,22365854	-0,11282273	0,9101711	1	protein_codir	receptor-type adenylate cyclase
TcG_04384	0					1	protein_codir	receptor-type adenylate cyclase
TcG_04385	148,8962489	0,275826236	0,15160166	1,81941432	0,06884824	0,20969394	protein_codir	hypothetical protein
TcG_04386	63,40673757	0,905257598	0,22959983	3,94276252	8,0548E-05	0,00092491	protein_codir	receptor-type adenylate cyclase
TcG_04387	201,8449424	0,563179325	0,12709354	4,43121908	9,3702E-06	0,00014437	protein_codir	esag4
TcG_04388	55,27215683	0,351552095	0,24684157	1,42420131	0,1543882	0,35556145	protein_codir	hypothetical protein
TcG_04389	52,85609422	0,331672498	0,24283417	1,36583951	0,17198934	0,37955591	protein_codir	mucin-associated surface protein (MASP)
TcG_04390	48,42533324	0,010935268	0,2484363	0,04401638	0,96489134	0,9843472	protein_codir	putative retrotransposon hot spot (RHS) protein
TcG_04391	106,964457	-0,158047909	0,1670642	-0,94603099	0,34413278	0,57834673	protein_codir	putative trans-sialidase
TcG_04392	81,55103313	0,051392437	0,19298241	0,26630632	0,7900033	0,89502471	protein_codir	putative trans-sialidase
TcG_04393	74,72241902	0,157430274	0,21232264	0,74146719	0,45841022	0,67743881	protein_codir	putative trans-sialidase
TcG_04394	87,94867537	-0,070343586	0,19157	-0,36719521	0,71347341	0,85278452	protein_codir	hypothetical protein
TcG_04395	76,54650593	0,01893601	0,20458273	0,09255918	0,92625377	0,96428935		
TcG_04396	249,3957399	-0,006875892	0,11395146	-0,06034053	0,95188442	0,97735058	protein_codir	hypothetical protein
TcG_04397	600,4263974	0,035009002	0,07747718	0,45186209	0,65136834	0,81533638	protein_codir	hypothetical protein
TcG_04398	190,5494755	-0,319964668	0,13052105	-2,45144119	0,01422854	0,06531375	protein_codir	nucleoside-diphosphate kinase
TcG_04399	623,3284982	0,02003234	0,07677214	0,26093242	0,79414462	0,89721693	protein_codir	putative subtilisin-like serine peptidase
TcG_04400	224,2092172	-0,200161828	0,12051344	-1,66090879	0,09673177	0,26308316	protein_codir	hypothetical protein
TcG_04401	103,1689855	-0,306539804	0,17066598	-1,79613894	0,07247242	0,21639612	protein_codir	hypothetical protein
TcG_04402	389,6717475	-0,239669208	0,09228254	-2,59712423	0,00940079	0,04698774	protein_codir	hypothetical protein
TcG_04403	531,9785608	-0,20413933	0,08463548	-2,411983	0,01586602	0,07061995	protein_codir	hypothetical protein
TcG_04404	1243,507632	-0,072242842	0,05674101	-1,27320338	0,20294588	0,42300656	protein_codir	putative AMP deaminase
TcG_04405	436,1600094	-0,158517007	0,08755923	-1,81039742	0,07023418	0,21231594	protein_codir	hypothetical protein
TcG_04406	280,8515614	-0,156452565	0,10849265	-1,44205683	0,14928634	0,34850525	protein_codir	putative katanin
TcG_04407	301,4238522	-0,197618536	0,10263084	-1,92552773	0,05416337	0,17509398	protein_codir	actin-like protein
TcG_04408	408,2642925	0,151987203	0,09094588	1,67118303	0,09468553	0,25903815	protein_codir	putative N(2), N(2)-dimethylguanosine tRNA methyltransferase
TcG_04409	192,0063511	-0,219479435	0,12821489	-1,71180926	0,08693183	0,24434549	protein_codir	glycosyl transferase-like protein
TcG_04410	648,7778686	-0,186426467	0,0742436	-2,51101062	0,01203861	0,05716365	protein_codir	hypothetical protein
TcG_04411	529,4755087	-0,355335313	0,08051803	-4,41311493	1,0189E-05	0,00015452	protein_codir	hypothetical protein
TcG_04412	473,7181555	-0,074606913	0,09058019	-0,82365595	0,41013508	0,63697386	protein_codir	hypothetical protein
TcG_04413	210,5474686	-0,002970291	0,12452385	-0,02385319	0,98096972	0,99270811	protein_codir	hypothetical protein
TcG_04414	178,6775308	-0,287448195	0,13201494	-2,17739137	0,02945138	0,11280122	protein_codir	hypothetical protein
TcG_04415	841,4439335	-0,089396263	0,06648793	-1,34454878	0,17877099	0,38979933	protein_codir	hypothetical protein
TcG_04416	1297,869733	0,032037762	0,06782893	0,47233185	0,63668994	0,8066089	protein_codir	hypothetical protein
TcG_04417	942,5146709	-0,256351269	0,06649277	-3,85532533	0,00011558	0,00126565	protein_codir	putative mitochondrial processing peptidase alpha subunit

TcG_04418	187,4725264	-0,211273356	0,13112138	-1,61128079	0,10711854	0,28208285	protein_codir hypothetical protein
TcG_04419	302,9758049	-0,323550208	0,10320216	-3,13511069	0,00171789	0,01215853	protein_codir hypothetical protein
TcG_04420	43,74877039	0,096657555	0,27045179	0,35739292	0,72079768	0,85670516	protein_codir hypothetical protein
TcG_04421	152,9315744	-0,161806538	0,14398005	-1,12381221	0,2610927	0,49358779	protein_codir hypothetical protein
TcG_04422	569,4908677	-0,001085806	0,08013659	-0,01354944	0,98918944	0,99554162	protein_codir hypothetical protein
TcG_04423	150,9888553	-0,064269942	0,14814363	-0,43383533	0,66440802	0,82462039	protein_codir hypothetical protein
TcG_04424	1114,953355	-0,258282431	0,06759219	-3,82118744	0,00013281	0,00141169	protein_codir hypothetical protein
TcG_04425	337,3114975	-0,353916532	0,10082862	-3,51008003	0,00044797	0,00394093	protein_codir hypothetical protein
TcG_04426	111,4374062	-0,320623946	0,16801717	-1,90828079	0,05635493	0,17996921	
TcG_04427	332,3078465	-0,187543881	0,09992749	-1,87679976	0,06054554	0,19098303	protein_codir cytoplasmic tRNA 2-thiolation protein 2
TcG_04428	414,3290188	-0,11257518	0,09082125	-1,23952472	0,21515124	0,4388705	protein_codir putative protein kinase
TcG_04429	445,057174	-0,195635486	0,08892536	-2,19999655	0,02780714	0,107967	protein_codir protein-tyrosine phosphatase
TcG_04430	811,7233353	-0,131959693	0,06775648	-1,94755825	0,05146784	0,1686864	protein_codir putative C2 domain protein
TcG_04431	344,7334555	-0,132447291	0,0993545	-1,33307788	0,18250625	0,39457313	protein_codir hypothetical protein
TcG_04432	166,5074382	-0,163360579	0,13604652	-1,20077002	0,22984042	0,45688956	protein_codir hypothetical protein
TcG_04433	904,0005261	-0,187386332	0,06429456	-2,91449731	0,00356262	0,02187414	protein_codir putative ubiquitin-like protein
TcG_04434	26,44397711	0,041566653	0,36163587	0,11494063	0,90849218	0,95550022	protein_codir trans-sialidase
TcG_04435	9,685436226	-0,445513255	0,5808435	-0,76701083	0,44307508	1	protein_codir trans-sialidase
TcG_04436	11,54986628	0,542135065	0,53338357	1,01640751	0,30943537	1	protein_codir hypothetical protein
TcG_04437	15,47898091	0,830605137	0,47526544	1,74766577	0,08052192	1	protein_codir hypothetical protein
TcG_04438	244,4139133	0,189610846	0,11620347	1,63171413	0,10273972	0,27408299	protein_codir putative protein kinase
TcG_04439	795,4992818	0,164439332	0,0714518	2,30140215	0,02136891	0,08857968	protein_codir WD repeat-containing protein 96
TcG_04440	173,6018088	0,235276956	0,1387012	1,69628635	0,08983166	0,24975399	protein_codir dynein light chain 2B, cytoplasmic
TcG_04441	354,5490494	0,268333081	0,10183761	2,63491134	0,00841593	0,04318287	protein_codir hypothetical protein
TcG_04442	393,9178554	0,171456322	0,0961608	1,78301682	0,07458357	0,22045189	protein_codir methylcrotonoyl-CoA carboxylase biotinylated subunit protein
TcG_04443	69,53006711	0,663595752	0,21448121	3,09395756	0,00197506	0,01360465	protein_codir phosphatidylethanolamine N-methyltransferase
TcG_04444	321,862846	0,077641015	0,10092982	0,76925741	0,44174051	0,66424472	protein_codir 200 kDa antigen p200
TcG_04445	236,2204558	0,031661767	0,12437534	0,25456626	0,79905812	0,89978495	protein_codir 200 kDa antigen p200
TcG_04446	194,7553602	0,330405707	0,13762065	2,40084405	0,01635731	0,07225152	protein_codir hypothetical protein
TcG_04447	575,5764109	0,318872164	0,07939444	4,01630365	5,9118E-05	0,00071274	protein_codir putative HEAT repeat-containing protein 7A
TcG_04448	240,2543286	0,397371776	0,1249667	3,17982128	0,00147366	0,01072476	protein_codir putative HEAT repeat-containing protein 7A
TcG_04449	225,1699776	0,461164218	0,11870538	3,88494777	0,00010235	0,00113696	protein_codir hypothetical protein
TcG_04450	202,8547135	0,353619879	0,1325524	2,66777421	0,00763556	0,0399393	protein_codir chaperone DNAJ protein
TcG_04451	1427,206239	0,337607504	0,05497651	6,14094129	8,2034E-10	3,3704E-08	protein_codir phosphoglycan beta 1,3 galactosyltransferase 5
TcG_04452	245,7376396	0,416930878	0,11609955	3,59114997	0,00032922	0,00302248	protein_codir hypothetical protein
TcG_04453	286,0237498	0,623439823	0,110756	5,6289485	1,8131E-08	5,4705E-07	protein_codir hypothetical protein
TcG_04454	225,6513545	0,344701214	0,1217979	2,83010812	0,00465323	0,02717354	protein_codir putative mu-adaptin 4, putative,adaptor complex AP-4 medium subunit
TcG_04455	785,6638088	0,377258654	0,06791097	5,55519502	2,773E-08	8,0927E-07	protein_codir hypothetical protein
TcG_04456	414,9137434	0,473833581	0,09974893	4,75026227	2,0315E-06	3,7781E-05	protein_codir hypothetical protein
TcG_04457	978,2717725	0,550450754	0,06480671	8,4937306	2,0011E-17	2,8623E-15	protein_codir hypothetical protein
TcG_04458	79,67600358	0,636108283	0,19834982	3,20700203	0,00134126	0,00989799	protein_codir hypothetical protein
TcG_04459	492,0574858	0,432070263	0,09367015	4,61267832	3,9751E-06	6,8536E-05	protein_codir hypothetical protein
TcG_04460	141,3440256	0,353102687	0,15036773	2,34826111	0,01886129	0,08016396	protein_codir hypothetical protein
TcG_04461	576,2890041	0,506967895	0,08165993	6,20828235	5,3567E-10	2,2901E-08	protein_codir hypothetical protein
TcG_04462	1588,725239	0,526127667	0,05522714	9,52661357	1,625E-21	3,4865E-19	protein_codir acetyl-CoA carboxylase
TcG_04463	734,1447614	-0,09117669	0,07313882	-1,2466251	0,212535	0,43575127	protein_codir putative serine/threonine-protein kinase a, putative,protein kinase
TcG_04464	2951,118003	0,190347903	0,0452697	4,20475332	2,6137E-05	0,00034968	protein_codir squalene synthase
TcG_04465	138,056029	1,104375918	0,16367316	6,7474467	1,5047E-11	9,1755E-10	protein_codir hypothetical protein
TcG_04466	327,5695447	0,378171495	0,10079717	3,75180678	0,00017556	0,00179215	protein_codir putative inositol polyphosphate 1-phosphatase
TcG_04467	285,1257826	0,093131233	0,1114968	0,83528163	0,40355916	0,63312391	protein_codir hypothetical protein
TcG_04468	333,9777974	0,034572611	0,0993077	0,34813626	0,72773785	0,85979918	protein_codir hypothetical protein
TcG_04469	359,4975527	0,559306678	0,10014185	5,58514418	2,3351E-08	6,9015E-07	protein_codir nucleoporin (NUP54/57)

TcG_04470	838,8940146	0,316137893	0,06943631	4,55291867	5,2907E-06	8,8198E-05	protein_codir	hypothetical protein
TcG_04471	160,0249341	0,459139079	0,14725535	3,1179789	0,00182096	0,01275552	protein_codir	hypothetical protein
TcG_04472	436,7351785	0,378375334	0,09280828	4,07695671	4,5629E-05	0,00057214	protein_codir	putative protein kinase
TcG_04473	270,5868287	0,375616027	0,11148903	3,36908496	0,00075418	0,00614916	protein_codir	ribosomal protein L7/L12
TcG_04474	320,0143381	0,344295053	0,10012649	3,43860116	0,00058473	0,00494501	protein_codir	hypothetical protein
TcG_04475	612,4103354	0,295967458	0,07847517	3,77147922	0,00016228	0,00168026	protein_codir	hypothetical protein
TcG_04476	260,330528	0,207752552	0,11365348	1,82794718	0,06755748	0,20694516	protein_codir	hypothetical protein
TcG_04477	228,0162831	0,271569573	0,11793158	2,30277237	0,02129165	0,08835426	protein_codir	hypothetical protein
TcG_04478	590,7286014	0,254524284	0,07849085	3,24272543	0,00118392	0,00894193	protein_codir	Smr domain containing protein
TcG_04479	460,780625	0,289732305	0,09026492	3,20979962	0,00132828	0,00983348	protein_codir	hypothetical protein
TcG_04480	514,1325615	0,260723531	0,08613004	3,02709172	0,00246919	0,01628232	protein_codir	kinetoplast-associated protein
TcG_04481	623,2548005	0,201849595	0,08872961	2,2748842	0,02291288	0,09363972	protein_codir	kinetoplast DNA-associated protein
TcG_04482	515,5832423	0,091107699	0,08295471	1,09828238	0,2720812	0,50504046	protein_codir	hypothetical protein
TcG_04483	464,6810569	0,297596702	0,09486542	3,1370408	0,00170662	0,01210835	protein_codir	ERGIC and golgi family 3
TcG_04484	283,0579316	0,570260922	0,10933169	5,21587951	1,8295E-07	4,5098E-06	protein_codir	transmembrane emp24 domain trafficking protein 2
TcG_04485	251,0952833	0,052595862	0,11204735	0,46940745	0,63877843	0,80773199	protein_codir	hypothetical protein
TcG_04486	82,72721949	0,63919809	0,20484431	3,12040937	0,001806	0,01266604	protein_codir	hypothetical protein
TcG_04487	78,83703108	0,108936741	0,19821874	0,54957841	0,58260857	0,77144033	protein_codir	hypothetical protein
TcG_04488	454,1244931	0,046996615	0,0869194	0,54069187	0,58871999	0,77564856	protein_codir	putative inositol 5'-phosphatase
TcG_04489	492,7301966	-0,068076203	0,08461191	-0,80457	0,42106786	0,647488	protein_codir	glutathione-S-transferase/glutaredoxin
TcG_04490	434,1372059	0,087482487	0,09184894	0,95246047	0,34086351	0,57510479	protein_codir	hypothetical protein
TcG_04491	355,0230726	0,000513113	0,09570427	0,00536144	0,99572221	0,99830716	protein_codir	hypothetical protein
TcG_04492	433,3765074	0,102016582	0,09721958	1,04934189	0,29402079	0,52708107	protein_codir	putative mitochondrial pyruvate carrier protein
TcG_04493	417,5388578	-0,004369114	0,09176248	-0,0476133	0,96202443	0,98254717	protein_codir	HEAT repeat-containing protein
TcG_04494	235,6554597	0,253872024	0,11657816	2,17769801	0,02942853	0,11275096	protein_codir	hypothetical protein
TcG_04495	633,8651297	-0,055765568	0,07970531	-0,69964685	0,48414788	0,69741854	protein_codir	hypothetical protein
TcG_04496	906,3744741	-0,4451154	0,06362746	-6,99564954	2,6403E-12	1,854E-10	protein_codir	hypothetical protein
TcG_04497	2220,937483	-0,336097467	0,05088114	-6,60554112	3,9607E-11	2,1645E-09	protein_codir	hypothetical protein
TcG_04498	1536,080763	-0,223986118	0,05853252	-3,82669558	0,00012987	0,00138813	protein_codir	flagellar associated protein
TcG_04499	488,0706938	0,188181356	0,08527248	2,20682393	0,02732636	0,10674418	protein_codir	hypothetical protein
TcG_04500	106,7401487	-0,425714568	0,19956555	-2,13320668	0,03290778	0,12255528	protein_codir	putative serine/threonine protein kinase, putative,protein kinase
TcG_04501	396,1583831	-0,15990074	0,09039353	-1,7689401	0,07690387	0,22464946	protein_codir	hypothetical protein
TcG_04502	258,0370089	0,562996336	0,11470358	4,90827179	9,1882E-07	1,8808E-05	protein_codir	COP-coated vesicle membrane protein p24 precursor
TcG_04503	365,3643715	-0,128657317	0,09732404	-1,32194799	0,18618547	0,40006473	protein_codir	putative pyrroline-5-carboxylate reductase
TcG_04504	859,3409165	-0,17780482	0,06576451	-2,70365925	0,00685806	0,03676884	protein_codir	putative tyrosyl-tRNA synthetase
TcG_04505	205,4724061	-0,018791266	0,1288662	-0,14581998	0,8840635	0,94348884	protein_codir	hypothetical protein
TcG_04506	762,9178261	-0,249676018	0,07365934	-3,3896043	0,00069994	0,00575139	protein_codir	putative DNA-J protein
TcG_04507	602,5400808	0,099464306	0,07631253	1,30338099	0,19244472	0,40883522	protein_codir	serine hydroxymethyltransferase (SHMT-S)
TcG_04508	206,8567776	0,009452004	0,13195338	0,07163139	0,94289526	0,97319113	protein_codir	hypothetical protein
TcG_04509	13,1713961	-0,087908427	0,4965134	-0,17705147	0,85946796	1	protein_codir	putative protein kinase
TcG_04510	162,4080062	0,355929682	0,14258653	2,49623641	0,01255189	0,05906833	protein_codir	BRCT domain-containing protein
TcG_04511	332,6103568	0,177678367	0,1031373	1,72273626	0,08493623	0,24025175	protein_codir	hypothetical protein
TcG_04512	1486,775289	0,207354274	0,06036319	3,43511132	0,00059231	0,00499819	protein_codir	ubiquitin/ribosomal protein S27a
TcG_04513	677,9129315	0,010433034	0,07238688	0,14412879	0,88539877	0,94437554	protein_codir	putative regulatory subunit of protein kinase a-like protein
TcG_04514	186,5536669	0,13154679	0,13402377	0,98151838	0,32633717	0,56072112	protein_codir	putative ankyrin repeat protein
TcG_04515	8,369077863	-0,44689956	0,61075629	-0,73171503	0,46434252	1		
TcG_04516	16,44957223	-0,089469305	0,42364565	-0,21118901	0,83273979	1	protein_codir	RNA-binding protein
TcG_04517	864,4166003	-0,281876516	0,06713843	-4,19843763	2,6876E-05	0,00035874	protein_codir	hypothetical protein
TcG_04518	292,6805105	0,170956467	0,11175659	1,52972161	0,12608565	0,31368443	protein_codir	hypothetical protein
TcG_04519	414,4546661	-0,003392519	0,09220332	-0,03679389	0,97064935	0,98735236	protein_codir	phosphatidyl serine synthase
TcG_04520	946,9097906	0,083894042	0,06987605	1,2006122	0,22990167	0,45688956	protein_codir	putative protein kinase
TcG_04521	610,640793	-0,01229748	0,0781966	-0,15726361	0,8750371	0,93987711	protein_codir	putative poly(A) polymerase

TcG_04522	395,7450721	0,085408492	0,09150724	0,93335227	0,35063811	0,58503646	protein_codir putative small GTP-binding protein Rab7
TcG_04523	145,1462596	0,386468091	0,15059629	2,56625243	0,0102804	0,05049118	protein_codir hypothetical protein
TcG_04524	299,4713914	0,669937018	0,1070276	6,25947905	3,8627E-10	1,7016E-08	protein_codir hypothetical protein
TcG_04525	986,1814134	0,018042381	0,06219544	0,29009169	0,77174609	0,88581833	protein_codir hypothetical protein
TcG_04526	273,975101	0,128920113	0,12042117	1,07057682	0,28435975	0,51809908	protein_codir putative dynein intermediate chain
TcG_04527	148,8708417	-0,408325432	0,16409447	-2,48835591	0,01283352	0,06012503	protein_codir myosin heavy chain
TcG_04528	0					1	protein_codir hypothetical protein
TcG_04529	129,4547068	-0,183544036	0,15481969	-1,18553421	0,2358063	0,46344584	protein_codir putative kinesin
TcG_04530	16,90590469	-0,586116395	0,4737142	-1,23727851	0,2159837	0,43986416	protein_codir kinesin-like protein
TcG_04531	75,45575882	0,168388827	0,20438283	0,82388931	0,41000245	0,63689009	protein_codir putative receptor-type adenylate cyclase
TcG_04532	1162,37616	-0,206221859	0,05880196	-3,50705766	0,00045309	0,00397992	protein_codir putative nucleosome assembly protein-like protein
TcG_04533	451,1438828	-0,239129254	0,08531077	-2,80303709	0,00506238	0,02899298	protein_codir transcription factor IIB
TcG_04534	129,0905835	-0,043038031	0,1552882	-0,27714939	0,7816654	0,89155102	protein_codir hypothetical protein
TcG_04535	1228,141683	-0,075924477	0,06394528	-1,18733517	0,23509544	0,46296424	protein_codir hypothetical protein
TcG_04536	624,4847705	-0,154625549	0,0744534	-2,07680971	0,03781913	0,1357832	protein_codir cyclic nucleotide-binding protein
TcG_04537	1194,709435	-0,01243411	0,06652977	-0,18689544	0,85174261	0,92822975	protein_codir putative protein kinase
TcG_04538	1122,535957	0,319751274	0,06904325	4,63117346	3,636E-06	6,3444E-05	protein_codir 60S acidic ribosomal protein P2
TcG_04539	455,4565713	-0,311418942	0,09005491	-3,45810052	0,000544	0,00466181	protein_codir oculocerebrorenal Lowe syndrome protein
TcG_04540	170,6140043	-0,219736717	0,13376808	-1,64266925	0,10045138	0,26959225	protein_codir oculocerebrorenal Lowe syndrome protein
TcG_04541	316,8553668	-0,159265467	0,10475916	-1,5203011	0,12843532	0,31738294	protein_codir putative heat shock protein DNAJ
TcG_04542	922,0952453	-0,024032619	0,07445705	-0,32277158	0,74686824	0,87215583	protein_codir putative nucleobase transporter
TcG_04543	0					1	protein_codir putative nucleobase transporter
TcG_04544	508,3714073	0,105559859	0,08226461	1,28317461	0,19943091	0,41820968	protein_codir ufm1-conjugating enzyme 1
TcG_04545	387,2203954	0,044783621	0,09761502	0,45877796	0,64639362	0,81201944	protein_codir putative magnesium and cobalt transport protein
TcG_04546	441,9667356	-0,033277339	0,09535787	-0,34897317	0,72710945	0,85927072	protein_codir hypothetical protein
TcG_04547	688,5556451	0,011567256	0,08064559	0,1434332	0,88594806	0,94465252	protein_codir hypothetical protein
TcG_04548	1884,127152	-0,115345226	0,0604808	-1,90713791	0,05650273	0,18024247	protein_codir topoisomerase
TcG_04549	75,3720321	0,200451115	0,20047274	0,99989211	0,31736272	0,55251157	protein_codir putative ubiquitin hydrolase, putative,cysteine peptidase, Clan CA, family C19
TcG_04550	638,8582227	-0,060785507	0,08014448	-0,75844912	0,44818216	0,66910023	protein_codir putative ubiquitin hydrolase
TcG_04551	802,1175016	-0,071924659	0,06810967	-1,05601246	0,29096252	0,52365178	protein_codir putative cation transporter
TcG_04552	564,8706424	-0,0677453	0,08126187	-0,83366656	0,40446891	0,63403826	protein_codir putative eukaryotic initiation factor 4a
TcG_04553	180,9877816	-0,132068235	0,13238242	-0,99762672	0,3184604	0,55357619	protein_codir putative actin-like protein
TcG_04554	287,5623721	-0,000824086	0,10966322	-0,00751469	0,9940042	0,99767711	protein_codir hypothetical protein
TcG_04555	77,82236704	0,729907418	0,20387299	3,58020655	0,00034332	0,00314197	protein_codir hypothetical protein
TcG_04556	124,2200805	-0,175749618	0,16004043	-1,09815763	0,27213566	0,50504046	protein_codir hypothetical protein
TcG_04557	594,9250255	-0,039496324	0,07787004	-0,50720825	0,61200871	0,79040608	protein_codir TBC1 domain family, member 19
TcG_04558	125,2161454	0,215628344	0,15908006	1,3554706	0,17526767	0,38452021	protein_codir hypothetical protein
TcG_04559	83,85973919	0,146274695	0,20191111	0,72445097	0,46878892	0,6853487	protein_codir hypothetical protein
TcG_04560	292,3316244	-0,216009376	0,10637611	-2,03061933	0,04229363	0,1469455	protein_codir putative actin-related protein 3
TcG_04561	397,0139652	0,148108435	0,09452474	1,56687487	0,11714394	0,29855471	protein_codir hypothetical protein
TcG_04562	1079,039334	0,010932859	0,06080392	0,17980518	0,85730552	0,93107816	protein_codir putative nucleolar RNA binding protein
TcG_04563	489,5629803	-0,191085986	0,0887865	-2,15219637	0,03138189	0,11816399	protein_codir pleckstrin domain-containing protein
TcG_04564	270,0704691	-0,053514714	0,128363	-0,41690141	0,67675052	0,83063385	protein_codir TMEM164 family protein
TcG_04565	289,3281296	0,043620438	0,10550593	0,41344063	0,67928381	0,83255921	protein_codir hypothetical protein
TcG_04566	533,121126	-0,143311356	0,07944098	-1,80399794	0,07123163	0,21408292	protein_codir E3 ubiquitin-protein ligase synoviolin
TcG_04567	638,3078548	0,059038977	0,07692313	0,76750614	0,44278065	0,66499591	protein_codir putative mismatch repair protein MSH3
TcG_04568	559,684991	-0,115905306	0,07886955	-1,46958253	0,14167485	0,33719081	protein_codir putative glutaminyl-tRNA synthetase
TcG_04569	578,5792235	0,077114203	0,08396088	0,91845394	0,35838126	0,59224152	protein_codir proliferative cell nuclear antigen (PCNA)
TcG_04570	425,6140184	0,195803193	0,08912725	2,19689474	0,02802796	0,10867871	protein_codir putative phosphomevalonate kinase protein
TcG_04571	119,2485776	-0,109354898	0,17277441	-0,63293459	0,52677636	0,72926645	protein_codir hypothetical protein
TcG_04572	1033,094249	-0,051265238	0,06037301	-0,84914159	0,39580251	0,62553102	protein_codir ribosomal protein S6
TcG_04573	336,4289367	-0,33054893	0,1041028	-3,1752165	0,00149725	0,01084872	protein_codir cyclic nucleotide phosphodiesterase

TcG_04574	188,3970659	0,043987775	0,13719282	0,32062738	0,74849279	0,87311203	protein_codir putative dolichyl-P-Man:GDP-Man7GlcNAc2-PP-dolichyl alpha-1,6-mannosyltransferase
TcG_04575	1004,155805	-0,177690768	0,06544966	-2,71492274	0,00662912	0,03577318	protein_codir putative RNA-binding protein
TcG_04576	379,7060868	-0,269869604	0,1010651	-2,67025525	0,00757936	0,03977949	protein_codir hypothetical protein
TcG_04577	242,3561687	0,359544725	0,118185	3,04221953	0,00234841	0,01565407	protein_codir hypothetical protein
TcG_04578	299,7739433	0,119711641	0,10446756	1,14592169	0,25182755	0,48186194	protein_codir hypothetical protein
TcG_04579	337,5485436	-0,069536496	0,10477298	-0,66368731	0,50689047	0,71367516	protein_codir ICAM-like surface protein
TcG_04580	354,8841232	-0,077165928	0,09638561	-0,80059593	0,42336561	0,64933994	protein_codir hypothetical protein
TcG_04581	305,5863851	-0,19890193	0,10905194	-1,82391924	0,06816428	0,20815798	protein_codir putative mitochondrial carrier protein
TcG_04582	351,4382051	-0,013933899	0,10845735	-0,12847353	0,89777424	0,95018119	protein_codir hypothetical protein
TcG_04583	470,9036084	-0,292100004	0,08658197	-3,37368184	0,0007417	0,00606018	protein_codir GMP-PDE, delta subunit family protein
TcG_04584	400,4133235	0,175117221	0,09692497	1,80672968	0,07080444	0,21351905	protein_codir putative TBC1 domain family member 22A
TcG_04585	1187,500877	-0,219503811	0,0644726	-3,40460623	0,0006626	0,00549129	protein_codir putative FtsJ cell division protein
TcG_04586	185,3854535	-0,005318176	0,14062863	-0,03781717	0,96983346	0,98703399	protein_codir small nuclear ribonucleoprotein
TcG_04587	245,7153974	-0,249526379	0,11717589	-2,12950273	0,03321269	0,12329453	protein_codir hypothetical protein
TcG_04588	654,4735597	-0,347889435	0,07465833	-4,65975348	3,1659E-06	5,6344E-05	protein_codir hypothetical protein
TcG_04589	500,6604235	-0,086654827	0,08429577	-1,02798546	0,30395667	0,53683566	protein_codir putative protein kinase
TcG_04590	191,3459915	0,199405792	0,12732149	1,5661597	0,11731124	0,29871451	protein_codir Variant surface glycoprotein
TcG_04591	611,7210021	-0,029610865	0,07912529	-0,37422759	0,708235	0,84943749	protein_codir hypothetical protein
TcG_04592	265,0450945	0,176477514	0,12107359	1,45760535	0,14494935	0,34175483	protein_codir oxidoreductase
TcG_04593	168,5974822	0,176539286	0,1375579	1,28338165	0,1993584	0,41820968	protein_codir hypothetical protein
TcG_04594	330,795697	-0,019205334	0,10229854	-0,18773811	0,85108195	0,92805389	protein_codir hypothetical protein
TcG_04595	321,2818082	0,035785366	0,11111108	0,32206839	0,74740089	0,87248228	protein_codir protein FAM18B1
TcG_04596	1151,862254	-0,099678913	0,06062923	-1,64407343	0,10016104	0,26899994	protein_codir hypothetical protein
TcG_04597	489,7413228	-0,006443099	0,08938875	-0,07207953	0,94253861	0,97319113	protein_codir putative endo/exonuclease Mre11
TcG_04598	272,7936956	0,221395955	0,10838026	2,04277011	0,0410752	0,14399312	protein_codir hypothetical protein
TcG_04599	1333,600694	0,028798673	0,05698752	0,50535049	0,61331268	0,79091991	protein_codir putative trypanothione synthetase
TcG_04600	92,54757405	0,236484198	0,18402614	1,28505766	0,19877214	0,41761447	protein_codir hypothetical protein
TcG_04601	218,0272398	-0,026042182	0,1204416	-0,21622248	0,82881433	0,91645761	protein_codir hypothetical protein
TcG_04602	400,9298432	-0,224455304	0,09463066	-2,37190889	0,01769645	0,07650415	protein_codir paraflagellar rod protein-like protein
TcG_04603	891,0741031	-0,283185948	0,06536994	-4,33205131	1,4773E-05	0,00021315	protein_codir putative dual specificity protein phosphatase
TcG_04604	209,4650928	0,017805317	0,1292549	0,13775352	0,89043522	0,94667588	protein_codir putative GTP-binding protein
TcG_04605	829,5249158	-0,148088453	0,06945364	-2,1321913	0,03299112	0,12282621	protein_codir NUP-1 protein
TcG_04606	4443,01032	0,276028432	0,03870658	7,13130528	9,9422E-13	7,5287E-11	protein_codir putative glycosomal phosphoenolpyruvate carboxykinase
TcG_04607	44,28682813	-0,075152461	0,26274067	-0,28603285	0,77485296	0,88753796	protein_codir putative protein kinase
TcG_04608	5,933726169	0,096678136	0,74218386	0,13026171	0,89635938	1	
TcG_04609	670,2862319	-0,024410774	0,07378673	-0,33082877	0,74077383	0,86780643	protein_codir hypothetical protein
TcG_04610	105,3085343	0,132809954	0,17400944	0,76323421	0,44532373	0,66703823	protein_codir hypothetical protein
TcG_04611	349,4614957	-0,102090265	0,09748987	-1,04718845	0,29501267	0,52793889	protein_codir hypothetical protein
TcG_04612	493,6392268	0,105734833	0,09089008	1,16332645	0,24469708	0,47286474	protein_codir putative enoyl-CoA hydratase/Enoyl-CoA isomerase/3-hydroxyacyl-CoA dehydrogenase
TcG_04613	569,0914437	0,076354043	0,08110794	0,94138804	0,34650604	0,58081872	protein_codir mitochondrial processing peptidase alpha subunit
TcG_04614	96,78925793	0,214802162	0,18048919	1,19011092	0,2340028	0,4617092	
TcG_04615	35,55940774	-0,046817522	0,30716773	-0,1524168	0,87885821	0,94148439	protein_codir hypothetical protein
TcG_04616	86,696898	0,10739369	0,19297716	0,55650984	0,57786237	0,76800198	protein_codir N-acetyltransferase complex ARD1 subunit
TcG_04617	493,6358516	0,003698791	0,08694073	0,04254382	0,96606518	0,98495028	protein_codir DEAD-box helicase-like protein
TcG_04618	130,5485101	0,094401204	0,15363045	0,61446937	0,53890521	0,73820598	
TcG_04619	386,2734109	0,009658336	0,09206583	0,10490684	0,91644973	0,95960113	protein_codir hypothetical protein
TcG_04620	227,1498882	-0,007889161	0,11722281	-0,06730056	0,94634243	0,97469438	protein_codir hypothetical protein
TcG_04621	542,7161649	-0,097934275	0,08208728	-1,19305056	0,23284957	0,46021753	protein_codir putative cytochrome P450
TcG_04622	468,0562736	-0,179749006	0,0854175	-2,10435796	0,03534723	0,12955807	protein_codir putative protein kinase
TcG_04623	398,0076671	-0,059177533	0,09590469	-0,61704522	0,5372049	0,73683627	protein_codir hypothetical protein
TcG_04624	110,5237252	0,081727417	0,17674925	0,46239187	0,64380033	0,810875	protein_codir hypothetical protein
TcG_04625	721,6895332	-0,042088501	0,07654256	-0,54987056	0,58240816	0,7714245	protein_codir CCCH zinc-finger protein

TcG_04626	216,6533673	-0,287594015	0,12213015	-2,35481584	0,01853189	0,07914134	protein_codir	hypothetical protein
TcG_04627	162,6563611	-0,34675703	0,14015832	-2,47403819	0,01335954	0,06221208	protein_codir	hypothetical protein
TcG_04628	213,8547683	-0,135467594	0,12803155	-1,05807981	0,29001906	0,52338953	protein_codir	hypothetical protein
TcG_04629	259,755152	0,067639589	0,11084485	0,61021861	0,541717	0,74008786	protein_codir	hypothetical protein
TcG_04630	264,6830025	0,054033384	0,10991325	0,4916003	0,62300193	0,79678777	protein_codir	hypothetical protein
TcG_04631	276,4102655	-0,286228923	0,10993006	-2,60373651	0,00922136	0,04633075	protein_codir	proteasome alpha 7 subunit
TcG_04632	154,9961024	-0,124876712	0,14727652	-0,84790648	0,39649006	0,6263613	protein_codir	hypothetical protein
TcG_04633	133,834533	0,097141216	0,15471539	0,62787042	0,53008883	0,73198176	protein_codir	hypothetical protein
TcG_04634	282,5274792	-0,014358429	0,10535974	-0,13628003	0,89159991	0,94710521	protein_codir	integral membrane protein
TcG_04635	145,7263488	0,178184017	0,146126	1,2193861	0,22269768	0,4488042	protein_codir	hypothetical protein
TcG_04636	187,1089738	0,147288485	0,13640894	1,07975681	0,28025049	0,51343804	protein_codir	hypothetical protein
TcG_04637	325,8002679	-0,068641272	0,10278108	-0,66783955	0,50423602	0,71207637	protein_codir	flap endonuclease-1 (FEN-1)
TcG_04638	181,1842453	-0,054164491	0,13268439	-0,40822052	0,68311178	0,83530693	protein_codir	putative protein kinase-like protein
TcG_04639	287,3078499	0,07079507	0,11236239	0,63006019	0,52865521	0,73102477	protein_codir	putative axoneme central apparatus protein
TcG_04640	559,739371	0,037307561	0,08540898	0,43681078	0,66224858	0,82307341	protein_codir	acyl carrier protein, mitochondrial precursor
TcG_04641	173,6738273	-0,271066344	0,13771532	-1,96830932	0,04903246	0,16319738	protein_codir	hypothetical protein
TcG_04642	86,11542651	-0,154891217	0,19145692	-0,80901344	0,41850742	0,64513397	protein_codir	putative ankyrin repeat protein
TcG_04643	219,8529208	-0,153285418	0,11839311	-1,29471567	0,19541833	0,41323541	protein_codir	hypothetical protein
TcG_04644	202,9359196	0,056449557	0,12634637	0,44678416	0,65503091	0,81771233	protein_codir	hypothetical protein
TcG_04645	305,8286647	-0,109809668	0,1020171	-1,0763849	0,28175515	0,51481079	protein_codir	gamma-tubulin
TcG_04646	453,9076364	-0,04893094	0,09049277	-0,54071656	0,58870296	0,77564856	protein_codir	calmodulin
TcG_04647	2275,830494	-0,185775328	0,11535906	-1,61040948	0,10730849	0,28223527	protein_codir	dynein, axonemal, heavy polypeptide 5
TcG_04648	154,9152447	0,056354402	0,14429171	0,39055884	0,69612335	0,84259143	protein_codir	hypothetical protein
TcG_04649	333,5745831	-0,169171937	0,09801904	-1,72590902	0,08436378	0,2392751	protein_codir	hypothetical protein
TcG_04650	467,167825	-0,099461316	0,08845586	-1,12441754	0,26083593	0,49331519	protein_codir	2OG-Fe(II) oxygenase
TcG_04651	517,9987752	-0,085264896	0,08148175	-1,04642936	0,29536284	0,52809782		
TcG_04652	229,026745	0,104477869	0,11849596	0,88169982	0,37793916	0,60858973	protein_codir	dehydrogenase-like protein
TcG_04653	561,9055066	0,224554435	0,07968951	2,81786702	0,00483438	0,02804765	protein_codir	transferase
TcG_04654	268,4828179	-0,045040162	0,11154493	-0,40378494	0,68637087	0,83720952	protein_codir	hypothetical protein
TcG_04655	180,9231679	0,214959555	0,1365868	1,57379446	0,11553498	0,29583886	protein_codir	frataxin-like, mitochondrial precursor
TcG_04656	942,7860612	-0,420289948	0,06870445	-6,11736142	9,5137E-10	3,8009E-08	protein_codir	hypothetical protein
TcG_04657	298,895143	-0,069052285	0,10836984	-0,63719099	0,52400045	0,72776714	protein_codir	hypothetical protein
TcG_04658	469,3162625	-0,201779945	0,08803761	-2,29197429	0,02190713	0,09019841	protein_codir	hypothetical protein
TcG_04659	50,08629366	0,288628189	0,24591067	1,17371155	0,24051058	0,46919609	protein_codir	hypothetical protein
TcG_04660	40,61253479	0,021297276	0,28150188	0,07565589	0,93969287	0,97207871	protein_codir	hypothetical protein
TcG_04661	344,8991356	0,049112969	0,09985832	0,49182653	0,62284198	0,79676839	protein_codir	hypothetical protein
TcG_04662	117,7453608	0,052484119	0,17422676	0,3012403	0,76323126	0,88040596	protein_codir	hypothetical protein
TcG_04663	939,5332996	0,084619143	0,0686967	1,23177885	0,21803171	0,44240199	protein_codir	DNA polymerase epsilon catalytic subunit A
TcG_04664	151,7403049	-0,384585887	0,14702141	-2,61584958	0,00890058	0,04512424	protein_codir	hypothetical protein
TcG_04665	572,1737993	-0,295218667	0,07848075	-3,76166971	0,00016878	0,00173362	protein_codir	oligoribonuclease
TcG_04666	221,0234491	0,031831045	0,12010767	0,26502093	0,79099334	0,89549041	protein_codir	hypothetical protein
TcG_04667	367,9093896	-0,239319579	0,10324518	-2,31797339	0,02045077	0,08556973	protein_codir	PIH1 domain-containing protein 1
TcG_04668	385,3661465	-0,054200193	0,09217652	-0,58800434	0,55652938	0,75124658	protein_codir	HAD-superfamily subfamily IIA hydrolase
TcG_04669	125,5741855	0,303005417	0,15923823	1,90284339	0,05706098	0,18172308	protein_codir	hypothetical protein
TcG_04670	295,7644912	-0,105340512	0,10620013	-0,99190566	0,32124355	0,5560344	protein_codir	hypothetical protein
TcG_04671	525,2745364	-0,232116505	0,08062173	-2,87908115	0,00398836	0,02400473	protein_codir	dynein heavy chain, cytosolic
TcG_04672	473,5580105	-0,184810067	0,09243356	-1,99938285	0,04556695	0,15486613	protein_codir	spindle assembly 6
TcG_04673	321,3158776	0,072090062	0,10792991	0,66793406	0,50417568	0,71207637	protein_codir	hypothetical protein
TcG_04674	410,4803146	-0,06401396	0,09538204	-0,67113219	0,50213632	0,71094291	protein_codir	zinc finger protein, predicted
TcG_04675	343,4472063	0,061399049	0,09680981	0,6342234	0,52593504	0,72890472	protein_codir	putative 3-demethylubiquinone-9 3-methyltransferase
TcG_04676	465,1480365	-0,06798626	0,08862067	-0,76716027	0,44298623	0,66499591	protein_codir	hypothetical protein
TcG_04677	190,4986038	0,211586847	0,13077967	1,61788787	0,10568676	0,27969092	protein_codir	hypothetical protein

TcG_04678	252,6246147	-0,053023075	0,118337	-0,44806845	0,6541038	0,81709489	protein_codir	putative synaptojanin (N-terminal domain), putative,inositol/phosphatidylinositol phosphatase
TcG_04679	106,7569687	0,014999614	0,16894674	0,0887831	0,92925429	0,96559105	protein_codir	hypothetical protein
TcG_04680	239,8306044	-0,172903544	0,11494202	-1,50426749	0,13251251	0,32357058		
TcG_04681	4601,981964	0,548067662	0,04242021	12,9199649	3,473E-38	3,0952E-35	protein_codir	prostaglandin F2alpha synthase
TcG_04682	177,7245598	0,3234415	0,13377541	2,41779482	0,01561488	0,06968952	protein_codir	hypothetical protein
TcG_04683	60,15733823	0,601254603	0,23482311	2,56045754	0,01045344	0,05114595	protein_codir	hypothetical protein
TcG_04684	45,12203095	0,560136385	0,26486158	2,11482683	0,03444471	0,12697308	protein_codir	hypothetical protein
TcG_04685	199,189825	-0,097743835	0,13330288	-0,73324623	0,46340826	0,68091923	protein_codir	hypothetical protein
TcG_04686	228,2034754	0,045828837	0,1183216	0,38732437	0,69851609	0,84414248	protein_codir	putative Bardet-Biedl syndrome 1 protein
TcG_04687	264,603056	0,115827635	0,10889789	1,06363524	0,28749397	0,52052968	protein_codir	DNA-directed RNA polymerase
TcG_04688	485,8439681	-0,207269131	0,08289224	-2,50046497	0,01240304	0,05858199	protein_codir	hypothetical protein
TcG_04689	362,8667961	-0,036290864	0,09594203	-0,37825823	0,70523877	0,84795521	protein_codir	Hsp70 subfamily B suppressor 1
TcG_04690	167,2748659	0,138031447	0,13510661	1,02164838	0,30694735	0,54005953	protein_codir	hypothetical protein
TcG_04691	2546,71689	-0,208564993	0,0449916	-4,63564287	3,5583E-06	6,245E-05	protein_codir	poly(A)-binding protein 2
TcG_04692	18,53978136	-0,15652521	0,40007976	-0,39123502	0,69562353	0,84234001		
TcG_04693	308,6570664	-0,049955764	0,10439103	-0,47854461	0,63226263	0,80290602	protein_codir	hypothetical protein
TcG_04694	282,7336359	0,13250796	0,10747601	1,23290731	0,21761035	0,44191689	protein_codir	hypothetical protein
TcG_04695	167,0805951	0,103233973	0,13609606	0,75853756	0,44812924	0,66910023	protein_codir	hydrolase of HD superfamily
TcG_04696	332,3384623	0,173332102	0,10580756	1,63818259	0,10138361	0,27146534	protein_codir	hypothetical protein
TcG_04697	187,0079521	0,298895104	0,1321952	2,2610132	0,02375844	0,09626617	protein_codir	hypothetical protein
TcG_04698	127,9673149	-0,0452699	0,16217499	-0,27914231	0,78013562	0,89059526	protein_codir	hypothetical protein
TcG_04699	196,4589097	-0,019006113	0,13043374	-0,1457147	0,88414662	0,94348884	protein_codir	TPR domain protein
TcG_04700	185,6157492	0,301987082	0,13548402	2,22894977	0,02581725	0,10219561	protein_codir	hypothetical protein
TcG_04701	453,7842814	0,000296567	0,09147823	0,00324194	0,99741331	0,99910416	protein_codir	putative protein kinase
TcG_04702	71,81497749	0,759815815	0,21145145	3,5933346	0,00032647	0,00300438	protein_codir	hypothetical protein
TcG_04703	233,5562645	0,116584316	0,11791201	0,98873993	0,3227904	0,55741474	protein_codir	chaperone protein DNAJ, putatative
TcG_04704	491,7559826	-0,055003566	0,08354104	-0,65840173	0,51028003	0,71705329	protein_codir	putative ATP-dependent RNA helicase
TcG_04705	295,6994842	-0,020302555	0,10346856	-0,19621956	0,84443832	0,92455702	protein_codir	putative Bem46-like serine peptidase, putative,Serine peptidase, Clan SC, Family S09X
TcG_04706	501,956066	-0,034300838	0,08238594	-0,41634335	0,67715878	0,83090609	protein_codir	GTP-binding protein
TcG_04707	222,4909	-0,158205456	0,12131921	-1,30404292	0,19221895	0,40855783	protein_codir	putative mitotubule-associated protein Gb4
TcG_04708	215,0129381	0,081166756	0,1278316	0,63495066	0,5254606	0,72866385	protein_codir	putative electon transport protein SCO1/SCO2
TcG_04709	2017,101742	-0,203691687	0,05200247	-3,916962	8,9672E-05	0,00101757	protein_codir	hypothetical protein
TcG_04710	1819,092071	-0,17739359	0,05379227	-3,29775263	0,00097462	0,00761426	protein_codir	microtubule-associated protein Gb4
TcG_04711	718,7703111	0,056688111	0,07059401	0,80301583	0,42196558	0,6481624	protein_codir	hypothetical protein
TcG_04712	303,9410365	-0,077882045	0,12137956	-0,64164054	0,5211066	0,72540161	protein_codir	hypothetical protein
TcG_04713	331,853171	0,30547216	0,1008428	3,0291915	0,00245209	0,01620647	protein_codir	putative deoxyhypusine hydroxylase
TcG_04714	710,4384813	-0,038017361	0,07349342	-0,51728929	0,60495423	0,78664418	protein_codir	hypothetical protein
TcG_04715	354,1383574	0,456364263	0,10155218	4,49388919	6,9934E-06	0,00011222	protein_codir	hypothetical protein
TcG_04716	468,9348686	0,127904591	0,08458489	1,51214471	0,13049705	0,32059772	protein_codir	hypothetical protein
TcG_04717	922,191505	0,288816634	0,06749971	4,27878309	1,8792E-05	0,00026263	protein_codir	hypothetical protein
TcG_04718	1127,706515	-0,031307851	0,05898794	-0,53075006	0,59559199	0,7806028	protein_codir	putative transmembrane transport protein
TcG_04719	249,0243282	0,354808584	0,11647797	3,04614325	0,00231797	0,0155058	protein_codir	engulfment and cell motility ELM family protein
TcG_04720	173,8819349	0,282137639	0,13477198	2,09344439	0,0363095	0,13216522	protein_codir	hypothetical protein
TcG_04721	123,350777	0,298342104	0,16428171	1,81603966	0,06936429	0,21075597	protein_codir	hypothetical protein
TcG_04722	878,6666818	0,07358008	0,07782402	0,94546747	0,34442027	0,57857811	protein_codir	hypothetical protein
TcG_04723	470,7229937	0,156634508	0,08592208	1,82298312	0,06830595	0,20840118	protein_codir	microtubule-associated protein, RP/EB family
TcG_04724	207,8598315	0,255471157	0,12478873	2,04722936	0,04063557	0,14271104	protein_codir	hypothetical protein
TcG_04725	170,2999479	0,254504284	0,13833144	1,83981513	0,06579538	0,20305723	protein_codir	putative O-6 methyl-guanine alkyl transferase
TcG_04726	2026,379887	0,083461587	0,09772728	0,85402545	0,39309089	0,62277465	protein_codir	hypothetical protein
TcG_04727	68,99768743	0,159321306	0,2131789	0,74735965	0,45484649	0,67475691	protein_codir	hypothetical protein
TcG_04728	116,070943	-0,31801012	0,1911881	-1,66333634	0,09624513	0,26237554	protein_codir	putative microtubule-associated protein Gb4
TcG_04729	7,698811434	0,983832137	0,64110118	1,53459729	0,12488278	1	protein_codir	hypothetical protein

TcG_04730	5,484526572	-0,007124402	0,74188662	-0,00960309	0,99233796	1	protein_codir	hypothetical protein
TcG_04731	229,2507535	0,070406119	0,11904477	0,59142553	0,55423534	0,74910997	protein_codir	putative surface protease GP63
TcG_04732	1,634630895	-0,912586743	1,35378519	-0,67410011	0,50024767	1	protein_codir	hypothetical protein
TcG_04733	10,32826159	-0,042974256	0,55046897	-0,07806844	0,93777361	1	protein_codir	hypothetical protein
TcG_04734	295,245571	0,309066951	0,10462142	2,95414604	0,00313536	0,019764	protein_codir	hypothetical protein
TcG_04735	122,0912876	0,486590809	0,15946092	3,05147366	0,00227721	0,0152935	protein_codir	hypothetical protein
TcG_04736	193,9511134	-0,438925714	0,1292271	-3,39654529	0,00068242	0,00562744		
TcG_04737	230,1771443	0,029017357	0,11931474	0,2432001	0,80785039	0,90504442	protein_codir	hypothetical protein
TcG_04738	108,1883564	0,186251133	0,17611293	1,05756649	0,29025313	0,52345679	protein_codir	hypothetical protein
TcG_04739	133,2627645	0,086833603	0,15439226	0,562422	0,57382853	0,76499121	protein_codir	hypothetical protein
TcG_04740	80,51170346	-0,132250189	0,20699594	-0,63890234	0,52288646	0,72683414	protein_codir	L1Tc protein
TcG_04741	1346,177759	0,067052872	0,05548827	1,20841529	0,22688756	0,45361852	protein_codir	putative protein kinase
TcG_04742	144,3522279	-0,160369049	0,14692982	-1,09146702	0,27506744	0,50763484	protein_codir	hypothetical protein
TcG_04743	36,37292535	-0,308870696	0,2937189	-1,05158605	0,29298951	0,52596474	protein_codir	hypothetical protein
TcG_04744	309,1975357	-0,109312976	0,10922819	-1,00077622	0,31693501	0,55193282	protein_codir	hypothetical protein
TcG_04745	1048,019002	-0,134770831	0,06175325	-2,18240855	0,02907939	0,11178296	protein_codir	cytochrome c oxidase subunit V
TcG_04746	862,5487346	0,023350868	0,0672062	0,34745108	0,72825247	0,86000745	protein_codir	putative fatty acid desaturase, putative,sphingolipid delta 4 desaturase
TcG_04747	8,19681122	0,716301627	0,61984363	1,15561667	0,24783799	1	protein_codir	hypothetical protein
TcG_04748	45,23552268	0,4308224	0,26063596	1,65296608	0,09833777	0,26564266	protein_codir	trans-sialidase
TcG_04749	144,0920973	-0,004924343	0,1504348	-0,03273407	0,97388665	0,98856236	protein_codir	programmed cell death protein 5
TcG_04750	264,1918331	0,134287132	0,11056438	1,21456055	0,22453374	0,45108253	protein_codir	hypothetical protein
TcG_04751	123,1838849	0,014155668	0,15992834	0,08851257	0,9294693	0,96572785	protein_codir	hypothetical protein
TcG_04752	300,0793523	0,234340411	0,10427157	2,24740475	0,02461417	0,09866823	protein_codir	S-acyltransferase
TcG_04753	1017,334057	0,020942114	0,06191833	0,33822155	0,73519624	0,86451601	protein_codir	ribosome biogenesis protein BMS1
TcG_04754	166,9711282	0,385996076	0,13688333	2,81989098	0,004804	0,0278993	protein_codir	hypothetical protein
TcG_04755	286,5712639	0,289629569	0,10723364	2,70092078	0,00691478	0,0369703	protein_codir	hypothetical protein
TcG_04756	362,6528069	-0,287269931	0,09484655	-3,02878624	0,00245538	0,01621897	protein_codir	hypothetical protein
TcG_04757	325,156024	-0,117356057	0,10306869	-1,13861983	0,25486175	0,48614229	protein_codir	putative C-terminal motor kinesin
TcG_04758	686,8479753	-0,07649037	0,07413841	-1,03172387	0,30220149	0,5353715	protein_codir	putative replication factor A, 51kDa subunit
TcG_04759	484,9779285	-0,062828388	0,08321173	-0,75504245	0,45022351	0,67090542	protein_codir	putative mitochondrial RNA binding complex 1 subunit
TcG_04760	881,0165141	0,114051052	0,0670532	1,70090405	0,08896101	0,24824235	protein_codir	hypothetical protein
TcG_04761	118,8888472	0,549261537	0,18178621	3,02146971	0,00251551	0,01650321	protein_codir	putative ATPase protein
TcG_04762	368,7150594	-0,110493656	0,09488926	-1,16444847	0,24424231	0,47241927	protein_codir	DNA topoisomerase 3-beta-1 isoform X1
TcG_04763	239,0187989	0,068010253	0,11889703	0,5720097	0,5673154	0,75908491	protein_codir	ADP-ribosylation factor GTPase activating protein
TcG_04764	583,6013786	-0,049098491	0,07859316	-0,62471711	0,53215674	0,7329332	protein_codir	putative protein kinase
TcG_04765	247,1682695	0,123267187	0,11713111	1,05238639	0,29262231	0,52538697	protein_codir	hypothetical protein
TcG_04766	213,5590149	0,222466615	0,12316984	1,80617768	0,07089059	0,21361206	protein_codir	hypothetical protein
TcG_04767	996,222499	-0,119553776	0,06320734	-1,89145395	0,05856377	0,18558088	protein_codir	putative proteasome regulatory non-ATP-ase subunit 2
TcG_04768	128,5386742	-0,068155122	0,16508405	-0,41285104	0,67971575	0,83282432	protein_codir	hypothetical protein
TcG_04769	763,4064909	-0,04530367	0,06935956	-0,65317126	0,51364588	0,71977518	protein_codir	hypothetical protein
TcG_04770	114,6125631	-0,015907843	0,17024908	-0,09343864	0,92555509	0,96364857	protein_codir	hypothetical protein
TcG_04771	246,0719757	0,157607766	0,11564186	1,36289548	0,17291545	0,38109158	protein_codir	hypothetical protein
TcG_04772	621,2200648	0,120153398	0,08132547	1,47743873	0,13955807	0,33409356	protein_codir	zinc finger protein kinase
TcG_04773	385,7540395	0,024818897	0,09483687	0,26170093	0,79355202	0,89680977	protein_codir	hypothetical protein
TcG_04774	375,2610803	-0,032109205	0,09649011	-0,33277197	0,73930642	0,86708727	protein_codir	putative serine/threonine protein kinase
TcG_04775	228,543947	0,047264048	0,12325339	0,38347057	0,70137088	0,84532228	protein_codir	hypothetical protein
TcG_04776	60,38241323	0,533211544	0,23470786	2,2718095	0,02309802	0,09416384	protein_codir	hypothetical protein
TcG_04777	305,8483369	-0,148907521	0,10171846	-1,46391842	0,14321624	0,33967316	protein_codir	DUF3342 domain containing protein
TcG_04778	481,246716	-0,034381048	0,08761292	-0,39241985	0,69474802	0,84207036	protein_codir	putative tRNA pseudouridine synthase
TcG_04779	175,7154301	-0,205767834	0,14664753	-1,40314563	0,16057342	0,36344905	protein_codir	hypothetical protein
TcG_04780	144,386571	-0,11547563	0,16508825	-0,69947817	0,48425325	0,69748361	protein_codir	putative helicase-like protein
TcG_04781	270,2994239	-0,261018505	0,11019932	-2,36860356	0,01785538	0,07704558	protein_codir	putative helicase-like protein

TcG_04782	239,3544073	0,198263687	0,11483878	1,72645246	0,08426605	0,23909571	protein_codir	hypothetical protein
TcG_04783	316,8718768	-0,068445129	0,10611377	-0,64501647	0,51891651	0,72371279	protein_codir	putative phosphonopyruvate decarboxylase
TcG_04784	280,7275815	0,034904072	0,10989343	0,31761744	0,75077514	0,87456599	protein_codir	putative prenyltransferase
TcG_04785	329,6419782	-0,103628413	0,10343925	-1,00182875	0,31642631	0,55146138	protein_codir	hypothetical protein
TcG_04786	163,2263937	0,024495751	0,13943291	0,17568127	0,86054435	0,93329372		
TcG_04787	1017,731294	0,273296419	0,06391081	4,27621605	1,901E-05	0,00026504	protein_codir	hypothetical protein
TcG_04788	470,7801235	-0,259095191	0,0954788	-2,71364105	0,00665482	0,03587845	protein_codir	transmembrane protein 222
TcG_04789	363,3418671	-0,018644325	0,09662908	-0,19294735	0,8470002	0,92596191	protein_codir	hypothetical protein
TcG_04790	149,7815513	-0,029372394	0,1444046	-0,20340345	0,8388197	0,9217152	protein_codir	putative adenylate kinase
TcG_04791	2223,688476	-0,176178528	0,04670173	-3,77241999	0,00016167	0,00167543	protein_codir	cell division control protein 48-like protein E
TcG_04792	231,624256	0,001948308	0,11953927	0,01629848	0,98699627	0,99474691	protein_codir	putative amino acid tansporter
TcG_04793	223,1152807	-0,052274935	0,11802562	-0,44291176	0,65782956	0,81952831	protein_codir	putative serine/cysteine trypsin-like peptidase
TcG_04794	458,0772874	0,113213037	0,08671089	1,30563806	0,19167567	0,40770228	protein_codir	hypothetical protein
TcG_04795	140,9018754	0,088799993	0,15468922	0,57405419	0,56593113	0,7582163	protein_codir	hypothetical protein
TcG_04796	809,1520299	-0,145560515	0,06787119	-2,14465824	0,03198018	0,11991016	protein_codir	putative coiled-coil domain-containing protein 104
TcG_04797	738,0565443	-0,058605167	0,07098534	-0,82559537	0,40903366	0,63660535	protein_codir	putative Ran GDP binding protein
TcG_04798	639,8937281	-0,165740089	0,07578231	-2,18705518	0,0287385	0,11061934	protein_codir	putative Ran GDP binding protein
TcG_04799	949,550612	-0,072466706	0,06428146	-1,12733455	0,25960106	0,4921081	protein_codir	translation elongation factor 1-beta
TcG_04800	452,4666924	-0,055708709	0,09557283	-0,58289272	0,55996552	0,75405439	protein_codir	glycosyltransferase
TcG_04801	1417,925199	0,180900949	0,06799214	2,6606158	0,00779979	0,04065154	protein_codir	vacuolar protein sorting-associated protein 13 family protein
TcG_04802	179,6893837	-0,146875162	0,14080469	-1,04311272	0,29689609	0,53010296	protein_codir	hypothetical protein
TcG_04803	676,8146504	-0,163074791	0,07508725	-2,17180415	0,02987044	0,11406688	protein_codir	hypothetical protein
TcG_04804	438,881863	0,216675823	0,09112574	2,3777675	0,0174178	0,07555323	protein_codir	hypothetical protein
TcG_04805	496,2289406	-0,042328856	0,08236534	-0,51391589	0,6073108	0,78793986	protein_codir	hypothetical protein
TcG_04806	306,0037857	0,147576737	0,1072074	1,3765536	0,1686503	0,37510151	protein_codir	hypothetical protein
TcG_04807	329,4539928	-0,218048788	0,09998818	-2,1807457	0,02920223	0,11204426	protein_codir	hypothetical protein
TcG_04808	117,7095257	-0,179692085	0,16797428	-1,06975954	0,28472756	0,5185246	protein_codir	hypothetical protein
TcG_04809	523,57573	0,047208492	0,08435429	0,55964543	0,57572131	0,76634962	protein_codir	hypothetical protein
TcG_04810	173,1109138	-0,158523565	0,13504546	-1,17385332	0,24045378	0,46916428	protein_codir	hypothetical protein
TcG_04811	17,50484158	0,050627421	0,43608283	0,11609588	0,90757655	0,95523092	protein_codir	hypothetical protein
TcG_04812	654,1975324	-0,018618032	0,07560567	-0,2462518	0,80548732	0,90403721	protein_codir	putative nima-related protein kinase
TcG_04813	237,5018214	0,114704264	0,11998192	0,95601293	0,3390657	0,57332217	protein_codir	putative serine/threonine protein kinase
TcG_04814	414,3776718	-0,086144398	0,0922595	-0,93371841	0,35044916	0,58488966	protein_codir	ELMO domain-containing protein 2
TcG_04815	493,1878796	-0,209905874	0,08378302	-2,50535114	0,01223299	0,05792047	protein_codir	leucine-richcontaining protein 23
TcG_04816	97,56290132	-0,111558989	0,17919035	-0,62257252	0,53356547	0,73410397		
TcG_04817	372,5301435	-0,269369288	0,09944808	-2,70864233	0,00675591	0,03632204	protein_codir	hypothetical protein
TcG_04818	941,8600699	-0,144413379	0,06498181	-2,22236638	0,02625856	0,10344496	protein_codir	hypothetical protein
TcG_04819	1336,116035	-0,113480782	0,05788857	-1,96033156	0,04995705	0,16541937	protein_codir	cohesin complex subunit SA-1/2
TcG_04820	200,9719425	-0,19501095	0,13432101	-1,4518276	0,14654955	0,344616	protein_codir	hypothetical protein
TcG_04821	474,2821616	-0,139181854	0,08735693	-1,59325491	0,11110302	0,28816646	protein_codir	29 kDa proteasome subunit TCPR29A
TcG_04822	2262,976239	-0,181962987	0,04963543	-3,66598989	0,00024638	0,00237622	protein_codir	clathrin heavy chain 1
TcG_04823	40,66577646	0,4044129	0,27690633	1,46046824	0,14416143	0,34078152	protein_codir	target of rapamycin (TOR) kinase 1
TcG_04824	124,2434987	0,523127064	0,1653314	3,16411205	0,00155557	0,0112152	protein_codir	hypothetical protein
TcG_04825	1703,41546	-0,425035587	0,05417596	-7,84546412	4,3136E-15	4,6275E-13	protein_codir	putative protein disulfide isomerase
TcG_04826	351,7709149	0,010769387	0,09671775	0,11134861	0,91133991	0,95684497	protein_codir	hypothetical protein
TcG_04827	92,71572075	-0,020128068	0,18410923	-0,10932677	0,91294331	0,95728087	protein_codir	hypothetical protein
TcG_04828	906,3793966	-0,060147038	0,06670293	-0,9017151	0,36720823	0,59939061	protein_codir	RNA-editing complex protein MP63
TcG_04829	358,3359991	-0,103716505	0,0968012	-1,07143817	0,28397246	0,51747482	protein_codir	ribosomal protein L1-like protein
TcG_04830	2025,518655	-0,152768761	0,04842616	-3,15467437	0,00160677	0,01153037	protein_codir	putative T-complex protein 1, theta subunit
TcG_04831	221,4625002	-0,015829217	0,12299038	-0,12870289	0,89759275	0,95018119	protein_codir	hypothetical protein
TcG_04832	1129,685572	-0,024386636	0,06551954	-0,37220402	0,70974095	0,85035063	protein_codir	putative nuclear pore complex protein (NUP155)
TcG_04833	184,6908831	-0,180826714	0,13743602	-1,31571559	0,18826952	0,40265419	protein_codir	putative DNA-binding protein

TcG_04834	154,5579737	-0,320791845	0,15770219	-2,0341623	0,04193523	0,14599208	protein_codir	hypothetical protein
TcG_04835	124,4186848	-0,019338194	0,15872664	-0,12183333	0,90303102	0,95313085	protein_codir	thymocyte nuclear protein 1
TcG_04836	254,4890105	0,087782402	0,11787874	0,7446839	0,45646284	0,67639993	protein_codir	calphotin-like protein
TcG_04837	545,1108383	-0,003904434	0,08778219	-0,04447866	0,96452287	0,98423128	protein_codir	hypothetical protein
TcG_04838	329,8459828	-0,025431095	0,10307932	-0,24671385	0,80512969	0,90385	protein_codir	hypothetical protein
TcG_04839	313,2667712	0,125755098	0,10973217	1,14601853	0,25178749	0,48186194	protein_codir	putative high mobility group (HMG) box domain-containing protein
TcG_04840	130,5472989	0,267596863	0,15522112	1,72397203	0,08471289	0,23991288	protein_codir	hypothetical protein
TcG_04841	549,5665125	0,012757135	0,08622988	0,14794334	0,88238749	0,94259095	protein_codir	hypothetical protein
TcG_04842	289,2275125	0,047712531	0,10947798	0,43581852	0,66296841	0,82354941	protein_codir	putative ATPase
TcG_04843	357,6690395	0,139262733	0,10249214	1,35876495	0,17422108	0,382804	protein_codir	SET and MYND domain-containing protein
TcG_04844	23,2875337	-0,684178272	0,3693619	-1,85232495	0,06397917	0,19872995	protein_codir	putative nuclear lim interactor-interacting factor
TcG_04845	932,2291425	-0,016165183	0,06612455	-0,24446567	0,80687019	0,9046141	protein_codir	putative beta-adaptin
TcG_04846	855,8047479	-0,139783125	0,06632059	-2,10768831	0,03505796	0,12878298	protein_codir	ATP synthase OSCP delta subunit-like protein
TcG_04847	414,6769315	0,51056336	0,09382381	5,44172501	5,2767E-08	1,4419E-06	protein_codir	putative histidine ammonia-lyase
TcG_04848	1350,613624	-0,045364546	0,06417186	-0,70692269	0,47961452	0,69390782	protein_codir	putative prolyl oligopeptidase
TcG_04849	162,6219557	0,067844455	0,13834235	0,49040989	0,62384388	0,79706621	protein_codir	hypothetical protein
TcG_04850	640,6664057	-0,011444015	0,07724251	-0,14815695	0,88221891	0,94249776	protein_codir	cpc1/kpl2
TcG_04851	80,76456944	0,194384699	0,20633334	0,94209061	0,34614625	0,58046758	protein_codir	cpc1/kpl2
TcG_04852	214,8379451	0,354274089	0,13456661	2,63270425	0,00847081	0,04340362	protein_codir	mitochondrial carrier protein
TcG_04853	345,3639641	0,108939107	0,09868253	1,10393512	0,26962128	0,50246617	protein_codir	putative replication factor C, subunit 5
TcG_04854	66,69327627	0,148229723	0,21965683	0,67482409	0,49978754	0,70879875	protein_codir	hypothetical protein
TcG_04855	337,1313778	0,085266523	0,10504954	0,81167917	0,41697575	0,643715	protein_codir	hypothetical protein
TcG_04856	404,7996376	0,217612218	0,09277785	2,34551917	0,0190006	0,08066726	protein_codir	f2o10.10 protein-like protein
TcG_04857	220,499416	0,053863125	0,12093466	0,4453903	0,65603772	0,81817578	protein_codir	hypothetical protein
TcG_04858	358,3414758	0,138491357	0,09520272	1,45469957	0,14575245	0,34329902	protein_codir	membrane-associated progesterone binding protein 2
TcG_04859	346,7944383	0,136042878	0,10334478	1,31639811	0,18804046	0,40240797	protein_codir	putative methyltransferase
TcG_04860	1146,235894	-0,124728627	0,0614252	-2,0305774	0,04229788	0,1469455	protein_codir	phosphoglyceromutase
TcG_04861	326,4066178	-0,040551281	0,10854671	-0,37358371	0,70871406	0,84966485	protein_codir	hypothetical protein
TcG_04862	189,866144	0,02397027	0,13189214	0,18174146	0,85578562	0,93021224	protein_codir	hypothetical protein
TcG_04863	113,1965046	0,155991199	0,16783788	0,92941595	0,35267357	0,58699554	protein_codir	hypothetical protein
TcG_04864	304,154721	0,116651488	0,10288069	1,13385206	0,25685661	0,48884829	protein_codir	putative mitochondrial substrate/solute carrier
TcG_04865	512,0004777	-0,037522119	0,08556717	-0,43851069	0,66101612	0,82226034	protein_codir	hypothetical protein
TcG_04866	282,5435491	-0,043270061	0,11052896	-0,39148167	0,69544123	0,84234001	protein_codir	coiled-coil domain protein
TcG_04867	296,1543685	-0,068046137	0,10319031	-0,65942373	0,50962371	0,71647861	protein_codir	hypothetical protein
TcG_04868	252,4332562	-0,03921195	0,11898154	-0,32956332	0,74172994	0,86857521	protein_codir	hypothetical protein
TcG_04869	191,2845219	0,141894406	0,12958965	1,09495171	0,27353779	0,50688937	protein_codir	putative protein-l-isoaspartate o-methyltransferase
TcG_04870	154,0984287	-0,080069126	0,15112121	-0,5298338	0,59622717	0,78066886	protein_codir	telomere-binding protein 1
TcG_04871	336,7152352	-0,065713086	0,10170467	-0,64611671	0,51820377	0,72327538	protein_codir	hypothetical protein
TcG_04872	115,7883562	0,175194597	0,16704524	1,04878534	0,29427693	0,52725669	protein_codir	hypothetical protein
TcG_04873	501,0136846	0,084705996	0,08391372	1,00944159	0,3127629	0,54688665	protein_codir	pre-rRNA-processing protein ESF1
TcG_04874	324,5491971	0,079009721	0,10347325	0,76357631	0,44511977	0,66698883	protein_codir	putative cop9 signalosome complex subunit
TcG_04875	526,1837941	0,100974529	0,08385011	1,20422661	0,228502	0,45564187	protein_codir	hypothetical protein
TcG_04876	303,2880487	-0,217161905	0,10568928	-2,05472022	0,03990604	0,14108982	protein_codir	putative ubiquitin fusion degardation protein
TcG_04877	254,0145314	-0,010255556	0,11534735	-0,08891019	0,92915328	0,96557269	protein_codir	putative protein kinase, putative,mitogen-activated protein kinase
TcG_04878	98,86326621	-0,106468492	0,17475635	-0,60923962	0,54236562	0,74032141	protein_codir	hypothetical protein
TcG_04879	49,42422616	-0,196216197	0,24881769	-0,78859426	0,43034919	0,65519392		
TcG_04880	73,55326972	0,163961718	0,20533176	0,798521	0,42456821	0,65015164	protein_codir	hypothetical protein
TcG_04881	38,44961284	0,506748704	0,28522513	1,77666222	0,07562383	0,22226731	protein_codir	structural maintenance of chromosome protein 4
TcG_04882	68,3172929	0,039301412	0,22175627	0,17722796	0,85932933	0,93231479	protein_codir	hypothetical protein
TcG_04883	238,9531188	0,537668491	0,11877007	4,52696953	5,9836E-06	9,8056E-05	protein_codir	hypothetical protein
TcG_04884	241,8807673	0,065064796	0,12114265	0,53709239	0,5912038	0,77687277	protein_codir	zinc finger domain-like protein
TcG_04885	201,4458806	0,177626311	0,12929761	1,3737788	0,16951037	0,37606704	protein_codir	Myb domain protein 40

TcG_04886	160,0191723	0,075124806	0,14437702	0,52033768	0,60282823	0,78518697	protein_codir	hypothetical protein
TcG_04887	382,9636912	-0,02739099	0,09303761	-0,29440773	0,76844636	0,88413302	protein_codir	putative 2-oxoisovalerate dehydrogenase alpha subunit
TcG_04888	294,8594548	-0,330196581	0,1050722	-3,14256856	0,00167472	0,01192585	protein_codir	cytochrome b5 domain containing 1
TcG_04889	535,1958956	-0,04320119	0,08130483	-0,53134842	0,59517736	0,78032419	protein_codir	putative arginine N-methyltransferase, type II
TcG_04890	472,1266127	-0,088475368	0,08579637	-1,03122511	0,30243527	0,5353728	protein_codir	hypothetical protein
TcG_04891	3764,93579	-0,128883499	0,04398779	-2,92998376	0,0033898	0,02101348	protein_codir	hypothetical protein
TcG_04892	218,6597978	-0,296172853	0,12282326	-2,41137428	0,01589253	0,07071077	protein_codir	hypothetical protein
TcG_04893	470,5400751	0,02123665	0,08531101	0,24893211	0,8034133	0,90293398	protein_codir	hypothetical protein
TcG_04894	451,7589118	-0,035052957	0,08692359	-0,40326171	0,68675571	0,83740347	protein_codir	hypothetical protein
TcG_04895	119,3685333	0,367377046	0,16219771	2,26499527	0,02351297	0,09551938	protein_codir	hypothetical protein
TcG_04896	752,1989241	0,058103882	0,07162442	0,81123003	0,41723358	0,64394142	protein_codir	hypothetical protein
TcG_04897	1505,003855	0,119509365	0,05313254	2,24926897	0,02449539	0,09826993	protein_codir	40S ribosomal protein S11
TcG_04898	337,509886	-0,22303487	0,1022451	-2,18137458	0,02915572	0,11200205	protein_codir	hypothetical protein
TcG_04899	1142,773433	-0,208951908	0,06638689	-3,14748763	0,0016468	0,0117559	protein_codir	putative RNA helicase
TcG_04900	813,6233171	-0,229270048	0,06902062	-3,32176166	0,00089451	0,00711799	protein_codir	hypothetical protein
TcG_04901	215,3430568	0,205266731	0,12537267	1,63725262	0,1015777	0,27185936	protein_codir	hypothetical protein
TcG_04902	28,71902655	-0,405737783	0,33692229	-1,20424738	0,22849398	0,45564187		
TcG_04903	162,0951304	-0,54893979	0,1472587	-3,72772405	0,00019322	0,00193986	protein_codir	hypothetical protein
TcG_04904	757,7275609	0,144798466	0,07149297	2,02535248	0,04283118	0,14829823	protein_codir	putative choline dehydrogenase
TcG_04905	779,9350213	0,027447177	0,0699611	0,39232054	0,69482139	0,84207119	protein_codir	putative serine/threonine-protein kinase
TcG_04906	432,5510079	-0,082051383	0,0898251	-0,9134572	0,36100214	0,59360925	protein_codir	hypothetical protein
TcG_04907	114,1347664	0,153813618	0,16785973	0,9163223	0,35949788	0,5926302	protein_codir	hypothetical protein
TcG_04908	249,5002727	0,324017066	0,11359084	2,85249294	0,00433778	0,02573882	protein_codir	peroxidase
TcG_04909	424,105893	0,004807715	0,08892446	0,05406516	0,95688325	0,98006094	protein_codir	hypothetical protein
TcG_04910	173,4359978	0,297597263	0,13770804	2,16107405	0,03068962	0,11619932	protein_codir	hypothetical protein
TcG_04911	221,1629521	-0,177018757	0,12361692	-1,43199453	0,15214539	0,35233988	protein_codir	hypothetical protein
TcG_04912	133,4132784	0,24096274	0,15164669	1,58897465	0,11206612	0,28988569	protein_codir	hypothetical protein
TcG_04913	174,8123299	0,002963082	0,14062325	0,02107107	0,98318896	0,99373875	protein_codir	hypothetical protein
TcG_04914	377,4105472	0,065109487	0,09348733	0,69645254	0,48614547	0,69827461	protein_codir	mitochondrial RNA binding complex 1 subunit
TcG_04915	163,1485985	0,036942314	0,13848041	0,26676923	0,78964684	0,89501549	protein_codir	calcineurin B subunit
TcG_04916	221,9009972	-0,002649027	0,11779973	-0,02248754	0,98205905	0,99328993	protein_codir	hypothetical protein
TcG_04917	251,1106827	-0,379223831	0,11217444	-3,38066179	0,00072311	0,00592085	protein_codir	putative protein kinase
TcG_04918	283,1806981	-0,153574117	0,11185856	-1,37293128	0,16977371	0,37606704	protein_codir	putative mitochondrial structure specific endonuclease I (SSE-1)
TcG_04919	221,0562847	0,044419738	0,11862033	0,37446984	0,70805479	0,84943749	protein_codir	hypothetical protein
TcG_04920	84,00758586	0,077456973	0,20264989	0,38222066	0,7022977	0,84624626	protein_codir	hypothetical protein
TcG_04921	440,2178019	0,204528436	0,0889239	2,30003907	0,02144601	0,08880394	protein_codir	hypothetical protein
TcG_04922	39,94387162	0,585581482	0,2806311	2,08665927	0,03691895	0,13350278	protein_codir	hypothetical protein
TcG_04923	545,9745816	-0,014004685	0,08511565	-0,16453713	0,86930832	0,9369122	protein_codir	pumilio protein 3
TcG_04924	108,6412594	0,161029561	0,16872365	0,9543983	0,33988207	0,57399583	protein_codir	hypothetical protein
TcG_04925	116,0960826	0,088282271	0,16339526	0,54029885	0,58899095	0,77564856	protein_codir	hypothetical protein
TcG_04926	420,0139272	-0,327780089	0,0969772	-3,37997066	0,00072494	0,00593157	protein_codir	proteasome alpha 2 subunit
TcG_04927	986,2533363	-0,112607213	0,06366033	-1,76887575	0,07691461	0,22464946	protein_codir	putative cytochrome c oxidase subunit VI
TcG_04928	1320,518986	0,189502677	0,05682027	3,33512478	0,00085261	0,00684571	protein_codir	60S ribosomal protein L32
TcG_04929	374,3758536	0,469103313	0,09580477	4,89645044	9,7583E-07	1,9905E-05	protein_codir	hypothetical protein
TcG_04930	346,0596217	-0,385384218	0,09898879	-3,89321065	9,8926E-05	0,00110526	protein_codir	proteasome regulatory non-ATPase subunit 11
TcG_04931	417,5961531	-0,235905211	0,09241449	-2,55268627	0,01068957	0,05205944	protein_codir	hypothetical protein
TcG_04932	484,5705509	-0,210863488	0,08700932	-2,42345877	0,0153735	0,06895755	protein_codir	hypothetical protein
TcG_04933	160,3221011	-0,013155233	0,13841393	-0,0950427	0,92428091	0,96301427	protein_codir	hypothetical protein
TcG_04934	624,9519257	-0,257141286	0,07815641	-3,29008565	0,00100157	0,00778804	protein_codir	hypothetical protein
TcG_04935	437,1389139	-0,150546736	0,08804704	-1,70984428	0,08729467	0,2450681	protein_codir	hypothetical protein
TcG_04936	457,5994504	-0,217457916	0,0860857	-2,52606312	0,01153488	0,05527009	protein_codir	hypothetical protein
TcG_04937	389,1595875	-0,17139201	0,09188557	-1,86527662	0,06214264	0,19469568	protein_codir	putative GTP-binding protein

TcG_04938	841,9848329	-0,33079985	0,06664502	-4,96360927	6,9195E-07	1,4576E-05	protein_codir	hypothetical protein
TcG_04939	119,6452337	-0,311872555	0,17183003	-1,81500614	0,06952297	0,21100455	protein_codir	putative Gamma-soluble NSF attachment protein (SNAP-gamma) (N-ethylmaleimide-sensitive factor attachment protein, gamma)
TcG_04940	286,2403075	-0,2476596	0,1105391	-2,24047051	0,02506039	0,0998795	protein_codir	putative Gamma-soluble NSF attachment protein (SNAP-gamma) (N-ethylmaleimide-sensitive factor attachment protein, gamma)
TcG_04941	691,1748801	-0,480344123	0,07212667	-6,6597292	2,7433E-11	1,558E-09	protein_codir	hypothetical protein
TcG_04942	591,7434572	-0,288385753	0,08143969	-3,54109594	0,00039847	0,00357603	protein_codir	hypothetical protein
TcG_04943	735,2746731	0,013327854	0,07854078	0,16969342	0,86525125	0,93506211	protein_codir	splicing factor ptsr1 interacting protein
TcG_04944	1002,582303	-0,329439582	0,06356274	-5,18290373	2,1846E-07	5,2403E-06	protein_codir	putative importin beta-1 subunit
TcG_04945	3531,229372	0,196083012	0,04213532	4,6536495	3,2611E-06	5,7861E-05	protein_codir	enolase
TcG_04946	2167,056737	-0,389332093	0,12795263	-3,04278303	0,00234401	0,01565287	protein_codir	putative calcium channel protein
TcG_04947	669,0701228	-0,416914881	0,07814345	-5,33525039	9,5413E-08	2,4898E-06	protein_codir	hypothetical protein
TcG_04948	593,399864	-0,2126446	0,07592367	-2,80076834	0,00509811	0,02912559	protein_codir	WD domain-containing protein
TcG_04949	193,4660825	-0,066462085	0,12729595	-0,52210683	0,60159595	0,78447842	protein_codir	hypothetical protein
TcG_04950	994,401376	-0,019820356	0,06561708	-0,30206093	0,76260562	0,88013366	protein_codir	ribosomal protein S25
TcG_04951	83,07002379	-0,195242583	0,2041273	-0,95647461	0,33883251	0,57327673	protein_codir	hypothetical protein
TcG_04952	222,1850953	0,054974569	0,12040211	0,4565914	0,64796476	0,81260383	protein_codir	hypothetical protein
TcG_04953	477,6732092	-0,378699724	0,08684286	-4,36074677	1,2962E-05	0,0001901	protein_codir	hypothetical protein
TcG_04954	159,7117523	0,213386293	0,13942426	1,53048178	0,12589752	0,31328365	protein_codir	hypothetical protein
TcG_04955	337,932055	-0,175160182	0,1004185	-1,74430199	0,08110645	0,23325476	protein_codir	putative conserved oligomeric Golgi complex subunit 6-like isoform X1
TcG_04956	906,8150638	-0,142468381	0,0648397	-2,19723998	0,02800331	0,10861947	protein_codir	DNA-directed RNA polymerase III subunit RPC1
TcG_04957	770,8604881	-0,331899774	0,07152874	-4,64009005	3,4826E-06	6,1321E-05	protein_codir	putative eukaryotic translation initiation factor 5
TcG_04958	433,8045986	-0,130207484	0,09023239	-1,44302378	0,14901377	0,3482337	protein_codir	putative bcs1 aaa-type ATPase
TcG_04959	270,1128224	0,142633496	0,11318935	1,26013178	0,20762183	0,42979431	protein_codir	putative deoxyhypusine synthase
TcG_04960	368,0113907	0,088106276	0,09816334	0,89754766	0,36942675	0,60112883	protein_codir	diguanylate cyclase
TcG_04961	83,26817513	-0,393252619	0,19321761	-2,03528353	0,04182235	0,14581239	protein_codir	hypothetical protein
TcG_04962	18,84002647	0,029664691	0,40563319	0,07313181	0,94170123	0,97296008	protein_codir	hypothetical protein
TcG_04963	244,0635461	-0,12941419	0,11549182	-1,12054855	0,26248007	0,49464771	protein_codir	hypothetical protein
TcG_04964	433,5420935	-0,113080348	0,09156584	-1,23496221	0,21684457	0,44089387	protein_codir	hypothetical protein
TcG_04965	305,8179025	0,020187534	0,10537217	0,19158316	0,84806873	0,92660555	protein_codir	acyltransferase
TcG_04966	552,5727646	-0,096334591	0,08239536	-1,16917502	0,24233313	0,47089863	protein_codir	putative ribonuclease II-like protein
TcG_04967	234,7373298	-0,013627886	0,11623994	-0,11723927	0,90667045	0,95471088	protein_codir	hypothetical protein
TcG_04968	360,9826677	0,153933668	0,09595164	1,60428383	0,1086515	0,28454709	protein_codir	putative Trichohyalin
TcG_04969	330,8856325	0,162613884	0,10203884	1,59364695	0,11101513	0,28803333	protein_codir	geranylgeranyltransferase type I beta subunit
TcG_04970	707,3257187	-0,045685595	0,07195042	-0,63495943	0,52545488	0,72866385	protein_codir	NET1-associated nuclear protein 1 (U3 small nucleolar RNA-associated protein 17)
TcG_04971	217,4148269	-0,066162016	0,12183152	-0,54306155	0,58708743	0,77559805	protein_codir	carbonic anhydrase-like protein
TcG_04972	532,3236404	-0,081758971	0,08118251	-1,0071008	0,31388634	0,54810657	protein_codir	carbonic anhydrase-like protein
TcG_04973	149,0923176	0,076337706	0,15220553	0,50154358	0,61598862	0,79256256	protein_codir	hypothetical protein
TcG_04974	309,3003583	-0,083818806	0,10354528	-0,80948939	0,4182337	0,64488364	protein_codir	hypothetical protein
TcG_04975	264,9701829	0,069941478	0,11010914	0,63520141	0,52529707	0,72866385	protein_codir	zinc finger, MYND-type containing 10
TcG_04976	568,8977582	-0,071186107	0,07999959	-0,88983086	0,37355671	0,60449626	protein_codir	putative protein kinase
TcG_04977	1083,997798	0,029194778	0,06043645	0,48306573	0,62904907	0,8011611	protein_codir	RAD50 DNA repair-like protein
TcG_04978	147,9468724	0,275666027	0,15389585	1,7912505	0,07325311	0,21800937	protein_codir	putative DNA repair protein RAD50
TcG_04979	1322,855775	0,395318323	0,05675447	6,96541298	3,2744E-12	2,2854E-10	protein_codir	ribosomal protein S26
TcG_04980	1184,433505	0,045267789	0,06118034	0,73990747	0,45935614	0,67791061	protein_codir	Rad51
TcG_04981	235,8734175	0,171556156	0,11966842	1,43359594	0,1516876	0,35187745	protein_codir	hypothetical protein
TcG_04982	235,2107456	-0,044527005	0,12074103	-0,36878105	0,71229093	0,85227746	protein_codir	hypothetical protein
TcG_04983	317,6697571	0,184417606	0,11216617	1,64414644	0,10014596	0,26899994	protein_codir	hypothetical protein
TcG_04984	198,9361081	0,089622283	0,12556408	0,71375732	0,47537726	0,69103267	protein_codir	hypothetical protein
TcG_04985	2885,650074	-0,069503248	0,11253939	-0,61759043	0,53684535	0,73660472	protein_codir	dynein, axonemal, heavy polypeptide 1
TcG_04986	843,7697267	-0,13821572	0,06828812	-2,02400827	0,0429693	0,14869842	protein_codir	putative protein kinase
TcG_04987	419,7489226	-0,180577858	0,08866761	-2,03657066	0,04169309	0,14545501	protein_codir	putative ER lumen retaining receptor protein
TcG_04988	97,47279544	-0,051847071	0,19082672	-0,27169713	0,78585491	0,89351472	protein_codir	H(+)-transporting two-sector ATPase
TcG_04989	181,539612	-0,072444053	0,13801179	-0,52491204	0,59964434	0,78310281	protein_codir	putative protein transport protein Sec13

TcG_04990	242,1411275	0,108434199	0,11940319	0,90813484	0,36380698	0,59568509	protein_codir	hypothetical protein
TcG_04991	355,7245966	0,145395106	0,10331691	1,40727304	0,15934644	0,36189689	protein_codir	e3 ubiquitin-protein ligase MIB2
TcG_04992	433,6476808	-0,369316783	0,09034944	-4,08764874	4,3577E-05	0,00054878	protein_codir	serine/threonine protein phosphatase catalytic subunit
TcG_04993	198,0527863	-0,036496646	0,12920819	-0,28246387	0,77758785	0,88900067	protein_codir	hypothetical protein
TcG_04994	260,6648954	-0,128944728	0,11814916	-1,09137232	0,27510909	0,50763484	protein_codir	hypothetical protein
TcG_04995	778,4411075	-0,192162176	0,06894087	-2,78734778	0,00531414	0,03003397	protein_codir	hypothetical protein
TcG_04996	573,3725118	-0,153640949	0,08087567	-1,89971774	0,05747017	0,18267473	protein_codir	U3 small nucleolar RNA-associated protein 3
TcG_04997	86,42377684	-0,079318736	0,19345032	-0,41002122	0,68179038	0,83421938	protein_codir	hypothetical protein
TcG_04998	284,7552067	-0,376743656	0,10555237	-3,56925829	0,00035799	0,00325311	protein_codir	hypothetical protein
TcG_04999	275,7459827	-0,175507851	0,11340067	-1,54767919	0,12169955	0,30612485	protein_codir	hypothetical protein
TcG_05000	395,648867	-0,163499552	0,09361547	-1,74650135	0,08072387	0,23253774	protein_codir	putative DNA repair protein
TcG_05001	263,3990943	0,232464718	0,11432158	2,03342817	0,04200928	0,14620594	protein_codir	hypothetical protein
TcG_05002	215,1388963	0,056651329	0,12273768	0,46156429	0,64439381	0,8110828	protein_codir	hypothetical protein
TcG_05003	477,9484528	0,139582573	0,08742855	1,59653305	0,11036983	0,28716481	protein_codir	ion transport protein
TcG_05004	241,692005	-0,017563993	0,11615919	-0,15120622	0,87981304	0,94162382	protein_codir	putative ion transport protein
TcG_05005	194,0576584	0,019245229	0,13531042	0,14223021	0,88689817	0,94531759	protein_codir	hypothetical protein
TcG_05006	1214,39008	-0,133383645	0,0571755	-2,33288101	0,01965439	0,082866	protein_codir	U3 small nucleolar RNA-associated protein 12
TcG_05007	56,69571997	0,354332367	0,23560034	1,50395526	0,13259289	0,32361438	protein_codir	hypothetical protein
TcG_05008	645,0781796	0,057966151	0,07417286	0,78150084	0,43450799	0,65873534	protein_codir	hypothetical protein
TcG_05009	255,8017973	0,018724726	0,11119081	0,16840174	0,86626724	0,9355306	protein_codir	23S rRNA methyltransferase
TcG_05010	203,5864138	-0,104990518	0,12599832	-0,8332692	0,40469292	0,63413202	protein_codir	hypothetical protein
TcG_05011	345,6491598	0,014015209	0,09954684	0,1407901	0,88803576	0,94594634	protein_codir	hypothetical protein
TcG_05012	697,9602595	0,048187401	0,07678999	0,6275219	0,53031719	0,73198176	protein_codir	putative run domain Beclin-1 interacting and cystein-rich containing protein-like
TcG_05013	257,3812353	0,019370678	0,1117342	0,17336391	0,86236539	0,93414012	protein_codir	hypothetical protein
TcG_05014	895,8708257	-0,364970419	0,07202321	-5,06739996	4,0329E-07	8,9733E-06	protein_codir	fatty acid elongase
TcG_05015	666,0453961	-0,19759293	0,07218251	-2,73740738	0,00619256	0,0338429	protein_codir	putative fatty acid elongase
TcG_05016	284,4367542	-0,378199484	0,10925162	-3,46172884	0,00053672	0,00460964	protein_codir	putative fatty acid elongase
TcG_05017	250,0234221	-0,01684915	0,1155522	-0,14581419	0,88406808	0,94348884	protein_codir	fatty acid elongase
TcG_05018	738,5552308	0,143322016	0,07136622	2,00826129	0,04461553	0,15255431	protein_codir	hypothetical protein
TcG_05019	148,4041918	0,459012618	0,1494057	3,07225645	0,00212447	0,01446822	protein_codir	putative guanin nucleotide-binding protein
TcG_05020	183,3196098	0,368964685	0,13313124	2,77143578	0,00558097	0,03120709	protein_codir	large subunit ribosomal protein L21
TcG_05021	933,2040745	0,29394512	0,0665996	4,41361703	1,0166E-05	0,00015437	protein_codir	hypothetical protein
TcG_05022	459,5639618	0,156599171	0,0895886	1,74798096	0,08046732	0,23202947	protein_codir	translation initiation factor IF-2
TcG_05023	367,7137555	-0,124575147	0,0944828	-1,31849546	0,18733784	0,40157193	protein_codir	hypothetical protein
TcG_05024	519,2483299	-0,103251324	0,08719026	-1,18420708	0,2363311	0,46361872	protein_codir	putative serine/threonine protein kinase
TcG_05025	590,0343808	0,157782647	0,07773571	2,02973178	0,04238381	0,14715578	protein_codir	putative DNA excision repair protein
TcG_05026	232,6129325	0,001919568	0,12726894	0,01508277	0,98796615	0,99514516	protein_codir	calpain-like cysteine peptidase
TcG_05027	637,2644211	0,248718121	0,07679596	3,23868764	0,00120081	0,00905178	protein_codir	calpain-like cysteine peptidase
TcG_05028	570,073496	0,282437065	0,08036403	3,51447119	0,00044063	0,00388815	protein_codir	calpain-like cysteine peptidase
TcG_05029	610,1604015	0,286863888	0,07863155	3,64820337	0,00026408	0,00251408	protein_codir	hypothetical protein
TcG_05030	498,0922882	-0,034789045	0,08209655	-0,42375772	0,67174251	0,82795838	protein_codir	hypothetical protein
TcG_05031	470,7411657	-0,035335535	0,0871302	-0,40554863	0,68507428	0,83655708	protein_codir	hypothetical protein
TcG_05032	371,4635772	-0,189567967	0,10110457	-1,87496927	0,06079695	0,19148679	protein_codir	putative protein phosphatase 2C
TcG_05033	367,6720317	0,076655721	0,09554184	0,80232616	0,42236432	0,64857693	protein_codir	glutathione synthetase
TcG_05034	1484,441476	-0,133946388	0,05564942	-2,4069682	0,01608557	0,07135047	protein_codir	putative mitochondrial DNA polymerase I protein C
TcG_05035	1155,78391	-0,254561858	0,05967262	-4,2659742	1,9903E-05	0,00027584	protein_codir	putative immunodominant antigen, putative,tc40 antigen-like
TcG_05036	19,76478898	-0,033502119	0,41300462	-0,08111803	0,93534809	0,96957529	protein_codir	hypothetical protein
TcG_05037	497,482927	-0,041404882	0,08301753	-0,49874867	0,61795645	0,793034	protein_codir	hypothetical protein
TcG_05038	110,1130327	-0,234575741	0,17249401	-1,35990656	0,17385949	0,38244467		
TcG_05039	594,8466962	0,229398341	0,0780468	2,93924066	0,00329017	0,02054207	protein_codir	ATP-binding cassette protein subfamily D, member 1
TcG_05040	470,2329322	-0,331410434	0,08366398	-3,96120788	7,4572E-05	0,00087095	protein_codir	vasohibin-1
TcG_05041	408,3190299	-0,01734969	0,09100216	-0,19065141	0,84879871	0,9269268	protein_codir	putative ribosome biogenesis protein

TcG_05042	295,841151	-0,175781071	0,10410088	-1,68856469	0,09130288	0,25234897	protein_codir	putative amino acid transporter
TcG_05043	460,3506012	-0,415955542	0,09385834	-4,43173784	9,3477E-06	0,00014421	protein_codir	putative mucin-associated surface protein (MASP)
TcG_05044	337,5341662	0,130214722	0,09902125	1,31501798	0,18850386	0,40280445	protein_codir	putative adenosine kinase
TcG_05045	257,746936	0,130349986	0,11634074	1,12041564	0,26253668	0,49467393	protein_codir	putative chaperone DNAJ protein
TcG_05046	113,5707225	0,205142578	0,18101835	1,1332695	0,2571011	0,48902898	protein_codir	hypothetical protein
TcG_05047	249,8010551	-0,10274786	0,1149953	-0,89349616	0,37159153	0,60280866	protein_codir	putative proteasome regulatory non-ATPase subunit
TcG_05048	1084,473588	-0,184247803	0,05951952	-3,09558611	0,00196424	0,01354682	protein_codir	putative ATP-dependent RNA helicase
TcG_05049	128,2088826	-0,029158148	0,15886572	-0,18353958	0,85437467	0,9293761	protein_codir	ARF GAP-like zinc finger-containing protein
TcG_05050	120,9474215	0,439578358	0,16278952	2,70028665	0,00692798	0,0370067	protein_codir	hypothetical protein
TcG_05051	297,8070203	0,054884854	0,10734882	0,5112758	0,60915795	0,788218	protein_codir	putative Transitional endoplasmic reticulum ATPase
TcG_05052	362,4982223	-0,229326452	0,09533023	-2,40560061	0,01614591	0,07152664	protein_codir	putative RNA-binding protein
TcG_05053	216,219661	0,097550635	0,12096204	0,80645657	0,41997964	0,64656135	protein_codir	RNA editing complex protein MP44
TcG_05054	349,4645592	-0,055448984	0,10095375	-0,54925138	0,58283295	0,77164925	protein_codir	hypothetical protein
TcG_05055	256,9050032	-0,096844065	0,11980118	-0,80837318	0,41887579	0,64539237	protein_codir	hypothetical protein
TcG_05056	154,1953374	-0,00830156	0,14662546	-0,05661745	0,95484993	0,97888932	protein_codir	putative NUDIX hydrolase
TcG_05057	604,1111843	0,060284255	0,07682678	0,78467759	0,43264263	0,65704515	protein_codir	hypothetical protein
TcG_05058	889,8340482	-0,241777714	0,06460917	-3,74215776	0,00018245	0,00185213	protein_codir	isocitrate dehydrogenase
TcG_05059	5068,624273	-0,064453006	0,03850625	-1,67383233	0,09416356	0,25797564	protein_codir	hypothetical protein
TcG_05060	3555,900981	0,048022344	0,04275584	1,12317634	0,26136261	0,49358779	protein_codir	cyclophilin A
TcG_05061	249,8191929	0,051505347	0,11202508	0,45976619	0,64568405	0,81199342	protein_codir	hypothetical protein
TcG_05062	120,2012311	-0,251858009	0,16270381	-1,54795399	0,12163337	0,30602481	protein_codir	putative protein kinase
TcG_05063	526,6529188	-0,058650484	0,08029225	-0,73046254	0,4651075	0,6821385	protein_codir	putative MYH7B protein
TcG_05064	107,4797909	-0,134557866	0,17131465	-0,78544285	0,43219397	0,65671849	protein_codir	putative protein kinase
TcG_05065	239,2300849	-0,129484831	0,11906264	-1,08753536	0,27680028	0,50904889	protein_codir	mitochondrial sodium/hydrogen exchanger NHA2
TcG_05066	497,8183581	-0,014480347	0,08280437	-0,17487419	0,86117849	0,93370896	protein_codir	putative glucosamine-6-phosphate isomerase, putative,glucosamine-6-phosphate deaminase
TcG_05067	376,2169919	-0,169852401	0,09408112	-1,8053824	0,07101486	0,2138197	protein_codir	putative C-terminal motor kinesin
TcG_05068	143,9829321	-0,430501258	0,14653442	-2,93788497	0,0033046	0,02060659	protein_codir	hypothetical protein
TcG_05069	295,2507267	-0,396282515	0,10976758	-3,61019642	0,00030597	0,00284732	protein_codir	putative pumilio-repeat, RNA-binding protein
TcG_05070	363,8907443	-0,144698631	0,10287276	-1,4065787	0,15955235	0,3621104	protein_codir	putative prefoldin
TcG_05071	746,2579455	-0,386715322	0,07030378	-5,50063354	3,7843E-08	1,0668E-06	protein_codir	putative serine/threonine protein kinase
TcG_05072	25,04347077	0,276833142	0,34541045	0,8014614	0,42286458	0,64891511	protein_codir	putative exportin 1, putative,RNA-nuclear export factor
TcG_05073	62,29184924	-0,445213774	0,22245263	-2,00138687	0,04535071	0,1543782	protein_codir	hypothetical protein
TcG_05074	586,3961183	-0,3628736	0,0789257	-4,59766066	4,2726E-06	7,3012E-05	protein_codir	poly-zinc finger protein 2
TcG_05075	97,0504518	-0,057452481	0,18392709	-0,31236551	0,75476276	0,87630838	protein_codir	hypothetical protein
TcG_05076	2054,759843	0,336110336	0,04988823	6,73726776	1,6139E-11	9,6886E-10	protein_codir	universal minicircle sequence binding protein (UMSBP)
TcG_05077	668,8242399	-0,150305191	0,07336293	-2,04878937	0,04048272	0,14239003	protein_codir	putative proteasome beta 5 subunit
TcG_05078	513,409445	-0,046644418	0,08625874	-0,54075006	0,58867987	0,77564856	protein_codir	putative tRNA pseudouridine synthase A
TcG_05079	320,3629554	-0,076169261	0,10092112	-0,75474055	0,45040467	0,67108906	protein_codir	hypothetical protein
TcG_05080	146,0946251	-0,236670907	0,14796705	-1,59948386	0,10971313	0,28632613	protein_codir	mitochondrial inner membrane protease ATP23
TcG_05081	559,3017316	-0,148181684	0,07952633	-1,86330342	0,06241959	0,19518638	protein_codir	hypothetical protein
TcG_05082	322,7652499	-0,060107154	0,10078711	-0,59637741	0,55092313	0,74654917	protein_codir	putative phosphatidylinositol N-acetylglucosaminyltransferase subunit C
TcG_05083	1548,703673	-0,030499499	0,0644494	-0,47323173	0,63604786	0,80601316	protein_codir	putative neurobeachin/beige protein
TcG_05084	459,13957	-0,073573195	0,08635038	-0,85203093	0,39419693	0,62410024	protein_codir	hypothetical protein
TcG_05085	670,282638	0,04631135	0,07381285	0,62741586	0,53038668	0,73199047	protein_codir	hypothetical protein
TcG_05086	433,3675874	0,284820757	0,09044121	3,14923631	0,00163698	0,01169851	protein_codir	putative mucin-associated surface protein (MASP)
TcG_05087	424,1313765	-0,061127246	0,0882227	-0,69287432	0,48838843	0,69978584	protein_codir	putative aldehyde dehydrogenase
TcG_05088	239,3751758	-0,032481282	0,11947558	-0,27186544	0,78572549	0,89351472	protein_codir	tetratricopeptide repeat protein 16
TcG_05089	547,2708357	0,040535882	0,07907599	0,51261935	0,60821761	0,78802211	protein_codir	putative exoribonuclease 2
TcG_05090	175,5942986	0,063614622	0,13929783	0,45668063	0,64790062	0,81260383	protein_codir	trichoplein, keratin filament binding protein
TcG_05091	274,3430346	-0,073321937	0,11333028	-0,6469757	0,51764767	0,72300087	protein_codir	hypothetical protein
TcG_05092	280,6069804	-0,083214598	0,10780822	-0,77187622	0,44018774	0,66320093	protein_codir	putative small GTP-binding protein RAB6
TcG_05093	440,9885189	0,107686891	0,09212347	1,16894091	0,24242744	0,47089863	protein_codir	hypothetical protein

TcG_05094	548,8655193	-0,054524484	0,08035912	-0,67851021	0,49744825	0,70725677	protein_codir	putative DEAD box RNA helicase
TcG_05095	25,5520738	0,221293348	0,34485812	0,64169389	0,52107196	0,72540161	protein_codir	hypothetical protein
TcG_05096	107,3286535	0,252652523	0,17992134	1,40423877	0,16024776	0,36306159	protein_codir	DREV methyltransferase
TcG_05097	511,6767417	0,075816843	0,08130985	0,9324435	0,35110737	0,58556643	protein_codir	putative DEAD box RNA helicase
TcG_05098	311,5226311	0,064154637	0,10191579	0,62948675	0,52903044	0,73107665	protein_codir	putative dynein heavy chain
TcG_05099	691,4668379	-0,230938161	0,07320864	-3,15452045	0,00160762	0,01153037	protein_codir	major vault protein
TcG_05100	398,7680486	0,161612421	0,09375919	1,72369691	0,08476257	0,23993628	protein_codir	putative condensin subunit 1
TcG_05101	164,4597077	0,417185523	0,13921836	2,99662719	0,00272984	0,01763435	protein_codir	endochitinase
TcG_05102	890,1145533	0,049226568	0,06816392	0,72217921	0,4701843	0,68645032	protein_codir	hypothetical protein
TcG_05103	154,3418836	0,318688906	0,14195278	2,24503467	0,02476591	0,09904653	protein_codir	hypothetical protein
TcG_05104	279,0662377	-0,1559264	0,1106682	-1,40895398	0,15884878	0,36122118	protein_codir	T-lymphocyte triggering factor
TcG_05105	275,7274512	0,156524563	0,10848832	1,442778	0,14908302	0,3482411	protein_codir	hypothetical protein
TcG_05106	339,1635195	-0,137972132	0,09943335	-1,38758409	0,16526373	0,3707155	protein_codir	hypothetical protein
TcG_05107	1118,948956	0,467390737	0,06511183	7,17827725	7,0595E-13	5,4528E-11	protein_codir	large subunit ribosomal protein L37Ae
TcG_05108	249,3245004	-0,238041689	0,1180442	-2,01654711	0,0437428	0,15052095	protein_codir	E3 ubiquitin-protein ligase RNF5
TcG_05109	261,3051123	-0,254384021	0,1112339	-2,28692884	0,02219998	0,0911442	protein_codir	putative dihydroxyacetone phosphate acyltransferase
TcG_05110	172,0535409	0,220940333	0,13663703	1,61698723	0,10588104	0,2800771	protein_codir	hypothetical protein
TcG_05111	218,8661208	-0,023776023	0,12404449	-0,19167334	0,84799809	0,92660555	protein_codir	fatty acid elongase
TcG_05112	467,2084945	-0,140918056	0,08722535	-1,61556318	0,10618879	0,28057088	protein_codir	BRCT domain-containing protein
TcG_05113	46,28505902	0,061615877	0,25319156	0,24335675	0,80772904	0,90504442	protein_codir	hypothetical protein
TcG_05114	571,8281864	-0,14395715	0,08999612	-1,59959284	0,10968894	0,28632613	protein_codir	small GTP-binding protein
TcG_05115	98,30364602	0,248496717	0,18407569	1,34997029	0,17702551	0,38718302	protein_codir	hypothetical protein
TcG_05116	825,7645112	-0,457373211	0,06919008	-6,61038675	3,8332E-11	2,1219E-09	protein_codir	paraflagellar rod component Par4
TcG_05117	550,2751872	-0,071774284	0,08432971	-0,85111501	0,39470547	0,62431497	protein_codir	hypothetical protein
TcG_05118	1230,075596	-0,107026483	0,05868766	-1,82366242	0,06820312	0,20822171	protein_codir	major vault protein
TcG_05119	121,4910575	-0,091613605	0,16964748	-0,54002338	0,58918091	0,77564856	protein_codir	hypothetical protein
TcG_05120	401,331898	-0,125912038	0,09168397	-1,37332666	0,16965082	0,37606704	protein_codir	putative protein kinase
TcG_05121	1427,451914	0,007595043	0,05675773	0,13381513	0,89354876	0,94865353	protein_codir	putative nucleolar RNA helicase II
TcG_05122	246,5212221	0,189041963	0,11457583	1,64992884	0,09895749	0,26663291	protein_codir	hypothetical protein
TcG_05123	243,5132195	-0,058892092	0,11808418	-0,49872972	0,61796981	0,793034	protein_codir	hypothetical protein
TcG_05124	125,9934171	-0,334936495	0,1547138	-2,164878	0,03039703	0,11546885	protein_codir	hypothetical protein
TcG_05125	546,4022267	-0,147098969	0,07848022	-1,8743444	0,06088297	0,19152596	protein_codir	putative phosphoprotein phosphatase
TcG_05126	136,1174841	0,281506258	0,15209144	1,8509014	0,06418374	0,19925852	protein_codir	hypothetical protein
TcG_05127	154,9426805	0,301780603	0,14766527	2,04368036	0,04098513	0,14372087	protein_codir	hypothetical protein
TcG_05128	329,8355963	0,098832372	0,10544804	0,93726131	0,34862416	0,58310373	protein_codir	putative NUDIX hydrolase, conserved
TcG_05129	311,6599803	0,075365085	0,10147584	0,74268993	0,45766943	0,67712399	protein_codir	hypothetical protein
TcG_05130	286,5660709	0,193600233	0,10710825	1,80751936	0,07068134	0,2133144	protein_codir	hypothetical protein
TcG_05131	373,6108504	0,325837263	0,09524323	3,42110676	0,00062367	0,00519443	protein_codir	putative dihydrolipoamide branched chain transacylase
TcG_05132	254,6806609	0,249532911	0,11963709	2,08574876	0,03700139	0,13367573	protein_codir	FIP1-like protein
TcG_05133	537,4015239	0,314934933	0,08950761	3,51852671	0,00043395	0,00383797	protein_codir	putative GTP-binding protein
TcG_05134	62,31818076	0,723136769	0,22538753	3,20841511	0,00133469	0,00986204	protein_codir	putative oligopeptidase B, putative,serine peptidase, clan SC, family S9A-like protein
TcG_05135	108,5541366	0,287036842	0,16998919	1,68855943	0,09130389	0,25234897	protein_codir	putative oligopeptidase B, putative,serine peptidase, clan SC, family S9A-like protein
TcG_05136	67,22560727	-0,063741702	0,22184673	-0,28732315	0,7738649	0,88691888	protein_codir	hypothetical protein
TcG_05137	93,11757117	0,078811979	0,18224798	0,43244363	0,66541901	0,82504261	protein_codir	putative oligopeptidase B, putative,serine peptidase, clan SC, family S9A-like protein
TcG_05138	253,5070594	0,41671988	0,11433504	3,64472585	0,00026768	0,00253789	protein_codir	hypothetical protein
TcG_05139	216,0988753	0,025931794	0,12971127	0,19991936	0,84154365	0,92278296	protein_codir	hypothetical protein
TcG_05140	673,8260141	0,344382297	0,07831409	4,39745018	1,0953E-05	0,00016417	protein_codir	putative ATP-dependent RNA helicase
TcG_05141	0,314371616	-2,1715226	2,91078434	-0,74602662	0,45565133	1	protein_codir	histone H4
TcG_05142	192,2783746	0,074923081	0,12866746	0,58230015	0,56036452	0,75431619	protein_codir	histone H4
TcG_05143	42,16330834	-0,704592108	0,28393596	-2,48151773	0,01308242	0,06114277	protein_codir	hypothetical protein
TcG_05144	73,83404763	0,181265902	0,21146034	0,85720994	0,39132887	0,62112715	protein_codir	hypothetical protein
TcG_05145	219,8435902	0,069706112	0,12161702	0,57316084	0,56653579	0,75847974	protein_codir	hypothetical protein

TcG_05146	347,4682471	-0,130578666	0,09742897	-1,3402448	0,18016577	0,39148549	protein_codir	hypothetical protein
TcG_05147	378,5771157	0,012683594	0,09279525	0,13668366	0,89128084	0,94697266	protein_codir	hypothetical protein
TcG_05148	223,9103167	0,350453197	0,12904317	2,71578268	0,00661193	0,03573033	protein_codir	putative TPR-repeat-containing chaperone protein DNAJ
TcG_05149	266,7083704	0,206876726	0,11384568	1,81716802	0,06919139	0,2104072	protein_codir	hypothetical protein
TcG_05150	474,844583	-0,150343266	0,09197705	-1,6345736	0,10213847	0,27291888	protein_codir	hypothetical protein
TcG_05151	248,6935524	0,14409153	0,12089687	1,19185492	0,23331814	0,46067211	protein_codir	hypothetical protein
TcG_05152	291,9320554	0,14692005	0,11124251	1,32071858	0,18659522	0,40057294	protein_codir	hypothetical protein
TcG_05153	248,4294711	0,335717233	0,1171734	2,8651319	0,00416836	0,02495843	protein_codir	hypothetical protein
TcG_05154	318,8941447	0,225983478	0,10084849	2,2408216	0,02503763	0,09982313	protein_codir	coiled-coil domain-containing protein 111
TcG_05155	545,133805	0,028039433	0,08193657	0,342209	0,73219361	0,86246393	protein_codir	hypothetical protein
TcG_05156	112,6778989	-0,07430772	0,16584988	-0,44804204	0,65412286	0,81709489	protein_codir	hypothetical protein
TcG_05157	334,0364211	0,031049568	0,10082708	0,3079487	0,75812137	0,87801518	protein_codir	hypothetical protein
TcG_05158	438,3053653	-0,182300526	0,0896837	-2,03270525	0,04208231	0,14632823	protein_codir	hypothetical protein
TcG_05159	157,4949693	0,121933304	0,14586136	0,83595343	0,40318111	0,63298674	protein_codir	hypothetical protein
TcG_05160	322,6343356	-0,010588992	0,10129461	-0,10453658	0,91674354	0,95973531	protein_codir	hypothetical protein
TcG_05161	1407,588486	-0,110105984	0,05491287	-2,00510339	0,044952	0,15340613	protein_codir	putative AAA family ATPase
TcG_05162	170,2613173	0,089244934	0,13408939	0,66556296	0,50569049	0,71267956	protein_codir	hypothetical protein
TcG_05163	726,6572579	0,018910784	0,07195689	0,26280712	0,79269924	0,89624823	protein_codir	putative nonsense mRNA reducing factor 1
TcG_05164	85,70012335	0,045151541	0,19010568	0,23750759	0,81226303	0,90759432	protein_codir	dual specificity phosphatase 12
TcG_05165	396,0098606	-0,10454731	0,09221752	-1,13370334	0,25691901	0,48885919	protein_codir	hypothetical protein
TcG_05166	319,3286376	-0,108092724	0,10602921	-1,01946174	0,30798381	0,54112123	protein_codir	amino acid aldolase
TcG_05167	279,6379484	0,119803435	0,10917145	1,09738795	0,27247184	0,5055018	protein_codir	hypothetical protein
TcG_05168	205,0803711	-0,162669979	0,12831417	-1,2677476	0,2048881	0,42614649	protein_codir	putative desumoylating isopeptidase 2
TcG_05169	166,4799289	-0,048304558	0,13978962	-0,34555182	0,72967956	0,86068906	protein_codir	hypothetical protein
TcG_05170	263,6765114	-0,065821282	0,11031664	-0,59665779	0,55073588	0,74654606	protein_codir	putative mitochondrial carrier protein
TcG_05171	229,7959415	-0,321124926	0,11854689	-2,70884307	0,00675183	0,03632204	protein_codir	hypothetical protein
TcG_05172	122,8209406	-0,093618444	0,16037426	-0,58374981	0,55938865	0,75387657	protein_codir	hypothetical protein
TcG_05173	245,4334194	-0,170020359	0,12040745	-1,41204191	0,15793762	0,35999711	protein_codir	hypothetical protein
TcG_05174	218,3176855	-0,120516087	0,12330432	-0,97738736	0,32837742	0,56286962	protein_codir	hypothetical protein
TcG_05175	156,3696521	0,088265702	0,14683714	0,60111294	0,54776477	0,74462074	protein_codir	hypothetical protein
TcG_05176	729,3959218	-0,608804951	0,07043598	-8,64337987	5,4574E-18	8,1296E-16	protein_codir	paraflagellar rod component
TcG_05177	737,757055	0,26988542	0,07346227	3,67379646	0,00023897	0,002315	protein_codir	hypothetical protein
TcG_05178	55,92536431	-0,367785488	0,23603588	-1,55817617	0,11919149	0,30178161	protein_codir	hypothetical protein
TcG_05179	441,4345207	-0,151917674	0,08679139	-1,75037715	0,08005326	0,23118071	protein_codir	hypothetical protein
TcG_05180	178,899412	-0,236504395	0,14848683	-1,59276347	0,11121326	0,28838784	protein_codir	hypothetical protein
TcG_05181	89,54521691	0,157859758	0,19105886	0,82623627	0,40867008	0,63660535	protein_codir	hypothetical protein
TcG_05182	232,197461	0,126456125	0,11719032	1,07906626	0,28055819	0,51365478	protein_codir	hypothetical protein
TcG_05183	216,3115397	0,04432451	0,12135857	0,36523594	0,71493529	0,85366926	protein_codir	hypothetical protein
TcG_05184	98,05093846	0,08008338	0,17647465	0,45379538	0,64997611	0,81420945	protein_codir	hypothetical protein
TcG_05185	41,23062984	0,243174617	0,28402453	0,85617469	0,39190116	0,62139829	protein_codir	hypothetical protein
TcG_05186	250,8847444	-0,106427888	0,11417366	-0,93215799	0,35125488	0,58572813	protein_codir	hypothetical protein
TcG_05187	194,1025325	-0,204746267	0,1306946	-1,56660077	0,11720804	0,2985867	protein_codir	ubiquinone biosynthesis protein-like protein
TcG_05188	78,11296648	0,033025825	0,19671165	0,16788952	0,8666702	0,93574468	protein_codir	hypothetical protein
TcG_05189	181,5500326	0,240108149	0,13168371	1,82337022	0,06824734	0,20830181	protein_codir	hypothetical protein
TcG_05190	72,88076415	-0,26467648	0,2029128	-1,30438533	0,19210223	0,40838468	protein_codir	putative heat shock protein 67B2
TcG_05191	145,6586447	0,097365383	0,1468039	0,66323432	0,5071805	0,71391	protein_codir	endoplasmic reticulum protein
TcG_05192	78,26033707	0,417619205	0,19911044	2,09742492	0,03595598	0,13124951	protein_codir	Heat shock protein 67Bb
TcG_05193	1020,445458	-0,094158523	0,06417548	-1,46720402	0,14232056	0,33831064	protein_codir	putative DNA J-binding protein
TcG_05194	263,1539477	0,060750157	0,11150956	0,54479775	0,58589262	0,77446115	protein_codir	cytochrome b5-like protein
TcG_05195	100,0132766	0,387551585	0,17759425	2,1822305	0,02909253	0,11179635	protein_codir	hypothetical protein
TcG_05196	927,9971639	-0,321569802	0,0639804	-5,02606755	5,0064E-07	1,0903E-05	protein_codir	hypothetical protein
TcG_05197	182,0730705	-0,437338412	0,1361635	-3,21186239	0,00131878	0,00978817	protein_codir	hypothetical protein

TcG_05198	115,5717768	-0,098330378	0,16982005	-0,57902692	0,56257101	0,75596703	protein_codir	cAMP-dependent protein kinase dimerization/docking domain
TcG_05199	78,12094104	0,217024722	0,21035242	1,03171961	0,30220349	0,5353715	protein_codir	putative GDP-mannose transporter
TcG_05200	478,5872171	-0,323072477	0,0870597	-3,71093012	0,0002065	0,00204092	protein_codir	BRCT domain-containing protein
TcG_05201	150,3657598	0,036335423	0,15002886	0,24218955	0,80863329	0,90526589	protein_codir	hypothetical protein
TcG_05202	126,6394427	-0,216099146	0,15765254	-1,37073052	0,17045898	0,37704042	protein_codir	hypothetical protein
TcG_05203	23,96568994	-0,609661716	0,38238785	-1,59435431	0,1108567	0,28778529		
TcG_05204	816,8145545	-0,266342276	0,06927786	-3,84455102	0,00012077	0,00130774	protein_codir	putative dual specificity protein phosphatase
TcG_05205	347,4269987	-0,015773167	0,1020194	-0,15460948	0,8771292	0,94102958	protein_codir	hypothetical protein
TcG_05206	305,1947534	-0,014034021	0,10626294	-0,13206882	0,89492986	0,9489813	protein_codir	hypothetical protein
TcG_05207	770,5059333	-0,282032564	0,06934183	-4,06727911	4,7565E-05	0,00059193	protein_codir	hypothetical protein
TcG_05208	608,8970342	-0,07714069	0,07646984	-1,0087727	0,31308366	0,5472184	protein_codir	putative MCAK-like kinesin
TcG_05209	367,3464385	-0,124184808	0,09644021	-1,287687	0,19785494	0,41642935	protein_codir	hypothetical protein
TcG_05210	8,222973371	0,015921491	0,60002187	0,02653485	0,97883074	1	protein_codir	hypothetical protein
TcG_05211	325,4981467	-0,042651772	0,09928908	-0,42957163	0,66750728	0,82581306	protein_codir	hypothetical protein
TcG_05212	308,4120615	-0,040902708	0,10884384	-0,37579258	0,7070711	0,84892495	protein_codir	putative mitochondrial glycoprotein
TcG_05213	445,0891066	0,040369745	0,09050797	0,44603526	0,65557178	0,81772823	protein_codir	MYND zinc finger (ZnF) domain-like protein
TcG_05214	177,9516757	-0,142281655	0,13168093	-1,08050313	0,27991819	0,51307265	protein_codir	putative vesicle transport protein SFT2B-like
TcG_05215	538,8978573	0,049256236	0,08863609	0,55571308	0,57840702	0,76824002	protein_codir	hypothetical protein
TcG_05216	539,3160928	0,303052962	0,08052776	3,7633353	0,00016766	0,00172363	protein_codir	methylthioadenosine phosphorylase
TcG_05217	540,3567405	-0,234113492	0,07941775	-2,94787369	0,00319968	0,02010383	protein_codir	hypothetical protein
TcG_05218	8,44833057	-0,335315714	0,59336817	-0,56510567	0,57200189	1		
TcG_05219	175,5567765	0,159469434	0,13538643	1,17788345	0,23884307	0,46688642	protein_codir	hypothetical protein
TcG_05220	550,1204632	-0,068930124	0,08393069	-0,8212744	0,41148998	0,63808037	protein_codir	hypothetical protein
TcG_05221	177,0878076	-0,045145495	0,13890003	-0,3250215	0,7451648	0,87118864	protein_codir	hypothetical protein
TcG_05222	1031,839742	-0,079155648	0,06515084	-1,21495973	0,22438145	0,45108253	protein_codir	splicing factor 3B subunit 3
TcG_05223	407,2938229	-0,096378033	0,09190811	-1,04863465	0,2943463	0,52725669	protein_codir	hypothetical protein
TcG_05224	565,9030015	0,005717048	0,08452885	0,06763428	0,94607677	0,97469438	protein_codir	putative paraflagellar rod protein
TcG_05225	596,1907103	0,002228102	0,07752985	0,02873864	0,97707304	0,99017719	protein_codir	putative ubiquitin-conjugating enzyme
TcG_05226	373,4987177	-0,099856007	0,09945973	-1,00398434	0,31538616	0,55022799	protein_codir	hypothetical protein
TcG_05227	207,7809776	0,129913058	0,13342652	0,97366741	0,33022171	0,56496584	protein_codir	putative glutaminyl cyclase
TcG_05228	322,8601344	-0,10043137	0,10453584	-0,96073627	0,33668479	0,57114185	protein_codir	putative glutaminyl cyclase
TcG_05229	173,4038065	0,063784249	0,14748793	0,43247098	0,66539914	0,82504261	protein_codir	putative ATPase
TcG_05230	1,145156382	-0,185704626	1,67630657	-0,11078202	0,9117892	1	protein_codir	ATPase
TcG_05231	618,9042595	0,070100141	0,07843542	0,8937307	0,37146599	0,60280866	protein_codir	uncharacterized protein
TcG_05232	628,9923235	0,160471869	0,08239165	1,94767159	0,05145427	0,1686864	protein_codir	hypothetical protein
TcG_05233	68,56956136	0,107265409	0,21526347	0,49829825	0,61827384	0,793034		
TcG_05234	835,3511161	-0,414991127	0,07218587	-5,7489249	8,9813E-09	2,8853E-07	protein_codir	hypothetical protein
TcG_05235	831,7348656	-0,213054181	0,06854667	-3,10816216	0,00188255	0,01309195	protein_codir	double-strand-break repair protein rad21
TcG_05236	766,3502386	-0,25740897	0,08046862	-3,19887374	0,00137966	0,01014901	protein_codir	putative methyltransferase
TcG_05237	317,7859184	-0,136402464	0,10498525	-1,29925366	0,19385689	0,41081411	protein_codir	hypothetical protein
TcG_05238	727,0084321	-0,102611789	0,07154396	-1,43424812	0,15150147	0,35179947	protein_codir	hypothetical protein
TcG_05239	5,761951631	-0,619258504	0,76812752	-0,80619231	0,42013197	1	protein_codir	protein kinase
TcG_05240	14,04431637	-0,002578749	0,49208229	-0,00524048	0,99581872	1	protein_codir	putative target of rapamycin (TOR) kinase 1
TcG_05241	8,714612151	0,531641238	0,5949535	0,89358452	0,37154423	1	protein_codir	hypothetical protein
TcG_05242	30,41492129	-0,020547434	0,33079921	-0,06211452	0,95047163	0,97694858	protein_codir	helicase-like protein
TcG_05243	409,230435	-0,09244091	0,096324	-0,9596872	0,33721267	0,57160877	protein_codir	hypothetical protein
TcG_05244	170,8562664	0,044131803	0,13437666	0,32841867	0,74259513	0,86906133	protein_codir	hypothetical protein
TcG_05245	562,326861	-0,062301681	0,08004952	-0,77828925	0,43639851	0,66000718	protein_codir	hypothetical protein
TcG_05246	479,7819621	-0,073674489	0,08494757	-0,86729365	0,38578114	0,61632504	protein_codir	hypothetical protein
TcG_05247	309,7272667	-0,048455591	0,10179629	-0,47600546	0,63407049	0,80419712	protein_codir	guanylate kinase
TcG_05248	216,8648284	-0,051289031	0,1234757	-0,41537754	0,67786554	0,83134859	protein_codir	hypothetical protein
TcG_05249	258,6428266	-0,147455769	0,11480171	-1,28443883	0,19898845	0,4178224	protein_codir	putative chloride channel protein

TcG_05250	175,0414725	-0,197187985	0,13971875	-1,41132084	0,15815003	0,36033949	protein_codir putative chloride channel protein
TcG_05251	282,0294433	0,161552819	0,10848588	1,48915991	0,13644527	0,32916375	protein_codir hypothetical protein
TcG_05252	274,9505725	-0,078229348	0,11229962	-0,69661275	0,48604517	0,69827461	protein_codir hypothetical protein
TcG_05253	396,7947764	0,094503312	0,09523623	0,99230419	0,32104916	0,5560344	protein_codir hypothetical protein
TcG_05254	297,1150675	-0,158677036	0,10448829	-1,51861071	0,12886051	0,31792545	protein_codir hypothetical protein
TcG_05255	348,5473639	-0,195048875	0,09842119	-1,98177722	0,04750418	0,15939283	protein_codir geranylgeranyl transferase type-2 subunit alpha
TcG_05256	539,0179086	-0,083177604	0,08614369	-0,96556813	0,33426033	0,56876783	protein_codir putative calcium-transporting ATPase
TcG_05257	260,8262258	0,021262312	0,11331266	0,18764286	0,85115662	0,92805389	protein_codir S-phase kinase-associated protein 1
TcG_05258	667,1031663	-0,177811687	0,07223827	-2,46146118	0,01383724	0,06379917	protein_codir hypothetical protein
TcG_05259	786,257785	-0,318466673	0,07051488	-4,51630475	6,2928E-06	0,00010197	protein_codir putative nicotinate phosphoribosyltransferase
TcG_05260	624,725237	0,006985	0,07470673	0,09349895	0,92550719	0,96364857	protein_codir putative WD40 repeat protein
TcG_05261	480,950877	-0,154843733	0,08728505	-1,77400068	0,07606304	0,22310484	protein_codir hypothetical protein
TcG_05262	231,0058376	0,269652404	0,11855581	2,27447655	0,02293735	0,09367365	protein_codir hypothetical protein
TcG_05263	483,1947442	-0,113252902	0,0844268	-1,34143309	0,17977988	0,39101365	protein_codir hypothetical protein
TcG_05264	663,439833	0,168671066	0,07318084	2,30485294	0,02117481	0,08804031	protein_codir 40S ribosomal protein S3
TcG_05265	695,4899006	0,172610224	0,07634044	2,2610589	0,02375561	0,09626617	protein_codir hypothetical protein
TcG_05266	334,5745462	-0,183865005	0,10571525	-1,73924765	0,08199121	0,2349617	protein_codir hypothetical protein
TcG_05267	152,39272	0,214596619	0,1420698	1,5105013	0,13091556	0,32142141	protein_codir hypothetical protein
TcG_05268	67,89291449	-0,414861725	0,21429688	-1,93592047	0,05287744	0,17175162	
TcG_05269	173,4426434	-0,09150562	0,1364288	-0,67072068	0,50239849	0,71099511	protein_codir hypothetical protein
TcG_05270	472,3735645	-0,195820076	0,08442561	-2,31943919	0,02037123	0,08529856	protein_codir hypothetical protein
TcG_05271	195,5211405	0,020958776	0,13549691	0,15468084	0,87707293	0,94102958	protein_codir hypothetical protein
TcG_05272	379,8512509	0,104308578	0,09549822	1,09225674	0,27472027	0,50755645	protein_codir hypothetical protein
TcG_05273	445,2673349	0,061856014	0,09238481	0,66954743	0,50314634	0,7116032	protein_codir hypothetical protein
TcG_05274	643,7807411	0,067128608	0,07594386	0,88392414	0,37673716	0,60741397	protein_codir hypothetical protein
TcG_05275	283,1388873	-0,076451095	0,10871469	-0,70322691	0,48191435	0,69593165	protein_codir hypothetical protein
TcG_05276	1100,507459	-0,071011795	0,06694104	-1,06081101	0,2887758	0,52204032	protein_codir cyclin 5
TcG_05277	44,55959471	0,041150074	0,26043436	0,15800555	0,87445243	0,93957209	
TcG_05278	710,256359	0,223792814	0,07438899	3,00841322	0,00262616	0,01706487	protein_codir hypothetical protein
TcG_05279	16,16012047	0,176020178	0,43677399	0,4030006	0,68694779	1	protein_codir putative glycine dehydrogenase, putative,glycine cleavage system P-protein
TcG_05280	35,44393695	-0,226621566	0,29279113	-0,7740042	0,43892831	0,66225073	protein_codir structural maintenance of chromosome protein 4
TcG_05281	23,64563269	0,296689139	0,35617056	0,83299736	0,40484622	0,63415119	protein_codir hypothetical protein
TcG_05282	8,549088568	0,273771581	0,62290467	0,43950799	0,66029349	1	protein_codir hypothetical protein
TcG_05283	16,44151283	-0,163726608	0,43187788	-0,37910394	0,70461068	1	protein_codir target of rapamycin (TOR) kinase 1
TcG_05284	431,4293776	-0,052404172	0,09138527	-0,5734422	0,56634532	0,75838888	protein_codir hypothetical protein
TcG_05285	376,5855236	-0,155959796	0,09784437	-1,59395787	0,11094547	0,28795122	protein_codir hypothetical protein
TcG_05286	216,1162375	0,034531414	0,12145809	0,28430724	0,77617494	0,88831084	protein_codir hypothetical protein
TcG_05287	529,8877723	-0,131699117	0,08037614	-1,63853489	0,10131016	0,27139411	protein_codir putative flavoprotein monooxygenase
TcG_05288	1037,693432	0,159066149	0,06304596	2,52301886	0,01163522	0,05563583	protein_codir hypothetical protein
TcG_05289	129,7442193	-0,043783345	0,15738779	-0,27818768	0,78086829	0,89087556	protein_codir hypothetical protein
TcG_05290	214,837253	0,011217819	0,12518636	0,08960896	0,92859796	0,96542881	protein_codir hypothetical protein
TcG_05291	513,3188928	-0,038771863	0,08617447	-0,44992285	0,65276607	0,81629225	protein_codir alanyl-tRNA synthetase
TcG_05292	396,2033295	-0,017434215	0,09147254	-0,19059506	0,84884286	0,9269268	protein_codir hypothetical protein
TcG_05293	387,2798299	-0,062465411	0,09388505	-0,66533926	0,50583352	0,71267956	protein_codir hypothetical protein
TcG_05294	178,412896	0,318601675	0,13909549	2,29052481	0,02199091	0,09043176	protein_codir hypothetical protein
TcG_05295	532,6747464	-0,487182192	0,08244923	-5,90887467	3,4445E-09	1,2167E-07	protein_codir putative protein kinase
TcG_05296	100,3129653	-0,254875978	0,18412311	-1,38426938	0,16627599	0,37183431	protein_codir ABC1 transporter
TcG_05297	183,0803558	-0,082860998	0,13465688	-0,61534918	0,53832414	0,7376728	protein_codir hypothetical protein
TcG_05298	457,5572743	-0,035040905	0,08673737	-0,40398856	0,68622114	0,83720952	protein_codir hypothetical protein
TcG_05299	1201,640421	-0,239758563	0,05971825	-4,01482912	5,9489E-05	0,00071572	protein_codir hypothetical protein
TcG_05300	429,2806766	-0,26395714	0,09115515	-2,89569088	0,00378325	0,02302139	protein_codir hypothetical protein
TcG_05301	119,1220974	-0,117506652	0,17117972	-0,68645194	0,49242816	0,7030527	protein_codir putative dolichol phosphate-mannose biosynthesis regulatory protein

TcG_05302	687,2513946	-0,276893593	0,07513983	-3,68504424	0,00022866	0,00223192	protein_codir	putative RAB GDP dissociation inhibitor alpha
TcG_05303	241,9150783	0,022203693	0,11338153	0,19583165	0,84474193	0,92480204	protein_codir	hypothetical protein
TcG_05304	414,2548968	0,083923435	0,08927518	0,94005338	0,34719018	0,58171301	protein_codir	hypothetical protein
TcG_05305	221,3130541	-0,022437636	0,12249201	-0,18317632	0,85465967	0,92942435	protein_codir	putative nucleotide sugar transporter
TcG_05306	416,2901633	0,027187073	0,09006165	0,3018718	0,7627498	0,88013366	protein_codir	hypothetical protein
TcG_05307	133,7067626	-0,115644832	0,1566162	-0,73839634	0,46027364	0,67839985	protein_codir	hypothetical protein
TcG_05308	269,6070186	-0,191379296	0,11554722	-1,65628646	0,09766382	0,26450047	protein_codir	hypothetical protein
TcG_05309	401,1156033	-0,068820044	0,09183651	-0,74937569	0,4536308	0,67403555	protein_codir	hypothetical protein
TcG_05310	295,8592285	-0,087153369	0,10748706	-0,81082661	0,41746525	0,64412737	protein_codir	putative myo-inositol-1(or 4)-monophosphatase 1
TcG_05311	678,1408014	-0,038386893	0,07248213	-0,52960493	0,59638588	0,78066886	protein_codir	putative trans-sialidase
TcG_05312	2047,722046	0,097938647	0,0481729	2,03306501	0,04204596	0,14624571	protein_codir	ATP-binding cassette protein subfamily G, member 4
TcG_05313	858,9188997	-0,05926089	0,06585876	-0,89981787	0,36821718	0,60027638	protein_codir	putative arginine kinase
TcG_05314	451,2152046	-0,038181501	0,09210395	-0,41454794	0,67847286	0,83182927	protein_codir	hypothetical protein
TcG_05315	631,1093728	0,212894437	0,07487643	2,84327711	0,00446522	0,02639873	protein_codir	putative MFS transporter
TcG_05316	46,83907634	0,075376865	0,26564429	0,28375112	0,77660112	0,88831084	protein_codir	hypothetical protein
TcG_05317	120,2531085	0,168494301	0,16808048	1,00246207	0,31612048	0,55115373	protein_codir	hypothetical protein
TcG_05318	242,5082284	0,208518489	0,11394328	1,83002009	0,06724693	0,20644488	protein_codir	hypothetical protein
TcG_05319	68,49678443	0,398618082	0,2103451	1,89506712	0,05808351	0,18416956	protein_codir	hypothetical protein
TcG_05320	324,2167035	0,17923809	0,10475862	1,71096264	0,08708801	0,24460647	protein_codir	cell cycle checkpoint protein
TcG_05321	1069,299898	0,326791879	0,06016814	5,43131088	5,5942E-08	1,525E-06	protein_codir	hypothetical protein
TcG_05322	21,96494395	0,037421873	0,37400928	0,100056	0,92029986	0,96146026	protein_codir	hypothetical protein
TcG_05323	9,150826649	1,017846182	0,607981	1,67414144	0,09410281	1	protein_codir	hypothetical protein
TcG_05324	58,08308083	-0,08095354	0,23416172	-0,34571637	0,72955588	0,86066942	protein_codir	hypothetical protein
TcG_05325	72,74637324	0,509043193	0,21049434	2,41832244	0,01559225	0,06964219	protein_codir	retrotransposon hot spot (RHS) protein
TcG_05326	197,6228984	-0,012580864	0,12516206	-0,1005166	0,91993421	0,96142502	protein_codir	hypothetical protein
TcG_05327	182,0468193	-0,008408587	0,13303543	-0,06320562	0,94960277	0,97657533	protein_codir	hypothetical protein
TcG_05328	474,8919268	-0,032056455	0,08509732	-0,37670348	0,70639398	0,84872764	protein_codir	BRCT domain-containing protein
TcG_05329	343,5824228	0,133075431	0,09900118	1,34418018	0,17889013	0,38979933	protein_codir	putative methyltransferase
TcG_05330	292,3383347	0,539030639	0,11229954	4,79993639	1,5872E-06	3,0395E-05	protein_codir	putative glycerol-3-phosphate dehydrogenase (FAD-dependent)
TcG_05331	431,4926943	0,066646271	0,09364699	0,71167556	0,47666571	0,69195279	protein_codir	hypothetical protein
TcG_05332	237,6149861	0,222976538	0,12635142	1,76473312	0,07760863	0,22597979	protein_codir	putative zeta tubulin
TcG_05333	263,0665325	0,043534126	0,11133382	0,39102338	0,69577996	0,84235178	protein_codir	hypothetical protein
TcG_05334	53,30849178	0,319735023	0,24800799	1,28921261	0,19732418	0,41574795	protein_codir	hypothetical protein
TcG_05335	202,172771	0,131377811	0,12368556	1,062192	0,28814853	0,52123168	protein_codir	nucleotidyl transferase domain-containing protein
TcG_05336	331,2344128	-0,012346213	0,10199765	-0,12104409	0,90365612	0,95335638	protein_codir	hypothetical protein
TcG_05337	110,6671393	0,182930601	0,17346918	1,05454236	0,29163468	0,52426368	protein_codir	hypothetical protein
TcG_05338	323,5155717	-0,063099572	0,10215733	-0,6176705	0,53679256	0,73660472	protein_codir	MORN repeat-containing protein
TcG_05339	293,517072	0,089684661	0,10440273	0,85902604	0,39032615	0,62040037	protein_codir	hypothetical protein
TcG_05340	192,3114566	0,079844095	0,12939727	0,61704621	0,53720425	0,73683627	protein_codir	RWD domain containing 1
TcG_05341	153,5308073	0,18411849	0,14519184	1,26810497	0,20476047	0,42606946	protein_codir	putative chaperone DNAJ protein
TcG_05342	4,155384206	0,911030477	0,87159257	1,0452481	0,29590832	1	protein_codir	hypothetical protein
TcG_05343	596,671774	0,015285755	0,08302367	0,18411321	0,85392464	0,92914828	protein_codir	cytidine triphosphate synthase
TcG_05344	223,7595916	0,222809933	0,12338594	1,80579683	0,07095008	0,21373573	protein_codir	cytidine triphosphate synthase
TcG_05345	440,6758122	-0,014415913	0,08948271	-0,16110277	0,87201246	0,93842991	protein_codir	hypothetical protein
TcG_05346	173,8425356	0,055141862	0,13966077	0,39482713	0,69297047	0,8410627	protein_codir	hypothetical protein
TcG_05347	251,3995655	0,358353102	0,1309822	2,73589167	0,00622115	0,03396713	protein_codir	hypothetical protein
TcG_05348	204,7013616	0,08396517	0,12371153	0,67871744	0,49731692	0,70725289	protein_codir	mitochondrial editosome-like complex associated TUTase
TcG_05349	318,044061	0,154626865	0,10417345	1,48432131	0,1377237	0,3311199	protein_codir	putative FG-GAP repeat protein
TcG_05350	134,5076362	0,053447504	0,15232879	0,35086937	0,72568635	0,8582017	protein_codir	hypothetical protein
TcG_05351	246,1640498	0,095419848	0,11564787	0,82508957	0,40932074	0,6367988	protein_codir	hypothetical protein
TcG_05352	504,2460204	0,06687024	0,08747395	0,76445887	0,44459384	0,66654558	protein_codir	kinesin heavy chain
TcG_05353	398,998735	-0,01521971	0,0936581	-0,16250287	0,87090986	0,93767881	protein_codir	rRNA biogenesis protein

TcG_05354	328,4794087	-0,225593241	0,10429151	-2,16310257	0,03053329	0,11579663	protein_codir	serine/threonine protein phosphatase 2a regulatory subunit
TcG_05355	203,3419433	0,063055572	0,13113532	0,48084354	0,6306277	0,80202553	protein_codir	hypothetical protein
TcG_05356	445,002551	0,43821523	0,09631301	4,549907	5,367E-06	8,9113E-05	protein_codir	hypothetical protein
TcG_05357	602,3954994	-0,019799245	0,07765002	-0,25498057	0,79873811	0,89959947	protein_codir	hypothetical protein
TcG_05358	141,5559562	0,139889758	0,1484479	0,94234921	0,34601388	0,58032959	protein_codir	hypothetical protein
TcG_05359	844,5053581	0,209828083	0,06970227	3,010348	0,00260949	0,01697557	protein_codir	pyruvate transporter 0
TcG_05360	146,2174515	0,548427219	0,16144851	3,39691728	0,0006815	0,0056238	protein_codir	putative amino acid transporter
TcG_05361	174,237376	-0,123880383	0,13799184	-0,89773703	0,36932576	0,60112883	protein_codir	putative glutamic acid/alanine-rich protein
TcG_05362	420,2911115	-7,07621E-05	0,09310965	-0,00075999	0,99939362	0,99981271	protein_codir	hypothetical protein
TcG_05363	216,881958	-0,111067393	0,12053889	-0,92142376	0,35682923	0,5907012	protein_codir	hypothetical protein
TcG_05364	269,6028301	-0,112355825	0,11374281	-0,98780598	0,32324768	0,55757366	protein_codir	putative periplasmic protein
TcG_05365	95,43503861	0,58477093	0,1896456	3,08349334	0,00204586	0,01401733	protein_codir	hypothetical protein
TcG_05366	645,7605335	0,231547192	0,07486492	3,09286647	0,00198233	0,01364665	protein_codir	putative mitochondrial DNA polymerase I protein B
TcG_05367	322,3093252	0,173633701	0,10553667	1,64524524	0,09991925	0,26859963	protein_codir	surfeit locus 1 family protein
TcG_05368	89,57611911	-0,21238515	0,18546218	-1,14516692	0,25214002	0,48230052	protein_codir	hypothetical protein
TcG_05369	192,6237795	0,164514973	0,13091151	1,2566884	0,20886649	0,4315134	protein_codir	hypothetical protein
TcG_05370	264,1625725	0,336551287	0,1100542	3,05805034	0,00222782	0,01500671	protein_codir	hypothetical protein
TcG_05371	500,7986997	0,070207838	0,08211826	0,8549601	0,39257324	0,62203959	protein_codir	calpain protease-like protein
TcG_05372	157,181383	0,171301382	0,14655755	1,16883354	0,2424707	0,47089863	protein_codir	putative 40S ribosomal protein S8
TcG_05373	746,3270172	-0,447234259	0,07389127	-6,05259966	1,4253E-09	5,4849E-08	protein_codir	regulatory subunit of protein kinase A
TcG_05374	347,3827347	0,233456739	0,09982122	2,3387485	0,01934845	0,08187406	protein_codir	putative histone acetyltransferase
TcG_05375	135,235175	-0,111069844	0,16122388	-0,68891682	0,49087561	0,70187819	protein_codir	variant surface glycoprotein 3054
TcG_05376	241,756477	0,118678313	0,11899248	0,99735975	0,31858992	0,55357619	protein_codir	putative ras-related protein rab-5
TcG_05377	91,12417999	0,499032551	0,18830507	2,65012813	0,00804612	0,04167296	protein_codir	hypothetical protein
TcG_05378	231,211181	-0,239094162	0,12158208	-1,96652467	0,04923804	0,16369351	protein_codir	hypothetical protein
TcG_05379	172,1259762	0,136416958	0,13539262	1,00756567	0,31366302	0,54796438	protein_codir	putative dynein heavy chain, cytosolic
TcG_05380	130,5599106	0,15671631	0,15783039	0,99294131	0,32073856	0,55596603	protein_codir	flagellar associated protein
TcG_05381	217,6228351	-0,109946601	0,12001452	-0,91611083	0,35960877	0,5926302	protein_codir	putative U4/U6 small nuclear ribonuclear protein
TcG_05382	418,5683647	0,07046827	0,09482262	0,74315888	0,4573855	0,67696325	protein_codir	N-acetyltransferase complex ARD1 subunit
TcG_05383	0,145251675	-1,420530545	4,08047286	-0,3481289	0,72774338	1		
TcG_05384	353,8579194	-0,143441887	0,09529875	-1,50518114	0,13227751	0,32319006	protein_codir	hypothetical protein
TcG_05385	824,360373	-0,004982192	0,07408057	-0,06725369	0,94637974	0,97469438	protein_codir	hypothetical protein
TcG_05386	289,5473171	-0,038858758	0,10631224	-0,36551535	0,71472675	0,8536143	protein_codir	hypothetical protein
TcG_05387	279,6567064	0,042543427	0,10680873	0,39831412	0,69039865	0,83922987	protein_codir	putative acyl-CoA synthetase
TcG_05388	502,4172065	-0,453459829	0,08499006	-5,33544569	9,531E-08	2,4898E-06	protein_codir	putative flagellar radial spoke component
TcG_05389	265,3857478	-0,28473548	0,11689501	-2,43582246	0,01485797	0,06732284	protein_codir	mitogen-activated protein kinase kinase 2
TcG_05390	964,3222314	-0,264201961	0,06931601	-3,81155732	0,00013809	0,00145848	protein_codir	hypothetical protein
TcG_05391	761,660082	-0,002159164	0,06903349	-0,03127705	0,97504859	0,98913519	protein_codir	hypothetical protein
TcG_05392	1,753889398	4,042872142	1,59674562	2,53194503	0,01134318	1	protein_codir	hypothetical protein
TcG_05393	1,278370512	0,676998756	1,58012652	0,42844592	0,6683265	1	protein_codir	hypothetical protein
TcG_05394	11,45818282	0,516499813	0,54373012	0,94991945	0,34215318	1	protein_codir	target of rapamycin (TOR) kinase 1
TcG_05395	5147,017029	-0,247817142	0,03992717	-6,20672986	5,4098E-10	2,3044E-08	protein_codir	putative epsilon tubulin
TcG_05396	257,3677675	-0,133701397	0,11083324	-1,20632941	0,2276905	0,45451795	protein_codir	surface antigen
TcG_05397	10,05469893	0,406832754	0,55065872	0,73881106	0,46002173	1	protein_codir	trans-sialidase
TcG_05398	314,2058021	-0,023462846	0,1013202	-0,23157127	0,81687103	0,90958077	protein_codir	hypothetical protein
TcG_05399	315,5785635	0,066292906	0,1023952	0,64742198	0,51735887	0,72279271	protein_codir	putative ADP-ribosylation factor GTPase activating protein
TcG_05400	95,24196211	0,287041507	0,18301918	1,56836848	0,11679516	0,2980592	protein_codir	hypothetical protein
TcG_05401	468,0083768	0,043874527	0,08399961	0,52231824	0,60144877	0,78437477	protein_codir	putative kinesin
TcG_05402	708,0913998	-0,190891564	0,0743808	-2,56640901	0,01027576	0,05048979	protein_codir	putative calcium motive p-type ATPase
TcG_05403	309,6980356	-0,114298732	0,10724122	-1,06580968	0,28650968	0,51971501	protein_codir	hypothetical protein
TcG_05404	409,1996141	0,445581213	0,09298963	4,79173029	1,6535E-06	3,1613E-05	protein_codir	putative intersectin-1-like
TcG_05405	23,13568723	0,206320093	0,36530533	0,56478808	0,57221792	0,76350239	protein_codir	60S ribosomal protein L32

TcG_05406	23,10455001	0,293515111	0,37579306	0,78105516	0,43477005	0,65898036	protein_codir 60S ribosomal protein L32
TcG_05407	998,9648994	0,358135166	0,0729518	4,90920249	9,1448E-07	1,8752E-05	protein_codir putative ankyrin repeat protein
TcG_05408	231,9743443	-0,061884777	0,1180621	-0,5241714	0,60015933	0,78350294	protein_codir putative calmodulin
TcG_05409	323,9197211	-0,018013433	0,10061985	-0,17902465	0,85791835	0,93156907	protein_codir NADH dehydrogenase (ubiquinone) 1 alpha subcomplex 9
TcG_05410	1956,524118	0,443504659	0,05284726	8,39219826	4,7714E-17	6,3541E-15	protein_codir 40S ribosomal protein S6
TcG_05411	210,7506063	0,169362981	0,12459258	1,3593344	0,17404065	0,38259333	protein_codir hypothetical protein
TcG_05412	457,3435614	0,150265883	0,08544861	1,7585526	0,07865353	0,2282765	protein_codir hypothetical protein
TcG_05413	48,11766002	0,427258499	0,25874061	1,65130053	0,09867722	0,26624926	protein_codir hypothetical protein
TcG_05414	1346,111004	-0,016084414	0,05901997	-0,27252495	0,78521841	0,89351472	protein_codir hypothetical protein
TcG_05415	413,8657429	0,010237309	0,08958267	0,11427778	0,90901759	0,95584124	protein_codir hypothetical protein
TcG_05416	249,0681709	-0,056415331	0,11299572	-0,49926964	0,61758944	0,793034	protein_codir UBX domain containing protein
TcG_05417	182,9354705	0,102600477	0,14482405	0,70844916	0,47866637	0,69314193	protein_codir ATP synthase mitochondrial F1 complex assembly factor 1
TcG_05418	242,8475328	0,051957548	0,12434835	0,41783867	0,67606507	0,83019501	protein_codir hypothetical protein
TcG_05419	46,93530707	0,389530666	0,25412303	1,53284282	0,12531459	0,31223544	protein_codir hypothetical protein
TcG_05420	86,51003223	-0,030057204	0,18971729	-0,15843155	0,87411676	0,9394728	protein_codir hypothetical protein
TcG_05421	0					1	protein_codir 60S ribosomal protein L5
TcG_05422	2181,200324	0,189103051	0,05572797	3,39332369	0,0006905	0,00568999	protein_codir 60S ribosomal protein L5
TcG_05423	131,8449207	0,256483301	0,16094638	1,59359476	0,11102683	0,28803333	protein_codir 60S ribosomal protein L5
TcG_05424	224,5593998	-0,062307438	0,11741782	-0,53064722	0,59566327	0,78060792	protein_codir hypothetical protein
TcG_05425	268,7476159	-0,123319865	0,10954311	-1,12576565	0,26026473	0,49261779	protein_codir COP-coated vesicle membrane protein gp25L precursor
TcG_05426	118,8918529	0,129728188	0,16993102	0,76341678	0,44521488	0,66703823	protein_codir hypothetical protein
TcG_05427	413,3564319	0,045337674	0,08921131	0,50820546	0,61130926	0,78979655	protein_codir hypothetical protein
TcG_05428	251,6130033	0,163506707	0,11736286	1,39317248	0,16356764	0,36826558	protein_codir putative rRNA processing protein
TcG_05429	259,6036913	-0,164537796	0,11314244	-1,4542535	0,14587604	0,34340242	protein_codir trypanin-like protein
TcG_05430	107,8661662	-0,116967436	0,17267177	-0,67739756	0,49815375	0,70742498	protein_codir DTW domain-containing protein
TcG_05431	209,283853	-0,301317441	0,12520891	-2,40651755	0,01610543	0,07141122	protein_codir putative DNA polymerase epsilon subunit b
TcG_05432	200,3489137	-0,027778086	0,12635738	-0,21983746	0,82599774	0,91511289	protein_codir hypothetical protein
TcG_05433	111,4097989	-0,306237219	0,1691086	-1,81089087	0,07015775	0,21217636	protein_codir hypothetical protein
TcG_05434	119,9888633	-0,1313411	0,16055563	-0,81804106	0,41333374	0,64005409	protein_codir hypothetical protein
TcG_05435	186,774711	-0,122506317	0,13216967	-0,92688678	0,35398532	0,58824927	protein_codir hypothetical protein
TcG_05436	119,705856	-0,05356015	0,16771686	-0,31934863	0,74946216	0,87356827	protein_codir hypothetical protein
TcG_05437	100,781734	0,232855788	0,17514344	1,32951476	0,1836782	0,39621963	protein_codir hypothetical protein
TcG_05438	7,517956349	0,094921073	0,65474212	0,14497475	0,88473081	1	protein_codir trans-sialidase
TcG_05439	8,338221842	1,157147672	0,64135172	1,80423258	0,07119485	1	protein_codir trans-sialidase
TcG_05440	7,731438765	-0,889592489	0,65715738	-1,35369778	0,17583282	1	protein_codir structural maintenance of chromosome protein 4
TcG_05441	0					1	protein_codir hypothetical protein
TcG_05442	127,291584	0,245605451	0,15711565	1,56321444	0,11800217	0,29981866	protein_codir hypothetical protein
TcG_05443	136,0155805	0,632019155	0,1554037	4,06695054	4,7632E-05	0,00059213	protein_codir cyclophilin
TcG_05444	164,8011547	0,588702877	0,14836471	3,96794404	7,2495E-05	0,00084927	protein_codir hypothetical protein
TcG_05445	445,6439165	0,092386878	0,08935325	1,03395096	0,30115909	0,53435138	protein_codir putative translation initiation factor IF-2
TcG_05446	283,9127093	0,148388831	0,11134538	1,33268965	0,18263367	0,39470131	protein_codir hypothetical protein
TcG_05447	159,7606441	0,620265993	0,14808463	4,18859139	2,8069E-05	0,00037231	protein_codir transferase
TcG_05448	113,4153944	0,362695575	0,17407771	2,08352678	0,03720323	0,13419572	protein_codir putative protein HID1 isoform 1
TcG_05449	188,6051021	0,301734127	0,12978685	2,3248436	0,02008032	0,08432426	protein_codir putative protein HID1 isoform 1
TcG_05450	158,9679278	0,291315082	0,14650569	1,98842159	0,04676508	0,15804895	protein_codir EF hand
TcG_05451	180,313259	0,287874223	0,1397965	2,05923767	0,03947148	0,13999295	protein_codir EF hand
TcG_05452	415,3945956	0,631829478	0,09352929	6,75541824	1,4242E-11	8,7772E-10	protein_codir mismatch repair protein MLH1
TcG_05453	706,962529	0,358633943	0,07737015	4,63530126	3,5642E-06	6,245E-05	protein_codir putative kinesin
TcG_05454	313,9473957	0,514268506	0,1033613	4,97544537	6,5098E-07	1,3814E-05	protein_codir hypothetical protein
TcG_05455	72,96947466	0,657244741	0,2062047	3,18734121	0,00143587	0,0104893	protein_codir hypothetical protein
TcG_05456	156,6170693	0,479058075	0,15407637	3,10922479	0,00187579	0,01305279	protein_codir citrate lyase subunit beta
TcG_05457	251,3677668	0,192227254	0,1150229	1,67120861	0,09468048	0,25903815	protein_codir putative protein kinase, putative,serine/threonine-protein kinase

TcG_05458	227,8916274	0,71955792	0,12384245	5,81026894	6,2373E-09	2,053E-07	protein_codir	hypothetical protein
TcG_05459	567,8549628	0,369771035	0,07921075	4,66819235	3,0386E-06	5,4413E-05	protein_codir	hypothetical protein
TcG_05460	232,2129847	0,381688396	0,11862583	3,21758249	0,00129276	0,00963829	protein_codir	hypothetical protein
TcG_05461	644,6678575	0,604699778	0,07702103	7,85109961	4,1241E-15	4,4655E-13	protein_codir	periodic tryptophan protein 1
TcG_05462	1838,889083	0,3229199	0,05808771	5,55917731	2,7105E-08	7,9302E-07	protein_codir	translationally controlled tumor protein (TCTP)
TcG_05463	457,3353479	0,704916952	0,08891767	7,92774849	2,2315E-15	2,486E-13	protein_codir	putative DNA repair protein
TcG_05464	755,1239167	0,765087735	0,07220796	10,5956147	3,1228E-26	1,0641E-23	protein_codir	hypothetical protein
TcG_05465	89,07099223	0,763064375	0,19538839	3,90537205	9,4081E-05	0,0010593	protein_codir	hypothetical protein
TcG_05466	536,6102442	0,584543994	0,09069942	6,44484791	1,1572E-10	5,6332E-09	protein_codir	putative ATP-dependent DEAD/H DNA helicase recQ
TcG_05467	132,2159266	0,655326713	0,16163282	4,05441603	5,026E-05	0,00061816	protein_codir	ATP-dependent DEAD/H DNA helicase recQ
TcG_05468	305,9884958	0,507100444	0,10550182	4,80655622	1,5355E-06	2,9651E-05	protein_codir	sigma-adaptin 3
TcG_05469	385,5457225	0,617379294	0,09466652	6,5216223	6,9551E-11	3,5499E-09	protein_codir	hypothetical protein
TcG_05470	631,6341119	-0,26675049	0,08083983	-3,29974076	0,00096774	0,00757965	protein_codir	calpain-like cysteine peptidase
TcG_05471	123,6544176	0,137047774	0,15949665	0,85925172	0,39020166	0,62040037	protein_codir	putative RNA-binding protein
TcG_05472	372,5952138	0,402835806	0,09504526	4,23835755	2,2516E-05	0,00030727	protein_codir	hypothetical protein
TcG_05473	138,0629396	0,152972505	0,1592262	0,96072445	0,33669073	0,57114185	protein_codir	hypothetical protein
TcG_05474	84,78766676	0,641683167	0,19475283	3,2948593	0,00098471	0,00767755	protein_codir	hypothetical protein
TcG_05475	925,4297214	0,08668347	0,06505156	1,33253479	0,18268452	0,39473757	protein_codir	succinate dehydrogenase (quinone)
TcG_05476	164,6777596	0,466160196	0,14259094	3,26921333	0,00107847	0,00830242	protein_codir	hypothetical protein
TcG_05477	219,0272173	0,076006289	0,12840595	0,59192185	0,55390292	0,74892277	protein_codir	ESAG-like protein
TcG_05478	476,3476773	0,317002877	0,08653126	3,66344941	0,00024884	0,00239458	protein_codir	fatty acyl-CoA reductase
TcG_05479	69,75139392	0,028896365	0,24077338	0,12001478	0,90447144	0,95369549	protein_codir	hypothetical protein
TcG_05480	105,1388071	0,047204715	0,17590744	0,26834974	0,78843012	0,89487471	protein_codir	hypothetical protein
TcG_05481	372,9809883	-0,094244109	0,09532425	-0,9886688	0,32282521	0,55741474	protein_codir	mitochondrial ATP-dependent zinc metallopeptidase
TcG_05482	415,0737863	-0,028328772	0,09431711	-0,30035664	0,76390513	0,88092016	protein_codir	SET and MYND domain-containing protein
TcG_05483	341,7828291	0,161568477	0,10026306	1,61144565	0,10708262	0,28208285	protein_codir	putative uracil phosphoribosyltransferase
TcG_05484	112,9414128	-0,393915596	0,17716981	-2,22337876	0,02619027	0,10328131	protein_codir	hypothetical protein
TcG_05485	95,08845774	0,274357684	0,18515175	1,48179906	0,13839377	0,33221448	protein_codir	hypothetical protein
TcG_05486	401,867097	-0,130099954	0,09245229	-1,40721187	0,15936457	0,36189689	protein_codir	hypothetical protein
TcG_05487	203,5033198	0,053470438	0,12700496	0,42101064	0,67374731	0,82902391	protein_codir	hypothetical protein
TcG_05488	276,6814183	-0,14158749	0,1192578	-1,18723885	0,23513342	0,46296424	protein_codir	hypothetical protein
TcG_05489	256,4084815	0,063741129	0,11838475	0,5384235	0,59028471	0,77623888	protein_codir	hypothetical protein
TcG_05490	520,8741657	-0,072148188	0,08088178	-0,8920203	0,37238205	0,60357583	protein_codir	myosin IF
TcG_05491	28,34137297	0,916004924	0,3403335	2,69149206	0,00711332	0,03774554	protein_codir	amastin
TcG_05492	335,9437214	-0,077076794	0,10525431	-0,73229109	0,46399092	0,68152242	protein_codir	tuzin
TcG_05493	383,2909339	-0,120798077	0,09297431	-1,29926293	0,19385371	0,41081411	protein_codir	hypothetical protein
TcG_05494	390,5323579	-0,001114176	0,09208234	-0,01209978	0,99034601	0,99603651	protein_codir	beta-lactamase domain-containing protein
TcG_05495	340,0139401	-0,030141842	0,10355754	-0,2910637	0,7710026	0,88514032	protein_codir	protein kinase Wee90
TcG_05496	137,2595459	-0,010124094	0,15085927	-0,06710953	0,94649451	0,97469438	protein_codir	hypothetical protein
TcG_05497	192,6749511	0,067867415	0,13198633	0,51420035	0,60711192	0,78777005	protein_codir	hypothetical protein
TcG_05498	332,2184826	-0,162717139	0,1016874	-1,60017011	0,10956085	0,28616785	protein_codir	hypothetical protein
TcG_05499	36,53978159	0,340849479	0,30090911	1,13273234	0,25732667	0,48915288	protein_codir	hypothetical protein
TcG_05500	303,3420602	-0,094478097	0,10713476	-0,88186222	0,37785132	0,60853286	protein_codir	hypothetical protein
TcG_05501	54,77818461	-0,037844623	0,24098059	-0,15704428	0,87520995	0,93987711	protein_codir	hypothetical protein
TcG_05502	76,40399222	-0,07990209	0,20643254	-0,38706151	0,69871067	0,84414248	protein_codir	hypothetical protein
TcG_05503	248,1637003	0,513750215	0,12112562	4,24146597	2,2206E-05	0,00030376	protein_codir	hypothetical protein
TcG_05504	0,324841555	-0,458748506	2,94047964	-0,15601146	0,87602399	1	protein_codir	hypothetical protein
TcG_05505	1606,84883	0,312728433	0,05488279	5,69811444	1,2114E-08	3,7628E-07	protein_codir	DNA-directed RNA polymerase subunit
TcG_05506	551,2970527	0,256235362	0,08198768	3,12529091	0,00177629	0,01252153	protein_codir	hypothetical protein
TcG_05507	5,529698517	0,088759935	0,74458166	0,11920779	0,90511073	1	protein_codir	hypothetical protein
TcG_05508	309,1525947	-0,037669884	0,1037111	-0,36321942	0,71644098	0,85441337	protein_codir	hypothetical protein
TcG_05509	252,7974334	0,159435587	0,12193925	1,3075002	0,19104289	0,40668168	protein_codir	putative RNA-binding protein

TcG_05510	1462,957399	0,100358705	0,05786876	1,73424655	0,08287435	0,23675955	protein_codir	60S ribosomal protein L13a
TcG_05511	222,3058774	0,063839868	0,12257007	0,52084386	0,60247554	0,78500693	protein_codir	serine/threonine-protein phosphatase PP1 beta
TcG_05512	816,3396826	0,047261782	0,0666843	0,70873927	0,47848629	0,69303613	protein_codir	putative 25 kDa translation elongation factor 1-beta
TcG_05513	125,0421806	0,00049416	0,15712108	0,00314509	0,99749058	0,99910416	protein_codir	hypothetical protein
TcG_05514	126,7444669	0,253364133	0,16400257	1,54487902	0,12237553	0,30729148	protein_codir	hypothetical protein
TcG_05515	192,7548157	0,119041817	0,12844669	0,92677992	0,35404081	0,58825712	protein_codir	serine/threonine-protein phosphatase PP1
TcG_05516	0,966128114	0,043593554	1,756675	0,02481595	0,98020177	1	protein_codir	serine/threonine-protein phosphatase PP1
TcG_05517	107,7934251	0,239663167	0,16875972	1,42014436	0,15556566	0,35697835	protein_codir	hypothetical protein
TcG_05518	247,3568756	0,383450672	0,12387768	3,09539752	0,00196549	0,01354682	protein_codir	3-beta-hydroxy-Delta(5)-steroid dehydrogenase
TcG_05519	289,0318683	0,150280411	0,10959721	1,37120658	0,17031057	0,37691908	protein_codir	hypothetical protein
TcG_05520	297,4798429	0,177012518	0,10777951	1,64235783	0,10051587	0,26970284	protein_codir	U3 small nucleolar RNA-associated protein 19
TcG_05521	313,6390663	0,192747344	0,11300792	1,70560918	0,08808087	0,24655634	protein_codir	protein phosphatase 2C
TcG_05522	195,8818224	-0,198805621	0,12914389	-1,53941171	0,12370382	0,3094866	protein_codir	superoxide dismutase, Fe-Mn family
TcG_05523	381,2282842	-0,15393722	0,17518371	-0,87871882	0,37955375	0,61070376	protein_codir	hypothetical protein
TcG_05524	84,86147672	0,437328208	0,20556818	2,127412	0,03338587	0,12381839	protein_codir	transmembrane protein 216
TcG_05525	140,6207542	0,062206907	0,14830228	0,41946022	0,67487982	0,82970688	protein_codir	hypothetical protein
TcG_05526	70,62885205	-0,137149818	0,223235	-0,61437418	0,5389681	0,73820598	protein_codir	hypothetical protein
TcG_05527	1030,051493	-0,170701818	0,06282962	-2,71690025	0,00658965	0,03564316	protein_codir	Flagellar attachment zone protein 1
TcG_05528	1070,023987	0,406308892	0,06069048	6,69477158	2,1601E-11	1,2513E-09	protein_codir	transketolase 1
TcG_05529	1277,260492	0,751408511	0,05855911	12,8316247	1,0905E-37	9,0249E-35	protein_codir	60S ribosomal protein L26
TcG_05530	63,78911659	0,761351886	0,25652593	2,96793349	0,00299809	0,01901253		
TcG_05531	156,6162033	0,674156742	0,14708329	4,58350343	4,5725E-06	7,7339E-05	protein_codir	conserved eukaryotic protein
TcG_05532	715,2546104	0,341663935	0,07131473	4,79093067	1,6601E-06	3,1687E-05	protein_codir	putative tubulin folding cofactor D
TcG_05533	436,135156	0,562559346	0,08870988	6,34156338	2,2745E-10	1,0294E-08	protein_codir	class III phosphatidylinositol 3-phosphate kinase
TcG_05534	439,9579181	0,645351164	0,09926016	6,50161303	7,9463E-11	3,9684E-09	protein_codir	hypothetical protein
TcG_05535	232,908687	0,299310246	0,11894363	2,51640419	0,01185591	0,05645812	protein_codir	hypothetical protein
TcG_05536	359,4664691	-0,143319666	0,0978126	-1,46524749	0,14285341	0,33915975	protein_codir	hypothetical protein
TcG_05537	422,7383293	0,320072827	0,09700353	3,29959989	0,00096823	0,00757965	protein_codir	hypothetical protein
TcG_05538	395,3570386	0,368192002	0,0940228	3,91598616	9,0035E-05	0,0010207	protein_codir	hypothetical protein
TcG_05539	260,9658648	0,555114099	0,11447668	4,84914563	1,2399E-06	2,4527E-05	protein_codir	hypothetical protein
TcG_05540	243,7586288	0,499108653	0,12147187	4,10884145	3,9765E-05	0,00050297	protein_codir	peptidyl-prolyl isomerase E (cyclophilin E)
TcG_05541	205,0792045	0,397147792	0,12874699	3,08471513	0,00203747	0,01396814	protein_codir	hypothetical protein
TcG_05542	359,2867483	0,400894604	0,10038867	3,99342473	6,5126E-05	0,00077389	protein_codir	hypothetical protein
TcG_05543	146,3568398	0,976671717	0,15308882	6,37977181	1,7735E-10	8,319E-09	protein_codir	DnaJ like protein subfamily C member 19
TcG_05544	200,4680366	0,544359699	0,13108715	4,15265483	3,2864E-05	0,00042639	protein_codir	alkyl and aryl transferase
TcG_05545	286,405006	0,355491398	0,1093573	3,25073306	0,00115108	0,00876625	protein_codir	hypothetical protein
TcG_05546	277,8537647	0,359657636	0,10989753	3,27266364	0,00106539	0,00821813	protein_codir	hypothetical protein
TcG_05547	15,57756055	-0,299389652	0,47020242	-0,63672504	0,52430396	1	protein_codir	cyclin-dependent protein kinase
TcG_05548	108,18762	0,695685486	0,17333224	4,01359548	5,9801E-05	0,00071873	protein_codir	putative cyclin 11
TcG_05549	12,61204464	0,181730368	0,5182412	0,35066754	0,72583778	1		
TcG_05550	590,5452162	0,516182453	0,07888479	6,54349785	6,0096E-11	3,1649E-09	protein_codir	hypothetical protein
TcG_05551	513,0959793	0,18531529	0,08370998	2,21377763	0,02684408	0,10532188	protein_codir	hypothetical protein
TcG_05552	639,1233433	0,419750333	0,0799496	5,25018649	1,5195E-07	3,8105E-06	protein_codir	hypothetical protein
TcG_05553	170,5536692	0,333222435	0,13684397	2,43505382	0,01488958	0,06738696	protein_codir	glycosyltransferase
TcG_05554	403,3166386	0,367035905	0,09048438	4,05634545	4,9847E-05	0,00061373	protein_codir	hypothetical protein
TcG_05555	195,086495	0,453118818	0,1316414	3,44206922	0,00057728	0,00489039	protein_codir	hypothetical protein
TcG_05556	513,0993641	0,320804583	0,08445902	3,79834622	0,00014566	0,00153564	protein_codir	lysophospholipase
TcG_05557	256,0187707	0,083887348	0,11496622	0,72966955	0,4655922	0,6821385	protein_codir	hypothetical protein
TcG_05558	318,6947514	0,399580124	0,10118793	3,94889103	7,8514E-05	0,00090604	protein_codir	putative short chain dehydrogenase/reductase
TcG_05559	255,2416479	0,323802577	0,11152243	2,90347487	0,00369047	0,0225575	protein_codir	putative short chain dehydrogenase/reductase
TcG_05560	114,5372679	-0,278937125	0,18346645	-1,52037129	0,12841769	0,31738294	protein_codir	hypothetical protein
TcG_05561	581,6787602	0,375556472	0,07901615	4,7529075	2,0051E-06	3,741E-05	protein_codir	putative RNA-binding protein

TcG_05562	746,403696	0,323430113	0,07267874	4,45013375	8,5817E-06	0,00013473	protein_codir	putative fatty acid transporter protein-like
TcG_05563	292,069109	-0,181190639	0,10681486	-1,69630557	0,08982803	0,24975399	protein_codir	trans-sialidase
TcG_05564	112,0330848	0,453725939	0,16788275	2,70263581	0,00687921	0,03684791	protein_codir	hypothetical protein
TcG_05565	222,2330746	0,294736531	0,12387996	2,37921068	0,01734976	0,07537094	protein_codir	hypothetical protein
TcG_05566	173,063619	-0,145952635	0,13484085	-1,08240667	0,27907187	0,51208848	protein_codir	hypothetical protein
TcG_05567	445,7451782	-0,504095108	0,09162415	-5,50177118	3,7599E-08	1,0625E-06	protein_codir	histone H2A
TcG_05568	227,8555335	-0,335382471	0,11788658	-2,84495895	0,00444171	0,02629622	protein_codir	putative NADH-ubiquinone oxidoreductase, mitochondrial
TcG_05569	312,4167684	-0,185622482	0,10395635	-1,785581	0,07416713	0,21993865	protein_codir	hypothetical protein
TcG_05570	372,4226337	-0,262231733	0,09440051	-2,7778636	0,00547176	0,03071502	protein_codir	hypothetical protein
TcG_05571	538,5621286	-0,104025019	0,08116509	-1,28164737	0,19996637	0,41887731	protein_codir	putative protein kinase
TcG_05572	207,2994084	0,016396605	0,12354697	0,13271555	0,89441834	0,94882077	protein_codir	protein kinase
TcG_05573	497,1845308	-0,244371822	0,08419233	-2,90254267	0,00370147	0,02258304	protein_codir	putative C-terminal motor kinesin
TcG_05574	337,8430058	-0,092358584	0,1015822	-0,90920045	0,36324432	0,59526856	protein_codir	DUF3508 domain containing protein
TcG_05575	353,2928915	-0,050654501	0,09858488	-0,5138161	0,60738057	0,78794214	protein_codir	putative peptidase t
TcG_05576	376,436819	-0,137808996	0,09603133	-1,435042	0,15127513	0,3515895	protein_codir	hypothetical protein
TcG_05577	258,7039483	0,068443704	0,11907134	0,5748126	0,56541804	0,75798662	protein_codir	hypothetical protein
TcG_05578	436,976217	0,431779901	0,09079395	4,75560213	1,9786E-06	3,7093E-05	protein_codir	hypothetical protein
TcG_05579	266,4586658	0,126448558	0,11876692	1,06467824	0,28702156	0,52024903	protein_codir	cell wall surface anchor family protein
TcG_05580	160,731478	-0,249090187	0,13901915	-1,79176892	0,07316999	0,21781796	protein_codir	ADP-ribosylation factor
TcG_05581	391,1611906	0,026981679	0,09203843	0,29315666	0,76940241	0,88436428	protein_codir	putative protein kinase
TcG_05582	170,4441548	-0,049464293	0,13570396	-0,36450146	0,71548358	0,85396046	protein_codir	hypothetical protein
TcG_05583	127,7099526	0,147635929	0,15946908	0,92579661	0,35455169	0,58843087	protein_codir	chaperone protein DNAJ
TcG_05584	176,5799388	0,094158706	0,14857779	0,63373339	0,52625483	0,72897997	protein_codir	hypothetical protein
TcG_05585	272,7003923	-0,149281563	0,10757263	-1,38772817	0,16521984	0,3707155	protein_codir	hypothetical protein
TcG_05586	92,84331386	-0,166045439	0,18030277	-0,92092564	0,35708925	0,59086491	protein_codir	hypothetical protein
TcG_05587	83,39495528	0,094886793	0,20169361	0,47045017	0,63803343	0,80751094	protein_codir	hypothetical protein
TcG_05588	217,4448882	-0,038601295	0,12601659	-0,30631915	0,75936166	0,8784787	protein_codir	hypothetical protein
TcG_05589	80,97404829	0,122505358	0,19708916	0,62157328	0,53422249	0,73457178	protein_codir	hypothetical protein
TcG_05590	83,93040586	0,227301124	0,19833262	1,14606022	0,25177023	0,48186194	protein_codir	hypothetical protein
TcG_05591	1273,915454	-0,031816747	0,05642618	-0,56386501	0,572846	0,76401448	protein_codir	hypothetical protein
TcG_05592	61,25812222	0,276342416	0,22560819	1,22487761	0,22062134	0,44570511	protein_codir	hypothetical protein
TcG_05593	26,05465704	-0,484142697	0,33498024	-1,44528735	0,14837719	0,3472224	protein_codir	putative retrotransposon hot spot (RHS) protein
TcG_05594	55,84553409	0,260438016	0,23644523	1,1014729	0,27069089	0,5038112	protein_codir	putative trans-sialidase
TcG_05595	14,17151195	0,068747158	0,46687841	0,14724853	0,88293586	1		
TcG_05596	46,08591824	0,164453152	0,25615888	0,64199668	0,52087534	0,72534395		
TcG_05597	49,1621871	0,177699713	0,25502221	0,69680092	0,48592739	0,69827461	protein_codir	hypothetical protein
TcG_05598	1350,079854	-0,026025814	0,0566241	-0,45962432	0,6457859	0,81201944	protein_codir	putative retrotransposon hot spot (RHS) protein
TcG_05599	75,5891652	0,452188417	0,21539031	2,09939069	0,03578247	0,13075061	protein_codir	hypothetical protein
TcG_05600	91,4379741	0,214038975	0,18404363	1,1629795	0,24483782	0,47301834	protein_codir	solanesyl-diphosphate synthase
TcG_05601	184,8911208	0,223912958	0,14390478	1,55598004	0,11971284	0,30290303	protein_codir	putative rab1 small GTP-binding protein
TcG_05602	439,168834	0,215854517	0,09074396	2,37872057	0,01737284	0,07541466	protein_codir	putative retrotransposon hot spot (RHS) protein
TcG_05603	123,1667774	-0,034054548	0,16153173	-0,21082265	0,83302566	0,91908998	protein_codir	hypothetical protein
TcG_05604	2139,610214	-0,143747862	0,05117374	-2,80901592	0,00496932	0,02867257	protein_codir	hypothetical protein
TcG_05605	617,4667025	-0,077346104	0,08436367	-0,91681763	0,35923821	0,5926302	protein_codir	putative tyrosyl-tRNA synthetase
TcG_05606	493,731212	0,254962408	0,09369637	2,72115566	0,00650541	0,03530291	protein_codir	hypothetical protein
TcG_05607	292,5416867	-0,007293419	0,10850955	-0,06721454	0,94641091	0,97469438	protein_codir	putative glycogen synthase kinase-3 alpha
TcG_05608	363,4829663	0,20674608	0,10471733	1,9743253	0,04834477	0,16157024	protein_codir	putative pyrroline-5-carboxylate reductase
TcG_05609	382,6965613	0,111896227	0,09171017	1,22010705	0,2224243	0,44840924	protein_codir	hypothetical protein
TcG_05610	245,9650403	0,381908418	0,11939977	3,19856906	0,00138111	0,01015329	protein_codir	hypothetical protein
TcG_05611	582,1190017	-0,009299622	0,07943463	-0,11707264	0,90680249	0,95476315	protein_codir	GTPase activating protein
TcG_05612	390,0688965	0,047480545	0,09526168	0,49842229	0,61818643	0,793034	protein_codir	peptidyl-prolyl cis-trans isomerase
TcG_05613	483,7437922	0,278720683	0,08350886	3,33761825	0,000845	0,00679399	protein_codir	hypothetical protein

TcG_05614	740,6157014	0,105773319	0,07159947	1,47729192	0,1395974	0,33409947	protein_codir putative kinesin
TcG_05615	681,5367305	-0,075679511	0,07475424	-1,01237746	0,31135761	0,54500518	protein_codir putative proteasome regulatory ATPase subunit 1
TcG_05616	601,1704651	-0,164085398	0,07787146	-2,10713148	0,03510618	0,12887841	protein_codir endoplasmatic reticulum retrieval protein
TcG_05617	276,7491792	0,504533654	0,10965937	4,60091705	4,2063E-06	7,22E-05	protein_codir SAM-binding protein
TcG_05618	281,3947431	0,269273771	0,10783783	2,49702524	0,012524	0,05898501	protein_codir N-acetyltransferase
TcG_05619	551,1189641	0,218724321	0,08329353	2,62594599	0,00864085	0,04408319	protein_codir ubiquitin-conjugating enzyme e2
TcG_05620	791,554154	-0,135300115	0,06954226	-1,94558131	0,05170505	0,16910378	protein_codir putative proteasome regulatory ATPase subunit 5
TcG_05621	523,6858786	-0,19901366	0,08064019	-2,46792159	0,01359001	0,0630572	protein_codir hypothetical protein
TcG_05622	145,2820306	-0,054913452	0,1528431	-0,35927988	0,71938572	0,85590939	protein_codir hypothetical protein
TcG_05623	398,0461797	0,144466794	0,10008896	1,44338394	0,14891234	0,34812317	protein_codir hypothetical protein
TcG_05624	850,5821767	-0,127110601	0,0670596	-1,89548704	0,05802791	0,18404362	protein_codir mitochondrial guide RNA binding complex subunit 1
TcG_05625	689,9626205	-0,070966195	0,07389916	-0,96031122	0,3368986	0,57141081	protein_codir glucoamylase-like protein
TcG_05626	501,0584216	0,115145895	0,08555908	1,34580566	0,1783652	0,3893254	protein_codir prefoldin
TcG_05627	92,39372845	0,177763528	0,18483636	0,96173462	0,33618293	0,57078138	protein_codir hypothetical protein
TcG_05628	171,7533971	0,182700891	0,13668897	1,33661767	0,18134747	0,39294274	protein_codir putative 3'a2rel-related protein
TcG_05629	407,7385053	0,098768651	0,09310412	1,06084086	0,28876223	0,52204032	protein_codir hypothetical protein
TcG_05630	232,7028065	0,112279198	0,11608825	0,96718826	0,33344993	0,56788929	protein_codir hypothetical protein
TcG_05631	615,1249715	0,288123355	0,07532404	3,82511828	0,00013071	0,00139447	protein_codir hypothetical protein
TcG_05632	546,1962479	0,033051297	0,08433459	0,39190677	0,6951271	0,84226548	protein_codir hypothetical protein
TcG_05633	315,0472616	-0,003695245	0,11910043	-0,0310263	0,97524857	0,98925144	protein_codir hypothetical protein
TcG_05634	1265,436	-0,301575233	0,05947681	-5,07046744	3,9684E-07	8,9104E-06	protein_codir hypothetical protein
TcG_05635	4,340198193	0,183728677	0,84453768	0,21754941	0,8277802	1	protein_codir hypothetical protein
TcG_05636	42,76812008	0,590883347	0,27649931	2,13701562	0,03259672	0,12178833	protein_codir hypothetical protein
TcG_05637	42,973248	0,219804668	0,27770465	0,79150517	0,42864926	0,65390759	protein_codir hypothetical protein
TcG_05638	116,5926092	0,251835539	0,16691203	1,50879199	0,13135195	0,32214765	protein_codir hypothetical protein
TcG_05639	110,6875328	0,303908868	0,17047133	1,7827565	0,07462595	0,22050912	protein_codir hypothetical protein
TcG_05640	75,22856543	-0,075434411	0,20498727	-0,36799559	0,71287652	0,85253792	protein_codir hypothetical protein
TcG_05641	405,7787018	-0,281069177	0,09493015	-2,96079988	0,00306841	0,01941596	protein_codir calmodulin-like protein containing EF hand domain
TcG_05642	169,7835981	0,176549381	0,13672029	1,29131803	0,19659342	0,41473623	protein_codir hypothetical protein
TcG_05643	160,7411308	0,098893152	0,14300618	0,6915306	0,48923216	0,70038845	protein_codir hypothetical protein
TcG_05644	283,6217223	0,016426345	0,1222912	0,13432156	0,8931483	0,94840219	protein_codir hypothetical protein
TcG_05645	828,6752655	0,015305492	0,07058113	0,21684963	0,82832553	0,91617944	protein_codir putative kinesin
TcG_05646	331,5693488	0,097459809	0,09964191	0,97810062	0,32802456	0,56261918	protein_codir zinc finger family protein
TcG_05647	111,4375956	0,489702002	0,1791521	2,73344276	0,0062676	0,03417244	protein_codir hypothetical protein
TcG_05648	194,1712948	0,617944214	0,13584597	4,54885944	5,3937E-06	8,9402E-05	protein_codir hypothetical protein
TcG_05649	434,9150962	-0,333684679	0,08708875	-3,83154757	0,00012734	0,00136734	protein_codir hypothetical protein
TcG_05650	473,1263504	-0,104044584	0,08776451	-1,1854973	0,23582088	0,46344584	protein_codir polyphosphate synthetase Protein
TcG_05651	519,5728529	-0,081937075	0,08258448	-0,99216067	0,32111915	0,5560344	protein_codir heat shock protein
TcG_05652	526,9855346	-0,074102269	0,08370125	-0,88531852	0,37598486	0,60653866	protein_codir putative ubiqitin hydrolase
TcG_05653	57,71495958	-0,412930863	0,2295181	-1,79912113	0,07199952	0,21566351	
TcG_05654	390,1661849	0,106457337	0,09429344	1,12900051	0,25889762	0,49149399	protein_codir putative minchromosome maintenance (MCM) complex subunit
TcG_05655	241,7162521	0,271036114	0,13045327	2,07764912	0,03774169	0,13558921	protein_codir hypothetical protein
TcG_05656	138,0894043	0,257605774	0,15364345	1,67664662	0,0936116	0,25683499	protein_codir putative integral membrane transport protein, putative,drug resistance protein
TcG_05657	385,0206502	0,078068403	0,09353241	0,8346669	0,40390529	0,63332611	protein_codir hypothetical protein
TcG_05658	304,8098606	-0,030454587	0,10262649	-0,29675171	0,76665607	0,88286227	protein_codir hypothetical protein
TcG_05659	481,5166016	0,443483256	0,08630658	5,13846423	2,7699E-07	6,4057E-06	protein_codir putative kinesin
TcG_05660	97,1695682	-0,153569359	0,17717657	-0,86675884	0,38607416	0,61652005	protein_codir hypothetical protein
TcG_05661	436,9071786	-0,093803575	0,0948925	-0,98852469	0,32289575	0,55745346	protein_codir putative protein kinase
TcG_05662	450,9430211	-0,275885671	0,08650975	-3,18907032	0,00142731	0,01043992	protein_codir hypothetical protein
TcG_05663	78,57575075	0,189959223	0,19690363	0,96473193	0,3346791	0,56906253	protein_codir hypothetical protein
TcG_05664	151,810161	-0,031400017	0,14317436	-0,21931314	0,82640613	0,91519226	protein_codir hypothetical protein
TcG_05665	389,4439739	0,103003837	0,09274975	1,11055646	0,26675931	0,49939561	protein_codir putative DNA photolyase

TcG_05666	83,37050396	0,003744324	0,19339341	0,01936118	0,98455298	0,99412361	protein_codir	ankyrin repeat protein
TcG_05667	210,7838611	0,003188929	0,12696619	0,02511637	0,97996215	0,9919484	protein_codir	hypothetical protein
TcG_05668	295,5598812	-0,00565889	0,10521875	-0,05378214	0,95710874	0,9801186	protein_codir	hypothetical protein
TcG_05669	731,7851279	-0,043121565	0,07030412	-0,61335761	0,53963991	0,73877679	protein_codir	hypothetical protein
TcG_05670	380,2901008	-0,23229218	0,10383538	-2,23711985	0,02527851	0,1005413	protein_codir	hypothetical protein
TcG_05671	151,6920898	0,052889295	0,14192528	0,37265591	0,70940455	0,85005287	protein_codir	protein kinase
TcG_05672	235,3988884	-0,236448429	0,11532647	-2,05025286	0,04033976	0,14202291	protein_codir	putative protein kinase
TcG_05673	219,3869266	-0,010484067	0,12756054	-0,08218895	0,93449646	0,96903929	protein_codir	hypothetical protein
TcG_05674	351,9959364	-0,170624892	0,09887421	-1,72567633	0,08440566	0,23933528	protein_codir	putative dolichyl-P-Man:GDP-Man5GlcNAc2-PP-dolichyl alpha-1,3-mannosyltransferase
TcG_05675	320,9031615	-0,191469338	0,10610593	-1,80451115	0,07115121	0,21392986	protein_codir	putative serine/threonine protein phosphatase
TcG_05676	266,966916	0,027699732	0,11038136	0,25094574	0,80185606	0,90177851	protein_codir	Poly polymerase and DNA-Ligase Zn-finger region family protein
TcG_05677	455,0770834	-0,109527489	0,09052131	-1,20996364	0,22629285	0,452742	protein_codir	putative Protein kinase domain
TcG_05678	295,2213936	0,013659048	0,10382322	0,13156062	0,89533183	0,9489813	protein_codir	hypothetical protein
TcG_05679	163,103109	-0,166129069	0,14148192	-1,17420706	0,2403121	0,46899211	protein_codir	stress-induced-phosphoprotein 1
TcG_05680	498,4471836	0,049978853	0,0924121	0,54082582	0,58862764	0,77564856	protein_codir	hypothetical protein
TcG_05681	305,9500436	0,023051794	0,10219839	0,22555928	0,82154422	0,91155059	protein_codir	transferase
TcG_05682	362,3533179	-0,130098237	0,09780433	-1,33018895	0,18345602	0,39588778	protein_codir	chaperone protein DnaJ
TcG_05683	234,3119575	0,401468422	0,1241941	3,23258845	0,00122674	0,00919937	protein_codir	hypothetical protein
TcG_05684	305,4106252	0,034489768	0,10876882	0,31709242	0,75117347	0,874683	protein_codir	hypothetical protein
TcG_05685	205,7990708	0,152557429	0,13114464	1,16327609	0,2447175	0,47286474	protein_codir	hypothetical protein
TcG_05686	1303,99673	0,04778755	0,05758316	0,82988756	0,40660236	0,63483385	protein_codir	putative DEAD box RNA helicase
TcG_05687	254,8461471	0,050274283	0,11041672	0,45531405	0,64888332	0,81328019	protein_codir	HAD family hydrolase
TcG_05688	611,5603446	0,149887926	0,07774638	1,92790882	0,05386647	0,17432874	protein_codir	COP-coated vesicle membrane protein
TcG_05689	279,0306035	0,172263489	0,10832204	1,59029036	0,11176937	0,28940906	protein_codir	putative G10 protein
TcG_05690	204,8300728	-0,118103681	0,12970383	-0,91056433	0,36252497	0,59502869	protein_codir	dynein light chain
TcG_05691	557,5323888	0,120791099	0,07811101	1,54640289	0,1220073	0,30663266	protein_codir	tRNA (cytosine34-C5)-methyltransferase
TcG_05692	361,1289796	-0,181258535	0,09538061	-1,90037096	0,05738445	0,18250241	protein_codir	putative tyrosine specific protein phosphatase
TcG_05693	241,3329956	0,319347718	0,116519	2,74073513	0,00613019	0,0335654	protein_codir	putative formin
TcG_05694	180,8098067	0,379912261	0,1340656	2,83377875	0,00460012	0,02697214	protein_codir	hypothetical protein
TcG_05695	1219,818337	-0,228797696	0,06394326	-3,5781363	0,00034605	0,00316196	protein_codir	hypothetical protein
TcG_05696	163,2931713	-0,056436931	0,14637015	-0,38557678	0,69981013	0,84436727	protein_codir	hypothetical protein
TcG_05697	365,0395356	0,034953352	0,09398792	0,37189195	0,7099733	0,8504252	protein_codir	putative vacuolar protein sorting-associated protein
TcG_05698	981,1116524	0,142833972	0,06998875	2,04081327	0,04126939	0,14449899	protein_codir	hypothetical protein
TcG_05699	588,153305	-0,152978741	0,08377909	-1,82597765	0,06785363	0,20764716	protein_codir	putative developmentally regulated GTP-binding protein 1
TcG_05700	169,495611	-0,059380291	0,13987694	-0,42451808	0,67118802	0,82771521	protein_codir	putative guanylate kinase
TcG_05701	243,9306234	-0,084424006	0,1131717	-0,74598161	0,45567852	0,67564517	protein_codir	hypothetical protein
TcG_05702	371,6116909	-0,006965394	0,09628872	-0,07233863	0,94233242	0,97319113	protein_codir	Bardet-Biedl syndrome 4 protein
TcG_05703	484,0614506	-0,416060408	0,08463176	-4,91612619	8,8274E-07	1,8263E-05	protein_codir	hypothetical protein
TcG_05704	331,4393411	-0,011326649	0,10041094	-0,11280294	0,91018679	0,95641431	protein_codir	putative kinteoplast poly(A) polymerase complex 1 subunit
TcG_05705	772,9988852	-0,031975209	0,06794763	-0,47058611	0,63793633	0,80751094	protein_codir	glucokinase
TcG_05706	135,9217527	0,365799327	0,15367759	2,38030367	0,01729838	0,07520412	protein_codir	putative endonuclease V
TcG_05707	261,1361079	-0,31371101	0,11376188	-2,75761089	0,00582255	0,03227752	protein_codir	putative short-chain dehydrogenase
TcG_05708	185,5372237	0,245654379	0,13027834	1,88561181	0,05934729	0,18756073	protein_codir	putative tyrosine aminotransferase, putative,L-tyrosine:2-oxoglutarate aminotransferase
TcG_05709	191,7794475	-0,034856279	0,13259759	-0,26287264	0,79264875	0,89624823	protein_codir	putative glyceraldehyde 3-phosphate dehydrogenase, cytosolic
TcG_05710	174,4601606	0,191946952	0,13591369	1,41227092	0,15787021	0,35991425	protein_codir	hypothetical protein
TcG_05711	194,5019828	0,040968474	0,13141242	0,31175496	0,75522676	0,87649576	protein_codir	hypothetical protein
TcG_05712	582,5223064	-0,02339311	0,07648078	-0,30586912	0,7597043	0,8786119	protein_codir	putative translation initiation factor
TcG_05713	488,8348494	0,017612688	0,08467358	0,20800688	0,8352236	0,92011986	protein_codir	hypothetical protein
TcG_05714	230,0620852	-0,11616169	0,1188324	-0,97752541	0,3283091	0,56286962	protein_codir	hypothetical protein
TcG_05715	162,9181824	-0,393588238	0,13956754	-2,8200557	0,00480153	0,02789897	protein_codir	putative protein kinase
TcG_05716	407,6487431	-0,174703226	0,10044154	-1,73935239	0,0819728	0,2349617	protein_codir	putative protein kinase
TcG_05717	810,1999808	-0,024570814	0,07224245	-0,34011602	0,73376916	0,8632666	protein_codir	protein kinase

TcG_05718	860,9044675	-0,10877301	0,06915527	-1,57288091	0,1157464	0,29616558	protein_codir putative ribose-phosphate pyrophosphokinase
TcG_05719	96,96423558	0,339203433	0,17691144	1,91736288	0,05519185	0,17742862	protein_codir hypothetical protein
TcG_05720	174,5912063	-0,113179451	0,14036207	-0,8063393	0,42004724	0,64656135	protein_codir hypothetical protein
TcG_05721	498,483815	0,056698627	0,08914448	0,63603074	0,52475639	0,7282975	protein_codir hypothetical protein
TcG_05722	236,8095321	0,262661543	0,11793089	2,22724969	0,02593059	0,10250148	protein_codir small ubiquitin protein
TcG_05723	228,8024097	-0,37852872	0,12339027	-3,06773559	0,00215687	0,01461377	protein_codir signal peptidase type I
TcG_05724	1451,689727	-0,033178241	0,06150534	-0,53943674	0,58958553	0,7758162	protein_codir hypothetical protein
TcG_05725	492,3230866	-0,353552685	0,08398016	-4,20995463	2,5542E-05	0,00034371	protein_codir hypothetical protein
TcG_05726	1052,496839	-0,026976877	0,07197765	-0,37479517	0,7078128	0,84937537	protein_codir hypothetical protein
TcG_05727	696,2468711	-0,290483138	0,07695492	-3,77471798	0,00016019	0,00166602	protein_codir hypothetical protein
TcG_05728	408,0961803	-0,003021321	0,09485758	-0,03185112	0,97459078	0,98893052	protein_codir hypothetical protein
TcG_05729	847,4722791	-0,052100884	0,06588173	-0,79082445	0,42904644	0,65398767	protein_codir hypothetical protein
TcG_05730	176,7840493	-0,047461522	0,13916053	-0,34105592	0,73306149	0,86278448	protein_codir hypothetical protein
TcG_05731	195,5802131	0,119464708	0,12772705	0,93531249	0,34962728	0,58410694	protein_codir hypothetical protein
TcG_05732	239,5213934	0,119166539	0,12034931	0,99017221	0,32208995	0,55680904	protein_codir hypothetical protein
TcG_05733	33,13644461	-0,359442796	0,33237712	-1,08143062	0,27950561	0,51247855	protein_codir putative protein kinase
TcG_05734	245,0197066	0,164733831	0,11649984	1,41402623	0,1573542	0,35923266	protein_codir putative protein kinase
TcG_05735	75,20305783	-0,042341204	0,20135224	-0,21028424	0,83344583	0,91912273	protein_codir protein kinase
TcG_05736	0,233855473	1,175456718	3,50839889	0,33504078	0,73759432	1	
TcG_05737	81,78625801	0,197141459	0,19486195	1,01169805	0,31168245	0,54549137	protein_codir hypothetical protein
TcG_05738	0					1	protein_codir ribosomal protein L27
TcG_05739	8,915847656	0,729264326	0,66935058	1,08951027	0,27592893	1	protein_codir ribosomal protein L27
TcG_05740	953,3746904	0,001891106	0,06613177	0,02859603	0,97718678	0,99017719	protein_codir hypothetical protein
TcG_05741	80,56891192	0,461084028	0,2053952	2,24486271	0,02477695	0,09905649	protein_codir ribosomal protein L27
TcG_05742	92,4310111	0,112666864	0,1804731	0,62428621	0,53243964	0,73316445	protein_codir ribosomal protein L27
TcG_05743	372,7680369	-0,169720384	0,09542472	-1,77857882	0,07530884	0,22154994	protein_codir hypothetical protein
TcG_05744	109,7065185	0,092568841	0,16768601	0,55203676	0,58092317	0,77044137	protein_codir hypothetical protein
TcG_05745	495,7276575	-0,106648884	0,08360696	-1,27559808	0,20209763	0,42219675	protein_codir trichohyalin
TcG_05746	147,1958921	-0,014919226	0,15551904	-0,09593183	0,92357471	0,96265225	protein_codir putative amino acid transporter
TcG_05747	31,88876637	0,357931466	0,30662834	1,1673137	0,24308371	0,47135864	protein_codir hypothetical protein
TcG_05748	132,4108867	0,123626386	0,15942153	0,77546858	0,43806282	0,66146173	protein_codir hypothetical protein
TcG_05749	617,544975	0,011153181	0,07618987	0,14638668	0,88361614	0,94348809	protein_codir methyltransferase
TcG_05750	369,6048072	0,279419745	0,10219824	2,73409555	0,00625519	0,03412082	protein_codir hypothetical protein
TcG_05751	288,3200782	-0,203632099	0,10732801	-1,89728763	0,05778999	0,18338943	protein_codir superoxide dismutase
TcG_05752	231,9399065	-0,198467118	0,1181988	-1,67909583	0,09313337	0,25587935	protein_codir hypothetical protein
TcG_05753	605,0811773	-0,170248844	0,07772923	-2,19028075	0,02850388	0,10991818	protein_codir dentin sialophosphoprotein precursor
TcG_05754	297,8339086	-0,034569352	0,10347197	-0,33409389	0,73830871	0,86642273	protein_codir hypothetical protein
TcG_05755	133,2595868	-0,245598609	0,1608637	-1,52674971	0,12682326	0,31497841	protein_codir hypothetical protein
TcG_05756	373,480174	0,363581323	0,09920522	3,66494136	0,0002474	0,00238264	protein_codir hypothetical protein
TcG_05757	1025,878947	-0,146536208	0,06174294	-2,3733274	0,01762863	0,07632393	protein_codir hypothetical protein
TcG_05758	313,6309422	-0,198989473	0,10139928	-1,96243473	0,04971189	0,16498481	protein_codir putative GPI transamidase component Tta1
TcG_05759	921,4478423	0,009559789	0,06428233	0,14871565	0,88177801	0,94218407	protein_codir adenosine monophosphate deaminase-like protein
TcG_05760	208,2081648	-0,033238908	0,13533107	-0,24561181	0,80598275	0,90423384	protein_codir hypothetical protein
TcG_05761	335,6127287	0,015251625	0,0994883	0,15330069	0,87816115	0,94142358	protein_codir hypothetical protein
TcG_05762	436,1996308	-0,1359274	0,09080864	-1,49685538	0,13443089	0,32602161	protein_codir hypothetical protein
TcG_05763	211,3190248	-0,190025861	0,1267134	-1,49965088	0,13370486	0,32523714	protein_codir hypothetical protein
TcG_05764	537,3374451	-0,080436631	0,08607386	-0,93450707	0,35004239	0,58454758	protein_codir hypothetical protein
TcG_05765	242,4992326	-0,11328724	0,1145737	-0,98877175	0,32277483	0,55741474	protein_codir hypothetical protein
TcG_05766	17,81998248	0,663512206	0,41942407	1,58196023	0,11365865	0,29269116	
TcG_05767	32,66402493	0,28425288	0,31039736	0,91577094	0,35978705	0,5926302	protein_codir syntaxin binding protein
TcG_05768	29,42741462	-0,239307598	0,34265844	-0,69838524	0,4849363	0,69795871	protein_codir hypothetical protein
TcG_05769	42,59352334	0,330790259	0,27262807	1,21333894	0,22500026	0,4515595	protein_codir putative syntaxin binding protein

TcG_05770	126,4989653	-0,063607822	0,16118621	-0,39462322	0,69312098	0,8410627	protein_codir	hypothetical protein
TcG_05771	5,085003381	-0,305218886	0,79104807	-0,38584114	0,69961433	1		
TcG_05772	444,4980683	-0,084795878	0,08594439	-0,98663662	0,32382081	0,55813567	protein_codir	hypothetical protein
TcG_05773	263,8859323	-0,156117865	0,11162786	-1,39855646	0,16194603	0,36568051	protein_codir	hypothetical protein
TcG_05774	268,1449619	-0,170021303	0,1100711	-1,54464976	0,122431	0,30729757	protein_codir	hypothetical protein
TcG_05775	347,7345128	-0,108543618	0,09756598	-1,11251498	0,26591679	0,49866613	protein_codir	hypothetical protein
TcG_05776	315,1890832	-0,003895458	0,10492657	-0,03712556	0,97038489	0,98717002	protein_codir	hypothetical protein
TcG_05777	193,3129031	0,17251819	0,13104766	1,31645384	0,18802176	0,40240797	protein_codir	ClpB chaperone
TcG_05778	261,9717111	0,210284538	0,11897855	1,7674156	0,07715865	0,22506549		
TcG_05779	543,2918399	0,087006871	0,0866197	1,00446977	0,31515224	0,54990268	protein_codir	variant-surface-glycoprotein phospholipase C
TcG_05780	324,3729322	0,078263948	0,10155836	0,77063025	0,44092613	0,66354606	protein_codir	variant-surface-glycoprotein phospholipase C
TcG_05781	1467,723532	-0,01202904	0,05478997	-0,21954821	0,82622303	0,91511289	protein_codir	hypothetical protein
TcG_05782	443,830968	0,012472221	0,08907837	0,14001402	0,88864891	0,94628587	protein_codir	putative heat shock 70 kDa protein, mitochondrial precursor
TcG_05783	208,5395753	0,10294884	0,12303707	0,83673027	0,4027442	0,6328518	protein_codir	putative DNA-J protein
TcG_05784	412,3536767	-0,142315212	0,09071327	-1,56884662	0,11668368	0,2979716	protein_codir	hypothetical protein
TcG_05785	335,1565022	-0,06774125	0,10148741	-0,66748424	0,50446287	0,71216119	protein_codir	hypothetical protein
TcG_05786	324,8104145	-0,189700498	0,10218015	-1,85652991	0,06337806	0,19765767	protein_codir	peptidyl-tRNA hydrolase, PTH2 family
TcG_05787	429,6200645	-0,232846474	0,08758814	-2,65842472	0,00785069	0,04088004	protein_codir	putative pre-mRNA cleavage complex II Clp1 protein
TcG_05788	235,186797	0,007554111	0,12412435	0,06085922	0,95147133	0,97719589	protein_codir	hypothetical protein
TcG_05789	477,4823965	-0,020603461	0,08623231	-0,23892973	0,81116008	0,90688996	protein_codir	hypothetical protein
TcG_05790	1840,005803	-0,090937558	0,05731889	-1,58651978	0,11262145	0,29073799	protein_codir	hypothetical protein
TcG_05791	166,0036402	0,289740455	0,14067525	2,05964054	0,03943292	0,13999295	protein_codir	hypothetical protein
TcG_05792	516,4042077	-0,150868077	0,0893771	-1,68799468	0,09141225	0,25252797	protein_codir	ADP-ribosylation factor-like protein
TcG_05793	501,4112037	-0,107879423	0,08251057	-1,30746182	0,19105591	0,40668168	protein_codir	putative zinc finger protein
TcG_05794	395,1739536	0,221320514	0,09595021	2,30661845	0,0210761	0,08780572	protein_codir	putative sphingosine 1-phosphate lyase
TcG_05795	528,7921044	-0,095547843	0,08081444	-1,18231145	0,23708215	0,46469865	protein_codir	putative zinc finger protein
TcG_05796	309,5974881	0,088415657	0,11401359	0,77548352	0,438054	0,66146173	protein_codir	hypothetical protein
TcG_05797	249,6651507	0,252740435	0,12014318	2,10366026	0,03540809	0,12974009	protein_codir	23S rRNA (cytosine1962-C5)-methyltransferase
TcG_05798	650,4798404	-0,066279896	0,0754998	-0,87788179	0,38000787	0,6107213	protein_codir	hypothetical protein
TcG_05799	426,9798928	-0,066945389	0,09053725	-0,73942367	0,45964977	0,67823513	protein_codir	ATP-dependent DEAD/H DNA helicase recQ family-like protein
TcG_05800	389,2437533	-0,196105114	0,1024901	-1,9134054	0,05569617	0,17850508	protein_codir	putative tubulin-tyrosine ligase-like protein
TcG_05801	810,1593886	-0,108784183	0,06632225	-1,64023674	0,10095595	0,27053938	protein_codir	hypothetical protein
TcG_05802	1197,122046	-0,287064837	0,06010474	-4,77607651	1,7875E-06	3,3674E-05	protein_codir	putative phospholipid-translocating P-type ATPase (flippase)
TcG_05803	583,6421285	-0,03149065	0,08307705	-0,37905353	0,70464811	0,84755533	protein_codir	zinc finger protein, conserved
TcG_05804	201,1195999	-0,311301772	0,12993181	-2,39588572	0,01658026	0,07301364	protein_codir	hypothetical protein
TcG_05805	598,6017393	-0,115282277	0,07770089	-1,48366742	0,13789717	0,3313307	protein_codir	hypothetical protein
TcG_05806	318,8300492	-0,074165194	0,10229236	-0,72503157	0,46843266	0,68500074	protein_codir	putative tRNA modification enzyme
TcG_05807	1629,829396	-0,02236317	0,0519345	-0,43060333	0,66675682	0,82543809	protein_codir	dnaJ-like protein subfamily C member 13 isoform X2
TcG_05808	146,1272273	0,306320614	0,14711244	2,08222094	0,03732229	0,13449956	protein_codir	zinc finger protein ZFP1
TcG_05809	116,5444225	0,444080241	0,17639147	2,51758343	0,0118163	0,05631577		
TcG_05810	11,77607546	0,598505395	0,52243424	1,14560905	0,25195695	1	protein_codir	hypothetical protein
TcG_05811	277,3212664	-0,154202529	0,1080604	-1,42700315	0,15357899	0,35431425	protein_codir	hypothetical protein
TcG_05812	251,5433289	0,076940965	0,11261649	0,68321226	0,49447273	0,70453824	protein_codir	putative protein farnesyltransferase
TcG_05813	175,3314122	-0,344435862	0,13453384	-2,56021721	0,01046068	0,05115973	protein_codir	tartrate-resistant acid phosphatase type 5 precursor
TcG_05814	346,6656451	-0,152667042	0,10267228	-1,4869353	0,1370319	0,33007767	protein_codir	hypothetical protein
TcG_05815	428,2953926	-0,149717797	0,09313374	-1,60755699	0,10793224	0,28317548	protein_codir	hypothetical protein
TcG_05816	232,0478091	-0,203465818	0,11829981	-1,71991669	0,08544759	0,24133259	protein_codir	hypothetical protein
TcG_05817	144,9826754	-0,292968171	0,1475483	-1,98557464	0,04708057	0,15847633	protein_codir	hypothetical protein
TcG_05818	84,91974743	-0,072031314	0,20230694	-0,35604965	0,72180338	0,85711748	protein_codir	putative cytochrome b
TcG_05819	495,5018342	0,050316653	0,09472322	0,53119658	0,59528256	0,78037381	protein_codir	hypothetical protein
TcG_05820	248,8358514	-0,310533941	0,12464144	-2,49141808	0,01272343	0,05968627	protein_codir	putative chaperone protein DNAj
TcG_05821	260,4653908	-0,05757132	0,11560788	-0,49798786	0,6184926	0,793034	protein_codir	hypothetical protein

TcG_05822	403,9858163	0,041946581	0,08994881	0,46633836	0,64097332	0,80940784	protein_codir	hypothetical protein
TcG_05823	216,9713581	-0,128214061	0,12107339	-1,05897801	0,28960979	0,52322143	protein_codir	hypothetical protein
TcG_05824	224,9652335	-0,180917978	0,11950759	-1,51386188	0,13006087	0,31986527	protein_codir	prefoldin
TcG_05825	228,6162632	-0,101172197	0,12510917	-0,80867131	0,41870424	0,64526567	protein_codir	putative GTP-binding protein
TcG_05826	399,60989	-0,212843231	0,10184806	-2,08981142	0,03663474	0,13305647	protein_codir	hypothetical protein
TcG_05827	7,579896876	0,689499823	0,65744583	1,04875534	0,29429074	1		
TcG_05828	157,4220333	0,035130653	0,14616876	0,2403431	0,81006429	0,90627702	protein_codir	hypothetical protein
TcG_05829	386,6135957	-0,219196318	0,09255821	-2,36819951	0,01787489	0,07704558	protein_codir	putative DNA ligase
TcG_05830	2007,445494	-0,023515303	0,04878896	-0,48197997	0,62982017	0,80168887	protein_codir	DNA ligase
TcG_05831	160,3913667	-0,179826906	0,14443667	-1,24502249	0,21312348	0,43664875	protein_codir	hypothetical protein
TcG_05832	211,6316465	-0,103200684	0,13356334	-0,77267223	0,43971638	0,66274932	protein_codir	hypothetical protein
TcG_05833	152,7543964	-0,148641393	0,14428628	-1,0301838	0,30292373	0,53561843	protein_codir	hypothetical protein
TcG_05834	381,6585655	-0,095993216	0,09182185	-1,04542895	0,29582476	0,5288421	protein_codir	hypothetical protein
TcG_05835	232,0015333	0,273229736	0,1233289	2,21545597	0,02672878	0,10501176	protein_codir	transcription factor jumonji, jmjC domain-containing protein
TcG_05836	3,037373347	-0,227161569	1,02611704	-0,22137978	0,82479673	1		
TcG_05837	393,0912468	-0,178158025	0,09256753	-1,92462765	0,05427596	0,17531119	protein_codir	hypothetical protein
TcG_05838	187,0199084	-0,029952524	0,13001013	-0,23038608	0,81779178	0,91026377	protein_codir	chaperone DnaJ protein
TcG_05839	210,0665348	-0,469916445	0,12781997	-3,67639297	0,00023656	0,0022935	protein_codir	putative small GTP-binding protein Rab11, putative,Rab11 GTPase
TcG_05840	57,47133773	0,230161763	0,23546518	0,97747686	0,32833313	0,56286962	protein_codir	putative mucin-like glycoprotein
TcG_05841	4,760328326	-0,237236425	0,79439028	-0,29863964	0,76521502	1		
TcG_05842	102,8606375	0,047094275	0,17400112	0,27065501	0,78665639	0,89379407	protein_codir	hypothetical protein
TcG_05843	123,2975655	0,102653376	0,15817344	0,6489925	0,51634322	0,72205516	protein_codir	hypothetical protein
TcG_05844	267,3435014	-0,139159152	0,11857932	-1,1735533	0,240574	0,4692408	protein_codir	prefoldin subunit 2
TcG_05845	199,122627	0,148219492	0,12937142	1,14568958	0,25192361	0,48196615	protein_codir	tRNA wybutosine-synthesizing protein 3
TcG_05846	421,0235911	0,005210225	0,09439774	0,05519438	0,95598361	0,97965912	protein_codir	hypothetical protein
TcG_05847	275,1208954	-0,057633463	0,10760499	-0,53560215	0,59223355	0,77769669	protein_codir	coiled-coil domain-containing protein 93
TcG_05848	221,0923307	0,110836499	0,12373075	0,8957878	0,37036611	0,6020008	protein_codir	putative Zinc finger DHHC domain containing transmembrane protein
TcG_05849	404,6710858	-0,057082508	0,09016446	-0,63309319	0,52667279	0,72926645	protein_codir	hypothetical protein
TcG_05850	162,9772197	0,060741542	0,1439641	0,42192146	0,67308234	0,82890021	protein_codir	hypothetical protein
TcG_05851	422,938342	0,093302211	0,09003999	1,03623082	0,30009447	0,53375722	protein_codir	hypothetical protein
TcG_05852	178,7526039	-0,075552043	0,13041193	-0,57933382	0,56236395	0,75577645	protein_codir	putative cyclin 1, putative,serine peptidase family S51, peptidase E
TcG_05853	414,8445218	0,117392223	0,09665823	1,2145083	0,22455368	0,45108253	protein_codir	putative ATP-dependent DNA helicase
TcG_05854	262,0358426	-0,16550331	0,11252171	-1,47085666	0,14132989	0,3366698	protein_codir	Derlin-2/3
TcG_05855	295,589147	0,224862368	0,10728722	2,09589139	0,03609183	0,131621	protein_codir	hypothetical protein
TcG_05856	104,6112991	-0,281020141	0,17117662	-1,64169702	0,10065281	0,26988277	protein_codir	phosphoric monoester hydrolase
TcG_05857	442,7351717	-0,286313128	0,09133385	-3,13479764	0,00171973	0,01216408	protein_codir	hypothetical protein
TcG_05858	478,0553813	0,008192547	0,0842252	0,09726955	0,92251234	0,96227026	protein_codir	putative leucine-rich repeat protein
TcG_05859	1138,995901	-0,236639452	0,07087507	-3,33882507	0,00084134	0,00676924	protein_codir	hypothetical protein
TcG_05860	136,7926043	0,127604732	0,15066429	0,84694743	0,39702443	0,62669278	protein_codir	hypothetical protein
TcG_05861	254,9606181	-0,014686817	0,1137983	-0,12906006	0,89731012	0,95018119	protein_codir	hypothetical protein
TcG_05862	226,2466573	0,188489561	0,12457653	1,51304236	0,1302689	0,32024092	protein_codir	acyltransferase
TcG_05863	545,2220581	0,159792622	0,08069636	1,98017128	0,04768429	0,15988335	protein_codir	hypothetical protein
TcG_05864	300,8666412	-0,036080652	0,1038231	-0,34752049	0,72820033	0,86000745	protein_codir	putative serine/threonine protein phosphatase
TcG_05865	717,3951558	-0,105168901	0,07151923	-1,47049815	0,14142689	0,3366698	protein_codir	hypothetical protein
TcG_05866	259,1596271	0,048969779	0,11229501	0,43608154	0,66277757	0,82347892	protein_codir	hypothetical protein
TcG_05867	146,4825006	-0,030703551	0,15324083	-0,20036143	0,84119793	0,92249117	protein_codir	hypothetical protein
TcG_05868	55,98493565	-0,334844838	0,22870745	-1,4640749	0,14317348	0,33967316	protein_codir	hypothetical protein
TcG_05869	147,4291702	-0,311048985	0,14966393	-2,07831633	0,03768023	0,13541042	protein_codir	hypothetical protein
TcG_05870	117,319678	-0,071645574	0,16248671	-0,44093191	0,65926229	0,82099665	protein_codir	hypothetical protein
TcG_05871	264,5929957	-0,026241089	0,11313607	-0,23194274	0,81658249	0,90954406	protein_codir	hypothetical protein
TcG_05872	247,7229828	-0,056356473	0,11216198	-0,50245611	0,61534672	0,79219196	protein_codir	hypothetical protein
TcG_05873	512,7077667	-0,082877003	0,08434684	-0,98257393	0,32581718	0,5602746	protein_codir	putative protein tyrosine phosphatase

TcG_05874	199,2686984	0,279174928	0,12649846	2,2069433	0,02731802	0,10674418	protein_codir	surface protease GP63
TcG_05875	347,3362861	-0,230486892	0,09710067	-2,3736899	0,01761133	0,07627847	protein_codir	UDP-galactopyranose mutase
TcG_05876	612,8844969	-0,407005287	0,07743816	-5,25587472	1,4732E-07	3,7106E-06	protein_codir	putative activator of 90 kDa heat shock protein ATPase 1-like
TcG_05877	751,2252726	-0,505694618	0,0700063	-7,22355838	5,0645E-13	4,0467E-11	protein_codir	putative RNA-binding protein
TcG_05878	529,505283	-0,033496979	0,10024809	-0,3341408	0,73827331	0,86642273	protein_codir	60S ribosomal protein L7
TcG_05879	201,0513259	-0,345993047	0,13291709	-2,60307414	0,0092392	0,04639162	protein_codir	putative calcium-dependent lipid binding protein, putative,synaptotagmin
TcG_05880	226,9159283	0,008365092	0,12218126	0,06846461	0,94541579	0,97469188	protein_codir	hypothetical protein
TcG_05881	141,7467017	0,121707785	0,15969295	0,76213625	0,44597869	0,66767142	protein_codir	hypothetical protein
TcG_05882	172,7026451	-0,001889904	0,13591965	-0,01390457	0,98890612	0,99552231	protein_codir	hypothetical protein
TcG_05883	182,0278111	-0,384308181	0,14122773	-2,72119499	0,00650464	0,03530291	protein_codir	putative glycogen synthase kinase-3 alpha
TcG_05884	503,7234611	-0,348603822	0,08366909	-4,16645902	3,0937E-05	0,00040547	protein_codir	hypothetical protein
TcG_05885	135,1807598	-0,016730849	0,15138926	-0,11051543	0,91200062	0,95710499	protein_codir	dephospho-CoA kinase
TcG_05886	39,22533606	-0,027449076	0,2851823	-0,09625098	0,92332124	0,96264894		
TcG_05887	560,3833539	-0,264801783	0,08236496	-3,21498103	0,00130453	0,00970109	protein_codir	mucin-associated surface protein (MASP)
TcG_05888	79,81280351	-0,030599073	0,1948105	-0,15707096	0,87518892	0,93987711	protein_codir	hypothetical protein
TcG_05889	109,9616892	-0,230258765	0,16627503	-1,38480664	0,1661116	0,3716614	protein_codir	hypothetical protein
TcG_05890	359,3373219	-0,18020038	0,09992265	-1,80339866	0,07132563	0,21430983	protein_codir	putative chaperone protein DNAj
TcG_05891	118,3695797	-0,23024897	0,17014817	-1,35322625	0,17598336	0,38565221	protein_codir	putative protein-like
TcG_05892	254,9904418	-0,153824276	0,1144613	-1,34389771	0,17898147	0,38979933	protein_codir	ATP synthase mitochondrial F1 complex assembly factor 2
TcG_05893	292,7107342	-0,266317525	0,10808193	-2,46403365	0,01373832	0,06346579	protein_codir	GPI-anchor transamidase subunit 8
TcG_05894	139,4982634	-0,386496803	0,14749705	-2,62036972	0,00878345	0,04463379	protein_codir	tubulin tyrosine ligase
TcG_05895	629,9280522	-0,306718406	0,07695669	-3,98559765	6,731E-05	0,00079822	protein_codir	hypothetical protein
TcG_05896	286,4555308	-0,22383085	0,11109709	-2,01473182	0,04393276	0,15104003	protein_codir	hypothetical protein
TcG_05897	315,5583103	-0,500886599	0,11166946	-4,4854394	7,2764E-06	0,00011612	protein_codir	UDP-galactose transporter
TcG_05898	259,2982856	-0,19954036	0,11805833	-1,69018447	0,09099267	0,251729	protein_codir	hypothetical protein
TcG_05899	68,86704698	-0,26683826	0,22114388	-1,20662736	0,22757569	0,45444535	protein_codir	hypothetical protein
TcG_05900	175,755782	-0,275490701	0,14197524	-1,94041372	0,05232943	0,17064137	protein_codir	phosphatidic acid phosphatase
TcG_05901	1233,374757	-0,481979518	0,05692806	-8,46646621	2,5295E-17	3,4889E-15	protein_codir	putative phosphatidic acid phosphatase
TcG_05902	185,0573538	0,018215227	0,13150854	0,13850983	0,8898375	0,94653573	protein_codir	hypothetical protein
TcG_05903	208,7825037	-0,203737838	0,12165711	-1,67468915	0,09399524	0,25757541	protein_codir	putative tubulin-specific chaperone
TcG_05904	435,6953245	-0,52645594	0,0869528	-6,05450276	1,4085E-09	5,4397E-08	protein_codir	pf20-like protein
TcG_05905	331,545056	-0,109284398	0,10435119	-1,04727505	0,29497274	0,52793889	protein_codir	uracil-DNA glycosylase
TcG_05906	306,522972	-0,320266595	0,11519976	-2,78009765	0,00543426	0,0305489	protein_codir	hypothetical protein
TcG_05907	310,8180586	-0,355348251	0,10306381	-3,44784694	0,00056507	0,00480992	protein_codir	hypothetical protein
TcG_05908	405,1942196	-0,280954109	0,09361686	-3,00110586	0,00269001	0,01740171	protein_codir	hypothetical protein
TcG_05909	1128,04497	-0,330897827	0,05960405	-5,55159998	2,8307E-08	8,2402E-07	protein_codir	cytosolic aconitase
TcG_05910	303,3464144	-0,407792873	0,10274719	-3,9688958	7,2206E-05	0,0008476	protein_codir	hypothetical protein
TcG_05911	401,0919842	-0,457891335	0,10312667	-4,4400867	8,9923E-06	0,00013966	protein_codir	Protein XRP2
TcG_05912	116,9105556	0,179345286	0,17213994	1,04185751	0,29747775	0,53089605	protein_codir	surface protease GP63
TcG_05913	304,6240853	-0,027189661	0,10757551	-0,25274955	0,80046176	0,90066523	protein_codir	hypothetical protein
TcG_05914	407,311276	-0,18363914	0,0931052	-1,97238334	0,04856586	0,16201672	protein_codir	hypothetical protein
TcG_05915	359,307813	-0,169105423	0,09625804	-1,75679271	0,07895315	0,22888182	protein_codir	putative target SNARE
TcG_05916	445,4165674	0,078936365	0,08827167	0,89424344	0,37119166	0,60275074	protein_codir	hypothetical protein
TcG_05917	166,8550089	0,112613383	0,1355046	0,83106688	0,40593585	0,63464372	protein_codir	hypothetical protein
TcG_05918	430,4112362	-0,162551842	0,08859829	-1,83470634	0,06654921	0,20484569	protein_codir	putative DEAD box RNA helicase
TcG_05919	341,4447285	0,000136362	0,09936163	0,00137238	0,998905	0,99976791	protein_codir	viral A-type inclusion protein
TcG_05920	369,3858759	0,042666435	0,09503443	0,44895768	0,65346219	0,81657908	protein_codir	hypothetical protein
TcG_05921	272,4023989	0,156097231	0,11051457	1,41245843	0,15781502	0,35991425	protein_codir	hypothetical protein
TcG_05922	1,0695558	-3,740338245	3,66987973	-1,01919913	0,30810844	1	protein_codir	membrane protein
TcG_05923	342,4988291	0,153628066	0,09839571	1,56132888	0,11844617	0,30062612	protein_codir	hypothetical protein
TcG_05924	10,42810318	0,021205875	0,53490663	0,03964407	0,96837689	1	protein_codir	surface protease GP63
TcG_05925	293,0216827	-0,002168263	0,1095394	-0,01979436	0,98420741	0,99398772	protein_codir	hypothetical protein

TcG_05926	515,5873742	0,118847529	0,08187324	1,45160405	0,14661173	0,34466504	protein_codir	DNA-directed DNA polymerase
TcG_05927	96,70559131	0,090888821	0,18629711	0,48787028	0,62564173	0,79875318	protein_codir	putative DNA polymerase kappa, putative,DNA polymerase IV
TcG_05928	58,3779798	0,060694925	0,22707399	0,2672914	0,78924481	0,89494301	protein_codir	putative DNA polymerase kappa, putative,DNA polymerase IV
TcG_05929	353,7228546	-0,174444828	0,09883102	-1,7650817	0,07755004	0,2259741	protein_codir	hypothetical protein
TcG_05930	133,5404876	-0,078031621	0,15316887	-0,5094483	0,61043803	0,78905613	protein_codir	putative DNA polymerase kappa, putative,DNA polymerase IV
TcG_05931	79,06313416	0,063268282	0,19557369	0,32350099	0,74631585	0,87165479	protein_codir	putative DNA polymerase kappa, putative,DNA polymerase IV
TcG_05932	387,5805923	1,005527245	0,09673074	10,3951155	2,6097E-25	7,9569E-23	protein_codir	hypothetical protein
TcG_05933	861,3021585	0,068124659	0,06551796	1,03978604	0,29843932	0,53212033	protein_codir	haloacid dehalogenase-like hydrolase
TcG_05934	569,1710522	-0,048930687	0,08176996	-0,59839444	0,54957678	0,7458664	protein_codir	hypothetical protein
TcG_05935	215,5026229	0,064466616	0,12085001	0,5334432	0,59372682	0,77886311	protein_codir	putative chaperone
TcG_05936	354,4910975	-0,174252505	0,09677452	-1,80060314	0,07176545	0,21511728	protein_codir	hypothetical protein
TcG_05937	447,7375102	-0,078406286	0,08926489	-0,87835526	0,37975096	0,6107213	protein_codir	hypothetical protein
TcG_05938	233,4373584	0,099195124	0,11877694	0,83513788	0,40364008	0,63312391	protein_codir	RNA editing complex protein
TcG_05939	493,6093427	0,154267396	0,08236876	1,87288724	0,06108396	0,19193429	protein_codir	calmodulin-like protein containing EF hand
TcG_05940	718,6442024	-0,231385691	0,07332753	-3,155509	0,00160218	0,0115083	protein_codir	cytochrome P450 reductase C
TcG_05941	450,843844	-0,072736925	0,08677376	-0,83823645	0,40189792	0,63220648	protein_codir	hypothetical protein
TcG_05942	288,5229979	0,167426331	0,11152886	1,50119292	0,13330567	0,32445352	protein_codir	hypothetical protein
TcG_05943	5622,19296	-0,364120298	0,03876047	-9,39411399	5,7701E-21	1,1331E-18	protein_codir	glucose-regulated protein 78
TcG_05944	146,9184576	0,366373063	0,14833692	2,46987111	0,01351617	0,06279103	protein_codir	hypothetical protein
TcG_05945	64,87813501	0,066640358	0,21415458	0,31117876	0,75566473	0,87682839	protein_codir	hypothetical protein
TcG_05946	108,2905913	0,363451394	0,17067112	2,1295424	0,03320941	0,12329453	protein_codir	putative serine/threonine protein phosphatase
TcG_05947	116,9551357	0,341601966	0,16609839	2,05662423	0,03972239	0,14050264	protein_codir	hypothetical protein
TcG_05948	153,514265	0,351406918	0,15357469	2,28818246	0,0221269	0,09094084	protein_codir	rab1 small GTP-binding protein
TcG_05949	139,1847291	0,223644796	0,1495739	1,49521266	0,13485895	0,32674107	protein_codir	putative trans-sialidase
TcG_05950	327,7117905	0,322433103	0,10878742	2,96388228	0,00303784	0,01924356	protein_codir	helicase-like protein
TcG_05951	189,8992021	-0,214679905	0,13001672	-1,65117148	0,09870356	0,26625832	protein_codir	surface antigen 2 (CA-2)
TcG_05952	101,2522412	-0,09112175	0,19746479	-0,46145821	0,6444699	0,8110828	protein_codir	acetyltransferase
TcG_05953	229,9614973	0,486804924	0,12691143	3,8357847	0,00012516	0,00134898	protein_codir	putative protein kinase
TcG_05954	182,511025	-0,046851437	0,13626979	-0,34381381	0,73098631	0,86165504	protein_codir	hypothetical protein
TcG_05955	112,7917107	0,350854672	0,17080915	2,05407428	0,0399685	0,14122448	protein_codir	hypothetical protein
TcG_05956	126,2081902	-0,231956805	0,1557682	-1,48911531	0,13645701	0,32916375	protein_codir	hypothetical protein
TcG_05957	135,3102545	0,121196153	0,15250489	0,79470336	0,42678607	0,65199676	protein_codir	U5 snRNP-specific 40 kDa protein
TcG_05958	348,6102375	-0,147403065	0,10099157	-1,45955814	0,14441155	0,34108791	protein_codir	hypothetical protein
TcG_05959	184,6147954	-0,005887626	0,13488882	-0,04364799	0,965185	0,98447341	protein_codir	hypothetical protein
TcG_05960	368,9508836	0,076397976	0,09384495	0,81408724	0,41559497	0,64239863	protein_codir	putative sodium/sulfate symporter
TcG_05961	56,62544985	0,226768367	0,2315085	0,97952502	0,32732063	0,56182768	protein_codir	hypothetical protein
TcG_05962	259,5388863	0,022869896	0,11374124	0,20106951	0,84064422	0,92246657	protein_codir	hypothetical protein
TcG_05963	227,3026921	-0,001435294	0,13089342	-0,01096537	0,99125108	0,99667057	protein_codir	putative oligomeric golgi complex component 8
TcG_05964	217,0950974	-0,006500374	0,12718927	-0,05110788	0,95923955	0,98117326	protein_codir	hypothetical protein
TcG_05965	2048,203003	0,013663968	0,06529136	0,20927682	0,83423215	0,91946477	protein_codir	Dynein heavy chain family protein
TcG_05966	1142,86691	0,301986097	0,06145386	4,91403005	8,9223E-07	1,8427E-05	protein_codir	60S ribosomal protein L14
TcG_05967	75,9580538	0,380058039	0,20508918	1,85313549	0,06386294	0,19852859	protein_codir	hypothetical protein
TcG_05968	198,2327525	0,900433651	0,13412265	6,71350918	1,9E-11	1,1062E-09	protein_codir	putative cytosolic malate dehydrogenase
TcG_05969	316,6833031	0,282982017	0,10112767	2,79826495	0,00513779	0,02928012	protein_codir	hypothetical protein
TcG_05970	289,0210748	-0,06099691	0,10497672	-0,58105176	0,56120557	0,75474494	protein_codir	hypothetical protein
TcG_05971	202,2069668	0,163603081	0,1271878	1,28631109	0,19833451	0,41692226	protein_codir	hypothetical protein
TcG_05972	451,0001841	-0,146525452	0,09095391	-1,61098579	0,10718282	0,28209578	protein_codir	hypothetical protein
TcG_05973	410,2944158	0,266114692	0,09066058	2,93528556	0,00333241	0,02072425	protein_codir	putative heat shock protein 70
TcG_05974	124,3484389	0,196049864	0,16144784	1,21432327	0,2246243	0,45108253	protein_codir	hypothetical protein
TcG_05975	287,1761441	0,301064367	0,10603883	2,83918972	0,00452283	0,02662676	protein_codir	hypothetical protein
TcG_05976	289,8472892	0,61937093	0,10952979	5,65481687	1,5601E-08	4,7693E-07	protein_codir	hypothetical protein
TcG_05977	440,79293	0,30809719	0,09329012	3,30257027	0,00095803	0,00753547	protein_codir	hypothetical protein

TcG_05978	71,68180801	-0,130462759	0,21354166	-0,61094756	0,5412343	0,73989536	protein_codir	heatshock protein hsp70
TcG_05979	19781,64098	0,063428808	0,0313954	2,02032173	0,04335003	0,14952468	protein_codir	heat shock cognate 70
TcG_05980	330,4619807	-0,032430057	0,0994551	-0,32607738	0,74436581	0,87043019	protein_codir	putative gamma-tubulin complex subunit
TcG_05981	359,5096496	-0,02594223	0,10116492	-0,25643505	0,79761493	0,89899335	protein_codir	a44l protein-like protein
TcG_05982	0					1	protein_codir	activated protein kinase C receptor
TcG_05983	1623,854479	0,023909661	0,05235789	0,45665825	0,6479167	0,81260383	protein_codir	activated protein kinase C receptor
TcG_05984	2,784529159	1,157373195	1,09994455	1,05221049	0,29270299	1	protein_codir	activated protein kinase C receptor
TcG_05985	412,0877388	-0,010631718	0,08969014	-0,11853831	0,90564114	0,95410701	protein_codir	hypothetical protein
TcG_05986	146,310235	0,174473644	0,14719491	1,18532387	0,23588942	0,46344584	protein_codir	hypothetical protein
TcG_05987	184,6503979	0,195624576	0,13375386	1,46257149	0,14358467	0,34000823	protein_codir	alkylated DNA repair protein alkB like protein 7
TcG_05988	20,37543199	0,0349487	0,4084386	0,08556659	0,93181096	0,96736431		
TcG_05989	11,13400988	0,142189395	0,5186285	0,27416425	0,7839584	1	protein_codir	hypothetical protein
TcG_05990	222,4619526	-0,27232313	0,12596362	-2,16191884	0,03062443	0,11604928	protein_codir	putative endosomal integral membrane protein
TcG_05991	100,167223	-0,165390916	0,18035667	-0,91702133	0,35913146	0,5926302	protein_codir	pre-mRNA-splicing factor ISY1
TcG_05992	1747,834975	0,105624836	0,05467974	1,93169982	0,05339657	0,17304969	protein_codir	histone-lysine N-methyltransferase
TcG_05993	215,3118664	0,597538046	0,12385328	4,82456364	1,4031E-06	2,7276E-05	protein_codir	glutamamyl carboxypeptidase
TcG_05994	189,4400492	-0,099423499	0,13639316	-0,72894783	0,46603357	0,68269882	protein_codir	hypothetical protein
TcG_05995	347,3823213	-0,210097139	0,09586516	-2,19159003	0,02840912	0,10982586	protein_codir	alpha-adaptin-like protein
TcG_05996	32,69665403	0,139014416	0,32536523	0,42725652	0,6691925	0,82630974	protein_codir	hypothetical protein
TcG_05997	877,7305907	-0,114116747	0,06603439	-1,72814114	0,08396292	0,23860545	protein_codir	putative cytochrome c1, heme protein, mitochondrial precursor
TcG_05998	164,5588133	-0,008093256	0,1431716	-0,05652836	0,9549209	0,97888932	protein_codir	hypothetical protein
TcG_05999	1657,215478	-0,213300718	0,05177565	-4,11971099	3,7935E-05	0,00048404	protein_codir	putative Golgi/lysosome glycoprotein
TcG_06000	734,4474071	0,056784042	0,07455692	0,76161997	0,44628685	0,66796015	protein_codir	pitrilysin-like metalloprotease
TcG_06001	311,5814811	-0,023425087	0,10350077	-0,22632766	0,82094658	0,91123655	protein_codir	Ca2+ binding protein, contains EF-hand motif (ISS)
TcG_06002	588,9097355	0,006958017	0,08059754	0,08633039	0,93120378	0,9669931	protein_codir	putative brefeldin A-inhibited guanine nucleotide-exchange protein 2
TcG_06003	672,4996326	0,008216547	0,08277048	0,09926904	0,92092466	0,96150609	protein_codir	putative brefeldin A-inhibited guanine nucleotide-exchange protein 2
TcG_06004	201,0085914	0,131789452	0,13495718	0,97652793	0,32880291	0,56320381	protein_codir	tRNA-methyl transferase
TcG_06005	219,0258299	0,3507176	0,12023736	2,91687706	0,00353555	0,02177509	protein_codir	putative 3-ketoacyl-CoA thiolase
TcG_06006	195,238561	0,231111666	0,12757101	1,81163162	0,07004314	0,21201645	protein_codir	putative ubiquitin ligase
TcG_06007	658,5793293	0,213348552	0,07548127	2,82650967	0,00470583	0,02741165	protein_codir	formin like proteiny 2 (FH2) domain protein
TcG_06008	226,8309455	0,330313108	0,12634574	2,61435895	0,00893951	0,0452482	protein_codir	putative membrane receptor protein
TcG_06009	907,3851595	-0,186604375	0,06808219	-2,74086928	0,00612769	0,0335654	protein_codir	hypothetical protein
TcG_06010	559,4835877	-0,148166235	0,08401087	-1,76365544	0,07779001	0,22628045	protein_codir	putative protein kinase
TcG_06011	92,57720071	-0,171448219	0,18097002	-0,94738467	0,3434428	0,57760608		
TcG_06012	275,0483215	-0,007140808	0,1219249	-0,05856726	0,95329679	0,97811695	protein_codir	hypothetical protein
TcG_06013	416,9582988	0,114752134	0,08871524	1,29348834	0,19584222	0,41381415	protein_codir	hypothetical protein
TcG_06014	441,5597587	0,309774575	0,09100876	3,40378845	0,00066458	0,00549989	protein_codir	oxidoreductase
TcG_06015	105,7115278	0,102370084	0,17208998	0,59486371	0,55193458	0,74737358	protein_codir	hypothetical protein
TcG_06016	408,9798768	0,25266984	0,09133365	2,76644855	0,00566705	0,03159696	protein_codir	putative phosphatidylglycerophosphate synthase
TcG_06017	323,5367755	0,042903808	0,10792425	0,3975363	0,69097202	0,83977781	protein_codir	protein phosphatase 2A
TcG_06018	472,0911778	0,12156387	0,08487439	1,43227978	0,15206377	0,3522917	protein_codir	hypothetical protein
TcG_06019	4,737414886	-0,015267199	0,86168198	-0,0177179	0,9858639	1		
TcG_06020	201,6365404	0,358818673	0,12620585	2,84312232	0,00446739	0,02639873	protein_codir	hypothetical protein
TcG_06021	481,9032916	0,087385317	0,08409227	1,03915998	0,29873034	0,5322319	protein_codir	putative protein kinase
TcG_06022	237,9863582	0,088882441	0,11521557	0,77144467	0,44044341	0,66339057	protein_codir	hypothetical protein
TcG_06023	332,040057	0,21507086	0,10388684	2,07024169	0,03842972	0,13725406	protein_codir	hypothetical protein
TcG_06024	499,8690143	-0,059049694	0,08494388	-0,69516121	0,48695428	0,69859488	protein_codir	putative serine/threonine protein kinase
TcG_06025	51,3463803	0,193990694	0,24810476	0,78189026	0,43427907	0,65866701		
TcG_06026	2,390449582	0,367507869	1,15406611	0,31844611	0,75014656	1	protein_codir	hypothetical protein
TcG_06027	207,394494	-0,589806839	0,1232997	-4,78352221	1,7225E-06	3,2716E-05	protein_codir	calpain-like cysteine peptidase
TcG_06028	625,3439826	-0,572411383	0,07491971	-7,64033095	2,1666E-14	2,0576E-12	protein_codir	putative vacuolar protein sorting complex subunit
TcG_06029	116,4642698	-0,623364398	0,17268948	-3,60974168	0,0003065	0,00285003	protein_codir	putative protein kinase A catalytic subunit

TcG_06030	274,8821709	-0,16736791	0,10680906	-1,56698228	0,11711883	0,29855471	protein_codir putative cAMP phosphodiesterase A
TcG_06031	174,145804	-0,205403813	0,13996946	-1,46749026	0,14224274	0,33819502	protein_codir 26S proteasome non-ATPase regulatory subunit 10
TcG_06032	392,7690609	-0,114223868	0,09309526	-1,22695692	0,21983878	0,44499055	protein_codir putative methyltransferase
TcG_06033	355,287272	-0,139608062	0,09827041	-1,42065206	0,15541794	0,35689154	protein_codir AN1-type zinc finger protein 2B
TcG_06034	92,61420078	-0,08398644	0,18081275	-0,46449401	0,64229385	0,8100665	protein_codir hypothetical protein
TcG_06035	446,0288107	-0,402684481	0,08996907	-4,47581015	7,6122E-06	0,00012049	protein_codir ras-related protein rab-5
TcG_06036	707,9203605	-0,399954091	0,07079631	-5,64936319	1,6104E-08	4,9101E-07	protein_codir 200 kDa antigen p200
TcG_06037	497,4065208	-0,618696408	0,0836202	-7,39888663	1,3733E-13	1,1963E-11	protein_codir hypothetical protein
TcG_06038	151,40386	0,146161927	0,14877463	0,98243849	0,32588387	0,5602746	protein_codir hypothetical protein
TcG_06039	216,2575768	-0,332056493	0,12057294	-2,75398853	0,00588738	0,032528	protein_codir hypothetical protein
TcG_06040	765,1505655	-0,309898537	0,06967622	-4,44769415	8,6797E-06	0,00013553	protein_codir hypothetical protein
TcG_06041	421,1500865	-0,267026018	0,08960902	-2,97990104	0,00288342	0,01844685	protein_codir hypothetical protein
TcG_06042	976,4879253	-0,317201464	0,06601496	-4,80499362	1,5476E-06	2,9739E-05	protein_codir uncharacterized protein
TcG_06043	927,8582744	-0,282325754	0,06352723	-4,44416877	8,8232E-06	0,00013759	protein_codir acid--amino-acid ligase
TcG_06044	260,3318914	-0,20551555	0,11373363	-1,80699014	0,07076382	0,21345212	protein_codir hypothetical protein
TcG_06045	92,07142263	-0,419920831	0,19012827	-2,20861862	0,02720118	0,10636276	protein_codir hypothetical protein
TcG_06046	274,4275938	-0,41125393	0,1072216	-3,83555117	0,00012528	0,001349	protein_codir hypothetical protein
TcG_06047	665,1734799	-0,217338469	0,07202067	-3,01772355	0,00254681	0,01668025	protein_codir putative protein kintoun-like
TcG_06048	313,6952951	-0,379912818	0,10413307	-3,64833986	0,00026394	0,00251408	protein_codir TTAGGG binding factor
TcG_06049	194,4720157	0,539250583	0,13045817	4,13351319	3,5726E-05	0,00045991	protein_codir putative mitochondrial carrier protein
TcG_06050	180,5453801	-0,361256886	0,13686408	-2,63953032	0,0083021	0,0426799	protein_codir putative acetyltransferase
TcG_06051	148,5982699	-0,509678424	0,14876069	-3,42616333	0,00061217	0,00512103	protein_codir hypothetical protein
TcG_06052	380,2243004	-0,284107189	0,09283022	-3,06050338	0,00220965	0,01489298	protein_codir putative cholinephosphate cytidylyltransferase A
TcG_06053	170,2237551	-0,245213736	0,13894405	-1,76483798	0,077591	0,22597979	protein_codir zinc finger protein family memeber
TcG_06054	86,42006474	-0,209762211	0,1890479	-1,10957178	0,2671836	0,4996109	protein_codir hypothetical protein
TcG_06055	639,3172185	-0,339502753	0,07632845	-4,44791895	8,6706E-06	0,00013553	protein_codir putative leucine-rich repeat-containing protein 72-like
TcG_06056	177,2181001	0,051180007	0,13225246	0,38698718	0,6987657	0,84414248	protein_codir putative heat shock protein
TcG_06057	371,4246867	-0,237775383	0,09543882	-2,49139058	0,01272442	0,05968627	protein_codir rev7
TcG_06058	239,0855254	-0,250476791	0,12178905	-2,05664462	0,03972042	0,14050264	protein_codir hypothetical protein
TcG_06059	144,1242623	-0,18426519	0,14828422	-1,24264871	0,21399731	0,43752213	protein_codir putative trans-sialidase
TcG_06060	19,17385281	-0,29740542	0,39585078	-0,75130689	0,45246799	0,67329805	protein_codir putative diacylglycerol acyltransferase
TcG_06061	145,9459947	-0,168027188	0,15137974	-1,10997146	0,26701132	0,49939561	protein_codir putative diacylglycerol acyltransferase
TcG_06062	28,95609837	-0,167660252	0,32441914	-0,51680136	0,60529483	0,78669877	
TcG_06063	249,6600482	-0,29728132	0,11548307	-2,57424168	0,01004601	0,04955004	protein_codir putative delta-1-pyrroline-5-carboxylate dehydrogenase
TcG_06064	23,88354569	-0,331256983	0,35813784	-0,9249427	0,35499572	0,58891471	protein_codir putative diacylglycerol acyltransferase
TcG_06065	6,178838067	-0,539122406	0,76881355	-0,70123947	0,48315358	1	protein_codir putative diacylglycerol acyltransferase
TcG_06066	579,1941093	-0,478572286	0,08170094	-5,85761083	4,6957E-09	1,6049E-07	protein_codir hypothetical protein
TcG_06067	534,116826	-0,195024029	0,08256135	-2,36217092	0,01816826	0,07793317	protein_codir putative DNA excision repair protein, putative,SNF2 family helicase-like protein
TcG_06068	466,1337884	-0,438279796	0,0866242	-5,05955348	4,2024E-07	9,2741E-06	protein_codir hypothetical protein
TcG_06069	365,9318557	-0,10335143	0,09638132	-1,0723181	0,28357718	0,51699846	protein_codir hypothetical protein
TcG_06070	102,5795687	-0,063671392	0,17626647	-0,36122238	0,71793322	0,85508792	protein_codir putative casein kinase
TcG_06071	705,0979319	-0,319181213	0,07206077	-4,42933393	9,4525E-06	0,00014525	protein_codir hypothetical protein
TcG_06072	960,1878215	-0,163972389	0,06502233	-2,52178565	0,01167608	0,05580821	protein_codir putative serine/threonine protein kinase
TcG_06073	245,6366333	-0,103598901	0,11633647	-0,89051096	0,37319159	0,60423908	protein_codir sTim1
TcG_06074	586,0677243	-0,252157224	0,07900005	-3,19186173	0,00141359	0,01035262	protein_codir putative mitochondrial RNA binding complex 1 subunit
TcG_06075	293,1658807	-0,125486386	0,10509718	-1,19400336	0,23247665	0,45964254	protein_codir putative glycogenin glucosyltransferase
TcG_06076	304,4004831	-0,260885277	0,10228783	-2,55050175	0,0107568	0,05234166	protein_codir hypothetical protein
TcG_06077	337,4409378	-0,523420658	0,10529222	-4,97112394	6,6566E-07	1,4099E-05	protein_codir putative serine/threonine protein kinase, putative,protein kinase
TcG_06078	100,9169668	-0,443750597	0,18238025	-2,4331067	0,01496989	0,06766691	protein_codir PIF1 helicase-like protein
TcG_06079	210,0840741	-0,248308292	0,13497009	-1,83972826	0,06580814	0,20305723	protein_codir hypothetical protein
TcG_06080	251,0352358	-0,307363089	0,11129125	-2,76179029	0,00574854	0,03189778	protein_codir putative nucleolar protein
TcG_06081	380,5604448	-0,174280889	0,09832713	-1,77245983	0,07631826	0,22348794	protein_codir putative outer dynein arm docking complex

TcG_06082	116,4557865	-0,406777365	0,1664357	-2,44405112	0,01452337	0,06622252	protein_codir	putative CAAX prenyl protease 2
TcG_06083	11,41272466	-0,014895882	0,53218183	-0,02799021	0,97766996	1	protein_codir	surface protease GP63
TcG_06084	5,500380076	0,047242913	0,7262749	0,06504825	0,94813558	1	protein_codir	hypothetical protein
TcG_06085	22,41004286	-0,901543902	0,40500715	-2,22599502	0,02601451	0,10279396	protein_codir	hypothetical protein
TcG_06086	14,62344913	0,467325494	0,46745575	0,99972136	0,31744537	1	protein_codir	hypothetical protein
TcG_06087	27,55090392	0,185269716	0,33179921	0,55837901	0,5765856	0,76688334	protein_codir	hypothetical protein
TcG_06088	50,91382202	0,087754365	0,24535382	0,35766456	0,72059436	0,85670516	protein_codir	trans-sialidase
TcG_06089	54,71488693	-0,64568263	0,23667586	-2,72813047	0,00636944	0,03466244	protein_codir	mucin-associated surface protein (MASP)
TcG_06090	31,38212729	-0,391365695	0,30950808	-1,26447651	0,20605905	0,42784948	protein_codir	90 kDa surface protein
TcG_06091	13,35497359	-0,0486998	0,48402684	-0,10061384	0,91985701	1	protein_codir	hypothetical protein
TcG_06092	6,571449484	0,407919782	0,68799633	0,59290982	0,55324151	1	protein_codir	hypothetical protein
TcG_06093	14,8287701	0,359999472	0,47995303	0,75007229	0,45321116	1	protein_codir	hypothetical protein
TcG_06094	218,4888582	-0,011361109	0,12581006	-0,09030366	0,92804591	0,96520107	protein_codir	HEAT repeat containing 2
TcG_06095	141,0555718	-0,058465199	0,14889949	-0,39264875	0,69457893	0,84195349	protein_codir	translation initiation factor EIF-2b alpha subunit
TcG_06096	156,1021492	-0,161955438	0,14272185	-1,13476277	0,25647473	0,48849519	protein_codir	putative PITH domain-containing protein 1-like
TcG_06097	368,9232952	-0,251597381	0,09477636	-2,6546428	0,00793924	0,04124846	protein_codir	putative proteasome beta-1 subunit
TcG_06098	178,8742237	0,019693076	0,13243933	0,14869507	0,88179425	0,94218407	protein_codir	hypothetical protein
TcG_06099	100,5249943	-0,357098268	0,17670483	-2,02087441	0,04329277	0,14937165	protein_codir	hypothetical protein
TcG_06100	184,7730643	-0,253395318	0,13259912	-1,91098786	0,05600614	0,17910217	protein_codir	hypothetical protein
TcG_06101	178,4669287	-0,20719479	0,13163805	-1,57397337	0,11549361	0,29583886	protein_codir	hypothetical protein
TcG_06102	321,2902717	0,084261009	0,10041679	0,83911276	0,40140604	0,63188728	protein_codir	hypothetical protein
TcG_06103	203,4948007	0,182365864	0,12361431	1,47528113	0,14013698	0,3345817	protein_codir	hypothetical protein
TcG_06104	256,98646	-0,084426388	0,12105794	-0,69740482	0,48554949	0,69826977	protein_codir	hypothetical protein
TcG_06105	304,216039	0,116272669	0,10340709	1,12441679	0,26083625	0,49331519	protein_codir	hypothetical protein
TcG_06106	484,3414244	-0,139199985	0,08549904	-1,62808825	0,10350617	0,27555665	protein_codir	hypothetical protein
TcG_06107	194,8710263	-0,047668154	0,12650121	-0,37681975	0,70630756	0,84872764	protein_codir	hypothetical protein
TcG_06108	354,4288165	0,186425626	0,09613131	1,93928101	0,05246713	0,17089963	protein_codir	putative leucine-rich repeat protein (LRRP)
TcG_06109	366,1515125	0,211362338	0,09647243	2,19090928	0,02845836	0,10986956	protein_codir	putative dolichyl-P-Man:GDP-Man5GlcNAc2-PP-dolichyl alpha-1,2-mannosyltransferase
TcG_06110	198,4187086	0,235560712	0,13097961	1,79845327	0,07210521	0,21592425	protein_codir	hypothetical protein
TcG_06111	210,9706704	0,112241405	0,13195619	0,85059601	0,39499381	0,62459373	protein_codir	hypothetical protein
TcG_06112	472,8678288	-0,109278498	0,08407559	-1,29976486	0,19368157	0,41080251	protein_codir	peptide hydrolase
TcG_06113	313,359219	-0,105756738	0,10591761	-0,99848116	0,3180461	0,55320253	protein_codir	hypothetical protein
TcG_06114	362,7869508	-0,036506683	0,10356532	-0,3524991	0,72446399	0,85798219	protein_codir	putative proteasome regulatory ATPase subunittcc1l8.3
TcG_06115	137,1323436	0,052761733	0,1518302	0,34750486	0,72821207	0,86000745	protein_codir	hypothetical protein
TcG_06116	228,6211021	0,100173483	0,12607121	0,79457855	0,4268587	0,65202174	protein_codir	putative hydroxyacylglutathione hydrolase, putative,glyoxalase II
TcG_06117	301,7139489	0,271828398	0,10445973	2,60223157	0,00926193	0,04644001	protein_codir	hypothetical protein
TcG_06118	462,9393433	-0,209972602	0,08410387	-2,49658655	0,01253951	0,05903402	protein_codir	hypothetical protein
TcG_06119	119,0529663	-0,089156991	0,16019827	-0,55654153	0,57784071	0,76800198	protein_codir	cysteine peptidase, putative,cysteine peptidase, clan CA, family C1, cathepsin L-like
TcG_06120	20,53406951	-0,409393115	0,38624298	-1,05993672	0,28917339	0,52267752	protein_codir	cysteine peptidase, putative,cysteine peptidase, clan CA, family C1, cathepsin L-like
TcG_06121	8668,470788	-0,096792906	0,03644941	-2,65554098	0,00791813	0,04118059	protein_codir	cruzipain
TcG_06122	120,5613388	-0,295114454	0,16151571	-1,82715638	0,06767626	0,20721383	protein_codir	cruzipain
TcG_06123	535,8016472	0,077295748	0,0837702	0,92271174	0,35615744	0,59016593	protein_codir	putative cysteinyl-tRNA synthetase
TcG_06124	333,1388505	0,040676544	0,10274607	0,3958939	0,6921833	0,84069156	protein_codir	helicase
TcG_06125	1069,426523	-0,345185934	0,0602339	-5,73075825	9,9983E-09	3,187E-07	protein_codir	metacaspase
TcG_06126	314,6799803	-0,371480382	0,10469018	-3,54837833	0,00038761	0,00348399	protein_codir	hypothetical protein
TcG_06127	268,2631013	-0,100341581	0,10922836	-0,91864038	0,3582837	0,59216476	protein_codir	hypothetical protein
TcG_06128	258,3357982	-0,079967007	0,11132668	-0,71830945	0,4725665	0,68836848	protein_codir	cleavage and polyadenylation specificity factor 30 kDa subunit
TcG_06129	272,3939196	-0,014149254	0,11031356	-0,12826396	0,89794008	0,95018119	protein_codir	hypothetical protein
TcG_06130	1067,910376	-0,114779096	0,06544281	-1,75388399	0,07945039	0,22984075	protein_codir	putative ubiquitin ligase
TcG_06131	593,7065647	-0,078430037	0,08028882	-0,97684874	0,32864404	0,56308329	protein_codir	putative ribonucleoside-diphosphate reductase small chain
TcG_06132	146,2068559	-0,250778504	0,15109454	-1,65974569	0,09696562	0,26341002	protein_codir	putative trans-sialidase
TcG_06133	105,4772572	0,086304472	0,17952818	0,48072939	0,63070884	0,80204067	protein_codir	L1Tc protein

TcG_06134	662,2756652	-0,05758971	0,07739188	-0,74413115	0,45679714	0,67661105	protein_codir	putative acyl-CoA binding protein
TcG_06135	1269,052403	-0,127281183	0,11082155	-1,14852372	0,25075243	0,48051896	protein_codir	hypothetical protein
TcG_06136	826,9979147	-0,083668054	0,06748906	-1,23972759	0,21507617	0,4388705	protein_codir	oligopeptidase b
TcG_06137	201,6104873	-0,270526226	0,13063708	-2,07082274	0,03837536	0,1371428	protein_codir	putative flagellar/basal body protein
TcG_06138	181,9225802	0,058786069	0,13670518	0,43002078	0,66718053	0,82567331	protein_codir	hypothetical protein
TcG_06139	156,856382	-0,068502023	0,14145561	-0,48426515	0,6281977	0,80069291	protein_codir	hypothetical protein
TcG_06140	70,32533981	0,135980509	0,21437239	0,63431914	0,52587257	0,72890472	protein_codir	hypothetical protein
TcG_06141	85,92070529	-0,169199058	0,19522362	-0,86669359	0,38610992	0,61652005	protein_codir	hypothetical protein
TcG_06142	59,55387913	0,25641007	0,23708236	1,08152317	0,27946446	0,51247855	protein_codir	hypothetical protein
TcG_06143	48,96347865	-0,03748162	0,24788153	-0,1512078	0,8798118	0,94162382	protein_codir	dynein heavy chain, cytosolic
TcG_06144	136,8493401	0,031948783	0,15870109	0,2013142	0,8404529	0,92245995	protein_codir	protein UXT
TcG_06145	185,7998134	-0,191325372	0,13667868	-1,39981868	0,16156762	0,36518191	protein_codir	hypothetical protein
TcG_06146	374,8575453	-0,28503479	0,09880237	-2,88489839	0,0039154	0,02366397	protein_codir	hypothetical protein
TcG_06147	375,1087901	0,054800369	0,09932812	0,5517105	0,58114672	0,77048234	protein_codir	DEAD/DEAH box helicase-like protein
TcG_06148	698,6399269	-0,14457026	0,0714692	-2,02283295	0,04309037	0,14907288	protein_codir	metal binding protein
TcG_06149	144,9591703	-0,395843882	0,14946015	-2,64849116	0,0080852	0,04181923	protein_codir	putative ras-related GTP-binding protein
TcG_06150	292,4664013	-0,045441596	0,10623371	-0,42775117	0,66883229	0,8263077	protein_codir	hypothetical protein
TcG_06151	2594,659977	-0,301838484	0,04409125	-6,84576863	7,6066E-12	4,9522E-10	protein_codir	hypothetical protein
TcG_06152	5,034613796	-0,033116619	0,77357091	-0,04281006	0,96585294	1		
TcG_06153	457,7835271	0,082553031	0,08839723	0,93388706	0,35036216	0,58488966	protein_codir	putative phospholipid transporting ATPase-like protein
TcG_06154	865,6210258	0,112658981	0,06581923	1,71164235	0,0869626	0,24437271	protein_codir	polyubiquitin
TcG_06155	825,0666323	0,063198529	0,0662459	0,95399917	0,34008406	0,57420781	protein_codir	polyubiquitin
TcG_06156	259,5664689	0,062467202	0,11012748	0,56722627	0,57056046	0,76219457	protein_codir	EF-hand protein 5
TcG_06157	403,7860248	0,177555094	0,0898448	1,97624236	0,04812733	0,16115702	protein_codir	putative diacylglycerol acyltransferase
TcG_06158	161,2179005	0,077591817	0,13826132	0,56119684	0,57466336	0,76564508	protein_codir	putative DNA-directed RNA polymerase II
TcG_06159	321,9805532	-0,093700447	0,10105419	-0,92722967	0,3538073	0,58808501	protein_codir	hypothetical protein
TcG_06160	631,0954308	0,063526132	0,07619155	0,83376868	0,40441135	0,63403381	protein_codir	hypothetical protein
TcG_06161	91,73321642	-0,021080361	0,19340388	-0,10899658	0,9132052	0,95741521	protein_codir	oxidoreductase
TcG_06162	227,3162691	-0,264775637	0,1199238	-2,20786567	0,02725364	0,10653193	protein_codir	hypothetical protein
TcG_06163	476,4181025	0,076690542	0,08574526	0,89439973	0,37110806	0,60269946	protein_codir	hypothetical protein
TcG_06164	579,8156212	-0,146545768	0,08137703	-1,80082463	0,07173053	0,21511728	protein_codir	hypothetical protein
TcG_06165	253,4412452	-0,025819598	0,11384578	-0,22679451	0,82058353	0,91114306	protein_codir	putative proteasome regulatory non-ATPase subunit 3
TcG_06166	335,0745112	0,009327446	0,09925979	0,09397003	0,92513296	0,96338221	protein_codir	ribosome biogenesis protein MAK21
TcG_06167	256,7369677	-0,131413456	0,11383433	-1,15442729	0,24832503	0,47760522	protein_codir	charged multivesicular body protein 2A
TcG_06168	360,505821	0,2581401	0,10189056	2,53350369	0,01129286	0,05438031	protein_codir	Trypanosoma vivax
TcG_06169	223,955875	-0,150428405	0,11964369	-1,2573033	0,20864383	0,43120717	protein_codir	choline kinase
TcG_06170	630,4387709	-0,076441064	0,07589897	-1,00714237	0,31386636	0,54810657	protein_codir	putative vacuolar protein sorting protein 16
TcG_06171	196,3667128	0,061062821	0,12981349	0,47038886	0,63807722	0,80751094	protein_codir	hypothetical protein
TcG_06172	100,67857	0,099889711	0,18220675	0,54822179	0,58353963	0,77214368	protein_codir	hypothetical protein
TcG_06173	81,95061865	-0,021015316	0,19766441	-0,10631816	0,91532992	0,9587752	protein_codir	hypothetical protein
TcG_06174	28,8406954	0,228885948	0,33518177	0,68287111	0,49468829	0,70453824	protein_codir	hypothetical protein
TcG_06175	2106,040274	0,003109039	0,05043846	0,06164024	0,95084933	0,9769901	protein_codir	60S acidic ribosomal protein P0
TcG_06176	57,96750467	0,20719669	0,23725427	0,87331069	0,38249375	0,61243403	protein_codir	60S acidic ribosomal protein P0
TcG_06177	181,8735164	-0,428331318	0,13347789	-3,20900565	0,00133195	0,00984809	protein_codir	hypothetical protein
TcG_06178	283,2021251	0,016146477	0,11100176	0,14546145	0,88434655	0,94355273	protein_codir	hypothetical protein
TcG_06179	405,9345056	-0,052256154	0,09094062	-0,57461841	0,5655494	0,75798662	protein_codir	hypothetical protein
TcG_06180	139,5096788	-0,078093628	0,1544536	-0,50561221	0,61312891	0,79081972	protein_codir	hypothetical protein
TcG_06181	213,4928298	-0,101635787	0,12227926	-0,83117767	0,40587327	0,63464372	protein_codir	transferase, transferring glycosyl group
TcG_06182	996,3039581	-0,142867627	0,06279126	-2,27527894	0,0228892	0,09357597	protein_codir	putative arginyl-tRNA synthetase
TcG_06183	835,1626055	-0,3743768	0,06851586	-5,46408976	4,6529E-08	1,2897E-06	protein_codir	hypothetical protein
TcG_06184	104,215058	-0,399732367	0,17885526	-2,23494889	0,0254207	0,10102821	protein_codir	hypothetical protein
TcG_06185	169,9687759	-0,439332809	0,1409262	-3,11746717	0,00182412	0,01276996	protein_codir	putative leucine-richprotein

TcG_06186	146,7631485	-0,053036817	0,14889795	-0,35619574	0,72169398	0,85711748	protein_codir ribonuclease III
TcG_06187	262,1766545	-0,297063544	0,10977061	-2,70622103	0,00680537	0,03657099	protein_codir hypothetical protein
TcG_06188	179,6191258	-0,137607254	0,13551165	-1,01546439	0,30988452	0,54323751	protein_codir ubiquitin hydrolase
TcG_06189	2,191671918	-1,051222325	1,20009708	-0,87594774	0,38105845	1	protein_codir hypothetical protein
TcG_06190	1,395491747	-1,793269671	1,60314818	-1,11859258	0,26331399	1	protein_codir hypothetical protein
TcG_06191	934,2810249	0,138116384	0,06414969	2,1530329	0,03131609	0,11795457	protein_codir putative lysyl-tRNA synthetase
TcG_06192	586,4976767	0,129649961	0,0797415	1,62587809	0,10397559	0,27636183	protein_codir putative acetylornithine deacetylase-like
TcG_06193	3432,066289	-0,149574666	0,10888777	-1,373659	0,16954757	0,37606704	protein_codir putative ubiquitin-protein ligase-like
TcG_06194	198,1193328	0,163581781	0,12649352	1,29320287	0,1959409	0,4138872	protein_codir hypothetical protein
TcG_06195	292,0020652	0,272485042	0,10906146	2,49845394	0,01247364	0,05881951	protein_codir hypothetical protein
TcG_06196	375,0556142	-0,056332991	0,09305364	-0,60538191	0,54492527	0,74188701	protein_codir hypothetical protein
TcG_06197	613,4249531	-0,293551843	0,07822732	-3,75254912	0,00017505	0,00178843	protein_codir paraflagellar rod protein-like protein
TcG_06198	510,0353226	0,052320525	0,08468858	0,61779907	0,5367078	0,73660472	protein_codir hypothetical protein
TcG_06199	169,4645843	0,146171969	0,13867836	1,05403591	0,29186647	0,52445086	protein_codir putative Golgi pH regulator C
TcG_06200	208,1133879	0,155895636	0,12530698	1,24410973	0,21345918	0,43698047	protein_codir putative NUDIX family hydrolase
TcG_06201	218,2144611	-0,001211677	0,12255304	-0,00988696	0,99211148	0,99678187	protein_codir ATP-dependent DEAD/H RNA helicase
TcG_06202	656,1163332	0,023588099	0,07660115	0,30793402	0,75813254	0,87801518	protein_codir putative ATP-dependent DEAD/H RNA helicase
TcG_06203	242,8887386	-0,159634251	0,11492232	-1,38906215	0,16481385	0,37020808	protein_codir hypothetical protein
TcG_06204	109,1363889	-0,021552566	0,18190477	-0,11848269	0,90568521	0,95410701	protein_codir hypothetical protein
TcG_06205	129,1616656	0,556690672	0,17184247	3,23954064	0,00119722	0,00903062	protein_codir hypothetical protein
TcG_06206	496,6414359	0,356666049	0,08986386	3,96895994	7,2187E-05	0,0008476	protein_codir hypothetical protein
TcG_06207	202,2350501	0,087158109	0,12866447	0,67740618	0,49814828	0,70742498	protein_codir hypothetical protein
TcG_06208	159,0204772	-0,14596105	0,14164333	-1,03048305	0,30278331	0,535498	protein_codir hypothetical protein
TcG_06209	207,4871919	-0,347378984	0,12326369	-2,8181777	0,00482971	0,02803456	protein_codir maoC-like dehydratase
TcG_06210	158,932496	-0,108971326	0,14100439	-0,77282223	0,43962759	0,66274932	protein_codir hypothetical protein
TcG_06211	632,3828053	-0,106840284	0,07673658	-1,39229932	0,16383178	0,36864536	protein_codir putative acyl-CoA dehydrogenase, mitochondrial precursor
TcG_06212	470,5179333	-0,155596585	0,09137011	-1,70292656	0,08858181	0,24742259	protein_codir pre-rRNA-processing protein TSR1
TcG_06213	897,5509153	-0,21474668	0,08180286	-2,62517334	0,00866049	0,04414448	protein_codir hypothetical protein
TcG_06214	901,3188679	-0,288303716	0,06643171	-4,33985077	1,4258E-05	0,00020675	protein_codir putative methionyl-tRNA synthetase
TcG_06215	28,08913431	0,266217091	0,34035843	0,78216687	0,43411651	0,65855826	
TcG_06216	2534,959054	-0,061257866	0,13051255	-0,4693638	0,63880962	0,80773199	protein_codir CCR4-NOT transcription complex subunit 1
TcG_06217	352,0523659	0,072574065	0,10168886	0,71368747	0,47542046	0,69103267	protein_codir putative small GTPase
TcG_06218	372,2057098	0,128086335	0,10008266	1,27980551	0,20061355	0,41986345	protein_codir hypothetical protein
TcG_06219	301,2322887	-0,215605875	0,11486836	-1,87698228	0,06052052	0,19095609	protein_codir hypothetical protein
TcG_06220	938,0634454	-0,137822275	0,06541209	-2,1069846	0,03511891	0,1288843	protein_codir 19S proteasome regulatory subunit
TcG_06221	954,4599071	0,049002539	0,06297207	0,77816304	0,4364729	0,66000718	protein_codir hypothetical protein
TcG_06222	545,2683477	-0,032260409	0,08359491	-0,38591358	0,69956068	0,84432352	protein_codir ATPase subunit 9
TcG_06223	208,3147177	-0,115984559	0,12312914	-0,9419749	0,34620549	0,5804829	protein_codir hypothetical protein
TcG_06224	1924,471934	0,258965392	0,05425379	4,7732225	1,813E-06	3,41E-05	protein_codir ribosomal proteins L36
TcG_06225	133,4341957	0,354126636	0,1543239	2,29469725	0,02175048	0,08968009	
TcG_06226	134,4151741	-0,041409508	0,15275547	-0,27108363	0,78632671	0,89358636	protein_codir hypothetical protein
TcG_06227	97,27485623	-0,208539798	0,18333618	-1,13747214	0,25534097	0,48673584	protein_codir hypothetical protein
TcG_06228	351,1851488	-0,39085043	0,10346011	-3,77778882	0,00015823	0,00164858	protein_codir putative dual specificity protein phosphatase
TcG_06229	830,0777978	-0,126767235	0,07526748	-1,6842231	0,09213856	0,2537919	protein_codir ATP-binding cassette sub-family E member 1
TcG_06230	405,2392367	0,111038855	0,09190983	1,20812823	0,22699794	0,4537609	protein_codir hypothetical protein
TcG_06231	88,69165182	-0,30432105	0,19270875	-1,57917606	0,11429568	0,29375105	protein_codir ATP-binding protein cassette, sub-family E, member 1
TcG_06232	255,5240547	-0,069584601	0,11058165	-0,62925995	0,52917889	0,73119459	protein_codir hypothetical protein
TcG_06233	139,6964831	0,049903852	0,15176087	0,32883214	0,74228257	0,86893659	protein_codir histone deacetylase-like protein HDO1
TcG_06234	142,1420707	0,117615533	0,15813129	0,74378407	0,45700712	0,6767839	protein_codir putative nucleotide-binding protein
TcG_06235	206,4389682	0,043105102	0,12536453	0,34383811	0,73096804	0,86165504	protein_codir hypothetical protein
TcG_06236	322,0107087	-0,171357119	0,10123352	-1,69269158	0,09051419	0,25095367	protein_codir putative phosphoglucomutase
TcG_06237	240,8607492	-0,132050202	0,12923248	-1,02180352	0,3068739	0,54005953	protein_codir putative DNA polymerase eta

TcG_06238	606,5005667	-0,112850737	0,07740382	-1,45794785	0,14485492	0,34175483	protein_codir putative RNA helicase
TcG_06239	143,7555979	-0,094687354	0,14732474	-0,64271185	0,52041109	0,72487171	protein_codir hypothetical protein
TcG_06240	174,4765544	-0,036667688	0,13477178	-0,27207246	0,78556631	0,89351472	protein_codir hypothetical protein
TcG_06241	5,024609892	1,113475993	0,83540259	1,33286156	0,18257724	1	protein_codir hypothetical protein
TcG_06242	48,03466658	0,619022488	0,26876993	2,30316869	0,02126935	0,08829335	protein_codir hypothetical protein
TcG_06243	35,67661767	-0,010334722	0,28945736	-0,03570378	0,97151855	0,98762955	protein_codir helicase-like protein
TcG_06244	18,95182809	0,10142104	0,40888951	0,24804021	0,8041033	0,90318531	protein_codir hypothetical protein
TcG_06245	427,3938453	0,089180324	0,08889827	1,00317277	0,31577751	0,55066198	protein_codir hypothetical protein
TcG_06246	393,8367222	0,008933771	0,09994311	0,08938856	0,92877311	0,96542881	protein_codir hypothetical protein
TcG_06247	571,0861047	0,366944364	0,07893225	4,64885244	3,3379E-06	5,9124E-05	protein_codir putative glycine synthase
TcG_06248	123,2148734	0,096105282	0,16934572	0,56750935	0,57036818	0,76219457	protein_codir hypothetical protein
TcG_06249	161,8319859	0,053413297	0,14209412	0,37590083	0,70699062	0,84892495	protein_codir hypothetical protein
TcG_06250	252,8628138	0,225714178	0,11777107	1,91655021	0,0552951	0,17766196	protein_codir hypothetical protein
TcG_06251	292,1750372	0,170739985	0,11522905	1,48174423	0,13840836	0,33221448	protein_codir hypothetical protein
TcG_06252	670,1896995	-0,078154808	0,07345405	-1,06399597	0,28733053	0,52042924	protein_codir putative glycyl tRNA synthetase
TcG_06253	474,8413727	-0,228778655	0,08579395	-2,666606	0,00766214	0,04002417	protein_codir charged multivesicular body protein 4
TcG_06254	311,7244048	0,189520953	0,10415537	1,81959846	0,06882018	0,20966357	protein_codir hypothetical protein
TcG_06255	568,7962231	0,053730382	0,07842074	0,68515524	0,49324596	0,70396005	protein_codir translation initiation factor 34
TcG_06256	193,188381	0,225180711	0,12685292	1,77513221	0,07587606	0,22272613	protein_codir hypothetical protein
TcG_06257	73,36912779	0,467533372	0,22506663	2,07731096	0,03777287	0,13565916	protein_codir hypothetical protein
TcG_06258	2158,722334	0,15531265	0,04975434	3,12159023	0,00179877	0,01263467	protein_codir adenosylhomocysteinase
TcG_06259	166,2168167	0,509907919	0,13943136	3,65705327	0,00025513	0,00244294	protein_codir hypothetical protein
TcG_06260	309,5427431	0,144214677	0,11284291	1,27801277	0,20124493	0,4207486	protein_codir hypothetical protein
TcG_06261	160,4507524	0,364107034	0,13876397	2,62393068	0,00869215	0,04424746	protein_codir putative oxidoreductase
TcG_06262	355,7808446	0,238007742	0,0959508	2,48051852	0,01311914	0,06126498	protein_codir putative replication factor C, subunit 4
TcG_06263	354,2558913	-0,18504324	0,0972747	-1,90227505	0,05713521	0,18190945	protein_codir golgi familyn subfamily A member 1
TcG_06264	465,1432243	0,031372048	0,08544642	0,36715462	0,71350369	0,85278452	protein_codir phosphoenolpyruvate mutase
TcG_06265	1379,782917	-0,219903716	0,05457702	-4,02923669	5,5958E-05	0,00068031	protein_codir putative 14-3-3 protein
TcG_06266	656,6448144	0,138954244	0,07313137	1,90006349	0,05742479	0,18258057	protein_codir putative DNA topoisomerase III
TcG_06267	576,554507	-0,063880791	0,08070946	-0,7914907	0,4286577	0,65390759	protein_codir putative nucleic acid binding protein
TcG_06268	97,4311621	0,402472293	0,17906672	2,24761081	0,02460102	0,09865953	protein_codir hypothetical protein
TcG_06269	317,0890642	0,106837565	0,10713217	0,99725004	0,31864316	0,55357619	protein_codir putative nucleotide binding protein
TcG_06270	276,361712	0,107392372	0,10757601	0,99829294	0,31813733	0,55327816	protein_codir tubulin-specific chaperone C
TcG_06271	723,6869477	0,014885143	0,0709794	0,20971074	0,83389344	0,91926636	protein_codir putative membrane-bound acid phosphatase 2
TcG_06272	221,8370988	0,182759517	0,12084654	1,51232727	0,13045063	0,32055163	protein_codir ADP-ribosylation factor GTPase activating protein
TcG_06273	195,0943007	-0,066663716	0,13718531	-0,48593918	0,62701029	0,79979536	protein_codir hypothetical protein
TcG_06274	166,0433285	0,139888069	0,14006034	0,99877004	0,3179061	0,55304205	protein_codir cyclophilin-like protein
TcG_06275	324,9340317	-0,137966933	0,11064037	-1,2469854	0,21240285	0,43561044	protein_codir EF hand family protein
TcG_06276	210,3695612	0,250873756	0,12928192	1,94051695	0,0523169	0,17064137	protein_codir hypothetical protein
TcG_06277	188,6236145	0,061352426	0,13028924	0,47089403	0,63771641	0,80751094	protein_codir hypothetical protein
TcG_06278	87,46757639	0,249856167	0,1859189	1,34389873	0,17898114	0,38979933	protein_codir hypothetical protein
TcG_06279	402,1587826	-0,177435761	0,09408547	-1,88589973	0,05930847	0,18756073	protein_codir putative ATP synthase
TcG_06280	133,4746273	0,281515138	0,15929408	1,76726678	0,07718356	0,22508148	protein_codir hypothetical protein
TcG_06281	464,4771995	-0,250521929	0,08859406	-2,82775078	0,00468763	0,02731935	protein_codir hypothetical protein
TcG_06282	69,41038667	0,041978272	0,21061407	0,19931372	0,84201735	0,9231054	protein_codir putative lipoate protein ligase
TcG_06283	26,66694536	0,487979147	0,34551433	1,41232679	0,15785376	0,35991425	protein_codir hypothetical protein
TcG_06284	21,62699099	-0,029293139	0,37724079	-0,07765104	0,93810564	0,97142888	protein_codir hypothetical protein
TcG_06285	19,47336926	0,124272195	0,39343855	0,31586177	0,75210743	0,87492919	protein_codir retrotransposon hot spot (RHS) protein
TcG_06286	360,0055387	0,004875772	0,10010754	0,04870534	0,96115412	0,98198472	protein_codir putative retrotransposon hot spot (RHS) protein
TcG_06287	22,25108128	-0,523600341	0,36539651	-1,43296481	0,15186789	0,35209328	protein_codir hypothetical protein
TcG_06288	45,98001961	0,31666038	0,26058767	1,21517789	0,22429825	0,45108253	protein_codir hypothetical protein
TcG_06289	61,61306311	0,250831009	0,23735358	1,05678209	0,29061105	0,52347942	

TcG_06290	762,2331072	-0,367994338	0,07029736	-5,23482455	1,6514E-07	4,0971E-06	protein_codir hypothetical protein
TcG_06291	163,3212698	0,072094227	0,13868931	0,51982543	0,60318526	0,78522521	protein_codir hypothetical protein
TcG_06292	69,10577535	0,338572679	0,21549282	1,57115528	0,11614659	0,29705835	protein_codir hypothetical protein
TcG_06293	111,7547027	-0,165411943	0,16631939	-0,99454393	0,31995813	0,55511154	protein_codir hypothetical protein
TcG_06294	295,7840962	-0,009081655	0,10454903	-0,08686504	0,93077879	0,96665499	protein_codir hypothetical protein
TcG_06295	218,6054347	0,199873201	0,12206355	1,63745192	0,10153608	0,27181077	protein_codir putative GTPase activator protein
TcG_06296	270,9185068	-0,188738394	0,11805264	-1,59876474	0,10987289	0,28645078	protein_codir hypothetical protein
TcG_06297	436,1246169	-0,015565914	0,09026164	-0,17245326	0,8630812	0,93446022	protein_codir hypothetical protein
TcG_06298	77,16661598	0,214891623	0,20783858	1,03393522	0,30116645	0,53435138	protein_codir hypothetical protein
TcG_06299	212,3169742	0,18295627	0,1223826	1,49495327	0,13492664	0,32683099	protein_codir hypothetical protein
TcG_06300	492,5821192	-0,335770022	0,09339835	-3,59503157	0,00032435	0,00298724	protein_codir endosomal integral membrane protein
TcG_06301	144,6244455	-0,258695728	0,1514768	-1,70782407	0,08766899	0,24564279	protein_codir hypothetical protein
TcG_06302	673,9752095	-0,147779941	0,07197805	-2,05312498	0,04006046	0,14150624	protein_codir hypothetical protein
TcG_06303	518,5366854	-0,212644076	0,08206726	-2,591095	0,00956711	0,0477161	protein_codir putative Zn-finger protein
TcG_06304	202,0413999	-0,050952694	0,12636085	-0,40323165	0,68677782	0,83740347	protein_codir putative ABC transporter
TcG_06305	630,453045	-0,25774856	0,07727973	-3,3352675	0,00085217	0,00684571	protein_codir putative subtilisin-like serine peptidase
TcG_06306	153,6266542	-0,21513428	0,14273042	-1,50727703	0,13173966	0,32262433	protein_codir thioredoxin
TcG_06307	182,6003337	0,082714135	0,13852045	0,59712579	0,5504234	0,74654606	protein_codir hypothetical protein
TcG_06308	131,5718443	-0,24351454	0,15418882	-1,57932686	0,1142611	0,29372735	protein_codir MORN Repeat Containing 1 protein
TcG_06309	158,4916621	0,103429935	0,13921089	0,742973	0,45749803	0,67704332	protein_codir putative mismatch repair protein MSH5
TcG_06310	348,7958859	-0,560471376	0,09797716	-5,720429	1,0626E-08	3,3636E-07	protein_codir putative paraflagellar rod protein 3
TcG_06311	1848,396413	-0,613927548	0,05394038	-11,3815954	5,1646E-30	2,678E-27	protein_codir putative paraflagellar rod protein 1D
TcG_06312	179,3592378	-0,194339081	0,14148524	-1,37356431	0,16957698	0,37606704	protein_codir putative diphthamide synthesis protein
TcG_06313	390,9861806	-0,32750891	0,09103987	-3,5974229	0,00032139	0,00296699	protein_codir topoisomerase (DNA) II binding protein 1
TcG_06314	310,1331887	0,472008102	0,10772368	4,38165596	1,1778E-05	0,00017473	protein_codir 40S ribosomal protein S15a
TcG_06315	246,262218	-0,182551038	0,12700085	-1,43740008	0,15060434	0,35066357	protein_codir putative serine/threonine protein phosphatase type 5
TcG_06316	193,9715701	-0,335192748	0,12834254	-2,61170417	0,00900922	0,04554136	protein_codir hypothetical protein
TcG_06317	249,5986741	-0,243502879	0,11320724	-2,15094802	0,0314803	0,11845755	protein_codir putative Tob55
TcG_06318	171,9951134	-0,27823701	0,14030259	-1,98312094	0,04735392	0,15911907	protein_codir putative dihydrolipoamide dehydrogenase
TcG_06319	2,367169927	-0,899807685	1,1209218	-0,80273903	0,42212559	1	
TcG_06320	541,8373511	-0,189145943	0,08079254	-2,34113141	0,0192254	0,08144259	protein_codir hypothetical protein
TcG_06321	306,2261265	-0,073051224	0,10696355	-0,68295438	0,49463567	0,70453824	protein_codir tRNA pseudouridine38-40 synthase
TcG_06322	227,6638451	0,062737975	0,1194072	0,525412	0,59929681	0,78310281	protein_codir hypothetical protein
TcG_06323	838,3166657	0,024279283	0,06665952	0,36422827	0,71568756	0,85396046	protein_codir ribosomal rRNA-processing protein 9
TcG_06324	410,5486131	0,066251202	0,08923465	0,7424381	0,45782194	0,67717669	protein_codir putative vacuolar fusion protein MON1 B-like
TcG_06325	327,1740947	-0,052570401	0,10002425	-0,52557657	0,59918244	0,78309393	protein_codir hypothetical protein
TcG_06326	78,00723784	-0,127398839	0,20960651	-0,6078	0,54332013	0,74086458	protein_codir hypothetical protein
TcG_06327	445,3252423	-0,108738192	0,08655351	-1,25631183	0,20900294	0,43156443	protein_codir hypothetical protein
TcG_06328	767,0017031	-0,201130067	0,07074996	-2,84282946	0,0044715	0,02639873	protein_codir hypothetical protein
TcG_06329	689,5474126	-0,230134074	0,07198526	-3,19696093	0,00138884	0,0102036	protein_codir hypothetical protein
TcG_06330	178,7873496	-0,05051432	0,13546303	-0,37290114	0,70922202	0,85005287	protein_codir hypothetical protein
TcG_06331	345,7279445	-0,152450808	0,09890936	-1,54131837	0,12323932	0,30852436	protein_codir hypothetical protein
TcG_06332	323,0467131	-0,203977376	0,10200697	-1,99964162	0,04553898	0,15486192	protein_codir hypothetical protein
TcG_06333	158,8729423	-0,128580059	0,13932891	-0,92285268	0,35608399	0,5901286	protein_codir hypothetical protein
TcG_06334	198,1542525	-0,165428253	0,14275227	-1,15884849	0,24651795	0,47515504	protein_codir hypothetical protein
TcG_06335	387,0345087	-0,237642433	0,09365624	-2,53739039	0,01116823	0,05391465	protein_codir hypothetical protein
TcG_06336	254,5364206	-0,047012779	0,11346081	-0,41435257	0,67861592	0,83185018	protein_codir hypothetical protein
TcG_06337	51,51211024	-0,078648393	0,26203764	-0,30014159	0,76406916	0,88102162	protein_codir hypothetical protein
TcG_06338	727,2350849	-0,009883052	0,07828677	-0,12624165	0,89954064	0,95117987	protein_codir putative lysosomal alpha-mannosidase precursor
TcG_06339	60,19162246	0,147905184	0,2253718	0,65627193	0,51164918	0,71775849	protein_codir hypothetical protein
TcG_06340	1095,103167	-0,125034076	0,06058175	-2,0638901	0,03902814	0,13908951	protein_codir putative malate dehydrogenase
TcG_06341	178,6521153	-0,144428228	0,13320662	-1,08424208	0,27825748	0,51099875	protein_codir putative malate dehydrogenase

TcG_06342	175,4720892	-0,098077102	0,13752088	-0,7131797	0,47573457	0,69116462	protein_codir	putative adenylate kinase
TcG_06343	141,1933333	0,14297099	0,15152775	0,94353008	0,34540984	0,57940037		
TcG_06344	172,8645869	-0,391522714	0,13549474	-2,88957867	0,00385758	0,02339999	protein_codir	putative adenylate kinase
TcG_06345	110,1811587	-0,408143348	0,17294238	-2,35999612	0,01827513	0,07821781	protein_codir	hypothetical protein
TcG_06346	193,4013386	-0,083151055	0,13468507	-0,61737394	0,53698811	0,73671334	protein_codir	hypothetical protein
TcG_06347	240,963173	-0,350506496	0,11858163	-2,95582446	0,00311835	0,01966747	protein_codir	hypothetical protein
TcG_06348	135,8085657	-0,136747153	0,15488181	-0,88291293	0,37728332	0,60795612	protein_codir	HPP family protein
TcG_06349	118,4088875	-0,450870148	0,16371523	-2,75399025	0,00588735	0,032528	protein_codir	hypothetical protein
TcG_06350	63,28015442	-0,205144653	0,2279713	-0,89987051	0,36818916	0,60027638		
TcG_06351	479,3377328	-0,200000421	0,08959955	-2,23215877	0,02560447	0,10159363	protein_codir	putative glucose-6-phosphate 1-dehydrogenase
TcG_06352	86,63338909	-0,243731017	0,19384274	-1,25736469	0,20862161	0,43120717	protein_codir	putative glucose-6-phosphate 1-dehydrogenase
TcG_06353	186,906287	-0,033972453	0,1360225	-0,24975613	0,80277594	0,90248031	protein_codir	glucose-6-phosphate dehydrogenase
TcG_06354	83,76658828	0,122753814	0,19157048	0,64077625	0,52166806	0,72540161	protein_codir	hypothetical protein
TcG_06355	883,2015072	-0,158567951	0,06511	-2,43538541	0,01487594	0,06735154	protein_codir	hypothetical protein
TcG_06356	257,0077472	-0,263861427	0,11169171	-2,36240831	0,01815663	0,07791213	protein_codir	putative protein kinase
TcG_06357	315,599089	-0,016899338	0,10218324	-0,16538268	0,86864278	0,93674123	protein_codir	ribosome recycling factor
TcG_06358	760,1084243	-0,123224847	0,06866749	-1,79451506	0,072731	0,21694934	protein_codir	putative eukaryotic translation initiation factor 4e
TcG_06359	1262,045669	0,183155258	0,05883323	3,11312574	0,00185117	0,01294368	protein_codir	putative aspartyl-tRNA synthetase
TcG_06360	469,7595495	0,126457617	0,08674169	1,45786431	0,14487795	0,34175483	protein_codir	hypothetical protein
TcG_06361	757,7672797	0,046412029	0,06947913	0,66799953	0,50413389	0,71207637	protein_codir	putative bystin
TcG_06362	344,9325998	-0,11170155	0,09842197	-1,13492499	0,25640675	0,488446	protein_codir	hypothetical protein
TcG_06363	171,4325862	-0,03926503	0,15336084	-0,25603035	0,7979274	0,89912341		
TcG_06364	456,7364841	-0,093245235	0,08766202	-1,06369023	0,28746905	0,52052968	protein_codir	hypothetical protein
TcG_06365	390,161018	0,109228976	0,0936852	1,16591499	0,24364882	0,47181875	protein_codir	hypothetical protein
TcG_06366	234,0582459	-0,231159882	0,11812009	-1,95699047	0,05034859	0,16631097	protein_codir	putative leucine-rich repeat protein (LRRP)
TcG_06367	362,3347913	-0,070309756	0,09953118	-0,70640937	0,47993359	0,69419608	protein_codir	ama1 protein
TcG_06368	440,5045591	0,140626245	0,08795663	1,59881352	0,10986204	0,28645078	protein_codir	putative N-alpha-acetyltransferase 25, NatB auxiliary subunit isoform X1
TcG_06369	260,5525568	-0,071897886	0,11586951	-0,6205074	0,53492379	0,73509987	protein_codir	putative glycosyltransferase family 28 protein
TcG_06370	77,29210839	-0,010723404	0,20430406	-0,05248748	0,95814027	0,98064539	protein_codir	nuclear cap binding protein
TcG_06371	358,2773496	0,083219276	0,09585008	0,86822328	0,38527211	0,61577634	protein_codir	putative LMBR1-like membrane protein
TcG_06372	426,8128579	-0,022679934	0,0884619	-0,25638082	0,7976568	0,89899335	protein_codir	hypothetical protein
TcG_06373	423,8660882	-0,08315304	0,0907261	-0,91652829	0,35938988	0,5926302	protein_codir	putative spliceosome-associated protein
TcG_06374	344,7522692	0,096489667	0,09951146	0,96963371	0,3322291	0,5663408	protein_codir	hypothetical protein
TcG_06375	633,2002873	-0,022881949	0,07670376	-0,29831588	0,76546208	0,8820133	protein_codir	fatty-acyl-CoA synthase
TcG_06376	289,2310341	0,022523943	0,10551179	0,21347323	0,83095787	0,91760178	protein_codir	putative intergrin alpha chain protein
TcG_06377	420,6333565	-0,262558549	0,09357176	-2,80595922	0,0050167	0,02884543	protein_codir	putative protein kinase
TcG_06378	178,3596164	-0,024182853	0,14210571	-0,1701751	0,86487244	0,93498506	protein_codir	putative nitrate reductase
TcG_06379	387,6048433	0,095078682	0,09434883	1,00773565	0,31358139	0,54790438	protein_codir	putative ribosome biogenesis regulatory protein (RRS1)
TcG_06380	503,9462715	-0,161645745	0,08366679	-1,93201812	0,05335727	0,17297071	protein_codir	putative histidyl-tRNA synthetase
TcG_06381	130,7581281	0,119655864	0,15578	0,76810801	0,44242302	0,66466715	protein_codir	trimeric LpxA-like protein
TcG_06382	123,9118042	0,295178765	0,16022767	1,84224587	0,06543919	0,20218093	protein_codir	hypothetical protein
TcG_06383	269,0429453	-0,032135651	0,11277911	-0,28494328	0,7756876	0,88823053	protein_codir	hypothetical protein
TcG_06384	573,5125619	-0,292876504	0,07836805	-3,73719276	0,00018609	0,00188297	protein_codir	phospholipase C, delta
TcG_06385	481,9073817	0,353073542	0,08579583	4,1152764	3,8672E-05	0,00049128		
TcG_06386	115,0071681	-0,374556834	0,1725838	-2,17028961	0,02998491	0,11442859	protein_codir	hypothetical protein
TcG_06387	264,8946093	-0,316750397	0,11990066	-2,64177365	0,00824732	0,04248706	protein_codir	nuclear movement protein
TcG_06388	205,454763	-0,159658743	0,12685212	-1,25862096	0,20816727	0,43076014	protein_codir	hypothetical protein
TcG_06389	154,3482772	-0,121118096	0,14256354	-0,84957276	0,39556266	0,62523725	protein_codir	4-oxalocrotonate decarboxylase
TcG_06390	95,6869645	-0,362479241	0,18236694	-1,98763679	0,04685187	0,15809563	protein_codir	hypothetical protein
TcG_06391	200,3759194	-0,214117869	0,12648162	-1,6928773	0,09047883	0,25095367	protein_codir	putative CDC16
TcG_06392	22,82632755	0,857324674	0,38557498	2,22349662	0,02618233	0,10328131	protein_codir	40S ribosomal protein S15
TcG_06393	517,2623951	-0,187902089	0,08411666	-2,2338273	0,02549444	0,10126108	protein_codir	transferase

TcG_06394	519,281754	-0,157489584	0,08257019	-1,90734189	0,05647633	0,18020786	protein_codir	hypothetical protein
TcG_06395	1000,910387	0,359758154	0,06927386	5,19327432	2,0663E-07	5,04E-06	protein_codir	40S ribosomal protein S15
TcG_06396	211,1635142	0,055083323	0,14076431	0,39131596	0,6955637	0,84234001	protein_codir	hypothetical protein
TcG_06397	469,431406	0,190958851	0,08510525	2,24379628	0,02484551	0,09925763	protein_codir	hypothetical protein
TcG_06398	318,1224429	0,277999344	0,10550289	2,63499263	0,00841391	0,04318287	protein_codir	hypothetical protein
TcG_06399	185,1457433	-0,07480368	0,13361075	-0,55986273	0,57557307	0,76628647	protein_codir	hypothetical protein
TcG_06400	321,8176863	0,150661536	0,10408965	1,44742096	0,14777906	0,34659275	protein_codir	hypothetical protein
TcG_06401	378,4742702	0,194019831	0,09660468	2,00838957	0,04460191	0,15255431	protein_codir	DNA primase small subunit
TcG_06402	958,2799343	-0,092602748	0,06481007	-1,42883274	0,15305231	0,35345109	protein_codir	hypothetical protein
TcG_06403	187,2045791	0,206318692	0,13557305	1,52182677	0,12805249	0,31693141	protein_codir	hypothetical protein
TcG_06404	125,8249538	-0,115179181	0,16117789	-0,71460907	0,47485064	0,69055096	protein_codir	hypothetical protein
TcG_06405	151,3764496	0,426132081	0,15344613	2,77707944	0,00548498	0,03075058	protein_codir	hypothetical protein
TcG_06406	220,8337828	0,193104611	0,11925726	1,61922726	0,10539837	0,2791828	protein_codir	putative methylenetetrahydrofolate dehydrogenase-like protein
TcG_06407	307,2500438	-0,203486501	0,10266485	-1,98204643	0,04747404	0,15933786	protein_codir	hypothetical protein
TcG_06408	380,9848365	-0,159508433	0,09383987	-1,69979376	0,08916973	0,24858529	protein_codir	UBX domain-containing protein 1
TcG_06409	477,1896193	0,206291186	0,08569573	2,40725157	0,01607309	0,07132243	protein_codir	putative carnitine O-palmitoyltransferase II
TcG_06410	1195,520063	0,028303821	0,05742639	0,49287133	0,62210351	0,79640439	protein_codir	hypothetical protein
TcG_06411	356,7890507	-0,081264988	0,10689959	-0,76019926	0,44713549	0,66845313	protein_codir	mitochondrial carrier domain-containing protein
TcG_06412	549,0789718	0,055081017	0,07901505	0,69709528	0,48574317	0,69827461	protein_codir	hypothetical protein
TcG_06413	180,6011276	-0,053792612	0,13461534	-0,39960239	0,68944939	0,8386805	protein_codir	hypothetical protein
TcG_06414	300,3751981	0,073013036	0,10972862	0,66539647	0,50579694	0,71267956	protein_codir	hypothetical protein
TcG_06415	1272,196325	-0,269272563	0,06086975	-4,42375033	9,7002E-06	0,00014827	protein_codir	translocon-associated protein subunit alpha
TcG_06416	52,14308395	-0,2929318	0,26484171	-1,10606371	0,26869894	0,50131175	protein_codir	hypothetical protein
TcG_06417	516,9541111	0,213134239	0,08674289	2,45708034	0,01400714	0,06442504	protein_codir	hypothetical protein
TcG_06418	457,1162561	0,895879444	0,09446703	9,48351448	2,4586E-21	5,0867E-19	protein_codir	hypothetical protein
TcG_06419	381,4814495	-0,150582773	0,09292731	-1,62043611	0,10513863	0,27863257	protein_codir	EF hand
TcG_06420	2400,806757	-0,002827127	0,05075577	-0,0557006	0,95558032	0,97933248	protein_codir	hypothetical protein
TcG_06421	1398,855885	-0,166357831	0,0571227	-2,91228959	0,0035879	0,02200603	protein_codir	hypothetical protein
TcG_06422	951,7058378	-0,0986613	0,06645	-1,48474483	0,13761143	0,33091865	protein_codir	hypothetical protein
TcG_06423	173,7560286	0,195154843	0,13532863	1,44208095	0,14927954	0,34850525	protein_codir	hypothetical protein
TcG_06424	219,9596556	2,95963E-05	0,1260847	0,00023473	0,99981271	0,99981271	protein_codir	hypothetical protein
TcG_06425	443,59343	-0,029831455	0,08845986	-0,33723155	0,73594235	0,86494473	protein_codir	putative conserved oligomeric golgi complex subunit
TcG_06426	252,2612624	-0,048107817	0,11741559	-0,40972256	0,68200948	0,83422317	protein_codir	hypothetical protein
TcG_06427	260,0359065	0,235493726	0,11749808	2,00423463	0,04504493	0,15363279	protein_codir	hypothetical protein
TcG_06428	11,99550087	-0,041411192	0,49534299	-0,08360105	0,93337363	1	protein_codir	hypothetical protein
TcG_06429	113,3464144	0,011657407	0,16469987	0,07077969	0,94357309	0,97337158	protein_codir	transferase
TcG_06430	146,9261216	-0,323123489	0,14649766	-2,20565635	0,02740807	0,10691916	protein_codir	hypothetical protein
TcG_06431	392,3044115	-0,085379206	0,09500514	-0,89867984	0,36882322	0,60086146	protein_codir	hypothetical protein
TcG_06432	229,3757145	0,004255271	0,11850808	0,03590701	0,9713565	0,98762955	protein_codir	apurinic/apyrimidinic endonuclease
TcG_06433	386,2411381	-0,076295713	0,09527177	-0,80082183	0,4232348	0,64922526	protein_codir	putative adaptin
TcG_06434	257,7202593	0,023804564	0,1108534	0,21473915	0,8299707	0,91738605	protein_codir	hypothetical protein
TcG_06435	274,7634404	-0,197895732	0,11290723	-1,7527286	0,0796486	0,23029916	protein_codir	hypothetical protein
TcG_06436	584,1978895	0,027872953	0,07865465	0,35437131	0,72306063	0,85733557	protein_codir	hypothetical protein
TcG_06437	622,9517117	-0,014122757	0,07442881	-0,18974852	0,8495062	0,92756374	protein_codir	putative glycosyl hydrolase
TcG_06438	275,199301	-0,052991629	0,11063208	-0,47898974	0,63194593	0,80290602	protein_codir	hypothetical protein
TcG_06439	295,2508948	-0,065938054	0,10975209	-0,60079084	0,5479793	0,74465027	protein_codir	protein OS-9-like protein isoform X2
TcG_06440	30,59125438	0,203354243	0,31629942	0,6429169	0,52027802	0,7247735		
TcG_06441	625,1433403	-0,021078147	0,07911014	-0,26644053	0,78989996	0,89502471	protein_codir	hypothetical protein
TcG_06442	340,5372732	-0,011030048	0,09795649	-0,1126015	0,91034649	0,95647184	protein_codir	hypothetical protein
TcG_06443	979,7585755	0,055563926	0,06359088	0,87377191	0,38224248	0,61220091	protein_codir	putative helicase
TcG_06444	435,7787194	-0,089588867	0,08914991	-1,0049238	0,31493354	0,54960386	protein_codir	putative aldehyde dehydrogenase
TcG_06445	252,3511562	0,012299581	0,11847471	0,10381609	0,9173153	0,96016036	protein_codir	vacuolar protein sorting-associated protein-like

TcG_06446	450,5222618	-0,119347509	0,09241524	-1,29142665	0,19655577	0,41473232	protein_codir	pre-mRNA-splicing factor CWC22
TcG_06447	360,0944179	-0,138370203	0,09748441	-1,41940853	0,15577995	0,35732854	protein_codir	putative kinesin-like protein
TcG_06448	387,8144776	-0,125602062	0,09348953	-1,34348801	0,17911401	0,39000468	protein_codir	hypothetical protein
TcG_06449	415,2300427	0,235579531	0,09009061	2,61491776	0,00892489	0,04519398	protein_codir	hypothetical protein
TcG_06450	372,9668138	0,147627994	0,09802348	1,50604731	0,13205503	0,32285072	protein_codir	hypothetical protein
TcG_06451	1851,609913	0,130104	0,05322314	2,44450085	0,01450527	0,06621675	protein_codir	hypothetical protein
TcG_06452	535,3749689	0,063933816	0,08119123	0,78744733	0,43102005	0,65575992	protein_codir	putative leucine-rich repeat protein (LRRP)
TcG_06453	665,6646895	-0,184693573	0,07700026	-2,39860961	0,01645745	0,07256541	protein_codir	hypothetical protein
TcG_06454	369,2717148	0,201478797	0,09648401	2,08820918	0,03677897	0,13335584	protein_codir	hypothetical protein
TcG_06455	302,6733649	0,033775235	0,10898528	0,3099064	0,75663214	0,87751151	protein_codir	hypothetical protein
TcG_06456	493,0833433	0,084531852	0,08691931	0,97253253	0,33078569	0,56523583	protein_codir	hypothetical protein
TcG_06457	306,5236815	-0,160189129	0,10546511	-1,51888268	0,12879203	0,31792545	protein_codir	putative ubiquitin activating E1 enzyme
TcG_06458	321,5733941	0,095747464	0,09976686	0,95971209	0,33720014	0,57160877	protein_codir	tRNA-splicing ligase RtcB
TcG_06459	123,9923483	0,167107742	0,16342287	1,0225481	0,30652156	0,53955618	protein_codir	hypothetical protein
TcG_06460	176,752047	-0,165887717	0,13280716	-1,24908714	0,2116332	0,43456948	protein_codir	hypothetical protein
TcG_06461	248,1664329	-0,226392862	0,11264139	-2,00985504	0,04444653	0,1522642	protein_codir	hypothetical protein
TcG_06462	43,59565342	0,132696446	0,26238685	0,50572826	0,61304743	0,79081972	protein_codir	procyclic form surface glycoprotein
TcG_06463	425,3266953	0,137378985	0,09065539	1,51539785	0,12967168	0,31936907	protein_codir	surface glycoprotein
TcG_06464	8,956040806	-0,136434708	0,63229719	-0,21577624	0,82916217	1	protein_codir	hypothetical protein
TcG_06465	422,3516477	0,167588877	0,08976165	1,86704324	0,06189555	0,19413152	protein_codir	putative surface glycoprotein
TcG_06466	299,5080724	0,15703604	0,10541891	1,4896383	0,13631936	0,32916375	protein_codir	putative procyclic form surface glycoprotein
TcG_06467	221,4281079	-0,113198283	0,1213909	-0,93251045	0,35107279	0,58556643	protein_codir	hypothetical protein
TcG_06468	174,4955065	-0,099425147	0,13459013	-0,7387254	0,46007376	0,67839985	protein_codir	hypothetical protein
TcG_06469	518,5762712	-0,477911123	0,08093593	-5,90480831	3,5306E-09	1,2396E-07	protein_codir	hypothetical protein
TcG_06470	164,1931738	0,233717898	0,14518192	1,60982789	0,10743544	0,28225554	protein_codir	hypothetical protein
TcG_06471	221,5283751	0,005228986	0,11871102	0,04404802	0,96486612	0,9843472	protein_codir	RNA-binding protein
TcG_06472	193,6417386	-0,388095796	0,13225939	-2,93435348	0,00334243	0,02076431	protein_codir	hypothetical protein
TcG_06473	193,0758974	-0,855866076	0,13301351	-6,4344295	1,2394E-10	6,0065E-09	protein_codir	hypothetical protein
TcG_06474	372,2297968	-0,549270709	0,09807294	-5,60063443	2,1357E-08	6,3938E-07	protein_codir	putative intraflagellar transport protein 57
TcG_06475	241,9021198	-0,319530482	0,11683799	-2,73481676	0,0062415	0,03406219	protein_codir	hypothetical protein
TcG_06476	165,6073314	-0,26495773	0,13613156	-1,94633586	0,05161441	0,16892784	protein_codir	hypothetical protein
TcG_06477	1845,854322	-0,267922799	0,05712242	-4,69032678	2,7277E-06	4,9303E-05	protein_codir	hypothetical protein
TcG_06478	97,43597155	0,074534048	0,17674512	0,42170357	0,6732414	0,82890021	protein_codir	hypothetical protein
TcG_06479	357,9863245	-0,105998841	0,09719785	-1,09054712	0,27547221	0,50773647	protein_codir	putative protein phosphatase 2A regulatory subunit
TcG_06480	258,9521415	-0,12307019	0,1142385	-1,07730921	0,28134215	0,51438064	protein_codir	putative sterol C-24 reductase
TcG_06481	59,73910549	-0,276937891	0,22992707	-1,2044597	0,22841195	0,45564187	protein_codir	amino acid transporter
TcG_06482	30,84948905	0,288480987	0,31706228	0,90985589	0,36289851	0,59520699	protein_codir	retrotransposon hot spot (RHS) protein
TcG_06483	0,467164841	2,094702395	2,70879376	0,77329711	0,43934656	1		
TcG_06484	625,0197556	0,081079926	0,07949415	1,01994838	0,30775294	0,54090194	protein_codir	putative CAAX prenyl protease 1
TcG_06485	183,5352109	0,238228348	0,13461731	1,76967093	0,07678198	0,22458873	protein_codir	putative amino acid permease-like protein
TcG_06486	294,1214922	-0,651108987	0,10942498	-5,95027732	2,6769E-09	9,753E-08	protein_codir	nuclear protein Tc22
TcG_06487	522,3328727	0,072067504	0,08416164	0,85629867	0,3918326	0,62139829	protein_codir	putative isopentenyl-diphosphate delta-isomerase
TcG_06488	279,5085276	-0,181609293	0,10925259	-1,66228824	0,096455	0,26270042	protein_codir	hypothetical protein
TcG_06489	359,7691008	-0,10091474	0,09560018	-1,05559151	0,29115488	0,52365178	protein_codir	putative GTP-binding protein
TcG_06490	191,0130061	0,024003098	0,13053055	0,18388874	0,85410074	0,92916537	protein_codir	hypothetical protein
TcG_06491	300,9704287	-0,058875732	0,10723893	-0,54901454	0,58299547	0,77177623	protein_codir	p21 antigen protein
TcG_06492	280,2093064	0,130019599	0,10834559	1,2000452	0,23012179	0,45716581	protein_codir	hypothetical protein
TcG_06493	389,2306548	-0,154780542	0,0995587	-1,55466621	0,12002559	0,3032308	protein_codir	WD repeat-containing protein 68
TcG_06494	162,008772	-0,076389234	0,13922091	-0,54869081	0,58321765	0,77198045	protein_codir	hypothetical protein
TcG_06495	192,5815956	0,162404771	0,12731102	1,27565366	0,20207798	0,42219675	protein_codir	tRNA (guanine-N(7)-)-methyltransferase
TcG_06496	208,4423715	-0,23977157	0,12948169	-1,85177976	0,06405746	0,19891977	protein_codir	hypothetical protein
TcG_06497	309,3300656	-0,159413927	0,10573698	-1,5076459	0,13164518	0,32246111	protein_codir	putative GTP-binding protein

TcG_06498	18,14623885	-0,374955943	0,42026797	-0,892183	0,37229485	0,6035725	protein_codir	hypothetical protein
TcG_06499	228,6410038	0,009601161	0,11768568	0,08158309	0,93497825	0,96927863	protein_codir	hypothetical protein
TcG_06500	102,0140988	0,160366713	0,17505049	0,91611691	0,35960558	0,5926302	protein_codir	GTP-binding protein
TcG_06501	274,0762186	0,178269377	0,11735333	1,51908242	0,12874175	0,31790323	protein_codir	putative map kinase
TcG_06502	113,9225854	-0,13671029	0,16886018	-0,80960643	0,41816641	0,6448657	protein_codir	hypothetical protein
TcG_06503	310,3534177	-0,023830316	0,10612146	-0,224557	0,82232392	0,91189174	protein_codir	hypothetical protein
TcG_06504	212,3267375	0,098661147	0,12292083	0,80263978	0,42218297	0,64838439	protein_codir	putative dolichyl-P-Man:GDP-Man1GlcNAc2-PP-dolichyl alpha-1,3-mannosyltransferase
TcG_06505	660,4378109	0,449374044	0,08159065	5,50766606	3,6362E-08	1,0326E-06		
TcG_06506	337,7466376	0,084747888	0,09954591	0,85134475	0,39457787	0,62431497	protein_codir	hypothetical protein
TcG_06507	106,2513226	-0,171381949	0,17278563	-0,9918762	0,32125792	0,5560344	protein_codir	hypothetical protein
TcG_06508	176,6201193	0,145631279	0,13563238	1,07372055	0,28294795	0,51617618	protein_codir	hypothetical protein
TcG_06509	82,19249285	-0,099544065	0,19816881	-0,50231955	0,61544276	0,79219196	protein_codir	putative p21-activated kinase 3
TcG_06510	41,3419035	0,048722213	0,27967581	0,17420961	0,86170073	0,93381907	protein_codir	hypothetical protein
TcG_06511	261,2503868	-0,016414269	0,11252295	-0,14586579	0,88402734	0,94348884	protein_codir	putative ankyrin repeat and zinc finger domain protein
TcG_06512	219,9252843	-0,26592148	0,12288754	-2,16394169	0,03046882	0,11560515	protein_codir	putative UDP-Gal or UDP-GlcNAc-dependent glycosyltransferase
TcG_06513	1155,489183	-0,097843843	0,06074329	-1,61077609	0,10722853	0,28209578	protein_codir	putative polyadenylate-binding protein 1
TcG_06514	146,0796393	-0,039839919	0,14874884	-0,26783348	0,7888275	0,89487471	protein_codir	putative SUMO1/Ulp2
TcG_06515	284,8188066	-0,023932234	0,10990377	-0,21775626	0,82761902	0,91588083	protein_codir	putative DNA repair protein RAD2
TcG_06516	706,1152385	-0,083834241	0,07194846	-1,16519856	0,24393863	0,47210867	protein_codir	putative target SNARE
TcG_06517	32,48485585	-0,376612678	0,33817346	-1,11366717	0,265422	0,49800474		
TcG_06518	219,3209828	-0,210159148	0,12206204	-1,72174043	0,08511655	0,2405856	protein_codir	putative protein kinase
TcG_06519	336,6748677	-0,099974938	0,09815085	-1,01858453	0,30840025	0,54138262	protein_codir	putative phosphatidylcholine:ceramide cholinephosphotransferase 2
TcG_06520	581,4466549	-0,047511002	0,08081551	-0,58789463	0,55660302	0,75125846	protein_codir	hypothetical protein
TcG_06521	607,6686844	-0,255794183	0,0768158	-3,3299684	0,00086856	0,00694487	protein_codir	3-hydroxyacyl-CoA dehydrogenase
TcG_06522	394,0257209	0,098440954	0,09570493	1,02858809	0,30367328	0,53649872	protein_codir	hypothetical protein
TcG_06523	320,4654519	0,193078645	0,10520338	1,83528941	0,06646282	0,20468852	protein_codir	sucraseferredoxin-like family protein
TcG_06524	153,8444194	-0,198177919	0,14120594	-1,40346732	0,16047753	0,36342703	protein_codir	hypothetical protein
TcG_06525	308,1440157	-0,05885574	0,10882032	-0,54085248	0,58860927	0,77564856	protein_codir	hypothetical protein
TcG_06526	323,4460241	-0,109744573	0,10010419	-1,09630351	0,27294597	0,50613829	protein_codir	hypothetical protein
TcG_06527	548,9815121	-0,18893378	0,07973608	-2,36948906	0,01781268	0,07692051	protein_codir	hypothetical protein
TcG_06528	321,9339089	0,196867851	0,10638147	1,85058412	0,06422941	0,19929349	protein_codir	hypothetical protein
TcG_06529	252,2975553	-0,026817711	0,11320587	-0,23689329	0,81273958	0,90759432	protein_codir	cytoplasmic tRNA 2-thiolation protein 1
TcG_06530	443,7566204	-0,229829793	0,0893687	-2,57170336	0,01011996	0,04982992		
TcG_06531	206,7424847	-0,223784884	0,12401897	-1,80444077	0,07116224	0,21392986	protein_codir	centrin
TcG_06532	791,2927153	-0,154998232	0,069539	-2,2289395	0,02581793	0,10219561	protein_codir	hypothetical protein
TcG_06533	428,8685465	-0,16784773	0,09315629	-1,80178636	0,07157902	0,21495971	protein_codir	ubiquinone biosynthesis protein
TcG_06534	469,5640299	-0,031709928	0,08675645	-0,36550515	0,71473436	0,8536143	protein_codir	hypothetical protein
TcG_06535	277,8204428	0,007387882	0,10934159	0,06756699	0,94613033	0,97469438	protein_codir	putative rab-like GTPase activating protein
TcG_06536	419,0516315	-0,038023742	0,09080846	-0,41872465	0,67541738	0,83012118	protein_codir	hypothetical protein
TcG_06537	303,0604715	-0,165980728	0,10658052	-1,55732711	0,11939284	0,30216464	protein_codir	putative rab-like GTPase activating protein
TcG_06538	1352,540589	-0,073727737	0,056213	-1,311578	0,18966256	0,40475786	protein_codir	hypothetical protein
TcG_06539	336,4827305	-0,13229675	0,09818976	-1,34735791	0,17786499	0,3883799	protein_codir	ubiquitin thioesterase OTU1
TcG_06540	331,3846091	-0,138021748	0,09799245	-1,40849366	0,15898494	0,36134294	protein_codir	hypothetical protein
TcG_06541	680,3404698	-0,10335711	0,07208207	-1,43388098	0,15160623	0,35179947	protein_codir	hypothetical protein
TcG_06542	546,0155986	-0,238564783	0,07915192	-3,01401135	0,00257818	0,01682863	protein_codir	hypothetical protein
TcG_06543	718,5896088	-0,349199015	0,07544589	-4,62846962	3,6838E-06	6,4084E-05	protein_codir	hypothetical protein
TcG_06544	314,9096927	0,150685299	0,1008719	1,49382831	0,1352205	0,32734323	protein_codir	hypothetical protein
TcG_06545	540,3422539	-0,000237822	0,07965562	-0,00298563	0,99761782	0,99910416	protein_codir	MmpL efflux pump
TcG_06546	248,9014214	-0,000296415	0,12015265	-0,00246699	0,99803163	0,99932542	protein_codir	hypothetical protein
TcG_06547	222,5891547	0,133283708	0,11825067	1,12712855	0,25968814	0,4921081	protein_codir	hypothetical protein
TcG_06548	281,9221152	-0,310601575	0,11520716	-2,69602671	0,0070172	0,03736274	protein_codir	hypothetical protein
TcG_06549	21,28647611	-0,209124248	0,38727922	-0,53998314	0,58920866	0,77564856		

TcG_06550	180,4166293	-0,209557768	0,13395138	-1,56443153	0,11771626	0,29924769	protein_codir	hypothetical protein
TcG_06551	149,9808791	-0,2417395	0,14483103	-1,66911403	0,09509478	0,25985098	protein_codir	hypothetical protein
TcG_06552	297,1685697	-0,307769734	0,10347337	-2,97438603	0,00293575	0,01868882	protein_codir	hypothetical protein
TcG_06553	250,7350462	-0,127540544	0,11744073	-1,0859992	0,27747934	0,50997393	protein_codir	putative histidine phosphatase
TcG_06554	764,9238319	0,053771199	0,07288111	0,73779339	0,46064001	0,6786591	protein_codir	activating signal cointegrator 1 complex subunit 3
TcG_06555	80,35198346	0,152136485	0,20037142	0,75927236	0,44768965	0,66876383	protein_codir	hypothetical protein
TcG_06556	432,3554629	-0,252350395	0,08758235	-2,88129296	0,00396047	0,02389898	protein_codir	hypothetical protein
TcG_06557	313,2314036	-0,070128236	0,10536761	-0,66555779	0,50569379	0,71267956	protein_codir	hypothetical protein
TcG_06558	158,7858652	0,032561208	0,14487943	0,22474693	0,82217615	0,91187534	protein_codir	hypothetical protein
TcG_06559	297,5455147	-0,197739588	0,11062019	-1,78755415	0,07384797	0,21927282	protein_codir	hypothetical protein
TcG_06560	1086,23554	0,154532491	0,06245269	2,47439275	0,01334629	0,06217535	protein_codir	40S ribosomal protein S12
TcG_06561	357,0394449	-0,323915947	0,0994369	-3,25750237	0,00112397	0,00857863	protein_codir	hypothetical protein
TcG_06562	107,3687677	0,28677573	0,16893239	1,69757692	0,08958764	0,24955883	protein_codir	hypothetical protein
TcG_06563	222,5553529	0,073078268	0,11863897	0,61597189	0,53791307	0,73738082	protein_codir	F-actin capping protein
TcG_06564	94,3493115	-0,119232742	0,19740414	-0,60400326	0,54584148	0,74285471	protein_codir	hypothetical protein
TcG_06565	85,36319184	0,29728287	0,19270655	1,54267135	0,12291054	0,30780546	protein_codir	putative actyltransferase-like
TcG_06566	109,8820304	-0,109320518	0,17178572	-0,6363772	0,5245306	0,72807135	protein_codir	hypothetical protein
TcG_06567	99,30141729	0,159160752	0,17844484	0,89193252	0,3724291	0,60357583	protein_codir	hypothetical protein
TcG_06568	181,5648956	-0,137734404	0,13480448	-1,02173463	0,30690652	0,54005953	protein_codir	hypothetical protein
TcG_06569	348,3951447	-0,113695727	0,1172406	-0,9697641	0,33216409	0,5663408	protein_codir	hypothetical protein
TcG_06570	1098,889016	-0,084142435	0,06091388	-1,38133429	0,1671762	0,37312723	protein_codir	importin-7
TcG_06571	132,1784658	-0,018067766	0,15077084	-0,11983594	0,90461311	0,95370277	protein_codir	putative zinc-binding protein
TcG_06572	220,3209114	-0,16478825	0,12123237	-1,35927595	0,17405916	0,38259333	protein_codir	hypothetical protein
TcG_06573	755,9516109	-0,27351644	0,0700274	-3,9058491	9,3895E-05	0,00105824	protein_codir	antigen 38
TcG_06574	503,2167837	-0,13036235	0,0823323	-1,58336817	0,11333757	0,29206609	protein_codir	proliferation-associated 2g4
TcG_06575	460,3865628	-0,410758059	0,09320893	-4,4068529	1,0488E-05	0,00015802	protein_codir	hypothetical protein
TcG_06576	41,93288749	0,028907841	0,2709598	0,10668683	0,91503743	0,95872886	protein_codir	hypothetical protein
TcG_06577	46,14449911	0,318768549	0,2733121	1,16631702	0,24348629	0,47180551	protein_codir	putative kinesin
TcG_06578	55,54282875	0,432269764	0,23322449	1,85344927	0,06381799	0,19849537	protein_codir	hypothetical protein
TcG_06579	24,76933192	0,217914951	0,34886579	0,62463836	0,53220844	0,7329332	protein_codir	putative trans-sialidase
TcG_06580	23,42665101	-0,324020838	0,37232266	-0,87026891	0,38415346	0,61449954	protein_codir	hypothetical protein
TcG_06581	50,77138134	-0,174187421	0,24412578	-0,71351507	0,47552709	0,69110096	protein_codir	protein ARV1
TcG_06582	257,9865906	-0,021522892	0,11656641	-0,1846406	0,85351094	0,92904714	protein_codir	putative glutathionylspermidine synthase
TcG_06583	576,3656764	-0,11291169	0,0783648	-1,44084711	0,14962788	0,34909155	protein_codir	pumilio protein 8
TcG_06584	123,5748907	-0,473820419	0,16192869	-2,92610544	0,00343235	0,02126586	protein_codir	hypothetical protein
TcG_06585	288,6991811	-0,355369512	0,10740983	-3,30853817	0,00093784	0,00740181	protein_codir	putative rac serine-threonine kinase
TcG_06586	373,0317994	-0,159926022	0,09506256	-1,68232399	0,09250603	0,25457836	protein_codir	putative protein kinase
TcG_06587	219,9003871	-0,445489583	0,12704807	-3,50646466	0,0004541	0,00398276	protein_codir	peptide hydrolase
TcG_06588	142,4076598	-0,22863585	0,15619845	-1,46375234	0,14326163	0,33971126	protein_codir	hypothetical protein
TcG_06589	706,2894468	-0,306963902	0,07130486	-4,30495047	1,6702E-05	0,00023686	protein_codir	putative structural maintenance of chromosome (SMC) family protein
TcG_06590	240,0894464	-0,321257438	0,11462875	-2,80259046	0,0050694	0,02900622	protein_codir	putative DNA repair protein
TcG_06591	315,8356001	-0,119448342	0,10342848	-1,15488826	0,24813619	0,47744549	protein_codir	DHHC containing zinc finger protein
TcG_06592	118,0978637	-0,415987856	0,16533765	-2,51598983	0,01186986	0,05647811	protein_codir	hypothetical protein
TcG_06593	78,83512939	0,099598507	0,20659466	0,48209622	0,62973759	0,80168887	protein_codir	hypothetical protein
TcG_06594	121,3387484	-0,256473316	0,17754532	-1,44455127	0,14858397	0,34749574	protein_codir	hypothetical protein
TcG_06595	128,1132906	0,174557448	0,15541339	1,1231815	0,26136042	0,49358779	protein_codir	hypothetical protein
TcG_06596	101,4770425	-0,160210056	0,17488406	-0,91609295	0,35961815	0,5926302	protein_codir	hypothetical protein
TcG_06597	209,6235669	-0,261487244	0,12449657	-2,10035703	0,03569745	0,13051139	protein_codir	putative DNA repair protein
TcG_06598	286,7950422	-0,391379471	0,11093445	-3,52802464	0,00041867	0,00371705	protein_codir	hypothetical protein
TcG_06599	241,8176652	-0,266947009	0,11996751	-2,22516088	0,02607044	0,10294891	protein_codir	putative membrane-bound acid phosphatase 2
TcG_06600	299,2065581	-0,402315915	0,10369783	-3,8796947	0,00010459	0,00115846	protein_codir	putative proteasome regulatory non-ATPase subunit 6
TcG_06601	315,6793145	-0,671641246	0,10294009	-6,52458357	6,8191E-11	3,4987E-09	protein_codir	ParcG domain containing protein

TcG_06602	194,8992346	0,133574235	0,12715218	1,05050687	0,29348513	0,52652814	protein_codir zf-DNL-domain-containing protein
TcG_06603	140,5143155	-0,245447655	0,14934814	-1,64345978	0,10028784	0,26921569	protein_codir putative chaperone DNAJ protein
TcG_06604	190,7773722	-0,342444176	0,13049111	-2,6242721	0,00868344	0,04422256	protein_codir putative vacuolar protein sorting-associated protein
TcG_06605	93,18225498	-0,436968916	0,18294254	-2,38855833	0,01691462	0,07411983	protein_codir polyribonucleotide 5-hydroxyl-kinase
TcG_06606	200,002852	-0,277584842	0,12751304	-2,17691337	0,02948703	0,11286401	protein_codir hypothetical protein
TcG_06607	110,315297	-0,356571897	0,16636494	-2,14331157	0,03208809	0,12027583	protein_codir hypothetical protein
TcG_06608	386,1527177	0,013841296	0,0925904	0,14948953	0,88116737	0,94206932	protein_codir putative replication factor A, 51kDa subunit
TcG_06609	1004,675434	-0,372866235	0,07099447	-5,25204617	1,5042E-07	3,7804E-06	protein_codir succinyl-CoA synthetase alpha subunit
TcG_06610	319,9980888	-0,312459402	0,10037452	-3,11293542	0,00185237	0,01294421	protein_codir hypothetical protein
TcG_06611	522,1420044	-0,353843453	0,08240239	-4,29409196	1,7541E-05	0,00024732	protein_codir putative L-ribulokinase
TcG_06612	334,5532595	0,082272357	0,09841903	0,83593955	0,40318891	0,63298674	protein_codir syntaxin
TcG_06613	213,9479223	-0,210147753	0,12381444	-1,69727979	0,08964378	0,24957742	protein_codir putative stress-induced protein sti1
TcG_06614	157,6702369	-0,038054532	0,14707662	-0,2587395	0,79583624	0,89816468	protein_codir putative RNA triphosphatase
TcG_06615	48,06339378	0,048945976	0,24894536	0,19661332	0,84413014	0,92439431	protein_codir putative trans-sialidase
TcG_06616	413,287863	-0,039353161	0,09229855	-0,42636814	0,66983961	0,82684442	protein_codir hypothetical protein
TcG_06617	489,4473598	0,066181369	0,08705635	0,76021303	0,44712726	0,66845313	protein_codir hypothetical protein
TcG_06618	250,9416492	0,162605249	0,11663956	1,39408318	0,16329249	0,36778903	protein_codir hypothetical protein
TcG_06619	385,2555085	0,222313473	0,09405273	2,36371091	0,01809292	0,07775394	protein_codir hypothetical protein
TcG_06620	342,1869817	0,114321267	0,09928984	1,15138942	0,24957206	0,47881138	protein_codir hypothetical protein
TcG_06621	210,5664762	0,101308412	0,12390803	0,81760971	0,41358008	0,64029481	protein_codir hypothetical protein
TcG_06622	558,755806	0,0485399	0,08172342	0,59395334	0,55254332	0,74786997	protein_codir hypothetical protein
TcG_06623	29,48054552	0,117807414	0,32679422	0,36049418	0,71847761	0,85543948	protein_codir hypothetical protein
TcG_06624	412,4974695	0,022848441	0,09012379	0,25352287	0,79986419	0,90034262	protein_codir nuclear transport factor 2 protein(NFT2)
TcG_06625	315,1452337	-0,279038073	0,10341118	-2,6983356	0,00696871	0,03719002	protein_codir putative protein kinase
TcG_06626	131,7043073	0,335474772	0,15813651	2,1214251	0,03388604	0,12531239	protein_codir protein disulfide-isomerase
TcG_06627	310,0386265	0,084433996	0,10197419	0,8279938	0,40767401	0,63596487	protein_codir protein disulfide isomerase
TcG_06628	82,73810853	0,101560773	0,19825731	0,51226749	0,60846381	0,78802389	protein_codir hypothetical protein
TcG_06629	358,8969588	-0,148939067	0,09573313	-1,55577346	0,11976197	0,30291726	protein_codir hypothetical protein
TcG_06630	72,01720804	-0,055658923	0,20761178	-0,26809136	0,788629	0,89487471	protein_codir hypothetical protein
TcG_06631	51,33692399	0,303108884	0,24515111	1,23641652	0,21630378	0,4403612	protein_codir hypothetical protein
TcG_06632	487,9658428	-0,1239531	0,0831644	-1,49045864	0,13610368	0,32893143	protein_codir cysteamine dioxygenase
TcG_06633	209,5809698	-0,195113765	0,12200693	-1,59920235	0,10977565	0,28632613	protein_codir hypothetical protein
TcG_06634	544,5818274	0,129205886	0,07958022	1,62359306	0,10446269	0,27740195	protein_codir putative trans-sialidase
TcG_06635	544,1741265	-0,415831386	0,08338572	-4,98684186	6,1374E-07	1,3095E-05	protein_codir putative cysteine protease
TcG_06636	526,2890007	0,08209817	0,08026406	1,02285095	0,30637833	0,53946798	protein_codir putative Monooxygenase
TcG_06637	65,63455286	0,222202	0,23955367	0,92756668	0,35363239	0,58808452	protein_codir hypothetical protein
TcG_06638	282,476087	-0,167672675	0,1137212	-1,47441877	0,14036887	0,33486575	protein_codir hypothetical protein
TcG_06639	487,9064952	0,051059324	0,08758036	0,5829997	0,5598935	0,75405439	protein_codir hypothetical protein
TcG_06640	179,7821091	0,101433091	0,13581127	0,74686802	0,45514323	0,67511068	protein_codir hypothetical protein
TcG_06641	293,0517331	0,038879343	0,11026576	0,35259671	0,72439081	0,85798219	protein_codir putative cis-prenyltransferase
TcG_06642	442,464756	0,074604392	0,09940082	0,75054101	0,45292893	0,67355117	protein_codir hypothetical protein
TcG_06643	52,45569848	0,040604007	0,2550552	0,15919694	0,87351371	0,93932295	protein_codir Atg8-like protein 1
TcG_06644	120,607518	-0,043481895	0,16044182	-0,27101347	0,78638067	0,89358636	protein_codir microtubule-associated protein 1A/1B, light chain 3
TcG_06645	161,2765952	0,099685024	0,14622209	0,68173711	0,4954052	0,70513079	protein_codir hypothetical protein
TcG_06646	468,8225783	0,325724766	0,08709789	3,73975491	0,0001842	0,00186551	protein_codir putative mercaptopyruvate sulfurtransferase
TcG_06647	63,45426173	0,256100218	0,22654268	1,13047227	0,25827728	0,49064584	
TcG_06648	30,52187734	-0,111608045	0,31486389	-0,35446442	0,72299087	0,85733557	protein_codir trans-sialidase
TcG_06649	51,04590781	0,458828616	0,25015715	1,83416149	0,06663003	0,20498553	protein_codir putative trans-sialidase
TcG_06650	23,1230285	0,111772875	0,36813573	0,30361865	0,76141844	0,87949151	protein_codir hypothetical protein
TcG_06651	62,64587595	0,072893816	0,23209224	0,31407262	0,75346589	0,87552748	protein_codir hypothetical protein
TcG_06652	3,062740066	-0,120316741	0,99227526	-0,12125339	0,90349034	1	protein_codir retrotransposon hot spot (RHS) protein
TcG_06653	329,2486403	0,100560554	0,10214913	0,98444843	0,32489508	0,5593216	protein_codir retrotransposon hot spot (RHS) protein

TcG_06654	173,138642	0,152838861	0,13676428	1,11753494	0,26376567	0,49602159	protein_codir	putative kinesin
TcG_06655	193,5362493	0,048049671	0,12968832	0,37050115	0,71100912	0,8514472	protein_codir	hypothetical protein
TcG_06656	95,44780657	0,092648365	0,18002289	0,51464769	0,60679923	0,78754071	protein_codir	putative kinesin
TcG_06657	99,89397817	-0,661047369	0,17758419	-3,72244498	0,0001973	0,00196895	protein_codir	PSP1 family protein
TcG_06658	3,984253535	-1,435996602	0,89575058	-1,60312104	0,10890793	1		
TcG_06659	744,3108712	-0,443495379	0,0704871	-6,29186544	3,1367E-10	1,3924E-08	protein_codir	hypothetical protein
TcG_06660	241,3436621	-0,392919463	0,11485765	-3,42092537	0,00062408	0,00519443	protein_codir	putative ATP-dependent DEAD/H RNA helicase
TcG_06661	194,2372244	-0,275761334	0,12579321	-2,19217983	0,02836652	0,10972667	protein_codir	hypothetical protein
TcG_06662	564,6238404	-0,617148803	0,080696	-7,647824	2,0441E-14	1,9573E-12	protein_codir	hypothetical protein
TcG_06663	714,4096919	-0,243162755	0,07076585	-3,43615956	0,00059002	0,00498615	protein_codir	putative nucleoside phosphatase, putative,guanosine diphosphatase
TcG_06664	187,6884175	-0,660402988	0,13103605	-5,03985739	4,6588E-07	1,0184E-05	protein_codir	putative leucine-rich repeat protein
TcG_06665	415,9507115	-0,470770371	0,09187755	-5,12388908	2,993E-07	6,8803E-06	protein_codir	hypothetical protein
TcG_06666	135,3775238	-0,535966835	0,15987466	-3,35241888	0,00080109	0,00647238	protein_codir	central apparatus associated protein C1a-18
TcG_06667	244,4489132	-0,635959227	0,11427486	-5,56517194	2,6189E-08	7,7013E-07	protein_codir	putative mitogen-activated protein kinase
TcG_06668	676,4913884	-0,268099855	0,0746221	-3,59276736	0,00032718	0,00300854	protein_codir	putative nucleolar protein
TcG_06669	190,1621565	-0,273022743	0,12814553	-2,13056785	0,03312476	0,12310099	protein_codir	hypothetical protein
TcG_06670	143,9127995	-0,208676554	0,1545591	-1,3501408	0,17697082	0,38718302	protein_codir	hypothetical protein
TcG_06671	120,4011533	-0,140671552	0,16387084	-0,85842945	0,39065537	0,62055474	protein_codir	putative golgi apparatus membrane protein
TcG_06672	166,6295135	0,238225398	0,13846472	1,72047727	0,08534572	0,24111571	protein_codir	hypothetical protein
TcG_06673	457,9745279	-0,133549716	0,08570778	-1,55819822	0,11918627	0,30178161	protein_codir	hypothetical protein
TcG_06674	361,1830469	-0,22890913	0,09797022	-2,33651743	0,01946429	0,08231055	protein_codir	putative dispersed gene family protein 1 (DGF-1)
TcG_06675	297,1525543	-0,377370216	0,12427547	-3,03656237	0,00239293	0,01587883	protein_codir	hypothetical protein
TcG_06676	620,0277479	-0,61597798	0,07583257	-8,1228683	4,5529E-16	5,275E-14	protein_codir	putative isocitrate dehydrogenase, mitochondrial precursor
TcG_06677	402,391755	-0,442298477	0,09050766	-4,88686215	1,0246E-06	2,0674E-05	protein_codir	putative leucine-rich repeat protein
TcG_06678	121,3148305	-0,278014241	0,16782098	-1,65661197	0,09759795	0,26438388	protein_codir	hypothetical protein
TcG_06679	651,2803657	-0,596464295	0,08329994	-7,16044072	8,0418E-13	6,1298E-11	protein_codir	putative protein transport protein Sec23
TcG_06680	467,1783782	-0,355039469	0,08436724	-4,20826235	2,5734E-05	0,00034509	protein_codir	putative trans-sialidase
TcG_06681	39,46214161	-0,596504306	0,28305715	-2,10736352	0,03508608	0,12884543	protein_codir	COP9 signalosome complex subunit 6
TcG_06682	196,6811863	-0,063599472	0,13142741	-0,48391331	0,62844739	0,80083497	protein_codir	COP9 signalosome complex subunit 6
TcG_06683	1273,191266	-0,244969018	0,05660982	-4,32732353	1,5093E-05	0,00021723	protein_codir	esag10
TcG_06684	501,7630351	0,161080584	0,08591354	1,87491505	0,06080441	0,19148679	protein_codir	ATP-binding cassette protein subfamily G, member 5
TcG_06685	688,6939094	0,2622986	0,07295878	3,59516161	0,00032419	0,00298724	protein_codir	hypothetical protein
TcG_06686	354,0588501	0,490648058	0,10492059	4,67637515	2,9199E-06	5,2531E-05	protein_codir	putative aldose 1-epimerase-like protein
TcG_06687	166,7873004	0,61790838	0,14036935	4,40201786	1,0725E-05	0,00016117	protein_codir	hypothetical protein
TcG_06688	447,2383656	0,154853579	0,08820447	1,75562063	0,07915321	0,22920996	protein_codir	permease-like protein
TcG_06689	119,0182957	0,481788431	0,16694771	2,88586422	0,00390341	0,02361611	protein_codir	trypanothione synthetase
TcG_06690	223,6600909	0,918804753	0,12520146	7,33861076	2,1582E-13	1,7861E-11	protein_codir	hypothetical protein
TcG_06691	729,8109016	0,055783575	0,07339614	0,76003421	0,44723414	0,66851435	protein_codir	ER to golgi family vesicle transport protein
TcG_06692	207,1961871	0,255374674	0,12278947	2,07977662	0,03754603	0,13501188	protein_codir	hypothetical protein
TcG_06693	461,2618171	0,203387339	0,08552877	2,37799897	0,01740687	0,0755341	protein_codir	5-AMP-activated protein kinase, regulatory beta subunit
TcG_06694	363,7637701	0,132104232	0,09697053	1,36231325	0,17309905	0,38135113	protein_codir	hypothetical protein
TcG_06695	289,2177021	0,53479223	0,11291254	4,73634053	2,1761E-06	4,0211E-05	protein_codir	putative 3-oxo-5-alpha-steroid 4-dehydrogenase
TcG_06696	235,6664346	0,53440719	0,12262201	4,35816702	1,3116E-05	0,00019211	protein_codir	hypothetical protein
TcG_06697	331,3502752	0,525146887	0,10173245	5,16203888	2,4427E-07	5,7877E-06	protein_codir	putative ribosomal RNA methyltransferase
TcG_06698	236,4734656	0,588394571	0,12836422	4,58378971	4,5662E-06	7,7339E-05	protein_codir	hypothetical protein
TcG_06699	384,9002703	0,264283874	0,09258182	2,85459794	0,00430913	0,02562917	protein_codir	acyl-CoA synthetase short-chain family member 2
TcG_06700	28,13547203	-0,106296202	0,33572155	-0,31662013	0,75153186	0,87492445		
TcG_06701	216,2425297	0,1976785	0,12649046	1,56279371	0,11810112	0,3000043	protein_codir	hypothetical protein
TcG_06702	547,7584246	0,452638498	0,08084254	5,59901351	2,1557E-08	6,4207E-07	protein_codir	putative 5'-3' exonuclease XRNC
TcG_06703	585,3053773	0,372749411	0,08018588	4,64856688	3,3425E-06	5,9124E-05	protein_codir	hypothetical protein
TcG_06704	290,3366304	0,439691383	0,10584459	4,1541223	3,2654E-05	0,00042413	protein_codir	putative oxidoreductase
TcG_06705	117,0925494	0,477737492	0,1795478	2,66078162	0,00779595	0,04064981	protein_codir	hypothetical protein

TcG_06706	179,7098531	0,40623331	0,13438994	3,02279549	0,00250451	0,01644365	protein_codir hypothetical protein
TcG_06707	365,7360004	0,279940032	0,09894209	2,82933204	0,00466453	0,0272258	protein_codir hypothetical protein
TcG_06708	262,0194904	0,419385145	0,11644705	3,6015095	0,00031637	0,00292994	protein_codir Protein X92
TcG_06709	660,773579	0,445288653	0,07655436	5,81663363	6,0045E-09	1,9933E-07	protein_codir putative ribonuclease
TcG_06710	181,4234754	0,490918765	0,13180258	3,72465215	0,00019558	0,00195686	protein_codir putative amino acid transporter
TcG_06711	236,0341364	0,008587823	0,11546696	0,07437472	0,94071222	0,97252537	protein_codir putative trans-sialidase
TcG_06712	868,4208901	0,710888519	0,06747012	10,5363453	5,8737E-26	1,9444E-23	protein_codir cathepsin B-like protease precursor
TcG_06713	56,97745239	0,354934357	0,24312133	1,45990631	0,14431583	0,34095497	protein_codir hypothetical protein
TcG_06714	97,55196839	0,386529439	0,18090772	2,13661113	0,03262963	0,121872	protein_codir hypothetical protein
TcG_06715	11,14044554	0,734882992	0,53887865	1,3637263	0,17265372	1	protein_codir hypothetical protein
TcG_06716	228,4087233	-0,051689308	0,12088457	-0,42759226	0,668948	0,8263077	protein_codir hypothetical protein
TcG_06717	428,8011118	-0,128528112	0,09690102	-1,32638554	0,18471203	0,39778319	protein_codir putative CCR4 associated factor
TcG_06718	122,8053646	-0,029819228	0,17416861	-0,17120897	0,86405945	0,93498506	protein_codir hypothetical protein
TcG_06719	229,470356	0,465925981	0,11796508	3,94969401	7,8251E-05	0,00090391	protein_codir hypothetical protein
TcG_06720	162,2315208	0,258237289	0,14166152	1,82291761	0,06831587	0,20840118	protein_codir hypothetical protein
TcG_06721	827,5766023	-0,073085094	0,0797791	-0,91609324	0,35961799	0,5926302	protein_codir hypothetical protein
TcG_06722	394,6446203	0,011701721	0,09215019	0,12698531	0,89895202	0,95069557	protein_codir hypothetical protein
TcG_06723	561,5580818	-0,042284185	0,08623797	-0,49031982	0,6239076	0,79706621	protein_codir putative serine/threonine protein phosphatase
TcG_06724	552,7112411	0,217385057	0,08049676	2,70054432	0,00692261	0,0369951	protein_codir hypothetical protein
TcG_06725	275,6373378	-0,070695276	0,10780464	-0,65577212	0,51197076	0,71812267	protein_codir hypothetical protein
TcG_06726	191,0299373	0,242000957	0,13764552	1,75814632	0,07872262	0,22841979	protein_codir putative ribosomal RNA processing protein 45
TcG_06727	182,0549847	-0,014234828	0,13323654	-0,10683877	0,91491689	0,95868925	protein_codir hypothetical protein
TcG_06728	47,30974776	0,279011779	0,25369708	1,09978317	0,27142661	0,50457365	protein_codir hypothetical protein
TcG_06729	285,0260922	-0,22195052	0,10881028	-2,0397937	0,04137088	0,14474996	protein_codir hypothetical protein
TcG_06730	871,8887107	-0,170602415	0,06702126	-2,545497	0,01091223	0,0528992	protein_codir alanine-tRNA ligase
TcG_06731	202,8113051	0,270025237	0,1273905	2,11966535	0,03403428	0,12574016	protein_codir putative dephospho-CoA kinase
TcG_06732	1291,946465	0,245525903	0,05728349	4,28615453	1,8179E-05	0,00025438	protein_codir 40S ribosomal protein L14
TcG_06733	242,4041304	0,02962964	0,11515749	0,25729669	0,79694974	0,89872949	protein_codir hypothetical protein
TcG_06734	723,5691558	-0,093140673	0,07310394	-1,27408551	0,20263311	0,42278178	protein_codir putative ATP-dependent DEAD/H RNA helicase
TcG_06735	232,4756703	-0,087511466	0,11707267	-0,747497	0,45476361	0,67475691	protein_codir putative serine/threonine protein phosphatase
TcG_06736	620,1589605	0,062489732	0,08361258	0,74737236	0,45483883	0,67475691	protein_codir L1Tc protein
TcG_06737	358,3719279	0,159037657	0,1062529	1,4967841	0,13444944	0,32602161	protein_codir putative kinetoplast DNA-associated protein
TcG_06738	87,20374628	0,185743473	0,18752919	0,99047768	0,3219407	0,55671715	
TcG_06739	492,7987749	0,016176072	0,08301954	0,19484656	0,84551307	0,92503539	protein_codir putative protein kinase, putative,cdc2-related kinase
TcG_06740	76,44317913	-0,051292691	0,20569864	-0,24935844	0,80308353	0,90265093	protein_codir hypothetical protein
TcG_06741	218,8768038	0,030054927	0,12884137	0,23327078	0,81555115	0,90916729	protein_codir hypothetical protein
TcG_06742	305,4856283	-0,102557453	0,10461154	-0,98036459	0,32690618	0,56146317	protein_codir hypothetical protein
TcG_06743	120,5500647	-0,346306167	0,16495821	-2,09935695	0,03578545	0,13075061	protein_codir hypothetical protein
TcG_06744	612,4414487	0,041924884	0,07980305	0,52535442	0,59933683	0,78310281	protein_codir putative ATP-dependent RNA helicase
TcG_06745	669,5224601	-0,04631238	0,07694316	-0,60190376	0,5472382	0,74407955	protein_codir hypothetical protein
TcG_06746	293,047504	-0,132278323	0,10643975	-1,24275304	0,21395885	0,43752213	protein_codir putative RNA-binding protein
TcG_06747	197,58539	-0,100271885	0,1254241	-0,79946263	0,42402221	0,64991682	protein_codir hypothetical protein
TcG_06748	54,13770083	0,05938488	0,23907366	0,24839574	0,80382823	0,90318531	protein_codir hypothetical protein
TcG_06749	310,9080599	0,035226864	0,11020327	0,31965351	0,749231	0,87347018	protein_codir UDP-glucuronic acid/UDP-N-acetylgalactosamine transporter
TcG_06750	598,4279431	-0,224135599	0,07933886	-2,82504195	0,00472744	0,02752369	protein_codir putative kinesin
TcG_06751	570,82339	0,085258205	0,09084684	0,93848285	0,34799633	0,58255822	protein_codir hypothetical protein
TcG_06752	386,1702245	0,126970744	0,09740543	1,30352837	0,19239444	0,40883522	protein_codir hypothetical protein
TcG_06753	288,4481114	0,018481419	0,10922772	0,16920081	0,86563869	0,93530634	protein_codir nitroreductase
TcG_06754	826,244664	0,162262419	0,07524541	2,15644285	0,0310491	0,11725388	protein_codir hypothetical protein
TcG_06755	578,7403371	0,222372159	0,08374019	2,65550096	0,00791907	0,04118059	protein_codir hypothetical protein
TcG_06756	63,96921877	0,030648394	0,22138596	0,13843874	0,88989368	0,94653573	protein_codir hypothetical protein
TcG_06757	826,6579789	0,169785224	0,07186377	2,36259818	0,01814733	0,07791213	protein_codir hypothetical protein

TcG_06758	485,2224892	-0,206331109	0,08852933	-2,33065262	0,01977168	0,08326962	protein_codir	hypothetical protein
TcG_06759	471,3583556	-0,084352697	0,08868302	-0,95117077	0,3415177	0,57595692	protein_codir	cyclin 4
TcG_06760	34,50390022	0,253471133	0,2991458	0,84731638	0,3968188	0,62662432	protein_codir	trans-sialidase
TcG_06761	77,73255841	0,342478964	0,20035058	1,70939846	0,08737717	0,24512151	protein_codir	hypothetical protein
TcG_06762	283,8173679	0,22077805	0,1072315	2,05889166	0,03950462	0,14000054	protein_codir	hypothetical protein
TcG_06763	111,0408472	0,110953979	0,18558433	0,59786285	0,54993145	0,7459906	protein_codir	hypothetical protein
TcG_06764	444,7073813	-0,044797398	0,09165298	-0,48877187	0,62500322	0,79811388	protein_codir	hypothetical protein
TcG_06765	215,5134153	0,182573858	0,12345117	1,47891555	0,13916289	0,33354183	protein_codir	hypothetical protein
TcG_06766	292,6731778	0,151781748	0,10936268	1,3878752	0,16517505	0,3707155	protein_codir	hypothetical protein
TcG_06767	577,5742229	-0,223864935	0,08468416	-2,64352799	0,0082047	0,04232397	protein_codir	hypothetical protein
TcG_06768	376,8751356	0,366490033	0,09515112	3,85166281	0,00011732	0,00127869	protein_codir	hypothetical protein
TcG_06769	236,0188721	-0,001929985	0,11565653	-0,01668721	0,98668615	0,9946703	protein_codir	glycerolphosphate mutase
TcG_06770	165,2693003	-0,163657407	0,13726353	-1,19228612	0,23314907	0,46049525	protein_codir	putative phosphoglycerate mutase
TcG_06771	219,6550183	-0,055519576	0,1212929	-0,45773144	0,6471454	0,81242026	protein_codir	zinc finger family protein
TcG_06772	445,9915274	0,086119397	0,08744185	0,98487619	0,32468489	0,55907719	protein_codir	hypothetical protein
TcG_06773	437,4581011	-0,049947587	0,08627722	-0,57891974	0,56264333	0,75597653	protein_codir	putative kinesin
TcG_06774	503,2363259	0,142467385	0,08743902	1,62933416	0,1032423	0,27498052	protein_codir	hypothetical protein
TcG_06775	333,0805567	-0,158581553	0,09911103	-1,60003942	0,10958984	0,28616785	protein_codir	4-nitrophenylphosphatase
TcG_06776	409,6346542	-0,029530454	0,09580651	-0,30823014	0,75790722	0,87801518	protein_codir	hypothetical protein
TcG_06777	275,3449755	0,755589679	0,11433137	6,60876949	3,8753E-11	2,1279E-09	protein_codir	hypothetical protein
TcG_06778	129,3463967	0,094985997	0,1527538	0,62182412	0,53405752	0,73443768	protein_codir	hypothetical protein
TcG_06779	22,19285771	-0,34320931	0,38241437	-0,89748016	0,36946275	0,60112883		
TcG_06780	318,7200753	0,106407281	0,10258651	1,03724439	0,29962197	0,53340814	protein_codir	hypothetical protein
TcG_06781	291,5778625	-0,043664142	0,11033826	-0,39572983	0,69230435	0,84069156	protein_codir	hypothetical protein
TcG_06782	243,0080898	-0,081690255	0,11648093	-0,70131873	0,48310412	0,6965212	protein_codir	hypothetical protein
TcG_06783	470,3122317	-0,147579077	0,0866331	-1,70349531	0,08847541	0,24718499	protein_codir	hypothetical protein
TcG_06784	418,5521098	0,059279969	0,0930706	0,6369355	0,52416686	0,72782805	protein_codir	transferase
TcG_06785	406,982462	-0,058731561	0,09165058	-0,64082039	0,52163938	0,72540161	protein_codir	hypothetical protein
TcG_06786	227,1477756	-0,25442199	0,11741155	-2,16692478	0,03024059	0,11517668	protein_codir	hypothetical protein
TcG_06787	252,6959078	-0,011066414	0,1111737	-0,09954165	0,92070822	0,96150609	protein_codir	hypothetical protein
TcG_06788	153,4419046	-0,056072385	0,14739334	-0,38042686	0,70362858	0,84707405	protein_codir	pre-rRNA-processing protein TSR3
TcG_06789	204,3868942	0,028986319	0,14096898	0,20562197	0,83708622	0,92068482	protein_codir	hypothetical protein
TcG_06790	375,3926235	-0,219765197	0,09489421	-2,31589671	0,02056391	0,08598104	protein_codir	flagellar associated protein
TcG_06791	199,9337386	0,325268151	0,12574939	2,58663802	0,00969173	0,04821315	protein_codir	hypothetical protein
TcG_06792	293,6663916	-0,401134374	0,10478713	-3,82808825	0,00012914	0,00138413	protein_codir	calpain-like cysteine peptidase
TcG_06793	441,3160334	-0,369309828	0,08847259	-4,17428508	2,9892E-05	0,00039446	protein_codir	COP-coated vesicle membrane protein p24 precursor
TcG_06794	339,14752	-0,208770852	0,09969728	-2,09404753	0,03625575	0,13205252	protein_codir	ras-related protein rab-2a
TcG_06795	258,2084012	0,098034024	0,11217729	0,87392038	0,38216162	0,61215602	protein_codir	hypothetical protein
TcG_06796	109,7388764	0,228864415	0,16900177	1,35421314	0,17566839	0,38518054	protein_codir	hypothetical protein
TcG_06797	228,8777347	-0,25517048	0,13013345	-1,96083701	0,04989804	0,16527121	protein_codir	pantetheine-phosphate adenylyltransferase
TcG_06798	211,8967114	0,341956862	0,12402452	2,7571715	0,00583038	0,03230547	protein_codir	putative deaminase
TcG_06799	176,694462	0,113763789	0,13733228	0,82838346	0,40745337	0,63572073	protein_codir	N5-glutamine methyltransferase MTQ2
TcG_06800	209,9146871	0,129890365	0,12676096	1,02468743	0,3055107	0,53859509	protein_codir	hypothetical protein
TcG_06801	536,5273844	-0,03889458	0,08282278	-0,46961208	0,63863219	0,80773199	protein_codir	hypothetical protein
TcG_06802	269,5725211	0,027297474	0,11357311	0,24035157	0,81005772	0,90627702	protein_codir	hypothetical protein
TcG_06803	307,7905076	0,045571964	0,10654592	0,42772134	0,66885401	0,8263077	protein_codir	putative phosphatidylinositol 3-kinase (tor2)
TcG_06804	338,6466358	-0,101035462	0,09892893	-1,02129343	0,30711544	0,5401912	protein_codir	hypothetical protein
TcG_06805	283,224298	0,167825461	0,11898252	1,41050517	0,15839057	0,36053303	protein_codir	cleavage stimulation factor subunit 2
TcG_06806	228,1413741	-0,161792038	0,12002154	-1,34802504	0,17765033	0,3879843	protein_codir	hypothetical protein
TcG_06807	1336,216923	0,037724292	0,05951437	0,63386862	0,52616657	0,72894486	protein_codir	karyopherin beta
TcG_06808	454,4565965	-0,124084221	0,08746771	-1,41862887	0,15600724	0,35756675	protein_codir	hypothetical protein
TcG_06809	129,3316759	-0,372652198	0,15622342	-2,38537986	0,01706149	0,07445643	protein_codir	hypothetical protein

TcG_06810	239,5797576	-0,415361877	0,11428143	-3,63455262	0,00027846	0,0026294	protein_codir	putative serine carboxypeptidase S28
TcG_06811	15,48027972	0,44638493	0,45339225	0,98454469	0,32484777	1	protein_codir	putative R-SNARE protein
TcG_06812	113,0347213	-0,060970064	0,17113353	-0,35627187	0,72163697	0,85711748	protein_codir	esag4
TcG_06813	52,93441093	0,027405474	0,2501737	0,10954579	0,91276961	0,95723874	protein_codir	adenylate cyclase
TcG_06814	41,25391384	0,117326599	0,2892819	0,40557877	0,68505213	0,83655708	protein_codir	hypothetical protein
TcG_06815	292,3135849	-0,049848929	0,11166202	-0,44642689	0,65528891	0,81772823	protein_codir	hypothetical protein
TcG_06816	253,4374476	-0,381011701	0,11432902	-3,33258953	0,00086042	0,00689405	protein_codir	hypothetical protein
TcG_06817	707,751604	-0,203994132	0,07821516	-2,60811497	0,00910424	0,04588155	protein_codir	myo-inositol-1-phosphate synthase
TcG_06818	2167,291299	0,2769779	0,0475596	5,82380694	5,7522E-09	1,9317E-07	protein_codir	putative delta-4 fatty acid desaturase
TcG_06819	358,0538336	0,014901263	0,09609465	0,15506861	0,87676723	0,94101205	protein_codir	putative DEAD/DEAH box helicase
TcG_06820	102,744925	0,188699907	0,17545005	1,07551927	0,28214231	0,51535564	protein_codir	small nuclear RNA activating protein 3
TcG_06821	604,3906796	-0,024597187	0,07839453	-0,31376154	0,75370216	0,87569126	protein_codir	putative nucleoporin interacting component (NUP93)
TcG_06822	464,1216539	0,006169644	0,0989568	0,06234684	0,95028663	0,97694613	protein_codir	putative chromatin assembly factor 1 subunit B
TcG_06823	421,4807255	-0,49679403	0,09247881	-5,37197678	7,7878E-08	2,0648E-06	protein_codir	hypothetical protein
TcG_06824	333,4930041	-0,082254578	0,09869171	-0,83344968	0,40459117	0,63405833	protein_codir	hypothetical protein
TcG_06825	397,6653613	-0,042348544	0,09386679	-0,45115578	0,65187728	0,81583489	protein_codir	hypothetical protein
TcG_06826	574,5587667	0,027922352	0,07808849	0,35757323	0,72066271	0,85670516	protein_codir	putative acid phosphatase
TcG_06827	262,6264906	-0,000719315	0,11617409	-0,0061917	0,99505977	0,99790206	protein_codir	hypothetical protein
TcG_06828	328,2267611	-0,093442665	0,1198731	-0,77951322	0,43567745	0,65939964	protein_codir	hypothetical protein
TcG_06829	217,2761315	-0,205507834	0,12651249	-1,62440745	0,10428888	0,2770674	protein_codir	hypothetical protein
TcG_06830	177,6623264	0,028136515	0,13425411	0,20957657	0,83399817	0,91929434	protein_codir	hypothetical protein
TcG_06831	109,8324303	-0,079028408	0,169815	-0,46537942	0,64165977	0,80983334	protein_codir	caltractin
TcG_06832	43,03794475	-0,076670502	0,26494852	-0,28937886	0,77229147	0,88635651	protein_codir	hypothetical protein
TcG_06833	215,0448027	-0,227541682	0,13194339	-1,72454027	0,08461036	0,23968107	protein_codir	hypothetical protein
TcG_06834	10,37531145	0,722775138	0,57336164	1,26059207	0,20745586	1	protein_codir	hypothetical protein
TcG_06835	591,7126492	-0,110583004	0,07800465	-1,4176463	0,15629405	0,35769079	protein_codir	putative dipeptidyl-peptidase 8-like serine peptidase
TcG_06836	614,0031195	-0,13677587	0,07922719	-1,72637542	0,0842799	0,23909571	protein_codir	hypothetical protein
TcG_06837	192,475353	-0,227469319	0,12625665	-1,80164233	0,0716017	0,21497208	protein_codir	hypothetical protein
TcG_06838	1401,293591	0,172805695	0,0576715	2,99637965	0,00273206	0,01763435		
TcG_06839	3217,637202	0,23157119	0,04490395	5,15703331	2,5089E-07	5,8962E-06	protein_codir	putative sterol 24-c-methyltransferase
TcG_06840	67,27635854	0,143548944	0,22439753	0,63970823	0,5223623	0,72619266	protein_codir	putative sterol 24-c-methyltransferase
TcG_06841	635,0526627	-0,111533547	0,07552536	-1,47676944	0,13973745	0,33429108	protein_codir	hypothetical protein
TcG_06842	293,3987715	-0,459518171	0,11602925	-3,96036477	7,4835E-05	0,00087312		
TcG_06843	148,2026522	0,260272502	0,14606395	1,78190793	0,07476425	0,22066725	protein_codir	hypothetical protein
TcG_06844	235,4421175	0,276105697	0,11832666	2,33341915	0,01962616	0,08277709	protein_codir	putative tyrosine aminotransferase
TcG_06845	178,7841322	0,031880928	0,13316367	0,2394116	0,81078644	0,90682225	protein_codir	putative haloacid dehalogenase hydrolase
TcG_06846	202,4953013	0,125403863	0,12436228	1,0083754	0,31327428	0,54745034	protein_codir	hypothetical protein
TcG_06847	618,4640736	-0,234914417	0,07564071	-3,10566113	0,00189854	0,01318735	protein_codir	histone H2B variant
TcG_06848	292,4798942	0,319708956	0,10571403	3,02428108	0,00249225	0,01640635	protein_codir	hypothetical protein
TcG_06849	289,5673458	-0,031426882	0,10915021	-0,28792324	0,77340549	0,88691888	protein_codir	hypothetical protein
TcG_06850	181,4677158	0,312291128	0,13077329	2,38803451	0,01693875	0,07419749	protein_codir	hypothetical protein
TcG_06851	328,2396105	-0,209895814	0,10216363	-2,05450616	0,03992673	0,14111991	protein_codir	putative phopshatase
TcG_06852	209,1464385	0,335003551	0,12937315	2,58943651	0,00961331	0,04786414	protein_codir	RNA-binding protein
TcG_06853	594,0999174	0,179190952	0,07991768	2,242194	0,02494884	0,0995719	protein_codir	putative major facilitator superfamily
TcG_06854	1117,364134	0,241111406	0,0649368	3,71301668	0,0002048	0,00202981	protein_codir	putative pantothenate kinase subunit
TcG_06855	125,2537829	-0,407932611	0,15836522	-2,57589778	0,00999802	0,04935537	protein_codir	hypothetical protein
TcG_06856	218,2663865	-0,01823362	0,12016292	-0,15174081	0,87939137	0,94162382	protein_codir	DNA-direcetd RNA polymerase II, subunit 9
TcG_06857	246,5103488	-0,031777948	0,11975126	-0,2653663	0,7907273	0,8952767	protein_codir	putative proteasome beta 3 subunit
TcG_06858	399,4866843	0,148464066	0,09306176	1,59532834	0,11063883	0,28754183	protein_codir	hypothetical protein
TcG_06859	284,8413322	-0,025503658	0,10745673	-0,23733885	0,81239392	0,90759432	protein_codir	hypothetical protein
TcG_06860	324,9310502	-0,410519001	0,10255101	-4,00307136	6,2525E-05	0,00074606	protein_codir	flagellar associated protein
TcG_06861	68,5067065	0,27745537	0,21222814	1,30734488	0,19109561	0,40668168	protein_codir	putative palmitoyltransferase ZDHHC12

TcG_06862	1262,237875	0,2700908	0,0593392	4,55164204	5,3229E-06	8,8608E-05	protein_codir	putative neurobeachin/beige protein
TcG_06863	509,7587775	-0,115923811	0,08152493	-1,42194303	0,15504279	0,35638871	protein_codir	hypothetical protein
TcG_06864	177,0114211	-0,056273166	0,14959966	-0,37615838	0,70679915	0,84892495	protein_codir	putative origin recognition complex subunit 1, putative,cell division cycle 6
TcG_06865	202,6130409	0,023306164	0,12495314	0,18651923	0,8520376	0,92831556	protein_codir	hypothetical protein
TcG_06866	227,4939087	-0,378035046	0,1243278	-3,04063161	0,00236082	0,01570879	protein_codir	putative pyruvate dehydrogenase (lipoamide) kinase
TcG_06867	71,18567586	0,186160102	0,20823559	0,8939879	0,37132837	0,60280376	protein_codir	putative PIMT protein
TcG_06868	127,2760287	-0,00343744	0,16107146	-0,02134109	0,98297357	0,99369442	protein_codir	hypothetical protein
TcG_06869	297,444179	-0,311435071	0,10328074	-3,01542241	0,00256622	0,01677888	protein_codir	ERGIC and golgi family 3
TcG_06870	191,5767122	-0,305935484	0,13261896	-2,30687591	0,02106174	0,08777745	protein_codir	gamma carbonic dehydratase
TcG_06871	615,6909944	0,252846549	0,07816184	3,23491046	0,00121681	0,00915451	protein_codir	neutral sphingomyelinase activation associated factor-like protein
TcG_06872	59,06468792	0,215772589	0,23255919	0,92781796	0,353502	0,58795208	protein_codir	neutral sphingomyelinase activation associated factor-like protein
TcG_06873	181,540029	0,636103265	0,1565406	4,06350348	4,8342E-05	0,00059838	protein_codir	hypothetical protein
TcG_06874	350,1737712	0,59346249	0,1046068	5,67326873	1,401E-08	4,3055E-07	protein_codir	hypothetical protein
TcG_06875	126,4915398	0,952341719	0,16474689	5,78063558	7,4419E-09	2,422E-07	protein_codir	hypothetical protein
TcG_06876	454,6452805	0,396385682	0,08813447	4,49751009	6,8754E-06	0,00011048	protein_codir	pre-mRNA-splicing factor SYF1
TcG_06877	323,5893055	0,296730518	0,10514973	2,8219807	0,00477281	0,0277599	protein_codir	Trypanosoma vivax
TcG_06878	553,9124252	0,561425175	0,08349528	6,72403521	1,7676E-11	1,0449E-09	protein_codir	hypothetical protein
TcG_06879	401,4376019	0,296729868	0,09693041	3,06126715	0,00220402	0,01486369	protein_codir	putative protoheme IX farnesyltransferase
TcG_06880	841,6060396	0,248138955	0,06748747	3,67681507	0,00023616	0,00229162	protein_codir	putative vacuolar proton translocating ATPase subunit A
TcG_06881	291,63404	0,725592978	0,11333022	6,40246706	1,5289E-10	7,2656E-09	protein_codir	hypothetical protein
TcG_06882	275,0258761	0,452669684	0,1113797	4,06420279	4,8197E-05	0,00059723	protein_codir	putative alanine racemase
TcG_06883	349,1790516	0,633305953	0,10333207	6,12884199	8,8521E-10	3,5617E-08	protein_codir	hypothetical protein
TcG_06884	294,0758772	0,398605298	0,1103241	3,61303934	0,00030263	0,00282535	protein_codir	Sulfate transporter
TcG_06885	403,746646	0,580029165	0,09141558	6,34497073	2,2247E-10	1,0108E-08	protein_codir	GAF domain-containing protein
TcG_06886	107,1433681	0,531339427	0,17327702	3,06641608	0,00216642	0,01466984	protein_codir	fukutin-related protein-like
TcG_06887	172,1172247	-3,405454576	0,1827211	-18,6374456	1,5969E-77	1,8501E-73	protein_codir	P21 protein
TcG_06888	517,6452667	0,218721822	0,08732209	2,50477092	0,01225307	0,05799187	protein_codir	hypothetical protein
TcG_06889	97,67200164	0,690931866	0,18675087	3,69975182	0,00021581	0,00212437	protein_codir	hypothetical protein
TcG_06890	2427,477497	0,342082884	0,04640764	7,37126172	1,6902E-13	1,4399E-11	protein_codir	short-chain dehydrogenase
TcG_06891	273,1716599	0,711177687	0,11493128	6,18785153	6,099E-10	2,551E-08	protein_codir	exosome component CSL4
TcG_06892	56,74487276	0,821976282	0,2529107	3,25006527	0,00115379	0,00877149		
TcG_06893	320,5417447	0,686314186	0,10737556	6,3917167	1,6403E-10	7,7256E-09	protein_codir	putative ankyrin repeat domain-containing protein 32-like isoform X1
TcG_06894	244,977008	0,518319028	0,11540755	4,49120564	7,0821E-06	0,00011333	protein_codir	vesicle transport v-SNARE 11
TcG_06895	233,2161329	0,218098842	0,11772114	1,85267358	0,06392916	0,19863082	protein_codir	vesicle transport v-SNARE 11
TcG_06896	506,3733574	0,423159725	0,08362527	5,06018956	4,1884E-07	9,2608E-06	protein_codir	protein containing C-terminal RING-finger
TcG_06897	269,3681345	0,491507391	0,10994613	4,47043832	7,8059E-06	0,00012321	protein_codir	hypothetical protein
TcG_06898	170,2899389	0,419150058	0,14440206	2,90265989	0,00370008	0,02258304	protein_codir	hypothetical protein
TcG_06899	384,1623629	0,536363751	0,09783817	5,48215247	4,2018E-08	1,1759E-06	protein_codir	hypothetical protein
TcG_06900	32,12048583	0,248904306	0,31051572	0,80158359	0,42279387	0,64889255	protein_codir	target of rapamycin (TOR) kinase 1
TcG_06901	36,06449627	0,265329782	0,29065578	0,91286602	0,36131301	0,59395184	protein_codir	target of rapamycin (TOR) kinase 1
TcG_06902	43,04806174	-0,042441631	0,26689477	-0,15902009	0,87365304	0,93932295	protein_codir	putative trans-sialidase
TcG_06903	29,92081762	0,293214402	0,32233993	0,90964343	0,36301058	0,59526856	protein_codir	phosphatidylinositol 3-related kinase
TcG_06904	38,6247561	0,10359589	0,27870491	0,37170458	0,71011282	0,85046176	protein_codir	structural maintenance of chromosome protein 4
TcG_06905	51,19927652	0,203489532	0,24453762	0,83213999	0,40532993	0,63430422	protein_codir	aspartyl-tRNA synthetase
TcG_06906	427,613682	0,410411765	0,08863743	4,63023066	3,6526E-06	6,3637E-05	protein_codir	putative mismatch repair protein MSH4
TcG_06907	211,5012903	0,144721349	0,12464867	1,16103404	0,24562806	0,47419697	protein_codir	hypothetical protein
TcG_06908	316,3792093	-0,021307247	0,1095266	-0,19453948	0,84575349	0,92503539	protein_codir	putative ABC transporter
TcG_06909	169,7283948	0,256909841	0,13960454	1,84026854	0,06572882	0,20296752	protein_codir	hypothetical protein
TcG_06910	392,6156758	-0,064543386	0,09269214	-0,69631997	0,48622847	0,69827461	protein_codir	hypothetical protein
TcG_06911	353,9846044	-0,140909828	0,0992051	-1,42038898	0,15549447	0,35689154	protein_codir	SCY1 family protein kinase
TcG_06912	310,9228246	0,00460685	0,10429066	0,04417318	0,96476636	0,9843472	protein_codir	hypothetical protein
TcG_06913	434,5418653	0,046692286	0,08891625	0,5251266	0,59949519	0,78310281	protein_codir	hypothetical protein

TcG_06914	519,534166	0,027167286	0,08454958	0,32131782	0,74796956	0,87282745	protein_codir hypothetical protein
TcG_06915	415,1398172	0,021582737	0,09191139	0,23482113	0,81434757	0,90826251	protein_codir hypothetical protein
TcG_06916	168,4040646	-0,160369048	0,13530524	-1,18523898	0,23592297	0,46344584	protein_codir hypothetical protein
TcG_06917	320,0742823	0,09570915	0,1125419	0,85043123	0,39508538	0,62462032	protein_codir putative 2-aminoethylphosphonate:pyruvateaminotransferase- likeprotein,putative
TcG_06918	557,7665646	-0,087000826	0,0821758	-1,05871585	0,28972921	0,52327398	protein_codir putative serine/threonine protein kinase
TcG_06919	416,6501429	-0,188471118	0,09012056	-2,09132205	0,0364992	0,13273063	protein_codir hypoxanthine-guanine phosphoribosyltransferase
TcG_06920	367,0584983	-0,275610911	0,09721134	-2,83517242	0,0045801	0,02688196	protein_codir putative hypoxanthine-guanine phosphoribosyltransferase
TcG_06921	230,5365839	0,133125828	0,13171236	1,01073151	0,31214495	0,54605336	protein_codir hypothetical protein
TcG_06922	1129,145726	-0,203817225	0,06050703	-3,36848852	0,00075582	0,00615814	protein_codir putative methionine aminopeptidase 2
TcG_06923	297,7499073	0,078381419	0,10808695	0,72517004	0,46834772	0,68500074	protein_codir hypothetical protein
TcG_06924	260,693069	0,240107774	0,1200994	1,99924206	0,04558217	0,15487244	protein_codir hypothetical protein
TcG_06925	842,3411903	-0,213752276	0,06617703	-3,23000727	0,00123787	0,00927682	protein_codir hypothetical protein
TcG_06926	124,1306959	-0,1094036	0,17440494	-0,62729646	0,53046492	0,73201127	protein_codir hypothetical protein
TcG_06927	127,3952008	-0,065212491	0,16848636	-0,38704908	0,69871987	0,84414248	protein_codir hypothetical protein
TcG_06928	515,4721812	-0,028365839	0,08284573	-0,34239351	0,73205477	0,86246393	protein_codir putative tyrosine protein kinase
TcG_06929	32,14884172	0,092491756	0,31183112	0,29660849	0,76676543	0,88290044	protein_codir putative retrotransposon hot spot (RHS) protein
TcG_06930	122,9647445	-0,198422284	0,16976317	-1,1688182	0,24247688	0,47089863	protein_codir putative retrotransposon hot spot (RHS) protein
TcG_06931	62,20486297	-0,58129934	0,2297367	-2,53028511	0,01139699	0,05485895	protein_codir amino acid transporter
TcG_06932	1112,105018	-0,826185504	0,06168109	-13,39447	6,5139E-41	7,3087E-38	protein_codir surface protein-2
TcG_06933	17,55916603	0,735752458	0,41939293	1,75432727	0,07937445	0,2296784	protein_codir hypothetical protein
TcG_06934	56,67268958	-0,051128017	0,24013377	-0,21291473	0,83139347	0,91799531	protein_codir hypothetical protein
TcG_06935	32,40020162	0,472609994	0,31346643	1,50768934	0,13163405	0,32246111	protein_codir putative trans-sialidase
TcG_06936	34,53192732	0,177671656	0,29840784	0,59539876	0,55157696	0,74708565	protein_codir helicase-like protein
TcG_06937	32,92858326	0,071345355	0,33808736	0,21102639	0,83286668	0,91908998	protein_codir hypothetical protein
TcG_06938	244,6746076	-0,301221586	0,11404132	-2,64133736	0,00825794	0,04250696	protein_codir hypothetical protein
TcG_06939	1381,169769	0,141108711	0,07062544	1,99798698	0,04571807	0,15510676	protein_codir phosphotransferase
TcG_06940	646,4689706	-0,138225026	0,07687171	-1,79812606	0,07215703	0,21601023	protein_codir hypothetical protein
TcG_06941	98,76129745	-0,185172662	0,18934473	-0,97796578	0,32809125	0,56265027	protein_codir cAMP specific 3,5 cyclic phosphodiesterase
TcG_06942	422,1400353	0,354832943	0,09305963	3,81296323	0,00013731	0,00145286	protein_codir hypothetical protein
TcG_06943	348,7728089	0,376046977	0,09680124	3,8847331	0,00010244	0,00113696	protein_codir putative formin
TcG_06944	813,4321172	0,046799556	0,07140401	0,65541918	0,51219791	0,71835431	protein_codir activating signal cointegrator 1 complex subunit 3-like 1
TcG_06945	359,0649234	0,082254163	0,1009974	0,81441861	0,41540518	0,64239863	protein_codir acetyltransferase
TcG_06946	142,6152409	-0,034913249	0,14724222	-0,23711439	0,81256805	0,90759432	protein_codir leucine-rich protein
TcG_06947	33,69844519	0,375571535	0,30635904	1,22591956	0,22022895	0,44530063	protein_codir hypothetical protein
TcG_06948	17,31731008	-0,236368846	0,42325907	-0,55844957	0,57653743	0,76688334	protein_codir hypothetical protein
TcG_06949	15,89250421	0,178250776	0,42854735	0,41594184	0,67745256	1	protein_codir hypothetical protein
TcG_06950	418,9089968	0,13299131	0,09248508	1,43797589	0,1504409	0,35042645	protein_codir putative SET and MYND domain-containing protein 1 isoform X2
TcG_06951	691,1476009	-0,021428499	0,07855701	-0,2727764	0,78502511	0,89351472	protein_codir hypothetical protein
TcG_06952	310,5430821	-0,193851298	0,10881094	-1,78154231	0,0748239	0,22070887	protein_codir putative tubulin tyrosine ligase
TcG_06953	129,4427599	-0,045043836	0,15794918	-0,2851793	0,77550678	0,88819905	protein_codir putative tubulin tyrosine ligase
TcG_06954	141,8868193	-0,113321537	0,14695364	-0,77113802	0,44062513	0,66350124	protein_codir putative folate/biopterin transporter
TcG_06955	199,9279006	0,194635355	0,12442481	1,56428091	0,11775161	0,29924769	protein_codir putative DNA replication factor
TcG_06956	134,4994718	0,195829943	0,16037888	1,2210457	0,22206872	0,44792621	protein_codir putative DNA replication factor
TcG_06957	138,0476331	-0,045197814	0,15707172	-0,28775272	0,77353603	0,88691888	protein_codir hypothetical protein
TcG_06958	139,5545299	0,096816942	0,14971884	0,64665839	0,51785306	0,72304718	protein_codir hypothetical protein
TcG_06959	134,6146503	0,45600279	0,15243885	2,99138167	0,00277718	0,01786587	protein_codir putative meiotic recombination protein DMC1
TcG_06960	465,9918044	0,201929625	0,09129333	2,21187712	0,02697516	0,10572874	protein_codir TPR Domain containing protein
TcG_06961	497,2081183	-0,115385813	0,08826563	-1,3072564	0,19112565	0,40668168	protein_codir protein FAM184A
TcG_06962	543,1691325	0,039880844	0,08282062	0,48153281	0,63013787	0,8017546	protein_codir hypothetical protein
TcG_06963	413,3634027	-0,082955256	0,09039607	-0,91768654	0,35878299	0,59256732	protein_codir putative proteasome alpha 1 subunit
TcG_06964	67,43631273	0,046273493	0,21657393	0,21366141	0,8308111	0,91760178	protein_codir hypothetical protein
TcG_06965	175,1137967	0,17459442	0,13573714	1,28626859	0,19834934	0,41692226	protein_codir hypothetical protein

TcG_06966	165,7311202	-0,025679905	0,13559125	-0,18939205	0,84978555	0,9276306	protein_codir	hypothetical protein
TcG_06967	178,5855675	-0,226707092	0,15135091	-1,49789055	0,13416169	0,32570991	protein_codir	hypothetical protein
TcG_06968	205,9692565	-0,130202688	0,12330801	-1,05591424	0,2910074	0,52365178	protein_codir	hypothetical protein
TcG_06969	564,8405838	-0,118703386	0,08116229	-1,46254367	0,14359229	0,34000823	protein_codir	adenosine monophosphate deaminase-like protein
TcG_06970	169,5975243	-0,253684416	0,13641991	-1,85958496	0,06294427	0,19649233	protein_codir	putative membrane-trafficking protein
TcG_06971	567,9295654	-0,11972078	0,07757066	-1,54337709	0,12273931	0,30767149	protein_codir	cyclophilin
TcG_06972	323,4071965	-0,344723064	0,10047007	-3,43110195	0,00060113	0,00505322	protein_codir	hypothetical protein
TcG_06973	153,0263882	-0,346247215	0,14457347	-2,39495672	0,01662233	0,07311552	protein_codir	putative glyceraldehyde-3-phosphate dehydrogenase
TcG_06974	133,3993878	-0,197209054	0,15913892	-1,23922576	0,2152619	0,4388705	protein_codir	putative regulator of chromosome condensation (RCC1)
TcG_06975	189,2227744	-0,139251377	0,12802377	-1,0876994	0,27672783	0,50904889	protein_codir	hypothetical protein
TcG_06976	382,2674719	-0,081148974	0,09296712	-0,87287823	0,38272945	0,61264209	protein_codir	glycine cleavage system H protein
TcG_06977	866,3052855	-0,227990583	0,06520039	-3,49676691	0,00047093	0,00410243	protein_codir	hypoxia up-regulated 1
TcG_06978	380,947078	-0,204119768	0,0961631	-2,12264132	0,03378392	0,12505447	protein_codir	putative MCAK-like kinesin
TcG_06979	10,54699541	0,712207848	0,54029869	1,3181743	0,18744531	1	protein_codir	trans-sialidase
TcG_06980	5,481284974	-0,022489002	0,77418163	-0,02904874	0,97682572	1	protein_codir	hypothetical protein
TcG_06981	0,441769291	0,396475077	2,50258318	0,15842633	0,87412086	1		
TcG_06982	157,298551	0,140166702	0,14757155	0,94982197	0,34220272	0,57657632	protein_codir	hypothetical protein
TcG_06983	227,3531758	0,525549473	0,12009012	4,37629236	1,2072E-05	0,00017839	protein_codir	hypothetical protein
TcG_06984	192,8458243	-0,061733002	0,12815077	-0,48172165	0,63000369	0,80168887	protein_codir	kelch domain-containing protein 3
TcG_06985	319,3885093	0,154352726	0,10039474	1,53745833	0,12418111	0,31021181	protein_codir	hypothetical protein
TcG_06986	122,7572151	0,136589798	0,15824107	0,86317538	0,38804105	0,61841988	protein_codir	hypothetical protein
TcG_06987	109,1433738	0,027653956	0,16996547	0,16270337	0,87075199	0,93767641	protein_codir	hypothetical protein
TcG_06988	266,9375019	0,224987978	0,10989402	2,04731784	0,04062689	0,14271104	protein_codir	hypothetical protein
TcG_06989	220,5978393	0,211217448	0,11939057	1,76913001	0,07687218	0,22464946	protein_codir	putative calpain-like cysteine peptidase
TcG_06990	185,4298662	0,39913435	0,13133077	3,03915324	0,00237244	0,01576862	protein_codir	hypothetical protein
TcG_06991	146,3164904	-0,251104927	0,15499717	-1,6200614	0,10521909	0,27877163	protein_codir	hypothetical protein
TcG_06992	93,76288559	-0,239508195	0,18162011	-1,31873171	0,18725882	0,40147681	protein_codir	hypothetical protein
TcG_06993	210,7358264	0,130060351	0,1278834	1,01702293	0,30914252	0,54234541	protein_codir	MORN-containing protein
TcG_06994	198,7044037	0,323501141	0,13412037	2,41202101	0,01586437	0,07061995	protein_codir	BTB/POZ domain containing protein
TcG_06995	217,8567884	0,189129074	0,12308946	1,53651725	0,12441157	0,31058661	protein_codir	hypothetical protein
TcG_06996	1058,80608	0,239635412	0,06750138	3,55008179	0,00038511	0,00346421	protein_codir	phosphoglycerate kinase
TcG_06997	191,6790247	-0,027602505	0,13440137	-0,20537369	0,83728018	0,9208095	protein_codir	hypothetical protein
TcG_06998	345,7897671	0,152673609	0,09825055	1,55392112	0,12020323	0,30354722	protein_codir	hypothetical protein
TcG_06999	114,8170497	0,168357469	0,17817596	0,94489439	0,3447128	0,57877968	protein_codir	hypothetical protein
TcG_07000	109,4928617	0,410608668	0,16806695	2,44312559	0,01456067	0,06633894	protein_codir	hypothetical protein
TcG_07001	256,8960633	0,187334865	0,11360127	1,64905611	0,09913614	0,26705216	protein_codir	putative DNA repair protein BRCA2
TcG_07002	81,75957266	0,193678373	0,19525239	0,99193854	0,32122751	0,5560344	protein_codir	anti-silencing protein ASF 1
TcG_07003	273,6209016	-0,073875256	0,1107764	-0,66688625	0,50484479	0,71221932	protein_codir	hypothetical protein
TcG_07004	934,3912199	0,304057744	0,0688712	4,41487514	1,0107E-05	0,00015367	protein_codir	phosphate-repressible phosphate permease
TcG_07005	844,1454724	0,007467252	0,06859712	0,10885664	0,9133162	0,95744494	protein_codir	histone-lysine N-methyltransferase
TcG_07006	221,3368067	0,157681358	0,12568728	1,25455299	0,20964108	0,43221692	protein_codir	WD40 repeat-containing protein
TcG_07007	143,7733511	0,188180493	0,155548	1,2097905	0,22635929	0,45279675	protein_codir	hypothetical protein
TcG_07008	458,336964	0,076525845	0,08726502	0,87693606	0,38052137	0,61079532	protein_codir	DNA-directed RNA polymerase III
TcG_07009	128,239535	0,145109096	0,15807406	0,91798171	0,35862844	0,59248098	protein_codir	hypothetical protein
TcG_07010	26,1060665	-0,05099898	0,36195203	-0,14089983	0,88794908	0,94594634	protein_codir	hypothetical protein
TcG_07011	250,1248542	-0,066090989	0,11584105	-0,57053166	0,56831715	0,76010481	protein_codir	hypothetical protein
TcG_07012	553,4060354	0,144730998	0,08160049	1,77365355	0,07612047	0,22310484	protein_codir	hypothetical protein
TcG_07013	234,7054564	0,36385874	0,11754345	3,09552548	0,00196464	0,01354682	protein_codir	putative histone deacetylase
TcG_07014	164,1827137	0,167071579	0,14378529	1,16195184	0,24525503	0,47374537	protein_codir	hypothetical protein
TcG_07015	913,2714358	0,179387464	0,06780218	2,6457477	0,00815106	0,04210351	protein_codir	putative heat shock protein HslVU, ATPase subunit HslU
TcG_07016	206,7363051	0,091418952	0,13508034	0,67677466	0,49854894	0,70769272	protein_codir	chaperone protein DNAJ
TcG_07017	82,84425904	0,123230773	0,19797712	0,62244957	0,53364629	0,73412799	protein_codir	hypothetical protein

TcG_07018	470,6470573	-0,008922829	0,0862239	-0,10348441	0,91757852	0,96017565	protein_codir	hypothetical protein
TcG_07019	275,0136958	-0,186523932	0,11344509	-1,64417804	0,10013943	0,26899994	protein_codir	hypothetical protein
TcG_07020	224,665059	0,418016816	0,12916992	3,23617778	0,00121142	0,00911988	protein_codir	hypothetical protein
TcG_07021	198,1115019	0,344074404	0,12901149	2,66700587	0,00765303	0,03999811	protein_codir	hypothetical protein
TcG_07022	450,1472463	0,060799299	0,08647734	0,70306622	0,48201448	0,69598951	protein_codir	putative protein kinase
TcG_07023	127,4999284	0,120780726	0,16273538	0,74219096	0,45797165	0,67721172	protein_codir	hypothetical protein
TcG_07024	636,8756661	0,223170434	0,0798145	2,79611405	0,00517211	0,02943552	protein_codir	ribonuclease Z
TcG_07025	379,6781705	0,169157687	0,09412703	1,79712118	0,07231638	0,21618229	protein_codir	putative mitochondrial DNA polymerase beta-PAK
TcG_07026	706,9252329	-0,208421073	0,07059786	-2,95222943	0,00315488	0,01986548	protein_codir	DNA polymerase beta
TcG_07027	129,7320178	0,342315421	0,1591015	2,15155365	0,03143253	0,11831619	protein_codir	histone-lysine N-methyltransferase ASHR2
TcG_07028	47,15168826	0,202370882	0,29002332	0,6977745	0,48531823	0,69814634		
TcG_07029	241,3567806	0,08051398	0,11882088	0,67760802	0,49802026	0,70742498	protein_codir	surface protein-2
TcG_07030	14,12397358	-0,07651501	0,46440063	-0,16476078	0,86913228	1	protein_codir	hypothetical protein
TcG_07031	87,1843576	-0,184738991	0,18450951	-1,00124375	0,31670898	0,55170279	protein_codir	hypothetical protein
TcG_07032	350,1275232	-0,112729524	0,10163412	-1,1091701	0,26735681	0,49977347	protein_codir	cell division protein kinase 2
TcG_07033	1599,938399	-0,178732645	0,05324965	-3,35650351	0,00078935	0,0063909	protein_codir	putative T-complex protein 1, delta subunit
TcG_07034	4702,087719	0,098892769	0,03917684	2,52426634	0,01159401	0,05548457	protein_codir	serine carboxypeptidase
TcG_07035	102,5364224	-0,028059255	0,18231689	-0,15390376	0,87768562	0,94120054	protein_codir	hypothetical protein
TcG_07036	110,4767444	0,078103822	0,16657035	0,4688939	0,63914548	0,80789216	protein_codir	hypothetical protein
TcG_07037	82,85723809	0,239991725	0,1940185	1,23695279	0,21610461	0,44003304	protein_codir	hypothetical protein
TcG_07038	72,128437	0,423021251	0,21563563	1,96174097	0,04979265	0,1649973	protein_codir	hypothetical protein
TcG_07039	92,56931963	0,192232179	0,18370202	1,04643478	0,29536034	0,52809782	protein_codir	Tbingi protein
TcG_07040	90,24688511	0,174577915	0,18398891	0,9488502	0,34269681	0,57679606	protein_codir	Tbingi protein
TcG_07041	60,05009123	0,202749623	0,23062458	0,87913276	0,3793293	0,6106388	protein_codir	trans-sialidase
TcG_07042	157,4343732	0,155167162	0,14398863	1,07763482	0,28119676	0,5142555	protein_codir	Tbingi protein
TcG_07043	15,43717601	0,625688715	0,472357	1,32460981	0,1853006	1	protein_codir	hypothetical protein
TcG_07044	15,31341553	0,368013802	0,44554428	0,82598704	0,40881145	1	protein_codir	hypothetical protein
TcG_07045	364,6843304	-0,193309475	0,0945468	-2,04459025	0,04089527	0,14344917	protein_codir	hypothetical protein
TcG_07046	1031,274979	-0,138784208	0,06134118	-2,26249666	0,02366673	0,09600938	protein_codir	hypothetical protein
TcG_07047	224,6464551	0,361099815	0,12064534	2,993069	0,00276187	0,01779703	protein_codir	hypothetical protein
TcG_07048	327,4474388	0,019225998	0,10044944	0,19139975	0,84821241	0,92660555	protein_codir	putative U4/U6 small nuclear ribonuclear protein
TcG_07049	468,0670804	-0,119429057	0,09352948	-1,27691348	0,20163279	0,42137762	protein_codir	hypothetical protein
TcG_07050	206,6768375	-0,004011216	0,12580394	-0,03188466	0,97456403	0,98893052	protein_codir	hypothetical protein
TcG_07051	1504,101463	-0,057597449	0,05486395	-1,04982317	0,29379942	0,52684725	protein_codir	hypothetical protein
TcG_07052	283,648988	-0,177327847	0,11211451	-1,58166724	0,11372555	0,29274034	protein_codir	hypothetical protein
TcG_07053	335,8598149	0,000427026	0,10275838	0,00415563	0,9966843	0,99883957	protein_codir	hypothetical protein
TcG_07054	476,5091083	-0,050033145	0,08343105	-0,59969454	0,54870983	0,74524792	protein_codir	PIF1 helicase-like protein
TcG_07055	185,2118571	0,247312915	0,15060503	1,64212916	0,10056324	0,26976747	protein_codir	hypothetical protein
TcG_07056	488,0473952	-0,098208511	0,08313846	-1,18126445	0,23749768	0,465277	protein_codir	putative thymidine kinase
TcG_07057	942,2241827	-0,074485612	0,06905189	-1,07869039	0,28072577	0,51382129	protein_codir	hypothetical protein
TcG_07058	288,4468645	0,255959698	0,10902622	2,34768948	0,01889026	0,08022822	protein_codir	hypothetical protein
TcG_07059	147,9991965	0,436136702	0,14984072	2,9106688	0,00360656	0,02210879	protein_codir	hypothetical protein
TcG_07060	509,1478034	0,279041194	0,08420733	3,31374001	0,00092057	0,0073003	protein_codir	hypothetical protein
TcG_07061	676,1354832	0,270890432	0,08547	3,16942104	0,00152743	0,0110398	protein_codir	L1Tc protein
TcG_07062	11,21307812	0,671763301	0,527706	1,2729878	0,20302237	1	protein_codir	hypothetical protein
TcG_07063	92,60373684	-0,072389636	0,18268659	-0,39625042	0,6919203	0,8405776	protein_codir	hypothetical protein
TcG_07064	56,39979938	-0,197253329	0,22996804	-0,85774236	0,39103475	0,62102798	protein_codir	hypothetical protein
TcG_07065	1090,553558	-0,258382725	0,06741382	-3,83278576	0,0001267	0,00136173	protein_codir	hypothetical protein
TcG_07066	1414,57521	-0,115858237	0,05558481	-2,08435076	0,03712827	0,13397323	protein_codir	stress-induced protein sti1
TcG_07067	236,1156589	-0,128079646	0,11886059	-1,07756189	0,28122932	0,5142555	protein_codir	EF-hand domain-containing family member C2
TcG_07068	418,4663466	-0,048426886	0,09348359	-0,51802551	0,60444047	0,7861526	protein_codir	putative phosphoribosylpyrophosphate synthetase
TcG_07069	354,3819113	0,034586642	0,09712036	0,35612143	0,72174963	0,85711748	protein_codir	putative suppressive immunomodulating factor

TcG_07070	234,2410765	0,045181505	0,11901277	0,37963577	0,70421581	0,84742878	protein_codir	hypothetical protein
TcG_07071	20,06977253	0,089812372	0,39702329	0,22621437	0,8210347	0,91124705	protein_codir	hypothetical protein
TcG_07072	282,150678	0,18957365	0,11373517	1,66679884	0,09555441	0,26079939	protein_codir	hypothetical protein
TcG_07073	112,3866878	0,2456868	0,16564434	1,48321885	0,13801628	0,33154812	protein_codir	thiopurine S-methyltransferase
TcG_07074	214,3150894	-0,01304044	0,12879845	-0,10124687	0,91935449	0,96120058	protein_codir	hypothetical protein
TcG_07075	222,0186045	-0,161576694	0,12363955	-1,3068366	0,19126821	0,40691031	protein_codir	pre-mRNA-processing factor SLU7
TcG_07076	600,7168989	-0,052352698	0,08518339	-0,61458811	0,53882677	0,73820598	protein_codir	WD repeat domain 35
TcG_07077	211,671903	0,06287743	0,1244408	0,50527987	0,61336228	0,79091991	protein_codir	putative MIX protein
TcG_07078	182,6850883	0,233688206	0,13780225	1,69582283	0,08991944	0,24989364	protein_codir	hypothetical protein
TcG_07079	241,4163932	0,724435805	0,12021958	6,02593863	1,6813E-09	6,3428E-08	protein_codir	putative poly(ADP-ribose) polymerase
TcG_07080	269,7444588	0,353398817	0,10958888	3,22476893	0,00126074	0,00941778	protein_codir	hypothetical protein
TcG_07081	418,9543378	-0,2422062	0,08956579	-2,70422672	0,00684636	0,0367401	protein_codir	hypothetical protein
TcG_07082	174,9795813	0,28979004	0,13419452	2,15947739	0,03081315	0,11659084	protein_codir	hypothetical protein
TcG_07083	318,0959046	-0,233241816	0,10470084	-2,22769762	0,02590069	0,10242678	protein_codir	hypothetical protein
TcG_07084	86,3744763	0,272167133	0,19307112	1,40967292	0,15863628	0,36087963	protein_codir	putative retrotransposon hot spot (RHS) protein
TcG_07085	35,78536472	-0,031414612	0,2858564	-0,10989648	0,91249147	0,95723874	protein_codir	hypothetical protein
TcG_07086	13,46790068	-1,9351E-05	0,54828735	-3,5294E-05	0,99997184	1	protein_codir	hypothetical protein
TcG_07087	84,82906979	0,091262651	0,19579154	0,46612153	0,64112851	0,80951558	protein_codir	putative trans-sialidase
TcG_07088	57,53669988	0,250017293	0,23481163	1,06475684	0,28698598	0,52024903	protein_codir	trans-sialidase
TcG_07089	27,07892155	-0,129677373	0,34500204	-0,37587422	0,70701041	0,84892495	protein_codir	hypothetical protein
TcG_07090	221,9046272	0,214390359	0,12127446	1,76781135	0,07709245	0,22498567	protein_codir	putative N-acyl-L-amino acid amidohydrolase
TcG_07091	58,1407042	0,245066793	0,23198351	1,05639749	0,29078665	0,52363291	protein_codir	aminoacylase
TcG_07092	26,28102297	0,508491637	0,34965946	1,4542482	0,1458775	0,34340242	protein_codir	aminoacylase
TcG_07093	111,9031575	0,113078697	0,16722013	0,67622659	0,49889679	0,70801301	protein_codir	putative aminoacylase, putative,N-acyl-L-amino acid amidohydrolase
TcG_07094	312,0384488	0,110962823	0,10680606	1,03891884	0,29884248	0,53234763	protein_codir	hypothetical protein
TcG_07095	177,0161758	0,282217302	0,1352244	2,08702949	0,03688547	0,13342337	protein_codir	RNA editing ligase
TcG_07096	128,4420467	0,20754362	0,15702796	1,32169851	0,18626857	0,40016829	protein_codir	hypothetical protein
TcG_07097	298,5955287	-0,001470272	0,1055222	-0,0139333	0,9888832	0,99552231	protein_codir	methyltransferase
TcG_07098	297,3158234	-0,042153322	0,10380972	-0,40606336	0,68469605	0,83644964	protein_codir	hypothetical protein
TcG_07099	210,1198622	-0,008092271	0,12686488	-0,06378654	0,9491402	0,97635962	protein_codir	putative soluble N-ethylmaleimide sensitive factor (NSF) attachment protein
TcG_07100	349,4600959	0,19750623	0,09638256	2,04919063	0,04044348	0,14229521	protein_codir	separase
TcG_07101	1579,828304	0,535880704	0,05336603	10,0416072	1,0003E-23	2,5755E-21	protein_codir	putative protein kinase
TcG_07102	321,2821617	0,038381033	0,10057084	0,38163183	0,70273447	0,84634944	protein_codir	hypothetical protein
TcG_07103	186,8651475	0,18860645	0,13497495	1,39734415	0,16231011	0,36607453	protein_codir	rar1
TcG_07104	1009,240167	0,093929613	0,06414209	1,46439898	0,14308497	0,33957034	protein_codir	40S ribosomal protein S11
TcG_07105	241,1635682	0,179088519	0,11908524	1,50386833	0,13261527	0,32361438	protein_codir	40S ribosomal protein S11
TcG_07106	305,733756	-0,13568625	0,10531793	-1,28834894	0,19762452	0,41622936	protein_codir	phosphatase-like protein
TcG_07107	168,3380239	0,018150198	0,13630439	0,1331593	0,8940674	0,94881323	protein_codir	putative GTPase activating protein of Rab-like GTPase
TcG_07108	327,3823722	0,524546147	0,10724228	4,8912253	1,0021E-06	2,0333E-05	protein_codir	putative cell division cycle protein
TcG_07109	595,1624088	0,01466778	0,08521753	0,17212163	0,86334191	0,93465514	protein_codir	hypothetical protein
TcG_07110	56,95868857	0,034367953	0,24280589	0,14154497	0,88743944	0,94569431	protein_codir	hypothetical protein
TcG_07111	387,3748124	0,160854265	0,09502966	1,69267435	0,09051747	0,25095367	protein_codir	hypothetical protein
TcG_07112	529,1815859	-0,214755466	0,07940589	-2,70452812	0,00684015	0,0367238	protein_codir	hypothetical protein
TcG_07113	150,738769	0,089781104	0,14332568	0,62641324	0,53104393	0,73237412	protein_codir	putative septum formation inhibitor, Maf-like protein
TcG_07114	330,4181571	0,114844305	0,09957372	1,15335959	0,24876282	0,47820907	protein_codir	hypothetical protein
TcG_07115	376,9168301	0,054132046	0,09398895	0,57594053	0,56465538	0,7574446	protein_codir	zinc-binding phosphatase
TcG_07116	349,7962062	0,072637125	0,10368024	0,70058794	0,48356021	0,69700529	protein_codir	hypothetical protein
TcG_07117	57,74165249	-0,012728293	0,23117835	-0,05505833	0,956092	0,97968355	protein_codir	dispersed gene family protein 1 (DGF-1)
TcG_07118	523,8472567	0,217690829	0,08695874	2,50338058	0,01230132	0,05814895	protein_codir	hypothetical protein
TcG_07119	244,0316741	0,231759308	0,12153844	1,90688072	0,05653604	0,18029907	protein_codir	hypothetical protein
TcG_07120	334,4157768	0,068311732	0,10176839	0,67124704	0,50206317	0,71093912	protein_codir	hypothetical protein
TcG_07121	134,7975852	-0,218700511	0,15660643	-1,39649763	0,16256469	0,36650604	protein_codir	glutaredoxin

TcG_07122	446,7271112	-0,016462787	0,08928946	-0,18437548	0,8537189	0,92909893	protein_codir hypothetical protein
TcG_07123	86,9265011	-0,081179962	0,20211485	-0,40165263	0,68793969	0,83793832	protein_codir hypothetical protein
TcG_07124	94,49116011	0,476501427	0,18807838	2,53352581	0,01129214	0,05438031	
TcG_07125	217,9033985	0,03586649	0,12288218	0,29187707	0,77038062	0,88468697	protein_codir hypothetical protein
TcG_07126	26,82839022	-0,525077249	0,33654126	-1,56021658	0,11870871	0,30069066	
TcG_07127	25,08573725	0,227900152	0,35123951	0,64884544	0,51643829	0,72205516	protein_codir hypothetical protein
TcG_07128	197,2565088	0,260942325	0,12911654	2,02098295	0,04328153	0,14937165	protein_codir hypothetical protein
TcG_07129	632,6183959	0,060883801	0,07549741	0,80643564	0,4199917	0,64656135	protein_codir hypothetical protein
TcG_07130	354,165229	-0,096760685	0,10630475	-0,91021978	0,36270661	0,59508238	protein_codir hypothetical protein
TcG_07131	181,0590475	0,118831847	0,13188442	0,90103022	0,36757225	0,59981579	protein_codir hypothetical protein
TcG_07132	547,8507698	0,114991209	0,08041414	1,42998737	0,15272065	0,35297158	protein_codir hypothetical protein
TcG_07133	475,4250095	-0,241108128	0,08447773	-2,85410275	0,00431586	0,02564518	protein_codir hypothetical protein
TcG_07134	1480,439517	-0,103315003	0,11901821	-0,86806046	0,38536123	0,61583383	protein_codir phosphatidylinositol 3-kinase
TcG_07135	119,6719729	0,657169405	0,16291891	4,03372078	5,4901E-05	0,00066971	protein_codir hypothetical protein
TcG_07136	104,0260506	0,322274515	0,17395168	1,8526669	0,06393012	0,19863082	protein_codir surface protease GP63
TcG_07137	25,63120061	0,128824896	0,35828696	0,35955787	0,71917779	0,85588296	protein_codir hypothetical protein
TcG_07138	20,75214846	-0,117258594	0,3805932	-0,3080943	0,75801058	0,87801518	protein_codir hypothetical protein
TcG_07139	102,7249394	-0,200582736	0,18883546	-1,06220905	0,28814079	0,52123168	protein_codir putative target of rapamycin (TOR) kinase 1
TcG_07140	696,7381091	0,260307602	0,07488235	3,47622084	0,00050853	0,0044002	protein_codir putative helicase
TcG_07141	251,479844	0,216355049	0,11338541	1,90813835	0,05637334	0,17997836	protein_codir methyltransferase
TcG_07142	379,2562863	-0,199554117	0,09365624	-2,13070821	0,03311319	0,12310099	protein_codir hypothetical protein
TcG_07143	443,7730716	-0,217257369	0,08832607	-2,45971962	0,01390456	0,06397865	protein_codir hypothetical protein
TcG_07144	485,60238	-0,041436751	0,08653939	-0,47881955	0,63206701	0,80290602	protein_codir hypothetical protein
TcG_07145	239,4055428	0,112693783	0,11562509	0,97464815	0,32973482	0,56434119	protein_codir hypothetical protein
TcG_07146	431,5866234	-0,09493997	0,08992647	-1,05575108	0,29108196	0,52365178	protein_codir 4-nitrophenylphosphatase/protein-tyrosine phosphatase
TcG_07147	339,6926492	0,132611328	0,09899121	1,33962732	0,18036654	0,39183409	protein_codir hypothetical protein
TcG_07148	372,2926892	-0,237337494	0,10065531	-2,35792314	0,0183775	0,07859789	protein_codir hypothetical protein
TcG_07149	393,3049424	-0,148489549	0,09713997	-1,52861431	0,12636009	0,31409915	protein_codir CCR4-NOT transcription complex subunit 2
TcG_07150	345,2246976	-0,142038193	0,0985385	-1,44144865	0,14945797	0,34883564	protein_codir putative elongation factor 1-gamma (EF-1-gamma)
TcG_07151	122,1057707	0,285193605	0,16049109	1,7770058	0,07556728	0,22215746	protein_codir hypothetical protein
TcG_07152	41,85886294	-0,227536315	0,28068179	-0,81065579	0,41756337	0,64419297	protein_codir pumilio protein 4
TcG_07153	61,71087815	0,03826364	0,2315706	0,16523532	0,86875877	0,93674123	protein_codir hypothetical protein
TcG_07154	153,0252624	-0,114501041	0,14730251	-0,77731903	0,43697057	0,66041495	protein_codir hypothetical protein
TcG_07155	112,1986383	-0,236584415	0,16497218	-1,43408672	0,15154752	0,35179947	protein_codir subtilisin-like serine peptidase
TcG_07156	584,4735974	-0,1538947	0,08449359	-1,82137729	0,06854952	0,20900389	protein_codir putative NADH-dependent fumarate reductase
TcG_07157	62,35622684	0,114613009	0,22231838	0,51553546	0,6061789	0,78708828	protein_codir hypothetical protein
TcG_07158	141,4665779	0,012303985	0,16239965	0,07576362	0,93960716	0,97207685	protein_codir putative glucosamine-fructose-6-phosphate aminotransferase
TcG_07159	1289,645109	0,146345037	0,05649771	2,59028278	0,00958971	0,0477877	protein_codir hypothetical protein
TcG_07160	121,0867762	-0,41422757	0,17458666	-2,37261861	0,01766249	0,07638582	protein_codir ribosomal RNA processing protein 40
TcG_07161	186,9568141	0,062976964	0,13610031	0,46272463	0,64356176	0,810875	protein_codir hypothetical protein
TcG_07162	267,5888775	-0,080195571	0,11788107	-0,68030918	0,49630871	0,70615654	protein_codir putative myosin heavy chain
TcG_07163	16,30653167	-0,276962965	0,42809929	-0,64695965	0,51765806	1	protein_codir hypothetical protein
TcG_07164	507,9042808	-0,107726379	0,08686248	-1,24019461	0,21490342	0,43882112	protein_codir hypothetical protein
TcG_07165	450,1246442	-0,19849022	0,08786375	-2,25906845	0,02387913	0,096533	protein_codir putative RNA pseudouridylate synthase
TcG_07166	184,5538689	-0,144804226	0,1322694	-1,09476742	0,27361854	0,50688937	protein_codir hypothetical protein
TcG_07167	370,1423334	0,060446445	0,10682743	0,56583264	0,57150756	0,76269221	protein_codir hypothetical protein
TcG_07168	663,380524	-0,192315743	0,07306533	-2,63210681	0,00848572	0,04342575	protein_codir putative AMP deaminase
TcG_07169	358,732022	-0,229387802	0,09615036	-2,38571968	0,01704574	0,07444098	protein_codir oxidoreductase
TcG_07170	135,9376409	0,08618856	0,14987987	0,57505096	0,56525684	0,75790599	protein_codir hypothetical protein
TcG_07171	329,2922546	-0,083888751	0,10461948	-0,80184641	0,42264181	0,64883106	protein_codir putative mitochondrial exoribonuclease DSS-1
TcG_07172	127,9125036	-0,412625735	0,15471668	-2,66697648	0,0076537	0,03999811	protein_codir ADP-ribosylation factor
TcG_07173	161,0067368	0,088721321	0,13845579	0,64079172	0,52165801	0,72540161	protein_codir hypothetical protein

TcG_07174	126,2569236	-0,5920481	0,15794873	-3,74835619	0,000178	0,0018106	protein_codir hypothetical protein
TcG_07175	729,4924835	-0,243155633	0,07345624	-3,31021067	0,00093226	0,00737164	protein_codir glycosyltransferase
TcG_07176	220,6466045	-0,322670419	0,12094095	-2,66799976	0,00763043	0,0399393	protein_codir hypothetical protein
TcG_07177	389,5259592	-0,155026265	0,09351124	-1,65783556	0,09735066	0,26402265	protein_codir phytanoyl-CoA dioxygenase
TcG_07178	23,91859528	-0,071110192	0,36660547	-0,19396926	0,84619995	0,925349	protein_codir dispersed gene family protein 1 (DGF-1)
TcG_07179	1205,389233	-0,05196146	0,06046458	-0,85937029	0,39013626	0,62038411	protein_codir putative retrotransposon hot spot (RHS) protein
TcG_07180	4,065419625	0,471695745	0,87386292	0,53978231	0,58934717	1	
TcG_07181	61,82090989	0,024382132	0,22021195	0,11072121	0,91183743	0,95702042	protein_codir retrotransposon hot spot (RHS) protein
TcG_07182	69,24775735	-0,20364945	0,21266157	-0,95762224	0,33825328	0,57289134	
TcG_07183	66,12280521	0,063013317	0,22148095	0,28450898	0,77602036	0,88831084	
TcG_07184	40,34035578	0,290309825	0,27724707	1,04711592	0,29504612	0,52793889	protein_codir hypothetical protein
TcG_07185	89,53363632	0,04037656	0,18563903	0,21750039	0,82781841	0,91588083	protein_codir putative kinesin
TcG_07186	27,24678947	-0,113331042	0,35464819	-0,31955906	0,74930261	0,87347018	protein_codir hypothetical protein
TcG_07187	66,07224819	-0,199273185	0,21209685	-0,93953865	0,34745426	0,58190301	protein_codir hypothetical protein
TcG_07188	53,38885339	-0,090167691	0,24016275	-0,37544411	0,7073302	0,84906006	protein_codir putative mitotic centromere-associated kinesin (MCAK)
TcG_07189	52,3290306	0,018820999	0,24018464	0,07836054	0,93754126	0,97124044	protein_codir hypothetical protein
TcG_07190	150,6517506	-0,140633369	0,15152626	-0,92811219	0,35334938	0,58778261	protein_codir retrotransposon hot spot (RHS) protein
TcG_07191	604,7524044	0,041873468	0,08119354	0,51572414	0,60604709	0,78700534	protein_codir putative retrotransposon hot spot (RHS) protein
TcG_07192	70,98246859	0,174935646	0,21050192	0,83104062	0,40595068	0,63464372	protein_codir hypothetical protein
TcG_07193	29,05968856	0,490928466	0,33504189	1,46527488	0,14284594	0,33915975	protein_codir hypothetical protein
TcG_07194	70,18429072	0,036458302	0,20926106	0,17422402	0,86168941	0,93381907	protein_codir trans-sialidase
TcG_07195	208,3901198	0,066082725	0,12898175	0,51234166	0,60841191	0,78802389	protein_codir putative trans-sialidase
TcG_07196	51,46860269	0,043359224	0,24280906	0,17857333	0,85827273	0,93174207	protein_codir putative trans-sialidase
TcG_07197	84,38733983	0,069925271	0,20382718	0,34306157	0,73155214	0,86212489	
TcG_07198	329,3612759	-0,001231356	0,09895076	-0,01244413	0,99007128	0,99600293	protein_codir hypothetical protein
TcG_07199	330,2463176	-0,187700026	0,10266859	-1,82821279	0,06751762	0,20689214	protein_codir hypothetical protein
TcG_07200	3258,88808	0,02980375	0,04720654	0,63134791	0,52781307	0,73026537	protein_codir glutamate dehydrogenase
TcG_07201	45,26845196	0,329116282	0,26270705	1,25278818	0,21028281	0,43289563	protein_codir putative glutamate dehydrogenase
TcG_07202	1810,475861	0,034660228	0,13476691	0,25718647	0,79703482	0,89872949	protein_codir transferase
TcG_07203	317,4407737	0,016667641	0,10250167	0,16260849	0,8708267	0,93767641	protein_codir solute carrier family 35, member F1/2
TcG_07204	141,8306141	-0,103347682	0,1500177	-0,68890325	0,49088416	0,70187819	protein_codir hypothetical protein
TcG_07205	121,4909749	0,425539432	0,16991764	2,50438641	0,0122664	0,05803123	protein_codir hypothetical protein
TcG_07206	368,0281008	0,166377527	0,09668938	1,72074254	0,08529755	0,2410384	protein_codir putative nitrilase
TcG_07207	248,2016741	-0,299050743	0,11457577	-2,61006959	0,00905238	0,04565994	protein_codir hypothetical protein
TcG_07208	158,2096911	-0,205085111	0,15409947	-1,33086186	0,18323447	0,39550543	protein_codir hypothetical protein
TcG_07209	282,817876	0,020713342	0,10796239	0,19185701	0,84785421	0,92660555	protein_codir hypothetical protein
TcG_07210	160,6089077	0,261816475	0,14581851	1,79549547	0,07257479	0,21660267	protein_codir hypothetical protein
TcG_07211	233,3421332	0,021179514	0,12230113	0,17317513	0,86251378	0,93414012	protein_codir hypothetical protein
TcG_07212	226,4019971	-0,109217203	0,12180877	-0,89662843	0,36991722	0,60152435	protein_codir serine/threonine protein kinase
TcG_07213	693,7811597	0,341914412	0,07496781	4,56081618	5,0955E-06	8,5067E-05	protein_codir 60S ribosomal protein L35
TcG_07214	307,2966255	0,543297938	0,10546486	5,15145931	2,5847E-07	6,0254E-06	protein_codir 60S ribosomal protein L35
TcG_07215	148,4687797	0,063960647	0,15027991	0,4256101	0,67039198	0,82708566	protein_codir hypothetical protein
TcG_07216	336,7896713	-0,103390743	0,10227828	-1,01087682	0,31207539	0,54601412	protein_codir putative sec1 family transport protein
TcG_07217	66,80741279	0,026313744	0,21984117	0,11969434	0,90472528	0,95370277	protein_codir zinc carboxypeptidase
TcG_07218	514,4294981	0,055433221	0,08641518	0,64147553	0,52121378	0,72540161	protein_codir hypothetical protein
TcG_07219	528,1053086	0,00791747	0,08646796	0,09156537	0,92704337	0,96441811	protein_codir hypothetical protein
TcG_07220	292,5848929	-0,127438313	0,1054461	-1,20856359	0,22683055	0,4535828	protein_codir minichromosome maintenance protein 10
TcG_07221	247,82636	0,212765729	0,11927026	1,78389583	0,07444059	0,220296	protein_codir putative peroxisomal membrane protein 4
TcG_07222	412,7098387	0,131224688	0,09166495	1,43156883	0,15226726	0,35241079	protein_codir hypothetical protein
TcG_07223	609,6764921	0,154214163	0,07664184	2,01214053	0,04420513	0,15183719	protein_codir hypothetical protein
TcG_07224	527,3801104	0,103729332	0,08109894	1,27904669	0,20088062	0,42026053	protein_codir protein kinase
TcG_07225	462,2763232	0,037329597	0,09064329	0,41182969	0,68046426	0,83338889	protein_codir hypothetical protein

TcG_07226	333,2990522	0,156995653	0,09964392	1,57556675	0,11512568	0,29549095	protein_codir	putative tubulin-tyrosine ligase
TcG_07227	455,2387418	0,054213167	0,0864846	0,62685343	0,53075531	0,732224	protein_codir	vesicular-fusion protein NsF
TcG_07228	215,2302133	-0,083127276	0,12159449	-0,68364345	0,49420034	0,70453824	protein_codir	hypothetical protein
TcG_07229	253,7878906	0,266707706	0,11948245	2,23219138	0,02560232	0,10159363	protein_codir	putative cytochrome c oxidase assembly factor, putative,electron transport protein SCO1/2
TcG_07230	819,9726842	-0,087087919	0,06752928	-1,28963205	0,19717844	0,41560128	protein_codir	hypothetical protein
TcG_07231	191,3006015	0,146067217	0,13176694	1,10852707	0,26763425	0,49992998	protein_codir	hypothetical protein
TcG_07232	955,9963883	0,182678829	0,06319287	2,89081375	0,00384246	0,02332044	protein_codir	ATP-dependent DEAD/H RNA helicase
TcG_07233	192,7119675	-0,002363312	0,12695038	-0,01861603	0,98514741	0,99415712	protein_codir	hypothetical protein
TcG_07234	210,6351724	-0,018965643	0,12524182	-0,15143219	0,8796348	0,94162382	protein_codir	RNA editing complex protein MP90
TcG_07235	277,0018201	0,041294929	0,10675906	0,38680492	0,69890064	0,84414248	protein_codir	RNA-editing complex protein
TcG_07236	140,3167894	0,038729956	0,15057573	0,25721247	0,79701476	0,89872949	protein_codir	hypothetical protein
TcG_07237	404,11266	0,091001167	0,09200941	0,98904196	0,32264261	0,55734864	protein_codir	protein AATF/BFR2
TcG_07238	749,4931153	0,04972178	0,07529244	0,66038207	0,50900868	0,71575087	protein_codir	putative helicase
TcG_07239	307,1662741	0,086641837	0,11459176	0,75609131	0,44959445	0,67044205	protein_codir	putative cyclophilin 15
TcG_07240	624,9020196	-0,162433591	0,07474049	-2,17330108	0,02975766	0,11367369	protein_codir	hypothetical protein
TcG_07241	86,16801341	0,266892961	0,21182348	1,25997815	0,20767724	0,42982289	protein_codir	putative protein kinase
TcG_07242	2919,069607	0,281116079	0,04280358	6,5675831	5,1138E-11	2,743E-09	protein_codir	cystathionine beta-synthase 6
TcG_07243	602,4272172	0,030732305	0,07984129	0,38491742	0,7002986	0,84438126	protein_codir	putative ubiquitin activating enzyme
TcG_07244	542,8076372	0,47431599	0,08118398	5,84248264	5,1429E-09	1,7423E-07	protein_codir	hypothetical protein
TcG_07245	218,6887336	0,177411263	0,12683423	1,39876486	0,16188351	0,36561059	protein_codir	hypothetical protein
TcG_07246	1900,684876	0,021732913	0,05018633	0,43304445	0,66498248	0,82489154	protein_codir	hypothetical protein
TcG_07247	426,014542	0,214596073	0,10803339	1,98638651	0,04699042	0,15840298	protein_codir	helicase-like protein
TcG_07248	333,6666765	0,035260443	0,09888599	0,35657672	0,72140871	0,85711748	protein_codir	hypothetical protein
TcG_07249	487,4285179	-0,01706715	0,08660868	-0,19706049	0,8437802	0,92438779	protein_codir	hypothetical protein
TcG_07250	2743,669074	-0,160619273	0,04653658	-3,45146255	0,00055756	0,00475689	protein_codir	hypothetical protein
TcG_07251	110,3101957	0,020926781	0,1664835	0,12569883	0,89997033	0,95137374	protein_codir	hypothetical protein
TcG_07252	438,0077915	-0,094395268	0,08676394	-1,08795509	0,27661493	0,50901191	protein_codir	Phosphoribosylamine--glycine ligase
TcG_07253	110,6949815	0,209282487	0,17556638	1,19204194	0,2332448	0,46060581	protein_codir	carbohydrate kinase, thermoresistant glucokinase
TcG_07254	664,1381886	-0,437452178	0,07406615	-5,90623593	3,5001E-09	1,2326E-07	protein_codir	flagellar associated protein
TcG_07255	67,02416884	0,13878183	0,21378558	0,64916367	0,51623259	0,72205516	protein_codir	putative solute carrier family 12 member 9-like
TcG_07256	461,9625829	-0,269639723	0,08920385	-3,02273647	0,002505	0,01644365	protein_codir	putative electron-transfer-flavoprotein, alpha polypeptide
TcG_07257	406,6396768	0,003900529	0,09090298	0,0429087	0,96577431	0,98490108	protein_codir	putative long-chain-fatty-acid-CoA ligase
TcG_07258	386,3762203	-0,559685106	0,09337974	-5,99364578	2,0519E-09	7,6196E-08	protein_codir	hypothetical protein
TcG_07259	389,6053005	-0,014709263	0,09624736	-0,1528277	0,87853415	0,94148439	protein_codir	hypothetical protein
TcG_07260	258,7064863	0,244638506	0,11799796	2,0732435	0,03814962	0,13654666	protein_codir	putative cystinosin
TcG_07261	53,08483718	0,334305869	0,2409779	1,3872885	0,16535381	0,37084577	protein_codir	hypothetical protein
TcG_07262	67,87679217	0,481329076	0,22401704	2,14862706	0,03166397	0,11895552	protein_codir	hypothetical protein
TcG_07263	282,2717181	0,239079963	0,11484266	2,08180439	0,03736034	0,13459481	protein_codir	hypothetical protein
TcG_07264	142,3795218	0,293651525	0,15325094	1,91614834	0,05534622	0,1777769	protein_codir	trans-sialidase
TcG_07265	357,5511093	0,272963297	0,09907041	2,75524545	0,00586481	0,03246522	protein_codir	Tbingi protein
TcG_07266	149,0281923	-0,191231739	0,15271449	-1,25221739	0,21049066	0,43302467	protein_codir	hypothetical protein
TcG_07267	732,3306599	-0,512381906	0,07064248	-7,25316959	4,0713E-13	3,3218E-11	protein_codir	putative UDP-Gal or UDP-GlcNAc-dependent glycosyltransferase
TcG_07268	97,144149	0,315922909	0,1798612	1,75648176	0,07900618	0,22895589	protein_codir	hypothetical protein
TcG_07269	31,03060104	0,17511171	0,31736759	0,55176305	0,58111071	0,77048234	protein_codir	hypothetical protein
TcG_07270	1049,689186	0,150509848	0,07133531	2,10989272	0,0348676	0,12819561	protein_codir	hypothetical protein
TcG_07271	250,9115743	-0,139966784	0,11713279	-1,19494104	0,23211006	0,45922596	protein_codir	hypothetical protein
TcG_07272	145,9384682	0,074725705	0,15462879	0,48325869	0,62891207	0,80107467	protein_codir	hypothetical protein
TcG_07273	600,9285334	0,033747141	0,079319	0,42546098	0,67050066	0,82713167	protein_codir	hypothetical protein
TcG_07274	257,5737119	-0,230773893	0,11724204	-1,9683545	0,04902726	0,16319738	protein_codir	hypothetical protein
TcG_07275	62,53342757	0,134180456	0,22624906	0,59306524	0,55313749	0,74832449	protein_codir	hypothetical protein
TcG_07276	42,80630211	0,138182974	0,26741151	0,51674281	0,60533571	0,78669877	protein_codir	hypothetical protein
TcG_07277	148,0118414	0,165351413	0,14490472	1,14110437	0,25382649	0,48488603		

TcG_07278	229,9518745	0,124718489	0,1169972	1,06599547	0,28642569	0,51965676	protein_codir	hypothetical protein
TcG_07279	200,4953505	-0,272733717	0,12832247	-2,12537769	0,03355511	0,12428693	protein_codir	hypothetical protein
TcG_07280	133,0161284	-0,046093388	0,1566584	-0,29422865	0,76858319	0,88420265	protein_codir	hypothetical protein
TcG_07281	207,2289782	-0,097610374	0,12360271	-0,78971061	0,42969679	0,65471686	protein_codir	hypothetical protein
TcG_07282	279,8574639	-0,104409323	0,10988948	-0,9501303	0,34204605	0,57651229	protein_codir	hypothetical protein
TcG_07283	126,7277301	0,026253169	0,16754611	0,15669221	0,87548743	0,93998678	protein_codir	hypothetical protein
TcG_07284	379,5626453	-0,063259493	0,10266834	-0,61615387	0,53779297	0,73738082	protein_codir	hypothetical protein
TcG_07285	226,3598428	0,160701134	0,12089615	1,32924942	0,18376569	0,39633458	protein_codir	putative ABC transporter
TcG_07286	46,23402384	-0,232910572	0,25813513	-0,90228157	0,36690731	0,59923712	protein_codir	putative ABC transporter
TcG_07287	193,583788	0,360354532	0,13749873	2,62078436	0,00877277	0,0445991	protein_codir	hypothetical protein
TcG_07288	297,9980296	0,310320544	0,10866222	2,8558273	0,00429249	0,02554326	protein_codir	microtubule-associated protein Gb4
TcG_07289	65,04327642	0,093928248	0,23111824	0,40640777	0,68444301	0,83622869		
TcG_07290	7,330900569	0,013276097	0,63202195	0,02100575	0,98324107	1		
TcG_07291	121,7467621	0,140012939	0,16808654	0,83298127	0,40485529	0,63415119	protein_codir	hypothetical protein
TcG_07292	166,8378948	0,155015062	0,14082896	1,10073283	0,27101295	0,50430483	protein_codir	methyltransferase
TcG_07293	136,820408	0,141485753	0,15635221	0,9049169	0,36550941	0,59769234	protein_codir	hypothetical protein
TcG_07294	404,4918339	0,073971748	0,09247482	0,79991235	0,42376158	0,64968926	protein_codir	hypothetical protein
TcG_07295	1072,577702	-0,062907204	0,06151462	-1,02263834	0,30647887	0,53955618	protein_codir	transferase
TcG_07296	47,88781081	-0,362682403	0,25502669	-1,42213508	0,15498704	0,35638871	protein_codir	putative protein kinase-like protein
TcG_07297	381,938904	-0,117579631	0,09384258	-1,25294546	0,21022556	0,43289563	protein_codir	putative serine/threonine protein phosphatase-like protein
TcG_07298	172,7218888	-0,161985468	0,13870384	-1,16785133	0,24286674	0,47117449	protein_codir	hypothetical protein
TcG_07299	335,1813521	0,134716054	0,10489566	1,28428621	0,19904183	0,41784719	protein_codir	putative SUMO1/Ulp2
TcG_07300	113,7179152	-0,611435168	0,16475598	-3,71115624	0,00020631	0,00204092	protein_codir	putative protein kinase
TcG_07301	234,1915475	0,577669616	0,11979753	4,82204953	1,4209E-06	2,7576E-05	protein_codir	hypothetical protein
TcG_07302	274,2895574	-0,21849635	0,11001893	-1,98598863	0,04703459	0,15845965	protein_codir	hypothetical protein
TcG_07303	269,0867506	0,26537264	0,111376	2,38267356	0,01718743	0,07486223	protein_codir	hypothetical protein
TcG_07304	359,9522946	0,262235824	0,11014754	2,38076881	0,01727655	0,07516565		
TcG_07305	225,5979755	0,369906305	0,12528711	2,95246902	0,00315244	0,01986087	protein_codir	hypothetical protein
TcG_07306	301,154448	0,08747581	0,10583819	0,82650513	0,40851761	0,63660535	protein_codir	hypothetical protein
TcG_07307	1906,053353	0,326628353	0,05255325	6,21518856	5,1263E-10	2,1998E-08	protein_codir	putative serine/threonine protein kinase
TcG_07308	404,1366793	0,209376026	0,09085293	2,30456008	0,02119122	0,08804031	protein_codir	hypothetical protein
TcG_07309	852,1020669	-0,123628239	0,07128872	-1,7341908	0,08288423	0,23675955	protein_codir	hypothetical protein
TcG_07310	281,7548374	0,283528977	0,10779797	2,63018845	0,00853375	0,04363287	protein_codir	hypothetical protein
TcG_07311	769,835204	0,247352777	0,07220993	3,42546739	0,00061374	0,00512676		
TcG_07312	230,5626519	-0,033869367	0,12905282	-0,26244578	0,79297778	0,89624823	protein_codir	hypothetical protein
TcG_07313	27,00559651	0,336030158	0,34232406	0,98161419	0,32628996	0,56072112	protein_codir	hypothetical protein
TcG_07314	146,4091107	0,123605253	0,14518893	0,85134074	0,3945801	0,62431497	protein_codir	hypothetical protein
TcG_07315	563,7190705	0,082136319	0,08060246	1,01902994	0,30818875	0,5412602	protein_codir	regulator of nonsense transcripts 1 isoform X2
TcG_07316	127,6338966	0,035120744	0,15385788	0,22826745	0,81943833	0,91087139	protein_codir	oligoribonuclease
TcG_07317	782,6398014	-0,422616714	0,07053993	-5,99116978	2,0834E-09	7,7118E-08	protein_codir	hypothetical protein
TcG_07318	319,807868	-0,305544079	0,12172438	-2,51013042	0,01206866	0,05728286		
TcG_07319	158,2497164	0,083757651	0,15482259	0,54099114	0,58851369	0,77564856	protein_codir	hypothetical protein
TcG_07320	237,6799594	0,080272148	0,11986459	0,66969027	0,50305526	0,71156125	protein_codir	hypothetical protein
TcG_07321	251,5631828	-0,037979113	0,11952254	-0,31775691	0,75066934	0,87453544	protein_codir	putative OSM3-like kinesin
TcG_07322	756,7573882	-0,018796303	0,06953225	-0,27032496	0,78691026	0,89392512	protein_codir	putative guanosine monophosphate reductase
TcG_07323	420,1071715	0,157154667	0,09544677	1,6465164	0,09965749	0,26802034	protein_codir	hypothetical protein
TcG_07324	311,3959451	0,142542294	0,10602643	1,34440344	0,17881796	0,38979933	protein_codir	putative cell division control protein
TcG_07325	117,8543009	-0,0246098	0,16412274	-0,14994753	0,88080601	0,94206932	protein_codir	hypothetical protein
TcG_07326	212,3331697	-0,128515508	0,12860212	-0,9993265	0,31763655	0,55273912	protein_codir	hypothetical protein
TcG_07327	142,7259296	0,483182438	0,14894432	3,24404739	0,00117844	0,00891925	protein_codir	hypothetical protein
TcG_07328	531,5246629	0,006192118	0,08221445	0,07531667	0,93996276	0,9721843	protein_codir	hypothetical protein
TcG_07329	283,6931414	0,281584673	0,11022317	2,55467766	0,01062862	0,05180613	protein_codir	Protein kinase domain

TcG_07330	305,9437071	0,272234075	0,10977995	2,47981594	0,01314502	0,06133638	protein_codir	hypothetical protein
TcG_07331	12,69267343	0,107149292	0,50339362	0,21285389	0,83144093	1	protein_codir	hypothetical protein
TcG_07332	73,49943207	0,277993701	0,22670273	1,22624768	0,22010548	0,44520638	protein_codir	hypothetical protein
TcG_07333	0					1		
TcG_07334	131,9090807	0,13819407	0,15886158	0,86990242	0,38435374	0,61456285	protein_codir	histone-lysine N-methyltransferase
TcG_07335	709,2894428	0,039816936	0,07688571	0,5178717	0,60454779	0,78620392	protein_codir	hypothetical protein
TcG_07336	150,7390128	-0,023204244	0,14615541	-0,15876418	0,87385467	0,93941728	protein_codir	hypothetical protein
TcG_07337	100,8310917	-0,14111681	0,17893683	-0,78864039	0,43032222	0,65519392	protein_codir	hypothetical protein
TcG_07338	425,479729	-0,001763325	0,08817628	-0,01999773	0,98404519	0,99398772	protein_codir	hypothetical protein
TcG_07339	212,5333154	-0,168243412	0,12140493	-1,38580383	0,16580681	0,37135854	protein_codir	putative ubiquitin ligase
TcG_07340	213,4962794	-0,030741616	0,12230579	-0,25135045	0,80154317	0,90153181	protein_codir	hypothetical protein
TcG_07341	428,5428925	-0,208203865	0,08735861	-2,38332382	0,01715709	0,07475822	protein_codir	serine/threonine-protein kinase HSL1,negative regulator of Swe1 kinase
TcG_07342	323,3370303	-0,094029551	0,10106496	-0,93038727	0,35217061	0,58641114	protein_codir	adenosinetriphosphatase
TcG_07343	1133,955828	-0,097436708	0,06156646	-1,58262648	0,11350662	0,29237537	protein_codir	putative nucleolar GTP-binding protein
TcG_07344	222,7270167	0,085189997	0,11981481	0,7110139	0,47707562	0,69213601	protein_codir	putative DNA-damage inducible protein DDI1-like protein
TcG_07345	194,5263341	-0,094067323	0,126681	-0,74255273	0,45775252	0,67716045	protein_codir	hypothetical protein
TcG_07346	683,894305	0,1693892	0,07784583	2,17595715	0,02955846	0,11306186	protein_codir	putative leucine-rich repeat protein
TcG_07347	215,4640167	-0,091244143	0,13300354	-0,68602795	0,49269548	0,70326104	protein_codir	lisH domain-containing protein C16orf63
TcG_07348	163,2271148	-0,126745892	0,13762824	-0,92092939	0,35708729	0,59086491	protein_codir	vesicular protein trafficking mediator
TcG_07349	523,5054433	-0,064407249	0,08189446	-0,7864665	0,43159424	0,65596889	protein_codir	putative peroxisomal targeting signal type 2 receptor
TcG_07350	230,4001221	0,108829961	0,11853311	0,91813974	0,35854571	0,59242878	protein_codir	hypothetical protein
TcG_07351	227,0761609	0,023989444	0,11963719	0,20051828	0,84107526	0,92246657	protein_codir	putative Trichohyalin
TcG_07352	78,35555359	0,128866105	0,2059938	0,62558243	0,53158886	0,73268958	protein_codir	hypothetical protein
TcG_07353	785,4425495	0,138374264	0,06816837	2,02988964	0,04236776	0,14714414	protein_codir	putative phosphoribosylpyrophosphate synthetase
TcG_07354	300,4398761	0,102952908	0,10375016	0,99231569	0,32104355	0,5560344	protein_codir	hypothetical protein
TcG_07355	210,4251833	0,151041624	0,12621642	1,19668756	0,23142836	0,45866044	protein_codir	hypothetical protein
TcG_07356	53,52301436	0,242399888	0,27150176	0,89281147	0,37195814	0,60323447	protein_codir	putative RNA editing complex protein MP46
TcG_07357	144,69152	0,102764485	0,15404043	0,6671267	0,5046912	0,71221932	protein_codir	RNA editing complex protein MP46
TcG_07358	17,45060354	0,612121321	0,43584227	1,40445606	0,16018309	0,36306159	protein_codir	hypothetical protein
TcG_07359	151,2386208	0,221670443	0,14566922	1,52173838	0,12807465	0,31693141	protein_codir	hypothetical protein
TcG_07360	319,8811901	0,117169643	0,10018236	1,16956362	0,24217662	0,47089863	protein_codir	cullin
TcG_07361	385,2349199	0,354038856	0,09351854	3,78576118	0,00015324	0,00160092	protein_codir	TFIIH basal transcription factor complex helicase subunit
TcG_07362	112,9325701	0,371815296	0,1786172	2,0816321	0,03737609	0,13460968	protein_codir	putative zinc-finger multi-pass transmembrane protein
TcG_07363	338,3218818	0,394053402	0,10296109	3,82720686	0,00012961	0,00138653	protein_codir	putative notchless-like protein
TcG_07364	262,2153854	0,206245789	0,11194428	1,84239683	0,06541712	0,20216666	protein_codir	fatty acid desaturase
TcG_07365	772,6200002	0,124665177	0,07191743	1,7334487	0,08301595	0,23681114	protein_codir	fatty acid desaturase
TcG_07366	162,1512091	0,36371799	0,13962128	2,60503264	0,00918655	0,04623605	protein_codir	cob(I)alamin adenosyltransferase
TcG_07367	1171,466613	0,431996327	0,05980111	7,22388488	5,0523E-13	4,0467E-11	protein_codir	hypothetical protein
TcG_07368	377,6298953	0,676601321	0,1007236	6,71740635	1,8499E-11	1,0825E-09	protein_codir	hypothetical protein
TcG_07369	1389,626034	0,597679398	0,05718774	10,4511799	1,4471E-25	4,6574E-23	protein_codir	60S ribosomal protein L12
TcG_07370	195,3107366	0,521432963	0,13295267	3,92194432	8,7837E-05	0,00099871	protein_codir	2-amino-3-ketobutyrate coenzyme A ligase
TcG_07371	527,2968505	0,401131203	0,08226132	4,87630375	1,0809E-06	2,1667E-05	protein_codir	hypothetical protein
TcG_07372	380,2520701	0,340055369	0,09411206	3,61330285	0,00030232	0,00282475	protein_codir	hypothetical protein
TcG_07373	1021,768997	0,758998787	0,06540136	11,6052445	3,8758E-31	2,2452E-28	protein_codir	putative 2-amino-3-ketobutyrate coenzyme A ligase
TcG_07374	42,94702577	0,811422089	0,28257135	2,87156527	0,00408444	0,02450667	protein_codir	2-amino-3-ketobutyrate coenzyme A ligase
TcG_07375	1022,18758	0,220706017	0,0637483	3,46214749	0,00053588	0,00460589	protein_codir	putative basal body component
TcG_07376	492,6277989	0,354026018	0,08495334	4,16729976	3,0823E-05	0,00040443	protein_codir	hypothetical protein
TcG_07377	190,2348527	0,189134524	0,13149019	1,43839271	0,15032266	0,35035975	protein_codir	glycerophosphoryl diester phosphodiesterase
TcG_07378	77,17534212	0,54208298	0,20139417	2,69165182	0,00710991	0,03774554	protein_codir	hypothetical protein
TcG_07379	129,3956902	0,66951189	0,16458158	4,06796366	4,7426E-05	0,00059083	protein_codir	acid--amino-acid ligase
TcG_07380	361,6483372	0,257293899	0,10332211	2,49021142	0,01276671	0,05986043	protein_codir	ubiquitin-conjugating enzyme E2
TcG_07381	1630,850573	0,301753575	0,0539216	5,5961536	2,1916E-08	6,5107E-07	protein_codir	hydroxymethylglutaryl-CoA synthase

TcG_07382	123,7476815	0,574356864	0,17537591	3,2750044	0,0010566	0,0081721	protein_codir tuzin
TcG_07383	188,1379417	0,284641003	0,12933213	2,20085299	0,02774643	0,10791211	protein_codir tuzin
TcG_07384	577,9346813	0,222095394	0,07694895	2,88626941	0,00389838	0,02360618	protein_codir amastin
TcG_07385	125,6952122	0,717197446	0,16554879	4,33224205	1,476E-05	0,00021315	protein_codir hypothetical protein
TcG_07386	113,5503978	0,483174416	0,16628094	2,90577155	0,00366349	0,02241033	protein_codir hypothetical protein
TcG_07387	234,8466863	0,074042087	0,12227803	0,60552242	0,54483193	0,74188701	protein_codir kelch-containing protein
TcG_07388	685,8976167	-0,194570522	0,07240603	-2,68721427	0,00720507	0,03817008	protein_codir eukaryotic translation initiation factor eIF-4E
TcG_07389	390,2722883	-0,401083892	0,09418153	-4,25862574	2,0569E-05	0,00028404	protein_codir flagellar inner dynein arm I1 intermediate chain IC140
TcG_07390	533,6343728	-0,359528098	0,08080612	-4,44926836	8,6163E-06	0,00013509	protein_codir programmed cell death 6-interacting protein
TcG_07391	137,5267193	-0,240811058	0,15162246	-1,58822819	0,11223475	0,29006286	protein_codir hypothetical protein
TcG_07392	422,0779509	-0,219548624	0,09243004	-2,3752951	0,01753492	0,07597593	protein_codir hypothetical protein
TcG_07393	521,6722854	-0,080664297	0,0807521	-0,99891274	0,31783696	0,55300482	protein_codir hypothetical protein
TcG_07394	200,1297677	-0,197784879	0,12893973	-1,53393276	0,1250462	0,31170078	protein_codir putative ABC transporter
TcG_07395	787,8696601	-0,106461815	0,06766542	-1,57335633	0,11563633	0,2960147	protein_codir putative eukaryotic translation release factor
TcG_07396	342,3972741	-0,108595912	0,10398379	-1,0443542	0,29632154	0,52932183	protein_codir putative RNA-binding protein
TcG_07397	451,2023667	-0,121894735	0,08669398	-1,40603452	0,15971387	0,36240598	protein_codir WASH complex subunit strumpellin
TcG_07398	203,2616099	0,078469577	0,12600062	0,62277135	0,53343478	0,73409852	protein_codir putative ubiquitin activating enzyme
TcG_07399	514,545866	-0,063436413	0,08459525	-0,74988147	0,4533261	0,67382444	protein_codir hypothetical protein
TcG_07400	376,8489507	-0,115889605	0,09698097	-1,1949727	0,2320977	0,45922596	protein_codir nuclear RNA export factor 1/2
TcG_07401	199,8720518	0,01235409	0,13456836	0,09180531	0,92685273	0,96430637	protein_codir hypothetical protein
TcG_07402	694,0798191	-0,256257685	0,07290667	-3,51487286	0,00043996	0,00388524	protein_codir putative phenylalanyl-tRNA synthetase
TcG_07403	368,3695003	-0,257547256	0,09848666	-2,61504716	0,00892151	0,04519398	protein_codir nuclear RNA export factor 1/2
TcG_07404	478,9851923	-0,212281226	0,08652015	-2,45354661	0,01414552	0,06498414	protein_codir 3-phosphoglycerate kinase, glycosomal
TcG_07405	301,9369156	-0,307396634	0,10443786	-2,94334489	0,00324686	0,02032316	protein_codir hypothetical protein
TcG_07406	353,1324504	0,127600863	0,10124343	1,2603372	0,20754775	0,4297852	protein_codir hypothetical protein
TcG_07407	166,8082238	0,058577846	0,13687297	0,42797233	0,66867126	0,8263077	protein_codir hypothetical protein
TcG_07408	248,4852313	0,172813893	0,11331636	1,52505689	0,12724491	0,31561967	protein_codir hypothetical protein
TcG_07409	254,7318655	0,353909667	0,11605064	3,04961409	0,00229136	0,01536322	protein_codir hypothetical protein
TcG_07410	245,9193065	0,017293488	0,11863286	0,14577316	0,88410046	0,94348884	protein_codir putative variant surface glycoprotein
TcG_07411	904,0455761	0,049278023	0,07182799	0,68605596	0,49267782	0,70326104	protein_codir putative mismatch repair protein MSH2
TcG_07412	275,6879769	0,003572702	0,10790698	0,03310909	0,97358759	0,98834538	protein_codir putative serine peptidase, putative,serine peptidase, clan SC, family S9D
TcG_07413	283,1356924	-0,230953635	0,10919848	-2,11498945	0,03443085	0,12696238	protein_codir hypothetical protein
TcG_07414	302,7311247	0,16602857	0,10450546	1,58870718	0,11212652	0,28989889	protein_codir hypothetical protein
TcG_07415	559,9273339	0,107633827	0,07897236	1,3629303	0,17290448	0,38109158	protein_codir putative WD40 repeat protein
TcG_07416	20146,94797	0,166882754	0,03254038	5,12848163	2,9209E-07	6,7279E-06	protein_codir Hsp90
TcG_07417	601,4595254	-0,147635876	0,07603295	-1,94173553	0,05216913	0,17026239	protein_codir ATP-binding cassette protein subfamily F, member 3
TcG_07418	116,5463096	-0,690463148	0,17150185	-4,0259807	5,6738E-05	0,00068769	protein_codir putative protein kinase
TcG_07419	0,770504906	-1,951463823	2,03471512	-0,95908454	0,33751616	1	
TcG_07420	0,4558669	-0,458748245	2,66621337	-0,17205984	0,86339048	1	
TcG_07421	0					1	
TcG_07422	0,169119941	-1,420531124	3,98996416	-0,35602604	0,72182107	1	
TcG_07423	0,155988004	0,503022807	4,08047286	0,12327562	0,90188885	1	
TcG_07424	299,9232835	-0,417485365	0,10670196	-3,91263074	9,1296E-05	0,00103297	protein_codir hypothetical protein
TcG_07425	328,0496598	-0,488821954	0,09955308	-4,91016408	9,1E-07	1,8694E-05	protein_codir hypothetical protein
TcG_07426	142,6804738	-0,085328825	0,15718404	-0,54285935	0,58722665	0,77563302	protein_codir hypothetical protein
TcG_07427	22,57242138	0,024983175	0,36642518	0,06818084	0,94564168	0,97469438	protein_codir hypothetical protein
TcG_07428	248,8440419	-0,392193802	0,1139435	-3,44200239	0,00057743	0,00489039	protein_codir RNA-binding protein RGGm
TcG_07429	208,1979047	-0,149746342	0,13631055	-1,09856749	0,27195676	0,50504046	protein_codir hypothetical protein
TcG_07430	451,3614374	0,716763853	0,09034503	7,93362795	2,1284E-15	2,3941E-13	protein_codir thiol transferase Tc52
TcG_07431	218,7362969	0,203966692	0,12109894	1,68429794	0,0921241	0,2537919	protein_codir putative adenosine deaminase-like protein
TcG_07432	22,47294413	-0,407505633	0,38535103	-1,057492	0,2902871	0,52345679	protein_codir hypothetical protein
TcG_07433	184,9081423	-0,216337569	0,13039625	-1,65907818	0,09710004	0,263528	protein_codir hypothetical protein

TcG_07434	798,0381338	0,020463875	0,07761907	0,26364494	0,79205352	0,89604169	protein_codir	putative myosin heavy chain
TcG_07435	226,1352817	-0,098158073	0,11911617	-0,8240533	0,40990927	0,63689009	protein_codir	tyrosine phosphatase
TcG_07436	210,6232587	-0,171960349	0,12130322	-1,4176074	0,15630542	0,35769079	protein_codir	tRNA wybutosine-synthesizing protein 2
TcG_07437	468,6926961	-0,284058828	0,08855043	-3,20787646	0,00133719	0,00987423	protein_codir	hypothetical protein
TcG_07438	485,9807008	-0,581474869	0,0887677	-6,55052329	5,7336E-11	3,044E-09	protein_codir	glycerol-3-phosphate acyl transferase
TcG_07439	388,359423	-0,036312664	0,09384265	-0,38695268	0,69879124	0,84414248	protein_codir	putative DNA primase large subunit
TcG_07440	214,9416409	-0,114729367	0,12802391	-0,89615579	0,37016956	0,60185021	protein_codir	cytochrome C oxidase assembly protein
TcG_07441	442,9102051	-0,053377034	0,08911347	-0,59897827	0,54918738	0,74559233	protein_codir	62 kDa protein Tc-1
TcG_07442	405,1992366	-0,219060834	0,09093811	-2,40890013	0,01600068	0,07105551	protein_codir	putative leucine-rich repeat protein (LRRP)
TcG_07443	207,6695984	-0,056021608	0,12719731	-0,44043077	0,65962514	0,82123543	protein_codir	putative acetyltransferase
TcG_07444	118,8889409	-0,232752497	0,16263899	-1,43109897	0,15240185	0,35265186	protein_codir	hypothetical protein
TcG_07445	648,1936928	-0,20297439	0,07472262	-2,7163714	0,00660018	0,03568349	protein_codir	ATP-binding cassette protein subfamily F, member 1
TcG_07446	202,9648615	0,185878141	0,12434533	1,49485426	0,13495248	0,32683099	protein_codir	hypothetical protein
TcG_07447	528,328407	-0,04948953	0,08237118	-0,60081123	0,54796572	0,74465027	protein_codir	putative U2 splicing auxiliary factor
TcG_07448	616,5989457	0,176096168	0,07788643	2,26093522	0,02376327	0,09626617	protein_codir	putative delta-1-pyrroline-5-carboxylate dehydrogenase
TcG_07449	1,054984978	1,009369208	1,70572808	0,59175271	0,5540162	1	protein_codir	metaciclina III
TcG_07450	395,6092531	0,039008843	0,10375641	0,37596562	0,70694245	0,84892495	protein_codir	putative protein kinase
TcG_07451	291,5275042	0,089367245	0,10660714	0,83828571	0,40187026	0,63220648	protein_codir	hypothetical protein
TcG_07452	553,4142871	-0,455417024	0,08549352	-5,32691873	9,9893E-08	2,5892E-06	protein_codir	putative long-chain-fatty-acid-CoA ligase
TcG_07453	148,6484912	0,047474868	0,14467081	0,32815789	0,74279228	0,86920426	protein_codir	hypothetical protein
TcG_07454	1099,807693	0,126588083	0,06529695	1,93865231	0,05254369	0,17109927		
TcG_07455	351,2720932	-0,075079177	0,10383965	-0,72302997	0,46966147	0,6861914	protein_codir	hypothetical protein
TcG_07456	66,24801163	0,048567416	0,21191585	0,22918255	0,81872703	0,91069234	protein_codir	structural maintenance of chromosome protein 4
TcG_07457	268,9455136	-0,235072518	0,12117828	-1,93988985	0,05239308	0,17080084	protein_codir	phosphatidylinositol 3-related kinase
TcG_07458	94,17783596	0,323938146	0,19798029	1,6362141	0,10179479	0,27218888	protein_codir	L1Tc protein
TcG_07459	106,0355508	0,14582946	0,17562735	0,83033456	0,40634965	0,63483385		
TcG_07460	91,41166151	0,083912516	0,1978763	0,42406553	0,67151803	0,82777091	protein_codir	hypothetical protein
TcG_07461	36,38426223	0,328515355	0,28585736	1,14922826	0,25046187	0,48012099	protein_codir	hypothetical protein
TcG_07462	138,3460343	-0,053027537	0,15527043	-0,3415173	0,73271418	0,86278448	protein_codir	hypothetical protein
TcG_07463	37,24237647	-0,015752373	0,28970282	-0,05437425	0,956637	0,97998198	protein_codir	hypothetical protein
TcG_07464	33,82707503	-0,055641608	0,30068154	-0,18505162	0,85318855	0,92887573		
TcG_07465	487,0851169	0,086083979	0,08305828	1,03642856	0,30000225	0,53367513	protein_codir	diphosphomevalonate decarboxylase
TcG_07466	228,6207486	-0,218681774	0,12605613	-1,73479677	0,08277681	0,23662771	protein_codir	hypothetical protein
TcG_07467	366,3479244	-0,304620001	0,10444226	-2,91663539	0,00353829	0,02177509	protein_codir	putative transporter, putative,major facilitator superfamily protein (MFS)
TcG_07468	221,6353368	-0,049622879	0,11921052	-0,41626259	0,67721786	0,83090609	protein_codir	hypothetical protein
TcG_07469	119,2850982	-0,423915095	0,16892207	-2,50953059	0,01208917	0,05735675	protein_codir	hypothetical protein
TcG_07470	447,9243779	-0,551262273	0,08888239	-6,20215379	5,5696E-10	2,3551E-08	protein_codir	hypothetical protein
TcG_07471	30,8238405	0,104457304	0,3111468	0,33571711	0,7370842	0,86558459		
TcG_07472	356,8718435	-0,219890209	0,09590132	-2,29287995	0,02185492	0,09007865	protein_codir	NADH dehydrogenase
TcG_07473	1225,919081	-0,416747632	0,05818458	-7,16251014	7,9213E-13	6,0779E-11	protein_codir	putative alpha glucosidase II subunit
TcG_07474	230,1634303	-0,334273183	0,11736747	-2,84809051	0,00439824	0,02607883	protein_codir	hypothetical protein
TcG_07475	11,23615066	-0,741021282	0,52998089	-1,39820378	0,16205188	1	protein_codir	hypothetical protein
TcG_07476	151,099804	0,016180911	0,14758763	0,10963596	0,91269809	0,95723874	protein_codir	putative ARF-like 2-binding protein
TcG_07477	83,0078768	-0,25914349	0,20564997	-1,26011927	0,20762634	0,42979431	protein_codir	hypothetical protein
TcG_07478	282,6135389	-0,211880007	0,11014667	-1,92361706	0,0544026	0,17562233	protein_codir	hypothetical protein
TcG_07479	378,2778336	-0,151310305	0,10184803	-1,48564785	0,13737229	0,33041216	protein_codir	putative receptor-type adenylate cyclase GRESAG 4
TcG_07480	527,4013571	-0,318827307	0,08174716	-3,90016372	9,6128E-05	0,00107816	protein_codir	serine/threonine protein phosphatase type 5
TcG_07481	588,6861014	-0,425366805	0,07860961	-5,41112985	6,2628E-08	1,6954E-06	protein_codir	putative lipase domain protein
TcG_07482	85,55740926	-0,000204319	0,20311438	-0,00100593	0,99919738	0,99981271	protein_codir	hypothetical protein
TcG_07483	108,3496795	0,05186048	0,17101314	0,30325436	0,76169602	0,87968602	protein_codir	hypothetical protein
TcG_07484	59,02252048	0,169718645	0,23640671	0,7179096	0,47281303	0,68862498	protein_codir	hypothetical protein
TcG_07485	69,17014051	0,211676141	0,21259408	0,9956822	0,31940458	0,55440022	protein_codir	hypothetical protein

TcG_07486	58,32031048	0,058212595	0,23668209	0,24595268	0,80571886	0,90420949	protein_codir hypothetical protein
TcG_07487	98,90329202	-0,030030353	0,17618597	-0,1704469	0,86465869	0,93498506	
TcG_07488	1505,636701	-0,210369518	0,06352385	-3,31166171	0,00092744	0,00734469	protein_codir hypothetical protein
TcG_07489	728,91616	-0,269589928	0,0700696	-3,84745932	0,00011935	0,00129717	protein_codir hypothetical protein
TcG_07490	54,56860103	-0,479456216	0,24181883	-1,98270832	0,04740002	0,15918163	
TcG_07491	500,8298132	-0,291556176	0,08594737	-3,39226397	0,00069318	0,00570799	protein_codir hypothetical protein
TcG_07492	211,4889828	-0,033328739	0,12509957	-0,26641769	0,78991754	0,89502471	protein_codir abhydrolase domain-containing protein 11
TcG_07493	198,5369252	-0,466661817	0,1295742	-3,60150256	0,00031638	0,00292994	protein_codir calmodulin
TcG_07494	358,7561894	0,052160184	0,09834215	0,530395	0,59583809	0,78066886	protein_codir hypothetical protein
TcG_07495	231,9392646	-0,443037319	0,12366932	-3,5824351	0,00034041	0,00311774	protein_codir outer arm dynein-like
TcG_07496	232,3779027	-0,248714912	0,11811726	-2,10566108	0,0352338	0,12918318	protein_codir putative adenylosuccinate synthetase
TcG_07497	7,150584345	1,123138699	0,67297779	1,668909	0,09513542	1	protein_codir hypothetical protein
TcG_07498	313,5753913	-0,078134345	0,10394589	-0,7516829	0,45224178	0,67313376	protein_codir hypothetical protein
TcG_07499	711,2205623	-0,220263772	0,07013581	-3,1405322	0,00168641	0,01199079	protein_codir putative nucleobase transporter
TcG_07500	53,5406895	0,220136124	0,24762607	0,88898604	0,37401058	0,60449626	protein_codir hypothetical protein
TcG_07501	3223,619272	0,082083018	0,04365412	1,88030419	0,06006663	0,18967893	protein_codir 40S ribosomal protein S4
TcG_07502	271,822792	0,200750475	0,11303444	1,77601157	0,07573101	0,22246944	protein_codir putative thiamine pyrophosphokinase
TcG_07503	2153,654373	-0,333912647	0,04765926	-7,00624911	2,4479E-12	1,74E-10	protein_codir aminopeptidase-like protein
TcG_07504	125,7318647	-0,229850163	0,15874281	-1,44794064	0,14763365	0,34639196	protein_codir putative DNA/RNA non-specific endonuclease protein-like
TcG_07505	5,377229641	-0,01545325	0,82768199	-0,01867052	0,98510395	1	
TcG_07506	18,4824624	0,248456977	0,40304072	0,61645627	0,53759342	0,73728189	
TcG_07507	25,35791522	0,556004893	0,34516641	1,61083139	0,10721648	0,28209578	protein_codir putative glycine dehydrogenase, putative,glycine cleavage system P-protein
TcG_07508	25,64035038	0,775097404	0,35155388	2,20477554	0,02746985	0,10708804	protein_codir hypothetical protein
TcG_07509	4,343341454	1,865803956	0,93971277	1,98550452	0,04708837	1	protein_codir phosphatidylinositol 3-related kinase
TcG_07510	79,61889254	0,156401816	0,21432863	0,72972901	0,46555584	0,6821385	protein_codir putative trans-sialidase
TcG_07511	352,3953358	-0,29229348	0,0982133	-2,976109	0,00291931	0,01863723	protein_codir hypothetical protein
TcG_07512	294,9643665	-0,152180625	0,10757049	-1,41470612	0,15715468	0,35894581	protein_codir hypothetical protein
TcG_07513	246,0594419	-0,123536456	0,11337233	-1,08965261	0,2758662	0,50821844	protein_codir putative katanin
TcG_07514	572,50984	-0,119774081	0,07700498	-1,5554069	0,11984919	0,30291726	protein_codir putative small nuclear RNA gene activation protein (SNAP) 50
TcG_07515	190,6986276	-0,269948595	0,13383991	-2,01695144	0,04370058	0,15042036	protein_codir putative dual specificity protein phosphatase
TcG_07516	272,932519	0,156438213	0,11188547	1,3981995	0,16205317	0,36577986	protein_codir rRNA-processing protein CGR1
TcG_07517	444,6496321	-0,260076336	0,08962958	-2,9016799	0,00371168	0,02263341	protein_codir chaperone protein DnaJ
TcG_07518	112,7872261	-0,17186933	0,16903703	-1,01675553	0,30926974	0,54241587	protein_codir protein kinase
TcG_07519	227,7104533	0,145403292	0,12439004	1,16893035	0,24243169	0,47089863	protein_codir preprotein translocase subunit YidC
TcG_07520	146,0672153	0,17824278	0,14966373	1,1909551	0,23367121	0,46121203	protein_codir hypothetical protein
TcG_07521	162,174392	0,033052009	0,14379765	0,22985083	0,81820768	0,91031113	protein_codir putative chaperone-like ATPase (ISS)
TcG_07522	182,6542054	0,073544947	0,13647332	0,53889614	0,58995852	0,77611666	protein_codir hypothetical protein
TcG_07523	413,4008428	-0,038252097	0,08947977	-0,42749438	0,66901928	0,8263077	protein_codir hypothetical protein
TcG_07524	170,2266802	0,117876382	0,14636066	0,80538297	0,42059872	0,64706635	protein_codir putative phosphatidylserine decarboxylase
TcG_07525	655,5185574	-0,045006152	0,08099452	-0,5556691	0,57843708	0,76824002	protein_codir methyltransferase
TcG_07526	467,9729827	0,083886296	0,08488766	0,98820364	0,32305293	0,55755865	protein_codir hypothetical protein
TcG_07527	264,4495757	0,284622677	0,10983561	2,59135152	0,00955998	0,04770108	protein_codir glycosyltransferase
TcG_07528	371,3564173	0,123568385	0,0959245	1,28818377	0,197682	0,41624814	protein_codir hypothetical protein
TcG_07529	160,9291539	0,02387162	0,14478865	0,16487218	0,86904459	0,93679156	protein_codir hypothetical protein
TcG_07530	79,63320323	0,344571425	0,1974131	1,74543346	0,08090945	0,23284074	protein_codir anaphase promoting complex subunit protein
TcG_07531	318,1644356	0,197803042	0,10339745	1,91303592	0,05574345	0,17855783	protein_codir hypothetical protein
TcG_07532	114,05901	0,103149586	0,16915633	0,6097885	0,54200192	0,74019653	protein_codir hypothetical protein
TcG_07533	1734,999949	-0,025913922	0,05047045	-0,51344744	0,60763837	0,78802211	protein_codir Wos2 protein
TcG_07534	144,313438	0,406030752	0,15211959	2,66915486	0,00760424	0,03986548	protein_codir receptor protein kinase
TcG_07535	1,130190449	3,392988792	1,77107258	1,9157819	0,05539286	1	protein_codir hypothetical protein
TcG_07536	4,829675185	0,074092839	0,80370824	0,09218873	0,92654809	1	protein_codir hypothetical protein
TcG_07537	10,19704203	1,128680189	0,58079034	1,94335222	0,05197362	1	protein_codir hypothetical protein

TcG_07538	1472,759805	0,18768018	0,05732133	3,27417701	0,0010597	0,0081834	protein_codir	cysteine peptidase, putative,cysteine peptidase, clan CA, family C1, cathepsin L-like
TcG_07539	47,80165348	0,209672325	0,25079423	0,83603329	0,40313618	0,63298674	protein_codir	hypothetical protein
TcG_07540	359,9296662	-0,054351337	0,09471939	-0,57381426	0,5660935	0,75832167	protein_codir	putative UDP-Gal or UDP-GlcNAc-dependent glycosyltransferase
TcG_07541	23,44856812	0,321197481	0,37211616	0,86316455	0,388047	0,61841988	protein_codir	hypothetical protein
TcG_07542	0,825598502	-0,285163302	1,96331478	-0,14524584	0,88451678	1	protein_codir	hypothetical protein
TcG_07543	0,27264935	1,353296468	3,06414982	0,4416548	0,65873902	1	protein_codir	cruzipain
TcG_07544	3,245828893	-0,547518028	0,99209403	-0,55188118	0,58102976	1	protein_codir	hypothetical protein
TcG_07545	13,31515364	0,219814237	0,50861039	0,4321859	0,66560631	1	protein_codir	hypothetical protein
TcG_07546	109,8844529	-0,078991273	0,17599346	-0,44883072	0,65355378	0,81657908	protein_codir	DUF866-domain-containing protein
TcG_07547	143,1886654	-0,014149632	0,14953498	-0,09462423	0,92461331	0,96318735	protein_codir	hypothetical protein
TcG_07548	434,3004841	0,003566805	0,08790467	0,04057583	0,96763405	0,98601654	protein_codir	hypothetical protein
TcG_07549	689,788384	0,046643668	0,07267758	0,64178894	0,52101023	0,72540161	protein_codir	hypothetical protein
TcG_07550	237,1143063	0,101817897	0,12260282	0,83046943	0,40627342	0,63480565	protein_codir	hypothetical protein
TcG_07551	47,74729082	0,095354539	0,26078726	0,36564109	0,71463291	0,8536143		
TcG_07552	72,12989146	-0,330451367	0,20603847	-1,60383335	0,10875079	0,28474275	protein_codir	hypothetical protein
TcG_07553	281,5283259	-0,54711721	0,12327738	-4,43809892	9,0757E-06	0,00014076	protein_codir	metacyclin II
TcG_07554	84,62981238	-0,091391336	0,19577081	-0,4668282	0,6406228	0,8092298	protein_codir	hypothetical protein
TcG_07555	623,9275424	-0,346195792	0,07710513	-4,48991886	7,125E-06	0,00011386	protein_codir	hypothetical protein
TcG_07556	1,69446454	0,006424212	1,34970833	0,0047597	0,99620232	1	protein_codir	hypothetical protein
TcG_07557	0,858842314	-0,037050539	1,90915581	-0,01940677	0,98451661	1	protein_codir	structural maintenance of chromosome protein 4
TcG_07558	3,02534475	0,145021434	1,0099788	0,14358859	0,88582535	1	protein_codir	putative trans-sialidase
TcG_07559	4,221236466	0,172291258	0,88950345	0,19369375	0,84641569	1	protein_codir	rab1 small GTP-binding protein
TcG_07560	21,63528949	0,169085279	0,40016274	0,42254128	0,67262997	0,828677	protein_codir	target of rapamycin (TOR) kinase 1
TcG_07561	0,884237863	1,464362917	2,02299933	0,72385734	0,46915332	1	protein_codir	hypothetical protein
TcG_07562	323,0368557	0,021588155	0,10082502	0,21411504	0,83045734	0,91760178	protein_codir	L1Tc protein
TcG_07563	411,2686638	0,222203997	0,09133497	2,43284688	0,01498063	0,06766691	protein_codir	hypothetical protein
TcG_07564	134,8667707	-0,202783206	0,15154773	-1,33808143	0,18086989	0,39227742	protein_codir	hypothetical protein
TcG_07565	358,2778985	-0,112094502	0,1040154	-1,07767216	0,28118009	0,5142555	protein_codir	hypothetical protein
TcG_07566	536,4576019	-0,019880329	0,08491212	-0,23412829	0,81488538	0,90851252	protein_codir	hypothetical protein
TcG_07567	835,5147302	0,292079346	0,0669095	4,36528959	1,2695E-05	0,00018666	protein_codir	101 kDa heat shock protein
TcG_07568	2,832958477	-0,714321726	1,05650585	-0,67611715	0,49896627	1	protein_codir	glycosylphosphatidylinositol-specific phospholipase C
TcG_07569	236,2442742	0,257997472	0,11608699	2,22244942	0,02625295	0,10344496	protein_codir	glycosylphosphatidylinositol-specific phospholipase C
TcG_07570	215,4098349	-0,037410243	0,13719896	-0,27267148	0,78510576	0,89351472	protein_codir	glycosylphosphatidylinositol-specific phospholipase C
TcG_07571	100,1014044	0,270917938	0,17979144	1,50684559	0,13185024	0,32280981	protein_codir	hypothetical protein
TcG_07572	112,1709616	0,009514857	0,16803167	0,05662538	0,95484362	0,97888932	protein_codir	hypothetical protein
TcG_07573	107,710162	0,03183132	0,16917258	0,18815886	0,85075212	0,92805389		
TcG_07574	155,6235705	-0,661817719	0,14637764	-4,52130346	6,146E-06	0,00010015	protein_codir	putative surface protein TolT
TcG_07575	2,780628603	-0,292774003	1,08683131	-0,26938311	0,78763488	1		
TcG_07576	0					1		
TcG_07577	291,6910394	-0,55412484	0,10563404	-5,2457035	1,5569E-07	3,8875E-06	protein_codir	putative surface protein TolT
TcG_07578	354,0297425	-0,114898542	0,09876395	-1,16336516	0,24468138	0,47286474	protein_codir	signal recognition particle receptor subunit beta
TcG_07579	579,8285037	-0,209145218	0,08460068	-2,47214569	0,01343048	0,06249217	protein_codir	putative dynein intermediate chain
TcG_07580	339,8090218	-0,169139232	0,10088563	-1,67654441	0,0936316	0,25683499	protein_codir	hypothetical protein
TcG_07581	243,7903637	0,062421776	0,1163666	0,53642347	0,59166592	0,77721083	protein_codir	ubiquitin activating enzyme
TcG_07582	496,8505221	-0,0915789	0,08385123	-1,09215926	0,27476311	0,50755645	protein_codir	hypothetical protein
TcG_07583	537,8226502	-0,058557804	0,0805597	-0,72688702	0,46729517	0,68394161	protein_codir	hypothetical protein
TcG_07584	226,507056	-0,118854241	0,11778438	-1,00908325	0,31293471	0,54710451	protein_codir	hypothetical protein
TcG_07585	220,4232069	0,139708385	0,11965659	1,16757784	0,24297709	0,47123077	protein_codir	putative DnaJ chaperone protein
TcG_07586	340,85208	-0,106872427	0,10010508	-1,06760239	0,28569991	0,51912745	protein_codir	putative minichromosome maintenance (MCM) complex subunit
TcG_07587	111,2641227	0,224031833	0,16708229	1,34084725	0,17997005	0,39120695	protein_codir	hypothetical protein
TcG_07588	284,1252081	0,132577311	0,10765018	1,23155683	0,21811467	0,44249284	protein_codir	putative beta-ketoacyl synthase family protein
TcG_07589	396,7474102	-0,053700157	0,09449018	-0,56831469	0,56982131	0,76182203	protein_codir	putative heterogeneous nuclear ribonucleoprotein H/F

TcG_07590	55,15157992	-0,10011696	0,23867209	-0,41947494	0,67486906	0,82970688	protein_codir	hypothetical protein
TcG_07591	482,0433962	-0,172760581	0,08388677	-2,05944961	0,03945119	0,13999295	protein_codir	putative glycosylphosphatidylinositol (GPI) anchor
TcG_07592	219,4915747	0,094765341	0,12162212	0,77917849	0,43587458	0,6595217	protein_codir	cystatin-like cysteine protease inhibitor domain-containing protein
TcG_07593	250,4079197	0,08907159	0,11312429	0,78737811	0,43106055	0,65575992	protein_codir	hypothetical protein
TcG_07594	824,0822939	0,005272368	0,06858364	0,07687501	0,93872298	0,97168077	protein_codir	RNA-binding protein
TcG_07595	453,8697443	0,140574466	0,09235049	1,52218424	0,12796292	0,31679026	protein_codir	hypothetical protein
TcG_07596	418,9113616	0,041037412	0,09568604	0,42887566	0,66801372	0,8261349	protein_codir	hypothetical protein
TcG_07597	335,3554693	0,249616161	0,10792333	2,3129028	0,02072798	0,08654213	protein_codir	cytochrome P450 reductase A
TcG_07598	204,1257531	0,200568459	0,12417508	1,61520701	0,10626587	0,28071053	protein_codir	putative leucine-rich repeat protein
TcG_07599	237,8417713	0,198057332	0,12021295	1,64755408	0,09944421	0,26775752	protein_codir	putative leucine-rich repeat protein
TcG_07600	304,5673279	0,080516984	0,1054113	0,76383634	0,44496478	0,66684283	protein_codir	dynein heavy chain
TcG_07601	311,4138295	0,010774178	0,10148219	0,10616817	0,91544893	0,95881317	protein_codir	CBS and cyclic nucleotide-binding protein domain-containing protein
TcG_07602	533,3127097	-0,001494	0,08136737	-0,01836117	0,98535073	0,9942757	protein_codir	phospholipid-transporting ATPase-like protein
TcG_07603	326,0627606	0,071944789	0,10250459	0,70186895	0,48276089	0,69628628	protein_codir	putative RNA helicase
TcG_07604	531,8141421	0,000173211	0,0805514	0,00215031	0,9982843	0,99947404	protein_codir	uncharacterized protein
TcG_07605	213,3648659	-0,008702045	0,1224406	-0,07107157	0,9433408	0,97337158	protein_codir	acyl-CoA binding protein
TcG_07606	349,8860863	0,039138741	0,09777034	0,40031304	0,68892596	0,83844588	protein_codir	adaptor complex subunit medium chain 3
TcG_07607	203,5944831	-0,120949833	0,13213221	-0,9153698	0,35999753	0,5926302	protein_codir	hypothetical protein
TcG_07608	616,7258086	-0,022961148	0,07888441	-0,29107333	0,77099524	0,88514032	protein_codir	hypothetical protein
TcG_07609	475,5827826	0,053056255	0,08454794	0,62752867	0,53031275	0,73198176	protein_codir	hypothetical protein
TcG_07610	421,0240903	-0,131951582	0,09030106	-1,46124074	0,14394939	0,34057537	protein_codir	kinesin-like protein
TcG_07611	999,0218823	0,298618119	0,0690704	4,32338757	1,5365E-05	0,00022046	protein_codir	antigenic protein
TcG_07612	277,6250657	0,213708925	0,11475086	1,86237313	0,06255051	0,19549778	protein_codir	antigenic protein
TcG_07613	223,9406973	0,55289839	0,12788944	4,32325283	1,5375E-05	0,00022046	protein_codir	hypothetical protein
TcG_07614	59,81182617	0,301565068	0,23171172	1,30146662	0,19309878	0,40990152	protein_codir	putative mitotic centromere-associated kinesin (MCAK)
TcG_07615	66,41656168	0,531053017	0,22116078	2,40120794	0,01634105	0,07223479	protein_codir	target of rapamycin (TOR) kinase 1
TcG_07616	616,7807557	0,359711699	0,08456583	4,25362945	2,1033E-05	0,00028976	protein_codir	putative mitochondrial DNA primase
TcG_07617	137,8621898	0,674838628	0,15660472	4,30918444	1,6386E-05	0,00023265	protein_codir	putative oxidoreductase
TcG_07618	207,0234729	0,650317481	0,12644555	5,14306324	2,7029E-07	6,2835E-06	protein_codir	spliceosomal U5 snRNP-specific protein
TcG_07619	727,1904205	0,093626463	0,06957657	1,34566082	0,17841193	0,38935404	protein_codir	hypothetical protein
TcG_07620	428,4787083	0,340854327	0,09310725	3,66087855	0,00025135	0,00241473	protein_codir	hypothetical protein
TcG_07621	176,3160193	0,229685779	0,13283254	1,72913796	0,08378441	0,23833197	protein_codir	LYT1p
TcG_07622	79,08814087	-0,166544812	0,19735685	-0,84387651	0,39873841	0,62871302	protein_codir	porin
TcG_07623	518,1959257	0,401190466	0,0877179	4,57364409	4,7931E-06	8,0713E-05	protein_codir	U3 small nucleolar RNA-associated protein 7
TcG_07624	208,8723944	0,54273116	0,12769692	4,25015076	2,1363E-05	0,00029395	protein_codir	hypothetical protein
TcG_07625	166,0242323	0,27964204	0,14058656	1,98910936	0,04668913	0,1578466	protein_codir	hypothetical protein
TcG_07626	509,165468	0,089700349	0,0808503	1,1094622	0,26723084	0,49961861	protein_codir	hypothetical protein
TcG_07627	322,6426223	0,199195938	0,10629422	1,87400545	0,06092967	0,19162085	protein_codir	hypothetical protein
TcG_07628	190,0169691	0,259134095	0,13264276	1,95362411	0,05074569	0,1673611	protein_codir	hypothetical protein
TcG_07629	630,8679013	0,190068765	0,07663278	2,48025396	0,01312888	0,06128576	protein_codir	putative kinesin
TcG_07630	1546,329254	0,315806998	0,05379507	5,87055627	4,3434E-09	1,4977E-07	protein_codir	ubiquitin-activating enzyme E1
TcG_07631	255,2522115	0,754788721	0,11850471	6,36927178	1,8993E-10	8,7669E-09	protein_codir	putative metallo-beta-lactamase-like protein
TcG_07632	3,526452684	0,543870309	1,85437289	0,29329069	0,76929997	1		
TcG_07633	0					1		
TcG_07634	0					1		
TcG_07635	0					1		
TcG_07636	0					1		
TcG_07637	0					1		
TcG_07638	0,116927736	0,503022807	4,08047286	0,12327562	0,90188885	1		
TcG_07639	0					1		
TcG_07640	0,446491354	-1,154452866	2,62789939	-0,43930634	0,66043958	1		
TcG_07641	0					1		

TcG_07642	1,862196699	1,410784796	1,33785307	1,054514	0,29164765	1	
TcG_07643	77,42434622	0,32232599	0,19692927	1,63676015	0,10168059	0,2719463	protein_codir hypothetical protein
TcG_07644	25,13723911	0,069561236	0,35686033	0,19492566	0,84545114	0,92503539	protein_codir surface protease GP63
TcG_07645	126,2713157	0,312713363	0,15524557	2,01431428	0,04397655	0,15114574	protein_codir rab1 small GTP-binding protein
TcG_07646	37,87912552	-0,066089246	0,28196691	-0,23438653	0,81468492	0,90837643	protein_codir target of rapamycin (TOR) kinase 1
TcG_07647	326,7983632	-0,27449251	0,09884443	-2,77701545	0,00548606	0,03075058	protein_codir beta galactofuranosyl glycosyltransferase
TcG_07648	46,52250826	0,193820105	0,26243287	0,73855117	0,46017958	0,67839985	protein_codir hypothetical protein
TcG_07649	23,20526572	0,913770312	0,3721637	2,45529135	0,01407704	0,06472089	protein_codir hypothetical protein
TcG_07650	24,0582296	0,496822579	0,35823263	1,38687137	0,16548099	0,3709156	protein_codir hypothetical protein
TcG_07651	101,9496755	0,197796412	0,1751019	1,12960745	0,25864167	0,49108856	protein_codir trans-sialidase
TcG_07652	91,99789387	0,237294835	0,19540619	1,21436705	0,22460759	0,45108253	protein_codir trans-sialidase
TcG_07653	66,80998864	-0,123219897	0,22346354	-0,55140939	0,58135306	0,77048234	protein_codir retrotransposon hot spot (RHS) protein
TcG_07654	43,47288509	-0,120070195	0,26419834	-0,45446991	0,64949065	0,81386531	protein_codir hypothetical protein
TcG_07655	29,93640905	0,477637971	0,31763868	1,50371476	0,13265483	0,32361438	protein_codir hypothetical protein
TcG_07656	3,311816371	0,310464997	0,96474127	0,32181167	0,74759537	1	protein_codir helicase-like protein
TcG_07657	41,58946121	0,200544698	0,27279733	0,73514171	0,4622532	0,67974471	protein_codir putative glycine dehydrogenase, putative,glycine cleavage system P-protein
TcG_07658	32,84879271	-0,178704838	0,30271454	-0,59034112	0,55496198	0,74965483	protein_codir putative retrotransposon hot spot (RHS) protein
TcG_07659	87,69227058	0,219310576	0,20399563	1,0750749	0,2823412	0,51557427	protein_codir hypothetical protein
TcG_07660	83,0536342	0,038218931	0,19982827	0,19125888	0,84832277	0,92662088	protein_codir target of rapamycin (TOR) kinase 1
TcG_07661	55,03571556	0,273507393	0,23367182	1,17047657	0,24180924	0,4704621	protein_codir protein kinase
TcG_07662	237,585556	-0,224720279	0,11560018	-1,94394399	0,05190221	0,16953452	
TcG_07663	41,66018942	-0,081277265	0,26758824	-0,30374004	0,76132595	0,87949151	protein_codir dispersed protein family protein 1
TcG_07664	28,90488454	0,241682063	0,32731745	0,73837207	0,46028838	0,67839985	protein_codir dispersed gene family protein 1 (DGF-1)
TcG_07665	174,6179072	-0,184018216	0,13403874	-1,37287341	0,1697917	0,37606704	protein_codir dispersed gene family protein 1 (DGF-1)
TcG_07666	157,0419164	0,201761067	0,14631771	1,37892443	0,16791805	0,37406241	protein_codir dispersed gene family protein 1 (DGF-1)
TcG_07667	139,7762779	-0,207542266	0,15382315	-1,34922645	0,17726424	0,38736015	protein_codir dispersed gene family protein 1 (DGF-1)
TcG_07668	289,5542759	-0,081316116	0,11683751	-0,69597609	0,48644381	0,69829488	protein_codir dispersed gene family protein 1 (DGF-1)
TcG_07669	339,7872121	-0,071338143	0,10048239	-0,70995667	0,477731	0,69260885	protein_codir dispersed gene family protein 1 (DGF-1)
TcG_07670	88,77652866	-0,018741645	0,20332076	-0,09217772	0,92655684	0,96430637	protein_codir dispersed gene family protein 1 (DGF-1)
TcG_07671	102,0205547	-0,339911263	0,17697331	-1,92069224	0,05477052	0,17656406	protein_codir dispersed protein family protein 1 (DGF-1)
TcG_07672	60,09457342	-0,301183514	0,23547244	-1,27906058	0,20087572	0,42026053	
TcG_07673	47,9425799	0,29083215	0,26544816	1,09562694	0,27324207	0,50652521	protein_codir retrotransposon hot spot (RHS) protein
TcG_07674	287,1720933	-0,102577505	0,11308913	-0,90705006	0,36438032	0,59637102	protein_codir retrotransposon hot spot (RHS) protein
TcG_07675	64,70002391	0,100350303	0,21878088	0,4586795	0,64646434	0,81201944	protein_codir hypothetical protein
TcG_07676	97,08307882	-0,053618932	0,17704792	-0,30284982	0,76200431	0,87977897	protein_codir dispersed gene family protein 1 (DGF-1)
TcG_07677	140,5273158	0,228932374	0,16153751	1,41720879	0,15642189	0,35787995	protein_codir SH3 domain protein
TcG_07678	214,2044777	0,047449533	0,12289124	0,38610996	0,69941523	0,84428265	protein_codir hypothetical protein
TcG_07679	7,044042876	0,130992241	0,66790016	0,19612548	0,84451195	1	protein_codir putative voltage-dependent anion-selective channel
TcG_07680	269,3377858	-0,302733484	0,11860569	-2,55243645	0,01069724	0,05207489	protein_codir stress-induced protein sti1
TcG_07681	127,3064477	0,002213776	0,16135195	0,01372017	0,98905323	0,99554162	protein_codir ARP2/3 complex subunit
TcG_07682	189,8382227	0,04887915	0,13596343	0,35950219	0,71921944	0,85588296	protein_codir WD-40containing protein
TcG_07683	310,9640913	0,132136004	0,1026169	1,28766314	0,19786325	0,41642935	protein_codir solute carrier family member b3
TcG_07684	174,0057464	-0,16904422	0,13539841	-1,24849484	0,2118499	0,43480831	protein_codir hypothetical protein
TcG_07685	695,9799641	-0,112661993	0,07592356	-1,48388719	0,13783885	0,33125927	protein_codir hypothetical protein
TcG_07686	126,3714485	-0,274559162	0,15569203	-1,76347599	0,07782024	0,22631158	protein_codir hypothetical protein
TcG_07687	369,3403243	-0,117049353	0,0965142	-1,21276826	0,22521843	0,45182011	protein_codir mitochondrial carrier protein
TcG_07688	302,5497717	0,206987352	0,10413336	1,98771415	0,04684331	0,15809563	protein_codir hypothetical protein
TcG_07689	106,0029468	-0,129971064	0,17165285	-0,75717392	0,44894567	0,66986279	protein_codir hypothetical protein
TcG_07690	376,0592712	-0,117880943	0,09395755	-1,25461921	0,20961703	0,43221692	protein_codir hypothetical protein
TcG_07691	1283,403315	0,000574918	0,05570386	0,01032096	0,99176521	0,99678187	protein_codir ATP-dependent Clp protease subunit, heat shock protein 78 (HSP78)
TcG_07692	776,0087838	-0,145524783	0,07039174	-2,06735592	0,03870063	0,13809223	protein_codir putative mannosyltransferase-like protein
TcG_07693	1828,717969	-0,191241729	0,05133413	-3,72543032	0,00019498	0,00195252	protein_codir oleate desaturase

TcG_07694	76,98959461	-0,143221991	0,19682339	-0,72766753	0,46681713	0,68341462	
TcG_07695	367,2401467	0,349203695	0,09439929	3,69921935	0,00021626	0,00212702	protein_codir hypothetical protein
TcG_07696	280,9505469	0,090956681	0,10871397	0,83666045	0,40278346	0,6328518	protein_codir hypothetical protein
TcG_07697	1039,829833	-0,061884317	0,06185092	-1,00054004	0,31704923	0,55204875	protein_codir polyadenylation/uridylation factor 1
TcG_07698	68,03503665	0,398966864	0,21512608	1,85457224	0,06365733	0,19815526	protein_codir hypothetical protein
TcG_07699	606,2600469	-0,443483064	0,0775923	-5,71555533	1,0935E-08	3,4426E-07	protein_codir putative surface antigen TASV, putative,mucin-like glycoprotein
TcG_07700	124,6192783	-0,624779931	0,15785796	-3,9578614	7,5624E-05	0,00087793	
TcG_07701	5,024617071	-0,91040768	0,78985217	-1,15263048	0,24906209	1	protein_codir trans-sialidase
TcG_07702	1,446957673	0,247178092	1,40991687	0,17531395	0,86083295	1	protein_codir hypothetical protein
TcG_07703	2,055056185	1,017330673	1,30466781	0,77976222	0,43553085	1	protein_codir hypothetical protein
TcG_07704	2,317617385	0,751021264	1,19466917	0,62864371	0,52958233	1	protein_codir hypothetical protein
TcG_07705	2,017927325	-0,971802005	1,33835658	-0,72611591	0,46776772	1	protein_codir hypothetical protein
TcG_07706	1599,299139	-0,12601899	0,05329587	-2,36451694	0,0180536	0,07764255	protein_codir trans-sialidase
TcG_07707	262,8535683	0,121162236	0,1133024	1,06937043	0,28490279	0,51876217	protein_codir hypothetical protein
TcG_07708	501,095628	0,05885007	0,08363357	0,70366562	0,48164103	0,69570871	protein_codir retrotransposon hot spot (RHS) protein
TcG_07709	181,4390375	0,221748984	0,13460347	1,64742394	0,09947094	0,26776726	protein_codir retrotransposon hot spot (RHS) protein
TcG_07710	82,0730578	-0,178664182	0,2000813	-0,89295793	0,3718797	0,60319168	
TcG_07711	24,21028441	0,258490074	0,35775716	0,72252942	0,46996905	0,68645032	protein_codir hypothetical protein
TcG_07712	24,35203232	-0,055535365	0,36079916	-0,15392321	0,87767028	0,94120054	protein_codir hypothetical protein
TcG_07713	62,14025239	-0,163262637	0,22587015	-0,72281635	0,46979272	0,68629662	protein_codir putative dynein assembly factor 1, axonemal-like
TcG_07714	148,2125236	-0,006973832	0,14290266	-0,04880127	0,96107767	0,98198472	protein_codir putative exosome-associated protein 4, putative,3' exoribonuclease
TcG_07715	882,100491	-0,06133392	0,0691555	-0,88689866	0,37513346	0,6057344	protein_codir hypothetical protein
TcG_07716	439,0798857	-0,625733233	0,08821831	-7,09300857	1,3123E-12	9,7462E-11	protein_codir hypothetical protein
TcG_07717	303,035802	0,20892431	0,10698377	1,95285985	0,0508362	0,16761191	protein_codir putative translation initiation factor 2 subunit
TcG_07718	118,2258216	0,201261826	0,16390695	1,22790297	0,2194834	0,4445842	protein_codir hypothetical protein
TcG_07719	339,2479277	-0,097552914	0,09879423	-0,9874353	0,32342929	0,55758888	protein_codir hypothetical protein
TcG_07720	759,1936552	0,00135087	0,0689262	0,01959879	0,98436343	0,99405863	protein_codir alkylated DNA repair protein alkB like protein 6
TcG_07721	672,2164897	0,065502948	0,07619275	0,85970051	0,38995415	0,62017966	protein_codir hypothetical protein
TcG_07722	177,9356972	-0,034575695	0,13320486	-0,25956782	0,79519717	0,89770577	protein_codir hypothetical protein
TcG_07723	57,62310006	-0,185758343	0,23697767	-0,78386432	0,43311973	0,65752733	protein_codir hypothetical protein
TcG_07724	239,6716709	0,079280044	0,11387096	0,69622705	0,48628665	0,69827461	protein_codir putative delta tubulin
TcG_07725	573,2626486	0,473271971	0,08794857	5,38123583	7,3976E-08	1,9794E-06	
TcG_07726	46514,22094	-0,061995485	0,03857924	-1,60696475	0,1080621	0,283452	
TcG_07727	23966,47076	0,402294307	0,11317659	3,55457188	0,0003786	0,00341886	
TcG_07728	8442,578004	-0,02541328	0,0337928	-0,75203239	0,45203158	0,67290735	
TcG_07729	19,30065688	0,446804143	0,41389739	1,07950461	0,28036284	0,51356267	protein_codir surface protease GP63
TcG_07730	35,38751324	-0,075189248	0,29257334	-0,25699282	0,79718432	0,89881058	protein_codir surface protease GP63
TcG_07731	177,6270762	0,248078283	0,13371059	1,85533755	0,06354804	0,19797461	protein_codir surface protease GP63
TcG_07732	358,1130939	0,104510693	0,09792802	1,06721952	0,28587272	0,51918774	protein_codir hypothetical protein
TcG_07733	308,6623348	-0,151935732	0,10218095	-1,48692812	0,1370338	0,33007767	protein_codir hypothetical protein
TcG_07734	433,4935992	0,064910368	0,09417946	0,68922003	0,49068481	0,70177438	protein_codir lipase-like protein
TcG_07735	321,9324417	-0,018039446	0,10274984	-0,17556666	0,8606344	0,93329372	protein_codir hypothetical protein
TcG_07736	596,1785311	0,402282338	0,07930054	5,07288241	3,9183E-07	8,8151E-06	protein_codir hypothetical protein
TcG_07737	338,7554479	0,201927378	0,10116642	1,99599208	0,04593479	0,15575078	protein_codir hypothetical protein
TcG_07738	693,6477208	-0,077601785	0,07192051	-1,07899387	0,28059046	0,51365478	protein_codir sperm-associated antigen 6 isoform X1
TcG_07739	147,7162569	0,483367836	0,1482001	3,26158916	0,0011079	0,00847826	protein_codir hypothetical protein
TcG_07740	548,8225222	0,179853581	0,08171296	2,20104102	0,02773312	0,10791211	protein_codir hypothetical protein
TcG_07741	566,2242848	-0,038243364	0,08051079	-0,47500919	0,63478042	0,80483322	protein_codir pumilio protein 9
TcG_07742	385,4361155	0,314855162	0,09241871	3,40683374	0,00065721	0,00545057	protein_codir exosome-associated protein 1
TcG_07743	1290,125644	-0,147397034	0,05988242	-2,46144086	0,01383802	0,06379917	protein_codir coatomer beta subunit
TcG_07744	13571,75092	-0,127880602	0,03411469	-3,7485493	0,00017786	0,0018106	protein_codir beta-tubulin
TcG_07745	189,0829842	0,374633333	0,13068882	2,86660575	0,004149	0,02486821	protein_codir putative deoxyhypusine synthase

TcG_07746	1633,527523	0,229677708	0,0666686	3,44506557	0,00057092	0,00485304	protein_codir	MDN1, midasin-like protein
TcG_07747	170,2949028	0,217789565	0,26974026	0,80740473	0,41943334	0,64604556	protein_codir	putative dynein heavy chain
TcG_07748	112,2332443	-0,282627888	0,16508301	-1,71203499	0,08689022	0,24434391	protein_codir	putative maoC-like dehydratase
TcG_07749	230,0758028	-0,282389943	0,12010247	-2,3512418	0,01871087	0,07964149	protein_codir	putative prolyl oligopeptidase, putative,serine peptidase clan SC, family S9A
TcG_07750	140,6518762	-0,287029672	0,14732638	-1,94825715	0,0513842	0,16868429	protein_codir	putative pyruvate dehydrogenase (lipoamide) kinase
TcG_07751	155,1946671	-0,061708769	0,14750066	-0,41836265	0,67568199	0,83017833	protein_codir	putative beta prime cop protein
TcG_07752	178,9379076	0,336918005	0,13900874	2,42371808	0,01536253	0,06893502	protein_codir	helicase-like protein
TcG_07753	0					1	protein_codir	SH3 domain protein
TcG_07754	29,9616675	0,481475564	0,32335213	1,48901311	0,13648392	0,32916375	protein_codir	SH3 domain protein
TcG_07755	135,4540138	0,465133879	0,18040535	2,57827097	0,00992961	0,04915852	protein_codir	trans-sialidase
TcG_07756	79,1178251	0,244370141	0,20133295	1,21376129	0,22483889	0,45131383	protein_codir	hypothetical protein
TcG_07757	304,3319383	0,010592021	0,10465892	0,10120515	0,91938761	0,96120058	protein_codir	hypothetical protein
TcG_07758	214,1742836	0,221896045	0,12613487	1,75919665	0,07854411	0,22801606		
TcG_07759	215,2161877	0,05402782	0,13469843	0,40110209	0,68834496	0,8382557	protein_codir	putative leucine-rich repeat protein (LRRP)
TcG_07760	321,188172	0,457697294	0,1033656	4,42794589	9,5135E-06	0,0001458	protein_codir	hypothetical protein
TcG_07761	289,7536435	0,157983609	0,10486836	1,50649452	0,13194027	0,32280981	protein_codir	hypothetical protein
TcG_07762	87,37807544	0,821147293	0,19380158	4,23705155	2,2647E-05	0,00030855	protein_codir	hypothetical protein
TcG_07763	131,90291	0,217656089	0,16187088	1,34462778	0,17874547	0,38979933	protein_codir	putative peroxisome targeting signal 1 receptor
TcG_07764	745,1451181	0,16350475	0,07603816	2,15029859	0,0315316	0,11857357	protein_codir	hypothetical protein
TcG_07765	759,8735849	-0,542454985	0,07090986	-7,64992326	2,011E-14	1,9416E-12	protein_codir	putative mitochondrial carrier protein
TcG_07766	374,9277311	-0,534741125	0,09991945	-5,35172191	8,7121E-08	2,2941E-06	protein_codir	hypothetical protein
TcG_07767	161,4714769	-0,55923017	0,15431628	-3,62392198	0,00029017	0,0027266	protein_codir	hypothetical protein
TcG_07768	389,1490759	-0,25145804	0,09733973	-2,58330337	0,00978592	0,04861137	protein_codir	hypothetical protein
TcG_07769	277,2508643	-0,068047275	0,11015359	-0,61774903	0,53674079	0,73660472	protein_codir	spermidine synthase
TcG_07770	394,183565	-0,368138784	0,09326878	-3,94707419	7,9112E-05	0,00091113	protein_codir	hypothetical protein
TcG_07771	263,9713249	-0,146237651	0,12326422	-1,18637551	0,23547404	0,46332981	protein_codir	hypothetical protein
TcG_07772	242,2533416	-0,393395921	0,11474535	-3,42842577	0,00060709	0,00508588	protein_codir	tyrosine phosphatase
TcG_07773	734,2700597	-0,338815655	0,07366895	-4,59916477	4,2419E-06	7,2594E-05	protein_codir	tetratricopeptide repeat protein 21B isoform a
TcG_07774	269,8081782	-0,329241764	0,117251	-2,8080081	0,0049849	0,02871955	protein_codir	EF hand
TcG_07775	378,0689871	-0,309897019	0,0990887	-3,12747098	0,00176317	0,012441	protein_codir	protein XRP2
TcG_07776	150,0837706	-0,190996911	0,14984634	-1,27461847	0,20244432	0,42261619	protein_codir	hypothetical protein
TcG_07777	545,4543322	-0,033705095	0,07941805	-0,42440092	0,67127345	0,82773246	protein_codir	putative huntingtin interacting protein (HIP)
TcG_07778	191,3878443	-0,567383265	0,14153929	-4,00866252	6,1064E-05	0,00073163	protein_codir	DNA primase small subunit
TcG_07779	477,0805337	-0,104434466	0,08690687	-1,20168245	0,22948658	0,45668697	protein_codir	hypothetical protein
TcG_07780	291,8994053	-0,19109126	0,10680499	-1,78916045	0,07358898	0,21867194	protein_codir	ADP-ribosylation factor
TcG_07781	3821,462834	0,237735215	0,04605341	5,16216342	2,4411E-07	5,7877E-06	protein_codir	60S ribosomal protein L11
TcG_07782	463,533493	-0,175241645	0,09134242	-1,91851323	0,05504597	0,17711984	protein_codir	hypothetical protein
TcG_07783	15,04194649	-0,909160437	0,45444584	-2,00059139	0,04543644	1		
TcG_07784	160,5397367	-0,02239065	0,14498922	-0,15442976	0,87727089	0,94102958	protein_codir	GP63 group II protein
TcG_07785	6,069964425	0,300482764	0,70772068	0,42457819	0,67114419	1	protein_codir	hypothetical protein
TcG_07786	11,14824988	-0,239321377	0,51922819	-0,46091754	0,64485777	1		
TcG_07787	312,1162293	0,130516172	0,10587812	1,23270201	0,21768696	0,44193467	protein_codir	sialidase
TcG_07788	106,293085	0,024584716	0,17680131	0,1390528	0,88940843	0,94653573	protein_codir	histone H3
TcG_07789	4241,691074	-0,152584967	0,03927646	-3,88489597	0,00010237	0,00113696	protein_codir	histone H3
TcG_07790	1560,043795	-0,013547754	0,05465609	-0,24787273	0,80423288	0,90318531	protein_codir	hypothetical protein
TcG_07791	593,083426	0,093064976	0,08022792	1,1600073	0,24604583	0,47463987	protein_codir	hypothetical protein
TcG_07792	185,5733476	-0,193592501	0,13130458	-1,47437734	0,14038002	0,33486575	protein_codir	calcyphosin
TcG_07793	301,816926	0,174477259	0,10319796	1,69070446	0,09089326	0,25157414	protein_codir	hypothetical protein
TcG_07794	585,3679648	0,02234559	0,07860291	0,28428449	0,77619237	0,88831084	protein_codir	hypothetical protein
TcG_07795	214,099747	-0,069336227	0,12108016	-0,57264732	0,56688351	0,75876992	protein_codir	hypothetical protein
TcG_07796	2626,422079	0,027639515	0,05265812	0,52488612	0,59966236	0,78310281	protein_codir	putative 5'-3' exonuclease
TcG_07797	628,5123458	-0,370512101	0,07650647	-4,84288577	1,2797E-06	2,5172E-05	protein_codir	hypothetical protein

TcG_07798	361,7969495	0,088756339	0,09828212	0,90307719	0,36648493	0,59871607	protein_codir hypothetical protein
TcG_07799	125,1480928	0,119251221	0,1593259	0,74847354	0,45417458	0,67419176	protein_codir hypothetical protein
TcG_07800	194,5861485	0,125811179	0,12732965	0,98807452	0,32311615	0,55757366	protein_codir putative ribonuclease H1
TcG_07801	264,9782156	0,311989839	0,1121834	2,78106968	0,00541801	0,03050198	protein_codir hypothetical protein
TcG_07802	369,5555826	0,024808709	0,09628779	0,25765167	0,79667575	0,89872949	protein_codir hypothetical protein
TcG_07803	615,4160632	0,169434742	0,07746384	2,18727526	0,02872244	0,11061934	protein_codir poly [ADP-ribose] polymerase
TcG_07804	734,3889263	-0,149751855	0,07062103	-2,12049958	0,03396394	0,12556036	protein_codir oligopeptidase B-like protein
TcG_07805	4,58692295	1,544420437	0,89512696	1,72536468	0,08446177	1	protein_codir retrotransposon hot spot (RHS) protein
TcG_07806	495,5347389	0,140507272	0,08458347	1,66116696	0,09667992	0,26300388	protein_codir syntaxin
TcG_07807	438,0915489	-0,229259785	0,08821449	-2,59889043	0,00935256	0,04681924	protein_codir hypothetical protein
TcG_07808	328,2618777	-0,073417839	0,10057827	-0,72995725	0,46541632	0,6821385	protein_codir hypothetical protein
TcG_07809	178,7572088	-0,049100579	0,13521579	-0,36312754	0,71650961	0,85441337	protein_codir putative lipase domain protein
TcG_07810	216,2172112	0,06836825	0,12423999	0,55029182	0,58211923	0,77119439	protein_codir putative poly(A) export protein
TcG_07811	144,1034658	-0,072465957	0,14862257	-0,48758379	0,62584468	0,79892425	protein_codir hypothetical protein
TcG_07812	169,3706416	0,111444967	0,13569783	0,821273	0,41149078	0,63808037	protein_codir exopolyphosphatase
TcG_07813	199,6984944	0,277127209	0,13420076	2,06501968	0,03892114	0,13877945	protein_codir putative p21-activated kinase 3
TcG_07814	153,1014371	0,212992705	0,14603114	1,45854309	0,14469091	0,34156252	protein_codir hypothetical protein
TcG_07815	184,0337339	0,233177978	0,13279644	1,75590535	0,07910457	0,22917254	protein_codir pseudouridylate synthase-like protein
TcG_07816	334,1197751	0,143888614	0,11440122	1,25775422	0,20848066	0,43110064	protein_codir putative N-acetyltransferase
TcG_07817	136,3385328	-0,1989105	0,15238237	-1,30533799	0,19177778	0,4078446	protein_codir thioredoxin
TcG_07818	555,5119659	0,221697153	0,08042437	2,7565917	0,00584072	0,03234733	protein_codir flagellar/basal body protein
TcG_07819	343,0671755	0,295809221	0,09934111	2,97771198	0,00290409	0,01856815	protein_codir hypothetical protein
TcG_07820	747,9903022	0,26450998	0,07063189	3,74490897	0,00018046	0,00183404	protein_codir putative DNA excision/repair protein SNF2
TcG_07821	522,9874189	0,021256498	0,08686403	0,24471002	0,80668097	0,90458824	protein_codir putative endo-beta-N-acetylglucosaminidase
TcG_07822	102,3035221	0,625850004	0,17820631	3,51194084	0,00044485	0,00392237	protein_codir hypothetical protein
TcG_07823	159,766161	0,156297694	0,14698613	1,06334997	0,28762327	0,52060666	protein_codir putative cyclophilin
TcG_07824	493,7249604	0,198337583	0,09115826	2,17574989	0,02957397	0,11308382	protein_codir hypothetical protein
TcG_07825	168,9506429	0,314754448	0,13935735	2,25861383	0,02390742	0,09661161	protein_codir protein-tyrosine phosphatase
TcG_07826	143,8462368	0,15868045	0,15505678	1,02336997	0,30613295	0,53918189	protein_codir hypothetical protein
TcG_07827	221,1711717	0,154241602	0,13159362	1,1721055	0,2411547	0,4698198	protein_codir putative CLC-type chloride channel
TcG_07828	427,2672411	0,121942589	0,09138697	1,33435418	0,18208781	0,39396253	protein_codir hypothetical protein
TcG_07829	18,78359141	0,055927647	0,40622527	0,13767643	0,89049615	0,94667588	protein_codir L1Tc protein
TcG_07830	218,1405126	0,284394937	0,12247083	2,32214429	0,02022517	0,08480955	protein_codir mucin TcSMUGS
TcG_07831	580,5667623	-0,331528438	0,08049248	-4,11875027	3,8093E-05	0,000485	protein_codir hypothetical protein
TcG_07832	277,8912048	-0,024970396	0,10651082	-0,23443998	0,81464342	0,90837643	protein_codir hypothetical protein
TcG_07833	729,074891	0,105307276	0,07631798	1,37984888	0,16763318	0,37364402	protein_codir hypothetical protein
TcG_07834	529,1437724	-0,398192409	0,08185581	-4,86455862	1,1471E-06	2,2836E-05	protein_codir hypothetical protein
TcG_07835	1290,111649	0,043135828	0,05706199	0,75594679	0,4496811	0,67044205	protein_codir hypothetical protein
TcG_07836	1168,962149	-0,033924183	0,05882211	-0,57672504	0,56412522	0,75700194	protein_codir putative protein kinase
TcG_07837	1204,092087	-0,02932407	0,05752872	-0,50972918	0,6102412	0,7890164	protein_codir histone H4
TcG_07838	305,5784683	-0,062098402	0,10958755	-0,56665563	0,57094817	0,7624407	protein_codir hypothetical protein
TcG_07839	78,28252652	-0,002170605	0,19610772	-0,01106843	0,99116885	0,99667057	protein_codir hypothetical protein
TcG_07840	580,893048	-0,111743161	0,0893173	-1,25108078	0,21090501	0,43348331	protein_codir hypothetical protein
TcG_07841	1306,182029	-0,040225861	0,17850908	-0,22534351	0,82171206	0,91160193	protein_codir hypothetical protein
TcG_07842	144,316428	-0,124987119	0,14943993	-0,8363703	0,40294661	0,63295092	protein_codir hypothetical protein
TcG_07843	14,00442725	0,462885342	0,47600181	0,9724445	0,33082946	1	protein_codir hypothetical protein
TcG_07844	8,602038012	0,420869475	0,59035787	0,71290567	0,47590413	1	protein_codir putative RNA-binding protein
TcG_07845	1482,211574	0,616042074	0,06738454	9,14218658	6,1202E-20	1,1079E-17	protein_codir inositol 1,4,5-trisphosphate receptor
TcG_07846	245,6024328	0,54161774	0,11542695	4,69229884	2,7015E-06	4,8906E-05	protein_codir GTP-binding protein
TcG_07847	310,9938415	0,524805755	0,10614679	4,94415096	7,6476E-07	1,5965E-05	protein_codir mitochondrial RNA binding protein
TcG_07848	620,7251537	0,315098275	0,08176042	3,85392174	0,00011624	0,00127053	protein_codir putative mitochondrial RNA binding protein
TcG_07849	88,0289246	0,441521019	0,19649652	2,24696606	0,0246422	0,09868451	protein_codir hypothetical protein

TcG_07850	185,2622272	0,498778257	0,13581485	3,67248697	0,0002402	0,00232302	protein_codir	hypothetical protein
TcG_07851	238,1050061	0,454910693	0,11854665	3,83739799	0,00012434	0,0013414	protein_codir	hypothetical protein
TcG_07852	223,495323	0,454658027	0,12229089	3,71784043	0,00020093	0,00199829	protein_codir	putative SET and MYND domain-containing protein 3
TcG_07853	128,3962874	0,626624609	0,17507775	3,5791219	0,00034475	0,00315255	protein_codir	hypothetical protein
TcG_07854	177,0858818	0,77666248	0,1385495	5,60566787	2,0745E-08	6,2268E-07	protein_codir	actin interacting protein-like protein
TcG_07855	332,4691042	0,36991098	0,10244707	3,61075222	0,00030531	0,00284732	protein_codir	hypothetical protein
TcG_07856	760,0980596	0,448475553	0,07030862	6,37867113	1,7863E-10	8,3452E-09	protein_codir	Na/H antiporter-like protein
TcG_07857	249,2724397	0,255327275	0,12141111	2,10299755	0,03546598	0,12985405	protein_codir	hypothetical protein
TcG_07858	177,8310452	0,228333513	0,13210385	1,72843955	0,08390945	0,23851199	protein_codir	hypothetical protein
TcG_07859	0,636551173	1,116862059	2,2097098	0,50543382	0,61325417	1		
TcG_07860	400,2719429	-0,062827871	0,10099368	-0,62209703	0,53387807	0,73435964	protein_codir	putative aspartyl-tRNA synthetase
TcG_07861	265,6944491	0,20372972	0,10883351	1,87193924	0,06121501	0,1921531	protein_codir	YbaK/aminoacyl-tRNA synthetase-associated domain-containing protein
TcG_07862	165,258078	0,325414905	0,1414347	2,30081385	0,02140216	0,08868576	protein_codir	hypothetical protein
TcG_07863	347,2196139	-0,154239917	0,09779371	-1,57719669	0,11475027	0,29472327	protein_codir	hypothetical protein
TcG_07864	529,745807	-0,279067327	0,08052595	-3,46555771	0,00052913	0,00455723	protein_codir	putative katanin
TcG_07865	275,734342	0,041749445	0,11425688	0,36539984	0,71481296	0,85362018	protein_codir	putative programmed cell death protein 2
TcG_07866	484,3358608	-0,237557675	0,08382163	-2,83408549	0,0045957	0,02695991	protein_codir	hypothetical protein
TcG_07867	248,5830016	0,104780676	0,11499018	0,91121412	0,36218257	0,59480293	protein_codir	hypothetical protein
TcG_07868	1256,57082	-0,233723745	0,05682561	-4,11300028	3,9055E-05	0,00049561	protein_codir	intraflagellar transport 172-like protein
TcG_07869	285,3796111	-0,120390364	0,10735691	-1,12140297	0,26211637	0,49428247	protein_codir	hypothetical protein
TcG_07870	591,3171168	-0,063384062	0,07565293	-0,8378269	0,40212793	0,63242218	protein_codir	hypothetical protein
TcG_07871	415,4127076	-0,178943791	0,08953445	-1,99860275	0,04565135	0,15492577	protein_codir	putative epsilon tubulin
TcG_07872	187,0189741	-0,098673678	0,13052795	-0,75595824	0,44967423	0,67044205	protein_codir	putative dynein arm light chain
TcG_07873	701,3876217	-0,187306162	0,0753155	-2,48695386	0,01288421	0,0603381	protein_codir	hypothetical protein
TcG_07874	1847,782993	-0,168014078	0,05476141	-3,06811096	0,00215417	0,01460396	protein_codir	putative kinesin
TcG_07875	38,67628683	0,24899914	0,27744053	0,89748652	0,36945936	0,60112883	protein_codir	protein kinase
TcG_07876	299,117785	0,045031984	0,10823108	0,41607256	0,67735691	0,83096283	protein_codir	hypothetical protein
TcG_07877	456,5521695	-0,058498589	0,09044863	-0,64676038	0,51778704	0,72304214	protein_codir	hypothetical protein
TcG_07878	465,0360446	0,059893318	0,08543193	0,7010648	0,48326257	0,69666296	protein_codir	putative ATP synthase
TcG_07879	780,621119	0,037550036	0,06976026	0,53827263	0,59038884	0,77624207	protein_codir	putative structural maintenance of chromosome protein 4
TcG_07880	186,0517456	-0,072416199	0,13536203	-0,53498163	0,59266256	0,77790738	protein_codir	putative structural maintenance of chromosome protein 4
TcG_07881	374,3165689	-0,088078718	0,09980485	-0,88250939	0,3775014	0,60822295		
TcG_07882	555,6316274	0,013261692	0,08336038	0,15908866	0,87359902	0,93932295	protein_codir	phytanoyl-CoA dioxygenase
TcG_07883	555,1266404	0,041675847	0,08873776	0,46965177	0,63860383	0,80773199	protein_codir	type 11 methyltransferase
TcG_07884	160,4748949	-0,24314921	0,14095389	-1,72502665	0,08452267	0,23954982	protein_codir	hypothetical protein
TcG_07885	455,4172932	0,06594039	0,09670303	0,68188544	0,49531139	0,70508389	protein_codir	vesicule-associated membrane protein
TcG_07886	64,26567598	0,140050807	0,24015805	0,58316099	0,55978493	0,75405439		
TcG_07887	454,1146677	-0,10629718	0,09165771	-1,1597189	0,24616327	0,47470834	protein_codir	hypothetical protein
TcG_07888	543,281701	-0,000654114	0,08977845	-0,00728586	0,99418677	0,99771745	protein_codir	hypothetical protein
TcG_07889	269,2775757	0,099725199	0,11215751	0,88915314	0,37392078	0,60449626	protein_codir	putative calpain-like cysteine peptidase
TcG_07890	680,1145412	-0,136315295	0,07736288	-1,76202446	0,07806516	0,22685302	protein_codir	putative adenylate kinase
TcG_07891	1110,870099	-0,262893521	0,06337187	-4,1484264	3,3477E-05	0,00043385	protein_codir	hypothetical protein
TcG_07892	65,12083199	0,325077288	0,21579198	1,50643825	0,13195471	0,32280981	protein_codir	hypothetical protein
TcG_07893	78,02288934	0,238666238	0,20112108	1,18667936	0,23535412	0,46319226	protein_codir	hypothetical protein
TcG_07894	547,1352588	-0,344765819	0,08018443	-4,29966041	1,7106E-05	0,00024199	protein_codir	putative surface protease GP63
TcG_07895	462,9290174	-0,095531365	0,08484943	-1,12589282	0,26021089	0,49261779	protein_codir	putative D-alanyl-glycyl endopeptidase-like protein
TcG_07896	370,8300469	-0,127823834	0,1012637	-1,26228685	0,20684559	0,4290213	protein_codir	hypothetical protein
TcG_07897	467,7246402	-0,197035265	0,08717611	-2,26019783	0,02380898	0,09631662	protein_codir	putative transcription elongation factor-like protein
TcG_07898	682,4773073	-0,127425375	0,0723322	-1,76166881	0,07812527	0,22697076	protein_codir	hypothetical protein
TcG_07899	783,0067884	0,015835407	0,06954603	0,22769677	0,81988198	0,91102214	protein_codir	putative GTP-binding elongation factor tu family protein
TcG_07900	1733,226628	-0,095261255	0,06288408	-1,51487084	0,12980512	0,31940564	protein_codir	putative neurobeachin/beige protein
TcG_07901	269,4632041	-0,32156228	0,10928613	-2,94238884	0,00325691	0,02036402	protein_codir	ubiquitin-conjugating enzyme

TcG_07902	422,1644244	-0,20965357	0,09094295	-2,30533062	0,02114806	0,08797898	protein_codir	hypothetical protein
TcG_07903	441,1325285	0,028393184	0,08973556	0,3164095	0,75169171	0,87492919	protein_codir	hypothetical protein
TcG_07904	181,2757684	-0,205346914	0,13790131	-1,48908605	0,13646471	0,32916375	protein_codir	putative E3 ubiquitin-protein ligase HECTD1-like isoform X6
TcG_07905	236,1372885	-0,121812118	0,118259	-1,03004521	0,30298878	0,53561843	protein_codir	putative E3 ubiquitin-protein ligase HECTD1-like isoform X6
TcG_07906	1704,623754	-0,160064218	0,0513504	-3,11709802	0,00182641	0,01277824	protein_codir	putative translation initiation factor IF-2
TcG_07907	780,5419711	-0,31212378	0,0738812	-4,22467114	2,3929E-05	0,00032426	protein_codir	mitochondrial guide RNA binding complex subunit 2
TcG_07908	142,6276417	-0,244933891	0,15325427	-1,59821899	0,10999425	0,28646723	protein_codir	putative kinesin
TcG_07909	140,5334441	0,192373023	0,16458896	1,16880877	0,24248068	0,47089863	protein_codir	multifunctional methyltransferase subunit TRM112
TcG_07910	226,9918122	-0,039847876	0,11921504	-0,33425207	0,73818935	0,86642273	protein_codir	hypothetical protein
TcG_07911	67,11239339	0,414706921	0,21408361	1,93712596	0,05272995	0,17142733	protein_codir	hypothetical protein
TcG_07912	453,7077102	0,033267759	0,0912616	0,36453184	0,7154609	0,85396046	protein_codir	putative proteasome beta 6 subunit, putative,20S proteasome beta 6 subunit
TcG_07913	118,0999497	-0,041569976	0,17118318	-0,24283914	0,80813001	0,90509218	protein_codir	putative GTP cyclohydrolase II
TcG_07914	680,5478141	-0,124310177	0,07500433	-1,65737336	0,09744401	0,26410072	protein_codir	cyclophilin
TcG_07915	291,5629904	0,045270343	0,10542827	0,42939473	0,66763599	0,82588411	protein_codir	hypothetical protein
TcG_07916	269,3843865	0,242950982	0,11054689	2,19771882	0,02796915	0,1085233	protein_codir	hypothetical protein
TcG_07917	173,4731758	-0,36321187	0,14259466	-2,54716314	0,01086027	0,05266933	protein_codir	hypothetical protein
TcG_07918	668,7972945	0,202398379	0,07355211	2,75176844	0,00592744	0,03270254	protein_codir	pumilio protein 5
TcG_07919	74,74677186	-0,191669982	0,20305541	-0,94392943	0,34520571	0,57929591	protein_codir	hypothetical protein
TcG_07920	135,4900875	0,06506616	0,15434924	0,42155154	0,67335238	0,82890021	protein_codir	39S ribosomal protein L46, mitochondrial
TcG_07921	41,55529712	-0,082985921	0,27427059	-0,30256952	0,76221795	0,87993793	protein_codir	hypothetical protein
TcG_07922	438,8198359	0,042598767	0,08869957	0,480259	0,63104324	0,80237785	protein_codir	hypothetical protein
TcG_07923	459,5219581	-0,151673191	0,0843549	-1,7980366	0,07217121	0,21601023	protein_codir	antigenic WD protein
TcG_07924	52,82798274	-0,003950665	0,24283258	-0,01626909	0,98701972	0,99474691	protein_codir	hypothetical protein
TcG_07925	29,39849971	0,180363912	0,31981938	0,56395554	0,57278439	0,76401448	protein_codir	hypothetical protein
TcG_07926	9,78610367	0,354998507	0,55229971	0,64276425	0,52037708	1	protein_codir	hypothetical protein
TcG_07927	11,18266619	-0,486028536	0,53590174	-0,90693592	0,36444068	1	protein_codir	dispersed gene family protein 1 (DGF-1)
TcG_07928	184,2123839	0,127677357	0,14337894	0,89048891	0,37320342	0,60423908	protein_codir	dispersed gene family protein 1 (DGF-1)
TcG_07929	38,5763931	-0,265713862	0,29519437	-0,90013189	0,36805007	0,60025733	protein_codir	dispersed protein family protein 1 (DGF-1)
TcG_07930	24,49900507	-0,148139565	0,3530673	-0,41957884	0,67479315	0,82970688	protein_codir	dispersed gene family protein 1 (DGF-1)
TcG_07931	239,6268401	0,159074776	0,11894756	1,33735217	0,18110771	0,39257511	protein_codir	dispersed gene family protein 1 (DGF-1)
TcG_07932	3290,573116	-0,081961721	0,04433605	-1,8486473	0,06450876	0,19999959	protein_codir	hypothetical protein
TcG_07933	184,5318159	0,313448686	0,14164387	2,21293499	0,02690213	0,10551177	protein_codir	calpain-like cysteine peptidase
TcG_07934	204,4460056	0,24225282	0,12461326	1,94403723	0,05189096	0,16953452	protein_codir	hypothetical protein
TcG_07935	138,1214353	0,304650649	0,15382735	1,98047133	0,04765059	0,1598378	protein_codir	calpain-like cysteine peptidase
TcG_07936	223,8344145	0,338944987	0,12061829	2,81006297	0,00495318	0,02860795	protein_codir	putative calpain-like cysteine peptidase
TcG_07937	997,0255406	-0,036914082	0,06417351	-0,57522303	0,56514047	0,75783767	protein_codir	putative nucleosome assembly protein
TcG_07938	534,4666938	-0,110855527	0,0816197	-1,3581957	0,1744016	0,38312797	protein_codir	calpain-like cysteine peptidase
TcG_07939	429,2797575	0,132322315	0,09223946	1,43455216	0,15141475	0,35179947	protein_codir	hypothetical protein
TcG_07940	67,35695493	-0,078514559	0,22354311	-0,35122781	0,72541744	0,85817259		
TcG_07941	355,0341862	-0,041761721	0,10765283	-0,3879296	0,69806813	0,84388746	protein_codir	calpain-like cysteine peptidase
TcG_07942	993,5590231	0,039007781	0,06834428	0,57075417	0,56816629	0,76007264	protein_codir	hypothetical protein
TcG_07943	185,4543241	0,104139582	0,13871821	0,75072756	0,45281663	0,67355117	protein_codir	putative calpain-like protein
TcG_07944	399,6230157	0,424860518	0,09809741	4,33100635	1,4843E-05	0,00021389	protein_codir	calpain-like cysteine peptidase, Clan CA, family C2
TcG_07945	454,2390949	-0,001979651	0,09547431	-0,02073491	0,98345712	0,99376391	protein_codir	calpain-like cysteine peptidase
TcG_07946	1217,000483	-0,196060955	0,06225034	-3,14955634	0,00163519	0,01169851	protein_codir	calpain
TcG_07947	620,5259363	0,015727844	0,07553912	0,20820793	0,83506662	0,92007617	protein_codir	calpain-like cysteine peptidase
TcG_07948	1763,752973	-0,14949978	0,05308838	-2,81605464	0,00486174	0,02817553	protein_codir	calpain-like cysteine peptidase
TcG_07949	163,9097888	0,064577147	0,14011132	0,46089885	0,64487118	0,81136925	protein_codir	protein disulfide isomerase
TcG_07950	453,0487585	-0,177523259	0,08713593	-2,03731415	0,04161857	0,14532633	protein_codir	C15orf24 protein
TcG_07951	150,7176946	0,12752945	0,14650296	0,87049058	0,38403237	0,614473	protein_codir	putative Qc-SNARE protein
TcG_07952	147,1047662	-0,208913946	0,14609573	-1,42997981	0,15272281	0,35297158	protein_codir	putative oxysterol-binding protein
TcG_07953	138,2528305	0,234115801	0,15316097	1,52856043	0,12637345	0,31409915	protein_codir	hypothetical protein

TcG_07954	298,8075535	-0,2133343	0,10660039	-2,00125246	0,04536519	0,1543782	protein_codir	putative sphingosine kinase A, B
TcG_07955	269,0962514	-0,175042137	0,11168532	-1,56727976	0,11704931	0,29851053	protein_codir	hypothetical protein
TcG_07956	358,7825709	-0,240167597	0,09919161	-2,42124916	0,01546727	0,06929768	protein_codir	hypothetical protein
TcG_07957	281,8810803	0,177317897	0,12431908	1,42631279	0,15377807	0,35463232	protein_codir	regulator of chromosome condensation
TcG_07958	131,6226283	0,128324021	0,16227363	0,79078791	0,42906777	0,65398767	protein_codir	hypothetical protein
TcG_07959	0					1	protein_codir	hypothetical protein
TcG_07960	539,7229567	-0,180615035	0,08246465	-2,19021152	0,0285089	0,10991818	protein_codir	regulator of chromosome condensation
TcG_07961	129,8499843	-0,079857012	0,15260139	-0,52330461	0,60076229	0,7835886	protein_codir	RNA-binding protein-19
TcG_07962	168,1243336	-0,109622602	0,14217864	-0,77102018	0,44069497	0,66350124	protein_codir	hypothetical protein
TcG_07963	203,4043083	0,038839381	0,13254709	0,29302326	0,76950437	0,88436428	protein_codir	RNA-binding protein 8A
TcG_07964	93,50131835	0,165194953	0,18482314	0,89380015	0,37142883	0,60280866	protein_codir	hypothetical protein
TcG_07965	253,7325471	-0,051767187	0,11255072	-0,45994539	0,64555542	0,81191979	protein_codir	hypothetical protein
TcG_07966	230,2430371	0,075736549	0,11943834	0,63410582	0,52601177	0,72890472	protein_codir	hypothetical protein
TcG_07967	2,290861052	0,031194511	1,14508469	0,0272421	0,97826664	1	protein_codir	hypothetical protein
TcG_07968	282,1516028	-0,2800997	0,10645654	-2,63111792	0,00851045	0,04353293	protein_codir	putative vesicle-associated membrane protein
TcG_07969	297,297202	0,01192558	0,10675252	0,11171239	0,91105145	0,95684497	protein_codir	hypothetical protein
TcG_07970	130,5869968	-0,104060824	0,15373002	-0,67690632	0,49846539	0,70766083	protein_codir	hypothetical protein
TcG_07971	89,56712389	0,23879836	0,19519401	1,22338979	0,22118251	0,4463718	protein_codir	hypothetical protein
TcG_07972	418,8807241	-0,259024829	0,08867312	-2,9211199	0,00348776	0,02158608	protein_codir	putative TPR-repeat protein
TcG_07973	1603,375812	-0,004563859	0,0521125	-0,08757705	0,93021285	0,96624046	protein_codir	putative chaperone DNAJ protein
TcG_07974	252,2308988	0,124839435	0,11133605	1,1212849	0,26216661	0,49428247	protein_codir	aldo-keto reductase
TcG_07975	257,0628212	0,282601604	0,11441912	2,46988087	0,01351581	0,06279103	protein_codir	26S proteasome regulatory subunit, ATPase 3, interacting protein
TcG_07976	437,4481573	-0,016077976	0,09450179	-0,17013408	0,86490469	0,93498506	protein_codir	hypothetical protein
TcG_07977	180,6845083	0,009958535	0,13114488	0,07593537	0,93947052	0,97202227	protein_codir	putative 3-oxoacyl-ACP reductase
TcG_07978	111,972215	-0,239986767	0,16599654	-1,44573358	0,14825194	0,34706951	protein_codir	hypothetical protein
TcG_07979	136,7589498	-0,382010576	0,17439895	-2,19044077	0,02849229	0,10991818	protein_codir	putative protein kinase
TcG_07980	322,9567821	-0,272576937	0,10199354	-2,67249222	0,00752901	0,0395606	protein_codir	putative splicing factor XB2
TcG_07981	396,2693008	0,212364223	0,09165492	2,31699757	0,02050386	0,08576093	protein_codir	putative tubulin-tyrosine ligase
TcG_07982	211,732364	0,260711964	0,13357712	1,95177112	0,05096538	0,1678945	protein_codir	hypothetical protein
TcG_07983	3302,333538	-0,080125588	0,08833262	-0,90708943	0,3643595	0,59637102	protein_codir	dynein heavy chain 7, axonemal isoform X1
TcG_07984	187,9037302	0,121689339	0,13932049	0,87344894	0,38241842	0,61239805	protein_codir	trans-sialidase-like protein
TcG_07985	5,281003649	0,559314339	0,77625096	0,72053288	0,47119696	1	protein_codir	hypothetical protein
TcG_07986	74,50399206	0,400345491	0,20530284	1,95002415	0,05117324	0,16825358	protein_codir	L1Tc protein
TcG_07987	132,7281193	-0,086321253	0,15419688	-0,55981192	0,57560773	0,76628647	protein_codir	putative thioredoxin-like protein
TcG_07988	174,5415175	-0,179220346	0,14275892	-1,25540557	0,20933157	0,4318721	protein_codir	ATPase family AAA domain-containing protein 1-B-like
TcG_07989	89,526244	-0,090126785	0,18470931	-0,48793851	0,62559339	0,79875318	protein_codir	hypothetical protein
TcG_07990	254,9914077	0,018747691	0,117071	0,16013949	0,8727712	0,93898478	protein_codir	putative alpha-ketoglutarate-dependent dioxygenase
TcG_07991	245,9985722	-0,032644945	0,12043377	-0,27106139	0,78634382	0,89358636	protein_codir	hypothetical protein
TcG_07992	106,5558104	0,420984265	0,17405184	2,41872915	0,01557483	0,06964219	protein_codir	putative alanine racemase 2
TcG_07993	579,7750914	-0,243983049	0,0762329	-3,20049554	0,00137191	0,01009848	protein_codir	ubiquitin-conjugating enzyme E2
TcG_07994	320,1502089	-0,010824747	0,10574137	-0,10237003	0,91846297	0,96084081	protein_codir	hypothetical protein
TcG_07995	219,841222	0,260780077	0,12259718	2,12712942	0,03340933	0,12386576	protein_codir	putative protein disulfide isomerase
TcG_07996	97,31820249	0,281724565	0,17764309	1,58590222	0,11276149	0,29103467	protein_codir	hypothetical protein
TcG_07997	50,71928927	0,027626948	0,25208738	0,10959275	0,91273236	0,95723874	protein_codir	hypothetical protein
TcG_07998	131,1442789	-0,155524014	0,16254671	-0,95679583	0,33867032	0,57327673	protein_codir	hypothetical protein
TcG_07999	722,5690375	-0,126733284	0,07662189	-1,65400876	0,09812573	0,26519355	protein_codir	metallo-peptidase, Clan ME, Family M16
TcG_08000	297,3694667	0,071586574	0,1107428	0,64642195	0,51800613	0,72317376	protein_codir	hypothetical protein
TcG_08001	127,7430113	0,382392615	0,15673837	2,43968725	0,01469998	0,06689473	protein_codir	hypothetical protein
TcG_08002	1108,700786	0,100642573	0,06606187	1,52345931	0,12764383	0,31640596	protein_codir	putative threonyl-tRNA synthetase
TcG_08003	498,636644	0,059558309	0,08250893	0,72184072	0,47039241	0,6865667	protein_codir	putative peroxisome targeting signal 1 receptor
TcG_08004	192,7009925	0,452419067	0,1288392	3,51150168	0,00044558	0,00392587	protein_codir	putative 60S ribosomal protein L2
TcG_08005	410,2335686	-0,13635835	0,08996059	-1,51575645	0,12958095	0,31926436	protein_codir	iron-sulfur cluster assembly protein

TcG_08006	366,8693972	-0,13020536	0,09637551	-1,35102121	0,17668864	0,38690504	protein_codir calmodulin-like protein containing EF hand domain
TcG_08007	195,106075	-0,044847802	0,12651433	-0,35448792	0,72297325	0,85733557	protein_codir hypothetical protein
TcG_08008	435,4430997	-0,207589292	0,09127457	-2,2743387	0,02294563	0,09367444	protein_codir putative transporter
TcG_08009	314,5703912	-0,196451533	0,10273803	-1,9121598	0,0558557	0,17886792	protein_codir putative transporter
TcG_08010	1118,465844	-0,30064663	0,22768136	-1,32047099	0,18667781	0,40067602	protein_codir hypothetical protein
TcG_08011	1712,975255	-0,29944178	0,06227692	-4,8082305	1,5227E-06	2,9453E-05	protein_codir hypothetical protein
TcG_08012	534,1961043	-0,289119478	0,08450518	-3,42132255	0,00062317	0,00519431	
TcG_08013	138,5732636	-0,093193832	0,14881855	-0,62622457	0,53116765	0,73245756	protein_codir hypothetical protein
TcG_08014	676,3841822	0,150184889	0,07823635	1,91963067	0,05490457	0,17679942	protein_codir putative transaldolase
TcG_08015	126,9202466	0,083407367	0,15750024	0,52956979	0,59641024	0,78066886	protein_codir hypothetical protein
TcG_08016	240,9969529	-0,084144386	0,11852391	-0,70993597	0,47774385	0,69260885	protein_codir putative ubiquitin hydrolase, putative,cysteine peptidase, Clan CA, family C19
TcG_08017	450,1185152	0,040684074	0,08929338	0,45562252	0,64866145	0,81328019	protein_codir putative ubiquitin hydrolase, putative,cysteine peptidase, Clan CA, family C19
TcG_08018	1947,724973	-0,415958769	0,15728017	-2,64469938	0,00817635	0,04219653	protein_codir hemagluttinin family protein
TcG_08019	481,7932897	-0,734561207	0,08387843	-8,75745015	1,9972E-18	3,0563E-16	protein_codir putative mitogen-activated protein kinase 3
TcG_08020	565,2924994	-0,663297615	0,07886981	-8,41003159	4,099E-17	5,5222E-15	protein_codir PIF1 helicase-like protein
TcG_08021	443,1872915	0,011516615	0,08824463	0,13050784	0,89616465	0,94960341	protein_codir ribonuclease mar1
TcG_08022	286,7201265	-0,316387325	0,10842027	-2,91815665	0,00352107	0,02173424	protein_codir putative ATP-dependent chaperone
TcG_08023	322,6302237	-0,544009848	0,10548378	-5,15728417	2,5056E-07	5,8962E-06	protein_codir hypothetical protein
TcG_08024	62,28039778	-0,031865934	0,22770654	-0,13994299	0,88870504	0,94628587	protein_codir hypothetical protein
TcG_08025	42,80964558	0,226109779	0,28217181	0,80131952	0,42294669	0,64895516	
TcG_08026	173,6317359	-0,178539161	0,13409455	-1,33144237	0,1830435	0,39529207	protein_codir hypothetical protein
TcG_08027	126,3250996	0,068944087	0,16122273	0,42763256	0,66891866	0,8263077	protein_codir cystathionine beta-synthase,cysteine synthase,serine sulfhydrylase
TcG_08028	699,039955	-0,054983629	0,07420279	-0,7409914	0,45869865	0,67743881	protein_codir hypothetical protein
TcG_08029	705,7710732	0,002097989	0,07418128	0,02828191	0,97743731	0,99022484	protein_codir exosome complex exonuclease RRP44 isoform X1
TcG_08030	131,3569159	0,259818414	0,17459779	1,48809683	0,13672536	0,32954025	protein_codir hypothetical protein
TcG_08031	290,223101	-0,017979947	0,1125605	-0,15973585	0,87308916	0,93915245	protein_codir hypothetical protein
TcG_08032	705,9271826	-0,030227072	0,07267777	-0,41590533	0,67747928	0,83096283	protein_codir putative casein kinase II, alpha chain
TcG_08033	452,4148273	0,068096873	0,08963551	0,75970864	0,44742877	0,66854651	protein_codir hypothetical protein
TcG_08034	121,7423152	-0,109946994	0,16611966	-0,66185419	0,50806468	0,7148072	protein_codir hypothetical protein
TcG_08035	303,6916652	0,141840547	0,10302458	1,37676413	0,16858518	0,37510151	protein_codir transcription elongation factor B, polypeptide 1
TcG_08036	300,9018353	0,051889755	0,10318497	0,50288093	0,61504799	0,79205726	protein_codir putative phosphoadenosine phosphosulfate reductase-like protein
TcG_08037	223,7026035	0,20470135	0,11909455	1,71881372	0,0856483	0,24179366	protein_codir putative surface protease GP63
TcG_08038	194,0822687	-0,090220301	0,1291204	-0,69873004	0,48472076	0,69780998	protein_codir hypothetical protein
TcG_08039	507,0725234	-0,093429624	0,08450928	-1,10555459	0,26891935	0,50149655	protein_codir hypothetical protein
TcG_08040	533,9872834	-0,09173416	0,08598478	-1,06686514	0,28603274	0,51918774	protein_codir hypothetical protein
TcG_08041	592,1181245	0,21266862	0,07766908	2,73813743	0,00617883	0,03381571	protein_codir glycerol-3-phosphate dehydrogenase (FAD-dependent), mitochondrial
TcG_08042	610,8871868	0,297260127	0,0768015	3,87049883	0,00010861	0,00119733	protein_codir putative myosin heavy chain
TcG_08043	0,586192964	-1,478472749	2,31699018	-0,63810057	0,52340822	1	
TcG_08044	166,6426449	0,341381279	0,14384888	2,37319389	0,017635	0,07632393	protein_codir putative protein kinase
TcG_08045	279,3274965	0,183623227	0,10837262	1,69436918	0,09019515	0,25041961	protein_codir hypothetical protein
TcG_08046	368,9558198	0,125266474	0,09883829	1,26738815	0,20501654	0,42629605	protein_codir methyltransferase/D-alanine--D-alanine ligase
TcG_08047	104,5909552	0,240324445	0,17289517	1,39000091	0,1645286	0,36983091	protein_codir hypothetical protein
TcG_08048	513,6992803	0,177280645	0,08697427	2,03831133	0,04151881	0,14510917	protein_codir hypothetical protein
TcG_08049	632,3341912	-0,020780005	0,07466916	-0,27829433	0,78078643	0,89087556	protein_codir hypothetical protein
TcG_08050	290,4691107	0,044710173	0,10783285	0,41462479	0,6784166	0,83182927	protein_codir putative protein kinase
TcG_08051	191,2634428	0,192040258	0,12961711	1,4815965	0,13844769	0,33224005	protein_codir putative dTDP-4-dehydrorhamnose reductase domain protein
TcG_08052	115,3247839	0,125189588	0,16888539	0,7412695	0,45853005	0,67743881	protein_codir hypothetical protein
TcG_08053	324,7317577	-0,122543734	0,10346704	-1,18437454	0,23626483	0,46360093	protein_codir phosphatidylinositol-4-phosphate 5-kinase
TcG_08054	770,6357237	-0,026067516	0,07450988	-0,34985313	0,72644893	0,85875291	protein_codir hypothetical protein
TcG_08055	207,6773208	0,302067875	0,12482595	2,41991249	0,01552424	0,06952604	protein_codir CDP-diacylglycerol--inositol 3-phosphatidyltransferase
TcG_08056	112,9180053	0,32463619	0,16442885	1,9743262	0,04834466	0,16157024	protein_codir hypothetical protein
TcG_08057	255,4167298	0,081129562	0,11909266	0,68123057	0,49572561	0,70550017	protein_codir hypothetical protein

TcG_08058	136,3908033	0,196675473	0,15706325	1,25220551	0,21049499	0,43302467	protein_codir	hypothetical protein
TcG_08059	365,1972446	0,364428618	0,09776737	3,72750746	0,00019338	0,00193986	protein_codir	putative protein kinase
TcG_08060	199,7374854	0,259833823	0,12977655	2,0021632	0,04526718	0,15425458	protein_codir	putative metallopeptidase, putative,metallo-peptidase, clan MP, family M67
TcG_08061	709,718129	-0,051462128	0,0719086	-0,71566026	0,47420115	0,68995285	protein_codir	hypothetical protein
TcG_08062	823,6089035	-0,21229855	0,06824593	-3,11078685	0,0018659	0,01301521	protein_codir	putative protein phosphatase 2A regulatory subunit
TcG_08063	877,6036459	-0,240774066	0,06645071	-3,62334843	0,00029081	0,00273044	protein_codir	putative calmodulin
TcG_08064	699,4081221	-0,057585209	0,07908564	-0,72813735	0,46652951	0,6831662	protein_codir	hypothetical protein
TcG_08065	718,5669699	-0,124649185	0,0742758	-1,67819367	0,0933093	0,25624117	protein_codir	putative PTP1-interacting protein, 39 kDa
TcG_08066	668,0794278	0,181032883	0,07670035	2,36026155	0,01826205	0,07819075	protein_codir	40S ribosomal protein S3
TcG_08067	261,0870608	-0,030148727	0,11484852	-0,26250861	0,79292935	0,89624823	protein_codir	putative 2Fe-2S iron-sulfur cluster binding domain containing protein
TcG_08068	304,8259432	-0,228616249	0,1038117	-2,20222053	0,02764973	0,10771682	protein_codir	putative NAD/FAD dependent dehydrogenase
TcG_08069	180,8411482	-0,17347074	0,13379028	-1,29658703	0,19477332	0,41217405	protein_codir	hypothetical protein
TcG_08070	248,4778492	0,008392646	0,1169924	0,07173668	0,94281146	0,97319113	protein_codir	hypothetical protein
TcG_08071	574,8524657	-0,252462603	0,07672067	-3,29067239	0,00099948	0,00777703	protein_codir	succinate dehydrogenase
TcG_08072	2122,024885	0,204003755	0,04778334	4,26934929	1,9604E-05	0,00027218	protein_codir	60S ribosomal protein L6
TcG_08073	1666,608359	-0,023643212	0,05106335	-0,46301724	0,64335201	0,8107327	protein_codir	glutamate dehydrogenase
TcG_08074	325,7222756	0,024106792	0,10506547	0,22944542	0,81852274	0,91055251	protein_codir	solanesyl diphosphate synthase
TcG_08075	329,5557066	-0,200982456	0,09941877	-2,0215746	0,04322032	0,1493122	protein_codir	SNW domain-containing protein 1
TcG_08076	101,7088718	0,12041053	0,17433072	0,69070171	0,48975301	0,70087481	protein_codir	tryparedoxin peroxidase
TcG_08077	1119,629091	-0,137941524	0,06609203	-2,08711278	0,03687794	0,13342337	protein_codir	tryparedoxin peroxidase
TcG_08078	269,8191488	0,280968257	0,11421317	2,46003374	0,0138924	0,06395065	protein_codir	putative developmentally regulated protein
TcG_08079	196,8671566	-0,02620795	0,13446992	-0,19489824	0,84547261	0,92503539	protein_codir	metacyclogenesis-specific protein
TcG_08080	345,1613419	0,064453536	0,10217694	0,63080313	0,52816926	0,73049648	protein_codir	tryparedoxin peroxidase
TcG_08081	443,3680201	0,147705442	0,08661983	1,70521502	0,08815433	0,24664479	protein_codir	kinetoplast DNA-associated protein
TcG_08082	225,0415032	-0,030948701	0,11869605	-0,2607391	0,79429371	0,89729787	protein_codir	hypothetical protein
TcG_08083	164,6587494	0,209907164	0,15242452	1,37712206	0,16847451	0,37494155	protein_codir	hypothetical protein
TcG_08084	666,7553956	0,063635839	0,07410985	0,85866907	0,39052312	0,62048833	protein_codir	protein tyrosine phosphatase
TcG_08085	8685,409508	-0,176939166	0,04096331	-4,31945492	1,5642E-05	0,00022373	protein_codir	histone H2B
TcG_08086	2257,720142	-0,155781456	0,05365498	-2,90339226	0,00369144	0,0225575	protein_codir	histone H2B
TcG_08087	136,5084279	-0,053945257	0,15243045	-0,35390079	0,72341323	0,85739757	protein_codir	putative mitochondrial carrier protein
TcG_08088	195,0959605	0,170621574	0,12773894	1,3357053	0,18164562	0,39329959	protein_codir	hypothetical protein
TcG_08089	236,6240329	0,344598146	0,12463798	2,76479254	0,0056959	0,03166637	protein_codir	glutaredoxin-like protein
TcG_08090	318,7530918	-0,043721312	0,10610056	-0,41207427	0,68028498	0,83325741	protein_codir	putative DNA replication licensing factor, putative,minichromosome maintenance protein-like protein
TcG_08091	278,9367303	-0,308562913	0,10982431	-2,80960477	0,00496024	0,02863443	protein_codir	hypothetical protein
TcG_08092	1256,209316	0,115598364	0,0595254	1,94200049	0,05213704	0,17020562	protein_codir	trypanothione reductase
TcG_08093	327,29751	-0,013071064	0,1014798	-0,12880459	0,89751227	0,95018119	protein_codir	putative ATP-dependent RNA helicase
TcG_08094	237,3262251	-0,156032702	0,11987767	-1,30159943	0,19305336	0,40988019	protein_codir	hypothetical protein
TcG_08095	0					1	protein_codir	microtubule-associated protein
TcG_08096	0,155988004	0,503022807	4,08047286	0,12327562	0,90188885	1		
TcG_08097	505,9629594	-0,374450169	0,08117266	-4,61300829	3,9688E-06	6,8529E-05	protein_codir	putative protein kinase
TcG_08098	981,6160221	-0,118911942	0,06267274	-1,89734707	0,05778215	0,18338943	protein_codir	putative structural maintenance of chromosome (SMC)
TcG_08099	285,9185189	0,087141635	0,10878522	0,80104296	0,42310677	0,64911481	protein_codir	anaphase-promoting complex subunit 3
TcG_08100	124,6724031	0,131890931	0,15639768	0,84330493	0,39905793	0,62898729	protein_codir	dynein light chain
TcG_08101	170,4279581	0,141736968	0,14591593	0,97136046	0,33136881	0,56560186	protein_codir	hypothetical protein
TcG_08102	204,4707521	0,134004575	0,12474163	1,07425707	0,28270748	0,51598124	protein_codir	hypothetical protein
TcG_08103	1,883678369	-0,321035105	1,28463101	-0,24990453	0,80266118	1	protein_codir	hypothetical protein
TcG_08104	465,6459178	0,198834551	0,09438654	2,10659866	0,03515238	0,12896628	protein_codir	L1Tc protein
TcG_08105	113,3009161	-0,033098617	0,16456523	-0,20112765	0,84059876	0,92246657	protein_codir	hypothetical protein
TcG_08106	194,7440521	-0,395753282	0,12919459	-3,06323407	0,00218959	0,01479216	protein_codir	putative protein phosphatase 1, regulatory subunit
TcG_08107	134,469536	-0,071487615	0,1556377	-0,45932068	0,6460039	0,81201944	protein_codir	prefoldin subunit
TcG_08108	778,4517776	-0,221509454	0,07298855	-3,034852	0,00240654	0,01595953	protein_codir	hypothetical protein
TcG_08109	268,8903239	0,074912087	0,1087589	0,68879043	0,49095516	0,70187819	protein_codir	hypothetical protein

TcG_08110	499,197316	-0,087713915	0,08609859	-1,01876131	0,3083163	0,54131726	protein_codir	hypothetical protein
TcG_08111	294,5037694	-0,043267442	0,10768545	-0,40179468	0,68783514	0,83789905	protein_codir	putative vacuolar ATP synthase
TcG_08112	154,7351214	0,212709069	0,14636401	1,45328807	0,14614379	0,34373161	protein_codir	hypothetical protein
TcG_08113	329,8904912	0,085907088	0,10029522	0,85654221	0,39169794	0,62139829	protein_codir	putative SPFH domain / Band 7 family protein
TcG_08114	382,3907651	-0,014971664	0,09269538	-0,16151467	0,87168806	0,93825509	protein_codir	succinate dehydrogenase subunit
TcG_08115	314,4420903	-0,015221563	0,10118563	-0,15043206	0,88042375	0,94196967	protein_codir	coiled-coil domain-containing protein 135
TcG_08116	261,2047087	0,134874492	0,11129655	1,21184788	0,22557062	0,45184321	protein_codir	putative methyltransferase
TcG_08117	464,0222689	0,305304084	0,09133632	3,34263627	0,00082987	0,00668625	protein_codir	putative transporter
TcG_08118	452,5175663	-0,022179923	0,09036564	-0,24544644	0,80611078	0,90423384	protein_codir	NADH:ubiquinone reductase (H(+)-translocating)/NADH dehydrogenase
TcG_08119	332,616554	-0,627276685	0,10637544	-5,89681856	3,7058E-09	1,2971E-07	protein_codir	trans-sialidase
TcG_08120	856,0590925	-0,540925885	0,06580864	-8,21967926	2,0405E-16	2,4123E-14	protein_codir	trans-sialidase
TcG_08121	50,36603635	0,268424352	0,26047824	1,03050586	0,3027726	0,535498	protein_codir	hypothetical protein
TcG_08122	14,40063667	-0,225435831	0,45189328	-0,49886963	0,61787123	1	protein_codir	hypothetical protein
TcG_08123	37,10418376	-0,177631209	0,29020621	-0,61208617	0,54048075	0,7394037	protein_codir	putative surface protease GP63
TcG_08124	265,1924095	0,486491903	0,11105053	4,38081551	1,1824E-05	0,00017518	protein_codir	membrane protein
TcG_08125	41,29917463	0,477953683	0,27835471	1,71706698	0,08596693	0,24251593	protein_codir	membrane protein
TcG_08126	194,0018252	0,443396312	0,1321976	3,35404214	0,0007964	0,00644352	protein_codir	hypothetical protein
TcG_08127	101,8389992	0,027851745	0,18706861	0,14888519	0,88164423	0,94218407	protein_codir	hypothetical protein
TcG_08128	151,2202623	-0,108412431	0,15656146	-0,69245926	0,48864896	0,69997689	protein_codir	dynein light chain
TcG_08129	1249,862789	0,233102095	0,0573299	4,0659776	4,7832E-05	0,00059397	protein_codir	40S ribosomal protein S14
TcG_08130	278,4748242	-0,028817792	0,10770636	-0,26755888	0,78903889	0,89494301	protein_codir	hypothetical protein
TcG_08131	1480,052842	-0,155157781	0,05565069	-2,78806552	0,00530238	0,02998214	protein_codir	P27 protein
TcG_08132	480,6450045	0,292861993	0,08869586	3,30186757	0,00096043	0,00753635	protein_codir	hypothetical protein
TcG_08133	297,0471493	-0,100105587	0,1032642	-0,96941233	0,3323395	0,5663408	protein_codir	hypothetical protein
TcG_08134	837,3781025	0,173449554	0,06669059	2,60080994	0,0093004	0,04660657	protein_codir	hypothetical protein
TcG_08135	1141,952003	0,300546438	0,0602088	4,99173641	5,9839E-07	1,2823E-05	protein_codir	ribosomal protein S20
TcG_08136	430,5293822	0,226240269	0,0889631	2,54307984	0,01098801	0,05315537	protein_codir	hypothetical protein
TcG_08137	0,91200019	-0,716536165	2,01847358	-0,35498912	0,72259774	1	protein_codir	ribosomal protein S20
TcG_08138	576,7177009	-0,062189635	0,0796411	-0,78087362	0,43487683	0,65905597	protein_codir	hypothetical protein
TcG_08139	22,69984223	0,485065806	0,37614975	1,28955503	0,1972052	0,41560128	protein_codir	surface protease GP63
TcG_08140	395,7813401	0,279124183	0,09404444	2,96800298	0,00299741	0,01901253	protein_codir	GP63 group II protein
TcG_08141	5,831149331	0,429067053	0,76493335	0,56092084	0,57485151	1	protein_codir	putative surface protease GP63
TcG_08142	127,1533204	0,319773251	0,15584419	2,05187789	0,04018153	0,14169142		
TcG_08143	535,9379129	0,018765536	0,08160878	0,22994506	0,81813446	0,91031113	protein_codir	putative amastin
TcG_08144	1129,258734	0,023382624	0,06995896	0,33423346	0,7382034	0,86642273	protein_codir	putative amastin
TcG_08145	1308,414089	0,054617007	0,0565935	0,96507555	0,33450697	0,56893684	protein_codir	putative 3-methylcrotonyl-CoA carboxylase
TcG_08146	1201,654935	0,312442093	0,06192894	5,0451711	4,5312E-07	9,924E-06	protein_codir	NAD(P)-dependent steroid dehydrogenase protein
TcG_08147	113,5902557	0,011755776	0,16924201	0,06946133	0,94462241	0,97413398	protein_codir	hypothetical protein
TcG_08148	333,3372297	-0,114028748	0,10210095	-1,11682362	0,26406975	0,49619385	protein_codir	palmitoyltransferase PFA3
TcG_08149	247,1101923	0,193339088	0,11532167	1,67652006	0,09363637	0,25683499	protein_codir	hypothetical protein
TcG_08150	173,4598218	0,143526637	0,13282626	1,08055922	0,27989323	0,51307265	protein_codir	generative cell specific-1
TcG_08151	46,41668289	0,090949302	0,26945048	0,33753624	0,7357127	0,86494473	protein_codir	putative Tartrateresistant acid phosphatase type 5
TcG_08152	671,2817527	-0,072412279	0,07777648	-0,93103053	0,35183777	0,58627821	protein_codir	putative zinc carboxypeptidase
TcG_08153	252,727611	-0,016082644	0,1177667	-0,13656359	0,89137576	0,94697266	protein_codir	hypothetical protein
TcG_08154	714,9445981	-0,280943996	0,07454148	-3,76896201	0,00016393	0,00169275	protein_codir	hypothetical protein
TcG_08155	273,4140969	0,098137553	0,10820874	0,90692811	0,36444481	0,59639232	protein_codir	hypothetical protein
TcG_08156	279,1821156	0,130811569	0,10919462	1,19796715	0,23092981	0,4583126	protein_codir	putative trans-splicing factor
TcG_08157	319,5843351	0,086306069	0,10368246	0,83240762	0,4051789	0,63430422	protein_codir	hypothetical protein
TcG_08158	543,6086833	0,003000672	0,08279606	0,03624173	0,97108962	0,98762678	protein_codir	hypothetical protein
TcG_08159	382,6741015	0,137920351	0,09277753	1,48657066	0,13712824	0,33016787	protein_codir	hypothetical protein
TcG_08160	262,8626861	-0,046897837	0,11375195	-0,41228162	0,68013302	0,83315935	protein_codir	dual specificity protein phosphatase or MAP kinase phosphatase
TcG_08161	296,2718252	0,302264373	0,10820509	2,79343942	0,00521508	0,02961858	protein_codir	putative hydrogenase

TcG_08162	558,626607	0,357271109	0,07908963	4,51729382	6,2635E-06	0,00010178	protein_codir kinetoplast DNA-associated protein
TcG_08163	9429,372477	0,152664662	0,03482956	4,3831919	1,1695E-05	0,00017372	protein_codir chaperonin HSP60, mitochondrial precursor
TcG_08164	662,3497148	-0,006618937	0,08092396	-0,08179206	0,93481207	0,96919308	protein_codir mismatch repair protein
TcG_08165	280,9597318	0,019460204	0,10716793	0,18158607	0,85590758	0,93025753	protein_codir hypothetical protein
TcG_08166	740,8569233	0,082744014	0,07746356	1,06816692	0,28544522	0,51904649	protein_codir putative brefeldin A-inhibited guanine nucleotide-exchange protein 1
TcG_08167	343,6595816	0,068255451	0,09860685	0,6921979	0,48881306	0,70004797	protein_codir PIF1 helicase-like protein
TcG_08168	522,6341108	-0,379544267	0,08155209	-4,65401004	3,2554E-06	5,7848E-05	protein_codir putative vacuolar protein sorting-associated protein 41
TcG_08169	106,1650349	0,048519463	0,17193926	0,28218955	0,77779817	0,88915339	protein_codir hypothetical protein
TcG_08170	387,3449688	-0,176173472	0,09955912	-1,76953625	0,07680443	0,2245977	protein_codir putative phosphomannomutase
TcG_08171	176,3712184	-0,245797337	0,13836363	-1,77645919	0,07565726	0,22230917	protein_codir alkylated DNA repair protein alkB like protein 4
TcG_08172	165,7043756	-0,111014989	0,13687771	-0,81105233	0,41733562	0,64401312	protein_codir calcineurin A2 subunit
TcG_08173	60,07570152	-0,308588109	0,22792668	-1,35389203	0,17577083	0,38533222	protein_codir hypothetical protein
TcG_08174	245,6906267	-0,137803192	0,11459442	-1,20252974	0,22915835	0,45626889	protein_codir hypothetical protein
TcG_08175	236,8521589	-0,168441801	0,11936831	-1,41110984	0,15821223	0,36041032	protein_codir hypothetical protein
TcG_08176	403,2875834	-0,285981624	0,09405751	-3,04049746	0,00236188	0,01570879	protein_codir hypothetical protein
TcG_08177	185,2381964	-0,084671276	0,13532663	-0,62568081	0,53152432	0,73268958	protein_codir putative amino acid transporter, putative,amino acid permease
TcG_08178	234,3260801	0,366426332	0,11874714	3,08576972	0,00203026	0,01392693	protein_codir hypothetical protein
TcG_08179	78,71607105	-0,046422576	0,20329744	-0,22834805	0,81937567	0,91087139	protein_codir leucine rich repeat containing 45
TcG_08180	175,0904831	-0,220921579	0,13796575	-1,60127841	0,10931527	0,2857042	protein_codir hypothetical protein
TcG_08181	79,21728146	0,180517642	0,20917478	0,86299909	0,38813797	0,61847343	protein_codir hypothetical protein
TcG_08182	221,9833853	-0,037726791	0,12229644	-0,30848642	0,75771223	0,87801518	protein_codir hypothetical protein
TcG_08183	39,62035326	-0,288042688	0,30656955	-0,93956718	0,34743962	0,58190301	
TcG_08184	228,9344976	0,057943086	0,11834862	0,48959662	0,62441937	0,79763206	protein_codir translation initiation factor 2A
TcG_08185	84,76298824	0,077439364	0,19607829	0,39494104	0,6928864	0,8410627	protein_codir translation initiation factor 2A
TcG_08186	66,59129972	0,012977427	0,21462998	0,06046419	0,95178594	0,97734573	protein_codir hypothetical protein
TcG_08187	178,1484197	-0,00497843	0,13927229	-0,03574602	0,97148487	0,98762955	protein_codir hypothetical protein
TcG_08188	118,2947248	0,333159626	0,16662166	1,99949772	0,04555453	0,15486613	protein_codir RING box protein
TcG_08189	77,61542629	0,231461771	0,2098765	1,10284747	0,27009341	0,50294153	protein_codir hypothetical protein
TcG_08190	273,5023004	0,067859254	0,10778117	0,62960212	0,52895494	0,73107665	protein_codir putative RNA-binding protein
TcG_08191	83,15878161	0,372640976	0,19476686	1,91326683	0,0557139	0,17851252	protein_codir putative meiotic recombination protein spo11
TcG_08192	116,6719654	0,28117106	0,16996973	1,654242	0,09807835	0,26514068	protein_codir hypothetical protein
TcG_08193	623,6452665	0,104172985	0,07467231	1,39506846	0,1629952	0,36733366	protein_codir hypothetical protein
TcG_08194	868,9732266	0,122323324	0,07660957	1,5967108	0,11033019	0,2871582	protein_codir CAD protein isoform X4
TcG_08195	177,7682935	0,558471385	0,13530205	4,12759003	3,6658E-05	0,00046931	protein_codir putative RNA methyltransferase
TcG_08196	195,5114874	0,404589172	0,14021755	2,88543881	0,00390868	0,02363571	protein_codir hypothetical protein
TcG_08197	227,2548682	0,616357646	0,12298034	5,01183879	5,3912E-07	1,1678E-05	protein_codir DNA repair and transcription factor protein
TcG_08198	152,0685022	0,616113147	0,1547736	3,98073806	6,8702E-05	0,00081388	protein_codir SET and MYND domain-containing protein
TcG_08199	196,229766	0,112209455	0,13784327	0,8140365	0,41562404	0,64239863	protein_codir hypothetical protein
TcG_08200	258,7120186	0,316181623	0,11460726	2,75882727	0,00580092	0,03217302	protein_codir ubiquitin-conjugating enzyme E2
TcG_08201	2542,767976	0,272033662	0,04502655	6,04162775	1,5257E-09	5,7766E-08	protein_codir putative cell division cycle protein
TcG_08202	445,8994171	0,50390324	0,09311386	5,41168874	6,2433E-08	1,694E-06	protein_codir hypothetical protein
TcG_08203	413,7349886	0,301461897	0,09434518	3,19530787	0,00139682	0,01024923	protein_codir putative protein kinase
TcG_08204	231,8471651	0,563225798	0,12401744	4,54150481	5,5854E-06	9,2447E-05	protein_codir transferase C1orf69 like protein, mitochondrial
TcG_08205	235,3504791	0,562434541	0,12044734	4,66954723	3,0186E-06	5,4139E-05	protein_codir hypothetical protein
TcG_08206	192,9587346	0,406950942	0,12798723	3,1796214	0,00147468	0,01072542	protein_codir hypothetical protein
TcG_08207	2454,126806	0,552679878	0,04814906	11,4785182	1,6915E-30	9,3325E-28	protein_codir cysteine peptidase inhibitor
TcG_08208	419,0457511	0,599236133	0,09853768	6,08128904	1,1922E-09	4,6823E-08	protein_codir hypothetical protein
TcG_08209	309,9587233	0,103171816	0,10337977	0,99798845	0,31828496	0,55336878	protein_codir hypothetical protein
TcG_08210	80,23452512	0,282252344	0,20010542	1,41051825	0,15838671	0,36053303	protein_codir surface protease GP63
TcG_08211	73,66658762	0,241283927	0,2054671	1,17431902	0,24026727	0,46899211	protein_codir surface protease GP63
TcG_08212	26,45842278	0,843343129	0,34740518	2,42754911	0,01520123	0,06829059	protein_codir mucin-associated surface protein (MASP)
TcG_08213	131,8151045	0,396819521	0,15544256	2,55283705	0,01068495	0,05205879	

TcG_08214	404,6507122	-0,129697044	0,09047308	-1,43354296	0,15170273	0,35187745	
TcG_08215	369,2572228	-0,09354664	0,09695893	-0,96480683	0,33464157	0,56906253	protein_codir kelch repeat-containing protein
TcG_08216	136,5867122	-0,009895819	0,14861019	-0,06658911	0,94690882	0,97492985	protein_codir small nuclear RNA activating protein 2
TcG_08217	342,4238156	-0,16840697	0,10076049	-1,67135917	0,09465076	0,25903815	protein_codir putative peroxisome assembly protein
TcG_08218	289,0120581	-0,107580245	0,11154996	-0,96441312	0,33483885	0,56925061	protein_codir arabinose efflux permease family protein
TcG_08219	285,652023	0,219002335	0,10778844	2,0317795	0,04217598	0,14660994	protein_codir hypothetical protein
TcG_08220	136,4429053	0,039862771	0,14939973	0,26681956	0,78960809	0,89501549	protein_codir hypothetical protein
TcG_08221	185,9167581	0,216805982	0,13333268	1,62605287	0,10393841	0,27632639	protein_codir arginine N-methyltransferase, type I
TcG_08222	197,3056042	-0,392864179	0,12822006	-3,06398384	0,00218411	0,01476375	protein_codir adenylate kinase
TcG_08223	176,1687306	-0,033770492	0,13239004	-0,25508332	0,79865874	0,89959947	protein_codir ribosomal protein L15 containing protein
TcG_08224	693,0051075	-0,054842312	0,08030843	-0,68289606	0,49467252	0,70453824	protein_codir putative myosin heavy chain
TcG_08225	24,94485722	0,083433517	0,35132395	0,23748314	0,81228199	0,90759432	protein_codir hypothetical protein
TcG_08226	182,3355894	-0,631015026	0,13733982	-4,59455244	4,3368E-06	7,4E-05	protein_codir putative trans-sialidase
TcG_08227	34,35802678	-0,441578253	0,30286703	-1,45799382	0,14484225	0,34175483	protein_codir putative complement regulatory protein
TcG_08228	28,68639436	0,451904629	0,34356061	1,31535633	0,18839017	0,40277302	protein_codir hypothetical protein
TcG_08229	37,71481658	0,295823995	0,29003262	1,01996803	0,30774362	0,54090194	protein_codir hypothetical protein
TcG_08230	96,28369245	-0,036280424	0,18541916	-0,19566707	0,84487076	0,92481739	protein_codir putative retrotransposon hot spot (RHS) protein
TcG_08231	187,0750174	-0,067819257	0,13486305	-0,50287499	0,61505217	0,79205726	protein_codir putative tubulin tyrosine ligase
TcG_08232	420,9794932	0,158741285	0,08805665	1,8027177	0,07143256	0,21457549	protein_codir hypothetical protein
TcG_08233	511,2771977	-0,061159428	0,08173295	-0,74828359	0,45428912	0,6742754	protein_codir hypothetical protein
TcG_08234	391,2426926	0,070210277	0,1043118	0,67308085	0,50089585	0,70971987	protein_codir tyrosine decarboxylase
TcG_08235	152,6316429	-0,135222296	0,14122258	-0,95751187	0,33830896	0,57289134	protein_codir hypothetical protein
TcG_08236	480,6701855	0,144282642	0,08432621	1,7110059	0,08708002	0,24460647	protein_codir putative fucose kinase
TcG_08237	435,8240404	-0,066964675	0,08944356	-0,74868074	0,45404965	0,67417907	protein_codir hypothetical protein
TcG_08238	700,1100265	0,030095473	0,07069997	0,42567871	0,67034198	0,82708566	protein_codir hypothetical protein
TcG_08239	513,9971633	-0,24831141	0,08733334	-2,84326006	0,00446546	0,02639873	protein_codir hypothetical protein
TcG_08240	407,3630191	0,089960138	0,09056184	0,99335587	0,32053656	0,55569903	protein_codir putative dihydroorotate dehydrogenase
TcG_08241	412,6006602	0,030632199	0,09441029	0,32445827	0,74559111	0,87142325	protein_codir putative aspartate carbamoyltransferase
TcG_08242	291,97256	0,153159069	0,11670651	1,31234384	0,18940415	0,40428085	protein_codir trans-sialidase
TcG_08243	35,60510525	-0,054091214	0,31205646	-0,17333791	0,86238583	0,93414012	protein_codir trans-sialidase
TcG_08244	69,85072639	0,119116228	0,21456494	0,55515234	0,57879047	0,76846166	protein_codir putative trans-sialidase
TcG_08245	39,24040646	0,388866743	0,27993513	1,38913162	0,16479273	0,37020808	protein_codir putative mucin-associated surface protein (MASP)
TcG_08246	196,8258115	-0,016566044	0,12929251	-0,12812841	0,89804735	0,95020791	protein_codir hypothetical protein
TcG_08247	69,04417364	0,154712174	0,20781705	0,74446332	0,45659623	0,67639993	protein_codir hypothetical protein
TcG_08248	81,170177	-0,001058777	0,19344196	-0,00547336	0,99563291	0,99830402	protein_codir mucin TcMUCII
TcG_08249	80,21863429	0,022415674	0,19305484	0,11611039	0,90756505	0,95523092	protein_codir hypothetical protein
TcG_08250	492,7712153	0,20062956	0,08565685	2,34224764	0,01916799	0,0812291	protein_codir putative transmembrane protein
TcG_08251	78,63206262	0,649635067	0,20148565	3,22422494	0,00126314	0,00942961	protein_codir hypothetical protein
TcG_08252	100,6376166	0,385555538	0,18657641	2,06647525	0,03878363	0,13834578	protein_codir hypothetical protein
TcG_08253	173,0975935	0,452761683	0,13628785	3,32209866	0,00089343	0,00711429	protein_codir putative ammonium transporter
TcG_08254	184,7503964	0,334225143	0,14780537	2,26125169	0,02374368	0,09626617	protein_codir hypothetical protein
TcG_08255	520,1225483	0,168755312	0,08577022	1,96752794	0,04912238	0,16340278	protein_codir hypothetical protein
TcG_08256	259,1670763	0,217412739	0,11374356	1,91142903	0,05594947	0,17901976	protein_codir viral A-type inclusion protein
TcG_08257	577,7241958	0,363109283	0,08111825	4,47629563	7,5949E-06	0,00012049	protein_codir putative cysteine proteinase
TcG_08258	372,0584662	0,19149058	0,09630771	1,98832039	0,04677627	0,15804895	protein_codir hypothetical protein
TcG_08259	20,60679095	1,233114358	0,41356201	2,98169157	0,00286661	0,01836975	
TcG_08260	1984,674687	0,550684594	0,05040984	10,9241478	8,8366E-28	3,6565E-25	protein_codir trans-sialidase
TcG_08261	1620,967411	0,669910075	0,05887212	11,3790721	5,3162E-30	2,678E-27	protein_codir trans-sialidase
TcG_08262	414,2504716	0,850762442	0,09187661	9,25983717	2,0474E-20	3,8261E-18	protein_codir putative retrotransposon hot spot (RHS) protein
TcG_08263	955,7815372	0,835544018	0,06801757	12,2842387	1,1008E-34	8,5023E-32	protein_codir putative retrotransposon hot spot (RHS) protein
TcG_08264	161,4172758	0,803069388	0,14754579	5,44284852	5,2435E-08	1,4362E-06	protein_codir retrotransposon hot spot (RHS) protein
TcG_08265	45,1905897	0,659464563	0,2762284	2,38738867	0,01696854	0,0742691	protein_codir target of rapamycin (TOR) kinase 1

TcG_08266	96,0260257	0,668952752	0,18112889	3,69324161	0,00022141	0,0021703	protein_codir target of rapamycin (TOR) kinase 1
TcG_08267	187,4799013	0,53785003	0,13582094	3,95999351	7,4952E-05	0,00087312	protein_codir hypothetical protein
TcG_08268	18,05972137	0,510750074	0,42328637	1,2066301	0,22757463	0,45444535	protein_codir structural maintenance of chromosome protein 4
TcG_08269	32,97561259	0,285784876	0,30841904	0,92661231	0,35412786	0,58831738	protein_codir putative glycine dehydrogenase, putative,glycine cleavage system P-protein
TcG_08270	0					1	
TcG_08271	0					1	
TcG_08272	0,145251675	-1,420530545	4,08047286	-0,3481289	0,72774338	1	
TcG_08273	465,620955	-0,272834177	0,0876963	-3,11112518	0,00186376	0,01300814	protein_codir putative ribonuclease
TcG_08274	394,8221638	-0,408672194	0,09248267	-4,4189058	9,9202E-06	0,00015123	protein_codir hypothetical protein
TcG_08275	306,3099611	0,002514651	0,10448144	0,02406792	0,98079843	0,99262148	protein_codir hypothetical protein
TcG_08276	1297,296672	-0,030224249	0,11735907	-0,25753653	0,79676462	0,89872949	protein_codir putative ubiquitin-protein ligase
TcG_08277	409,5808661	-0,114513011	0,09076431	-1,26165248	0,20707386	0,42926423	protein_codir putative legume-like lectin
TcG_08278	377,2615676	-0,115174455	0,09484666	-1,21432268	0,22462453	0,45108253	protein_codir hypothetical protein
TcG_08279	169,4487567	0,021223411	0,152046	0,13958547	0,88898752	0,9464367	protein_codir putative target of rapamycin (TOR) kinase 1
TcG_08280	1607,018633	-0,188133764	0,05903816	-3,18664682	0,00143932	0,01050127	protein_codir serine/threonine-protein kinase mTOR
TcG_08281	2191,351816	0,280421056	0,05400552	5,19245128	2,0754E-07	5,0517E-06	protein_codir 40S ribosomal protein S12
TcG_08282	2,576486619	1,583743932	1,22522341	1,29261644	0,19614375	1	protein_codir hexose transporter
TcG_08283	39,4156402	-0,029783732	0,27742842	-0,10735645	0,9145062	0,95834559	protein_codir hypothetical protein
TcG_08284	486,716373	-0,434183412	0,09193837	-4,7225483	2,3291E-06	4,2765E-05	protein_codir putative GTP-binding protein
TcG_08285	194,0802765	-0,130578889	0,13945057	-0,93638118	0,34907697	0,58360833	protein_codir putative leucine-rich repeat protein
TcG_08286	151,2847785	0,029839839	0,14472264	0,20618639	0,83664532	0,92063564	protein_codir oxidoreductase (with NAD(+) or NADP(+) as acceptor)
TcG_08287	1156,898525	-0,272724764	0,05897873	-4,62412079	3,7619E-06	6,5248E-05	protein_codir metallo-peptidase, Clan MA(E), Family M1
TcG_08288	185,384857	-0,020349135	0,12826316	-0,15865144	0,8739435	0,93941728	protein_codir hypothetical protein
TcG_08289	107,0994676	-0,144111525	0,17186079	-0,83853638	0,40172952	0,63220648	protein_codir GINS complex subunit 4
TcG_08290	220,8949343	-0,048760481	0,12376542	-0,393975	0,6935995	0,84125796	protein_codir hypothetical protein
TcG_08291	224,1372712	0,002921893	0,12903172	0,02264477	0,98193363	0,99328993	protein_codir putative acyltransferase
TcG_08292	325,7784985	-0,1622672	0,10350242	-1,56776234	0,11693661	0,29835444	protein_codir hypothetical protein
TcG_08293	226,9781161	-0,146573502	0,12203434	-1,20108411	0,22971858	0,45688956	protein_codir putative cysteine peptidase, Clan CA, family C19
TcG_08294	216,1316114	0,056625285	0,12201816	0,46407258	0,64259575	0,81030848	protein_codir enoyl-CoA hydratase, mitochondrial precursor
TcG_08295	88,78547701	-0,340591906	0,18615813	-1,82958381	0,0673122	0,20653688	protein_codir putative protein kinase
TcG_08296	217,3572898	-0,520798157	0,12427678	-4,19063131	2,7818E-05	0,00037046	protein_codir hypothetical protein
TcG_08297	85,5751731	-0,552525578	0,2048067	-2,69779051	0,00698014	0,03723381	protein_codir hypothetical protein
TcG_08298	266,770168	-0,341953725	0,11129746	-3,07243055	0,00212323	0,01446822	protein_codir template-activating factor I
TcG_08299	127,9001545	-0,274619438	0,15878459	-1,72950937	0,08371797	0,23820148	protein_codir putative ubiquinone biosynthesis protein COQ7
TcG_08300	0,233855473	1,175456718	3,50839889	0,33504078	0,73759432	1	
TcG_08301	88,58135262	0,141281306	0,18616469	0,75890496	0,44790941	0,66893782	protein_codir mucin-like glycoprotein
TcG_08302	183,2666936	0,046195585	0,13031867	0,3544817	0,72297791	0,85733557	protein_codir hypothetical protein
TcG_08303	246,6945151	-0,019499194	0,11751828	-0,16592478	0,86821615	0,93669357	protein_codir hypothetical protein
TcG_08304	329,4033372	0,117537902	0,10097938	1,16397919	0,24443245	0,47270812	protein_codir hypothetical protein
TcG_08305	150,9099221	0,042256022	0,14384081	0,29376936	0,76893415	0,88434297	protein_codir hypothetical protein
TcG_08306	395,6729228	-0,222339488	0,09150389	-2,4298365	0,01510564	0,0681253	protein_codir hypothetical protein
TcG_08307	358,644987	0,057299215	0,0955701	0,59955169	0,54880505	0,74524792	protein_codir malate dehydrogenase (quinone)
TcG_08308	283,2875988	0,09868666	0,11144288	0,88553581	0,37586771	0,60653866	protein_codir hypothetical protein
TcG_08309	273,437659	0,137386795	0,11645871	1,17970393	0,23811799	0,46594072	protein_codir hypothetical protein
TcG_08310	145,192245	-0,016499171	0,14541318	-0,11346407	0,90966265	0,9562972	protein_codir hypothetical protein
TcG_08311	463,4833321	0,217503691	0,08612113	2,52555533	0,01155156	0,05530428	protein_codir putative signal recognition particle receptor like protein
TcG_08312	185,2534316	0,248232028	0,13550269	1,83193436	0,0669612	0,2057858	protein_codir hypothetical protein
TcG_08313	429,7700268	-0,139965124	0,08914285	-1,57012166	0,11638681	0,29741014	protein_codir hypothetical protein
TcG_08314	1118,472699	-0,172260841	0,06168375	-2,79264559	0,00522789	0,02966228	protein_codir putative cysteine synthase
TcG_08315	291,3004337	0,019088308	0,11636153	0,16404312	0,8696972	0,93715697	protein_codir hypothetical protein
TcG_08316	462,1532799	-0,429688397	0,08844792	-4,85809472	1,1852E-06	2,3513E-05	protein_codir hypothetical protein
TcG_08317	59,99524225	0,177923742	0,22608914	0,7869628	0,43130364	0,65595747	protein_codir hypothetical protein

TcG_08318	187,7647022	0,055507045	0,12891348	0,43057596	0,66677672	0,82543809	protein_codir	1-alkyl-2-acetylglycerophosphocholine esterase
TcG_08319	106,6381996	-0,462351822	0,1771326	-2,61020172	0,00904888	0,04565994	protein_codir	hypothetical protein
TcG_08320	267,9494088	-0,16602057	0,10995448	-1,50990269	0,13106826	0,32172814	protein_codir	Tectonic-1
TcG_08321	250,7194669	0,113684902	0,115026	0,98834092	0,32298571	0,55752569	protein_codir	hypothetical protein
TcG_08322	370,3205689	-0,05291066	0,09595332	-0,55142086	0,58134521	0,77048234	protein_codir	AP-endonuclease
TcG_08323	366,7505658	-0,115485967	0,0965158	-1,1965499	0,23148204	0,45868837	protein_codir	dispersed gene family protein 1 (DGF-1)
TcG_08324	580,4522422	0,179457489	0,08089723	2,21833901	0,02653173	0,10434371	protein_codir	dispersed gene family protein 1 (DGF-1)
TcG_08325	524,3997628	-0,027735642	0,08561423	-0,32396066	0,74596782	0,87149696	protein_codir	hypothetical protein
TcG_08326	139,3496228	-0,182141833	0,14973996	-1,21638764	0,22383729	0,45039577	protein_codir	ubiquitin hydrolase
TcG_08327	306,3995348	-0,001379271	0,10337799	-0,01334202	0,98935493	0,99554162	protein_codir	flagellar calcium-binding protein
TcG_08328	78,5906699	-0,294050199	0,20185851	-1,45671441	0,14519523	0,34219527	protein_codir	flagellar calcium-binding protein
TcG_08329	0,116927736	0,503022807	4,08047286	0,12327562	0,90188885	1	protein_codir	flagellar calcium-binding protein
TcG_08330	3372,937219	-0,578513287	0,04785269	-12,0894613	1,2007E-33	8,1832E-31	protein_codir	putative flagellar calcium-binding protein
TcG_08331	4,823169719	0,113463929	0,84860903	0,13370578	0,89363524	1	protein_codir	serine/threonine protein phosphatase
TcG_08332	412,6010788	0,403297533	0,09152873	4,40623978	1,0518E-05	0,00015826	protein_codir	myosin heavy chain kinase A
TcG_08333	283,714602	0,498708812	0,11283791	4,41969201	9,8842E-06	0,00015088	protein_codir	putative lipase
TcG_08334	562,4080727	0,140741692	0,08200024	1,71635706	0,08609671	0,24272461	protein_codir	phospholipase A1
TcG_08335	44,00594329	-0,310528091	0,2783135	-1,1157493	0,26452946	0,49689337	protein_codir	lipase
TcG_08336	283,9630617	0,398812943	0,10841025	3,6787382	0,00023439	0,00227823	protein_codir	putative surface protease GP63, putative,metallopeptidase
TcG_08337	3,801685932	1,295818984	0,93076018	1,39221576	0,16385707	1		
TcG_08338	154,2865741	0,739259689	0,1458432	5,06886645	4,0019E-07	8,951E-06	protein_codir	adrenodoxin precursor
TcG_08339	135,523247	0,436562528	0,1541364	2,83231301	0,00462126	0,02705503	protein_codir	hypothetical protein
TcG_08340	269,1785943	-0,100977801	0,11160909	-0,90474528	0,36560035	0,59769234	protein_codir	hypothetical protein
TcG_08341	601,6966049	0,049103078	0,07829883	0,62712401	0,53057795	0,73208004	protein_codir	hypothetical protein
TcG_08342	2118,849998	-0,193514056	0,0468486	-4,13062612	3,6178E-05	0,00046469	protein_codir	putative calreticulin
TcG_08343	144,7451981	0,493110052	0,14719116	3,35013372	0,00080773	0,00652147	protein_codir	hypothetical protein
TcG_08344	1516,440707	0,244495575	0,05808964	4,20893572	2,5658E-05	0,0003448	protein_codir	RNA polymerase II 215kD subunit, isoform A
TcG_08345	139,0902888	0,233503459	0,15380208	1,5182074	0,12896212	0,3181084		
TcG_08346	291,5408844	0,058806426	0,11295446	0,52062064	0,60263107	0,785033		
TcG_08347	204,5910908	0,148666157	0,12500643	1,18926806	0,2343342	0,46208093	protein_codir	putative retrotransposon hot spot (RHS) protein
TcG_08348	44,96258136	0,802347691	0,26768816	2,99732235	0,00272363	0,01760933	protein_codir	dispersed protein family protein 1
TcG_08349	3,625586053	0,807010055	0,93852312	0,85987233	0,38985942	1	protein_codir	dispersed gene family protein 1 (DGF-1)
TcG_08350	11,49873069	0,637003713	0,51621974	1,23397783	0,21721116	1	protein_codir	dispersed gene family protein 1 (DGF-1)
TcG_08351	7,089559312	-0,274320913	0,65316015	-0,41999028	0,67449256	1	protein_codir	dispersed gene family protein 1 (DGF-1)
TcG_08352	23,59787649	-0,037897519	0,37870858	-0,1000704	0,92028843	0,96146026	protein_codir	dispersed gene family protein 1 (DGF-1)
TcG_08353	24,00397923	0,570499247	0,35690845	1,598447	0,10994353	0,28646723	protein_codir	dispersed gene family protein 1 (DGF-1)
TcG_08354	16,09057609	0,000109551	0,45130852	0,00024274	0,99980632	1	protein_codir	dispersed gene family protein 1 (DGF-1)
TcG_08355	11,23421585	-0,005334001	0,52001124	-0,01025747	0,99181586	1	protein_codir	dispersed gene family protein 1 (DGF-1)
TcG_08356	5,689593905	-0,06157691	0,71700125	-0,08588118	0,93156088	1	protein_codir	dispersed gene family protein 1 (DGF-1)
TcG_08357	3,955893586	1,074298199	0,90718927	1,18420514	0,23633187	1	protein_codir	dispersed gene family protein 1 (DGF-1)
TcG_08358	6,913673954	1,307620516	0,73921175	1,76893903	0,07690405	1	protein_codir	dispersed protein family protein 1
TcG_08359	5,714192763	0,268066105	0,74557856	0,35954106	0,71919037	1	protein_codir	hypothetical protein
TcG_08360	11,9527401	-0,582202537	0,50087107	-1,16238005	0,24508112	1	protein_codir	retrotransposon hot spot (RHS) protein
TcG_08361	30,55941631	-0,078299288	0,31857883	-0,24577681	0,80585501	0,90423384	protein_codir	hypothetical protein
TcG_08362	1,43553101	-1,163011499	1,60050122	-0,72665455	0,4674376	1	protein_codir	retrotransposon hot spot (RHS) protein
TcG_08363	18,16973903	-0,222200923	0,41033251	-0,5415143	0,58815314	0,77564856	protein_codir	hypothetical protein
TcG_08364	0,911733799	-0,897817394	3,94638757	-0,2275036	0,82003217	1		
TcG_08365	6,911307529	1,08868834	0,68123202	1,59811681	0,11001698	1	protein_codir	hypothetical protein
TcG_08366	182,7827854	0,090941574	0,13552127	0,67105017	0,50218857	0,71094291	protein_codir	trans-sialidase
TcG_08367	57,08012213	-0,142096813	0,24024491	-0,5914665	0,5542079	0,74910997	protein_codir	trans-sialidase
TcG_08368	54,93117388	0,264600738	0,23597238	1,12132082	0,26215132	0,49428247	protein_codir	trans-sialidase
TcG_08369	434,8244486	0,219210623	0,09064314	2,41839166	0,01558929	0,06964219	protein_codir	hypothetical protein

TcG_08370	304,1208342	-0,344367339	0,10459333	-3,29244082	0,00099322	0,00773868	protein_codir	putative GTPase
TcG_08371	403,6587512	-0,191620163	0,09295211	-2,06149337	0,039256	0,13947254	protein_codir	hypothetical protein
TcG_08372	196,3539061	-0,244690926	0,13173687	-1,85742176	0,06325117	0,19731505	protein_codir	putative ATPase alpha subunit
TcG_08373	2341,738421	-0,132004855	0,04573718	-2,88616096	0,00389973	0,02360618	protein_codir	ATPase alpha subunit
TcG_08374	22,35681402	0,354679259	0,36853539	0,96240217	0,33584762	0,57037974	protein_codir	trans-sialidase
TcG_08375	29,68353351	0,995036807	0,33271282	2,99067767	0,00278359	0,01788724	protein_codir	selenocysteine-tRNA-specific elongation factor
TcG_08376	1,367995811	-0,413796123	1,45601517	-0,28419767	0,7762589	1	protein_codir	hypothetical protein
TcG_08377	129,506945	-0,031227088	0,15855187	-0,19695187	0,8438652	0,92438779	protein_codir	putative p22 protein precursor
TcG_08378	146,1799857	-0,335444062	0,16178441	-2,07340169	0,03813491	0,13653618	protein_codir	hypothetical protein
TcG_08379	1312,670475	-0,09872578	0,05549088	-1,77913522	0,0752176	0,2214666	protein_codir	putative I/6 autoantigen
TcG_08380	108,148184	-0,00031209	0,17117255	-0,00182325	0,99854526	0,99956197	protein_codir	putative cyclophilin
TcG_08381	397,7256755	-0,617465286	0,09895538	-6,2398357	4,3803E-10	1,9151E-08	protein_codir	hypothetical protein
TcG_08382	154,1511842	-0,095100374	0,15224679	-0,62464615	0,53220332	0,7329332	protein_codir	peptidyl-prolyl cis-trans isomerase
TcG_08383	193,9819115	0,047201119	0,13336362	0,353928	0,72339285	0,85739757	protein_codir	putative aquaporin
TcG_08384	314,6515361	0,17906718	0,11360885	1,57617279	0,11498599	0,2951978	protein_codir	centrin
TcG_08385	19,35171762	-0,073836202	0,39870954	-0,18518795	0,85308162	0,92887573	protein_codir	centrin
TcG_08386	143,7128887	-0,095782788	0,15946008	-0,60066937	0,54806022	0,74467289	protein_codir	hypothetical protein
TcG_08387	124,7396872	0,17845807	0,15644175	1,14073175	0,25398157	0,48503774	protein_codir	hypothetical protein
TcG_08388	295,9424548	0,126483358	0,10679269	1,18438213	0,23626183	0,46360093	protein_codir	putative mak-16-like RNA binding protein
TcG_08389	371,1204304	-0,238741937	0,09559188	-2,49751269	0,0125068	0,058944	protein_codir	intraflagellar transport protein-like protein
TcG_08390	425,2963669	0,135509187	0,08951875	1,51375201	0,13008875	0,31986592	protein_codir	farnesyl diphosphate synthase precursor
TcG_08391	326,1379071	0,100806399	0,10393283	0,96991877	0,33208698	0,5663408	protein_codir	hypothetical protein
TcG_08392	357,8843472	0,016718132	0,09828189	0,17010389	0,86492843	0,93498506	protein_codir	napsin-A
TcG_08393	42,09824152	-0,892654174	0,31371684	-2,84541364	0,00443538	0,02627213	protein_codir	hypothetical protein
TcG_08394	135,4896631	-0,490857481	0,16501523	-2,9746193	0,00293352	0,01868488	protein_codir	hypothetical protein
TcG_08395	568,5978917	0,148871682	0,07894449	1,88577665	0,05932506	0,18756073	protein_codir	hypothetical protein
TcG_08396	235,6577107	-0,039596578	0,11979408	-0,33053868	0,74099297	0,86797539	protein_codir	hypothetical protein
TcG_08397	892,4344291	-0,163616523	0,06861325	-2,38461993	0,01709677	0,07455146	protein_codir	putative GTP binding protein
TcG_08398	232,3219925	-0,3251994	0,12689419	-2,56276041	0,01038437	0,05091548	protein_codir	protein phosphatase 2C
TcG_08399	134,2464701	0,018360785	0,15247871	0,1204154	0,90415409	0,95353444	protein_codir	hypothetical protein
TcG_08400	75,95624722	-0,273876067	0,20401064	-1,34245971	0,17944698	0,39065629	protein_codir	central apparatus associated protein C1a-18
TcG_08401	449,2694083	-0,00201274	0,08763961	-0,0229661	0,98167731	0,99325066	protein_codir	transcription elongation regulator-like protein
TcG_08402	278,0263194	-0,123923964	0,11130173	-1,11340558	0,26553428	0,49806938	protein_codir	putative acyl-CoA oxidase
TcG_08403	337,5284753	-0,180950573	0,10309764	-1,75513785	0,07923573	0,2293916	protein_codir	hypothetical protein
TcG_08404	110,8339064	-0,277914308	0,16590721	-1,67511894	0,0939109	0,25750928		
TcG_08405	471,3989905	-0,219515804	0,08614006	-2,54835905	0,0108231	0,05259918	protein_codir	hypothetical protein
TcG_08406	336,1713972	-0,139399753	0,09762218	-1,42795162	0,15330578	0,3538244	protein_codir	hypothetical protein
TcG_08407	179,3634657	0,030301168	0,13320005	0,22748616	0,82004573	0,91102214	protein_codir	rRNA small subunit pseudouridine methyltransferase Nep1
TcG_08408	224,587686	0,197662604	0,11888318	1,66266245	0,09638002	0,26258838	protein_codir	hypothetical protein
TcG_08409	577,8503642	0,103932256	0,07903099	1,31508233	0,18848223	0,40280445	protein_codir	hypothetical protein
TcG_08410	48,84250248	-0,223454264	0,2512998	-0,88919395	0,37389886	0,60449626	protein_codir	putative esterase
TcG_08411	63,57504376	0,013470367	0,23176167	0,05812163	0,95365174	0,97828779	protein_codir	hypothetical protein
TcG_08412	179,5324459	0,081845361	0,13213119	0,61942499	0,53563642	0,73555572	protein_codir	retrotransposon hot spot (RHS) protein
TcG_08413	282,6267421	0,23147323	0,11123231	2,08098917	0,0374349	0,13473772	protein_codir	retrotransposon hot spot (RHS) protein
TcG_08414	31,41108968	0,006097887	0,31767358	0,01919545	0,98468519	0,99412361	protein_codir	hypothetical protein
TcG_08415	31,91919919	-0,700856281	0,31600851	-2,21783988	0,02656575	0,10444208	protein_codir	subtilisin-like serine peptidase
TcG_08416	41,41031355	0,255988489	0,27124616	0,94374971	0,34529756	0,57929591	protein_codir	dynein
TcG_08417	47,44962148	-0,186478865	0,25491485	-0,73153394	0,46445308	0,68173811	protein_codir	putative kinesin
TcG_08418	22,78555548	0,425416263	0,39805054	1,06874937	0,28518262	0,51877427		
TcG_08419	26,10801823	0,164681862	0,34554341	0,47658805	0,6336555	0,80419712	protein_codir	hypothetical protein
TcG_08420	51,18267122	0,554512078	0,24768794	2,23875286	0,025172	0,10025535	protein_codir	trans-sialidase
TcG_08421	18,73583189	-0,641421976	0,41449353	-1,54748368	0,12174666	0,30617685	protein_codir	N-acetyltransferase complex ARD1 subunit

TcG_08422	13,96233562	-0,068812773	0,48199231	-0,14276737	0,88647391	1	protein_codir hypothetical protein
TcG_08423	18,76933589	0,251330556	0,40286499	0,62385802	0,53272083	0,73337733	protein_codir retrotransposon hot spot protein (RHS)
TcG_08424	79,14439482	0,443120181	0,20384194	2,17384205	0,029717	0,11359325	protein_codir retrotransposon hot spot (RHS) protein
TcG_08425	71,65766584	0,38447328	0,21553801	1,78378409	0,07445876	0,220296	protein_codir retrotransposon hot spot (RHS) protein
TcG_08426	44,05223046	0,657222112	0,28827521	2,27984261	0,02261703	0,09262667	protein_codir retrotransposon hot spot (RHS) protein
TcG_08427	47,29092609	0,226061321	0,25717193	0,87902798	0,37938611	0,6106388	protein_codir putative trans-sialidase
TcG_08428	96,83942791	0,150000871	0,18147663	0,82655751	0,40848791	0,63660535	protein_codir trans-sialidase
TcG_08429	105,6588713	0,275117676	0,17411485	1,58009313	0,11408554	0,29340623	protein_codir rab1 small GTP-binding protein
TcG_08430	38,38384593	0,125909398	0,28842162	0,43654633	0,6624404	0,82314827	protein_codir hypothetical protein
TcG_08431	45,73527456	0,37947434	0,25904336	1,46490666	0,14294639	0,33931097	protein_codir putative trans-sialidase
TcG_08432	52,44963811	0,223881804	0,24049232	0,93093118	0,35188916	0,58627953	protein_codir putative trans-sialidase
TcG_08433	30,75995652	0,467855546	0,31523585	1,48414449	0,13777059	0,33116392	protein_codir putative trans-sialidase
TcG_08434	56,0127074	0,277950419	0,23642165	1,17565555	0,23973255	0,46823016	
TcG_08435	1113,521189	0,065232835	0,0617963	1,05561067	0,29114612	0,52365178	protein_codir putative retrotransposon hot spot (RHS) protein
TcG_08436	34,77604755	-0,069938957	0,30728056	-0,22760619	0,81995241	0,91102214	protein_codir hypothetical protein
TcG_08437	11,13882494	1,00743902	0,58465321	1,72313948	0,08486331	1	protein_codir zinc finger protein family memeber
TcG_08438	339,0698853	-0,493778578	0,10230434	-4,82656508	1,3891E-06	2,7049E-05	protein_codir zinc finger protein family memeber
TcG_08439	654,1515442	-0,366372809	0,0733976	-4,99161807	5,9876E-07	1,2823E-05	protein_codir hypothetical protein
TcG_08440	2640,899141	-0,196104954	0,04508932	-4,349255	1,366E-05	0,00019908	protein_codir putative heat shock protein
TcG_08441	893,9011321	-0,409629895	0,06910061	-5,92802168	3,0661E-09	1,1066E-07	protein_codir putative pyruvate dehydrogenase E1 component alpha subunit
TcG_08442	282,0272075	-0,262954369	0,11241439	-2,3391522	0,01932756	0,08181552	protein_codir hypothetical protein
TcG_08443	1059,959776	0,017333257	0,06853805	0,25289978	0,80034566	0,90062207	protein_codir 60S ribosomal protein L34
TcG_08444	241,418713	-0,477118457	0,12017498	-3,97019808	7,1813E-05	0,00084529	protein_codir hypothetical protein
TcG_08445	603,3766824	-0,583705009	0,08405956	-6,94394553	3,813E-12	2,6296E-10	protein_codir pumilio protein 2
TcG_08446	11,73113492	-0,50784166	0,51294975	-0,99004174	0,32215372	1	protein_codir hypothetical protein
TcG_08447	317,2286574	-0,002267434	0,10828568	-0,02093937	0,98329402	0,99375824	protein_codir hypothetical protein
TcG_08448	85,1607257	-0,058008017	0,20731165	-0,27981069	0,77962275	0,89036069	protein_codir hypothetical protein
TcG_08449	575,9950344	0,363962749	0,08049028	4,52182211	6,131E-06	0,00010005	protein_codir NADH-cytochrome b5 reductase
TcG_08450	226,5775529	-0,166423922	0,13473672	-1,23517863	0,21676403	0,44089387	protein_codir translation initiation factor 2D
TcG_08451	225,9485478	0,019095969	0,12932886	0,14765435	0,88261556	0,94267772	protein_codir translation initiation factor 2D
TcG_08452	115,1245183	-0,408986618	0,16713653	-2,44702108	0,01440424	0,06584288	protein_codir hypothetical protein
TcG_08453	59,84830461	-0,093128702	0,23012833	-0,40468161	0,68571156	0,83697127	protein_codir hypothetical protein
TcG_08454	269,755351	-0,311615064	0,10967483	-2,84126323	0,00449352	0,02649462	protein_codir putative protein kinase
TcG_08455	36,7652816	-0,077548982	0,29802313	-0,26021128	0,7947008	0,89753819	
TcG_08456	118,8184989	-0,144273357	0,16414081	-0,87896091	0,37942247	0,6106388	protein_codir ribosomal protein L22/L17-like protein
TcG_08457	293,9650448	-0,312055693	0,10361083	-3,01180569	0,00259699	0,01693001	protein_codir hypothetical protein
TcG_08458	419,5963162	0,046026425	0,09223438	0,49901593	0,61776817	0,793034	protein_codir hypothetical protein
TcG_08459	68,55082166	0,557608968	0,21993057	2,53538631	0,01123234	0,05420153	protein_codir hypothetical protein
TcG_08460	262,9880367	-0,147450235	0,1185071	-1,24423128	0,21341446	0,43698047	protein_codir IQ and ubiquitin-like domain-containing protein
TcG_08461	126,2308782	-0,259501293	0,15950349	-1,62693173	0,1037516	0,27606061	protein_codir hypothetical protein
TcG_08462	258,9962848	-0,047141157	0,11302469	-0,41708724	0,6766146	0,83060571	protein_codir hypothetical protein
TcG_08463	88,60571476	-0,027806165	0,18396295	-0,1511509	0,87985668	0,94162382	protein_codir histone H2A
TcG_08464	264,4043088	-0,125828011	0,11254813	-1,11799291	0,26357002	0,49589514	protein_codir putative tubulin tyrosine ligase
TcG_08465	249,5118304	-0,160942003	0,11460587	-1,40430861	0,16022697	0,36306159	protein_codir putative 3-Beta-hydroxysteroid-delta(8), delta(7)-isomerase
TcG_08466	97,43714026	-0,016134047	0,18565193	-0,08690482	0,93074717	0,96665499	protein_codir hypothetical protein
TcG_08467	93,20746793	-0,066157068	0,19274413	-0,34323778	0,73141959	0,86207806	protein_codir hypothetical protein
TcG_08468	640,8910956	-0,330128648	0,08893249	-3,71212655	0,00020553	0,00203523	protein_codir recombination initiation protein NBS1
TcG_08469	416,5240273	-0,22537129	0,09201036	-2,449412	0,01430897	0,06560494	protein_codir putative recombination initiation protein NBS1
TcG_08470	307,72085	-0,011323158	0,10263075	-0,1103291	0,91214838	0,95717336	protein_codir tRNA (guanine-N(1)-)-methyltransferase
TcG_08471	379,6134504	-0,173903972	0,09274481	-1,87508026	0,06078168	0,19148679	protein_codir protein kinase
TcG_08472	149,2337237	-0,149188339	0,1469289	-1,0153778	0,30992577	0,54323751	protein_codir putative proteasome 26S non-ATPase subunit 9
TcG_08473	273,108227	0,011417515	0,11331181	0,10076191	0,91973947	0,9613082	protein_codir hypothetical protein

TcG_08474	78,66873576	0,294884891	0,20503759	1,4381992	0,15037754	0,35041718	
TcG_08475	100,3627749	0,042135939	0,17778829	0,23700064	0,8126563	0,90759432	protein_codir protein tyrosine phosphatase PRL
TcG_08476	273,6347276	-0,051159311	0,11062186	-0,46247018	0,64374418	0,810875	protein_codir histone acetyltransferase-like protein
TcG_08477	68,480213	0,111656962	0,2160343	0,5168483	0,60526206	0,78669877	protein_codir hypothetical protein
TcG_08478	266,3612613	0,03097158	0,11635136	0,26619009	0,79009281	0,89502471	protein_codir putative trans-sialidase
TcG_08479	112,2467912	0,230359618	0,17342525	1,32829345	0,18408118	0,39686725	protein_codir rab1 small GTP-binding protein
TcG_08480	80,9050571	0,23055281	0,20066489	1,14894441	0,2505789	0,48026587	protein_codir putative target of rapamycin (TOR) kinase 1
TcG_08481	134,2350064	0,118613605	0,15906739	0,74568145	0,45585987	0,67582757	protein_codir target of rapamycin (TOR) kinase 1
TcG_08482	45,2907533	0,055016425	0,25765495	0,21352753	0,83091552	0,91760178	
TcG_08483	68,48508523	0,202263334	0,21318464	0,94877068	0,34273726	0,57679606	
TcG_08484	211,2131308	-0,200580044	0,12449696	-1,61112408	0,10715268	0,28208838	protein_codir ESAG-like protein
TcG_08485	248,1015213	-0,074658999	0,11365935	-0,6568663	0,51126689	0,71768359	protein_codir protein tyrosine phosphatase
TcG_08486	248,0565922	-0,097494725	0,11519795	-0,84632344	0,39737234	0,62698568	protein_codir putative protein kinase
TcG_08487	60,47371572	-0,272653874	0,24348246	-1,11980908	0,26279513	0,49491943	protein_codir hypothetical protein
TcG_08488	133,5542123	-0,126011893	0,15254104	-0,82608518	0,40875577	0,63660535	protein_codir hypothetical protein
TcG_08489	277,1413048	0,072051422	0,10872416	0,66269929	0,50752316	0,71430556	protein_codir protein KRI1
TcG_08490	0					1	protein_codir hypothetical protein
TcG_08491	97,2277992	0,050811308	0,1843121	0,2756808	0,78279324	0,89239816	protein_codir hypothetical protein
TcG_08492	3,734870516	-0,931341111	0,91225233	-1,02092489	0,30729002	1	
TcG_08493	7,804201902	-0,260038481	0,63075331	-0,41226653	0,68014408	1	
TcG_08494	70,40174479	0,122267109	0,22292807	0,54845992	0,58337614	0,77201553	protein_codir hypothetical protein
TcG_08495	82,45533281	-0,116288343	0,21726728	-0,53523172	0,59248963	0,7778347	protein_codir hypothetical protein
TcG_08496	22,56737243	0,145194112	0,36337359	0,39957255	0,68947138	0,8386805	protein_codir oligosaccharyl transferase subunit
TcG_08497	177,2233559	-0,165230676	0,13481974	-1,22556737	0,22036152	0,44541323	protein_codir putative oligosaccharyl transferase subunit
TcG_08498	179,0476016	-0,142260632	0,13220152	-1,07608924	0,28188735	0,51497112	protein_codir oligosaccharyl transferase subunit
TcG_08499	134,425495	0,029812848	0,15137792	0,19694317	0,84387201	0,92438779	protein_codir Rhodanese-like protein
TcG_08500	2,043031965	2,077746071	1,40248569	1,48147399	0,13848031	1	
TcG_08501	305,6053997	-0,104772188	0,10611111	-0,98738191	0,32345545	0,55758888	protein_codir NADH-dependent fumarate reductase-like protein
TcG_08502	2,344298642	-0,096464523	1,13763205	-0,08479413	0,93242506	1	
TcG_08503	817,3548961	-0,076786579	0,07450297	-1,03065123	0,3027044	0,535498	protein_codir gamma-glutamylcysteine synthetase
TcG_08504	338,9414891	-0,395663811	0,10113397	-3,91227391	9,1431E-05	0,00103309	protein_codir putative stress-inducible protein STI1-like
TcG_08505	350,1691618	-0,761456971	0,09677945	-7,86796112	3,6047E-15	3,94E-13	protein_codir hypothetical protein
TcG_08506	241,5746179	-0,341204839	0,1150061	-2,96684118	0,00300876	0,01906977	protein_codir calmodulin
TcG_08507	167,7413913	-0,395972512	0,13786187	-2,87224107	0,00407572	0,02446699	protein_codir putative membrane transporter protein
TcG_08508	279,1001689	-0,556266233	0,10891384	-5,10739692	3,2663E-07	7,4348E-06	protein_codir hypothetical protein
TcG_08509	88,22438187	-0,152501884	0,19550965	-0,78002227	0,43537777	0,65939964	protein_codir putative to be involved in ER-Golgi transport
TcG_08510	643,3372575	-0,2848718	0,08040227	-3,54308148	0,00039548	0,00355197	protein_codir nucleolar complex protein 2
TcG_08511	437,5671187	-0,350512819	0,09062813	-3,86759424	0,00010991	0,00120937	protein_codir putative Unc104-like kinesin
TcG_08512	143,1792751	-0,178283534	0,14703435	-1,21252986	0,22530962	0,45182011	protein_codir synaptobrevin-type transport protein
TcG_08513	132,630383	-0,178398534	0,16062642	-1,11064256	0,26672224	0,49939561	protein_codir hypothetical protein
TcG_08514	203,7199348	-0,406828465	0,12569508	-3,2366299	0,0012095	0,00911137	protein_codir putative vesicle-fusing ATPase
TcG_08515	279,5804175	-0,306502628	0,10973155	-2,79320421	0,00521887	0,02962561	protein_codir putative protein kinase
TcG_08516	565,5499758	-0,297737516	0,07860093	-3,78796425	0,00015189	0,00158967	protein_codir putative kinesin
TcG_08517	40,08963976	-0,088398882	0,2728016	-0,32404092	0,74590706	0,87149696	protein_codir plasma membrane Ca2 ATPase
TcG_08518	390,4281804	-0,04743933	0,09409524	-0,50416294	0,61414688	0,79140316	protein_codir hypothetical protein
TcG_08519	134,5838081	-0,229085872	0,15261082	-1,50111157	0,13332671	0,32445352	protein_codir hypothetical protein
TcG_08520	224,3613575	-0,079248645	0,12100866	-0,65490059	0,51253176	0,71856159	protein_codir hypothetical protein
TcG_08521	153,3006224	-0,264836723	0,1450926	-1,82529448	0,0679566	0,20781967	protein_codir Ubiquitin-fold modifier 1
TcG_08522	75,79265449	-0,564898111	0,19890316	-2,84006605	0,00451042	0,02658073	protein_codir putative ATP-dependent chaperone
TcG_08523	251,7732487	-0,334567446	0,1134076	-2,95013245	0,00317638	0,0199791	protein_codir putative protein kinase
TcG_08524	376,1634165	-0,3059164	0,09633973	-3,17539188	0,00149634	0,01084872	protein_codir kinesin K39
TcG_08525	429,3867654	-0,060517774	0,09048317	-0,66882904	0,50360454	0,71199051	protein_codir putative protein SERAC1-like

TcG_08526	284,9899581	-0,03783165	0,10775189	-0,35109965	0,72551359	0,85817259	protein_codir variant surface glycoprotein 3275
TcG_08527	46,72984231	-0,016857535	0,27617161	-0,06104007	0,9513273	0,97713458	protein_codir variant surface glycoprotein 3275
TcG_08528	186,2209281	-0,085835535	0,12906785	-0,66504197	0,50602365	0,71271456	protein_codir methyltransferase
TcG_08529	219,975162	-0,008097519	0,12495547	-0,06480324	0,94833066	0,97570906	protein_codir charged multivesicular body protein 6
TcG_08530	9,971958403	0,464933021	0,56865087	0,81760715	0,41358154	1	protein_codir RNA-binding protein
TcG_08531	415,2927073	0,061033774	0,09076522	0,67243569	0,50130637	0,71012783	protein_codir hypothetical protein
TcG_08532	420,849665	-0,03861242	0,0923841	-0,41795525	0,67597983	0,83017833	protein_codir small nuclear ribonucleoprotein SmD2
TcG_08533	135,9619173	0,068786653	0,15066549	0,45655214	0,64799299	0,81260383	protein_codir putative serine protease PepD
TcG_08534	309,9644879	-0,068687976	0,10537701	-0,65183078	0,51451034	0,7202291	protein_codir hypothetical protein
TcG_08535	599,7283644	-0,119490233	0,07682092	-1,5554388	0,1198416	0,30291726	protein_codir transcription elongation factor SPT6
TcG_08536	227,3768127	0,015371151	0,11976387	0,12834548	0,89787558	0,95018119	protein_codir putative sedoheptulose-1,7-bisphosphatase
TcG_08537	82,17292665	0,186973732	0,19337814	0,96688142	0,33360331	0,56806702	protein_codir hypothetical protein
TcG_08538	60,58836677	-0,097999877	0,22720191	-0,43133386	0,66622564	0,82519673	protein_codir hypothetical protein
TcG_08539	20,77027323	-0,16977127	0,37907215	-0,44786004	0,65425421	0,81709489	protein_codir hypothetical protein
TcG_08540	15,50750594	-0,614323293	0,43795776	-1,40269986	0,16070636	1	protein_codir hypothetical protein
TcG_08541	8,762821932	-1,151081792	0,61218505	-1,88028405	0,06006938	1	protein_codir hypothetical protein
TcG_08542	331,037351	-0,254306949	0,10659434	-2,38574528	0,01704455	0,07444098	protein_codir putative tyrosyl-DNA Phosphodiesterase (Tdp1)
TcG_08543	301,1925146	-0,064976323	0,11202669	-0,58000752	0,56190955	0,7553645	protein_codir putative ras-related protein Rab21
TcG_08544	3,663657027	-1,552397251	1,02296639	-1,51754473	0,12912921	1	
TcG_08545	6,49705686	-0,543377077	0,69815683	-0,77830231	0,43639081	1	protein_codir putative profilin
TcG_08546	323,2453483	0,21555094	0,10093107	2,13562515	0,03270998	0,12205405	protein_codir trans-sialidase
TcG_08547	31,26529658	0,050360483	0,30869109	0,163142	0,87040663	0,93739833	protein_codir putative dispersed gene family protein 1 (DGF-1)
TcG_08548	28,81367667	0,088586269	0,34779475	0,25470848	0,79894827	0,8997487	protein_codir hypothetical protein
TcG_08549	38,72793538	0,070585675	0,27834932	0,25358667	0,7998149	0,90034262	protein_codir hypothetical protein
TcG_08550	375,3317407	0,185745893	0,09326223	1,99165195	0,04640926	0,15699202	protein_codir putative retrotransposon hot spot (RHS) protein
TcG_08551	59,68832745	0,188951384	0,2324539	0,81285529	0,41630103	0,64327337	protein_codir trans-sialidase
TcG_08552	96,47474427	0,222924019	0,18147856	1,22837663	0,21930561	0,44436426	protein_codir trans-sialidase
TcG_08553	355,900455	0,049223583	0,09722186	0,5063016	0,61264494	0,79081972	protein_codir dispersed gene family protein 1 (DGF-1)
TcG_08554	142,6275442	0,118937666	0,14986965	0,7936074	0,42742403	0,65262714	protein_codir putative dispersed gene family protein 1 (DGF-1)
TcG_08555	88,32611466	-0,09886564	0,18690629	-0,52895834	0,59683435	0,78090602	protein_codir hypothetical protein
TcG_08556	403,0521207	0,178856091	0,09097293	1,96603634	0,04929441	0,16383393	protein_codir putative retrotransposon hot spot (RHS) protein
TcG_08557	92,15756713	-0,283775959	0,20076018	-1,41350717	0,15750666	0,35943906	protein_codir trans-sialidase
TcG_08558	204,2540059	-0,222012779	0,1254723	-1,76941671	0,07682436	0,2245993	protein_codir trans-sialidase-like protein
TcG_08559	20,34819463	-0,212900425	0,38540482	-0,55240727	0,58066935	0,77019291	protein_codir hypothetical protein
TcG_08560	145,6438743	-0,060920204	0,15819678	-0,3850913	0,70016978	0,84438126	protein_codir retrotransposon hot spot (RHS) protein
TcG_08561	27,62612552	-0,090303652	0,33210436	-0,27191347	0,78568855	0,89351472	protein_codir hypothetical protein
TcG_08562	10,83799649	-0,128898691	0,53706512	-0,2400057	0,81032583	1	protein_codir retrotransposon hot spot (RHS) protein
TcG_08563	15,72368329	0,360555358	0,4889943	0,73734062	0,46091524	1	protein_codir hypothetical protein
TcG_08564	19,79780489	-0,427148876	0,38550668	-1,1080194	0,26785344	0,50021759	
TcG_08565	24,501131	0,757937789	0,37059553	2,04518869	0,04083626	0,14328555	protein_codir putative trans-sialidase
TcG_08566	17,47888748	0,132159261	0,4657445	0,28375914	0,77659497	0,88831084	protein_codir hypothetical protein
TcG_08567	0,911733799	-0,897817394	3,94638757	-0,2275036	0,82003217	1	
TcG_08568	28,97596789	-0,169910691	0,3209411	-0,52941393	0,59651833	0,78066886	
TcG_08569	18,13279894	-0,431501011	0,40980344	-1,05294629	0,29236561	0,52508882	protein_codir hypothetical protein
TcG_08570	12,29481393	-0,378533542	0,51465196	-0,73551365	0,46202673	1	protein_codir trans-sialidase
TcG_08571	122,6127233	-0,214058215	0,16323693	-1,3113345	0,18974478	0,40485875	
TcG_08572	147,397268	-0,3255106	0,145652	-2,23485163	0,02542709	0,10102821	protein_codir putative retrotransposon hot spot (RHS) protein
TcG_08573	78,68609045	-0,066450198	0,19751716	-0,33642747	0,73654854	0,86530636	protein_codir putative retrotransposon hot spot (RHS) protein
TcG_08574	13,38068672	0,214751858	0,4814637	0,44603956	0,65556867	1	protein_codir hypothetical protein
TcG_08575	26,22762599	-0,288085967	0,34918856	-0,82501548	0,4093628	0,6367988	
TcG_08576	16,04678568	0,037176478	0,4499879	0,08261662	0,93415639	1	
TcG_08577	65,19870338	-0,077388525	0,22480004	-0,34425494	0,73065456	0,86143928	protein_codir putative dispersed gene family protein 1 (DGF-1)

TcG_08578	90,33449275	-0,074697321	0,18850731	-0,39625689	0,69191553	0,8405776	protein_codir	dispersed gene family protein 1 (DGF-1)
TcG_08579	108,6013523	0,214403431	0,18577642	1,1540939	0,24846167	0,47778871	protein_codir	hypothetical protein
TcG_08580	552,5157444	0,226888293	0,07920997	2,86439057	0,00417812	0,0249911	protein_codir	hypothetical protein
TcG_08581	110,3315867	0,595598152	0,17360514	3,4307634	0,00060189	0,00505322	protein_codir	hypothetical protein
TcG_08582	318,0871843	0,469684717	0,10199615	4,60492608	4,1261E-06	7,0928E-05	protein_codir	putative beta propeller protein
TcG_08583	1830,957403	0,319487042	0,04997657	6,39273621	1,6294E-10	7,7056E-09	protein_codir	tryparedoxin peroxidase
TcG_08584	865,7987129	0,681165764	0,06896502	9,87697497	5,2391E-23	1,3196E-20	protein_codir	cyclophilin
TcG_08585	226,4763764	0,544458725	0,12384533	4,39627993	1,1012E-05	0,00016484	protein_codir	putative agmatinase
TcG_08586	1338,616788	0,363492981	0,05703856	6,3727586	1,8566E-10	8,6387E-09	protein_codir	hypothetical protein
TcG_08587	251,4288958	0,437894506	0,11468378	3,81827742	0,00013439	0,00142583	protein_codir	hypothetical protein
TcG_08588	452,5355629	0,274820466	0,08673392	3,16854662	0,00153203	0,01106616	protein_codir	GDP-mannose pyrophosphorylase
TcG_08589	54,75355032	0,509880013	0,2384454	2,13835124	0,03248825	0,1215063	protein_codir	hypothetical protein
TcG_08590	52,24328645	0,773350876	0,24933556	3,10164699	0,00192447	0,01333464	protein_codir	hypothetical protein
TcG_08591	42,84525489	0,578954724	0,28799732	2,01027818	0,04440176	0,15224585	protein_codir	hypothetical protein
TcG_08592	27,77398764	0,548241216	0,34641364	1,58262017	0,11350806	0,29237537	protein_codir	selenocysteine-tRNA-specific elongation factor
TcG_08593	45,2935929	0,749664341	0,26234879	2,85751014	0,00426979	0,02542127	protein_codir	putative trans-sialidase
TcG_08594	33,20360556	0,500312254	0,30960081	1,61599145	0,10609616	0,28051806	protein_codir	hypothetical protein
TcG_08595	14,78711597	0,49313406	0,49200116	1,00230263	0,31619745	1	protein_codir	hypothetical protein
TcG_08596	16,8617507	1,129725154	0,43821515	2,57801481	0,00993697	0,04915852	protein_codir	hypothetical protein
TcG_08597	104,7914047	0,599757678	0,17473059	3,43247099	0,00059811	0,00503245	protein_codir	RNA-binding protein
TcG_08598	90,4098322	0,876872929	0,21554051	4,06825108	4,7367E-05	0,00059074	protein_codir	Tbingi protein
TcG_08599	65,59536584	0,754625382	0,22002668	3,42969943	0,00060425	0,0050694	protein_codir	hypothetical protein
TcG_08600	31,69142971	0,347362031	0,32159276	1,08013012	0,28008424	0,51321462	protein_codir	Tbingi protein
TcG_08601	127,1516733	0,842107316	0,1624308	5,18440652	2,167E-07	5,209E-06	protein_codir	Tbingi protein
TcG_08602	46,63098654	0,975704624	0,28374203	3,4387032	0,00058451	0,00494501	protein_codir	multidrug resistance-associated protein
TcG_08603	311,9036292	-0,185565728	0,10306283	-1,80051075	0,07178003	0,21511728	protein_codir	putative ATPase
TcG_08604	347,1842576	0,014356482	0,09821613	0,14617235	0,88378533	0,94348884	protein_codir	hypothetical protein
TcG_08605	157,8933675	0,26149531	0,14885197	1,75674743	0,07896087	0,22888182	protein_codir	hypothetical protein
TcG_08606	704,3843517	-0,163452916	0,07050539	-2,31830397	0,02043281	0,08552547	protein_codir	putative GTPase activating protein
TcG_08607	45,71636286	-0,112715819	0,2620969	-0,430054	0,66715636	0,82567331	protein_codir	putative GTPase activating protein
TcG_08608	118,2369674	-0,073050047	0,16521578	-0,44214933	0,65838115	0,8201273	protein_codir	putative GTPase activating protein
TcG_08609	410,4129428	-0,194596852	0,09119559	-2,13384056	0,03285583	0,12244056	protein_codir	rib72 protein-like protein
TcG_08610	170,5443473	-0,349188371	0,13611546	-2,56538365	0,01030618	0,05059635	protein_codir	ATP-binding cassette protein subfamily G, member 1
TcG_08611	637,7421003	-0,134041418	0,07480812	-1,79180305	0,07316452	0,21781796	protein_codir	putative 40S ribosomal protein S8
TcG_08612	137,3634184	-0,021570109	0,15061717	-0,14321148	0,88612316	0,94475227	protein_codir	hypothetical protein
TcG_08613	393,9110076	-0,171018529	0,09597687	-1,78187228	0,07477006	0,22066725	protein_codir	hypothetical protein
TcG_08614	161,8417469	-0,270548431	0,15173842	-1,78299232	0,07458755	0,22045189	protein_codir	hypothetical protein
TcG_08615	744,5169283	-0,104028656	0,07332987	-1,41863965	0,1560041	0,35756675	protein_codir	protein transport protein Sec23-like protein
TcG_08616	157,81399	-0,038371807	0,14284336	-0,26862857	0,78821553	0,89483476	protein_codir	hypothetical protein
TcG_08617	127,9559587	0,272226807	0,15529646	1,75294916	0,07961073	0,23024712	protein_codir	hypothetical protein
TcG_08618	240,3216871	-0,284761064	0,11651725	-2,4439391	0,01452788	0,06622252	protein_codir	hypothetical protein
TcG_08619	125,5525332	0,309203525	0,1574011	1,96443056	0,04948018	0,16435705	protein_codir	hypothetical protein
TcG_08620	20,71083401	0,475521558	0,40146738	1,18445878	0,2362315	0,46360093	protein_codir	hypothetical protein
TcG_08621	225,2932142	0,417746507	0,12240338	3,41286741	0,00064283	0,00534279	protein_codir	hypothetical protein
TcG_08622	176,6730453	0,388383348	0,13563187	2,86351095	0,00418974	0,02504766	protein_codir	hypothetical protein
TcG_08623	303,7914223	0,325414798	0,11534944	2,8211215	0,00478561	0,0278204	protein_codir	hypothetical protein
TcG_08624	433,9558273	0,284261484	0,08711424	3,26308876	0,00110205	0,00844545	protein_codir	hypothetical protein
TcG_08625	6,018054118	0,533514919	0,70465218	0,7571323	0,44897061	1		
TcG_08626	491,4958552	0,118613889	0,08547988	1,38762343	0,16525174	0,3707155	protein_codir	transferase
TcG_08627	34,71640831	0,066330607	0,29295382	0,22642001	0,82087476	0,91123655	protein_codir	hypothetical protein
TcG_08628	151,128788	-0,232718743	0,15102384	-1,54094042	0,12333129	0,3086879	protein_codir	hypothetical protein
TcG_08629	415,2517819	0,141299006	0,09326578	1,51501445	0,12976875	0,31940564	protein_codir	hypothetical protein

TcG_08630	253,6756052	0,03811978	0,12069798	0,31582783	0,7521332	0,87492919	protein_codir	hypothetical protein
TcG_08631	345,715906	0,161963911	0,09805775	1,65171969	0,09859171	0,26608048	protein_codir	hypothetical protein
TcG_08632	798,0343393	0,113016662	0,08185081	1,38076409	0,16735151	0,37337465	protein_codir	hypothetical protein
TcG_08633	51,74309351	0,208967699	0,25360717	0,82398182	0,40994989	0,63689009	protein_codir	hypothetical protein
TcG_08634	187,9838469	0,083067228	0,237947	0,34909971	0,72701446	0,85927072	protein_codir	hypothetical protein
TcG_08635	15,78018786	-0,112182222	0,43193876	-0,25971789	0,79508139	1	protein_codir	hypothetical protein
TcG_08636	260,4123191	-0,384150975	0,12726794	-3,01844253	0,00254078	0,01665013	protein_codir	proton motive ATPase
TcG_08637	654,2100237	-0,164463428	0,07748826	-2,12243016	0,03380163	0,12508007	protein_codir	hypothetical protein
TcG_08638	145,8225033	-0,038333478	0,14764011	-0,25964135	0,79514044	0,89770577	protein_codir	chaperone DnaJ protein
TcG_08639	386,7549341	-0,298920161	0,09634933	-3,10246239	0,00191918	0,01330677	protein_codir	electron transfer protein
TcG_08640	397,7890048	-0,668373114	0,1020417	-6,54999964	5,7537E-11	3,044E-09	protein_codir	hypothetical protein
TcG_08641	194,4994719	-0,266836994	0,13218063	-2,01872998	0,04351529	0,1499162	protein_codir	hypothetical protein
TcG_08642	399,2560948	-0,466976136	0,09547101	-4,89128712	1,0018E-06	2,0333E-05	protein_codir	WDdomain 60
TcG_08643	362,1290029	-0,37653923	0,10000104	-3,76535311	0,00016631	0,00171433	protein_codir	DNA primase small subunit
TcG_08644	203,9424585	-0,560622624	0,12640711	-4,43505619	9,2048E-06	0,00014239	protein_codir	calcium-binding protein CML
TcG_08645	259,4037299	-0,426260994	0,11445192	-3,72436745	0,00019581	0,00195738	protein_codir	putative chaperone DNAJ protein
TcG_08646	69,07668476	-0,745112008	0,21630186	-3,4447786	0,00057153	0,00485463	protein_codir	hypothetical protein
TcG_08647	123,6796001	0,64484167	0,17830828	3,61644272	0,00029868	0,00279749	protein_codir	membrane associated protein
TcG_08648	53,75702041	0,769883145	0,25789292	2,98528216	0,00283317	0,01816552	protein_codir	hypothetical protein
TcG_08649	78,40267445	0,269636193	0,20327975	1,32642921	0,18469757	0,39778319		
TcG_08650	199,6016791	0,11177788	0,1248868	0,89503359	0,37076913	0,60232513	protein_codir	hypothetical protein
TcG_08651	291,378191	0,074989888	0,10691294	0,70141081	0,48304668	0,6965212	protein_codir	hypothetical protein
TcG_08652	112,3036501	0,30636643	0,17299705	1,77093446	0,0765716	0,22408653	protein_codir	monothiol glutaredoxin
TcG_08653	105,284732	0,470187501	0,18492439	2,54259324	0,01100332	0,05320515	protein_codir	glycoside hydrolase family protein
TcG_08654	268,8839881	0,766201655	0,11199571	6,84134807	7,8451E-12	5,0779E-10	protein_codir	UMP-CMP kinase 2, mitochondrial
TcG_08655	40,25221263	0,664091441	0,29033734	2,28730981	0,02217775	0,09108521		
TcG_08656	199,6081774	0,005160313	0,1267627	0,04070845	0,96752832	0,98599553	protein_codir	hypothetical protein
TcG_08657	377,7851955	0,048775324	0,09319032	0,52339477	0,60069956	0,7835886	protein_codir	hypothetical protein
TcG_08658	144,6838907	0,037054892	0,15450329	0,23983238	0,8104602	0,90663241	protein_codir	hypothetical protein
TcG_08659	71,37954127	0,499902999	0,20583486	2,42866056	0,01515471	0,06816091	protein_codir	hypothetical protein
TcG_08660	780,268538	0,084344922	0,06966651	1,21069533	0,22601219	0,45243566	protein_codir	putative MCAK-like kinesin
TcG_08661	371,5232378	0,24701664	0,10167504	2,42947182	0,01512084	0,06816091	protein_codir	hypothetical protein
TcG_08662	365,0485819	-0,030857924	0,0962623	-0,32056084	0,74854322	0,87311203	protein_codir	hypothetical protein
TcG_08663	102,1460261	-0,120249179	0,17233576	-0,69776105	0,48532664	0,69814634	protein_codir	hypothetical protein
TcG_08664	67,8746938	-0,679860085	0,24016252	-2,83083345	0,00464269	0,02713935	protein_codir	trans-sialidase
TcG_08665	51,61033868	0,080638655	0,25427755	0,31712849	0,75114611	0,874683		
TcG_08666	90,37208765	0,199651659	0,19293081	1,03483554	0,30074572	0,53419983	protein_codir	hypothetical protein
TcG_08667	122,1674471	-0,293046046	0,1615759	-1,81367421	0,0697279	0,21142447	protein_codir	hypothetical protein
TcG_08668	368,1086903	-0,00596794	0,09503322	-0,06279846	0,94992699	0,97674192	protein_codir	hypothetical protein
TcG_08669	222,9145579	0,05942053	0,13316749	0,44620899	0,65544629	0,81772823	protein_codir	putative aquaporin-like protein
TcG_08670	343,7784943	0,18398537	0,10104354	1,82085244	0,06862929	0,20919204	protein_codir	putative aquaporin-like protein
TcG_08671	395,0191754	-0,038134377	0,09401048	-0,40563963	0,6850074	0,83655708	protein_codir	hypothetical protein
TcG_08672	199,5606232	0,170763536	0,12869421	1,32689367	0,18454386	0,39756882	protein_codir	hypothetical protein
TcG_08673	396,1135658	-0,216651937	0,09717707	-2,22940945	0,02578667	0,10217661	protein_codir	putative histone deacetylase
TcG_08674	429,2655018	-0,264805895	0,09264962	-2,85814323	0,00426128	0,0253967	protein_codir	hypothetical protein
TcG_08675	160,9480545	0,022961733	0,13930828	0,16482677	0,86908033	0,93679156	protein_codir	hypothetical protein
TcG_08676	205,9365046	0,37423537	0,12642991	2,96002234	0,00307617	0,01944379	protein_codir	hypothetical protein
TcG_08677	359,8404468	-0,196874368	0,09954031	-1,9778356	0,04794726	0,16064056	protein_codir	mitochondrial edited mRNA stability factor 1
TcG_08678	214,6731258	0,191957328	0,12573856	1,52663854	0,12685092	0,31497959	protein_codir	putative poly(A) polymerase
TcG_08679	266,614245	0,143927654	0,11303785	1,27326955	0,20292241	0,42300656	protein_codir	hypothetical protein
TcG_08680	133,4863419	0,313019872	0,15252948	2,05219258	0,04015095	0,14165314	protein_codir	hypothetical protein
TcG_08681	190,668091	-0,212901258	0,13394261	-1,58949615	0,11194842	0,28971061	protein_codir	hypothetical protein

TcG_08682	455,6319503	-0,051749242	0,08630542	-0,59960594	0,54876889	0,74524792	protein_codir	hypothetical protein
TcG_08683	159,2672744	0,004597578	0,1421559	0,0323418	0,97419947	0,98878235	protein_codir	hypothetical protein
TcG_08684	160,9240645	-0,096125363	0,13986426	-0,6872761	0,49190876	0,70263551	protein_codir	monooxygenase
TcG_08685	105,2127074	-0,01467064	0,17617209	-0,08327448	0,93363329	0,96857766	protein_codir	putative vesicle-associated membrane protein 713
TcG_08686	371,4528457	0,066625568	0,09417585	0,70745919	0,47928116	0,69382977	protein_codir	hypothetical protein
TcG_08687	81,45463482	-0,167222919	0,20256555	-0,82552498	0,40907361	0,63660535	protein_codir	hypothetical protein
TcG_08688	114,6245154	-0,113594528	0,16859766	-0,673761	0,50046327	0,70928042	protein_codir	hypothetical protein
TcG_08689	161,072697	-0,063406504	0,13737417	-0,46156059	0,64439646	0,8110828	protein_codir	hypothetical protein
TcG_08690	629,4006232	0,310946843	0,07871106	3,95048479	7,7993E-05	0,00090182	protein_codir	hypothetical protein
TcG_08691	586,2494803	-0,041335864	0,07923848	-0,521664	0,60190429	0,78475506	protein_codir	hypothetical protein
TcG_08692	157,588534	0,133736915	0,14515944	0,92131048	0,35688835	0,5907012	protein_codir	SFT2 domain containing 2
TcG_08693	123,3071785	0,211863289	0,16026611	1,32194692	0,18618583	0,40006473	protein_codir	hypothetical protein
TcG_08694	224,6323769	-0,183160924	0,12233413	-1,49721852	0,13433641	0,32588393	protein_codir	surface protein-2
TcG_08695	156,8360514	-0,413381531	0,14010414	-2,95053049	0,00317229	0,01996422	protein_codir	clathrin assembly sigma-adaptin protein complex 4
TcG_08696	336,84191	-0,094450558	0,1014959	-0,93058492	0,35206832	0,58640936	protein_codir	hypothetical protein
TcG_08697	420,4191137	-0,533929505	0,08822218	-6,05210029	1,4297E-09	5,4849E-08	protein_codir	n-myristoyl transferase
TcG_08698	834,0910093	-0,423380835	0,07154251	-5,91789178	3,2609E-09	1,1626E-07	protein_codir	hypothetical protein
TcG_08699	221,6449574	-0,352606587	0,12094191	-2,91550373	0,00355115	0,02181527	protein_codir	hypothetical protein
TcG_08700	156,2730573	0,019472157	0,14079448	0,138302	0,89000175	0,94653573	protein_codir	hypothetical protein
TcG_08701	284,1502944	-0,300221571	0,10661566	-2,81592377	0,00486372	0,02817553	protein_codir	putative syntaxin 5
TcG_08702	394,1735147	-0,326265451	0,09230556	-3,53462416	0,00040836	0,00363939	protein_codir	hypothetical protein
TcG_08703	503,9458126	-0,241790759	0,09208484	-2,62573894	0,00864611	0,0440906	protein_codir	putative protein transport protein Sec13
TcG_08704	1603,776965	-0,574214999	0,05315074	-10,803518	3,3126E-27	1,3235E-24	protein_codir	lipoprotein, type 6
TcG_08705	959,2092443	-0,459953446	0,06918058	-6,64859156	2,9591E-11	1,6643E-09	protein_codir	pyruvate kinase
TcG_08706	281,653137	-0,214122517	0,11671892	-1,83451428	0,06657769	0,20487891	protein_codir	signal recognition particle subunit SRP19
TcG_08707	99,0931054	0,208422253	0,18888112	1,10345734	0,26982861	0,50269083	protein_codir	putative rab1 small GTP-binding protein
TcG_08708	4,599786664	0,32432668	0,83488476	0,3884688	0,69766914	1	protein_codir	rab1 small GTP-binding protein
TcG_08709	28,14911097	-0,174718463	0,34546491	-0,50574879	0,61303301	0,79081972	protein_codir	target of rapamycin (TOR) kinase 1
TcG_08710	369,5105757	-0,112784097	0,10151951	-1,11095977	0,26658567	0,49937939	protein_codir	BRCT domain-containing protein
TcG_08711	226,7810141	-0,144128233	0,12274854	-1,17417469	0,24032506	0,46899211	protein_codir	hypothetical protein
TcG_08712	182,2787415	-0,044102652	0,13751503	-0,32071149	0,74842904	0,87311203	protein_codir	hypothetical protein
TcG_08713	90,19291841	-0,247557531	0,18642729	-1,32790392	0,18420985	0,39707076	protein_codir	hypothetical protein
TcG_08714	563,7796642	-0,119114265	0,0801268	-1,48657216	0,13712784	0,33016787	protein_codir	hypothetical protein
TcG_08715	59,92906989	0,361588886	0,23867944	1,51495617	0,1297835	0,31940564	protein_codir	ADP-ribosylation factor-like 2
TcG_08716	139,2578865	0,02579124	0,15294042	0,16863586	0,86608307	0,93550699	protein_codir	coatomer zeta subunit
TcG_08717	396,8717063	-0,165017289	0,09090951	-1,81518188	0,06949596	0,21100111	protein_codir	ubiquinol-cytochrome c reductase subunit 7
TcG_08718	84,16402168	0,041861675	0,22216555	0,18842559	0,85054304	0,92805389	protein_codir	hypothetical protein
TcG_08719	120,2274279	-0,028753239	0,16512554	-0,17412957	0,86176364	0,93381907	protein_codir	Trk system potassium uptake protein
TcG_08720	248,3816103	0,186494962	0,11752508	1,58685242	0,11254607	0,29060816	protein_codir	putative prohibitin
TcG_08721	258,4640842	0,040207725	0,1139011	0,3530056	0,72408424	0,85788322	protein_codir	putative trichohyalin-like
TcG_08722	115,3422533	-0,173654949	0,16430461	-1,05690859	0,29055331	0,52345679	protein_codir	hypothetical protein
TcG_08723	237,2219318	0,26982745	0,11900392	2,26738281	0,02336685	0,09495908	protein_codir	putative 2-oxoisovalerate dehydrogenase beta subunit, mitochondrial precursor
TcG_08724	60,15810235	0,420264624	0,22872952	1,83738689	0,0661528	0,20395064	protein_codir	hypothetical protein
TcG_08725	41,5839939	0,15221762	0,28073977	0,54220184	0,58767947	0,77564856	protein_codir	hypothetical protein
TcG_08726	73,52838833	0,207343507	0,20422638	1,01526309	0,30998043	0,54325114	protein_codir	retrotransposon hot spot protein (RHS)
TcG_08727	23,16032039	0,714669608	0,38325116	1,86475523	0,06221572	0,19481928	protein_codir	putative retrotransposon hot spot (RHS) protein
TcG_08728	0					1	protein_codir	retrotransposon hot spot (RHS) protein
TcG_08729	21,76636191	-0,02934181	0,37858492	-0,07750391	0,93822268	0,97142888	protein_codir	retrotransposon hot spot (RHS) protein
TcG_08730	28,85875564	0,092447171	0,3298322	0,28028547	0,7792585	0,89003243	protein_codir	hypothetical protein
TcG_08731	37,00670247	0,353486504	0,28457618	1,24215071	0,21418096	0,43780886	protein_codir	retrotransposon hot spot (RHS) protein
TcG_08732	50,53175797	0,687784369	0,26316591	2,61350102	0,00896198	0,04534215	protein_codir	hypothetical protein
TcG_08733	318,8330665	0,367738696	0,1021663	3,59941305	0,00031894	0,00294908	protein_codir	hypothetical protein

TcG_08734	27,54586434	-0,039250023	0,34120177	-0,11503464	0,90841767	0,95550022		
TcG_08735	157,7048856	0,310706289	0,14349717	2,16524337	0,03036905	0,11540041	protein_codir	putative trans-sialidase
TcG_08736	7,709099069	0,893984906	0,68597323	1,30323584	0,19249426	1	protein_codir	dispersed protein family protein 1
TcG_08737	171,8509432	0,281401296	0,14703545	1,91383299	0,0556415	0,17847796	protein_codir	dispersed gene family protein 1 (DGF-1)
TcG_08738	37,59139444	0,420085542	0,28589724	1,4693585	0,14173558	0,33726605	protein_codir	dispersed gene family protein 1 (DGF-1)
TcG_08739	930,4418577	0,262149142	0,06364013	4,11924253	3,8012E-05	0,0004845	protein_codir	putative 3,2-trans-enoyl-CoA isomerase, mitochondrial precursor
TcG_08740	502,8001968	0,386941121	0,08387244	4,6134476	3,9604E-06	6,8486E-05	protein_codir	putative aminopeptidase, putative,metallo-peptidase, clan MA(E), family M1
TcG_08741	9,551526552	0,056283516	0,57293826	0,09823662	0,92174441	1		
TcG_08742	156,257168	0,457822968	0,1652592	2,77033275	0,00559991	0,03128922	protein_codir	putative p-nitrophenylphosphatase
TcG_08743	62,94697719	0,151184282	0,22004827	0,68705053	0,49205088	0,70263551	protein_codir	hypothetical protein
TcG_08744	666,0777572	0,374536727	0,08166773	4,58610413	4,5159E-06	7,6606E-05	protein_codir	hypothetical protein
TcG_08745	540,4796582	0,438096769	0,08331334	5,25842292	1,453E-07	3,6692E-06	protein_codir	putative pterin-4-alpha-carbinolamine dehydratase
TcG_08746	257,8927276	0,440306633	0,11254903	3,91213166	9,1485E-05	0,00103309	protein_codir	hypothetical protein
TcG_08747	298,4102925	0,524016559	0,1084985	4,82971264	1,3673E-06	2,6759E-05	protein_codir	putative metal-ion transporter
TcG_08748	83,58177758	0,741081251	0,20079484	3,69073858	0,0002236	0,00218622	protein_codir	hypothetical protein
TcG_08749	2008,827279	0,538917277	0,05193538	10,3766891	3,1656E-25	9,1693E-23	protein_codir	hypothetical protein
TcG_08750	27,13154626	0,42170081	0,33667357	1,25255097	0,21036917	0,43299648	protein_codir	hypothetical protein
TcG_08751	336,141158	-0,006429506	0,10239623	-0,06279045	0,94993337	0,97674192	protein_codir	putative dual specificity protein phosphatase
TcG_08752	307,5496249	0,046458099	0,10243178	0,45355163	0,65015158	0,81434121	protein_codir	Thioredoxin domain-containing protein
TcG_08753	158,7969824	-0,062408377	0,14735499	-0,42352402	0,67191298	0,82808039	protein_codir	vitamin-K-epoxide reductase (warfarin-sensitive)
TcG_08754	293,9280483	0,20732049	0,11054062	1,87551407	0,06072204	0,19138343	protein_codir	nuclear lim interactor-interacting factor
TcG_08755	977,7406298	-0,149268798	0,06400565	-2,33211906	0,01969443	0,0830046	protein_codir	WD repeat domain 65
TcG_08756	254,4661226	0,100170713	0,12574095	0,7966435	0,42565811	0,6514762	protein_codir	hypothetical protein
TcG_08757	658,2781098	-0,305909366	0,07791352	-3,92626789	8,6274E-05	0,00098286	protein_codir	hypothetical protein
TcG_08758	420,565338	0,138177471	0,09479192	1,45769254	0,14492531	0,34175483	protein_codir	putative ras-related protein rab-14
TcG_08759	620,6733799	0,059495713	0,07641777	0,77855857	0,43623979	0,65991308	protein_codir	putative protein kinase
TcG_08760	392,4247164	-0,175530539	0,09363777	-1,87456975	0,06085194	0,19150771	protein_codir	hypothetical protein
TcG_08761	444,3247759	0,182040528	0,09331242	1,95087131	0,05107236	0,16807367	protein_codir	trans-sialidase
TcG_08762	15,27111369	0,809381794	0,45627354	1,77389598	0,07608036	1	protein_codir	trans-sialidase
TcG_08763	464,620904	-0,088144093	0,0906793	-0,9720421	0,3310296	0,56526293	protein_codir	trans-sialidase-like protein
TcG_08764	221,761904	0,292590691	0,12943185	2,26057713	0,02378546	0,09628871	protein_codir	hypothetical protein
TcG_08765	288,4798643	0,191368512	0,1093897	1,74941983	0,08021848	0,23148475	protein_codir	retrotransposon hot spot (RHS) protein
TcG_08766	45,17270855	0,178978239	0,26935976	0,66445796	0,50639725	0,71315407	protein_codir	hypothetical protein
TcG_08767	180,9283975	-0,022121073	0,1378552	-0,16046601	0,87251399	0,93879524	protein_codir	dispersed gene family protein 1 (DGF-1)
TcG_08768	70,96272571	0,02113092	0,22168567	0,09531929	0,92406122	0,96287196	protein_codir	dispersed gene family protein 1 (DGF-1)
TcG_08769	185,0718735	0,164476815	0,13408694	1,22664307	0,21995678	0,44507763	protein_codir	dispersed gene family protein 1 (DGF-1)
TcG_08770	42,63554633	0,161190984	0,26473374	0,60887965	0,54260421	0,74045253		
TcG_08771	86,29843487	-0,04941847	0,20249511	-0,24404772	0,80719386	0,90472556	protein_codir	selenocysteine-tRNA-specific elongation factor
TcG_08772	36,26998408	0,521180839	0,29914883	1,7422125	0,08147127	0,23410866	protein_codir	hypothetical protein
TcG_08773	328,5537827	0,164710419	0,1077547	1,52856824	0,12637151	0,31409915	protein_codir	hypothetical protein
TcG_08774	194,4563458	0,11486804	0,13331059	0,86165724	0,38887616	0,61923024	protein_codir	hypothetical protein
TcG_08775	418,5820556	0,006417037	0,0898061	0,07145435	0,94303615	0,97319113	protein_codir	putative ATP-dependent RNA helicase
TcG_08776	364,3598254	-0,069824241	0,09720876	-0,71829162	0,4725775	0,68836848	protein_codir	putative methionyl-tRNA formyltransferase
TcG_08777	596,7112579	0,067640032	0,07891139	0,85716434	0,39135407	0,62112715	protein_codir	putative heat shock protein Hsp20
TcG_08778	551,9548737	-0,200085819	0,07910065	-2,52950906	0,01142222	0,05493477	protein_codir	vesicular transport-associated repeat protein
TcG_08779	264,8432219	-0,220025414	0,10903313	-2,01796843	0,04359455	0,1501446	protein_codir	hypothetical protein
TcG_08780	333,8107126	0,089442415	0,10558239	0,84713387	0,39692051	0,62669278	protein_codir	putative sphingosine-1-phosphate phosphatase
TcG_08781	129,1123881	0,292114975	0,17658799	1,65421771	0,09808329	0,26514068	protein_codir	hypothetical protein
TcG_08782	227,9323783	0,097562295	0,11880378	0,82120532	0,41152933	0,63808037	protein_codir	putative actin 2
TcG_08783	272,3334323	-0,102278405	0,10764504	-0,95014504	0,34203856	0,57651229	protein_codir	hypothetical protein
TcG_08784	540,6805272	0,046380217	0,08074934	0,57437272	0,56571561	0,75808247	protein_codir	putative C-14 sterol reductase
TcG_08785	123,685781	0,038151537	0,16095016	0,23703944	0,81262619	0,90759432	protein_codir	hypothetical protein

TcG_08786	30,42557388	-0,082202997	0,31249109	-0,26305709	0,79250657	0,89624823	protein_codir hypothetical protein
TcG_08787	240,1454202	-0,223923419	0,11442577	-1,95693164	0,05035551	0,16631097	protein_codir GP63 group II protein
TcG_08788	102,6981268	0,161904203	0,18193497	0,88990151	0,37351878	0,60449626	
TcG_08789	46,57918261	-0,466195875	0,25696824	-1,8142159	0,06964449	0,21127643	protein_codir surface protease GP63
TcG_08790	353,1936561	-0,458383887	0,09889481	-4,63506533	3,5682E-06	6,245E-05	protein_codir ubiquinone biosynthesis protein
TcG_08791	182,0152398	-0,525439737	0,13120342	-4,00477169	6,2077E-05	0,00074147	protein_codir hypothetical protein
TcG_08792	581,7458071	-0,455439865	0,07751428	-5,87556079	4,2141E-09	1,4618E-07	protein_codir hypothetical protein
TcG_08793	193,1348491	-0,501784907	0,12916901	-3,88471585	0,00010245	0,00113696	protein_codir putative oxidoreductase
TcG_08794	314,693324	-0,513968054	0,10941674	-4,6973438	2,6357E-06	4,7788E-05	protein_codir hypothetical protein
TcG_08795	82,11085503	-0,309123438	0,19245613	-1,60620211	0,10822951	0,28376264	protein_codir hypothetical protein
TcG_08796	498,1511751	-0,555722371	0,08536316	-6,5100957	7,5103E-11	3,7832E-09	protein_codir Viral A-type inclusion protein
TcG_08797	250,2969221	-0,528928387	0,11432865	-4,62638519	3,721E-06	6,4635E-05	protein_codir hypothetical protein
TcG_08798	186,6516584	0,323498168	0,13700346	2,36124081	0,0182139	0,07804225	protein_codir L1Tc protein
TcG_08799	47,10849976	0,093341044	0,2654808	0,35159245	0,72514393	0,85817259	protein_codir trans-sialidase
TcG_08800	87,33595131	0,286838872	0,18821685	1,52398086	0,12751349	0,31621818	protein_codir trans-sialidase
TcG_08801	44,15086193	0,41898746	0,26374309	1,58861969	0,11214628	0,28989889	protein_codir trans-sialidase
TcG_08802	150,0626623	0,458766556	0,15255988	3,00712462	0,00263732	0,01711818	protein_codir hypothetical protein
TcG_08803	54,05939085	-0,002274392	0,24442171	-0,0093052	0,99257563	0,99705057	protein_codir hypothetical protein
TcG_08804	168,0288229	0,377179616	0,13891797	2,71512482	0,00662508	0,03577318	protein_codir retrotransposon hot spot (RHS) protein
TcG_08805	188,7835612	0,170474325	0,13460151	1,26651124	0,20533011	0,4267849	protein_codir retrotransposon hot spot (RHS) protein
TcG_08806	103,1259217	0,457350715	0,17750302	2,57657991	0,00997831	0,04927909	protein_codir trans-sialidase
TcG_08807	451,3178178	0,266454559	0,08739009	3,0490248	0,00229586	0,01538449	protein_codir trans-sialidase
TcG_08808	49,35845993	0,608051498	0,24932101	2,43882973	0,01473491	0,06699852	protein_codir hypothetical protein
TcG_08809	62,63116248	0,365646549	0,22993246	1,59023458	0,11178194	0,28940906	protein_codir trans-sialidase
TcG_08810	85,31914823	0,235401505	0,19977085	1,17835763	0,23865406	0,46667443	protein_codir putative trans-sialidase
TcG_08811	64,58689253	0,082757964	0,21667458	0,38194588	0,70250151	0,84624626	protein_codir trans-sialidase
TcG_08812	61,28558236	0,44915819	0,22353338	2,00935627	0,04449936	0,15235509	
TcG_08813	68,03518479	0,110012411	0,24182953	0,45491719	0,64916881	0,81355001	protein_codir hypothetical protein
TcG_08814	11,19197879	0,271735902	0,51951741	0,52305446	0,60093635	1	protein_codir hypothetical protein
TcG_08815	99,60130516	0,21632877	0,17849664	1,2119487	0,22553202	0,45184321	protein_codir mucin-like glycoprotein
TcG_08816	29,13242318	-0,491390477	0,32313946	-1,52067618	0,12834112	0,31731972	
TcG_08817	152,0990766	-0,195289457	0,15334071	-1,27356564	0,2028174	0,42300656	protein_codir mucin-associated surface protein (MASP)
TcG_08818	19,90730545	0,514994199	0,40412874	1,27433205	0,20254576	0,42275179	protein_codir surface protease GP63
TcG_08819	12,43064501	0,109209017	0,49105266	0,22239777	0,82400425	1	protein_codir putative dynein heavy chain
TcG_08820	6,872605492	-0,681235916	0,65521249	-1,03971754	0,29847115	1	protein_codir hypothetical protein
TcG_08821	7,514628213	1,059121103	0,65805787	1,60946498	0,10751471	1	protein_codir hypothetical protein
TcG_08822	492,9173509	-0,326369409	0,0906251	-3,60131373	0,00031661	0,00292994	protein_codir hypothetical protein
TcG_08823	908,5539028	-0,109504811	0,06829463	-1,60341748	0,10884251	0,28485416	protein_codir putative glutamine amidotransferase
TcG_08824	5,431477598	-0,925434077	0,74901932	-1,23552765	0,21663419	1	protein_codir hypothetical protein
TcG_08825	692,4184587	0,03265557	0,07536851	0,43327867	0,66481234	0,82476879	protein_codir putative methyltransferase, putative,mRNA cap methyltransferase-like protein
TcG_08826	348,7763068	-0,225058542	0,1009638	-2,22910125	0,02580717	0,10219561	protein_codir heat shock protein DnaJ
TcG_08827	278,2819229	0,317458965	0,10935087	2,90312241	0,00369462	0,02256504	protein_codir putative DNA repair protein
TcG_08828	423,9204224	0,20161604	0,090031	2,2394069	0,02512945	0,1001203	protein_codir transcription factor-like protein
TcG_08829	605,755313	-0,133152798	0,07728201	-1,72294678	0,08489815	0,24020268	protein_codir hypothetical protein
TcG_08830	303,0020575	0,026994165	0,10618037	0,25422933	0,79931839	0,89990311	protein_codir hypothetical protein
TcG_08831	20,60051076	-0,569897196	0,3919609	-1,45396442	0,14595617	0,34342977	
TcG_08832	6,871921718	-0,653986047	0,69102416	-0,94640114	0,34394402	1	
TcG_08833	622,1639396	0,307021679	0,07942223	3,86568954	0,00011078	0,00121769	protein_codir putative retrotransposon hot spot (RHS) protein
TcG_08834	72,74266178	0,117189395	0,21701559	0,54000449	0,58919394	0,77564856	protein_codir hypothetical protein
TcG_08835	1,79734577	0,764304816	1,42666968	0,53572654	0,59214756	1	protein_codir hypothetical protein
TcG_08836	227,9129657	0,108179875	0,1189638	0,90935123	0,36316476	0,59526856	protein_codir surface protease GP63
TcG_08837	365,4842472	0,136612593	0,10374453	1,31681731	0,18789987	0,40225571	protein_codir surface protease GP63

TcG_08838	167,756718	0,206349127	0,13696983	1,50652972	0,13193124	0,32280981	protein_codir SET and MYND domain-containing protein
TcG_08839	305,7718914	0,00562038	0,10510575	0,05347357	0,95735459	0,98019709	protein_codir hypothetical protein
TcG_08840	296,0147853	0,147920969	0,10549936	1,40210303	0,16088448	0,36392183	protein_codir Pin2-interacting protein X1
TcG_08841	416,765844	0,067926598	0,09029796	0,7522495	0,45190103	0,67279945	protein_codir putative kinesin
TcG_08842	478,8623071	-0,163414041	0,08341065	-1,95915078	0,05009513	0,16573449	protein_codir axonemal p66.0
TcG_08843	288,5364973	-0,023329068	0,10717614	-0,21767036	0,82768596	0,91588083	protein_codir putative alpha-ketoglutarate-dependent dioxygenase
TcG_08844	594,0241919	0,064620444	0,0766389	0,84318069	0,3991274	0,62898729	protein_codir putative deoxyribose-phosphate aldolase
TcG_08845	643,3559909	-0,11479478	0,07327597	-1,56660875	0,11720617	0,2985867	protein_codir hypothetical protein
TcG_08846	156,6507464	0,025054204	0,14354832	0,17453499	0,86144504	0,93381907	protein_codir hypothetical protein
TcG_08847	634,2459129	-0,165183855	0,07504772	-2,20105098	0,02773241	0,10791211	protein_codir hypothetical protein
TcG_08848	46,92051965	0,933349817	0,26926053	3,46634468	0,00052759	0,00454808	protein_codir Tbingi protein
TcG_08849	190,8174549	0,672869326	0,13482155	4,9908143	6,0125E-07	1,2853E-05	protein_codir hypothetical protein
TcG_08850	65,41042068	0,313584967	0,23500666	1,33436627	0,18208385	0,39396253	protein_codir hypothetical protein
TcG_08851	78,27880674	0,736370719	0,2305615	3,19381468	0,00140406	0,01028935	protein_codir dispersed gene family protein 1 (DGF-1)
TcG_08852	28,91132761	1,118180448	0,34267818	3,26306288	0,00110215	0,00844545	protein_codir trans-sialidase
TcG_08853	18,99945013	0,587307841	0,40703817	1,44288149	0,14905386	0,3482411	protein_codir hypothetical protein
TcG_08854	138,4744625	0,381752414	0,16043284	2,3795154	0,01733542	0,07533689	protein_codir dispersed gene family protein 1 (DGF-1)
TcG_08855	10,80063377	1,254586872	0,57530359	2,18073883	0,02920274	1	protein_codir hypothetical protein
TcG_08856	0					1	
TcG_08857	0,155988004	0,503022807	4,08047286	0,12327562	0,90188885	1	
TcG_08858	0					1	
TcG_08859	3,274687446	0,585012994	0,99996608	0,58503284	0,55852564	1	
TcG_08860	94,21451483	0,440443686	0,1902308	2,31531217	0,02059585	0,08605249	protein_codir putative retrotransposon hot spot (RHS) protein
TcG_08861	77,03321531	0,737475545	0,20211305	3,64882696	0,00026344	0,00251212	protein_codir hypothetical protein
TcG_08862	151,3297519	0,43241897	0,14813392	2,91910831	0,00351034	0,02167955	
TcG_08863	19,96106081	0,447260765	0,39688141	1,12693805	0,25976867	0,4921081	protein_codir hypothetical protein
TcG_08864	15,8119034	0,448068948	0,4496585	0,99646498	0,31902427	1	protein_codir helicase-like protein
TcG_08865	36,91192493	0,011761052	0,30556374	0,03848968	0,96929726	0,9867567	protein_codir putative trans-sialidase
TcG_08866	35,58504844	0,804173799	0,30609249	2,62722486	0,00860844	0,04395655	protein_codir trans-sialidase
TcG_08867	158,5612274	0,371736842	0,14498849	2,56390582	0,01035016	0,05076926	protein_codir putative target of rapamycin (TOR) kinase 1
TcG_08868	154,5284004	0,363294676	0,14725471	2,46711755	0,01362056	0,0631484	protein_codir target of rapamycin (TOR) kinase 1
TcG_08869	1309,191492	0,062572455	0,05886976	1,06289635	0,28782896	0,52089758	protein_codir kinetoplast-associated protein Tcp16
TcG_08870	286,3542407	0,129377451	0,11188091	1,15638543	0,24752354	0,47677601	protein_codir hypothetical protein
TcG_08871	346,8626754	-0,193412937	0,10090424	-1,916797	0,05526373	0,17761041	protein_codir hypothetical protein
TcG_08872	574,9206835	-0,317661078	0,08552632	-3,71419081	0,00020385	0,00202214	
TcG_08873	2858,009191	-0,190614178	0,04346976	-4,38498318	1,1599E-05	0,00017252	protein_codir hypothetical protein
TcG_08874	502,7874916	-0,229839348	0,08279343	-2,77605783	0,00550224	0,03081149	protein_codir hypothetical protein
TcG_08875	267,9497934	0,045452546	0,12223547	0,37184415	0,71000889	0,8504252	protein_codir putative small GTPase
TcG_08876	935,334954	0,02021104	0,07119786	0,28387146	0,77650889	0,88831084	protein_codir hypothetical protein
TcG_08877	399,3250235	-0,26098317	0,09349846	-2,79130971	0,00524952	0,02975585	protein_codir putative choline/ethanolamine phosphotransferase (CEPT)
TcG_08878	350,4280086	0,070473425	0,09858123	0,7148767	0,47468524	0,69039708	protein_codir hypothetical protein
TcG_08879	624,8970661	-0,464716444	0,07712529	-6,02547423	1,6861E-09	6,3428E-08	protein_codir kinetoplast-associated protein
TcG_08880	34,12179182	0,404062421	0,30288113	1,33406272	0,1821833	0,39402198	
TcG_08881	14,83847406	0,196893942	0,45674253	0,43108301	0,66640802	1	protein_codir hypothetical protein
TcG_08882	2,175611591	0,380381178	1,25076775	0,30411815	0,76103788	1	
TcG_08883	115,2107696	-0,340467524	0,16544721	-2,05786199	0,03960338	0,14023374	protein_codir putative beta galactofuranosyl glycosyltransferase
TcG_08884	14,79198947	0,293686892	0,44791687	0,65567276	0,5120347	1	
TcG_08885	16,87748549	0,197351797	0,42074639	0,46905167	0,6390327	0,80783774	
TcG_08886	75,1465587	-0,09644214	0,20013834	-0,48187738	0,62989305	0,80168887	protein_codir trans-sialidase
TcG_08887	46,89901959	-0,229064309	0,25284908	-0,90593292	0,36497136	0,59700101	protein_codir putative trans-sialidase
TcG_08888	38,83701578	-0,033862772	0,2840803	-0,11920141	0,90511579	0,95390315	protein_codir trans-sialidase
TcG_08889	31,89011376	0,529441708	0,31281467	1,69250921	0,09054893	0,25098083	protein_codir retrotransposon hot spot (RHS) protein

TcG_08890	156,5673099	0,299133767	0,15375486	1,94552393	0,05171195	0,16910378	protein_codir putative retrotransposon hot spot (RHS) protein
TcG_08891	102,1535892	0,135770119	0,1844208	0,73619744	0,46161055	0,6794505	protein_codir retrotransposon hot spot protein (RHS)
TcG_08892	10,11572312	-0,041588633	0,54353682	-0,07651484	0,93900951	1	
TcG_08893	68,34968	0,303682112	0,21343299	1,42284524	0,15478102	0,35609471	protein_codir dispersed gene family protein 1 (DGF-1)
TcG_08894	66,17151848	0,073997546	0,24016734	0,30810828	0,75799994	0,87801518	protein_codir dispersed gene family protein 1 (DGF-1)
TcG_08895	74,86957375	0,337427685	0,20305879	1,66172408	0,09656811	0,26284825	protein_codir dispersed gene family protein 1 (DGF-1)
TcG_08896	29,43344159	0,18992309	0,3290613	0,5771663	0,56382712	0,75680428	protein_codir dispersed gene family protein 1 (DGF-1)
TcG_08897	247,3481037	-0,157241769	0,11749992	-1,33822869	0,1808219	0,39227742	protein_codir putative nuclear lim interactor-interacting factor
TcG_08898	1331,982454	0,046494405	0,06030716	0,77095996	0,44073067	0,66350124	protein_codir kinesin K39
TcG_08899	215,7869807	-0,154913368	0,12167591	-1,27316385	0,20295991	0,42300656	protein_codir putative zinc finger protein
TcG_08900	163,6633282	-0,088256885	0,1386565	-0,63651458	0,52444108	0,72803431	protein_codir putative NADH-ubiquinone oxidoreductase complex I subunit
TcG_08901	263,6052318	-0,030179202	0,10972795	-0,2750366	0,78328812	0,89263342	protein_codir protein G2
TcG_08902	1882,50453	0,187505856	0,04952498	3,78608655	0,00015304	0,00160027	protein_codir 60S ribosomal protein L30
TcG_08903	278,931904	0,104012042	0,10738665	0,96857512	0,33275722	0,56687622	protein_codir hypothetical protein
TcG_08904	154,9209334	-0,036416956	0,14087971	-0,25849681	0,79602351	0,89828854	protein_codir putative GTP-ase activating protein
TcG_08905	132,9898596	0,408017866	0,15664758	2,60468666	0,00919583	0,04624324	protein_codir putative cruzipain precursor
TcG_08906	107,5375496	0,103204532	0,16706165	0,61776315	0,53673148	0,73660472	protein_codir Tbingi protein
TcG_08907	97,54285365	0,277820513	0,18064356	1,53794864	0,12406117	0,31011278	
TcG_08908	80,95598383	-0,089077682	0,20020382	-0,44493498	0,65636675	0,81840993	
TcG_08909	735,201991	-0,063968218	0,07073595	-0,90432404	0,3658236	0,59797295	protein_codir putative ABC transporter
TcG_08910	1100,619909	-0,240581523	0,06050409	-3,9762852	7E-05	0,00082589	protein_codir hypothetical protein
TcG_08911	2613,399429	-0,072797519	0,04507431	-1,6150557	0,10629863	0,28071304	protein_codir hypothetical protein
TcG_08912	2,863320144	1,256494767	1,12781103	1,11410045	0,2652361	1	
TcG_08913	365,364486	0,403414042	0,09585217	4,20871041	2,5683E-05	0,0003448	protein_codir 60S ribosomal protein L44
TcG_08914	227,9088812	-0,043645933	0,12615844	-0,34596126	0,72937183	0,86056342	protein_codir hypothetical protein
TcG_08915	83,46474419	0,148953584	0,18933798	0,78670738	0,43145318	0,65596889	protein_codir hypothetical protein
TcG_08916	198,1236255	-0,159545834	0,12513033	-1,27503728	0,20229605	0,42238277	protein_codir zinc finger family protein
TcG_08917	1050,738429	-0,088441245	0,06388648	-1,38434988	0,16625135	0,37183431	protein_codir coatomer subunit beta'
TcG_08918	459,7039179	-0,618304377	0,08708045	-7,10038082	1,2441E-12	9,2997E-11	protein_codir putative mucin-associated surface protein (MASP)
TcG_08919	0					1	protein_codir putative surface protein TolT
TcG_08920	129,7014978	-0,504026798	0,15767884	-3,1965406	0,00139086	0,010212	
TcG_08921	212,7366873	-0,618411957	0,13670934	-4,52355297	6,081E-06	9,9372E-05	protein_codir putative surface protein TolT
TcG_08922	440,3341366	-0,352658544	0,09095385	-3,87733482	0,00010561	0,00116752	protein_codir putative surface protein TolT
TcG_08923	5,305065414	-0,042738063	0,74132223	-0,05765113	0,95402652	1	
TcG_08924	1239,142673	-0,137743328	0,05676214	-2,42667608	0,01523785	0,0684286	
TcG_08925	2553,355155	-0,102726712	0,04556349	-2,254584	0,02415945	0,09739437	protein_codir putative retrotransposon hot spot (RHS) protein
TcG_08926	45,85697427	0,158253002	0,2575121	0,61454588	0,53885466	0,73820598	protein_codir hypothetical protein
TcG_08927	67,47567937	0,272616925	0,23064152	1,18199413	0,23720803	0,46486675	protein_codir hypothetical protein
TcG_08928	50,63101452	0,263984085	0,26977784	0,97852399	0,32781523	0,56234339	protein_codir hypothetical protein
TcG_08929	71,76668716	-0,012899525	0,20864769	-0,06182443	0,95070265	0,97695803	protein_codir trans-sialidase
TcG_08930	110,0557078	0,085448738	0,17145691	0,49836859	0,61822427	0,793034	protein_codir putative kinesin
TcG_08931	24,12920707	-0,06012712	0,35016463	-0,171711	0,86366473	0,93486334	protein_codir subtilisin-like serine peptidase
TcG_08932	7,571538168	0,14323659	0,64330124	0,22265866	0,82380118	1	protein_codir subtilisin-like serine peptidase
TcG_08933	6,654250566	-0,659698075	0,68500997	-0,96304887	0,335523	1	protein_codir putative kinesin
TcG_08934	0,757668191	0,484897547	3,8652831	0,12544943	0,90016776	1	
TcG_08935	8,889879364	0,510426786	0,58224074	0,87665934	0,3806717	1	protein_codir hypothetical protein
TcG_08936	18,79046427	0,702840808	0,41766539	1,68278443	0,09241683	0,2543933	protein_codir retrotransposon hot spot (RHS) protein
TcG_08937	59,97986671	0,531540046	0,22755234	2,33590238	0,01949633	0,08234943	protein_codir hypothetical protein
TcG_08938	25,94568043	0,181571093	0,37272676	0,48714262	0,62615726	0,79923522	protein_codir target of rapamycin (TOR) kinase 1
TcG_08939	13,68391986	0,221079357	0,48499933	0,45583435	0,6485091	1	protein_codir trans-sialidase
TcG_08940	12,25350898	0,118698452	0,52571009	0,2257869	0,82136717	1	protein_codir helicase-like protein
TcG_08941	181,3965139	0,231126724	0,13207682	1,74994168	0,08012838	0,23133999	protein_codir mucin-associated surface protein (MASP)

TcG_08942	91,57285905	0,246356706	0,19988882	1,23246868	0,21777406	0,44195661	protein_codir	mucin TcMUCII
TcG_08943	36,31852754	0,231112302	0,28781589	0,80298661	0,42198248	0,6481624	protein_codir	hypothetical protein
TcG_08944	11,85313944	0,172064222	0,52514425	0,32765135	0,74317529	1	protein_codir	surface protease GP63
TcG_08945	82,70782624	-0,136940803	0,19483608	-0,70285135	0,48214839	0,6960961	protein_codir	putative trans-sialidase
TcG_08946	36,02253465	-0,104152572	0,28582139	-0,3643974	0,71556127	0,85396046	protein_codir	putative trans-sialidase
TcG_08947	55,13691968	-0,050749625	0,24559175	-0,20664222	0,83628928	0,92052325	protein_codir	putative trans-sialidase
TcG_08948	324,9966001	-0,177324651	0,10204142	-1,73777126	0,08225113	0,23553177	protein_codir	hypothetical protein
TcG_08949	699,6443432	-0,077222683	0,07636959	-1,01117054	0,31193481	0,54585059	protein_codir	putative peptidylprolyl isomerase-like
TcG_08950	594,7200332	0,22820047	0,07996062	2,85391082	0,00431847	0,02564518	protein_codir	putative choline/carnitine O-acetyltransferase
TcG_08951	227,4804025	0,476789719	0,12095226	3,94196613	8,0816E-05	0,00092615	protein_codir	putative protein kinase
TcG_08952	454,3786504	-0,311750277	0,0866473	-3,5979226	0,00032077	0,00296366	protein_codir	hypothetical protein
TcG_08953	917,1264115	-0,101739145	0,06621957	-1,53639089	0,12444254	0,310597	protein_codir	inosine-5'-monophosphate dehydrogenase
TcG_08954	286,4323631	-0,168228324	0,11198683	-1,5022152	0,13304154	0,32423628	protein_codir	ATP-dependent zinc metallopeptidase-like protein
TcG_08955	180,2434305	0,19579687	0,13340543	1,46768292	0,14219037	0,33813991	protein_codir	protein kinase
TcG_08956	190,2310969	0,107192293	0,12859771	0,83354742	0,40453607	0,63405775	protein_codir	potassium voltage-gated channel
TcG_08957	81,75454923	0,078176325	0,19577972	0,39930757	0,68966659	0,8386805	protein_codir	hypothetical protein
TcG_08958	12,05171762	0,549563921	0,50698367	1,08398743	0,27837037	1		
TcG_08959	592,8298472	-0,006829494	0,08434357	-0,08097231	0,93546397	0,96960866	protein_codir	hypothetical protein
TcG_08960	641,7425188	0,155139841	0,07479205	2,0742826	0,03805307	0,13629906	protein_codir	putative pre-mRNA-splicing factor ATP-dependent RNA helicase DEAH3 isoform X1
TcG_08961	220,2936699	0,305914472	0,1329125	2,30162311	0,02135644	0,08855965	protein_codir	putative choline ethanolamine kinase
TcG_08962	206,2917282	-0,112220637	0,12791885	-0,87727994	0,38033461	0,61073866	protein_codir	hypothetical protein
TcG_08963	241,2333208	0,305025313	0,1163546	2,62151478	0,008754	0,04452318	protein_codir	hypothetical protein
TcG_08964	87,00069271	0,247031402	0,20191697	1,22343059	0,22116711	0,4463718	protein_codir	hypothetical protein
TcG_08965	176,3096748	0,32873035	0,13534363	2,42885723	0,0151465	0,06816091	protein_codir	hypothetical protein
TcG_08966	579,7265036	0,26012795	0,08387196	3,10148899	0,0019255	0,01333464	protein_codir	hypothetical protein
TcG_08967	436,3106435	0,350086724	0,08867276	3,94807534	7,8782E-05	0,00090823	protein_codir	60S ribosomal protein L2
TcG_08968	165,4415118	-0,275280681	0,13848341	-1,98782422	0,04683113	0,15809563	protein_codir	putative 2,4-dienoyl-CoA reductase FADH1
TcG_08969	65,12959745	-0,312936379	0,21520048	-1,45416208	0,14590137	0,34340242	protein_codir	2,4-dienoyl-CoA reductase FADH1
TcG_08970	691,9690336	-0,057093268	0,07443849	-0,76698584	0,44308994	0,66506542	protein_codir	putative condensin subunit 1
TcG_08971	250,1824371	-0,185613776	0,11683039	-1,5887457	0,11211782	0,28989889	protein_codir	putative protein kinase
TcG_08972	248,4261269	-0,225297144	0,1134371	-1,9860975	0,0470225	0,15845965	protein_codir	hypothetical protein
TcG_08973	915,7835749	-0,023256525	0,0709469	-0,32780184	0,74306149	0,86930139	protein_codir	putative zinc transporter
TcG_08974	229,1244607	-0,138087681	0,11704909	-1,17974163	0,23810299	0,46594072	protein_codir	presenilin-like aspartic peptidase
TcG_08975	310,6264488	-0,157953427	0,10119362	-1,56090303	0,11854663	0,30067454	protein_codir	putative radical SAM domain protein
TcG_08976	198,2199558	0,0279019	0,12742304	0,2189706	0,82667295	0,91523915	protein_codir	GINS complex subunit 1
TcG_08977	307,9883019	-0,176183029	0,10950149	-1,60895554	0,10762606	0,28260879	protein_codir	hypothetical protein
TcG_08978	472,4122181	-0,399286777	0,08336208	-4,78978905	1,6696E-06	3,1815E-05	protein_codir	hypothetical protein
TcG_08979	37,79415923	-0,476508668	0,2959167	-1,61027974	0,1073368	0,28223527	protein_codir	cAMP specific phosphodiesterase
TcG_08980	6,079441694	1,006721174	0,72829351	1,38230145	0,16687916	1	protein_codir	hypothetical protein
TcG_08981	31,23991535	0,464288377	0,32516693	1,42784623	0,15333612	0,3538244		
TcG_08982	642,9096277	0,803214058	0,0786074	10,2180465	1,6462E-24	4,5413E-22	protein_codir	ribosome biogenesis protein YTM1
TcG_08983	245,4591585	0,363265971	0,11434886	3,17682185	0,00148898	0,01080913	protein_codir	hypothetical protein
TcG_08984	504,9314529	0,500118778	0,08699593	5,74876089	8,99E-09	2,8853E-07	protein_codir	putative nucleolar protein
TcG_08985	163,0183691	0,372618932	0,14174876	2,62872791	0,00857049	0,04380137	protein_codir	hypothetical protein
TcG_08986	436,5319662	0,267922154	0,09695083	2,76348501	0,00571877	0,03176304	protein_codir	hypothetical protein
TcG_08987	385,6505841	0,299982339	0,10341381	2,90079576	0,00372216	0,02266548	protein_codir	hypothetical protein
TcG_08988	1922,887868	0,471471874	0,05158654	9,13943663	6,2779E-20	1,119E-17	protein_codir	DNA-directed RNA polymerase II subunit RPB2
TcG_08989	218,474402	0,365963994	0,13367906	2,73763132	0,00618834	0,03384056	protein_codir	hypothetical protein
TcG_08990	601,8891141	0,233861879	0,08087634	2,89159816	0,00383288	0,02328543	protein_codir	putative ubiquitin hydrolase
TcG_08991	258,2662378	-0,289499926	0,1158622	-2,49865717	0,01246648	0,05880973	protein_codir	putative mucin-associated surface protein (MASP)
TcG_08992	232,7188867	-0,088113316	0,11909097	-0,73988245	0,45937132	0,67791061	protein_codir	trans-sialidase
TcG_08993	79,37934202	-0,034787946	0,19468337	-0,17868987	0,85818123	0,93174207	protein_codir	trans-sialidase

TcG_08994	50,80970084	0,446911982	0,24569816	1,81894719	0,06891949	0,20974552	
TcG_08995	33,21463804	0,395657555	0,3037504	1,30257459	0,19272004	0,40931596	protein_codir hypothetical protein
TcG_08996	68,81584497	0,286153022	0,21204892	1,34946702	0,17718701	0,38726442	protein_codir hypothetical protein
TcG_08997	99,87720413	0,170010978	0,1852565	0,91770587	0,35877287	0,59256732	protein_codir hypothetical protein
TcG_08998	113,918927	0,08133478	0,16504545	0,49280233	0,62215226	0,79640439	protein_codir hypothetical protein
TcG_08999	93,16983356	0,173619628	0,19283566	0,90035022	0,3679339	0,60015236	protein_codir dispersed gene family protein 1 (DGF-1)
TcG_09000	10,21731398	0,262856989	0,59404947	0,44248333	0,65813949	1	protein_codir dispersed gene family protein 1 (DGF-1)
TcG_09001	23,57839989	-0,043561111	0,35302215	-0,12339484	0,90179444	0,95260671	protein_codir dispersed gene family protein 1 (DGF-1)
TcG_09002	5,720386279	0,906852099	0,76626227	1,18347482	0,23662102	1	protein_codir dispersed gene family protein 1 (DGF-1)
TcG_09003	14,17772084	1,090063189	0,50738955	2,14837531	0,03168395	1	protein_codir dispersed gene family protein 1 (DGF-1)
TcG_09004	2,00506609	2,288244956	1,44181423	1,58705949	0,11249917	1	protein_codir hypothetical protein
TcG_09005	2,248872395	0,884725734	1,22598606	0,72164421	0,47051325	1	protein_codir hypothetical protein
TcG_09006	180,4423734	0,121098988	0,13215949	0,91630945	0,35950461	0,5926302	protein_codir hypothetical protein
TcG_09007	69,86047099	-0,153905104	0,20773991	-0,74085477	0,4587815	0,67747164	protein_codir UPF0505 protein C16orf62
TcG_09008	41,88866564	0,07541643	0,26746395	0,28196858	0,7779676	0,88925638	protein_codir UPF0505 protein C16orf62
TcG_09009	250,3469928	-0,031039112	0,11800036	-0,26304253	0,7925178	0,89624823	protein_codir UPF0505 protein C16orf62
TcG_09010	366,6178428	0,03755005	0,09646783	0,38924944	0,69709163	0,84314685	protein_codir 1-acyl-sn-glycerol-3-phosphate acyltransferase
TcG_09011	197,9498082	0,065314615	0,13092667	0,49886408	0,61787514	0,793034	protein_codir hypothetical protein
TcG_09012	315,4910741	-0,350611318	0,10950677	-3,20173178	0,00136604	0,01006164	protein_codir putative heat shock protein-like protein
TcG_09013	700,4684504	-0,174713736	0,07301006	-2,39300905	0,01671083	0,07339335	protein_codir hypothetical protein
TcG_09014	160,4663839	0,063697609	0,14937008	0,42644156	0,66978612	0,82684442	protein_codir hypothetical protein
TcG_09015	149,1754174	-0,281565365	0,15602351	-1,80463427	0,07113193	0,21392986	protein_codir hypothetical protein
TcG_09016	799,2629788	-0,435884688	0,07025169	-6,20461472	5,4831E-10	2,327E-08	protein_codir hypothetical protein
TcG_09017	36,86604356	-0,269988649	0,28828579	-0,93653125	0,34899973	0,58360833	protein_codir dispersed gene family protein 1 (DGF-1)
TcG_09018	94,50464159	-0,241982076	0,18001093	-1,34426325	0,17886328	0,38979933	protein_codir dispersed gene family protein 1 (DGF-1)
TcG_09019	138,1598156	-0,048144821	0,16145887	-0,29818628	0,76556099	0,88203954	protein_codir dispersed gene family protein 1 (DGF-1)
TcG_09020	38,03694851	-0,372513147	0,28072867	-1,32695085	0,18452494	0,39756882	
TcG_09021	145,4823528	-0,143566124	0,1450834	-0,98954203	0,32239802	0,55717533	protein_codir hypothetical protein
TcG_09022	252,9198249	-0,105711595	0,11472841	-0,92140732	0,35683781	0,5907012	protein_codir retrotransposon hot spot protein (RHS)
TcG_09023	71,75384754	-0,065384422	0,21400392	-0,30552909	0,75996322	0,87873591	protein_codir retrotransposon hot spot (RHS) protein
TcG_09024	38,77326961	0,014631328	0,27926174	0,05239288	0,95821565	0,98064539	
TcG_09025	16,97996369	-0,864269879	0,42618189	-2,02793667	0,04256671	0,14761387	protein_codir hypothetical protein
TcG_09026	185,3127296	-0,445847295	0,13519194	-3,29788363	0,00097416	0,00761426	protein_codir putative trans-sialidase
TcG_09027	120,2464483	-0,059613076	0,16216876	-0,36759901	0,71317225	0,85271556	
TcG_09028	77,8547322	0,167672818	0,20075853	0,83519648	0,40360709	0,63312391	protein_codir hypothetical protein
TcG_09029	205,7126636	0,150931074	0,12575922	1,20015911	0,23007755	0,45715632	protein_codir dispersed gene family protein 1 (DGF-1)
TcG_09030	278,2365582	0,180318917	0,12040506	1,49760247	0,13423656	0,32570991	protein_codir dispersed gene family protein 1 (DGF-1)
TcG_09031	3,502164851	0,662918286	1,01069487	0,65590348	0,51188623	1	protein_codir dispersed protein family protein 1
TcG_09032	38,39318305	0,217810471	0,27872083	0,78146465	0,43452926	0,65873534	protein_codir putative retrotransposon hot spot (RHS) protein
TcG_09033	370,7711199	-0,474093712	0,09727037	-4,87397881	1,0937E-06	2,1886E-05	protein_codir putative surface protease GP63
TcG_09034	289,5291698	-0,235441757	0,10694731	-2,20147443	0,02770246	0,10788593	protein_codir mucin-associated surface protein (MASP)
TcG_09035	24,86140973	0,317805299	0,3671314	0,86564457	0,38668511	0,61692835	
TcG_09036	596,7683463	-0,242457031	0,08107581	-2,99049766	0,00278523	0,01788786	protein_codir hypothetical protein
TcG_09037	977,7798555	-0,104598844	0,06602316	-1,58427502	0,11313115	0,29159899	protein_codir cytochrome c oxidase subunit IX
TcG_09038	150,2730934	-0,287244718	0,14767459	-1,94511947	0,0517606	0,1692151	protein_codir putative acetyltransferase
TcG_09039	45,05650037	-0,140425355	0,26286432	-0,53421232	0,59319465	0,7784294	protein_codir putative U1A small nuclear ribonucleoprotein
TcG_09040	1376,482802	-0,082286053	0,05572405	-1,47667035	0,13976402	0,33429108	protein_codir putative eukaryotic translation initiation factor 3 subunit 8
TcG_09041	147,242453	-0,196557194	0,15826334	-1,24196286	0,21425026	0,43787327	protein_codir hypothetical protein
TcG_09042	166,7185544	-0,227586795	0,13931513	-1,63361143	0,10234047	0,27333256	protein_codir hypothetical protein
TcG_09043	266,9089319	-0,129317021	0,11734985	-1,10197858	0,27047098	0,50348278	protein_codir elks delta-like protein
TcG_09044	24,19503149	0,28102328	0,38976075	0,72101483	0,47090039	0,68692532	protein_codir hypothetical protein
TcG_09045	72,25852025	0,360556662	0,21011471	1,71599916	0,0861622	0,24277121	protein_codir helicase-like protein

TcG_09046	20,54590384	0,438538031	0,42382663	1,0347109	0,30080394	0,53419983	protein_codir	trans-sialidase
TcG_09047	32,94247851	-0,123252855	0,32546274	-0,37870035	0,70491039	0,84764835	protein_codir	target of rapamycin (TOR) kinase 1
TcG_09048	22,69556589	-0,251271238	0,36763894	-0,68347286	0,4943081	0,70453824	protein_codir	protein kinase
TcG_09049	13,61347854	0,402308609	0,47317871	0,85022551	0,39519972	1	protein_codir	hypothetical protein
TcG_09050	3,443893954	0,223307397	0,95727722	0,23327349	0,81554904	1	protein_codir	subtilisin-like serine peptidase
TcG_09051	4,290275418	-0,970338611	0,86466926	-1,12220783	0,26177409	1	protein_codir	hypothetical protein
TcG_09052	113,987764	0,027955984	0,1766125	0,15828995	0,87422833	0,93950556	protein_codir	retrotransposon hot spot (RHS) protein
TcG_09053	33,25863441	-0,338450424	0,30565414	-1,10729867	0,26816482	0,50071839	protein_codir	retrotransposon hot spot (RHS) protein
TcG_09054	504,9799223	-0,220588974	0,08158826	-2,70368524	0,00685752	0,03676884	protein_codir	Trypanosoma vivax
TcG_09055	387,6082181	-0,046080204	0,09202495	-0,50073599	0,61655694	0,79265743	protein_codir	hypothetical protein
TcG_09056	306,8773682	-0,07862985	0,10499524	-0,7488897	0,45392369	0,67407843	protein_codir	hypothetical protein
TcG_09057	443,1514734	-0,039588172	0,08748627	-0,45250726	0,65090359	0,81484268	protein_codir	putative protein kinase
TcG_09058	131,7236644	0,053869385	0,15341129	0,35114356	0,72548065	0,85817259	protein_codir	amino acid transporter
TcG_09059	789,7884086	0,027701339	0,07068002	0,39192604	0,69511286	0,84226548	protein_codir	putative GPR1/FUN34/yaaH family protein
TcG_09060	178,8451593	-0,163482974	0,14103706	-1,15914907	0,24639543	0,47507696	protein_codir	GPR1/FUN34/yaaH family protein
TcG_09061	30,0433246	0,183713848	0,3305417	0,55579628	0,57835013	0,76824002	protein_codir	putative glycine dehydrogenase, putative,glycine cleavage system P-protein
TcG_09062	82,28148227	0,11324648	0,19500107	0,580748	0,56141031	0,75484505	protein_codir	hypothetical protein
TcG_09063	3208,894351	0,767329466	0,04389577	17,4807168	2,0096E-68	7,761E-65	protein_codir	glutamamyl carboxypeptidase
TcG_09064	100,8882951	0,180497806	0,17693404	1,02014179	0,30766122	0,54090194	protein_codir	hypothetical protein
TcG_09065	43,03661267	0,138333449	0,26702633	0,51805173	0,60442218	0,7861526		
TcG_09066	103,0336685	0,341728153	0,1746487	1,95666011	0,05038744	0,16632163	protein_codir	hypothetical protein
TcG_09067	280,6787097	0,444573691	0,1122691	3,95989375	7,4983E-05	0,00087312	protein_codir	putative dihydrouridine synthase (Dus)
TcG_09068	568,3299879	0,272465666	0,08427717	3,23297109	0,0012251	0,00919301	protein_codir	hypothetical protein
TcG_09069	39,09710907	0,510500468	0,2987103	1,70901529	0,08744812	0,24520181		
TcG_09070	451,9366267	0,473301052	0,09136672	5,18023496	2,2161E-07	5,3048E-06	protein_codir	hypersensitive-induced response protein 1-like
TcG_09071	5810,138457	0,570329851	0,03654698	15,6053883	6,6901E-55	1,5502E-51	protein_codir	putative amino acid transporter
TcG_09072	241,0052918	-0,128579542	0,11694174	-1,09951798	0,2715422	0,50466601	protein_codir	putative trans-sialidase
TcG_09073	60,14085041	-0,066943548	0,22888204	-0,29248056	0,76991922	0,88436428	protein_codir	retrotransposon hot spot protein (RHS)
TcG_09074	38,0393222	0,305878244	0,2954244	1,03538586	0,30048875	0,53419983	protein_codir	retrotransposon hot spot (RHS) protein
TcG_09075	25,83484007	0,498503101	0,35984551	1,38532532	0,16595301	0,37161415	protein_codir	retrotransposon hot spot (RHS) protein
TcG_09076	60,97110838	-0,020217417	0,22641379	-0,08929411	0,92884818	0,96542881		
TcG_09077	29,35378023	0,037367506	0,32745406	0,11411526	0,90914642	0,95584124	protein_codir	hypothetical protein
TcG_09078	28,78037908	0,171807574	0,33086658	0,51926541	0,60357567	0,78564517		
TcG_09079	48,12211492	0,235431706	0,25902688	0,9089084	0,36339848	0,59543697	protein_codir	dispersed gene family protein 1 (DGF-1)
TcG_09080	22,01238365	0,933478499	0,3966984	2,35311887	0,01861668	0,07932802	protein_codir	dispersed gene family protein 1 (DGF-1)
TcG_09081	14,47045336	0,423601576	0,45730491	0,92630007	0,35429006	1	protein_codir	dispersed gene family protein 1 (DGF-1)
TcG_09082	4,724169384	1,223372846	0,84998582	1,43928618	0,15006946	1	protein_codir	dispersed protein family protein 1
TcG_09083	7,703366996	0,789253116	0,65603577	1,20306416	0,2289515	1	protein_codir	dispersed gene family protein 1 (DGF-1)
TcG_09084	4,196853406	0,477837317	0,83652454	0,57121733	0,56785233	1	protein_codir	dispersed protein family protein 1
TcG_09085	49,09744879	-0,31488245	0,25601816	-1,22992233	0,21872619	0,44350408	protein_codir	trans-sialidase
TcG_09086	32,58766811	-0,089748376	0,32567067	-0,27558016	0,78287055	0,89239848	protein_codir	trans-sialidase
TcG_09087	370,7051524	-0,533177644	0,09923677	-5,37278329	7,753E-08	2,0602E-06	protein_codir	hypothetical protein
TcG_09088	243,3193244	-0,467189793	0,11575848	-4,03590128	5,4393E-05	0,00066547	protein_codir	putative folate/biopterin transporter
TcG_09089	154,9398653	-0,366679019	0,14351458	-2,55499498	0,01061893	0,05180251	protein_codir	putative folate/biopterin transporter
TcG_09090	134,6849381	-0,503069415	0,15476312	-3,25057692	0,00115171	0,00876625	protein_codir	putative thymidylate kinase
TcG_09091	369,890055	-0,297490008	0,09859837	-3,0171898	0,0025513	0,01670021	protein_codir	hypothetical protein
TcG_09092	359,2959274	-0,237490859	0,09447322	-2,51384312	0,01194235	0,05676835	protein_codir	putative myosin heavy chain
TcG_09093	334,457428	-0,423482635	0,09967922	-4,24845475	2,1525E-05	0,00029535	protein_codir	hypothetical protein
TcG_09094	188,3668586	0,050704463	0,13056477	0,38834721	0,69775911	0,843778	protein_codir	amastigote surface protein 4
TcG_09095	31,75071735	-0,124946149	0,30937668	-0,40386415	0,68631262	0,83720952	protein_codir	hypothetical protein
TcG_09096	14,95969379	-0,214451527	0,46779847	-0,45842716	0,64664558	1		
TcG_09097	90,5961013	-0,523992174	0,18871996	-2,77655934	0,00549376	0,03077887	protein_codir	putative mucin-associated surface protein (MASP)

TcG_09098	7,246195079	1,104476051	0,70896255	1,55787644	0,11926254	1	protein_codir hypothetical protein
TcG_09099	11,67930426	-0,173774045	0,50720757	-0,34260933	0,73189238	1	protein_codir hypothetical protein
TcG_09100	3,569033754	-0,204199865	1,00183777	-0,20382528	0,83849004	1	protein_codir hypothetical protein
TcG_09101	7,965051821	-0,293546299	0,63697683	-0,46084298	0,64491127	1	protein_codir hypothetical protein
TcG_09102	5,28279549	-0,030592714	0,78973914	-0,03873775	0,96909948	1	protein_codir hypothetical protein
TcG_09103	4,949237922	0,521363869	0,77492463	0,672793	0,50107899	1	protein_codir hypothetical protein
TcG_09104	7,14463492	-0,303608472	0,65565028	-0,46306466	0,64331802	1	protein_codir hypothetical protein
TcG_09105	13,80541203	0,305561848	0,48516096	0,62981541	0,52881536	1	protein_codir hypothetical protein
TcG_09106	8,674286678	-0,180178409	0,59976555	-0,30041474	0,76386083	1	protein_codir putative syntaxin binding protein
TcG_09107	9,546833321	0,206195591	0,56386226	0,36568433	0,71460064	1	protein_codir hypothetical protein
TcG_09108	14,09312714	0,416161216	0,46362777	0,89761926	0,36938857	1	protein_codir peptide hydrolase
TcG_09109	3,334341267	0,834952321	1,03236507	0,80877622	0,41864388	1	protein_codir putative surface protease GP63
TcG_09110	3,179820978	0,080200147	0,97264505	0,08245572	0,93428433	1	protein_codir hypothetical protein
TcG_09111	46,17704862	-0,400786509	0,25686468	-1,56030214	0,1186885	0,30069066	protein_codir putative mucin-associated surface protein (MASP)
TcG_09112	11,49634036	-0,192343212	0,51167232	-0,37591092	0,70698312	1	
TcG_09113	70,47504076	-0,360227573	0,22537679	-1,5983348	0,10996849	0,28646723	protein_codir putative trans-sialidase
TcG_09114	6,60678937	-0,768947294	0,69070762	-1,11327467	0,26559048	1	protein_codir hypothetical protein
TcG_09115	7,430966975	-0,144599301	0,6244894	-0,23154805	0,81688906	1	protein_codir hypothetical protein
TcG_09116	15,50613268	0,246426836	0,44479048	0,55402902	0,57955898	1	protein_codir hypothetical protein
TcG_09117	126,3200556	-0,159373437	0,16101956	-0,98977688	0,32228319	0,55705998	protein_codir mucin-associated surface protein (MASP)
TcG_09118	0,155721614	0,503022807	4,08047286	0,12327562	0,90188885	1	protein_codir putative mucin-associated surface protein (MASP)
TcG_09119	27,93439012	-0,104190841	0,33732053	-0,30887785	0,75741444	0,87801518	protein_codir mucin TcMUCII
TcG_09120	29,28791034	-0,042696719	0,32076958	-0,13310713	0,89410866	0,94881323	protein_codir hypothetical protein
TcG_09121	407,2418653	-0,049018255	0,0924362	-0,53029284	0,59590891	0,78066886	
TcG_09122	69,35851137	0,336064058	0,21205268	1,58481403	0,11300859	0,29141277	protein_codir putative trans-sialidase
TcG_09123	17,76287426	-0,747416799	0,40912648	-1,82685998	0,06772083	0,2072955	protein_codir hypothetical protein
TcG_09124	10,84090036	0,123219711	0,52273314	0,23572202	0,81364838	1	protein_codir hypothetical protein
TcG_09125	16,57440532	0,024371757	0,41809523	0,05829236	0,95351575	1	protein_codir hypothetical protein
TcG_09126	8,491189384	-0,364970156	0,58930329	-0,61932483	0,53570239	1	protein_codir hypothetical protein
TcG_09127	54,13115667	0,030187595	0,24101415	0,12525238	0,90032375	0,95157371	protein_codir putative trans-sialidase
TcG_09128	12,53309553	0,200124354	0,49391737	0,4051778	0,68534682	1	protein_codir hypothetical protein
TcG_09129	45,2013663	-0,359626382	0,2664311	-1,34979132	0,17708293	0,38718302	protein_codir putative Unc104-like kinesin
TcG_09130	1211,547503	-0,478084377	0,05755437	-8,30665664	9,8436E-17	1,2397E-14	protein_codir putative Unc104-like kinesin
TcG_09131	551,8141354	-0,276623071	0,08258908	-3,34939023	0,0008099	0,00653444	protein_codir RNA-binding protein
TcG_09132	391,6338629	-0,557290362	0,09623478	-5,79094535	6,9991E-09	2,2843E-07	protein_codir cAMP-specific phosphodiesterase
TcG_09133	22,25569319	0,411910841	0,38267495	1,07639877	0,28174895	0,51481079	protein_codir hypothetical protein
TcG_09134	12,75328629	0,326992293	0,49430859	0,66151448	0,50828243	1	protein_codir trans-sialidase
TcG_09135	15,5393586	0,30730169	0,47097178	0,65248429	0,5140888	1	protein_codir hypothetical protein
TcG_09136	0,545831481	2,353994432	2,35718398	0,99864688	0,31796578	1	protein_codir retrotransposon hot spot (RHS) protein
TcG_09137	511,854134	0,201577382	0,08334669	2,41854096	0,01558289	0,06964219	protein_codir putative retrotransposon hot spot (RHS) protein
TcG_09138	98,0643238	-0,041754422	0,17488072	-0,23875943	0,81129213	0,90695008	protein_codir retrotransposon hot spot (RHS) protein
TcG_09139	67,72484443	-0,143849136	0,21896135	-0,65696132	0,51120579	0,71768359	protein_codir putative retrotransposon hot spot (RHS) protein
TcG_09140	33,49781214	-0,200994689	0,31131911	-0,64562271	0,51852373	0,72346049	protein_codir hypothetical protein
TcG_09141	103,9308535	0,269966899	0,18042801	1,49625825	0,13458637	0,32614886	protein_codir trans-sialidase
TcG_09142	128,1847278	-0,144287694	0,15813034	-0,91246055	0,36152633	0,59419539	protein_codir sialidase
TcG_09143	75,27883342	-0,404535015	0,2010584	-2,01202744	0,04421704	0,15183719	protein_codir trans-sialidase
TcG_09144	68,94486895	0,013159219	0,21267733	0,0618741	0,95066309	0,97695803	protein_codir retrotransposon hot spot (RHS) protein
TcG_09145	179,9858387	-0,181242882	0,13430233	-1,34951405	0,17717191	0,38726442	protein_codir putative retrotransposon hot spot (RHS) protein
TcG_09146	71,23882195	-0,323014523	0,21992375	-1,46875691	0,14189874	0,33758496	protein_codir putative retrotransposon hot spot (RHS) protein
TcG_09147	214,8810196	0,028751232	0,12351921	0,23276729	0,81594211	0,90921145	protein_codir hypothetical protein
TcG_09148	461,555938	-0,55489148	0,08500141	-6,52802645	6,6642E-11	3,4469E-09	protein_codir hypothetical protein
TcG_09149	2247,071064	-0,157040761	0,06165155	-2,5472314	0,01085814	0,05266933	protein_codir hypothetical protein

TcG_09150	387,8022112	0,041667519	0,09178608	0,45396339	0,64985517	0,81414598	protein_codir	putative calpain-like cysteine peptidase
TcG_09151	640,3150961	-0,233811413	0,07711109	-3,03213732	0,00242829	0,01606747	protein_codir	putative adenylosuccinate lyase
TcG_09152	62,47811769	0,533664087	0,23157229	2,30452483	0,0211932	0,08804031	protein_codir	hypothetical protein
TcG_09153	400,0647168	0,502981861	0,09265788	5,42837662	5,6869E-08	1,5467E-06	protein_codir	citrate transporter
TcG_09154	227,8295264	0,501533674	0,13291362	3,77338063	0,00016105	0,00167198	protein_codir	putative citrate transporter
TcG_09155	139,1852837	0,320468758	0,15143466	2,11621803	0,03432627	0,12665737	protein_codir	hypothetical protein
TcG_09156	73,50613771	0,596583615	0,2098619	2,84274383	0,0044727	0,02639873	protein_codir	hypothetical protein
TcG_09157	310,9249619	0,630622257	0,10838103	5,81856689	5,9354E-09	1,9818E-07	protein_codir	hypothetical protein
TcG_09158	24,01964135	-0,049746848	0,37811663	-0,13156482	0,89532851	0,9489813		
TcG_09159	117,050893	0,677750528	0,16613871	4,0794258	4,5147E-05	0,00056671		
TcG_09160	114,7023241	0,802827171	0,16890506	4,75312671	2,0029E-06	3,741E-05	protein_codir	CrcB-like protein
TcG_09161	520,0892444	0,462300527	0,08969548	5,15411203	2,5484E-07	5,9647E-06	protein_codir	hypothetical protein
TcG_09162	400,3554678	0,295536773	0,09612022	3,07465765	0,00210744	0,0143713	protein_codir	putative serine/threonine protein phosphatase catalytic subunit
TcG_09163	246,3605858	0,4950718	0,12059007	4,10541115	4,036E-05	0,00050993	protein_codir	putative dihydrolipoamide dehydrogenase
TcG_09164	231,1341432	0,297639706	0,11770632	2,52866372	0,01144977	0,05500699	protein_codir	putative dihydrolipoamide dehydrogenase, putative,acetoin dehydrogenase e3 component
TcG_09165	174,7182303	-0,027390408	0,13763239	-0,19901135	0,84225387	0,9231054	protein_codir	hypothetical protein
TcG_09166	356,3168409	-0,278083934	0,10491014	-2,65068701	0,00803282	0,04162268	protein_codir	putative trans-sialidase
TcG_09167	30,48946217	-0,280619888	0,31509131	-0,89059863	0,37314453	0,60423908		
TcG_09168	190,9644336	-0,19878585	0,12737153	-1,56067722	0,11859993	0,30069066	protein_codir	hypothetical protein
TcG_09169	884,4629508	-0,05901304	0,06722159	-0,87788824	0,38000437	0,6107213	protein_codir	Ribonucleoside-diphosphate reductase large chain 1
TcG_09170	172,1123375	-0,146592523	0,14711893	-0,99642189	0,3190452	0,55410215	protein_codir	hypothetical protein
TcG_09171	533,8218773	0,297336866	0,08320208	3,57367101	0,00035201	0,00320798	protein_codir	putative ubiquitin-protein ligase-like
TcG_09172	136,3731923	0,310567637	0,15574803	1,99403893	0,0461478	0,15624442	protein_codir	hypothetical protein
TcG_09173	321,0628183	-0,207526656	0,09950555	-2,08557877	0,0370168	0,13368971	protein_codir	minichromosome maintenance protein 2
TcG_09174	63,37019784	0,086442583	0,21952755	0,39376645	0,69375347	0,84130498	protein_codir	minichromosome maintenance (MCM) complex subunit
TcG_09175	163,8507149	0,37540513	0,14157062	2,65171636	0,00800838	0,04155176	protein_codir	hypothetical protein
TcG_09176	76,589431	-0,023102772	0,20244494	-0,11411879	0,90914363	0,95584124	protein_codir	hypothetical protein
TcG_09177	324,6643192	-0,037157573	0,10293303	-0,36098785	0,71810853	0,85508792	protein_codir	hypothetical protein
TcG_09178	211,1190114	0,139612352	0,12161442	1,14799178	0,25097196	0,4807806	protein_codir	hypothetical protein
TcG_09179	336,8603127	-0,103664918	0,09953319	-1,04151103	0,29763844	0,53101924	protein_codir	hypothetical protein
TcG_09180	432,1776519	0,110526755	0,0895197	1,23466407	0,21695555	0,44089387	protein_codir	hypothetical protein
TcG_09181	319,4373918	-0,017983883	0,10171048	-0,17681445	0,85965414	0,93257986	protein_codir	hypothetical protein
TcG_09182	340,7341396	0,121898131	0,10199995	1,19508034	0,23205564	0,45922596	protein_codir	hypothetical protein
TcG_09183	2763,90537	0,221114719	0,04990638	4,43059028	9,3975E-06	0,00014459	protein_codir	60S ribosomal protein L6
TcG_09184	481,7259228	0,028559	0,08781917	0,32520235	0,74502793	0,87111652	protein_codir	proton-dependent oligopeptide transporter, POT family
TcG_09185	299,6008932	-0,130612866	0,10578701	-1,23467774	0,21695046	0,44089387	protein_codir	tetratricopeptidedomain 4
TcG_09186	13,06371519	-0,121140522	0,47922494	-0,25278426	0,80043493	1	protein_codir	amino acid transporter
TcG_09187	284,607523	0,305820239	0,11664395	2,62182685	0,00874599	0,04450197	protein_codir	hypothetical protein
TcG_09188	298,1348518	0,261617936	0,11551463	2,26480355	0,02352474	0,09553369	protein_codir	adenylate cyclase
TcG_09189	1204,273126	0,104146694	0,05735124	1,81594478	0,06937884	0,21075597	protein_codir	esag4
TcG_09190	287,1071013	0,136331976	0,11690892	1,16613839	0,2435585	0,47180551	protein_codir	hypothetical protein
TcG_09191	201,6900961	0,030270634	0,1301328	0,23261341	0,81606161	0,90921145	protein_codir	hypothetical protein
TcG_09192	664,1857296	0,022438685	0,07235979	0,31009881	0,75648582	0,87742964		
TcG_09193	681,4548088	-0,261338815	0,07324689	-3,5679167	0,00035983	0,00326724	protein_codir	co-chaperone protein
TcG_09194	621,70025	-0,325539594	0,0766283	-4,2482948	2,154E-05	0,00029535	protein_codir	small G-protein
TcG_09195	1044,794768	-0,072789357	0,06120734	-1,18922604	0,23435073	0,46208093	protein_codir	putative small G-protein
TcG_09196	1337,531955	-0,198752875	0,05517863	-3,60198975	0,00031579	0,0029296	protein_codir	putative flagellum transition zone component
TcG_09197	181,2888613	0,057534921	0,13114553	0,4387105	0,66087132	0,82224396	protein_codir	mucin-associated surface protein (MASP)
TcG_09198	46,95055766	0,309750547	0,26133465	1,18526399	0,23591308	0,46344584	protein_codir	hypothetical protein
TcG_09199	17,20721377	-0,086079801	0,42045623	-0,20472951	0,83778346	0,92127555	protein_codir	trans-sialidase
TcG_09200	7,909382939	-0,019103248	0,61601493	-0,03101102	0,97526076	1	protein_codir	hypothetical protein
TcG_09201	3,862398681	2,386004076	1,09766175	2,17371524	0,02972653	1	protein_codir	putative mucin-like glycoprotein

TcG_09202	4,78056709	-0,878999129	0,84582287	-1,03922365	0,29870073	1	
TcG_09203	33,49006203	-0,250216983	0,31468437	-0,79513635	0,42653419	0,65178385	protein_codir trans-sialidase
TcG_09204	15,91546502	-0,133522415	0,45257797	-0,29502632	0,76797377	1	protein_codir putative trans-sialidase
TcG_09205	6,276141594	1,287502451	0,75679782	1,7012502	0,08889601	1	
TcG_09206	163,3319105	-0,22520414	0,13977022	-1,61124546	0,10712623	0,28208285	protein_codir mucin-associated surface protein (MASP)
TcG_09207	7,605408369	-0,209156296	0,64185444	-0,3258625	0,74452839	1	protein_codir putative mucin TcMUCII
TcG_09208	0					1	protein_codir hypothetical protein
TcG_09209	0,548227089	-0,494168331	2,31937726	-0,21306078	0,83127955	1	protein_codir hypothetical protein
TcG_09210	0,155721614	0,503022807	4,08047286	0,12327562	0,90188885	1	protein_codir hypothetical protein
TcG_09211	2,999236596	-0,40995836	1,04615044	-0,39187324	0,69515188	1	protein_codir hypothetical protein
TcG_09212	1,307866945	-2,837383317	1,8208754	-1,55825232	0,11917345	1	protein_codir hypothetical protein
TcG_09213	24,61073311	0,374665329	0,36135472	1,03683529	0,29981263	0,53358358	protein_codir hypothetical protein
TcG_09214	1,484095545	-0,606595853	1,46760796	-0,41332282	0,67937012	1	protein_codir hypothetical protein
TcG_09215	79,35569496	0,171646894	0,19924524	0,86148555	0,38897068	0,61928495	protein_codir trans-sialidase
TcG_09216	11,93723914	0,058204336	0,51529408	0,11295363	0,91006732	1	protein_codir target of rapamycin (TOR) kinase 1
TcG_09217	85,47655849	0,251142299	0,1965093	1,2780174	0,20124329	0,4207486	protein_codir mucin-associated surface protein (MASP)
TcG_09218	58,19273215	0,328871301	0,23181337	1,41868997	0,15598942	0,35756675	protein_codir protein kinase, putative,serine/threonine protein kinase
TcG_09219	184,6041662	0,444201792	0,14776884	3,00605865	0,00264658	0,01716868	protein_codir hypothetical protein
TcG_09220	118,2394403	0,164679267	0,16598457	0,99213598	0,3211312	0,5560344	protein_codir hypothetical protein
TcG_09221	128,8696925	0,222368506	0,15779176	1,40925296	0,15876038	0,36109105	protein_codir hypothetical protein
TcG_09222	99,58713406	0,312480252	0,17914772	1,74426032	0,08111371	0,23325476	protein_codir hypothetical protein
TcG_09223	41,55723668	0,401873751	0,27223049	1,47622609	0,13988321	0,33450709	protein_codir hypothetical protein
TcG_09224	57,69364772	0,189005234	0,23668986	0,79853541	0,42455985	0,65015164	protein_codir hypothetical protein
TcG_09225	57,47265419	0,298517665	0,23729243	1,25801599	0,20838598	0,43098178	protein_codir hypothetical protein
TcG_09226	38,40803301	0,154405067	0,27748097	0,55645282	0,57790134	0,76800198	
TcG_09227	52,45362243	0,115940495	0,25808787	0,44922877	0,65326663	0,81656567	protein_codir trans-sialidase
TcG_09228	66,04939246	0,292660514	0,22518499	1,29964483	0,19372273	0,41080251	protein_codir putative trans-sialidase
TcG_09229	22,34011891	0,276654026	0,37464346	0,73844616	0,46024337	0,67839985	protein_codir putative mismatch repair protein MSH4
TcG_09230	197,5188943	-0,309428119	0,13676239	-2,26252343	0,02366508	0,09600938	protein_codir hypothetical protein
TcG_09231	246,384767	-0,18499208	0,11948438	-1,54825327	0,12156133	0,30590998	protein_codir hypothetical protein
TcG_09232	4,933384418	0,47868015	0,82959918	0,57700171	0,5639383	1	protein_codir elongation factor 1-gamma (EF-1-gamma)
TcG_09233	2576,877828	0,07869749	0,05366491	1,46646095	0,14252275	0,33858286	protein_codir putative elongation factor 1-gamma (EF-1-gamma)
TcG_09234	182,0573801	0,076014075	0,1306416	0,58185198	0,56066639	0,75442638	protein_codir hypothetical protein
TcG_09235	211,8994095	0,012923573	0,12661643	0,10206869	0,91870215	0,96094146	protein_codir SLA/LP autoantigen-like protein
TcG_09236	504,1508432	-0,102370354	0,0865672	-1,1825536	0,23698611	0,46458901	protein_codir hypothetical protein
TcG_09237	41,90146496	-0,881485828	0,28320869	-3,11249572	0,00185513	0,0129557	protein_codir calmodulin
TcG_09238	317,6748885	-0,351042869	0,11018097	-3,18605717	0,00144226	0,01051607	protein_codir calmodulin
TcG_09239	28,78376868	-0,717534112	0,32456029	-2,21078833	0,0270505	0,10588077	
TcG_09240	158,1028805	-0,595937841	0,14150318	-4,21148029	2,537E-05	0,00034179	protein_codir calmodulin
TcG_09241	11,92179449	-0,661167833	0,50991362	-1,29662712	0,19475952	1	protein_codir hypothetical protein
TcG_09242	10,55495849	0,73117019	0,56904138	1,28491567	0,19882175	1	protein_codir trans-sialidase
TcG_09243	33,92990976	0,142670754	0,30191909	0,47254632	0,63653689	0,80653066	protein_codir trans-sialidase
TcG_09244	38,716297	-0,620771104	0,28283241	-2,19483723	0,02817528	0,10910386	protein_codir trans-sialidase
TcG_09245	1,503743219	-1,142985752	1,53110717	-0,74650931	0,45535981	1	protein_codir hypothetical protein
TcG_09246	1,606306956	-0,384103396	1,33355373	-0,28802994	0,77332382	1	protein_codir hypothetical protein
TcG_09247	4,199975293	-0,75942801	0,83579159	-0,90863322	0,36354376	1	protein_codir hypothetical protein
TcG_09248	6,661709287	0,764186967	0,70092637	1,09025285	0,27560178	1	protein_codir hypothetical protein
TcG_09249	25,33386555	0,024797258	0,35543685	0,06976558	0,94438024	0,97397094	protein_codir hypothetical protein
TcG_09250	10,26017344	-0,115174382	0,55163253	-0,20878823	0,83461356	1	protein_codir retrotransposon hot spot (RHS) protein
TcG_09251	21,83678743	-0,185951331	0,36832151	-0,50486145	0,61365615	0,79121079	protein_codir target of rapamycin (TOR) kinase 1
TcG_09252	18,67937595	-0,145591688	0,40903263	-0,3559415	0,72188438	0,85711748	protein_codir target of rapamycin (TOR) kinase 1
TcG_09253	12,19841905	0,774481884	0,51523818	1,50315312	0,13279956	1	protein_codir trans-sialidase

TcG_09254	5,817115925	0,100311161	0,75349435	0,13312795	0,89409219	1	protein_codir	SH3 domain protein
TcG_09255	29,49504642	0,175394683	0,32472406	0,54013454	0,58910425	0,77564856	protein_codir	hypothetical protein
TcG_09256	19,51644327	-0,019727735	0,39037307	-0,05053559	0,95969559	0,98146642	protein_codir	dispersed gene family protein 1 (DGF-1)
TcG_09257	1,353043202	2,546690421	1,69130819	1,50575184	0,13213089	1	protein_codir	dispersed protein family protein 1 (DGF-1)
TcG_09258	33,77412806	-0,070434374	0,3054705	-0,23057668	0,81764369	0,91018637	protein_codir	L1Tc protein
TcG_09259	36,5465315	0,044456884	0,2876929	0,15452896	0,87719268	0,94102958		
TcG_09260	204,4844471	0,109969303	0,13213897	0,83222459	0,40528219	0,63430422		
TcG_09261	385,1243379	0,215830728	0,09962085	2,16652164	0,03027135	0,11518024		
TcG_09262	1,998546041	-0,639690917	1,23572235	-0,51766557	0,60469162	1		
TcG_09263	618,2772287	0,39987637	0,08355523	4,78577288	1,7033E-06	3,2405E-05		
TcG_09264	110,6739754	-0,227425524	0,16826195	-1,35161588	0,17649823	0,38656115		
TcG_09265	137,6127174	-0,035229674	0,15219378	-0,23147907	0,81694264	0,90958077		
TcG_09266	727,8577501	-0,004155624	0,08785087	-0,04730317	0,96227161	0,98256823		
TcG_09267	28,533577	-0,011479731	0,32415142	-0,03541472	0,97174905	0,98777616	protein_codir	hypothetical protein
TcG_09268	207,5551362	-0,115857531	0,12438663	-0,93143078	0,35163078	0,58601758	protein_codir	cyclophilin
TcG_09269	429,5842768	-0,077224112	0,08923419	-0,86540943	0,38681411	0,61704919	protein_codir	ribosome biogenesis protein
TcG_09270	64,88766516	0,050238315	0,22944633	0,21895454	0,82668546	0,91523915	protein_codir	hypothetical protein
TcG_09271	220,0409562	0,019719128	0,12074302	0,16331485	0,87027055	0,93739833	protein_codir	hypothetical protein
TcG_09272	209,9545532	0,057784648	0,12914473	0,447441	0,65455668	0,81729644	protein_codir	putative 40S ribosomal protein S33
TcG_09273	446,2495949	0,477931549	0,09398542	5,08516719	3,673E-07	8,3117E-06	protein_codir	40S ribosomal protein S33
TcG_09274	531,1312428	0,25886012	0,0822759	3,14624463	0,00165382	0,01179872	protein_codir	40S ribosomal protein S33
TcG_09275	154,4558578	-0,073464346	0,14287026	-0,51420322	0,60710991	0,78777005	protein_codir	putative CDP-diacylglycerol synthetase
TcG_09276	248,7351458	0,092113882	0,11529291	0,79895533	0,42431632	0,65009738	protein_codir	proline oxidase, mitochondrial precursor-like protein
TcG_09277	139,1090108	0,029128706	0,14923513	0,19518665	0,84524683	0,92503539	protein_codir	hypothetical protein
TcG_09278	814,0046592	-0,16211963	0,06726293	-2,41023727	0,01594215	0,07084992	protein_codir	metallo-peptidase, Clan MA(E), Family M3
TcG_09279	41,9176033	0,406044352	0,2751782	1,47556877	0,14005969	0,3345817	protein_codir	putative RNA-binding protein 5
TcG_09280	706,7202647	0,018620094	0,07157116	0,26016196	0,79473884	0,89753819	protein_codir	putative RNA helicase
TcG_09281	251,2582138	-0,122751011	0,11533877	-1,06426498	0,28720868	0,52042536	protein_codir	RNA-binding protein
TcG_09282	191,13388	-0,097638129	0,13377252	-0,72988181	0,46546243	0,6821385	protein_codir	putative endonuclease III
TcG_09283	117,4576424	0,208753988	0,16906189	1,23477847	0,21691296	0,44089387	protein_codir	N-Acetyl-D-glucosaminylphosphatidylinositol de-N-acetylase
TcG_09284	191,7631008	-0,142459741	0,12795173	-1,1133866	0,26554243	0,49806938	protein_codir	hypothetical protein
TcG_09285	96,1370666	0,00701358	0,18281737	0,03836386	0,96939757	0,98677212	protein_codir	hypothetical protein
TcG_09286	250,4985054	-0,067823238	0,11368793	-0,5965738	0,55079197	0,74654606	protein_codir	hypothetical protein
TcG_09287	999,3910444	0,065237621	0,06827338	0,95553519	0,33930712	0,57339735	protein_codir	hypothetical protein
TcG_09288	154,3964751	-0,364946597	0,1420401	-2,56932086	0,01018981	0,05011982	protein_codir	ribosome biogenesis protein NSA1
TcG_09289	162,7729622	-0,213200884	0,13999532	-1,52291433	0,12778014	0,31654541	protein_codir	hypothetical protein
TcG_09290	62,60952307	0,131933611	0,22665685	0,58208525	0,56050926	0,7544215	protein_codir	L1Tc protein
TcG_09291	18,47352222	0,304919944	0,4144236	0,73576877	0,46187143	0,67953581	protein_codir	hypothetical protein
TcG_09292	5,617063692	0,072552009	0,75435344	0,09617774	0,92337941	1	protein_codir	hypothetical protein
TcG_09293	83,86433267	-0,166262906	0,19358413	-0,85886642	0,39041422	0,62040037	protein_codir	putative mucin-associated surface protein (MASP)
TcG_09294	12,10778039	-1,075584148	0,50623851	-2,12465887	0,03361509	1	protein_codir	hypothetical protein
TcG_09295	4,690990349	0,330144978	0,90346324	0,36542159	0,71479672	1	protein_codir	hypothetical protein
TcG_09296	7,287174069	-0,293941047	0,66696925	-0,44071155	0,65942184	1	protein_codir	hypothetical protein
TcG_09297	7,021697229	0,124408762	0,65976179	0,18856618	0,85043284	1	protein_codir	hypothetical protein
TcG_09298	23,96341057	0,086775312	0,35832954	0,24216622	0,80865136	0,90526589		
TcG_09299	23,96655022	-0,28647688	0,35860158	-0,79887232	0,42436445	0,65009738	protein_codir	90 kDa surface protein
TcG_09300	29,428645	-0,445908307	0,32168328	-1,38617187	0,16569443	0,37125037	protein_codir	serine/threonine protein phosphatase
TcG_09301	43,9410056	0,259125845	0,2728626	0,94965687	0,34228663	0,57657632	protein_codir	target of rapamycin (TOR) kinase 1
TcG_09302	2,087835796	0,077562919	1,25627481	0,06174041	0,95076956	1	protein_codir	hypothetical protein
TcG_09303	12,40108901	0,141385074	0,4946201	0,28584579	0,77499624	1	protein_codir	putative trans-sialidase
TcG_09304	11,10326806	0,39859723	0,52284922	0,76235598	0,44584757	1	protein_codir	putative GAG protein
TcG_09305	34,25368561	-0,573025629	0,32481505	-1,76415972	0,07770509	0,22617359	protein_codir	retrotransposon hot spot (RHS) protein

TcG_09306	48,2428531	-0,037807148	0,24850192	-0,15214027	0,87907631	0,94148439	protein_codir putative retrotransposon hot spot (RHS) protein
TcG_09307	8,692283298	-0,441397424	0,63505645	-0,69505226	0,48702255	1	protein_codir hypothetical protein
TcG_09308	58,49045152	0,149711373	0,2276337	0,65768546	0,51074027	0,71743898	protein_codir hypothetical protein
TcG_09309	21,62722848	0,05543385	0,37062858	0,14956712	0,88110615	0,94206932	
TcG_09310	20,60985401	-0,154170173	0,37742611	-0,40847777	0,68292295	0,83516417	protein_codir complement regulatory protein
TcG_09311	11,93309179	-0,05854687	0,50473475	-0,11599532	0,90765625	1	
TcG_09312	271,542316	-0,264091064	0,11188809	-2,36031437	0,01825945	0,07819075	protein_codir hypothetical protein
TcG_09313	743,9621887	0,029275092	0,06966871	0,42020433	0,67433619	0,82930253	protein_codir putative ubiquitin-protein ligase-like
TcG_09314	321,7136749	0,097510641	0,10468319	0,93148329	0,35160362	0,58601758	protein_codir ADP/ATP translocase
TcG_09315	445,0448371	-0,020999909	0,08780734	-0,23915892	0,81098235	0,90685309	protein_codir putative ADP,ATP carrier protein 1, mitochondrial precursor, putative,ADP/ATP translocase 1
TcG_09316	617,4961563	-0,109082179	0,07526265	-1,44935341	0,14723891	0,34588606	protein_codir RET1 protein
TcG_09317	225,7400492	0,298918335	0,12268403	2,43648931	0,01483061	0,06732284	protein_codir 50S ribosomal protein L16
TcG_09318	83,72912631	-0,240408704	0,19163469	-1,25451556	0,20965467	0,43221692	protein_codir hypothetical protein
TcG_09319	253,5355702	-0,115279583	0,11342975	-1,01630816	0,30948266	0,54262502	protein_codir hypothetical protein
TcG_09320	505,6091685	-0,070477022	0,08986909	-0,78421872	0,43291178	0,65736775	protein_codir putative peroxin 14
TcG_09321	653,6364866	-0,117998923	0,07982862	-1,47815303	0,13936682	0,33382345	protein_codir putative proteasome alpha 5 subunit
TcG_09322	424,3471059	-0,172571359	0,09256606	-1,86430499	0,06227889	0,19491172	protein_codir putative GPI transamidase component GAA1
TcG_09323	817,1667604	0,013078605	0,06852763	0,19085157	0,84864188	0,92688206	protein_codir vacuolar ATP synthase
TcG_09324	630,7812648	-0,163957436	0,07920228	-2,07011011	0,03844203	0,13725406	protein_codir putative ATP synthase F1 subunit gamma protein
TcG_09325	605,5218625	-0,089692657	0,07747708	-1,1576669	0,247	0,47584668	protein_codir putative centromere/microtubule binding protein cbf5
TcG_09326	205,5517779	-0,391037292	0,12728558	-3,07212569	0,0021254	0,01446822	protein_codir putative RNA polymerase II
TcG_09327	96,92060441	-0,067847716	0,183353	-0,37003875	0,71135362	0,85161231	protein_codir hypothetical protein
TcG_09328	237,6244327	-0,550266056	0,11536254	-4,76988507	1,8433E-06	3,4614E-05	protein_codir putative amino acid transporter
TcG_09329	9,475468839	0,078953812	0,60217755	0,13111384	0,89568525	1	protein_codir neutral sphingomyelinase activation associated factor-like protein
TcG_09330	31,180264	0,387380231	0,32004559	1,21039078	0,22612898	0,45249229	protein_codir trans-sialidase
TcG_09331	27,99657406	0,369641332	0,35485855	1,04165825	0,29757015	0,53097917	protein_codir trans-sialidase
TcG_09332	16,00408095	0,670789411	0,43898159	1,52805817	0,1264981	1	protein_codir putative trans-sialidase
TcG_09333	21,01102242	-0,200328786	0,37268919	-0,5375224	0,59090682	0,77668571	protein_codir trans-sialidase
TcG_09334	53,59498616	0,074471933	0,23640437	0,31501927	0,75274702	0,87534451	
TcG_09335	8,663701341	-0,295705906	0,59498017	-0,49700128	0,61918815	1	protein_codir putative retrotransposon hot spot (RHS) protein
TcG_09336	4,252097993	0,694873056	0,88926191	0,78140427	0,43456476	1	protein_codir dispersed protein family protein 1
TcG_09337	1,5111471	0,984593028	1,48733116	0,66198642	0,50797993	1	protein_codir dispersed gene family protein 1 (DGF-1)
TcG_09338	5,035346221	1,352345369	0,8472803	1,59610152	0,11046613	1	protein_codir hypothetical protein
TcG_09339	0,469265227	-2,470320505	2,56600864	-0,96270935	0,3356934	1	protein_codir dispersed gene family protein 1 (DGF-1)
TcG_09340	198,6617444	-0,022606587	0,13380489	-0,16895188	0,86583449	0,93535321	protein_codir dispersed gene family protein 1 (DGF-1)
TcG_09341	69,28373152	0,102507275	0,22833033	0,44894287	0,65347287	0,81657908	protein_codir dispersed gene family protein 1 (DGF-1)
TcG_09342	45,71271742	-0,042862382	0,25754196	-0,16642874	0,86781956	0,9363529	protein_codir dispersed gene family protein 1 (DGF-1)
TcG_09343	11,28197376	1,016244565	0,55953142	1,81624218	0,06933323	1	
TcG_09344	166,9198471	-0,122672756	0,13651418	-0,89860817	0,3688614	0,60086146	protein_codir glyceraldehyde 3-phosphate dehydrogenase, cytosolic
TcG_09345	105,4550559	0,013184718	0,17853313	0,07385026	0,94112954	0,97278319	protein_codir hypothetical protein
TcG_09346	86,06475899	0,125112012	0,19961327	0,62677203	0,53080868	0,732224	protein_codir hypothetical protein
TcG_09347	87,68837023	0,211388269	0,19245195	1,09839505	0,27203202	0,50504046	protein_codir hypothetical protein
TcG_09348	212,0098828	0,179501711	0,1288064	1,39357762	0,16344519	0,36806142	protein_codir hypothetical protein
TcG_09349	282,2739784	-0,035473671	0,10655004	-0,33292969	0,73918735	0,86708727	protein_codir hypothetical protein
TcG_09350	177,5145396	0,194757276	0,1380362	1,41091449	0,15826983	0,36047066	protein_codir hypothetical protein
TcG_09351	82,95449646	-0,354834195	0,19243172	-1,84394859	0,06519063	0,20157424	protein_codir putative mucin-associated surface protein (MASP)
TcG_09352	290,1785364	0,210857093	0,10780363	1,95593692	0,05047259	0,16650779	protein_codir hypothetical protein
TcG_09353	514,685084	-0,128043963	0,08256718	-1,55078532	0,12095314	0,30497564	protein_codir flagellar associated protein
TcG_09354	4331,571398	0,094949675	0,04246542	2,23592919	0,02535641	0,10081653	protein_codir 40S ribosomal protein SA
TcG_09355	132,4103695	-0,506511674	0,15323616	-3,30543179	0,0009483	0,00746401	protein_codir hypothetical protein
TcG_09356	469,4353679	-0,138846407	0,0902786	-1,5379769	0,12405426	0,31011278	protein_codir hypothetical protein
TcG_09357	289,1430214	-0,329746356	0,10891041	-3,0276844	0,00246435	0,01625968	protein_codir putative dynein heavy chain

TcG_09358	376,4457859	0,015682451	0,09586076	0,16359616	0,87004907	0,93736177	protein_codir hypothetical protein
TcG_09359	220,016637	0,047842898	0,12700153	0,3767112	0,70638824	0,84872764	protein_codir hypothetical protein
TcG_09360	142,0806925	0,053012218	0,15319041	0,34605442	0,72930181	0,86056342	protein_codir hypothetical protein
TcG_09361	541,4814066	-0,008991424	0,08811681	-0,10203982	0,91872507	0,96094146	protein_codir hypothetical protein
TcG_09362	200,7903891	0,024507187	0,12633992	0,19397817	0,84619298	0,925349	protein_codir hypothetical protein
TcG_09363	804,5328168	-0,248560138	0,06810616	-3,6495986	0,00026265	0,00250665	protein_codir putative mitochondrial carrier protein
TcG_09364	343,0833392	-0,072849005	0,10162826	-0,71681839	0,47348616	0,68925879	protein_codir putative amino acid permease
TcG_09365	699,491444	0,130244299	0,07701318	1,69119504	0,09079956	0,25143492	protein_codir putative p21-activated kinase 3
TcG_09366	232,9416578	0,055090122	0,11836796	0,46541415	0,6416349	0,80983334	protein_codir charged multivesicular body protein 1
TcG_09367	81,25524341	0,252991142	0,20592608	1,2285532	0,21923937	0,44430774	protein_codir putative MIP18 family protein
TcG_09368	216,8579222	-0,019276544	0,12669347	-0,15215105	0,8790678	0,94148439	protein_codir zinc finger, MYND domain containing 12
TcG_09369	110,1489425	0,43118903	0,16692939	2,58306238	0,00979276	0,04861137	
TcG_09370	208,9409233	0,183581144	0,12869411	1,42649225	0,1537263	0,35458351	protein_codir hypothetical protein
TcG_09371	39,25741958	0,553951147	0,29148557	1,90044106	0,05737526	0,18250241	protein_codir hypothetical protein
TcG_09372	384,7622763	0,469507195	0,10063343	4,66551927	3,0784E-06	5,4956E-05	protein_codir putative phosphatidylinositol-4-phosphate 5-kinase-like protein
TcG_09373	315,2707831	0,459613349	0,11015949	4,17225387	3,016E-05	0,00039728	protein_codir exonuclease 3-5 domain containing 2
TcG_09374	150,0388636	0,492858317	0,14926779	3,3018398	0,00096053	0,00753635	protein_codir hypothetical protein
TcG_09375	51,82204297	0,125166344	0,24391418	0,5131573	0,60784129	0,78802211	protein_codir hypothetical protein
TcG_09376	331,0534765	0,393989822	0,10442581	3,77291615	0,00016135	0,0016736	protein_codir hypothetical protein
TcG_09377	72,58141418	0,077548292	0,20525014	0,37782333	0,70556184	0,84825563	protein_codir hypothetical protein
TcG_09378	10,55189984	0,303155086	0,54028176	0,56110554	0,57472559	1	
TcG_09379	42,0040733	0,500776651	0,27250752	1,83766179	0,06611226	0,20393414	protein_codir retrotransposon hot spot (RHS) protein
TcG_09380	205,5506893	0,114404536	0,12867938	0,88906659	0,37396729	0,60449626	protein_codir retrotransposon hot spot (RHS) protein
TcG_09381	505,4980243	0,511493734	0,08523063	6,00129028	1,9576E-09	7,2927E-08	protein_codir dispersed gene family protein 1 (DGF-1)
TcG_09382	153,7807312	0,416308733	0,15793801	2,63589959	0,00839146	0,04309548	protein_codir hypothetical protein
TcG_09383	123,2136929	0,273447853	0,17311359	1,57958628	0,11420164	0,29363964	protein_codir hypothetical protein
TcG_09384	386,8901163	0,026134861	0,09506353	0,27491993	0,78337775	0,89263342	protein_codir protein kinase
TcG_09385	26,07363606	0,105708493	0,3478869	0,3038588	0,76123547	0,87949151	protein_codir retrotransposon hot spot (RHS) protein
TcG_09386	64,38297499	0,407649775	0,24426985	1,66885016	0,09514708	0,25993257	protein_codir retrotransposon hot spot (RHS) protein
TcG_09387	28,68637329	0,805126421	0,3523429	2,28506498	0,02230902	0,09149461	protein_codir retrotransposon hot spot protein (RHS)
TcG_09388	41,17864255	0,142995893	0,26712845	0,53530762	0,59243716	0,7778347	protein_codir hypothetical protein
TcG_09389	26,30307062	0,43803186	0,35947269	1,21854004	0,22301882	0,44921697	
TcG_09390	47,83341869	0,085073952	0,2528016	0,33652458	0,73647532	0,86530636	protein_codir dispersed protein family protein 1 (DGF-1)
TcG_09391	49,11700602	0,247260679	0,2715616	0,91051413	0,36255143	0,59502869	protein_codir dispersed gene family protein 1 (DGF-1)
TcG_09392	25,42185621	0,385730238	0,36429564	1,05883847	0,28967335	0,52325467	protein_codir dispersed gene family protein 1 (DGF-1)
TcG_09393	43,01033463	0,349058995	0,27189311	1,28380965	0,19920857	0,41804572	protein_codir dispersed gene family protein 1 (DGF-1)
TcG_09394	14,35049728	0,961628523	0,49396832	1,94674129	0,05156576	1	protein_codir dispersed gene family protein 1 (DGF-1)
TcG_09395	74,89455075	0,1978551	0,22876255	0,86489286	0,3870976	0,61733142	protein_codir dispersed gene family protein 1 (DGF-1)
TcG_09396	2,688929916	-0,20906618	1,08200741	-0,19322066	0,84678616	1	
TcG_09397	456,2873919	-0,060679936	0,0910104	-0,66673625	0,50494062	0,71221932	protein_codir putative diacylglycerol acyltransferase
TcG_09398	4,767518824	0,876895346	0,80606588	1,08787056	0,27665225	1	
TcG_09399	236,7455644	0,107108986	0,13216102	0,81044311	0,41768555	0,64429567	protein_codir hypothetical protein
TcG_09400	375,5221582	-0,157224403	0,09626548	-1,63323756	0,10241904	0,27335338	protein_codir putative choline/carnitine O-acetyltransferase
TcG_09401	73,54214398	0,169581321	0,21656261	0,78305911	0,4335924	0,65797008	protein_codir hypothetical protein
TcG_09402	301,0646016	-0,169628634	0,1045254	-1,622846	0,10462233	0,27763498	protein_codir hypothetical protein
TcG_09403	27,85041667	-0,077530328	0,32884463	-0,23576583	0,81361438	0,90788175	protein_codir trans-sialidase
TcG_09404	5,004229794	-0,076356028	0,76661407	-0,09960165	0,92066058	1	
TcG_09405	56,79032737	-0,527367917	0,23652092	-2,22968822	0,02576815	0,10213814	protein_codir mucin-associated surface protein (MASP)
TcG_09406	66,89828497	-0,577970221	0,21368942	-2,70472086	0,00683618	0,03671951	protein_codir hypothetical protein
TcG_09407	54,82635111	-0,155516023	0,23686979	-0,65654645	0,51147259	0,71768359	protein_codir mucin TcMUCII
TcG_09408	14,22126011	-0,089649559	0,45908792	-0,19527754	0,84517568	1	protein_codir hypothetical protein
TcG_09409	11,0154327	0,071549273	0,51554279	0,13878435	0,88962056	1	protein_codir thimet oligopeptidase

TcG_09410	61,88059662	-0,656437137	0,23665032	-2,77386968	0,00553939	0,03100451	protein_codir	mucin-associated surface protein (MASP)
TcG_09411	41,19699377	-0,701495998	0,27276823	-2,57176573	0,01011813	0,04982992	protein_codir	mucin TcMUCII
TcG_09412	21,84337048	-0,517509323	0,36982253	-1,39934505	0,16170954	0,36537202	protein_codir	hypothetical protein
TcG_09413	2,385281305	0,448684709	1,13011882	0,39702437	0,69134949	1	protein_codir	surface protease GP63
TcG_09414	363,8051798	-0,121539573	0,09807844	-1,23920789	0,21526852	0,4388705		
TcG_09415	31,57046429	-0,350408793	0,31672963	-1,10633412	0,26858193	0,50117414	protein_codir	mucin-associated surface protein (MASP)
TcG_09416	4,590509104	-0,213509134	0,8292173	-0,25748273	0,79680615	1		
TcG_09417	14,16653209	0,37288793	0,50135755	0,74375648	0,45702381	1	protein_codir	hypothetical protein
TcG_09418	2,617609495	0,288962292	1,15817328	0,24949832	0,80297534	1	protein_codir	hypothetical protein
TcG_09419	7,102002748	0,099527453	0,67066902	0,14840025	0,8820269	1	protein_codir	hypothetical protein
TcG_09420	2,58733185	-0,427258986	1,1222003	-0,38073327	0,70340118	1	protein_codir	hypothetical protein
TcG_09421	2,367900518	-0,12065428	1,16571242	-0,10350261	0,91756408	1	protein_codir	hypothetical protein
TcG_09422	5,783879866	-2,198708296	0,8214075	-2,67675704	0,00743385	1	protein_codir	hypothetical protein
TcG_09423	2,647105927	-1,135682259	1,11942518	-1,0145227	0,3103334	1	protein_codir	hypothetical protein
TcG_09424	0					1	protein_codir	trans-sialidase
TcG_09425	2,00928237	-0,1984599	1,19163919	-0,16654362	0,86772916	1	protein_codir	trans-sialidase
TcG_09426	37,85927948	-0,461552588	0,28769112	-1,60433377	0,1086405	0,28454709	protein_codir	gp85-like protein
TcG_09427	0,51990315	0,821404694	2,48035172	0,3311646	0,74052016	1	protein_codir	trans-sialidase
TcG_09428	0,155721614	0,503022807	4,08047286	0,12327562	0,90188885	1	protein_codir	hypothetical protein
TcG_09429	0					1	protein_codir	trans-sialidase
TcG_09430	4,471489928	0,145591235	0,82712782	0,17602024	0,86027804	1	protein_codir	hypothetical protein
TcG_09431	43,31781342	0,078652821	0,26943635	0,29191615	0,77035073	0,88468697	protein_codir	hypothetical protein
TcG_09432	20,06297036	-0,36273372	0,41486791	-0,87433544	0,38193561	0,61205441	protein_codir	hypothetical protein
TcG_09433	22,29319798	0,078827048	0,36796206	0,21422602	0,8303708	0,91760178	protein_codir	L1Tc protein
TcG_09434	4,352741519	0,686423891	0,85508049	0,80275938	0,42211382	1	protein_codir	RNaseH
TcG_09435	8,453811546	-0,522059898	0,62066543	-0,84112933	0,40027548	1	protein_codir	hypothetical protein
TcG_09436	25,48613098	-0,272380056	0,36667613	-0,74283554	0,45758126	0,67708001	protein_codir	hypothetical protein
TcG_09437	25,04859944	0,085917405	0,34441738	0,24945723	0,80300712	0,90265093	protein_codir	hypothetical protein
TcG_09438	163,3078504	-0,202654683	0,13740877	-1,47483073	0,14025806	0,33471264	protein_codir	SNF1-related protein kinase
TcG_09439	553,5376183	-0,012458494	0,07935109	-0,1570047	0,87524114	0,93987711	protein_codir	putative eukaryotic translation initiation factor 6 (eIF-6)
TcG_09440	298,9298078	0,215002398	0,10719369	2,00573747	0,04488427	0,15326528	protein_codir	Nu1 protein
TcG_09441	601,6473555	0,083157918	0,07612465	1,09239142	0,27466109	0,50755645	protein_codir	putative DEAH-box RNA helicase
TcG_09442	70,62024835	-0,040493032	0,21083957	-0,19205613	0,84769824	0,92655017		
TcG_09443	461,8384057	-0,065188876	0,09356464	-0,6967256	0,48597454	0,69827461	protein_codir	putative mitogen-activated protein kinase kinase 5
TcG_09444	490,8388944	-0,133175644	0,08886606	-1,49861092	0,1339746	0,32557556	protein_codir	hypothetical protein
TcG_09445	38,65754565	-0,202454967	0,27674504	-0,73150485	0,46447085	0,68173811	protein_codir	retrotransposon hot spot (RHS) protein
TcG_09446	129,2187348	0,184447948	0,16788632	1,09864788	0,27192168	0,50504046	protein_codir	putative retrotransposon hot spot (RHS) protein
TcG_09447	30,67303135	0,335826896	0,31414733	1,06901081	0,2850648	0,51877427	protein_codir	retrotransposon hot spot (RHS) protein
TcG_09448	38,38798196	-0,208772437	0,28446303	-0,73391765	0,46299893	0,6805767	protein_codir	hypothetical protein
TcG_09449	8,985554939	-0,446267751	0,57133151	-0,78110124	0,43474295	1	protein_codir	dispersed gene family protein 1 (DGF-1)
TcG_09450	42,78205713	-0,037765217	0,2615748	-0,14437636	0,88520328	0,94429291	protein_codir	dispersed gene family protein 1 (DGF-1)
TcG_09451	0					1	protein_codir	dispersed gene family protein 1 (DGF-1)
TcG_09452	18,49793411	0,261884597	0,40169023	0,6519566	0,51442917	0,7202291	protein_codir	dispersed gene family protein 1 (DGF-1)
TcG_09453	58,71175965	0,408314368	0,22721727	1,79702174	0,07233217	0,21618229	protein_codir	dispersed gene family protein 1 (DGF-1)
TcG_09454	469,1786862	-0,21122739	0,08584421	-2,46058996	0,01387088	0,06390283	protein_codir	hypothetical protein
TcG_09455	34,45913214	-0,283186024	0,3233597	-0,87576164	0,38115963	0,61114247	protein_codir	hypothetical protein
TcG_09456	1291,739614	0,065281648	0,06398507	1,02026379	0,30760337	0,54090194	protein_codir	hypothetical protein
TcG_09457	77,66025654	-0,076728982	0,20250296	-0,37890302	0,70475988	0,84755533	protein_codir	putative vacuolar protein sorting-associated protein 13A isoform X4
TcG_09458	174,6319317	-0,065045008	0,13918054	-0,46734269	0,64025472	0,80885303	protein_codir	hypothetical protein
TcG_09459	0,311976008	1,510501932	3,1463032	0,48008785	0,63116493	1		
TcG_09460	0					1		
TcG_09461	0,27291574	1,35431792	3,0630933	0,4421406	0,65838746	1		

TcG_09462	15,70409872	0,383476183	0,47779448	0,80259651	0,42220798	1		
TcG_09463	2168,967013	-0,724541828	0,047636	-15,2099627	3,0372E-52	5,8648E-49	protein_codir	hypothetical protein
TcG_09464	882,1482769	-0,047452363	0,06863222	-0,69140067	0,48931378	0,70041877	protein_codir	hypothetical protein
TcG_09465	4612,919992	0,423549988	0,04217511	10,0426525	9,8976E-24	2,5755E-21	protein_codir	c71 surface protein
TcG_09466	2696,951156	0,412744904	0,04531501	9,10834948	8,3645E-20	1,4683E-17	protein_codir	trans-sialidase
TcG_09467	1017,274757	0,321457217	0,06437037	4,99386898	5,9182E-07	1,2721E-05		
TcG_09468	206,9826626	-0,223992669	0,13248361	-1,6907199	0,09089031	0,25157414	protein_codir	hypothetical protein
TcG_09469	1079,400626	-0,169596046	0,05924877	-2,86243994	0,00420393	0,02510974	protein_codir	trans-sialidase
TcG_09470	94,4131571	-0,072308931	0,19557132	-0,36973178	0,71158235	0,8517815		
TcG_09471	263,1219983	0,108785308	0,11293432	0,96326171	0,3354162	0,56989766	protein_codir	hypothetical protein
TcG_09472	253,3691628	0,199052363	0,1110705	1,79212636	0,07311273	0,21779928	protein_codir	surface protease GP63
TcG_09473	562,6692017	0,056665027	0,08408789	0,67387858	0,50038851	0,70926123		
TcG_09474	1058,617754	-0,038973339	0,06490526	-0,600465	0,54819638	0,74477055	protein_codir	amastigote surface protein 4
TcG_09475	78,71704222	0,368917745	0,2054053	1,79604783	0,07248691	0,21639612	protein_codir	hypothetical protein
TcG_09476	225,0101402	0,422893543	0,12068122	3,50421993	0,00045795	0,00400738	protein_codir	hypothetical protein
TcG_09477	285,9147304	0,499793119	0,10770539	4,64037223	3,4778E-06	6,1321E-05	protein_codir	hypothetical protein
TcG_09478	422,961485	0,351472794	0,09871292	3,56055522	0,00037007	0,00334711	protein_codir	cullin-4B
TcG_09479	166,8490712	0,445067502	0,13721969	3,24346673	0,00118085	0,00893034	protein_codir	hypothetical protein
TcG_09480	99,01737922	0,323195805	0,18199292	1,77587024	0,07575431	0,22248147	protein_codir	hypothetical protein
TcG_09481	116,6609031	0,433892477	0,16587125	2,61583895	0,00890085	0,04512424	protein_codir	putative dihydroceramide synthase
TcG_09482	73,88592691	0,408115322	0,20361912	2,00430741	0,04503714	0,15363279	protein_codir	putative UDP-Gal or UDP-GlcNAc-dependent glycosyltransferase
TcG_09483	2161,7466	0,884035798	0,05274495	16,7605776	4,74E-63	1,3729E-59	protein_codir	putative amino acid transporter
TcG_09484	361,3024478	-0,08523748	0,10059488	-0,84733413	0,39680891	0,62662432	protein_codir	U2 small nuclear ribonucleoprotein B
TcG_09485	4516,746487	-0,349728507	0,03911263	-8,94157507	3,8366E-19	6,1738E-17	protein_codir	putative guanine deaminase
TcG_09486	321,5906824	-0,12280142	0,10305486	-1,19161216	0,23341335	0,46078159	protein_codir	hypothetical protein
TcG_09487	1106,225611	-0,395481432	0,0603241	-6,5559446	5,5291E-11	2,9521E-09	protein_codir	putative high mobility group protein
TcG_09488	236,3320988	-0,083904815	0,12955705	-0,64762832	0,51722537	0,72269334	protein_codir	hypothetical protein
TcG_09489	255,8783939	-0,070243964	0,11278313	-0,62282332	0,53340063	0,73409852	protein_codir	hypothetical protein
TcG_09490	334,1306496	-0,116879169	0,10150611	-1,15144964	0,2495473	0,47881138	protein_codir	hypothetical protein
TcG_09491	108,3567097	-0,186138711	0,17272585	-1,07765404	0,28118818	0,5142555		
TcG_09492	349,0951125	-0,075155362	0,10495857	-0,71604789	0,47396178	0,68969118	protein_codir	hypothetical protein
TcG_09493	892,6883667	0,250388753	0,07262539	3,44767531	0,00056543	0,00480992	protein_codir	membrane associated protein
TcG_09494	3,864907503	1,342389844	0,94467672	1,42100447	0,15531546	1	protein_codir	dispersed gene family protein 1 (DGF-1)
TcG_09495	25,12275547	-0,219291139	0,34814839	-0,62987837	0,52877416	0,73107129		
TcG_09496	65,52147354	0,083462541	0,21428327	0,3894963	0,69690904	0,84301401	protein_codir	retrotransposon hot spot (RHS) protein
TcG_09497	93,82058892	0,125461466	0,19956458	0,62867603	0,52956117	0,73154832	protein_codir	putative retrotransposon hot spot (RHS) protein
TcG_09498	101,9168613	0,389070272	0,18508557	2,10211019	0,03554362	0,13003108	protein_codir	retrotransposon hot spot (RHS) protein
TcG_09499	6,794122413	0,272422231	0,65677934	0,41478502	0,67829929	1	protein_codir	hypothetical protein
TcG_09500	2,541209741	-0,646441816	1,08438055	-0,59613926	0,5510822	1	protein_codir	trans-sialidase
TcG_09501	5,359417573	-0,081411051	0,76247292	-0,10677238	0,91496956	1		
TcG_09502	4,391240175	0,566577942	0,83758685	0,67644082	0,49876081	1	protein_codir	hypothetical protein
TcG_09503	9,834969894	0,358079279	0,58047387	0,61687407	0,53731779	1	protein_codir	hypothetical protein
TcG_09504	81,08105862	0,215331864	0,19630877	1,09690397	0,27268338	0,50573229	protein_codir	trans-sialidase-like protein
TcG_09505	16,30276141	0,261728583	0,45874599	0,57053051	0,56831793	1	protein_codir	trans-sialidase
TcG_09506	2,254995564	-0,824150881	1,18826596	-0,69357443	0,48794914	1		
TcG_09507	377,7887883	0,153991108	0,09571337	1,60887779	0,10764307	0,28260879	protein_codir	putative retrotransposon hot spot (RHS) protein
TcG_09508	118,0445688	0,177087304	0,18129922	0,97676814	0,32868395	0,56308329	protein_codir	putative surface antigen 2 (CA-2)
TcG_09509	155,2252826	-0,085632385	0,15575473	-0,54978996	0,58246345	0,7714245	protein_codir	hypothetical protein
TcG_09510	672,3165483	-0,176695349	0,07691305	-2,29733912	0,02159943	0,08937536	protein_codir	hypothetical protein
TcG_09511	59,88273668	0,129376861	0,2361952	0,54775399	0,58386083	0,77248049	protein_codir	hypothetical protein
TcG_09512	468,951486	-0,039104191	0,08762894	-0,44624748	0,65541849	0,81772823	protein_codir	hypothetical protein
TcG_09513	4,622616017	0,365999362	0,84837809	0,43141067	0,66616979	1	protein_codir	hypothetical protein

TcG_09514	13,68286296	0,149221973	0,48767454	0,3059868	0,75961469	1	protein_codir beta galactofuranosyl glycosyltransferase
TcG_09515	23,77257577	0,290963269	0,36558702	0,79587965	0,42610199	0,6517508	protein_codir beta galactofuranosyl glycosyltransferase
TcG_09516	16,80320353	0,051167085	0,43597971	0,11736116	0,90657386	0,95471088	
TcG_09517	4,879848907	0,047928012	0,77902381	0,06152317	0,95094256	1	protein_codir hypothetical protein
TcG_09518	244,2116519	-0,410776401	0,12441071	-3,30177692	0,00096074	0,00753635	protein_codir putative trans-sialidase
TcG_09519	6,235576378	0,193090009	0,71780334	0,26900127	0,78792871	1	protein_codir structural maintenance of chromosome protein 4
TcG_09520	17,11678457	0,208484793	0,42325266	0,49257764	0,62231105	0,79651965	protein_codir SH3 domain protein
TcG_09521	6,61787579	1,724032652	0,7553754	2,28235212	0,02246856	1	protein_codir hypothetical protein
TcG_09522	23,0598944	0,479628781	0,36581802	1,31111305	0,18981957	0,40494376	protein_codir hypothetical protein
TcG_09523	386,2691144	0,28875465	0,096364	2,99649926	0,00273099	0,01763435	protein_codir hypothetical protein
TcG_09524	142,1194415	0,552277578	0,15221276	3,62832631	0,00028526	0,00268705	protein_codir GINS complex subunit 3
TcG_09525	413,1669405	-0,012822964	0,0910981	-0,14075995	0,88805958	0,94594634	protein_codir hypothetical protein
TcG_09526	335,9099992	0,136611745	0,10070965	1,35649117	0,17494294	0,38402594	protein_codir hypothetical protein
TcG_09527	34,39503035	0,798985925	0,30993657	2,57790141	0,00994024	0,04915852	protein_codir hypothetical protein
TcG_09528	290,2889549	0,746951465	0,11559552	6,46176816	1,0349E-10	5,0802E-09	protein_codir hypothetical protein
TcG_09529	26,50397052	0,796289206	0,34698757	2,29486379	0,02174093	0,08967264	
TcG_09530	282,4779353	-0,010540331	0,11125538	-0,09473997	0,92452137	0,96317819	protein_codir expression site-associated gene (ESAG-like) protein
TcG_09531	105,2325635	0,317257856	0,17493613	1,81356391	0,06974489	0,21142447	
TcG_09532	16,21331828	0,903567553	0,46200654	1,95574626	0,05049505	1	protein_codir hypothetical protein
TcG_09533	20,28952483	0,682929985	0,3891948	1,75472534	0,0793063	0,22953854	protein_codir myosin light chain kinase
TcG_09534	26,68506616	0,095074019	0,33618706	0,28280095	0,77732943	0,88888065	protein_codir hypothetical protein
TcG_09535	134,9060336	0,702373721	0,15673767	4,48120555	7,4223E-06	0,00011796	protein_codir hypothetical protein
TcG_09536	43,37628294	0,50542449	0,28226263	1,79061779	0,07335465	0,21814348	protein_codir putative surface protease GP63
TcG_09537	33,9443498	-0,255094848	0,29651201	-0,86031877	0,38961335	0,62006322	protein_codir solanesyl-diphosphate synthase
TcG_09538	24,38027465	-0,002935607	0,35329107	-0,00830932	0,9933702	0,99751132	protein_codir putative target of rapamycin (TOR) kinase 1
TcG_09539	61,90040408	0,199125903	0,23270639	0,85569588	0,39216602	0,62156436	protein_codir target of rapamycin (TOR) kinase 1
TcG_09540	155,5865893	0,31092442	0,14847433	2,09412912	0,03624848	0,13205252	protein_codir L1Tc protein
TcG_09541	85,49445002	-0,110598944	0,20611062	-0,53659992	0,591544	0,77721083	protein_codir hypothetical protein
TcG_09542	106,0230991	0,207695593	0,18028497	1,15204054	0,24930441	0,47853561	protein_codir hypothetical protein
TcG_09543	75,1720213	0,03611903	0,20348284	0,17750406	0,85911248	0,93216681	protein_codir hypothetical protein
TcG_09544	199,6801633	0,204109763	0,13617948	1,49882907	0,13391798	0,32557556	protein_codir hypothetical protein
TcG_09545	82,42462651	0,383312232	0,2046574	1,87294589	0,06107586	0,19193429	protein_codir hypothetical protein
TcG_09546	1061,595127	-1,200647833	0,06853988	-17,5175059	1,0534E-68	6,1025E-65	protein_codir trans-sialidase
TcG_09547	78,67527524	-1,23930662	0,20087507	-6,16953929	6,8489E-10	2,8441E-08	protein_codir trans-sialidase
TcG_09548	46,68525498	-0,097522882	0,2530958	-0,38532004	0,70000032	0,84436727	protein_codir hypothetical protein
TcG_09549	149,0373597	0,16562831	0,14608021	1,13381756	0,25687109	0,48884829	protein_codir dispersed gene family protein 1 (DGF-1)
TcG_09550	297,2597802	0,088100583	0,11065762	0,79615468	0,42594213	0,6517508	protein_codir retrotransposon hot spot (RHS) protein
TcG_09551	60,22409614	-0,030817322	0,22547344	-0,13667828	0,8912851	0,94697266	protein_codir retrotransposon hot spot (RHS) protein
TcG_09552	17,09661351	0,108881783	0,45730312	0,23809543	0,81180708	0,90735065	protein_codir hypothetical protein
TcG_09553	17,42453448	-0,679328608	0,415348	-1,63556489	0,10193068	0,27248936	protein_codir hypothetical protein
TcG_09554	19,48755581	-0,104188636	0,41531688	-0,2508654	0,80191818	0,90177851	protein_codir putative trans-sialidase
TcG_09555	34,07550228	-0,64056269	0,314219	-2,03858673	0,04149129	0,14505676	protein_codir hypothetical protein
TcG_09556	19,93327683	0,009872589	0,38998033	0,02531561	0,97980323	0,9919484	protein_codir hypothetical protein
TcG_09557	17,00658667	-0,413571545	0,41681218	-0,99222519	0,32108768	0,5560344	protein_codir hypothetical protein
TcG_09558	1150,647927	-0,099635811	0,06997666	-1,42384351	0,15449178	0,35571179	protein_codir exo-alpha-sialidase
TcG_09559	26,25342196	-0,345650284	0,37506911	-0,92156424	0,35675592	0,5907012	protein_codir putative trans-sialidase
TcG_09560	107,8599059	0,033844858	0,1706332	0,19834861	0,84277233	0,92351841	protein_codir putative trans-sialidase
TcG_09561	23,53296684	0,35319936	0,38570663	0,91572022	0,35981366	0,5926302	protein_codir hypothetical protein
TcG_09562	28,20101288	0,268373364	0,33200767	0,80833483	0,41889786	0,64539237	protein_codir hypothetical protein
TcG_09563	476,6877616	-0,101780959	0,08547369	-1,19078705	0,23373719	0,46126369	protein_codir retrotransposon hot spot (RHS) protein
TcG_09564	338,6640595	-0,110572097	0,09819883	-1,1260022	0,26016459	0,49261779	protein_codir putative R-SNARE protein
TcG_09565	280,318292	-0,166286346	0,10816594	-1,53732636	0,12421341	0,3102256	protein_codir hypothetical protein

TcG_09566	126,362461	0,32288775	0,17002609	1,89904829	0,05755813	0,18280385	protein_codir	hypothetical protein
TcG_09567	408,061203	0,044606718	0,08996058	0,49584739	0,62000208	0,79444195	protein_codir	translation initiation factor 3 subunit K
TcG_09568	494,1216109	-0,160066935	0,08687025	-1,84259779	0,06538776	0,20212982	protein_codir	small nuclear ribonucleoprotein component-like protein
TcG_09569	250,2975849	0,180722182	0,11430618	1,581036	0,11386981	0,29298147	protein_codir	hypothetical protein
TcG_09570	246,1905078	0,050596045	0,11862664	0,42651504	0,66973259	0,82684442	protein_codir	hypothetical protein
TcG_09571	138,2901206	-0,10793397	0,14964975	-0,72124392	0,47075946	0,68692532	protein_codir	dispersed gene family protein 1 (DGF-1)
TcG_09572	524,2926036	-0,238086689	0,0853263	-2,79030833	0,00526579	0,02983345	protein_codir	dispersed gene family protein 1 (DGF-1)
TcG_09573	30,35449285	0,08376735	0,3139052	0,26685557	0,78958037	0,89501549	protein_codir	dispersed protein family protein 1 (DGF-1)
TcG_09574	85,91709867	-0,118993647	0,18791786	-0,63322158	0,52658895	0,72926645	protein_codir	dispersed protein family protein 1 (DGF-1)
TcG_09575	148,8138936	-0,248384358	0,14381047	-1,72716468	0,08413809	0,23892743		
TcG_09576	97,35739824	0,058161292	0,17795376	0,32683374	0,74379364	0,86984891	protein_codir	hypothetical protein
TcG_09577	141,0607968	-0,129508487	0,15687303	-0,82556248	0,40905233	0,63660535	protein_codir	hypothetical protein
TcG_09578	30,79286908	0,531798141	0,31540106	1,68610131	0,09177629	0,25317144	protein_codir	trans-sialidase
TcG_09579	43,51382136	0,826839143	0,26750096	3,0909764	0,00199499	0,01371337	protein_codir	rab1 small GTP-binding protein
TcG_09580	70,81957678	0,475290268	0,21803385	2,17989213	0,02926546	0,1122004	protein_codir	target of rapamycin (TOR) kinase 1
TcG_09581	81,84375999	0,281922446	0,19513104	1,44478525	0,14851822	0,34741208	protein_codir	protein kinase, putative,serine/threonine protein kinase
TcG_09582	65,79344862	0,954177098	0,23134966	4,12439381	3,7171E-05	0,00047535	protein_codir	hypothetical protein
TcG_09583	44,63359341	0,817071747	0,27309289	2,99191881	0,0027723	0,01784437	protein_codir	hypothetical protein
TcG_09584	230,7149632	0,189114878	0,12229033	1,54644176	0,12199792	0,30663266	protein_codir	putative trans-sialidase
TcG_09585	63,73830464	0,303791786	0,2286171	1,32882354	0,18390619	0,39656377		
TcG_09586	64,92010493	0,319710775	0,21453679	1,4902375	0,1361618	0,32900325	protein_codir	hypothetical protein
TcG_09587	85,92348179	0,299904174	0,19535992	1,53513666	0,12475026	0,31110872	protein_codir	putative retrotransposon hot spot (RHS) protein
TcG_09588	18,33099024	-0,312398855	0,41321537	-0,75601945	0,44963754	0,67044205	protein_codir	retrotransposon hot spot (RHS) protein
TcG_09589	102,3700318	-0,022413007	0,17784039	-0,12602878	0,89970914	0,95123628	protein_codir	retrotransposon hot spot (RHS) protein
TcG_09590	281,2857685	0,153035457	0,12126568	1,26198485	0,20695424	0,4291629		
TcG_09591	149,4025759	0,226165187	0,14655209	1,543241	0,12277231	0,30768765	protein_codir	mucin TcMUCII
TcG_09592	45,82120365	0,17123539	0,25478505	0,67207785	0,50153413	0,71036363		
TcG_09593	179,9029365	-0,536352598	0,13383834	-4,00746603	6,1374E-05	0,00073458	protein_codir	mucin-associated surface protein (MASP)
TcG_09594	95,57581039	-0,181933818	0,18714158	-0,97217208	0,33096495	0,56523583	protein_codir	hypothetical protein
TcG_09595	50,34782758	-0,262808633	0,2672637	-0,98333083	0,32544464	0,55987308	protein_codir	hypothetical protein
TcG_09596	42,59317698	-0,357956042	0,26830994	-1,33411397	0,18216651	0,39402198	protein_codir	hypothetical protein
TcG_09597	70,91817544	-0,090889986	0,20927051	-0,43431817	0,66405741	0,82434295	protein_codir	mucin TcMUCII
TcG_09598	69,00026951	-0,143641255	0,21411448	-0,67086192	0,5023085	0,71099511		
TcG_09599	367,5358546	-0,460487305	0,09720473	-4,73729323	2,1659E-06	4,0087E-05	protein_codir	mucin-associated surface protein (MASP)
TcG_09600	138,9720228	0,214209827	0,14862336	1,44129317	0,14950188	0,34886782	protein_codir	surface protease GP63
TcG_09601	7,405273724	-0,476300063	0,63695898	-0,74777196	0,45459772	1	protein_codir	hypothetical protein
TcG_09602	1,746836569	-0,168221425	1,31454996	-0,12796883	0,89817364	1	protein_codir	hypothetical protein
TcG_09603	2,504475928	-0,241211711	1,07263072	-0,22487862	0,82207371	1	protein_codir	hypothetical protein
TcG_09604	0,116927736	0,503022807	4,08047286	0,12327562	0,90188885	1	protein_codir	trans-sialidase
TcG_09605	11,09080035	-0,158675058	0,52589111	-0,30172607	0,76286089	1	protein_codir	hypothetical protein
TcG_09606	20,06260282	0,471020376	0,40402519	1,16581932	0,2436875	0,47181875	protein_codir	hypothetical protein
TcG_09607	161,5462563	0,23117905	0,14494964	1,59489216	0,11073635	0,28760174	protein_codir	retrotransposon hot spot (RHS) protein
TcG_09608	19,87154654	0,48138907	0,41099177	1,17128639	0,24148368	0,47019585	protein_codir	trans-sialidase
TcG_09609	17,50958064	0,508048232	0,42244	1,20265181	0,22911109	0,45626889	protein_codir	trans-sialidase
TcG_09610	105,8600408	0,143735414	0,18056529	0,79603016	0,42601451	0,6517508	protein_codir	surface protein-2
TcG_09611	34,18825864	0,547356588	0,29815502	1,8358121	0,06638545	0,204559		
TcG_09612	24,04805575	0,459698913	0,39324954	1,16897507	0,24241368	0,47089863	protein_codir	hypothetical protein
TcG_09613	34,41106832	0,170462442	0,30979801	0,55023737	0,58215658	0,77119439	protein_codir	beta galactofuranosyl glycosyltransferase
TcG_09614	16,0880496	0,508467435	0,44704966	1,13738468	0,25537751	1	protein_codir	beta galactofuranosyl glycosyltransferase
TcG_09615	23,46488096	-0,388869814	0,37063063	-1,04921121	0,29408092	0,52710729		
TcG_09616	42,47841592	0,354669587	0,27212153	1,30334994	0,19245532	0,40883522	protein_codir	putative serine/threonine protein phosphatase
TcG_09617	49,58374481	0,46108537	0,25269085	1,8247015	0,06804609	0,20790663	protein_codir	target of rapamycin (TOR) kinase 1

TcG_09618	31,25618736	0,191083662	0,31166457	0,61310678	0,53980573	0,7389165	protein_codir hypothetical protein
TcG_09619	34,1563123	0,278881379	0,29872416	0,93357491	0,35052322	0,58492899	protein_codir trans-sialidase
TcG_09620	26,88768566	-0,032680496	0,35419677	-0,0922665	0,92648631	0,96430637	protein_codir hypothetical protein
TcG_09621	16,37593565	0,360064198	0,44885761	0,80217912	0,42244935	1	
TcG_09622	160,4539173	-0,41750694	0,14049455	-2,97169492	0,00296161	0,01882238	protein_codir mucin-associated surface protein (MASP)
TcG_09623	207,3275623	-0,162983832	0,12806486	-1,27266631	0,20313648	0,42329843	
TcG_09624	745,7122652	-0,248423264	0,07128795	-3,48478621	0,00049253	0,0042777	protein_codir hypothetical protein
TcG_09625	308,7848082	-0,361223425	0,10773862	-3,35277562	0,00080006	0,00646856	protein_codir hypothetical protein
TcG_09626	991,7532282	-0,055787583	0,0635681	-0,87760339	0,38015899	0,6107213	protein_codir phospholipid:diacylglycerol acyltransferase
TcG_09627	130,2977437	0,122860268	0,15652468	0,78492586	0,43249704	0,65696635	protein_codir DNA-directed RNA polymerase III subunit
TcG_09628	704,682119	-0,31096414	0,07699187	-4,03892179	5,3697E-05	0,00065765	protein_codir putative eukaryotic translation initiation factor 2 subunit
TcG_09629	399,7055137	-0,110669585	0,09536292	-1,1605096	0,24584139	0,47432445	protein_codir hypothetical protein
TcG_09630	31,83473218	-0,035309807	0,30869936	-0,11438251	0,90893458	0,95584124	protein_codir trans-sialidase
TcG_09631	279,8430034	-0,086521225	0,1100098	-0,78648653	0,43158251	0,65596889	protein_codir trans-sialidase-like protein
TcG_09632	63,2125668	0,127834174	0,21853012	0,58497279	0,55856601	0,75321786	protein_codir hypothetical protein
TcG_09633	265,5998105	-0,344978624	0,11525843	-2,99308789	0,0027617	0,01779703	protein_codir mucin-associated surface protein (MASP)
TcG_09634	56,98016058	0,131782792	0,23690246	0,55627447	0,57802324	0,76800198	protein_codir putative trans-sialidase
TcG_09635	101,1975405	0,095945533	0,1916012	0,50075643	0,61654255	0,79265743	protein_codir putative trans-sialidase
TcG_09636	37,46592847	0,296608979	0,28397219	1,04450011	0,29625407	0,5292829	
TcG_09637	42,10819348	0,301591646	0,27716215	1,08814153	0,27653263	0,50896062	
TcG_09638	35,19635655	-0,154014825	0,29358896	-0,52459339	0,59986588	0,7832803	protein_codir hypothetical protein
TcG_09639	97,78407578	-0,172480822	0,17908391	-0,96312851	0,33548304	0,56992763	protein_codir retrotransposon hot spot (RHS) protein
TcG_09640	297,8277098	0,166257394	0,10608004	1,56728257	0,11704866	0,29851053	protein_codir retrotransposon hot spot (RHS) protein
TcG_09641	390,1813139	-0,050378224	0,09288559	-0,54236859	0,58756462	0,77564856	protein_codir putative retrotransposon hot spot (RHS) protein
TcG_09642	159,6779905	0,075180228	0,13921957	0,54001191	0,58918882	0,77564856	protein_codir putative rab1 small GTP-binding protein
TcG_09643	45,51647451	0,0304544	0,2640637	0,11532975	0,90818376	0,95543603	protein_codir hypothetical protein
TcG_09644	121,7332681	0,185479881	0,17327217	1,07045397	0,28441502	0,5181183	protein_codir structural maintenance of chromosome protein 4
TcG_09645	370,4888451	-0,241866967	0,09367011	-2,58211482	0,00981969	0,04870331	protein_codir solute carrier family 35, member E3
TcG_09646	226,6075523	-0,058764926	0,13058027	-0,45002915	0,65268942	0,8162845	protein_codir hypothetical protein
TcG_09647	139,3958106	-0,679040499	0,14852841	-4,57178854	4,8358E-06	8,1199E-05	protein_codir putative eukaryotic translation initiation factor 4E (eIF4E) interacting protein
TcG_09648	116,4146163	0,039735788	0,16714952	0,237726	0,81209361	0,90758335	protein_codir hypothetical protein
TcG_09649	112,3110327	-0,210874754	0,16843766	-1,2519454	0,21058976	0,43314272	protein_codir hypothetical protein
TcG_09650	180,4231802	-0,420026298	0,13683951	-3,06948125	0,00214431	0,0145627	protein_codir putative oxidoreductase-like protein
TcG_09651	184,9387648	-0,440460298	0,13247614	-3,3248274	0,00088473	0,00706445	protein_codir putative mitogen-activated protein kinase
TcG_09652	106,5825385	-0,700534085	0,17710503	-3,95547259	7,6384E-05	0,00088498	protein_codir hypothetical protein
TcG_09653	123,9447652	0,142865056	0,16877617	0,84647648	0,39728699	0,62698529	protein_codir putative retrotransposon hot spot (RHS) protein
TcG_09654	92,23858895	0,104409385	0,18206867	0,57346157	0,56633221	0,75838888	protein_codir hypothetical protein
TcG_09655	97,31886757	-0,037027972	0,20624261	-0,17953599	0,85751686	0,93122039	
TcG_09656	709,9962515	-0,040015124	0,07756161	-0,51591407	0,60591443	0,78692126	protein_codir surface protein-2
TcG_09657	0,311709618	1,504707249	2,92454247	0,51451031	0,60689526	1	
TcG_09658	108,1273679	0,185732153	0,17952327	1,03458541	0,30086257	0,53419983	protein_codir hypothetical protein
TcG_09659	69,0827392	0,167358382	0,21258189	0,78726546	0,43112648	0,65577411	protein_codir hypothetical protein
TcG_09660	26,43336405	-0,367471981	0,38462801	-0,95539579	0,33937758	0,5734328	protein_codir hypothetical protein
TcG_09661	24,76957137	0,178553166	0,35729146	0,49974093	0,61725751	0,793034	protein_codir hypothetical protein
TcG_09662	83,93026647	0,019877021	0,20562291	0,09666735	0,92299057	0,96257611	protein_codir hypothetical protein
TcG_09663	89,52280415	0,277240148	0,19709651	1,40662132	0,15953971	0,3621104	protein_codir hypothetical protein
TcG_09664	184,2473174	0,337605889	0,13675692	2,46865677	0,01356212	0,06295698	protein_codir putative retrotransposon hot spot (RHS) protein
TcG_09665	277,1543843	0,129942675	0,11085839	1,17215015	0,24113678	0,4698198	protein_codir hypothetical protein
TcG_09666	100,5089945	0,120626799	0,18218194	0,66212272	0,50789257	0,71473866	protein_codir hypothetical protein
TcG_09667	34,17198295	-0,15149121	0,30856544	-0,49095326	0,62345951	0,79702106	protein_codir hypothetical protein
TcG_09668	28,80399258	0,055213185	0,33313274	0,16573929	0,86836212	0,93674123	protein_codir hypothetical protein
TcG_09669	123,0511737	0,251133731	0,16269853	1,54355251	0,12269678	0,30767149	protein_codir surface protein-2

TcG_09670	37,50544866	-0,096051491	0,28884749	-0,33253358	0,73948639	0,86708727	protein_codir	trans-sialidase
TcG_09671	12,33444434	0,336884457	0,50260313	0,67027927	0,50267978	1	protein_codir	hypothetical protein
TcG_09672	17,26829711	0,339064252	0,42958025	0,78929199	0,42994137	0,65483116	protein_codir	hypothetical protein
TcG_09673	14,30031693	0,464039439	0,50741552	0,91451566	0,36044595	1	protein_codir	hypothetical protein
TcG_09674	24,58652316	0,109981268	0,34870641	0,31539789	0,75245957	0,87512513		
TcG_09675	47,06125047	-0,185999882	0,25479074	-0,73001036	0,46538385	0,6821385		
TcG_09676	200,61702	-0,030262793	0,13222802	-0,22886823	0,81897133	0,91079764	protein_codir	retrotransposon hot spot (RHS) protein
TcG_09677	101,2634682	0,070743126	0,17649293	0,40082696	0,68854753	0,83839548	protein_codir	retrotransposon hot spot (RHS) protein
TcG_09678	124,651245	-0,122134612	0,1572466	-0,7767075	0,43733136	0,66071414	protein_codir	putative retrotransposon hot spot (RHS) protein
TcG_09679	15,98175873	0,029637678	0,46897509	0,0631967	0,94960987	1	protein_codir	retrotransposon hot spot (RHS) protein
TcG_09680	16,89633139	0,666570129	0,44481224	1,49854269	0,13399231	0,32557556		
TcG_09681	0					1		
TcG_09682	1784,54606	0,492168804	0,05200464	9,46393948	2,9655E-21	6,0279E-19	protein_codir	hypothetical protein
TcG_09683	541,7631145	0,47504353	0,08447871	5,62323381	1,8742E-08	5,64E-07	protein_codir	dynein heavy chain, cytosolic
TcG_09684	1843,432094	0,611507136	0,05575961	10,9668477	5,5162E-28	2,4581E-25	protein_codir	putative tryptophanyl-tRNA synthetase
TcG_09685	797,7084888	0,610502948	0,0706351	8,64305288	5,4731E-18	8,1296E-16	protein_codir	tryptophanyl-tRNA synthetase
TcG_09686	611,644016	0,746529803	0,07905167	9,44356834	3,603E-21	7,1972E-19	protein_codir	HUS1 checkpoint protein
TcG_09687	2563,989267	0,613473792	0,045564	13,4640036	2,5473E-41	3,2792E-38	protein_codir	putative helicase
TcG_09688	0					1	protein_codir	hypothetical protein
TcG_09689	9,689680376	0,900161175	0,57121979	1,57585783	0,11505857	1	protein_codir	putative helicase
TcG_09690	62,35472552	0,506366617	0,24790058	2,04261977	0,04109009	0,14400176	protein_codir	putative helicase
TcG_09691	749,8041375	0,480162953	0,06969756	6,88923602	5,6093E-12	3,7566E-10	protein_codir	putative NAD(P)-dependent oxidoreductase
TcG_09692	634,3574954	0,542395473	0,07705533	7,0390388	1,9357E-12	1,4017E-10	protein_codir	ATP-binding cassette protein subfamily C, member 2
TcG_09693	182,1100367	0,492424092	0,13179148	3,73638777	0,00018668	0,00188735	protein_codir	hypothetical protein
TcG_09694	259,1517344	0,035770822	0,11313381	0,31618154	0,75186472	0,87492919	protein_codir	hypothetical protein
TcG_09695	1066,932047	0,483292126	0,06215366	7,77576254	7,4994E-15	7,8989E-13	protein_codir	putative glycosyl transferase
TcG_09696	363,7410249	0,284592546	0,09577341	2,97151929	0,0029633	0,01882282	protein_codir	hypothetical protein
TcG_09697	418,0447776	0,27565524	0,09152885	3,01167601	0,0025981	0,01693001	protein_codir	(H)-ATPase G subunit
TcG_09698	91,3608603	0,161195792	0,1894686	0,85077839	0,39489247	0,62451872	protein_codir	hypothetical protein
TcG_09699	167,9780854	0,426771971	0,15741319	2,71115766	0,00670487	0,03611467	protein_codir	hypothetical protein
TcG_09700	116,0275125	0,439895429	0,17970343	2,44789673	0,01436928	0,06577737	protein_codir	hypothetical protein
TcG_09701	29,55343828	-0,137197832	0,31740533	-0,43224804	0,66556114	0,82504261	protein_codir	hypothetical protein
TcG_09702	33,21159577	-0,22288425	0,31528671	-0,70692561	0,47961271	0,69390782	protein_codir	target of rapamycin (TOR) kinase 1
TcG_09703	4,218758954	1,097153914	0,87247826	1,25751433	0,20856745	1	protein_codir	target of rapamycin (TOR) kinase 1
TcG_09704	38,67604091	-0,164887128	0,28262937	-0,58340407	0,55962132	0,75401472	protein_codir	hypothetical protein
TcG_09705	58,58831461	0,215732589	0,24608709	0,87665138	0,38067602	0,61095891	protein_codir	retrotransposon hot spot protein (RHS)
TcG_09706	127,1515476	0,074101992	0,15812804	0,4686202	0,63934114	0,80805132	protein_codir	retrotransposon hot spot (RHS) protein
TcG_09707	281,7934322	0,140423304	0,11280523	1,24482971	0,21319435	0,43671672	protein_codir	retrotransposon hot spot (RHS) protein
TcG_09708	104,3166818	0,069818227	0,17224007	0,40535414	0,68521721	0,83655708	protein_codir	trans-sialidase
TcG_09709	73,71758919	0,142305964	0,20275027	0,70187804	0,48275523	0,69628628	protein_codir	trans-sialidase
TcG_09710	72,06704016	0,320912256	0,20710049	1,54954854	0,12124991	0,30552446	protein_codir	trans-sialidase
TcG_09711	57,20895656	-0,086794227	0,25152961	-0,34506565	0,73004502	0,86096321	protein_codir	hypothetical protein
TcG_09712	45,37393988	-0,110996086	0,26231662	-0,42313782	0,67219471	0,82829035	protein_codir	trans-sialidase
TcG_09713	30,12111838	-0,771214635	0,32153237	-2,39855989	0,01645968	0,07256541	protein_codir	mucin TcMUCII
TcG_09714	8,742357064	0,097583328	0,59619789	0,16367607	0,86998616	1		
TcG_09715	48,54967828	0,79282971	0,2606063	3,04225071	0,00234816	0,01565407	protein_codir	dispersed gene family protein 1 (DGF-1)
TcG_09716	48,87539166	0,346776284	0,25856075	1,34117915	0,1798623	0,39111947	protein_codir	dispersed gene family protein 1 (DGF-1)
TcG_09717	29,09378883	0,536292236	0,34173212	1,5693352	0,11656986	0,2978122	protein_codir	dispersed gene family protein 1 (DGF-1)
TcG_09718	10,56215864	0,196051746	0,53370539	0,36734076	0,71336485	1	protein_codir	dispersed gene family protein 1 (DGF-1)
TcG_09719	111,742307	-0,067187095	0,17684714	-0,37991621	0,70400761	0,84742878	protein_codir	hypothetical protein
TcG_09720	72,13661314	0,229701825	0,20885335	1,09982351	0,27140903	0,50457365	protein_codir	trans-sialidase
TcG_09721	18,72732113	-0,082128121	0,40253142	-0,2040291	0,83833076	0,92144011	protein_codir	trans-sialidase

TcG_09722	5,740243319	-0,487814086	0,78152629	-0,62418129	0,53250853	1	protein_codir	hypothetical protein
TcG_09723	121,6316185	0,196182034	0,16809712	1,16707549	0,24317988	0,47146622	protein_codir	trans-sialidase
TcG_09724	17,45665262	-0,384235213	0,42333985	-0,90762826	0,36407466	0,59603914	protein_codir	mucin-associated surface protein (MASP)
TcG_09725	248,2822996	0,396876602	0,11594706	3,42291219	0,00061954	0,00517147	protein_codir	hypothetical protein
TcG_09726	917,9035866	0,449024167	0,06510553	6,89686725	5,3162E-12	3,6019E-10	protein_codir	putative NADP-dependent alcohol hydrogenase
TcG_09727	12,61739903	0,389904431	0,5152421	0,75674025	0,4492055	1		
TcG_09728	75,23601576	0,55221174	0,21494013	2,56914208	0,01019506	0,05011982	protein_codir	hypothetical protein
TcG_09729	45,43793168	0,184800035	0,25951404	0,71210034	0,47640264	0,69175791	protein_codir	beta galactofuranosyl glycosyltransferase
TcG_09730	55,79700927	0,363459001	0,23892682	1,52121472	0,12820596	0,31717445	protein_codir	hypothetical protein
TcG_09731	49,2297723	0,007059648	0,24628503	0,02866454	0,97713214	0,99017719		
TcG_09732	11,09733656	0,054925984	0,53698065	0,10228671	0,9185291	1	protein_codir	hypothetical protein
TcG_09733	236,7768648	0,218952585	0,11526454	1,89956587	0,05749011	0,182688	protein_codir	exo-alpha-sialidase
TcG_09734	87,65314248	0,262305951	0,1910354	1,37307512	0,16972899	0,37606704	protein_codir	dispersed gene family protein 1 (DGF-1)
TcG_09735	90,42292919	0,102910732	0,18811654	0,54705841	0,58433861	0,77293608	protein_codir	dispersed gene family protein 1 (DGF-1)
TcG_09736	208,3845462	0,097502385	0,13083588	0,74522665	0,45613471	0,67606203	protein_codir	dispersed gene family protein 1 (DGF-1)
TcG_09737	80,83350254	0,241041924	0,19358155	1,24516991	0,2130693	0,43661495	protein_codir	putative dispersed gene family protein 1 (DGF-1)
TcG_09738	25,68865915	-0,184667703	0,33803508	-0,54629745	0,58486149	0,77353941		
TcG_09739	61,04219739	0,121500848	0,22862242	0,53144765	0,59510861	0,78032236		
TcG_09740	2,565046697	-1,283820586	1,19367316	-1,07552102	0,28214152	1		
TcG_09741	9,479345559	-0,633980056	0,59364828	-1,06793884	0,2855481	1	protein_codir	mucin-associated surface protein (MASP)
TcG_09742	11,78512327	-0,427374515	0,51111834	-0,83615571	0,40306731	1	protein_codir	trans-sialidase
TcG_09743	26,67907629	0,211228967	0,34623368	0,61007631	0,54181126	0,74008786	protein_codir	putative trans-sialidase
TcG_09744	88,90332868	0,008922415	0,18560994	0,04807079	0,96165983	0,98226138	protein_codir	retrotransposon hot spot (RHS) protein
TcG_09745	27,33154674	-0,24098784	0,33367302	-0,72222753	0,4701546	0,68645032	protein_codir	retrotransposon hot spot (RHS) protein
TcG_09746	9,883561214	-0,204290842	1,04159807	-0,19613212	0,84450675	1		
TcG_09747	27,27666963	0,074018811	0,32915188	0,22487738	0,82207467	0,91187534	protein_codir	hypothetical protein
TcG_09748	19,85267525	-0,739291045	0,40236005	-1,8373868	0,06615281	0,20395064	protein_codir	trans-sialidase
TcG_09749	40,50820882	-0,217154728	0,27081286	-0,8018627	0,42263239	0,64883106	protein_codir	putative trans-sialidase
TcG_09750	2,233256515	-1,73338103	1,23482459	-1,40374677	0,16039427	1	protein_codir	hypothetical protein
TcG_09751	1,396881362	-0,487302077	1,59500973	-0,30551668	0,75997267	1	protein_codir	hypothetical protein
TcG_09752	38,81122912	-0,121960468	0,28928848	-0,42158771	0,67332598	0,82890021	protein_codir	putative retrotransposon hot spot (RHS) protein
TcG_09753	44,86828967	0,125745692	0,26148236	0,48089551	0,63059077	0,80202553	protein_codir	retrotransposon hot spot (RHS) protein
TcG_09754	17,83736764	0,74169534	0,4279258	1,73323353	0,08305417	0,23681114	protein_codir	retrotransposon hot spot (RHS) protein
TcG_09755	27,59635285	0,474570615	0,35366201	1,34187616	0,17963615	0,39078528	protein_codir	retrotransposon hot spot (RHS) protein
TcG_09756	3,173424923	-0,520046965	1,04814496	-0,49615939	0,61978196	1	protein_codir	hypothetical protein
TcG_09757	5,740369497	0,215183286	0,7321804	0,29389381	0,76883905	1	protein_codir	hypothetical protein
TcG_09758	6,185592779	1,538468096	0,75924981	2,02630027	0,04273402	1	protein_codir	hypothetical protein
TcG_09759	11,72142779	0,491233007	0,51961428	0,94538011	0,34446485	1		
TcG_09760	8,169240208	0,463278601	0,61011553	0,75932931	0,44765559	1	protein_codir	trans-sialidase
TcG_09761	31,51881989	0,101708058	0,30504346	0,33342154	0,73881611	0,86691548	protein_codir	hypothetical protein
TcG_09762	17,53177865	0,71708136	0,42934746	1,67016559	0,09488661	0,25952696	protein_codir	trans-sialidase
TcG_09763	50,95660295	0,250098999	0,24531373	1,01950675	0,30796245	0,54112123	protein_codir	retrotransposon hot spot (RHS) protein
TcG_09764	97,49081231	-0,369953091	0,17499927	-2,11402653	0,034513	0,12714392	protein_codir	retrotransposon hot spot (RHS) protein
TcG_09765	74,20201932	-0,144199935	0,21649612	-0,66606246	0,50537118	0,7124885	protein_codir	retrotransposon hot spot protein (RHS)
TcG_09766	82,60700777	0,167417895	0,20604862	0,81251644	0,41649536	0,64343944	protein_codir	putative retrotransposon hot spot (RHS) protein
TcG_09767	140,2497712	0,591807418	0,15181739	3,89815314	9,6929E-05	0,00108609	protein_codir	retrotransposon hot spot (RHS) protein
TcG_09768	14,10271705	0,756596797	0,48214974	1,56921539	0,11659776	1	protein_codir	hypothetical protein
TcG_09769	15,23377977	1,423651069	0,47913327	2,97130494	0,00296537	1		
TcG_09770	12,03124787	1,170561571	0,5377373	2,17682793	0,02949341	1	protein_codir	trans-sialidase
TcG_09771	7,632924125	0,359544253	0,66319964	0,54213578	0,58772497	1	protein_codir	hypothetical protein
TcG_09772	54,27627409	0,733094707	0,24774304	2,95909305	0,00308546	0,01949189	protein_codir	subtilisin-like serine peptidase
TcG_09773	67,6580768	0,310687349	0,22416863	1,38595373	0,16576103	0,37132779	protein_codir	putative kinesin

TcG_09774	392,8382238	-0,121130249	0,09370714	-1,29264701	0,19613317	0,41414233	protein_codir putative retrotransposon hot spot (RHS) protein
TcG_09775	9,576479849	1,088516298	0,58959063	1,84622388	0,0648597	1	protein_codir retrotransposon hot spot (RHS) protein
TcG_09776	144,6650167	0,081826671	0,14602051	0,56037795	0,57522167	0,76621273	protein_codir retrotransposon hot spot (RHS) protein
TcG_09777	31,51662428	0,233816547	0,31858288	0,7339269	0,46299329	0,6805767	protein_codir putative kinesin
TcG_09778	23,83103715	0,503875428	0,37841129	1,33155495	0,18300648	0,3952858	protein_codir putative kinesin
TcG_09779	4,925622722	0,504843403	0,78536088	0,6428171	0,52034279	1	
TcG_09780	24,44592774	1,155472917	0,37519618	3,07965003	0,00207244	0,01417431	protein_codir hypothetical protein
TcG_09781	6,097667183	-0,505244983	0,74803323	-0,67543121	0,49940184	1	protein_codir putative ATP-dependent DEAD/H RNA helicase
TcG_09782	32,39376527	0,744901082	0,31265838	2,38247599	0,01719665	0,07487427	protein_codir retrotransposon hot spot (RHS) protein
TcG_09783	42,94841486	0,253206917	0,26692017	0,94862413	0,34281181	0,57679606	protein_codir retrotransposon hot spot (RHS) protein
TcG_09784	9,204011588	-0,019363671	0,56154908	-0,0344826	0,97249232	1	protein_codir hypothetical protein
TcG_09785	561,7568778	0,086722024	0,07971658	1,08787936	0,27664837	0,50901191	protein_codir putative beta galactofuranosyl glycosyltransferase
TcG_09786	27,35805467	-0,134013301	0,33447082	-0,40067262	0,68866117	0,83839548	protein_codir hypothetical protein
TcG_09787	35,28792158	-0,020023445	0,30526009	-0,0655947	0,94770051	0,97539448	protein_codir hypothetical protein
TcG_09788	28,17919482	0,354727971	0,36700373	0,96655141	0,33376833	0,56822639	protein_codir hypothetical protein
TcG_09789	97,34311235	-0,443359381	0,20399913	-2,17333954	0,02975477	0,11367369	protein_codir trans-sialidase
TcG_09790	50,88248657	-0,304165521	0,25123771	-1,21066824	0,22602258	0,45243566	protein_codir trans-sialidase
TcG_09791	83,48441172	-0,183168766	0,18899913	-0,96915135	0,33246968	0,56646966	protein_codir trans-sialidase
TcG_09792	88,81986587	-0,209972036	0,19182899	-1,09457925	0,27370101	0,50688937	protein_codir retrotransposon hot spot (RHS) protein
TcG_09793	53,96720407	-0,275843436	0,25263016	-1,09188639	0,27488304	0,50755645	protein_codir trans-sialidase
TcG_09794	269,8201334	-0,193094484	0,10796176	-1,78854511	0,0736881	0,21891035	protein_codir sialidase-like protein
TcG_09795	24,31193253	0,45180052	0,35824272	1,26115759	0,20725208	0,42947998	protein_codir hypothetical protein
TcG_09796	105,9899004	-0,232880146	0,17184177	-1,35520108	0,1753535	0,38463568	protein_codir kinesin
TcG_09797	18,72706845	0,070708391	0,4228861	0,16720434	0,86720927	0,93578156	protein_codir subtilisin-like serine peptidase
TcG_09798	21,70857136	-0,244355618	0,39105992	-0,62485467	0,53206645	0,7329332	protein_codir putative trans-sialidase
TcG_09799	25,56126991	-0,390193391	0,35104739	-1,11151202	0,26634802	0,4990149	protein_codir hypothetical protein
TcG_09800	505,7650642	-0,112180965	0,08393506	-1,33652095	0,18137906	0,39294274	protein_codir putative retrotransposon hot spot (RHS) protein
TcG_09801	35,73127129	-0,16738842	0,31599377	-0,52972063	0,59630564	0,78066886	protein_codir retrotransposon hot spot (RHS) protein
TcG_09802	43,49610484	0,017947282	0,2763353	0,06494748	0,94821581	0,97570906	protein_codir hypothetical protein
TcG_09803	40,35235385	-0,095225643	0,27119567	-0,35113261	0,72548887	0,85817259	
TcG_09804	40,39916834	0,172827074	0,27303051	0,63299547	0,5267366	0,72926645	protein_codir hypothetical protein
TcG_09805	81,99390358	-0,556891364	0,19416526	-2,86813075	0,00412905	0,02476147	protein_codir putative trans-sialidase
TcG_09806	15,44722608	-0,756385075	0,43988702	-1,71949851	0,08552364	1	protein_codir trans-sialidase
TcG_09807	19,79145939	-0,104519816	0,38846391	-0,26905927	0,78788408	0,8945928	protein_codir trans-sialidase
TcG_09808	162,5487382	-0,512707334	0,13867815	-3,69710244	0,00021807	0,00214119	protein_codir hypothetical protein
TcG_09809	550,4085212	-0,666677391	0,08058095	-8,27338734	1,3021E-16	1,6049E-14	protein_codir hypothetical protein
TcG_09810	202,2099809	-0,499723299	0,13724304	-3,64115574	0,00027142	0,00256915	protein_codir putative vacuolar ATP synthase subunit c
TcG_09811	354,2760431	-0,380221905	0,09974615	-3,81189559	0,00013791	0,00145782	protein_codir hypothetical protein
TcG_09812	302,1355832	-0,315780641	0,10360507	-3,04792665	0,00230426	0,01543189	protein_codir peroxisomal enoyl-CoA hydratase
TcG_09813	70,14318847	-0,474084515	0,21889065	-2,16585091	0,03032258	0,11529945	protein_codir putative RNA-binding protein
TcG_09814	55,01668191	-0,065351451	0,23383501	-0,27947676	0,77987897	0,89039	protein_codir dispersed gene family protein 1 (DGF-1)
TcG_09815	18,46359217	-0,120158545	0,39637249	-0,30314552	0,76177897	0,87969412	protein_codir hypothetical protein
TcG_09816	21,69544316	0,086700589	0,37291575	0,23249377	0,81615452	0,90922753	protein_codir hypothetical protein
TcG_09817	36,24902756	0,322219452	0,33430896	0,96383732	0,33512749	0,56965773	protein_codir trans-sialidase
TcG_09818	78,31691879	-0,378527838	0,20315999	-1,86320069	0,06243404	0,19518638	protein_codir trans-sialidase
TcG_09819	54,19360492	0,00713946	0,23475977	0,03041177	0,97573866	0,98966192	protein_codir putative trans-sialidase
TcG_09820	8,811952327	0,128872593	0,58286645	0,22110141	0,82501347	1	protein_codir hypothetical protein
TcG_09821	299,9812046	-0,134669149	0,10967588	-1,22788297	0,2194909	0,4445842	
TcG_09822	9,480563945	-0,143738971	0,59440452	-0,24182012	0,80891955	1	protein_codir putative trans-sialidase
TcG_09823	24,4792029	0,717826142	0,35822694	2,00383073	0,0450882	0,15371346	protein_codir protein kinase, putative,serine/threonine protein kinase
TcG_09824	29,7768862	-0,213382295	0,32438955	-0,65779646	0,51066893	0,71742576	
TcG_09825	0,155721614	0,503022807	4,08047286	0,12327562	0,90188885	1	

TcG_09826	56,08415342	0,673873255	0,25814216	2,61047346	0,0090417	0,0456458	protein_codir	hypothetical protein
TcG_09827	2,676217546	0,21637967	1,06994402	0,20223457	0,83973335	1		
TcG_09828	37,47567982	0,850007153	0,29151202	2,9158563	0,00354714	0,0218022	protein_codir	hypothetical protein
TcG_09829	9,002453502	0,616542628	0,58659324	1,05105648	0,29323265	1		
TcG_09830	7,687375824	0,77322459	0,63581004	1,21612517	0,22393724	1		
TcG_09831	27,77784596	1,438585967	0,62766332	2,29197075	0,02190733	0,09019841	protein_codir	putative retrotransposon hot spot (RHS) protein
TcG_09832	150,9687081	0,540309407	0,15202008	3,55419754	0,00037913	0,00342107	protein_codir	putative retrotransposon hot spot (RHS) protein
TcG_09833	32,12667367	0,459348843	0,31776462	1,44556322	0,14829975	0,34711129	protein_codir	hypothetical protein
TcG_09834	24,57737377	0,782722457	0,38125881	2,05299508	0,04007305	0,14150759	protein_codir	hypothetical protein
TcG_09835	13,79499693	0,72700659	0,49754034	1,4612013	0,14396021	1	protein_codir	trans-sialidase
TcG_09836	7,112306187	0,170294072	0,65233895	0,26105152	0,79405278	1		
TcG_09837	45,85814368	0,34727285	0,27032964	1,28462734	0,19892254	0,4178224	protein_codir	hypothetical protein
TcG_09838	441,9949756	-0,207005869	0,08618611	-2,40184725	0,01631252	0,07216625	protein_codir	hypothetical protein
TcG_09839	120,4563867	-0,00946823	0,16366068	-0,05785281	0,95386587	0,97835429	protein_codir	HD superfamily hydrolase
TcG_09840	282,2415933	0,261941801	0,11150425	2,34916429	0,0188156	0,07999911	protein_codir	hypothetical protein
TcG_09841	100,1649068	0,207093336	0,18154738	1,14071237	0,25398964	0,48503774		
TcG_09842	335,5990746	0,12922175	0,10999775	1,17476723	0,24008786	0,46876608	protein_codir	hypothetical protein
TcG_09843	318,9922467	-0,162600876	0,10382893	-1,566046	0,11733785	0,29871451	protein_codir	putative flagellar antigen
TcG_09844	9,443246824	-0,186399751	0,58319438	-0,31961857	0,7492575	1	protein_codir	hypothetical protein
TcG_09845	54,6690978	-0,025055399	0,23283568	-0,10760979	0,91430523	0,95822165	protein_codir	retrotransposon hot spot (RHS) protein
TcG_09846	37,40755816	0,187429108	0,29567928	0,63389328	0,52615047	0,72894486	protein_codir	putative retrotransposon hot spot (RHS) protein
TcG_09847	54,06497026	0,109097263	0,23894928	0,45657079	0,64797958	0,81260383	protein_codir	retrotransposon hot spot (RHS) protein
TcG_09848	26,17800285	-0,163441555	0,35088512	-0,46579791	0,64136016	0,8096316	protein_codir	retrotransposon hot spot (RHS) protein
TcG_09849	195,1573248	0,052186978	0,12898484	0,40459777	0,6857732	0,83697127	protein_codir	putative retrotransposon hot spot (RHS) protein
TcG_09850	24,17275432	-0,030230604	0,35490152	-0,08518026	0,93211808	0,96743887	protein_codir	retrotransposon hot spot (RHS) protein
TcG_09851	16,54647642	0,373641378	0,42537896	0,87837296	0,37974135	1	protein_codir	trans-sialidase
TcG_09852	10,05154447	0,24034963	0,54293599	0,44268502	0,65799358	1	protein_codir	hypothetical protein
TcG_09853	17,03638164	0,294393548	0,42440963	0,69365426	0,48789905	0,69945302	protein_codir	trans-sialidase
TcG_09854	15,12125526	0,805085836	0,48500344	1,65995903	0,0969227	1	protein_codir	hypothetical protein
TcG_09855	15,99171596	0,047770549	0,44061122	0,10841882	0,91366347	1	protein_codir	hypothetical protein
TcG_09856	19,59780498	0,437732368	0,39477956	1,10880202	0,2675156	0,49992998	protein_codir	retrotransposon hot spot (RHS) protein
TcG_09857	102,199584	0,236434724	0,17650397	1,33954339	0,18039384	0,39183409	protein_codir	hypothetical protein
TcG_09858	62,22716077	-0,29950535	0,22384875	-1,3379809	0,18090266	0,39227742	protein_codir	trans-sialidase
TcG_09859	91,61193274	-0,233186602	0,18417245	-1,26613185	0,20546588	0,42684735	protein_codir	trans-sialidase
TcG_09860	18,47242493	0,722226036	0,41467229	1,74167906	0,08156462	0,2342607	protein_codir	hypothetical protein
TcG_09861	12,30526765	0,294356993	0,49451269	0,59524659	0,55167865	1	protein_codir	trans-sialidase
TcG_09862	8,620300384	0,823727281	0,62712064	1,3135069	0,1890122	1	protein_codir	trans-sialidase
TcG_09863	23,3987098	0,414646307	0,36310413	1,14194875	0,25347532	0,48445481	protein_codir	putative elongation factor 1-gamma (EF-1-gamma)
TcG_09864	138,33865	0,104878674	0,17635717	0,5946947	0,55204756	0,74737358	protein_codir	putative mucin-associated surface protein (MASP)
TcG_09865	196,6819702	-0,274572553	0,12854222	-2,13604957	0,03267537	0,1219642	protein_codir	putative trans-sialidase
TcG_09866	13,90442649	0,281729497	0,52973251	0,5318335	0,59484132	1	protein_codir	hypothetical protein
TcG_09867	9,899317521	0,191558723	0,55848772	0,34299541	0,73160192	1	protein_codir	hypothetical protein
TcG_09868	538,6912525	0,15867128	0,09635893	1,64666919	0,09962606	0,26802034	protein_codir	hypothetical protein
TcG_09869	142,9721671	0,291879159	0,15088506	1,9344471	0,05305818	0,17209745	protein_codir	Arf/Sar family, other
TcG_09870	91,80722108	0,083142827	0,18258117	0,4553746	0,64883976	0,81328019	protein_codir	hypothetical protein
TcG_09871	245,6932443	-0,079893472	0,11312001	-0,70627179	0,48001913	0,69423314	protein_codir	membrane protein YIP1
TcG_09872	335,4046094	0,039481068	0,09913735	0,39824616	0,69044874	0,83922987	protein_codir	hypothetical protein
TcG_09873	66,0778962	0,187529086	0,21652747	0,86607527	0,38644888	0,61672132		
TcG_09874	88,10376798	-0,04961593	0,18691758	-0,26544283	0,79066835	0,8952767	protein_codir	retrotransposon hot spot (RHS) protein
TcG_09875	35,0007416	0,22585738	0,30120374	0,74984918	0,45334555	0,67382444	protein_codir	hypothetical protein
TcG_09876	75,47302882	-0,006135613	0,20646643	-0,02971724	0,97629256	0,9898771	protein_codir	putative trans-sialidase
TcG_09877	50,16662506	-0,045693241	0,26863852	-0,17009192	0,86493785	0,93498506	protein_codir	trans-sialidase

TcG_09878	85,64338563	0,024799885	0,20201063	0,12276525	0,90229299	0,95278588	protein_codir	hypothetical protein
TcG_09879	83,90004895	0,17546029	0,20406773	0,85981398	0,38989159	0,6201653	protein_codir	hypothetical protein
TcG_09880	353,5450235	-0,086344325	0,09705102	-0,88967973	0,37363788	0,60449626	protein_codir	putative retrotransposon hot spot (RHS) protein
TcG_09881	263,4547419	-0,018357721	0,11332325	-0,1619943	0,87131034	0,93802282	protein_codir	complement regulatory protein
TcG_09882	32,83362398	0,168053555	0,31198264	0,53866315	0,5901193	0,77623888	protein_codir	hypothetical protein
TcG_09883	15,20171783	0,536506355	0,46718331	1,14838511	0,25080962	1	protein_codir	hypothetical protein
TcG_09884	56,56397574	-0,015692756	0,24411059	-0,06428544	0,94874295	0,97603763	protein_codir	putative trans-sialidase
TcG_09885	51,46629793	-0,393014897	0,26171254	-1,50170451	0,13317344	0,32441177	protein_codir	trans-sialidase
TcG_09886	95,38710979	0,216352683	0,18497668	1,1696214	0,24215336	0,47089863	protein_codir	putative calpain-like cysteine peptidase
TcG_09887	115,0109847	0,363675524	0,16842859	2,15922678	0,03083258	0,11662626	protein_codir	putative calpain-like cysteine peptidase
TcG_09888	498,3470078	0,030241863	0,08663924	0,34905502	0,72704801	0,85927072	protein_codir	putative calpain-like cysteine peptidase
TcG_09889	0,155988004	0,503022807	4,08047286	0,12327562	0,90188885	1	protein_codir	hypothetical protein
TcG_09890	4,162009418	-0,603220409	0,83210911	-0,72492946	0,4684953	1	protein_codir	hypothetical protein
TcG_09891	0,389843477	1,884304201	2,66693245	0,70654366	0,47985011	1	protein_codir	hypothetical protein
TcG_09892	4,972741877	0,057681257	0,82525224	0,06989531	0,94427699	1	protein_codir	hypothetical protein
TcG_09893	1,106910793	0,187511471	1,68231129	0,11146063	0,91125109	1	protein_codir	hypothetical protein
TcG_09894	0,806916499	1,32353367	2,06474826	0,64101455	0,52151323	1	protein_codir	hypothetical protein
TcG_09895	1,953462496	0,94151317	1,27604172	0,73783886	0,46061237	1	protein_codir	surface protease GP63
TcG_09896	4,609508534	0,660230663	0,88347775	0,74730876	0,4548772	1	protein_codir	hypothetical protein
TcG_09897	7,591318811	1,472468561	0,67736939	2,17380441	0,02971983	1	protein_codir	retrotransposon hot spot (RHS) protein
TcG_09898	0,794106782	-1,999049109	2,09320552	-0,95501808	0,33956856	1	protein_codir	retrotransposon hot spot (RHS) protein
TcG_09899	27,94444783	0,649097813	0,33963567	1,91115913	0,05598413	0,17908122	protein_codir	retrotransposon hot spot (RHS) protein
TcG_09900	30,51402155	0,355001426	0,31982655	1,10998112	0,26700716	0,49939561	protein_codir	trans-sialidase
TcG_09901	15,32922462	0,469964862	0,45066744	1,04281966	0,29703182	1	protein_codir	trans-sialidase
TcG_09902	32,89076461	0,241404041	0,30252414	0,79796621	0,4248901	0,65055856	protein_codir	hypothetical protein
TcG_09903	640,0636235	0,068233679	0,08776842	0,7774286	0,43690594	0,66040735	protein_codir	hypothetical protein
TcG_09904	379,8778147	-0,147260881	0,09428001	-1,56195238	0,11829921	0,30044161	protein_codir	putative nucleic acid binding protein
TcG_09905	500,7043673	0,014910452	0,08439785	0,17666863	0,85976868	0,93261679	protein_codir	putative 3-methylcrotonoyl-CoA carboxylase beta subunit
TcG_09906	543,7145713	0,184789509	0,08312766	2,22296051	0,02621846	0,1033573	protein_codir	cytochrome b5-like
TcG_09907	40,36804291	0,137289067	0,27562609	0,49809895	0,6184143	0,793034	protein_codir	hypothetical protein
TcG_09908	182,6350347	-0,344917988	0,13525046	-2,55021681	0,01076559	0,05234166	protein_codir	putative phosphatidylinositol 3-kinase
TcG_09909	524,1008301	-0,022492939	0,08213331	-0,2738589	0,78419306	0,89320299	protein_codir	vacuolar transporter chaperone
TcG_09910	221,0682525	-0,11676931	0,1200557	-0,97262612	0,33073916	0,56523583	protein_codir	putative dynein heavy chain
TcG_09911	394,9843059	-0,398265432	0,09879595	-4,03119191	5,5495E-05	0,00067538	protein_codir	putative protein kinase
TcG_09912	109,0707572	-0,537947468	0,16998821	-3,1646163	0,00155288	0,01120275	protein_codir	hypothetical protein
TcG_09913	277,1456753	-0,429927145	0,10758464	-3,99617586	6,4374E-05	0,00076575	protein_codir	hypothetical protein
TcG_09914	347,0230924	-0,356368522	0,101502	-3,51095068	0,00044651	0,00393103	protein_codir	cleft lip and palate transmembrane 1 family protein
TcG_09915	429,0322868	-0,167760758	0,08825519	-1,90085997	0,05732036	0,1823987	protein_codir	putative ascorbate-dependent peroxidase
TcG_09916	967,5635036	-0,125819081	0,06511642	-1,93221739	0,05333268	0,17293939	protein_codir	mitochondrial RNA binding protein
TcG_09917	230,1318303	0,160691857	0,12688653	1,2664217	0,20536215	0,4267849	protein_codir	hypothetical protein
TcG_09918	239,3795184	0,171360731	0,12516338	1,36909634	0,17096916	0,37780826	protein_codir	putative U4/U6 small nuclear ribonuclear protein
TcG_09919	261,6943468	0,182545048	0,11569098	1,57786762	0,11459603	0,29439236	protein_codir	RNA polymerase B subunit RPB8
TcG_09920	82,23768243	-0,027562396	0,19438088	-0,14179582	0,88724129	0,94559631	protein_codir	calmodulin
TcG_09921	285,6893102	-0,135295159	0,11428055	-1,18388619	0,23645812	0,46378936	protein_codir	putative phopshatase
TcG_09922	241,0820953	-0,175501701	0,11631509	-1,50884728	0,13133781	0,32214765	protein_codir	putative RNA polymerase I
TcG_09923	233,9833912	-0,2221726	0,12105531	-1,83529832	0,0664615	0,20468852	protein_codir	hypothetical protein
TcG_09924	286,7014654	0,007765265	0,10691255	0,07263193	0,94209902	0,97317753	protein_codir	hypothetical protein
TcG_09925	13,56267466	-0,338878515	0,46882834	-0,72282003	0,46979046	1	protein_codir	hypothetical protein
TcG_09926	78,08807149	-0,242824377	0,20410472	-1,18970484	0,23416242	0,46189082	protein_codir	vesicle transport through interaction with t-SNARE 1
TcG_09927	66,55921775	0,245010918	0,23495534	1,04279784	0,29704193	0,53028164	protein_codir	cytidine deaminase-like protein
TcG_09928	246,4300744	0,015363775	0,11541909	0,13311295	0,89410405	0,94881323	protein_codir	tRNA-dihydrouridine synthase 3
TcG_09929	54,57785309	0,231126235	0,24080044	0,95982482	0,33714339	0,57160877	protein_codir	hypothetical protein

TcG_09930	418,5059114	-0,009424159	0,09154447	-0,10294624	0,91800564	0,96050307	protein_codir putative FG-GAP repeat protein
TcG_09931	38,81235861	-0,021723132	0,28412697	-0,07645572	0,93905654	0,97168077	
TcG_09932	98,17953664	-0,016748606	0,18320855	-0,09141825	0,92716026	0,96445311	protein_codir putative O-sialoglycoprotein endopeptidase
TcG_09933	86,29507442	0,092967929	0,19513629	0,47642561	0,63377119	0,80419712	protein_codir retrotransposon hot spot protein (RHS)
TcG_09934	18,82834089	0,476184269	0,41089588	1,15889277	0,2464999	0,47515504	protein_codir putative retrotransposon hot spot (RHS) protein
TcG_09935	2,142907056	4,292992325	1,58003862	2,71701733	0,00658732	1	protein_codir glutamine synthetase
TcG_09936	20,13688909	0,859704513	0,39258922	2,18983221	0,02853641	0,10998763	protein_codir hypothetical protein
TcG_09937	96,35253903	-0,125103546	0,17582125	-0,71153827	0,47675074	0,69195279	protein_codir trans-sialidase
TcG_09938	154,241504	-0,290959334	0,15030747	-1,93576097	0,05289698	0,17176694	protein_codir putative trans-sialidase
TcG_09939	21,90871622	1,063864035	0,41525159	2,56197464	0,01040789	0,05100924	
TcG_09940	15,5633133	0,90811035	0,46920503	1,93542329	0,05293837	1	protein_codir hypothetical protein
TcG_09941	158,5237265	0,705399483	0,14461885	4,8776455	1,0736E-06	2,1558E-05	protein_codir putative beta galactofuranosyl glycosyltransferase
TcG_09942	82,65223731	0,183468783	0,19375913	0,94689102	0,34369431	0,57794518	protein_codir hypothetical protein
TcG_09943	51,70342271	-0,216341966	0,23868108	-0,90640601	0,36472099	0,59668524	protein_codir hypothetical protein
TcG_09944	53,58530663	-0,003481945	0,23938345	-0,01454547	0,9883948	0,99535351	protein_codir putative dispersed gene family protein 1 (DGF-1)
TcG_09945	52,67280042	0,223685587	0,2549693	0,87730401	0,38032153	0,61073866	protein_codir dispersed gene family protein 1 (DGF-1)
TcG_09946	62,23807882	0,044725341	0,22482701	0,19893224	0,84231575	0,9231054	protein_codir dispersed gene family protein 1 (DGF-1)
TcG_09947	54,44664693	0,355456509	0,24759213	1,43565353	0,15110095	0,35146671	protein_codir dispersed gene family protein 1 (DGF-1)
TcG_09948	23,47060889	0,645473567	0,37451543	1,72348993	0,08479996	0,23998348	protein_codir hypothetical protein
TcG_09949	489,7444988	0,355367056	0,08387441	4,23689483	2,2663E-05	0,00030855	protein_codir hypothetical protein
TcG_09950	385,1357549	-0,079811781	0,10355502	-0,77071858	0,44087376	0,66354606	protein_codir hypothetical protein
TcG_09951	556,3151894	-0,246403567	0,07874599	-3,12909356	0,00175346	0,01239229	protein_codir hypothetical protein
TcG_09952	526,0338644	-0,073835407	0,08062387	-0,91580079	0,35977139	0,5926302	protein_codir putative katanin, putative,serine peptidase, Clan SJ, family S16
TcG_09953	159,7736283	0,210152353	0,14091882	1,49130082	0,13588253	0,32846548	protein_codir putative protein kinase
TcG_09954	491,4317972	-0,04712165	0,09190434	-0,51272496	0,60814373	0,78802211	protein_codir hypothetical protein
TcG_09955	3,896017786	0,518378621	0,89168972	0,58134417	0,56100852	1	protein_codir hypothetical protein
TcG_09956	17,97165128	0,131164773	0,44261395	0,29634125	0,76696948	0,88304764	protein_codir hypothetical protein
TcG_09957	22,49247606	-0,176304572	0,38909373	-0,45311594	0,65046525	0,81446995	
TcG_09958	65,18994651	0,29035065	0,22787508	1,27416586	0,20260464	0,42278178	protein_codir trans-sialidase-like protein
TcG_09959	42,55985583	0,53360845	0,26970814	1,97846622	0,04787614	0,16045499	protein_codir hypothetical protein
TcG_09960	46,5925475	0,231514249	0,26114515	0,88653476	0,37532943	0,60590314	protein_codir putative retrotransposon hot spot (RHS) protein
TcG_09961	150,8170713	0,158833064	0,14754377	1,07651488	0,28169705	0,51481079	protein_codir retrotransposon hot spot (RHS) protein
TcG_09962	2,966090704	0,314319422	1,07191319	0,29323216	0,7693447	1	protein_codir complement regulatory protein
TcG_09963	15,73043095	-0,043853854	0,44951947	-0,09755718	0,92228392	1	protein_codir hypothetical protein
TcG_09964	7,432631927	-0,029033785	0,64235367	-0,04519906	0,96394864	1	protein_codir retrotransposon hot spot (RHS) protein
TcG_09965	19,62301353	0,960177129	0,4014548	2,39174405	0,01676853	0,07353328	protein_codir retrotransposon hot spot protein (RHS)
TcG_09966	8,858686955	0,740276582	0,62490827	1,1846164	0,23616915	1	protein_codir retrotransposon hot spot protein (RHS)
TcG_09967	18,99541228	0,553848574	0,46638376	1,1875383	0,23501536	0,46291872	
TcG_09968	7,510871821	1,280764233	0,66388065	1,92920855	0,05370498	1	protein_codir putative trans-sialidase
TcG_09969	5,98857101	1,138277024	0,74797767	1,52180617	0,12805766	1	protein_codir selenocysteine-tRNA-specific elongation factor
TcG_09970	5,38622932	1,345809959	0,79569849	1,69135669	0,0907687	1	protein_codir trans-sialidase
TcG_09971	5,250743991	0,478880673	0,74783167	0,64035891	0,52193929	1	protein_codir trans-sialidase
TcG_09972	75,98610392	0,330400537	0,20693288	1,59665555	0,11034251	0,2871582	protein_codir rab1 small GTP-binding protein
TcG_09973	0,155988004	0,503022807	4,08047286	0,12327562	0,90188885	1	protein_codir hypothetical protein
TcG_09974	6,744569872	0,871922638	0,67741857	1,28712539	0,19805059	1	protein_codir serine/threonine protein phosphatase
TcG_09975	19,85201391	0,751262896	0,39757399	1,8896178	0,05880909	0,18626631	protein_codir 90 kDa surface protein
TcG_09976	6,429649967	1,030597111	0,7113107	1,44887053	0,14737374	1	
TcG_09977	317,416279	0,13643546	0,1099885	1,24045207	0,21480823	0,43870407	protein_codir surface protease GP63
TcG_09978	7,740585153	0,259145992	0,65312193	0,39678042	0,69152939	1	protein_codir hypothetical protein
TcG_09979	58,88332355	0,102328834	0,23043418	0,44406968	0,6569922	0,81910165	protein_codir hypothetical protein
TcG_09980	0,155988004	0,503022807	4,08047286	0,12327562	0,90188885	1	protein_codir hypothetical protein
TcG_09981	4,434088145	0,612219309	0,88954488	0,68823881	0,49130241	1	protein_codir hypothetical protein

TcG_09982	47,61663192	-0,28888186	0,25857338	-1,11721424	0,26390273	0,49619385	protein_codir	trans-sialidase
TcG_09983	15,84061585	0,940712956	0,4693368	2,00434518	0,0450331	1	protein_codir	putative syntaxin binding protein
TcG_09984	6,335101057	0,066778297	0,68652105	0,09727057	0,92251152	1	protein_codir	syntaxin binding protein
TcG_09985	11,98202282	0,215170139	0,54616175	0,39396779	0,69360482	1	protein_codir	hypothetical protein
TcG_09986	6,025746725	0,20368999	0,71879681	0,28337631	0,77688838	1	protein_codir	putative surface protease GP63
TcG_09987	7,910830863	-0,144189334	0,61214923	-0,23554605	0,81378494	1	protein_codir	hypothetical protein
TcG_09988	78,94685077	-0,444006375	0,19663709	-2,25799911	0,02394572	0,09666727	protein_codir	putative mucin-associated surface protein (MASP)
TcG_09989	125,0204653	0,201258404	0,17300534	1,16330748	0,24470477	0,47286474	protein_codir	retrotransposon hot spot protein (RHS)
TcG_09990	6,900466997	0,235173091	0,67949665	0,34609897	0,72926834	1	protein_codir	retrotransposon hot spot (RHS) protein
TcG_09991	48,83069586	-0,174204199	0,25496862	-0,68323781	0,49445659	0,70453824	protein_codir	hypothetical protein
TcG_09992	208,7400239	0,206840529	0,13629388	1,51760688	0,12911353	0,31841409	protein_codir	dispersed gene family protein 1 (DGF-1)
TcG_09993	102,3268967	-0,072387087	0,1765289	-0,41005799	0,68176341	0,83421938	protein_codir	dispersed gene family protein 1 (DGF-1)
TcG_09994	8,558291692	-0,51098823	0,5953923	-0,85823789	0,39076112	1	protein_codir	dispersed protein family protein 1 (DGF-1)
TcG_09995	118,2565666	-0,08567293	0,16772024	-0,51080852	0,60948514	0,7885533		
TcG_09996	267,1890707	0,090643576	0,11248715	0,80581273	0,42035084	0,64685679	protein_codir	surface protease GP63
TcG_09997	461,1482282	0,244832425	0,08623269	2,83920679	0,00452258	0,02662676	protein_codir	putative surface protease GP63
TcG_09998	278,369411	0,171374992	0,10693918	1,60254634	0,10903485	0,28516428	protein_codir	L1Tc protein
TcG_09999	250,6508934	0,334652663	0,1214745	2,75492105	0,00587063	0,03248191	protein_codir	L1Tc protein
TcG_10000	390,6782529	0,185462907	0,09154341	2,0259558	0,04276931	0,1481391	protein_codir	hypothetical protein
TcG_10001	2,57981012	0,653275025	1,08198091	0,60377685	0,54599201	1	protein_codir	surface protease GP63
TcG_10002	84,56582682	0,54714925	0,1988696	2,75129664	0,00593599	0,0327341	protein_codir	surface protease GP63
TcG_10003	169,547938	0,536893469	0,14274858	3,7611125	0,00016916	0,00173594	protein_codir	putative surface protease GP63
TcG_10004	108,4416907	0,507223283	0,17392043	2,91641	0,00354085	0,02177509	protein_codir	surface protease GP63
TcG_10005	3,093763781	-1,022689546	1,01664447	-1,00594611	0,3144415	1		
TcG_10006	4,401414956	-0,423831661	0,81797432	-0,51814788	0,60435509	1		
TcG_10007	3,260399715	-2,210448912	1,18834312	-1,86010999	0,06286997	1		
TcG_10008	306,1291159	0,064280331	0,10579373	0,60760056	0,54345243	0,74086458	protein_codir	hypothetical protein
TcG_10009	269,1777757	-0,057398575	0,1093243	-0,52503036	0,59956209	0,78310281	protein_codir	hypothetical protein
TcG_10010	222,3854069	0,165874022	0,12119189	1,36868915	0,17109646	0,37787336	protein_codir	hypothetical protein
TcG_10011	259,3501945	0,073278775	0,11361791	0,64495798	0,51895442	0,72371279	protein_codir	hypothetical protein
TcG_10012	31,25891886	-0,094450454	0,30900865	-0,30565634	0,75986632	0,87871156	protein_codir	retrotransposon hot spot (RHS) protein
TcG_10013	47,87306534	0,258081765	0,26164695	0,98637406	0,32394959	0,55819154	protein_codir	retrotransposon hot spot (RHS) protein
TcG_10014	12,67908208	0,191391418	0,50919849	0,375868	0,70701503	1	protein_codir	retrotransposon hot spot (RHS) protein
TcG_10015	27,0674636	-0,000696685	0,33742223	-0,00206473	0,99835259	0,99947404		
TcG_10016	83,88759628	-0,239407453	0,20070352	-1,19284135	0,23293151	0,46030095	protein_codir	trans-sialidase
TcG_10017	100,6356016	-0,052953388	0,18418078	-0,28750768	0,77372362	0,88691888	protein_codir	trans-sialidase
TcG_10018	118,3736867	-0,108896797	0,16583143	-0,65667165	0,51139207	0,71768359		
TcG_10019	85,25528474	-0,134159906	0,20115871	-0,66693562	0,50481326	0,71221932	protein_codir	putative retrotransposon hot spot (RHS) protein
TcG_10020	147,6229634	0,095246215	0,14959289	0,6367028	0,52431845	0,7279513	protein_codir	putative retrotransposon hot spot (RHS) protein
TcG_10021	98,84617743	-0,407056977	0,17943335	-2,26856919	0,02329454	0,09469971	protein_codir	hypothetical protein
TcG_10022	152,0334551	0,034964075	0,15332638	0,22803692	0,81961754	0,9109832	protein_codir	hypothetical protein
TcG_10023	307,5133267	-0,216466188	0,11062593	-1,95673999	0,05037805	0,16632163	protein_codir	hypothetical protein
TcG_10024	99,43960695	0,017747152	0,18465108	0,09611183	0,92343176	0,96264894	protein_codir	hypothetical protein
TcG_10025	525,6865722	-0,04320519	0,08709577	-0,49606531	0,61984834	0,7943328	protein_codir	pentatricopeptide repeat-containing protein
TcG_10026	180,4153496	-0,15256775	0,14737608	-1,03522737	0,30056274	0,53419983	protein_codir	putative f-actin capping protein beta subunit
TcG_10027	50,79836117	0,222127858	0,2445796	0,90820273	0,36377112	0,59568509	protein_codir	putative aminoacylase, putative,N-acyl-L-amino acid amidohydrolase
TcG_10028	80,20714098	0,131529215	0,20119781	0,65373083	0,51328524	0,71944383	protein_codir	retrotransposon hot spot (RHS) protein
TcG_10029	97,59809393	0,233848783	0,18107482	1,29144841	0,19654823	0,41473232	protein_codir	retrotransposon hot spot protein (RHS)
TcG_10030	79,25296729	0,162765945	0,19953563	0,81572372	0,41465817	0,64165922	protein_codir	retrotransposon hot spot (RHS) protein
TcG_10031	4,711288394	0,218594505	0,85699769	0,25507012	0,79866894	1	protein_codir	trans-sialidase
TcG_10032	3,127067161	0,175756215	0,99454935	0,17671945	0,85972876	1	protein_codir	hypothetical protein
TcG_10033	3,903715056	0,30627638	0,9023629	0,33941597	0,73429639	1	protein_codir	hypothetical protein

TcG_10034	9,499668633	-0,773450927	0,59642542	-1,29681081	0,19469629	1	
TcG_10035	72,63370923	-0,358641029	0,20979673	-1,70946907	0,0873641	0,24512151	protein_codir trans-sialidase
TcG_10036	36,40329149	0,17632348	0,30894547	0,57072686	0,56818481	0,76007264	protein_codir hypothetical protein
TcG_10037	84,66546741	-0,037380312	0,20019249	-0,18672184	0,85187873	0,92822975	protein_codir hypothetical protein
TcG_10038	83,75736077	-0,343673002	0,19852231	-1,73115555	0,08342402	0,23748176	protein_codir retrotransposon hot spot (RHS) protein
TcG_10039	35,93129672	0,38921881	0,28820971	1,35047084	0,176865	0,38714489	protein_codir retrotransposon hot spot (RHS) protein
TcG_10040	99,72791859	0,222773332	0,18607721	1,19720911	0,23122506	0,45851772	protein_codir retrotransposon hot spot (RHS) protein
TcG_10041	653,5110461	-0,078544551	0,07581803	-1,03596142	0,30022014	0,53389878	protein_codir retrotransposon hot spot (RHS) protein
TcG_10042	225,2163326	-0,122251053	0,12209411	-1,0012854	0,31668885	0,55170279	protein_codir retrotransposon hot spot (RHS) protein
TcG_10043	30,49204543	-0,048334479	0,31607338	-0,1529217	0,87846002	0,94148439	protein_codir hypothetical protein
TcG_10044	21,47225245	-0,013033231	0,38756784	-0,03362826	0,97317359	0,98816458	protein_codir hypothetical protein
TcG_10045	37,73898596	0,307169992	0,28253636	1,08718749	0,27695396	0,50916988	protein_codir hypothetical protein
TcG_10046	26,10927237	0,222835967	0,34446611	0,64690244	0,51769509	0,72300087	protein_codir hypothetical protein
TcG_10047	47,60931769	0,38285512	0,25461923	1,50363787	0,13267464	0,32361438	protein_codir trans-sialidase
TcG_10048	27,66061944	-0,186786722	0,32630297	-0,57243342	0,56702838	0,75887615	
TcG_10049	73,02705928	-0,067353107	0,23996925	-0,2806739	0,77896053	0,88986755	protein_codir kinesin
TcG_10050	31,9251721	0,076846716	0,30706092	0,25026538	0,80238213	0,90221267	protein_codir putative trans-sialidase
TcG_10051	53,12994135	0,116884125	0,267065	0,43766171	0,66163153	0,82276086	
TcG_10052	5,212354173	0,452447075	0,78339125	0,57754931	0,56356844	1	protein_codir hypothetical protein
TcG_10053	1,99505604	-0,042424584	1,22353636	-0,03467374	0,9723399	1	
TcG_10054	7,52532766	-0,423334879	0,61961493	-0,68322253	0,49446624	1	protein_codir hypothetical protein
TcG_10055	4,892281564	-0,209054526	0,79957907	-0,26145573	0,79374109	1	protein_codir hypothetical protein
TcG_10056	3,38541208	1,550889228	1,03114605	1,50404419	0,13256999	1	protein_codir hypothetical protein
TcG_10057	3,187908632	1,101660221	1,01557161	1,08476863	0,27802414	1	protein_codir hypothetical protein
TcG_10058	4,623466706	-1,48744954	0,82978815	-1,79256542	0,07304244	1	protein_codir mucin TcMUCII
TcG_10059	1,874767025	0,535751315	1,25676915	0,42629254	0,66989469	1	protein_codir hypothetical protein
TcG_10060	13,67993457	0,280048603	0,48779625	0,57410979	0,56589351	1	protein_codir surface protease GP63
TcG_10061	129,0046812	-0,28854185	0,1563536	-1,84544428	0,06497294	0,20106209	protein_codir mucin-associated surface protein (MASP)
TcG_10062	41,91278089	-0,268992259	0,26565307	-1,01256974	0,31126572	0,54492666	protein_codir mucin TcMUCII
TcG_10063	80,3085693	-0,336448041	0,19275673	-1,74545416	0,08090585	0,23284074	protein_codir mucin TcMUCII
TcG_10064	39,75054149	-0,248036118	0,27852762	-0,89052612	0,37318345	0,60423908	protein_codir hypothetical protein
TcG_10065	4,42950845	-1,928360203	0,94334599	-2,04417067	0,04093669	1	protein_codir putative mucin TcMUCII
TcG_10066	0					1	protein_codir putative mucin TcMUCII
TcG_10067	0,45613329	-0,458748245	2,66575971	-0,17208912	0,86336747	1	protein_codir hypothetical protein
TcG_10068	0,729610643	1,464569989	2,01319231	0,72748638	0,46692806	1	protein_codir hypothetical protein
TcG_10069	5,551776848	1,006319564	0,78162384	1,28747296	0,19792949	1	protein_codir hypothetical protein
TcG_10070	5,28949204	-1,868098617	0,85820661	-2,17674694	0,02949945	1	protein_codir mucin TcMUCII
TcG_10071	0,587287357	-0,37147687	2,17226456	-0,17100904	0,86421666	1	
TcG_10072	1,822042039	1,082875749	1,3822556	0,78341209	0,43338516	1	protein_codir hypothetical protein
TcG_10073	0,718312701	-0,525171849	2,07113411	-0,25356728	0,79982988	1	
TcG_10074	0,628743233	-3,095698115	2,25348788	-1,37373631	0,16952356	1	protein_codir hypothetical protein
TcG_10075	54,73354161	-0,384073829	0,23294087	-1,64880396	0,0991878	0,26712922	protein_codir mucin TcMUCII
TcG_10076	13,62234802	0,107603024	0,49122469	0,21905052	0,82661069	1	
TcG_10077	3,243115442	1,172523492	1,10834895	1,05790103	0,29010057	1	protein_codir mucin-associated surface protein (MASP)
TcG_10078	296,9120123	-0,007730329	0,10592769	-0,07297742	0,94182409	0,97298029	protein_codir mucin-associated surface protein (MASP)
TcG_10079	0,663081438	-1,806860419	2,31229633	-0,78141387	0,43455912	1	protein_codir putative mucin-associated surface protein (MASP)
TcG_10080	172,6299703	0,323992495	0,13786366	2,35009349	0,0187687	0,07982897	protein_codir putative trans-sialidase
TcG_10081	116,8921356	0,329238251	0,17117975	1,92334815	0,05443634	0,1756823	protein_codir putative trans-sialidase
TcG_10082	59,75295463	0,171938825	0,2232023	0,77032729	0,44110577	0,66354606	protein_codir putative trans-sialidase
TcG_10083	32,56010486	0,179127081	0,31307593	0,5721522	0,56721887	0,7590434	protein_codir hypothetical protein
TcG_10084	86,28716501	0,012925357	0,199489	0,06479233	0,94833935	0,97570906	protein_codir retrotransposon hot spot (RHS) protein
TcG_10085	171,2988459	0,277583553	0,14171649	1,95872449	0,05014506	0,16573473	protein_codir retrotransposon hot spot (RHS) protein

TcG_10086	23,02602941	0,306626574	0,36270647	0,84538491	0,39789597	0,62755549	protein_codir hypothetical protein
TcG_10087	73,55382546	0,249883802	0,21202713	1,17854636	0,23857886	0,46660613	protein_codir helicase-like protein
TcG_10088	179,2971391	-0,043593269	0,13266183	-0,32860446	0,74245468	0,86898474	protein_codir UDP-Gal or UDP-GlcNAc-dependent glycosyltransferase
TcG_10089	129,589669	0,332267682	0,15748806	2,10979597	0,03487593	0,12819561	protein_codir hypothetical protein
TcG_10090	250,8993727	0,297288776	0,11800336	2,51932478	0,01175801	0,05610723	protein_codir putative citrate transporter
TcG_10091	87,94481056	0,492502235	0,20277388	2,42882478	0,01514785	0,06816091	protein_codir hypothetical protein
TcG_10092	57,863897	0,249525463	0,24060378	1,03708038	0,2996984	0,53346223	protein_codir hypothetical protein
TcG_10093	59,57727295	0,291301161	0,24408663	1,1934335	0,23269964	0,45999966	
TcG_10094	114,8936994	0,186495291	0,16958251	1,0997319	0,27144895	0,50457365	protein_codir putative UDP-Gal or UDP-GlcNAc-dependent glycosyltransferase
TcG_10095	97,43681923	0,27691525	0,1835469	1,50868937	0,13137818	0,32214765	protein_codir UDP-Gal or UDP-GlcNAc-dependent glycosyltransferase
TcG_10096	65,53857738	0,411995026	0,2233283	1,84479539	0,06506731	0,20126059	protein_codir putative ubiquitin-activating enzyme e1
TcG_10097	83,10768772	0,039163554	0,18923937	0,20695247	0,83604698	0,92052325	protein_codir rab1 small GTP-binding protein
TcG_10098	80,16673879	-0,122450652	0,20226493	-0,60539736	0,54491501	0,74188701	protein_codir sialidase
TcG_10099	78,91434485	-0,543110744	0,197465	-2,7504153	0,00595198	0,03279118	protein_codir sialidase
TcG_10100	3,533936635	-0,224446157	0,96100915	-0,23355257	0,81533235	1	
TcG_10101	171,957652	-0,058700042	0,13952786	-0,4207048	0,67397065	0,82902391	
TcG_10102	83,55578299	-0,316532266	0,19843197	-1,59516771	0,11067474	0,28757064	protein_codir dispersed gene family protein 1 (DGF-1)
TcG_10103	92,80640762	-0,231918988	0,18294494	-1,26769828	0,20490572	0,42614649	protein_codir dispersed gene family protein 1 (DGF-1)
TcG_10104	93,32956952	0,181514763	0,19117522	0,94946803	0,34238263	0,57657632	protein_codir hypothetical protein
TcG_10105	390,1618658	0,008963915	0,09162667	0,09783085	0,92206661	0,96209148	
TcG_10106	44,61017238	-0,134335164	0,28188017	-0,47656834	0,63366953	0,80419712	
TcG_10107	160,2535022	0,314403031	0,13990194	2,24731006	0,02462022	0,09866823	
TcG_10108	53,67689718	0,591745616	0,25391867	2,33045333	0,0197822	0,08328365	protein_codir putative retrotransposon hot spot (RHS) protein
TcG_10109	81,02943099	0,473255509	0,20263491	2,33550827	0,01951688	0,0824062	protein_codir retrotransposon hot spot (RHS) protein
TcG_10110	263,7123659	0,439245201	0,11139559	3,94311107	8,0431E-05	0,00092448	protein_codir protein ARV1
TcG_10111	0					1	
TcG_10112	25,18820759	0,507815463	0,35445006	1,43268552	0,15194773	0,35209328	protein_codir hypothetical protein
TcG_10113	35,03595625	0,356957215	0,29166512	1,22385979	0,22100512	0,4463248	protein_codir hypothetical protein
TcG_10114	77,20817729	0,499361956	0,20117268	2,48225534	0,01305537	0,06104822	protein_codir subtilisin-like serine peptidase
TcG_10115	63,72975292	0,623004065	0,22269299	2,79759176	0,00514851	0,02932679	protein_codir putative kinesin
TcG_10116	36,15169655	0,224228029	0,2896103	0,77424052	0,43878857	0,66212613	
TcG_10117	27,89759519	0,373884332	0,33091187	1,12986073	0,25853491	0,49096632	
TcG_10118	54,27935173	-0,069284112	0,23573853	-0,29390237	0,7688325	0,88431385	protein_codir dispersed gene family protein 1 (DGF-1)
TcG_10119	49,56322073	0,159838495	0,26707056	0,59848789	0,54951444	0,7458664	protein_codir dispersed gene family protein 1 (DGF-1)
TcG_10120	48,43664509	0,461165665	0,25665217	1,79685082	0,07235931	0,21618229	protein_codir dispersed gene family protein 1 (DGF-1)
TcG_10121	21,75665915	0,562728211	0,37717243	1,49196539	0,13570822	0,32824957	
TcG_10122	27,4495987	0,318565386	0,34473236	0,92409481	0,35543696	0,58930918	protein_codir putative retrotransposon hot spot (RHS) protein
TcG_10123	141,4780465	0,165993721	0,14893518	1,11453665	0,26504903	0,4975089	protein_codir putative retrotransposon hot spot (RHS) protein
TcG_10124	222,7632475	-0,332624	0,12230399	-2,71964964	0,00653511	0,03543828	protein_codir hypothetical protein
TcG_10125	595,735189	-0,082160858	0,08011316	-1,02556003	0,30509902	0,53795118	protein_codir hypothetical protein
TcG_10126	458,933618	-0,145055792	0,08890701	-1,631545	0,10277537	0,27411498	protein_codir myosin XXI
TcG_10127	187,5985158	-0,137467632	0,12937591	-1,06254426	0,28798867	0,52110523	protein_codir putative ubiquitin-conjugating enzyme
TcG_10128	314,2032449	-0,229449392	0,10288212	-2,2302163	0,02573309	0,10203407	protein_codir autophagin-2
TcG_10129	29,6994304	0,219841765	0,32974999	0,66669225	0,50496873	0,71221932	protein_codir trans-sialidase
TcG_10130	28,22058036	0,8049609	0,33816932	2,38034873	0,01729626	0,07520412	protein_codir trans-sialidase
TcG_10131	34,378304	0,141675787	0,30113281	0,47047609	0,63801491	0,80751094	protein_codir hypothetical protein
TcG_10132	180,2874222	0,257381207	0,13211827	1,9481121	0,05140155	0,16868429	protein_codir putative surface protease GP63
TcG_10133	50,05529771	0,010456605	0,25005866	0,04181661	0,96664489	0,98535525	protein_codir mucin-associated surface protein (MASP)
TcG_10134	10,03848359	0,229976059	0,55795759	0,4121748	0,68021131	1	protein_codir hypothetical protein
TcG_10135	16,19688933	0,58578681	0,43273161	1,35369545	0,17583356	1	protein_codir hypothetical protein
TcG_10136	78,85483819	0,121184944	0,19939359	0,6077675	0,54334169	0,74086458	protein_codir elongation factor 1-gamma (EF-1-gamma)
TcG_10137	67,52200868	0,124611502	0,22130248	0,56308226	0,57337887	0,76454916	protein_codir hypothetical protein

TcG_10138	22,03856653	0,363481646	0,38435625	0,94568943	0,34430702	0,57855563	protein_codir trans-sialidase
TcG_10139	15,35135263	-0,020376658	0,44108894	-0,04619626	0,96315383	1	protein_codir trans-sialidase
TcG_10140	0,301239679	-0,45874858	3,01362118	-0,15222503	0,87900945	1	protein_codir putative retrotransposon hot spot (RHS) protein
TcG_10141	4,682106067	-1,204265855	0,83233863	-1,44684605	0,14794005	1	protein_codir hypothetical protein
TcG_10142	15,49374939	0,212667445	0,43653819	0,48716802	0,62613926	1	protein_codir telomerase reverse transcriptase
TcG_10143	3,151372695	0,43960455	1,02148856	0,4303568	0,66693611	1	protein_codir putative retrotransposon hot spot (RHS) protein
TcG_10144	6,542550674	-0,275887125	0,67854051	-0,40658903	0,68430986	1	protein_codir cleavage and polyadenylation specificity factor
TcG_10145	565,0782821	-0,18452852	0,07837117	-2,35454599	0,01854535	0,07916746	protein_codir hypothetical protein
TcG_10146	415,4577881	0,117714702	0,09164792	1,28442309	0,19899396	0,4178224	protein_codir hypothetical protein
TcG_10147	264,2008743	-0,075469323	0,10984465	-0,68705509	0,49204801	0,70263551	protein_codir hypothetical protein
TcG_10148	82,36279449	-0,025395376	0,1924516	-0,13195721	0,89501814	0,9489813	protein_codir hypothetical protein
TcG_10149	277,0837061	-0,143625788	0,11374288	-1,26272334	0,20668862	0,4287725	protein_codir hypothetical protein
TcG_10150	97,38080255	-0,181560218	0,19213511	-0,94496117	0,3446787	0,57877968	protein_codir Placental protein 25 (PP25)
TcG_10151	284,6134819	-0,125837534	0,11312812	-1,11234532	0,2659897	0,49866613	protein_codir mitochondrial import inner membrane translocase subunit Tim17
TcG_10152	638,066829	-0,365667988	0,0770671	-4,74480006	2,0871E-06	3,8752E-05	protein_codir mitochondrial RNA-binding protein 2
TcG_10153	275,6442227	-0,258638415	0,10791139	-2,39676669	0,01654045	0,07286604	protein_codir hypothetical protein
TcG_10154	84,19253452	0,395779964	0,19580162	2,0213314	0,04324547	0,14934189	protein_codir dispersed gene family protein 1 (DGF-1)
TcG_10155	92,05299268	-0,130372262	0,18085583	-0,72086293	0,47099385	0,68692532	protein_codir dispersed gene family protein 1 (DGF-1)
TcG_10156	80,87652991	0,095795405	0,20149173	0,47543095	0,63447984	0,80462822	protein_codir dispersed gene family protein 1 (DGF-1)
TcG_10157	81,01074682	-0,046448965	0,19623219	-0,2367041	0,81288635	0,90759432	protein_codir dispersed gene family protein 1 (DGF-1)
TcG_10158	88,85977031	0,19391778	0,18850705	1,02870309	0,30361922	0,53649872	protein_codir dispersed gene family protein 1 (DGF-1)
TcG_10159	25,2006257	-0,237929204	0,34844874	-0,68282413	0,49471798	0,70453824	protein_codir hypothetical protein
TcG_10160	308,2613915	-0,335408122	0,11013902	-3,04531589	0,00232436	0,01553955	protein_codir putative ras-related GTP-binding protein
TcG_10161	451,5640784	-0,40653139	0,08965061	-4,53461935	5,7707E-06	9,4837E-05	protein_codir putative FYVE, RhoGEF and PH domain-containing protein 2
TcG_10162	95,89531047	-0,349277834	0,17743586	-1,96847378	0,04901355	0,16319738	protein_codir hypothetical protein
TcG_10163	321,8556352	-0,44806331	0,10305637	-4,34774995	1,3754E-05	0,0002002	protein_codir metallo-peptidase, Clan MA(E), Family M41
TcG_10164	4431,955337	0,028715494	0,03873857	0,74126374	0,45853354	0,67743881	protein_codir 60S ribosomal protein L10a
TcG_10165	334,3894811	0,111851986	0,09911794	1,12847363	0,25911995	0,49163663	protein_codir hypothetical protein
TcG_10166	120,4127746	0,070235726	0,16532727	0,42482844	0,67096174	0,82752425	protein_codir hypothetical protein
TcG_10167	115,5600583	-0,317960211	0,16385457	-1,94050255	0,05231864	0,17064137	protein_codir hypothetical protein
TcG_10168	397,1604987	-0,44458708	0,09666347	-4,59932881	4,2385E-06	7,2594E-05	protein_codir hypothetical protein
TcG_10169	23,58923292	-0,642734544	0,37492965	-1,71428037	0,08647725	0,24359965	protein_codir hypothetical protein
TcG_10170	432,6002678	-0,119415832	0,09267545	-1,28853794	0,19755877	0,41616653	protein_codir hypothetical protein
TcG_10171	80,56293753	0,065499324	0,22225927	0,29469783	0,76822472	0,88396579	protein_codir hypothetical protein
TcG_10172	29,01520666	-0,183467439	0,34042524	-0,53893607	0,58993097	0,77611666	protein_codir retrotransposon hot spot (RHS) protein
TcG_10173	183,0702488	0,158837742	0,13360521	1,18885891	0,23449519	0,4622871	protein_codir putative retrotransposon hot spot (RHS) protein
TcG_10174	63,10124045	-0,29184222	0,21927495	-1,33094189	0,18320813	0,39550543	protein_codir retrotransposon hot spot (RHS) protein
TcG_10175	30,44290836	0,015166889	0,30992576	0,04893717	0,96096937	0,98198472	
TcG_10176	327,4469321	0,058584846	0,10051412	0,58285192	0,55999299	0,75405439	protein_codir hypothetical protein
TcG_10177	179,0791729	0,033237087	0,13733576	0,24201334	0,80876982	0,90526589	protein_codir hypothetical protein
TcG_10178	105,1004672	0,436814447	0,17721967	2,46481926	0,01370824	0,06340026	protein_codir hypothetical protein
TcG_10179	652,6242868	0,26152752	0,07641779	3,4223381	0,00062085	0,00517867	protein_codir hypothetical protein
TcG_10180	314,7613046	0,161098743	0,20473931	0,78684814	0,43137077	0,65596889	protein_codir surface protease GP63
TcG_10181	263,018818	0,268017879	0,11230546	2,386508	0,01700924	0,07439374	protein_codir putative SH3 domain protein
TcG_10182	303,7010386	0,133381926	0,10400835	1,28241559	0,1996969	0,41846415	protein_codir target of rapamycin (TOR) kinase 1
TcG_10183	144,9476717	0,370917998	0,14630656	2,535211	0,01123796	0,05420609	protein_codir protein kinase
TcG_10184	1265,366799	-0,129923924	0,05594038	-2,32254265	0,02020373	0,08475035	protein_codir trans-sialidase
TcG_10185	1040,130859	0,164451833	0,06741389	2,43943545	0,01471023	0,06691509	
TcG_10186	632,3462425	-0,054327102	0,07735801	-0,70228152	0,48250362	0,69628628	protein_codir DnaJ chaperone protein
TcG_10187	828,6637193	0,061578585	0,06757967	0,91119978	0,36219012	0,59480293	protein_codir putative DnaJ chaperone protein
TcG_10188	128,4105743	0,1265324	0,16028127	0,7894397	0,42985506	0,65483116	protein_codir amino acid transporter
TcG_10189	346,0142937	-0,071552887	0,10786743	-0,66334097	0,5071122	0,7139006	protein_codir ubiquitin hydrolase, putative,cysteine peptidase, Clan CA, family C19

TcG_10190	538,8686929	-0,021343598	0,08213114	-0,25987218	0,79496237	0,89770313	protein_codir	trans-sialidase
TcG_10191	666,7515604	0,035312976	0,07406863	0,47676022	0,63353287	0,80419712	protein_codir	rab1 small GTP-binding protein
TcG_10192	11,1244285	-0,457627087	0,53048513	-0,86265771	0,38832569	1	protein_codir	hypothetical protein
TcG_10193	4,030156071	-0,744380613	0,8527493	-0,87291847	0,38270752	1	protein_codir	dispersed gene family protein 1 (DGF-1)
TcG_10194	48,21914982	0,426421428	0,25554972	1,66864367	0,09518802	0,25998312	protein_codir	dispersed gene family protein 1 (DGF-1)
TcG_10195	3,025207081	0,194454462	0,97398951	0,19964739	0,84175636	1	protein_codir	dispersed gene family protein 1 (DGF-1)
TcG_10196	17,11659838	-0,667068422	0,44192721	-1,50945317	0,13118301	0,32194162	protein_codir	dispersed gene family protein 1 (DGF-1)
TcG_10197	5,462185589	0,201413388	0,75880474	0,26543507	0,79067432	1	protein_codir	dispersed gene family protein 1 (DGF-1)
TcG_10198	21,03037455	0,246362102	0,3821815	0,6446207	0,51917302	0,7237563	protein_codir	putative mucin TcMUCII
TcG_10199	16,51526378	0,695564087	0,47339069	1,46932354	0,14174506	1		
TcG_10200	19,61333668	0,160948681	0,39543574	0,40701602	0,68399622	0,83594728		
TcG_10201	95,82882163	-0,514360545	0,1813701	-2,83597208	0,00456864	0,02684194	protein_codir	mucin-associated surface protein (MASP)
TcG_10202	15,85955858	0,04946111	0,47053331	0,10511713	0,91628287	1	protein_codir	peptide hydrolase
TcG_10203	6,368668348	0,458498334	0,72188013	0,6351447	0,52533405	1	protein_codir	hypothetical protein
TcG_10204	4,370442343	0,196117886	0,83700217	0,23430989	0,81474441	1	protein_codir	mucin TcMUCII
TcG_10205	0					1	protein_codir	hypothetical protein
TcG_10206	2,906205957	-0,334828494	1,00934722	-0,33172776	0,74009484	1	protein_codir	hypothetical protein
TcG_10207	0					1	protein_codir	hypothetical protein
TcG_10208	18,6268964	-0,008363614	0,464663	-0,01799931	0,9856394	0,99432608	protein_codir	hypothetical protein
TcG_10209	9,577968418	-0,561701444	0,57191982	-0,98213321	0,32603423	1		
TcG_10210	12,91879005	0,071448019	0,47557291	0,15023568	0,88057868	1	protein_codir	surface protease GP63
TcG_10211	3,450925058	-0,370354184	0,95063848	-0,38958468	0,69684369	1	protein_codir	hypothetical protein
TcG_10212	10,62230978	0,374859814	0,52912685	0,70844981	0,47866597	1	protein_codir	hypothetical protein
TcG_10213	50,06945463	-0,606938018	0,24465653	-2,48077589	0,01310968	0,06124545	protein_codir	putative mucin-associated surface protein (MASP)
TcG_10214	72,80203511	0,026793893	0,21270596	0,12596682	0,89975819	0,95123628	protein_codir	putative trans-sialidase
TcG_10215	18,23348408	0,045249788	0,40374297	0,11207573	0,91076336	0,9566731		
TcG_10216	11,909641	0,121964908	0,53977124	0,22595666	0,82123513	1	protein_codir	hypothetical protein
TcG_10217	9,339488293	-0,694295446	0,57689461	-1,20350482	0,22878103	1	protein_codir	trans-sialidase
TcG_10218	6,220448673	0,127508735	0,73557265	0,17334621	0,8623793	1	protein_codir	Sialidase 85-1.3
TcG_10219	199,7568159	0,91909357	0,12959384	7,09210821	1,3208E-12	9,7473E-11	protein_codir	hypothetical protein
TcG_10220	25,08827236	0,322197871	0,34829126	0,9250817	0,35492341	0,58887909	protein_codir	rab1 small GTP-binding protein
TcG_10221	15,71234589	0,4594474	0,44207485	1,03929777	0,29866627	1	protein_codir	target of rapamycin (TOR) kinase 1
TcG_10222	6,661520451	1,502853662	0,72923984	2,06084965	0,03931739	1	protein_codir	trans-sialidase
TcG_10223	18,2927101	1,510382697	0,44963091	3,3591612	0,00078179	0,00633861	protein_codir	putative phosphatidylinositol-4-phosphate 5-kinase-like
TcG_10224	15,90781119	0,747740181	0,44135477	1,69419306	0,0902286	1	protein_codir	surface protease GP63
TcG_10225	5,179166191	-0,208277954	0,74881832	-0,27814217	0,78090322	1	protein_codir	hypothetical protein
TcG_10226	3,23728135	-0,394905837	0,96171683	-0,4106259	0,68134687	1	protein_codir	hypothetical protein
TcG_10227	565,3719797	-0,161397477	0,08543239	-1,88918358	0,05886723	0,1863995	protein_codir	putative retrotransposon hot spot (RHS) protein
TcG_10228	601,0191107	-0,177520304	0,08017831	-2,21406901	0,02682403	0,10527887	protein_codir	trans-sialidase
TcG_10229	58,08607323	0,042810039	0,22607288	0,18936388	0,84980763	0,9276306	protein_codir	hypothetical protein
TcG_10230	41,43628423	0,017533245	0,27657328	0,06339457	0,94945231	0,97650727	protein_codir	hypothetical protein
TcG_10231	29,09183647	-0,043873225	0,32656537	-0,13434745	0,89312784	0,94840219	protein_codir	hypothetical protein
TcG_10232	40,7710585	0,229714472	0,27692495	0,82951888	0,40681086	0,63504589	protein_codir	hypothetical protein
TcG_10233	0					1	protein_codir	retrotransposon hot spot (RHS) protein
TcG_10234	47,21182608	0,272465981	0,25344423	1,07505299	0,282351	0,51557427	protein_codir	retrotransposon hot spot (RHS) protein
TcG_10235	40,57860286	0,166472896	0,27309868	0,60957049	0,54214637	0,74019653	protein_codir	retrotransposon hot spot (RHS) protein
TcG_10236	45,41045976	0,04196869	0,26744469	0,15692474	0,87530416	0,93987711	protein_codir	retrotransposon hot spot (RHS) protein
TcG_10237	38,99374609	0,55544428	0,28514614	1,94792848	0,05142352	0,16868429	protein_codir	putative retrotransposon hot spot (RHS) protein
TcG_10238	21,65610798	0,161650796	0,41828213	0,38646355	0,6991534	0,84414248	protein_codir	dispersed gene family protein 1 (DGF-1)
TcG_10239	43,87362973	0,497088005	0,27001074	1,84099343	0,06562252	0,20269329	protein_codir	dispersed gene family protein 1 (DGF-1)
TcG_10240	2,668285196	1,155414421	1,124274	1,02769824	0,30409179	1	protein_codir	dispersed gene family protein 1 (DGF-1)
TcG_10241	1,5641518	0,447239627	1,37153266	0,32608748	0,74435817	1	protein_codir	hypothetical protein

TcG_10242	8,235961079	-0,18727789	0,61032071	-0,30685161	0,75895632	1	protein_codir	hypothetical protein
TcG_10243	10,98666775	-0,530377031	0,54236096	-0,97790414	0,32812174	1		
TcG_10244	3,762941388	-1,092498685	0,91210897	-1,19777211	0,23100575	1	protein_codir	hypothetical protein
TcG_10245	6,451284878	-0,183222986	0,68620048	-0,26701087	0,78946079	1	protein_codir	hypothetical protein
TcG_10246	4,802376486	1,067988987	0,85278703	1,25235135	0,21044186	1	protein_codir	hypothetical protein
TcG_10247	16,26104619	0,23328737	0,42841576	0,54453499	0,58607338	1	protein_codir	surface protease GP63
TcG_10248	195,8193981	-0,042048775	0,12552668	-0,33497878	0,73764109	0,866063	protein_codir	mucin-associated surface protein (MASP)
TcG_10249	71,10314582	0,616017246	0,22041452	2,79481245	0,00519298	0,02952203	protein_codir	90 kDa surface protein
TcG_10250	43,54687981	0,294704647	0,26233388	1,1233953	0,26126964	0,49358779	protein_codir	protein kinase
TcG_10251	44,32549937	0,24391817	0,26121423	0,93378592	0,35041433	0,58488966	protein_codir	protein kinase
TcG_10252	84,40211888	0,355843324	0,19457866	1,82878904	0,06743122	0,20673673	protein_codir	putative target of rapamycin (TOR) kinase 1
TcG_10253	31,67808439	0,364589955	0,33306983	1,0946352	0,27367649	0,50688937	protein_codir	hypothetical protein
TcG_10254	8,499631763	1,041644672	0,62253119	1,67324095	0,09427987	1	protein_codir	putative GAG protein
TcG_10255	19,14350787	0,343936934	0,39446056	0,87191717	0,38325357	0,6133963	protein_codir	putative trans-sialidase
TcG_10256	328,7231082	0,603958399	0,10190785	5,92651519	3,0943E-09	1,1134E-07	protein_codir	hypothetical protein
TcG_10257	57,87605726	0,305787661	0,23827228	1,2833539	0,19936812	0,41820968	protein_codir	hypothetical protein
TcG_10258	37,59468354	0,648634521	0,3002917	2,16001479	0,03077152	0,11647138	protein_codir	trans-sialidase
TcG_10259	42,86819358	0,409179764	0,27532245	1,48618379	0,13723051	0,33025591	protein_codir	putative target of rapamycin (TOR) kinase 1
TcG_10260	33,91940497	0,956207075	0,3151063	3,03455395	0,00240892	0,01595953	protein_codir	hypothetical protein
TcG_10261	41,15456318	0,255954079	0,27568146	0,9284414	0,35317865	0,58758298	protein_codir	retrotransposon hot spot (RHS) protein
TcG_10262	417,2804376	0,473231007	0,09027749	5,24196037	1,5888E-07	3,9502E-06	protein_codir	putative retrotransposon hot spot (RHS) protein
TcG_10263	487,0478797	0,527820195	0,08963901	5,88828704	3,9022E-09	1,3618E-07	protein_codir	hypothetical protein
TcG_10264	26,59412809	0,578046466	0,34671264	1,66722063	0,09547054	0,26063188	protein_codir	trans-sialidase
TcG_10265	132,2705652	0,671468055	0,15866422	4,23200678	2,3162E-05	0,0003146	protein_codir	trans-sialidase
TcG_10266	16,73800402	0,041125876	0,42175783	0,09751064	0,92232089	1	protein_codir	trans-sialidase
TcG_10267	18,72571884	-0,583277452	0,41568597	-1,40316849	0,1605666	0,36344905	protein_codir	trans-sialidase
TcG_10268	12,08696911	-0,440438015	0,52962568	-0,83160246	0,40563337	1	protein_codir	surface protease GP63
TcG_10269	51,43560341	-0,577968011	0,24896631	-2,32147074	0,02026145	0,08493096	protein_codir	surface protease GP63
TcG_10270	6,702400104	-0,862153795	0,70662175	-1,2201065	0,22242451	1	protein_codir	hypothetical protein
TcG_10271	4,810469414	-0,794706364	0,81309818	-0,97738058	0,32838077	1	protein_codir	hypothetical protein
TcG_10272	10,95482032	-0,503202939	0,53718195	-0,9367458	0,34888933	1		
TcG_10273	38,38109977	-1,018905806	0,31217684	-3,26387374	0,001099	0,00843247	protein_codir	putative mucin TcMUCII
TcG_10274	121,7319195	-0,424230606	0,16193862	-2,61970005	0,00880071	0,04470192	protein_codir	mucin-associated surface protein (MASP)
TcG_10275	49,02623982	-0,028508194	0,27008004	-0,10555461	0,91593574	0,95914962	protein_codir	putative trans-sialidase
TcG_10276	96,51348466	-0,27684124	0,18101315	-1,52939846	0,12616569	0,31381617	protein_codir	hypothetical protein
TcG_10277	191,6916674	-0,054697662	0,12770984	-0,42829639	0,66843534	0,8262554	protein_codir	hypothetical protein
TcG_10278	116,8183191	0,289728828	0,16685795	1,73638013	0,08249664	0,23611811	protein_codir	hypothetical protein
TcG_10279	244,6814403	-0,157437184	0,11520832	-1,3665435	0,17176844	0,37914062	protein_codir	hypothetical protein
TcG_10280	0					1		
TcG_10281	24,25173971	0,224771228	0,35540575	0,63243555	0,52710231	0,72963051	protein_codir	hypothetical protein
TcG_10282	18,44036703	0,650627269	0,42008515	1,54879855	0,12143016	0,30577913	protein_codir	hypothetical protein
TcG_10283	60,87400586	-0,102070597	0,22822706	-0,44723266	0,65470708	0,81739615	protein_codir	selenocysteine-tRNA-specific elongation factor
TcG_10284	24,12300269	0,427300571	0,36562589	1,16868248	0,24253158	0,47091854	protein_codir	hypothetical protein
TcG_10285	43,91046505	0,009142128	0,26612936	0,0343522	0,9725963	0,9878584	protein_codir	serine/threonine protein phosphatase
TcG_10286	83,77599627	-0,160515635	0,20436626	-0,78543119	0,4322008	0,65671849	protein_codir	trans-sialidase
TcG_10287	30,24440321	0,007763717	0,32878867	0,02361309	0,98116123	0,9928152	protein_codir	trans-sialidase
TcG_10288	160,3253514	0,065766852	0,14145598	0,46492804	0,64198299	0,80988839	protein_codir	trans-sialidase
TcG_10289	95,0724476	-0,038387849	0,18441566	-0,20815938	0,83510452	0,92007617		
TcG_10290	50,82099319	0,129653441	0,24986708	0,51888966	0,60383769	0,78580967	protein_codir	dispersed gene family protein 1 (DGF-1)
TcG_10291	25,44987907	-0,122187235	0,34869567	-0,35041225	0,72602933	0,85851968	protein_codir	Tbingi protein
TcG_10292	44,48588743	-0,124458298	0,28765762	-0,43266123	0,6652609	0,82504261	protein_codir	hypothetical protein
TcG_10293	16,4648393	0,134774396	0,42589884	0,31644696	0,75166327	1	protein_codir	hypothetical protein

TcG_10294	14,85339725	-0,155738003	0,45767974	-0,34027725	0,73364775	1	protein_codir	hypothetical protein
TcG_10295	17,77709708	0,393570093	0,41401194	0,95062498	0,34179478	0,57634032	protein_codir	hypothetical protein
TcG_10296	38,96318648	0,339664167	0,28859376	1,17696296	0,23921029	0,4673677	protein_codir	trans-sialidase
TcG_10297	18,00743188	0,095852172	0,40523924	0,23653231	0,81301964	0,90759432	protein_codir	putative elongation factor 1-gamma (EF-1-gamma)
TcG_10298	61,65753168	-0,452067593	0,22709248	-1,99067624	0,04651649	0,15730884	protein_codir	mucin-associated surface protein (MASP)
TcG_10299	168,5267666	0,202743347	0,14438152	1,40421952	0,16025349	0,36306159	protein_codir	dispersed gene family protein 1 (DGF-1)
TcG_10300	83,33336002	0,025403209	0,1942566	0,13077141	0,89595614	0,9494693	protein_codir	dispersed gene family protein 1 (DGF-1)
TcG_10301	34,75132335	0,167948115	0,29088866	0,57736219	0,56369481	0,75677498	protein_codir	putative dispersed gene family protein 1 (DGF-1)
TcG_10302	76,43912529	-0,136309915	0,20126277	-0,67727336	0,49823253	0,70742498	protein_codir	hypothetical protein
TcG_10303	100,3267173	-0,094717086	0,17300241	-0,54748997	0,58404216	0,77263216	protein_codir	hypothetical protein
TcG_10304	113,5594922	-0,0805699	0,1634748	-0,49285823	0,62211276	0,79640439	protein_codir	hypothetical protein
TcG_10305	127,097042	0,025393596	0,16356692	0,15524897	0,87662504	0,94094661	protein_codir	hypothetical protein
TcG_10306	76,39964011	0,013053323	0,21126901	0,06178532	0,9507338	0,97695803		
TcG_10307	71,06424542	0,192423722	0,22000759	0,87462311	0,38177901	0,61196619	protein_codir	trans-sialidase
TcG_10308	44,06744848	-0,073051298	0,2646722	-0,27600669	0,78254292	0,89228839	protein_codir	rab1 small GTP-binding protein
TcG_10309	32,27726709	0,060026706	0,30481738	0,19692678	0,84388483	0,92438779	protein_codir	target of rapamycin (TOR) kinase 1
TcG_10310	10,17661386	-0,171182599	0,54366837	-0,31486584	0,75286352	1	protein_codir	hypothetical protein
TcG_10311	33,89669188	-0,538093827	0,30026841	-1,79204276	0,07312612	0,21779928		
TcG_10312	43,53731244	-0,202654132	0,26402783	-0,76754837	0,44275554	0,66499591	protein_codir	amastigote surface protein 4
TcG_10313	20,09659062	0,344554919	0,38323769	0,89906324	0,36861898	0,60076234	protein_codir	trans-sialidase
TcG_10314	83,20754885	0,107207195	0,19349314	0,55406198	0,57953643	0,76901886	protein_codir	mucin-associated surface protein (MASP)
TcG_10315	32,12029635	-0,163931818	0,30360744	-0,53994664	0,58923383	0,77564856	protein_codir	mucin TcMUCII
TcG_10316	11,85238307	-0,067310875	0,52195105	-0,12896013	0,8973892	1	protein_codir	mucin TcMUCII
TcG_10317	3,797072771	-0,000472488	0,88521044	-0,00053376	0,99957412	1	protein_codir	hypothetical protein
TcG_10318	4,865967068	-0,619011601	0,8042775	-0,76964928	0,44150796	1	protein_codir	hypothetical protein
TcG_10319	6,509136624	-1,34449598	0,7565847	-1,77705943	0,07555846	1	protein_codir	hypothetical protein
TcG_10320	424,4153281	0,375956626	0,10046935	3,74200335	0,00018256	0,00185213	protein_codir	structural maintenance of chromosome protein 4
TcG_10321	1291,766694	0,322867545	0,06947411	4,64730713	3,363E-06	5,9395E-05	protein_codir	hypothetical protein
TcG_10322	488,1789352	0,154884193	0,0834593	1,85580511	0,06348134	0,19782002	protein_codir	protein kinase, putative,serine/threonine protein kinase
TcG_10323	109,1314982	-0,108328994	0,18337535	-0,59075002	0,55468793	0,74941774	protein_codir	Zinc finger (ISS)
TcG_10324	1184,924886	-0,207134991	0,06039847	-3,42947416	0,00060475	0,00506994	protein_codir	leucine--tRNA ligase
TcG_10325	303,5626591	0,562684805	0,10700808	5,2583396	1,4536E-07	3,6692E-06		
TcG_10326	282,7114573	0,676927154	0,11189896	6,04945005	1,4534E-09	5,5575E-08	protein_codir	cyclophilin type peptidyl-prolyl cis-trans isomerase
TcG_10327	424,4204507	0,650659895	0,09821986	6,6245247	3,4837E-11	1,9498E-09	protein_codir	putative vacuolar ATP synthase 16 kDa proteolipid subunit
TcG_10328	547,8093247	0,489449219	0,08590686	5,69744047	1,2162E-08	3,7676E-07	protein_codir	putative monoglyceride lipase
TcG_10329	908,0684377	0,500028294	0,06697536	7,46585478	8,2761E-14	7,5501E-12	protein_codir	leucine richcontaining 34
TcG_10330	711,6169217	0,631080048	0,07305425	8,63851273	5,695E-18	8,3521E-16	protein_codir	putative protein kinase A regulatory subunit
TcG_10331	620,4560726	0,659732872	0,08000442	8,24620548	1,635E-16	1,9733E-14	protein_codir	hypothetical protein
TcG_10332	454,418255	0,439998917	0,09140964	4,81348487	1,4832E-06	2,8737E-05	protein_codir	hypothetical protein
TcG_10333	294,9615196	0,368149074	0,10654768	3,45525178	0,00054978	0,00470439	protein_codir	hypothetical protein
TcG_10334	929,7031986	0,649828789	0,07172445	9,0600737	1,3036E-19	2,2543E-17	protein_codir	TCP17 protein
TcG_10335	88,43539721	0,561224287	0,19042805	2,94717233	0,00320694	0,02013857		
TcG_10336	21,52160579	-0,112127886	0,37406095	-0,29975833	0,7643615	0,88127101	protein_codir	putative mucin-associated surface protein (MASP)
TcG_10337	22,73049146	0,345015803	0,39307548	0,87773422	0,38008797	0,6107213	protein_codir	RNA-binding protein
TcG_10338	0,797274563	0,599188768	1,99573116	0,30023521	0,76399775	1	protein_codir	trans-sialidase
TcG_10339	15,13363215	0,562160879	0,45217816	1,24322874	0,21378356	1	protein_codir	hypothetical protein
TcG_10340	17,04887045	-0,636231884	0,43878409	-1,44998851	0,14706172	0,34553988	protein_codir	trans-sialidase
TcG_10341	71,0419437	0,056943964	0,20897439	0,27249255	0,78524332	0,89351472	protein_codir	putative trans-sialidase
TcG_10342	3,837729476	-0,449885239	0,87824488	-0,5122549	0,60847263	1	protein_codir	hypothetical protein
TcG_10343	3,188901367	-1,534119302	1,00334147	-1,52901016	0,12626193	1	protein_codir	hypothetical protein
TcG_10344	54,94609395	0,236772173	0,25010022	0,94670919	0,34378699	0,57799373	protein_codir	hypothetical protein
TcG_10345	29,28184696	0,295970217	0,32207114	0,91895913	0,35811695	0,5919736		

TcG_10346	40,74062924	0,322772027	0,27386187	1,17859425	0,23855978	0,46660613	protein_codir	dispersed gene family protein 1 (DGF-1)
TcG_10347	262,2133425	0,033495492	0,12007865	0,27894627	0,78028606	0,89067206	protein_codir	dispersed gene family protein 1 (DGF-1)
TcG_10348	166,3862395	-0,032970819	0,14035589	-0,23490869	0,8142796	0,90826251	protein_codir	dispersed gene family protein 1 (DGF-1)
TcG_10349	5,784607081	0,462750887	0,72306556	0,63998469	0,52218255	1	protein_codir	hypothetical protein
TcG_10350	21,15116223	0,613267992	0,42340923	1,44840489	0,14750385	0,34622763		
TcG_10351	29,78468576	0,411026518	0,32446673	1,26677553	0,20523556	0,4266749		
TcG_10352	8,880723964	0,140036759	0,60238018	0,23247239	0,81617113	1	protein_codir	putative trans-sialidase
TcG_10353	15,13956886	0,379913363	0,45817976	0,82917972	0,40700272	1		
TcG_10354	12,72219306	-0,63017219	0,5129296	-1,22857442	0,21923141	1	protein_codir	retrotransposon hot spot (RHS) protein
TcG_10355	0,611155623	-0,424209629	2,24295747	-0,18912959	0,84999125	1	protein_codir	hypothetical protein
TcG_10356	25,27716489	0,214047686	0,35794432	0,59799158	0,54984555	0,74596143	protein_codir	retrotransposon hot spot (RHS) protein
TcG_10357	214,6713843	0,175111036	0,1226253	1,42801721	0,1532869	0,3538244	protein_codir	putative retrotransposon hot spot (RHS) protein
TcG_10358	264,3484607	0,216087539	0,10946599	1,97401527	0,04838001	0,1616294	protein_codir	HPP family protein
TcG_10359	20,28750727	0,466240366	0,3894829	1,19707533	0,23127719	0,45851772	protein_codir	chitin-binding like protein
TcG_10360	448,2781416	-0,245351274	0,0918137	-2,67227305	0,00753393	0,03956851	protein_codir	trans-sialidase
TcG_10361	30,61610673	-1,41002058	0,32873888	-4,28918103	1,7933E-05	0,00025154	protein_codir	trans-sialidase
TcG_10362	45,22455581	-0,46191067	0,26042904	-1,77365271	0,07612061	0,22310484	protein_codir	hypothetical protein
TcG_10363	38,11285153	-0,840078214	0,29326034	-2,86461584	0,00417515	0,02498623	protein_codir	hypothetical protein
TcG_10364	27,48296409	-0,512095701	0,33026749	-1,55054834	0,12100996	0,30498617	protein_codir	mucin-associated surface protein (MASP)
TcG_10365	45,84971191	-0,445432422	0,25956256	-1,71608885	0,08614578	0,24277121	protein_codir	regulator of sigma E protease
TcG_10366	8,460820315	-0,206068416	0,60492145	-0,34065318	0,73336469	1	protein_codir	hypothetical protein
TcG_10367	34,99617864	-0,182871564	0,31405145	-0,58229809	0,56036591	0,75431619	protein_codir	hypothetical protein
TcG_10368	12,86608772	-1,141864063	0,52994474	-2,15468515	0,03118648	1	protein_codir	hypothetical protein
TcG_10369	7,250784746	-0,656200054	0,6630027	-0,98973965	0,32230139	1	protein_codir	hypothetical protein
TcG_10370	22,48864813	0,078437255	0,36062773	0,21750201	0,82781714	0,91588083	protein_codir	L1Tc protein
TcG_10371	537,1712861	-0,089543715	0,08114423	-1,10351309	0,26980441	0,50269083	protein_codir	hypothetical protein
TcG_10372	527,0410383	-0,032445903	0,08055322	-0,40278838	0,68710391	0,83753665	protein_codir	methyltransferase
TcG_10373	141,0895941	0,021038813	0,15018568	0,14008534	0,88859256	0,94628587	protein_codir	hypothetical protein
TcG_10374	36,80754949	0,24253389	0,29424663	0,82425376	0,40979539	0,63689009	protein_codir	hypothetical protein
TcG_10375	31,82412513	-0,238714855	0,30986215	-0,77039051	0,44106828	0,66354606	protein_codir	trans-sialidase
TcG_10376	12,2520143	-0,322836237	0,54365621	-0,59382425	0,55262967	1	protein_codir	putative trans-sialidase
TcG_10377	47,41702825	-0,11905471	0,25308239	-0,47041879	0,63805584	0,80751094	protein_codir	hypothetical protein
TcG_10378	66,44079208	-0,1857162	0,22565723	-0,82300133	0,41050724	0,63729557	protein_codir	surface glycoprotein Tc85-11
TcG_10379	27,08505825	-0,151121636	0,33876959	-0,44608973	0,65553243	0,81772823	protein_codir	trans-sialidase
TcG_10380	15,09502163	-0,290949133	0,46025848	-0,6321429	0,5272935	1	protein_codir	hypothetical protein
TcG_10381	7,860188277	-0,506080715	0,61512657	-0,82272614	0,41066374	1	protein_codir	hypothetical protein
TcG_10382	19,95773068	0,200220159	0,39601688	0,50558491	0,61314807	0,79081972	protein_codir	hypothetical protein
TcG_10383	13,89132105	-0,176250533	0,48364596	-0,36442056	0,71554398	1	protein_codir	hypothetical protein
TcG_10384	29,13520567	0,272111916	0,32677795	0,83271198	0,40500719	0,63428133	protein_codir	hypothetical protein
TcG_10385	25,89449028	-0,072859582	0,35680401	-0,20420057	0,83819676	0,92144011	protein_codir	hypothetical protein
TcG_10386	31,36112012	-0,000276255	0,30562105	-0,00090391	0,99927878	0,99981271	protein_codir	dispersed gene family protein 1 (DGF-1)
TcG_10387	18,32855982	-0,368924164	0,41496529	-0,88904825	0,37397715	0,60449626	protein_codir	hypothetical protein
TcG_10388	56,58542159	0,157471097	0,2428573	0,64840997	0,51671983	0,72224827	protein_codir	hypothetical protein
TcG_10389	70,80204473	0,131072772	0,22189416	0,5906995	0,55472178	0,74941774	protein_codir	retrotransposon hot spot (RHS) protein
TcG_10390	76,80880718	0,048649846	0,21134247	0,23019437	0,81794074	0,91031113		
TcG_10391	117,2475105	0,471878218	0,17866175	2,6411821	0,00826173	0,04250696	protein_codir	surface protein-2
TcG_10392	46,24425606	-0,360467352	0,26069789	-1,38270144	0,16675643	0,3726929	protein_codir	trans-sialidase
TcG_10393	25,56805165	0,220014901	0,34496529	0,63778851	0,52361136	0,72766717		
TcG_10394	288,6703173	-0,18269451	0,10593092	-1,72465704	0,0845893	0,23968002	protein_codir	mucin-associated surface protein (MASP)
TcG_10395	901,905335	-0,024588861	0,06717274	-0,36605418	0,71432465	0,85349203	protein_codir	hypothetical protein
TcG_10396	1906,372887	-0,062468594	0,05333395	-1,1712726	0,24148922	0,47019585	protein_codir	putative zinc finger protein
TcG_10397	236,6402803	-0,343843934	0,12256447	-2,80541276	0,00502522	0,02888005		

TcG_10398	478,9311022	-0,029507291	0,08482569	-0,34785796	0,72794686	0,85990949	protein_codir	hypothetical protein
TcG_10399	172,417745	0,086545919	0,13305116	0,65047098	0,51538804	0,7212569	protein_codir	hypothetical protein
TcG_10400	462,6223059	-0,078002545	0,08970255	-0,86956885	0,38453607	0,61476955	protein_codir	chaperone protein DNAJ
TcG_10401	206,5016982	-0,055425505	0,12485663	-0,44391318	0,65710535	0,81915458	protein_codir	Hrf1 family protein
TcG_10402	146,7289693	0,217044944	0,14785171	1,4679908	0,14210672	0,33801036	protein_codir	pre-mRNA-splicing factor 38A
TcG_10403	307,6028397	0,01012872	0,10483238	0,09661824	0,92302957	0,96257611	protein_codir	hypothetical protein
TcG_10404	29,96062456	0,341730269	0,33260821	1,02742584	0,30421999	0,53710252	protein_codir	hypothetical protein
TcG_10405	13,17192508	-0,224661289	0,46828407	-0,47975429	0,63140212	1	protein_codir	hypothetical protein
TcG_10406	27,13656263	0,064139107	0,34319771	0,18688676	0,85174942	0,92822975		
TcG_10407	739,4930464	0,067380311	0,06976179	0,96586275	0,33411286	0,56860042	protein_codir	NADH-dependent fumarate reductase-like protein
TcG_10408	417,0972899	-0,282748545	0,08942095	-3,16199441	0,00156693	0,01128303	protein_codir	hypothetical protein
TcG_10409	714,4755333	-0,197849382	0,07206459	-2,74544502	0,00604289	0,03319721	protein_codir	hypothetical protein
TcG_10410	300,6982605	-0,108494755	0,10602511	-1,02329304	0,30616931	0,53918189	protein_codir	hypothetical protein
TcG_10411	160,6889348	0,023902013	0,14002067	0,17070347	0,86445694	0,93498506	protein_codir	GDP-mannose 4,6 dehydratase
TcG_10412	65,66837329	0,038695148	0,23051527	0,16786371	0,8666905	0,93574468	protein_codir	putative GDP-mannose 4,6 dehydratase
TcG_10413	391,9271965	-0,145217271	0,09472424	-1,53305284	0,12526284	0,31217363	protein_codir	putative kynureninase
TcG_10414	707,2418714	0,091678181	0,07819548	1,17242303	0,24102726	0,46972948	protein_codir	hypothetical protein
TcG_10415	51,87949298	0,04873769	0,24294348	0,20061329	0,84100097	0,92246657	protein_codir	regulator of sigma E protease
TcG_10416	0,456961293	0,528046641	2,47447195	0,21339771	0,83101677	1		
TcG_10417	162,7549228	0,072544173	0,14031905	0,51699446	0,60516003	0,78669877	protein_codir	hypothetical protein
TcG_10418	359,7759571	0,013792473	0,09948505	0,13863865	0,8897357	0,94653573	protein_codir	hypothetical protein
TcG_10419	17,70490437	0,000316241	0,43527912	0,00072653	0,99942032	0,99981271	protein_codir	casein kinase
TcG_10420	2678,792027	-0,036038114	0,04388022	-0,82128373	0,41148467	0,63808037	protein_codir	casein kinase
TcG_10421	4,044493071	0,048021959	0,84435963	0,05687382	0,95464571	1	protein_codir	casein kinase
TcG_10422	176,3139957	0,38041379	0,13311812	2,85771614	0,00426702	0,02541783	protein_codir	Zinc finger protein CTH1
TcG_10423	33,59580438	-0,185648104	0,29948401	-0,6198932	0,5353281	0,73545133	protein_codir	trans-sialidase
TcG_10424	17,55854039	-0,216058831	0,46728398	-0,46237158	0,64381488	0,810875	protein_codir	trans-sialidase
TcG_10425	22,98960935	0,098347943	0,36783892	0,26736688	0,7891867	0,89494301	protein_codir	trans-sialidase
TcG_10426	52,66307457	-0,554104835	0,24607787	-2,25174591	0,02433833	0,09782343	protein_codir	mucin-associated surface protein (MASP)
TcG_10427	225,8159272	-0,403004313	0,11883155	-3,39139144	0,00069539	0,00572213	protein_codir	mucin-associated surface protein (MASP)
TcG_10428	262,2127267	0,030699789	0,11321283	0,27116882	0,78626119	0,89358636	protein_codir	hypothetical protein
TcG_10429	72,88509754	-0,142337216	0,21300423	-0,66823658	0,50398259	0,71207637	protein_codir	hypothetical protein
TcG_10430	61,30986098	-0,163231513	0,237208	-0,68813663	0,49136675	0,70214296	protein_codir	hypothetical protein
TcG_10431	94,62372909	-0,267115477	0,1874022	-1,42535931	0,15405336	0,35505516	protein_codir	hypothetical protein
TcG_10432	138,7867611	-0,046319287	0,1491352	-0,31058588	0,75611546	0,87716571	protein_codir	hypothetical protein
TcG_10433	28,76712055	0,364805694	0,32283182	1,13001776	0,25846874	0,49092112	protein_codir	dispersed gene family protein 1 (DGF-1)
TcG_10434	61,12493643	0,139820833	0,23588965	0,59273831	0,5533563	0,74853311	protein_codir	dispersed gene family protein 1 (DGF-1)
TcG_10435	171,3289424	0,309920127	0,1449686	2,13784313	0,03252948	0,12161553	protein_codir	dispersed gene family protein 1 (DGF-1)
TcG_10436	127,8558495	0,294801355	0,28713812	1,02668833	0,30456725	0,53734066	protein_codir	dispersed gene family protein 1 (DGF-1)
TcG_10437	106,4401965	-0,238740926	0,17115234	-1,39490307	0,16304507	0,3673746	protein_codir	hypothetical protein
TcG_10438	1073,839328	0,09320655	0,06165351	1,51178012	0,13058981	0,32075758	protein_codir	hypothetical protein
TcG_10439	25,29860961	-0,246679573	0,39670706	-0,62181795	0,53406158	0,73443768	protein_codir	trans-sialidase
TcG_10440	19,24299629	-0,925689211	0,4125762	-2,24368059	0,02485296	0,09925763	protein_codir	trans-sialidase
TcG_10441	7,234364421	0,147401828	0,63634757	0,23163729	0,81681974	1	protein_codir	hypothetical protein
TcG_10442	3,099138335	0,499873265	0,99540357	0,50218151	0,61553985	1	protein_codir	surface protease GP63
TcG_10443	7,059487593	0,692125373	0,6591528	1,05002266	0,2937077	1	protein_codir	surface protease GP63
TcG_10444	0,924672238	1,929221891	1,87788406	1,02733812	0,30426128	1	protein_codir	surface protease GP63
TcG_10445	1,472324391	0,553420904	1,48675084	0,37223514	0,70971779	1	protein_codir	hypothetical protein
TcG_10446	8,606078846	-0,080225397	0,65194504	-0,12305546	0,90206318	1	protein_codir	putative syntaxin binding protein
TcG_10447	132,0478277	0,152386012	0,15295966	0,99624969	0,31912884	0,55410215	protein_codir	dispersed gene family protein 1 (DGF-1)
TcG_10448	81,94482206	0,05820392	0,20006321	0,29092765	0,77110665	0,88517207	protein_codir	dispersed gene family protein 1 (DGF-1)
TcG_10449	37,43851554	0,240941231	0,33594436	0,71720575	0,47324715	0,68908401	protein_codir	dispersed protein family protein 1 (DGF-1)

TcG_10450	65,21194152	0,341803809	0,43848004	0,77951965	0,43567367	0,65939964	protein_codir dispersed gene family protein 1 (DGF-1)
TcG_10451	47,9709588	-0,015506593	0,25817432	-0,06006249	0,95210586	0,9773959	protein_codir hypothetical protein
TcG_10452	176,6933155	0,233638416	0,13513718	1,72889812	0,08382733	0,23833704	protein_codir hypothetical protein
TcG_10453	50,66547361	0,020228648	0,24553111	0,08238731	0,93433872	0,96900069	protein_codir putative profilin
TcG_10454	3,34525742	1,370893071	1,00138616	1,36899542	0,17100071	1	
TcG_10455	10,74928541	0,376428786	0,54200617	0,69451015	0,48736233	1	protein_codir hypothetical protein
TcG_10456	18,22183637	0,761771627	0,44461797	1,71331724	0,08665419	0,24397946	protein_codir hypothetical protein
TcG_10457	47,71636995	-0,111504289	0,26657475	-0,41828526	0,67573856	0,83017833	protein_codir putative mucin TcMUCII
TcG_10458	26,19503461	0,086081415	0,35166399	0,24478314	0,80662435	0,90458824	protein_codir hypothetical protein
TcG_10459	38,8075662	-0,185013535	0,27752731	-0,66664983	0,50499583	0,71221932	protein_codir putative trans-sialidase
TcG_10460	4,020083013	1,395969645	0,91994362	1,51745129	0,12915278	1	protein_codir putative trans-sialidase
TcG_10461	32,12988506	-0,31132723	0,31686665	-0,98251815	0,32584465	0,5602746	protein_codir trans-sialidase
TcG_10462	13,21287823	0,977856864	0,51187552	1,91034112	0,05608931	1	protein_codir hypothetical protein
TcG_10463	5,62089516	0,492471384	0,74401898	0,66190702	0,50803081	1	protein_codir hypothetical protein
TcG_10464	9,865675701	0,956595754	1,11139918	0,86071303	0,38939612	1	protein_codir hypothetical protein
TcG_10465	21,61626975	0,356322089	0,37170646	0,9586115	0,33775449	0,57235974	protein_codir hypothetical protein
TcG_10466	33,36242988	0,022709613	0,30432261	0,07462348	0,94051429	0,97240751	protein_codir retrotransposon hot spot (RHS) protein
TcG_10467	896,274401	0,182698478	0,06517037	2,8033977	0,00505673	0,02897803	protein_codir putative retrotransposon hot spot (RHS) protein
TcG_10468	124,1125314	0,012337756	0,15870096	0,07774216	0,93803316	0,97142888	protein_codir retrotransposon hot spot (RHS) protein
TcG_10469	17,53902498	0,856626552	0,4292088	1,9958271	0,04595275	0,1557661	protein_codir hypothetical protein
TcG_10470	11,12908106	0,081857239	0,53725407	0,15236225	0,87890123	1	protein_codir hypothetical protein
TcG_10471	8,882433902	-0,041119305	0,61517279	-0,06684188	0,94670758	1	protein_codir hypothetical protein
TcG_10472	20,46418277	-0,143196483	0,37790987	-0,37891703	0,70474948	0,84755533	protein_codir trans-sialidase
TcG_10473	62,65082854	0,196543018	0,22275869	0,88231357	0,37760726	0,60830892	protein_codir hypothetical protein
TcG_10474	90,9483343	0,383123851	0,18357335	2,08703416	0,03688505	0,13342337	protein_codir hypothetical protein
TcG_10475	141,4989985	0,212176446	0,15276287	1,38892679	0,16485501	0,37022876	protein_codir dispersed gene family protein 1 (DGF-1)
TcG_10476	45,16491679	-0,25203944	0,25813596	-0,97638254	0,32887493	0,56324389	protein_codir dispersed gene family protein 1 (DGF-1)
TcG_10477	15,09031006	0,123014885	0,47830862	0,25718726	0,79703422	1	protein_codir dispersed protein family protein 1 (DGF-1)
TcG_10478	22,78234069	0,424304136	0,38278151	1,10847604	0,26765628	0,49992998	protein_codir hypothetical protein
TcG_10479	34,87469957	0,259605647	0,30284232	0,85723041	0,39131756	0,62112715	
TcG_10480	237,3121753	0,29205473	0,11552098	2,5281532	0,01146643	0,05505597	protein_codir putative trans-sialidase
TcG_10481	82,66354416	0,597570692	0,19456151	3,07137158	0,00213078	0,01448779	protein_codir hypothetical protein
TcG_10482	38,06052872	0,613303432	0,30973284	1,98010465	0,04769177	0,15988335	
TcG_10483	51,99071795	0,782266669	0,25476394	3,07055497	0,00213661	0,01451895	protein_codir protein kinase, putative,serine/threonine protein kinase
TcG_10484	59,51129723	0,813022268	0,23667982	3,43511445	0,0005923	0,00499819	protein_codir target of rapamycin (TOR) kinase 1
TcG_10485	56,4370686	0,615214333	0,24949342	2,46585398	0,0136687	0,06329128	protein_codir hypothetical protein
TcG_10486	70,20791264	0,498885933	0,21126295	2,3614454	0,01820385	0,07802806	protein_codir surface protease GP63
TcG_10487	125,6053125	0,137717848	0,15612575	0,8820957	0,37772506	0,60841409	protein_codir hypothetical protein
TcG_10488	1640,681264	0,401941552	0,05725682	7,01997713	2,219E-12	1,5969E-10	protein_codir 40S ribosomal protein S13
TcG_10489	273,1436209	0,011538657	0,11137147	0,10360514	0,91748271	0,96017565	protein_codir hypothetical protein
TcG_10490	223,3679946	-0,016219693	0,12106486	-0,13397524	0,89342216	0,94860604	protein_codir hypothetical protein
TcG_10491	300,9324687	-0,034055653	0,11080712	-0,30734175	0,75858326	0,87801518	protein_codir hypothetical protein
TcG_10492	128,9948552	-0,155567394	0,16482933	-0,94380892	0,3452673	0,57929591	protein_codir hypothetical protein
TcG_10493	76,26409823	0,37669555	0,21160113	1,78021523	0,07504074	0,22117071	protein_codir dispersed gene family protein 1 (DGF-1)
TcG_10494	18,84447333	0,047102307	0,43109383	0,10926231	0,91299444	0,95728087	protein_codir dispersed gene family protein 1 (DGF-1)
TcG_10495	14,86013124	-0,338101101	0,45112628	-0,74946	0,45358	1	protein_codir dispersed gene family protein 1 (DGF-1)
TcG_10496	7,980619689	-1,131401449	0,62018922	-1,82428428	0,06810911	1	protein_codir dispersed protein family protein 1
TcG_10497	18,77325441	0,184727769	0,40342316	0,45790075	0,64702375	0,81242026	
TcG_10498	4,887770061	0,54238588	0,83685467	0,64812434	0,51690453	1	protein_codir hypothetical protein
TcG_10499	139,5909881	0,127124723	0,15182179	0,83732858	0,4024079	0,63269072	protein_codir dispersed gene family protein 1 (DGF-1)
TcG_10500	16,48343474	0,101958508	0,425234	0,23977036	0,81050829	1	protein_codir dispersed gene family protein 1 (DGF-1)
TcG_10501	166,6059955	-0,056407409	0,13749264	-0,41025767	0,68161694	0,83418336	

TcG_10502	116,276293	0,228485375	0,16307123	1,40113844	0,16117268	0,36450257	protein_codir	retrotransposon hot spot (RHS) protein
TcG_10503	238,1185347	0,282903757	0,1155832	2,44761998	0,01438032	0,06580191	protein_codir	putative retrotransposon hot spot (RHS) protein
TcG_10504	17,55736116	0,963864599	0,43998414	2,19068034	0,02847493	0,10989692	protein_codir	hypothetical protein
TcG_10505	21,87854223	0,575110799	0,38127207	1,50840001	0,13145218	0,32226089	protein_codir	hypothetical protein
TcG_10506	122,0859854	0,375336842	0,17057778	2,20038535	0,02777957	0,10793286	protein_codir	surface protein-2
TcG_10507	89,99447877	0,725663281	0,19465687	3,72790989	0,00019307	0,00193986	protein_codir	hypothetical protein
TcG_10508	147,3282274	0,285254344	0,14858001	1,91987027	0,05487429	0,17679942	protein_codir	hypothetical protein
TcG_10509	67,26737797	-0,071363845	0,21057152	-0,33890549	0,73468093	0,86407606		
TcG_10510	25,99558968	0,42933735	0,35208593	1,21941071	0,22268835	0,4488042		
TcG_10511	2,843317745	-1,74348979	1,12125803	-1,55494074	0,11996018	1	protein_codir	surface protease GP63
TcG_10512	135,6926703	-0,419781966	0,15295548	-2,74447158	0,00606084	0,03328004	protein_codir	mucin-associated surface protein (MASP)
TcG_10513	18,44279771	-0,832015637	0,41138919	-2,02245379	0,04312949	0,14915711		
TcG_10514	27,96247869	-0,098239031	0,32673667	-0,30066729	0,76366821	0,88082229	protein_codir	mucin TcMUCII
TcG_10515	42,03126474	-0,218432664	0,26563556	-0,82230205	0,41090501	0,63757656		
TcG_10516	38,40152345	-0,46590708	0,28826809	-1,61622842	0,10604494	0,28044662		
TcG_10517	6,829935448	-0,715960773	0,74991219	-0,95472615	0,3397162	1	protein_codir	putative surface protease GP63
TcG_10518	11,72726744	0,035449679	0,51336531	0,06905351	0,94494702	1	protein_codir	putative surface protease GP63
TcG_10519	12,24230909	-0,555544881	0,52155273	-1,0651749	0,28679679	1		
TcG_10520	105,1498297	0,162709522	0,18138479	0,89704063	0,36969724	0,60125101	protein_codir	putative trans-sialidase
TcG_10521	74,34108114	0,4610442	0,20300684	2,27107713	0,02314231	0,09420641	protein_codir	hypothetical protein
TcG_10522	113,3819774	0,208464754	0,1738742	1,19894013	0,23055123	0,45778346		
TcG_10523	35,31261281	0,466920378	0,2972192	1,57096303	0,11619124	0,29710697	protein_codir	hypothetical protein
TcG_10524	12,84389306	0,33073306	0,48379805	0,683618	0,49421641	1	protein_codir	retrotransposon hot spot (RHS) protein
TcG_10525	0,155988004	0,503022807	4,08047286	0,12327562	0,90188885	1	protein_codir	retrotransposon hot spot (RHS) protein
TcG_10526	153,5517869	0,347082167	0,15050555	2,30610879	0,02110455	0,0878356	protein_codir	putative retrotransposon hot spot (RHS) protein
TcG_10527	245,6905468	0,100103631	0,11306808	0,88533941	0,3759736	0,60653866	protein_codir	sister chromatid cohesion protein DCC1
TcG_10528	487,0876182	-0,2999077	0,08710414	-3,44309348	0,0005751	0,00487783	protein_codir	hypothetical protein
TcG_10529	54,59921069	0,148921571	0,23919241	0,62260156	0,53354638	0,73410397	protein_codir	hypothetical protein
TcG_10530	31,398907	-0,276152156	0,31854572	-0,8669153	0,38598842	0,61649598		
TcG_10531	53,99347831	-0,217951476	0,24114805	-0,90380775	0,36609735	0,59825161		
TcG_10532	104,3406764	-0,459294645	0,1784048	-2,57445229	0,01003989	0,04954097	protein_codir	trans-sialidase
TcG_10533	51,9837075	-0,196168939	0,24551493	-0,79901023	0,42428448	0,65009738	protein_codir	trans-sialidase
TcG_10534	50,74525442	-0,409139719	0,24460251	-1,67267182	0,09439192	0,25854014	protein_codir	trans-sialidase
TcG_10535	210,4920805	-0,406089212	0,12425571	-3,26817337	0,00108244	0,00832193		
TcG_10536	32,35926194	-0,771409478	0,31520528	-2,44732413	0,01439213	0,06582995	protein_codir	hypothetical protein
TcG_10537	41,53013321	-0,282708356	0,26734211	-1,05747785	0,29029356	0,52345679	protein_codir	trans-sialidase
TcG_10538	31,01884436	0,232604389	0,33438263	0,69562342	0,48666469	0,69843889	protein_codir	hypothetical protein
TcG_10539	11,14313029	-0,658035512	0,53881346	-1,22126778	0,22198465	1	protein_codir	hypothetical protein
TcG_10540	6,13588619	-1,431841283	0,71851232	-1,99278599	0,04628489	1	protein_codir	hypothetical protein
TcG_10541	36,15866912	-0,443979747	0,28661526	-1,54904434	0,12137106	0,30569676	protein_codir	hypothetical protein
TcG_10542	17,70574599	0,066880512	0,40841048	0,16375807	0,8699216	0,9373116	protein_codir	hypothetical protein
TcG_10543	73,38729561	-0,803014392	0,2105949	-3,81307611	0,00013725	0,00145286	protein_codir	mucin-associated surface protein (MASP)
TcG_10544	92,10153138	-0,282324777	0,18085426	-1,56106234	0,11850904	0,30064501		
TcG_10545	19,93650517	-0,22819122	0,38365284	-0,59478569	0,55198673	0,74737358	protein_codir	putative trans-sialidase
TcG_10546	2,6100924	-0,827813517	1,16337538	-0,71156183	0,47673615	1	protein_codir	putative trans-sialidase
TcG_10547	14,67699838	0,030657734	0,48218782	0,06358048	0,94930427	1	protein_codir	putative trans-sialidase
TcG_10548	37,16207315	-0,399857057	0,29074294	-1,37529412	0,16904028	0,37547942	protein_codir	putative trans-sialidase
TcG_10549	170,549831	-0,007424504	0,13854718	-0,05358827	0,9572632	0,98019014	protein_codir	RNA editing complex protein MP90
TcG_10550	164,9369151	-0,139721861	0,14369228	-0,9723686	0,33086721	0,56523583	protein_codir	beta galactofuranosyl glycosyltransferase
TcG_10551	65,93493843	0,175394667	0,2194994	0,79906674	0,42425171	0,65009738		
TcG_10552	37,48330552	0,165039814	0,28084259	0,58765949	0,55676087	0,75138398	protein_codir	hypothetical protein
TcG_10553	352,7068061	-0,361265131	0,09519475	-3,79501094	0,00014764	0,0015522	protein_codir	sialidase-like protein

TcG_10554	28,86245882	-0,134121469	0,32519734	-0,41243102	0,68002353	0,83311332	protein_codir trans-sialidase
TcG_10555	6,439738533	-0,232992645	0,67246738	-0,34647427	0,72898632	1	protein_codir trans-sialidase
TcG_10556	14,78086431	0,382931611	0,489724	0,78193352	0,43425364	1	protein_codir trans-sialidase
TcG_10557	19,23205789	-0,579388119	0,44401708	-1,30487799	0,1919344	0,40810276	protein_codir putative trans-sialidase
TcG_10558	15,84292318	0,135114193	0,46527853	0,29039421	0,77151467	1	protein_codir hypothetical protein
TcG_10559	755,5274297	0,329934565	0,06981906	4,72556604	2,2948E-06	4,2202E-05	protein_codir putative retrotransposon hot spot (RHS) protein
TcG_10560	6,718663877	-0,017307704	0,67152397	-0,02577377	0,97943778	1	protein_codir retrotransposon hot spot (RHS) protein
TcG_10561	11,69594419	-0,249988928	0,4987172	-0,5012639	0,61618541	1	protein_codir retrotransposon hot spot (RHS) protein
TcG_10562	9,310207085	0,163719601	0,62203901	0,26319828	0,79239775	1	protein_codir hypothetical protein
TcG_10563	10,18648617	0,748007234	0,59230797	1,26286876	0,20663635	1	protein_codir hypothetical protein
TcG_10564	23,97868759	-0,185328373	0,36474762	-0,50810029	0,61138301	0,78979655	protein_codir mucin-associated surface protein (MASP)
TcG_10565	2,699975142	-1,077701816	1,15487506	-0,93317611	0,35072905	1	protein_codir hypothetical protein
TcG_10566	1,002806097	2,083335566	1,93166526	1,0785179	0,2808027	1	protein_codir hypothetical protein
TcG_10567	10,54804298	0,571781134	0,54524026	1,04867739	0,29432662	1	protein_codir surface protease GP63
TcG_10568	5,360609887	0,464699193	0,77677451	0,59824207	0,54967842	1	protein_codir hypothetical protein
TcG_10569	10,9314355	0,324310802	0,53687209	0,60407462	0,54579403	1	
TcG_10570	9,123764019	0,204042706	0,57602613	0,35422474	0,72317046	1	
TcG_10571	1,153102055	-1,933583056	1,77710801	-1,08805038	0,27657287	1	protein_codir putative trans-sialidase
TcG_10572	1,526783483	-1,898148536	1,63100346	-1,16379185	0,24450838	1	protein_codir trans-sialidase
TcG_10573	8,772372954	-0,33415065	0,58840789	-0,56788948	0,57011002	1	protein_codir hypothetical protein
TcG_10574	5,11088109	-0,909623875	0,79153178	-1,14919439	0,25047584	1	protein_codir hypothetical protein
TcG_10575	2,412382129	-0,262511708	1,1585162	-0,22659304	0,8207402	1	protein_codir dispersed gene family protein 1 (DGF-1)
TcG_10576	3,466452539	0,132527965	1,02843786	0,12886337	0,89746576	1	protein_codir dispersed gene family protein 1 (DGF-1)
TcG_10577	68,77609545	0,015786347	0,23624108	0,06682304	0,94672258	0,97482473	protein_codir dispersed gene family protein 1 (DGF-1)
TcG_10578	25,11354748	-0,787407431	0,34867005	-2,2583168	0,02392592	0,096621	protein_codir dispersed gene family protein 1 (DGF-1)
TcG_10579	53,69161424	-0,121790863	0,24221506	-0,50282119	0,61509	0,79205726	protein_codir mucin-associated surface protein (MASP)
TcG_10580	5,360240848	0,688173403	0,78762436	0,87373301	0,38226367	1	protein_codir surface protease GP63
TcG_10581	6,502915406	0,302987483	0,70694954	0,42858431	0,66822576	1	protein_codir surface protease GP63
TcG_10582	7,750037968	0,773897869	0,6299326	1,22854076	0,21924404	1	protein_codir hypothetical protein
TcG_10583	42,42998603	-0,001094554	0,26298961	-0,00416197	0,99667924	0,99883957	protein_codir putative mucin-associated surface protein (MASP)
TcG_10584	37,36059068	-0,427069699	0,29208295	-1,46215212	0,14369953	0,34019263	protein_codir mucin TcMUCII
TcG_10585	12,23971744	0,158156503	0,49388756	0,32022775	0,74879569	1	protein_codir mucin TcMUCII
TcG_10586	13,50649013	-0,031584586	0,49285292	-0,06408522	0,94890237	1	protein_codir hypothetical protein
TcG_10587	11,76204485	-0,597786354	0,54050222	-1,10598316	0,26873381	1	
TcG_10588	47,08558291	0,16213883	0,25414941	0,63796657	0,52349544	0,72759335	protein_codir hypothetical protein
TcG_10589	101,8747472	0,213563087	0,17741585	1,20374301	0,22868892	0,45574613	protein_codir hypothetical protein
TcG_10590	166,9386727	-0,106335666	0,13838844	-0,76838547	0,44225821	0,66466715	protein_codir hypothetical protein
TcG_10591	829,2836214	0,129671345	0,072161	1,79697279	0,07233994	0,21618229	protein_codir hypothetical protein
TcG_10592	110,0455477	0,118328404	0,3567815	0,3316551	0,7401497	0,86756146	protein_codir hypothetical protein
TcG_10593	19,0834516	0,384924971	0,43806583	0,87869208	0,37956825	0,61070376	protein_codir protein ARV1
TcG_10594	24,83712273	0,023479276	0,34269878	0,06851287	0,94537738	0,97469188	protein_codir hypothetical protein
TcG_10595	14,41674507	0,089260913	0,4608525	0,19368651	0,84642135	1	protein_codir putative mitotic centromere-associated kinesin (MCAK)
TcG_10596	40,44845539	-0,220689156	0,28160277	-0,78368959	0,43322227	0,65758067	protein_codir hypothetical protein
TcG_10597	15,26832365	-0,080731788	0,47886675	-0,16858925	0,86611973	1	protein_codir putative kinesin
TcG_10598	0,417901025	0,396008574	2,54186689	0,15579438	0,8761951	1	protein_codir putative kinesin
TcG_10599	21,02561864	0,401147263	0,37900758	1,05841487	0,28986634	0,52327695	protein_codir ATP-dependent DEAD/H RNA helicase
TcG_10600	61,69291914	0,663020461	0,22708732	2,91967194	0,003504	0,02165192	protein_codir trans-sialidase
TcG_10601	26,44435506	0,403872552	0,34339301	1,1761234	0,23954557	0,46794384	protein_codir hypothetical protein
TcG_10602	24,25830736	0,00019815	0,36166068	0,00054789	0,99956285	0,99981271	protein_codir hypothetical protein
TcG_10603	48,68516985	0,338571516	0,26444061	1,28033104	0,20042874	0,41961824	protein_codir retrotransposon hot spot (RHS) protein
TcG_10604	23,18168222	0,28204831	0,38608898	0,7305267	0,4650683	0,6821385	protein_codir putative retrotransposon hot spot (RHS) protein
TcG_10605	434,6275374	0,183045747	0,09158307	1,99868534	0,04564241	0,15492577	protein_codir putative retrotransposon hot spot (RHS) protein

TcG_10606	27,90422196	0,358944347	0,33801025	1,06193333	0,28826595	0,52128131	protein_codir mitotic centromere-associated kinesin (MCAK)
TcG_10607	19,74161996	0,556644511	0,39265341	1,41764847	0,15629342	0,35769079	protein_codir hypothetical protein
TcG_10608	67,1176014	0,029116689	0,2210544	0,1317173	0,8952079	0,9489813	protein_codir hypothetical protein
TcG_10609	26,14467452	0,211969092	0,34893296	0,60747798	0,54353375	0,74086847	
TcG_10610	125,3239528	0,00944141	0,16723628	0,05645551	0,95497893	0,97888932	protein_codir ATP-dependent DEAD/H RNA helicase
TcG_10611	1,732403416	2,007034156	1,48262248	1,35370547	0,17583036	1	protein_codir dispersed gene family protein 1 (DGF-1)
TcG_10612	12,31460478	0,777110014	0,515804	1,50659942	0,13191337	1	protein_codir dispersed gene family protein 1 (DGF-1)
TcG_10613	87,86761352	0,01648955	0,1881911	0,08762131	0,93017767	0,96624046	protein_codir dispersed gene family protein 1 (DGF-1)
TcG_10614	5,690788123	0,8458761	0,77464819	1,09194872	0,27485564	1	
TcG_10615	20,09265714	0,001020864	0,40025333	0,00255055	0,99796496	0,99932542	protein_codir hypothetical protein
TcG_10616	10,55430538	-0,853623836	0,58769727	-1,45248903	0,14636568	1	protein_codir trans-sialidase
TcG_10617	8,126478805	0,358614383	0,6097001	0,58818161	0,5564104	1	protein_codir Sialidase 85-1.3
TcG_10618	12,69563052	0,77441363	0,50430681	1,53560018	0,12463647	1	protein_codir hypothetical protein
TcG_10619	34,01973554	-0,227265538	0,29720024	-0,76468828	0,44445719	0,66642694	protein_codir mucin-associated surface protein (MASP)
TcG_10620	9,202135437	0,167540288	0,58109844	0,28831654	0,77310445	1	protein_codir hypothetical protein
TcG_10621	15,9264138	0,11083188	0,44372758	0,2497746	0,80276166	1	protein_codir trans-sialidase
TcG_10622	6,766455665	-0,314859755	0,67570173	-0,46597447	0,64123378	1	protein_codir trans-sialidase
TcG_10623	25,85338693	0,312543677	0,34783622	0,8985369	0,36889938	0,60086146	protein_codir putative trans-sialidase
TcG_10624	325,6852002	0,071954753	0,10061051	0,71518127	0,47449704	0,69021001	protein_codir retrotransposon hot spot (RHS) protein
TcG_10625	79,63845917	0,637475823	0,19708393	3,23453981	0,00121839	0,00916045	protein_codir retrotransposon hot spot (RHS) protein
TcG_10626	0					1	protein_codir retrotransposon hot spot (RHS) protein
TcG_10627	8,749303858	-0,328390374	0,58031	-0,56588785	0,57147002	1	protein_codir hypothetical protein
TcG_10628	10,75044706	0,201187056	0,53010051	0,37952624	0,70429712	1	
TcG_10629	17,5350447	0,745230365	0,41874166	1,77969004	0,0751267	0,2212552	protein_codir trans-sialidase
TcG_10630	8,911181784	-0,032369122	0,58408003	-0,05541898	0,95580467	1	protein_codir trans-sialidase
TcG_10631	7,124747439	0,481815787	0,72916267	0,66077956	0,50875369	1	protein_codir surface glycoprotein Tc-85/20
TcG_10632	71,27901127	0,043294747	0,21353479	0,20275266	0,83932836	0,92192691	protein_codir retrotransposon hot spot (RHS) protein
TcG_10633	93,75870796	0,139942444	0,18519797	0,75563704	0,44986684	0,67063268	protein_codir retrotransposon hot spot (RHS) protein
TcG_10634	0					1	protein_codir dispersed gene family protein 1 (DGF-1)
TcG_10635	36,97780978	0,303657442	0,28900985	1,05068196	0,29340468	0,52646533	protein_codir dispersed gene family protein 1 (DGF-1)
TcG_10636	284,4189413	0,318732305	0,10598681	3,00728283	0,00263594	0,01711818	protein_codir dispersed gene family protein 1 (DGF-1)
TcG_10637	23,79764371	-0,760817009	0,36777179	-2,06872043	0,03857233	0,13767683	protein_codir putative mucin TcMUCII
TcG_10638	78,06829497	-0,124849435	0,19975783	-0,62500396	0,53196846	0,7329332	protein_codir mucin-associated surface protein (MASP)
TcG_10639	3,916396115	-0,363102416	0,8699856	-0,41736601	0,67641072	1	protein_codir surface protease GP63
TcG_10640	3,862327759	0,285371602	0,94088667	0,30330072	0,7616607	1	protein_codir surface protease GP63
TcG_10641	4,955543098	-0,048839355	0,79774259	-0,06122195	0,95118245	1	protein_codir hypothetical protein
TcG_10642	3,27272296	-1,549243739	1,03790683	-1,49266167	0,13552577	1	protein_codir putative syntaxin binding protein
TcG_10643	6,337440835	0,254043086	0,6994694	0,363194	0,71645997	1	protein_codir putative syntaxin binding protein
TcG_10644	34,78904955	0,027821362	0,29472413	0,09439798	0,92479302	0,96320831	protein_codir hypothetical protein
TcG_10645	16,37191622	-0,685717113	0,44478531	-1,5416811	0,12315111	1	protein_codir mucin TcMUCII
TcG_10646	137,5742466	-0,111619164	0,15315198	-0,72881304	0,46611603	0,68273329	
TcG_10647	270,5477141	-0,142966379	0,1090595	-1,31090259	0,18989067	0,404961	protein_codir mucin-associated surface protein (MASP)
TcG_10648	186,8043024	0,231898168	0,1387286	1,67159602	0,09460401	0,2589986	protein_codir adenylate cyclase
TcG_10649	107,3672891	0,031475943	0,16761006	0,18779268	0,85103917	0,92805389	protein_codir receptor-type adenylate cyclase
TcG_10650	166,29919	0,095909944	0,16037438	0,59803783	0,54981469	0,74596143	protein_codir trans-sialidase
TcG_10651	91,14395994	-0,140269659	0,19210321	-0,73017864	0,465281	0,6821385	protein_codir hypothetical protein
TcG_10652	569,8475759	-0,399199102	0,08269246	-4,82751516	1,3825E-06	2,6965E-05	protein_codir putative proteasome regulatory ATPase subunit 2
TcG_10653	336,5246866	0,008860426	0,0996062	0,08895456	0,92911803	0,96557269	protein_codir putative NADH-cytochrome b5 reductase
TcG_10654	1,067721804	-0,098571876	1,87112848	-0,05268044	0,95798652	1	
TcG_10655	262,6834862	0,114137939	0,11330756	1,00732854	0,31377692	0,54808072	protein_codir regulator of sigma E protease
TcG_10656	92,05526018	-0,287517054	0,1810851	-1,58774547	0,11234391	0,29018174	protein_codir mucin-like glycoprotein
TcG_10657	143,8168535	-0,425346286	0,14880726	-2,85837062	0,00425823	0,0253967	protein_codir mucin-associated surface protein (MASP)

TcG_10658	34,93287132	0,268507643	0,30342852	0,88491234	0,37620391	0,60672307	protein_codir	trans-sialidase
TcG_10659	364,1153486	0,147906143	0,10934945	1,35260066	0,17618324	0,38601722	protein_codir	retrotransposon hot spot protein (RHS)
TcG_10660	6,871990233	-0,276270418	0,68396923	-0,40392229	0,68626987	1	protein_codir	retrotransposon hot spot (RHS) protein
TcG_10661	14,20760167	0,148604487	0,46336371	0,32070808	0,74843162	1	protein_codir	retrotransposon hot spot (RHS) protein
TcG_10662	8,236205134	0,42234617	0,67634467	0,62445405	0,53232944	1	protein_codir	hypothetical protein
TcG_10663	21,18295192	0,425448875	0,37753005	1,12692718	0,25977327	0,4921081		
TcG_10664	18,03451761	-0,503424392	0,40472431	-1,24386991	0,21354745	0,43698047	protein_codir	putative ABC transporter
TcG_10665	325,2074496	-0,154926648	0,09999411	-1,54935781	0,12129573	0,30557346	protein_codir	hypothetical protein
TcG_10666	145,8214385	0,052101038	0,14808939	0,35182154	0,7249721	0,85817259	protein_codir	hypothetical protein
TcG_10667	135,5094563	0,433984142	0,16260197	2,66899681	0,00760782	0,0398662	protein_codir	hypothetical protein
TcG_10668	198,8696427	0,041470624	0,12671312	0,32727963	0,7434564	0,86954228	protein_codir	D-alanyl-glycyl endopeptidase-like protein
TcG_10669	62,1798771	-0,560673741	0,22358177	-2,50768989	0,01215233	0,05763277	protein_codir	D-alanyl-glycyl endopeptidase-like protein
TcG_10670	274,0574946	-0,236422834	0,11265463	-2,0986517	0,03584762	0,1308952	protein_codir	D-alanyl-glycyl endopeptidase-like protein
TcG_10671	73,96240435	-0,154209719	0,20412758	-0,75545752	0,44997451	0,67070689	protein_codir	dispersed gene family protein 1 (DGF-1)
TcG_10672	85,48742663	-0,058226278	0,21804524	-0,2670376	0,78944021	0,89501549	protein_codir	dispersed gene family protein 1 (DGF-1)
TcG_10673	134,9548291	-0,330012566	0,15584045	-2,117631	0,03420633	0,12625503	protein_codir	dispersed gene family protein 1 (DGF-1)
TcG_10674	119,4689847	-0,392993951	0,16158346	-2,43214227	0,01500981	0,06774588	protein_codir	dispersed gene family protein 1 (DGF-1)
TcG_10675	116,611109	-0,648233694	0,1630155	-3,97651578	6,9932E-05	0,00082589	protein_codir	hypothetical protein
TcG_10676	1094,318315	-0,051762251	0,06984775	-0,74107259	0,45864943	0,67743881	protein_codir	surface protein-2
TcG_10677	178,523991	0,029765488	0,13545419	0,21974579	0,82606914	0,91511289	protein_codir	putative 50S ribosomal protein L17
TcG_10678	70,7245194	-0,165629932	0,21723031	-0,76246233	0,44578412	0,66755264	protein_codir	hypothetical protein
TcG_10679	138,3076725	0,099802588	0,14898481	0,66988432	0,50293154	0,71148496	protein_codir	hypothetical protein
TcG_10680	469,4898115	-0,182180815	0,08561062	-2,12801645	0,03333572	0,123672	protein_codir	putative eukaryotic translation initiation factor 1A
TcG_10681	175,9906012	-0,23941644	0,13504651	-1,77284438	0,0762545	0,22338422	protein_codir	anti-silencing protein a-like protein
TcG_10682	123,8108467	0,316131159	0,16029667	1,97216299	0,048591	0,16205393	protein_codir	trafficking protein particle complex 2
TcG_10683	276,7600918	-0,284768699	0,10781941	-2,64116347	0,00826218	0,04250696	protein_codir	putative 6-phosphogluconolactonase
TcG_10684	27,30324585	0,094891377	0,349524	0,27148744	0,78601616	0,89352269	protein_codir	hypothetical protein
TcG_10685	310,8576422	0,107907048	0,10267638	1,05094328	0,29328464	0,52633145	protein_codir	subtilisin-like serine peptidase
TcG_10686	23,81394493	-0,072749698	0,36564371	-0,19896335	0,84229141	0,9231054		
TcG_10687	135,3389386	-0,068880513	0,15932563	-0,43232538	0,66550494	0,82504261	protein_codir	hypothetical protein
TcG_10688	82,59139519	-0,063477844	0,19737307	-0,3216135	0,74774552	0,87270873	protein_codir	retrotransposon hot spot (RHS) protein
TcG_10689	14,51892985	-0,076501724	0,46844478	-0,16331002	0,87027435	1		
TcG_10690	730,42944	0,18605139	0,07012031	2,65331664	0,00797051	0,04139233	protein_codir	putative retrotransposon hot spot (RHS) protein
TcG_10691	193,5436163	-0,035382364	0,13137291	-0,26932771	0,78767751	0,89453088	protein_codir	sialidase-like protein
TcG_10692	37,66758274	-0,342413427	0,29136007	-1,17522427	0,239905	0,46848801	protein_codir	trans-sialidase
TcG_10693	9,993975879	-0,574132293	0,55621962	-1,03220432	0,30197641	1	protein_codir	hypothetical protein
TcG_10694	57,31538348	0,106665744	0,22957594	0,46462075	0,64220307	0,8100665	protein_codir	hypothetical protein
TcG_10695	20,22558448	-1,368084548	0,42167418	-3,2444115	0,00117694	0,00891823	protein_codir	hypothetical protein
TcG_10696	9,610648232	-0,421220996	0,5751326	-0,73238936	0,46393095	1	protein_codir	putative trans-sialidase
TcG_10697	32,15411796	-0,591731803	0,30371726	-1,94829821	0,05137929	0,16868429	protein_codir	target of rapamycin (TOR) kinase 1
TcG_10698	43,40592354	-0,366761959	0,26579884	-1,37984787	0,16763349	0,37364402	protein_codir	putative profilin
TcG_10699	190,811656	-0,022094172	0,12923162	-0,17096568	0,86425075	0,93498506	protein_codir	mucin-associated surface protein (MASP)
TcG_10700	0					1		
TcG_10701	6,887632761	0,607291641	0,6684277	0,90853752	0,3635943	1	protein_codir	hypothetical protein
TcG_10702	2,029660636	0,417092374	1,26020504	0,33097183	0,74066576	1	protein_codir	structural maintenance of chromosome protein 4
TcG_10703	32,57702166	0,259377048	0,31515266	0,82302034	0,41049643	0,63729557	protein_codir	putative trans-sialidase
TcG_10704	7,683655443	-0,194837682	0,61985155	-0,31432959	0,75327073	1	protein_codir	hypothetical protein
TcG_10705	3,408530509	-1,095952621	0,93539698	-1,17164438	0,24133986	1	protein_codir	hypothetical protein
TcG_10706	0					1	protein_codir	hypothetical protein
TcG_10707	2,901723286	1,24078653	1,06822815	1,16153701	0,24542358	1	protein_codir	hypothetical protein
TcG_10708	23,62666379	0,758432355	0,36788475	2,06160313	0,03924554	0,13947254	protein_codir	putative profilin
TcG_10709	12,31680315	0,367766511	0,49064696	0,74955424	0,45352322	1	protein_codir	hypothetical protein

TcG_10710	48,28599888	1,187249485	0,26475702	4,48429843	7,3154E-06	0,00011658	protein_codir	hypothetical protein
TcG_10711	153,1540343	0,010679498	0,14805772	0,07213064	0,94249794	0,97319113	protein_codir	surface protease GP63
TcG_10712	221,5015395	0,129910621	0,11976837	1,08468225	0,27806241	0,51072149	protein_codir	surface protease GP63
TcG_10713	0,701286704	2,70705338	2,05473234	1,31747252	0,18768029	1	protein_codir	putative trans-sialidase
TcG_10714	41,22955388	-0,036023575	0,28712989	-0,1254609	0,90015868	0,95148603	protein_codir	hypothetical protein
TcG_10715	31,73734274	0,531579043	0,31666849	1,67866098	0,09321814	0,25605152	protein_codir	putative trans-sialidase
TcG_10716	18,45512942	-0,096267857	0,45049998	-0,21369114	0,83078792	0,91760178	protein_codir	target of rapamycin (TOR) kinase 1
TcG_10717	10,15642317	0,851182727	0,59678499	1,42628038	0,15378742	1	protein_codir	hypothetical protein
TcG_10718	66,00110673	-0,159526681	0,21425987	-0,74454764	0,45654523	0,67639993	protein_codir	putative protein tyrosine phosphatase-like protein
TcG_10719	402,3608909	-0,047880619	0,09272443	-0,51637543	0,60559223	0,78688931	protein_codir	Alpha/beta hydrolase fold protein
TcG_10720	40,11054366	-0,106835672	0,27274551	-0,3917046	0,69527649	0,84234001	protein_codir	hypothetical protein
TcG_10721	83,46304467	0,00874646	0,19228261	0,04548752	0,96371872	0,98367061	protein_codir	hypothetical protein
TcG_10722	77,27009422	0,010738148	0,2049047	0,05240557	0,95820553	0,98064539	protein_codir	hypothetical protein
TcG_10723	17,76887845	0,335194353	0,43511113	0,77036493	0,44108345	0,66354606	protein_codir	hypothetical protein
TcG_10724	34,19144192	0,613907032	0,29839396	2,05737087	0,03965056	0,14035791		
TcG_10725	15,8346289	-0,092199067	0,4331539	-0,21285522	0,83143989	1	protein_codir	hypothetical protein
TcG_10726	61,93451037	0,526800468	0,2278041	2,31251527	0,0207493	0,08659994	protein_codir	hypothetical protein
TcG_10727	51,57369092	0,175950417	0,24653076	0,71370573	0,47540916	0,69103267	protein_codir	dispersed gene family protein 1 (DGF-1)
TcG_10728	24,43577683	0,374083148	0,36033315	1,03815911	0,29919599	0,53281351	protein_codir	dispersed gene family protein 1 (DGF-1)
TcG_10729	1,005188382	0,099414818	1,66782921	0,05960731	0,95246839	1	protein_codir	hypothetical protein
TcG_10730	2,713524462	-1,586854187	1,11749322	-1,42001236	0,15560408	1	protein_codir	hypothetical protein
TcG_10731	2,305431873	-2,03158436	1,36964589	-1,48329168	0,13799693	1	protein_codir	hypothetical protein
TcG_10732	2,757803563	1,549938951	1,17937367	1,31420515	0,18877718	1		
TcG_10733	12,69428042	-1,941707438	0,5405825	-3,59187993	0,0003283	1	protein_codir	mucin TcMUCII
TcG_10734	5,95333191	0,345998623	0,70635834	0,48983441	0,62425108	1		
TcG_10735	172,2935683	-0,200200344	0,13816807	-1,44896247	0,14734806	0,3460723	protein_codir	mucin-associated surface protein (MASP)
TcG_10736	433,5350442	-0,101288644	0,09321982	-1,08655692	0,27723267	0,50960142	protein_codir	AAA family ATPase-like protein
TcG_10737	427,7188314	0,031429738	0,08882255	0,35384863	0,72345232	0,85739757	protein_codir	RING finger protein
TcG_10738	235,329586	0,072243498	0,11818026	0,61129918	0,54100154	0,73976676	protein_codir	hypothetical protein
TcG_10739	208,171289	0,109958156	0,13434945	0,81844886	0,41310093	0,63986462	protein_codir	hypothetical protein
TcG_10740	52,64941658	0,071703991	0,24420815	0,29361834	0,76904956	0,88436428	protein_codir	hypothetical protein
TcG_10741	0					1	protein_codir	mitochondrial heat shock
TcG_10742	376,5593081	-0,255260766	0,10203976	-2,5015815	0,012364	0,0584214	protein_codir	Flagellar attachment zone protein 1
TcG_10743	147,5965486	0,21525767	0,15196048	1,41653719	0,15661828	0,35818781	protein_codir	hypothetical protein
TcG_10744	1133,730478	0,191282818	0,05958988	3,20998838	0,0013274	0,00983331	protein_codir	L1Tc protein
TcG_10745	629,7669968	0,538030477	0,07686023	7,00011573	2,5575E-12	1,8068E-10	protein_codir	coiled-coil domain containing 39
TcG_10746	1159,00896	0,496418207	0,06028475	8,2345575	1,8022E-16	2,1526E-14	protein_codir	putative ribonuclease inhibitor
TcG_10747	63,94362312	0,533560696	0,22303042	2,39232252	0,01674212	0,07346244	protein_codir	hypothetical protein
TcG_10748	0					1		
TcG_10749	5,907975894	-1,557215467	0,7554932	-2,06119058	0,03928486	1	protein_codir	trans-sialidase
TcG_10750	12,4462596	-0,433822522	0,50065276	-0,86651379	0,38620847	1	protein_codir	trans-sialidase
TcG_10751	8,405504964	-0,138632646	0,59319082	-0,23370666	0,81521272	1		
TcG_10752	5,192207249	-1,388486485	0,83003438	-1,67280599	0,0943655	1	protein_codir	dispersed gene family protein 1 (DGF-1)
TcG_10753	20,73386341	0,157714939	0,37769865	0,41756819	0,67626286	0,8302619	protein_codir	dispersed gene family protein 1 (DGF-1)
TcG_10754	34,55891058	-0,596909488	0,33404767	-1,7868991	0,0739538	0,21947457	protein_codir	mucin TcMUCII
TcG_10755	3,643071939	-0,774209739	0,93038724	-0,8321371	0,40533156	1	protein_codir	trans-sialidase
TcG_10756	3,166334251	-0,949811846	1,04495013	-0,90895424	0,36337428	1	protein_codir	hypothetical protein
TcG_10757	9,570722025	-0,309709465	0,59730427	-0,51851206	0,60410105	1	protein_codir	telomerase reverse transcriptase
TcG_10758	11,91917584	0,40036479	0,51745059	0,77372564	0,43909306	1	protein_codir	hypothetical protein
TcG_10759	0,938070566	0,848909746	1,82527637	0,46508559	0,64187016	1	protein_codir	putative surface protease GP63
TcG_10760	31,872519	-0,163471578	0,32514691	-0,50276221	0,61513146	0,79205726	protein_codir	mucin-associated surface protein (MASP)
TcG_10761	54,28733416	0,287458046	0,23622838	1,21686497	0,22365559	0,45017916	protein_codir	mucin-associated surface protein (MASP)

TcG_10762	69,1454665	-0,108161702	0,21150411	-0,5113929	0,60907597	0,788218	protein_codir mucin TcMUCII
TcG_10763	81,08854453	-0,117841196	0,1922853	-0,61284559	0,53997844	0,73897829	protein_codir putative syntaxin binding protein
TcG_10764	13,90432821	0,403019516	0,4657769	0,86526299	0,38689446	1	protein_codir syntaxin binding protein
TcG_10765	9,967093944	0,936513834	0,57528663	1,62790823	0,10354435	1	protein_codir hypothetical protein
TcG_10766	33,97969747	0,044158466	0,29709914	0,14863209	0,88184395	0,94218407	protein_codir surface protease GP63
TcG_10767	25,38926689	0,734916258	0,38092243	1,92930687	0,05369278	0,17386375	protein_codir surface protease GP63
TcG_10768	32,50285063	0,033425094	0,31400386	0,10644803	0,91522688	0,95875395	protein_codir regulator of sigma E protease
TcG_10769	121,8806676	-0,571508603	0,16881948	-3,38532373	0,00071094	0,00582944	protein_codir cytochrome b5
TcG_10770	477,5557004	-0,413815367	0,08541675	-4,844663	1,2683E-06	2,5033E-05	protein_codir succinate dehydrogenase flavoprotein subunit
TcG_10771	181,262384	-0,229406539	0,13595621	-1,68735613	0,09153489	0,25268603	protein_codir putative ADP-ribosylation factor-like protein 3A
TcG_10772	21,1961487	-0,003503581	0,37275769	-0,00939908	0,99250073	0,99705057	
TcG_10773	65,10934798	0,081261133	0,22425734	0,36235663	0,71708554	0,85492417	protein_codir RNA editing complex protein MP90
TcG_10774	67,45276337	0,129987962	0,21723094	0,59838604	0,54958238	0,7458664	protein_codir target of rapamycin (TOR) kinase 1
TcG_10775	266,3580324	0,067709127	0,11131254	0,60827941	0,54300218	0,74066673	protein_codir beta galactofuranosyl glycosyltransferase
TcG_10776	76,62358276	0,000937611	0,20925215	0,00448077	0,99642487	0,99883878	protein_codir hypothetical protein
TcG_10777	90,2993382	-0,002893372	0,18595685	-0,01555937	0,98758592	0,99514441	protein_codir putative trans-sialidase
TcG_10778	39,66903088	-0,522406519	0,2754383	-1,89663714	0,05787585	0,18361159	protein_codir trans-sialidase
TcG_10779	9,191390642	-0,251410916	0,59699385	-0,42112815	0,6736615	1	protein_codir hypothetical protein
TcG_10780	26,18943153	0,006181086	0,33994711	0,01818249	0,98549327	0,99432608	protein_codir hypothetical protein
TcG_10781	4,492511709	0,163387439	0,84053584	0,19438486	0,84587455	1	protein_codir retrotransposon hot spot (RHS) protein
TcG_10782	623,5850948	0,052851329	0,07857844	0,67259328	0,50120608	0,71007259	protein_codir putative retrotransposon hot spot (RHS) protein
TcG_10783	75,34999419	-0,410139637	0,19891772	-2,06185571	0,03922148	0,13943541	protein_codir c71 surface protein
TcG_10784	39,81468771	0,068406008	0,28207548	0,24250959	0,80838533	0,90518531	protein_codir hypothetical protein
TcG_10785	28,73169316	0,086842863	0,32372243	0,26826335	0,78849661	0,89487471	protein_codir hypothetical protein
TcG_10786	8,255683828	0,176133946	0,62470529	0,28194726	0,77798395	1	protein_codir hypothetical protein
TcG_10787	24,36947318	0,268944979	0,35251274	0,76293691	0,44550102	0,66721495	protein_codir hypothetical protein
TcG_10788	19,29818196	-0,059547072	0,42990456	-0,13851231	0,88983554	0,94653573	
TcG_10789	204,3518697	0,554978766	0,12742993	4,35516813	1,3296E-05	0,00019451	protein_codir putative short chain dehydrogenase
TcG_10790	10,97824202	-0,177345135	0,55297663	-0,32071	0,74843017	1	
TcG_10791	101,3452284	0,644732476	0,17723327	3,63776211	0,00027502	0,00259898	protein_codir hypothetical protein
TcG_10792	172,6367199	0,310401532	0,1351244	2,29715376	0,02161	0,08938717	protein_codir hypothetical protein
TcG_10793	78,42685971	0,256965171	0,20965309	1,22566843	0,22032348	0,44541323	
TcG_10794	126,6366982	-0,051872119	0,15826713	-0,32775042	0,74310038	0,86930139	protein_codir putative UDP-Gal or UDP-GlcNAc-dependent glycosyltransferase
TcG_10795	30,68033335	0,394216516	0,36089093	1,09234255	0,27468257	0,50755645	protein_codir hypothetical protein
TcG_10796	14,08576528	0,305085107	1,09465515	0,27870431	0,78047176	1	protein_codir putative mucin TcMUCII
TcG_10797	10,44843901	0,008664318	0,55155769	0,01570882	0,98746669	1	protein_codir hypothetical protein
TcG_10798	7,64457284	0,438781583	0,62983434	0,6966619	0,48601441	1	protein_codir hypothetical protein
TcG_10799	1,188139541	-0,820597086	1,5976945	-0,51361326	0,6075224	1	protein_codir hypothetical protein
TcG_10800	2,864763405	0,334126016	1,02302842	0,32660482	0,7439668	1	protein_codir hypothetical protein
TcG_10801	13,8457286	0,128433761	0,4832841	0,26575209	0,79043014	1	protein_codir hypothetical protein
TcG_10802	33,23409443	-0,497228663	0,29923285	-1,66167809	0,09657734	0,26284825	protein_codir hypothetical protein
TcG_10803	633,2102346	0,153898026	0,07415497	2,07535689	0,03795347	0,13614561	protein_codir hypothetical protein
TcG_10804	393,3986319	-0,057600994	0,09463624	-0,60865681	0,54275194	0,74049976	protein_codir hypothetical protein
TcG_10805	48,62663251	0,189574972	0,2664415	0,71150692	0,47677017	0,69195279	protein_codir hypothetical protein
TcG_10806	503,810166	-0,14688327	0,0871375	-1,68564926	0,09186338	0,25335136	protein_codir profilin
TcG_10807	347,9053247	-0,125412593	0,10200676	-1,22945379	0,21890171	0,44370106	protein_codir miltefosine transporter beta subunit
TcG_10808	202,5877152	-0,187806985	0,1271748	-1,47676252	0,13973931	0,33429108	protein_codir ras-related rab-4
TcG_10809	18,2741627	0,152604178	0,42444221	0,35954053	0,71919076	0,85588296	
TcG_10810	3,534231857	-0,025422789	0,91492645	-0,0277867	0,97783227	1	protein_codir surface protease GP63
TcG_10811	21,96485738	0,064467496	0,37769089	0,17068851	0,8644687	0,93498506	protein_codir hypothetical protein
TcG_10812	18,33654371	-0,570304896	0,40663645	-1,40249329	0,16076799	0,36372934	
TcG_10813	244,8033234	-0,104802672	0,11835792	-0,88547243	0,37590188	0,60653866	protein_codir dispersed gene family protein 1 (DGF-1)

TcG_10814	235,7587287	-0,072768617	0,1192374	-0,61028347	0,54167404	0,74008786	protein_codir	hypothetical protein
TcG_10815	200,1920671	-0,158645636	0,12621986	-1,25689918	0,20879014	0,4314326	protein_codir	solute carrier family 50 (sugar transporter)
TcG_10816	56,63901413	0,354108144	0,23207918	1,52580745	0,12705782	0,31529063	protein_codir	glyceraldehyde 3-phosphate dehydrogenase
TcG_10817	183,7828612	0,207429673	0,14632481	1,41759738	0,15630834	0,35769079	protein_codir	putative dispersed gene family protein 1 (DGF-1)
TcG_10818	6,927507707	0,68362204	0,68555619	0,99717871	0,31867777	1	protein_codir	hypothetical protein
TcG_10819	6,222094379	-0,845986813	0,69761227	-1,21268912	0,2252487	1	protein_codir	hypothetical protein
TcG_10820	9,114451066	-0,252091692	0,57526216	-0,43822054	0,66122642	1	protein_codir	hypothetical protein
TcG_10821	78,12150925	-0,42125681	0,214703	-1,96204431	0,04975733	0,16499381	protein_codir	putative 90 kDa surface protein
TcG_10822	34,54475362	-0,102521022	0,30387246	-0,33738175	0,73582914	0,86494473	protein_codir	putative mucin TcMUCII
TcG_10823	9,653797826	0,004820148	0,58274369	0,00827147	0,9934004	1	protein_codir	hypothetical protein
TcG_10824	6,539349334	-0,160104515	0,73674024	-0,21731474	0,82796307	1	protein_codir	hypothetical protein
TcG_10825	37,00402438	0,35593177	0,29510552	1,20611697	0,22777239	0,45460309	protein_codir	hypothetical protein
TcG_10826	2,685606415	0,388638495	1,07817805	0,36045855	0,71850425	1	protein_codir	trans-sialidase
TcG_10827	4,277635939	0,074755693	0,82465453	0,09065092	0,92776997	1	protein_codir	trans-sialidase
TcG_10828	3,980585542	-0,101560846	0,85395224	-0,11893036	0,90533053	1	protein_codir	hypothetical protein
TcG_10829	48,48237329	0,383528737	0,25541148	1,50161121	0,13319755	0,32441177	protein_codir	hypothetical protein
TcG_10830	41,34599235	0,229501017	0,27311904	0,84029667	0,40074207	0,63106768	protein_codir	putative trans-sialidase
TcG_10831	13,81008975	0,167128233	0,46134879	0,36226004	0,71715772	1	protein_codir	hypothetical protein
TcG_10832	57,17472415	-0,277965556	0,23200327	-1,19811052	0,23087399	0,4583126	protein_codir	putative mucin TcMUCII
TcG_10833	4,270978222	-1,102864511	0,8845956	-1,24674429	0,21249127	1	protein_codir	mucin-associated surface protein (MASP)
TcG_10834	5,040204184	-0,79899811	0,77696286	-1,02836076	0,30378016	1	protein_codir	hypothetical protein
TcG_10835	3,81428332	-1,360265994	0,94406682	-1,44085775	0,14962487	1	protein_codir	hypothetical protein
TcG_10836	0,626347624	-0,249464434	2,22309332	-0,11221501	0,91065293	1	protein_codir	hypothetical protein
TcG_10837	6,822906241	-0,33815665	0,65577312	-0,51566104	0,60609117	1	protein_codir	mucin TcMUCII
TcG_10838	2,195392299	3,290417954	1,5087313	2,18091714	0,02918955	1		
TcG_10839	493,3581578	0,158610979	0,08917452	1,77865799	0,07529585	0,22154994	protein_codir	putative dihydroorotase
TcG_10840	813,3845683	0,110412129	0,06738976	1,63841102	0,10133598	0,27140053	protein_codir	orotidine-5-phosphate decarboxylase/orotate phosphoribosyltransferase
TcG_10841	73,0723486	0,303446864	0,2182427	1,39041013	0,16440437	0,36971838		
TcG_10842	256,5809881	0,110953604	0,12209246	0,90876704	0,36347311	0,59544737	protein_codir	putative trans-sialidase
TcG_10843	14,46329785	0,952700314	0,48562296	1,96181069	0,04978453	1	protein_codir	hypothetical protein
TcG_10844	18,35867555	-0,085609703	0,44314659	-0,19318597	0,84681333	0,92593235	protein_codir	hypothetical protein
TcG_10845	36,65384524	0,379588571	0,29564344	1,28394047	0,19916279	0,41802537		
TcG_10846	142,2237407	-0,296826322	0,14777789	-2,00859758	0,04457983	0,15254043	protein_codir	putative mucin-associated surface protein (MASP)
TcG_10847	124,5479294	-0,12870345	0,15809223	-0,81410355	0,41558563	0,64239863		
TcG_10848	10,84394415	-0,04833918	0,52063499	-0,09284658	0,92602544	1	protein_codir	hypothetical protein
TcG_10849	11,47418117	-0,275313731	0,51268438	-0,53700433	0,59126462	1	protein_codir	hypothetical protein
TcG_10850	5,900242614	0,103405634	0,72163015	0,1432945	0,8860576	1	protein_codir	hypothetical protein
TcG_10851	9,367789545	0,183745015	0,57009747	0,32230456	0,74722198	1	protein_codir	hypothetical protein
TcG_10852	25,79357415	-0,265631128	0,3471421	-0,76519422	0,4441559	0,6664064	protein_codir	hypothetical protein
TcG_10853	24,63261994	-0,725571413	0,35165002	-2,06333391	0,03908091	0,13912475		
TcG_10854	28,70340044	-0,255289199	0,32633577	-0,78228996	0,43404419	0,65855826	protein_codir	hypothetical protein
TcG_10855	52,41538408	-0,179501924	0,25193218	-0,71250098	0,4761546	0,6915792	protein_codir	hypothetical protein
TcG_10856	26,67077753	0,265381301	0,34545221	0,76821423	0,44235992	0,66466715		
TcG_10857	19,65888076	-0,444823697	0,39655718	-1,12171389	0,26198411	0,49428247	protein_codir	hypothetical protein
TcG_10858	11,09106491	-0,568387825	0,52215477	-1,08854283	0,27635554	1	protein_codir	N-acetyltransferase complex ARD1 subunit
TcG_10859	42,40903583	0,558413978	0,27465174	2,03317112	0,04203524	0,14624571	protein_codir	trans-sialidase
TcG_10860	41,78793634	-0,052582731	0,27019401	-0,19461102	0,84569748	0,92503539	protein_codir	putative myosin heavy chain
TcG_10861	43,68808	0,081313233	0,26277577	0,30943961	0,75698714	0,8777475	protein_codir	subtilisin-like serine peptidase
TcG_10862	19,767828	0,616338384	0,39159389	1,57392236	0,1155054	0,29583886	protein_codir	subtilisin-like serine peptidase
TcG_10863	25,5846115	0,424310209	0,34409531	1,23311824	0,21753165	0,44185163	protein_codir	putative kinesin
TcG_10864	25,86593287	0,064763541	0,34454517	0,18796822	0,85090157	0,92805389	protein_codir	subtilisin-like serine peptidase
TcG_10865	44,21560562	-0,037899904	0,26789543	-0,14147276	0,88749648	0,94569431	protein_codir	dynein

TcG_10866	42,07757558	0,068786639	0,26906634	0,25564936	0,7982216	0,89936745	protein_codir putative myosin heavy chain
TcG_10867	35,33615862	0,398718542	0,30122921	1,32363837	0,18562317	0,39952259	protein_codir hypothetical protein
TcG_10868	45,882752	-0,151816223	0,25844464	-0,58742261	0,55691992	0,75151108	protein_codir trans-sialidase
TcG_10869	20,26077144	-0,067327672	0,37832615	-0,17796198	0,85875283	0,93207022	protein_codir hypothetical protein
TcG_10870	20,94683296	0,291429782	0,41504379	0,70216635	0,48257543	0,69628628	protein_codir hypothetical protein
TcG_10871	25,5264932	-0,420815723	0,35548945	-1,1837643	0,23650638	0,4638055	protein_codir retrotransposon hot spot protein (RHS)
TcG_10872	193,2450552	0,069537995	0,13030957	0,53363691	0,59359277	0,77886311	protein_codir retrotransposon hot spot (RHS) protein
TcG_10873	36,05214793	0,346816381	0,29273734	1,18473572	0,23612196	0,46360093	protein_codir trans-sialidase
TcG_10874	34,20861311	0,077005407	0,30433293	0,25303015	0,80024492	0,90062207	protein_codir putative trans-sialidase
TcG_10875	4,992485638	-0,528681218	0,801937	-0,6592553	0,50973184	1	
TcG_10876	20,43028636	0,379856869	0,41184671	0,92232585	0,35635864	0,59041487	protein_codir hypothetical protein
TcG_10877	2,054172417	-0,135232202	1,2991438	-0,10409333	0,91709529	1	protein_codir hypothetical protein
TcG_10878	0,534000759	0,261756182	2,52038867	0,10385548	0,91728404	1	protein_codir hypothetical protein
TcG_10879	0,770238516	-1,951474499	2,03486913	-0,9590172	0,33755008	1	protein_codir hypothetical protein
TcG_10880	30,01665455	0,086965365	0,32195326	0,27011799	0,78706949	0,8939307	protein_codir putative trans-sialidase
TcG_10881	41,07951157	-0,148867169	0,27932983	-0,53294404	0,59407232	0,77918921	protein_codir putative kinesin
TcG_10882	14,75118232	-0,100806886	0,44701448	-0,22551145	0,82158142	1	protein_codir putative kinesin
TcG_10883	43,01052257	0,021835185	0,26518017	0,08234094	0,9343756	0,96900069	protein_codir hypothetical protein
TcG_10884	47,195063	0,061906828	0,2508076	0,24682995	0,80503983	0,90385	protein_codir trans-sialidase
TcG_10885	25,6721612	0,044063614	0,34716542	0,12692397	0,89900057	0,95069557	protein_codir hypothetical protein
TcG_10886	84,39002604	0,040220495	0,19453465	0,20675234	0,83620328	0,92052325	
TcG_10887	29,51967196	-0,128947354	0,329592	-0,39123327	0,69562482	0,84234001	protein_codir trans-sialidase
TcG_10888	9,602982244	-0,940799747	0,57560758	-1,63444642	0,10216515	1	protein_codir hypothetical protein
TcG_10889	16,14365856	0,412749328	0,42990627	0,96009145	0,33700919	1	protein_codir hypothetical protein
TcG_10890	30,70243421	-0,055506662	0,32014866	-0,17337778	0,86235449	0,93414012	protein_codir beta galactofuranosyl glycosyltransferase
TcG_10891	22,93553453	0,267104272	0,36283617	0,73615668	0,46163535	0,6794505	protein_codir serine/threonine protein phosphatase
TcG_10892	8,879949959	-0,800549459	0,63119155	-1,2683146	0,20468563	1	protein_codir SH3 domain protein
TcG_10893	19,32651123	-0,962082781	0,40227325	-2,39161513	0,01677442	0,07353328	protein_codir mucin TcMUCII
TcG_10894	37,73755458	-0,152568949	0,29752217	-0,51279859	0,60809222	0,78802211	protein_codir hypothetical protein
TcG_10895	16,67650479	0,243045461	0,4224815	0,57528073	0,56510145	1	protein_codir putative syntaxin binding protein
TcG_10896	8,362728627	-0,439415556	0,63537389	-0,6915858	0,48919748	1	protein_codir syntaxin binding protein
TcG_10897	6,543478502	0,446065313	0,68149538	0,65453901	0,5127646	1	protein_codir hypothetical protein
TcG_10898	7,711692488	0,902212919	0,64486837	1,39906524	0,16179342	1	protein_codir hypothetical protein
TcG_10899	4,525083239	-0,72339664	0,80686789	-0,89654905	0,3699596	1	protein_codir surface protease GP63
TcG_10900	18,75359833	-0,176843718	0,40666429	-0,43486414	0,66366104	0,82413471	protein_codir putative elongation factor 1-gamma (EF-1-gamma)
TcG_10901	21,32899696	-0,325266452	0,39471197	-0,82406027	0,40990531	0,63689009	protein_codir hypothetical protein
TcG_10902	373,1971867	-0,205446775	0,09693072	-2,1195218	0,03404639	0,12574482	protein_codir putative dynamin-like protein
TcG_10903	70,68127976	0,01561242	0,2164064	0,07214398	0,94248732	0,97319113	protein_codir hypothetical protein
TcG_10904	0,781802848	0,328756738	1,95376118	0,16826864	0,86637194	1	
TcG_10905	0,145251675	-1,420530545	4,08047286	-0,3481289	0,72774338	1	
TcG_10906	215,6862683	0,95846583	0,12401957	7,72834349	1,0896E-14	1,1171E-12	protein_codir dispersed gene family protein 1 (DGF-1)
TcG_10907	90,46217833	1,076723184	0,21323635	5,04943545	4,4312E-07	9,7437E-06	protein_codir dispersed gene family protein 1 (DGF-1)
TcG_10908	44,33611363	1,098633578	0,27748039	3,95931968	7,5164E-05	0,00087434	protein_codir dispersed gene family protein 1 (DGF-1)
TcG_10909	64,13833385	0,821214947	0,22399642	3,66619679	0,00024618	0,00237622	protein_codir dispersed gene family protein 1 (DGF-1)
TcG_10910	460,7172017	-0,017934251	0,08658773	-0,2071223	0,83591435	0,92052325	protein_codir putative trans-sialidase
TcG_10911	46,22170356	-0,049708996	0,25974129	-0,19137888	0,84822877	0,92660555	protein_codir hypothetical protein
TcG_10912	437,4389609	0,005735295	0,09642029	0,05948224	0,95256801	0,97754234	protein_codir trans-sialidase-like protein
TcG_10913	246,7634456	-0,049301721	0,11713794	-0,420886	0,67383833	0,82902391	protein_codir putative cell cycle sequence binding phosphoprotein (RBP33)
TcG_10914	62,10605459	0,32840671	0,2272484	1,44514418	0,14841739	0,34724634	protein_codir hypothetical protein
TcG_10915	36,42072013	0,334462294	0,28958099	1,15498707	0,24809572	0,47744549	protein_codir hypothetical protein
TcG_10916	6,28317093	-0,613628151	0,68774016	-0,89223835	0,37226519	1	
TcG_10917	29,16716886	-0,508905516	0,34173095	-1,48919937	0,13643488	0,32916375	

TcG_10918	12,1037477	0,450185625	0,50945624	0,88365906	0,37688029	1	protein_codir	hypothetical protein
TcG_10919	77,22436779	0,019518067	0,21602431	0,09035125	0,92800809	0,96520107	protein_codir	hypothetical protein
TcG_10920	100,5987042	-0,304747014	0,17278532	-1,76373213	0,07777709	0,22628045	protein_codir	putative kinesin
TcG_10921	183,1540122	-0,466311798	0,13058525	-3,57093774	0,00035571	0,00323485	protein_codir	putative kinesin
TcG_10922	17,99554137	-0,330870298	0,42658986	-0,77561688	0,43797523	0,66146173	protein_codir	putative kinesin
TcG_10923	694,2450681	0,59324233	0,07862763	7,54496025	4,5243E-14	4,2273E-12		
TcG_10924	264,2379408	-0,286933957	0,11025243	-2,6025183	0,00925419	0,04643526	protein_codir	putative aspartyl aminopeptidase, putative,metallo-peptidase, clan MH, family M20
TcG_10925	0,719407094	0,307042706	2,08080436	0,14755962	0,88269032	1	protein_codir	putative trans-sialidase
TcG_10926	1,225911917	1,431829341	1,65346989	0,86595429	0,38651523	1	protein_codir	Sialidase 85-1.3
TcG_10927	5,709172999	0,14983635	0,73924598	0,20268808	0,83937884	1	protein_codir	hypothetical protein
TcG_10928	46,86471216	-0,436008632	0,25445648	-1,71348997	0,08662244	0,24394933	protein_codir	mucin-associated surface protein (MASP)
TcG_10929	7,687810898	0,898428866	0,6605182	1,36018791	0,17377047	1	protein_codir	hypothetical protein
TcG_10930	18,41260468	0,369293797	0,42056393	0,87809193	0,37989383	0,6107213	protein_codir	hypothetical protein
TcG_10931	2,787595217	0,422108342	1,03388769	0,40827292	0,68307332	1	protein_codir	hypothetical protein
TcG_10932	7,057337001	-0,707034025	0,68580091	-1,03096105	0,30255909	1		
TcG_10933	770,6119657	-0,491331263	0,07131368	-6,88972001	5,5902E-12	3,7566E-10	protein_codir	viral A-type inclusion protein
TcG_10934	98,08296091	0,314995092	0,18747101	1,68023362	0,09291187	0,25545252	protein_codir	putative antigenic protein
TcG_10935	229,7845571	0,380695269	0,12166745	3,12898206	0,00175413	0,01239229	protein_codir	putative transmembrane amino acid transporter
TcG_10936	167,7125944	0,342673734	0,14639914	2,34068129	0,01924859	0,08151103	protein_codir	hypothetical protein
TcG_10937	1039,166252	0,245641596	0,06097593	4,02850119	5,6134E-05	0,00068172	protein_codir	acetyl-CoA C-acetyltransferase
TcG_10938	43,59697816	0,195135949	0,27122686	0,71945657	0,47185965	0,68784171	protein_codir	putative dispersed gene family protein 1 (DGF-1)
TcG_10939	34,415979	-0,068591757	0,29994964	-0,22867758	0,81911952	0,91086657	protein_codir	dispersed gene family protein 1 (DGF-1)
TcG_10940	18,88761382	-0,098518211	0,39939459	-0,24666887	0,8051645	0,90385	protein_codir	dispersed gene family protein 1 (DGF-1)
TcG_10941	0,116927736	0,503022807	4,08047286	0,12327562	0,90188885	1	protein_codir	dispersed gene family protein 1 (DGF-1)
TcG_10942	23,87332003	0,876466298	0,36435803	2,40550837	0,01614998	0,07152664	protein_codir	dispersed gene family protein 1 (DGF-1)
TcG_10943	23,74324272	0,673821651	0,3685541	1,82828424	0,0675069	0,20689214	protein_codir	dispersed gene family protein 1 (DGF-1)
TcG_10944	3,702079414	0,683009548	0,92387362	0,73928894	0,45973157	1	protein_codir	dispersed gene family protein 1 (DGF-1)
TcG_10945	132,3709357	0,011670003	0,1592658	0,07327376	0,94158828	0,97296008	protein_codir	dispersed gene family protein 1 (DGF-1)
TcG_10946	10,68388837	0,250984126	0,54105287	0,46388096	0,64273303	1		
TcG_10947	147,9456012	0,165442961	0,1505235	1,09911716	0,27171697	0,50482886	protein_codir	surface protein-2
TcG_10948	30,1228615	0,382164631	0,34042641	1,12260572	0,26160499	0,49388225	protein_codir	hypothetical protein
TcG_10949	79,90876144	-0,030150702	0,19816503	-0,15214946	0,87906905	0,94148439	protein_codir	trans-sialidase
TcG_10950	36,36901327	0,005821667	0,29310955	0,01986174	0,98415366	0,99398772	protein_codir	trans-sialidase
TcG_10951	12,69810555	0,391129893	0,53219785	0,73493324	0,46238015	1	protein_codir	dispersed protein family protein 1
TcG_10952	41,95433495	0,543645264	0,27419044	1,98272871	0,04739774	0,15918163	protein_codir	dispersed gene family protein 1 (DGF-1)
TcG_10953	88,0129896	0,311352981	0,20952532	1,48599213	0,1372812	0,33026163	protein_codir	dispersed gene family protein 1 (DGF-1)
TcG_10954	7,781290295	0,413479553	0,62344948	0,66321261	0,5071944	1	protein_codir	dispersed gene family protein 1 (DGF-1)
TcG_10955	17,90796516	0,237816706	0,41990576	0,56635733	0,5711509	0,76245585	protein_codir	dispersed gene family protein 1 (DGF-1)
TcG_10956	2,291366834	1,783120001	1,25707915	1,4184628	0,15605569	1	protein_codir	dispersed gene family protein 1 (DGF-1)
TcG_10957	4,473636207	1,170084922	0,89970477	1,30052097	0,19342247	1	protein_codir	dispersed gene family protein 1 (DGF-1)
TcG_10958	4,879020905	-0,080135392	0,77156702	-0,10386057	0,91728	1	protein_codir	hypothetical protein
TcG_10959	1,516681657	0,525586926	1,53137196	0,3432131	0,73143815	1	protein_codir	hypothetical protein
TcG_10960	1,44191339	3,77000623	1,68047911	2,24341154	0,02487029	1	protein_codir	hypothetical protein
TcG_10961	4,278477266	0,250314221	0,85473877	0,29285465	0,76963326	1	protein_codir	hypothetical protein
TcG_10962	0,338239883	-2,234864625	3,05001116	-0,73273982	0,46371713	1	protein_codir	hypothetical protein
TcG_10963	2,730148169	1,124057401	1,21903839	0,92208532	0,35648407	1	protein_codir	hypothetical protein
TcG_10964	3,820128542	-0,051287314	0,89697115	-0,05717833	0,95440314	1	protein_codir	hypothetical protein
TcG_10965	43,65891131	0,108996697	0,27692755	0,39359283	0,69388168	0,84137238	protein_codir	mucin-associated surface protein (MASP)
TcG_10966	5,476777849	0,764947431	0,79474904	0,96250186	0,33579757	1	protein_codir	hypothetical protein
TcG_10967	75,94281562	-0,24335618	0,20077024	-1,21211281	0,2254692	0,45182011	protein_codir	mucin TcMUCI
TcG_10968	78,75131904	-0,28544332	0,20581697	-1,38687943	0,16547853	0,3709156	protein_codir	mucin-associated surface protein (MASP)
TcG_10969	21,50201119	-0,190503111	0,37967574	-0,50175213	0,61584189	0,79256256	protein_codir	trans-sialidase

TcG_10970	44,41915452	0,282905493	0,27020371	1,04700819	0,2950958	0,52794626	protein_codir amastigote surface protein 4
TcG_10971	102,0917407	-0,391543059	0,1719985	-2,27643304	0,0228201	0,09335937	protein_codir trans-sialidase
TcG_10972	739,7973038	0,107336965	0,06984599	1,53676624	0,12435056	0,31050121	protein_codir trans-sialidase
TcG_10973	78,26715585	-1,226772894	0,20896644	-5,8706695	4,3404E-09	1,4977E-07	protein_codir trans-sialidase-like protein
TcG_10974	34,26581984	-0,561879314	0,30071198	-1,86849658	0,06169289	0,1936007	protein_codir mucin-associated surface protein (MASP)
TcG_10975	61,96898004	-0,380981513	0,24413843	-1,5605143	0,11863839	0,30069066	protein_codir mucin TcMUCII
TcG_10976	20,75793891	-0,411994572	0,38074244	-1,08208209	0,27921605	0,51214997	
TcG_10977	26,67373028	0,122185899	0,36242656	0,33713285	0,73601675	0,86494473	protein_codir hypothetical protein
TcG_10978	29,08924759	-0,023771599	0,35402762	-0,06714617	0,94646534	0,97469438	protein_codir trans-sialidase
TcG_10979	13,69156615	0,204639446	0,47131201	0,43419103	0,66414973	1	protein_codir hypothetical protein
TcG_10980	12,51914892	0,237577844	0,48713787	0,48770145	0,62576133	1	protein_codir hypothetical protein
TcG_10981	5,190921837	-0,635035317	0,82162918	-0,77289771	0,43958292	1	
TcG_10982	16,07635066	-0,741291019	0,44642939	-1,66048884	0,09681615	1	protein_codir trans-sialidase
TcG_10983	12,18188844	-0,31267012	0,50885269	-0,61446098	0,53891075	1	protein_codir hypothetical protein
TcG_10984	24,41700376	0,371431411	0,37432972	0,99225733	0,32107201	0,5560344	protein_codir hypothetical protein
TcG_10985	22,10403313	-0,092424794	0,3780418	-0,244483	0,80685677	0,9046141	
TcG_10986	46,51077472	0,069830854	0,26291748	0,2655999	0,79054736	0,89524795	protein_codir trans-sialidase
TcG_10987	40,23980117	0,273665955	0,29891941	0,91551751	0,35992001	0,5926302	protein_codir hypothetical protein
TcG_10988	23,19810094	-0,028894108	0,36404144	-0,07937038	0,93673803	0,9705819	protein_codir hypothetical protein
TcG_10989	39,02116863	0,3303827	0,27702573	1,19260655	0,2330235	0,46040421	protein_codir target of rapamycin (TOR) kinase 1
TcG_10990	17,1269748	0,25459357	0,43089602	0,59084688	0,55462302	0,74941774	protein_codir putative target of rapamycin (TOR) kinase 1
TcG_10991	4,96729817	-0,247120673	0,84620688	-0,2920334	0,77026109	1	protein_codir hypothetical protein
TcG_10992	19,38426396	-0,205490112	0,39420005	-0,52128383	0,60216907	0,78475506	protein_codir mitochondrial processing peptide beta subunit
TcG_10993	170,7938595	0,229924788	0,1390965	1,65298758	0,09833339	0,26564266	protein_codir putative glycine dehydrogenase, putative,glycine cleavage system P-protein
TcG_10994	1,543644813	-0,40452982	1,41296887	-0,28629776	0,77465007	1	protein_codir hypothetical protein
TcG_10995	1,79187078	4,040520836	1,61347389	2,5042369	0,01227158	1	protein_codir hypothetical protein
TcG_10996	3,883769681	0,104868836	0,86785971	0,12083616	0,90382081	1	protein_codir hypothetical protein
TcG_10997	9,558086923	0,254617492	0,56852569	0,44785573	0,65425732	1	protein_codir hypothetical protein
TcG_10998	19,3647797	0,452393947	0,39614852	1,14198066	0,25346206	0,48445481	
TcG_10999	42,94376095	0,44106648	0,27204673	1,6212894	0,10495559	0,27832809	protein_codir putative trans-sialidase
TcG_11000	204,12794	0,104325132	0,12571475	0,82985596	0,40662023	0,63483385	protein_codir hypothetical protein
TcG_11001	391,5004312	0,027528027	0,10443474	0,2635907	0,79209532	0,89604169	protein_codir putative SNF2 DNA repair protein
TcG_11002	610,5910931	-0,212979419	0,07779828	-2,73758529	0,00618921	0,03384056	protein_codir putative protein kinase-like protein
TcG_11003	56,79216314	-0,02783978	0,2396867	-0,11615071	0,9075331	0,95523092	protein_codir Unc104-like kinesin
TcG_11004	1,491213216	-3,141177727	1,66544742	-1,8860864	0,05928331	1	protein_codir mucin-associated surface protein (MASP)
TcG_11005	2,983254435	-2,655016334	1,1854422	-2,23968434	0,02511142	1	protein_codir hypothetical protein
TcG_11006	3,121692607	-1,658779735	1,92038005	-0,8637768	0,38771052	1	protein_codir hypothetical protein
TcG_11007	0					1	protein_codir hypothetical protein
TcG_11008	3,472287046	-0,854625614	0,92412851	-0,92479087	0,3550747	1	protein_codir hypothetical protein
TcG_11009	6,371186117	0,067122411	0,71222599	0,09424314	0,92491602	1	protein_codir hypothetical protein
TcG_11010	5,395931786	-1,258736133	0,77895694	-1,61592518	0,10611049	1	protein_codir hypothetical protein
TcG_11011	31,90998009	0,073444835	0,30720648	0,2390732	0,81104882	0,90685309	protein_codir putative mucin-associated surface protein (MASP)
TcG_11012	2,614745947	-2,4312683	1,23379844	-1,97055549	0,04877474	1	protein_codir trans-sialidase
TcG_11013	6,651739559	-0,400915894	0,68676244	-0,58377668	0,55937057	1	protein_codir trans-sialidase
TcG_11014	41,78524609	0,035818168	0,29663583	0,12074795	0,90389068	0,95349059	protein_codir trans-sialidase
TcG_11015	1,45756528	0,913576117	1,57547373	0,57987391	0,56199965	1	protein_codir hypothetical protein
TcG_11016	0,116927736	0,503022807	4,08047286	0,12327562	0,90188885	1	protein_codir hypothetical protein
TcG_11017	6,583611438	-0,590445914	0,68682204	-0,85967817	0,38996647	1	protein_codir hypothetical protein
TcG_11018	2,333393334	-0,933551265	1,21390174	-0,76905011	0,44186356	1	protein_codir hypothetical protein
TcG_11019	49,4113618	-0,452331333	0,24620606	-1,83720634	0,06617944	0,20397845	protein_codir mucin-associated surface protein (MASP)
TcG_11020	1,229162527	-2,961037463	1,79736122	-1,64743594	0,09946847	1	protein_codir hypothetical protein
TcG_11021	2,493766598	-2,881733226	1,38251965	-2,08440671	0,03712319	1	protein_codir surface protease GP63

TcG_11022	0,638385169	-2,974621413	2,29321366	-1,29714098	0,19458268	1	protein_codir	syntaxin binding protein
TcG_11023	2,010110373	0,10533212	1,21650644	0,08658575	0,9310008	1	protein_codir	putative syntaxin binding protein
TcG_11024	5,180977916	-0,593992877	0,80354979	-0,73921104	0,45977886	1	protein_codir	hypothetical protein
TcG_11025	4,633896322	-0,43465373	0,79612283	-0,54596315	0,58509127	1	protein_codir	hypothetical protein
TcG_11026	574,272402	0,653240734	0,08013973	8,1512716	3,6012E-16	4,2145E-14	protein_codir	putative retrotransposon hot spot (RHS) protein
TcG_11027	93,46405582	0,70441665	0,19239966	3,66121576	0,00025102	0,00241355	protein_codir	target of rapamycin (TOR) kinase 1
TcG_11028	52,40601787	-0,075188228	0,25665135	-0,29295863	0,76955377	0,88436428	protein_codir	putative rab1 small GTP-binding protein
TcG_11029	71,13941095	0,699679304	0,21290042	3,28641581	0,00101471	0,00787282	protein_codir	putative mitotic centromere-associated kinesin (MCAK)
TcG_11030	178,1806793	0,479700814	0,13779427	3,48128277	0,00049902	0,00433081	protein_codir	hypothetical protein
TcG_11031	194,8493104	0,355161661	0,1406267	2,52556352	0,01155129	0,05530428	protein_codir	hypothetical protein
TcG_11032	44,85817996	0,58368091	0,26815668	2,1766413	0,02950734	0,11290359	protein_codir	dispersed gene family protein 1 (DGF-1)
TcG_11033	103,1229943	0,680575421	0,18078432	3,76457097	0,00016684	0,00171818	protein_codir	dispersed gene family protein 1 (DGF-1)
TcG_11034	31,00893829	0,085526621	0,3089344	0,27684396	0,78189993	0,89173074	protein_codir	trans-sialidase
TcG_11035	20,5930944	-0,037573181	0,37693663	-0,09968037	0,92059809	0,96150609	protein_codir	hypothetical protein
TcG_11036	23,42136862	0,216501596	0,36392529	0,5949067	0,55190584	0,74737358	protein_codir	hypothetical protein
TcG_11037	6,932752317	-1,262333795	0,67696719	-1,86468978	0,0622249	1	protein_codir	hypothetical protein
TcG_11038	80,38222455	-0,332913387	0,2022384	-1,64614332	0,09973425	0,26816456	protein_codir	putative retrotransposon hot spot (RHS) protein
TcG_11039	29,78076377	0,04743435	0,31591736	0,15014797	0,88064787	0,94206932	protein_codir	hypothetical protein
TcG_11040	161,2410426	-0,133507364	0,1383264	-0,96516188	0,33446374	0,56893684	protein_codir	mucin-associated surface protein (MASP)
TcG_11041	55,04527363	-0,534696754	0,23854882	-2,24145634	0,02499653	0,09969754		
TcG_11042	16,88601321	-0,158507697	0,41648302	-0,38058621	0,70351031	0,84701969	protein_codir	hypothetical protein
TcG_11043	15,2337548	-0,28818115	0,48237216	-0,59742493	0,55022372	1	protein_codir	hypothetical protein
TcG_11044	6,583040814	0,073592529	0,66368195	0,11088523	0,91170735	1		
TcG_11045	44,66679484	-0,707423775	0,26716912	-2,64785012	0,00810054	0,04187992	protein_codir	mucin-associated surface protein (MASP)
TcG_11046	22,43808464	0,422618759	0,39638878	1,06617235	0,28634574	0,51959307	protein_codir	hypothetical protein
TcG_11047	90,92335326	-0,224298045	0,18872013	-1,18852207	0,23462779	0,46246982	protein_codir	trans-sialidase
TcG_11048	57,41850623	0,278842098	0,23320686	1,19568564	0,23181926	0,4591995		
TcG_11049	87,84362406	0,105978647	0,19753928	0,53649405	0,59161714	0,77721083	protein_codir	mucin-associated surface protein (MASP)
TcG_11050	113,2477815	-0,013327675	0,18613699	-0,07160143	0,9429191	0,97319113	protein_codir	mucin TcMUCII
TcG_11051	36,10856733	0,108435785	0,28769707	0,37690959	0,7062408	0,84872764		
TcG_11052	4,406294507	0,598881744	0,83305042	0,71890216	0,4722012	1	protein_codir	hypothetical protein
TcG_11053	19,8772005	-0,402593991	0,4076622	-0,98756763	0,32336445	0,55758888	protein_codir	putative surface protease GP63
TcG_11054	0,469265227	-2,470320505	2,56600864	-0,96270935	0,3356934	1		
TcG_11055	55,72014483	-0,35621418	0,23304787	-1,52850218	0,1263879	0,31409915	protein_codir	mucin-associated surface protein (MASP)
TcG_11056	33,5182594	-0,481405085	0,29965086	-1,60655334	0,10815239	0,28362461	protein_codir	serine-alanine-and proline-rich protein
TcG_11057	12,11458227	-0,168727067	0,51156136	-0,32982762	0,74153022	1	protein_codir	serine-alanine-and proline-rich protein
TcG_11058	27,70206476	0,479577799	0,34429959	1,39290841	0,16364749	0,36837377	protein_codir	hypothetical protein
TcG_11059	43,10967142	-0,107326562	0,2619323	-0,40974924	0,68198991	0,83422317	protein_codir	mucin TcMUCII
TcG_11060	3,925045382	0,493737454	0,90379685	0,54629251	0,58486488	1	protein_codir	mucin-associated surface protein (MASP)
TcG_11061	3,635829923	0,096276859	0,90314341	0,10660196	0,91510476	1	protein_codir	hypothetical protein
TcG_11062	15,58430448	0,253145681	0,48114905	0,52612737	0,59879972	1	protein_codir	hypothetical protein
TcG_11063	4,354409015	-7,87795E-05	0,81246678	-9,6963E-05	0,99992263	1	protein_codir	hypothetical protein
TcG_11064	28,78564844	-0,243717448	0,32329095	-0,75386412	0,45093082	0,67161389	protein_codir	mucin TcMUCII
TcG_11065	155,083134	-0,293796798	0,1456624	-2,01697078	0,04369856	0,15042036	protein_codir	mucin-associated surface protein (MASP)
TcG_11066	53,89207783	0,447986062	0,24705418	1,813311	0,06978387	0,21143198		
TcG_11067	90,2258239	-0,243184661	0,20003345	-1,21571995	0,22409162	0,45075096	protein_codir	mucin-associated surface protein (MASP)
TcG_11068	15,47166449	-0,183043954	0,45695554	-0,40057279	0,68873468	1		
TcG_11069	9,209721657	-0,591195111	0,59612953	-0,99172257	0,32133288	1	protein_codir	surface protease GP63
TcG_11070	7,85061543	-0,344327072	0,62958017	-0,54691537	0,58443687	1	protein_codir	hypothetical protein
TcG_11071	2,669169029	0,119745214	1,11434106	0,10745832	0,91442539	1	protein_codir	hypothetical protein
TcG_11072	0,846538379	1,74528242	1,91060067	0,91347316	0,36099375	1	protein_codir	hypothetical protein
TcG_11073	19,05123306	-0,212924976	0,40459251	-0,52627019	0,59870049	0,78264063	protein_codir	mucin TcMUCII

TcG_11074	3,579917337	-2,057766597	1,01298536	-2,03138828	0,04221562	1		
TcG_11075	70,41911701	-0,116005634	0,21059271	-0,55085304	0,58173442	0,77089958	protein_codir	putative trans-sialidase
TcG_11076	215,186752	0,311259209	0,12155336	2,56067954	0,01044677	0,05113556	protein_codir	trans-sialidase
TcG_11077	55,7871325	0,283999826	0,24222226	1,17247614	0,24100595	0,46972948	protein_codir	trans-sialidase
TcG_11078	142,5050635	0,046942135	0,14937851	0,31424958	0,75333149	0,87552748	protein_codir	putative mucin-associated surface protein (MASP)
TcG_11079	20,10239649	-0,044987357	0,39879674	-0,11280773	0,91018299	0,95641431	protein_codir	hypothetical protein
TcG_11080	5,931311315	-0,766957059	0,72401754	-1,0593073	0,28945985	1		
TcG_11081	28,75240375	0,403425288	0,3590512	1,12358706	0,26118825	0,49358779	protein_codir	hypothetical protein
TcG_11082	25,10361296	0,363038921	0,3475489	1,04456932	0,29622207	0,5292829	protein_codir	hypothetical protein
TcG_11083	178,7237836	-0,140314418	0,13596	-1,03202717	0,30205939	0,53534644	protein_codir	calmodulin
TcG_11084	205,7992404	-0,307884546	0,12917833	-2,38340712	0,01715321	0,07475822	protein_codir	hypothetical protein
TcG_11085	222,6330616	-0,377348045	0,13304456	-2,83625316	0,00456462	0,02683193	protein_codir	NUDIX hydrolase
TcG_11086	180,526584	0,353640572	0,13738865	2,57401595	0,01005256	0,04956128	protein_codir	L1Tc protein
TcG_11087	112,6622849	0,49313555	0,1704554	2,89304731	0,00381524	0,02320386	protein_codir	hypothetical protein
TcG_11088	84,36575127	0,523334308	0,19758878	2,64860331	0,00808251	0,04181923	protein_codir	hypothetical protein
TcG_11089	1006,152272	0,005302163	0,06205629	0,0854412	0,93191064	0,96736431	protein_codir	hypothetical protein
TcG_11090	541,549532	0,036390237	0,08543548	0,42593821	0,67015287	0,82696679	protein_codir	hypothetical protein
TcG_11091	285,1760946	-0,320364957	0,10724259	-2,98729215	0,00281461	0,01805649		
TcG_11092	560,5096512	-0,352200312	0,07959709	-4,42478877	9,6537E-06	0,00014775	protein_codir	ubiquitin-conjugating enzyme E2
TcG_11093	23,47207512	0,208082784	0,35347029	0,58868535	0,55607237	0,75089213	protein_codir	dispersed protein family protein 1 (DGF-1)
TcG_11094	5,802749742	0,132432825	0,74501377	0,17775889	0,85891233	1	protein_codir	dispersed gene family protein 1 (DGF-1)
TcG_11095	14,94562219	0,209110977	0,4497392	0,46496053	0,64195972	1	protein_codir	dispersed gene family protein 1 (DGF-1)
TcG_11096	0,155721614	0,503022807	4,08047286	0,12327562	0,90188885	1	protein_codir	dispersed protein family protein 1
TcG_11097	88,27049483	0,08850358	0,19140106	0,46239859	0,64379551	0,810875		
TcG_11098	621,0404378	0,009503881	0,07481351	0,12703428	0,89891327	0,95069557	protein_codir	UDP-Gal or UDP-GlcNAc-dependent glycosyltransferase
TcG_11099	821,4892896	-0,084770053	0,0753026	-1,12572549	0,26028173	0,49261779		
TcG_11100	530,8414145	0,231393017	0,08314643	2,78295778	0,00538658	0,03036931	protein_codir	hypothetical protein
TcG_11101	1000,92529	0,115686958	0,06498405	1,78023618	0,07503732	0,22117071	protein_codir	putative citrate transporter
TcG_11102	93,04036056	0,017165642	0,18653935	0,09202156	0,92668091	0,96430637	protein_codir	ATPase
TcG_11103	26,58659471	-0,241421278	0,33672119	-0,71697678	0,47338842	0,6892031	protein_codir	hypothetical protein
TcG_11104	17,93152584	-0,745504995	0,41824436	-1,78246277	0,0746738	0,22059424	protein_codir	hypothetical protein
TcG_11105	72,53056975	-0,0800467	0,20693521	-0,38682011	0,69888939	0,84414248		
TcG_11106	57,71145931	-0,402874421	0,23567406	-1,70945591	0,08736653	0,24512151	protein_codir	putative mucin TcMUCII
TcG_11107	220,7290552	-0,602056409	0,12012797	-5,01179204	5,3925E-07	1,1678E-05	protein_codir	putative mucin-associated surface protein (MASP)
TcG_11108	18,07690654	0,467874375	0,42605422	1,09815688	0,27213599	0,50504046	protein_codir	hypothetical protein
TcG_11109	5,691953503	1,167091438	0,76678319	1,52206185	0,12799358	1	protein_codir	structural maintenance of chromosome protein 4
TcG_11110	16,13162907	1,259193247	0,4636899	2,71559342	0,00661571	1	protein_codir	hypothetical protein
TcG_11111	6,921378043	0,422900073	0,67408822	0,62736607	0,53041931	1	protein_codir	target of rapamycin (TOR) kinase 1
TcG_11112	167,1802221	0,262048767	0,1460293	1,7944944	0,07273429	0,21694934	protein_codir	target of rapamycin (TOR) kinase 1
TcG_11113	329,1058362	-0,288169294	0,10268815	-2,80625644	0,00501208	0,02883314		
TcG_11114	45,78821577	-0,635461838	0,25825767	-2,46057297	0,01387154	0,06390283		
TcG_11115	88,48551216	0,11309104	0,20795862	0,54381511	0,58656871	0,77508954	protein_codir	dispersed gene family protein 1 (DGF-1)
TcG_11116	82,45216578	-0,360836279	0,1956002	-1,84476434	0,06507183	0,20126059		
TcG_11117	20,20873067	-0,112810484	0,41258417	-0,27342417	0,78452718	0,89340789		
TcG_11118	530,6510718	0,159105779	0,08167755	1,94797442	0,05141802	0,16868429	protein_codir	putative retrotransposon hot spot (RHS) protein
TcG_11119	8,066272762	-0,106863812	0,62553502	-0,17083586	0,86435283	1	protein_codir	hypothetical protein
TcG_11120	0,548227089	-0,494168331	2,31937726	-0,21306078	0,83127955	1		
TcG_11121	8,321563086	-0,636862472	0,6159649	-1,03392657	0,30117049	1	protein_codir	hypothetical protein
TcG_11122	5,487399131	0,222682123	0,74044605	0,30074051	0,76361238	1	protein_codir	hypothetical protein
TcG_11123	13,10224142	-0,540269707	0,47958138	-1,12654437	0,25993517	1	protein_codir	hypothetical protein
TcG_11124	10,01455794	-0,047366616	0,54898116	-0,08628095	0,93124308	1	protein_codir	hypothetical protein
TcG_11125	19,44298525	0,113932942	0,40094072	0,28416406	0,77628466	0,88831084	protein_codir	trans-sialidase

TcG_11126	39,96933677	0,022782906	0,27481755	0,08290193	0,93392952	0,96871149	protein_codir hypothetical protein
TcG_11127	27,90356186	-0,297597709	0,32996704	-0,90190131	0,3671093	0,59939061	protein_codir hypothetical protein
TcG_11128	35,991361	0,446045835	0,30273241	1,47339968	0,1406433	0,33542471	protein_codir putative trans-sialidase
TcG_11129	21,24076765	0,4749714	0,37741646	1,25848087	0,2082179	0,43078797	protein_codir hypothetical protein
TcG_11130	24,79238769	0,210188683	0,35532383	0,59154119	0,55415786	0,74910997	protein_codir hypothetical protein
TcG_11131	0					1	protein_codir hypothetical protein
TcG_11132	255,4539567	-0,000854428	0,11477816	-0,00744417	0,99406047	0,99767711	protein_codir mucin-associated surface protein (MASP)
TcG_11133	466,8241359	-0,151691979	0,08756373	-1,73236087	0,08320933	0,23704532	
TcG_11134	0,27264935	1,353296468	3,06414982	0,4416548	0,65873902	1	
TcG_11135	274,5523704	-0,011560618	0,11102931	-0,10412222	0,91707236	0,95999281	protein_codir hypothetical protein
TcG_11136	65,74969927	-0,044625886	0,21436964	-0,20817261	0,8350942	0,92007617	protein_codir hypothetical protein
TcG_11137	80,07070257	-0,03048009	0,20127658	-0,15143386	0,87963348	0,94162382	
TcG_11138	174,9962671	0,150605197	0,13818421	1,08988716	0,27576286	0,50810885	protein_codir hypothetical protein
TcG_11139	62,25211534	-0,292468961	0,21795776	-1,34186074	0,17964115	0,39078528	protein_codir hypothetical protein
TcG_11140	119,4138295	0,142158408	0,16243919	0,87514844	0,38149314	0,61159258	protein_codir hypothetical protein
TcG_11141	12,86512385	0,114988991	0,48661765	0,23630255	0,81319791	1	
TcG_11142	12,50203021	-0,283008645	0,48752625	-0,5804993	0,56157796	1	protein_codir hypothetical protein
TcG_11143	8,550505756	-0,132341984	0,61526925	-0,21509605	0,82969243	1	protein_codir hypothetical protein
TcG_11144	0,314371616	-2,1715226	2,91078434	-0,74602662	0,45565133	1	
TcG_11145	78,91527885	-0,267418707	0,20003069	-1,33688839	0,18125907	0,3928297	protein_codir mucin-associated surface protein (MASP)
TcG_11146	43,92325396	0,255549296	0,2641647	0,96738623	0,33335099	0,56780426	protein_codir hypothetical protein
TcG_11147	33,23017235	0,292699885	0,29904196	0,97879203	0,32768275	0,56219937	
TcG_11148	55,37356081	0,195917447	0,24044055	0,81482698	0,41517136	0,64212726	protein_codir helicase-like protein
TcG_11149	29,2919836	0,27595433	0,32175052	0,85766554	0,39107717	0,62102798	protein_codir trans-sialidase
TcG_11150	29,08609046	0,273319021	0,33312507	0,8204697	0,4119484	0,63850624	protein_codir target of rapamycin (TOR) kinase 1
TcG_11151	42,6523304	0,100998081	0,27659542	0,36514734	0,71500142	0,85366926	protein_codir putative target of rapamycin (TOR) kinase 1
TcG_11152	540,754441	0,058624475	0,08560525	0,68482334	0,49345541	0,70398758	protein_codir RNA-binding protein NOB1
TcG_11153	520,133018	-0,069039203	0,08295508	-0,83224807	0,40526893	0,63430422	protein_codir gamma carbonic dehydratase
TcG_11154	16,75416887	-0,494655268	0,41940606	-1,17941852	0,23823157	1	protein_codir hypothetical protein
TcG_11155	20,81598927	0,543321785	0,4004371	1,35682178	0,17483784	0,38394073	protein_codir putative mucin-associated surface protein (MASP)
TcG_11156	2,22458025	-1,664132002	1,20532094	-1,38065468	0,16738516	1	protein_codir hypothetical protein
TcG_11157	1,786596117	0,120829688	1,36690226	0,08839673	0,92956136	1	protein_codir hypothetical protein
TcG_11158	2,19987497	0,409414592	1,16221094	0,35227219	0,72463414	1	protein_codir hypothetical protein
TcG_11159	3,520400639	0,123047513	0,90626755	0,13577394	0,892	1	protein_codir hypothetical protein
TcG_11160	9,940651438	-0,102264004	0,59231287	-0,17265201	0,86292497	1	protein_codir hypothetical protein
TcG_11161	2,769869939	1,118744228	1,0981705	1,01873455	0,308329	1	protein_codir hypothetical protein
TcG_11162	33,64563989	0,28797848	0,30752458	0,93644052	0,34904642	0,58360833	
TcG_11163	3,280011443	-0,551926832	0,96778278	-0,57030033	0,56847402	1	protein_codir hypothetical protein
TcG_11164	31,02912533	-0,985607485	0,32052171	-3,07501003	0,00210495	0,01437083	protein_codir mucin TcMUCII
TcG_11165	11,78474332	-0,054814595	0,50743601	-0,10802267	0,91397771	1	protein_codir surface protease GP63
TcG_11166	12,63566822	0,242135209	0,49172924	0,49241573	0,62242548	1	protein_codir surface protease GP63
TcG_11167	64,40103068	0,140687059	0,21947936	0,64100361	0,52152034	0,72540161	
TcG_11168	5,491575089	0,041371069	0,74609488	0,05545014	0,95577985	1	protein_codir hypothetical protein
TcG_11169	74,72539088	0,154830595	0,20134732	0,76897272	0,4419095	0,6644126	protein_codir receptor-type adenylate cyclase
TcG_11170	172,1541871	0,239550938	0,14493339	1,65283474	0,0983645	0,26565294	protein_codir hypothetical protein
TcG_11171	219,5017487	-0,10697671	0,12189287	-0,87762892	0,38014513	0,6107213	protein_codir esag4
TcG_11172	58,71187166	-0,448742347	0,24277584	-1,8483814	0,06454719	0,2000652	protein_codir trans-sialidase
TcG_11173	16,63940598	-0,228282502	0,43607527	-0,52349335	0,60063098	1	protein_codir putative trans-sialidase
TcG_11174	31,19161938	-0,292906795	0,31313776	-0,93539276	0,34958593	0,58410694	protein_codir trans-sialidase
TcG_11175	10,20534186	-0,282208106	0,54951033	-0,51356287	0,60755764	1	protein_codir surface protease GP63
TcG_11176	33,54023406	-0,160918829	0,31382934	-0,51275903	0,60811989	0,78802211	
TcG_11177	4,648320463	-0,100476489	0,8299754	-0,1210596	0,90364383	1	

TcG_11178	28,66333843	0,186339963	0,32204039	0,57862297	0,5628436	0,75615793	protein_codir putative trans-sialidase
TcG_11179	48,93110792	-0,033666751	0,25418539	-0,13244959	0,89462869	0,94884365	
TcG_11180	54,16585565	0,151107692	0,23810939	0,63461459	0,52567981	0,72887348	protein_codir mucin-associated surface protein (MASP)
TcG_11181	196,5260085	0,13462794	0,13095845	1,02802023	0,30394031	0,53683566	protein_codir UDP-Gal or UDP-GlcNAc-dependent glycosyltransferase
TcG_11182	45,91468578	-0,020432761	0,26365043	-0,07749944	0,93822624	0,97142888	protein_codir UDP-Gal or UDP-GlcNAc-dependent glycosyltransferase
TcG_11183	11,19527993	-0,044786995	0,5240383	-0,08546512	0,93189163	1	protein_codir hypothetical protein
TcG_11184	1,381633531	1,638078331	1,58051226	1,03642242	0,30000512	1	protein_codir hypothetical protein
TcG_11185	17,28088266	0,230733789	0,42659862	0,54086858	0,58859817	0,77564856	protein_codir hypothetical protein
TcG_11186	6,290805191	1,261071469	0,7413187	1,7011192	0,08892061	1	protein_codir hypothetical protein
TcG_11187	12,79936161	0,731163423	0,52790059	1,38503999	0,16604024	1	protein_codir hypothetical protein
TcG_11188	9,344076999	-0,181861034	0,55999813	-0,32475293	0,74536807	1	protein_codir surface protease GP63
TcG_11189	6,903118216	-0,594317462	0,65909868	-0,90171241	0,36720966	1	protein_codir surface protease GP63
TcG_11190	84,57722825	0,334354795	0,18971097	1,76244313	0,07799446	0,22670441	protein_codir hypothetical protein
TcG_11191	106,82271	-0,063850113	0,17718384	-0,36036081	0,71857733	0,8554703	protein_codir mucin TcMUCI
TcG_11192	43,07522898	0,024046896	0,26164251	0,09190745	0,92677157	0,96430637	
TcG_11193	111,3412986	0,017463469	0,17588791	0,09928749	0,92091001	0,96150609	protein_codir hypothetical protein
TcG_11194	92,59796073	0,191027839	0,18490603	1,03310767	0,30155352	0,53487432	protein_codir hypothetical protein
TcG_11195	18,3265413	0,308036908	0,41835246	0,73630954	0,46154234	0,6794505	
TcG_11196	73,26713024	0,312891513	0,22590376	1,38506552	0,16603243	0,37164834	protein_codir retrotransposon hot spot (RHS) protein
TcG_11197	19,25595335	0,141704001	0,39858282	0,35551959	0,72220037	0,85728556	protein_codir retrotransposon hot spot (RHS) protein
TcG_11198	182,0594104	0,076399328	0,13184823	0,57944902	0,56228624	0,75575967	protein_codir putative retrotransposon hot spot (RHS) protein
TcG_11199	10,32099918	-0,180644791	0,60430373	-0,29893046	0,76499311	1	protein_codir hypothetical protein
TcG_11200	11,85127881	0,337241039	0,51697802	0,65233148	0,51418735	1	protein_codir hypothetical protein
TcG_11201	5,564526997	0,837927255	0,74570594	1,12366981	0,26115313	1	protein_codir hypothetical protein
TcG_11202	41,05732164	0,000132309	0,29693059	0,00044559	0,99964447	0,99981271	
TcG_11203	10,46678604	0,076139127	0,53992386	0,14101827	0,88785551	1	
TcG_11204	17,78447651	0,543573876	0,42300952	1,28501571	0,19878679	0,41761447	protein_codir dispersed gene family protein 1 (DGF-1)
TcG_11205	58,7365481	0,285733734	0,24398336	1,17111977	0,24155064	0,47019585	protein_codir dispersed gene family protein 1 (DGF-1)
TcG_11206	5,060954875	1,313556254	0,83559959	1,57199246	0,1159523	1	protein_codir dispersed gene family protein 1 (DGF-1)
TcG_11207	17,84180146	0,84654605	0,44314669	1,91030659	0,05609375	0,17928336	protein_codir dispersed protein family protein 1 (DGF-1)
TcG_11208	1028,494898	0,401617764	0,06344104	6,3305669	2,4426E-10	1,1012E-08	protein_codir 60S ribosomal protein L34
TcG_11209	290,5707827	0,240097324	0,11207558	2,14228054	0,03217091	0,12046936	protein_codir hypothetical protein
TcG_11210	429,7595348	0,048185143	0,09092849	0,52992348	0,59616499	0,78066886	protein_codir hypothetical protein
TcG_11211	279,8914664	-0,077542079	0,1080583	-0,71759485	0,47300713	0,6888211	protein_codir hypothetical protein
TcG_11212	64,01768865	0,385427671	0,22136511	1,7411401	0,08165902	0,23447372	protein_codir hypothetical protein
TcG_11213	10,14537653	-0,18502652	0,57250213	-0,32318922	0,74655194	1	
TcG_11214	32,60877975	0,080154088	0,31291771	0,25615069	0,79783448	0,89910615	protein_codir dynein
TcG_11215	74,46437958	0,114764117	0,19973735	0,57457516	0,56557866	0,75798662	protein_codir hypothetical protein
TcG_11216	80,20778819	-0,182877871	0,19664643	-0,92998319	0,35237979	0,58667513	protein_codir putative trans-sialidase
TcG_11217	58,41952585	0,2496748	0,22859937	1,09219376	0,27474795	0,50755645	protein_codir putative kinesin
TcG_11218	293,4455595	-0,301912794	0,10833115	-2,78694353	0,00532078	0,0300568	protein_codir putative retrotransposon hot spot (RHS) protein
TcG_11219	475,8541895	0,183877381	0,09022191	2,03805687	0,04154425	0,14512555	protein_codir oxidoreductase-like protein
TcG_11220	620,2383423	-0,014461723	0,07684222	-0,18820022	0,8507197	0,92805389	protein_codir hypothetical protein
TcG_11221	194,9552943	0,183125507	0,12577574	1,45596841	0,14540135	0,34261136	protein_codir hypothetical protein
TcG_11222	19,45214127	-0,250384865	0,40915857	-0,61195068	0,54057039	0,73943903	protein_codir putative trans-sialidase
TcG_11223	57,01847794	-0,074622798	0,23388383	-0,31905924	0,74968159	0,87373613	protein_codir hypothetical protein
TcG_11224	196,0209612	-0,018508085	0,13327021	-0,13887639	0,88954783	0,94653573	protein_codir putative trans-sialidase
TcG_11225	16,13915607	0,343106126	0,42793884	0,80176439	0,42268926	1	protein_codir trans-sialidase
TcG_11226	1045,84025	0,277108477	0,06191032	4,47596552	7,6067E-06	0,00012049	protein_codir putative retrotransposon hot spot (RHS) protein
TcG_11227	26,40226091	-0,172100951	0,34544968	-0,49819398	0,61834732	0,793034	protein_codir mucin TcMUCII
TcG_11228	9,085649603	-0,388135723	0,5952125	-0,65209605	0,51433921	1	protein_codir hypothetical protein
TcG_11229	4,980950138	-0,721201371	0,77525361	-0,93027799	0,35222717	1	protein_codir hypothetical protein

TcG_11230	4,017161802	-0,592375946	0,87009868	-0,68081467	0,49598877	1	protein_codir mucin-associated surface protein (MASP)
TcG_11231	25,44256919	0,584525182	0,35016847	1,66926846	0,09506419	0,25983367	protein_codir dispersed gene family protein 1 (DGF-1)
TcG_11232	240,4285425	-0,098854429	0,11448816	-0,86344673	0,3878919	0,61834281	protein_codir dispersed gene family protein 1 (DGF-1)
TcG_11233	121,226241	-0,133484024	0,16745332	-0,79714171	0,42536873	0,65120536	protein_codir dispersed gene family protein 1 (DGF-1)
TcG_11234	177,3611303	0,003300707	0,1466092	0,02251364	0,98203823	0,99328993	protein_codir trans-sialidase
TcG_11235	11,72587782	-0,077390785	0,52005315	-0,14881322	0,88170102	1	protein_codir hypothetical protein
TcG_11236	111,5264166	-0,10705385	0,17592001	-0,60853709	0,54283131	0,74052085	protein_codir mucin-associated surface protein (MASP)
TcG_11237	123,253397	0,048415187	0,16251113	0,29791921	0,76576482	0,88218666	protein_codir trans-sialidase
TcG_11238	11,110795	-0,472860927	0,52792648	-0,89569465	0,37041587	1	protein_codir trans-sialidase
TcG_11239	50,42055217	-0,249645009	0,24867869	-1,0038858	0,31543366	0,550228	protein_codir trans-sialidase
TcG_11240	19,36987263	0,578289917	0,42842854	1,34979316	0,17708234	0,38718302	protein_codir hypothetical protein
TcG_11241	53,51449019	-0,791275087	0,24467014	-3,23404844	0,00122049	0,00916558	protein_codir mucin-associated surface protein (MASP)
TcG_11242	30,83257009	-0,184322094	0,32907092	-0,56012878	0,5753916	0,76625291	protein_codir hypothetical protein
TcG_11243	708,7597807	-0,014239005	0,07241815	-0,19662205	0,84412332	0,92439431	protein_codir hypothetical protein
TcG_11244	104,5514892	-0,410908684	0,17337888	-2,37000432	0,01778788	0,07685644	protein_codir hypothetical protein
TcG_11245	6,812653359	0,828898995	0,6830458	1,21353355	0,22492589	1	protein_codir trans-sialidase
TcG_11246	34,75355809	-0,341245603	0,29403	-1,16058089	0,24581238	0,47432445	protein_codir mucin-associated surface protein (MASP)
TcG_11247	33,8831192	-0,280776352	0,30635301	-0,91651245	0,35939818	0,5926302	protein_codir hypothetical protein
TcG_11248	24,84424188	0,250179392	0,36261197	0,68993694	0,49023384	0,70130255	protein_codir trans-sialidase
TcG_11249	28,66659515	0,112394676	0,32490137	0,34593475	0,72939175	0,86056342	protein_codir trans-sialidase
TcG_11250	3,143662101	0,639441139	0,99046835	0,64559472	0,51854186	1	protein_codir calpain-like cysteine peptidase
TcG_11251	127,62114	-0,424344238	0,16076771	-2,63948671	0,00830317	0,0426799	
TcG_11252	232,4539375	-0,384421593	0,12251806	-3,13767294	0,00170295	0,01208968	protein_codir putative calpain-like cysteine peptidase
TcG_11253	516,4614248	-0,317854927	0,08988352	-3,53629797	0,00040578	0,00362198	protein_codir putative calpain-like cysteine peptidase, putative,cysteine peptidase, Clan CA, family C2
TcG_11254	7,739549134	-0,762851425	0,64646392	-1,18003713	0,23798545	1	protein_codir mucin-associated surface protein (MASP)
TcG_11255	16,85093499	0,50582847	0,43290268	1,16845769	0,2426222	0,47101555	
TcG_11256	24,12401138	0,476427391	0,36063458	1,32108073	0,18647445	0,40038787	protein_codir hypothetical protein
TcG_11257	7,134280889	0,062319201	0,7048383	0,08841631	0,9295458	1	protein_codir hypothetical protein
TcG_11258	14,63212917	0,114318871	0,47810552	0,23910803	0,81102181	1	protein_codir hypothetical protein
TcG_11259	28,05377027	-0,088739503	0,33124688	-0,26789537	0,78877986	0,89487471	protein_codir dispersed gene family protein 1 (DGF-1)
TcG_11260	56,67445721	0,443935286	0,23784911	1,86645762	0,06197737	0,19433553	protein_codir dispersed gene family protein 1 (DGF-1)
TcG_11261	17,89832577	0,011614929	0,44344595	0,02619243	0,97910385	0,99133944	protein_codir dispersed gene family protein 1 (DGF-1)
TcG_11262	44,63323033	0,242405163	0,26163246	0,92651028	0,35418086	0,58832106	protein_codir hypothetical protein
TcG_11263	11,19574886	-0,187578452	0,51425121	-0,36476035	0,7152903	1	protein_codir hypothetical protein
TcG_11264	12,84510713	0,26165888	0,48907682	0,53500569	0,59264592	1	protein_codir dispersed gene family protein 1 (DGF-1)
TcG_11265	19,74691258	0,5383015	0,4003097	1,34471262	0,17871806	0,38979933	protein_codir dispersed gene family protein 1 (DGF-1)
TcG_11266	82,11069663	0,381653516	0,1994447	1,91358068	0,05567376	0,17850508	protein_codir dispersed gene family protein 1 (DGF-1)
TcG_11267	29,66626421	0,202065474	0,32013602	0,6311863	0,52791872	0,73032433	protein_codir mucin TcMUC
TcG_11268	38,68821203	-1,131015196	0,28596644	-3,95506272	7,6515E-05	0,00088561	protein_codir mucin TcMUCII
TcG_11269	3,161010319	-1,133238814	0,9918204	-1,1425847	0,25321106	1	protein_codir trans-sialidase
TcG_11270	108,6608254	0,008371888	0,16861671	0,04965041	0,96040098	0,98188578	protein_codir putative trans-sialidase
TcG_11271	28,36165482	-0,357043913	0,35460201	-1,00688631	0,31398941	0,54820394	protein_codir hypothetical protein
TcG_11272	3,820435255	-1,016733698	0,9006251	-1,12892001	0,25893158	1	protein_codir hypothetical protein
TcG_11273	16,54727659	-0,218347539	0,4403414	-0,49585967	0,61999343	1	protein_codir trans-sialidase
TcG_11274	6,472981771	-0,025372381	0,72921954	-0,03479389	0,9722441	1	protein_codir trans-sialidase
TcG_11275	10,11803892	-0,256313358	0,58537385	-0,43786267	0,66148584	1	protein_codir trans-sialidase
TcG_11276	5,477404238	0,265474061	0,80489075	0,32982621	0,74153129	1	protein_codir trans-sialidase
TcG_11277	33,4585927	-0,568980494	0,30312738	-1,87703431	0,06051339	0,19095609	
TcG_11278	12,65256366	-0,329450758	0,51162496	-0,64393018	0,51962071	1	protein_codir mucin-associated surface protein (MASP)
TcG_11279	12,20386682	0,515676142	0,51433732	1,00260301	0,31605244	1	protein_codir hypothetical protein
TcG_11280	8,613336305	0,134292902	0,60470733	0,22207917	0,82425225	1	protein_codir hypothetical protein
TcG_11281	16,41842191	0,361215775	0,45901317	0,78693989	0,43131706	1	protein_codir hypothetical protein

TcG_11282	6,215033797	-0,013102932	0,70746739	-0,0185209	0,98522331	1	protein_codir	hypothetical protein
TcG_11283	1,965053826	-0,125323857	1,25038377	-0,10022831	0,92016307	1	protein_codir	hypothetical protein
TcG_11284	18,89597436	0,354259054	0,40437999	0,87605486	0,38100021	0,61104814	protein_codir	hypothetical protein
TcG_11285	24,29729654	-0,230362618	0,35155784	-0,65526235	0,51229886	0,71838578	protein_codir	helicase-like protein
TcG_11286	23,31410309	-0,400100809	0,37111209	-1,07811312	0,28098328	0,51421138	protein_codir	trans-sialidase
TcG_11287	324,8882006	-0,020189972	0,1006654	-0,20056516	0,8410386	0,92246657	protein_codir	putative retrotransposon hot spot (RHS) protein
TcG_11288	56,72046575	0,864767986	0,2405009	3,59569547	0,00032353	0,00298437	protein_codir	putative retrotransposon hot spot (RHS) protein
TcG_11289	15,12034979	0,557662863	0,45071851	1,23727527	0,21598491	1	protein_codir	hypothetical protein
TcG_11290	8,565120222	0,792851528	0,65669609	1,20733402	0,22730353	1	protein_codir	hypothetical protein
TcG_11291	3,520400639	0,123047513	0,90626755	0,13577394	0,892	1		
TcG_11292	168,8140228	0,385828493	0,15084959	2,55770321	0,0105366	0,05144417	protein_codir	RNA-binding protein
TcG_11293	139,3146858	-0,08288986	0,14833029	-0,55881951	0,57628491	0,76670634	protein_codir	glutathione-S-transferase/glutaredoxin
TcG_11294	447,6317203	0,273723543	0,08856759	3,09056107	0,00199779	0,01372043	protein_codir	trifunctional enzyme alpha subunit, mitochondrial precursor-like protein
TcG_11295	0,459623291	-2,652712423	2,53013933	-1,0484452	0,29443354	1	protein_codir	trans-sialidase
TcG_11296	0					1	protein_codir	hypothetical protein
TcG_11297	0,311976008	1,510501932	3,1463032	0,48008785	0,63116493	1	protein_codir	hypothetical protein
TcG_11298	38,15107207	-0,087484653	0,28688131	-0,30495069	0,76040371	0,87906978	protein_codir	trans-sialidase
TcG_11299	16,45083972	-0,635322491	0,46667009	-1,36139535	0,17338878	1	protein_codir	trans-sialidase
TcG_11300	35,94180271	-0,14296486	0,30118626	-0,47467258	0,63502037	0,80504935	protein_codir	mucin-like glycoprotein
TcG_11301	9,998054491	-1,038943837	0,55608513	-1,86831796	0,06171777	1	protein_codir	putative trans-sialidase
TcG_11302	38,87545706	-0,124835014	0,28919492	-0,43166392	0,66598569	0,82519673	protein_codir	trans-sialidase
TcG_11303	50,48845255	-0,424516365	0,25584347	-1,65928164	0,09705905	0,26348213	protein_codir	hypothetical protein
TcG_11304	9,44612411	-0,416332892	0,59176538	-0,70354385	0,48171689	1	protein_codir	trans-sialidase
TcG_11305	10,51189095	0,912546325	0,5809262	1,57084724	0,11621814	1	protein_codir	trans-sialidase
TcG_11306	36,57263297	0,039903225	0,28499251	0,140015	0,88864814	0,94628587	protein_codir	hypothetical protein
TcG_11307	144,3256388	0,038404	0,14715089	0,26098381	0,79410499	0,89721693	protein_codir	putative retrotransposon hot spot (RHS) protein
TcG_11308	59,09877403	0,071462134	0,22601731	0,31617991	0,75186595	0,87492919	protein_codir	retrotransposon hot spot (RHS) protein
TcG_11309	12,91663815	0,912717804	0,49808318	1,8324606	0,06688283	1	protein_codir	hypothetical protein
TcG_11310	4,838939136	0,438567748	0,81542081	0,53784223	0,59068597	1	protein_codir	trans-sialidase
TcG_11311	291,063412	0,10864056	0,1045072	1,03955092	0,29854859	0,5322319	protein_codir	hypothetical protein
TcG_11312	0					1	protein_codir	thimet oligopeptidase
TcG_11313	0					1	protein_codir	hypothetical protein
TcG_11314	4,509302626	-0,043169867	0,80378534	-0,0537082	0,95716765	1	protein_codir	hypothetical protein
TcG_11315	258,9630799	-0,079212636	0,11525791	-0,68726423	0,49191624	0,70263551		
TcG_11316	24,07583861	-0,460438216	0,36487949	-1,2618912	0,20698794	0,4291629	protein_codir	putative trans-sialidase
TcG_11317	0					1	protein_codir	hypothetical protein
TcG_11318	1,215901867	-2,719168274	1,77865398	-1,52877867	0,12631932	1	protein_codir	hypothetical protein
TcG_11319	0,614516902	-2,923786983	2,20971919	-1,32314866	0,18578595	1	protein_codir	hypothetical protein
TcG_11320	2,127038109	0,257488977	1,19170666	0,21606741	0,8289352	1	protein_codir	hypothetical protein
TcG_11321	26,4795225	-0,401393917	0,33612825	-1,19416894	0,23241189	0,45964254	protein_codir	hypothetical protein
TcG_11322	40,28152047	-0,04895627	0,28276016	-0,17313709	0,86254367	0,93414012	protein_codir	hypothetical protein
TcG_11323	27,67363541	-0,185380399	0,340157	-0,54498482	0,58576396	0,77437942	protein_codir	hypothetical protein
TcG_11324	16,51725166	-0,097820762	0,41935788	-0,2332632	0,81555703	1	protein_codir	mucin TcMUCII
TcG_11325	11,10042247	-0,664115136	0,52917663	-1,2549971	0,20947981	1	protein_codir	hypothetical protein
TcG_11326	6,616832915	0,184068198	0,69053747	0,26655787	0,7898096	1	protein_codir	hypothetical protein
TcG_11327	0,927587303	-0,166402636	1,89853715	-0,08764782	0,9301566	1	protein_codir	hypothetical protein
TcG_11328	12,94159993	-0,66098201	0,47596115	-1,38873102	0,16491456	1	protein_codir	hypothetical protein
TcG_11329	14,79699462	-0,506751294	0,46113347	-1,09892543	0,27180059	1	protein_codir	hypothetical protein
TcG_11330	8,277974255	-1,004759304	0,6114416	-1,64326291	0,10032855	1	protein_codir	hypothetical protein
TcG_11331	175,643804	-0,319839901	0,13235094	-2,41660475	0,01566602	0,06983705	protein_codir	mucin-associated surface protein (MASP)
TcG_11332	63,50020279	-0,504852547	0,22148074	-2,27944219	0,02264079	0,09269124	protein_codir	mucin TcMUCII
TcG_11333	20,88128244	-0,141263568	0,3903097	-0,36192687	0,71740668	0,85508792	protein_codir	hypothetical protein

TcG_11334	0,494227887	-1,278901833	2,54834049	-0,50185673	0,6157683	1	protein_codir	hypothetical protein
TcG_11335	36,46028733	0,382458172	0,2975645	1,28529502	0,19868921	0,41756089	protein_codir	protein kinase
TcG_11336	37,55943923	0,697384414	0,30861793	2,25970156	0,02383978	0,09640757	protein_codir	putative trans-sialidase
TcG_11337	30,94274646	0,056524738	0,31278754	0,18071288	0,85659295	0,93065321	protein_codir	trans-sialidase
TcG_11338	24,55860978	0,353296756	0,3605823	0,97979506	0,32718729	0,56176525	protein_codir	hypothetical protein
TcG_11339	374,3492411	0,030746559	0,09482474	0,32424618	0,74575166	0,87149696	protein_codir	retrotransposon hot spot (RHS) protein
TcG_11340	44,71213713	0,219899418	0,28210701	0,77948939	0,43569149	0,65939964	protein_codir	hypothetical protein
TcG_11341	56,99735694	-0,016704635	0,24834456	-0,06726395	0,94637158	0,97469438		
TcG_11342	81,74242555	0,256980697	0,19196249	1,33870264	0,1806675	0,39206215	protein_codir	ATP-dependent DEAD/H RNA helicase
TcG_11343	13,97380637	-0,547449383	0,47025297	-1,16415934	0,24435944	1	protein_codir	hypothetical protein
TcG_11344	25,83189101	-0,209925831	0,35235028	-0,59578733	0,55131731	0,74682127	protein_codir	trans-sialidase
TcG_11345	47,05993008	0,297948052	0,26263625	1,13445135	0,25660527	0,48858318	protein_codir	trans-sialidase
TcG_11346	0,756278576	-1,907568997	3,81645378	-0,49982762	0,61719646	1	protein_codir	hypothetical protein
TcG_11347	0,469265227	-2,470320505	2,56600864	-0,96270935	0,3356934	1	protein_codir	hypothetical protein
TcG_11348	39,64671818	-0,27255822	0,27805115	-0,98024489	0,32696525	0,56146723	protein_codir	mucin-associated surface protein (MASP)
TcG_11349	7,624456302	0,122698472	0,65240156	0,18807201	0,8508202	1	protein_codir	trans-sialidase
TcG_11350	72,26942455	0,056579816	0,20297217	0,27875652	0,78043169	0,89067206	protein_codir	putative trans-sialidase
TcG_11351	3,311714648	-0,910748674	0,95544528	-0,95321908	0,34047908	1	protein_codir	surface protease GP63
TcG_11352	4,890627327	1,555001257	0,87074045	1,78583785	0,07412552	1	protein_codir	surface protease GP63
TcG_11353	1,535281757	0,946096683	1,45296879	0,65114728	0,51495142	1	protein_codir	syntaxin binding protein
TcG_11354	6,087616811	-0,635095988	0,73901985	-0,85937609	0,39013306	1	protein_codir	putative syntaxin binding protein
TcG_11355	1,632248611	0,334596411	1,39398018	0,24002953	0,81030736	1	protein_codir	hypothetical protein
TcG_11356	5,457563417	0,267857577	0,73507393	0,36439543	0,71556275	1	protein_codir	hypothetical protein
TcG_11357	14,75614251	-0,274560997	0,46059845	-0,59609622	0,55111095	1	protein_codir	trans-sialidase
TcG_11358	10,4334826	0,200017719	0,54603801	0,36630732	0,71413577	1		
TcG_11359	19,16565323	0,201092091	0,40444777	0,49720163	0,61904687	0,79365686	protein_codir	hypothetical protein
TcG_11360	83,99273228	-0,995041151	0,19637958	-5,06692781	4,0429E-07	8,9733E-06	protein_codir	mucin-associated surface protein (MASP)
TcG_11361	118,850105	-0,561502546	0,16306907	-3,44334171	0,00057457	0,00487692	protein_codir	mucin-associated surface protein (MASP)
TcG_11362	52,57156126	-0,335540593	0,24497708	-1,36968158	0,17078632	0,37747622	protein_codir	trans-sialidase
TcG_11363	82,51006461	-0,149996201	0,20348208	-0,73714697	0,46103298	0,6791517	protein_codir	trans-sialidase
TcG_11364	1,657197946	0,587019982	1,37692573	0,42632654	0,66986992	1	protein_codir	target of rapamycin (TOR) kinase 1
TcG_11365	1,474719999	-0,7496187	1,42341671	-0,52663334	0,59844823	1	protein_codir	hypothetical protein
TcG_11366	1,741135511	0,043361943	2,66169192	0,01629112	0,98700214	1	protein_codir	surface protease GP63
TcG_11367	2,801808223	0,239215874	1,10268914	0,21693863	0,82825617	1	protein_codir	hypothetical protein
TcG_11368	0,756840188	-0,42123777	2,11738708	-0,19894226	0,84230791	1	protein_codir	hypothetical protein
TcG_11369	586,8929928	0,30090301	0,08201616	3,66882578	0,00024367	0,00235457	protein_codir	ubiquitin hydrolase, putative,cysteine peptidase, Clan CA, family C19
TcG_11370	10,05965298	-0,830824377	0,56297839	-1,47576601	0,14000672	1	protein_codir	hypothetical protein
TcG_11371	3,094720505	-0,634030434	0,9821439	-0,64555757	0,51856592	1	protein_codir	hypothetical protein
TcG_11372	35,71976337	-0,27961188	0,28760781	-0,9721985	0,33095181	0,56523583	protein_codir	retrotransposon hot spot (RHS) protein
TcG_11373	15,73187604	0,094818216	0,44049501	0,21525378	0,82956946	1	protein_codir	putative retrotransposon hot spot (RHS) protein
TcG_11374	22,14185265	-0,371875801	0,36496764	-1,01892814	0,30823708	0,5412602	protein_codir	retrotransposon hot spot protein (RHS)
TcG_11375	19,83496937	0,094090681	0,4163786	0,22597386	0,82122175	0,91136735	protein_codir	retrotransposon hot spot protein (RHS)
TcG_11376	5,802778925	-0,483261452	0,74838026	-0,64574319	0,51844569	1	protein_codir	retrotransposon hot spot (RHS) protein
TcG_11377	15,83893232	-0,495434742	0,44116883	-1,12300487	0,26143543	1	protein_codir	hypothetical protein
TcG_11378	46,0657645	0,085107683	0,25619932	0,33219324	0,73974335	0,86729518	protein_codir	trans-sialidase
TcG_11379	21,87293912	0,16404768	0,37471102	0,43779785	0,66153283	0,82272643	protein_codir	ATP-dependent DEAD/H RNA helicase
TcG_11380	7,428875535	0,145802525	0,66078692	0,22064984	0,82536509	1		
TcG_11381	24,91793657	0,012198298	0,34876177	0,03497602	0,97209886	0,98777616	protein_codir	putative trans-sialidase
TcG_11382	8,955469896	0,298464419	0,57462002	0,5194118	0,60347361	1	protein_codir	hypothetical protein
TcG_11383	14,36548046	-1,467086188	0,47475193	-3,09021636	0,00200011	1	protein_codir	subtilisin-like serine peptidase
TcG_11384	4,115278879	-1,467813328	0,88871545	-1,65161226	0,09861362	1	protein_codir	subtilisin-like serine peptidase
TcG_11385	74,77057948	0,170200214	0,20488845	0,83069698	0,40614483	0,63469032		

TcG_11386	159,5006932	0,190472517	0,1520381	1,25279466	0,21028044	0,43289563	protein_codir putative mitochondrial carrier protein
TcG_11387	386,3976303	-0,263490777	0,09464941	-2,78386061	0,00537161	0,03031441	protein_codir cell morphogenesis protein Las1
TcG_11388	225,3599366	-0,102071904	0,126647	-0,80595595	0,42026825	0,64681561	protein_codir hypothetical protein
TcG_11389	298,0517642	0,031155592	0,10806739	0,28829782	0,77311878	0,88691888	protein_codir hypothetical protein
TcG_11390	126,9888074	0,037064625	0,15933854	0,23261557	0,81605994	0,90921145	protein_codir trans-sialidase
TcG_11391	26,82274198	-0,004557052	0,37594633	-0,01212155	0,99032864	0,99603651	protein_codir hypothetical protein
TcG_11392	54,29451287	0,05594189	0,24443881	0,22885846	0,81897893	0,91079764	protein_codir hypothetical protein
TcG_11393	288,5635926	-0,293633368	0,10934077	-2,68548843	0,00724239	0,03833271	protein_codir mucin-associated surface protein (MASP)
TcG_11394	53,46790005	0,334363529	0,24331464	1,37420226	0,1693789	0,37606421	protein_codir hypothetical protein
TcG_11395	403,0871275	-0,146612896	0,09493488	-1,54435227	0,12250302	0,30735575	protein_codir putative retrotransposon hot spot (RHS) protein
TcG_11396	16,7034944	0,397128579	0,44857793	0,88530565	0,3759918	1	protein_codir hypothetical protein
TcG_11397	42,6381922	0,343550598	0,26641822	1,28951617	0,1972187	0,41560128	protein_codir trans-sialidase
TcG_11398	42,03712528	0,001912594	0,27121506	0,00705195	0,99437341	0,99773191	protein_codir hypothetical protein
TcG_11399	34,93044037	0,15336985	0,30108011	0,50939881	0,61047271	0,78905613	protein_codir hypothetical protein
TcG_11400	7,213830436	0,465802476	0,6662645	0,69912546	0,48447363	1	protein_codir dispersed gene family protein 1 (DGF-1)
TcG_11401	70,19761249	0,304886614	0,2302895	1,32392757	0,1855271	0,39939	protein_codir dispersed gene family protein 1 (DGF-1)
TcG_11402	61,85905598	0,066428226	0,22358909	0,29709958	0,76639048	0,88273189	
TcG_11403	29,07448313	-0,282318653	0,33984369	-0,83073089	0,40612567	0,63469032	protein_codir putative trans-sialidase
TcG_11404	11,80812744	0,067025241	0,5789988	0,11576059	0,90784229	1	protein_codir trans-sialidase
TcG_11405	207,811501	0,345154201	0,12810673	2,69427055	0,00705429	0,03752032	protein_codir putative trans-sialidase
TcG_11406	1624,633414	0,184833112	0,06129994	3,01522516	0,00256789	0,01678033	protein_codir L1Tc protein
TcG_11407	41,98601421	0,167201554	0,2714482	0,61596119	0,53792014	0,73738082	protein_codir trans-sialidase
TcG_11408	31,73750481	0,333243822	0,31700275	1,05123322	0,29315149	0,526174	protein_codir mucin TcMUCII
TcG_11409	459,0405522	0,043171656	0,09253083	0,46656509	0,64081107	0,80929115	protein_codir retrotransposon hot spot (RHS) protein
TcG_11410	12,06244719	0,667109937	0,52100019	1,28044086	0,20039013	1	protein_codir hypothetical protein
TcG_11411	7,376352163	0,744813703	0,64460134	1,15546409	0,24790043	1	protein_codir subtilisin-like serine peptidase
TcG_11412	32,91870937	-0,042678045	0,303162	-0,14077637	0,88804661	0,94594634	protein_codir subtilisin-like serine peptidase
TcG_11413	39,33930596	0,271086235	0,30943934	0,87605614	0,38099952	0,61104814	protein_codir putative kinesin
TcG_11414	8,452088794	0,043650629	0,59532431	0,07332244	0,94154954	1	protein_codir dispersed gene family protein 1 (DGF-1)
TcG_11415	20,43890007	-0,006865303	0,38191823	-0,01797584	0,98565812	0,99432608	protein_codir dispersed gene family protein 1 (DGF-1)
TcG_11416	30,13634221	0,113064513	0,31910877	0,3543134	0,72310402	0,85733557	protein_codir dispersed gene family protein 1 (DGF-1)
TcG_11417	10,54229568	0,306762243	0,53379986	0,57467652	0,56551009	1	protein_codir dispersed gene family protein 1 (DGF-1)
TcG_11418	107,3130728	-0,723387644	0,16843298	-4,29480995	1,7484E-05	0,00024704	protein_codir mucin TcMUCII
TcG_11419	81,4018163	-0,65785116	0,19849357	-3,31421902	0,00091899	0,00729279	protein_codir putative mucin-associated surface protein (MASP)
TcG_11420	6,784862266	0,241451953	0,69450109	0,34766245	0,7280937	1	protein_codir surface protease GP63
TcG_11421	8,443259353	0,030547114	0,60198172	0,05074426	0,95952931	1	protein_codir surface protease GP63
TcG_11422	3,803027469	-0,385811406	0,91640031	-0,4210075	0,6737496	1	protein_codir hypothetical protein
TcG_11423	11,21757164	0,204922436	0,51972436	0,39429061	0,69336649	1	
TcG_11424	48,84801153	-0,290756746	0,26379541	-1,10220548	0,27037235	0,50338005	protein_codir trans-sialidase
TcG_11425	102,4410659	-0,574301396	0,1770346	-3,24400658	0,00117861	0,00891925	protein_codir mucin-associated surface protein (MASP)
TcG_11426	40,87752699	-0,51917749	0,27154943	-1,91190784	0,05588802	0,17890697	
TcG_11427	5,087689834	-0,708488053	0,81103906	-0,87355602	0,38236009	1	
TcG_11428	16,72553607	0,158129962	0,4370233	0,36183417	0,71747596	1	protein_codir hypothetical protein
TcG_11429	13,92051478	0,156095078	0,4975354	0,31373662	0,75372108	1	protein_codir hypothetical protein
TcG_11430	393,4550196	0,106417969	0,09566296	1,11242607	0,265955	0,49866613	protein_codir trans-sialidase-like protein
TcG_11431	114,365762	0,000570534	0,16942609	0,00336745	0,99731317	0,99910416	protein_codir beta galactofuranosyl glycosyltransferase
TcG_11432	13,42502165	0,583922672	0,4999103	1,1680549	0,24278462	1	protein_codir hypothetical protein
TcG_11433	63,26259008	0,379625095	0,24774055	1,53234943	0,12543623	0,31237885	protein_codir trans-sialidase
TcG_11434	93,10485475	-0,029665892	0,18638124	-0,1591678	0,87353667	0,93932295	protein_codir putative mucin-associated surface protein (MASP)
TcG_11435	147,1765449	-0,191467804	0,14737932	-1,29914977	0,19389253	0,41081411	protein_codir trans-sialidase
TcG_11436	42,78689803	-0,425304195	0,26321178	-1,61582509	0,10613213	0,28054915	protein_codir trans-sialidase
TcG_11437	341,272344	-0,195154111	0,10079143	-1,93621734	0,05284109	0,17168167	

TcG_11438	411,4590707	0,81392351	0,09425618	8,63522687	5,8611E-18	8,4883E-16	protein_codir dispersed gene family protein 1 (DGF-1)
TcG_11439	17,27007895	0,891000199	0,42649085	2,08914257	0,03669489	0,13323316	protein_codir hypothetical protein
TcG_11440	85,05524858	0,875679881	0,20274428	4,31913493	1,5664E-05	0,00022378	protein_codir dispersed gene family protein 1 (DGF-1)
TcG_11441	45,72562796	-0,118475275	0,26340775	-0,44977901	0,6528698	0,81633385	protein_codir hypothetical protein
TcG_11442	17,31218171	0,070981711	0,41775746	0,1699113	0,8650799	0,93501039	protein_codir hypothetical protein
TcG_11443	90,60348713	-0,014451293	0,18502259	-0,07810556	0,93774408	0,97127686	protein_codir retrotransposon hot spot (RHS) protein
TcG_11444	329,1897843	0,080924756	0,0987998	0,81907812	0,41274184	0,6393939	protein_codir putative retrotransposon hot spot (RHS) protein
TcG_11445	68,48717956	0,208159171	0,21256028	0,97929478	0,32743435	0,56193962	protein_codir retrotransposon hot spot (RHS) protein
TcG_11446	33,99866449	-0,110316572	0,30988176	-0,35599569	0,72184379	0,85711748	
TcG_11447	73,58996425	-0,05837566	0,20184657	-0,28920809	0,77242214	0,88641867	protein_codir putative retrotransposon hot spot (RHS) protein
TcG_11448	32,61173171	-0,058226461	0,31658512	-0,1839204	0,8540759	0,92916537	protein_codir trans-sialidase
TcG_11449	31,85102042	0,189680459	0,3129814	0,60604387	0,54448562	0,74164242	protein_codir trans-sialidase
TcG_11450	0,573889029	1,026419886	2,17405032	0,47212333	0,63683876	1	protein_codir retrotransposon hot spot (RHS) protein
TcG_11451	141,458148	0,172334652	0,1519383	1,13424101	0,25669347	0,4886708	protein_codir hypothetical protein
TcG_11452	58,71787219	0,413202886	0,22896625	1,80464542	0,07113019	0,21392986	protein_codir hypothetical protein
TcG_11453	333,616653	-0,010357717	0,10067644	-0,10288124	0,91805722	0,96050307	protein_codir putative trans-sialidase
TcG_11454	238,5169301	-0,133526536	0,11922542	-1,11995019	0,26273499	0,49488661	protein_codir 85 kDa surface glycoprotein
TcG_11455	15,70077782	-0,038004667	0,45612674	-0,08332041	0,93359677	1	protein_codir hypothetical protein
TcG_11456	28,03946168	0,124945798	0,33938248	0,3681563	0,71275669	0,85248988	protein_codir trans-sialidase
TcG_11457	62,22121719	0,012094507	0,2201651	0,05493381	0,9561912	0,97969855	protein_codir trans-sialidase
TcG_11458	49,27339636	0,141167489	0,24917762	0,56653357	0,57103112	0,7624407	protein_codir hypothetical protein
TcG_11459	48,2060885	0,470297633	0,25878852	1,81730488	0,06917044	0,21039873	protein_codir hypothetical protein
TcG_11460	46,38956814	0,38351889	0,26212601	1,46310886	0,1434376	0,33991982	protein_codir mucin-associated surface protein (MASP)
TcG_11461	48,81059101	0,475996517	0,2497669	1,90576302	0,05668096	0,18066179	protein_codir hypothetical protein
TcG_11462	45,27293119	0,575803075	0,26193386	2,19827662	0,0279294	0,10840537	protein_codir hypothetical protein
TcG_11463	0					1	protein_codir hypothetical protein
TcG_11464	15,24748108	0,641430621	0,45536939	1,40859407	0,15895523	1	protein_codir hypothetical protein
TcG_11465	39,15361462	0,436238183	0,28373714	1,53747299	0,12417752	0,31021181	protein_codir hypothetical protein
TcG_11466	12,11213808	-0,953761584	0,53819989	-1,77213263	0,07637254	1	protein_codir hypothetical protein
TcG_11467	51,38203022	0,068760348	0,24456567	0,2811529	0,77859313	0,88953555	protein_codir hypothetical protein
TcG_11468	24,48340179	-0,977669895	0,37559506	-2,60298923	0,00924149	0,04639162	protein_codir hypothetical protein
TcG_11469	18,77781524	-0,492797332	0,398126	-1,2377924	0,21579305	0,43955314	protein_codir thimet oligopeptidase
TcG_11470	42,42495262	-0,721895624	0,28251576	-2,55524016	0,01061146	0,05178784	
TcG_11471	2,506691713	-1,260271447	1,22717924	-1,02696608	0,30443644	1	
TcG_11472	306,3896014	-0,131774512	0,10742886	-1,22662119	0,219965	0,44507763	protein_codir putative retrotransposon hot spot (RHS) protein
TcG_11473	58,15438971	0,042680797	0,23181607	0,18411492	0,8539233	0,92914828	protein_codir hypothetical protein
TcG_11474	65,65703611	0,10333549	0,21380461	0,48331741	0,62887038	0,80107467	protein_codir trans-sialidase
TcG_11475	131,1299213	0,106335253	0,16367825	0,64966024	0,51591171	0,72181537	protein_codir trans-sialidase-like protein
TcG_11476	47,67583251	0,067460396	0,25176298	0,26795201	0,78873626	0,89487471	protein_codir hypothetical protein
TcG_11477	140,0006584	0,155695666	0,16303224	0,95499927	0,33957807	0,57368788	protein_codir hypothetical protein
TcG_11478	318,5334464	-0,072759766	0,11116074	-0,65454554	0,5127604	0,71879516	protein_codir retrotransposon hot spot (RHS) protein
TcG_11479	135,2898263	-0,057392449	0,15400887	-0,37265676	0,70940392	0,85005287	protein_codir putative retrotransposon hot spot (RHS) protein
TcG_11480	58,2044643	-0,162550301	0,23807115	-0,68278034	0,49474565	0,70453824	
TcG_11481	1,39895475	0,329276146	1,57769194	0,2087075	0,83467658	1	protein_codir hypothetical protein
TcG_11482	2,830292166	-2,389620952	1,2859069	-1,85831568	0,0631242	1	protein_codir hypothetical protein
TcG_11483	9,660988389	0,068360966	0,55462333	0,12325656	0,90190393	1	
TcG_11484	11,23738823	0,716695005	0,54095453	1,32487108	0,18521392	1	
TcG_11485	9,513770941	-2,318866099	0,64366784	-3,60258188	0,00031507	1	protein_codir mucin TcMUCII
TcG_11486	2,055773518	-0,498565891	1,2881513	-0,38703985	0,6987267	1	
TcG_11487	20,18063525	-0,19580434	0,39176324	-0,49980273	0,61721399	0,793034	protein_codir mucin-associated surface protein (MASP)
TcG_11488	83,90810139	0,14980952	0,19665202	0,76180007	0,44617934	0,66788551	protein_codir dispersed gene family protein 1 (DGF-1)
TcG_11489	25,92000123	0,04631987	0,35614695	0,13005831	0,89652029	0,94988308	

TcG_11490	190,9253936	-0,060887851	0,13273922	-0,45870281	0,6464476	0,81201944		
TcG_11491	510,741018	0,02232475	0,08202195	0,2721802	0,78548346	0,89351472	protein_codir	putative retrotransposon hot spot (RHS) protein
TcG_11492	61,31566783	0,23488228	0,22660904	1,03650889	0,2999648	0,53367513	protein_codir	putative trans-sialidase
TcG_11493	125,8396649	0,582588041	0,16305073	3,57304775	0,00035285	0,00321141	protein_codir	dispersed gene family protein 1 (DGF-1)
TcG_11494	283,8489134	0,597088091	0,1194896	4,99698811	5,8233E-07	1,2564E-05	protein_codir	dispersed gene family protein 1 (DGF-1)
TcG_11495	17,31521929	-0,341556184	0,44210751	-0,77256362	0,43978068	0,66276001	protein_codir	hypothetical protein
TcG_11496	7,655669232	-0,429576032	0,6293058	-0,6826189	0,49484769	1	protein_codir	hypothetical protein
TcG_11497	10,58576946	0,162622065	0,53289499	0,30516719	0,76023882	1	protein_codir	mucin-associated surface protein (MASP)
TcG_11498	14,74211348	-0,359327037	0,44689834	-0,80404648	0,42137013	1	protein_codir	hypothetical protein
TcG_11499	6,63654073	0,398145072	0,73240974	0,54360974	0,58671006	1	protein_codir	hypothetical protein
TcG_11500	49,40601757	-0,318264542	0,25474214	-1,24935963	0,21153357	0,43452566		
TcG_11501	62,63562358	0,125574046	0,22107841	0,56800682	0,57003033	0,76192564	protein_codir	mucin TcMUCII
TcG_11502	171,8194996	-0,304731872	0,13908753	-2,1909359	0,02845643	0,10986956	protein_codir	mucin-associated surface protein (MASP)
TcG_11503	95,24977544	0,134710581	0,18683761	0,72100355	0,47090733	0,68692532	protein_codir	trans-sialidase
TcG_11504	2,296872839	0,597938551	2,85763666	0,20924233	0,83425907	1	protein_codir	hypothetical protein
TcG_11505	2,32222405	0,401511375	1,17434351	0,34190283	0,73242402	1	protein_codir	hypothetical protein
TcG_11506	1,904787291	-1,062261353	1,32783807	-0,79999314	0,42371477	1	protein_codir	hypothetical protein
TcG_11507	4,75170133	-1,172498368	0,82205171	-1,42630731	0,15377966	1	protein_codir	hypothetical protein
TcG_11508	17,20239521	0,590794364	0,41999382	1,40667393	0,1595241	0,3621104	protein_codir	mucin TcMUCII
TcG_11509	1,679971819	0,197485257	1,33355388	0,14808945	0,88227218	1		
TcG_11510	40,61870255	0,195603279	0,27766479	0,70445834	0,48114739	0,69536181		
TcG_11511	40,52580868	0,560805885	0,30348515	1,84788574	0,06461887	0,20023383	protein_codir	putative trans-sialidase
TcG_11512	46,2919952	-0,334613844	0,26704192	-1,25303864	0,21019164	0,43289563	protein_codir	trans-sialidase
TcG_11513	72,22769138	-0,400694388	0,21249163	-1,88569489	0,05933608	0,18756073	protein_codir	retrotransposon hot spot protein (RHS)
TcG_11514	37,9945976	-0,140664084	0,28051235	-0,50145416	0,61605153	0,79256256	protein_codir	putative retrotransposon hot spot (RHS) protein
TcG_11515	71,7250035	0,165186492	0,22388127	0,73783078	0,46061729	0,6786591	protein_codir	trans-sialidase
TcG_11516	76,35888214	0,469042646	0,21868605	2,14482197	0,03196708	0,11991016	protein_codir	trans-sialidase
TcG_11517	30,73746224	-0,529893166	0,37041665	-1,43053281	0,15256415	0,35281603		
TcG_11518	23,26676506	0,054132503	0,37693313	0,14361302	0,88580606	0,94458804	protein_codir	hypothetical protein
TcG_11519	5,339247898	0,817051318	0,77556787	1,05348784	0,29211747	1	protein_codir	hypothetical protein
TcG_11520	6,775273911	0,377197479	0,66832621	0,56439127	0,57248788	1	protein_codir	hypothetical protein
TcG_11521	0,324841555	-0,458748506	2,94047964	-0,15601146	0,87602399	1	protein_codir	putative trans-sialidase
TcG_11522	0,807744502	1,687520936	1,94531987	0,86747735	0,38568051	1	protein_codir	helicase-like protein
TcG_11523	0,116927736	0,503022807	4,08047286	0,12327562	0,90188885	1	protein_codir	hypothetical protein
TcG_11524	0,350783209	1,758746969	2,99002236	0,58820529	0,55639451	1	protein_codir	hypothetical protein
TcG_11525	8,766508783	0,812928032	0,61255049	1,32712005	0,18446898	1	protein_codir	hypothetical protein
TcG_11526	6,27067816	0,338683023	0,71036685	0,476772	0,63352449	1	protein_codir	dispersed gene family protein 1 (DGF-1)
TcG_11527	23,56474341	0,317114633	0,40026399	0,7922637	0,42820693	0,65356415	protein_codir	dispersed gene family protein 1 (DGF-1)
TcG_11528	10,28087766	0,293676782	0,55706169	0,52718897	0,59806236	1	protein_codir	dispersed gene family protein 1 (DGF-1)
TcG_11529	9,244980512	1,118781818	0,59981187	1,86522121	0,0621504	1	protein_codir	hypothetical protein
TcG_11530	12,57857905	-0,244708621	0,53959761	-0,45350205	0,65018727	1	protein_codir	retrotransposon hot spot (RHS) protein
TcG_11531	30,32649776	0,22126257	0,33094258	0,66858297	0,50376154	0,7120387	protein_codir	retrotransposon hot spot (RHS) protein
TcG_11532	29,24019791	0,101440475	0,33038382	0,30703826	0,75881425	0,87810846	protein_codir	hypothetical protein
TcG_11533	7,802868052	-0,260266928	0,64448666	-0,40383602	0,68633331	1		
TcG_11534	58,24011374	0,127129236	0,23609309	0,53847079	0,59025206	0,77623888		
TcG_11535	3,727781677	-0,007444812	0,88599944	-0,00840273	0,99329567	1		
TcG_11536	27,74059817	-0,045098287	0,32725892	-0,13780614	0,89039363	0,94667588	protein_codir	hypothetical protein
TcG_11537	49,70262966	0,064930111	0,26652786	0,24361473	0,80752922	0,90492635	protein_codir	retrotransposon hot spot (RHS) protein
TcG_11538	23,2559068	0,254417503	0,36361939	0,69968078	0,48412668	0,69741854	protein_codir	retrotransposon hot spot (RHS) protein
TcG_11539	26,10643373	-0,077733012	0,35935385	-0,21631328	0,82874355	0,91645761	protein_codir	retrotransposon hot spot (RHS) protein
TcG_11540	41,65140033	0,501380676	0,28730204	1,74513444	0,08096147	0,2329326	protein_codir	hypothetical protein
TcG_11541	19,55322422	-0,538619833	0,40874809	-1,31773052	0,18759388	0,40204636		

TcG_11542	29,54747708	-0,336247822	0,34036748	-0,98789643	0,32320337	0,55757366	
TcG_11543	18,75063252	-0,306059949	0,3957282	-0,7734095	0,43928007	0,66263171	protein_codir hypothetical protein
TcG_11544	17,88209771	-0,809156571	0,40982574	-1,97439179	0,04833721	0,16157024	protein_codir hypothetical protein
TcG_11545	12,21698206	-0,429695257	0,50287521	-0,85447691	0,3928408	1	protein_codir hypothetical protein
TcG_11546	141,549784	-0,015413022	0,14736794	-0,10458871	0,91670217	0,95973531	protein_codir surface protein-2
TcG_11547	543,432778	0,780663826	0,0842839	9,26231206	2,0005E-20	3,7997E-18	protein_codir dispersed gene family protein 1 (DGF-1)
TcG_11548	269,533506	0,733632504	0,11533484	6,36089224	2,0059E-10	9,2006E-09	protein_codir dispersed gene family protein 1 (DGF-1)
TcG_11549	147,1224971	0,245521189	0,16460563	1,49157225	0,13581132	0,32836351	protein_codir hypothetical protein
TcG_11550	71,25729771	-0,025600167	0,21523294	-0,11894168	0,90532156	0,95390315	protein_codir hypothetical protein
TcG_11551	347,8695842	0,142977475	0,09970091	1,43406393	0,15155402	0,35179947	protein_codir putative syntaxin binding protein
TcG_11552	94,77147229	0,17847633	0,18041246	0,9892683	0,32253189	0,55724873	protein_codir hypothetical protein
TcG_11553	190,3290925	0,026866468	0,12761616	0,21052559	0,83325748	0,91908998	protein_codir surface protease GP63
TcG_11554	189,0707325	-0,185445938	0,14047626	-1,32012299	0,18679396	0,40078489	protein_codir mucin-associated surface protein (MASP)
TcG_11555	138,7102426	-0,002558389	0,15075053	-0,01697101	0,98645974	0,99463124	protein_codir elongation factor 1-gamma (EF-1-gamma)
TcG_11556	49,28713869	-0,321312499	0,25199585	-1,27507059	0,20228426	0,42238277	protein_codir hypothetical protein
TcG_11557	42,1816695	-0,073396454	0,27126082	-0,27057522	0,78671776	0,89379407	protein_codir trans-sialidase
TcG_11558	22,25575115	0,489959301	0,36663088	1,33638306	0,1814241	0,39296684	protein_codir trans-sialidase
TcG_11559	117,5562983	-0,167529361	0,16195583	-1,03441389	0,30094271	0,53419983	protein_codir putative calpain-like cysteine peptidase
TcG_11560	35,69453103	0,365327235	0,31366074	1,16472093	0,24413197	0,47228469	protein_codir calpain-like cysteine peptidase, putative,cysteine peptidase, Clan CA, family C2
TcG_11561	3,599220455	0,294305929	0,95921079	0,30682091	0,75897969	1	protein_codir calpain-like cysteine peptidase
TcG_11562	20,14281699	0,368375311	0,41219605	0,89368956	0,37148801	0,60280866	protein_codir trans-sialidase
TcG_11563	2,554718739	0,415385103	1,14210972	0,36369982	0,71608218	1	protein_codir mucin TcMUC
TcG_11564	0,94826079	1,85906385	1,94893086	0,95388907	0,34013979	1	protein_codir hypothetical protein
TcG_11565	1,170285541	0,784823943	1,70795871	0,45950991	0,64586804	1	protein_codir hypothetical protein
TcG_11566	0,428370964	1,990401553	2,58761618	0,76920278	0,44177294	1	protein_codir hypothetical protein
TcG_11567	1,05232298	3,269962262	1,84561937	1,77174249	0,07643731	1	
TcG_11568	44,66815333	0,108201612	0,27164104	0,39832572	0,6903901	0,83922987	protein_codir hypothetical protein
TcG_11569	287,1500195	0,035823784	0,11495661	0,31162876	0,75532268	0,87651928	protein_codir regulator of sigma E protease
TcG_11570	167,757995	-0,253904691	0,13449444	-1,88784523	0,05904673	0,18686573	protein_codir trans-sialidase
TcG_11571	42,96315328	-0,369893192	0,26133096	-1,4154205	0,15694524	0,35858166	protein_codir mucin-associated surface protein (MASP)
TcG_11572	21,02222634	0,42515422	0,3845072	1,10571198	0,2688512	0,50149655	protein_codir hypothetical protein
TcG_11573	2,465286939	-0,406808424	1,17753492	-0,34547462	0,72973759	1	
TcG_11574	17,88278763	-0,256348848	0,41981807	-0,6106189	0,54145191	0,74003324	protein_codir putative trans-sialidase
TcG_11575	9,590076338	0,774706242	0,59318255	1,30601658	0,19154692	1	protein_codir trans-sialidase
TcG_11576	40,48715747	0,241325507	0,27852567	0,86643901	0,38624946	0,61657291	protein_codir trans-sialidase
TcG_11577	7,571451602	0,218833306	0,65604211	0,33356595	0,73870712	1	protein_codir hypothetical protein
TcG_11578	8,315497876	0,026800606	0,60765383	0,04410506	0,96482066	1	protein_codir hypothetical protein
TcG_11579	132,2547378	0,339268746	0,16547803	2,05023434	0,04034157	0,14202291	protein_codir GP63 group II protein
TcG_11580	421,9847195	-0,109412908	0,08996818	-1,2161289	0,22393582	0,45051579	protein_codir XPA-interacting protein
TcG_11581	329,0515031	-0,142057721	0,10018946	-1,41789082	0,15622264	0,35769079	protein_codir hypothetical protein
TcG_11582	259,503118	0,21807148	0,11393879	1,91393538	0,05562841	0,17847796	protein_codir hypothetical protein
TcG_11583	70,52586547	0,068804866	0,21176146	0,32491684	0,74524401	0,87119333	protein_codir hypothetical protein
TcG_11584	7,691592105	0,187250658	0,61647703	0,30374312	0,7613236	1	protein_codir trans-sialidase
TcG_11585	7,769725964	0,222275117	0,62049721	0,35822098	0,72017795	1	protein_codir putative trans-sialidase
TcG_11586	15,97540913	0,288728015	0,44907036	0,64294605	0,52025911	1	protein_codir hypothetical protein
TcG_11587	120,9871718	0,069004165	0,16480379	0,41870497	0,67543176	0,83012118	protein_codir putative trans-sialidase
TcG_11588	35,15228713	0,235514219	0,29880049	0,7881989	0,43058037	0,65538512	
TcG_11589	24,15172656	0,811039679	0,3740893	2,16803761	0,03015583	0,1149294	protein_codir putative retrotransposon hot spot (RHS) protein
TcG_11590	68,90309236	-0,269670106	0,21469894	-1,25603835	0,20910207	0,43162731	protein_codir retrotransposon hot spot (RHS) protein
TcG_11591	24,12294997	0,362842254	0,35174596	1,03154633	0,30228469	0,5353728	protein_codir retrotransposon hot spot (RHS) protein
TcG_11592	24,01486317	0,181296755	0,35699884	0,5078357	0,61156857	0,7899257	protein_codir retrotransposon hot spot (RHS) protein
TcG_11593	267,4462324	0,144892972	0,10956543	1,32243335	0,18602389	0,39987989	protein_codir retrotransposon hot spot (RHS) protein

TcG_11594	74,90815754	-0,165416884	0,20078579	-0,82384758	0,41002617	0,63689009	protein_codir	ATP-dependent DEAD/H RNA helicase
TcG_11595	44,85420919	0,17622153	0,27878717	0,63210057	0,52732116	0,72984626	protein_codir	putative kinesin
TcG_11596	95,88275895	0,169321855	0,17707211	0,95623108	0,3389555	0,57332217	protein_codir	hypothetical protein
TcG_11597	29,60103422	-0,411067133	0,34238288	-1,20060655	0,22990386	0,45688956	protein_codir	mitotic centromere-associated kinesin (MCAK)
TcG_11598	34,89897437	0,277159466	0,29642175	0,93501731	0,34977938	0,5842768	protein_codir	hypothetical protein
TcG_11599	56,15382647	-0,368323724	0,24867427	-1,48114931	0,13856679	0,33238817	protein_codir	mucin-associated surface protein (MASP)
TcG_11600	7,137231068	0,210790121	0,6690736	0,31504773	0,75272541	1		
TcG_11601	11,14301428	0,437429463	0,5285651	0,82757916	0,40790887	1	protein_codir	surface protease GP63
TcG_11602	7,036485107	0,867195046	0,67931523	1,27657236	0,20175327	1	protein_codir	putative surface protease GP63
TcG_11603	14,65420179	0,22063424	0,45864787	0,48105366	0,63047836	1	protein_codir	retrotransposon hot spot (RHS) protein
TcG_11604	10,8369217	0,05310241	0,53355309	0,09952601	0,92072064	1	protein_codir	retrotransposon hot spot (RHS) protein
TcG_11605	12,589806	-0,236262524	0,48443542	-0,48770696	0,62575742	1	protein_codir	hypothetical protein
TcG_11606	22,46226454	-0,069134551	0,36729433	-0,18822657	0,85069905	0,92805389		
TcG_11607	109,2478754	0,070185314	0,1709379	0,41058954	0,68137354	0,83397357		
TcG_11608	1,236381856	2,394359416	1,71154662	1,3989449	0,16182951	1	protein_codir	hypothetical protein
TcG_11609	1,331863933	-2,075534761	1,65243388	-1,25604709	0,2090989	1	protein_codir	hypothetical protein
TcG_11610	1,747664571	0,189667504	1,35260962	0,14022339	0,88848349	1	protein_codir	hypothetical protein
TcG_11611	0,857807489	2,992029954	2,04862197	1,46050857	0,14415035	1	protein_codir	hypothetical protein
TcG_11612	8,849749158	0,465252898	0,62658986	0,74251584	0,45777486	1	protein_codir	trans-sialidase
TcG_11613	15,76210256	-0,219847894	0,4621072	-0,47575085	0,63425189	1	protein_codir	trans-sialidase
TcG_11614	24,46699228	-0,24628326	0,36099784	-0,68222919	0,49509404	0,70486109	protein_codir	trans-sialidase
TcG_11615	93,97549023	0,015676352	0,18176432	0,08624549	0,93127127	0,9669931	protein_codir	mucin-associated surface protein (MASP)
TcG_11616	26,22879999	0,198258947	0,38206876	0,51890908	0,60382415	0,78580967	protein_codir	dispersed gene family protein 1 (DGF-1)
TcG_11617	89,43414681	-0,096138854	0,19066453	-0,50423042	0,61409946	0,79140316	protein_codir	dispersed gene family protein 1 (DGF-1)
TcG_11618	27,85604628	0,502488555	0,33057691	1,52003525	0,12850212	0,31744681	protein_codir	dispersed gene family protein 1 (DGF-1)
TcG_11619	3,126381204	0,071657331	0,99899993	0,07172907	0,94281752	1	protein_codir	thimet oligopeptidase
TcG_11620	24,30046673	0,068710527	0,36346664	0,18904218	0,85005975	0,9278184		
TcG_11621	3,31122776	1,246289347	1,02564441	1,2151281	0,22431724	1	protein_codir	hypothetical protein
TcG_11622	2,375413301	-1,085260902	1,19497499	-0,90818712	0,36377937	1	protein_codir	hypothetical protein
TcG_11623	215,6789033	-0,18167815	0,12748615	-1,42508143	0,15413366	0,35516958	protein_codir	putative surface protease GP63
TcG_11624	11,00449131	1,132308023	0,54550646	2,07570049	0,03792166	1	protein_codir	hypothetical protein
TcG_11625	15,60290265	-0,262495015	0,43762033	-0,59982363	0,54862378	1	protein_codir	hypothetical protein
TcG_11626	23,07544419	0,027574034	0,6483698	0,04252825	0,96607759	0,98495028	protein_codir	hypothetical protein
TcG_11627	0					1	protein_codir	hypothetical protein
TcG_11628	8,341239527	-0,091258931	0,66500528	-0,13723038	0,89084869	1	protein_codir	hypothetical protein
TcG_11629	7,421082752	0,374465898	0,68116555	0,54974286	0,58249576	1	protein_codir	dispersed protein family protein 1 (DGF-1)
TcG_11630	36,53617477	0,013585811	0,29352088	0,04628567	0,96308256	0,98319451	protein_codir	dispersed gene family protein 1 (DGF-1)
TcG_11631	10,93224368	-0,15940736	0,54616515	-0,29186659	0,77038863	1	protein_codir	dispersed protein family protein 1 (DGF-1)
TcG_11632	0,350783209	1,758746969	2,99002236	0,58820529	0,55639451	1		
TcG_11633	472,0553574	-0,250981531	0,09265432	-2,70879481	0,00675281	0,03632204	protein_codir	retrotransposon hot spot (RHS) protein
TcG_11634	228,9658918	-0,427479192	0,12094864	-3,53438615	0,00040872	0,00363987	protein_codir	retrotransposon hot spot (RHS) protein
TcG_11635	5,798886697	0,553750015	0,77655348	0,71308677	0,47579206	1	protein_codir	hypothetical protein
TcG_11636	20,1211731	-0,646678622	0,39715755	-1,62826721	0,10346824	0,27551896	protein_codir	hypothetical protein
TcG_11637	19,60021427	0,140646125	0,38876819	0,36177374	0,71752111	0,85508792	protein_codir	hypothetical protein
TcG_11638	5,092005654	-0,841760051	0,78233798	-1,07595448	0,28194762	1	protein_codir	hypothetical protein
TcG_11639	4,8754714	-1,438492703	0,84553648	-1,70127812	0,08889078	1	protein_codir	hypothetical protein
TcG_11640	69,56925044	-0,522397905	0,2148278	-2,43170535	0,01502793	0,06780123	protein_codir	mucin-associated surface protein (MASP)
TcG_11641	29,22408493	-0,020003629	0,32512938	-0,06152514	0,950941	0,97699764	protein_codir	90 kDa surface protein
TcG_11642	11,72443405	-0,191007095	0,5407638	-0,35321723	0,72392559	1	protein_codir	hypothetical protein
TcG_11643	1,173085208	-1,451105745	1,872188	-0,77508549	0,43828915	1	protein_codir	prolyl-tRNA synthetase, putative,bifunctional aminoacyl-tRNA synthetase
TcG_11644	1,337413998	-2,059721941	1,64788132	-1,24992129	0,2113283	1	protein_codir	ubiquitin-protein ligase-like
TcG_11645	1,293296123	0,758805081	1,58202635	0,47964124	0,63148252	1	protein_codir	reverse transcriptase endonuclease

TcG_11646	0,598023686	1,027640128	2,24558912	0,45762607	0,64722111	1	protein_codir	reverse transcriptase endonuclease
TcG_11647	0					1	protein_codir	hypothetical protein
TcG_11648	0,27291574	1,35431792	3,0630933	0,4421406	0,65838746	1	protein_codir	dispersed protein family protein 1
TcG_11649	14,53401511	-0,140679906	0,47113073	-0,29860057	0,76524483	1	protein_codir	dispersed gene family protein 1 (DGF-1)
TcG_11650	1,270995463	-1,145780573	1,6187242	-0,7078294	0,47905121	1	protein_codir	dispersed protein family protein 1
TcG_11651	0,402975414	0,247428563	2,72600293	0,09076607	0,92767847	1	protein_codir	putative dispersed gene family protein 1 (DGF-1)
TcG_11652	0,155988004	0,503022807	4,08047286	0,12327562	0,90188885	1	protein_codir	dispersed gene family protein 1 (DGF-1)
TcG_11653	0					1		
TcG_11654	75,50523751	-0,259993528	0,20261749	-1,28317413	0,19943108	0,41820968	protein_codir	mucin TcMUCII
TcG_11655	68,91617715	0,138362369	0,23022306	0,60099266	0,54784487	0,74464227	protein_codir	mucin-associated surface protein (MASP)
TcG_11656	11,71263648	0,168759592	0,50432514	0,33462459	0,73790829	1	protein_codir	hypothetical protein
TcG_11657	12,74693345	-0,698971251	0,48568329	-1,4391503	0,15010794	1	protein_codir	hypothetical protein
TcG_11658	6,321509582	0,099444559	0,69245184	0,14361224	0,88580668	1		
TcG_11659	94,62502109	-0,56561976	0,18211363	-3,1058618	0,00189725	0,01318631	protein_codir	mucin-associated surface protein (MASP)
TcG_11660	34,3878179	-0,287626865	0,30286581	-0,94968417	0,34227275	0,57657632	protein_codir	hypothetical protein
TcG_11661	10,33980793	-0,018964366	0,53012116	-0,03577365	0,97146285	1	protein_codir	hypothetical protein
TcG_11662	20,7199211	0,434642312	0,38418794	1,13132731	0,25791735	0,49019529	protein_codir	hypothetical protein
TcG_11663	43,68494929	0,285175703	0,28042575	1,01693835	0,30918276	0,54234541	protein_codir	putative trans-sialidase
TcG_11664	3,981284823	0,002871564	0,86947956	0,00330262	0,99736489	1	protein_codir	structural maintenance of chromosome protein 4
TcG_11665	13,43134634	0,411547373	0,48343643	0,85129574	0,39460509	1	protein_codir	hypothetical protein
TcG_11666	7,539562937	-0,548000348	0,64002026	-0,85622344	0,3918742	1	protein_codir	hypothetical protein
TcG_11667	3,930788946	-0,33541728	0,87246931	-0,38444594	0,70064796	1	protein_codir	hypothetical protein
TcG_11668	0,300145286	-1,420535481	3,2277253	-0,44010421	0,65986164	1	protein_codir	hypothetical protein
TcG_11669	0,652611499	-3,135370342	2,25081891	-1,39299093	0,16362253	1	protein_codir	hypothetical protein
TcG_11670	3,716539566	-1,379415283	1,00176622	-1,37698322	0,16851744	1	protein_codir	hypothetical protein
TcG_11671	6,194558121	-0,009389498	0,68076841	-0,0137925	0,98899553	1	protein_codir	hypothetical protein
TcG_11672	6,055205379	-0,392453	0,72242753	-0,54324204	0,58696317	1	protein_codir	hypothetical protein
TcG_11673	30,71746717	-0,420006148	0,32395849	-1,29648137	0,19480969	0,41217405	protein_codir	mucin TcMUCII
TcG_11674	58,78049394	-0,164973663	0,24773652	-0,66592388	0,50545976	0,71252668	protein_codir	mucin-associated surface protein (MASP)
TcG_11675	53,67306321	0,452446676	0,24331374	1,85951961	0,06295352	0,19649233		
TcG_11676	14,89767255	0,267988867	0,45274814	0,591916	0,55390684	1	protein_codir	hypothetical protein
TcG_11677	208,4405484	0,025327138	0,12239232	0,20693405	0,83606137	0,92052325	protein_codir	Alpha-(1,3)-fucosyltransferase, family GT10
TcG_11678	943,2706963	-0,091587328	0,0681438	-1,3440303	0,17893859	0,38979933	protein_codir	MGT2 magnesium transporter
TcG_11679	77,7724227	0,036522105	0,20187849	0,18091132	0,85643718	0,93057125		
TcG_11680	124,575857	0,099923167	0,16988613	0,58817731	0,55641328	0,75122326	protein_codir	mucin-associated surface protein (MASP)
TcG_11681	19,35583711	0,216978972	0,40944916	0,52992897	0,59616118	0,78066886	protein_codir	hypothetical protein
TcG_11682	27,74340804	0,060559994	0,3329402	0,18189451	0,85566551	0,93016895	protein_codir	hypothetical protein
TcG_11683	21,7570422	-0,185635161	0,3718669	-0,49919786	0,61764	0,793034	protein_codir	trans-sialidase
TcG_11684	59,46443888	0,003893228	0,22817609	0,01706238	0,98638685	0,99463124	protein_codir	putative trans-sialidase
TcG_11685	43,1743701	-0,200823462	0,26224009	-0,76579999	0,44379532	0,66603789	protein_codir	putative retrotransposon hot spot (RHS) protein
TcG_11686	52,34332676	-0,36721731	0,24333419	-1,5091069	0,13127147	0,32209047		
TcG_11687	9,411037262	-0,362877987	0,57787484	-0,62795256	0,53003501	1	protein_codir	hypothetical protein
TcG_11688	2,178135921	-0,416460897	1,18254335	-0,35217389	0,72470785	1	protein_codir	hypothetical protein
TcG_11689	1,372731198	-0,84402288	1,54699324	-0,54558925	0,58534832	1	protein_codir	hypothetical protein
TcG_11690	6,283423932	0,091566515	0,76100762	0,12032273	0,9042275	1	protein_codir	hypothetical protein
TcG_11691	227,8670832	-0,221507905	0,12368227	-1,79094309	0,07330243	0,21810014	protein_codir	mucin-associated surface protein (MASP)
TcG_11692	34,4104159	-0,053934961	0,29577779	-0,1823496	0,85530838	0,92986796	protein_codir	hypothetical protein
TcG_11693	12,0389743	0,165704304	0,52246145	0,31716083	0,75112157	1	protein_codir	mucin-associated surface protein (MASP)
TcG_11694	4,988602358	0,63186134	0,83737799	0,75457123	0,45050629	1	protein_codir	mucin TcMUCII
TcG_11695	5,091068175	0,281054147	0,78965544	0,35591998	0,72190049	1		
TcG_11696	2,433868111	0,647631852	1,21958845	0,53102491	0,59540152	1	protein_codir	hypothetical protein
TcG_11697	2,560424525	-1,066877494	1,08680119	-0,98166758	0,32626364	1		

TcG_11698	6,117749035	1,556351773	0,76226296	2,0417518	0,04117616	1	protein_codir	mucin-associated surface protein (MASP)
TcG_11699	266,1327386	0,270268296	0,11130935	2,4280827	0,01517888	0,06821665	protein_codir	retrotransposon hot spot (RHS) protein
TcG_11700	2,954981053	0,895342489	1,05855311	0,84581726	0,3976547	1		
TcG_11701	168,4760793	-0,224850858	0,13568341	-1,65717279	0,09748455	0,26413843	protein_codir	retrotransposon hot spot (RHS) protein
TcG_11702	7,257135816	-0,050022238	0,67246154	-0,07438676	0,94070264	1	protein_codir	hypothetical protein
TcG_11703	4,974213905	0,650759018	0,79624567	0,81728421	0,41376603	1	protein_codir	hypothetical protein
TcG_11704	8,483832321	-0,502904145	0,60329144	-0,83360066	0,40450605	1		
TcG_11705	236,266054	-0,286254367	0,12049747	-2,37560483	0,01752021	0,0759406	protein_codir	putative mucin-associated surface protein (MASP)
TcG_11706	39,9472765	-0,136499035	0,28312393	-0,48211762	0,62972239	0,80168887	protein_codir	elongation factor 1-alpha (EF-1-alpha)
TcG_11707	58,91508489	-0,195038298	0,22935652	-0,85037173	0,39511845	0,62462032		
TcG_11708	117,5233293	0,213601738	0,16649332	1,28294476	0,19951143	0,41830246	protein_codir	mucin TcMUCII
TcG_11709	23,68824596	-0,287774532	0,35339591	-0,81431201	0,41546623	0,64239863		
TcG_11710	0					1	protein_codir	thimet oligopeptidase
TcG_11711	7,756397141	-0,554190372	0,62860758	-0,88161579	0,37798461	1		
TcG_11712	1,945778901	-0,678011498	1,25820268	-0,53887304	0,58997446	1		
TcG_11713	9,560640084	0,138911057	0,55825397	0,24883129	0,80349129	1	protein_codir	trans-sialidase
TcG_11714	43,50511786	0,284769552	0,26277019	1,08372092	0,27848856	0,51134206	protein_codir	trans-sialidase
TcG_11715	14,77698199	-0,352437286	0,45273117	-0,77846923	0,43629244	1	protein_codir	trans-sialidase
TcG_11716	55,91367042	0,122125591	0,25126455	0,48604385	0,62693608	0,79978874	protein_codir	dispersed gene family protein 1 (DGF-1)
TcG_11717	74,92105977	0,42951454	0,21546809	1,99340209	0,04621743	0,15643447	protein_codir	dispersed gene family protein 1 (DGF-1)
TcG_11718	114,4708585	0,307655584	0,17888766	1,7198256	0,08546415	0,24133259	protein_codir	stage-specific surface protein GP82
TcG_11719	191,0716294	0,109419756	0,12725818	0,85982494	0,38988555	0,6201653	protein_codir	trans-sialidase
TcG_11720	470,7151897	0,053365435	0,09470585	0,56348615	0,57310389	0,76427044		
TcG_11721	297,230676	-0,023425606	0,11352828	-0,20634159	0,8365241	0,92063564	protein_codir	retrotransposon hot spot (RHS) protein
TcG_11722	102,7034157	0,293577001	0,17997067	1,63124911	0,10283777	0,27421826	protein_codir	hypothetical protein
TcG_11723	202,3754356	0,09091632	0,12621275	0,72034178	0,47131459	0,68730659	protein_codir	putative retrotransposon hot spot (RHS) protein
TcG_11724	94,5412365	-0,235745166	0,18099712	-1,30248021	0,19275228	0,40931596	protein_codir	retrotransposon hot spot (RHS) protein
TcG_11725	39,29729969	-0,222061576	0,28679995	-0,77427341	0,43876912	0,66212613	protein_codir	hypothetical protein
TcG_11726	90,57638028	-0,091394628	0,18871036	-0,48431167	0,62816469	0,80069291	protein_codir	hypothetical protein
TcG_11727	298,5170312	0,124913303	0,10562941	1,18256174	0,23698288	0,46458901	protein_codir	putative UDP-Gal or UDP-GlcNAc-dependent glycosyltransferase
TcG_11728	37,6804285	0,227490694	0,29112223	0,78142674	0,43455155	0,65873534		
TcG_11729	31,74612401	-0,092735733	0,30614082	-0,30291855	0,76195194	0,87977897		
TcG_11730	26,09720169	0,063736031	0,34124631	0,18677427	0,85183762	0,92822975	protein_codir	hypothetical protein
TcG_11731	47,53159213	-0,080156915	0,26355922	-0,30413247	0,76102697	0,87939833	protein_codir	retrotransposon hot spot (RHS) protein
TcG_11732	280,3985675	0,043494485	0,12019593	0,36186321	0,71745425	0,85508792	protein_codir	retrotransposon hot spot (RHS) protein
TcG_11733	18,14577461	0,148246041	0,40821269	0,36315883	0,71648624	0,85441337	protein_codir	retrotransposon hot spot (RHS) protein
TcG_11734	4,538540783	0,631848879	0,88351783	0,71515125	0,47451559	1	protein_codir	putative retrotransposon hot spot (RHS) protein
TcG_11735	11,37755536	0,887205813	0,53056394	1,67219395	0,09448608	1	protein_codir	retrotransposon hot spot protein (RHS)
TcG_11736	19,69187481	0,32954229	0,40140709	0,82096778	0,41166462	0,63815176	protein_codir	retrotransposon hot spot (RHS) protein
TcG_11737	7,571045645	0,296697406	0,63690868	0,46583979	0,64133018	1	protein_codir	retrotransposon hot spot (RHS) protein
TcG_11738	11,37599551	0,177511716	0,50944374	0,34844224	0,72750809	1	protein_codir	retrotransposon hot spot protein (RHS)
TcG_11739	119,4190774	0,107392302	0,15935314	0,67392649	0,50035805	0,70926123	protein_codir	putative retrotransposon hot spot (RHS) protein
TcG_11740	85,61377785	-0,010771894	0,18808543	-0,05727128	0,9543291	0,97874276	protein_codir	putative retrotransposon hot spot (RHS) protein
TcG_11741	36,02483471	0,184872377	0,29574517	0,625107	0,53190084	0,7329332	protein_codir	retrotransposon hot spot (RHS) protein
TcG_11742	50,44413587	-0,043973926	0,25377533	-0,17327896	0,86243216	0,93414012	protein_codir	hypothetical protein
TcG_11743	22,35165054	0,475326684	0,38238196	1,24306776	0,21384286	0,43745922	protein_codir	hypothetical protein
TcG_11744	50,42178485	-0,127536991	0,26009301	-0,49035147	0,62388521	0,79706621	protein_codir	trans-sialidase
TcG_11745	42,74470583	0,256968747	0,26837494	0,95749905	0,33831543	0,57289134	protein_codir	ATP-dependent DEAD/H RNA helicase
TcG_11746	9,982042602	-0,359309027	0,56251256	-0,63875735	0,5229808	1		
TcG_11747	35,9046889	0,319107778	0,2930934	1,08875799	0,27626062	0,5087364	protein_codir	dispersed gene family protein 1 (DGF-1)
TcG_11748	79,55976997	-0,236662941	0,20282448	-1,16683615	0,24327654	0,47157471	protein_codir	dispersed gene family protein 1 (DGF-1)
TcG_11749	44,8676664	-0,317902768	0,2629327	-1,20906518	0,22663781	0,45327561	protein_codir	trans-sialidase

TcG_11750	54,21962152	0,082330384	0,24942014	0,33008716	0,74133411	0,86827896	protein_codir	putative mucin-associated surface protein (MASP)
TcG_11751	266,6869549	0,102628233	0,12723678	0,80659249	0,4199013	0,64656135	protein_codir	structural maintenance of chromosome protein 4
TcG_11752	32,5978902	0,396128064	0,30637627	1,29294631	0,19602963	0,41399914	protein_codir	hypothetical protein
TcG_11753	2,44474867	1,292537927	1,20063035	1,07654943	0,28168161	1	protein_codir	hypothetical protein
TcG_11754	1,431430128	2,612905746	1,65497652	1,57881741	0,11437794	1	protein_codir	hypothetical protein
TcG_11755	4,865205194	0,136807776	0,82985312	0,16485782	0,86905589	1	protein_codir	hypothetical protein
TcG_11756	4,394504108	-0,557321679	0,83553113	-0,66702683	0,504755	1		
TcG_11757	0,155988004	0,503022807	4,08047286	0,12327562	0,90188885	1		
TcG_11758	59,60293898	-0,32744792	0,23405681	-1,39901046	0,16180985	0,36551548	protein_codir	hypothetical protein
TcG_11759	17,92334326	0,423570693	0,41360333	1,02409885	0,30578859	0,53883629	protein_codir	hypothetical protein
TcG_11760	40,93669807	-0,369892941	0,28237986	-1,30991262	0,1902254	0,40551085	protein_codir	hypothetical protein
TcG_11761	14,16645714	-0,38563125	0,49971035	-0,77170956	0,44028647	1	protein_codir	hypothetical protein
TcG_11762	0,874862318	0,728287021	1,85947535	0,39166264	0,6953075	1	protein_codir	trans-sialidase
TcG_11763	4,849422749	-0,154965234	0,81993472	-0,18899704	0,85009514	1	protein_codir	hypothetical protein
TcG_11764	3,110334553	-0,301688578	1,09576688	-0,27532186	0,78306897	1	protein_codir	hypothetical protein
TcG_11765	1,204439259	-0,005291937	1,69072387	-0,00312998	0,99750264	1	protein_codir	hypothetical protein
TcG_11766	36,13278629	0,14938009	0,29108944	0,51317591	0,60782827	0,78802211	protein_codir	mucin-associated surface protein (MASP)
TcG_11767	32,9633378	-0,956646357	0,30829923	-3,10298	0,00191583	0,01329147	protein_codir	mucin TcMUCII
TcG_11768	0,783636844	-3,302230925	2,03495429	-1,62275435	0,10464193	1	protein_codir	hypothetical protein
TcG_11769	1,399248139	-3,05906022	1,69180526	-1,80816333	0,07058108	1	protein_codir	hypothetical protein
TcG_11770	5,973188951	-0,923772689	0,76122357	-1,21353664	0,22492472	1	protein_codir	hypothetical protein
TcG_11771	45,15253804	0,441769048	0,25925678	1,70398264	0,08838433	0,24704966	protein_codir	surface protease GP63
TcG_11772	13,40387522	0,216156203	0,49000909	0,44112692	0,65912112	1	protein_codir	hypothetical protein
TcG_11773	7,682764847	0,212356485	0,63527634	0,33427419	0,73817267	1	protein_codir	putative syntaxin binding protein
TcG_11774	18,83774687	-0,53071713	0,39491304	-1,34388353	0,17898605	0,38979933	protein_codir	putative syntaxin binding protein
TcG_11775	17,67777028	0,280537529	0,41041521	0,68354565	0,49426212	0,70453824	protein_codir	hypothetical protein
TcG_11776	57,8921318	-0,311483717	0,22645897	-1,37545319	0,16899099	0,37544192	protein_codir	trans-sialidase
TcG_11777	25,2013386	0,670762192	0,35054753	1,91347001	0,05568791	0,17850508	protein_codir	nucleoside deoxyribosyltransferase
TcG_11778	7,969083227	0,82174055	0,65146517	1,26137297	0,2071745	1	protein_codir	hypothetical protein
TcG_11779	38,50028063	0,013555832	0,28067057	0,04829802	0,96147874	0,98216299	protein_codir	trans-sialidase
TcG_11780	109,0897724	0,286977316	0,1780449	1,61182554	0,10699991	0,28208285	protein_codir	trans-sialidase
TcG_11781	4,969979988	0,49276346	0,78489085	0,62781144	0,53012747	1	protein_codir	trans-sialidase
TcG_11782	16,85697455	-0,424375029	0,42839257	-0,99062182	0,32187028	0,55667847	protein_codir	structural maintenance of chromosome protein 4
TcG_11783	4,207727403	0,703952516	0,86439339	0,81438906	0,41542211	1		
TcG_11784	875,3700087	-0,44488547	0,06588355	-6,75260362	1,4522E-11	8,9019E-10	protein_codir	hypothetical protein
TcG_11785	181,8579619	-0,541133678	0,13203844	-4,09830421	4,1619E-05	0,00052527	protein_codir	type IB topoisomerase small subunit
TcG_11786	20,14274316	-0,607660855	0,38908388	-1,56177338	0,11834138	0,30048286	protein_codir	hypothetical protein
TcG_11787	1848,301497	0,274339347	0,06581942	4,16806084	3,072E-05	0,00040354	protein_codir	L1Tc protein
TcG_11788	7,024681384	0,08292207	0,65833338	0,12595757	0,89976552	1	protein_codir	hypothetical protein
TcG_11789	18,41599938	0,67222413	0,42255454	1,59085766	0,11164161	0,28930434	protein_codir	elongation factor 1-gamma (EF-1-gamma)
TcG_11790	57,65064303	-0,128102095	0,23609021	-0,54259809	0,58740656	0,77564856	protein_codir	putative mucin-associated surface protein (MASP)
TcG_11791	20,26312016	-0,027408936	0,38749157	-0,07073428	0,94360924	0,97337158	protein_codir	mucin TcMUCII
TcG_11792	0					1	protein_codir	hypothetical protein
TcG_11793	30,7789874	0,413716875	0,34820417	1,18814452	0,23477648	0,46268417	protein_codir	hypothetical protein
TcG_11794	11,47553606	-0,199011793	0,58465594	-0,3403913	0,73356187	1	protein_codir	target of rapamycin (TOR) kinase 1
TcG_11795	5,330598632	0,115852149	0,75059509	0,15434707	0,87733609	1	protein_codir	hypothetical protein
TcG_11796	34,9090982	0,365906359	0,29838785	1,22627768	0,2200942	0,44520638	protein_codir	selenocysteine-tRNA-specific elongation factor
TcG_11797	0,753745299	1,464639247	1,99808055	0,73302312	0,46354433	1	protein_codir	retrotransposon hot spot (RHS) protein
TcG_11798	7,518067593	0,435843608	0,6603951	0,65997401	0,50927051	1	protein_codir	retrotransposon hot spot (RHS) protein
TcG_11799	17,06947198	0,257886985	0,42477073	0,60712042	0,543771	0,74093036	protein_codir	retrotransposon hot spot (RHS) protein
TcG_11800	3,025497991	-1,30247146	1,12864954	-1,15400876	0,24849658	1	protein_codir	hypothetical protein
TcG_11801	2,010909544	0,114243885	1,27041979	0,08992609	0,92834595	1	protein_codir	hypothetical protein

TcG_11802	1,631536006	-0,013507203	1,33750958	-0,01009877	0,99194248	1	protein_codir trans-sialidase
TcG_11803	250,6988076	0,293068125	0,11599778	2,52649764	0,01152062	0,0552246	protein_codir hypothetical protein
TcG_11804	316,3635343	0,565578216	0,10489994	5,39159693	6,9834E-08	1,8773E-06	protein_codir hypothetical protein
TcG_11805	45,92075209	0,8644604	0,27447	3,14956247	0,00163515	0,01169851	
TcG_11806	3,430298389	-0,341929743	0,92447405	-0,36986408	0,71148377	1	protein_codir hypothetical protein
TcG_11807	0,155721614	0,503022807	4,08047286	0,12327562	0,90188885	1	protein_codir hypothetical protein
TcG_11808	14,73764915	-0,245861544	0,46304515	-0,53096667	0,59544187	1	protein_codir hypothetical protein
TcG_11809	35,04194889	0,311550298	0,30498775	1,02151743	0,30700936	0,54008661	protein_codir elongation factor 1-gamma (EF-1-gamma)
TcG_11810	45,14345305	0,419505847	0,26166059	1,6032443	0,10888073	0,28488982	protein_codir mucin-associated surface protein (MASP)
TcG_11811	119,6864059	-0,365078718	0,16212728	-2,25180312	0,02433472	0,09782343	
TcG_11812	68,94309242	-0,047367731	0,22517671	-0,21035804	0,83338824	0,91912273	protein_codir putative trans-sialidase
TcG_11813	1086,667202	0,136423793	0,05968995	2,28554028	0,02228117	0,09141275	protein_codir putative retrotransposon hot spot (RHS) protein
TcG_11814	16,04511726	0,527948045	0,44074267	1,19786007	0,2309715	1	protein_codir mucin TcMUCII
TcG_11815	5,522132448	0,404383724	0,74521603	0,5426396	0,58737797	1	protein_codir hypothetical protein
TcG_11816	0,300973289	-0,458748581	3,01449841	-0,15218073	0,87904439	1	protein_codir hypothetical protein
TcG_11817	0,507359824	-2,754314599	2,63041628	-1,04710217	0,29505246	1	protein_codir hypothetical protein
TcG_11818	1,046644258	-1,603153737	1,76336528	-0,90914444	0,36327389	1	protein_codir hypothetical protein
TcG_11819	423,8562649	0,047058596	0,09051903	0,51987517	0,60315058	0,78522521	protein_codir trans-sialidase
TcG_11820	40,59354646	0,201671853	0,27555028	0,73188768	0,46423713	0,68170486	protein_codir hypothetical protein
TcG_11821	40,04102898	-0,177585285	0,27435339	-0,64728664	0,51744645	0,7228279	protein_codir hypothetical protein
TcG_11822	4,762796761	1,078586638	0,8230869	1,31041648	0,19005498	1	
TcG_11823	22,71610847	-0,195861783	0,37035321	-0,52885131	0,5969086	0,78091497	protein_codir putative surface protease GP63
TcG_11824	13,4048127	-0,220030668	0,49194912	-0,44726306	0,65468514	1	protein_codir hypothetical protein
TcG_11825	244,5216196	0,11789698	0,1207874	0,97607017	0,32902969	0,56342566	protein_codir putative ferric reductase
TcG_11826	230,6524921	0,076387723	0,12399953	0,61603234	0,53787317	0,73738082	protein_codir ferric reductase
TcG_11827	98,83242984	-0,231644275	0,17512954	-1,32270247	0,18593434	0,39987989	protein_codir amastigote surface protein 4
TcG_11828	6,896731108	-1,002104353	0,68824424	-1,45603014	0,14538429	1	protein_codir trans-sialidase
TcG_11829	5,747642888	-0,862264072	0,72049124	-1,19677246	0,23139525	1	protein_codir hypothetical protein
TcG_11830	6,01174463	0,13468282	0,70517952	0,19099083	0,84853278	1	protein_codir mucin-associated surface protein (MASP)
TcG_11831	16,35935584	0,135836688	0,43193235	0,31448602	0,75315193	1	protein_codir trans-sialidase
TcG_11832	91,63607124	-0,100637352	0,19667336	-0,51169794	0,60886243	0,788218	protein_codir trans-sialidase
TcG_11833	148,6390539	-0,052765966	0,14368123	-0,36724326	0,71343757	0,85278452	protein_codir trans-sialidase
TcG_11834	12,11423129	-0,341718813	0,50443905	-0,67742339	0,49813736	1	
TcG_11835	179,3186581	-0,231882981	0,14321874	-1,6190827	0,10542947	0,27920134	protein_codir hypothetical protein
TcG_11836	187,209992	0,205230719	0,13534792	1,51631972	0,12943853	0,3191072	protein_codir target of rapamycin (TOR) kinase 1
TcG_11837	306,5270753	0,192662577	0,10788013	1,78589494	0,07411627	0,21984411	protein_codir SH3 domain protein
TcG_11838	248,6701898	0,148769797	0,11598576	1,28265567	0,19961274	0,41843915	protein_codir structural maintenance of chromosome protein 4
TcG_11839	106,0014196	0,331472837	0,18679704	1,77450794	0,07597917	0,2229723	protein_codir hypothetical protein
TcG_11840	10,45171372	-0,072817337	0,54230177	-0,13427457	0,89318546	1	protein_codir putative amino acid transporter
TcG_11841	3,447857167	-1,071258598	0,94239649	-1,13673873	0,25564752	1	protein_codir syntaxin binding protein
TcG_11842	5,319134256	-1,087868759	0,78185014	-1,39140316	0,16410321	1	protein_codir hypothetical protein
TcG_11843	2,740966401	0,709331333	1,10868124	0,63979736	0,52230435	1	protein_codir putative syntaxin binding protein
TcG_11844	3,546269401	-0,505931459	0,92847042	-0,54490854	0,58581642	1	protein_codir surface protease GP63
TcG_11845	3,135854161	-0,220507721	0,97602438	-0,2259244	0,82126022	1	protein_codir hypothetical protein
TcG_11846	7,286394761	-0,846963438	0,67924158	-1,2469252	0,21242493	1	protein_codir hypothetical protein
TcG_11847	9,011474258	0,241037073	0,58575314	0,41149941	0,68070637	1	protein_codir hypothetical protein
TcG_11848	25,59478722	-0,658054297	0,36001362	-1,82785944	0,06757065	0,20694516	protein_codir trans-sialidase
TcG_11849	65,46205976	-0,685551273	0,22316534	-3,07194329	0,0021267	0,01446856	protein_codir putative trans-sialidase
TcG_11850	53,05686531	-0,178022098	0,24161768	-0,73679252	0,46124853	0,67929148	protein_codir putative trans-sialidase
TcG_11851	31,24108662	-0,16035813	0,31460399	-0,50971423	0,61025168	0,7890164	protein_codir trans-sialidase
TcG_11852	6,638565467	-0,387625056	0,67529469	-0,57400875	0,56596188	1	protein_codir surface protease GP63
TcG_11853	24,34487731	0,384801862	0,35562868	1,08203272	0,27923799	0,51214997	protein_codir mucin-like glycoprotein

TcG_11854	17,42999766	-0,016587177	0,44016133	-0,03768431	0,96993938	0,98703399	protein_codir	hypothetical protein
TcG_11855	14,53282304	0,336649091	0,46645818	0,72171333	0,47047074	1	protein_codir	trans-sialidase
TcG_11856	13,22818216	0,027621649	0,47287525	0,05841213	0,95342036	1	protein_codir	Sialidase 85-1.3
TcG_11857	23,78213311	0,209630891	0,35895068	0,58401029	0,55921339	0,75381574		
TcG_11858	28,11896853	0,47384726	0,35865239	1,32118807	0,18643866	0,40038787	protein_codir	hypothetical protein
TcG_11859	26,52170707	0,436209145	0,35976725	1,21247598	0,22533023	0,45182011	protein_codir	hypothetical protein
TcG_11860	49,90049109	0,458549233	0,2500647	1,8337224	0,06669521	0,20510851	protein_codir	hypothetical protein
TcG_11861	45,95655711	0,618072152	0,26152398	2,36334789	0,01811066	0,0778013	protein_codir	hypothetical protein
TcG_11862	50,57390068	0,324048965	0,26732357	1,21219751	0,22543678	0,45182011	protein_codir	trans-sialidase
TcG_11863	18,42546288	-0,743609625	0,41954998	-1,77239819	0,07632848	0,22348794	protein_codir	hypothetical protein
TcG_11864	5,087407936	-0,812932369	0,76173352	-1,0672136	0,28587539	1	protein_codir	trans-sialidase
TcG_11865	10,77605844	-0,48062296	0,53566954	-0,89723781	0,36959203	1	protein_codir	putative trans-sialidase
TcG_11866	4,387688838	-2,403863207	0,94920657	-2,53249743	0,01132532	1		
TcG_11867	18,80331718	-0,160741876	0,39983321	-0,40202232	0,6876676	0,83778305	protein_codir	hypothetical protein
TcG_11868	8,518338285	0,298991491	0,61723886	0,4844016	0,62810088	1	protein_codir	mucin-associated surface protein (MASP)
TcG_11869	46,628073	-0,476994308	0,26582178	-1,79441393	0,07274712	0,21694934	protein_codir	hypothetical protein
TcG_11870	2,903695016	-1,214438831	1,10508781	-1,09895234	0,27178885	1	protein_codir	hypothetical protein
TcG_11871	11,03765828	-0,030329859	0,52549471	-0,05771677	0,95397423	1	protein_codir	trans-sialidase
TcG_11872	3,956422054	-0,25085572	0,86899343	-0,2886739	0,77283094	1	protein_codir	hypothetical protein
TcG_11873	24,61273078	0,522502501	0,35276557	1,4811607	0,13856376	0,33238817	protein_codir	hypothetical protein
TcG_11874	123,5104589	0,048707118	0,15852743	0,30724726	0,75865517	0,87801518	protein_codir	retrotransposon hot spot (RHS) protein
TcG_11875	27,61489167	0,273208139	0,35142737	0,77742419	0,43690854	0,66040735	protein_codir	putative retrotransposon hot spot (RHS) protein
TcG_11876	196,4229137	0,1368205	0,12943223	1,05708211	0,29047412	0,52345679	protein_codir	retrotransposon hot spot (RHS) protein
TcG_11877	525,4137824	-0,029426301	0,08727053	-0,33718486	0,73597755	0,86494473	protein_codir	protein kinase
TcG_11878	543,826191	0,275319777	0,08024748	3,43088387	0,00060162	0,00505322	protein_codir	glycine dehydrogenase
TcG_11879	615,938107	0,136009182	0,08455925	1,6084482	0,10773705	0,28272756	protein_codir	glycine dehydrogenase
TcG_11880	147,0974169	0,191677176	0,14596885	1,31313756	0,1891366	0,40378416	protein_codir	glycine dehydrogenase, putative,glycine cleavage system P-protein
TcG_11881	1419,306825	0,05345898	0,05538716	0,96518728	0,33445102	0,56893684	protein_codir	putative glycine dehydrogenase, putative,glycine cleavage system P-protein
TcG_11882	1,966594433	0,456441531	1,29805302	0,35163551	0,72511163	1	protein_codir	putative GDP-mannose 4,6 dehydratase
TcG_11883	185,0969903	-0,150569136	0,13184086	-1,14205214	0,25343235	0,48445481	protein_codir	hypothetical protein
TcG_11884	180,1519629	-0,443703117	0,13208184	-3,3593046	0,00078139	0,00633861	protein_codir	putative SNARE protein
TcG_11885	38,63177588	0,146720758	0,28480712	0,51515832	0,60644239	0,78734217	protein_codir	retrotransposon hot spot (RHS) protein
TcG_11886	13,77132937	0,291395196	0,46745047	0,62337127	0,53304057	1	protein_codir	retrotransposon hot spot (RHS) protein
TcG_11887	23,0035086	0,330127447	0,37243221	0,8864095	0,3753969	0,60592763	protein_codir	retrotransposon hot spot (RHS) protein
TcG_11888	34,23624736	-0,076848263	0,29594267	-0,2596728	0,79511617	0,89770577	protein_codir	trans-sialidase
TcG_11889	5,468437479	-0,439070164	0,72675171	-0,60415429	0,54574107	1	protein_codir	trans-sialidase
TcG_11890	4,962070325	-0,887781761	0,78764579	-1,12713326	0,25968614	1	protein_codir	trans-sialidase
TcG_11891	15,24823221	-0,601047516	0,4550778	-1,32075773	0,18658216	1	protein_codir	Sialidase 85-1.3
TcG_11892	30,56753925	-0,746120806	0,35716538	-2,08900649	0,03670714	0,13323587	protein_codir	putative elongation factor 1-gamma (EF-1-gamma)
TcG_11893	110,8283771	-0,50656884	0,16826514	-3,01053935	0,00260784	0,01697441	protein_codir	putative mucin-associated surface protein (MASP)
TcG_11894	79,50414119	0,076619283	0,20332612	0,37682951	0,70630031	0,84872764	protein_codir	dispersed gene family protein 1 (DGF-1)
TcG_11895	31,96048618	0,278950186	0,32572221	0,85640517	0,39177371	0,62139829	protein_codir	dispersed protein family protein 1 (DGF-1)
TcG_11896	18,83934792	0,802737666	0,41229553	1,9469958	0,05153524	0,16876407	protein_codir	hypothetical protein
TcG_11897	32,72410575	-0,219872668	0,30846052	-0,71280652	0,47596549	0,6913912	protein_codir	trans-sialidase
TcG_11898	5,142535026	-0,236642184	0,8255111	-0,28666142	0,77437158	1	protein_codir	hypothetical protein
TcG_11899	2,534101082	-1,797222286	1,17301584	-1,53213812	0,12548835	1	protein_codir	hypothetical protein
TcG_11900	94,66883299	-0,248626336	0,17948935	-1,38518709	0,16599527	0,37163694	protein_codir	retrotransposon hot spot (RHS) protein
TcG_11901	280,284424	-0,207827512	0,10681207	-1,9457306	0,05168711	0,16910378	protein_codir	retrotransposon hot spot (RHS) protein
TcG_11902	38,4410133	-0,220215132	0,28253552	-0,77942459	0,43572964	0,65939964	protein_codir	hypothetical protein
TcG_11903	27,62842011	-0,673507421	0,34130986	-1,97330195	0,04846117	0,16176063	protein_codir	retrotransposon hot spot (RHS) protein
TcG_11904	220,3038287	-0,015425071	0,12058635	-0,12791723	0,89821448	0,95029796	protein_codir	retrotransposon hot spot (RHS) protein
TcG_11905	0,467431231	2,090445105	2,51848818	0,83003967	0,40651635	1	protein_codir	dispersed gene family protein 1 (DGF-1)

TcG_11906	0					1	protein_codir	hypothetical protein
TcG_11907	0					1	protein_codir	trans-sialidase
TcG_11908	0					1	protein_codir	putative trans-sialidase
TcG_11909	59,56071776	-0,612733945	0,22429019	-2,73188033	0,0062974	0,03430263	protein_codir	mucin TcMUCII
TcG_11910	3,818499185	-0,309894614	0,92656286	-0,33445611	0,7380354	1	protein_codir	hypothetical protein
TcG_11911	10,80935725	-0,045222037	0,57934007	-0,07805784	0,93778204	1		
TcG_11912	6,039407131	-0,798168613	0,71474027	-1,1167254	0,26411175	1	protein_codir	hypothetical protein
TcG_11913	5,127102708	0,004023847	0,76063528	0,00529011	0,99577912	1	protein_codir	hypothetical protein
TcG_11914	2,836572823	-1,462150619	1,11767371	-1,30820883	0,19080249	1	protein_codir	hypothetical protein
TcG_11915	3,141588713	0,255261391	0,97385794	0,26211358	0,79323388	1	protein_codir	hypothetical protein
TcG_11916	2,365513922	-1,656857661	1,20751874	-1,37211755	0,17002685	1	protein_codir	hypothetical protein
TcG_11917	8,066045704	0,677227471	0,63508984	1,06634908	0,28626587	1	protein_codir	mucin TcMUCII
TcG_11918	2,410561457	0,950844156	1,17518712	0,80910022	0,4184575	1	protein_codir	hypothetical protein
TcG_11919	2,750102555	-0,560591225	1,08784725	-0,51532164	0,60632828	1	protein_codir	hypothetical protein
TcG_11920	26,67925219	0,13279587	0,33554119	0,39576623	0,69227749	0,84069156	protein_codir	putative mucin TcMUCII
TcG_11921	16,94506278	0,486565058	0,43294642	1,12384589	0,26107841	0,49358779	protein_codir	hypothetical protein
TcG_11922	87,37879147	-0,263505374	0,19848761	-1,32756589	0,18432156	0,39723765	protein_codir	mucin-associated surface protein (MASP)
TcG_11923	30,47176916	-0,003479713	0,33217129	-0,01047566	0,99164179	0,99678187	protein_codir	putative trans-sialidase
TcG_11924	995,3278746	-0,064249374	0,06462046	-0,99425746	0,32009754	0,55518714	protein_codir	L1Tc protein
TcG_11925	1647,268381	0,154977901	0,06095586	2,54246104	0,01100749	0,05320515	protein_codir	L1Tc protein
TcG_11926	236,9608382	0,135446574	0,12183763	1,11169734	0,2662683	0,49894624	protein_codir	membrane associated protein
TcG_11927	11,80559606	0,24142519	0,52622396	0,45878791	0,64638647	1	protein_codir	complement regulatory protein
TcG_11928	184,8320448	-0,356360663	0,13470109	-2,64556631	0,00815543	0,04210731	protein_codir	sialidase-like protein
TcG_11929	8,366400062	-0,339013426	0,60192512	-0,56321529	0,5732883	1	protein_codir	putative elongation factor 1-gamma (EF-1-gamma)
TcG_11930	14,79583297	-0,347110953	0,44385031	-0,78204509	0,43418808	1	protein_codir	putative mucin TcMUCII
TcG_11931	24,54331644	-0,434230167	0,34772575	-1,24877196	0,21174849	0,43467719	protein_codir	hypothetical protein
TcG_11932	23,33028333	-0,377875153	0,36321416	-1,04036459	0,29817055	0,5318048	protein_codir	trans-sialidase
TcG_11933	72,88658847	-0,304238092	0,21261073	-1,43096304	0,15244081	0,35267157	protein_codir	mucin-associated surface protein (MASP)
TcG_11934	18,88080109	-0,627138408	0,39653536	-1,58154471	0,11375354	0,29274735	protein_codir	mucin TcMUCII
TcG_11935	14,42420978	-0,585245933	0,45908676	-1,27480463	0,2023784	1	protein_codir	mucin TcMUCII
TcG_11936	11,22699652	-0,519494788	0,51753068	-1,00379515	0,31547736	1	protein_codir	hypothetical protein
TcG_11937	11,394167	0,053429932	0,52904777	0,10099264	0,9195563	1	protein_codir	hypothetical protein
TcG_11938	14,19532902	-0,110561446	0,47663325	-0,23196335	0,81656648	1	protein_codir	hypothetical protein
TcG_11939	29,9256508	0,3151812	0,32384709	0,97324079	0,33043364	0,56507811	protein_codir	hypothetical protein
TcG_11940	32,69690434	0,159172528	0,30691028	0,51862886	0,60401958	0,78595809	protein_codir	trans-sialidase
TcG_11941	45,66851668	0,445633462	0,26516798	1,68057039	0,09284639	0,25533309	protein_codir	hypothetical protein
TcG_11942	25,56362989	-0,014164725	0,35472391	-0,03993169	0,96814759	0,98636633	protein_codir	putative amino acid transporter
TcG_11943	529,2018689	0,281485751	0,0860692	3,27045872	0,00107373	0,00827145	protein_codir	hypothetical protein
TcG_11944	242,8331133	0,610812711	0,11651814	5,24221109	1,5866E-07	3,9502E-06	protein_codir	hypothetical protein
TcG_11945	486,0998096	0,319041075	0,08724593	3,65680191	0,00025538	0,00244331	protein_codir	terbinafine resistance locus protein (yip1)
TcG_11946	377,9447651	0,567286849	0,09578472	5,92251939	3,1705E-09	1,1372E-07	protein_codir	hypothetical protein
TcG_11947	331,1482636	0,695323579	0,10166973	6,839042	7,9725E-12	5,1316E-10	protein_codir	hypothetical protein
TcG_11948	53,68782216	-0,139556002	0,23524129	-0,59324619	0,5530164	0,74829844	protein_codir	putative trans-sialidase
TcG_11949	144,3458187	-0,078742542	0,14913356	-0,52800014	0,59749924	0,78133478	protein_codir	amastigote surface protein 4
TcG_11950	30,09902947	0,187737689	0,32135561	0,58420541	0,55908212	0,75372649	protein_codir	hypothetical protein
TcG_11951	44,39562319	0,128615802	0,27016124	0,47607051	0,63402414	0,80419712	protein_codir	hypothetical protein
TcG_11952	0					1	protein_codir	hypothetical protein
TcG_11953	39,87162009	-0,119634145	0,28698887	-0,41685988	0,6767809	0,83063385	protein_codir	hypothetical protein
TcG_11954	56,15828726	0,126750853	0,23031507	0,55033676	0,58208841	0,77119439	protein_codir	hypothetical protein
TcG_11955	198,3450297	-0,393056073	0,13091379	-3,00240394	0,00267856	0,01733842	protein_codir	mucin-associated surface protein (MASP)
TcG_11956	7,749904998	-0,480386891	0,63865338	-0,75218719	0,4519385	1	protein_codir	surface protease GP63
TcG_11957	13,96265936	-0,361782235	0,49578865	-0,72971061	0,4655671	1	protein_codir	surface protease GP63

TcG_11958	7,35188978	-0,903815789	0,64021664	-1,41173429	0,15802821	1	protein_codir syntaxin binding protein
TcG_11959	4,99061132	0,741768506	0,82379107	0,90043281	0,36788997	1	protein_codir putative syntaxin binding protein
TcG_11960	2,055451297	0,968325021	1,23962167	0,78114561	0,43471686	1	protein_codir hypothetical protein
TcG_11961	0,545831481	2,353994432	2,35718398	0,99864688	0,31796578	1	protein_codir hypothetical protein
TcG_11962	0,951189179	-0,226581371	1,79471565	-0,12624918	0,89953469	1	protein_codir hypothetical protein
TcG_11963	26,97418238	0,043295025	0,33577138	0,12894197	0,89740356	0,95018119	protein_codir mucin TcMUCII
TcG_11964	38,99828717	-0,039982955	0,27612634	-0,1447995	0,88486918	0,94402342	
TcG_11965	23,2342257	0,260359645	0,36083179	0,72155406	0,47056869	0,68673749	protein_codir putative target of rapamycin (TOR) kinase 1
TcG_11966	36,8434693	-0,216167438	0,2993732	-0,72206678	0,47025342	0,68645032	protein_codir hypothetical protein
TcG_11967	28,21470173	0,616931196	0,34182805	1,80479981	0,07110601	0,21392986	protein_codir hypothetical protein
TcG_11968	18,89003047	1,026714746	0,42403575	2,42129289	0,01546541	0,06929768	protein_codir amino acid transporter
TcG_11969	62,0230257	0,34943008	0,23203736	1,50592161	0,1320873	0,32286148	protein_codir hypothetical protein
TcG_11970	13,64535691	0,290214368	0,46570997	0,62316545	0,5331758	1	protein_codir trans-sialidase
TcG_11971	2,305391551	-0,023622143	1,26027736	-0,01874361	0,98504564	1	
TcG_11972	0,6499495	-0,306228688	2,20024638	-0,13917927	0,88930849	1	protein_codir trans-sialidase
TcG_11973	1,81283116	-1,574011129	1,40973685	-1,11652833	0,26419605	1	protein_codir hypothetical protein
TcG_11974	2,030024437	-0,182602287	1,29789463	-0,14069115	0,88811394	1	
TcG_11975	0,689722372	0,936118941	2,17568091	0,43026481	0,66700302	1	
TcG_11976	43,68638591	-0,456609875	0,27639443	-1,65202272	0,09852992	0,26597568	protein_codir mucin-associated surface protein (MASP)
TcG_11977	23,06939268	0,427401927	0,36799867	1,16142248	0,24547013	0,47408183	protein_codir dispersed gene family protein 1 (DGF-1)
TcG_11978	12,72587432	1,092370874	0,52098607	2,0967372	0,03601685	1	protein_codir dispersed gene family protein 1 (DGF-1)
TcG_11979	9,674588265	0,825101173	0,56855551	1,45122361	0,14671761	1	protein_codir hypothetical protein
TcG_11980	4,828957853	0,751372795	0,85618346	0,87758387	0,38016958	1	protein_codir hypothetical protein
TcG_11981	4,654949414	-0,096203243	0,80147639	-0,12003254	0,90445737	1	
TcG_11982	4,301808374	0,130219046	0,85041045	0,15312494	0,87829975	1	protein_codir hypothetical protein
TcG_11983	1019,346026	0,018963223	0,0622703	0,30453077	0,76072355	0,87926408	protein_codir retrotransposon hot spot protein (RHS)
TcG_11984	36,75625431	-0,414704294	0,29962682	-1,38406934	0,16633722	0,37189947	protein_codir hypothetical protein
TcG_11985	41,38711283	-0,319420265	0,2732357	-1,1690283	0,24239223	0,47089863	protein_codir hypothetical protein
TcG_11986	10,72674101	0,387328723	0,54840317	0,70628462	0,48001116	1	protein_codir hypothetical protein
TcG_11987	4,391267173	-0,329101551	0,83785892	-0,39278874	0,69447552	1	protein_codir hypothetical protein
TcG_11988	7,831240068	0,589696896	0,62305375	0,94646231	0,34391284	1	protein_codir syntaxin binding protein
TcG_11989	6,892046343	-0,306315556	0,66776316	-0,45871886	0,64643607	1	protein_codir hypothetical protein
TcG_11990	0,910335238	-1,510920849	3,7967832	-0,39794762	0,69066879	1	protein_codir syntaxin binding protein
TcG_11991	3,719904583	0,035404621	0,91109386	0,03885947	0,96900243	1	protein_codir hypothetical protein
TcG_11992	3,487622861	2,04129422	1,09223894	1,86890812	0,0616356	1	protein_codir surface protease GP63
TcG_11993	100,5011892	-0,294280904	0,17435724	-1,68780433	0,0914488	0,25256871	protein_codir retrotransposon hot spot (RHS) protein
TcG_11994	17,17380275	0,3567821	0,44371984	0,80407065	0,42135617	0,647488	protein_codir hypothetical protein
TcG_11995	49,56206398	0,123738874	0,24542522	0,50418157	0,61413379	0,79140316	protein_codir trans-sialidase
TcG_11996	18,08324337	0,173876353	0,4109634	0,4230945	0,67222632	0,82829035	protein_codir putative mucin TcMUCII
TcG_11997	2,006776092	-0,995362907	1,26000175	-0,78996947	0,4295456	1	protein_codir hypothetical protein
TcG_11998	5,584634559	0,11501689	0,74724538	0,15392118	0,87767188	1	protein_codir hypothetical protein
TcG_11999	1,307995666	-2,05315139	1,65750018	-1,23870357	0,2154553	1	
TcG_12000	97,00188279	-0,867912594	0,18062819	-4,80496751	1,5478E-06	2,9739E-05	protein_codir mucin-associated surface protein (MASP)
TcG_12001	206,0351781	0,040697845	0,12287204	0,33122136	0,74047729	0,86763449	protein_codir mucin-associated surface protein (MASP)
TcG_12002	116,3255078	-0,03977373	0,16316471	-0,24376429	0,80741338	0,90488406	protein_codir trans-sialidase
TcG_12003	24,2818338	0,017885416	0,34942743	0,05118492	0,95917817	0,98117326	protein_codir trans-sialidase
TcG_12004	103,4847802	0,098265876	0,18137272	0,54178973	0,58796337	0,77564856	protein_codir trans-sialidase
TcG_12005	124,5126949	0,048820033	0,15770973	0,30955626	0,75689842	0,87773247	protein_codir trans-sialidase
TcG_12006	199,7829844	-0,163413985	0,1287456	-1,26927824	0,20434185	0,42546605	protein_codir trans-sialidase
TcG_12007	11,73474238	0,199105262	0,51191439	0,3889425	0,69731868	1	protein_codir hypothetical protein
TcG_12008	11,7111182	-0,34762694	0,50623512	-0,68669069	0,49227767	1	protein_codir syntaxin binding protein
TcG_12009	4,946749667	-2,065554446	0,84907642	-2,43270734	0,01498641	1	protein_codir hypothetical protein

TcG_12010	6,234486713	-0,462981512	0,68103577	-0,67981967	0,49661865	1	protein_codir	hypothetical protein
TcG_12011	16,46318768	0,091790503	0,42960176	0,21366417	0,83080896	1		
TcG_12012	91,45987033	-0,743942052	0,18442845	-4,03377045	5,4889E-05	0,00066971	protein_codir	mucin-associated surface protein (MASP)
TcG_12013	41,66792803	-0,695682618	0,27078785	-2,56910569	0,01019614	0,05011982	protein_codir	trans-sialidase
TcG_12014	35,24400385	-0,055926153	0,30196582	-0,1852069	0,85306676	0,92887573		
TcG_12015	98,62956563	0,073459786	0,18006254	0,40796818	0,68329703	0,83535712	protein_codir	L1Tc protein
TcG_12016	55,33735293	0,181103997	0,25740682	0,70357109	0,48169992	0,69570871	protein_codir	retrotransposon hot spot protein (RHS)
TcG_12017	24,28283082	-0,375140678	0,34942059	-1,07360781	0,2829985	0,51618712	protein_codir	hypothetical protein
TcG_12018	26,51821781	-0,340229963	0,34993254	-0,972273	0,33091475	0,56523583	protein_codir	glutamine synthetase
TcG_12019	5,64856014	-0,358217302	0,72011921	-0,49744167	0,61887763	1	protein_codir	hypothetical protein
TcG_12020	7,138305225	0,730148217	0,71603386	1,01971186	0,30786514	1	protein_codir	hypothetical protein
TcG_12021	5,824839842	0,02556646	0,71417412	0,03579864	0,97144292	1	protein_codir	hypothetical protein
TcG_12022	7,003958563	1,143170681	0,69077728	1,65490487	0,0979438	1	protein_codir	hypothetical protein
TcG_12023	0,615344905	-1,716476007	2,23353637	-0,76850148	0,44218931	1	protein_codir	hypothetical protein
TcG_12024	414,5860828	0,740676802	0,09259863	7,99878796	1,2565E-15	1,4414E-13	protein_codir	hypothetical protein
TcG_12025	265,381775	0,299222278	0,11425974	2,61879017	0,00882422	0,04478205	protein_codir	hypothetical protein
TcG_12026	0,747731033	-0,555671401	3,73591897	-0,14873754	0,88176074	1	protein_codir	hypothetical protein
TcG_12027	0,442035682	0,397306084	2,50212989	0,15878715	0,87383657	1	protein_codir	hypothetical protein
TcG_12028	7,587534227	0,696191449	0,66241579	1,05098861	0,29326382	1	protein_codir	surface protease GP63
TcG_12029	14,26142159	-0,417110298	0,46091334	-0,90496469	0,36548409	1	protein_codir	putative target of rapamycin (TOR) kinase 1
TcG_12030	26,69526994	0,323190741	0,35267797	0,91639049	0,35946212	0,5926302	protein_codir	hypothetical protein
TcG_12031	3,666355907	1,783148954	1,03557517	1,72189234	0,08508902	1	protein_codir	structural maintenance of chromosome protein 4
TcG_12032	14,34336204	0,388613855	0,48160887	0,8069076	0,41971972	1	protein_codir	dispersed gene family protein 1 (DGF-1)
TcG_12033	3,207698352	0,850817698	1,00117567	0,84981859	0,39542595	1	protein_codir	amastigote surface protein
TcG_12034	39,11737657	0,339849682	0,28067722	1,21082032	0,22596428	0,45243566	protein_codir	putative dispersed gene family protein 1 (DGF-1)
TcG_12035	24,67930313	0,240556505	0,36375413	0,66131622	0,50840954	0,71511873		
TcG_12036	31,05181286	-0,112349219	0,32276735	-0,34808111	0,72777927	0,85979918		
TcG_12037	91,07557253	-0,150774006	0,1855739	-0,81247421	0,41651958	0,64343944	protein_codir	trans-sialidase-like protein
TcG_12038	30,27628736	-0,563321966	0,31432398	-1,79216989	0,07310576	0,21779928	protein_codir	dispersed gene family protein 1 (DGF-1)
TcG_12039	53,81530721	-0,393275723	0,24305564	-1,61804811	0,10565223	0,27966341	protein_codir	putative dispersed gene family protein 1 (DGF-1)
TcG_12040	10,27998578	-0,572652551	0,53845795	-1,06350468	0,28755314	1		
TcG_12041	58,05047484	0,375704737	0,23770697	1,58053735	0,11398386	0,29320982	protein_codir	dispersed gene family protein 1 (DGF-1)
TcG_12042	42,34472307	0,044833774	0,26771162	0,1674704	0,86699993	0,93578156	protein_codir	phosphatidylinositol 3-related kinase
TcG_12043	43,30363096	0,421850364	0,27340816	1,54293262	0,12284712	0,30774201	protein_codir	putative target of rapamycin (TOR) kinase 1
TcG_12044	58,07399788	-0,048976438	0,22668874	-0,21605148	0,82894762	0,91651752	protein_codir	mucin-associated surface protein (MASP)
TcG_12045	51,17371072	0,056855029	0,24557722	0,23151589	0,81691404	0,90958077	protein_codir	trans-sialidase
TcG_12046	56,48050205	0,231720141	0,23900262	0,9695297	0,33228097	0,5663408	protein_codir	hypothetical protein
TcG_12047	64,14630444	0,233615522	0,22274522	1,04880153	0,29426947	0,52725669	protein_codir	hypothetical protein
TcG_12048	20,58885426	0,473113488	0,45528572	1,03915732	0,29873158	0,5322319		
TcG_12049	64,79446064	-0,132387968	0,21672118	-0,61086771	0,54128716	0,73989536	protein_codir	mucin-associated surface protein (MASP)
TcG_12050	33,70695296	-0,064444892	0,30905518	-0,20852229	0,83482118	0,92002646	protein_codir	mucin-associated surface protein (MASP)
TcG_12051	20,37493297	-0,192477493	0,38376738	-0,5015473	0,615986	0,79256256	protein_codir	regulator of sigma E protease
TcG_12052	8,853348089	0,439300926	0,60028518	0,73182037	0,46427821	1	protein_codir	trans-sialidase
TcG_12053	56,92607616	-0,107742649	0,2326226	-0,46316501	0,6432461	0,8106874	protein_codir	trans-sialidase
TcG_12054	5,627622031	0,952339397	0,75377688	1,26342346	0,20643704	1	protein_codir	dispersed gene family protein 1 (DGF-1)
TcG_12055	17,00036735	0,196643642	0,41879873	0,46954211	0,63868219	0,80773199	protein_codir	dispersed gene family protein 1 (DGF-1)
TcG_12056	0,911300909	-1,12574557	1,89660393	-0,5935586	0,55280738	1	protein_codir	hypothetical protein
TcG_12057	2,928124829	0,516673819	1,03365589	0,49985089	0,61718007	1	protein_codir	hypothetical protein
TcG_12058	7,942414358	-0,998899756	0,63868088	-1,56400448	0,11781652	1	protein_codir	mucin TcMUCII
TcG_12059	5,473468023	0,406025337	0,74539501	0,54471164	0,58595186	1	protein_codir	hypothetical protein
TcG_12060	65,07368094	0,02767771	0,21352117	0,12962513	0,89686302	0,95008274	protein_codir	trans-sialidase
TcG_12061	56,33889433	-0,005032439	0,23087388	-0,02179735	0,98260961	0,99358657	protein_codir	trans-sialidase

TcG_12062	21,43100333	0,133112118	0,37238051	0,35746263	0,7207455	0,85670516	protein_codir	hypothetical protein
TcG_12063	26,62377677	-0,161951424	0,35064415	-0,46186832	0,64417575	0,8110828	protein_codir	dispersed gene family protein 1 (DGF-1)
TcG_12064	29,45511306	0,703201899	0,34518264	2,0371879	0,04163122	0,14532668	protein_codir	dispersed gene family protein 1 (DGF-1)
TcG_12065	6,182892066	-0,983217852	0,72062095	-1,36440364	0,17244056	1	protein_codir	hypothetical protein
TcG_12066	125,2365074	0,105451116	0,17315822	0,60898705	0,54253302	0,74045253	protein_codir	mucin-associated surface protein (MASP)
TcG_12067	62,18688695	0,144423146	0,23531722	0,6137381	0,53938841	0,73860703	protein_codir	putative trans-sialidase
TcG_12068	82,15076132	0,183753481	0,19816397	0,92727998	0,35378119	0,58808501	protein_codir	putative retrotransposon hot spot (RHS) protein
TcG_12069	22,42393242	0,273839048	0,36490267	0,75044409	0,45298729	0,67355117	protein_codir	hypothetical protein
TcG_12070	24,91626589	0,531172048	0,38907473	1,36521858	0,17218436	0,37984159	protein_codir	hypothetical protein
TcG_12071	12,1049357	0,274897772	0,49975788	0,5500619	0,58227692	1	protein_codir	hypothetical protein
TcG_12072	38,16045297	0,119408258	0,29328157	0,40714545	0,68390116	0,83594728	protein_codir	trans-sialidase
TcG_12073	39,66193769	-0,279850665	0,27952979	-1,00114792	0,3167553	0,55170279	protein_codir	putative kinesin
TcG_12074	258,2914214	0,052698711	0,11483008	0,45892775	0,64628605	0,81201944	protein_codir	retrotransposon hot spot (RHS) protein
TcG_12075	289,1967621	0,024712302	0,10603264	0,23306317	0,81571235	0,90917204	protein_codir	retrotransposon hot spot (RHS) protein
TcG_12076	18,02281986	-0,033724579	0,40654734	-0,08295363	0,93388841	0,96871149	protein_codir	retrotransposon hot spot protein (RHS)
TcG_12077	7,347679996	0,446447792	0,65845586	0,67802235	0,49775752	1	protein_codir	hypothetical protein
TcG_12078	15,42423348	0,329043861	0,44416812	0,74080926	0,4588091	1		
TcG_12079	13,54886517	0,266655088	0,48822518	0,54617234	0,58494748	1	protein_codir	retrotransposon hot spot protein (RHS)
TcG_12080	167,8463242	-0,059979169	0,14361528	-0,4176378	0,67621196	0,8302619	protein_codir	putative retrotransposon hot spot (RHS) protein
TcG_12081	11,36584779	-0,178423813	0,50801365	-0,35121854	0,7254244	1	protein_codir	hypothetical protein
TcG_12082	21,38667932	-0,245492351	0,39099606	-0,627864	0,53009304	0,73198176	protein_codir	hypothetical protein
TcG_12083	3,014517571	0,5839229	1,83056609	0,31898488	0,74973798	1	protein_codir	hypothetical protein
TcG_12084	17,35712147	-0,267861407	0,42683968	-0,6275457	0,53030159	0,73198176	protein_codir	hypothetical protein
TcG_12085	4,317615634	-0,509095679	0,85161083	-0,5978032	0,54997126	1	protein_codir	hypothetical protein
TcG_12086	35,74663247	0,321542261	0,30382767	1,05830473	0,28991654	0,52328602		
TcG_12087	1,04451504	0,170764434	1,72482873	0,0990037	0,92113533	1	protein_codir	hypothetical protein
TcG_12088	4,237481279	-0,084857931	0,82076629	-0,10338866	0,91765451	1	protein_codir	mucin TcMUCII
TcG_12089	5,750728765	-0,240020102	0,70536064	-0,34027998	0,7336457	1	protein_codir	hypothetical protein
TcG_12090	1,068116916	0,120305675	1,64311699	0,07321796	0,94163267	1		
TcG_12091	10,87967879	-0,331292811	0,53021299	-0,62482967	0,53208285	1	protein_codir	complement regulatory protein
TcG_12092	17,6897861	-0,236612266	0,41627246	-0,56840721	0,5697585	0,76182203		
TcG_12093	23,0730766	-0,154822476	0,35948912	-0,43067361	0,66670571	0,82543809	protein_codir	hypothetical protein
TcG_12094	29,99899991	0,416242966	0,33970071	1,22532263	0,22045368	0,44546587	protein_codir	retrotransposon hot spot (RHS) protein
TcG_12095	7,738700694	-0,191880402	0,67283387	-0,28518244	0,77550438	1		
TcG_12096	2560,473855	0,285679275	0,05284184	5,40630835	6,4337E-08	1,7335E-06	protein_codir	L1Tc protein
TcG_12097	72,35143191	-0,068792328	0,22390148	-0,30724373	0,75865786	0,87801518	protein_codir	L1Tc protein
TcG_12098	34,51295734	-0,216422625	0,29168379	-0,74197688	0,45810134	0,67724412	protein_codir	putative trans-sialidase
TcG_12099	174,418656	-0,148743242	0,13296273	-1,11868373	0,26327509	0,49548251	protein_codir	gp85-like protein
TcG_12100	62,57860132	0,389402747	0,22029815	1,76761694	0,07712496	0,22502388	protein_codir	surface protease GP63
TcG_12101	366,9557013	-0,277362062	0,09519227	-2,91370358	0,00357169	0,02191821	protein_codir	hypothetical protein
TcG_12102	489,5580946	-0,315007871	0,0870688	-3,61791912	0,00029698	0,00278384	protein_codir	vacuolar type H+ ATPase subunit
TcG_12103	49,14259506	-0,194750994	0,28755534	-0,67726441	0,49823821	0,70742498	protein_codir	complement regulatory protein
TcG_12104	9,275897934	0,44271784	0,58869497	0,75203265	0,45203142	1	protein_codir	hypothetical protein
TcG_12105	23,0926325	0,126542219	0,36140301	0,35014157	0,72623245	0,85867223	protein_codir	trans-sialidase
TcG_12106	32,08637209	0,290051293	0,30892422	0,93890757	0,3477782	0,5822772	protein_codir	hypothetical protein
TcG_12107	56,97161532	0,363092224	0,24850843	1,46108612	0,14399181	0,34060619	protein_codir	hypothetical protein
TcG_12108	166,3553351	-0,042480427	0,15274076	-0,2781211	0,7809194	0,89087556	protein_codir	hypothetical protein
TcG_12109	35,3327504	-0,237133774	0,29986902	-0,79079117	0,42906586	0,65398767	protein_codir	putative RNA-binding protein
TcG_12110	16,77732418	-0,279230884	0,41850651	-0,66720799	0,50463929	0,71221932	protein_codir	zinc finger protein ZFP1
TcG_12111	2,48691715	0,739371386	1,13094872	0,65376208	0,5132651	1	protein_codir	hypothetical protein
TcG_12112	3,008893688	1,224934736	1,08125031	1,1328873	0,25726159	1	protein_codir	hypothetical protein
TcG_12113	7,300545333	0,733740101	0,67264775	1,0908237	0,27535047	1	protein_codir	hypothetical protein

TcG_12114	2,86488775	0,238386248	1,06549847	0,22373213	0,82296574	1		
TcG_12115	39,32199621	0,095485893	0,28752113	0,33210044	0,73981342	0,86729518	protein_codir	hypothetical protein
TcG_12116	25,61032278	0,127412829	0,33933719	0,37547558	0,70730681	0,84906006	protein_codir	hypothetical protein
TcG_12117	62,27144716	-0,017509911	0,22712832	-0,07709259	0,93854989	0,97167715	protein_codir	trans-sialidase
TcG_12118	4,830640856	0,17128935	0,81476149	0,2102325	0,83348621	1	protein_codir	hypothetical protein
TcG_12119	1,444295674	1,632165986	1,52265453	1,0719214	0,28375534	1	protein_codir	hypothetical protein
TcG_12120	2,474791206	-0,165514506	1,12834442	-0,14668793	0,88337835	1	protein_codir	surface protease GP63
TcG_12121	4,552640575	0,604081965	0,84464869	0,71518724	0,47449335	1	protein_codir	hypothetical protein
TcG_12122	51,03895654	0,272875548	0,24379531	1,11928138	0,26302012	0,49518217	protein_codir	mucin-associated surface protein (MASP)
TcG_12123	33,99685353	0,518172885	0,33027228	1,56892637	0,1166651	0,2979716	protein_codir	hypothetical protein
TcG_12124	27,73928879	0,739173774	0,33381316	2,2143338	0,02680582	0,10524307	protein_codir	hypothetical protein
TcG_12125	2,397792063	-1,749627287	1,24405284	-1,40639307	0,15960744	1	protein_codir	putative trans-sialidase
TcG_12126	0,652611499	-3,135370342	2,25081891	-1,39299093	0,16362253	1		
TcG_12127	663,1993536	0,441380776	0,07742138	5,70101952	1,1909E-08	3,7092E-07	protein_codir	ATP-binding cassette protein subfamily C, member 1
TcG_12128	19,50322936	0,368128957	0,40268746	0,91418033	0,3606221	0,59332116	protein_codir	putative dispersed gene family protein 1 (DGF-1)
TcG_12129	55,02407046	0,558537117	0,23926203	2,33441604	0,01957395	0,08261703	protein_codir	dispersed gene family protein 1 (DGF-1)
TcG_12130	189,9167131	0,494219823	0,13859145	3,56601954	0,00036244	0,00328582	protein_codir	helicase-like protein
TcG_12131	14,39986105	0,606696272	0,45756997	1,32590928	0,18486975	1	protein_codir	hypothetical protein
TcG_12132	36,42996873	0,599004112	0,29008377	2,06493492	0,03892916	0,13877945	protein_codir	hypothetical protein
TcG_12133	20,73444977	0,221005059	0,38961784	0,56723547	0,57055421	0,76219457	protein_codir	putative trans-sialidase
TcG_12134	91,2898069	0,351528978	0,19003691	1,84979321	0,06434336	0,19959362	protein_codir	target of rapamycin (TOR) kinase 1
TcG_12135	66,6969262	-0,227634876	0,21222848	-1,07259346	0,28345356	0,51685441	protein_codir	putative retrotransposon hot spot (RHS) protein
TcG_12136	10,9612177	-0,224790949	0,54944348	-0,40912479	0,68244808	1	protein_codir	retrotransposon hot spot (RHS) protein
TcG_12137	64,34021312	0,218955584	0,21479078	1,01939005	0,30801783	0,54112123	protein_codir	retrotransposon hot spot (RHS) protein
TcG_12138	16,38707635	-0,350648861	0,4826056	-0,72657437	0,46748673	1	protein_codir	hypothetical protein
TcG_12139	3,444202499	0,42728352	0,94062566	0,45425458	0,6496456	1	protein_codir	putative syntaxin binding protein
TcG_12140	1,340499875	0,676716533	1,5031782	0,45019049	0,65257309	1	protein_codir	hypothetical protein
TcG_12141	3,362914183	-0,122690126	0,94904659	-0,12927724	0,89713828	1	protein_codir	surface protease GP63
TcG_12142	3,026062082	-0,812131705	0,98951063	-0,82074076	0,41179395	1	protein_codir	hypothetical protein
TcG_12143	4,480965364	0,111764329	0,81548953	0,13705182	0,89098983	1	protein_codir	surface protease GP63
TcG_12144	68,34930778	-0,250505807	0,22008878	-1,13820343	0,25503555	0,48638803	protein_codir	putative complement regulatory protein
TcG_12145	12,79719177	-0,535587906	0,49117853	-1,09041393	0,27553085	1	protein_codir	hypothetical protein
TcG_12146	14,66309678	-0,895694569	0,45970482	-1,94841241	0,05136564	1	protein_codir	hypothetical protein
TcG_12147	6,328019396	-0,917361571	0,69438276	-1,32111801	0,18646202	1	protein_codir	hypothetical protein
TcG_12148	26,37180081	0,10643198	0,3635841	0,29273002	0,76972852	0,88436428	protein_codir	retrotransposon hot spot protein (RHS)
TcG_12149	792,927901	-0,22715264	0,06797211	-3,34185058	0,00083222	0,00670054	protein_codir	putative retrotransposon hot spot (RHS) protein
TcG_12150	243,2056556	0,113526187	0,11597404	0,97889306	0,32763282	0,56219696	protein_codir	hypothetical protein
TcG_12151	30,24105281	0,33208895	0,32337294	1,02695343	0,30444239	0,53722719	protein_codir	hypothetical protein
TcG_12152	19,79172365	-0,348135129	0,40696891	-0,85543421	0,39231081	0,6217088	protein_codir	mitotic centromere-associated kinesin (MCAK)
TcG_12153	3,675855862	0,236486961	0,88761661	0,26642917	0,7899087	1	protein_codir	trans-sialidase
TcG_12154	13,61339323	0,850106835	0,51132531	1,66255575	0,09640139	1	protein_codir	cytochrome P450
TcG_12155	20,63543067	0,507576976	0,39069442	1,29916617	0,19388691	0,41081411	protein_codir	trans-sialidase
TcG_12156	36,56746842	0,157144375	0,29679287	0,52947489	0,59647606	0,78066886	protein_codir	trans-sialidase
TcG_12157	56,85031085	0,000409507	0,2355657	0,0017384	0,99861296	0,99956197	protein_codir	mucin-associated surface protein (MASP)
TcG_12158	771,4578476	0,020474725	0,06779067	0,30202864	0,76263023	0,88013366	protein_codir	retrotransposon hot spot (RHS) protein
TcG_12159	338,7296886	0,050518424	0,09845996	0,51308599	0,60789117	0,78802211	protein_codir	retrotransposon hot spot (RHS) protein
TcG_12160	36,81154261	0,378967933	0,28824178	1,31475714	0,18859154	0,40291749	protein_codir	trans-sialidase
TcG_12161	40,35104919	0,346873155	0,28136386	1,23282767	0,21764007	0,44191689	protein_codir	dispersed gene family protein 1 (DGF-1)
TcG_12162	76,99089741	0,399728377	0,1988674	2,01002465	0,04442858	0,1522642	protein_codir	dispersed gene family protein 1 (DGF-1)
TcG_12163	4,33166214	-0,700195575	0,8282432	-0,84539852	0,39788837	1	protein_codir	hypothetical protein
TcG_12164	2,357013197	-0,453021535	1,17793325	-0,38459016	0,70054109	1	protein_codir	hypothetical protein
TcG_12165	0					1	protein_codir	trans-sialidase

TcG_12166	3,881984954	-0,330446555	0,9582236	-0,34485328	0,73020467	1	protein_codir	hypothetical protein
TcG_12167	6,797470369	0,041762611	0,66676396	0,06263478	0,95005734	1	protein_codir	hypothetical protein
TcG_12168	34,16239768	0,16443271	0,3031444	0,54242371	0,58752665	0,77564856	protein_codir	hypothetical protein
TcG_12169	8,115618066	0,898782069	0,62403532	1,44027436	0,14978979	1	protein_codir	hypothetical protein
TcG_12170	24,97407107	0,52849782	0,36353247	1,45378435	0,1460061	0,34347751	protein_codir	trans-sialidase
TcG_12171	13,538397	0,927071757	0,50512847	1,83531876	0,06645847	1	protein_codir	dispersed gene family protein 1 (DGF-1)
TcG_12172	31,1512251	1,012372629	0,36601612	2,76592359	0,00567618	0,03160252	protein_codir	dispersed gene family protein 1 (DGF-1)
TcG_12173	2,055731012	1,016541084	1,28783097	0,78934356	0,42991123	1	protein_codir	trans-sialidase
TcG_12174	2,970361889	-0,543932525	1,06413479	-0,51115003	0,60924601	1	protein_codir	trans-sialidase
TcG_12175	3,659375969	0,935577728	0,92459275	1,01188088	0,31159501	1	protein_codir	putative trans-sialidase
TcG_12176	24,2790462	0,772318293	0,36794556	2,09900155	0,03581676	0,13082378	protein_codir	hypothetical protein
TcG_12177	7,945428316	-0,248991574	0,67727083	-0,36763959	0,71314199	1	protein_codir	hypothetical protein
TcG_12178	2,357361426	0,813893593	1,25735405	0,64730661	0,51743352	1		
TcG_12179	5,346323414	0,136361748	0,75684148	0,18017214	0,85701743	1	protein_codir	hypothetical protein
TcG_12180	14,19629104	-0,355611854	0,47402843	-0,75019098	0,45313969	1		
TcG_12181	1,600297002	1,858791288	1,51435572	1,22744694	0,21965465	1		
TcG_12182	41,59991848	0,646242998	0,27849664	2,32046962	0,02031549	0,08512667	protein_codir	putative kinesin
TcG_12183	49,27797783	0,526210146	0,25304133	2,0795423	0,03756753	0,1350473	protein_codir	putative trans-sialidase
TcG_12184	108,9274554	0,415683777	0,18110236	2,29529741	0,02171609	0,08963398	protein_codir	hypothetical protein
TcG_12185	19,22450747	0,683288297	0,43088681	1,58577214	0,11279101	0,29104601	protein_codir	putative kinesin
TcG_12186	21,54351949	-0,210641763	0,39189702	-0,53749263	0,59092737	0,77668571	protein_codir	mitotic centromere-associated kinesin (MCAK)
TcG_12187	25,52714271	0,008203521	0,36981715	0,02218264	0,98230227	0,99336249	protein_codir	subtilisin-like serine peptidase
TcG_12188	12,71409511	0,285127428	0,49484939	0,57619032	0,56448655	1	protein_codir	kinesin
TcG_12189	53,6696709	0,017489769	0,24254589	0,07210911	0,94251507	0,97319113	protein_codir	putative kinesin
TcG_12190	30,24415206	0,306903549	0,32328311	0,9493337	0,34245092	0,57660752		
TcG_12191	720,9945997	0,436710108	0,07460359	5,85374126	4,8064E-09	1,6365E-07	protein_codir	hypothetical protein
TcG_12192	36,61566192	0,666503093	0,30287977	2,20055338	0,02776766	0,10793286	protein_codir	hypothetical protein
TcG_12193	6,21936506	0,665447799	0,71089082	0,93607595	0,34923408	1	protein_codir	hypothetical protein
TcG_12194	1,370069199	0,499899786	1,50799279	0,33150012	0,74026675	1	protein_codir	hypothetical protein
TcG_12195	8,703007146	1,251608437	0,63210251	1,98007192	0,04769545	1	protein_codir	hypothetical protein
TcG_12196	4,577386113	-0,122928246	0,80566694	-0,15257948	0,8787299	1	protein_codir	hypothetical protein
TcG_12197	5,768918245	-0,276267166	0,75504256	-0,36589615	0,71444257	1	protein_codir	hypothetical protein
TcG_12198	183,1269522	0,217453726	0,14566297	1,4928552	0,13547509	0,32775411	protein_codir	retrotransposon hot spot (RHS) protein
TcG_12199	309,1865743	0,004233032	0,10885155	0,03888811	0,96897959	0,9866934	protein_codir	retrotransposon hot spot (RHS) protein
TcG_12200	11,13499537	0,712399584	0,52613743	1,35401807	0,17573061	1	protein_codir	hypothetical protein
TcG_12201	21,09884462	0,338770833	0,39513847	0,85734713	0,39125307	0,62112715	protein_codir	hypothetical protein
TcG_12202	67,48336951	0,114135946	0,21026146	0,54282864	0,58724779	0,77563302	protein_codir	L1Tc protein
TcG_12203	39,69820164	0,881790579	0,29369669	3,00238518	0,00267873	0,01733842	protein_codir	hypothetical protein
TcG_12204	213,6542388	0,158233511	0,13193755	1,19930611	0,23040894	0,45757936	protein_codir	putative mucin-associated surface protein (MASP)
TcG_12205	33,45477111	-0,161696998	0,29641402	-0,54551062	0,58540238	0,77407806	protein_codir	hypothetical protein
TcG_12206	90,32678291	0,115409019	0,19166493	0,60213947	0,5470813	0,74395352	protein_codir	dispersed gene family protein 1 (DGF-1)
TcG_12207	53,85099432	0,401550723	0,23972625	1,67503862	0,09392665	0,25750928	protein_codir	dispersed gene family protein 1 (DGF-1)
TcG_12208	0,29050335	-2,096028338	3,222359	-0,65046394	0,51539259	1	protein_codir	putative dolichyl-P-Man:GDP-Man7GlcNAc2-PP-dolichyl alpha-1,6-mannosyltransferase
TcG_12209	322,69577	0,165443933	0,10442446	1,58434087	0,11311617	0,29159899	protein_codir	putative ribosomal protein L11
TcG_12210	129,7045609	0,007426658	0,15273851	0,04862335	0,96121946	0,98198472	protein_codir	hypothetical protein
TcG_12211	236,5144162	-0,086483177	0,12186172	-0,7096829	0,4779008	0,69263931	protein_codir	putative esterase
TcG_12212	11,59187734	-0,456946002	0,51323241	-0,8903296	0,37328893	1	protein_codir	surface protease GP63
TcG_12213	49,6442161	0,238749715	0,25017125	0,95434513	0,33990897	0,57399583	protein_codir	surface protease GP63
TcG_12214	77,33805524	-0,051172403	0,19935187	-0,25669386	0,79741511	0,89898331		
TcG_12215	135,6959246	-0,182405742	0,15102794	-1,20776158	0,22713898	0,45396451		
TcG_12216	106,6050359	-0,282820742	0,17424129	-1,62315572	0,10455612	0,27752285	protein_codir	hypothetical protein
TcG_12217	44,80081929	-0,185011662	0,2612934	-0,70806098	0,47890739	0,69340428		

TcG_12218	21,94897083	-0,11794975	0,36872371	-0,31988653	0,74905435	0,87339734	protein_codir	mucin TcMUCII
TcG_12219	2,478547598	-0,661557748	1,08761595	-0,60826411	0,54301232	1	protein_codir	hypothetical protein
TcG_12220	2,293453897	0,516435088	1,21189924	0,42613698	0,67000803	1		
TcG_12221	6,365435725	1,559372451	0,74025971	2,10652076	0,03515914	1	protein_codir	hypothetical protein
TcG_12222	13,77894162	0,756155207	0,52745673	1,43358719	0,1516901	1	protein_codir	hypothetical protein
TcG_12223	12,85470405	1,512213651	0,53011281	2,85262613	0,00433596	1		
TcG_12224	14,85054162	0,853986847	0,47326182	1,80447021	0,07115763	1	protein_codir	trans-sialidase
TcG_12225	28,68586692	0,727425386	0,3296856	2,20642148	0,0273545	0,1067821		
TcG_12226	27,37396343	-0,193824562	0,33258603	-0,58278022	0,56004126	0,75405439	protein_codir	hypothetical protein
TcG_12227	27,07163582	-0,267486447	0,33069715	-0,80885622	0,41859785	0,64518754	protein_codir	retrotransposon hot spot (RHS) protein
TcG_12228	112,5006382	0,101780172	0,16718013	0,60880543	0,54265341	0,74045253	protein_codir	putative retrotransposon hot spot (RHS) protein
TcG_12229	22,23567621	-0,189884502	0,37466701	-0,5068087	0,61228905	0,79067999	protein_codir	trans-sialidase
TcG_12230	51,98378004	-0,050790292	0,23903651	-0,21247923	0,83173318	0,91819539	protein_codir	putative trans-sialidase
TcG_12231	136,5636843	0,009405344	0,15611458	0,06024642	0,95195938	0,97735058		
TcG_12232	1,766317742	-0,735401488	1,35238373	-0,54378168	0,58659172	1	protein_codir	trans-sialidase
TcG_12233	0,389577086	1,883588912	2,66752822	0,70611771	0,48011494	1	protein_codir	hypothetical protein
TcG_12234	6,780764408	-0,10573823	0,65894326	-0,16046636	0,87251372	1	protein_codir	hypothetical protein
TcG_12235	0,573889029	1,026419886	2,17405032	0,47212333	0,63683876	1		
TcG_12236	60,84453001	-0,512922119	0,22793967	-2,25025383	0,02443284	0,09812092	protein_codir	sialidase-like protein
TcG_12237	1,28049973	-0,676364055	1,54583357	-0,43754002	0,66171976	1	protein_codir	trans-sialidase
TcG_12238	39,95143003	-0,144595264	0,27374154	-0,5282182	0,5973479	0,78122505	protein_codir	trans-sialidase
TcG_12239	0,927320913	-0,18393331	1,80800719	-0,10173262	0,91896891	1	protein_codir	hypothetical protein
TcG_12240	0,155721614	0,503022807	4,08047286	0,12327562	0,90188885	1	protein_codir	hypothetical protein
TcG_12241	2,546069473	-1,197400426	1,16582884	-1,02708081	0,30438241	1	protein_codir	surface protease GP63
TcG_12242	2,96337294	0,385652076	1,02225719	0,37725543	0,70598379	1	protein_codir	hypothetical protein
TcG_12243	3,750164241	0,19157414	0,93402158	0,20510676	0,83748871	1	protein_codir	hypothetical protein
TcG_12244	246,9365751	-0,00049378	0,11741537	-0,00420541	0,99664458	0,99883957	protein_codir	L1Tc protein
TcG_12245	19,10280351	-0,40620721	0,39604139	-1,02566857	0,30504784	0,53795118		
TcG_12246	41,20986885	-0,185949213	0,2881367	-0,64535067	0,51869996	0,72361924	protein_codir	hypothetical protein
TcG_12247	17,18628725	-0,853504618	0,43835346	-1,94706943	0,05152641	0,16876407	protein_codir	trans-sialidase
TcG_12248	16,91534752	-0,354259723	0,41744845	-0,84863107	0,39608661	0,62589464	protein_codir	SH3 domain protein
TcG_12249	91,18643239	0,306620706	0,19972756	1,53519477	0,12473599	0,31110872	protein_codir	retrotransposon hot spot (RHS) protein
TcG_12250	34,41291034	-0,410791747	0,33711541	-1,21854931	0,2230153	0,44921697	protein_codir	hypothetical protein
TcG_12251	59,43644137	-0,018390007	0,2352456	-0,07817365	0,93768992	0,97127686		
TcG_12252	28,35881683	0,314271945	0,32825735	0,95739499	0,33836793	0,57289651	protein_codir	cytochrome P450
TcG_12253	37,45095101	-0,263700914	0,3006183	-0,87719516	0,38038064	0,61073866	protein_codir	hypothetical protein
TcG_12254	16,33191821	0,088330114	0,42477914	0,20794363	0,83527299	1	protein_codir	hypothetical protein
TcG_12255	11,39946442	-0,711965706	0,51130637	-1,39244442	0,16378786	1	protein_codir	putative retrotransposon hot spot (RHS) protein
TcG_12256	0,389843477	1,884304201	2,66693245	0,70654366	0,47985011	1	protein_codir	retrotransposon hot spot protein (RHS)
TcG_12257	12,86162208	0,677989827	0,49866381	1,35961306	0,1739524	1	protein_codir	putative retrotransposon hot spot (RHS) protein
TcG_12258	252,1937044	-0,030389428	0,11423049	-0,26603604	0,79021145	0,89504251	protein_codir	exo-alpha-sialidase
TcG_12259	28,43943283	0,003870286	0,3569601	0,01084235	0,99134923	0,99668276		
TcG_12260	22,86409899	0,26183913	0,37452926	0,69911528	0,48447999	0,69763673	protein_codir	putative retrotransposon hot spot (RHS) protein
TcG_12261	38,35256011	0,71828358	0,29323436	2,4495205	0,01430466	0,06560494	protein_codir	helicase-like protein
TcG_12262	41,75104816	0,490551139	0,27582355	1,77849624	0,07532239	0,22154994	protein_codir	trans-sialidase
TcG_12263	74,00478433	0,223970715	0,21666463	1,03372072	0,30126675	0,53444748	protein_codir	target of rapamycin (TOR) kinase 1
TcG_12264	58,79601979	-0,038253778	0,23428499	-0,16327882	0,87029891	0,93739833	protein_codir	putative trans-sialidase
TcG_12265	19,08017287	0,177166823	0,40209255	0,44061205	0,65949388	0,82116024	protein_codir	serine/threonine protein phosphatase
TcG_12266	12,18044048	0,689578091	0,51333513	1,34332925	0,17916538	1	protein_codir	hypothetical protein
TcG_12267	29,60929841	0,558456798	0,33730634	1,6556368	0,09779539	0,26473304	protein_codir	putative trans-sialidase
TcG_12268	7,535531209	-0,301530527	0,62495112	-0,48248658	0,62946033	1	protein_codir	structural maintenance of chromosome protein 4
TcG_12269	1,111379789	-0,344676894	1,65274402	-0,20854826	0,8348009	1	protein_codir	hypothetical protein

TcG_12270	0,624986841	-1,473189537	2,27966173	-0,64623164	0,51812935	1	protein_codir	hypothetical protein
TcG_12271	0,301239679	-0,45874858	3,01362118	-0,15222503	0,87900945	1	protein_codir	hypothetical protein
TcG_12272	2,852863529	0,890589411	1,06386184	0,83712882	0,40252016	1	protein_codir	hypothetical protein
TcG_12273	19,30053508	0,255692769	0,40933277	0,62465747	0,53219589	0,7329332	protein_codir	trans-sialidase
TcG_12274	128,8222896	-0,184995166	0,28085475	-0,65868626	0,51009726	0,71697037		
TcG_12275	8,088686609	-0,680779378	0,63411804	-1,07358463	0,28300889	1	protein_codir	hypothetical protein
TcG_12276	15,86913558	-0,180435442	0,44482133	-0,40563577	0,68501024	1	protein_codir	surface protease GP63
TcG_12277	1,618610891	-1,528040853	1,42755771	-1,07038815	0,28444463	1	protein_codir	hypothetical protein
TcG_12278	1,301972388	0,634511042	1,51054352	0,42005479	0,67444543	1	protein_codir	hypothetical protein
TcG_12279	1,572492521	2,745833125	1,64155574	1,67270173	0,09438603	1	protein_codir	hypothetical protein
TcG_12280	4,70396911	0,053481637	0,8279513	0,06459515	0,94849635	1	protein_codir	hypothetical protein
TcG_12281	52,59380407	0,313052376	0,2408553	1,29975288	0,19368568	0,41080251	protein_codir	hypothetical protein
TcG_12282	231,0061926	0,093963884	0,11811388	0,79553632	0,42630159	0,65178385	protein_codir	rab1 small GTP-binding protein
TcG_12283	443,0160423	-0,028080014	0,08845985	-0,3174323	0,7509156	0,87456599	protein_codir	hypothetical protein
TcG_12284	3,833034412	-1,291025184	0,92585863	-1,39440854	0,16319427	1	protein_codir	hypothetical protein
TcG_12285	22,03235756	-0,017320233	0,37961713	-0,04562553	0,96360872	0,98364499	protein_codir	putative trans-sialidase
TcG_12286	413,5807692	-0,267318158	0,11081007	-2,41239952	0,01584791	0,07059355	protein_codir	calpain-like cysteine peptidase
TcG_12287	87,44324946	-0,431395806	0,20640911	-2,09000373	0,03661746	0,13303542	protein_codir	calpain-like cysteine peptidase
TcG_12288	26,99477614	0,188126765	0,3385862	0,55562443	0,57846763	0,76824002	protein_codir	trans-sialidase
TcG_12289	20,57097978	0,215423173	0,39457183	0,54596694	0,58508866	0,77375154	protein_codir	putative trans-sialidase
TcG_12290	21,14501655	-0,679865963	0,37518109	-1,81210085	0,06997062	0,21188697	protein_codir	mucin TcMUCII
TcG_12291	4,563577231	-1,099943241	0,8615909	-1,27664212	0,20172862	1	protein_codir	hypothetical protein
TcG_12292	6,384056392	-0,590736499	0,69452245	-0,85056502	0,39501103	1	protein_codir	hypothetical protein
TcG_12293	44,20017761	0,109514364	0,27409577	0,39954781	0,68948961	0,8386805	protein_codir	NUP-1 protein
TcG_12294	103,4836431	-0,16682296	0,18759341	-0,88927942	0,37385293	0,60449626	protein_codir	NUP-1 protein
TcG_12295	556,5774129	-0,234065563	0,07983622	-2,93182181	0,0033698	0,02091189	protein_codir	NUP-1 protein
TcG_12296	48,56162923	0,012461563	0,25126338	0,04959562	0,96044464	0,98188578	protein_codir	hypothetical protein
TcG_12297	78,69959616	-0,137785596	0,19761131	-0,69725564	0,48564283	0,69826977	protein_codir	mucin-associated surface protein (MASP)
TcG_12298	61,44978536	0,107387316	0,22437806	0,47859989	0,6322233	0,80290602	protein_codir	hypothetical protein
TcG_12299	75,22315485	0,204934936	0,2023338	1,01285567	0,31112911	0,54476981	protein_codir	hypothetical protein
TcG_12300	75,44225789	-0,047150303	0,21741475	-0,21686801	0,82831121	0,91617944	protein_codir	hypothetical protein
TcG_12301	11,54563922	0,5754869	0,53328803	1,07912961	0,28052995	1	protein_codir	hypothetical protein
TcG_12302	6,156528366	-1,364514757	0,74175172	-1,83958422	0,0658293	1	protein_codir	hypothetical protein
TcG_12303	3,223643631	-1,399410097	1,01693676	-1,37610336	0,16878964	1	protein_codir	hypothetical protein
TcG_12304	3,774699218	-1,752995192	0,94913599	-1,84693787	0,06475615	1	protein_codir	surface protease GP63
TcG_12305	25,70298222	-1,226622149	0,38805138	-3,16097869	0,0015724	0,01131542	protein_codir	mucin-associated surface protein (MASP)
TcG_12306	30,06487627	-0,585620088	0,32838387	-1,78333999	0,07453098	0,22045189	protein_codir	mucin TcMUCII
TcG_12307	34,30640254	-0,445621727	0,29673671	-1,50174113	0,13316398	0,32441177		
TcG_12308	7,659778165	0,470271297	0,62756657	0,74935684	0,45364216	1		
TcG_12309	8,348210517	0,222125593	0,61818341	0,35931989	0,7193558	1	protein_codir	hypothetical protein
TcG_12310	8,440877419	-0,206906317	0,58861878	-0,35151158	0,72520459	1	protein_codir	hypothetical protein
TcG_12311	14,90834748	-0,184144128	0,45880605	-0,40135506	0,68815873	1	protein_codir	hypothetical protein
TcG_12312	14,08198801	0,740103481	0,50059697	1,47844178	0,13928957	1	protein_codir	hypothetical protein
TcG_12313	35,20923164	-0,033727244	0,30371888	-0,11104757	0,91157862	0,95692216	protein_codir	retrotransposon hot spot (RHS) protein
TcG_12314	18,11758215	0,165246571	0,41935363	0,39405065	0,69354364	0,84125796	protein_codir	retrotransposon hot spot (RHS) protein
TcG_12315	13,81914343	0,123040338	0,47152676	0,26094031	0,79413854	1	protein_codir	retrotransposon hot spot (RHS) protein
TcG_12316	55,78969069	-0,117125981	0,23585439	-0,49660294	0,61946908	0,79399097	protein_codir	retrotransposon hot spot (RHS) protein
TcG_12317	47,14149077	-0,278633696	0,2634439	-1,05765855	0,29021114	0,52345679	protein_codir	hypothetical protein
TcG_12318	61,67497004	-0,600890225	0,22234737	-2,70248408	0,00688235	0,03684791	protein_codir	mucin TcMUCII
TcG_12319	12,01311731	-0,135027253	0,49282708	-0,27398505	0,78409611	1		
TcG_12320	8,677670228	-0,39862552	0,61116939	-0,6522341	0,51425016	1	protein_codir	putative surface protease GP63
TcG_12321	6,253905775	-0,571877148	0,73026495	-0,78310913	0,43356303	1	protein_codir	surface protease GP63

TcG_12322	63,08094929	0,705183644	0,23277371	3,02948143	0,00244974	0,01620016	protein_codir	putative retrotransposon hot spot (RHS) protein
TcG_12323	40,41877219	0,767677881	0,29143966	2,63408861	0,00843635	0,04326849	protein_codir	hypothetical protein
TcG_12324	17,13800854	0,348877689	0,4225402	0,82566744	0,40899277	0,63660535	protein_codir	putative amino acid transporter
TcG_12325	33,14875467	0,408904139	0,31193049	1,31088222	0,18989756	0,404961	protein_codir	putative GAG protein
TcG_12326	36,95161358	0,04944046	0,28645054	0,17259684	0,86296833	0,93442534	protein_codir	trans-sialidase
TcG_12327	19,01017594	0,109679488	0,41208605	0,26615676	0,79011848	0,89502471	protein_codir	putative protein tyrosine phosphatase
TcG_12328	36,2544683	0,441552863	0,29640554	1,48969166	0,13630533	0,32916375	protein_codir	target of rapamycin (TOR) kinase 1
TcG_12329	12,76721359	0,132993362	0,50433506	0,26370041	0,79201078	1	protein_codir	serine/threonine protein phosphatase
TcG_12330	72,05075092	0,395907709	0,22321366	1,7736715	0,0761175	0,22310484	protein_codir	trans-sialidase
TcG_12331	126,5960905	-0,162840386	0,15429047	-1,05541443	0,29123583	0,52370919	protein_codir	sialidase
TcG_12332	57,96333378	0,527463301	0,24036463	2,19442977	0,02820453	0,10918064	protein_codir	rab1 small GTP-binding protein
TcG_12333	76,31274309	0,18944366	0,20168071	0,93932463	0,3475641	0,58200284	protein_codir	trans-sialidase
TcG_12334	36,27626338	0,367098842	0,30990275	1,18456143	0,2361909	0,46360093	protein_codir	hypothetical protein
TcG_12335	40,83444687	0,800121948	0,27581576	2,90092901	0,00372058	0,02266548	protein_codir	structural maintenance of chromosome protein 4
TcG_12336	65,06659384	0,736519222	0,2245204	3,28041112	0,00103656	0,00803316	protein_codir	hypothetical protein
TcG_12337	175,6896146	0,094734899	0,13554949	0,69889529	0,48461747	0,69774799	protein_codir	putative retrotransposon hot spot (RHS) protein
TcG_12338	66,39387479	0,075415636	0,22939553	0,32875809	0,74233854	0,86893659	protein_codir	retrotransposon hot spot (RHS) protein
TcG_12339	26,25322325	-0,025540404	0,34162158	-0,07476227	0,94040386	0,97238011	protein_codir	putative retrotransposon hot spot (RHS) protein
TcG_12340	17,55378019	0,043653398	0,44158989	0,09885507	0,92125334	0,96166313	protein_codir	retrotransposon hot spot (RHS) protein
TcG_12341	13,7047864	0,25949437	0,50689868	0,51192552	0,60870314	1	protein_codir	hypothetical protein
TcG_12342	12,9511565	0,190491325	0,50296209	0,37873893	0,70488174	1	protein_codir	retrotransposon hot spot (RHS) protein
TcG_12343	33,54366456	-0,427709458	0,30622569	-1,39671317	0,16249984	0,36643114	protein_codir	retrotransposon hot spot (RHS) protein
TcG_12344	29,62673853	-0,195738469	0,33646219	-0,58175472	0,56073191	0,75442638	protein_codir	trans-sialidase
TcG_12345	31,41074641	-0,033937334	0,30841641	-0,11003738	0,91237973	0,95723874		
TcG_12346	28,21366286	0,305164596	0,34014252	0,89716685	0,36962989	0,60122588	protein_codir	mucin-associated surface protein (MASP)
TcG_12347	17,92860714	0,46617581	0,42256724	1,10319914	0,2699407	0,5027758	protein_codir	hypothetical protein
TcG_12348	49,46604926	0,087751084	0,25797466	0,34015389	0,73374064	0,8632666	protein_codir	putative trans-sialidase
TcG_12349	21,85906228	-0,440037878	0,3852757	-1,14213763	0,25339681	0,48445481	protein_codir	dispersed gene family protein 1 (DGF-1)
TcG_12350	32,28669759	-0,685733622	0,30378265	-2,25731659	0,0239883	0,09677174	protein_codir	putative dispersed gene family protein 1 (DGF-1)
TcG_12351	22,89248276	0,083514608	0,38046522	0,21950655	0,82625548	0,91511289		
TcG_12352	4,681845248	1,133100131	0,87922243	1,28875253	0,19748413	1	protein_codir	putative elongation factor 1-gamma (EF-1-gamma)
TcG_12353	58,74031969	-0,351386157	0,2258866	-1,55558653	0,11980644	0,30291726	protein_codir	mucin-associated surface protein (MASP)
TcG_12354	28,01643194	0,604408294	0,33816593	1,78731281	0,07388694	0,21933234	protein_codir	dispersed gene family protein 1 (DGF-1)
TcG_12355	23,56348139	-0,263094712	0,37691105	-0,69802865	0,48515928	0,69814081	protein_codir	dispersed protein family protein 1 (DGF-1)
TcG_12356	49,5046317	0,199667426	0,26946113	0,74098786	0,4587008	0,67743881	protein_codir	dispersed gene family protein 1 (DGF-1)
TcG_12357	76,0174514	0,058685099	0,19997759	0,29345838	0,76917181	0,88436428	protein_codir	hypothetical protein
TcG_12358	311,2946216	0,243687495	0,10732189	2,27062246	0,02316984	0,09425765	protein_codir	GP63 group I member a protein
TcG_12359	50,36466002	0,26659235	0,24984279	1,06704041	0,28595359	0,51918774	protein_codir	serine/threonine protein phosphatase
TcG_12360	151,6120866	0,136800151	0,14869089	0,92003051	0,35755682	0,59135536	protein_codir	putative rab1 small GTP-binding protein
TcG_12361	12,39230207	-0,112664117	0,50741712	-0,22203452	0,82428701	1	protein_codir	hypothetical protein
TcG_12362	50,41000258	0,015376859	0,28395823	0,05415183	0,9568142	0,98006094	protein_codir	structural maintenance of chromosome protein 4
TcG_12363	229,998417	0,398494567	0,12171308	3,27404881	0,00106018	0,0081834	protein_codir	Tbingi protein
TcG_12364	326,2830306	0,072010165	0,10130895	0,71079763	0,47720965	0,69224377	protein_codir	UDP-Gal or UDP-GlcNAc-dependent glycosyltransferase
TcG_12365	33,69105837	0,403696847	0,30334199	1,33083076	0,18324471	0,39550543	protein_codir	hypothetical protein
TcG_12366	231,4480187	0,741018323	0,12422326	5,96521417	2,4431E-09	8,9576E-08	protein_codir	Tbingi protein
TcG_12367	216,5268814	-0,535704029	0,12863334	-4,16458165	3,1192E-05	0,0004079	protein_codir	putative complement regulatory protein
TcG_12368	211,938631	-0,056707863	0,12634792	-0,44882309	0,65355928	0,81657908	protein_codir	hypothetical protein
TcG_12369	879,7043039	-0,112742083	0,06530179	-1,7264777	0,08426151	0,23909571	protein_codir	putative cytochrome-b5 reductase
TcG_12370	24,36857969	-0,152596543	0,35626606	-0,42832186	0,6684168	0,8262554	protein_codir	hypothetical protein
TcG_12371	36,99955706	-0,282218517	0,28610811	-0,98640516	0,32393433	0,55819154	protein_codir	subtilisin-like serine peptidase
TcG_12372	13,48922944	-0,889941312	0,49817746	-1,78639417	0,07403546	1	protein_codir	subtilisin-like serine peptidase
TcG_12373	78,86516955	-0,265030436	0,21065514	-1,25812473	0,20834665	0,43097738	protein_codir	subtilisin-like serine peptidase

TcG_12374	31,15651013	0,026330231	0,31188624	0,08442255	0,93272048	0,96789069	protein_codir	putative kinesin
TcG_12375	0					1	protein_codir	hypothetical protein
TcG_12376	5,140221256	0,749601517	0,84199405	0,89026938	0,37332126	1	protein_codir	hypothetical protein
TcG_12377	0,300973289	-0,458748581	3,01449841	-0,15218073	0,87904439	1	protein_codir	hypothetical protein
TcG_12378	1,994090369	-0,25447292	1,24292529	-0,2047371	0,83777753	1	protein_codir	trans-sialidase
TcG_12379	4,129349489	-0,592279752	0,89112185	-0,66464507	0,50627753	1	protein_codir	hypothetical protein
TcG_12380	9,362616956	-0,342019504	0,57707554	-0,59267718	0,55339722	1	protein_codir	hypothetical protein
TcG_12381	10,5481103	-0,126195739	0,5387843	-0,23422312	0,81481177	1	protein_codir	surface protease GP63
TcG_12382	12,37204217	-0,413540737	0,4927806	-0,83919849	0,40135793	1	protein_codir	putative mucin-associated surface protein (MASP)
TcG_12383	1,2131022	-0,268819298	1,53568487	-0,17504848	0,86104154	1		
TcG_12384	63,38441844	0,031408274	0,4681672	0,06708773	0,94651186	0,97469438	protein_codir	hypothetical protein
TcG_12385	74,33364995	0,080905279	0,20922521	0,38668991	0,69898578	0,84414248	protein_codir	retrotransposon hot spot protein (RHS)
TcG_12386	90,71963227	-0,029264459	0,18344772	-0,15952479	0,87325543	0,9392441	protein_codir	putative retrotransposon hot spot (RHS) protein
TcG_12387	92,26283234	-0,185653805	0,18614995	-0,99733471	0,31860207	0,55357619	protein_codir	retrotransposon hot spot (RHS) protein
TcG_12388	247,5583999	-0,058759287	0,11476709	-0,51198724	0,60865994	0,78817478	protein_codir	retrotransposon hot spot (RHS) protein
TcG_12389	7,248759796	-0,327499292	0,69313345	-0,47249096	0,63657639	1	protein_codir	hypothetical protein
TcG_12390	10,49863769	0,356274178	0,54657152	0,65183451	0,51450794	1	protein_codir	syntaxin binding protein
TcG_12391	0,428370964	1,990401553	2,58761618	0,76920278	0,44177294	1	protein_codir	putative syntaxin binding protein
TcG_12392	0					1	protein_codir	putative syntaxin binding protein
TcG_12393	7,375963907	0,89639944	0,71411786	1,2552542	0,2093865	1	protein_codir	putative syntaxin binding protein
TcG_12394	25,00177374	-0,154191129	0,34420911	-0,44795773	0,65418371	0,81709489	protein_codir	dispersed gene family protein 1 (DGF-1)
TcG_12395	52,54996899	0,170493573	0,25169119	0,6773919	0,49815733	0,70742498	protein_codir	dispersed gene family protein 1 (DGF-1)
TcG_12396	15,44677212	-0,095640151	0,46544769	-0,20547991	0,83719719	1	protein_codir	hypothetical protein
TcG_12397	1,164804055	-0,787737267	1,67011707	-0,4716659	0,63716528	1	protein_codir	thimet oligopeptidase
TcG_12398	0,300145286	-1,420535481	3,2277253	-0,44010421	0,65986164	1	protein_codir	hypothetical protein
TcG_12399	8,488347146	0,849993583	0,63324062	1,34229164	0,17950145	1	protein_codir	putative elongation factor 1-gamma (EF-1-gamma)
TcG_12400	3,25542156	0,786011844	0,97708456	0,80444608	0,4211394	1	protein_codir	surface protease GP63
TcG_12401	3,406245636	-0,752308016	1,01609878	-0,74038866	0,4590642	1	protein_codir	surface protease GP63
TcG_12402	256,9068755	-0,011122457	0,11353353	-0,09796627	0,92195908	0,96209148	protein_codir	putative nuclear receptor binding factor
TcG_12403	21,61815353	-0,242742986	0,40103488	-0,60529145	0,54498536	0,74188701		
TcG_12404	315,7759391	-0,078763554	0,10668603	-0,73827433	0,46034776	0,67840107	protein_codir	kinetoplast DNA-associated protein
TcG_12405	134,8764223	-0,265580548	0,15198642	-1,74739654	0,08056858	0,2321481	protein_codir	mucin-associated surface protein (MASP)
TcG_12406	69,08466905	0,336124148	0,21398118	1,57081173	0,11622639	0,29713127	protein_codir	hypothetical protein
TcG_12407	37,05423344	0,581553485	0,29640071	1,96205158	0,04975648	0,16499381	protein_codir	hypothetical protein
TcG_12408	3,943842718	1,484492968	0,935843	1,58626283	0,1126797	1	protein_codir	mucin TcMUCII
TcG_12409	7,874962831	0,090368166	0,61723596	0,14640781	0,88359946	1	protein_codir	hypothetical protein
TcG_12410	7,948832332	-0,244998115	0,64489144	-0,37990598	0,70401521	1	protein_codir	hypothetical protein
TcG_12411	5,585944815	-0,026123336	0,75491902	-0,03460416	0,97239539	1	protein_codir	hypothetical protein
TcG_12412	13,53933673	0,106619641	0,47639045	0,22380726	0,82290728	1	protein_codir	hypothetical protein
TcG_12413	64,47419489	-0,513291752	0,24004301	-2,1383324	0,03248977	0,1215063	protein_codir	mucin-associated surface protein (MASP)
TcG_12414	44,47551067	0,14577364	0,27955302	0,52145257	0,60205154	0,78475506	protein_codir	putative trans-sialidase
TcG_12415	32,27352875	-0,004053334	0,30370132	-0,01334645	0,98935139	0,99554162	protein_codir	putative profilin
TcG_12416	37,1836887	0,044333075	0,2808037	0,15787924	0,87455196	0,9395919	protein_codir	rab1 small GTP-binding protein
TcG_12417	22,77452769	-0,006493199	0,37331437	-0,01739338	0,98612279	0,99461455	protein_codir	hypothetical protein
TcG_12418	8,543252867	-0,505508203	0,59161482	-0,85445494	0,39285296	1	protein_codir	selenocysteine-tRNA-specific elongation factor
TcG_12419	10,54563714	-0,375785238	0,54513631	-0,68934179	0,49060821	1	protein_codir	putative trans-sialidase
TcG_12420	112,1730773	0,276412112	0,16995937	1,62634226	0,10387687	0,27628957		
TcG_12421	100,7976614	-0,038794397	0,18412462	-0,21069642	0,83312417	0,91908998	protein_codir	trans-sialidase
TcG_12422	226,1133887	-0,176026433	0,13532643	-1,30075424	0,19334259	0,41026872	protein_codir	putative complement regulatory protein
TcG_12423	22,47675948	0,229078022	0,37443611	0,61179468	0,54067361	0,73949291	protein_codir	retrotransposon hot spot (RHS) protein
TcG_12424	27,5961205	0,260347018	0,33372451	0,78012555	0,43531698	0,65939964		
TcG_12425	9,458788951	0,112176663	0,56759322	0,19763567	0,84333013	1	protein_codir	hypothetical protein

TcG_12426	84,76269171	-0,019763155	0,19082032	-0,10356945	0,91751103	0,96017565	protein_codir retrotransposon hot spot (RHS) protein
TcG_12427	24,70226289	0,21236493	0,34828527	0,60974422	0,54203126	0,74019653	protein_codir putative retrotransposon hot spot (RHS) protein
TcG_12428	300,2751525	0,187537115	0,12146474	1,5439634	0,1225972	0,30751486	protein_codir helicase-like protein
TcG_12429	18,66944338	0,002740607	0,41109816	0,00666655	0,9946809	0,99778366	protein_codir hypothetical protein
TcG_12430	7,992891352	0,557953939	0,64277749	0,8680359	0,38537468	1	protein_codir trans-sialidase
TcG_12431	6,243950658	0,175788683	0,69485279	0,25298694	0,8002783	1	protein_codir hypothetical protein
TcG_12432	19,50636901	-0,107328615	0,40033878	-0,26809447	0,7886266	0,89487471	protein_codir retrotransposon hot spot (RHS) protein
TcG_12433	2,540736529	0,587791799	1,09269447	0,53792878	0,59062622	1	protein_codir retrotransposon hot spot (RHS) protein
TcG_12434	5,461379922	-0,866152266	0,83471689	-1,03765993	0,29942841	1	protein_codir hypothetical protein
TcG_12435	8,686987493	0,696159968	0,59407394	1,17184061	0,24126106	1	protein_codir hypothetical protein
TcG_12436	7,536114546	0,797003618	0,65960586	1,20830282	0,22693081	1	protein_codir hypothetical protein
TcG_12437	23,91349456	0,762853306	0,37666966	2,02525819	0,04284086	0,14829823	protein_codir surface protein-2
TcG_12438	49,20670043	-0,454987083	0,25103053	-1,8124771	0,06991251	0,21176636	protein_codir trans-sialidase-like protein
TcG_12439	36,59155185	0,397894473	0,28770256	1,38300638	0,16666291	0,37255575	protein_codir dispersed gene family protein 1 (DGF-1)
TcG_12440	8,17231994	-0,264078507	0,60632057	-0,43554271	0,66316854	1	
TcG_12441	23,50760378	-0,103203905	0,36300999	-0,28430045	0,77618014	0,88831084	protein_codir hypothetical protein
TcG_12442	58,33609117	-0,072609456	0,22874606	-0,31742385	0,75092202	0,87456599	protein_codir hypothetical protein
TcG_12443	1,619310172	-1,335565474	1,51434989	-0,88193982	0,37780935	1	
TcG_12444	57,96401779	-0,164558148	0,23266659	-0,7072702	0,47939858	0,69385534	protein_codir putative mucin-associated surface protein (MASP)
TcG_12445	5,517838964	-0,313724667	0,72867221	-0,43054293	0,66680075	1	
TcG_12446	11,10412271	0,475580682	0,54398713	0,87424988	0,38198219	1	protein_codir hypothetical protein
TcG_12447	7,654618964	-0,715122796	0,65790221	-1,08697431	0,27704816	1	protein_codir retrotransposon hot spot protein (RHS)
TcG_12448	4,157622326	-0,832279799	0,88476453	-0,94067944	0,34686916	1	protein_codir retrotransposon hot spot (RHS) protein
TcG_12449	13,39443334	0,015805837	0,47213823	0,03347714	0,9732941	1	protein_codir retrotransposon hot spot (RHS) protein
TcG_12450	0,469265227	-2,470320505	2,56600864	-0,96270935	0,3356934	1	protein_codir hypothetical protein
TcG_12451	3,870549344	-0,685445198	0,90926751	-0,75384328	0,45094333	1	protein_codir putative syntaxin binding protein
TcG_12452	1,963104432	1,195522899	1,33511169	0,89544785	0,37054773	1	protein_codir putative syntaxin binding protein
TcG_12453	2,44725318	-1,807872815	1,2424786	-1,45505348	0,14565446	1	protein_codir hypothetical protein
TcG_12454	4,053422403	-0,029261634	0,84964452	-0,03443985	0,9725264	1	protein_codir surface protease GP63
TcG_12455	1,968690443	-1,665599866	3,84746697	-0,43290817	0,66508149	1	protein_codir surface protease GP63
TcG_12456	29,2076321	-0,210427606	0,33968807	-0,61947306	0,53560476	0,73555572	protein_codir hypothetical protein
TcG_12457	33,0050481	-0,464727912	0,30348018	-1,53132868	0,12568818	0,31289713	protein_codir hypothetical protein
TcG_12458	7,43608415	-0,213783819	0,64183093	-0,33308432	0,73907064	1	protein_codir trans-sialidase
TcG_12459	5,505226483	-0,1654656	0,80845205	-0,20466965	0,83783023	1	protein_codir hypothetical protein
TcG_12460	8,983343883	-0,555073312	0,57801269	-0,96031337	0,33689752	1	protein_codir hypothetical protein
TcG_12461	57,28274151	0,328130627	0,23930586	1,37117673	0,17031987	0,37691908	protein_codir retrotransposon hot spot (RHS) protein
TcG_12462	261,0494273	0,205489778	0,11410777	1,80083948	0,07172818	0,21511728	protein_codir retrotransposon hot spot (RHS) protein
TcG_12463	62,8069445	0,409806134	0,22021972	1,86089665	0,06275877	0,19604291	protein_codir putative kinesin
TcG_12464	91,32919698	0,205805586	0,18164851	1,13298803	0,25721928	0,48902898	protein_codir subtilisin-like serine peptidase
TcG_12465	43,13143366	-0,191801756	0,26282129	-0,72978014	0,46552459	0,6821385	protein_codir mucin TcMUCII
TcG_12466	8,90322411	0,274111868	0,63193912	0,43376309	0,66446049	1	protein_codir hypothetical protein
TcG_12467	11,76997469	0,097690895	0,51738427	0,1888169	0,85023633	1	
TcG_12468	119,8015402	0,226621357	0,16342491	1,38670019	0,1655332	0,37096087	protein_codir hypothetical protein
TcG_12469	25,36295146	-0,057509825	0,35732517	-0,16094535	0,87213644	0,93847616	protein_codir helicase-like protein
TcG_12470	60,86066349	-0,617310186	0,22191114	-2,78179	0,005406	0,03044916	
TcG_12471	300,3813401	-0,577033825	0,10936445	-5,27624691	1,3186E-07	3,3502E-06	protein_codir putative UDP-Gal or UDP-GlcNAc-dependent glycosyltransferase
TcG_12472	0					1	protein_codir hypothetical protein
TcG_12473	137,0186824	0,267244275	0,15135734	1,76565121	0,07745438	0,22575761	protein_codir dispersed gene family protein 1 (DGF-1)
TcG_12474	42,35412642	0,513855858	0,29634975	1,73395069	0,08292683	0,23681114	protein_codir retrotransposon hot spot (RHS) protein
TcG_12475	48,06268533	0,197709952	0,26894351	0,73513561	0,46225691	0,67974471	
TcG_12476	11,40133996	0,22646719	0,51387492	0,44070489	0,65942665	1	protein_codir hypothetical protein
TcG_12477	7,075888098	-0,96079579	0,66279392	-1,44961467	0,147166	1	protein_codir trans-sialidase

TcG_12478	1,695177145	0,349483648	1,31365382	0,26603938	0,79020888	1		
TcG_12479	23,02795025	0,106957611	0,37213896	0,2874131	0,77379603	0,88691888	protein_codir	hypothetical protein
TcG_12480	421,7812136	0,162887051	0,08966794	1,81655844	0,06928475	0,21058057	protein_codir	hypothetical protein
TcG_12481	189,526393	-0,101398683	0,13271956	-0,76400708	0,44486303	0,66677659	protein_codir	hypothetical protein
TcG_12482	252,4121072	-0,04078837	0,11496297	-0,35479573	0,72274263	0,85733557	protein_codir	hypothetical protein
TcG_12483	156,8976586	0,734090783	0,14823646	4,95216093	7,3394E-07	1,5433E-05	protein_codir	dispersed protein family protein 1
TcG_12484	720,9548785	0,203154271	0,07181235	2,82896005	0,00466995	0,02723375	protein_codir	cytochrome c oxidase subunit 10
TcG_12485	2,327232323	3,369180977	1,49203491	2,25811136	0,02393872	1	protein_codir	hypothetical protein
TcG_12486	5,11741015	-0,797685493	0,78539745	-1,01564564	0,30979816	1	protein_codir	hypothetical protein
TcG_12487	2,530040522	-1,157820416	1,11421208	-1,03913827	0,29874043	1	protein_codir	hypothetical protein
TcG_12488	5,188482168	-0,825285486	0,80317989	-1,02752259	0,30417445	1	protein_codir	trans-sialidase
TcG_12489	19,8288726	-0,195535435	0,40793845	-0,47932582	0,63170685	0,80290602	protein_codir	dispersed gene family protein 1 (DGF-1)
TcG_12490	21,72401296	0,164052295	0,3738555	0,43881204	0,66079774	0,82224396	protein_codir	dispersed gene family protein 1 (DGF-1)
TcG_12491	9,249368085	-0,249466892	0,59311233	-0,42060649	0,67404246	1	protein_codir	putative dispersed gene family protein 1 (DGF-1)
TcG_12492	3,604686498	-0,620940938	0,92397409	-0,67203285	0,50156279	1	protein_codir	hypothetical protein
TcG_12493	2,589911945	-0,53549244	1,14149878	-0,46911346	0,63898854	1	protein_codir	hypothetical protein
TcG_12494	2,094944455	1,339563545	1,28211595	1,04480686	0,29611224	1		
TcG_12495	2,484530553	-0,553786755	1,16424989	-0,4756597	0,63431683	1		
TcG_12496	151,6217893	-0,148310503	0,14363526	-1,03254938	0,30181483	0,53501019	protein_codir	putative mucin-associated surface protein (MASP)
TcG_12497	53,84709742	0,093169446	0,24292549	0,38353097	0,70132612	0,84532228	protein_codir	trans-sialidase
TcG_12498	9,790292952	0,272630415	0,54833021	0,49720116	0,61904721	1	protein_codir	trans-sialidase
TcG_12499	14,3731786	0,042371639	0,46701582	0,09072849	0,92770833	1	protein_codir	hypothetical protein
TcG_12500	16,40312542	0,018534991	0,42753159	0,0433535	0,96541974	1	protein_codir	hypothetical protein
TcG_12501	26,66488816	-0,098324529	0,34033264	-0,28890714	0,77265244	0,88659514	protein_codir	trans-sialidase
TcG_12502	163,2327475	0,197605321	0,14007653	1,41069543	0,15833444	0,36053303	protein_codir	glycine dehydrogenase
TcG_12503	268,3931726	0,015874742	0,1153534	0,13761833	0,89054207	0,94667588	protein_codir	putative retrotransposon hot spot (RHS) protein
TcG_12504	32,81137423	0,213047801	0,30063335	0,70866322	0,47853349	0,69303613	protein_codir	dispersed gene family protein 1 (DGF-1)
TcG_12505	21,47690627	-0,461255044	0,38771552	-1,18967392	0,23417458	0,46189082	protein_codir	putative dispersed protein family protein 1 (DGF-1)
TcG_12506	62,9921243	0,613117245	0,24700429	2,48221295	0,01305692	0,06104822	protein_codir	putative trans-sialidase
TcG_12507	57,48674686	0,295515543	0,23694988	1,24716475	0,21233709	0,43557676	protein_codir	hypothetical protein
TcG_12508	32,03905804	0,267410715	0,31955763	0,83681529	0,4026964	0,6328518	protein_codir	putative dispersed gene family protein 1 (DGF-1)
TcG_12509	26,876603	-0,100410054	0,34309888	-0,29265632	0,76978486	0,88436428		
TcG_12510	11,66193809	0,749298002	0,5200846	1,4407233	0,14966287	1	protein_codir	hypothetical protein
TcG_12511	19,25634374	0,331264355	0,39522417	0,83816826	0,40193621	0,63220648	protein_codir	hypothetical protein
TcG_12512	68,53352532	-0,126355995	0,2157746	-0,58559254	0,55814936	0,75281939	protein_codir	retrotransposon hot spot (RHS) protein
TcG_12513	98,71039923	0,264984579	0,19240992	1,37718772	0,16845422	0,37494155	protein_codir	retrotransposon hot spot protein (RHS)
TcG_12514	85,539649	-0,007899845	0,19250033	-0,04103809	0,96726553	0,98581443	protein_codir	hypothetical protein
TcG_12515	88,36115564	0,248057928	0,19628169	1,2637854	0,20630706	0,4282877		
TcG_12516	91,52605662	0,019333885	0,19153634	0,10094108	0,91959723	0,9613082		
TcG_12517	105,1389756	0,11215598	0,17367121	0,64579489	0,5184122	0,723392	protein_codir	putative kinesin
TcG_12518	57,72780177	0,400551975	0,24264853	1,65074962	0,09878971	0,26636667	protein_codir	subtilisin-like serine peptidase
TcG_12519	127,3930612	0,118690713	0,16158407	0,73454464	0,46261687	0,68018769	protein_codir	putative kinesin
TcG_12520	44,14022658	-0,194335691	0,26372434	-0,73688948	0,46118956	0,67929148	protein_codir	amastigote surface protein 4
TcG_12521	44,8300914	0,263425136	0,28663832	0,91901578	0,35808732	0,5919736	protein_codir	target of rapamycin (TOR) kinase 1
TcG_12522	46,95482892	0,201946075	0,25637258	0,78770545	0,43086902	0,65564072	protein_codir	target of rapamycin (TOR) kinase 1
TcG_12523	99,84697889	0,423987409	0,18009199	2,35428243	0,0185585	0,07916746	protein_codir	hypothetical protein
TcG_12524	36,29281069	0,744101702	0,30029353	2,47791451	0,01321528	0,06161458	protein_codir	hypothetical protein
TcG_12525	63,61740408	0,358883803	0,21819451	1,64478841	0,10001345	0,2687905	protein_codir	structural maintenance of chromosome protein 4
TcG_12526	25,87869824	0,110387358	0,35695584	0,30924654	0,75713399	0,87782993	protein_codir	trans-sialidase
TcG_12527	21,48429422	0,049740247	0,38271453	0,12996697	0,89659256	0,94988308	protein_codir	trans-sialidase
TcG_12528	123,318759	-0,128810397	0,16291854	-0,79064294	0,42915238	0,65398767	protein_codir	amastigote surface protein 4
TcG_12529	390,9578545	-0,409724382	0,09942151	-4,121084	3,7709E-05	0,0004817	protein_codir	hypothetical protein

TcG_12530	34,34007448	0,636737081	0,3093488	2,05831436	0,03955997	0,14012283	protein_codir dispersed gene family protein 1 (DGF-1)
TcG_12531	86,01311647	-0,150495762	0,19530163	-0,77058117	0,44095522	0,66354606	protein_codir hypothetical protein
TcG_12532	534,5113988	-0,294776483	0,08058304	-3,65804607	0,00025415	0,00243551	protein_codir hypothetical protein
TcG_12533	51,71368421	-0,165673	0,24101572	-0,687395	0,49183384	0,70263551	protein_codir trans-sialidase
TcG_12534	2,319704097	1,329479437	1,21132965	1,09753727	0,2724066	1	protein_codir hypothetical protein
TcG_12535	12,99608223	0,337981204	0,48876947	0,69149409	0,48925509	1	protein_codir hypothetical protein
TcG_12536	23,03846889	0,015300572	0,37908869	0,04036146	0,96780496	0,98610397	protein_codir hypothetical protein
TcG_12537	15,43432884	0,215069288	0,45049669	0,47740481	0,63307389	1	protein_codir retrotransposon hot spot (RHS) protein
TcG_12538	140,9828179	0,08106634	0,15369956	0,52743379	0,59789239	0,78176066	
TcG_12539	81,77212871	0,152002814	0,19626568	0,77447477	0,43865008	0,66208961	protein_codir putative retrotransposon hot spot (RHS) protein
TcG_12540	66,7417721	-0,302448662	0,21890123	-1,38166725	0,16707389	0,37307211	protein_codir mucin-associated surface protein (MASP)
TcG_12541	8,236960309	-0,477511826	0,61781475	-0,77290454	0,43957888	1	protein_codir hypothetical protein
TcG_12542	48,5175316	0,024634188	0,25176344	0,09784657	0,92205413	0,96209148	protein_codir putative retrotransposon hot spot (RHS) protein
TcG_12543	198,4963128	-0,042251398	0,13538901	-0,31207406	0,75498424	0,87636394	protein_codir mucin-associated surface protein (MASP)
TcG_12544	6,866282329	-0,441808505	0,67453992	-0,65497755	0,51248221	1	protein_codir hypothetical protein
TcG_12545	3,561869839	-0,329362797	0,91498548	-0,35996506	0,71887327	1	protein_codir hypothetical protein
TcG_12546	64,46824762	0,193850968	0,2254468	0,85985237	0,38987043	0,6201653	protein_codir hypothetical protein
TcG_12547	39,90926313	0,199163859	0,28045382	0,71014851	0,47761205	0,69260885	protein_codir trans-sialidase
TcG_12548	30,59755306	-0,064026115	0,31356754	-0,20418605	0,83820811	0,92144011	
TcG_12549	10,41459847	-0,378818737	0,53578952	-0,70702901	0,47954845	1	protein_codir hypothetical protein
TcG_12550	3,900234066	-0,718406721	0,88930356	-0,8078307	0,41918805	1	protein_codir thimet oligopeptidase
TcG_12551	2,903093081	-0,251663433	1,03496088	-0,24316227	0,8078797	1	
TcG_12552	67,27801246	0,337771843	0,21102801	1,60060197	0,10946511	0,28603129	protein_codir putative retrotransposon hot spot (RHS) protein
TcG_12553	30,45407946	-0,103777824	0,33730511	-0,30766751	0,75833534	0,87801518	protein_codir mucin-associated surface protein (MASP)
TcG_12554	147,218085	0,112049942	0,14930174	0,75049321	0,45295771	0,67355117	protein_codir mucin-associated surface protein (MASP)
TcG_12555	349,0143643	0,521721137	0,09938693	5,24939357	1,526E-07	3,8186E-06	protein_codir putative retrotransposon hot spot (RHS) protein
TcG_12556	193,9624122	0,552354296	0,13056058	4,23063592	2,3303E-05	0,00031615	
TcG_12557	17,04720644	0,285885917	0,42861025	0,66700673	0,50476784	0,71221932	protein_codir dispersed gene family protein 1 (DGF-1)
TcG_12558	16,76272256	0,063083981	0,42583322	0,14814246	0,88223034	1	protein_codir dispersed gene family protein 1 (DGF-1)
TcG_12559	2,20014136	0,408830624	1,17964814	0,34656997	0,72891441	1	protein_codir dispersed protein family protein 1
TcG_12560	327,7381747	0,305220314	0,10155227	3,00554901	0,00265102	0,01718786	protein_codir surface protease GP63
TcG_12561	141,7561263	0,290704099	0,15722455	1,84897396	0,06446157	0,19990679	
TcG_12562	162,9818521	0,159257738	0,14180531	1,1230732	0,26140641	0,49358779	
TcG_12563	354,6235147	0,257405482	0,09642993	2,66935256	0,00759976	0,03986005	protein_codir surface protease GP63
TcG_12564	65,09125948	0,150491377	0,21702245	0,69343691	0,48803541	0,69945302	protein_codir hypothetical protein
TcG_12565	150,4608302	0,205072069	0,1483691	1,38217506	0,16691796	0,37291003	protein_codir retrotransposon hot spot (RHS) protein
TcG_12566	17,91016514	0,047481773	0,41087229	0,11556334	0,90799862	0,95541477	protein_codir putative trans-sialidase
TcG_12567	12,38246529	-0,4979846	0,49791049	-1,00014884	0,31723849	1	protein_codir putative trans-sialidase
TcG_12568	20,35765639	-0,025283916	0,38404888	-0,06583515	0,94750907	0,97539448	protein_codir hypothetical protein
TcG_12569	39,90088822	-0,074084251	0,2836041	-0,26122419	0,79391962	0,89713769	protein_codir surface protease GP63
TcG_12570	154,2714561	0,08103268	0,14351664	0,5646222	0,57233076	0,76350307	protein_codir complement regulatory protein
TcG_12571	25,85530538	-0,002708131	0,33607122	-0,00805821	0,99357055	0,99761751	protein_codir hypothetical protein
TcG_12572	17,75589828	-0,324382955	0,42694217	-0,75978195	0,44738494	0,66854651	protein_codir hypothetical protein
TcG_12573	66,41118714	-0,252172255	0,21423873	-1,17706193	0,23917079	0,4673677	protein_codir ATPase
TcG_12574	14,81386245	-0,01187851	0,45237495	-0,02625811	0,97905147	1	protein_codir retrotransposon hot spot (RHS) protein
TcG_12575	22,69418613	0,093773999	0,37070077	0,25296413	0,80029593	0,90062207	protein_codir hypothetical protein
TcG_12576	9,522664262	-1,058561065	0,58270682	-1,81662721	0,06927421	1	protein_codir hypothetical protein
TcG_12577	14,2754681	-0,476562316	0,46042934	-1,03503898	0,30065071	1	protein_codir trans-sialidase
TcG_12578	29,58253439	-0,037912165	0,31562381	-0,1201182	0,90438951	0,95369549	protein_codir trans-sialidase
TcG_12579	96,03307684	0,410157163	0,18608711	2,20411373	0,02751635	0,10723323	protein_codir L1Tc protein
TcG_12580	69,85175416	0,323095993	0,22070066	1,46395574	0,14320604	0,33967316	protein_codir L1Tc protein
TcG_12581	2706,790555	0,163986783	0,05458861	3,00404737	0,00266414	0,01726326	protein_codir L1Tc protein

TcG_12582	1081,482729	0,232903364	0,07503499	3,10393004	0,00190968	0,01325681	protein_codir	L1Tc protein
TcG_12583	16,07502241	0,385115342	0,43532445	0,8846628	0,37633853	1	protein_codir	hypothetical protein
TcG_12584	8,406738793	1,081249463	0,64968028	1,66427934	0,09605662	1	protein_codir	trans-sialidase
TcG_12585	11,48239966	0,428954681	0,51282857	0,83644849	0,40290264	1	protein_codir	trans-sialidase
TcG_12586	69,43588824	0,189074954	0,21044078	0,89847107	0,36893446	0,60086146	protein_codir	putative trans-sialidase
TcG_12587	52,82314385	-0,238148318	0,24920581	-0,95562909	0,33925966	0,57339735	protein_codir	putative protein kinase, putative,casein kinase I
TcG_12588	485,8140209	-0,032367443	0,09208784	-0,35148445	0,72522494	0,85817259	protein_codir	putative myosin heavy chain
TcG_12589	592,7064521	0,129052436	0,07988028	1,6155731	0,10618664	0,28057088	protein_codir	retrotransposon hot spot (RHS) protein
TcG_12590	9,100441857	0,224058174	0,5661864	0,39573217	0,69230262	1	protein_codir	hypothetical protein
TcG_12591	11,10717347	0,553585684	0,55061862	1,00538861	0,31470976	1	protein_codir	trans-sialidase
TcG_12592	556,7543453	-0,011011934	0,08305853	-0,13258041	0,89452522	0,94882077	protein_codir	retrotransposon hot spot (RHS) protein
TcG_12593	12,04052607	0,864130659	0,51915927	1,66448086	0,09601637	1	protein_codir	hypothetical protein
TcG_12594	123,8442676	0,173630841	0,16810915	1,03284586	0,30167604	0,53500973	protein_codir	retrotransposon hot spot (RHS) protein
TcG_12595	20,78633448	-0,557837964	0,38116773	-1,46349734	0,14333134	0,33978217	protein_codir	putative trans-sialidase
TcG_12596	13,423397	-0,246595408	0,47039684	-0,52422846	0,60011965	1	protein_codir	putative trans-sialidase
TcG_12597	15,41725383	0,237392591	0,45707822	0,51936973	0,60350294	1	protein_codir	hypothetical protein
TcG_12598	1,131647384	-2,750683068	3,9593398	-0,69473276	0,48722279	1	protein_codir	hypothetical protein
TcG_12599	45,56238644	0,508124935	0,28144954	1,80538558	0,07101437	0,2138197	protein_codir	putative mucin-associated surface protein (MASP)
TcG_12600	35,66754597	0,291170729	0,28837833	1,0096831	0,31264714	0,54676676	protein_codir	dispersed gene family protein 1 (DGF-1)
TcG_12601	35,32887735	0,23924187	0,29734672	0,80458891	0,42105695	0,647488	protein_codir	dispersed gene family protein 1 (DGF-1)
TcG_12602	703,5252655	-0,169746577	0,07188181	-2,3614676	0,01820276	0,07802806	protein_codir	retrotransposon hot spot (RHS) protein
TcG_12603	24,53766488	0,402802813	0,35930621	1,1210572	0,26226352	0,49432001	protein_codir	hypothetical protein
TcG_12604	100,9770893	0,107091315	0,17767036	0,60275283	0,54667313	0,74357301		
TcG_12605	159,6597769	0,225385691	0,14319499	1,57397747	0,11549266	0,29583886	protein_codir	L1Tc protein
TcG_12606	0					1	protein_codir	L1Tc protein
TcG_12607	302,855773	0,396873668	0,10403473	3,81481913	0,00013628	0,00144462	protein_codir	L1Tc protein
TcG_12608	14,60359891	0,633268573	0,4546502	1,39286988	0,16365914	1	protein_codir	dispersed gene family protein 1 (DGF-1)
TcG_12609	52,15334163	-0,030027114	0,24385016	-0,12313756	0,90199817	0,95270504	protein_codir	trans-sialidase
TcG_12610	21,55203823	0,049956181	0,37668723	0,13261979	0,89449408	0,94882077		
TcG_12611	5,281794705	-0,764207922	0,7790066	-0,98100314	0,32659119	1	protein_codir	hypothetical protein
TcG_12612	11,91882323	0,112634686	0,53104241	0,21210111	0,83202815	1	protein_codir	dispersed gene family protein 1 (DGF-1)
TcG_12613	37,64122103	-0,034067705	0,30973988	-0,10998811	0,9124188	0,95723874	protein_codir	dispersed gene family protein 1 (DGF-1)
TcG_12614	17,62512381	-0,354028005	0,41354586	-0,8560792	0,39195397	0,62139829	protein_codir	mucin-associated surface protein (MASP)
TcG_12615	41,49468912	-0,758719543	0,2859711	-2,65313363	0,00797483	0,04139622	protein_codir	mucin TcMUCII
TcG_12616	5,364572526	-0,15396625	0,75360579	-0,20430609	0,83811431	1	protein_codir	putative trans-sialidase
TcG_12617	2,70857759	-0,432667471	1,09150568	-0,39639507	0,6918136	1	protein_codir	hypothetical protein
TcG_12618	28,84581811	-0,745370828	0,33568577	-2,22044213	0,02638877	0,10388729	protein_codir	hypothetical protein
TcG_12619	61,96777738	-0,396690023	0,2215169	-1,79078894	0,07332717	0,21811774	protein_codir	hypothetical protein
TcG_12620	33,64929813	-0,612740398	0,300344	-2,04012866	0,04133751	0,14469378	protein_codir	mitotic centromere-associated kinesin (MCAK)
TcG_12621	52,56822008	0,276553185	0,25952161	1,06562682	0,28659237	0,51971501	protein_codir	hypothetical protein
TcG_12622	39,22378697	0,694764171	0,31484692	2,20667293	0,02733692	0,10674942	protein_codir	dispersed protein family protein 1
TcG_12623	18,23139193	0,603752355	0,43827936	1,37755143	0,16834183	0,37486227	protein_codir	dispersed gene family protein 1 (DGF-1)
TcG_12624	1,360160873	0,283683357	1,59621509	0,17772251	0,8589409	1	protein_codir	dispersed gene family protein 1 (DGF-1)
TcG_12625	2,54008835	0,611396707	1,1655263	0,52456706	0,59988419	1	protein_codir	dispersed gene family protein 1 (DGF-1)
TcG_12626	2,041224967	1,257677982	1,29303262	0,97265758	0,33072351	1	protein_codir	hypothetical protein
TcG_12627	6,814012237	0,627001977	0,75461098	0,83089432	0,40603334	1	protein_codir	dispersed gene family protein 1 (DGF-1)
TcG_12628	5,767747584	0,97696391	0,75894144	1,28727179	0,19799957	1	protein_codir	hypothetical protein
TcG_12629	7,369283826	-0,067745647	0,6349947	-0,10668695	0,91503734	1	protein_codir	hypothetical protein
TcG_12630	5,808317794	0,334120802	0,74526466	0,44832503	0,65391864	1	protein_codir	hypothetical protein
TcG_12631	13,10031742	0,911161466	0,49536161	1,83938653	0,06585835	1	protein_codir	retrotransposon hot spot (RHS) protein
TcG_12632	40,16680302	0,08874232	0,28967541	0,30635089	0,75933749	0,8784787	protein_codir	trans-sialidase
TcG_12633	24,75091589	0,635854713	0,37122172	1,71287046	0,08673637	0,24409221	protein_codir	trans-sialidase

TcG_12634	522,0460654	0,742869374	0,08433943	8,80809096	1,273E-18	2,0164E-16	protein_codir dispersed protein family protein 1 (DGF-1)
TcG_12635	158,7985809	0,485309927	0,14557268	3,33379805	0,00085669	0,00686892	protein_codir dispersed gene family protein 1 (DGF-1)
TcG_12636	20,49522412	-0,005927079	0,39110762	-0,0151546	0,98790884	0,99514516	protein_codir hypothetical protein
TcG_12637	10,65399599	-0,756561128	0,55114394	-1,37271059	0,16984234	1	protein_codir hypothetical protein
TcG_12638	46,75122624	0,170333612	0,25447388	0,66935597	0,50326843	0,71168901	protein_codir hypothetical protein
TcG_12639	48,22148542	-0,241664292	0,25697864	-0,94040615	0,34700927	0,5815781	protein_codir hypothetical protein
TcG_12640	4652,084563	0,444646292	0,04127715	10,772213	4,6567E-27	1,7984E-24	protein_codir putative hexose transporter
TcG_12641	5,528146715	0,72931619	0,7895796	0,92367659	0,35565473	1	protein_codir retrotransposon hot spot protein (RHS)
TcG_12642	9,279785138	0,148939627	0,64222879	0,23191054	0,8166075	1	
TcG_12643	7,949311531	-0,374633971	0,60622712	-0,61797626	0,53659099	1	protein_codir dispersed protein family protein 1
TcG_12644	30,52064061	-0,161848436	0,31653341	-0,51131548	0,60913016	0,788218	protein_codir hypothetical protein
TcG_12645	10,9285252	-0,873605186	0,55182868	-1,58310943	0,11339652	1	protein_codir hypothetical protein
TcG_12646	5,813055365	0,273054515	0,71705711	0,38079884	0,70335252	1	protein_codir hypothetical protein
TcG_12647	5,557616564	-0,813069744	0,76332733	-1,06516524	0,28680116	1	
TcG_12648	43,37227208	-0,122596678	0,30461205	-0,40246824	0,68733946	0,83764801	protein_codir dispersed gene family protein 1 (DGF-1)
TcG_12649	50,47774519	0,007367778	0,24534655	0,03003008	0,97604306	0,98978872	protein_codir dispersed gene family protein 1 (DGF-1)
TcG_12650	4,64947188	1,725505377	0,96783074	1,78285861	0,07460932	1	protein_codir hypothetical protein
TcG_12651	6,646093693	0,019831011	0,70543386	0,02811179	0,97757299	1	protein_codir putative leucine-rich repeat protein
TcG_12652	4,803255655	0,23543903	0,79682101	0,29547292	0,76763264	1	protein_codir mucin-associated surface protein (MASP)
TcG_12653	5,413648052	-0,645224882	0,77298195	-0,83472179	0,40387437	1	protein_codir hypothetical protein
TcG_12654	0,863297986	-1,045201145	1,92159747	-0,54392304	0,58649443	1	protein_codir mucin TcMUCII
TcG_12655	3,716878848	-0,35518552	0,88245422	-0,40249739	0,68731801	1	protein_codir hypothetical protein
TcG_12656	3,38368756	0,273544784	0,97592812	0,28029194	0,77925353	1	protein_codir hypothetical protein
TcG_12657	3,437558041	0,321082464	0,94272829	0,34058855	0,73341335	1	protein_codir hypothetical protein
TcG_12658	42,56167561	0,333737524	0,30321488	1,10066342	0,27104317	0,50430483	protein_codir kinesin
TcG_12659	30,12438336	0,81249364	0,33355089	2,4358911	0,01485516	0,06732284	protein_codir subtilisin-like serine peptidase
TcG_12660	16,9158923	0,890148373	0,42967679	2,07166968	0,03829626	0,13697102	protein_codir retrotransposon hot spot (RHS) protein
TcG_12661	34,80691668	1,046962684	0,31843657	3,28782172	0,00100966	0,00784041	protein_codir SH3 domain protein
TcG_12662	44,44004012	1,041116015	0,27208766	3,82639925	0,00013003	0,00138852	protein_codir surface protease GP63
TcG_12663	17,13216113	0,040564622	0,4173456	0,09719672	0,92257017	0,96227026	protein_codir hypothetical protein
TcG_12664	28,80711392	0,147457687	0,3192523	0,46188449	0,64416416	0,8110828	protein_codir trans-sialidase
TcG_12665	112,1251986	0,721137856	0,17166197	4,20091799	2,6583E-05	0,00035524	protein_codir dispersed gene family protein 1 (DGF-1)
TcG_12666	127,1839566	0,80807928	0,16816896	4,80516298	1,5463E-06	2,9739E-05	protein_codir dispersed gene family protein 1 (DGF-1)
TcG_12667	1,460356001	-0,357719607	1,40870826	-0,25393449	0,79954617	1	protein_codir hypothetical protein
TcG_12668	0,350783209	1,758746969	2,99002236	0,58820529	0,55639451	1	protein_codir trans-sialidase
TcG_12669	0,4558669	-0,458748245	2,66621337	-0,17205984	0,86339048	1	protein_codir hypothetical protein
TcG_12670	5,365183408	-0,847943059	0,78104477	-1,08565231	0,27763285	1	protein_codir hypothetical protein
TcG_12671	2,934552166	-0,820278824	1,07772162	-0,7611231	0,44658354	1	protein_codir hypothetical protein
TcG_12672	5,426881713	-0,043341875	0,748956	-0,05786972	0,9538524	1	
TcG_12673	97,31646707	-0,111495371	0,17967612	-0,62053528	0,53490544	0,73509987	protein_codir putative trans-sialidase
TcG_12674	111,0053441	-0,410978649	0,16787714	-2,44809176	0,01436151	0,06576776	protein_codir hypothetical protein
TcG_12675	26,18128036	-0,59190491	0,34151617	-1,73316802	0,08306581	0,23681114	protein_codir mucin TcMUCII
TcG_12676	31,12828659	-0,154683993	0,3434014	-0,4504466	0,65238845	0,8161724	
TcG_12677	34,28003458	0,655997803	0,3031389	2,16401725	0,03046303	0,11560515	protein_codir SH3 domain protein
TcG_12678	26,35672877	-0,009845291	0,36278898	-0,02713779	0,97834983	0,99085144	protein_codir hypothetical protein
TcG_12679	32,70135071	0,266444589	0,31123428	0,8560901	0,39194794	0,62139829	protein_codir putative GAG protein
TcG_12680	40,91203058	0,513556062	0,28779945	1,78442333	0,0743549	0,22015933	protein_codir putative amino acid transporter
TcG_12681	38,61303897	0,12521544	0,28400519	0,44089138	0,65929163	0,82099665	protein_codir hypothetical protein
TcG_12682	20,95447845	0,083973241	0,38458095	0,21834998	0,82715644	0,9155856	protein_codir putative retrotransposon hot spot (RHS) protein
TcG_12683	25,02596013	-0,361243307	0,36331887	-0,99428722	0,32008305	0,55518714	protein_codir retrotransposon hot spot (RHS) protein
TcG_12684	1,312875217	2,262617112	1,73159786	1,3066643	0,19132675	1	
TcG_12685	8,799017691	0,045342573	0,59564437	0,07612357	0,93932079	1	protein_codir target of rapamycin (TOR) kinase 1

TcG_12686	15,63580876	0,354555241	0,46251282	0,76658467	0,44332849	1	protein_codir	putative trans-sialidase
TcG_12687	13,8688299	-0,656396154	0,47430204	-1,38392014	0,16638291	1	protein_codir	helicase-like protein
TcG_12688	4,584241706	0,438672361	0,82029215	0,53477576	0,59280492	1	protein_codir	hypothetical protein
TcG_12689	3,433415003	1,521561575	1,05535688	1,44175075	0,1493727	1	protein_codir	hypothetical protein
TcG_12690	2,113271668	-1,315955307	1,28091444	-1,02735613	0,3042528	1	protein_codir	hypothetical protein
TcG_12691	41,98587966	0,05214204	0,27861271	0,18714882	0,85154395	0,92821415	protein_codir	hypothetical protein
TcG_12692	29,36515788	0,023594219	0,3293191	0,07164546	0,94288406	0,97319113	protein_codir	trans-sialidase
TcG_12693	2,177087773	0,664187737	1,28535649	0,51673426	0,60534168	1	protein_codir	hypothetical protein
TcG_12694	32,06278642	0,058739389	0,31850527	0,18442203	0,85368239	0,92909893	protein_codir	dispersed gene family protein 1 (DGF-1)
TcG_12695	213,5008164	-0,024954287	0,12374068	-0,201666	0,84017785	0,92224543	protein_codir	sialidase-like protein
TcG_12696	59,2591252	0,563603462	0,23302952	2,41859252	0,01558068	0,06964219	protein_codir	trans-sialidase-like protein
TcG_12697	50,05319456	0,081731798	0,27077064	0,30184882	0,76276731	0,88013366	protein_codir	putative kinesin
TcG_12698	63,32271775	-0,087515163	0,21753427	-0,40230518	0,68745944	0,83768363	protein_codir	subtilisin-like serine peptidase
TcG_12699	51,61601214	0,039202444	0,25153062	0,15585555	0,87614688	0,94060765	protein_codir	putative kinesin
TcG_12700	769,4864688	-0,034125921	0,07334903	-0,46525387	0,64174966	0,80983762	protein_codir	putative glutamine synthetase
TcG_12701	5,40861033	0,292991079	0,79356976	0,36920646	0,71197384	1	protein_codir	hypothetical protein
TcG_12702	3,501048187	0,495590597	0,9451321	0,52436119	0,60002734	1	protein_codir	hypothetical protein
TcG_12703	4,537642073	-0,124418555	0,8315811	-0,14961686	0,8810669	1	protein_codir	trans-sialidase
TcG_12704	38,98807207	0,163321222	0,27881686	0,58576522	0,5580333	0,75275047	protein_codir	retrotransposon hot spot (RHS) protein
TcG_12705	9,550652334	0,379306657	0,60533205	0,62660924	0,53091541	1	protein_codir	hypothetical protein
TcG_12706	3,660102249	0,036728519	0,90495787	0,04058589	0,96762603	1	protein_codir	trans-sialidase
TcG_12707	29,249465	0,231923792	0,32661005	0,71009386	0,47764593	0,69260885	protein_codir	trans-sialidase
TcG_12708	39,04088587	0,015956137	0,28499229	0,05598796	0,9553514	0,97918447	protein_codir	dispersed gene family protein 1 (DGF-1)
TcG_12709	2,258523343	-1,473995237	1,24262168	-1,18619791	0,23554415	1	protein_codir	hypothetical protein
TcG_12710	104,0721311	-1,155348184	0,1767867	-6,53526652	6,3496E-11	3,299E-09		
TcG_12711	5,851476465	-0,617322911	0,74554456	-0,82801612	0,40766137	1	protein_codir	mucin TcMUCII
TcG_12712	24,59876096	-0,121693151	0,35577555	-0,34205035	0,732313	0,86251687	protein_codir	mucin TcMUCII
TcG_12713	41,98266294	-0,152932911	0,26582501	-0,57531423	0,5650788	0,75783767	protein_codir	mucin TcMUCII
TcG_12714	71,24218924	0,035732852	0,21628742	0,16521003	0,86877867	0,93674123	protein_codir	trans-sialidase-like protein
TcG_12715	24,1796245	0,302375276	0,34908642	0,86619031	0,38638581	0,6167056	protein_codir	putative kinesin
TcG_12716	9,754183343	0,058130726	0,56172718	0,10348569	0,9175775	1	protein_codir	putative trans-sialidase
TcG_12717	46,00091781	0,277480788	0,26037643	1,06569088	0,2865634	0,51971501	protein_codir	hypothetical protein
TcG_12718	21,82983777	0,650071634	0,39711861	1,63697098	0,10163653	0,2719463	protein_codir	putative kinesin
TcG_12719	91,32548156	-0,319025353	0,19884832	-1,60436532	0,10863355	0,28454709	protein_codir	putative trans-sialidase
TcG_12720	12,17491958	-0,135687709	0,51094691	-0,26556127	0,79057712	1	protein_codir	hypothetical protein
TcG_12721	1,18464954	0,302518536	1,7208047	0,17580062	0,86045058	1		
TcG_12722	79,58622177	0,045307392	0,21852669	0,20733116	0,83575124	0,92052325	protein_codir	trans-sialidase
TcG_12723	51,4604724	-0,002353818	0,25698921	-0,00915921	0,99269211	0,99708113	protein_codir	hypothetical protein
TcG_12724	442,0402247	-0,104556758	0,08741276	-1,19612691	0,23164704	0,45893683		
TcG_12725	21,89086602	0,23145829	0,36996351	0,62562464	0,53156117	0,73268958	protein_codir	trans-sialidase
TcG_12726	5,82340997	-0,357404733	0,71316651	-0,50115187	0,61626424	1	protein_codir	hypothetical protein
TcG_12727	15,45103262	0,260971532	0,44376911	0,58807954	0,5564789	1	protein_codir	retrotransposon hot spot (RHS) protein
TcG_12728	155,3075757	0,314648602	0,14203664	2,21526363	0,02674197	0,10502797	protein_codir	putative dispersed gene family protein 1 (DGF-1)
TcG_12729	132,4489334	-0,1300937	0,15419307	-0,84370653	0,39883342	0,62877725	protein_codir	dispersed gene family protein 1 (DGF-1)
TcG_12730	2,469871333	-0,602061194	1,09820552	-0,5482227	0,583539	1	protein_codir	hypothetical protein
TcG_12731	163,3739217	-0,247306389	0,13673268	-1,80868533	0,0704999	0,21293323	protein_codir	trans-sialidase-like protein
TcG_12732	35,65179863	0,344217204	0,3017902	1,14058444	0,2540429	0,48505949	protein_codir	hypothetical protein
TcG_12733	35,17996879	0,201865977	0,30700166	0,65754034	0,51083354	0,71748302	protein_codir	hypothetical protein
TcG_12734	19,6692948	-0,018152667	0,38592035	-0,04703734	0,96248347	0,9826695	protein_codir	hypothetical protein
TcG_12735	10,76197107	0,68813338	0,53194192	1,29362504	0,19579497	1	protein_codir	hypothetical protein
TcG_12736	1,213368591	-0,268484184	1,53561106	-0,17483866	0,86120641	1		
TcG_12737	7,255106488	0,137449699	0,64240892	0,21395982	0,83057839	1	protein_codir	putative trans-sialidase

TcG_12738	16,08988669	-0,375403923	0,42898589	-0,87509621	0,38152156	1	protein_codir trans-sialidase
TcG_12739	13,78606492	-0,597133314	0,46365549	-1,28788148	0,19778722	1	protein_codir trans-sialidase
TcG_12740	8,522769438	0,754938945	0,60294911	1,2520774	0,21054166	1	protein_codir putative trans-sialidase
TcG_12741	13,88624757	0,34630601	0,4663607	0,74257117	0,45774135	1	protein_codir putative GAG protein
TcG_12742	39,7151218	0,486167184	0,28398831	1,71192672	0,08691017	0,24434391	protein_codir hypothetical protein
TcG_12743	38,12923321	0,311348459	0,29183348	1,06687026	0,28603042	0,51918774	protein_codir target of rapamycin (TOR) kinase 1
TcG_12744	3,170978213	-0,004743017	0,99345561	-0,00477426	0,9961907	1	protein_codir hypothetical protein
TcG_12745	2,348616646	-0,321158477	1,1174968	-0,28739096	0,77381298	1	protein_codir hypothetical protein
TcG_12746	6,13632812	-0,984732519	0,7509502	-1,31131535	0,18975125	1	protein_codir hypothetical protein
TcG_12747	6,691674074	0,151831993	0,67157012	0,22608509	0,82113524	1	
TcG_12748	0,155721614	0,503022807	4,08047286	0,12327562	0,90188885	1	
TcG_12749	110,16893	0,210969288	0,17527972	1,20361491	0,22873845	0,45574613	protein_codir hypothetical protein
TcG_12750	74,32523582	-0,046784275	0,20607343	-0,22702721	0,82040258	0,91114306	protein_codir retrotransposon hot spot (RHS) protein
TcG_12751	74,3781024	0,080006693	0,20784503	0,38493436	0,70028605	0,84438126	protein_codir hypothetical protein
TcG_12752	53,82053199	-0,031402496	0,23731005	-0,13232687	0,89472576	0,94885974	protein_codir hypothetical protein
TcG_12753	56,71555982	-0,221589441	0,23788446	-0,93150026	0,35159485	0,58601758	protein_codir mucin-associated surface protein (MASP)
TcG_12754	31,86232226	0,686429897	0,31647363	2,16899557	0,03008302	0,11472741	protein_codir mucin TcMUCII
TcG_12755	7,770825565	-0,575797536	0,64433593	-0,8936294	0,37152021	1	
TcG_12756	135,6531976	-0,030878461	0,15188662	-0,20329942	0,83890101	0,92171713	protein_codir retrotransposon hot spot (RHS) protein
TcG_12757	32,06984159	0,485653925	0,31888358	1,52298193	0,12776323	0,31654541	protein_codir putative retrotransposon hot spot (RHS) protein
TcG_12758	89,07725923	0,122707917	0,1868919	0,65657162	0,5114564	0,71768359	
TcG_12759	17,85880525	0,415174788	0,41923796	0,99030819	0,32202351	0,55677725	protein_codir retrotransposon hot spot (RHS) protein
TcG_12760	31,67008066	-0,003233176	0,32070329	-0,01008152	0,99195625	0,99678187	protein_codir kinesin
TcG_12761	53,03991115	-0,291576134	0,2562001	-1,13807971	0,2550872	0,48638803	protein_codir subtilisin-like serine peptidase
TcG_12762	25,67545557	-0,06499814	0,34718522	-0,18721459	0,85149238	0,92821415	protein_codir putative trans-sialidase
TcG_12763	27,70509642	0,00560781	0,32425262	0,01729457	0,98620162	0,99461455	protein_codir mitotic centromere-associated kinesin (MCAK)
TcG_12764	17,31465021	0,151726595	0,41703487	0,36382233	0,71599069	0,85423418	
TcG_12765	21,64872122	0,114466722	0,38227035	0,29943918	0,76460497	0,88129227	protein_codir hypothetical protein
TcG_12766	30,70674195	-0,08868823	0,30885548	-0,28715123	0,77399653	0,88691888	protein_codir hypothetical protein
TcG_12767	1,081386522	-1,191968622	1,87835297	-0,63458181	0,5257012	1	protein_codir hypothetical protein
TcG_12768	160,785276	0,035218958	0,13946419	0,25253048	0,80063106	0,90076825	protein_codir trans-sialidase
TcG_12769	85,48823537	0,086047545	0,20119281	0,42768698	0,66887903	0,8263077	protein_codir trans-sialidase
TcG_12770	37,36757087	-0,107239813	0,29044567	-0,36922504	0,71195999	0,85196948	protein_codir mucin TcMUCII
TcG_12771	13,75679884	-0,185842501	0,46786332	-0,39721537	0,69120865	1	protein_codir hypothetical protein
TcG_12772	41,81653526	0,014360282	0,26617297	0,05395094	0,95697425	0,9800675	protein_codir hypothetical protein
TcG_12773	40,56598506	-0,283878679	0,27680121	-1,02556878	0,30509489	0,53795118	protein_codir hypothetical protein
TcG_12774	18,36686683	0,392123923	0,41197977	0,95180383	0,34119648	0,57558276	protein_codir hypothetical protein
TcG_12775	6,696115229	-1,559532084	0,7376201	-2,11427548	0,03449175	1	
TcG_12776	5,813601469	0,298032671	0,71783634	0,41518192	0,67800873	1	protein_codir hypothetical protein
TcG_12777	17,11356195	-0,300737424	0,4327414	-0,69495875	0,48708115	0,69860389	protein_codir hypothetical protein
TcG_12778	13,57564911	-0,827209395	0,46985197	-1,76057449	0,07831045	1	protein_codir mucin TcMUCII
TcG_12779	6,468379964	-0,314753336	0,68639665	-0,45855896	0,64655091	1	protein_codir RNaseH
TcG_12780	21,03480229	-0,388269205	0,37641819	-1,03148363	0,30231408	0,5353728	protein_codir L1Tc protein
TcG_12781	8,096761513	-0,470012679	0,61606321	-0,7629293	0,44550556	1	protein_codir hypothetical protein
TcG_12782	50,41992305	-0,505605715	0,25138608	-2,01127171	0,04429677	0,15197581	protein_codir mucin TcMUCI
TcG_12783	166,9457144	0,083565961	0,1377322	0,60672784	0,54403155	0,7411982	protein_codir dispersed gene family protein 1 (DGF-1)
TcG_12784	6,070102158	-0,485562887	0,70374509	-0,68996984	0,49021315	1	
TcG_12785	28,68570271	-0,596435756	0,32007088	-1,86344898	0,06239912	0,19518257	protein_codir putative trans-sialidase
TcG_12786	7,720506487	0,241243523	0,64958039	0,37138363	0,71035182	1	protein_codir hypothetical protein
TcG_12787	4,618859051	0,887026542	0,93508579	0,94860445	0,34282183	1	protein_codir hypothetical protein
TcG_12788	11,40950755	0,588353603	0,5268997	1,11663302	0,26415127	1	protein_codir hypothetical protein
TcG_12789	221,3100507	-0,386335554	0,12038753	-3,20909942	0,00133151	0,00984809	protein_codir cAMP phosphodiesterase

TcG_12790	177,2456237	-0,417037372	0,14741948	-2,82891634	0,00467059	0,02723375	protein_codir	putative cAMP specific phosphodiesterase
TcG_12791	17,04234841	1,22690736	0,44366522	2,76539	0,00568548	0,03163878	protein_codir	retrotransposon hot spot (RHS) protein
TcG_12792	23,17872699	0,900333031	0,38014243	2,36840975	0,01786474	0,07704558	protein_codir	retrotransposon hot spot (RHS) protein
TcG_12793	18,95555555	-0,117050519	0,41881129	-0,27948272	0,7798744	0,89039	protein_codir	hypothetical protein
TcG_12794	80,22322544	0,005911302	0,20422527	0,02894501	0,97690845	0,99017719	protein_codir	putative retrotransposon hot spot (RHS) protein
TcG_12795	10,03318038	-0,481644766	0,55667766	-0,86521302	0,38692188	1	protein_codir	retrotransposon hot spot (RHS) protein
TcG_12796	7,786503558	0,084323007	0,64753013	0,13022252	0,89639038	1		
TcG_12797	89,9236845	0,277549472	0,19289517	1,4388617	0,15018971	0,35019078	protein_codir	hypothetical protein
TcG_12798	161,8204654	0,225300049	0,14038257	1,60490039	0,10851573	0,28438435	protein_codir	hypothetical protein
TcG_12799	55,94327035	-0,09777727	0,23555933	-0,41508553	0,67807929	0,83152272		
TcG_12800	24,06531322	-0,251007165	0,36445829	-0,688713	0,49100389	0,70187819		
TcG_12801	18,55566695	0,985232309	0,41572123	2,36993504	0,01779121	0,07685644	protein_codir	hypothetical protein
TcG_12802	5,759335876	0,243661358	0,71820057	0,33926645	0,73440901	1	protein_codir	putative GAG protein
TcG_12803	26,86219736	0,836933128	0,34136242	2,45174361	0,01421659	0,06528475	protein_codir	putative trans-sialidase
TcG_12804	704,8550778	0,415421771	0,07708098	5,38942017	7,0685E-08	1,8957E-06	protein_codir	putative mannosyl-oligosaccharide 1,2-alpha-mannosidase IB
TcG_12805	1103,856724	0,38516735	0,06335495	6,07951483	1,2055E-09	4,7184E-08	protein_codir	putative mannosyl-oligosaccharide 1,2-alpha-mannosidase IB
TcG_12806	59,58177494	-0,546750319	0,23174255	-2,3593005	0,01830942	0,07833566	protein_codir	mucin-associated surface protein (MASP)
TcG_12807	281,3443332	0,206239821	0,10809948	1,90787062	0,05640794	0,18003923	protein_codir	hypothetical protein
TcG_12808	20,66924334	-0,113048963	0,39327218	-0,28745731	0,77376219	0,88691888		
TcG_12809	20,06609592	-0,176374794	0,38496098	-0,45816279	0,64683549	0,81238331	protein_codir	trans-sialidase
TcG_12810	58,98388722	-0,265113891	0,22939276	-1,15572041	0,24779554	0,47722059	protein_codir	putative trans-sialidase
TcG_12811	49,08617417	-0,272637983	0,26356747	-1,03441441	0,30094247	0,53419983		
TcG_12812	1643,829484	0,239401793	0,06420113	3,72893415	0,00019229	0,00193393	protein_codir	L1Tc protein
TcG_12813	7,22000937	0,175069424	0,65036763	0,26918533	0,78778707	1	protein_codir	hypothetical protein
TcG_12814	4,021484118	0,567208406	0,88634436	0,63994135	0,52221073	1		
TcG_12815	144,3376838	-0,113539847	0,1476948	-0,76874643	0,44204386	0,66452837	protein_codir	putative trans-sialidase
TcG_12816	15,72280029	0,056417289	0,46505196	0,12131395	0,90344237	1	protein_codir	hypothetical protein
TcG_12817	43,99296788	-0,007519257	0,26750901	-0,02810843	0,97757567	0,99022484	protein_codir	trans-sialidase
TcG_12818	7,705949276	0,843638758	0,6647918	1,26902702	0,20443143	1	protein_codir	surface protease GP63
TcG_12819	6,876982647	0,958365517	0,68543276	1,39819043	0,16205589	1	protein_codir	hypothetical protein
TcG_12820	24,83264257	-0,217156457	0,35621129	-0,60962823	0,54210811	0,74019653	protein_codir	trans-sialidase
TcG_12821	26,93075947	0,003574309	0,33552097	0,01065301	0,99150029	0,99674814	protein_codir	hypothetical protein
TcG_12822	27,63256122	0,375557854	0,3461751	1,08487831	0,27797556	0,51065198	protein_codir	target of rapamycin (TOR) kinase 1
TcG_12823	0					1	protein_codir	hypothetical protein
TcG_12824	0,701553094	2,707439836	2,05454854	1,31777847	0,18757782	1	protein_codir	trans-sialidase
TcG_12825	0,76867091	1,535796318	2,06738331	0,74286965	0,4575606	1	protein_codir	trans-sialidase
TcG_12826	296,5154378	-0,300708364	0,10696872	-2,81118029	0,00493601	0,02852301	protein_codir	putative trans-sialidase
TcG_12827	68,47382968	-0,426457331	0,21762232	-1,95962131	0,05004007	0,16559961	protein_codir	mucin-associated surface protein (MASP)
TcG_12828	2,50170326	-0,92124696	1,12361419	-0,81989616	0,41227531	1	protein_codir	hypothetical protein
TcG_12829	701,2759114	0,013261271	0,07181732	0,18465284	0,85350134	0,92904714		
TcG_12830	4080,448349	0,572103634	0,04112739	13,9105267	5,4675E-44	7,9184E-41	protein_codir	tyrosine aminotransferase
TcG_12831	348,2038596	0,214278621	0,09761385	2,19516607	0,02815169	0,10906105	protein_codir	putative retrotransposon hot spot (RHS) protein
TcG_12832	34,34382836	-0,256120587	0,30765283	-0,83249871	0,40512751	0,63430422	protein_codir	mucin-associated surface protein (MASP)
TcG_12833	66,87757032	0,306648378	0,21203821	1,44619394	0,14812281	0,3469074	protein_codir	mucin TcMUCII
TcG_12834	15,87185517	0,071448769	0,4356702	0,16399737	0,86973321	1		
TcG_12835	24,76766299	-0,465018714	0,3666242	-1,26837976	0,20466236	0,42594183	protein_codir	retrotransposon hot spot (RHS) protein
TcG_12836	33,92717516	0,109534531	0,32109891	0,34112396	0,73301026	0,86278448	protein_codir	trans-sialidase
TcG_12837	145,6789624	-0,084866667	0,16060977	-0,52840291	0,59721972	0,78114559	protein_codir	putative retrotransposon hot spot (RHS) protein
TcG_12838	160,7853792	-0,213930232	0,14371727	-1,48854922	0,13660611	0,32938989	protein_codir	putative retrotransposon hot spot (RHS) protein
TcG_12839	11,14169566	0,447436535	0,51885241	0,86235802	0,38849054	1	protein_codir	serine/threonine protein phosphatase
TcG_12840	18,04173877	0,065378959	0,41498673	0,1575447	0,87481558	0,93978798	protein_codir	hypothetical protein
TcG_12841	18,12151744	0,401846271	0,41431322	0,96990936	0,33209167	0,5663408	protein_codir	putative trans-sialidase

TcG_12842	38,65769963	0,476605547	0,28637179	1,66428947	0,0960546	0,2620411	protein_codir	retrotransposon hot spot (RHS) protein
TcG_12843	48,07803167	-0,008778367	0,2648354	-0,0331465	0,97355776	0,98834538	protein_codir	putative retrotransposon hot spot (RHS) protein
TcG_12844	7,050305545	0,188046398	0,64262753	0,29262114	0,76981176	1	protein_codir	putative trans-sialidase
TcG_12845	4,356779744	1,968956541	1,048926	1,87711672	0,0605021	1	protein_codir	putative trans-sialidase
TcG_12846	3,627438036	0,504958363	0,98606053	0,51209672	0,60858332	1	protein_codir	hypothetical protein
TcG_12847	6,197952544	0,69833447	0,7301574	0,95641634	0,33886193	1		
TcG_12848	25,76565616	-0,249239671	0,35381317	-0,70443865	0,48115964	0,69536181	protein_codir	hypothetical protein
TcG_12849	141,2051592	0,11218748	0,15864732	0,70715015	0,47947317	0,69387661	protein_codir	retrotransposon hot spot (RHS) protein
TcG_12850	28,39900096	0,161260461	0,33700835	0,47850584	0,63229022	0,80290602	protein_codir	trans-sialidase
TcG_12851	36,11282171	-0,322026918	0,30738945	-1,04761863	0,29481435	0,52793185	protein_codir	trans-sialidase
TcG_12852	5,239841162	0,217996984	0,76744968	0,28405378	0,77636916	1	protein_codir	mucin TcMUCII
TcG_12853	1,124778117	-1,132218905	1,7223639	-0,65736335	0,51094731	1	protein_codir	hypothetical protein
TcG_12854	15,36515566	-0,459717227	0,43785461	-1,04993124	0,29374973	1		
TcG_12855	52,37418279	-0,166780427	0,24074256	-0,692775	0,48845076	0,69978861	protein_codir	trans-sialidase
TcG_12856	50,51325399	-0,024349597	0,24485698	-0,09944416	0,92078562	0,96150609	protein_codir	trans-sialidase
TcG_12857	23,29359865	-0,621074327	0,36495148	-1,70179971	0,08879292	0,24795246	protein_codir	hypothetical protein
TcG_12858	0,441769291	0,396475077	2,50258318	0,15842633	0,87412086	1	protein_codir	hypothetical protein
TcG_12859	2,584252469	0,316121531	1,12482785	0,28103992	0,77867978	1	protein_codir	hypothetical protein
TcG_12860	1,888993355	0,327709614	1,24489805	0,26324213	0,79236396	1	protein_codir	hypothetical protein
TcG_12861	2,934234674	-0,880348259	1,05135893	-0,83734321	0,40239968	1	protein_codir	hypothetical protein
TcG_12862	5,149370799	-0,87225536	0,76912781	-1,13408376	0,25675942	1	protein_codir	hypothetical protein
TcG_12863	23,27191985	0,978311654	0,38360566	2,55030554	0,01076285	0,05234166		
TcG_12864	154,413736	-0,37740383	0,14472626	-2,60770804	0,00911507	0,0458962	protein_codir	trans-sialidase
TcG_12865	81,93901465	-0,142697704	0,20009354	-0,71315499	0,47574986	0,69116462	protein_codir	trans-sialidase
TcG_12866	227,268494	-0,508192487	0,13078739	-3,88563816	0,00010206	0,00113696	protein_codir	proton motive P-type ATPase 1
TcG_12867	8,760672212	0,142944247	0,61455152	0,23259929	0,81607258	1	protein_codir	hypothetical protein
TcG_12868	7,155655626	-0,000350956	0,64172323	-0,0005469	0,99956364	1	protein_codir	hypothetical protein
TcG_12869	22,71407631	0,012985412	0,36929826	0,0351624	0,97195025	0,98777616	protein_codir	trans-sialidase
TcG_12870	40,70938389	0,192937847	0,27274414	0,70739501	0,47932104	0,69382977	protein_codir	rab1 small GTP-binding protein
TcG_12871	10,35501503	0,910867275	0,5606317	1,62471597	0,10422309	1	protein_codir	hypothetical protein
TcG_12872	35,08484326	0,109824803	0,30583276	0,35910085	0,71951965	0,85597645	protein_codir	hypothetical protein
TcG_12873	11,5068652	0,062814295	0,51582546	0,12177432	0,90307775	1	protein_codir	hypothetical protein
TcG_12874	10,8123851	0,536130797	0,53177179	1,00819714	0,31335983	1	protein_codir	hypothetical protein
TcG_12875	43,33664704	0,730339487	0,27747348	2,63210552	0,00848575	0,04342575	protein_codir	multidrug resistance-associated protein
TcG_12876	27,3633175	1,257106196	0,36072576	3,48493603	0,00049225	0,0042777	protein_codir	multidrug resistance-associated protein
TcG_12877	206,3173695	0,376866051	0,12466002	3,02315101	0,00250157	0,01644365	protein_codir	mucin-associated surface protein (MASP)
TcG_12878	71,97077605	0,451045482	0,21178522	2,12973066	0,03319386	0,12329453		
TcG_12879	13,67306008	-0,691084147	0,4702852	-1,4695001	0,14169719	1	protein_codir	trans-sialidase
TcG_12880	7,262026922	-0,591810294	0,67770502	-0,87325647	0,3825233	1	protein_codir	trans-sialidase
TcG_12881	45,91946827	-0,025942809	0,26266946	-0,09876599	0,92132407	0,96166313	protein_codir	putative retrotransposon hot spot (RHS) protein
TcG_12882	30,09764857	-0,037172793	0,31603659	-0,1176218	0,90636733	0,95471088	protein_codir	retrotransposon hot spot (RHS) protein
TcG_12883	102,8658016	-0,0950208	0,17433771	-0,54503872	0,58572689	0,77437942	protein_codir	retrotransposon hot spot (RHS) protein
TcG_12884	35,03610045	-0,435328995	0,30577612	-1,42368541	0,15453756	0,35574651	protein_codir	mucin-associated surface protein (MASP)
TcG_12885	3,073868963	1,64891126	1,10325884	1,49458241	0,13502346	1	protein_codir	hypothetical protein
TcG_12886	4,663032756	-0,564989044	0,80093028	-0,70541601	0,48055138	1	protein_codir	hypothetical protein
TcG_12887	99,5534428	0,039704931	0,19474363	0,20388308	0,83844487	0,92147811	protein_codir	proton motive ATPase
TcG_12888	826,7365048	-0,302659847	0,0672906	-4,49780283	6,8659E-06	0,00011048	protein_codir	proton motive ATPase
TcG_12889	5,886090101	-0,142274251	0,72693298	-0,19571853	0,84483048	1	protein_codir	hypothetical protein
TcG_12890	5,568093265	-0,861932763	0,81706473	-1,05491368	0,2914648	1	protein_codir	hypothetical protein
TcG_12891	1,20250354	-1,476588472	1,73130204	-0,85287745	0,39372727	1	protein_codir	hypothetical protein
TcG_12892	3,927694057	0,014445748	0,87831965	0,01644703	0,98687776	1	protein_codir	hypothetical protein
TcG_12893	22,13037144	0,232485948	0,37159464	0,62564398	0,53154848	0,73268958		

TcG_12894	0,987600772	1,979939579	1,83033272	1,08173752	0,27936917	1	
TcG_12895	109,6809627	-0,599339021	0,17778682	-3,37111048	0,00074866	0,00611272	protein_codir mucin TcMUCII
TcG_12896	0,97939772	-1,359815763	1,94279564	-0,69992733	0,48397269	1	
TcG_12897	4,872119068	-1,306188556	0,80556522	-1,62145599	0,10491988	1	protein_codir hypothetical protein
TcG_12898	5,716699041	0,584651396	0,76192817	0,76733139	0,44288451	1	protein_codir hypothetical protein
TcG_12899	39,04560794	-0,005839959	0,28484477	-0,02050225	0,98364272	0,99376391	protein_codir trans-sialidase
TcG_12900	183,4581462	-0,100225692	0,13531624	-0,74067748	0,45888902	0,67754405	protein_codir sialidase
TcG_12901	20,83777891	0,468649449	0,37794705	1,23998704	0,21498019	0,4388705	
TcG_12902	39,33883707	-0,474321183	0,27788216	-1,70691483	0,08783788	0,24598889	protein_codir putative mucin TcMUCII
TcG_12903	1,911460515	2,115717721	1,43511445	1,47425017	0,14041425	1	protein_codir hypothetical protein
TcG_12904	0,481095949	0,532926224	2,57274452	0,20714308	0,83589812	1	protein_codir hypothetical protein
TcG_12905	1,288840451	1,428846426	1,58567405	0,90109719	0,36753665	1	protein_codir hypothetical protein
TcG_12906	23,07409463	0,117346373	0,37258682	0,31495042	0,7527993	0,87534451	protein_codir dispersed gene family protein 1 (DGF-1)
TcG_12907	4,47942659	0,820260171	0,84679028	0,9686698	0,33270996	1	protein_codir dispersed gene family protein 1 (DGF-1)
TcG_12908	19,24861679	-0,392430583	0,39153289	-1,00229277	0,31620221	0,55115373	protein_codir mucin-associated surface protein (MASP)
TcG_12909	22,60287087	-0,488635765	0,38516062	-1,26865452	0,20456431	0,42581424	protein_codir hypothetical protein
TcG_12910	34,32659003	-0,540327432	0,29700108	-1,81927768	0,06886908	0,20970227	protein_codir surface protease GP63
TcG_12911	5,500362089	-0,174788234	0,74819801	-0,23361227	0,815286	1	protein_codir mucin TcMUCII
TcG_12912	0,884770644	1,463065956	1,93727958	0,75521673	0,45011895	1	protein_codir hypothetical protein
TcG_12913	2,142394778	-1,855279794	1,34814204	-1,37617531	0,16876736	1	protein_codir trans-sialidase
TcG_12914	6,828694439	-0,628629032	0,6818846	-0,92189944	0,35658103	1	
TcG_12915	51,01283505	-0,178490183	0,25068391	-0,71201293	0,47645677	0,69175791	protein_codir trans-sialidase
TcG_12916	17,34866661	-0,117706439	0,40983291	-0,28720592	0,77395465	0,88691888	
TcG_12917	2,294488722	-0,633365366	1,14792913	-0,55174605	0,58112235	1	protein_codir hypothetical protein
TcG_12918	10,55199287	0,13720008	0,53232381	0,25773801	0,79660911	1	protein_codir hypothetical protein
TcG_12919	1271,031726	0,175360399	0,05955641	2,94444222	0,00323537	0,02026372	protein_codir kinesin-like protein
TcG_12920	17,17875549	0,875827762	0,43046773	2,03459563	0,04189158	0,14588392	protein_codir kinesin-like protein
TcG_12921	85,40987375	-0,007110453	0,20432028	-0,03480053	0,9722388	0,98777616	protein_codir putative trans-sialidase
TcG_12922	21,82399908	0,246892136	0,36856659	0,66987118	0,50293991	0,71148496	
TcG_12923	15,4117772	-0,054828253	0,46715445	-0,11736644	0,90656968	1	protein_codir hypothetical protein
TcG_12924	15,36179431	-0,071332657	0,4452591	-0,16020483	0,87271973	1	protein_codir hypothetical protein
TcG_12925	10,48829683	-0,116359177	0,56064275	-0,20754603	0,83558345	1	protein_codir trans-sialidase
TcG_12926	10,70805721	-1,023517266	0,54487146	-1,87845636	0,06031876	1	protein_codir mucin-associated surface protein (MASP)
TcG_12927	1,47762139	0,485643037	1,55382137	0,31254753	0,75462444	1	
TcG_12928	8,041635711	0,68880248	0,62504342	1,10200741	0,27045845	1	protein_codir hypothetical protein
TcG_12929	5,22082171	0,650397223	0,82425543	0,78907241	0,43006968	1	protein_codir hypothetical protein
TcG_12930	7,405839649	0,295851771	0,63369903	0,4668648	0,64059661	1	protein_codir hypothetical protein
TcG_12931	10,21456824	0,19009647	0,55149715	0,34469166	0,73032619	1	protein_codir putative ATP-dependent DEAD/H RNA helicase
TcG_12932	136,584387	0,171816252	0,15416352	1,11450656	0,26506193	0,4975089	protein_codir retrotransposon hot spot (RHS) protein
TcG_12933	153,6186824	0,265261529	0,1423242	1,86378376	0,06235208	0,19508808	protein_codir retrotransposon hot spot protein (RHS)
TcG_12934	32,19014342	-0,335323955	0,30758833	-1,09017124	0,27563772	0,50795906	protein_codir hypothetical protein
TcG_12935	22,17390811	-0,076054743	0,3793407	-0,20049191	0,84109588	0,92246657	protein_codir hypothetical protein
TcG_12936	7,196745222	-0,195586884	0,65878851	-0,29688873	0,76655146	1	protein_codir hypothetical protein
TcG_12937	47,78448595	0,788430048	0,25801681	3,05573127	0,00224512	0,01511447	protein_codir hypothetical protein
TcG_12938	36,73352089	0,122138698	0,28974813	0,42153403	0,67336517	0,82890021	protein_codir putative target of rapamycin (TOR) kinase 1
TcG_12939	28,13367315	0,461877856	0,34460898	1,34029546	0,18014931	0,39148549	protein_codir target of rapamycin (TOR) kinase 1
TcG_12940	20,36238536	-0,216192502	0,40047916	-0,53983458	0,58931112	0,77564856	
TcG_12941	44,13042799	0,402524646	0,27836244	1,44604512	0,14816455	0,34693501	protein_codir retrotransposon hot spot (RHS) protein
TcG_12942	55,94492021	-0,24555859	0,23501659	-1,0448564	0,29608935	0,52923344	protein_codir retrotransposon hot spot protein (RHS)
TcG_12943	71,93789915	0,362023974	0,21558547	1,67925964	0,09310145	0,25585233	protein_codir putative retrotransposon hot spot (RHS) protein
TcG_12944	2,413338854	0,108828121	1,11492387	0,09761036	0,9222417	1	protein_codir hypothetical protein
TcG_12945	7,228441931	-1,200070648	0,66599447	-1,80192283	0,07155755	1	protein_codir hypothetical protein

TcG_12946	4,057229896	-0,245585448	0,87146574	-0,28180735	0,77809124	1	protein_codir	hypothetical protein
TcG_12947	90,13957791	-0,005321285	0,18600459	-0,02860835	0,97717695	0,99017719	protein_codir	protein kinase, putative,serine/threonine protein kinase
TcG_12948	30,62971227	0,370536622	0,32932688	1,12513325	0,26053257	0,49290176	protein_codir	hypothetical protein
TcG_12949	53,75074838	0,428280985	0,24465781	1,7505306	0,0800268	0,23118071	protein_codir	helicase-like protein
TcG_12950	7,553800141	0,241919956	0,66493186	0,36382668	0,71598743	1	protein_codir	hypothetical protein
TcG_12951	1,790618899	-0,579702448	1,28389255	-0,45151944	0,65161521	1		
TcG_12952	29,29787414	8,22048E-05	0,31869144	0,00025794	0,99979419	0,99981271	protein_codir	putative trans-sialidase
TcG_12953	18,80417614	0,165842512	0,39867234	0,41598701	0,67741951	0,83096283	protein_codir	putative GAG protein
TcG_12954	28,56793972	-0,141835277	0,32303252	-0,4390743	0,6606077	0,82219366	protein_codir	trans-sialidase
TcG_12955	25,94642878	0,113006269	0,33981576	0,33255159	0,73947279	0,86708727	protein_codir	putative trans-sialidase
TcG_12956	32,56826598	-0,21564053	0,31945287	-0,67503081	0,49965619	0,70874347	protein_codir	trans-sialidase
TcG_12957	17,68364221	0,318016214	0,4179918	0,76081927	0,44676502	0,6682442	protein_codir	mitotic centromere-associated kinesin (MCAK)
TcG_12958	6,708291349	-0,296764037	0,72717064	-0,40810784	0,68319451	1	protein_codir	hypothetical protein
TcG_12959	0					1	protein_codir	structural maintenance of chromosome protein 4
TcG_12960	15,58768993	0,126539395	0,45053038	0,28086762	0,77881194	1	protein_codir	trans-sialidase
TcG_12961	28,49402588	-0,092520602	0,32221595	-0,28713849	0,77400628	0,88691888	protein_codir	trans-sialidase
TcG_12962	4,888813833	-0,036728522	0,81079803	-0,04529922	0,96386881	1	protein_codir	hypothetical protein
TcG_12963	4,655244571	1,131760436	0,87970335	1,28652509	0,19825987	1	protein_codir	hypothetical protein
TcG_12964	3,692142257	0,42789037	0,90861891	0,47092391	0,63769507	1	protein_codir	hypothetical protein
TcG_12965	22,83588148	0,11216271	0,39351388	0,2850286	0,77562223	0,88823053	protein_codir	putative retrotransposon hot spot (RHS) protein
TcG_12966	49,94638352	0,294683896	0,26259367	1,12220489	0,26177533	0,49412333	protein_codir	retrotransposon hot spot (RHS) protein
TcG_12967	10,09849924	0,467245111	0,55020665	0,84921748	0,39576029	1	protein_codir	hypothetical protein
TcG_12968	37,03745569	-0,167249226	0,30136042	-0,55498073	0,57890784	0,76847231	protein_codir	hypothetical protein
TcG_12969	6,966883348	0,011426068	0,66191375	0,01726217	0,98622746	1	protein_codir	trans-sialidase
TcG_12970	713,5688021	-0,019904026	0,07064145	-0,28176131	0,77812654	0,88925638	protein_codir	host cell signaling surface protein
TcG_12971	21,69459936	0,012830886	0,3734916	0,03435388	0,97259496	0,9878584	protein_codir	putative dispersed gene family protein 1 (DGF-1)
TcG_12972	20,67868572	0,18349229	0,38526221	0,47627897	0,63387564	0,80419712	protein_codir	dispersed gene family protein 1 (DGF-1)
TcG_12973	8,093906016	0,185002114	0,60458515	0,30599844	0,75960583	1	protein_codir	structural maintenance of chromosome protein 4
TcG_12974	11,80671958	0,390514809	0,51673531	0,75573471	0,44980827	1	protein_codir	putative mucin-associated surface protein (MASP)
TcG_12975	81,56430348	0,650365399	0,19852867	3,2759268	0,00105316	0,0081509	protein_codir	dispersed gene family protein 1 (DGF-1)
TcG_12976	7,477848507	0,262023596	0,6273849	0,41764409	0,67620736	1	protein_codir	hypothetical protein
TcG_12977	7,738569214	-0,137995833	0,64202889	-0,21493711	0,82981635	1	protein_codir	hypothetical protein
TcG_12978	11,39001064	0,387445612	0,53274451	0,72726345	0,46706458	1	protein_codir	hypothetical protein
TcG_12979	12,31962509	0,192160124	0,50614979	0,37965071	0,70420472	1	protein_codir	putative leucine-rich repeat protein
TcG_12980	104,9337255	0,281894746	0,18019564	1,56438162	0,11772797	0,29924769	protein_codir	retrotransposon hot spot (RHS) protein
TcG_12981	47,09107989	0,107737711	0,27349897	0,39392364	0,69363741	0,84125796	protein_codir	retrotransposon hot spot protein (RHS)
TcG_12982	7,083398014	0,781828534	0,68845012	1,1356357	0,25610906	1	protein_codir	hypothetical protein
TcG_12983	53,08560166	0,111640598	0,23866003	0,46778087	0,6399413	0,80854525	protein_codir	hypothetical protein
TcG_12984	3,989794588	-0,137513199	0,85443567	-0,16094038	0,87214036	1	protein_codir	hypothetical protein
TcG_12985	122,4526533	0,12069516	0,1639571	0,73613865	0,46164632	0,6794505	protein_codir	putative trans-sialidase
TcG_12986	79,36012214	0,011230011	0,21338977	0,05262676	0,9580293	0,98064539	protein_codir	trans-sialidase-like protein
TcG_12987	24,35005756	-0,346838569	0,34975713	-0,99165545	0,32136563	0,55605469	protein_codir	trans-sialidase
TcG_12988	210,8791412	0,080986374	0,1327405	0,61011051	0,54178861	0,74008786	protein_codir	hypothetical protein
TcG_12989	116,8766654	-0,058077757	0,1702521	-0,34112799	0,73300723	0,86278448	protein_codir	complement regulatory protein
TcG_12990	50,93129883	0,167643469	0,24654925	0,67995936	0,49653019	0,70638492	protein_codir	putative trans-sialidase
TcG_12991	126,1018636	-0,486400537	0,16567574	-2,93585851	0,00332626	0,02069713	protein_codir	sialidase
TcG_12992	66,76322927	-0,180317099	0,21928486	-0,82229618	0,41090835	0,63757656	protein_codir	trans-sialidase
TcG_12993	4,233733899	-1,028019828	0,85579263	-1,20124874	0,22965473	1		
TcG_12994	204,5878204	0,144388828	0,12544953	1,15097147	0,24974397	0,47898256	protein_codir	hypothetical protein
TcG_12995	114,1294699	-0,039209667	0,17854564	-0,21960585	0,82617814	0,91511289	protein_codir	hypothetical protein
TcG_12996	4,291085369	-0,056152311	0,85299937	-0,06582925	0,94751377	1	protein_codir	hypothetical protein
TcG_12997	8,509435952	-0,102766213	0,60890764	-0,16877143	0,86597643	1	protein_codir	hypothetical protein

TcG_12998	13,58400064	0,191460809	0,47711977	0,40128458	0,68821061	1	
TcG_12999	16,59054397	1,365656641	0,47521843	2,8737451	0,00405636	1	protein_codir retrotransposon hot spot (RHS) protein
TcG_13000	22,66297293	0,553020582	0,38795368	1,42548094	0,15401822	0,3550448	protein_codir retrotransposon hot spot (RHS) protein
TcG_13001	36,32181811	0,110440614	0,2864838	0,38550387	0,69986414	0,84436727	protein_codir trans-sialidase
TcG_13002	23,70056724	-0,147471256	0,3625162	-0,40679908	0,68415556	0,83596566	protein_codir N-acetyltransferase complex ARD1 subunit
TcG_13003	35,3246647	-0,176962847	0,29659174	-0,59665468	0,55073796	0,74654606	protein_codir hypothetical protein
TcG_13004	0,913094583	0,185694647	1,88114384	0,09871369	0,9213656	1	protein_codir hypothetical protein
TcG_13005	0,145251675	-1,420530545	4,08047286	-0,3481289	0,72774338	1	protein_codir hypothetical protein
TcG_13006	0					1	protein_codir hypothetical protein
TcG_13007	0					1	protein_codir hypothetical protein
TcG_13008	8,967829661	-0,218921682	0,57287205	-0,3821476	0,70235188	1	protein_codir dispersed protein family protein 1
TcG_13009	15,58195376	-0,054625816	0,43303202	-0,12614729	0,89961534	1	protein_codir hypothetical protein
TcG_13010	21,67087777	-0,227152644	0,37612262	-0,60393243	0,54588857	0,74285471	protein_codir hypothetical protein
TcG_13011	34,2263353	-0,270770225	0,29749799	-0,91015815	0,36273911	0,59508238	protein_codir retrotransposon hot spot (RHS) protein
TcG_13012	36,26751591	0,180067066	0,32072295	0,56144117	0,57449683	0,76562855	protein_codir hypothetical protein
TcG_13013	39,4685246	-0,205692565	0,28567524	-0,72002237	0,47151122	0,6875068	protein_codir retrotransposon hot spot (RHS) protein
TcG_13014	23,99450717	0,122007434	0,35760226	0,34118195	0,73296661	0,86278448	protein_codir hypothetical protein
TcG_13015	7,611673583	-0,635322251	0,62239689	-1,02076706	0,30736481	1	protein_codir mucin-associated surface protein (MASP)
TcG_13016	2,476280711	0,172977383	1,09409726	0,15810055	0,87437756	1	protein_codir hypothetical protein
TcG_13017	2,01264365	-0,956079645	1,25850266	-0,75969617	0,44743622	1	protein_codir hypothetical protein
TcG_13018	3,325756426	-0,09388737	0,99761096	-0,09411221	0,92502002	1	protein_codir hypothetical protein
TcG_13019	4,423953176	-0,015701776	0,90675067	-0,01731653	0,9861841	1	protein_codir mucin-associated surface protein (MASP)
TcG_13020	10,5335268	-0,308907344	0,55701183	-0,5545795	0,57918231	1	protein_codir hypothetical protein
TcG_13021	6,362501831	-0,626915618	0,72636785	-0,86308283	0,38809193	1	protein_codir trans-sialidase
TcG_13022	7,247018769	0,38934191	0,65790958	0,59178635	0,55399366	1	protein_codir trans-sialidase
TcG_13023	89,49017059	-0,030070751	0,1896012	-0,15860001	0,87398402	0,93941728	
TcG_13024	27,08959367	-0,006320974	0,33633209	-0,01879385	0,98500556	0,99412989	protein_codir putative trans-sialidase
TcG_13025	5,001485382	-0,049185618	0,83268696	-0,05906856	0,9528975	1	protein_codir hypothetical protein
TcG_13026	4,455749638	0,010535299	0,83821785	0,01256869	0,9899719	1	protein_codir hypothetical protein
TcG_13027	20,43918233	-0,180625833	0,38463043	-0,4696088	0,63863454	0,80773199	protein_codir hypothetical protein
TcG_13028	5,858167742	-0,175718245	0,72933266	-0,24093017	0,80960923	1	protein_codir hypothetical protein
TcG_13029	12,67901173	-0,370127226	0,48664732	-0,76056564	0,44691655	1	protein_codir putative elongation factor 1-gamma (EF-1-gamma)
TcG_13030	11,12375107	0,238840308	0,55040896	0,43393245	0,66433749	1	protein_codir trans-sialidase
TcG_13031	6,971749576	0,680442137	0,66914932	1,01687638	0,30921224	1	protein_codir trans-sialidase
TcG_13032	2,930464607	0,968828152	1,05312216	0,919958	0,35759471	1	protein_codir hypothetical protein
TcG_13033	52,55599015	0,012416223	0,24541624	0,05059251	0,95965023	0,98146642	protein_codir putative trans-sialidase
TcG_13034	84,95278014	0,104755532	0,22758421	0,4602935	0,64530557	0,81178179	protein_codir trans-sialidase
TcG_13035	23,31384594	-0,424780376	0,64832994	-0,65519167	0,51234436	0,71838578	protein_codir hypothetical protein
TcG_13036	1,709137084	0,146436551	1,30651869	0,11208148	0,9107588	1	protein_codir mucin-associated surface protein (MASP)
TcG_13037	1,214936196	-2,863964133	1,7557499	-1,6311914	0,10284994	1	protein_codir hypothetical protein
TcG_13038	1,751838346	-0,511482485	1,41044979	-0,36263785	0,71687543	1	protein_codir hypothetical protein
TcG_13039	1,274669951	-0,810979684	1,70872447	-0,47461115	0,63506416	1	
TcG_13040	0,701020314	2,706632999	2,17304287	1,24554975	0,21292974	1	protein_codir mucin-associated surface protein (MASP)
TcG_13041	8,530682542	-0,31107896	0,59268372	-0,52486503	0,59967702	1	
TcG_13042	1,993249043	-0,716809443	1,24208234	-0,577103	0,56386988	1	protein_codir hypothetical protein
TcG_13043	38,81581161	-0,133158382	0,28894203	-0,46084809	0,6449076	0,81136925	protein_codir surface protease GP63
TcG_13044	8,984781842	-0,283322985	0,60290458	-0,46993006	0,63840499	1	protein_codir putative dispersed gene family protein 1 (DGF-1)
TcG_13045	6,137013726	-0,268611989	0,75142808	-0,35746866	0,72074098	1	protein_codir putative dispersed gene family protein 1 (DGF-1)
TcG_13046	6,945655096	-0,133363456	0,71267373	-0,18713115	0,8515578	1	protein_codir dispersed gene family protein 1 (DGF-1)
TcG_13047	3,688974476	-0,06875094	0,92858249	-0,07403859	0,94097968	1	
TcG_13048	53,38806095	0,50271977	0,2407236	2,08836925	0,03676454	0,13335584	protein_codir hypothetical protein
TcG_13049	23,39634501	-1,033518888	0,36354245	-2,84291115	0,00447035	0,02639873	protein_codir mucin-associated surface protein (MASP)

TcG_13050	2,916366999	1,123264913	1,05361401	1,06610666	0,28637543	1	
TcG_13051	4,221598944	-0,336952349	0,8758913	-0,38469653	0,70046226	1	protein_codir hypothetical protein
TcG_13052	48,48574089	0,163658632	0,2532321	0,64627917	0,51809857	0,72321567	protein_codir dispersed gene family protein 1 (DGF-1)
TcG_13053	28,70566256	0,185150044	0,32607394	0,56781614	0,57015982	0,76201081	protein_codir retrotransposon hot spot (RHS) protein
TcG_13054	10,70701578	0,035318545	0,5491362	0,06431655	0,94871818	1	protein_codir hypothetical protein
TcG_13055	0,846271989	1,744895683	1,99714161	0,87369653	0,38228354	1	
TcG_13056	37,02820283	0,023431478	0,29418024	0,07965008	0,93651557	0,97043819	
TcG_13057	5,628555132	-0,273791094	0,77891025	-0,35150532	0,72520928	1	protein_codir hypothetical protein
TcG_13058	12,92316633	-0,675140417	0,47872705	-1,41028257	0,15845626	1	protein_codir hypothetical protein
TcG_13059	21,04665403	0,453117386	0,38948537	1,16337459	0,24467755	0,47286474	protein_codir hypothetical protein
TcG_13060	74,05248303	-0,597714447	0,21059123	-2,83826852	0,0045359	0,02669017	protein_codir mucin-associated surface protein (MASP)
TcG_13061	39,64634122	0,479503557	0,28033443	1,71046972	0,08717905	0,24480282	protein_codir dispersed gene family protein 1 (DGF-1)
TcG_13062	10,12631611	-1,363143609	0,59113583	-2,30597359	0,02111211	1	protein_codir hypothetical protein
TcG_13063	7,316363214	-0,143124695	0,70209893	-0,2038526	0,83846868	1	protein_codir hypothetical protein
TcG_13064	9,184232714	-0,05639064	0,57958726	-0,09729448	0,92249254	1	protein_codir hypothetical protein
TcG_13065	45,30100621	0,033101598	0,26552113	0,12466653	0,90078756	0,95174891	protein_codir trans-sialidase
TcG_13066	132,1776138	-0,317449154	0,15471519	-2,05182924	0,04018626	0,14169142	protein_codir putative NADH-dependent fumarate reductase
TcG_13067	43,25564806	0,275501659	0,26782238	1,02867304	0,30363334	0,53649872	protein_codir hypothetical protein
TcG_13068	164,4147175	-0,096941674	0,1496477	-0,64779927	0,51711479	0,72262597	protein_codir mucin-associated surface protein (MASP)
TcG_13069	316,8881708	0,334574765	0,10418191	3,21144771	0,00132068	0,00979603	protein_codir hypothetical protein
TcG_13070	343,9653191	0,103510728	0,09940459	1,04130729	0,29773295	0,53110608	protein_codir putative retrotransposon hot spot (RHS) protein
TcG_13071	542,0911533	-0,05343663	0,07886709	-0,677553	0,49805515	0,70742498	protein_codir putative mitotubule-associated protein Gb4
TcG_13072	60,16218263	-0,053630111	0,22803494	-0,23518375	0,81406612	0,90821089	protein_codir hypothetical protein
TcG_13073	3,927758834	-0,956498139	0,89705421	-1,06626571	0,28630355	1	
TcG_13074	4,606311987	-0,985164164	0,81008504	-1,21612437	0,22393755	1	
TcG_13075	16,0751679	-0,591132782	0,44400896	-1,33135327	0,1830728	1	protein_codir trans-sialidase
TcG_13076	4,769279928	-0,389995359	0,80400004	-0,48506883	0,62762752	1	protein_codir hypothetical protein
TcG_13077	13,06152171	0,567721588	0,48493693	1,17071223	0,24171447	1	
TcG_13078	199,4984273	1,13242442	0,13337961	8,4902361	2,0622E-17	2,9137E-15	protein_codir phospholipase A1
TcG_13079	135,6090242	0,056024077	0,1578006	0,35503083	0,72256649	0,85733557	protein_codir retrotransposon hot spot (RHS) protein
TcG_13080	37,98447026	0,317783701	0,28723153	1,10636773	0,26856739	0,50117414	protein_codir putative retrotransposon hot spot (RHS) protein
TcG_13081	22,70792686	-0,017676108	0,37362043	-0,04731034	0,96226589	0,98256823	protein_codir retrotransposon hot spot (RHS) protein
TcG_13082	69,91674663	-0,104682953	0,21266976	-0,49223244	0,62255504	0,79666529	protein_codir hypothetical protein
TcG_13083	38,82825457	0,020480013	0,2762468	0,07413665	0,94090165	0,97263442	protein_codir hypothetical protein
TcG_13084	52,34505321	0,058917856	0,25984839	0,22673935	0,82062643	0,91114306	protein_codir hypothetical protein
TcG_13085	126,7505793	-0,081966096	0,15584921	-0,52593207	0,59893541	0,78285939	protein_codir putative retrotransposon hot spot (RHS) protein
TcG_13086	615,8546905	0,050376349	0,07641026	0,65928779	0,50971098	0,71651437	protein_codir trans-sialidase-like protein
TcG_13087	1112,072228	0,065348228	0,06328518	1,03259921	0,3017915	0,53501019	protein_codir putative amino acid transporter PAT1
TcG_13088	292,9619464	0,679616434	0,1114219	6,09948729	1,0641E-09	4,2077E-08	protein_codir hypothetical protein
TcG_13089	1013,503828	0,021437273	0,06472156	0,33122303	0,74047602	0,86763449	protein_codir amino acid transporter
TcG_13090	614,5737746	0,276523566	0,09112609	3,03451596	0,00240922	0,01595953	protein_codir structural maintenance of chromosome protein 4
TcG_13091	25,39605075	-0,019574204	0,37439646	-0,05228202	0,95830398	0,98064917	protein_codir hypothetical protein
TcG_13092	8,802428333	-0,300839783	0,6235939	-0,482429	0,62950122	1	protein_codir putative target of rapamycin (TOR) kinase 1
TcG_13093	12,05957489	0,047072485	0,50498146	0,09321626	0,92573175	1	protein_codir putative target of rapamycin (TOR) kinase 1
TcG_13094	48,35904369	-0,089802194	0,25668384	-0,34985527	0,72644732	0,85875291	protein_codir dispersed gene family protein 1 (DGF-1)
TcG_13095	13,27298503	1,07562589	0,50939768	2,11156418	0,03472385	1	protein_codir mucin TcMUCII
TcG_13096	10,15659898	0,441546108	0,56727565	0,77836252	0,43635533	1	protein_codir hypothetical protein
TcG_13097	1025,549081	0,178357048	0,06199558	2,87693165	0,00401563	0,02413124	protein_codir trans-sialidase-like protein
TcG_13098	1283,500685	-0,299175554	0,05630631	-5,31335739	1,0762E-07	2,771E-06	protein_codir putative P-type H+-ATPase
TcG_13099	4256,306814	0,015660118	0,04065154	0,38522819	0,70006836	0,84436727	protein_codir stress-70 protein, mitochondrial
TcG_13100	85,82926149	0,035200635	0,18895725	0,18628888	0,85221823	0,92842505	protein_codir hypothetical protein
TcG_13101	21,01073338	-0,310352949	0,40164758	-0,77269967	0,43970014	0,66274932	protein_codir dispersed gene family protein 1 (DGF-1)

TcG_13102	79,50069516	0,598137803	0,20310844	2,94491856	0,0032304	0,02026362	protein_codir rab1 small GTP-binding protein
TcG_13103	22,39767501	0,31026199	0,3745465	0,82836709	0,40746264	0,63572073	protein_codir hypothetical protein
TcG_13104	13,10047784	-0,145940624	0,48103669	-0,30338772	0,7615944	1	protein_codir helicase-like protein
TcG_13105	30,01305874	-0,075309989	0,32302827	-0,23313745	0,81565467	0,90917204	protein_codir trans-sialidase
TcG_13106	53,60762717	0,061780049	0,24090803	0,25644661	0,797606	0,89899335	protein_codir surface protein-2
TcG_13107	96,43942543	0,017259963	0,1774736	0,09725369	0,92252493	0,96227026	protein_codir Sialidase 85-1.3
TcG_13108	16,50172411	0,07311846	0,41998801	0,17409654	0,86178959	1	
TcG_13109	33,25192008	0,528766823	0,31081977	1,70120073	0,0889053	0,24814666	protein_codir dispersed gene family protein 1 (DGF-1)
TcG_13110	48,61248608	0,082576108	0,25774489	0,32037923	0,74868087	0,87318467	protein_codir dispersed gene family protein 1 (DGF-1)
TcG_13111	73,66296513	-0,352113951	0,2062771	-1,70699485	0,08782301	0,24598889	protein_codir hypothetical protein
TcG_13112	86,18229164	0,096489877	0,20155131	0,47873605	0,63212642	0,80290602	protein_codir dispersed gene family protein 1 (DGF-1)
TcG_13113	43,18531628	0,078790108	0,26379845	0,2986754	0,76518773	0,88178486	protein_codir putative trans-sialidase
TcG_13114	30,86916807	-0,060706836	0,31193483	-0,19461384	0,84569527	0,92503539	
TcG_13115	40,70793661	0,132929944	0,27338075	0,48624471	0,62679368	0,79969514	protein_codir trans-sialidase
TcG_13116	4,990592074	-0,679652554	0,79477432	-0,85515163	0,39246721	1	protein_codir hypothetical protein
TcG_13117	12,37573899	-0,785306588	0,51251449	-1,53226222	0,12545774	1	protein_codir hypothetical protein
TcG_13118	450,4590803	-0,080649217	0,08541269	-0,94422995	0,34505216	0,57921969	protein_codir trans-sialidase
TcG_13119	256,8643166	-0,03382489	0,11447058	-0,29548982	0,76761973	0,88350974	protein_codir trans-sialidase
TcG_13120	47,33403612	-0,029426263	0,27212955	-0,10813329	0,91388996	0,95787309	protein_codir hypothetical protein
TcG_13121	88,41018526	0,306710189	0,18920094	1,62108172	0,10500011	0,27838245	protein_codir putative beta galactofuranosyl glycosyltransferase
TcG_13122	47,09453053	0,195032924	0,25719452	0,75830902	0,44826601	0,66910718	protein_codir putative beta galactofuranosyl glycosyltransferase
TcG_13123	259,856742	0,063991065	0,11264144	0,56809526	0,56997029	0,76192564	protein_codir dispersed gene family protein 1 (DGF-1)
TcG_13124	15,44903131	-1,284079577	0,45882041	-2,79865399	0,00513161	1	protein_codir hypothetical protein
TcG_13125	40,68393547	0,191854107	0,27597374	0,69518971	0,48693642	0,69859488	protein_codir dispersed gene family protein 1 (DGF-1)
TcG_13126	19,42362322	0,487294782	0,40759758	1,19552914	0,23188036	0,45922596	protein_codir hypothetical protein
TcG_13127	10,8272527	0,81727659	0,54389669	1,50263204	0,13293396	1	
TcG_13128	19,3176759	0,189975107	0,40458998	0,4695497	0,63867677	0,80773199	protein_codir putative trans-sialidase
TcG_13129	23,24189357	0,24583727	0,36387337	0,67561215	0,49928692	0,70839313	protein_codir putative trans-sialidase
TcG_13130	9,279088337	0,198698373	0,57068161	0,34817728	0,72770705	1	protein_codir hypothetical protein
TcG_13131	94,47268899	-0,344621265	0,18384639	-1,87450654	0,06086064	0,19150771	protein_codir mucin TcMUCII
TcG_13132	3,766560046	-0,113991416	0,89057393	-0,1279977	0,89815079	1	protein_codir mucin TcMUCII
TcG_13133	2,409733519	-1,205487415	1,22260679	-0,98599764	0,32413427	1	protein_codir hypothetical protein
TcG_13134	5,632522149	-0,714043074	0,73753361	-0,96814987	0,33296952	1	protein_codir hypothetical protein
TcG_13135	173,7590824	0,136582696	0,13776845	0,99139316	0,32149364	0,55611008	
TcG_13136	199,6214135	0,319310094	0,12652828	2,52362636	0,01161513	0,05556272	protein_codir surface protease GP63
TcG_13137	54,38391345	0,102244954	0,242018	0,42246839	0,67268317	0,828677	protein_codir trans-sialidase
TcG_13138	213,3254934	-0,000974107	0,12561043	-0,00775499	0,99381248	0,99767711	
TcG_13139	5,615749218	-0,324152384	0,72859272	-0,44490204	0,65639055	1	protein_codir putative trans-sialidase
TcG_13140	5,270993599	-0,202881178	0,73762664	-0,27504589	0,78328098	1	protein_codir putative trans-sialidase
TcG_13141	8,066791868	0,361705057	0,60661436	0,59626853	0,55099585	1	protein_codir hypothetical protein
TcG_13142	21,0620895	-0,396877058	0,38361613	-1,03456821	0,3008706	0,53419983	protein_codir hypothetical protein
TcG_13143	19,48049771	0,190779543	0,39806592	0,47926621	0,63174926	0,80290602	protein_codir dispersed gene family protein 1 (DGF-1)
TcG_13144	24,85972003	0,683742697	0,35958573	1,90147341	0,05724003	0,18219314	protein_codir hypothetical protein
TcG_13145	23,39424077	0,159136474	0,36260993	0,43886409	0,66076002	0,82224396	protein_codir syntaxin binding protein
TcG_13146	26,24271412	-0,186816603	0,34632784	-0,53942127	0,58959621	0,7758162	protein_codir hypothetical protein
TcG_13147	43,33833757	0,304261353	0,27550116	1,10439228	0,26942301	0,50225824	protein_codir surface protease GP63
TcG_13148	67,21617107	0,562921359	0,21297443	2,64314062	0,00821409	0,04235357	protein_codir hypothetical protein
TcG_13149	8,986700434	-0,342948077	0,60420348	-0,56760361	0,57030416	1	protein_codir hypothetical protein
TcG_13150	61,7227286	0,120883021	0,23482382	0,51478176	0,60670553	0,78750732	protein_codir putative trans-sialidase
TcG_13151	63,2350185	0,095702725	0,22892562	0,41805162	0,67590938	0,83017833	protein_codir putative trans-sialidase
TcG_13152	103,4995252	0,147314275	0,1727273	0,85287197	0,39373031	0,62353189	protein_codir hypothetical protein
TcG_13153	123,911547	0,151403404	0,1786673	0,84740412	0,39676991	0,62662432	protein_codir retrotransposon hot spot (RHS) protein

TcG_13154	182,696291	0,223851418	0,1401765	1,59692542	0,11028233	0,28713059	protein_codir	retrotransposon hot spot (RHS) protein
TcG_13155	62,9993256	0,030641198	0,22153336	0,13831415	0,88999214	0,94653573	protein_codir	receptor-type adenylate cyclase
TcG_13156	26,75908421	0,216965397	0,34465341	0,62951762	0,52901023	0,73107665	protein_codir	hypothetical protein
TcG_13157	172,1648777	-0,285896175	0,13697688	-2,08718559	0,03687136	0,13342337	protein_codir	trans-sialidase-like protein
TcG_13158	124,8914122	-0,094595227	0,15733582	-0,60123136	0,5476859	0,7446009		
TcG_13159	56,74226277	0,642830056	0,24575421	2,61574381	0,00890333	0,04512424	protein_codir	target of rapamycin (TOR) kinase 1
TcG_13160	89,53454008	0,480784941	0,18775712	2,56067486	0,01044691	0,05113556	protein_codir	target of rapamycin (TOR) kinase 1
TcG_13161	53,44617953	0,523596359	0,25035457	2,09141921	0,0364905	0,13273063	protein_codir	trans-sialidase
TcG_13162	2,231100299	0,810200723	1,18904684	0,68138672	0,49562683	1	protein_codir	hypothetical protein
TcG_13163	3,268957557	0,466117791	0,9660463	0,48250047	0,62945047	1	protein_codir	mucin TcMUC
TcG_13164	3,79449325	-0,224578199	0,96311447	-0,23317913	0,81562231	1	protein_codir	dispersed protein family protein 1
TcG_13165	102,7640258	0,301239877	0,18106956	1,66366935	0,09617852	0,26231742	protein_codir	putative dispersed gene family protein 1 (DGF-1)
TcG_13166	73,58841266	-0,096516086	0,22269141	-0,43340732	0,66471889	0,82476879	protein_codir	hypothetical protein
TcG_13167	44,28351326	0,19646824	0,26287556	0,74738115	0,45483352	0,67475691	protein_codir	hypothetical protein
TcG_13168	4,444906377	0,402100514	0,86238162	0,46626749	0,64102405	1	protein_codir	hypothetical protein
TcG_13169	28,43615116	0,480507344	0,36003442	1,334615	0,18200239	0,39392483	protein_codir	retrotransposon hot spot (RHS) protein
TcG_13170	18,57827526	-0,38113089	0,44072759	-0,86477657	0,38716144	0,61734825		
TcG_13171	24,38890805	0,296352023	0,3501237	0,84642092	0,39731797	0,62698529	protein_codir	putative trans-sialidase
TcG_13172	505,8416649	-0,031266197	0,08460008	-0,36957646	0,71169809	0,85183204		
TcG_13173	5,665435145	-0,752452507	0,76440488	-0,98436382	0,32493666	1	protein_codir	hypothetical protein
TcG_13174	75,46281374	0,471912144	0,21888782	2,15595437	0,03108723	0,11735961	protein_codir	hypothetical protein
TcG_13175	7,498069283	-0,311897938	0,64197518	-0,48584112	0,62707982	1	protein_codir	hypothetical protein
TcG_13176	8,579622305	-0,354499358	0,59815776	-0,59265194	0,55341411	1	protein_codir	hypothetical protein
TcG_13177	11,10232684	0,377143666	0,51894058	0,72675693	0,46737487	1	protein_codir	retrotransposon hot spot (RHS) protein
TcG_13178	10,65320477	0,12351603	0,55847616	0,22116617	0,82496305	1		
TcG_13179	80,81249324	0,067728406	0,1950613	0,347216	0,72842905	0,86012831	protein_codir	retrotransposon hot spot protein (RHS)
TcG_13180	101,5683826	0,12529745	0,17941361	0,69837205	0,48494455	0,69795871	protein_codir	retrotransposon hot spot (RHS) protein
TcG_13181	81,69256593	0,139828107	0,19362686	0,72215244	0,47020076	0,68645032	protein_codir	hypothetical protein
TcG_13182	59,3140669	0,067877653	0,23194022	0,2926515	0,76978855	0,88436428	protein_codir	dispersed gene family protein 1 (DGF-1)
TcG_13183	94,1470328	-0,204867417	0,18040204	-1,13561583	0,25611738	0,48805526	protein_codir	dispersed gene family protein 1 (DGF-1)
TcG_13184	19,61711994	0,270979778	0,40160673	0,67473914	0,49984151	0,70879875	protein_codir	hypothetical protein
TcG_13185	37,27644583	0,16325866	0,28275191	0,5773919	0,56367475	0,75677498		
TcG_13186	52,41374781	0,263510214	0,24213158	1,08829343	0,27646559	0,50891807	protein_codir	dispersed gene family protein 1 (DGF-1)
TcG_13187	165,1256053	0,215967009	0,13796821	1,56533895	0,11750345	0,29894489	protein_codir	putative trans-sialidase
TcG_13188	25,21488888	-0,036016856	0,35565042	-0,10127039	0,91933582	0,96120058		
TcG_13189	53,95159807	-0,077634803	0,2458546	-0,31577527	0,75217309	0,87492919	protein_codir	trans-sialidase
TcG_13190	107,9126359	0,568236732	0,19535009	2,9088123	0,00362805	0,02222874	protein_codir	hypothetical protein
TcG_13191	149,2654602	-0,228388288	0,15240222	-1,49858902	0,13398028	0,32557556		
TcG_13192	4,069755616	0,651417679	0,98282861	0,66279886	0,50745938	1	protein_codir	dispersed protein family protein 1 (DGF-1)
TcG_13193	464,4685293	0,15431599	0,08506393	1,81411785	0,06965958	0,21127643	protein_codir	methyltransferase
TcG_13194	3,028150978	-0,229304072	1,02861146	-0,22292584	0,82359323	1	protein_codir	hypothetical protein
TcG_13195	10,73439111	0,500882668	0,54214467	0,92389116	0,35554299	1	protein_codir	trans-sialidase
TcG_13196	2,06017336	0,557614503	1,20663222	0,46212466	0,64399193	1	protein_codir	trans-sialidase
TcG_13197	53,52953399	-0,085539985	0,23912962	-0,35771389	0,72055744	0,85670516	protein_codir	mucin-associated surface protein (MASP)
TcG_13198	10,8425405	0,601531333	0,57468819	1,04670906	0,29523378	1	protein_codir	elongation factor 1-alpha (EF-1-alpha)
TcG_13199	38,28113055	0,196258069	0,28237377	0,69502939	0,48703689	0,69860389	protein_codir	subtilisin-like serine peptidase
TcG_13200	29,45232936	0,18502357	0,32951817	0,56149733	0,57445855	0,76562855	protein_codir	subtilisin-like serine peptidase
TcG_13201	26,4328065	0,129980713	0,34116827	0,38098711	0,70321281	0,84674949	protein_codir	putative kinesin
TcG_13202	468,7205804	-0,580617294	0,08399389	-6,91261351	4,758E-12	3,2428E-10	protein_codir	hypothetical protein
TcG_13203	4,979608601	0,542883704	0,81056579	0,66975897	0,50301146	1	protein_codir	hypothetical protein
TcG_13204	6,60664269	0,066831357	0,66867531	0,0999459	0,92038727	1	protein_codir	hypothetical protein
TcG_13205	2,328799928	1,5721751	1,22995768	1,27823512	0,20116654	1		

TcG_13206	45,34172008	-0,009235088	0,26370001	-0,03502119	0,97206284	0,98777616	protein_codir	hypothetical protein
TcG_13207	41,96083417	0,207874082	0,27423829	0,75800532	0,4484478	0,6692923	protein_codir	hypothetical protein
TcG_13208	251,8412912	0,384267345	0,11983891	3,20653234	0,00134345	0,00990785	protein_codir	rab1 small GTP-binding protein
TcG_13209	10,68691234	-0,432737861	0,54348902	-0,7962219	0,42590307	1	protein_codir	trans-sialidase
TcG_13210	8,33346473	0,280887604	0,61129341	0,45949719	0,64587717	1	protein_codir	hypothetical protein
TcG_13211	27,92519453	-0,416536068	0,32477228	-1,28254811	0,19965044	0,41844247	protein_codir	hypothetical protein
TcG_13212	21,24711595	-0,295405861	0,37686714	-0,78384617	0,43313038	0,65752733	protein_codir	hypothetical protein
TcG_13213	33,07781458	-0,218725708	0,31367931	-0,69729084	0,4856208	0,69826977	protein_codir	hypothetical protein
TcG_13214	264,1568008	0,159935024	0,11055544	1,44664998	0,14799498	0,3468885	protein_codir	putative retrotransposon hot spot (RHS) protein
TcG_13215	92,52052079	0,29265528	0,18338135	1,5958836	0,11051478	0,28741286	protein_codir	putative retrotransposon hot spot (RHS) protein
TcG_13216	36,4998907	-0,313861235	0,29119426	-1,07784142	0,28110453	0,5142555	protein_codir	dispersed gene family protein 1 (DGF-1)
TcG_13217	12,39152517	-0,14321476	0,48619164	-0,29456442	0,76832664	1	protein_codir	hypothetical protein
TcG_13218	23,79478715	-0,022831834	0,38275578	-0,05965118	0,95243346	0,97749648	protein_codir	hypothetical protein
TcG_13219	17,1729536	-0,076941565	0,45943812	-0,16746883	0,86700117	0,93578156		
TcG_13220	27,17292303	0,0467703	0,35202558	0,13286052	0,89430369	0,94882077	protein_codir	trans-sialidase
TcG_13221	12,37801829	0,045668641	0,5135969	0,08891923	0,9291461	1	protein_codir	trans-sialidase
TcG_13222	10,97466059	0,234366478	0,52265606	0,44841435	0,65385419	1	protein_codir	hypothetical protein
TcG_13223	94,03111811	-0,548346111	0,18339717	-2,98993774	0,00279034	0,01791076	protein_codir	mucin-associated surface protein (MASP)
TcG_13224	59,23512952	0,166067647	0,22872698	0,72605184	0,46780699	0,68434493	protein_codir	hypothetical protein
TcG_13225	0,233855473	1,175456718	3,50839889	0,33504078	0,73759432	1		
TcG_13226	29,78797971	-0,103164444	0,31977438	-0,32261635	0,74698581	0,87217349	protein_codir	hypothetical protein
TcG_13227	21,62796068	0,208241146	0,38543376	0,54027738	0,58900575	0,77564856	protein_codir	90 kDa surface protein
TcG_13228	33,83646003	0,039618546	0,32222176	0,12295428	0,9021433	0,95271464	protein_codir	hypothetical protein
TcG_13229	13,74143599	0,22280698	0,48648545	0,4579931	0,6469574	1	protein_codir	hypothetical protein
TcG_13230	37,71075844	-0,010946063	0,28800241	-0,03800684	0,96968223	0,98697516	protein_codir	trans-sialidase
TcG_13231	30,78222432	0,311329112	0,32548576	0,95650609	0,33881661	0,57327673	protein_codir	hypothetical protein
TcG_13232	21,60744722	0,198477118	0,3838913	0,51701385	0,60514649	0,78669877	protein_codir	surface protease GP63
TcG_13233	15,49218896	-0,229162577	0,43904628	-0,5219554	0,60170138	1		
TcG_13234	44,91194594	-0,177311757	0,26556613	-0,66767459	0,50434133	0,71207637	protein_codir	hypothetical protein
TcG_13235	48,68725916	0,193446313	0,25293094	0,76481869	0,44437952	0,66642694	protein_codir	hypothetical protein
TcG_13236	37,65181931	0,144236908	0,2879014	0,50099412	0,61637526	0,79265743	protein_codir	hypothetical protein
TcG_13237	40,71447753	0,303923626	0,29476908	1,03105667	0,30251424	0,5354308		
TcG_13238	51,23094259	-0,277170533	0,2481159	-1,11710105	0,26395112	0,49619385	protein_codir	mucin TcMUCII
TcG_13239	79,63152847	-0,422475409	0,19567798	-2,15903395	0,03084753	0,11664475	protein_codir	mucin-associated surface protein (MASP)
TcG_13240	10,61666883	-0,041658747	0,53298697	-0,07816091	0,93770006	1	protein_codir	hypothetical protein
TcG_13241	17,91162024	-0,530113193	0,40501259	-1,30888077	0,19057474	0,40595678	protein_codir	hypothetical protein
TcG_13242	24,42610903	-0,943111203	0,37314438	-2,52746995	0,01148877	0,05514036	protein_codir	hypothetical protein
TcG_13243	27,07530533	0,105759746	0,34363663	0,30776622	0,75826023	0,87801518	protein_codir	putative trans-sialidase
TcG_13244	2,814926486	1,265498015	1,10664426	1,14354546	0,25281219	1	protein_codir	hypothetical protein
TcG_13245	12,67839429	-0,178322913	0,49847825	-0,35773459	0,72054194	1	protein_codir	hypothetical protein
TcG_13246	16,93711224	0,770829923	0,44509488	1,73183284	0,08330333	0,23722921		
TcG_13247	78,4838538	-0,077789283	0,19924938	-0,39041166	0,69623217	0,84263511	protein_codir	trans-sialidase
TcG_13248	181,5324443	-0,059007913	0,13256952	-0,4451092	0,65624084	0,81834101	protein_codir	putative surface protease GP63, putative,metallopeptidase
TcG_13249	27,76628364	0,176729698	0,33164403	0,53288973	0,59410992	0,77918921	protein_codir	dispersed gene family protein 1 (DGF-1)
TcG_13250	28,9422553	0,142991813	0,35272155	0,40539574	0,68518664	0,83655708	protein_codir	dispersed protein family protein 1
TcG_13251	19,27777463	-1,742186882	0,43126695	-4,03969488	5,3521E-05	0,00065688	protein_codir	mucin-associated surface protein (MASP)
TcG_13252	7,213986572	-1,158428601	0,73539119	-1,57525493	0,11519761	1	protein_codir	putative mucin TcMUCII
TcG_13253	128,9540048	-0,371517596	0,15798437	-2,35160975	0,01869237	0,0795939	protein_codir	putative trans-sialidase
TcG_13254	18,99361385	0,300642942	0,40889622	0,73525488	0,46218428	0,67974471	protein_codir	hypothetical protein
TcG_13255	172,7999791	-0,503308744	0,1372968	-3,6658446	0,00024652	0,00237622	protein_codir	putative trans-sialidase
TcG_13256	103,0105115	0,22115134	0,17380516	1,27240953	0,20322765	0,42341226	protein_codir	putative retrotransposon hot spot (RHS) protein
TcG_13257	29,62689549	0,242821426	0,33383593	0,72736757	0,46700082	0,68359715		

TcG_13258	70,46277977	-0,16265513	0,21061049	-0,77230306	0,43993495	0,66290627	protein_codir trans-sialidase
TcG_13259	57,17311602	0,02866011	0,23015697	0,12452419	0,90090025	0,95174891	
TcG_13260	81,38886517	-0,275643417	0,19393367	-1,42132832	0,15522133	0,35661201	protein_codir sialidase
TcG_13261	135,5837401	-0,298053675	0,15331242	-1,94409341	0,05188419	0,16953452	protein_codir hypothetical protein
TcG_13262	52,41981721	-0,471397898	0,2772578	-1,70021512	0,08909047	0,24848391	protein_codir hypothetical protein
TcG_13263	5,366715003	0,410986591	0,77067282	0,53328284	0,59383781	1	protein_codir hypothetical protein
TcG_13264	8,305374038	-0,33180129	0,64600232	-0,51362245	0,60751598	1	protein_codir hypothetical protein
TcG_13265	9,515033121	-0,461238979	0,5762861	-0,80036458	0,4234996	1	protein_codir hypothetical protein
TcG_13266	48,68392707	0,152256583	0,25454602	0,59814952	0,54974017	0,74596143	protein_codir hypothetical protein
TcG_13267	831,2031741	0,189386709	0,06745981	2,8074007	0,00499431	0,02874518	protein_codir L1Tc protein
TcG_13268	52,12190841	0,241974541	0,25788207	0,9383147	0,34808271	0,58261865	
TcG_13269	18,5686796	0,143074372	0,4024685	0,3554921	0,72222096	0,85728556	protein_codir trans-sialidase
TcG_13270	4,194227417	-1,205794245	0,85672757	-1,40744185	0,15929641	1	protein_codir peptide hydrolase
TcG_13271	15,3340959	-0,166808036	0,44497352	-0,37487182	0,7077558	1	protein_codir surface protease GP63
TcG_13272	23,02191211	-0,01302253	0,37860541	-0,03439605	0,97256133	0,9878584	protein_codir hypothetical protein
TcG_13273	39,99159674	0,103240536	0,31034467	0,33266412	0,73938784	0,86708727	protein_codir hypothetical protein
TcG_13274	119,5047991	0,158173408	0,15989088	0,98925851	0,32253668	0,55724873	protein_codir dispersed gene family protein 1 (DGF-1)
TcG_13275	1,853698425	-0,646231476	1,37691683	-0,46933225	0,63883217	1	protein_codir hypothetical protein
TcG_13276	42,37443721	0,080697665	0,26689477	0,30235761	0,76237947	0,88003672	
TcG_13277	51,15759448	0,13285974	0,25356805	0,52396089	0,60030574	0,78350294	protein_codir putative retrotransposon hot spot (RHS) protein
TcG_13278	21,35946171	0,908390221	0,41439717	2,19207633	0,028374	0,10972667	protein_codir structural maintenance of chromosome protein 4
TcG_13279	57,64266525	0,396527584	0,25190438	1,57411944	0,11545984	0,29583886	protein_codir hypothetical protein
TcG_13280	33,34894241	0,524598537	0,30641511	1,71205177	0,08688713	0,24434391	protein_codir hypothetical protein
TcG_13281	11,78582439	-0,014331031	0,50879296	-0,02816673	0,97752918	1	protein_codir putative trans-sialidase
TcG_13282	3,609703717	0,615747579	0,93446281	0,65893214	0,50993935	1	protein_codir trans-sialidase
TcG_13283	1,119187729	2,222337238	1,76973891	1,25574299	0,20920917	1	protein_codir hypothetical protein
TcG_13284	3,63871799	0,541080708	0,92158871	0,58711734	0,5571249	1	protein_codir hypothetical protein
TcG_13285	5,480782995	-0,360634102	0,78989782	-0,45655792	0,64798883	1	protein_codir hypothetical protein
TcG_13286	90,64784413	0,165870408	0,18480497	0,89754298	0,36942925	0,60112883	
TcG_13287	28,50109113	-0,235550647	0,34041207	-0,69195739	0,48896409	0,70017772	protein_codir hypothetical protein
TcG_13288	8,323724874	-0,014852165	0,65076907	-0,02282248	0,98179187	1	protein_codir hypothetical protein
TcG_13289	6,926104483	0,354012286	0,66674475	0,53095624	0,5954491	1	protein_codir hypothetical protein
TcG_13290	4,953242717	0,019142237	0,81666221	0,0234396	0,98129962	1	
TcG_13291	9,618710495	-2,130211123	0,63394908	-3,36022434	0,00077879	1	protein_codir mucin TcMUCII
TcG_13292	22,27867466	-0,331793346	0,36605822	-0,90639501	0,36472681	0,59668524	protein_codir mucin-associated surface protein (MASP)
TcG_13293	45,86865182	0,18504675	0,26095432	0,70911548	0,47825282	0,69283432	protein_codir hypothetical protein
TcG_13294	34,90232342	-0,22422534	0,32213848	-0,69605264	0,48639586	0,69829488	protein_codir trans-sialidase
TcG_13295	640,2624514	-0,267294739	0,07451531	-3,58711168	0,00033436	0,00306723	protein_codir UDP-Gal or UDP-GlcNAc-dependent glycosyltransferase
TcG_13296	120,167003	-0,36071773	0,15971647	-2,25848801	0,02391525	0,09661161	
TcG_13297	2,176479916	-1,124932816	1,25869209	-0,89373154	0,37146555	1	protein_codir hypothetical protein
TcG_13298	5,735845661	-0,852204965	0,76494605	-1,11407198	0,26524831	1	protein_codir hypothetical protein
TcG_13299	112,3516908	-0,232544228	0,16713741	-1,3913356	0,16412368	0,3691588	protein_codir dispersed gene family protein 1 (DGF-1)
TcG_13300	7,365178632	-0,144097345	0,65150076	-0,22117755	0,82495419	1	protein_codir hypothetical protein
TcG_13301	8,760841191	0,285579143	0,5802125	0,4921975	0,62257973	1	protein_codir hypothetical protein
TcG_13302	8,788192963	-0,485857738	0,59812224	-0,81230509	0,41661659	1	protein_codir hypothetical protein
TcG_13303	82,93633736	0,320380636	0,19323165	1,65801329	0,09731478	0,26398714	protein_codir hypothetical protein
TcG_13304	154,6297338	0,13413809	0,14708378	0,91198424	0,36177701	0,59446156	protein_codir hypothetical protein
TcG_13305	31,03138099	0,174380418	0,31296756	0,55718369	0,57740193	0,76770471	protein_codir hypothetical protein
TcG_13306	18,81191598	-0,273187283	0,42487257	-0,6429864	0,52023292	0,7247735	protein_codir hypothetical protein
TcG_13307	50,66605594	-0,088465323	0,24483682	-0,36132361	0,71785755	0,85508792	protein_codir trans-sialidase
TcG_13308	17,66783495	0,472056969	0,41832581	1,12844332	0,25913274	0,49163663	protein_codir hypothetical protein
TcG_13309	129,689281	-0,455544249	0,15914767	-2,86239979	0,00420446	0,02510974	protein_codir putative mucin-associated surface protein (MASP)

TcG_13310	754,9451704	-0,002520195	0,06997272	-0,03601683	0,97126894	0,98762955	protein_codir retrotransposon hot spot (RHS) protein
TcG_13311	41,82144976	0,207966051	0,27906417	0,74522664	0,45613472	0,67606203	protein_codir trans-sialidase
TcG_13312	100,0773783	-0,048032369	0,19095429	-0,25153856	0,80139775	0,90145576	protein_codir trans-sialidase
TcG_13313	41,01731771	0,144488783	0,28397742	0,50880377	0,61088977	0,78940095	protein_codir trans-sialidase
TcG_13314	33,72603268	-0,045182767	0,29604944	-0,15261899	0,87869875	0,94148439	protein_codir trans-sialidase
TcG_13315	27,62009902	0,715858606	0,34487674	2,07569406	0,03792226	0,13611129	protein_codir hypothetical protein
TcG_13316	1,953586841	0,920018746	1,35303769	0,67996535	0,4965264	1	protein_codir hypothetical protein
TcG_13317	1,420693798	1,672303167	1,56663018	1,06745242	0,28576759	1	protein_codir hypothetical protein
TcG_13318	6,994374972	-0,973072831	0,6801156	-1,43074624	0,15250296	1	protein_codir hypothetical protein
TcG_13319	11,58235073	-0,085393948	0,50010811	-0,17075098	0,86441958	1	protein_codir hypothetical protein
TcG_13320	23,13577427	0,037002209	0,38753907	0,09547994	0,92393362	0,96282559	protein_codir hypothetical protein
TcG_13321	39,5862928	0,350794748	0,29298074	1,19733042	0,23117779	0,45851772	protein_codir trans-sialidase
TcG_13322	12,08143835	-0,219944294	0,50087353	-0,43912142	0,66057356	1	protein_codir trans-sialidase
TcG_13323	33,47760983	-0,42697268	0,29801516	-1,43272132	0,15193749	0,35209328	protein_codir trans-sialidase
TcG_13324	11,12340877	0,56501859	0,52379186	1,07870822	0,28071782	1	
TcG_13325	22,10523903	-0,245807349	0,38902681	-0,63185195	0,52748362	0,72991427	protein_codir mucin-associated surface protein (MASP)
TcG_13326	171,212429	-0,630755836	0,14104918	-4,47188574	7,7533E-06	0,00012255	protein_codir mucin TcMUCI
TcG_13327	22,07077149	0,287722603	0,39130222	0,7352951	0,46215979	0,67974471	protein_codir hypothetical protein
TcG_13328	25,2035519	-0,325074959	0,34986537	-0,929143	0,35281498	0,58714657	
TcG_13329	7,959672633	-0,139857316	0,64002091	-0,21851992	0,82702404	1	protein_codir hypothetical protein
TcG_13330	29,41168194	-0,710191344	0,32836998	-2,16277795	0,03055826	0,11585341	protein_codir mucin TcMUCII
TcG_13331	291,312581	-0,2169763	0,11387241	-1,90543339	0,05672376	0,18074848	
TcG_13332	96,44137051	-0,476716926	0,18346254	-2,59844284	0,00936476	0,04684808	protein_codir putative trans-sialidase
TcG_13333	4,810680039	-1,033824675	0,80455909	-1,28495804	0,19880695	1	
TcG_13334	4,436869825	-0,163149108	0,83034308	-0,19648397	0,84423138	1	
TcG_13335	0,892716318	-1,109608292	1,96410539	-0,56494336	0,57211229	1	protein_codir trans-sialidase
TcG_13336	25,94733961	-0,122442602	0,33862127	-0,36159159	0,71765725	0,85508792	protein_codir hypothetical protein
TcG_13337	7,516638072	-0,431669696	0,63642085	-0,67827711	0,49759601	1	protein_codir hypothetical protein
TcG_13338	13,41192995	0,238728758	0,50702007	0,47084676	0,63775017	1	protein_codir dispersed gene family protein 1 (DGF-1)
TcG_13339	165,5666502	-0,294573442	0,13892673	-2,12035105	0,03397645	0,12556656	protein_codir hypothetical protein
TcG_13340	36,43331768	0,271291864	0,29336202	0,92476818	0,35508651	0,588981	protein_codir hypothetical protein
TcG_13341	41,94431727	0,734809243	0,28284853	2,59788956	0,00937987	0,04690338	protein_codir dispersed gene family protein 1 (DGF-1)
TcG_13342	3,451464335	1,252305522	1,01403161	1,23497681	0,21683914	1	protein_codir hypothetical protein
TcG_13343	4,379675843	0,199491236	0,83760941	0,23816738	0,81175128	1	protein_codir hypothetical protein
TcG_13344	3,637916986	-0,64177615	0,89916089	-0,71375007	0,47538174	1	protein_codir hypothetical protein
TcG_13345	2,582077007	-0,113917978	1,06934772	-0,10653034	0,91516158	1	protein_codir hypothetical protein
TcG_13346	37,92462422	0,14119178	0,28492901	0,49553318	0,62022381	0,79463818	protein_codir 90 kDa surface protein
TcG_13347	34,58636499	0,585072786	0,32263864	1,8133996	0,06977021	0,21143198	protein_codir protein kinase, putative,serine/threonine protein kinase
TcG_13348	27,79710661	-0,407435298	0,33891631	-1,2021708	0,22929736	0,45646722	protein_codir mucin TcMUCII
TcG_13349	52,83060852	-0,245789234	0,24982087	-0,98386188	0,32518343	0,55965169	protein_codir mucin-associated surface protein (MASP)
TcG_13350	20,61045762	-0,053855211	0,86551382	-0,0622234	0,95038493	0,97694613	protein_codir dispersed gene family protein 1 (DGF-1)
TcG_13351	62,81698209	-0,205462929	0,22899103	-0,89725316	0,36958384	0,60122588	protein_codir putative trans-sialidase
TcG_13352	71,09067881	0,005874885	0,20702569	0,02837756	0,97736102	0,99022484	protein_codir trans-sialidase
TcG_13353	203,8297236	0,086195304	0,13097097	0,65812526	0,51045765	0,7172159	protein_codir putative retrotransposon hot spot (RHS) protein
TcG_13354	135,062878	0,076998917	0,15383103	0,50054215	0,61669338	0,79274487	protein_codir retrotransposon hot spot (RHS) protein
TcG_13355	48,32246594	-0,268275535	0,2537983	-1,0570423	0,29049228	0,52345679	
TcG_13356	3,031654238	0,988631769	1,04774189	0,94358332	0,34538262	1	protein_codir putative D-isomer specific 2-hydroxyacid dehydrogenase-protein
TcG_13357	109,8004809	-0,235920839	0,17467	-1,35066608	0,17680242	0,38708104	protein_codir putative D-isomer specific 2-hydroxyacid dehydrogenase-protein
TcG_13358	12,3052384	1,007269579	0,52370053	1,92336941	0,05443367	1	protein_codir dispersed gene family protein 1 (DGF-1)
TcG_13359	26,83930082	-0,058626879	0,33387096	-0,17559742	0,86061023	0,93329372	protein_codir trans-sialidase
TcG_13360	266,5879745	-0,016731982	0,11176916	-0,14970124	0,88100033	0,94206932	protein_codir trans-sialidase
TcG_13361	24,29254079	-0,112266659	0,36768216	-0,30533616	0,76011014	0,87881809	

TcG_13362	69,18536835	0,045535849	0,21627791	0,21054323	0,83324372	0,91908998	protein_codir putative dispersed gene family protein 1 (DGF-1)
TcG_13363	6,272412266	0,012206273	0,68504053	0,01781832	0,98578379	1	protein_codir dispersed protein family protein 1
TcG_13364	82,11530136	-0,12468765	0,19122629	-0,6520424	0,51437382	0,7202291	protein_codir trans-sialidase
TcG_13365	149,1025164	-0,413064393	0,15146309	-2,727162	0,00638817	0,03474803	protein_codir trans-sialidase
TcG_13366	17,63465472	-0,301966861	0,40680171	-0,74229496	0,45790864	0,67721172	protein_codir mucin-associated surface protein (MASP)
TcG_13367	306,8301613	0,264477262	0,10498999	2,51907125	0,01176648	0,05610813	protein_codir putative retrotransposon hot spot (RHS) protein
TcG_13368	13,30179775	0,390257483	0,47853361	0,81552785	0,41477023	1	protein_codir hypothetical protein
TcG_13369	9,014768281	0,362699176	0,59460756	0,60998077	0,54187455	1	protein_codir hypothetical protein
TcG_13370	14,34497373	0,875903785	0,49204963	1,78011268	0,07505752	1	protein_codir trans-sialidase
TcG_13371	8,833829109	-0,208887096	0,57663037	-0,36225476	0,71716166	1	protein_codir putative mitotic centromere-associated kinesin (MCAK)
TcG_13372	19,59593353	-0,001603237	0,40206516	-0,00398751	0,99681844	0,9988876	protein_codir subtilisin-like serine peptidase
TcG_13373	69,01215098	0,236533296	0,21689456	1,09054507	0,27547311	0,50773647	protein_codir hypothetical protein
TcG_13374	11,57346391	-0,341385521	0,50913263	-0,67052375	0,50252397	1	protein_codir trans-sialidase
TcG_13375	10,04079736	-0,233192079	0,54677891	-0,42648331	0,66975571	1	
TcG_13376	10,03740477	-0,227583832	0,55575169	-0,40950633	0,68216812	1	
TcG_13377	10,19320265	-0,999228337	0,54983748	-1,81731579	0,06916877	1	protein_codir trans-sialidase
TcG_13378	7,319658393	0,010702211	0,65394216	0,01636568	0,98694266	1	protein_codir trans-sialidase
TcG_13379	1,987234776	-1,772471914	1,32885252	-1,33383644	0,18225747	1	protein_codir putative trans-sialidase
TcG_13380	11,21481661	0,52287895	0,55569504	0,94094586	0,3467326	1	
TcG_13381	80,26013517	0,010786904	0,20890783	0,05163475	0,95881973	0,98091703	protein_codir hypothetical protein
TcG_13382	51,03767145	0,133024865	0,24437851	0,54433947	0,58620789	0,77478949	protein_codir rab1 small GTP-binding protein
TcG_13383	109,4471444	0,162425146	0,18012261	0,90174767	0,36719093	0,59939061	protein_codir protein kinase
TcG_13384	192,9978631	-0,425102719	0,13179308	-3,22553137	0,00125739	0,00940486	protein_codir putative trans-sialidase
TcG_13385	8,749198759	0,475654859	0,59075327	0,80516669	0,4207235	1	protein_codir hypothetical protein
TcG_13386	5,846776192	0,053276319	0,73839816	0,07215121	0,94248157	1	protein_codir hypothetical protein
TcG_13387	8,988272768	-1,037260213	0,59548396	-1,74187766	0,08152985	1	protein_codir hypothetical protein
TcG_13388	16,99813048	-0,318541788	0,42499899	-0,74951186	0,45354875	0,67403555	protein_codir hypothetical protein
TcG_13389	21,3488557	-0,058065663	0,38069816	-0,15252415	0,87877354	0,94148439	protein_codir rab1 small GTP-binding protein
TcG_13390	7,189109648	1,072451761	0,70430731	1,52270429	0,1278327	1	protein_codir dispersed gene family protein 1 (DGF-1)
TcG_13391	28,59979216	0,256477646	0,3368647	0,76136695	0,44643792	0,66804047	protein_codir dispersed gene family protein 1 (DGF-1)
TcG_13392	53,47410018	-0,070113329	0,24029535	-0,2917798	0,77045499	0,88468697	protein_codir dispersed gene family protein 1 (DGF-1)
TcG_13393	0,600419294	-1,762951393	2,24734199	-0,78446066	0,43276985	1	
TcG_13394	3,596598779	-0,213342063	0,94707439	-0,22526431	0,82177366	1	protein_codir hypothetical protein
TcG_13395	13,46846075	-0,39971848	0,47729881	-0,83745962	0,40233426	1	protein_codir putative mucin-associated surface protein (MASP)
TcG_13396	183,8834475	0,187504883	0,13710371	1,36761347	0,1714331	0,37854467	protein_codir putative retrotransposon hot spot (RHS) protein
TcG_13397	3,931761113	1,1948089	0,97332581	1,22755288	0,21961486	1	protein_codir hypothetical protein
TcG_13398	23,37573506	-0,531590326	0,36149536	-1,47053155	0,14141785	0,3366698	protein_codir dispersed gene family protein 1 (DGF-1)
TcG_13399	46,88881679	-0,202078772	0,26197256	-0,77137382	0,44048539	0,66339057	protein_codir hypothetical protein
TcG_13400	81,71693198	0,192104837	0,19733526	0,97349473	0,33030748	0,56502915	protein_codir dispersed gene family protein 1 (DGF-1)
TcG_13401	14,97827083	0,376157403	0,4966273	0,75742393	0,44879593	1	protein_codir hypothetical protein
TcG_13402	4,43199677	0,029000008	0,80577056	0,0359904	0,97129001	1	
TcG_13403	54,24408629	-0,156503219	0,2476973	-0,63183255	0,5274963	0,72991427	protein_codir retrotransposon hot spot (RHS) protein
TcG_13404	2,986886417	-0,904948889	1,07133302	-0,8446943	0,39828155	1	protein_codir putative retrotransposon hot spot (RHS) protein
TcG_13405	16,89213815	0,158528089	0,42320831	0,37458643	0,70796807	0,84943749	protein_codir retrotransposon hot spot (RHS) protein
TcG_13406	3,12192122	-1,448723638	1,03069759	-1,40557585	0,15985011	1	protein_codir hypothetical protein
TcG_13407	2,749629342	0,610079618	1,03525492	0,58930376	0,55565752	1	protein_codir hypothetical protein
TcG_13408	9,36364277	-0,077294829	0,55962545	-0,13811886	0,89014648	1	
TcG_13409	3,490292038	-1,46071061	0,98259615	-1,48658288	0,13712501	1	protein_codir hypothetical protein
TcG_13410	5,229982105	-0,617584479	0,81547473	-0,75733123	0,44885145	1	
TcG_13411	77,76214422	1,07681548	0,22031777	4,88755621	1,021E-06	2,0644E-05	protein_codir putative kinesin
TcG_13412	76,23693271	0,1394853	0,20564247	0,67829034	0,49758762	0,70728133	protein_codir trans-sialidase
TcG_13413	11,4084594	0,809452031	0,52135046	1,55260634	0,12051721	1	protein_codir surface protein-2

TcG_13414	30,05708716	-0,171765682	0,33041081	-0,51985492	0,6031647	0,78522521	protein_codir	trans-sialidase
TcG_13415	27,71884149	-0,301986825	0,33168903	-0,91045164	0,36258437	0,59502869	protein_codir	hypothetical protein
TcG_13416	29,07526338	-0,150334447	0,3292263	-0,45662952	0,64793736	0,81260383	protein_codir	trans-sialidase
TcG_13417	16,96247028	0,289952687	0,4340572	0,66800571	0,50412995	0,71207637	protein_codir	hypothetical protein
TcG_13418	16,59836626	0,156420829	0,44356121	0,35264767	0,72435259	1	protein_codir	hypothetical protein
TcG_13419	3,409766883	0,579713969	0,97519687	0,5944584	0,55220556	1		
TcG_13420	46,1967833	0,212120977	0,2597915	0,8165047	0,41421154	0,6411339	protein_codir	protein kinase
TcG_13421	41,92981782	-0,283059936	0,27099241	-1,04453085	0,29623985	0,5292829	protein_codir	beta galactofuranosyl glycosyltransferase
TcG_13422	0,428637354	1,990244214	2,43930454	0,81590641	0,41455366	1	protein_codir	hypothetical protein
TcG_13423	2,096038848	1,604734614	1,3083557	1,22652778	0,22000013	1		
TcG_13424	20,82897061	-0,242782386	0,38961037	-0,62314149	0,53319155	0,73393813	protein_codir	retrotransposon hot spot (RHS) protein
TcG_13425	12,71036318	0,39890216	0,53867547	0,74052409	0,45898205	1	protein_codir	retrotransposon hot spot protein (RHS)
TcG_13426	36,82113921	0,56151453	0,3111099	1,80487515	0,07109422	0,21392986	protein_codir	retrotransposon hot spot (RHS) protein
TcG_13427	37,56759485	0,416899264	0,2933802	1,42102045	0,15531081	0,35674684	protein_codir	retrotransposon hot spot (RHS) protein
TcG_13428	30,35308696	0,458905759	0,32001417	1,43401698	0,15156742	0,35179947	protein_codir	retrotransposon hot spot (RHS) protein
TcG_13429	136,0342246	0,128171859	0,16418631	0,78064889	0,43500903	0,6591701	protein_codir	retrotransposon hot spot (RHS) protein
TcG_13430	42,80158985	-0,098871438	0,26856426	-0,36814815	0,71276277	0,85248988	protein_codir	putative elongation factor 1-gamma (EF-1-gamma)
TcG_13431	235,451921	-0,387298421	0,1162457	-3,33172249	0,0008631	0,00691079	protein_codir	mucin-associated surface protein (MASP)
TcG_13432	234,8574175	0,539570814	0,11895719	4,53584017	5,7375E-06	9,4424E-05	protein_codir	ceramide synthase
TcG_13433	131,3106136	0,660690752	0,15928759	4,14778544	3,3571E-05	0,00043458	protein_codir	dispersed gene family protein 1 (DGF-1)
TcG_13434	66,61787368	-0,382497364	0,22087099	-1,73176826	0,08331483	0,23722921	protein_codir	hypothetical protein
TcG_13435	57,14543238	-0,475119694	0,23813637	-1,99515807	0,04602564	0,15592196	protein_codir	mucin-associated surface protein (MASP)
TcG_13436	6,535956772	0,882783588	0,69432595	1,2714253	0,20357739	1	protein_codir	hypothetical protein
TcG_13437	3,30931904	-0,309409131	0,95396195	-0,32434116	0,74567976	1	protein_codir	hypothetical protein
TcG_13438	19,25505485	-0,14281846	0,4246497	-0,33632064	0,73662909	0,86531325	protein_codir	hypothetical protein
TcG_13439	18,11416073	0,128446932	0,40662729	0,31588369	0,75209079	0,87492919	protein_codir	hypothetical protein
TcG_13440	61,39532858	-0,327396308	0,22908894	-1,42912315	0,15296884	0,35332874	protein_codir	trans-sialidase
TcG_13441	73,66994832	-0,21516428	0,21233578	-1,0133209	0,31090691	0,54446303	protein_codir	hypothetical protein
TcG_13442	14,10558683	-0,759477456	0,46767351	-1,62394798	0,10438691	1	protein_codir	putative mucin TcMUCII
TcG_13443	17,52238948	-0,556626833	0,41607801	-1,33779439	0,18096347	0,39233585	protein_codir	hypothetical protein
TcG_13444	240,0137346	0,240373962	0,11723846	2,05029955	0,04033521	0,14202291	protein_codir	trans-sialidase-like protein
TcG_13445	0,27291574	1,35431792	3,0630933	0,4421406	0,65838746	1	protein_codir	hypothetical protein
TcG_13446	2,416014176	-0,533294628	1,14645249	-0,46516941	0,64181014	1	protein_codir	hypothetical protein
TcG_13447	5,308959474	-0,312603568	0,73301379	-0,42646342	0,6697702	1		
TcG_13448	1,33182361	0,802102879	1,57384252	0,50964621	0,61029934	1	protein_codir	hypothetical protein
TcG_13449	1,373279486	-0,47024359	1,54567887	-0,3042311	0,76095183	1	protein_codir	mucin-associated surface protein (MASP)
TcG_13450	2,711787812	0,756994269	1,15165488	0,65731	0,51098161	1	protein_codir	hypothetical protein
TcG_13451	281,4778911	0,196880344	0,11864976	1,65934043	0,09704721	0,26348213	protein_codir	trans-sialidase
TcG_13452	121,0427361	0,205042836	0,1626461	1,2606686	0,20742827	0,42962689	protein_codir	dispersed gene family protein 1 (DGF-1)
TcG_13453	22,8934234	0,140240218	0,41465311	0,33821094	0,73520423	0,86451601	protein_codir	trans-sialidase
TcG_13454	29,13594961	-0,05271655	0,3438799	-0,15329931	0,87816225	0,94142358	protein_codir	trans-sialidase
TcG_13455	4,390090017	0,051194352	0,84956777	0,06025929	0,95194912	1		
TcG_13456	36,77835456	0,418831483	0,28633467	1,46273411	0,14354015	0,34000823	protein_codir	trans-sialidase
TcG_13457	12,7479936	0,748168493	0,49526254	1,51065026	0,13087758	1	protein_codir	putative trans-sialidase
TcG_13458	15,88933466	-0,492198324	0,43483048	-1,13193152	0,25766323	1	protein_codir	hypothetical protein
TcG_13459	3,970257585	0,962103484	0,89676188	1,07286394	0,28333217	1		
TcG_13460	47,33011206	0,369152296	0,25664103	1,43839936	0,15032077	0,35035975	protein_codir	hypothetical protein
TcG_13461	42,27024784	0,132533478	0,2679965	0,49453436	0,62092885	0,79536558	protein_codir	hypothetical protein
TcG_13462	228,0354582	0,495981698	0,12584748	3,94113323	8,1098E-05	0,00092845	protein_codir	hypothetical protein
TcG_13463	573,9867194	-0,040059419	0,08270614	-0,48435847	0,62813149	0,80069291	protein_codir	protein kinase
TcG_13464	1368,579194	0,124620703	0,05758502	2,16411682	0,03045539	0,11560515	protein_codir	hypothetical protein
TcG_13465	2294,471873	0,439535672	0,05019239	8,75701811	2,0048E-18	3,0563E-16	protein_codir	40S ribosomal protein S8

TcG_13466	493,6988985	0,068955219	0,08893381	0,77535438	0,43813028	0,66147738	protein_codir fibrillarin
TcG_13467	92,50648026	-0,137553509	0,18125748	-0,75888459	0,4479216	0,66893782	protein_codir hypothetical protein
TcG_13468	481,0606611	-0,362236511	0,08840622	-4,09740998	4,178E-05	0,00052673	protein_codir hypothetical protein
TcG_13469	7,166501432	-0,800776404	0,67436005	-1,18746122	0,23504575	1	
TcG_13470	131,4783819	-0,173570142	0,15353908	-1,13046233	0,25828146	0,49064584	protein_codir putative ABC transporter
TcG_13471	3049,413219	0,311916431	0,04683351	6,66011181	2,7362E-11	1,558E-09	protein_codir putative 40S ribosomal protein S23
TcG_13472	133,649359	-0,001191049	0,15236618	-0,00781701	0,99376299	0,99767711	protein_codir hypothetical protein
TcG_13473	421,607906	-0,076349469	0,0924591	-0,82576479	0,40893753	0,63660535	protein_codir hypothetical protein
TcG_13474	120,2150572	0,019966535	0,16406203	0,12170114	0,90313571	0,95315453	protein_codir hypothetical protein
TcG_13475	41,75877445	0,199455213	0,28389269	0,70257255	0,48232217	0,69626024	protein_codir mucin-associated surface protein (MASP)
TcG_13476	290,6452623	-0,518145663	0,10610368	-4,88339014	1,0428E-06	2,0975E-05	protein_codir hypothetical protein
TcG_13477	390,241258	0,22134496	0,09216445	2,40163061	0,01632218	0,07217893	protein_codir hypothetical protein
TcG_13478	24,77541582	-0,353323826	0,36879911	-0,95803871	0,33804324	0,57268153	protein_codir mucin-associated surface protein (MASP)
TcG_13479	253,7310929	0,395350017	0,11689052	3,38222478	0,00071901	0,00589143	protein_codir hypothetical protein
TcG_13480	4,567044897	-0,270942274	0,79088959	-0,34257914	0,7319151	1	protein_codir mucin-associated surface protein (MASP)
TcG_13481	318,0860795	-0,569845195	0,10408003	-5,47506751	4,3734E-08	1,221E-06	protein_codir rab1 small GTP-binding protein
TcG_13482	135,6978819	-0,013570772	0,15169988	-0,08945803	0,92871791	0,96542881	protein_codir hypothetical protein
TcG_13483	330,0816881	-0,133511475	0,10296469	-1,29667247	0,1947439	0,41217405	protein_codir putative ADP-ribosylation factor 3
TcG_13484	127,4571366	-0,420216646	0,15762104	-2,66599341	0,00767612	0,0400791	protein_codir hypothetical protein
TcG_13485	217,8222116	0,264408492	0,12276227	2,15382532	0,03125387	0,11783514	protein_codir hypothetical protein
TcG_13486	1208,965073	0,1619201	0,06245253	2,5926907	0,00952284	0,04755672	protein_codir basic transcription factor 3a
TcG_13487	75,15110897	-0,273163014	0,20370359	-1,3409828	0,17992604	0,39118466	protein_codir hypothetical protein
TcG_13488	449,6668778	-0,129142534	0,09128839	-1,41466553	0,15716659	0,35894581	protein_codir hypothetical protein
TcG_13489	8,603901191	-0,268842799	0,59177537	-0,45429873	0,64961383	1	protein_codir mucin-associated surface protein (MASP)
TcG_13490	271,9318631	-0,227532798	0,10913056	-2,08495955	0,03707297	0,13385088	protein_codir ATP binding protein-like protein
TcG_13491	152,9864134	-0,306551235	0,14937484	-2,052228	0,04014751	0,14165314	protein_codir hypothetical protein
TcG_13492	134,2401958	-0,376842777	0,15689789	-2,40183451	0,01631309	0,07216625	protein_codir uncharacterized protein
TcG_13493	4,561330164	0,592987942	0,84263705	0,70372878	0,48160169	1	protein_codir hypothetical protein
TcG_13494	2,422599001	0,225699007	1,16149272	0,19431806	0,84592685	1	protein_codir mucin-associated surface protein (MASP)
TcG_13495	646,523959	-0,232828447	0,07567723	-3,07659824	0,00209377	0,01431177	protein_codir hypothetical protein
TcG_13496	8,739784083	-0,460223631	0,6323033	-0,72785264	0,46670379	1	protein_codir mucin-associated surface protein (MASP)
TcG_13497	0,445396961	-2,390354933	2,60655946	-0,91705368	0,35911451	1	
TcG_13498	0,155988004	0,503022807	4,08047286	0,12327562	0,90188885	1	protein_codir hypothetical protein
TcG_13499	0,233855473	1,175456718	3,50839889	0,33504078	0,73759432	1	
TcG_13500	28,42210343	-0,092433393	0,38183266	-0,24207828	0,80871951	0,90526589	protein_codir calpain-like cysteine peptidase
TcG_13501	67,81475192	-0,425807205	0,21706121	-1,96169184	0,04979837	0,1649973	protein_codir glycerol uptake protein
TcG_13502	4,11929265	-0,428406453	0,86790247	-0,49361128	0,62158073	1	protein_codir mucin-associated surface protein (MASP)
TcG_13503	0					1	
