# Supplementary material for: P21 Ablation Unveils Strain-Specific Transcriptional Reprogramming in Trypanosoma cruzi Amastigotes
Source: Int J Microbiol. 2025 Jul 4;2025:9919200. doi: 10.1155/ijm/9919200 (PMC12253989; doi:10.1155/ijm/9919200)
Supplement: Supporting Information 9 — Table S8: Comprehensive table listing all differentially expressed genes by Y strain (Cas9 vs. P21−/−) amastigotes along with the fold change values. [file 9919200.f9.pdf]

Y (Cas9 vs. P21−/−) gene_id	baseMean	log2FoldChange	lfcSE	stat	pvalue	padj	type	description
TcG_00002	67,32149148	0,016750716	0,229124367	0,07310753	0,94172055	0,972960076	protein_codin	rhoptry protein
TcG_00003	509,6049534	-0,136734814	0,086018163	-1,58960397	0,111924102	0,289710614	protein_codin	hypothetical protein
TcG_00004	267,4062127	-0,160914656	0,111262446	-1,44626207	0,148103709	0,346907403	protein_codin	hypothetical protein
TcG_00005	285,2653049	-0,243088835	0,113897193	-2,13428293	0,032819628	0,122344983	protein_codin	hypothetical protein
TcG_00006	167,9781029	-0,34758614	0,137557616	-2,52684039	0,01150938	0,055193573	protein_codin	hypothetical protein
TcG_00007	211,4145772	-0,012539236	0,123758168	-0,10132047	0,919296067	0,961200581	protein_codin	COMPASS component SWD2
TcG_00008	285,6265811	-0,045589697	0,106127518	-0,4295747	0,667505041	0,825813061	protein_codin	protein SHQ1
TcG_00009	250,169546	-0,224223152	0,114397639	-1,96003304	0,049991928	0,165487566	protein_codin	hypothetical protein
TcG_00010	17,13858077	-1,12654426	0,433397264	-2,59933403	0,009340483	0,04678722	protein_codin	kinetoplast DNA-associated protein
TcG_00011	381,5212046	-0,784635582	0,092674131	-8,46660848	2,52642E-17	3,48891E-15	protein_codin	kinetoplast DNA-associated protein 3
TcG_00012	219,7292782	-0,360192885	0,120863539	-2,98016167	0,002880963	0,018441346	protein_codin	putative enolase
TcG_00013	121,7052966	-0,028946324	0,17071923	-0,16955514	0,865360001	0,935092424	protein_codin	tRNA wybutosine-synthesizing protein 3
TcG_00014	116,6092249	0,08502413	0,17061452	0,498340527	0,618244047	0,793033999	protein_codin	hypothetical protein
TcG_00015	185,2586073	-0,043732979	0,136563476	-0,32023921	0,748787003	0,873220555	protein_codin	hypothetical protein
TcG_00016	212,3144838	0,052715133	0,121793787	0,432822844	0,665143476	0,825002924	protein_codin	hypothetical protein
TcG_00017	389,628074	-0,304488684	0,100539693	-3,02854201	0,002457369	0,016222836	protein_codin	hypothetical protein
TcG_00018	550,6400845	-0,326620783	0,081450151	-4,01006969	6,07008E-05	0,000728391	protein_codin	hypothetical protein
TcG_00019	424,6790982	-0,227861692	0,095914567	-2,37567348	0,017516956	0,075940595	protein_codin	putative enoyl-CoA hydratase/isomerase family protein
TcG_00020	323,3864505	-0,131615677	0,104960018	-1,25396012	0,2098565	0,432479084	protein_codin	putative enoyl-CoA hydratase/isomerase family protein
TcG_00021	209,0430728	-0,198711355	0,124895346	-1,5910229	0,111604423	0,289272673	protein_codin	transducin family protein / WD-40 repeat family protein
TcG_00022	199,5705799	-0,119552106	0,125414293	-0,95325742	0,340459659	0,574674476	protein_codin	hypothetical protein
TcG_00023	716,4718685	0,043837232	0,07121874	0,615529454	0,538205124	0,737596944	protein_codin	hypothetical protein
TcG_00024	636,9325052	-0,223414946	0,073972632	-3,02023791	0,002525762	0,016561109	protein_codin	hypothetical protein
TcG_00025	118,5878322	0,175497811	0,163425869	1,073867996	0,282881849	0,516148762	protein_codin	hypothetical protein
TcG_00026	155,6971989	-0,255311919	0,140932708	-1,8115874	0,070049973	0,212016453	protein_codin	hypothetical protein
TcG_00027	559,5453515	-0,153161969	0,079777411	-1,91986639	0,054874779	0,17679942	protein_codin	hypothetical protein
TcG_00028	77,84822553	0,040440973	0,200193111	0,202009812	0,839909054	0,9222125	protein_codin	TBC1 domain family member 14
TcG_00029	104,0511625	0,362756097	0,1802533	2,012479641	0,044169401	0,151763546	protein_codin	hypothetical protein
TcG_00030	64,62576017	-0,648959906	0,215450322	-3,01210924	0,002594392	0,016924903	protein_codin	hypothetical protein
TcG_00031	503,8819187	-0,097116455	0,086287553	-1,12549785	0,260378129	0,492690022	protein_codin	putative CMP-sialic acid transporter
TcG_00032	280,3619162	-0,016608951	0,110927907	-0,14972744	0,880979659	0,942069315	protein_codin	putative ATP-dependent DEAD/H RNA helicase
TcG_00033	314,6297706	-0,251245915	0,103447417	-2,42873067	0,015151783	0,068160911	protein_codin	mediator of RNA polymerase II transcription subunit 24
TcG_00034	369,7774674	0,09246622	0,099718797	0,927269708	0,353786518	0,58808501	protein_codin	putative RNA-binding protein
TcG_00035	259,4989666	0,140377723	0,118332434	1,18629963	0,235503993	0,463329811	protein_codin	exosome-associated protein 2
TcG_00036	493,9625354	-0,224823747	0,090085941	-2,49565852	0,012572357	0,059110386	protein_codin	hypothetical protein
TcG_00037	215,3064973	-0,015120931	0,123723631	-0,12221538	0,902728444	0,952972786	protein_codin	hypothetical protein
TcG_00038	415,6655508	-0,132446978	0,090633412	-1,46134825	0,143919895	0,340575143	protein_codin	hypothetical protein
TcG_00039	161,434705	-0,000776225	0,139227716	-0,00557522	0,995551644	0,99830402	protein_codin	hypothetical protein
TcG_00040	241,5815004	-0,276914899	0,114850622	-2,41108749	0,015905032	0,070739234	protein_codin	hypothetical protein
TcG_00041	159,2213754	-0,11763716	0,149100444	-0,78897927	0,430124122	0,655023406	protein_codin	hypothetical protein
TcG_00042	251,7106092	-0,222358387	0,118554889	-1,87557332	0,060713893	0,191383433	protein_codin	hypothetical protein
TcG_00043	148,0234246	-0,109739434	0,148059819	-0,74118309	0,458582436	0,677438813	protein_codin	hypothetical protein
TcG_00044	793,3015992	0,019014994	0,072237963	0,263227162	0,792375497	0,896248225	protein_codin	hypothetical protein
TcG_00045	93,79136634	-0,224419035	0,181734508	-1,23487299	0,216877778	0,440893872	protein_codin	hypothetical protein
TcG_00046	2485,403916	-0,267736895	0,046464556	-5,76217487	8,30369E-09	2,68733E-07	protein_codin	hypothetical protein
TcG_00047	421,6799283	-0,198413881	0,09501995	-2,08812865	0,036786231	0,133355843	protein_codin	hypothetical protein
TcG_00048	300,2903321	0,147291364	0,103901199	1,417609851	0,156304703	0,357690793	protein_codin	tRNA pseudouridine13 synthase
TcG_00049	261,4273843	-0,220784122	0,111797035	-1,97486564	0,048283398	0,161539546	protein_codin	putative chloride channel protein
TcG_00050	385,3621804	-0,277344502	0,099838029	-2,77794448	0,005470397	0,030715015	protein_codin	putative lipase
TcG_00051	328,4962183	-0,238856009	0,108051489	-2,21057582	0,027065225	0,105902631	protein_codin	hypothetical protein
TcG_00052	483,1698054	-0,130464442	0,083562816	-1,56127387	0,118459145	0,300626124	protein_codin	hypothetical protein
TcG_00053	173,5816604	-0,410791994	0,139351672	-2,94787991	0,003199613	0,020103832	protein_codin	hypothetical protein
TcG_00054	355,5802612	-0,375502851	0,103049018	-3,64392461	0,000268512	0,00254373	protein_codin	putative CYC2-like cyclin, putative,cyclin 6
TcG_00055	995,4364559	-0,240701117	0,065309186	-3,68556297	0,000228198	0,002229256	protein_codin	putative dihydrolipoamide dehydrogenase
TcG_00056	461,0359266	-0,307972918	0,100585499	-3,06180236	0,002200087	0,014854431	protein_codin	putative chaperone DNAJ protein
TcG_00057	136,9346284	-0,153582994	0,153872697	-0,99811726	0,318222504	0,553343228	protein_codin	hypothetical protein

TcG_00058	114,2515281	-0,062596072	0,164007094	-0,38166686	0,702708487	0,846349439	protein_codin hypothetical protein
TcG_00059	1097,639708	-0,403511726	0,060457314	-6,67432441	2,48374E-11	1,43167E-09	protein_codin putative chaperonin alpha subunit
TcG_00060	143,2736187	-0,153025824	0,145777729	-1,04972018	0,293846782	0,526850638	protein_codin hypothetical protein
TcG_00061	1057,605709	-0,118523093	0,061779101	-1,9184982	0,05504787	0,177119842	protein_codin putative glucosamine-6-phosphate isomerase, putative,glucosamine-6-phosphate deaminase
TcG_00062	195,9015663	-0,466090652	0,13042851	-3,57353351	0,000352196	0,003207975	
TcG_00063	502,433043	-0,454551979	0,08206416	-5,53898288	3,04233E-08	8,79202E-07	protein_codin putative mitogen-activated protein kinase, putative,kinase
TcG_00064	325,3679422	-0,029433792	0,103720612	-0,28377958	0,776579302	0,888310845	protein_codin hypothetical protein
TcG_00065	360,7541536	-0,313236924	0,108628647	-2,88355726	0,003932112	0,0237402	protein_codin putative dynein light intermediate chain
TcG_00066	5,38257034	1,286492518	0,803716335	1,60067982	0,109447853	1	protein_codin hypothetical protein
TcG_00067	218,0378946	-0,235339427	0,126012063	-1,86759442	0,061818624	0,193942751	protein_codin Rab family, other
TcG_00068	359,8524031	-0,135339045	0,09460551	-1,43056197	0,152555793	0,352816028	protein_codin hypothetical protein
TcG_00069	438,5080417	-0,119276175	0,086356645	-1,38120436	0,167216134	0,373144478	protein_codin putative conserved oligomeric Golgi complex subunit 5-like
TcG_00070	375,6503268	0,039544339	0,094331045	0,419208114	0,675064043	0,829845305	protein_codin putative mitochondrial RNA binding protein
TcG_00071	337,9397658	-0,001181101	0,098019752	-0,01204962	0,990386029	0,996036512	protein_codin putative NADH dehydrogenase subunit NI8M
TcG_00072	452,7995244	-0,073063527	0,087128479	-0,83857228	0,401709373	0,63220648	protein_codin hypothetical protein
TcG_00073	1696,382468	-0,236278556	0,055040955	-4,29277716	1,76452E-05	0,000248405	protein_codin hypothetical protein
TcG_00074	713,9666735	-0,19925636	0,071743938	-2,77732678	0,005480805	0,030750584	protein_codin delta-1-pyrroline-5-carboxylate synthetase
TcG_00075	351,5835263	-0,071796267	0,10360348	-0,69299088	0,488315274	0,699767565	protein_codin hypothetical protein
TcG_00076	223,4634915	-0,029827549	0,12601375	-0,23670075	0,812888955	0,907594318	protein_codin hypothetical protein
TcG_00077	280,6662301	-0,14150613	0,113763292	-1,24386458	0,213549408	0,436980474	protein_codin hypothetical protein
TcG_00078	448,6111135	-0,047354476	0,087696176	-0,53998337	0,589208503	0,775648564	protein_codin putative ATP-binding cassette protein subfamily B, member 3
TcG_00079	445,7349032	-0,360214769	0,09137396	-3,94220377	8,07363E-05	0,00092615	protein_codin hypothetical protein
TcG_00080	189,9670902	-0,048173719	0,129455773	-0,37212492	0,709799843	0,850350634	protein_codin hypothetical protein
TcG_00081	387,0587369	-0,28331541	0,097983833	-2,89145056	0,003834679	0,023285426	protein_codin hypothetical protein
TcG_00082	644,0197328	-0,131159429	0,075667331	-1,7333693	0,08303005	0,236811141	protein_codin hypothetical protein
TcG_00083	47,70027137	-0,163374593	0,253371952	-0,64480141	0,519055886	0,7237563	protein_codin hypothetical protein
TcG_00084	53,69766374	-0,018669679	0,235874122	-0,07915103	0,936912498	0,970675865	protein_codin hypothetical protein
TcG_00085	67,47093987	-0,427842864	0,219666965	-1,94768869	0,051452224	0,168686401	protein_codin hypothetical protein
TcG_00086	60,07118313	-0,079599661	0,229796747	-0,34639159	0,729048444	0,860563425	protein_codin hypothetical protein
TcG_00087	352,5601867	-0,256860662	0,097031217	-2,64719614	0,008116226	0,041942283	protein_codin putative protein phosphatase 2A catalytic subunit
TcG_00088	255,0195716	-0,23083238	0,111876434	-2,06327975	0,039086057	0,139124749	protein_codin putative chaperone DNAJ protein
TcG_00089	315,985289	-0,094578373	0,106851076	-0,88514198	0,376080062	0,606607768	protein_codin GTP-binding protein
TcG_00090	2415,132875	-0,494459058	0,046379873	-10,6610696	1,54807E-26	5,78577E-24	protein_codin leucine-rich repeat protein
TcG_00091	463,856426	-0,304386448	0,084949955	-3,58312666	0,000339506	0,003111958	protein_codin CBS domain-containing protein
TcG_00092	224,0229706	-0,304468377	0,123400054	-2,46732774	0,01361257	0,063136604	protein_codin hypothetical protein
TcG_00093	163,7547416	-0,287436621	0,140431167	-2,04681501	0,040676252	0,142810621	protein_codin Rab5-interacting family protein
TcG_00094	76,14741636	-0,249677903	0,199468653	-1,25171499	0,210673737	0,433161652	protein_codin cytochrome-c oxidase
TcG_00095	119,1453952	-0,222511311	0,165039345	-1,34823191	0,177583805	0,387912135	protein_codin hypothetical protein
TcG_00096	217,3550353	-0,242640804	0,127850786	-1,89784365	0,057716681	0,183257183	protein_codin hypothetical protein
TcG_00097	179,8146801	-0,338841506	0,145021706	-2,3364882	0,019465812	0,082310546	protein_codin hypothetical protein
TcG_00098	32,40286867	-0,093735528	0,319781985	-0,29312323	0,769427962	0,884364283	protein_codin hypothetical protein
TcG_00099	110,4894224	0,773930063	0,173531753	4,459875782	8,20072E-06	0,00012927	protein_codin hypothetical protein
TcG_00100	71,30189977	0,487338875	0,22358457	2,179662372	0,029282498	0,112228587	protein_codin trans-sialidase
TcG_00101	41,06241506	-0,257649362	0,270230608	-0,95344256	0,340365888	0,574599909	protein_codin hypothetical protein
TcG_00102	179,3247205	-0,301026311	0,133564031	-2,25379773	0,024208895	0,097525821	protein_codin putative class 3 lipase
TcG_00103	229,2272901	-0,098906039	0,120222757	-0,82268982	0,410684404	0,6373998	protein_codin TATA binding protein
TcG_00104	336,4429842	-0,236801631	0,100517564	-2,35582341	0,018481701	0,078985242	protein_codin putative cation transporter
TcG_00105	230,4317945	-0,288712314	0,119200592	-2,42207114	0,015432329	0,069194645	protein_codin hypothetical protein
TcG_00106	120,2883349	-0,19549	0,160558592	-1,21756175	0,223390559	0,449809352	protein_codin hypothetical protein
TcG_00107	381,903406	-0,050125992	0,093536119	-0,53589984	0,592027772	0,777514596	protein_codin putative phosphatidic acid phosphatase protein
TcG_00108	269,0780953	-0,314781202	0,10906463	-2,88618962	0,003899371	0,023606182	protein_codin putative GUP1
TcG_00109	96,25456123	-0,304164871	0,178343646	-1,70549879	0,088101439	0,246556345	protein_codin glycerol uptake protein
TcG_00110	160,3132682	0,052984211	0,149349435	0,354766735	0,722764351	0,857335574	protein_codin hypothetical protein
TcG_00111	36,95259042	0,238571395	0,289321757	0,824588504	0,40960525	0,636866847	protein_codin putative glycerol uptake protein
TcG_00112	129,5727643	-0,162460667	0,15814881	-1,02726455	0,30429591	0,537107314	protein_codin putative glycerol uptake protein
TcG_00113	263,9427432	-0,159244317	0,113366597	-1,40468464	0,160115075	0,363061588	protein_codin hypothetical protein
TcG_00114	216,0938671	0,00192035	0,129241193	0,014858655	0,988144945	0,995188398	protein_codin putative protein kinase

TcG_00115	171,5943205	-0,242183672	0,13832432	-1,75083942	0,079973575	0,231123433	protein_codin putative RNA-binding protein
TcG_00116	176,2032225	-0,102267568	0,131141147	-0,77982823	0,43549199	0,659399643	protein_codin hypothetical protein
TcG_00117	263,6632248	-0,254133731	0,109971577	-2,31090377	0,020838171	0,086908225	protein_codin putative peroxisome assembly protein
TcG_00118	344,0459717	-0,170502532	0,097560613	-1,74765745	0,080523361	0,232133283	protein_codin hypothetical protein
TcG_00119	1,525078208	0,329011134	1,39775154	0,235385993	0,813909157	1	
TcG_00120	0,611155623	-0,424209629	2,242957467	-0,18912959	0,849991249	1	
TcG_00121	0					1	
TcG_00122	11,13185034	-0,700882823	0,520214062	-1,34729696	0,177884612	1	protein_codin putative RNA-binding protein 4
TcG_00123	171,5659236	-0,108460539	0,144887654	-0,74858372	0,454108147	0,674179522	protein_codin hypothetical protein
TcG_00124	663,8937395	-0,200367505	0,078944394	-2,53808403	0,011146122	0,053830332	protein_codin kinesin-14
TcG_00125	118,1901889	-0,056604069	0,160866482	-0,35186987	0,724935853	0,858172586	protein_codin hypothetical protein
TcG_00126	159,4182693	-0,2866039	0,14369301	-1,99455701	0,046091216	0,156098459	protein_codin U3 small nucleolar RNA-associated protein 24
TcG_00127	249,7387186	-0,360024725	0,116549366	-3,08903203	0,002008098	0,01378307	protein_codin putative actin-related protein 2
TcG_00128	165,8986728	-0,426189179	0,145159613	-2,93600384	0,003324702	0,020697126	protein_codin Cytochrome c oxidase assembly protein COX19
TcG_00129	407,7502408	-0,382248206	0,093881372	-4,07160863	4,66896E-05	0,000583544	protein_codin hypothetical protein
TcG_00130	341,6351049	-0,254468869	0,103932403	-2,44840744	0,014348929	0,065736139	protein_codin hypothetical protein
TcG_00131	251,1908048	-0,196246971	0,112394845	-1,74604958	0,080802336	0,232705908	protein_codin hypothetical protein
TcG_00132	1223,144238	-0,405705634	0,063783742	-6,36064334	2,0091E-10	9,20059E-09	protein_codin hypothetical protein
TcG_00133	1465,793522	-0,484264165	0,058227987	-8,31669078	9,04533E-17	1,15164E-14	protein_codin hypothetical protein
TcG_00134	226,4090568	-0,326111504	0,117830749	-2,76762651	0,005646612	0,031498147	protein_codin cleavage stimulation factor subunit 1
TcG_00135	1349,58184	-0,042887872	0,062209611	-0,6894091	0,490565858	0,701690868	protein_codin hypothetical protein
TcG_00136	421,3516826	-0,370832539	0,090216874	-4,11045652	3,94878E-05	0,000500006	protein_codin proteasome regulatory non-ATPase subunit
TcG_00137	159,8698725	-0,385074046	0,139792746	-2,75460679	0,005876271	0,032497602	protein_codin putative mitochondrial carrier protein
TcG_00138	492,2277161	-0,613589422	0,094357548	-6,50281227	7,88322E-11	3,95389E-09	protein_codin hypothetical protein
TcG_00139	155,8390725	-0,314735944	0,140338674	-2,2426886	0,024916903	0,099478716	protein_codin anaphase promoting complex subunit 10
TcG_00140	536,4304544	-0,112974161	0,083580698	-1,35167765	0,176478457	0,386561145	protein_codin hypothetical protein
TcG_00141	269,7251449	-0,057446827	0,129454723	-0,44375999	0,657216115	0,819204509	protein_codin hypothetical protein
TcG_00142	298,7924131	-0,108190927	0,104103536	-1,03926275	0,298682556	0,532231897	protein_codin putative sphingosine kinase A, B
TcG_00143	1255,838737	-0,381107985	0,061148912	-6,23245729	4,59175E-10	2E-08	protein_codin hypothetical protein
TcG_00144	331,0376348	-0,236116275	0,101174046	-2,33376329	0,01960812	0,082731129	protein_codin putative tRNA pseudouridine synthase A-like protein
TcG_00145	615,0623991	-0,296915774	0,077728489	-3,81990926	0,000133501	0,001417727	protein_codin WD repeat-containing protein wat1
TcG_00146	272,1063719	-0,471081417	0,110269433	-4,27209431	1,93646E-05	0,000269337	protein_codin putative zinc finger protein
TcG_00147	132,3742507	0,177648167	0,156104295	1,138009476	0,255116525	0,486388031	protein_codin putative FKBP-type peptidyl-prolyl cis-trans isomerase
TcG_00148	400,4341877	-0,460410577	0,098499502	-4,67424269	2,9504E-06	5,29975E-05	protein_codin macrophage infectivity potentiator, precursor
TcG_00149	82,95832345	-0,409605483	0,204739597	-2,00061683	0,045433698	0,154549274	protein_codin dynein light chain
TcG_00150	337,4879073	-0,511329911	0,10568846	-4,83808651	1,31095E-06	2,57E-05	protein_codin transcription factor IIA alpha-beta subunit
TcG_00151	249,0818305	-0,277615943	0,111641405	-2,48667547	0,012894297	0,060360937	protein_codin hypothetical protein
TcG_00152	112,1628379	0,03142457	0,169820512	0,185045783	0,853193126	0,928875734	protein_codin putative ADP-ribosylation factor family
TcG_00153	675,3000987	-0,223338159	0,07884545	-2,83260683	0,004617014	0,027043842	protein_codin hypothetical protein
TcG_00154	531,3106499	-0,279286352	0,084119888	-3,32009893	0,000899856	0,007155612	protein_codin vps53-like domain protein
TcG_00155	108,2963988	-0,238913193	0,1670266	-1,43039008	0,152605092	0,352840272	protein_codin hypothetical protein
TcG_00156	618,9603478	-0,373922554	0,079063319	-4,72940622	2,25177E-06	4,1477E-05	protein_codin ATP-binding cassette protein subfamily F, member 2
TcG_00157	607,3674027	-0,137201233	0,079026742	-1,73613678	0,082539652	0,236182863	protein_codin putative signal recognition particle protein
TcG_00158	86,04288517	-0,257207218	0,190698554	-1,34876334	0,177412993	0,387612095	protein_codin hypothetical protein
TcG_00159	135,5100919	-0,144137167	0,153754017	-0,93745302	0,348525581	0,583103728	protein_codin putative GDP-mannose 4,6 dehydratase
TcG_00160	184,6914314	0,12192153	0,130849242	0,931771009	0,35145488	0,585977298	protein_codin hypothetical protein
TcG_00161	520,0826113	-0,098604359	0,085784489	-1,14944274	0,250373464	0,480030938	protein_codin putative transmembrane protein
TcG_00162	410,1316241	-0,017500285	0,090749021	-0,19284269	0,847082169	0,925964149	protein_codin hypothetical protein
TcG_00163	129,0937241	0,070205106	0,15283719	0,459345703	0,645985933	0,812019442	protein_codin hypothetical protein
TcG_00164	289,2797514	-0,068170324	0,106977439	-0,63724019	0,5239684	0,72776714	protein_codin hypothetical protein
TcG_00165	288,1552045	0,308784889	0,10731612	2,877339287	0,004010441	0,024112597	protein_codin hypothetical protein
TcG_00166	224,7216449	0,10296281	0,124650512	0,826011931	0,408797324	0,636605346	protein_codin hypothetical protein
TcG_00167	403,5487922	-0,015716303	0,097118364	-0,16182627	0,871442665	0,938078112	protein_codin putative chaperonin HSP60, mitochondrial precursor
TcG_00168	170,4471292	-0,229133563	0,14353068	-1,59640826	0,110397673	0,287172752	protein_codin putative rab11B GTPase
TcG_00169	37,94769869	-0,58572458	0,29332256	-1,99686168	0,045840211	0,15547561	protein_codin putative iron superoxide dismutase
TcG_00170	507,2577705	-0,206352126	0,0861559	-2,3951015	0,016615765	0,073115521	protein_codin putative protein kinase
TcG_00171	182,9293073	-0,103455881	0,134460225	-0,76941625	0,441646247	0,664189177	protein_codin hypothetical protein

TcG_00172	138,7592286	-0,133944061	0,151398313	-0,88471303	0,376311425	0,606811993	protein_codin	hypothetical protein
TcG_00173	302,4960867	-0,117175389	0,104487977	-1,12142461	0,262107165	0,494282475	protein_codin	hypothetical protein
TcG_00174	260,4833169	-0,013923615	0,11863333	-0,11736681	0,906569386	0,954710879	protein_codin	hypothetical protein
TcG_00175	433,9316528	-0,081785825	0,089378293	-0,91505244	0,360164103	0,592820188	protein_codin	hypothetical protein
TcG_00176	390,1327477	-0,159417503	0,098174304	-1,62382106	0,104414005	0,277336235	protein_codin	hypothetical protein
TcG_00177	752,0259309	0,137645868	0,069912554	1,968829055	0,048972725	0,163181662	protein_codin	putative pumilio/PUF RNA binding protein 7
TcG_00178	305,0119979	-0,214645829	0,110037616	-1,95065866	0,051097664	0,168091294	protein_codin	CCCH zinc-finger 2
TcG_00179	247,8625687	0,073152116	0,11942424	0,612539931	0,540180587	0,739113156	protein_codin	methyltransferase
TcG_00180	663,2070347	-0,067218451	0,076912756	-0,87395712	0,382141606	0,61215602	protein_codin	hypothetical protein
TcG_00181	845,4875699	0,065400998	0,078709077	0,830920658	0,406018453	0,634664031	protein_codin	hypothetical protein
TcG_00182	343,396954	-0,082956046	0,109658731	-0,75649284	0,449353761	0,670299044	protein_codin	hypothetical protein
TcG_00183	268,0443621	-0,293260196	0,108548264	-2,70165717	0,006899486	0,036905562	protein_codin	putative protein phosphatase 2C
TcG_00184	234,5823053	0,246175862	0,12568211	1,958718401	0,050145774	0,165734726	protein_codin	putative mitochondrial carrier protein
TcG_00185	293,1845698	-0,030859682	0,11238246	-0,27459518	0,783627271	0,892734077	protein_codin	putative coatomer epsilon subunit
TcG_00186	330,9582944	-0,109987957	0,099488446	-1,10553497	0,268927848	0,501496548	protein_codin	DNA polymerase alpha subunit B
TcG_00187	492,2623549	-0,287504193	0,087253441	-3,29504704	0,000984052	0,007677555	protein_codin	hypothetical protein
TcG_00188	255,7512955	-0,122831367	0,111622376	-1,10041886	0,271149664	0,504340987	protein_codin	putative NAD synthase
TcG_00189	328,2795477	-0,044750545	0,100930169	-0,44338126	0,657489989	0,819281459	protein_codin	hypothetical protein
TcG_00190	5,352566293	0,405108401	0,786785904	0,514890264	0,606629705	1		
TcG_00191	307,1436927	-0,128155764	0,102668164	-1,24825223	0,211938703	0,434913534	protein_codin	hypothetical protein
TcG_00192	1062,404178	-0,064355532	0,062188204	-1,03485112	0,300738446	0,534199829	protein_codin	putative chromosomal passenger protein
TcG_00193	216,4036187	-0,004690917	0,121033586	-0,03875715	0,969084007	0,986713007	protein_codin	hypothetical protein
TcG_00194	326,9329721	-0,087086257	0,102241567	-0,85176959	0,39434199	0,624244609	protein_codin	hypothetical protein
TcG_00195	194,8295786	0,041616012	0,126438132	0,329141304	0,742048886	0,868773079	protein_codin	hypothetical protein
TcG_00196	295,0804906	-0,107101677	0,111173715	-0,96337229	0,335360725	0,569886969	protein_codin	hypothetical protein
TcG_00197	259,2197562	0,308493243	0,111688916	2,762075715	0,005743516	0,031885181	protein_codin	putative PIF1 helicase-like protein
TcG_00198	518,7122629	0,13724806	0,081363918	1,686841837	0,091633768	0,252898723	protein_codin	putative phosphomannose isomerase
TcG_00199	301,4909214	0,090846917	0,107910471	0,841873053	0,399859015	0,629794256	protein_codin	stromal membrane-associated protein
TcG_00200	137,6298484	0,07764834	0,149859372	0,518141368	0,604359639	0,786152595	protein_codin	hypothetical protein
TcG_00201	271,3021916	-0,248817886	0,110468165	-2,25239449	0,024297353	0,09778018	protein_codin	hypothetical protein
TcG_00202	318,024922	-0,332222327	0,10109365	-3,28628285	0,00101519	0,00787282	protein_codin	putative cell cycle sequence binding phosphoprotein (RBP33)
TcG_00203	299,943365	0,146013329	0,107281627	1,361028284	0,173504751	0,38209961	protein_codin	hypothetical protein
TcG_00204	209,222193	-0,2004311	0,12325095	-1,62620329	0,103906417	0,276304738	protein_codin	oligoribonuclease
TcG_00205	171,0413114	0,128477385	0,138180958	0,929776341	0,352486899	0,586769139	protein_codin	hypothetical protein
TcG_00206	481,7373385	-0,24842096	0,085883505	-2,89253402	0,003821478	0,023229613	protein_codin	putative ATP-dependent zinc metallopeptidase
TcG_00207	220,0499113	0,278562358	0,122639774	2,271386751	0,023123576	0,094177886	protein_codin	hypothetical protein
TcG_00208	284,9768816	0,071335835	0,119728121	0,595815209	0,551298682	0,746821273	protein_codin	hypothetical protein
TcG_00209	355,8716871	0,075170969	0,102919105	0,730388875	0,465152516	0,682138496	protein_codin	hypothetical protein
TcG_00210	434,4490554	0,026507883	0,088484972	0,299574977	0,764501373	0,881292267	protein_codin	putative TRAF3-interacting protein 1-like
TcG_00211	283,4652461	0,343373028	0,126299317	2,718724353	0,00655342	0,035480336	protein_codin	putative phosphatidylinositol 3-related kinase
TcG_00212	929,6831412	0,298644513	0,073140311	4,083172599	4,4425E-05	0,000558252	protein_codin	putative phosphatidylinositol 3-related kinase
TcG_00213	139,9872196	0,194620048	0,151199965	1,287169926	0,198035067	0,416639602	protein_codin	hypothetical protein
TcG_00214	284,6818873	0,196068551	0,105638841	1,8560271	0,063449695	0,197817088	protein_codin	hypothetical protein
TcG_00215	740,6295641	-0,025968265	0,070293233	-0,36942766	0,711808985	0,851876759	protein_codin	hypothetical protein
TcG_00216	298,0428711	0,095762752	0,104477156	0,916590342	0,359357347	0,592630203	protein_codin	hypothetical protein
TcG_00217	212,9120131	0,016194975	0,130037947	0,124540374	0,900887435	0,951748911	protein_codin	hypothetical protein
TcG_00218	437,1449208	0,567260811	0,093233441	6,084306275	1,16997E-09	4,61063E-08	protein_codin	putative DEAD/DEAH box helicase-like protein
TcG_00219	253,8087021	0,047504055	0,113525306	0,418444636	0,675622057	0,83017833	protein_codin	hypothetical protein
TcG_00220	112,3622879	-0,065518332	0,163980151	-0,39955038	0,689487706	0,838680499	protein_codin	hypothetical protein
TcG_00221	135,4297045	0,184578974	0,154189804	1,197089361	0,231271726	0,458517724	protein_codin	hypothetical protein
TcG_00222	897,1733842	-0,782750953	0,067074921	-11,6698005	1,81849E-31	1,1089E-28	protein_codin	trypomastigote small surface antigen
TcG_00223	47,85485859	0,057806059	0,25297399	0,228505939	0,819252939	0,910871391	protein_codin	hypothetical protein
TcG_00224	1,867924756	-0,764781126	1,296065736	-0,59007896	0,555137712	1		
TcG_00225	37,42259348	-0,119853022	0,296739383	-0,40389995	0,686286294	0,837209523	protein_codin	hypothetical protein
TcG_00226	53,72495219	-0,00931021	0,247521114	-0,0376138	0,969995603	0,987033994	protein_codin	hypothetical protein
TcG_00227	0					1	protein_codin	hypothetical protein
TcG_00228	3,046413349	0,812560764	0,997828332	0,814329216	0,415456377	1	protein_codin	hypothetical protein

TcG_00229	16,78029875	0,05995677	0,431888915	0,138824517	0,88958882	0,946535728	protein_codin	hypothetical protein
TcG_00230	33,79256316	-0,253204731	0,318386133	-0,79527562	0,42645319	0,651783849	protein_codin	hypothetical protein
TcG_00231	43,67388121	-0,173650327	0,264149052	-0,65739523	0,510926819	0,717505232	protein_codin	hypothetical protein
TcG_00232	58,41115106	-0,179578698	0,229761761	-0,78158653	0,434457604	0,658735343		
TcG_00233	194,0877733	0,528133391	0,146623582	3,601967591	0,000315818	0,002929595	protein_codin	hypothetical protein
TcG_00234	660,0055877	-0,348901004	0,073049775	-4,77620913	1,78631E-06	3,36745E-05	protein_codin	hypothetical protein
TcG_00235	590,9543704	-0,238947258	0,081320215	-2,93835006	0,003299642	0,020586782	protein_codin	RNA guanylyltransferase
TcG_00236	296,8273296	0,263561991	0,103578004	2,544574925	0,010941084	0,052972585	protein_codin	hypothetical protein
TcG_00237	1238,274487	0,030830429	0,061322358	0,502759995	0,615133023	0,792057258	protein_codin	cleavage and polyadenylation specificity factor-like protein
TcG_00238	298,7945539	0,087891226	0,109312451	0,804036735	0,42137576	0,647488005	protein_codin	hypothetical protein
TcG_00239	359,4049928	-0,167676605	0,095119963	-1,7627909	0,077935764	0,226590654	protein_codin	hypothetical protein
TcG_00240	367,1607598	-0,095372709	0,09366348	-1,01824861	0,308559824	0,541498656	protein_codin	hypothetical protein
TcG_00241	230,612419	0,238602437	0,124569335	1,915418723	0,055439125	0,178025972	protein_codin	putative polypeptide deformylase-like protein
TcG_00242	664,0803014	-0,307844132	0,073405828	-4,19372877	2,74406E-05	0,000365854	protein_codin	transferase
TcG_00243	190,5007087	0,052358829	0,136532888	0,383488771	0,701357395	0,845322279	protein_codin	RNA polymerase-like protein
TcG_00244	331,454585	-0,041428567	0,104943897	-0,39476871	0,693013585	0,841062696	protein_codin	hypothetical protein
TcG_00245	224,6712527	-0,030294907	0,121941211	-0,24843862	0,803795052	0,903185306	protein_codin	hypothetical protein
TcG_00246	260,0629772	0,187644517	0,117015656	1,603584704	0,108805625	0,284821954	protein_codin	ubiquitin hydrolase
TcG_00247	398,4256081	0,002823208	0,100148016	0,028190357	0,977510328	0,990224843	protein_codin	Bardet-Biedl syndrome 9
TcG_00248	632,7704389	-0,047711842	0,078582731	-0,60715428	0,543748531	0,740930357	protein_codin	putative ADP-ribosylation factor GTPase activating protein 1
TcG_00249	576,9484864	-0,202493053	0,078340577	-2,58477868	0,009744152	0,048453111	protein_codin	hypothetical protein
TcG_00250	485,8214867	-0,121074024	0,082998895	-1,4587426	0,144635967	0,341502407	protein_codin	hypothetical protein
TcG_00251	316,8707991	-0,208776172	0,101939545	-2,04803908	0,04055617	0,142561829	protein_codin	proteasome regulatory non-ATPase subunit
TcG_00252	197,0101555	0,259740574	0,130681911	1,987578631	0,046858312	0,158095631	protein_codin	hypothetical protein
TcG_00253	262,1343684	0,007877274	0,11140751	0,070706853	0,943631066	0,973371575	protein_codin	LmrCD-specific DARPin
TcG_00254	1547,625722	-0,006557018	0,056793214	-0,11545425	0,908085084	0,955418977	protein_codin	putative ABC transporter
TcG_00255	231,6073581	0,053018436	0,131317401	0,40374265	0,686401973	0,837209523	protein_codin	hypothetical protein
TcG_00256	382,5180784	0,034750407	0,098926346	0,351275555	0,725381632	0,858172586	protein_codin	putative phosphatidylinositol-4-phosphate 5-kinase
TcG_00257	218,6803104	-0,079269423	0,126087269	-0,62868697	0,529554012	0,731548321		
TcG_00258	155,3032501	0,078386149	0,153885765	0,509378815	0,610486723	0,789056132	protein_codin	hypothetical protein
TcG_00259	498,4985171	-0,214487892	0,085119566	-2,51984241	0,011740739	0,056047878	protein_codin	polyadenylation/uridylation factor 2
TcG_00260	803,4692183	-0,196265938	0,069301246	-2,83206938	0,004624781	0,027057142	protein_codin	hypothetical protein
TcG_00261	254,2757745	0,182692473	0,111850556	1,633362226	0,102392837	0,27334981	protein_codin	putative mitochondrial carrier protein
TcG_00262	205,3152598	0,143227713	0,12478322	1,147812287	0,251046064	0,480843065	protein_codin	putative mitochondrial carrier protein
TcG_00263	886,8265237	-0,117603841	0,067772398	-1,73527637	0,082691865	0,236443225	protein_codin	putative exportin 1
TcG_00264	881,5334106	-0,184922119	0,07140162	-2,58988688	0,009600747	0,047822124	protein_codin	E3 ubiquitin-protein ligase TRIP12
TcG_00265	363,5123437	-0,064506305	0,09427831	-0,68421152	0,493841611	0,704246612	protein_codin	hypothetical protein
TcG_00266	349,7809271	-0,1849449	0,098752996	-1,87280293	0,061095603	0,191934289	protein_codin	hypothetical protein
TcG_00267	741,8820286	-0,395441884	0,072069518	-5,48695058	4,08931E-08	1,14997E-06	protein_codin	putative dynein
TcG_00268	178,3997654	-0,036169175	0,145939389	-0,24783696	0,804260556	0,903185306	protein_codin	vacuolar sorting protein
TcG_00269	384,8135038	0,022020462	0,096429055	0,228359199	0,819367006	0,910871391	protein_codin	hypothetical protein
TcG_00270	266,7221123	-0,02610419	0,110113908	-0,23706533	0,812606108	0,907594318	protein_codin	septum formation protein
TcG_00271	417,1956261	0,076367636	0,091247115	0,836932058	0,402630759	0,632851796	protein_codin	putative protein kinase
TcG_00272	471,6713959	0,103597932	0,089221938	1,161126217	0,245590574	0,474196968	protein_codin	hypothetical protein
TcG_00273	1337,93585	-0,078995985	0,056527438	-1,39748038	0,162269162	0,36605345	protein_codin	T-complex protein 1 subunit epsilon
TcG_00274	880,0040259	-0,034384664	0,06824815	-0,50381825	0,614389099	0,791539208	protein_codin	putative jouberin-like
TcG_00275	460,8007885	-0,058823874	0,085622803	-0,68701178	0,492075307	0,702635507	protein_codin	hypothetical protein
TcG_00276	11,64973494	-0,152345018	0,500556727	-0,30435116	0,760860376	1	protein_codin	hypothetical protein
TcG_00277	11,82541525	-0,034134906	0,496949213	-0,06868892	0,945237236	1	protein_codin	hypothetical protein
TcG_00278	711,5908686	-0,134988228	0,074376242	-1,81493746	0,069533521	0,211004551	protein_codin	calpain-like cysteine peptidase
TcG_00279	634,293495	-0,226847744	0,079527974	-2,85242705	0,004338677	0,025738818	protein_codin	putative ubiquitin-conjugating enzyme protein
TcG_00280	1395,91648	-0,194468307	0,054953173	-3,53880032	0,00040195	0,003593112	protein_codin	4SNc-Tudor domain protein
TcG_00281	423,7929785	0,001120512	0,09360005	0,01197128	0,990448529	0,996036512	protein_codin	putative CLIP-associating protein 1-like
TcG_00282	255,5827523	0,103046199	0,113115929	0,910978674	0,36230661	0,594909919	protein_codin	hypothetical protein
TcG_00283	226,135009	-0,163230396	0,119378542	-1,36733448	0,17152049	0,378665472	protein_codin	hypothetical protein
TcG_00284	515,5561183	-0,073966672	0,086880885	-0,85135725	0,394570931	0,624314965	protein_codin	putative vacuolar proton-ATPase-like protein
TcG_00285	333,9147555	-0,1243735	0,106694932	-1,16569268	0,243738722	0,471839071	protein_codin	protein phosphatase methylesterase 1

TcG_00286	186,830462	-0,062085644	0,137717702	-0,45081818	0,652120597	0,815925403	protein_codin hypothetical protein
TcG_00287	459,3763346	-0,095446604	0,087210018	-1,09444541	0,273759676	0,506916989	protein_codin putative ethanolamine-phosphate cytidylyltransferase
TcG_00288	229,3233181	0,053444269	0,119392551	0,44763487	0,654416733	0,817209772	protein_codin 60S ribosomal protein L18a
TcG_00289	352,9163912	0,238017148	0,102462457	2,322969354	0,020180799	0,084684801	protein_codin 60S ribosomal protein L18a
TcG_00290	732,597013	-0,075957703	0,070373078	-1,07935741	0,280428428	0,513601607	protein_codin putative phenylalanyl-tRNA synthetase alpha chain
TcG_00291	655,3716556	0,030508508	0,08447266	0,361164293	0,717976633	0,855087918	protein_codin hypothetical protein
TcG_00292	604,6548037	0,17848357	0,077925101	2,290450285	0,021995227	0,090431762	protein_codin transcription factor subunit 2
TcG_00293	1042,434278	-0,05236696	0,063883547	-0,81972531	0,412372718	0,639078426	protein_codin polypyrimidine binding protein
TcG_00294	74,68398931	-0,088284318	0,209755743	-0,42089107	0,673834629	0,829023911	
TcG_00295	724,1058445	-0,120096875	0,084370665	-1,42344349	0,154607632	0,355766439	protein_codin hypothetical protein
TcG_00296	178,4684818	0,062722786	0,133705521	0,46911141	0,638990004	0,807837741	protein_codin CYC2-like cyclin 6
TcG_00297	254,2266166	-0,031234012	0,119983884	-0,2603184	0,794618184	0,897538186	protein_codin putative serine/threonine protein kinase
TcG_00298	258,0279649	0,117051089	0,116035721	1,008750479	0,313094316	0,547218396	protein_codin putative serine/threonine protein kinase
TcG_00299	97,8375089	0,178806702	0,181031786	0,987708879	0,323295245	0,557573663	protein_codin serine/threonine protein kinase
TcG_00300	233,8961511	0,298145096	0,123351558	2,4170355	0,015647491	0,069781305	protein_codin hypothetical protein
TcG_00301	480,5829494	-0,138533991	0,089217722	-1,55276315	0,12047973	0,303930104	protein_codin putative MAP protein kinase
TcG_00302	160,0177867	0,061561361	0,144433792	0,426225473	0,669943558	0,826884634	protein_codin putative ubiquitin-protein ligase
TcG_00303	234,3347235	0,104925453	0,126216761	0,831311561	0,405797645	0,634643717	protein_codin glycosyl transferase family 2
TcG_00304	425,84453	0,509028072	0,095272987	5,342837324	9,15029E-08	2,39853E-06	protein_codin hypothetical protein
TcG_00305	142,898068	0,135244438	0,146074888	0,925856868	0,354520369	0,588430867	protein_codin cyclin 9
TcG_00306	1753,373114	0,210995076	0,055997544	3,76793443	0,000164604	0,00169822	protein_codin putative RNA-binding protein
TcG_00307	193,628478	-0,133036732	0,138317907	-0,96181858	0,336140745	0,57078138	protein_codin holo-[acyl-carrier-protein] synthase
TcG_00308	1010,192256	0,131766845	0,065155394	2,022347454	0,043140469	0,149157111	protein_codin RNA binding protein-like protein
TcG_00309	249,0532403	0,334042249	0,121502091	2,749271614	0,005972787	0,032870019	protein_codin hypothetical protein
TcG_00310	362,7694791	0,053258599	0,09606176	0,554420402	0,579291167	0,768893053	protein_codin putative GDP-L-fucose synthetase
TcG_00311	449,5828898	-0,111912538	0,090688467	-1,23403274	0,217190704	0,441236455	protein_codin Leucine-rich repeat-containing protein ODA7
TcG_00312	324,0167645	0,023913722	0,10708604	0,223313158	0,823291787	0,912790301	protein_codin Pab1p-dependent poly(A) ribonuclease subunit
TcG_00313	150,5052851	0,209004627	0,147434593	1,417609149	0,156304908	0,357690793	protein_codin U6 snRNA-associated Sm-like protein LSm4
TcG_00314	630,942921	0,057889704	0,076391375	0,757804186	0,448568216	0,6693858	protein_codin putative ubiquitin carrier protein 4
TcG_00315	208,4842182	0,24544391	0,126071385	1,946864546	0,051550976	0,168767903	protein_codin putative U3 small nucleolar ribonucleoprotein IMP3
TcG_00316	946,3506735	-0,258214294	0,06502038	-3,97128247	7,14868E-05	0,000842569	protein_codin putative OSM3-like kinesin
TcG_00317	201,039642	0,02016594	0,127617239	0,158018932	0,87444188	0,939572087	protein_codin hypothetical protein
TcG_00318	188,5372567	-0,034301611	0,130134452	-0,26358594	0,792098997	0,896041689	protein_codin centrin
TcG_00319	202,4824479	-0,062353231	0,126677033	-0,49222207	0,622562365	0,796665292	protein_codin A kinase (PRKA) anchor protein 7
TcG_00320	349,063662	0,050482898	0,10131947	0,498254655	0,618304564	0,793033999	protein_codin ammecr1
TcG_00321	183,7757615	0,116942125	0,136988643	0,853662921	0,393291785	0,623007741	protein_codin putative Mg transporter
TcG_00322	213,5599175	-0,150118867	0,128323859	-1,16984377	0,242063845	0,470878393	protein_codin hypothetical protein
TcG_00323	200,5833126	0,126718008	0,131997544	0,960002772	0,33705382	0,571590392	protein_codin nucleoporin SEH1
TcG_00324	397,4134718	-0,284444581	0,096593633	-2,94475499	0,003232105	0,020263619	protein_codin hypothetical protein
TcG_00325	325,5594173	-0,000982613	0,099005999	-0,00992478	0,992081302	0,996781867	protein_codin hypothetical protein
TcG_00326	236,6489185	-0,247793813	0,116671677	-2,12385576	0,033682207	0,124717817	protein_codin hypothetical protein
TcG_00327	427,525864	-0,147353657	0,090935593	-1,62041783	0,105142552	0,278632572	protein_codin putative ATP-dependent RNA helicase-like protein
TcG_00328	86,1902272	0,594677417	0,199903168	2,974827375	0,002931535	0,018682487	protein_codin hypothetical protein
TcG_00329	95,6580229	0,135012281	0,186668693	0,723272223	0,469512655	0,686147025	protein_codin hypothetical protein
TcG_00330	1025,950502	-0,061841036	0,062297341	-0,99267536	0,320868185	0,556034397	protein_codin co-chaperone GrpE
TcG_00331	900,8107665	-0,059459536	0,068619174	-0,86651489	0,386207863	0,616572913	protein_codin kinesin
TcG_00332	115,122161	0,350239532	0,164389709	2,130544146	0,033126715	0,123100992	protein_codin hypothetical protein
TcG_00333	154,9105821	-0,204316176	0,14126787	-1,44630322	0,148092172	0,346907403	protein_codin hypothetical protein
TcG_00334	129,9315872	-0,045657666	0,171814347	-0,26573838	0,790440705	0,895214664	protein_codin hypothetical protein
TcG_00335	1127,896549	0,237085544	0,060254575	3,93473101	8,32899E-05	0,000950736	protein_codin putative 4-methyl-5(beta-hydroxyethyl)-thiazole monophosphate synthesis protein
TcG_00336	173,1832162	0,080661293	0,135166865	0,596753452	0,550672	0,746546058	protein_codin putative metallo-beta-lactamase superfamily
TcG_00337	560,6311869	0,058696132	0,078602685	0,746744624	0,455217728	0,675134741	protein_codin hypothetical protein
TcG_00338	161,9098926	-0,228095859	0,138212393	-1,65032856	0,098875754	0,266474642	protein_codin antigen 2
TcG_00339	185,437212	0,033386469	0,130661928	0,25551796	0,798323074	0,899394315	protein_codin mitochondrial binding protein TBRGG1
TcG_00340	802,9913681	-0,119256194	0,066915421	-1,782193	0,074717764	0,220667247	protein_codin mitochondrial oligo-U binding protein TBRGG1
TcG_00341	191,671989	0,081403977	0,128999794	0,631039588	0,52801464	0,730369821	protein_codin hypothetical protein
TcG_00342	313,1937677	-0,146144242	0,104389381	-1,39999146	0,161515875	0,365136182	protein_codin rac serine-threonine kinase-like protein

TcG_00343	176,478676	-0,037280989	0,134329607	-0,27753367	0,781370353	0,891302246	protein_codin hypothetical protein
TcG_00344	145,233977	0,184136692	0,28675762	0,642133562	0,520786466	0,725307368	protein_codin hypothetical protein
TcG_00345	354,8763498	-0,016397753	0,099246182	-0,16522301	0,868768453	0,936741226	protein_codin SAM-dependent methyltransferase
TcG_00346	720,3415432	-0,103484654	0,075314877	-1,37402673	0,169433383	0,37606421	protein_codin hypothetical protein
TcG_00347	350,7082939	-0,175930393	0,104203828	-1,68832946	0,091348003	0,252410676	protein_codin hypothetical protein
TcG_00348	392,2323568	0,0529218	0,096467781	0,548595604	0,583283003	0,771980452	protein_codin hypothetical protein
TcG_00349	43,08768326	0,119462789	0,270588363	0,441492708	0,65885634	0,820630999	protein_codin amastin
TcG_00350	130,1234718	0,391181779	0,177104819	2,20875853	0,027191441	0,106360579	protein_codin amastin
TcG_00351	557,6798163	-0,040836319	0,079772987	-0,51190661	0,608716373	0,788174776	protein_codin putative dTDP-glucose 4,6-dehydratase
TcG_00352	702,0430139	-0,1781456	0,072163625	-2,46863431	0,013562974	0,062956979	protein_codin putative adenosine kinase
TcG_00353	391,4460282	-0,059920925	0,095108618	-0,63002624	0,528677417	0,73102477	protein_codin hypothetical protein
TcG_00354	634,2589178	-0,083012959	0,074485592	-1,11448343	0,265071848	0,497508899	protein_codin putative ubiquitin-protein ligase
TcG_00355	486,1510105	0,083639968	0,086134351	0,971040789	0,331527967	0,565780384	protein_codin hypothetical protein
TcG_00356	671,7039103	0,475799334	0,074969837	6,346543506	2,20206E-10	1,00445E-08	protein_codin Aspartate ammonia-lyase
TcG_00357	322,6120202	0,111190114	0,100176788	1,109938894	0,267025359	0,499395611	protein_codin p22 protein precursor
TcG_00358	258,4432896	0,026308977	0,111524576	0,235902955	0,813507975	0,907881422	
TcG_00359	158,3318617	0,10762684	0,140720808	0,764825337	0,444375558	0,666426941	protein_codin hypothetical protein
TcG_00360	683,597611	0,009492265	0,073814543	0,128596134	0,897677226	0,950181186	protein_codin p22 protein precursor
TcG_00361	284,6740477	-0,031319217	0,11112064	-0,28184878	0,778059466	0,889256383	protein_codin hypothetical protein
TcG_00362	292,2484585	0,155207433	0,104976072	1,478502957	0,139273206	0,33366819	protein_codin hypothetical protein
TcG_00363	376,3626721	0,077357343	0,093826135	0,824475424	0,409669474	0,636866847	protein_codin putative protein kinase
TcG_00364	895,2703224	-0,135255073	0,068075467	-1,9868402	0,046940109	0,158279423	protein_codin putative ubiquitin conjugation factor E4 B
TcG_00365	235,5360402	-0,101744879	0,122589446	-0,82996442	0,406558898	0,634833846	protein_codin hypothetical protein
TcG_00366	266,177088	0,092488891	0,116941448	0,790899143	0,429002849	0,653987669	protein_codin putative DnaJ chaperone protein
TcG_00367	432,969857	-0,170645648	0,095351794	-1,78964277	0,073511359	0,218497333	protein_codin succinate dehydrogenase subunit
TcG_00368	99,5040323	0,153993988	0,175798878	0,875966847	0,381048057	0,611048137	protein_codin ribosome-associated protein
TcG_00369	221,2122631	0,124237213	0,120149982	1,034017746	0,301127867	0,534351377	protein_codin hypothetical protein
TcG_00370	62,52936991	0,059278282	0,24647079	0,240508345	0,809936195	0,906277019	protein_codin hypothetical protein
TcG_00371	426,5170215	0,404795518	0,094438055	4,286360189	1,81624E-05	0,000254378	protein_codin putative DREV methyltransferase
TcG_00372	825,0749668	0,081684861	0,068806045	1,187175649	0,235158344	0,462964244	protein_codin putative RNA-binding protein
TcG_00373	503,1522597	0,080279223	0,085038768	0,944030886	0,345153865	0,579295913	protein_codin hypothetical protein
TcG_00374	252,9749784	0,180571801	0,114509027	1,576921969	0,114813481	0,294820254	protein_codin hypothetical protein
TcG_00375	359,07912	0,088427007	0,105788658	0,835883629	0,403220373	0,632986744	protein_codin hypothetical protein
TcG_00376	198,8268462	0,089551974	0,12720159	0,704016155	0,481422713	0,695568469	protein_codin hypothetical protein
TcG_00377	337,2405266	-0,067974234	0,097604473	-0,6964254	0,486162463	0,698274608	protein_codin ER membrane DUF1077 domain-containing protein
TcG_00378	91,22577804	0,013898794	0,189200185	0,073460785	0,941439449	0,97292993	protein_codin hypothetical protein
TcG_00379	212,6941863	0,129946385	0,130542988	0,995429834	0,319527255	0,554530074	protein_codin hypothetical protein
TcG_00380	903,4109958	-0,224345337	0,064991581	-3,45191384	0,000556625	0,004752758	protein_codin putative importin alpha
TcG_00381	386,5907465	-0,053400789	0,092490402	-0,57736573	0,563692417	0,756774978	protein_codin putative nucleolar MIF4G domain-containing protein 1-like
TcG_00382	126,4825092	0,012111606	0,169464783	0,071469749	0,9430239	0,973191135	protein_codin putative nucleolar MIF4G domain-containing protein 1-like
TcG_00383	413,7899103	-0,093030129	0,089133774	-1,04371357	0,296617931	0,529769593	protein_codin hypothetical protein
TcG_00384	190,3642152	-0,27651723	0,132549321	-2,08614595	0,036965404	0,13358739	protein_codin NUDIX hydrolase
TcG_00385	100,8087523	0,022466466	0,174781663	0,128540178	0,897721505	0,950181186	protein_codin hypothetical protein
TcG_00386	419,2196112	-0,429090538	0,091290265	-4,70028799	2,59795E-06	4,71784E-05	protein_codin putative ubiquitin hydrolase
TcG_00387	125,0107467	-0,668561071	0,164759054	-4,05781081	4,95349E-05	0,000611194	protein_codin putative ubiquitin hydrolase, putative,cysteine peptidase, Clan CA, family C19
TcG_00388	196,6257214	0,16051633	0,130826555	1,226939975	0,219845152	0,444990554	
TcG_00389	509,3042268	-0,164004404	0,097019066	-1,69043479	0,090944804	0,251656676	protein_codin hypothetical protein
TcG_00390	502,3451344	-0,070280917	0,083897323	-0,83770154	0,402198347	0,632447075	protein_codin zinc finger, c2h2 type domain containing protein
TcG_00391	178,3968818	0,011062508	0,131945191	0,083841691	0,9331823	0,968196484	protein_codin putative calcium-binding protein
TcG_00392	592,4997479	0,11887404	0,07993656	1,487104773	0,136987141	0,330077667	protein_codin hypothetical protein
TcG_00393	243,670428	0,389505307	0,114500818	3,401768768	0,000669513	0,005528847	protein_codin Pyridoxal kinase
TcG_00394	120,7268668	-0,098955501	0,168255419	-0,58812668	0,556447266	0,75122326	protein_codin transferase
TcG_00395	433,2488966	-0,014993335	0,088749266	-0,16894039	0,865843531	0,935353208	protein_codin hypothetical protein
TcG_00396	413,5785906	-0,012519399	0,089730999	-0,13952144	0,889038111	0,9464367	protein_codin hypothetical protein
TcG_00397	354,3787825	-0,093534964	0,096487384	-0,96940098	0,332345165	0,566340797	protein_codin putative vacuolar assembly protein vps41
TcG_00398	1,235553853	2,181924197	1,746321218	1,249440352	0,211504057	1	protein_codin vacuolar assembly protein vps41
TcG_00399	2053,153326	0,279003115	0,052045433	5,360760772	8,28722E-08	2,18715E-06	protein_codin U3 small nuclear ribonucleoprotein (snRNP)

TcG_00400	2025,465091	0,5454744	0,052723046	10,34603346	4,36184E-25	1,23259E-22	protein_codin	putative dTDP-glucose 4,6-dehydratase
TcG_00401	904,7831264	0,076278474	0,071488426	1,067004522	0,285969794	0,519187736	protein_codin	hypothetical protein
TcG_00402	0					1	protein_codin	vacuolar assembly protein vps41
TcG_00403	244,9223501	0,259507496	0,114612221	2,264221859	0,023560473	0,095645283	protein_codin	hypothetical protein
TcG_00404	75,32328115	0,05777992	0,206650661	0,279601915	0,779782938	0,890390004	protein_codin	hypothetical protein
TcG_00405	248,6501451	0,081958819	0,116240185	0,705081628	0,480759439	0,695043531	protein_codin	putative ABC transporter
TcG_00406	185,9598785	-0,274804036	0,129508965	-2,121892	0,033846808	0,125207254	protein_codin	hypothetical protein
TcG_00407	249,2809483	-0,071623749	0,123109385	-0,58178951	0,56070847	0,754426375	protein_codin	putative GTPase activating protein
TcG_00408	346,558723	-0,060270437	0,097213747	-0,61997854	0,535271915	0,735451331	protein_codin	RNA helicase
TcG_00409	379,107597	0,003766926	0,094900158	0,03969357	0,968337428	0,98638627	protein_codin	hypothetical protein
TcG_00410	94,86442783	0,255695538	0,193753097	1,319697812	0,186935931	0,400932932	protein_codin	putative heat shock protein HslVU, ATPase subunit HslU
TcG_00411	240,1271737	0,027368023	0,122719297	0,223013204	0,82352523	0,912961757	protein_codin	pleiotropic regulator 1
TcG_00412	266,8182985	0,089670963	0,118524595	0,756559963	0,449313536	0,670299044	protein_codin	hypothetical protein
TcG_00413	296,2791926	0,235556601	0,104858138	2,246431271	0,024676397	0,098756732	protein_codin	200 kDa antigen p200
TcG_00414	390,7953492	-0,453449703	0,093710985	-4,83881055	1,30618E-06	2,56499E-05	protein_codin	putative radial spoke protein 3
TcG_00415	325,1651706	0,043001651	0,099247053	0,43327887	0,664812187	0,824768789	protein_codin	hypothetical protein
TcG_00416	369,1349541	0,131326861	0,099522509	1,319569433	0,186978814	0,400950683	protein_codin	hypothetical protein
TcG_00417	91,14090045	0,131972882	0,183596912	0,718818639	0,472252672	0,688328023	protein_codin	phosphatidylinositol glycan, class P
TcG_00418	546,7847368	0,020468827	0,082425227	0,248332071	0,803877487	0,903185306	protein_codin	hypothetical protein
TcG_00419	252,0900292	0,019121934	0,125127833	0,152819187	0,878540867	0,941484386	protein_codin	hypothetical protein
TcG_00420	382,3264798	-0,233997978	0,099416058	-2,35372417	0,018586397	0,079228108	protein_codin	hypothetical protein
TcG_00421	134,5159758	-0,260498583	0,154677476	-1,68414038	0,092154542	0,2537919	protein_codin	hypothetical protein
TcG_00422	314,1422237	0,115792823	0,102061302	1,134541895	0,25656731	0,488583182	protein_codin	WD domain containing protein
TcG_00423	271,6799051	-0,132111109	0,107970036	-1,2235905	0,221106747	0,446371802	protein_codin	hypothetical protein
TcG_00424	181,7673258	-0,166791735	0,132755855	-1,2563795	0,20897841	0,431564429	protein_codin	hypothetical protein
TcG_00425	300,1804419	0,053012084	0,105029813	0,504733683	0,6137459	0,791238455	protein_codin	hypothetical protein
TcG_00426	335,5728487	0,133612705	0,101213181	1,32011171	0,186797724	0,400784893	protein_codin	putative MCAK-like kinesin
TcG_00427	305,2872613	0,144530456	0,10217433	1,414547627	0,157201174	0,35895404	protein_codin	60S acidic ribosomal protein P0
TcG_00428	189,6775266	0,194479777	0,136057381	1,42939527	0,152890657	0,353289022	protein_codin	putative CDC16
TcG_00429	95,80858277	0,302341787	0,1821521	1,659831469	0,096948361	0,263410017	protein_codin	Inosine triphosphate pyrophosphatase family protein isoform 1
TcG_00430	224,650959	0,088405934	0,12303475	0,718544431	0,472421663	0,688368479	protein_codin	putative serine/threonine protein kinase, putative,protein kinase
TcG_00431	623,3312878	-0,234989238	0,076563039	-3,06922559	0,002146145	0,014566627	protein_codin	hypothetical protein
TcG_00432	484,5164035	0,06576163	0,084082438	0,782108984	0,434150527	0,658558262	protein_codin	putative myosin heavy chain
TcG_00433	233,3821416	0,207497488	0,121437095	1,708682908	0,087509702	0,245315124	protein_codin	hypothetical protein
TcG_00434	469,4085707	0,00447972	0,088903748	0,050388427	0,959812859	0,981499716	protein_codin	syntaxin 1B/2/3
TcG_00435	121,9330616	0,115642072	0,165212269	0,699960557	0,483951937	0,697309681	protein_codin	hypothetical protein
TcG_00436	281,787217	-0,106725206	0,116973301	-0,91238945	0,36156374	0,594195389	protein_codin	hypothetical protein
TcG_00437	470,6427867	0,096132354	0,085717984	1,121495747	0,262076901	0,494282475	protein_codin	hypothetical protein
TcG_00438	324,5595424	-0,156144803	0,114385769	-1,36507193	0,172230443	0,379870915	protein_codin	putative cell cycle sequence binding phosphoprotein (RBP45)
TcG_00439	493,3603627	0,00158049	0,08425944	0,018757425	0,985034618	0,994129886	protein_codin	putative ABC1 protein
TcG_00440	498,1140277	0,025233393	0,086058452	0,293212259	0,769359914	0,884364283	protein_codin	putative phosphoribosylpyrophosphate synthetase
TcG_00441	2622,570741	0,114351239	0,047800771	2,39224673	0,016745581	0,073462439	protein_codin	60S ribosomal protein L18
TcG_00442	329,9572697	-0,30310525	0,110220876	-2,74998042	0,005959883	0,032819011	protein_codin	hypothetical protein
TcG_00443	309,7436675	-0,089572769	0,105563227	-0,84852246	0,396147072	0,625904811	protein_codin	methyltransferase
TcG_00444	248,0932471	-0,03167488	0,118452152	-0,26740653	0,789156168	0,894943008	protein_codin	putative amino acid permease-like protein
TcG_00445	376,9574927	0,106138703	0,095570428	1,110581015	0,266748738	0,499395611	protein_codin	putative chaperone DNAJ protein
TcG_00446	436,9086101	-0,199965886	0,092497698	-2,16184716	0,030629959	0,116049283	protein_codin	hypothetical protein
TcG_00447	724,2439691	-0,106309007	0,072053146	-1,47542492	0,140098341	0,334581697	protein_codin	metallo-peptidase, Clan MA(E), Family M3
TcG_00448	256,6640547	-0,092701199	0,113653707	-0,81564607	0,414702593	0,641659221	protein_codin	hypothetical protein
TcG_00449	728,1342939	-0,076098416	0,069760248	-1,09085643	0,275336059	0,507736472	protein_codin	hypothetical protein
TcG_00450	103,3055865	0,284102977	0,182969173	1,55273685	0,120486015	0,303930104	protein_codin	putative cytoplasmic l-asparaginase i-like protein
TcG_00451	778,4934171	0,216599488	0,071273117	3,0390068	0,002373595	0,015768619	protein_codin	60S ribosomal protein L22
TcG_00452	214,612156	0,001765164	0,123183368	0,014329566	0,988567052	0,995440454	protein_codin	hypothetical protein
TcG_00453	363,2102732	0,041389843	0,096849068	0,427364393	0,669113938	0,826307698	protein_codin	hypothetical protein
TcG_00454	670,9706459	-0,100027572	0,073753076	-1,3562495	0,175019799	0,384121876	protein_codin	putative ATP-dependent RNA helicase
TcG_00455	286,6652114	-0,094066044	0,107629167	-0,87398283	0,382127606	0,61215602	protein_codin	ADP-ribosylation factor-like protein
TcG_00456	330,7915822	0,151927787	0,108807778	1,396295281	0,162625594	0,366572011	protein_codin	putative gamma-tubulin complex subunit

TcG_00457	134,7736211	0,306662068	0,156291843	1,962111788	0,049749471	0,164993809	protein_codin putative GTPase activating protein of Rab-like GTPase
TcG_00458	1311,881198	0,100876533	0,070093198	1,439177213	0,15010032	0,350052799	protein_codin ubiquitin-protein ligase
TcG_00459	340,8795539	-0,193084554	0,09782782	-1,97371827	0,048413787	0,16166191	protein_codin proteasome regulatory ATPase subunit
TcG_00460	256,1439909	-0,079974148	0,112657617	-0,70988674	0,477774376	0,692608846	protein_codin putative kinesin
TcG_00461	276,2588011	0,152398021	0,110771756	1,375784095	0,168888487	0,375286155	protein_codin hypothetical protein
TcG_00462	327,3683425	-0,378028523	0,098440706	-3,84016468	0,000122952	0,001328843	protein_codin hypothetical protein
TcG_00463	409,6787409	-0,317082931	0,101977545	-3,10934068	0,001875054	0,013052791	protein_codin putative C2 domain protein
TcG_00464	577,165096	0,080337724	0,084532044	0,950381894	0,341918241	0,576464601	protein_codin hypothetical protein
TcG_00465	203,8636324	0,243490358	0,133759517	1,820359129	0,068704332	0,209365701	protein_codin hypothetical protein
TcG_00466	567,7739074	0,004472819	0,078639199	0,056877724	0,954642599	0,978889315	protein_codin putative trypanothione synthetase
TcG_00467	909,6857869	0,124477841	0,065545894	1,89909441	0,057552064	0,182803854	protein_codin putative ADP-ribosylation factor-like 2, arl2
TcG_00468	391,5655172	-0,190064285	0,091590175	-2,07516018	0,037971692	0,136145608	protein_codin crooked neck
TcG_00469	723,8946175	-0,257759577	0,071496953	-3,60518269	0,000311933	0,002898204	protein_codin putative dispersed gene family protein 1 (DGF-1)
TcG_00470	250,3631421	-0,211566994	0,112017011	-1,88870416	0,058931481	0,186551949	protein_codin putative immunodominant antigen, putative,tc40 antigen-like
TcG_00471	6,230421776	-0,417581614	0,721402141	-0,57884721	0,562692276	1	
TcG_00472	5,440191641	-0,980253655	0,775123516	-1,26464187	0,205999736	1	protein_codin putative protein kinase
TcG_00473	440,7044174	0,096650229	0,088592725	1,090949946	0,275294908	0,507736472	protein_codin hypothetical protein
TcG_00474	164,896286	-0,025216649	0,135631364	-0,18592048	0,852507118	0,928652451	protein_codin hypothetical protein
TcG_00475	133,5930857	0,045935087	0,160400636	0,286377215	0,774589224	0,887499085	protein_codin hypothetical protein
TcG_00476	573,5128393	-0,654732846	0,079255603	-8,26102917	1,44422E-16	1,76134E-14	protein_codin putative paraflagellar rod component
TcG_00477	74,14989203	0,229982196	0,20827227	1,104238196	0,269489825	0,50230198	protein_codin putative oxidoreductase
TcG_00478	3,181517306	-0,63410607	0,965820084	-0,65654678	0,511472377	1	protein_codin hypothetical protein
TcG_00479	0,389577086	1,883588912	2,667528218	0,706117708	0,48011494	1	protein_codin target of rapamycin (TOR) kinase 1
TcG_00480	116,6789372	0,003098897	0,163643212	0,018936908	0,984891436	0,994129886	protein_codin trans-sialidase
TcG_00481	94,70979656	0,189886591	0,183363639	1,035573859	0,300400993	0,534138413	protein_codin helicase-like protein
TcG_00482	474,9215284	-0,0273957	0,092080588	-0,29751874	0,766070503	0,882451069	protein_codin putative tyrosine specific protein phosphatase
TcG_00483	652,8162588	0,168096475	0,075129548	2,237421616	0,025258797	0,100497398	protein_codin putative NADH dehydrogenase
TcG_00484	802,9396812	-0,066528636	0,067811659	-0,98107961	0,326553484	0,561009588	protein_codin putative phosphoribosylpyrophosphate synthetase
TcG_00485	410,1606046	0,054214531	0,092412663	0,586656952	0,557434125	0,75211736	protein_codin putative ATP-dependent chaperone
TcG_00486	394,6308443	0,044182776	0,096636532	0,45720573	0,64752318	0,812603825	protein_codin putative selenophosphate synthetase
TcG_00487	260,969041	0,015533543	0,116248338	0,133623782	0,893700078	0,94872724	protein_codin branch point binding protein
TcG_00488	202,2459113	0,196038428	0,130407537	1,503275286	0,13276807	0,323682997	protein_codin macrocin-O-methyltransferase domain-containing protein
TcG_00489	397,8847737	-0,170389814	0,095434699	-1,78540737	0,074195264	0,219965797	protein_codin hypothetical protein
TcG_00490	363,5268889	0,052206721	0,104137096	0,501326839	0,616141119	0,792562564	protein_codin hypothetical protein
TcG_00491	513,7696577	-0,956771236	0,090074261	-10,622027	2,35393E-26	8,52271E-24	protein_codin putative trans-sialidase
TcG_00492	9,442952925	-0,146892782	0,581877459	-0,25244625	0,800696156	1	protein_codin hypothetical protein
TcG_00493	66,25419693	0,533438056	0,225652971	2,363975331	0,018080016	0,077727299	protein_codin hypothetical protein
TcG_00494	74,57245657	-0,070988331	0,204954194	-0,34636193	0,729070728	0,860563425	protein_codin hypothetical protein
TcG_00495	82,01856895	-0,062959116	0,19752108	-0,31874631	0,749918891	0,873924791	protein_codin hypothetical protein
TcG_00496	200,048079	-0,098741795	0,128696313	-0,76724649	0,442934975	0,664995914	protein_codin hypothetical protein
TcG_00497	139,5457995	-0,055686805	0,148964442	-0,37382616	0,708533656	0,849536473	protein_codin hypothetical protein
TcG_00498	374,845694	-0,303781815	0,108485421	-2,80020867	0,005106958	0,029161763	protein_codin DHHC-type zinc finger family protein
TcG_00499	203,2136739	-0,026796266	0,13364071	-0,20050975	0,84108193	0,922466573	protein_codin nuclear lim interactor-interacting factor
TcG_00500	276,2035581	-0,013260777	0,115308536	-0,11500256	0,908443091	0,955500221	protein_codin hypothetical protein
TcG_00501	249,6504171	-0,049445461	0,116546849	-0,42425395	0,671380618	0,827770911	protein_codin hypothetical protein
TcG_00502	144,711337	-0,098999032	0,15033499	-0,65852289	0,510202193	0,717030884	protein_codin hypothetical protein
TcG_00503	317,322234	-0,137493796	0,103942494	-1,32278716	0,185906169	0,399879894	protein_codin hypothetical protein
TcG_00504	163,1544683	-0,017833898	0,142641422	-0,12502608	0,900502905	0,951676244	protein_codin ankyrin repeat protein
TcG_00505	354,4923627	-0,279563621	0,100122571	-2,79221375	0,005234876	0,02968736	protein_codin hypothetical protein
TcG_00506	370,8743952	-0,154406763	0,1007692	-1,53228132	0,125453029	0,312378851	protein_codin hypothetical protein
TcG_00507	295,1156624	0,140667447	0,109914314	1,279791881	0,20061834	0,419863455	protein_codin hypothetical protein
TcG_00508	184,7487604	0,016453634	0,137872817	0,11933922	0,905006613	0,953903154	protein_codin syntaxin
TcG_00509	385,3455341	-0,105154851	0,096307781	-1,09186247	0,274893555	0,507556451	protein_codin putative acyl-CoA dehydrogenase
TcG_00510	229,919335	-0,221435443	0,123462227	-1,7935481	0,072885327	0,217249652	protein_codin putative transporter
TcG_00511	0,467697622	2,091063019	2,518049959	0,83042952	0,406295981	1	
TcG_00512	246,900817	0,288161302	0,115469969	2,495551917	0,012576135	0,059110386	protein_codin putative galactokinase-like protein
TcG_00513	213,0362435	0,007422728	0,124449974	0,05964427	0,952438959	0,977496481	protein_codin hypothetical protein

TcG_00514	156,9883893	-0,051345015	0,141183623	-0,36367543	0,716100391	0,854275943	protein_codin	hypothetical protein
TcG_00515	328,3833022	0,276211746	0,100569046	2,746488686	0,006023696	0,03310747	protein_codin	hypothetical protein
TcG_00516	478,4584848	-0,070548967	0,086124777	-0,81914833	0,412701793	0,6393939	protein_codin	hypothetical protein
TcG_00517	273,162215	-0,000514351	0,109941688	-0,0046784	0,996267194	0,998767129	protein_codin	hypothetical protein
TcG_00518	30,26646853	0,424379993	0,329065383	1,289652497	0,19717134	0,415601281		
TcG_00519	259,6291175	-0,102689984	0,115205946	-0,89136011	0,372736012	0,60390427	protein_codin	putative transporter
TcG_00520	194,8630578	0,158741855	0,134727379	1,178244963	0,238698962	0,466683458	protein_codin	putative transporter
TcG_00521	199,5922592	0,139041604	0,12457227	1,116152127	0,264357023	0,496649986	protein_codin	hypothetical protein
TcG_00522	273,1079006	0,221125909	0,120910939	1,828832941	0,067424638	0,206736727	protein_codin	putative cysteine desulfurase
TcG_00523	452,7177509	0,031158996	0,089342767	0,348757897	0,727271074	0,859374061	protein_codin	hypothetical protein
TcG_00524	154,0988764	0,159076522	0,142332808	1,11763777	0,263721731	0,496021594	protein_codin	hypothetical protein
TcG_00525	356,4832808	0,107226584	0,096195403	1,114674719	0,264989838	0,497508899	protein_codin	putative protein kinase-like protein
TcG_00526	127,409156	0,174482684	0,154330572	1,130577571	0,258232932	0,490645845	protein_codin	trafficking protein particle complex subunit 1
TcG_00527	222,7355618	0,151835577	0,126008041	1,204967363	0,228215901	0,455331399	protein_codin	tRNA/rRNA methyltransferase YsgA
TcG_00528	248,5488222	0,304255035	0,113624463	2,677724725	0,007412411	0,039018716	protein_codin	hypothetical protein
TcG_00529	97,84863207	-0,159241795	0,189008476	-0,8425114	0,399501764	0,629402697	protein_codin	putative dynein light chain
TcG_00530	178,1406228	0,235267771	0,138702684	1,696202009	0,08984763	0,249753993	protein_codin	poly(ADP-ribose) glycohydrolase
TcG_00531	428,6999539	-0,220669049	0,090588728	-2,43594379	0,014852992	0,067322838	protein_codin	hypothetical protein
TcG_00532	429,5212799	-0,024801213	0,090754589	-0,27327778	0,784639691	0,893448203	protein_codin	putative dolichol kinase
TcG_00533	849,9984793	0,22559866	0,074531458	3,026891822	0,002470824	0,016283824	protein_codin	tRNA wybutosine-synthesizing protein 4
TcG_00534	740,2015622	0,039791726	0,070327468	0,565806327	0,571525445	0,762692214	protein_codin	hypothetical protein
TcG_00535	189,6008465	0,110339638	0,128802756	0,856655882	0,391635092	0,621398293	protein_codin	Suppression of tumorigenicity 5
TcG_00536	329,3137231	-0,228730096	0,108209974	-2,11376168	0,034535628	0,127186835	protein_codin	hypothetical protein
TcG_00537	253,2567201	0,160366929	0,113860036	1,408456686	0,158995884	0,361342941	protein_codin	ATP/GTP nucleotide-binding protein
TcG_00538	603,7963529	0,248873331	0,076333639	3,26033626	0,001112802	0,008504567	protein_codin	putative chaperone DNAJ protein
TcG_00539	194,7318616	0,002954763	0,130750427	0,022598498	0,981970542	0,993289929	protein_codin	zinc finger protein, predicted
TcG_00540	164,4827867	0,395951571	0,143234272	2,764363346	0,005703399	0,031692845	protein_codin	hypothetical protein
TcG_00541	128,3317429	0,233842218	0,156070073	1,498315556	0,134051284	0,325601296	protein_codin	lactoylglutathione lyase-like protein
TcG_00542	449,7639016	-0,192476335	0,085341523	-2,2553656	0,02411039	0,097230414	protein_codin	putative phospholipase A1
TcG_00543	175,0857094	0,056549475	0,135768229	0,416514787	0,677033349	0,83085567	protein_codin	putative phospholipase A2-like protein
TcG_00544	160,9998627	-0,23352696	0,143024501	-1,63277592	0,102516134	0,273549501	protein_codin	putative prenyl protein specific carboxyl methyltransferase
TcG_00545	310,1889415	-0,105709123	0,107026125	-0,98769458	0,32330225	0,557573663	protein_codin	NADH:ubiquinone reductase (H(+)-translocating)
TcG_00546	322,6311299	0,092286608	0,099859612	0,924163493	0,3554012	0,589309183	protein_codin	putative nucleic acid binding protein
TcG_00547	1994,225053	-0,061549948	0,052206247	-1,17897669	0,238407459	0,466428373	protein_codin	putative ubiquitin-activating enzyme e1
TcG_00548	350,1495545	0,026935522	0,100288025	0,268581637	0,788251645	0,89483476	protein_codin	hypothetical protein
TcG_00549	347,7752357	-0,223142682	0,09716185	-2,296608	0,021641142	0,08947769	protein_codin	putative glycerol kinase, glycosomal
TcG_00550	166,6322581	-0,028139062	0,139514394	-0,20169289	0,840156818	0,922245432	protein_codin	hypothetical protein
TcG_00551	387,3538915	-0,348884897	0,09364588	-3,72557658	0,000194869	0,00195252		
TcG_00552	1775,9602	-0,167425796	0,060900708	-2,74916009	0,00597482	0,032870019	protein_codin	putative ATP-dependent RNA helicase
TcG_00553	154,0866478	0,063979425	0,152110147	0,420612474	0,674038084	0,829023911	protein_codin	MFS transporter, OPA family, solute carrier family 37 (glycerol-3-phosphate transporter), member 1/2
TcG_00554	346,2429057	-0,066461666	0,099912668	-0,6651976	0,505924116	0,712679562	protein_codin	hypothetical protein
TcG_00555	376,9031292	0,140255747	0,096951393	1,446660467	0,147992042	0,346888496	protein_codin	hypothetical protein
TcG_00556	172,3388018	0,002054034	0,135660199	0,015141018	0,987919677	0,995145161	protein_codin	dynein light chain-like protein
TcG_00557	69,27891504	0,102821082	0,208501975	0,493142007	0,621912252	0,796361113	protein_codin	hypothetical protein
TcG_00558	315,887352	0,223076279	0,111100151	2,007884561	0,04465556	0,152619268	protein_codin	inositol-trisphosphate 3-kinase
TcG_00559	273,1923137	-0,044709444	0,108768997	-0,41104952	0,681036227	0,833648782	protein_codin	hypothetical protein
TcG_00560	147,7153688	-0,114085984	0,144298139	-0,79062686	0,429161768	0,653987669	protein_codin	hypothetical protein
TcG_00561	254,6599734	0,400409145	0,117432645	3,409691959	0,000650363	0,005401509	protein_codin	riboflavin kinase
TcG_00562	6,144213236	-0,241898584	0,702977917	-0,34410552	0,730766929	1		
TcG_00563	69,55647919	0,235845538	0,220652815	1,068853517	0,28513568	0,518774269	protein_codin	putative protein kinase
TcG_00564	358,8224245	-0,099369358	0,096051002	-1,03454785	0,300880118	0,534199829	protein_codin	hypothetical protein
TcG_00565	419,6993613	-0,222553465	0,092024073	-2,41842658	0,015587791	0,069642191	protein_codin	hypothetical protein
TcG_00566	602,8982091	-0,176622683	0,08136214	-2,1708215	0,029944668	0,114312661	protein_codin	putative RNA-binding protein
TcG_00567	456,7649629	-0,133685849	0,088751816	-1,5062886	0,131993102	0,32282246	protein_codin	protein phosphatase 2 (formerly 2A), regulatory subunit B
TcG_00568	267,2093022	0,038780388	0,116470502	0,332963173	0,739162082	0,867087269	protein_codin	hypothetical protein
TcG_00569	1206,507559	0,093495262	0,058073603	1,609944231	0,107410032	0,282252807	protein_codin	putative cystathione gamma lyase
TcG_00570	487,0791448	-0,12217782	0,08778514	-1,39178248	0,163988276	0,368925857	protein_codin	hypothetical protein

TcG_00571	266,566184	-0,2017744	0,110943908	-1,81870644	0,068956228	0,209802221	protein_codin putative replication factor C, subunit 3
TcG_00572	441,283475	-0,1070042	0,086246612	-1,24067715	0,21472504	0,438611479	protein_codin peroxin 19
TcG_00573	120,0392542	-0,020506188	0,170230673	-0,12046118	0,904117831	0,953534435	protein_codin hypothetical protein
TcG_00574	133,2814614	-0,232911021	0,157858679	-1,47544007	0,140094269	0,334581697	protein_codin 60S ribosomal subunit protein L31
TcG_00575	967,5883271	0,392230433	0,064659337	6,06610662	1,31048E-09	5,1122E-08	protein_codin 60S ribosomal subunit protein L31
TcG_00576	0,27291574	1,35431792	3,063093298	0,442140604	0,658387464	1	protein_codin large subunit ribosomal protein L31e
TcG_00577	344,2988804	0,06120435	0,099859296	0,61290588	0,539938575	0,738978292	protein_codin leucine rich repeat protein
TcG_00578	339,0873158	0,03407457	0,098865253	0,344656682	0,730352488	0,861170764	protein_codin hypothetical protein
TcG_00579	236,3784725	0,032582213	0,11570149	0,281605819	0,778245778	0,889256383	protein_codin putative transport protein particle (TRAPP) subunit
TcG_00580	143,451427	0,055537041	0,151513565	0,366548311	0,71395597	0,853212364	protein_codin putative mitochondrial carrier protein
TcG_00581	0					1	protein_codin putative mitochondrial carrier protein
TcG_00582	437,8794559	0,141608679	0,090288136	1,568408499	0,116785829	0,298059197	protein_codin putative 6-phosphogluconate dehydrogenase,decarboxylating
TcG_00583	267,5471691	0,015040539	0,109408101	0,137471891	0,890657809	0,946712053	protein_codin 16S rRNA (cytidine1402-2-O)-methyltransferase
TcG_00584	920,5005762	0,136891692	0,070152466	1,951345394	0,051015973	0,168013376	protein_codin hypothetical protein
TcG_00585	182,3585884	0,130878325	0,133024979	0,983862769	0,325182992	0,559651692	protein_codin hypothetical protein
TcG_00586	1031,362773	-0,002744378	0,06366325	-0,04310773	0,965615659	0,984825971	protein_codin ATP-dependent RNA helicase
TcG_00587	265,150503	0,061423884	0,116083045	0,52913743	0,596710118	0,78083165	protein_codin hypothetical protein
TcG_00588	294,3160924	-0,129442565	0,110848558	-1,16774244	0,242910674	0,471180824	protein_codin dihydroxyacetone kinase 1-like protein
TcG_00589	124,3623656	0,238339634	0,164542166	1,44850186	0,147476746	0,346227632	protein_codin hypothetical protein
TcG_00590	499,7186449	-0,02056639	0,085044718	-0,24183031	0,808911654	0,905337174	protein_codin hypothetical protein
TcG_00591	339,3789483	0,086861244	0,104797068	0,828851849	0,407188245	0,635463767	protein_codin hypothetical protein
TcG_00592	283,6589153	-0,173050293	0,106480809	-1,62517824	0,104124587	0,276694372	protein_codin hypothetical protein
TcG_00593	291,3101457	-0,121224983	0,107689644	-1,1256884	0,260297437	0,492617789	protein_codin putative replication factor A protein 3
TcG_00594	552,7702473	0,243013797	0,079577403	3,053804085	0,002259596	0,015185428	protein_codin putative DNA-repair protein
TcG_00595	260,7430342	0,281576585	0,112922888	2,493529791	0,012647997	0,059375889	protein_codin putative ascorbate-dependent peroxidase
TcG_00596	262,4580512	0,253749549	0,123062775	2,061952121	0,039212295	0,139435414	protein_codin acyl transferase-like protein
TcG_00597	136,2399666	-0,049155709	0,160151437	-0,30693267	0,75889462	0,878113759	protein_codin hypothetical protein
TcG_00598	292,3464651	-0,038477233	0,112433935	-0,34222082	0,732184718	0,86246393	protein_codin hypothetical protein
TcG_00599	433,5835357	0,088131248	0,091556746	0,962586069	0,335755292	0,570306525	protein_codin hypothetical protein
TcG_00600	135,7249444	-0,117407601	0,154292282	-0,7609428	0,446691233	0,668220093	protein_codin zinc finger protein
TcG_00601	55,24940191	0,086553098	0,239725352	0,361051084	0,71806126	0,855087918	protein_codin hypothetical protein
TcG_00602	335,4539486	0,092088266	0,102056559	0,902325799	0,366883825	0,599237121	protein_codin hypothetical protein
TcG_00603	1532,649324	-0,116902888	0,052366889	-2,23238177	0,025589741	0,101593633	protein_codin putative structural maintenance of chromosome (SMC) family protein
TcG_00604	257,109681	0,031340036	0,110921729	0,282541897	0,777528025	0,889000669	protein_codin RNA-binding protein PNO1
TcG_00605	340,5753824	0,088744244	0,103300543	0,859087876	0,390292036	0,620400374	protein_codin hypothetical protein
TcG_00606	289,8527839	0,077908467	0,10551252	0,738381257	0,460282801	0,678399847	protein_codin cellular retinaldehyde-binding protein/triple function domain-containing protein
TcG_00607	171,8615835	0,144689162	0,1356713	1,06646846	0,286211929	0,519431612	protein_codin putative membrane transporter protein
TcG_00608	337,574317	0,216491653	0,104027825	2,081093724	0,037425328	0,134737721	protein_codin putative membrane transporter protein
TcG_00609	681,2186083	0,045082674	0,075650442	0,595934041	0,551219291	0,746821273	protein_codin hypothetical protein
TcG_00610	465,4553053	0,035349053	0,089737245	0,393917302	0,693642096	0,841257963	protein_codin putative DNA repair protein RAD2
TcG_00611	223,8258412	0,30251194	0,128444329	2,35519889	0,018512794	0,07908895	protein_codin methyltransferase like 6
TcG_00612	183,4311707	0,251812838	0,133615024	1,884614688	0,059481878	0,187934834	protein_codin peptidyl-prolyl cis-trans isomerase (cyclophilin)
TcG_00613	303,2206882	-0,26428281	0,10849497	-2,4358992	0,014854823	0,067322838	protein_codin iron-sulfur cluster assembly protein
TcG_00614	153,8817578	0,40840422	0,146387581	2,789882981	0,005272709	0,029843484	protein_codin hypothetical protein
TcG_00615	649,6793865	0,096865245	0,07753677	1,249281409	0,211562165	0,43452566	protein_codin hypothetical protein
TcG_00616	533,1292062	0,204920475	0,083245195	2,461649274	0,013829983	0,063799168	protein_codin hypothetical protein
TcG_00617	209,5603106	0,008868512	0,13029062	0,068067158	0,945732174	0,974694377	protein_codin NADH dehydrogenase
TcG_00618	135,9122692	0,475079053	0,15209535	3,123560675	0,00178677	0,012576865	protein_codin putative galactokinase
TcG_00619	130,3128496	0,273866737	0,159413334	1,717966312	0,08580276	0,242170714	protein_codin ribulose-phosphate 3-epimerase
TcG_00620	119,67721	0,137904611	0,160002401	0,861890882	0,388747567	0,619110558	protein_codin Gim5A protein
TcG_00621	109,3733519	0,058383898	0,166349416	0,350971463	0,725609758	0,858198719	protein_codin hypothetical protein
TcG_00622	1343,630384	0,08946588	0,057591822	1,553447634	0,12031623	0,303704071	protein_codin Gim5A protein
TcG_00623	511,0919682	0,079709691	0,086083947	0,925953018	0,354470397	0,588430867	protein_codin hypothetical protein
TcG_00624	37,42188149	0,104226972	0,293301871	0,355357338	0,722321905	0,857285556	protein_codin hypothetical protein
TcG_00625	1202,219899	0,013822643	0,059599422	0,231925786	0,816595656	0,909544056	protein_codin hypothetical protein
TcG_00626	260,6697995	-0,002429619	0,113668901	-0,02137453	0,982946893	0,99369442	protein_codin hypothetical protein
TcG_00627	213,3707795	-0,015106149	0,124529739	-0,12130556	0,90344902	0,953224692	protein_codin hypothetical protein

TcG_00628	607,7414655	0,258625906	0,079230267	3,264231165	0,001097616	0,008432469	protein_codin anaphase-promoting complex subunit 2
TcG_00629	21,63738457	0,274810197	0,391409943	0,70210326	0,482614769	0,696286283	protein_codin 60S ribosomal protein L27A/L29
TcG_00630	6,669653127	-0,183873557	0,663119867	-0,27728555	0,781560854	1	protein_codin 60S ribosomal protein L27A/L29
TcG_00631	34,86608889	0,287609074	0,302834401	0,949723919	0,342252553	0,576576323	protein_codin 60S ribosomal protein L27A/L29
TcG_00632	269,3310784	0,281995508	0,116669687	2,417041786	0,015647221	0,069781305	protein_codin 60S ribosomal protein L23
TcG_00633	558,4358239	0,307023242	0,084741071	3,623074822	0,000291122	0,002731121	protein_codin 60S ribosomal protein L23
TcG_00634	182,939512	-0,118713149	0,136410181	-0,87026604	0,384155031	0,614499543	protein_codin hypothetical protein
TcG_00635	162,399856	-0,033873735	0,13812374	-0,24524195	0,806269096	0,904301428	protein_codin hypothetical protein
TcG_00636	110,9506603	-0,197762446	0,170765985	-1,15809039	0,246827151	0,475592778	protein_codin hypothetical protein
TcG_00637	299,3767095	0,225157715	0,107067997	2,102941327	0,035470899	0,129854051	protein_codin protein DENND6A
TcG_00638	514,8047402	-0,1581605	0,08436447	-1,87472878	0,060830045	0,19150771	protein_codin putative proteasome beta 2 subunit
TcG_00639	191,7952936	-0,258255996	0,134160118	-1,92498337	0,054231439	0,175265119	protein_codin putative 30S Ribosomal protein S17
TcG_00640	1057,882019	-0,052717631	0,063502235	-0,8301697	0,406442843	0,634833846	protein_codin T-complex protein 1 subunit eta
TcG_00641	188,5486933	-0,405113248	0,12877418	-3,14591983	0,001655654	0,011804557	protein_codin putative nucleoside diphosphate kinase
TcG_00642	298,7537395	-0,165313813	0,103802037	-1,59258738	0,111252788	0,288425777	protein_codin Sof1-like protein
TcG_00643	576,7023788	-0,24418001	0,077858566	-3,13619967	0,001711526	0,012128284	protein_codin calmodulin
TcG_00644	238,0491957	-0,150236048	0,115601208	-1,29960622	0,193735968	0,410802512	protein_codin hypothetical protein
TcG_00645	362,3561396	-0,0381148	0,101814365	-0,37435582	0,708139609	0,849437494	protein_codin hypothetical protein
TcG_00646	377,6525238	-0,143709265	0,097413521	-1,47524967	0,140145432	0,334581697	protein_codin hypothetical protein
TcG_00647	130,1505245	-0,121620895	0,154958166	-0,78486277	0,432534035	0,656966351	protein_codin unc-50 related protein
TcG_00648	278,4075935	-0,081105292	0,110756607	-0,73228401	0,463995237	0,681522416	protein_codin hypothetical protein
TcG_00649	50,54448476	-0,234061183	0,249690318	-0,93740593	0,348549794	0,583103728	protein_codin hypothetical protein
TcG_00650	64,06199472	0,306712868	0,231904086	1,322585009	0,185973422	0,399879894	protein_codin hypothetical protein
TcG_00651	862,0305516	0,083687949	0,080531113	1,039200209	0,298711633	0,532231897	protein_codin pre-mRNA-processing-splicing factor 8-like
TcG_00652	482,9740528	0,043797326	0,084012193	0,521321066	0,602143128	0,784755061	protein_codin U5 snRNA-associated splicing factor
TcG_00653	40,04176581	-0,289657056	0,285821977	-1,01341772	0,310860679	0,544463034	protein_codin protein kinase A catalytic subunit isoform 1
TcG_00654	170,0682499	-0,138286425	0,135100335	-1,02358314	0,306032211	0,539104333	protein_codin protein kinase-A catalytic subunit
TcG_00655	344,2270673	-0,220856197	0,104672425	-2,10997496	0,034860512	0,128195613	protein_codin zinc finger protein, predicted
TcG_00656	804,7547948	-0,107577165	0,069966202	-1,53755902	0,124156473	0,310211809	protein_codin putative 4E-interacting protein
TcG_00657	128,2296385	0,272858967	0,159650474	1,70910214	0,087432031	0,245201813	protein_codin putative mitochondrial carrier protein
TcG_00658	364,3227907	0,313026124	0,096932476	3,229321443	0,001240843	0,009293091	protein_codin putative DNAJ-domain protein
TcG_00659	346,9269847	0,284775993	0,098726751	2,884486617	0,003920524	0,023682583	protein_codin WASH complex subunit 7-like
TcG_00660	250,6620215	0,180320632	0,114552566	1,574130008	0,115457396	0,295838856	protein_codin hypothetical protein
TcG_00661	82,88026241	0,305913924	0,204132493	1,498604753	0,133976199	0,32557556	protein_codin putative UPF0598 protein C8orf82
TcG_00662	128,1297825	0,133183473	0,154503281	0,862010642	0,388681662	0,619090697	protein_codin hypothetical protein
TcG_00663	94,0683513	0,288124832	0,184026945	1,56566655	0,117426701	0,298815234	protein_codin transferase
TcG_00664	183,753479	0,22410988	0,132366773	1,69309771	0,09043687	0,250953674	protein_codin putative glycogenin glucosyltransferase
TcG_00665	97,49431155	-0,161107478	0,177180503	-0,90928446	0,36319999	0,595268564	protein_codin protein kinase
TcG_00666	890,7966841	0,184005054	0,071307501	2,580444554	0,00986732	0,048897677	protein_codin hypothetical protein
TcG_00667	279,1855763	0,282482173	0,109316558	2,584074885	0,009764059	0,04853127	protein_codin translation factor SUI1
TcG_00668	186,7413969	0,589647485	0,140827565	4,187017547	2,82644E-05	0,000374253	protein_codin hypothetical protein
TcG_00669	346,3898213	0,505637039	0,102172906	4,948836812	7,46583E-07	1,56418E-05	protein_codin hypothetical protein
TcG_00670	162,1359893	0,17045463	0,14271759	1,19434913	0,232341427	0,45960522	protein_codin secreted protein
TcG_00671	122,0528288	0,758528913	0,160729727	4,719282041	2,36678E-06	4,32517E-05	protein_codin putative UDP-Gal or UDP-GlcNAc-dependent glycosyltransferase
TcG_00672	578,1718276	0,457645472	0,078761343	5,810534152	6,22738E-09	2,05298E-07	protein_codin hypothetical protein
TcG_00673	84,24572135	0,159421542	0,19208752	0,829942218	0,406571454	0,634833846	protein_codin hypothetical protein
TcG_00674	478,0042141	0,336275865	0,095012409	3,539283644	0,000401215	0,003590658	protein_codin hypothetical protein
TcG_00675	946,152465	0,413139184	0,068750465	6,009256594	1,86376E-09	6,96565E-08	protein_codin putative mitochondrial DNA-directed RNA polymerase
TcG_00676	1704,657715	0,341418887	0,056503491	6,042438791	1,51802E-09	5,76648E-08	protein_codin hypothetical protein
TcG_00677	234,6610825	0,477771472	0,118684956	4,025543669	5,68438E-05	0,000688185	protein_codin hypothetical protein
TcG_00678	319,4585327	0,449044982	0,102317239	4,388751964	1,14003E-05	0,000170126	protein_codin hypothetical protein
TcG_00679	404,1524132	0,486876506	0,097950821	4,970621974	6,67385E-07	1,41101E-05	protein_codin hypothetical protein
TcG_00680	238,9437324	0,317758895	0,117971063	2,693532521	0,007069925	0,03757438	protein_codin putative formin
TcG_00681	341,7006764	0,42408233	0,100032553	4,239443238	2,24075E-05	0,000306148	protein_codin putative formin
TcG_00682	482,7977311	0,362138666	0,087967921	4,116712788	3,84315E-05	0,000488767	protein_codin putative pre-mRNA splicing factor
TcG_00683	355,821934	0,211567194	0,096731435	2,187160708	0,028730794	0,11061934	protein_codin hypothetical protein
TcG_00684	192,7889437	0,176090872	0,139682369	1,260652103	0,207434218	0,429626894	protein_codin putative DnaJ chaperone protein

TcG_00685	638,045209	0,277947981	0,079280506	3,505880532	0,000455099	0,003988489	protein_codin	hypothetical protein
TcG_00686	605,0149734	0,449972097	0,079742838	5,642790093	1,67316E-08	5,07468E-07	protein_codin	putative helicase-like protein
TcG_00687	299,1247647	0,42786925	0,11472232	3,729607713	0,000191778	0,001930607	protein_codin	hypothetical protein
TcG_00688	163,7650193	0,033019944	0,138533895	0,23835282	0,811607457	0,907215051	protein_codin	dynein light chain
TcG_00689	0					1	protein_codin	dynein light chain
TcG_00690	1392,095	0,195451607	0,055001645	3,553559324	0,000380055	0,003424046	protein_codin	putative nucleoside transporter 1
TcG_00691	156,9007795	0,372184426	0,149120596	2,495861986	0,012565148	0,059106701	protein_codin	hypothetical protein
TcG_00692	781,6100424	0,492684712	0,07274102	6,773134485	1,26022E-11	7,89236E-10	protein_codin	hypothetical protein
TcG_00693	18,50975724	1,42342162	0,42995558	3,310624837	0,000930879	0,007366917	protein_codin	dynein light chain
TcG_00694	243,307555	0,278945214	0,116471818	2,394958879	0,016622229	0,073115521	protein_codin	hypothetical protein
TcG_00695	272,8872834	0,352731443	0,112001293	3,149351518	0,001636332	0,011698514	protein_codin	putative DNA mismatch repair protein MSH2
TcG_00696	435,0827006	0,500093798	0,090437789	5,529699549	3,2078E-08	9,19939E-07	protein_codin	putative replication factor C, subunit 1
TcG_00697	845,7734264	0,006422931	0,072664324	0,08839181	0,929565271	0,965740964	protein_codin	hypothetical protein
TcG_00698	211,2667044	0,466741181	0,132323167	3,527282435	0,000419849	0,00372463	protein_codin	putative pre-mRNA splicing factor ATP-dependent RNA helicase
TcG_00699	342,3454958	0,324428207	0,103965037	3,120551058	0,00180513	0,012666041	protein_codin	hypothetical protein
TcG_00700	242,5651579	0,670931403	0,115623578	5,802721332	6,52472E-09	2,13546E-07	protein_codin	putative GTPase activating protein
TcG_00701	305,6620928	0,485524663	0,103781417	4,678339104	2,89208E-06	5,21114E-05	protein_codin	hypothetical protein
TcG_00702	282,4784095	0,34811117	0,111021853	3,135519362	0,001715501	0,012149019	protein_codin	putative cell division cycle protein
TcG_00703	162,4788995	0,7973018	0,143749313	5,546473816	2,91488E-08	8,46412E-07	protein_codin	putative protein transport protein Sec31
TcG_00704	458,7272289	0,39238208	0,08599995	4,562584969	5,05276E-06	8,45973E-05	protein_codin	putative minchromosome maintenance (MCM) complex subunit
TcG_00705	423,646738	0,341046089	0,088687014	3,845501975	0,000120306	0,001303895	protein_codin	hypothetical protein
TcG_00706	415,3431876	0,413534217	0,098185432	4,21176757	2,5338E-05	0,000341754	protein_codin	putative mitochondrial DNA polymerase I protein A
TcG_00707	205,4489682	0,486871884	0,123918927	3,928954974	8,53158E-05	0,000972903	protein_codin	putative transmembrane protein
TcG_00708	109,9206617	0,809760629	0,170390374	4,752384828	2,01031E-06	3,74461E-05	protein_codin	hypothetical protein
TcG_00709	224,6134993	0,566779789	0,120111535	4,718779014	2,37264E-06	4,32905E-05	protein_codin	hypothetical protein
TcG_00710	978,8736931	0,494139499	0,064169483	7,700537352	1,35495E-14	1,34175E-12	protein_codin	triosephosphate isomerase
TcG_00711	274,3589485	0,092882404	0,107391698	0,864893704	0,387097133	0,617331417	protein_codin	putative 33 kDa inner dynein arm light chain, axonemal
TcG_00712	522,9049527	0,33853633	0,081142104	4,172141374	3,0175E-05	0,000397282	protein_codin	hypothetical protein
TcG_00713	668,421863	0,351643523	0,084564276	4,158298759	3,20627E-05	0,000417391	protein_codin	putative synaptojanin (N-terminal domain), putative,inositol/phosphatidylinositol phosphatase
TcG_00714	338,6896628	0,367328077	0,102991705	3,566579246	0,000361671	0,003281383	protein_codin	phosphotransferase
TcG_00715	231,7090826	0,326364752	0,120763438	2,70251292	0,006881751	0,036847913	protein_codin	Trypanosoma vivax
TcG_00716	371,1891199	0,812395037	0,097385642	8,342041181	7,3019E-17	9,39998E-15	protein_codin	hypothetical protein
TcG_00717	498,0573521	0,308137175	0,085346855	3,610410435	0,000305713	0,00284732	protein_codin	malic enzyme
TcG_00718	1666,482447	0,335188295	0,053245107	6,29519434	3,07015E-10	1,37339E-08	protein_codin	malic enzyme
TcG_00719	1037,960186	0,449997901	0,06109099	7,366027345	1,75788E-13	1,46524E-11	protein_codin	putative DNA repair and recombination protein RAD54
TcG_00720	63,75265484	0,860236976	0,223287438	3,852599063	0,000116871	0,001276214	protein_codin	hypothetical protein
TcG_00721	446,8904844	0,347991812	0,092723532	3,753004288	0,000174728	0,001786758	protein_codin	hypothetical protein
TcG_00722	492,1775957	0,491532323	0,083973992	5,853387626	4,8166E-09	1,63651E-07	protein_codin	hypothetical protein
TcG_00723	590,7933568	0,334772646	0,086855308	3,85437174	0,000116027	0,001269397	protein_codin	putative signal recognition particle
TcG_00724	113,5754798	0,510304837	0,164821063	3,096114229	0,001960748	0,013538276	protein_codin	hypothetical protein
TcG_00725	175,8725151	0,314902387	0,13576005	2,3195512	0,020365168	0,085298564	protein_codin	Trypanosoma vivax
TcG_00726	43,19584862	0,095833114	0,272530247	0,35164212	0,725106673	0,858172586	protein_codin	hypothetical protein
TcG_00727	126,4739457	0,278038582	0,167630597	1,658638615	0,097188636	0,263706682	protein_codin	putative aldo/keto reductase
TcG_00728	218,922597	0,485650545	0,120954925	4,015136604	5,94114E-05	0,00071553	protein_codin	kelch repeat protein
TcG_00729	553,9338389	0,454504388	0,081161703	5,599985825	2,14369E-08	6,40125E-07	protein_codin	putative sugar transporter
TcG_00730	405,3064669	0,378323486	0,093117133	4,062877283	4,84715E-05	0,00059935	protein_codin	putative protein kinase
TcG_00731	403,8938786	0,446328313	0,090311036	4,942123724	7,72761E-07	1,60851E-05	protein_codin	hypothetical protein
TcG_00732	263,7897986	0,272652371	0,117072708	2,328914875	0,019863576	0,083504858	protein_codin	hypothetical protein
TcG_00733	384,3151448	0,452300506	0,099695302	4,536828701	5,71065E-06	9,4116E-05	protein_codin	hypothetical protein
TcG_00734	478,3043336	0,088428214	0,086072513	1,027368805	0,304246835	0,537102518	protein_codin	putative MCAK-like kinesin
TcG_00735	300,54188	0,392689143	0,10665051	3,682018444	0,000231395	0,002254784	protein_codin	putative mitochondrial carrier protein
TcG_00736	405,3556135	0,196296378	0,093601429	2,097151499	0,035980166	0,131296443	protein_codin	ATPase subunit 9
TcG_00737	966,3153331	0,238093994	0,064014924	3,719351349	0,000199735	0,001989794	protein_codin	putative ubiquitin hydrolase
TcG_00738	356,2106587	0,529641477	0,099596509	5,317871893	1,04988E-07	2,70911E-06	protein_codin	transferase
TcG_00739	360,588993	0,429888345	0,100511432	4,277009458	1,89421E-05	0,000264413	protein_codin	putative DNA helicase
TcG_00740	788,2128699	0,503557416	0,073388464	6,861533702	6,81251E-12	4,51027E-10	protein_codin	hypothetical protein
TcG_00741	165,251312	0,392934919	0,140072743	2,805220418	0,005028218	0,028882963	protein_codin	hexosyltransferase

TcG_00742	161,0786723	0,484877025	0,14016448	3,459343087	0,000541495	0,004647228	protein_codin	tRNA guanosine-2-O-methyltransferase TRM13
TcG_00743	50,01883718	0,465326142	0,257343471	1,808190979	0,070576782	0,213109877	protein_codin	tRNA guanosine-2-O-methyltransferase TRM13
TcG_00744	286,1694946	0,373735483	0,107503557	3,476494103	0,000508015	0,004399003	protein_codin	hypothetical protein
TcG_00745	587,6284368	0,384434958	0,083818216	4,586532309	4,50669E-06	7,65608E-05	protein_codin	putative chaperone DNAJ protein
TcG_00746	202,2984799	0,588233697	0,127304183	4,620694182	3,82458E-06	6,62356E-05	protein_codin	hypothetical protein
TcG_00747	138,1952969	0,250168027	0,151111497	1,655519479	0,097819168	0,264735549	protein_codin	hypothetical protein
TcG_00748	707,6967019	0,237367766	0,074798568	3,173426597	0,00150651	0,010909015	protein_codin	hypothetical protein
TcG_00749	243,1602959	0,153609647	0,115061612	1,335020809	0,181869536	0,393710845	protein_codin	hypothetical protein
TcG_00750	166,0096969	0,546577224	0,14853568	3,679770563	0,000233444	0,002270933	protein_codin	hypothetical protein
TcG_00751	201,1932613	0,459865707	0,130100797	3,534687854	0,000408257	0,00363939	protein_codin	putative ubiquitin-activating enzyme
TcG_00752	416,2880015	0,315009132	0,090969207	3,462810569	0,000534565	0,00459797	protein_codin	hypothetical protein
TcG_00753	212,0509419	0,228315084	0,123192794	1,853315249	0,063837187	0,198501784	protein_codin	putative protein kinase
TcG_00754	328,369709	0,488944204	0,195084495	2,50632016	0,01219951	0,057832865	protein_codin	putative ubiquitin carboxyl-terminal hydrolase, putative,cysteine peptidase, Clan CA, family C12
TcG_00755	345,7789821	0,476089032	0,099586665	4,780650402	1,74729E-06	3,30373E-05	protein_codin	hypothetical protein
TcG_00756	307,973813	0,683753028	0,104541516	6,540492764	6,13165E-11	3,21454E-09	protein_codin	putative actin
TcG_00757	421,989663	0,934827331	0,095556506	9,782979385	1,3323E-22	3,1502E-20	protein_codin	hypothetical protein
TcG_00758	409,0504952	0,615034556	0,091242929	6,740627061	1,57705E-11	9,51648E-10	protein_codin	hypothetical protein
TcG_00759	405,6066779	0,350650724	0,092987515	3,770944113	0,000162631	0,001682361	protein_codin	hypothetical protein
TcG_00760	302,4758906	0,615229348	0,108924034	5,648242385	1,62097E-08	4,92927E-07	protein_codin	putative aspartate aminotransferase, mitochondrial
TcG_00761	20,16109446	1,005587945	0,408103253	2,464052755	0,013737589	0,063465785		
TcG_00762	148,3528343	0,877381599	0,150519203	5,829034324	5,5749E-09	1,87764E-07	protein_codin	hypothetical protein
TcG_00763	442,7438087	0,331278133	0,087429465	3,789090232	0,0001512	0,001583909	protein_codin	putative ethanolamine phosphotransferase
TcG_00764	758,7600499	0,631572105	0,071714282	8,806782781	1,28789E-18	2,01642E-16	protein_codin	methyltransferase
TcG_00765	752,6092909	0,651736106	0,070874591	9,195624244	3,72824E-20	6,8564E-18	protein_codin	putative fumarate hydratase
TcG_00766	293,8093148	0,429563784	0,109653756	3,917456186	8,94883E-05	0,001016482	protein_codin	putative leucine-rich repeat protein (LRRP)
TcG_00767	460,3223474	0,370087641	0,089628662	4,129121558	3,64152E-05	0,000466983	protein_codin	hypothetical protein
TcG_00768	591,6732422	0,523531224	0,079562759	6,580103978	4,70119E-11	2,54524E-09	protein_codin	hypothetical protein
TcG_00769	386,3424746	0,131808151	0,093066687	1,416276388	0,156694598	0,358291615	protein_codin	putative TFIIF-stimulated CTD phosphatase
TcG_00770	427,8612951	0,179247635	0,093007007	1,927248712	0,053948642	0,174497199	protein_codin	dynein intermediate-chain-like protein
TcG_00771	206,1447731	0,558266351	0,134345222	4,155461154	3,24632E-05	0,000422131	protein_codin	putative protein phosphatase
TcG_00772	493,4279595	0,606427746	0,092197454	6,577489053	4,78459E-11	2,57834E-09	protein_codin	ATP-dependent RNA helicase
TcG_00773	295,6992598	0,472333319	0,110824353	4,261999341	2,02606E-05	0,000280453	protein_codin	putative WD40 repeat protein
TcG_00774	102,7151573	0,529812766	0,189073887	2,802146695	0,005076378	0,02903007	protein_codin	hypothetical protein
TcG_00775	180,2768149	0,523654396	0,135935923	3,85221497	0,000117054	0,001277015	protein_codin	putative transcription elongation factor
TcG_00776	128,1859645	0,624799759	0,160784399	3,885947655	0,000101931	0,001136649	protein_codin	putative transcription elongation factor
TcG_00777	459,5197738	0,090892036	0,091170471	0,996945993	0,318790725	0,553749526	protein_codin	serine/threonine-protein phosphatase 4 regulatory subunit 4
TcG_00778	150,4141547	0,300757585	0,151469497	1,985598361	0,047077939	0,158476334	protein_codin	ATPase domain protein
TcG_00779	289,982436	0,473895196	0,107460879	4,409932251	1,03403E-05	0,0001564	protein_codin	hypothetical protein
TcG_00780	334,1124533	0,022555262	0,105447019	0,213901376	0,830623967	0,917601778	protein_codin	hypothetical protein
TcG_00781	96,17192585	0,225527172	0,187371178	1,203638544	0,228729315	0,455746126	protein_codin	putative glycosomal membrane protein
TcG_00782	747,781634	0,2915477	0,071603398	4,071702048	4,66709E-05	0,000583544	protein_codin	putative ankyrin repeat family protein
TcG_00783	417,4386912	0,164425311	0,091513537	1,796732125	0,072378156	0,216182862	protein_codin	guanine nucleotide-binding protein beta subunit-like protein
TcG_00784	315,6180348	0,39338593	0,10468256	3,75789367	0,00017135	0,001755311	protein_codin	hypothetical protein
TcG_00785	318,0467486	-0,056302389	0,100287658	-0,56140895	0,574518782	0,76562855	protein_codin	enkurin
TcG_00786	254,1707454	0,472829697	0,113687377	4,15903428	3,19596E-05	0,000416517	protein_codin	hypothetical protein
TcG_00787	174,370533	0,332032257	0,138671708	2,39437635	0,016648656	0,07320354	protein_codin	excreted/secreted protein 66
TcG_00788	304,3666875	0,150235036	0,109621363	1,370490495	0,17053384	0,377134009	protein_codin	hypothetical protein
TcG_00789	370,4271674	0,314032374	0,097756105	3,21240678	0,001316279	0,0097759	protein_codin	hypothetical protein
TcG_00790	183,0003769	0,191935373	0,130514708	1,470603394	0,141398405	0,3366698	protein_codin	hypothetical protein
TcG_00791	1182,362046	0,7177622	0,060871704	11,79139328	4,32329E-32	2,78276E-29	protein_codin	60S ribosomal protein L17
TcG_00792	206,965825	0,264460805	0,12436039	2,126567834	0,033456007	0,123999137	protein_codin	hypothetical protein
TcG_00793	227,8567029	0,122966499	0,126418831	0,972691318	0,330706742	0,565235831	protein_codin	putative copper homeostasis protein
TcG_00794	622,5876827	0,19584104	0,076000546	2,576837278	0,009970888	0,049279089	protein_codin	putative clathrin coat assembly protein
TcG_00795	252,0204116	0,134222827	0,11208639	1,19749442	0,231113901	0,458507818	protein_codin	hypothetical protein
TcG_00796	291,3824236	0,04292635	0,10582703	0,405627469	0,685016342	0,836557076	protein_codin	putative pyruvate dehydrogenase (lipoamide) kinase
TcG_00797	11,77652167	0,655911311	0,513019721	1,278530404	0,201062472	1	protein_codin	putative trans-sialidase
TcG_00798	6,447853186	-0,360461708	0,693202278	-0,51999498	0,603067071	1	protein_codin	trans-sialidase

TcG_00799	16,5382623	-0,077095658	0,445273605	-0,17314222	0,862539643	1	
TcG_00800	7,544788332	0,644062923	0,647443844	0,994778047	0,319844225	1	protein_codin dispersed gene family protein 1 (DGF-1)
TcG_00801	0,27264935	1,353296468	3,06414982	0,441654798	0,658739024	1	protein_codin dispersed gene family protein 1 (DGF-1)
TcG_00802	5,181714925	-0,536891778	0,809385429	-0,66333265	0,507117533	1	protein_codin dispersed gene family protein 1 (DGF-1)
TcG_00803	12,69893477	0,075243285	0,49531819	0,151908989	0,879258718	1	protein_codin dispersed gene family protein 1 (DGF-1)
TcG_00804	11,87530708	-0,081548256	0,53104832	-0,1535609	0,877955972	1	protein_codin nuclear lim interactor-interacting factor
TcG_00805	212,5950541	-0,369461437	0,126045851	-2,93116697	0,003376912	0,02094481	protein_codin hypothetical protein
TcG_00806	227,3190627	-0,047972262	0,119265313	-0,40223146	0,687513688	0,837683625	protein_codin putative acyltransferase
TcG_00807	346,5925491	-0,236306986	0,099885282	-2,36578385	0,017991945	0,077492446	protein_codin putative acyltransferase
TcG_00808	328,634868	-0,37203637	0,107010756	-3,47662595	0,000507766	0,004399003	protein_codin hypothetical protein
TcG_00809	137,5451437	0,142641207	0,14921924	0,955916989	0,339114175	0,57332217	protein_codin hypothetical protein
TcG_00810	94,70911282	-0,132695768	0,189043518	-0,70193239	0,482721331	0,696286283	protein_codin cAMP-phosphodiesterase D
TcG_00811	427,0950532	-0,070914333	0,091194151	-0,77761931	0,436793475	0,660405742	protein_codin heat shock protein 20
TcG_00812	1339,268592	0,102117414	0,057985016	1,761100042	0,078221472	0,227193275	protein_codin 60S ribosomal protein L13
TcG_00813	31,54437081	0,149063698	0,307908454	0,484116939	0,62830288	0,800738881	protein_codin 60S ribosomal protein L13
TcG_00814	567,4948805	-0,452272049	0,078954499	-5,72826189	1,01465E-08	3,22074E-07	protein_codin hypothetical protein
TcG_00815	1058,290018	-0,373109069	0,061119158	-6,10461728	1,03047E-09	4,08872E-08	protein_codin hypothetical protein
TcG_00816	222,5847564	-0,244037352	0,122920254	-1,98533069	0,047107692	0,158521557	protein_codin putative protein kinase
TcG_00817	587,059353	-0,214371651	0,079990418	-2,67996665	0,00736295	0,038849178	protein_codin vacuolar protein sorting-associated protein 4
TcG_00818	581,687456	-0,090137986	0,079957371	-1,12732553	0,259604871	0,492108097	protein_codin ATP-dependent phosphofructokinase
TcG_00819	129,7919995	-0,231326596	0,163748847	-1,41269145	0,157746466	0,359844567	protein_codin hypothetical protein
TcG_00820	151,8253903	-0,117907601	0,142618799	-0,82673253	0,408388679	0,636605346	protein_codin hypothetical protein
TcG_00821	357,1917372	0,069178053	0,103801139	0,666447921	0,505124841	0,712274476	protein_codin tRNA (cytosine34-C5)-methyltransferase
TcG_00822	544,5661394	-0,122136866	0,079359964	-1,53902371	0,123798508	0,309656631	protein_codin RNA polymerase-associated protein CTR9
TcG_00823	536,0700226	-0,211757971	0,082001783	-2,58235811	0,00981277	0,048689831	protein_codin hypothetical protein
TcG_00824	114,35706	-0,189500027	0,16918166	-1,12009793	0,262672033	0,494848484	
TcG_00825	645,4804833	-0,284932156	0,074438997	-3,82772696	0,000129332	0,001384882	protein_codin putative protein kinase
TcG_00826	243,7259344	-0,146207093	0,118299401	-1,2359073	0,216493025	0,440591636	protein_codin hypothetical protein
TcG_00827	91,23891082	0,052797777	0,19941615	0,264761789	0,791192975	0,895628901	protein_codin hypothetical protein
TcG_00828	195,9973337	-0,305105197	0,127805423	-2,38726331	0,016974329	0,074269099	protein_codin hypothetical protein
TcG_00829	182,6537911	-0,485132385	0,135043024	-3,59242833	0,000327611	0,00301007	protein_codin hypothetical protein
TcG_00830	104,3829992	0,171655499	0,171763469	0,999371402	0,317614808	0,552739124	protein_codin poly(A) polymerase
TcG_00831	292,5358236	0,338747015	0,10729591	3,157128866	0,001593309	0,011451662	protein_codin putative poly(A) polymerase, putative,polynucleotide adenylyltransferase
TcG_00832	430,5562151	-0,078832435	0,088692043	-0,88883324	0,374092711	0,604496256	protein_codin hypothetical protein
TcG_00833	337,2331585	-0,051910879	0,102645508	-0,50572967	0,613046435	0,790819724	protein_codin hypothetical protein
TcG_00834	1820,662883	-0,129541748	0,056662641	-2,28619326	0,022242955	0,091288303	protein_codin hypothetical protein
TcG_00835	210,5033366	-0,094830564	0,122666349	-0,77307726	0,439476659	0,662646613	protein_codin hypothetical protein
TcG_00836	737,1647764	-0,217964095	0,073615459	-2,9608468	0,003067945	0,019415962	protein_codin condensin complex subunit 3
TcG_00837	112,3075739	0,195306458	0,171527582	1,13863004	0,254857493	0,486142289	protein_codin peptidyl-prolyl cis-trans isomerase
TcG_00838	502,4527091	-0,144578679	0,083621431	-1,72896681	0,083815036	0,238337043	protein_codin putative helicase
TcG_00839	256,4512667	-0,158063898	0,115399743	-1,36970754	0,170778214	0,377476218	protein_codin putative serine/threonine-protein kinase Nek1, putative,protein kinase
TcG_00840	232,2351139	0,093806991	0,118138832	0,794040271	0,427171988	0,652414269	protein_codin putative cAMP phosphodiesterase A
TcG_00841	489,8738963	-0,223326804	0,09585721	-2,3297862	0,019817454	0,083367872	protein_codin hypothetical protein
TcG_00842	460,1546825	-0,097834023	0,087797461	-1,11431495	0,265144097	0,497563898	protein_codin hypothetical protein
TcG_00843	155,8501075	-0,481434689	0,145539052	-3,30794164	0,000939844	0,007412547	protein_codin parallel beta-helix repeat-containing protein
TcG_00844	213,1193239	-0,058821038	0,12430054	-0,47321627	0,63605889	0,806013157	protein_codin protein kinase Wee570
TcG_00845	454,9591591	-0,194579689	0,089042536	-2,18524425	0,028870943	0,11105536	protein_codin hypothetical protein
TcG_00846	126,4472827	-0,088226257	0,162880028	-0,54166406	0,588049956	0,775648564	protein_codin putative actin-like protein
TcG_00847	395,1413366	-0,351181432	0,091466359	-3,83946007	0,000123305	0,00133142	protein_codin tetratricopeptide repeat protein 26 isoform X1
TcG_00848	94,0108025	0,109294685	0,185586538	0,588914939	0,555918336	0,750771633	protein_codin putative trypsin-like cysteine/serine peptidase
TcG_00849	154,564106	0,028233261	0,141477363	0,199560275	0,841824499	0,923003562	protein_codin putative mitochondrial carrier protein
TcG_00850	148,1262073	-0,109233993	0,149077161	-0,73273459	0,46372032	0,681291355	protein_codin hypothetical protein
TcG_00851	518,8359054	-0,276758744	0,081178325	-3,40926896	0,000651372	0,005406017	protein_codin putative inosine-adenosine-guanosine-nucleoside hydrolase
TcG_00852	410,4281104	-0,244795853	0,090039087	-2,71877317	0,006552452	0,035480336	protein_codin ribonuclease inhibitor-like protein
TcG_00853	80,00619353	0,27960274	0,201918342	1,384731758	0,166134504	0,371661396	protein_codin hypothetical protein
TcG_00854	22,28046457	-0,899926808	0,377204141	-2,38578189	0,017042856	0,074440982	protein_codin hypothetical protein
TcG_00855	33,14855609	-0,222458995	0,331245567	-0,67158331	0,501849006	0,710722721	protein_codin RNA-binding protein 6

TcG_00856	5,247198576	-0,304628128	0,856761683	-0,3555576	0,722171901	1	
TcG_00857	152,4343352	0,140647091	0,147127846	0,955951537	0,339096719	0,57332217	protein_codin hypothetical protein
TcG_00858	205,7630275	-0,191148293	0,131686661	-1,45153877	0,146629895	0,344665037	protein_codin hypothetical protein
TcG_00859	114,7444675	-0,221220392	0,168328347	-1,31421948	0,188772356	0,403229446	protein_codin hypothetical protein
TcG_00860	879,2478088	-0,343870275	0,06630913	-5,18586621	2,15013E-07	5,17909E-06	protein_codin eukaryotic initiation factor 2 alpha subunit
TcG_00861	286,1183807	-0,10206455	0,107871795	-0,94616531	0,344064279	0,578315499	protein_codin putative protein kinase
TcG_00862	272,3339569	-0,591249387	0,109909837	-5,37940371	7,4733E-08	1,99506E-06	protein_codin hypothetical protein
TcG_00863	483,0077796	-0,330016641	0,086288973	-3,82455173	0,00013101	0,001396398	protein_codin hypothetical protein
TcG_00864	397,1611431	-0,088824013	0,090604585	-0,98034788	0,326914429	0,561463175	protein_codin hypothetical protein
TcG_00865	575,4220923	-0,187359128	0,080543193	-2,32619443	0,02000818	0,084051769	protein_codin hypothetical protein
TcG_00866	99,81770943	-0,151773236	0,187357573	-0,8100726	0,417898449	0,644538263	protein_codin hypothetical protein
TcG_00867	161,971995	-0,172878474	0,142502633	-1,21315986	0,225068706	0,45161864	protein_codin hypothetical protein
TcG_00868	144,0029497	-0,29541345	0,145661898	-2,02807635	0,042552457	0,147613203	protein_codin hypothetical protein
TcG_00869	253,2758493	0,045960269	0,12253625	0,37507488	0,707604778	0,84921369	protein_codin putative inorganic pyrophosphatase
TcG_00870	260,9088035	-0,233000739	0,118106805	-1,9727969	0,048518706	0,161906027	protein_codin putative peter pan protein
TcG_00871	357,9043109	-0,253175367	0,096170095	-2,63257894	0,008473933	0,043403618	protein_codin putative CTD nuclear envelope phosphatase 1A-like
TcG_00872	899,7691845	-0,233060866	0,071430031	-3,26278546	0,00110323	0,008448132	protein_codin hypothetical protein
TcG_00873	65,4493322	-0,112850646	0,214901432	-0,52512747	0,59949458	0,783102809	protein_codin hypothetical protein
TcG_00874	332,3315382	-0,117859819	0,098623347	-1,19504988	0,232067541	0,459225958	protein_codin hypothetical protein
TcG_00875	358,2198474	-0,351519095	0,096896684	-3,62777217	0,000285877	0,002690638	protein_codin hypothetical protein
TcG_00876	308,630301	-0,128496737	0,106452661	-1,20707868	0,227401846	0,454333124	protein_codin hypothetical protein
TcG_00877	802,4323401	-0,138183	0,069628659	-1,98457075	0,047192249	0,15875794	protein_codin hypothetical protein
TcG_00878	633,8811214	-0,06460841	0,078339262	-0,8247258	0,409527281	0,636866847	protein_codin hypothetical protein
TcG_00879	184,5636196	-0,083927555	0,144707274	-0,57998159	0,561927034	0,755364499	protein_codin hypothetical protein
TcG_00880	599,8139765	-0,283192169	0,076229593	-3,71498989	0,000203212	0,002017491	protein_codin aminoalcohol phosphotransferase
TcG_00881	902,1099037	-5,00389E-05	0,068438684	-0,00073115	0,999416627	0,99981271	protein_codin hypothetical protein
TcG_00882	805,4031867	-0,491880329	0,076009413	-6,47130812	9,71581E-11	4,7901E-09	protein_codin putative 6-phosphofructo-2-kinase/fructose-2,6-biphosphatase
TcG_00883	425,9023384	-0,21116695	0,087679975	-2,40838286	0,016023367	0,071129017	protein_codin hypothetical protein
TcG_00884	338,1338468	-0,442492146	0,099836712	-4,43215865	9,32943E-06	0,000144121	protein_codin putative serine/threonine protein kinase, putative,protein kinase
TcG_00885	645,8270132	0,141779263	0,078903811	1,79686204	0,072357524	0,216182287	protein_codin tubulin-specific chaperone E
TcG_00886	270,639504	0,019575532	0,112022724	0,174746081	0,861279157	0,93373073	protein_codin hypothetical protein
TcG_00887	144,8184714	-0,132323324	0,145618003	-0,90870168	0,363507616	0,595447368	protein_codin hypothetical protein
TcG_00888	337,1020447	-0,300952485	0,107524227	-2,7989272	0,00512727	0,02923452	protein_codin hypothetical protein
TcG_00889	189,6978766	0,037762751	0,139758506	0,270200016	0,78700638	0,893930698	protein_codin EbsC protein
TcG_00890	864,9404681	-0,378859092	0,070508971	-5,37320408	7,73497E-08	2,06017E-06	protein_codin hypothetical protein
TcG_00891	454,0992394	-0,229031206	0,088014914	-2,60218634	0,009263151	0,04644001	protein_codin GPI inositol deacylase 2
TcG_00892	1040,857572	-0,182804517	0,06611437	-2,76497406	0,005692732	0,031663945	protein_codin ATP-dependent DEAD/H RNA helicase
TcG_00893	706,0157225	-0,220677256	0,074160532	-2,97566979	0,002923495	0,018641502	protein_codin ATP-dependent DEAD/H RNA helicase
TcG_00894	67,45764874	-0,23351861	0,213159133	-1,09551304	0,273291935	0,506536612	protein_codin hypothetical protein
TcG_00895	528,3964242	-0,237821949	0,088614854	-2,68377072	0,007279701	0,038495033	protein_codin hypothetical protein
TcG_00896	381,6482071	-0,220164424	0,09173303	-2,40005616	0,016392557	0,072326144	protein_codin hypothetical protein
TcG_00897	278,6266782	-0,186632668	0,107678654	-1,73323738	0,083053486	0,236811141	protein_codin hypothetical protein
TcG_00898	245,5697523	-0,063534414	0,122144155	-0,52015927	0,602952572	0,785186973	protein_codin hypothetical protein
TcG_00899	573,6117019	-0,099377247	0,079446139	-1,25087574	0,210979817	0,433560156	protein_codin RNA editing complex protein MP99
TcG_00900	441,0152327	-0,092156162	0,091212869	-1,01034167	0,312331623	0,546297432	protein_codin putative arginine N-methyltransferase
TcG_00901	597,2081334	0,015609236	0,07554477	0,206622322	0,836304826	0,920523248	protein_codin putative DNA repair helicase
TcG_00902	167,9402852	-0,117130064	0,142283338	-0,82321701	0,410384595	0,637275958	protein_codin hypothetical protein
TcG_00903	230,2001985	-0,075514676	0,11657063	-0,6478019	0,517113086	0,722625968	protein_codin hypothetical protein
TcG_00904	176,5210858	-0,049710757	0,135144293	-0,36783467	0,712996512	0,852593414	protein_codin hypothetical protein
TcG_00905	213,8586036	-0,060980311	0,127030766	-0,48004364	0,631196362	0,802484479	protein_codin putative quinone oxidoreductase
TcG_00906	610,0757941	-0,380636668	0,077131495	-4,9349059	8,01894E-07	1,66501E-05	protein_codin hypothetical protein
TcG_00907	538,8231633	-0,330050358	0,083135474	-3,97003037	7,18635E-05	0,00084529	protein_codin putative 26S protease regulatory subunit
TcG_00908	983,07977	-0,04301556	0,07211972	-0,59644657	0,550876938	0,746549166	protein_codin hypothetical protein
TcG_00909	694,4225457	-0,121533801	0,073329999	-1,65735446	0,097447832	0,264100721	protein_codin U2AF65-like splicing factor
TcG_00910	1138,144508	-0,227208817	0,058930579	-3,8555334	0,000115478	0,001265654	protein_codin protein-tyrosine phosphatase
TcG_00911	535,8439953	-0,348910812	0,090284879	-3,86455426	0,000111292	0,001221053	protein_codin hypothetical protein
TcG_00912	438,614951	-0,166290105	0,088781059	-1,87303583	0,061063437	0,191934289	protein_codin putative 6-phosphofructo-2-kinase/fructose-2,6-biphosphatase

TcG_00913	368,710397	-0,172730416	0,097007748	-1,78058371	0,074980483	0,221105594	protein_codin	hypothetical protein
TcG_00914	4,061597169	-1,640345947	0,987873364	-1,66048201	0,096817524	1	protein_codin	hypothetical protein
TcG_00915	589,7001521	-0,058303747	0,076875956	-0,7584133	0,448203598	0,66910023	protein_codin	conserved TLD domain protein
TcG_00916	914,203089	0,160442782	0,06646002	2,414124785	0,015773059	0,070287179	protein_codin	60S acidic ribosomal protein P2
TcG_00917	304,9046471	-0,195489798	0,104849287	-1,8644838	0,062253795	0,194885832	protein_codin	putative PTP1-interacting protein, 39 kDa
TcG_00918	467,8814482	-0,022705803	0,093509316	-0,24281862	0,808145914	0,905092175	protein_codin	putative MFS transporter
TcG_00919	287,0304797	-0,120711567	0,108233655	-1,11528679	0,264727543	0,497184845	protein_codin	putative calcium channel protein
TcG_00920	48,35702861	-0,164314196	0,256203132	-0,64134343	0,521299578	0,725401608	protein_codin	hypothetical protein
TcG_00921	365,4011672	-0,122447842	0,094461369	-1,29627428	0,194881007	0,412249652	protein_codin	protein kinase, putative,serine/threonine protein kinase
TcG_00922	61,40573229	0,21874196	0,221867741	0,98591151	0,324176538	0,558416499	protein_codin	hypothetical protein
TcG_00923	239,2188102	-0,018226018	0,118935722	-0,15324259	0,878206974	0,941423575	protein_codin	putative retrotransposon hot spot (RHS) protein
TcG_00924	27,31240633	0,316438085	0,342550783	0,923769846	0,355606158	0,589421022	protein_codin	retrotransposon hot spot (RHS) protein
TcG_00925	36,61193044	0,134268621	0,293301164	0,457784141	0,647107531	0,812420258	protein_codin	hypothetical protein
TcG_00926	57,98136785	-0,148927051	0,229179691	-0,64982656	0,515804258	0,72175219	protein_codin	hypothetical protein
TcG_00927	18,68822965	-0,045960226	0,407175123	-0,11287582	0,910129006	0,956414305		
TcG_00928	148,6401618	-0,01313758	0,143032109	-0,09185057	0,926816767	0,964306367	protein_codin	putative trans-sialidase
TcG_00929	860,3954681	0,04675294	0,071126274	0,657323055	0,510973215	0,717505232	protein_codin	putative AGP2beta-2
TcG_00930	243,2817219	0,151633749	0,121084008	1,252302024	0,210459832	0,433024666	protein_codin	putative ribosomal P protein AGP2beta-1
TcG_00931	1537,13346	0,17118722	0,063880786	2,679792031	0,007366791	0,038849178	protein_codin	60S acidic ribosomal protein P2 beta (H6.4)
TcG_00932	597,3208956	-0,044194761	0,076709707	-0,57612997	0,564527335	0,757366107	protein_codin	putative damage-specific DNA binding protein
TcG_00933	176,9148203	-0,048233041	0,142286818	-0,33898461	0,734621326	0,864076059	protein_codin	putative damage-specific DNA binding protein
TcG_00934	998,8964689	-0,076276096	0,065700098	-1,16097386	0,245652529	0,474196968	protein_codin	putative protein kinase, putative,polo-like protein kinase
TcG_00935	706,4929827	-0,274754573	0,072879417	-3,76998865	0,000163255	0,001687308	protein_codin	hypothetical protein
TcG_00936	811,8457784	-0,142574895	0,073570872	-1,93792585	0,05263227	0,171281863	protein_codin	tetratricopeptide repeat domain 39B
TcG_00937	839,9997955	-0,381894124	0,06774602	-5,63714477	1,72893E-08	5,23012E-07	protein_codin	hypothetical protein
TcG_00938	151,3418114	0,311082742	0,162996717	1,908521518	0,056323845	0,17991951	protein_codin	hypothetical protein
TcG_00939	197,2005665	-0,079544327	0,129655489	-0,61350528	0,539542294	0,738730443	protein_codin	putative vacuolar ATP synthase
TcG_00940	358,5122096	0,394996584	0,097010705	4,071680375	4,66752E-05	0,000583544	protein_codin	ribosomal protein L15
TcG_00941	408,0549863	0,041910238	0,08993366	0,466012814	0,641206328	0,80952561	protein_codin	putative leucine-rich repeat protein
TcG_00942	124,8072208	0,728069507	0,160771099	4,528609376	5,93731E-06	9,74359E-05	protein_codin	cyclin
TcG_00943	696,7366152	-0,111386444	0,070732307	-1,5747605	0,115311737	0,295771926	protein_codin	hypothetical protein
TcG_00944	453,8545881	-0,032826373	0,094822349	-0,34618814	0,729201324	0,860563425	protein_codin	putative dynein heavy chain
TcG_00945	565,1720577	-0,015253537	0,089536609	-0,1703609	0,864726319	0,934985064	protein_codin	ATP synthase, epsilon chain
TcG_00946	1221,519745	0,253757108	0,059653731	4,253834618	2,10141E-05	0,000289765	protein_codin	40S ribosomal protein S14
TcG_00947	1075,146734	-0,151901761	0,063497097	-2,39226309	0,016744834	0,073462439	protein_codin	putative protein kinase
TcG_00948	513,3010578	-0,02448159	0,083284391	-0,29395172	0,768794797	0,884313847	protein_codin	zinc finger-domain protein
TcG_00949	447,503342	0,111426118	0,088051565	1,265464367	0,205704914	0,427190739	protein_codin	mago nashi-like protein
TcG_00950	270,2467402	0,297043846	0,108753557	2,731348336	0,006307576	0,034341909	protein_codin	cytochrome P450-like protein
TcG_00951	375,9449275	-0,021656177	0,095119829	-0,22767258	0,819900792	0,911022136	protein_codin	putative peptide chain release factor 1
TcG_00952	171,2162625	0,033458038	0,141851172	0,235867194	0,813535726	0,907881422	protein_codin	S-adenosylmethionine synthetase
TcG_00953	1819,402845	-0,285916476	0,052882263	-5,4066611	6,42105E-08	1,73351E-06	protein_codin	S-adenosylmethionine synthetase
TcG_00954	235,4979252	0,301107162	0,123177002	2,444507969	0,014504988	0,066216747	protein_codin	hypothetical protein
TcG_00955	423,9197037	-0,05599307	0,091553615	-0,61158776	0,540810538	0,739592882	protein_codin	lorien protein
TcG_00956	422,969251	-0,04036211	0,089642207	-0,45025788	0,652524508	0,816254475	protein_codin	putative chromosomal passenger protein
TcG_00957	364,2200719	0,088391607	0,108405508	0,815379299	0,41485523	0,64180968	protein_codin	putative casein kinase 1 isoform 2
TcG_00958	178,9023987	0,067813799	0,132213492	0,512911339	0,608013341	0,788022108	protein_codin	zinc-binding protein (Yippee)
TcG_00959	460,5667261	0,139948219	0,088848727	1,575129139	0,115226639	0,295619097	protein_codin	tetratricopeptide repeat domain 5
TcG_00960	106,7861703	0,254136477	0,168945653	1,504249879	0,132517039	0,323570582	protein_codin	putative zinc-binding protein (Yippee)
TcG_00961	547,0367749	0,039919815	0,079116405	0,504570638	0,613860437	0,791298067	protein_codin	putative DNA ligase I
TcG_00962	982,0601948	-0,275238227	0,062700007	-4,38976391	1,13474E-05	0,00016964	protein_codin	viral life cyclerelated protein
TcG_00963	816,6324049	-0,145419867	0,066684857	-2,18070299	0,029205392	0,112044261	protein_codin	hypothetical protein
TcG_00964	826,6296832	-0,148697236	0,070733874	-2,10220688	0,035535157	0,130031079	protein_codin	hypothetical protein
TcG_00965	663,0003431	-0,229032679	0,073310519	-3,12414484	0,001783227	0,012559552	protein_codin	putative CAS/CSE/importin domain protein
TcG_00966	708,5570629	-0,102139625	0,073932817	-1,38151945	0,167119303	0,373072109	protein_codin	putative PAS-domain containing phosphoglycerate kinase
TcG_00967	388,8363728	-0,287312567	0,09530211	-3,01475557	0,002571864	0,016796854	protein_codin	putative calmodulin
TcG_00968	368,2613144	-0,116796819	0,096457311	-1,21086538	0,225947004	0,452435657	protein_codin	hypothetical protein
TcG_00969	303,6068966	0,239680509	0,104433026	2,295064297	0,02172944	0,089657154	protein_codin	hypothetical protein

TcG_00970	2349,963462	0,233854789	0,045741425	5,112538304	3,17859E-07	7,24943E-06	protein_codin putative 60S ribosomal protein L9
TcG_00971	183,9452987	-0,29435053	0,14460658	-2,03552653	0,041797922	0,145776859	protein_codin hypothetical protein
TcG_00972	684,0077247	-0,178446854	0,073491263	-2,428137	0,015176609	0,06821665	protein_codin hypothetical protein
TcG_00973	751,4078604	-0,23774024	0,071699034	-3,31580813	0,000913785	0,007261398	protein_codin flagellar component
TcG_00974	140,6158884	-0,16415965	0,155295765	-1,05707744	0,290476245	0,523456792	protein_codin putative cationic amino acid transporter
TcG_00975	522,746723	-0,147374589	0,084702203	-1,73991447	0,081874037	0,234916442	protein_codin putative UV excision repair RAD23-like protein
TcG_00976	424,3301547	-0,151207469	0,099737916	-1,51604801	0,129507212	0,319181145	protein_codin hypothetical protein
TcG_00977	277,4821826	-0,143200049	0,109335214	-1,30973401	0,190285833	0,405520849	protein_codin hypothetical protein
TcG_00978	313,7571258	0,003479211	0,10371357	0,03354634	0,973238912	0,988164581	protein_codin putative serine/threonine protein phosphatase
TcG_00979	242,9018721	-0,055746034	0,115043608	-0,48456438	0,627985389	0,800686554	protein_codin hypothetical protein
TcG_00980	266,9231859	-0,208491639	0,109053784	-1,91182397	0,055898777	0,178906968	protein_codin ankyrin repeat protein
TcG_00981	505,0562282	-0,192141121	0,083319271	-2,3060826	0,021106018	0,087835603	protein_codin putative ATP-dependent RNA helicase
TcG_00982	99,50621788	-0,119029094	0,17570633	-0,67743202	0,498131888	0,707424985	protein_codin ATP-dependent RNA helicase
TcG_00983	750,303631	-0,250889559	0,071208689	-3,52329977	0,000426209	0,003775273	protein_codin putative glutamyl-tRNA synthetase
TcG_00984	93,19531082	-0,04696959	0,181524409	-0,25875082	0,795827504	0,898164685	protein_codin hypothetical protein
TcG_00985	105,1421144	-0,193906037	0,177271077	-1,09383911	0,274025549	0,507085133	protein_codin hypothetical protein
TcG_00986	273,1488731	-0,34982968	0,113771556	-3,07484307	0,002106133	0,014370825	protein_codin hypothetical protein
TcG_00987	420,5524759	-0,362874885	0,09031054	-4,01807899	5,86745E-05	0,000708867	protein_codin hypothetical protein
TcG_00988	676,8698433	-0,040011521	0,074765697	-0,5351588	0,592540054	0,7778347	protein_codin hydroxymethylglutaryl CoA reductase
TcG_00989	170,1096908	-0,020212496	0,133847124	-0,1510118	0,879966405	0,941654269	protein_codin putative RNA-binding protein
TcG_00990	286,2244522	0,003749436	0,110498781	0,033931918	0,972931441	0,988112173	protein_codin putative amino acid permease
TcG_00991	308,2823354	-0,11875359	0,10798328	-1,09974054	0,271445186	0,504573652	protein_codin hypothetical protein
TcG_00992	335,9665818	-0,641235853	0,098649139	-6,50016675	8,0231E-11	3,98951E-09	protein_codin meiosis-specific nuclear structural 1
TcG_00993	231,2218837	-0,153234732	0,120730806	-1,26922645	0,20436031	0,425466046	protein_codin hypothetical protein
TcG_00994	675,8092987	-0,379320661	0,073099631	-5,18909131	2,11323E-07	5,12117E-06	protein_codin translocon-associated protein subunit beta
TcG_00995	351,6963268	-0,098919306	0,101485105	-0,97471748	0,329700422	0,56434119	protein_codin hypothetical protein
TcG_00996	1591,657445	-0,098390503	0,053719136	-1,83157272	0,067015104	0,205896843	protein_codin valyl-tRNA synthetase
TcG_00997	249,1645512	-0,121077624	0,116728	-1,0372629	0,29961335	0,533408141	protein_codin putative S-adenosylmethionine decarboxylase proenzyme
TcG_00998	138,7663252	-0,282107594	0,161854367	-1,74297178	0,081338545	0,23384327	
TcG_00999	627,6182396	-0,062841747	0,07851354	-0,80039375	0,4234827	0,649433562	protein_codin S-adenosylmethionine decarboxylase
TcG_01000	356,8599886	-0,05095193	0,097262119	-0,52386202	0,600374516	0,783502945	protein_codin hypothetical protein
TcG_01001	3185,775487	0,110332693	0,04375492	2,521606552	0,011682029	0,055813603	protein_codin putative RNA-binding protein
TcG_01002	606,1697128	0,339221998	0,076343992	4,443335885	8,85747E-06	0,000137934	protein_codin putative homoserine kinase
TcG_01003	361,2953364	-0,220873133	0,096967207	-2,27781268	0,02273774	0,093055266	protein_codin hypothetical protein
TcG_01004	243,6511902	0,198907818	0,125050493	1,590620028	0,111695114	0,289313569	protein_codin tRNA (guanine-N(1)-)-methyltransferase TRM10
TcG_01005	964,3855796	-0,058367249	0,064132974	-0,91009734	0,362771178	0,595082383	protein_codin putative kinesin
TcG_01006	1026,457495	0,066017275	0,069524084	0,949559798	0,342335974	0,576576323	protein_codin protein kinase-like protein
TcG_01007	353,9920636	-0,347257814	0,098081304	-3,54050976	0,000399355	0,003581212	protein_codin protein fam63a
TcG_01008	55,57717523	-0,146882427	0,236508205	-0,6210458	0,534569491	0,734918657	
TcG_01009	1125,881026	-0,123613171	0,059975157	-2,06107292	0,039296084	0,139572174	protein_codin putative eukaryotic translation initiation factor 3 subunit 7-like protein
TcG_01010	365,7612549	0,141096546	0,094211917	1,49765073	0,134224019	0,325709912	protein_codin hypothetical protein
TcG_01011	215,4741845	0,075523284	0,123616097	0,610950239	0,541232524	0,739895358	protein_codin hypothetical protein
TcG_01012	315,4323226	0,152164552	0,105485674	1,442513914	0,149157449	0,34834473	protein_codin ribosomal RNA assembly protein
TcG_01013	149,8870475	0,395916011	0,145686314	2,717592345	0,00657588	0,03558531	protein_codin U6 snRNA-associated Sm-like protein LSm5p
TcG_01014	141,1274478	0,434388258	0,151590391	2,865539533	0,004162993	0,024939212	protein_codin hypothetical protein
TcG_01015	597,9461717	0,063714623	0,079667381	0,799757969	0,423851039	0,649740426	protein_codin hypothetical protein
TcG_01016	185,2537988	0,318513734	0,139213408	2,287953002	0,022140258	0,090963486	protein_codin hypothetical protein
TcG_01017	590,7905772	-0,030230289	0,076237787	-0,39652632	0,691716798	0,840508134	protein_codin hypothetical protein
TcG_01018	390,4079473	0,210064219	0,092324065	2,275292141	0,022888411	0,093575968	protein_codin putative DNA topoisomerase III
TcG_01019	230,3477133	-0,189721787	0,126069215	-1,50490178	0,13234933	0,323297352	protein_codin putative calpain-like cysteine peptidase
TcG_01020	5760,473614	-0,21735872	0,039250678	-5,53770613	3,06459E-08	8,83242E-07	protein_codin glycosomal glyceraldehyde-3-phosphate dehydrogenase
TcG_01021	312,0027794	0,118008916	0,102506248	1,151236326	0,249635024	0,478852879	protein_codin hypothetical protein
TcG_01022	373,9804884	-0,070846924	0,104596174	-0,67733762	0,498191765	0,707424985	protein_codin hypothetical protein
TcG_01023	198,2618078	0,149874978	0,134963449	1,11048568	0,266789796	0,499395611	
TcG_01024	146,5513476	-0,030452846	0,157796486	-0,19298811	0,846968279	0,92596191	protein_codin hypothetical protein
TcG_01025	297,4616977	0,003547561	0,105447331	0,033642969	0,973161858	0,988164581	protein_codin hypothetical protein
TcG_01026	302,0036529	-0,146553709	0,103179211	-1,42038021	0,15549702	0,356891537	protein_codin putative mitogen-activated protein kinase 5, putative,protein kinase

TcG_01027	800,6528916	0,150313423	0,073484817	2,045503124	0,040805282	0,143220234	protein_codin putative aldehyde dehydrogenase
TcG_01028	375,5639309	-0,049467608	0,094384141	-0,52410932	0,600202507	0,783502945	protein_codin hypothetical protein
TcG_01029	338,3954292	0,178560762	0,100071638	1,784329363	0,074370162	0,220159333	protein_codin putative mitochondrial protein
TcG_01030	206,2694174	-0,440239179	0,124585248	-3,53363811	0,000409882	0,003647382	protein_codin hypothetical protein
TcG_01031	443,8819764	0,103167326	0,089542841	1,152156054	0,249256953	0,478523787	protein_codin hypothetical protein
TcG_01032	268,1295784	-0,088399981	0,11507508	-0,76819395	0,442371966	0,664667154	protein_codin hypothetical protein
TcG_01033	176,606953	0,004636162	0,132779027	0,03491637	0,972146427	0,987776155	protein_codin hypothetical protein
TcG_01034	744,8112685	0,039296403	0,070916857	0,554119357	0,579497163	0,769018862	protein_codin hypothetical protein
TcG_01035	470,3823953	-0,696917886	0,092586141	-7,52723767	5,1825E-14	4,76543E-12	protein_codin hypothetical protein
TcG_01036	377,5063157	-0,155519651	0,108238496	-1,43682383	0,150768058	0,350762794	protein_codin succinate dehydrogenase cytochrome B subunit
TcG_01037	263,2004972	-0,02083389	0,113989	-0,18277106	0,854977658	0,929595641	protein_codin putative GTPase activating protein of Rab-like GTPase
TcG_01038	627,1756866	-0,158390205	0,078782586	-2,01047229	0,044381226	0,152220509	protein_codin trichohyalin
TcG_01039	352,501682	-0,084992458	0,096994101	-0,87626419	0,380886428	0,611048137	protein_codin putative chaperonin HSP60/CNP60
TcG_01040	207,9168299	-0,192419779	0,127751735	-1,50620091	0,132015606	0,32282246	protein_codin hypothetical protein
TcG_01041	371,7941009	-0,067746929	0,094573638	-0,71634053	0,473781107	0,689514873	protein_codin hypothetical protein
TcG_01042	135,7229299	0,176424932	0,153857224	1,146679546	0,251514087	0,481580269	protein_codin hypothetical protein
TcG_01043	260,115918	0,36123197	0,122205692	2,955934085	0,003117237	0,019667473	protein_codin hypothetical protein
TcG_01044	322,337083	0,085323795	0,108309573	0,787777039	0,430827134	0,655640721	protein_codin 39S ribosomal protein L28
TcG_01045	399,0598486	0,168969947	0,102102044	1,654912477	0,097942253	0,265006759	protein_codin putative superoxide dismutase
TcG_01046	320,9447575	-0,128858833	0,105405247	-1,22250871	0,221515311	0,446965586	protein_codin hypothetical protein
TcG_01047	1457,923313	0,022394488	0,054398618	0,411673844	0,680578499	0,833394663	protein_codin putative small glutamine-rich tetratricopeptide repeat protein
TcG_01048	90,17064247	0,090525703	0,184100481	0,491718992	0,622918011	0,796768389	protein_codin hypothetical protein
TcG_01049	365,2448974	-0,000353778	0,094715781	-0,00373515	0,997019789	0,999002964	protein_codin hypothetical protein
TcG_01050	460,0685405	-0,007279383	0,090515378	-0,08042151	0,935902022	0,969975922	protein_codin GRAM domain containing protein
TcG_01051	476,7389194	-0,272604298	0,093782438	-2,90677343	0,003651776	0,022362302	protein_codin hypothetical protein
TcG_01052	234,7921736	-0,194126523	0,116948433	-1,65993266	0,096928	0,263410017	protein_codin hypothetical protein
TcG_01053	465,7505029	0,133746105	0,085165992	1,570416797	0,116318177	0,297300331	protein_codin SH3 domain protein
TcG_01054	291,2361925	4,78738E-05	0,107783548	0,000444166	0,999645607	0,99981271	protein_codin hypothetical protein
TcG_01055	205,8890609	0,213251115	0,132335435	1,611443788	0,10708303	0,282082846	protein_codin hypothetical protein
TcG_01056	623,9866632	-0,049912032	0,07542757	-0,66172132	0,508149837	0,714840216	protein_codin hypothetical protein
TcG_01057	392,3228228	-0,026246919	0,09280258	-0,28282531	0,777310751	0,888880652	protein_codin hypothetical protein
TcG_01058	432,0314302	-0,132408023	0,098953359	-1,33808518	0,180868673	0,392277422	protein_codin hypothetical protein
TcG_01059	144,3821154	-0,15574886	0,147138299	-1,05852019	0,289818352	0,523276948	protein_codin hypothetical protein
TcG_01060	198,8559585	0,502616396	0,128852716	3,900704694	9,59131E-05	0,001076792	protein_codin hypothetical protein
TcG_01061	415,9289093	-0,070013287	0,091536863	-0,76486438	0,444352304	0,666426941	protein_codin hypothetical protein
TcG_01062	374,0356152	-0,281490416	0,097765587	-2,87923823	0,003986371	0,024004729	protein_codin putative replication factor C, subunit 2
TcG_01063	321,597778	-0,039344539	0,101307963	-0,38836571	0,697745414	0,843778001	protein_codin putative RNA-binding protein
TcG_01064	428,5581728	0,034112585	0,089815514	0,37980727	0,704088485	0,847428783	protein_codin C-1-tetrahydrofolate synthase, cytoplasmic
TcG_01065	293,7955953	0,054519054	0,107816293	0,505666187	0,613091009	0,790819724	protein_codin hypothetical protein
TcG_01066	277,7778943	0,096989113	0,11180094	0,867516072	0,38565931	0,616225177	protein_codin putative chaperone DNAJ protein
TcG_01067	1023,114411	-0,161445374	0,061308636	-2,6333219	0,008455417	0,043347106	protein_codin putative reticulon domain protein
TcG_01068	367,7774577	-0,045868776	0,101180886	-0,4533344	0,650307965	0,814449042	protein_codin hypothetical protein
TcG_01069	494,6804655	-0,214011316	0,085804676	-2,49416844	0,012625261	0,059293182	protein_codin hypothetical protein
TcG_01070	303,8523507	-0,059541734	0,107204827	-0,55540162	0,578619986	0,768354288	protein_codin hypothetical protein
TcG_01071	16,52643405	-0,127500882	0,433339867	-0,29422837	0,768583405	1	
TcG_01072	0,155988004	0,503022807	4,080472857	0,123275616	0,901888849	1	protein_codin mitochondrial heat shock
TcG_01073	454,0753689	-0,376764144	0,095241026	-3,95590179	7,62465E-05	0,000884276	protein_codin putative ATP-dependent RNA helicase
TcG_01074	606,1625714	-0,219588266	0,078076071	-2,81249126	0,004915936	0,028435364	protein_codin hypothetical protein
TcG_01075	400,5861207	-0,111947697	0,094254616	-1,1877158	0,234945396	0,462859608	protein_codin hypothetical protein
TcG_01076	316,9865451	-0,361095447	0,102609212	-3,51913282	0,00043296	0,003832142	protein_codin putative kinesin
TcG_01077	1159,954989	-0,012479735	0,063799133	-0,19560979	0,844915598	0,924817395	protein_codin 60S ribosomal protein L17
TcG_01078	347,6422443	-0,224121196	0,097554417	-2,29739671	0,021596149	0,089375364	protein_codin hypothetical protein
TcG_01079	316,0067289	0,365535944	0,10488125	3,485236354	0,000491703	0,004276926	protein_codin putative 40S ribosomal protein S2
TcG_01080	110,8427583	-0,087838322	0,167789201	-0,52350403	0,600623549	0,783588603	protein_codin putative 40S ribosomal protein S2
TcG_01081	278,663622	-0,172122489	0,109473264	-1,57227877	0,115885915	0,296457101	protein_codin hypothetical protein
TcG_01082	587,4306175	0,162186773	0,078290944	2,071590458	0,038303651	0,136971017	protein_codin prostaglandin F synthase
TcG_01083	51,88880068	0,487721807	0,269266586	1,811297175	0,070094863	0,212096913	protein_codin aldo-keto reductase

TcG_01084	690,1769802	-0,51172311	0,072648831	-7,04378999	1,8708E-12	1,37184E-10	protein_codin	hypothetical protein
TcG_01085	194,0874269	-0,233021396	0,140142681	-1,66274396	0,096363699	0,262588384	protein_codin	putative replication termination factor
TcG_01086	127,6611412	0,18260693	0,158453717	1,152430712	0,249144126	0,478493188	protein_codin	hypothetical protein
TcG_01087	214,6925741	-0,401309334	0,124350921	-3,2272325	0,001249938	0,00935516	protein_codin	hypothetical protein
TcG_01088	482,149549	-0,189076471	0,086571865	-2,18404064	0,028959262	0,111358118	protein_codin	putative ribosome biogenesis protein
TcG_01089	383,5478505	-0,283658062	0,100128198	-2,83294884	0,004612077	0,027028589	protein_codin	putative syntaxin
TcG_01090	1480,429433	-0,371422686	0,053512564	-6,94085006	3,89748E-12	2,67196E-10	protein_codin	nucleic acid binding protein Tc38
TcG_01091	248,5400671	-0,002228353	0,117108485	-0,01902811	0,984818682	0,994129886	protein_codin	putative 40S ribosomal protein S2
TcG_01092	338,0694952	-0,536650205	0,098512016	-5,4475609	5,10652E-08	1,40199E-06	protein_codin	putative peroxin 13
TcG_01093	690,2084612	-0,613145469	0,07320221	-8,37605128	5,47331E-17	7,20611E-15	protein_codin	putative chaperone protein DNAj
TcG_01094	483,6745686	-0,52333173	0,083987442	-6,23107119	4,63257E-10	2,01022E-08	protein_codin	hypothetical protein
TcG_01095	396,2683389	-0,281737238	0,094911603	-2,96841724	0,002993377	0,019003433	protein_codin	putative fibrillarin
TcG_01096	748,3940211	-0,199457986	0,074525632	-2,6763676	0,007442497	0,039141519	protein_codin	hypothetical protein
TcG_01097	195,0782898	-0,368229086	0,138345584	-2,66166129	0,007775608	0,040561995	protein_codin	syntaxin 7
TcG_01098	275,0235984	-0,571120225	0,10969954	-5,20622262	1,92723E-07	4,74075E-06	protein_codin	putative protein kinase
TcG_01099	603,0416714	-0,586855639	0,075974448	-7,72438176	1,12398E-14	1,14231E-12	protein_codin	putative protein kinase
TcG_01100	1153,264663	-0,364379318	0,059487083	-6,12535187	9,04834E-10	3,62748E-08	protein_codin	putative aminopeptidase, putative,metallo-peptidase, Clan MG, Family M24
TcG_01101	422,5760594	-0,154587511	0,091312547	-1,69294928	0,090465121	0,250953674	protein_codin	hypothetical protein
TcG_01102	373,1716004	-0,652588187	0,09813083	-6,65018511	2,92725E-11	1,65439E-09	protein_codin	putative protein kinase
TcG_01103	391,8490578	-0,468902381	0,091921885	-5,10109621	3,37692E-07	7,67156E-06	protein_codin	transcription factor
TcG_01104	1896,987977	-0,477767446	0,051061958	-9,35662212	8,23266E-21	1,58973E-18	protein_codin	putative ADP,ATP carrier protein 1, mitochondrial precursor, putative,ADP/ATP translocase 1
TcG_01105	444,6855294	-0,175016078	0,091226338	-1,9184819	0,055049935	0,177119842	protein_codin	putative polyprenyl synthase
TcG_01106	600,6170817	-0,272683257	0,075426368	-3,61522457	0,000300087	0,002806144	protein_codin	hypothetical protein
TcG_01107	162,7260661	-0,500060129	0,148881525	-3,35877894	0,000782877	0,006342943	protein_codin	hypothetical protein
TcG_01108	571,2647831	-0,294994689	0,079675915	-3,70243241	0,000213542	0,002105618	protein_codin	hypothetical protein
TcG_01109	219,3100651	-0,595476922	0,126141602	-4,72070207	2,35032E-06	4,30187E-05	protein_codin	hypothetical protein
TcG_01110	525,3858721	-0,505236657	0,081713965	-6,18299033	6,28986E-10	2,62138E-08	protein_codin	C-terminal kinesin KIFC1
TcG_01111	57,64586566	-0,315244769	0,23537366	-1,3393375	0,180460825	0,391853641		
TcG_01112	421,3302459	-0,1676408	0,09925426	-1,68900357	0,091218746	0,252249621	protein_codin	hypothetical protein
TcG_01113	443,4758736	-0,216578616	0,086162588	-2,51360389	0,011950457	0,056768347	protein_codin	putative sarcoplasmic reticulum glycoprotein, putative,sarcalumenin precursor
TcG_01114	241,7260871	0,103620403	0,119035413	0,870500637	0,384026871	0,614472999	protein_codin	hypothetical protein
TcG_01115	502,6812417	-0,314858093	0,088963008	-3,53920241	0,000401338	0,003590658	protein_codin	RNA-binding protein
TcG_01116	884,5943424	-0,36963478	0,066911171	-5,52426115	3,30875E-08	9,44216E-07	protein_codin	putative RNA-binding protein
TcG_01117	161,0862939	-0,371585625	0,151599534	-2,45110004	0,014242036	0,065349796	protein_codin	membrane protein
TcG_01118	407,0657215	-0,399678709	0,091072785	-4,38856358	1,14102E-05	0,000170126	protein_codin	putative intraflagellar transport protein component
TcG_01119	487,4572102	-0,306925826	0,084177037	-3,64619422	0,000266153	0,00253173	protein_codin	intraflagellar transport protein 80
TcG_01120	165,3153008	-0,609053677	0,139645052	-4,36144115	1,29209E-05	0,000189735	protein_codin	calcyphosin
TcG_01121	230,1879807	-0,21974242	0,118520074	-1,85405234	0,063731673	0,198333377	protein_codin	putative lipoic acid synthetase, mitochondrial precursor
TcG_01122	442,9712431	-0,154634431	0,094815758	-1,63089379	0,102912732	0,274291906	protein_codin	putative protein kinase
TcG_01123	532,3941143	-0,224855574	0,088267588	-2,5474308	0,010851937	0,05266933	protein_codin	hypothetical protein
TcG_01124	110,5351704	-1,29284286	0,170734177	-7,57225577	3,66798E-14	3,45506E-12	protein_codin	mucin-associated surface protein (MASP)
TcG_01125	160,9150114	-0,306569802	0,142916975	-2,1450902	0,031945636	0,119897033	protein_codin	hypothetical protein
TcG_01126	562,915463	-0,20170336	0,082754213	-2,43737875	0,014794175	0,067217768	protein_codin	hypothetical protein
TcG_01127	1093,843398	-0,464765676	0,060306516	-7,70672402	1,29089E-14	1,28933E-12	protein_codin	hypothetical protein
TcG_01128	876,6160545	-0,076709723	0,065793288	-1,16592019	0,243646714	0,471818752	protein_codin	40S ribosomal protein S13
TcG_01129	234,3269824	-0,088380627	0,120113857	-0,73580708	0,461848111	0,679535811	protein_codin	synaptobrevin-type transport protein
TcG_01130	299,4138476	-0,129539809	0,11235553	-1,15294555	0,248932733	0,478376953	protein_codin	hypothetical protein
TcG_01131	170,0629955	-0,084017307	0,138656138	-0,60594004	0,544554568	0,741649138	protein_codin	RNA polymerase III C11 subunit
TcG_01132	326,1379482	-0,145804225	0,10051064	-1,45063472	0,146881602	0,345186661	protein_codin	hypothetical protein
TcG_01133	598,773181	-0,189877339	0,076596521	-2,47892901	0,013177753	0,061464351	protein_codin	hypothetical protein
TcG_01134	667,6836	-0,21171755	0,07407517	-2,85814463	0,004261261	0,025396702	protein_codin	putative nucleosome assembly protein
TcG_01135	394,0932476	-0,254149012	0,094183066	-2,69845763	0,006966161	0,037190019	protein_codin	hypothetical protein
TcG_01136	95,32477783	-0,226410223	0,182670059	-1,239449	0,215179263	0,438870501	protein_codin	coiled-coil domain-containing protein
TcG_01137	161,1718842	-0,20417033	0,142091485	-1,43689349	0,15074826	0,350762794	protein_codin	nuclear cap binding complex subunit CBP30
TcG_01138	191,5681468	-0,298729925	0,126910745	-2,35385841	0,018579687	0,079228108	protein_codin	hypothetical protein
TcG_01139	288,4661519	-0,336405277	0,109351183	-3,0763753	0,002095339	0,014314034	protein_codin	hypothetical protein
TcG_01140	867,7276173	-0,06696028	0,067144036	-0,99726326	0,318636742	0,553576194	protein_codin	hypothetical protein

TcG_01141	227,8011366	0,018595517	0,120020325	0,154936398	0,876871459	0,941029583	protein_codin putative arginase
TcG_01142	559,1908655	-0,658344039	0,078244435	-8,41394075	3,96461E-17	5,404E-15	protein_codin hypothetical protein
TcG_01143	404,8576068	-0,715879598	0,093049486	-7,69353632	1,43123E-14	1,40527E-12	protein_codin putative leucine-rich repeat protein (LRRP)
TcG_01144	609,8940238	-0,203119671	0,078631012	-2,58320051	0,009788842	0,048611369	protein_codin putative methionine aminopeptidase, putative,metallo-peptidase, Clan MG, Family M24
TcG_01145	418,7572666	-0,352545733	0,09336652	-3,77593311	0,00015941	0,001659408	protein_codin spermatogenesis-associated protein 4
TcG_01146	126,6662869	-0,138412951	0,161245423	-0,85839925	0,390672039	0,620554736	protein_codin hypothetical protein
TcG_01147	575,2216383	-0,454821349	0,082113346	-5,53894547	3,04298E-08	8,79202E-07	protein_codin transferase
TcG_01148	858,4544088	-0,483825232	0,066025905	-7,32780912	2,33946E-13	1,92234E-11	protein_codin putative protein kinase, putative,serine/threonine protein kinase
TcG_01149	57,40220348	0,011537601	0,234427542	0,049216065	0,960747109	0,981984718	protein_codin hypothetical protein
TcG_01150	198,1350943	-0,280539943	0,127716848	-2,1965774	0,028050642	0,108730256	protein_codin hypothetical protein
TcG_01151	762,0273637	-0,482585319	0,074690448	-6,46113836	1,03918E-10	5,08016E-09	protein_codin hypothetical protein
TcG_01152	809,3259526	-0,510899644	0,069272384	-7,37522826	1,64064E-13	1,40803E-11	protein_codin histone H3 variant
TcG_01153	445,2371478	-0,186485682	0,088352629	-2,1106976	0,034798312	0,128032152	protein_codin putative extracellular receptor
TcG_01154	1076,082773	-0,238697434	0,06358915	-3,7537447	0,000174212	0,00178306	protein_codin putative RNA helicase
TcG_01155	373,0296635	-0,022223038	0,096606407	-0,23003689	0,818063103	0,910311132	protein_codin putative DNA-directed RNA polymerase, alpha subunit
TcG_01156	301,2619816	-0,412414368	0,107583557	-3,8334331	0,000126367	0,001359415	protein_codin U3 snoRNA-associated protein UTP11
TcG_01157	441,9439478	-0,375804397	0,098960366	-3,79752434	0,000146148	0,001539342	protein_codin putative kinesin
TcG_01158	3388,493187	-0,47795558	0,042174114	-11,3329132	9,01553E-30	4,35225E-27	protein_codin putative kinesin
TcG_01159	1774,610662	-0,112794919	0,054145654	-2,08317586	0,037235197	0,134227439	protein_codin putative malate dehydrogenase
TcG_01160	224,5553372	-0,134761383	0,12281413	-1,09727914	0,272519388	0,505509066	protein_codin S-adenosyl-L-methionine-dependent methyltransferase
TcG_01161	233,0950042	-0,294921337	0,119738737	-2,46304031	0,013776443	0,063616526	protein_codin hypothetical protein
TcG_01162	179,4919873	-0,314226142	0,13086991	-2,40105723	0,01634778	0,072236985	protein_codin hypothetical protein
TcG_01163	252,5646703	-0,629280336	0,119422687	-5,26935333	1,36905E-07	3,47086E-06	protein_codin hypothetical protein
TcG_01164	188,4769478	-0,422972876	0,129588543	-3,26396815	0,001098635	0,008432469	protein_codin hypothetical protein
TcG_01165	249,2904191	-0,224769637	0,117085827	-1,91969978	0,054895833	0,17679942	protein_codin ESCRT-II complex subunit VPS36
TcG_01166	209,6289259	-0,027417433	0,130579291	-0,20996769	0,833692888	0,919248114	protein_codin hypothetical protein
TcG_01167	434,6273439	-0,192093825	0,087661827	-2,19130529	0,028429708	0,109868777	protein_codin putative transporter
TcG_01168	175,285427	-0,114237931	0,133395358	-0,85638611	0,391784248	0,621398293	protein_codin putative transporter
TcG_01169	261,547647	0,578071572	0,114097959	5,066449729	4,05304E-07	8,97868E-06	protein_codin hypothetical protein
TcG_01170	27,52628479	0,654750465	0,332577072	1,96871799	0,048985484	0,163181662	protein_codin hypothetical protein
TcG_01171	727,622286	-0,128756006	0,070542191	-1,82523401	0,067965723	0,20781967	protein_codin putative cyclophilin
TcG_01172	86,89725654	-0,156611674	0,192141908	-0,81508337	0,415024589	0,641985967	protein_codin p53 and DNA damage-regulated protein 1
TcG_01173	153,2509122	-0,058652482	0,150536156	-0,38962388	0,696814688	0,842987885	protein_codin hypothetical protein
TcG_01174	161,7989498	-0,435519162	0,142264672	-3,06133039	0,002203558	0,014863688	protein_codin hypothetical protein
TcG_01175	198,7985087	-0,156217902	0,129545337	-1,20589368	0,227858489	0,4546966	protein_codin UDP-GlcNAc:PI a1-6 GlcNAc-transferase
TcG_01176	56,75447041	0,245720463	0,238268576	1,031275158	0,302411804	0,535372803	protein_codin hypothetical protein
TcG_01177	794,3246001	-0,014994469	0,075263381	-0,19922662	0,84208548	0,923105402	protein_codin DNA polymerase delta catalytic subunit
TcG_01178	1189,875223	-0,032507682	0,058725569	-0,55355244	0,579885181	0,769328949	protein_codin putative DNA-dependent ATPase
TcG_01179	505,1719365	-0,455476199	0,088297319	-5,15843746	2,49019E-07	5,87604E-06	protein_codin putative protein kinase
TcG_01180	284,0681678	-0,03953736	0,108032733	-0,36597575	0,714383171	0,853492027	protein_codin TPR Domain containing protein
TcG_01181	563,9275687	-0,208272896	0,082422452	-2,52689517	0,011507585	0,055193573	protein_codin hypothetical protein
TcG_01182	306,0817574	-0,121744513	0,111481653	-1,09205873	0,274807288	0,507556451	protein_codin autophagocytosis associated protein
TcG_01183	1843,808561	0,126732063	0,05036105	2,516469803	0,011853706	0,056458121	protein_codin putative peptidase M20/M25/M40
TcG_01184	768,7550783	0,115539636	0,070171262	1,646537809	0,099653083	0,268020344	protein_codin hypothetical protein
TcG_01185	1081,185435	-0,312922749	0,059222894	-5,28381386	1,26522E-07	3,22172E-06	protein_codin putative short-chain dehydrogenase
TcG_01186	197,4460353	-0,327348481	0,13475781	-2,42916149	0,01513379	0,068160911	protein_codin hypothetical protein
TcG_01187	206,8054975	-0,182617622	0,1236067	-1,47740876	0,139566099	0,334093557	protein_codin hypothetical protein
TcG_01188	304,4382602	0,102315751	0,104953375	0,974868616	0,329625435	0,56434119	protein_codin putative ribulose-5-phosphate 3-epimerase
TcG_01189	191,5283414	0,442599016	0,128865853	3,434571734	0,000593491	0,005000863	protein_codin hypothetical protein
TcG_01190	295,582635	-0,20531864	0,106097699	-1,93518467	0,052967638	0,17194818	protein_codin RAB-interacting protein
TcG_01191	1870,664138	-0,245771544	0,050566388	-4,86037369	1,17164E-06	2,32842E-05	protein_codin hypothetical protein
TcG_01192	248,6088359	0,00199215	0,111969383	0,017791921	0,98580485	0,994387514	protein_codin putative RAB-interacting protein
TcG_01193	195,5553713	-0,129349789	0,135930966	-0,95158442	0,341307788	0,575686712	protein_codin hypothetical protein
TcG_01194	240,6644126	0,226252229	0,117285929	1,929065414	0,053722742	0,173912179	protein_codin hypothetical protein
TcG_01195	483,1394493	-0,063868228	0,097721338	-0,65357504	0,513385627	0,719497505	protein_codin transmembrane protein NRF-6
TcG_01196	690,2551336	0,015916151	0,075851302	0,209833595	0,833797552	0,919248114	protein_codin hypothetical protein
TcG_01197	1428,215131	-0,090633215	0,060646633	-1,4944476	0,135058669	0,327019799	protein_codin hypothetical protein

TcG_01198	269,5571983	-0,17349424	0,118265178	-1,46699344	0,142377839	0,338328548	protein_codin hypothetical protein
TcG_01199	23,88757249	-0,181566942	0,354234748	-0,51256107	0,60825839	0,788022108	protein_codin RNA-binding protein
TcG_01200	43,86118275	0,032622067	0,268922055	0,121306775	0,903448054	0,953224692	protein_codin RNA-binding protein
TcG_01201	4,553959778	0,357144171	0,847409073	0,421454269	0,673423398	1	
TcG_01202	342,9525477	-0,074818735	0,097137721	-0,77023359	0,441161344	0,663546065	protein_codin RNA-binding protein
TcG_01203	150,1224793	0,38780628	0,144184831	2,689646875	0,007152766	0,037927666	protein_codin hypothetical protein
TcG_01204	970,3888988	-0,053386789	0,06303119	-0,84699002	0,397000682	0,626692779	protein_codin hypothetical protein
TcG_01205	445,3310973	-0,085846731	0,093774972	-0,91545461	0,359953021	0,592630203	protein_codin putative amino acid permease-like protein
TcG_01206	334,4307179	0,08384739	0,102497593	0,818042525	0,413332905	0,640054093	protein_codin putative protein kinase
TcG_01207	352,0023591	-0,09911296	0,100539786	-0,98580835	0,324227169	0,558420691	protein_codin hypothetical protein
TcG_01208	574,2073735	-0,319398488	0,083037823	-3,84642175	0,000119855	0,001301386	protein_codin putative mitogen activated protein kinase
TcG_01209	434,1531551	-0,120542653	0,087693738	-1,37458678	0,169259591	0,375894502	protein_codin hypothetical protein
TcG_01210	202,8878809	0,088899722	0,127839787	0,695399483	0,486804985	0,698553697	protein_codin putative DNA repair protein
TcG_01211	223,6946656	0,329146389	0,122485247	2,687232917	0,007204669	0,038170078	protein_codin hypothetical protein
TcG_01212	264,4405743	-0,085337703	0,109873073	-0,77669351	0,437339614	0,660714144	protein_codin hypothetical protein
TcG_01213	389,5574155	0,032659147	0,091296183	0,35772741	0,720547319	0,85670516	protein_codin RNA-binding protein
TcG_01214	342,2062523	-0,129708695	0,098461865	-1,31734957	0,187721476	0,402096695	protein_codin putative vesicle-associated membrane protein, putative,syntaxin-like protein
TcG_01215	261,6223488	-0,151179354	0,125282739	-1,20670537	0,227545632	0,454445346	protein_codin hypothetical protein
TcG_01216	357,5874094	0,157074482	0,09748601	1,611251521	0,107124913	0,282082846	protein_codin hypothetical protein
TcG_01217	288,1510902	-0,004133784	0,107220236	-0,03855414	0,969245868	0,986756701	protein_codin hypothetical protein
TcG_01218	232,2186231	0,128119247	0,119873065	1,06879095	0,285163878	0,518774269	protein_codin MFS transporter, PPP family, 3-phenylpropionic acid transporter
TcG_01219	207,9316172	0,033187475	0,125503473	0,264434711	0,791444968	0,89576419	protein_codin hypothetical protein
TcG_01220	590,078334	0,101241216	0,076952001	1,315641118	0,188294526	0,402654186	protein_codin putative ABC transporter
TcG_01221	350,7671331	-0,175936593	0,097183406	-1,81035632	0,070240548	0,212315938	protein_codin putative NAD(P)-dependent oxidoreductase
TcG_01222	103,361738	-0,086811762	0,179563992	-0,48345863	0,628770126	0,801070011	protein_codin hypothetical protein
TcG_01223	214,3127614	-0,087744307	0,123379019	-0,71117689	0,476974626	0,692136011	protein_codin hypothetical protein
TcG_01224	229,1256787	0,07993469	0,119248874	0,670318196	0,50265497	0,711255555	protein_codin hypothetical protein
TcG_01225	275,2054734	-0,065568254	0,108096993	-0,60656872	0,544137167	0,741254934	protein_codin hypothetical protein
TcG_01226	628,0509545	-0,121940282	0,078871707	-1,54605861	0,122090414	0,306775003	protein_codin hypothetical protein
TcG_01227	394,3339704	0,08220694	0,092697945	0,886825923	0,375172629	0,605734403	protein_codin putative amidinotransferase
TcG_01228	131,4354162	0,18205605	0,165032553	1,103152355	0,269961008	0,502775797	protein_codin hypothetical protein
TcG_01229	202,5505805	-0,097470079	0,228076625	-0,42735672	0,669119525	0,826307698	protein_codin hypothetical protein
TcG_01230	160,8827365	-0,067266234	0,140537633	-0,47863503	0,632198291	0,802906018	protein_codin hypothetical protein
TcG_01231	55,78582746	0,175304861	0,241250847	0,726649722	0,467440561	0,684067997	protein_codin hypothetical protein
TcG_01232	664,7936723	0,048969605	0,073618909	0,665177006	0,505937286	0,712679562	protein_codin hypothetical protein
TcG_01233	375,4139412	0,026343418	0,093558511	0,281571588	0,778272029	0,889256383	protein_codin hypothetical protein
TcG_01234	335,3143148	-0,006803916	0,103886789	-0,06549357	0,947781029	0,975394476	protein_codin hypothetical protein
TcG_01235	283,9965044	-0,005712201	0,109755401	-0,05204483	0,958492972	0,980755946	protein_codin putative 33 kDa inner dynein arm light chain, axonemal
TcG_01236	815,2696185	-0,313700147	0,069346635	-4,52365348	6,07812E-06	9,93718E-05	protein_codin hypothetical protein
TcG_01237	346,0383357	-0,115430051	0,105769008	-1,09134096	0,275122882	0,507634843	protein_codin hypothetical protein
TcG_01238	755,0358862	-0,320071238	0,07639583	-4,18964277	2,79394E-05	0,000371222	protein_codin putative pumilio protein 6
TcG_01239	296,6209366	-0,075790987	0,105463976	-0,71864337	0,472360682	0,688368479	
TcG_01240	270,3553279	0,461724742	0,123457612	3,739945497	0,00018406	0,001865506	
TcG_01241	1027,615494	0,13616798	0,063490314	2,144704767	0,03197646	0,11991016	protein_codin putative trans-sialidase
TcG_01242	337,1593657	0,275499561	0,110313846	2,497415977	0,012510211	0,058944003	protein_codin putative trans-sialidase
TcG_01243	284,2734317	0,041541902	0,107048423	0,388066454	0,697966856	0,843887456	protein_codin hypothetical protein
TcG_01244	224,9169119	0,173043536	0,127714899	1,354920505	0,175442886	0,384758901	protein_codin hypothetical protein
TcG_01245	169,2011116	0,177929033	0,137729387	1,291874134	0,196400737	0,41463173	protein_codin hypothetical protein
TcG_01246	271,0965199	0,186306089	0,122371015	1,522469105	0,127891581	0,316681311	protein_codin hypothetical protein
TcG_01247	138,0721765	0,310429013	0,161886732	1,917569209	0,055165658	0,177393647	protein_codin hypothetical protein
TcG_01248	198,4892607	0,25411364	0,136919733	1,855931461	0,063463328	0,197817088	protein_codin trans-sialidase
TcG_01249	230,4322566	0,166427167	0,129342537	1,286716426	0,198193146	0,4168208	protein_codin amino acid transporter
TcG_01250	301,4229769	-0,141092085	0,106692934	-1,32241264	0,186030781	0,399879894	protein_codin putative N-acetyltransferase subunit Nat1
TcG_01251	973,3324905	-0,069501995	0,061988702	-1,12120423	0,26220094	0,494282475	protein_codin putative N-acetyltransferase subunit Nat1
TcG_01252	422,8991115	0,041624297	0,090898591	0,457920155	0,647009806	0,812420258	protein_codin heat shock protein
TcG_01253	395,4681955	0,065432918	0,091072053	0,718474176	0,472464965	0,688368479	protein_codin hypothetical protein
TcG_01254	352,5612918	-0,117043958	0,100471784	-1,16494356	0,244041839	0,47218917	protein_codin calmodulin-like protein containing EF hand domain

TcG_01255	415,8541685	-0,082290039	0,089724043	-0,91714591	0,359066186	0,592630203	protein_codin	hypothetical protein
TcG_01256	208,3626016	-0,232791493	0,130774758	-1,780095	0,075060414	0,221172421	protein_codin	hypothetical protein
TcG_01257	2421,204616	-0,104654257	0,045344416	-2,30798554	0,020999941	0,08755139	protein_codin	fructose-bisphosphate aldolase (ald)
TcG_01258	2557,729853	0,128090024	0,046728264	2,741168044	0,006122119	0,033552918	protein_codin	small subunit ribosomal protein S9e
TcG_01259	282,7893144	0,166165718	0,108031905	1,538117069	0,124019993	0,310112782	protein_codin	hypothetical protein
TcG_01260	381,0625419	0,003567848	0,095260928	0,037453423	0,970123477	0,987041075	protein_codin	putative protein phosphatase 2C
TcG_01261	295,6582803	0,156217637	0,109978668	1,4204358	0,155480847	0,356891537	protein_codin	hypothetical protein
TcG_01262	205,2311444	0,018482382	0,126277322	0,14636343	0,883634495	0,94348809	protein_codin	hypothetical protein
TcG_01263	673,4262252	-0,145401824	0,073816014	-1,96978699	0,048862788	0,162913457	protein_codin	GPI alpha-mannosyltransferase III
TcG_01264	403,1816197	-0,034018817	0,092963216	-0,36593847	0,714410986	0,853492027	protein_codin	hypothetical protein
TcG_01265	402,1314284	-0,267049436	0,095081537	-2,8086361	0,004975185	0,028677857	protein_codin	hypothetical protein
TcG_01266	46,3740635	0,316454238	0,256243244	1,234975927	0,216839464	0,440893872		
TcG_01267	965,3378087	0,334832983	0,063144039	5,302685585	1,14111E-07	2,9121E-06	protein_codin	hypothetical protein
TcG_01268	453,6507961	-0,077751131	0,087411456	-0,88948445	0,373742779	0,604496256	protein_codin	protein MEMO1
TcG_01269	295,2959651	-0,086860149	0,106975463	-0,81196329	0,416812695	0,643630898	protein_codin	putative endonuclease/exonuclease/phosphatase
TcG_01270	379,8184522	-0,095804329	0,096284436	-0,99501366	0,319729623	0,554798174	protein_codin	hypothetical protein
TcG_01271	412,9360557	0,408846373	0,089859459	4,549842366	5,36861E-06	8,91128E-05	protein_codin	HORMA domain containing protein
TcG_01272	112,9820374	0,18301658	0,171343073	1,068129435	0,28546213	0,51904649	protein_codin	ribosomal protein L24
TcG_01273	199,5841836	0,080565407	0,130042926	0,619529329	0,535567707	0,735555719	protein_codin	hypothetical protein
TcG_01274	0,300145286	-1,420535481	3,227725303	-0,44010421	0,659861636	1		
TcG_01275	91,1344933	0,536157873	0,193317702	2,773454616	0,005546458	0,031029096	protein_codin	ribosomal protein L24
TcG_01276	950,0894148	-0,002027962	0,064068869	-0,03165284	0,974748905	0,988974186	protein_codin	putative nucleoplasmin-like protein (NLP)
TcG_01277	469,1922628	-0,147473222	0,084788724	-1,73930228	0,081981606	0,234961703	protein_codin	putative RNA editing complex protein MP61
TcG_01278	295,3074561	-0,196590949	0,105993714	-1,85474159	0,063633136	0,198133167	protein_codin	flagellar associated protein
TcG_01279	686,4419559	0,081118552	0,074522966	1,088504075	0,276372639	0,508827807	protein_codin	hypothetical protein
TcG_01280	515,2600327	-0,295347421	0,083977074	-3,51700063	0,000436453	0,003857164	protein_codin	putative 3-transmembrane protein A13
TcG_01281	601,9597947	0,067958422	0,076234355	0,891440897	0,372692686	0,60390427	protein_codin	hypothetical protein
TcG_01282	641,5830918	-0,242143575	0,078151643	-3,09838113	0,00194581	0,013451167	protein_codin	intraflagellar transport 122-like protein
TcG_01283	134,8367807	0,280016503	0,152915248	1,831187585	0,067072549	0,206018705	protein_codin	40S ribosomal protein S10
TcG_01284	351,3168237	0,218395885	0,099670923	2,191169484	0,02843953	0,109869556	protein_codin	40S ribosomal protein S10
TcG_01285	60,8578588	0,293393546	0,234173595	1,252889105	0,210246067	0,432895627	protein_codin	40S ribosomal protein S10
TcG_01286	208,8566834	-0,013122914	0,123896725	-0,10591817	0,915647278	0,958934228	protein_codin	hypothetical protein
TcG_01287	318,9452104	-0,03596168	0,106641169	-0,33722136	0,735950039	0,864944729	protein_codin	hypothetical protein
TcG_01288	2156,613681	-0,184444627	0,102018545	-1,80795194	0,070613981	0,213166646	protein_codin	putative dynein heavy chain
TcG_01289	241,2014629	0,368451265	0,117771489	3,128526856	0,00175685	0,012403936	protein_codin	40S ribosomal protein S18
TcG_01290	295,205036	0,02142017	0,11412427	0,187691631	0,851118391	0,928053891	protein_codin	40S ribosomal protein S18
TcG_01291	394,7245668	0,131013856	0,091665706	1,429257038	0,152930369	0,35331032	protein_codin	MP67 protein
TcG_01292	292,4401583	-0,227197408	0,103961421	-2,18540115	0,028859447	0,111048008	protein_codin	cyclic nucleotide-binding protein
TcG_01293	230,1459297	-0,152453727	0,115865823	-1,31577822	0,18824849	0,402654186	protein_codin	hypothetical protein
TcG_01294	278,6864428	-0,143689509	0,108033956	-1,33004025	0,183505011	0,395919751	protein_codin	1,2-Dihydroxy-3-keto-5-methylthiopentene dioxygenase
TcG_01295	150,4992597	0,077459358	0,151914254	0,50988868	0,610129453	0,789016404	protein_codin	hypothetical protein
TcG_01296	157,762352	-0,247887556	0,158875558	-1,56026238	0,118697891	0,300690664	protein_codin	hypothetical protein
TcG_01297	67,19062458	0,156180458	0,216225749	0,722302769	0,470108351	0,686450317	protein_codin	hypothetical protein
TcG_01298	528,684321	-0,112209341	0,081181591	-1,38220179	0,166909751	0,372910035	protein_codin	putative RNA-binding protein
TcG_01299	222,2038266	-0,213100701	0,121661217	-1,75159107	0,079844149	0,230806964	protein_codin	putative cell differentiation protein
TcG_01300	869,7207831	-0,044076462	0,075538632	-0,58349563	0,559559696	0,754014719	protein_codin	phosphatidylinositol 3-kinase tor2
TcG_01301	300,7090575	-0,019598416	0,10865008	-0,18038106	0,856853426	0,930840697	protein_codin	putative proteasome beta 7 subunit
TcG_01302	245,2704369	0,219861492	0,120471683	1,825005568	0,068000189	0,207821206	protein_codin	putative quinonoid dihydropteridine reductase
TcG_01303	175,6301974	0,15446065	0,136032689	1,135467149	0,256179639	0,488093618	protein_codin	hypothetical protein
TcG_01304	1065,708009	-0,025217647	0,065237271	-0,38655276	0,699087335	0,844142481	protein_codin	coatomer protein
TcG_01305	280,7150829	0,153681709	0,108074429	1,421998805	0,155026598	0,35638871	protein_codin	alkylated DNA repair protein
TcG_01306	111,9802151	0,226549255	0,166476201	1,360850702	0,173560875	0,38215057	protein_codin	hypothetical protein
TcG_01307	195,9600536	0,034695043	0,126780059	0,27366325	0,784343422	0,893286433	protein_codin	snoRNP protein GAR1
TcG_01308	0,467697622	2,091063019	2,518049959	0,83042952	0,406295981	1	protein_codin	D-alanyl-glycyl endopeptidase-like protein
TcG_01309	141,2503519	-0,132223094	0,146976145	-0,89962282	0,368321011	0,600361175	protein_codin	DCN1-like protein 2
TcG_01310	709,1163748	-0,054992222	0,073318415	-0,75004652	0,453226687	0,673820659	protein_codin	putative Unc104-like kinesin
TcG_01311	279,0360104	-0,041360524	0,113458679	-0,36454262	0,715452851	0,853960462	protein_codin	hypothetical protein

TcG_01312	173,3669324	0,225877281	0,136531219	1,654400231	0,09804622	0,265140679	protein_codin	hypothetical protein
TcG_01313	130,0374457	0,254684314	0,153668377	1,657363209	0,097446065	0,264100721	protein_codin	hypothetical protein
TcG_01314	202,9069152	0,348064975	0,125170767	2,780720949	0,005423834	0,030506564	protein_codin	hypothetical protein
TcG_01315	278,8270912	0,255290817	0,108941028	2,343385427	0,019109631	0,081041061	protein_codin	hypothetical protein
TcG_01316	206,0685224	0,303719103	0,128444096	2,364601508	0,01804948	0,077642551	protein_codin	putative serine/threonine protein phosphatase
TcG_01317	489,4327696	0,243861366	0,087013446	2,802571056	0,005069705	0,029006222	protein_codin	hypothetical protein
TcG_01318	249,2507175	0,303069612	0,119267077	2,541100351	0,01105042	0,053390394	protein_codin	hypothetical protein
TcG_01319	467,9027129	0,174361459	0,084782748	2,05656767	0,039727831	0,140502639	protein_codin	cytoplasmic dynein 2 heavy chain 1 isoform X1
TcG_01320	894,5769921	0,279178469	0,153221341	1,822059953	0,068445889	0,208742846	protein_codin	cytoplasmic dynein 2 heavy chain 1 isoform X1
TcG_01321	189,7180835	0,222223163	0,130362189	1,704659645	0,08825792	0,246875003	protein_codin	hypothetical protein
TcG_01322	198,255202	0,006574213	0,125049789	0,052572762	0,95807232	0,98064539	protein_codin	hypothetical protein
TcG_01323	918,0943976	-0,0783659	0,064055903	-1,22339857	0,221179194	0,446371802	protein_codin	hypothetical protein
TcG_01324	176,9675631	0,125710946	0,137532812	0,914043306	0,360694091	0,593355351	protein_codin	hypothetical protein
TcG_01325	463,7048272	0,012988555	0,085458794	0,151986169	0,879197845	0,941527519	protein_codin	hypothetical protein
TcG_01326	937,7053408	-0,034410911	0,07322645	-0,46992461	0,63840888	0,807731988	protein_codin	hypothetical protein
TcG_01327	132,9942146	-0,086229142	0,152157814	-0,5667086	0,570912175	0,762440697	protein_codin	hypothetical protein
TcG_01328	179,5400848	-0,15500001	0,14311875	-1,08301679	0,278800969	0,511753489	protein_codin	hypothetical protein
TcG_01329	218,1546774	0,101592418	0,122922755	0,82647365	0,408535459	0,636605346	protein_codin	chaperone protein DNAJ
TcG_01330	392,1794756	-0,249484591	0,0923438	-2,70169291	0,006898745	0,036905562	protein_codin	putative chaperone protein DNAj
TcG_01331	16,80514785	0,356313241	0,426726054	0,834992937	0,403721685	0,63312391		
TcG_01332	467,9305823	-0,133346719	0,088829298	-1,50115696	0,133314968	0,324453521	protein_codin	ubiquitin hydrolase
TcG_01333	264,2507808	0,024762042	0,109351374	0,226444722	0,820855548	0,911236551	protein_codin	hypothetical protein
TcG_01334	180,7812267	0,136687941	0,134211184	1,018454175	0,308462168	0,541409283	protein_codin	hypothetical protein
TcG_01335	167,4440129	-0,170212716	0,13810059	-1,23252707	0,217752262	0,441956607	protein_codin	putative geranylgeranyl transferase type II beta subunit
TcG_01336	174,1144949	-0,275168149	0,136142715	-2,02117425	0,04326173	0,149353517	protein_codin	hypothetical protein
TcG_01337	473,5131304	0,255417324	0,083987793	3,041124359	0,002356965	0,015694133	protein_codin	dihydroflavonol-4-reductase
TcG_01338	364,2505502	-0,085630662	0,095777416	-0,89405902	0,371290314	0,60280376	protein_codin	hypothetical protein
TcG_01339	302,4008427	0,072943914	0,104722148	0,696547155	0,486086237	0,698274608	protein_codin	hypothetical protein
TcG_01340	194,7280745	0,113843709	0,129939099	0,87613128	0,38095867	0,611048137	protein_codin	hypothetical protein
TcG_01341	364,9866531	0,14222807	0,097309869	1,461599638	0,143850954	0,340481542	protein_codin	50S ribosomal protein L7Ae
TcG_01342	519,4003644	-0,128069221	0,090904246	-1,40883652	0,158883514	0,361229278	protein_codin	putative adaptor gamma-1 chain
TcG_01343	205,2580717	0,154568824	0,129454998	1,193996569	0,23247931	0,459642541	protein_codin	putative ubiquitin-protein ligase
TcG_01344	689,2146368	0,081461094	0,077711867	1,048245236	0,294525634	0,527496367	protein_codin	putative ubiquitin-protein ligase
TcG_01345	158,9200617	0,05275222	0,141031905	0,374044584	0,708371146	0,849437494	protein_codin	hypothetical protein
TcG_01346	1187,610949	0,005731663	0,074917381	0,07650645	0,939016182	0,971680773	protein_codin	phosphatidylinositol 3-kinase-like protein
TcG_01347	345,0356707	0,504411509	0,100343659	5,026839909	4,98628E-07	1,08797E-05	protein_codin	ESAG-like protein
TcG_01348	220,5554552	0,043528095	0,122573597	0,355118033	0,722501168	0,857335574	protein_codin	hypothetical protein
TcG_01349	139,6563727	-0,255513411	0,148224714	-1,72382462	0,084739509	0,239929606	protein_codin	hypothetical protein
TcG_01350	348,4431807	-0,105769755	0,099358579	-1,06452565	0,287090643	0,52029285	protein_codin	transferase
TcG_01351	166,6026137	-0,491115003	0,140248025	-3,50176057	0,000462195	0,004040885	protein_codin	hypothetical protein
TcG_01352	148,7831549	0,094296199	0,142802373	0,660326554	0,509044294	0,715750872	protein_codin	hypothetical protein
TcG_01353	1591,872223	-0,177806456	0,063647809	-2,79359903	0,005212507	0,029618491	protein_codin	dynein-1-alpha heavy chain, flagellar inner arm I1 complex protein
TcG_01354	371,3450423	0,139758998	0,09611287	1,454113255	0,145914909	0,343402425	protein_codin	putative TPR-repeat-containing chaperone protein DNAJ,putative
TcG_01355	146,7806843	-0,058679811	0,145644277	-0,40289816	0,687023144	0,83752632	protein_codin	small nuclear ribonucleoprotein SmD3
TcG_01356	182,0657957	0,288492749	0,141449047	2,039552447	0,041394922	0,144763529	protein_codin	Aquaporin 2
TcG_01357	129,1013343	-0,054773201	0,162752658	-0,33654259	0,736461741	0,865306361	protein_codin	hypothetical protein
TcG_01358	202,5655707	0,112916996	0,13109295	0,861350642	0,389044952	0,619284946	protein_codin	hypothetical protein
TcG_01359	261,9244845	-0,268987262	0,117350854	-2,29216281	0,02189625	0,090198414	protein_codin	TPR protein
TcG_01360	135,7670888	0,362044092	0,15165602	2,387271489	0,016973951	0,074269099	protein_codin	ribosomal protein L14
TcG_01361	110,1621189	-0,082013436	0,169016717	-0,4852386	0,627507102	0,800252866	protein_codin	hypothetical protein
TcG_01362	164,6364441	0,133420889	0,139259829	0,958071612	0,338026649	0,572681532	protein_codin	hypothetical protein
TcG_01363	435,4813865	0,263145545	0,088431063	2,975713937	0,002923074	0,018641502	protein_codin	hypothetical protein
TcG_01364	402,4983759	0,351206149	0,092559807	3,794369916	0,000148019	0,001554802	protein_codin	putative phosphomannomutase-like protein
TcG_01365	109,1991449	0,205788449	0,173713235	1,184644618	0,236157988	0,463600927	protein_codin	hypothetical protein
TcG_01366	123,1760587	0,148804007	0,164997297	0,901857243	0,367132707	0,599390612	protein_codin	putative actin
TcG_01367	345,1787685	-0,242243644	0,103299444	-2,34506241	0,019023895	0,080736574	protein_codin	hypothetical protein
TcG_01368	119,0967518	0,144495051	0,162882136	0,88711417	0,375017436	0,605652636	protein_codin	hypothetical protein

TcG_01369	476,049188	0,21774801	0,08828586	2,466397342	0,013647984	0,063250219	protein_codin putative serine-palmitoyl-CoA transferase
TcG_01370	185,8649863	0,116963942	0,131469084	0,889668792	0,373643756	0,604496256	protein_codin hypothetical protein
TcG_01371	131,2171445	0,272056223	0,159644595	1,70413676	0,08835554	0,247049662	protein_codin hypothetical protein
TcG_01372	111,9975438	-0,24326103	0,171547843	-1,41803608	0,156180232	0,357690793	protein_codin hypothetical protein
TcG_01373	151,6910636	0,27657431	0,143255943	1,930630619	0,053528749	0,173429553	protein_codin inositol-pentakisphosphate 2-kinase
TcG_01374	414,4846583	0,339257513	0,098810959	3,433399649	0,000596063	0,005018883	protein_codin hypothetical protein
TcG_01375	121,5315365	0,161313817	0,164163762	0,98263962	0,325784837	0,560274604	protein_codin ribosomal protein L13
TcG_01376	557,3028604	0,027623761	0,079595375	0,347052339	0,728551999	0,860185821	protein_codin vacuolar H+-ATPase
TcG_01377	382,5365593	-0,472420579	0,098417783	-4,80015468	1,58543E-06	3,03948E-05	protein_codin endosomal integral membrane protein
TcG_01378	17,23875482	-0,529481555	0,413382017	-1,2808529	0,20024534	0,419331186	protein_codin endosomal integral membrane protein
TcG_01379	844,5236009	-0,265961006	0,067020184	-3,9683718	7,23654E-05	0,000848608	protein_codin endomembrane protein
TcG_01380	4689,435406	0,587023589	0,043841184	13,38977502	6,93906E-41	7,30872E-38	protein_codin ribosomal protein L21E (60S)
TcG_01381	2961,373568	-0,074261318	0,043130834	-1,72176865	0,085111436	0,240585596	protein_codin beta-fructofuranosidase-like protein
TcG_01382	68,11160931	0,179057166	0,215285081	0,831721201	0,405566331	0,634470903	protein_codin endomembrane protein
TcG_01383	284,047078	0,114198284	0,106963032	1,067642546	0,285681784	0,519127451	protein_codin beta-fructofuranosidase-like protein
TcG_01384	468,0791802	-0,056986104	0,087356854	-0,65233695	0,514183831	0,720093541	protein_codin hypothetical protein
TcG_01385	243,1542608	0,050715288	0,117707913	0,430857079	0,666572294	0,825438095	protein_codin patatin family phospholipase
TcG_01386	150,2770156	-0,026196762	0,144411683	-0,18140334	0,856050997	0,930268068	protein_codin LYR motif containing protein 1
TcG_01387	135,8262299	-0,002764078	0,163062891	-0,01695099	0,986475714	0,994631244	protein_codin putative adenosine kinase
TcG_01388	321,0446156	-0,170565148	0,102753019	-1,65995268	0,096923973	0,263410017	protein_codin hypothetical protein
TcG_01389	206,3726066	-0,213276659	0,125680541	-1,69697438	0,089701505	0,249607897	protein_codin putative phosphatidylinositol 4-kinase
TcG_01390	210,5017949	-0,144879701	0,122330403	-1,18433111	0,236282019	0,463600927	protein_codin hypothetical protein
TcG_01391	247,7102526	0,069271341	0,116023919	0,597043625	0,550478255	0,746546058	protein_codin hypothetical protein
TcG_01392	122,8847438	-0,055544692	0,171093982	-0,32464434	0,745450267	0,871346529	protein_codin hypothetical protein
TcG_01393	297,2945405	-0,051797303	0,106417311	-0,48673756	0,626444322	0,799398063	protein_codin putative ankyrin
TcG_01394	116,0075673	-0,076181934	0,164472289	-0,46319009	0,643228122	0,810687402	protein_codin pre-rRNA-processing protein TSR4
TcG_01395	204,7117745	0,350137633	0,130651681	2,679932097	0,00736371	0,038849178	protein_codin hypothetical protein
TcG_01396	387,7617796	0,13647737	0,095248338	1,432858289	0,15189834	0,35209328	protein_codin hypothetical protein
TcG_01397	141,5397532	-0,098367251	0,154258099	-0,63767965	0,523682234	0,727678384	protein_codin hypothetical protein
TcG_01398	0					1	
TcG_01399	0					1	
TcG_01400	0					1	
TcG_01401	2,021113092	0,529151953	1,208953793	0,437694109	0,661608041	1	protein_codin hypothetical protein
TcG_01402	0					1	
TcG_01403	453,5967159	0,558878797	0,09224641	6,058542507	1,3736E-09	5,3226E-08	protein_codin hypothetical protein
TcG_01404	1199,446017	0,200534627	0,059499795	3,370341493	0,000750751	0,006125492	protein_codin putative mitochondrial RNA binding complex 1 subunit
TcG_01405	813,4406208	0,237941029	0,067998292	3,499220668	0,00046662	0,00407098	protein_codin putative mitochondrial RNA binding protein
TcG_01406	951,9071845	0,70292774	0,067637567	10,39256393	2,68049E-25	7,96311E-23	protein_codin putative FtsJ cell division protein
TcG_01407	872,0777588	0,26989323	0,066399803	4,064669137	4,81006E-05	0,000596675	protein_codin transferase
TcG_01408	636,2912319	0,229188943	0,080303258	2,854042899	0,00431667	0,025645176	protein_codin hypothetical protein
TcG_01409	259,5838458	0,573412505	0,113568693	5,049036743	4,44043E-07	9,74372E-06	protein_codin c2 domain protein
TcG_01410	10,1258996	0,922886997	0,566950479	1,627808831	0,103565428	1	protein_codin hypothetical protein
TcG_01411	88,10770259	0,995798392	0,195349492	5,097522309	3,44128E-07	7,80248E-06	protein_codin hypothetical protein
TcG_01412	605,3422458	0,150897485	0,078797493	1,91500362	0,055492042	0,178108821	protein_codin putative ATP-dependent zinc metallopeptidase
TcG_01413	492,0633511	0,239135529	0,086453113	2,766071916	0,005673602	0,031602524	protein_codin putative small GTP-binding rab protein
TcG_01414	201,7853323	0,371938749	0,226648632	1,641036813	0,100789774	0,270187487	protein_codin hypothetical protein
TcG_01415	386,5147732	0,107313389	0,094686816	1,133350911	0,25706692	0,489028976	protein_codin hypothetical protein
TcG_01416	685,2439143	0,138819824	0,08496302	1,633885229	0,102282952	0,273241937	protein_codin putative RNA-binding protein
TcG_01417	42,64649042	-0,021861343	0,290865982	-0,0751595	0,940087804	0,97222684	
TcG_01418	219,9683656	0,087503346	0,124646206	0,702013716	0,48267061	0,696286283	protein_codin hypothetical protein
TcG_01419	343,8953255	0,294229742	0,098815907	2,977554427	0,002905581	0,01856815	protein_codin hypothetical protein
TcG_01420	621,0979501	0,01667083	0,075830651	0,219842902	0,825993506	0,915112889	protein_codin hypothetical protein
TcG_01421	120,0199056	0,349166529	0,161157725	2,166613669	0,030264324	0,115180241	protein_codin divalent cation tolerance protein
TcG_01422	180,693099	0,231192906	0,13401582	1,725116532	0,084506474	0,239549816	protein_codin serine/threonine protein kinase
TcG_01423	285,3818932	0,365368051	0,112733898	3,240977724	0,001191205	0,008991075	protein_codin hypothetical protein
TcG_01424	510,0521995	0,087243179	0,090723596	0,961637135	0,336231911	0,57078138	protein_codin endosomal P24B protein
TcG_01425	24,14893906	-0,022153635	0,364766075	-0,06073381	0,951571202	0,977211838	protein_codin trans-sialidase

TcG_01426	648,0309207	0,215189276	0,076414225	2,81608921	0,004861217	0,028175533	protein_codin putative monoglyceride lipase
TcG_01427	898,9819425	0,176983709	0,064434504	2,746722601	0,006019402	0,03309957	protein_codin putative leucine-rich repeat protein (LRRP)
TcG_01428	891,1250665	0,505117337	0,069903692	7,225903553	4,97781E-13	4,03307E-11	protein_codin hypothetical protein
TcG_01429	313,9096673	0,421016022	0,108720848	3,872449757	0,000107747	0,00118891	protein_codin hypothetical protein
TcG_01430	124,6464164	0,082297071	0,160524814	0,512675076	0,608178627	0,788022108	protein_codin receptor-type adenylate cyclase
TcG_01431	64,09504024	0,033052267	0,21954364	0,150549871	0,880330807	0,941957216	protein_codin receptor-type adenylate cyclase
TcG_01432	71,37692433	0,423786167	0,20644448	2,052785168	0,040093418	0,141536361	protein_codin adenylyl cyclase
TcG_01433	282,4756218	0,305806939	0,10908575	2,803362851	0,005057274	0,028978028	protein_codin adenylyl cyclase
TcG_01434	43,7169251	0,375351466	0,265677576	1,412808231	0,157712117	0,35983706	protein_codin receptor-type adenylate cyclase
TcG_01435	82,35031438	0,484729583	0,20196663	2,400047888	0,016392927	0,072326144	protein_codin hypothetical protein
TcG_01436	18,46729698	0,526714504	0,413063546	1,275141582	0,202259134	0,422382768	protein_codin adenylyl cyclase
TcG_01437	2761,995502	0,365293051	0,109882044	3,324410777	0,000886056	0,007065274	protein_codin hypothetical protein
TcG_01438	1317,692861	0,27675012	0,061295546	4,515011906	6,33132E-06	0,000102451	protein_codin vacuolar H+-PPase
TcG_01439	115,0093039	0,218063041	0,166585456	1,309016085	0,190528902	0,405933774	protein_codin hypothetical protein
TcG_01440	190,6026145	-0,01942467	0,133374146	-0,14564045	0,884205236	0,943488844	protein_codin hypothetical protein
TcG_01441	165,7558189	0,098660724	0,138562956	0,712028143	0,476447349	0,691757908	protein_codin hypothetical protein
TcG_01442	51,1233289	0,297318445	0,241790409	1,229653591	0,218826853	0,443626932	protein_codin hypothetical protein
TcG_01443	334,6168731	0,205756339	0,099932865	2,058945657	0,039499445	0,140000543	protein_codin putative kinesin-like protein
TcG_01444	933,783354	0,084667925	0,068286685	1,239889223	0,21501637	0,438870501	protein_codin hypothetical protein
TcG_01445	59,28844042	0,338806744	0,247527248	1,368765445	0,171072606	0,377873357	protein_codin hypothetical protein
TcG_01446	424,290319	0,140208267	0,089528493	1,56607424	0,117331238	0,298714513	protein_codin hypothetical protein
TcG_01447	533,4157262	0,199735101	0,084715234	2,357723538	0,018387381	0,078611143	protein_codin hypothetical protein
TcG_01448	918,0194137	0,154344749	0,065170181	2,368333884	0,017868402	0,07704558	protein_codin hypothetical protein
TcG_01449	850,1234475	0,248116733	0,068288784	3,633345307	0,00027977	0,002639591	protein_codin dynein heavy chain, cytosolic
TcG_01450	1068,452427	0,370749367	0,062649082	5,917873868	3,2613E-09	1,16263E-07	protein_codin ATP-binding cassette protein subfamily C, member 1
TcG_01451	246,5024237	0,508661353	0,120176838	4,232607231	2,30998E-05	0,000314124	protein_codin hypothetical protein
TcG_01452	671,9326053	0,148942753	0,073871373	2,016244531	0,043774412	0,150585017	protein_codin hypothetical protein
TcG_01453	1476,959337	0,33703275	0,061824704	5,451425233	4,99677E-08	1,3782E-06	protein_codin putative protein phosphatase 2C
TcG_01454	488,0638353	0,435021045	0,086633845	5,02137523	5,13028E-07	1,11519E-05	protein_codin rac serine-threonine kinase
TcG_01455	9,519045281	-0,211347506	0,61806648	-0,34194947	0,732388918	1	
TcG_01456	26,18579618	0,280048863	0,346692944	0,807772029	0,41922183	0,645805627	protein_codin hypothetical protein
TcG_01457	316,8239979	0,678726588	0,102955652	6,592417002	4,32723E-11	2,35377E-09	protein_codin hypothetical protein
TcG_01458	678,5286125	0,474536294	0,073756339	6,433837384	1,24422E-10	6,00647E-09	protein_codin hypothetical protein
TcG_01459	524,7665771	0,227911806	0,084490393	2,697487827	0,006986484	0,03725053	protein_codin zinc-finger protein Lsd1
TcG_01460	967,7252933	0,392290972	0,068924763	5,691582485	1,25867E-08	3,87845E-07	protein_codin hypothetical protein
TcG_01461	1284,888689	0,721966788	0,064790492	11,14309786	7,738E-29	3,5861E-26	protein_codin hypothetical protein
TcG_01462	201,9665892	0,728648679	0,130306495	5,591806285	2,24719E-08	6,65882E-07	protein_codin hypothetical protein
TcG_01463	705,8346	0,488382552	0,072435725	6,74228841	1,55911E-11	9,45752E-10	protein_codin hypothetical protein
TcG_01464	677,1158929	0,374787276	0,073963091	5,067220332	4,03667E-07	8,97332E-06	protein_codin hypothetical protein
TcG_01465	119,0843777	0,30479744	0,160094963	1,903854029	0,056929198	0,181353226	
TcG_01466	729,5024435	0,182722276	0,071524336	2,554686801	0,010628338	0,051806127	protein_codin hypothetical protein
TcG_01467	457,8096507	0,386209531	0,088413433	4,36822232	1,25262E-05	0,000184642	protein_codin putative protein kinase
TcG_01468	296,6018402	0,429328353	0,10510156	4,084890395	4,40976E-05	0,00055474	protein_codin hypothetical protein
TcG_01469	212,6524669	0,337228028	0,125298104	2,691405676	0,007115162	0,037745541	protein_codin hypothetical protein
TcG_01470	1909,248188	0,176277874	0,050243362	3,508480905	0,000450674	0,003961688	protein_codin cytochrome c oxidase VIII (COX VIII)
TcG_01471	94,93351265	0,45647935	0,194114294	2,351600902	0,018692819	0,0795939	protein_codin hypothetical protein
TcG_01472	250,0731418	0,341204767	0,121674836	2,804234443	0,005043622	0,028941577	protein_codin hypothetical protein
TcG_01473	257,9805189	0,537966715	0,11253184	4,780573349	1,74796E-06	3,30373E-05	protein_codin putative diphthine synthase
TcG_01474	205,2891574	0,31679668	0,129903834	2,438701539	0,014740137	0,066998519	protein_codin hypothetical protein
TcG_01475	899,5417653	0,4910212	0,066641209	7,368131626	1,73036E-13	1,45275E-11	protein_codin thiolase protein-like protein
TcG_01476	30,39098055	-0,095328023	0,346759934	-0,27491072	0,783384831	0,892633424	protein_codin dispersed protein family protein 1
TcG_01477	49,16402018	-0,109701504	0,25094817	-0,43714805	0,662003981	0,822959026	protein_codin hypothetical protein
TcG_01478	464,5444974	-0,156452958	0,089874539	-1,74079289	0,081719889	0,234532235	protein_codin putative protein kinase
TcG_01479	321,4198657	-0,072590585	0,103020019	-0,70462601	0,481043006	0,695361812	protein_codin putative nuclear transcription factor
TcG_01480	312,2054704	0,111130285	0,102991952	1,079019113	0,280579207	0,513654778	protein_codin putative 3,2-trans-enoyl-CoA isomerase, mitochondrial precursor
TcG_01481	1268,106749	-0,230103293	0,060833131	-3,78253246	0,000155241	0,001620379	protein_codin transcription factor-like protein
TcG_01482	372,6801718	-0,26317211	0,096665867	-2,72249263	0,006479148	0,035193346	protein_codin ribosomal protein L3-like protein

TcG_01483	177,8733785	-0,187827047	0,135412191	-1,38707635	0,165418484	0,370915599	protein_codin hypothetical protein
TcG_01484	224,1671597	-0,103343898	0,123292815	-0,83819887	0,401919023	0,63220648	protein_codin hypothetical protein
TcG_01485	1095,545245	-0,203428544	0,062095198	-3,27607528	0,001052605	0,008150898	protein_codin putative tryptophanyl-tRNA synthetase
TcG_01486	216,6911067	-0,335710789	0,128553394	-2,61145021	0,009015911	0,04555532	protein_codin putative syntaxin
TcG_01487	69,11886014	0,138209962	0,223447796	0,618533566	0,536223678	0,736274892	protein_codin hypothetical protein
TcG_01488	249,5599316	-0,198315987	0,120393272	-1,64723479	0,099509796	0,267809638	protein_codin small GTP-binding protein
TcG_01489	164,1902521	0,103237225	0,151652877	0,680746897	0,496031656	0,705849026	protein_codin WD repeat domain 34
TcG_01490	936,252735	-0,380742109	0,064354487	-5,91632574	3,29213E-09	1,16644E-07	protein_codin putative membrane associated protein
TcG_01491	112,5303299	-0,36463822	0,180319623	-2,02217715	0,043158054	0,149173393	protein_codin putative membrane associated protein
TcG_01492	835,671827	-0,400977677	0,067305023	-5,95761893	2,55939E-09	9,3543E-08	protein_codin putative proteasome regulatory non-ATPase subunit
TcG_01493	404,8574102	-0,205213878	0,094698657	-2,16701995	0,030233333	0,115176685	protein_codin IQ calmodulin-binding protein motif family protein
TcG_01494	303,0835303	-0,180548107	0,107165986	-1,68475198	0,092036434	0,253707857	protein_codin putative DNA-directed RNA polymerase II subunit 3
TcG_01495	563,8785542	-0,283371023	0,081771414	-3,46540446	0,000529435	0,004557228	protein_codin tetratricopeptide repeat protein 30A
TcG_01496	113,5198051	0,07298331	0,168070923	0,434241145	0,664113338	0,824342954	protein_codin transmembrane protein 180
TcG_01497	62,1549931	0,23477186	0,230122415	1,020204222	0,307631616	0,540901939	protein_codin hypothetical protein
TcG_01498	243,6687317	-0,087360041	0,119431597	-0,73146507	0,464495133	0,681738107	protein_codin putative protein phosphatase inhibitor
TcG_01499	602,4646342	-0,026387735	0,082258994	-0,32078844	0,74837072	0,873112027	protein_codin hypothetical protein
TcG_01500	650,410562	0,006816518	0,080364541	0,084819965	0,932404521	0,967649479	protein_codin hypothetical protein
TcG_01501	948,9081815	-0,075395244	0,063873739	-1,18037938	0,23784936	0,465651011	protein_codin SNF2 DNA repair protein
TcG_01502	263,958483	-0,078374877	0,113197451	-0,69237317	0,488703012	0,699976894	protein_codin hypothetical protein
TcG_01503	774,5767991	-0,371194328	0,0731288	-5,07589797	3,8567E-07	8,69334E-06	protein_codin putative protein transport protein Sec24A
TcG_01504	158,8893823	0,218917148	0,143324562	1,527422411	0,126656007	0,314697941	protein_codin hypothetical protein
TcG_01505	436,4796059	-0,272226532	0,088352419	-3,08114408	0,002062068	0,01411171	protein_codin RIO kinase 1
TcG_01506	160,2890105	-0,175150619	0,140807284	-1,24390311	0,213535225	0,436980474	protein_codin putative thymine-7-hydroxylase
TcG_01507	193,985143	0,106839098	0,138186106	0,773153696	0,439431426	0,662646613	protein_codin putative tubulin-tyrosine ligase
TcG_01508	860,6701561	-0,455015174	0,066466936	-6,84573713	7,60831E-12	4,95224E-10	protein_codin host cell surface-exposed lipoprotein
TcG_01509	121,4886101	-0,006539967	0,174905737	-0,03739138	0,970172947	0,987041075	protein_codin Smad nuclear-interacting protein 1
TcG_01510	211,591471	-0,455008143	0,121335188	-3,75000979	0,000176828	0,001803455	protein_codin hypothetical protein
TcG_01511	1111,00185	-0,08998796	0,060414038	-1,48952069	0,136350308	0,329163749	protein_codin Hsc70-interacting protein
TcG_01512	326,3010608	-0,216732015	0,100857929	-2,14888426	0,031643576	0,118917442	protein_codin ADP-ribosylation factor GTPase activating protein
TcG_01513	503,8580768	-0,245191538	0,088024902	-2,78547925	0,005344866	0,030178176	protein_codin UBX domain protein 7
TcG_01514	532,8126528	-0,214666629	0,082676562	-2,59646295	0,009418906	0,047057977	protein_codin hypothetical protein
TcG_01515	173,2342563	0,168876231	0,135859155	1,243024292	0,213858882	0,437459217	protein_codin hypothetical protein
TcG_01516	451,3598892	-0,251767461	0,098831154	-2,54745039	0,010851328	0,05266933	protein_codin hypothetical protein
TcG_01517	351,9784152	0,001318739	0,09841075	0,013400359	0,989308381	0,995541618	protein_codin putative RNA-binding protein
TcG_01518	160,5120757	0,033673984	0,147996715	0,227531968	0,820010114	0,911022136	protein_codin DNA-directed RNA polymerase
TcG_01519	242,2656253	-0,17804227	0,123707518	-1,43921949	0,150088345	0,350052799	protein_codin hypothetical protein
TcG_01520	317,4928174	-0,408303787	0,107538825	-3,7968035	0,000146574	0,001542421	protein_codin hypothetical protein
TcG_01521	548,2098878	-0,097526582	0,078557349	-1,24146988	0,214432219	0,438167847	protein_codin hypothetical protein
TcG_01522	301,5319198	-0,142551816	0,116974674	-1,21865538	0,22297502	0,449216975	protein_codin putative serine/threonine protein phosphatase
TcG_01523	194,0542014	0,038624114	0,12800831	0,301731299	0,762856907	0,880149385	protein_codin putative DNA repair protein
TcG_01524	70,11205917	-0,166275237	0,211745974	-0,78525808	0,432302269	0,656786532	protein_codin hypothetical protein
TcG_01525	73,34905704	0,181459261	0,206269825	0,879717918	0,37901214	0,610232721	protein_codin hypothetical protein
TcG_01526	206,7026915	-0,497632236	0,125589414	-3,96237405	7,42082E-05	0,00086846	protein_codin hypothetical protein
TcG_01527	275,4876597	-0,112974988	0,110326741	-1,02400367	0,305833542	0,538836287	protein_codin uncharacterized protein
TcG_01528	143,4385672	-0,410221529	0,15561559	-2,63612102	0,008385982	0,043086467	protein_codin hypothetical protein
TcG_01529	721,2850054	-0,448828957	0,074230036	-6,04646015	1,48063E-09	5,64295E-08	protein_codin cofilin/actin depolymerizing factor
TcG_01530	210,949941	0,148003762	0,121619541	1,216940638	0,223626802	0,450179159	protein_codin hypothetical protein
TcG_01531	526,3147952	-0,194941717	0,082350776	-2,36721165	0,01792268	0,077222822	protein_codin megakaryocyte stimulating factor
TcG_01532	397,3100007	0,098758111	0,090556367	1,090570603	0,275461871	0,507736472	protein_codin exonuclease
TcG_01533	290,9908633	-0,369111742	0,104270539	-3,53994277	0,000400214	0,003586139	protein_codin hypothetical protein
TcG_01534	41,13183494	-0,356830027	0,270906636	-1,31716975	0,18778173	0,402151409	protein_codin hypothetical protein
TcG_01535	286,9435498	-0,130990512	0,110469861	-1,18575792	0,235717914	0,463445836	protein_codin hypothetical protein
TcG_01536	259,548361	-0,207538567	0,112414315	-1,8461934	0,064864129	0,200779001	protein_codin hypothetical protein
TcG_01537	204,269867	-0,505591192	0,125178687	-4,03895587	5,36897E-05	0,000657652	protein_codin hypothetical protein
TcG_01538	553,5370726	-0,050812658	0,085141939	-0,5967994	0,55064132	0,746546058	protein_codin putative DNA repair helicase
TcG_01539	276,6914972	-0,443428999	0,110357241	-4,01812328	5,86635E-05	0,000708867	protein_codin hypothetical protein

TcG_01540	948,9616153	-0,284832274	0,069963919	-4,07113094	4,67854E-05	0,000584112	protein_codin zinc finger protein
TcG_01541	2980,184544	-0,450709758	0,053888205	-8,36379235	6,07338E-17	7,90631E-15	protein_codin ATP-binding cassette protein subfamily A, member 10
TcG_01542	79,93586361	0,070979939	0,200404192	0,354183905	0,723201065	0,857362891	protein_codin Golgi vesicular membrane trafficking protein
TcG_01543	171,589966	-0,071396977	0,14554187	-0,4905597	0,623737894	0,797066211	protein_codin aprataxin
TcG_01544	147,4965085	-0,253959317	0,149819047	-1,69510701	0,090055122	0,250210705	protein_codin putative phosphate transporter
TcG_01545	118,4864421	-0,328911342	0,16351671	-2,01148459	0,044274296	0,151943719	protein_codin putative phosphate transporter
TcG_01546	536,9900846	-0,420929046	0,081292362	-5,17796552	2,24319E-07	5,35867E-06	protein_codin putative flagellar radial spoke protein-like
TcG_01547	120,4539852	-0,172031976	0,166873154	-1,03091463	0,302580855	0,535466899	protein_codin hypothetical protein
TcG_01548	396,9895345	-0,061267506	0,091012006	-0,67318048	0,500832476	0,709716863	protein_codin nuclear cap binding protein
TcG_01549	221,3041248	-0,298847119	0,122619377	-2,43719325	0,014801767	0,067225899	protein_codin hypothetical protein
TcG_01550	226,3318621	-0,174306204	0,12328645	-1,41383099	0,157411532	0,359292752	protein_codin hypothetical protein
TcG_01551	406,0181793	-0,136870024	0,09479427	-1,4438639	0,148777263	0,347877572	protein_codin putative intergrin alpha chain protein
TcG_01552	51,04865711	0,025762946	0,241852896	0,106523205	0,915167245	0,958753949	protein_codin hypothetical protein
TcG_01553	240,7591021	0,16530804	0,11624693	1,4220422	0,155014	0,35638871	protein_codin putative elongation factor ts
TcG_01554	250,6484421	-0,145263468	0,120658264	-1,20392473	0,228618672	0,455742591	protein_codin NAD-dependent epimerase/dehydratase family protein
TcG_01555	181,6919567	-0,390155756	0,132742568	-2,93919095	0,003290703	0,020542069	protein_codin hypothetical protein
TcG_01556	322,1217936	-0,170586824	0,106968283	-1,59474211	0,110769917	0,287624443	protein_codin putative peroxisomal targeting signal type 2 receptor
TcG_01557	97,26409785	-0,073637954	0,195066554	-0,37750169	0,70580081	0,848454886	protein_codin hypothetical protein
TcG_01558	631,2828225	-0,244846891	0,076860491	-3,18560145	0,001444534	0,010526023	protein_codin putative U3 small nucleolar ribonucleoprotein MPP10
TcG_01559	2618,987143	-0,435369193	0,045850675	-9,49537152	2,19428E-21	4,62234E-19	protein_codin putative glucose regulated protein 94
TcG_01560	109,5044029	0,118462623	0,184690109	0,641412925	0,521254438	0,725401608	protein_codin hypothetical protein
TcG_01561	551,7785928	-0,353672275	0,082037213	-4,31112007	1,6243E-05	0,000231451	protein_codin putative U-box domain protein
TcG_01562	356,196017	-0,106061539	0,097191124	-1,09126775	0,275155085	0,507634843	protein_codin hypersensitive-induced reaction protein 1
TcG_01563	917,87366	0,039533237	0,064543674	0,612503664	0,540204575	0,739113156	protein_codin putative dolicholphosphate-mannose synthase
TcG_01564	255,412579	-0,191914459	0,113472677	-1,69128344	0,090782681	0,251434916	protein_codin putative nucleoporin (NUP54/57)
TcG_01565	0					1	protein_codin mucin-associated surface protein (MASP)
TcG_01566	5,415518995	0,303046299	0,77315724	0,391959465	0,695088164	1	protein_codin hypothetical protein
TcG_01567	343,7799738	-0,140693793	0,102159305	-1,37719999	0,168450424	0,374941548	protein_codin L1Tc protein
TcG_01568	61,31899467	-0,119759189	0,224486473	-0,53348065	0,593700903	0,778863106	protein_codin mucin-associated surface protein (MASP)
TcG_01569	50,3000527	0,38310184	0,245541579	1,560232046	0,118705055	0,300690664	protein_codin hypothetical protein
TcG_01570	31,90289371	0,051228752	0,342627495	0,149517341	0,881145427	0,942069315	protein_codin receptor-type adenylate cyclase
TcG_01571	12,40320674	0,515944371	0,540579565	0,954428181	0,339866947	1	protein_codin putative receptor-type adenylate cyclase
TcG_01572	133,9324625	0,212711177	0,150828097	1,410288802	0,158454424	0,360607533	protein_codin putative receptor-type adenylate cyclase
TcG_01573	67,66689055	0,358909875	0,214285925	1,674911099	0,093951676	0,257516942	protein_codin receptor-type adenylate cyclase
TcG_01574	456,7370635	-0,446657516	0,091404868	-4,88658345	1,02601E-06	2,06736E-05	protein_codin hypothetical protein
TcG_01575	754,5509593	-0,095973106	0,07598841	-1,26299664	0,206590387	0,428645456	protein_codin hypothetical protein
TcG_01576	46,18339428	0,133892367	0,262988801	0,509118132	0,610669423	0,789204232	protein_codin receptor-type adenylate cyclase
TcG_01577	28,56783279	-0,045947916	0,319097297	-0,14399344	0,885505652	0,94437554	protein_codin surface protease GP63
TcG_01578	157,3454787	0,211989982	0,147503553	1,4371856	0,150665264	0,350664472	protein_codin hypothetical protein
TcG_01579	482,9915439	-0,266184162	0,091764169	-2,90074181	0,003722805	0,022665484	protein_codin hypothetical protein
TcG_01580	766,1587391	-0,038155749	0,069055103	-0,55254062	0,580578014	0,770159935	protein_codin hypothetical protein
TcG_01581	727,3525017	0,127947641	0,072299293	1,769694222	0,076778093	0,224588734	protein_codin hypothetical protein
TcG_01582	272,3046121	-0,162347895	0,112080043	-1,44849958	0,147477382	0,346227632	protein_codin putative cell cycle associated protein MOB1
TcG_01583	429,2473962	-0,139267145	0,090140506	-1,54500071	0,122346091	0,307284155	protein_codin putative glucosamine-fructose-6-phosphate aminotransferase
TcG_01584	200,710035	-0,218649698	0,123943668	-1,76410543	0,07771423	0,226173593	protein_codin hypothetical protein
TcG_01585	384,8664803	0,017957303	0,099815425	0,179905088	0,85722708	0,931078155	protein_codin 2,4-dienoyl-CoA reductase-like protein
TcG_01586	339,6709388	0,039840448	0,098717318	0,403581143	0,686520755	0,837266259	protein_codin hypothetical protein
TcG_01587	283,3627566	-0,132030982	0,121393334	-1,08762959	0,276758663	0,509048895	protein_codin peptidyl-tRNA hydrolase, PTH2 family
TcG_01588	567,3102875	0,04275762	0,07918654	0,539960703	0,589224133	0,775648564	protein_codin putative protein phosphatase
TcG_01589	154,6156345	-0,040272714	0,143555473	-0,28053764	0,779065049	0,889899207	protein_codin hypothetical protein
TcG_01590	630,1020885	-0,062397657	0,07548332	-0,82664167	0,408440191	0,636605346	protein_codin hypothetical protein
TcG_01591	176,2189222	0,075777731	0,139308303	0,543957027	0,586471045	0,775048879	protein_codin hypothetical protein
TcG_01592	408,2801651	-0,015984961	0,097338455	-0,16422041	0,869557634	0,937093735	protein_codin putative acyl-CoA dehydrogenase
TcG_01593	318,7795621	0,013069967	0,104729445	0,124797442	0,900683913	0,951748911	protein_codin hypothetical protein
TcG_01594	549,3136881	0,057918971	0,079245821	0,730877285	0,464854112	0,682092166	protein_codin putative arginine N-methyltransferase, type III
TcG_01595	1017,137443	0,002015508	0,062499726	0,032248262	0,974274069	0,988782354	protein_codin dihydrofolate reductase-thymidylate synthetase
TcG_01596	225,1624823	0,07516002	0,131004241	0,573722036	0,566155914	0,758321667	protein_codin hypothetical protein

TcG_01597	231,6517404	0,226627013	0,121760598	1,861250823	0,062708764	0,195939521	protein_codin exosome-associated protein 3
TcG_01598	463,7938851	-0,188185245	0,088990529	-2,11466598	0,034458426	0,126983246	protein_codin putative lipin
TcG_01599	277,4451208	0,086134219	0,112242702	0,767392596	0,442848129	0,664995914	protein_codin putative Qb-SNARE protein
TcG_01600	887,0314858	0,2042302	0,069361805	2,94441876	0,003235619	0,020263719	protein_codin hypothetical protein
TcG_01601	548,2974691	-0,143134806	0,082610272	-1,73265143	0,083157642	0,236956331	protein_codin hypothetical protein
TcG_01602	88,46359772	-0,066894381	0,183632544	-0,36428391	0,715646014	0,853960462	protein_codin hypothetical protein
TcG_01603	558,1536432	0,124291474	0,083512045	1,488305954	0,136670226	0,329475912	protein_codin hypothetical protein
TcG_01604	10,07190723	0,319932617	0,553302844	0,578223338	0,563113344	1	protein_codin putative RNA-binding protein
TcG_01605	480,3304475	-0,138047848	0,084634651	-1,63110317	0,102868551	0,274237237	protein_codin hypothetical protein
TcG_01606	225,2001932	0,143358229	0,120688194	1,1878397	0,23489657	0,462842119	protein_codin adiponectin receptor
TcG_01607	612,5970195	-0,029597865	0,077381814	-0,38249123	0,702097033	0,846109447	protein_codin hypothetical protein
TcG_01608	55,26915194	0,04873064	0,247629742	0,196788315	0,843993195	0,924394313	protein_codin sodium stibogluconate resistance protein
TcG_01609	456,7035661	0,017484752	0,087039648	0,200882609	0,840790366	0,922466573	protein_codin hypothetical protein
TcG_01610	541,8517817	-0,008101949	0,084152147	-0,09627739	0,923300272	0,962648944	protein_codin hypothetical protein
TcG_01611	247,9804899	-0,187909051	0,114956938	-1,63460382	0,102132125	0,272918878	protein_codin putative serine-threonine dehydratase
TcG_01612	355,1707564	0,020158763	0,09602717	0,209927704	0,833724099	0,919248114	protein_codin putative protein AF-9
TcG_01613	888,4935516	-0,031431734	0,067836124	-0,46334803	0,643114926	0,810687402	protein_codin hypothetical protein
TcG_01614	721,188832	0,018742714	0,070390322	0,266268334	0,790032559	0,895024708	protein_codin hypothetical protein
TcG_01615	1272,302701	0,045338953	0,068650342	0,660433022	0,508975987	0,715750872	protein_codin hypothetical protein
TcG_01616	270,562308	0,104939089	0,113333686	0,925930253	0,354482228	0,588430867	protein_codin hypothetical protein
TcG_01617	247,1452527	-0,032619039	0,120103619	-0,27159081	0,785936666	0,893519989	protein_codin hypothetical protein
TcG_01618	623,0687172	-0,025974567	0,082664385	-0,31421715	0,75335612	0,875527481	protein_codin hypothetical protein
TcG_01619	290,3471493	-0,464379905	0,106326118	-4,36750552	1,25674E-05	0,000185013	protein_codin hypothetical protein
TcG_01620	946,5909078	0,003319393	0,064210756	0,051695279	0,958771499	0,980917031	protein_codin lanosterol synthase
TcG_01621	1307,696698	-0,09214085	0,064636688	-1,42551936	0,154007128	0,355044797	protein_codin putative protein kinase
TcG_01622	1144,662551	-0,1981825	0,067534008	-2,93455856	0,003340225	0,020761722	protein_codin hypothetical protein
TcG_01623	1790,743958	0,105116896	0,05019597	2,094130167	0,036248386	0,132052525	protein_codin hypothetical protein
TcG_01624	455,6335972	0,050783941	0,085611516	0,593190535	0,553053646	0,748298441	protein_codin Phosphoethanolamine/phosphocholine phosphatase
TcG_01625	223,8044069	0,185530689	0,124843267	1,486108893	0,13725032	0,330255911	protein_codin hypothetical protein
TcG_01626	176,4185444	0,298999074	0,132895977	2,249873029	0,024457006	0,098183949	protein_codin hypothetical protein
TcG_01627	835,2631226	-0,200499411	0,071252291	-2,81393633	0,004893892	0,028321994	protein_codin hypothetical protein
TcG_01628	2682,838916	0,196150838	0,047707292	4,111548334	3,93015E-05	0,000498191	protein_codin putative 60S ribosomal protein L23a
TcG_01629	434,2896889	0,100064519	0,089182924	1,122014334	0,261856345	0,494195735	protein_codin deoxyuridine triphosphatase
TcG_01630	538,6695748	0,010679911	0,081551968	0,130958343	0,895808261	0,949399425	protein_codin hypothetical protein
TcG_01631	601,7989487	0,051885706	0,079603549	0,651801423	0,514529284	0,720229102	protein_codin Basic immunoglobulin-like variable motif-containing protein
TcG_01632	128,6496151	0,154445616	0,162746291	0,948996224	0,342622533	0,576796063	protein_codin hypothetical protein
TcG_01633	383,5271549	0,199416413	0,093414294	2,134752668	0,032781218	0,122280488	protein_codin hypothetical protein
TcG_01634	199,8271523	0,269591043	0,133449152	2,020178019	0,043364925	0,149531555	protein_codin 23S rRNA (guanosine2251-2-O)-methyltransferase
TcG_01635	421,5229081	0,099398217	0,091070446	1,091443177	0,275077922	0,507634843	protein_codin hypothetical protein
TcG_01636	379,0864793	-0,12721786	0,095234695	-1,33583522	0,181603139	0,393281116	protein_codin hypothetical protein
TcG_01637	663,8283602	-0,199605664	0,073566122	-2,7132824	0,006662031	0,035900598	protein_codin chromosome-associated protein H
TcG_01638	630,8733826	-0,165254689	0,079061018	-2,09021708	0,036598304	0,133007512	protein_codin putative inorganic polyphosphate/ATP-NAD kinase, putative,poly(p)/ATP NAD kinase
TcG_01639	116,4426733	-0,32738348	0,160886211	-2,03487595	0,041863357	0,14582948	
TcG_01640	228,9815237	-0,160033583	0,122987098	-1,30122253	0,193182295	0,410003677	protein_codin hypothetical protein
TcG_01641	687,3222074	-0,243086176	0,07312071	-3,32445042	0,00088593	0,007065274	protein_codin hypothetical protein
TcG_01642	442,9072808	-0,126828217	0,086244996	-1,47055741	0,14141085	0,3366698	protein_codin hypothetical protein
TcG_01643	195,3646031	-0,10802841	0,130654612	-0,82682432	0,408336646	0,636605346	protein_codin hypothetical protein
TcG_01644	296,1685326	0,291590078	0,10601679	2,750414146	0,005951999	0,032791182	protein_codin 60S ribosomal protein L19
TcG_01645	320,5986926	-0,076071341	0,101549245	-0,74910789	0,453792177	0,674035553	protein_codin hypothetical protein
TcG_01646	785,6688387	0,157366636	0,068549784	2,295654729	0,021695631	0,089581461	protein_codin 60S ribosomal protein L19
TcG_01647	377,761181	-0,125016999	0,09660414	-1,29411638	0,195625225	0,413446525	protein_codin hypothetical protein
TcG_01648	232,4633366	-0,012989216	0,116490913	-0,11150412	0,911216601	0,956844965	protein_codin hypothetical protein
TcG_01649	75,06379363	-0,118421494	0,200288755	-0,59125383	0,554350357	0,749178028	protein_codin calpain-like cysteine peptidase
TcG_01650	52,79764198	-0,442125123	0,241669436	-1,8294623	0,067330381	0,206536881	protein_codin putative calpain-like cysteine peptidase, putative,cysteine peptidase, Clan CA, family C2
TcG_01651	418,3310793	-0,620695112	0,091378564	-6,79256803	1,10155E-11	6,97407E-10	protein_codin putative radial spoke protein 3
TcG_01652	335,3391593	-0,216990419	0,097523261	-2,22501193	0,026080438	0,102953305	protein_codin hypothetical protein
TcG_01653	278,5668617	0,070619602	0,109699397	0,643755606	0,519733925	0,724276793	protein_codin hypothetical protein

TcG_01654	40,64156929	-0,591722114	0,274195391	-2,15803085	0,030925433	0,116901164		
TcG_01655	16,91146704	-0,666546261	0,436473329	-1,52711796	0,126731681	0,314818451	protein_codin	hypothetical protein
TcG_01656	77,91056886	-0,619975637	0,197416875	-3,14043892	0,001686949	0,011990791	protein_codin	mucin TcMUCI
TcG_01657	38,60477568	-0,115250174	0,289366425	-0,39828454	0,690420453	0,83922987	protein_codin	hypothetical protein
TcG_01658	23,7597744	-0,056524242	0,380289835	-0,14863464	0,88184194	0,942184067	protein_codin	hypothetical protein
TcG_01659	41,03286646	0,119364885	0,282816829	0,422057221	0,672983252	0,828900214	protein_codin	RNaseH
TcG_01660	53,8243127	0,118986562	0,238311293	0,499290489	0,617574754	0,793033999	protein_codin	hypothetical protein
TcG_01661	34,71579219	0,160709884	0,30868706	0,520623976	0,602628743	0,785033003	protein_codin	hypothetical protein
TcG_01662	90,06258073	-0,334293441	0,185637893	-1,80078235	0,071737191	0,215117279		
TcG_01663	254,314423	-0,084780005	0,114806163	-0,73846214	0,460233664	0,678399847	protein_codin	hypothetical protein
TcG_01664	271,4608469	-0,198787288	0,110397469	-1,80065077	0,071757941	0,215117279	protein_codin	PHD and RING finger domain-containing protein 1-like
TcG_01665	278,2612799	-0,114038665	0,108177155	-1,05418436	0,291798518	0,524450861	protein_codin	WD40 repeat-containing protein SMU1
TcG_01666	370,7138959	-0,171600737	0,098730801	-1,73806689	0,082199028	0,235498994	protein_codin	putative dynein assembly factor 3, axonemal
TcG_01667	518,7282097	-0,147890446	0,083136458	-1,77888797	0,07525813	0,221529648	protein_codin	hypothetical protein
TcG_01668	238,9131534	-0,240348295	0,118493424	-2,02836822	0,042522681	0,147593704	protein_codin	glycoprotein 96-92
TcG_01669	243,8556463	-0,092848496	0,114297287	-0,81234209	0,416595367	0,643470726	protein_codin	hypothetical protein
TcG_01670	452,4718157	-0,168045721	0,086470332	-1,94339165	0,051968855	0,169704385	protein_codin	hypothetical protein
TcG_01671	283,0214816	-0,194039831	0,110198176	-1,76082616	0,07826783	0,227270948	protein_codin	copper-transporting ATPase-like protein
TcG_01672	79,47137026	-0,099607407	0,199466309	-0,49936958	0,617519045	0,793033999	protein_codin	hypothetical protein
TcG_01673	166,4473847	0,28764955	0,138868741	2,071377243	0,038323556	0,136999913	protein_codin	hypothetical protein
TcG_01674	2394,130286	-0,000144631	0,048859986	-0,0029601	0,997638184	0,999104158	protein_codin	putative copper-transporting ATPase-like protein
TcG_01675	63,63644697	0,111458243	0,222542314	0,500840675	0,616483257	0,792657426	protein_codin	hypothetical protein
TcG_01676	324,4478082	-0,401092314	0,10867308	-3,69081573	0,000223536	0,002186223	protein_codin	hypothetical protein
TcG_01677	208,6860573	-0,096887587	0,121791208	-0,79552201	0,426309911	0,651783849	protein_codin	hypothetical protein
TcG_01678	295,594545	-0,26248218	0,10739013	-2,44419278	0,014517667	0,06622252	protein_codin	ubiquilin
TcG_01679	231,1927703	0,132085511	0,118269319	1,11681975	0,264071402	0,496193848	protein_codin	putative NADH-cytochrome b5 reductase
TcG_01680	264,7321979	-0,098375503	0,114216993	-0,86130357	0,389070872	0,619284946	protein_codin	NADH-ubiquinone oxidoreductase 20 kDa subunit
TcG_01681	258,4345564	-0,013464125	0,119175684	-0,11297712	0,910048696	0,956414305	protein_codin	diagnostic antigen
TcG_01682	72,21250733	0,142519774	0,211391497	0,674198235	0,500185292	0,709146695	protein_codin	hypothetical protein
TcG_01683	57,03164153	-0,136971845	0,230811415	-0,59343618	0,55288928	0,748250812	protein_codin	hypothetical protein
TcG_01684	137,8880919	-0,078640524	0,150115005	-0,52386851	0,600369999	0,783502945	protein_codin	putative calcium uniporter protein, mitochondrial
TcG_01685	420,5698346	-0,064737212	0,092789022	-0,6976818	0,485376213	0,698146344	protein_codin	multidrug resistance protein, MATE family
TcG_01686	257,4910959	-0,218247605	0,113085631	-1,92993224	0,053615234	0,173661198	protein_codin	hypothetical protein
TcG_01687	619,7832304	-0,097171223	0,080913629	-1,20092529	0,229780183	0,456889557	protein_codin	hypothetical protein
TcG_01688	330,6416767	-0,004389178	0,105728527	-0,04151366	0,966886405	0,985514725	protein_codin	hypothetical protein
TcG_01689	440,5748104	-0,354757403	0,092235729	-3,84620372	0,000119962	0,001301386	protein_codin	hypothetical protein
TcG_01690	218,8537258	-0,112281948	0,124093238	-0,90481924	0,365561159	0,597692344	protein_codin	hypothetical protein
TcG_01691	582,7168728	-0,169540137	0,077295879	-2,19339168	0,028279171	0,109433025	protein_codin	hypothetical protein
TcG_01692	574,3284132	-0,539752136	0,07985761	-6,75893174	1,39013E-11	8,61285E-10	protein_codin	hypothetical protein
TcG_01693	225,2606362	-0,154563011	0,124241928	-1,24404872	0,213481631	0,436980474	protein_codin	hypothetical protein
TcG_01694	897,0623094	-0,334142434	0,064544832	-5,17690455	2,25598E-07	5,37813E-06	protein_codin	oxoglutarate dehydrogenase (succinyl-transferring)
TcG_01695	841,0696781	-0,226824991	0,067502131	-3,36026415	0,00077868	0,006322204	protein_codin	putative phosphatidylinositol (3,5) kinase
TcG_01696	3,670854085	0,429013326	0,945198561	0,453886986	0,649910171	1		
TcG_01697	24,78740552	0,335700244	0,345974243	0,97030415	0,331894909	0,566239791		
TcG_01698	877,6515974	0,114477014	0,064567831	1,77297288	0,076233201	0,223378317	protein_codin	putative retrotransposon hot spot (RHS) protein
TcG_01699	142,9583379	0,237354105	0,146739918	1,617515588	0,105767034	0,279839427	protein_codin	putative retrotransposon hot spot (RHS) protein
TcG_01700	50,66736359	0,061236294	0,249515718	0,245420589	0,806130789	0,904233839		
TcG_01701	292,4968083	-0,13357137	0,104518199	-1,27797237	0,201259169	0,420748598	protein_codin	isovaleryl-CoA dehydrogenase
TcG_01702	353,7505586	-0,087914927	0,095362093	-0,92190643	0,356577385	0,590608375	protein_codin	hypothetical protein
TcG_01703	801,9521241	0,441381873	0,07281965	6,061301759	1,35024E-09	5,24963E-08	protein_codin	putative 1,2-Dihydroxy-3-keto-5-methylthiopentene dioxygenase
TcG_01704	411,9842456	0,034736392	0,091493112	0,379661281	0,704196865	0,847428783	protein_codin	hypothetical protein
TcG_01705	240,1314556	0,11564989	0,114892714	1,006590283	0,314131706	0,548369737	protein_codin	hypothetical protein
TcG_01706	120,8130306	0,018076637	0,159840294	0,113091862	0,909957727	0,956414305	protein_codin	hypothetical protein
TcG_01707	173,3987982	-0,259839465	0,138773076	-1,87240546	0,06115053	0,19200272	protein_codin	hypothetical protein
TcG_01708	206,5596321	0,054071111	0,123677816	0,437193286	0,661971179	0,822959026	protein_codin	TatD related deoxyribonuclease
TcG_01709	439,2119278	-0,311485965	0,094719705	-3,28850227	0,00100722	0,007826725	protein_codin	hypothetical protein
TcG_01710	302,5121064	0,040333953	0,103122295	0,391127379	0,695703084	0,842346738	protein_codin	hypothetical protein

TcG_01711	434,5706762	-0,042806061	0,088843979	-0,48181161	0,629939779	0,801688869	protein_codin	hypothetical protein
TcG_01712	190,8129466	-0,056706839	0,134803963	-0,42066151	0,674002269	0,829023911	protein_codin	hypothetical protein
TcG_01713	384,8170239	-0,08658525	0,101725386	-0,85116658	0,394676822	0,624314965	protein_codin	hypothetical protein
TcG_01714	74,95051894	-0,21217304	0,200935512	-1,05592604	0,291002006	0,523651784	protein_codin	hypothetical protein
TcG_01715	415,2480836	-0,082008012	0,089834204	-0,91288182	0,3613047	0,593951838	protein_codin	putative vesicular transport protein
TcG_01716	239,7461449	0,016558263	0,121269276	0,136541287	0,891393388	0,946972656	protein_codin	putative cysteine desulfurase
TcG_01717	425,4285824	-0,376155792	0,089107402	-4,22137535	2,42816E-05	0,000328269	protein_codin	putative vesicular-fusion ATPase-like protein
TcG_01718	285,3365674	-0,293063758	0,107117649	-2,73590542	0,00622089	0,033967129	protein_codin	cholestenol Delta-isomerase
TcG_01719	562,7731374	-0,341884458	0,079326601	-4,30983371	1,63377E-05	0,000232256	protein_codin	hypothetical protein
TcG_01720	491,1646174	-0,272766685	0,08951098	-3,04729863	0,002309082	0,015455241	protein_codin	putative mitochondrial RNA binding protein 1
TcG_01721	200,0977923	0,039649322	0,126618145	0,313140916	0,754173604	0,875975477	protein_codin	pre-rRNA-processing protein TSR2
TcG_01722	60,52017255	-0,07539993	0,227526086	-0,33139027	0,740349716	0,867634494	protein_codin	hypothetical protein
TcG_01723	501,9505562	-0,317603109	0,085893711	-3,69762939	0,000217622	0,002138568	protein_codin	putative intraflagellar transport protein IFT88
TcG_01724	312,8546885	-0,05781259	0,107383853	-0,53837321	0,590319417	0,776238879	protein_codin	hypothetical protein
TcG_01725	131,6418348	-0,34086098	0,157106823	-2,16961284	0,030036188	0,114586524	protein_codin	putative transporter
TcG_01726	151,5964407	0,09116258	0,141717608	0,643269253	0,520049404	0,724629271	protein_codin	hypothetical protein
TcG_01727	440,2668894	-0,179264495	0,089555668	-2,0017102	0,045315909	0,154329841	protein_codin	hypothetical protein
TcG_01728	206,1957095	-0,055242525	0,126430828	-0,43693872	0,662155787	0,82305943	protein_codin	hypothetical protein
TcG_01729	129,166712	0,036653667	0,153247858	0,239178986	0,810966796	0,906853087	protein_codin	protein phosphatase 2C
TcG_01730	415,0905397	-0,151984271	0,091463125	-1,66169997	0,096572947	0,262848254	protein_codin	histone h1
TcG_01731	136,3347986	-0,072744696	0,152684995	-0,47643644	0,633763476	0,804197117	protein_codin	putative E3 ubiquitin-protein ligase
TcG_01732	329,9691701	-0,27963962	0,100042843	-2,79519864	0,005186783	0,029501263	protein_codin	putative translation initiation factor eIF2B delta subunit
TcG_01733	116,9135523	-0,265771603	0,172246712	-1,54297055	0,122837922	0,307742007	protein_codin	hypothetical protein
TcG_01734	184,5438571	-0,188490371	0,128490323	-1,4669616	0,142386501	0,338328548	protein_codin	hypothetical protein
TcG_01735	641,0109879	-0,52493071	0,07762465	-6,76242287	1,35703E-11	8,45298E-10	protein_codin	hypothetical protein
TcG_01736	320,2979987	0,091563916	0,103607666	0,883756188	0,376827842	0,607475633	protein_codin	putative dynein heavy chain
TcG_01737	193,2297348	0,275494841	0,12924085	2,131639032	0,033036532	0,122955755	protein_codin	glutamine-dependent carbamoyl-phosphate synthetase
TcG_01738	133,2580619	-0,300480517	0,155988334	-1,92630122	0,054066774	0,174830489	protein_codin	hypothetical protein
TcG_01739	127,0240079	-0,312460663	0,155032049	-2,01545851	0,043856629	0,150823065	protein_codin	hypothetical protein
TcG_01740	409,6266973	-0,311108781	0,096740807	-3,2159002	0,00130036	0,009676285	protein_codin	putative eukaryotic translation initiation factor 3 subunit
TcG_01741	1195,746374	-0,303036904	0,058083099	-5,21729913	1,81551E-07	4,48497E-06	protein_codin	T-complex protein 1 subunit beta
TcG_01742	402,532482	-0,469292088	0,094817798	-4,94940927	7,44391E-07	1,56241E-05	protein_codin	putative cation transporter protein
TcG_01743	39,28911182	-0,012608499	0,280158277	-0,04500491	0,964103394	0,983976561	protein_codin	surface protease GP63
TcG_01744	247,2735985	0,000380114	0,114659101	0,003315164	0,997354886	0,999104158	protein_codin	hypothetical protein
TcG_01745	477,3443413	0,086595041	0,084270835	1,027580203	0,304147341	0,537090549	protein_codin	cytochrome oxidase assembly protein
TcG_01746	222,9900202	0,077007195	0,119590116	0,643926084	0,519623367	0,724276793	protein_codin	putative serine/threonine-protein phosphatase 2A, catalytic subunit
TcG_01747	263,5128552	0,188177551	0,110856715	1,697484467	0,089605105	0,249558834	protein_codin	peptide methionine sulfoxide reductase B8
TcG_01748	462,3237492	-0,057215312	0,0876515	-0,65275907	0,513911614	0,719973393	protein_codin	molybdenum cofactor biosynthesis protein
TcG_01749	140,7013923	-0,11055848	0,147173932	-0,75120966	0,452526488	0,673298046	protein_codin	hypothetical protein
TcG_01750	562,1042658	-0,353822088	0,08412658	-4,20582995	2,60126E-05	0,000348418	protein_codin	putative coatomer gamma subunit
TcG_01751	28,6259899	0,048907804	0,328993109	0,148659053	0,881822673	0,942184067	protein_codin	hypothetical protein
TcG_01752	190,8836622	0,042669072	0,131770273	0,323814097	0,746078783	0,871496958	protein_codin	hypothetical protein
TcG_01753	237,278525	-0,009156576	0,122059944	-0,07501705	0,94020115	0,972257276	protein_codin	hypothetical protein
TcG_01754	195,2958882	0,135280193	0,128449862	1,053175073	0,292260761	0,524981887	protein_codin	hypothetical protein
TcG_01755	375,5256526	0,017153132	0,096516993	0,177721369	0,858941803	0,932070224	protein_codin	putative leucine-rich repeat protein (LRRP)
TcG_01756	872,9949321	0,071649243	0,065609692	1,092052734	0,274809923	0,507556451	protein_codin	putative splicing factor 3B subunit 1
TcG_01757	163,7475033	-0,272215489	0,136573955	-1,99317277	0,046242531	0,156473705	protein_codin	hypothetical protein
TcG_01758	196,3607301	0,160108659	0,128028117	1,250574192	0,211089871	0,43370939	protein_codin	40S ribosomal protein S17
TcG_01759	372,403477	0,313782922	0,094710727	3,313066346	0,000922791	0,007312897	protein_codin	40S ribosomal protein S17
TcG_01760	393,333931	0,179258222	0,09797842	1,829568407	0,067314501	0,206536881	protein_codin	40S ribosomal protein S17
TcG_01761	452,9458763	-0,010950779	0,088980375	-0,1230696	0,902051983	0,952705039	protein_codin	serine/arginine repetitive matrix protein 1
TcG_01762	231,9570681	0,615255005	0,118583962	5,18834922	2,12166E-07	5,12117E-06	protein_codin	putative DNA repair and recombination protein,mitochondrial precursor
TcG_01763	214,3406324	0,018241032	0,121711989	0,149870464	0,880866815	0,942069315	protein_codin	hypothetical protein
TcG_01764	591,6910718	-0,101359604	0,080047587	-1,26624183	0,205426514	0,426842108	protein_codin	putative acyl-CoA dehydrogenase
TcG_01765	548,1000623	-0,150994455	0,085385288	-1,76838959	0,076995795	0,224812905	protein_codin	putative eukaryotic translation initiation factor
TcG_01766	188,2410437	0,321183518	0,130274947	2,465428122	0,013684963	0,06332028	protein_codin	hypothetical protein
TcG_01767	2,308126441	0,612868731	1,140842778	0,537207004	0,591124633	1	protein_codin	putative membrane-bound acid phosphatase

TcG_01768	1891,915848	0,52887236	0,049818259	10,6160345	2,50999E-26	8,81233E-24	protein_codin putative membrane-bound acid phosphatase
TcG_01769	265,8136707	0,195900541	0,111457049	1,757632586	0,078810045	0,22861622	protein_codin putative MRP protein
TcG_01770	738,3986218	0,503048851	0,071731532	7,012938869	2,33363E-12	1,66898E-10	protein_codin ribosomal protein S29
TcG_01771	282,8445834	0,029797772	0,109663945	0,27171895	0,78583813	0,893514718	protein_codin putative FG-GAP repeat protein
TcG_01772	161,7015634	0,083104981	0,150666728	0,551581506	0,58123511	0,770482335	protein_codin hypothetical protein
TcG_01773	739,9888763	-0,039837698	0,078784122	-0,50565644	0,613097854	0,790819724	protein_codin GDP-mannose pyrophosphorylase
TcG_01774	891,4191249	-0,207330826	0,06692233	-3,09808142	0,001947779	0,013456749	protein_codin vacuolar ATPase subunit B
TcG_01775	883,1710604	-0,165211239	0,064405637	-2,56516738	0,010312605	0,050606453	protein_codin 2-oxoglutarate dehydrogenase, E2 component, dihydrolipoamide succinyltransferase
TcG_01776	169,2254737	-0,265852399	0,142559304	-1,86485477	0,062201763	0,194819283	protein_codin putative dolichyl-P-Man:GDP-Man5GlcNAc2-PP-dolichyl alpha-1,2-mannosyltranslocase
TcG_01777	340,6856938	0,111724097	0,09686007	1,15345877	0,248722131	0,478209066	protein_codin putative tRNA methyltransferase complex subunit
TcG_01778	508,0139682	0,071172451	0,082479648	0,862909252	0,388187364	0,618473432	protein_codin putative glutamic acid-rich protein precursor
TcG_01779	439,1188132	-0,243465248	0,09394627	-2,59153714	0,009554823	0,047695896	protein_codin putative DNA replication licensing factor
TcG_01780	10,12296909	0,556834564	0,55729939	0,999165932	0,317714316	1	protein_codin cation transporter
TcG_01781	259,6699177	0,423319371	0,115971163	3,650212358	0,000262024	0,002502725	protein_codin putative cation transporter
TcG_01782	6,753434972	0,173159259	0,68185086	0,253954742	0,799530523	1	protein_codin cation transporter
TcG_01783	381,8631546	-0,060378329	0,094732829	-0,6373538	0,523894414	0,72776714	protein_codin putative ZIP Zn transporter
TcG_01784	22,19213698	-0,010194181	0,378910447	-0,02690393	0,978536356	0,990851444	protein_codin ZIP Zn transporter
TcG_01785	2373,063181	-0,168012991	0,049952161	-3,36347792	0,00076967	0,006253433	protein_codin putative cation transporter
TcG_01786	1795,465564	0,322521866	0,054510787	5,916661287	3,28542E-09	1,16644E-07	protein_codin putative X-pro, dipeptidyl-peptidase,serine peptidase,Clan SC, family S15
TcG_01787	233,8708938	0,320340268	0,121885763	2,628200867	0,008583781	0,043849951	protein_codin putative elongation factor G2-like protein
TcG_01788	340,5024343	0,387542161	0,100725746	3,84749855	0,00011933	0,001297166	protein_codin elongation factor G2-like protein
TcG_01789	316,1613103	0,138485909	0,110401245	1,254387197	0,209701304	0,432236133	protein_codin ribose 5-phosphate isomerase
TcG_01790	518,3815882	0,079247555	0,081462922	0,972805212	0,330650122	0,565235831	protein_codin hypothetical protein
TcG_01791	727,0607347	-0,127977886	0,078502168	-1,63024652	0,103049406	0,274529872	protein_codin leucine rich
TcG_01792	335,3979647	0,215191761	0,103709049	2,074956457	0,037990571	0,136145608	protein_codin Small Surface Antigen
TcG_01793	271,8029063	0,103637443	0,109602511	0,945575449	0,344365171	0,578569442	protein_codin hypothetical protein
TcG_01794	236,3891453	-0,274344226	0,11461387	-2,39363897	0,01668216	0,073295225	protein_codin hypothetical protein
TcG_01795	153,2022787	0,14185479	0,14603896	0,971348946	0,331374543	0,565601864	protein_codin putative NADH dehydrogenase subunit NB6M
TcG_01796	147,5059575	-0,032887527	0,151194069	-0,21751863	0,827804189	0,915880831	protein_codin DPCD protein
TcG_01797	181,1843687	0,071223891	0,140673746	0,506305496	0,612642205	0,790819724	protein_codin DNA-directed RNA polymerase I/III subunit
TcG_01798	338,1225626	0,065825273	0,100258142	0,656557881	0,511465239	0,717683589	protein_codin putative translation factor (SUA5)
TcG_01799	599,5071481	-0,048427442	0,075701163	-0,63971859	0,522355565	0,726192662	protein_codin mitochondrial DEAD box protein
TcG_01800	189,6885929	-0,288909143	0,134852682	-2,14240562	0,032160856	0,120469363	protein_codin centrosomal protein 76kDa
TcG_01801	72,7457629	0,240576921	0,222097597	1,08320362	0,278718053	0,511682359	protein_codin hypothetical protein
TcG_01802	203,5650359	-0,214316026	0,127797881	-1,67699202	0,093544042	0,2567641	protein_codin glycerophosphoryl diester phosphodiesterase
TcG_01803	222,0524215	0,134379561	0,125036809	1,074720016	0,2825001	0,51568397	protein_codin L1Tc protein
TcG_01804	5,254969218	-0,519660765	0,810121256	-0,64146047	0,521223553	1	protein_codin hypothetical protein
TcG_01805	0					1	protein_codin hypothetical protein
TcG_01806	0,888260645	-0,268975824	1,768148023	-0,15212291	0,879089995	1	protein_codin hypothetical protein
TcG_01807	15,04434802	-0,569016982	0,452237631	-1,25822564	0,208310165	1	protein_codin hypothetical protein
TcG_01808	46,07625854	0,193666652	0,258516947	0,749144898	0,453769877	0,674035553	protein_codin helicase-like protein
TcG_01809	15,18230185	-0,178425067	0,43582575	-0,40939542	0,682249498	1	protein_codin putative trans-sialidase
TcG_01810	16,106836	0,495512376	0,442551372	1,119671991	0,262853565	1	protein_codin putative glycogen synthase kinase-3 alpha
TcG_01811	351,6854964	-0,306357013	0,095401235	-3,21124787	0,001321599	0,009796574	protein_codin putative cullin 2
TcG_01812	301,0922514	-0,144380054	0,111173606	-1,29869003	0,194050326	0,411017747	protein_codin putative endonuclease III
TcG_01813	136,7438485	-0,004707622	0,149055116	-0,0315831	0,974804523	0,988974186	protein_codin type 11 methyltransferase
TcG_01814	1050,351357	0,029709254	0,060236884	0,493207014	0,621866323	0,796361113	protein_codin hypothetical protein
TcG_01815	140,2521384	0,181612773	0,15116826	1,201394881	0,229598061	0,456821116	protein_codin hypothetical protein
TcG_01816	141,7925483	-0,065005782	0,148847531	-0,43672731	0,662309118	0,823073415	protein_codin hypothetical protein
TcG_01817	252,3489409	0,182023256	0,117996987	1,54260935	0,122925588	0,30780546	protein_codin hypothetical protein
TcG_01818	185,9163249	0,185842283	0,134702664	1,37964816	0,167695001	0,37370923	protein_codin hypothetical protein
TcG_01819	393,5399068	-0,077043981	0,096807072	-0,79585075	0,426118791	0,651750801	protein_codin mannosyl-oligosaccharide glucosidase
TcG_01820	367,5548145	-0,026843235	0,101732213	-0,26386171	0,791886483	0,896041689	protein_codin mannosyl-oligosaccharide glucosidase
TcG_01821	308,6405746	-0,096430818	0,117317919	-0,82196155	0,411098782	0,637786622	protein_codin trichohyalin
TcG_01822	257,5841426	-0,049425341	0,110734037	-0,4463428	0,655349641	0,817728228	protein_codin trichohyalin
TcG_01823	337,198098	0,125963683	0,100181511	1,257354588	0,208625266	0,431207169	protein_codin putative 2,3-bisphosphoglycerate-independent phosphoglycerate mutase
TcG_01824	146,8168421	-0,211029905	0,153708916	-1,37291909	0,169777502	0,376067037	protein_codin hypothetical protein

TcG_01825	924,925312	-0,11407022	0,070276622	-1,62316025	0,104555152	0,277522847	protein_codin putative cyclin dependent kinase-binding protein
TcG_01826	127,9308446	0,249739565	0,17103229	1,46018956	0,144237984	0,340840563	protein_codin hypothetical protein
TcG_01827	1449,000782	0,16826855	0,06011142	2,799277567	0,005121709	0,029231586	protein_codin putative glycosomal membrane protein
TcG_01828	813,127454	0,108150873	0,072423111	1,493319906	0,135353467	0,327596673	protein_codin hypothetical protein
TcG_01829	417,8223765	0,163613758	0,091523998	1,787659648	0,073830936	0,219272823	protein_codin histone acetyltransferase
TcG_01830	1239,783796	-0,01290053	0,061268955	-0,21055573	0,833233958	0,919089983	protein_codin DNA topoisomerase 2
TcG_01831	631,3243851	-0,053753517	0,076384366	-0,70372407	0,481604629	0,695708707	protein_codin translation initiation factor eIF-2B subunit epsilon
TcG_01832	262,5877607	-0,154123181	0,125163976	-1,23137013	0,218184463	0,442556931	protein_codin hypothetical protein
TcG_01833	511,481417	-0,121345811	0,083078527	-1,46061582	0,144120902	0,340781521	protein_codin putative eukaryotic translation initiation factor
TcG_01834	321,0303868	0,105655246	0,100960952	1,046496133	0,29533203	0,528097822	protein_codin hypothetical protein
TcG_01835	342,4221091	0,112982441	0,098035799	1,152461067	0,24913166	0,478493188	protein_codin hypothetical protein
TcG_01836	388,9923834	-0,064445236	0,096512242	-0,66774158	0,504298566	0,712076368	protein_codin hypothetical protein
TcG_01837	257,9897979	-0,090255719	0,122671006	-0,73575429	0,461880244	0,679535811	protein_codin hypothetical protein
TcG_01838	137,1329395	-0,119395254	0,150003729	-0,79594857	0,426061927	0,651750801	protein_codin surface protease GP63
TcG_01839	344,2742251	0,226078022	0,10040371	2,251689925	0,024341874	0,09782343	protein_codin hypothetical protein
TcG_01840	1414,371152	0,037017171	0,057418411	0,644691668	0,519127017	0,7237563	protein_codin aminopeptidase
TcG_01841	400,1596029	0,142301272	0,092354309	1,540818977	0,123360849	0,308695205	protein_codin hypothetical protein
TcG_01842	410,2370368	0,022849249	0,100761882	0,226764807	0,820606631	0,911143056	protein_codin hypothetical protein
TcG_01843	702,9201794	0,118731691	0,076681483	1,548374985	0,12153204	0,30590272	protein_codin hypothetical protein
TcG_01844	6,819920763	-0,540917265	1,255307339	-0,43090425	0,666537995	1	protein_codin hypothetical protein
TcG_01845	327,2242952	0,093858213	0,108393677	0,865901186	0,386544353	0,616788717	protein_codin hypothetical protein
TcG_01846	1164,925627	0,226387006	0,062110131	3,644928796	0,000267466	0,002537893	protein_codin hypothetical protein
TcG_01847	737,5814193	0,28803188	0,0763197	3,774017457	0,00016064	0,00166921	protein_codin hypothetical protein
TcG_01848	364,5806724	0,261416531	0,097030619	2,694165334	0,007056515	0,037520323	protein_codin putative membrane transporter
TcG_01849	157,6924497	-0,062683006	0,14254447	-0,43974351	0,660122885	0,821678528	protein_codin ankyrin
TcG_01850	166,8959778	0,234056366	0,139033858	1,683448687	0,092288265	0,254099771	protein_codin hypothetical protein
TcG_01851	373,5447175	-0,007875617	0,102339454	-0,07695583	0,938658687	0,971680773	protein_codin putative anion-transporting ATPase-like
TcG_01852	436,9071821	-0,115944739	0,086609576	-1,33870578	0,180666475	0,392062146	protein_codin hypothetical protein
TcG_01853	3455,059727	-0,174819233	0,043340034	-4,03366627	5,49133E-05	0,000669711	protein_codin PUF nine target 1
TcG_01854	512,8200551	-0,133507421	0,088609934	-1,50668683	0,131890949	0,322809808	protein_codin hypothetical protein
TcG_01855	398,7230431	-0,473891961	0,092546631	-5,12057495	3,04606E-07	6,98843E-06	protein_codin hypothetical protein
TcG_01856	273,3859567	-0,1052239	0,113713329	-0,92534359	0,35478721	0,588737412	protein_codin hypothetical protein
TcG_01857	272,8053804	-0,017860247	0,116114737	-0,1538155	0,877755209	0,941200542	protein_codin 40S ribosomal protein S21
TcG_01858	2145,412623	0,074214364	0,049707893	1,493009662	0,135434658	0,327724717	protein_codin 40S ribosomal protein S21
TcG_01859	249,197306	0,126862528	0,118907643	1,066899691	0,286017134	0,519187736	protein_codin hypothetical protein
TcG_01860	293,8621843	0,318236188	0,114063991	2,789979418	0,005271139	0,029843484	protein_codin putative helicase ARIP4 isoform X3
TcG_01861	136,2490785	-0,075224684	0,158724013	-0,47393386	0,635547072	0,805540791	protein_codin hypothetical protein
TcG_01862	742,6504442	-0,175861507	0,073725294	-2,38536189	0,017062327	0,074456429	protein_codin hypothetical protein
TcG_01863	202,5637778	0,005422656	0,123666614	0,04384899	0,965024776	0,984396642	protein_codin putative serine incorporator
TcG_01864	475,0859209	-0,054752342	0,08476866	-0,64590312	0,518342096	0,723381296	protein_codin putative serine incorporator
TcG_01865	1691,968305	-0,243879242	0,060486993	-4,03192865	5,5321E-05	0,000673974	protein_codin hypothetical protein
TcG_01866	1877,879071	-0,344753592	0,05590456	-6,16682418	6,96752E-10	2,88306E-08	protein_codin hypothetical protein
TcG_01867	485,1106587	-0,062193646	0,084795972	-0,73345048	0,463283721	0,680908942	protein_codin hypothetical protein
TcG_01868	671,6119818	-0,194352933	0,075334364	-2,57987089	0,009883726	0,048958037	protein_codin hypothetical protein
TcG_01869	501,5750174	-0,101653219	0,082916201	-1,22597535	0,22020795	0,445300625	protein_codin hypothetical protein
TcG_01870	1406,652429	0,054072099	0,056552644	0,95613742	0,339002811	0,57332217	protein_codin hypothetical protein
TcG_01871	894,4450916	0,167156869	0,066483595	2,514257359	0,011928335	0,056733043	protein_codin ARM repeat-containing protein
TcG_01872	1210,556253	-0,104300882	0,058787558	-1,77419995	0,076030082	0,223065214	protein_codin putative kinesin
TcG_01873	349,4203443	-0,070062929	0,100710126	-0,69568902	0,486623602	0,698438886	protein_codin hypothetical protein
TcG_01874	375,0487556	-0,111536101	0,095252751	-1,17094887	0,241619331	0,470250557	protein_codin hypothetical protein
TcG_01875	601,8032581	-0,186182152	0,080786496	-2,30461973	0,02118788	0,088040307	protein_codin hypothetical protein
TcG_01876	473,8630828	-0,028456132	0,085528968	-0,33270754	0,73935506	0,867087269	protein_codin 60S ribosomal protein L24
TcG_01877	806,3909511	0,016960305	0,067851714	0,249961333	0,802617251	0,902389468	protein_codin putative ATPase
TcG_01878	320,2226098	-0,095716589	0,112461247	-0,85110731	0,394709746	0,624314965	protein_codin hypothetical protein
TcG_01879	477,2155671	-0,111732142	0,083901397	-1,33170776	0,182956238	0,395250975	protein_codin hypothetical protein
TcG_01880	252,5998433	0,163141392	0,117553617	1,387804098	0,165196706	0,370715499	protein_codin hypothetical protein
TcG_01881	266,5173455	-0,356509501	0,114211089	-3,12149639	0,001799345	0,01263467	protein_codin 40S ribosomal protein S5

TcG_01882	306,7263284	-0,25058553	0,103386288	-2,42377916	0,015359943	0,068935024	protein_codin 40S ribosomal protein S5
TcG_01883	146,9212713	0,198947295	0,154044377	1,291493387	0,196532647	0,414732323	protein_codin hypothetical protein
TcG_01884	129,094261	0,028112067	0,169933604	0,165429709	0,868605771	0,936741226	protein_codin beta-adaptin 1
TcG_01885	320,5832489	-0,172283299	0,102569771	-1,67966934	0,093021668	0,255693723	protein_codin putative beta-adaptin 1
TcG_01886	3847,24079	0,213773325	0,041593468	5,139588796	2,7534E-07	6,38019E-06	protein_codin pyruvate phosphate dikinase 1
TcG_01887	98,99801681	-0,011069492	0,181114763	-0,06111866	0,951264705	0,97713458	protein_codin inositol-1,4,5-trisphosphate (IP3) 5-phosphatase
TcG_01888	212,156531	-0,02675784	0,122530914	-0,21837624	0,827135978	0,915585603	protein_codin putative inositol-1,4,5-trisphosphate (IP3) 5-phosphatase
TcG_01889	424,5078097	-0,042891072	0,08904051	-0,4817029	0,630017016	0,801688869	protein_codin hypothetical protein
TcG_01890	741,0472965	-0,154569664	0,071668172	-2,15674072	0,031025873	0,117204356	protein_codin hypothetical protein
TcG_01891	49,96327857	0,255962757	0,248177353	1,031370323	0,302367192	0,535372803	protein_codin hypothetical protein
TcG_01892	8,130433007	0,440002598	0,602418803	0,730393201	0,465149873	1	
TcG_01893	1440,586908	-0,398738339	0,055352794	-7,2035811	5,86514E-13	4,59145E-11	protein_codin putative SEC61-like (pretranslocation process) protein
TcG_01894	433,4877269	-0,416306037	0,088451887	-4,70658177	2,51905E-06	4,58174E-05	protein_codin putative zinc finger domain protein
TcG_01895	1357,242299	-0,180632302	0,054866898	-3,29219091	0,000994101	0,007740359	protein_codin putative lanosterol 14-alpha-demethylase
TcG_01896	76,4572348	0,25022739	0,199301715	1,255520501	0,209289871	0,431872099	protein_codin 60S ribosomal protein L28
TcG_01897	95,85883855	-0,024409809	0,1805651	-0,13518564	0,892465098	0,947937351	protein_codin hypothetical protein
TcG_01898	395,6228571	0,15012282	0,095916355	1,565143092	0,117549362	0,298974288	protein_codin 60S ribosomal protein L28
TcG_01899	32,4080152	0,075927582	0,306203656	0,247964321	0,804162013	0,903185306	protein_codin 60S ribosomal protein L28
TcG_01900	345,3032591	0,051253027	0,097873797	0,523664434	0,600511957	0,783588603	protein_codin phosphatidylinositol-4-phosphate 5-kinase
TcG_01901	1083,844032	-0,320616259	0,064572796	-4,96519089	6,86335E-07	1,44843E-05	protein_codin putative protein transport protein Sec31
TcG_01902	274,4177467	-0,032225684	0,113848206	-0,28305834	0,777132119	0,888830477	protein_codin putative eukaryotic release factor 3
TcG_01903	747,5169038	-0,014728436	0,069842709	-0,21088008	0,83298085	0,919089983	protein_codin putative eukaryotic release factor 3
TcG_01904	266,3040575	-0,158840641	0,110825142	-1,43325457	0,1517851	0,351998032	protein_codin preprotein translocase subunit YidC
TcG_01905	454,6834881	0,153387303	0,088388126	1,735383576	0,082672887	0,236443225	protein_codin methyltransferase
TcG_01906	891,6385589	0,412549012	0,065829866	6,26689735	3,68313E-10	1,62873E-08	protein_codin 40S ribosomal protein S15a
TcG_01907	318,0757165	-0,074928852	0,100033964	-0,74903412	0,453836643	0,674035553	protein_codin hypothetical protein
TcG_01908	283,7737511	-0,661390223	0,111183588	-5,94863178	2,70393E-09	9,82061E-08	protein_codin S-phase kinase-associated protein
TcG_01909	355,9388866	-0,735210207	0,098809013	-7,44072005	1,00138E-13	9,06405E-12	
TcG_01910	40,64958409	-0,901634392	0,277814704	-3,24545238	0,001172642	0,00889151	
TcG_01911	473,240423	0,086481547	0,0880993	0,981637166	0,326278631	0,560721119	protein_codin ABC1 transporter
TcG_01912	239,262866	0,018658735	0,120583004	0,154737682	0,877028122	0,941029583	protein_codin heat shock protein-like protein
TcG_01913	245,0680518	0,090441724	0,11547658	0,783204041	0,433507302	0,657927116	protein_codin tatD related deoxyribonuclease
TcG_01914	196,6712088	-0,420157199	0,13394191	-3,13686133	0,001707669	0,012108354	protein_codin putative protein kinase
TcG_01915	221,9310747	-0,274820823	0,122446185	-2,24442127	0,024805309	0,099135672	protein_codin putative acyl-CoA thioesterase
TcG_01916	294,4006412	-0,15781007	0,104646392	-1,50803164	0,131546429	0,322355525	protein_codin putative protein kinase
TcG_01917	210,3600646	-0,107230988	0,128409977	-0,83506742	0,403679752	0,63312391	protein_codin hypothetical protein
TcG_01918	488,4977199	-0,258154891	0,08419665	-3,06609457	0,002168747	0,014677047	protein_codin putative retrotransposon hot spot (RHS) protein
TcG_01919	873,530498	-0,023281467	0,068347236	-0,34063509	0,733378312	0,862982036	protein_codin hypothetical protein
TcG_01920	457,2282831	0,042752862	0,086398105	0,494835647	0,62071614	0,795181025	protein_codin hypothetical protein
TcG_01921	192,5069444	-0,017627531	0,131197944	-0,13435829	0,893119263	0,948402185	protein_codin putative small GTP-binding protein RAB6
TcG_01922	176,5355876	-0,34453522	0,143723063	-2,39721596	0,016520184	0,072804432	protein_codin hypothetical protein
TcG_01923	177,408189	-0,172528403	0,134060661	-1,2869428	0,198114225	0,416730466	protein_codin hypothetical protein
TcG_01924	392,3919917	-0,208598553	0,092836234	-2,24695191	0,024643101	0,098684512	protein_codin hypothetical protein
TcG_01925	257,945759	0,193936497	0,112285807	1,727168397	0,084137425	0,238927435	protein_codin tRNA pseudouridine13 synthase
TcG_01926	427,0673579	0,074776323	0,089499884	0,835490722	0,403441469	0,63312391	protein_codin hypothetical protein
TcG_01927	573,8341953	-0,023918667	0,078659287	-0,30407938	0,761067413	0,879398329	protein_codin phosphatidylinositol-phospholipase C
TcG_01928	76,29399232	0,040291452	0,205161783	0,196388682	0,844305954	0,92449946	protein_codin putative trans-sialidase
TcG_01929	0,286047678	-0,458748634	3,065513525	-0,14964822	0,881042165	1	protein_codin hypothetical protein
TcG_01930	0					1	
TcG_01931	1,001587711	-2,491122606	3,853773934	-0,64641119	0,518013094	1	
TcG_01932	0					1	
TcG_01933	1,248952181	0,97832668	1,668608237	0,586312987	0,557665205	1	
TcG_01934	0					1	
TcG_01935	0,324841555	-0,458748506	2,940479636	-0,15601146	0,876023992	1	
TcG_01936	0,714418641	1,39408784	2,101031487	0,663525439	0,506994092	1	
TcG_01937	171,9210003	-0,02235972	0,157070994	-0,14235423	0,886800213	0,945300144	protein_codin putative ubiquitin-conjugating enzyme e2
TcG_01938	111,9986126	-0,19619228	0,170132841	-1,15317113	0,248840152	0,478278367	protein_codin hypothetical protein

TcG_01939	201,8037417	0,060656551	0,126543017	0,479335427	0,631700023	0,802906018	protein_codin hypothetical protein
TcG_01940	624,8072846	0,307680177	0,084797503	3,628410817	0,000285171	0,002687053	protein_codin putative dihydroxyacetone phosphate acyltransferase
TcG_01941	203,641785	0,14952396	0,127987892	1,168266452	0,242699303	0,47105644	protein_codin hypothetical protein
TcG_01942	106,0409666	0,127963336	0,176203282	0,726225613	0,467700472	0,684316061	protein_codin hypothetical protein
TcG_01943	245,9758138	-0,013875817	0,113618594	-0,12212628	0,902799007	0,952972786	protein_codin SBDS-like protein
TcG_01944	139,774208	0,271219723	0,151505998	1,790158324	0,073428463	0,218306946	protein_codin sperm flagellar protein 1
TcG_01945	286,1939271	0,037524935	0,106527687	0,352255235	0,724646857	0,85802335	protein_codin hypothetical protein
TcG_01946	152,7207963	0,174391961	0,141200113	1,23506956	0,216804617	0,440893872	protein_codin putative tRNA-dihydrouridine synthase 4
TcG_01947	143,4667585	0,226491527	0,155421793	1,457270073	0,145041844	0,341903318	protein_codin hypothetical protein
TcG_01948	128,1820884	0,021470044	0,156568892	0,137128414	0,890929294	0,946913751	protein_codin hypothetical protein
TcG_01949	97,97276654	0,577870161	0,181784408	3,178876384	0,001478471	0,010739541	protein_codin hypothetical protein
TcG_01950	301,0021636	-0,169405028	0,106162924	-1,59570801	0,110554	0,287450325	protein_codin hypothetical protein
TcG_01951	351,0055135	-0,09434827	0,10065574	-0,93733621	0,348585643	0,583103728	protein_codin hypothetical protein
TcG_01952	318,6946585	-0,200324654	0,10288706	-1,9470345	0,051530599	0,168764068	protein_codin hypothetical protein
TcG_01953	232,2687267	0,061523634	0,116736546	0,527029759	0,598172923	0,78203921	protein_codin hypothetical protein
TcG_01954	228,278739	0,448764783	0,120192213	3,73372595	0,000188668	0,001905758	protein_codin hypothetical protein
TcG_01955	585,7189305	0,014826565	0,087081306	0,170261164	0,864804752	0,934985064	protein_codin ATP-dependent RNA helicase-like protein
TcG_01956	165,8769674	0,432264728	0,141664469	3,051327763	0,002278317	0,015293501	protein_codin hypothetical protein
TcG_01957	121,4727219	0,015354105	0,160526649	0,095648327	0,923799882	0,962772819	protein_codin hypothetical protein
TcG_01958	257,9427046	-0,17873662	0,115564634	-1,54663771	0,121950632	0,30662327	protein_codin inositol 5-phosphatase-like protein
TcG_01959	497,6976103	0,027649381	0,086447444	0,319840348	0,74908936	0,873397335	protein_codin hypothetical protein
TcG_01960	219,2066235	-0,062854052	0,124310997	-0,5056194	0,613123857	0,790819724	protein_codin hypothetical protein
TcG_01961	132,7037	-0,083891517	0,158666825	-0,52872752	0,596994483	0,780939153	protein_codin chaperone DnaJ protein
TcG_01962	114,7338676	0,22227224	0,17255596	1,288116848	0,197705291	0,416248138	protein_codin hypothetical protein
TcG_01963	412,9220216	-0,079651716	0,089472044	-0,89024139	0,373336286	0,604369737	protein_codin DNA polymerase I-like protein A
TcG_01964	851,6426269	0,321983857	0,074596228	4,316355721	1,58626E-05	0,000226335	protein_codin hypothetical protein
TcG_01965	120,1440517	-0,118857352	0,172270956	-0,68994423	0,490229259	0,701302546	protein_codin hypothetical protein
TcG_01966	137,4531395	0,262172698	0,159674422	1,641920445	0,100606492	0,269821023	protein_codin hypothetical protein
TcG_01967	196,2789518	-0,099829827	0,126244875	-0,7907634	0,429082071	0,653987669	protein_codin hypothetical protein
TcG_01968	320,5505811	-0,13636507	0,103496207	-1,3175852	0,187642543	0,402050738	protein_codin hypothetical protein
TcG_01969	236,2486701	-0,260268216	0,116650074	-2,23118774	0,025668696	0,101813595	protein_codin hypothetical protein
TcG_01970	467,9313176	0,062699478	0,08469218	0,740321928	0,459104678	0,677776096	protein_codin hypothetical protein
TcG_01971	475,8427265	-0,070382426	0,084905032	-0,8289547	0,407130041	0,635458528	protein_codin hypothetical protein
TcG_01972	1274,556974	-0,007691835	0,064637956	-0,11899874	0,905276356	0,953903154	protein_codin hypothetical protein
TcG_01973	149,1157407	0,067654678	0,152551993	0,443486033	0,65741422	0,819281459	protein_codin hypothetical protein
TcG_01974	1725,012711	0,283778141	0,121810639	2,329666303	0,019823795	0,083367872	protein_codin hypothetical protein
TcG_01975	144,9790827	0,076008473	0,151311935	0,502329658	0,61543565	0,79219196	protein_codin ESCRT-II complex subunit VPS22
TcG_01976	677,7321198	0,341522327	0,072070066	4,738754163	2,15036E-06	3,98625E-05	protein_codin D-isomer specific 2-hydroxyacid dehydrogenase-protein
TcG_01977	693,7379804	0,283487938	0,074797765	3,790058928	0,000150612	0,001579172	protein_codin D-isomer specific 2-hydroxyacid dehydrogenase-protein
TcG_01978	635,4812253	0,177567376	0,079934676	2,221406077	0,02632347	0,103665439	protein_codin hypothetical protein
TcG_01979	372,7408063	0,101948709	0,09484133	1,074939683	0,282401734	0,515585642	protein_codin hypothetical protein
TcG_01980	79,88499207	0,582884036	0,202384448	2,880083144	0,003975703	0,023953456	protein_codin hypothetical protein
TcG_01981	358,8753778	0,14165169	0,100040002	1,415950488	0,156790001	0,358297624	protein_codin putative tyrosine phosphatase isoform
TcG_01982	516,4771197	-0,239406438	0,080879597	-2,96003499	0,003076041	0,019443794	protein_codin hypothetical protein
TcG_01983	358,3663192	0,211491763	0,098687338	2,143048608	0,032109195	0,120316021	protein_codin hypothetical protein
TcG_01984	243,1627038	-0,046548344	0,116238109	-0,40045683	0,688820074	0,838445877	protein_codin hypothetical protein
TcG_01985	162,9546796	-0,178823688	0,151481657	-1,1804973	0,237802482	0,465651011	protein_codin hypothetical protein
TcG_01986	288,1054092	0,141523537	0,109153171	1,296559105	0,194782931	0,412174055	protein_codin hypothetical protein
TcG_01987	12,28976675	0,547070667	0,504281869	1,084850954	0,277987673	1	protein_codin hypothetical protein
TcG_01988	25,78774804	0,072082778	0,355363604	0,202842319	0,839258279	0,921926912	protein_codin hypothetical protein
TcG_01989	293,6519277	0,073129495	0,104773506	0,697976978	0,485191592	0,698140808	protein_codin tetratricopeptidedomain 39C
TcG_01990	5,618020417	0,218010379	0,72150459	0,302160765	0,762529512	1	protein_codin hypothetical protein
TcG_01991	92,53307032	0,13788183	0,185797143	0,742109526	0,458020978	0,677211722	protein_codin hypothetical protein
TcG_01992	515,9199067	-0,033208482	0,084132793	-0,39471508	0,693053173	0,841062696	protein_codin hypothetical protein
TcG_01993	332,0239614	-0,09571644	0,099567082	-0,96132615	0,336388204	0,570804865	
TcG_01994	716,3312151	-0,030492871	0,077639482	-0,39274954	0,694504475	0,841951328	protein_codin flagellar associated protein
TcG_01995	204,9188075	0,118804727	0,126890889	0,936274682	0,34913178	0,583615756	protein_codin putative kinesin-like protein

TcG_01996	11,67291943	-0,149248029	0,509828344	-0,29274172	0,769719575	1	protein_codin	putative kinesin-like protein
TcG_01997	34,93976588	0,643093828	0,294832911	2,181214525	0,029167553	0,112010364	protein_codin	kinesin-like protein
TcG_01998	16,32472672	0,360016619	0,436752942	0,824302674	0,409767599	1	protein_codin	putative kinesin-like protein
TcG_01999	138,9995697	-0,00106232	0,148480783	-0,0071546	0,994291507	0,997731905	protein_codin	hypothetical protein
TcG_02000	437,8998151	0,009173212	0,095430386	0,096124646	0,923421577	0,962648944	protein_codin	putative ATP dependent DEAD-box helicase
TcG_02001	174,9144387	0,092049727	0,141082298	0,652454123	0,514108259	0,720089936	protein_codin	ubiquitin-conjugating enzyme E2
TcG_02002	430,0703526	-0,314197616	0,089645142	-3,504904	0,000456772	0,004000121	protein_codin	putative hydroxymethylglutaryl-CoA lyase, putative,3-hydroxy-3-methylglutarate-CoA lyase
TcG_02003	411,8532587	0,130956447	0,089672392	1,460387577	0,144183585	0,340781521	protein_codin	hypothetical protein
TcG_02004	263,1385802	0,055662732	0,11481986	0,484783135	0,627830187	0,800576772	protein_codin	putative pyroglutamyl-peptidase I (PGP), putative,cysteine peptidase, Clan CF, family C15
TcG_02005	422,3703941	-0,090514164	0,090906372	-0,99568558	0,319402937	0,554400221	protein_codin	hypothetical protein
TcG_02006	311,4463742	-0,077755141	0,104578804	-0,74350765	0,457174391	0,676783905	protein_codin	hypothetical protein
TcG_02007	554,0576736	0,004121519	0,082283656	0,050089159	0,960051339	0,981656942	protein_codin	hypothetical protein
TcG_02008	472,7141265	0,085953727	0,086492904	0,993766226	0,320336692	0,555518772	protein_codin	putative ATP-dependent RNA helicase
TcG_02009	346,8606183	-0,110753939	0,100744214	-1,09935782	0,271612022	0,504714817	protein_codin	putative glycogenin glucosyltransferase
TcG_02010	146,7601056	0,175466463	0,152520142	1,150447813	0,249959477	0,479316534	protein_codin	hypothetical protein
TcG_02011	63,11641749	-0,425366791	0,218219498	-1,94926115	0,051264245	0,168400211	protein_codin	folate/pteridine transporter
TcG_02012	531,1930143	0,225576503	0,180196765	1,251834369	0,210630224	0,433149054	protein_codin	NADPH cytochrome P450 reductase B
TcG_02013	283,737946	-0,359410899	0,106358186	-3,37924998	0,000726839	0,005942946	protein_codin	putative RNA binding protein
TcG_02014	220,559352	0,062711585	0,121454453	0,516338289	0,605618158	0,786889311	protein_codin	hypothetical protein
TcG_02015	220,7819747	0,099659469	0,126441561	0,788186009	0,430587912	0,655385122	protein_codin	Low complexity protein
TcG_02016	200,9428912	0,066263319	0,127064316	0,52149432	0,602022462	0,784755061	protein_codin	RING finger protein 32
TcG_02017	115,972855	0,329215434	0,167405901	1,966570069	0,049232797	0,163693509	protein_codin	hypothetical protein
TcG_02018	300,3226185	-0,028749801	0,105785372	-0,27177482	0,785795166	0,893514718	protein_codin	hypothetical protein
TcG_02019	517,6821625	-0,046500834	0,083304747	-0,55820149	0,576706802	0,766888498	protein_codin	hypothetical protein
TcG_02020	243,783614	0,06841937	0,12297846	0,556352475	0,577969921	0,768001978	protein_codin	hypothetical protein
TcG_02021	207,7786676	0,318170394	0,139956965	2,273344483	0,023005429	0,093852429	protein_codin	hypothetical protein
TcG_02022	366,4517177	-0,103473533	0,097252967	-1,06396274	0,287345579	0,520429245	protein_codin	E3 ubiquitin-protein ligase TRIP12
TcG_02023	180,8307569	0,049652793	0,132719388	0,374118611	0,708316073	0,849437494	protein_codin	fumarylpyruvate hydrolase
TcG_02024	369,3683626	0,118605902	0,098619739	1,20265885	0,229108364	0,456268888	protein_codin	hypothetical protein
TcG_02025	119,640635	0,180623599	0,166341883	1,085857605	0,277541996	0,510008178	protein_codin	hypothetical protein
TcG_02026	579,6559161	-0,059745819	0,081625556	-0,73194992	0,464199137	0,681704862	protein_codin	putative phosphoinositide-binding protein
TcG_02027	68,8164557	-0,183224167	0,235455746	-0,77816817	0,436469878	0,660007183	protein_codin	ubiquitin hydrolase
TcG_02028	590,2804554	-0,18514881	0,07903185	-2,34271133	0,019144189	0,081157912	protein_codin	hypothetical protein
TcG_02029	190,3344696	0,276957504	0,13521283	2,048307875	0,040529842	0,142512518	protein_codin	hypothetical protein
TcG_02030	445,5757519	0,010939395	0,094511094	0,115747205	0,907852893	0,955360827	protein_codin	hypothetical protein
TcG_02031	144,9925183	0,357890139	0,149503304	2,393861075	0,016672062	0,073278647	protein_codin	hypothetical protein
TcG_02032	609,2093166	0,051634059	0,075382811	0,684958002	0,493370424	0,703987578	protein_codin	metallo-peptidase, Clan MG, Family M24
TcG_02033	478,29628	0,050516833	0,085037152	0,594056023	0,552474643	0,747864379	protein_codin	putative ribonuclease
TcG_02034	345,0372301	0,230161232	0,102151493	2,253136257	0,024250559	0,097659707	protein_codin	hypothetical protein
TcG_02035	1267,562634	0,037849488	0,059042636	0,641053497	0,521487926	0,725401608	protein_codin	transportin 1
TcG_02036	115,8886609	-0,047860274	0,163550696	-0,29263265	0,769802951	0,884364283	protein_codin	hypothetical protein
TcG_02037	207,6729408	0,036693206	0,124233774	0,29535612	0,76772185	0,883509739	protein_codin	hypothetical protein
TcG_02038	296,5635341	0,076235518	0,112379969	0,678372832	0,49753533	0,707281334	protein_codin	hypothetical protein
TcG_02039	120,2764242	0,037151202	0,158102054	0,234982411	0,814222385	0,908262507	protein_codin	hypothetical protein
TcG_02040	103,8342	0,428483206	0,173775021	2,465735318	0,013673233	0,063291281	protein_codin	hypothetical protein
TcG_02041	409,6843924	0,111882944	0,092125338	1,214464402	0,224570436	0,451082534	protein_codin	hypothetical protein
TcG_02042	954,2878663	-0,178676376	0,070232567	-2,5440673	0,010956997	0,053027473	protein_codin	ATP-dependent RNA helicase-like protein
TcG_02043	76,63093785	0,077068377	0,197306145	0,390603025	0,69609069	0,842591429	protein_codin	hypothetical protein
TcG_02044	177,2681989	0,047443188	0,133847517	0,354456986	0,722996434	0,857335574	protein_codin	biotin carboxylase
TcG_02045	592,966222	0,019365366	0,079736315	0,242867584	0,808107978	0,905092175	protein_codin	hypothetical protein
TcG_02046	141,6134808	0,298425637	0,152543546	1,956330791	0,050426199	0,166402149	protein_codin	hypothetical protein
TcG_02047	17,31934386	-0,4268385	0,419457948	-1,01759545	0,30887025	0,541961338	protein_codin	ADP-ribosylation factor 1
TcG_02048	0					1	protein_codin	hypothetical protein
TcG_02049	123,226668	0,102475452	0,161316329	0,635245379	0,525268391	0,728663849	protein_codin	ADP-ribosylation factor 1
TcG_02050	48,06652477	-0,11686146	0,251619048	-0,46443805	0,642333935	0,810066503	protein_codin	ADP-ribosylation factor 1
TcG_02051	298,2054017	0,446039577	0,107174763	4,161796717	3,15753E-05	0,000411973	protein_codin	protein kinase
TcG_02052	161,943145	-0,410062283	0,137112136	-2,99070742	0,00278332	0,017887238	protein_codin	hypothetical protein

TcG_02053	131,4199604	-0,18865509	0,151818773	-1,24263348	0,214002928	0,437522132	protein_codin putative trans-sialidase
TcG_02054	1180,556015	-0,23035683	0,058870622	-3,9129335	9,11817E-05	0,001032679	protein_codin kinetoplastid membrane protein KMP-11
TcG_02055	283,7442188	0,154580714	0,110467862	1,399327474	0,161714805	0,365372022	protein_codin hypothetical protein
TcG_02056	706,6608038	-0,32636007	0,076049016	-4,29144367	1,77515E-05	0,000249598	protein_codin putative RNA-binding protein
TcG_02057	1169,943075	0,271280779	0,059445384	4,563529742	5,03007E-06	8,43392E-05	protein_codin 60S ribosomal protein L12
TcG_02058	349,3972399	0,054221131	0,096812156	0,560065313	0,575434889	0,766252913	protein_codin serine/threonine protein phosphatase 2B catalytic subunit
TcG_02059	619,0790766	0,219146713	0,078223946	2,801529779	0,005086095	0,029071284	protein_codin hypothetical protein
TcG_02060	215,9690644	-0,085217816	0,123378708	-0,69070116	0,489753362	0,700874809	protein_codin putative short-chain dehydrogenase
TcG_02061	343,0008998	0,083660813	0,101378893	0,825229107	0,409241531	0,636781142	protein_codin hypothetical protein
TcG_02062	228,7513827	0,110390552	0,120094663	0,919196154	0,357992982	0,59193759	protein_codin short-chain dehydrogenase
TcG_02063	26,76048531	0,109878755	0,349880394	0,31404662	0,753485631	0,875527481	protein_codin serine/threonine protein phosphatase
TcG_02064	45,03871182	0,313453192	0,267326675	1,172547379	0,240977362	0,469729485	protein_codin rab1 small GTP-binding protein
TcG_02065	23,53879409	-0,099437529	0,365782321	-0,27184892	0,785738188	0,893514718	protein_codin putative mitotic centromere-associated kinesin (MCAK)
TcG_02066	0					1	protein_codin hypothetical protein
TcG_02067	30,08078647	0,338313578	0,320190639	1,056600466	0,290693968	0,523547382	protein_codin hypothetical protein
TcG_02068	426,5951044	-0,042384887	0,090548771	-0,46808904	0,639720917	0,808354951	protein_codin protein kinase domain
TcG_02069	274,9568871	0,334573482	0,116924107	2,861458518	0,004216967	0,025171447	protein_codin hypothetical protein
TcG_02070	395,3682484	-0,181296806	0,101043147	-1,79425138	0,072773055	0,216970822	protein_codin putative vesicle-associated membrane protein
TcG_02071	552,8605247	0,101049094	0,080749491	1,251389856	0,210792277	0,433328481	protein_codin hypothetical protein
TcG_02072	582,2557485	0,009809145	0,084757371	0,115732059	0,907864898	0,955360827	protein_codin hypothetical protein
TcG_02073	389,9525271	0,078318516	0,101267318	0,77338393	0,439295198	0,662631709	protein_codin hypothetical protein
TcG_02074	266,0537392	0,086239824	0,122906485	0,701670251	0,482884829	0,696378346	protein_codin hypothetical protein
TcG_02075	110,7040479	0,19475611	0,168613319	1,155045822	0,248071662	0,477445495	protein_codin hypothetical protein
TcG_02076	672,3525302	-0,305234301	0,073089056	-4,17619705	2,96423E-05	0,000391603	protein_codin putative reiske iron-sulfur protein precursor
TcG_02077	287,7930273	-0,02001695	0,106395494	-0,1881372	0,850769103	0,928053891	protein_codin hypothetical protein
TcG_02078	257,6258591	0,120518629	0,126093524	0,955787622	0,339179543	0,573349021	protein_codin putative chaperone DNAJ protein
TcG_02079	326,5591396	0,455153643	0,103243793	4,40853275	1,04073E-05	0,00015707	protein_codin hypothetical protein
TcG_02080	357,8678318	0,062297354	0,098934662	0,629681775	0,528902809	0,731076654	protein_codin hypothetical protein
TcG_02081	355,837947	-0,009573322	0,09982754	-0,0958986	0,923601103	0,962652247	protein_codin putative metacaspase 5
TcG_02082	427,1218092	-0,107333778	0,08821187	-1,21677251	0,223690784	0,450179159	protein_codin hypothetical protein
TcG_02083	437,3734022	-0,028274513	0,089886253	-0,31455882	0,753096653	0,875514531	protein_codin hypothetical protein
TcG_02084	408,7576243	0,037484424	0,089102443	0,420689064	0,673982148	0,829023911	protein_codin hypothetical protein
TcG_02085	751,1082974	-0,04269908	0,06875892	-0,62099696	0,534601626	0,734918657	protein_codin putative chaperone DNAJ protein
TcG_02086	346,0592135	-0,216185318	0,096112108	-2,24930367	0,024493182	0,098269935	protein_codin calmodulin
TcG_02087	168,5634068	0,11626022	0,147282842	0,789367037	0,429897516	0,654831163	protein_codin RNA helicase
TcG_02088	218,4015658	0,020407334	0,123568817	0,165149549	0,868826274	0,936741226	protein_codin thiopurine S-methyltransferase
TcG_02089	179,2111678	0,007239155	0,132455007	0,054653692	0,956414362	0,979840552	protein_codin putative antigenic protein
TcG_02090	399,5122508	-0,059806088	0,094131305	-0,63534749	0,525201805	0,728663849	protein_codin putative kinesin
TcG_02091	434,0266958	0,008695521	0,087488432	0,099390524	0,920828206	0,96150609	protein_codin mismatch repair protein
TcG_02092	1751,275302	0,538263625	0,059560238	9,037298132	1,60592E-19	2,69655E-17	protein_codin 60S ribosomal protein L26
TcG_02093	326,0110572	-0,00914317	0,107093421	-0,08537564	0,931962755	0,967364315	protein_codin hypothetical protein
TcG_02094	282,2135111	0,082670399	0,108714872	0,760433211	0,446995678	0,668416614	protein_codin putative leucine-rich repeat protein (LRRP)
TcG_02095	245,7842412	0,046945407	0,11455017	0,409823988	0,681935068	0,834223169	protein_codin putative protein kinase
TcG_02096	284,5351376	-0,238990507	0,107229927	-2,22876685	0,025829422	0,102206176	protein_codin putative cyclophilin
TcG_02097	30,76625916	0,505620594	0,316891276	1,595564889	0,110585972	0,287468941	protein_codin hypothetical protein
TcG_02098	346,2987079	-0,338225995	0,09733762	-3,47477157	0,000511289	0,00441415	protein_codin putative casein kinase II, alpha chain
TcG_02099	724,1524894	-0,146038451	0,074805343	-1,95224626	0,050908974	0,167756364	protein_codin putative cysteine peptidase, Clan CA, family C19
TcG_02100	206,4063485	0,084503681	0,127654747	0,661970532	0,507990107	0,714789092	protein_codin putative UDP-glucoronosyl and UDP-glucosyl transferase
TcG_02101	7,834701238	0,343570926	0,625257706	0,549486912	0,582671344	1	
TcG_02102	295,2388137	-0,017926258	0,105537412	-0,1698569	0,865122677	0,935010385	protein_codin putative small GTP-binding protein Rab18
TcG_02103	228,05036	-0,213209653	0,12422499	-1,71631854	0,086103757	0,242724606	protein_codin putative calpain cysteine peptidase
TcG_02104	210,0419294	-0,050869518	0,134090447	-0,37936721	0,7044152	0,847555325	protein_codin hypothetical protein
TcG_02105	134,5591008	-0,118473652	0,158111852	-0,74930279	0,453674726	0,674035553	protein_codin hypothetical protein
TcG_02106	91,59175362	0,23730999	0,185279132	1,280824167	0,200255433	0,419331186	protein_codin hypothetical protein
TcG_02107	8,278525022	-0,844904795	0,64363969	-1,31269841	0,189284595	1	protein_codin hypothetical protein
TcG_02108	141,5068906	-0,001503242	0,152518373	-0,00985614	0,992136069	0,996781867	protein_codin hypothetical protein
TcG_02109	223,2807973	-0,036016167	0,130216447	-0,27658693	0,782097301	0,891868044	protein_codin hypothetical protein

TcG_02110	363,5912825	0,013744597	0,095469416	0,143968584	0,885525278	0,94437554	protein_codin putative prohibitin
TcG_02111	428,9968562	-0,100016182	0,091702851	-1,09065509	0,275424678	0,507736472	protein_codin putative eukaryotic translation initiation factor 4 gamma
TcG_02112	152,7932565	0,052541375	0,143115016	0,367126917	0,713524351	0,852784519	protein_codin putative U1 small nuclear ribonucleoprotein
TcG_02113	536,9051158	-0,208867641	0,07999574	-2,61098455	0,009028197	0,045597513	protein_codin putative kinesin
TcG_02114	97,81924548	-0,178388884	0,177966437	-1,00237374	0,316163118	0,551153727	protein_codin hypothetical protein
TcG_02115	334,0775259	-0,080194104	0,098613372	-0,81321733	0,416093462	0,643038396	protein_codin putative protein transport protein Sec23A
TcG_02116	674,1227238	-0,253005163	0,085074957	-2,97390881	0,002940324	0,018707633	protein_codin WDdomain 65
TcG_02117	724,3067934	-0,0836571	0,077716076	-1,07644523	0,281728183	0,514810791	protein_codin putative DNA polymerase I alpha catalytic subunit
TcG_02118	309,2861181	0,242388912	0,105547115	2,296499634	0,02164733	0,08947769	protein_codin putative endoplasmic reticulum oxidoreductin
TcG_02119	32,5016105	0,747506294	0,310065955	2,410797705	0,015917675	0,070768295	protein_codin hypothetical protein
TcG_02120	391,8701608	-0,25328947	0,097124582	-2,60788223	0,009110429	0,045892798	protein_codin hypothetical protein
TcG_02121	163,5070116	-0,349833602	0,138005561	-2,53492396	0,011247174	0,054227947	protein_codin hypothetical protein
TcG_02122	689,4215415	0,019522658	0,074374416	0,262491582	0,792942474	0,896248225	protein_codin putative protein kinase
TcG_02123	328,7865376	-0,06927708	0,100158872	-0,69167192	0,489143384	0,700347906	protein_codin hypothetical protein
TcG_02124	226,4491221	-0,138073075	0,121865987	-1,13299107	0,257218002	0,489028976	protein_codin hypothetical protein
TcG_02125	346,5225993	0,064599189	0,101868014	0,634145948	0,525985579	0,728904717	protein_codin putative mitochondrial DNA topoisomerase II
TcG_02126	114,5056144	-0,20429517	0,163840508	-1,24691489	0,212428705	0,435610439	protein_codin putative aspartate aminotransferase, mitochondrial
TcG_02127	662,6605446	-0,327874785	0,08185787	-4,00541556	6,19085E-05	0,000740219	protein_codin putative kinesin
TcG_02128	371,0414507	-0,421638741	0,093570307	-4,50611689	6,60247E-06	0,000106689	protein_codin putative OSM3-like kinesin
TcG_02129	148,7793892	0,223065055	0,149064653	1,496431591	0,134541222	0,326111428	protein_codin hypothetical protein
TcG_02130	498,8049941	0,148706931	0,085815854	1,732860824	0,083120409	0,236908501	protein_codin hypothetical protein
TcG_02131	15,27476713	-0,659949043	0,441170012	-1,4959064	0,134678049	1	protein_codin paraflagellar rod protein 3
TcG_02132	3305,326781	-0,573856848	0,043936829	-13,0609527	5,50347E-39	5,3136E-36	protein_codin paraflagellar rod protein 2C
TcG_02133	361,0348561	-0,383139338	0,097108831	-3,94546338	7,96458E-05	0,000916362	protein_codin hypothetical protein
TcG_02134	326,2530722	-0,392635642	0,114612662	-3,42576146	0,000613079	0,005124912	protein_codin hypothetical protein
TcG_02135	114,8518781	0,072988224	0,16365633	0,445984727	0,655608278	0,817728228	protein_codin trafficking protein particle complex subunit 3
TcG_02136	348,9088833	0,068676086	0,09864545	0,696191117	0,48630915	0,698274608	protein_codin essential for mitotic growth 1
TcG_02137	704,3923334	-0,078622247	0,072785423	-1,08019221	0,280056598	0,513214617	protein_codin hypothetical protein
TcG_02138	166,8808044	-0,259831675	0,144560718	-1,79738783	0,072274072	0,216182287	protein_codin putative ADP-ribosylation factor
TcG_02139	252,7935302	-0,189932196	0,111789797	-1,69901191	0,08931694	0,248935788	protein_codin hypothetical protein
TcG_02140	556,0515656	-0,190325409	0,08269068	-2,30165491	0,021354641	0,088559651	protein_codin fatty acid amide hydrolase 2
TcG_02141	1169,56193	0,027125007	0,070409561	0,385246078	0,700055108	0,844367272	protein_codin putative DNA-directed rna polymerase I largest subunit
TcG_02142	974,6286027	0,156050013	0,066280736	2,354379612	0,018553653	0,079167463	protein_codin hypothetical protein
TcG_02143	355,5630147	-0,200311415	0,098100372	-2,0419027	0,041161184	0,144207281	protein_codin hypothetical protein
TcG_02144	407,7838993	-0,426328217	0,090942039	-4,68791138	2,76007E-06	4,98103E-05	protein_codin cytochrome c
TcG_02145	80,10026288	-0,239675271	0,196098284	-1,22222013	0,221624392	0,447107819	protein_codin glycosyl transferase family 2
TcG_02146	69,39193665	-0,10403093	0,211832928	-0,49109896	0,623356459	0,797021059	protein_codin glycosyl transferase family 2
TcG_02147	597,6345155	-0,521868304	0,077646524	-6,72107753	1,80386E-11	1,06089E-09	protein_codin hypothetical protein
TcG_02148	353,6319729	-0,215476892	0,101136109	-2,1305634	0,033125128	0,123100992	protein_codin putative diacylglycerol kinase
TcG_02149	140,1595437	-0,180308376	0,150642637	-1,1969279	0,231334657	0,45855318	protein_codin hypothetical protein
TcG_02150	978,5413119	-0,353795021	0,06861245	-5,15642597	2,51708E-07	5,90342E-06	protein_codin hypothetical protein
TcG_02151	253,9242166	-0,132311265	0,11355757	-1,16514702	0,243959485	0,472108668	protein_codin glycosyltransferase
TcG_02152	470,2804125	-0,309869331	0,091405815	-3,39003959	0,000698825	0,005746338	protein_codin hypothetical protein
TcG_02153	75,34368343	-0,292569066	0,215130556	-1,35996054	0,173842413	0,382444674	protein_codin putative small nuclear ribonucleoprotein
TcG_02154	1373,3351	-0,212819964	0,057201142	-3,72055447	0,000198786	0,001982042	protein_codin hypothetical protein
TcG_02155	774,7749601	-0,23466283	0,080428972	-2,91764055	0,003526907	0,021747067	protein_codin cullin
TcG_02156	1095,192207	-0,133769999	0,059434921	-2,25069701	0,024404734	0,098042043	protein_codin hypothetical protein
TcG_02157	180,0382579	-0,395087202	0,132048885	-2,99197681	0,002771773	0,01784437	protein_codin cyclophilin
TcG_02158	796,9824646	-0,05598088	0,067276308	-0,83210393	0,405350286	0,634304216	protein_codin hypothetical protein
TcG_02159	621,6120081	-0,293043901	0,077867466	-3,76336762	0,00016764	0,001723632	protein_codin putative coatomer delta subunit
TcG_02160	218,5956528	0,081233582	0,119688708	0,678707152	0,497323433	0,707252891	protein_codin hypothetical protein
TcG_02161	392,6547278	-0,030430267	0,090669647	-0,33561691	0,737159771	0,865585599	protein_codin putative NEDD4-like E3 ubiquitin-protein ligase WWP1
TcG_02162	591,8103342	-0,483441093	0,076428308	-6,32541925	2,52546E-10	1,13411E-08	protein_codin hypothetical protein
TcG_02163	531,6987706	0,012202964	0,087837067	0,138927268	0,889507623	0,946535728	protein_codin putative coenzyme Q-binding protein COQ10 B, mitochondrial-like
TcG_02164	93,73259157	0,267329606	0,180281169	1,482848192	0,138114749	0,331647146	protein_codin hypothetical protein
TcG_02165	191,4193672	-0,157417895	0,128540395	-1,22465701	0,22070448	0,445795346	protein_codin tyrosyl or methionyl-tRNA synthetase
TcG_02166	538,9387674	-0,045731588	0,089615292	-0,5103101	0,609834231	0,788828782	protein_codin hypothetical protein

TcG_02167	568,3527892	-0,088078064	0,08048633	-1,09432327	0,273813222	0,506935122	protein_codin	hypothetical protein
TcG_02168	214,5810354	-0,303828863	0,122267423	-2,48495351	0,012956835	0,060629198	protein_codin	hypothetical protein
TcG_02169	700,6322623	-0,052154609	0,075070259	-0,69474396	0,487215771	0,698710474	protein_codin	putative surface antigen TASV, putative,mucin-like glycoprotein
TcG_02170	263,979158	0,033159233	0,112235154	0,295444264	0,767654524	0,883509739		
TcG_02171	112,5825861	-0,423334859	0,16551049	-2,55775243	0,010535106	0,051444171	protein_codin	hypothetical protein
TcG_02172	2609,36697	-0,066186976	0,048092107	-1,37625443	0,168742875	0,375106475	protein_codin	putative amino acid transporter
TcG_02173	95,53111063	-0,249721127	0,178669721	-1,39766898	0,162212494	0,365996875	protein_codin	hypothetical protein
TcG_02174	532,3056291	-0,318582956	0,082075679	-3,88157565	0,000103782	0,001150638	protein_codin	hypothetical protein
TcG_02175	374,995263	-0,441853984	0,094693203	-4,6661637	3,06875E-06	5,48682E-05	protein_codin	coiled-coil domain protein
TcG_02176	542,4923766	-0,315359123	0,081508807	-3,86901901	0,000109274	0,00120347	protein_codin	Importin-beta, N-terminal domain-containing protein
TcG_02177	97,60441261	-0,074089611	0,184088346	-0,40246769	0,687339865	0,837648015	protein_codin	hypothetical protein
TcG_02178	660,442704	-0,744788421	0,077295765	-9,63556573	5,65795E-22	1,28535E-19	protein_codin	putative flagellar protofilament ribbon protein
TcG_02179	68,0528417	-0,357869589	0,223373039	-1,60211631	0,109129891	0,285348436	protein_codin	hypothetical protein
TcG_02180	155,8597015	-0,560056485	0,279213086	-2,00583895	0,044873433	0,153265281	protein_codin	putative small GTP-binding protein Rab7
TcG_02181	447,1662324	-0,573686034	0,085996899	-6,67100841	2,54052E-11	1,45715E-09	protein_codin	hypothetical protein
TcG_02182	121,2857789	-0,349261922	0,163115669	-2,14119173	0,03225858	0,120758612	protein_codin	hypothetical protein
TcG_02183	426,4222978	-0,569948103	0,089021843	-6,40233997	1,53013E-10	7,26563E-09	protein_codin	flagella associated protein
TcG_02184	292,7823994	-0,391235901	0,106226149	-3,68304702	0,000230463	0,002247593	protein_codin	zinc finger family protein
TcG_02185	565,5262983	-0,234222927	0,077487071	-3,02273561	0,00250501	0,016443652	protein_codin	hypothetical protein
TcG_02186	111,1242357	-0,12637194	0,168281311	-0,75095647	0,452678851	0,67343826	protein_codin	hypothetical protein
TcG_02187	1174,166593	-0,670017982	0,061216509	-10,9450538	7,01761E-28	3,01133E-25	protein_codin	putative ataxin-2 isoform X1
TcG_02188	124,2925853	0,104722753	0,161314262	0,649184713	0,516218991	0,722055158	protein_codin	hypothetical protein
TcG_02189	413,3399523	-0,647788731	0,093266487	-6,94556803	3,76941E-12	2,61511E-10	protein_codin	hypothetical protein
TcG_02190	287,3541274	-0,291747134	0,108961075	-2,67753538	0,007416602	0,039023048	protein_codin	putative nucleoside diphosphate kinase
TcG_02191	995,4091758	-0,527200624	0,063514833	-8,30043311	1,03733E-16	1,29231E-14	protein_codin	putative eukaryotic translation initiation factor 4 gamma
TcG_02192	581,9077035	-0,24555387	0,083488288	-2,94117744	0,003269672	0,020432803	protein_codin	hypothetical protein
TcG_02193	372,2377801	-0,345493211	0,094967685	-3,63800813	0,000274755	0,002598619	protein_codin	hypothetical protein
TcG_02194	163,8298786	-0,215136588	0,141975226	-1,51531077	0,129693723	0,31936907	protein_codin	chaperone protein DNAJ
TcG_02195	487,9023776	-0,325061341	0,087157475	-3,72958648	0,000191794	0,001930607	protein_codin	hypothetical protein
TcG_02196	623,8568488	-0,485409202	0,074903716	-6,4804422	9,14542E-11	4,52815E-09	protein_codin	putative RNA-binding protein
TcG_02197	281,1693953	-0,581512319	0,111795918	-5,20155233	1,97631E-07	4,85117E-06	protein_codin	putative carrier protein
TcG_02198	417,353824	0,181949238	0,097490642	1,866325155	0,061995887	0,194341004	protein_codin	nucleoside phosphorylase
TcG_02199	134,5715614	-0,324731326	0,154889746	-2,09653211	0,036035014	0,131455187	protein_codin	hypothetical protein
TcG_02200	207,5444537	-0,227423122	0,138657415	-1,64018002	0,100967742	0,270539375	protein_codin	putative ARP2/3 complex subunit
TcG_02201	121,393054	-0,288605883	0,17701794	-1,63037647	0,103021955	0,274519865	protein_codin	hypothetical protein
TcG_02202	367,8554427	-0,552636511	0,09499284	-5,81766491	5,96754E-09	1,98678E-07	protein_codin	hypothetical protein
TcG_02203	398,5553476	-0,508508358	0,099326892	-5,11954364	3,06276E-07	6,99904E-06	protein_codin	putative translation initiation factor eif-2b beta subunit, putative,eIF-2B GDP-GTP exchange factor
TcG_02204	399,7377541	-0,283612933	0,099670738	-2,84549848	0,004434197	0,026272135		
TcG_02205	752,9542167	-0,527971701	0,071107603	-7,42496834	1,12807E-13	9,97695E-12	protein_codin	hypothetical protein
TcG_02206	953,5732041	-0,623419111	0,069012192	-9,03346344	1,66323E-19	2,75288E-17	protein_codin	hypothetical protein
TcG_02207	200,6401226	-0,464537711	0,12391189	-3,74893572	0,000177587	0,001809603	protein_codin	hypothetical protein
TcG_02208	846,1663474	-0,502426685	0,065480093	-7,6729684	1,68061E-14	1,63626E-12	protein_codin	hypothetical protein
TcG_02209	359,9414596	-0,60877231	0,103836362	-5,86280469	4,55113E-09	1,56004E-07	protein_codin	putative small GTP-binding protein Rab11, putative,Rab11 GTPase
TcG_02210	868,7761685	-0,679912892	0,067460633	-10,0786616	6,86549E-24	1,84985E-21	protein_codin	hypothetical protein
TcG_02211	706,6864953	-0,744488068	0,075473193	-9,86427149	5,94645E-23	1,46586E-20	protein_codin	hypothetical protein
TcG_02212	70,07475985	-0,358527561	0,205359682	-1,74585175	0,080836716	0,232747064		
TcG_02213	591,1130042	-0,245970996	0,078868114	-3,1187635	0,001816117	0,012729297	protein_codin	hypothetical protein
TcG_02214	359,8995466	-0,548118033	0,105531734	-5,1938693	2,05968E-07	5,03448E-06	protein_codin	hypothetical protein
TcG_02215	377,5009048	-0,611353887	0,095946316	-6,37183282	1,86782E-10	8,65625E-09	protein_codin	nuclear transport factor 2
TcG_02216	361,0986432	-0,383801849	0,095912154	-4,00159765	6,29162E-05	0,000749946	protein_codin	adenosine deaminase
TcG_02217	623,7413575	-0,415644129	0,082012261	-5,06807305	4,01863E-07	8,97107E-06	protein_codin	putative protein kinase
TcG_02218	345,1689948	-0,484977987	0,106302063	-4,56226319	5,06052E-06	8,46048E-05	protein_codin	hypothetical protein
TcG_02219	1318,701317	-0,559225597	0,057184753	-9,77927803	1,38193E-22	3,2022E-20	protein_codin	hypothetical protein
TcG_02220	1732,227346	-0,168370575	0,059972058	-2,80748371	0,004993021	0,028745179	protein_codin	hypothetical protein
TcG_02221	1465,045414	-0,532499925	0,055781944	-9,54609842	1,34673E-21	2,944E-19	protein_codin	putative glutamic acid rich protein
TcG_02222	416,650006	-0,541056593	0,089946439	-6,01531979	1,79532E-09	6,73158E-08	protein_codin	hypothetical protein
TcG_02223	418,8700046	-0,559750207	0,090390267	-6,1925938	5,91821E-10	2,48436E-08	protein_codin	WD domain-containing protein

TcG_02224	61,86857636	-0,084197947	0,225661971	-0,37311536	0,709062589	0,84999474	
TcG_02225	331,7030416	-0,227091857	0,099997969	-2,27096469	0,023149116	0,094206411	protein_codin hypothetical protein
TcG_02226	299,6541951	-0,44813755	0,10312136	-4,34572961	1,38813E-05	0,000201793	protein_codin hypothetical protein
TcG_02227	1167,588803	-0,586229134	0,059898309	-9,78707314	1,27946E-22	3,08829E-20	protein_codin hypothetical protein
TcG_02228	152,0426521	-0,404186746	0,147392958	-2,74223918	0,006102189	0,033475361	protein_codin putative deaminase
TcG_02229	370,3699447	-0,262598573	0,098633904	-2,66235607	0,007759575	0,040496591	protein_codin hypothetical protein
TcG_02230	34,99578563	-0,52366266	0,289771819	-1,80715524	0,070738078	0,213430046	protein_codin hypothetical protein
TcG_02231	388,8812859	-0,474590671	0,091964861	-5,16056532	2,46205E-07	5,8215E-06	protein_codin hypothetical protein
TcG_02232	285,1479213	-0,419322198	0,108482577	-3,8653414	0,000110934	0,001218275	protein_codin putative plectin-like protein
TcG_02233	522,0726404	-0,257135558	0,081938661	-3,13814695	0,001700196	0,012077543	protein_codin hypothetical protein
TcG_02234	299,595877	-0,30186219	0,103504238	-2,91642349	0,003540696	0,021775091	protein_codin hypothetical protein
TcG_02235	212,0626118	-0,799740812	0,130340221	-6,13579452	8,47346E-10	3,44469E-08	protein_codin hypothetical protein
TcG_02236	262,258221	-0,482326036	0,116814613	-4,12898713	3,64365E-05	0,000466983	protein_codin hypothetical protein
TcG_02237	340,7905185	-0,542244119	0,101950756	-5,31868658	1,04519E-07	2,70303E-06	protein_codin putative citrate transporter
TcG_02238	352,5083495	-0,711060463	0,099838635	-7,12209721	1,06297E-12	7,99713E-11	protein_codin insect stage-specific antigen
TcG_02239	30,91824916	-0,8102685	0,319746873	-2,53409358	0,011273862	0,054334012	
TcG_02240	721,586816	-0,808773167	0,077553733	-10,4285523	1,83665E-25	5,7512E-23	protein_codin hypothetical protein
TcG_02241	481,4391609	-0,465832254	0,085457699	-5,45102737	5,00797E-08	1,3782E-06	protein_codin endonuclease G
TcG_02242	749,9321405	0,054522885	0,06989298	0,780091003	0,435337312	0,659399643	protein_codin putative conserved RIO1-domain protein
TcG_02243	263,9994847	0,02843906	0,110258815	0,257930034	0,796460905	0,898607075	protein_codin putative ESAG8-associated protein
TcG_02244	187,701615	-0,3769741	0,128337479	-2,93736563	0,003310136	0,020630035	protein_codin hypothetical protein
TcG_02245	155,2425727	0,301412358	0,14342035	2,101601046	0,035588238	0,130153197	protein_codin hypothetical protein
TcG_02246	434,7495789	-0,318512066	0,089364261	-3,56419963	0,000364968	0,003306115	protein_codin putative kinesin
TcG_02247	649,073845	-0,258918886	0,0744903	-3,47587383	0,000509192	0,004402609	protein_codin putative p1/s1 nuclease
TcG_02248	426,953786	-0,118219442	0,09036416	-1,30825586	0,190786541	0,406258566	protein_codin SAM domain-containing protein
TcG_02249	240,5338774	0,090868926	0,119471272	0,760592271	0,446900638	0,668360757	protein_codin pseudouridylate synthase
TcG_02250	268,8114283	-0,027108481	0,109275018	-0,24807574	0,804075804	0,903185306	protein_codin hypothetical protein
TcG_02251	328,6222575	-0,068191496	0,100950429	-0,67549486	0,499361414	0,708412066	protein_codin AAA+-type ATPase
TcG_02252	341,3728939	-0,125993221	0,099756835	-1,26300339	0,20658796	0,428645456	protein_codin putative conserved flavoprotein
TcG_02253	75,27816536	0,104615297	0,20460606	0,511301065	0,609140259	0,788218003	protein_codin hypothetical protein
TcG_02254	765,5122817	-0,076975741	0,068274737	-1,12744104	0,259556056	0,492108097	protein_codin putative epsilon-adaptin, putative,AP-1/4 adapter complex gamma/epsilon subunit
TcG_02255	270,5717049	0,040141939	0,108485831	0,370020112	0,711367505	0,851612308	protein_codin hypothetical protein
TcG_02256	432,1198052	0,041601172	0,088403752	0,470581515	0,637939611	0,807510942	protein_codin putative pseudouridine synthase A-like protein
TcG_02257	501,8445717	0,059078165	0,091055282	0,648816447	0,516457025	0,722055158	protein_codin putative protein kinase
TcG_02258	266,7694304	0,264735482	0,112950328	2,343822167	0,01908727	0,080975872	protein_codin hypothetical protein
TcG_02259	279,5869599	-0,104237495	0,110675556	-0,94182942	0,346279982	0,580523783	protein_codin hypothetical protein
TcG_02260	217,2841513	0,476122665	0,121344712	3,923719938	8,71921E-05	0,000992345	protein_codin sec4-like phosphatidylinositol transfer-like protein
TcG_02261	159,9773471	-0,146168491	0,141239028	-1,03490157	0,300714884	0,534199829	protein_codin hypothetical protein
TcG_02262	331,0396369	-0,15767362	0,103815124	-1,5187924	0,12881476	0,317925448	protein_codin adenosinetriphosphatase
TcG_02263	270,5696243	-0,041939011	0,11065572	-0,37900446	0,704684552	0,847555325	protein_codin hypothetical protein
TcG_02264	276,0626956	0,013500784	0,110217992	0,122491653	0,902509656	0,952909103	protein_codin putative small GTP-binding protein Rab28
TcG_02265	266,8028065	0,021827412	0,117804987	0,185284275	0,853006071	0,928875734	protein_codin putative aldehyde dehydrogenase family
TcG_02266	155,4523431	0,00718359	0,145358132	0,049419937	0,96058464	0,981942266	protein_codin hypothetical protein
TcG_02267	212,4182289	-0,288628972	0,139422368	-2,07017695	0,038435777	0,137254056	protein_codin hypothetical protein
TcG_02268	160,6427608	-0,076932013	0,137826669	-0,55817944	0,576721861	0,766888498	protein_codin hypothetical protein
TcG_02269	258,3242194	0,116148555	0,11889585	0,976893267	0,328621995	0,563083286	protein_codin hypothetical protein
TcG_02270	1190,318932	-0,2592362	0,063855555	-4,05972825	4,91299E-05	0,000606843	protein_codin XMAP215 family protein
TcG_02271	603,3378408	0,02163243	0,078773343	0,274616119	0,783611181	0,892734077	protein_codin hypothetical protein
TcG_02272	354,7336127	-0,00292459	0,1005885	-0,0290748	0,976804938	0,990177192	protein_codin putative protein kinase, putative,cdc2
TcG_02273	234,2455019	-0,2177113	0,117366705	-1,85496646	0,063601017	0,198086393	protein_codin putative DNA-J protein
TcG_02274	588,8629485	0,111374469	0,080605474	1,381723401	0,167056646	0,373072109	protein_codin putative queuine tRNA-ribosyltransferase
TcG_02275	111,1743193	-0,369138503	0,167347787	-2,20581646	0,027396853	0,106911399	protein_codin hypothetical protein
TcG_02276	750,4528191	-0,242170419	0,245566804	-0,9861692	0,32405009	0,558281686	protein_codin hypothetical protein
TcG_02277	1386,932061	-0,288867375	0,063900419	-4,52058655	6,16685E-06	0,00010035	protein_codin hydin-like protein
TcG_02278	425,6109898	-0,231009919	0,090776682	-2,54481562	0,010933545	0,052972585	protein_codin putative splicing factor 3a
TcG_02279	312,9141266	0,088954858	0,108955462	0,816433211	0,414252408	0,641133904	protein_codin hypothetical protein
TcG_02280	331,7932563	-0,173528371	0,099682275	-1,74081472	0,081716061	0,234532235	protein_codin hypothetical protein

TcG_02281	294,6833328	0,040075464	0,110557993	0,36248364	0,716990647	0,854899006	protein_codin	hypothetical protein
TcG_02282	172,217035	-0,003362407	0,1346511	-0,02497125	0,980077893	0,9919789	protein_codin	hypothetical protein
TcG_02283	252,3879254	0,37660086	0,119042186	3,163591599	0,001558353	0,011228283	protein_codin	putative kinase
TcG_02284	674,4709516	-0,093687092	0,073805028	-1,2693863	0,20430332	0,425466046	protein_codin	hypothetical protein
TcG_02285	326,1178339	0,056996422	0,101344706	0,562401567	0,573842451	0,764991214	protein_codin	putative RNA pseudouridylate synthase
TcG_02286	289,8711464	-0,080623263	0,105896954	-0,76133694	0,446455843	0,668040475	protein_codin	hypothetical protein
TcG_02287	145,1325588	-0,165971665	0,153331801	-1,08243472	0,279059407	0,512088477	protein_codin	hypothetical protein
TcG_02288	374,6022835	-0,097248891	0,099920711	-0,9732606	0,3304238	0,565078112	protein_codin	Zinc finger Transcription Factor family member (ztf-7)
TcG_02289	218,9210539	-0,09925357	0,120202015	-0,82572302	0,408961233	0,636605346	protein_codin	hypothetical protein
TcG_02290	98,491767	-0,103306498	0,176934595	-0,58386828	0,559308933	0,753856829	protein_codin	hypothetical protein
TcG_02291	670,5409427	-0,083144417	0,079948221	-1,03997832	0,298349971	0,532042907	protein_codin	cyclosome subunit-like protein
TcG_02292	162,0225351	-0,191621494	0,138819889	-1,38036051	0,167475671	0,37350782	protein_codin	intraflagellar transport (IFT) protein
TcG_02293	267,4233426	-0,007027172	0,110803605	-0,06342007	0,949432006	0,976507274	protein_codin	mannosyltransferase
TcG_02294	148,1004926	-0,009172541	0,150015034	-0,06114415	0,951244411	0,97713458	protein_codin	hypothetical protein
TcG_02295	342,322876	-0,07245896	0,101312949	-0,7151994	0,474485842	0,690210007	protein_codin	calpain-like cysteine peptidase
TcG_02296	66,97310266	0,022923928	0,21188116	0,108192385	0,913843085	0,957873088	protein_codin	putative ferric reductase transmembrane protein
TcG_02297	281,2541972	-0,205535647	0,109758631	-1,8726149	0,061121583	0,191963854	protein_codin	hypothetical protein
TcG_02298	176,1915186	-0,34638502	0,148669166	-2,32990491	0,019811178	0,083367872	protein_codin	coiled-coil domain-containing protein 25
TcG_02299	281,2339481	-0,145613867	0,107134364	-1,3591705	0,174092567	0,382594174	protein_codin	single-stranded nucleic acid binding protein R3H domain-containing protein
TcG_02300	217,5853674	-0,182209317	0,119456469	-1,5253198	0,127179353	0,315524621	protein_codin	hypothetical protein
TcG_02301	23,76260455	0,220709949	0,363019678	0,60798343	0,543198464	0,740847252	protein_codin	hypothetical protein
TcG_02302	67,59283794	0,110573477	0,227203973	0,486670526	0,626491836	0,799398063	protein_codin	structural maintenance of chromosome protein 4
TcG_02303	70,22272025	0,16453358	0,211064441	0,779541923	0,435660552	0,659399643	protein_codin	target of rapamycin (TOR) kinase 1
TcG_02304	61,67970575	0,021706924	0,222500035	0,097559192	0,92228233	0,962229903	protein_codin	hypothetical protein
TcG_02305	163,9024307	-0,098262717	0,141472855	-0,69456941	0,487325188	0,698780895	protein_codin	putative phosphomannomutase-like protein
TcG_02306	408,5833932	-0,231650816	0,089861365	-2,57786887	0,009941171	0,04915852	protein_codin	hypothetical protein
TcG_02307	213,2306713	-0,054200218	0,126143337	-0,42967166	0,667434499	0,825813061	protein_codin	hypothetical protein
TcG_02308	240,4714955	-0,180179879	0,12216901	-1,47484112	0,140255264	0,334712637	protein_codin	hypothetical protein
TcG_02309	462,9344045	-0,289928848	0,086191206	-3,36378688	0,000768809	0,006250821	protein_codin	putative protein kinase
TcG_02310	131,2604927	-0,026857875	0,159563386	-0,16832104	0,866330723	0,935530596	protein_codin	putative nucleoside diphosphate-linked moiety X motif 22-like
TcG_02311	357,6919215	-0,048761888	0,099616744	-0,4894949	0,624491364	0,797636086	protein_codin	hypothetical protein
TcG_02312	235,3979435	0,003116322	0,115589775	0,026960188	0,978491488	0,990851444	protein_codin	putative WD repeat-containing protein 27-like
TcG_02313	227,5636628	0,037213466	0,118976057	0,31278113	0,754446952	0,876205131	protein_codin	putative WD repeat-containing protein 27-like
TcG_02314	294,3830689	-0,04327208	0,106347154	-0,40689457	0,684085421	0,835965655	protein_codin	hypothetical protein
TcG_02315	150,3803599	-0,072437884	0,150608291	-0,48096877	0,630538696	0,802025529	protein_codin	hypothetical protein
TcG_02316	199,9763823	-0,074358783	0,128696393	-0,57778452	0,563409611	0,75668455	protein_codin	hypothetical protein
TcG_02317	181,5304919	0,049487486	0,13692503	0,361420307	0,71778527	0,855087918	protein_codin	putative RNA-binding protein
TcG_02318	51,18493556	0,53402902	0,262034047	2,038013866	0,041548546	0,145125552	protein_codin	hypothetical protein
TcG_02319	1953,011738	-0,178881279	0,048702418	-3,67294448	0,000239772	0,002320797	protein_codin	putative kinesin
TcG_02320	268,7140168	0,321460983	0,111694103	2,878047929	0,004001443	0,024070988	protein_codin	protein ARV1
TcG_02321	193,6553745	0,447368551	0,131019002	3,414531827	0,000638917	0,005314067	protein_codin	retrotransposon hot spot (RHS) protein
TcG_02322	270,923598	0,446485598	0,114461009	3,900765882	9,58888E-05	0,001076792	protein_codin	retrotransposon hot spot (RHS) protein
TcG_02323	82,75950782	0,216449722	0,191785717	1,128601889	0,259065814	0,491636632	protein_codin	hypothetical protein
TcG_02324	24,34531674	0,155669055	0,363181347	0,42862624	0,668195247	0,826134898	protein_codin	trans-sialidase
TcG_02325	129,3656188	-0,080151555	0,168633221	-0,4753011	0,634572378	0,804657499	protein_codin	trans-sialidase-like protein
TcG_02326	20,15221542	-0,235098255	0,414546622	-0,56712139	0,570631714	0,762201872	protein_codin	trans-sialidase
TcG_02327	44,51135021	-0,046502028	0,260494393	-0,17851451	0,85831893	0,931742071	protein_codin	hypothetical protein
TcG_02328	63,20004929	0,245166231	0,231411715	1,059437423	0,289400614	0,522925064	protein_codin	exo-alpha-sialidase
TcG_02329	268,9982819	-0,034113287	0,108373378	-0,31477552	0,752932098	0,875411067	protein_codin	putative SpoU type methylase
TcG_02330	230,2995901	0,068334904	0,119330216	0,572653825	0,566879108	0,758769917	protein_codin	hypothetical protein
TcG_02331	328,3188758	-0,073551115	0,098920897	-0,74353466	0,457158047	0,676783905	protein_codin	serine peptidase
TcG_02332	342,7756001	-0,000686572	0,103034954	-0,00666348	0,99468335	0,997783662	protein_codin	CNH domain-containing protein
TcG_02333	277,9059647	0,067413282	0,109839555	0,61374322	0,539385023	0,738607031	protein_codin	hypothetical protein
TcG_02334	79,88077675	0,23525687	0,210511765	1,117547372	0,263760357	0,496021594	protein_codin	hypothetical protein
TcG_02335	81,49297782	0,268949034	0,19969768	1,346780968	0,178050786	0,388712344	protein_codin	putative ribonuclease H1/H2 small subunit
TcG_02336	258,0871109	0,024658777	0,114655987	0,215067509	0,829714685	0,917190568	protein_codin	hypothetical protein
TcG_02337	189,0862341	0,104776781	0,131743508	0,795308869	0,426433856	0,651783849	protein_codin	hypothetical protein

TcG_02338	213,7347184	-0,01231337	0,120869155	-0,10187355	0,918857045	0,960992753	protein_codin	hypothetical protein
TcG_02339	466,8217594	-0,085193862	0,087777958	-0,97056099	0,33176694	0,566104827	protein_codin	putative arginine N-methyltransferase
TcG_02340	195,2099965	0,165958543	0,126847554	1,308330657	0,190761181	0,406258566	protein_codin	hypothetical protein
TcG_02341	335,3947827	0,164650442	0,103008284	1,598419421	0,109949665	0,286467226	protein_codin	hypothetical protein
TcG_02342	51,39582729	-0,324759491	0,245386896	-1,32345898	0,185682788	0,399576669		
TcG_02343	882,282761	-0,456049225	0,066556708	-6,85204	7,28042E-12	4,79267E-10	protein_codin	hypothetical protein
TcG_02344	840,8040683	0,089416078	0,068235102	1,310411723	0,19005659	0,405225552	protein_codin	puromycin-sensitive aminopeptidase-like protein
TcG_02345	322,3245157	0,035797638	0,09991639	0,358275936	0,720136825	0,856446854	protein_codin	metallo-peptidase, Clan MA(E), Family M1
TcG_02346	203,3162766	-0,168015728	0,125181714	-1,34217468	0,179539363	0,390710568	protein_codin	SNARE associated golgi family protein
TcG_02347	337,6848832	-0,021859071	0,100950437	-0,2165327	0,828572539	0,916365162	protein_codin	hypothetical protein
TcG_02348	578,9538332	0,129959306	0,07711166	1,685339233	0,09192314	0,253455855	protein_codin	hypothetical protein
TcG_02349	145,5682305	-0,169545595	0,147094156	-1,15263312	0,249061004	0,478493188	protein_codin	hypothetical protein
TcG_02350	739,3320081	-0,063113031	0,079566773	-0,79320838	0,427656426	0,652895949	protein_codin	calcium/potassium channel (CAKC)
TcG_02351	205,7114039	0,182806899	0,12738356	1,43509021	0,151261393	0,351589501	protein_codin	ABC transporter
TcG_02352	200,7744934	-0,041833379	0,124531886	-0,33592504	0,736927395	0,865488171	protein_codin	hypothetical protein
TcG_02353	148,4853994	0,168979579	0,144010313	1,173385262	0,240641344	0,469293152	protein_codin	hypothetical protein
TcG_02354	349,9867107	-0,16901545	0,10553451	-1,60151831	0,109262168	0,285629847	protein_codin	hypothetical protein
TcG_02355	318,8075023	0,039582203	0,102375288	0,386638255	0,699024033	0,844142481	protein_codin	hypothetical protein
TcG_02356	213,9509676	0,046849661	0,12141832	0,38585331	0,699605311	0,844323521	protein_codin	hypothetical protein
TcG_02357	205,124559	0,326780425	0,127716927	2,5586305	0,010508537	0,051350447	protein_codin	hypothetical protein
TcG_02358	197,6008373	-0,230812888	0,124949104	-1,84725525	0,064710156	0,200379284	protein_codin	hypothetical protein
TcG_02359	1201,318138	-0,235801941	0,060443025	-3,90122664	9,57065E-05	0,001076559	protein_codin	hypothetical protein
TcG_02360	393,3230819	-0,291138039	0,090502144	-3,21691868	0,001295753	0,009654403	protein_codin	putative chaperone DNAJ protein
TcG_02361	240,6324591	-0,025285688	0,124814406	-0,20258629	0,839458403	0,921979814	protein_codin	hypothetical protein
TcG_02362	305,4118467	-0,1203642	0,105686394	-1,13888076	0,254752892	0,486142289	protein_codin	hypothetical protein
TcG_02363	439,9161159	-0,17091918	0,086070788	-1,98579778	0,047055783	0,158476334	protein_codin	hypothetical protein
TcG_02364	271,4334975	0,076845042	0,121336797	0,633320173	0,526524582	0,729266445	protein_codin	hypothetical protein
TcG_02365	98,24698587	0,039217208	0,181743743	0,215782988	0,82915691	0,916661446	protein_codin	membrane protein
TcG_02366	323,6965432	-0,09781549	0,102237452	-0,95674812	0,338694403	0,573276734	protein_codin	hypothetical protein
TcG_02367	159,8901254	0,168302915	0,140492409	1,197950245	0,230936388	0,458312605	protein_codin	hypothetical protein
TcG_02368	246,9069926	-0,025241232	0,115275816	-0,21896381	0,826678241	0,915239152	protein_codin	bardet-Biedl syndrome 5 protein
TcG_02369	278,2926084	0,171209312	0,107635278	1,590643098	0,111689919	0,289313569	protein_codin	putative kinase
TcG_02370	946,6119542	-0,000396174	0,062855649	-0,00630292	0,994971035	0,997899447	protein_codin	putative cytochrome c oxidase subunit IV
TcG_02371	60,32774548	0,163150167	0,223387821	0,730344951	0,465179358	0,682138496	protein_codin	hypothetical protein
TcG_02372	49,82915827	0,385518895	0,255199859	1,510654812	0,130876421	0,321393432	protein_codin	hypothetical protein
TcG_02373	49,77667059	0,150913735	0,257989971	0,584959696	0,558574818	0,753217858	protein_codin	hypothetical protein
TcG_02374	53,35939981	-0,56565956	0,249033278	-2,27142157	0,02312147	0,094177886		
TcG_02375	1502,146311	-0,321478107	0,057788586	-5,56300348	2,65171E-08	7,77789E-07	protein_codin	Ser/Thr protein phosphatase
TcG_02376	268,2287926	0,0331557	0,115334858	0,287473372	0,773749888	0,886918879	protein_codin	hypothetical protein
TcG_02377	856,0179048	-0,213835078	0,070688864	-3,02501791	0,002486184	0,016375741	protein_codin	DNA repair protein RAD50
TcG_02378	15,26584337	0,070580287	0,450270571	0,156750832	0,875441224	1	protein_codin	putative alanine aminotransferase
TcG_02379	95,85440229	0,028052538	0,183828724	0,152601493	0,878712547	0,941484386	protein_codin	alanine transaminase
TcG_02380	34,37715681	0,634819988	0,300948264	2,109399067	0,03491015	0,128280684		
TcG_02381	51,00684486	0,092998891	0,260941838	0,356397011	0,721543268	0,857117481	protein_codin	putative alanine aminotransferase
TcG_02382	384,7935069	0,226754717	0,093344162	2,429232976	0,015130806	0,068160911	protein_codin	hypothetical protein
TcG_02383	350,7087934	0,11674831	0,101833909	1,146458099	0,251605655	0,481675995	protein_codin	hypothetical protein
TcG_02384	725,2754654	0,013881071	0,072458328	0,191573167	0,848076563	0,926605553	protein_codin	arylsulfatase G
TcG_02385	242,1383735	-0,061605937	0,133449677	-0,46164171	0,644338282	0,811082798	protein_codin	hypothetical protein
TcG_02386	721,0759469	-0,033334351	0,071674162	-0,46508184	0,641872851	0,809837619	protein_codin	hypothetical protein
TcG_02387	249,10163	-0,015868531	0,115990852	-0,13680847	0,891182189	0,946972656	protein_codin	hypothetical protein
TcG_02388	1835,565324	0,162535376	0,053572569	3,033929115	0,002413911	0,015981473	protein_codin	cytosolic glucose-6-phosphate isomerase
TcG_02389	141,8242844	0,028600756	0,15442697	0,185205706	0,853067694	0,928875734	protein_codin	ATP synthase subunit
TcG_02390	65,40923456	0,256583609	0,228456564	1,123117691	0,261387513	0,493587786	protein_codin	hypothetical protein
TcG_02391	102,0971701	0,039552473	0,171392852	0,23077084	0,817492836	0,91010589	protein_codin	peroxiredoxin-like protein
TcG_02392	10,83530102	0,496211946	0,535180972	0,927185329	0,353830319	1	protein_codin	UDP-Gal or UDP-GlcNAc-dependent glycosyltransferase
TcG_02393	0					1		
TcG_02394	208,2801553	0,105519568	0,128228892	0,822900102	0,410564805	0,637299548	protein_codin	hypothetical protein

TcG_02395	777,8923214	0,051157768	0,073770443	0,693472426	0,488013124	0,699453024	protein_codin putative N-acetylglucosamine-6-phosphate deacetylase-like protein
TcG_02396	510,3210801	0,15137501	0,081795166	1,850659615	0,064218537	0,19929349	protein_codin pumilio protein 6
TcG_02397	251,2966393	-0,128874106	0,112375715	-1,14681456	0,251458272	0,481552981	protein_codin putative L-ribulokinase
TcG_02398	221,6044809	-0,149102424	0,122451849	-1,21764126	0,223360328	0,449809352	protein_codin hypothetical protein
TcG_02399	273,4045529	-0,111074514	0,113381718	-0,97965101	0,327258417	0,561804121	protein_codin putative stress-inducible protein STI1-like
TcG_02400	355,1034739	-0,08141377	0,095417036	-0,85324145	0,393525421	0,623292622	protein_codin putative leucine carboxyl methyltransferase
TcG_02401	415,9892808	-0,044414741	0,092895916	-0,47811295	0,632569816	0,803173028	protein_codin putative trans-sialidase
TcG_02402	271,3701509	-0,013787075	0,107455605	-0,12830485	0,897907726	0,950181186	protein_codin putative DNA damage repair protein
TcG_02403	381,5981187	-0,152136073	0,094203138	-1,61497883	0,10631528	0,280713043	protein_codin hypothetical protein
TcG_02404	157,4051002	-0,125432786	0,142866854	-0,87796982	0,379960096	0,610721305	protein_codin putative mRNA capping methyltransferase
TcG_02405	266,5715192	0,093441602	0,111795279	0,835827803	0,403251783	0,632986744	protein_codin putative ubiquitin regulatory protein (ISS)
TcG_02406	66,68369849	-0,074021232	0,213784064	-0,34624298	0,729160113	0,860563425	protein_codin hypothetical protein
TcG_02407	248,1775845	-0,074499329	0,114807148	-0,64890846	0,516397546	0,722055158	protein_codin putative fructose-6-phosphate2-kinase/fructose-2,6-bisphos phatase,putative
TcG_02408	221,7670265	-0,181550867	0,118243963	-1,53539227	0,124687501	0,311108715	protein_codin hypothetical protein
TcG_02409	136,5709428	-0,130050235	0,153693777	-0,84616461	0,397460921	0,627040065	protein_codin ARP2/3 complex subunit
TcG_02410	104,8060399	-0,41877878	0,171938107	-2,43563679	0,014865603	0,067331068	protein_codin hypothetical protein
TcG_02411	36,52443149	0,532233511	0,290753451	1,830532054	0,067170418	0,206264634	protein_codin elongation factor 2
TcG_02412	8453,844183	-0,011664699	0,034187212	-0,34120064	0,732952541	0,862784475	protein_codin elongation factor 2
TcG_02413	0,325107945	-0,458748506	2,939702538	-0,1560527	0,875991485	1	protein_codin elongation factor 2
TcG_02414	220,0739435	0,067714808	0,120689301	0,5610672	0,574751727	0,765674774	protein_codin hypothetical protein
TcG_02415	101,4893309	-0,053058133	0,19061413	-0,27835362	0,780740919	0,890875557	protein_codin mitochondrial inner membrane signal peptidase
TcG_02416	292,8680385	-0,169047347	0,112419315	-1,50372155	0,132653078	0,323614384	protein_codin putative SET domain protein
TcG_02417	252,6282851	0,079810698	0,117513894	0,679159673	0,497036696	0,707018681	protein_codin dolicholphosphate-mannose synthase
TcG_02418	457,998603	0,195545136	0,090809753	2,153349488	0,031291222	0,117937573	protein_codin peptidyl-prolyl cis-trans isomerase
TcG_02419	255,5444542	-0,009633147	0,112777465	-0,0854173	0,931929639	0,967364315	protein_codin hypothetical protein
TcG_02420	808,9156911	0,151407163	0,067386516	2,246846575	0,024649834	0,098684512	protein_codin putative eukaryotic translation initiation factor 3 (eIF-3) interacting protein
TcG_02421	398,0336066	-0,112781313	0,090478556	-1,24649772	0,212581727	0,435769973	protein_codin hypothetical protein
TcG_02422	179,6053425	0,129417843	0,131578385	0,983579806	0,325322158	0,559807296	protein_codin hypothetical protein
TcG_02423	239,9041851	0,156771093	0,115632527	1,355769845	0,175172409	0,384384001	protein_codin hypothetical protein
TcG_02424	204,0664157	0,087784148	0,125976222	0,696831091	0,485908506	0,698274608	protein_codin MutT 8-oxo-dGTP pyrophosphohydrolase-like protein
TcG_02425	199,6121479	-0,167216195	0,128789368	-1,29836956	0,194160373	0,411175667	protein_codin dynein light chain
TcG_02426	276,7176076	0,195632795	0,110634415	1,768281554	0,077013846	0,224812905	protein_codin putative dolicholphosphate-mannose synthase
TcG_02427	331,6173244	-0,031445768	0,100611114	-0,31254766	0,754624349	0,876235488	protein_codin putative proteasome beta 2 subunit
TcG_02428	155,9291507	-0,009047082	0,145293716	-0,06226754	0,95034978	0,976946126	protein_codin hypothetical protein
TcG_02429	199,5109736	0,058287812	0,124904034	0,466660765	0,640742605	0,809291154	protein_codin hypothetical protein
TcG_02430	284,2222059	-0,064253505	0,107080256	-0,60004997	0,548472932	0,745058904	protein_codin putative ribonuclease HII
TcG_02431	186,2552304	0,120500103	0,129140359	0,933094067	0,350771402	0,585174581	protein_codin uncharacterized protein
TcG_02432	272,7291915	0,147149303	0,108727979	1,353371081	0,175937111	0,385623794	protein_codin hypothetical protein
TcG_02433	407,4224029	0,061802212	0,091602173	0,674680627	0,499878696	0,708798748	protein_codin hypothetical protein
TcG_02434	244,8164468	0,053613996	0,117705798	0,455491545	0,648755649	0,813280193	protein_codin putative phosphatidylinositol-4-phosphate 5-kinase
TcG_02435	356,9724231	0,673342172	0,103202754	6,524459312	6,82473E-11	3,49873E-09	protein_codin putative GPI inositol deacylase precursor
TcG_02436	137,3467496	0,169329424	0,150241506	1,127048235	0,259722088	0,492108097	protein_codin hypothetical protein
TcG_02437	273,2900804	0,091277283	0,110319624	0,827389361	0,408016409	0,636413316	protein_codin hypothetical protein
TcG_02438	91,90681802	0,170664625	0,184622978	0,92439536	0,35528051	0,589218435	protein_codin hypothetical protein
TcG_02439	137,6145542	-0,115927577	0,154086969	-0,75235159	0,451839648	0,672794521	protein_codin hypothetical protein
TcG_02440	237,9260618	-0,189225999	0,117196093	-1,61461013	0,106395151	0,280804733	protein_codin hypothetical protein
TcG_02441	169,107721	0,235475771	0,138924675	1,694988818	0,090077542	0,250212995	protein_codin hypothetical protein
TcG_02442	110,594228	0,236508139	0,171635504	1,377967455	0,168213337	0,374648158	protein_codin hypothetical protein
TcG_02443	363,794196	-0,139650192	0,096505635	-1,44706776	0,147877948	0,346754484	protein_codin hypothetical protein
TcG_02444	4,837605351	-0,446168928	0,804620086	-0,55450881	0,579230676	1	
TcG_02445	454,194309	-0,369636267	0,085210464	-4,33792106	1,43837E-05	0,000208248	protein_codin hypothetical protein
TcG_02446	310,1697895	-0,150262761	0,108843177	-1,38054369	0,167419309	0,373454007	protein_codin putative vacuolar protein sorting-associated protein
TcG_02447	166,4359928	0,171904131	0,141799883	1,212300938	0,225397205	0,451820114	protein_codin putative dihydrouridine synthase
TcG_02448	1157,790392	-0,294898641	0,06005525	-4,91045567	9,0865E-07	1,86938E-05	protein_codin cytochrome-c oxidase
TcG_02449	104,2629686	-0,464039716	0,187880558	-2,46986553	0,013516385	0,062791032	protein_codin hypothetical protein
TcG_02450	473,9009847	-0,222331773	0,084878791	-2,61940316	0,008808378	0,044721238	protein_codin putative TPR-repeat-containing chaperone protein DNAJ
TcG_02451	248,1696859	0,152643024	0,113971576	1,33930782	0,180470484	0,391853641	protein_codin putative mitochondrial carrier protein

TcG_02452	227,1077466	-0,254908914	0,118298403	-2,15479591	0,031177808	0,117586617	protein_codin	hypothetical protein
TcG_02453	172,2700993	-0,26254197	0,13621946	-1,92734555	0,05393658	0,174497199	protein_codin	protein phosphatase 2C-like
TcG_02454	164,5657098	-0,327751324	0,13857119	-2,36521982	0,018019372	0,077552914	protein_codin	serine/threonine protein kinase TbPK6
TcG_02455	119,0060707	0,223343446	0,179335843	1,245392116	0,21298765	0,436524838	protein_codin	small nuclear ribonucleoprotein
TcG_02456	49,3463326	0,065997464	0,25505752	0,258755217	0,795824113	0,898164685	protein_codin	hypothetical protein
TcG_02457	1054,898159	-0,047671238	0,07153588	-0,66639619	0,505157895	0,712274476	protein_codin	putative ubiquitin-like protein
TcG_02458	456,1334738	-0,232297353	0,087070478	-2,66792326	0,007632169	0,039939297	protein_codin	cdc2-related protein kinase 3
TcG_02459	420,1071984	-0,350849311	0,094676546	-3,70576797	0,000210751	0,002079866	protein_codin	hypothetical protein
TcG_02460	407,0123789	-0,210089357	0,089936465	-2,33597526	0,019492531	0,082349427	protein_codin	putative Mitochondrial elongation factor G
TcG_02461	276,9616745	-0,275334376	0,1070769	-2,57137043	0,010129691	0,04985667	protein_codin	putative Mitochondrial elongation factor G
TcG_02462	223,0972067	-0,018695178	0,121532954	-0,15382806	0,877745312	0,941200542	protein_codin	putative cytosolic factor SEC14, putative,phosphatidylinositol/phosphatidylcholine transfer protein
TcG_02463	382,1552248	-0,244838887	0,095019884	-2,57671213	0,009974498	0,049279089	protein_codin	hypothetical protein
TcG_02464	1124,893567	0,172408525	0,060804765	2,835444329	0,004576199	0,026872702	protein_codin	ubiquitin/ribosomal protein S27a
TcG_02465	796,6019093	-0,323560626	0,06865183	-4,71306625	2,44017E-06	4,44525E-05	protein_codin	putative DnaJ chaperone protein
TcG_02466	281,3256782	-0,253748128	0,111087496	-2,28421862	0,022358686	0,091665864	protein_codin	hypothetical protein
TcG_02467	273,2409148	0,053519817	0,112420601	0,476067696	0,63402615	0,804197117	protein_codin	putative ribonuclease H
TcG_02468	388,548557	-0,246448272	0,092378617	-2,66780648	0,007634822	0,039939297	protein_codin	putative GPI transamidase component Tta2
TcG_02469	323,8298057	-0,002113542	0,099951509	-0,02114567	0,983129451	0,993738755	protein_codin	putative ATP-dependent DEAD/H RNA helicase
TcG_02470	143,1464867	0,121490103	0,149588145	0,812163977	0,416697546	0,643542758	protein_codin	putative MP24
TcG_02471	356,7227553	-0,009988545	0,100325452	-0,09956142	0,920692522	0,96150609	protein_codin	RNA editing complex protein MP18
TcG_02472	276,8304518	-0,016757715	0,117652859	-0,14243356	0,886737557	0,945300144	protein_codin	Protein C21orf2
TcG_02473	704,5574714	-0,136839307	0,077935054	-1,75581207	0,079120503	0,229172538	protein_codin	putative protein kinase, putative,mitogen-activated protein kinase
TcG_02474	73,75682737	-0,854327053	0,205259692	-4,16217644	3,15229E-05	0,000411752	protein_codin	hypothetical protein
TcG_02475	994,8730464	-0,040226314	0,063335819	-0,63512739	0,525345332	0,728663849	protein_codin	hypothetical protein
TcG_02476	724,3444628	-0,271537603	0,0718528	-3,77908172	0,000157408	0,001641518	protein_codin	putative TPR-repeat-containing chaperone protein DNAJ
TcG_02477	19,46956173	-0,172063977	0,390953635	-0,44011351	0,659854898	0,821433205	protein_codin	hypothetical protein
TcG_02478	3,609060267	-0,405950519	0,910053487	-0,44607325	0,655544333	1		
TcG_02479	356,7990988	-0,199089641	0,098202596	-2,02733582	0,042628081	0,147738241	protein_codin	hypothetical protein
TcG_02480	325,1731356	-0,022093949	0,101773627	-0,21708914	0,828138877	0,916147907	protein_codin	zinc transporter
TcG_02481	247,4851831	0,118518324	0,112363614	1,054774939	0,291528269	0,524153713	protein_codin	hypothetical protein
TcG_02482	303,9995336	-0,124175587	0,106318078	-1,16796305	0,242821668	0,471165943	protein_codin	DNA repair and transcription factor protein
TcG_02483	413,7017384	-0,082564616	0,097729342	-0,84482934	0,39820613	0,627959198	protein_codin	hypothetical protein
TcG_02484	280,468041	0,026464743	0,110401329	0,239713989	0,810551989	0,906647552	protein_codin	putative ADP-ribosylation factor-like protein
TcG_02485	1179,972772	-0,255316733	0,063670743	-4,00995371	6,07307E-05	0,000728391	protein_codin	putative 40S ribosomal protein S9
TcG_02486	592,4783298	-0,313885258	0,079678516	-3,93939638	8,16869E-05	0,000934279	protein_codin	putative zinc finger protein family member
TcG_02487	70,48408338	0,43606043	0,215707144	2,021539124	0,043223987	0,149312197	protein_codin	hypothetical protein
TcG_02488	65,69589882	-0,048119476	0,224305076	-0,21452692	0,83013618	0,917481425	protein_codin	hypothetical protein
TcG_02489	378,8990887	0,181435565	0,095047046	1,908902722	0,056274642	0,179811914	protein_codin	surface protein-2
TcG_02490	140,1334208	0,031920346	0,150932444	0,211487636	0,832506779	0,918874301		
TcG_02491	669,7756139	-0,102167677	0,073505635	-1,38992987	0,164550175	0,369830907	protein_codin	lysine decarboxylase domain-containing protein
TcG_02492	291,2117561	-0,213262029	0,111365428	-1,91497516	0,055495671	0,178108821	protein_codin	NADH-cytochrome b5 reductase
TcG_02493	2136,91317	-0,169893493	0,054083132	-3,14133975	0,001681768	0,011968652	protein_codin	putative eukaryotic initiation factor 4a
TcG_02494	359,4354499	-0,154902145	0,098427834	-1,57376363	0,115542105	0,295838856	protein_codin	kinectin
TcG_02495	558,4496472	0,222903359	0,085431005	2,609162317	0,009076418	0,045761261	protein_codin	hypothetical protein
TcG_02496	201,3470983	-0,02645149	0,124407452	-0,21261982	0,831623508	0,918161803	protein_codin	hypothetical protein
TcG_02497	48,72992078	-0,718421216	0,255788276	-2,80865577	0,004974881	0,028677857		
TcG_02498	411,5908138	-0,332202693	0,089205882	-3,72399986	0,000196091	0,001958544	protein_codin	putative heat shock protein HslVU, ATPase subunit HslU
TcG_02499	239,3195628	-0,132373885	0,123530429	-1,07158929	0,283904545	0,517432446	protein_codin	dimethylaniline monooxygenase (N-oxide forming)
TcG_02500	268,7398637	-0,381155134	0,10869763	-3,50656342	0,000453933	0,003982757	protein_codin	hypothetical protein
TcG_02501	277,3562271	-0,08269738	0,108475504	-0,76235995	0,445845204	0,667557835	protein_codin	putative mitochondrial carrier protein
TcG_02502	616,8680179	-0,147187132	0,080005505	-1,83971254	0,065810451	0,203057226	protein_codin	putative ATP-dependent RNA helicase
TcG_02503	126,9514171	-0,325613241	0,15877532	-2,05077994	0,040288382	0,142008273		
TcG_02504	638,4701048	-0,492841922	0,080313744	-6,13645804	8,43816E-10	3,44241E-08	protein_codin	hypothetical protein
TcG_02505	10,14887979	0,139101812	0,542214612	0,256543828	0,797530943	1	protein_codin	hypothetical protein
TcG_02506	226,0684382	-0,058074645	0,116958506	-0,49654058	0,619513063	0,793990967	protein_codin	putative Kelch repeat protein
TcG_02507	260,9702819	-0,208331074	0,109263982	-1,90667656	0,056562484	0,180333775	protein_codin	hypothetical protein
TcG_02508	470,2399102	0,014650158	0,085384388	0,17157888	0,863768613	0,934863339	protein_codin	putative protein phosphatase 1 catalitic subunit

TcG_02509	445,115559	-0,308651163	0,091099531	-3,38806535	0,000703875	0,005779656	protein_codin hypothetical protein
TcG_02510	2366,369205	0,19181023	0,048449523	3,958970501	7,52735E-05	0,000874743	protein_codin 60S ribosomal protein L13a
TcG_02511	256,7505615	-0,385367689	0,110152404	-3,4984955	0,000467891	0,004078996	protein_codin hypothetical protein
TcG_02512	228,532884	0,213228878	0,118491761	1,799524936	0,071935683	0,215528013	protein_codin membrane-associated progesterone binding protein 2
TcG_02513	510,6926849	-0,298628967	0,081717973	-3,65438537	0,000257799	0,002464403	protein_codin hypothetical protein
TcG_02514	1132,405207	-0,270610416	0,060685502	-4,45922677	8,22559E-06	0,000129486	protein_codin putative serine/threonine protein kinase, putative,protein kinase
TcG_02515	785,5908436	-0,241108544	0,06784965	-3,55357094	0,000380039	0,003424046	protein_codin putative protein phosphatase 2C
TcG_02516	387,8831818	0,005656352	0,097455748	0,058040207	0,953716602	0,978287787	protein_codin WD40 repeat protein
TcG_02517	1122,861888	-0,106712641	0,061949553	-1,72257321	0,084965734	0,240276542	protein_codin hypothetical protein
TcG_02518	451,6888613	-0,293792281	0,092811487	-3,16547328	0,001548309	0,011176764	protein_codin hypothetical protein
TcG_02519	593,1547553	0,083615829	0,07726455	1,08220172	0,279162905	0,512149969	protein_codin putative replication Factor A 28 kDa subunit
TcG_02520	1319,622107	-0,247361423	0,058596787	-4,22141613	2,42772E-05	0,000328269	protein_codin ribonucleoprotein p18
TcG_02521	628,0014297	0,046071022	0,077807942	0,592112076	0,553775541	0,748837934	protein_codin hypothetical protein
TcG_02522	475,7381268	-0,017376117	0,083633901	-0,20776404	0,83541321	0,920241248	protein_codin hypothetical protein
TcG_02523	276,9566741	-0,125918005	0,115179513	-1,09323266	0,274291662	0,507334482	protein_codin putative ecotin
TcG_02524	496,7374595	-0,013651915	0,082848309	-0,16478206	0,869115525	0,936791559	protein_codin hypothetical protein
TcG_02525	345,9865645	-0,077126911	0,101053127	-0,76323132	0,445325454	0,66703823	protein_codin hypothetical protein
TcG_02526	546,3793209	0,057155617	0,080586444	0,709246042	0,478171806	0,692834318	protein_codin hypothetical protein
TcG_02527	1543,382148	0,171861815	0,055424039	3,100853321	0,001929639	0,013355312	protein_codin 60S acidic ribosomal protein
TcG_02528	191,2923876	0,616817213	0,130394059	4,730408881	2,24068E-06	4,13384E-05	protein_codin putative cytoplasmic l-asparaginase i-like protein
TcG_02529	580,2813001	0,104502877	0,082068972	1,273354285	0,202892352	0,423006561	protein_codin hypothetical protein
TcG_02530	621,6335111	-0,110827401	0,080911489	-1,36973626	0,170769245	0,377476218	protein_codin hypothetical protein
TcG_02531	551,6104826	-0,036356939	0,083424326	-0,4358074	0,662976471	0,823549415	protein_codin hypothetical protein
TcG_02532	115,812527	-0,002434306	0,172312348	-0,01412729	0,988728432	0,995516435	protein_codin hypothetical protein
TcG_02533	1931,100549	-0,216904027	0,05441257	-3,98628527	6,71158E-05	0,000796725	protein_codin putative lysosomal/endosomal membrane protein p67, putative,lysosomal membrane glycoprotein
TcG_02534	308,103388	-0,021215948	0,103180104	-0,20562053	0,83708734	0,920684823	protein_codin hypothetical protein
TcG_02535	45,12080401	0,36377024	0,276225372	1,316932751	0,187861167	0,402247179	protein_codin hypothetical protein
TcG_02536	0,572794636	0,398549884	2,349194663	0,169653835	0,865282382	1	protein_codin hypothetical protein
TcG_02537	85,86639932	-0,056665177	0,193762298	-0,29244687	0,769944979	0,884364283	protein_codin hypothetical protein
TcG_02538	23,54654434	-0,336187042	0,375479642	-0,89535358	0,370598107	0,602208929	protein_codin calpain-like cysteine peptidase
TcG_02539	719,5095454	-0,039826031	0,081466769	-0,48886228	0,624939201	0,798113883	protein_codin hypothetical protein
TcG_02540	470,8195349	0,028864487	0,084725606	0,340681977	0,733343008	0,862982036	protein_codin hypothetical protein
TcG_02541	361,1727138	0,012275994	0,095465012	0,128591553	0,89768085	0,950181186	protein_codin hypothetical protein
TcG_02542	848,6598704	0,137571207	0,070992447	1,937828783	0,052644115	0,171281863	protein_codin putative katanin-like protein, putative,serine peptidase, Clan SJ, family S16
TcG_02543	257,5308309	-0,130753802	0,111689557	-1,17068959	0,24172357	0,470374417	protein_codin ecotin
TcG_02544	888,5410212	-0,201156552	0,067479357	-2,98100873	0,002873006	0,018400579	protein_codin hypothetical protein
TcG_02545	350,8890692	-0,25669122	0,099234278	-2,58671929	0,009689449	0,048213155	protein_codin hypothetical protein
TcG_02546	341,5733459	-0,287591999	0,099827214	-2,88089777	0,003965442	0,02391651	protein_codin hypothetical protein
TcG_02547	726,1948085	-0,224965732	0,072053467	-3,12220552	0,001795016	0,012622094	protein_codin hypothetical protein
TcG_02548	604,9542835	0,068249732	0,076877997	0,887766789	0,374666211	0,605254143	protein_codin putative coiled-coil domain-containing protein 13 isoform X1
TcG_02549	441,2267349	-0,43154232	0,094360208	-4,57335065	4,79986E-06	8,07129E-05	protein_codin peptide hydrolase
TcG_02550	310,867581	-0,726818022	0,10805818	-6,72617308	1,74183E-11	1,03492E-09	protein_codin hypothetical protein
TcG_02551	1211,400203	-0,062564809	0,061074367	-1,02440373	0,305644623	0,538749217	protein_codin hypothetical protein
TcG_02552	696,4720514	-0,050235433	0,074899633	-0,67070333	0,502409544	0,710995111	protein_codin hypothetical protein
TcG_02553	472,7857421	-0,059361255	0,084759096	-0,70035262	0,483707119	0,697045336	protein_codin hypothetical protein
TcG_02554	121,1644641	-0,218243896	0,158627705	-1,37582458	0,168875949	0,375286155	protein_codin hypothetical protein
TcG_02555	573,7158576	-0,094084233	0,078713377	-1,19527628	0,231979104	0,459225958	protein_codin hypothetical protein
TcG_02556	132,2842171	0,327602789	0,152391365	2,149746401	0,03157528	0,118699284	protein_codin hypothetical protein
TcG_02557	46,83423653	-0,123379921	0,259180122	-0,47603929	0,634046387	0,804197117	protein_codin hypothetical protein
TcG_02558	1399,299235	0,072146668	0,068495113	1,053311176	0,292198398	0,524951255	protein_codin myosin heavy chain
TcG_02559	139,6054832	-0,011821742	0,17436993	-0,06779691	0,945947306	0,974694377	protein_codin flagellar attachment zone protein
TcG_02560	462,6120061	-0,122407264	0,086810977	-1,41004362	0,158526803	0,360701402	protein_codin putative tetratricopeptide repeat protein 18-like
TcG_02561	777,7911788	-0,036330911	0,070758952	-0,51344615	0,607639269	0,788022108	protein_codin putative la RNA binding protein
TcG_02562	452,1382092	0,055431298	0,086441598	0,64125721	0,521355585	0,725401608	protein_codin dihydrolipoamide acetyltransferase
TcG_02563	229,5153439	0,075796054	0,121441984	0,624133854	0,532539683	0,733215064	protein_codin hypothetical protein
TcG_02564	320,3741529	0,052619773	0,100955287	0,521218591	0,602214504	0,784755061	protein_codin hypothetical protein
TcG_02565	322,4936345	-0,053047942	0,100172001	-0,52956856	0,596411096	0,780668859	protein_codin hypothetical protein

TcG_02566	363,9958061	0,045838227	0,10166566	0,45087227	0,652081611	0,815925403	protein_codin hypothetical protein
TcG_02567	187,4177539	-0,231702372	0,130484075	-1,7757138	0,075780102	0,222500827	protein_codin thioesterase superfamily protein
TcG_02568	165,0683313	0,112283619	0,141823519	0,79171367	0,42852765	0,653907586	protein_codin thioesterase superfamily protein
TcG_02569	486,2745585	-0,168452688	0,085610637	-1,96766071	0,049107089	0,163398833	protein_codin putative chaperone DNAJ protein
TcG_02570	255,0646905	0,127029862	0,113560787	1,118606746	0,263307944	0,495482515	protein_codin hypothetical protein
TcG_02571	138,740483	0,292263033	0,157238794	1,858720905	0,063066709	0,19679259	protein_codin hypothetical protein
TcG_02572	785,9160623	-0,087343254	0,067856604	-1,28717398	0,198033656	0,416639602	protein_codin vacuolar sorting-associated-like protein
TcG_02573	387,57235	0,068213383	0,093448521	0,729956793	0,465416595	0,682138496	protein_codin hypothetical protein
TcG_02574	238,5653839	0,030396149	0,119518894	0,254320867	0,79924768	0,89990311	protein_codin hypothetical protein
TcG_02575	116,6861722	0,014704862	0,164640728	0,089314851	0,928831692	0,965428814	protein_codin hypothetical protein
TcG_02576	325,783523	-0,091429577	0,101503776	-0,90075049	0,367720995	0,599974011	protein_codin putative ubiquitin-conjugating enzyme variant Kua
TcG_02577	1908,147136	-0,153177486	0,050912622	-3,00863479	0,002624244	0,017062002	protein_codin hypothetical protein
TcG_02578	265,5179341	0,091151881	0,1181293	0,771628047	0,440334758	0,663336173	protein_codin ferredoxin NADP+ reductase-like protein
TcG_02579	441,7753592	0,20024894	0,094780605	2,112762834	0,034621072	0,127420503	protein_codin putative PIWI-like protein 1
TcG_02580	487,5173456	0,044513856	0,082463597	0,539800078	0,589334914	0,775648564	protein_codin putative ubiquitin hydrolase
TcG_02581	227,1412848	0,027534718	0,117462786	0,234412268	0,814664936	0,908376426	protein_codin hypothetical protein
TcG_02582	710,9339072	-0,373098544	0,071437973	-5,22269216	1,7634E-07	4,36556E-06	protein_codin Flagellar Associated Protein
TcG_02583	247,2865655	0,048853253	0,113514424	0,430370439	0,666926195	0,825534925	protein_codin hypothetical protein
TcG_02584	259,5020597	-0,100887031	0,11494365	-0,87770861	0,380101873	0,610721305	protein_codin hypothetical protein
TcG_02585	175,8044258	-0,01031217	0,140328805	-0,07348577	0,941419567	0,97292993	protein_codin hypothetical protein
TcG_02586	547,4134416	0,04071903	0,083036867	0,490372906	0,623870046	0,797066211	protein_codin mitochondrial processing peptidase alpha subunit
TcG_02587	277,7318885	0,01374974	0,11393961	0,120675683	0,90394792	0,953490587	protein_codin Vps51/Vps67 protein
TcG_02588	141,5970626	0,053023556	0,147814049	0,358717976	0,719806079	0,856197241	protein_codin elongation factor 1-alpha (ef-1-alpha)
TcG_02589	23331,22048	0,178566484	0,036222827	4,929667286	8,23698E-07	1,70722E-05	protein_codin elongation factor-1 alpha
TcG_02590	46,31726177	-0,177683449	0,25961996	-0,68439826	0,493723713	0,704208167	protein_codin elongation factor 1-alpha (ef-1-alpha)
TcG_02591	292,1444738	0,281960912	0,106042936	2,658931598	0,007838888	0,040836939	protein_codin hypothetical protein
TcG_02592	173,4235701	0,14214771	0,136332481	1,042654763	0,297108216	0,530318255	protein_codin hypothetical protein
TcG_02593	619,1923565	0,219376532	0,074542685	2,942965243	0,003250849	0,020337116	protein_codin hypothetical protein
TcG_02594	177,1551047	0,237892086	0,142273417	1,672076845	0,09450917	0,258800104	protein_codin hypothetical protein
TcG_02595	100,591624	-0,100347637	0,189063303	-0,53076211	0,595583641	0,780602803	
TcG_02596	386,8474488	-0,118003838	0,094596706	-1,24744131	0,212235729	0,435445928	protein_codin putative protein kinase
TcG_02597	426,9584356	0,45381019	0,091130148	4,979803072	6,3649E-07	1,35558E-05	protein_codin pentatricopeptidecontaining protein
TcG_02598	1051,702846	-0,044620466	0,06042604	-0,73843107	0,460252538	0,678399847	protein_codin putative hexokinase
TcG_02599	180,5982638	0,001164911	0,14037326	0,008298668	0,993378697	0,997511318	protein_codin putative actin-like protein
TcG_02600	237,9747651	0,159540312	0,117594608	1,356697515	0,174877343	0,383954688	protein_codin acyl-CoA dehydrogenase
TcG_02601	104,8184277	0,264698807	0,17411564	1,520247158	0,128448869	0,317382939	protein_codin calmodulin
TcG_02602	250,5688663	0,093507126	0,112472857	0,831375042	0,405761794	0,634643717	protein_codin hypothetical protein
TcG_02603	560,2858746	-0,098404036	0,07838542	-1,25538698	0,209338314	0,431872099	protein_codin putative major vault protein
TcG_02604	379,5970511	0,090961387	0,095479238	0,952682378	0,340751024	0,575004248	protein_codin hypothetical protein
TcG_02605	370,056416	-0,013127681	0,098772799	-0,13290786	0,894266254	0,948820769	protein_codin TPR repeat-containing protein
TcG_02606	156,1423823	0,130946505	0,149049193	0,878545547	0,379647732	0,610721305	protein_codin TPRcontaining protein
TcG_02607	131,0740964	0,337674757	0,16176189	2,087480291	0,036844741	0,133423365	protein_codin hypothetical protein
TcG_02608	718,6288593	0,095214466	0,07691395	1,237934935	0,215740187	0,439522738	protein_codin hypothetical protein
TcG_02609	249,6629575	0,232726963	0,114753299	2,028063376	0,04255378	0,147613203	protein_codin putative leucine-rich repeat protein (LRRP)
TcG_02610	138,8588718	0,187780572	0,149791708	1,253611255	0,209983338	0,432663517	protein_codin putative serine/threonine protein kinase
TcG_02611	343,590777	0,147735034	0,098278607	1,503226778	0,132780574	0,323682997	protein_codin hypothetical protein
TcG_02612	382,7004832	-0,277812693	0,096457408	-2,88015922	0,003974744	0,023953456	protein_codin putative serine/threonine protein kinase
TcG_02613	1146,202786	-0,06910081	0,064562455	-1,07029403	0,28448698	0,518167922	protein_codin topoisomerase IAmt
TcG_02614	854,4687061	-0,195504111	0,069072349	-2,83042513	0,004648619	0,027160312	protein_codin putative calpain
TcG_02615	331,0348158	-0,098665736	0,101237257	-0,97459906	0,329759179	0,56434119	protein_codin hypothetical protein
TcG_02616	148,4907364	-0,100594678	0,147421258	-0,68236209	0,495010021	0,704828082	protein_codin hypothetical protein
TcG_02617	115,3106701	0,01139306	0,165289649	0,06892785	0,94504705	0,974485148	protein_codin hypothetical protein
TcG_02618	41,39458524	0,329883277	0,282782844	1,166560435	0,243387929	0,471711701	protein_codin MFS transporter, FLVCR family, disrupted in renal carcinoma protein 2
TcG_02619	263,7260146	-0,133773884	0,110168656	-1,21426447	0,22464675	0,451082534	protein_codin hypothetical protein
TcG_02620	199,9947199	0,009238867	0,130509449	0,070790793	0,94356426	0,973371575	protein_codin WD repeat domain 31
TcG_02621	245,9123316	0,099509709	0,118163676	0,842134509	0,399712667	0,629649348	protein_codin hypothetical protein
TcG_02622	159,2717232	-0,172081332	0,138855802	-1,23928082	0,21524152	0,438870501	protein_codin syntaxin

TcG_02623	1241,416743	0,100571938	0,058235864	1,72697597	0,084171979	0,238965095	protein_codin	hypothetical protein
TcG_02624	119,1444922	-0,19374006	0,159522198	-1,21450219	0,224556013	0,451082534	protein_codin	hypothetical protein
TcG_02625	565,7500603	0,048249753	0,079727627	0,605182354	0,545057839	0,741898511	protein_codin	hypothetical protein
TcG_02626	176,5833371	-0,08538408	0,139954897	-0,61008283	0,541806937	0,740087861	protein_codin	methyltransferase domain-containing protein
TcG_02627	349,7030846	0,05258556	0,105851141	0,496787842	0,61933867	0,79394311	protein_codin	helicase-like protein
TcG_02628	116,0115976	0,089899506	0,163570827	0,549605987	0,582589652	0,771440332	protein_codin	hypothetical protein
TcG_02629	61,91322609	-0,059805491	0,222134931	-0,26923047	0,787752338	0,894530881	protein_codin	retrotransposon hot spot (RHS) protein
TcG_02630	17,31415502	0,386371162	0,429317752	0,899965493	0,368138615	0,600276384		
TcG_02631	604,2745928	-0,626474292	0,079500307	-7,88014932	3,2699E-15	3,6081E-13	protein_codin	hypothetical protein
TcG_02632	47,81730482	0,305538307	0,257760653	1,185356661	0,235876459	0,463445836		
TcG_02633	139,4823325	-0,216855344	0,153144962	-1,41601357	0,156771532	0,358297624	protein_codin	putative trans-sialidase
TcG_02634	166,8783739	-0,2781146	0,148034315	-1,87871711	0,06028313	0,19031072	protein_codin	putative complement regulatory protein
TcG_02635	217,0940523	0,02732357	0,121277737	0,225297495	0,821747848	0,911601931	protein_codin	methyltransferase-like protein
TcG_02636	132,2750794	-0,238447437	0,1567994	-1,52071652	0,128330992	0,317319718	protein_codin	hypothetical protein
TcG_02637	220,9080906	0,055492349	0,122166186	0,454236566	0,649658567	0,813987689	protein_codin	hypothetical protein
TcG_02638	115,4750871	-0,034109245	0,165623884	-0,205944	0,836834657	0,920655075	protein_codin	putative serine/threonine-protein phosphatase 2A, catalytic subunit
TcG_02639	1943,30393	0,226803823	0,056023996	4,048333578	5,15836E-05	0,000633773	protein_codin	ribosomal protein S19
TcG_02640	469,7279037	-0,05136871	0,085733524	-0,59916714	0,549061434	0,745508704	protein_codin	signal recognition particle subunit SRP68
TcG_02641	506,6048995	-0,237222377	0,084297841	-2,81409789	0,004891433	0,028321911	protein_codin	metallo-peptidase, Clan MC, Family M14
TcG_02642	1007,712334	0,172623782	0,062349086	2,768665789	0,005628634	0,031428122	protein_codin	putative regulatory subunit of protein kinase a-like protein
TcG_02643	94,7580979	0,456740789	0,183096547	2,494535243	0,01261222	0,059255954	protein_codin	ubiquitin-related modifier
TcG_02644	293,9426041	0,041024735	0,106345324	0,385769057	0,699667714	0,844323521	protein_codin	putative selenocysteine-tRNA-specific elongation factor
TcG_02645	276,3918593	0,067452542	0,107644058	0,626625785	0,530904562	0,732269078	protein_codin	putative serine acetyltransferase
TcG_02646	142,8550265	0,202965586	0,154051564	1,317517201	0,187665319	0,402050738	protein_codin	hypothetical protein
TcG_02647	220,4397338	0,226343916	0,130245605	1,737823822	0,082241862	0,235531775	protein_codin	hypothetical protein
TcG_02648	0					1	protein_codin	putative ribosomal protein L13
TcG_02649	2651,488186	0,302221088	0,044587839	6,77810582	1,21762E-11	7,66701E-10	protein_codin	putative ribosomal protein L3
TcG_02650	4,293738421	-0,298123665	1,630335922	-0,18286027	0,854907658	1	protein_codin	ribosomal protein L13
TcG_02651	199,4314642	0,025350296	0,133811838	0,189447333	0,84974223	0,927630599	protein_codin	patatin-like phospholipase
TcG_02652	78,70068584	0,212313676	0,205990158	1,030698156	0,30268239	0,535497997		
TcG_02653	231,0437662	0,322305293	0,120343984	2,678200299	0,007401894	0,038981065	protein_codin	hypothetical protein
TcG_02654	441,2474312	0,051770756	0,087430759	0,592134357	0,553760622	0,748837934	protein_codin	putative tyrosine phosphatase
TcG_02655	161,6540269	-0,131285648	0,144338983	-0,90956473	0,363052105	0,595268564	protein_codin	hypothetical protein
TcG_02656	176,2458892	0,071393461	0,13768585	0,518524313	0,604092501	0,785964707	protein_codin	hypothetical protein
TcG_02657	182,7891093	0,038118798	0,133265818	0,286035826	0,774850682	0,887537958	protein_codin	hypothetical protein
TcG_02658	76,47460563	0,096106256	0,208235327	0,461527146	0,64442045	0,811082798	protein_codin	hypothetical protein
TcG_02659	171,5368497	0,189898352	0,137127609	1,384829459	0,166104621	0,371661396	protein_codin	hypothetical protein
TcG_02660	280,8479695	-0,183461302	0,107391207	-1,70834566	0,087572223	0,245431005	protein_codin	putative nucleoside diphosphate kinase
TcG_02661	265,8092639	-0,120243697	0,116007674	-1,03651502	0,299961937	0,533675126	protein_codin	testis specific, 14
TcG_02662	307,3428451	-0,200673821	0,108615865	-1,84755533	0,064666697	0,200328436	protein_codin	putative casein kinase
TcG_02663	171,2811869	-0,056564833	0,138960161	-0,40705791	0,683965453	0,835947285	protein_codin	hypothetical protein
TcG_02664	364,5047538	-0,180462838	0,094443525	-1,9108016	0,056030083	0,179129287	protein_codin	hypothetical protein
TcG_02665	142,9890151	-0,003991019	0,14792117	-0,02698072	0,978475115	0,990851444	protein_codin	hypothetical protein
TcG_02666	248,3439573	0,139167458	0,114058594	1,220140044	0,222411791	0,448409237	protein_codin	putative ADP,ATP carrier protein 1, mitochondrial precursor, putative,ADP/ATP translocase 1
TcG_02667	421,5831619	-0,27564555	0,098301327	-2,8040878	0,005045916	0,028941577	protein_codin	hypothetical protein
TcG_02668	258,7600039	0,178553573	0,114991587	1,552753367	0,120482067	0,303930104	protein_codin	putative GDP-mannose transporter
TcG_02669	285,5019484	0,045303914	0,118777526	0,381418229	0,702892937	0,846452299	protein_codin	putative exosome subunit rrp6p-like protein
TcG_02670	89,4122913	0,246953734	0,204519741	1,207481161	0,227246896	0,454101852	protein_codin	hypothetical protein
TcG_02671	42,85146127	-0,036890839	0,280254353	-0,13163342	0,895274251	0,948981304	protein_codin	hypothetical protein
TcG_02672	151,0364502	0,166843522	0,158846749	1,050342693	0,293560583	0,526581966	protein_codin	putative phosphatidylinositol-4-phosphate 5-kinase-like
TcG_02673	378,8767459	0,17598009	0,095908413	1,834876462	0,066523996	0,204822487	protein_codin	putative suppressive immunomodulating factor
TcG_02674	194,4067279	-0,207903216	0,125803021	-1,65260909	0,098410447	0,265715087	protein_codin	retrotransposon hot spot (RHS) protein
TcG_02675	257,7982183	0,201923097	0,116232159	1,737239489	0,082344907	0,235742055	protein_codin	putative trans-sialidase
TcG_02676	274,9117409	0,106375059	0,115629167	0,919967353	0,35758982	0,59135536	protein_codin	hypothetical protein
TcG_02677	126,3462318	-0,131625067	0,169789176	-0,77522649	0,438205838	0,661505255	protein_codin	hypothetical protein
TcG_02678	250,2001595	-0,023538091	0,11431732	-0,20590135	0,836867969	0,920655075	protein_codin	hypothetical protein
TcG_02679	234,0087239	0,035973854	0,116690073	0,30828547	0,757865122	0,878015178	protein_codin	hypothetical protein

TcG_02680	289,4677481	0,053995395	0,108379575	0,498206377	0,618338589	0,793033999	protein_codin	putative sugar transporter
TcG_02681	316,3796251	-0,210173361	0,102657581	-2,04732432	0,040626252	0,142711038	protein_codin	dihydroflavonol-4-reductase
TcG_02682	513,5516922	0,076380825	0,082061656	0,930773631	0,351970672	0,586331015	protein_codin	hypothetical protein
TcG_02683	185,8931374	0,00301	0,14557902	0,020676055	0,98350407	0,99376391	protein_codin	hypothetical protein
TcG_02684	7,32144312	0,20787449	0,64424125	0,322665601	0,746948506	1	protein_codin	hypothetical protein
TcG_02685	10,76027728	0,532908764	0,544054928	0,979512796	0,327326669	1	protein_codin	hypothetical protein
TcG_02686	432,3609462	0,001164015	0,089369225	0,013024791	0,989608014	0,995709809	protein_codin	putative phospholipid-transporting ATPase IIB
TcG_02687	4,703973838	-0,685031025	0,807923335	-0,84789112	0,396498608	1	protein_codin	hypothetical protein
TcG_02688	29,95901825	0,584666797	0,318864235	1,833591645	0,066714633	0,205108511	protein_codin	hypothetical protein
TcG_02689	1773,690922	0,057340294	0,064508672	0,888877296	0,374069028	0,604496256	protein_codin	putative RNA editing associated helicase 2,putative
TcG_02690	208,9770355	-0,030280955	0,124761208	-0,2427113	0,808229054	0,905097798	protein_codin	hypothetical protein
TcG_02691	301,163666	-0,152742213	0,10314819	-1,48080362	0,138658912	0,332540294	protein_codin	DNA cross-link repair 1A protein
TcG_02692	205,577194	0,069211548	0,127860176	0,541306531	0,588296319	0,775648564	protein_codin	acetyl-CoA C-acetyltransferase
TcG_02693	103,8575149	0,247907442	0,171799719	1,443002606	0,149019737	0,348233698	protein_codin	hypothetical protein
TcG_02694	234,7387051	0,060793473	0,119096566	0,510455293	0,609732528	0,788785291	protein_codin	BRCA1-associated protein
TcG_02695	198,6577038	0,185948644	0,129577023	1,435043338	0,151274748	0,351589501	protein_codin	hypothetical protein
TcG_02696	148,5730733	0,238249864	0,147850226	1,611427124	0,107086659	0,282082846	protein_codin	putative rRNA maturation factor
TcG_02697	205,1811743	-0,088518274	0,126586017	-0,69927371	0,484380993	0,69758088	protein_codin	hypothetical protein
TcG_02698	93,95683217	0,181289404	0,180976277	1,001730206	0,316473914	0,551461387	protein_codin	hypothetical protein
TcG_02699	284,0963365	0,233610644	0,10976327	2,128313446	0,033311104	0,12362026	protein_codin	hypothetical protein
TcG_02700	263,6311918	0,392497632	0,112090933	3,501600179	0,000462473	0,004040885	protein_codin	putative vacuolar transport protein 4A
TcG_02701	324,104584	0,044119466	0,104834369	0,420849251	0,673865165	0,829023911	protein_codin	hypothetical protein
TcG_02702	272,0469378	0,655468651	0,112560251	5,823269271	5,77075E-09	1,93237E-07	protein_codin	hypothetical protein
TcG_02703	108,529289	0,028682627	0,168359732	0,170365125	0,864722998	0,934985064	protein_codin	Chain A, Structural Analysis Of A Probable Eukaryotic D-Amino Acid Trna Deacylase
TcG_02704	122,9487189	0,147594041	0,161347316	0,914759813	0,360317736	0,592955808	protein_codin	hypothetical protein
TcG_02705	321,4769467	0,083506485	0,101911141	0,819404866	0,41255546	0,639276121	protein_codin	aldose 1-epimerase-like protein
TcG_02706	18,44305635	-0,231854788	0,397545758	-0,58321535	0,55974834	0,754054394	protein_codin	hypothetical protein
TcG_02707	200,7521301	0,034275602	0,135859577	0,252286978	0,800819253	0,9008925	protein_codin	hydroxyacylglutathione hydrolase
TcG_02708	547,5057108	0,037538631	0,086225841	0,435352452	0,663306618	0,823782878	protein_codin	putative cleavage and polyadenylation specificity factor
TcG_02709	786,5048257	-0,216420561	0,070018852	-3,09088986	0,001995576	0,013713374	protein_codin	DNA topoisomerase IB, large subunit
TcG_02710	350,3905643	-0,064365562	0,09879144	-0,65152975	0,51470458	0,720387445	protein_codin	hypothetical protein
TcG_02711	306,6802516	0,117641508	0,113916767	1,032697033	0,301745703	0,535010187	protein_codin	hypothetical protein
TcG_02712	1947,51938	-0,032061688	0,050305581	-0,63733859	0,523904323	0,72776714	protein_codin	linear amidine hydrolase
TcG_02713	143,0334129	-0,046839397	0,151917001	-0,30832228	0,757837112	0,878015178	protein_codin	hypothetical protein
TcG_02714	279,1894612	0,148958306	0,109750637	1,357243208	0,174703946	0,383719416	protein_codin	hypothetical protein
TcG_02715	563,3552289	-0,089710801	0,078536475	-1,14228199	0,253336826	0,484454814	protein_codin	ruvB-like 1
TcG_02716	117,8558216	0,252180025	0,165511709	1,523638582	0,12759902	0,316362559	protein_codin	hypothetical protein
TcG_02717	507,2364504	0,05754538	0,081600656	0,705207327	0,480681222	0,695017177	protein_codin	peroxisome biosynthesis protein-like protein
TcG_02718	559,5796188	-0,387821876	0,089055574	-4,35482991	1,3317E-05	0,000194566	protein_codin	putative phosphatase 2C
TcG_02719	417,480548	-0,266058599	0,096118018	-2,76804083	0,005639439	0,031473285	protein_codin	putative DNA repair protein
TcG_02720	3256,869284	0,28318067	0,044994452	6,293679773	3,10027E-10	1,38153E-08	protein_codin	eukaryotic initiation factor 5a
TcG_02721	659,9239994	-0,482247046	0,072958417	-6,60988915	3,84608E-11	2,12194E-09	protein_codin	putative mucin-associated surface protein (MASP)
TcG_02722	237,197664	-0,305669249	0,120002915	-2,54718185	0,010859682	0,05266933	protein_codin	secretory carrier membrane protein 3
TcG_02723	153,691677	-0,187380627	0,148322541	-1,2633321	0,206469855	0,428548861	protein_codin	hypothetical protein
TcG_02724	270,8635363	-0,290009965	0,11574832	-2,50552202	0,012227079	0,057916162	protein_codin	putative ribose-phosphate pyrophosphokinase
TcG_02725	904,0217825	-0,211273484	0,064082569	-3,29689471	0,000977601	0,007632406	protein_codin	epsin
TcG_02726	88,60343877	0,229321223	0,203241353	1,128319702	0,259184926	0,491636632	protein_codin	hypothetical protein
TcG_02727	1309,667505	-0,18670684	0,063939341	-2,92006201	0,003499617	0,021636376	protein_codin	hypothetical protein
TcG_02728	905,1756421	-0,053685352	0,063694879	-0,84285194	0,399311257	0,629188116	protein_codin	putative RNA polymerase I second largest subunit
TcG_02729	595,698081	-0,076560072	0,085539567	-0,89502525	0,370773593	0,602325132	protein_codin	regulatory associated protein of mTOR
TcG_02730	207,4132258	-0,403189986	0,123239067	-3,27160855	0,001069375	0,008243368	protein_codin	hypothetical protein
TcG_02731	88,33146136	0,136696111	0,19073478	0,716681619	0,47357057	0,689295054	protein_codin	hypothetical protein
TcG_02732	537,623748	-0,131116673	0,085910962	-1,52619259	0,126961909	0,315187633		
TcG_02733	98,53383015	-0,311453578	0,174552025	-1,78430229	0,074374558	0,220159333	protein_codin	putative CYC2-like cyclin 6
TcG_02734	222,3982339	-0,280287334	0,119978682	-2,33614281	0,0194838	0,082349427	protein_codin	putative complement regulatory protein
TcG_02735	68,68194465	-0,116400843	0,208291701	-0,55883572	0,57627384	0,766706337	protein_codin	putative tyrosine phosphatase
TcG_02736	140,4180054	-0,262141722	0,149965135	-1,74801778	0,08046095	0,23202947	protein_codin	hypothetical protein

TcG_02737	221,9785165	-0,131031383	0,119707492	-1,09459634	0,273693518	0,50688937	protein_codin hypothetical protein
TcG_02738	852,8930014	-0,484375943	0,06708978	-7,21981717	5,20575E-13	4,13108E-11	protein_codin kinesin
TcG_02739	88,36467343	-0,615723084	0,18893222	-3,25896284	0,001118203	0,008540212	
TcG_02740	459,8746203	-0,008891758	0,094377337	-0,09421496	0,924938397	0,963266182	protein_codin hypothetical protein
TcG_02741	223,3541799	-0,016864562	0,138889605	-0,12142422	0,903355035	0,953224692	protein_codin guanine nucleotide releasing protein
TcG_02742	626,4127201	-0,119374256	0,074607414	-1,60003208	0,109591467	0,286167848	protein_codin ATP-binding cassette protein subfamily B, member 1
TcG_02743	258,5868221	-0,141240709	0,115270872	-1,225294	0,220464466	0,445465872	protein_codin RNA-binding protein
TcG_02744	921,5902247	-0,175082155	0,068079446	-2,57173295	0,010119091	0,049829924	protein_codin hypothetical protein
TcG_02745	581,0916583	-0,338341504	0,077999962	-4,33771369	1,43973E-05	0,000208248	protein_codin U-rich RNA-binding protein UBP-2
TcG_02746	243,258721	-0,370147188	0,12061364	-3,06886673	0,002148724	0,014575596	
TcG_02747	381,60141	-0,232761422	0,103247069	-2,2544119	0,024170267	0,097404076	protein_codin RNA-binding protein
TcG_02748	663,5232304	-0,020666659	0,077329772	-0,26725358	0,789273922	0,894943008	protein_codin putative myosin heavy chain
TcG_02749	425,2010106	-0,156727378	0,093890378	-1,6692592	0,095066022	0,259833672	protein_codin putative hepatocyte growth factor-like
TcG_02750	141,0016636	0,227567392	0,153845722	1,479192198	0,139088954	0,333433607	protein_codin hypothetical protein
TcG_02751	231,8182177	0,110126584	0,121220838	0,908478984	0,363625212	0,595555797	protein_codin putative small GTP-binding protein
TcG_02752	279,606504	-0,020191976	0,11199822	-0,18028836	0,856926193	0,930840697	protein_codin transcription factor
TcG_02753	861,1848927	-0,079022743	0,068394673	-1,15539324	0,247929429	0,477399096	protein_codin U3 small nucleolar RNA-associated protein 13
TcG_02754	215,8575255	0,122458492	0,130816636	0,936107938	0,349217616	0,583675029	protein_codin putative 60S ribosomal protein L7a
TcG_02755	542,0662784	-0,137977643	0,083603034	-1,65039037	0,098863119	0,266474642	protein_codin hypothetical protein
TcG_02756	252,3098645	-0,470849088	0,113046389	-4,1650962	3,11221E-05	0,000407436	protein_codin COP9 signalosome complex subunit 7, variant
TcG_02757	327,7626559	-0,033585873	0,100533304	-0,33407708	0,738321392	0,866422734	protein_codin putative sigma-adaptin 3, putative,adaptor complex AP-3 small subunit
TcG_02758	174,174939	-0,0294234	0,14293015	-0,2058586	0,836901368	0,920655075	protein_codin nucleolus protein required for cell viability
TcG_02759	126,5911142	0,288153042	0,157941591	1,824427882	0,06808741	0,207978047	protein_codin hypothetical protein
TcG_02760	264,0554022	-0,128492681	0,110780579	-1,15988454	0,246095818	0,474657257	protein_codin hypothetical protein
TcG_02761	544,2210245	-0,236860997	0,081493775	-2,90649189	0,003655064	0,022370612	protein_codin hypothetical protein
TcG_02762	88,10150362	-0,33273724	0,196048255	-1,69722113	0,089654862	0,249577422	protein_codin B9 protein domain 2
TcG_02763	440,1580502	-0,316993166	0,092516648	-3,42633648	0,000611782	0,00512103	protein_codin repressor activator protein 1
TcG_02764	154,8331314	-0,310630858	0,140453113	-2,21163384	0,026991976	0,105758887	protein_codin hypothetical protein
TcG_02765	213,5415374	-0,057170096	0,122037719	-0,46846251	0,639453873	0,808105647	protein_codin putative ATP-binding protein
TcG_02766	390,9201313	-0,256445341	0,10077576	-2,54471254	0,010936773	0,052972585	protein_codin hypothetical protein
TcG_02767	436,7730351	-0,083304172	0,089847308	-0,92717493	0,353835716	0,58808501	protein_codin hypothetical protein
TcG_02768	610,8201714	-0,188731895	0,078444763	-2,40592089	0,016131757	0,07150059	protein_codin hypothetical protein
TcG_02769	171,4037455	-0,010905573	0,142639183	-0,07645566	0,939056587	0,971680773	
TcG_02770	719,2355337	-0,229916076	0,078033665	-2,94637035	0,003215272	0,020179923	protein_codin hypothetical protein
TcG_02771	213,8648197	-0,31600697	0,13147653	-2,40352381	0,01623791	0,071888583	protein_codin hypothetical protein
TcG_02772	299,7227473	-0,185518329	0,106509795	-1,74179596	0,081544155	0,234259999	protein_codin dual specificity phosphatase
TcG_02773	364,1017789	-0,131432588	0,094675844	-1,38823783	0,165064637	0,370627691	protein_codin hypothetical protein
TcG_02774	333,0203212	-0,28989568	0,102007048	-2,84191814	0,0044843	0,02645372	protein_codin mitochondrial carrier protein
TcG_02775	754,6237272	-0,224185268	0,072088389	-3,10986651	0,001871719	0,013040131	protein_codin WD repeat-containing protein 19 isoform X2
TcG_02776	236,6258639	-0,168554775	0,116539281	-1,44633444	0,148083422	0,346907403	protein_codin ubiquitin carboxyl-terminal hydrolase
TcG_02777	579,4579362	-0,164740014	0,084108458	-1,95866168	0,050152421	0,165734726	protein_codin hypothetical protein
TcG_02778	546,6565528	-0,13427287	0,079138226	-1,69668788	0,089755684	0,249678118	protein_codin putative cleavage and polyadenylation specificity factor
TcG_02779	155,8859389	-0,258300028	0,143441784	-1,80073073	0,071745332	0,215117279	protein_codin hypothetical protein
TcG_02780	458,9315523	-0,31054578	0,085631873	-3,62652093	0,000287266	0,002701509	protein_codin hypothetical protein
TcG_02781	128,5363017	-0,210243132	0,153118674	-1,3730731	0,169729623	0,376067037	protein_codin putative DNA-directed RNA polymerase subunit
TcG_02782	568,7432636	0,033309235	0,081434662	0,409030192	0,682517504	0,834756445	protein_codin putative elongation factor
TcG_02783	418,2623285	-0,258301557	0,09497845	-2,71958067	0,006536475	0,035438277	protein_codin electron transfer flavoprotein
TcG_02784	335,9494181	-0,00934486	0,103781664	-0,09004346	0,928252682	0,965329465	protein_codin hypothetical protein
TcG_02785	390,6344527	-0,139337398	0,104463216	-1,33384175	0,182255725	0,394105045	
TcG_02786	148,8160584	-0,057921335	0,148639982	-0,38967534	0,696776636	0,842987885	protein_codin putative Qc-SNARE protein
TcG_02787	654,9611881	0,01244447	0,074342788	0,167393098	0,867060755	0,93578156	protein_codin ADP-ribosylation factor guanine nucleotide-exchange factor 1(brefeldin A-inhibited)
TcG_02788	41,91898959	0,07889482	0,265736493	0,296891175	0,766549594	0,882827395	protein_codin putative protein phosphatase 2C
TcG_02789	70,11281438	-0,406953921	0,210319796	-1,93492923	0,052998981	0,172001733	protein_codin protein phosphatase 2C
TcG_02790	216,5716925	-0,0610077	0,124252853	-0,49099637	0,623429012	0,797021059	protein_codin hypothetical protein
TcG_02791	99,66684476	0,165597512	0,183892551	0,900512347	0,367847658	0,600096165	protein_codin hypothetical protein
TcG_02792	398,0385137	-0,2957653	0,095944094	-3,08268376	0,00205143	0,014047205	protein_codin coiled-coil protein required for normal flagellar motility
TcG_02793	69,80237091	-0,064053029	0,214119462	-0,29914623	0,764828475	0,881458541	protein_codin hypothetical protein

TcG_02794	79,93532579	0,02299106	0,195944406	0,11733461	0,9065949	0,954710879	protein_codin hypothetical protein
TcG_02795	126,7031782	0,324500877	0,161662023	2,007279581	0,044719902	0,152794098	protein_codin hypothetical protein
TcG_02796	1717,76318	0,412496768	0,054707665	7,540017793	4,69907E-14	4,35548E-12	protein_codin ribosomal protein L35A
TcG_02797	195,6329755	0,233899538	0,128125057	1,825556554	0,067917085	0,207786466	protein_codin hypothetical protein
TcG_02798	179,9881838	0,025557718	0,136322825	0,187479375	0,851284794	0,928106297	protein_codin putative GTP-binding protein
TcG_02799	181,2176499	-0,003832909	0,132634343	-0,02889831	0,976945692	0,990177192	protein_codin hypothetical protein
TcG_02800	58,44522754	0,182621299	0,240502912	0,759330925	0,447654623	0,668763828	protein_codin hypothetical protein
TcG_02801	492,7028248	0,040403289	0,087925069	0,459519563	0,645861107	0,812019442	protein_codin hypothetical protein
TcG_02802	99,70992449	-0,074848608	0,174550753	-0,42880714	0,668063586	0,826134898	protein_codin hypothetical protein
TcG_02803	515,4053672	-0,065593749	0,082490024	-0,7951719	0,426513512	0,651783849	protein_codin putative TPR-repeat protein
TcG_02804	346,2740465	0,311783033	0,098073645	3,179070514	0,001477481	0,010739084	protein_codin putative dolichyl-P-Man:GDP-Man1GlcNAc2-PP-dolichyl alpha-1,3-mannosyltransferase
TcG_02805	300,5998468	0,980671517	0,108376268	9,048766297	1,44593E-19	2,4636E-17	protein_codin protein G6
TcG_02806	144,9695502	-0,067720221	0,148213368	-0,45691034	0,647735489	0,812603825	protein_codin hypothetical protein
TcG_02807	255,3253869	-0,012657257	0,112578116	-0,11243089	0,910481768	0,956471837	protein_codin centrin
TcG_02808	145,2399635	0,223473599	0,147439533	1,515696601	0,129596088	0,319264357	protein_codin hypothetical protein
TcG_02809	163,594454	0,192146518	0,140002954	1,372446169	0,169924586	0,376273905	protein_codin hypothetical protein
TcG_02810	470,5953704	-0,202687017	0,089214405	-2,27190908	0,023092002	0,094163844	protein_codin hypothetical protein
TcG_02811	481,6747076	-0,03369174	0,088179532	-0,38208118	0,702401148	0,84624626	protein_codin hypothetical protein
TcG_02812	1237,06387	-0,036939896	0,057379222	-0,64378525	0,5197147	0,724276793	protein_codin putative asparaginyl-tRNA synthetase
TcG_02813	127,0616323	0,107253488	0,160408232	0,668628331	0,503732596	0,712038696	protein_codin clathrin coat assembly protein AP17
TcG_02814	138,4630783	-0,124450663	0,148801429	-0,83635395	0,40295581	0,632950923	protein_codin hypothetical protein
TcG_02815	254,4614597	0,230149692	0,111542882	2,063329268	0,039081355	0,139124749	protein_codin hypothetical protein
TcG_02816	454,1648852	-0,110908506	0,087088497	-1,27351498	0,202835359	0,423006561	protein_codin putative G-actin binding protein, putative,twinfilin
TcG_02817	189,1585445	0,031764496	0,13014111	0,244077344	0,807170919	0,904725559	protein_codin hypothetical protein
TcG_02818	273,8956866	0,204732645	0,112174418	1,825127775	0,06798175	0,20781967	protein_codin hypothetical protein
TcG_02819	265,9079282	0,085694426	0,108953862	0,786520319	0,43156272	0,655968892	protein_codin putative sarcoplasmic reticulum glycoprotein
TcG_02820	293,5807581	0,010513662	0,106173719	0,09902321	0,921119842	0,961623219	protein_codin ATP-dependent RNA helicase-like protein
TcG_02821	757,7320406	0,25671571	0,069034163	3,718676329	0,000200269	0,001993404	protein_codin hypothetical protein
TcG_02822	407,3636479	0,163730877	0,09627206	1,700710218	0,088997415	0,248284145	protein_codin glycosyl hydrolase
TcG_02823	173,7462458	0,229271746	0,13759747	1,666249723	0,095663686	0,261036144	protein_codin hypothetical protein
TcG_02824	247,7929771	0,074292432	0,122347708	0,607223736	0,543702442	0,740930357	protein_codin 2,5-phosphodiesterase
TcG_02825	240,4662693	0,24599036	0,118669094	2,072910079	0,038180649	0,136615503	protein_codin hypothetical protein
TcG_02826	185,4437883	0,024177933	0,131922717	0,183273461	0,854583457	0,929424347	protein_codin metal ion transporter, MIT family
TcG_02827	517,3335113	-0,026217161	0,082287085	-0,31860603	0,750025282	0,873960868	protein_codin putative thioredoxin
TcG_02828	403,8446011	-0,017034329	0,090619129	-0,18797719	0,850894531	0,928053891	protein_codin putative dual specificity protein phosphatase
TcG_02829	469,9062003	0,049288456	0,084274164	0,584858441	0,558642905	0,753222006	protein_codin hypothetical protein
TcG_02830	349,9662141	0,296016314	0,097291262	3,042578628	0,002345605	0,015654073	protein_codin putative 8-oxoguanine DNA glycosylase
TcG_02831	50,13188772	0,226326984	0,244323128	0,926342856	0,354267831	0,588324037	protein_codin alpha-1,2-mannosyltransferase
TcG_02832	202,8278392	0,018285461	0,123856517	0,147634229	0,88263144	0,942677716	protein_codin hypothetical protein
TcG_02833	765,535652	-0,180119015	0,073036928	-2,46613625	0,013657937	0,063271035	protein_codin putative protein kinase
TcG_02834	348,4029043	0,06697638	0,097046289	0,690148805	0,490100614	0,701285132	protein_codin hypothetical protein
TcG_02835	518,6289939	0,145956328	0,082131941	1,777095813	0,075552477	0,222157463	protein_codin putative multidrug resistance-associated protein
TcG_02836	148,6454419	0,26609567	0,15340171	1,734633008	0,082805829	0,236652278	protein_codin NAD-dependent deacetylase
TcG_02837	512,7868652	0,038220049	0,087996789	0,434334594	0,664045486	0,824342954	protein_codin DoxX
TcG_02838	156,6574648	-0,17829885	0,146848948	-1,21416498	0,224684732	0,451082534	protein_codin hypothetical protein
TcG_02839	342,2769979	0,092955271	0,103739989	0,896040872	0,370230934	0,601865526	protein_codin putative cardiolipin synthetase
TcG_02840	231,4385819	0,298007717	0,122491434	2,432886183	0,014979009	0,067666914	protein_codin hypothetical protein
TcG_02841	365,1040041	-0,029140065	0,094575578	-0,30811406	0,757995543	0,878015178	protein_codin hypothetical protein
TcG_02842	240,3054357	0,175607939	0,118985902	1,475871817	0,139978307	0,334581697	protein_codin hypothetical protein
TcG_02843	506,7700552	-0,151809098	0,08404401	-1,8063048	0,070870743	0,213607812	protein_codin hypothetical protein
TcG_02844	65,36355111	-0,36421141	0,213737997	-1,70400872	0,088379458	0,247049662	
TcG_02845	260,986442	-0,047525111	0,111570502	-0,42596484	0,670133467	0,826966786	protein_codin putative protein kinase
TcG_02846	607,4179229	0,015254878	0,083359877	0,183000242	0,854797829	0,929487343	protein_codin hypothetical protein
TcG_02847	626,2914395	0,176765851	0,077919712	2,26856397	0,023294855	0,094699715	protein_codin permease, family protein
TcG_02848	23,55612306	0,294904079	0,383729621	0,768520496	0,442178019	0,664643815	protein_codin amino acid transporter
TcG_02849	8,350172884	0,413321011	0,62537836	0,660913516	0,508667778	1	protein_codin hypothetical protein
TcG_02850	1,372717874	-1,041142804	1,517271893	-0,68619396	0,492590808	1	protein_codin RNA-binding protein

TcG_02851	0					1	protein_codin target of rapamycin (TOR) kinase 1
TcG_02852	8,863219599	-1,472472368	0,613172965	-2,40139806	0,016332559	1	protein_codin target of rapamycin (TOR) kinase 1
TcG_02853	143,7662261	-0,370548519	0,153248856	-2,41795292	0,015608096	0,06968609	protein_codin putative tRNA isopentenyltransferase
TcG_02854	335,4279996	-0,486326899	0,099899651	-4,86815412	1,12645E-06	2,24632E-05	protein_codin putative signal peptide peptidase, putative,aspartic peptidase, clan AD, family A22B
TcG_02855	305,2790755	-0,754459013	0,110726209	-6,81373472	9,5097E-12	6,05381E-10	protein_codin leucine zipper-EF-hand containing transmembrane protein 2
TcG_02856	232,6848292	-0,187155914	0,128623539	-1,45506736	0,145650614	0,343128917	protein_codin putative ABC transporter
TcG_02857	124,8286091	-0,177386765	0,159529696	-1,1119357	0,266165796	0,498834829	protein_codin hypothetical protein
TcG_02858	799,7139898	-0,37137189	0,06719448	-5,52682144	3,26085E-08	9,32843E-07	protein_codin generic methyltransferase
TcG_02859	173,3455457	-0,129867208	0,145505808	-0,8925225	0,372112936	0,603401046	protein_codin hypothetical protein
TcG_02860	822,0668739	-0,166312457	0,066554647	-2,49888571	0,012458448	0,058809729	protein_codin putative Unc104-like kinesin
TcG_02861	241,4443215	-0,083245954	0,117067886	-0,71109129	0,477027668	0,692136011	protein_codin hypothetical protein
TcG_02862	497,4495615	-0,4541885	0,089331961	-5,08427774	3,69028E-07	8,33441E-06	protein_codin hypothetical protein
TcG_02863	408,5659454	-0,527484424	0,092401624	-5,70860555	1,13906E-08	3,57645E-07	protein_codin hypothetical protein
TcG_02864	278,8025782	-0,001706283	0,106521454	-0,01601821	0,987219861	0,994862078	protein_codin putative oxidoreductase
TcG_02865	254,4838016	-0,099011334	0,111128428	-0,89096314	0,372948943	0,604164772	protein_codin hypothetical protein
TcG_02866	167,185667	-0,308535562	0,137854803	-2,23811978	0,025213245	0,100385105	protein_codin hypothetical protein
TcG_02867	626,9249555	-0,130320542	0,077490392	-1,68176387	0,092614636	0,254816713	protein_codin hypothetical protein
TcG_02868	363,3642745	-0,129342116	0,098433567	-1,31400415	0,188844808	0,403235522	protein_codin putative KU70 protein
TcG_02869	303,8667143	0,096599291	0,103271395	0,935392528	0,349586046	0,584106942	protein_codin hypothetical protein
TcG_02870	4508,581108	0,11284276	0,040615737	2,778301461	0,00546439	0,030703403	protein_codin putative 60S ribosomal protein L4
TcG_02871	332,0277194	-0,334451952	0,100467707	-3,32894978	0,000871741	0,006965513	protein_codin ankyrin
TcG_02872	348,4413242	-0,201827501	0,095494884	-2,1134902	0,034558833	0,127231852	protein_codin hypothetical protein
TcG_02873	62,8001801	-0,074905388	0,23843452	-0,31415497	0,753403344	0,875527481	protein_codin hypothetical protein
TcG_02874	188,361135	-0,273805902	0,132967046	-2,05920121	0,039474967	0,13999295	protein_codin tryparedoxin
TcG_02875	1942,92564	-0,265720033	0,049857592	-5,32958015	9,84401E-08	2,55723E-06	protein_codin tryparedoxin
TcG_02876	375,8366785	-0,311253095	0,096243717	-3,2340095	0,001220654	0,009165584	protein_codin hypothetical protein
TcG_02877	925,1221865	-0,10132497	0,066404241	-1,52588099	0,127039505	0,315290628	protein_codin hypothetical protein
TcG_02878	147,6799989	-0,198462238	0,145362682	-1,36529015	0,172161872	0,379841586	protein_codin hypothetical protein
TcG_02879	0					1	protein_codin serine/threonine protein phosphatase
TcG_02880	2,981815551	-1,161417546	1,036427848	-1,12059662	0,262459603	1	protein_codin serine/threonine protein phosphatase
TcG_02881	223,6229292	-0,238431546	0,12076802	-1,97429373	0,048348353	0,161570239	protein_codin putative serine/threonine protein phosphatase
TcG_02882	18,76962249	-0,258609413	0,405916978	-0,63709928	0,52406018	0,72776714	protein_codin serine/threonine protein phosphatase
TcG_02883	16,92913009	-0,7804292	0,448601419	-1,73969401	0,081912762	0,234961703	protein_codin serine/threonine protein phosphatase
TcG_02884	387,8211132	-0,51694903	0,09391993	-5,50414623	3,70962E-08	1,05085E-06	protein_codin hypothetical protein
TcG_02885	420,1208444	-0,372755241	0,090224119	-4,13143675	3,60503E-05	0,000463572	protein_codin hypothetical protein
TcG_02886	241,8767673	-0,045567265	0,114131466	-0,39925243	0,689707215	0,838680499	protein_codin hypothetical protein
TcG_02887	1251,186387	-0,073655843	0,062360127	-1,18113684	0,237548367	0,465297613	protein_codin hypothetical protein
TcG_02888	110,178659	-0,035585752	0,167988683	-0,21183422	0,832236369	0,918663355	protein_codin hypothetical protein
TcG_02889	468,849183	-0,10085578	0,092309679	-1,09258077	0,27457791	0,507556451	protein_codin putative lipase domain protein
TcG_02890	730,8925839	-0,310350749	0,073656044	-4,21351366	2,51428E-05	0,000339516	protein_codin putative lipase domain protein
TcG_02891	231,1777621	-0,186279793	0,117759861	-1,58186152	0,113681187	0,292691163	protein_codin hypothetical protein
TcG_02892	245,9339793	0,032695498	0,114888872	0,284583678	0,775963118	0,888310845	protein_codin hypothetical protein
TcG_02893	795,2051949	-0,321322683	0,06933495	-4,6343537	3,58054E-06	6,25704E-05	protein_codin carnitine/choline acetyltransferase
TcG_02894	193,9491001	-0,22960504	0,133744403	-1,71674503	0,086025768	0,24262282	protein_codin DNA-directed RNA polymerase
TcG_02895	290,8735535	-0,183420142	0,10566955	-1,73578994	0,082600983	0,236299997	protein_codin putative serine/threonine protein kinase, putative,protein kinase
TcG_02896	66,9864449	-0,121831752	0,217785173	-0,55941252	0,57588022	0,766473088	protein_codin putative RNA-binding protein
TcG_02897	4,25402074	0,06071667	0,878142079	0,069142194	0,944876435	1	
TcG_02898	503,9250538	0,009681458	0,087395406	0,110777657	0,911792663	0,957020424	protein_codin protein LTV1
TcG_02899	13,10815951	-0,428210927	0,516641087	-0,82883638	0,407197001	1	protein_codin putative RNA-binding protein
TcG_02900	214,2536746	0,041031419	0,126726698	0,32377881	0,7461055	0,871496958	protein_codin RNA binding protein
TcG_02901	512,9382489	-0,117397178	0,082431703	-1,42417509	0,154395794	0,355561453	protein_codin hypothetical protein
TcG_02902	111,4013423	-0,098768048	0,171372226	-0,57633638	0,56438784	0,757266649	protein_codin hypothetical protein
TcG_02903	296,8252916	-0,122085648	0,11582879	-1,05401816	0,291874603	0,524450861	protein_codin putative asparagine synthase
TcG_02904	128,775387	-0,371372943	0,154733944	-2,40007417	0,01639175	0,072326144	protein_codin hypothetical protein
TcG_02905	894,8373981	-0,296642077	0,065877563	-4,50293034	6,70229E-06	0,000108001	protein_codin hypothetical protein
TcG_02906	554,3096535	-0,257975514	0,078791523	-3,27415317	0,001059791	0,008183398	protein_codin hypothetical protein
TcG_02907	704,1906255	-0,039614161	0,073716527	-0,53738507	0,591001657	0,776695236	protein_codin putative phosphatidylinositol 4-kinase alpha

TcG_02908	20,98188809	-0,264960824	0,399028552	-0,6640147	0,506680903	0,713466814	protein_codin hypothetical protein
TcG_02909	13,75797134	-0,391633114	0,474124513	-0,82601322	0,408796594	1	protein_codin hypothetical protein
TcG_02910	22,91562571	-0,509908403	0,380897902	-1,338701	0,18066803	0,392062146	protein_codin hypothetical protein
TcG_02911	20,62398766	-0,277287547	0,382924464	-0,72413119	0,468985199	0,685549145	protein_codin hypothetical protein
TcG_02912	107,917004	-0,198192132	0,175814013	-1,12728291	0,259622886	0,492108097	protein_codin hypothetical protein
TcG_02913	1556,213634	-0,0884903	0,054566578	-1,62169413	0,104868856	0,278161759	protein_codin clathrin assembly protein AP19-like protein
TcG_02914	955,1973111	-0,00157469	0,07057976	-0,02231079	0,982200039	0,993345815	protein_codin protein G7
TcG_02915	344,1560659	-0,096360341	0,097844093	-0,98483556	0,324704852	0,55907719	protein_codin hypothetical protein
TcG_02916	328,531924	-0,457097544	0,099533921	-4,59237954	4,3822E-06	7,4665E-05	protein_codin hypothetical protein
TcG_02917	45,90287678	0,065284882	0,252982511	0,258060852	0,796359944	0,898580669	protein_codin hypothetical protein
TcG_02918	126,6562103	-0,234792809	0,160219086	-1,46544844	0,142798611	0,33915975	protein_codin hypothetical protein
TcG_02919	80,10321514	-0,273613772	0,195764003	-1,39767152	0,162211729	0,365996875	
TcG_02920	380,8194717	0,080512162	0,097474219	0,825984169	0,408813072	0,636605346	protein_codin putative membrane-bound acid phosphatase 2
TcG_02921	390,5558626	0,08600156	0,095153733	0,903816988	0,366092452	0,598251607	protein_codin putative short chain 3-hydroxyacyl-CoA dehydrogenase
TcG_02922	10,73271923	0,778458227	0,547780979	1,421112191	0,155284147	1	protein_codin putative glyceraldehyde 3-phosphate dehydrogenase, cytosolic
TcG_02923	622,6287659	-0,376185023	0,083490504	-4,50572227	6,61476E-06	0,000106739	protein_codin transportin2-like protein
TcG_02924	469,0446657	-0,383258235	0,091503402	-4,18845886	2,80855E-05	0,00037231	protein_codin mitochondrial ATP-dependent zinc metallopeptidase
TcG_02925	812,4680027	-0,172206055	0,07443971	-2,3133628	0,020702701	0,086467733	protein_codin hypothetical protein
TcG_02926	404,5833219	-0,141848397	0,102079043	-1,38959372	0,164652284	0,369988628	protein_codin hypothetical protein
TcG_02927	32,60698664	0,018266818	0,308183087	0,059272617	0,952734971	0,977627081	
TcG_02928	153,5380963	0,260373958	0,149004441	1,747424148	0,080563792	0,232148104	protein_codin hypothetical protein
TcG_02929	431,5227451	0,154795881	0,100147131	1,545684629	0,122180751	0,306935426	protein_codin hypothetical protein
TcG_02930	959,9181358	-0,005346393	0,063522243	-0,08416568	0,932924708	0,968015911	protein_codin putative dihydrolipoamide acetyltransferase precursor
TcG_02931	292,5714449	-0,016656853	0,112970322	-0,14744451	0,882781179	0,942750736	protein_codin hypothetical protein
TcG_02932	168,3012364	0,372308107	0,152303592	2,444512983	0,014504786	0,066216747	protein_codin tRNA methyltransferase
TcG_02933	324,7772968	-0,214678966	0,102845621	-2,08739045	0,036852855	0,133423365	protein_codin COP9 signalosome complex subunit 12
TcG_02934	301,4634	0,164941016	0,106803921	1,544334834	0,122507238	0,307355751	protein_codin putative phosphatidylinositol-4-phosphate 5-kinase
TcG_02935	296,8310169	0,184773976	0,106039881	1,742495127	0,081421843	0,234024678	protein_codin putative protein kinase
TcG_02936	489,5234997	-0,212372812	0,085886445	-2,47271629	0,013409055	0,06241756	protein_codin putative UDP-glucose/GDP-mannose dehydrogenase family, NAD binding domain
TcG_02937	342,7472119	0,229387349	0,097707441	2,347695794	0,018889942	0,080228219	protein_codin oligoribonuclease
TcG_02938	196,9292261	0,105907307	0,131713491	0,804073344	0,421354618	0,647488005	protein_codin putative folylpolyglutamate synthase
TcG_02939	158,1164204	-0,20519822	0,140596078	-1,45948752	0,144430974	0,341087905	protein_codin putative chromatin binding protein
TcG_02940	6,376970003	-1,080268983	0,711463759	-1,51837528	0,12891982	1	protein_codin hypothetical protein
TcG_02941	1,136480117	-0,044216644	1,664540954	-0,02656387	0,978807593	1	
TcG_02942	0,483491558	-2,708304379	2,490004568	-1,08767045	0,276740619	1	
TcG_02943	810,0665279	-0,117123495	0,074394803	-1,5743505	0,115406441	0,295838856	protein_codin putative cullin-like protein
TcG_02944	251,5648553	-0,084330363	0,115325195	-0,73123972	0,464632741	0,681853697	protein_codin hypothetical protein
TcG_02945	61,54516533	0,208409061	0,220575813	0,944840954	0,34474008	0,578779679	protein_codin RNA-binding protein
TcG_02946	225,6976488	-0,124826704	0,118243257	-1,05567714	0,291115742	0,523651784	protein_codin putative clathrin coat assembly protein
TcG_02947	197,2540516	0,283832822	0,129632823	2,189513549	0,028559535	0,110040163	protein_codin putative exosome complex exonuclease RRP41A
TcG_02948	1128,106627	-0,064325846	0,060232687	-1,06795578	0,285540459	0,519107446	protein_codin superkiller viralicidic activity 2-like 2
TcG_02949	255,6445885	0,022321177	0,121718986	0,183382869	0,854497617	0,929422586	protein_codin putative GTP-binding protein
TcG_02950	325,6035835	0,174131798	0,10360982	1,680649555	0,092831004	0,255333088	protein_codin bromodomain protein
TcG_02951	143,9841769	0,156201157	0,146868692	1,063542916	0,287535814	0,520529678	protein_codin hypothetical protein
TcG_02952	66,55429458	-0,217614819	0,21843779	-0,99623247	0,319137206	0,554102154	protein_codin hypothetical protein
TcG_02953	1809,231143	-0,080109721	0,053475962	-1,49805105	0,134119986	0,325699887	protein_codin putative succinyl-CoA ligase beta-chain
TcG_02954	334,8976833	-0,00439408	0,103104899	-0,04261757	0,966006389	0,984950279	
TcG_02955	636,4248088	0,359882059	0,077621793	4,636353346	3,5461E-06	6,23446E-05	protein_codin DNA dependent protein kinase catalytic subunit
TcG_02956	291,8696623	-0,026317168	0,111731997	-0,23553833	0,813790932	0,907991308	protein_codin hypothetical protein
TcG_02957	470,7259465	0,044997229	0,08650431	0,520173265	0,602942817	0,785186973	protein_codin hypothetical protein
TcG_02958	87,09310506	0,582398359	0,199660173	2,916948086	0,003534746	0,021775091	protein_codin PHD finger-like domain-containing protein 5A
TcG_02959	279,1608	-0,004784505	0,113987516	-0,04197394	0,966519473	0,985314089	protein_codin hypothetical protein
TcG_02960	146,957651	0,051757609	0,14980644	0,345496555	0,7297211	0,860689063	protein_codin hypothetical protein
TcG_02961	379,3905409	0,206010442	0,095603305	2,154846439	0,031173853	0,117586617	protein_codin hypothetical protein
TcG_02962	239,2580975	0,265792846	0,117588515	2,260363999	0,023798669	0,09630855	protein_codin hypothetical protein
TcG_02963	227,9730886	0,054180131	0,120102982	0,45111395	0,651907424	0,815834891	protein_codin putative ATP-binding cassette protein
TcG_02964	687,6771972	0,054596488	0,072283442	0,755311123	0,450062326	0,670751493	protein_codin hypothetical protein

TcG_02965	283,9370367	0,196246513	0,111189289	1,764976781	0,077567668	0,225974102	protein_codin 40S ribosomal protein S24E
TcG_02966	281,2150082	0,526230744	0,118814225	4,429021392	9,46616E-06	0,000145265	protein_codin 40S ribosomal protein S24E
TcG_02967	177,5655561	-0,04460216	0,135159171	-0,32999729	0,741402012	0,868278957	protein_codin hypothetical protein
TcG_02968	523,2207807	-0,324206536	0,086878401	-3,73172769	0,000190171	0,001919269	protein_codin putative terminal uridylyltransferase 3
TcG_02969	53,71613451	0,216787	0,24832629	0,872992545	0,382667141	0,612626986	protein_codin hypothetical protein
TcG_02970	803,3946886	-0,042997902	0,074422758	-0,57775207	0,563431522	0,75668455	protein_codin putative serine carboxypeptidase S28
TcG_02971	1017,208453	-0,004388399	0,066795116	-0,0656994	0,947617148	0,975394476	protein_codin hypothetical protein
TcG_02972	561,4259511	0,125468456	0,079754325	1,573186859	0,115675557	0,296049703	protein_codin ATP-dependent RNA helicase DHX8
TcG_02973	326,1734651	-0,015534516	0,104266773	-0,14898818	0,881562963	0,942184067	protein_codin hypothetical protein
TcG_02974	68,19844286	-0,239252814	0,218293445	-1,09601465	0,273072364	0,506291632	protein_codin 40S ribosomal protein S24e
TcG_02975	99,47416043	0,269801968	0,179824586	1,500361959	0,133520668	0,324857299	protein_codin 40S ribosomal protein S24e
TcG_02976	429,8564917	-0,127552745	0,089302353	-1,42832457	0,153198459	0,353718083	protein_codin hypothetical protein
TcG_02977	995,4366501	-0,015157573	0,065952459	-0,22982574	0,818227182	0,910311132	protein_codin nucleoside-diphosphate kinase
TcG_02978	204,8506016	0,147572149	0,124479249	1,18551606	0,235813468	0,463445836	protein_codin putative meiotic recombination protein spo11
TcG_02979	122,1708383	0,29488564	0,163005133	1,809057393	0,070442087	0,212814087	protein_codin hypothetical protein
TcG_02980	80,75957974	0,178491866	0,202119618	0,883100155	0,37718216	0,607956124	protein_codin hypothetical protein
TcG_02981	520,2127623	-0,065683244	0,085811242	-0,76543868	0,444010369	0,666274334	protein_codin putative serine/threonine protein phosphatase type 5
TcG_02982	336,52862	-0,167504018	0,100747833	-1,66260667	0,096391196	0,262588384	protein_codin hypothetical protein
TcG_02983	383,262424	0,039912876	0,097821648	0,408016801	0,68326134	0,835357122	protein_codin primary-amine oxidase
TcG_02984	32,21621464	0,149949701	0,311140359	0,481935874	0,629851501	0,801688869	protein_codin hypothetical protein
TcG_02985	6,248051541	-0,556853019	0,684009251	-0,81410159	0,415586754	1	protein_codin hypothetical protein
TcG_02986	53,74787833	-0,231611025	0,239670326	-0,96637339	0,333857375	0,568249089	protein_codin hypothetical protein
TcG_02987	256,2076703	0,121219792	0,110864595	1,093404003	0,274216457	0,507334482	protein_codin ubiquitin-conjugating enzyme E2
TcG_02988	693,7679751	-0,095541959	0,077134053	-1,23864824	0,215475794	0,439138531	protein_codin hypothetical protein
TcG_02989	507,6406146	0,090884888	0,101712848	0,89354383	0,371566011	0,602808656	protein_codin hypothetical protein
TcG_02990	383,725016	0,033800058	0,094673771	0,357016076	0,721079771	0,856864638	protein_codin phosphoacetylglucosamine mutase
TcG_02991	533,5566658	-0,126478382	0,086597944	-1,46052407	0,144146096	0,340781521	protein_codin putative RNA-binding protein
TcG_02992	220,28933	0,128881526	0,125153314	1,029789162	0,30310899	0,535746873	protein_codin hypothetical protein
TcG_02993	322,6500948	-0,117120428	0,102825393	-1,13902242	0,254693804	0,486142077	protein_codin chaperone protein DNAJ
TcG_02994	375,1362137	-0,099584823	0,095907285	-1,03834472	0,299109595	0,532741548	protein_codin putative 6-phosphofructo-2-kinase/fructose-2,6-biphosphatase
TcG_02995	11,84070708	0,334892343	0,542502466	0,617310269	0,537030095	1	protein_codin hypothetical protein
TcG_02996	191,3206986	0,187942369	0,12699102	1,479965824	0,138882367	0,333007266	protein_codin chaperone protein DNAJ
TcG_02997	148,6358122	-0,167169083	0,144768908	-1,15473057	0,248200778	0,477445495	protein_codin hypothetical protein
TcG_02998	104,4286369	0,112677159	0,182984365	0,615774791	0,538043168	0,737462219	protein_codin hypothetical protein
TcG_02999	260,8619297	0,160632484	0,114254593	1,40591708	0,159748749	0,362414138	protein_codin hypothetical protein
TcG_03000	150,9355868	-0,005940116	0,151443134	-0,03922341	0,968712272	0,986594619	protein_codin putative protein phosphatase inhibitor
TcG_03001	3,527675798	0,982717658	0,956626692	1,027273926	0,304291496	1	protein_codin putative Protein kinase domain
TcG_03002	1,079552525	1,856485987	1,900001118	0,977097313	0,328520978	1	
TcG_03003	1121,844493	0,027567955	0,06293464	0,438041027	0,661356543	0,822595481	protein_codin hypothetical protein
TcG_03004	292,4605527	0,034164179	0,108965935	0,313530822	0,753877408	0,875807044	protein_codin putative malonyl-CoA decarboxylase, mitochondrial precursor
TcG_03005	732,2502035	0,238098131	0,076261033	3,122146666	0,001795375	0,012622094	protein_codin vacuolar protein sorting protein 18
TcG_03006	661,3803998	0,171578728	0,079210199	2,166119138	0,030302084	0,115259339	protein_codin hypothetical protein
TcG_03007	217,1530048	0,084246391	0,122340298	0,688623393	0,491060296	0,701878188	protein_codin centrin
TcG_03008	561,9603505	-0,037465028	0,077441547	-0,48378461	0,628538738	0,800863282	protein_codin putative DNA polymerase sigma
TcG_03009	996,8466729	-0,232220398	0,068238935	-3,4030484	0,000666385	0,005510874	protein_codin putative protein kinase
TcG_03010	158,9717041	-0,102835039	0,146017023	-0,70426747	0,481266223	0,695429092	protein_codin putative Qa-SNARE protein
TcG_03011	350,0070869	0,237166637	0,097508216	2,432273368	0,015004378	0,067745876	protein_codin putative serine/threonine protein phosphatase
TcG_03012	227,0843504	0,254106678	0,122460088	2,07501629	0,037985025	0,136145608	protein_codin hypothetical protein
TcG_03013	414,2542856	-0,020548151	0,091654822	-0,22419061	0,822608987	0,912120559	protein_codin hypothetical protein
TcG_03014	502,3859099	-0,089240124	0,08196822	-1,08871614	0,276279081	0,508736401	protein_codin putative vacuolar-type Ca2+-ATPase
TcG_03015	672,5826854	-0,026927724	0,0719924	-0,37403566	0,708377789	0,849437494	protein_codin hypothetical protein
TcG_03016	70,11154966	0,026565098	0,205785624	0,129091128	0,897285542	0,950181186	protein_codin hypothetical protein
TcG_03017	346,4288407	0,101989588	0,097252507	1,04870909	0,294312029	0,527256689	protein_codin hypothetical protein
TcG_03018	142,5496079	0,243555525	0,148185218	1,643588536	0,100261224	0,269206613	protein_codin hypothetical protein
TcG_03019	197,0053704	0,055752403	0,141241533	0,39473094	0,693041466	0,841062696	protein_codin hypothetical protein
TcG_03020	0,300973289	-0,458748581	3,014498415	-0,15218073	0,879044389	1	protein_codin hypothetical protein
TcG_03021	122,475066	-0,019862041	0,165873449	-0,11974214	0,90468742	0,953702767	protein_codin putative electron transfer flavoprotein-ubiquinone oxidoreductase

TcG_03022	49,62591384	0,069622591	0,254972414	0,273059307	0,784807627	0,893463807	protein_codin electron transfer flavoprotein-ubiquinone oxidoreductase
TcG_03023	816,3025958	-0,094241023	0,067165383	-1,40311898	0,160581363	0,363449048	protein_codin hypothetical protein
TcG_03024	417,1882151	0,075950579	0,092493468	0,821145329	0,411563493	0,638080373	protein_codin NUC156 family protein
TcG_03025	121,9233252	-0,022841639	0,165935964	-0,13765333	0,890514402	0,94667588	protein_codin hypothetical protein
TcG_03026	700,8757391	0,097740974	0,071176949	1,373211062	0,169686742	0,376067037	protein_codin hypothetical protein
TcG_03027	329,9201108	0,020357781	0,114549862	0,177719824	0,858943016	0,932070224	protein_codin hypothetical protein
TcG_03028	227,0719797	-0,155085317	0,125142437	-1,23927039	0,21524538	0,438870501	protein_codin hypothetical protein
TcG_03029	342,9250977	0,175385241	0,098275439	1,784629436	0,074321444	0,220159333	protein_codin hypothetical protein
TcG_03030	217,1481427	0,052473884	0,121592292	0,43155601	0,666064136	0,825196732	protein_codin putative mitochondrial carrier protein
TcG_03031	224,4784116	0,062788494	0,129013146	0,486682916	0,626483054	0,799398063	protein_codin putative QA-SNARE protein
TcG_03032	5000,3046	0,202117389	0,045088865	4,482645326	7,37234E-06	0,00011733	protein_codin putative 60S ribosomal protein L7a
TcG_03033	209,065741	0,437854499	0,138807182	3,154408086	0,00160824	0,011530366	protein_codin putative 60S ribosomal protein L7a
TcG_03034	213,8974925	-0,160071818	0,123219991	-1,29907345	0,193918725	0,410814106	protein_codin hypothetical protein
TcG_03035	257,6214656	0,167100873	0,113258864	1,475388922	0,140108012	0,334581697	protein_codin hypothetical protein
TcG_03036	278,8227719	0,178785114	0,115089722	1,553441185	0,12031777	0,303704071	protein_codin hypothetical protein
TcG_03037	676,6341058	0,227163344	0,151293791	1,501471695	0,133233602	0,324431383	protein_codin hypothetical protein
TcG_03038	70,53583412	0,357607744	0,213212293	1,677237923	0,093495966	0,256692953	protein_codin hypothetical protein
TcG_03039	94,5263414	-0,160283086	0,181529277	-0,88295997	0,377257898	0,607956124	protein_codin hypothetical protein
TcG_03040	20,74614295	-0,132103072	0,398379201	-0,33160133	0,740190316	0,867561457	
TcG_03041	526,1910627	-0,045713594	0,084614434	-0,54025764	0,589019367	0,775648564	protein_codin protein kinase, putative,NIMA/Nek Serine/threonine-protein kinase family
TcG_03042	539,7633479	-0,234143707	0,079821556	-2,9333393	0,003353372	0,020821098	protein_codin small GTP-binding protein RAB6
TcG_03043	109,1125395	0,152402052	0,166153719	0,917235275	0,359019366	0,592630203	protein_codin hypothetical protein
TcG_03044	528,2727386	-0,646836778	0,086985496	-7,43614518	1,03666E-13	9,31064E-12	protein_codin hypothetical protein
TcG_03045	323,2016709	0,137421994	0,110024037	1,249017927	0,211658517	0,434569481	protein_codin ribosomal RNA processing protein 41B
TcG_03046	107,8355274	0,445389691	0,182016898	2,446968915	0,014406326	0,065842878	protein_codin hypothetical protein
TcG_03047	695,4869549	-0,162362253	0,072943002	-2,22587842	0,026022326	0,102793957	protein_codin putative histone deacetylase
TcG_03048	184,6857358	0,268236661	0,1318003	2,035174885	0,041833279	0,145812387	protein_codin cytochrome b-domain protein
TcG_03049	232,9277744	0,264686723	0,119293911	2,218778145	0,026501822	0,104261496	protein_codin hypothetical protein
TcG_03050	470,6318618	-0,121301523	0,089075084	-1,3617896	0,173264295	0,381642608	protein_codin hypothetical protein
TcG_03051	718,4568435	0,157564514	0,069966006	2,252015269	0,024321307	0,09782343	protein_codin hypothetical protein
TcG_03052	230,2748216	0,135308878	0,126564528	1,069090052	0,285029094	0,518774269	protein_codin hypothetical protein
TcG_03053	144,4535652	0,386223854	0,150802621	2,561121632	0,010433482	0,051113033	protein_codin hypothetical protein
TcG_03054	1144,222068	0,105060283	0,068448028	1,534891302	0,12481053	0,311180289	protein_codin hypothetical protein
TcG_03055	827,651195	-0,240595608	0,07237738	-3,32418232	0,000886782	0,007066201	protein_codin hypothetical protein
TcG_03056	1357,138272	-0,051136254	0,055583677	-0,9199869	0,357579607	0,59135536	protein_codin exportin T (tRNA exportin)-like protein
TcG_03057	2031,563581	0,097932364	0,060824291	1,610086394	0,107378997	0,282235268	protein_codin phosphatidylinositol kinase related protein
TcG_03058	367,0119588	-0,034810059	0,097438797	-0,3572505	0,720904284	0,856743978	protein_codin hypothetical protein
TcG_03059	7,53518199	0,612890047	0,647810056	0,946095297	0,344099982	1	protein_codin SMUG L protein
TcG_03060	29,34535036	0,489856816	0,333039534	1,47086687	0,141327124	0,3366698	
TcG_03061	532,09376	0,439631187	0,088958353	4,941988852	7,73296E-07	1,60851E-05	protein_codin hypothetical protein
TcG_03062	100,6134214	-0,667805856	0,174718724	-3,82217683	0,000132279	0,001408039	
TcG_03063	591,5544932	-0,397218053	0,079416485	-5,00170782	5,68247E-07	1,2283E-05	protein_codin putative surface antigen TASV, putative,mucin-like glycoprotein
TcG_03064	152,3851724	-0,054219608	0,153528475	-0,35315669	0,723970976	0,857836749	protein_codin hypothetical protein
TcG_03065	134,7452775	-0,055772094	0,15814688	-0,3526601	0,724343279	0,857982193	protein_codin hypothetical protein
TcG_03066	90,13801254	-0,100688922	0,187287983	-0,5376155	0,590842528	0,776685709	protein_codin putative target of rapamycin (TOR) kinase 1
TcG_03067	172,0042021	-0,061950146	0,137766854	-0,44967381	0,652945657	0,816340604	protein_codin trans-sialidase
TcG_03068	100,0031938	0,175074113	0,183043847	0,956459969	0,338839899	0,573276734	protein_codin hypothetical protein
TcG_03069	1112,259138	0,029672508	0,062830251	0,472264684	0,636737875	0,806608902	protein_codin phosphoric diester hydrolase
TcG_03070	878,9312667	-0,154150428	0,067145263	-2,29577518	0,021688739	0,089581461	protein_codin hypothetical protein
TcG_03071	1141,761271	-0,047525502	0,0597624	-0,79524084	0,426473418	0,651783849	protein_codin hypothetical protein
TcG_03072	971,7966046	-0,145561649	0,064941104	-2,24144094	0,024997527	0,099697539	protein_codin hypothetical protein
TcG_03073	323,4707591	-0,045899212	0,100766067	-0,45550266	0,648747654	0,813280193	protein_codin hypothetical protein
TcG_03074	763,2535862	0,018107437	0,070357065	0,257364864	0,796897124	0,898729487	protein_codin UDP-GlcNAc:polypeptide N-acetylglucosaminyltransferase
TcG_03075	372,5904665	-0,044411993	0,092743143	-0,4788709	0,632030471	0,802906018	protein_codin hypothetical protein
TcG_03076	627,2931353	-0,424908484	0,07921743	-5,36382568	8,14775E-08	2,15525E-06	protein_codin casein kinase II, alpha chain
TcG_03077	41,37327266	-0,058514357	0,287147388	-0,20377813	0,838526885	0,921480839	
TcG_03078	896,972065	-0,027442384	0,06984731	-0,39289107	0,694399938	0,841912691	protein_codin casein kinase II, alpha chain

TcG_03079	90,00987874	0,224565232	0,199846327	1,123689568	0,261144743	0,493587786	protein_codin casein kinase II, alpha chain
TcG_03080	696,1311619	-0,352095804	0,07194285	-4,89410419	9,87545E-07	2,01084E-05	protein_codin proteasome regulatory non-ATP-ase subunit 6
TcG_03081	395,3863394	-0,067230299	0,094813356	-0,70908047	0,478274544	0,692834318	protein_codin putative ribosomal RNA methyltransferase
TcG_03082	330,255499	-0,23269188	0,101569809	-2,29095517	0,021966007	0,090407869	protein_codin putative ubiquitin-conjugating enzyme e2
TcG_03083	802,0439234	-0,059389209	0,069055402	-0,86002263	0,389776568	0,620165295	protein_codin putative RNA-editing complex protein MP81
TcG_03084	1025,903611	0,030608025	0,061347607	0,498927765	0,617830273	0,793033999	protein_codin hypothetical protein
TcG_03085	273,7958182	0,223718421	0,108664451	2,058800447	0,039513359	0,140000543	protein_codin hypothetical protein
TcG_03086	390,8376981	-0,03921705	0,103439408	-0,37913065	0,704590844	0,847555325	protein_codin serine peptidase, Clan S-, family S54
TcG_03087	3143,090859	0,207899791	0,047159475	4,408441632	1,04117E-05	0,00015707	protein_codin putative voltage-dependent anion-selective channel
TcG_03088	832,0997972	0,181355509	0,066693445	2,71924039	0,006543204	0,035458165	protein_codin hypothetical protein
TcG_03089	1329,497128	-0,002900213	0,061356532	-0,0472682	0,962299474	0,98256823	protein_codin hypothetical protein
TcG_03090	1281,261903	-0,085336842	0,060759817	-1,40449472	0,160171582	0,363061588	protein_codin hypothetical protein
TcG_03091	260,8092717	0,171237167	0,110430187	1,550637296	0,120988629	0,304986165	protein_codin hypothetical protein
TcG_03092	237,7442779	0,256314882	0,121167058	2,115384222	0,034397213	0,126878738	protein_codin hypothetical protein
TcG_03093	0					1	protein_codin putative glycerate kinase
TcG_03094	2278,229578	0,780000361	0,051425781	15,16749672	5,80488E-52	9,60791E-49	protein_codin putative glycerate kinase
TcG_03095	2,589718447	0,810428993	1,100101319	0,736685775	0,461313459	1	protein_codin putative glycerate kinase
TcG_03096	194,7859718	-0,086479259	0,12927494	-0,66895609	0,503523489	0,711962795	protein_codin hypothetical protein
TcG_03097	490,1977877	0,044185751	0,088231652	0,500792512	0,616517156	0,792657426	protein_codin putative imidazolonepropionase
TcG_03098	1579,813923	-0,103942287	0,063637246	-1,63335616	0,102394111	0,27334981	protein_codin hypothetical protein
TcG_03099	471,9487852	-0,0382714	0,172205175	-0,22224303	0,824124699	0,913451522	protein_codin hypothetical protein
TcG_03100	45,2686816	-0,093343657	0,256736266	-0,363578	0,716173161	0,854275943	protein_codin hypothetical protein
TcG_03101	44,27361363	-0,087481597	0,280386194	-0,31200394	0,75503753	0,876363937	protein_codin hypothetical protein
TcG_03102	143,5036533	0,573739834	0,165943551	3,457439773	0,000545334	0,004669801	protein_codin hypothetical protein
TcG_03103	305,3020781	-0,142836826	0,104988735	-1,36049668	0,173672801	0,382251724	protein_codin hypothetical protein
TcG_03104	860,6156854	0,216923921	0,07174826	3,023403237	0,002499489	0,016443652	protein_codin SMUG S protein
TcG_03105	248,504666	0,289348236	0,124282716	2,328145423	0,019904383	0,083646058	protein_codin mucin TcSMUGS
TcG_03106	47,31307242	-0,145478179	0,281940612	-0,51598873	0,60586228	0,786921256	
TcG_03107	41,84346846	0,371481352	0,282689898	1,314094895	0,188814272	0,403235522	protein_codin hypothetical protein
TcG_03108	50,45289687	0,281983319	0,246967965	1,141780956	0,25354508	0,484508214	protein_codin hypothetical protein
TcG_03109	22,37037849	-0,459069216	0,382016521	-1,2016999	0,229479819	0,456686974	protein_codin hypothetical protein
TcG_03110	22,86532153	0,339956809	0,367000591	0,926311338	0,354284205	0,588324037	protein_codin hypothetical protein
TcG_03111	493,9464342	-0,464464308	0,084712875	-5,48280661	4,18631E-08	1,1744E-06	protein_codin zinc finger protein
TcG_03112	142,957373	-0,003676387	0,146210761	-0,02514444	0,979939755	0,991948403	protein_codin putative ras-related protein rab
TcG_03113	305,6319378	-0,14091214	0,105734178	-1,33270191	0,182629646	0,394701309	protein_codin ribosome biogenesis protein ENP2
TcG_03114	1429,079784	-0,245734445	0,05538965	-4,43646865	9,14466E-06	0,000141644	protein_codin 70 kDa heat shock protein
TcG_03115	208,797826	-0,093194738	0,128038506	-0,72786493	0,466696274	0,683324027	protein_codin hypothetical protein
TcG_03116	581,2345787	-0,039423471	0,080172555	-0,49173275	0,622908281	0,796768389	protein_codin hypothetical protein
TcG_03117	290,974237	-0,226246715	0,107420422	-2,10617973	0,035188743	0,129058809	protein_codin hypothetical protein
TcG_03118	356,4129271	-0,009265997	0,09816758	-0,09438958	0,924799692	0,963208309	protein_codin putative chaperone protein DNAj
TcG_03119	337,8745695	-0,172098812	0,104005569	-1,65470767	0,09798381	0,265057302	protein_codin hypothetical protein
TcG_03120	283,2278241	-0,254774057	0,105306558	-2,41935605	0,015548013	0,069605596	protein_codin hypothetical protein
TcG_03121	25,78173477	-0,292944385	0,348994814	-0,83939467	0,401247876	0,631724133	
TcG_03122	167,3975718	0,057199182	0,136814719	0,418077694	0,675890313	0,83017833	protein_codin hypothetical protein
TcG_03123	211,476981	0,07477221	0,1287385	0,580806911	0,561370597	0,754845051	protein_codin tRNA pseudouridine synthase 9
TcG_03124	156,0003044	0,184549294	0,146312713	1,261334647	0,207188303	0,42942463	protein_codin hypothetical protein
TcG_03125	297,8509385	-0,255744091	0,103763198	-2,46468976	0,013713193	0,06340026	protein_codin hypothetical protein
TcG_03126	269,6297003	-0,204287399	0,109815059	-1,86028584	0,062845096	0,196259644	protein_codin hypothetical protein
TcG_03127	203,2381332	-0,209546336	0,128804205	-1,62685943	0,103766956	0,276060609	protein_codin dynein arm light chain, axonemal
TcG_03128	691,6816733	0,132015615	0,077346119	1,706816274	0,087856207	0,245988886	protein_codin telomerase-associated protein
TcG_03129	270,5358094	0,174866231	0,13005502	1,344555797	0,178768726	0,389799325	protein_codin putative telomerase-associated protein
TcG_03130	467,1666681	-0,114900578	0,084198458	-1,36463993	0,172366248	0,38004187	protein_codin hypothetical protein
TcG_03131	172,0705987	0,094416824	0,144714018	0,652437305	0,514119105	0,720089936	protein_codin hypothetical protein
TcG_03132	529,8697185	-0,085754053	0,081683723	-1,04983037	0,29379611	0,526847251	protein_codin hypothetical protein
TcG_03133	203,0022764	0,000934283	0,123117212	0,007588564	0,99394526	0,997677114	protein_codin hypothetical protein
TcG_03134	323,0906945	-0,079909812	0,108469697	-0,73670172	0,461303761	0,679291481	protein_codin putative RNA-binding protein
TcG_03135	96,90314462	0,076818692	0,177798414	0,432054992	0,665701446	0,825042607	protein_codin electron transfer protein

TcG_03136	408,9409049	0,032051047	0,089364511	0,358655209	0,71985304	0,856197241	protein_codin nicalin
TcG_03137	299,7077064	-0,154760606	0,106162329	-1,45777328	0,144903047	0,341754828	protein_codin hypothetical protein
TcG_03138	43,67851317	0,311493302	0,280589754	1,110137837	0,266939635	0,499395611	protein_codin aldo/keto reductase
TcG_03139	131,3671101	0,093496198	0,16287637	0,574031686	0,56594636	0,758216295	protein_codin aldo/keto reductase
TcG_03140	926,2806584	-0,404026635	0,064908165	-6,22458879	4,82821E-10	2,07954E-08	protein_codin dynein, axonemal, heavy polypeptide 1
TcG_03141	83,94735764	-0,21172762	0,191293259	-1,10682217	0,268370821	0,500941571	protein_codin hypothetical protein
TcG_03142	258,0500553	-0,063416004	0,112999211	-0,5612075	0,574656093	0,765645084	protein_codin hypothetical protein
TcG_03143	613,8811149	-0,153773874	0,076840562	-2,00120704	0,045370083	0,154378203	protein_codin putative protein kinase C substrate protein, heavy chain
TcG_03144	275,1344586	-0,253764588	0,11032716	-2,30010985	0,021441998	0,088803943	protein_codin hypothetical protein
TcG_03145	63,41343762	0,12695027	0,23016003	0,551573919	0,58124031	0,770482335	protein_codin putative protein kinase
TcG_03146	515,1265989	-0,148665537	0,092161701	-1,61309455	0,106723968	0,281471408	protein_codin nonsense-mediated mRNA decay protein 3
TcG_03147	178,4950533	0,114220239	0,135938391	0,840235328	0,400776454	0,631067681	protein_codin hypothetical protein
TcG_03148	470,0942685	-0,211945981	0,086079882	-2,46220111	0,013808721	0,063740178	protein_codin hypothetical protein
TcG_03149	827,421861	-0,16656295	0,073053387	-2,2800168	0,022606692	0,09261709	protein_codin putative chaperone DNAJ protein
TcG_03150	439,6583395	-0,144814577	0,09329131	-1,55228367	0,120594363	0,304137198	protein_codin hypothetical protein
TcG_03151	93,32282842	0,054868632	0,181991816	0,301489556	0,763041213	0,880274369	protein_codin hypothetical protein
TcG_03152	103,6247979	0,002609826	0,17375526	0,015020129	0,988016122	0,995145161	protein_codin hypothetical protein
TcG_03153	460,1178736	-0,0858466	0,085415434	-1,00504787	0,314873801	0,549582384	protein_codin heat shock 70 protein-like protein
TcG_03154	515,0574082	0,257548007	0,09158843	2,812014643	0,004923226	0,028463323	protein_codin putative 40S ribosomal protein S16
TcG_03155	115,8235271	0,255580664	0,173483314	1,47322908	0,140689276	0,335465313	protein_codin 40S ribosomal protein S16
TcG_03156	355,8913785	-0,132944118	0,097735391	-1,36024543	0,173752272	0,382354002	protein_codin hypothetical protein
TcG_03157	599,6801956	0,151851364	0,076520508	1,984453178	0,047205343	0,15875794	protein_codin RNA editing complex protein MP100
TcG_03158	342,6230406	0,059597941	0,107579279	0,553990895	0,579585077	0,769018862	protein_codin hypothetical protein
TcG_03159	315,9876353	0,159040822	0,106280473	1,496425618	0,134542778	0,326111428	protein_codin hypothetical protein
TcG_03160	745,2888407	-0,00147981	0,069331297	-0,02134404	0,982971216	0,99369442	protein_codin hypothetical protein
TcG_03161	1069,959393	-0,193423562	0,063312405	-3,05506576	0,002250112	0,015130467	protein_codin putative asparagine synthetase a
TcG_03162	223,1380978	0,20614802	0,121868897	1,691555641	0,09073073	0,251364477	protein_codin hypothetical protein
TcG_03163	278,0140039	0,265166227	0,108859262	2,435862804	0,014856318	0,067322838	protein_codin glutathione peroxidase-like protein
TcG_03164	572,1170519	-0,197512587	0,078408216	-2,51902921	0,011767889	0,056108134	protein_codin trypanothione/tryparedoxin dependent peroxidase 2
TcG_03165	585,6342128	-0,114103313	0,081499098	-1,40005615	0,161496505	0,365136182	protein_codin putative eukaryotic initiation factor 4a
TcG_03166	94,01586825	-0,20633968	0,18798529	-1,09763737	0,272362867	0,505380553	protein_codin hypothetical protein
TcG_03167	268,8809499	-0,017172782	0,110480939	-0,1554366	0,876477129	0,940875013	protein_codin hypothetical protein
TcG_03168	423,4463002	-0,337718976	0,088151924	-3,8311016	0,000127571	0,001368551	protein_codin hypothetical protein
TcG_03169	226,5282015	0,371711225	0,122100304	3,044310399	0,002332144	0,015582594	protein_codin hypothetical protein
TcG_03170	611,0162406	-0,365060537	0,079729704	-4,57872685	4,67815E-06	7,90102E-05	protein_codin putative protein kinase
TcG_03171	316,7121528	-0,154353195	0,101895631	-1,51481663	0,129818849	0,319405645	protein_codin putative ubiquinone biosynthesis methyltransferase
TcG_03172	28,29898157	0,215654126	0,330215888	0,653070108	0,513711082	0,71977949	
TcG_03173	254,969102	0,250016776	0,119469094	2,092731829	0,0363731	0,132355132	protein_codin hypothetical protein
TcG_03174	374,3896444	-0,100954333	0,095379723	-1,0584465	0,28985193	0,523276948	protein_codin hypothetical protein
TcG_03175	628,8118287	0,086450422	0,079165358	1,092023387	0,274822822	0,507556451	protein_codin U3 small nucleolar RNA-associated protein 21
TcG_03176	401,9196547	-0,147697105	0,100356746	-1,47172075	0,141096302	0,336297419	protein_codin hypothetical protein
TcG_03177	228,5081127	0,346365913	0,119999231	2,886401115	0,003896752	0,023606182	protein_codin GINS complex subunit 2
TcG_03178	200,419445	-0,057215357	0,127101903	-0,45015342	0,652599818	0,816260551	protein_codin putative UDP-N-acetylglucosamine-dolichyl-phosphate N-acetylglucosaminephosphotransferase
TcG_03179	166,3170491	-0,029840515	0,13970622	-0,21359475	0,830863092	0,917601778	protein_codin hypothetical protein
TcG_03180	422,1058729	-0,392551412	0,091295821	-4,29977417	1,70972E-05	0,00024199	protein_codin flagellar associated protein
TcG_03181	205,2393217	0,061830763	0,125916017	0,491047637	0,623392755	0,797021059	protein_codin DNA-directed RNA polymerase
TcG_03182	190,1280909	-0,102423404	0,134059597	-0,76401396	0,444858922	0,666776586	protein_codin hypothetical protein
TcG_03183	205,8524209	0,249657543	0,130164196	1,918020088	0,055108464	0,17725893	protein_codin hypothetical protein
TcG_03184	283,8267027	-0,176872728	0,111570068	-1,58530626	0,112896766	0,291249765	protein_codin hypothetical protein
TcG_03185	165,0159732	-0,130079045	0,137288258	-0,9474885	0,343389916	0,577600982	protein_codin hypothetical protein
TcG_03186	284,834832	0,141171423	0,106570747	1,324673303	0,185279532	0,398931175	protein_codin putative glycosyl hydrolase-like protein
TcG_03187	299,8650774	0,227923054	0,109558124	2,080384789	0,037490253	0,134895054	protein_codin putative glycosyl hydrolase-like protein
TcG_03188	239,9146198	-0,225736026	0,113770516	-1,98413469	0,047240827	0,158831173	protein_codin hypothetical protein
TcG_03189	183,9618888	-0,064556576	0,131623815	-0,49046273	0,623806497	0,797066211	protein_codin putative spermatogenesis-associated protein 17-like
TcG_03190	9,359158266	-0,059023084	0,564617611	-0,10453639	0,916743695	1	protein_codin hypothetical protein
TcG_03191	10,10679842	-0,582497728	0,557127545	-1,04553748	0,295774629	1	protein_codin hypothetical protein
TcG_03192	105,4894185	0,197068057	0,184601169	1,067534178	0,285730689	0,519127451	protein_codin putative phosphoglycerate mutase family member 5

TcG_03193	38,57688431	0,255321093	0,284423524	0,897679241	0,36935658	0,601128826		
TcG_03194	355,4355923	-0,179310572	0,102549481	-1,74852734	0,080372756	0,231872198	protein_codin	hypothetical protein
TcG_03195	325,081436	0,174667269	0,103514553	1,687369206	0,091532381	0,252686034	protein_codin	Cut9 interacting protein Scn1
TcG_03196	604,6659794	0,018201745	0,076962455	0,236501619	0,813043451	0,907594318	protein_codin	hypothetical protein
TcG_03197	491,7399191	-0,155433238	0,084145177	-1,84720317	0,064717701	0,200379284	protein_codin	putative zinc carboxypeptidase, putative,metallo-peptidase, Clan MC, Family M14
TcG_03198	156,3636159	-0,077257834	0,14262874	-0,54167087	0,588045258	0,775648564	protein_codin	putative Golgi SNARE protein-like
TcG_03199	165,5620125	-0,197480848	0,139884144	-1,41174576	0,158024834	0,360125044	protein_codin	hypothetical protein
TcG_03200	149,940214	0,159009708	0,143422819	1,108677887	0,267569165	0,499929984	protein_codin	hypothetical protein
TcG_03201	554,9696346	0,093081976	0,078068776	1,192307362	0,233140749	0,460495255	protein_codin	hslvu complex proteolytic subunit-like
TcG_03202	354,7740911	-0,038650083	0,096552715	-0,40030032	0,688935331	0,838445877	protein_codin	parafibromin
TcG_03203	202,771108	-0,03857702	0,128420511	-0,30039609	0,763875042	0,880920161	protein_codin	hypothetical protein
TcG_03204	386,8493104	-0,137658701	0,095777975	-1,43726886	0,150641615	0,350664472	protein_codin	hypothetical protein
TcG_03205	255,758032	0,158512452	0,116572563	1,359774962	0,173901148	0,382463686	protein_codin	putative glycosyl transferase-like
TcG_03206	339,8156201	-0,263876349	0,107495611	-2,45476393	0,014097713	0,064790206	protein_codin	hypothetical protein
TcG_03207	389,3451103	0,073566266	0,097585069	0,753868054	0,450928455	0,67161389	protein_codin	putative telomerase reverse transcriptase
TcG_03208	530,759955	-0,205644712	0,081308753	-2,52918297	0,011432841	0,054963027	protein_codin	hypothetical protein
TcG_03209	166,7273316	0,176699798	0,139160552	1,26975494	0,204171936	0,425303137	protein_codin	hypothetical protein
TcG_03210	527,443117	0,308430335	0,083543986	3,691831695	0,000222645	0,002180526	protein_codin	60S ribosomal protein L22
TcG_03211	970,6523101	-0,223082074	0,062798679	-3,55233707	0,000381825	0,003437319	protein_codin	hypothetical protein
TcG_03212	295,8735122	0,047326476	0,114594989	0,412989049	0,679614637	0,832788491	protein_codin	hypothetical protein
TcG_03213	311,9011508	0,186122849	0,109388306	1,701487632	0,088851458	0,248056144	protein_codin	hypothetical protein
TcG_03214	374,9510933	-0,211354933	0,096058857	-2,20026492	0,027788105	0,107932859	protein_codin	hypothetical protein
TcG_03215	249,5439841	-0,065181693	0,115066278	-0,56647085	0,571073741	0,762440697	protein_codin	putative actin-like protein
TcG_03216	196,6866422	-0,342884162	0,126338256	-2,7140169	0,006647278	0,03585445	protein_codin	hypothetical protein
TcG_03217	273,0306273	-0,299250168	0,109133043	-2,74206748	0,00610538	0,033477013	protein_codin	flagella associated protein
TcG_03218	493,4415073	-0,090109782	0,083998345	-1,07275664	0,283380322	0,516802206	protein_codin	hypothetical protein
TcG_03219	534,6139762	-0,040520426	0,095552568	-0,42406423	0,67151897	0,827770911	protein_codin	hypothetical protein
TcG_03220	223,3852767	-0,213679288	0,120801316	-1,768849	0,076919076	0,224649463	protein_codin	putative variant surface glycoprotein
TcG_03221	140,4916922	0,166959736	0,156919034	1,063986512	0,28733481	0,520429245	protein_codin	hypothetical protein
TcG_03222	311,2735323	-0,303499938	0,104017857	-2,91776765	0,003525469	0,021747067	protein_codin	hypothetical protein
TcG_03223	499,9422113	0,105026636	0,08220175	1,277669092	0,201366128	0,420896257	protein_codin	putative biotin/lipoate protein ligase
TcG_03224	115,3456704	-0,067929058	0,175502509	-0,38705463	0,698715766	0,844142481	protein_codin	hypothetical protein
TcG_03225	108,1136421	0,173557529	0,180110782	0,963615429	0,335238769	0,569763294	protein_codin	hypothetical protein
TcG_03226	413,5553455	0,590575827	0,094845437	6,226718406	4,76306E-10	2,05914E-08		
TcG_03227	143,380101	0,46815916	0,151020335	3,099974328	0,001935374	0,013387012		
TcG_03228	455,8016309	0,001771583	0,086178466	0,020557137	0,983598933	0,99376391	protein_codin	short-chain dehydrogenase
TcG_03229	49,80351062	-0,407907894	0,266409358	-1,531132	0,125736773	0,312950859		
TcG_03230	411,1252636	0,083342714	0,089177891	0,934567006	0,350011489	0,584547582	protein_codin	putative vacuolar protein sorting-associated protein-like
TcG_03231	382,7993903	-0,164404711	0,09213853	-1,78432096	0,074371526	0,220159333	protein_codin	Sec14 cytosolic factor
TcG_03232	273,3756198	-0,043944301	0,106905199	-0,41105859	0,681029571	0,833648782	protein_codin	putative SNF7-like protein
TcG_03233	185,4181573	-0,144725055	0,130143423	-1,11204279	0,266119754	0,49882923	protein_codin	putative glycosyl transferase-like protein
TcG_03234	399,8332643	-0,088791753	0,096940043	-0,91594506	0,359695714	0,592630203	protein_codin	putative protein NipSnap 3A-like
TcG_03235	1057,974586	-0,245163558	0,061655539	-3,9763428	6,99832E-05	0,000825888	protein_codin	putative alpha-ketoglutarate dehydrogenase complex subunit Kgd1
TcG_03236	1532,190856	0,375193227	0,053297587	7,039591283	1,92805E-12	1,40169E-10	protein_codin	mucin-like glycoprotein
TcG_03237	27,38463115	-0,342619656	0,327144407	-1,04730403	0,294959378	0,527938889	protein_codin	dispersed gene family protein 1 (DGF-1)
TcG_03238	4,827022198	-0,608563712	0,789350855	-0,77096732	0,440726303	1	protein_codin	dispersed gene family protein 1 (DGF-1)
TcG_03239	505,6716884	-0,404081138	0,082972556	-4,87005774	1,11566E-06	2,22862E-05	protein_codin	transferase
TcG_03240	801,357914	-0,112165279	0,070646727	-1,58769252	0,112355885	0,29018174	protein_codin	hydantoinase/oxoprolinase
TcG_03241	315,3571394	-0,170875064	0,102422785	-1,66833057	0,095250122	0,260091425	protein_codin	alkaline phosphatase
TcG_03242	23,49098882	-0,338110911	0,37350777	-0,90523126	0,365342882	0,59752437	protein_codin	putative trans-sialidase
TcG_03243	71,28664303	-0,340385785	0,204901572	-1,66121608	0,096670058	0,263003884	protein_codin	hypothetical protein
TcG_03244	345,1246169	-0,363810167	0,097526581	-3,73036933	0,000191199	0,001927968	protein_codin	hypothetical protein
TcG_03245	462,2565422	-0,125195122	0,092747952	-1,34984244	0,177066527	0,387183019	protein_codin	hypothetical protein
TcG_03246	241,943767	-0,505903332	0,115284542	-4,38830154	1,14239E-05	0,000170126	protein_codin	dynein light chain
TcG_03247	134,848484	-0,055292668	0,153560541	-0,36007081	0,718794182	0,855640542	protein_codin	putative mitochondrial carrier protein
TcG_03248	434,3296128	-0,224937459	0,089824159	-2,50419778	0,012272941	0,058038486	protein_codin	putative UTP-glucose-1-phosphate uridylyltransferase 2
TcG_03249	198,0018589	-0,300936253	0,141072236	-2,1332068	0,032907766	0,122555283	protein_codin	hypothetical protein

TcG_03250	399,5697361	-0,258108456	0,093317375	-2,76592066	0,005676234	0,031602524	protein_codin hypothetical protein
TcG_03251	440,9915864	-0,382824527	0,087488731	-4,37570099	1,21043E-05	0,00017865	protein_codin putative mannosyltransferase-II
TcG_03252	134,2100436	-0,177255604	0,153500393	-1,15475668	0,24819008	0,477445495	protein_codin hypothetical protein
TcG_03253	169,0177272	-0,159507751	0,134574102	-1,18527821	0,235907465	0,463445836	protein_codin hypothetical protein
TcG_03254	987,4724509	-0,218237703	0,066089971	-3,3021304	0,000959534	0,007536348	protein_codin hypothetical protein
TcG_03255	425,979181	-0,071499181	0,090061203	-0,79389547	0,427256288	0,652457013	protein_codin hypothetical protein
TcG_03256	180,1229274	-0,279548254	0,139839347	-1,99906722	0,045601082	0,154891273	protein_codin putative ARP2/3 complex subunit
TcG_03257	150,4858111	0,013318675	0,14844957	0,089718515	0,928510902	0,965428814	protein_codin hypothetical protein
TcG_03258	166,6591105	-0,071749433	0,140311913	-0,51135667	0,609101329	0,788218003	protein_codin hypothetical protein
TcG_03259	244,7283766	-0,022243311	0,114260282	-0,19467229	0,845649507	0,925035391	protein_codin hypothetical protein
TcG_03260	214,2654979	-0,302511753	0,1270406	-2,38122107	0,017255353	0,075101623	protein_codin putative dolichyl-P-Man:GDP-ManGlcNAc2-PP-dolichyl beta-1,4-mannosyltransferase
TcG_03261	194,3372759	-0,254999507	0,126979938	-2,00818736	0,044623386	0,152554309	protein_codin putative cyclophilin type peptidyl-prolyl cis-trans isomerase
TcG_03262	112,7049811	-0,358991163	0,165689759	-2,16664666	0,030261807	0,115180241	protein_codin putative cyclophilin type peptidyl-prolyl cis-trans isomerase
TcG_03263	388,2032459	-0,179854305	0,092193945	-1,95082556	0,0510778	0,168073669	protein_codin hypothetical protein
TcG_03264	278,1006008	-0,356226586	0,111773649	-3,18703548	0,001437391	0,010493772	protein_codin hypothetical protein
TcG_03265	665,0380017	-0,32342404	0,075025177	-4,31087343	1,62611E-05	0,000231451	protein_codin hypothetical protein
TcG_03266	136,9186523	0,058178435	0,157132038	0,370251897	0,711194811	0,851581551	protein_codin putative serine/threonine protein phosphatase
TcG_03267	464,1395617	-0,297322833	0,09375665	-3,1712186	0,001518009	0,010978557	protein_codin mitochondrial ATP-dependent zinc metallopeptidase
TcG_03268	319,0269924	-0,341509466	0,101438	-3,36668177	0,000760784	0,00619427	protein_codin hypothetical protein
TcG_03269	127,0684068	-0,310652834	0,160575582	-1,93462063	0,053036866	0,172076486	protein_codin hypothetical protein
TcG_03270	480,3247549	-0,180435737	0,084468329	-2,1361348	0,032668428	0,121964201	protein_codin periodic tryptophan protein 2-like protein
TcG_03271	1917,440873	-0,180973491	0,057558408	-3,14417122	0,00166558	0,011868022	protein_codin hypothetical protein
TcG_03272	95,3774696	-0,270231209	0,183538072	-1,47234417	0,140927962	0,335965302	protein_codin putative ethanolamine phosphotransferase
TcG_03273	184,9441547	-0,181564859	0,129427539	-1,40283019	0,160667482	0,363572938	protein_codin putative ethanolamine phosphotransferase
TcG_03274	232,2528001	-0,451044151	0,126126144	-3,57613525	0,000348711	0,003181235	protein_codin 30S ribosomal protein S8
TcG_03275	272,395356	-0,10560545	0,110649311	-0,95441579	0,339873216	0,573995826	protein_codin DNA-directed RNA polymerase II
TcG_03276	241,1673395	-0,30712251	0,121850665	-2,52048285	0,011719395	0,055969048	protein_codin DNA-directed RNA polymerase II/III subunit
TcG_03277	988,6571866	-0,26279041	0,066335849	-3,96151425	7,44759E-05	0,000870715	protein_codin hypothetical protein
TcG_03278	67,40860025	-0,007640493	0,219715462	-0,03477449	0,972259561	0,987776155	protein_codin hypothetical protein
TcG_03279	595,8560531	0,034862242	0,080667683	0,432171104	0,665617059	0,825042607	protein_codin hypothetical protein
TcG_03280	167,4362029	0,466853071	0,140806416	3,315566736	0,000914575	0,007262691	protein_codin putative alcohol dehydrogenase
TcG_03281	769,6778117	-0,447469517	0,071571759	-6,25204023	4,05125E-10	1,77795E-08	protein_codin putative elongation factor Tu
TcG_03282	110,0554284	-0,208426591	0,173500121	-1,20130516	0,229632848	0,456821116	protein_codin hypothetical protein
TcG_03283	248,3740609	-0,220334104	0,123444575	-1,78488285	0,074280322	0,220159333	protein_codin hypothetical protein
TcG_03284	1,67259677	-1,391992493	1,422377546	-0,97863784	0,327758954	1	protein_codin hypothetical protein
TcG_03285	1,805124943	-0,580506831	1,353470994	-0,42890231	0,667994322	1	protein_codin hypothetical protein
TcG_03286	238,0362234	0,028343852	0,116556863	0,243176173	0,807868924	0,905044416	protein_codin hypothetical protein
TcG_03287	246,1312125	-0,371561503	0,11366855	-3,26881538	0,001079987	0,008308589	protein_codin putative citrate synthase
TcG_03288	431,0858739	-0,379481559	0,08934677	-4,24728909	2,16373E-05	0,000296323	protein_codin hypothetical protein
TcG_03289	374,751945	-0,4336017	0,094732706	-4,57710668	4,71451E-06	7,95085E-05	protein_codin serine/threonine-protein phosphatase 4 regulatory subunit 1
TcG_03290	394,301875	-0,469341697	0,091401303	-5,13495628	2,8221E-07	6,51331E-06	protein_codin hypothetical protein
TcG_03291	161,4852348	-0,30657352	0,138091436	-2,22007627	0,02641359	0,10394968	protein_codin putative serine/threonine protein kinase
TcG_03292	12,52223296	0,182328564	0,485945822	0,37520348	0,707509142	1	protein_codin hypothetical protein
TcG_03293	26,27173312	-0,313055229	0,339997479	-0,9207575	0,357177047	0,590925784	protein_codin trans-sialidase
TcG_03294	27,11167573	-0,382387903	0,349463553	-1,09421397	0,273861144	0,506942836	protein_codin trans-sialidase
TcG_03295	24,29326484	0,112932378	0,363714126	0,31049764	0,756182554	0,877165706	protein_codin hypothetical protein
TcG_03296	19,44363962	0,134010798	0,416267001	0,321934714	0,747502154	0,872512589	protein_codin hypothetical protein
TcG_03297	71,27155603	0,08301621	0,207898356	0,399311528	0,689663674	0,838680499	protein_codin beta galactofuranosyl glycosyltransferase
TcG_03298	32,97669814	-0,10725234	0,31270077	-0,34298713	0,731608141	0,862124891	
TcG_03299	36,47726863	0,406592842	0,289441558	1,404749355	0,160095824	0,363061588	protein_codin protein kinase, putative,serine/threonine protein kinase
TcG_03300	52,02845242	0,023643541	0,240651903	0,098247889	0,921735456	0,962005855	protein_codin hypothetical protein
TcG_03301	11,59118391	0,470437783	0,523323703	0,898942242	0,368683426	1	protein_codin RNA-binding protein
TcG_03302	1391,909453	0,421714195	0,056768548	7,428659144	1,09704E-13	9,77716E-12	protein_codin hypothetical protein
TcG_03303	1387,120894	-0,130556137	0,056612128	-2,30615136	0,021102176	0,087835603	protein_codin chaperonin TCP20
TcG_03304	3504,267816	-0,271021648	0,088417313	-3,06525542	0,002174842	0,0147097	protein_codin dynein heavy chain 9, axonemal isoform 2
TcG_03305	1287,427909	-0,030459213	0,057453238	-0,5301566	0,596003359	0,780668859	protein_codin putative mitochondrial DNA polymerase I protein D
TcG_03306	1706,25488	-0,124665677	0,051204798	-2,43464834	0,014906269	0,067436168	protein_codin squalene monooxygenase-like protein

TcG_03307	567,6175476	-0,351712113	0,077868062	-4,51676983	6,27901E-06	0,000101889	protein_codin putative MCAK-like kinesin
TcG_03308	1116,845461	-0,010491715	0,062721498	-0,16727462	0,867153974	0,93578156	protein_codin protein p166
TcG_03309	622,75027	-0,445054577	0,076556227	-5,81343407	6,12041E-09	2,02603E-07	protein_codin centromere protein J
TcG_03310	657,3936136	-0,008676324	0,074327959	-0,11673029	0,907073785	0,954962006	protein_codin ubiquitin-conjugating enzyme-like
TcG_03311	489,8158809	-0,260699288	0,086585446	-3,0108904	0,002604829	0,01696433	protein_codin putative phosphoprotein phosphatase
TcG_03312	395,343193	0,139511231	0,092500172	1,508226716	0,131496512	0,322301372	protein_codin putative ras-family member, GTP-binding protein
TcG_03313	764,9128984	0,221316157	0,071601084	3,090961003	0,001995098	0,013713374	protein_codin hypothetical protein
TcG_03314	409,9344721	0,128400046	0,089466858	1,435168831	0,151238993	0,351589501	protein_codin hypothetical protein
TcG_03315	183,1344683	0,155348931	0,131464841	1,181676638	0,237334032	0,465035023	protein_codin phospholipid-transporting ATPase 1-like protein
TcG_03316	682,7009537	-0,085373149	0,071936426	-1,18678608	0,235312013	0,46318807	protein_codin IQ motif containing with AAA domain 1
TcG_03317	282,2155558	0,355552018	0,106273062	3,345645752	0,000820912	0,00661871	protein_codin putative DNA cross-link repair 1A protein
TcG_03318	323,7762624	-0,165781824	0,10323671	-1,6058418	0,108308674	0,283905949	protein_codin Ran-binding protein 1
TcG_03319	1604,303687	0,094212232	0,068720782	1,370942377	0,17039292	0,376966272	protein_codin putative ubiquitin-protein ligase
TcG_03320	189,2930168	-0,166200986	0,13110514	-1,26769237	0,204907829	0,426146492	protein_codin calmodulin
TcG_03321	312,502013	-0,104456911	0,102513621	-1,01895641	0,308223661	0,541260203	protein_codin putative mucin-associated surface protein (MASP)
TcG_03322	266,8942519	0,067666598	0,117757987	0,574624272	0,565545433	0,757986619	protein_codin hypothetical protein
TcG_03323	473,0434682	0,100633113	0,092047869	1,09326934	0,27427556	0,507334482	protein_codin hypothetical protein
TcG_03324	89,25716887	-0,34127397	0,191562943	-1,78152394	0,074826897	0,220708868	protein_codin ATP-binding cassette transporter ABCA1
TcG_03325	1690,71961	-0,132638948	0,062380555	-2,12628675	0,033479391	0,124046122	protein_codin ABC1 transporter
TcG_03326	421,9117325	-0,103859065	0,098262522	-1,05695501	0,290532122	0,523456792	protein_codin putative leucine-rich repeat protein (LRRP)
TcG_03327	186,3698778	-0,121592698	0,133076829	-0,913703	0,360872929	0,593536952	protein_codin hypothetical protein
TcG_03328	366,6154571	-0,022486613	0,096610751	-0,23275477	0,815951838	0,90921145	protein_codin hypothetical protein
TcG_03329	29,74257697	-0,45418884	0,326156167	-1,39255022	0,163755847	0,368546084	
TcG_03330	517,7376281	0,136922741	0,083651619	1,636821174	0,10166784	0,271946299	protein_codin hypothetical protein
TcG_03331	237,8580785	-0,111995666	0,121220128	-0,92390321	0,35553671	0,589390231	protein_codin hypothetical protein
TcG_03332	383,1641438	-0,315204922	0,09309233	-3,38593869	0,000709352	0,005820507	protein_codin putative endomembrane protein
TcG_03333	632,7872907	0,061120637	0,075796447	0,806378651	0,420024553	0,646561352	protein_codin DNA-directed RNA polymerase
TcG_03334	1084,522795	-0,380637774	0,06228533	-6,11119459	9,88881E-10	3,93717E-08	protein_codin hypothetical protein
TcG_03335	680,139799	-0,283045068	0,072669747	-3,89495049	9,82188E-05	0,00109842	protein_codin protein dpy-30
TcG_03336	491,3608372	-0,224182503	0,083647266	-2,6800936	0,007360158	0,038849178	protein_codin peptidyl-prolyl isomerase E (cyclophilin E)
TcG_03337	20,11647829	-0,115759947	0,415276171	-0,27875413	0,780433519	0,89067206	protein_codin surface protein-2
TcG_03338	247,770521	-0,018854675	0,115504719	-0,16323727	0,870331619	0,937398327	protein_codin hypothetical protein
TcG_03339	221,2346863	-0,057682016	0,124011787	-0,46513333	0,641835976	0,809837619	protein_codin rabGTPase-activating protein
TcG_03340	134,1911028	-0,044694794	0,156253397	-0,28604046	0,774847132	0,887537958	protein_codin hypothetical protein
TcG_03341	117,6076285	-0,15262024	0,167578536	-0,91073859	0,362433125	0,595028691	protein_codin hypothetical protein
TcG_03342	49,87592454	0,252873578	0,24875625	1,016551655	0,309366762	0,542503906	protein_codin hypothetical protein
TcG_03343	461,8933305	0,24566221	0,088060004	2,78971383	0,005275465	0,029844499	protein_codin putative zinc-finger protein ZPR1
TcG_03344	295,3760402	0,045769129	0,106064192	0,431522911	0,666088197	0,825196732	protein_codin hypothetical protein
TcG_03345	336,3092832	-0,104463562	0,102906168	-1,01513412	0,310041899	0,543276685	protein_codin hypothetical protein
TcG_03346	251,4652255	0,212610744	0,120387988	1,766046155	0,077388106	0,225621186	protein_codin otubain
TcG_03347	226,5993538	-0,014602276	0,119289726	-0,12241017	0,902574184	0,952909103	protein_codin myo-inositol-1(or 4)-monophosphatase 1
TcG_03348	285,4383172	-0,004924792	0,110439044	-0,04459286	0,964431838	0,984225075	protein_codin hypothetical protein
TcG_03349	65,76435725	0,231263585	0,217772696	1,061949408	0,288258653	0,521281309	protein_codin hypothetical protein
TcG_03350	541,0141605	0,128231454	0,083011642	1,544740604	0,122409019	0,307297575	protein_codin protein SDA1
TcG_03351	88,58872818	0,394565763	0,203445817	1,939414476	0,052450889	0,170899631	protein_codin putative L-gulonolactone oxidase
TcG_03352	94,66660444	0,444464413	0,186340457	2,385227662	0,017068555	0,074456429	protein_codin putative L-gulonolactone oxidase
TcG_03353	65,3737962	-0,146473957	0,213868355	-0,68487906	0,493420244	0,703987578	protein_codin hypothetical protein
TcG_03354	106,5712761	0,2029014	0,168720235	1,202590788	0,229134714	0,456268888	protein_codin nicotinamide mononucleotide adenylyltransferase
TcG_03355	156,682806	0,037632241	0,143314999	0,262584107	0,792871151	0,896248225	protein_codin hypothetical protein
TcG_03356	346,2556997	-0,205696376	0,10032522	-2,05029578	0,040335577	0,142022914	protein_codin hypothetical protein
TcG_03357	269,0775471	0,118079671	0,110600076	1,067627392	0,285688622	0,519127451	protein_codin hypothetical protein
TcG_03358	362,0672948	-0,092024823	0,1028693	-0,89458004	0,371011625	0,602627321	protein_codin hypothetical protein
TcG_03359	252,576984	0,167752341	0,112005483	1,497715436	0,1342072	0,325709912	protein_codin hypothetical protein
TcG_03360	150,7018601	0,719691261	0,148572605	4,844037434	1,27227E-06	2,50689E-05	protein_codin Appr-1-p processing domain-containing protein
TcG_03361	183,0283971	0,060353173	0,13611492	0,443398661	0,657477404	0,819281459	protein_codin putative choline/carnitine O-acetyltransferase
TcG_03362	135,9187438	-0,091731877	0,159298224	-0,57584997	0,564716597	0,757444604	protein_codin hypothetical protein
TcG_03363	1177,283028	-0,064640346	0,060672048	-1,0654057	0,28669238	0,519815009	protein_codin putative translation initiation factor

TcG_03364	263,0744486	-0,154398946	0,11698224	-1,31984946	0,186885284	0,400898518	protein_codin hypothetical protein
TcG_03365	254,0484096	-0,13696414	0,118008139	-1,160633	0,24579118	0,474324447	protein_codin organic solute carrier partner 1
TcG_03366	384,0450349	-0,070830792	0,094280916	-0,75127391	0,452487825	0,673298046	protein_codin nat10 protein
TcG_03367	203,1663921	0,039633931	0,128973221	0,307303573	0,758612317	0,878015178	protein_codin hypothetical protein
TcG_03368	417,9522166	0,018354884	0,090746003	0,202266585	0,839708321	0,922079481	protein_codin tetratricopeptide repeat domain 27
TcG_03369	457,5485952	0,049382728	0,088334645	0,559041452	0,576133431	0,766706337	protein_codin hypothetical protein
TcG_03370	111,676482	0,124293229	0,175948291	0,706419077	0,479927561	0,694196081	protein_codin hypothetical protein
TcG_03371	150,0974473	0,117997822	0,156620009	0,75340196	0,451208405	0,671940949	protein_codin hypothetical protein
TcG_03372	307,9384311	-0,091810837	0,114035378	-0,80510836	0,420757151	0,647224157	protein_codin hypothetical protein
TcG_03373	167,498154	0,536121359	0,138312467	3,876160781	0,000106118	0,001172048	protein_codin hypothetical protein
TcG_03374	369,8383183	-0,165308424	0,098128128	-1,68461813	0,092062271	0,253718713	protein_codin Na/H hydrogen antiporter 1
TcG_03375	129,3757775	-0,190287645	0,156984321	-1,21214427	0,225457159	0,451820114	protein_codin hypothetical protein
TcG_03376	213,5321046	0,063095442	0,125853671	0,501339705	0,616132067	0,792562564	protein_codin hypothetical protein
TcG_03377	175,9605504	0,015691368	0,140887647	0,111375048	0,911318946	0,956844965	protein_codin hypothetical protein
TcG_03378	459,3636377	0,228958846	0,087934056	2,603756217	0,009220832	0,046330746	protein_codin UDP-sugar pyrophosphorylase
TcG_03379	335,9614257	-0,143734647	0,099275923	-1,44782987	0,147664636	0,346394507	protein_codin putative 5'-3' exonuclease
TcG_03380	414,7582609	-0,005025708	0,088605912	-0,05671978	0,95476842	0,978889315	protein_codin hypothetical protein
TcG_03381	192,0934386	-0,078523803	0,13006566	-0,60372432	0,546026941	0,742955741	protein_codin membrane transporter protein
TcG_03382	308,7608451	0,036416434	0,102475104	0,355368595	0,722313473	0,857285556	protein_codin tRNA (cytosine34-C5)-methyltransferase
TcG_03383	586,1907777	-0,28121351	0,079469945	-3,53861464	0,000402233	0,003593112	protein_codin putative kinesin
TcG_03384	553,9367132	-0,138332904	0,083301651	-1,66062619	0,096788545	0,2631758	protein_codin putative ubiquitin hydrolase
TcG_03385	284,7307246	-0,207353793	0,107043454	-1,93709925	0,052733213	0,17142733	protein_codin hypothetical protein
TcG_03386	4,981502165	0,671464693	0,846100876	0,793598862	0,427428995	1	
TcG_03387	90,10658218	0,098106909	0,182922109	0,536331609	0,591729389	0,777210826	protein_codin hypothetical protein
TcG_03388	181,467238	0,055393576	0,137446974	0,403017795	0,686935133	0,837507151	protein_codin hydrolase-like protein
TcG_03389	386,0886854	0,412633947	0,095365687	4,326859674	1,5125E-05	0,000217418	protein_codin putative hydrolase, alpha/beta fold family
TcG_03390	111,4921574	0,183648865	0,171302351	1,0720744	0,283686616	0,517116604	protein_codin hydrolase-like protein
TcG_03391	106,6009681	0,114193904	0,176995874	0,645178337	0,518811623	0,723687872	protein_codin hypothetical protein
TcG_03392	478,2097372	-0,439209822	0,084649225	-5,18858647	2,11896E-07	5,12117E-06	protein_codin UDP-GlcNAc:polypeptide N-acetylglucosaminyltransferase
TcG_03393	448,7368653	0,096970146	0,08772955	1,105330482	0,269016414	0,501580975	protein_codin putative SNF2/RAD54 related DNA helicase
TcG_03394	695,5865023	0,26219694	0,074206718	3,533331597	0,000410357	0,003648811	protein_codin putative protein kinase
TcG_03395	354,7985708	0,076464918	0,095188903	0,803296562	0,421803345	0,648059084	protein_codin putative MYH7B protein
TcG_03396	299,1181721	0,11776723	0,10543245	1,116992254	0,263997637	0,496193848	protein_codin putative MYH7B protein
TcG_03397	216,6590198	0,161912202	0,125083616	1,29443174	0,195516333	0,413333203	protein_codin hypothetical protein
TcG_03398	272,2750267	0,058709012	0,109783928	0,534768733	0,592809787	0,778012482	protein_codin hypothetical protein
TcG_03399	828,4925896	-0,298760582	0,067162281	-4,44833883	8,65369E-06	0,000135489	protein_codin putative seryl-tRNA synthetase
TcG_03400	300,4411329	0,196691265	0,104223626	1,887204199	0,059132866	0,187087216	protein_codin putative protein kinase, putative,cyclin-dependent protein kinase
TcG_03401	183,749039	-0,039457034	0,131771601	-0,29943503	0,764608136	0,881292267	protein_codin hypothetical protein
TcG_03402	175,7568738	0,029872516	0,132313928	0,225770005	0,821380308	0,911456014	protein_codin putative NGG1 interacting factor 3
TcG_03403	372,928712	0,07988368	0,099153053	0,805660313	0,420438739	0,646906139	protein_codin putative cell cycle sequence binding phosphoprotein (RBP45)
TcG_03404	308,9852753	0,13250575	0,103637668	1,278548156	0,201056217	0,420551963	protein_codin hypothetical protein
TcG_03405	357,5184787	0,212867081	0,097640742	2,18010513	0,02924967	0,112176985	protein_codin exocyst complex component 6
TcG_03406	116,7138961	0,196617205	0,162978067	1,206402857	0,227662195	0,454517947	protein_codin hypothetical protein
TcG_03407	187,5417443	0,261419554	0,130993922	1,995661717	0,045970757	0,15578157	protein_codin cytoplasmic translation machinery associated protein
TcG_03408	251,2287549	0,228578252	0,112324873	2,034974499	0,041853439	0,14582948	protein_codin putative DEAD-boc ATP-dependent (RNA) helicase
TcG_03409	274,7555394	0,39387623	0,110766007	3,555930577	0,000376644	0,003403894	protein_codin hypothetical protein
TcG_03410	75,724576	0,253671714	0,20037653	1,265975184	0,205521967	0,426887327	protein_codin hypothetical protein
TcG_03411	489,5497094	-0,307573394	0,083130194	-3,69989989	0,000215685	0,002124367	protein_codin putative acidocalcisomal pyrophosphatase
TcG_03412	244,6026293	0,379213429	0,120097271	3,157552422	0,001590996	0,011442137	protein_codin hypothetical protein
TcG_03413	279,1412149	0,184389185	0,108660116	1,696935279	0,089708897	0,249607897	protein_codin pterin-4-alpha-carbinolamine dehydratase
TcG_03414	152,5924621	0,60068808	0,143741115	4,178957978	2,92848E-05	0,000387321	protein_codin putative DNA repair protein
TcG_03415	456,7510898	-0,135912001	0,088535244	-1,53511749	0,124754971	0,311108715	protein_codin putative proteasome alpha 7 subunit
TcG_03416	39,1960669	0,401739225	0,280584287	1,431795167	0,152202451	0,352394143	protein_codin putative protein kinase
TcG_03417	128,059694	0,184898748	0,154092374	1,199921469	0,230169841	0,457182887	protein_codin putative protein kinase, putative,serine/threonine protein kinase
TcG_03418	601,2213658	-0,162398212	0,079560346	-2,04119539	0,04123141	0,144409647	protein_codin carboxy-lyase
TcG_03419	239,1015418	0,17125555	0,122468569	1,39836328	0,162004004	0,365740139	protein_codin putative NADH-cytochrome b5 reductase
TcG_03420	239,6558719	-0,013737183	0,115501834	-0,11893476	0,905327039	0,953903154	protein_codin Protein tipD

TcG_03421	266,534958	-0,054858752	0,110053485	-0,49847356	0,618150303	0,793033999	protein_codin	hypothetical protein
TcG_03422	124,163665	0,113357423	0,156389402	0,724840824	0,468549687	0,685085396	protein_codin	hypothetical protein
TcG_03423	582,5448278	-0,124252782	0,07989771	-1,55514823	0,119910772	0,303006805	protein_codin	hypothetical protein
TcG_03424	196,5705955	0,123059931	0,126939302	0,969439159	0,332326122	0,566340797	protein_codin	hypothetical protein
TcG_03425	497,3911232	-0,06133014	0,082755633	-0,74109928	0,458633245	0,677438813	protein_codin	hypothetical protein
TcG_03426	473,2751193	-0,025919656	0,08538139	-0,303575	0,761451698	0,879491513	protein_codin	putative PIF1 helicase-like protein
TcG_03427	887,6216191	-0,319524878	0,067685476	-4,7207303	2,34999E-06	4,30187E-05	protein_codin	putative DNA repair and recombination protein,mitochondrial precursor
TcG_03428	1396,058519	-0,287782809	0,05418767	-5,31085405	1,09113E-07	2,80306E-06	protein_codin	putative 14-3-3 protein
TcG_03429	413,2463869	0,253720211	0,091586956	2,770265789	0,005601057	0,03128922	protein_codin	hypothetical protein
TcG_03430	338,2012257	-0,190353211	0,098280475	-1,9368365	0,052765332	0,17148363	protein_codin	hypothetical protein
TcG_03431	653,8466413	-0,081858742	0,078456439	-1,0433655	0,296779044	0,529975648	protein_codin	hypothetical protein
TcG_03432	283,3249228	-0,24987789	0,112170311	-2,22766513	0,025902854	0,102426779	protein_codin	hypothetical protein
TcG_03433	1178,077795	-0,067803391	0,066208408	-1,02409034	0,305792605	0,538836287	protein_codin	putative tubulin-tyrsoine ligase-like protein
TcG_03434	412,9367633	0,139919402	0,092090736	1,519364571	0,128670755	0,317795644	protein_codin	hypothetical protein
TcG_03435	818,4279101	0,034061104	0,067039458	0,50807547	0,611400417	0,789796547	protein_codin	ribosome biogenesis protein ERB1
TcG_03436	126,4433189	0,147350073	0,164553519	0,895453795	0,370544555	0,60220637	protein_codin	hypothetical protein
TcG_03437	177,1054858	-0,182929697	0,134255607	-1,36254791	0,173025035	0,381260566	protein_codin	putative chromatin binding protein
TcG_03438	314,7909878	-0,089032688	0,104432468	-0,85253838	0,393915345	0,623739673	protein_codin	hypothetical protein
TcG_03439	556,9873402	-0,146844541	0,08108082	-1,81108851	0,070127152	0,212139212	protein_codin	hypothetical protein
TcG_03440	236,2765582	0,113192445	0,116490488	0,971688301	0,331205639	0,565480184	protein_codin	hypothetical protein
TcG_03441	285,9511381	0,057490362	0,111430945	0,515928152	0,605904591	0,786921256	protein_codin	DNA ligase
TcG_03442	309,6067926	-0,03272289	0,101516689	-0,32234	0,747195132	0,872329988	protein_codin	putative pumilio-repeat, RNA-binding protein
TcG_03443	145,9397141	0,052423548	0,153004593	0,342627283	0,731878874	0,862356213	protein_codin	pumilio
TcG_03444	0,262179411	-0,45874873	3,156715507	-0,1453247	0,884454515	1		
TcG_03445	0,574155419	1,027852573	2,270469696	0,452704819	0,650761309	1		
TcG_03446	801,8692991	-0,176978898	0,067734521	-2,61283162	0,008979553	0,045411219	protein_codin	hypothetical protein
TcG_03447	278,4229043	-0,255523185	0,110931653	-2,30342897	0,021254719	0,08826422	protein_codin	tetratricopeptide repeat (TPR) protein
TcG_03448	308,1708417	0,060025881	0,101351621	0,592253787	0,553680656	0,748837934	protein_codin	putative protein kinase
TcG_03449	702,0938307	0,346061107	0,076221064	4,540229308	5,61931E-06	9,27426E-05	protein_codin	putative amino acid permease/transporter
TcG_03450	611,9553036	0,120384541	0,076418325	1,575336035	0,1151789	0,295562069	protein_codin	hypothetical protein
TcG_03451	653,4416059	-0,116997071	0,074940289	-1,5612039	0,11847565	0,300626124	protein_codin	putative transcription modulator/accessory protein
TcG_03452	339,1040711	0,386376625	0,100423738	3,847463088	0,000119347	0,001297166	protein_codin	putative nucleoside transporter-like
TcG_03453	442,7314373	0,344415864	0,105610746	3,261182009	0,001109488	0,008484837	protein_codin	helicase-like protein
TcG_03454	53,74354909	0,478118073	0,237681662	2,011590081	0,044263166	0,151943719	protein_codin	putative cytochrome P450
TcG_03455	394,7270064	0,259535594	0,094999267	2,7319747	0,006295598	0,034302627	protein_codin	putative trans-sialidase
TcG_03456	55,74168719	0,229080366	0,240459905	0,952675941	0,340754287	0,575004248	protein_codin	hypothetical protein
TcG_03457	24,15302199	0,100314948	0,368363861	0,272325704	0,785371594	0,893514718	protein_codin	trans-sialidase
TcG_03458	272,4929929	-0,232232414	0,116196688	-1,99861475	0,045650053	0,154925766	protein_codin	sialidase-like protein
TcG_03459	72,79332342	0,161912943	0,201976712	0,801641644	0,422760281	0,648892545	protein_codin	hypothetical protein
TcG_03460	154,7627749	0,239955906	0,141660087	1,693885069	0,090287125	0,250614908	protein_codin	putative retrotransposon hot spot (RHS) protein
TcG_03461	125,1717089	0,172144465	0,158677922	1,08486715	0,277980499	0,510651984	protein_codin	putative retrotransposon hot spot (RHS) protein
TcG_03462	323,2324655	0,117112045	0,102817304	1,139030507	0,25469043	0,486142077	protein_codin	retrotransposon hot spot (RHS) protein
TcG_03463	28,96317568	0,261821496	0,343994458	0,761121261	0,446584643	0,66814691	protein_codin	retrotransposon hot spot (RHS) protein
TcG_03464	44,19625698	0,074579721	0,262540662	0,284069222	0,776357333	0,888310845	protein_codin	hypothetical protein
TcG_03465	14,13223674	-0,193820672	0,454832655	-0,42613623	0,670008583	1		
TcG_03466	22,87846144	0,917366757	0,399521839	2,29616173	0,021666636	0,08952555		
TcG_03467	45,15771661	0,121942712	0,265865194	0,458663695	0,646475689	0,812019442	protein_codin	trans-sialidase
TcG_03468	72,66462536	0,080806336	0,208254263	0,38801768	0,69800295	0,843887456	protein_codin	hypothetical protein
TcG_03469	33,83053245	0,239283962	0,326295409	0,733335361	0,463353913	0,680919231	protein_codin	trans-sialidase
TcG_03470	39,57753573	-0,002443288	0,283836997	-0,00860807	0,993131841	0,99743633	protein_codin	hypothetical protein
TcG_03471	612,6121374	0,171385482	0,077893575	2,200251849	0,027789031	0,107932859	protein_codin	putative calcium/potassium channel (CAKC)
TcG_03472	244,5825974	0,306247017	0,115524285	2,650931934	0,008027002	0,041611115	protein_codin	hypothetical protein
TcG_03473	376,5444695	0,095588077	0,093203296	1,025586878	0,305086355	0,537951182	protein_codin	hypothetical protein
TcG_03474	832,8221658	-0,117452029	0,072554211	-1,61881754	0,105486527	0,279288598	protein_codin	putative eukaryotic initiation factor 4a
TcG_03475	133,6324614	0,540495152	0,166673116	3,242845427	0,001183424	0,008941933	protein_codin	hypothetical protein
TcG_03476	529,813692	0,285917158	0,081008431	3,529474094	0,000416386	0,003699581	protein_codin	putative protein kinase
TcG_03477	329,6622777	0,272409159	0,100555857	2,709033229	0,006747958	0,036322045	protein_codin	hypothetical protein

TcG_03478	737,6416947	0,132007556	0,07511955	1,757299625	0,078866752	0,22872345	protein_codin	hypothetical protein
TcG_03479	581,179439	0,106793157	0,076587639	1,394391549	0,163199399	0,367650833	protein_codin	hypothetical protein
TcG_03480	446,8442726	0,148994499	0,0918657	1,62187301	0,104830542	0,278123805	protein_codin	putative ubiquitin activating enzyme
TcG_03481	175,8961265	0,202042146	0,14232319	1,41960102	0,155723868	0,357270641	protein_codin	hypothetical protein
TcG_03482	126,5761499	0,345502459	0,156700058	2,204864916	0,027463575	0,107088045	protein_codin	hypothetical protein
TcG_03483	296,3540285	0,301594897	0,107863533	2,796078419	0,005172685	0,029435523	protein_codin	hypothetical protein
TcG_03484	1116,046299	-0,077300671	0,059765192	-1,29340621	0,195870603	0,413814152	protein_codin	hypothetical protein
TcG_03485	344,9001522	0,007726702	0,096904706	0,079735059	0,936447975	0,970438187	protein_codin	hypothetical protein
TcG_03486	679,5681872	0,272806343	0,073405256	3,716441565	0,000202048	0,00200766	protein_codin	hypothetical protein
TcG_03487	413,0685529	-0,065287014	0,089904679	-0,72618038	0,467728197	0,684316061	protein_codin	putative mitochondrial processing peptide beta subunit
TcG_03488	473,7126685	0,090525479	0,086003273	1,052581798	0,292532703	0,525307486	protein_codin	putative heat shock protein 70 (hsp70)
TcG_03489	222,3137099	0,25627219	0,123549852	2,074241171	0,038056918	0,136299059	protein_codin	hypothetical protein
TcG_03490	335,8889728	-0,186014294	0,098216224	-1,89392635	0,058234787	0,184598697	protein_codin	hypothetical protein
TcG_03491	415,624939	0,100550273	0,094886946	1,059685003	0,289287928	0,522802984	protein_codin	putative DNA-damage inducible protein DDI1-like protein
TcG_03492	512,7825072	0,117135438	0,081459566	1,437958045	0,150445959	0,350426454	protein_codin	putative GTPase activating protein
TcG_03493	450,9486594	0,359297423	0,088564061	4,056921262	4,97238E-05	0,000612873	protein_codin	REL1 protein
TcG_03494	870,5125643	-0,103548416	0,069826935	-1,48292941	0,138093166	0,331647146	protein_codin	hypothetical protein
TcG_03495	341,6314194	0,135901462	0,099589477	1,364616689	0,172373557	0,38004187	protein_codin	putative tricarboxylate carrier
TcG_03496	355,8732668	0,218622685	0,100820505	2,168434724	0,030125626	0,114852091	protein_codin	U4/U6 small nuclear ribonucleoprotein PRP3
TcG_03497	555,3370722	0,195548662	0,085373882	2,290497468	0,021992495	0,090431762	protein_codin	hypothetical protein
TcG_03498	400,9060607	-0,0798378	0,091767662	-0,86999928	0,384300798	0,614562845	protein_codin	hypothetical protein
TcG_03499	323,5038841	-0,186839193	0,105486242	-1,77121859	0,076524359	0,224004858	protein_codin	putative long-chain-fatty-acid-CoA ligase
TcG_03500	429,7691613	0,231732924	0,094820902	2,443901283	0,014529402	0,06622252	protein_codin	putative fatty acyl CoA syntetase 1
TcG_03501	413,3396722	0,109438096	0,092711707	1,180412903	0,237836032	0,465651011	protein_codin	putative fatty acyl CoA synthetase 2
TcG_03502	749,1344764	0,059659361	0,071426386	0,835256616	0,403573239	0,63312391	protein_codin	putative fatty acyl CoA syntetase 1
TcG_03503	2466,299415	-0,021716052	0,055703106	-0,38985352	0,696644862	0,842958473	protein_codin	hypothetical protein
TcG_03504	252,4768461	0,449908482	0,115502247	3,89523576	9,81033E-05	0,001098188	protein_codin	alpha/beta-hydrolase
TcG_03505	78,88215711	0,470086872	0,197607082	2,378896884	0,017364533	0,07540685		
TcG_03506	192,2202889	0,156940121	0,131031773	1,197725695	0,231023823	0,458407605	protein_codin	hypothetical protein
TcG_03507	142,522106	0,104291721	0,166076191	0,627975149	0,530020219	0,731981763	protein_codin	BRCA1-associated protein
TcG_03508	3044,739601	0,452983955	0,047339829	9,568770362	1,08185E-21	2,41044E-19	protein_codin	putative ribosomal protein S7
TcG_03509	121,3614741	0,330652322	0,165172454	2,001861165	0,045299665	0,154319883	protein_codin	hypothetical protein
TcG_03510	323,8404121	0,041041901	0,102276936	0,401282068	0,688212464	0,838182446	protein_codin	hypothetical protein
TcG_03511	602,0119076	0,10540366	0,079686348	1,322731722	0,18592461	0,399879894	protein_codin	hypothetical protein
TcG_03512	159,3072208	0,216301908	0,143683393	1,505406457	0,13221961	0,323116727	protein_codin	hypothetical protein
TcG_03513	136,6935098	0,357481109	0,1597695	2,237480304	0,025254965	0,100497398	protein_codin	hypothetical protein
TcG_03514	0,467431231	2,090445105	2,518488183	0,830039672	0,406516354	1	protein_codin	hypothetical protein
TcG_03515	45,32657911	0,573512753	0,263452629	2,176910342	0,029487258	0,112864013	protein_codin	hypothetical protein
TcG_03516	101,1769749	0,633267026	0,179595495	3,526074114	0,000421769	0,003738802	protein_codin	hypothetical protein
TcG_03517	18,93491283	0,167192886	0,403527322	0,414328538	0,678633516	0,831850182		
TcG_03518	230,3180594	0,47859935	0,118878325	4,02595973	5,67434E-05	0,000687687	protein_codin	metal ion binding protein
TcG_03519	176,7460568	0,455919505	0,140441894	3,246321262	0,001169068	0,008876033	protein_codin	zinc finger protein
TcG_03520	188,4085819	0,456677874	0,138462079	3,29821622	0,000973012	0,007611961	protein_codin	zinc finger protein
TcG_03521	42,923806	0,557968813	0,267147073	2,088620351	0,036741912	0,133320322	protein_codin	peptidyl-prolyl isomerase E (cyclophilin E)
TcG_03522	454,9134869	0,173788212	0,08848785	1,963978243	0,049532615	0,164484058	protein_codin	electron transfer protein
TcG_03523	145,3954854	0,41815431	0,157798946	2,649918266	0,008051125	0,041680219	protein_codin	putative phosphatidylinositol 3-kinase
TcG_03524	154,5861964	0,472732895	0,14555731	3,247744103	0,001163238	0,00883756	protein_codin	hypothetical protein
TcG_03525	770,1722742	0,547002493	0,070414642	7,768306139	7,95426E-15	8,30253E-13	protein_codin	putative RNA helicase
TcG_03526	681,2757763	0,140025303	0,072853816	1,922003677	0,054605295	0,17612944	protein_codin	hypothetical protein
TcG_03527	209,3250573	0,336559074	0,124791707	2,696966677	0,006997427	0,037291717	protein_codin	mitochondrial carrier protein
TcG_03528	235,1724257	0,571541351	0,122556946	4,663475785	3,10913E-06	5,5419E-05	protein_codin	hypothetical protein
TcG_03529	358,7923116	0,438606378	0,099270543	4,418293342	9,94833E-06	0,00015146	protein_codin	acetyltransferase-like protein
TcG_03530	443,0225982	0,424575129	0,092094589	4,610207105	4,02268E-06	6,92523E-05	protein_codin	hypothetical protein
TcG_03531	791,2384474	0,554273035	0,074995996	7,390701661	1,46056E-13	1,26284E-11	protein_codin	putative DNA polymerase zeta catalytic subunit
TcG_03532	169,8659466	0,555522849	0,146525846	3,791295967	0,000149863	0,00157275	protein_codin	PhnP protein
TcG_03533	72,84095192	0,525057698	0,205179649	2,55901451	0,010496936	0,051315401	protein_codin	hypothetical protein
TcG_03534	2147,539288	0,240262288	0,103045905	2,331604419	0,019721511	0,083088519	protein_codin	1-beta dynein

TcG_03535	558,2017286	0,483771941	0,084943729	5,695204901	1,23224E-08	3,80712E-07	protein_codin putative lathosterol oxidase
TcG_03536	134,65235	0,340351259	0,157743906	2,157619058	0,030957462	0,116984068	protein_codin hypothetical protein
TcG_03537	205,1760591	0,550582462	0,125695567	4,380285451	1,18524E-05	0,000175379	protein_codin hypothetical protein
TcG_03538	573,3644479	0,329268763	0,079457709	4,14394986	3,41374E-05	0,000440932	protein_codin putative exonuclease
TcG_03539	139,7229952	0,313434138	0,154574382	2,027723697	0,042588456	0,147645077	protein_codin hypothetical protein
TcG_03540	702,2466727	0,410931673	0,079111983	5,194303806	2,05487E-07	5,03335E-06	protein_codin hypothetical protein
TcG_03541	216,4916773	0,51127193	0,123415464	4,14268936	3,43257E-05	0,000442377	protein_codin hypothetical protein
TcG_03542	571,6234795	0,523671405	0,080344316	6,517840081	7,1327E-11	3,61994E-09	protein_codin hypothetical protein
TcG_03543	307,5650957	0,619192205	0,104208928	5,941834504	2,8185E-09	1,02047E-07	protein_codin hypothetical protein
TcG_03544	452,7125395	0,712357295	0,091820016	7,758191795	8,61488E-15	8,91179E-13	protein_codin hypothetical protein
TcG_03545	213,6272185	0,340293045	0,124974552	2,722898696	0,00647119	0,035166606	protein_codin hypothetical protein
TcG_03546	1003,127687	0,271861006	0,062930444	4,320023639	1,56012E-05	0,000223431	protein_codin putative T-complex protein 1, gamma subunit
TcG_03547	401,1449222	0,854193009	0,094827109	9,007898845	2,10042E-19	3,42753E-17	protein_codin putative S-adenosyl-methyltransferase mraW-like protein
TcG_03548	46,1629356	0,798890683	0,264933101	3,015443066	0,002566041	0,016778881	protein_codin transmembrane protein 234
TcG_03549	368,6933895	0,534508135	0,096988561	5,511043036	3,56713E-08	1,01545E-06	protein_codin hypothetical protein
TcG_03550	408,9370487	0,379024188	0,09522908	3,980130716	6,88774E-05	0,000815131	protein_codin putative coronin
TcG_03551	103,9613337	0,289384962	0,186176832	1,554355388	0,120099668	0,303351811	protein_codin hypothetical protein
TcG_03552	591,8191619	0,499056109	0,081217004	6,144724431	8,01025E-10	3,30273E-08	protein_codin hypothetical protein
TcG_03553	129,8255039	0,943234466	0,164598553	5,730514944	1,00126E-08	3,18698E-07	protein_codin hypothetical protein
TcG_03554	528,0322411	0,486907193	0,084456384	5,765191124	8,15654E-09	2,6471E-07	protein_codin putative cytosolic leucyl aminopeptidase, putative,metallo-peptidase, Clan MF, Family M17
TcG_03555	500,9837713	0,433535441	0,087103906	4,977221555	6,45035E-07	1,37126E-05	protein_codin hypothetical protein
TcG_03556	285,055025	0,44717236	0,107866424	4,145612157	3,38907E-05	0,000438234	protein_codin hypothetical protein
TcG_03557	559,7886217	0,200833306	0,080373052	2,498764217	0,01246272	0,058809729	protein_codin hypothetical protein
TcG_03558	171,4364506	0,195295684	0,136201481	1,433873428	0,151608386	0,351799471	
TcG_03559	521,5929615	0,349435129	0,081849906	4,269218458	1,96159E-05	0,00027218	protein_codin hypothetical protein
TcG_03560	361,3683627	0,566479141	0,099305048	5,704434475	1,1673E-08	3,64946E-07	protein_codin hypothetical protein
TcG_03561	72,73076724	0,606646899	0,226018623	2,684057134	0,007273468	0,038479632	protein_codin hypothetical protein
TcG_03562	265,8557276	0,453412147	0,109433872	4,143252341	3,42415E-05	0,000441784	protein_codin hypothetical protein
TcG_03563	118,6954198	0,571196075	0,172803201	3,305471621	0,000948167	0,007464012	protein_codin hypothetical protein
TcG_03564	198,9106477	0,490318971	0,135620585	3,615372781	0,000299916	0,002806144	protein_codin putative Bis(5-adenosyl)-triphosphatase
TcG_03565	308,3693173	0,352798839	0,102714625	3,434747853	0,000593105	0,005000863	protein_codin OB fold-containing protein
TcG_03566	144,9834326	0,537315136	0,15469025	3,47349064	0,000513735	0,004431971	protein_codin methyltransferase
TcG_03567	133,6699076	0,561989538	0,154176476	3,645105608	0,000267282	0,002537893	protein_codin methyltransferase
TcG_03568	300,8547348	0,348317941	0,110496664	3,152293722	0,001619932	0,011607011	protein_codin Leucine-rich, ribonuclease inhibitor subtype
TcG_03569	426,5233207	-0,039340272	0,089972077	-0,43724979	0,661930204	0,822959026	protein_codin MGT2 magnesium transporter
TcG_03570	256,4025887	-0,056280966	0,121475106	-0,46331275	0,643140206	0,810687402	protein_codin putative AAA ATPase
TcG_03571	318,2322831	-0,055697173	0,108195281	-0,51478376	0,606704136	0,787507317	protein_codin putative AAA ATPase
TcG_03572	496,7665873	-0,238751727	0,087054814	-2,7425448	0,006096513	0,033460068	protein_codin putative heat shock protein DNAJ
TcG_03573	1135,020399	0,037502171	0,06499062	0,577039757	0,563912599	0,756804282	protein_codin hypothetical protein
TcG_03574	126,2134607	0,112212013	0,16420711	0,683356605	0,494381534	0,70453824	protein_codin hypothetical protein
TcG_03575	173,2254105	0,281802339	0,13481204	2,090335089	0,036587709	0,133007512	protein_codin mucin-associated surface protein (MASP)
TcG_03576	113,7744747	0,229138784	0,177619196	1,290056422	0,197031069	0,415508184	protein_codin structural maintenance of chromosome protein 4
TcG_03577	1487,267863	-0,17104541	0,056074503	-3,05032412	0,002285945	0,01533582	protein_codin cytochrome c oxidase VII
TcG_03578	197,9567161	0,046633097	0,129057003	0,361337204	0,717847385	0,855087918	protein_codin hypothetical protein
TcG_03579	191,1314978	-0,278818207	0,133628339	-2,08652004	0,036931542	0,133506659	protein_codin LMBR1 domain-containing protein 1
TcG_03580	1742,845607	-0,390225474	0,052651216	-7,41151872	1,24861E-13	1,09594E-11	protein_codin ATPase beta subunit
TcG_03581	1470,63732	0,154685811	0,052962731	2,920653958	0,003492976	0,021606842	protein_codin ribosomal protein S25
TcG_03582	767,0913424	-0,129131303	0,069228813	-1,86528264	0,062141798	0,19469568	protein_codin putative aminopeptidase, putative,metallo-peptidase, Clan MG, Family M24
TcG_03583	290,6185787	0,008035681	0,104844188	0,076644028	0,938906732	0,971680773	protein_codin hypothetical protein
TcG_03584	595,6474001	0,074523752	0,076497033	0,974204476	0,329955023	0,564592954	protein_codin hypothetical protein
TcG_03585	536,375585	0,03638045	0,080318931	0,452949876	0,650584835	0,814531651	protein_codin hypothetical protein
TcG_03586	532,9659433	0,074585247	0,083014325	0,898462369	0,368939099	0,600861456	protein_codin hypothetical protein
TcG_03587	351,1499267	-0,170721326	0,103093617	-1,65598348	0,097725164	0,264604756	protein_codin hypothetical protein
TcG_03588	506,0819086	0,147515791	0,086113589	1,71303731	0,086705676	0,244065102	protein_codin putative cullin 4B
TcG_03589	417,6798566	0,167317684	0,091982803	1,819010507	0,068909827	0,209745518	protein_codin nuclear protein localization protein 4
TcG_03590	371,3545444	0,078136687	0,095571238	0,81757533	0,413599719	0,640294808	protein_codin hypothetical protein
TcG_03591	151,8323292	-0,057779455	0,142726634	-0,40482602	0,685605401	0,836942807	protein_codin hypothetical protein

TcG_03592	164,4983742	0,142099784	0,145398218	0,97731448	0,328413486	0,562869623	protein_codin putative GCN5-like protein
TcG_03593	389,0852695	-0,117650952	0,093312155	-1,26083201	0,207369379	0,429626894	protein_codin putative serine/threonine-protein phosphatase 2A, catalytic subunit
TcG_03594	494,5738807	-0,061037298	0,084351218	-0,72360898	0,469305828	0,685931288	protein_codin structure-specific endonuclease subunit SLX1
TcG_03595	621,9027535	-0,113275904	0,079494704	-1,42494908	0,154171922	0,355187093	protein_codin putative protein transport protein Sec24C
TcG_03596	441,1022838	0,118302469	0,096187316	1,229917565	0,218727976	0,443504083	protein_codin hypothetical protein
TcG_03597	1114,249183	-0,054928816	0,067876333	-0,80924843	0,418372261	0,645011445	protein_codin hypothetical protein
TcG_03598	220,9374005	0,04329266	0,122882352	0,352309824	0,724605922	0,85802335	protein_codin hypothetical protein
TcG_03599	271,8253366	-0,051144036	0,111187033	-0,45998202	0,645529126	0,81191979	protein_codin hypothetical protein
TcG_03600	190,7602199	0,435602867	0,13660439	3,188791132	0,001428691	0,010443413	protein_codin mucin-associated surface protein (MASP)
TcG_03601	25,16726652	0,021564489	0,359533058	0,059979155	0,952172236	0,977395901	protein_codin trans-sialidase
TcG_03602	232,785925	0,199530493	0,1255646	1,589066454	0,11204539	0,289885692	protein_codin structural maintenance of chromosome protein 4
TcG_03603	138,264176	0,250339098	0,161648674	1,548661625	0,121463083	0,305795585	protein_codin helicase-like protein
TcG_03604	332,3093348	0,164954391	0,103137265	1,599367519	0,109738965	0,286326125	protein_codin hypothetical protein
TcG_03605	271,6781181	0,246523079	0,111476458	2,211436242	0,027005642	0,105776664	protein_codin hypothetical protein
TcG_03606	543,542553	0,32154852	0,085540558	3,759018274	0,000170581	0,001748987	protein_codin GPN-loop GTPase 3
TcG_03607	371,9478482	-0,173628184	0,096592315	-1,79753622	0,072250533	0,216182287	protein_codin putative splicing factor 3 subunit
TcG_03608	391,7365936	0,006342407	0,093467656	0,067856706	0,945899703	0,974694377	protein_codin putative DNA polymerase delta subunit 2
TcG_03609	1324,47081	-0,155876161	0,058716873	-2,65470813	0,007937706	0,041248461	protein_codin GTP-binding nuclear protein rtb2
TcG_03610	403,1396546	-0,135753057	0,092763714	-1,46342843	0,143350184	0,339782167	protein_codin clusterin-associated protein 1
TcG_03611	331,5490835	0,144799727	0,1053815	1,374052622	0,169425347	0,37606421	protein_codin hypothetical protein
TcG_03612	245,8869684	-0,092850622	0,115416384	-0,8044839	0,421117565	0,647488005	protein_codin hypothetical protein
TcG_03613	305,0794176	-0,154786812	0,109266835	-1,41659463	0,156601478	0,358187807	protein_codin putative C-14 sterol reductase
TcG_03614	187,7082099	-0,214002359	0,129638131	-1,65076708	0,098786145	0,266366674	protein_codin putative peptide chain release factor 1
TcG_03615	594,511032	-0,385400781	0,07844936	-4,91273325	8,98154E-07	1,8516E-05	protein_codin protein associated with the ribbon compartment of flagellar microtubule
TcG_03616	204,632378	-0,073293226	0,131723085	-0,55641899	0,577924461	0,768001978	protein_codin putative cyclin
TcG_03617	0					1	
TcG_03618	28,39344468	-0,005386405	0,321956125	-0,01673025	0,986651818	0,994670296	
TcG_03619	8,936665733	0,493935596	0,586143015	0,842687848	0,399403045	1	protein_codin hypothetical protein
TcG_03620	4,393760128	0,022594003	0,920993726	0,0245322	0,980428099	1	protein_codin hypothetical protein
TcG_03621	1,483089552	1,66635831	1,524686313	1,092918783	0,27442946	1	protein_codin putative mucin-associated surface protein (MASP)
TcG_03622	3,832556471	0,512187105	0,907522684	0,564379396	0,572495958	1	protein_codin putative mucin-associated surface protein (MASP)
TcG_03623	32,61876189	0,034193721	0,304156397	0,112421509	0,910489202	0,956471837	
TcG_03624	34,35934448	-0,290316219	0,319289943	-0,90925576	0,363215138	0,595268564	protein_codin trans-sialidase
TcG_03625	79,95923026	0,391714811	0,200880554	1,9499887	0,051177466	0,168253584	protein_codin SH3 domain protein
TcG_03626	75,18732688	0,261627131	0,199762353	1,309691881	0,190300091	0,405520849	protein_codin rab1 small GTP-binding protein
TcG_03627	125,2149914	0,113684385	0,160287942	0,709251012	0,478168722	0,692834318	protein_codin protein kinase, putative,serine/threonine protein kinase
TcG_03628	390,2355917	-0,504237687	0,092120001	-5,47370473	4,40723E-08	1,22451E-06	protein_codin hypothetical protein
TcG_03629	403,9802776	-0,206801522	0,099217174	-2,0843319	0,037129987	0,133973227	protein_codin elongation factor 2-like protein
TcG_03630	246,7562643	-0,300257109	0,115899513	-2,59066757	0,009578997	0,047754847	protein_codin hypothetical protein
TcG_03631	254,114176	-0,192314738	0,110960175	-1,73318705	0,083062428	0,236811141	protein_codin protein phosphatase 2C
TcG_03632	338,6801273	-0,215726077	0,103327172	-2,0877962	0,036816222	0,133422819	protein_codin putative helicase-like protein
TcG_03633	320,2058482	-0,094270263	0,10459692	-0,90127188	0,36744378	0,59969061	protein_codin putative pseudouridylate synthase-like protein
TcG_03634	209,7915655	-0,419451415	0,126765562	-3,30887513	0,000936716	0,00739795	protein_codin hypothetical protein
TcG_03635	256,5333251	-0,542801294	0,112406613	-4,8289089	1,37283E-06	2,68223E-05	protein_codin nuclear lim interactor-interacting factor-like protein
TcG_03636	212,4319012	-0,432975984	0,125520775	-3,44943682	0,000561757	0,004788861	protein_codin hypothetical protein
TcG_03637	346,6532118	-0,201314379	0,099714184	-2,01891417	0,043496141	0,149894792	protein_codin putative aminopeptidase P1
TcG_03638	219,9555388	-0,063409458	0,128867514	-0,49205153	0,622682913	0,796731555	protein_codin putative trans-sialidase
TcG_03639	197,0694542	-0,016689929	0,131107572	-0,1272995	0,898703355	0,950695567	protein_codin 3,2-trans-enoyl-CoA isomerase
TcG_03640	703,0180852	-0,118958485	0,083088964	-1,43170017	0,152229646	0,352394143	protein_codin putative dynein heavy chain
TcG_03641	282,3062908	-0,313191393	0,112557687	-2,7824967	0,005394241	0,030397705	protein_codin putative protein kinase
TcG_03642	1031,372806	-0,255355503	0,073231397	-3,48696752	0,000488531	0,004252529	protein_codin chaperonin containing t-complex protein
TcG_03643	583,5786372	-0,161868538	0,082213976	-1,96886887	0,048968151	0,163181662	protein_codin putative kinesin
TcG_03644	120,2799677	-0,064122671	0,163870977	-0,39129974	0,69557569	0,842340007	protein_codin acylphosphatase
TcG_03645	447,5346193	-0,283439986	0,088228096	-3,21258191	0,001315476	0,0097759	protein_codin putative kinesin
TcG_03646	311,0066595	0,057112643	0,10197907	0,560042792	0,57545025	0,766252913	protein_codin hypothetical protein
TcG_03647	113,6956219	-0,043084061	0,175529003	-0,24545266	0,806105959	0,904233839	protein_codin hypothetical protein
TcG_03648	360,041104	0,558118542	0,097842692	5,704243516	1,16861E-08	3,64946E-07	protein_codin urocanate hydratase isoform X1

TcG_03649	301,1524206	-0,438136731	0,109072806	-4,01691994	5,89637E-05	0,000711619	protein_codin putative intraflagellar transport protein IFT88
TcG_03650	153,5714302	0,039011693	0,141894741	0,274934028	0,783366924	0,892633424	protein_codin hypothetical protein
TcG_03651	120,9038054	-0,206353403	0,178142944	-1,15835855	0,246717745	0,475461044	protein_codin putative mucin-like glycoprotein
TcG_03652	142,5111893	-0,140549962	0,14947312	-0,94030259	0,347062371	0,581582967	protein_codin hypothetical protein
TcG_03653	254,3712028	0,25467687	0,115177576	2,211167131	0,027024266	0,105813836	protein_codin carboxypeptidase
TcG_03654	200,7324873	-0,107291755	0,134618519	-0,79700591	0,425447601	0,651240046	protein_codin hypothetical protein
TcG_03655	354,2588248	-0,234581904	0,099053545	-2,36823329	0,017873262	0,07704558	protein_codin putative ras-related GTP-binding protein
TcG_03656	77,2750863	-0,352820202	0,198008482	-1,78184388	0,074774694	0,220667247	protein_codin putative ras-related GTP-binding protein
TcG_03657	144,2060227	-0,245855529	0,14463816	-1,69979713	0,089169092	0,248585286	protein_codin putative actin-like protein
TcG_03658	239,1110647	-0,392367344	0,117788812	-3,33110875	0,000865008	0,006921259	protein_codin hypothetical protein
TcG_03659	311,9425301	-0,077887029	0,10689147	-0,72865523	0,466212584	0,682788396	protein_codin Pre-mRNA-processing factor 17
TcG_03660	436,2005102	-0,701003038	0,088328777	-7,93629276	2,08314E-15	2,3662E-13	protein_codin CCR4-NOT transcription complex subunit 3
TcG_03661	135,8460182	-0,549757386	0,15082297	-3,64505079	0,000267339	0,002537893	protein_codin hypothetical protein
TcG_03662	264,2652476	-0,633077538	0,111945303	-5,65523983	1,55629E-08	4,76929E-07	protein_codin hypothetical protein
TcG_03663	76,7421093	-0,996428093	0,203426222	-4,89822839	9,67046E-07	1,97605E-05	protein_codin hypothetical protein
TcG_03664	214,1479513	-0,319445327	0,129854806	-2,46001929	0,013892955	0,063950646	protein_codin hypothetical protein
TcG_03665	96,10557165	-0,163609654	0,188662587	-0,86720773	0,385828202	0,616325044	protein_codin hypothetical protein
TcG_03666	115,3921876	-0,16673912	0,161875972	-1,03004243	0,302990089	0,535618427	protein_codin hypothetical protein
TcG_03667	16,1430399	-0,472639453	0,434742265	-1,08717162	0,27696097	1	protein_codin putative D-alanyl-glycyl endopeptidase-like protein
TcG_03668	101,7042437	-0,194045385	0,175026442	-1,10866325	0,267575479	0,499929984	protein_codin D-alanyl-glycyl endopeptidase-like protein
TcG_03669	80,78030869	-0,049144867	0,192724556	-0,25500054	0,798722679	0,899599469	protein_codin putative D-alanyl-glycyl endopeptidase-like protein
TcG_03670	501,2141599	-0,168522941	0,084769406	-1,98801607	0,046809913	0,158095631	protein_codin methyltransferase/D-alanine--D-alanine ligase
TcG_03671	185,1182741	-0,356837276	0,129648889	-2,75233577	0,005917182	0,03266149	protein_codin putative protein kinase
TcG_03672	1825,440721	-0,220175059	0,051274847	-4,29401687	1,75469E-05	0,000247322	protein_codin putative 3-oxo-5-alpha-steroid 4-dehydrogenase
TcG_03673	280,791873	-0,049351498	0,113592144	-0,43446224	0,663952807	0,824342954	protein_codin apurinic/apyrimidinic endonuclease
TcG_03674	185,2109637	-0,32670692	0,132269032	-2,47001823	0,013510617	0,062791032	
TcG_03675	175,2880415	-0,404595793	0,150030932	-2,69674918	0,007001999	0,037298923	protein_codin putative mitochondrial RNA binding complex 1 subunit
TcG_03676	346,197377	-0,168631693	0,098980875	-1,70367956	0,088440968	0,24714835	protein_codin hypothetical protein
TcG_03677	834,2916002	-0,302232551	0,068063188	-4,44047006	8,97626E-06	0,000139596	protein_codin putative dynein heavy chain
TcG_03678	618,5017755	-0,382632756	0,07547795	-5,06946406	3,98937E-07	8,94021E-06	protein_codin putative 2-oxoisovalerate dehydrogenase beta subunit, mitochondrial precursor
TcG_03679	120,6790384	-0,226225157	0,164758667	-1,37306984	0,169730637	0,376067037	protein_codin U6 snRNA-associated Sm-like protein LSm8p
TcG_03680	78,74107144	0,294072905	0,197156675	1,49156961	0,13581201	0,328363512	protein_codin L1Tc protein
TcG_03681	1,082480914	-0,385994369	1,751814201	-0,22033979	0,825606536	1	protein_codin hypothetical protein
TcG_03682	645,8946004	0,170362666	0,075360323	2,260641396	0,023781472	0,096288708	protein_codin putative target of rapamycin (TOR) kinase 1
TcG_03683	63,15159882	-0,437075104	0,224169012	-1,94975702	0,051205087	0,168253584	protein_codin hypothetical protein
TcG_03684	18,04975425	-0,404594757	0,403304496	-1,00319922	0,315764747	0,550661979	protein_codin hypothetical protein
TcG_03685	124,519583	-0,010293933	0,156754212	-0,06566926	0,947641148	0,975394476	protein_codin type 11 methyltransferase
TcG_03686	116,1107309	-0,148134297	0,162491056	-0,91164585	0,361955177	0,594585663	protein_codin putative leucine-rich repeat protein 1 (LRRP1)
TcG_03687	137,9935345	-0,622060555	0,157993113	-3,93726374	8,2416E-05	0,000941688	
TcG_03688	1850,987794	-0,281838031	0,049084578	-5,74188558	9,36281E-09	2,99662E-07	protein_codin actin
TcG_03689	1146,424535	-0,410194785	0,061996245	-6,61644563	3,67938E-11	2,04949E-09	protein_codin hypothetical protein
TcG_03690	193,8718126	-0,309974444	0,128671469	-2,40903788	0,015994638	0,071055513	protein_codin cAMP-dependent protein kinase regulator
TcG_03691	281,680643	-0,28086551	0,114972035	-2,44290282	0,01456966	0,066353805	protein_codin putative tRNA nucleotidyltransferase
TcG_03692	1909,65375	-0,319203977	0,059889155	-5,3299129	9,82599E-08	2,55723E-06	protein_codin hypothetical protein
TcG_03693	1279,457394	-0,329037635	0,0567011	-5,80302036	6,51309E-09	2,13546E-07	protein_codin putative RNA-binding protein
TcG_03694	441,0226782	-0,453715524	0,091721248	-4,94667846	7,54905E-07	1,57876E-05	protein_codin putative fructose-1,6-bisphosphatase, cytosolic
TcG_03695	89,79097722	-0,141459353	0,184369088	-0,76726177	0,442925892	0,664995914	protein_codin hypothetical protein
TcG_03696	275,3911738	-0,258448364	0,111604261	-2,31575714	0,020571533	0,085981883	protein_codin hypothetical protein
TcG_03697	139,5401137	0,047410002	0,154936734	0,305995879	0,759607784	0,878587979	protein_codin cytochrome c oxidase assembly factor
TcG_03698	202,0616049	-0,034326468	0,129905019	-0,26424282	0,791592821	0,895818952	protein_codin Rho-like GTP binding protein
TcG_03699	591,0795905	-0,249907637	0,080345609	-3,11040815	0,00186829	0,013024072	protein_codin hypothetical protein
TcG_03700	1350,962733	-0,345600863	0,065146303	-5,30499578	1,12676E-07	2,88818E-06	protein_codin P-ATPase family transporter: proton
TcG_03701	343,4244393	-0,325653972	0,097673352	-3,3341128	0,000855719	0,006865904	protein_codin ankyrin
TcG_03702	764,7500955	-0,351688742	0,070406265	-4,99513419	5,87949E-07	1,26617E-05	protein_codin hypothetical protein
TcG_03703	253,1325061	-0,12342524	0,11692612	-1,05558314	0,291158708	0,523651784	protein_codin putative transcription factor 25-like
TcG_03704	158,5231186	-0,18357594	0,141406104	-1,29821793	0,194212458	0,411210808	protein_codin putative transcription factor 25-like
TcG_03705	73,61212017	-0,080749988	0,209007871	-0,38634903	0,699238194	0,844156894	protein_codin hypothetical protein

TcG_03706	134,2551617	-0,494408195	0,155254232	-3,18450705	0,001450008	0,010559265	protein_codin	hypothetical protein
TcG_03707	177,7168466	-0,13167893	0,135368433	-0,97274473	0,330680187	0,565235831	protein_codin	hypothetical protein
TcG_03708	591,630262	-0,162507674	0,081982136	-1,98223274	0,047453198	0,159314042	protein_codin	putative chloride channel protein
TcG_03709	321,9670959	-0,203941759	0,101698331	-2,00535993	0,044924582	0,153357752	protein_codin	hypothetical protein
TcG_03710	518,3819244	-0,245681342	0,082078544	-2,99324685	0,002760264	0,01779703	protein_codin	hypothetical protein
TcG_03711	416,3014673	-0,283116513	0,088740227	-3,19039656	0,001420777	0,010398687	protein_codin	hypothetical protein
TcG_03712	224,5500067	-0,215446163	0,120597231	-1,78649344	0,074019398	0,219612995	protein_codin	hypothetical protein
TcG_03713	148,8051786	-0,295240616	0,148832588	-1,98370948	0,047288237	0,158944447	protein_codin	adenylate kinase
TcG_03714	432,4804881	-0,071965879	0,089498102	-0,80410509	0,421336285	0,647488005	protein_codin	60S ribosomal protein L10
TcG_03715	421,7587794	-0,104051706	0,089223594	-1,16619048	0,243537442	0,471805512	protein_codin	putative 60S ribosomal protein L10
TcG_03716	551,5159903	-0,283903604	0,081102106	-3,50057005	0,000464264	0,004053478	protein_codin	putative chaperone protein DNAj
TcG_03717	266,6778969	-0,378238741	0,117380482	-3,22233079	0,001271522	0,009486065	protein_codin	zinc finger protein, predicted
TcG_03718	116,7366155	-0,169238332	0,166992023	-1,01345159	0,310844507	0,544463034	protein_codin	hypothetical protein
TcG_03719	67,3407328	-0,834632751	0,218372913	-3,82205255	0,000132345	0,001408039	protein_codin	hypothetical protein
TcG_03720	386,639758	-0,506700656	0,092860403	-5,45658474	4,8538E-08	1,34215E-06	protein_codin	hypothetical protein
TcG_03721	156,6966265	-0,122999622	0,148728426	-0,82700816	0,408232435	0,636605346	protein_codin	uncharacterized protein
TcG_03722	404,048619	-0,131743797	0,091997366	-1,4320388	0,152132718	0,352339884	protein_codin	putative rhomboid-like protein, putative,serine peptidase, Clan S-, family S54
TcG_03723	251,8021082	-0,132215409	0,114787569	-1,1518269	0,249392213	0,478624844	protein_codin	putative tyrosine phospatase-like protein
TcG_03724	503,1947534	-0,29798094	0,08330859	-3,57683332	0,000347782	0,003175256	protein_codin	hypothetical protein
TcG_03725	324,697057	-0,157627738	0,105192836	-1,49846458	0,134012588	0,32557556	protein_codin	putative metalloprotease-like protein, putative,polypeptide deformylase
TcG_03726	1061,548192	-0,153931736	0,065156937	-2,36247655	0,01815329	0,077912126	protein_codin	pescadillo
TcG_03727	173,1547736	-0,403495369	0,142241039	-2,83670151	0,00455822	0,026807889	protein_codin	hypothetical protein
TcG_03728	421,5903242	-0,533555574	0,090946149	-5,86671978	4,44501E-09	1,52819E-07	protein_codin	hypothetical protein
TcG_03729	193,1118079	-0,254726379	0,130457258	-1,95256579	0,050871068	0,167679146	protein_codin	putative heat shock protein
TcG_03730	1745,641044	-0,108005307	0,05193116	-2,07977842	0,037545861	0,135011876	protein_codin	nascent polypeptide associated complex subunit
TcG_03731	13,52677732	-0,00417161	0,485629788	-0,0085901	0,993146173	1		
TcG_03732	15,51693205	-0,065939456	0,448495201	-0,14702377	0,883113258	1	protein_codin	putative 60S ribosomal protein L10
TcG_03733	298,1273083	-0,135818541	0,110011261	-1,23458762	0,21698402	0,440893872	protein_codin	putative WAS protein family 3-like
TcG_03734	217,2984222	-0,345289737	0,122285384	-2,82363866	0,004748188	0,027630592	protein_codin	uncharacterized protein
TcG_03735	791,405576	-0,350360305	0,06800622	-5,1518862	2,5788E-07	6,02377E-06	protein_codin	hypothetical protein
TcG_03736	524,4364238	-0,362263429	0,083216371	-4,35327119	1,34121E-05	0,000195709	protein_codin	hypothetical protein
TcG_03737	214,1226796	-0,367244366	0,123509349	-2,9734135	0,002945074	0,018727567	protein_codin	putative Mg transporter
TcG_03738	65,65477153	-0,322410698	0,216511732	-1,48911422	0,136457295	0,329163749	protein_codin	hypothetical protein
TcG_03739	74,52963213	0,09384567	0,236947733	0,396060636	0,692060297	0,84065953	protein_codin	hypothetical protein
TcG_03740	247,6979017	0,449538153	0,115889542	3,879022597	0,000104877	0,001160559	protein_codin	peptide methionine sulfoxide reductase
TcG_03741	373,1570893	-0,027380248	0,095142773	-0,28778064	0,773514656	0,886918879	protein_codin	Ras like protein family, member T1
TcG_03742	542,0703896	-0,216906019	0,084034521	-2,58115374	0,009847072	0,048818218	protein_codin	putative proteasome regulatory non-ATPase subunit
TcG_03743	258,4615793	0,034982661	0,11041469	0,316829767	0,751372776	0,874827151	protein_codin	hypothetical protein
TcG_03744	353,8998658	0,045950037	0,100138061	0,458866852	0,646329784	0,812019442	protein_codin	putative carnitine O-palmitoyltransferase
TcG_03745	255,412959	0,114107492	0,11418052	0,999360412	0,31762013	0,552739124		
TcG_03746	113,6050391	0,070587776	0,164476956	0,42916514	0,66780305	0,826002577	protein_codin	hypothetical protein
TcG_03747	383,4838591	0,088192029	0,094781755	0,930474748	0,352125333	0,586411136	protein_codin	hypothetical protein
TcG_03748	269,9618509	0,182415005	0,109936573	1,659274988	0,097060393	0,263482126	protein_codin	RNA-editing complex protein MP42
TcG_03749	225,9337644	0,05792079	0,126791169	0,456820377	0,647800158	0,812603825	protein_codin	lipoate-protein ligase
TcG_03750	942,136823	-0,327106999	0,071292129	-4,58826245	4,46951E-06	7,60407E-05	protein_codin	putative cation transporting ATPase
TcG_03751	178,8542221	-0,15527701	0,134453742	-1,1548731	0,248142398	0,477445495	protein_codin	Sel1 domain-containing protein
TcG_03752	142,1535952	-0,036626068	0,148040204	-0,24740623	0,804593856	0,903472031	protein_codin	hypothetical protein
TcG_03753	174,5114967	0,145243085	0,135254103	1,073853453	0,282888368	0,516148762	protein_codin	hypothetical protein
TcG_03754	143,9817557	0,026533877	0,15465415	0,171569121	0,863776285	0,934863339	protein_codin	RNA-editing complex protein
TcG_03755	319,7247177	0,027582818	0,104319955	0,264405964	0,791467117	0,89576419	protein_codin	hypothetical protein
TcG_03756	303,7847047	-0,079978177	0,110614331	-0,7230363	0,469657581	0,686191399	protein_codin	peptidyl-prolyl cis-trans isomerase
TcG_03757	517,3207562	-0,014794507	0,083238617	-0,1777361	0,858930232	0,932070224	protein_codin	PIF1 helicase-like protein
TcG_03758	242,2534827	-0,106369809	0,116424421	-0,91363829	0,360906942	0,593536952	protein_codin	rabGTPase-activating protein
TcG_03759	1379,119919	-0,252081407	0,068555044	-3,6770658	0,000235932	0,002291291	protein_codin	putative RNA-binding protein
TcG_03760	46,66177247	-0,731840871	0,272843271	-2,68227568	0,007312317	0,038649867		
TcG_03761	122,6760936	-0,017705288	0,15886385	-0,11144944	0,911259953	0,956844965	protein_codin	MIP18 family protein
TcG_03762	1725,62259	0,128358833	0,052607665	2,439926441	0,014690253	0,066876727	protein_codin	nucleolar RNA-binding protein

TcG_03763	908,4751594	0,172496572	0,064397792	2,678610043	0,007392844	0,038951108	protein_codin nucleolar RNA-binding protein
TcG_03764	34,96035138	0,332906568	0,292911136	1,136544593	0,255728714	0,487394781	protein_codin nucleolar RNA-binding protein
TcG_03765	317,6456523	-0,019182874	0,109593178	-0,17503712	0,861050465	0,933657528	protein_codin hypothetical protein
TcG_03766	560,1990656	-0,047565003	0,078952257	-0,60245273	0,546872821	0,743757308	protein_codin vesicular transport-associated repeat protein
TcG_03767	397,229871	0,001501323	0,091217612	0,0164587	0,986868451	0,994746907	protein_codin hypothetical protein
TcG_03768	2766,206322	-0,10131815	0,046156034	-2,19512254	0,028154809	0,109061055	protein_codin hypothetical protein
TcG_03769	202,053469	-0,225014421	0,129789628	-1,73368569	0,082973868	0,236811141	protein_codin radial spoke head protein 9
TcG_03770	627,499673	-0,05307158	0,074349597	-0,71381127	0,47534389	0,691032669	protein_codin vacuole import and degradation
TcG_03771	63,87075355	0,070270575	0,217734116	0,322735713	0,746895403	0,87215583	protein_codin hypothetical protein
TcG_03772	630,1915148	0,061179335	0,078467517	0,779677214	0,435580894	0,659399643	protein_codin putative DNA-directed RNA polymerase III largest subunit
TcG_03773	308,6264227	-0,033236116	0,106980205	-0,31067538	0,756047417	0,877165706	protein_codin hypothetical protein
TcG_03774	295,5870568	0,178295215	0,109557783	1,627408026	0,103650468	0,2758774	protein_codin hypothetical protein
TcG_03775	365,4325928	-0,087250595	0,099559952	-0,87636236	0,380833071	0,611048137	protein_codin hypothetical protein
TcG_03776	244,8078567	0,183198706	0,11462955	1,598180459	0,110002821	0,286467226	protein_codin glycosyltransferase
TcG_03777	135,3484258	0,108717858	0,160664855	0,676674795	0,498612312	0,707695975	protein_codin hypothetical protein
TcG_03778	53,56311149	-0,344604695	0,243372528	-1,41595561	0,156788502	0,358297624	protein_codin hypothetical protein
TcG_03779	172,9459455	-0,199295403	0,147647045	-1,34980963	0,177077055	0,387183019	protein_codin putative serine/threonine protein kinase, putative,protein kinase
TcG_03780	603,2941266	-0,156010478	0,078956875	-1,9758948	0,048166694	0,161242217	protein_codin hypothetical protein
TcG_03781	331,3397869	-0,126471408	0,103602205	-1,2207405	0,222184288	0,448081317	protein_codin putative protein kinase
TcG_03782	1043,984668	-0,128430057	0,065372679	-1,96458304	0,049462517	0,16434549	protein_codin putative small GTP-binding protein Rab11, putative,Rab11 GTPase
TcG_03783	40,66410968	-0,163691747	0,281530657	-0,58143489	0,560947387	0,754572904	
TcG_03784	32,39535319	-0,817972783	0,306335741	-2,67018397	0,00758097	0,039779492	
TcG_03785	370,3562082	-0,171022154	0,098824584	-1,73056284	0,083529763	0,237724353	protein_codin splicing factor TSR1
TcG_03786	53,04831721	-0,431658843	0,237575689	-1,81693188	0,069227544	0,210461907	protein_codin hypothetical protein
TcG_03787	56,86743383	-0,466110706	0,228857306	-2,03668702	0,041681416	0,145455009	protein_codin hypothetical protein
TcG_03788	99,0169395	-0,22161219	0,183072239	-1,21051772	0,226080296	0,45247302	protein_codin hypothetical protein
TcG_03789	64,3872776	0,198359638	0,222343685	0,892130744	0,372322858	0,603572496	protein_codin microtubial binding protein
TcG_03790	310,3909015	0,063050392	0,103773215	0,60757867	0,543466951	0,740864584	protein_codin hypothetical protein
TcG_03791	197,9429266	0,304968145	0,127822382	2,385874376	0,01703857	0,074440982	protein_codin hypothetical protein
TcG_03792	398,5868775	0,192349125	0,097987994	1,962986665	0,049647724	0,164819061	protein_codin hypothetical protein
TcG_03793	518,6658748	-0,041841252	0,084741342	-0,49375253	0,621480963	0,795984793	protein_codin hypothetical protein
TcG_03794	295,6714326	0,068789673	0,104790227	0,656451225	0,511533841	0,717683589	protein_codin hypothetical protein
TcG_03795	467,8195527	0,042614486	0,093151831	0,45747341	0,64733081	0,81256498	protein_codin putative translation initiation factor IF-2
TcG_03796	493,8112214	0,122285662	0,084443306	1,448139197	0,147578125	0,346331813	protein_codin hypothetical protein
TcG_03797	168,2222688	0,15098275	0,141282296	1,068660086	0,285222863	0,518774269	protein_codin hypothetical protein
TcG_03798	323,4265696	-0,032976592	0,09961017	-0,33105647	0,740601828	0,867692666	protein_codin hypothetical protein
TcG_03799	361,2566638	0,210636523	0,098405632	2,140492552	0,032314982	0,120930679	protein_codin cation transport protein ChaC
TcG_03800	227,2212796	0,09603346	0,118378075	0,811243637	0,41722577	0,643941425	protein_codin putative protein kinase
TcG_03801	161,2252702	0,064297368	0,148838273	0,431994856	0,665745152	0,825042607	protein_codin hypothetical protein
TcG_03802	243,7167797	0,30993439	0,125194379	2,475625441	0,013300303	0,061986046	protein_codin serine/threonine protein kinase
TcG_03803	288,9626441	-0,054058772	0,105491883	-0,51244485	0,608339707	0,788023892	protein_codin adaptor medium chain 1
TcG_03804	79,09997864	-0,179522346	0,208095523	-0,86269201	0,388306824	0,618578697	protein_codin hypothetical protein
TcG_03805	130,7575768	-0,078728869	0,152520918	-0,51618407	0,605725852	0,786921256	protein_codin hypothetical protein
TcG_03806	581,3818783	0,105564923	0,076992017	1,371115169	0,170339057	0,376919082	protein_codin putative dynein heavy chain, cytosolic
TcG_03807	1038,457814	0,297735639	0,140581775	2,117882204	0,034185045	0,126216677	protein_codin cytoplasmic dynein 1 heavy chain 1
TcG_03808	746,2322284	-0,026249915	0,072545563	-0,36184039	0,717471305	0,855087918	protein_codin hypothetical protein
TcG_03809	296,795723	-0,245487954	0,106182594	-2,31194158	0,020780903	0,086700591	protein_codin hypothetical protein
TcG_03810	205,035911	0,240625983	0,133208313	1,806388639	0,070857655	0,213607812	protein_codin hypothetical protein
TcG_03811	274,8396417	0,010245969	0,111410729	0,091965732	0,926725266	0,964306367	protein_codin hypothetical protein
TcG_03812	142,7062987	0,06179711	0,1528145	0,404392974	0,685923766	0,837066859	protein_codin hypothetical protein
TcG_03813	257,3661599	-0,072089905	0,111179242	-0,64841155	0,516718801	0,722248269	
TcG_03814	404,858165	-0,124510416	0,094665386	-1,31526867	0,188419625	0,402773021	protein_codin transporter protein
TcG_03815	247,6369687	0,003460566	0,115607873	0,029933651	0,976119968	0,989788723	protein_codin trichohyalin
TcG_03816	546,5994801	-0,01368004	0,08011602	-0,17075287	0,864418089	0,934985064	protein_codin hypothetical protein
TcG_03817	344,4423843	0,243317861	0,103514042	2,350578292	0,018744264	0,07975433	protein_codin hypothetical protein
TcG_03818	90,09623814	0,03985333	0,196564173	0,202749715	0,839330663	0,921926912	protein_codin hypothetical protein
TcG_03819	487,6849588	0,015878595	0,09931751	0,159877095	0,872977892	0,93911995	protein_codin solute carrier family 35 (UDP-galactose transporter), member B1

TcG_03820	314,1220152	0,075331112	0,101325639	0,743455579	0,457205906	0,676783905	protein_codin hypothetical protein
TcG_03821	613,0680528	0,29066872	0,078949153	3,681720578	0,000231665	0,002255524	protein_codin hypothetical protein
TcG_03822	282,5463188	-0,170996586	0,106311546	-1,60844793	0,107737112	0,282727561	protein_codin G-protein (beta)-like protein
TcG_03823	542,7829661	0,070055985	0,079884532	0,876965582	0,380505333	0,610795316	protein_codin hypothetical protein
TcG_03824	181,0012568	-0,030218458	0,134497236	-0,22467717	0,822230426	0,911875343	protein_codin hypothetical protein
TcG_03825	381,8735091	0,096068822	0,097829496	0,982002628	0,326098554	0,560560511	protein_codin hypothetical protein
TcG_03826	828,0106532	0,114882351	0,066493566	1,727721307	0,084038202	0,238760815	protein_codin putative kinesin
TcG_03827	66,8924551	0,306643714	0,222772823	1,376486191	0,16867116	0,375101512	protein_codin protein kinase
TcG_03828	278,2251789	0,220865481	0,108282429	2,03971672	0,041378549	0,144749959	protein_codin hypothetical protein
TcG_03829	302,4126061	0,239518528	0,109822611	2,18095824	0,029186505	0,112044261	protein_codin a44l protein-like protein
TcG_03830	4357,660001	-0,220841969	0,043739351	-5,04904542	4,44023E-07	9,74372E-06	protein_codin histone H2A
TcG_03831	1424,456047	0,019443202	0,063059882	0,308329186	0,75783186	0,878015178	protein_codin histone H2A
TcG_03832	1606,757197	-0,34121796	0,057991122	-5,8839689	4,00543E-09	1,3936E-07	protein_codin histone H2A
TcG_03833	533,9736327	0,088244023	0,085512163	1,031947033	0,302096931	0,535346443	protein_codin trans-sialidase
TcG_03834	682,4906958	0,211641999	0,079699005	2,65551621	0,007918713	0,04118059	protein_codin 3-hydroxyacyl-CoA dehydrogenase
TcG_03835	253,4180295	-0,117064204	0,115502432	-1,01352154	0,310811111	0,544463034	protein_codin hypothetical protein
TcG_03836	231,1103504	-0,013629415	0,124445335	-0,1095213	0,912789026	0,957238745	protein_codin hypothetical protein
TcG_03837	405,0363862	0,034682444	0,094201793	0,368171806	0,712745131	0,852489876	protein_codin putative serine palmitoyltransferase
TcG_03838	231,8176021	0,088919977	0,119440066	0,744473609	0,456590001	0,676399932	protein_codin 3-keto-dihydrosphingosine reductase
TcG_03839	118,7474085	0,421200404	0,167404945	2,516057121	0,011867595	0,056478114	protein_codin hypothetical protein
TcG_03840	318,5943743	-0,13950746	0,10165483	-1,37236431	0,169950056	0,376273905	protein_codin hypothetical protein
TcG_03841	237,8837229	0,05524656	0,1164862	0,474275583	0,635303401	0,805320044	protein_codin enoyl-CoA hydratase/isomerase family protein
TcG_03842	608,8875255	-0,071875347	0,076672399	-0,93743444	0,348535132	0,583103728	protein_codin putative ATP-dependent DEAD-box RNA helicase
TcG_03843	671,6619772	-0,109813689	0,07723162	-1,42187474	0,155062618	0,35638871	protein_codin hypothetical protein
TcG_03844	90,09341337	-0,111315921	0,199216763	-0,55876785	0,57632017	0,766706337	protein_codin hypothetical protein
TcG_03845	191,6381731	0,002010244	0,1315723	0,015278625	0,987809895	0,995145161	protein_codin hypothetical protein
TcG_03846	126,373812	0,545761547	0,160398045	3,402544884	0,000667614	0,0055171	protein_codin 40S ribosomal protein S3a
TcG_03847	1903,095711	0,356028727	0,055592416	6,404267978	1,51093E-10	7,23371E-09	protein_codin 40S ribosomal protein S3A
TcG_03848	282,6621547	0,238780765	0,110899542	2,153126699	0,031308722	0,117954569	protein_codin hypothetical protein
TcG_03849	93,08769259	-0,068361189	0,178982116	-0,38194424	0,702502721	0,84624626	protein_codin hypothetical protein
TcG_03850	200,2360446	-0,07796971	0,126184411	-0,61790287	0,536639364	0,73660472	protein_codin hypothetical protein
TcG_03851	289,5254309	0,160623804	0,105595163	1,521128423	0,128227613	0,31717445	protein_codin hypothetical protein
TcG_03852	240,4830141	0,259957435	0,118110378	2,200970316	0,027738125	0,107912108	protein_codin hypothetical protein
TcG_03853	207,6047963	-0,142145705	0,123786703	-1,14831158	0,250839958	0,480607203	protein_codin putative protein kinase
TcG_03854	389,6469286	0,206921061	0,107507979	1,924704223	0,054266372	0,175311191	protein_codin putative phosphatidylinositol-4-phosphate 5-kinase-like
TcG_03855	338,5302286	0,1227037	0,09885937	1,24119443	0,214533932	0,438298384	protein_codin EF hand domain containing protein
TcG_03856	563,8324149	0,129734617	0,081341061	1,594946205	0,110724266	0,287601745	protein_codin hypothetical protein
TcG_03857	330,6958341	-0,004881671	0,099787566	-0,04892064	0,960982541	0,981984718	protein_codin putative SNF-7-like protein
TcG_03858	30,69400209	0,435005323	0,351168558	1,238736534	0,215443084	0,439138531	protein_codin hypothetical protein
TcG_03859	77,19778406	0,231927075	0,207285667	1,118876564	0,263192803	0,495426777	protein_codin hypothetical protein
TcG_03860	762,4482876	0,203902193	0,069429488	2,936824103	0,003315921	0,02065498	protein_codin 60S ribosomal protein L18a
TcG_03861	837,066329	-0,033154485	0,071478097	-0,46384118	0,642761535	0,810429334	protein_codin hypothetical protein
TcG_03862	275,1031896	0,086500998	0,109110833	0,792781025	0,427905414	0,653190003	protein_codin hypothetical protein
TcG_03863	156,2395086	-0,025093588	0,14887556	-0,16855411	0,866147376	0,935506992	protein_codin conserved RING finger protein
TcG_03864	2157,736363	0,191348816	0,056214407	3,403910603	0,000664285	0,005499891	protein_codin hypothetical protein
TcG_03865	566,9280012	-0,143332635	0,081928871	-1,7494765	0,080208687	0,231484747	protein_codin hypothetical protein
TcG_03866	19,48075232	-0,644191831	0,411375596	-1,56594566	0,117361338	0,298714513	
TcG_03867	203,5611119	-0,059498944	0,122593846	-0,48533386	0,627439544	0,800252866	protein_codin TPR-repeat-containing protein
TcG_03868	253,439167	0,200580916	0,120576691	1,663513197	0,096209751	0,262340829	protein_codin putative vacuolar ATP synthase subunit d
TcG_03869	162,9905886	0,135191325	0,142811968	0,946638624	0,343822955	0,577993726	protein_codin hypothetical protein
TcG_03870	158,7069155	-0,000974264	0,145090182	-0,00671488	0,994642339	0,997783662	protein_codin hypothetical protein
TcG_03871	231,0199282	-0,028624633	0,11613394	-0,24647948	0,805311088	0,903926978	protein_codin hypothetical protein
TcG_03872	336,9087197	0,110713432	0,10359109	1,068754393	0,285180355	0,518774269	protein_codin hypothetical protein
TcG_03873	499,2497542	0,087241839	0,092854118	0,939558106	0,347444274	0,581903013	protein_codin putative proteasome activator protein pa26
TcG_03874	421,0185086	-0,185313487	0,08981311	-2,06332336	0,039081916	0,139124749	protein_codin conserved CBS domain protein
TcG_03875	185,1668829	0,124083308	0,132865788	0,933899611	0,350355679	0,584889659	protein_codin putative rRNA dimethyltransferase
TcG_03876	151,7680376	0,026456994	0,145873763	0,181369109	0,856077864	0,930268068	protein_codin putative actin

TcG_03877	177,2929506	-0,149796355	0,136114767	-1,10051509	0,27110776	0,504340987	protein_codin	hypothetical protein
TcG_03878	277,5300783	-0,226124359	0,108975872	-2,07499472	0,037987024	0,136145608	protein_codin	hypothetical protein
TcG_03879	552,8816549	0,270980992	0,081865806	3,310063216	0,000932749	0,007371644	protein_codin	putative aspartate aminotransferase
TcG_03880	187,4772499	0,066671651	0,130743241	0,509943388	0,610091124	0,789016404	protein_codin	zinc finger protein
TcG_03881	243,3222855	-0,036017865	0,114920278	-0,31341609	0,753964558	0,875820471	protein_codin	hypothetical protein
TcG_03882	102,6566682	-0,20890347	0,172350974	-1,21208175	0,225481089	0,451820114	protein_codin	hypothetical protein
TcG_03883	143,0371608	-0,091361613	0,147167944	-0,62079832	0,534732328	0,735011123	protein_codin	hypothetical protein
TcG_03884	173,9711044	0,305479021	0,134367377	2,273461222	0,0229984	0,093852429	protein_codin	putative serine protease PepD
TcG_03885	179,664439	0,337510715	0,137842713	2,448520542	0,014344425	0,065736139	protein_codin	Adenine phosphoribosyltransferase
TcG_03886	373,7875787	0,021584985	0,098030519	0,220186376	0,825726006	0,915051316	protein_codin	Adenine phosphoribosyltransferase
TcG_03887	264,9299547	0,200478924	0,110758241	1,810058747	0,070286677	0,212399959	protein_codin	E2F target protein 1
TcG_03888	176,3624615	-0,101423839	0,140936365	-0,71964279	0,471744958	0,687761053	protein_codin	hypothetical protein
TcG_03889	100,4469833	0,037579121	0,183772847	0,204486796	0,837973109	0,921396644	protein_codin	putative 60S ribosomal protein L7
TcG_03890	26,08092813	0,172443519	0,36586393	0,471332385	0,637403392	0,807275437	protein_codin	putative 60S ribosomal protein L7
TcG_03891	117,4767632	0,069109732	0,17517034	0,39452873	0,69319072	0,841062696	protein_codin	putative 60S ribosomal protein L7
TcG_03892	78,78750323	0,141031678	0,199730323	0,706110497	0,480119424	0,694291519	protein_codin	hypothetical protein
TcG_03893	207,651913	0,038710177	0,125871284	0,307537795	0,758434059	0,878015178	protein_codin	putative HIRA-interacting protein 5
TcG_03894	259,9104386	-0,102461135	0,112016042	-0,91470055	0,360348856	0,592955808	protein_codin	eukaryotic peptide chain release factor subunit 1
TcG_03895	342,5201809	0,181201688	0,101617087	1,783181289	0,074556797	0,220451886	protein_codin	hypothetical protein
TcG_03896	276,1277957	0,04430432	0,112152132	0,395037698	0,692815067	0,841062696	protein_codin	putative silent information regulator 2
TcG_03897	188,0936304	-0,056597382	0,12997371	-0,43545254	0,663233981	0,823780972	protein_codin	hypothetical protein
TcG_03898	328,9948474	0,034786392	0,106267202	0,327348335	0,743404439	0,86954228	protein_codin	hypothetical protein
TcG_03899	353,1097117	-0,251478321	0,101038991	-2,48892352	0,012813053	0,060053409	protein_codin	hypothetical protein
TcG_03900	368,7911722	-0,081011883	0,102400572	-0,79112725	0,428869741	0,653987669	protein_codin	hypothetical protein
TcG_03901	433,9343962	0,029079403	0,093015371	0,312630083	0,75456172	0,876235488	protein_codin	hypothetical protein
TcG_03902	280,9216741	-0,222286937	0,108434252	-2,04996975	0,040367383	0,142070625	protein_codin	ras-like small GTPase
TcG_03903	181,1461543	-0,108589082	0,135024623	-0,80421689	0,421271726	0,647488005	protein_codin	hypothetical protein
TcG_03904	141,9897825	-0,174525192	0,165015811	-1,05762709	0,290225487	0,523456792	protein_codin	hypothetical protein
TcG_03905	355,8286482	-0,14659866	0,098627255	-1,48639096	0,137175739	0,330213611	protein_codin	6-phosphofructo-2-kinase/fructose-2, 6-biphosphatase-1-like protein
TcG_03906	169,3402005	-0,010269121	0,135947928	-0,07553717	0,939787327	0,972089632	protein_codin	putative C-1-tetrahydrofolate synthase, cytoplasmic
TcG_03907	119,617789	-0,259404312	0,160680393	-1,61441173	0,10643815	0,280845457	protein_codin	putative mitotic cyclin
TcG_03908	182,2311124	-0,059022612	0,133233834	-0,44300018	0,657765605	0,819528311	protein_codin	pyrimidine-specific ribonucleoside hydrolase
TcG_03909	456,1185106	-0,041501408	0,091576629	-0,45318777	0,65041354	0,814469949	protein_codin	Vacuolar protein sorting protein
TcG_03910	518,6042282	0,105538748	0,085232398	1,238246846	0,215624546	0,439364402	protein_codin	hypothetical protein
TcG_03911	274,0603928	-0,131418504	0,109450951	-1,20070682	0,229864947	0,456889557	protein_codin	TPR Domain containing protein
TcG_03912	359,1750236	-0,153320894	0,097835214	-1,56713405	0,117083363	0,298531655	protein_codin	RET2, RNA editing complex MP57
TcG_03913	125,9849571	-0,035879327	0,173933029	-0,20628242	0,836570308	0,920635641	protein_codin	Thermonuclease precursor
TcG_03914	196,532864	0,166544387	0,132693592	1,255104971	0,209440656	0,43200631	protein_codin	oxidoreductase
TcG_03915	201,2420162	-0,260031783	0,124187406	-2,09386597	0,036271921	0,132069919	protein_codin	putative pseudouridylate synthase I
TcG_03916	395,4518655	-0,144297023	0,09276464	-1,55551753	0,119822863	0,302917264	protein_codin	putative nucleotide-binding protein
TcG_03917	149,5103148	-0,030486103	0,15104982	-0,20182813	0,840051089	0,922245432	protein_codin	hypothetical protein
TcG_03918	380,7677192	-0,102209393	0,097664769	-1,0465329	0,295315064	0,528097822	protein_codin	hypothetical protein
TcG_03919	545,2783415	-0,265700419	0,083655648	-3,1761205	0,001492589	0,010828516	protein_codin	ATPase subunit 9
TcG_03920	941,0807035	-0,290665244	0,065234247	-4,45571545	8,36139E-06	0,000131445	protein_codin	hypothetical protein
TcG_03921	326,5802956	-0,140237102	0,102438911	-1,36898276	0,171004665	0,377814654	protein_codin	hypothetical protein
TcG_03922	132,0852408	0,309123715	0,159565919	1,937279061	0,05271124	0,17142733	protein_codin	cytoplasmic protein
TcG_03923	203,9704316	-0,220278134	0,131937838	-1,66955997	0,095006456	0,259793438	protein_codin	putative DNA polymerase sigma-like protein
TcG_03924	314,1062966	-0,080851073	0,109363804	-0,73928548	0,459733663	0,678272535	protein_codin	putative leucine-rich repeat protein (LRRP)
TcG_03925	550,6982173	-0,359205584	0,083731083	-4,28999088	1,7868E-05	0,000250932	protein_codin	hypothetical protein
TcG_03926	408,2060794	-0,063232938	0,093559655	-0,67585689	0,499131508	0,708259357	protein_codin	hypothetical protein
TcG_03927	434,3347625	-0,136920301	0,095224702	-1,43786536	0,15047226	0,350426454	protein_codin	hypothetical protein
TcG_03928	342,6316283	0,028422325	0,098628832	0,288174609	0,773213083	0,886918879	protein_codin	hypothetical protein
TcG_03929	102,588974	-0,169501176	0,197345469	-0,85890584	0,390392467	0,620400374	protein_codin	hypothetical protein
TcG_03930	307,2717245	-0,013556279	0,106556247	-0,12722182	0,898764838	0,950695567	protein_codin	putative spliced leader RNA PSE-promoter transcription factor PPB1
TcG_03931	345,3357409	-0,20329064	0,105665889	-1,92390034	0,054367076	0,175556562	protein_codin	hypothetical protein
TcG_03932	273,4299805	0,121330318	0,107769709	1,125829505	0,260237693	0,492617789	protein_codin	hypothetical protein
TcG_03933	28,65673562	0,075056643	0,330481402	0,227113063	0,820335827	0,911143056	protein_codin	10 kDa heat shock protein

TcG_03934	2,292272937	0,530677983	1,243475007	0,426770124	0,669546769	1	
TcG_03935	228,5949943	0,0466903	0,121653946	0,38379602	0,701129638	0,845295316	protein_codin hypothetical protein
TcG_03936	396,8431543	0,158376484	0,098363062	1,610121536	0,107371326	0,282235268	protein_codin 10 kDa heat shock protein
TcG_03937	1,486996936	0,707428819	1,472878587	0,480303553	0,631011563	1	
TcG_03938	675,3908293	-0,009840827	0,073778361	-0,13338365	0,893889973	0,948813234	protein_codin thiolester hydrolase
TcG_03939	144,6025124	0,268639434	0,150936803	1,779813999	0,075106406	0,22125167	protein_codin 10 kDa heat shock protein
TcG_03940	0,155988004	0,503022807	4,080472857	0,123275616	0,901888849	1	protein_codin 10 kDa heat shock protein
TcG_03941	693,8266382	-0,134723054	0,071545038	-1,88305239	0,059693269	0,188551312	protein_codin hypothetical protein
TcG_03942	1141,197942	-0,350389762	0,060839619	-5,75923658	8,44952E-09	2,72691E-07	protein_codin putative protein disulfide isomerase
TcG_03943	179,3597317	-0,202692259	0,131322066	-1,54347449	0,122715693	0,307671493	protein_codin putative exosome complex exonuclease, putative,ribosomal RNA processing protein 4
TcG_03944	133,8644693	0,015585517	0,155477367	0,100242993	0,920151414	0,961460255	protein_codin hypothetical protein
TcG_03945	343,5999051	-0,319666061	0,096842608	-3,3008824	0,000963813	0,0075553	protein_codin putative phosphatidylinositol 3-kinase 2
TcG_03946	147,2419957	0,108059084	0,149886481	0,720939498	0,47094674	0,686925321	protein_codin hypothetical protein
TcG_03947	623,7005608	0,099628997	0,080609434	1,235947112	0,216478223	0,440591636	protein_codin putative j-binding protein
TcG_03948	100,1588654	-0,37165647	0,183369983	-2,02681194	0,042681649	0,14787966	
TcG_03949	174,1339461	0,135815432	0,136716413	0,993409855	0,320510261	0,555699026	protein_codin hypothetical protein
TcG_03950	104,8235885	0,142080297	0,179493916	0,791560518	0,428616976	0,653907586	protein_codin ADP-ribosylation factor-like protein 16
TcG_03951	140,1368495	0,026012164	0,155567983	0,167207697	0,867206629	0,93578156	protein_codin putative leucine-rich repeat protein (LRRP)
TcG_03952	163,5916326	0,041042945	0,142122183	0,288786342	0,772744885	0,886613412	protein_codin hypothetical protein
TcG_03953	191,9824305	-0,383093262	0,137768368	-2,78070553	0,005424091	0,030506564	protein_codin hypothetical protein
TcG_03954	271,3296948	0,01557133	0,11165961	0,139453558	0,889091753	0,9464367	protein_codin hypothetical protein
TcG_03955	206,1287541	0,004890754	0,125050572	0,039110209	0,968802522	0,986599808	protein_codin putative chaperone protein DNAj
TcG_03956	19,00167521	0,232242668	0,399252839	0,581693215	0,560773342	0,754426375	protein_codin hypothetical protein
TcG_03957	243,3540091	-0,049528162	0,11482508	-0,43133575	0,666224256	0,825196732	protein_codin hypothetical protein
TcG_03958	171,1014796	-0,363028837	0,139375505	-2,60468177	0,009195963	0,046243239	protein_codin putative inosine-guanine nucleoside hydrolase
TcG_03959	365,6561493	0,076160383	0,095196251	0,800035527	0,423690214	0,649665804	protein_codin putative histone acetyltransferase
TcG_03960	216,6144319	0,008638498	0,122366779	0,070595123	0,943719992	0,973376643	protein_codin 60S ribosomal protein
TcG_03961	256,0341313	0,153314668	0,111394283	1,376324379	0,168721228	0,375106475	protein_codin hypothetical protein
TcG_03962	135,7913756	0,222977412	0,166113701	1,342318006	0,179492908	0,390682853	protein_codin hypothetical protein
TcG_03963	994,2544218	-0,065256984	0,072711901	-0,89747322	0,369466457	0,601128826	protein_codin hypothetical protein
TcG_03964	442,0264608	0,125913255	0,08969157	1,403847146	0,160364371	0,363241761	protein_codin hypothetical protein
TcG_03965	256,0764563	-0,037285128	0,113177124	-0,3294405	0,741822762	0,868596111	protein_codin putative coiled-coil domain-containing protein 96
TcG_03966	529,5888589	0,200196914	0,08801619	2,274546461	0,022933151	0,093673646	protein_codin hypothetical protein
TcG_03967	647,5954128	0,160236763	0,075072701	2,134421191	0,032808318	0,122342188	protein_codin hypothetical protein
TcG_03968	293,2016763	0,213952225	0,109732281	1,949765597	0,051204064	0,168253584	protein_codin adenosine 5'-monophosphoramidase
TcG_03969	448,2978637	-0,241586927	0,090162075	-2,67947392	0,007373795	0,03886842	protein_codin translocon-associated protein subunit gamma
TcG_03970	294,2352104	-0,123592744	0,108528784	-1,13880152	0,254785947	0,486142289	protein_codin hypothetical protein
TcG_03971	297,8812186	0,22182427	0,104117416	2,130520314	0,033128681	0,123100992	protein_codin hypothetical protein
TcG_03972	540,3877198	-0,077063527	0,088745302	-0,8683674	0,385193235	0,615735212	protein_codin hypothetical protein
TcG_03973	172,9184012	0,244984672	0,144214592	1,698750931	0,089366123	0,249012963	protein_codin hypothetical protein
TcG_03974	138,2534443	0,009044943	0,154877739	0,058400538	0,953429586	0,97816658	protein_codin hypothetical protein
TcG_03975	477,434247	-0,113118469	0,085973883	-1,3157306	0,188264481	0,402654186	protein_codin putative proteasome alpha 3 subunit
TcG_03976	842,2175928	0,213774116	0,066933566	3,193825281	0,001404011	0,010289351	protein_codin putative amino acid permease
TcG_03977	190,434251	-0,058800954	0,131841138	-0,44599854	0,655598304	0,817728228	protein_codin hypothetical protein
TcG_03978	145,7099611	-0,059001602	0,173618948	-0,33983389	0,733981627	0,863428889	protein_codin putative inositol polyphosphate kinase-like protein
TcG_03979	332,4937753	0,235892554	0,101502358	2,324010582	0,020124927	0,084480943	protein_codin putative threonine synthase
TcG_03980	417,0294459	0,039671762	0,090445369	0,438626787	0,660931985	0,822243957	protein_codin hypothetical protein
TcG_03981	448,9387855	-0,080928983	0,088250131	-0,91704094	0,359121187	0,592630203	protein_codin hypothetical protein
TcG_03982	661,2310442	-0,96413818	0,079503103	-12,127051	7,5935E-34	5,49865E-31	protein_codin hypothetical protein
TcG_03983	420,9721426	-0,400327985	0,089090932	-4,49347621	7,00699E-06	0,000112286	protein_codin hypothetical protein
TcG_03984	121,4794252	0,016821533	0,166871108	0,100805547	0,919704823	0,961308205	protein_codin hypothetical protein
TcG_03985	544,9211443	0,020154037	0,083615874	0,241031226	0,809530915	0,905942739	protein_codin U3 small nucleolar RNA-associated protein 25
TcG_03986	440,6670094	0,139240163	0,094348056	1,475813807	0,139993883	0,334581697	protein_codin hypothetical protein
TcG_03987	227,7273675	-0,149485196	0,131926532	-1,13309426	0,257174673	0,489028976	protein_codin hypothetical protein
TcG_03988	288,5265002	0,019394509	0,111327214	0,174211749	0,861699052	0,933819072	protein_codin putative apoptosis-inducing factor A-like
TcG_03989	356,885273	0,216451166	0,095886589	2,257366426	0,023985188	0,09677174	protein_codin hypothetical protein
TcG_03990	733,1126788	-0,186208903	0,069152364	-2,69273374	0,007086884	0,037647244	protein_codin nuclear distribution protein C like protein

TcG_03991	235,135777	0,047860058	0,129904509	0,368424914	0,712556423	0,852489876	protein_codin putative UDP-glucoronosyl and UDP-glucosyl transferase
TcG_03992	55,46713156	-0,023358786	0,233278954	-0,10013242	0,920239198	0,961460255	protein_codin putative UDP-glucoronosyl and UDP-glucosyl transferase
TcG_03993	443,0317178	-0,060258853	0,086040969	-0,70035071	0,483708312	0,697045336	protein_codin putative metallopeptidase, putative,metallo-peptidase, clan MP, family M67
TcG_03994	406,9070768	0,111967845	0,097169395	1,152295379	0,249199715	0,478493188	
TcG_03995	15,58637342	0,461007616	0,439469077	1,049010363	0,294173349	1	
TcG_03996	983,4087811	-0,075044846	0,062321469	-1,20415721	0,228528822	0,455641875	protein_codin putative adaptor complex protein (AP) 3 delta subunit 1
TcG_03997	375,1450782	-0,10053743	0,096000173	-1,04726301	0,294978293	0,527938889	protein_codin putative myosin heavy chain
TcG_03998	198,3555933	0,079355358	0,135437056	0,58592058	0,557928887	0,752697261	protein_codin putative queuine tRNA-ribosyltransferase
TcG_03999	200,0640429	0,28891511	0,130392241	2,215738509	0,026709416	0,104971266	protein_codin hypothetical protein
TcG_04000	107,7909453	-0,058494787	0,169555197	-0,34498965	0,730102155	0,860963213	protein_codin putative amino acid permease
TcG_04001	349,3855736	-0,056448884	0,097111064	-0,5812817	0,561050614	0,754624148	protein_codin transmembrane protein 67
TcG_04002	179,171037	-0,028089516	0,131454676	-0,21368214	0,830794941	0,917601778	protein_codin hypothetical protein
TcG_04003	335,9135466	-0,140738621	0,10873095	-1,29437498	0,19553593	0,413333203	protein_codin putative vesicle-associated membrane protein
TcG_04004	218,5725766	-0,046064302	0,122349712	-0,37649702	0,706547427	0,848735976	protein_codin hypothetical protein
TcG_04005	277,4839813	0,166461018	0,109782821	1,516275649	0,129449666	0,319107199	protein_codin phosphoglycerate mutase protein
TcG_04006	413,1783522	0,098876913	0,089071196	1,110088535	0,266960877	0,499395611	protein_codin thyroid hormone receptor interactor 4
TcG_04007	491,0702498	-0,061623544	0,08288505	-0,74348201	0,45718991	0,676783905	protein_codin putative ATP-dependent DEAD/H RNA helicase
TcG_04008	413,3354515	-0,093560633	0,091107709	-1,02692334	0,304456563	0,537227191	protein_codin putative adaptor complex protein (AP) 3 delta subunit 1
TcG_04009	102,0558132	-0,132263738	0,197965514	-0,66811505	0,504060158	0,712076368	protein_codin putative dual-specificity protein phosphatase
TcG_04010	263,3314821	-0,030246733	0,110730569	-0,27315612	0,784733206	0,893463807	protein_codin hypothetical protein
TcG_04011	164,7616948	0,454766048	0,148850207	3,055192585	0,00224916	0,015130467	protein_codin hypothetical protein
TcG_04012	367,0160108	0,115831834	0,094384967	1,227227576	0,219737068	0,444990554	protein_codin hypothetical protein
TcG_04013	182,8013021	0,179307959	0,13898281	1,290144865	0,197000365	0,415508184	protein_codin hypothetical protein
TcG_04014	433,5031844	0,320114996	0,089844846	3,562975604	0,000366675	0,00331898	protein_codin putative DNA repair protein
TcG_04015	93,27556301	0,065206784	0,181495487	0,359274961	0,719389404	0,855909389	protein_codin phosphatidylinositol glycan, class H
TcG_04016	121,0540192	0,043946962	0,159290117	0,275892581	0,782630573	0,892300513	protein_codin hypothetical protein
TcG_04017	389,3156246	-0,046079852	0,09769669	-0,47166236	0,637167805	0,807065287	protein_codin hypothetical protein
TcG_04018	244,218781	0,128009857	0,115303124	1,110202857	0,266911622	0,499395611	protein_codin gamma carbonic dehydratase
TcG_04019	569,6135987	-0,203555601	0,081228536	-2,50596172	0,012211884	0,057867848	protein_codin putative eukaryotic translation initiation factor 3 subunit
TcG_04020	153,315956	-0,053549403	0,142211258	-0,37654827	0,706509336	0,848735976	protein_codin hypothetical protein
TcG_04021	473,5107387	-0,026298095	0,085889235	-0,30618616	0,759462909	0,878508113	protein_codin ubiquitin-activating enzyme-like protein
TcG_04022	287,3113779	-0,003251311	0,107712907	-0,03018497	0,975919536	0,989758731	protein_codin aarF domain-containing kinase
TcG_04023	550,1105596	0,002901296	0,081108707	0,035770461	0,971465387	0,98762955	protein_codin hypothetical protein
TcG_04024	1094,115143	-0,325885254	0,063366859	-5,1428343	2,70624E-07	6,28347E-06	protein_codin sarcoplasmic/endoplasmic reticulum calcium ATPase 3
TcG_04025	142,6350114	-0,143139333	0,155285358	-0,92178255	0,356642013	0,590630983	protein_codin ADG1 protein
TcG_04026	361,5452112	0,048611231	0,096470147	0,503899216	0,614332202	0,791539208	protein_codin putative ADG2
TcG_04027	43,8500313	0,14183655	0,26674591	0,531729051	0,594913668	0,780155037	protein_codin hypothetical protein
TcG_04028	129,9146254	-0,095247135	0,157934101	-0,6030815	0,546454472	0,743362863	protein_codin hypothetical protein
TcG_04029	325,9936309	0,054649793	0,100935947	0,541430429	0,588210939	0,775648564	protein_codin putative ribosomal protein L2
TcG_04030	372,6573373	0,030810098	0,093973368	0,327859886	0,743017605	0,869301389	protein_codin putative iron superoxide dismutase
TcG_04031	1353,250685	0,292580043	0,068465695	4,2733816	1,92531E-05	0,000268108	protein_codin putative DNA repair and recombination protein RAD54
TcG_04032	692,5585843	-0,175562618	0,072158114	-2,4330267	0,014973197	0,067666914	protein_codin protein kinase
TcG_04033	183,5429262	0,148800321	0,13186745	1,128408267	0,259147538	0,491636632	protein_codin hypothetical protein
TcG_04034	49,0123572	0,537797732	0,248332885	2,165632359	0,030339292	0,115325142	protein_codin putative protein kinase
TcG_04035	27,67931902	0,137426525	0,332631484	0,413149482	0,679497098	0,832732534	protein_codin hypothetical protein
TcG_04036	793,7531165	-0,080899731	0,069073839	-1,17120653	0,241515772	0,470195853	protein_codin hypothetical protein
TcG_04037	189,0810982	0,103387905	0,130049297	0,794990108	0,426619256	0,651827866	protein_codin hypothetical protein
TcG_04038	165,0075691	-0,046740382	0,136833109	-0,34158678	0,732661884	0,862784475	protein_codin hypothetical protein
TcG_04039	242,4425409	-0,08914218	0,119479916	-0,74608506	0,455616034	0,675638982	protein_codin hypothetical protein
TcG_04040	295,5193113	0,015872154	0,104955318	0,151227727	0,879796079	0,941623821	protein_codin hypothetical protein
TcG_04041	474,9822646	-0,333817931	0,085812337	-3,89009251	0,000100206	0,001118484	protein_codin putative rab-GDP dissociation inhibitor
TcG_04042	62,34660214	-0,342603085	0,226476589	-1,51275276	0,130342472	0,320353815	protein_codin putative protein kinase
TcG_04043	257,4578921	-0,09631148	0,112533019	-0,85585085	0,392080285	0,621513502	protein_codin WD40 repeat-containing protein
TcG_04044	345,3888324	0,028171254	0,102644011	0,274455895	0,783734293	0,892768216	protein_codin putative C-8 sterol isomerase
TcG_04045	352,0341974	-0,094147794	0,099270894	-0,94839273	0,34292956	0,576910393	protein_codin putative leucine-rich repeat protein
TcG_04046	201,6723039	-0,136622994	0,123431296	-1,10687482	0,268348056	0,500941571	protein_codin hypothetical protein
TcG_04047	154,9923053	0,11188574	0,140101223	0,798606447	0,424518648	0,65015164	protein_codin hypothetical protein

TcG_04048	1541,071644	-0,0830051	0,058311664	-1,42347337	0,154598977	0,355766439	protein_codin UDP-glucose:glycoprotein glucosyltransferase
TcG_04049	846,3509584	-0,03785556	0,065597241	-0,57709074	0,563878163	0,756804282	protein_codin hypothetical protein
TcG_04050	197,9813038	0,247556071	0,12888493	1,920752647	0,054762899	0,176564058	protein_codin retinol dehydrogenase 14
TcG_04051	608,143539	0,09083539	0,083532998	1,087419249	0,276851568	0,509062413	protein_codin hypothetical protein
TcG_04052	256,5329358	-0,100019875	0,114943837	-0,87016301	0,384211329	0,614504757	protein_codin hypothetical protein
TcG_04053	414,1589611	-0,051432438	0,092656131	-0,55508942	0,5788335	0,768461663	protein_codin importin 7
TcG_04054	101,4084915	-0,319043475	0,174004614	-1,83353457	0,066723112	0,205108511	protein_codin hypothetical protein
TcG_04055	345,1693877	-0,337121615	0,096867824	-3,48022287	0,000500997	0,004344723	protein_codin hypothetical protein
TcG_04056	349,5385692	-0,408453624	0,095880564	-4,26002527	2,04404E-05	0,000282604	protein_codin putative UDP-GlcNAc:PI a1-6 GlcNAc-transferase
TcG_04057	815,1448164	-0,431391476	0,072082236	-5,98471278	2,16772E-09	7,99847E-08	protein_codin putative protein kinase
TcG_04058	262,4050753	-0,100737637	0,119138017	-0,84555409	0,397801548	0,627491999	protein_codin hypothetical protein
TcG_04059	448,1398872	-0,00174157	0,090727075	-0,01919571	0,984684982	0,994123614	protein_codin hypothetical protein
TcG_04060	177,5265844	0,094199423	0,135841373	0,69345164	0,488026165	0,699453024	protein_codin hypothetical protein
TcG_04061	215,9490555	0,031243952	0,127851042	0,244377764	0,806938261	0,904614097	protein_codin hypothetical protein
TcG_04062	716,2854283	-0,258162129	0,070533487	-3,66013565	0,000252082	0,002419734	protein_codin hypothetical protein
TcG_04063	37,69869646	0,21038925	0,284284559	0,740065694	0,459260134	0,677910606	protein_codin putative fumarate hydratase
TcG_04064	628,5188768	-0,509483031	0,075697118	-6,73054726	1,69026E-11	1,00945E-09	protein_codin fumarate hydratase
TcG_04065	333,5713832	-0,337157416	0,107064752	-3,14909818	0,001637752	0,011698514	protein_codin putative protein farnesyltransferase alpha subunit
TcG_04066	120,8973683	-0,341717646	0,15989181	-2,13718041	0,03258332	0,121777532	protein_codin diacylglycerol kinase-like protein
TcG_04067	101,2463357	0,154614341	0,17895922	0,863964099	0,387607612	0,617974652	protein_codin hypothetical protein
TcG_04068	179,6293336	-0,287332723	0,139295719	-2,06275344	0,03913606	0,139259948	protein_codin protein phosphatase 2 (formerly 2A), regulatory subunit B
TcG_04069	198,0735531	-0,261671482	0,126863692	-2,06261916	0,039148826	0,139262602	protein_codin hypothetical protein
TcG_04070	128,2979248	-0,001597314	0,156539559	-0,0102039	0,991858605	0,996781867	protein_codin hypothetical protein
TcG_04071	139,5578033	-0,115474746	0,148216642	-0,77909433	0,435924149	0,659521701	protein_codin hypothetical protein
TcG_04072	245,8136851	-0,000726872	0,114453242	-0,00635082	0,99493281	0,997899447	protein_codin hypothetical protein
TcG_04073	250,4223615	-0,099048833	0,113285681	-0,87432792	0,381939701	0,612054409	protein_codin hypothetical protein
TcG_04074	108,5919193	0,070204374	0,17992777	0,390180872	0,696402805	0,842753592	protein_codin hypothetical protein
TcG_04075	206,5184088	-0,423599229	0,122714952	-3,45189582	0,000556663	0,004752758	protein_codin hypothetical protein
TcG_04076	1,82305276	-3,517156309	1,708001692	-2,05922297	0,039472884	1	
TcG_04077	2,470151047	-0,52547107	1,179390325	-0,44554467	0,655926188	1	
TcG_04078	147,8503232	-0,05250281	0,148570396	-0,35338675	0,723798517	0,85772012	protein_codin putative DnaJ chaperone protein
TcG_04079	1356,793533	-0,130507033	0,06037858	-2,16147902	0,030658356	0,116118897	protein_codin R27-2 protein
TcG_04080	436,0357176	0,097717455	0,089315556	1,094069819	0,273924358	0,506978852	protein_codin hypothetical protein
TcG_04081	348,2487849	0,324518198	0,099635871	3,257041816	0,001125798	0,0085869	protein_codin putative protein kinase
TcG_04082	526,4117089	-0,048645167	0,089491719	-0,54357171	0,586736233	0,775222488	protein_codin putative UDP-galactose 4-epimerase
TcG_04083	227,6440988	0,053243474	0,118487497	0,449359429	0,653172393	0,816535967	protein_codin hypothetical protein
TcG_04084	372,3542311	-0,001872835	0,094338866	-0,01985221	0,984161265	0,993987719	protein_codin putative cell division cycle protein 45 (CDC45)
TcG_04085	625,6231905	0,031723724	0,075185146	0,421941378	0,673067807	0,828900214	protein_codin hypothetical protein
TcG_04086	477,1743176	0,099670691	0,085085033	1,171424479	0,241428198	0,470195853	protein_codin hypothetical protein
TcG_04087	1403,267296	0,375752649	0,054552484	6,887910886	5,66177E-12	3,76996E-10	protein_codin putative succinyl-CoA:3-ketoacid-coenzyme A transferase
TcG_04088	416,9004706	-0,045299912	0,094140316	-0,48119566	0,630377443	0,801971346	protein_codin hypothetical protein
TcG_04089	601,8334051	-0,18043588	0,079014884	-2,28356824	0,022396918	0,09179013	protein_codin hypothetical protein
TcG_04090	433,1877487	0,060222341	0,095929159	0,627779305	0,530148525	0,731981763	protein_codin Golgi reassembly stacking protein
TcG_04091	1544,527467	0,011593817	0,056822441	0,204035881	0,83832546	0,92144011	protein_codin putative heat shock protein
TcG_04092	282,2036112	-0,06949702	0,109525878	-0,63452603	0,525737588	0,728873483	protein_codin putative GTPase
TcG_04093	307,5520084	0,218665583	0,103981915	2,102919373	0,035472818	0,129854051	protein_codin putative diphthamide synthesis protein
TcG_04094	1620,516598	-0,19021794	0,051261031	-3,71077083	0,000206629	0,002040925	protein_codin putative OSM3-like kinesin
TcG_04095	594,1570892	-0,077135335	0,083651913	-0,92209887	0,356477005	0,590526534	protein_codin hypothetical protein
TcG_04096	635,0712467	-0,070441099	0,074256039	-0,94862451	0,342811622	0,576796063	protein_codin hypothetical protein
TcG_04097	164,8419524	0,277068869	0,138274055	2,003766137	0,045095118	0,153713455	protein_codin putative retrotransposon hot spot (RHS) protein
TcG_04098	757,1929285	0,002825416	0,071501821	0,0395153	0,968479555	0,986444319	protein_codin hypothetical protein
TcG_04099	258,8032577	-0,044005254	0,117275652	-0,37522924	0,707489987	0,849163885	protein_codin putative dynein heavy chain
TcG_04100	264,528269	-0,032998535	0,111658206	-0,29553166	0,767587771	0,883509739	protein_codin putative meiotic recombination protein DMC1
TcG_04101	112,0924895	0,390192348	0,173800027	2,245064945	0,024763964	0,09904653	protein_codin helicase-like protein
TcG_04102	199,4599925	-0,013825851	0,124298878	-0,1112307	0,91143341	0,956856423	protein_codin radial spoke protein Ci-RSP9
TcG_04103	485,631837	-0,048295563	0,085135885	-0,56727622	0,570526529	0,762194568	protein_codin putative mitochondrial RNA binding complex 1 subunit
TcG_04104	259,741819	0,079667737	0,111707624	0,713180836	0,475733863	0,691164619	protein_codin UDP-sugar pyrophosphorylase

TcG_04105	1023,863919	0,011190427	0,064714674	0,172919471	0,862714727	0,934238043	protein_codin ribosomal rRNA-processing protein 12
TcG_04106	974,9700001	0,043148174	0,064555636	0,668387403	0,503886334	0,712076368	protein_codin carboxypeptidase
TcG_04107	646,4466715	-0,237587655	0,073861857	-3,2166488	0,001296973	0,009657278	protein_codin putative Unc104-like kinesin
TcG_04108	423,2963907	-0,145851708	0,090626645	-1,60936894	0,107535693	0,282454895	protein_codin putative aminopeptidase, putative,metallo-peptidase, Clan MF, Family M17
TcG_04109	276,3211765	0,176158048	0,111126499	1,585202892	0,112920244	0,291249765	protein_codin hypothetical protein
TcG_04110	800,6704268	0,062725556	0,070640245	0,887957793	0,374563456	0,605172528	protein_codin FGF receptor activating protein
TcG_04111	195,856907	0,257202071	0,130315706	1,973684363	0,048417644	0,16166191	protein_codin adenylate kinase
TcG_04112	315,2130528	-0,219430939	0,104780826	-2,09418982	0,036243075	0,132052525	protein_codin putative tubulin binding cofactor c
TcG_04113	1889,719458	0,094964257	0,060154286	1,578678148	0,114409899	0,293979396	protein_codin cytoplasmic dynein 2 heavy chain 1 isoform X1
TcG_04114	313,0358077	0,163220792	0,107479754	1,518618957	0,128858437	0,317925448	protein_codin putative tubulin tyrosine ligase
TcG_04115	77,78075344	-0,081694065	0,206025187	-0,39652465	0,691718027	0,840508134	protein_codin hypothetical protein
TcG_04116	156,2700216	0,205830542	0,148988191	1,381522523	0,167118358	0,373072109	protein_codin GDP-mannose 4,6 dehydratase
TcG_04117	429,8712215	-0,049161431	0,090612994	-0,54254284	0,587444604	0,775648564	protein_codin putative iron superoxide dismutase
TcG_04118	151,3179817	-0,107166876	0,147798363	-0,72508838	0,46839781	0,685000736	protein_codin putative glutathione peroxidase
TcG_04119	173,2359449	-0,230996709	0,134869752	-1,71273919	0,086760532	0,244100905	protein_codin component of TRAPP complex
TcG_04120	727,8626288	-0,109232867	0,073874335	-1,4786308	0,139239015	0,33365527	protein_codin hypothetical protein
TcG_04121	378,0047855	-0,440015533	0,100020134	-4,3992696	1,08616E-05	0,000163008	protein_codin putative amino acid transporter, putative,amino acid permease
TcG_04122	28,94069961	-0,218403661	0,317261551	-0,68840255	0,491199318	0,701990292	
TcG_04123	206,7764187	0,079086311	0,127593632	0,619829606	0,535369975	0,735451331	protein_codin As/Sb Reductase
TcG_04124	483,7917366	-0,1752137	0,087172726	-2,0099601	0,044435412	0,152264203	protein_codin hypothetical protein
TcG_04125	461,3862537	-0,240866206	0,086055698	-2,7989571	0,005126795	0,02923452	protein_codin hypothetical protein
TcG_04126	517,0349929	-0,281383163	0,083604254	-3,36565603	0,000763619	0,006212982	protein_codin hypothetical protein
TcG_04127	811,8539517	-0,145132361	0,073483338	-1,97503766	0,048263874	0,161520868	protein_codin hypothetical protein
TcG_04128	35,04451583	-0,272115542	0,301225131	-0,90336268	0,366333439	0,598552985	
TcG_04129	192,7263393	0,075222664	0,130090625	0,578232786	0,563106966	0,756424036	protein_codin exonuclease
TcG_04130	735,6007383	-0,225746276	0,077242886	-2,92255105	0,003471767	0,021498607	protein_codin hypothetical protein
TcG_04131	333,9318777	-0,216760608	0,110650065	-1,95897408	0,050115823	0,165734726	protein_codin putative RNA-binding protein
TcG_04132	436,6912118	-0,243499936	0,092725224	-2,62603773	0,008638524	0,044083188	protein_codin proteasome regulatory non-ATP-ase subunit 7
TcG_04133	531,6344536	-0,139180834	0,082405549	-1,68897406	0,091224401	0,252249621	protein_codin hypothetical protein
TcG_04134	151,2366918	-0,153988962	0,145645361	-1,0572871	0,290380579	0,523456792	protein_codin hypothetical protein
TcG_04135	432,6561331	-0,319296659	0,091876153	-3,47529417	0,000510293	0,004408844	protein_codin TPR Domain containing protein
TcG_04136	238,0744633	-0,222517567	0,117649675	-1,89135726	0,05857667	0,185580885	protein_codin putative zinc finger protein
TcG_04137	214,1424334	-0,123395057	0,1286716	-0,95899217	0,337562688	0,572118388	protein_codin putative phosphatidate cytidylyltransferase-like protein
TcG_04138	199,3770689	-0,111264795	0,12537726	-0,88744	0,374842055	0,605453793	protein_codin hypothetical protein
TcG_04139	291,0525665	-0,237634599	0,109818263	-2,16388961	0,030472823	0,115605149	protein_codin putative SNAP protein
TcG_04140	699,2560806	-0,525678594	0,072940018	-7,20699846	5,71987E-13	4,50819E-11	protein_codin putative outer dynein arm docking complex
TcG_04141	797,1428168	-0,184130726	0,068314727	-2,69532989	0,007031897	0,037423777	protein_codin putative ubiquitin hydrolase
TcG_04142	619,350208	-0,23128592	0,076126822	-3,03816597	0,002380228	0,015803624	protein_codin putative intergrin alpha chain protein
TcG_04143	31,2608538	0,370298982	0,321332437	1,152385938	0,249162517	0,478493188	protein_codin hypothetical protein
TcG_04144	503,4234177	-0,213242581	0,084331899	-2,52861118	0,011451481	0,05500699	protein_codin hypothetical protein
TcG_04145	870,4989858	-0,355154719	0,066420157	-5,34709247	8,93784E-08	2,34816E-06	protein_codin nucleoside diphosphate kinase
TcG_04146	490,4702987	-0,335840133	0,087435432	-3,84100731	0,00012253	0,001325526	protein_codin putative minichromosome maintenance (MCM) complex subunit
TcG_04147	406,300225	-0,30314347	0,09168776	-3,30625887	0,000945507	0,007452138	protein_codin hypothetical protein
TcG_04148	211,265947	-0,234488848	0,120985643	-1,93815434	0,052604396	0,171248815	protein_codin tubulin binding cofactor A-like protein
TcG_04149	311,4692552	-0,182835157	0,106439342	-1,71774039	0,085843979	0,242228043	protein_codin putative cyclic nucleotide-binding protein
TcG_04150	140,1514693	-0,11716063	0,150899969	-0,77641255	0,437505438	0,660878488	protein_codin hypothetical protein
TcG_04151	617,8014876	-0,12200172	0,075615195	-1,61345509	0,106645674	0,281328956	protein_codin hypothetical protein
TcG_04152	484,4308993	-0,305900658	0,088445804	-3,45862262	0,000542945	0,004656226	protein_codin hypothetical protein
TcG_04153	309,4057963	0,069903326	0,109951279	0,635766373	0,524928708	0,728449396	protein_codin hypothetical protein
TcG_04154	602,2442811	-0,086372957	0,079321311	-1,08889977	0,276198087	0,508736401	protein_codin hypothetical protein
TcG_04155	313,6276758	-0,031871847	0,104556026	-0,30483032	0,760495385	0,879088051	protein_codin putative DNA repair helicase
TcG_04156	1078,857942	-0,014048571	0,060663642	-0,2315814	0,816863157	0,909580769	protein_codin 60S ribosomal protein L2
TcG_04157	209,5790318	0,122044876	0,126956107	0,961315522	0,336393545	0,570804865	protein_codin surface protease GP63
TcG_04158	499,4279992	0,070116138	0,085030536	0,824599512	0,409598998	0,636866847	protein_codin L1Tc protein
TcG_04159	185,9795259	0,12365659	0,133159309	0,928636463	0,353077518	0,587499085	
TcG_04160	96,93671433	0,354096425	0,18259199	1,939276883	0,052467632	0,170899631	protein_codin hypothetical protein
TcG_04161	116,7320545	0,128632539	0,169287128	0,759848318	0,447345257	0,668546514	protein_codin hypothetical protein

TcG_04162	277,713639	0,105162215	0,106947637	0,983305637	0,325457036	0,559873085	protein_codin hypothetical protein
TcG_04163	147,2717968	0,046613992	0,146586426	0,317996647	0,75048748	0,874411499	
TcG_04164	515,4239603	0,103835494	0,082670106	1,25602227	0,209107896	0,431627308	protein_codin 2-hydroxyglutarate dehydrogenase
TcG_04165	710,2560966	-0,104530127	0,073540937	-1,42138693	0,155204303	0,356612014	protein_codin putative guanylate cyclase
TcG_04166	670,5564089	-0,193308302	0,072085889	-2,68163857	0,007326256	0,038705886	protein_codin hypothetical protein
TcG_04167	236,9951688	0,554169735	0,115911961	4,780953837	1,74465E-06	3,30373E-05	protein_codin hypothetical protein
TcG_04168	459,0715427	0,066649125	0,08715805	0,764692712	0,444454547	0,666426941	protein_codin adiponectin receptor protein 1
TcG_04169	1,860274304	0,788757284	1,357219571	0,581156727	0,561134829	1	
TcG_04170	215,8561964	0,360136405	0,127168378	2,831965073	0,00462629	0,027057142	protein_codin adiponectin receptor protein 1
TcG_04171	451,5724149	-0,135651544	0,090241637	-1,50320349	0,132786577	0,323682997	protein_codin putative phosphorylated CTD-interacting factor 1-like
TcG_04172	465,7871856	-0,191234463	0,088728526	-2,15527601	0,031140242	0,117521447	protein_codin hypothetical protein
TcG_04173	675,5606962	0,06732557	0,073547478	0,915402847	0,359980184	0,592630203	protein_codin hypothetical protein
TcG_04174	206,5322561	0,085355488	0,12578271	0,67859476	0,497394664	0,707256775	protein_codin hypothetical protein
TcG_04175	638,9464634	-0,204197926	0,076013797	-2,68632715	0,007224232	0,038254089	protein_codin hypothetical protein
TcG_04176	342,5413437	0,23327308	0,098303111	2,372997941	0,017644362	0,076335915	protein_codin putative G-actin binding protein, putative,CAP/Srv2p
TcG_04177	324,2391543	-0,313124966	0,100193076	-3,12521561	0,001776748	0,012521533	protein_codin hypothetical protein
TcG_04178	476,088892	0,016951356	0,08379274	0,20230101	0,83968141	0,922079481	protein_codin putative beta-adaptin
TcG_04179	253,0744537	-0,299144319	0,113225916	-2,6420128	0,008241494	0,042475957	protein_codin hypothetical protein
TcG_04180	320,5408892	-0,137616526	0,101138668	-1,36067172	0,173617455	0,382202514	protein_codin hypothetical protein
TcG_04181	633,4463872	-0,124015723	0,077368369	-1,6029254	0,108951125	0,285009649	protein_codin hypothetical protein
TcG_04182	382,5705065	0,043234851	0,093770677	0,461070055	0,644748345	0,811345099	protein_codin hypothetical protein
TcG_04183	1340,776508	-0,278652861	0,057465455	-4,84904993	1,24054E-06	2,45272E-05	protein_codin isoleucine--tRNA ligase
TcG_04184	303,4773965	-0,207659299	0,114373444	-1,81562513	0,069427896	0,210849699	protein_codin hypothetical protein
TcG_04185	338,7075216	-0,145377715	0,098386597	-1,47761707	0,139510303	0,334093557	protein_codin hypothetical protein
TcG_04186	605,53484	0,0657522	0,078938352	0,832956332	0,404869358	0,634151194	protein_codin cyclin L1
TcG_04187	630,5621254	-0,247828236	0,078143454	-3,17145229	0,001516788	0,010976579	protein_codin hypothetical protein
TcG_04188	469,2157934	-0,043713657	0,086354506	-0,50621165	0,612708078	0,790819724	protein_codin hypothetical protein
TcG_04189	541,928164	-0,018176937	0,080124962	-0,22685735	0,820534667	0,911143056	protein_codin putative RNA helicase
TcG_04190	365,6756368	0,093229735	0,096964693	0,961481263	0,336310242	0,570804865	protein_codin hypothetical protein
TcG_04191	192,3217263	-0,053324696	0,12963035	-0,41135966	0,680808831	0,833546561	protein_codin hypothetical protein
TcG_04192	613,7884913	-0,055994139	0,076532022	-0,73164327	0,464386328	0,681738107	protein_codin hypothetical protein
TcG_04193	596,8333622	0,039536661	0,075555231	0,523281583	0,600778315	0,783588603	protein_codin hypothetical protein
TcG_04194	326,8971918	-0,197667392	0,099484147	-1,98692354	0,046930871	0,158279423	protein_codin hypothetical protein
TcG_04195	581,9335594	0,003803256	0,077848314	0,048854693	0,961035095	0,981984718	protein_codin hypothetical protein
TcG_04196	409,8963965	-0,256176417	0,094358488	-2,71492712	0,006629036	0,035773183	protein_codin hypothetical protein
TcG_04197	379,3126327	0,115606043	0,098194889	1,177312219	0,239070912	0,467252966	protein_codin tRNA pseudouridine13 synthase
TcG_04198	820,9793925	-0,03094672	0,067434914	-0,45891243	0,646297054	0,812019442	protein_codin hypothetical protein
TcG_04199	177,5612151	0,119464875	0,136187915	0,877206133	0,380374686	0,610738657	protein_codin hypothetical protein
TcG_04200	679,0811714	0,136090615	0,077744266	1,750490701	0,080033677	0,231180714	protein_codin tRNA (adenine-N(1)-)-methyltransferase non-catalytic subunit
TcG_04201	132,6235988	0,242320428	0,154490317	1,568515311	0,11676092	0,298059197	protein_codin putative leucine-rich repeat protein (LRRP)
TcG_04202	765,1091144	0,081537627	0,07424787	1,09818137	0,2721253	0,505040461	protein_codin hypothetical protein
TcG_04203	289,1761921	0,065881231	0,10914638	0,603604358	0,546106715	0,742977031	protein_codin hypothetical protein
TcG_04204	4453,562967	-0,325958197	0,042283168	-7,70893507	1,26872E-14	1,27821E-12	protein_codin hypothetical protein
TcG_04205	582,0516281	0,100761926	0,082124322	1,226943788	0,219843719	0,444990554	protein_codin kinetoplast-associated protein
TcG_04206	364,0489641	-0,284456627	0,096191303	-2,95719694	0,003104498	0,019601477	protein_codin hypothetical protein
TcG_04207	517,9844142	-0,262585806	0,080647458	-3,25597127	0,001130052	0,008613672	protein_codin proteasome regulatory non-ATPase subunit 8
TcG_04208	310,2627367	-0,059509782	0,105120333	-0,56611105	0,571318292	0,762591444	protein_codin hypothetical protein
TcG_04209	813,3791219	-0,410339505	0,06876525	-5,96725095	2,41284E-09	8,87466E-08	protein_codin hypothetical protein
TcG_04210	767,663726	-0,306764138	0,072189819	-4,24941002	2,14334E-05	0,000294576	protein_codin hypothetical protein
TcG_04211	364,022943	-0,649423593	0,095055519	-6,83204511	8,37125E-12	5,35853E-10	protein_codin hypothetical protein
TcG_04212	488,3526916	-0,629191917	0,085389398	-7,36850164	1,72556E-13	1,45275E-11	protein_codin hypothetical protein
TcG_04213	745,4858556	-0,397794367	0,071263108	-5,58205188	2,37697E-08	7,00754E-07	protein_codin hypothetical protein
TcG_04214	706,9048841	-0,339519427	0,076942307	-4,41264944	1,02113E-05	0,000154652	protein_codin hypothetical protein
TcG_04215	865,3820967	-0,348578313	0,06808529	-5,11973017	3,05973E-07	6,99904E-06	protein_codin Intraflagellar Transport Protein 140
TcG_04216	548,2342623	-0,48000604	0,07831955	-6,12881512	8,85359E-10	3,56173E-08	protein_codin putative leucine-rich repeat protein (LRRP)
TcG_04217	277,0982478	-0,127543873	0,109179473	-1,16820378	0,242724577	0,47105644	protein_codin hypothetical protein
TcG_04218	276,5832128	-0,254311106	0,114310065	-2,22474815	0,02609815	0,102988134	protein_codin B9 protein domain 1

TcG_04219	1208,354337	-0,229144443	0,066432983	-3,44925717	0,000562131	0,004788861	protein_codin	hypothetical protein
TcG_04220	500,4986822	-0,388220442	0,093117602	-4,1691413	3,05749E-05	0,00040209	protein_codin	putative protein kinase, putative,serine/threonine-protein kinase Nek1
TcG_04221	1060,484126	-0,143515933	0,063710607	-2,25262228	0,024282975	0,09775627	protein_codin	putative protein kinase
TcG_04222	1011,582069	-0,225067981	0,062338991	-3,61038861	0,000305739	0,00284732	protein_codin	hypothetical protein
TcG_04223	1081,733183	-0,391652655	0,060093632	-6,51737372	7,1549E-11	3,61994E-09	protein_codin	hypothetical protein
TcG_04224	809,5792503	-0,415970885	0,067139858	-6,19558781	5,80679E-10	2,44645E-08	protein_codin	structure-specific recognition protein 1
TcG_04225	526,3291514	-0,178083282	0,080478313	-2,21281082	0,026910691	0,105511766	protein_codin	hypothetical protein
TcG_04226	573,4182588	-0,208149107	0,07850766	-2,65132226	0,00801773	0,041581657	protein_codin	hypothetical protein
TcG_04227	190,6741591	0,055316741	0,129026697	0,42872322	0,668124661	0,826134898	protein_codin	hypothetical protein
TcG_04228	353,0683704	-0,126907595	0,097989651	-1,29511223	0,195281518	0,41302148	protein_codin	putative U2 small nuclear ribonucleoprotein 40K
TcG_04229	185,5720548	0,0464099	0,131578908	0,352715344	0,724301855	0,857982193	protein_codin	hypothetical protein
TcG_04230	265,2264075	-0,359374792	0,11142859	-3,22515784	0,001259032	0,009411064	protein_codin	hypothetical protein
TcG_04231	547,4708236	-0,458527125	0,082807597	-5,53725918	3,07242E-08	8,83301E-07	protein_codin	hypothetical protein
TcG_04232	837,6471163	-0,301974505	0,072074988	-4,18972676	2,79291E-05	0,000371222	protein_codin	U3 small nucleolar RNA-associated protein 14
TcG_04233	159,2572472	-0,495084533	0,14331097	-3,45461713	0,000551075	0,004711994	protein_codin	U3 small nucleolar RNA-associated protein 14
TcG_04234	775,4309	-0,413302977	0,075496866	-5,47443886	4,389E-08	1,22238E-06	protein_codin	vacuolar protein 8
TcG_04235	309,2836659	-0,345265081	0,106221615	-3,25042205	0,001152339	0,008766247	protein_codin	hypothetical protein
TcG_04236	414,9395519	-0,598181764	0,097435432	-6,13926325	8,29051E-10	3,39413E-08	protein_codin	putative protein kinase, putative,serine/threonine protein kinase
TcG_04237	294,4035254	-0,154402336	0,111958461	-1,37910377	0,167862761	0,374011144	protein_codin	putative mitochondrial carrier protein
TcG_04238	0,689722372	0,936118941	2,175680909	0,430264814	0,667003019	1		
TcG_04239	19,90112191	-0,750340246	0,384598461	-1,95097048	0,051060557	0,168073669	protein_codin	Dullard-like phosphatase domain-containing protein
TcG_04240	28,00252663	0,133396067	0,332948005	0,400651348	0,688676835	0,838395483	protein_codin	hypothetical protein
TcG_04241	30,73291059	-0,100942939	0,31418953	-0,3212804	0,747997905	0,872827448	protein_codin	hypothetical protein
TcG_04242	40,01483246	-0,605852589	0,281694	-2,15074722	0,031496157	0,118478725	protein_codin	beta galactofuranosyl glycosyltransferase
TcG_04243	59,56513408	-0,009168874	0,230644753	-0,03975323	0,968289863	0,98638627	protein_codin	beta galactofuranosyl glycosyltransferase
TcG_04244	13,24066071	0,445463712	0,477165317	0,933562638	0,350529545	1	protein_codin	beta galactofuranosyl glycosyltransferase
TcG_04245	51,97840504	0,05434531	0,245669001	0,221213544	0,824926164	0,914252395	protein_codin	hypothetical protein
TcG_04246	62,69089015	0,126509144	0,221752174	0,570497873	0,568340065	0,760104812		
TcG_04247	115,5079828	-0,13552662	0,161961609	-0,83678237	0,402714906	0,632851796	protein_codin	cyclophilin
TcG_04248	439,6904859	0,085847576	0,086584462	0,991489396	0,321446668	0,556110081	protein_codin	hypothetical protein
TcG_04249	56,56017464	0,20298107	0,246200469	0,824454441	0,409681392	0,636866847	protein_codin	hypothetical protein
TcG_04250	556,2471278	-0,134529342	0,083320809	-1,61459477	0,106398479	0,280804733	protein_codin	trichohyalin
TcG_04251	481,0299616	0,142025838	0,083814432	1,69452724	0,09016514	0,250396288	protein_codin	hypothetical protein
TcG_04252	236,1153411	-0,180689436	0,115451383	-1,56506948	0,11756662	0,298974288	protein_codin	hypothetical protein
TcG_04253	553,2218631	-0,160850113	0,078106775	-2,05936186	0,039459587	0,13999295	protein_codin	hypothetical protein
TcG_04254	656,680762	-0,042163754	0,073984265	-0,56990165	0,568744408	0,760557792	protein_codin	hypothetical protein
TcG_04255	177,8827852	-0,284471671	0,132455714	-2,14767384	0,031739675	0,119162631	protein_codin	putative leucine-rich repeat protein (LRRP)
TcG_04256	875,0257495	-0,144206105	0,069222265	-2,08323298	0,037229993	0,134227439	protein_codin	alkyldihydroxyacetonephosphate synthase
TcG_04257	242,2532965	0,109245055	0,124445244	0,877856408	0,380021648	0,610721305	protein_codin	putative lysyl-tRNA synthetase
TcG_04258	300,7009725	0,117046896	0,10420498	1,123237068	0,261336823	0,493587786	protein_codin	glutamine amidotransferase class-I
TcG_04259	0,445396961	-2,390354933	2,606559457	-0,91705368	0,359114513	1		
TcG_04260	523,7175737	0,013510649	0,083797046	0,16123061	0,871911773	0,938408714	protein_codin	protein YIPF2
TcG_04261	352,2318354	-0,023553814	0,099794258	-0,23602374	0,813414246	0,907881422	protein_codin	putative leucine-rich repeat protein
TcG_04262	185,5286417	0,347281805	0,132300744	2,6249422	0,008666368	0,044155029	protein_codin	SET domain containing protein
TcG_04263	661,3594217	0,119948484	0,07702246	1,557318281	0,119394937	0,302164644	protein_codin	putative 2-hydroxy-3-oxopropionate reductase
TcG_04264	307,0882031	0,284426595	0,104344244	2,72584844	0,006413644	0,03487024	protein_codin	putative polynucleotide kinase 3'-phosphatase
TcG_04265	269,0316499	0,297585374	0,111344468	2,672655232	0,007525355	0,039559329	protein_codin	putative serine peptidase
TcG_04266	162,88433	0,285535294	0,141392433	2,019452438	0,043440216	0,149746606	protein_codin	hypothetical protein
TcG_04267	361,1158202	-0,078600506	0,101647442	-0,77326595	0,439365002	0,662646613	protein_codin	putative ribosomal RNA adenine dimethylase family protein
TcG_04268	127,3806	0,058541561	0,157061241	0,372730796	0,709348814	0,850052867	protein_codin	hypothetical protein
TcG_04269	226,6368133	0,144207295	0,122213003	1,17996688	0,238013388	0,465893413	protein_codin	hypothetical protein
TcG_04270	176,8046305	0,232609969	0,137916658	1,686598066	0,091680664	0,252967891	protein_codin	solute carrier family 25 (mitochondrial adenine nucleotide translocator), member 4/5/6/31
TcG_04271	174,5594795	0,418995682	0,137733884	3,042066843	0,002349597	0,015654073	protein_codin	hypothetical protein
TcG_04272	288,1367512	0,036165551	0,107591471	0,336137713	0,73676702	0,86538754	protein_codin	hypothetical protein
TcG_04273	626,1460927	-0,170582237	0,075101723	-2,27134917	0,023125849	0,094177886	protein_codin	putative cysteine peptidase, Clan CA, family C19
TcG_04274	120,2687412	0,252649413	0,16687652	1,513990183	0,130028329	0,319853126	protein_codin	hypothetical protein
TcG_04275	470,6971271	0,028459801	0,089957859	0,316368141	0,751723096	0,87492919	protein_codin	putative Zn-finger protein

TcG_04276	244,4672831	0,130551674	0,114384643	1,141339176	0,253728805	0,484779343	protein_codin	putative AUT2/APG4/ATG4 cysteine peptidase, putative,cysteine peptidase, Clan CA, family C54
TcG_04277	163,9600819	0,073928123	0,148450644	0,497997994	0,618485456	0,793033999	protein_codin	hypothetical protein
TcG_04278	211,1352631	0,077457586	0,135094678	0,573357788	0,566402465	0,758388878	protein_codin	hypothetical protein
TcG_04279	64,17674149	0,094066002	0,221246403	0,425163984	0,670717137	0,82731063	protein_codin	hypothetical protein
TcG_04280	68,58532561	0,063445472	0,208586422	0,304168756	0,760999327	0,879398329	protein_codin	hypothetical protein
TcG_04281	630,2840467	0,000975365	0,075891246	0,012852145	0,989745754	0,995761923	protein_codin	hypothetical protein
TcG_04282	233,7342629	-0,146583739	0,116669384	-1,25640279	0,208969972	0,431564429	protein_codin	hypothetical protein
TcG_04283	478,4494524	0,027915403	0,086161128	0,323990693	0,745945081	0,871496958	protein_codin	hypothetical protein
TcG_04284	314,5673145	-0,044233222	0,102722657	-0,43060824	0,666753249	0,825438095	protein_codin	putative KU80 protein
TcG_04285	75,22721547	-0,155293879	0,199862908	-0,777002	0,437157589	0,660611429	protein_codin	KU80 protein
TcG_04286	420,4751496	-0,221877504	0,088269168	-2,5136467	0,011949007	0,056768347	protein_codin	putative kinesin
TcG_04287	578,6959657	-0,023506703	0,07959834	-0,29531649	0,767752119	0,883509739	protein_codin	putative endosomal integral membrane protein
TcG_04288	207,0519043	-0,220964784	0,130584679	-1,69211875	0,090623334	0,25112699	protein_codin	mitogen-activated protein kinase, putative,protein kinase
TcG_04289	179,8693245	-0,093146879	0,13603257	-0,68473954	0,493508296	0,703987578	protein_codin	hypothetical protein
TcG_04290	326,5290523	-0,300663676	0,104537549	-2,87613092	0,004025827	0,024180006	protein_codin	putative phosphatase 2C
TcG_04291	107,362373	0,141022508	0,169501519	0,831983738	0,405418124	0,634324697	protein_codin	putative rab6 GTPase activating protein
TcG_04292	100,5001714	0,042459491	0,190601895	0,222765314	0,823718165	0,913088276	protein_codin	rab6 GTPase activating protein
TcG_04293	317,3496578	-0,085320171	0,101188933	-0,8431769	0,399129515	0,62898729	protein_codin	hypothetical protein
TcG_04294	133,335917	-0,090383488	0,160050141	-0,56471983	0,572264347	0,763502386	protein_codin	PAP2 family protein
TcG_04295	256,7871835	0,080078432	0,117045837	0,684163012	0,493872236	0,704246612	protein_codin	WD-40 repeat protein
TcG_04296	127,0012827	0,214125029	0,162080539	1,321102651	0,186467139	0,400387872	protein_codin	putative diacylglycerol kinase
TcG_04297	0					1	protein_codin	hypothetical protein
TcG_04298	758,0477136	0,05199701	0,068938944	0,754247273	0,450700756	0,671443868	protein_codin	quiescin sulfhydryl oxidase
TcG_04299	48,6899285	0,111032506	0,269740578	0,411627005	0,680612835	0,833394663	protein_codin	dispersed gene family protein 1 (DGF-1)
TcG_04300	16,64561935	-0,362540707	0,43878609	-0,82623564	0,408670433	1	protein_codin	hypothetical protein
TcG_04301	28,02354903	-0,236569487	0,333284159	-0,70981317	0,477819999	0,692608846	protein_codin	trans-sialidase
TcG_04302	0,145251675	-1,420530545	4,080472857	-0,3481289	0,727743378	1	protein_codin	trans-sialidase
TcG_04303	2,81099027	1,598708411	1,120807323	1,426390048	0,153755785	1	protein_codin	hypothetical protein
TcG_04304	76,77093871	0,417953095	0,21132976	1,97772947	0,047959234	0,160640556	protein_codin	target of rapamycin (TOR) kinase 1
TcG_04305	11,60028412	0,513044201	0,512825601	1,000426265	0,317104264	1	protein_codin	hypothetical protein
TcG_04306	143,8156401	0,650740098	0,162648187	4,000905937	6,31004E-05	0,000751369	protein_codin	helicase-like protein
TcG_04307	0,116927736	0,503022807	4,080472857	0,123275616	0,901888849	1		
TcG_04308	0					1		
TcG_04309	240,348523	-0,213378092	0,113737763	-1,87605317	0,060647979	0,191254077	protein_codin	hypothetical protein
TcG_04310	109,2076823	-0,268210998	0,176117815	-1,52290669	0,127782053	0,316545406	protein_codin	hypothetical protein
TcG_04311	56,64482672	-0,472363921	0,23505587	-2,00958147	0,044475504	0,152318413	protein_codin	hypothetical protein
TcG_04312	32,86692864	-0,011031984	0,30416051	-0,03627027	0,971066856	0,987626781	protein_codin	hypothetical protein
TcG_04313	356,6489166	-0,356627779	0,10986308	-3,24611125	0,001169931	0,008876766	protein_codin	protein phosphatase 2 (formerly 2A), regulatory subunit B
TcG_04314	411,8901647	0,127225751	0,089648283	1,419165502	0,155850771	0,357420236	protein_codin	hypothetical protein
TcG_04315	192,955108	0,120972223	0,127995844	0,945126183	0,34459446	0,578779679	protein_codin	hypothetical protein
TcG_04316	11,35533753	0,055045444	0,505435229	0,108907019	0,91327624	1		
TcG_04317	589,044143	-0,2910739	0,078758783	-3,69576431	0,000219226	0,002150682	protein_codin	hypothetical protein
TcG_04318	711,7545696	0,00598445	0,073007573	0,081970267	0,934670358	0,969132877		
TcG_04319	220,2624261	-0,074837155	0,122826411	-0,60929204	0,542330882	0,740321408	protein_codin	putative P-type ATPase
TcG_04320	394,8951421	-0,046455731	0,093197647	-0,49846463	0,618156592	0,793033999	protein_codin	hypothetical protein
TcG_04321	282,7513448	0,022156205	0,107193937	0,206692704	0,836249856	0,920523248	protein_codin	hypothetical protein
TcG_04322	111,5837738	0,264860267	0,172813219	1,53263893	0,125364844	0,312293503	protein_codin	hypothetical protein
TcG_04323	375,6712874	0,143209921	0,097917573	1,46255587	0,143588946	0,340008225	protein_codin	hypothetical protein
TcG_04324	156,2274646	0,473871627	0,147844936	3,205193496	0,001349717	0,009947724	protein_codin	helicase-like protein
TcG_04325	4,294612603	1,634505107	0,92243172	1,771952407	0,076402458	1	protein_codin	putative GAG protein
TcG_04326	29,28584483	0,321552353	0,352664319	0,911780229	0,36188442	0,594553728	protein_codin	hypothetical protein
TcG_04327	53,90186797	0,342128427	0,243059237	1,407592782	0,159251686	0,361853311	protein_codin	trans-sialidase
TcG_04328	64,49136684	0,091145155	0,217980879	0,418133716	0,675849355	0,83017833	protein_codin	rab1 small GTP-binding protein
TcG_04329	37,78378621	0,253281299	0,311972389	0,811870885	0,416865723	0,643630898	protein_codin	rab1 small GTP-binding protein
TcG_04330	70,10127661	0,418314314	0,217585311	1,922530124	0,054539087	0,175964874	protein_codin	target of rapamycin (TOR) kinase 1
TcG_04331	22,57149687	0,468169241	0,365169471	1,282060189	0,199821533	0,418649599	protein_codin	target of rapamycin (TOR) kinase 1
TcG_04332	7,812869155	0,33297405	0,65787331	0,506137041	0,612760449	1	protein_codin	RNA editing complex protein MP90

TcG_04333	299,2753288	0,12612149	0,105520499	1,195232125	0,23199635	0,459225958	protein_codin hypothetical protein
TcG_04334	119,9975794	-0,052976371	0,169721762	-0,31213658	0,754936727	0,876363937	protein_codin hypothetical protein
TcG_04335	697,2211921	0,043777865	0,074286055	0,589314708	0,55565018	0,750496967	protein_codin hypothetical protein
TcG_04336	299,8849346	0,238673501	0,111105451	2,148170933	0,031700179	0,119052925	protein_codin putative protein kinase
TcG_04337	1008,539047	0,210346903	0,076422155	2,752433556	0,005915415	0,03266149	protein_codin hypothetical protein
TcG_04338	203,1691362	0,198083724	0,12475681	1,587758815	0,112340888	0,29018174	protein_codin putative chaperone protein DNAj
TcG_04339	416,353659	0,125917655	0,089473882	1,407311858	0,159334935	0,361896891	protein_codin putative NADH-dependent fumarate reductase
TcG_04340	175,8557035	0,322894362	0,136510866	2,365338171	0,018013614	0,077552914	protein_codin fructosamine kinase
TcG_04341	707,6775244	-0,117085376	0,073210841	-1,59929013	0,109756152	0,286326125	protein_codin hypothetical protein
TcG_04342	381,6330287	0,12839818	0,093280708	1,376470897	0,168675892	0,375101512	protein_codin hypothetical protein
TcG_04343	331,9535659	-0,143872783	0,100332111	-1,43396548	0,151582115	0,351799471	protein_codin hypothetical protein
TcG_04344	624,8995201	-0,028784837	0,091172244	-0,3157193	0,752215576	0,87492919	protein_codin methyltransferase
TcG_04345	730,9168915	-0,029287976	0,073328009	-0,39941048	0,68959077	0,838680499	protein_codin hypothetical protein
TcG_04346	66,82273441	0,446835556	0,222429323	2,008887815	0,044549032	0,152480086	protein_codin hypothetical protein
TcG_04347	0					1	protein_codin hypothetical protein
TcG_04348	457,6861757	0,529394343	0,086377911	6,128816218	8,85353E-10	3,56173E-08	protein_codin lysine-specific demethylase NO66
TcG_04349	1094,154184	0,301905594	0,066487223	4,54080618	5,60395E-06	9,26211E-05	protein_codin putative ATP-dependent RNA helicase
TcG_04350	333,2857051	0,403786342	0,103308627	3,908544262	9,28539E-05	0,001047523	protein_codin putative chaperone DNAJ protein
TcG_04351	325,1182973	0,495548053	0,104225535	4,75457434	1,98865E-06	3,72221E-05	protein_codin hypothetical protein
TcG_04352	128,1247671	0,201361285	0,156519535	1,28649299	0,198271064	0,416908991	protein_codin mitotic centromere-associated kinesin (MCAK)
TcG_04353	439,724992	0,195025506	0,099418181	1,96166841	0,049801102	0,164997303	protein_codin hypothetical protein
TcG_04354	589,0947754	0,505600598	0,078732298	6,421768622	1,347E-10	6,47566E-09	protein_codin hypothetical protein
TcG_04355	79,3079501	0,552148496	0,198379502	2,78329409	0,005381001	0,030352616	protein_codin hypothetical protein
TcG_04356	96,87321536	0,217136443	0,193959604	1,11949312	0,262929824	0,495092629	protein_codin putative amino acid permease
TcG_04357	102,5117648	0,308348801	0,177261579	1,739512885	0,081944588	0,234961703	protein_codin amino acid permease
TcG_04358	511,8537085	0,323890711	0,085239434	3,799775478	0,000144827	0,001528204	protein_codin putative amino acid permease
TcG_04359	166,7552344	0,36972493	0,146164083	2,529519714	0,011421876	0,054934774	protein_codin amino acid permease
TcG_04360	272,3087293	0,57154278	0,110610649	5,167158704	2,37679E-07	5,65453E-06	protein_codin hypothetical protein
TcG_04361	1346,742628	0,318556015	0,055707604	5,718357838	1,07558E-08	3,39556E-07	protein_codin hypothetical protein
TcG_04362	398,3662914	0,285018648	0,09576989	2,976077853	0,002919607	0,018637229	protein_codin hypothetical protein
TcG_04363	778,5246149	0,510337865	0,070870998	7,200940903	5,97985E-13	4,64983E-11	protein_codin calpain-like protein
TcG_04364	690,3313269	0,47664058	0,081731433	5,831790314	5,48358E-09	1,85227E-07	protein_codin putative calpain-like cysteine peptidase
TcG_04365	1520,718381	-0,147809546	0,056876022	-2,59880249	0,009354958	0,046819242	protein_codin cytoskeleton-associated protein CAP5.5
TcG_04366	646,9888358	0,519602008	0,079467683	6,538532282	6,21255E-11	3,24228E-09	protein_codin calpain-like cysteine peptidase
TcG_04367	145,6391491	0,064940829	0,149770779	0,43360146	0,664577875	0,824742851	protein_codin hypothetical protein
TcG_04368	108,5457536	0,242813088	0,178625842	1,359339085	0,174039164	0,382593335	protein_codin hypothetical protein
TcG_04369	323,0188689	0,443074238	0,102010971	4,343397893	1,40296E-05	0,000203692	protein_codin hypothetical protein
TcG_04370	425,2646467	0,1229182	0,089069297	1,380028853	0,167577761	0,373644017	protein_codin chaperone protein DnaJ
TcG_04371	88,89888062	0,2195367	0,187138827	1,173122132	0,240746832	0,469419859	protein_codin putative UDP-Gal or UDP-GlcNAc-dependent glycosyltransferase
TcG_04372	573,9402092	0,413500933	0,079643577	5,191893034	2,08167E-07	5,05622E-06	protein_codin tRNA guanosine-2-O-methyltransferase TRM13
TcG_04373	208,3240562	-0,234555527	0,126546345	-1,85351483	0,063808603	0,198495368	protein_codin hypothetical protein
TcG_04374	639,3270469	0,402738178	0,075935744	5,303670686	1,13497E-07	2,90282E-06	protein_codin glycosomal ABC transporter member 1
TcG_04375	603,3417072	0,073963101	0,076526897	0,96649811	0,333794992	0,568226385	protein_codin 3',5'-cyclic-nucleotide phosphodiesterase
TcG_04376	551,0161531	0,402797813	0,082365665	4,890360741	1,00651E-06	2,03872E-05	protein_codin putative mevalonate kinase
TcG_04377	396,3915254	0,129172143	0,092926065	1,390052876	0,164512821	0,369830907	protein_codin putative UDP-Gal or UDP-GlcNAc-dependent glycosyltransferase
TcG_04378	867,3914869	0,539352845	0,069309374	7,781816769	7,14904E-15	7,59897E-13	protein_codin putative C-5 sterol desaturase
TcG_04379	25,86208702	0,53860847	0,353756918	1,522538338	0,127874247	0,316681311	protein_codin putative C-5 sterol desaturase
TcG_04380	169,8969819	0,135126984	0,136260265	0,991682966	0,321352203	0,556054685	protein_codin hypothetical protein
TcG_04381	340,8724082	0,308803729	0,110125544	2,804106282	0,005045627	0,028941577	protein_codin hypothetical protein
TcG_04382	214,7056816	0,454788658	0,124266563	3,659783034	0,000252429	0,002421061	protein_codin hypothetical protein
TcG_04383	0,625814844	-0,250879225	2,223658541	-0,11282273	0,910171098	1	protein_codin receptor-type adenylate cyclase
TcG_04384	0					1	protein_codin receptor-type adenylate cyclase
TcG_04385	148,8962489	0,275826236	0,151601663	1,819414323	0,068848243	0,20969394	protein_codin hypothetical protein
TcG_04386	63,40673757	0,905257598	0,229599828	3,942762519	8,05484E-05	0,00092491	protein_codin receptor-type adenylate cyclase
TcG_04387	201,8449424	0,563179325	0,127093541	4,431219084	9,37018E-06	0,000144366	protein_codin esag4
TcG_04388	55,27215683	0,351552095	0,246841574	1,424201314	0,154388205	0,355561453	protein_codin hypothetical protein
TcG_04389	52,85609422	0,331672498	0,242834166	1,365839507	0,171989343	0,379555911	protein_codin mucin-associated surface protein (MASP)

TcG_04390	48,42533324	0,010935268	0,248436302	0,044016384	0,964891344	0,984347197	protein_codin putative retrotransposon hot spot (RHS) protein
TcG_04391	106,964457	-0,158047909	0,167064198	-0,94603099	0,344132779	0,578346734	protein_codin putative trans-sialidase
TcG_04392	81,55103313	0,051392437	0,192982414	0,266306323	0,790003304	0,895024708	protein_codin putative trans-sialidase
TcG_04393	74,72241902	0,157430274	0,212322644	0,741467187	0,45841022	0,677438813	protein_codin putative trans-sialidase
TcG_04394	87,94867537	-0,070343586	0,191569999	-0,36719521	0,713473413	0,852784519	protein_codin hypothetical protein
TcG_04395	76,54650593	0,01893601	0,204582733	0,092559181	0,926253773	0,964289354	
TcG_04396	249,3957399	-0,006875892	0,113951464	-0,06034053	0,951884422	0,977350584	protein_codin hypothetical protein
TcG_04397	600,4263974	0,035009002	0,077477184	0,451862089	0,651368337	0,815336383	protein_codin hypothetical protein
TcG_04398	190,5494755	-0,319964668	0,130521046	-2,45144119	0,014228543	0,06531375	protein_codin nucleoside-diphosphate kinase
TcG_04399	623,3284982	0,02003234	0,076772138	0,260932423	0,794144621	0,897216926	protein_codin putative subtilisin-like serine peptidase
TcG_04400	224,2092172	-0,200161828	0,120513438	-1,66090879	0,096731767	0,263083159	protein_codin hypothetical protein
TcG_04401	103,1689855	-0,306539804	0,170665975	-1,79613894	0,072472423	0,216396122	protein_codin hypothetical protein
TcG_04402	389,6717475	-0,239669208	0,092282535	-2,59712423	0,009400792	0,046987737	protein_codin hypothetical protein
TcG_04403	531,9785608	-0,20413933	0,084635476	-2,411983	0,015866021	0,070619947	protein_codin hypothetical protein
TcG_04404	1243,507632	-0,072242842	0,056741007	-1,27320338	0,202945881	0,423006561	protein_codin putative AMP deaminase
TcG_04405	436,1600094	-0,158517007	0,087559232	-1,81039742	0,07023418	0,212315938	protein_codin hypothetical protein
TcG_04406	280,8515614	-0,156452565	0,108492648	-1,44205683	0,149286342	0,348505251	protein_codin putative katanin
TcG_04407	301,4238522	-0,197618536	0,102630844	-1,92552773	0,05416337	0,175093976	protein_codin actin-like protein
TcG_04408	408,2642925	0,151987203	0,090945875	1,671183026	0,094685532	0,259038152	protein_codin putative N(2), N(2)-dimethylguanosine tRNA methyltransferase
TcG_04409	192,0063511	-0,219479435	0,128214889	-1,71180926	0,086931825	0,244345494	protein_codin glycosyl transferase-like protein
TcG_04410	648,7778686	-0,186426467	0,0742436	-2,51101062	0,012038607	0,05716365	protein_codin hypothetical protein
TcG_04411	529,4755087	-0,355335313	0,080518028	-4,41311493	1,01894E-05	0,000154521	protein_codin hypothetical protein
TcG_04412	473,7181555	-0,074606913	0,090580191	-0,82365595	0,410135077	0,636973861	protein_codin hypothetical protein
TcG_04413	210,5474686	-0,002970291	0,12452385	-0,02385319	0,980969715	0,992708107	protein_codin hypothetical protein
TcG_04414	178,6775308	-0,287448195	0,132014942	-2,17739137	0,029451379	0,112801216	protein_codin hypothetical protein
TcG_04415	841,4439335	-0,089396263	0,066487928	-1,34454878	0,178770993	0,389799325	protein_codin hypothetical protein
TcG_04416	1297,869733	0,032037762	0,067828925	0,472331848	0,636689941	0,806608902	protein_codin hypothetical protein
TcG_04417	942,5146709	-0,256351269	0,066492772	-3,85532533	0,000115576	0,001265654	protein_codin putative mitochondrial processing peptidase alpha subunit
TcG_04418	187,4725264	-0,211273356	0,131121377	-1,61128079	0,107118536	0,282082846	protein_codin hypothetical protein
TcG_04419	302,9758049	-0,323550208	0,103202164	-3,13511069	0,001717893	0,012158525	protein_codin hypothetical protein
TcG_04420	43,74877039	0,096657555	0,270451791	0,357392918	0,720797678	0,85670516	protein_codin hypothetical protein
TcG_04421	152,9315744	-0,161806538	0,14398005	-1,12381221	0,261092699	0,493587786	protein_codin hypothetical protein
TcG_04422	569,4908677	-0,001085806	0,080136592	-0,01354944	0,989189439	0,995541618	protein_codin hypothetical protein
TcG_04423	150,9888553	-0,064269942	0,148143634	-0,43383533	0,664408024	0,824620392	protein_codin hypothetical protein
TcG_04424	1114,953355	-0,258282431	0,067592191	-3,82118744	0,000132811	0,001411692	protein_codin hypothetical protein
TcG_04425	337,3114975	-0,353916532	0,100828622	-3,51008003	0,000447972	0,003940928	protein_codin hypothetical protein
TcG_04426	111,4374062	-0,320623946	0,168017175	-1,90828079	0,056354935	0,179969205	
TcG_04427	332,3078465	-0,187543881	0,099927486	-1,87679976	0,060545544	0,19098303	protein_codin cytoplasmic tRNA 2-thiolation protein 2
TcG_04428	414,3290188	-0,11257518	0,090821246	-1,23952472	0,215151241	0,438870501	protein_codin putative protein kinase
TcG_04429	445,057174	-0,195635486	0,08892536	-2,19999655	0,02780714	0,107966999	protein_codin protein-tyrosine phosphatase
TcG_04430	811,7233353	-0,131959693	0,06775648	-1,94755825	0,051467843	0,168686401	protein_codin putative C2 domain protein
TcG_04431	344,7334555	-0,132447291	0,099354504	-1,33307788	0,182506249	0,394573129	protein_codin hypothetical protein
TcG_04432	166,5074382	-0,163360579	0,136046517	-1,20077002	0,229840424	0,456889557	protein_codin hypothetical protein
TcG_04433	904,0005261	-0,187386332	0,064294563	-2,91449731	0,003562619	0,021874139	protein_codin putative ubiquitin-like protein
TcG_04434	26,44397711	0,041566653	0,361635867	0,114940626	0,908492183	0,955500221	protein_codin trans-sialidase
TcG_04435	9,685436226	-0,445513255	0,580843501	-0,76701083	0,443075077	1	protein_codin trans-sialidase
TcG_04436	11,54986628	0,542135065	0,533383567	1,016407513	0,30943537	1	protein_codin hypothetical protein
TcG_04437	15,47898091	0,830605137	0,475265439	1,747665767	0,08052192	1	protein_codin hypothetical protein
TcG_04438	244,4139133	0,189610846	0,116203471	1,63171413	0,102739722	0,274082988	protein_codin putative protein kinase
TcG_04439	795,4992818	0,164439332	0,071451802	2,30140215	0,021368911	0,088579677	protein_codin WD repeat-containing protein 96
TcG_04440	173,6018088	0,235276956	0,138701202	1,696286355	0,089831663	0,249753993	protein_codin dynein light chain 2B, cytoplasmic
TcG_04441	354,5490494	0,268333081	0,101837613	2,63491134	0,008415926	0,043182871	protein_codin hypothetical protein
TcG_04442	393,9178554	0,171456322	0,096160799	1,78301682	0,074583565	0,220451886	protein_codin methylcrotonoyl-CoA carboxylase biotinylated subunit protein
TcG_04443	69,53006711	0,663595752	0,214481207	3,093957563	0,001975057	0,013604646	protein_codin phosphatidylethanolamine N-methyltransferase
TcG_04444	321,862846	0,077641015	0,100929823	0,769257414	0,441740513	0,664244721	protein_codin 200 kDa antigen p200
TcG_04445	236,2204558	0,031661767	0,124375343	0,254566265	0,799058118	0,89978495	protein_codin 200 kDa antigen p200
TcG_04446	194,7553602	0,330405707	0,137620645	2,400844048	0,016357306	0,072251524	protein_codin hypothetical protein

TcG_04447	575,5764109	0,318872164	0,079394436	4,01630365	5,91181E-05	0,000712739	protein_codin putative HEAT repeat-containing protein 7A
TcG_04448	240,2543286	0,397371776	0,124966701	3,179821282	0,001473659	0,010724758	protein_codin putative HEAT repeat-containing protein 7A
TcG_04449	225,1699776	0,461164218	0,118705384	3,884947767	0,000102352	0,001136955	protein_codin hypothetical protein
TcG_04450	202,8547135	0,353619879	0,132552402	2,667774214	0,007635555	0,039939297	protein_codin chaperone DNAJ protein
TcG_04451	1427,206239	0,337607504	0,054976507	6,140941291	8,20339E-10	3,37037E-08	protein_codin phosphoglycan beta 1,3 galactosyltransferase 5
TcG_04452	245,7376396	0,416930878	0,116099545	3,591149971	0,000329222	0,003022478	protein_codin hypothetical protein
TcG_04453	286,0237498	0,623439823	0,110756001	5,628948495	1,81312E-08	5,47051E-07	protein_codin hypothetical protein
TcG_04454	225,6513545	0,344701214	0,121797896	2,830108123	0,004653228	0,027173536	protein_codin putative mu-adaptin 4, putative,adaptor complex AP-4 medium subunit
TcG_04455	785,6638088	0,377258654	0,067910965	5,555195015	2,77302E-08	8,09275E-07	protein_codin hypothetical protein
TcG_04456	414,9137434	0,473833581	0,099748931	4,750262268	2,03153E-06	3,77806E-05	protein_codin hypothetical protein
TcG_04457	978,2717725	0,550450754	0,064806712	8,4937306	2,00108E-17	2,86228E-15	protein_codin hypothetical protein
TcG_04458	79,67600358	0,636108283	0,198349823	3,207002028	0,001341261	0,00989799	protein_codin hypothetical protein
TcG_04459	492,0574858	0,432070263	0,093670148	4,612678319	3,97513E-06	6,85355E-05	protein_codin hypothetical protein
TcG_04460	141,3440256	0,353102687	0,150367728	2,348261109	0,018861294	0,080163959	protein_codin hypothetical protein
TcG_04461	576,2890041	0,506967895	0,081659929	6,208282353	5,35668E-10	2,29013E-08	protein_codin hypothetical protein
TcG_04462	1588,725239	0,526127667	0,055227145	9,52661357	1,62498E-21	3,48648E-19	protein_codin acetyl-CoA carboxylase
TcG_04463	734,1447614	-0,09117669	0,073138821	-1,2466251	0,212534995	0,435751275	protein_codin putative serine/threonine-protein kinase a, putative,protein kinase
TcG_04464	2951,118003	0,190347903	0,045269696	4,204753321	2,61367E-05	0,000349677	protein_codin squalene synthase
TcG_04465	138,056029	1,104375918	0,163673159	6,747446697	1,50469E-11	9,17547E-10	protein_codin hypothetical protein
TcG_04466	327,5695447	0,378171495	0,100797167	3,751806778	0,000175565	0,001792153	protein_codin putative inositol polyphosphate 1-phosphatase
TcG_04467	285,1257826	0,093131233	0,111496805	0,835281631	0,403559158	0,63312391	protein_codin hypothetical protein
TcG_04468	333,9777974	0,034572611	0,099307698	0,348136262	0,727737853	0,859799184	protein_codin hypothetical protein
TcG_04469	359,4975527	0,559306678	0,100141851	5,585144182	2,33506E-08	6,90154E-07	protein_codin nucleoporin (NUP54/57)
TcG_04470	838,8940146	0,316137893	0,069436315	4,552918674	5,29067E-06	8,81982E-05	protein_codin hypothetical protein
TcG_04471	160,0249341	0,459139079	0,147255352	3,117978901	0,001820958	0,012755516	protein_codin hypothetical protein
TcG_04472	436,7351785	0,378375334	0,092808279	4,076956713	4,5629E-05	0,00057214	protein_codin putative protein kinase
TcG_04473	270,5868287	0,375616027	0,111489034	3,369084962	0,000754182	0,006149156	protein_codin ribosomal protein L7/L12
TcG_04474	320,0143381	0,344295053	0,100126487	3,438601157	0,000584728	0,004945007	protein_codin hypothetical protein
TcG_04475	612,4103354	0,295967458	0,078475166	3,771479225	0,000162283	0,001680257	protein_codin hypothetical protein
TcG_04476	260,330528	0,207752552	0,113653476	1,827947185	0,067557479	0,20694516	protein_codin hypothetical protein
TcG_04477	228,0162831	0,271569573	0,117931576	2,302772365	0,021291654	0,088354264	protein_codin hypothetical protein
TcG_04478	590,7286014	0,254524284	0,078490853	3,242725426	0,001183922	0,008941933	protein_codin Smr domain containing protein
TcG_04479	460,780625	0,289732305	0,09026492	3,209799619	0,001328275	0,009833481	protein_codin hypothetical protein
TcG_04480	514,1325615	0,260723531	0,08613004	3,027091718	0,00246919	0,016282321	protein_codin kinetoplast-associated protein
TcG_04481	623,2548005	0,201849595	0,088729613	2,274884204	0,022912877	0,093639716	protein_codin kinetoplast DNA-associated protein
TcG_04482	515,5832423	0,091107699	0,082954712	1,098282384	0,272081202	0,505040461	protein_codin hypothetical protein
TcG_04483	464,6810569	0,297596702	0,094865423	3,137040803	0,001706624	0,012108354	protein_codin ERGIC and golgi family 3
TcG_04484	283,0579316	0,570260922	0,10933169	5,215879507	1,82947E-07	4,50984E-06	protein_codin transmembrane emp24 domain trafficking protein 2
TcG_04485	251,0952833	0,052595862	0,112047353	0,469407448	0,638778426	0,807731988	protein_codin hypothetical protein
TcG_04486	82,72721949	0,63919809	0,204844306	3,12040937	0,001805999	0,012666041	protein_codin hypothetical protein
TcG_04487	78,83703108	0,108936741	0,198218743	0,549578409	0,582608571	0,771440332	protein_codin hypothetical protein
TcG_04488	454,1244931	0,046996615	0,086919405	0,540691867	0,588719985	0,775648564	protein_codin putative inositol 5'-phosphatase
TcG_04489	492,7301966	-0,068076203	0,084611908	-0,80457	0,421067858	0,647488005	protein_codin glutathione-S-transferase/glutaredoxin
TcG_04490	434,1372059	0,087482487	0,091848942	0,952460468	0,340863506	0,575104788	protein_codin hypothetical protein
TcG_04491	355,0230726	0,000513113	0,095704274	0,005361445	0,995722207	0,998307155	protein_codin hypothetical protein
TcG_04492	433,3765074	0,102016582	0,097219585	1,049341886	0,294020794	0,527081065	protein_codin putative mitochondrial pyruvate carrier protein
TcG_04493	417,5388578	-0,004369114	0,091762477	-0,0476133	0,962024435	0,98254717	protein_codin HEAT repeat-containing protein
TcG_04494	235,6554597	0,253872024	0,116578158	2,177698015	0,029428527	0,112750963	protein_codin hypothetical protein
TcG_04495	633,8651297	-0,055765568	0,079705309	-0,69964685	0,484147879	0,697418541	protein_codin hypothetical protein
TcG_04496	906,3744741	-0,4451154	0,063627458	-6,99564954	2,64033E-12	1,85399E-10	protein_codin hypothetical protein
TcG_04497	2220,937483	-0,336097467	0,050881141	-6,60554112	3,96068E-11	2,16455E-09	protein_codin hypothetical protein
TcG_04498	1536,080763	-0,223986118	0,058532515	-3,82669558	0,000129875	0,001388129	protein_codin flagellar associated protein
TcG_04499	488,0706938	0,188181356	0,085272483	2,206823926	0,027326363	0,106744181	protein_codin hypothetical protein
TcG_04500	106,7401487	-0,425714568	0,199565552	-2,13320668	0,032907775	0,122555283	protein_codin putative serine/threonine protein kinase, putative,protein kinase
TcG_04501	396,1583831	-0,15990074	0,09039353	-1,7689401	0,076903872	0,224649463	protein_codin hypothetical protein
TcG_04502	258,0370089	0,562996336	0,114703578	4,908271786	9,18825E-07	1,88083E-05	protein_codin COP-coated vesicle membrane protein p24 precursor
TcG_04503	365,3643715	-0,128657317	0,097324039	-1,32194799	0,186185472	0,400064726	protein_codin putative pyrroline-5-carboxylate reductase

TcG_04504	859,3409165	-0,17780482	0,065764508	-2,70365925	0,006858058	0,036768837	protein_codin putative tyrosyl-tRNA synthetase
TcG_04505	205,4724061	-0,018791266	0,128866196	-0,14581998	0,884063505	0,943488844	protein_codin hypothetical protein
TcG_04506	762,9178261	-0,249676018	0,073659341	-3,3896043	0,000699936	0,005751387	protein_codin putative DNA-J protein
TcG_04507	602,5400808	0,099464306	0,076312534	1,303380991	0,192444723	0,408835223	protein_codin serine hydroxymethyltransferase (SHMT-S)
TcG_04508	206,8567776	0,009452004	0,131953379	0,071631389	0,942895259	0,973191135	protein_codin hypothetical protein
TcG_04509	13,1713961	-0,087908427	0,496513405	-0,17705147	0,859467963	1	protein_codin putative protein kinase
TcG_04510	162,4080062	0,355929682	0,142586528	2,496236411	0,012551892	0,059068325	protein_codin BRCT domain-containing protein
TcG_04511	332,6103568	0,177678367	0,1031373	1,722736257	0,084936231	0,24025175	protein_codin hypothetical protein
TcG_04512	1486,775289	0,207354274	0,060363189	3,43511132	0,00059231	0,004998186	protein_codin ubiquitin/ribosomal protein S27a
TcG_04513	677,9129315	0,010433034	0,072386882	0,144128789	0,885398772	0,94437554	protein_codin putative regulatory subunit of protein kinase a-like protein
TcG_04514	186,5536669	0,13154679	0,134023766	0,981518381	0,326337175	0,560721119	protein_codin putative ankyrin repeat protein
TcG_04515	8,369077863	-0,44689956	0,610756292	-0,73171503	0,464342519	1	
TcG_04516	16,44957223	-0,089469305	0,423645653	-0,21118901	0,832739788	1	protein_codin RNA-binding protein
TcG_04517	864,4166003	-0,281876516	0,067138431	-4,19843763	2,68763E-05	0,000358743	protein_codin hypothetical protein
TcG_04518	292,6805105	0,170956467	0,111756587	1,52972161	0,126085652	0,313684425	protein_codin hypothetical protein
TcG_04519	414,4546661	-0,003392519	0,09220332	-0,03679389	0,970649347	0,987352355	protein_codin phosphatidyl serine synthase
TcG_04520	946,9097906	0,083894042	0,069876053	1,200612199	0,229901667	0,456889557	protein_codin putative protein kinase
TcG_04521	610,640793	-0,01229748	0,078196602	-0,15726361	0,875037101	0,939877107	protein_codin putative poly(A) polymerase
TcG_04522	395,7450721	0,085408492	0,091507242	0,933352274	0,350638114	0,585036461	protein_codin putative small GTP-binding protein Rab7
TcG_04523	145,1462596	0,386468091	0,150596288	2,566252434	0,010280398	0,05049118	protein_codin hypothetical protein
TcG_04524	299,4713914	0,669937018	0,1070276	6,259479048	3,86266E-10	1,70162E-08	protein_codin hypothetical protein
TcG_04525	986,1814134	0,018042381	0,062195443	0,290091688	0,771746095	0,885818333	protein_codin hypothetical protein
TcG_04526	273,975101	0,128920113	0,120421169	1,070576823	0,284359749	0,518099081	protein_codin putative dynein intermediate chain
TcG_04527	148,8708417	-0,408325432	0,164094465	-2,48835591	0,012833523	0,06012503	protein_codin myosin heavy chain
TcG_04528	0					1	protein_codin hypothetical protein
TcG_04529	129,4547068	-0,183544036	0,154819688	-1,18553421	0,235806299	0,463445836	protein_codin putative kinesin
TcG_04530	16,90590469	-0,586116395	0,473714198	-1,23727851	0,215983705	0,439864159	protein_codin kinesin-like protein
TcG_04531	75,45575882	0,168388827	0,204382827	0,823889313	0,410002453	0,636890088	protein_codin putative receptor-type adenylate cyclase
TcG_04532	1162,37616	-0,206221859	0,058801959	-3,50705766	0,000453091	0,003979918	protein_codin putative nucleosome assembly protein-like protein
TcG_04533	451,1438828	-0,239129254	0,085310771	-2,80303709	0,005062385	0,028992975	protein_codin transcription factor IIB
TcG_04534	129,0905835	-0,043038031	0,155288205	-0,27714939	0,781665399	0,891551025	protein_codin hypothetical protein
TcG_04535	1228,141683	-0,075924477	0,063945277	-1,18733517	0,235095439	0,462964244	protein_codin hypothetical protein
TcG_04536	624,4847705	-0,154625549	0,074453402	-2,07680971	0,037819126	0,135783201	protein_codin cyclic nucleotide-binding protein
TcG_04537	1194,709435	-0,01243411	0,066529766	-0,18689544	0,851742615	0,928229751	protein_codin putative protein kinase
TcG_04538	1122,535957	0,319751274	0,069043252	4,631173459	3,63599E-06	6,34437E-05	protein_codin 60S acidic ribosomal protein P2
TcG_04539	455,4565713	-0,311418942	0,090054913	-3,45810052	0,000543998	0,004661809	protein_codin oculocerebrorenal Lowe syndrome protein
TcG_04540	170,6140043	-0,219736717	0,133768083	-1,64266925	0,100451383	0,269592246	protein_codin oculocerebrorenal Lowe syndrome protein
TcG_04541	316,8553668	-0,159265467	0,104759161	-1,5203011	0,128435318	0,317382939	protein_codin putative heat shock protein DNAJ
TcG_04542	922,0952453	-0,024032619	0,074457048	-0,32277158	0,746868239	0,87215583	protein_codin putative nucleobase transporter
TcG_04543	0					1	protein_codin putative nucleobase transporter
TcG_04544	508,3714073	0,105559859	0,082264611	1,283174609	0,19943091	0,418209675	protein_codin ufm1-conjugating enzyme 1
TcG_04545	387,2203954	0,044783621	0,097615023	0,458777961	0,646393623	0,812019442	protein_codin putative magnesium and cobalt transport protein
TcG_04546	441,9667356	-0,033277339	0,095357873	-0,34897317	0,727109452	0,859270718	protein_codin hypothetical protein
TcG_04547	688,5556451	0,011567256	0,080645593	0,1434332	0,885948064	0,944652519	protein_codin hypothetical protein
TcG_04548	1884,127152	-0,115345226	0,060480799	-1,90713791	0,056502731	0,180242466	protein_codin topoisomerase
TcG_04549	75,3720321	0,200451115	0,200472744	0,999892113	0,317362722	0,55251157	protein_codin putative ubiquitin hydrolase, putative,cysteine peptidase, Clan CA, family C19
TcG_04550	638,8582227	-0,060785507	0,080144475	-0,75844912	0,44818216	0,66910023	protein_codin putative ubiquitin hydrolase
TcG_04551	802,1175016	-0,071924659	0,068109669	-1,05601246	0,290962524	0,523651784	protein_codin putative cation transporter
TcG_04552	564,8706424	-0,0677453	0,081261866	-0,83366656	0,40446891	0,634038261	protein_codin putative eukaryotic initiation factor 4a
TcG_04553	180,9877816	-0,132068235	0,132382415	-0,99762672	0,318460398	0,553576194	protein_codin putative actin-like protein
TcG_04554	287,5623721	-0,000824086	0,109663224	-0,00751469	0,994004198	0,997677114	protein_codin hypothetical protein
TcG_04555	77,82236704	0,729907418	0,203872991	3,580206552	0,000343323	0,003141972	protein_codin hypothetical protein
TcG_04556	124,2200805	-0,175749618	0,160040429	-1,09815763	0,272135663	0,505040461	protein_codin hypothetical protein
TcG_04557	594,9250255	-0,039496324	0,077870035	-0,50720825	0,612008709	0,790406076	protein_codin TBC1 domain family, member 19
TcG_04558	125,2161454	0,215628344	0,15908006	1,355470601	0,175267669	0,384520207	protein_codin hypothetical protein
TcG_04559	83,85973919	0,146274695	0,201911105	0,724450967	0,46878892	0,685348697	protein_codin hypothetical protein
TcG_04560	292,3316244	-0,216009376	0,106376105	-2,03061933	0,042293625	0,146945505	protein_codin putative actin-related protein 3

TcG_04561	397,0139652	0,148108435	0,094524737	1,566874869	0,117143943	0,298554712	protein_codin hypothetical protein
TcG_04562	1079,039334	0,010932859	0,060803918	0,179805175	0,857305521	0,931078155	protein_codin putative nucleolar RNA binding protein
TcG_04563	489,5629803	-0,191085986	0,088786501	-2,15219637	0,031381891	0,118163988	protein_codin pleckstrin domain-containing protein
TcG_04564	270,0704691	-0,053514714	0,128362998	-0,41690141	0,676750524	0,830633848	protein_codin TMEM164 family protein
TcG_04565	289,3281296	0,043620438	0,105505931	0,413440629	0,679283813	0,832559214	protein_codin hypothetical protein
TcG_04566	533,121126	-0,143311356	0,079440975	-1,80399794	0,07123163	0,214082923	protein_codin E3 ubiquitin-protein ligase synoviolin
TcG_04567	638,3078548	0,059038977	0,076923134	0,767506138	0,442780645	0,664995914	protein_codin putative mismatch repair protein MSH3
TcG_04568	559,684991	-0,115905306	0,078869545	-1,46958253	0,141674855	0,337190811	protein_codin putative glutaminyl-tRNA synthetase
TcG_04569	578,5792235	0,077114203	0,083960882	0,918453941	0,358381262	0,59224152	protein_codin proliferative cell nuclear antigen (PCNA)
TcG_04570	425,6140184	0,195803193	0,089127253	2,196894739	0,028027965	0,108678714	protein_codin putative phosphomevalonate kinase protein
TcG_04571	119,2485776	-0,109354898	0,172774406	-0,63293459	0,526776358	0,729266446	protein_codin hypothetical protein
TcG_04572	1033,094249	-0,051265238	0,060373015	-0,84914159	0,395802513	0,625531021	protein_codin ribosomal protein S6
TcG_04573	336,4289367	-0,33054893	0,104102801	-3,1752165	0,001497247	0,010848724	protein_codin cyclic nucleotide phosphodiesterase
TcG_04574	188,3970659	0,043987775	0,137192823	0,320627375	0,748492789	0,873112027	protein_codin putative dolichyl-P-Man:GDP-Man7GlcNAc2-PP-dolichyl alpha-1,6-mannosyltransferase
TcG_04575	1004,155805	-0,177690768	0,065449659	-2,71492274	0,006629123	0,035773183	protein_codin putative RNA-binding protein
TcG_04576	379,7060868	-0,269869604	0,101065096	-2,67025525	0,007579361	0,039779492	protein_codin hypothetical protein
TcG_04577	242,3561687	0,359544725	0,118185003	3,042219529	0,002348406	0,015654073	protein_codin hypothetical protein
TcG_04578	299,7739433	0,119711641	0,104467557	1,145921695	0,251827554	0,481861939	protein_codin hypothetical protein
TcG_04579	337,5485436	-0,069536496	0,104772978	-0,66368731	0,506890465	0,713675165	protein_codin ICAM-like surface protein
TcG_04580	354,8841232	-0,077165928	0,096385611	-0,80059593	0,423365607	0,649339942	protein_codin hypothetical protein
TcG_04581	305,5863851	-0,19890193	0,109051939	-1,82391924	0,068164282	0,208157978	protein_codin putative mitochondrial carrier protein
TcG_04582	351,4382051	-0,013933899	0,108457352	-0,12847353	0,897774245	0,950181186	protein_codin hypothetical protein
TcG_04583	470,9036084	-0,292100004	0,086581965	-3,37368184	0,0007417	0,006060184	protein_codin GMP-PDE, delta subunit family protein
TcG_04584	400,4133235	0,175117221	0,09692497	1,806729683	0,070804437	0,213519055	protein_codin putative TBC1 domain family member 22A
TcG_04585	1187,500877	-0,219503811	0,064472599	-3,40460623	0,000662595	0,005491294	protein_codin putative FtsJ cell division protein
TcG_04586	185,3854535	-0,005318176	0,140628631	-0,03781717	0,969833458	0,987033994	protein_codin small nuclear ribonucleoprotein
TcG_04587	245,7153974	-0,249526379	0,11717589	-2,12950273	0,033212689	0,123294526	protein_codin hypothetical protein
TcG_04588	654,4735597	-0,347889435	0,074658335	-4,65975348	3,16588E-06	5,6344E-05	protein_codin hypothetical protein
TcG_04589	500,6604235	-0,086654827	0,084295771	-1,02798546	0,303956666	0,536835661	protein_codin putative protein kinase
TcG_04590	191,3459915	0,199405792	0,127321493	1,566159698	0,117311235	0,298714513	protein_codin Variant surface glycoprotein
TcG_04591	611,7210021	-0,029610865	0,079125286	-0,37422759	0,708234997	0,849437494	protein_codin hypothetical protein
TcG_04592	265,0450945	0,176477514	0,121073592	1,457605347	0,144949355	0,341754828	protein_codin oxidoreductase
TcG_04593	168,5974822	0,176539286	0,137557901	1,283381651	0,199358399	0,418209675	protein_codin hypothetical protein
TcG_04594	330,795697	-0,019205334	0,102298536	-0,18773811	0,851081954	0,928053891	protein_codin hypothetical protein
TcG_04595	321,2818082	0,035785366	0,11111108	0,322068387	0,747400887	0,872482285	protein_codin protein FAM18B1
TcG_04596	1151,862254	-0,099678913	0,060629234	-1,64407343	0,100161036	0,268999944	protein_codin hypothetical protein
TcG_04597	489,7413228	-0,006443099	0,089388748	-0,07207953	0,942538613	0,973191135	protein_codin putative endo/exonuclease Mre11
TcG_04598	272,7936956	0,221395955	0,108380259	2,042770114	0,0410752	0,143993123	protein_codin hypothetical protein
TcG_04599	1333,600694	0,028798673	0,056987522	0,505350494	0,613312683	0,79091991	protein_codin putative trypanothione synthetase
TcG_04600	92,54757405	0,236484198	0,184026138	1,285057657	0,198772137	0,417614468	protein_codin hypothetical protein
TcG_04601	218,0272398	-0,026042182	0,120441604	-0,21622248	0,828814332	0,916457611	protein_codin hypothetical protein
TcG_04602	400,9298432	-0,224455304	0,09463066	-2,37190889	0,017696454	0,076504147	protein_codin paraflagellar rod protein-like protein
TcG_04603	891,0741031	-0,283185948	0,065369943	-4,33205131	1,47727E-05	0,000213146	protein_codin putative dual specificity protein phosphatase
TcG_04604	209,4650928	0,017805317	0,129254898	0,137753521	0,890435219	0,94667588	protein_codin putative GTP-binding protein
TcG_04605	829,5249158	-0,148088453	0,069453643	-2,1321913	0,032991123	0,122826206	protein_codin NUP-1 protein
TcG_04606	4443,01032	0,276028432	0,038706579	7,131305279	9,94215E-13	7,52874E-11	protein_codin putative glycosomal phosphoenolpyruvate carboxykinase
TcG_04607	44,28682813	-0,075152461	0,262740667	-0,28603285	0,774852964	0,887537958	protein_codin putative protein kinase
TcG_04608	5,933726169	0,096678136	0,742183864	0,130261705	0,896359377	1	
TcG_04609	670,2862319	-0,024410774	0,073786733	-0,33082877	0,740773828	0,867806428	protein_codin hypothetical protein
TcG_04610	105,3085343	0,132809954	0,174009436	0,763234211	0,445323729	0,66703823	protein_codin hypothetical protein
TcG_04611	349,4614957	-0,102090265	0,097489869	-1,04718845	0,295012669	0,527938889	protein_codin hypothetical protein
TcG_04612	493,6392268	0,105734833	0,090890079	1,163326451	0,244697078	0,472864744	protein_codin putative enoyl-CoA hydratase/Enoyl-CoA isomerase/3-hydroxyacyl-CoA dehydrogenase
TcG_04613	569,0914437	0,076354043	0,081107938	0,941388042	0,346506039	0,580818717	protein_codin mitochondrial processing peptidase alpha subunit
TcG_04614	96,78925793	0,214802162	0,180489195	1,190110923	0,234002798	0,461709199	
TcG_04615	35,55940774	-0,046817522	0,307167728	-0,1524168	0,878858209	0,941484386	protein_codin hypothetical protein
TcG_04616	86,696898	0,10739369	0,192977162	0,556509841	0,577862369	0,768001978	protein_codin N-acetyltransferase complex ARD1 subunit
TcG_04617	493,6358516	0,003698791	0,086940733	0,042543821	0,966065179	0,984950279	protein_codin DEAD-box helicase-like protein

TcG_04618	130,5485101	0,094401204	0,153630449	0,614469367	0,53890521	0,738205979	
TcG_04619	386,2734109	0,009658336	0,092065831	0,104906843	0,916449729	0,959601135	protein_codin hypothetical protein
TcG_04620	227,1498882	-0,007889161	0,117222812	-0,06730056	0,946342433	0,974694377	protein_codin hypothetical protein
TcG_04621	542,7161649	-0,097934275	0,08208728	-1,19305056	0,232849574	0,460217531	protein_codin putative cytochrome P450
TcG_04622	468,0562736	-0,179749006	0,085417505	-2,10435796	0,035347234	0,129558067	protein_codin putative protein kinase
TcG_04623	398,0076671	-0,059177533	0,095904695	-0,61704522	0,537204902	0,736836273	protein_codin hypothetical protein
TcG_04624	110,5237252	0,081727417	0,17674925	0,462391872	0,643800327	0,810875005	protein_codin hypothetical protein
TcG_04625	721,6895332	-0,042088501	0,07654256	-0,54987056	0,582408156	0,771424497	protein_codin CCCH zinc-finger protein
TcG_04626	216,6533673	-0,287594015	0,122130151	-2,35481584	0,018531887	0,079141337	protein_codin hypothetical protein
TcG_04627	162,6563611	-0,34675703	0,140158317	-2,47403819	0,013359541	0,062212077	protein_codin hypothetical protein
TcG_04628	213,8547683	-0,135467594	0,128031546	-1,05807981	0,290019058	0,523389534	protein_codin hypothetical protein
TcG_04629	259,755152	0,067639589	0,110844848	0,610218614	0,541717001	0,740087861	protein_codin hypothetical protein
TcG_04630	264,6830025	0,054033384	0,109913245	0,491600295	0,623001935	0,796787771	protein_codin hypothetical protein
TcG_04631	276,4102655	-0,286228923	0,109930065	-2,60373651	0,009221362	0,046330746	protein_codin proteasome alpha 7 subunit
TcG_04632	154,9961024	-0,124876712	0,147276516	-0,84790648	0,396490058	0,626361305	protein_codin hypothetical protein
TcG_04633	133,834533	0,097141216	0,154715389	0,627870418	0,530088831	0,731981763	protein_codin hypothetical protein
TcG_04634	282,5274792	-0,014358429	0,105359744	-0,13628003	0,891599911	0,947105214	protein_codin integral membrane protein
TcG_04635	145,7263488	0,178184017	0,146126003	1,2193861	0,222697683	0,448804202	protein_codin hypothetical protein
TcG_04636	187,1089738	0,147288485	0,136408943	1,079756815	0,280250486	0,513438035	protein_codin hypothetical protein
TcG_04637	325,8002679	-0,068641272	0,102781083	-0,66783955	0,50423602	0,712076368	protein_codin flap endonuclease-1 (FEN-1)
TcG_04638	181,1842453	-0,054164491	0,13268439	-0,40822052	0,683111783	0,835306925	protein_codin putative protein kinase-like protein
TcG_04639	287,3078499	0,07079507	0,112362393	0,630060186	0,528655207	0,73102477	protein_codin putative axoneme central apparatus protein
TcG_04640	559,739371	0,037307561	0,085408976	0,436810777	0,662248582	0,823073415	protein_codin acyl carrier protein, mitochondrial precursor
TcG_04641	173,6738273	-0,271066344	0,137715318	-1,96830932	0,049032458	0,163197375	protein_codin hypothetical protein
TcG_04642	86,11542651	-0,154891217	0,191456915	-0,80901344	0,418507417	0,645133972	protein_codin putative ankyrin repeat protein
TcG_04643	219,8529208	-0,153285418	0,118393113	-1,29471567	0,195418334	0,413235411	protein_codin hypothetical protein
TcG_04644	202,9359196	0,056449557	0,126346371	0,446784158	0,65503091	0,817712329	protein_codin hypothetical protein
TcG_04645	305,8286647	-0,109809668	0,102017103	-1,0763849	0,281755155	0,514810791	protein_codin gamma-tubulin
TcG_04646	453,9076364	-0,04893094	0,090492772	-0,54071656	0,588702964	0,775648564	protein_codin calmodulin
TcG_04647	2275,830494	-0,185775328	0,115359062	-1,61040948	0,107308492	0,282235268	protein_codin dynein, axonemal, heavy polypeptide 5
TcG_04648	154,9152447	0,056354402	0,144291706	0,390558844	0,696123352	0,842591429	protein_codin hypothetical protein
TcG_04649	333,5745831	-0,169171937	0,098019036	-1,72590902	0,084363782	0,2392751	protein_codin hypothetical protein
TcG_04650	467,167825	-0,099461316	0,088455856	-1,12441754	0,260835934	0,493315185	protein_codin 2OG-Fe(II) oxygenase
TcG_04651	517,9987752	-0,085264896	0,08148175	-1,04642936	0,295362842	0,528097822	
TcG_04652	229,026745	0,104477869	0,118495963	0,881699816	0,37793916	0,608589731	protein_codin dehydrogenase-like protein
TcG_04653	561,9055066	0,224554435	0,079689508	2,817867016	0,004834383	0,028047651	protein_codin transferase
TcG_04654	268,4828179	-0,045040162	0,111544929	-0,40378494	0,686370872	0,837209523	protein_codin hypothetical protein
TcG_04655	180,9231679	0,214959555	0,136586804	1,573794459	0,115534976	0,295838856	protein_codin frataxin-like, mitochondrial precursor
TcG_04656	942,7860612	-0,420289948	0,068704449	-6,11736142	9,51374E-10	3,8009E-08	protein_codin hypothetical protein
TcG_04657	298,895143	-0,069052285	0,10836984	-0,63719099	0,524000447	0,72776714	protein_codin hypothetical protein
TcG_04658	469,3162625	-0,201779945	0,088037613	-2,29197429	0,021907127	0,090198414	protein_codin hypothetical protein
TcG_04659	50,08629366	0,288628189	0,245910666	1,173711551	0,240510582	0,469196094	protein_codin hypothetical protein
TcG_04660	40,61253479	0,021297276	0,281501875	0,075655894	0,939692867	0,97207871	protein_codin hypothetical protein
TcG_04661	344,8991356	0,049112969	0,099858316	0,491826534	0,622841977	0,796768389	protein_codin hypothetical protein
TcG_04662	117,7453608	0,052484119	0,174226755	0,301240295	0,763231265	0,880405957	protein_codin hypothetical protein
TcG_04663	939,5332996	0,084619143	0,068696701	1,231778846	0,218031708	0,44240199	protein_codin DNA polymerase epsilon catalytic subunit A
TcG_04664	151,7403049	-0,384585887	0,147021407	-2,61584958	0,008900576	0,045124243	protein_codin hypothetical protein
TcG_04665	572,1737993	-0,295218667	0,078480752	-3,76166971	0,000168783	0,001733615	protein_codin oligoribonuclease
TcG_04666	221,0234491	0,031831045	0,120107668	0,265020926	0,790993341	0,89549041	protein_codin hypothetical protein
TcG_04667	367,9093896	-0,239319579	0,10324518	-2,31797339	0,020450767	0,085569733	protein_codin PIH1 domain-containing protein 1
TcG_04668	385,3661465	-0,054200193	0,092176519	-0,58800434	0,556529379	0,751246579	protein_codin HAD-superfamily subfamily IIA hydrolase
TcG_04669	125,5741855	0,303005417	0,159238232	1,902843385	0,057060984	0,181723079	protein_codin hypothetical protein
TcG_04670	295,7644912	-0,105340512	0,106200132	-0,99190566	0,321243548	0,556034397	protein_codin hypothetical protein
TcG_04671	525,2745364	-0,232116505	0,080621731	-2,87908115	0,003988357	0,024004729	protein_codin dynein heavy chain, cytosolic
TcG_04672	473,5580105	-0,184810067	0,092433556	-1,99938285	0,045566946	0,154866131	protein_codin spindle assembly 6
TcG_04673	321,3158776	0,072090062	0,107929908	0,667934062	0,504175684	0,712076368	protein_codin hypothetical protein
TcG_04674	410,4803146	-0,06401396	0,095382045	-0,67113219	0,502136323	0,710942905	protein_codin zinc finger protein, predicted

TcG_04675	343,4472063	0,061399049	0,096809814	0,634223395	0,525935041	0,728904717	protein_codin putative 3-demethylubiquinone-9 3-methyltransferase
TcG_04676	465,1480365	-0,06798626	0,088620673	-0,76716027	0,44298623	0,664995914	protein_codin hypothetical protein
TcG_04677	190,4986038	0,211586847	0,130779673	1,617887874	0,105686764	0,27969092	protein_codin hypothetical protein
TcG_04678	252,6246147	-0,053023075	0,118336996	-0,44806845	0,654103796	0,817094887	protein_codin putative synaptojanin (N-terminal domain), putative,inositol/phosphatidylinositol phosphatase
TcG_04679	106,7569687	0,014999614	0,168946735	0,088783097	0,929254292	0,965591051	protein_codin hypothetical protein
TcG_04680	239,8306044	-0,172903544	0,11494202	-1,50426749	0,132512506	0,323570582	
TcG_04681	4601,981964	0,548067662	0,042420213	12,91996493	3,473E-38	3,09524E-35	protein_codin prostaglandin F2alpha synthase
TcG_04682	177,7245598	0,3234415	0,133775413	2,417794818	0,015614879	0,069689517	protein_codin hypothetical protein
TcG_04683	60,15733823	0,601254603	0,234823111	2,560457536	0,010453444	0,051145946	protein_codin hypothetical protein
TcG_04684	45,12203095	0,560136385	0,264861584	2,114826831	0,034444709	0,126973083	protein_codin hypothetical protein
TcG_04685	199,189825	-0,097743835	0,133302881	-0,73324623	0,463408263	0,680919231	protein_codin hypothetical protein
TcG_04686	228,2034754	0,045828837	0,118321595	0,387324368	0,698516088	0,844142481	protein_codin putative Bardet-Biedl syndrome 1 protein
TcG_04687	264,603056	0,115827635	0,108897892	1,063635236	0,287493974	0,520529678	protein_codin DNA-directed RNA polymerase
TcG_04688	485,8439681	-0,207269131	0,082892235	-2,50046497	0,01240304	0,05858199	protein_codin hypothetical protein
TcG_04689	362,8667961	-0,036290864	0,095942034	-0,37825823	0,705238769	0,847955207	protein_codin Hsp70 subfamily B suppressor 1
TcG_04690	167,2748659	0,138031447	0,135106608	1,021648376	0,306947353	0,540059534	protein_codin hypothetical protein
TcG_04691	2546,71689	-0,208564993	0,044991601	-4,63564287	3,5583E-06	6,24497E-05	protein_codin poly(A)-binding protein 2
TcG_04692	18,53978136	-0,15652521	0,400079755	-0,39123502	0,695623527	0,842340007	
TcG_04693	308,6570664	-0,049955764	0,104391028	-0,47854461	0,632262629	0,802906018	protein_codin hypothetical protein
TcG_04694	282,7336359	0,13250796	0,107476012	1,232907305	0,21761035	0,441916894	protein_codin hypothetical protein
TcG_04695	167,0805951	0,103233973	0,13609606	0,758537557	0,448129238	0,66910023	protein_codin hydrolase of HD superfamily
TcG_04696	332,3384623	0,173332102	0,10580756	1,638182586	0,101383611	0,271465338	protein_codin hypothetical protein
TcG_04697	187,0079521	0,298895104	0,132195205	2,261013204	0,023758439	0,096266166	protein_codin hypothetical protein
TcG_04698	127,9673149	-0,0452699	0,162174985	-0,27914231	0,780135616	0,890595256	protein_codin hypothetical protein
TcG_04699	196,4589097	-0,019006113	0,130433743	-0,1457147	0,884146619	0,943488844	protein_codin TPR domain protein
TcG_04700	185,6157492	0,301987082	0,135484023	2,228949773	0,025817247	0,102195608	protein_codin hypothetical protein
TcG_04701	453,7842814	0,000296567	0,091478229	0,003241938	0,997413312	0,999104158	protein_codin putative protein kinase
TcG_04702	71,81497749	0,759815815	0,211451451	3,593334595	0,000326473	0,003004381	protein_codin hypothetical protein
TcG_04703	233,5562645	0,116584316	0,117912013	0,988739932	0,3227904	0,557414741	protein_codin chaperone protein DNAJ, putatative
TcG_04704	491,7559826	-0,055003566	0,083541042	-0,65840173	0,510280027	0,717053292	protein_codin putative ATP-dependent RNA helicase
TcG_04705	295,6994842	-0,020302555	0,103468558	-0,19621956	0,844438321	0,92455702	protein_codin putative Bem46-like serine peptidase, putative,Serine peptidase, Clan SC, Family S09X
TcG_04706	501,956066	-0,034300838	0,08238594	-0,41634335	0,677158777	0,830906086	protein_codin GTP-binding protein
TcG_04707	222,4909	-0,158205456	0,121319209	-1,30404292	0,192218949	0,408557832	protein_codin putative mitotubule-associated protein Gb4
TcG_04708	215,0129381	0,081166756	0,127831596	0,634950659	0,525460596	0,728663849	protein_codin putative electon transport protein SCO1/SCO2
TcG_04709	2017,101742	-0,203691687	0,052002467	-3,916962	8,96719E-05	0,001017569	protein_codin hypothetical protein
TcG_04710	1819,092071	-0,17739359	0,053792267	-3,29775263	0,00097462	0,007614256	protein_codin microtubule-associated protein Gb4
TcG_04711	718,7703111	0,056688111	0,070594014	0,803015831	0,421965585	0,648162398	protein_codin hypothetical protein
TcG_04712	303,9410365	-0,077882045	0,121379557	-0,64164054	0,521106601	0,725401608	protein_codin hypothetical protein
TcG_04713	331,853171	0,30547216	0,100842802	3,029191502	0,002452092	0,016206467	protein_codin putative deoxyhypusine hydroxylase
TcG_04714	710,4384813	-0,038017361	0,073493423	-0,51728929	0,604954227	0,786644184	protein_codin hypothetical protein
TcG_04715	354,1383574	0,456364263	0,101552184	4,493889192	6,9934E-06	0,000112224	protein_codin hypothetical protein
TcG_04716	468,9348686	0,127904591	0,084584888	1,512144713	0,130497052	0,32059772	protein_codin hypothetical protein
TcG_04717	922,191505	0,288816634	0,067499714	4,278783089	1,87918E-05	0,000262632	protein_codin hypothetical protein
TcG_04718	1127,706515	-0,031307851	0,058987937	-0,53075006	0,595591989	0,780602803	protein_codin putative transmembrane transport protein
TcG_04719	249,0243282	0,354808584	0,116477971	3,046143248	0,002317974	0,015505799	protein_codin engulfment and cell motility ELM family protein
TcG_04720	173,8819349	0,282137639	0,134771977	2,093444393	0,036309503	0,132165222	protein_codin hypothetical protein
TcG_04721	123,350777	0,298342104	0,164281712	1,816039658	0,069364289	0,210755973	protein_codin hypothetical protein
TcG_04722	878,6666818	0,07358008	0,07782402	0,945467473	0,344420269	0,578578113	protein_codin hypothetical protein
TcG_04723	470,7229937	0,156634508	0,085922083	1,82298312	0,068305947	0,208401176	protein_codin microtubule-associated protein, RP/EB family
TcG_04724	207,8598315	0,255471157	0,124788732	2,047229359	0,04063557	0,142711038	protein_codin hypothetical protein
TcG_04725	170,2999479	0,254504284	0,138331445	1,839815126	0,065795384	0,203057226	protein_codin putative O-6 methyl-guanine alkyl transferase
TcG_04726	2026,379887	0,083461587	0,097727283	0,854025455	0,393090886	0,622774648	protein_codin hypothetical protein
TcG_04727	68,99768743	0,159321306	0,213178897	0,74735965	0,454846495	0,674756913	protein_codin hypothetical protein
TcG_04728	116,070943	-0,31801012	0,191188103	-1,66333634	0,096245128	0,262375542	protein_codin putative microtubule-associated protein Gb4
TcG_04729	7,698811434	0,983832137	0,641101181	1,534597295	0,124882778	1	protein_codin hypothetical protein
TcG_04730	5,484526572	-0,007124402	0,741886619	-0,00960309	0,992337962	1	protein_codin hypothetical protein
TcG_04731	229,2507535	0,070406119	0,119044775	0,591425531	0,554235338	0,749109966	protein_codin putative surface protease GP63

TcG_04732	1,634630895	-0,912586743	1,353785192	-0,67410011	0,500247671	1	protein_codin hypothetical protein
TcG_04733	10,32826159	-0,042974256	0,550468975	-0,07806844	0,937773609	1	protein_codin hypothetical protein
TcG_04734	295,245571	0,309066951	0,104621419	2,954146038	0,003135356	0,019764001	protein_codin hypothetical protein
TcG_04735	122,0912876	0,486590809	0,159460924	3,051473657	0,00227721	0,015293501	protein_codin hypothetical protein
TcG_04736	193,9511134	-0,438925714	0,129227105	-3,39654529	0,000682423	0,005627437	
TcG_04737	230,1771443	0,029017357	0,119314741	0,243200097	0,807850392	0,905044416	protein_codin hypothetical protein
TcG_04738	108,1883564	0,186251133	0,176112931	1,057566486	0,290253129	0,523456792	protein_codin hypothetical protein
TcG_04739	133,2627645	0,086833603	0,154392257	0,562422003	0,573828532	0,764991214	protein_codin hypothetical protein
TcG_04740	80,51170346	-0,132250189	0,206995937	-0,63890234	0,522886464	0,726834142	protein_codin L1Tc protein
TcG_04741	1346,177759	0,067052872	0,055488268	1,208415286	0,226887563	0,453618516	protein_codin putative protein kinase
TcG_04742	144,3522279	-0,160369049	0,146929817	-1,09146702	0,275067437	0,507634843	protein_codin hypothetical protein
TcG_04743	36,37292535	-0,308870696	0,293718898	-1,05158605	0,29298951	0,525964745	protein_codin hypothetical protein
TcG_04744	309,1975357	-0,109312976	0,109228191	-1,00077622	0,316935011	0,551932818	protein_codin hypothetical protein
TcG_04745	1048,019002	-0,134770831	0,061753254	-2,18240855	0,029079394	0,111782964	protein_codin cytochrome c oxidase subunit V
TcG_04746	862,5487346	0,023350868	0,067206204	0,347451076	0,728252467	0,86000745	protein_codin putative fatty acid desaturase, putative,sphingolipid delta 4 desaturase
TcG_04747	8,19681122	0,716301627	0,619843626	1,155616669	0,247837987	1	protein_codin hypothetical protein
TcG_04748	45,23552268	0,4308224	0,260635959	1,652966079	0,098337767	0,265642661	protein_codin trans-sialidase
TcG_04749	144,0920973	-0,004924343	0,150434795	-0,03273407	0,973886653	0,988562359	protein_codin programmed cell death protein 5
TcG_04750	264,1918331	0,134287132	0,110564378	1,214560547	0,224533744	0,451082534	protein_codin hypothetical protein
TcG_04751	123,1838849	0,014155668	0,159928337	0,088512566	0,929469297	0,965727852	protein_codin hypothetical protein
TcG_04752	300,0793523	0,234340411	0,104271565	2,24740475	0,024614172	0,09866823	protein_codin S-acyltransferase
TcG_04753	1017,334057	0,020942114	0,061918332	0,338221552	0,735196236	0,864516011	protein_codin ribosome biogenesis protein BMS1
TcG_04754	166,9711282	0,385996076	0,136883333	2,819890979	0,004803997	0,027899302	protein_codin hypothetical protein
TcG_04755	286,5712639	0,289629569	0,107233641	2,700920782	0,006914781	0,036970304	protein_codin hypothetical protein
TcG_04756	362,6528069	-0,287269931	0,094846552	-3,02878624	0,002455384	0,01621897	protein_codin hypothetical protein
TcG_04757	325,156024	-0,117356057	0,103068692	-1,13861983	0,254861752	0,486142289	protein_codin putative C-terminal motor kinesin
TcG_04758	686,8479753	-0,07649037	0,074138413	-1,03172387	0,302201492	0,535371497	protein_codin putative replication factor A, 51kDa subunit
TcG_04759	484,9779285	-0,062828388	0,083211729	-0,75504245	0,450223514	0,670905419	protein_codin putative mitochondrial RNA binding complex 1 subunit
TcG_04760	881,0165141	0,114051052	0,067053196	1,700904045	0,088961007	0,248242347	protein_codin hypothetical protein
TcG_04761	118,8888472	0,549261537	0,181786213	3,021469711	0,002515508	0,016503214	protein_codin putative ATPase protein
TcG_04762	368,7150594	-0,110493656	0,094889262	-1,16444847	0,244242314	0,472419274	protein_codin DNA topoisomerase 3-beta-1 isoform X1
TcG_04763	239,0187989	0,068010253	0,118897027	0,572009699	0,567315404	0,759084914	protein_codin ADP-ribosylation factor GTPase activating protein
TcG_04764	583,6013786	-0,049098491	0,078593158	-0,62471711	0,532156738	0,732933197	protein_codin putative protein kinase
TcG_04765	247,1682695	0,123267187	0,117131111	1,052386387	0,292622312	0,525386968	protein_codin hypothetical protein
TcG_04766	213,5590149	0,222466615	0,12316984	1,806177682	0,07089059	0,213612061	protein_codin hypothetical protein
TcG_04767	996,222499	-0,119553776	0,063207342	-1,89145395	0,058563772	0,185580885	protein_codin putative proteasome regulatory non-ATP-ase subunit 2
TcG_04768	128,5386742	-0,068155122	0,16508405	-0,41285104	0,679715754	0,832824315	protein_codin hypothetical protein
TcG_04769	763,4064909	-0,04530367	0,069359558	-0,65317126	0,513645879	0,719775176	protein_codin hypothetical protein
TcG_04770	114,6125631	-0,015907843	0,170249084	-0,09343864	0,925555092	0,963648571	protein_codin hypothetical protein
TcG_04771	246,0719757	0,157607766	0,115641859	1,362895475	0,172915455	0,381091585	protein_codin hypothetical protein
TcG_04772	621,2200648	0,120153398	0,081325469	1,477438735	0,139558069	0,334093557	protein_codin zinc finger protein kinase
TcG_04773	385,7540395	0,024818897	0,09483687	0,261700928	0,793552025	0,89680977	protein_codin hypothetical protein
TcG_04774	375,2610803	-0,032109205	0,096490113	-0,33277197	0,739306416	0,867087269	protein_codin putative serine/threonine protein kinase
TcG_04775	228,543947	0,047264048	0,12325339	0,383470574	0,701370885	0,845322279	protein_codin hypothetical protein
TcG_04776	60,38241323	0,533211544	0,234707859	2,271809503	0,023098019	0,094163844	protein_codin hypothetical protein
TcG_04777	305,8483369	-0,148907521	0,101718456	-1,46391842	0,143216241	0,339673155	protein_codin DUF3342 domain containing protein
TcG_04778	481,246716	-0,034381048	0,087612918	-0,39241985	0,694748025	0,842070364	protein_codin putative tRNA pseudouridine synthase
TcG_04779	175,7154301	-0,205767834	0,146647525	-1,40314563	0,160573417	0,363449048	protein_codin hypothetical protein
TcG_04780	144,386571	-0,11547563	0,165088255	-0,69947817	0,484253253	0,697483614	protein_codin putative helicase-like protein
TcG_04781	270,2994239	-0,261018505	0,110199321	-2,36860356	0,017855381	0,07704558	protein_codin putative helicase-like protein
TcG_04782	239,3544073	0,198263687	0,114838776	1,726452457	0,084266046	0,239095707	protein_codin hypothetical protein
TcG_04783	316,8718768	-0,068445129	0,106113769	-0,64501647	0,518916513	0,723712794	protein_codin putative phosphonopyruvate decarboxylase
TcG_04784	280,7275815	0,034904072	0,109893435	0,317617441	0,750775143	0,87456599	protein_codin putative prenyltransferase
TcG_04785	329,6419782	-0,103628413	0,103439249	-1,00182875	0,316426311	0,551461377	protein_codin hypothetical protein
TcG_04786	163,2263937	0,024495751	0,139432911	0,175681267	0,860544353	0,933293722	
TcG_04787	1017,731294	0,273296419	0,063910807	4,276216053	1,90097E-05	0,000265037	protein_codin hypothetical protein
TcG_04788	470,7801235	-0,259095191	0,0954788	-2,71364105	0,006654823	0,035878446	protein_codin transmembrane protein 222

TcG_04789	363,3418671	-0,018644325	0,096629078	-0,19294735	0,8470002	0,92596191	protein_codin hypothetical protein
TcG_04790	149,7815513	-0,029372394	0,144404602	-0,20340345	0,838819704	0,921715202	protein_codin putative adenylate kinase
TcG_04791	2223,688476	-0,176178528	0,046701727	-3,77241999	0,000161672	0,001675429	protein_codin cell division control protein 48-like protein E
TcG_04792	231,624256	0,001948308	0,119539266	0,01629848	0,98699627	0,994746907	protein_codin putative amino acid transporter
TcG_04793	223,1152807	-0,052274935	0,118025619	-0,44291176	0,657829561	0,819528311	protein_codin putative serine/cysteine trypsin-like peptidase
TcG_04794	458,0772874	0,113213037	0,086710889	1,305638062	0,19167567	0,40770228	protein_codin hypothetical protein
TcG_04795	140,9018754	0,088799993	0,154689217	0,574054186	0,565931134	0,758216295	protein_codin hypothetical protein
TcG_04796	809,1520299	-0,145560515	0,067871194	-2,14465824	0,031980182	0,11991016	protein_codin putative coiled-coil domain-containing protein 104
TcG_04797	738,0565443	-0,058605167	0,070985339	-0,82559537	0,409033665	0,636605346	protein_codin putative Ran GDP binding protein
TcG_04798	639,8937281	-0,165740089	0,075782308	-2,18705518	0,028738496	0,11061934	protein_codin putative Ran GDP binding protein
TcG_04799	949,550612	-0,072466706	0,064281456	-1,12733455	0,25960106	0,492108097	protein_codin translation elongation factor 1-beta
TcG_04800	452,4666924	-0,055708709	0,095572833	-0,58289272	0,559965519	0,754054394	protein_codin glycosyltransferase
TcG_04801	1417,925199	0,180900949	0,067992135	2,660615798	0,00779979	0,040651538	protein_codin vacuolar protein sorting-associated protein 13 family protein
TcG_04802	179,6893837	-0,146875162	0,140804689	-1,04311272	0,296896092	0,530102962	protein_codin hypothetical protein
TcG_04803	676,8146504	-0,163074791	0,075087245	-2,17180415	0,029870438	0,114066875	protein_codin hypothetical protein
TcG_04804	438,881863	0,216675823	0,09112574	2,3777675	0,017417804	0,075553232	protein_codin hypothetical protein
TcG_04805	496,2289406	-0,042328856	0,082365339	-0,51391589	0,6073108	0,787939858	protein_codin hypothetical protein
TcG_04806	306,0037857	0,147576737	0,107207403	1,376553605	0,168650303	0,375101512	protein_codin hypothetical protein
TcG_04807	329,4539928	-0,218048788	0,099988177	-2,1807457	0,029202231	0,112044261	protein_codin hypothetical protein
TcG_04808	117,7095257	-0,179692085	0,167974277	-1,06975954	0,284727559	0,5185246	protein_codin hypothetical protein
TcG_04809	523,57573	0,047208492	0,084354289	0,559645431	0,57572131	0,766349621	protein_codin hypothetical protein
TcG_04810	173,1109138	-0,158523565	0,135045463	-1,17385332	0,240453782	0,469164283	protein_codin hypothetical protein
TcG_04811	17,50484158	0,050627421	0,436082835	0,116095882	0,907576552	0,955230917	protein_codin hypothetical protein
TcG_04812	654,1975324	-0,018618032	0,075605671	-0,2462518	0,805487322	0,904037209	protein_codin putative nima-related protein kinase
TcG_04813	237,5018214	0,114704264	0,119981917	0,956012934	0,3390657	0,57332217	protein_codin putative serine/threonine protein kinase
TcG_04814	414,3776718	-0,086144398	0,092259504	-0,93371841	0,350449164	0,584889659	protein_codin ELMO domain-containing protein 2
TcG_04815	493,1878796	-0,209905874	0,083783016	-2,50535114	0,012232988	0,057920474	protein_codin leucine-richcontaining protein 23
TcG_04816	97,56290132	-0,111558989	0,179190352	-0,62257252	0,533565473	0,734103975	
TcG_04817	372,5301435	-0,269369288	0,099448083	-2,70864233	0,006755913	0,036322045	protein_codin hypothetical protein
TcG_04818	941,8600699	-0,144413379	0,064981805	-2,22236638	0,026258556	0,103444959	protein_codin hypothetical protein
TcG_04819	1336,116035	-0,113480782	0,057888566	-1,96033156	0,049957049	0,16541937	protein_codin cohesin complex subunit SA-1/2
TcG_04820	200,9719425	-0,19501095	0,13432101	-1,4518276	0,146549547	0,344616005	protein_codin hypothetical protein
TcG_04821	474,2821616	-0,139181854	0,087356928	-1,59325491	0,111103017	0,288166456	protein_codin 29 kDa proteasome subunit TCPR29A
TcG_04822	2262,976239	-0,181962987	0,049635431	-3,66598989	0,000246384	0,002376225	protein_codin clathrin heavy chain 1
TcG_04823	40,66577646	0,4044129	0,276906329	1,460468244	0,144161429	0,340781521	protein_codin target of rapamycin (TOR) kinase 1
TcG_04824	124,2434987	0,523127064	0,165331397	3,164112049	0,001555569	0,011215197	protein_codin hypothetical protein
TcG_04825	1703,41546	-0,425035587	0,054175965	-7,84546412	4,31355E-15	4,62748E-13	protein_codin putative protein disulfide isomerase
TcG_04826	351,7709149	0,010769387	0,096717749	0,11134861	0,91133991	0,956844965	protein_codin hypothetical protein
TcG_04827	92,71572075	-0,020128068	0,18410923	-0,10932677	0,912943314	0,957280869	protein_codin hypothetical protein
TcG_04828	906,3793966	-0,060147038	0,066702929	-0,9017151	0,367208231	0,599390612	protein_codin RNA-editing complex protein MP63
TcG_04829	358,3359991	-0,103716505	0,096801205	-1,07143817	0,283972458	0,517474818	protein_codin ribosomal protein L1-like protein
TcG_04830	2025,518655	-0,152768761	0,048426158	-3,15467437	0,001606773	0,011530366	protein_codin putative T-complex protein 1, theta subunit
TcG_04831	221,4625002	-0,015829217	0,122990381	-0,12870289	0,897592748	0,950181186	protein_codin hypothetical protein
TcG_04832	1129,685572	-0,024386636	0,065519538	-0,37220402	0,709740951	0,850350634	protein_codin putative nuclear pore complex protein (NUP155)
TcG_04833	184,6908831	-0,180826714	0,137436019	-1,31571559	0,188269518	0,402654186	protein_codin putative DNA-binding protein
TcG_04834	154,5579737	-0,320791845	0,157702188	-2,0341623	0,041935234	0,145992075	protein_codin hypothetical protein
TcG_04835	124,4186848	-0,019338194	0,158726636	-0,12183333	0,903031018	0,953130853	protein_codin thymocyte nuclear protein 1
TcG_04836	254,4890105	0,087782402	0,117878743	0,744683898	0,456462835	0,676399932	protein_codin calphotin-like protein
TcG_04837	545,1108383	-0,003904434	0,08778219	-0,04447866	0,964522865	0,984231277	protein_codin hypothetical protein
TcG_04838	329,8459828	-0,025431095	0,103079317	-0,24671385	0,805129688	0,903850005	protein_codin hypothetical protein
TcG_04839	313,2667712	0,125755098	0,109732168	1,146018529	0,251787485	0,481861939	protein_codin putative high mobility group (HMG) box domain-containing protein
TcG_04840	130,5472989	0,267596863	0,155221116	1,723972031	0,084712892	0,239912875	protein_codin hypothetical protein
TcG_04841	549,5665125	0,012757135	0,086229875	0,147943337	0,882387487	0,942590948	protein_codin hypothetical protein
TcG_04842	289,2275125	0,047712531	0,109477979	0,43581852	0,662968406	0,823549415	protein_codin putative ATPase
TcG_04843	357,6690395	0,139262733	0,102492145	1,358764948	0,174221084	0,382803997	protein_codin SET and MYND domain-containing protein
TcG_04844	23,2875337	-0,684178272	0,369361904	-1,85232495	0,063979174	0,19872995	protein_codin putative nuclear lim interactor-interacting factor
TcG_04845	932,2291425	-0,016165183	0,066124555	-0,24446567	0,806870188	0,904614097	protein_codin putative beta-adaptin

TcG_04846	855,8047479	-0,139783125	0,066320587	-2,10768831	0,035057959	0,128782979	protein_codin ATP synthase OSCP delta subunit-like protein
TcG_04847	414,6769315	0,51056336	0,093823808	5,441725011	5,27671E-08	1,44189E-06	protein_codin putative histidine ammonia-lyase
TcG_04848	1350,613624	-0,045364546	0,064171863	-0,70692269	0,47961452	0,693907821	protein_codin putative prolyl oligopeptidase
TcG_04849	162,6219557	0,067844455	0,138342348	0,490409888	0,623843881	0,797066211	protein_codin hypothetical protein
TcG_04850	640,6664057	-0,011444015	0,07724251	-0,14815695	0,882218905	0,942497763	protein_codin cpc1/kpl2
TcG_04851	80,76456944	0,194384699	0,206333337	0,942090605	0,346146254	0,580467578	protein_codin cpc1/kpl2
TcG_04852	214,8379451	0,354274089	0,134566611	2,63270425	0,008470807	0,043403618	protein_codin mitochondrial carrier protein
TcG_04853	345,3639641	0,108939107	0,098682527	1,103935115	0,269621285	0,502466174	protein_codin putative replication factor C, subunit 5
TcG_04854	66,69327627	0,148229723	0,219656834	0,674824087	0,499787535	0,708798748	protein_codin hypothetical protein
TcG_04855	337,1313778	0,085266523	0,10504954	0,811679168	0,416975751	0,643714997	protein_codin hypothetical protein
TcG_04856	404,7996376	0,217612218	0,092777847	2,345519175	0,019000601	0,080667265	protein_codin f2o10.10 protein-like protein
TcG_04857	220,499416	0,053863125	0,12093466	0,445390301	0,65603772	0,818175783	protein_codin hypothetical protein
TcG_04858	358,3414758	0,138491357	0,095202721	1,454699573	0,145752451	0,343299023	protein_codin membrane-associated progesterone binding protein 2
TcG_04859	346,7944383	0,136042878	0,103344783	1,316398108	0,188040457	0,402407967	protein_codin putative methyltransferase
TcG_04860	1146,235894	-0,124728627	0,061425202	-2,0305774	0,042297882	0,146945505	protein_codin phosphoglyceromutase
TcG_04861	326,4066178	-0,040551281	0,108546706	-0,37358371	0,708714059	0,849664847	protein_codin hypothetical protein
TcG_04862	189,866144	0,02397027	0,131892135	0,181741464	0,855785625	0,930212238	protein_codin hypothetical protein
TcG_04863	113,1965046	0,155991199	0,167837877	0,929415945	0,352673567	0,58699554	protein_codin hypothetical protein
TcG_04864	304,154721	0,116651488	0,102880695	1,133852065	0,256856607	0,488848291	protein_codin putative mitochondrial substrate/solute carrier
TcG_04865	512,0004777	-0,037522119	0,08556717	-0,43851069	0,661016123	0,822260339	protein_codin hypothetical protein
TcG_04866	282,5435491	-0,043270061	0,110528957	-0,39148167	0,695441232	0,842340007	protein_codin coiled-coil domain protein
TcG_04867	296,1543685	-0,068046137	0,103190306	-0,65942373	0,509623706	0,716478613	protein_codin hypothetical protein
TcG_04868	252,4332562	-0,03921195	0,118981536	-0,32956332	0,74172994	0,868575205	protein_codin hypothetical protein
TcG_04869	191,2845219	0,141894406	0,129589648	1,094951709	0,273537793	0,50688937	protein_codin putative protein-l-isoaspartate o-methyltransferase
TcG_04870	154,0984287	-0,080069126	0,151121212	-0,5298338	0,596227168	0,780668859	protein_codin telomere-binding protein 1
TcG_04871	336,7152352	-0,065713086	0,101704669	-0,64611671	0,518203774	0,72327538	protein_codin hypothetical protein
TcG_04872	115,7883562	0,175194597	0,167045238	1,048785339	0,294276926	0,527256689	protein_codin hypothetical protein
TcG_04873	501,0136846	0,084705996	0,083913717	1,009441593	0,3127629	0,546886652	protein_codin pre-rRNA-processing protein ESF1
TcG_04874	324,5491971	0,079009721	0,103473248	0,763576311	0,445119771	0,666988834	protein_codin putative cop9 signalosome complex subunit
TcG_04875	526,1837941	0,100974529	0,083850106	1,204226613	0,228502002	0,455641875	protein_codin hypothetical protein
TcG_04876	303,2880487	-0,217161905	0,105689282	-2,05472022	0,039906036	0,141089817	protein_codin putative ubiquitin fusion degardation protein
TcG_04877	254,0145314	-0,010255556	0,115347354	-0,08891019	0,929153282	0,96557269	protein_codin putative protein kinase, putative,mitogen-activated protein kinase
TcG_04878	98,86326621	-0,106468492	0,17475635	-0,60923962	0,542365623	0,740321408	protein_codin hypothetical protein
TcG_04879	49,42422616	-0,196216197	0,248817685	-0,78859426	0,43034919	0,655193917	
TcG_04880	73,55326972	0,163961718	0,205331755	0,798520998	0,424568213	0,65015164	protein_codin hypothetical protein
TcG_04881	38,44961284	0,506748704	0,285225125	1,776662217	0,075623832	0,222267306	protein_codin structural maintenance of chromosome protein 4
TcG_04882	68,3172929	0,039301412	0,221756269	0,177227965	0,85932933	0,932314788	protein_codin hypothetical protein
TcG_04883	238,9531188	0,537668491	0,118770071	4,526969534	5,98356E-06	9,80558E-05	protein_codin hypothetical protein
TcG_04884	241,8807673	0,065064796	0,121142652	0,537092389	0,591203798	0,776872769	protein_codin zinc finger domain-like protein
TcG_04885	201,4458806	0,177626311	0,129297607	1,373778797	0,169510366	0,376067037	protein_codin Myb domain protein 40
TcG_04886	160,0191723	0,075124806	0,144377024	0,520337684	0,602828235	0,785186973	protein_codin hypothetical protein
TcG_04887	382,9636912	-0,02739099	0,093037605	-0,29440773	0,768446362	0,884133024	protein_codin putative 2-oxoisovalerate dehydrogenase alpha subunit
TcG_04888	294,8594548	-0,330196581	0,105072196	-3,14256856	0,001674725	0,011925853	protein_codin cytochrome b5 domain containing 1
TcG_04889	535,1958956	-0,04320119	0,081304825	-0,53134842	0,59517736	0,780324192	protein_codin putative arginine N-methyltransferase, type II
TcG_04890	472,1266127	-0,088475368	0,085796367	-1,03122511	0,302435266	0,535372803	protein_codin hypothetical protein
TcG_04891	3764,93579	-0,128883499	0,043987786	-2,92998376	0,003389797	0,021013478	protein_codin hypothetical protein
TcG_04892	218,6597978	-0,296172853	0,122823261	-2,41137428	0,01589253	0,070710773	protein_codin hypothetical protein
TcG_04893	470,5400751	0,02123665	0,085311011	0,248932111	0,803413296	0,902933984	protein_codin hypothetical protein
TcG_04894	451,7589118	-0,035052957	0,086923592	-0,40326171	0,686755705	0,837403474	protein_codin hypothetical protein
TcG_04895	119,3685333	0,367377046	0,16219771	2,264995272	0,02351297	0,095519378	protein_codin hypothetical protein
TcG_04896	752,1989241	0,058103882	0,071624422	0,811230033	0,417233581	0,643941425	protein_codin hypothetical protein
TcG_04897	1505,003855	0,119509365	0,053132536	2,249268973	0,024495389	0,098269935	protein_codin 40S ribosomal protein S11
TcG_04898	337,509886	-0,22303487	0,102245103	-2,18137458	0,029155722	0,112002054	protein_codin hypothetical protein
TcG_04899	1142,773433	-0,208951908	0,066386888	-3,14748763	0,001646801	0,011755905	protein_codin putative RNA helicase
TcG_04900	813,6233171	-0,229270048	0,06902062	-3,32176166	0,000894511	0,007117995	protein_codin hypothetical protein
TcG_04901	215,3430568	0,205266731	0,125372669	1,63725262	0,101577696	0,271859365	protein_codin hypothetical protein
TcG_04902	28,71902655	-0,405737783	0,336922288	-1,20424738	0,228493978	0,455641875	

TcG_04903	162,0951304	-0,54893979	0,147258698	-3,72772405	0,000193217	0,001939856	protein_codin hypothetical protein
TcG_04904	757,7275609	0,144798466	0,071492971	2,02535248	0,042831183	0,148298235	protein_codin putative choline dehydrogenase
TcG_04905	779,9350213	0,027447177	0,069961101	0,392320539	0,694821389	0,842071194	protein_codin putative serine/threonine-protein kinase
TcG_04906	432,5510079	-0,082051383	0,089825099	-0,9134572	0,361002136	0,593609245	protein_codin hypothetical protein
TcG_04907	114,1347664	0,153813618	0,167859735	0,9163223	0,359497875	0,592630203	protein_codin hypothetical protein
TcG_04908	249,5002727	0,324017066	0,113590839	2,85249294	0,004337778	0,025738818	protein_codin peroxidase
TcG_04909	424,105893	0,004807715	0,088924456	0,054065156	0,956883253	0,980060942	protein_codin hypothetical protein
TcG_04910	173,4359978	0,297597263	0,137708036	2,16107405	0,03068962	0,116199325	protein_codin hypothetical protein
TcG_04911	221,1629521	-0,177018757	0,123616922	-1,43199453	0,152145386	0,352339884	protein_codin hypothetical protein
TcG_04912	133,4132784	0,24096274	0,151646686	1,588974652	0,112066116	0,289885692	protein_codin hypothetical protein
TcG_04913	174,8123299	0,002963082	0,140623248	0,021071067	0,983188965	0,993738755	protein_codin hypothetical protein
TcG_04914	377,4105472	0,065109487	0,093487328	0,696452539	0,48614547	0,698274608	protein_codin mitochondrial RNA binding complex 1 subunit
TcG_04915	163,1485985	0,036942314	0,138480413	0,266769235	0,789646843	0,895015489	protein_codin calcineurin B subunit
TcG_04916	221,9009972	-0,002649027	0,117799731	-0,02248754	0,982059048	0,993289929	protein_codin hypothetical protein
TcG_04917	251,1106827	-0,379223831	0,112174437	-3,38066179	0,000723115	0,005920855	protein_codin putative protein kinase
TcG_04918	283,1806981	-0,153574117	0,11185856	-1,37293128	0,169773711	0,376067037	protein_codin putative mitochondrial structure specific endonuclease I (SSE-1)
TcG_04919	221,0562847	0,044419738	0,118620334	0,374469843	0,708054789	0,849437494	protein_codin hypothetical protein
TcG_04920	84,00758586	0,077456973	0,202649887	0,382220657	0,702297701	0,84624626	protein_codin hypothetical protein
TcG_04921	440,2178019	0,204528436	0,088923896	2,300039069	0,021446007	0,088803943	protein_codin hypothetical protein
TcG_04922	39,94387162	0,585581482	0,280631097	2,086659275	0,036918945	0,133502777	protein_codin hypothetical protein
TcG_04923	545,9745816	-0,014004685	0,08511565	-0,16453713	0,869308319	0,936912203	protein_codin pumilio protein 3
TcG_04924	108,6412594	0,161029561	0,168723646	0,954398302	0,339882066	0,573995826	protein_codin hypothetical protein
TcG_04925	116,0960826	0,088282271	0,16339526	0,540298848	0,588990953	0,775648564	protein_codin hypothetical protein
TcG_04926	420,0139272	-0,327780089	0,096977199	-3,37997066	0,000724936	0,005931571	protein_codin proteasome alpha 2 subunit
TcG_04927	986,2533363	-0,112607213	0,06366033	-1,76887575	0,076914611	0,224649463	protein_codin putative cytochrome c oxidase subunit VI
TcG_04928	1320,518986	0,189502677	0,056820266	3,335124776	0,000852611	0,006845707	protein_codin 60S ribosomal protein L32
TcG_04929	374,3758536	0,469103313	0,095804771	4,896450445	9,75832E-07	1,99049E-05	protein_codin hypothetical protein
TcG_04930	346,0596217	-0,385384218	0,098988792	-3,89321065	9,89262E-05	0,001105264	protein_codin proteasome regulatory non-ATPase subunit 11
TcG_04931	417,5961531	-0,235905211	0,092414494	-2,55268627	0,010689573	0,052059435	protein_codin hypothetical protein
TcG_04932	484,5705509	-0,210863488	0,087009315	-2,42345877	0,015373498	0,06895755	protein_codin hypothetical protein
TcG_04933	160,3221011	-0,013155233	0,138413929	-0,0950427	0,924280911	0,963014266	protein_codin hypothetical protein
TcG_04934	624,9519257	-0,257141286	0,078156411	-3,29008565	0,001001569	0,007788038	protein_codin hypothetical protein
TcG_04935	437,1389139	-0,150546736	0,088047044	-1,70984428	0,087294671	0,245068104	protein_codin hypothetical protein
TcG_04936	457,5994504	-0,217457916	0,086085701	-2,52606312	0,011534875	0,055270085	protein_codin hypothetical protein
TcG_04937	389,1595875	-0,17139201	0,091885572	-1,86527662	0,06214264	0,19469568	protein_codin putative GTP-binding protein
TcG_04938	841,9848329	-0,33079985	0,066645022	-4,96360927	6,91951E-07	1,45763E-05	protein_codin hypothetical protein
TcG_04939	119,6452337	-0,311872555	0,171830027	-1,81500614	0,069522966	0,211004551	protein_codin putative Gamma-soluble NSF attachment protein (SNAP-gamma) (N-ethylmaleimide-sensitive factor attachment protein, gamma)
TcG_04940	286,2403075	-0,2476596	0,110539103	-2,24047051	0,025060393	0,099879503	protein_codin putative Gamma-soluble NSF attachment protein (SNAP-gamma) (N-ethylmaleimide-sensitive factor attachment protein, gamma)
TcG_04941	691,1748801	-0,480344123	0,072126675	-6,6597292	2,74333E-11	1,55805E-09	protein_codin hypothetical protein
TcG_04942	591,7434572	-0,288385753	0,081439689	-3,54109594	0,000398469	0,003576032	protein_codin hypothetical protein
TcG_04943	735,2746731	0,013327854	0,078540779	0,169693424	0,865251246	0,935062115	protein_codin splicing factor ptsr1 interacting protein
TcG_04944	1002,582303	-0,329439582	0,063562744	-5,18290373	2,18458E-07	5,24027E-06	protein_codin putative importin beta-1 subunit
TcG_04945	3531,229372	0,196083012	0,04213532	4,653649505	3,26111E-06	5,7861E-05	protein_codin enolase
TcG_04946	2167,056737	-0,389332093	0,12795263	-3,04278303	0,002344013	0,015652871	protein_codin putative calcium channel protein
TcG_04947	669,0701228	-0,416914881	0,078143452	-5,33525039	9,54128E-08	2,48976E-06	protein_codin hypothetical protein
TcG_04948	593,399864	-0,2126446	0,075923666	-2,80076834	0,00509811	0,029125594	protein_codin WD domain-containing protein
TcG_04949	193,4660825	-0,066462085	0,127295951	-0,52210683	0,601595954	0,784478416	protein_codin hypothetical protein
TcG_04950	994,401376	-0,019820356	0,065617079	-0,30206093	0,762605615	0,880133662	protein_codin ribosomal protein S25
TcG_04951	83,07002379	-0,195242583	0,204127304	-0,95647461	0,338832508	0,573276734	protein_codin hypothetical protein
TcG_04952	222,1850953	0,054974569	0,120402112	0,456591402	0,647964761	0,812603825	protein_codin hypothetical protein
TcG_04953	477,6732092	-0,378699724	0,086842861	-4,36074677	1,29619E-05	0,000190097	protein_codin hypothetical protein
TcG_04954	159,7117523	0,213386293	0,139424262	1,530481777	0,125897521	0,31328365	protein_codin hypothetical protein
TcG_04955	337,932055	-0,175160182	0,100418496	-1,74430199	0,081106445	0,233254758	protein_codin putative conserved oligomeric Golgi complex subunit 6-like isoform X1
TcG_04956	906,8150638	-0,142468381	0,0648397	-2,19723998	0,028003311	0,108619473	protein_codin DNA-directed RNA polymerase III subunit RPC1
TcG_04957	770,8604881	-0,331899774	0,071528736	-4,64009005	3,48257E-06	6,13208E-05	protein_codin putative eukaryotic translation initiation factor 5
TcG_04958	433,8045986	-0,130207484	0,09023239	-1,44302378	0,149013772	0,348233698	protein_codin putative bcs1 aaa-type ATPase
TcG_04959	270,1128224	0,142633496	0,113189349	1,260131782	0,207621827	0,429794312	protein_codin putative deoxyhypusine synthase

TcG_04960	368,0113907	0,088106276	0,09816334	0,897547658	0,369426755	0,601128826	protein_codin diguanylate cyclase
TcG_04961	83,26817513	-0,393252619	0,19321761	-2,03528353	0,041822352	0,145812387	protein_codin hypothetical protein
TcG_04962	18,84002647	0,029664691	0,405633193	0,073131814	0,941701225	0,972960076	protein_codin hypothetical protein
TcG_04963	244,0635461	-0,12941419	0,115491818	-1,12054855	0,262480074	0,494647712	protein_codin hypothetical protein
TcG_04964	433,5420935	-0,113080348	0,091565836	-1,23496221	0,216844568	0,440893872	protein_codin hypothetical protein
TcG_04965	305,8179025	0,020187534	0,105372172	0,191583162	0,848068733	0,926605553	protein_codin acyltransferase
TcG_04966	552,5727646	-0,096334591	0,082395356	-1,16917502	0,242333125	0,470898628	protein_codin putative ribonuclease II-like protein
TcG_04967	234,7373298	-0,013627886	0,11623994	-0,11723927	0,906670447	0,954710879	protein_codin hypothetical protein
TcG_04968	360,9826677	0,153933668	0,095951642	1,604283829	0,108651504	0,28454709	protein_codin putative Trichohyalin
TcG_04969	330,8856325	0,162613884	0,102038839	1,593646946	0,111015133	0,288033328	protein_codin geranylgeranyltransferase type I beta subunit
TcG_04970	707,3257187	-0,045685595	0,071950415	-0,63495943	0,525454876	0,728663849	protein_codin NET1-associated nuclear protein 1 (U3 small nucleolar RNA-associated protein 17)
TcG_04971	217,4148269	-0,066162016	0,121831525	-0,54306155	0,587087428	0,775598055	protein_codin carbonic anhydrase-like protein
TcG_04972	532,3236404	-0,081758971	0,08118251	-1,0071008	0,313886338	0,548106574	protein_codin carbonic anhydrase-like protein
TcG_04973	149,0923176	0,076337706	0,152205531	0,501543577	0,615988617	0,792562564	protein_codin hypothetical protein
TcG_04974	309,3003583	-0,083818806	0,10354528	-0,80948939	0,418233702	0,644883639	protein_codin hypothetical protein
TcG_04975	264,9701829	0,069941478	0,110109136	0,635201406	0,525297066	0,728663849	protein_codin zinc finger, MYND-type containing 10
TcG_04976	568,8977582	-0,071186107	0,079999594	-0,88983086	0,373556713	0,604496256	protein_codin putative protein kinase
TcG_04977	1083,997798	0,029194778	0,06043645	0,483065727	0,629049068	0,801161097	protein_codin RAD50 DNA repair-like protein
TcG_04978	147,9468724	0,275666027	0,153895855	1,791250499	0,073253106	0,218009371	protein_codin putative DNA repair protein RAD50
TcG_04979	1322,855775	0,395318323	0,05675447	6,965412977	3,27442E-12	2,28539E-10	protein_codin ribosomal protein S26
TcG_04980	1184,433505	0,045267789	0,061180338	0,739907471	0,459356141	0,677910606	protein_codin Rad51
TcG_04981	235,8734175	0,171556156	0,119668416	1,433595945	0,151687601	0,351877447	protein_codin hypothetical protein
TcG_04982	235,2107456	-0,044527005	0,120741032	-0,36878105	0,712290928	0,852277464	protein_codin hypothetical protein
TcG_04983	317,6697571	0,184417606	0,112166168	1,644146441	0,100145957	0,268999944	protein_codin hypothetical protein
TcG_04984	198,9361081	0,089622283	0,125564083	0,713757316	0,475377258	0,691032669	protein_codin hypothetical protein
TcG_04985	2885,650074	-0,069503248	0,112539386	-0,61759043	0,536845353	0,73660472	protein_codin dynein, axonemal, heavy polypeptide 1
TcG_04986	843,7697267	-0,13821572	0,06828812	-2,02400827	0,042969301	0,148698422	protein_codin putative protein kinase
TcG_04987	419,7489226	-0,180577858	0,088667612	-2,03657066	0,041693085	0,145455009	protein_codin putative ER lumen retaining receptor protein
TcG_04988	97,47279544	-0,051847071	0,190826716	-0,27169713	0,785854909	0,893514718	protein_codin H(+)-transporting two-sector ATPase
TcG_04989	181,539612	-0,072444053	0,138011794	-0,52491204	0,599644336	0,783102809	protein_codin putative protein transport protein Sec13
TcG_04990	242,1411275	0,108434199	0,119403192	0,908134844	0,363806983	0,595685091	protein_codin hypothetical protein
TcG_04991	355,7245966	0,145395106	0,103316913	1,407273039	0,159346441	0,361896891	protein_codin e3 ubiquitin-protein ligase MIB2
TcG_04992	433,6476808	-0,369316783	0,090349442	-4,08764874	4,35767E-05	0,000548783	protein_codin serine/threonine protein phosphatase catalytic subunit
TcG_04993	198,0527863	-0,036496646	0,129208193	-0,28246387	0,777587846	0,889000669	protein_codin hypothetical protein
TcG_04994	260,6648954	-0,128944728	0,118149164	-1,09137232	0,275109086	0,507634843	protein_codin hypothetical protein
TcG_04995	778,4411075	-0,192162176	0,068940868	-2,78734778	0,005314141	0,030033972	protein_codin hypothetical protein
TcG_04996	573,3725118	-0,153640949	0,080875672	-1,89971774	0,057470171	0,182674734	protein_codin U3 small nucleolar RNA-associated protein 3
TcG_04997	86,42377684	-0,079318736	0,193450317	-0,41002122	0,681790379	0,834219382	protein_codin hypothetical protein
TcG_04998	284,7552067	-0,376743656	0,105552366	-3,56925829	0,000357993	0,003253107	protein_codin hypothetical protein
TcG_04999	275,7459827	-0,175507851	0,113400666	-1,54767919	0,121699555	0,306124847	protein_codin hypothetical protein
TcG_05000	395,648867	-0,163499552	0,093615474	-1,74650135	0,080723873	0,232537742	protein_codin putative DNA repair protein
TcG_05001	263,3990943	0,232464718	0,114321578	2,033428169	0,042009284	0,146205936	protein_codin hypothetical protein
TcG_05002	215,1388963	0,056651329	0,122737678	0,46156429	0,644393808	0,811082798	protein_codin hypothetical protein
TcG_05003	477,9484528	0,139582573	0,087428552	1,59653305	0,110369833	0,287164807	protein_codin ion transport protein
TcG_05004	241,692005	-0,017563993	0,11615919	-0,15120622	0,879813041	0,941623821	protein_codin putative ion transport protein
TcG_05005	194,0576584	0,019245229	0,135310419	0,142230211	0,886898169	0,945317589	protein_codin hypothetical protein
TcG_05006	1214,39008	-0,133383645	0,057175503	-2,33288101	0,019654392	0,082866005	protein_codin U3 small nucleolar RNA-associated protein 12
TcG_05007	56,69571997	0,354332367	0,235600338	1,503955257	0,132592888	0,323614384	protein_codin hypothetical protein
TcG_05008	645,0781796	0,057966151	0,074172859	0,781500836	0,434507985	0,658735343	protein_codin hypothetical protein
TcG_05009	255,8017973	0,018724726	0,111190809	0,168401744	0,866267236	0,935530596	protein_codin 23S rRNA methyltransferase
TcG_05010	203,5864138	-0,104990518	0,125998318	-0,8332692	0,404692921	0,634132023	protein_codin hypothetical protein
TcG_05011	345,6491598	0,014015209	0,099546836	0,140790102	0,888035762	0,945946339	protein_codin hypothetical protein
TcG_05012	697,9602595	0,048187401	0,076789992	0,627521899	0,530317186	0,731981763	protein_codin putative run domain Beclin-1 interacting and cystein-rich containing protein-like
TcG_05013	257,3812353	0,019370678	0,1117342	0,17336391	0,862365391	0,934140118	protein_codin hypothetical protein
TcG_05014	895,8708257	-0,364970419	0,072023212	-5,06739996	4,03286E-07	8,97332E-06	protein_codin fatty acid elongase
TcG_05015	666,0453961	-0,19759293	0,072182508	-2,73740738	0,006192556	0,0338429	protein_codin putative fatty acid elongase
TcG_05016	284,4367542	-0,378199484	0,10925162	-3,46172884	0,000536718	0,004609644	protein_codin putative fatty acid elongase

TcG_05017	250,0234221	-0,01684915	0,115552203	-0,14581419	0,884068076	0,943488844	protein_codin fatty acid elongase
TcG_05018	738,5552308	0,143322016	0,071366219	2,008261287	0,044615533	0,152554309	protein_codin hypothetical protein
TcG_05019	148,4041918	0,459012618	0,149405698	3,072256446	0,002124471	0,014468219	protein_codin putative guanin nucleotide-binding protein
TcG_05020	183,3196098	0,368964685	0,133131241	2,77143578	0,005580968	0,031207092	protein_codin large subunit ribosomal protein L21
TcG_05021	933,2040745	0,29394512	0,066599598	4,413617028	1,01658E-05	0,000154365	protein_codin hypothetical protein
TcG_05022	459,5639618	0,156599171	0,089588602	1,747980965	0,080467324	0,23202947	protein_codin translation initiation factor IF-2
TcG_05023	367,7137555	-0,124575147	0,094482804	-1,31849546	0,187337845	0,401571928	protein_codin hypothetical protein
TcG_05024	519,2483299	-0,103251324	0,087190261	-1,18420708	0,236331101	0,463618717	protein_codin putative serine/threonine protein kinase
TcG_05025	590,0343808	0,157782647	0,077735713	2,02973178	0,042383811	0,147155778	protein_codin putative DNA excision repair protein
TcG_05026	232,6129325	0,001919568	0,127268945	0,015082765	0,987966151	0,995145161	protein_codin calpain-like cysteine peptidase
TcG_05027	637,2644211	0,248718121	0,076795958	3,238687641	0,00120081	0,00905178	protein_codin calpain-like cysteine peptidase
TcG_05028	570,073496	0,282437065	0,080364029	3,514471188	0,00044063	0,003888153	protein_codin calpain-like cysteine peptidase
TcG_05029	610,1604015	0,286863888	0,07863155	3,64820337	0,000264081	0,002514082	protein_codin hypothetical protein
TcG_05030	498,0922882	-0,034789045	0,082096546	-0,42375772	0,671742515	0,82795838	protein_codin hypothetical protein
TcG_05031	470,7411657	-0,035335535	0,087130204	-0,40554863	0,685074281	0,836557076	protein_codin hypothetical protein
TcG_05032	371,4635772	-0,189567967	0,101104573	-1,87496927	0,060796952	0,191486794	protein_codin putative protein phosphatase 2C
TcG_05033	367,6720317	0,076655721	0,095541845	0,802326156	0,422364316	0,648576934	protein_codin glutathione synthetase
TcG_05034	1484,441476	-0,133946388	0,055649422	-2,4069682	0,01608557	0,071350467	protein_codin putative mitochondrial DNA polymerase I protein C
TcG_05035	1155,78391	-0,254561858	0,05967262	-4,2659742	1,99032E-05	0,000275835	protein_codin putative immunodominant antigen, putative,tc40 antigen-like
TcG_05036	19,76478898	-0,033502119	0,413004615	-0,08111803	0,93534809	0,969575286	protein_codin hypothetical protein
TcG_05037	497,482927	-0,041404882	0,083017529	-0,49874867	0,617956453	0,793033999	protein_codin hypothetical protein
TcG_05038	110,1130327	-0,234575741	0,172494013	-1,35990656	0,173859494	0,382444674	
TcG_05039	594,8466962	0,229398341	0,078046805	2,939240659	0,003290175	0,020542069	protein_codin ATP-binding cassette protein subfamily D, member 1
TcG_05040	470,2329322	-0,331410434	0,083663984	-3,96120788	7,45716E-05	0,000870954	protein_codin vasohibin-1
TcG_05041	408,3190299	-0,01734969	0,09100216	-0,19065141	0,84879871	0,926926801	protein_codin putative ribosome biogenesis protein
TcG_05042	295,841151	-0,175781071	0,104100881	-1,68856469	0,091302882	0,252348969	protein_codin putative amino acid tansporter
TcG_05043	460,3506012	-0,415955542	0,093858337	-4,43173784	9,34766E-06	0,00014421	protein_codin putative mucin-associated surface protein (MASP)
TcG_05044	337,5341662	0,130214722	0,099021248	1,315017982	0,188503859	0,402804447	protein_codin putative adenosine kinase
TcG_05045	257,746936	0,130349986	0,116340741	1,120415638	0,262536684	0,494673934	protein_codin putative chaperone DNAJ protein
TcG_05046	113,5707225	0,205142578	0,181018353	1,133269496	0,257101097	0,489028976	protein_codin hypothetical protein
TcG_05047	249,8010551	-0,10274786	0,114995301	-0,89349616	0,371591526	0,602808656	protein_codin putative proteasome regulatory non-ATPase subunit
TcG_05048	1084,473588	-0,184247803	0,059519521	-3,09558611	0,001964243	0,013546817	protein_codin putative ATP-dependent RNA helicase
TcG_05049	128,2088826	-0,029158148	0,158865723	-0,18353958	0,854374666	0,929376104	protein_codin ARF GAP-like zinc finger-containing protein
TcG_05050	120,9474215	0,439578358	0,162789516	2,700286655	0,006927975	0,037006696	protein_codin hypothetical protein
TcG_05051	297,8070203	0,054884854	0,10734882	0,511275799	0,609157949	0,788218003	protein_codin putative Transitional endoplasmic reticulum ATPase
TcG_05052	362,4982223	-0,229326452	0,095330227	-2,40560061	0,016145906	0,071526641	protein_codin putative RNA-binding protein
TcG_05053	216,219661	0,097550635	0,120962044	0,806456572	0,419979639	0,646561352	protein_codin RNA editing complex protein MP44
TcG_05054	349,4645592	-0,055448984	0,100953746	-0,54925138	0,582832949	0,771649246	protein_codin hypothetical protein
TcG_05055	256,9050032	-0,096844065	0,119801184	-0,80837318	0,418875785	0,645392368	protein_codin hypothetical protein
TcG_05056	154,1953374	-0,00830156	0,146625465	-0,05661745	0,954849932	0,978889315	protein_codin putative NUDIX hydrolase
TcG_05057	604,1111843	0,060284255	0,076826783	0,784677587	0,432642627	0,657045153	protein_codin hypothetical protein
TcG_05058	889,8340482	-0,241777714	0,064609172	-3,74215776	0,000182447	0,001852127	protein_codin isocitrate dehydrogenase
TcG_05059	5068,624273	-0,064453006	0,03850625	-1,67383233	0,094163556	0,257975636	protein_codin hypothetical protein
TcG_05060	3555,900981	0,048022344	0,042755836	1,123176345	0,261362607	0,493587786	protein_codin cyclophilin A
TcG_05061	249,8191929	0,051505347	0,112025085	0,459766193	0,645684051	0,811993424	protein_codin hypothetical protein
TcG_05062	120,2012311	-0,251858009	0,162703808	-1,54795399	0,121633374	0,306024815	protein_codin putative protein kinase
TcG_05063	526,6529188	-0,058650484	0,080292255	-0,73046254	0,465107502	0,682138496	protein_codin putative MYH7B protein
TcG_05064	107,4797909	-0,134557866	0,17131465	-0,78544285	0,432193967	0,656718489	protein_codin putative protein kinase
TcG_05065	239,2300849	-0,129484831	0,11906264	-1,08753536	0,276800279	0,509048895	protein_codin mitochondrial sodium/hydrogen exchanger NHA2
TcG_05066	497,8183581	-0,014480347	0,082804367	-0,17487419	0,86117849	0,933708964	protein_codin putative glucosamine-6-phosphate isomerase, putative,glucosamine-6-phosphate deaminase
TcG_05067	376,2169919	-0,169852401	0,094081122	-1,8053824	0,071014863	0,213819698	protein_codin putative C-terminal motor kinesin
TcG_05068	143,9829321	-0,430501258	0,146534416	-2,93788497	0,003304596	0,020606593	protein_codin hypothetical protein
TcG_05069	295,2507267	-0,396282515	0,109767578	-3,61019642	0,000305965	0,00284732	protein_codin putative pumilio-repeat, RNA-binding protein
TcG_05070	363,8907443	-0,144698631	0,102872759	-1,4065787	0,159552354	0,362110397	protein_codin putative prefoldin
TcG_05071	746,2579455	-0,386715322	0,070303779	-5,50063354	3,78429E-08	1,06678E-06	protein_codin putative serine/threonine protein kinase
TcG_05072	25,04347077	0,276833142	0,345410449	0,801461399	0,422864583	0,648915106	protein_codin putative exportin 1, putative,RNA-nuclear export factor
TcG_05073	62,29184924	-0,445213774	0,222452631	-2,00138687	0,045350715	0,154378203	protein_codin hypothetical protein

TcG_05074	586,3961183	-0,3628736	0,078925703	-4,59766066	4,27261E-06	7,30125E-05	protein_codin poly-zinc finger protein 2
TcG_05075	97,0504518	-0,057452481	0,183927092	-0,31236551	0,754762756	0,876308377	protein_codin hypothetical protein
TcG_05076	2054,759843	0,336110336	0,049888226	6,737267758	1,61393E-11	9,68857E-10	protein_codin universal minicircle sequence binding protein (UMSBP)
TcG_05077	668,8242399	-0,150305191	0,07336293	-2,04878937	0,040482716	0,142390027	protein_codin putative proteasome beta 5 subunit
TcG_05078	513,409445	-0,046644418	0,086258738	-0,54075006	0,588679869	0,775648564	protein_codin putative tRNA pseudouridine synthase A
TcG_05079	320,3629554	-0,076169261	0,10092112	-0,75474055	0,450404671	0,671089058	protein_codin hypothetical protein
TcG_05080	146,0946251	-0,236670907	0,147967049	-1,59948386	0,109713133	0,286326125	protein_codin mitochondrial inner membrane protease ATP23
TcG_05081	559,3017316	-0,148181684	0,07952633	-1,86330342	0,062419589	0,195186384	protein_codin hypothetical protein
TcG_05082	322,7652499	-0,060107154	0,100787107	-0,59637741	0,550923129	0,746549166	protein_codin putative phosphatidylinositol N-acetylglucosaminyltransferase subunit C
TcG_05083	1548,703673	-0,030499499	0,064449396	-0,47323173	0,63604786	0,806013157	protein_codin putative neurobeachin/beige protein
TcG_05084	459,13957	-0,073573195	0,08635038	-0,85203093	0,394196926	0,624100243	protein_codin hypothetical protein
TcG_05085	670,282638	0,04631135	0,073812846	0,627415858	0,530386676	0,731990473	protein_codin hypothetical protein
TcG_05086	433,3675874	0,284820757	0,090441215	3,149236308	0,001636978	0,011698514	protein_codin putative mucin-associated surface protein (MASP)
TcG_05087	424,1313765	-0,061127246	0,088222704	-0,69287432	0,488388427	0,699785841	protein_codin putative aldehyde dehydrogenase
TcG_05088	239,3751758	-0,032481282	0,119475584	-0,27186544	0,785725488	0,893514718	protein_codin tetratricopeptide repeat protein 16
TcG_05089	547,2708357	0,040535882	0,079075988	0,512619353	0,608217613	0,788022108	protein_codin putative exoribonuclease 2
TcG_05090	175,5942986	0,063614622	0,139297835	0,456680625	0,647900619	0,812603825	protein_codin trichoplein, keratin filament binding protein
TcG_05091	274,3430346	-0,073321937	0,11333028	-0,6469757	0,517647668	0,723000875	protein_codin hypothetical protein
TcG_05092	280,6069804	-0,083214598	0,107808216	-0,77187622	0,440187742	0,663200934	protein_codin putative small GTP-binding protein RAB6
TcG_05093	440,9885189	0,107686891	0,092123468	1,168940909	0,24242744	0,470898628	protein_codin hypothetical protein
TcG_05094	548,8655193	-0,054524484	0,080359121	-0,67851021	0,497448253	0,707256775	protein_codin putative DEAD box RNA helicase
TcG_05095	25,5520738	0,221293348	0,34485812	0,641693889	0,521071958	0,725401608	protein_codin hypothetical protein
TcG_05096	107,3286535	0,252652523	0,179921341	1,404238772	0,16024776	0,363061588	protein_codin DREV methyltransferase
TcG_05097	511,6767417	0,075816843	0,081309852	0,932443503	0,351107371	0,585566432	protein_codin putative DEAD box RNA helicase
TcG_05098	311,5226311	0,064154637	0,101915786	0,629486753	0,529030439	0,731076654	protein_codin putative dynein heavy chain
TcG_05099	691,4668379	-0,230938161	0,073208643	-3,15452045	0,001607621	0,011530366	protein_codin major vault protein
TcG_05100	398,7680486	0,161612421	0,093759187	1,723696907	0,084762574	0,239936276	protein_codin putative condensin subunit 1
TcG_05101	164,4597077	0,417185523	0,13921836	2,996627192	0,002729843	0,017634347	protein_codin endochitinase
TcG_05102	890,1145533	0,049226568	0,068163923	0,722179209	0,470184304	0,686450317	protein_codin hypothetical protein
TcG_05103	154,3418836	0,318688906	0,141952777	2,245034671	0,024765907	0,09904653	protein_codin hypothetical protein
TcG_05104	279,0662377	-0,1559264	0,110668199	-1,40895398	0,158848777	0,361221182	protein_codin T-lymphocyte triggering factor
TcG_05105	275,7274512	0,156524563	0,108488321	1,442777996	0,14908302	0,348241102	protein_codin hypothetical protein
TcG_05106	339,1635195	-0,137972132	0,099433348	-1,38758409	0,165263728	0,370715499	protein_codin hypothetical protein
TcG_05107	1118,948956	0,467390737	0,065111826	7,178277254	7,05953E-13	5,45278E-11	protein_codin large subunit ribosomal protein L37Ae
TcG_05108	249,3245004	-0,238041689	0,118044199	-2,01654711	0,043742797	0,150520953	protein_codin E3 ubiquitin-protein ligase RNF5
TcG_05109	261,3051123	-0,254384021	0,111233903	-2,28692884	0,022199977	0,0911442	protein_codin putative dihydroxyacetone phosphate acyltransferase
TcG_05110	172,0535409	0,220940333	0,13663703	1,616987234	0,105881036	0,280077098	protein_codin hypothetical protein
TcG_05111	218,8661208	-0,023776023	0,124044492	-0,19167334	0,847998088	0,926605553	protein_codin fatty acid elongase
TcG_05112	467,2084945	-0,140918056	0,087225345	-1,61556318	0,106188789	0,28057088	protein_codin BRCT domain-containing protein
TcG_05113	46,28505902	0,061615877	0,253191565	0,243356754	0,807729042	0,905044416	protein_codin hypothetical protein
TcG_05114	571,8281864	-0,14395715	0,08999612	-1,59959284	0,109688938	0,286326125	protein_codin small GTP-binding protein
TcG_05115	98,30364602	0,248496717	0,184075692	1,349970293	0,177025512	0,387183019	protein_codin hypothetical protein
TcG_05116	825,7645112	-0,457373211	0,069190083	-6,61038675	3,83317E-11	2,12194E-09	protein_codin paraflagellar rod component Par4
TcG_05117	550,2751872	-0,071774284	0,084329713	-0,85111501	0,394705469	0,624314965	protein_codin hypothetical protein
TcG_05118	1230,075596	-0,107026483	0,058687661	-1,82366242	0,068203123	0,208221708	protein_codin major vault protein
TcG_05119	121,4910575	-0,091613605	0,16964748	-0,54002338	0,589180911	0,775648564	protein_codin hypothetical protein
TcG_05120	401,331898	-0,125912038	0,091683969	-1,37332666	0,16965082	0,376067037	protein_codin putative protein kinase
TcG_05121	1427,451914	0,007595043	0,056757733	0,133815128	0,893548764	0,948653531	protein_codin putative nucleolar RNA helicase II
TcG_05122	246,5212221	0,189041963	0,114575828	1,64992884	0,098957491	0,266632905	protein_codin hypothetical protein
TcG_05123	243,5132195	-0,058892092	0,118084185	-0,49872972	0,617969806	0,793033999	protein_codin hypothetical protein
TcG_05124	125,9934171	-0,334936495	0,154713796	-2,164878	0,030397029	0,115468846	protein_codin hypothetical protein
TcG_05125	546,4022267	-0,147098969	0,078480225	-1,8743444	0,060882972	0,191525959	protein_codin putative phosphoprotein phosphatase
TcG_05126	136,1174841	0,281506258	0,152091439	1,850901401	0,064183739	0,199258522	protein_codin hypothetical protein
TcG_05127	154,9426805	0,301780603	0,147665265	2,043680362	0,040985134	0,143720874	protein_codin hypothetical protein
TcG_05128	329,8355963	0,098832372	0,105448045	0,937261307	0,348624161	0,583103728	protein_codin putative NUDIX hydrolase, conserved
TcG_05129	311,6599803	0,075365085	0,10147584	0,742689932	0,457669427	0,677123992	protein_codin hypothetical protein
TcG_05130	286,5660709	0,193600233	0,107108248	1,807519363	0,070681338	0,213314401	protein_codin hypothetical protein

TcG_05131	373,6108504	0,325837263	0,095243231	3,421106763	0,000623668	0,005194428	protein_codin	putative dihydrolipoamide branched chain transacylase
TcG_05132	254,6806609	0,249532911	0,11963709	2,085748758	0,037001387	0,133675732	protein_codin	FIP1-like protein
TcG_05133	537,4015239	0,314934933	0,089507615	3,51852671	0,00043395	0,003837974	protein_codin	putative GTP-binding protein
TcG_05134	62,31818076	0,723136769	0,225387534	3,208415113	0,001334687	0,009862043	protein_codin	putative oligopeptidase B, putative,serine peptidase, clan SC, family S9A-like protein
TcG_05135	108,5541366	0,287036842	0,169989185	1,688559432	0,091303891	0,252348969	protein_codin	putative oligopeptidase B, putative,serine peptidase, clan SC, family S9A-like protein
TcG_05136	67,22560727	-0,063741702	0,221846731	-0,28732315	0,773864896	0,886918879	protein_codin	hypothetical protein
TcG_05137	93,11757117	0,078811979	0,182247981	0,432443632	0,665419012	0,825042607	protein_codin	putative oligopeptidase B, putative,serine peptidase, clan SC, family S9A-like protein
TcG_05138	253,5070594	0,41671988	0,114335041	3,644725854	0,000267677	0,002537893	protein_codin	hypothetical protein
TcG_05139	216,0988753	0,025931794	0,129711271	0,199919356	0,841543652	0,92278296	protein_codin	hypothetical protein
TcG_05140	673,8260141	0,344382297	0,078314087	4,397450176	1,0953E-05	0,000164168	protein_codin	putative ATP-dependent RNA helicase
TcG_05141	0,314371616	-2,1715226	2,910784339	-0,74602662	0,455651335	1	protein_codin	histone H4
TcG_05142	192,2783746	0,074923081	0,12866746	0,582300149	0,560364525	0,754316187	protein_codin	histone H4
TcG_05143	42,16330834	-0,704592108	0,283935955	-2,48151773	0,01308242	0,061142767	protein_codin	hypothetical protein
TcG_05144	73,83404763	0,181265902	0,211460336	0,857209942	0,391328869	0,621127152	protein_codin	hypothetical protein
TcG_05145	219,8435902	0,069706112	0,121617017	0,573160844	0,566535794	0,758479744	protein_codin	hypothetical protein
TcG_05146	347,4682471	-0,130578666	0,097428967	-1,3402448	0,180165771	0,391485489	protein_codin	hypothetical protein
TcG_05147	378,5771157	0,012683594	0,092795246	0,136683662	0,891280844	0,946972656	protein_codin	hypothetical protein
TcG_05148	223,9103167	0,350453197	0,129043167	2,715782678	0,00661193	0,035730329	protein_codin	putative TPR-repeat-containing chaperone protein DNAJ
TcG_05149	266,7083704	0,206876726	0,113845678	1,817168019	0,06919139	0,210407204	protein_codin	hypothetical protein
TcG_05150	474,844583	-0,150343266	0,091977055	-1,6345736	0,102138465	0,272918878	protein_codin	hypothetical protein
TcG_05151	248,6935524	0,14409153	0,12089687	1,191854924	0,233318135	0,460672105	protein_codin	hypothetical protein
TcG_05152	291,9320554	0,14692005	0,11124251	1,320718585	0,186595215	0,400572941	protein_codin	hypothetical protein
TcG_05153	248,4294711	0,335717233	0,117173395	2,8651319	0,004168356	0,024958434	protein_codin	hypothetical protein
TcG_05154	318,8941447	0,225983478	0,100848492	2,240821596	0,025037633	0,099823132	protein_codin	coiled-coil domain-containing protein 111
TcG_05155	545,133805	0,028039433	0,081936573	0,342208998	0,732193614	0,86246393	protein_codin	hypothetical protein
TcG_05156	112,6778989	-0,07430772	0,165849884	-0,44804204	0,654122859	0,817094887	protein_codin	hypothetical protein
TcG_05157	334,0364211	0,031049568	0,100827079	0,307948698	0,758121369	0,878015178	protein_codin	hypothetical protein
TcG_05158	438,3053653	-0,182300526	0,089683699	-2,03270525	0,04208231	0,146328226	protein_codin	hypothetical protein
TcG_05159	157,4949693	0,121933304	0,145861361	0,835953425	0,403181105	0,632986744	protein_codin	hypothetical protein
TcG_05160	322,6343356	-0,010588992	0,101294611	-0,10453658	0,916743542	0,959735311	protein_codin	hypothetical protein
TcG_05161	1407,588486	-0,110105984	0,054912871	-2,00510339	0,044951995	0,153406132	protein_codin	putative AAA family ATPase
TcG_05162	170,2613173	0,089244934	0,134089394	0,665562964	0,505690486	0,712679562	protein_codin	hypothetical protein
TcG_05163	726,6572579	0,018910784	0,071956894	0,262807125	0,792699244	0,896248225	protein_codin	putative nonsense mRNA reducing factor 1
TcG_05164	85,70012335	0,045151541	0,190105676	0,237507591	0,812263031	0,907594318	protein_codin	dual specificity phosphatase 12
TcG_05165	396,0098606	-0,10454731	0,092217519	-1,13370334	0,256919008	0,488859194	protein_codin	hypothetical protein
TcG_05166	319,3286376	-0,108092724	0,106029211	-1,01946174	0,307983808	0,541121233	protein_codin	amino acid aldolase
TcG_05167	279,6379484	0,119803435	0,109171451	1,097387949	0,272471839	0,505501798	protein_codin	hypothetical protein
TcG_05168	205,0803711	-0,162669979	0,128314168	-1,2677476	0,204888099	0,426146492	protein_codin	putative desumoylating isopeptidase 2
TcG_05169	166,4799289	-0,048304558	0,139789619	-0,34555182	0,729679557	0,860689063	protein_codin	hypothetical protein
TcG_05170	263,6765114	-0,065821282	0,110316639	-0,59665779	0,550735877	0,746546058	protein_codin	putative mitochondrial carrier protein
TcG_05171	229,7959415	-0,321124926	0,118546892	-2,70884307	0,006751827	0,036322045	protein_codin	hypothetical protein
TcG_05172	122,8209406	-0,093618444	0,16037426	-0,58374981	0,559388651	0,753876574	protein_codin	hypothetical protein
TcG_05173	245,4334194	-0,170020359	0,120407445	-1,41204191	0,157937623	0,359997107	protein_codin	hypothetical protein
TcG_05174	218,3176855	-0,120516087	0,123304323	-0,97738736	0,328377418	0,562869623	protein_codin	hypothetical protein
TcG_05175	156,3696521	0,088265702	0,146837136	0,60111294	0,547764766	0,744620741	protein_codin	hypothetical protein
TcG_05176	729,3959218	-0,608804951	0,070435982	-8,64337987	5,45741E-18	8,1296E-16	protein_codin	paraflagellar rod component
TcG_05177	737,757055	0,26988542	0,073462268	3,673796458	0,000238973	0,002315002	protein_codin	hypothetical protein
TcG_05178	55,92536431	-0,367785488	0,236035883	-1,55817617	0,119191492	0,301781605	protein_codin	hypothetical protein
TcG_05179	441,4345207	-0,151917674	0,086791395	-1,75037715	0,080053256	0,231180714	protein_codin	hypothetical protein
TcG_05180	178,899412	-0,236504395	0,148486828	-1,59276347	0,111213264	0,288387841	protein_codin	hypothetical protein
TcG_05181	89,54521691	0,157859758	0,191058859	0,826236266	0,40867008	0,636605346	protein_codin	hypothetical protein
TcG_05182	232,197461	0,126456125	0,117190324	1,079066259	0,280558191	0,513654778	protein_codin	hypothetical protein
TcG_05183	216,3115397	0,04432451	0,121358567	0,365235937	0,714935294	0,853669261	protein_codin	hypothetical protein
TcG_05184	98,05093846	0,08008338	0,17647465	0,453795378	0,649976111	0,814209452	protein_codin	hypothetical protein
TcG_05185	41,23062984	0,243174617	0,284024533	0,856174694	0,391901158	0,621398293	protein_codin	hypothetical protein
TcG_05186	250,8847444	-0,106427888	0,114173658	-0,93215799	0,35125488	0,585728129	protein_codin	hypothetical protein
TcG_05187	194,1025325	-0,204746267	0,130694604	-1,56660077	0,117208037	0,2985867	protein_codin	ubiquinone biosynthesis protein-like protein

TcG_05188	78,11296648	0,033025825	0,196711651	0,16788952	0,866670195	0,93574468	protein_codin hypothetical protein
TcG_05189	181,5500326	0,240108149	0,131683706	1,82337022	0,068247337	0,208301805	protein_codin hypothetical protein
TcG_05190	72,88076415	-0,26467648	0,202912801	-1,30438533	0,192102235	0,408384677	protein_codin putative heat shock protein 67B2
TcG_05191	145,6586447	0,097365383	0,146803898	0,663234316	0,507180497	0,713910003	protein_codin endoplasmic reticulum protein
TcG_05192	78,26033707	0,417619205	0,199110443	2,09742492	0,035955977	0,131249512	protein_codin Heat shock protein 67Bb
TcG_05193	1020,445458	-0,094158523	0,06417548	-1,46720402	0,142320563	0,338310637	protein_codin putative DNA J-binding protein
TcG_05194	263,1539477	0,060750157	0,111509559	0,544797753	0,585892624	0,774461146	protein_codin cytochrome b5-like protein
TcG_05195	100,0132766	0,387551585	0,177594248	2,182230499	0,029092525	0,111796352	protein_codin hypothetical protein
TcG_05196	927,9971639	-0,321569802	0,063980398	-5,02606755	5,0064E-07	1,0903E-05	protein_codin hypothetical protein
TcG_05197	182,0730705	-0,437338412	0,136163496	-3,21186239	0,001318775	0,009788168	protein_codin hypothetical protein
TcG_05198	115,5717768	-0,098330378	0,169820046	-0,57902692	0,562571008	0,755967027	protein_codin cAMP-dependent protein kinase dimerization/docking domain
TcG_05199	78,12094104	0,217024722	0,210352424	1,031719615	0,302203486	0,535371497	protein_codin putative GDP-mannose transporter
TcG_05200	478,5872171	-0,323072477	0,087059704	-3,71093012	0,000206499	0,002040925	protein_codin BRCT domain-containing protein
TcG_05201	150,3657598	0,036335423	0,150028864	0,242189549	0,808633293	0,905265886	protein_codin hypothetical protein
TcG_05202	126,6394427	-0,216099146	0,157652538	-1,37073052	0,170458977	0,377040418	protein_codin hypothetical protein
TcG_05203	23,96568994	-0,609661716	0,38238785	-1,59435431	0,1108567	0,287785285	
TcG_05204	816,8145545	-0,266342276	0,069277862	-3,84455102	0,000120773	0,001307738	protein_codin putative dual specificity protein phosphatase
TcG_05205	347,4269987	-0,015773167	0,102019404	-0,15460948	0,877129197	0,941029583	protein_codin hypothetical protein
TcG_05206	305,1947534	-0,014034021	0,10626294	-0,13206882	0,894929858	0,948981304	protein_codin hypothetical protein
TcG_05207	770,5059333	-0,282032564	0,069341827	-4,06727911	4,75653E-05	0,000591935	protein_codin hypothetical protein
TcG_05208	608,8970342	-0,07714069	0,076469843	-1,0087727	0,313083656	0,547218396	protein_codin putative MCAK-like kinesin
TcG_05209	367,3464385	-0,124184808	0,096440212	-1,287687	0,19785494	0,416429354	protein_codin hypothetical protein
TcG_05210	8,222973371	0,015921491	0,600021869	0,026534852	0,978830736	1	protein_codin hypothetical protein
TcG_05211	325,4981467	-0,042651772	0,099289081	-0,42957163	0,667507278	0,825813061	protein_codin hypothetical protein
TcG_05212	308,4120615	-0,040902708	0,108843842	-0,37579258	0,707071103	0,848924953	protein_codin putative mitochondrial glycoprotein
TcG_05213	445,0891066	0,040369745	0,090507969	0,446035259	0,655571777	0,817728228	protein_codin MYND zinc finger (ZnF) domain-like protein
TcG_05214	177,9516757	-0,142281655	0,131680928	-1,08050313	0,279918195	0,513072647	protein_codin putative vesicle transport protein SFT2B-like
TcG_05215	538,8978573	0,049256236	0,088636094	0,555713075	0,578407017	0,768240018	protein_codin hypothetical protein
TcG_05216	539,3160928	0,303052962	0,08052776	3,7633353	0,000167662	0,001723632	protein_codin methylthioadenosine phosphorylase
TcG_05217	540,3567405	-0,234113492	0,079417749	-2,94787369	0,003199678	0,020103832	protein_codin hypothetical protein
TcG_05218	8,44833057	-0,335315714	0,593368165	-0,56510567	0,572001893	1	
TcG_05219	175,5567765	0,159469434	0,135386429	1,177883454	0,238843072	0,466886424	protein_codin hypothetical protein
TcG_05220	550,1204632	-0,068930124	0,083930685	-0,8212744	0,411489984	0,638080373	protein_codin hypothetical protein
TcG_05221	177,0878076	-0,045145495	0,138900029	-0,3250215	0,745164802	0,871188637	protein_codin hypothetical protein
TcG_05222	1031,839742	-0,079155648	0,065150841	-1,21495973	0,224381452	0,451082534	protein_codin splicing factor 3B subunit 3
TcG_05223	407,2938229	-0,096378033	0,091908114	-1,04863465	0,294346303	0,527256689	protein_codin hypothetical protein
TcG_05224	565,9030015	0,005717048	0,084528852	0,067634275	0,94607677	0,974694377	protein_codin putative paraflagellar rod protein
TcG_05225	596,1907103	0,002228102	0,077529845	0,028738641	0,977073038	0,990177192	protein_codin putative ubiquitin-conjugating enzyme
TcG_05226	373,4987177	-0,099856007	0,099459725	-1,00398434	0,31538616	0,550227985	protein_codin hypothetical protein
TcG_05227	207,7809776	0,129913058	0,133426525	0,973667405	0,330221706	0,564965843	protein_codin putative glutaminyl cyclase
TcG_05228	322,8601344	-0,10043137	0,104535837	-0,96073627	0,33668479	0,571141848	protein_codin putative glutaminyl cyclase
TcG_05229	173,4038065	0,063784249	0,147487931	0,432470977	0,665399142	0,825042607	protein_codin putative ATPase
TcG_05230	1,145156382	-0,185704626	1,676306574	-0,11078202	0,911789205	1	protein_codin ATPase
TcG_05231	618,9042595	0,070100141	0,078435417	0,893730705	0,371465993	0,602808656	protein_codin uncharacterized protein
TcG_05232	628,9923235	0,160471869	0,082391647	1,947671591	0,051454271	0,168686401	protein_codin hypothetical protein
TcG_05233	68,56956136	0,107265409	0,215263468	0,498298249	0,618273842	0,793033999	
TcG_05234	835,3511161	-0,414991127	0,072185867	-5,7489249	8,98127E-09	2,88526E-07	protein_codin hypothetical protein
TcG_05235	831,7348656	-0,213054181	0,068546675	-3,10816216	0,001882547	0,013091953	protein_codin double-strand-break repair protein rad21
TcG_05236	766,3502386	-0,25740897	0,080468625	-3,19887374	0,001379656	0,010149011	protein_codin putative methyltransferase
TcG_05237	317,7859184	-0,136402464	0,104985246	-1,29925366	0,193856893	0,410814106	protein_codin hypothetical protein
TcG_05238	727,0084321	-0,102611789	0,071543959	-1,43424812	0,151501471	0,351799471	protein_codin hypothetical protein
TcG_05239	5,761951631	-0,619258504	0,768127523	-0,80619231	0,420131971	1	protein_codin protein kinase
TcG_05240	14,04431637	-0,002578749	0,492082294	-0,00524048	0,995818719	1	protein_codin putative target of rapamycin (TOR) kinase 1
TcG_05241	8,714612151	0,531641238	0,594953503	0,893584516	0,371544234	1	protein_codin hypothetical protein
TcG_05242	30,41492129	-0,020547434	0,33079921	-0,06211452	0,950471634	0,976948576	protein_codin helicase-like protein
TcG_05243	409,230435	-0,09244091	0,096324	-0,9596872	0,337212666	0,57160877	protein_codin hypothetical protein
TcG_05244	170,8562664	0,044131803	0,134376656	0,328418673	0,742595126	0,869061326	protein_codin hypothetical protein

TcG_05245	562,326861	-0,062301681	0,080049519	-0,77828925	0,436398509	0,660007183	protein_codin	hypothetical protein
TcG_05246	479,7819621	-0,073674489	0,084947572	-0,86729365	0,385781136	0,616325044	protein_codin	hypothetical protein
TcG_05247	309,7272667	-0,048455591	0,101796291	-0,47600546	0,634070487	0,804197117	protein_codin	guanylate kinase
TcG_05248	216,8648284	-0,051289031	0,123475696	-0,41537754	0,677865543	0,831348595	protein_codin	hypothetical protein
TcG_05249	258,6428266	-0,147455769	0,114801706	-1,28443883	0,198988453	0,417822396	protein_codin	putative chloride channel protein
TcG_05250	175,0414725	-0,197187985	0,139718752	-1,41132084	0,158150033	0,360339486	protein_codin	putative chloride channel protein
TcG_05251	282,0294433	0,161552819	0,108485877	1,489159906	0,136445266	0,329163749	protein_codin	hypothetical protein
TcG_05252	274,9505725	-0,078229348	0,112299621	-0,69661275	0,486045174	0,698274608	protein_codin	hypothetical protein
TcG_05253	396,7947764	0,094503312	0,095236232	0,992304191	0,32104916	0,556034397	protein_codin	hypothetical protein
TcG_05254	297,1150675	-0,158677036	0,10448829	-1,51861071	0,128860513	0,317925448	protein_codin	hypothetical protein
TcG_05255	348,5473639	-0,195048875	0,098421191	-1,98177722	0,04750418	0,159392826	protein_codin	geranylgeranyl transferase type-2 subunit alpha
TcG_05256	539,0179086	-0,083177604	0,086143692	-0,96556813	0,334260329	0,568767832	protein_codin	putative calcium-transporting ATPase
TcG_05257	260,8262258	0,021262312	0,113312661	0,187642862	0,851156624	0,928053891	protein_codin	S-phase kinase-associated protein 1
TcG_05258	667,1031663	-0,177811687	0,072238266	-2,46146118	0,013837237	0,063799168	protein_codin	hypothetical protein
TcG_05259	786,257785	-0,318466673	0,070514877	-4,51630475	6,29281E-06	0,00010197	protein_codin	putative nicotinate phosphoribosyltransferase
TcG_05260	624,725237	0,006985	0,074706728	0,093498946	0,925507187	0,963648571	protein_codin	putative WD40 repeat protein
TcG_05261	480,950877	-0,154843733	0,087285047	-1,77400068	0,076063039	0,223104838	protein_codin	hypothetical protein
TcG_05262	231,0058376	0,269652404	0,118555808	2,274476548	0,02293735	0,093673646	protein_codin	hypothetical protein
TcG_05263	483,1947442	-0,113252902	0,084426799	-1,34143309	0,179779881	0,391013648	protein_codin	hypothetical protein
TcG_05264	663,439833	0,168671066	0,073180836	2,304852939	0,021174811	0,088040307	protein_codin	40S ribosomal protein S3
TcG_05265	695,4899006	0,172610224	0,076340437	2,261058897	0,02375561	0,096266166	protein_codin	hypothetical protein
TcG_05266	334,5745462	-0,183865005	0,105715252	-1,73924765	0,081991211	0,234961703	protein_codin	hypothetical protein
TcG_05267	152,39272	0,214596619	0,142069801	1,510501303	0,130915557	0,321421412	protein_codin	hypothetical protein
TcG_05268	67,89291449	-0,414861725	0,214296884	-1,93592047	0,052877442	0,171751623		
TcG_05269	173,4426434	-0,09150562	0,136428804	-0,67072068	0,502398487	0,710995111	protein_codin	hypothetical protein
TcG_05270	472,3735645	-0,195820076	0,084425613	-2,31943919	0,020371235	0,085298564	protein_codin	hypothetical protein
TcG_05271	195,5211405	0,020958776	0,135496908	0,154680844	0,877072933	0,941029583	protein_codin	hypothetical protein
TcG_05272	379,8512509	0,104308578	0,095498223	1,092256737	0,27472027	0,507556451	protein_codin	hypothetical protein
TcG_05273	445,2673349	0,061856014	0,092384813	0,66954743	0,503146336	0,711603204	protein_codin	hypothetical protein
TcG_05274	643,7807411	0,067128608	0,075943857	0,88392414	0,376737165	0,60741397	protein_codin	hypothetical protein
TcG_05275	283,1388873	-0,076451095	0,108714689	-0,70322691	0,48191435	0,695931654	protein_codin	hypothetical protein
TcG_05276	1100,507459	-0,071011795	0,066941043	-1,06081101	0,288775799	0,52204032	protein_codin	cyclin 5
TcG_05277	44,55959471	0,041150074	0,260434363	0,158005548	0,874452427	0,939572087		
TcG_05278	710,256359	0,223792814	0,074388988	3,008413215	0,002626158	0,017064872	protein_codin	hypothetical protein
TcG_05279	16,16012047	0,176020178	0,43677399	0,403000596	0,686947786	1	protein_codin	putative glycine dehydrogenase, putative,glycine cleavage system P-protein
TcG_05280	35,44393695	-0,226621566	0,292791132	-0,7740042	0,438928306	0,66225073	protein_codin	structural maintenance of chromosome protein 4
TcG_05281	23,64563269	0,296689139	0,356170564	0,83299736	0,404846218	0,634151194	protein_codin	hypothetical protein
TcG_05282	8,549088568	0,273771581	0,622904669	0,439507993	0,660293492	1	protein_codin	hypothetical protein
TcG_05283	16,44151283	-0,163726608	0,431877883	-0,37910394	0,704610678	1	protein_codin	target of rapamycin (TOR) kinase 1
TcG_05284	431,4293776	-0,052404172	0,091385273	-0,5734422	0,566345321	0,758388878	protein_codin	hypothetical protein
TcG_05285	376,5855236	-0,155959796	0,097844365	-1,59395787	0,110945472	0,287951218	protein_codin	hypothetical protein
TcG_05286	216,1162375	0,034531414	0,12145809	0,284307235	0,776174942	0,888310845	protein_codin	hypothetical protein
TcG_05287	529,8877723	-0,131699117	0,080376145	-1,63853489	0,101310163	0,271394115	protein_codin	putative flavoprotein monooxygenase
TcG_05288	1037,693432	0,159066149	0,063045961	2,523018861	0,011635216	0,055635829	protein_codin	hypothetical protein
TcG_05289	129,7442193	-0,043783345	0,157387793	-0,27818768	0,780868294	0,890875557	protein_codin	hypothetical protein
TcG_05290	214,837253	0,011217819	0,12518636	0,08960896	0,928597964	0,965428814	protein_codin	hypothetical protein
TcG_05291	513,3188928	-0,038771863	0,086174469	-0,44992285	0,652766069	0,816292247	protein_codin	alanyl-tRNA synthetase
TcG_05292	396,2033295	-0,017434215	0,091472545	-0,19059506	0,848842858	0,926926801	protein_codin	hypothetical protein
TcG_05293	387,2798299	-0,062465411	0,093885052	-0,66533926	0,505833524	0,712679562	protein_codin	hypothetical protein
TcG_05294	178,412896	0,318601675	0,139095492	2,290524806	0,021990912	0,090431762	protein_codin	hypothetical protein
TcG_05295	532,6747464	-0,487182192	0,082449234	-5,90887467	3,44453E-09	1,21672E-07	protein_codin	putative protein kinase
TcG_05296	100,3129653	-0,254875978	0,184123106	-1,38426938	0,166275986	0,371834313	protein_codin	ABC1 transporter
TcG_05297	183,0803558	-0,082860998	0,134656876	-0,61534918	0,538324144	0,737672801	protein_codin	hypothetical protein
TcG_05298	457,5572743	-0,035040905	0,08673737	-0,40398856	0,686221135	0,837209523	protein_codin	hypothetical protein
TcG_05299	1201,640421	-0,239758563	0,059718249	-4,01482912	5,94889E-05	0,00071572	protein_codin	hypothetical protein
TcG_05300	429,2806766	-0,26395714	0,091155151	-2,89569088	0,003783249	0,023021386	protein_codin	hypothetical protein
TcG_05301	119,1220974	-0,117506652	0,171179723	-0,68645194	0,492428163	0,703052704	protein_codin	putative dolichol phosphate-mannose biosynthesis regulatory protein

TcG_05302	687,2513946	-0,276893593	0,075139829	-3,68504424	0,000228663	0,00223192	protein_codin putative RAB GDP dissociation inhibitor alpha
TcG_05303	241,9150783	0,022203693	0,113381532	0,195831654	0,844741934	0,924802045	protein_codin hypothetical protein
TcG_05304	414,2548968	0,083923435	0,089275181	0,940053378	0,347190181	0,581713007	protein_codin hypothetical protein
TcG_05305	221,3130541	-0,022437636	0,122492012	-0,18317632	0,854659675	0,929424347	protein_codin putative nucleotide sugar transporter
TcG_05306	416,2901633	0,027187073	0,090061655	0,301871796	0,762749797	0,880133662	protein_codin hypothetical protein
TcG_05307	133,7067626	-0,115644832	0,156616204	-0,73839634	0,460273638	0,678399847	protein_codin hypothetical protein
TcG_05308	269,6070186	-0,191379296	0,11554722	-1,65628646	0,097663821	0,264500475	protein_codin hypothetical protein
TcG_05309	401,1156033	-0,068820044	0,091836505	-0,74937569	0,453630799	0,674035553	protein_codin hypothetical protein
TcG_05310	295,8592285	-0,087153369	0,10748706	-0,81082661	0,41746525	0,644127366	protein_codin putative myo-inositol-1(or 4)-monophosphatase 1
TcG_05311	678,1408014	-0,038386893	0,072482129	-0,52960493	0,596385876	0,780668859	protein_codin putative trans-sialidase
TcG_05312	2047,722046	0,097938647	0,048172905	2,033065005	0,042045956	0,146245706	protein_codin ATP-binding cassette protein subfamily G, member 4
TcG_05313	858,9188997	-0,05926089	0,065858761	-0,89981787	0,368217181	0,600276384	protein_codin putative arginine kinase
TcG_05314	451,2152046	-0,038181501	0,092103945	-0,41454794	0,678472865	0,831829271	protein_codin hypothetical protein
TcG_05315	631,1093728	0,212894437	0,074876429	2,843277115	0,004465223	0,02639873	protein_codin putative MFS transporter
TcG_05316	46,83907634	0,075376865	0,265644293	0,283751118	0,776601117	0,888310845	protein_codin hypothetical protein
TcG_05317	120,2531085	0,168494301	0,168080476	1,002462067	0,316120478	0,551153727	protein_codin hypothetical protein
TcG_05318	242,5082284	0,208518489	0,113943278	1,830020094	0,067246934	0,20644488	protein_codin hypothetical protein
TcG_05319	68,49678443	0,398618082	0,2103451	1,895067115	0,058083511	0,184169555	protein_codin hypothetical protein
TcG_05320	324,2167035	0,17923809	0,104758623	1,710962635	0,087088011	0,244606471	protein_codin cell cycle checkpoint protein
TcG_05321	1069,299898	0,326791879	0,060168141	5,431310884	5,59416E-08	1,52503E-06	protein_codin hypothetical protein
TcG_05322	21,96494395	0,037421873	0,374009277	0,100056003	0,920299865	0,961460255	protein_codin hypothetical protein
TcG_05323	9,150826649	1,017846182	0,607980997	1,67414144	0,094102805	1	protein_codin hypothetical protein
TcG_05324	58,08308083	-0,08095354	0,234161719	-0,34571637	0,729555877	0,860669422	protein_codin hypothetical protein
TcG_05325	72,74637324	0,509043193	0,210494343	2,418322444	0,015592253	0,069642191	protein_codin retrotransposon hot spot (RHS) protein
TcG_05326	197,6228984	-0,012580864	0,125162062	-0,1005166	0,919934208	0,961425016	protein_codin hypothetical protein
TcG_05327	182,0468193	-0,008408587	0,133035431	-0,06320562	0,949602767	0,976575329	protein_codin hypothetical protein
TcG_05328	474,8919268	-0,032056455	0,085097316	-0,37670348	0,706393976	0,848727637	protein_codin BRCT domain-containing protein
TcG_05329	343,5824228	0,133075431	0,099001185	1,344180181	0,178890129	0,389799325	protein_codin putative methyltransferase
TcG_05330	292,3383347	0,539030639	0,112299538	4,799936388	1,58716E-06	3,03948E-05	protein_codin putative glycerol-3-phosphate dehydrogenase (FAD-dependent)
TcG_05331	431,4926943	0,066646271	0,093646986	0,711675558	0,476665707	0,691952794	protein_codin hypothetical protein
TcG_05332	237,6149861	0,222976538	0,126351422	1,764733116	0,07760863	0,225979791	protein_codin putative zeta tubulin
TcG_05333	263,0665325	0,043534126	0,11133382	0,391023378	0,695779956	0,842351784	protein_codin hypothetical protein
TcG_05334	53,30849178	0,319735023	0,248007985	1,289212614	0,197324181	0,415747947	protein_codin hypothetical protein
TcG_05335	202,172771	0,131377811	0,123685559	1,062191997	0,288148532	0,521231677	protein_codin nucleotidyl transferase domain-containing protein
TcG_05336	331,2344128	-0,012346213	0,10199765	-0,12104409	0,903656115	0,953356379	protein_codin hypothetical protein
TcG_05337	110,6671393	0,182930601	0,173469183	1,054542356	0,291634679	0,524263676	protein_codin hypothetical protein
TcG_05338	323,5155717	-0,063099572	0,102157334	-0,6176705	0,536792561	0,73660472	protein_codin MORN repeat-containing protein
TcG_05339	293,517072	0,089684661	0,104402727	0,859026045	0,390326147	0,620400374	protein_codin hypothetical protein
TcG_05340	192,3114566	0,079844095	0,12939727	0,617046211	0,537204246	0,736836273	protein_codin RWD domain containing 1
TcG_05341	153,5308073	0,18411849	0,145191837	1,268104971	0,204760467	0,426069462	protein_codin putative chaperone DNAJ protein
TcG_05342	4,155384206	0,911030477	0,871592567	1,045248102	0,295908317	1	protein_codin hypothetical protein
TcG_05343	596,671774	0,015285755	0,083023671	0,184113212	0,853924642	0,929148282	protein_codin cytidine triphosphate synthase
TcG_05344	223,7595916	0,222809933	0,123385936	1,805796833	0,07095008	0,213735734	protein_codin cytidine triphosphate synthase
TcG_05345	440,6758122	-0,014415913	0,089482713	-0,16110277	0,872012461	0,938429906	protein_codin hypothetical protein
TcG_05346	173,8425356	0,055141862	0,139660773	0,394827128	0,692970473	0,841062696	protein_codin hypothetical protein
TcG_05347	251,3995655	0,358353102	0,130982197	2,73589167	0,00622115	0,033967129	protein_codin hypothetical protein
TcG_05348	204,7013616	0,08396517	0,123711526	0,678717437	0,497316915	0,707252891	protein_codin mitochondrial editosome-like complex associated TUTase
TcG_05349	318,044061	0,154626865	0,104173445	1,484321311	0,137723699	0,331119896	protein_codin putative FG-GAP repeat protein
TcG_05350	134,5076362	0,053447504	0,152328785	0,350869366	0,725686354	0,858201705	protein_codin hypothetical protein
TcG_05351	246,1640498	0,095419848	0,115647865	0,825089572	0,40932074	0,636798799	protein_codin hypothetical protein
TcG_05352	504,2460204	0,06687024	0,087473954	0,764458868	0,444593839	0,666545577	protein_codin kinesin heavy chain
TcG_05353	398,998735	-0,01521971	0,093658096	-0,16250287	0,870909864	0,937678811	protein_codin rRNA biogenesis protein
TcG_05354	328,4794087	-0,225593241	0,104291514	-2,16310257	0,030533292	0,115796634	protein_codin serine/threonine protein phosphatase 2a regulatory subunit
TcG_05355	203,3419433	0,063055572	0,131135322	0,480843539	0,630627703	0,802025529	protein_codin hypothetical protein
TcG_05356	445,002551	0,43821523	0,096313008	4,549906997	5,36696E-06	8,91128E-05	protein_codin hypothetical protein
TcG_05357	602,3954994	-0,019799245	0,077650015	-0,25498057	0,79873811	0,899599469	protein_codin hypothetical protein
TcG_05358	141,5559562	0,139889758	0,148447896	0,942349209	0,346013882	0,580329594	protein_codin hypothetical protein

TcG_05359	844,5053581	0,209828083	0,069702268	3,010348002	0,002609485	0,016975573	protein_codin pyruvate transporter 0
TcG_05360	146,2174515	0,548427219	0,161448506	3,396917282	0,000681496	0,005623795	protein_codin putative amino acid transporter
TcG_05361	174,237376	-0,123880383	0,137991838	-0,89773703	0,369325762	0,601128826	protein_codin putative glutamic acid/alanine-rich protein
TcG_05362	420,2911115	-7,07621E-05	0,093109646	-0,00075999	0,999393618	0,99981271	protein_codin hypothetical protein
TcG_05363	216,881958	-0,111067393	0,120538886	-0,92142376	0,35682923	0,590701202	protein_codin hypothetical protein
TcG_05364	269,6028301	-0,112355825	0,113742807	-0,98780598	0,323247676	0,557573663	protein_codin putative periplasmic protein
TcG_05365	95,43503861	0,58477093	0,189645595	3,083493341	0,002045857	0,014017326	protein_codin hypothetical protein
TcG_05366	645,7605335	0,231547192	0,074864917	3,092866474	0,001982333	0,013646649	protein_codin putative mitochondrial DNA polymerase I protein B
TcG_05367	322,3093252	0,173633701	0,105536668	1,645245239	0,099919248	0,26859963	protein_codin surfeit locus 1 family protein
TcG_05368	89,57611911	-0,21238515	0,185462177	-1,14516692	0,25214002	0,482300523	protein_codin hypothetical protein
TcG_05369	192,6237795	0,164514973	0,130911508	1,256688397	0,208866491	0,431513404	protein_codin hypothetical protein
TcG_05370	264,1625725	0,336551287	0,110054201	3,058050345	0,002227821	0,015006709	protein_codin hypothetical protein
TcG_05371	500,7986997	0,070207838	0,082118262	0,854960102	0,392573236	0,622039594	protein_codin calpain protease-like protein
TcG_05372	157,181383	0,171301382	0,146557551	1,168833545	0,242470702	0,470898628	protein_codin putative 40S ribosomal protein S8
TcG_05373	746,3270172	-0,447234259	0,073891267	-6,05259966	1,42527E-09	5,48491E-08	protein_codin regulatory subunit of protein kinase A
TcG_05374	347,3827347	0,233456739	0,099821225	2,338748497	0,019348454	0,081874063	protein_codin putative histone acetyltransferase
TcG_05375	135,235175	-0,111069844	0,161223883	-0,68891682	0,490875615	0,701878188	protein_codin variant surface glycoprotein 3054
TcG_05376	241,756477	0,118678313	0,118992483	0,997359748	0,318589922	0,553576194	protein_codin putative ras-related protein rab-5
TcG_05377	91,12417999	0,499032551	0,188305065	2,65012813	0,008046125	0,041672957	protein_codin hypothetical protein
TcG_05378	231,211181	-0,239094162	0,121582081	-1,96652467	0,049238035	0,163693509	protein_codin hypothetical protein
TcG_05379	172,1259762	0,136416958	0,135392623	1,007565665	0,313663019	0,547964376	protein_codin putative dynein heavy chain, cytosolic
TcG_05380	130,5599106	0,15671631	0,157830386	0,99294131	0,320738557	0,555966026	protein_codin flagellar associated protein
TcG_05381	217,6228351	-0,109946601	0,12001452	-0,91611083	0,35960877	0,592630203	protein_codin putative U4/U6 small nuclear ribonuclear protein
TcG_05382	418,5683647	0,07046827	0,094822618	0,743158879	0,457385496	0,676963255	protein_codin N-acetyltransferase complex ARD1 subunit
TcG_05383	0,145251675	-1,420530545	4,080472857	-0,3481289	0,727743378	1	
TcG_05384	353,8579194	-0,143441887	0,095298754	-1,50518114	0,132277514	0,323190062	protein_codin hypothetical protein
TcG_05385	824,360373	-0,004982192	0,074080568	-0,06725369	0,946379744	0,974694377	protein_codin hypothetical protein
TcG_05386	289,5473171	-0,038858758	0,106312244	-0,36551535	0,714726748	0,853614301	protein_codin hypothetical protein
TcG_05387	279,6567064	0,042543427	0,106808734	0,398314125	0,69039865	0,83922987	protein_codin putative acyl-CoA synthetase
TcG_05388	502,4172065	-0,453459829	0,084990056	-5,33544569	9,53102E-08	2,48976E-06	protein_codin putative flagellar radial spoke component
TcG_05389	265,3857478	-0,28473548	0,116895006	-2,43582246	0,014857975	0,067322838	protein_codin mitogen-activated protein kinase kinase 2
TcG_05390	964,3222314	-0,264201961	0,069316014	-3,81155732	0,000138094	0,001458484	protein_codin hypothetical protein
TcG_05391	761,660082	-0,002159164	0,06903349	-0,03127705	0,975048591	0,989135188	protein_codin hypothetical protein
TcG_05392	1,753889398	4,042872142	1,596745621	2,531945032	0,011343177	1	protein_codin hypothetical protein
TcG_05393	1,278370512	0,676998756	1,580126519	0,428445917	0,668326501	1	protein_codin hypothetical protein
TcG_05394	11,45818282	0,516499813	0,543730118	0,949919449	0,342153184	1	protein_codin target of rapamycin (TOR) kinase 1
TcG_05395	5147,017029	-0,247817142	0,039927167	-6,20672986	5,40985E-10	2,30436E-08	protein_codin putative epsilon tubulin
TcG_05396	257,3677675	-0,133701397	0,11083324	-1,20632941	0,227690503	0,454517947	protein_codin surface antigen
TcG_05397	10,05469893	0,406832754	0,550658718	0,738811065	0,460021732	1	protein_codin trans-sialidase
TcG_05398	314,2058021	-0,023462846	0,101320196	-0,23157127	0,816871028	0,909580769	protein_codin hypothetical protein
TcG_05399	315,5785635	0,066292906	0,102395204	0,647421981	0,517358874	0,722792706	protein_codin putative ADP-ribosylation factor GTPase activating protein
TcG_05400	95,24196211	0,287041507	0,183019176	1,568368481	0,116795163	0,298059197	protein_codin hypothetical protein
TcG_05401	468,0083768	0,043874527	0,083999607	0,522318243	0,601448769	0,784374768	protein_codin putative kinesin
TcG_05402	708,0913998	-0,190891564	0,074380804	-2,56640901	0,010275758	0,050489794	protein_codin putative calcium motive p-type ATPase
TcG_05403	309,6980356	-0,114298732	0,107241222	-1,06580968	0,286509684	0,519715008	protein_codin hypothetical protein
TcG_05404	409,1996141	0,445581213	0,092989627	4,791730295	1,65349E-06	3,16128E-05	protein_codin putative intersectin-1-like
TcG_05405	23,13568723	0,206320093	0,365305327	0,564788076	0,572217918	0,763502386	protein_codin 60S ribosomal protein L32
TcG_05406	23,10455001	0,293515111	0,375793064	0,78105516	0,434770053	0,658980355	protein_codin 60S ribosomal protein L32
TcG_05407	998,9648994	0,358135166	0,072951802	4,90920249	9,14475E-07	1,87524E-05	protein_codin putative ankyrin repeat protein
TcG_05408	231,9743443	-0,061884777	0,118062103	-0,5241714	0,600159333	0,783502945	protein_codin putative calmodulin
TcG_05409	323,9197211	-0,018013433	0,100619849	-0,17902465	0,85791835	0,931569072	protein_codin NADH dehydrogenase (ubiquinone) 1 alpha subcomplex 9
TcG_05410	1956,524118	0,443504659	0,052847257	8,392198259	4,77135E-17	6,35413E-15	protein_codin 40S ribosomal protein S6
TcG_05411	210,7506063	0,169362981	0,124592581	1,359334396	0,174040649	0,382593335	protein_codin hypothetical protein
TcG_05412	457,3435614	0,150265883	0,085448614	1,758552602	0,07865353	0,228276501	protein_codin hypothetical protein
TcG_05413	48,11766002	0,427258499	0,258740606	1,651300527	0,098677225	0,266249261	protein_codin hypothetical protein
TcG_05414	1346,111004	-0,016084414	0,059019968	-0,27252495	0,785218407	0,893514718	protein_codin hypothetical protein
TcG_05415	413,8657429	0,010237309	0,089582671	0,114277782	0,909017595	0,955841241	protein_codin hypothetical protein

TcG_05416	249,0681709	-0,056415331	0,112995716	-0,49926964	0,617589439	0,793033999	protein_codin UBX domain containing protein
TcG_05417	182,9354705	0,102600477	0,144824049	0,708449164	0,478666371	0,693141929	protein_codin ATP synthase mitochondrial F1 complex assembly factor 1
TcG_05418	242,8475328	0,051957548	0,124348347	0,417838672	0,676065074	0,830195013	protein_codin hypothetical protein
TcG_05419	46,93530707	0,389530666	0,254123033	1,532842822	0,125314587	0,312235442	protein_codin hypothetical protein
TcG_05420	86,51003223	-0,030057204	0,189717293	-0,15843155	0,874116757	0,939472797	protein_codin hypothetical protein
TcG_05421	0					1	protein_codin 60S ribosomal protein L5
TcG_05422	2181,200324	0,189103051	0,055727973	3,393323686	0,0006905	0,005689994	protein_codin 60S ribosomal protein L5
TcG_05423	131,8449207	0,256483301	0,160946376	1,593594759	0,111026829	0,288033328	protein_codin 60S ribosomal protein L5
TcG_05424	224,5593998	-0,062307438	0,117417817	-0,53064722	0,595663268	0,780607921	protein_codin hypothetical protein
TcG_05425	268,7476159	-0,123319865	0,109543105	-1,12576565	0,260264727	0,492617789	protein_codin COP-coated vesicle membrane protein gp25L precursor
TcG_05426	118,8918529	0,129728188	0,169931016	0,763416776	0,445214878	0,66703823	protein_codin hypothetical protein
TcG_05427	413,3564319	0,045337674	0,089211308	0,508205459	0,611309263	0,789796547	protein_codin hypothetical protein
TcG_05428	251,6130033	0,163506707	0,11736286	1,393172476	0,163567641	0,368265582	protein_codin putative rRNA processing protein
TcG_05429	259,6036913	-0,164537796	0,113142445	-1,4542535	0,145876037	0,343402425	protein_codin trypanin-like protein
TcG_05430	107,8661662	-0,116967436	0,172671771	-0,67739756	0,498153746	0,707424985	protein_codin DTW domain-containing protein
TcG_05431	209,283853	-0,301317441	0,12520891	-2,40651755	0,01610543	0,071411218	protein_codin putative DNA polymerase epsilon subunit b
TcG_05432	200,3489137	-0,027778086	0,126357381	-0,21983746	0,825997742	0,915112889	protein_codin hypothetical protein
TcG_05433	111,4097989	-0,306237219	0,1691086	-1,81089087	0,070157746	0,212176364	protein_codin hypothetical protein
TcG_05434	119,9888633	-0,1313411	0,160555632	-0,81804106	0,413333741	0,640054093	protein_codin hypothetical protein
TcG_05435	186,774711	-0,122506317	0,132169666	-0,92688678	0,353985319	0,58824927	protein_codin hypothetical protein
TcG_05436	119,705856	-0,05356015	0,167716861	-0,31934863	0,749462158	0,873568266	protein_codin hypothetical protein
TcG_05437	100,781734	0,232855788	0,17514344	1,329514757	0,183678201	0,39621963	protein_codin hypothetical protein
TcG_05438	7,517956349	0,094921073	0,654742118	0,144974748	0,88473081	1	protein_codin trans-sialidase
TcG_05439	8,338221842	1,157147672	0,641351722	1,80423258	0,071194854	1	protein_codin trans-sialidase
TcG_05440	7,731438765	-0,889592489	0,657157381	-1,35369778	0,175832816	1	protein_codin structural maintenance of chromosome protein 4
TcG_05441	0					1	protein_codin hypothetical protein
TcG_05442	127,291584	0,245605451	0,157115649	1,563214435	0,118002166	0,299818661	protein_codin hypothetical protein
TcG_05443	136,0155805	0,632019155	0,155403698	4,066950541	4,76323E-05	0,000592133	protein_codin cyclophilin
TcG_05444	164,8011547	0,588702877	0,148364713	3,967944038	7,24954E-05	0,000849273	protein_codin hypothetical protein
TcG_05445	445,6439165	0,092386878	0,089353249	1,033950958	0,30115909	0,534351377	protein_codin putative translation initiation factor IF-2
TcG_05446	283,9127093	0,148388831	0,111345377	1,332689649	0,182633671	0,394701309	protein_codin hypothetical protein
TcG_05447	159,7606441	0,620265993	0,148084627	4,188591389	2,80691E-05	0,00037231	protein_codin transferase
TcG_05448	113,4153944	0,362695575	0,174077712	2,083526784	0,037203233	0,134195721	protein_codin putative protein HID1 isoform 1
TcG_05449	188,6051021	0,301734127	0,12978685	2,324843601	0,020080325	0,084324263	protein_codin putative protein HID1 isoform 1
TcG_05450	158,9679278	0,291315082	0,146505693	1,988421587	0,046765083	0,158048946	protein_codin EF hand
TcG_05451	180,313259	0,287874223	0,139796502	2,05923767	0,039471476	0,13999295	protein_codin EF hand
TcG_05452	415,3945956	0,631829478	0,093529291	6,755418239	1,42424E-11	8,77725E-10	protein_codin mismatch repair protein MLH1
TcG_05453	706,962529	0,358633943	0,077370148	4,635301262	3,56418E-06	6,24497E-05	protein_codin putative kinesin
TcG_05454	313,9473957	0,514268506	0,103361301	4,975445367	6,50978E-07	1,38136E-05	protein_codin hypothetical protein
TcG_05455	72,96947466	0,657244741	0,206204701	3,187341207	0,001435873	0,010489295	protein_codin hypothetical protein
TcG_05456	156,6170693	0,479058075	0,154076372	3,109224793	0,001875789	0,013052791	protein_codin citrate lyase subunit beta
TcG_05457	251,3677668	0,192227254	0,115022896	1,671208611	0,09468048	0,259038152	protein_codin putative protein kinase, putative,serine/threonine-protein kinase
TcG_05458	227,8916274	0,71955792	0,123842446	5,810268944	6,23726E-09	2,05298E-07	protein_codin hypothetical protein
TcG_05459	567,8549628	0,369771035	0,079210754	4,668192353	3,03861E-06	5,44133E-05	protein_codin hypothetical protein
TcG_05460	232,2129847	0,381688396	0,118625831	3,217582494	0,001292758	0,009638288	protein_codin hypothetical protein
TcG_05461	644,6678575	0,604699778	0,07702103	7,85109961	4,12405E-15	4,46554E-13	protein_codin periodic tryptophan protein 1
TcG_05462	1838,889083	0,3229199	0,058087714	5,559177308	2,71049E-08	7,93024E-07	protein_codin translationally controlled tumor protein (TCTP)
TcG_05463	457,3353479	0,704916952	0,088917673	7,927748494	2,23155E-15	2,48603E-13	protein_codin putative DNA repair protein
TcG_05464	755,1239167	0,765087735	0,072207961	10,59561468	3,12283E-26	1,06415E-23	protein_codin hypothetical protein
TcG_05465	89,07099223	0,763064375	0,195388394	3,905372053	9,40805E-05	0,001059297	protein_codin hypothetical protein
TcG_05466	536,6102442	0,584543994	0,090699424	6,444847907	1,15716E-10	5,63315E-09	protein_codin putative ATP-dependent DEAD/H DNA helicase recQ
TcG_05467	132,2159266	0,655326713	0,161632824	4,054416031	5,02597E-05	0,000618162	protein_codin ATP-dependent DEAD/H DNA helicase recQ
TcG_05468	305,9884958	0,507100444	0,105501823	4,806556216	1,53552E-06	2,9651E-05	protein_codin sigma-adaptin 3
TcG_05469	385,5457225	0,617379294	0,094666521	6,521622299	6,9551E-11	3,54986E-09	protein_codin hypothetical protein
TcG_05470	631,6341119	-0,26675049	0,080839832	-3,29974076	0,000967742	0,007579652	protein_codin calpain-like cysteine peptidase
TcG_05471	123,6544176	0,137047774	0,159496654	0,859251715	0,390201657	0,620400374	protein_codin putative RNA-binding protein
TcG_05472	372,5952138	0,402835806	0,095045263	4,238357552	2,25161E-05	0,000307269	protein_codin hypothetical protein

TcG_05473	138,0629396	0,152972505	0,159226201	0,960724454	0,336690732	0,571141848	protein_codin	hypothetical protein
TcG_05474	84,78766676	0,641683167	0,194752828	3,294859302	0,00098471	0,007677555	protein_codin	hypothetical protein
TcG_05475	925,4297214	0,08668347	0,065051563	1,332534786	0,182684519	0,394737567	protein_codin	succinate dehydrogenase (quinone)
TcG_05476	164,6777596	0,466160196	0,142590938	3,269213327	0,00107847	0,008302424	protein_codin	hypothetical protein
TcG_05477	219,0272173	0,076006289	0,128405951	0,591921853	0,55390292	0,748922771	protein_codin	ESAG-like protein
TcG_05478	476,3476773	0,317002877	0,086531255	3,663449414	0,000248841	0,002394582	protein_codin	fatty acyl-CoA reductase
TcG_05479	69,75139392	0,028896365	0,24077338	0,120014782	0,904471438	0,953695494	protein_codin	hypothetical protein
TcG_05480	105,1388071	0,047204715	0,175907438	0,26834974	0,788430123	0,894874711	protein_codin	hypothetical protein
TcG_05481	372,9809883	-0,094244109	0,095324247	-0,9886688	0,322825212	0,557414741	protein_codin	mitochondrial ATP-dependent zinc metallopeptidase
TcG_05482	415,0737863	-0,028328772	0,094317114	-0,30035664	0,763905131	0,880920161	protein_codin	SET and MYND domain-containing protein
TcG_05483	341,7828291	0,161568477	0,100263063	1,611445647	0,107082625	0,282082846	protein_codin	putative uracil phosphoribosyltransferase
TcG_05484	112,9414128	-0,393915596	0,177169812	-2,22337876	0,026190271	0,103281306	protein_codin	hypothetical protein
TcG_05485	95,08845774	0,274357684	0,185151747	1,481799057	0,13839377	0,33221448	protein_codin	hypothetical protein
TcG_05486	401,867097	-0,130099954	0,092452286	-1,40721187	0,159364573	0,361896891	protein_codin	hypothetical protein
TcG_05487	203,5033198	0,053470438	0,127004956	0,421010645	0,673747309	0,829023911	protein_codin	hypothetical protein
TcG_05488	276,6814183	-0,14158749	0,119257797	-1,18723885	0,23513342	0,462964244	protein_codin	hypothetical protein
TcG_05489	256,4084815	0,063741129	0,118384747	0,538423496	0,590284709	0,776238879	protein_codin	hypothetical protein
TcG_05490	520,8741657	-0,072148188	0,080881779	-0,8920203	0,372382053	0,603575828	protein_codin	myosin IF
TcG_05491	28,34137297	0,916004924	0,340333504	2,691492058	0,007113319	0,037745541	protein_codin	amastin
TcG_05492	335,9437214	-0,077076794	0,105254311	-0,73229109	0,463990919	0,681522416	protein_codin	tuzin
TcG_05493	383,2909339	-0,120798077	0,092974312	-1,29926293	0,193853712	0,410814106	protein_codin	hypothetical protein
TcG_05494	390,5323579	-0,001114176	0,092082343	-0,01209978	0,99034601	0,996036512	protein_codin	beta-lactamase domain-containing protein
TcG_05495	340,0139401	-0,030141842	0,103557544	-0,2910637	0,771002603	0,885140325	protein_codin	protein kinase Wee90
TcG_05496	137,2595459	-0,010124094	0,150859269	-0,06710953	0,946494511	0,974694377	protein_codin	hypothetical protein
TcG_05497	192,6749511	0,067867415	0,131986326	0,514200348	0,607111925	0,787770048	protein_codin	hypothetical protein
TcG_05498	332,2184826	-0,162717139	0,101687401	-1,60017011	0,109560852	0,286167848	protein_codin	hypothetical protein
TcG_05499	36,53978159	0,340849479	0,300909109	1,132732341	0,257326672	0,489152883	protein_codin	hypothetical protein
TcG_05500	303,3420602	-0,094478097	0,107134759	-0,88186222	0,377851317	0,608532856	protein_codin	hypothetical protein
TcG_05501	54,77818461	-0,037844623	0,24098059	-0,15704428	0,875209952	0,939877107	protein_codin	hypothetical protein
TcG_05502	76,40399222	-0,07990209	0,20643254	-0,38706151	0,698710674	0,844142481	protein_codin	hypothetical protein
TcG_05503	248,1637003	0,513750215	0,121125625	4,241465967	2,22065E-05	0,000303759	protein_codin	hypothetical protein
TcG_05504	0,324841555	-0,458748506	2,940479636	-0,15601146	0,876023992	1	protein_codin	hypothetical protein
TcG_05505	1606,84883	0,312728433	0,054882793	5,698114438	1,2114E-08	3,7628E-07	protein_codin	DNA-directed RNA polymerase subunit
TcG_05506	551,2970527	0,256235362	0,081987684	3,125290914	0,001776293	0,012521533	protein_codin	hypothetical protein
TcG_05507	5,529698517	0,088759935	0,744581659	0,119207792	0,905110734	1	protein_codin	hypothetical protein
TcG_05508	309,1525947	-0,037669884	0,103711097	-0,36321942	0,716440983	0,854413373	protein_codin	hypothetical protein
TcG_05509	252,7974334	0,159435587	0,121939245	1,307500199	0,191042887	0,406681682	protein_codin	putative RNA-binding protein
TcG_05510	1462,957399	0,100358705	0,057868764	1,734246553	0,082874346	0,23675955	protein_codin	60S ribosomal protein L13a
TcG_05511	222,3058774	0,063839868	0,122570069	0,520843864	0,602475544	0,785006933	protein_codin	serine/threonine-protein phosphatase PP1 beta
TcG_05512	816,3396826	0,047261782	0,066684299	0,70873927	0,478486291	0,69303613	protein_codin	putative 25 kDa translation elongation factor 1-beta
TcG_05513	125,0421806	0,00049416	0,157121077	0,003145091	0,997490585	0,999104158	protein_codin	hypothetical protein
TcG_05514	126,7444669	0,253364133	0,164002572	1,54487902	0,122375529	0,307291478	protein_codin	hypothetical protein
TcG_05515	192,7548157	0,119041817	0,128446695	0,926779917	0,354040814	0,588257117	protein_codin	serine/threonine-protein phosphatase PP1
TcG_05516	0,966128114	0,043593554	1,756674999	0,024815947	0,980201771	1	protein_codin	serine/threonine-protein phosphatase PP1
TcG_05517	107,7934251	0,239663167	0,168759722	1,42014436	0,155565658	0,356978355	protein_codin	hypothetical protein
TcG_05518	247,3568756	0,383450672	0,123877683	3,095397521	0,001965493	0,013546817	protein_codin	3-beta-hydroxy-Delta(5)-steroid dehydrogenase
TcG_05519	289,0318683	0,150280411	0,109597207	1,37120658	0,170310568	0,376919082	protein_codin	hypothetical protein
TcG_05520	297,4798429	0,177012518	0,107779508	1,642357826	0,10051587	0,269702841	protein_codin	U3 small nucleolar RNA-associated protein 19
TcG_05521	313,6390663	0,192747344	0,113007919	1,705609177	0,08808087	0,246556345	protein_codin	protein phosphatase 2C
TcG_05522	195,8818224	-0,198805621	0,129143893	-1,53941171	0,123703817	0,309486597	protein_codin	superoxide dismutase, Fe-Mn family
TcG_05523	381,2282842	-0,15393722	0,175183706	-0,87871882	0,379553751	0,610703762	protein_codin	hypothetical protein
TcG_05524	84,86147672	0,437328208	0,205568177	2,127412002	0,033385866	0,123818387	protein_codin	transmembrane protein 216
TcG_05525	140,6207542	0,062206907	0,148302282	0,419460217	0,674879824	0,82970688	protein_codin	hypothetical protein
TcG_05526	70,62885205	-0,137149818	0,223234998	-0,61437418	0,538968097	0,738205979	protein_codin	hypothetical protein
TcG_05527	1030,051493	-0,170701818	0,062829623	-2,71690025	0,006589647	0,035643158	protein_codin	Flagellar attachment zone protein 1
TcG_05528	1070,023987	0,406308892	0,060690479	6,694771576	2,16009E-11	1,25134E-09	protein_codin	transketolase 1
TcG_05529	1277,260492	0,751408511	0,058559109	12,83162467	1,09053E-37	9,02492E-35	protein_codin	60S ribosomal protein L26

TcG_05530	63,78911659	0,761351886	0,256525926	2,967933487	0,002998092	0,019012531	
TcG_05531	156,6162033	0,674156742	0,147083285	4,583503431	4,57249E-06	7,73386E-05	protein_codin conserved eukaryotic protein
TcG_05532	715,2546104	0,341663935	0,071314732	4,79093067	1,66009E-06	3,16868E-05	protein_codin putative tubulin folding cofactor D
TcG_05533	436,135156	0,562559346	0,088709883	6,341563379	2,27445E-10	1,02937E-08	protein_codin class III phosphatidylinositol 3-phosphate kinase
TcG_05534	439,9579181	0,645351164	0,099260162	6,501613026	7,94633E-11	3,96837E-09	protein_codin hypothetical protein
TcG_05535	232,908687	0,299310246	0,118943629	2,516404187	0,011855913	0,056458121	protein_codin hypothetical protein
TcG_05536	359,4664691	-0,143319666	0,0978126	-1,46524749	0,142853408	0,33915975	protein_codin hypothetical protein
TcG_05537	422,7383293	0,320072827	0,097003527	3,299599887	0,000968228	0,007579652	protein_codin hypothetical protein
TcG_05538	395,3570386	0,368192002	0,094022805	3,915986155	9,00354E-05	0,001020695	protein_codin hypothetical protein
TcG_05539	260,9658648	0,555114099	0,114476681	4,849145631	1,23994E-06	2,45272E-05	protein_codin hypothetical protein
TcG_05540	243,7586288	0,499108653	0,12147187	4,108841453	3,97649E-05	0,000502965	protein_codin peptidyl-prolyl isomerase E (cyclophilin E)
TcG_05541	205,0792045	0,397147792	0,128746991	3,084715129	0,002037472	0,013968136	protein_codin hypothetical protein
TcG_05542	359,2867483	0,400894604	0,100388672	3,99342473	6,51258E-05	0,000773894	protein_codin hypothetical protein
TcG_05543	146,3568398	0,976671717	0,153088817	6,379771807	1,77352E-10	8,31903E-09	protein_codin DnaJ like protein subfamily C member 19
TcG_05544	200,4680366	0,544359699	0,131087153	4,152654829	3,2864E-05	0,000426386	protein_codin alkyl and aryl transferase
TcG_05545	286,405006	0,355491398	0,109357302	3,250733058	0,001151079	0,008766247	protein_codin hypothetical protein
TcG_05546	277,8537647	0,359657636	0,109897526	3,272663638	0,001065392	0,008218129	protein_codin hypothetical protein
TcG_05547	15,57756055	-0,299389652	0,470202417	-0,63672504	0,524303962	1	protein_codin cyclin-dependent protein kinase
TcG_05548	108,18762	0,695685486	0,173332238	4,01359548	5,98008E-05	0,000718726	protein_codin putative cyclin 11
TcG_05549	12,61204464	0,181730368	0,518241198	0,350667543	0,725837778	1	
TcG_05550	590,5452162	0,516182453	0,07888479	6,543497848	6,00963E-11	3,16489E-09	protein_codin hypothetical protein
TcG_05551	513,0959793	0,18531529	0,083709984	2,213777632	0,02684408	0,105321878	protein_codin hypothetical protein
TcG_05552	639,1233433	0,419750333	0,079949604	5,250186487	1,51945E-07	3,81047E-06	protein_codin hypothetical protein
TcG_05553	170,5536692	0,333222435	0,136843971	2,435053815	0,014889575	0,06738696	protein_codin glycosyltransferase
TcG_05554	403,3166386	0,367035905	0,09048438	4,05634545	4,98465E-05	0,000613732	protein_codin hypothetical protein
TcG_05555	195,086495	0,453118818	0,131641402	3,442069224	0,000577283	0,004890386	protein_codin hypothetical protein
TcG_05556	513,0993641	0,320804583	0,084459016	3,798346216	0,000145665	0,001535643	protein_codin lysophospholipase
TcG_05557	256,0187707	0,083887348	0,114966218	0,729669547	0,4655922	0,682138496	protein_codin hypothetical protein
TcG_05558	318,6947514	0,399580124	0,101187934	3,948891031	7,85141E-05	0,00090604	protein_codin putative short chain dehydrogenase/reductase
TcG_05559	255,2416479	0,323802577	0,111522431	2,903474873	0,003690466	0,022557499	protein_codin putative short chain dehydrogenase/reductase
TcG_05560	114,5372679	-0,278937125	0,183466451	-1,52037129	0,128417686	0,317382939	protein_codin hypothetical protein
TcG_05561	581,6787602	0,375556472	0,079016154	4,752907499	2,00512E-06	3,74096E-05	protein_codin putative RNA-binding protein
TcG_05562	746,403696	0,323430113	0,07267874	4,450133749	8,58168E-06	0,000134725	protein_codin putative fatty acid transporter protein-like
TcG_05563	292,069109	-0,181190639	0,106814858	-1,69630557	0,089828026	0,249753993	protein_codin trans-sialidase
TcG_05564	112,0330848	0,453725939	0,167882752	2,702635812	0,006879207	0,036847913	protein_codin hypothetical protein
TcG_05565	222,2330746	0,294736531	0,123879963	2,379210675	0,017349757	0,075370935	protein_codin hypothetical protein
TcG_05566	173,063619	-0,145952635	0,13484085	-1,08240667	0,279071866	0,512088477	protein_codin hypothetical protein
TcG_05567	445,7451782	-0,504095108	0,09162415	-5,50177118	3,75995E-08	1,06251E-06	protein_codin histone H2A
TcG_05568	227,8555335	-0,335382471	0,117886576	-2,84495895	0,004441715	0,026296222	protein_codin putative NADH-ubiquinone oxidoreductase, mitochondrial
TcG_05569	312,4167684	-0,185622482	0,103956349	-1,785581	0,074167126	0,219938655	protein_codin hypothetical protein
TcG_05570	372,4226337	-0,262231733	0,094400508	-2,7778636	0,005471758	0,030715015	protein_codin hypothetical protein
TcG_05571	538,5621286	-0,104025019	0,081165086	-1,28164737	0,199966374	0,418877311	protein_codin putative protein kinase
TcG_05572	207,2994084	0,016396605	0,123546971	0,132715553	0,894418342	0,948820769	protein_codin protein kinase
TcG_05573	497,1845308	-0,244371822	0,084192327	-2,90254267	0,003701467	0,022583044	protein_codin putative C-terminal motor kinesin
TcG_05574	337,8430058	-0,092358584	0,101582202	-0,90920045	0,363244325	0,595268564	protein_codin DUF3508 domain containing protein
TcG_05575	353,2928915	-0,050654501	0,098584884	-0,5138161	0,607380569	0,787942142	protein_codin putative peptidase t
TcG_05576	376,436819	-0,137808996	0,096031333	-1,435042	0,151275131	0,351589501	protein_codin hypothetical protein
TcG_05577	258,7039483	0,068443704	0,119071336	0,5748126	0,565418045	0,757986619	protein_codin hypothetical protein
TcG_05578	436,976217	0,431779901	0,09079395	4,755602132	1,97856E-06	3,70932E-05	protein_codin hypothetical protein
TcG_05579	266,4586658	0,126448558	0,118766922	1,064678244	0,287021558	0,520249026	protein_codin cell wall surface anchor family protein
TcG_05580	160,731478	-0,249090187	0,139019147	-1,79176892	0,073169989	0,217817959	protein_codin ADP-ribosylation factor
TcG_05581	391,1611906	0,026981679	0,092038428	0,293156664	0,769402406	0,884364283	protein_codin putative protein kinase
TcG_05582	170,4441548	-0,049464293	0,135703964	-0,36450146	0,715483584	0,853960462	protein_codin hypothetical protein
TcG_05583	127,7099526	0,147635929	0,159469076	0,925796608	0,35455169	0,588430867	protein_codin chaperone protein DNAJ
TcG_05584	176,5799388	0,094158706	0,14857779	0,633733389	0,526254831	0,72897997	protein_codin hypothetical protein
TcG_05585	272,7003923	-0,149281563	0,107572626	-1,38772817	0,165219835	0,370715499	protein_codin hypothetical protein
TcG_05586	92,84331386	-0,166045439	0,180302765	-0,92092564	0,357089253	0,590864907	protein_codin hypothetical protein

TcG_05587	83,39495528	0,094886793	0,20169361	0,47045017	0,638033427	0,807510942	protein_codin	hypothetical protein
TcG_05588	217,4448882	-0,038601295	0,126016592	-0,30631915	0,75936166	0,878478701	protein_codin	hypothetical protein
TcG_05589	80,97404829	0,122505358	0,197089162	0,621573284	0,534222493	0,734571778	protein_codin	hypothetical protein
TcG_05590	83,93040586	0,227301124	0,198332618	1,146060223	0,251770234	0,481861939	protein_codin	hypothetical protein
TcG_05591	1273,915454	-0,031816747	0,056426177	-0,56386501	0,572846003	0,76401448	protein_codin	hypothetical protein
TcG_05592	61,25812222	0,276342416	0,225608187	1,224877608	0,220621339	0,445705114	protein_codin	hypothetical protein
TcG_05593	26,05465704	-0,484142697	0,334980235	-1,44528735	0,148377189	0,347222402	protein_codin	putative retrotransposon hot spot (RHS) protein
TcG_05594	55,84553409	0,260438016	0,236445233	1,101472897	0,270690894	0,503811197	protein_codin	putative trans-sialidase
TcG_05595	14,17151195	0,068747158	0,466878408	0,147248528	0,882935858	1		
TcG_05596	46,08591824	0,164453152	0,256158884	0,641996677	0,520875341	0,725343954		
TcG_05597	49,1621871	0,177699713	0,255022213	0,69680092	0,485927391	0,698274608	protein_codin	hypothetical protein
TcG_05598	1350,079854	-0,026025814	0,056624102	-0,45962432	0,6457859	0,812019442	protein_codin	putative retrotransposon hot spot (RHS) protein
TcG_05599	75,5891652	0,452188417	0,215390312	2,099390691	0,035782475	0,130750609	protein_codin	hypothetical protein
TcG_05600	91,4379741	0,214038975	0,184043634	1,162979504	0,244837818	0,473018336	protein_codin	solanesyl-diphosphate synthase
TcG_05601	184,8911208	0,223912958	0,143904776	1,555980037	0,11971284	0,302903027	protein_codin	putative rab1 small GTP-binding protein
TcG_05602	439,168834	0,215854517	0,090743957	2,378720568	0,01737284	0,075414658	protein_codin	putative retrotransposon hot spot (RHS) protein
TcG_05603	123,1667774	-0,034054548	0,161531728	-0,21082265	0,833025661	0,919089983	protein_codin	hypothetical protein
TcG_05604	2139,610214	-0,143747862	0,051173744	-2,80901592	0,004969319	0,028672574	protein_codin	hypothetical protein
TcG_05605	617,4667025	-0,077346104	0,084363674	-0,91681763	0,359238212	0,592630203	protein_codin	putative tyrosyl-tRNA synthetase
TcG_05606	493,731212	0,254962408	0,09369637	2,721155657	0,006505412	0,035302906	protein_codin	hypothetical protein
TcG_05607	292,5416867	-0,007293419	0,108509546	-0,06721454	0,946410914	0,974694377	protein_codin	putative glycogen synthase kinase-3 alpha
TcG_05608	363,4829663	0,20674608	0,104717333	1,9743253	0,048344766	0,161570239	protein_codin	putative pyrroline-5-carboxylate reductase
TcG_05609	382,6965613	0,111896227	0,091710172	1,220107053	0,222424295	0,448409237	protein_codin	hypothetical protein
TcG_05610	245,9650403	0,381908418	0,119399772	3,198569064	0,001381114	0,010153295	protein_codin	hypothetical protein
TcG_05611	582,1190017	-0,009299622	0,079434632	-0,11707264	0,906802491	0,954763146	protein_codin	GTPase activating protein
TcG_05612	390,0688965	0,047480545	0,09526168	0,498422292	0,618186428	0,793033999	protein_codin	peptidyl-prolyl cis-trans isomerase
TcG_05613	483,7437922	0,278720683	0,083508856	3,337618249	0,000844998	0,006793992	protein_codin	hypothetical protein
TcG_05614	740,6157014	0,105773319	0,071599471	1,477291919	0,139597403	0,334099466	protein_codin	putative kinesin
TcG_05615	681,5367305	-0,075679511	0,074754243	-1,01237746	0,311357613	0,545005183	protein_codin	putative proteasome regulatory ATPase subunit 1
TcG_05616	601,1704651	-0,164085398	0,077871457	-2,10713148	0,035106185	0,128878408	protein_codin	endoplasmatic reticulum retrieval protein
TcG_05617	276,7491792	0,504533654	0,109659368	4,60091705	4,20635E-06	7,21996E-05	protein_codin	SAM-binding protein
TcG_05618	281,3947431	0,269273771	0,107837825	2,497025239	0,012524004	0,058985005	protein_codin	N-acetyltransferase
TcG_05619	551,1189641	0,218724321	0,083293534	2,625945986	0,008640853	0,044083188	protein_codin	ubiquitin-conjugating enzyme e2
TcG_05620	791,554154	-0,135300115	0,069542257	-1,94558131	0,051705053	0,169103777	protein_codin	putative proteasome regulatory ATPase subunit 5
TcG_05621	523,6858786	-0,19901366	0,080640188	-2,46792159	0,013590008	0,063057203	protein_codin	hypothetical protein
TcG_05622	145,2820306	-0,054913452	0,152843103	-0,35927988	0,719385724	0,855909389	protein_codin	hypothetical protein
TcG_05623	398,0461797	0,144466794	0,100088958	1,443383943	0,148912345	0,348123169	protein_codin	hypothetical protein
TcG_05624	850,5821767	-0,127110601	0,067059599	-1,89548704	0,058027907	0,184043618	protein_codin	mitochondrial guide RNA binding complex subunit 1
TcG_05625	689,9626205	-0,070966195	0,073899163	-0,96031122	0,336898605	0,571410809	protein_codin	glucoamylase-like protein
TcG_05626	501,0584216	0,115145895	0,085559081	1,345805657	0,178365201	0,389325398	protein_codin	prefoldin
TcG_05627	92,39372845	0,177763528	0,184836363	0,961734617	0,336182929	0,57078138	protein_codin	hypothetical protein
TcG_05628	171,7533971	0,182700891	0,136688969	1,336617668	0,181347469	0,39294274	protein_codin	putative 3'a2rel-related protein
TcG_05629	407,7385053	0,098768651	0,093104117	1,060840857	0,288762231	0,52204032	protein_codin	hypothetical protein
TcG_05630	232,7028065	0,112279198	0,116088255	0,967188263	0,333449928	0,567889294	protein_codin	hypothetical protein
TcG_05631	615,1249715	0,288123355	0,075324038	3,825118277	0,000130709	0,001394473	protein_codin	hypothetical protein
TcG_05632	546,1962479	0,033051297	0,084334591	0,391906774	0,695127098	0,842265483	protein_codin	hypothetical protein
TcG_05633	315,0472616	-0,003695245	0,119100432	-0,0310263	0,975248568	0,989251436	protein_codin	hypothetical protein
TcG_05634	1265,436	-0,301575233	0,059476811	-5,07046744	3,9684E-07	8,91044E-06	protein_codin	hypothetical protein
TcG_05635	4,340198193	0,183728677	0,84453768	0,217549413	0,827780203	1	protein_codin	hypothetical protein
TcG_05636	42,76812008	0,590883347	0,276499311	2,13701562	0,032596721	0,12178833	protein_codin	hypothetical protein
TcG_05637	42,973248	0,219804668	0,277704653	0,791505166	0,428649263	0,653907586	protein_codin	hypothetical protein
TcG_05638	116,5926092	0,251835539	0,166912033	1,508791993	0,131351948	0,322147648	protein_codin	hypothetical protein
TcG_05639	110,6875328	0,303908868	0,170471328	1,782756501	0,07462595	0,220509118	protein_codin	hypothetical protein
TcG_05640	75,22856543	-0,075434411	0,204987271	-0,36799559	0,712876525	0,852537925	protein_codin	hypothetical protein
TcG_05641	405,7787018	-0,281069177	0,09493015	-2,96079988	0,003068412	0,019415962	protein_codin	calmodulin-like protein containing EF hand domain
TcG_05642	169,7835981	0,176549381	0,136720295	1,291318026	0,196593423	0,414736234	protein_codin	hypothetical protein
TcG_05643	160,7411308	0,098893152	0,143006183	0,691530604	0,489232155	0,700388453	protein_codin	hypothetical protein

TcG_05644	283,6217223	0,016426345	0,122291197	0,134321563	0,893148303	0,948402185	protein_codin hypothetical protein
TcG_05645	828,6752655	0,015305492	0,070581128	0,216849634	0,82832553	0,916179436	protein_codin putative kinesin
TcG_05646	331,5693488	0,097459809	0,099641905	0,978100618	0,328024562	0,562619183	protein_codin zinc finger family protein
TcG_05647	111,4375956	0,489702002	0,179152097	2,733442759	0,006267602	0,034172439	protein_codin hypothetical protein
TcG_05648	194,1712948	0,617944214	0,135845968	4,548859441	5,39375E-06	8,94019E-05	protein_codin hypothetical protein
TcG_05649	434,9150962	-0,333684679	0,087088748	-3,83154757	0,00012734	0,001367339	protein_codin hypothetical protein
TcG_05650	473,1263504	-0,104044584	0,087764505	-1,1854973	0,235820881	0,463445836	protein_codin polyphosphate synthetase Protein
TcG_05651	519,5728529	-0,081937075	0,082584482	-0,99216067	0,321119153	0,556034397	protein_codin heat shock protein
TcG_05652	526,9855346	-0,074102269	0,083701252	-0,88531852	0,375984863	0,606538655	protein_codin putative ubiqitin hydrolase
TcG_05653	57,71495958	-0,412930863	0,2295181	-1,79912113	0,071999523	0,215663513	
TcG_05654	390,1661849	0,106457337	0,094293436	1,129000509	0,258897619	0,491493988	protein_codin putative minchromosome maintenance (MCM) complex subunit
TcG_05655	241,7162521	0,271036114	0,130453267	2,077649116	0,037741689	0,135589211	protein_codin hypothetical protein
TcG_05656	138,0894043	0,257605774	0,153643452	1,67664662	0,093611605	0,256834988	protein_codin putative integral membrane transport protein, putative,drug resistance protein
TcG_05657	385,0206502	0,078068403	0,093532405	0,834666896	0,403905286	0,633326112	protein_codin hypothetical protein
TcG_05658	304,8098606	-0,030454587	0,10262649	-0,29675171	0,766656073	0,882862267	protein_codin hypothetical protein
TcG_05659	481,5166016	0,443483256	0,086306577	5,138464229	2,76993E-07	6,40567E-06	protein_codin putative kinesin
TcG_05660	97,1695682	-0,153569359	0,177176571	-0,86675884	0,38607416	0,616520055	protein_codin hypothetical protein
TcG_05661	436,9071786	-0,093803575	0,094892496	-0,98852469	0,322895749	0,557453457	protein_codin putative protein kinase
TcG_05662	450,9430211	-0,275885671	0,086509748	-3,18907032	0,001427312	0,010439919	protein_codin hypothetical protein
TcG_05663	78,57575075	0,189959223	0,196903635	0,964731925	0,3346791	0,569062527	protein_codin hypothetical protein
TcG_05664	151,810161	-0,031400017	0,143174355	-0,21931314	0,82640613	0,91519226	protein_codin hypothetical protein
TcG_05665	389,4439739	0,103003837	0,092749753	1,110556462	0,266759312	0,499395611	protein_codin putative DNA photolyase
TcG_05666	83,37050396	0,003744324	0,193393413	0,019361177	0,984552981	0,994123614	protein_codin ankyrin repeat protein
TcG_05667	210,7838611	0,003188929	0,126966192	0,025116367	0,979962146	0,991948403	protein_codin hypothetical protein
TcG_05668	295,5598812	-0,00565889	0,105218745	-0,05378214	0,957108736	0,980118597	protein_codin hypothetical protein
TcG_05669	731,7851279	-0,043121565	0,070304116	-0,61335761	0,539639906	0,738776787	protein_codin hypothetical protein
TcG_05670	380,2901008	-0,23229218	0,103835376	-2,23711985	0,025278508	0,100541296	protein_codin hypothetical protein
TcG_05671	151,6920898	0,052889295	0,141925281	0,372655911	0,709404555	0,850052867	protein_codin protein kinase
TcG_05672	235,3988884	-0,236448429	0,115326472	-2,05025286	0,040339762	0,142022914	protein_codin putative protein kinase
TcG_05673	219,3869266	-0,010484067	0,127560537	-0,08218895	0,93449646	0,96903929	protein_codin hypothetical protein
TcG_05674	351,9959364	-0,170624892	0,098874215	-1,72567633	0,084405659	0,239335283	protein_codin putative dolichyl-P-Man:GDP-Man5GlcNAc2-PP-dolichyl alpha-1,3-mannosyltransferase
TcG_05675	320,9031615	-0,191469338	0,106105932	-1,80451115	0,071151213	0,21392986	protein_codin putative serine/threonine protein phosphatase
TcG_05676	266,966916	0,027699732	0,110381359	0,250945743	0,801856058	0,90177851	protein_codin Poly polymerase and DNA-Ligase Zn-finger region family protein
TcG_05677	455,0770834	-0,109527489	0,090521306	-1,20996364	0,226292846	0,452741998	protein_codin putative Protein kinase domain
TcG_05678	295,2213936	0,013659048	0,103823222	0,131560621	0,895331835	0,948981304	protein_codin hypothetical protein
TcG_05679	163,103109	-0,166129069	0,141481919	-1,17420706	0,240312097	0,468992108	protein_codin stress-induced-phosphoprotein 1
TcG_05680	498,4471836	0,049978853	0,092412105	0,540825823	0,588627642	0,775648564	protein_codin hypothetical protein
TcG_05681	305,9500436	0,023051794	0,102198387	0,225559277	0,821544217	0,911550594	protein_codin transferase
TcG_05682	362,3533179	-0,130098237	0,097804328	-1,33018895	0,183456024	0,395887781	protein_codin chaperone protein DnaJ
TcG_05683	234,3119575	0,401468422	0,124194103	3,232588449	0,001226742	0,00919937	protein_codin hypothetical protein
TcG_05684	305,4106252	0,034489768	0,108768819	0,317092424	0,751173474	0,874683002	protein_codin hypothetical protein
TcG_05685	205,7990708	0,152557429	0,131144645	1,163276087	0,244717504	0,472864744	protein_codin hypothetical protein
TcG_05686	1303,99673	0,04778755	0,057583163	0,82988756	0,406602359	0,634833846	protein_codin putative DEAD box RNA helicase
TcG_05687	254,8461471	0,050274283	0,110416718	0,455314054	0,648883317	0,813280193	protein_codin HAD family hydrolase
TcG_05688	611,5603446	0,149887926	0,077746377	1,927908818	0,053866469	0,174328745	protein_codin COP-coated vesicle membrane protein
TcG_05689	279,0306035	0,172263489	0,108322036	1,590290357	0,111769371	0,289409059	protein_codin putative G10 protein
TcG_05690	204,8300728	-0,118103681	0,129703831	-0,91056433	0,362524974	0,595028691	protein_codin dynein light chain
TcG_05691	557,5323888	0,120791099	0,078111015	1,546402886	0,122007297	0,306632657	protein_codin tRNA (cytosine34-C5)-methyltransferase
TcG_05692	361,1289796	-0,181258535	0,095380607	-1,90037096	0,057384455	0,182502414	protein_codin putative tyrosine specific protein phosphatase
TcG_05693	241,3329956	0,319347718	0,116519001	2,740735126	0,00613019	0,0335654	protein_codin putative formin
TcG_05694	180,8098067	0,379912261	0,134065605	2,833778746	0,004600117	0,026972143	protein_codin hypothetical protein
TcG_05695	1219,818337	-0,228797696	0,063943259	-3,5781363	0,000346053	0,003161964	protein_codin hypothetical protein
TcG_05696	163,2931713	-0,056436931	0,146370149	-0,38557678	0,699810133	0,844367272	protein_codin hypothetical protein
TcG_05697	365,0395356	0,034953352	0,093987923	0,371891947	0,709973301	0,850425202	protein_codin putative vacuolar protein sorting-associated protein
TcG_05698	981,1116524	0,142833972	0,069988751	2,040813269	0,041269391	0,144498993	protein_codin hypothetical protein
TcG_05699	588,153305	-0,152978741	0,083779088	-1,82597765	0,067853629	0,20764716	protein_codin putative developmentally regulated GTP-binding protein 1
TcG_05700	169,495611	-0,059380291	0,139876941	-0,42451808	0,671188022	0,827715213	protein_codin putative guanylate kinase

TcG_05701	243,9306234	-0,084424006	0,113171699	-0,74598161	0,455678524	0,675645172	protein_codin hypothetical protein
TcG_05702	371,6116909	-0,006965394	0,096288724	-0,07233863	0,942332425	0,973191135	protein_codin Bardet-Biedl syndrome 4 protein
TcG_05703	484,0614506	-0,416060408	0,084631759	-4,91612619	8,82735E-07	1,82632E-05	protein_codin hypothetical protein
TcG_05704	331,4393411	-0,011326649	0,100410938	-0,11280294	0,910186788	0,956414305	protein_codin putative kinteoplast poly(A) polymerase complex 1 subunit
TcG_05705	772,9988852	-0,031975209	0,067947626	-0,47058611	0,637936327	0,807510942	protein_codin glucokinase
TcG_05706	135,9217527	0,365799327	0,153677589	2,380303665	0,017298376	0,075204123	protein_codin putative endonuclease V
TcG_05707	261,1361079	-0,31371101	0,113761884	-2,75761089	0,005822546	0,032277521	protein_codin putative short-chain dehydrogenase
TcG_05708	185,5372237	0,245654379	0,130278342	1,885611806	0,059347285	0,187560733	protein_codin putative tyrosine aminotransferase, putative,L-tyrosine:2-oxoglutarate aminotransferase
TcG_05709	191,7794475	-0,034856279	0,132597595	-0,26287264	0,792648747	0,896248225	protein_codin putative glyceraldehyde 3-phosphate dehydrogenase, cytosolic
TcG_05710	174,4601606	0,191946952	0,13591369	1,412270917	0,157870206	0,359914248	protein_codin hypothetical protein
TcG_05711	194,5019828	0,040968474	0,131412421	0,311754961	0,755226755	0,876495761	protein_codin hypothetical protein
TcG_05712	582,5223064	-0,02339311	0,076480783	-0,30586912	0,759704299	0,8786119	protein_codin putative translation initiation factor
TcG_05713	488,8348494	0,017612688	0,084673583	0,20800688	0,835223595	0,920119861	protein_codin hypothetical protein
TcG_05714	230,0620852	-0,11616169	0,118832399	-0,97752541	0,328309105	0,562869623	protein_codin hypothetical protein
TcG_05715	162,9181824	-0,393588238	0,139567541	-2,8200557	0,004801531	0,027898968	protein_codin putative protein kinase
TcG_05716	407,6487431	-0,174703226	0,100441536	-1,73935239	0,081972798	0,234961703	protein_codin putative protein kinase
TcG_05717	810,1999808	-0,024570814	0,072242449	-0,34011602	0,733769161	0,863266603	protein_codin protein kinase
TcG_05718	860,9044675	-0,10877301	0,069155274	-1,57288091	0,115746396	0,29616558	protein_codin putative ribose-phosphate pyrophosphokinase
TcG_05719	96,96423558	0,339203433	0,176911442	1,917362883	0,055191846	0,177428616	protein_codin hypothetical protein
TcG_05720	174,5912063	-0,113179451	0,140362068	-0,8063393	0,420047237	0,646561352	protein_codin hypothetical protein
TcG_05721	498,483815	0,056698627	0,089144476	0,636030736	0,524756389	0,728297499	protein_codin hypothetical protein
TcG_05722	236,8095321	0,262661543	0,117930892	2,227249692	0,025930591	0,102501475	protein_codin small ubiquitin protein
TcG_05723	228,8024097	-0,37852872	0,123390269	-3,06773559	0,002156874	0,014613765	protein_codin signal peptidase type I
TcG_05724	1451,689727	-0,033178241	0,061505341	-0,53943674	0,589585534	0,775816202	protein_codin hypothetical protein
TcG_05725	492,3230866	-0,353552685	0,083980165	-4,20995463	2,55422E-05	0,000343707	protein_codin hypothetical protein
TcG_05726	1052,496839	-0,026976877	0,071977654	-0,37479517	0,707812804	0,849375365	protein_codin hypothetical protein
TcG_05727	696,2468711	-0,290483138	0,076954925	-3,77471798	0,000160189	0,001666021	protein_codin hypothetical protein
TcG_05728	408,0961803	-0,003021321	0,094857584	-0,03185112	0,974590776	0,988930525	protein_codin hypothetical protein
TcG_05729	847,4722791	-0,052100884	0,065881732	-0,79082445	0,429046443	0,653987669	protein_codin hypothetical protein
TcG_05730	176,7840493	-0,047461522	0,139160528	-0,34105592	0,733061486	0,862784475	protein_codin hypothetical protein
TcG_05731	195,5802131	0,119464708	0,127727052	0,935312495	0,349627278	0,584106942	protein_codin hypothetical protein
TcG_05732	239,5213934	0,119166539	0,120349306	0,990172211	0,322089953	0,556809041	protein_codin hypothetical protein
TcG_05733	33,13644461	-0,359442796	0,332377122	-1,08143062	0,279505608	0,512478553	protein_codin putative protein kinase
TcG_05734	245,0197066	0,164733831	0,116499841	1,41402623	0,157354201	0,359232665	protein_codin putative protein kinase
TcG_05735	75,20305783	-0,042341204	0,20135224	-0,21028424	0,833445832	0,91912273	protein_codin protein kinase
TcG_05736	0,233855473	1,175456718	3,508398891	0,335040785	0,737594321	1	
TcG_05737	81,78625801	0,197141459	0,194861954	1,01169805	0,311682448	0,545491366	protein_codin hypothetical protein
TcG_05738	0					1	protein_codin ribosomal protein L27
TcG_05739	8,915847656	0,729264326	0,669350577	1,089510268	0,275928929	1	protein_codin ribosomal protein L27
TcG_05740	953,3746904	0,001891106	0,066131766	0,02859603	0,977186778	0,990177192	protein_codin hypothetical protein
TcG_05741	80,56891192	0,461084028	0,205395202	2,244862707	0,024776948	0,099056493	protein_codin ribosomal protein L27
TcG_05742	92,4310111	0,112666864	0,180473095	0,624286206	0,532439641	0,73316445	protein_codin ribosomal protein L27
TcG_05743	372,7680369	-0,169720384	0,095424719	-1,77857882	0,07530884	0,221549936	protein_codin hypothetical protein
TcG_05744	109,7065185	0,092568841	0,167686009	0,552036758	0,580923169	0,770441373	protein_codin hypothetical protein
TcG_05745	495,7276575	-0,106648884	0,083606965	-1,27559808	0,202097633	0,422196751	protein_codin trichohyalin
TcG_05746	147,1958921	-0,014919226	0,155519038	-0,09593183	0,923574714	0,962652247	protein_codin putative amino acid transporter
TcG_05747	31,88876637	0,357931466	0,306628344	1,167313699	0,243083708	0,471358635	protein_codin hypothetical protein
TcG_05748	132,4108867	0,123626386	0,159421528	0,775468581	0,438062824	0,661461733	protein_codin hypothetical protein
TcG_05749	617,544975	0,011153181	0,076189866	0,146386678	0,883616144	0,94348809	protein_codin methyltransferase
TcG_05750	369,6048072	0,279419745	0,102198237	2,73409555	0,006255189	0,034120819	protein_codin hypothetical protein
TcG_05751	288,3200782	-0,203632099	0,107328007	-1,89728763	0,057789987	0,183389426	protein_codin superoxide dismutase
TcG_05752	231,9399065	-0,198467118	0,118198804	-1,67909583	0,093133369	0,25587935	protein_codin hypothetical protein
TcG_05753	605,0811773	-0,170248844	0,077729233	-2,19028075	0,028503882	0,109918176	protein_codin dentin sialophosphoprotein precursor
TcG_05754	297,8339086	-0,034569352	0,103471969	-0,33409389	0,738308714	0,866422734	protein_codin hypothetical protein
TcG_05755	133,2595868	-0,245598609	0,160863701	-1,52674971	0,12682326	0,314978413	protein_codin hypothetical protein
TcG_05756	373,480174	0,363581323	0,099205222	3,664941361	0,000247395	0,002382645	protein_codin hypothetical protein
TcG_05757	1025,878947	-0,146536208	0,061742939	-2,3733274	0,01762863	0,076323929	protein_codin hypothetical protein

TcG_05758	313,6309422	-0,198989473	0,101399282	-1,96243473	0,049711893	0,164984815	protein_codin	putative GPI transamidase component Tta1
TcG_05759	921,4478423	0,009559789	0,064282331	0,148715653	0,881778009	0,942184067	protein_codin	adenosine monophosphate deaminase-like protein
TcG_05760	208,2081648	-0,033238908	0,13533107	-0,24561181	0,80598275	0,904233839	protein_codin	hypothetical protein
TcG_05761	335,6127287	0,015251625	0,0994883	0,153300695	0,878161153	0,941423575	protein_codin	hypothetical protein
TcG_05762	436,1996308	-0,1359274	0,090808639	-1,49685538	0,134430892	0,326021613	protein_codin	hypothetical protein
TcG_05763	211,3190248	-0,190025861	0,126713399	-1,49965088	0,13370486	0,325237142	protein_codin	hypothetical protein
TcG_05764	537,3374451	-0,080436631	0,08607386	-0,93450707	0,350042389	0,584547582	protein_codin	hypothetical protein
TcG_05765	242,4992326	-0,11328724	0,114573702	-0,98877175	0,32277483	0,557414741	protein_codin	hypothetical protein
TcG_05766	17,81998248	0,663512206	0,419424074	1,581960232	0,113658649	0,292691163		
TcG_05767	32,66402493	0,28425288	0,310397356	0,915770944	0,359787046	0,592630203	protein_codin	syntaxin binding protein
TcG_05768	29,42741462	-0,239307598	0,34265844	-0,69838524	0,484936302	0,697958705	protein_codin	hypothetical protein
TcG_05769	42,59352334	0,330790259	0,272628074	1,213338941	0,225000261	0,451559504	protein_codin	putative syntaxin binding protein
TcG_05770	126,4989653	-0,063607822	0,161186215	-0,39462322	0,693120978	0,841062696	protein_codin	hypothetical protein
TcG_05771	5,085003381	-0,305218886	0,79104807	-0,38584114	0,699614328	1		
TcG_05772	444,4980683	-0,084795878	0,085944386	-0,98663662	0,323820813	0,558135666	protein_codin	hypothetical protein
TcG_05773	263,8859323	-0,156117865	0,111627861	-1,39855646	0,161946032	0,365680515	protein_codin	hypothetical protein
TcG_05774	268,1449619	-0,170021303	0,110071103	-1,54464976	0,122431003	0,307297575	protein_codin	hypothetical protein
TcG_05775	347,7345128	-0,108543618	0,097565984	-1,11251498	0,265916793	0,498666135	protein_codin	hypothetical protein
TcG_05776	315,1890832	-0,003895458	0,104926565	-0,03712556	0,970384891	0,987170019	protein_codin	hypothetical protein
TcG_05777	193,3129031	0,17251819	0,131047656	1,316453841	0,188021761	0,402407967	protein_codin	ClpB chaperone
TcG_05778	261,9717111	0,210284538	0,118978546	1,7674156	0,077158652	0,225065495		
TcG_05779	543,2918399	0,087006871	0,086619701	1,004469766	0,315152237	0,549902684	protein_codin	variant-surface-glycoprotein phospholipase C
TcG_05780	324,3729322	0,078263948	0,101558365	0,770630245	0,440926128	0,663546065	protein_codin	variant-surface-glycoprotein phospholipase C
TcG_05781	1467,723532	-0,01202904	0,054789969	-0,21954821	0,826223028	0,915112889	protein_codin	hypothetical protein
TcG_05782	443,830968	0,012472221	0,089078366	0,140014024	0,88864891	0,94628587	protein_codin	putative heat shock 70 kDa protein, mitochondrial precursor
TcG_05783	208,5395753	0,10294884	0,123037069	0,836730269	0,402744201	0,632851796	protein_codin	putative DNA-J protein
TcG_05784	412,3536767	-0,142315212	0,090713274	-1,56884662	0,116683683	0,2979716	protein_codin	hypothetical protein
TcG_05785	335,1565022	-0,06774125	0,101487415	-0,66748424	0,504462873	0,712161185	protein_codin	hypothetical protein
TcG_05786	324,8104145	-0,189700498	0,102180146	-1,85652991	0,063378064	0,19765767	protein_codin	peptidyl-tRNA hydrolase, PTH2 family
TcG_05787	429,6200645	-0,232846474	0,087588139	-2,65842472	0,007850689	0,040880036	protein_codin	putative pre-mRNA cleavage complex II Clp1 protein
TcG_05788	235,186797	0,007554111	0,124124349	0,060859216	0,95147133	0,977195889	protein_codin	hypothetical protein
TcG_05789	477,4823965	-0,020603461	0,086232306	-0,23892973	0,811160075	0,906889958	protein_codin	hypothetical protein
TcG_05790	1840,005803	-0,090937558	0,057318893	-1,58651978	0,11262145	0,290737993	protein_codin	hypothetical protein
TcG_05791	166,0036402	0,289740455	0,140675253	2,059640544	0,039432917	0,13999295	protein_codin	hypothetical protein
TcG_05792	516,4042077	-0,150868077	0,089377104	-1,68799468	0,09141225	0,252527974	protein_codin	ADP-ribosylation factor-like protein
TcG_05793	501,4112037	-0,107879423	0,082510573	-1,30746182	0,191055914	0,406681682	protein_codin	putative zinc finger protein
TcG_05794	395,1739536	0,221320514	0,095950205	2,306618453	0,0210761	0,087805715	protein_codin	putative sphingosine 1-phosphate lyase
TcG_05795	528,7921044	-0,095547843	0,080814445	-1,18231145	0,237082146	0,464698654	protein_codin	putative zinc finger protein
TcG_05796	309,5974881	0,088415657	0,114013586	0,775483521	0,438053999	0,661461733	protein_codin	hypothetical protein
TcG_05797	249,6651507	0,252740435	0,120143181	2,10366026	0,035408093	0,129740092	protein_codin	23S rRNA (cytosine1962-C5)-methyltransferase
TcG_05798	650,4798404	-0,066279896	0,075499797	-0,87788179	0,380007874	0,610721305	protein_codin	hypothetical protein
TcG_05799	426,9798928	-0,066945389	0,090537255	-0,73942367	0,459649772	0,678235133	protein_codin	ATP-dependent DEAD/H DNA helicase recQ family-like protein
TcG_05800	389,2437533	-0,196105114	0,102490102	-1,9134054	0,055696174	0,178505081	protein_codin	putative tubulin-tyrosine ligase-like protein
TcG_05801	810,1593886	-0,108784183	0,066322245	-1,64023674	0,100955952	0,270539375	protein_codin	hypothetical protein
TcG_05802	1197,122046	-0,287064837	0,06010474	-4,77607651	1,78748E-06	3,36745E-05	protein_codin	putative phospholipid-translocating P-type ATPase (flippase)
TcG_05803	583,6421285	-0,03149065	0,083077053	-0,37905353	0,704648112	0,847555325	protein_codin	zinc finger protein, conserved
TcG_05804	201,1195999	-0,311301772	0,129931812	-2,39588572	0,016580259	0,073013638	protein_codin	hypothetical protein
TcG_05805	598,6017393	-0,115282277	0,077700889	-1,48366742	0,137897174	0,331330705	protein_codin	hypothetical protein
TcG_05806	318,8300492	-0,074165194	0,102292364	-0,72503157	0,468432663	0,685000736	protein_codin	putative tRNA modification enzyme
TcG_05807	1629,829396	-0,02236317	0,051934502	-0,43060333	0,666756817	0,825438095	protein_codin	dnaJ-like protein subfamily C member 13 isoform X2
TcG_05808	146,1272273	0,306320614	0,147112445	2,082220944	0,037322293	0,134499561	protein_codin	zinc finger protein ZFP1
TcG_05809	116,5444225	0,444080241	0,17639147	2,517583429	0,011816298	0,056315767		
TcG_05810	11,77607546	0,598505395	0,522434241	1,14560905	0,251956951	1	protein_codin	hypothetical protein
TcG_05811	277,3212664	-0,154202529	0,108060399	-1,42700315	0,153578988	0,354314247	protein_codin	hypothetical protein
TcG_05812	251,5433289	0,076940965	0,112616488	0,683212257	0,494472729	0,70453824	protein_codin	putative protein farnesyltransferase
TcG_05813	175,3314122	-0,344435862	0,134533844	-2,56021721	0,010460676	0,051159726	protein_codin	tartrate-resistant acid phosphatase type 5 precursor
TcG_05814	346,6656451	-0,152667042	0,102672283	-1,4869353	0,137031899	0,330077667	protein_codin	hypothetical protein

TcG_05815	428,2953926	-0,149717797	0,093133742	-1,60755699	0,107932239	0,283175481	protein_codin hypothetical protein
TcG_05816	232,0478091	-0,203465818	0,11829981	-1,71991669	0,085447586	0,24133259	protein_codin hypothetical protein
TcG_05817	144,9826754	-0,292968171	0,147548304	-1,98557464	0,047080575	0,158476334	protein_codin hypothetical protein
TcG_05818	84,91974743	-0,072031314	0,202306936	-0,35604965	0,721803383	0,857117481	protein_codin putative cytochrome b
TcG_05819	495,5018342	0,050316653	0,094723224	0,531196583	0,59528256	0,780373811	protein_codin hypothetical protein
TcG_05820	248,8358514	-0,310533941	0,124641442	-2,49141808	0,012723431	0,059686266	protein_codin putative chaperone protein DNAj
TcG_05821	260,4653908	-0,05757132	0,115607877	-0,49798786	0,618492596	0,793033999	protein_codin hypothetical protein
TcG_05822	403,9858163	0,041946581	0,08994881	0,466338363	0,640973323	0,809407838	protein_codin hypothetical protein
TcG_05823	216,9713581	-0,128214061	0,121073393	-1,05897801	0,289609792	0,523221434	protein_codin hypothetical protein
TcG_05824	224,9652335	-0,180917978	0,119507585	-1,51386188	0,130060874	0,31986527	protein_codin prefoldin
TcG_05825	228,6162632	-0,101172197	0,125109171	-0,80867131	0,418704235	0,645265665	protein_codin putative GTP-binding protein
TcG_05826	399,60989	-0,212843231	0,101848056	-2,08981142	0,036634743	0,133056469	protein_codin hypothetical protein
TcG_05827	7,579896876	0,689499823	0,657445826	1,048755343	0,294290736	1	
TcG_05828	157,4220333	0,035130653	0,146168759	0,2403431	0,810064285	0,906277019	protein_codin hypothetical protein
TcG_05829	386,6135957	-0,219196318	0,092558214	-2,36819951	0,017874894	0,07704558	protein_codin putative DNA ligase
TcG_05830	2007,445494	-0,023515303	0,048788963	-0,48197997	0,629820174	0,801688869	protein_codin DNA ligase
TcG_05831	160,3913667	-0,179826906	0,144436672	-1,24502249	0,213123482	0,436648748	protein_codin hypothetical protein
TcG_05832	211,6316465	-0,103200684	0,13356334	-0,77267223	0,439716385	0,662749321	protein_codin hypothetical protein
TcG_05833	152,7543964	-0,148641393	0,144286285	-1,0301838	0,302923733	0,535618427	protein_codin hypothetical protein
TcG_05834	381,6585655	-0,095993216	0,091821845	-1,04542895	0,29582476	0,528842104	protein_codin hypothetical protein
TcG_05835	232,0015333	0,273229736	0,123328895	2,215455966	0,026728784	0,105011762	protein_codin transcription factor jumonji, jmjC domain-containing protein
TcG_05836	3,037373347	-0,227161569	1,026117044	-0,22137978	0,824796733	1	
TcG_05837	393,0912468	-0,178158025	0,092567528	-1,92462765	0,054275957	0,175311191	protein_codin hypothetical protein
TcG_05838	187,0199084	-0,029952524	0,130010132	-0,23038608	0,817791779	0,910263767	protein_codin chaperone DnaJ protein
TcG_05839	210,0665348	-0,469916445	0,127819971	-3,67639297	0,000236555	0,002293495	protein_codin putative small GTP-binding protein Rab11, putative,Rab11 GTPase
TcG_05840	57,47133773	0,230161763	0,23546518	0,977476855	0,328333131	0,562869623	protein_codin putative mucin-like glycoprotein
TcG_05841	4,760328326	-0,237236425	0,794390282	-0,29863964	0,765215019	1	
TcG_05842	102,8606375	0,047094275	0,174001121	0,270655007	0,786656385	0,893794073	protein_codin hypothetical protein
TcG_05843	123,2975655	0,102653376	0,158173438	0,648992503	0,516343222	0,722055158	protein_codin hypothetical protein
TcG_05844	267,3435014	-0,139159152	0,11857932	-1,1735533	0,240573997	0,469240796	protein_codin prefoldin subunit 2
TcG_05845	199,122627	0,148219492	0,129371423	1,145689585	0,251923615	0,481966149	protein_codin tRNA wybutosine-synthesizing protein 3
TcG_05846	421,0235911	0,005210225	0,094397737	0,05519438	0,955983606	0,979659124	protein_codin hypothetical protein
TcG_05847	275,1208954	-0,057633463	0,107604989	-0,53560215	0,592233545	0,777696685	protein_codin coiled-coil domain-containing protein 93
TcG_05848	221,0923307	0,110836499	0,123730752	0,895787801	0,370366106	0,602000801	protein_codin putative Zinc finger DHHC domain containing transmembrane protein
TcG_05849	404,6710858	-0,057082508	0,090164464	-0,63309319	0,52667279	0,729266446	protein_codin hypothetical protein
TcG_05850	162,9772197	0,060741542	0,143964095	0,421921464	0,673082343	0,828900214	protein_codin hypothetical protein
TcG_05851	422,938342	0,093302211	0,090039988	1,03623082	0,300094472	0,533757223	protein_codin hypothetical protein
TcG_05852	178,7526039	-0,075552043	0,130411932	-0,57933382	0,562363951	0,755776445	protein_codin putative cyclin 1, putative,serine peptidase family S51, peptidase E
TcG_05853	414,8445218	0,117392223	0,09665823	1,214508302	0,224553681	0,451082534	protein_codin putative ATP-dependent DNA helicase
TcG_05854	262,0358426	-0,16550331	0,112521712	-1,47085666	0,141329886	0,3366698	protein_codin Derlin-2/3
TcG_05855	295,589147	0,224862368	0,107287224	2,095891388	0,036091827	0,131620997	protein_codin hypothetical protein
TcG_05856	104,6112991	-0,281020141	0,171176617	-1,64169702	0,10065281	0,269882772	protein_codin phosphoric monoester hydrolase
TcG_05857	442,7351717	-0,286313128	0,091333847	-3,13479764	0,001719727	0,012164076	protein_codin hypothetical protein
TcG_05858	478,0553813	0,008192547	0,084225199	0,097269547	0,922512339	0,96227026	protein_codin putative leucine-rich repeat protein
TcG_05859	1138,995901	-0,236639452	0,070875067	-3,33882507	0,000841335	0,006769243	protein_codin hypothetical protein
TcG_05860	136,7926043	0,127604732	0,15066429	0,846947426	0,397024426	0,626692779	protein_codin hypothetical protein
TcG_05861	254,9606181	-0,014686817	0,113798305	-0,12906006	0,897310121	0,950181186	protein_codin hypothetical protein
TcG_05862	226,2466573	0,188489561	0,124576526	1,513042364	0,130268898	0,32024092	protein_codin acyltransferase
TcG_05863	545,2220581	0,159792622	0,080696364	1,980171277	0,047684286	0,159883351	protein_codin hypothetical protein
TcG_05864	300,8666412	-0,036080652	0,103823095	-0,34752049	0,728200331	0,86000745	protein_codin putative serine/threonine protein phosphatase
TcG_05865	717,3951558	-0,105168901	0,071519234	-1,47049815	0,141426887	0,3366698	protein_codin hypothetical protein
TcG_05866	259,1596271	0,048969779	0,112295005	0,436081538	0,662777571	0,823478921	protein_codin hypothetical protein
TcG_05867	146,4825006	-0,030703551	0,153240831	-0,20036143	0,841197926	0,922491166	protein_codin hypothetical protein
TcG_05868	55,98493565	-0,334844838	0,22870745	-1,4640749	0,143173484	0,339673155	protein_codin hypothetical protein
TcG_05869	147,4291702	-0,311048985	0,149663927	-2,07831633	0,037680233	0,135410416	protein_codin hypothetical protein
TcG_05870	117,319678	-0,071645574	0,162486709	-0,44093191	0,659262295	0,820996653	protein_codin hypothetical protein
TcG_05871	264,5929957	-0,026241089	0,113136066	-0,23194274	0,81658249	0,909544056	protein_codin hypothetical protein

TcG_05872	247,7229828	-0,056356473	0,112161983	-0,50245611	0,61534672	0,79219196	protein_codin hypothetical protein
TcG_05873	512,7077667	-0,082877003	0,084346836	-0,98257393	0,325817179	0,560274604	protein_codin putative protein tyrosine phosphatase
TcG_05874	199,2686984	0,279174928	0,12649846	2,206943296	0,027318021	0,106744181	protein_codin surface protease GP63
TcG_05875	347,3362861	-0,230486892	0,097100675	-2,3736899	0,017611334	0,076278474	protein_codin UDP-galactopyranose mutase
TcG_05876	612,8844969	-0,407005287	0,077438164	-5,25587472	1,47323E-07	3,71061E-06	protein_codin putative activator of 90 kDa heat shock protein ATPase 1-like
TcG_05877	751,2252726	-0,505694618	0,070006303	-7,22355838	5,06445E-13	4,04667E-11	protein_codin putative RNA-binding protein
TcG_05878	529,505283	-0,033496979	0,100248095	-0,3341408	0,738273312	0,866422734	protein_codin 60S ribosomal protein L7
TcG_05879	201,0513259	-0,345993047	0,132917092	-2,60307414	0,009239197	0,046391618	protein_codin putative calcium-dependent lipid binding protein, putative,synaptotagmin
TcG_05880	226,9159283	0,008365092	0,122181261	0,068464609	0,945415792	0,974691882	protein_codin hypothetical protein
TcG_05881	141,7467017	0,121707785	0,159692948	0,762136252	0,445978688	0,667671415	protein_codin hypothetical protein
TcG_05882	172,7026451	-0,001889904	0,135919648	-0,01390457	0,988906115	0,995522309	protein_codin hypothetical protein
TcG_05883	182,0278111	-0,384308181	0,141227726	-2,72119499	0,006504638	0,035302906	protein_codin putative glycogen synthase kinase-3 alpha
TcG_05884	503,7234611	-0,348603822	0,083669087	-4,16645902	3,09367E-05	0,000405467	protein_codin hypothetical protein
TcG_05885	135,1807598	-0,016730849	0,15138926	-0,11051543	0,912000616	0,957104994	protein_codin dephospho-CoA kinase
TcG_05886	39,22533606	-0,027449076	0,285182299	-0,09625098	0,923321243	0,962648944	
TcG_05887	560,3833539	-0,264801783	0,08236496	-3,21498103	0,001304531	0,009701092	protein_codin mucin-associated surface protein (MASP)
TcG_05888	79,81280351	-0,030599073	0,194810498	-0,15707096	0,87518892	0,939877107	protein_codin hypothetical protein
TcG_05889	109,9616892	-0,230258765	0,16627503	-1,38480664	0,1661116	0,371661396	protein_codin hypothetical protein
TcG_05890	359,3373219	-0,18020038	0,099922653	-1,80339866	0,071325627	0,214309834	protein_codin putative chaperone protein DNAj
TcG_05891	118,3695797	-0,23024897	0,17014817	-1,35322625	0,175983362	0,385652211	protein_codin putative protein-like
TcG_05892	254,9904418	-0,153824276	0,114461298	-1,34389771	0,178981466	0,389799325	protein_codin ATP synthase mitochondrial F1 complex assembly factor 2
TcG_05893	292,7107342	-0,266317525	0,108081935	-2,46403365	0,013738321	0,063465785	protein_codin GPI-anchor transamidase subunit 8
TcG_05894	139,4982634	-0,386496803	0,14749705	-2,62036972	0,008783448	0,044633787	protein_codin tubulin tyrosine ligase
TcG_05895	629,9280522	-0,306718406	0,076956691	-3,98559765	6,73105E-05	0,000798218	protein_codin hypothetical protein
TcG_05896	286,4555308	-0,22383085	0,111097094	-2,01473182	0,043932756	0,151040033	protein_codin hypothetical protein
TcG_05897	315,5583103	-0,500886599	0,111669461	-4,4854394	7,27638E-06	0,000116121	protein_codin UDP-galactose transporter
TcG_05898	259,2982856	-0,19954036	0,118058332	-1,69018447	0,090992669	0,251729001	protein_codin hypothetical protein
TcG_05899	68,86704698	-0,26683826	0,221143883	-1,20662736	0,227575686	0,454445346	protein_codin hypothetical protein
TcG_05900	175,755782	-0,275490701	0,141975239	-1,94041372	0,05232943	0,170641367	protein_codin phosphatidic acid phosphatase
TcG_05901	1233,374757	-0,481979518	0,056928062	-8,46646621	2,5295E-17	3,48891E-15	protein_codin putative phosphatidic acid phosphatase
TcG_05902	185,0573538	0,018215227	0,131508545	0,138509833	0,889837499	0,946535728	protein_codin hypothetical protein
TcG_05903	208,7825037	-0,203737838	0,121657108	-1,67468915	0,093995237	0,257575407	protein_codin putative tubulin-specific chaperone
TcG_05904	435,6953245	-0,52645594	0,086952795	-6,05450276	1,40852E-09	5,43971E-08	protein_codin pf20-like protein
TcG_05905	331,545056	-0,109284398	0,104351191	-1,04727505	0,294972742	0,527938889	protein_codin uracil-DNA glycosylase
TcG_05906	306,522972	-0,320266595	0,115199765	-2,78009765	0,005434255	0,0305489	protein_codin hypothetical protein
TcG_05907	310,8180586	-0,355348251	0,103063813	-3,44784694	0,000565074	0,00480992	protein_codin hypothetical protein
TcG_05908	405,1942196	-0,280954109	0,093616861	-3,00110586	0,00269001	0,017401708	protein_codin hypothetical protein
TcG_05909	1128,04497	-0,330897827	0,059604047	-5,55159998	2,83067E-08	8,24023E-07	protein_codin cytosolic aconitase
TcG_05910	303,3464144	-0,407792873	0,102747185	-3,9688958	7,22064E-05	0,000847603	protein_codin hypothetical protein
TcG_05911	401,0919842	-0,457891335	0,103126665	-4,4400867	8,99226E-06	0,000139657	protein_codin Protein XRP2
TcG_05912	116,9105556	0,179345286	0,172139937	1,041857512	0,297477745	0,53089605	protein_codin surface protease GP63
TcG_05913	304,6240853	-0,027189661	0,107575509	-0,25274955	0,800461756	0,900665233	protein_codin hypothetical protein
TcG_05914	407,311276	-0,18363914	0,093105198	-1,97238334	0,048565863	0,162016724	protein_codin hypothetical protein
TcG_05915	359,307813	-0,169105423	0,09625804	-1,75679271	0,078953148	0,228881818	protein_codin putative target SNARE
TcG_05916	445,4165674	0,078936365	0,088271674	0,894243437	0,371191656	0,60275074	protein_codin hypothetical protein
TcG_05917	166,8550089	0,112613383	0,135504598	0,831066883	0,405935847	0,634643717	protein_codin hypothetical protein
TcG_05918	430,4112362	-0,162551842	0,088598289	-1,83470634	0,066549212	0,204845689	protein_codin putative DEAD box RNA helicase
TcG_05919	341,4447285	0,000136362	0,09936163	0,001372377	0,998905002	0,999767913	protein_codin viral A-type inclusion protein
TcG_05920	369,3858759	0,042666435	0,095034426	0,448957676	0,653462188	0,81657908	protein_codin hypothetical protein
TcG_05921	272,4023989	0,156097231	0,110514567	1,412458428	0,157815023	0,359914248	protein_codin hypothetical protein
TcG_05922	1,0695558	-3,740338245	3,669879733	-1,01919913	0,308108438	1	protein_codin membrane protein
TcG_05923	342,4988291	0,153628066	0,098395712	1,561328876	0,118446174	0,300626124	protein_codin hypothetical protein
TcG_05924	10,42810318	0,021205875	0,534906635	0,039644068	0,968376894	1	protein_codin surface protease GP63
TcG_05925	293,0216827	-0,002168263	0,109539402	-0,01979436	0,984207415	0,993987719	protein_codin hypothetical protein
TcG_05926	515,5873742	0,118847529	0,081873242	1,451604046	0,146611733	0,344665037	protein_codin DNA-directed DNA polymerase
TcG_05927	96,70559131	0,090888821	0,186297107	0,487870278	0,625641729	0,798753175	protein_codin putative DNA polymerase kappa, putative,DNA polymerase IV
TcG_05928	58,3779798	0,060694925	0,22707399	0,267291401	0,789244807	0,894943008	protein_codin putative DNA polymerase kappa, putative,DNA polymerase IV

TcG_05929	353,7228546	-0,174444828	0,098831022	-1,7650817	0,077550036	0,225974102	protein_codin hypothetical protein
TcG_05930	133,5404876	-0,078031621	0,153168871	-0,5094483	0,610438026	0,789056132	protein_codin putative DNA polymerase kappa, putative,DNA polymerase IV
TcG_05931	79,06313416	0,063268282	0,195573687	0,323500995	0,746315854	0,871654787	protein_codin putative DNA polymerase kappa, putative,DNA polymerase IV
TcG_05932	387,5805923	1,005527245	0,096730743	10,39511546	2,60971E-25	7,95686E-23	protein_codin hypothetical protein
TcG_05933	861,3021585	0,068124659	0,065517959	1,039786038	0,298439317	0,532120334	protein_codin haloacid dehalogenase-like hydrolase
TcG_05934	569,1710522	-0,048930687	0,081769957	-0,59839444	0,549576776	0,745866398	protein_codin hypothetical protein
TcG_05935	215,5026229	0,064466616	0,120850011	0,533443198	0,593726821	0,778863106	protein_codin putative chaperone
TcG_05936	354,4910975	-0,174252505	0,09677452	-1,80060314	0,071765453	0,215117279	protein_codin hypothetical protein
TcG_05937	447,7375102	-0,078406286	0,08926489	-0,87835526	0,379750958	0,610721305	protein_codin hypothetical protein
TcG_05938	233,4373584	0,099195124	0,118776942	0,835137879	0,403640081	0,63312391	protein_codin RNA editing complex protein
TcG_05939	493,6093427	0,154267396	0,082368758	1,87288724	0,061083958	0,191934289	protein_codin calmodulin-like protein containing EF hand
TcG_05940	718,6442024	-0,231385691	0,073327533	-3,155509	0,001602183	0,011508305	protein_codin cytochrome P450 reductase C
TcG_05941	450,843844	-0,072736925	0,086773757	-0,83823645	0,401897921	0,63220648	protein_codin hypothetical protein
TcG_05942	288,5229979	0,167426331	0,111528857	1,501192918	0,133305671	0,324453521	protein_codin hypothetical protein
TcG_05943	5622,19296	-0,364120298	0,038760473	-9,39411399	5,77014E-21	1,1331E-18	protein_codin glucose-regulated protein 78
TcG_05944	146,9184576	0,366373063	0,148336915	2,469871114	0,013516174	0,062791032	protein_codin hypothetical protein
TcG_05945	64,87813501	0,066640358	0,21415458	0,311178765	0,755664725	0,876828393	protein_codin hypothetical protein
TcG_05946	108,2905913	0,363451394	0,170671124	2,129542397	0,033209411	0,123294526	protein_codin putative serine/threonine protein phosphatase
TcG_05947	116,9551357	0,341601966	0,166098386	2,056624231	0,039722386	0,140502639	protein_codin hypothetical protein
TcG_05948	153,514265	0,351406918	0,153574692	2,288182462	0,022126898	0,090940843	protein_codin rab1 small GTP-binding protein
TcG_05949	139,1847291	0,223644796	0,149573904	1,495212662	0,13485895	0,326741069	protein_codin putative trans-sialidase
TcG_05950	327,7117905	0,322433103	0,108787419	2,963882284	0,003037845	0,019243559	protein_codin helicase-like protein
TcG_05951	189,8992021	-0,214679905	0,130016723	-1,65117148	0,098703564	0,266258323	protein_codin surface antigen 2 (CA-2)
TcG_05952	101,2522412	-0,09112175	0,197464794	-0,46145821	0,644469898	0,811082798	protein_codin acetyltransferase
TcG_05953	229,9614973	0,486804924	0,126911431	3,835784703	0,000125164	0,001348977	protein_codin putative protein kinase
TcG_05954	182,511025	-0,046851437	0,136269791	-0,34381381	0,730986311	0,861655042	protein_codin hypothetical protein
TcG_05955	112,7917107	0,350854672	0,170809145	2,054074283	0,039968503	0,141224481	protein_codin hypothetical protein
TcG_05956	126,2081902	-0,231956805	0,155768196	-1,48911531	0,136457007	0,329163749	protein_codin hypothetical protein
TcG_05957	135,3102545	0,121196153	0,152504894	0,794703364	0,426786075	0,651996765	protein_codin U5 snRNP-specific 40 kDa protein
TcG_05958	348,6102375	-0,147403065	0,100991567	-1,45955814	0,14441155	0,341087905	protein_codin hypothetical protein
TcG_05959	184,6147954	-0,005887626	0,134888822	-0,04364799	0,965185	0,984473405	protein_codin hypothetical protein
TcG_05960	368,9508836	0,076397976	0,09384495	0,814087241	0,415594975	0,64239863	protein_codin putative sodium/sulfate symporter
TcG_05961	56,62544985	0,226768367	0,231508499	0,979525019	0,327320633	0,561827682	protein_codin hypothetical protein
TcG_05962	259,5388863	0,022869896	0,113741243	0,201069508	0,840644224	0,922466573	protein_codin hypothetical protein
TcG_05963	227,3026921	-0,001435294	0,130893424	-0,01096537	0,991251079	0,996670572	protein_codin putative oligomeric golgi complex component 8
TcG_05964	217,0950974	-0,006500374	0,127189267	-0,05110788	0,959239553	0,981173255	protein_codin hypothetical protein
TcG_05965	2048,203003	0,013663968	0,065291361	0,209276818	0,834232148	0,91946477	protein_codin Dynein heavy chain family protein
TcG_05966	1142,86691	0,301986097	0,061453856	4,914030049	8,92231E-07	1,84267E-05	protein_codin 60S ribosomal protein L14
TcG_05967	75,9580538	0,380058039	0,205089181	1,853135487	0,063862942	0,198528586	protein_codin hypothetical protein
TcG_05968	198,2327525	0,900433651	0,134122651	6,713509177	1,89999E-11	1,10619E-09	protein_codin putative cytosolic malate dehydrogenase
TcG_05969	316,6833031	0,282982017	0,101127671	2,798264952	0,005137795	0,029280124	protein_codin hypothetical protein
TcG_05970	289,0210748	-0,06099691	0,104976724	-0,58105176	0,561205567	0,754744945	protein_codin hypothetical protein
TcG_05971	202,2069668	0,163603081	0,127187803	1,28631109	0,198334514	0,416922256	protein_codin hypothetical protein
TcG_05972	451,0001841	-0,146525452	0,090953907	-1,61098579	0,107182819	0,282095776	protein_codin hypothetical protein
TcG_05973	410,2944158	0,266114692	0,09066058	2,935285565	0,003332409	0,020724254	protein_codin putative heat shock protein 70
TcG_05974	124,3484389	0,196049864	0,161447835	1,214323275	0,224624301	0,451082534	protein_codin hypothetical protein
TcG_05975	287,1761441	0,301064367	0,106038834	2,839189723	0,004522826	0,026626757	protein_codin hypothetical protein
TcG_05976	289,8472892	0,61937093	0,109529795	5,654816872	1,56013E-08	4,76929E-07	protein_codin hypothetical protein
TcG_05977	440,79293	0,30809719	0,093290124	3,302570272	0,000958031	0,007535468	protein_codin hypothetical protein
TcG_05978	71,68180801	-0,130462759	0,213541663	-0,61094756	0,541234296	0,739895358	protein_codin heatshock protein hsp70
TcG_05979	19781,64098	0,063428808	0,0313954	2,020321732	0,043350026	0,14952468	protein_codin heat shock cognate 70
TcG_05980	330,4619807	-0,032430057	0,099455098	-0,32607738	0,744365813	0,870430189	protein_codin putative gamma-tubulin complex subunit
TcG_05981	359,5096496	-0,02594223	0,101164916	-0,25643505	0,797614928	0,898993352	protein_codin a44l protein-like protein
TcG_05982	0					1	protein_codin activated protein kinase C receptor
TcG_05983	1623,854479	0,023909661	0,052357886	0,456658254	0,647916701	0,812603825	protein_codin activated protein kinase C receptor
TcG_05984	2,784529159	1,157373195	1,099944551	1,05221049	0,292702988	1	protein_codin activated protein kinase C receptor
TcG_05985	412,0877388	-0,010631718	0,089690145	-0,11853831	0,905641145	0,954107005	protein_codin hypothetical protein

TcG_05986	146,310235	0,174473644	0,147194914	1,185323866	0,235889421	0,463445836	protein_codin hypothetical protein
TcG_05987	184,6503979	0,195624576	0,133753856	1,462571492	0,143584669	0,340008225	protein_codin alkylated DNA repair protein alkB like protein 7
TcG_05988	20,37543199	0,0349487	0,4084386	0,085566594	0,931810955	0,967364315	
TcG_05989	11,13400988	0,142189395	0,518628499	0,274164253	0,783958398	1	protein_codin hypothetical protein
TcG_05990	222,4619526	-0,27232313	0,125963623	-2,16191884	0,030624433	0,116049283	protein_codin putative endosomal integral membrane protein
TcG_05991	100,167223	-0,165390916	0,180356673	-0,91702133	0,359131462	0,592630203	protein_codin pre-mRNA-splicing factor ISY1
TcG_05992	1747,834975	0,105624836	0,054679736	1,931699821	0,053396567	0,173049685	protein_codin histone-lysine N-methyltransferase
TcG_05993	215,3118664	0,597538046	0,123853283	4,824563645	1,4031E-06	2,72757E-05	protein_codin glutamamyl carboxypeptidase
TcG_05994	189,4400492	-0,099423499	0,136393161	-0,72894783	0,466033574	0,682698822	protein_codin hypothetical protein
TcG_05995	347,3823213	-0,210097139	0,095865165	-2,19159003	0,028409122	0,109825856	protein_codin alpha-adaptin-like protein
TcG_05996	32,69665403	0,139014416	0,325365226	0,427256524	0,669192496	0,826309736	protein_codin hypothetical protein
TcG_05997	877,7305907	-0,114116747	0,06603439	-1,72814114	0,083962923	0,23860545	protein_codin putative cytochrome c1, heme protein, mitochondrial precursor
TcG_05998	164,5588133	-0,008093256	0,143171597	-0,05652836	0,954920903	0,978889315	protein_codin hypothetical protein
TcG_05999	1657,215478	-0,213300718	0,051775651	-4,11971099	3,79348E-05	0,000484045	protein_codin putative Golgi/lysosome glycoprotein
TcG_06000	734,4474071	0,056784042	0,074556924	0,761619967	0,446286854	0,667960146	protein_codin pitrilysin-like metalloprotease
TcG_06001	311,5814811	-0,023425087	0,103500766	-0,22632766	0,820946583	0,911236551	protein_codin Ca2+ binding protein, contains EF-hand motif (ISS)
TcG_06002	588,9097355	0,006958017	0,08059754	0,086330392	0,931203779	0,966993095	protein_codin putative brefeldin A-inhibited guanine nucleotide-exchange protein 2
TcG_06003	672,4996326	0,008216547	0,082770485	0,099269043	0,920924657	0,96150609	protein_codin putative brefeldin A-inhibited guanine nucleotide-exchange protein 2
TcG_06004	201,0085914	0,131789452	0,134957177	0,97652793	0,328802914	0,563203809	protein_codin tRNA-methyl transferase
TcG_06005	219,0258299	0,3507176	0,120237361	2,916877056	0,003535551	0,021775091	protein_codin putative 3-ketoacyl-CoA thiolase
TcG_06006	195,238561	0,231111666	0,127571005	1,811631616	0,070043136	0,212016453	protein_codin putative ubiquitin ligase
TcG_06007	658,5793293	0,213348552	0,075481274	2,826509671	0,004705832	0,027411651	protein_codin formin like proteiny 2 (FH2) domain protein
TcG_06008	226,8309455	0,330313108	0,126345737	2,614358946	0,008939506	0,045248196	protein_codin putative membrane receptor protein
TcG_06009	907,3851595	-0,186604375	0,068082187	-2,74086928	0,006127688	0,0335654	protein_codin hypothetical protein
TcG_06010	559,4835877	-0,148166235	0,084010874	-1,76365544	0,077790007	0,226280448	protein_codin putative protein kinase
TcG_06011	92,57720071	-0,171448219	0,180970017	-0,94738467	0,343442802	0,577606083	
TcG_06012	275,0483215	-0,007140808	0,121924902	-0,05856726	0,953296786	0,978116946	protein_codin hypothetical protein
TcG_06013	416,9582988	0,114752134	0,088715245	1,293488337	0,195842215	0,413814152	protein_codin hypothetical protein
TcG_06014	441,5597587	0,309774575	0,091008763	3,403788453	0,000664582	0,005499891	protein_codin oxidoreductase
TcG_06015	105,7115278	0,102370084	0,172089982	0,594863707	0,551934579	0,747373577	protein_codin hypothetical protein
TcG_06016	408,9798768	0,25266984	0,091333649	2,766448546	0,005667053	0,031596955	protein_codin putative phosphatidylglycerophosphate synthase
TcG_06017	323,5367755	0,042903808	0,107924252	0,397536299	0,690972024	0,839777811	protein_codin protein phosphatase 2A
TcG_06018	472,0911778	0,12156387	0,084874388	1,432279779	0,152063768	0,352291704	protein_codin hypothetical protein
TcG_06019	4,737414886	-0,015267199	0,86168198	-0,0177179	0,985863897	1	
TcG_06020	201,6365404	0,358818673	0,126205851	2,843122323	0,004467392	0,02639873	protein_codin hypothetical protein
TcG_06021	481,9032916	0,087385317	0,084092265	1,039159982	0,298730339	0,532231897	protein_codin putative protein kinase
TcG_06022	237,9863582	0,088882441	0,115215575	0,771444668	0,440443408	0,663390574	protein_codin hypothetical protein
TcG_06023	332,040057	0,21507086	0,103886836	2,070241694	0,038429717	0,137254056	protein_codin hypothetical protein
TcG_06024	499,8690143	-0,059049694	0,084943885	-0,69516121	0,486954277	0,698594881	protein_codin putative serine/threonine protein kinase
TcG_06025	51,3463803	0,193990694	0,248104759	0,781890258	0,434279071	0,658667014	
TcG_06026	2,390449582	0,367507869	1,154066113	0,318446114	0,750146563	1	protein_codin hypothetical protein
TcG_06027	207,394494	-0,589806839	0,123299697	-4,78352221	1,7225E-06	3,27162E-05	protein_codin calpain-like cysteine peptidase
TcG_06028	625,3439826	-0,572411383	0,07491971	-7,64033095	2,16664E-14	2,0576E-12	protein_codin putative vacuolar protein sorting complex subunit
TcG_06029	116,4642698	-0,623364398	0,172689476	-3,60974168	0,000306502	0,002850027	protein_codin putative protein kinase A catalytic subunit
TcG_06030	274,8821709	-0,16736791	0,106809063	-1,56698228	0,117118834	0,298554712	protein_codin putative cAMP phosphodiesterase A
TcG_06031	174,145804	-0,205403813	0,139969456	-1,46749026	0,142242737	0,338195024	protein_codin 26S proteasome non-ATPase regulatory subunit 10
TcG_06032	392,7690609	-0,114223868	0,093095256	-1,22695692	0,219838784	0,444990554	protein_codin putative methyltransferase
TcG_06033	355,287272	-0,139608062	0,098270411	-1,42065206	0,155417935	0,356891537	protein_codin AN1-type zinc finger protein 2B
TcG_06034	92,61420078	-0,08398644	0,180812752	-0,46449401	0,642293851	0,810066503	protein_codin hypothetical protein
TcG_06035	446,0288107	-0,402684481	0,089969071	-4,47581015	7,61221E-06	0,000120485	protein_codin ras-related protein rab-5
TcG_06036	707,9203605	-0,399954091	0,070796314	-5,64936319	1,61043E-08	4,91013E-07	protein_codin 200 kDa antigen p200
TcG_06037	497,4065208	-0,618696408	0,083620204	-7,39888663	1,37331E-13	1,19633E-11	protein_codin hypothetical protein
TcG_06038	151,40386	0,146161927	0,148774634	0,982438487	0,325883874	0,560274604	protein_codin hypothetical protein
TcG_06039	216,2575768	-0,332056493	0,12057294	-2,75398853	0,005887383	0,032528002	protein_codin hypothetical protein
TcG_06040	765,1505655	-0,309898537	0,069676225	-4,44769415	8,6797E-06	0,00013553	protein_codin hypothetical protein
TcG_06041	421,1500865	-0,267026018	0,089609022	-2,97990104	0,002883415	0,018446851	protein_codin hypothetical protein
TcG_06042	976,4879253	-0,317201464	0,066014961	-4,80499362	1,54756E-06	2,97387E-05	protein_codin uncharacterized protein

TcG_06043	927,8582744	-0,282325754	0,063527235	-4,44416877	8,82323E-06	0,000137585	protein_codin acid--amino-acid ligase
TcG_06044	260,3318914	-0,20551555	0,113733631	-1,80699014	0,070763816	0,213452115	protein_codin hypothetical protein
TcG_06045	92,07142263	-0,419920831	0,190128267	-2,20861862	0,027201179	0,106362761	protein_codin hypothetical protein
TcG_06046	274,4275938	-0,41125393	0,107221599	-3,83555117	0,000125283	0,001349005	protein_codin hypothetical protein
TcG_06047	665,1734799	-0,217338469	0,072020669	-3,01772355	0,002546811	0,016680246	protein_codin putative protein kintoun-like
TcG_06048	313,6952951	-0,379912818	0,104133067	-3,64833986	0,00026394	0,002514082	protein_codin TTAGGG binding factor
TcG_06049	194,4720157	0,539250583	0,130458174	4,133513186	3,5726E-05	0,000459912	protein_codin putative mitochondrial carrier protein
TcG_06050	180,5453801	-0,361256886	0,136864079	-2,63953032	0,0083021	0,042679904	protein_codin putative acetyltransferase
TcG_06051	148,5982699	-0,509678424	0,148760691	-3,42616333	0,000612172	0,00512103	protein_codin hypothetical protein
TcG_06052	380,2243004	-0,284107189	0,092830216	-3,06050338	0,002209653	0,014892982	protein_codin putative cholinephosphate cytidylyltransferase A
TcG_06053	170,2237551	-0,245213736	0,138944049	-1,76483798	0,077590999	0,225979791	protein_codin zinc finger protein family memeber
TcG_06054	86,42006474	-0,209762211	0,189047897	-1,10957178	0,267183597	0,499610903	protein_codin hypothetical protein
TcG_06055	639,3172185	-0,339502753	0,076328449	-4,44791895	8,67062E-06	0,00013553	protein_codin putative leucine-rich repeat-containing protein 72-like
TcG_06056	177,2181001	0,051180007	0,132252459	0,386987183	0,698765696	0,844142481	protein_codin putative heat shock protein
TcG_06057	371,4246867	-0,237775383	0,095438822	-2,49139058	0,012724415	0,059686266	protein_codin rev7
TcG_06058	239,0855254	-0,250476791	0,121789048	-2,05664462	0,039720423	0,140502639	protein_codin hypothetical protein
TcG_06059	144,1242623	-0,18426519	0,148284216	-1,24264871	0,213997311	0,437522132	protein_codin putative trans-sialidase
TcG_06060	19,17385281	-0,29740542	0,395850784	-0,75130689	0,452467986	0,673298046	protein_codin putative diacylglycerol acyltransferase
TcG_06061	145,9459947	-0,168027188	0,151379736	-1,10997146	0,267011323	0,499395611	protein_codin putative diacylglycerol acyltransferase
TcG_06062	28,95609837	-0,167660252	0,324419139	-0,51680136	0,605294831	0,786698766	
TcG_06063	249,6600482	-0,29728132	0,115483065	-2,57424168	0,010046007	0,049550038	protein_codin putative delta-1-pyrroline-5-carboxylate dehydrogenase
TcG_06064	23,88354569	-0,331256983	0,358137843	-0,9249427	0,354995715	0,588914713	protein_codin putative diacylglycerol acyltransferase
TcG_06065	6,178838067	-0,539122406	0,768813551	-0,70123947	0,483153583	1	protein_codin putative diacylglycerol acyltransferase
TcG_06066	579,1941093	-0,478572286	0,081700936	-5,85761083	4,69573E-09	1,60486E-07	protein_codin hypothetical protein
TcG_06067	534,116826	-0,195024029	0,082561354	-2,36217092	0,018168264	0,077933175	protein_codin putative DNA excision repair protein, putative,SNF2 family helicase-like protein
TcG_06068	466,1337884	-0,438279796	0,086624205	-5,05955348	4,20239E-07	9,27408E-06	protein_codin hypothetical protein
TcG_06069	365,9318557	-0,10335143	0,096381316	-1,0723181	0,283577182	0,516998462	protein_codin hypothetical protein
TcG_06070	102,5795687	-0,063671392	0,176266466	-0,36122238	0,717933216	0,855087918	protein_codin putative casein kinase
TcG_06071	705,0979319	-0,319181213	0,07206077	-4,42933393	9,45246E-06	0,000145247	protein_codin hypothetical protein
TcG_06072	960,1878215	-0,163972389	0,065022334	-2,52178565	0,011676083	0,05580821	protein_codin putative serine/threonine protein kinase
TcG_06073	245,6366333	-0,103598901	0,116336469	-0,89051096	0,373191586	0,604239079	protein_codin sTim1
TcG_06074	586,0677243	-0,252157224	0,079000046	-3,19186173	0,00141359	0,010352625	protein_codin putative mitochondrial RNA binding complex 1 subunit
TcG_06075	293,1658807	-0,125486386	0,10509718	-1,19400336	0,232476654	0,459642541	protein_codin putative glycogenin glucosyltransferase
TcG_06076	304,4004831	-0,260885277	0,102287825	-2,55050175	0,010756798	0,052341661	protein_codin hypothetical protein
TcG_06077	337,4409378	-0,523420658	0,105292216	-4,97112394	6,65659E-07	1,40993E-05	protein_codin putative serine/threonine protein kinase, putative,protein kinase
TcG_06078	100,9169668	-0,443750597	0,182380245	-2,4331067	0,014969889	0,067666914	protein_codin PIF1 helicase-like protein
TcG_06079	210,0840741	-0,248308292	0,134970092	-1,83972826	0,065808143	0,203057226	protein_codin hypothetical protein
TcG_06080	251,0352358	-0,307363089	0,111291248	-2,76179029	0,005748539	0,031897783	protein_codin putative nucleolar protein
TcG_06081	380,5604448	-0,174280889	0,098327131	-1,77245983	0,076318258	0,223487944	protein_codin putative outer dynein arm docking complex
TcG_06082	116,4557865	-0,406777365	0,166435703	-2,44405112	0,014523369	0,06622252	protein_codin putative CAAX prenyl protease 2
TcG_06083	11,41272466	-0,014895882	0,532181832	-0,02799021	0,977669959	1	protein_codin surface protease GP63
TcG_06084	5,500380076	0,047242913	0,726274904	0,065048252	0,948135582	1	protein_codin hypothetical protein
TcG_06085	22,41004286	-0,901543902	0,40500715	-2,22599502	0,026014515	0,102793957	protein_codin hypothetical protein
TcG_06086	14,62344913	0,467325494	0,467455745	0,999721361	0,317445372	1	protein_codin hypothetical protein
TcG_06087	27,55090392	0,185269716	0,331799211	0,558379014	0,5765856	0,766883339	protein_codin hypothetical protein
TcG_06088	50,91382202	0,087754365	0,24535382	0,357664556	0,720594361	0,85670516	protein_codin trans-sialidase
TcG_06089	54,71488693	-0,64568263	0,236675862	-2,72813047	0,006369441	0,034662444	protein_codin mucin-associated surface protein (MASP)
TcG_06090	31,38212729	-0,391365695	0,309508079	-1,26447651	0,206059048	0,427849485	protein_codin 90 kDa surface protein
TcG_06091	13,35497359	-0,0486998	0,484026842	-0,10061384	0,919857008	1	protein_codin hypothetical protein
TcG_06092	6,571449484	0,407919782	0,687996335	0,592909819	0,553241506	1	protein_codin hypothetical protein
TcG_06093	14,8287701	0,359999472	0,479953033	0,750072294	0,453211165	1	protein_codin hypothetical protein
TcG_06094	218,4888582	-0,011361109	0,125810057	-0,09030366	0,928045909	0,965201068	protein_codin HEAT repeat containing 2
TcG_06095	141,0555718	-0,058465199	0,148899492	-0,39264875	0,694578928	0,84195349	protein_codin translation initiation factor EIF-2b alpha subunit
TcG_06096	156,1021492	-0,161955438	0,142721847	-1,13476277	0,256474731	0,488495189	protein_codin putative PITH domain-containing protein 1-like
TcG_06097	368,9232952	-0,251597381	0,09477636	-2,6546428	0,007939243	0,041248461	protein_codin putative proteasome beta-1 subunit
TcG_06098	178,8742237	0,019693076	0,132439334	0,148695069	0,881794253	0,942184067	protein_codin hypothetical protein
TcG_06099	100,5249943	-0,357098268	0,176704829	-2,02087441	0,043292767	0,149371651	protein_codin hypothetical protein

TcG_06100	184,7730643	-0,253395318	0,132599125	-1,91098786	0,056006141	0,179102166	protein_codin	hypothetical protein
TcG_06101	178,4669287	-0,20719479	0,131638053	-1,57397337	0,115493605	0,295838856	protein_codin	hypothetical protein
TcG_06102	321,2902717	0,084261009	0,10041679	0,839112757	0,401406038	0,631887277	protein_codin	hypothetical protein
TcG_06103	203,4948007	0,182365864	0,123614313	1,475281127	0,140136978	0,334581697	protein_codin	hypothetical protein
TcG_06104	256,98646	-0,084426388	0,121057937	-0,69740482	0,485549488	0,698269767	protein_codin	hypothetical protein
TcG_06105	304,216039	0,116272669	0,103407091	1,124416789	0,260836253	0,493315185	protein_codin	hypothetical protein
TcG_06106	484,3414244	-0,139199985	0,085499042	-1,62808825	0,103506174	0,275556649	protein_codin	hypothetical protein
TcG_06107	194,8710263	-0,047668154	0,12650121	-0,37681975	0,706307564	0,848727637	protein_codin	hypothetical protein
TcG_06108	354,4288165	0,186425626	0,096131311	1,93928101	0,05246713	0,170899631	protein_codin	putative leucine-rich repeat protein (LRRP)
TcG_06109	366,1515125	0,211362338	0,096472428	2,19090928	0,028458358	0,109869556	protein_codin	putative dolichyl-P-Man:GDP-Man5GlcNAc2-PP-dolichyl alpha-1,2-mannosyltransferase
TcG_06110	198,4187086	0,235560712	0,130979612	1,798453267	0,072105208	0,215924254	protein_codin	hypothetical protein
TcG_06111	210,9706704	0,112241405	0,131956186	0,850596007	0,394993808	0,62459373	protein_codin	hypothetical protein
TcG_06112	472,8678288	-0,109278498	0,08407559	-1,29976486	0,193681571	0,410802512	protein_codin	peptide hydrolase
TcG_06113	313,359219	-0,105756738	0,10591761	-0,99848116	0,318046096	0,553202532	protein_codin	hypothetical protein
TcG_06114	362,7869508	-0,036506683	0,10356532	-0,3524991	0,72446399	0,857982193	protein_codin	putative proteasome regulatory ATPase subunittcc1l8.3
TcG_06115	137,1323436	0,052761733	0,151830202	0,347504861	0,728212067	0,86000745	protein_codin	hypothetical protein
TcG_06116	228,6211021	0,100173483	0,126071214	0,794578551	0,426858699	0,652021739	protein_codin	putative hydroxyacylglutathione hydrolase, putative,glyoxalase II
TcG_06117	301,7139489	0,271828398	0,104459727	2,602231571	0,009261929	0,04644001	protein_codin	hypothetical protein
TcG_06118	462,9393433	-0,209972602	0,084103874	-2,49658655	0,012539506	0,059034019	protein_codin	hypothetical protein
TcG_06119	119,0529663	-0,089156991	0,160198271	-0,55654153	0,57784071	0,768001978	protein_codin	cysteine peptidase, putative,cysteine peptidase, clan CA, family C1, cathepsin L-like
TcG_06120	20,53406951	-0,409393115	0,386242979	-1,05993672	0,289173391	0,52267752	protein_codin	cysteine peptidase, putative,cysteine peptidase, clan CA, family C1, cathepsin L-like
TcG_06121	8668,470788	-0,096792906	0,036449411	-2,65554098	0,007918131	0,04118059	protein_codin	cruzipain
TcG_06122	120,5613388	-0,295114454	0,161515706	-1,82715638	0,06767626	0,207213833	protein_codin	cruzipain
TcG_06123	535,8016472	0,077295748	0,083770201	0,922711743	0,356157444	0,590165926	protein_codin	putative cysteinyl-tRNA synthetase
TcG_06124	333,1388505	0,040676544	0,102746073	0,395893905	0,692183298	0,84069156	protein_codin	helicase
TcG_06125	1069,426523	-0,345185934	0,060233902	-5,73075825	9,99827E-09	3,18698E-07	protein_codin	metacaspase
TcG_06126	314,6799803	-0,371480382	0,104690185	-3,54837833	0,000387611	0,003483989	protein_codin	hypothetical protein
TcG_06127	268,2631013	-0,100341581	0,109228359	-0,91864038	0,358283704	0,592164764	protein_codin	hypothetical protein
TcG_06128	258,3357982	-0,079967007	0,111326681	-0,71830945	0,472566505	0,688368479	protein_codin	cleavage and polyadenylation specificity factor 30 kDa subunit
TcG_06129	272,3939196	-0,014149254	0,110313556	-0,12826396	0,897940083	0,950181186	protein_codin	hypothetical protein
TcG_06130	1067,910376	-0,114779096	0,065442809	-1,75388399	0,079450387	0,229840745	protein_codin	putative ubiquitin ligase
TcG_06131	593,7065647	-0,078430037	0,080288824	-0,97684874	0,328644042	0,563083286	protein_codin	putative ribonucleoside-diphosphate reductase small chain
TcG_06132	146,2068559	-0,250778504	0,151094536	-1,65974569	0,096965624	0,263410017	protein_codin	putative trans-sialidase
TcG_06133	105,4772572	0,086304472	0,179528177	0,480729395	0,630708837	0,802040674	protein_codin	L1Tc protein
TcG_06134	662,2756652	-0,05758971	0,077391883	-0,74413115	0,456797137	0,676611049	protein_codin	putative acyl-CoA binding protein
TcG_06135	1269,052403	-0,127281183	0,110821553	-1,14852372	0,250752426	0,480518955	protein_codin	hypothetical protein
TcG_06136	826,9979147	-0,083668054	0,067489063	-1,23972759	0,215076171	0,438870501	protein_codin	oligopeptidase b
TcG_06137	201,6104873	-0,270526226	0,130637075	-2,07082274	0,038375364	0,137142805	protein_codin	putative flagellar/basal body protein
TcG_06138	181,9225802	0,058786069	0,136705182	0,43002078	0,667180526	0,825673314	protein_codin	hypothetical protein
TcG_06139	156,856382	-0,068502023	0,14145561	-0,48426515	0,628197703	0,800692914	protein_codin	hypothetical protein
TcG_06140	70,32533981	0,135980509	0,21437239	0,634319136	0,52587257	0,728904717	protein_codin	hypothetical protein
TcG_06141	85,92070529	-0,169199058	0,195223617	-0,86669359	0,386109919	0,616520055	protein_codin	hypothetical protein
TcG_06142	59,55387913	0,25641007	0,237082363	1,081523172	0,279464459	0,512478553	protein_codin	hypothetical protein
TcG_06143	48,96347865	-0,03748162	0,247881527	-0,1512078	0,879811797	0,941623821	protein_codin	dynein heavy chain, cytosolic
TcG_06144	136,8493401	0,031948783	0,158701089	0,201314203	0,840452898	0,922459954	protein_codin	protein UXT
TcG_06145	185,7998134	-0,191325372	0,136678682	-1,39981868	0,161567624	0,365181913	protein_codin	hypothetical protein
TcG_06146	374,8575453	-0,28503479	0,098802367	-2,88489839	0,0039154	0,023663967	protein_codin	hypothetical protein
TcG_06147	375,1087901	0,054800369	0,099328125	0,551710496	0,581146718	0,770482335	protein_codin	DEAD/DEAH box helicase-like protein
TcG_06148	698,6399269	-0,14457026	0,071469204	-2,02283295	0,043090373	0,149072875	protein_codin	metal binding protein
TcG_06149	144,9591703	-0,395843882	0,149460148	-2,64849116	0,008085196	0,041819234	protein_codin	putative ras-related GTP-binding protein
TcG_06150	292,4664013	-0,045441596	0,106233714	-0,42775117	0,668832289	0,826307698	protein_codin	hypothetical protein
TcG_06151	2594,659977	-0,301838484	0,044091248	-6,84576863	7,60663E-12	4,95224E-10	protein_codin	hypothetical protein
TcG_06152	5,034613796	-0,033116619	0,77357091	-0,04281006	0,965852942	1		
TcG_06153	457,7835271	0,082553031	0,088397232	0,933887057	0,350362156	0,584889659	protein_codin	putative phospholipid transporting ATPase-like protein
TcG_06154	865,6210258	0,112658981	0,06581923	1,711642348	0,086962599	0,244372707	protein_codin	polyubiquitin
TcG_06155	825,0666323	0,063198529	0,066245896	0,953999166	0,340084064	0,574207807	protein_codin	polyubiquitin
TcG_06156	259,5664689	0,062467202	0,110127483	0,567226273	0,57056046	0,762194568	protein_codin	EF-hand protein 5

TcG_06157	403,7860248	0,177555094	0,089844797	1,976242364	0,048127334	0,16115702	protein_codin	putative diacylglycerol acyltransferase
TcG_06158	161,2179005	0,077591817	0,138261322	0,56119684	0,574663357	0,765645084	protein_codin	putative DNA-directed RNA polymerase II
TcG_06159	321,9805532	-0,093700447	0,101054194	-0,92722967	0,353807303	0,58808501	protein_codin	hypothetical protein
TcG_06160	631,0954308	0,063526132	0,076191554	0,833768681	0,404411347	0,634033811	protein_codin	hypothetical protein
TcG_06161	91,73321642	-0,021080361	0,193403882	-0,10899658	0,913205205	0,957415212	protein_codin	oxidoreductase
TcG_06162	227,3162691	-0,264775637	0,119923798	-2,20786567	0,027253638	0,106531934	protein_codin	hypothetical protein
TcG_06163	476,4181025	0,076690542	0,085745264	0,894399733	0,371108055	0,602699457	protein_codin	hypothetical protein
TcG_06164	579,8156212	-0,146545768	0,081377035	-1,80082463	0,071730526	0,215117279	protein_codin	hypothetical protein
TcG_06165	253,4412452	-0,025819598	0,113845778	-0,22679451	0,820583531	0,911143056	protein_codin	putative proteasome regulatory non-ATPase subunit 3
TcG_06166	335,0745112	0,009327446	0,099259793	0,093970031	0,925132963	0,963382214	protein_codin	ribosome biogenesis protein MAK21
TcG_06167	256,7369677	-0,131413456	0,113834329	-1,15442729	0,248325032	0,477605217	protein_codin	charged multivesicular body protein 2A
TcG_06168	360,505821	0,2581401	0,101890556	2,533503687	0,011292855	0,054380308	protein_codin	Trypanosoma vivax
TcG_06169	223,955875	-0,150428405	0,119643689	-1,2573033	0,208643828	0,431207169	protein_codin	choline kinase
TcG_06170	630,4387709	-0,076441064	0,075898966	-1,00714237	0,313866362	0,548106574	protein_codin	putative vacuolar protein sorting protein 16
TcG_06171	196,3667128	0,061062821	0,129813493	0,470388862	0,63807722	0,807510942	protein_codin	hypothetical protein
TcG_06172	100,67857	0,099889711	0,182206753	0,548221786	0,583539625	0,772143684	protein_codin	hypothetical protein
TcG_06173	81,95061865	-0,021015316	0,197664411	-0,10631816	0,915329924	0,958775201	protein_codin	hypothetical protein
TcG_06174	28,8406954	0,228885948	0,33518177	0,682871112	0,494688289	0,70453824	protein_codin	hypothetical protein
TcG_06175	2106,040274	0,003109039	0,050438455	0,061640242	0,95084933	0,976990097	protein_codin	60S acidic ribosomal protein P0
TcG_06176	57,96750467	0,20719669	0,237254268	0,873310694	0,382493753	0,612434027	protein_codin	60S acidic ribosomal protein P0
TcG_06177	181,8735164	-0,428331318	0,133477895	-3,20900565	0,001331949	0,009848091	protein_codin	hypothetical protein
TcG_06178	283,2021251	0,016146477	0,111001761	0,14546145	0,884346551	0,943552734	protein_codin	hypothetical protein
TcG_06179	405,9345056	-0,052256154	0,09094062	-0,57461841	0,565549401	0,757986619	protein_codin	hypothetical protein
TcG_06180	139,5096788	-0,078093628	0,154453603	-0,50561221	0,613128908	0,790819724	protein_codin	hypothetical protein
TcG_06181	213,4928298	-0,101635787	0,122279256	-0,83117767	0,405873266	0,634643717	protein_codin	transferase, transferring glycosyl group
TcG_06182	996,3039581	-0,142867627	0,062791258	-2,27527894	0,022889202	0,093575968	protein_codin	putative arginyl-tRNA synthetase
TcG_06183	835,1626055	-0,3743768	0,068515858	-5,46408976	4,65288E-08	1,28967E-06	protein_codin	hypothetical protein
TcG_06184	104,215058	-0,399732367	0,178855261	-2,23494889	0,025420705	0,101028215	protein_codin	hypothetical protein
TcG_06185	169,9687759	-0,439332809	0,140926202	-3,11746717	0,001824123	0,012769961	protein_codin	putative leucine-richprotein
TcG_06186	146,7631485	-0,053036817	0,148897952	-0,35619574	0,72169398	0,857117481	protein_codin	ribonuclease III
TcG_06187	262,1766545	-0,297063544	0,109770614	-2,70622103	0,006805373	0,036570991	protein_codin	hypothetical protein
TcG_06188	179,6191258	-0,137607254	0,135511649	-1,01546439	0,309884515	0,543237513	protein_codin	ubiquitin hydrolase
TcG_06189	2,191671918	-1,051222325	1,200097084	-0,87594774	0,381058446	1	protein_codin	hypothetical protein
TcG_06190	1,395491747	-1,793269671	1,603148185	-1,11859258	0,263313989	1	protein_codin	hypothetical protein
TcG_06191	934,2810249	0,138116384	0,064149686	2,1530329	0,031316093	0,117954569	protein_codin	putative lysyl-tRNA synthetase
TcG_06192	586,4976767	0,129649961	0,079741502	1,625878095	0,103975591	0,276361825	protein_codin	putative acetylornithine deacetylase-like
TcG_06193	3432,066289	-0,149574666	0,10888777	-1,373659	0,16954757	0,376067037	protein_codin	putative ubiquitin-protein ligase-like
TcG_06194	198,1193328	0,163581781	0,126493518	1,29320287	0,195940903	0,413887202	protein_codin	hypothetical protein
TcG_06195	292,0020652	0,272485042	0,109061463	2,498453943	0,012473635	0,05881951	protein_codin	hypothetical protein
TcG_06196	375,0556142	-0,056332991	0,093053642	-0,60538191	0,544925271	0,741887014	protein_codin	hypothetical protein
TcG_06197	613,4249531	-0,293551843	0,078227315	-3,75254912	0,000175046	0,001788428	protein_codin	paraflagellar rod protein-like protein
TcG_06198	510,0353226	0,052320525	0,084688578	0,617799067	0,536707799	0,73660472	protein_codin	hypothetical protein
TcG_06199	169,4645843	0,146171969	0,138678358	1,054035915	0,291866474	0,524450861	protein_codin	putative Golgi pH regulator C
TcG_06200	208,1133879	0,155895636	0,125306982	1,244109728	0,213459181	0,436980474	protein_codin	putative NUDIX family hydrolase
TcG_06201	218,2144611	-0,001211677	0,122553045	-0,00988696	0,992111479	0,996781867	protein_codin	ATP-dependent DEAD/H RNA helicase
TcG_06202	656,1163332	0,023588099	0,076601146	0,307934016	0,758132542	0,878015178	protein_codin	putative ATP-dependent DEAD/H RNA helicase
TcG_06203	242,8887386	-0,159634251	0,114922324	-1,38906215	0,164813851	0,37020808	protein_codin	hypothetical protein
TcG_06204	109,1363889	-0,021552566	0,181904767	-0,11848269	0,90568521	0,954107005	protein_codin	hypothetical protein
TcG_06205	129,1616656	0,556690672	0,171842472	3,239540639	0,001197224	0,009030624	protein_codin	hypothetical protein
TcG_06206	496,6414359	0,356666049	0,089863857	3,968959935	7,2187E-05	0,000847603	protein_codin	hypothetical protein
TcG_06207	202,2350501	0,087158109	0,128664472	0,677406181	0,498148278	0,707424985	protein_codin	hypothetical protein
TcG_06208	159,0204772	-0,14596105	0,14164333	-1,03048305	0,302783305	0,535497997	protein_codin	hypothetical protein
TcG_06209	207,4871919	-0,347378984	0,12326369	-2,8181777	0,004829707	0,028034563	protein_codin	maoC-like dehydratase
TcG_06210	158,932496	-0,108971326	0,141004388	-0,77282223	0,439627595	0,662749321	protein_codin	hypothetical protein
TcG_06211	632,3828053	-0,106840284	0,076736577	-1,39229932	0,163831776	0,36864536	protein_codin	putative acyl-CoA dehydrogenase, mitochondrial precursor
TcG_06212	470,5179333	-0,155596585	0,091370109	-1,70292656	0,088581814	0,247422588	protein_codin	pre-rRNA-processing protein TSR1
TcG_06213	897,5509153	-0,21474668	0,081802857	-2,62517334	0,008660487	0,044144478	protein_codin	hypothetical protein

TcG_06214	901,3188679	-0,288303716	0,066431712	-4,33985077	1,4258E-05	0,000206749	protein_codin	putative methionyl-tRNA synthetase
TcG_06215	28,08913431	0,266217091	0,340358432	0,782166875	0,434116509	0,658558262		
TcG_06216	2534,959054	-0,061257866	0,130512547	-0,4693638	0,638809616	0,807731988	protein_codin	CCR4-NOT transcription complex subunit 1
TcG_06217	352,0523659	0,072574065	0,10168886	0,71368747	0,475420456	0,691032669	protein_codin	putative small GTPase
TcG_06218	372,2057098	0,128086335	0,100082657	1,279805506	0,200613547	0,419863455	protein_codin	hypothetical protein
TcG_06219	301,2322887	-0,215605875	0,114868359	-1,87698228	0,060520522	0,190956093	protein_codin	hypothetical protein
TcG_06220	938,0634454	-0,137822275	0,065412094	-2,1069846	0,035118914	0,128884302	protein_codin	19S proteasome regulatory subunit
TcG_06221	954,4599071	0,049002539	0,062972072	0,778163042	0,436472902	0,660007183	protein_codin	hypothetical protein
TcG_06222	545,2683477	-0,032260409	0,083594906	-0,38591358	0,699560676	0,844323521	protein_codin	ATPase subunit 9
TcG_06223	208,3147177	-0,115984559	0,12312914	-0,9419749	0,34620549	0,580482895	protein_codin	hypothetical protein
TcG_06224	1924,471934	0,258965392	0,054253786	4,773222497	1,81301E-06	3,40999E-05	protein_codin	ribosomal proteins L36
TcG_06225	133,4341957	0,354126636	0,154323903	2,294697255	0,02175048	0,089680094		
TcG_06226	134,4151741	-0,041409508	0,152755474	-0,27108363	0,786326714	0,893586355	protein_codin	hypothetical protein
TcG_06227	97,27485623	-0,208539798	0,183336181	-1,13747214	0,255340966	0,48673584	protein_codin	hypothetical protein
TcG_06228	351,1851488	-0,39085043	0,103460106	-3,77778882	0,000158227	0,001648577	protein_codin	putative dual specificity protein phosphatase
TcG_06229	830,0777978	-0,126767235	0,075267483	-1,6842231	0,09213856	0,2537919	protein_codin	ATP-binding cassette sub-family E member 1
TcG_06230	405,2392367	0,111038855	0,091909825	1,208128233	0,226997941	0,453760895	protein_codin	hypothetical protein
TcG_06231	88,69165182	-0,30432105	0,192708754	-1,57917606	0,11429568	0,293751054	protein_codin	ATP-binding protein cassette, sub-family E, member 1
TcG_06232	255,5240547	-0,069584601	0,110581647	-0,62925995	0,52917889	0,731194587	protein_codin	hypothetical protein
TcG_06233	139,6964831	0,049903852	0,151760871	0,328832143	0,742282567	0,86893659	protein_codin	histone deacetylase-like protein HDO1
TcG_06234	142,1420707	0,117615533	0,158131288	0,743784069	0,45700712	0,676783905	protein_codin	putative nucleotide-binding protein
TcG_06235	206,4389682	0,043105102	0,125364527	0,343838112	0,730968037	0,861655042	protein_codin	hypothetical protein
TcG_06236	322,0107087	-0,171357119	0,101233516	-1,69269158	0,090514189	0,250953674	protein_codin	putative phosphoglucomutase
TcG_06237	240,8607492	-0,132050202	0,129232479	-1,02180352	0,306873902	0,540059534	protein_codin	putative DNA polymerase eta
TcG_06238	606,5005667	-0,112850737	0,077403823	-1,45794785	0,144854917	0,341754828	protein_codin	putative RNA helicase
TcG_06239	143,7555979	-0,094687354	0,147324737	-0,64271185	0,520411089	0,72487171	protein_codin	hypothetical protein
TcG_06240	174,4765544	-0,036667688	0,134771776	-0,27207246	0,785566307	0,893514718	protein_codin	hypothetical protein
TcG_06241	5,024609892	1,113475993	0,835402585	1,33286156	0,182577239	1	protein_codin	hypothetical protein
TcG_06242	48,03466658	0,619022488	0,26876993	2,303168693	0,021269354	0,088293347	protein_codin	hypothetical protein
TcG_06243	35,67661767	-0,010334722	0,28945736	-0,03570378	0,971518555	0,98762955	protein_codin	helicase-like protein
TcG_06244	18,95182809	0,10142104	0,408889512	0,248040209	0,804103297	0,903185306	protein_codin	hypothetical protein
TcG_06245	427,3938453	0,089180324	0,08889827	1,003172775	0,315777506	0,550661979	protein_codin	hypothetical protein
TcG_06246	393,8367222	0,008933771	0,09994311	0,089388565	0,928773111	0,965428814	protein_codin	hypothetical protein
TcG_06247	571,0861047	0,366944364	0,078932246	4,648852443	3,33787E-06	5,91238E-05	protein_codin	putative glycine synthase
TcG_06248	123,2148734	0,096105282	0,169345725	0,567509348	0,570368178	0,762194568	protein_codin	hypothetical protein
TcG_06249	161,8319859	0,053413297	0,142094117	0,375900832	0,706990622	0,848924953	protein_codin	hypothetical protein
TcG_06250	252,8628138	0,225714178	0,117771074	1,916550212	0,055295098	0,177661955	protein_codin	hypothetical protein
TcG_06251	292,1750372	0,170739985	0,115229053	1,481744231	0,138408363	0,33221448	protein_codin	hypothetical protein
TcG_06252	670,1896995	-0,078154808	0,073454045	-1,06399597	0,287330526	0,520429245	protein_codin	putative glycyl tRNA synthetase
TcG_06253	474,8413727	-0,228778655	0,085793948	-2,666606	0,007662144	0,040024166	protein_codin	charged multivesicular body protein 4
TcG_06254	311,7244048	0,189520953	0,104155371	1,81959846	0,068820176	0,209663572	protein_codin	hypothetical protein
TcG_06255	568,7962231	0,053730382	0,078420741	0,685155244	0,493245963	0,703960055	protein_codin	translation initiation factor 34
TcG_06256	193,188381	0,225180711	0,126852924	1,77513221	0,07587606	0,222726128	protein_codin	hypothetical protein
TcG_06257	73,36912779	0,467533372	0,225066628	2,077310958	0,037772868	0,135659161	protein_codin	hypothetical protein
TcG_06258	2158,722334	0,15531265	0,049754336	3,121590229	0,001798771	0,01263467	protein_codin	adenosylhomocysteinase
TcG_06259	166,2168167	0,509907919	0,139431362	3,65705327	0,000255131	0,002442936	protein_codin	hypothetical protein
TcG_06260	309,5427431	0,144214677	0,112842908	1,278012766	0,201244925	0,420748598	protein_codin	hypothetical protein
TcG_06261	160,4507524	0,364107034	0,138763968	2,623930683	0,008692147	0,04424746	protein_codin	putative oxidoreductase
TcG_06262	355,7808446	0,238007742	0,095950802	2,480518519	0,013119145	0,061264979	protein_codin	putative replication factor C, subunit 4
TcG_06263	354,2558913	-0,18504324	0,097274703	-1,90227505	0,057135206	0,181909452	protein_codin	golgi familyn subfamily A member 1
TcG_06264	465,1432243	0,031372048	0,085446419	0,36715462	0,713503687	0,852784519	protein_codin	phosphoenolpyruvate mutase
TcG_06265	1379,782917	-0,219903716	0,054577016	-4,02923669	5,59583E-05	0,000680307	protein_codin	putative 14-3-3 protein
TcG_06266	656,6448144	0,138954244	0,073131369	1,900063492	0,057424788	0,182580569	protein_codin	putative DNA topoisomerase III
TcG_06267	576,554507	-0,063880791	0,080709465	-0,7914907	0,428657701	0,653907586	protein_codin	putative nucleic acid binding protein
TcG_06268	97,4311621	0,402472293	0,179066719	2,247610809	0,024601018	0,098659533	protein_codin	hypothetical protein
TcG_06269	317,0890642	0,106837565	0,107132174	0,997250037	0,318643159	0,553576194	protein_codin	putative nucleotide binding protein
TcG_06270	276,361712	0,107392372	0,107576011	0,998292939	0,318137331	0,553278162	protein_codin	tubulin-specific chaperone C

TcG_06271	723,6869477	0,014885143	0,0709794	0,209710741	0,833893442	0,919266357	protein_codin putative membrane-bound acid phosphatase 2
TcG_06272	221,8370988	0,182759517	0,120846539	1,512327273	0,130450626	0,320551634	protein_codin ADP-ribosylation factor GTPase activating protein
TcG_06273	195,0943007	-0,066663716	0,137185308	-0,48593918	0,627010292	0,799795358	protein_codin hypothetical protein
TcG_06274	166,0433285	0,139888069	0,140060337	0,998770045	0,3179061	0,553042054	protein_codin cyclophilin-like protein
TcG_06275	324,9340317	-0,137966933	0,110640375	-1,2469854	0,212402849	0,435610439	protein_codin EF hand family protein
TcG_06276	210,3695612	0,250873756	0,12928192	1,940516947	0,052316897	0,170641367	protein_codin hypothetical protein
TcG_06277	188,6236145	0,061352426	0,130289242	0,470894033	0,637716409	0,807510942	protein_codin hypothetical protein
TcG_06278	87,46757639	0,249856167	0,185918895	1,34389873	0,178981137	0,389799325	protein_codin hypothetical protein
TcG_06279	402,1587826	-0,177435761	0,094085469	-1,88589973	0,059308468	0,187560733	protein_codin putative ATP synthase
TcG_06280	133,4746273	0,281515138	0,159294081	1,767266782	0,07718356	0,225081482	protein_codin hypothetical protein
TcG_06281	464,4771995	-0,250521929	0,088594063	-2,82775078	0,004687628	0,027319348	protein_codin hypothetical protein
TcG_06282	69,41038667	0,041978272	0,210614066	0,199313718	0,842017349	0,923105402	protein_codin putative lipoate protein ligase
TcG_06283	26,66694536	0,487979147	0,345514331	1,412326791	0,157853761	0,359914248	protein_codin hypothetical protein
TcG_06284	21,62699099	-0,029293139	0,377240793	-0,07765104	0,938105642	0,971428881	protein_codin hypothetical protein
TcG_06285	19,47336926	0,124272195	0,393438547	0,315861768	0,752107432	0,87492919	protein_codin retrotransposon hot spot (RHS) protein
TcG_06286	360,0055387	0,004875772	0,100107541	0,048705341	0,961154119	0,981984718	protein_codin putative retrotransposon hot spot (RHS) protein
TcG_06287	22,25108128	-0,523600341	0,365396511	-1,43296481	0,151867894	0,35209328	protein_codin hypothetical protein
TcG_06288	45,98001961	0,31666038	0,260587674	1,21517789	0,224298251	0,451082534	protein_codin hypothetical protein
TcG_06289	61,61306311	0,250831009	0,237353576	1,056782092	0,290611049	0,523479418	
TcG_06290	762,2331072	-0,367994338	0,070297358	-5,23482455	1,65142E-07	4,09707E-06	protein_codin hypothetical protein
TcG_06291	163,3212698	0,072094227	0,138689305	0,519825425	0,603185257	0,785225212	protein_codin hypothetical protein
TcG_06292	69,10577535	0,338572679	0,215492819	1,571155276	0,116146586	0,297058354	protein_codin hypothetical protein
TcG_06293	111,7547027	-0,165411943	0,166319393	-0,99454393	0,319958129	0,555111543	protein_codin hypothetical protein
TcG_06294	295,7840962	-0,009081655	0,104549025	-0,08686504	0,930778791	0,96665499	protein_codin hypothetical protein
TcG_06295	218,6054347	0,199873201	0,122063554	1,637451916	0,101536078	0,271810768	protein_codin putative GTPase activator protein
TcG_06296	270,9185068	-0,188738394	0,118052637	-1,59876474	0,109872886	0,286450778	protein_codin hypothetical protein
TcG_06297	436,1246169	-0,015565914	0,090261636	-0,17245326	0,863081202	0,934460219	protein_codin hypothetical protein
TcG_06298	77,16661598	0,214891623	0,207838576	1,033935216	0,30116645	0,534351377	protein_codin hypothetical protein
TcG_06299	212,3169742	0,18295627	0,122382602	1,494953271	0,134926638	0,326830988	protein_codin hypothetical protein
TcG_06300	492,5821192	-0,335770022	0,093398351	-3,59503157	0,000324352	0,002987238	protein_codin endosomal integral membrane protein
TcG_06301	144,6244455	-0,258695728	0,151476802	-1,70782407	0,087668991	0,245642789	protein_codin hypothetical protein
TcG_06302	673,9752095	-0,147779941	0,071978054	-2,05312498	0,040060458	0,14150624	protein_codin hypothetical protein
TcG_06303	518,5366854	-0,212644076	0,082067263	-2,591095	0,009567108	0,047716104	protein_codin putative Zn-finger protein
TcG_06304	202,0413999	-0,050952694	0,12636085	-0,40323165	0,686777819	0,837403474	protein_codin putative ABC transporter
TcG_06305	630,453045	-0,25774856	0,077279726	-3,3352675	0,000852174	0,006845707	protein_codin putative subtilisin-like serine peptidase
TcG_06306	153,6266542	-0,21513428	0,142730417	-1,50727703	0,131739662	0,322624334	protein_codin thioredoxin
TcG_06307	182,6003337	0,082714135	0,138520454	0,597125791	0,5504234	0,746546058	protein_codin hypothetical protein
TcG_06308	131,5718443	-0,24351454	0,154188817	-1,57932686	0,114261104	0,293727347	protein_codin MORN Repeat Containing 1 protein
TcG_06309	158,4916621	0,103429935	0,139210894	0,742973	0,457498027	0,677043319	protein_codin putative mismatch repair protein MSH5
TcG_06310	348,7958859	-0,560471376	0,097977158	-5,720429	1,06255E-08	3,36359E-07	protein_codin putative paraflagellar rod protein 3
TcG_06311	1848,396413	-0,613927548	0,053940377	-11,3815954	5,16462E-30	2,678E-27	protein_codin putative paraflagellar rod protein 1D
TcG_06312	179,3592378	-0,194339081	0,141485243	-1,37356431	0,169576982	0,376067037	protein_codin putative diphthamide synthesis protein
TcG_06313	390,9861806	-0,32750891	0,091039869	-3,5974229	0,000321386	0,002966992	protein_codin topoisomerase (DNA) II binding protein 1
TcG_06314	310,1331887	0,472008102	0,107723679	4,381655962	1,17781E-05	0,000174726	protein_codin 40S ribosomal protein S15a
TcG_06315	246,262218	-0,182551038	0,127000854	-1,43740008	0,150604345	0,350663573	protein_codin putative serine/threonine protein phosphatase type 5
TcG_06316	193,9715701	-0,335192748	0,128342541	-2,61170417	0,009009217	0,045541359	protein_codin hypothetical protein
TcG_06317	249,5986741	-0,243502879	0,113207236	-2,15094802	0,031480303	0,118457547	protein_codin putative Tob55
TcG_06318	171,9951134	-0,27823701	0,140302593	-1,98312094	0,047353922	0,159119067	protein_codin putative dihydrolipoamide dehydrogenase
TcG_06319	2,367169927	-0,899807685	1,120921804	-0,80273903	0,422125587	1	
TcG_06320	541,8373511	-0,189145943	0,080792536	-2,34113141	0,019225399	0,081442586	protein_codin hypothetical protein
TcG_06321	306,2261265	-0,073051224	0,10696355	-0,68295438	0,494635672	0,70453824	protein_codin tRNA pseudouridine38-40 synthase
TcG_06322	227,6638451	0,062737975	0,119407197	0,525412002	0,599296812	0,783102809	protein_codin hypothetical protein
TcG_06323	838,3166657	0,024279283	0,066659524	0,364228266	0,715687561	0,853960462	protein_codin ribosomal rRNA-processing protein 9
TcG_06324	410,5486131	0,066251202	0,089234647	0,742438102	0,457821941	0,67717669	protein_codin putative vacuolar fusion protein MON1 B-like
TcG_06325	327,1740947	-0,052570401	0,100024247	-0,52557657	0,599182437	0,783093933	protein_codin hypothetical protein
TcG_06326	78,00723784	-0,127398839	0,209606515	-0,6078	0,543320131	0,740864584	protein_codin hypothetical protein
TcG_06327	445,3252423	-0,108738192	0,086553505	-1,25631183	0,209002936	0,431564429	protein_codin hypothetical protein

TcG_06328	767,0017031	-0,201130067	0,070749959	-2,84282946	0,004471499	0,02639873	protein_codin hypothetical protein
TcG_06329	689,5474126	-0,230134074	0,071985263	-3,19696093	0,001388837	0,010203595	protein_codin hypothetical protein
TcG_06330	178,7873496	-0,05051432	0,135463033	-0,37290114	0,709222021	0,850052867	protein_codin hypothetical protein
TcG_06331	345,7279445	-0,152450808	0,098909356	-1,54131837	0,12323932	0,308524365	protein_codin hypothetical protein
TcG_06332	323,0467131	-0,203977376	0,102006966	-1,99964162	0,045538976	0,154861924	protein_codin hypothetical protein
TcG_06333	158,8729423	-0,128580059	0,139328912	-0,92285268	0,356083985	0,590128602	protein_codin hypothetical protein
TcG_06334	198,1542525	-0,165428253	0,142752271	-1,15884849	0,246517949	0,475155042	protein_codin hypothetical protein
TcG_06335	387,0345087	-0,237642433	0,093656236	-2,53739039	0,011168234	0,053914648	protein_codin hypothetical protein
TcG_06336	254,5364206	-0,047012779	0,113460812	-0,41435257	0,678615921	0,831850182	protein_codin hypothetical protein
TcG_06337	51,51211024	-0,078648393	0,262037637	-0,30014159	0,764069157	0,881021622	protein_codin hypothetical protein
TcG_06338	727,2350849	-0,009883052	0,078286773	-0,12624165	0,89954064	0,951179871	protein_codin putative lysosomal alpha-mannosidase precursor
TcG_06339	60,19162246	0,147905184	0,225371798	0,656271925	0,511649178	0,717758491	protein_codin hypothetical protein
TcG_06340	1095,103167	-0,125034076	0,060581751	-2,0638901	0,039028138	0,139089513	protein_codin putative malate dehydrogenase
TcG_06341	178,6521153	-0,144428228	0,133206625	-1,08424208	0,278257477	0,510998753	protein_codin putative malate dehydrogenase
TcG_06342	175,4720892	-0,098077102	0,137520883	-0,7131797	0,475734566	0,691164619	protein_codin putative adenylate kinase
TcG_06343	141,1933333	0,14297099	0,15152775	0,943530082	0,345409836	0,57940037	
TcG_06344	172,8645869	-0,391522714	0,135494741	-2,88957867	0,003857585	0,023399988	protein_codin putative adenylate kinase
TcG_06345	110,1811587	-0,408143348	0,172942381	-2,35999612	0,018275126	0,07821781	protein_codin hypothetical protein
TcG_06346	193,4013386	-0,083151055	0,134685074	-0,61737394	0,536988106	0,736713344	protein_codin hypothetical protein
TcG_06347	240,963173	-0,350506496	0,118581635	-2,95582446	0,003118345	0,019667473	protein_codin hypothetical protein
TcG_06348	135,8085657	-0,136747153	0,15488181	-0,88291293	0,377283319	0,607956124	protein_codin HPP family protein
TcG_06349	118,4088875	-0,450870148	0,16371523	-2,75399025	0,005887352	0,032528002	protein_codin hypothetical protein
TcG_06350	63,28015442	-0,205144653	0,227971302	-0,89987051	0,368189163	0,600276384	
TcG_06351	479,3377328	-0,200000421	0,08959955	-2,23215877	0,025604472	0,101593633	protein_codin putative glucose-6-phosphate 1-dehydrogenase
TcG_06352	86,63338909	-0,243731017	0,193842741	-1,25736469	0,20862161	0,431207169	protein_codin putative glucose-6-phosphate 1-dehydrogenase
TcG_06353	186,906287	-0,033972453	0,136022497	-0,24975613	0,802775944	0,902480311	protein_codin glucose-6-phosphate dehydrogenase
TcG_06354	83,76658828	0,122753814	0,191570479	0,640776252	0,521668064	0,725401608	protein_codin hypothetical protein
TcG_06355	883,2015072	-0,158567951	0,065110003	-2,43538541	0,014875936	0,067351539	protein_codin hypothetical protein
TcG_06356	257,0077472	-0,263861427	0,111691711	-2,36240831	0,018156632	0,077912126	protein_codin putative protein kinase
TcG_06357	315,599089	-0,016899338	0,102183237	-0,16538268	0,868642785	0,936741226	protein_codin ribosome recycling factor
TcG_06358	760,1084243	-0,123224847	0,068667491	-1,79451506	0,072730997	0,21694934	protein_codin putative eukaryotic translation initiation factor 4e
TcG_06359	1262,045669	0,183155258	0,058833235	3,11312574	0,001851171	0,012943676	protein_codin putative aspartyl-tRNA synthetase
TcG_06360	469,7595495	0,126457617	0,086741692	1,457864307	0,144877949	0,341754828	protein_codin hypothetical protein
TcG_06361	757,7672797	0,046412029	0,069479134	0,667999526	0,504133895	0,712076368	protein_codin putative bystin
TcG_06362	344,9325998	-0,11170155	0,098421967	-1,13492499	0,256406747	0,488446	protein_codin hypothetical protein
TcG_06363	171,4325862	-0,03926503	0,153360842	-0,25603035	0,797927402	0,899123407	
TcG_06364	456,7364841	-0,093245235	0,08766202	-1,06369023	0,28746905	0,520529678	protein_codin hypothetical protein
TcG_06365	390,161018	0,109228976	0,093685197	1,165914995	0,243648816	0,471818752	protein_codin hypothetical protein
TcG_06366	234,0582459	-0,231159882	0,118120085	-1,95699047	0,050348589	0,166310971	protein_codin putative leucine-rich repeat protein (LRRP)
TcG_06367	362,3347913	-0,070309756	0,099531176	-0,70640937	0,479933593	0,694196081	protein_codin ama1 protein
TcG_06368	440,5045591	0,140626245	0,087956627	1,598813523	0,109862043	0,286450778	protein_codin putative N-alpha-acetyltransferase 25, NatB auxiliary subunit isoform X1
TcG_06369	260,5525568	-0,071897886	0,115869508	-0,6205074	0,534923787	0,735099868	protein_codin putative glycosyltransferase family 28 protein
TcG_06370	77,29210839	-0,010723404	0,20430406	-0,05248748	0,958140275	0,98064539	protein_codin nuclear cap binding protein
TcG_06371	358,2773496	0,083219276	0,095850085	0,868223284	0,38527211	0,615776337	protein_codin putative LMBR1-like membrane protein
TcG_06372	426,8128579	-0,022679934	0,088461898	-0,25638082	0,797656798	0,898993352	protein_codin hypothetical protein
TcG_06373	423,8660882	-0,08315304	0,090726103	-0,91652829	0,359389878	0,592630203	protein_codin putative spliceosome-associated protein
TcG_06374	344,7522692	0,096489667	0,099511461	0,96963371	0,332229104	0,566340797	protein_codin hypothetical protein
TcG_06375	633,2002873	-0,022881949	0,076703756	-0,29831588	0,765462085	0,882013298	protein_codin fatty-acyl-CoA synthase
TcG_06376	289,2310341	0,022523943	0,105511795	0,213473229	0,830957868	0,917601778	protein_codin putative intergrin alpha chain protein
TcG_06377	420,6333565	-0,262558549	0,093571762	-2,80595922	0,005016704	0,028845426	protein_codin putative protein kinase
TcG_06378	178,3596164	-0,024182853	0,142105712	-0,1701751	0,864872435	0,934985064	protein_codin putative nitrate reductase
TcG_06379	387,6048433	0,095078682	0,094348833	1,007735647	0,313581387	0,547904381	protein_codin putative ribosome biogenesis regulatory protein (RRS1)
TcG_06380	503,9462715	-0,161645745	0,083666785	-1,93201812	0,05335727	0,172970713	protein_codin putative histidyl-tRNA synthetase
TcG_06381	130,7581281	0,119655864	0,155779997	0,76810801	0,442423018	0,664667154	protein_codin trimeric LpxA-like protein
TcG_06382	123,9118042	0,295178765	0,160227671	1,842245868	0,065439194	0,202180934	protein_codin hypothetical protein
TcG_06383	269,0429453	-0,032135651	0,112779114	-0,28494328	0,775687596	0,888230528	protein_codin hypothetical protein
TcG_06384	573,5125619	-0,292876504	0,078368049	-3,73719276	0,000186086	0,001882965	protein_codin phospholipase C, delta

TcG_06385	481,9073817	0,353073542	0,085795827	4,115276397	3,86716E-05	0,000491282	
TcG_06386	115,0071681	-0,374556834	0,172583803	-2,17028961	0,029984914	0,114428593	protein_codin hypothetical protein
TcG_06387	264,8946093	-0,316750397	0,119900657	-2,64177365	0,008247315	0,042487059	protein_codin nuclear movement protein
TcG_06388	205,454763	-0,159658743	0,126852124	-1,25862096	0,208167271	0,430760136	protein_codin hypothetical protein
TcG_06389	154,3482772	-0,121118096	0,142563535	-0,84957276	0,395562663	0,625237246	protein_codin 4-oxalocrotonate decarboxylase
TcG_06390	95,6869645	-0,362479241	0,182366941	-1,98763679	0,046851875	0,158095631	protein_codin hypothetical protein
TcG_06391	200,3759194	-0,214117869	0,126481623	-1,6928773	0,090478826	0,250953674	protein_codin putative CDC16
TcG_06392	22,82632755	0,857324674	0,385574984	2,223496623	0,026182331	0,103281306	protein_codin 40S ribosomal protein S15
TcG_06393	517,2623951	-0,187902089	0,084116659	-2,2338273	0,025494439	0,101261078	protein_codin transferase
TcG_06394	519,281754	-0,157489584	0,082570191	-1,90734189	0,056476329	0,180207862	protein_codin hypothetical protein
TcG_06395	1000,910387	0,359758154	0,069273859	5,193274317	2,06627E-07	5,03997E-06	protein_codin 40S ribosomal protein S15
TcG_06396	211,1635142	0,055083323	0,140764314	0,391315962	0,695563702	0,842340007	protein_codin hypothetical protein
TcG_06397	469,431406	0,190958851	0,085105254	2,243796279	0,024845512	0,099257632	protein_codin hypothetical protein
TcG_06398	318,1224429	0,277999344	0,105502893	2,63499263	0,008413911	0,043182871	protein_codin hypothetical protein
TcG_06399	185,1457433	-0,07480368	0,133610751	-0,55986273	0,575573073	0,766286474	protein_codin hypothetical protein
TcG_06400	321,8176863	0,150661536	0,104089646	1,447420959	0,147779059	0,346592749	protein_codin hypothetical protein
TcG_06401	378,4742702	0,194019831	0,096604679	2,008389574	0,04460191	0,152554309	protein_codin DNA primase small subunit
TcG_06402	958,2799343	-0,092602748	0,064810069	-1,42883274	0,153052313	0,353451086	protein_codin hypothetical protein
TcG_06403	187,2045791	0,206318692	0,135573047	1,521826771	0,128052492	0,316931411	protein_codin hypothetical protein
TcG_06404	125,8249538	-0,115179181	0,161177889	-0,71460907	0,474850641	0,690550964	protein_codin hypothetical protein
TcG_06405	151,3764496	0,426132081	0,153446126	2,777079438	0,005484977	0,030750584	protein_codin hypothetical protein
TcG_06406	220,8337828	0,193104611	0,119257263	1,619227264	0,105398374	0,279182798	protein_codin putative methylenetetrahydrofolate dehydrogenase-like protein
TcG_06407	307,2500438	-0,203486501	0,102664851	-1,98204643	0,047474044	0,159337856	protein_codin hypothetical protein
TcG_06408	380,9848365	-0,159508433	0,093839874	-1,69979376	0,089169726	0,248585286	protein_codin UBX domain-containing protein 1
TcG_06409	477,1896193	0,206291186	0,085695732	2,407251572	0,016073094	0,071322429	protein_codin putative carnitine O-palmitoyltransferase II
TcG_06410	1195,520063	0,028303821	0,057426391	0,492871327	0,622103508	0,79640439	protein_codin hypothetical protein
TcG_06411	356,7890507	-0,081264988	0,106899588	-0,76019926	0,447135487	0,66845313	protein_codin mitochondrial carrier domain-containing protein
TcG_06412	549,0789718	0,055081017	0,079015048	0,69709528	0,485743168	0,698274608	protein_codin hypothetical protein
TcG_06413	180,6011276	-0,053792612	0,134615341	-0,39960239	0,689449395	0,838680499	protein_codin hypothetical protein
TcG_06414	300,3751981	0,073013036	0,109728618	0,665396474	0,50579694	0,712679562	protein_codin hypothetical protein
TcG_06415	1272,196325	-0,269272563	0,060869747	-4,42375033	9,70021E-06	0,000148267	protein_codin translocon-associated protein subunit alpha
TcG_06416	52,14308395	-0,2929318	0,264841705	-1,10606371	0,268698943	0,501311747	protein_codin hypothetical protein
TcG_06417	516,9541111	0,213134239	0,086742886	2,457080338	0,014007136	0,06442504	protein_codin hypothetical protein
TcG_06418	457,1162561	0,895879444	0,09446703	9,483514478	2,4586E-21	5,08667E-19	protein_codin hypothetical protein
TcG_06419	381,4814495	-0,150582773	0,092927312	-1,62043611	0,105138628	0,278632572	protein_codin EF hand
TcG_06420	2400,806757	-0,002827127	0,050755775	-0,0557006	0,955580325	0,979332476	protein_codin hypothetical protein
TcG_06421	1398,855885	-0,166357831	0,057122695	-2,91228959	0,003587898	0,02200603	protein_codin hypothetical protein
TcG_06422	951,7058378	-0,0986613	0,066450004	-1,48474483	0,137611431	0,330918646	protein_codin hypothetical protein
TcG_06423	173,7560286	0,195154843	0,135328633	1,442080945	0,14927954	0,348505251	protein_codin hypothetical protein
TcG_06424	219,9596556	2,95963E-05	0,126084702	0,000234733	0,99981271	0,99981271	protein_codin hypothetical protein
TcG_06425	443,59343	-0,029831455	0,088459858	-0,33723155	0,735942354	0,864944729	protein_codin putative conserved oligomeric golgi complex subunit
TcG_06426	252,2612624	-0,048107817	0,117415594	-0,40972256	0,682009482	0,834223169	protein_codin hypothetical protein
TcG_06427	260,0359065	0,235493726	0,117498082	2,004234632	0,045044932	0,15363279	protein_codin hypothetical protein
TcG_06428	11,99550087	-0,041411192	0,495342986	-0,08360105	0,933373635	1	protein_codin hypothetical protein
TcG_06429	113,3464144	0,011657407	0,16469987	0,070779695	0,943573092	0,973371575	protein_codin transferase
TcG_06430	146,9261216	-0,323123489	0,146497658	-2,20565635	0,02740807	0,106919158	protein_codin hypothetical protein
TcG_06431	392,3044115	-0,085379206	0,095005142	-0,89867984	0,368823217	0,600861456	protein_codin hypothetical protein
TcG_06432	229,3757145	0,004255271	0,118508082	0,035907014	0,971356503	0,98762955	protein_codin apurinic/apyrimidinic endonuclease
TcG_06433	386,2411381	-0,076295713	0,095271769	-0,80082183	0,423234798	0,649225258	protein_codin putative adaptin
TcG_06434	257,7202593	0,023804564	0,110853399	0,214739145	0,829970701	0,917386047	protein_codin hypothetical protein
TcG_06435	274,7634404	-0,197895732	0,11290723	-1,7527286	0,079648604	0,230299157	protein_codin hypothetical protein
TcG_06436	584,1978895	0,027872953	0,078654655	0,354371308	0,723060634	0,857335574	protein_codin hypothetical protein
TcG_06437	622,9517117	-0,014122757	0,074428813	-0,18974852	0,849506201	0,92756374	protein_codin putative glycosyl hydrolase
TcG_06438	275,199301	-0,052991629	0,110632075	-0,47898974	0,631945926	0,802906018	protein_codin hypothetical protein
TcG_06439	295,2508948	-0,065938054	0,109752095	-0,60079084	0,547979303	0,74465027	protein_codin protein OS-9-like protein isoform X2
TcG_06440	30,59125438	0,203354243	0,316299422	0,642916898	0,520278024	0,724773499	
TcG_06441	625,1433403	-0,021078147	0,079110141	-0,26644053	0,789899958	0,895024708	protein_codin hypothetical protein

TcG_06442	340,5372732	-0,011030048	0,097956491	-0,1126015	0,910346494	0,956471837	protein_codin hypothetical protein
TcG_06443	979,7585755	0,055563926	0,063590881	0,873771911	0,38224248	0,612200909	protein_codin putative helicase
TcG_06444	435,7787194	-0,089588867	0,08914991	-1,0049238	0,314933541	0,549603857	protein_codin putative aldehyde dehydrogenase
TcG_06445	252,3511562	0,012299581	0,11847471	0,103816085	0,917315301	0,960160365	protein_codin vacuolar protein sorting-associated protein-like
TcG_06446	450,5222618	-0,119347509	0,092415244	-1,29142665	0,196555773	0,414732323	protein_codin pre-mRNA-splicing factor CWC22
TcG_06447	360,0944179	-0,138370203	0,09748441	-1,41940853	0,155779946	0,357328541	protein_codin putative kinesin-like protein
TcG_06448	387,8144776	-0,125602062	0,09348953	-1,34348801	0,179114007	0,390004677	protein_codin hypothetical protein
TcG_06449	415,2300427	0,235579531	0,090090608	2,614917761	0,008924894	0,045193979	protein_codin hypothetical protein
TcG_06450	372,9668138	0,147627994	0,098023477	1,506047312	0,132055029	0,32285072	protein_codin hypothetical protein
TcG_06451	1851,609913	0,130104	0,053223135	2,444500847	0,014505274	0,066216747	protein_codin hypothetical protein
TcG_06452	535,3749689	0,063933816	0,081191228	0,787447326	0,431020052	0,655759925	protein_codin putative leucine-rich repeat protein (LRRP)
TcG_06453	665,6646895	-0,184693573	0,077000264	-2,39860961	0,01645745	0,072565414	protein_codin hypothetical protein
TcG_06454	369,2717148	0,201478797	0,096484011	2,088209176	0,03677897	0,133355843	protein_codin hypothetical protein
TcG_06455	302,6733649	0,033775235	0,108985278	0,309906397	0,756632138	0,877511506	protein_codin hypothetical protein
TcG_06456	493,0833433	0,084531852	0,086919305	0,972532533	0,330785688	0,565235831	protein_codin hypothetical protein
TcG_06457	306,5236815	-0,160189129	0,10546511	-1,51888268	0,128792029	0,317925448	protein_codin putative ubiquitin activating E1 enzyme
TcG_06458	321,5733941	0,095747464	0,099766863	0,959712089	0,337200137	0,57160877	protein_codin tRNA-splicing ligase RtcB
TcG_06459	123,9923483	0,167107742	0,163422866	1,022548103	0,306521558	0,539556179	protein_codin hypothetical protein
TcG_06460	176,752047	-0,165887717	0,132807161	-1,24908714	0,211633203	0,434569481	protein_codin hypothetical protein
TcG_06461	248,1664329	-0,226392862	0,112641388	-2,00985504	0,044446533	0,152264203	protein_codin hypothetical protein
TcG_06462	43,59565342	0,132696446	0,26238685	0,505728262	0,613047425	0,790819724	protein_codin procyclic form surface glycoprotein
TcG_06463	425,3266953	0,137378985	0,090655391	1,515397851	0,129671681	0,31936907	protein_codin surface glycoprotein
TcG_06464	8,956040806	-0,136434708	0,63229719	-0,21577624	0,829162173	1	protein_codin hypothetical protein
TcG_06465	422,3516477	0,167588877	0,089761647	1,867043244	0,061895549	0,19413152	protein_codin putative surface glycoprotein
TcG_06466	299,5080724	0,15703604	0,105418906	1,489638304	0,136319365	0,329163749	protein_codin putative procyclic form surface glycoprotein
TcG_06467	221,4281079	-0,113198283	0,1213909	-0,93251045	0,351072786	0,585566432	protein_codin hypothetical protein
TcG_06468	174,4955065	-0,099425147	0,13459013	-0,7387254	0,460073761	0,678399847	protein_codin hypothetical protein
TcG_06469	518,5762712	-0,477911123	0,080935925	-5,90480831	3,53057E-09	1,23955E-07	protein_codin hypothetical protein
TcG_06470	164,1931738	0,233717898	0,145181916	1,609827888	0,107435435	0,282255545	protein_codin hypothetical protein
TcG_06471	221,5283751	0,005228986	0,118711023	0,044048023	0,964866124	0,984347197	protein_codin RNA-binding protein
TcG_06472	193,6417386	-0,388095796	0,132259388	-2,93435348	0,003342433	0,020764306	protein_codin hypothetical protein
TcG_06473	193,0758974	-0,855866076	0,133013514	-6,4344295	1,23938E-10	6,00647E-09	protein_codin hypothetical protein
TcG_06474	372,2297968	-0,549270709	0,098072944	-5,60063443	2,13569E-08	6,39382E-07	protein_codin putative intraflagellar transport protein 57
TcG_06475	241,9021198	-0,319530482	0,116837986	-2,73481676	0,006241501	0,034062191	protein_codin hypothetical protein
TcG_06476	165,6073314	-0,26495773	0,136131557	-1,94633586	0,051614409	0,168927837	protein_codin hypothetical protein
TcG_06477	1845,854322	-0,267922799	0,057122416	-4,69032678	2,72769E-06	4,93027E-05	protein_codin hypothetical protein
TcG_06478	97,43597155	0,074534048	0,17674512	0,421703572	0,673241397	0,828900214	protein_codin hypothetical protein
TcG_06479	357,9863245	-0,105998841	0,097197855	-1,09054712	0,275472209	0,507736472	protein_codin putative protein phosphatase 2A regulatory subunit
TcG_06480	258,9521415	-0,12307019	0,114238502	-1,07730921	0,281342149	0,514380643	protein_codin putative sterol C-24 reductase
TcG_06481	59,73910549	-0,276937891	0,229927071	-1,2044597	0,228411948	0,455641875	protein_codin amino acid transporter
TcG_06482	30,84948905	0,288480987	0,317062283	0,909855895	0,362898512	0,595206988	protein_codin retrotransposon hot spot (RHS) protein
TcG_06483	0,467164841	2,094702395	2,708793758	0,773297114	0,439346563	1	
TcG_06484	625,0197556	0,081079926	0,079494146	1,019948382	0,307752942	0,540901939	protein_codin putative CAAX prenyl protease 1
TcG_06485	183,5352109	0,238228348	0,134617315	1,769670926	0,076781976	0,224588734	protein_codin putative amino acid permease-like protein
TcG_06486	294,1214922	-0,651108987	0,109424982	-5,95027732	2,67689E-09	9,75295E-08	protein_codin nuclear protein Tc22
TcG_06487	522,3328727	0,072067504	0,084161644	0,856298667	0,391832598	0,621398293	protein_codin putative isopentenyl-diphosphate delta-isomerase
TcG_06488	279,5085276	-0,181609293	0,109252589	-1,66228824	0,096454995	0,262700418	protein_codin hypothetical protein
TcG_06489	359,7691008	-0,10091474	0,095600181	-1,05559151	0,291154879	0,523651784	protein_codin putative GTP-binding protein
TcG_06490	191,0130061	0,024003098	0,130530548	0,183888739	0,854100739	0,929165368	protein_codin hypothetical protein
TcG_06491	300,9704287	-0,058875732	0,107238932	-0,54901454	0,582995474	0,77177623	protein_codin p21 antigen protein
TcG_06492	280,2093064	0,130019599	0,108345585	1,200045199	0,230121787	0,457165813	protein_codin hypothetical protein
TcG_06493	389,2306548	-0,154780542	0,099558697	-1,55466621	0,120025586	0,303230799	protein_codin WD repeat-containing protein 68
TcG_06494	162,008772	-0,076389234	0,139220911	-0,54869081	0,583217654	0,771980452	protein_codin hypothetical protein
TcG_06495	192,5815956	0,162404771	0,127311022	1,27565366	0,202077977	0,422196751	protein_codin tRNA (guanine-N(7)-)-methyltransferase
TcG_06496	208,4423715	-0,23977157	0,129481688	-1,85177976	0,064057455	0,198919774	protein_codin hypothetical protein
TcG_06497	309,3300656	-0,159413927	0,105736981	-1,5076459	0,131645178	0,322461107	protein_codin putative GTP-binding protein
TcG_06498	18,14623885	-0,374955943	0,420267975	-0,892183	0,372294852	0,603572496	protein_codin hypothetical protein

TcG_06499	228,6410038	0,009601161	0,117685685	0,081583087	0,934978251	0,969278634	protein_codin hypothetical protein
TcG_06500	102,0140988	0,160366713	0,175050489	0,916116914	0,359605577	0,592630203	protein_codin GTP-binding protein
TcG_06501	274,0762186	0,178269377	0,117353328	1,519082417	0,128741752	0,317903227	protein_codin putative map kinase
TcG_06502	113,9225854	-0,13671029	0,168860182	-0,80960643	0,418166409	0,644865701	protein_codin hypothetical protein
TcG_06503	310,3534177	-0,023830316	0,106121456	-0,224557	0,822323919	0,911891742	protein_codin hypothetical protein
TcG_06504	212,3267375	0,098661147	0,122920828	0,802639783	0,422182967	0,648384393	protein_codin putative dolichyl-P-Man:GDP-Man1GlcNAc2-PP-dolichyl alpha-1,3-mannosyltransferase
TcG_06505	660,4378109	0,449374044	0,081590648	5,507666058	3,63622E-08	1,03258E-06	
TcG_06506	337,7466376	0,084747888	0,09954591	0,851344752	0,394577872	0,624314965	protein_codin hypothetical protein
TcG_06507	106,2513226	-0,171381949	0,172785625	-0,9918762	0,32125792	0,556034397	protein_codin hypothetical protein
TcG_06508	176,6201193	0,145631279	0,135632385	1,073720553	0,282947947	0,516176179	protein_codin hypothetical protein
TcG_06509	82,19249285	-0,099544065	0,198168807	-0,50231955	0,615442761	0,79219196	protein_codin putative p21-activated kinase 3
TcG_06510	41,3419035	0,048722213	0,279675807	0,174209608	0,861700735	0,933819072	protein_codin hypothetical protein
TcG_06511	261,2503868	-0,016414269	0,112529946	-0,14586579	0,884027341	0,943488844	protein_codin putative ankyrin repeat and zinc finger domain protein
TcG_06512	219,9252843	-0,26592148	0,122887545	-2,16394169	0,030468825	0,115605149	protein_codin putative UDP-Gal or UDP-GlcNAc-dependent glycosyltransferase
TcG_06513	1155,489183	-0,097843843	0,060743292	-1,61077609	0,107228534	0,282095776	protein_codin putative polyadenylate-binding protein 1
TcG_06514	146,0796393	-0,039839919	0,148748841	-0,26783348	0,788827501	0,894874711	protein_codin putative SUMO1/Ulp2
TcG_06515	284,8188066	-0,023932234	0,109903771	-0,21775626	0,827619025	0,915880831	protein_codin putative DNA repair protein RAD2
TcG_06516	706,1152385	-0,083834241	0,071948459	-1,16519856	0,243938628	0,472108668	protein_codin putative target SNARE
TcG_06517	32,48485585	-0,376612678	0,338173457	-1,11366717	0,265421997	0,498004739	
TcG_06518	219,3209828	-0,210159148	0,122062039	-1,72174043	0,085116551	0,240585596	protein_codin putative protein kinase
TcG_06519	336,6748677	-0,099974938	0,09815085	-1,01858453	0,308400251	0,541382622	protein_codin putative phosphatidylcholine:ceramide cholinephosphotransferase 2
TcG_06520	581,4466549	-0,047511002	0,080815507	-0,58789463	0,556603023	0,75125846	protein_codin hypothetical protein
TcG_06521	607,6686844	-0,255794183	0,076815799	-3,3299684	0,000868558	0,006944871	protein_codin 3-hydroxyacyl-CoA dehydrogenase
TcG_06522	394,0257209	0,098440954	0,095704932	1,02858809	0,303673277	0,536498717	protein_codin hypothetical protein
TcG_06523	320,4654519	0,193078645	0,105203377	1,835289414	0,066462819	0,204688521	protein_codin sucraseferredoxin-like family protein
TcG_06524	153,8444194	-0,198177919	0,141205938	-1,40346732	0,16047753	0,363427025	protein_codin hypothetical protein
TcG_06525	308,1440157	-0,05885574	0,10882032	-0,54085248	0,588609266	0,775648564	protein_codin hypothetical protein
TcG_06526	323,4460241	-0,109744573	0,100104188	-1,09630351	0,272945972	0,506138289	protein_codin hypothetical protein
TcG_06527	548,9815121	-0,18893378	0,079736085	-2,36948906	0,017812682	0,076920512	protein_codin hypothetical protein
TcG_06528	321,9339089	0,196867851	0,106381465	1,850584123	0,064229406	0,19929349	protein_codin hypothetical protein
TcG_06529	252,2975553	-0,026817711	0,113205872	-0,23689329	0,812739576	0,907594318	protein_codin cytoplasmic tRNA 2-thiolation protein 1
TcG_06530	443,7566204	-0,229829793	0,089368702	-2,57170336	0,010119956	0,049829924	
TcG_06531	206,7424847	-0,223784884	0,12401897	-1,80444077	0,071162237	0,21392986	protein_codin centrin
TcG_06532	791,2927153	-0,154998232	0,069539004	-2,2289395	0,025817931	0,102195608	protein_codin hypothetical protein
TcG_06533	428,8685465	-0,16784773	0,093156288	-1,80178636	0,071579024	0,214959714	protein_codin ubiquinone biosynthesis protein
TcG_06534	469,5640299	-0,031709928	0,086756445	-0,36550515	0,714734363	0,853614301	protein_codin hypothetical protein
TcG_06535	277,8204428	0,007387882	0,109341592	0,06756699	0,946130333	0,974694377	protein_codin putative rab-like GTPase activating protein
TcG_06536	419,0516315	-0,038023742	0,090808462	-0,41872465	0,675417375	0,830121185	protein_codin hypothetical protein
TcG_06537	303,0604715	-0,165980728	0,106580517	-1,55732711	0,119392842	0,302164644	protein_codin putative rab-like GTPase activating protein
TcG_06538	1352,540589	-0,073727737	0,056213003	-1,311578	0,18966256	0,40475786	protein_codin hypothetical protein
TcG_06539	336,4827305	-0,13229675	0,09818976	-1,34735791	0,177864988	0,388379901	protein_codin ubiquitin thioesterase OTU1
TcG_06540	331,3846091	-0,138021748	0,097992452	-1,40849366	0,158984942	0,361342941	protein_codin hypothetical protein
TcG_06541	680,3404698	-0,10335711	0,07208207	-1,43388098	0,15160623	0,351799471	protein_codin hypothetical protein
TcG_06542	546,0155986	-0,238564783	0,079151919	-3,01401135	0,002578182	0,016828626	protein_codin hypothetical protein
TcG_06543	718,5896088	-0,349199015	0,075445891	-4,62846962	3,68378E-06	6,40845E-05	protein_codin hypothetical protein
TcG_06544	314,9096927	0,150685299	0,1008719	1,493828312	0,135220499	0,327343232	protein_codin hypothetical protein
TcG_06545	540,3422539	-0,000237822	0,079655618	-0,00298563	0,997617818	0,999104158	protein_codin MmpL efflux pump
TcG_06546	248,9014214	-0,000296415	0,120152652	-0,00246699	0,998031631	0,999325424	protein_codin hypothetical protein
TcG_06547	222,5891547	0,133283708	0,118250672	1,127128547	0,259688135	0,492108097	protein_codin hypothetical protein
TcG_06548	281,9221152	-0,310601575	0,115207158	-2,69602671	0,007017204	0,037362741	protein_codin hypothetical protein
TcG_06549	21,28647611	-0,209124248	0,38727922	-0,53998314	0,589208662	0,775648564	
TcG_06550	180,4166293	-0,209557768	0,133951383	-1,56443153	0,117716261	0,299247687	protein_codin hypothetical protein
TcG_06551	149,9808791	-0,2417395	0,144831027	-1,66911403	0,095094783	0,259850981	protein_codin hypothetical protein
TcG_06552	297,1685697	-0,307769734	0,103473366	-2,97438603	0,002935755	0,018688822	protein_codin hypothetical protein
TcG_06553	250,7350462	-0,127540544	0,117440735	-1,0859992	0,277479344	0,509973935	protein_codin putative histidine phosphatase
TcG_06554	764,9238319	0,053771199	0,072881106	0,737793394	0,460640008	0,678659097	protein_codin activating signal cointegrator 1 complex subunit 3
TcG_06555	80,35198346	0,152136485	0,200371425	0,759272362	0,447689647	0,668763828	protein_codin hypothetical protein

TcG_06556	432,3554629	-0,252350395	0,087582345	-2,88129296	0,003960473	0,023898982	protein_codin hypothetical protein
TcG_06557	313,2314036	-0,070128236	0,105367613	-0,66555779	0,505693791	0,712679562	protein_codin hypothetical protein
TcG_06558	158,7858652	0,032561208	0,144879433	0,224746933	0,822176152	0,911875343	protein_codin hypothetical protein
TcG_06559	297,5455147	-0,197739588	0,110620195	-1,78755415	0,073847968	0,219272823	protein_codin hypothetical protein
TcG_06560	1086,23554	0,154532491	0,062452693	2,474392751	0,013346289	0,062175352	protein_codin 40S ribosomal protein S12
TcG_06561	357,0394449	-0,323915947	0,099436903	-3,25750237	0,001123973	0,008578625	protein_codin hypothetical protein
TcG_06562	107,3687677	0,28677573	0,168932393	1,697576916	0,089587643	0,249558834	protein_codin hypothetical protein
TcG_06563	222,5553529	0,073078268	0,118638966	0,615971892	0,537913071	0,73738082	protein_codin F-actin capping protein
TcG_06564	94,3493115	-0,119232742	0,197404138	-0,60400326	0,545841477	0,742854706	protein_codin hypothetical protein
TcG_06565	85,36319184	0,29728287	0,192706548	1,542671348	0,122910537	0,30780546	protein_codin putative actyltransferase-like
TcG_06566	109,8820304	-0,109320518	0,171785725	-0,6363772	0,524530599	0,728071346	protein_codin hypothetical protein
TcG_06567	99,30141729	0,159160752	0,178444837	0,891932518	0,372429104	0,603575828	protein_codin hypothetical protein
TcG_06568	181,5648956	-0,137734404	0,134804478	-1,02173463	0,306906517	0,540059534	protein_codin hypothetical protein
TcG_06569	348,3951447	-0,113695727	0,117240602	-0,9697641	0,332164089	0,566340797	protein_codin hypothetical protein
TcG_06570	1098,889016	-0,084142435	0,060913883	-1,38133429	0,1671762	0,37312723	protein_codin importin-7
TcG_06571	132,1784658	-0,018067766	0,150770839	-0,11983594	0,90461311	0,953702767	protein_codin putative zinc-binding protein
TcG_06572	220,3209114	-0,16478825	0,121232374	-1,35927595	0,174059163	0,382593335	protein_codin hypothetical protein
TcG_06573	755,9516109	-0,27351644	0,070027395	-3,9058491	9,38951E-05	0,001058238	protein_codin antigen 38
TcG_06574	503,2167837	-0,13036235	0,082332304	-1,58336817	0,113337573	0,292066085	protein_codin proliferation-associated 2g4
TcG_06575	460,3865628	-0,410758059	0,093208934	-4,4068529	1,04883E-05	0,000158021	protein_codin hypothetical protein
TcG_06576	41,93288749	0,028907841	0,270959797	0,106686827	0,915037434	0,958728858	protein_codin hypothetical protein
TcG_06577	46,14449911	0,318768549	0,273312096	1,16631702	0,243486294	0,471805512	protein_codin putative kinesin
TcG_06578	55,54282875	0,432269764	0,233224492	1,853449269	0,063817991	0,198495368	protein_codin hypothetical protein
TcG_06579	24,76933192	0,217914951	0,348865786	0,624638356	0,53220844	0,732933197	protein_codin putative trans-sialidase
TcG_06580	23,42665101	-0,324020838	0,372322662	-0,87026891	0,384153465	0,614499543	protein_codin hypothetical protein
TcG_06581	50,77138134	-0,174187421	0,244125777	-0,71351507	0,475527091	0,691100963	protein_codin protein ARV1
TcG_06582	257,9865906	-0,021522892	0,116566407	-0,1846406	0,853510937	0,929047136	protein_codin putative glutathionylspermidine synthase
TcG_06583	576,3656764	-0,11291169	0,078364796	-1,44084711	0,149627882	0,349091551	protein_codin pumilio protein 8
TcG_06584	123,5748907	-0,473820419	0,161928689	-2,92610544	0,003432346	0,02126586	protein_codin hypothetical protein
TcG_06585	288,6991811	-0,355369512	0,107409827	-3,30853817	0,000937844	0,007401812	protein_codin putative rac serine-threonine kinase
TcG_06586	373,0317994	-0,159926022	0,095062558	-1,68232399	0,092506032	0,254578358	protein_codin putative protein kinase
TcG_06587	219,9003871	-0,445489583	0,127048074	-3,50646466	0,000454102	0,003982757	protein_codin peptide hydrolase
TcG_06588	142,4076598	-0,22863585	0,156198452	-1,46375234	0,143261629	0,339711264	protein_codin hypothetical protein
TcG_06589	706,2894468	-0,306963902	0,071304863	-4,30495047	1,67023E-05	0,000236858	protein_codin putative structural maintenance of chromosome (SMC) family protein
TcG_06590	240,0894464	-0,321257438	0,114628749	-2,80259046	0,0050694	0,029006222	protein_codin putative DNA repair protein
TcG_06591	315,8356001	-0,119448342	0,103428483	-1,15488826	0,248136186	0,477445495	protein_codin DHHC containing zinc finger protein
TcG_06592	118,0978637	-0,415987856	0,165337654	-2,51598983	0,011869861	0,056478114	protein_codin hypothetical protein
TcG_06593	78,83512939	0,099598507	0,206594664	0,482096222	0,629737594	0,801688869	protein_codin hypothetical protein
TcG_06594	121,3387484	-0,256473316	0,17754532	-1,44455127	0,14858397	0,347495736	protein_codin hypothetical protein
TcG_06595	128,1132906	0,174557448	0,155413393	1,1231815	0,261360418	0,493587786	protein_codin hypothetical protein
TcG_06596	101,4770425	-0,160210056	0,174884062	-0,91609295	0,359618146	0,592630203	protein_codin hypothetical protein
TcG_06597	209,6235669	-0,261487244	0,124496569	-2,10035703	0,035697446	0,130511395	protein_codin putative DNA repair protein
TcG_06598	286,7950422	-0,391379471	0,11093445	-3,52802464	0,000418673	0,003717048	protein_codin hypothetical protein
TcG_06599	241,8176652	-0,266947009	0,119967509	-2,22516088	0,02607044	0,102948915	protein_codin putative membrane-bound acid phosphatase 2
TcG_06600	299,2065582	-0,402315915	0,103697828	-3,8796947	0,000104588	0,001158463	protein_codin putative proteasome regulatory non-ATPase subunit 6
TcG_06601	315,6793145	-0,671641246	0,102940094	-6,52458357	6,81907E-11	3,49873E-09	protein_codin ParcG domain containing protein
TcG_06602	194,8992346	0,133574235	0,127152177	1,050506874	0,293485132	0,526528142	protein_codin zf-DNL-domain-containing protein
TcG_06603	140,5143155	-0,245447655	0,149348136	-1,64345978	0,100287839	0,269215688	protein_codin putative chaperone DNAJ protein
TcG_06604	190,7773722	-0,342444176	0,130491109	-2,6242721	0,008683438	0,044222557	protein_codin putative vacuolar protein sorting-associated protein
TcG_06605	93,18225498	-0,436968916	0,182942535	-2,38855833	0,016914623	0,074119827	protein_codin polyribonucleotide 5-hydroxyl-kinase
TcG_06606	200,002852	-0,277584842	0,12751304	-2,17691337	0,029487032	0,112864013	protein_codin hypothetical protein
TcG_06607	110,315297	-0,356571897	0,166364938	-2,14331157	0,032088088	0,12027583	protein_codin hypothetical protein
TcG_06608	386,1527177	0,013841296	0,092590403	0,149489528	0,881167372	0,942069315	protein_codin putative replication factor A, 51kDa subunit
TcG_06609	1004,675434	-0,372866235	0,07099447	-5,25204617	1,50419E-07	3,78037E-06	protein_codin succinyl-CoA synthetase alpha subunit
TcG_06610	319,9980888	-0,312459402	0,100374521	-3,11293542	0,001852365	0,012944213	protein_codin hypothetical protein
TcG_06611	522,1420044	-0,353843453	0,082402393	-4,29409196	1,7541E-05	0,000247322	protein_codin putative L-ribulokinase
TcG_06612	334,5532595	0,082272357	0,098419026	0,835939554	0,403188909	0,632986744	protein_codin syntaxin

TcG_06613	213,9479223	-0,210147753	0,123814444	-1,69727979	0,089643777	0,249577422	protein_codin putative stress-induced protein sti1
TcG_06614	157,6702369	-0,038054532	0,147076623	-0,2587395	0,795836238	0,898164685	protein_codin putative RNA triphosphatase
TcG_06615	48,06339378	0,048945976	0,248945365	0,196613324	0,844130143	0,924394313	protein_codin putative trans-sialidase
TcG_06616	413,287863	-0,039353161	0,09229855	-0,42636814	0,669839613	0,826844423	protein_codin hypothetical protein
TcG_06617	489,4473598	0,066181369	0,087056347	0,760213034	0,447127255	0,66845313	protein_codin hypothetical protein
TcG_06618	250,9416492	0,162605249	0,11663956	1,394083181	0,163292487	0,367789028	protein_codin hypothetical protein
TcG_06619	385,2555085	0,222313473	0,094052734	2,363710908	0,018092925	0,077753942	protein_codin hypothetical protein
TcG_06620	342,1869817	0,114321267	0,099289836	1,151389425	0,249572062	0,478811379	protein_codin hypothetical protein
TcG_06621	210,5664762	0,101308412	0,123908034	0,817609712	0,41358008	0,640294808	protein_codin hypothetical protein
TcG_06622	558,755806	0,0485399	0,081723422	0,593953342	0,55254332	0,747869965	protein_codin hypothetical protein
TcG_06623	29,48054552	0,117807414	0,326794216	0,360494184	0,718477605	0,855439475	protein_codin hypothetical protein
TcG_06624	412,4974695	0,022848441	0,090123789	0,253522867	0,799864193	0,900342615	protein_codin nuclear transport factor 2 protein(NFT2)
TcG_06625	315,1452337	-0,279038073	0,103411182	-2,6983356	0,006968715	0,037190019	protein_codin putative protein kinase
TcG_06626	131,7043073	0,335474772	0,158136515	2,121425104	0,033886045	0,125312388	protein_codin protein disulfide-isomerase
TcG_06627	310,0386265	0,084433996	0,101974189	0,827993801	0,40767401	0,635964869	protein_codin protein disulfide isomerase
TcG_06628	82,73810853	0,101560773	0,198257306	0,512267493	0,608463813	0,788023892	protein_codin hypothetical protein
TcG_06629	358,8969588	-0,148939067	0,095733133	-1,55577346	0,11976197	0,302917264	protein_codin hypothetical protein
TcG_06630	72,01720804	-0,055658923	0,207611777	-0,26809136	0,788629	0,894874711	protein_codin hypothetical protein
TcG_06631	51,33692399	0,303108884	0,245151111	1,236416523	0,216303777	0,440361196	protein_codin hypothetical protein
TcG_06632	487,9658428	-0,1239531	0,083164401	-1,49045864	0,136103684	0,328931431	protein_codin cysteamine dioxygenase
TcG_06633	209,5809698	-0,195113765	0,122006927	-1,59920235	0,109775647	0,286326125	protein_codin hypothetical protein
TcG_06634	544,5818274	0,129205886	0,079580215	1,623593064	0,104462687	0,277401946	protein_codin putative trans-sialidase
TcG_06635	544,1741265	-0,415831386	0,083385717	-4,98684186	6,13743E-07	1,30954E-05	protein_codin putative cysteine protease
TcG_06636	526,2890007	0,08209817	0,08026406	1,022850946	0,306378326	0,539467976	protein_codin putative Monooxygenase
TcG_06637	65,63455286	0,222202	0,239553667	0,927566682	0,353632387	0,588084518	protein_codin hypothetical protein
TcG_06638	282,476087	-0,167672675	0,113721202	-1,47441877	0,140368873	0,33486575	protein_codin hypothetical protein
TcG_06639	487,9064952	0,051059324	0,087580361	0,582999702	0,559893501	0,754054394	protein_codin hypothetical protein
TcG_06640	179,7821091	0,101433091	0,135811266	0,746868017	0,455143234	0,675110678	protein_codin hypothetical protein
TcG_06641	293,0517331	0,038879343	0,110265758	0,352596706	0,724390807	0,857982193	protein_codin putative cis-prenyltransferase
TcG_06642	442,464756	0,074604392	0,09940082	0,750541014	0,452928932	0,673551167	protein_codin hypothetical protein
TcG_06643	52,45569848	0,040604007	0,255055195	0,15919694	0,873513714	0,939322955	protein_codin Atg8-like protein 1
TcG_06644	120,607518	-0,043481895	0,160441821	-0,27101347	0,786380673	0,893586355	protein_codin microtubule-associated protein 1A/1B, light chain 3
TcG_06645	161,2765952	0,099685024	0,146222088	0,681737112	0,495405196	0,705130786	protein_codin hypothetical protein
TcG_06646	468,8225783	0,325724766	0,087097891	3,73975491	0,0001842	0,001865506	protein_codin putative mercaptopyruvate sulfurtransferase
TcG_06647	63,45426173	0,256100218	0,22654268	1,130472269	0,258277277	0,490645845	
TcG_06648	30,52187734	-0,111608045	0,314863888	-0,35446442	0,722990866	0,857335574	protein_codin trans-sialidase
TcG_06649	51,04590781	0,458828616	0,250157153	1,834161486	0,066630027	0,204985525	protein_codin putative trans-sialidase
TcG_06650	23,1230285	0,111772875	0,368135732	0,303618654	0,761418438	0,879491513	protein_codin hypothetical protein
TcG_06651	62,64587595	0,072893816	0,232092236	0,314072617	0,753465887	0,875527481	protein_codin hypothetical protein
TcG_06652	3,062740066	-0,120316741	0,992275262	-0,12125339	0,903490336	1	protein_codin retrotransposon hot spot (RHS) protein
TcG_06653	329,2486403	0,100560554	0,102149134	0,984448426	0,324895078	0,559321601	protein_codin retrotransposon hot spot (RHS) protein
TcG_06654	173,138642	0,152838861	0,13676428	1,11753494	0,263765669	0,496021594	protein_codin putative kinesin
TcG_06655	193,5362493	0,048049671	0,129688318	0,370501152	0,711009118	0,851447198	protein_codin hypothetical protein
TcG_06656	95,44780657	0,092648365	0,18002289	0,514647693	0,606799231	0,787540707	protein_codin putative kinesin
TcG_06657	99,89397817	-0,661047369	0,177584188	-3,72244498	0,000197303	0,001968951	protein_codin PSP1 family protein
TcG_06658	3,984253535	-1,435996602	0,895750582	-1,60312104	0,108907934	1	
TcG_06659	744,3108712	-0,443495379	0,070487105	-6,29186544	3,13673E-10	1,39242E-08	protein_codin hypothetical protein
TcG_06660	241,3436621	-0,392919463	0,114857654	-3,42092537	0,000624085	0,005194428	protein_codin putative ATP-dependent DEAD/H RNA helicase
TcG_06661	194,2372244	-0,275761334	0,125793208	-2,19217983	0,028366524	0,109726674	protein_codin hypothetical protein
TcG_06662	564,6238404	-0,617148803	0,080696	-7,647824	2,04409E-14	1,95726E-12	protein_codin hypothetical protein
TcG_06663	714,4096919	-0,243162755	0,070765851	-3,43615956	0,000590023	0,004986149	protein_codin putative nucleoside phosphatase, putative,guanosine diphosphatase
TcG_06664	187,6884175	-0,660402988	0,131036047	-5,03985739	4,65879E-07	1,01843E-05	protein_codin putative leucine-rich repeat protein
TcG_06665	415,9507115	-0,470770371	0,091877549	-5,12388908	2,99297E-07	6,88027E-06	protein_codin hypothetical protein
TcG_06666	135,3775238	-0,535966835	0,159874662	-3,35241888	0,000801087	0,006472382	protein_codin central apparatus associated protein C1a-18
TcG_06667	244,4489132	-0,635959227	0,114274857	-5,56517194	2,61894E-08	7,70128E-07	protein_codin putative mitogen-activated protein kinase
TcG_06668	676,4913884	-0,268099855	0,074622103	-3,59276736	0,000327185	0,003008541	protein_codin putative nucleolar protein
TcG_06669	190,1621565	-0,273022743	0,128145529	-2,13056785	0,033124761	0,123100992	protein_codin hypothetical protein

TcG_06670	143,9127995	-0,208676554	0,154559105	-1,3501408	0,176970822	0,387183019	protein_codin hypothetical protein
TcG_06671	120,4011533	-0,140671552	0,163870836	-0,85842945	0,390655369	0,620554736	protein_codin putative golgi apparatus membrane protein
TcG_06672	166,6295135	0,238225398	0,138464717	1,720477273	0,085345721	0,24111571	protein_codin hypothetical protein
TcG_06673	457,9745279	-0,133549716	0,085707783	-1,55819822	0,119186266	0,301781605	protein_codin hypothetical protein
TcG_06674	361,1830469	-0,22890913	0,097970221	-2,33651743	0,01946429	0,082310546	protein_codin putative dispersed gene family protein 1 (DGF-1)
TcG_06675	297,1525543	-0,377370216	0,12427547	-3,03656237	0,002392926	0,015878832	protein_codin hypothetical protein
TcG_06676	620,0277479	-0,61597798	0,07583257	-8,1228683	4,55293E-16	5,27503E-14	protein_codin putative isocitrate dehydrogenase, mitochondrial precursor
TcG_06677	402,391755	-0,442298477	0,090507664	-4,88686215	1,02456E-06	2,06736E-05	protein_codin putative leucine-rich repeat protein
TcG_06678	121,3148305	-0,278014241	0,167820979	-1,65661197	0,097597951	0,264383882	protein_codin hypothetical protein
TcG_06679	651,2803657	-0,596464295	0,083299942	-7,16044072	8,04181E-13	6,12976E-11	protein_codin putative protein transport protein Sec23
TcG_06680	467,1783782	-0,355039469	0,084367237	-4,20826235	2,57342E-05	0,000345088	protein_codin putative trans-sialidase
TcG_06681	39,46214161	-0,596504306	0,283057147	-2,10736352	0,035086081	0,128845433	protein_codin COP9 signalosome complex subunit 6
TcG_06682	196,6811863	-0,063599472	0,131427408	-0,48391331	0,628447394	0,800834966	protein_codin COP9 signalosome complex subunit 6
TcG_06683	1273,191266	-0,244969018	0,056609823	-4,32732353	1,50932E-05	0,00021723	protein_codin esag10
TcG_06684	501,7630351	0,161080584	0,085913537	1,874915046	0,060804412	0,191486794	protein_codin ATP-binding cassette protein subfamily G, member 5
TcG_06685	688,6939094	0,2622986	0,072958779	3,595161606	0,00032419	0,002987238	protein_codin hypothetical protein
TcG_06686	354,0588501	0,490648058	0,104920594	4,676375152	2,9199E-06	5,2531E-05	protein_codin putative aldose 1-epimerase-like protein
TcG_06687	166,7873004	0,61790838	0,140369349	4,402017863	1,07249E-05	0,000161165	protein_codin hypothetical protein
TcG_06688	447,2383656	0,154853579	0,088204465	1,75562063	0,079153207	0,229209962	protein_codin permease-like protein
TcG_06689	119,0182957	0,481788431	0,166947713	2,885864219	0,003903405	0,023616111	protein_codin trypanothione synthetase
TcG_06690	223,6600909	0,918804753	0,125201456	7,338610756	2,15822E-13	1,78608E-11	protein_codin hypothetical protein
TcG_06691	729,8109016	0,055783575	0,073396137	0,760034211	0,447234135	0,668514346	protein_codin ER to golgi family vesicle transport protein
TcG_06692	207,1961871	0,255374674	0,122789472	2,079776617	0,037546027	0,135011876	protein_codin hypothetical protein
TcG_06693	461,2618171	0,203387339	0,085528775	2,377998968	0,017406875	0,075534101	protein_codin 5-AMP-activated protein kinase, regulatory beta subunit
TcG_06694	363,7637701	0,132104232	0,096970526	1,362313245	0,173099049	0,381351129	protein_codin hypothetical protein
TcG_06695	289,2177021	0,53479223	0,112912538	4,736340531	2,17612E-06	4,02113E-05	protein_codin putative 3-oxo-5-alpha-steroid 4-dehydrogenase
TcG_06696	235,6664346	0,53440719	0,122622008	4,358167021	1,31156E-05	0,000192108	protein_codin hypothetical protein
TcG_06697	331,3502752	0,525146887	0,101732455	5,162038878	2,44275E-07	5,78766E-06	protein_codin putative ribosomal RNA methyltransferase
TcG_06698	236,4734656	0,588394571	0,128364216	4,583789713	4,56623E-06	7,73386E-05	protein_codin hypothetical protein
TcG_06699	384,9002703	0,264283874	0,092581821	2,854597939	0,004309134	0,025629174	protein_codin acyl-CoA synthetase short-chain family member 2
TcG_06700	28,13547203	-0,106296202	0,335721554	-0,31662013	0,751531859	0,874924449	
TcG_06701	216,2425297	0,1976785	0,126490463	1,56279371	0,118101124	0,300004302	protein_codin hypothetical protein
TcG_06702	547,7584246	0,452638498	0,080842544	5,599013514	2,15575E-08	6,4207E-07	protein_codin putative 5'-3' exonuclease XRNC
TcG_06703	585,3053773	0,372749411	0,080185877	4,648566884	3,34249E-06	5,91238E-05	protein_codin hypothetical protein
TcG_06704	290,3366304	0,439691383	0,105844593	4,154122299	3,26539E-05	0,000424134	protein_codin putative oxidoreductase
TcG_06705	117,0925494	0,477737492	0,179547803	2,660781616	0,00779595	0,040649811	protein_codin hypothetical protein
TcG_06706	179,7098531	0,40623331	0,134389942	3,022795493	0,002504514	0,016443652	protein_codin hypothetical protein
TcG_06707	365,7360004	0,279940032	0,098942092	2,829332044	0,004664528	0,027225802	protein_codin hypothetical protein
TcG_06708	262,0194904	0,419385145	0,116447047	3,601509505	0,000316375	0,002929937	protein_codin Protein X92
TcG_06709	660,773579	0,445288653	0,076554358	5,816633635	6,00445E-09	1,99334E-07	protein_codin putative ribonuclease
TcG_06710	181,4234754	0,490918765	0,131802581	3,724652145	0,000195585	0,001956861	protein_codin putative amino acid transporter
TcG_06711	236,0341364	0,008587823	0,11546696	0,07437472	0,940712223	0,97252537	protein_codin putative trans-sialidase
TcG_06712	868,4208901	0,710888519	0,067470124	10,53634529	5,87374E-26	1,94437E-23	protein_codin cathepsin B-like protease precursor
TcG_06713	56,97745239	0,354934357	0,243121326	1,459906307	0,144315826	0,340954966	protein_codin hypothetical protein
TcG_06714	97,55196839	0,386529439	0,180907716	2,136611132	0,032629633	0,121871996	protein_codin hypothetical protein
TcG_06715	11,14044554	0,734882992	0,538878654	1,363726297	0,172653724	1	protein_codin hypothetical protein
TcG_06716	228,4087233	-0,051689308	0,120884573	-0,42759226	0,668948003	0,826307698	protein_codin hypothetical protein
TcG_06717	428,8011118	-0,128528112	0,096901021	-1,32638554	0,184712027	0,397783186	protein_codin putative CCR4 associated factor
TcG_06718	122,8053646	-0,029819228	0,174168607	-0,17120897	0,864059453	0,934985064	protein_codin hypothetical protein
TcG_06719	229,470356	0,465925981	0,117965083	3,949694011	7,82512E-05	0,000903906	protein_codin hypothetical protein
TcG_06720	162,2315208	0,258237289	0,141661525	1,822917614	0,06831587	0,208401176	protein_codin hypothetical protein
TcG_06721	827,5766023	-0,073085094	0,0797791	-0,91609324	0,359617994	0,592630203	protein_codin hypothetical protein
TcG_06722	394,6446203	0,011701721	0,09215019	0,126985312	0,898952023	0,950695567	protein_codin hypothetical protein
TcG_06723	561,5580818	-0,042284185	0,086237967	-0,49031982	0,623907601	0,797066211	protein_codin putative serine/threonine protein phosphatase
TcG_06724	552,7112411	0,217385057	0,080496756	2,700544321	0,006922611	0,036995099	protein_codin hypothetical protein
TcG_06725	275,6373378	-0,070695276	0,107804639	-0,65577212	0,51197076	0,718122666	protein_codin hypothetical protein
TcG_06726	191,0299373	0,242000957	0,137645516	1,758146324	0,078722615	0,22841979	protein_codin putative ribosomal RNA processing protein 45

TcG_06727	182,0549847	-0,014234828	0,133236535	-0,10683877	0,91491689	0,958689254	protein_codin hypothetical protein
TcG_06728	47,30974776	0,279011779	0,253697081	1,099783165	0,271426609	0,504573652	protein_codin hypothetical protein
TcG_06729	285,0260922	-0,22195052	0,108810278	-2,0397937	0,041370878	0,144749959	protein_codin hypothetical protein
TcG_06730	871,8887107	-0,170602415	0,06702126	-2,545497	0,01091223	0,052899205	protein_codin alanine-tRNA ligase
TcG_06731	202,8113051	0,270025237	0,127390504	2,119665351	0,034034277	0,125740157	protein_codin putative dephospho-CoA kinase
TcG_06732	1291,946465	0,245525903	0,057283493	4,286154533	1,81793E-05	0,000254378	protein_codin 40S ribosomal protein L14
TcG_06733	242,4041304	0,02962964	0,115157486	0,257296694	0,796949744	0,898729487	protein_codin hypothetical protein
TcG_06734	723,5691558	-0,093140673	0,073103942	-1,27408551	0,202633112	0,422781782	protein_codin putative ATP-dependent DEAD/H RNA helicase
TcG_06735	232,4756703	-0,087511466	0,117072666	-0,747497	0,454763614	0,674756913	protein_codin putative serine/threonine protein phosphatase
TcG_06736	620,1589605	0,062489732	0,083612581	0,74737236	0,454838825	0,674756913	protein_codin L1Tc protein
TcG_06737	358,3719279	0,159037657	0,106252904	1,496784099	0,134449445	0,326021613	protein_codin putative kinetoplast DNA-associated protein
TcG_06738	87,20374628	0,185743473	0,187529186	0,990477676	0,321940696	0,556717149	
TcG_06739	492,7987749	0,016176072	0,08301954	0,194846563	0,845513068	0,925035391	protein_codin putative protein kinase, putative,cdc2-related kinase
TcG_06740	76,44317913	-0,051292691	0,20569864	-0,24935844	0,803083533	0,902650932	protein_codin hypothetical protein
TcG_06741	218,8768038	0,030054927	0,128841373	0,233270776	0,81555115	0,909167288	protein_codin hypothetical protein
TcG_06742	305,4856283	-0,102557453	0,104611543	-0,98036459	0,326906182	0,561463175	protein_codin hypothetical protein
TcG_06743	120,5500647	-0,346306167	0,164958211	-2,09935695	0,035785446	0,130750609	protein_codin hypothetical protein
TcG_06744	612,4414487	0,041924884	0,079803048	0,525354421	0,599336832	0,783102809	protein_codin putative ATP-dependent RNA helicase
TcG_06745	669,5224601	-0,04631238	0,076943165	-0,60190376	0,547238204	0,744079549	protein_codin hypothetical protein
TcG_06746	293,047504	-0,132278323	0,10643975	-1,24275304	0,213958853	0,437522132	protein_codin putative RNA-binding protein
TcG_06747	197,58539	-0,100271885	0,125424104	-0,79946263	0,424022205	0,649916824	protein_codin hypothetical protein
TcG_06748	54,13770083	0,05938488	0,239073661	0,248395745	0,803828226	0,903185306	protein_codin hypothetical protein
TcG_06749	310,9080599	0,035226864	0,110203274	0,319653514	0,749231003	0,873470177	protein_codin UDP-glucuronic acid/UDP-N-acetylgalactosamine transporter
TcG_06750	598,4279431	-0,224135599	0,079338857	-2,82504195	0,004727443	0,027523694	protein_codin putative kinesin
TcG_06751	570,82339	0,085258205	0,090846845	0,938482847	0,347996328	0,582558222	protein_codin hypothetical protein
TcG_06752	386,1702245	0,126970744	0,097405433	1,303528367	0,192394438	0,408835223	protein_codin hypothetical protein
TcG_06753	288,4481114	0,018481419	0,10922772	0,169200808	0,865638694	0,935306343	protein_codin nitroreductase
TcG_06754	826,244664	0,162262419	0,075245407	2,156442846	0,031049102	0,117253878	protein_codin hypothetical protein
TcG_06755	578,7403371	0,222372159	0,083740192	2,65550096	0,007919071	0,04118059	protein_codin hypothetical protein
TcG_06756	63,96921877	0,030648394	0,221385956	0,138438744	0,889893678	0,946535728	protein_codin hypothetical protein
TcG_06757	826,6579789	0,169785224	0,071863775	2,362598179	0,018147334	0,077912126	protein_codin hypothetical protein
TcG_06758	485,2224892	-0,206331109	0,088529328	-2,33065262	0,019771685	0,083269625	protein_codin hypothetical protein
TcG_06759	471,3583556	-0,084352697	0,088683021	-0,95117077	0,341517696	0,575956918	protein_codin cyclin 4
TcG_06760	34,50390022	0,253471133	0,299145797	0,847316376	0,396818801	0,626624319	protein_codin trans-sialidase
TcG_06761	77,73255841	0,342478964	0,200350575	1,709398457	0,087377167	0,245121514	protein_codin hypothetical protein
TcG_06762	283,8173679	0,22077805	0,107231505	2,058891655	0,039504619	0,140000543	protein_codin hypothetical protein
TcG_06763	111,0408472	0,110953979	0,185584334	0,597862854	0,549931447	0,745990604	protein_codin hypothetical protein
TcG_06764	444,7073813	-0,044797398	0,09165298	-0,48877187	0,625003216	0,798113883	protein_codin hypothetical protein
TcG_06765	215,5134153	0,182573858	0,123451172	1,478915551	0,139162887	0,333541829	protein_codin hypothetical protein
TcG_06766	292,6731778	0,151781748	0,109362678	1,387875196	0,165175052	0,370715499	protein_codin hypothetical protein
TcG_06767	577,5742229	-0,223864935	0,084684155	-2,64352799	0,008204698	0,042323969	protein_codin hypothetical protein
TcG_06768	376,8751356	0,366490033	0,095151121	3,851662805	0,000117318	0,001278694	protein_codin hypothetical protein
TcG_06769	236,0188721	-0,001929985	0,115656527	-0,01668721	0,986686148	0,994670296	protein_codin glycerolphosphate mutase
TcG_06770	165,2693003	-0,163657407	0,137263534	-1,19228612	0,233149073	0,460495255	protein_codin putative phosphoglycerate mutase
TcG_06771	219,6550183	-0,055519576	0,121292903	-0,45773144	0,647145396	0,812420258	protein_codin zinc finger family protein
TcG_06772	445,9915274	0,086119397	0,087441851	0,984876189	0,324684891	0,55907719	protein_codin hypothetical protein
TcG_06773	437,4581011	-0,049947587	0,086277223	-0,57891974	0,562643331	0,755976532	protein_codin putative kinesin
TcG_06774	503,2363259	0,142467385	0,087439022	1,629334157	0,103242299	0,274980523	protein_codin hypothetical protein
TcG_06775	333,0805567	-0,158581553	0,099111029	-1,60003942	0,109589839	0,286167848	protein_codin 4-nitrophenylphosphatase
TcG_06776	409,6346542	-0,029530454	0,095806511	-0,30823014	0,757907222	0,878015178	protein_codin hypothetical protein
TcG_06777	275,3449755	0,755589679	0,114331371	6,608769488	3,87528E-11	2,12791E-09	protein_codin hypothetical protein
TcG_06778	129,3463967	0,094985997	0,152753798	0,621824124	0,534057522	0,73443768	protein_codin hypothetical protein
TcG_06779	22,19285771	-0,34320931	0,38241437	-0,89748016	0,369462755	0,601128826	
TcG_06780	318,7200753	0,106407281	0,102586509	1,037244394	0,299621973	0,533408141	protein_codin hypothetical protein
TcG_06781	291,5778625	-0,043664142	0,110338262	-0,39572983	0,692304348	0,84069156	protein_codin hypothetical protein
TcG_06782	243,0080898	-0,081690255	0,116480925	-0,70131873	0,483104125	0,696521203	protein_codin hypothetical protein
TcG_06783	470,3122317	-0,147579077	0,086633099	-1,70349531	0,088475415	0,24718499	protein_codin hypothetical protein

TcG_06784	418,5521098	0,059279969	0,093070601	0,636935496	0,52416686	0,727828049	protein_codin transferase
TcG_06785	406,982462	-0,058731561	0,091650581	-0,64082039	0,521639384	0,725401608	protein_codin hypothetical protein
TcG_06786	227,1477756	-0,25442199	0,117411547	-2,16692478	0,03024059	0,115176685	protein_codin hypothetical protein
TcG_06787	252,6959078	-0,011066414	0,111173702	-0,09954165	0,920708216	0,96150609	protein_codin hypothetical protein
TcG_06788	153,4419046	-0,056072385	0,147393336	-0,38042686	0,703628576	0,847074053	protein_codin pre-rRNA-processing protein TSR3
TcG_06789	204,3868942	0,028986319	0,140968981	0,205621969	0,837086215	0,920684823	protein_codin hypothetical protein
TcG_06790	375,3926235	-0,219765197	0,094894214	-2,31589671	0,020563909	0,085981036	protein_codin flagellar associated protein
TcG_06791	199,9337386	0,325268151	0,125749389	2,586638023	0,009691735	0,048213155	protein_codin hypothetical protein
TcG_06792	293,6663916	-0,401134374	0,104787128	-3,82808825	0,000129142	0,00138413	protein_codin calpain-like cysteine peptidase
TcG_06793	441,3160334	-0,369309828	0,088472594	-4,17428508	2,98923E-05	0,000394456	protein_codin COP-coated vesicle membrane protein p24 precursor
TcG_06794	339,14752	-0,208770852	0,099697285	-2,09404753	0,036255747	0,132052525	protein_codin ras-related protein rab-2a
TcG_06795	258,2084012	0,098034024	0,112177295	0,873920377	0,382161617	0,61215602	protein_codin hypothetical protein
TcG_06796	109,7388764	0,228864415	0,169001768	1,354213139	0,175668388	0,385180535	protein_codin hypothetical protein
TcG_06797	228,8777347	-0,25517048	0,130133447	-1,96083701	0,04989804	0,165271208	protein_codin pantetheine-phosphate adenylyltransferase
TcG_06798	211,8967114	0,341956862	0,124024516	2,757171499	0,005830376	0,032305471	protein_codin putative deaminase
TcG_06799	176,694462	0,113763789	0,13733228	0,828383461	0,40745337	0,635720733	protein_codin N5-glutamine methyltransferase MTQ2
TcG_06800	209,9146871	0,129890365	0,126760963	1,024687428	0,305510697	0,538595091	protein_codin hypothetical protein
TcG_06801	536,5273844	-0,03889458	0,082822785	-0,46961208	0,638632191	0,807731988	protein_codin hypothetical protein
TcG_06802	269,5725211	0,027297474	0,113573106	0,240351566	0,810057722	0,906277019	protein_codin hypothetical protein
TcG_06803	307,7905076	0,045571964	0,10654592	0,427721339	0,668854012	0,826307698	protein_codin putative phosphatidylinositol 3-kinase (tor2)
TcG_06804	338,6466358	-0,101035462	0,098928926	-1,02129343	0,307115437	0,540191203	protein_codin hypothetical protein
TcG_06805	283,224298	0,167825461	0,118982521	1,410505173	0,15839057	0,360533035	protein_codin cleavage stimulation factor subunit 2
TcG_06806	228,1413741	-0,161792038	0,120021538	-1,34802504	0,17765033	0,387984302	protein_codin hypothetical protein
TcG_06807	1336,216923	0,037724292	0,059514371	0,63386862	0,526166566	0,728944857	protein_codin karyopherin beta
TcG_06808	454,4565965	-0,124084221	0,087467712	-1,41862887	0,156007244	0,357566753	protein_codin hypothetical protein
TcG_06809	129,3316759	-0,372652198	0,15622342	-2,38537986	0,017061494	0,074456429	protein_codin hypothetical protein
TcG_06810	239,5797576	-0,415361877	0,114281432	-3,63455262	0,000278463	0,002629403	protein_codin putative serine carboxypeptidase S28
TcG_06811	15,48027972	0,44638493	0,45339225	0,984544687	0,324847771	1	protein_codin putative R-SNARE protein
TcG_06812	113,0347213	-0,060970064	0,171133534	-0,35627187	0,721636975	0,857117481	protein_codin esag4
TcG_06813	52,93441093	0,027405474	0,2501737	0,109545785	0,912769609	0,957238745	protein_codin adenylate cyclase
TcG_06814	41,25391384	0,117326599	0,289281904	0,405578772	0,685052129	0,836557076	protein_codin hypothetical protein
TcG_06815	292,3135849	-0,049848929	0,111662021	-0,44642689	0,655288912	0,817728228	protein_codin hypothetical protein
TcG_06816	253,4374476	-0,381011701	0,114329022	-3,33258953	0,000860418	0,006894053	protein_codin hypothetical protein
TcG_06817	707,751604	-0,203994132	0,078215161	-2,60811497	0,009104237	0,045881552	protein_codin myo-inositol-1-phosphate synthase
TcG_06818	2167,291299	0,2769779	0,047559595	5,82380694	5,7522E-09	1,93174E-07	protein_codin putative delta-4 fatty acid desaturase
TcG_06819	358,0538336	0,014901263	0,096094645	0,155068608	0,87676723	0,941012055	protein_codin putative DEAD/DEAH box helicase
TcG_06820	102,744925	0,188699907	0,175450048	1,075519267	0,282142311	0,515355638	protein_codin small nuclear RNA activating protein 3
TcG_06821	604,3906796	-0,024597187	0,078394526	-0,31376154	0,753702159	0,875691257	protein_codin putative nucleoporin interacting component (NUP93)
TcG_06822	464,1216539	0,006169644	0,098956802	0,062346836	0,950286631	0,976946126	protein_codin putative chromatin assembly factor 1 subunit B
TcG_06823	421,4807255	-0,49679403	0,092478812	-5,37197678	7,78781E-08	2,06475E-06	protein_codin hypothetical protein
TcG_06824	333,4930041	-0,082254578	0,098691715	-0,83344968	0,404591167	0,634058334	protein_codin hypothetical protein
TcG_06825	397,6653613	-0,042348544	0,093866788	-0,45115578	0,651877276	0,815834891	protein_codin hypothetical protein
TcG_06826	574,5587667	0,027922352	0,078088486	0,357573233	0,720662713	0,85670516	protein_codin putative acid phosphatase
TcG_06827	262,6264906	-0,000719315	0,116174095	-0,0061917	0,995059767	0,997902057	protein_codin hypothetical protein
TcG_06828	328,2267611	-0,093442665	0,119873098	-0,77951322	0,435677455	0,659399643	protein_codin hypothetical protein
TcG_06829	217,2761315	-0,205507834	0,126512492	-1,62440745	0,104288878	0,277067403	protein_codin hypothetical protein
TcG_06830	177,6623264	0,028136515	0,134254109	0,209576566	0,833998171	0,91929434	protein_codin hypothetical protein
TcG_06831	109,8324303	-0,079028408	0,169815002	-0,46537942	0,641659767	0,80983334	protein_codin caltractin
TcG_06832	43,03794475	-0,076670502	0,264948522	-0,28937886	0,772291467	0,886356507	protein_codin hypothetical protein
TcG_06833	215,0448027	-0,227541682	0,131943386	-1,72454027	0,084610356	0,239681073	protein_codin hypothetical protein
TcG_06834	10,37531145	0,722775138	0,573361643	1,260592066	0,207455859	1	protein_codin hypothetical protein
TcG_06835	591,7126492	-0,110583004	0,07800465	-1,4176463	0,156294055	0,357690793	protein_codin putative dipeptidyl-peptidase 8-like serine peptidase
TcG_06836	614,0031195	-0,13677587	0,079227188	-1,72637542	0,084279895	0,239095707	protein_codin hypothetical protein
TcG_06837	192,475353	-0,227469319	0,126256646	-1,80164233	0,071601696	0,214972079	protein_codin hypothetical protein
TcG_06838	1401,293591	0,172805695	0,057671495	2,996379654	0,002732061	0,017634347	
TcG_06839	3217,637202	0,23157119	0,044903955	5,157033313	2,50893E-07	5,89624E-06	protein_codin putative sterol 24-c-methyltransferase
TcG_06840	67,27635854	0,143548944	0,224397525	0,639708231	0,522362303	0,726192662	protein_codin putative sterol 24-c-methyltransferase

TcG_06841	635,0526627	-0,111533547	0,075525362	-1,47676944	0,139737451	0,334291079	protein_codin	hypothetical protein
TcG_06842	293,3987715	-0,459518171	0,116029254	-3,96036477	7,48354E-05	0,00087312		
TcG_06843	148,2026522	0,260272502	0,146063946	1,781907925	0,074764248	0,220667247	protein_codin	hypothetical protein
TcG_06844	235,4421175	0,276105697	0,118326661	2,333419151	0,019626157	0,082777086	protein_codin	putative tyrosine aminotransferase
TcG_06845	178,7841322	0,031880928	0,133163674	0,239411599	0,810786437	0,906822247	protein_codin	putative haloacid dehalogenase hydrolase
TcG_06846	202,4953013	0,125403863	0,124362279	1,008375404	0,313274276	0,547450341	protein_codin	hypothetical protein
TcG_06847	618,4640736	-0,234914417	0,075640711	-3,10566113	0,001898542	0,013187351	protein_codin	histone H2B variant
TcG_06848	292,4798942	0,319708956	0,105714035	3,024281081	0,002492248	0,016406353	protein_codin	hypothetical protein
TcG_06849	289,5673458	-0,031426882	0,109150208	-0,28792324	0,773405495	0,886918879	protein_codin	hypothetical protein
TcG_06850	181,4677158	0,312291128	0,13077329	2,388034507	0,016938751	0,074197494	protein_codin	hypothetical protein
TcG_06851	328,2396105	-0,209895814	0,102163634	-2,05450616	0,039926728	0,141119912	protein_codin	putative phopshatase
TcG_06852	209,1464385	0,335003551	0,129373147	2,589436511	0,009613315	0,047864145	protein_codin	RNA-binding protein
TcG_06853	594,0999174	0,179190952	0,079917684	2,242194003	0,024948837	0,099571898	protein_codin	putative major facilitator superfamily
TcG_06854	1117,364134	0,241111406	0,064936796	3,71301668	0,000204803	0,002029814	protein_codin	putative pantothenate kinase subunit
TcG_06855	125,2537829	-0,407932611	0,158365217	-2,57589778	0,00999802	0,049355372	protein_codin	hypothetical protein
TcG_06856	218,2663865	-0,01823362	0,120162924	-0,15174081	0,879391367	0,941623821	protein_codin	DNA-direcetd RNA polymerase II, subunit 9
TcG_06857	246,5103488	-0,031777948	0,11975126	-0,2653663	0,790727297	0,895276699	protein_codin	putative proteasome beta 3 subunit
TcG_06858	399,4866843	0,148464066	0,093061762	1,595328343	0,110638831	0,287541833	protein_codin	hypothetical protein
TcG_06859	284,8413322	-0,025503658	0,107456733	-0,23733885	0,812393923	0,907594318	protein_codin	hypothetical protein
TcG_06860	324,9310502	-0,410519001	0,102551008	-4,00307136	6,25254E-05	0,000746055	protein_codin	flagellar associated protein
TcG_06861	68,5067065	0,27745537	0,212228138	1,307344882	0,191095608	0,406681682	protein_codin	putative palmitoyltransferase ZDHHC12
TcG_06862	1262,237875	0,2700908	0,059339201	4,551642044	5,32288E-06	8,86077E-05	protein_codin	putative neurobeachin/beige protein
TcG_06863	509,7587775	-0,115923811	0,081524933	-1,42194303	0,155042789	0,35638871	protein_codin	hypothetical protein
TcG_06864	177,0114211	-0,056273166	0,149599659	-0,37615838	0,706799151	0,848924953	protein_codin	putative origin recognition complex subunit 1, putative,cell division cycle 6
TcG_06865	202,6130409	0,023306164	0,124953145	0,186519228	0,852037602	0,928315559	protein_codin	hypothetical protein
TcG_06866	227,4939087	-0,378035046	0,124327803	-3,04063161	0,002360825	0,015708786	protein_codin	putative pyruvate dehydrogenase (lipoamide) kinase
TcG_06867	71,18567586	0,186160102	0,208235595	0,8939879	0,371328365	0,60280376	protein_codin	putative PIMT protein
TcG_06868	127,2760287	-0,00343744	0,161071457	-0,02134109	0,982973567	0,99369442	protein_codin	hypothetical protein
TcG_06869	297,444179	-0,311435071	0,103280744	-3,01542241	0,002566216	0,016778881	protein_codin	ERGIC and golgi family 3
TcG_06870	191,5767122	-0,305935484	0,13261896	-2,30687591	0,021061739	0,087777448	protein_codin	gamma carbonic dehydratase
TcG_06871	615,6909944	0,252846549	0,078161838	3,23491046	0,001216809	0,009154511	protein_codin	neutral sphingomyelinase activation associated factor-like protein
TcG_06872	59,06468792	0,215772589	0,232559185	0,927817963	0,353502004	0,587952085	protein_codin	neutral sphingomyelinase activation associated factor-like protein
TcG_06873	181,540029	0,636103265	0,156540598	4,063503482	4,83416E-05	0,000598382	protein_codin	hypothetical protein
TcG_06874	350,1737712	0,59346249	0,1046068	5,673268731	1,40098E-08	4,30551E-07	protein_codin	hypothetical protein
TcG_06875	126,4915398	0,952341719	0,164746887	5,780635582	7,44189E-09	2,42196E-07	protein_codin	hypothetical protein
TcG_06876	454,6452805	0,396385682	0,088134473	4,497510092	6,87539E-06	0,000110483	protein_codin	pre-mRNA-splicing factor SYF1
TcG_06877	323,5893055	0,296730518	0,105149734	2,821980696	0,004772805	0,027759899	protein_codin	Trypanosoma vivax
TcG_06878	553,9124252	0,561425175	0,083495276	6,724035213	1,7676E-11	1,04487E-09	protein_codin	hypothetical protein
TcG_06879	401,4376019	0,296729868	0,096930406	3,061267148	0,002204023	0,014863688	protein_codin	putative protoheme IX farnesyltransferase
TcG_06880	841,6060396	0,248138955	0,067487472	3,676815073	0,000236164	0,002291623	protein_codin	putative vacuolar proton translocating ATPase subunit A
TcG_06881	291,63404	0,725592978	0,113330217	6,402467056	1,52886E-10	7,26563E-09	protein_codin	hypothetical protein
TcG_06882	275,0258761	0,452669684	0,111379699	4,064202793	4,81969E-05	0,000597229	protein_codin	putative alanine racemase
TcG_06883	349,1790516	0,633305953	0,103332074	6,128841986	8,8521E-10	3,56173E-08	protein_codin	hypothetical protein
TcG_06884	294,0758772	0,398605298	0,110324096	3,613039339	0,000302629	0,002825348	protein_codin	Sulfate transporter
TcG_06885	403,746646	0,580029165	0,091415578	6,344970729	2,22468E-10	1,01079E-08	protein_codin	GAF domain-containing protein
TcG_06886	107,1433681	0,531339427	0,173277016	3,06641608	0,002166416	0,014669841	protein_codin	fukutin-related protein-like
TcG_06887	172,1172247	-3,405454576	0,1827211	-18,6374456	1,59686E-77	1,85012E-73	protein_codin	P21 protein
TcG_06888	517,6452667	0,218721822	0,087322086	2,504770921	0,012253072	0,05799187	protein_codin	hypothetical protein
TcG_06889	97,67200164	0,690931866	0,186750869	3,699751819	0,00021581	0,002124367	protein_codin	hypothetical protein
TcG_06890	2427,477497	0,342082884	0,046407643	7,371261716	1,69021E-13	1,43991E-11	protein_codin	short-chain dehydrogenase
TcG_06891	273,1716599	0,711177687	0,114931278	6,187851529	6,09897E-10	2,551E-08	protein_codin	exosome component CSL4
TcG_06892	56,74487276	0,821976282	0,2529107	3,250065272	0,001153785	0,008771493		
TcG_06893	320,5417447	0,686314186	0,107375564	6,391716702	1,64034E-10	7,72558E-09	protein_codin	putative ankyrin repeat domain-containing protein 32-like isoform X1
TcG_06894	244,977008	0,518319028	0,115407547	4,491205644	7,08211E-06	0,000113333	protein_codin	vesicle transport v-SNARE 11
TcG_06895	233,2161329	0,218098842	0,117721138	1,852673575	0,06392916	0,198630821	protein_codin	vesicle transport v-SNARE 11
TcG_06896	506,3733574	0,423159725	0,083625271	5,060189561	4,1884E-07	9,26084E-06	protein_codin	protein containing C-terminal RING-finger
TcG_06897	269,3681345	0,491507391	0,109946129	4,470438323	7,80595E-06	0,000123215	protein_codin	hypothetical protein

TcG_06898	170,2899389	0,419150058	0,144402057	2,902659888	0,003700082	0,022583044	protein_codin	hypothetical protein
TcG_06899	384,1623629	0,536363751	0,097838167	5,482152471	4,20182E-08	1,1759E-06	protein_codin	hypothetical protein
TcG_06900	32,12048583	0,248904306	0,310515721	0,801583588	0,422793874	0,648892545	protein_codin	target of rapamycin (TOR) kinase 1
TcG_06901	36,06449627	0,265329782	0,290655777	0,912866018	0,361313012	0,593951838	protein_codin	target of rapamycin (TOR) kinase 1
TcG_06902	43,04806174	-0,042441631	0,266894768	-0,15902009	0,873653043	0,939322955	protein_codin	putative trans-sialidase
TcG_06903	29,92081762	0,293214402	0,322339931	0,909643434	0,363010585	0,595268564	protein_codin	phosphatidylinositol 3-related kinase
TcG_06904	38,6247561	0,10359589	0,278704909	0,371704575	0,710112818	0,850461764	protein_codin	structural maintenance of chromosome protein 4
TcG_06905	51,19927652	0,203489532	0,244537618	0,832139994	0,40532993	0,634304216	protein_codin	aspartyl-tRNA synthetase
TcG_06906	427,613682	0,410411765	0,088637434	4,630230661	3,65259E-06	6,36374E-05	protein_codin	putative mismatch repair protein MSH4
TcG_06907	211,5012903	0,144721349	0,12464867	1,161034038	0,245628058	0,474196968	protein_codin	hypothetical protein
TcG_06908	316,3792093	-0,021307247	0,109526596	-0,19453948	0,845753487	0,925035391	protein_codin	putative ABC transporter
TcG_06909	169,7283948	0,256909841	0,139604539	1,840268542	0,065728821	0,202967517	protein_codin	hypothetical protein
TcG_06910	392,6156758	-0,064543386	0,092692137	-0,69631997	0,486228469	0,698274608	protein_codin	hypothetical protein
TcG_06911	353,9846044	-0,140909828	0,099205098	-1,42038898	0,15549447	0,356891537	protein_codin	SCY1 family protein kinase
TcG_06912	310,9228246	0,00460685	0,104290656	0,044173182	0,964766359	0,984347197	protein_codin	hypothetical protein
TcG_06913	434,5418653	0,046692286	0,088916245	0,525126601	0,599495185	0,783102809	protein_codin	hypothetical protein
TcG_06914	519,534166	0,027167286	0,084549577	0,321317817	0,747969556	0,872827448	protein_codin	hypothetical protein
TcG_06915	415,1398172	0,021582737	0,091911394	0,234821129	0,814347568	0,908262507	protein_codin	hypothetical protein
TcG_06916	168,4040646	-0,160369048	0,135305242	-1,18523898	0,235922971	0,463445836	protein_codin	hypothetical protein
TcG_06917	320,0742823	0,09570915	0,112541904	0,850431234	0,395085377	0,624620319	protein_codin	putative 2-aminoethylphosphonate:pyruvateaminotransferase- likeprotein,putative
TcG_06918	557,7665646	-0,087000826	0,082175804	-1,05871585	0,289729206	0,523273981	protein_codin	putative serine/threonine protein kinase
TcG_06919	416,6501429	-0,188471118	0,090120561	-2,09132205	0,036499204	0,132730628	protein_codin	hypoxanthine-guanine phosphoribosyltransferase
TcG_06920	367,0584983	-0,275610911	0,09721134	-2,83517242	0,004580096	0,026881961	protein_codin	putative hypoxanthine-guanine phosphoribosyltransferase
TcG_06921	230,5365839	0,133125828	0,131712356	1,010731508	0,312144953	0,546053363	protein_codin	hypothetical protein
TcG_06922	1129,145726	-0,203817225	0,060507027	-3,36848852	0,000755815	0,006158142	protein_codin	putative methionine aminopeptidase 2
TcG_06923	297,7499073	0,078381419	0,108086951	0,725170042	0,468347719	0,685000736	protein_codin	hypothetical protein
TcG_06924	260,693069	0,240107774	0,120099401	1,999242061	0,04558217	0,154872439	protein_codin	hypothetical protein
TcG_06925	842,3411903	-0,213752276	0,066177026	-3,23000727	0,001237871	0,009276824	protein_codin	hypothetical protein
TcG_06926	124,1306959	-0,1094036	0,174404938	-0,62729646	0,530464923	0,732011267	protein_codin	hypothetical protein
TcG_06927	127,3952008	-0,065212491	0,16848636	-0,38704908	0,698719872	0,844142481	protein_codin	hypothetical protein
TcG_06928	515,4721812	-0,028365839	0,082845725	-0,34239351	0,732054768	0,86246393	protein_codin	putative tyrosine protein kinase
TcG_06929	32,14884172	0,092491756	0,311831117	0,296608488	0,76676543	0,882900444	protein_codin	putative retrotransposon hot spot (RHS) protein
TcG_06930	122,9647445	-0,198422284	0,169763171	-1,1688182	0,242476885	0,470898628	protein_codin	putative retrotransposon hot spot (RHS) protein
TcG_06931	62,20486297	-0,58129934	0,229736696	-2,53028511	0,011396987	0,054858951	protein_codin	amino acid transporter
TcG_06932	1112,105018	-0,826185504	0,06168109	-13,39447	6,51394E-41	7,30872E-38	protein_codin	surface protein-2
TcG_06933	17,55916603	0,735752458	0,419392933	1,754327269	0,079374446	0,229678404	protein_codin	hypothetical protein
TcG_06934	56,67268958	-0,051128017	0,240133771	-0,21291473	0,831393471	0,917995307	protein_codin	hypothetical protein
TcG_06935	32,40020162	0,472609994	0,313466429	1,507689342	0,131634055	0,322461107	protein_codin	putative trans-sialidase
TcG_06936	34,53192732	0,177671656	0,298407838	0,595398756	0,551576957	0,747085647	protein_codin	helicase-like protein
TcG_06937	32,92858326	0,071345355	0,338087356	0,211026392	0,832866677	0,919089983	protein_codin	hypothetical protein
TcG_06938	244,6746076	-0,301221586	0,114041315	-2,64133736	0,008257944	0,042506957	protein_codin	hypothetical protein
TcG_06939	1381,169769	0,141108711	0,070625441	1,997986984	0,045718071	0,155106757	protein_codin	phosphotransferase
TcG_06940	646,4689706	-0,138225026	0,076871711	-1,79812606	0,072157033	0,216010232	protein_codin	hypothetical protein
TcG_06941	98,76129745	-0,185172662	0,189344725	-0,97796578	0,328091249	0,562650269	protein_codin	cAMP specific 3,5 cyclic phosphodiesterase
TcG_06942	422,1400353	0,354832943	0,093059629	3,812963231	0,000137311	0,001452858	protein_codin	hypothetical protein
TcG_06943	348,7728089	0,376046977	0,096801239	3,884733104	0,000102442	0,001136955	protein_codin	putative formin
TcG_06944	813,4321172	0,046799556	0,071404008	0,655419181	0,512197905	0,718354307	protein_codin	activating signal cointegrator 1 complex subunit 3-like 1
TcG_06945	359,0649234	0,082254163	0,100997402	0,814418608	0,415405182	0,64239863	protein_codin	acetyltransferase
TcG_06946	142,6152409	-0,034913249	0,147242216	-0,23711439	0,812568046	0,907594318	protein_codin	leucine-rich protein
TcG_06947	33,69844519	0,375571535	0,306359037	1,22591956	0,220228947	0,445300625	protein_codin	hypothetical protein
TcG_06948	17,31731008	-0,236368846	0,423259074	-0,55844957	0,576537434	0,766883339	protein_codin	hypothetical protein
TcG_06949	15,89250421	0,178250776	0,428547352	0,415941845	0,677452559	1	protein_codin	hypothetical protein
TcG_06950	418,9089968	0,13299131	0,092485077	1,437975886	0,150440896	0,350426454	protein_codin	putative SET and MYND domain-containing protein 1 isoform X2
TcG_06951	691,1476009	-0,021428499	0,078557014	-0,2727764	0,785025106	0,893514718	protein_codin	hypothetical protein
TcG_06952	310,5430821	-0,193851298	0,108810943	-1,78154231	0,074823899	0,220708868	protein_codin	putative tubulin tyrosine ligase
TcG_06953	129,4427599	-0,045043836	0,157949177	-0,2851793	0,775506779	0,888199045	protein_codin	putative tubulin tyrosine ligase
TcG_06954	141,8868193	-0,113321537	0,146953638	-0,77113802	0,44062513	0,663501236	protein_codin	putative folate/biopterin transporter

TcG_06955	199,9279006	0,194635355	0,12442481	1,564280906	0,117751614	0,299247687	protein_codin putative DNA replication factor
TcG_06956	134,4994718	0,195829943	0,160378881	1,2210457	0,222068717	0,447926211	protein_codin putative DNA replication factor
TcG_06957	138,0476331	-0,045197814	0,157071717	-0,28775272	0,773536031	0,886918879	protein_codin hypothetical protein
TcG_06958	139,5545299	0,096816942	0,149718836	0,646658392	0,517853057	0,723047183	protein_codin hypothetical protein
TcG_06959	134,6146503	0,45600279	0,152438853	2,991381666	0,002777181	0,017865866	protein_codin putative meiotic recombination protein DMC1
TcG_06960	465,9918044	0,201929625	0,091293329	2,211877118	0,026975157	0,105728743	protein_codin TPR Domain containing protein
TcG_06961	497,2081183	-0,115385813	0,088265633	-1,3072564	0,191125648	0,406681682	protein_codin protein FAM184A
TcG_06962	543,1691325	0,039880844	0,082820617	0,481532807	0,630137869	0,801754596	protein_codin hypothetical protein
TcG_06963	413,3634027	-0,082955256	0,090396069	-0,91768654	0,358782994	0,592567323	protein_codin putative proteasome alpha 1 subunit
TcG_06964	67,43631273	0,046273493	0,216573932	0,213661415	0,830811103	0,917601778	protein_codin hypothetical protein
TcG_06965	175,1137967	0,17459442	0,135737141	1,286268586	0,198349342	0,416922256	protein_codin hypothetical protein
TcG_06966	165,7311202	-0,025679905	0,135591248	-0,18939205	0,849785552	0,927630599	protein_codin hypothetical protein
TcG_06967	178,5855675	-0,226707092	0,151350906	-1,49789055	0,13416169	0,325709912	protein_codin hypothetical protein
TcG_06968	205,9692565	-0,130202688	0,123308014	-1,05591424	0,291007396	0,523651784	protein_codin hypothetical protein
TcG_06969	564,8405838	-0,118703386	0,081162285	-1,46254367	0,143592288	0,340008225	protein_codin adenosine monophosphate deaminase-like protein
TcG_06970	169,5975243	-0,253684416	0,136419912	-1,85958496	0,062944269	0,196492326	protein_codin putative membrane-trafficking protein
TcG_06971	567,9295654	-0,11972078	0,077570661	-1,54337709	0,12273931	0,307671493	protein_codin cyclophilin
TcG_06972	323,4071965	-0,344723064	0,100470073	-3,43110195	0,000601135	0,00505322	protein_codin hypothetical protein
TcG_06973	153,0263882	-0,346247215	0,144573475	-2,39495672	0,016622327	0,073115521	protein_codin putative glyceraldehyde-3-phosphate dehydrogenase
TcG_06974	133,3993878	-0,197209054	0,159138924	-1,23922576	0,215261903	0,438870501	protein_codin putative regulator of chromosome condensation (RCC1)
TcG_06975	189,2227744	-0,139251377	0,128023769	-1,0876994	0,276727832	0,509048895	protein_codin hypothetical protein
TcG_06976	382,2674719	-0,081148974	0,092967118	-0,87287823	0,382729451	0,612642086	protein_codin glycine cleavage system H protein
TcG_06977	866,3052855	-0,227990583	0,065200395	-3,49676691	0,000470933	0,004102429	protein_codin hypoxia up-regulated 1
TcG_06978	380,947078	-0,204119768	0,096163099	-2,12264132	0,03378392	0,125054471	protein_codin putative MCAK-like kinesin
TcG_06979	10,54699541	0,712207848	0,54029869	1,318174302	0,187445306	1	protein_codin trans-sialidase
TcG_06980	5,481284974	-0,022489002	0,774181633	-0,02904874	0,976825717	1	protein_codin hypothetical protein
TcG_06981	0,441769291	0,396475077	2,50258318	0,158426333	0,874120865	1	
TcG_06982	157,298551	0,140166702	0,147571552	0,949821967	0,342202722	0,576576323	protein_codin hypothetical protein
TcG_06983	227,3531758	0,525549473	0,12009012	4,376292356	1,20715E-05	0,000178393	protein_codin hypothetical protein
TcG_06984	192,8458243	-0,061733002	0,128150772	-0,48172165	0,630003692	0,801688869	protein_codin kelch domain-containing protein 3
TcG_06985	319,3885093	0,154352726	0,100394738	1,537458327	0,124181113	0,310211809	protein_codin hypothetical protein
TcG_06986	122,7572151	0,136589798	0,158241073	0,863175379	0,388041049	0,618419884	protein_codin hypothetical protein
TcG_06987	109,1433738	0,027653956	0,169965472	0,162703373	0,870751991	0,937676411	protein_codin hypothetical protein
TcG_06988	266,9375019	0,224987978	0,109894015	2,04731784	0,040626887	0,142711038	protein_codin hypothetical protein
TcG_06989	220,5978393	0,211217448	0,119390574	1,769130013	0,07687218	0,224649463	protein_codin putative calpain-like cysteine peptidase
TcG_06990	185,4298662	0,39913435	0,131330775	3,039153242	0,002372441	0,015768619	protein_codin hypothetical protein
TcG_06991	146,3164904	-0,251104927	0,154997166	-1,6200614	0,105219087	0,278771632	protein_codin hypothetical protein
TcG_06992	93,76288559	-0,239508195	0,181620107	-1,31873171	0,187258822	0,401476815	protein_codin hypothetical protein
TcG_06993	210,7358264	0,130060351	0,127883401	1,017022927	0,309142523	0,542345412	protein_codin MORN-containing protein
TcG_06994	198,7044037	0,323501141	0,134120366	2,412021015	0,015864367	0,070619947	protein_codin BTB/POZ domain containing protein
TcG_06995	217,8567884	0,189129074	0,123089457	1,536517254	0,124411569	0,310586607	protein_codin hypothetical protein
TcG_06996	1058,80608	0,239635412	0,067501378	3,550081794	0,000385111	0,003464209	protein_codin phosphoglycerate kinase
TcG_06997	191,6790247	-0,027602505	0,134401371	-0,20537369	0,837280177	0,920809505	protein_codin hypothetical protein
TcG_06998	345,7897671	0,152673609	0,098250553	1,553921117	0,120203234	0,303547225	protein_codin hypothetical protein
TcG_06999	114,8170497	0,168357469	0,178175964	0,944894389	0,344712797	0,578779679	protein_codin hypothetical protein
TcG_07000	109,4928617	0,410608668	0,168066951	2,44312559	0,014560669	0,066338936	protein_codin hypothetical protein
TcG_07001	256,8960633	0,187334865	0,113601268	1,649056107	0,099136141	0,267052157	protein_codin putative DNA repair protein BRCA2
TcG_07002	81,75957266	0,193678373	0,195252393	0,991938536	0,321227509	0,556034397	protein_codin anti-silencing protein ASF 1
TcG_07003	273,6209016	-0,073875256	0,110776397	-0,66688625	0,504844792	0,71221932	protein_codin hypothetical protein
TcG_07004	934,3912199	0,304057744	0,068871199	4,414875141	1,01068E-05	0,000153672	protein_codin phosphate-repressible phosphate permease
TcG_07005	844,1454724	0,007467252	0,068597125	0,108856636	0,913316202	0,957444943	protein_codin histone-lysine N-methyltransferase
TcG_07006	221,3368067	0,157681358	0,125687284	1,254552989	0,209641078	0,432216915	protein_codin WD40 repeat-containing protein
TcG_07007	143,7733511	0,188180493	0,155548	1,209790502	0,226359292	0,452796746	protein_codin hypothetical protein
TcG_07008	458,336964	0,076525845	0,087265023	0,876936058	0,38052137	0,610795316	protein_codin DNA-directed RNA polymerase III
TcG_07009	128,239535	0,145109096	0,15807406	0,917981714	0,358628439	0,592480978	protein_codin hypothetical protein
TcG_07010	26,1060665	-0,05099898	0,361952029	-0,14089983	0,887949075	0,945946339	protein_codin hypothetical protein
TcG_07011	250,1248542	-0,066090989	0,115841054	-0,57053166	0,568317155	0,760104812	protein_codin hypothetical protein

TcG_07012	553,4060354	0,144730998	0,08160049	1,773653546	0,076120475	0,223104838	protein_codin hypothetical protein
TcG_07013	234,7054564	0,36385874	0,117543449	3,095525476	0,001964645	0,013546817	protein_codin putative histone deacetylase
TcG_07014	164,1827137	0,167071579	0,143785287	1,161951844	0,245255026	0,473745371	protein_codin hypothetical protein
TcG_07015	913,2714358	0,179387464	0,067802181	2,645747704	0,008151058	0,042103506	protein_codin putative heat shock protein HslVU, ATPase subunit HslU
TcG_07016	206,7363051	0,091418952	0,13508034	0,676774661	0,498548938	0,707692721	protein_codin chaperone protein DNAJ
TcG_07017	82,84425904	0,123230773	0,197977119	0,622449571	0,533646294	0,734127993	protein_codin hypothetical protein
TcG_07018	470,6470573	-0,008922829	0,086223898	-0,10348441	0,917578521	0,960175645	protein_codin hypothetical protein
TcG_07019	275,0136958	-0,186523932	0,113445094	-1,64417804	0,100139432	0,268999944	protein_codin hypothetical protein
TcG_07020	224,665059	0,418016816	0,129169918	3,236177777	0,001211419	0,009119884	protein_codin hypothetical protein
TcG_07021	198,1115019	0,344074404	0,129011491	2,667005871	0,007653033	0,039998106	protein_codin hypothetical protein
TcG_07022	450,1472463	0,060799299	0,086477345	0,703066216	0,482014485	0,695989509	protein_codin putative protein kinase
TcG_07023	127,4999284	0,120780726	0,162735379	0,742190956	0,457971647	0,677211722	protein_codin hypothetical protein
TcG_07024	636,8756661	0,223170434	0,079814496	2,796114054	0,005172114	0,029435523	protein_codin ribonuclease Z
TcG_07025	379,6781705	0,169157687	0,094127034	1,797121177	0,072316384	0,216182287	protein_codin putative mitochondrial DNA polymerase beta-PAK
TcG_07026	706,9252329	-0,208421073	0,070597858	-2,95222943	0,003154884	0,019865483	protein_codin DNA polymerase beta
TcG_07027	129,7320178	0,342315421	0,159101504	2,151553655	0,031432526	0,118316193	protein_codin histone-lysine N-methyltransferase ASHR2
TcG_07028	47,15168826	0,202370882	0,290023325	0,697774504	0,485318226	0,698146344	
TcG_07029	241,3567806	0,08051398	0,118820878	0,677608017	0,498020261	0,707424985	protein_codin surface protein-2
TcG_07030	14,12397358	-0,07651501	0,464400631	-0,16476078	0,869132276	1	protein_codin hypothetical protein
TcG_07031	87,1843576	-0,184738991	0,184509506	-1,00124375	0,316708978	0,551702786	protein_codin hypothetical protein
TcG_07032	350,1275232	-0,112729524	0,101634117	-1,1091701	0,267356808	0,499773472	protein_codin cell division protein kinase 2
TcG_07033	1599,938399	-0,178732645	0,053249652	-3,35650351	0,000789347	0,006390899	protein_codin putative T-complex protein 1, delta subunit
TcG_07034	4702,087719	0,098892769	0,039176836	2,52426634	0,011594005	0,05548457	protein_codin serine carboxypeptidase
TcG_07035	102,5364224	-0,028059255	0,182316888	-0,15390376	0,877685617	0,941200542	protein_codin hypothetical protein
TcG_07036	110,4767444	0,078103822	0,166570354	0,468893897	0,639145479	0,807892158	protein_codin hypothetical protein
TcG_07037	82,85723809	0,239991725	0,194018501	1,236952788	0,216104611	0,440033044	protein_codin hypothetical protein
TcG_07038	72,128437	0,423021251	0,21563563	1,961740972	0,049792649	0,164997303	protein_codin hypothetical protein
TcG_07039	92,56931963	0,192232179	0,183702016	1,046434781	0,295360342	0,528097822	protein_codin Tbingi protein
TcG_07040	90,24688511	0,174577915	0,183988912	0,948850196	0,342696808	0,576796063	protein_codin Tbingi protein
TcG_07041	60,05009123	0,202749623	0,230624581	0,879132756	0,379329299	0,6106388	protein_codin trans-sialidase
TcG_07042	157,4343732	0,155167162	0,14398863	1,077634822	0,281196756	0,514255504	protein_codin Tbingi protein
TcG_07043	15,43717601	0,625688715	0,472356997	1,324609815	0,185300599	1	protein_codin hypothetical protein
TcG_07044	15,31341553	0,368013802	0,445544283	0,825987036	0,408811446	1	protein_codin hypothetical protein
TcG_07045	364,6843304	-0,193309475	0,094546804	-2,04459025	0,040895271	0,143449171	protein_codin hypothetical protein
TcG_07046	1031,274979	-0,138784208	0,061341177	-2,26249666	0,023666735	0,096009381	protein_codin hypothetical protein
TcG_07047	224,6464551	0,361099815	0,120645336	2,993069	0,002761873	0,01779703	protein_codin hypothetical protein
TcG_07048	327,4474388	0,019225998	0,100449441	0,191399753	0,848212413	0,926605553	protein_codin putative U4/U6 small nuclear ribonuclear protein
TcG_07049	468,0670804	-0,119429057	0,093529482	-1,27691348	0,201632793	0,421377623	protein_codin hypothetical protein
TcG_07050	206,6768375	-0,004011216	0,125803945	-0,03188466	0,974564032	0,988930525	protein_codin hypothetical protein
TcG_07051	1504,101463	-0,057597449	0,054863953	-1,04982317	0,293799421	0,526847251	protein_codin hypothetical protein
TcG_07052	283,648988	-0,177327847	0,112114509	-1,58166724	0,113725554	0,29274034	protein_codin hypothetical protein
TcG_07053	335,8598149	0,000427026	0,10275838	0,004155631	0,996684296	0,998839568	protein_codin hypothetical protein
TcG_07054	476,5091083	-0,050033145	0,083431049	-0,59969454	0,548709829	0,745247925	protein_codin PIF1 helicase-like protein
TcG_07055	185,2118571	0,247312915	0,150605032	1,642129162	0,100563239	0,269767467	protein_codin hypothetical protein
TcG_07056	488,0473952	-0,098208511	0,083138463	-1,18126445	0,237497684	0,465276998	protein_codin putative thymidine kinase
TcG_07057	942,2241827	-0,074485612	0,069051891	-1,07869039	0,280725771	0,513821293	protein_codin hypothetical protein
TcG_07058	288,4468645	0,255959698	0,109026215	2,347689476	0,018890262	0,080228219	protein_codin hypothetical protein
TcG_07059	147,9991965	0,436136702	0,149840718	2,9106688	0,003606561	0,022108791	protein_codin hypothetical protein
TcG_07060	509,1478034	0,279041194	0,084207328	3,313740015	0,000920571	0,007300295	protein_codin hypothetical protein
TcG_07061	676,1354832	0,270890432	0,085470005	3,169421042	0,00152743	0,011039799	protein_codin L1Tc protein
TcG_07062	11,21307812	0,671763301	0,527705999	1,272987802	0,20302237	1	protein_codin hypothetical protein
TcG_07063	92,60373684	-0,072389636	0,182686586	-0,39625042	0,6919203	0,840577603	protein_codin hypothetical protein
TcG_07064	56,39979938	-0,197253329	0,22996804	-0,85774236	0,391034746	0,621027978	protein_codin hypothetical protein
TcG_07065	1090,553558	-0,258382725	0,067413819	-3,83278576	0,0001267	0,001361734	protein_codin hypothetical protein
TcG_07066	1414,57521	-0,115858237	0,055584808	-2,08435076	0,037128273	0,133973227	protein_codin stress-induced protein sti1
TcG_07067	236,1156589	-0,128079646	0,118860593	-1,07756189	0,281229317	0,514255504	protein_codin EF-hand domain-containing family member C2
TcG_07068	418,4663466	-0,048426886	0,093483593	-0,51802551	0,604440473	0,786152595	protein_codin putative phosphoribosylpyrophosphate synthetase

TcG_07069	354,3819113	0,034586642	0,09712036	0,356121435	0,721749625	0,857117481	protein_codin putative suppressive immunomodulating factor
TcG_07070	234,2410765	0,045181505	0,119012771	0,379635769	0,704215806	0,847428783	protein_codin hypothetical protein
TcG_07071	20,06977253	0,089812372	0,397023286	0,226214368	0,821034696	0,911247053	protein_codin hypothetical protein
TcG_07072	282,150678	0,18957365	0,11373517	1,666798837	0,095554412	0,260799391	protein_codin hypothetical protein
TcG_07073	112,3866878	0,2456868	0,165644335	1,483218848	0,138016275	0,331548116	protein_codin thiopurine S-methyltransferase
TcG_07074	214,3150894	-0,01304044	0,128798446	-0,10124687	0,919354489	0,961200581	protein_codin hypothetical protein
TcG_07075	222,0186045	-0,161576694	0,123639554	-1,3068366	0,191268213	0,406910305	protein_codin pre-mRNA-processing factor SLU7
TcG_07076	600,7168989	-0,052352698	0,085183389	-0,61458811	0,538826766	0,738205979	protein_codin WD repeat domain 35
TcG_07077	211,671903	0,06287743	0,124440798	0,505279868	0,613362281	0,79091991	protein_codin putative MIX protein
TcG_07078	182,6850883	0,233688206	0,137802252	1,695822834	0,089919437	0,249893643	protein_codin hypothetical protein
TcG_07079	241,4163932	0,724435805	0,120219579	6,02593863	1,68131E-09	6,34275E-08	protein_codin putative poly(ADP-ribose) polymerase
TcG_07080	269,7444588	0,353398817	0,109588881	3,22476893	0,001260744	0,009417781	protein_codin hypothetical protein
TcG_07081	418,9543378	-0,2422062	0,089565789	-2,70422672	0,006846356	0,036740101	protein_codin hypothetical protein
TcG_07082	174,9795813	0,28979004	0,134194524	2,159477388	0,03081315	0,11659084	protein_codin hypothetical protein
TcG_07083	318,0959046	-0,233241816	0,104700842	-2,22769762	0,025900686	0,102426779	protein_codin hypothetical protein
TcG_07084	86,3744763	0,272167133	0,193071122	1,409672925	0,158636283	0,360879634	protein_codin putative retrotransposon hot spot (RHS) protein
TcG_07085	35,78536472	-0,031414612	0,285856401	-0,10989648	0,912491474	0,957238745	protein_codin hypothetical protein
TcG_07086	13,46790068	-1,9351E-05	0,548287346	-3,5294E-05	0,99997184	1	protein_codin hypothetical protein
TcG_07087	84,82906979	0,091262651	0,195791536	0,466121532	0,641128512	0,809515577	protein_codin putative trans-sialidase
TcG_07088	57,53669988	0,250017293	0,234811634	1,064756836	0,286985983	0,520249026	protein_codin trans-sialidase
TcG_07089	27,07892155	-0,129677373	0,345002042	-0,37587422	0,707010408	0,848924953	protein_codin hypothetical protein
TcG_07090	221,9046272	0,214390359	0,121274456	1,767811348	0,077092447	0,224985666	protein_codin putative N-acyl-L-amino acid amidohydrolase
TcG_07091	58,1407042	0,245066793	0,231983506	1,056397489	0,290786653	0,523632913	protein_codin aminoacylase
TcG_07092	26,28102297	0,508491637	0,349659457	1,454248204	0,145877505	0,343402425	protein_codin aminoacylase
TcG_07093	111,9031575	0,113078697	0,167220128	0,676226591	0,498896792	0,708013013	protein_codin putative aminoacylase, putative,N-acyl-L-amino acid amidohydrolase
TcG_07094	312,0384488	0,110962823	0,106806055	1,038918842	0,298842483	0,532347633	protein_codin hypothetical protein
TcG_07095	177,0161758	0,282217302	0,135224396	2,087029494	0,036885469	0,133423365	protein_codin RNA editing ligase
TcG_07096	128,4420467	0,20754362	0,15702796	1,321698506	0,186268566	0,400168294	protein_codin hypothetical protein
TcG_07097	298,5955287	-0,001470272	0,105522199	-0,0139333	0,988883196	0,995522309	protein_codin methyltransferase
TcG_07098	297,3158234	-0,042153322	0,103809717	-0,40606336	0,684696049	0,836449644	protein_codin hypothetical protein
TcG_07099	210,1198622	-0,008092271	0,126864878	-0,06378654	0,949140199	0,976359615	protein_codin putative soluble N-ethylmaleimide sensitive factor (NSF) attachment protein
TcG_07100	349,4600959	0,19750623	0,096382556	2,049190625	0,040443478	0,142295214	protein_codin separase
TcG_07101	1579,828304	0,535880704	0,053366029	10,04160719	1,00031E-23	2,57546E-21	protein_codin putative protein kinase
TcG_07102	321,2821617	0,038381033	0,10057084	0,381631827	0,702734473	0,846349439	protein_codin hypothetical protein
TcG_07103	186,8651475	0,18860645	0,134974945	1,397344149	0,162310105	0,366074534	protein_codin rar1
TcG_07104	1009,240167	0,093929613	0,064142091	1,46439898	0,143084967	0,339570345	protein_codin 40S ribosomal protein S11
TcG_07105	241,1635682	0,179088519	0,119085239	1,503868326	0,132615274	0,323614384	protein_codin 40S ribosomal protein S11
TcG_07106	305,733756	-0,13568625	0,105317935	-1,28834894	0,197624523	0,416229363	protein_codin phosphatase-like protein
TcG_07107	168,3380239	0,018150198	0,136304394	0,133159299	0,894067399	0,948813234	protein_codin putative GTPase activating protein of Rab-like GTPase
TcG_07108	327,3823722	0,524546147	0,107242279	4,891225295	1,0021E-06	2,03334E-05	protein_codin putative cell division cycle protein
TcG_07109	595,1624088	0,01466778	0,085217526	0,172121631	0,863341907	0,934655143	protein_codin hypothetical protein
TcG_07110	56,95868857	0,034367953	0,242805893	0,141544972	0,887439435	0,94569431	protein_codin hypothetical protein
TcG_07111	387,3748124	0,160854265	0,095029658	1,692674353	0,09051747	0,250953674	protein_codin hypothetical protein
TcG_07112	529,1815859	-0,214755466	0,079405891	-2,70452812	0,006840148	0,036723797	protein_codin hypothetical protein
TcG_07113	150,738769	0,089781104	0,143325681	0,626413237	0,53104393	0,732374118	protein_codin putative septum formation inhibitor, Maf-like protein
TcG_07114	330,4181571	0,114844305	0,09957372	1,15335959	0,248762821	0,478209066	protein_codin hypothetical protein
TcG_07115	376,9168301	0,054132046	0,093988951	0,575940529	0,56465538	0,757444604	protein_codin zinc-binding phosphatase
TcG_07116	349,7962062	0,072637125	0,103680238	0,700587941	0,483560207	0,697005294	protein_codin hypothetical protein
TcG_07117	57,74165249	-0,012728293	0,231178349	-0,05505833	0,956091997	0,979683548	protein_codin dispersed gene family protein 1 (DGF-1)
TcG_07118	523,8472567	0,217690829	0,086958743	2,503380583	0,012301319	0,05814895	protein_codin hypothetical protein
TcG_07119	244,0316741	0,231759308	0,12153844	1,906880719	0,056536035	0,180299066	protein_codin hypothetical protein
TcG_07120	334,4157768	0,068311732	0,101768392	0,671247038	0,502063168	0,710939118	protein_codin hypothetical protein
TcG_07121	134,7975852	-0,218700511	0,156606432	-1,39649763	0,162564694	0,366506041	protein_codin glutaredoxin
TcG_07122	446,7271112	-0,016462787	0,089289459	-0,18437548	0,8537189	0,929098927	protein_codin hypothetical protein
TcG_07123	86,9265011	-0,081179962	0,202114855	-0,40165263	0,687939693	0,837938319	protein_codin hypothetical protein
TcG_07124	94,49116011	0,476501427	0,188078379	2,533525805	0,011292143	0,054380308	
TcG_07125	217,9033985	0,03586649	0,122882177	0,291877069	0,770380617	0,884686967	protein_codin hypothetical protein

TcG_07126	26,82839022	-0,525077249	0,336541257	-1,56021658	0,118708709	0,300690664	
TcG_07127	25,08573725	0,227900152	0,351239508	0,648845436	0,516438286	0,722055158	protein_codin hypothetical protein
TcG_07128	197,2565088	0,260942325	0,129116539	2,020982954	0,04328153	0,149371651	protein_codin hypothetical protein
TcG_07129	632,6183959	0,060883801	0,075497408	0,806435645	0,419991701	0,646561352	protein_codin hypothetical protein
TcG_07130	354,165229	-0,096760685	0,106304749	-0,91021978	0,362706614	0,595082383	protein_codin hypothetical protein
TcG_07131	181,0590475	0,118831847	0,131884419	0,901030223	0,367572251	0,599815788	protein_codin hypothetical protein
TcG_07132	547,8507698	0,114991209	0,080414143	1,429987365	0,152720645	0,352971576	protein_codin hypothetical protein
TcG_07133	475,4250095	-0,241108128	0,084477732	-2,85410275	0,004315857	0,025645176	protein_codin hypothetical protein
TcG_07134	1480,439517	-0,103315003	0,119018211	-0,86806046	0,385361235	0,61583383	protein_codin phosphatidylinositol 3-kinase
TcG_07135	119,6719729	0,657169405	0,162918913	4,03372078	5,49005E-05	0,000669711	protein_codin hypothetical protein
TcG_07136	104,0260506	0,322274515	0,173951677	1,852666904	0,063930117	0,198630821	protein_codin surface protease GP63
TcG_07137	25,63120061	0,128824896	0,358286956	0,359557872	0,719177792	0,855882956	protein_codin hypothetical protein
TcG_07138	20,75214846	-0,117258594	0,380593196	-0,3080943	0,758010578	0,878015178	protein_codin hypothetical protein
TcG_07139	102,7249394	-0,200582736	0,188835461	-1,06220905	0,288140791	0,521231677	protein_codin putative target of rapamycin (TOR) kinase 1
TcG_07140	696,7381091	0,260307602	0,074882355	3,476220843	0,000508533	0,0044002	protein_codin putative helicase
TcG_07141	251,479844	0,216355049	0,11338541	1,908138351	0,056373337	0,179978364	protein_codin methyltransferase
TcG_07142	379,2562863	-0,199554117	0,093656239	-2,13070821	0,033113189	0,123100992	protein_codin hypothetical protein
TcG_07143	443,7730716	-0,217257369	0,088326071	-2,45971962	0,01390456	0,063978645	protein_codin hypothetical protein
TcG_07144	485,60238	-0,041436751	0,086539388	-0,47881955	0,632067007	0,802906018	protein_codin hypothetical protein
TcG_07145	239,4055428	0,112693783	0,115625093	0,97464815	0,329734821	0,56434119	protein_codin hypothetical protein
TcG_07146	431,5866234	-0,09493997	0,089926472	-1,05575108	0,291081955	0,523651784	protein_codin 4-nitrophenylphosphatase/protein-tyrosine phosphatase
TcG_07147	339,6926492	0,132611328	0,098991209	1,339627317	0,180366538	0,391834087	protein_codin hypothetical protein
TcG_07148	372,2926892	-0,237337494	0,100655314	-2,35792314	0,018377497	0,07859789	protein_codin hypothetical protein
TcG_07149	393,3049424	-0,148489549	0,097139971	-1,52861431	0,126360086	0,31409915	protein_codin CCR4-NOT transcription complex subunit 2
TcG_07150	345,2246976	-0,142038193	0,098538504	-1,44144865	0,149457975	0,348835635	protein_codin putative elongation factor 1-gamma (EF-1-gamma)
TcG_07151	122,1057707	0,285193605	0,160491094	1,777005804	0,075567285	0,222157463	protein_codin hypothetical protein
TcG_07152	41,85886294	-0,227536315	0,280681787	-0,81065579	0,41756337	0,644192971	protein_codin pumilio protein 4
TcG_07153	61,71087815	0,03826364	0,231570595	0,165235315	0,86875877	0,936741226	protein_codin hypothetical protein
TcG_07154	153,0252624	-0,114501041	0,147302505	-0,77731903	0,436970569	0,66041495	protein_codin hypothetical protein
TcG_07155	112,1986383	-0,236584415	0,164972181	-1,43408672	0,151547516	0,351799471	protein_codin subtilisin-like serine peptidase
TcG_07156	584,4735974	-0,1538947	0,084493587	-1,82137729	0,068549525	0,209003893	protein_codin putative NADH-dependent fumarate reductase
TcG_07157	62,35622684	0,114613009	0,222318383	0,515535456	0,6061789	0,787088281	protein_codin hypothetical protein
TcG_07158	141,4665779	0,012303985	0,162399654	0,075763616	0,939607163	0,972076845	protein_codin putative glucosamine-fructose-6-phosphate aminotransferase
TcG_07159	1289,645109	0,146345037	0,056497707	2,590282777	0,009589712	0,047787701	protein_codin hypothetical protein
TcG_07160	121,0867762	-0,41422757	0,174586665	-2,37261861	0,017662491	0,076385824	protein_codin ribosomal RNA processing protein 40
TcG_07161	186,9568141	0,062976964	0,136100308	0,462724626	0,643561765	0,810875005	protein_codin hypothetical protein
TcG_07162	267,5888775	-0,080195571	0,117881065	-0,68030918	0,496308712	0,706156543	protein_codin putative myosin heavy chain
TcG_07163	16,30653167	-0,276962965	0,428099286	-0,64695965	0,517658056	1	protein_codin hypothetical protein
TcG_07164	507,9042808	-0,107726379	0,08686248	-1,24019461	0,214903422	0,438821122	protein_codin hypothetical protein
TcG_07165	450,1246442	-0,19849022	0,087863747	-2,25906845	0,023879127	0,096532996	protein_codin putative RNA pseudouridylate synthase
TcG_07166	184,5538689	-0,144804226	0,132269396	-1,09476742	0,273618542	0,50688937	protein_codin hypothetical protein
TcG_07167	370,1423334	0,060446445	0,106827427	0,565832637	0,571507558	0,762692214	protein_codin hypothetical protein
TcG_07168	663,380524	-0,192315743	0,073065327	-2,63210681	0,008485718	0,043425752	protein_codin putative AMP deaminase
TcG_07169	358,732022	-0,229387802	0,096150358	-2,38571968	0,017045738	0,074440982	protein_codin oxidoreductase
TcG_07170	135,9376409	0,08618856	0,149879866	0,575050955	0,565256836	0,757905995	protein_codin hypothetical protein
TcG_07171	329,2922546	-0,083888751	0,104619475	-0,80184641	0,422641808	0,648831056	protein_codin putative mitochondrial exoribonuclease DSS-1
TcG_07172	127,9125036	-0,412625735	0,154716676	-2,66697648	0,007653703	0,039998106	protein_codin ADP-ribosylation factor
TcG_07173	161,0067368	0,088721321	0,138455786	0,640791719	0,521658014	0,725401608	protein_codin hypothetical protein
TcG_07174	126,2569236	-0,5920481	0,15794873	-3,74835619	0,000177997	0,001810603	protein_codin hypothetical protein
TcG_07175	729,4924835	-0,243155633	0,073456241	-3,31021067	0,000932258	0,007371644	protein_codin glycosyltransferase
TcG_07176	220,6466045	-0,322670419	0,120940947	-2,66799976	0,007630431	0,039939297	protein_codin hypothetical protein
TcG_07177	389,5259592	-0,155026265	0,093511244	-1,65783556	0,097350661	0,26402265	protein_codin phytanoyl-CoA dioxygenase
TcG_07178	23,91859528	-0,071110192	0,366605466	-0,19396926	0,84619995	0,925348997	protein_codin dispersed gene family protein 1 (DGF-1)
TcG_07179	1205,389233	-0,05196146	0,060464576	-0,85937029	0,390136255	0,620384114	protein_codin putative retrotransposon hot spot (RHS) protein
TcG_07180	4,065419625	0,471695745	0,873862924	0,539782307	0,58934717	1	
TcG_07181	61,82090989	0,024382132	0,220211948	0,110721206	0,91183743	0,957020424	protein_codin retrotransposon hot spot (RHS) protein
TcG_07182	69,24775735	-0,20364945	0,212661571	-0,95762224	0,33825328	0,572891338	

TcG_07183	66,12280521	0,063013317	0,221480946	0,284508978	0,776020355	0,888310845		
TcG_07184	40,34035578	0,290309825	0,277247074	1,047115923	0,295046116	0,527938889	protein_codin	hypothetical protein
TcG_07185	89,53363632	0,04037656	0,185639028	0,217500386	0,827818406	0,915880831	protein_codin	putative kinesin
TcG_07186	27,24678947	-0,113331042	0,35464819	-0,31955906	0,749302614	0,873470177	protein_codin	hypothetical protein
TcG_07187	66,07224819	-0,199273185	0,212096847	-0,93953865	0,347454259	0,581903013	protein_codin	hypothetical protein
TcG_07188	53,38885339	-0,090167691	0,24016275	-0,37544411	0,707330202	0,849060062	protein_codin	putative mitotic centromere-associated kinesin (MCAK)
TcG_07189	52,3290306	0,018820999	0,240184644	0,078360541	0,93754126	0,971240437	protein_codin	hypothetical protein
TcG_07190	150,6517506	-0,140633369	0,15152626	-0,92811219	0,353349379	0,587782615	protein_codin	retrotransposon hot spot (RHS) protein
TcG_07191	604,7524044	0,041873468	0,08119354	0,515724138	0,606047093	0,787005338	protein_codin	putative retrotransposon hot spot (RHS) protein
TcG_07192	70,98246859	0,174935646	0,210501919	0,831040624	0,405950681	0,634643717	protein_codin	hypothetical protein
TcG_07193	29,05968856	0,490928466	0,335041891	1,465274878	0,142845938	0,33915975	protein_codin	hypothetical protein
TcG_07194	70,18429072	0,036458302	0,209261055	0,17422402	0,861689409	0,933819072	protein_codin	trans-sialidase
TcG_07195	208,3901198	0,066082725	0,128981752	0,512341663	0,608411912	0,788023892	protein_codin	putative trans-sialidase
TcG_07196	51,46860269	0,043359224	0,242809062	0,178573335	0,858272734	0,931742071	protein_codin	putative trans-sialidase
TcG_07197	84,38733983	0,069925271	0,203827176	0,343061572	0,73155214	0,862124891		
TcG_07198	329,3612759	-0,001231356	0,098950758	-0,01244413	0,990071275	0,996002934	protein_codin	hypothetical protein
TcG_07199	330,2463176	-0,187700026	0,102668589	-1,82821279	0,067517623	0,206892139	protein_codin	hypothetical protein
TcG_07200	3258,88808	0,02980375	0,04720654	0,631347911	0,527813066	0,730265367	protein_codin	glutamate dehydrogenase
TcG_07201	45,26845196	0,329116282	0,262707046	1,252788176	0,210282806	0,432895627	protein_codin	putative glutamate dehydrogenase
TcG_07202	1810,475861	0,034660228	0,134766915	0,257186475	0,797034824	0,898729487	protein_codin	transferase
TcG_07203	317,4407737	0,016667641	0,102501669	0,162608488	0,870826703	0,937676411	protein_codin	solute carrier family 35, member F1/2
TcG_07204	141,8306141	-0,103347682	0,150017702	-0,68890325	0,490884157	0,701878188	protein_codin	hypothetical protein
TcG_07205	121,4909749	0,425539432	0,169917641	2,504386415	0,012266398	0,058031234	protein_codin	hypothetical protein
TcG_07206	368,0281008	0,166377527	0,096689379	1,720742539	0,085297553	0,241038403	protein_codin	putative nitrilase
TcG_07207	248,2016741	-0,299050743	0,114575774	-2,61006959	0,00905238	0,045659939	protein_codin	hypothetical protein
TcG_07208	158,2096911	-0,205085111	0,154099473	-1,33086186	0,183234468	0,395505431	protein_codin	hypothetical protein
TcG_07209	282,817876	0,020713342	0,107962391	0,191857012	0,847854211	0,926605553	protein_codin	hypothetical protein
TcG_07210	160,6089077	0,261816475	0,14581851	1,795495475	0,072574793	0,216602667	protein_codin	hypothetical protein
TcG_07211	233,3421332	0,021179514	0,122301133	0,173175126	0,862513775	0,934140118	protein_codin	hypothetical protein
TcG_07212	226,4019971	-0,109217203	0,121808767	-0,89662843	0,369917225	0,601524346	protein_codin	serine/threonine protein kinase
TcG_07213	693,7811597	0,341914412	0,074967813	4,560816179	5,09552E-06	8,50672E-05	protein_codin	60S ribosomal protein L35
TcG_07214	307,2966255	0,543297938	0,10546486	5,151459314	2,58467E-07	6,02536E-06	protein_codin	60S ribosomal protein L35
TcG_07215	148,4687797	0,063960647	0,150279907	0,425610103	0,670391977	0,827085662	protein_codin	hypothetical protein
TcG_07216	336,7896713	-0,103390743	0,102278281	-1,01087682	0,312075392	0,546014118	protein_codin	putative sec1 family transport protein
TcG_07217	66,80741279	0,026313744	0,219841167	0,119694344	0,904725281	0,953702767	protein_codin	zinc carboxypeptidase
TcG_07218	514,4294981	0,055433221	0,086415177	0,641475526	0,521213777	0,725401608	protein_codin	hypothetical protein
TcG_07219	528,1053086	0,00791747	0,086467957	0,091565366	0,92704337	0,964418109	protein_codin	hypothetical protein
TcG_07220	292,5848929	-0,127438313	0,105446097	-1,20856359	0,226830551	0,453582804	protein_codin	minichromosome maintenance protein 10
TcG_07221	247,82636	0,212765729	0,119270265	1,783895834	0,074440593	0,220296002	protein_codin	putative peroxisomal membrane protein 4
TcG_07222	412,7098387	0,131224688	0,091664952	1,431568826	0,152267256	0,352410794	protein_codin	hypothetical protein
TcG_07223	609,6764921	0,154214163	0,076641845	2,012140532	0,044205125	0,151837188	protein_codin	hypothetical protein
TcG_07224	527,3801104	0,103729332	0,081098941	1,279046688	0,200880615	0,420260529	protein_codin	protein kinase
TcG_07225	462,2763232	0,037329597	0,090643288	0,411829689	0,680464259	0,83338889	protein_codin	hypothetical protein
TcG_07226	333,2990522	0,156995653	0,099643923	1,575566753	0,115125683	0,295490954	protein_codin	putative tubulin-tyrosine ligase
TcG_07227	455,2387418	0,054213167	0,086484598	0,626853432	0,530755315	0,732224	protein_codin	vesicular-fusion protein NsF
TcG_07228	215,2302133	-0,083127276	0,121594489	-0,68364345	0,494200342	0,70453824	protein_codin	hypothetical protein
TcG_07229	253,7878906	0,266707706	0,119482455	2,232191385	0,025602317	0,101593633	protein_codin	putative cytochrome c oxidase assembly factor, putative,electron transport protein SCO1/2
TcG_07230	819,9726842	-0,087087919	0,067529276	-1,28963205	0,197178441	0,415601281	protein_codin	hypothetical protein
TcG_07231	191,3006015	0,146067217	0,131766937	1,108527074	0,267634253	0,499929984	protein_codin	hypothetical protein
TcG_07232	955,9963883	0,182678829	0,063192874	2,890813751	0,003842458	0,023320437	protein_codin	ATP-dependent DEAD/H RNA helicase
TcG_07233	192,7119675	-0,002363312	0,126950384	-0,01861603	0,985147413	0,994157123	protein_codin	hypothetical protein
TcG_07234	210,6351724	-0,018965643	0,125241825	-0,15143219	0,879634802	0,941623821	protein_codin	RNA editing complex protein MP90
TcG_07235	277,0018201	0,041294929	0,106759059	0,386804917	0,698900637	0,844142481	protein_codin	RNA-editing complex protein
TcG_07236	140,3167894	0,038729956	0,150575731	0,257212469	0,797014759	0,898729487	protein_codin	hypothetical protein
TcG_07237	404,11266	0,091001167	0,09200941	0,98904196	0,322642613	0,557348637	protein_codin	protein AATF/BFR2
TcG_07238	749,4931153	0,04972178	0,075292444	0,66038207	0,509008675	0,715750872	protein_codin	putative helicase
TcG_07239	307,1662741	0,086641837	0,114591764	0,756091312	0,449594451	0,670442053	protein_codin	putative cyclophilin 15

TcG_07240	624,9020196	-0,162433591	0,074740491	-2,17330108	0,029757664	0,113673689	protein_codin hypothetical protein
TcG_07241	86,16801341	0,266892961	0,211823483	1,259978154	0,207677243	0,42982289	protein_codin putative protein kinase
TcG_07242	2919,069607	0,281116079	0,042803582	6,567583102	5,11385E-11	2,74301E-09	protein_codin cystathionine beta-synthase 6
TcG_07243	602,4272172	0,030732305	0,079841295	0,384917421	0,700298599	0,844381264	protein_codin putative ubiquitin activating enzyme
TcG_07244	542,8076372	0,47431599	0,081183979	5,842482639	5,14285E-09	1,74225E-07	protein_codin hypothetical protein
TcG_07245	218,6887336	0,177411263	0,12683423	1,398764855	0,161883509	0,365610591	protein_codin hypothetical protein
TcG_07246	1900,684876	0,021732913	0,050186332	0,433044451	0,664982477	0,824891539	protein_codin hypothetical protein
TcG_07247	426,014542	0,214596073	0,108033392	1,986386514	0,046990423	0,158402979	protein_codin helicase-like protein
TcG_07248	333,6666765	0,035260443	0,098885994	0,356576719	0,721408709	0,857117481	protein_codin hypothetical protein
TcG_07249	487,4285179	-0,01706715	0,086608684	-0,19706049	0,8437802	0,924387794	protein_codin hypothetical protein
TcG_07250	2743,669074	-0,160619273	0,046536583	-3,45146255	0,000557557	0,00475689	protein_codin hypothetical protein
TcG_07251	110,3101957	0,020926781	0,1664835	0,12569883	0,899970328	0,951373743	protein_codin hypothetical protein
TcG_07252	438,0077915	-0,094395268	0,086763938	-1,08795509	0,276614934	0,509011914	protein_codin Phosphoribosylamine--glycine ligase
TcG_07253	110,6949815	0,209282487	0,175566379	1,19204194	0,233244801	0,460605806	protein_codin carbohydrate kinase, thermoresistant glucokinase
TcG_07254	664,1381886	-0,437452178	0,074066154	-5,90623593	3,50013E-09	1,2326E-07	protein_codin flagellar associated protein
TcG_07255	67,02416884	0,13878183	0,213785577	0,649163669	0,516232592	0,722055158	protein_codin putative solute carrier family 12 member 9-like
TcG_07256	461,9625829	-0,269639723	0,089203847	-3,02273647	0,002505003	0,016443652	protein_codin putative electron-transfer-flavoprotein, alpha polypeptide
TcG_07257	406,6396768	0,003900529	0,090902978	0,042908701	0,965774313	0,984901081	protein_codin putative long-chain-fatty-acid-CoA ligase
TcG_07258	386,3762203	-0,559685106	0,093379744	-5,99364578	2,05188E-09	7,61958E-08	protein_codin hypothetical protein
TcG_07259	389,6053005	-0,014709263	0,096247361	-0,1528277	0,878534152	0,941484386	protein_codin hypothetical protein
TcG_07260	258,7064863	0,244638506	0,117997961	2,073243497	0,038149624	0,136546662	protein_codin putative cystinosin
TcG_07261	53,08483718	0,334305869	0,240977901	1,387288497	0,165353809	0,370845768	protein_codin hypothetical protein
TcG_07262	67,87679217	0,481329076	0,224017041	2,148627062	0,031663975	0,118955516	protein_codin hypothetical protein
TcG_07263	282,2717181	0,239079963	0,114842664	2,08180439	0,037360341	0,134594809	protein_codin hypothetical protein
TcG_07264	142,3795218	0,293651525	0,153250935	1,916148339	0,055346217	0,177776897	protein_codin trans-sialidase
TcG_07265	357,5511093	0,272963297	0,09907041	2,755245451	0,005864812	0,032465223	protein_codin Tbingi protein
TcG_07266	149,0281923	-0,191231739	0,152714489	-1,25221739	0,21049066	0,433024666	protein_codin hypothetical protein
TcG_07267	732,3306599	-0,512381906	0,070642482	-7,25316959	4,0713E-13	3,32184E-11	protein_codin putative UDP-Gal or UDP-GlcNAc-dependent glycosyltransferase
TcG_07268	97,144149	0,315922909	0,179861195	1,756481759	0,079006183	0,228955888	protein_codin hypothetical protein
TcG_07269	31,03060104	0,17511171	0,31736759	0,551763051	0,581110705	0,770482335	protein_codin hypothetical protein
TcG_07270	1049,689186	0,150509848	0,071335308	2,10989272	0,034867597	0,128195613	protein_codin hypothetical protein
TcG_07271	250,9115743	-0,139966784	0,117132794	-1,19494104	0,232110065	0,459225958	protein_codin hypothetical protein
TcG_07272	145,9384682	0,074725705	0,154628787	0,483258689	0,628912069	0,801074673	protein_codin hypothetical protein
TcG_07273	600,9285334	0,033747141	0,079319004	0,425460979	0,670500662	0,827131672	protein_codin hypothetical protein
TcG_07274	257,5737119	-0,230773893	0,117242038	-1,9683545	0,049027264	0,163197375	protein_codin hypothetical protein
TcG_07275	62,53342757	0,134180456	0,226249064	0,593065243	0,55313749	0,748324493	protein_codin hypothetical protein
TcG_07276	42,80630211	0,138182974	0,267411507	0,516742811	0,605335707	0,786698766	protein_codin hypothetical protein
TcG_07277	148,0118414	0,165351413	0,144904723	1,141104369	0,253826493	0,484886027	
TcG_07278	229,9518745	0,124718489	0,116997204	1,065995469	0,28642569	0,519656757	protein_codin hypothetical protein
TcG_07279	200,4953505	-0,272733717	0,128322471	-2,12537769	0,03355511	0,124286926	protein_codin hypothetical protein
TcG_07280	133,0161284	-0,046093388	0,156658396	-0,29422865	0,768583192	0,884202647	protein_codin hypothetical protein
TcG_07281	207,2289782	-0,097610374	0,123602713	-0,78971061	0,429696791	0,654716863	protein_codin hypothetical protein
TcG_07282	279,8574639	-0,104409323	0,109889478	-0,9501303	0,342046047	0,576512293	protein_codin hypothetical protein
TcG_07283	126,7277301	0,026253169	0,167546107	0,156692208	0,875487428	0,93998678	protein_codin hypothetical protein
TcG_07284	379,5626453	-0,063259493	0,102668336	-0,61615387	0,537792968	0,73738082	protein_codin hypothetical protein
TcG_07285	226,3598428	0,160701134	0,120896147	1,329249422	0,183765695	0,396334576	protein_codin putative ABC transporter
TcG_07286	46,23402384	-0,232910572	0,258135132	-0,90228157	0,366907314	0,599237121	protein_codin putative ABC transporter
TcG_07287	193,583788	0,360354532	0,137498734	2,620784358	0,008772773	0,044599101	protein_codin hypothetical protein
TcG_07288	297,9980296	0,310320544	0,108662223	2,8558273	0,004292485	0,025543265	protein_codin microtubule-associated protein Gb4
TcG_07289	65,04327642	0,093928248	0,231118238	0,406407773	0,684443009	0,836228694	
TcG_07290	7,330900569	0,013276097	0,632021949	0,021005753	0,983241066	1	
TcG_07291	121,7467621	0,140012939	0,168086539	0,832981274	0,404855291	0,634151194	protein_codin hypothetical protein
TcG_07292	166,8378948	0,155015062	0,140828963	1,10073283	0,271012954	0,50430483	protein_codin methyltransferase
TcG_07293	136,820408	0,141485753	0,156352206	0,904916898	0,365509414	0,597692344	protein_codin hypothetical protein
TcG_07294	404,4918339	0,073971748	0,092474817	0,799912347	0,423761584	0,649689256	protein_codin hypothetical protein
TcG_07295	1072,577702	-0,062907204	0,061514616	-1,02263834	0,306478875	0,539556179	protein_codin transferase
TcG_07296	47,88781081	-0,362682403	0,255026691	-1,42213508	0,15498704	0,35638871	protein_codin putative protein kinase-like protein

TcG_07297	381,938904	-0,117579631	0,093842577	-1,25294546	0,210225557	0,432895627	protein_codin putative serine/threonine protein phosphatase-like protein
TcG_07298	172,7218888	-0,161985468	0,138703843	-1,16785133	0,242866739	0,471174488	protein_codin hypothetical protein
TcG_07299	335,1813521	0,134716054	0,104895663	1,284286208	0,199041831	0,417847192	protein_codin putative SUMO1/Ulp2
TcG_07300	113,7179152	-0,611435168	0,164755976	-3,71115624	0,000206315	0,002040925	protein_codin putative protein kinase
TcG_07301	234,1915475	0,577669616	0,119797528	4,822049528	1,42091E-06	2,75756E-05	protein_codin hypothetical protein
TcG_07302	274,2895574	-0,21849635	0,110018933	-1,98598863	0,047034587	0,158459645	protein_codin hypothetical protein
TcG_07303	269,0867506	0,26537264	0,111375996	2,382673564	0,017187428	0,074862232	protein_codin hypothetical protein
TcG_07304	359,9522946	0,262235824	0,110147538	2,380768813	0,017276551	0,075165647	
TcG_07305	225,5979755	0,369906305	0,125287108	2,952469021	0,003152437	0,019860867	protein_codin hypothetical protein
TcG_07306	301,154448	0,08747581	0,105838193	0,826505134	0,408517607	0,636605346	protein_codin hypothetical protein
TcG_07307	1906,053353	0,326628353	0,052553249	6,215188563	5,12631E-10	2,19975E-08	protein_codin putative serine/threonine protein kinase
TcG_07308	404,1366793	0,209376026	0,090852926	2,304560076	0,021191224	0,088040307	protein_codin hypothetical protein
TcG_07309	852,1020669	-0,123628239	0,071288718	-1,7341908	0,082884234	0,23675955	protein_codin hypothetical protein
TcG_07310	281,7548374	0,283528977	0,10779797	2,630188452	0,008533755	0,04363287	protein_codin hypothetical protein
TcG_07311	769,835204	0,247352777	0,072209935	3,425467394	0,000613743	0,005126764	
TcG_07312	230,5626519	-0,033869367	0,129052816	-0,26244578	0,79297778	0,896248225	protein_codin hypothetical protein
TcG_07313	27,00559651	0,336030158	0,342324064	0,981614188	0,326289955	0,560721119	protein_codin hypothetical protein
TcG_07314	146,4091107	0,123605253	0,145188931	0,851340744	0,394580098	0,624314965	protein_codin hypothetical protein
TcG_07315	563,7190705	0,082136319	0,08060246	1,019029936	0,308188755	0,541260203	protein_codin regulator of nonsense transcripts 1 isoform X2
TcG_07316	127,6338966	0,035120744	0,153857876	0,228267445	0,819438331	0,910871391	protein_codin oligoribonuclease
TcG_07317	782,6398014	-0,422616714	0,070539933	-5,99116978	2,08337E-09	7,7118E-08	protein_codin hypothetical protein
TcG_07318	319,807868	-0,305544079	0,121724384	-2,51013042	0,012068658	0,057282864	
TcG_07319	158,2497164	0,083757651	0,154822592	0,540991145	0,588513686	0,775648564	protein_codin hypothetical protein
TcG_07320	237,6799594	0,080272148	0,119864588	0,669690266	0,503055258	0,711561252	protein_codin hypothetical protein
TcG_07321	251,5631828	-0,037979113	0,119522542	-0,31775691	0,750669341	0,874535443	protein_codin putative OSM3-like kinesin
TcG_07322	756,7573882	-0,018796303	0,069532251	-0,27032496	0,786910264	0,893925122	protein_codin putative guanosine monophosphate reductase
TcG_07323	420,1071715	0,157154667	0,095446767	1,646516399	0,099657487	0,268020344	protein_codin hypothetical protein
TcG_07324	311,3959451	0,142542294	0,106026428	1,344403436	0,178817963	0,389799325	protein_codin putative cell division control protein
TcG_07325	117,8543009	-0,0246098	0,164122738	-0,14994753	0,880806009	0,942069315	protein_codin hypothetical protein
TcG_07326	212,3331697	-0,128515508	0,128602122	-0,9993265	0,317636552	0,552739124	protein_codin hypothetical protein
TcG_07327	142,7259296	0,483182438	0,148944322	3,244047395	0,001178441	0,008919254	protein_codin hypothetical protein
TcG_07328	531,5246629	0,006192118	0,082214446	0,075316667	0,939962761	0,972184302	protein_codin hypothetical protein
TcG_07329	283,6931414	0,281584673	0,110223171	2,554677656	0,010628618	0,051806127	protein_codin Protein kinase domain
TcG_07330	305,9437071	0,272234075	0,109779952	2,479815935	0,013145022	0,061336377	protein_codin hypothetical protein
TcG_07331	12,69267343	0,107149292	0,503393622	0,212853893	0,831440926	1	protein_codin hypothetical protein
TcG_07332	73,49943207	0,277993701	0,226702734	1,226247678	0,220105484	0,44520638	protein_codin hypothetical protein
TcG_07333	0					1	
TcG_07334	131,9090807	0,13819407	0,158861577	0,869902415	0,384353735	0,614562845	protein_codin histone-lysine N-methyltransferase
TcG_07335	709,2894428	0,039816936	0,076885715	0,517871696	0,604547791	0,786203919	protein_codin hypothetical protein
TcG_07336	150,7390128	-0,023204244	0,146155406	-0,15876418	0,873854668	0,939417284	protein_codin hypothetical protein
TcG_07337	100,8310917	-0,14111681	0,178936828	-0,78864039	0,430322216	0,655193917	protein_codin hypothetical protein
TcG_07338	425,479729	-0,001763325	0,088176281	-0,01999773	0,984045187	0,993987719	protein_codin hypothetical protein
TcG_07339	212,5333154	-0,168243412	0,121404926	-1,38580383	0,165806812	0,371358539	protein_codin putative ubiquitin ligase
TcG_07340	213,4962794	-0,030741616	0,122305793	-0,25135045	0,801543171	0,901531811	protein_codin hypothetical protein
TcG_07341	428,5428925	-0,208203865	0,087358614	-2,38332382	0,017157095	0,074758218	protein_codin serine/threonine-protein kinase HSL1,negative regulator of Swe1 kinase
TcG_07342	323,3370303	-0,094029551	0,101064959	-0,93038727	0,35217061	0,586411136	protein_codin adenosinetriphosphatase
TcG_07343	1133,955828	-0,097436708	0,061566459	-1,58262648	0,113506625	0,292375371	protein_codin putative nucleolar GTP-binding protein
TcG_07344	222,7270167	0,085189997	0,119814814	0,711013896	0,477075625	0,692136011	protein_codin putative DNA-damage inducible protein DDI1-like protein
TcG_07345	194,5263341	-0,094067323	0,126681	-0,74255273	0,457752515	0,67716045	protein_codin hypothetical protein
TcG_07346	683,894305	0,1693892	0,077845835	2,175957153	0,029558465	0,113061859	protein_codin putative leucine-rich repeat protein
TcG_07347	215,4640167	-0,091244143	0,133003536	-0,68602795	0,492695482	0,70326104	protein_codin lisH domain-containing protein C16orf63
TcG_07348	163,2271148	-0,126745892	0,137628241	-0,92092939	0,357087293	0,590864907	protein_codin vesicular protein trafficking mediator
TcG_07349	523,5054433	-0,064407249	0,08189446	-0,7864665	0,43159424	0,655968892	protein_codin putative peroxisomal targeting signal type 2 receptor
TcG_07350	230,4001221	0,108829961	0,118533113	0,918139739	0,358545712	0,592428783	protein_codin hypothetical protein
TcG_07351	227,0761609	0,023989444	0,11963719	0,200518284	0,84107526	0,922466573	protein_codin putative Trichohyalin
TcG_07352	78,35555359	0,128866105	0,205993804	0,625582433	0,531588864	0,732689576	protein_codin hypothetical protein
TcG_07353	785,4425495	0,138374264	0,068168368	2,029889638	0,042367759	0,14714414	protein_codin putative phosphoribosylpyrophosphate synthetase

TcG_07354	300,4398761	0,102952908	0,103750155	0,992315694	0,32104355	0,556034397	protein_codin hypothetical protein
TcG_07355	210,4251833	0,151041624	0,126216423	1,19668756	0,231428358	0,458660443	protein_codin hypothetical protein
TcG_07356	53,52301436	0,242399888	0,271501763	0,892811468	0,371958144	0,60323447	protein_codin putative RNA editing complex protein MP46
TcG_07357	144,69152	0,102764485	0,154040432	0,667126704	0,504691204	0,71221932	protein_codin RNA editing complex protein MP46
TcG_07358	17,45060354	0,612121321	0,435842274	1,404456056	0,160183089	0,363061588	protein_codin hypothetical protein
TcG_07359	151,2386208	0,221670443	0,14566922	1,521738377	0,128074648	0,316931411	protein_codin hypothetical protein
TcG_07360	319,8811901	0,117169643	0,100182359	1,169563622	0,242176624	0,470898628	protein_codin cullin
TcG_07361	385,2349199	0,354038856	0,093518539	3,785761182	0,000153239	0,001600924	protein_codin TFIIH basal transcription factor complex helicase subunit
TcG_07362	112,9325701	0,371815296	0,1786172	2,081632096	0,037376087	0,134609682	protein_codin putative zinc-finger multi-pass transmembrane protein
TcG_07363	338,3218818	0,394053402	0,102961093	3,827206862	0,000129606	0,001386529	protein_codin putative notchless-like protein
TcG_07364	262,2153854	0,206245789	0,111944281	1,842396832	0,065417125	0,20216666	protein_codin fatty acid desaturase
TcG_07365	772,6200002	0,124665177	0,071917431	1,733448699	0,083015948	0,236811141	protein_codin fatty acid desaturase
TcG_07366	162,1512091	0,36371799	0,139621279	2,605032643	0,009186551	0,046236045	protein_codin cob(I)alamin adenosyltransferase
TcG_07367	1171,466613	0,431996327	0,059801109	7,223884884	5,0523E-13	4,04667E-11	protein_codin hypothetical protein
TcG_07368	377,6298953	0,676601321	0,100723596	6,717406349	1,84988E-11	1,08246E-09	protein_codin hypothetical protein
TcG_07369	1389,626034	0,597679398	0,057187744	10,45117987	1,44714E-25	4,65736E-23	protein_codin 60S ribosomal protein L12
TcG_07370	195,3107366	0,521432963	0,132952669	3,921944321	8,78373E-05	0,000998707	protein_codin 2-amino-3-ketobutyrate coenzyme A ligase
TcG_07371	527,2968505	0,401131203	0,082261324	4,876303746	1,08092E-06	2,1667E-05	protein_codin hypothetical protein
TcG_07372	380,2520701	0,340055369	0,094112058	3,613302849	0,000302321	0,002824753	protein_codin hypothetical protein
TcG_07373	1021,768997	0,758998787	0,065401361	11,60524454	3,87578E-31	2,24524E-28	protein_codin putative 2-amino-3-ketobutyrate coenzyme A ligase
TcG_07374	42,94702577	0,811422089	0,282571355	2,871565273	0,004084444	0,024506665	protein_codin 2-amino-3-ketobutyrate coenzyme A ligase
TcG_07375	1022,18758	0,220706017	0,0637483	3,462147486	0,000535883	0,004605894	protein_codin putative basal body component
TcG_07376	492,6277989	0,354026018	0,084953336	4,16729976	3,08229E-05	0,000404433	protein_codin hypothetical protein
TcG_07377	190,2348527	0,189134524	0,131490185	1,438392715	0,150322659	0,350359751	protein_codin glycerophosphoryl diester phosphodiesterase
TcG_07378	77,17534212	0,54208298	0,201394169	2,691651818	0,007109913	0,037745541	protein_codin hypothetical protein
TcG_07379	129,3956902	0,66951189	0,164581581	4,067963658	4,74258E-05	0,000590833	protein_codin acid--amino-acid ligase
TcG_07380	361,6483372	0,257293899	0,10332211	2,490211417	0,012766713	0,059860435	protein_codin ubiquitin-conjugating enzyme E2
TcG_07381	1630,850573	0,301753575	0,053921603	5,596153604	2,1916E-08	6,51072E-07	protein_codin hydroxymethylglutaryl-CoA synthase
TcG_07382	123,7476815	0,574356864	0,175375906	3,275004396	0,001056603	0,0081721	protein_codin tuzin
TcG_07383	188,1379417	0,284641003	0,129332129	2,200852991	0,027746433	0,107912108	protein_codin tuzin
TcG_07384	577,9346813	0,222095394	0,076948948	2,886269409	0,003898383	0,023606182	protein_codin amastin
TcG_07385	125,6952122	0,717197446	0,165548794	4,332242048	1,47599E-05	0,000213146	protein_codin hypothetical protein
TcG_07386	113,5503978	0,483174416	0,166280937	2,905771554	0,003663488	0,022410334	protein_codin hypothetical protein
TcG_07387	234,8466863	0,074042087	0,122278027	0,605522422	0,544831931	0,741887014	protein_codin kelch-containing protein
TcG_07388	685,8976167	-0,194570522	0,072406032	-2,68721427	0,007205072	0,038170078	protein_codin eukaryotic translation initiation factor eIF-4E
TcG_07389	390,2722883	-0,401083892	0,094181531	-4,25862574	2,05688E-05	0,00028404	protein_codin flagellar inner dynein arm I1 intermediate chain IC140
TcG_07390	533,6343728	-0,359528098	0,080806117	-4,44926836	8,61633E-06	0,000135086	protein_codin programmed cell death 6-interacting protein
TcG_07391	137,5267193	-0,240811058	0,151622456	-1,58822819	0,112234749	0,290062861	protein_codin hypothetical protein
TcG_07392	422,0779509	-0,219548624	0,092430041	-2,3752951	0,017534925	0,075975931	protein_codin hypothetical protein
TcG_07393	521,6722854	-0,080664297	0,080752096	-0,99891274	0,317836964	0,553004816	protein_codin hypothetical protein
TcG_07394	200,1297677	-0,197784879	0,128939732	-1,53393276	0,125046195	0,311700779	protein_codin putative ABC transporter
TcG_07395	787,8696601	-0,106461815	0,067665419	-1,57335633	0,115636333	0,296014704	protein_codin putative eukaryotic translation release factor
TcG_07396	342,3972741	-0,108595912	0,103983794	-1,0443542	0,296321544	0,529321832	protein_codin putative RNA-binding protein
TcG_07397	451,2023667	-0,121894735	0,086693985	-1,40603452	0,159713875	0,362405984	protein_codin WASH complex subunit strumpellin
TcG_07398	203,2616099	0,078469577	0,126000621	0,622771355	0,533434785	0,734098517	protein_codin putative ubiquitin activating enzyme
TcG_07399	514,545866	-0,063436413	0,084595254	-0,74988147	0,453326097	0,673824437	protein_codin hypothetical protein
TcG_07400	376,8489507	-0,115889605	0,096980965	-1,1949727	0,232097698	0,459225958	protein_codin nuclear RNA export factor 1/2
TcG_07401	199,8720518	0,01235409	0,134568362	0,091805307	0,926852728	0,964306367	protein_codin hypothetical protein
TcG_07402	694,0798191	-0,256257685	0,072906673	-3,51487286	0,000439965	0,003885236	protein_codin putative phenylalanyl-tRNA synthetase
TcG_07403	368,3695003	-0,257547256	0,098486658	-2,61504716	0,008921514	0,045193979	protein_codin nuclear RNA export factor 1/2
TcG_07404	478,9851923	-0,212281226	0,086520152	-2,45354661	0,01414552	0,06498414	protein_codin 3-phosphoglycerate kinase, glycosomal
TcG_07405	301,9369156	-0,307396634	0,104437857	-2,94334489	0,003246865	0,020323163	protein_codin hypothetical protein
TcG_07406	353,1324504	0,127600863	0,101243432	1,260337198	0,207547747	0,4297852	protein_codin hypothetical protein
TcG_07407	166,8082238	0,058577846	0,136872975	0,427972331	0,668671265	0,826307698	protein_codin hypothetical protein
TcG_07408	248,4852313	0,172813893	0,113316358	1,525056895	0,127244907	0,315619674	protein_codin hypothetical protein
TcG_07409	254,7318655	0,353909677	0,11605064	3,049614087	0,002291356	0,015363223	protein_codin hypothetical protein
TcG_07410	245,9193065	0,017293488	0,118632861	0,145773165	0,884100461	0,943488844	protein_codin putative variant surface glycoprotein

TcG_07411	904,0455761	0,049278023	0,071827993	0,686055956	0,492677824	0,70326104	protein_codin putative mismatch repair protein MSH2
TcG_07412	275,6879769	0,003572702	0,107906983	0,033109094	0,973587591	0,988345381	protein_codin putative serine peptidase, putative,serine peptidase, clan SC, family S9D
TcG_07413	283,1356924	-0,230953635	0,109198481	-2,11498945	0,034430846	0,126962376	protein_codin hypothetical protein
TcG_07414	302,7311247	0,16602857	0,104505457	1,588707182	0,112126517	0,289898887	protein_codin hypothetical protein
TcG_07415	559,9273339	0,107633827	0,078972363	1,362930297	0,172904479	0,381091585	protein_codin putative WD40 repeat protein
TcG_07416	20146,94797	0,166882754	0,032540383	5,128481632	2,92088E-07	6,7279E-06	protein_codin Hsp90
TcG_07417	601,4595254	-0,147635876	0,076032948	-1,94173553	0,052169126	0,170262391	protein_codin ATP-binding cassette protein subfamily F, member 3
TcG_07418	116,5463096	-0,690463148	0,171501852	-4,0259807	5,67383E-05	0,000687687	protein_codin putative protein kinase
TcG_07419	0,770504906	-1,951463823	2,034715119	-0,95908454	0,337516156	1	
TcG_07420	0,4558669	-0,458748245	2,666213369	-0,17205984	0,863390485	1	
TcG_07421	0					1	
TcG_07422	0,169119941	-1,420531124	3,989964164	-0,35602604	0,721821067	1	
TcG_07423	0,155988004	0,503022807	4,080472857	0,123275616	0,901888849	1	
TcG_07424	299,9232835	-0,417485365	0,106701959	-3,91263074	9,12961E-05	0,001032965	protein_codin hypothetical protein
TcG_07425	328,0496598	-0,488821954	0,099553079	-4,91016408	9,10002E-07	1,86938E-05	protein_codin hypothetical protein
TcG_07426	142,6804738	-0,085328825	0,15718404	-0,54285935	0,587226647	0,775633024	protein_codin hypothetical protein
TcG_07427	22,57242138	0,024983175	0,366425177	0,068180836	0,945641682	0,974694377	protein_codin hypothetical protein
TcG_07428	248,8440419	-0,392193802	0,113943501	-3,44200239	0,000577425	0,004890386	protein_codin RNA-binding protein RGGm
TcG_07429	208,1979047	-0,149746342	0,136310553	-1,09856749	0,271956763	0,505040461	protein_codin hypothetical protein
TcG_07430	451,3614374	0,716763853	0,09034503	7,933627952	2,12835E-15	2,39408E-13	protein_codin thiol transferase Tc52
TcG_07431	218,7362969	0,203966692	0,121098938	1,684297938	0,092124104	0,2537919	protein_codin putative adenosine deaminase-like protein
TcG_07432	22,47294413	-0,407505633	0,38535103	-1,057492	0,290287102	0,523456792	protein_codin hypothetical protein
TcG_07433	184,9081423	-0,216337569	0,130396248	-1,65907818	0,097100039	0,263528005	protein_codin hypothetical protein
TcG_07434	798,0381338	0,020463875	0,077619069	0,263644943	0,792053523	0,896041689	protein_codin putative myosin heavy chain
TcG_07435	226,1352817	-0,098158073	0,11911617	-0,8240533	0,409909273	0,636890088	protein_codin tyrosine phosphatase
TcG_07436	210,6232587	-0,171960349	0,121303224	-1,4176074	0,156305418	0,357690793	protein_codin tRNA wybutosine-synthesizing protein 2
TcG_07437	468,6926961	-0,284058828	0,088550426	-3,20787646	0,001337189	0,009874235	protein_codin hypothetical protein
TcG_07438	485,9807008	-0,581474869	0,088767697	-6,55052329	5,73358E-11	3,04396E-09	protein_codin glycerol-3-phosphate acyl transferase
TcG_07439	388,359423	-0,036312664	0,093842649	-0,38695268	0,698791242	0,844142481	protein_codin putative DNA primase large subunit
TcG_07440	214,9416409	-0,114729367	0,128023908	-0,89615579	0,370169564	0,601850206	protein_codin cytochrome C oxidase assembly protein
TcG_07441	442,9102051	-0,053377034	0,089113474	-0,59897827	0,549187378	0,745592332	protein_codin 62 kDa protein Tc-1
TcG_07442	405,1992366	-0,219060834	0,090938114	-2,40890013	0,016000676	0,071055513	protein_codin putative leucine-rich repeat protein (LRRP)
TcG_07443	207,6695984	-0,056021608	0,127197307	-0,44043077	0,65962514	0,821235426	protein_codin putative acetyltransferase
TcG_07444	118,8889409	-0,232752497	0,162638994	-1,43109897	0,152401852	0,352651859	protein_codin hypothetical protein
TcG_07445	648,1936928	-0,20297439	0,074722621	-2,7163714	0,006600183	0,035683492	protein_codin ATP-binding cassette protein subfamily F, member 1
TcG_07446	202,9648615	0,185878141	0,124345326	1,494854263	0,134952481	0,326830988	protein_codin hypothetical protein
TcG_07447	528,328407	-0,04948953	0,082371179	-0,60081123	0,547965722	0,74465027	protein_codin putative U2 splicing auxiliary factor
TcG_07448	616,5989457	0,176096168	0,077886428	2,260935218	0,023763269	0,096266166	protein_codin putative delta-1-pyrroline-5-carboxylate dehydrogenase
TcG_07449	1,054984978	1,009369208	1,705728077	0,591752708	0,554016196	1	protein_codin metaciclina III
TcG_07450	395,6092531	0,039008843	0,103756409	0,375965621	0,706942455	0,848924953	protein_codin putative protein kinase
TcG_07451	291,5275042	0,089367245	0,106607144	0,838285712	0,401870258	0,63220648	protein_codin hypothetical protein
TcG_07452	553,4142871	-0,455417024	0,085493518	-5,32691873	9,98928E-08	2,58917E-06	protein_codin putative long-chain-fatty-acid-CoA ligase
TcG_07453	148,6484912	0,047474868	0,14467081	0,328157895	0,742792282	0,86920426	protein_codin hypothetical protein
TcG_07454	1099,807693	0,126588083	0,06529695	1,938652309	0,052543689	0,171099265	
TcG_07455	351,2720932	-0,075079177	0,103839647	-0,72302997	0,46966147	0,686191399	protein_codin hypothetical protein
TcG_07456	66,24801163	0,048567416	0,211915854	0,22918255	0,818727035	0,910692341	protein_codin structural maintenance of chromosome protein 4
TcG_07457	268,9455136	-0,235072518	0,121178281	-1,93988985	0,052393078	0,170800843	protein_codin phosphatidylinositol 3-related kinase
TcG_07458	94,17783596	0,323938146	0,197980292	1,636214099	0,101794787	0,272188876	protein_codin L1Tc protein
TcG_07459	106,0355508	0,14582946	0,175627351	0,830334561	0,406349653	0,634833846	
TcG_07460	91,41166151	0,083912516	0,197876298	0,424065526	0,671518026	0,827770911	protein_codin hypothetical protein
TcG_07461	36,38426223	0,328515355	0,28585736	1,149228255	0,250461873	0,480120989	protein_codin hypothetical protein
TcG_07462	138,3460343	-0,053027537	0,155270426	-0,3415173	0,73271418	0,862784475	protein_codin hypothetical protein
TcG_07463	37,24237647	-0,015752373	0,28970282	-0,05437425	0,956636996	0,979981984	protein_codin hypothetical protein
TcG_07464	33,82707503	-0,055641608	0,300681545	-0,18505162	0,853188546	0,928875734	
TcG_07465	487,0851169	0,086083979	0,083058285	1,036428559	0,300002252	0,533675126	protein_codin diphosphomevalonate decarboxylase
TcG_07466	228,6207486	-0,218681774	0,126056134	-1,73479677	0,082776809	0,23662771	protein_codin hypothetical protein
TcG_07467	366,3479244	-0,304620001	0,104442263	-2,91663539	0,003538291	0,021775091	protein_codin putative transporter, putative,major facilitator superfamily protein (MFS)

TcG_07468	221,6353368	-0,049622879	0,119210519	-0,41626259	0,677217864	0,830906086	protein_codin hypothetical protein
TcG_07469	119,2850982	-0,423915095	0,168922067	-2,50953059	0,012089174	0,057356747	protein_codin hypothetical protein
TcG_07470	447,9243779	-0,551262273	0,088882393	-6,20215379	5,56956E-10	2,35507E-08	protein_codin hypothetical protein
TcG_07471	30,8238405	0,104457304	0,311146799	0,335717109	0,737084203	0,865584591	
TcG_07472	356,8718435	-0,219890209	0,095901318	-2,29287995	0,021854918	0,090078646	protein_codin NADH dehydrogenase
TcG_07473	1225,919081	-0,416747632	0,058184578	-7,16251014	7,9213E-13	6,07789E-11	protein_codin putative alpha glucosidase II subunit
TcG_07474	230,1634303	-0,334273183	0,117367472	-2,84809051	0,004398241	0,026078825	protein_codin hypothetical protein
TcG_07475	11,23615066	-0,741021282	0,52998089	-1,39820378	0,162051883	1	protein_codin hypothetical protein
TcG_07476	151,099804	0,016180911	0,14758763	0,10963596	0,91269809	0,957238745	protein_codin putative ARF-like 2-binding protein
TcG_07477	83,0078768	-0,25914349	0,20564997	-1,26011927	0,207626339	0,429794312	protein_codin hypothetical protein
TcG_07478	282,6135389	-0,211880007	0,110146666	-1,92361706	0,054402601	0,175622326	protein_codin hypothetical protein
TcG_07479	378,2778336	-0,151310305	0,101848029	-1,48564785	0,13737229	0,330412156	protein_codin putative receptor-type adenylate cyclase GRESAG 4
TcG_07480	527,4013571	-0,318827307	0,08174716	-3,90016372	9,61277E-05	0,001078156	protein_codin serine/threonine protein phosphatase type 5
TcG_07481	588,6861014	-0,425366805	0,07860961	-5,41112985	6,26283E-08	1,69535E-06	protein_codin putative lipase domain protein
TcG_07482	85,55740926	-0,000204319	0,203114377	-0,00100593	0,999197383	0,99981271	protein_codin hypothetical protein
TcG_07483	108,3496795	0,05186048	0,171013138	0,303254364	0,761696022	0,879686016	protein_codin hypothetical protein
TcG_07484	59,02252048	0,169718645	0,236406706	0,717909603	0,472813026	0,688624981	protein_codin hypothetical protein
TcG_07485	69,17014051	0,211676141	0,21259408	0,9956822	0,319404581	0,554400221	protein_codin hypothetical protein
TcG_07486	58,32031048	0,058212595	0,236682092	0,245952679	0,805718864	0,904209488	protein_codin hypothetical protein
TcG_07487	98,90329202	-0,030030353	0,176185973	-0,1704469	0,864658692	0,934985064	
TcG_07488	1505,636701	-0,210369518	0,063523855	-3,31166171	0,000927436	0,007344686	protein_codin hypothetical protein
TcG_07489	728,91616	-0,269589928	0,070069598	-3,84745932	0,000119349	0,001297166	protein_codin hypothetical protein
TcG_07490	54,56860103	-0,479456216	0,241818835	-1,98270832	0,04740002	0,159181631	
TcG_07491	500,8298132	-0,291556176	0,085947373	-3,39226397	0,000693176	0,005707989	protein_codin hypothetical protein
TcG_07492	211,4889828	-0,033328739	0,125099572	-0,26641769	0,789917542	0,895024708	protein_codin abhydrolase domain-containing protein 11
TcG_07493	198,5369252	-0,466661817	0,129574201	-3,60150256	0,000316383	0,002929937	protein_codin calmodulin
TcG_07494	358,7561894	0,052160184	0,09834215	0,530394998	0,595838094	0,780668859	protein_codin hypothetical protein
TcG_07495	231,9392646	-0,443037319	0,123669322	-3,5824351	0,000340406	0,003117744	protein_codin outer arm dynein-like
TcG_07496	232,3779027	-0,248714912	0,118117257	-2,10566108	0,035233803	0,129183176	protein_codin putative adenylosuccinate synthetase
TcG_07497	7,150584345	1,123138699	0,672977794	1,668909003	0,095135415	1	protein_codin hypothetical protein
TcG_07498	313,5753913	-0,078134345	0,103945886	-0,7516829	0,452241775	0,673133763	protein_codin hypothetical protein
TcG_07499	711,2205623	-0,220263772	0,070135811	-3,1405322	0,001686412	0,011990791	protein_codin putative nucleobase transporter
TcG_07500	53,5406895	0,220136124	0,247626074	0,888986045	0,37401058	0,604496256	protein_codin hypothetical protein
TcG_07501	3223,619272	0,082083018	0,043654117	1,880304189	0,060066633	0,189678933	protein_codin 40S ribosomal protein S4
TcG_07502	271,822792	0,200750475	0,113034441	1,776011568	0,07573101	0,222469444	protein_codin putative thiamine pyrophosphokinase
TcG_07503	2153,654373	-0,333912647	0,04765926	-7,00624911	2,44792E-12	1,73997E-10	protein_codin aminopeptidase-like protein
TcG_07504	125,7318647	-0,229850163	0,158742808	-1,44794064	0,147633652	0,346391959	protein_codin putative DNA/RNA non-specific endonuclease protein-like
TcG_07505	5,377229641	-0,01545325	0,827681987	-0,01867052	0,985103949	1	
TcG_07506	18,4824624	0,248456977	0,403040717	0,616456271	0,537593424	0,737281891	
TcG_07507	25,35791522	0,556004893	0,345166414	1,610831387	0,107216478	0,282095776	protein_codin putative glycine dehydrogenase, putative,glycine cleavage system P-protein
TcG_07508	25,64035038	0,775097404	0,351553883	2,204775545	0,027469849	0,107088045	protein_codin hypothetical protein
TcG_07509	4,343341454	1,865803956	0,939712774	1,985504516	0,047088368	1	protein_codin phosphatidylinositol 3-related kinase
TcG_07510	79,61889254	0,156401816	0,214328625	0,729729014	0,465555842	0,682138496	protein_codin putative trans-sialidase
TcG_07511	352,3953358	-0,29229348	0,098213298	-2,976109	0,002919311	0,018637229	protein_codin hypothetical protein
TcG_07512	294,9643665	-0,152180625	0,107570486	-1,41470612	0,157154679	0,358945805	protein_codin hypothetical protein
TcG_07513	246,0594419	-0,123536456	0,113372331	-1,08965261	0,275866199	0,508218443	protein_codin putative katanin
TcG_07514	572,50984	-0,119774081	0,077004982	-1,5554069	0,119849192	0,302917264	protein_codin putative small nuclear RNA gene activation protein (SNAP) 50
TcG_07515	190,6986276	-0,269948595	0,133839908	-2,01695144	0,043700582	0,150420362	protein_codin putative dual specificity protein phosphatase
TcG_07516	272,932519	0,156438213	0,111885473	1,398199496	0,162053168	0,365779857	protein_codin rRNA-processing protein CGR1
TcG_07517	444,6496321	-0,260076336	0,089629575	-2,9016799	0,003711676	0,022633409	protein_codin chaperone protein DnaJ
TcG_07518	112,7872261	-0,17186933	0,169037025	-1,01675553	0,309269743	0,542415871	protein_codin protein kinase
TcG_07519	227,7104533	0,145403292	0,124390039	1,168930352	0,242431693	0,470898628	protein_codin preprotein translocase subunit YidC
TcG_07520	146,0672153	0,17824278	0,149663727	1,190955104	0,23367121	0,461212035	protein_codin hypothetical protein
TcG_07521	162,174392	0,033052009	0,143797647	0,229850833	0,818207683	0,910311132	protein_codin putative chaperone-like ATPase (ISS)
TcG_07522	182,6542054	0,073544947	0,136473324	0,53889614	0,589958521	0,77611666	protein_codin hypothetical protein
TcG_07523	413,4008428	-0,038252097	0,089479765	-0,42749438	0,669019278	0,826307698	protein_codin hypothetical protein
TcG_07524	170,2266802	0,117876382	0,146360658	0,805382972	0,420598715	0,647066354	protein_codin putative phosphatidylserine decarboxylase

TcG_07525	655,5185574	-0,045006152	0,08099452	-0,5556691	0,578437082	0,768240018	protein_codin methyltransferase
TcG_07526	467,9729827	0,083886296	0,084887661	0,98820364	0,323052926	0,557558647	protein_codin hypothetical protein
TcG_07527	264,4495757	0,284622677	0,109835611	2,591351516	0,009559979	0,047701082	protein_codin glycosyltransferase
TcG_07528	371,3564173	0,123568385	0,0959245	1,288183774	0,197681999	0,416248138	protein_codin hypothetical protein
TcG_07529	160,9291539	0,02387162	0,144788652	0,164872177	0,869044592	0,936791559	protein_codin hypothetical protein
TcG_07530	79,63320323	0,344571425	0,197413097	1,745433461	0,080909445	0,232840744	protein_codin anaphase promoting complex subunit protein
TcG_07531	318,1644356	0,197803042	0,103397454	1,913035917	0,055743455	0,178557828	protein_codin hypothetical protein
TcG_07532	114,05901	0,103149586	0,169156331	0,609788503	0,542001918	0,740196534	protein_codin hypothetical protein
TcG_07533	1734,999949	-0,025913922	0,050470447	-0,51344744	0,607638371	0,788022108	protein_codin Wos2 protein
TcG_07534	144,313438	0,406030752	0,152119593	2,669154865	0,007604238	0,039865476	protein_codin receptor protein kinase
TcG_07535	1,130190449	3,392988792	1,771072575	1,915781905	0,055392862	1	protein_codin hypothetical protein
TcG_07536	4,829675185	0,074092839	0,803708238	0,092188727	0,926548094	1	protein_codin hypothetical protein
TcG_07537	10,19704203	1,128680189	0,580790336	1,943352222	0,051973616	1	protein_codin hypothetical protein
TcG_07538	1472,759805	0,18768018	0,05732133	3,274177005	0,001059702	0,008183398	protein_codin cysteine peptidase, putative,cysteine peptidase, clan CA, family C1, cathepsin L-like
TcG_07539	47,80165348	0,209672325	0,250794231	0,836033287	0,403136177	0,632986744	protein_codin hypothetical protein
TcG_07540	359,9296662	-0,054351337	0,094719389	-0,57381426	0,566093496	0,758321667	protein_codin putative UDP-Gal or UDP-GlcNAc-dependent glycosyltransferase
TcG_07541	23,44856812	0,321197481	0,372116163	0,863164551	0,388047001	0,618419884	protein_codin hypothetical protein
TcG_07542	0,825598502	-0,285163302	1,963314784	-0,14524584	0,884516776	1	protein_codin hypothetical protein
TcG_07543	0,27264935	1,353296468	3,06414982	0,441654798	0,658739024	1	protein_codin cruzipain
TcG_07544	3,245828893	-0,547518028	0,992094032	-0,55188118	0,581029761	1	protein_codin hypothetical protein
TcG_07545	13,31515364	0,219814237	0,508610388	0,432185899	0,665606307	1	protein_codin hypothetical protein
TcG_07546	109,8844529	-0,078991273	0,175993465	-0,44883072	0,653553779	0,81657908	protein_codin DUF866-domain-containing protein
TcG_07547	143,1886654	-0,014149632	0,149534984	-0,09462423	0,924613306	0,963187355	protein_codin hypothetical protein
TcG_07548	434,3004841	0,003566805	0,087904668	0,040575833	0,96763405	0,986016544	protein_codin hypothetical protein
TcG_07549	689,788384	0,046643668	0,072677582	0,641788941	0,521010231	0,725401608	protein_codin hypothetical protein
TcG_07550	237,1143063	0,101817897	0,122602823	0,830469431	0,406273424	0,63480565	protein_codin hypothetical protein
TcG_07551	47,74729082	0,095354539	0,260787261	0,365641092	0,714632908	0,853614301	
TcG_07552	72,12989146	-0,330451367	0,206038469	-1,60383335	0,108750792	0,284742752	protein_codin hypothetical protein
TcG_07553	281,5283259	-0,54711721	0,123277381	-4,43809892	9,07569E-06	0,000140764	protein_codin metacyclin II
TcG_07554	84,62981238	-0,091391336	0,195770811	-0,4668282	0,640622796	0,809229799	protein_codin hypothetical protein
TcG_07555	623,9275424	-0,346195792	0,077105133	-4,48991886	7,12503E-06	0,000113863	protein_codin hypothetical protein
TcG_07556	1,69446454	0,006424212	1,349708328	0,004759704	0,99620232	1	protein_codin hypothetical protein
TcG_07557	0,858842314	-0,037050539	1,909155807	-0,01940677	0,984516613	1	protein_codin structural maintenance of chromosome protein 4
TcG_07558	3,02534475	0,145021434	1,009978799	0,143588593	0,885825349	1	protein_codin putative trans-sialidase
TcG_07559	4,221236466	0,172291258	0,889503452	0,193693748	0,846415687	1	protein_codin rab1 small GTP-binding protein
TcG_07560	21,63528949	0,169085279	0,400162742	0,422541283	0,672629975	0,828677003	protein_codin target of rapamycin (TOR) kinase 1
TcG_07561	0,884237863	1,464362917	2,022999332	0,723857341	0,469153322	1	protein_codin hypothetical protein
TcG_07562	323,0368557	0,021588155	0,100825025	0,214115045	0,830457344	0,917601778	protein_codin L1Tc protein
TcG_07563	411,2686638	0,222203997	0,09133497	2,432846877	0,014980635	0,067666914	protein_codin hypothetical protein
TcG_07564	134,8667707	-0,202783206	0,151547731	-1,33808143	0,180869892	0,392277422	protein_codin hypothetical protein
TcG_07565	358,2778985	-0,112094502	0,104015401	-1,07767216	0,281180087	0,514255504	protein_codin hypothetical protein
TcG_07566	536,4576019	-0,019880329	0,084912118	-0,23412829	0,814885385	0,908512516	protein_codin hypothetical protein
TcG_07567	835,5147302	0,292079346	0,0669095	4,365289591	1,26954E-05	0,000186662	protein_codin 101 kDa heat shock protein
TcG_07568	2,832958477	-0,714321726	1,056505848	-0,67611715	0,498966267	1	protein_codin glycosylphosphatidylinositol-specific phospholipase C
TcG_07569	236,2442742	0,257997472	0,116086994	2,222449417	0,026252949	0,103444959	protein_codin glycosylphosphatidylinositol-specific phospholipase C
TcG_07570	215,4098349	-0,037410243	0,137198957	-0,27267148	0,785105757	0,893514718	protein_codin glycosylphosphatidylinositol-specific phospholipase C
TcG_07571	100,1014044	0,270917938	0,179791439	1,506845594	0,13185024	0,322809808	protein_codin hypothetical protein
TcG_07572	112,1709616	0,009514857	0,168031667	0,056625381	0,954843616	0,978889315	protein_codin hypothetical protein
TcG_07573	107,710162	0,03183132	0,16917258	0,188158859	0,850752122	0,928053891	
TcG_07574	155,6235705	-0,661817719	0,146377638	-4,52130346	6,146E-06	0,000100151	protein_codin putative surface protein TolT
TcG_07575	2,780628603	-0,292774003	1,086831314	-0,26938311	0,78763488	1	
TcG_07576	0					1	
TcG_07577	291,6910394	-0,55412484	0,105634037	-5,2457035	1,55687E-07	3,88748E-06	protein_codin putative surface protein TolT
TcG_07578	354,0297425	-0,114898542	0,098763953	-1,16336516	0,244681378	0,472864744	protein_codin signal recognition particle receptor subunit beta
TcG_07579	579,8285037	-0,209145218	0,084600685	-2,47214569	0,013430477	0,062492172	protein_codin putative dynein intermediate chain
TcG_07580	339,8090218	-0,169139232	0,100885625	-1,67654441	0,093631604	0,256834988	protein_codin hypothetical protein
TcG_07581	243,7903637	0,062421776	0,116366602	0,536423468	0,591665916	0,777210826	protein_codin ubiquitin activating enzyme

TcG_07582	496,8505221	-0,0915789	0,083851233	-1,09215926	0,274763108	0,507556451	protein_codin	hypothetical protein
TcG_07583	537,8226502	-0,058557804	0,080559705	-0,72688702	0,467295168	0,683941614	protein_codin	hypothetical protein
TcG_07584	226,507056	-0,118854241	0,117784376	-1,00908325	0,312934712	0,547104508	protein_codin	hypothetical protein
TcG_07585	220,4232069	0,139708385	0,119656592	1,167577841	0,242977093	0,471230766	protein_codin	putative DnaJ chaperone protein
TcG_07586	340,85208	-0,106872427	0,100105084	-1,06760239	0,285699907	0,519127451	protein_codin	putative minichromosome maintenance (MCM) complex subunit
TcG_07587	111,2641227	0,224031833	0,167082293	1,34084725	0,179970052	0,391206945	protein_codin	hypothetical protein
TcG_07588	284,1252081	0,132577311	0,107650177	1,231556835	0,218114674	0,44249284	protein_codin	putative beta-ketoacyl synthase family protein
TcG_07589	396,7474102	-0,053700157	0,094490179	-0,56831469	0,569821308	0,761822026	protein_codin	putative heterogeneous nuclear ribonucleoprotein H/F
TcG_07590	55,15157992	-0,10011696	0,238672087	-0,41947494	0,674869064	0,82970688	protein_codin	hypothetical protein
TcG_07591	482,0433962	-0,172760581	0,083886773	-2,05944961	0,039451188	0,13999295	protein_codin	putative glycosylphosphatidylinositol (GPI) anchor
TcG_07592	219,4915747	0,094765341	0,121622121	0,779178494	0,435874577	0,659521701	protein_codin	cystatin-like cysteine protease inhibitor domain-containing protein
TcG_07593	250,4079197	0,08907159	0,11312429	0,787378114	0,431060555	0,655759925	protein_codin	hypothetical protein
TcG_07594	824,0822939	0,005272368	0,068583636	0,076875012	0,938722976	0,971680773	protein_codin	RNA-binding protein
TcG_07595	453,8697443	0,140574466	0,092350494	1,522184243	0,127962922	0,31679026	protein_codin	hypothetical protein
TcG_07596	418,9113616	0,041037412	0,095686037	0,428875658	0,668013715	0,826134898	protein_codin	hypothetical protein
TcG_07597	335,3554693	0,249616161	0,107923325	2,312902802	0,020727984	0,086542133	protein_codin	cytochrome P450 reductase A
TcG_07598	204,1257531	0,200568459	0,124175079	1,615207012	0,106265873	0,280710534	protein_codin	putative leucine-rich repeat protein
TcG_07599	237,8417713	0,198057332	0,120212947	1,647554085	0,099444209	0,26775752	protein_codin	putative leucine-rich repeat protein
TcG_07600	304,5673279	0,080516984	0,105411302	0,763836343	0,444964776	0,666842827	protein_codin	dynein heavy chain
TcG_07601	311,4138295	0,010774178	0,101482186	0,106168166	0,915448928	0,958813169	protein_codin	CBS and cyclic nucleotide-binding protein domain-containing protein
TcG_07602	533,3127097	-0,001494	0,081367371	-0,01836117	0,985350729	0,994275697	protein_codin	phospholipid-transporting ATPase-like protein
TcG_07603	326,0627606	0,071944789	0,10250459	0,701868951	0,482760894	0,696286283	protein_codin	putative RNA helicase
TcG_07604	531,8141421	0,000173211	0,080551404	0,002150314	0,998284299	0,99947404	protein_codin	uncharacterized protein
TcG_07605	213,3648659	-0,008702045	0,122440601	-0,07107157	0,943340797	0,973371575	protein_codin	acyl-CoA binding protein
TcG_07606	349,8860863	0,039138741	0,097770336	0,400313044	0,688925961	0,838445877	protein_codin	adaptor complex subunit medium chain 3
TcG_07607	203,5944831	-0,120949833	0,132132209	-0,9153698	0,359997529	0,592630203	protein_codin	hypothetical protein
TcG_07608	616,7258086	-0,022961148	0,078884411	-0,29107333	0,770995235	0,885140325	protein_codin	hypothetical protein
TcG_07609	475,5827826	0,053056255	0,084547938	0,627528673	0,530312747	0,731981763	protein_codin	hypothetical protein
TcG_07610	421,0240903	-0,131951582	0,090301056	-1,46124074	0,143949386	0,34057537	protein_codin	kinesin-like protein
TcG_07611	999,0218823	0,298618119	0,069070402	4,323387573	1,53651E-05	0,000220457	protein_codin	antigenic protein
TcG_07612	277,6250657	0,213708925	0,114750864	1,862373125	0,062550515	0,195497778	protein_codin	antigenic protein
TcG_07613	223,9406973	0,55289839	0,127889442	4,323252825	1,53745E-05	0,000220457	protein_codin	hypothetical protein
TcG_07614	59,81182617	0,301565068	0,231711718	1,301466623	0,193098782	0,409901518	protein_codin	putative mitotic centromere-associated kinesin (MCAK)
TcG_07615	66,41656168	0,531053017	0,221160779	2,40120794	0,016341048	0,072234788	protein_codin	target of rapamycin (TOR) kinase 1
TcG_07616	616,7807557	0,359711699	0,084565829	4,253629447	2,10333E-05	0,000289765	protein_codin	putative mitochondrial DNA primase
TcG_07617	137,8621898	0,674838628	0,156604721	4,309184438	1,63858E-05	0,000232654	protein_codin	putative oxidoreductase
TcG_07618	207,0234729	0,650317481	0,126445554	5,14306324	2,70295E-07	6,28347E-06	protein_codin	spliceosomal U5 snRNP-specific protein
TcG_07619	727,1904205	0,093626463	0,069576569	1,345660824	0,178411926	0,389354036	protein_codin	hypothetical protein
TcG_07620	428,4787083	0,340854327	0,093107248	3,660878549	0,000251352	0,002414729	protein_codin	hypothetical protein
TcG_07621	176,3160193	0,229685779	0,132832535	1,729137964	0,083784406	0,238331973	protein_codin	LYT1p
TcG_07622	79,08814087	-0,166544812	0,197356852	-0,84387651	0,398738413	0,628713018	protein_codin	porin
TcG_07623	518,1959257	0,401190466	0,087717902	4,573644087	4,79314E-06	8,07129E-05	protein_codin	U3 small nucleolar RNA-associated protein 7
TcG_07624	208,8723944	0,54273116	0,12769692	4,250150764	2,13627E-05	0,000293952	protein_codin	hypothetical protein
TcG_07625	166,0242323	0,27964204	0,140586559	1,989109355	0,046689134	0,157846602	protein_codin	hypothetical protein
TcG_07626	509,165468	0,089700349	0,080850298	1,109462201	0,267230842	0,499618612	protein_codin	hypothetical protein
TcG_07627	322,6426223	0,199195938	0,106294215	1,874005445	0,060929675	0,191620849	protein_codin	hypothetical protein
TcG_07628	190,0169691	0,259134095	0,132642761	1,953624108	0,050745687	0,167361097	protein_codin	hypothetical protein
TcG_07629	630,8679013	0,190068765	0,076632783	2,480253963	0,013128884	0,061285755	protein_codin	putative kinesin
TcG_07630	1546,329254	0,315806998	0,053795072	5,870556265	4,34335E-09	1,49768E-07	protein_codin	ubiquitin-activating enzyme E1
TcG_07631	255,2522115	0,754788721	0,118504712	6,36927178	1,89928E-10	8,76695E-09	protein_codin	putative metallo-beta-lactamase-like protein
TcG_07632	3,526452684	0,543870309	1,854372888	0,293290693	0,769299967	1		
TcG_07633	0					1		
TcG_07634	0					1		
TcG_07635	0					1		
TcG_07636	0					1		
TcG_07637	0					1		
TcG_07638	0,116927736	0,503022807	4,080472857	0,123275616	0,901888849	1		

TcG_07639	0					1	
TcG_07640	0,446491354	-1,154452866	2,627899391	-0,43930634	0,66043958	1	
TcG_07641	0					1	
TcG_07642	1,862196699	1,410784796	1,337853068	1,054514005	0,291647652	1	
TcG_07643	77,42434622	0,32232599	0,196929275	1,636760154	0,101680594	0,271946299	protein_codin hypothetical protein
TcG_07644	25,13723911	0,069561236	0,356860331	0,194925662	0,845451144	0,925035391	protein_codin surface protease GP63
TcG_07645	126,2713157	0,312713363	0,155245567	2,014314277	0,043976548	0,151145738	protein_codin rab1 small GTP-binding protein
TcG_07646	37,87912552	-0,066089246	0,281966914	-0,23438653	0,814684916	0,908376426	protein_codin target of rapamycin (TOR) kinase 1
TcG_07647	326,7983632	-0,27449251	0,09884443	-2,77701545	0,005486057	0,030750584	protein_codin beta galactofuranosyl glycosyltransferase
TcG_07648	46,52250826	0,193820105	0,262432872	0,73855117	0,460179584	0,678399847	protein_codin hypothetical protein
TcG_07649	23,20526572	0,913770312	0,372163699	2,455291352	0,014077045	0,064720888	protein_codin hypothetical protein
TcG_07650	24,0582296	0,496822579	0,35823263	1,386871373	0,165480989	0,370915599	protein_codin hypothetical protein
TcG_07651	101,9496755	0,197796412	0,175101901	1,129607449	0,258641671	0,491088561	protein_codin trans-sialidase
TcG_07652	91,99789387	0,237294835	0,195406187	1,214367052	0,224607591	0,451082534	protein_codin trans-sialidase
TcG_07653	66,80998864	-0,123219897	0,223463543	-0,55140939	0,581353062	0,770482335	protein_codin retrotransposon hot spot (RHS) protein
TcG_07654	43,47288509	-0,120070195	0,264198339	-0,45446991	0,649490646	0,813865306	protein_codin hypothetical protein
TcG_07655	29,93640905	0,477637971	0,31763868	1,503714756	0,132654828	0,323614384	protein_codin hypothetical protein
TcG_07656	3,311816371	0,310464997	0,964741271	0,321811667	0,747595375	1	protein_codin helicase-like protein
TcG_07657	41,58946121	0,200544698	0,272797334	0,735141706	0,462253197	0,679744706	protein_codin putative glycine dehydrogenase, putative,glycine cleavage system P-protein
TcG_07658	32,84879271	-0,178704838	0,302714539	-0,59034112	0,55496198	0,749654833	protein_codin putative retrotransposon hot spot (RHS) protein
TcG_07659	87,69227058	0,219310576	0,203995625	1,075074899	0,282341196	0,515574266	protein_codin hypothetical protein
TcG_07660	83,0536342	0,038218931	0,199828267	0,191258885	0,848322772	0,926620876	protein_codin target of rapamycin (TOR) kinase 1
TcG_07661	55,03571556	0,273507393	0,233671822	1,170476571	0,241809237	0,470462103	protein_codin protein kinase
TcG_07662	237,585556	-0,224720279	0,115600182	-1,94394399	0,051902205	0,169534522	
TcG_07663	41,66018942	-0,081277265	0,267588245	-0,30374004	0,761325948	0,879491513	protein_codin dispersed protein family protein 1
TcG_07664	28,90488454	0,241682063	0,32731745	0,73837207	0,460288382	0,678399847	protein_codin dispersed gene family protein 1 (DGF-1)
TcG_07665	174,6179072	-0,184018216	0,134038736	-1,37287341	0,169791703	0,376067037	protein_codin dispersed gene family protein 1 (DGF-1)
TcG_07666	157,0419164	0,201761067	0,146317711	1,378924434	0,167918054	0,374062406	protein_codin dispersed gene family protein 1 (DGF-1)
TcG_07667	139,7762779	-0,207542266	0,153823153	-1,34922645	0,177264242	0,387360148	protein_codin dispersed gene family protein 1 (DGF-1)
TcG_07668	289,5542759	-0,081316116	0,116837515	-0,69597609	0,486443806	0,698294875	protein_codin dispersed gene family protein 1 (DGF-1)
TcG_07669	339,7872121	-0,071338143	0,100482389	-0,70995667	0,477731004	0,692608846	protein_codin dispersed gene family protein 1 (DGF-1)
TcG_07670	88,77652866	-0,018741645	0,203320765	-0,09217772	0,926556839	0,964306367	protein_codin dispersed gene family protein 1 (DGF-1)
TcG_07671	102,0205547	-0,339911263	0,17697331	-1,92069224	0,054770518	0,176564058	protein_codin dispersed protein family protein 1 (DGF-1)
TcG_07672	60,09457342	-0,301183514	0,235472439	-1,27906058	0,200875724	0,420260529	
TcG_07673	47,9425799	0,29083215	0,265448155	1,095626941	0,273242067	0,506525214	protein_codin retrotransposon hot spot (RHS) protein
TcG_07674	287,1720933	-0,102577505	0,113089133	-0,90705006	0,364380324	0,596371017	protein_codin retrotransposon hot spot (RHS) protein
TcG_07675	64,70002391	0,100350303	0,218780879	0,458679496	0,64646434	0,812019442	protein_codin hypothetical protein
TcG_07676	97,08307882	-0,053618932	0,177047923	-0,30284982	0,762004313	0,879778971	protein_codin dispersed gene family protein 1 (DGF-1)
TcG_07677	140,5273158	0,228932374	0,161537506	1,41720879	0,156421892	0,357879946	protein_codin SH3 domain protein
TcG_07678	214,2044777	0,047449533	0,122891243	0,386109964	0,699415231	0,844282649	protein_codin hypothetical protein
TcG_07679	7,044042876	0,130992241	0,667900165	0,196125481	0,844511953	1	protein_codin putative voltage-dependent anion-selective channel
TcG_07680	269,3377858	-0,302733484	0,118605689	-2,55243645	0,010697242	0,052074894	protein_codin stress-induced protein sti1
TcG_07681	127,3064477	0,002213776	0,16135195	0,013720168	0,989053233	0,995541618	protein_codin ARP2/3 complex subunit
TcG_07682	189,8382227	0,04887915	0,13596343	0,359502186	0,719219443	0,855882956	protein_codin WD-40containing protein
TcG_07683	310,9640913	0,132136004	0,102616903	1,287663144	0,197863248	0,416429354	protein_codin solute carrier family member b3
TcG_07684	174,0057464	-0,16904422	0,135398413	-1,24849484	0,211849897	0,43480831	protein_codin hypothetical protein
TcG_07685	695,9799641	-0,112661993	0,075923557	-1,48388719	0,137838851	0,331259268	protein_codin hypothetical protein
TcG_07686	126,3714485	-0,274559162	0,155692033	-1,76347599	0,077820244	0,226311583	protein_codin hypothetical protein
TcG_07687	369,3403243	-0,117049353	0,096514196	-1,21276826	0,225218435	0,451820114	protein_codin mitochondrial carrier protein
TcG_07688	302,5497717	0,206987352	0,104133359	1,987714154	0,046843313	0,158095631	protein_codin hypothetical protein
TcG_07689	106,0029468	-0,129971064	0,171652855	-0,75717392	0,448945675	0,669862793	protein_codin hypothetical protein
TcG_07690	376,0592712	-0,117880943	0,093957547	-1,25461921	0,209617027	0,432216915	protein_codin hypothetical protein
TcG_07691	1283,403315	0,000574918	0,055703864	0,010320965	0,991765208	0,996781867	protein_codin ATP-dependent Clp protease subunit, heat shock protein 78 (HSP78)
TcG_07692	776,0087838	-0,145524783	0,070391741	-2,06735592	0,038700628	0,138092233	protein_codin putative mannosyltransferase-like protein
TcG_07693	1828,717969	-0,191241729	0,051334131	-3,72543032	0,000194982	0,00195252	protein_codin oleate desaturase
TcG_07694	76,98959461	-0,143221991	0,196823392	-0,72766753	0,466817133	0,683414621	
TcG_07695	367,2401467	0,349203695	0,094399294	3,699219348	0,000216264	0,002127021	protein_codin hypothetical protein

TcG_07696	280,9505469	0,090956681	0,108713973	0,836660447	0,402783458	0,632851796	protein_codin hypothetical protein
TcG_07697	1039,829833	-0,061884317	0,061850915	-1,00054004	0,31704923	0,55204875	protein_codin polyadenylation/uridylation factor 1
TcG_07698	68,03503665	0,398966864	0,215126084	1,854572244	0,063657335	0,198155261	protein_codin hypothetical protein
TcG_07699	606,2600469	-0,443483064	0,077592296	-5,71555533	1,09346E-08	3,44263E-07	protein_codin putative surface antigen TASV, putative,mucin-like glycoprotein
TcG_07700	124,6192783	-0,624779931	0,157857961	-3,9578614	7,56238E-05	0,000877933	
TcG_07701	5,024617071	-0,91040768	0,789852167	-1,15263048	0,249062089	1	protein_codin trans-sialidase
TcG_07702	1,446957673	0,247178092	1,409916865	0,175313948	0,860832952	1	protein_codin hypothetical protein
TcG_07703	2,055056185	1,017330673	1,304667809	0,779762224	0,435530845	1	protein_codin hypothetical protein
TcG_07704	2,317617385	0,751021264	1,19466917	0,628643714	0,529582335	1	protein_codin hypothetical protein
TcG_07705	2,017927325	-0,971802005	1,338356581	-0,72611591	0,467767717	1	protein_codin hypothetical protein
TcG_07706	1599,299139	-0,12601899	0,053295871	-2,36451694	0,018053602	0,077642551	protein_codin trans-sialidase
TcG_07707	262,8535683	0,121162236	0,113302401	1,069370426	0,284902788	0,518762172	protein_codin hypothetical protein
TcG_07708	501,095628	0,05885007	0,083633573	0,703665624	0,481641033	0,695708707	protein_codin retrotransposon hot spot (RHS) protein
TcG_07709	181,4390375	0,221748984	0,134603473	1,647423939	0,099470938	0,267767261	protein_codin retrotransposon hot spot (RHS) protein
TcG_07710	82,0730578	-0,178664182	0,200081297	-0,89295793	0,3718797	0,603191685	
TcG_07711	24,21028441	0,258490074	0,357757163	0,722529416	0,469969047	0,686450317	protein_codin hypothetical protein
TcG_07712	24,35203232	-0,055535365	0,360799155	-0,15392321	0,877670282	0,941200542	protein_codin hypothetical protein
TcG_07713	62,14025239	-0,163262637	0,22587015	-0,72281635	0,469792723	0,68629662	protein_codin putative dynein assembly factor 1, axonemal-like
TcG_07714	148,2125236	-0,006973832	0,142902657	-0,04880127	0,961077666	0,981984718	protein_codin putative exosome-associated protein 4, putative,3' exoribonuclease
TcG_07715	882,100491	-0,06133392	0,0691555	-0,88689866	0,375133462	0,605734403	protein_codin hypothetical protein
TcG_07716	439,0798857	-0,625733233	0,088218311	-7,09300857	1,31227E-12	9,74615E-11	protein_codin hypothetical protein
TcG_07717	303,035802	0,20892431	0,106983771	1,952859851	0,050836204	0,167611913	protein_codin putative translation initiation factor 2 subunit
TcG_07718	118,2258216	0,201261826	0,163906946	1,227902968	0,219483395	0,444584196	protein_codin hypothetical protein
TcG_07719	339,2479277	-0,097552914	0,098794234	-0,9874353	0,323429286	0,557588877	protein_codin hypothetical protein
TcG_07720	759,1936552	0,00135087	0,068926196	0,019598792	0,984363428	0,994058631	protein_codin alkylated DNA repair protein alkB like protein 6
TcG_07721	672,2164897	0,065502948	0,076192752	0,859700511	0,389954153	0,620179659	protein_codin hypothetical protein
TcG_07722	177,9356972	-0,034575695	0,133204862	-0,25956782	0,795197165	0,897705774	protein_codin hypothetical protein
TcG_07723	57,62310006	-0,185758343	0,236977674	-0,78386432	0,433119729	0,657527329	protein_codin hypothetical protein
TcG_07724	239,6716709	0,079280044	0,113870962	0,696227053	0,486286648	0,698274608	protein_codin putative delta tubulin
TcG_07725	573,2626486	0,473271971	0,087948565	5,381235833	7,39762E-08	1,97942E-06	
TcG_07726	46514,22094	-0,061995485	0,038579244	-1,60696475	0,1080621	0,283452002	
TcG_07727	23966,47076	0,402294307	0,113176585	3,554571876	0,000378595	0,003418863	
TcG_07728	8442,578004	-0,02541328	0,0337928	-0,75203239	0,452031579	0,672907346	
TcG_07729	19,30065688	0,446804143	0,413897394	1,079504606	0,280362842	0,513562669	protein_codin surface protease GP63
TcG_07730	35,38751324	-0,075189248	0,292573344	-0,25699282	0,797184317	0,898810578	protein_codin surface protease GP63
TcG_07731	177,6270762	0,248078283	0,133710593	1,855337547	0,06354804	0,197974613	protein_codin surface protease GP63
TcG_07732	358,1130939	0,104510693	0,097928019	1,06721952	0,28587272	0,519187736	protein_codin hypothetical protein
TcG_07733	308,6623348	-0,151935732	0,102180953	-1,48692812	0,137033798	0,330077667	protein_codin hypothetical protein
TcG_07734	433,4935992	0,064910368	0,094179457	0,689220032	0,490684812	0,701774378	protein_codin lipase-like protein
TcG_07735	321,9324417	-0,018039446	0,10274984	-0,17556666	0,860634397	0,933293722	protein_codin hypothetical protein
TcG_07736	596,1785311	0,402282338	0,079300545	5,072882411	3,91835E-07	8,81514E-06	protein_codin hypothetical protein
TcG_07737	338,7554479	0,201927378	0,101166423	1,995992077	0,045934785	0,155750782	protein_codin hypothetical protein
TcG_07738	693,6477208	-0,077601785	0,071920506	-1,07899387	0,280590462	0,513654778	protein_codin sperm-associated antigen 6 isoform X1
TcG_07739	147,7162569	0,483367836	0,148200099	3,261589164	0,001107896	0,008478257	protein_codin hypothetical protein
TcG_07740	548,8225222	0,179853581	0,081712962	2,201041024	0,02773312	0,107912108	protein_codin hypothetical protein
TcG_07741	566,2242848	-0,038243364	0,080510787	-0,47500919	0,634780421	0,80483322	protein_codin pumilio protein 9
TcG_07742	385,4361155	0,314855162	0,092418705	3,406833738	0,000657211	0,005450574	protein_codin exosome-associated protein 1
TcG_07743	1290,125644	-0,147397034	0,05988242	-2,46144086	0,013838021	0,063799168	protein_codin coatomer beta subunit
TcG_07744	13571,75092	-0,127880602	0,034114691	-3,7485493	0,00017786	0,001810603	protein_codin beta-tubulin
TcG_07745	189,0829842	0,374633333	0,130688823	2,866605755	0,004148995	0,024868214	protein_codin putative deoxyhypusine synthase
TcG_07746	1633,527523	0,229677708	0,066668603	3,445065566	0,000570921	0,004853037	protein_codin MDN1, midasin-like protein
TcG_07747	170,2949028	0,217789565	0,269740262	0,807404735	0,41943334	0,646045557	protein_codin putative dynein heavy chain
TcG_07748	112,2332443	-0,282627888	0,165083009	-1,71203499	0,08689022	0,244343914	protein_codin putative maoC-like dehydratase
TcG_07749	230,0758028	-0,282389943	0,120102468	-2,3512418	0,01871087	0,079641492	protein_codin putative prolyl oligopeptidase, putative,serine peptidase clan SC, family S9A
TcG_07750	140,6518762	-0,287029672	0,14732638	-1,94825715	0,051384202	0,168684291	protein_codin putative pyruvate dehydrogenase (lipoamide) kinase
TcG_07751	155,1946671	-0,061708769	0,147500664	-0,41836265	0,675681991	0,83017833	protein_codin putative beta prime cop protein
TcG_07752	178,9379076	0,336918005	0,139008744	2,423718078	0,015362526	0,068935024	protein_codin helicase-like protein

TcG_07753	0					1	protein_codin SH3 domain protein
TcG_07754	29,9616675	0,481475564	0,323352132	1,489013112	0,136483916	0,329163749	protein_codin SH3 domain protein
TcG_07755	135,4540138	0,465133879	0,180405351	2,578270973	0,00992961	0,04915852	protein_codin trans-sialidase
TcG_07756	79,1178251	0,244370141	0,20133295	1,213761288	0,224838893	0,451313828	protein_codin hypothetical protein
TcG_07757	304,3319383	0,010592021	0,104658917	0,10120515	0,919387609	0,961200581	protein_codin hypothetical protein
TcG_07758	214,1742836	0,221896045	0,126134873	1,759196649	0,078544113	0,22801606	
TcG_07759	215,2161877	0,05402782	0,134698426	0,40110209	0,688344962	0,8382557	protein_codin putative leucine-rich repeat protein (LRRP)
TcG_07760	321,188172	0,457697294	0,103365602	4,427945886	9,51348E-06	0,000145798	protein_codin hypothetical protein
TcG_07761	289,7536435	0,157983609	0,104868359	1,506494521	0,131940273	0,322809808	protein_codin hypothetical protein
TcG_07762	87,37807544	0,821147293	0,193801582	4,23705155	2,26474E-05	0,00030855	protein_codin hypothetical protein
TcG_07763	131,90291	0,217656089	0,161870885	1,344627782	0,178745467	0,389799325	protein_codin putative peroxisome targeting signal 1 receptor
TcG_07764	745,1451181	0,16350475	0,076038161	2,150298589	0,031531604	0,118573568	protein_codin hypothetical protein
TcG_07765	759,8735849	-0,542454985	0,07090986	-7,64992326	2,01099E-14	1,94161E-12	protein_codin putative mitochondrial carrier protein
TcG_07766	374,9277311	-0,534741125	0,099919453	-5,35172191	8,71212E-08	2,29406E-06	protein_codin hypothetical protein
TcG_07767	161,4714769	-0,55923017	0,154316283	-3,62392198	0,000290169	0,002726602	protein_codin hypothetical protein
TcG_07768	389,1490759	-0,25145804	0,097339725	-2,58330337	0,009785923	0,048611369	protein_codin hypothetical protein
TcG_07769	277,2508643	-0,068047275	0,110153593	-0,61774903	0,536740789	0,73660472	protein_codin spermidine synthase
TcG_07770	394,183565	-0,368138784	0,093268777	-3,94707419	7,9112E-05	0,000911125	protein_codin hypothetical protein
TcG_07771	263,9713249	-0,146237651	0,123264219	-1,18637551	0,235474038	0,463329811	protein_codin hypothetical protein
TcG_07772	242,2533416	-0,393395921	0,114745352	-3,42842577	0,000607093	0,005085881	protein_codin tyrosine phosphatase
TcG_07773	734,2700597	-0,338815655	0,073668953	-4,59916477	4,24188E-06	7,25945E-05	protein_codin tetratricopeptide repeat protein 21B isoform a
TcG_07774	269,8081782	-0,329241764	0,117251002	-2,8080081	0,004984897	0,028719552	protein_codin EF hand
TcG_07775	378,0689871	-0,309897019	0,099088695	-3,12747098	0,001763172	0,012440995	protein_codin protein XRP2
TcG_07776	150,0837706	-0,190996911	0,149846338	-1,27461847	0,202444316	0,422616188	protein_codin hypothetical protein
TcG_07777	545,4543322	-0,033705095	0,079418054	-0,42440092	0,67127345	0,82773246	protein_codin putative huntingtin interacting protein (HIP)
TcG_07778	191,3878443	-0,567383265	0,141539295	-4,00866252	6,10636E-05	0,000731627	protein_codin DNA primase small subunit
TcG_07779	477,0805337	-0,104434466	0,086906875	-1,20168245	0,229486584	0,456686974	protein_codin hypothetical protein
TcG_07780	291,8994053	-0,19109126	0,106804988	-1,78916045	0,073588979	0,218671945	protein_codin ADP-ribosylation factor
TcG_07781	3821,462834	0,237735215	0,046053407	5,162163424	2,44112E-07	5,78766E-06	protein_codin 60S ribosomal protein L11
TcG_07782	463,533493	-0,175241645	0,091342422	-1,91851323	0,055045966	0,177119842	protein_codin hypothetical protein
TcG_07783	15,04194649	-0,909160437	0,45444584	-2,00059139	0,045436442	1	
TcG_07784	160,5397367	-0,02239065	0,144989217	-0,15442976	0,87727089	0,941029583	protein_codin GP63 group II protein
TcG_07785	6,069964425	0,300482764	0,707720677	0,424578191	0,671144195	1	protein_codin hypothetical protein
TcG_07786	11,14824988	-0,239321377	0,519228187	-0,46091754	0,64485777	1	
TcG_07787	312,1162293	0,130516172	0,105878121	1,232702008	0,217686962	0,441934667	protein_codin sialidase
TcG_07788	106,293085	0,024584716	0,176801307	0,139052797	0,889408429	0,946535728	protein_codin histone H3
TcG_07789	4241,691074	-0,152584967	0,039276461	-3,88489597	0,000102374	0,001136955	protein_codin histone H3
TcG_07790	1560,043795	-0,013547754	0,05465609	-0,24787273	0,804232879	0,903185306	protein_codin hypothetical protein
TcG_07791	593,083426	0,093064976	0,080227923	1,160007298	0,246045835	0,474639867	protein_codin hypothetical protein
TcG_07792	185,5733476	-0,193592501	0,131304583	-1,47437734	0,140380023	0,33486575	protein_codin calcyphosin
TcG_07793	301,816926	0,174477259	0,103197965	1,690704457	0,090893263	0,251574138	protein_codin hypothetical protein
TcG_07794	585,3679648	0,02234559	0,078602915	0,284284493	0,776192369	0,888310845	protein_codin hypothetical protein
TcG_07795	214,099747	-0,069336227	0,121080157	-0,57264732	0,566883515	0,758769917	protein_codin hypothetical protein
TcG_07796	2626,422079	0,027639515	0,052658119	0,524886116	0,599662362	0,783102809	protein_codin putative 5'-3' exonuclease
TcG_07797	628,5123458	-0,370512101	0,076506471	-4,84288577	1,27967E-06	2,51719E-05	protein_codin hypothetical protein
TcG_07798	361,7969495	0,088756339	0,098282118	0,903077191	0,366484929	0,598716072	protein_codin hypothetical protein
TcG_07799	125,1480928	0,119251221	0,159325901	0,748473541	0,454174579	0,674191758	protein_codin hypothetical protein
TcG_07800	194,5861485	0,125811179	0,127329646	0,988074522	0,323116153	0,557573663	protein_codin putative ribonuclease H1
TcG_07801	264,9782156	0,311989839	0,112183395	2,781069679	0,005418011	0,030501979	protein_codin hypothetical protein
TcG_07802	369,5555826	0,024808709	0,096287787	0,257651667	0,796675751	0,898729487	protein_codin hypothetical protein
TcG_07803	615,4160632	0,169434742	0,077463841	2,187275256	0,028722436	0,11061934	protein_codin poly [ADP-ribose] polymerase
TcG_07804	734,3889263	-0,149751855	0,070621025	-2,12049958	0,033963937	0,12556036	protein_codin oligopeptidase B-like protein
TcG_07805	4,58692295	1,544420437	0,895126958	1,725364679	0,084461773	1	protein_codin retrotransposon hot spot (RHS) protein
TcG_07806	495,5347389	0,140507272	0,084583474	1,661166961	0,096679919	0,263003884	protein_codin syntaxin
TcG_07807	438,0915489	-0,229259785	0,088214487	-2,59889043	0,009352562	0,046819242	protein_codin hypothetical protein
TcG_07808	328,2618777	-0,073417839	0,100578272	-0,72995725	0,465416316	0,682138496	protein_codin hypothetical protein
TcG_07809	178,7572088	-0,049100579	0,135215794	-0,36312754	0,716509609	0,854413373	protein_codin putative lipase domain protein

TcG_07810	216,2172112	0,06836825	0,12423999	0,550291819	0,582119235	0,771194386	protein_codin putative poly(A) export protein
TcG_07811	144,1034658	-0,072465957	0,148622572	-0,48758379	0,625844679	0,798924246	protein_codin hypothetical protein
TcG_07812	169,3706416	0,111444967	0,135697834	0,821273001	0,411490783	0,638080373	protein_codin exopolyphosphatase
TcG_07813	199,6984944	0,277127209	0,134200759	2,06501968	0,038921138	0,138779454	protein_codin putative p21-activated kinase 3
TcG_07814	153,1014371	0,212992705	0,146031137	1,458543089	0,144690907	0,341562521	protein_codin hypothetical protein
TcG_07815	184,0337339	0,233177978	0,13279644	1,755905347	0,079104572	0,229172538	protein_codin pseudouridylate synthase-like protein
TcG_07816	334,1197751	0,143888614	0,114401217	1,257754224	0,208480655	0,431100637	protein_codin putative N-acetyltransferase
TcG_07817	136,3385328	-0,1989105	0,152382373	-1,30533799	0,191777784	0,407844604	protein_codin thioredoxin
TcG_07818	555,5119659	0,221697153	0,080424371	2,756591701	0,005840723	0,032347333	protein_codin flagellar/basal body protein
TcG_07819	343,0671755	0,295809221	0,099341113	2,977711977	0,002904087	0,01856815	protein_codin hypothetical protein
TcG_07820	747,9903022	0,26450998	0,070631885	3,744908967	0,000180459	0,001834035	protein_codin putative DNA excision/repair protein SNF2
TcG_07821	522,9874189	0,021256498	0,086864026	0,244710022	0,806680967	0,904588239	protein_codin putative endo-beta-N-acetylglucosaminidase
TcG_07822	102,3035221	0,625850004	0,178206306	3,511940837	0,000444847	0,003922373	protein_codin hypothetical protein
TcG_07823	159,766161	0,156297694	0,146986127	1,063349971	0,287623272	0,520606659	protein_codin putative cyclophilin
TcG_07824	493,7249604	0,198337583	0,091158264	2,175749893	0,029573967	0,113083823	protein_codin hypothetical protein
TcG_07825	168,9506429	0,314754448	0,139357354	2,258613834	0,023907417	0,096611612	protein_codin protein-tyrosine phosphatase
TcG_07826	143,8462368	0,15868045	0,155056778	1,023369967	0,306132954	0,539181888	protein_codin hypothetical protein
TcG_07827	221,1711717	0,154241602	0,131593617	1,172105502	0,241154701	0,469819803	protein_codin putative CLC-type chloride channel
TcG_07828	427,2672411	0,121942589	0,091386973	1,334354176	0,18208781	0,393962535	protein_codin hypothetical protein
TcG_07829	18,78359141	0,055927647	0,406225273	0,137676433	0,890496145	0,94667588	protein_codin L1Tc protein
TcG_07830	218,1405126	0,284394937	0,122470829	2,322144288	0,020225168	0,084809552	protein_codin mucin TcSMUGS
TcG_07831	580,5667623	-0,331528438	0,080492483	-4,11875027	3,80933E-05	0,000484998	protein_codin hypothetical protein
TcG_07832	277,8912048	-0,024970396	0,106510825	-0,23443998	0,81464342	0,908376426	protein_codin hypothetical protein
TcG_07833	729,074891	0,105307276	0,076317977	1,37984888	0,167633179	0,373644017	protein_codin hypothetical protein
TcG_07834	529,1437724	-0,398192409	0,081855815	-4,86455862	1,14713E-06	2,28361E-05	protein_codin hypothetical protein
TcG_07835	1290,111649	0,043135828	0,05706199	0,755946793	0,449681097	0,670442053	protein_codin hypothetical protein
TcG_07836	1168,962149	-0,033924183	0,058822109	-0,57672504	0,564125216	0,75700194	protein_codin putative protein kinase
TcG_07837	1204,092087	-0,02932407	0,057528724	-0,50972918	0,610241204	0,789016404	protein_codin histone H4
TcG_07838	305,5784683	-0,062098402	0,109587549	-0,56665563	0,570948168	0,762440697	protein_codin hypothetical protein
TcG_07839	78,28252652	-0,002170605	0,196107723	-0,01106843	0,99116885	0,996670572	protein_codin hypothetical protein
TcG_07840	580,893048	-0,111743161	0,089317303	-1,25108078	0,210905007	0,433483308	protein_codin hypothetical protein
TcG_07841	1306,182029	-0,040225861	0,178509076	-0,22534351	0,821712056	0,911601931	protein_codin hypothetical protein
TcG_07842	144,316428	-0,124987119	0,14943993	-0,8363703	0,402946613	0,632950923	protein_codin hypothetical protein
TcG_07843	14,00442725	0,462885342	0,476001808	0,972444503	0,330829461	1	protein_codin hypothetical protein
TcG_07844	8,602038012	0,420869475	0,590357874	0,712905668	0,475904131	1	protein_codin putative RNA-binding protein
TcG_07845	1482,211574	0,616042074	0,067384544	9,142186584	6,12021E-20	1,10795E-17	protein_codin inositol 1,4,5-trisphosphate receptor
TcG_07846	245,6024328	0,54161774	0,115426949	4,69229884	2,70152E-06	4,8906E-05	protein_codin GTP-binding protein
TcG_07847	310,9938415	0,524805755	0,10614679	4,944150959	7,64764E-07	1,5965E-05	protein_codin mitochondrial RNA binding protein
TcG_07848	620,7251537	0,315098275	0,081760424	3,853921741	0,000116241	0,001270533	protein_codin putative mitochondrial RNA binding protein
TcG_07849	88,0289246	0,441521019	0,196496524	2,246966057	0,024642196	0,098684512	protein_codin hypothetical protein
TcG_07850	185,2622272	0,498778257	0,135814847	3,672486975	0,000240201	0,002323016	protein_codin hypothetical protein
TcG_07851	238,1050061	0,454910693	0,118546654	3,837397986	0,000124345	0,001341396	protein_codin hypothetical protein
TcG_07852	223,495323	0,454658027	0,122290893	3,717840427	0,000200933	0,001998293	protein_codin putative SET and MYND domain-containing protein 3
TcG_07853	128,3962874	0,626624609	0,17507775	3,579121903	0,000344751	0,00315255	protein_codin hypothetical protein
TcG_07854	177,0858818	0,77666248	0,1385495	5,605667867	2,07453E-08	6,22683E-07	protein_codin actin interacting protein-like protein
TcG_07855	332,4691042	0,36991098	0,102447069	3,610752218	0,00030531	0,00284732	protein_codin hypothetical protein
TcG_07856	760,0980596	0,448475553	0,070308618	6,378671131	1,78631E-10	8,34524E-09	protein_codin Na/H antiporter-like protein
TcG_07857	249,2724397	0,255327275	0,121411114	2,102997551	0,035465984	0,129854051	protein_codin hypothetical protein
TcG_07858	177,8310452	0,228333513	0,132103846	1,728439555	0,083909449	0,238511991	protein_codin hypothetical protein
TcG_07859	0,636551173	1,116862059	2,209709801	0,505433817	0,613254172	1	
TcG_07860	400,2719429	-0,062827871	0,100993683	-0,62209703	0,533878066	0,734359643	protein_codin putative aspartyl-tRNA synthetase
TcG_07861	265,6944491	0,20372972	0,108833511	1,87193924	0,06121501	0,192153104	protein_codin YbaK/aminoacyl-tRNA synthetase-associated domain-containing protein
TcG_07862	165,258078	0,325414905	0,141434695	2,300813845	0,021402156	0,088685756	protein_codin hypothetical protein
TcG_07863	347,2196139	-0,154239917	0,097793711	-1,57719669	0,114750274	0,29472327	protein_codin hypothetical protein
TcG_07864	529,745807	-0,279067327	0,08052595	-3,46555771	0,000529133	0,004557228	protein_codin putative katanin
TcG_07865	275,734342	0,041749445	0,114256877	0,365399841	0,71481296	0,853620177	protein_codin putative programmed cell death protein 2
TcG_07866	484,3358608	-0,237557675	0,083821634	-2,83408549	0,004595704	0,02695991	protein_codin hypothetical protein

TcG_07867	248,5830016	0,104780676	0,11499018	0,911214117	0,362182567	0,594802935	protein_codin hypothetical protein
TcG_07868	1256,57082	-0,233723745	0,056825609	-4,11300028	3,9055E-05	0,000495609	protein_codin intraflagellar transport 172-like protein
TcG_07869	285,3796111	-0,120390364	0,107356915	-1,12140297	0,262116371	0,494282475	protein_codin hypothetical protein
TcG_07870	591,3171168	-0,063384062	0,075652933	-0,8378269	0,402127929	0,632422178	protein_codin hypothetical protein
TcG_07871	415,4127076	-0,178943791	0,089534446	-1,99860275	0,045651352	0,154925766	protein_codin putative epsilon tubulin
TcG_07872	187,0189741	-0,098673678	0,130527947	-0,75595824	0,449674232	0,670442053	protein_codin putative dynein arm light chain
TcG_07873	701,3876217	-0,187306162	0,075315495	-2,48695386	0,012884211	0,060338103	protein_codin hypothetical protein
TcG_07874	1847,782993	-0,168014078	0,054761409	-3,06811096	0,002154166	0,014603961	protein_codin putative kinesin
TcG_07875	38,67628683	0,24899914	0,277440535	0,897486519	0,369459364	0,601128826	protein_codin protein kinase
TcG_07876	299,117785	0,045031984	0,108231082	0,416072561	0,677356908	0,830962833	protein_codin hypothetical protein
TcG_07877	456,5521695	-0,058498589	0,090448627	-0,64676038	0,517787036	0,723042136	protein_codin hypothetical protein
TcG_07878	465,0360446	0,059893318	0,085431928	0,701064805	0,483262573	0,696662956	protein_codin putative ATP synthase
TcG_07879	780,621119	0,037550036	0,069760255	0,538272633	0,590388842	0,77624207	protein_codin putative structural maintenance of chromosome protein 4
TcG_07880	186,0517456	-0,072416199	0,13536203	-0,53498163	0,592662561	0,777907379	protein_codin putative structural maintenance of chromosome protein 4
TcG_07881	374,3165689	-0,088078718	0,09980485	-0,88250939	0,377501402	0,608222952	
TcG_07882	555,6316274	0,013261692	0,083360381	0,159088662	0,873599021	0,939322955	protein_codin phytanoyl-CoA dioxygenase
TcG_07883	555,1266404	0,041675847	0,088737762	0,46965177	0,638603831	0,807731988	protein_codin type 11 methyltransferase
TcG_07884	160,4748949	-0,24314921	0,140953886	-1,72502665	0,084522669	0,239549816	protein_codin hypothetical protein
TcG_07885	455,4172932	0,06594039	0,096703032	0,681885441	0,495311392	0,705083891	protein_codin vesicule-associated membrane protein
TcG_07886	64,26567598	0,140050807	0,240158052	0,583160987	0,559784931	0,754054394	
TcG_07887	454,1146677	-0,10629718	0,091657711	-1,1597189	0,246163273	0,474708335	protein_codin hypothetical protein
TcG_07888	543,281701	-0,000654114	0,089778454	-0,00728586	0,994186773	0,99771745	protein_codin hypothetical protein
TcG_07889	269,2775757	0,099725199	0,112157506	0,88915314	0,373920783	0,604496256	protein_codin putative calpain-like cysteine peptidase
TcG_07890	680,1145412	-0,136315295	0,077362884	-1,76202446	0,078065164	0,226853021	protein_codin putative adenylate kinase
TcG_07891	1110,870099	-0,262893521	0,063371866	-4,1484264	3,34768E-05	0,000433851	protein_codin hypothetical protein
TcG_07892	65,12083199	0,325077288	0,215791977	1,506438247	0,131954708	0,322809808	protein_codin hypothetical protein
TcG_07893	78,02288934	0,238666238	0,201121084	1,186679357	0,23535412	0,463192261	protein_codin hypothetical protein
TcG_07894	547,1352588	-0,344765819	0,08018443	-4,29966041	1,7106E-05	0,00024199	protein_codin putative surface protease GP63
TcG_07895	462,9290174	-0,095531365	0,084849431	-1,12589282	0,260210889	0,492617789	protein_codin putative D-alanyl-glycyl endopeptidase-like protein
TcG_07896	370,8300469	-0,127823834	0,101263698	-1,26228685	0,206845588	0,429021301	protein_codin hypothetical protein
TcG_07897	467,7246402	-0,197035265	0,087176115	-2,26019783	0,023808976	0,096316617	protein_codin putative transcription elongation factor-like protein
TcG_07898	682,4773073	-0,127425375	0,072332197	-1,76166881	0,078125271	0,226970759	protein_codin hypothetical protein
TcG_07899	783,0067884	0,015835407	0,069546031	0,227696775	0,81988198	0,911022136	protein_codin putative GTP-binding elongation factor tu family protein
TcG_07900	1733,226628	-0,095261255	0,062884077	-1,51487084	0,129805117	0,319405645	protein_codin putative neurobeachin/beige protein
TcG_07901	269,4632041	-0,32156228	0,109286127	-2,94238884	0,003256907	0,020364017	protein_codin ubiquitin-conjugating enzyme
TcG_07902	422,1644244	-0,20965357	0,090942951	-2,30533062	0,021148064	0,087978984	protein_codin hypothetical protein
TcG_07903	441,1325285	0,028393184	0,089735562	0,316409499	0,751691708	0,87492919	protein_codin hypothetical protein
TcG_07904	181,2757684	-0,205346914	0,137901308	-1,48908605	0,136464711	0,329163749	protein_codin putative E3 ubiquitin-protein ligase HECTD1-like isoform X6
TcG_07905	236,1372885	-0,121812118	0,118259001	-1,03004521	0,302988782	0,535618427	protein_codin putative E3 ubiquitin-protein ligase HECTD1-like isoform X6
TcG_07906	1704,623754	-0,160064218	0,051350396	-3,11709802	0,001826408	0,012778241	protein_codin putative translation initiation factor IF-2
TcG_07907	780,5419711	-0,31212378	0,073881201	-4,22467114	2,3929E-05	0,000324259	protein_codin mitochondrial guide RNA binding complex subunit 2
TcG_07908	142,6276417	-0,244933891	0,153254274	-1,59821899	0,109994248	0,286467226	protein_codin putative kinesin
TcG_07909	140,5334441	0,192373023	0,164588962	1,168808774	0,242480685	0,470898628	protein_codin multifunctional methyltransferase subunit TRM112
TcG_07910	226,9918122	-0,039847876	0,119215044	-0,33425207	0,738189354	0,866422734	protein_codin hypothetical protein
TcG_07911	67,11239339	0,414706921	0,214083611	1,937125959	0,052729948	0,17142733	protein_codin hypothetical protein
TcG_07912	453,7077102	0,033267759	0,0912616	0,364531842	0,715460901	0,853960462	protein_codin putative proteasome beta 6 subunit, putative,20S proteasome beta 6 subunit
TcG_07913	118,0999497	-0,041569976	0,171183177	-0,24283914	0,808130013	0,905092175	protein_codin putative GTP cyclohydrolase II
TcG_07914	680,5478141	-0,124310177	0,075004329	-1,65737336	0,097444014	0,264100721	protein_codin cyclophilin
TcG_07915	291,5629904	0,045270343	0,10542827	0,429394726	0,667635992	0,825884113	protein_codin hypothetical protein
TcG_07916	269,3843865	0,242950982	0,110546891	2,197718822	0,027969149	0,108523297	protein_codin hypothetical protein
TcG_07917	173,4731758	-0,36321187	0,142594663	-2,54716314	0,010860265	0,05266933	protein_codin hypothetical protein
TcG_07918	668,7972945	0,202398379	0,073552111	2,751768438	0,005927442	0,032702543	protein_codin pumilio protein 5
TcG_07919	74,74677186	-0,191669982	0,203055415	-0,94392943	0,345205711	0,579295913	protein_codin hypothetical protein
TcG_07920	135,4900875	0,06506616	0,154349239	0,42155154	0,673352384	0,828900214	protein_codin 39S ribosomal protein L46, mitochondrial
TcG_07921	41,55529712	-0,082985921	0,27427059	-0,30256952	0,762217945	0,879937935	protein_codin hypothetical protein
TcG_07922	438,8198359	0,042598767	0,088699571	0,480258995	0,631043242	0,802377854	protein_codin hypothetical protein
TcG_07923	459,5219581	-0,151673191	0,084354896	-1,7980366	0,072171207	0,216010232	protein_codin antigenic WD protein

TcG_07924	52,82798274	-0,003950665	0,242832576	-0,01626909	0,987019717	0,994746907	protein_codin hypothetical protein
TcG_07925	29,39849971	0,180363912	0,319819383	0,563955537	0,572784392	0,76401448	protein_codin hypothetical protein
TcG_07926	9,78610367	0,354998507	0,552299709	0,642764248	0,520377085	1	protein_codin hypothetical protein
TcG_07927	11,18266619	-0,486028536	0,535901738	-0,90693592	0,364440684	1	protein_codin dispersed gene family protein 1 (DGF-1)
TcG_07928	184,2123839	0,127677357	0,143378942	0,890488907	0,373203422	0,604239079	protein_codin dispersed gene family protein 1 (DGF-1)
TcG_07929	38,5763931	-0,265713862	0,295194365	-0,90013189	0,368050067	0,60025733	protein_codin dispersed protein family protein 1 (DGF-1)
TcG_07930	24,49900507	-0,148139565	0,353067297	-0,41957884	0,674793152	0,82970688	protein_codin dispersed gene family protein 1 (DGF-1)
TcG_07931	239,6268401	0,159074776	0,118947559	1,337352165	0,181107711	0,392575105	protein_codin dispersed gene family protein 1 (DGF-1)
TcG_07932	3290,573116	-0,081961721	0,044336051	-1,8486473	0,064508759	0,199999593	protein_codin hypothetical protein
TcG_07933	184,5318159	0,313448686	0,141643875	2,212934987	0,026902128	0,105511766	protein_codin calpain-like cysteine peptidase
TcG_07934	204,4460056	0,24225282	0,124613262	1,944037233	0,051890961	0,169534522	protein_codin hypothetical protein
TcG_07935	138,1214353	0,304650649	0,153827346	1,980471331	0,047650592	0,159837798	protein_codin calpain-like cysteine peptidase
TcG_07936	223,8344145	0,338944987	0,120618289	2,810062972	0,004953181	0,028607952	protein_codin putative calpain-like cysteine peptidase
TcG_07937	997,0255406	-0,036914082	0,064173512	-0,57522303	0,56514047	0,757837672	protein_codin putative nucleosome assembly protein
TcG_07938	534,4666938	-0,110855527	0,081619701	-1,3581957	0,174401596	0,383127966	protein_codin calpain-like cysteine peptidase
TcG_07939	429,2797575	0,132322315	0,092239459	1,434552163	0,151414755	0,351799471	protein_codin hypothetical protein
TcG_07940	67,35695493	-0,078514559	0,223543113	-0,35122781	0,725417445	0,858172586	
TcG_07941	355,0341862	-0,041761721	0,107652833	-0,3879296	0,698068132	0,843887456	protein_codin calpain-like cysteine peptidase
TcG_07942	993,5590231	0,039007781	0,068344278	0,570754169	0,568166294	0,760072642	protein_codin hypothetical protein
TcG_07943	185,4543241	0,104139582	0,138718208	0,750727564	0,452816631	0,673551167	protein_codin putative calpain-like protein
TcG_07944	399,6230157	0,424860518	0,098097413	4,331006352	1,48429E-05	0,000213893	protein_codin calpain-like cysteine peptidase, Clan CA, family C2
TcG_07945	454,2390949	-0,001979651	0,095474307	-0,02073491	0,98345712	0,99376391	protein_codin calpain-like cysteine peptidase
TcG_07946	1217,000483	-0,196060955	0,062250341	-3,14955634	0,001635186	0,011698514	protein_codin calpain
TcG_07947	620,5259363	0,015727844	0,075539123	0,208207925	0,835066621	0,92007617	protein_codin calpain-like cysteine peptidase
TcG_07948	1763,752973	-0,14949978	0,05308838	-2,81605464	0,00486174	0,028175533	protein_codin calpain-like cysteine peptidase
TcG_07949	163,9097888	0,064577147	0,140111322	0,460898849	0,644871178	0,811369249	protein_codin protein disulfide isomerase
TcG_07950	453,0487585	-0,177523259	0,087135928	-2,03731415	0,041618571	0,145326329	protein_codin C15orf24 protein
TcG_07951	150,7176946	0,12752945	0,146502963	0,87049058	0,384032365	0,614472999	protein_codin putative Qc-SNARE protein
TcG_07952	147,1047662	-0,208913946	0,146095731	-1,42997981	0,152722813	0,352971576	protein_codin putative oxysterol-binding protein
TcG_07953	138,2528305	0,234115801	0,153160972	1,528560428	0,126373452	0,31409915	protein_codin hypothetical protein
TcG_07954	298,8075535	-0,2133343	0,106600393	-2,00125246	0,04536519	0,154378203	protein_codin putative sphingosine kinase A, B
TcG_07955	269,0962514	-0,175042137	0,111685317	-1,56727976	0,117049313	0,298510531	protein_codin hypothetical protein
TcG_07956	358,7825709	-0,240167597	0,099191608	-2,42124916	0,015467272	0,069297684	protein_codin hypothetical protein
TcG_07957	281,8810803	0,177317897	0,124319082	1,426312795	0,153778073	0,354632315	protein_codin regulator of chromosome condensation
TcG_07958	131,6226283	0,128324021	0,162273626	0,790787909	0,429067767	0,653987669	protein_codin hypothetical protein
TcG_07959	0					1	protein_codin hypothetical protein
TcG_07960	539,7229567	-0,180615035	0,082464654	-2,19021152	0,0285089	0,109918176	protein_codin regulator of chromosome condensation
TcG_07961	129,8499843	-0,079857012	0,152601392	-0,52330461	0,600762295	0,783588603	protein_codin RNA-binding protein-19
TcG_07962	168,1243336	-0,109622602	0,142178642	-0,77102018	0,44069497	0,663501236	protein_codin hypothetical protein
TcG_07963	203,4043083	0,038839381	0,132547093	0,293023257	0,769504375	0,884364283	protein_codin RNA-binding protein 8A
TcG_07964	93,50131835	0,165194953	0,184823143	0,893800151	0,371428828	0,602808656	protein_codin hypothetical protein
TcG_07965	253,7325471	-0,051767187	0,112550725	-0,45994539	0,64555542	0,81191979	protein_codin hypothetical protein
TcG_07966	230,2430371	0,075736549	0,119438345	0,634105816	0,526011768	0,728904717	protein_codin hypothetical protein
TcG_07967	2,290861052	0,031194511	1,145084692	0,0272421	0,978266637	1	protein_codin hypothetical protein
TcG_07968	282,1516028	-0,2800997	0,106456536	-2,63111792	0,00851045	0,043532925	protein_codin putative vesicle-associated membrane protein
TcG_07969	297,297202	0,01192558	0,106752524	0,111712393	0,911051453	0,956844965	protein_codin hypothetical protein
TcG_07970	130,5869968	-0,104060824	0,153730022	-0,67690632	0,498465391	0,707660829	protein_codin hypothetical protein
TcG_07971	89,56712389	0,23879836	0,19519401	1,223389794	0,221182506	0,446371802	protein_codin hypothetical protein
TcG_07972	418,8807241	-0,259024829	0,088673125	-2,9211199	0,003487756	0,021586078	protein_codin putative TPR-repeat protein
TcG_07973	1603,375812	-0,004563859	0,052112498	-0,08757705	0,930212847	0,966240456	protein_codin putative chaperone DNAJ protein
TcG_07974	252,2308988	0,124839435	0,111336053	1,121284904	0,262166609	0,494282475	protein_codin aldo-keto reductase
TcG_07975	257,0628212	0,282601604	0,114419124	2,469880866	0,013515806	0,062791032	protein_codin 26S proteasome regulatory subunit, ATPase 3, interacting protein
TcG_07976	437,4481573	-0,016077976	0,094501793	-0,17013408	0,864904688	0,934985064	protein_codin hypothetical protein
TcG_07977	180,6845083	0,009958535	0,131144881	0,075935368	0,939470518	0,972022274	protein_codin putative 3-oxoacyl-ACP reductase
TcG_07978	111,972215	-0,239986767	0,165996537	-1,44573358	0,148251943	0,347069512	protein_codin hypothetical protein
TcG_07979	136,7589498	-0,382010576	0,174398953	-2,19044077	0,028492286	0,109918176	protein_codin putative protein kinase
TcG_07980	322,9567821	-0,272576937	0,101993538	-2,67249222	0,007529012	0,039560605	protein_codin putative splicing factor XB2

TcG_07981	396,2693008	0,212364223	0,091654918	2,316997573	0,020503864	0,085760927	protein_codin putative tubulin-tyrosine ligase
TcG_07982	211,732364	0,260711964	0,133577119	1,951771123	0,050965384	0,167894495	protein_codin hypothetical protein
TcG_07983	3302,333538	-0,080125588	0,088332622	-0,90708943	0,364359505	0,596371017	protein_codin dynein heavy chain 7, axonemal isoform X1
TcG_07984	187,9037302	0,121689339	0,139320495	0,873448942	0,382418425	0,612398047	protein_codin trans-sialidase-like protein
TcG_07985	5,281003649	0,559314339	0,776250962	0,720532877	0,471196965	1	protein_codin hypothetical protein
TcG_07986	74,50399206	0,400345491	0,205302837	1,950024149	0,051173241	0,168253584	protein_codin L1Tc protein
TcG_07987	132,7281193	-0,086321253	0,154196883	-0,55981192	0,575607732	0,766286474	protein_codin putative thioredoxin-like protein
TcG_07988	174,5415175	-0,179220346	0,142758921	-1,25540557	0,209331567	0,431872099	protein_codin ATPase family AAA domain-containing protein 1-B-like
TcG_07989	89,526244	-0,090126785	0,184709307	-0,48793851	0,625593395	0,798753175	protein_codin hypothetical protein
TcG_07990	254,9914077	0,018747691	0,117071004	0,160139487	0,872771196	0,938984779	protein_codin putative alpha-ketoglutarate-dependent dioxygenase
TcG_07991	245,9985722	-0,032644945	0,120433772	-0,27106139	0,78634382	0,893586355	protein_codin hypothetical protein
TcG_07992	106,5558104	0,420984265	0,174051842	2,418729153	0,015574832	0,069642191	protein_codin putative alanine racemase 2
TcG_07993	579,7750914	-0,243983049	0,076232898	-3,20049554	0,001371915	0,010098479	protein_codin ubiquitin-conjugating enzyme E2
TcG_07994	320,1502089	-0,010824747	0,105741366	-0,10237003	0,918462973	0,960840813	protein_codin hypothetical protein
TcG_07995	219,841222	0,260780077	0,122597184	2,127129424	0,033409331	0,123865763	protein_codin putative protein disulfide isomerase
TcG_07996	97,31820249	0,281724565	0,177643086	1,585902222	0,112761492	0,291034672	protein_codin hypothetical protein
TcG_07997	50,71928927	0,027626948	0,25208738	0,109592745	0,912732365	0,957238745	protein_codin hypothetical protein
TcG_07998	131,1442789	-0,155524014	0,16254671	-0,95679583	0,338670318	0,573276734	protein_codin hypothetical protein
TcG_07999	722,5690375	-0,126733284	0,076621894	-1,65400876	0,098125732	0,265193546	protein_codin metallo-peptidase, Clan ME, Family M16
TcG_08000	297,3694667	0,071586574	0,110742795	0,64642195	0,518006129	0,723173757	protein_codin hypothetical protein
TcG_08001	127,7430113	0,382392615	0,156738375	2,43968725	0,014699982	0,066894734	protein_codin hypothetical protein
TcG_08002	1108,700786	0,100642573	0,066061871	1,523459314	0,127643832	0,316405956	protein_codin putative threonyl-tRNA synthetase
TcG_08003	498,636644	0,059558309	0,082508934	0,721840723	0,470392409	0,686566699	protein_codin putative peroxisome targeting signal 1 receptor
TcG_08004	192,7009925	0,452419067	0,1288392	3,511501677	0,000445583	0,003925871	protein_codin putative 60S ribosomal protein L2
TcG_08005	410,2335686	-0,13635835	0,089960594	-1,51575645	0,129580947	0,319264357	protein_codin iron-sulfur cluster assembly protein
TcG_08006	366,8693972	-0,13020536	0,096375512	-1,35102121	0,17668864	0,386905042	protein_codin calmodulin-like protein containing EF hand domain
TcG_08007	195,106075	-0,044847802	0,126514329	-0,35448792	0,722973254	0,857335574	protein_codin hypothetical protein
TcG_08008	435,4430997	-0,207589292	0,091274572	-2,2743387	0,02294563	0,093674443	protein_codin putative transporter
TcG_08009	314,5703912	-0,196451533	0,102738031	-1,9121598	0,0558557	0,178867923	protein_codin putative transporter
TcG_08010	1118,465844	-0,30064663	0,227681359	-1,32047099	0,186677814	0,40067602	protein_codin hypothetical protein
TcG_08011	1712,975255	-0,29944178	0,062276919	-4,8082305	1,52272E-06	2,94528E-05	protein_codin hypothetical protein
TcG_08012	534,1961043	-0,289119478	0,08450518	-3,42132255	0,000623174	0,00519431	
TcG_08013	138,5732636	-0,093193832	0,148818548	-0,62622457	0,531167651	0,732457558	protein_codin hypothetical protein
TcG_08014	676,3841822	0,150184889	0,078236346	1,919630671	0,054904567	0,17679942	protein_codin putative transaldolase
TcG_08015	126,9202466	0,083407367	0,157500236	0,52956979	0,596410244	0,780668859	protein_codin hypothetical protein
TcG_08016	240,9969529	-0,084144386	0,118523909	-0,70993597	0,477743846	0,692608846	protein_codin putative ubiquitin hydrolase, putative,cysteine peptidase, Clan CA, family C19
TcG_08017	450,1185152	0,040684074	0,08929338	0,455622516	0,648661449	0,813280193	protein_codin putative ubiquitin hydrolase, putative,cysteine peptidase, Clan CA, family C19
TcG_08018	1947,724973	-0,415958769	0,15728017	-2,64469938	0,008176352	0,042196533	protein_codin hemagluttinin family protein
TcG_08019	481,7932897	-0,734561207	0,083878434	-8,75745015	1,99717E-18	3,05632E-16	protein_codin putative mitogen-activated protein kinase 3
TcG_08020	565,2924994	-0,663297615	0,078869812	-8,41003159	4,099E-17	5,52222E-15	protein_codin PIF1 helicase-like protein
TcG_08021	443,1872915	0,011516615	0,088244627	0,130507836	0,896164655	0,949603411	protein_codin ribonuclease mar1
TcG_08022	286,7201265	-0,316387325	0,108420268	-2,91815665	0,003521074	0,021734238	protein_codin putative ATP-dependent chaperone
TcG_08023	322,6302237	-0,544009848	0,105483784	-5,15728417	2,50557E-07	5,89624E-06	protein_codin hypothetical protein
TcG_08024	62,28039778	-0,031865934	0,227706542	-0,13994299	0,888705037	0,94628587	protein_codin hypothetical protein
TcG_08025	42,80964558	0,226109779	0,28217181	0,801319518	0,422946694	0,648955158	
TcG_08026	173,6317359	-0,178539161	0,134094547	-1,33144237	0,183043497	0,39529207	protein_codin hypothetical protein
TcG_08027	126,3250996	0,068944087	0,161222725	0,42763256	0,668918657	0,826307698	protein_codin cystathionine beta-synthase,cysteine synthase,serine sulfhydrylase
TcG_08028	699,039955	-0,054983629	0,074202789	-0,7409914	0,458698653	0,677438813	protein_codin hypothetical protein
TcG_08029	705,7710732	0,002097989	0,074181284	0,028281912	0,977437307	0,990224843	protein_codin exosome complex exonuclease RRP44 isoform X1
TcG_08030	131,3569159	0,259818414	0,174597787	1,488096834	0,136725358	0,329540254	protein_codin hypothetical protein
TcG_08031	290,223101	-0,017979947	0,112560501	-0,15973585	0,87308916	0,939152447	protein_codin hypothetical protein
TcG_08032	705,9271826	-0,030227072	0,072677769	-0,41590533	0,677479279	0,830962833	protein_codin putative casein kinase II, alpha chain
TcG_08033	452,4148273	0,068096873	0,089635513	0,759708637	0,447428765	0,668546514	protein_codin hypothetical protein
TcG_08034	121,7423152	-0,109946994	0,166119662	-0,66185419	0,508064675	0,714807204	protein_codin hypothetical protein
TcG_08035	303,6916652	0,141840547	0,103024581	1,376764134	0,168585183	0,375101512	protein_codin transcription elongation factor B, polypeptide 1
TcG_08036	300,9018353	0,051889755	0,103184972	0,502880932	0,615047988	0,792057258	protein_codin putative phosphoadenosine phosphosulfate reductase-like protein
TcG_08037	223,7026035	0,20470135	0,119094553	1,718813716	0,085648297	0,241793658	protein_codin putative surface protease GP63

TcG_08038	194,0822687	-0,090220301	0,129120398	-0,69873004	0,484720758	0,697809978	protein_codin hypothetical protein
TcG_08039	507,0725234	-0,093429624	0,084509281	-1,10555459	0,268919353	0,501496548	protein_codin hypothetical protein
TcG_08040	533,9872834	-0,09173416	0,085984776	-1,06686514	0,286032739	0,519187736	protein_codin hypothetical protein
TcG_08041	592,1181245	0,21266862	0,077669082	2,738137425	0,006178825	0,033815714	protein_codin glycerol-3-phosphate dehydrogenase (FAD-dependent), mitochondrial
TcG_08042	610,8871868	0,297260127	0,076801503	3,870498826	0,000108613	0,001197325	protein_codin putative myosin heavy chain
TcG_08043	0,586192964	-1,478472749	2,316990182	-0,63810057	0,523408217	1	
TcG_08044	166,6426449	0,341381279	0,143848878	2,373193887	0,017635004	0,076323929	protein_codin putative protein kinase
TcG_08045	279,3274965	0,183623227	0,10837262	1,694369179	0,090195152	0,250419611	protein_codin hypothetical protein
TcG_08046	368,9558198	0,125266474	0,098838288	1,267388146	0,205016536	0,426296049	protein_codin methyltransferase/D-alanine--D-alanine ligase
TcG_08047	104,5909552	0,240324445	0,172895171	1,390000906	0,164528602	0,369830907	protein_codin hypothetical protein
TcG_08048	513,6992803	0,177280645	0,086974272	2,038311334	0,041518807	0,14510917	protein_codin hypothetical protein
TcG_08049	632,3341912	-0,020780005	0,074669163	-0,27829433	0,780786426	0,890875557	protein_codin hypothetical protein
TcG_08050	290,4691107	0,044710173	0,107832851	0,414624793	0,678416596	0,831829271	protein_codin putative protein kinase
TcG_08051	191,2634428	0,192040258	0,129617111	1,4815965	0,138447691	0,332240047	protein_codin putative dTDP-4-dehydrorhamnose reductase domain protein
TcG_08052	115,3247839	0,125189588	0,16888539	0,741269499	0,458530052	0,677438813	protein_codin hypothetical protein
TcG_08053	324,7317577	-0,122543734	0,103467045	-1,18437454	0,236264833	0,463600927	protein_codin phosphatidylinositol-4-phosphate 5-kinase
TcG_08054	770,6357237	-0,026067516	0,074509884	-0,34985313	0,726448926	0,858752908	protein_codin hypothetical protein
TcG_08055	207,6773208	0,302067875	0,12482595	2,41991249	0,015524243	0,069526043	protein_codin CDP-diacylglycerol--inositol 3-phosphatidyltransferase
TcG_08056	112,9180053	0,32463619	0,164428852	1,974326198	0,048344664	0,161570239	protein_codin hypothetical protein
TcG_08057	255,4167298	0,081129562	0,119092662	0,68123057	0,495725608	0,705500171	protein_codin hypothetical protein
TcG_08058	136,3908033	0,196675473	0,157063254	1,252205515	0,210494987	0,433024666	protein_codin hypothetical protein
TcG_08059	365,1972446	0,364428618	0,097767374	3,727507465	0,000193383	0,001939856	protein_codin putative protein kinase
TcG_08060	199,7374854	0,259833823	0,129776545	2,002163199	0,045267182	0,15425458	protein_codin putative metallopeptidase, putative,metallo-peptidase, clan MP, family M67
TcG_08061	709,718129	-0,051462128	0,071908601	-0,71566026	0,474201152	0,68995285	protein_codin hypothetical protein
TcG_08062	823,6089035	-0,21229855	0,068245933	-3,11078685	0,001865896	0,013015213	protein_codin putative protein phosphatase 2A regulatory subunit
TcG_08063	877,6036459	-0,240774066	0,066450707	-3,62334843	0,000290814	0,002730443	protein_codin putative calmodulin
TcG_08064	699,4081221	-0,057585209	0,079085641	-0,72813735	0,466529513	0,683166195	protein_codin hypothetical protein
TcG_08065	718,5669699	-0,124649185	0,074275805	-1,67819367	0,093309297	0,256241174	protein_codin putative PTP1-interacting protein, 39 kDa
TcG_08066	668,0794278	0,181032883	0,076700348	2,360261553	0,018262054	0,078190747	protein_codin 40S ribosomal protein S3
TcG_08067	261,0870608	-0,030148727	0,114848524	-0,26250861	0,792929346	0,896248225	protein_codin putative 2Fe-2S iron-sulfur cluster binding domain containing protein
TcG_08068	304,8259432	-0,228616249	0,103811697	-2,20222053	0,027649735	0,107716821	protein_codin putative NAD/FAD dependent dehydrogenase
TcG_08069	180,8411482	-0,17347074	0,133790278	-1,29658703	0,194773316	0,412174055	protein_codin hypothetical protein
TcG_08070	248,4778492	0,008392646	0,116992396	0,07173668	0,942811465	0,973191135	protein_codin hypothetical protein
TcG_08071	574,8524657	-0,252462603	0,076720674	-3,29067239	0,000999482	0,007777034	protein_codin succinate dehydrogenase
TcG_08072	2122,024885	0,204003755	0,047783337	4,269349289	1,96044E-05	0,00027218	protein_codin 60S ribosomal protein L6
TcG_08073	1666,608359	-0,023643212	0,051063351	-0,46301724	0,643352013	0,810732698	protein_codin glutamate dehydrogenase
TcG_08074	325,7222756	0,024106792	0,105065473	0,229445421	0,818522737	0,910552513	protein_codin solanesyl diphosphate synthase
TcG_08075	329,5557066	-0,200982456	0,099418768	-2,0215746	0,043220318	0,149312197	protein_codin SNW domain-containing protein 1
TcG_08076	101,7088718	0,12041053	0,174330724	0,690701713	0,489753013	0,700874809	protein_codin tryparedoxin peroxidase
TcG_08077	1119,629091	-0,137941524	0,066092032	-2,08711278	0,036877941	0,133423365	protein_codin tryparedoxin peroxidase
TcG_08078	269,8191488	0,280968257	0,114213172	2,460033736	0,013892396	0,063950646	protein_codin putative developmentally regulated protein
TcG_08079	196,8671566	-0,02620795	0,134469917	-0,19489824	0,845472614	0,925035391	protein_codin metacyclogenesis-specific protein
TcG_08080	345,1613419	0,064453536	0,102176943	0,630803128	0,528169258	0,730496481	protein_codin tryparedoxin peroxidase
TcG_08081	443,3680201	0,147705442	0,086619834	1,705215017	0,08815433	0,246644789	protein_codin kinetoplast DNA-associated protein
TcG_08082	225,0415032	-0,030948701	0,118696049	-0,2607391	0,794293709	0,897297866	protein_codin hypothetical protein
TcG_08083	164,6587494	0,209907164	0,152424516	1,377122064	0,168474512	0,374941548	protein_codin hypothetical protein
TcG_08084	666,7553956	0,063635839	0,074109853	0,858669065	0,390523122	0,620488329	protein_codin protein tyrosine phosphatase
TcG_08085	8685,409508	-0,176939166	0,040963309	-4,31945492	1,56415E-05	0,000223731	protein_codin histone H2B
TcG_08086	2257,720142	-0,155781456	0,053654981	-2,90339226	0,00369144	0,022557499	protein_codin histone H2B
TcG_08087	136,5084279	-0,053945257	0,15243045	-0,35390079	0,723413234	0,857397569	protein_codin putative mitochondrial carrier protein
TcG_08088	195,0959605	0,170621574	0,127738936	1,335705297	0,181645617	0,393299593	protein_codin hypothetical protein
TcG_08089	236,6240329	0,344598146	0,124637976	2,764792537	0,005695901	0,031666367	protein_codin glutaredoxin-like protein
TcG_08090	318,7530918	-0,043721312	0,106100562	-0,41207427	0,680284984	0,833257408	protein_codin putative DNA replication licensing factor, putative,minichromosome maintenance protein-like protein
TcG_08091	278,9367303	-0,308562913	0,109824313	-2,80960477	0,004960237	0,028634433	protein_codin hypothetical protein
TcG_08092	1256,209316	0,115598364	0,059525404	1,942000495	0,05213704	0,170205621	protein_codin trypanothione reductase
TcG_08093	327,29751	-0,013071064	0,101479803	-0,12880459	0,897512274	0,950181186	protein_codin putative ATP-dependent RNA helicase
TcG_08094	237,3262251	-0,156032702	0,119877666	-1,30159943	0,193053356	0,409880188	protein_codin hypothetical protein

TcG_08095	0					1	protein_codin microtubule-associated protein
TcG_08096	0,155988004	0,503022807	4,080472857	0,123275616	0,901888849	1	
TcG_08097	505,9629594	-0,374450169	0,081172663	-4,61300829	3,96882E-06	6,85288E-05	protein_codin putative protein kinase
TcG_08098	981,6160221	-0,118911942	0,062672741	-1,89734707	0,057782146	0,183389426	protein_codin putative structural maintenance of chromosome (SMC)
TcG_08099	285,9185189	0,087141635	0,10878522	0,801042964	0,423106773	0,649114814	protein_codin anaphase-promoting complex subunit 3
TcG_08100	124,6724031	0,131890931	0,156397676	0,843304929	0,399057927	0,62898729	protein_codin dynein light chain
TcG_08101	170,4279581	0,141736968	0,145915934	0,971360457	0,331368812	0,565601864	protein_codin hypothetical protein
TcG_08102	204,4707521	0,134004575	0,124741628	1,074257067	0,282707482	0,515981235	protein_codin hypothetical protein
TcG_08103	1,883678369	-0,321035105	1,284631007	-0,24990453	0,802661182	1	protein_codin hypothetical protein
TcG_08104	465,6459178	0,198834551	0,094386536	2,106598665	0,035152383	0,128966278	protein_codin L1Tc protein
TcG_08105	113,3009161	-0,033098617	0,164565227	-0,20112765	0,840598763	0,922466573	protein_codin hypothetical protein
TcG_08106	194,7440521	-0,395753282	0,129194594	-3,06323407	0,002189587	0,014792162	protein_codin putative protein phosphatase 1, regulatory subunit
TcG_08107	134,469536	-0,071487615	0,155637701	-0,45932068	0,646003899	0,812019442	protein_codin prefoldin subunit
TcG_08108	778,4517776	-0,221509454	0,072988552	-3,034852	0,002406537	0,015959534	protein_codin hypothetical protein
TcG_08109	268,8903239	0,074912087	0,108758896	0,688790432	0,490955158	0,701878188	protein_codin hypothetical protein
TcG_08110	499,197316	-0,087713915	0,086098592	-1,01876131	0,308316296	0,541317261	protein_codin hypothetical protein
TcG_08111	294,5037694	-0,043267442	0,107685452	-0,40179468	0,687835137	0,837899053	protein_codin putative vacuolar ATP synthase
TcG_08112	154,7351214	0,212709069	0,14636401	1,453288072	0,146143788	0,343731613	protein_codin hypothetical protein
TcG_08113	329,8904912	0,085907088	0,100295218	0,856542207	0,391697937	0,621398293	protein_codin putative SPFH domain / Band 7 family protein
TcG_08114	382,3907651	-0,014971664	0,092695378	-0,16151467	0,871688057	0,938255094	protein_codin succinate dehydrogenase subunit
TcG_08115	314,4420903	-0,015221563	0,101185633	-0,15043206	0,88042375	0,941969671	protein_codin coiled-coil domain-containing protein 135
TcG_08116	261,2047087	0,134874492	0,111296553	1,211847883	0,225570615	0,451843214	protein_codin putative methyltransferase
TcG_08117	464,0222689	0,305304084	0,091336316	3,342636271	0,000829866	0,00668625	protein_codin putative transporter
TcG_08118	452,5175663	-0,022179923	0,090365637	-0,24544644	0,806110778	0,904233839	protein_codin NADH:ubiquinone reductase (H(+)-translocating)/NADH dehydrogenase
TcG_08119	332,616554	-0,627276685	0,106375443	-5,89681856	3,70577E-09	1,29713E-07	protein_codin trans-sialidase
TcG_08120	856,0590925	-0,540925885	0,065808637	-8,21967926	2,04048E-16	2,41235E-14	protein_codin trans-sialidase
TcG_08121	50,36603635	0,268424352	0,260478238	1,030505863	0,302772601	0,535497997	protein_codin hypothetical protein
TcG_08122	14,40063667	-0,225435831	0,451893276	-0,49886963	0,617871231	1	protein_codin hypothetical protein
TcG_08123	37,10418376	-0,177631209	0,290206211	-0,61208617	0,540480745	0,739403698	protein_codin putative surface protease GP63
TcG_08124	265,1924095	0,486491903	0,111050534	4,380815505	1,18236E-05	0,000175177	protein_codin membrane protein
TcG_08125	41,29917463	0,477953683	0,278354711	1,717066979	0,085966935	0,242515926	protein_codin membrane protein
TcG_08126	194,0018252	0,443396312	0,132197597	3,354042136	0,000796402	0,006443518	protein_codin hypothetical protein
TcG_08127	101,8389992	0,027851745	0,187068611	0,148885186	0,881644231	0,942184067	protein_codin hypothetical protein
TcG_08128	151,2202623	-0,108412431	0,156561458	-0,69245926	0,488648963	0,699976894	protein_codin dynein light chain
TcG_08129	1249,862789	0,233102095	0,057329901	4,065977603	4,78315E-05	0,000593972	protein_codin 40S ribosomal protein S14
TcG_08130	278,4748242	-0,028817792	0,107706356	-0,26755888	0,78903889	0,894943008	protein_codin hypothetical protein
TcG_08131	1480,052842	-0,155157781	0,055650694	-2,78806552	0,005302382	0,029982136	protein_codin P27 protein
TcG_08132	480,6450045	0,292861993	0,088695863	3,301867573	0,000960434	0,007536348	protein_codin hypothetical protein
TcG_08133	297,0471493	-0,100105587	0,103264198	-0,96941233	0,332339501	0,566340797	protein_codin hypothetical protein
TcG_08134	837,3781025	0,173449554	0,066690592	2,600809941	0,009300396	0,046606571	protein_codin hypothetical protein
TcG_08135	1141,952003	0,300546438	0,060208796	4,991736405	5,98389E-07	1,28229E-05	protein_codin ribosomal protein S20
TcG_08136	430,5293822	0,226240269	0,088963101	2,543079844	0,010988012	0,053155367	protein_codin hypothetical protein
TcG_08137	0,91200019	-0,716536165	2,018473582	-0,35498912	0,72259774	1	protein_codin ribosomal protein S20
TcG_08138	576,7177009	-0,062189635	0,079641101	-0,78087362	0,434876827	0,659055973	protein_codin hypothetical protein
TcG_08139	22,69984223	0,485065806	0,376149754	1,289555027	0,197205199	0,415601281	protein_codin surface protease GP63
TcG_08140	395,7813401	0,279124183	0,094044442	2,968002978	0,002997414	0,019012531	protein_codin GP63 group II protein
TcG_08141	5,831149331	0,429067053	0,764933349	0,560920835	0,574851506	1	protein_codin putative surface protease GP63
TcG_08142	127,1533204	0,319773251	0,155844192	2,051877886	0,040181531	0,141691423	
TcG_08143	535,9379129	0,018765536	0,081608783	0,229945057	0,818134464	0,910311132	protein_codin putative amastin
TcG_08144	1129,258734	0,023382624	0,069958957	0,334233462	0,738203395	0,866422734	protein_codin putative amastin
TcG_08145	1308,414089	0,054617007	0,056593504	0,96507555	0,334506972	0,568936844	protein_codin putative 3-methylcrotonyl-CoA carboxylase
TcG_08146	1201,654935	0,312442093	0,061928939	5,045171101	4,53116E-07	9,924E-06	protein_codin NAD(P)-dependent steroid dehydrogenase protein
TcG_08147	113,5902557	0,011755776	0,169242012	0,069461332	0,944622411	0,974133979	protein_codin hypothetical protein
TcG_08148	333,3372297	-0,114028748	0,102100946	-1,11682362	0,264069746	0,496193848	protein_codin palmitoyltransferase PFA3
TcG_08149	247,1101923	0,193339088	0,115321667	1,67652006	0,09363637	0,256834988	protein_codin hypothetical protein
TcG_08150	173,4598218	0,143526637	0,132826257	1,080559224	0,279893229	0,513072647	protein_codin generative cell specific-1
TcG_08151	46,41668289	0,090949302	0,269450479	0,337536241	0,735712697	0,864944729	protein_codin putative Tartrateresistant acid phosphatase type 5

TcG_08152	671,2817527	-0,072412279	0,077776482	-0,93103053	0,351837772	0,586278214	protein_codin	putative zinc carboxypeptidase
TcG_08153	252,727611	-0,016082644	0,117766704	-0,13656359	0,89137576	0,946972656	protein_codin	hypothetical protein
TcG_08154	714,9445981	-0,280943996	0,074541477	-3,76896201	0,000163928	0,001692752	protein_codin	hypothetical protein
TcG_08155	273,4140969	0,098137553	0,108208745	0,906928113	0,364444814	0,596392318	protein_codin	hypothetical protein
TcG_08156	279,1821156	0,130811569	0,109194621	1,197967151	0,230929805	0,458312605	protein_codin	putative trans-splicing factor
TcG_08157	319,5843351	0,086306069	0,103682459	0,832407623	0,405178901	0,634304216	protein_codin	hypothetical protein
TcG_08158	543,6086833	0,003000672	0,082796056	0,036241726	0,971089616	0,987626781	protein_codin	hypothetical protein
TcG_08159	382,6741015	0,137920351	0,092777528	1,486570662	0,137128241	0,330167872	protein_codin	hypothetical protein
TcG_08160	262,8626861	-0,046897837	0,113751948	-0,41228162	0,680133019	0,833159353	protein_codin	dual specificity protein phosphatase or MAP kinase phosphatase
TcG_08161	296,2718252	0,302264373	0,108205093	2,793439423	0,00521508	0,029618584	protein_codin	putative hydrogenase
TcG_08162	558,626607	0,357271109	0,079089633	4,517293823	6,2635E-06	0,00010178	protein_codin	kinetoplast DNA-associated protein
TcG_08163	9429,372477	0,152664662	0,034829564	4,383191896	1,16953E-05	0,00017372	protein_codin	chaperonin HSP60, mitochondrial precursor
TcG_08164	662,3497148	-0,006618937	0,080923955	-0,08179206	0,934812069	0,969193077	protein_codin	mismatch repair protein
TcG_08165	280,9597318	0,019460204	0,107167935	0,181586072	0,85590758	0,930257525	protein_codin	hypothetical protein
TcG_08166	740,8569233	0,082744014	0,077463562	1,068166921	0,285445223	0,51904649	protein_codin	putative brefeldin A-inhibited guanine nucleotide-exchange protein 1
TcG_08167	343,6595816	0,068255451	0,098606846	0,692197897	0,488813059	0,700047974	protein_codin	PIF1 helicase-like protein
TcG_08168	522,6341108	-0,379544267	0,081552095	-4,65401004	3,25541E-06	5,78484E-05	protein_codin	putative vacuolar protein sorting-associated protein 41
TcG_08169	106,1650349	0,048519463	0,171939265	0,282189549	0,777798172	0,889153391	protein_codin	hypothetical protein
TcG_08170	387,3449688	-0,176173472	0,09955912	-1,76953625	0,076804426	0,224597699	protein_codin	putative phosphomannomutase
TcG_08171	176,3712184	-0,245797337	0,138363627	-1,77645919	0,075657262	0,222309166	protein_codin	alkylated DNA repair protein alkB like protein 4
TcG_08172	165,7043756	-0,111014989	0,136877713	-0,81105233	0,417335618	0,644013116	protein_codin	calcineurin A2 subunit
TcG_08173	60,07570152	-0,308588109	0,227926676	-1,35389203	0,175770828	0,385332225	protein_codin	hypothetical protein
TcG_08174	245,6906267	-0,137803192	0,114594415	-1,20252974	0,229158351	0,456268888	protein_codin	hypothetical protein
TcG_08175	236,8521589	-0,168441801	0,119368313	-1,41110984	0,158212229	0,360410319	protein_codin	hypothetical protein
TcG_08176	403,2875834	-0,285981624	0,094057511	-3,04049746	0,002361877	0,015708786	protein_codin	hypothetical protein
TcG_08177	185,2381964	-0,084671276	0,135326631	-0,62568081	0,531524323	0,732689576	protein_codin	putative amino acid transporter, putative,amino acid permease
TcG_08178	234,3260801	0,366426332	0,118747141	3,085769723	0,00203026	0,013926934	protein_codin	hypothetical protein
TcG_08179	78,71607105	-0,046422576	0,203297444	-0,22834805	0,819375669	0,910871391	protein_codin	leucine rich repeat containing 45
TcG_08180	175,0904831	-0,220921579	0,137965752	-1,60127841	0,109315269	0,285704199	protein_codin	hypothetical protein
TcG_08181	79,21728146	0,180517642	0,209174777	0,862999087	0,388137969	0,618473432	protein_codin	hypothetical protein
TcG_08182	221,9833853	-0,037726791	0,122296438	-0,30848642	0,757712229	0,878015178	protein_codin	hypothetical protein
TcG_08183	39,62035326	-0,288042688	0,306569549	-0,93956718	0,347439615	0,581903013		
TcG_08184	228,9344976	0,057943086	0,118348623	0,489596624	0,624419366	0,797632058	protein_codin	translation initiation factor 2A
TcG_08185	84,76298824	0,077439364	0,196078289	0,394941042	0,6928864	0,841062696	protein_codin	translation initiation factor 2A
TcG_08186	66,59129972	0,012977427	0,214629977	0,060464188	0,951785938	0,97734573	protein_codin	hypothetical protein
TcG_08187	178,1484197	-0,00497843	0,139272288	-0,03574602	0,971484875	0,98762955	protein_codin	hypothetical protein
TcG_08188	118,2947248	0,333159626	0,166621658	1,999497722	0,045554528	0,154866131	protein_codin	RING box protein
TcG_08189	77,61542629	0,231461771	0,209876503	1,102847473	0,270093408	0,502941535	protein_codin	hypothetical protein
TcG_08190	273,5023004	0,067859254	0,107781172	0,629602116	0,528954939	0,731076654	protein_codin	putative RNA-binding protein
TcG_08191	83,15878161	0,372640976	0,194766862	1,913266825	0,055713902	0,17851252	protein_codin	putative meiotic recombination protein spo11
TcG_08192	116,6719654	0,28117106	0,169969727	1,654241995	0,098078353	0,265140679	protein_codin	hypothetical protein
TcG_08193	623,6452665	0,104172985	0,074672311	1,395068463	0,162995195	0,367333657	protein_codin	hypothetical protein
TcG_08194	868,9732266	0,122323324	0,076609568	1,596710799	0,110330187	0,287158202	protein_codin	CAD protein isoform X4
TcG_08195	177,7682935	0,558471385	0,135302048	4,127590029	3,66585E-05	0,00046931	protein_codin	putative RNA methyltransferase
TcG_08196	195,5114874	0,404589172	0,140217554	2,885438814	0,003908684	0,023635708	protein_codin	hypothetical protein
TcG_08197	227,2548682	0,616357646	0,122980342	5,011838788	5,39124E-07	1,16781E-05	protein_codin	DNA repair and transcription factor protein
TcG_08198	152,0685022	0,616113147	0,154773597	3,980738058	6,87016E-05	0,000813882	protein_codin	SET and MYND domain-containing protein
TcG_08199	196,229766	0,112209455	0,137843272	0,814036502	0,41562404	0,64239863	protein_codin	hypothetical protein
TcG_08200	258,7120186	0,316181623	0,114607256	2,75882727	0,005800919	0,032173023	protein_codin	ubiquitin-conjugating enzyme E2
TcG_08201	2542,767976	0,272033662	0,045026551	6,041627754	1,52567E-09	5,77661E-08	protein_codin	putative cell division cycle protein
TcG_08202	445,8994171	0,50390324	0,093113862	5,411688736	6,24331E-08	1,69403E-06	protein_codin	hypothetical protein
TcG_08203	413,7349886	0,301461897	0,09434518	3,195307866	0,001396818	0,010249226	protein_codin	putative protein kinase
TcG_08204	231,8471651	0,563225798	0,124017439	4,541504813	5,58541E-06	9,24465E-05	protein_codin	transferase C1orf69 like protein, mitochondrial
TcG_08205	235,3504791	0,562434541	0,12044734	4,669547228	3,01864E-06	5,41393E-05	protein_codin	hypothetical protein
TcG_08206	192,9587346	0,406950942	0,127987232	3,1796214	0,001474676	0,01072542	protein_codin	hypothetical protein
TcG_08207	2454,126806	0,552679878	0,048149062	11,47851817	1,69155E-30	9,33251E-28	protein_codin	cysteine peptidase inhibitor
TcG_08208	419,0457511	0,599236133	0,098537683	6,08128904	1,1922E-09	4,68232E-08	protein_codin	hypothetical protein

TcG_08209	309,9587233	0,103171816	0,103379769	0,997988454	0,318284958	0,553368775	protein_codin	hypothetical protein
TcG_08210	80,23452512	0,282252344	0,200105418	1,410518248	0,158386712	0,360533035	protein_codin	surface protease GP63
TcG_08211	73,66658762	0,241283927	0,205467103	1,17431902	0,240267268	0,468992108	protein_codin	surface protease GP63
TcG_08212	26,45842278	0,843343129	0,347405177	2,427549115	0,015201228	0,06829059	protein_codin	mucin-associated surface protein (MASP)
TcG_08213	131,8151045	0,396819521	0,155442558	2,552837048	0,010684947	0,052058789		
TcG_08214	404,6507122	-0,129697044	0,090473078	-1,43354296	0,151702732	0,351877447		
TcG_08215	369,2572228	-0,09354664	0,096958932	-0,96480683	0,334641573	0,569062527	protein_codin	kelch repeat-containing protein
TcG_08216	136,5867122	-0,009895819	0,148610189	-0,06658911	0,946908819	0,974929848	protein_codin	small nuclear RNA activating protein 2
TcG_08217	342,4238156	-0,16840697	0,100760491	-1,67135917	0,094650756	0,259038152	protein_codin	putative peroxisome assembly protein
TcG_08218	289,0120581	-0,107580245	0,11154996	-0,96441312	0,334838849	0,569250609	protein_codin	arabinose efflux permease family protein
TcG_08219	285,652023	0,219002335	0,107788436	2,031779504	0,042175983	0,146609943	protein_codin	hypothetical protein
TcG_08220	136,4429053	0,039862771	0,149399734	0,26681956	0,789608093	0,895015489	protein_codin	hypothetical protein
TcG_08221	185,9167581	0,216805982	0,133332677	1,626052871	0,103938408	0,276326388	protein_codin	arginine N-methyltransferase, type I
TcG_08222	197,3056042	-0,392864179	0,128220056	-3,06398384	0,002184107	0,014763748	protein_codin	adenylate kinase
TcG_08223	176,1687306	-0,033770492	0,132390043	-0,25508332	0,798658744	0,899599469	protein_codin	ribosomal protein L15 containing protein
TcG_08224	693,0051075	-0,054842312	0,080308432	-0,68289606	0,494672524	0,70453824	protein_codin	putative myosin heavy chain
TcG_08225	24,94485722	0,083433517	0,351323952	0,237483145	0,812281994	0,907594318	protein_codin	hypothetical protein
TcG_08226	182,3355894	-0,631015026	0,137339825	-4,59455244	4,33679E-06	7,40001E-05	protein_codin	putative trans-sialidase
TcG_08227	34,35802678	-0,441578253	0,302867025	-1,45799382	0,144842245	0,341754828	protein_codin	putative complement regulatory protein
TcG_08228	28,68639436	0,451904629	0,343560613	1,315356333	0,188390174	0,402773021	protein_codin	hypothetical protein
TcG_08229	37,71481658	0,295823995	0,290032615	1,019968028	0,307743624	0,540901939	protein_codin	hypothetical protein
TcG_08230	96,28369245	-0,036280424	0,185419164	-0,19566707	0,844870764	0,924817395	protein_codin	putative retrotransposon hot spot (RHS) protein
TcG_08231	187,0750174	-0,067819257	0,134863054	-0,50287499	0,615052166	0,792057258	protein_codin	putative tubulin tyrosine ligase
TcG_08232	420,9794932	0,158741285	0,088056652	1,802717702	0,071432561	0,214575486	protein_codin	hypothetical protein
TcG_08233	511,2771977	-0,061159428	0,081732953	-0,74828359	0,45428912	0,674275396	protein_codin	hypothetical protein
TcG_08234	391,2426926	0,070210277	0,104311803	0,673080853	0,500895854	0,709719869	protein_codin	tyrosine decarboxylase
TcG_08235	152,6316429	-0,135222296	0,141222579	-0,95751187	0,338308956	0,572891338	protein_codin	hypothetical protein
TcG_08236	480,6701855	0,144282642	0,084326209	1,711005904	0,087080023	0,244606471	protein_codin	putative fucose kinase
TcG_08237	435,8240404	-0,066964675	0,089443565	-0,74868074	0,454049654	0,674179071	protein_codin	hypothetical protein
TcG_08238	700,1100265	0,030095473	0,070699973	0,425678709	0,670341978	0,827085662	protein_codin	hypothetical protein
TcG_08239	513,9971633	-0,24831141	0,087333344	-2,84326006	0,004465462	0,02639873	protein_codin	hypothetical protein
TcG_08240	407,3630191	0,089960138	0,090561843	0,993355868	0,32053656	0,555699026	protein_codin	putative dihydroorotate dehydrogenase
TcG_08241	412,6006602	0,030632199	0,09441029	0,324458265	0,745591114	0,871423247	protein_codin	putative aspartate carbamoyltransferase
TcG_08242	291,97256	0,153159069	0,116706509	1,312343842	0,189404146	0,404280846	protein_codin	trans-sialidase
TcG_08243	35,60510525	-0,054091214	0,312056462	-0,17333791	0,862385829	0,934140118	protein_codin	trans-sialidase
TcG_08244	69,85072639	0,119116228	0,214564939	0,55515234	0,578790465	0,768461663	protein_codin	putative trans-sialidase
TcG_08245	39,24040646	0,388866743	0,279935133	1,389131616	0,164792731	0,37020808	protein_codin	putative mucin-associated surface protein (MASP)
TcG_08246	196,8258115	-0,016566044	0,12929251	-0,12812841	0,898047355	0,950207913	protein_codin	hypothetical protein
TcG_08247	69,04417364	0,154712174	0,207817054	0,744463317	0,456596226	0,676399932	protein_codin	hypothetical protein
TcG_08248	81,170177	-0,001058777	0,193441959	-0,00547336	0,995632915	0,99830402	protein_codin	mucin TcMUCII
TcG_08249	80,21863429	0,022415674	0,193054842	0,116110394	0,907565051	0,955230917	protein_codin	hypothetical protein
TcG_08250	492,7712153	0,20062956	0,085656852	2,342247639	0,019167992	0,081229098	protein_codin	putative transmembrane protein
TcG_08251	78,63206262	0,649635067	0,201485653	3,224224936	0,001263141	0,009429611	protein_codin	hypothetical protein
TcG_08252	100,6376166	0,385555538	0,186576413	2,066475247	0,038783627	0,138345784	protein_codin	hypothetical protein
TcG_08253	173,0975935	0,452761683	0,13628785	3,322098662	0,000893431	0,007114289	protein_codin	putative ammonium transporter
TcG_08254	184,7503964	0,334225143	0,14780537	2,261251687	0,023743676	0,096266166	protein_codin	hypothetical protein
TcG_08255	520,1225483	0,168755312	0,085770224	1,96752794	0,049122378	0,163402777	protein_codin	hypothetical protein
TcG_08256	259,1670763	0,217412739	0,113743558	1,911429034	0,055949468	0,179019758	protein_codin	viral A-type inclusion protein
TcG_08257	577,7241958	0,363109283	0,081118253	4,476295632	7,59493E-06	0,000120485	protein_codin	putative cysteine proteinase
TcG_08258	372,0584662	0,19149058	0,096307708	1,988320387	0,046776267	0,158048946	protein_codin	hypothetical protein
TcG_08259	20,60679095	1,233114358	0,41356201	2,981691571	0,002866606	0,018369746		
TcG_08260	1984,674687	0,550684594	0,050409845	10,92414781	8,8366E-28	3,65646E-25	protein_codin	trans-sialidase
TcG_08261	1620,967411	0,669910075	0,058872118	11,37907209	5,31624E-30	2,678E-27	protein_codin	trans-sialidase
TcG_08262	414,2504716	0,850762442	0,091876609	9,259837167	2,04744E-20	3,82607E-18	protein_codin	putative retrotransposon hot spot (RHS) protein
TcG_08263	955,7815372	0,835544018	0,068017566	12,28423871	1,10076E-34	8,5023E-32	protein_codin	putative retrotransposon hot spot (RHS) protein
TcG_08264	161,4172758	0,803069388	0,147545791	5,442848521	5,24352E-08	1,4362E-06	protein_codin	retrotransposon hot spot (RHS) protein
TcG_08265	45,1905897	0,659464563	0,276228404	2,387388675	0,016968541	0,074269099	protein_codin	target of rapamycin (TOR) kinase 1

TcG_08266	96,0260257	0,668952752	0,18112889	3,693241609	0,000221413	0,002170302	protein_codin target of rapamycin (TOR) kinase 1
TcG_08267	187,4799013	0,53785003	0,135820937	3,95999351	7,49518E-05	0,00087312	protein_codin hypothetical protein
TcG_08268	18,05972137	0,510750074	0,42328637	1,206630097	0,227574631	0,454445346	protein_codin structural maintenance of chromosome protein 4
TcG_08269	32,97561259	0,285784876	0,308419038	0,926612307	0,354127862	0,588317381	protein_codin putative glycine dehydrogenase, putative,glycine cleavage system P-protein
TcG_08270	0					1	
TcG_08271	0					1	
TcG_08272	0,145251675	-1,420530545	4,080472857	-0,3481289	0,727743378	1	
TcG_08273	465,620955	-0,272834177	0,087696303	-3,11112518	0,001863759	0,01300814	protein_codin putative ribonuclease
TcG_08274	394,8221638	-0,408672194	0,092482667	-4,4189058	9,92019E-06	0,000151231	protein_codin hypothetical protein
TcG_08275	306,3099611	0,002514651	0,104481443	0,024067918	0,980798434	0,992621476	protein_codin hypothetical protein
TcG_08276	1297,296672	-0,030224249	0,117359073	-0,25753653	0,796764616	0,898729487	protein_codin putative ubiquitin-protein ligase
TcG_08277	409,5808661	-0,114513011	0,090764305	-1,26165248	0,207073862	0,429264227	protein_codin putative legume-like lectin
TcG_08278	377,2615676	-0,115174455	0,094846664	-1,21432268	0,224624529	0,451082534	protein_codin hypothetical protein
TcG_08279	169,4487567	0,021223411	0,152045996	0,139585467	0,888987524	0,9464367	protein_codin putative target of rapamycin (TOR) kinase 1
TcG_08280	1607,018633	-0,188133764	0,05903816	-3,18664682	0,001439324	0,010501266	protein_codin serine/threonine-protein kinase mTOR
TcG_08281	2191,351816	0,280421056	0,054005525	5,192451281	2,07543E-07	5,05167E-06	protein_codin 40S ribosomal protein S12
TcG_08282	2,576486619	1,583743932	1,225223414	1,292616443	0,19614375	1	protein_codin hexose transporter
TcG_08283	39,4156402	-0,029783732	0,277428423	-0,10735645	0,914506199	0,958345588	protein_codin hypothetical protein
TcG_08284	486,716373	-0,434183412	0,091938374	-4,7225483	2,32908E-06	4,2765E-05	protein_codin putative GTP-binding protein
TcG_08285	194,0802765	-0,130578889	0,13945057	-0,93638118	0,349076966	0,58360833	protein_codin putative leucine-rich repeat protein
TcG_08286	151,2847785	0,029839839	0,144722642	0,20618639	0,836645319	0,920635641	protein_codin oxidoreductase (with NAD(+) or NADP(+) as acceptor)
TcG_08287	1156,898525	-0,272724764	0,058978728	-4,62412079	3,76191E-06	6,52477E-05	protein_codin metallo-peptidase, Clan MA(E), Family M1
TcG_08288	185,384857	-0,020349135	0,128263159	-0,15865144	0,873943497	0,939417284	protein_codin hypothetical protein
TcG_08289	107,0994676	-0,144111525	0,171860791	-0,83853638	0,401729525	0,63220648	protein_codin GINS complex subunit 4
TcG_08290	220,8949343	-0,048760481	0,123765418	-0,393975	0,693599495	0,841257963	protein_codin hypothetical protein
TcG_08291	224,1372712	0,002921893	0,129031724	0,022644767	0,981933634	0,993289929	protein_codin putative acyltransferase
TcG_08292	325,7784985	-0,1622672	0,103502423	-1,56776234	0,116936607	0,298354443	protein_codin hypothetical protein
TcG_08293	226,9781161	-0,146573502	0,122034336	-1,20108411	0,229718575	0,456889557	protein_codin putative cysteine peptidase, Clan CA, family C19
TcG_08294	216,1316114	0,056625285	0,122018165	0,46407258	0,642595746	0,81030848	protein_codin enoyl-CoA hydratase, mitochondrial precursor
TcG_08295	88,78547701	-0,340591906	0,186158133	-1,82958381	0,067312196	0,206536881	protein_codin putative protein kinase
TcG_08296	217,3572898	-0,520798157	0,124276778	-4,19063131	2,78179E-05	0,000370458	protein_codin hypothetical protein
TcG_08297	85,5751731	-0,552525578	0,204806702	-2,69779051	0,006980135	0,037233815	protein_codin hypothetical protein
TcG_08298	266,770168	-0,341953725	0,111297463	-3,07243055	0,002123232	0,014468219	protein_codin template-activating factor I
TcG_08299	127,9001545	-0,274619438	0,158784591	-1,72950937	0,083717971	0,238201477	protein_codin putative ubiquinone biosynthesis protein COQ7
TcG_08300	0,233855473	1,175456718	3,508398891	0,335040785	0,737594321	1	
TcG_08301	88,58135262	0,141281306	0,186164689	0,758904962	0,447909411	0,668937821	protein_codin mucin-like glycoprotein
TcG_08302	183,2666936	0,046195585	0,130318673	0,354481702	0,722977915	0,857335574	protein_codin hypothetical protein
TcG_08303	246,6945151	-0,019499194	0,117518277	-0,16592478	0,868216148	0,936693574	protein_codin hypothetical protein
TcG_08304	329,4033372	0,117537902	0,100979384	1,163979188	0,244432446	0,472708116	protein_codin hypothetical protein
TcG_08305	150,9099221	0,042256022	0,143840808	0,293769362	0,768934148	0,884342966	protein_codin hypothetical protein
TcG_08306	395,6729228	-0,222339488	0,091503889	-2,4298365	0,015105636	0,068125299	protein_codin hypothetical protein
TcG_08307	358,644987	0,057299215	0,095570099	0,599551694	0,548805049	0,745247925	protein_codin malate dehydrogenase (quinone)
TcG_08308	283,2875988	0,09868666	0,111442878	0,88553581	0,375867715	0,606538655	protein_codin hypothetical protein
TcG_08309	273,437659	0,137386795	0,116458707	1,179703935	0,238117988	0,465940722	protein_codin hypothetical protein
TcG_08310	145,192245	-0,016499171	0,145413177	-0,11346407	0,909662648	0,956297199	protein_codin hypothetical protein
TcG_08311	463,4833321	0,217503691	0,086121135	2,52555533	0,011551559	0,055304282	protein_codin putative signal recognition particle receptor like protein
TcG_08312	185,2534316	0,248232028	0,135502687	1,831934359	0,066961201	0,205785802	protein_codin hypothetical protein
TcG_08313	429,7700268	-0,139965124	0,089142853	-1,57012166	0,116386811	0,297410144	protein_codin hypothetical protein
TcG_08314	1118,472699	-0,172260841	0,061683746	-2,79264559	0,005227894	0,02966228	protein_codin putative cysteine synthase
TcG_08315	291,3004337	0,019088308	0,116361532	0,164043116	0,869697202	0,937156974	protein_codin hypothetical protein
TcG_08316	462,1532799	-0,429688397	0,088447925	-4,85809472	1,18521E-06	2,35134E-05	protein_codin hypothetical protein
TcG_08317	59,99524225	0,177923742	0,226089138	0,786962802	0,431303642	0,655957468	protein_codin hypothetical protein
TcG_08318	187,7647022	0,055507045	0,128913479	0,43057596	0,666776724	0,825438095	protein_codin 1-alkyl-2-acetylglycerophosphocholine esterase
TcG_08319	106,6381996	-0,462351822	0,177132602	-2,61020172	0,009048885	0,045659939	protein_codin hypothetical protein
TcG_08320	267,9494088	-0,16602057	0,109954483	-1,50990269	0,131068255	0,321728137	protein_codin Tectonic-1
TcG_08321	250,7194669	0,113684902	0,115025999	0,988340924	0,322985709	0,55752569	protein_codin hypothetical protein
TcG_08322	370,3205689	-0,05291066	0,095953317	-0,55142086	0,581345207	0,770482335	protein_codin AP-endonuclease

TcG_08323	366,7505658	-0,115485967	0,096515797	-1,1965499	0,231482037	0,458688366	protein_codin dispersed gene family protein 1 (DGF-1)
TcG_08324	580,4522422	0,179457489	0,080897234	2,218339005	0,026531727	0,104343715	protein_codin dispersed gene family protein 1 (DGF-1)
TcG_08325	524,3997628	-0,027735642	0,085614229	-0,32396066	0,745967822	0,871496958	protein_codin hypothetical protein
TcG_08326	139,3496228	-0,182141833	0,149739957	-1,21638764	0,223837289	0,450395767	protein_codin ubiquitin hydrolase
TcG_08327	306,3995348	-0,001379271	0,103377985	-0,01334202	0,989354927	0,995541618	protein_codin flagellar calcium-binding protein
TcG_08328	78,5906699	-0,294050199	0,20185851	-1,45671441	0,145195231	0,342195271	protein_codin flagellar calcium-binding protein
TcG_08329	0,116927736	0,503022807	4,080472857	0,123275616	0,901888849	1	protein_codin flagellar calcium-binding protein
TcG_08330	3372,937219	-0,578513287	0,047852694	-12,0894613	1,20071E-33	8,18322E-31	protein_codin putative flagellar calcium-binding protein
TcG_08331	4,823169719	0,113463929	0,84860903	0,133705776	0,893635238	1	protein_codin serine/threonine protein phosphatase
TcG_08332	412,6010788	0,403297533	0,09152873	4,406239777	1,05181E-05	0,000158263	protein_codin myosin heavy chain kinase A
TcG_08333	283,714602	0,498708812	0,112837911	4,419692009	9,88417E-06	0,00015088	protein_codin putative lipase
TcG_08334	562,4080727	0,140741692	0,08200024	1,716357064	0,08609671	0,242724606	protein_codin phospholipase A1
TcG_08335	44,00594329	-0,310528091	0,278313498	-1,1157493	0,26452946	0,496893372	protein_codin lipase
TcG_08336	283,9630617	0,398812943	0,108410254	3,678738197	0,000234391	0,002278231	protein_codin putative surface protease GP63, putative,metallopeptidase
TcG_08337	3,801685932	1,295818984	0,930760178	1,392215755	0,163857073	1	
TcG_08338	154,2865741	0,739259689	0,145843197	5,068866449	4,00192E-07	8,95101E-06	protein_codin adrenodoxin precursor
TcG_08339	135,523247	0,436562528	0,154136399	2,832313012	0,004621259	0,027055029	protein_codin hypothetical protein
TcG_08340	269,1785943	-0,100977801	0,111609095	-0,90474528	0,365600349	0,597692344	protein_codin hypothetical protein
TcG_08341	601,6966049	0,049103078	0,078298833	0,62712401	0,53057795	0,732080044	protein_codin hypothetical protein
TcG_08342	2118,849998	-0,193514056	0,046848601	-4,13062612	3,61777E-05	0,000464694	protein_codin putative calreticulin
TcG_08343	144,7451981	0,493110052	0,147191155	3,350133719	0,000807726	0,006521469	protein_codin hypothetical protein
TcG_08344	1516,440707	0,244495575	0,058089643	4,208935724	2,56576E-05	0,000344804	protein_codin RNA polymerase II 215kD subunit, isoform A
TcG_08345	139,0902888	0,233503459	0,153802081	1,518207405	0,128962121	0,318108397	
TcG_08346	291,5408844	0,058806426	0,112954464	0,520620643	0,602631066	0,785033003	
TcG_08347	204,5910908	0,148666157	0,125006432	1,189268059	0,2343342	0,462080934	protein_codin putative retrotransposon hot spot (RHS) protein
TcG_08348	44,96258136	0,802347691	0,267688156	2,997322346	0,002723626	0,017609333	protein_codin dispersed protein family protein 1
TcG_08349	3,625586053	0,807010055	0,938523115	0,859872328	0,389859424	1	protein_codin dispersed gene family protein 1 (DGF-1)
TcG_08350	11,49873069	0,637003713	0,516219737	1,233977834	0,217211164	1	protein_codin dispersed gene family protein 1 (DGF-1)
TcG_08351	7,089559312	-0,274320913	0,653160149	-0,41999028	0,674492556	1	protein_codin dispersed gene family protein 1 (DGF-1)
TcG_08352	23,59787649	-0,037897519	0,378708576	-0,1000704	0,920288435	0,961460255	protein_codin dispersed gene family protein 1 (DGF-1)
TcG_08353	24,00397923	0,570499247	0,356908453	1,598447003	0,109943531	0,286467226	protein_codin dispersed gene family protein 1 (DGF-1)
TcG_08354	16,09057609	0,000109551	0,451308515	0,00024274	0,999806322	1	protein_codin dispersed gene family protein 1 (DGF-1)
TcG_08355	11,23421585	-0,005334001	0,520011237	-0,01025747	0,991815864	1	protein_codin dispersed gene family protein 1 (DGF-1)
TcG_08356	5,689593905	-0,06157691	0,717001248	-0,08588118	0,931560876	1	protein_codin dispersed gene family protein 1 (DGF-1)
TcG_08357	3,955893586	1,074298199	0,907189275	1,184205137	0,236331869	1	protein_codin dispersed gene family protein 1 (DGF-1)
TcG_08358	6,913673954	1,307620516	0,73921175	1,76893903	0,07690405	1	protein_codin dispersed protein family protein 1
TcG_08359	5,714192763	0,268066105	0,745578555	0,359541061	0,719190366	1	protein_codin hypothetical protein
TcG_08360	11,9527401	-0,582202537	0,500871068	-1,16238005	0,245081123	1	protein_codin retrotransposon hot spot (RHS) protein
TcG_08361	30,55941631	-0,078299288	0,31857883	-0,24577681	0,805855012	0,904233839	protein_codin hypothetical protein
TcG_08362	1,43553101	-1,163011499	1,600501219	-0,72665455	0,4674376	1	protein_codin retrotransposon hot spot (RHS) protein
TcG_08363	18,16973903	-0,222200923	0,410332508	-0,5415143	0,588153141	0,775648564	protein_codin hypothetical protein
TcG_08364	0,911733799	-0,897817394	3,946387567	-0,2275036	0,820032167	1	
TcG_08365	6,911307529	1,08868834	0,681232016	1,598116815	0,110016982	1	protein_codin hypothetical protein
TcG_08366	182,7827854	0,090941574	0,135521273	0,671050174	0,502188567	0,710942905	protein_codin trans-sialidase
TcG_08367	57,08012213	-0,142096813	0,240244905	-0,5914665	0,554207897	0,749109966	protein_codin trans-sialidase
TcG_08368	54,93117388	0,264600738	0,235972375	1,121320825	0,262151324	0,494282475	protein_codin trans-sialidase
TcG_08369	434,8244486	0,219210623	0,090643144	2,418391658	0,015589287	0,069642191	protein_codin hypothetical protein
TcG_08370	304,1208342	-0,344367339	0,104593327	-3,29244082	0,000993218	0,007738684	protein_codin putative GTPase
TcG_08371	403,6587512	-0,191620163	0,092952112	-2,06149337	0,039255995	0,139472543	protein_codin hypothetical protein
TcG_08372	196,3539061	-0,244690926	0,131736869	-1,85742176	0,063251173	0,19731505	protein_codin putative ATPase alpha subunit
TcG_08373	2341,738421	-0,132004855	0,045737177	-2,88616096	0,003899727	0,023606182	protein_codin ATPase alpha subunit
TcG_08374	22,35681402	0,354679259	0,368535389	0,962402171	0,335847623	0,570379737	protein_codin trans-sialidase
TcG_08375	29,68353351	0,995036807	0,332712822	2,990677671	0,002783591	0,017887238	protein_codin selenocysteine-tRNA-specific elongation factor
TcG_08376	1,367995811	-0,413796123	1,456015173	-0,28419767	0,776258899	1	protein_codin hypothetical protein
TcG_08377	129,506945	-0,031227088	0,158551869	-0,19695187	0,843865199	0,924387794	protein_codin putative p22 protein precursor
TcG_08378	146,1799857	-0,335444062	0,161784406	-2,07340169	0,038134912	0,136536183	protein_codin hypothetical protein
TcG_08379	1312,670475	-0,09872578	0,05549088	-1,77913522	0,075217595	0,221466596	protein_codin putative I/6 autoantigen

TcG_08380	108,148184	-0,00031209	0,171172553	-0,00182325	0,998545259	0,999561967	protein_codin putative cyclophilin
TcG_08381	397,7256755	-0,617465286	0,098955376	-6,2398357	4,38031E-10	1,9151E-08	protein_codin hypothetical protein
TcG_08382	154,1511842	-0,095100374	0,152246795	-0,62464615	0,532203325	0,732933197	protein_codin peptidyl-prolyl cis-trans isomerase
TcG_08383	193,9819115	0,047201119	0,133363619	0,353927996	0,723392845	0,857397569	protein_codin putative aquaporin
TcG_08384	314,6515361	0,17906718	0,113608851	1,576172789	0,114985988	0,295197796	protein_codin centrin
TcG_08385	19,35171762	-0,073836202	0,398709536	-0,18518795	0,853081618	0,928875734	protein_codin centrin
TcG_08386	143,7128887	-0,095782788	0,159460084	-0,60066937	0,548060223	0,744672892	protein_codin hypothetical protein
TcG_08387	124,7396872	0,17845807	0,156441749	1,140731754	0,253981568	0,485037736	protein_codin hypothetical protein
TcG_08388	295,9424548	0,126483358	0,106792694	1,184382129	0,236261831	0,463600927	protein_codin putative mak-16-like RNA binding protein
TcG_08389	371,1204304	-0,238741937	0,095591882	-2,49751269	0,012506799	0,058944003	protein_codin intraflagellar transport protein-like protein
TcG_08390	425,2963669	0,135509187	0,08951875	1,513752013	0,130088748	0,319865923	protein_codin farnesyl diphosphate synthase precursor
TcG_08391	326,1379071	0,100806399	0,103932826	0,969918769	0,332086984	0,566340797	protein_codin hypothetical protein
TcG_08392	357,8843472	0,016718132	0,098281891	0,170103891	0,864928433	0,934985064	protein_codin napsin-A
TcG_08393	42,09824152	-0,892654174	0,31371684	-2,84541364	0,004435378	0,026272135	protein_codin hypothetical protein
TcG_08394	135,4896631	-0,490857481	0,165015228	-2,9746193	0,002933524	0,018684884	protein_codin hypothetical protein
TcG_08395	568,5978917	0,148871682	0,078944493	1,885776649	0,059325059	0,187560733	protein_codin hypothetical protein
TcG_08396	235,6577107	-0,039596578	0,119794084	-0,33053868	0,740992971	0,867975387	protein_codin hypothetical protein
TcG_08397	892,4344291	-0,163616523	0,06861325	-2,38461993	0,017096774	0,07455146	protein_codin putative GTP binding protein
TcG_08398	232,3219925	-0,3251994	0,126894187	-2,56276041	0,010384368	0,050915483	protein_codin protein phosphatase 2C
TcG_08399	134,2464701	0,018360785	0,152478709	0,120415401	0,904154092	0,953534435	protein_codin hypothetical protein
TcG_08400	75,95624722	-0,273876067	0,204010641	-1,34245971	0,179446984	0,390656287	protein_codin central apparatus associated protein C1a-18
TcG_08401	449,2694083	-0,00201274	0,087639612	-0,0229661	0,981677311	0,993250661	protein_codin transcription elongation regulator-like protein
TcG_08402	278,0263194	-0,123923964	0,111301728	-1,11340558	0,265534281	0,49806938	protein_codin putative acyl-CoA oxidase
TcG_08403	337,5284753	-0,180950573	0,103097642	-1,75513785	0,079235732	0,229391601	protein_codin hypothetical protein
TcG_08404	110,8339064	-0,277914308	0,165907208	-1,67511894	0,093910897	0,25750928	
TcG_08405	471,3989905	-0,219515804	0,086140061	-2,54835905	0,010823101	0,052599183	protein_codin hypothetical protein
TcG_08406	336,1713972	-0,139399753	0,097622182	-1,42795162	0,153305784	0,353824404	protein_codin hypothetical protein
TcG_08407	179,3634657	0,030301168	0,133200053	0,227486156	0,820045733	0,911022136	protein_codin rRNA small subunit pseudouridine methyltransferase Nep1
TcG_08408	224,587686	0,197662604	0,118883183	1,662662447	0,096380023	0,262588384	protein_codin hypothetical protein
TcG_08409	577,8503642	0,103932256	0,079030988	1,315082333	0,188482233	0,402804447	protein_codin hypothetical protein
TcG_08410	48,84250248	-0,223454264	0,251299804	-0,88919395	0,373898856	0,604496256	protein_codin putative esterase
TcG_08411	63,57504376	0,013470367	0,231761666	0,058121635	0,953651742	0,978287787	protein_codin hypothetical protein
TcG_08412	179,5324459	0,081845361	0,13213119	0,619424989	0,535636423	0,735555719	protein_codin retrotransposon hot spot (RHS) protein
TcG_08413	282,6267421	0,23147323	0,111232309	2,080989166	0,037434898	0,134737721	protein_codin retrotransposon hot spot (RHS) protein
TcG_08414	31,41108968	0,006097887	0,317673582	0,019195448	0,984685189	0,994123614	protein_codin hypothetical protein
TcG_08415	31,91919919	-0,700856281	0,316008512	-2,21783988	0,026565752	0,104442077	protein_codin subtilisin-like serine peptidase
TcG_08416	41,41031355	0,255988489	0,271246164	0,943749712	0,345297564	0,579295913	protein_codin dynein
TcG_08417	47,44962148	-0,186478865	0,25491485	-0,73153394	0,464453079	0,681738107	protein_codin putative kinesin
TcG_08418	22,78555548	0,425416263	0,39805054	1,068749367	0,28518262	0,518774269	
TcG_08419	26,10801823	0,164681862	0,345543414	0,476588049	0,633655496	0,804197117	protein_codin hypothetical protein
TcG_08420	51,18267122	0,554512078	0,247687938	2,238752856	0,025172001	0,100255348	protein_codin trans-sialidase
TcG_08421	18,73583189	-0,641421976	0,414493531	-1,54748368	0,121746656	0,306176852	protein_codin N-acetyltransferase complex ARD1 subunit
TcG_08422	13,96233562	-0,068812773	0,481992305	-0,14276737	0,886473909	1	protein_codin hypothetical protein
TcG_08423	18,76933589	0,251330556	0,402864994	0,623858015	0,532720835	0,733377328	protein_codin retrotransposon hot spot protein (RHS)
TcG_08424	79,14439482	0,443120181	0,20384194	2,173842053	0,029716999	0,113593254	protein_codin retrotransposon hot spot (RHS) protein
TcG_08425	71,65766584	0,38447328	0,215538014	1,783784091	0,074458756	0,220296002	protein_codin retrotransposon hot spot (RHS) protein
TcG_08426	44,05223046	0,657222112	0,288275213	2,279842605	0,022617025	0,092626671	protein_codin retrotransposon hot spot (RHS) protein
TcG_08427	47,29092609	0,226061321	0,257171929	0,879027977	0,379386107	0,6106388	protein_codin putative trans-sialidase
TcG_08428	96,83942791	0,150000871	0,181476628	0,826557514	0,408487906	0,636605346	protein_codin trans-sialidase
TcG_08429	105,6588713	0,275117676	0,174114849	1,580093126	0,114085541	0,293406233	protein_codin rab1 small GTP-binding protein
TcG_08430	38,38384593	0,125909398	0,288421619	0,436546325	0,662440396	0,823148266	protein_codin hypothetical protein
TcG_08431	45,73527456	0,37947434	0,259043358	1,464906658	0,142946386	0,339310966	protein_codin putative trans-sialidase
TcG_08432	52,44963811	0,223881804	0,240492325	0,93093118	0,351889164	0,586279531	protein_codin putative trans-sialidase
TcG_08433	30,75995652	0,467855546	0,315235847	1,484144492	0,137770592	0,331163917	protein_codin putative trans-sialidase
TcG_08434	56,0127074	0,277950419	0,236421645	1,175655549	0,239732548	0,468230159	
TcG_08435	1113,521189	0,065232835	0,061796301	1,055610673	0,291146123	0,523651784	protein_codin putative retrotransposon hot spot (RHS) protein
TcG_08436	34,77604755	-0,069938957	0,30728056	-0,22760619	0,81995241	0,911022136	protein_codin hypothetical protein

TcG_08437	11,13882494	1,00743902	0,584653206	1,723139478	0,084863305	1	protein_codin	zinc finger protein family memeber
TcG_08438	339,0698853	-0,493778578	0,102304345	-4,82656508	1,38908E-06	2,70485E-05	protein_codin	zinc finger protein family memeber
TcG_08439	654,1515442	-0,366372809	0,073397605	-4,99161807	5,98756E-07	1,28229E-05	protein_codin	hypothetical protein
TcG_08440	2640,899141	-0,196104954	0,045089321	-4,349255	1,36601E-05	0,000199076	protein_codin	putative heat shock protein
TcG_08441	893,9011321	-0,409629895	0,069100607	-5,92802168	3,06606E-09	1,10665E-07	protein_codin	putative pyruvate dehydrogenase E1 component alpha subunit
TcG_08442	282,0272075	-0,262954369	0,11241439	-2,3391522	0,019327558	0,081815522	protein_codin	hypothetical protein
TcG_08443	1059,959776	0,017333257	0,068538048	0,252899781	0,800345659	0,900622067	protein_codin	60S ribosomal protein L34
TcG_08444	241,418713	-0,477118457	0,120174976	-3,97019808	7,18129E-05	0,00084529	protein_codin	hypothetical protein
TcG_08445	603,3766824	-0,583705009	0,08405956	-6,94394553	3,81298E-12	2,62959E-10	protein_codin	pumilio protein 2
TcG_08446	11,73113492	-0,50784166	0,512949745	-0,99004174	0,322153716	1	protein_codin	hypothetical protein
TcG_08447	317,2286574	-0,002267434	0,108285678	-0,02093937	0,983294017	0,993758242	protein_codin	hypothetical protein
TcG_08448	85,1607257	-0,058008017	0,207311651	-0,27981069	0,779622748	0,890360686	protein_codin	hypothetical protein
TcG_08449	575,9950344	0,363962749	0,080490285	4,521822109	6,13096E-06	0,000100047	protein_codin	NADH-cytochrome b5 reductase
TcG_08450	226,5775529	-0,166423922	0,134736724	-1,23517863	0,216764031	0,440893872	protein_codin	translation initiation factor 2D
TcG_08451	225,9485478	0,019095969	0,129328861	0,147654351	0,882615559	0,942677716	protein_codin	translation initiation factor 2D
TcG_08452	115,1245183	-0,408986618	0,167136533	-2,44702108	0,014404242	0,065842878	protein_codin	hypothetical protein
TcG_08453	59,84830461	-0,093128702	0,23012833	-0,40468161	0,685711565	0,836971272	protein_codin	hypothetical protein
TcG_08454	269,755351	-0,311615064	0,109674831	-2,84126323	0,00449352	0,02649462	protein_codin	putative protein kinase
TcG_08455	36,7652816	-0,077548982	0,298023132	-0,26021128	0,7947008	0,897538186		
TcG_08456	118,8184989	-0,144273357	0,16414081	-0,87896091	0,379422468	0,6106388	protein_codin	ribosomal protein L22/L17-like protein
TcG_08457	293,9650448	-0,312055693	0,103610832	-3,01180569	0,002596988	0,016930009	protein_codin	hypothetical protein
TcG_08458	419,5963162	0,046026425	0,092234381	0,499015925	0,617768165	0,793033999	protein_codin	hypothetical protein
TcG_08459	68,55082166	0,557608968	0,219930574	2,535386312	0,011232338	0,054201528	protein_codin	hypothetical protein
TcG_08460	262,9880367	-0,147450235	0,118507095	-1,24423128	0,213414455	0,436980474	protein_codin	IQ and ubiquitin-like domain-containing protein
TcG_08461	126,2308782	-0,259501293	0,159503492	-1,62693173	0,103751598	0,276060609	protein_codin	hypothetical protein
TcG_08462	258,9962848	-0,047141157	0,11302469	-0,41708724	0,676614595	0,830605711	protein_codin	hypothetical protein
TcG_08463	88,60571476	-0,027806165	0,183962946	-0,1511509	0,879856679	0,941623821	protein_codin	histone H2A
TcG_08464	264,4043088	-0,125828011	0,112548129	-1,11799291	0,263570019	0,495895136	protein_codin	putative tubulin tyrosine ligase
TcG_08465	249,5118304	-0,160942003	0,114605865	-1,40430861	0,160226972	0,363061588	protein_codin	putative 3-Beta-hydroxysteroid-delta(8), delta(7)-isomerase
TcG_08466	97,43714026	-0,016134047	0,185651929	-0,08690482	0,930747168	0,96665499	protein_codin	hypothetical protein
TcG_08467	93,20746793	-0,066157068	0,192744134	-0,34323778	0,731419588	0,862078062	protein_codin	hypothetical protein
TcG_08468	640,8910956	-0,330128648	0,088932487	-3,71212655	0,000205525	0,002035226	protein_codin	recombination initiation protein NBS1
TcG_08469	416,5240273	-0,22537129	0,092010364	-2,449412	0,014308967	0,065604944	protein_codin	putative recombination initiation protein NBS1
TcG_08470	307,72085	-0,011323158	0,102630749	-0,1103291	0,912148378	0,957173364	protein_codin	tRNA (guanine-N(1)-)-methyltransferase
TcG_08471	379,6134504	-0,173903972	0,092744815	-1,87508026	0,060781682	0,191486794	protein_codin	protein kinase
TcG_08472	149,2337237	-0,149188339	0,146928895	-1,0153778	0,309925769	0,543237513	protein_codin	putative proteasome 26S non-ATPase subunit 9
TcG_08473	273,108227	0,011417515	0,113311815	0,100761908	0,919739466	0,961308205	protein_codin	hypothetical protein
TcG_08474	78,66873576	0,294884891	0,205037586	1,438199195	0,150377544	0,35041718		
TcG_08475	100,3627749	0,042135939	0,177788295	0,237000636	0,812656297	0,907594318	protein_codin	protein tyrosine phosphatase PRL
TcG_08476	273,6347276	-0,051159311	0,11062186	-0,46247018	0,643744184	0,810875005	protein_codin	histone acetyltransferase-like protein
TcG_08477	68,480213	0,111656962	0,216034303	0,516848298	0,605262062	0,786698766	protein_codin	hypothetical protein
TcG_08478	266,3612613	0,03097158	0,116351362	0,266190093	0,790092813	0,895024708	protein_codin	putative trans-sialidase
TcG_08479	112,2467912	0,230359618	0,173425245	1,328293451	0,184081182	0,396867246	protein_codin	rab1 small GTP-binding protein
TcG_08480	80,9050571	0,23055281	0,200664895	1,148944413	0,250578903	0,480265867	protein_codin	putative target of rapamycin (TOR) kinase 1
TcG_08481	134,2350064	0,118613605	0,159067394	0,745681447	0,455859869	0,675827568	protein_codin	target of rapamycin (TOR) kinase 1
TcG_08482	45,2907533	0,055016425	0,257654953	0,213527526	0,830915521	0,917601778		
TcG_08483	68,48508523	0,202263334	0,213184638	0,948770678	0,342737258	0,576796063		
TcG_08484	211,2131308	-0,200580044	0,124496956	-1,61112408	0,10715268	0,282088378	protein_codin	ESAG-like protein
TcG_08485	248,1015213	-0,074658999	0,113659353	-0,6568663	0,511266887	0,717683589	protein_codin	protein tyrosine phosphatase
TcG_08486	248,0565922	-0,097494725	0,115197949	-0,84632344	0,397372335	0,626985684	protein_codin	putative protein kinase
TcG_08487	60,47371572	-0,272653874	0,243482463	-1,11980908	0,262795127	0,494919432	protein_codin	hypothetical protein
TcG_08488	133,5542123	-0,126011893	0,152541041	-0,82608518	0,408755775	0,636605346	protein_codin	hypothetical protein
TcG_08489	277,1413048	0,072051422	0,108724157	0,662699294	0,507523162	0,714305558	protein_codin	protein KRI1
TcG_08490	0					1	protein_codin	hypothetical protein
TcG_08491	97,2277992	0,050811308	0,184312102	0,275680799	0,782793244	0,892398163	protein_codin	hypothetical protein
TcG_08492	3,734870516	-0,931341111	0,912252328	-1,02092489	0,307290024	1		
TcG_08493	7,804201902	-0,260038481	0,630753309	-0,41226653	0,680144075	1		

TcG_08494	70,40174479	0,122267109	0,222928067	0,548459918	0,583376144	0,772015535	protein_codin	hypothetical protein
TcG_08495	82,45533281	-0,116288343	0,217267284	-0,53523172	0,592489633	0,7778347	protein_codin	hypothetical protein
TcG_08496	22,56737243	0,145194112	0,363373586	0,399572553	0,689471375	0,838680499	protein_codin	oligosaccharyl transferase subunit
TcG_08497	177,2233559	-0,165230676	0,134819742	-1,22556737	0,220361525	0,445413229	protein_codin	putative oligosaccharyl transferase subunit
TcG_08498	179,0476016	-0,142260632	0,132201519	-1,07608924	0,281887348	0,514971116	protein_codin	oligosaccharyl transferase subunit
TcG_08499	134,425495	0,029812848	0,151377922	0,196943173	0,843872008	0,924387794	protein_codin	Rhodanese-like protein
TcG_08500	2,043031965	2,077746071	1,402485694	1,481473985	0,138480312	1		
TcG_08501	305,6053997	-0,104772188	0,106111108	-0,98738191	0,32345545	0,557588877	protein_codin	NADH-dependent fumarate reductase-like protein
TcG_08502	2,344298642	-0,096464523	1,137632047	-0,08479413	0,932425058	1		
TcG_08503	817,3548961	-0,076786579	0,074502971	-1,03065123	0,302704404	0,535497997	protein_codin	gamma-glutamylcysteine synthetase
TcG_08504	338,9414891	-0,395663811	0,101133975	-3,91227391	9,14311E-05	0,001033085	protein_codin	putative stress-inducible protein STI1-like
TcG_08505	350,1691618	-0,761456971	0,096779453	-7,86796112	3,60468E-15	3,93998E-13	protein_codin	hypothetical protein
TcG_08506	241,5746179	-0,341204839	0,115006102	-2,96684118	0,003008763	0,019069766	protein_codin	calmodulin
TcG_08507	167,7413913	-0,395972512	0,137861865	-2,87224107	0,004075719	0,024466986	protein_codin	putative membrane transporter protein
TcG_08508	279,1001689	-0,556266233	0,108913844	-5,10739692	3,26627E-07	7,43478E-06	protein_codin	hypothetical protein
TcG_08509	88,22438187	-0,152501884	0,195509654	-0,78002227	0,435377768	0,659399643	protein_codin	putative to be involved in ER-Golgi transport
TcG_08510	643,3372575	-0,2848718	0,080402272	-3,54308148	0,00039548	0,003551967	protein_codin	nucleolar complex protein 2
TcG_08511	437,5671187	-0,350512819	0,090628126	-3,86759424	0,000109914	0,001209371	protein_codin	putative Unc104-like kinesin
TcG_08512	143,1792751	-0,178283534	0,147034345	-1,21252986	0,225309618	0,451820114	protein_codin	synaptobrevin-type transport protein
TcG_08513	132,630383	-0,178398534	0,160626416	-1,11064256	0,266722235	0,499395611	protein_codin	hypothetical protein
TcG_08514	203,7199348	-0,406828465	0,125695083	-3,2366299	0,001209502	0,009111369	protein_codin	putative vesicle-fusing ATPase
TcG_08515	279,5804175	-0,306502628	0,10973155	-2,79320421	0,005218873	0,029625609	protein_codin	putative protein kinase
TcG_08516	565,5499758	-0,297737516	0,078600931	-3,78796425	0,000151887	0,001589665	protein_codin	putative kinesin
TcG_08517	40,08963976	-0,088398882	0,272801604	-0,32404092	0,745907056	0,871496958	protein_codin	plasma membrane Ca2 ATPase
TcG_08518	390,4281804	-0,04743933	0,094095236	-0,50416294	0,614146882	0,791403156	protein_codin	hypothetical protein
TcG_08519	134,5838081	-0,229085872	0,152610822	-1,50111157	0,133326706	0,324453521	protein_codin	hypothetical protein
TcG_08520	224,3613575	-0,079248645	0,121008663	-0,65490059	0,512531759	0,718561587	protein_codin	hypothetical protein
TcG_08521	153,3006224	-0,264836723	0,145092601	-1,82529448	0,067956603	0,20781967	protein_codin	Ubiquitin-fold modifier 1
TcG_08522	75,79265449	-0,564898111	0,19890316	-2,84006605	0,004510419	0,026580732	protein_codin	putative ATP-dependent chaperone
TcG_08523	251,7732487	-0,334567446	0,113407602	-2,95013245	0,003176377	0,019979103	protein_codin	putative protein kinase
TcG_08524	376,1634165	-0,3059164	0,096339731	-3,17539188	0,001496343	0,010848724	protein_codin	kinesin K39
TcG_08525	429,3867654	-0,060517774	0,090483174	-0,66882904	0,503604541	0,711990507	protein_codin	putative protein SERAC1-like
TcG_08526	284,9899581	-0,03783165	0,107751889	-0,35109965	0,725513592	0,858172586	protein_codin	variant surface glycoprotein 3275
TcG_08527	46,72984231	-0,016857535	0,276171607	-0,06104007	0,951327297	0,97713458	protein_codin	variant surface glycoprotein 3275
TcG_08528	186,2209281	-0,085835535	0,129067846	-0,66504197	0,506023647	0,71271456	protein_codin	methyltransferase
TcG_08529	219,975162	-0,008097519	0,124955468	-0,06480324	0,948330664	0,97570906	protein_codin	charged multivesicular body protein 6
TcG_08530	9,971958403	0,464933021	0,568650874	0,81760715	0,413581544	1	protein_codin	RNA-binding protein
TcG_08531	415,2927073	0,061033774	0,09076522	0,672435695	0,501306365	0,710127833	protein_codin	hypothetical protein
TcG_08532	420,849665	-0,03861242	0,092384099	-0,41795525	0,675979834	0,83017833	protein_codin	small nuclear ribonucleoprotein SmD2
TcG_08533	135,9619173	0,068786653	0,150665492	0,45655214	0,647992986	0,812603825	protein_codin	putative serine protease PepD
TcG_08534	309,9644879	-0,068687976	0,105377005	-0,65183078	0,51451034	0,720229102	protein_codin	hypothetical protein
TcG_08535	599,7283644	-0,119490233	0,076820915	-1,5554388	0,119841599	0,302917264	protein_codin	transcription elongation factor SPT6
TcG_08536	227,3768127	0,015371151	0,119763868	0,128345477	0,897875577	0,950181186	protein_codin	putative sedoheptulose-1,7-bisphosphatase
TcG_08537	82,17292665	0,186973732	0,193378141	0,966881423	0,333603314	0,568067019	protein_codin	hypothetical protein
TcG_08538	60,58836677	-0,097999877	0,227201913	-0,43133386	0,666225637	0,825196732	protein_codin	hypothetical protein
TcG_08539	20,77027323	-0,16977127	0,379072153	-0,44786004	0,654254209	0,817094887	protein_codin	hypothetical protein
TcG_08540	15,50750594	-0,614323293	0,437957763	-1,40269986	0,160706358	1	protein_codin	hypothetical protein
TcG_08541	8,762821932	-1,151081792	0,612185053	-1,88028405	0,060069376	1	protein_codin	hypothetical protein
TcG_08542	331,037351	-0,254306949	0,106594342	-2,38574528	0,017044552	0,074440982	protein_codin	putative tyrosyl-DNA Phosphodiesterase (Tdp1)
TcG_08543	301,1925146	-0,064976323	0,11202669	-0,58000752	0,561909545	0,755364499	protein_codin	putative ras-related protein Rab21
TcG_08544	3,663657027	-1,552397251	1,022966386	-1,51754473	0,129129209	1		
TcG_08545	6,49705686	-0,543377077	0,698156835	-0,77830231	0,436390814	1	protein_codin	putative profilin
TcG_08546	323,2453483	0,21555094	0,100931074	2,135625155	0,032709979	0,122054049	protein_codin	trans-sialidase
TcG_08547	31,26529658	0,050360483	0,308691089	0,163142003	0,870406629	0,937398327	protein_codin	putative dispersed gene family protein 1 (DGF-1)
TcG_08548	28,81367667	0,088586269	0,347794746	0,254708475	0,79894827	0,899748703	protein_codin	hypothetical protein
TcG_08549	38,72793538	0,070585675	0,278349316	0,253586666	0,799814899	0,900342615	protein_codin	hypothetical protein
TcG_08550	375,3317407	0,185745893	0,093262225	1,99165195	0,046409259	0,156992023	protein_codin	putative retrotransposon hot spot (RHS) protein

TcG_08551	59,68832745	0,188951384	0,232453902	0,812855291	0,41630103	0,643273371	protein_codin	trans-sialidase
TcG_08552	96,47474427	0,222924019	0,181478557	1,228376635	0,219305615	0,444364263	protein_codin	trans-sialidase
TcG_08553	355,900455	0,049223583	0,09722186	0,506301598	0,612644942	0,790819724	protein_codin	dispersed gene family protein 1 (DGF-1)
TcG_08554	142,6275442	0,118937666	0,149869654	0,793607396	0,427424026	0,652627143	protein_codin	putative dispersed gene family protein 1 (DGF-1)
TcG_08555	88,32611466	-0,09886564	0,186906289	-0,52895834	0,59683435	0,780906017	protein_codin	hypothetical protein
TcG_08556	403,0521207	0,178856091	0,090972932	1,966036345	0,049294412	0,163833925	protein_codin	putative retrotransposon hot spot (RHS) protein
TcG_08557	92,15756713	-0,283775959	0,200760184	-1,41350717	0,157506655	0,35943906	protein_codin	trans-sialidase
TcG_08558	204,2540059	-0,222012779	0,125472297	-1,76941671	0,076824359	0,224599299	protein_codin	trans-sialidase-like protein
TcG_08559	20,34819463	-0,212900425	0,385404822	-0,55240727	0,580669352	0,770192915	protein_codin	hypothetical protein
TcG_08560	145,6438743	-0,060920204	0,158196784	-0,3850913	0,700169777	0,844381264	protein_codin	retrotransposon hot spot (RHS) protein
TcG_08561	27,62612552	-0,090303652	0,332104364	-0,27191347	0,78568855	0,893514718	protein_codin	hypothetical protein
TcG_08562	10,83799649	-0,128898691	0,537065116	-0,2400057	0,810325835	1	protein_codin	retrotransposon hot spot (RHS) protein
TcG_08563	15,72368329	0,360555358	0,488994296	0,73734062	0,460915236	1	protein_codin	hypothetical protein
TcG_08564	19,79780489	-0,427148876	0,385506678	-1,1080194	0,26785344	0,500217594		
TcG_08565	24,501131	0,757937789	0,370595531	2,045188694	0,040836259	0,143285552	protein_codin	putative trans-sialidase
TcG_08566	17,47888748	0,132159261	0,465744503	0,283759143	0,776594968	0,888310845	protein_codin	hypothetical protein
TcG_08567	0,911733799	-0,897817394	3,946387567	-0,2275036	0,820032167	1		
TcG_08568	28,97596789	-0,169910691	0,320941102	-0,52941393	0,596518334	0,780668859		
TcG_08569	18,13279894	-0,431501011	0,409803439	-1,05294629	0,292365609	0,525088815	protein_codin	hypothetical protein
TcG_08570	12,29481393	-0,378533542	0,514651959	-0,73551365	0,462026728	1	protein_codin	trans-sialidase
TcG_08571	122,6127233	-0,214058215	0,163236928	-1,3113345	0,189744779	0,404858749		
TcG_08572	147,397268	-0,3255106	0,145651996	-2,23485163	0,025427091	0,101028215	protein_codin	putative retrotransposon hot spot (RHS) protein
TcG_08573	78,68609045	-0,066450198	0,197517158	-0,33642747	0,736548535	0,865306361	protein_codin	putative retrotransposon hot spot (RHS) protein
TcG_08574	13,38068672	0,214751858	0,481463698	0,446039564	0,655568667	1	protein_codin	hypothetical protein
TcG_08575	26,22762599	-0,288085967	0,349188561	-0,82501548	0,409362805	0,636798799		
TcG_08576	16,04678568	0,037176478	0,449987898	0,082616618	0,934156387	1		
TcG_08577	65,19870338	-0,077388525	0,224800041	-0,34425494	0,730654565	0,861439278	protein_codin	putative dispersed gene family protein 1 (DGF-1)
TcG_08578	90,33449275	-0,074697321	0,188507313	-0,39625689	0,691915528	0,840577603	protein_codin	dispersed gene family protein 1 (DGF-1)
TcG_08579	108,6013523	0,214403431	0,185776418	1,154093898	0,248461675	0,477788708	protein_codin	hypothetical protein
TcG_08580	552,5157444	0,226888293	0,079209971	2,864390572	0,004178125	0,024991097	protein_codin	hypothetical protein
TcG_08581	110,3315867	0,595598152	0,173605137	3,430763403	0,000601885	0,00505322	protein_codin	hypothetical protein
TcG_08582	318,0871843	0,469684717	0,101996147	4,604926076	4,12612E-06	7,09277E-05	protein_codin	putative beta propeller protein
TcG_08583	1830,957403	0,319487042	0,049976572	6,39273621	1,62943E-10	7,70555E-09	protein_codin	tryparedoxin peroxidase
TcG_08584	865,7987129	0,681165764	0,068965019	9,876974974	5,23906E-23	1,31956E-20	protein_codin	cyclophilin
TcG_08585	226,4763764	0,544458725	0,123845327	4,396279931	1,10122E-05	0,000164841	protein_codin	putative agmatinase
TcG_08586	1338,616788	0,363492981	0,057038561	6,372758596	1,85658E-10	8,63869E-09	protein_codin	hypothetical protein
TcG_08587	251,4288958	0,437894506	0,114683785	3,81827742	0,000134387	0,001425829	protein_codin	hypothetical protein
TcG_08588	452,5355629	0,274820466	0,086733919	3,168546616	0,001532032	0,01106616	protein_codin	GDP-mannose pyrophosphorylase
TcG_08589	54,75355032	0,509880013	0,238445399	2,138351236	0,032488247	0,121506303	protein_codin	hypothetical protein
TcG_08590	52,24328645	0,773350876	0,249335556	3,101646985	0,001924473	0,013334636	protein_codin	hypothetical protein
TcG_08591	42,84525489	0,578954724	0,287997318	2,010278178	0,044401755	0,152245852	protein_codin	hypothetical protein
TcG_08592	27,77398764	0,548241216	0,346413641	1,582620172	0,113508063	0,292375371	protein_codin	selenocysteine-tRNA-specific elongation factor
TcG_08593	45,2935929	0,749664341	0,262348794	2,857510142	0,00426979	0,025421267	protein_codin	putative trans-sialidase
TcG_08594	33,20360556	0,500312254	0,309600806	1,615991448	0,106096162	0,280518059	protein_codin	hypothetical protein
TcG_08595	14,78711597	0,49313406	0,492001163	1,00230263	0,316197453	1	protein_codin	hypothetical protein
TcG_08596	16,8617507	1,129725154	0,438215154	2,578014806	0,009936974	0,04915852	protein_codin	hypothetical protein
TcG_08597	104,7914047	0,599757678	0,174730589	3,432470991	0,000598108	0,005032446	protein_codin	RNA-binding protein
TcG_08598	90,4098322	0,876872929	0,215540514	4,068251083	4,73673E-05	0,00059074	protein_codin	Tbingi protein
TcG_08599	65,59536584	0,754625382	0,220026681	3,429699428	0,00060425	0,005069401	protein_codin	hypothetical protein
TcG_08600	31,69142971	0,347362031	0,321592764	1,080130119	0,280084241	0,513214617	protein_codin	Tbingi protein
TcG_08601	127,1516733	0,842107316	0,162430803	5,184406518	2,16704E-07	5,20898E-06	protein_codin	Tbingi protein
TcG_08602	46,63098654	0,975704624	0,283742029	3,4387032	0,000584508	0,004945007	protein_codin	multidrug resistance-associated protein
TcG_08603	311,9036292	-0,185565728	0,103062827	-1,80051075	0,071780028	0,215117279	protein_codin	putative ATPase
TcG_08604	347,1842576	0,014356482	0,098216127	0,14617235	0,883785333	0,943488844	protein_codin	hypothetical protein
TcG_08605	157,8933675	0,26149531	0,148851967	1,756747433	0,078960869	0,228881818	protein_codin	hypothetical protein
TcG_08606	704,3843517	-0,163452916	0,070505386	-2,31830397	0,020432807	0,085525469	protein_codin	putative GTPase activating protein
TcG_08607	45,71636286	-0,112715819	0,262096897	-0,430054	0,667156359	0,825673314	protein_codin	putative GTPase activating protein

TcG_08608	118,2369674	-0,073050047	0,165215781	-0,44214933	0,658381149	0,820127297	protein_codin putative GTPase activating protein
TcG_08609	410,4129428	-0,194596852	0,091195591	-2,13384056	0,032855834	0,122440558	protein_codin rib72 protein-like protein
TcG_08610	170,5443473	-0,349188371	0,136115458	-2,56538365	0,010306178	0,050596347	protein_codin ATP-binding cassette protein subfamily G, member 1
TcG_08611	637,7421003	-0,134041418	0,07480812	-1,79180305	0,07316452	0,217817959	protein_codin putative 40S ribosomal protein S8
TcG_08612	137,3634184	-0,021570109	0,150617174	-0,14321148	0,88612316	0,944752272	protein_codin hypothetical protein
TcG_08613	393,9110076	-0,171018529	0,095976873	-1,78187228	0,074770061	0,220667247	protein_codin hypothetical protein
TcG_08614	161,8417469	-0,270548431	0,151738416	-1,78299232	0,074587553	0,220451886	protein_codin hypothetical protein
TcG_08615	744,5169283	-0,104028656	0,073329867	-1,41863965	0,156004101	0,357566753	protein_codin protein transport protein Sec23-like protein
TcG_08616	157,81399	-0,038371807	0,14284336	-0,26862857	0,788215526	0,89483476	protein_codin hypothetical protein
TcG_08617	127,9559587	0,272226807	0,155296465	1,752949157	0,079610735	0,230247122	protein_codin hypothetical protein
TcG_08618	240,3216871	-0,284761064	0,116517251	-2,4439391	0,014527879	0,06622252	protein_codin hypothetical protein
TcG_08619	125,5525332	0,309203525	0,157401097	1,964430561	0,049480182	0,164357048	protein_codin hypothetical protein
TcG_08620	20,71083401	0,475521558	0,401467376	1,184458779	0,236231504	0,463600927	protein_codin hypothetical protein
TcG_08621	225,2932142	0,417746507	0,12240338	3,412867409	0,000642832	0,005342792	protein_codin hypothetical protein
TcG_08622	176,6730453	0,388383348	0,135631871	2,863510951	0,004189743	0,02504766	protein_codin hypothetical protein
TcG_08623	303,7914223	0,325414798	0,115349445	2,821121497	0,004785607	0,027820395	protein_codin hypothetical protein
TcG_08624	433,9558273	0,284261484	0,087114236	3,263088759	0,00110205	0,008445448	protein_codin hypothetical protein
TcG_08625	6,018054118	0,533514919	0,704652175	0,757132296	0,448970607	1	
TcG_08626	491,4958552	0,118613889	0,085479883	1,387623432	0,165251743	0,370715499	protein_codin transferase
TcG_08627	34,71640831	0,066330607	0,292953816	0,226420014	0,820874763	0,911236551	protein_codin hypothetical protein
TcG_08628	151,128788	-0,232718743	0,151023843	-1,54094042	0,123331288	0,308687903	protein_codin hypothetical protein
TcG_08629	415,2517819	0,141299006	0,093265782	1,515014446	0,129768746	0,319405645	protein_codin hypothetical protein
TcG_08630	253,6756052	0,03811978	0,120697977	0,315827826	0,752133197	0,87492919	protein_codin hypothetical protein
TcG_08631	345,715906	0,161963911	0,098057747	1,651719689	0,098591707	0,266080484	protein_codin hypothetical protein
TcG_08632	798,0343393	0,113016662	0,081850812	1,380764088	0,167351509	0,373374654	protein_codin hypothetical protein
TcG_08633	51,74309351	0,208967699	0,253607172	0,823981818	0,409949889	0,636890088	protein_codin hypothetical protein
TcG_08634	187,9838469	0,083067228	0,237946998	0,349099707	0,727014456	0,859270718	protein_codin hypothetical protein
TcG_08635	15,78018786	-0,112182222	0,431938759	-0,25971789	0,795081395	1	protein_codin hypothetical protein
TcG_08636	260,4123191	-0,384150975	0,127267944	-3,01844253	0,002540776	0,016650129	protein_codin proton motive ATPase
TcG_08637	654,2100237	-0,164463428	0,077488264	-2,12243016	0,033801632	0,125080071	protein_codin hypothetical protein
TcG_08638	145,8225033	-0,038333478	0,147640112	-0,25964135	0,795140437	0,897705774	protein_codin chaperone DnaJ protein
TcG_08639	386,7549341	-0,298920161	0,096349326	-3,10246239	0,001919179	0,013306769	protein_codin electron transfer protein
TcG_08640	397,7890048	-0,668373114	0,102041702	-6,54999964	5,75372E-11	3,04396E-09	protein_codin hypothetical protein
TcG_08641	194,4994719	-0,266836994	0,132180627	-2,01872998	0,043515293	0,1499162	protein_codin hypothetical protein
TcG_08642	399,2560948	-0,466976136	0,095471013	-4,89128712	1,00179E-06	2,03334E-05	protein_codin WDdomain 60
TcG_08643	362,1290029	-0,37653923	0,100001041	-3,76535311	0,000166314	0,001714335	protein_codin DNA primase small subunit
TcG_08644	203,9424585	-0,560622624	0,126407107	-4,43505619	9,20483E-06	0,000142386	protein_codin calcium-binding protein CML
TcG_08645	259,4037299	-0,426260994	0,114451917	-3,72436745	0,000195806	0,00195738	protein_codin putative chaperone DNAJ protein
TcG_08646	69,07668476	-0,745112008	0,216301857	-3,4447786	0,000571527	0,00485463	protein_codin hypothetical protein
TcG_08647	123,6796001	0,64484167	0,178308277	3,61644272	0,000298679	0,002797493	protein_codin membrane associated protein
TcG_08648	53,75702041	0,769883145	0,257892924	2,985282156	0,002833169	0,018165518	protein_codin hypothetical protein
TcG_08649	78,40267445	0,269636193	0,203279746	1,326429214	0,184697568	0,397783186	
TcG_08650	199,6016791	0,11177788	0,124886799	0,895033589	0,370769133	0,602325132	protein_codin hypothetical protein
TcG_08651	291,378191	0,074989888	0,106912935	0,701410808	0,483046679	0,696521203	protein_codin hypothetical protein
TcG_08652	112,3036501	0,30636643	0,172997046	1,770934462	0,076571601	0,224086528	protein_codin monothiol glutaredoxin
TcG_08653	105,284732	0,470187501	0,184924389	2,542593239	0,011003324	0,053205151	protein_codin glycoside hydrolase family protein
TcG_08654	268,8839881	0,766201655	0,111995713	6,841348073	7,84514E-12	5,07786E-10	protein_codin UMP-CMP kinase 2, mitochondrial
TcG_08655	40,25221263	0,664091441	0,290337338	2,287309811	0,022177746	0,091085207	
TcG_08656	199,6081774	0,005160313	0,126762695	0,040708453	0,967528322	0,985995527	protein_codin hypothetical protein
TcG_08657	377,7851955	0,048775324	0,093190316	0,523394771	0,600699562	0,783588603	protein_codin hypothetical protein
TcG_08658	144,6838907	0,037054892	0,154503289	0,239832384	0,810460201	0,906632411	protein_codin hypothetical protein
TcG_08659	71,37954127	0,499902999	0,205834857	2,428660563	0,015154713	0,068160911	protein_codin hypothetical protein
TcG_08660	780,268538	0,084344922	0,069666513	1,210695326	0,226012194	0,452435657	protein_codin putative MCAK-like kinesin
TcG_08661	371,5232378	0,24701664	0,101675038	2,429471821	0,01512084	0,068160911	protein_codin hypothetical protein
TcG_08662	365,0485819	-0,030857924	0,096262301	-0,32056084	0,748543221	0,873112027	protein_codin hypothetical protein
TcG_08663	102,1460261	-0,120249179	0,172335757	-0,69776105	0,485326639	0,698146344	protein_codin hypothetical protein
TcG_08664	67,8746938	-0,679860085	0,240162517	-2,83083345	0,004642689	0,027139351	protein_codin trans-sialidase

TcG_08665	51,61033868	0,080638655	0,25427755	0,317128489	0,751146109	0,874683002	
TcG_08666	90,37208765	0,199651659	0,19293081	1,034835541	0,300745722	0,534199829	protein_codin hypothetical protein
TcG_08667	122,1674471	-0,293046046	0,161575901	-1,81367421	0,069727897	0,211424465	protein_codin hypothetical protein
TcG_08668	368,1086903	-0,00596794	0,095033217	-0,06279846	0,949926992	0,976741925	protein_codin hypothetical protein
TcG_08669	222,9145579	0,05942053	0,133167485	0,446208993	0,655446287	0,817728228	protein_codin putative aquaporin-like protein
TcG_08670	343,7784943	0,18398537	0,101043536	1,82085244	0,06862929	0,209192044	protein_codin putative aquaporin-like protein
TcG_08671	395,0191754	-0,038134377	0,094010482	-0,40563963	0,685007404	0,836557076	protein_codin hypothetical protein
TcG_08672	199,5606232	0,170763536	0,128694212	1,326893674	0,184543857	0,397568823	protein_codin hypothetical protein
TcG_08673	396,1135658	-0,216651937	0,09717907	-2,22940945	0,025786674	0,102176609	protein_codin putative histone deacetylase
TcG_08674	429,2655018	-0,264805895	0,092649624	-2,85814323	0,00426128	0,025396702	protein_codin hypothetical protein
TcG_08675	160,9480545	0,022961733	0,139308276	0,164826769	0,869080334	0,936791559	protein_codin hypothetical protein
TcG_08676	205,9365046	0,37423537	0,126429914	2,960022343	0,003076167	0,019443794	protein_codin hypothetical protein
TcG_08677	359,8404468	-0,196874368	0,099540309	-1,9778356	0,047947256	0,160640556	protein_codin mitochondrial edited mRNA stability factor 1
TcG_08678	214,6731258	0,191957328	0,125738558	1,526638536	0,126850919	0,314979586	protein_codin putative poly(A) polymerase
TcG_08679	266,614245	0,143927654	0,113037851	1,273269551	0,202922408	0,423006561	protein_codin hypothetical protein
TcG_08680	133,4863419	0,313019872	0,152529482	2,052192585	0,04015095	0,141653138	protein_codin hypothetical protein
TcG_08681	190,668091	-0,212901258	0,133942606	-1,58949615	0,111948422	0,289710614	protein_codin hypothetical protein
TcG_08682	455,6319503	-0,051749242	0,086305419	-0,59960594	0,548768889	0,745247925	protein_codin hypothetical protein
TcG_08683	159,2672744	0,004597578	0,142155897	0,032341802	0,974199473	0,988782354	protein_codin hypothetical protein
TcG_08684	160,9240645	-0,096125363	0,13986426	-0,6872761	0,491908759	0,702635507	protein_codin monooxygenase
TcG_08685	105,2127074	-0,01467064	0,176172088	-0,08327448	0,933633289	0,968577658	protein_codin putative vesicle-associated membrane protein 713
TcG_08686	371,4528457	0,066625568	0,094175846	0,707459195	0,479281162	0,693829774	protein_codin hypothetical protein
TcG_08687	81,45463482	-0,167222919	0,202565546	-0,82552498	0,409073606	0,636605346	protein_codin hypothetical protein
TcG_08688	114,6245154	-0,113594528	0,16859766	-0,673761	0,500463271	0,709280423	protein_codin hypothetical protein
TcG_08689	161,072697	-0,063406504	0,137374171	-0,46156059	0,64439646	0,811082798	protein_codin hypothetical protein
TcG_08690	629,4006232	0,310946843	0,078711059	3,950484792	7,79931E-05	0,000901824	protein_codin hypothetical protein
TcG_08691	586,2494803	-0,041335864	0,079238483	-0,521664	0,601904291	0,784755061	protein_codin hypothetical protein
TcG_08692	157,588534	0,133736915	0,145159441	0,921310483	0,356888349	0,590701202	protein_codin SFT2 domain containing 2
TcG_08693	123,3071785	0,211863289	0,160266109	1,321946917	0,186185828	0,400064726	protein_codin hypothetical protein
TcG_08694	224,6323769	-0,183160924	0,12233413	-1,49721852	0,134336409	0,325883927	protein_codin surface protein-2
TcG_08695	156,8360514	-0,413381531	0,140104138	-2,95053049	0,003172287	0,019964216	protein_codin clathrin assembly sigma-adaptin protein complex 4
TcG_08696	336,84191	-0,094450558	0,101495904	-0,93058492	0,352068316	0,586409361	protein_codin hypothetical protein
TcG_08697	420,4191137	-0,533929505	0,088222184	-6,05210029	1,42969E-09	5,48491E-08	protein_codin n-myristoyl transferase
TcG_08698	834,0910093	-0,423380835	0,071542511	-5,91789178	3,26094E-09	1,16263E-07	protein_codin hypothetical protein
TcG_08699	221,6449574	-0,352606587	0,120941909	-2,91550373	0,003551149	0,021815275	protein_codin hypothetical protein
TcG_08700	156,2730573	0,019472157	0,140794476	0,138301999	0,890001746	0,946535728	protein_codin hypothetical protein
TcG_08701	284,1502944	-0,300221571	0,10661566	-2,81592377	0,004863721	0,028175533	protein_codin putative syntaxin 5
TcG_08702	394,1735147	-0,326265451	0,092305557	-3,53462416	0,000408356	0,00363939	protein_codin hypothetical protein
TcG_08703	503,9458126	-0,241790759	0,092084843	-2,62573894	0,00864611	0,044090595	protein_codin putative protein transport protein Sec13
TcG_08704	1603,776965	-0,574214999	0,053150742	-10,803518	3,31263E-27	1,32345E-24	protein_codin lipoprotein, type 6
TcG_08705	959,2092443	-0,459953446	0,069180584	-6,64859156	2,95911E-11	1,66428E-09	protein_codin pyruvate kinase
TcG_08706	281,653137	-0,214122517	0,116718915	-1,83451428	0,06657769	0,204878915	protein_codin signal recognition particle subunit SRP19
TcG_08707	99,0931054	0,208422253	0,188881116	1,103457337	0,269828609	0,502690828	protein_codin putative rab1 small GTP-binding protein
TcG_08708	4,599786664	0,32432668	0,834884758	0,3884688	0,697669139	1	protein_codin rab1 small GTP-binding protein
TcG_08709	28,14911097	-0,174718463	0,345464914	-0,50574879	0,61303301	0,790819724	protein_codin target of rapamycin (TOR) kinase 1
TcG_08710	369,5105757	-0,112784097	0,101519515	-1,11095977	0,266585668	0,499379393	protein_codin BRCT domain-containing protein
TcG_08711	226,7810141	-0,144128233	0,122748543	-1,17417469	0,24032506	0,468992108	protein_codin hypothetical protein
TcG_08712	182,2787415	-0,044102652	0,137515033	-0,32071149	0,748429036	0,873112027	protein_codin hypothetical protein
TcG_08713	90,19291841	-0,247557531	0,186427292	-1,32790392	0,18420985	0,397070758	protein_codin hypothetical protein
TcG_08714	563,7796642	-0,119114265	0,080126797	-1,48657216	0,137127845	0,330167872	protein_codin hypothetical protein
TcG_08715	59,92906989	0,361588886	0,238679437	1,514956172	0,129783504	0,319405645	protein_codin ADP-ribosylation factor-like 2
TcG_08716	139,2578865	0,02579124	0,152940421	0,168635864	0,86608307	0,935506992	protein_codin coatomer zeta subunit
TcG_08717	396,8717063	-0,165017289	0,090909506	-1,81518188	0,069495964	0,211001109	protein_codin ubiquinol-cytochrome c reductase subunit 7
TcG_08718	84,16402168	0,041861675	0,222165548	0,188425592	0,850543039	0,928053891	protein_codin hypothetical protein
TcG_08719	120,2274279	-0,028753239	0,165125541	-0,17412957	0,861763638	0,933819072	protein_codin Trk system potassium uptake protein
TcG_08720	248,3816103	0,186494962	0,117525083	1,58685242	0,112546073	0,290608158	protein_codin putative prohibitin
TcG_08721	258,4640842	0,040207725	0,113901098	0,3530056	0,724084242	0,857883222	protein_codin putative trichohyalin-like

TcG_08722	115,3422533	-0,173654949	0,164304606	-1,05690859	0,290553308	0,523456792	protein_codin	hypothetical protein
TcG_08723	237,2219318	0,26982745	0,119003923	2,267382814	0,02336685	0,094959077	protein_codin	putative 2-oxoisovalerate dehydrogenase beta subunit, mitochondrial precursor
TcG_08724	60,15810235	0,420264624	0,228729521	1,837386894	0,0661528	0,203950639	protein_codin	hypothetical protein
TcG_08725	41,5839939	0,15221762	0,280739772	0,542201837	0,58767947	0,775648564	protein_codin	hypothetical protein
TcG_08726	73,52838833	0,207343507	0,204226381	1,015263091	0,309980433	0,543251142	protein_codin	retrotransposon hot spot protein (RHS)
TcG_08727	23,16032039	0,714669608	0,383251161	1,86475523	0,062215721	0,194819283	protein_codin	putative retrotransposon hot spot (RHS) protein
TcG_08728	0					1	protein_codin	retrotransposon hot spot (RHS) protein
TcG_08729	21,76636191	-0,02934181	0,378584917	-0,07750391	0,938222684	0,971428881	protein_codin	retrotransposon hot spot (RHS) protein
TcG_08730	28,85875564	0,092447171	0,329832198	0,280285466	0,779258501	0,890032432	protein_codin	hypothetical protein
TcG_08731	37,00670247	0,353486504	0,28457618	1,242150707	0,214180963	0,437808863	protein_codin	retrotransposon hot spot (RHS) protein
TcG_08732	50,53175797	0,687784369	0,263165908	2,613501023	0,008961982	0,045342148	protein_codin	hypothetical protein
TcG_08733	318,8330665	0,367738696	0,102166295	3,599413045	0,000318936	0,002949079	protein_codin	hypothetical protein
TcG_08734	27,54586434	-0,039250023	0,341201769	-0,11503464	0,908417665	0,955500221		
TcG_08735	157,7048856	0,310706289	0,143497167	2,165243369	0,030369053	0,115400408	protein_codin	putative trans-sialidase
TcG_08736	7,709099069	0,893984906	0,685973235	1,303235841	0,192494257	1	protein_codin	dispersed protein family protein 1
TcG_08737	171,8509432	0,281401296	0,14703545	1,913832993	0,055641498	0,178477962	protein_codin	dispersed gene family protein 1 (DGF-1)
TcG_08738	37,59139444	0,420085542	0,285897242	1,469358499	0,141735578	0,337266051	protein_codin	dispersed gene family protein 1 (DGF-1)
TcG_08739	930,4418577	0,262149142	0,063640133	4,119242527	3,8012E-05	0,000484496	protein_codin	putative 3,2-trans-enoyl-CoA isomerase, mitochondrial precursor
TcG_08740	502,8001968	0,386941121	0,083872443	4,613447599	3,96044E-06	6,84861E-05	protein_codin	putative aminopeptidase, putative,metallo-peptidase, clan MA(E), family M1
TcG_08741	9,551526552	0,056283516	0,572938257	0,098236618	0,921744406	1		
TcG_08742	156,257168	0,457822968	0,165259198	2,770332755	0,005599905	0,03128922	protein_codin	putative p-nitrophenylphosphatase
TcG_08743	62,94697719	0,151184282	0,220048271	0,687050533	0,492050885	0,702635507	protein_codin	hypothetical protein
TcG_08744	666,0777572	0,374536727	0,081667733	4,586104134	4,51593E-06	7,66056E-05	protein_codin	hypothetical protein
TcG_08745	540,4796582	0,438096769	0,083313339	5,258422917	1,45296E-07	3,6692E-06	protein_codin	putative pterin-4-alpha-carbinolamine dehydratase
TcG_08746	257,8927276	0,440306633	0,112549032	3,912131665	9,1485E-05	0,001033085	protein_codin	hypothetical protein
TcG_08747	298,4102925	0,524016559	0,108498496	4,829712637	1,3673E-06	2,67594E-05	protein_codin	putative metal-ion transporter
TcG_08748	83,58177758	0,741081251	0,200794837	3,690738584	0,000223604	0,002186223	protein_codin	hypothetical protein
TcG_08749	2008,827279	0,538917277	0,051935379	10,37668907	3,16564E-25	9,16928E-23	protein_codin	hypothetical protein
TcG_08750	27,13154626	0,42170081	0,336673572	1,252550973	0,210369167	0,432996476	protein_codin	hypothetical protein
TcG_08751	336,141158	-0,006429506	0,102396235	-0,06279045	0,949933368	0,976741925	protein_codin	putative dual specificity protein phosphatase
TcG_08752	307,5496249	0,046458099	0,102431777	0,453551628	0,650151576	0,814341207	protein_codin	Thioredoxin domain-containing protein
TcG_08753	158,7969824	-0,062408377	0,14735499	-0,42352402	0,671912979	0,828080393	protein_codin	vitamin-K-epoxide reductase (warfarin-sensitive)
TcG_08754	293,9280483	0,20732049	0,110540621	1,875514075	0,060722035	0,191383433	protein_codin	nuclear lim interactor-interacting factor
TcG_08755	977,7406298	-0,149268798	0,064005651	-2,33211906	0,019694429	0,083004602	protein_codin	WD repeat domain 65
TcG_08756	254,4661226	0,100170713	0,125740954	0,796643495	0,425658109	0,651476202	protein_codin	hypothetical protein
TcG_08757	658,2781098	-0,305909366	0,077913524	-3,92626789	8,62741E-05	0,000982863	protein_codin	hypothetical protein
TcG_08758	420,565338	0,138177471	0,094791918	1,45769254	0,144925309	0,341754828	protein_codin	putative ras-related protein rab-14
TcG_08759	620,6733799	0,059495713	0,076417774	0,778558566	0,436239795	0,659913077	protein_codin	putative protein kinase
TcG_08760	392,4247164	-0,175530539	0,093637774	-1,87456975	0,060851939	0,19150771	protein_codin	hypothetical protein
TcG_08761	444,3247759	0,182040528	0,093312423	1,950871307	0,051072357	0,168073669	protein_codin	trans-sialidase
TcG_08762	15,27111369	0,809381794	0,456273536	1,773895984	0,076080357	1	protein_codin	trans-sialidase
TcG_08763	464,620904	-0,088144093	0,090679295	-0,9720421	0,331029604	0,565262931	protein_codin	trans-sialidase-like protein
TcG_08764	221,761904	0,292590691	0,129431855	2,260577133	0,023785455	0,096288708	protein_codin	hypothetical protein
TcG_08765	288,4798643	0,191368512	0,109389701	1,749419829	0,080218476	0,231484747	protein_codin	retrotransposon hot spot (RHS) protein
TcG_08766	45,17270855	0,178978239	0,269359763	0,66445796	0,506397247	0,713154067	protein_codin	hypothetical protein
TcG_08767	180,9283975	-0,022121073	0,137855197	-0,16046601	0,872513994	0,938795239	protein_codin	dispersed gene family protein 1 (DGF-1)
TcG_08768	70,96272571	0,02113092	0,221685669	0,095319289	0,924061222	0,96287196	protein_codin	dispersed gene family protein 1 (DGF-1)
TcG_08769	185,0718735	0,164476815	0,13408694	1,226643065	0,219956776	0,445077631	protein_codin	dispersed gene family protein 1 (DGF-1)
TcG_08770	42,63554633	0,161190984	0,264733736	0,608879648	0,542604215	0,740452532		
TcG_08771	86,29843487	-0,04941847	0,202495113	-0,24404772	0,807193863	0,904725559	protein_codin	selenocysteine-tRNA-specific elongation factor
TcG_08772	36,26998408	0,521180839	0,299148834	1,742212504	0,081471267	0,234108655	protein_codin	hypothetical protein
TcG_08773	328,5537827	0,164710419	0,107754705	1,528568242	0,126371514	0,31409915	protein_codin	hypothetical protein
TcG_08774	194,4563458	0,11486804	0,133310595	0,861657243	0,388876161	0,619230236	protein_codin	hypothetical protein
TcG_08775	418,5820556	0,006417037	0,089806099	0,071454353	0,943036153	0,973191135	protein_codin	putative ATP-dependent RNA helicase
TcG_08776	364,3598254	-0,069824241	0,097208764	-0,71829162	0,472577497	0,688368479	protein_codin	putative methionyl-tRNA formyltransferase
TcG_08777	596,7112579	0,067640032	0,078911392	0,857164342	0,391354066	0,621127152	protein_codin	putative heat shock protein Hsp20
TcG_08778	551,9548737	-0,200085819	0,079100653	-2,52950906	0,011422223	0,054934774	protein_codin	vesicular transport-associated repeat protein

TcG_08779	264,8432219	-0,220025414	0,10903313	-2,01796843	0,043594549	0,150144604	protein_codin hypothetical protein
TcG_08780	333,8107126	0,089442415	0,105582386	0,847133871	0,396920508	0,626692779	protein_codin putative sphingosine-1-phosphate phosphatase
TcG_08781	129,1123881	0,292114975	0,176587987	1,65421771	0,098083286	0,265140679	protein_codin hypothetical protein
TcG_08782	227,9323783	0,097562295	0,118803779	0,821205317	0,411529329	0,638080373	protein_codin putative actin 2
TcG_08783	272,3334323	-0,102278405	0,107645045	-0,95014504	0,342038562	0,576512293	protein_codin hypothetical protein
TcG_08784	540,6805272	0,046380217	0,080749338	0,574372721	0,565715609	0,758082471	protein_codin putative C-14 sterol reductase
TcG_08785	123,685781	0,038151537	0,160950164	0,237039444	0,81262619	0,907594318	protein_codin hypothetical protein
TcG_08786	30,42557388	-0,082202997	0,312491088	-0,26305709	0,792506574	0,896248225	protein_codin hypothetical protein
TcG_08787	240,1454202	-0,223923419	0,114425775	-1,95693164	0,050355506	0,166310971	protein_codin GP63 group II protein
TcG_08788	102,6981268	0,161904203	0,18193497	0,889901506	0,373518775	0,604496256	
TcG_08789	46,57918261	-0,466195875	0,256968244	-1,8142159	0,069644493	0,211276425	protein_codin surface protease GP63
TcG_08790	353,1936561	-0,458383887	0,098894806	-4,63506533	3,56825E-06	6,24497E-05	protein_codin ubiquinone biosynthesis protein
TcG_08791	182,0152398	-0,525439737	0,131203419	-4,00477169	6,20774E-05	0,000741473	protein_codin hypothetical protein
TcG_08792	581,7458071	-0,455439865	0,07751428	-5,87556079	4,21414E-09	1,46183E-07	protein_codin hypothetical protein
TcG_08793	193,1348491	-0,501784907	0,129169012	-3,88471585	0,00010245	0,001136955	protein_codin putative oxidoreductase
TcG_08794	314,693324	-0,513968054	0,109416742	-4,6973438	2,63567E-06	4,77885E-05	protein_codin hypothetical protein
TcG_08795	82,11085503	-0,309123438	0,192456127	-1,60620211	0,10822951	0,283762639	protein_codin hypothetical protein
TcG_08796	498,1511751	-0,555722371	0,085363165	-6,5100957	7,51029E-11	3,78323E-09	protein_codin Viral A-type inclusion protein
TcG_08797	250,2969221	-0,528928387	0,114328653	-4,62638519	3,72103E-06	6,46355E-05	protein_codin hypothetical protein
TcG_08798	186,6516584	0,323498168	0,137003463	2,361240807	0,018213899	0,078042247	protein_codin L1Tc protein
TcG_08799	47,10849976	0,093341044	0,265480801	0,351592447	0,72514393	0,858172586	protein_codin trans-sialidase
TcG_08800	87,33595131	0,286838872	0,188216847	1,52398086	0,127513493	0,316218177	protein_codin trans-sialidase
TcG_08801	44,15086193	0,41898746	0,263743087	1,588619687	0,112146281	0,289898887	protein_codin trans-sialidase
TcG_08802	150,0626623	0,458766556	0,152559875	3,007124617	0,002637316	0,017118177	protein_codin hypothetical protein
TcG_08803	54,05939085	-0,002274392	0,244421713	-0,0093052	0,992575634	0,997050572	protein_codin hypothetical protein
TcG_08804	168,0288229	0,377179616	0,138917965	2,715124822	0,006625079	0,035773183	protein_codin retrotransposon hot spot (RHS) protein
TcG_08805	188,7835612	0,170474325	0,13460151	1,266511235	0,20533011	0,426784904	protein_codin retrotransposon hot spot (RHS) protein
TcG_08806	103,1259217	0,457350715	0,17750302	2,576579911	0,009978314	0,049279089	protein_codin trans-sialidase
TcG_08807	451,3178178	0,266454559	0,087390092	3,049024799	0,002295855	0,015384488	protein_codin trans-sialidase
TcG_08808	49,35845993	0,608051498	0,249321013	2,438829729	0,014734909	0,066998519	protein_codin hypothetical protein
TcG_08809	62,63116248	0,365646549	0,22993246	1,59023458	0,111781938	0,289409059	protein_codin trans-sialidase
TcG_08810	85,31914823	0,235401505	0,19977085	1,178357627	0,238654063	0,466674425	protein_codin putative trans-sialidase
TcG_08811	64,58689253	0,082757964	0,216674581	0,381945883	0,702501506	0,84624626	protein_codin trans-sialidase
TcG_08812	61,28536236	0,44915819	0,223533376	2,00935627	0,044499364	0,152355093	
TcG_08813	68,03518479	0,110012411	0,24182953	0,454917192	0,649168814	0,813550014	protein_codin hypothetical protein
TcG_08814	11,19197879	0,271735902	0,519517414	0,523054464	0,600936353	1	protein_codin hypothetical protein
TcG_08815	99,60130516	0,21632877	0,178496639	1,211948702	0,225532018	0,451843214	protein_codin mucin-like glycoprotein
TcG_08816	29,13242318	-0,491390477	0,323139457	-1,52067618	0,128341119	0,317319718	
TcG_08817	152,0990766	-0,195289457	0,153340709	-1,27356564	0,202817397	0,423006561	protein_codin mucin-associated surface protein (MASP)
TcG_08818	19,90730545	0,514994199	0,404128735	1,274332049	0,202545763	0,422751794	protein_codin surface protease GP63
TcG_08819	12,43064501	0,109209017	0,491052663	0,222397769	0,824004248	1	protein_codin putative dynein heavy chain
TcG_08820	6,872605492	-0,681235916	0,655212487	-1,03971754	0,298471148	1	protein_codin hypothetical protein
TcG_08821	7,514628213	1,059121103	0,658057873	1,609464983	0,107514706	1	protein_codin hypothetical protein
TcG_08822	492,9173509	-0,326369409	0,090625098	-3,60131373	0,000316613	0,002929937	protein_codin hypothetical protein
TcG_08823	908,5539028	-0,109504811	0,068294635	-1,60341748	0,108842515	0,284854162	protein_codin putative glutamine amidotransferase
TcG_08824	5,431477598	-0,925434077	0,749019319	-1,23552765	0,216634194	1	protein_codin hypothetical protein
TcG_08825	692,4184587	0,03265557	0,075368515	0,433278665	0,664812336	0,824768789	protein_codin putative methyltransferase, putative,mRNA cap methyltransferase-like protein
TcG_08826	348,7763068	-0,225058542	0,100963804	-2,22910125	0,025807169	0,102195608	protein_codin heat shock protein DnaJ
TcG_08827	278,2819229	0,317458965	0,109350871	2,903122409	0,003694622	0,022565045	protein_codin putative DNA repair protein
TcG_08828	423,9204224	0,20161604	0,090030999	2,239406904	0,025129453	0,100120302	protein_codin transcription factor-like protein
TcG_08829	605,755313	-0,133152798	0,077282015	-1,72294678	0,084898151	0,240202679	protein_codin hypothetical protein
TcG_08830	303,0020575	0,026994165	0,106180373	0,254229329	0,799318393	0,89990311	protein_codin hypothetical protein
TcG_08831	20,60051076	-0,569897196	0,391960896	-1,45396442	0,145956171	0,343429773	
TcG_08832	6,871921718	-0,653986047	0,691024155	-0,94640114	0,343944025	1	
TcG_08833	622,1639396	0,307021679	0,079422229	3,865689543	0,000110776	0,001217693	protein_codin putative retrotransposon hot spot (RHS) protein
TcG_08834	72,74266178	0,117189395	0,217015594	0,54000449	0,589193936	0,775648564	protein_codin hypothetical protein
TcG_08835	1,79734577	0,764304816	1,426669685	0,535726541	0,592147557	1	protein_codin hypothetical protein

TcG_08836	227,9129657	0,108179875	0,118963797	0,909351228	0,363164757	0,595268564	protein_codin	surface protease GP63
TcG_08837	365,4842472	0,136612593	0,10374453	1,316817308	0,18789987	0,402255708	protein_codin	surface protease GP63
TcG_08838	167,756718	0,206349127	0,136969835	1,506529722	0,131931243	0,322809808	protein_codin	SET and MYND domain-containing protein
TcG_08839	305,7718914	0,00562038	0,10510575	0,053473571	0,957354588	0,980197088	protein_codin	hypothetical protein
TcG_08840	296,0147853	0,147920969	0,105499357	1,402103034	0,160884481	0,363921827	protein_codin	Pin2-interacting protein X1
TcG_08841	416,765844	0,067926598	0,090297964	0,752249499	0,451901031	0,672799454	protein_codin	putative kinesin
TcG_08842	478,8623071	-0,163414041	0,083410651	-1,95915078	0,050095132	0,165734494	protein_codin	axonemal p66.0
TcG_08843	288,5364973	-0,023329068	0,107176136	-0,21767036	0,827685958	0,915880831	protein_codin	putative alpha-ketoglutarate-dependent dioxygenase
TcG_08844	594,0241919	0,064620444	0,076638903	0,84318069	0,399127396	0,62898729	protein_codin	putative deoxyribose-phosphate aldolase
TcG_08845	643,3559909	-0,11479478	0,073275973	-1,56660875	0,117206172	0,2985867	protein_codin	hypothetical protein
TcG_08846	156,6507464	0,025054204	0,143548321	0,174534987	0,861445037	0,933819072	protein_codin	hypothetical protein
TcG_08847	634,2459129	-0,165183855	0,075047719	-2,20105098	0,027732415	0,107912108	protein_codin	hypothetical protein
TcG_08848	46,92051965	0,933349817	0,269260533	3,466344682	0,000527586	0,004548078	protein_codin	Tbingi protein
TcG_08849	190,8174549	0,672869326	0,134821551	4,990814296	6,01253E-07	1,28526E-05	protein_codin	hypothetical protein
TcG_08850	65,41042068	0,313584967	0,235006665	1,334366269	0,182083849	0,393962535	protein_codin	hypothetical protein
TcG_08851	78,27880674	0,736370719	0,230561505	3,193814684	0,001404062	0,010289351	protein_codin	dispersed gene family protein 1 (DGF-1)
TcG_08852	28,91132761	1,118180448	0,34267818	3,26306288	0,001102151	0,008445448	protein_codin	trans-sialidase
TcG_08853	18,99945013	0,587307841	0,407038169	1,442881494	0,149053857	0,348241102	protein_codin	hypothetical protein
TcG_08854	138,4744625	0,381752414	0,16043284	2,379515401	0,017335419	0,075336894	protein_codin	dispersed gene family protein 1 (DGF-1)
TcG_08855	10,80063377	1,254586872	0,575303586	2,180738834	0,029202739	1	protein_codin	hypothetical protein
TcG_08856	0					1		
TcG_08857	0,155988004	0,503022807	4,080472857	0,123275616	0,901888849	1		
TcG_08858	0					1		
TcG_08859	3,274687446	0,585012994	0,99996608	0,585032838	0,558525637	1		
TcG_08860	94,21451483	0,440443686	0,1902308	2,315312172	0,020595854	0,086052494	protein_codin	putative retrotransposon hot spot (RHS) protein
TcG_08861	77,03321531	0,737475545	0,20211305	3,648826958	0,00026344	0,002512116	protein_codin	hypothetical protein
TcG_08862	151,3297519	0,43241897	0,148133925	2,919108307	0,003510342	0,021679545		
TcG_08863	19,96106081	0,447260765	0,396881412	1,126938052	0,259768674	0,492108097	protein_codin	hypothetical protein
TcG_08864	15,8119034	0,448068948	0,4496585	0,99646498	0,319024274	1	protein_codin	helicase-like protein
TcG_08865	36,91192493	0,011761052	0,305563738	0,038489684	0,969297256	0,986756701	protein_codin	putative trans-sialidase
TcG_08866	35,58504844	0,804173799	0,30609249	2,62722486	0,008608443	0,043956552	protein_codin	trans-sialidase
TcG_08867	158,5612274	0,371736842	0,144988494	2,56390582	0,010350163	0,050769256	protein_codin	putative target of rapamycin (TOR) kinase 1
TcG_08868	154,5284004	0,363294676	0,147254709	2,467117548	0,013620564	0,063148399	protein_codin	target of rapamycin (TOR) kinase 1
TcG_08869	1309,191492	0,062572455	0,058869762	1,062896354	0,287828956	0,520897577	protein_codin	kinetoplast-associated protein Tcp16
TcG_08870	286,3542407	0,129377451	0,111880907	1,156385434	0,247523537	0,47677601	protein_codin	hypothetical protein
TcG_08871	346,8626754	-0,193412937	0,100904236	-1,916797	0,055263726	0,17761041	protein_codin	hypothetical protein
TcG_08872	574,9206835	-0,317661078	0,085526322	-3,71419081	0,000203855	0,002022143		
TcG_08873	2858,009191	-0,190614178	0,043469763	-4,38498318	1,15995E-05	0,000172518	protein_codin	hypothetical protein
TcG_08874	502,7874916	-0,229839348	0,082793429	-2,77605783	0,005502242	0,030811492	protein_codin	hypothetical protein
TcG_08875	267,9497934	0,045452546	0,122235473	0,371844152	0,710008888	0,850425202	protein_codin	putative small GTPase
TcG_08876	935,334954	0,02021104	0,071197858	0,283871463	0,776508887	0,888310845	protein_codin	hypothetical protein
TcG_08877	399,3250235	-0,26098317	0,093498464	-2,79130971	0,005249522	0,02975585	protein_codin	putative choline/ethanolamine phosphotransferase (CEPT)
TcG_08878	350,4280086	0,070473425	0,098581231	0,7148767	0,474685235	0,69039708	protein_codin	hypothetical protein
TcG_08879	624,8970661	-0,464716444	0,077125289	-6,02547423	1,68614E-09	6,34275E-08	protein_codin	kinetoplast-associated protein
TcG_08880	34,12179182	0,404062421	0,302881127	1,334062721	0,182183302	0,394021978		
TcG_08881	14,83847406	0,196893942	0,456742528	0,431083006	0,666408017	1	protein_codin	hypothetical protein
TcG_08882	2,175611591	0,380381178	1,250767749	0,304118154	0,761037877	1		
TcG_08883	115,2107696	-0,340467524	0,16544721	-2,05786199	0,039603383	0,140233741	protein_codin	putative beta galactofuranosyl glycosyltransferase
TcG_08884	14,79198947	0,293686892	0,447916874	0,655672757	0,512034701	1		
TcG_08885	16,87748549	0,197351797	0,42074639	0,469051671	0,639032703	0,807837741		
TcG_08886	75,1465587	-0,09644214	0,200138341	-0,48187738	0,629893054	0,801688869	protein_codin	trans-sialidase
TcG_08887	46,89901959	-0,229064309	0,252849085	-0,90593292	0,36497136	0,597001013	protein_codin	putative trans-sialidase
TcG_08888	38,83701578	-0,033862772	0,284080297	-0,11920141	0,90511579	0,953903154	protein_codin	trans-sialidase
TcG_08889	31,89011376	0,529441708	0,312814669	1,692509207	0,090548926	0,250980828	protein_codin	retrotransposon hot spot (RHS) protein
TcG_08890	156,5673099	0,299133767	0,153754864	1,94552393	0,051711953	0,169103777	protein_codin	putative retrotransposon hot spot (RHS) protein
TcG_08891	102,1535892	0,135770119	0,184420797	0,736197441	0,461610549	0,679450496	protein_codin	retrotransposon hot spot protein (RHS)
TcG_08892	10,11572312	-0,041588633	0,54353682	-0,07651484	0,939009508	1		

TcG_08893	68,34968	0,303682112	0,213432989	1,422845237	0,154781024	0,356094708	protein_codin dispersed gene family protein 1 (DGF-1)
TcG_08894	66,17151848	0,073997546	0,240167339	0,308108282	0,757999939	0,878015178	protein_codin dispersed gene family protein 1 (DGF-1)
TcG_08895	74,86957375	0,337427685	0,203058793	1,661724078	0,096568111	0,262848254	protein_codin dispersed gene family protein 1 (DGF-1)
TcG_08896	29,43344159	0,18992309	0,329061296	0,577166297	0,563827122	0,756804282	protein_codin dispersed gene family protein 1 (DGF-1)
TcG_08897	247,3481037	-0,157241769	0,117499923	-1,33822869	0,180821898	0,392277422	protein_codin putative nuclear lim interactor-interacting factor
TcG_08898	1331,982454	0,046494405	0,060307159	0,770959956	0,440730668	0,663501236	protein_codin kinesin K39
TcG_08899	215,7869807	-0,154913368	0,121675908	-1,27316385	0,202959906	0,423006561	protein_codin putative zinc finger protein
TcG_08900	163,6633282	-0,088256885	0,138656501	-0,63651458	0,524441078	0,728034307	protein_codin putative NADH-ubiquinone oxidoreductase complex I subunit
TcG_08901	263,6052318	-0,030179202	0,109727949	-0,2750366	0,783288118	0,892633424	protein_codin protein G2
TcG_08902	1882,50453	0,187505856	0,049524979	3,786086555	0,000153038	0,001600273	protein_codin 60S ribosomal protein L30
TcG_08903	278,931904	0,104012042	0,107386655	0,968575118	0,332757223	0,566876221	protein_codin hypothetical protein
TcG_08904	154,9209334	-0,036416956	0,14087971	-0,25849681	0,796023511	0,898288536	protein_codin putative GTP-ase activating protein
TcG_08905	132,9898596	0,408017866	0,156647581	2,604686662	0,009195831	0,046243239	protein_codin putative cruzipain precursor
TcG_08906	107,5375496	0,103204532	0,167061651	0,617763148	0,536731479	0,73660472	protein_codin Tbingi protein
TcG_08907	97,54285365	0,277820513	0,180643557	1,537948642	0,124061173	0,310112782	
TcG_08908	80,95598383	-0,089077682	0,20020382	-0,44493498	0,656366746	0,818409935	
TcG_08909	735,201991	-0,063968218	0,070735947	-0,90432404	0,365823604	0,59797295	protein_codin putative ABC transporter
TcG_08910	1100,619909	-0,240581523	0,060504091	-3,9762852	7,00002E-05	0,000825888	protein_codin hypothetical protein
TcG_08911	2613,399429	-0,072797519	0,045074309	-1,6150557	0,106298633	0,280713043	protein_codin hypothetical protein
TcG_08912	2,863320144	1,256494767	1,127811027	1,114100446	0,265236098	1	
TcG_08913	365,364486	0,403414042	0,095852174	4,208710406	2,56832E-05	0,000344804	protein_codin 60S ribosomal protein L44
TcG_08914	227,9088812	-0,043645933	0,126158439	-0,34596126	0,729371826	0,860563425	protein_codin hypothetical protein
TcG_08915	83,46474419	0,148953584	0,189337976	0,786707381	0,431453182	0,655968892	protein_codin hypothetical protein
TcG_08916	198,1236255	-0,159545834	0,125130328	-1,27503728	0,202296045	0,422382768	protein_codin zinc finger family protein
TcG_08917	1050,738429	-0,088441245	0,063886483	-1,38434988	0,166251346	0,371834313	protein_codin coatomer subunit beta'
TcG_08918	459,7039179	-0,618304377	0,087080453	-7,10038082	1,24414E-12	9,29972E-11	protein_codin putative mucin-associated surface protein (MASP)
TcG_08919	0					1	protein_codin putative surface protein TolT
TcG_08920	129,7014978	-0,504026798	0,157678835	-3,1965406	0,001390863	0,010211998	
TcG_08921	212,7366873	-0,618411957	0,136709343	-4,52355297	6,08101E-06	9,93718E-05	protein_codin putative surface protein TolT
TcG_08922	440,3341366	-0,352658544	0,090953854	-3,87733482	0,000105607	0,001167521	protein_codin putative surface protein TolT
TcG_08923	5,305065414	-0,042738063	0,741322233	-0,05765113	0,954026523	1	
TcG_08924	1239,142673	-0,137743328	0,05676214	-2,42667608	0,015237854	0,068428595	
TcG_08925	2553,355155	-0,102726712	0,045563488	-2,254584	0,024159453	0,09739437	protein_codin putative retrotransposon hot spot (RHS) protein
TcG_08926	45,85697427	0,158253002	0,257512101	0,614545885	0,538854662	0,738205979	protein_codin hypothetical protein
TcG_08927	67,47567937	0,272616925	0,230641521	1,181994133	0,237208029	0,464866749	protein_codin hypothetical protein
TcG_08928	50,63101452	0,263984085	0,269777836	0,978523992	0,327815231	0,562343392	protein_codin hypothetical protein
TcG_08929	71,76668716	-0,012899525	0,208647688	-0,06182443	0,950702646	0,976958027	protein_codin trans-sialidase
TcG_08930	110,0557078	0,085448738	0,171456909	0,498368592	0,61822427	0,793033999	protein_codin putative kinesin
TcG_08931	24,12920707	-0,06012712	0,350164629	-0,171711	0,863664734	0,934863339	protein_codin subtilisin-like serine peptidase
TcG_08932	7,571538168	0,14323659	0,643301238	0,222658658	0,82380118	1	protein_codin subtilisin-like serine peptidase
TcG_08933	6,654250566	-0,659698075	0,685009966	-0,96304887	0,335522998	1	protein_codin putative kinesin
TcG_08934	0,757668191	0,484897547	3,865283102	0,125449426	0,900167761	1	
TcG_08935	8,889879364	0,510426786	0,582240744	0,87665934	0,380671698	1	protein_codin hypothetical protein
TcG_08936	18,79046427	0,702840808	0,417665386	1,682784429	0,092416831	0,2543933	protein_codin retrotransposon hot spot (RHS) protein
TcG_08937	59,97986671	0,531540046	0,227552337	2,335902382	0,01949633	0,082349427	protein_codin hypothetical protein
TcG_08938	25,94568043	0,181571093	0,372726764	0,487142622	0,626157264	0,799235216	protein_codin target of rapamycin (TOR) kinase 1
TcG_08939	13,68391986	0,221079357	0,484999334	0,455834352	0,6485091	1	protein_codin trans-sialidase
TcG_08940	12,25350898	0,118698452	0,52571009	0,225786901	0,821367166	1	protein_codin helicase-like protein
TcG_08941	181,3965139	0,231126724	0,132076815	1,749941684	0,080128377	0,231339989	protein_codin mucin-associated surface protein (MASP)
TcG_08942	91,57285905	0,246356706	0,199888817	1,232468675	0,217774061	0,441956607	protein_codin mucin TcMUCII
TcG_08943	36,31852754	0,231112302	0,287815886	0,802986606	0,421982476	0,648162398	protein_codin hypothetical protein
TcG_08944	11,85313944	0,172064222	0,52514425	0,327651349	0,743175292	1	protein_codin surface protease GP63
TcG_08945	82,70782624	-0,136940803	0,19483608	-0,70285135	0,48214839	0,696096105	protein_codin putative trans-sialidase
TcG_08946	36,02253465	-0,104152572	0,285821388	-0,3643974	0,715561273	0,853960462	protein_codin putative trans-sialidase
TcG_08947	55,13691968	-0,050749625	0,245591748	-0,20664222	0,836289283	0,920523248	protein_codin putative trans-sialidase
TcG_08948	324,9966001	-0,177324651	0,102041422	-1,73777126	0,082251127	0,235531775	protein_codin hypothetical protein
TcG_08949	699,6443432	-0,077222683	0,076369593	-1,01117054	0,311934815	0,545850591	protein_codin putative peptidylprolyl isomerase-like

TcG_08950	594,7200332	0,22820047	0,079960617	2,853910817	0,004318465	0,025645176	protein_codin putative choline/carnitine O-acetyltransferase
TcG_08951	227,4804025	0,476789719	0,120952261	3,941966134	8,08164E-05	0,000926151	protein_codin putative protein kinase
TcG_08952	454,3786504	-0,311750277	0,086647299	-3,5979226	0,000320769	0,00296366	protein_codin hypothetical protein
TcG_08953	917,1264115	-0,101739145	0,06621957	-1,53639089	0,12444254	0,310597	protein_codin inosine-5'-monophosphate dehydrogenase
TcG_08954	286,4323631	-0,168228324	0,111986834	-1,5022152	0,133041541	0,324236284	protein_codin ATP-dependent zinc metallopeptidase-like protein
TcG_08955	180,2434305	0,19579687	0,13340543	1,467682916	0,142190373	0,338139915	protein_codin protein kinase
TcG_08956	190,2310969	0,107192293	0,128597715	0,833547417	0,404536067	0,634057748	protein_codin potassium voltage-gated channel
TcG_08957	81,75454923	0,078176325	0,195779721	0,39930757	0,68966659	0,838680499	protein_codin hypothetical protein
TcG_08958	12,05171762	0,549563921	0,506983666	1,08398743	0,278370372	1	
TcG_08959	592,8298472	-0,006829494	0,084343571	-0,08097231	0,935463974	0,96960866	protein_codin hypothetical protein
TcG_08960	641,7425188	0,155139841	0,074792047	2,074282597	0,038053073	0,136299059	protein_codin putative pre-mRNA-splicing factor ATP-dependent RNA helicase DEAH3 isoform X1
TcG_08961	220,2936699	0,305914472	0,132912496	2,301623107	0,021356436	0,088559651	protein_codin putative choline ethanolamine kinase
TcG_08962	206,2917282	-0,112220637	0,127918846	-0,87727994	0,380334606	0,610738657	protein_codin hypothetical protein
TcG_08963	241,2333208	0,305025313	0,116354604	2,621514781	0,008753997	0,04452318	protein_codin hypothetical protein
TcG_08964	87,00069271	0,247031402	0,201916973	1,223430589	0,221167106	0,446371802	protein_codin hypothetical protein
TcG_08965	176,3096748	0,32873035	0,135343628	2,428857227	0,015146496	0,068160911	protein_codin hypothetical protein
TcG_08966	579,7265036	0,26012795	0,083871956	3,101488994	0,0019255	0,013334636	protein_codin hypothetical protein
TcG_08967	436,3106435	0,350086724	0,088672757	3,948075337	7,8782E-05	0,000908227	protein_codin 60S ribosomal protein L2
TcG_08968	165,4415118	-0,275280681	0,138483412	-1,98782422	0,046831134	0,158095631	protein_codin putative 2,4-dienoyl-CoA reductase FADH1
TcG_08969	65,12959745	-0,312936379	0,21520048	-1,45416208	0,145901374	0,343402425	protein_codin 2,4-dienoyl-CoA reductase FADH1
TcG_08970	691,9690336	-0,057093268	0,074438491	-0,76698584	0,443089937	0,665065425	protein_codin putative condensin subunit 1
TcG_08971	250,1824371	-0,185613776	0,116830387	-1,5887457	0,112117817	0,289898887	protein_codin putative protein kinase
TcG_08972	248,4261269	-0,225297144	0,113437101	-1,9860975	0,047022498	0,158459645	protein_codin hypothetical protein
TcG_08973	915,7835749	-0,023256525	0,0709469	-0,32780184	0,743061492	0,869301389	protein_codin putative zinc transporter
TcG_08974	229,1244607	-0,138087681	0,117049087	-1,17974163	0,23810299	0,465940722	protein_codin presenilin-like aspartic peptidase
TcG_08975	310,6264488	-0,157953427	0,101193619	-1,56090303	0,118546633	0,300674537	protein_codin putative radical SAM domain protein
TcG_08976	198,2199558	0,0279019	0,127423042	0,218970598	0,826672951	0,915239152	protein_codin GINS complex subunit 1
TcG_08977	307,9883019	-0,176183029	0,10950149	-1,60895554	0,107626064	0,282608788	protein_codin hypothetical protein
TcG_08978	472,4122181	-0,399286777	0,08336208	-4,78978905	1,66957E-06	3,18151E-05	protein_codin hypothetical protein
TcG_08979	37,79415923	-0,476508668	0,295916701	-1,61027974	0,107336801	0,282235268	protein_codin cAMP specific phosphodiesterase
TcG_08980	6,079441694	1,006721174	0,728293509	1,382301451	0,166879161	1	protein_codin hypothetical protein
TcG_08981	31,23991535	0,464288377	0,325166932	1,427846228	0,153336124	0,353824404	
TcG_08982	642,9096277	0,803214058	0,078607399	10,2180465	1,64625E-24	4,5413E-22	protein_codin ribosome biogenesis protein YTM1
TcG_08983	245,4591585	0,363265971	0,114348864	3,176821854	0,001488985	0,010809133	protein_codin hypothetical protein
TcG_08984	504,9314529	0,500118778	0,086995926	5,748760888	8,98998E-09	2,88526E-07	protein_codin putative nucleolar protein
TcG_08985	163,0183691	0,372618932	0,141748764	2,628727907	0,00857049	0,043801369	protein_codin hypothetical protein
TcG_08986	436,5319662	0,267922154	0,096950826	2,763485011	0,005718772	0,031763035	protein_codin hypothetical protein
TcG_08987	385,6505841	0,299982339	0,103413809	2,900795755	0,003722164	0,022665484	protein_codin hypothetical protein
TcG_08988	1922,887868	0,471471874	0,051586536	9,139436629	6,27785E-20	1,119E-17	protein_codin DNA-directed RNA polymerase II subunit RPB2
TcG_08989	218,474402	0,365963994	0,133679065	2,737631318	0,006188341	0,033840562	protein_codin hypothetical protein
TcG_08990	601,8891141	0,233861879	0,080876341	2,891598155	0,003832878	0,023285426	protein_codin putative ubiquitin hydrolase
TcG_08991	258,2662378	-0,289499926	0,115862204	-2,49865717	0,012466485	0,058809729	protein_codin putative mucin-associated surface protein (MASP)
TcG_08992	232,7188867	-0,088113316	0,119090966	-0,73988245	0,459371325	0,677910606	protein_codin trans-sialidase
TcG_08993	79,37934202	-0,034787946	0,194683374	-0,17868987	0,858181226	0,931742071	protein_codin trans-sialidase
TcG_08994	50,80970084	0,446911982	0,245698163	1,818947186	0,068919488	0,209745518	
TcG_08995	33,21463804	0,395657555	0,303750402	1,302574588	0,192720038	0,409315964	protein_codin hypothetical protein
TcG_08996	68,81584497	0,286153022	0,212048919	1,349467018	0,177187007	0,387264416	protein_codin hypothetical protein
TcG_08997	99,87720413	0,170010978	0,1852565	0,917705872	0,358772872	0,592567323	protein_codin hypothetical protein
TcG_08998	113,918927	0,08133478	0,165045446	0,49280233	0,622152264	0,79640439	protein_codin hypothetical protein
TcG_08999	93,16983356	0,173619628	0,192835658	0,900350223	0,367933901	0,600152356	protein_codin dispersed gene family protein 1 (DGF-1)
TcG_09000	10,21731398	0,262856989	0,594049472	0,44248333	0,658139491	1	protein_codin dispersed gene family protein 1 (DGF-1)
TcG_09001	23,57839989	-0,043561111	0,353022146	-0,12339484	0,901794444	0,952606713	protein_codin dispersed gene family protein 1 (DGF-1)
TcG_09002	5,720386279	0,906852099	0,766262267	1,18347482	0,23662102	1	protein_codin dispersed gene family protein 1 (DGF-1)
TcG_09003	14,17772084	1,090063189	0,507389554	2,148375307	0,031683953	1	protein_codin dispersed gene family protein 1 (DGF-1)
TcG_09004	2,00506609	2,288244956	1,441814232	1,587059487	0,112499172	1	protein_codin hypothetical protein
TcG_09005	2,248872395	0,884725734	1,225986056	0,721644206	0,470513253	1	protein_codin hypothetical protein
TcG_09006	180,4423734	0,121098988	0,132159488	0,916309451	0,359504613	0,592630203	protein_codin hypothetical protein

TcG_09007	69,86047099	-0,153905104	0,207739911	-0,74085477	0,458781504	0,677471643	protein_codin UPF0505 protein C16orf62
TcG_09008	41,88866564	0,07541643	0,267463947	0,281968582	0,777967601	0,889256383	protein_codin UPF0505 protein C16orf62
TcG_09009	250,3469928	-0,031039112	0,118000359	-0,26304253	0,792517802	0,896248225	protein_codin UPF0505 protein C16orf62
TcG_09010	366,6178428	0,03755005	0,096467833	0,389249442	0,697091632	0,843146847	protein_codin 1-acyl-sn-glycerol-3-phosphate acyltransferase
TcG_09011	197,9498082	0,065314615	0,130926675	0,498864081	0,61787514	0,793033999	protein_codin hypothetical protein
TcG_09012	315,4910741	-0,350611318	0,109506774	-3,20173178	0,001366041	0,010061637	protein_codin putative heat shock protein-like protein
TcG_09013	700,4684504	-0,174713736	0,073010061	-2,39300905	0,016710829	0,073393352	protein_codin hypothetical protein
TcG_09014	160,4663839	0,063697609	0,149370077	0,426441562	0,669786122	0,826844423	protein_codin hypothetical protein
TcG_09015	149,1754174	-0,281565365	0,156023506	-1,80463427	0,071131932	0,21392986	protein_codin hypothetical protein
TcG_09016	799,2629788	-0,435884688	0,070251693	-6,20461472	5,48311E-10	2,32701E-08	protein_codin hypothetical protein
TcG_09017	36,86604356	-0,269988649	0,288285789	-0,93653125	0,348999732	0,58360833	protein_codin dispersed gene family protein 1 (DGF-1)
TcG_09018	94,50464159	-0,241982076	0,180010929	-1,34426325	0,178863276	0,389799325	protein_codin dispersed gene family protein 1 (DGF-1)
TcG_09019	138,1598156	-0,048144821	0,161458874	-0,29818628	0,765560991	0,882039543	protein_codin dispersed gene family protein 1 (DGF-1)
TcG_09020	38,03694851	-0,372513147	0,280728671	-1,32695085	0,184524943	0,397568823	
TcG_09021	145,4823528	-0,143566124	0,145083402	-0,98954203	0,322398018	0,557175334	protein_codin hypothetical protein
TcG_09022	252,9198249	-0,105711595	0,114728408	-0,92140732	0,356837808	0,590701202	protein_codin retrotransposon hot spot protein (RHS)
TcG_09023	71,75384754	-0,065384422	0,214003919	-0,30552909	0,759963219	0,878735913	protein_codin retrotransposon hot spot (RHS) protein
TcG_09024	38,77326961	0,014631328	0,279261745	0,052392883	0,958215645	0,98064539	
TcG_09025	16,97996369	-0,864269879	0,426181888	-2,02793667	0,042566712	0,147613866	protein_codin hypothetical protein
TcG_09026	185,3127296	-0,445847295	0,135191943	-3,29788363	0,000974165	0,007614256	protein_codin putative trans-sialidase
TcG_09027	120,2464483	-0,059613076	0,162168762	-0,36759901	0,713172254	0,852715555	
TcG_09028	77,8547322	0,167672818	0,200758529	0,835196485	0,403607089	0,63312391	protein_codin hypothetical protein
TcG_09029	205,7126636	0,150931074	0,12575922	1,200159109	0,230077553	0,457156324	protein_codin dispersed gene family protein 1 (DGF-1)
TcG_09030	278,2365582	0,180318917	0,120405061	1,497602472	0,134236564	0,325709912	protein_codin dispersed gene family protein 1 (DGF-1)
TcG_09031	3,502164851	0,662918286	1,010694874	0,655903481	0,511886228	1	protein_codin dispersed protein family protein 1
TcG_09032	38,39318305	0,217810471	0,278720825	0,781464645	0,434529263	0,658735343	protein_codin putative retrotransposon hot spot (RHS) protein
TcG_09033	370,7711199	-0,474093712	0,097270368	-4,87397881	1,09373E-06	2,18859E-05	protein_codin putative surface protease GP63
TcG_09034	289,5291698	-0,235441757	0,106947305	-2,20147443	0,027702455	0,107885931	protein_codin mucin-associated surface protein (MASP)
TcG_09035	24,86140973	0,317805299	0,367131397	0,865644566	0,386685108	0,616928348	
TcG_09036	596,7683463	-0,242457031	0,081075814	-2,99049766	0,002785233	0,017887863	protein_codin hypothetical protein
TcG_09037	977,7798555	-0,104598844	0,066023161	-1,58427502	0,113131147	0,291598993	protein_codin cytochrome c oxidase subunit IX
TcG_09038	150,2730934	-0,287244718	0,147674589	-1,94511947	0,051760601	0,169215102	protein_codin putative acetyltransferase
TcG_09039	45,05650037	-0,140425355	0,262864316	-0,53421232	0,593194649	0,778429403	protein_codin putative U1A small nuclear ribonucleoprotein
TcG_09040	1376,482802	-0,082286053	0,05572405	-1,47667035	0,139764024	0,334291079	protein_codin putative eukaryotic translation initiation factor 3 subunit 8
TcG_09041	147,242453	-0,196557194	0,158263343	-1,24196286	0,214250265	0,43787327	protein_codin hypothetical protein
TcG_09042	166,7185544	-0,227586795	0,139315134	-1,63361143	0,102340467	0,27333256	protein_codin hypothetical protein
TcG_09043	266,9089319	-0,129317021	0,11734985	-1,10197858	0,270470985	0,503482781	protein_codin elks delta-like protein
TcG_09044	24,19503149	0,28102328	0,389760748	0,721014833	0,470900389	0,686925321	protein_codin hypothetical protein
TcG_09045	72,25852025	0,360556662	0,210114707	1,715999165	0,086162195	0,242771205	protein_codin helicase-like protein
TcG_09046	20,54590384	0,438538031	0,423826627	1,034710901	0,300803944	0,534199829	protein_codin trans-sialidase
TcG_09047	32,94247851	-0,123252855	0,325462745	-0,37870035	0,704910394	0,847648347	protein_codin target of rapamycin (TOR) kinase 1
TcG_09048	22,69556589	-0,251271238	0,367638941	-0,68347286	0,494308095	0,70453824	protein_codin protein kinase
TcG_09049	13,61347854	0,402308609	0,473178709	0,85022551	0,395199721	1	protein_codin hypothetical protein
TcG_09050	3,443893954	0,223307397	0,957277223	0,233273488	0,815549044	1	protein_codin subtilisin-like serine peptidase
TcG_09051	4,290275418	-0,970338611	0,864669259	-1,12220783	0,261774085	1	protein_codin hypothetical protein
TcG_09052	113,987764	0,027955984	0,176612502	0,158289949	0,874228328	0,939505557	protein_codin retrotransposon hot spot (RHS) protein
TcG_09053	33,25863441	-0,338450424	0,30565414	-1,10729867	0,268164818	0,500718385	protein_codin retrotransposon hot spot (RHS) protein
TcG_09054	504,9799223	-0,220588974	0,08158826	-2,70368524	0,006857521	0,036768837	protein_codin Trypanosoma vivax
TcG_09055	387,6082181	-0,046080204	0,092024949	-0,50073599	0,616556942	0,792657426	protein_codin hypothetical protein
TcG_09056	306,8773682	-0,07862985	0,104995235	-0,7488897	0,45392369	0,674078426	protein_codin hypothetical protein
TcG_09057	443,1514734	-0,039588172	0,087486269	-0,45250726	0,65090359	0,814842679	protein_codin putative protein kinase
TcG_09058	131,7236644	0,053869385	0,153411287	0,351143559	0,725480651	0,858172586	protein_codin amino acid transporter
TcG_09059	789,7884086	0,027701339	0,070680017	0,391926039	0,695112863	0,842265483	protein_codin putative GPR1/FUN34/yaaH family protein
TcG_09060	178,8451593	-0,163482974	0,141037058	-1,15914907	0,246395429	0,475076958	protein_codin GPR1/FUN34/yaaH family protein
TcG_09061	30,0433246	0,183713848	0,330541699	0,555796285	0,578350125	0,768240018	protein_codin putative glycine dehydrogenase, putative,glycine cleavage system P-protein
TcG_09062	82,28148227	0,11324648	0,195001067	0,580748	0,561410306	0,754845051	protein_codin hypothetical protein
TcG_09063	3208,894351	0,767329466	0,043895767	17,48071681	2,00957E-68	7,76096E-65	protein_codin glutamamyl carboxypeptidase

TcG_09064	100,8882951	0,180497806	0,176934037	1,020141792	0,307661219	0,540901939	protein_codin hypothetical protein
TcG_09065	43,03661267	0,138333449	0,267026325	0,518051728	0,604422178	0,786152595	
TcG_09066	103,0336685	0,341728153	0,174648704	1,956660114	0,050387442	0,166321626	protein_codin hypothetical protein
TcG_09067	280,6787097	0,444573691	0,112269096	3,959893749	7,49831E-05	0,00087312	protein_codin putative dihydrouridine synthase (Dus)
TcG_09068	568,3299879	0,272465666	0,084277174	3,232971085	0,0012251	0,009193008	protein_codin hypothetical protein
TcG_09069	39,09710907	0,510500468	0,298710299	1,709015288	0,087448118	0,245201813	
TcG_09070	451,9366267	0,473301052	0,091366715	5,180234955	2,21607E-07	5,30482E-06	protein_codin hypersensitive-induced response protein 1-like
TcG_09071	5810,138457	0,570329851	0,036546982	15,60538826	6,69015E-55	1,55024E-51	protein_codin putative amino acid transporter
TcG_09072	241,0052918	-0,128579542	0,116941736	-1,09951798	0,271542196	0,504666007	protein_codin putative trans-sialidase
TcG_09073	60,14085041	-0,066943548	0,228882038	-0,29248056	0,769919218	0,884364283	protein_codin retrotransposon hot spot protein (RHS)
TcG_09074	38,0393222	0,305878244	0,295424398	1,035385856	0,300488748	0,534199829	protein_codin retrotransposon hot spot (RHS) protein
TcG_09075	25,83484007	0,498503101	0,359845512	1,385325324	0,165953013	0,37161415	protein_codin retrotransposon hot spot (RHS) protein
TcG_09076	60,97110838	-0,020217417	0,226413788	-0,08929411	0,928848178	0,965428814	
TcG_09077	29,35378023	0,037367506	0,32745406	0,114115261	0,909146425	0,955841241	protein_codin hypothetical protein
TcG_09078	28,78037908	0,171807574	0,330866584	0,519265414	0,603575669	0,785645174	
TcG_09079	48,12211492	0,235431706	0,25902688	0,908908396	0,363398482	0,595436969	protein_codin dispersed gene family protein 1 (DGF-1)
TcG_09080	22,01238365	0,933478499	0,396698404	2,353118867	0,018616682	0,079328016	protein_codin dispersed gene family protein 1 (DGF-1)
TcG_09081	14,47045336	0,423601576	0,457304914	0,926300074	0,354290058	1	protein_codin dispersed gene family protein 1 (DGF-1)
TcG_09082	4,724169384	1,223372846	0,849985821	1,439286181	0,150069457	1	protein_codin dispersed protein family protein 1
TcG_09083	7,703366996	0,789253116	0,656035767	1,203064155	0,228951495	1	protein_codin dispersed gene family protein 1 (DGF-1)
TcG_09084	4,196853406	0,477837317	0,836524542	0,571217331	0,567852331	1	protein_codin dispersed protein family protein 1
TcG_09085	49,09744879	-0,31488245	0,256018159	-1,22992233	0,218726193	0,443504083	protein_codin trans-sialidase
TcG_09086	32,58766811	-0,089748376	0,32567067	-0,27558016	0,782870549	0,892398482	protein_codin trans-sialidase
TcG_09087	370,7051524	-0,533177644	0,099236767	-5,37278329	7,75305E-08	2,06025E-06	protein_codin hypothetical protein
TcG_09088	243,3193244	-0,467189793	0,115758479	-4,03590128	5,43931E-05	0,000665468	protein_codin putative folate/biopterin transporter
TcG_09089	154,9398653	-0,366679019	0,143514575	-2,55499498	0,010618933	0,051802508	protein_codin putative folate/biopterin transporter
TcG_09090	134,6849381	-0,503069415	0,154763117	-3,25057692	0,001151711	0,008766247	protein_codin putative thymidylate kinase
TcG_09091	369,890055	-0,297490008	0,098598374	-3,0171898	0,0025513	0,016700205	protein_codin hypothetical protein
TcG_09092	359,2959274	-0,237490859	0,094473222	-2,51384312	0,011942354	0,056768347	protein_codin putative myosin heavy chain
TcG_09093	334,457428	-0,423482635	0,099679215	-4,24845475	2,1525E-05	0,000295345	protein_codin hypothetical protein
TcG_09094	188,3668586	0,050704463	0,130564768	0,388347205	0,697759108	0,843778001	protein_codin amastigote surface protein 4
TcG_09095	31,75071735	-0,124946149	0,309376679	-0,40386415	0,686312622	0,837209523	protein_codin hypothetical protein
TcG_09096	14,95969379	-0,214451527	0,467798475	-0,45842716	0,646645582	1	
TcG_09097	90,5961013	-0,523992174	0,188719962	-2,77655934	0,005493761	0,030778873	protein_codin putative mucin-associated surface protein (MASP)
TcG_09098	7,246195079	1,104476051	0,708962548	1,55787644	0,119262541	1	protein_codin hypothetical protein
TcG_09099	11,67930426	-0,173774045	0,507207572	-0,34260933	0,731892385	1	protein_codin hypothetical protein
TcG_09100	3,569033754	-0,204199865	1,001837773	-0,20382528	0,838490036	1	protein_codin hypothetical protein
TcG_09101	7,965051821	-0,293546299	0,636976832	-0,46084298	0,644911266	1	protein_codin hypothetical protein
TcG_09102	5,28279549	-0,030592714	0,789739142	-0,03873775	0,969099479	1	protein_codin hypothetical protein
TcG_09103	4,949237922	0,521363869	0,774924634	0,672793	0,501078992	1	protein_codin hypothetical protein
TcG_09104	7,14463492	-0,303608472	0,65565028	-0,46306466	0,643318022	1	protein_codin hypothetical protein
TcG_09105	13,80541203	0,305561848	0,485160958	0,629815411	0,528815361	1	protein_codin hypothetical protein
TcG_09106	8,674286678	-0,180178409	0,599765549	-0,30041474	0,763860825	1	protein_codin putative syntaxin binding protein
TcG_09107	9,546833321	0,206195591	0,56386226	0,365684328	0,714600642	1	protein_codin hypothetical protein
TcG_09108	14,09312714	0,416161216	0,463627768	0,897619264	0,369388565	1	protein_codin peptide hydrolase
TcG_09109	3,334341267	0,834952321	1,032365071	0,808776221	0,418643876	1	protein_codin putative surface protease GP63
TcG_09110	3,179820978	0,080200147	0,972645051	0,082455719	0,93428433	1	protein_codin hypothetical protein
TcG_09111	46,17704862	-0,400786509	0,25686468	-1,56030214	0,118688498	0,300690664	protein_codin putative mucin-associated surface protein (MASP)
TcG_09112	11,49634036	-0,192343212	0,511672316	-0,37591092	0,70698312	1	
TcG_09113	70,47504076	-0,360227573	0,225376794	-1,5983348	0,109968486	0,286467226	protein_codin putative trans-sialidase
TcG_09114	6,60678937	-0,768947294	0,690707615	-1,11327467	0,265590481	1	protein_codin hypothetical protein
TcG_09115	7,430966975	-0,144599301	0,624489401	-0,23154805	0,816889064	1	protein_codin hypothetical protein
TcG_09116	15,50613268	0,246426836	0,444790483	0,554029021	0,579558985	1	protein_codin hypothetical protein
TcG_09117	126,3200556	-0,159373437	0,161019559	-0,98977688	0,322283188	0,557059976	protein_codin mucin-associated surface protein (MASP)
TcG_09118	0,155721614	0,503022807	4,080472857	0,123275616	0,901888849	1	protein_codin putative mucin-associated surface protein (MASP)
TcG_09119	27,93439012	-0,104190841	0,33732053	-0,30887785	0,757414444	0,878015178	protein_codin mucin TcMUCII
TcG_09120	29,28791034	-0,042696719	0,320769582	-0,13310713	0,894108656	0,948813234	protein_codin hypothetical protein

TcG_09121	407,2418653	-0,049018255	0,092436199	-0,53029284	0,59590891	0,780668859	
TcG_09122	69,35851137	0,336064058	0,212052676	1,584814035	0,113008593	0,291412765	protein_codin putative trans-sialidase
TcG_09123	17,76287426	-0,747416799	0,409126484	-1,82685998	0,067720826	0,207295505	protein_codin hypothetical protein
TcG_09124	10,84090036	0,123219711	0,522733143	0,235722017	0,813648385	1	protein_codin hypothetical protein
TcG_09125	16,57440532	0,024371757	0,418095227	0,058292358	0,953515755	1	protein_codin hypothetical protein
TcG_09126	8,491189384	-0,364970156	0,589303288	-0,61932483	0,535702393	1	protein_codin hypothetical protein
TcG_09127	54,13115667	0,030187595	0,241014146	0,125252379	0,900323752	0,951573708	protein_codin putative trans-sialidase
TcG_09128	12,53309553	0,200124354	0,493917373	0,405177799	0,685346823	1	protein_codin hypothetical protein
TcG_09129	45,2013663	-0,359626382	0,266431096	-1,34979132	0,177082929	0,387183019	protein_codin putative Unc104-like kinesin
TcG_09130	1211,547503	-0,478084377	0,057554368	-8,30665664	9,84363E-17	1,23965E-14	protein_codin putative Unc104-like kinesin
TcG_09131	551,8141354	-0,276623071	0,082589084	-3,34939023	0,000809896	0,006534443	protein_codin RNA-binding protein
TcG_09132	391,6338629	-0,557290362	0,096234782	-5,79094535	6,99914E-09	2,28428E-07	protein_codin cAMP-specific phosphodiesterase
TcG_09133	22,25569319	0,411910841	0,382674946	1,07639877	0,281748952	0,514810791	protein_codin hypothetical protein
TcG_09134	12,75328629	0,326992293	0,494308593	0,661514482	0,50828243	1	protein_codin trans-sialidase
TcG_09135	15,5393586	0,30730169	0,470971783	0,652484291	0,514088803	1	protein_codin hypothetical protein
TcG_09136	0,545831481	2,353994432	2,357183978	0,998646883	0,31796578	1	protein_codin retrotransposon hot spot (RHS) protein
TcG_09137	511,854134	0,201577382	0,08334669	2,418540956	0,015582891	0,069642191	protein_codin putative retrotransposon hot spot (RHS) protein
TcG_09138	98,0643238	-0,041754422	0,174880722	-0,23875943	0,811292131	0,90695008	protein_codin retrotransposon hot spot (RHS) protein
TcG_09139	67,72484443	-0,143849136	0,218961348	-0,65696132	0,511205788	0,717683589	protein_codin putative retrotransposon hot spot (RHS) protein
TcG_09140	33,49781214	-0,200994689	0,311319113	-0,64562271	0,518523727	0,723460489	protein_codin hypothetical protein
TcG_09141	103,9308535	0,269966899	0,180428011	1,496258248	0,134586371	0,326148858	protein_codin trans-sialidase
TcG_09142	128,1847278	-0,144287694	0,158130336	-0,91246055	0,361526325	0,594195389	protein_codin sialidase
TcG_09143	75,27883342	-0,404535015	0,201058398	-2,01202744	0,044217044	0,151837188	protein_codin trans-sialidase
TcG_09144	68,94486895	0,013159219	0,212677333	0,061874101	0,950663093	0,976958027	protein_codin retrotransposon hot spot (RHS) protein
TcG_09145	179,9858387	-0,181242882	0,13430233	-1,34951405	0,177171909	0,387264416	protein_codin putative retrotransposon hot spot (RHS) protein
TcG_09146	71,23882195	-0,323014523	0,219923747	-1,46875691	0,141898736	0,33758496	protein_codin putative retrotransposon hot spot (RHS) protein
TcG_09147	214,8810196	0,028751232	0,123519212	0,232767291	0,815942113	0,90921145	protein_codin hypothetical protein
TcG_09148	461,555938	-0,55489148	0,085001414	-6,52802645	6,66419E-11	3,44693E-09	protein_codin hypothetical protein
TcG_09149	2247,071064	-0,157040761	0,061651549	-2,5472314	0,010858141	0,05266933	protein_codin hypothetical protein
TcG_09150	387,8022112	0,041667519	0,091786076	0,453963394	0,649855175	0,814145983	protein_codin putative calpain-like cysteine peptidase
TcG_09151	640,3150961	-0,233811413	0,07711109	-3,03213732	0,002428287	0,016067467	protein_codin putative adenylosuccinate lyase
TcG_09152	62,47811769	0,533664087	0,231572288	2,30452483	0,0211932	0,088040307	protein_codin hypothetical protein
TcG_09153	400,0647168	0,502981861	0,092657878	5,428376618	5,68689E-08	1,54667E-06	protein_codin citrate transporter
TcG_09154	227,8295264	0,501533674	0,132913618	3,773380634	0,00016105	0,001671979	protein_codin putative citrate transporter
TcG_09155	139,1852837	0,320468758	0,15143466	2,116218035	0,034326268	0,12665737	protein_codin hypothetical protein
TcG_09156	73,50613771	0,596583615	0,209861898	2,842743832	0,0044727	0,02639873	protein_codin hypothetical protein
TcG_09157	310,9249619	0,630622257	0,108381027	5,818566886	5,93543E-09	1,98178E-07	protein_codin hypothetical protein
TcG_09158	24,01964135	-0,049746848	0,378116632	-0,13156482	0,895328511	0,948981304	
TcG_09159	117,050893	0,677750528	0,166138707	4,079425804	4,51471E-05	0,000566711	
TcG_09160	114,7023241	0,802827171	0,168905064	4,753126711	2,00295E-06	3,74096E-05	protein_codin CrcB-like protein
TcG_09161	520,0892444	0,462300527	0,089695475	5,154112031	2,54836E-07	5,9647E-06	protein_codin hypothetical protein
TcG_09162	400,3554678	0,295536773	0,096120221	3,074657646	0,002107443	0,014371298	protein_codin putative serine/threonine protein phosphatase catalytic subunit
TcG_09163	246,3605858	0,4950718	0,120590066	4,105411146	4,03596E-05	0,000509931	protein_codin putative dihydrolipoamide dehydrogenase
TcG_09164	231,1341432	0,297639706	0,117706322	2,528663723	0,011449767	0,05500699	protein_codin putative dihydrolipoamide dehydrogenase, putative,acetoin dehydrogenase e3 component
TcG_09165	174,7182303	-0,027390408	0,137632393	-0,19901135	0,842253867	0,923105402	protein_codin hypothetical protein
TcG_09166	356,3168409	-0,278083934	0,104910136	-2,65068701	0,008032824	0,041622676	protein_codin putative trans-sialidase
TcG_09167	30,48946217	-0,280619888	0,31509131	-0,89059863	0,373144535	0,604239079	
TcG_09168	190,9644336	-0,19878585	0,127371533	-1,56067722	0,118599929	0,300690664	protein_codin hypothetical protein
TcG_09169	884,4629508	-0,05901304	0,067221587	-0,87788824	0,380004374	0,610721305	protein_codin Ribonucleoside-diphosphate reductase large chain 1
TcG_09170	172,1123375	-0,146592523	0,147118931	-0,99642189	0,3190452	0,554102154	protein_codin hypothetical protein
TcG_09171	533,8218773	0,297336866	0,083202081	3,57367101	0,000352011	0,003207975	protein_codin putative ubiquitin-protein ligase-like
TcG_09172	136,3731923	0,310567637	0,155748031	1,994038927	0,046147801	0,156244424	protein_codin hypothetical protein
TcG_09173	321,0628183	-0,207526656	0,099505547	-2,08557877	0,037016796	0,133689714	protein_codin minichromosome maintenance protein 2
TcG_09174	63,37019784	0,086442583	0,219527546	0,393766452	0,693753475	0,841304978	protein_codin minichromosome maintenance (MCM) complex subunit
TcG_09175	163,8507149	0,37540513	0,14157062	2,651716362	0,008008379	0,041551757	protein_codin hypothetical protein
TcG_09176	76,589431	-0,023102772	0,202444943	-0,11411879	0,909143626	0,955841241	protein_codin hypothetical protein
TcG_09177	324,6643192	-0,037157573	0,102933029	-0,36098785	0,718108531	0,855087918	protein_codin hypothetical protein

TcG_09178	211,1190114	0,139612352	0,121614418	1,147991782	0,250971957	0,480780603	protein_codin	hypothetical protein
TcG_09179	336,8603127	-0,103664918	0,099533193	-1,04151103	0,297638437	0,531019238	protein_codin	hypothetical protein
TcG_09180	432,1776519	0,110526755	0,089519698	1,234664074	0,216955552	0,440893872	protein_codin	hypothetical protein
TcG_09181	319,4373918	-0,017983883	0,101710483	-0,17681445	0,859654141	0,932579857	protein_codin	hypothetical protein
TcG_09182	340,7341396	0,121898131	0,101999947	1,195080336	0,232055644	0,459225958	protein_codin	hypothetical protein
TcG_09183	2763,90537	0,221114719	0,049906379	4,430590281	9,39755E-06	0,000144595	protein_codin	60S ribosomal protein L6
TcG_09184	481,7259228	0,028559	0,087819169	0,325202345	0,745027934	0,871116524	protein_codin	proton-dependent oligopeptide transporter, POT family
TcG_09185	299,6008932	-0,130612866	0,10578701	-1,23467774	0,216950464	0,440893872	protein_codin	tetratricopeptidedomain 4
TcG_09186	13,06371519	-0,121140522	0,479224944	-0,25278426	0,800434934	1	protein_codin	amino acid transporter
TcG_09187	284,607523	0,305820239	0,11664395	2,621826847	0,008745985	0,04450197	protein_codin	hypothetical protein
TcG_09188	298,1348518	0,261617936	0,115514627	2,26480355	0,023524737	0,095533687	protein_codin	adenylate cyclase
TcG_09189	1204,273126	0,104146694	0,057351245	1,815944778	0,069378843	0,210755973	protein_codin	esag4
TcG_09190	287,1071013	0,136331976	0,116908917	1,166138386	0,243558499	0,471805512	protein_codin	hypothetical protein
TcG_09191	201,6900961	0,030270634	0,130132798	0,232613409	0,816061614	0,90921145	protein_codin	hypothetical protein
TcG_09192	664,1857296	0,022438685	0,072359791	0,310098806	0,75648582	0,877429644		
TcG_09193	681,4548088	-0,261338815	0,073246894	-3,5679167	0,000359831	0,003267242	protein_codin	co-chaperone protein
TcG_09194	621,70025	-0,325539594	0,076628297	-4,2482948	2,15404E-05	0,000295345	protein_codin	small G-protein
TcG_09195	1044,794768	-0,072789357	0,061207336	-1,18922604	0,234350731	0,462080934	protein_codin	putative small G-protein
TcG_09196	1337,531955	-0,198752875	0,055178634	-3,60198975	0,000315791	0,002929595	protein_codin	putative flagellum transition zone component
TcG_09197	181,2888613	0,057534921	0,131145531	0,4387105	0,660871318	0,822243957	protein_codin	mucin-associated surface protein (MASP)
TcG_09198	46,95055766	0,309750547	0,261334646	1,185263995	0,235913085	0,463445836	protein_codin	hypothetical protein
TcG_09199	17,20721377	-0,086079801	0,420456233	-0,20472951	0,837783459	0,921275547	protein_codin	trans-sialidase
TcG_09200	7,909382939	-0,019103248	0,616014934	-0,03101102	0,975260755	1	protein_codin	hypothetical protein
TcG_09201	3,862398681	2,386004076	1,097661752	2,173715238	0,029726528	1	protein_codin	putative mucin-like glycoprotein
TcG_09202	4,78056709	-0,878999129	0,845822871	-1,03922365	0,298700735	1		
TcG_09203	33,49006203	-0,250216983	0,314684371	-0,79513635	0,426534192	0,651783849	protein_codin	trans-sialidase
TcG_09204	15,91546502	-0,133522415	0,452577971	-0,29502632	0,76797377	1	protein_codin	putative trans-sialidase
TcG_09205	6,276141594	1,287502451	0,756797824	1,701250202	0,088896015	1		
TcG_09206	163,3319105	-0,22520414	0,139770225	-1,61124546	0,107126232	0,282082846	protein_codin	mucin-associated surface protein (MASP)
TcG_09207	7,605408369	-0,209156296	0,641854443	-0,3258625	0,744528386	1	protein_codin	putative mucin TcMUCII
TcG_09208	0					1	protein_codin	hypothetical protein
TcG_09209	0,548227089	-0,494168331	2,319377258	-0,21306078	0,831279554	1	protein_codin	hypothetical protein
TcG_09210	0,155721614	0,503022807	4,080472857	0,123275616	0,901888849	1	protein_codin	hypothetical protein
TcG_09211	2,999236596	-0,40995836	1,046150437	-0,39187324	0,695151877	1	protein_codin	hypothetical protein
TcG_09212	1,307866945	-2,837383317	1,820875404	-1,55825232	0,119173447	1	protein_codin	hypothetical protein
TcG_09213	24,61073311	0,374665329	0,361354724	1,036835288	0,299812627	0,533583578	protein_codin	hypothetical protein
TcG_09214	1,484095545	-0,606595853	1,467607956	-0,41332282	0,679370116	1	protein_codin	hypothetical protein
TcG_09215	79,35569496	0,171646894	0,199245239	0,861485547	0,388970678	0,619284946	protein_codin	trans-sialidase
TcG_09216	11,93723914	0,058204336	0,515294083	0,112953627	0,91006732	1	protein_codin	target of rapamycin (TOR) kinase 1
TcG_09217	85,47655849	0,251142299	0,196509296	1,278017398	0,201243292	0,420748598	protein_codin	mucin-associated surface protein (MASP)
TcG_09218	58,19273215	0,328871301	0,231813368	1,418689971	0,155989422	0,357566753	protein_codin	protein kinase, putative,serine/threonine protein kinase
TcG_09219	184,6041662	0,444201792	0,147768837	3,006058654	0,002646579	0,017168684	protein_codin	hypothetical protein
TcG_09220	118,2394403	0,164679267	0,165984572	0,992135983	0,321131195	0,556034397	protein_codin	hypothetical protein
TcG_09221	128,8696925	0,222368506	0,157791761	1,409252961	0,158760382	0,361091046	protein_codin	hypothetical protein
TcG_09222	99,58713406	0,312480252	0,179147716	1,744260318	0,081113708	0,233254758	protein_codin	hypothetical protein
TcG_09223	41,55723668	0,401873751	0,27223049	1,476226088	0,139883208	0,33450709	protein_codin	hypothetical protein
TcG_09224	57,69364772	0,189005234	0,23668986	0,798535409	0,424559854	0,65015164	protein_codin	hypothetical protein
TcG_09225	57,47265419	0,298517665	0,237292426	1,258015985	0,208385975	0,430981776	protein_codin	hypothetical protein
TcG_09226	38,40803301	0,154405067	0,277480968	0,556452821	0,577901338	0,768001978		
TcG_09227	52,45362243	0,115940495	0,258087865	0,449228772	0,653266634	0,816565672	protein_codin	trans-sialidase
TcG_09228	66,04939246	0,292660514	0,225184995	1,29964483	0,193722727	0,410802512	protein_codin	putative trans-sialidase
TcG_09229	22,34011891	0,276654026	0,374643462	0,73844616	0,460243373	0,678399847	protein_codin	putative mismatch repair protein MSH4
TcG_09230	197,5188943	-0,309428119	0,136762393	-2,26252343	0,023665083	0,096009381	protein_codin	hypothetical protein
TcG_09231	246,384767	-0,18499208	0,119484378	-1,54825327	0,12156133	0,305909984	protein_codin	hypothetical protein
TcG_09232	4,933384418	0,47868015	0,829599184	0,577001712	0,563938299	1	protein_codin	elongation factor 1-gamma (EF-1-gamma)
TcG_09233	2576,877828	0,07869749	0,053664906	1,466460951	0,142522752	0,338582859	protein_codin	putative elongation factor 1-gamma (EF-1-gamma)
TcG_09234	182,0573801	0,076014075	0,130641602	0,581851978	0,560666389	0,754426375	protein_codin	hypothetical protein

TcG_09235	211,8994095	0,012923573	0,126616426	0,102068689	0,918702154	0,960941465	protein_codin SLA/LP autoantigen-like protein
TcG_09236	504,1508432	-0,102370354	0,0865672	-1,1825536	0,23698611	0,464589014	protein_codin hypothetical protein
TcG_09237	41,90146496	-0,881485828	0,283208688	-3,11249572	0,001855127	0,012955698	protein_codin calmodulin
TcG_09238	317,6748885	-0,351042869	0,11018097	-3,18605717	0,001442261	0,01051607	protein_codin calmodulin
TcG_09239	28,78376868	-0,717534112	0,324560295	-2,21078833	0,027050498	0,105880769	
TcG_09240	158,1028805	-0,595937841	0,141503177	-4,21148029	2,53703E-05	0,000341791	protein_codin calmodulin
TcG_09241	11,92179449	-0,661167833	0,509913623	-1,29662712	0,194759516	1	protein_codin hypothetical protein
TcG_09242	10,55495849	0,73117019	0,569041384	1,28491567	0,198821755	1	protein_codin trans-sialidase
TcG_09243	33,92990976	0,142670754	0,301919087	0,472546321	0,636536887	0,806530662	protein_codin trans-sialidase
TcG_09244	38,716297	-0,620771104	0,28283241	-2,19483723	0,028175276	0,109103859	protein_codin trans-sialidase
TcG_09245	1,503743219	-1,142985752	1,531107169	-0,74650931	0,455359811	1	protein_codin hypothetical protein
TcG_09246	1,606306956	-0,384103396	1,333553731	-0,28802994	0,773323823	1	protein_codin hypothetical protein
TcG_09247	4,199975293	-0,75942801	0,835791595	-0,90863322	0,363543764	1	protein_codin hypothetical protein
TcG_09248	6,661709287	0,764186967	0,700926365	1,090252849	0,275601779	1	protein_codin hypothetical protein
TcG_09249	25,33386555	0,024797258	0,35543685	0,069765581	0,944380243	0,973970936	protein_codin hypothetical protein
TcG_09250	10,26017344	-0,115174382	0,551632535	-0,20878823	0,834613559	1	protein_codin retrotransposon hot spot (RHS) protein
TcG_09251	21,83678743	-0,185951331	0,36832151	-0,50486145	0,613656153	0,791210793	protein_codin target of rapamycin (TOR) kinase 1
TcG_09252	18,67937595	-0,145591688	0,409032631	-0,3559415	0,721884376	0,857117481	protein_codin target of rapamycin (TOR) kinase 1
TcG_09253	12,19841905	0,774481884	0,515238182	1,503153125	0,132799562	1	protein_codin trans-sialidase
TcG_09254	5,817115925	0,100311161	0,753494354	0,133127953	0,894092189	1	protein_codin SH3 domain protein
TcG_09255	29,49504642	0,175394683	0,324724064	0,54013454	0,589104252	0,775648564	protein_codin hypothetical protein
TcG_09256	19,51644327	-0,019727735	0,390373066	-0,05053559	0,959695586	0,981466419	protein_codin dispersed gene family protein 1 (DGF-1)
TcG_09257	1,353043202	2,546690421	1,69130819	1,505751841	0,13213089	1	protein_codin dispersed protein family protein 1 (DGF-1)
TcG_09258	33,77412806	-0,070434374	0,305470502	-0,23057668	0,817643687	0,910186372	protein_codin L1Tc protein
TcG_09259	36,5465315	0,044456884	0,2876929	0,154528956	0,877192682	0,941029583	
TcG_09260	204,4844471	0,109969303	0,132138973	0,832224592	0,405282185	0,634304216	
TcG_09261	385,1243379	0,215830728	0,09962085	2,166521644	0,030271348	0,115180241	
TcG_09262	1,998546041	-0,639690917	1,235722351	-0,51766557	0,604691623	1	
TcG_09263	618,2772287	0,39987637	0,083555233	4,785772882	1,70331E-06	3,24048E-05	
TcG_09264	110,6739754	-0,227425524	0,16826195	-1,35161588	0,176498227	0,386561145	
TcG_09265	137,6127174	-0,035229674	0,152193779	-0,23147907	0,816942645	0,909580769	
TcG_09266	727,8577501	-0,004155624	0,087850871	-0,04730317	0,962271605	0,98256823	
TcG_09267	28,533577	-0,011479731	0,324151416	-0,03541472	0,971749049	0,987776155	protein_codin hypothetical protein
TcG_09268	207,5551362	-0,115857531	0,124386626	-0,93143078	0,351630778	0,586017576	protein_codin cyclophilin
TcG_09269	429,5842768	-0,077224112	0,089234193	-0,86540943	0,386814109	0,61704919	protein_codin ribosome biogenesis protein
TcG_09270	64,88766516	0,050238315	0,229446326	0,218954542	0,826685459	0,915239152	protein_codin hypothetical protein
TcG_09271	220,0409562	0,019719128	0,120743021	0,163314846	0,870270546	0,937398327	protein_codin hypothetical protein
TcG_09272	209,9545532	0,057784648	0,129144732	0,447440999	0,654556679	0,817296442	protein_codin putative 40S ribosomal protein S33
TcG_09273	446,2495949	0,477931549	0,093985415	5,085167189	3,67302E-07	8,31165E-06	protein_codin 40S ribosomal protein S33
TcG_09274	531,1312428	0,25886012	0,082275904	3,146244629	0,001653816	0,011798715	protein_codin 40S ribosomal protein S33
TcG_09275	154,4558578	-0,073464346	0,142870255	-0,51420322	0,607109914	0,787770048	protein_codin putative CDP-diacylglycerol synthetase
TcG_09276	248,7351458	0,092113882	0,115292907	0,798955326	0,424316317	0,650097383	protein_codin proline oxidase, mitochondrial precursor-like protein
TcG_09277	139,1090108	0,029128706	0,149235133	0,195186652	0,845246828	0,925035391	protein_codin hypothetical protein
TcG_09278	814,0046592	-0,16211963	0,067262934	-2,41023727	0,015942149	0,070849919	protein_codin metallo-peptidase, Clan MA(E), Family M3
TcG_09279	41,9176033	0,406044352	0,275178196	1,475568772	0,140059693	0,334581697	protein_codin putative RNA-binding protein 5
TcG_09280	706,7202647	0,018620094	0,071571161	0,260161961	0,794738845	0,897538186	protein_codin putative RNA helicase
TcG_09281	251,2582138	-0,122751011	0,115338768	-1,06426498	0,287208677	0,520425356	protein_codin RNA-binding protein
TcG_09282	191,13388	-0,097638129	0,133772519	-0,72988181	0,465462431	0,682138496	protein_codin putative endonuclease III
TcG_09283	117,4576424	0,208753988	0,169061895	1,234778471	0,216912962	0,440893872	protein_codin N-Acetyl-D-glucosaminylphosphatidylinositol de-N-acetylase
TcG_09284	191,7631008	-0,142459741	0,127951729	-1,1133866	0,265542427	0,49806938	protein_codin hypothetical protein
TcG_09285	96,1370666	0,00701358	0,182817366	0,038363861	0,969397574	0,986772122	protein_codin hypothetical protein
TcG_09286	250,4985054	-0,067823238	0,113687925	-0,5965738	0,550791965	0,746546058	protein_codin hypothetical protein
TcG_09287	999,3910444	0,065237621	0,068273384	0,955535192	0,339307117	0,573397354	protein_codin hypothetical protein
TcG_09288	154,3964751	-0,364946597	0,142040102	-2,56932086	0,010189806	0,050119824	protein_codin ribosome biogenesis protein NSA1
TcG_09289	162,7729622	-0,213200884	0,139995323	-1,52291433	0,127780139	0,316545406	protein_codin hypothetical protein
TcG_09290	62,60952307	0,131933611	0,226656852	0,582085253	0,560509258	0,754421499	protein_codin L1Tc protein
TcG_09291	18,47352222	0,304919944	0,414423599	0,735768774	0,461871427	0,679535811	protein_codin hypothetical protein

TcG_09292	5,617063692	0,072552009	0,754353442	0,09617774	0,92337941	1	protein_codin hypothetical protein
TcG_09293	83,86433267	-0,166262906	0,193584126	-0,85886642	0,390414218	0,620400374	protein_codin putative mucin-associated surface protein (MASP)
TcG_09294	12,10778039	-1,075584148	0,506238513	-2,12465887	0,033615087	1	protein_codin hypothetical protein
TcG_09295	4,690990349	0,330144978	0,903463244	0,365421594	0,714796725	1	protein_codin hypothetical protein
TcG_09296	7,287174069	-0,293941047	0,666969245	-0,44071155	0,659421837	1	protein_codin hypothetical protein
TcG_09297	7,021697229	0,124408762	0,659761795	0,188566181	0,850432841	1	protein_codin hypothetical protein
TcG_09298	23,96341057	0,086775312	0,358329542	0,242166225	0,808651365	0,905265886	
TcG_09299	23,96655022	-0,28647688	0,358601584	-0,79887232	0,424364449	0,650097383	protein_codin 90 kDa surface protein
TcG_09300	29,428645	-0,445908307	0,321683275	-1,38617187	0,165694429	0,371250368	protein_codin serine/threonine protein phosphatase
TcG_09301	43,9410056	0,259125845	0,272862603	0,949656868	0,342286632	0,576576323	protein_codin target of rapamycin (TOR) kinase 1
TcG_09302	2,087835796	0,077562919	1,256274807	0,061740407	0,950769561	1	protein_codin hypothetical protein
TcG_09303	12,40108901	0,141385074	0,494620104	0,28584579	0,774996236	1	protein_codin putative trans-sialidase
TcG_09304	11,10326806	0,39859723	0,522849222	0,762355979	0,445847572	1	protein_codin putative GAG protein
TcG_09305	34,25368561	-0,573025629	0,324815051	-1,76415972	0,077705092	0,226173593	protein_codin retrotransposon hot spot (RHS) protein
TcG_09306	48,2428531	-0,037807148	0,248501922	-0,15214027	0,879076307	0,941484386	protein_codin putative retrotransposon hot spot (RHS) protein
TcG_09307	8,692283298	-0,441397424	0,635056452	-0,69505226	0,487022549	1	protein_codin hypothetical protein
TcG_09308	58,49045152	0,149711373	0,227633696	0,657685462	0,510740266	0,717438981	protein_codin hypothetical protein
TcG_09309	21,62722848	0,05543385	0,370628584	0,149567119	0,881106151	0,942069315	
TcG_09310	20,60985401	-0,154170173	0,377426107	-0,40847777	0,682922951	0,835164166	protein_codin complement regulatory protein
TcG_09311	11,93309179	-0,05854687	0,504734752	-0,11599532	0,90765625	1	
TcG_09312	271,542316	-0,264091064	0,111888089	-2,36031437	0,018259454	0,078190747	protein_codin hypothetical protein
TcG_09313	743,9621887	0,029275092	0,069668707	0,420204333	0,67433619	0,829302525	protein_codin putative ubiquitin-protein ligase-like
TcG_09314	321,7136749	0,097510641	0,104683189	0,931483289	0,351603625	0,586017576	protein_codin ADP/ATP translocase
TcG_09315	445,0448371	-0,020999909	0,087807342	-0,23915892	0,810982352	0,906853087	protein_codin putative ADP,ATP carrier protein 1, mitochondrial precursor, putative,ADP/ATP translocase 1
TcG_09316	617,4961563	-0,109082179	0,07526265	-1,44935341	0,147238913	0,34588606	protein_codin RET1 protein
TcG_09317	225,7400492	0,298918335	0,122684033	2,436489311	0,014830608	0,067322838	protein_codin 50S ribosomal protein L16
TcG_09318	83,72912631	-0,240408704	0,191634693	-1,25451556	0,209654675	0,432216915	protein_codin hypothetical protein
TcG_09319	253,5355702	-0,115279583	0,113429752	-1,01630816	0,309482664	0,542625021	protein_codin hypothetical protein
TcG_09320	505,6091685	-0,070477022	0,08986909	-0,78421872	0,432911784	0,65736775	protein_codin putative peroxin 14
TcG_09321	653,6364866	-0,117998923	0,079828625	-1,47815303	0,139366824	0,333823448	protein_codin putative proteasome alpha 5 subunit
TcG_09322	424,3471059	-0,172571359	0,092566055	-1,86430499	0,062278887	0,194911718	protein_codin putative GPI transamidase component GAA1
TcG_09323	817,1667604	0,013078605	0,068527628	0,19085157	0,848641884	0,926882058	protein_codin vacuolar ATP synthase
TcG_09324	630,7812648	-0,163957436	0,079202278	-2,07011011	0,038442034	0,137254056	protein_codin putative ATP synthase F1 subunit gamma protein
TcG_09325	605,5218625	-0,089692657	0,077477085	-1,1576669	0,246999996	0,475846684	protein_codin putative centromere/microtubule binding protein cbf5
TcG_09326	205,5517779	-0,391037292	0,127285577	-3,07212569	0,002125402	0,014468219	protein_codin putative RNA polymerase II
TcG_09327	96,92060441	-0,067847716	0,183353	-0,37003875	0,711353616	0,851612308	protein_codin hypothetical protein
TcG_09328	237,6244327	-0,550266056	0,11536254	-4,76988507	1,84331E-06	3,46136E-05	protein_codin putative amino acid transporter
TcG_09329	9,475468839	0,078953812	0,602177546	0,131113843	0,89568525	1	protein_codin neutral sphingomyelinase activation associated factor-like protein
TcG_09330	31,180264	0,387380231	0,32004559	1,210390777	0,226128979	0,452492289	protein_codin trans-sialidase
TcG_09331	27,99657406	0,369641332	0,354858545	1,04165825	0,297570152	0,530979174	protein_codin trans-sialidase
TcG_09332	16,00408095	0,670789411	0,438981593	1,528058174	0,126498095	1	protein_codin putative trans-sialidase
TcG_09333	21,01102242	-0,200328786	0,372689187	-0,5375224	0,590906816	0,776685709	protein_codin trans-sialidase
TcG_09334	53,59498616	0,074471933	0,236404372	0,315019273	0,752747021	0,875344511	
TcG_09335	8,663701341	-0,295705906	0,594980166	-0,49700128	0,619188146	1	protein_codin putative retrotransposon hot spot (RHS) protein
TcG_09336	4,252097993	0,694873056	0,889261911	0,781404271	0,43456476	1	protein_codin dispersed protein family protein 1
TcG_09337	1,5111471	0,984593028	1,48733116	0,661986419	0,507979926	1	protein_codin dispersed gene family protein 1 (DGF-1)
TcG_09338	5,035346221	1,352345369	0,847280299	1,596101515	0,110466131	1	protein_codin hypothetical protein
TcG_09339	0,469265227	-2,470320505	2,566008638	-0,96270935	0,335693404	1	protein_codin dispersed gene family protein 1 (DGF-1)
TcG_09340	198,6617444	-0,022606587	0,133804887	-0,16895188	0,865834492	0,935353208	protein_codin dispersed gene family protein 1 (DGF-1)
TcG_09341	69,28373152	0,102507275	0,228330333	0,448942869	0,65347287	0,81657908	protein_codin dispersed gene family protein 1 (DGF-1)
TcG_09342	45,71271742	-0,042862382	0,257541956	-0,16642874	0,867819561	0,936352899	protein_codin dispersed gene family protein 1 (DGF-1)
TcG_09343	11,28197376	1,016244565	0,559531419	1,816242181	0,06933323	1	
TcG_09344	166,9198471	-0,122672756	0,136514177	-0,89860817	0,368861404	0,600861456	protein_codin glyceraldehyde 3-phosphate dehydrogenase, cytosolic
TcG_09345	105,4550559	0,013184718	0,178533129	0,073850259	0,941129535	0,972783192	protein_codin hypothetical protein
TcG_09346	86,06475899	0,125112012	0,199613266	0,626772029	0,530808681	0,732224	protein_codin hypothetical protein
TcG_09347	87,68837023	0,211388269	0,19245195	1,09839505	0,272032023	0,505040461	protein_codin hypothetical protein
TcG_09348	212,0098828	0,179501711	0,128806396	1,393577618	0,163445191	0,368061416	protein_codin hypothetical protein

TcG_09349	282,2739784	-0,035473671	0,106550036	-0,33292969	0,739187354	0,867087269	protein_codin	hypothetical protein
TcG_09350	177,5145396	0,194757276	0,138036201	1,410914486	0,15826983	0,360470661	protein_codin	hypothetical protein
TcG_09351	82,95449646	-0,354834195	0,192431718	-1,84394859	0,065190633	0,201574239	protein_codin	putative mucin-associated surface protein (MASP)
TcG_09352	290,1785364	0,210857093	0,107803626	1,955936923	0,050472585	0,166507793	protein_codin	hypothetical protein
TcG_09353	514,685084	-0,128043963	0,082567175	-1,55078532	0,12095314	0,304975643	protein_codin	flagellar associated protein
TcG_09354	4331,571398	0,094949675	0,042465421	2,235929189	0,02535641	0,100816529	protein_codin	40S ribosomal protein SA
TcG_09355	132,4103695	-0,506511674	0,15323616	-3,30543179	0,000948302	0,007464012	protein_codin	hypothetical protein
TcG_09356	469,4353679	-0,138846407	0,090278604	-1,5379769	0,124054263	0,310112782	protein_codin	hypothetical protein
TcG_09357	289,1430214	-0,329746356	0,108910412	-3,0276844	0,002464353	0,016259678	protein_codin	putative dynein heavy chain
TcG_09358	376,4457859	0,015682451	0,095860757	0,163596157	0,87004907	0,937361775	protein_codin	hypothetical protein
TcG_09359	220,016637	0,047842898	0,127001527	0,376711204	0,706388236	0,848727637	protein_codin	hypothetical protein
TcG_09360	142,0806925	0,053012218	0,153190408	0,346054424	0,729301814	0,860563425	protein_codin	hypothetical protein
TcG_09361	541,4814066	-0,008991424	0,088116808	-0,10203982	0,918725065	0,960941465	protein_codin	hypothetical protein
TcG_09362	200,7903891	0,024507187	0,126339924	0,19397817	0,846192975	0,925348997	protein_codin	hypothetical protein
TcG_09363	804,5328168	-0,248560138	0,068106158	-3,6495986	0,00026265	0,002506646	protein_codin	putative mitochondrial carrier protein
TcG_09364	343,0833392	-0,072849005	0,101628259	-0,71681839	0,473486163	0,689258787	protein_codin	putative amino acid permease
TcG_09365	699,491444	0,130244299	0,077013175	1,691195036	0,090799559	0,251434916	protein_codin	putative p21-activated kinase 3
TcG_09366	232,9416578	0,055090122	0,118367956	0,46541415	0,641634903	0,80983334	protein_codin	charged multivesicular body protein 1
TcG_09367	81,25524341	0,252991142	0,205926077	1,228553204	0,21923937	0,444307738	protein_codin	putative MIP18 family protein
TcG_09368	216,8579222	-0,019276544	0,126693468	-0,15215105	0,879067801	0,941484386	protein_codin	zinc finger, MYND domain containing 12
TcG_09369	110,1489425	0,43118903	0,166929391	2,583062378	0,009792762	0,048611369		
TcG_09370	208,9409233	0,183581144	0,128694106	1,426492247	0,153726303	0,354583506	protein_codin	hypothetical protein
TcG_09371	39,25741958	0,553951147	0,291485571	1,90044106	0,057375263	0,182502414	protein_codin	hypothetical protein
TcG_09372	384,7622763	0,469507195	0,100633427	4,665519272	3,07839E-06	5,49556E-05	protein_codin	putative phosphatidylinositol-4-phosphate 5-kinase-like protein
TcG_09373	315,2707831	0,459613349	0,110159488	4,172253873	3,01601E-05	0,000397282	protein_codin	exonuclease 3-5 domain containing 2
TcG_09374	150,0388636	0,492858317	0,149267786	3,301839796	0,000960529	0,007536348	protein_codin	hypothetical protein
TcG_09375	51,82204297	0,125166344	0,243914184	0,513157301	0,607841292	0,788022108	protein_codin	hypothetical protein
TcG_09376	331,0534765	0,393989822	0,104425809	3,772916152	0,000161351	0,001673596	protein_codin	hypothetical protein
TcG_09377	72,58141418	0,077548292	0,205250142	0,377823331	0,705561841	0,848255628	protein_codin	hypothetical protein
TcG_09378	10,55189984	0,303155086	0,540281759	0,561105537	0,574725594	1		
TcG_09379	42,0040733	0,500776651	0,272507517	1,837661786	0,066112258	0,203934138	protein_codin	retrotransposon hot spot (RHS) protein
TcG_09380	205,5506893	0,114404536	0,128679379	0,889066587	0,373967295	0,604496256	protein_codin	retrotransposon hot spot (RHS) protein
TcG_09381	505,4980243	0,511493734	0,085230627	6,001290283	1,95756E-09	7,29269E-08	protein_codin	dispersed gene family protein 1 (DGF-1)
TcG_09382	153,7807312	0,416308733	0,157938009	2,635899585	0,008391456	0,043095483	protein_codin	hypothetical protein
TcG_09383	123,2136929	0,273447853	0,173113591	1,57958628	0,114201643	0,293639644	protein_codin	hypothetical protein
TcG_09384	386,8901163	0,026134861	0,095063535	0,274919934	0,783377752	0,892633424	protein_codin	protein kinase
TcG_09385	26,07363606	0,105708493	0,347886897	0,303858795	0,761235471	0,879491513	protein_codin	retrotransposon hot spot (RHS) protein
TcG_09386	64,38297499	0,407649775	0,244269847	1,668850164	0,095147078	0,259932575	protein_codin	retrotransposon hot spot (RHS) protein
TcG_09387	28,68637329	0,805126421	0,352342899	2,285064983	0,022309018	0,091494612	protein_codin	retrotransposon hot spot protein (RHS)
TcG_09388	41,17864255	0,142995893	0,267128448	0,535307616	0,592437161	0,7778347	protein_codin	hypothetical protein
TcG_09389	26,30307062	0,43803186	0,359472685	1,218540039	0,223018819	0,449216975		
TcG_09390	47,83341869	0,085073952	0,252801602	0,336524575	0,736475323	0,865306361	protein_codin	dispersed protein family protein 1 (DGF-1)
TcG_09391	49,11700602	0,247260679	0,271561604	0,910514135	0,362551431	0,595028691	protein_codin	dispersed gene family protein 1 (DGF-1)
TcG_09392	25,42185621	0,385730238	0,364295639	1,058838471	0,28967335	0,523254666	protein_codin	dispersed gene family protein 1 (DGF-1)
TcG_09393	43,01033463	0,349058995	0,271893107	1,283809653	0,199208565	0,418045723	protein_codin	dispersed gene family protein 1 (DGF-1)
TcG_09394	14,35049728	0,961628523	0,49396832	1,946741286	0,051565759	1	protein_codin	dispersed gene family protein 1 (DGF-1)
TcG_09395	74,89455075	0,1978551	0,228762554	0,864892862	0,387097596	0,617331417	protein_codin	dispersed gene family protein 1 (DGF-1)
TcG_09396	2,688929916	-0,20906618	1,082007407	-0,19322066	0,846786163	1		
TcG_09397	456,2873919	-0,060679936	0,091010404	-0,66673625	0,50494062	0,71221932	protein_codin	putative diacylglycerol acyltransferase
TcG_09398	4,767518824	0,876895346	0,806065883	1,087870564	0,276652253	1		
TcG_09399	236,7455644	0,107108986	0,132161017	0,810443112	0,417685548	0,644295668	protein_codin	hypothetical protein
TcG_09400	375,5221582	-0,157224403	0,096265483	-1,63323756	0,102419042	0,273353381	protein_codin	putative choline/carnitine O-acetyltransferase
TcG_09401	73,54214398	0,169581321	0,216562606	0,783059109	0,433592402	0,657970081	protein_codin	hypothetical protein
TcG_09402	301,0646016	-0,169628634	0,104525404	-1,622846	0,10462233	0,277634978	protein_codin	hypothetical protein
TcG_09403	27,85041667	-0,077530328	0,328844631	-0,23576583	0,813614384	0,907881754	protein_codin	trans-sialidase
TcG_09404	5,004229794	-0,076356028	0,766614071	-0,09960165	0,920660583	1		
TcG_09405	56,79032737	-0,527367917	0,236520924	-2,22968822	0,025768149	0,102138135	protein_codin	mucin-associated surface protein (MASP)

TcG_09406	66,89828497	-0,577970221	0,213689416	-2,70472086	0,006836181	0,036719514	protein_codin hypothetical protein
TcG_09407	54,82635111	-0,155516023	0,236869795	-0,65654645	0,511472592	0,717683589	protein_codin mucin TcMUCII
TcG_09408	14,22126011	-0,089649559	0,459087922	-0,19527754	0,845175681	1	protein_codin hypothetical protein
TcG_09409	11,0154327	0,071549273	0,515542791	0,138784354	0,889620558	1	protein_codin thimet oligopeptidase
TcG_09410	61,88059662	-0,656437137	0,236650316	-2,77386968	0,005539386	0,031004507	protein_codin mucin-associated surface protein (MASP)
TcG_09411	41,19699377	-0,701495998	0,272768235	-2,57176573	0,010118133	0,049829924	protein_codin mucin TcMUCII
TcG_09412	21,84337048	-0,517509323	0,369822527	-1,39934505	0,161709536	0,365372022	protein_codin hypothetical protein
TcG_09413	2,385281305	0,448684709	1,130118823	0,397024366	0,691349493	1	protein_codin surface protease GP63
TcG_09414	363,8051798	-0,121539573	0,098078437	-1,23920789	0,215268519	0,438870501	
TcG_09415	31,57046429	-0,350408793	0,316729628	-1,10633412	0,26858193	0,501174141	protein_codin mucin-associated surface protein (MASP)
TcG_09416	4,590509104	-0,213509134	0,829217298	-0,25748273	0,796806145	1	
TcG_09417	14,16653209	0,37288793	0,501357553	0,743756483	0,457023812	1	protein_codin hypothetical protein
TcG_09418	2,617609495	0,288962292	1,158173275	0,249498324	0,802975337	1	protein_codin hypothetical protein
TcG_09419	7,102002748	0,099527453	0,670669022	0,148400254	0,8820269	1	protein_codin hypothetical protein
TcG_09420	2,58733185	-0,427258986	1,122200299	-0,38073327	0,703401182	1	protein_codin hypothetical protein
TcG_09421	2,367900518	-0,12065428	1,165712419	-0,10350261	0,917564077	1	protein_codin hypothetical protein
TcG_09422	5,783879866	-2,198708296	0,821407496	-2,67675704	0,007433852	1	protein_codin hypothetical protein
TcG_09423	2,647105927	-1,135682259	1,119425185	-1,0145227	0,310333404	1	protein_codin hypothetical protein
TcG_09424	0					1	protein_codin trans-sialidase
TcG_09425	2,00928237	-0,1984599	1,191639194	-0,16654362	0,867729162	1	protein_codin trans-sialidase
TcG_09426	37,85927948	-0,461552588	0,287691125	-1,60433377	0,108640501	0,28454709	protein_codin gp85-like protein
TcG_09427	0,51990315	0,821404694	2,48035172	0,331164604	0,740520155	1	protein_codin trans-sialidase
TcG_09428	0,155721614	0,503022807	4,080472857	0,123275616	0,901888849	1	protein_codin hypothetical protein
TcG_09429	0					1	protein_codin trans-sialidase
TcG_09430	4,471489928	0,145591235	0,827127825	0,176020237	0,860278044	1	protein_codin hypothetical protein
TcG_09431	43,31781342	0,078652821	0,269436345	0,291916152	0,770350734	0,884686967	protein_codin hypothetical protein
TcG_09432	20,06297036	-0,36273372	0,414867915	-0,87433544	0,381935606	0,612054409	protein_codin hypothetical protein
TcG_09433	22,29319798	0,078827048	0,367962059	0,214226023	0,830370804	0,917601778	protein_codin L1Tc protein
TcG_09434	4,352741519	0,686423891	0,855080495	0,802759384	0,422113822	1	protein_codin RNaseH
TcG_09435	8,453811546	-0,522059898	0,620665429	-0,84112933	0,400275484	1	protein_codin hypothetical protein
TcG_09436	25,48613098	-0,272380056	0,366676125	-0,74283554	0,457581258	0,677080007	protein_codin hypothetical protein
TcG_09437	25,04859944	0,085917405	0,344417381	0,249457228	0,803007122	0,902650932	protein_codin hypothetical protein
TcG_09438	163,3078504	-0,202654683	0,137408774	-1,47483073	0,140258057	0,334712637	protein_codin SNF1-related protein kinase
TcG_09439	553,5376183	-0,012458494	0,079351087	-0,1570047	0,875241144	0,939877107	protein_codin putative eukaryotic translation initiation factor 6 (eIF-6)
TcG_09440	298,9298078	0,215002398	0,107193689	2,00573747	0,044884265	0,153265281	protein_codin Nu1 protein
TcG_09441	601,6473555	0,083157918	0,076124653	1,092391417	0,274661094	0,507556451	protein_codin putative DEAH-box RNA helicase
TcG_09442	70,62024835	-0,040493032	0,210839568	-0,19205613	0,847698235	0,926550165	
TcG_09443	461,8384057	-0,065188876	0,093564635	-0,6967256	0,485974537	0,698274608	protein_codin putative mitogen-activated protein kinase kinase 5
TcG_09444	490,8388944	-0,133175644	0,088866057	-1,49861092	0,133974599	0,32557556	protein_codin hypothetical protein
TcG_09445	38,65754565	-0,202454967	0,276765039	-0,73150485	0,464470846	0,681738107	protein_codin retrotransposon hot spot (RHS) protein
TcG_09446	129,2187348	0,184447948	0,167886318	1,098647882	0,271921684	0,505040461	protein_codin putative retrotransposon hot spot (RHS) protein
TcG_09447	30,67303135	0,335826896	0,314147333	1,069010813	0,285064797	0,518774269	protein_codin retrotransposon hot spot (RHS) protein
TcG_09448	38,38798196	-0,208772437	0,284463027	-0,73391765	0,462998927	0,680576702	protein_codin hypothetical protein
TcG_09449	8,985554939	-0,446267751	0,571331512	-0,78110124	0,434742954	1	protein_codin dispersed gene family protein 1 (DGF-1)
TcG_09450	42,78205713	-0,037765217	0,261574801	-0,14437636	0,885203283	0,944292905	protein_codin dispersed gene family protein 1 (DGF-1)
TcG_09451	0					1	protein_codin dispersed gene family protein 1 (DGF-1)
TcG_09452	18,49793411	0,261884597	0,401690234	0,651956595	0,514429173	0,720229102	protein_codin dispersed gene family protein 1 (DGF-1)
TcG_09453	58,71175965	0,408314368	0,227217266	1,797021744	0,072332168	0,216182287	protein_codin dispersed gene family protein 1 (DGF-1)
TcG_09454	469,1786862	-0,21122739	0,085844206	-2,46058996	0,013870879	0,06390283	protein_codin hypothetical protein
TcG_09455	34,45913214	-0,283186024	0,323359704	-0,87576164	0,381159629	0,611142466	protein_codin hypothetical protein
TcG_09456	1291,739614	0,065281648	0,063985068	1,020263787	0,307603373	0,540901939	protein_codin hypothetical protein
TcG_09457	77,66025654	-0,076728982	0,202502957	-0,37890302	0,704759883	0,847555325	protein_codin putative vacuolar protein sorting-associated protein 13A isoform X4
TcG_09458	174,6319317	-0,065045008	0,139180541	-0,46734269	0,640254719	0,808853034	protein_codin hypothetical protein
TcG_09459	0,311976008	1,510501932	3,146303199	0,480087848	0,631164929	1	
TcG_09460	0					1	
TcG_09461	0,27291574	1,35431792	3,063093298	0,442140604	0,658387464	1	
TcG_09462	15,70409872	0,383476183	0,477794479	0,802596514	0,422207984	1	

TcG_09463	2168,967013	-0,724541828	0,047636003	-15,2099627	3,03716E-52	5,86476E-49	protein_codin hypothetical protein
TcG_09464	882,1482769	-0,047452363	0,06863222	-0,69140067	0,489313783	0,700418766	protein_codin hypothetical protein
TcG_09465	4612,919992	0,423549988	0,042175111	10,04265254	9,89762E-24	2,57546E-21	protein_codin c71 surface protein
TcG_09466	2696,951156	0,412744904	0,045315005	9,108349479	8,36447E-20	1,46835E-17	protein_codin trans-sialidase
TcG_09467	1017,274757	0,321457217	0,064370375	4,993868977	5,91816E-07	1,27213E-05	
TcG_09468	206,9826626	-0,223992669	0,132483606	-1,6907199	0,090890311	0,251574138	protein_codin hypothetical protein
TcG_09469	1079,400626	-0,169596046	0,05924877	-2,86243994	0,004203929	0,02510974	protein_codin trans-sialidase
TcG_09470	94,4131571	-0,072308931	0,195571318	-0,36973178	0,71158235	0,851781497	
TcG_09471	263,1219983	0,108785308	0,112934322	0,963261708	0,335416204	0,56989766	protein_codin hypothetical protein
TcG_09472	253,3691628	0,199052363	0,111070496	1,792126357	0,073112728	0,217799278	protein_codin surface protease GP63
TcG_09473	562,6692017	0,056665027	0,084087889	0,673878583	0,500388509	0,709261227	
TcG_09474	1058,617754	-0,038973339	0,064905263	-0,600465	0,54819638	0,744770551	protein_codin amastigote surface protein 4
TcG_09475	78,71704222	0,368917745	0,205405301	1,796047827	0,072486911	0,216396122	protein_codin hypothetical protein
TcG_09476	225,0101402	0,422893543	0,120681222	3,504219933	0,000457947	0,004007382	protein_codin hypothetical protein
TcG_09477	285,9147304	0,499793119	0,107705394	4,64037223	3,47782E-06	6,13208E-05	protein_codin hypothetical protein
TcG_09478	422,961485	0,351472794	0,098712918	3,560555221	0,000370071	0,00334711	protein_codin cullin-4B
TcG_09479	166,8490712	0,445067502	0,137219691	3,243466726	0,001180846	0,008930341	protein_codin hypothetical protein
TcG_09480	99,01737922	0,323195805	0,181992917	1,775870241	0,075754307	0,22248147	protein_codin hypothetical protein
TcG_09481	116,6609031	0,433892477	0,16587125	2,615838949	0,008900853	0,045124243	protein_codin putative dihydroceramide synthase
TcG_09482	73,88592691	0,408115322	0,203619125	2,004307415	0,04503714	0,15363279	protein_codin putative UDP-Gal or UDP-GlcNAc-dependent glycosyltransferase
TcG_09483	2161,7466	0,884035798	0,052744948	16,76057761	4,73997E-63	1,37293E-59	protein_codin putative amino acid transporter
TcG_09484	361,3024478	-0,08523748	0,100594885	-0,84733413	0,396808905	0,626624319	protein_codin U2 small nuclear ribonucleoprotein B
TcG_09485	4516,746487	-0,349728507	0,039112629	-8,94157507	3,83662E-19	6,17377E-17	protein_codin putative guanine deaminase
TcG_09486	321,5906824	-0,12280142	0,103054856	-1,19161216	0,233413355	0,460781586	protein_codin hypothetical protein
TcG_09487	1106,225611	-0,395481432	0,060324096	-6,5559446	5,52908E-11	2,95207E-09	protein_codin putative high mobility group protein
TcG_09488	236,3320988	-0,083904815	0,129557049	-0,64762832	0,517225374	0,722693341	protein_codin hypothetical protein
TcG_09489	255,8783939	-0,070243964	0,112783131	-0,62282332	0,533400634	0,734098517	protein_codin hypothetical protein
TcG_09490	334,1306496	-0,116879169	0,101506106	-1,15144964	0,2495473	0,478811379	protein_codin hypothetical protein
TcG_09491	108,3567097	-0,186138711	0,17272585	-1,07765404	0,281188175	0,514255504	
TcG_09492	349,0951125	-0,075155362	0,104958569	-0,71604789	0,473961778	0,689691177	protein_codin hypothetical protein
TcG_09493	892,6883667	0,250388753	0,072625387	3,447675309	0,000565433	0,00480992	protein_codin membrane associated protein
TcG_09494	3,864907503	1,342389844	0,944676718	1,421004475	0,155315459	1	protein_codin dispersed gene family protein 1 (DGF-1)
TcG_09495	25,12275547	-0,219291139	0,348148385	-0,62987837	0,528774163	0,731071295	
TcG_09496	65,52147354	0,083462541	0,214283268	0,389496305	0,696909044	0,843014009	protein_codin retrotransposon hot spot (RHS) protein
TcG_09497	93,82058892	0,125461466	0,199564577	0,628676029	0,529561174	0,731548321	protein_codin putative retrotransposon hot spot (RHS) protein
TcG_09498	101,9168613	0,389070272	0,185085574	2,102110195	0,035543624	0,130031079	protein_codin retrotransposon hot spot (RHS) protein
TcG_09499	6,794122413	0,272422231	0,65677934	0,414785018	0,678299288	1	protein_codin hypothetical protein
TcG_09500	2,541209741	-0,646441816	1,084380552	-0,59613926	0,551082201	1	protein_codin trans-sialidase
TcG_09501	5,359417573	-0,081411051	0,762472925	-0,10677238	0,914969557	1	
TcG_09502	4,391240175	0,566577942	0,837586855	0,676440824	0,498760805	1	protein_codin hypothetical protein
TcG_09503	9,834969894	0,358079279	0,580473866	0,616874074	0,537317788	1	protein_codin hypothetical protein
TcG_09504	81,08105862	0,215331864	0,196308766	1,096903965	0,272683375	0,505732285	protein_codin trans-sialidase-like protein
TcG_09505	16,30276141	0,261728583	0,458745989	0,57053051	0,568317935	1	protein_codin trans-sialidase
TcG_09506	2,254995564	-0,824150881	1,188265956	-0,69357443	0,487949135	1	
TcG_09507	377,7887883	0,153991108	0,095713366	1,608877787	0,107643068	0,282608788	protein_codin putative retrotransposon hot spot (RHS) protein
TcG_09508	118,0445688	0,177087304	0,181299222	0,976768141	0,328683952	0,563083286	protein_codin putative surface antigen 2 (CA-2)
TcG_09509	155,2252826	-0,085632385	0,155754727	-0,54978996	0,582463447	0,771424497	protein_codin hypothetical protein
TcG_09510	672,3165483	-0,176695349	0,076913046	-2,29733912	0,021599432	0,089375364	protein_codin hypothetical protein
TcG_09511	59,88273668	0,129376861	0,236195195	0,547753991	0,583860834	0,772480487	protein_codin hypothetical protein
TcG_09512	468,951486	-0,039104191	0,087628935	-0,44624748	0,655418492	0,817728228	protein_codin hypothetical protein
TcG_09513	4,622616017	0,365999362	0,848378088	0,431410673	0,666169791	1	protein_codin hypothetical protein
TcG_09514	13,68286296	0,149221973	0,487674536	0,305986804	0,759614693	1	protein_codin beta galactofuranosyl glycosyltransferase
TcG_09515	23,77257577	0,290963269	0,36558702	0,795879648	0,426101992	0,651750801	protein_codin beta galactofuranosyl glycosyltransferase
TcG_09516	16,80320353	0,051167085	0,435979709	0,117361161	0,90657386	0,954710879	
TcG_09517	4,879848907	0,047928012	0,779023807	0,061523168	0,950942564	1	protein_codin hypothetical protein
TcG_09518	244,2116519	-0,410776401	0,124410707	-3,30177692	0,000960744	0,007536348	protein_codin putative trans-sialidase
TcG_09519	6,235576378	0,193090009	0,717803342	0,269001268	0,787928707	1	protein_codin structural maintenance of chromosome protein 4

TcG_09520	17,11678457	0,208484793	0,423252657	0,492577636	0,622311053	0,796519649	protein_codin SH3 domain protein
TcG_09521	6,61787579	1,724032652	0,755375403	2,282352119	0,02246856	1	protein_codin hypothetical protein
TcG_09522	23,0598944	0,479628781	0,365818019	1,311113054	0,189819571	0,404943758	protein_codin hypothetical protein
TcG_09523	386,2691144	0,28875465	0,096363998	2,99649926	0,002730989	0,017634347	protein_codin hypothetical protein
TcG_09524	142,1194415	0,552277578	0,152212764	3,628326313	0,000285265	0,002687053	protein_codin GINS complex subunit 3
TcG_09525	413,1669405	-0,012822964	0,091098101	-0,14075995	0,888059583	0,945946339	protein_codin hypothetical protein
TcG_09526	335,9099992	0,136611745	0,100709646	1,356491171	0,174942943	0,384025944	protein_codin hypothetical protein
TcG_09527	34,39503035	0,798985925	0,309936572	2,577901408	0,009940235	0,04915852	protein_codin hypothetical protein
TcG_09528	290,2889549	0,746951465	0,115595522	6,461768164	1,03487E-10	5,08016E-09	protein_codin hypothetical protein
TcG_09529	26,50397052	0,796289206	0,346987568	2,294863791	0,021740931	0,089672635	
TcG_09530	282,4779353	-0,010540331	0,111255385	-0,09473997	0,924521373	0,963178187	protein_codin expression site-associated gene (ESAG-like) protein
TcG_09531	105,2325635	0,317257856	0,174936132	1,81356391	0,069744891	0,211424465	
TcG_09532	16,21331828	0,903567553	0,462006536	1,955746256	0,050495053	1	protein_codin hypothetical protein
TcG_09533	20,28952483	0,682929985	0,389194805	1,754725336	0,0793063	0,229538544	protein_codin myosin light chain kinase
TcG_09534	26,68506616	0,095074019	0,33618706	0,282800946	0,777329429	0,888880652	protein_codin hypothetical protein
TcG_09535	134,9060336	0,702373721	0,156737671	4,481205547	7,42226E-06	0,000117962	protein_codin hypothetical protein
TcG_09536	43,37628294	0,50542449	0,282262633	1,790617785	0,073354651	0,218143477	protein_codin putative surface protease GP63
TcG_09537	33,9443498	-0,255094848	0,296512009	-0,86031877	0,389613347	0,62006322	protein_codin solanesyl-diphosphate synthase
TcG_09538	24,38027465	-0,002935607	0,353291065	-0,00830932	0,993370201	0,997511318	protein_codin putative target of rapamycin (TOR) kinase 1
TcG_09539	61,90040408	0,199125903	0,232706395	0,855695881	0,392166019	0,621564364	protein_codin target of rapamycin (TOR) kinase 1
TcG_09540	155,5865893	0,31092442	0,148474331	2,094129119	0,03624848	0,132052525	protein_codin L1Tc protein
TcG_09541	85,49445002	-0,110598944	0,206110625	-0,53659992	0,591544001	0,777210826	protein_codin hypothetical protein
TcG_09542	106,0230991	0,207695593	0,180284969	1,152040544	0,249304413	0,478535608	protein_codin hypothetical protein
TcG_09543	75,1720213	0,03611903	0,203482843	0,177504058	0,859112478	0,932166807	protein_codin hypothetical protein
TcG_09544	199,6801633	0,204109763	0,13617948	1,498829068	0,133917981	0,32557556	protein_codin hypothetical protein
TcG_09545	82,42462651	0,383312232	0,204657398	1,87294589	0,061075857	0,191934289	protein_codin hypothetical protein
TcG_09546	1061,595127	-1,200647833	0,068539885	-17,5175059	1,05343E-68	6,10254E-65	protein_codin trans-sialidase
TcG_09547	78,67527524	-1,23930662	0,200875067	-6,16953929	6,84893E-10	2,84415E-08	protein_codin trans-sialidase
TcG_09548	46,68525498	-0,097522882	0,253095796	-0,38532004	0,700000317	0,844367272	protein_codin hypothetical protein
TcG_09549	149,0373597	0,16562831	0,146080213	1,133817556	0,256871085	0,488848291	protein_codin dispersed gene family protein 1 (DGF-1)
TcG_09550	297,2597802	0,088100583	0,110657621	0,796154684	0,425942133	0,651750801	protein_codin retrotransposon hot spot (RHS) protein
TcG_09551	60,22409614	-0,030817322	0,225473442	-0,13667828	0,891285099	0,946972656	protein_codin retrotransposon hot spot (RHS) protein
TcG_09552	17,09661351	0,108881783	0,457303121	0,23809543	0,811807079	0,907350648	protein_codin hypothetical protein
TcG_09553	17,42453448	-0,679328608	0,415348001	-1,63556489	0,101930684	0,272489364	protein_codin hypothetical protein
TcG_09554	19,48755581	-0,104188636	0,415316884	-0,2508654	0,801918176	0,90177851	protein_codin putative trans-sialidase
TcG_09555	34,07550228	-0,64056269	0,314219004	-2,03858673	0,041491291	0,145056759	protein_codin hypothetical protein
TcG_09556	19,93327683	0,009872589	0,389980325	0,025315607	0,979803225	0,991948403	protein_codin hypothetical protein
TcG_09557	17,00658667	-0,413571545	0,416812179	-0,99222519	0,321087685	0,556034397	protein_codin hypothetical protein
TcG_09558	1150,647927	-0,099635811	0,069976659	-1,42384351	0,154491778	0,355711793	protein_codin exo-alpha-sialidase
TcG_09559	26,25342196	-0,345650284	0,375069113	-0,92156424	0,356755917	0,590701202	protein_codin putative trans-sialidase
TcG_09560	107,8599059	0,033844858	0,170633204	0,198348605	0,842772325	0,923518411	protein_codin putative trans-sialidase
TcG_09561	23,53296684	0,35319936	0,385706631	0,915720216	0,359813659	0,592630203	protein_codin hypothetical protein
TcG_09562	28,20101288	0,268373364	0,332007673	0,808334825	0,41889786	0,645392368	protein_codin hypothetical protein
TcG_09563	476,6877616	-0,101780959	0,085473687	-1,19078705	0,233737194	0,461263691	protein_codin retrotransposon hot spot (RHS) protein
TcG_09564	338,6640595	-0,110572097	0,098198829	-1,1260022	0,260164587	0,492617789	protein_codin putative R-SNARE protein
TcG_09565	280,318292	-0,166286346	0,108165937	-1,53732636	0,12421341	0,310225603	protein_codin hypothetical protein
TcG_09566	126,362461	0,32288775	0,170026087	1,899048291	0,057558127	0,182803854	protein_codin hypothetical protein
TcG_09567	408,061203	0,044606718	0,089960577	0,495847395	0,620002083	0,794441953	protein_codin translation initiation factor 3 subunit K
TcG_09568	494,1216109	-0,160066935	0,086870252	-1,84259779	0,065387757	0,202129817	protein_codin small nuclear ribonucleoprotein component-like protein
TcG_09569	250,2975849	0,180722182	0,114306177	1,581036001	0,113869806	0,292981474	protein_codin hypothetical protein
TcG_09570	246,1905078	0,050596045	0,118626639	0,426515038	0,669732593	0,826844423	protein_codin hypothetical protein
TcG_09571	138,2901206	-0,10793397	0,149649748	-0,72124392	0,470759457	0,686925321	protein_codin dispersed gene family protein 1 (DGF-1)
TcG_09572	524,2926036	-0,238086689	0,085326301	-2,79030833	0,005265787	0,029833451	protein_codin dispersed gene family protein 1 (DGF-1)
TcG_09573	30,35449285	0,08376735	0,313905202	0,266855566	0,78958037	0,895015489	protein_codin dispersed protein family protein 1 (DGF-1)
TcG_09574	85,91709867	-0,118993647	0,187917864	-0,63322158	0,526588954	0,729266446	protein_codin dispersed protein family protein 1 (DGF-1)
TcG_09575	148,8138936	-0,248384358	0,143810466	-1,72716468	0,084138092	0,238927435	
TcG_09576	97,35739824	0,058161292	0,177953758	0,326833736	0,743793642	0,869848908	protein_codin hypothetical protein

TcG_09577	141,0607968	-0,129508487	0,156873029	-0,82556248	0,409052327	0,636605346	protein_codin hypothetical protein
TcG_09578	30,79286908	0,531798141	0,315401059	1,686101313	0,091776287	0,253171444	protein_codin trans-sialidase
TcG_09579	43,51382136	0,826839143	0,267500957	3,090976397	0,001994995	0,013713374	protein_codin rab1 small GTP-binding protein
TcG_09580	70,81957678	0,475290268	0,218033847	2,179892133	0,029265458	0,112200397	protein_codin target of rapamycin (TOR) kinase 1
TcG_09581	81,84375999	0,281922446	0,195131039	1,444785246	0,148518216	0,347412085	protein_codin protein kinase, putative,serine/threonine protein kinase
TcG_09582	65,79344862	0,954177098	0,231349658	4,124393814	3,71712E-05	0,000475349	protein_codin hypothetical protein
TcG_09583	44,63359341	0,817071747	0,273092888	2,991918809	0,0027723	0,01784437	protein_codin hypothetical protein
TcG_09584	230,7149632	0,189114878	0,122290333	1,54644176	0,121997915	0,306632657	protein_codin putative trans-sialidase
TcG_09585	63,73830464	0,303791786	0,2286171	1,328823544	0,183906193	0,396563773	
TcG_09586	64,92010493	0,319710775	0,214536793	1,490237503	0,136161799	0,329003253	protein_codin hypothetical protein
TcG_09587	85,92348179	0,299904174	0,195359919	1,535136659	0,124750263	0,311108715	protein_codin putative retrotransposon hot spot (RHS) protein
TcG_09588	18,33099024	-0,312398855	0,413215368	-0,75601945	0,449637535	0,670442053	protein_codin retrotransposon hot spot (RHS) protein
TcG_09589	102,3700318	-0,022413007	0,177840388	-0,12602878	0,899709142	0,951236277	protein_codin retrotransposon hot spot (RHS) protein
TcG_09590	281,2857685	0,153035457	0,121265684	1,261984852	0,206954237	0,429162896	
TcG_09591	149,4025759	0,226165187	0,146552085	1,543241	0,122772313	0,307687652	protein_codin mucin TcMUCII
TcG_09592	45,82120365	0,17123539	0,254785051	0,672077855	0,501534134	0,710363627	
TcG_09593	179,9029365	-0,536352598	0,133838339	-4,00746603	6,13737E-05	0,000734582	protein_codin mucin-associated surface protein (MASP)
TcG_09594	95,57581039	-0,181933818	0,187141578	-0,97217208	0,330964947	0,565235831	protein_codin hypothetical protein
TcG_09595	50,34782758	-0,262808633	0,267263697	-0,98333083	0,325444642	0,559873085	protein_codin hypothetical protein
TcG_09596	42,59317698	-0,357956042	0,268309943	-1,33411397	0,182166509	0,394021978	protein_codin hypothetical protein
TcG_09597	70,91817544	-0,090889986	0,209270513	-0,43431817	0,664057412	0,824342954	protein_codin mucin TcMUCII
TcG_09598	69,00026951	-0,143641255	0,214114484	-0,67086192	0,502308495	0,710995111	
TcG_09599	367,5358546	-0,460487305	0,097204729	-4,73729323	2,16592E-06	4,00868E-05	protein_codin mucin-associated surface protein (MASP)
TcG_09600	138,9720228	0,214209827	0,148623356	1,441293166	0,149501877	0,348867825	protein_codin surface protease GP63
TcG_09601	7,405273724	-0,476300063	0,63695898	-0,74777196	0,454597719	1	protein_codin hypothetical protein
TcG_09602	1,746836569	-0,168221425	1,314549958	-0,12796883	0,898173637	1	protein_codin hypothetical protein
TcG_09603	2,504475928	-0,241211711	1,072630722	-0,22487862	0,822073706	1	protein_codin hypothetical protein
TcG_09604	0,116927736	0,503022807	4,080472857	0,123275616	0,901888849	1	protein_codin trans-sialidase
TcG_09605	11,09080035	-0,158675058	0,525891113	-0,30172607	0,762860894	1	protein_codin hypothetical protein
TcG_09606	20,06260282	0,471020376	0,404025192	1,165819323	0,243687503	0,471818752	protein_codin hypothetical protein
TcG_09607	161,5462563	0,23117905	0,144949643	1,594892161	0,110736353	0,287601745	protein_codin retrotransposon hot spot (RHS) protein
TcG_09608	19,87154654	0,48138907	0,410991773	1,17128639	0,24148368	0,470195853	protein_codin trans-sialidase
TcG_09609	17,50958064	0,508048232	0,422440001	1,202651809	0,22911109	0,456268888	protein_codin trans-sialidase
TcG_09610	105,8600408	0,143735414	0,180565287	0,796030158	0,426014506	0,651750801	protein_codin surface protein-2
TcG_09611	34,18825864	0,547356588	0,298155017	1,835812098	0,066385452	0,204559001	
TcG_09612	24,04805575	0,459698913	0,393249544	1,16897507	0,242413676	0,470898628	protein_codin hypothetical protein
TcG_09613	34,41106832	0,170462442	0,309798011	0,550237367	0,582156577	0,771194386	protein_codin beta galactofuranosyl glycosyltransferase
TcG_09614	16,0880496	0,508467435	0,44704966	1,137384681	0,255377508	1	protein_codin beta galactofuranosyl glycosyltransferase
TcG_09615	23,46488096	-0,388869814	0,370630631	-1,04921121	0,294080919	0,52710729	
TcG_09616	42,47841592	0,354669587	0,272121534	1,303349944	0,192455317	0,408835223	protein_codin putative serine/threonine protein phosphatase
TcG_09617	49,58374481	0,46108537	0,252690848	1,824701504	0,068046086	0,207906634	protein_codin target of rapamycin (TOR) kinase 1
TcG_09618	31,25618736	0,191083662	0,31166457	0,613106784	0,539805735	0,738916498	protein_codin hypothetical protein
TcG_09619	34,1563123	0,278881379	0,298724159	0,933574905	0,350523215	0,584928989	protein_codin trans-sialidase
TcG_09620	26,88768566	-0,032680496	0,354196773	-0,0922665	0,926486305	0,964306367	protein_codin hypothetical protein
TcG_09621	16,37593565	0,360064198	0,448857606	0,802179117	0,422449354	1	
TcG_09622	160,4539173	-0,41750694	0,14049455	-2,97169492	0,002961608	0,018822376	protein_codin mucin-associated surface protein (MASP)
TcG_09623	207,3275623	-0,162983832	0,12806486	-1,27266631	0,203136479	0,423298425	
TcG_09624	745,7122652	-0,248423264	0,07128795	-3,48478621	0,00049253	0,004277704	protein_codin hypothetical protein
TcG_09625	308,7848082	-0,361223425	0,107738622	-3,35277562	0,000800055	0,006468557	protein_codin hypothetical protein
TcG_09626	991,7532282	-0,055787583	0,063568104	-0,87760339	0,380158989	0,610721305	protein_codin phospholipid:diacylglycerol acyltransferase
TcG_09627	130,2977437	0,122860268	0,15652468	0,784925855	0,432497042	0,656966351	protein_codin DNA-directed RNA polymerase III subunit
TcG_09628	704,682119	-0,31096414	0,07699187	-4,03892179	5,36975E-05	0,000657652	protein_codin putative eukaryotic translation initiation factor 2 subunit
TcG_09629	399,7055137	-0,110669585	0,095362921	-1,1605096	0,245841387	0,474324447	protein_codin hypothetical protein
TcG_09630	31,83473218	-0,035309807	0,308699356	-0,11438251	0,908934579	0,955841241	protein_codin trans-sialidase
TcG_09631	279,8430034	-0,086521225	0,110009798	-0,78648653	0,431582507	0,655968892	protein_codin trans-sialidase-like protein
TcG_09632	63,2125668	0,127834174	0,218530119	0,58497279	0,558566013	0,753217858	protein_codin hypothetical protein
TcG_09633	265,5998105	-0,344978624	0,115258434	-2,99308789	0,002761702	0,01779703	protein_codin mucin-associated surface protein (MASP)

TcG_09634	56,98016058	0,131782792	0,236902465	0,556274467	0,578023239	0,768001978	protein_codin putative trans-sialidase
TcG_09635	101,1975405	0,095945533	0,191601201	0,500756427	0,616542555	0,792657426	protein_codin putative trans-sialidase
TcG_09636	37,46592847	0,296608979	0,283972186	1,04450011	0,296254069	0,529282905	
TcG_09637	42,10819348	0,301591646	0,27716215	1,088141528	0,276532633	0,508960617	
TcG_09638	35,19635655	-0,154014825	0,293588956	-0,52459339	0,599865884	0,783280304	protein_codin hypothetical protein
TcG_09639	97,78407578	-0,172480822	0,179083912	-0,96312851	0,335483036	0,569927632	protein_codin retrotransposon hot spot (RHS) protein
TcG_09640	297,8277098	0,166257394	0,106080038	1,567282566	0,117048659	0,298510531	protein_codin retrotransposon hot spot (RHS) protein
TcG_09641	390,1813139	-0,050378224	0,092885586	-0,54236859	0,587564615	0,775648564	protein_codin putative retrotransposon hot spot (RHS) protein
TcG_09642	159,6779905	0,075180228	0,139219575	0,540011907	0,589188821	0,775648564	protein_codin putative rab1 small GTP-binding protein
TcG_09643	45,51647451	0,0304544	0,264063699	0,115329747	0,908183761	0,955436035	protein_codin hypothetical protein
TcG_09644	121,7332681	0,185479881	0,173272169	1,07045397	0,284415017	0,5181183	protein_codin structural maintenance of chromosome protein 4
TcG_09645	370,4888451	-0,241866967	0,093670106	-2,58211482	0,009819691	0,048703313	protein_codin solute carrier family 35, member E3
TcG_09646	226,6075523	-0,058764926	0,130580266	-0,45002915	0,65268942	0,816284502	protein_codin hypothetical protein
TcG_09647	139,3958106	-0,679040499	0,148528413	-4,57178854	4,83579E-06	8,11992E-05	protein_codin putative eukaryotic translation initiation factor 4E (eIF4E) interacting protein
TcG_09648	116,4146163	0,039735788	0,167149522	0,237726003	0,812093614	0,907583352	protein_codin hypothetical protein
TcG_09649	112,3110327	-0,210874754	0,16843766	-1,2519454	0,21058976	0,433142723	protein_codin hypothetical protein
TcG_09650	180,4231802	-0,420026298	0,136839506	-3,06948125	0,002144309	0,014562696	protein_codin putative oxidoreductase-like protein
TcG_09651	184,9387648	-0,440460298	0,13247614	-3,3248274	0,000884733	0,007064449	protein_codin putative mitogen-activated protein kinase
TcG_09652	106,5825385	-0,700534085	0,177105028	-3,95547259	7,63835E-05	0,000884979	protein_codin hypothetical protein
TcG_09653	123,9447652	0,142865056	0,168776168	0,846476478	0,39728699	0,626985292	protein_codin putative retrotransposon hot spot (RHS) protein
TcG_09654	92,23858895	0,104409385	0,18206867	0,573461572	0,566332211	0,758388878	protein_codin hypothetical protein
TcG_09655	97,31886757	-0,037027972	0,206242612	-0,17953599	0,857516859	0,931220389	
TcG_09656	709,9962515	-0,040015124	0,077561606	-0,51591407	0,605914425	0,786921256	protein_codin surface protein-2
TcG_09657	0,311709618	1,504707249	2,924542472	0,514510308	0,606895255	1	
TcG_09658	108,1273679	0,185732153	0,179523267	1,034585413	0,300862569	0,534199829	protein_codin hypothetical protein
TcG_09659	69,0827392	0,167358382	0,212581892	0,787265463	0,431126483	0,655774114	protein_codin hypothetical protein
TcG_09660	26,43336405	-0,367471981	0,384628011	-0,95539579	0,339377584	0,573432797	protein_codin hypothetical protein
TcG_09661	24,76957137	0,178553166	0,357291458	0,499740931	0,617257507	0,793033999	protein_codin hypothetical protein
TcG_09662	83,93026647	0,019877021	0,205622905	0,096667348	0,922990571	0,962576111	protein_codin hypothetical protein
TcG_09663	89,52280415	0,277240148	0,197096506	1,406621322	0,159539707	0,362110397	protein_codin hypothetical protein
TcG_09664	184,2473174	0,337605889	0,136756917	2,468656772	0,013562123	0,062956979	protein_codin putative retrotransposon hot spot (RHS) protein
TcG_09665	277,1543843	0,129942675	0,110858387	1,172150154	0,241136777	0,469819803	protein_codin hypothetical protein
TcG_09666	100,5089945	0,120626799	0,182181935	0,662122723	0,507892574	0,714738657	protein_codin hypothetical protein
TcG_09667	34,17198295	-0,15149121	0,308565444	-0,49095326	0,623459507	0,797021059	protein_codin hypothetical protein
TcG_09668	28,80399258	0,055213185	0,333132738	0,165739294	0,868362121	0,936741226	protein_codin hypothetical protein
TcG_09669	123,0511737	0,251133731	0,162698534	1,543552515	0,122696776	0,307671493	protein_codin surface protein-2
TcG_09670	37,50544866	-0,096051491	0,288847494	-0,33253358	0,739486389	0,867087269	protein_codin trans-sialidase
TcG_09671	12,33444434	0,336884457	0,502603127	0,67027927	0,50267978	1	protein_codin hypothetical protein
TcG_09672	17,26829711	0,339064252	0,429580251	0,789291993	0,429941365	0,654831163	protein_codin hypothetical protein
TcG_09673	14,30031693	0,464039439	0,507415517	0,914515664	0,360445951	1	protein_codin hypothetical protein
TcG_09674	24,58652316	0,109981268	0,348706413	0,315397892	0,752459567	0,87512513	
TcG_09675	47,06125047	-0,185999882	0,254790744	-0,73001036	0,465383855	0,682138496	
TcG_09676	200,61702	-0,030262793	0,132228019	-0,22886823	0,818971333	0,910797645	protein_codin retrotransposon hot spot (RHS) protein
TcG_09677	101,2634682	0,070743126	0,176492931	0,400826962	0,688547527	0,838395483	protein_codin retrotransposon hot spot (RHS) protein
TcG_09678	124,651245	-0,122134612	0,157246599	-0,7767075	0,437331364	0,660714144	protein_codin putative retrotransposon hot spot (RHS) protein
TcG_09679	15,98175873	0,029637678	0,468975091	0,063196699	0,949609873	1	protein_codin retrotransposon hot spot (RHS) protein
TcG_09680	16,89633139	0,666570129	0,444812239	1,49854269	0,13399231	0,32557556	
TcG_09681	0					1	
TcG_09682	1784,54606	0,492168804	0,052004644	9,463939478	2,96555E-21	6,02787E-19	protein_codin hypothetical protein
TcG_09683	541,7631145	0,47504353	0,084478708	5,623233808	1,87415E-08	5,63999E-07	protein_codin dynein heavy chain, cytosolic
TcG_09684	1843,432094	0,611507136	0,055759609	10,96684773	5,51621E-28	2,45811E-25	protein_codin putative tryptophanyl-tRNA synthetase
TcG_09685	797,7084888	0,610502948	0,070635105	8,643052881	5,47306E-18	8,1296E-16	protein_codin tryptophanyl-tRNA synthetase
TcG_09686	611,644016	0,746529803	0,079051665	9,44356834	3,60296E-21	7,19722E-19	protein_codin HUS1 checkpoint protein
TcG_09687	2563,989267	0,613473792	0,045563995	13,46400357	2,54731E-41	3,27924E-38	protein_codin putative helicase
TcG_09688	0					1	protein_codin hypothetical protein
TcG_09689	9,689680376	0,900161175	0,571219786	1,575857834	0,11505857	1	protein_codin putative helicase
TcG_09690	62,35472552	0,506366617	0,247900576	2,042619771	0,041090092	0,144001757	protein_codin putative helicase

TcG_09691	749,8041375	0,480162953	0,069697562	6,889236024	5,60928E-12	3,7566E-10	protein_codin putative NAD(P)-dependent oxidoreductase
TcG_09692	634,3574954	0,542395473	0,077055332	7,039038798	1,9357E-12	1,40169E-10	protein_codin ATP-binding cassette protein subfamily C, member 2
TcG_09693	182,1100367	0,492424092	0,131791485	3,736387772	0,000186683	0,001887352	protein_codin hypothetical protein
TcG_09694	259,1517344	0,035770822	0,113133808	0,316181539	0,751864719	0,87492919	protein_codin hypothetical protein
TcG_09695	1066,932047	0,483292126	0,062153663	7,775762538	7,49943E-15	7,89894E-13	protein_codin putative glycosyl transferase
TcG_09696	363,7410249	0,284592546	0,095773413	2,971519293	0,002963303	0,01882282	protein_codin hypothetical protein
TcG_09697	418,0447776	0,27565524	0,091528849	3,011676014	0,002598097	0,016930009	protein_codin (H)-ATPase G subunit
TcG_09698	91,3608603	0,161195792	0,189468603	0,850778388	0,394892469	0,624518721	protein_codin hypothetical protein
TcG_09699	167,9780854	0,426771971	0,157413188	2,711157665	0,006704874	0,036114675	protein_codin hypothetical protein
TcG_09700	116,0275125	0,439895429	0,179703426	2,447896728	0,014369283	0,065777368	protein_codin hypothetical protein
TcG_09701	29,55343828	-0,137197832	0,317405328	-0,43224804	0,665561145	0,825042607	protein_codin hypothetical protein
TcG_09702	33,21159577	-0,22288425	0,31528671	-0,70692561	0,479612708	0,693907821	protein_codin target of rapamycin (TOR) kinase 1
TcG_09703	4,218758954	1,097153914	0,872478256	1,257514335	0,208567452	1	protein_codin target of rapamycin (TOR) kinase 1
TcG_09704	38,67604091	-0,164887128	0,282629375	-0,58340407	0,559621316	0,754014719	protein_codin hypothetical protein
TcG_09705	58,58831461	0,215732589	0,246087092	0,876651382	0,380676022	0,610958912	protein_codin retrotransposon hot spot protein (RHS)
TcG_09706	127,1515476	0,074101992	0,158128038	0,468620195	0,63934114	0,808051319	protein_codin retrotransposon hot spot (RHS) protein
TcG_09707	281,7934322	0,140423304	0,112805232	1,244829709	0,213194353	0,436716721	protein_codin retrotransposon hot spot (RHS) protein
TcG_09708	104,3166818	0,069818227	0,172240072	0,405354141	0,685217215	0,836557076	protein_codin trans-sialidase
TcG_09709	73,71758919	0,142305964	0,202750273	0,701878038	0,482755226	0,696286283	protein_codin trans-sialidase
TcG_09710	72,06704016	0,320912256	0,207100486	1,549548542	0,121249912	0,305524463	protein_codin trans-sialidase
TcG_09711	57,20895656	-0,086794227	0,251529605	-0,34506565	0,730045015	0,860963213	protein_codin hypothetical protein
TcG_09712	45,37393988	-0,110996086	0,262316625	-0,42313782	0,672194707	0,828290346	protein_codin trans-sialidase
TcG_09713	30,12111838	-0,771214635	0,321532366	-2,39855989	0,016459685	0,072565414	protein_codin mucin TcMUCII
TcG_09714	8,742357064	0,097583328	0,596197893	0,16367607	0,869986157	1	
TcG_09715	48,54967828	0,79282971	0,260606303	3,042250711	0,002348162	0,015654073	protein_codin dispersed gene family protein 1 (DGF-1)
TcG_09716	48,87539166	0,346776284	0,258560749	1,341179146	0,179862295	0,391119474	protein_codin dispersed gene family protein 1 (DGF-1)
TcG_09717	29,09378883	0,536292236	0,341732116	1,569335195	0,116569856	0,297812204	protein_codin dispersed gene family protein 1 (DGF-1)
TcG_09718	10,56215864	0,196051746	0,533705389	0,367340765	0,71336485	1	protein_codin dispersed gene family protein 1 (DGF-1)
TcG_09719	111,742307	-0,067187095	0,176847141	-0,37991621	0,704007615	0,847428783	protein_codin hypothetical protein
TcG_09720	72,13661314	0,229701825	0,20885335	1,099823512	0,271409026	0,504573652	protein_codin trans-sialidase
TcG_09721	18,72732113	-0,082128121	0,402531416	-0,2040291	0,838330761	0,92144011	protein_codin trans-sialidase
TcG_09722	5,740243319	-0,487814086	0,781526288	-0,62418129	0,532508534	1	protein_codin hypothetical protein
TcG_09723	121,6316185	0,196182034	0,168097125	1,167075491	0,243179885	0,471466222	protein_codin trans-sialidase
TcG_09724	17,45665262	-0,384235213	0,423339852	-0,90762826	0,364074658	0,59603914	protein_codin mucin-associated surface protein (MASP)
TcG_09725	248,2822996	0,396876602	0,115947059	3,422912189	0,000619541	0,00517147	protein_codin hypothetical protein
TcG_09726	917,9035866	0,449024167	0,065105526	6,896867252	5,31618E-12	3,60195E-10	protein_codin putative NADP-dependent alcohol hydrogenase
TcG_09727	12,61739903	0,389904431	0,515242097	0,756740245	0,449205499	1	
TcG_09728	75,23601576	0,55221174	0,214940133	2,569142081	0,010195065	0,050119824	protein_codin hypothetical protein
TcG_09729	45,43793168	0,184800035	0,259514038	0,712100343	0,476402642	0,691757908	protein_codin beta galactofuranosyl glycosyltransferase
TcG_09730	55,79700927	0,363459001	0,238926824	1,52121472	0,128205963	0,31717445	protein_codin hypothetical protein
TcG_09731	49,2297723	0,007059648	0,246285028	0,028664543	0,977132136	0,990177192	
TcG_09732	11,09733656	0,054925984	0,536980654	0,10228671	0,918529104	1	protein_codin hypothetical protein
TcG_09733	236,7768648	0,218952585	0,115264539	1,899565872	0,057490114	0,182688005	protein_codin exo-alpha-sialidase
TcG_09734	87,65314248	0,262305951	0,191035397	1,373075125	0,169728993	0,376067037	protein_codin dispersed gene family protein 1 (DGF-1)
TcG_09735	90,42292919	0,102910732	0,188116536	0,547058406	0,584338607	0,772936077	protein_codin dispersed gene family protein 1 (DGF-1)
TcG_09736	208,3845462	0,097502385	0,13083588	0,745226654	0,456134712	0,676062028	protein_codin dispersed gene family protein 1 (DGF-1)
TcG_09737	80,83350254	0,241041924	0,193581552	1,245169912	0,2130693	0,436614947	protein_codin putative dispersed gene family protein 1 (DGF-1)
TcG_09738	25,68865915	-0,184667703	0,338035081	-0,54629745	0,58486149	0,773539409	
TcG_09739	61,04219739	0,121500848	0,228622419	0,531447652	0,595108608	0,780322355	
TcG_09740	2,565046697	-1,283820586	1,193673165	-1,07552102	0,282141524	1	
TcG_09741	9,479345559	-0,633980056	0,593648279	-1,06793884	0,2855481	1	protein_codin mucin-associated surface protein (MASP)
TcG_09742	11,78512327	-0,427374515	0,511118336	-0,83615571	0,403067312	1	protein_codin trans-sialidase
TcG_09743	26,67907629	0,211228967	0,346233681	0,610076311	0,541811258	0,740087861	protein_codin putative trans-sialidase
TcG_09744	88,90332868	0,008922415	0,185609935	0,048070785	0,961659829	0,982261375	protein_codin retrotransposon hot spot (RHS) protein
TcG_09745	27,33154674	-0,24098784	0,333673017	-0,72222753	0,470154598	0,686450317	protein_codin retrotransposon hot spot (RHS) protein
TcG_09746	9,883561214	-0,204290842	1,041598065	-0,19613212	0,844506753	1	
TcG_09747	27,27666963	0,074018811	0,329151879	0,224877377	0,822074669	0,911875343	protein_codin hypothetical protein

TcG_09748	19,85267525	-0,739291045	0,40236005	-1,8373868	0,066152814	0,203950639	protein_codin trans-sialidase
TcG_09749	40,50820882	-0,217154728	0,270812857	-0,8018627	0,422632386	0,648831056	protein_codin putative trans-sialidase
TcG_09750	2,233256515	-1,73338103	1,234824589	-1,40374677	0,16039427	1	protein_codin hypothetical protein
TcG_09751	1,396881362	-0,487302077	1,595009726	-0,30551668	0,759972668	1	protein_codin hypothetical protein
TcG_09752	38,81122912	-0,121960468	0,289288482	-0,42158771	0,67332598	0,828900214	protein_codin putative retrotransposon hot spot (RHS) protein
TcG_09753	44,86828967	0,125745692	0,261482359	0,480895508	0,630590765	0,802025529	protein_codin retrotransposon hot spot (RHS) protein
TcG_09754	17,83736764	0,74169534	0,427925798	1,733233527	0,08305417	0,236811141	protein_codin retrotransposon hot spot (RHS) protein
TcG_09755	27,59635285	0,474570615	0,353662008	1,341876156	0,179636154	0,390785281	protein_codin retrotransposon hot spot (RHS) protein
TcG_09756	3,173424923	-0,520046965	1,048144958	-0,49615939	0,619781959	1	protein_codin hypothetical protein
TcG_09757	5,740369497	0,215183286	0,732180395	0,293893811	0,768839048	1	protein_codin hypothetical protein
TcG_09758	6,185592779	1,538468096	0,759249811	2,026300269	0,042734024	1	protein_codin hypothetical protein
TcG_09759	11,72142779	0,491233007	0,519614283	0,945380108	0,344464854	1	
TcG_09760	8,169240208	0,463278601	0,610115526	0,759329309	0,447655589	1	protein_codin trans-sialidase
TcG_09761	31,51881989	0,101708058	0,305043455	0,333421537	0,738816108	0,866915478	protein_codin hypothetical protein
TcG_09762	17,53177865	0,71708136	0,429347464	1,670165588	0,094886606	0,259526965	protein_codin trans-sialidase
TcG_09763	50,95660295	0,250098999	0,245313726	1,019506747	0,307962452	0,541121233	protein_codin retrotransposon hot spot (RHS) protein
TcG_09764	97,49081231	-0,369953091	0,174999266	-2,11402653	0,034513002	0,127143923	protein_codin retrotransposon hot spot (RHS) protein
TcG_09765	74,20201932	-0,144199935	0,216496117	-0,66606246	0,505371181	0,712488501	protein_codin retrotransposon hot spot protein (RHS)
TcG_09766	82,60700777	0,167417895	0,206048625	0,812516438	0,416495358	0,643439442	protein_codin putative retrotransposon hot spot (RHS) protein
TcG_09767	140,2497712	0,591807418	0,151817386	3,898153138	9,69291E-05	0,001086094	protein_codin retrotransposon hot spot (RHS) protein
TcG_09768	14,10271705	0,756596797	0,482149743	1,569215389	0,11659776	1	protein_codin hypothetical protein
TcG_09769	15,23377977	1,423651069	0,479133275	2,971304945	0,002965372	1	
TcG_09770	12,03124787	1,170561571	0,5377373	2,176827928	0,029493408	1	protein_codin trans-sialidase
TcG_09771	7,632924125	0,359544253	0,663199639	0,542135778	0,587724973	1	protein_codin hypothetical protein
TcG_09772	54,27627409	0,733094707	0,247743039	2,959093053	0,003085459	0,019491892	protein_codin subtilisin-like serine peptidase
TcG_09773	67,6580768	0,310687349	0,224168631	1,385953726	0,165761034	0,371327791	protein_codin putative kinesin
TcG_09774	392,8382238	-0,121130249	0,093707136	-1,29264701	0,196133174	0,414142327	protein_codin putative retrotransposon hot spot (RHS) protein
TcG_09775	9,576479849	1,088516298	0,589590628	1,846223881	0,064859705	1	protein_codin retrotransposon hot spot (RHS) protein
TcG_09776	144,6650167	0,081826671	0,146020507	0,560377945	0,575221672	0,766212726	protein_codin retrotransposon hot spot (RHS) protein
TcG_09777	31,51662428	0,233816547	0,31858288	0,733926904	0,462993289	0,680576702	protein_codin putative kinesin
TcG_09778	23,83103715	0,503875428	0,378411292	1,331554949	0,183006477	0,395285803	protein_codin putative kinesin
TcG_09779	4,925622722	0,504843403	0,785360882	0,642817098	0,520342788	1	
TcG_09780	24,44592774	1,155472917	0,375196177	3,079650027	0,00207244	0,014174313	protein_codin hypothetical protein
TcG_09781	6,097667183	-0,505244983	0,748033225	-0,67543121	0,499401845	1	protein_codin putative ATP-dependent DEAD/H RNA helicase
TcG_09782	32,39376527	0,744901082	0,312658379	2,382475993	0,017196653	0,074874267	protein_codin retrotransposon hot spot (RHS) protein
TcG_09783	42,94841486	0,253206917	0,266920173	0,948624128	0,342811815	0,576796063	protein_codin retrotransposon hot spot (RHS) protein
TcG_09784	9,204011588	-0,019363671	0,561549081	-0,0344826	0,972492319	1	protein_codin hypothetical protein
TcG_09785	561,7568778	0,086722024	0,079716582	1,087879356	0,276648371	0,509011914	protein_codin putative beta galactofuranosyl glycosyltransferase
TcG_09786	27,35805467	-0,134013301	0,334470824	-0,40067262	0,688661173	0,838395483	protein_codin hypothetical protein
TcG_09787	35,28792158	-0,020023445	0,305260088	-0,0655947	0,947700507	0,975394476	protein_codin hypothetical protein
TcG_09788	28,17919482	0,354727971	0,367003728	0,966551411	0,333768335	0,568226385	protein_codin hypothetical protein
TcG_09789	97,34311235	-0,443359381	0,203999133	-2,17333954	0,029754772	0,113673689	protein_codin trans-sialidase
TcG_09790	50,88248657	-0,304165521	0,251237713	-1,21066824	0,226022577	0,452435657	protein_codin trans-sialidase
TcG_09791	83,48441172	-0,183168766	0,188999134	-0,96915135	0,33246968	0,566469664	protein_codin trans-sialidase
TcG_09792	88,81986587	-0,209972036	0,191828993	-1,09457925	0,27370101	0,50688937	protein_codin retrotransposon hot spot (RHS) protein
TcG_09793	53,96720407	-0,275843436	0,252630162	-1,09188639	0,27488304	0,507556451	protein_codin trans-sialidase
TcG_09794	269,8201334	-0,193094484	0,107961764	-1,78854511	0,073688104	0,218910353	protein_codin sialidase-like protein
TcG_09795	24,31193253	0,45180052	0,358242716	1,261157589	0,207252075	0,429479976	protein_codin hypothetical protein
TcG_09796	105,9899004	-0,232880146	0,171841766	-1,35520108	0,1753535	0,384635679	protein_codin kinesin
TcG_09797	18,72706845	0,070708391	0,422886101	0,167204339	0,86720927	0,93578156	protein_codin subtilisin-like serine peptidase
TcG_09798	21,70875136	-0,244355618	0,391059921	-0,62485467	0,532066446	0,732933197	protein_codin putative trans-sialidase
TcG_09799	25,56126991	-0,390193391	0,351047387	-1,11151202	0,266348017	0,499014898	protein_codin hypothetical protein
TcG_09800	505,7650642	-0,112180965	0,083935059	-1,33652095	0,181379059	0,39294274	protein_codin putative retrotransposon hot spot (RHS) protein
TcG_09801	35,73127129	-0,16738842	0,315993774	-0,52972063	0,596305645	0,780668859	protein_codin retrotransposon hot spot (RHS) protein
TcG_09802	43,49610484	0,017947282	0,276335297	0,064947482	0,948215815	0,97570906	protein_codin hypothetical protein
TcG_09803	40,35235385	-0,095225643	0,271195672	-0,35113261	0,725488868	0,858172586	
TcG_09804	40,39916834	0,172827074	0,273030508	0,632995467	0,526736603	0,729266446	protein_codin hypothetical protein

TcG_09805	81,99390358	-0,556891364	0,194165264	-2,86813075	0,004129049	0,024761469	protein_codin putative trans-sialidase
TcG_09806	15,44722608	-0,756385075	0,43988702	-1,71949851	0,085523639	1	protein_codin trans-sialidase
TcG_09807	19,79145939	-0,104519816	0,388463914	-0,26905927	0,787884076	0,894592797	protein_codin trans-sialidase
TcG_09808	162,5487382	-0,512707334	0,138678152	-3,69710244	0,000218074	0,002141195	protein_codin hypothetical protein
TcG_09809	550,4085212	-0,666677391	0,080580948	-8,27338734	1,30206E-16	1,60486E-14	protein_codin hypothetical protein
TcG_09810	202,2099809	-0,499723299	0,137243045	-3,64115574	0,000271417	0,002569147	protein_codin putative vacuolar ATP synthase subunit c
TcG_09811	354,2760431	-0,380221905	0,099746149	-3,81189559	0,000137905	0,001457818	protein_codin hypothetical protein
TcG_09812	302,1355832	-0,315780641	0,103605066	-3,04792665	0,002304262	0,015431894	protein_codin peroxisomal enoyl-CoA hydratase
TcG_09813	70,14318847	-0,474084515	0,21889065	-2,16585091	0,030322581	0,115299452	protein_codin putative RNA-binding protein
TcG_09814	55,01668191	-0,065351451	0,23383501	-0,27947676	0,779878971	0,890390004	protein_codin dispersed gene family protein 1 (DGF-1)
TcG_09815	18,46359217	-0,120158545	0,396372495	-0,30314552	0,761778967	0,87969412	protein_codin hypothetical protein
TcG_09816	21,69544316	0,086700589	0,372915746	0,232493774	0,816154522	0,909227529	protein_codin hypothetical protein
TcG_09817	36,24902756	0,322219452	0,334308959	0,963837323	0,335127492	0,569657734	protein_codin trans-sialidase
TcG_09818	78,31691879	-0,378527838	0,203159992	-1,86320069	0,062434036	0,195186384	protein_codin trans-sialidase
TcG_09819	54,19360492	0,00713946	0,234759772	0,03041177	0,975738658	0,989661918	protein_codin putative trans-sialidase
TcG_09820	8,811952327	0,128872593	0,582866451	0,221101408	0,825013474	1	protein_codin hypothetical protein
TcG_09821	299,9812046	-0,134669149	0,109675883	-1,22788297	0,219490903	0,444584196	
TcG_09822	9,480563945	-0,143738971	0,594404522	-0,24182012	0,80891955	1	protein_codin putative trans-sialidase
TcG_09823	24,4792029	0,717826142	0,358226936	2,003830729	0,045088196	0,153713455	protein_codin protein kinase, putative,serine/threonine protein kinase
TcG_09824	29,7768862	-0,213382295	0,324389547	-0,65779646	0,510668931	0,717425759	
TcG_09825	0,155721614	0,503022807	4,080472857	0,123275616	0,901888849	1	
TcG_09826	56,08415342	0,673873255	0,258142159	2,610473462	0,009041699	0,045645804	protein_codin hypothetical protein
TcG_09827	2,676217546	0,21637967	1,069944019	0,202234572	0,839733347	1	
TcG_09828	37,47567982	0,850007153	0,291512018	2,915856298	0,003547138	0,021802199	protein_codin hypothetical protein
TcG_09829	9,002453502	0,616542628	0,586593242	1,051056481	0,29323265	1	
TcG_09830	7,687375824	0,77322459	0,635810038	1,21612517	0,223937244	1	
TcG_09831	27,77784596	1,438585967	0,627663318	2,291970751	0,021907331	0,090198414	protein_codin putative retrotransposon hot spot (RHS) protein
TcG_09832	150,9687081	0,540309407	0,152020084	3,554197536	0,000379134	0,003421067	protein_codin putative retrotransposon hot spot (RHS) protein
TcG_09833	32,12667367	0,459348843	0,317764618	1,445563216	0,14829975	0,347111294	protein_codin hypothetical protein
TcG_09834	24,57737377	0,782722457	0,381258807	2,052995083	0,040073054	0,141507592	protein_codin hypothetical protein
TcG_09835	13,79499693	0,72700659	0,497540339	1,4612013	0,143960206	1	protein_codin trans-sialidase
TcG_09836	7,112306187	0,170294072	0,652338951	0,261051515	0,794052781	1	
TcG_09837	45,85814368	0,34727285	0,270329643	1,284627339	0,19892254	0,417822396	protein_codin hypothetical protein
TcG_09838	441,9949756	-0,207005869	0,086186109	-2,40184725	0,016312519	0,072166255	protein_codin hypothetical protein
TcG_09839	120,4563867	-0,00946823	0,163660683	-0,05785281	0,953865873	0,978354285	protein_codin HD superfamily hydrolase
TcG_09840	282,2415933	0,261941801	0,111504249	2,349164295	0,018815602	0,079999108	protein_codin hypothetical protein
TcG_09841	100,1649068	0,207093336	0,181547375	1,140712368	0,253989638	0,485037736	
TcG_09842	335,5990746	0,12922175	0,109997748	1,174767226	0,240087856	0,468766076	protein_codin hypothetical protein
TcG_09843	318,9922467	-0,162600876	0,103828927	-1,566046	0,117337848	0,298714513	protein_codin putative flagellar antigen
TcG_09844	9,443246824	-0,186399751	0,583194379	-0,31961857	0,749257498	1	protein_codin hypothetical protein
TcG_09845	54,6690978	-0,025055399	0,232835682	-0,10760979	0,914305229	0,958221654	protein_codin retrotransposon hot spot (RHS) protein
TcG_09846	37,40755816	0,187429108	0,29567928	0,633893278	0,526150473	0,728944857	protein_codin putative retrotransposon hot spot (RHS) protein
TcG_09847	54,06497026	0,109097263	0,238949281	0,456570793	0,647979577	0,812603825	protein_codin retrotransposon hot spot (RHS) protein
TcG_09848	26,17800285	-0,163441555	0,350885117	-0,46579791	0,641360161	0,8096316	protein_codin retrotransposon hot spot (RHS) protein
TcG_09849	195,1573248	0,052186978	0,128984838	0,404597768	0,685773199	0,836971272	protein_codin putative retrotransposon hot spot (RHS) protein
TcG_09850	24,17275432	-0,030230604	0,354901519	-0,08518026	0,932118083	0,967438871	protein_codin retrotransposon hot spot (RHS) protein
TcG_09851	16,54647642	0,373641378	0,42537896	0,878372964	0,379741351	1	protein_codin trans-sialidase
TcG_09852	10,05154447	0,24034963	0,542935987	0,442685023	0,657993577	1	protein_codin hypothetical protein
TcG_09853	17,03638164	0,294393548	0,424409627	0,693654264	0,487899054	0,699453024	protein_codin trans-sialidase
TcG_09854	15,12125526	0,805085836	0,485003439	1,659959026	0,096922696	1	protein_codin hypothetical protein
TcG_09855	15,99171596	0,047770549	0,440611221	0,10841882	0,913663473	1	protein_codin hypothetical protein
TcG_09856	19,59780498	0,437732368	0,394779555	1,108802017	0,2675156	0,499929984	protein_codin retrotransposon hot spot (RHS) protein
TcG_09857	102,199584	0,236434724	0,176503968	1,33954339	0,180393839	0,391834087	protein_codin hypothetical protein
TcG_09858	62,22716077	-0,29950535	0,223848749	-1,3379809	0,180902664	0,392277422	protein_codin trans-sialidase
TcG_09859	91,61193274	-0,233186602	0,184172448	-1,26613185	0,20546588	0,426847354	protein_codin trans-sialidase
TcG_09860	18,47242493	0,722226036	0,414672286	1,741679056	0,081564619	0,234260704	protein_codin hypothetical protein
TcG_09861	12,30526765	0,294356993	0,494512693	0,595246587	0,551678654	1	protein_codin trans-sialidase

TcG_09862	8,620300384	0,823727281	0,627120637	1,313506896	0,189012199	1	protein_codin trans-sialidase
TcG_09863	23,3987098	0,414646307	0,363104129	1,141948752	0,253475322	0,484454814	protein_codin putative elongation factor 1-gamma (EF-1-gamma)
TcG_09864	138,33865	0,104878674	0,176357168	0,594694702	0,552047564	0,747373577	protein_codin putative mucin-associated surface protein (MASP)
TcG_09865	196,6819702	-0,274572553	0,12854222	-2,13604957	0,032675374	0,121964201	protein_codin putative trans-sialidase
TcG_09866	13,90442649	0,281729497	0,529732513	0,531833502	0,594841317	1	protein_codin hypothetical protein
TcG_09867	9,899317521	0,191558723	0,55848772	0,342995407	0,731601916	1	protein_codin hypothetical protein
TcG_09868	538,6912525	0,15867128	0,096358929	1,646669193	0,09962606	0,268020344	protein_codin hypothetical protein
TcG_09869	142,9721671	0,291879159	0,150885056	1,934447099	0,05305818	0,172097446	protein_codin Arf/Sar family, other
TcG_09870	91,80722108	0,083142827	0,182581169	0,455374603	0,648839763	0,813280193	protein_codin hypothetical protein
TcG_09871	245,6932443	-0,079893472	0,113120009	-0,70627179	0,48001913	0,694233135	protein_codin membrane protein YIP1
TcG_09872	335,4046094	0,039481068	0,099137346	0,398246162	0,690448742	0,83922987	protein_codin hypothetical protein
TcG_09873	66,0778962	0,187529086	0,216527468	0,866075273	0,386448884	0,616721319	
TcG_09874	88,10376798	-0,04961593	0,186917575	-0,26544283	0,790668346	0,895276699	protein_codin retrotransposon hot spot (RHS) protein
TcG_09875	35,0007416	0,22585738	0,301203743	0,749849179	0,453345545	0,673824437	protein_codin hypothetical protein
TcG_09876	75,47302882	-0,006135613	0,206466435	-0,02971724	0,976292561	0,989877099	protein_codin putative trans-sialidase
TcG_09877	50,16662506	-0,045693241	0,268638519	-0,17009192	0,864937849	0,934985064	protein_codin trans-sialidase
TcG_09878	85,64338563	0,024799885	0,202010631	0,122765248	0,902292994	0,952785876	protein_codin hypothetical protein
TcG_09879	83,90004895	0,17546029	0,204067735	0,859813975	0,389891594	0,620165295	protein_codin hypothetical protein
TcG_09880	353,5450235	-0,086344325	0,09705102	-0,88967973	0,37363788	0,604496256	protein_codin putative retrotransposon hot spot (RHS) protein
TcG_09881	263,4547419	-0,018357721	0,113323253	-0,1619943	0,871310342	0,938022823	protein_codin complement regulatory protein
TcG_09882	32,83362398	0,168053555	0,311982644	0,538663155	0,590119301	0,776238879	protein_codin hypothetical protein
TcG_09883	15,20171783	0,536506355	0,467183307	1,148385113	0,250809617	1	protein_codin hypothetical protein
TcG_09884	56,56397574	-0,015692756	0,244110593	-0,06428544	0,948742949	0,976037632	protein_codin putative trans-sialidase
TcG_09885	51,46629793	-0,393014897	0,261712536	-1,50170451	0,133173438	0,324411766	protein_codin trans-sialidase
TcG_09886	95,38710979	0,216352683	0,18497668	1,169621401	0,242153361	0,470898628	protein_codin putative calpain-like cysteine peptidase
TcG_09887	115,0109847	0,363675524	0,168428591	2,159226776	0,030832578	0,116626264	protein_codin putative calpain-like cysteine peptidase
TcG_09888	498,3470078	0,030241863	0,086639244	0,349055017	0,727048006	0,859270718	protein_codin putative calpain-like cysteine peptidase
TcG_09889	0,155988004	0,503022807	4,080472857	0,123275616	0,901888849	1	protein_codin hypothetical protein
TcG_09890	4,162009418	-0,603220409	0,832109107	-0,72492946	0,468495304	1	protein_codin hypothetical protein
TcG_09891	0,389843477	1,884304201	2,666932449	0,706543655	0,479850114	1	protein_codin hypothetical protein
TcG_09892	4,972741877	0,057681257	0,82525224	0,069895305	0,94427699	1	protein_codin hypothetical protein
TcG_09893	1,106910793	0,187511471	1,682311295	0,111460626	0,911251087	1	protein_codin hypothetical protein
TcG_09894	0,806916499	1,32353367	2,064748261	0,641014547	0,521513232	1	protein_codin hypothetical protein
TcG_09895	1,953462496	0,94151317	1,276041717	0,737838863	0,460612374	1	protein_codin surface protease GP63
TcG_09896	4,609508534	0,660230663	0,883477749	0,747308762	0,454877204	1	protein_codin hypothetical protein
TcG_09897	7,591318811	1,472468561	0,677369387	2,173804411	0,029719827	1	protein_codin retrotransposon hot spot (RHS) protein
TcG_09898	0,794106782	-1,999049109	2,093205517	-0,95501808	0,339568555	1	protein_codin retrotransposon hot spot (RHS) protein
TcG_09899	27,94444783	0,649097813	0,339635671	1,911159127	0,055984135	0,179081221	protein_codin retrotransposon hot spot (RHS) protein
TcG_09900	30,51402155	0,355001426	0,319826546	1,109981115	0,267007164	0,499395611	protein_codin trans-sialidase
TcG_09901	15,32922462	0,469964862	0,450667436	1,042819661	0,297031824	1	protein_codin trans-sialidase
TcG_09902	32,89076461	0,241404041	0,302524137	0,797966214	0,424890097	0,650558565	protein_codin hypothetical protein
TcG_09903	640,0636235	0,068233679	0,087768419	0,777428599	0,436905945	0,660407355	protein_codin hypothetical protein
TcG_09904	379,8778147	-0,147260881	0,094280007	-1,56195238	0,118299207	0,300441608	protein_codin putative nucleic acid binding protein
TcG_09905	500,7043673	0,014910452	0,084397849	0,176668629	0,859768684	0,932616794	protein_codin putative 3-methylcrotonoyl-CoA carboxylase beta subunit
TcG_09906	543,7145713	0,184789509	0,083127662	2,222960507	0,026218463	0,103357303	protein_codin cytochrome b5-like
TcG_09907	40,36804291	0,137289067	0,275626095	0,498098945	0,618414304	0,793033999	protein_codin hypothetical protein
TcG_09908	182,6350347	-0,344917988	0,135250456	-2,55021681	0,010765594	0,052341661	protein_codin putative phosphatidylinositol 3-kinase
TcG_09909	524,1008301	-0,022492939	0,082133313	-0,2738589	0,784193059	0,893202987	protein_codin vacuolar transporter chaperone
TcG_09910	221,0682525	-0,11676931	0,120055701	-0,97262612	0,330739158	0,565235831	protein_codin putative dynein heavy chain
TcG_09911	394,9843059	-0,398265432	0,098795949	-4,03119191	5,54947E-05	0,00067538	protein_codin putative protein kinase
TcG_09912	109,0707572	-0,537947468	0,169988212	-3,1646163	0,001552876	0,011202752	protein_codin hypothetical protein
TcG_09913	277,1456753	-0,429927145	0,107584641	-3,99617586	6,43739E-05	0,000765746	protein_codin hypothetical protein
TcG_09914	347,0230924	-0,356368522	0,101502002	-3,51095068	0,000446507	0,003931028	protein_codin cleft lip and palate transmembrane 1 family protein
TcG_09915	429,0322868	-0,167760758	0,08825519	-1,90085997	0,057320356	0,182398695	protein_codin putative ascorbate-dependent peroxidase
TcG_09916	967,5635036	-0,125819081	0,065116421	-1,93221739	0,05333268	0,172939388	protein_codin mitochondrial RNA binding protein
TcG_09917	230,1318303	0,160691857	0,126886532	1,266421696	0,205362147	0,426784904	protein_codin hypothetical protein
TcG_09918	239,3795184	0,171360731	0,125163383	1,369096345	0,170969161	0,377808258	protein_codin putative U4/U6 small nuclear ribonuclear protein

TcG_09919	261,6943468	0,182545048	0,115690978	1,577867618	0,114596026	0,294392362	protein_codin RNA polymerase B subunit RPB8
TcG_09920	82,23768243	-0,027562396	0,194380877	-0,14179582	0,887241286	0,945596314	protein_codin calmodulin
TcG_09921	285,6893102	-0,135295159	0,114280546	-1,18388619	0,236458119	0,463789364	protein_codin putative phopshatase
TcG_09922	241,0820953	-0,175501701	0,116315085	-1,50884728	0,131337814	0,322147648	protein_codin putative RNA polymerase I
TcG_09923	233,9833912	-0,2221726	0,121055306	-1,83529832	0,0664615	0,204688521	protein_codin hypothetical protein
TcG_09924	286,7014654	0,007765265	0,106912548	0,072631929	0,942099018	0,973177534	protein_codin hypothetical protein
TcG_09925	13,56267466	-0,338878515	0,468828339	-0,72282003	0,469790459	1	protein_codin hypothetical protein
TcG_09926	78,08807149	-0,242824377	0,204104724	-1,18970484	0,234162424	0,461890819	protein_codin vesicle transport through interaction with t-SNARE 1
TcG_09927	66,55921775	0,245010918	0,234955337	1,042797841	0,297041932	0,530281636	protein_codin cytidine deaminase-like protein
TcG_09928	246,4300744	0,015363775	0,115419086	0,133112953	0,894104051	0,948813234	protein_codin tRNA-dihydrouridine synthase 3
TcG_09929	54,57785309	0,231126235	0,240800437	0,959824818	0,33714339	0,57160877	protein_codin hypothetical protein
TcG_09930	418,5059114	-0,009424159	0,09154447	-0,10294624	0,918005637	0,96050307	protein_codin putative FG-GAP repeat protein
TcG_09931	38,81235861	-0,021723132	0,284126968	-0,07645572	0,939056541	0,971680773	
TcG_09932	98,17953664	-0,016748606	0,183208546	-0,09141825	0,927160257	0,96445311	protein_codin putative O-sialoglycoprotein endopeptidase
TcG_09933	86,29507442	0,092967929	0,195136294	0,476425614	0,63377119	0,804197117	protein_codin retrotransposon hot spot protein (RHS)
TcG_09934	18,82834089	0,476184269	0,410895885	1,158892768	0,246499897	0,475155042	protein_codin putative retrotransposon hot spot (RHS) protein
TcG_09935	2,142907056	4,292992325	1,580038625	2,71701733	0,006587316	1	protein_codin glutamine synthetase
TcG_09936	20,13688909	0,859704513	0,392589217	2,189832214	0,028536408	0,109987631	protein_codin hypothetical protein
TcG_09937	96,35253903	-0,125103546	0,175821247	-0,71153827	0,476750744	0,691952794	protein_codin trans-sialidase
TcG_09938	154,241504	-0,290959334	0,15030747	-1,93576097	0,052896983	0,171766942	protein_codin putative trans-sialidase
TcG_09939	21,90871622	1,063864035	0,415251586	2,561974645	0,010407892	0,051009235	
TcG_09940	15,5633133	0,90811035	0,469205033	1,935423292	0,052938373	1	protein_codin hypothetical protein
TcG_09941	158,5237265	0,705399483	0,14461885	4,877645503	1,0736E-06	2,15575E-05	protein_codin putative beta galactofuranosyl glycosyltransferase
TcG_09942	82,65223731	0,183468783	0,193759132	0,946891021	0,343694314	0,577945184	protein_codin hypothetical protein
TcG_09943	51,70342271	-0,216341966	0,23868108	-0,90640601	0,364720992	0,596685239	protein_codin hypothetical protein
TcG_09944	53,58530663	-0,003481945	0,239383446	-0,01454547	0,988394803	0,995353515	protein_codin putative dispersed gene family protein 1 (DGF-1)
TcG_09945	52,67280042	0,223685587	0,254969297	0,877304012	0,380321533	0,610738657	protein_codin dispersed gene family protein 1 (DGF-1)
TcG_09946	62,23807882	0,044725341	0,224827014	0,198932236	0,842315753	0,923105402	protein_codin dispersed gene family protein 1 (DGF-1)
TcG_09947	54,44664693	0,355456509	0,247592126	1,435653529	0,151100955	0,351466706	protein_codin dispersed gene family protein 1 (DGF-1)
TcG_09948	23,47060889	0,645473567	0,374515427	1,723489932	0,084799964	0,239983485	protein_codin hypothetical protein
TcG_09949	489,7444988	0,355367056	0,08387441	4,236894827	2,26632E-05	0,00030855	protein_codin hypothetical protein
TcG_09950	385,1357549	-0,079811781	0,103555023	-0,77071858	0,440873756	0,663546065	protein_codin hypothetical protein
TcG_09951	556,3151894	-0,246403567	0,078745989	-3,12909356	0,001753465	0,012392288	protein_codin hypothetical protein
TcG_09952	526,0338644	-0,073835407	0,080623874	-0,91580079	0,359771391	0,592630203	protein_codin putative katanin, putative,serine peptidase, Clan SJ, family S16
TcG_09953	159,7736283	0,210152353	0,140918821	1,491300822	0,135882533	0,328465477	protein_codin putative protein kinase
TcG_09954	491,4317972	-0,04712165	0,091904342	-0,51272496	0,60814373	0,788022108	protein_codin hypothetical protein
TcG_09955	3,896017786	0,518378621	0,891689721	0,581344171	0,561008516	1	protein_codin hypothetical protein
TcG_09956	17,97165128	0,131164773	0,44261395	0,296341255	0,766969483	0,883047643	protein_codin hypothetical protein
TcG_09957	22,49247606	-0,176304572	0,389093728	-0,45311594	0,650465255	0,814469949	
TcG_09958	65,18994651	0,29035065	0,227875082	1,274165859	0,202604642	0,422781782	protein_codin trans-sialidase-like protein
TcG_09959	42,55985583	0,53360845	0,269708143	1,978466217	0,047876136	0,160454993	protein_codin hypothetical protein
TcG_09960	46,5925475	0,231514249	0,261145147	0,886534755	0,375329434	0,605903139	protein_codin putative retrotransposon hot spot (RHS) protein
TcG_09961	150,8170713	0,158833064	0,14754377	1,076514881	0,281697049	0,514810791	protein_codin retrotransposon hot spot (RHS) protein
TcG_09962	2,966090704	0,314319422	1,071913191	0,293232161	0,769344703	1	protein_codin complement regulatory protein
TcG_09963	15,73043095	-0,043853854	0,44951947	-0,09755718	0,922283924	1	protein_codin hypothetical protein
TcG_09964	7,432631927	-0,029033785	0,642353671	-0,04519906	0,96394864	1	protein_codin retrotransposon hot spot (RHS) protein
TcG_09965	19,62301353	0,960177129	0,4014548	2,391744049	0,016768532	0,073533278	protein_codin retrotransposon hot spot protein (RHS)
TcG_09966	8,858686955	0,740276582	0,624908266	1,184616401	0,236169149	1	protein_codin retrotransposon hot spot protein (RHS)
TcG_09967	18,99541228	0,553848574	0,466383755	1,187538304	0,235015357	0,462918722	
TcG_09968	7,510871821	1,280764233	0,663880654	1,929208551	0,053704977	1	protein_codin putative trans-sialidase
TcG_09969	5,98857101	1,138277024	0,747977667	1,521806165	0,128057656	1	protein_codin selenocysteine-tRNA-specific elongation factor
TcG_09970	5,38622932	1,345809959	0,795698489	1,691356686	0,0907687	1	protein_codin trans-sialidase
TcG_09971	5,250743991	0,478880673	0,747831669	0,640358911	0,521939289	1	protein_codin trans-sialidase
TcG_09972	75,98610392	0,330400537	0,206932884	1,596655548	0,110342509	0,287158202	protein_codin rab1 small GTP-binding protein
TcG_09973	0,155988004	0,503022807	4,080472857	0,123275616	0,901888849	1	protein_codin hypothetical protein
TcG_09974	6,744569872	0,871922638	0,67741857	1,287125386	0,198050589	1	protein_codin serine/threonine protein phosphatase
TcG_09975	19,85201391	0,751262896	0,397573994	1,889617802	0,058809095	0,186266312	protein_codin 90 kDa surface protein

TcG_09976	6,429649967	1,030597111	0,711310702	1,448870525	0,147373744	1	
TcG_09977	317,416279	0,13643546	0,109988498	1,240452071	0,214808231	0,438704066	protein_codin surface protease GP63
TcG_09978	7,740585153	0,259145992	0,653121926	0,39678042	0,691529391	1	protein_codin hypothetical protein
TcG_09979	58,88332355	0,102328834	0,23043418	0,444069685	0,656992203	0,819101654	protein_codin hypothetical protein
TcG_09980	0,155988004	0,503022807	4,080472857	0,123275616	0,901888849	1	protein_codin hypothetical protein
TcG_09981	4,434088145	0,612219309	0,889544884	0,688238806	0,49130241	1	protein_codin hypothetical protein
TcG_09982	47,61663192	-0,28888186	0,258573379	-1,11721424	0,263902734	0,496193848	protein_codin trans-sialidase
TcG_09983	15,84061585	0,940712956	0,469336802	2,004345179	0,045033097	1	protein_codin putative syntaxin binding protein
TcG_09984	6,335101057	0,066778297	0,686521053	0,097270574	0,922511524	1	protein_codin syntaxin binding protein
TcG_09985	11,98202282	0,215170139	0,546161748	0,393967794	0,693604817	1	protein_codin hypothetical protein
TcG_09986	6,025746725	0,20368999	0,718796806	0,283376315	0,776888383	1	protein_codin putative surface protease GP63
TcG_09987	7,910830863	-0,144189334	0,612149233	-0,23554605	0,813784941	1	protein_codin hypothetical protein
TcG_09988	78,94685077	-0,444006375	0,196637091	-2,25799911	0,023945715	0,096667267	protein_codin putative mucin-associated surface protein (MASP)
TcG_09989	125,0204653	0,201258404	0,173005339	1,163307478	0,244704773	0,472864744	protein_codin retrotransposon hot spot protein (RHS)
TcG_09990	6,900466997	0,235173091	0,679496647	0,346098972	0,729268336	1	protein_codin retrotransposon hot spot (RHS) protein
TcG_09991	48,83069586	-0,174204199	0,254968617	-0,68323781	0,494456585	0,70453824	protein_codin hypothetical protein
TcG_09992	208,7400239	0,206840529	0,136293879	1,517606878	0,129113532	0,318414087	protein_codin dispersed gene family protein 1 (DGF-1)
TcG_09993	102,3268967	-0,072387087	0,176528905	-0,41005799	0,68176341	0,834219382	protein_codin dispersed gene family protein 1 (DGF-1)
TcG_09994	8,558291692	-0,51098823	0,595392295	-0,85823789	0,390761115	1	protein_codin dispersed protein family protein 1 (DGF-1)
TcG_09995	118,2565666	-0,08567293	0,167720242	-0,51080852	0,609485139	0,788553303	
TcG_09996	267,1890707	0,090643576	0,112487148	0,805812729	0,420350837	0,646856794	protein_codin surface protease GP63
TcG_09997	461,1482282	0,244832425	0,086232685	2,83920679	0,004522584	0,026626757	protein_codin putative surface protease GP63
TcG_09998	278,369411	0,171374992	0,10693918	1,602546343	0,109034848	0,285164278	protein_codin L1Tc protein
TcG_09999	250,6508934	0,334652663	0,121474502	2,754921052	0,00587063	0,032481909	protein_codin L1Tc protein
TcG_10000	390,6782529	0,185462907	0,091543412	2,025955796	0,042769315	0,148139097	protein_codin hypothetical protein
TcG_10001	2,57981012	0,653275025	1,081980909	0,60377685	0,545992013	1	protein_codin surface protease GP63
TcG_10002	84,56582682	0,54714925	0,198869596	2,75129664	0,005935986	0,032734097	protein_codin surface protease GP63
TcG_10003	169,547938	0,536893469	0,14274858	3,761112504	0,000169159	0,001735943	protein_codin putative surface protease GP63
TcG_10004	108,4416907	0,507223283	0,173920431	2,916410001	0,003540849	0,021775091	protein_codin surface protease GP63
TcG_10005	3,093763781	-1,022689546	1,016644471	-1,00594611	0,314441496	1	
TcG_10006	4,401414956	-0,423831661	0,817974319	-0,51814788	0,604355095	1	
TcG_10007	3,260399715	-2,210448912	1,18834312	-1,86010999	0,062869966	1	
TcG_10008	306,1291159	0,064280331	0,105793733	0,60760056	0,543452429	0,740864584	protein_codin hypothetical protein
TcG_10009	269,1777757	-0,057398575	0,109324297	-0,52503036	0,599562086	0,783102809	protein_codin hypothetical protein
TcG_10010	222,3854069	0,165874022	0,121191888	1,368689147	0,171096464	0,377873357	protein_codin hypothetical protein
TcG_10011	259,3501945	0,073278775	0,113617906	0,644957977	0,518954418	0,723712794	protein_codin hypothetical protein
TcG_10012	31,25891886	-0,094450454	0,309008651	-0,30565634	0,759866318	0,878711564	protein_codin retrotransposon hot spot (RHS) protein
TcG_10013	47,87306534	0,258081765	0,26164695	0,986374063	0,323949588	0,558191541	protein_codin retrotransposon hot spot (RHS) protein
TcG_10014	12,67908208	0,191391418	0,50919849	0,375867999	0,707015032	1	protein_codin retrotransposon hot spot (RHS) protein
TcG_10015	27,0674636	-0,000696685	0,337422228	-0,00206473	0,998352587	0,99947404	
TcG_10016	83,88759628	-0,239407453	0,200703516	-1,19284135	0,232931512	0,460300955	protein_codin trans-sialidase
TcG_10017	100,6356016	-0,052953388	0,184180777	-0,28750768	0,773723622	0,886918879	protein_codin trans-sialidase
TcG_10018	118,3736867	-0,108896797	0,165831427	-0,65667165	0,511392068	0,717683589	
TcG_10019	85,25528474	-0,134159906	0,201158706	-0,66693562	0,504813258	0,71221932	protein_codin putative retrotransposon hot spot (RHS) protein
TcG_10020	147,6229634	0,095246215	0,149592895	0,636702799	0,52431845	0,727951296	protein_codin putative retrotransposon hot spot (RHS) protein
TcG_10021	98,84617743	-0,407056977	0,179433353	-2,26856919	0,023294537	0,094699715	protein_codin hypothetical protein
TcG_10022	152,0334551	0,034964075	0,153326379	0,228036917	0,819617541	0,910983196	protein_codin hypothetical protein
TcG_10023	307,5133267	-0,216466188	0,110625934	-1,95673999	0,050378046	0,166321626	protein_codin hypothetical protein
TcG_10024	99,43960695	0,017747152	0,184651077	0,096111826	0,923431759	0,962648944	protein_codin hypothetical protein
TcG_10025	525,6865722	-0,04320519	0,087095772	-0,49606531	0,619848337	0,794332798	protein_codin pentatricopeptide repeat-containing protein
TcG_10026	180,4153496	-0,15256775	0,147376079	-1,03522737	0,300562741	0,534199829	protein_codin putative f-actin capping protein beta subunit
TcG_10027	50,79836117	0,222127858	0,244579598	0,908202728	0,363771123	0,595685091	protein_codin putative aminoacylase, putative,N-acyl-L-amino acid amidohydrolase
TcG_10028	80,20714098	0,131529215	0,201197814	0,653730832	0,513285236	0,719443835	protein_codin retrotransposon hot spot (RHS) protein
TcG_10029	97,59809393	0,233848783	0,181074816	1,291448412	0,196548233	0,414732323	protein_codin retrotransposon hot spot protein (RHS)
TcG_10030	79,25296729	0,162765945	0,199535629	0,815723719	0,41465817	0,641659221	protein_codin retrotransposon hot spot (RHS) protein
TcG_10031	4,711288394	0,218594505	0,856997693	0,255070121	0,798668941	1	protein_codin trans-sialidase
TcG_10032	3,127067161	0,175756215	0,994549351	0,17671945	0,859728762	1	protein_codin hypothetical protein

TcG_10033	3,903715056	0,30627638	0,902362898	0,339415972	0,734296388	1	protein_codin	hypothetical protein
TcG_10034	9,499668633	-0,773450927	0,596425417	-1,29681081	0,194696291	1		
TcG_10035	72,63370923	-0,358641029	0,209796735	-1,70946907	0,087364096	0,245121514	protein_codin	trans-sialidase
TcG_10036	36,40329149	0,17632348	0,30894547	0,570726865	0,568184805	0,760072642	protein_codin	hypothetical protein
TcG_10037	84,66546741	-0,037380312	0,200192495	-0,18672184	0,851878728	0,928229751	protein_codin	hypothetical protein
TcG_10038	83,75736077	-0,343673002	0,198522311	-1,73115555	0,083424024	0,237481756	protein_codin	retrotransposon hot spot (RHS) protein
TcG_10039	35,93129672	0,38921881	0,28820971	1,35047084	0,176865001	0,38714489	protein_codin	retrotransposon hot spot (RHS) protein
TcG_10040	99,72791859	0,222773332	0,186077211	1,197209111	0,23122506	0,458517724	protein_codin	retrotransposon hot spot (RHS) protein
TcG_10041	653,5110461	-0,078544551	0,075818027	-1,03596142	0,30022014	0,533898778	protein_codin	retrotransposon hot spot (RHS) protein
TcG_10042	225,2163326	-0,122251053	0,122094113	-1,0012854	0,316688848	0,551702786	protein_codin	retrotransposon hot spot (RHS) protein
TcG_10043	30,49204543	-0,048334479	0,316073382	-0,1529217	0,878460022	0,941484386	protein_codin	hypothetical protein
TcG_10044	21,47225245	-0,013033231	0,387567841	-0,03362826	0,973173588	0,988164581	protein_codin	hypothetical protein
TcG_10045	37,73898596	0,307169992	0,282536357	1,087187488	0,276953959	0,509169878	protein_codin	hypothetical protein
TcG_10046	26,10927237	0,222835967	0,344466109	0,646902442	0,517695085	0,723000875	protein_codin	hypothetical protein
TcG_10047	47,60931769	0,38285512	0,254619232	1,503637868	0,132674635	0,323614384	protein_codin	trans-sialidase
TcG_10048	27,66061944	-0,186786722	0,326302967	-0,57243342	0,567028383	0,758876151		
TcG_10049	73,02705928	-0,067353107	0,239969253	-0,2806739	0,778960525	0,889867546	protein_codin	kinesin
TcG_10050	31,9251721	0,076846716	0,307060917	0,250265376	0,80238213	0,902212671	protein_codin	putative trans-sialidase
TcG_10051	53,12994135	0,116884125	0,267065004	0,437661707	0,661631532	0,82276086		
TcG_10052	5,212354173	0,452447075	0,783391252	0,577549308	0,56356844	1	protein_codin	hypothetical protein
TcG_10053	1,99505604	-0,042424584	1,223536361	-0,03467374	0,9723399	1		
TcG_10054	7,52532766	-0,423334879	0,619614927	-0,68322253	0,494466237	1	protein_codin	hypothetical protein
TcG_10055	4,892281564	-0,209054526	0,799579065	-0,26145573	0,793741087	1	protein_codin	hypothetical protein
TcG_10056	3,38541208	1,550889228	1,031146052	1,504044189	0,132569989	1	protein_codin	hypothetical protein
TcG_10057	3,187908632	1,101660221	1,015571612	1,084768625	0,278024145	1	protein_codin	hypothetical protein
TcG_10058	4,623466706	-1,48744954	0,829788149	-1,79256542	0,07304244	1	protein_codin	mucin TcMUCII
TcG_10059	1,874767025	0,535751315	1,256769154	0,42629254	0,669894693	1	protein_codin	hypothetical protein
TcG_10060	13,67993457	0,280048603	0,487796253	0,574109788	0,565893511	1	protein_codin	surface protease GP63
TcG_10061	129,0046812	-0,28854185	0,156353597	-1,84544428	0,064972937	0,201062086	protein_codin	mucin-associated surface protein (MASP)
TcG_10062	41,91278089	-0,268992259	0,265653069	-1,01256974	0,31126572	0,544926659	protein_codin	mucin TcMUCII
TcG_10063	80,3085693	-0,336448041	0,192756733	-1,74545416	0,080905846	0,232840744	protein_codin	mucin TcMUCII
TcG_10064	39,75054149	-0,248036118	0,278527617	-0,89052612	0,373183451	0,604239079	protein_codin	hypothetical protein
TcG_10065	4,42950845	-1,928360203	0,943345989	-2,04417067	0,04093669	1	protein_codin	putative mucin TcMUCII
TcG_10066	0					1	protein_codin	putative mucin TcMUCII
TcG_10067	0,45613329	-0,458748245	2,665759712	-0,17208912	0,863367466	1	protein_codin	hypothetical protein
TcG_10068	0,729610643	1,464569989	2,013192312	0,727486381	0,466928055	1	protein_codin	hypothetical protein
TcG_10069	5,551776848	1,006319564	0,781623841	1,287472964	0,197929487	1	protein_codin	hypothetical protein
TcG_10070	5,28949204	-1,868098617	0,858206612	-2,17674694	0,029499454	1	protein_codin	mucin TcMUCII
TcG_10071	0,587287357	-0,37147687	2,17226456	-0,17100904	0,86421666	1		
TcG_10072	1,822042039	1,082875749	1,3822556	0,78341209	0,433385159	1	protein_codin	hypothetical protein
TcG_10073	0,718312701	-0,525171849	2,071134114	-0,25356728	0,799829875	1		
TcG_10074	0,628743233	-3,095698115	2,25348788	-1,37373631	0,169523561	1	protein_codin	hypothetical protein
TcG_10075	54,73354161	-0,384073829	0,23294087	-1,64880396	0,099187804	0,267129219	protein_codin	mucin TcMUCII
TcG_10076	13,62234802	0,107603024	0,491224689	0,219050521	0,826610693	1		
TcG_10077	3,243115442	1,172523492	1,108348947	1,05790103	0,290100566	1	protein_codin	mucin-associated surface protein (MASP)
TcG_10078	296,9120123	-0,007730329	0,10592769	-0,07297742	0,941824089	0,972980285	protein_codin	mucin-associated surface protein (MASP)
TcG_10079	0,663081438	-1,806860419	2,312296325	-0,78141387	0,434559116	1	protein_codin	putative mucin-associated surface protein (MASP)
TcG_10080	172,6299703	0,323992495	0,137863662	2,350093491	0,018768696	0,07982897	protein_codin	putative trans-sialidase
TcG_10081	116,8921356	0,329238251	0,171179747	1,923348151	0,054436341	0,175682298	protein_codin	putative trans-sialidase
TcG_10082	59,75295463	0,171938825	0,223202302	0,770327292	0,441105771	0,663546065	protein_codin	putative trans-sialidase
TcG_10083	32,56010486	0,179127081	0,313075926	0,572152203	0,567218865	0,759043402	protein_codin	hypothetical protein
TcG_10084	86,28716501	0,012925357	0,199489004	0,064792326	0,948339352	0,97570906	protein_codin	retrotransposon hot spot (RHS) protein
TcG_10085	171,2988459	0,277583553	0,141716486	1,958724494	0,05014506	0,165734726	protein_codin	retrotransposon hot spot (RHS) protein
TcG_10086	23,02602941	0,306626574	0,362706466	0,845384912	0,397895966	0,627555494	protein_codin	hypothetical protein
TcG_10087	73,55382546	0,249883802	0,21202713	1,17854636	0,238578862	0,466606126	protein_codin	helicase-like protein
TcG_10088	179,2971391	-0,043593269	0,132661832	-0,32860446	0,742454679	0,868984737	protein_codin	UDP-Gal or UDP-GlcNAc-dependent glycosyltransferase
TcG_10089	129,589669	0,332267682	0,157488064	2,109795965	0,034875934	0,128195613	protein_codin	hypothetical protein

TcG_10090	250,8993727	0,297288776	0,118003355	2,519324775	0,011758015	0,056107232	protein_codin putative citrate transporter
TcG_10091	87,94481056	0,492502235	0,202773885	2,428824779	0,015147851	0,068160911	protein_codin hypothetical protein
TcG_10092	57,863897	0,249525463	0,240603784	1,03708038	0,299698398	0,533462228	protein_codin hypothetical protein
TcG_10093	59,57727295	0,291301161	0,244086629	1,193433505	0,23269964	0,459999663	
TcG_10094	114,8936994	0,186495291	0,169582506	1,099731896	0,271448953	0,504573652	protein_codin putative UDP-Gal or UDP-GlcNAc-dependent glycosyltransferase
TcG_10095	97,43681923	0,27691525	0,183546896	1,508689368	0,131378184	0,322147648	protein_codin UDP-Gal or UDP-GlcNAc-dependent glycosyltransferase
TcG_10096	65,53857738	0,411995026	0,223328304	1,844795394	0,065067308	0,201260593	protein_codin putative ubiquitin-activating enzyme e1
TcG_10097	83,10768772	0,039163554	0,189239368	0,206952467	0,836046981	0,920523248	protein_codin rab1 small GTP-binding protein
TcG_10098	80,16673879	-0,122450652	0,202264926	-0,60539736	0,544915006	0,741887014	protein_codin sialidase
TcG_10099	78,91434485	-0,543110744	0,197464996	-2,7504153	0,005951978	0,032791182	protein_codin sialidase
TcG_10100	3,533936635	-0,224446157	0,961009145	-0,23355257	0,815332352	1	
TcG_10101	171,957652	-0,058700042	0,139527863	-0,4207048	0,673970653	0,829023911	
TcG_10102	83,55578299	-0,316532266	0,198431967	-1,59516771	0,110674737	0,287570643	protein_codin dispersed gene family protein 1 (DGF-1)
TcG_10103	92,80640762	-0,231918988	0,182944942	-1,26769828	0,204905721	0,426146492	protein_codin dispersed gene family protein 1 (DGF-1)
TcG_10104	93,32956952	0,181514763	0,191175225	0,949468028	0,342382626	0,576576323	protein_codin hypothetical protein
TcG_10105	390,1618658	0,008963915	0,091626675	0,097830849	0,922066611	0,962091476	
TcG_10106	44,61017238	-0,134335164	0,281880168	-0,47656834	0,633669531	0,804197117	
TcG_10107	160,2535022	0,314403031	0,139901937	2,247310058	0,024620219	0,09866823	
TcG_10108	53,67689718	0,591745616	0,253918672	2,330453334	0,019782204	0,083283653	protein_codin putative retrotransposon hot spot (RHS) protein
TcG_10109	81,02943099	0,473255509	0,202634911	2,335508265	0,019516885	0,082406205	protein_codin retrotransposon hot spot (RHS) protein
TcG_10110	263,7123659	0,439245201	0,111395594	3,943111073	8,04314E-05	0,000924482	protein_codin protein ARV1
TcG_10111	0					1	
TcG_10112	25,18820759	0,507815463	0,354450057	1,432685517	0,15194773	0,35209328	protein_codin hypothetical protein
TcG_10113	35,03595625	0,356957215	0,291665122	1,223859791	0,221005124	0,446324799	protein_codin hypothetical protein
TcG_10114	77,20817729	0,499361956	0,201172678	2,482255341	0,013055369	0,061048224	protein_codin subtilisin-like serine peptidase
TcG_10115	63,72975292	0,623004065	0,222692987	2,797591757	0,005148514	0,029326787	protein_codin putative kinesin
TcG_10116	36,15169655	0,224228029	0,289610301	0,774240518	0,438788572	0,662126125	
TcG_10117	27,89759519	0,373884332	0,330911875	1,12986073	0,258534913	0,490966318	
TcG_10118	54,27935173	-0,069284112	0,235738525	-0,29390237	0,768832503	0,884313847	protein_codin dispersed gene family protein 1 (DGF-1)
TcG_10119	49,56322073	0,159838495	0,267070558	0,598487891	0,549514437	0,745866398	protein_codin dispersed gene family protein 1 (DGF-1)
TcG_10120	48,43664509	0,461165665	0,256652171	1,796850825	0,072359305	0,216182287	protein_codin dispersed gene family protein 1 (DGF-1)
TcG_10121	21,75665915	0,562728211	0,37717243	1,491965389	0,135708219	0,328249567	
TcG_10122	27,4495987	0,318565386	0,344732362	0,924094809	0,355436956	0,589309183	protein_codin putative retrotransposon hot spot (RHS) protein
TcG_10123	141,4780465	0,165993721	0,148935183	1,114536652	0,26504903	0,497508899	protein_codin putative retrotransposon hot spot (RHS) protein
TcG_10124	222,7632475	-0,332624	0,122303989	-2,71964964	0,006535112	0,035438277	protein_codin hypothetical protein
TcG_10125	595,735189	-0,082160858	0,080113163	-1,02556003	0,305099017	0,537951182	protein_codin hypothetical protein
TcG_10126	458,933618	-0,145055792	0,088907012	-1,631545	0,102775372	0,274114978	protein_codin myosin XXI
TcG_10127	187,5985158	-0,137467632	0,129375911	-1,06254426	0,287988675	0,521105229	protein_codin putative ubiquitin-conjugating enzyme
TcG_10128	314,2032449	-0,229449392	0,102882125	-2,2302163	0,025733086	0,102034065	protein_codin autophagin-2
TcG_10129	29,6994304	0,219841765	0,329749994	0,666692249	0,504968731	0,71221932	protein_codin trans-sialidase
TcG_10130	28,22058036	0,8049609	0,338169315	2,380348729	0,017296261	0,075204123	protein_codin trans-sialidase
TcG_10131	34,378304	0,141675787	0,30113281	0,470476091	0,638014912	0,807510942	protein_codin hypothetical protein
TcG_10132	180,2874222	0,257381207	0,132118273	1,948112103	0,051401551	0,168684291	protein_codin putative surface protease GP63
TcG_10133	50,05529771	0,010456605	0,25005866	0,041816609	0,966644894	0,985355248	protein_codin mucin-associated surface protein (MASP)
TcG_10134	10,03848359	0,229976059	0,55795759	0,412174801	0,680211306	1	protein_codin hypothetical protein
TcG_10135	16,19688933	0,58578681	0,43273161	1,353695446	0,175833562	1	protein_codin hypothetical protein
TcG_10136	78,85483819	0,121184944	0,199393591	0,607767498	0,543341689	0,740864584	protein_codin elongation factor 1-gamma (EF-1-gamma)
TcG_10137	67,52200868	0,124611502	0,221302485	0,563082255	0,573378874	0,764549158	protein_codin hypothetical protein
TcG_10138	22,03856653	0,363481646	0,384356254	0,945689428	0,344307016	0,578555633	protein_codin trans-sialidase
TcG_10139	15,35135263	-0,020376658	0,441088944	-0,04619626	0,963153826	1	protein_codin trans-sialidase
TcG_10140	0,301239679	-0,45874858	3,013621183	-0,15222503	0,879009452	1	protein_codin putative retrotransposon hot spot (RHS) protein
TcG_10141	4,682106067	-1,204265855	0,83233863	-1,44684605	0,147940047	1	protein_codin hypothetical protein
TcG_10142	15,49374939	0,212667445	0,436538186	0,487168023	0,626139264	1	protein_codin telomerase reverse transcriptase
TcG_10143	3,151372695	0,43960455	1,021488564	0,4303568	0,666936115	1	protein_codin putative retrotransposon hot spot (RHS) protein
TcG_10144	6,542550674	-0,275887125	0,678540509	-0,40658903	0,684309858	1	protein_codin cleavage and polyadenylation specificity factor
TcG_10145	565,0782821	-0,18452852	0,078371168	-2,35454599	0,018545349	0,079167463	protein_codin hypothetical protein
TcG_10146	415,4577881	0,117714702	0,091647918	1,284423095	0,198993956	0,417822396	protein_codin hypothetical protein

TcG_10147	264,2008743	-0,075469323	0,109844645	-0,68705509	0,492048012	0,702635507	protein_codin hypothetical protein
TcG_10148	82,36279449	-0,025395376	0,192451602	-0,13195721	0,895018139	0,948981304	protein_codin hypothetical protein
TcG_10149	277,0837061	-0,143625788	0,113742879	-1,26272334	0,206688624	0,428772498	protein_codin hypothetical protein
TcG_10150	97,38080255	-0,181560218	0,192135109	-0,94496117	0,344678698	0,578779679	protein_codin Placental protein 25 (PP25)
TcG_10151	284,6134819	-0,125837534	0,113128119	-1,11234532	0,265989704	0,498666135	protein_codin mitochondrial import inner membrane translocase subunit Tim17
TcG_10152	638,066829	-0,365667988	0,077067102	-4,74480006	2,08712E-06	3,87523E-05	protein_codin mitochondrial RNA-binding protein 2
TcG_10153	275,6442227	-0,258638415	0,107911386	-2,39676669	0,016540452	0,072866037	protein_codin hypothetical protein
TcG_10154	84,19253452	0,395779964	0,195801621	2,021331399	0,043245471	0,149341885	protein_codin dispersed gene family protein 1 (DGF-1)
TcG_10155	92,05299268	-0,130372262	0,180855827	-0,72086293	0,470993851	0,686925321	protein_codin dispersed gene family protein 1 (DGF-1)
TcG_10156	80,87652991	0,095795405	0,201491731	0,475430951	0,634479838	0,804628219	protein_codin dispersed gene family protein 1 (DGF-1)
TcG_10157	81,01074682	-0,046448965	0,196232191	-0,2367041	0,812886351	0,907594318	protein_codin dispersed gene family protein 1 (DGF-1)
TcG_10158	88,85977031	0,19391778	0,188507046	1,028703085	0,30361922	0,536498717	protein_codin dispersed gene family protein 1 (DGF-1)
TcG_10159	25,2006257	-0,237929204	0,348448736	-0,68282413	0,494717982	0,70453824	protein_codin hypothetical protein
TcG_10160	308,2613915	-0,335408122	0,110139025	-3,04531589	0,00232436	0,01553955	protein_codin putative ras-related GTP-binding protein
TcG_10161	451,5640784	-0,40653139	0,08965061	-4,53461935	5,77075E-06	9,48367E-05	protein_codin putative FYVE, RhoGEF and PH domain-containing protein 2
TcG_10162	95,89531047	-0,349277834	0,177435858	-1,96847378	0,04901355	0,163197375	protein_codin hypothetical protein
TcG_10163	321,8556352	-0,44806331	0,103056366	-4,34774995	1,37541E-05	0,000200195	protein_codin metallo-peptidase, Clan MA(E), Family M41
TcG_10164	4431,955337	0,028715494	0,038738566	0,741263742	0,458533541	0,677438813	protein_codin 60S ribosomal protein L10a
TcG_10165	334,3894811	0,111851986	0,099117944	1,128473629	0,259119948	0,491636632	protein_codin hypothetical protein
TcG_10166	120,4127746	0,070235726	0,165327268	0,42482844	0,670961743	0,827524245	protein_codin hypothetical protein
TcG_10167	115,5600583	-0,317960211	0,16385457	-1,94050255	0,052318644	0,170641367	protein_codin hypothetical protein
TcG_10168	397,1604987	-0,44458708	0,096663469	-4,59932881	4,23854E-06	7,25945E-05	protein_codin hypothetical protein
TcG_10169	23,58923292	-0,642734544	0,374929652	-1,71428037	0,086477246	0,243599653	protein_codin hypothetical protein
TcG_10170	432,6002678	-0,119415832	0,092675449	-1,28853794	0,19755877	0,41616653	protein_codin hypothetical protein
TcG_10171	80,56293753	0,065499324	0,222259265	0,294697835	0,768224717	0,883965794	protein_codin hypothetical protein
TcG_10172	29,01520666	-0,183467439	0,34042524	-0,53893607	0,589930971	0,77611666	protein_codin retrotransposon hot spot (RHS) protein
TcG_10173	183,0702488	0,158837742	0,133605207	1,188858915	0,23449519	0,462287097	protein_codin putative retrotransposon hot spot (RHS) protein
TcG_10174	63,10124045	-0,29184222	0,219274952	-1,33094189	0,183208131	0,395505431	protein_codin retrotransposon hot spot (RHS) protein
TcG_10175	30,44290836	0,015166889	0,309925756	0,048937169	0,960969368	0,981984718	
TcG_10176	327,4469321	0,058584846	0,100514118	0,582851916	0,559992992	0,754054394	protein_codin hypothetical protein
TcG_10177	179,0791729	0,033237087	0,137335763	0,242013343	0,808769825	0,905265886	protein_codin hypothetical protein
TcG_10178	105,1004672	0,436814447	0,177219667	2,464819257	0,013708238	0,06340026	protein_codin hypothetical protein
TcG_10179	652,6242868	0,26152752	0,076417792	3,422338096	0,000620851	0,005178672	protein_codin hypothetical protein
TcG_10180	314,7613046	0,161098743	0,204739307	0,786848143	0,431370767	0,655968892	protein_codin surface protease GP63
TcG_10181	263,018818	0,268017879	0,11230546	2,386507996	0,017009237	0,074393743	protein_codin putative SH3 domain protein
TcG_10182	303,7010386	0,133381926	0,104008347	1,282415591	0,199696898	0,418464145	protein_codin target of rapamycin (TOR) kinase 1
TcG_10183	144,9476717	0,370917998	0,146306559	2,535211005	0,011237961	0,054206086	protein_codin protein kinase
TcG_10184	1265,366799	-0,129923924	0,055940382	-2,32254265	0,020203734	0,08475035	protein_codin trans-sialidase
TcG_10185	1040,130859	0,164451833	0,06741389	2,439435449	0,014710231	0,066915089	
TcG_10186	632,3462425	-0,054327102	0,077358012	-0,70228152	0,482503618	0,696286283	protein_codin DnaJ chaperone protein
TcG_10187	828,6637193	0,061578585	0,067579675	0,911199784	0,362190118	0,594802935	protein_codin putative DnaJ chaperone protein
TcG_10188	128,4105743	0,1265324	0,160281272	0,789439702	0,429855059	0,654831163	protein_codin amino acid transporter
TcG_10189	346,0142937	-0,071552887	0,107867431	-0,66334097	0,5071122	0,713900601	protein_codin ubiquitin hydrolase, putative,cysteine peptidase, Clan CA, family C19
TcG_10190	538,8686929	-0,021343598	0,082131139	-0,25987218	0,794962374	0,897703126	protein_codin trans-sialidase
TcG_10191	666,7515604	0,035312976	0,074068629	0,476760223	0,633532874	0,804197117	protein_codin rab1 small GTP-binding protein
TcG_10192	11,1244285	-0,457627087	0,530485127	-0,86265771	0,388325688	1	protein_codin hypothetical protein
TcG_10193	4,030156071	-0,744380613	0,852749303	-0,87291847	0,38270752	1	protein_codin dispersed gene family protein 1 (DGF-1)
TcG_10194	48,21914982	0,426421428	0,255549724	1,668643666	0,09518802	0,25998312	protein_codin dispersed gene family protein 1 (DGF-1)
TcG_10195	3,025207081	0,194454462	0,973989515	0,199647387	0,841756364	1	protein_codin dispersed gene family protein 1 (DGF-1)
TcG_10196	17,11659838	-0,667068422	0,441927206	-1,50945317	0,131183014	0,321941622	protein_codin dispersed gene family protein 1 (DGF-1)
TcG_10197	5,462185589	0,201413388	0,758804738	0,265435068	0,790674324	1	protein_codin dispersed gene family protein 1 (DGF-1)
TcG_10198	21,03037455	0,246362102	0,382181497	0,644620696	0,51917302	0,7237563	protein_codin putative mucin TcMUCII
TcG_10199	16,51526378	0,695564087	0,473390692	1,469323538	0,141745055	1	
TcG_10200	19,61333668	0,160948681	0,395435743	0,407016016	0,683996225	0,835947285	
TcG_10201	95,82882163	-0,514360545	0,181370102	-2,83597208	0,004568644	0,026841942	protein_codin mucin-associated surface protein (MASP)
TcG_10202	15,85955858	0,04946111	0,470533315	0,105117127	0,916282869	1	protein_codin peptide hydrolase
TcG_10203	6,368668348	0,458498334	0,721880128	0,635144696	0,525334049	1	protein_codin hypothetical protein

TcG_10204	4,370442343	0,196117886	0,837002173	0,234309888	0,81474441	1	protein_codin	mucin TcMUCII
TcG_10205	0					1	protein_codin	hypothetical protein
TcG_10206	2,906205957	-0,334828494	1,009347224	-0,33172776	0,740094837	1	protein_codin	hypothetical protein
TcG_10207	0					1	protein_codin	hypothetical protein
TcG_10208	18,6268964	-0,008363614	0,464662999	-0,01799931	0,985639403	0,99432608	protein_codin	hypothetical protein
TcG_10209	9,577968418	-0,561701444	0,571919818	-0,98213321	0,326034229	1		
TcG_10210	12,91879005	0,071448019	0,475572907	0,150235681	0,880578676	1	protein_codin	surface protease GP63
TcG_10211	3,450925058	-0,370354184	0,950638482	-0,38958468	0,696843686	1	protein_codin	hypothetical protein
TcG_10212	10,62230978	0,374859814	0,529126849	0,708449807	0,478665972	1	protein_codin	hypothetical protein
TcG_10213	50,06945463	-0,606938018	0,244656529	-2,48077589	0,013109677	0,06124545	protein_codin	putative mucin-associated surface protein (MASP)
TcG_10214	72,80203511	0,026793893	0,212705956	0,125966822	0,899758188	0,951236277	protein_codin	putative trans-sialidase
TcG_10215	18,23348408	0,045249788	0,403742972	0,112075729	0,910763361	0,956673101		
TcG_10216	11,909641	0,121964908	0,539771244	0,225956661	0,821235128	1	protein_codin	hypothetical protein
TcG_10217	9,339488293	-0,694295446	0,576894613	-1,20350482	0,228781028	1	protein_codin	trans-sialidase
TcG_10218	6,220448673	0,127508735	0,735572654	0,173346214	0,8623793	1	protein_codin	Sialidase 85-1.3
TcG_10219	199,7568159	0,91909357	0,129593845	7,092108206	1,32084E-12	9,74731E-11	protein_codin	hypothetical protein
TcG_10220	25,08827236	0,322197871	0,348291261	0,925081696	0,354923413	0,588879088	protein_codin	rab1 small GTP-binding protein
TcG_10221	15,71234589	0,4594474	0,442074846	1,039297766	0,298666274	1	protein_codin	target of rapamycin (TOR) kinase 1
TcG_10222	6,661520451	1,502853662	0,729239837	2,060849649	0,039317386	1	protein_codin	trans-sialidase
TcG_10223	18,2927101	1,510382697	0,449630907	3,359161203	0,000781794	0,006338608	protein_codin	putative phosphatidylinositol-4-phosphate 5-kinase-like
TcG_10224	15,90781119	0,747740181	0,441354767	1,694193056	0,090228604	1	protein_codin	surface protease GP63
TcG_10225	5,179166191	-0,208277954	0,748818318	-0,27814217	0,780903223	1	protein_codin	hypothetical protein
TcG_10226	3,23728135	-0,394905837	0,961716833	-0,4106259	0,681346872	1	protein_codin	hypothetical protein
TcG_10227	565,3719797	-0,161397477	0,085432395	-1,88918358	0,058867234	0,186399502	protein_codin	putative retrotransposon hot spot (RHS) protein
TcG_10228	601,0191107	-0,177520304	0,080178307	-2,21406901	0,026824032	0,105278875	protein_codin	trans-sialidase
TcG_10229	58,08607323	0,042810039	0,226072879	0,189363884	0,849807628	0,927630599	protein_codin	hypothetical protein
TcG_10230	41,43628423	0,017533245	0,276573277	0,063394573	0,949452309	0,976507274	protein_codin	hypothetical protein
TcG_10231	29,09183647	-0,043873225	0,326565374	-0,13434745	0,893127835	0,948402185	protein_codin	hypothetical protein
TcG_10232	40,7710585	0,229714472	0,276924947	0,82951888	0,406810858	0,635045891	protein_codin	hypothetical protein
TcG_10233	0					1	protein_codin	retrotransposon hot spot (RHS) protein
TcG_10234	47,21182608	0,272465981	0,253444232	1,075052994	0,282351003	0,515574266	protein_codin	retrotransposon hot spot (RHS) protein
TcG_10235	40,57860286	0,166472896	0,273098681	0,609570489	0,542146366	0,740196534	protein_codin	retrotransposon hot spot (RHS) protein
TcG_10236	45,41045976	0,04196869	0,267444694	0,156924743	0,875304159	0,939877107	protein_codin	retrotransposon hot spot (RHS) protein
TcG_10237	38,99374609	0,55544428	0,285146136	1,947928483	0,051423521	0,168684291	protein_codin	putative retrotransposon hot spot (RHS) protein
TcG_10238	21,65610798	0,161650796	0,418282132	0,386463545	0,699153396	0,844142481	protein_codin	dispersed gene family protein 1 (DGF-1)
TcG_10239	43,87362973	0,497088005	0,270010743	1,840993431	0,065622521	0,20269329	protein_codin	dispersed gene family protein 1 (DGF-1)
TcG_10240	2,668285196	1,155414421	1,124274005	1,027698244	0,304091794	1	protein_codin	dispersed gene family protein 1 (DGF-1)
TcG_10241	1,5641518	0,447239627	1,371532655	0,32608748	0,744358169	1	protein_codin	hypothetical protein
TcG_10242	8,235961079	-0,18727789	0,610320705	-0,30685161	0,758956324	1	protein_codin	hypothetical protein
TcG_10243	10,98666775	-0,530377031	0,542360964	-0,97790414	0,328121738	1		
TcG_10244	3,762941388	-1,092498685	0,912108971	-1,19777211	0,231005748	1	protein_codin	hypothetical protein
TcG_10245	6,451284878	-0,183222986	0,686200482	-0,26701087	0,789460794	1	protein_codin	hypothetical protein
TcG_10246	4,802376486	1,067988987	0,852787027	1,252351353	0,210441864	1	protein_codin	hypothetical protein
TcG_10247	16,26104619	0,23328737	0,428415755	0,544534992	0,586073376	1	protein_codin	surface protease GP63
TcG_10248	195,8193981	-0,042048775	0,125526683	-0,33497878	0,737641093	0,866063002	protein_codin	mucin-associated surface protein (MASP)
TcG_10249	71,10314582	0,616017246	0,220414521	2,794812447	0,005192983	0,029522033	protein_codin	90 kDa surface protein
TcG_10250	43,54687981	0,294704647	0,262333878	1,123395304	0,261269644	0,493587786	protein_codin	protein kinase
TcG_10251	44,32549937	0,24391817	0,26121423	0,933785922	0,350414333	0,584889659	protein_codin	protein kinase
TcG_10252	84,40211888	0,355843324	0,194578662	1,828789037	0,067431218	0,206736727	protein_codin	putative target of rapamycin (TOR) kinase 1
TcG_10253	31,67808439	0,364589955	0,333069826	1,094635197	0,273676489	0,50688937	protein_codin	hypothetical protein
TcG_10254	8,499631763	1,041644672	0,622531186	1,673240949	0,094279872	1	protein_codin	putative GAG protein
TcG_10255	19,14350787	0,343936934	0,394460559	0,871917169	0,383253567	0,613396301	protein_codin	putative trans-sialidase
TcG_10256	328,7231082	0,603958399	0,101907846	5,926515195	3,09431E-09	1,11337E-07	protein_codin	hypothetical protein
TcG_10257	57,87605726	0,305787661	0,238272281	1,283353898	0,199368118	0,418209675	protein_codin	hypothetical protein
TcG_10258	37,59468354	0,648634521	0,300291704	2,160014789	0,030771525	0,116471377	protein_codin	trans-sialidase
TcG_10259	42,86819358	0,409179764	0,275322451	1,486183792	0,137230513	0,330255911	protein_codin	putative target of rapamycin (TOR) kinase 1
TcG_10260	33,91940497	0,956207075	0,315106302	3,034553951	0,002408917	0,015959534	protein_codin	hypothetical protein

TcG_10261	41,15456318	0,255954079	0,27568146	0,928441396	0,353178654	0,587582982	protein_codin retrotransposon hot spot (RHS) protein
TcG_10262	417,2804376	0,473231007	0,090277487	5,241960372	1,58879E-07	3,95017E-06	protein_codin putative retrotransposon hot spot (RHS) protein
TcG_10263	487,0478797	0,527820195	0,089639006	5,888287042	3,90219E-09	1,36177E-07	protein_codin hypothetical protein
TcG_10264	26,59412809	0,578046466	0,34671264	1,66722063	0,095470542	0,26063188	protein_codin trans-sialidase
TcG_10265	132,2705652	0,671468055	0,15866422	4,23200678	2,31615E-05	0,000314595	protein_codin trans-sialidase
TcG_10266	16,73800402	0,041125876	0,42175783	0,097510639	0,922320886	1	protein_codin trans-sialidase
TcG_10267	18,72571884	-0,583277452	0,415685968	-1,40316849	0,160566602	0,363449048	protein_codin trans-sialidase
TcG_10268	12,08696911	-0,440438015	0,529625678	-0,83160246	0,405633375	1	protein_codin surface protease GP63
TcG_10269	51,43560341	-0,577968011	0,248966313	-2,32147074	0,020261452	0,084930962	protein_codin surface protease GP63
TcG_10270	6,702400104	-0,862153795	0,706621755	-1,2201065	0,222424505	1	protein_codin hypothetical protein
TcG_10271	4,810469414	-0,794706364	0,813098176	-0,97738058	0,328380775	1	protein_codin hypothetical protein
TcG_10272	10,95482032	-0,503202939	0,537181954	-0,9367458	0,348889331	1	
TcG_10273	38,38109977	-1,018905806	0,312176845	-3,26387374	0,001099001	0,008432469	protein_codin putative mucin TcMUCII
TcG_10274	121,7319195	-0,424230606	0,161938618	-2,61970005	0,008800714	0,044701915	protein_codin mucin-associated surface protein (MASP)
TcG_10275	49,02623982	-0,028508194	0,270080037	-0,10555461	0,915935736	0,959149624	protein_codin putative trans-sialidase
TcG_10276	96,51348466	-0,27684124	0,181013155	-1,52939846	0,126165694	0,313816172	protein_codin hypothetical protein
TcG_10277	191,6916674	-0,054697662	0,127709837	-0,42829639	0,668435344	0,826255403	protein_codin hypothetical protein
TcG_10278	116,8183191	0,289728828	0,166857949	1,736380131	0,082496643	0,236118109	protein_codin hypothetical protein
TcG_10279	244,6814403	-0,157437184	0,115208322	-1,3665435	0,171768438	0,379140621	protein_codin hypothetical protein
TcG_10280	0					1	
TcG_10281	24,25173971	0,224771228	0,355405747	0,632435546	0,527102313	0,729630514	protein_codin hypothetical protein
TcG_10282	18,44036703	0,650627269	0,42008515	1,548798546	0,121430155	0,30577913	protein_codin hypothetical protein
TcG_10283	60,87400586	-0,102070597	0,228227062	-0,44723266	0,654707081	0,817396146	protein_codin selenocysteine-tRNA-specific elongation factor
TcG_10284	24,12300269	0,427300571	0,365625889	1,168682478	0,242531584	0,47091854	protein_codin hypothetical protein
TcG_10285	43,91046505	0,009142128	0,266129364	0,034352197	0,972596302	0,987858399	protein_codin serine/threonine protein phosphatase
TcG_10286	83,77599627	-0,160515635	0,20436626	-0,78543119	0,432200801	0,656718489	protein_codin trans-sialidase
TcG_10287	30,24440321	0,007763717	0,328788674	0,023613092	0,981161229	0,992815197	protein_codin trans-sialidase
TcG_10288	160,3253514	0,065766852	0,141455982	0,464928038	0,641982991	0,809888386	protein_codin trans-sialidase
TcG_10289	95,0724476	-0,038387849	0,184415659	-0,20815938	0,835104523	0,92007617	
TcG_10290	50,82099319	0,129653441	0,249867075	0,518889657	0,60383769	0,785809669	protein_codin dispersed gene family protein 1 (DGF-1)
TcG_10291	25,44987907	-0,122187235	0,348695669	-0,35041225	0,726029331	0,858519681	protein_codin Tbingi protein
TcG_10292	44,48588743	-0,124458298	0,287657618	-0,43266123	0,665260902	0,825042607	protein_codin hypothetical protein
TcG_10293	16,4648393	0,134774196	0,425898843	0,316446965	0,751663274	1	protein_codin hypothetical protein
TcG_10294	14,85339725	-0,155738003	0,457679737	-0,34027725	0,733647746	1	protein_codin hypothetical protein
TcG_10295	17,77709708	0,393570093	0,414011941	0,95062498	0,341794783	0,576340323	protein_codin hypothetical protein
TcG_10296	38,96318648	0,339664167	0,288593761	1,176962957	0,239210292	0,467367696	protein_codin trans-sialidase
TcG_10297	18,00743188	0,095852172	0,405239242	0,236532305	0,813019643	0,907594318	protein_codin putative elongation factor 1-gamma (EF-1-gamma)
TcG_10298	61,65753168	-0,452067593	0,227092475	-1,99067624	0,046516493	0,157308841	protein_codin mucin-associated surface protein (MASP)
TcG_10299	168,5267666	0,202743347	0,144381519	1,404219515	0,160253492	0,363061588	protein_codin dispersed gene family protein 1 (DGF-1)
TcG_10300	83,33336002	0,025403209	0,194256601	0,130771408	0,895956142	0,9494693	protein_codin dispersed gene family protein 1 (DGF-1)
TcG_10301	34,75132335	0,167948115	0,290888661	0,577362192	0,563694809	0,756774978	protein_codin putative dispersed gene family protein 1 (DGF-1)
TcG_10302	76,43912529	-0,136309915	0,201262773	-0,67727336	0,498232528	0,707424985	protein_codin hypothetical protein
TcG_10303	100,3267173	-0,094717086	0,173002414	-0,54748997	0,58404216	0,772632162	protein_codin hypothetical protein
TcG_10304	113,5594922	-0,0805699	0,163474798	-0,49285823	0,622112761	0,79640439	protein_codin hypothetical protein
TcG_10305	127,097042	0,025393596	0,163566918	0,155248973	0,876625042	0,940946612	protein_codin hypothetical protein
TcG_10306	76,39964011	0,013053323	0,211269013	0,061785319	0,950733795	0,976958027	
TcG_10307	71,06424542	0,192423722	0,22000759	0,874623107	0,381779011	0,611966189	protein_codin trans-sialidase
TcG_10308	44,06744848	-0,073051298	0,264672197	-0,27600669	0,782542925	0,89228839	protein_codin rab1 small GTP-binding protein
TcG_10309	32,27726709	0,060026706	0,304817379	0,196926782	0,843884835	0,924387794	protein_codin target of rapamycin (TOR) kinase 1
TcG_10310	10,17661386	-0,171182599	0,543668372	-0,31486584	0,752863519	1	protein_codin hypothetical protein
TcG_10311	33,89669188	-0,538093827	0,300268409	-1,79204276	0,073126117	0,217799278	
TcG_10312	43,53731244	-0,202654132	0,264027831	-0,76754837	0,442755545	0,664995914	protein_codin amastigote surface protein 4
TcG_10313	20,09659062	0,344554919	0,38323769	0,899063238	0,368618978	0,600762341	protein_codin trans-sialidase
TcG_10314	83,20754885	0,107207195	0,193493143	0,554061984	0,579536426	0,769018862	protein_codin mucin-associated surface protein (MASP)
TcG_10315	32,12029635	-0,163931818	0,303607444	-0,53994664	0,589233833	0,775648564	protein_codin mucin TcMUCII
TcG_10316	11,85238307	-0,067310875	0,521951052	-0,12896013	0,897389197	1	protein_codin mucin TcMUCII
TcG_10317	3,797072771	-0,000472488	0,885210439	-0,00053376	0,999574123	1	protein_codin hypothetical protein

TcG_10318	4,865967068	-0,619011601	0,804277504	-0,76964928	0,441507965	1	protein_codin	hypothetical protein
TcG_10319	6,509136624	-1,34449598	0,7565847	-1,77705943	0,075558461	1	protein_codin	hypothetical protein
TcG_10320	424,4153281	0,375956626	0,100469345	3,742003351	0,000182559	0,001852127	protein_codin	structural maintenance of chromosome protein 4
TcG_10321	1291,766694	0,322867545	0,069474114	4,647307129	3,36296E-06	5,93953E-05	protein_codin	hypothetical protein
TcG_10322	488,1789352	0,154884193	0,083459299	1,855805108	0,063481342	0,197820019	protein_codin	protein kinase, putative,serine/threonine protein kinase
TcG_10323	109,1314982	-0,108328994	0,183375355	-0,59075002	0,55468793	0,749417743	protein_codin	Zinc finger (ISS)
TcG_10324	1184,924886	-0,207134991	0,06039847	-3,42947416	0,000604752	0,00506994	protein_codin	leucine--tRNA ligase
TcG_10325	303,5626591	0,562684805	0,107008076	5,258339597	1,45362E-07	3,6692E-06		
TcG_10326	282,7114573	0,676927154	0,111898957	6,049450053	1,45341E-09	5,5575E-08	protein_codin	cyclophilin type peptidyl-prolyl cis-trans isomerase
TcG_10327	424,4204507	0,650659895	0,098219861	6,624524697	3,48367E-11	1,94985E-09	protein_codin	putative vacuolar ATP synthase 16 kDa proteolipid subunit
TcG_10328	547,8093247	0,489449219	0,08590686	5,697440467	1,21619E-08	3,7676E-07	protein_codin	putative monoglyceride lipase
TcG_10329	908,0684377	0,500028294	0,066975358	7,465854784	8,27606E-14	7,55011E-12	protein_codin	leucine richcontaining 34
TcG_10330	711,6169217	0,631080048	0,073054248	8,638512729	5,69495E-18	8,35211E-16	protein_codin	putative protein kinase A regulatory subunit
TcG_10331	620,4560726	0,659732872	0,080004418	8,246205477	1,63503E-16	1,97328E-14	protein_codin	hypothetical protein
TcG_10332	454,418255	0,439998917	0,09140964	4,813484872	1,48321E-06	2,87365E-05	protein_codin	hypothetical protein
TcG_10333	294,9615196	0,368149074	0,106547684	3,455251778	0,000549779	0,004704388	protein_codin	hypothetical protein
TcG_10334	929,7031986	0,649828789	0,071724448	9,060073699	1,30362E-19	2,2543E-17	protein_codin	TCP17 protein
TcG_10335	88,43539721	0,561224287	0,190428053	2,947172329	0,003206944	0,020138567		
TcG_10336	21,52160579	-0,112127886	0,374060954	-0,29975833	0,764361504	0,88127101	protein_codin	putative mucin-associated surface protein (MASP)
TcG_10337	22,73049146	0,345015803	0,393075482	0,877734223	0,380087968	0,610721305	protein_codin	RNA-binding protein
TcG_10338	0,797274563	0,599188768	1,995731163	0,300235211	0,763997748	1	protein_codin	trans-sialidase
TcG_10339	15,13363215	0,562160879	0,452178158	1,243228735	0,213783557	1	protein_codin	hypothetical protein
TcG_10340	17,04887045	-0,636231884	0,438784088	-1,44998851	0,147061725	0,345539878	protein_codin	trans-sialidase
TcG_10341	71,0419437	0,056943964	0,208974386	0,272492551	0,785243318	0,893514718	protein_codin	putative trans-sialidase
TcG_10342	3,837729476	-0,449885239	0,878244877	-0,5122549	0,608472627	1	protein_codin	hypothetical protein
TcG_10343	3,188901367	-1,534119302	1,003341472	-1,52901016	0,126261926	1	protein_codin	hypothetical protein
TcG_10344	54,94609395	0,236772173	0,250100217	0,946709189	0,343786987	0,577993726	protein_codin	hypothetical protein
TcG_10345	29,28184696	0,295970217	0,322071143	0,918959131	0,358116949	0,591973602		
TcG_10346	40,74062924	0,322772027	0,273861871	1,178594251	0,238559782	0,466606126	protein_codin	dispersed gene family protein 1 (DGF-1)
TcG_10347	262,2133425	0,033495492	0,12007865	0,27894627	0,780286059	0,89067206	protein_codin	dispersed gene family protein 1 (DGF-1)
TcG_10348	166,3862395	-0,032970819	0,140355891	-0,23490869	0,814279601	0,908262507	protein_codin	dispersed gene family protein 1 (DGF-1)
TcG_10349	5,784607081	0,462750887	0,723065562	0,639984687	0,522182555	1	protein_codin	hypothetical protein
TcG_10350	21,15116223	0,613267992	0,423409226	1,448404887	0,147503849	0,346227632		
TcG_10351	29,78468576	0,411026518	0,324466733	1,266775532	0,205235564	0,426674904		
TcG_10352	8,880723964	0,140036759	0,602380181	0,232472387	0,816171131	1	protein_codin	putative trans-sialidase
TcG_10353	15,13956886	0,379913363	0,458179756	0,829179721	0,407002718	1		
TcG_10354	12,72219306	-0,63017219	0,512929603	-1,22857442	0,219231411	1	protein_codin	retrotransposon hot spot (RHS) protein
TcG_10355	0,611155623	-0,424209629	2,242957467	-0,18912959	0,849991249	1	protein_codin	hypothetical protein
TcG_10356	25,27716489	0,214047686	0,357944316	0,597991576	0,549845554	0,745961428	protein_codin	retrotransposon hot spot (RHS) protein
TcG_10357	214,6713843	0,175111036	0,122625297	1,428017215	0,153286904	0,353824404	protein_codin	putative retrotransposon hot spot (RHS) protein
TcG_10358	264,3484607	0,216087539	0,109465992	1,974015273	0,048380006	0,161629398	protein_codin	HPP family protein
TcG_10359	20,28750727	0,466240366	0,389482896	1,19707533	0,231277195	0,458517724	protein_codin	chitin-binding like protein
TcG_10360	448,2781416	-0,245351274	0,091813699	-2,67227305	0,007533932	0,03956851	protein_codin	trans-sialidase
TcG_10361	30,61610673	-1,41002058	0,328738883	-4,28918103	1,79333E-05	0,000251544	protein_codin	trans-sialidase
TcG_10362	45,22455581	-0,46191067	0,260429039	-1,77365271	0,076120613	0,223104838	protein_codin	hypothetical protein
TcG_10363	38,11285153	-0,840078214	0,293260339	-2,86461584	0,004175154	0,024986227	protein_codin	hypothetical protein
TcG_10364	27,48296409	-0,512095701	0,330267485	-1,55054834	0,12100996	0,304986165	protein_codin	mucin-associated surface protein (MASP)
TcG_10365	45,84971191	-0,445432422	0,259562564	-1,71608885	0,086145782	0,242771205	protein_codin	regulator of sigma E protease
TcG_10366	8,460820315	-0,206068416	0,604921449	-0,34065318	0,733364689	1	protein_codin	hypothetical protein
TcG_10367	34,99617864	-0,182871564	0,314051455	-0,58229809	0,560365909	0,754316187	protein_codin	hypothetical protein
TcG_10368	12,86608772	-1,141864063	0,529944741	-2,15468515	0,031186481	1	protein_codin	hypothetical protein
TcG_10369	7,250784746	-0,656200054	0,663002697	-0,98973965	0,322301392	1	protein_codin	hypothetical protein
TcG_10370	22,48864813	0,078437255	0,360627727	0,217502007	0,827817143	0,915880831	protein_codin	L1Tc protein
TcG_10371	537,1712861	-0,089543715	0,081144225	-1,10351309	0,269804409	0,502690828	protein_codin	hypothetical protein
TcG_10372	527,0410383	-0,032445903	0,080553225	-0,40278838	0,687103909	0,837536654	protein_codin	methyltransferase
TcG_10373	141,0895941	0,021038813	0,150185681	0,140085343	0,888592561	0,94628587	protein_codin	hypothetical protein
TcG_10374	36,80754949	0,24253389	0,294246628	0,82425376	0,409795386	0,636890088	protein_codin	hypothetical protein

TcG_10375	31,82412513	-0,238714855	0,309862146	-0,77039051	0,441068283	0,663546065	protein_codin trans-sialidase
TcG_10376	12,2520143	-0,322836237	0,543656206	-0,59382425	0,552629671	1	protein_codin putative trans-sialidase
TcG_10377	47,41702825	-0,11905471	0,253082385	-0,47041879	0,63805584	0,807510942	protein_codin hypothetical protein
TcG_10378	66,44079208	-0,1857162	0,22565723	-0,82300133	0,410507239	0,637295575	protein_codin surface glycoprotein Tc85-11
TcG_10379	27,08505825	-0,151121636	0,338769594	-0,44608973	0,655532433	0,817728228	protein_codin trans-sialidase
TcG_10380	15,09502163	-0,290949133	0,460258482	-0,6321429	0,527293503	1	protein_codin hypothetical protein
TcG_10381	7,860188277	-0,506080715	0,615126574	-0,82272614	0,410663744	1	protein_codin hypothetical protein
TcG_10382	19,95773068	0,200220159	0,396016876	0,505584915	0,613148074	0,790819724	protein_codin hypothetical protein
TcG_10383	13,89132105	-0,176250533	0,483645959	-0,36442056	0,715543982	1	protein_codin hypothetical protein
TcG_10384	29,13520567	0,272111916	0,326777955	0,832711976	0,405007189	0,634281332	protein_codin hypothetical protein
TcG_10385	25,89449028	-0,072859582	0,356804007	-0,20420057	0,838196765	0,92144011	protein_codin hypothetical protein
TcG_10386	31,36112012	-0,000276255	0,305621045	-0,00090391	0,999278783	0,99981271	protein_codin dispersed gene family protein 1 (DGF-1)
TcG_10387	18,32855982	-0,368924164	0,414965287	-0,88904825	0,373977147	0,604496256	protein_codin hypothetical protein
TcG_10388	56,58542159	0,157471097	0,242857305	0,648409966	0,516719826	0,722248269	protein_codin hypothetical protein
TcG_10389	70,80204473	0,131072772	0,221894163	0,590699502	0,554721782	0,749417743	protein_codin retrotransposon hot spot (RHS) protein
TcG_10390	76,80880718	0,048649846	0,211342472	0,230194365	0,81794074	0,910311132	
TcG_10391	117,2475105	0,471878218	0,17866175	2,641182104	0,00826173	0,042506957	protein_codin surface protein-2
TcG_10392	46,24425606	-0,360467352	0,260697893	-1,38270144	0,166756431	0,372692904	protein_codin trans-sialidase
TcG_10393	25,56805165	0,220014901	0,344965292	0,637788514	0,523611357	0,727667169	
TcG_10394	288,6703173	-0,18269451	0,105930922	-1,72465704	0,084589298	0,23968002	protein_codin mucin-associated surface protein (MASP)
TcG_10395	901,905335	-0,024588861	0,067172736	-0,36605418	0,714324646	0,853492027	protein_codin hypothetical protein
TcG_10396	1906,372887	-0,062468594	0,05333395	-1,1712726	0,241489221	0,470195853	protein_codin putative zinc finger protein
TcG_10397	236,6402803	-0,343843934	0,122564472	-2,80541276	0,005025218	0,028880049	
TcG_10398	478,9311022	-0,029507291	0,084825687	-0,34785796	0,727946857	0,859909491	protein_codin hypothetical protein
TcG_10399	172,417745	0,086545919	0,133051162	0,650470975	0,515388044	0,721256901	protein_codin hypothetical protein
TcG_10400	462,6223059	-0,078002545	0,089702552	-0,86956885	0,384536069	0,614769546	protein_codin chaperone protein DNAJ
TcG_10401	206,5016982	-0,055425505	0,124856632	-0,44391318	0,657105353	0,819154576	protein_codin Hrf1 family protein
TcG_10402	146,7289693	0,217044944	0,147851706	1,4679908	0,142106722	0,338010363	protein_codin pre-mRNA-splicing factor 38A
TcG_10403	307,6028397	0,01012872	0,104832378	0,096618242	0,923029569	0,962576111	protein_codin hypothetical protein
TcG_10404	29,96062456	0,341730269	0,332608209	1,027425841	0,304219989	0,537102518	protein_codin hypothetical protein
TcG_10405	13,17192508	-0,224661289	0,468284069	-0,47975429	0,631402122	1	protein_codin hypothetical protein
TcG_10406	27,13656263	0,064139107	0,343197706	0,186886758	0,851749422	0,928229751	
TcG_10407	739,4930464	0,067380311	0,069761786	0,96586275	0,334112864	0,568600417	protein_codin NADH-dependent fumarate reductase-like protein
TcG_10408	417,0972899	-0,282748545	0,08942095	-3,16199441	0,001566926	0,011283034	protein_codin hypothetical protein
TcG_10409	714,4755333	-0,197849382	0,072064595	-2,74544502	0,006042889	0,033197209	protein_codin hypothetical protein
TcG_10410	300,6982605	-0,108494755	0,106025108	-1,02329304	0,306169311	0,539181888	protein_codin hypothetical protein
TcG_10411	160,6889348	0,023902013	0,140020665	0,170703468	0,864456935	0,934985064	protein_codin GDP-mannose 4,6 dehydratase
TcG_10412	65,66837329	0,038695148	0,230515272	0,167863707	0,866690503	0,93574468	protein_codin putative GDP-mannose 4,6 dehydratase
TcG_10413	391,9271965	-0,145217271	0,094724244	-1,53305284	0,125262836	0,312173633	protein_codin putative kynureninase
TcG_10414	707,2418714	0,091678181	0,078195479	1,172423035	0,241027256	0,469729485	protein_codin hypothetical protein
TcG_10415	51,87949298	0,04873769	0,242943479	0,200613287	0,841000967	0,922466573	protein_codin regulator of sigma E protease
TcG_10416	0,456961293	0,528046641	2,474471948	0,213397708	0,831016768	1	
TcG_10417	162,7549228	0,072544173	0,140319053	0,516994458	0,605160028	0,786698766	protein_codin hypothetical protein
TcG_10418	359,7759571	0,013792473	0,099485054	0,138638645	0,889735704	0,946535728	protein_codin hypothetical protein
TcG_10419	17,70490437	0,000316241	0,435279122	0,000726525	0,999420317	0,99981271	protein_codin casein kinase
TcG_10420	2678,792027	-0,036038114	0,043880225	-0,82128373	0,411484674	0,638080373	protein_codin casein kinase
TcG_10421	4,044493071	0,048021959	0,844359627	0,056873821	0,954645708	1	protein_codin casein kinase
TcG_10422	176,3139957	0,38041379	0,133118117	2,857716143	0,004267019	0,025417832	protein_codin Zinc finger protein CTH1
TcG_10423	33,59580438	-0,185648104	0,299484013	-0,6198932	0,5353281	0,735451331	protein_codin trans-sialidase
TcG_10424	17,55854039	-0,216058831	0,46728398	-0,46237158	0,643814877	0,810875005	protein_codin trans-sialidase
TcG_10425	22,98960935	0,098347943	0,367838918	0,267366878	0,789186699	0,894943008	protein_codin trans-sialidase
TcG_10426	52,66307457	-0,554104835	0,246077869	-2,25174591	0,024338334	0,09782343	protein_codin mucin-associated surface protein (MASP)
TcG_10427	225,8159272	-0,403004313	0,118831554	-3,39139144	0,000695387	0,005722127	protein_codin mucin-associated surface protein (MASP)
TcG_10428	262,2127267	0,030699789	0,113212826	0,271168823	0,786261191	0,893586355	protein_codin hypothetical protein
TcG_10429	72,88509754	-0,142337216	0,213004228	-0,66823658	0,503982591	0,712076368	protein_codin hypothetical protein
TcG_10430	61,30986098	-0,163231513	0,237208	-0,68813663	0,491366747	0,702142961	protein_codin hypothetical protein
TcG_10431	94,62372909	-0,267115477	0,187402204	-1,42535931	0,154053363	0,355055156	protein_codin hypothetical protein

TcG_10432	138,7867611	-0,046319287	0,149135201	-0,31058588	0,756115463	0,877165706	protein_codin	hypothetical protein
TcG_10433	28,76712055	0,364805694	0,322831824	1,13001776	0,258468741	0,49092112	protein_codin	dispersed gene family protein 1 (DGF-1)
TcG_10434	61,12493643	0,139820833	0,23588965	0,592738313	0,553356296	0,748533106	protein_codin	dispersed gene family protein 1 (DGF-1)
TcG_10435	171,3289424	0,309920127	0,144968601	2,137843131	0,032529477	0,121615528	protein_codin	dispersed gene family protein 1 (DGF-1)
TcG_10436	127,8558495	0,294801355	0,287138118	1,026688329	0,304567247	0,537340661	protein_codin	dispersed gene family protein 1 (DGF-1)
TcG_10437	106,4401965	-0,238740926	0,17115234	-1,39490307	0,16304507	0,367374598	protein_codin	hypothetical protein
TcG_10438	1073,839328	0,09320655	0,061653509	1,511780119	0,130589809	0,320757584	protein_codin	hypothetical protein
TcG_10439	25,29860961	-0,246679573	0,396707061	-0,62181795	0,534061579	0,73443768	protein_codin	trans-sialidase
TcG_10440	19,24299629	-0,925689211	0,412576199	-2,24368059	0,02485296	0,099257632	protein_codin	trans-sialidase
TcG_10441	7,234364421	0,147401828	0,63634757	0,231637293	0,81681974	1	protein_codin	hypothetical protein
TcG_10442	3,099138335	0,499873265	0,995403568	0,502181508	0,615539849	1	protein_codin	surface protease GP63
TcG_10443	7,059487593	0,692125373	0,659152799	1,050022656	0,293707697	1	protein_codin	surface protease GP63
TcG_10444	0,924672238	1,929221891	1,877884065	1,027338124	0,304261277	1	protein_codin	surface protease GP63
TcG_10445	1,472324391	0,553420904	1,486750844	0,372235137	0,709717787	1	protein_codin	hypothetical protein
TcG_10446	8,606078846	-0,080225397	0,651945043	-0,12305546	0,902063181	1	protein_codin	putative syntaxin binding protein
TcG_10447	132,0478277	0,152386012	0,152959658	0,996249691	0,319128841	0,554102154	protein_codin	dispersed gene family protein 1 (DGF-1)
TcG_10448	81,94482206	0,05820392	0,200063214	0,290927649	0,771106655	0,88517207	protein_codin	dispersed gene family protein 1 (DGF-1)
TcG_10449	37,43851554	0,240941231	0,335944365	0,717205754	0,47324715	0,689084011	protein_codin	dispersed protein family protein 1 (DGF-1)
TcG_10450	65,21194152	0,341803809	0,438480044	0,779519647	0,435673669	0,659399643	protein_codin	dispersed gene family protein 1 (DGF-1)
TcG_10451	47,9709588	-0,015506593	0,25817432	-0,06006249	0,952105864	0,977395901	protein_codin	hypothetical protein
TcG_10452	176,6933155	0,233638416	0,13513718	1,728898121	0,08382733	0,238337043	protein_codin	hypothetical protein
TcG_10453	50,66547361	0,020228648	0,245531112	0,082387312	0,934338725	0,969000687	protein_codin	putative profilin
TcG_10454	3,34525742	1,370893071	1,001386163	1,36899542	0,171000706	1		
TcG_10455	10,74928541	0,376428786	0,542006165	0,694510155	0,487362335	1	protein_codin	hypothetical protein
TcG_10456	18,22183637	0,761771627	0,444617967	1,713317237	0,086654193	0,243979461	protein_codin	hypothetical protein
TcG_10457	47,71636995	-0,111504289	0,266574752	-0,41828526	0,675738565	0,83017833	protein_codin	putative mucin TcMUCII
TcG_10458	26,19503461	0,086081415	0,351663986	0,24478314	0,806624349	0,904588239	protein_codin	hypothetical protein
TcG_10459	38,8075662	-0,185013535	0,277527312	-0,66664983	0,504995832	0,71221932	protein_codin	putative trans-sialidase
TcG_10460	4,020083013	1,395969645	0,919943625	1,517451295	0,129152782	1	protein_codin	putative trans-sialidase
TcG_10461	32,12988506	-0,31132723	0,316866647	-0,98251815	0,325844648	0,560274604	protein_codin	trans-sialidase
TcG_10462	13,21287823	0,977856864	0,511875524	1,910341125	0,056089306	1	protein_codin	hypothetical protein
TcG_10463	5,62089516	0,492471384	0,74401898	0,661907017	0,508030815	1	protein_codin	hypothetical protein
TcG_10464	9,865675701	0,956595754	1,111399184	0,860713026	0,389396117	1	protein_codin	hypothetical protein
TcG_10465	21,61626975	0,356322089	0,37170646	0,958611505	0,337754495	0,572359745	protein_codin	hypothetical protein
TcG_10466	33,36242988	0,022709613	0,304322608	0,074623484	0,940514289	0,972407509	protein_codin	retrotransposon hot spot (RHS) protein
TcG_10467	896,274401	0,182698478	0,065170374	2,803397698	0,005056727	0,028978028	protein_codin	putative retrotransposon hot spot (RHS) protein
TcG_10468	124,1125314	0,012337756	0,15870096	0,077742161	0,938033156	0,971428881	protein_codin	retrotransposon hot spot (RHS) protein
TcG_10469	17,53902498	0,856626552	0,429208799	1,995827101	0,045952746	0,155766095	protein_codin	hypothetical protein
TcG_10470	11,12908106	0,081857239	0,537254071	0,15236225	0,87890123	1	protein_codin	hypothetical protein
TcG_10471	8,882433902	-0,041119305	0,615172787	-0,06684188	0,946707583	1	protein_codin	hypothetical protein
TcG_10472	20,46418277	-0,143196483	0,377909866	-0,37891703	0,704749479	0,847555325	protein_codin	trans-sialidase
TcG_10473	62,65082854	0,196543018	0,222758695	0,882313567	0,377607262	0,608308918	protein_codin	hypothetical protein
TcG_10474	90,9483343	0,383123851	0,18357335	2,087034157	0,036885047	0,133423365	protein_codin	hypothetical protein
TcG_10475	141,4989985	0,212176446	0,152762873	1,388926786	0,164855014	0,370228764	protein_codin	dispersed gene family protein 1 (DGF-1)
TcG_10476	45,16491679	-0,25203944	0,258135955	-0,97638254	0,32887493	0,563243894	protein_codin	dispersed gene family protein 1 (DGF-1)
TcG_10477	15,09031006	0,123014885	0,478308622	0,257187262	0,797034217	1	protein_codin	dispersed protein family protein 1 (DGF-1)
TcG_10478	22,78234069	0,424304136	0,382781514	1,108476036	0,267656283	0,499929984	protein_codin	hypothetical protein
TcG_10479	34,87469957	0,259605647	0,302842322	0,857230407	0,391317561	0,621127152		
TcG_10480	237,3121753	0,29205473	0,115520978	2,528153196	0,011466431	0,055055974	protein_codin	putative trans-sialidase
TcG_10481	82,66354416	0,597570692	0,19456151	3,071371577	0,002130778	0,01448779	protein_codin	hypothetical protein
TcG_10482	38,06052872	0,613303432	0,309732838	1,980104645	0,047691771	0,159883351		
TcG_10483	51,99071795	0,782266669	0,254763936	3,070554973	0,002136613	0,014518947	protein_codin	protein kinase, putative,serine/threonine protein kinase
TcG_10484	59,51129723	0,813022268	0,236679819	3,435114453	0,000592304	0,004998186	protein_codin	target of rapamycin (TOR) kinase 1
TcG_10485	56,4370686	0,615214333	0,249493416	2,465853979	0,013668704	0,063291281	protein_codin	hypothetical protein
TcG_10486	70,20791264	0,498885933	0,211262955	2,361445398	0,018203852	0,078028056	protein_codin	surface protease GP63
TcG_10487	125,6053125	0,137717848	0,156125745	0,8820957	0,377725057	0,608414085	protein_codin	hypothetical protein
TcG_10488	1640,681264	0,401941552	0,057256818	7,01997713	2,21905E-12	1,59689E-10	protein_codin	40S ribosomal protein S13

TcG_10489	273,1436209	0,011538657	0,111371475	0,103605137	0,917482711	0,960175645	protein_codin	hypothetical protein
TcG_10490	223,3679946	-0,016219693	0,121064861	-0,13397524	0,893422157	0,94860604	protein_codin	hypothetical protein
TcG_10491	300,9324687	-0,034055653	0,110807116	-0,30734175	0,758583261	0,878015178	protein_codin	hypothetical protein
TcG_10492	128,9948552	-0,155567394	0,164829333	-0,94380892	0,345267301	0,579295913	protein_codin	hypothetical protein
TcG_10493	76,26409823	0,37669555	0,211601128	1,780215225	0,075040745	0,221170712	protein_codin	dispersed gene family protein 1 (DGF-1)
TcG_10494	18,84447333	0,047102307	0,431093835	0,109262307	0,912994442	0,957280869	protein_codin	dispersed gene family protein 1 (DGF-1)
TcG_10495	14,86013124	-0,338101101	0,45112628	-0,74946	0,45358	1	protein_codin	dispersed gene family protein 1 (DGF-1)
TcG_10496	7,980619689	-1,131401449	0,620189224	-1,82428428	0,068109106	1	protein_codin	dispersed protein family protein 1
TcG_10497	18,77325441	0,184727769	0,403423161	0,457900754	0,647023745	0,812420258		
TcG_10498	4,887770061	0,54238588	0,836854674	0,648124336	0,516904535	1	protein_codin	hypothetical protein
TcG_10499	139,5909881	0,127124723	0,151821789	0,837328582	0,402407898	0,632690718	protein_codin	dispersed gene family protein 1 (DGF-1)
TcG_10500	16,48343474	0,101958508	0,425233995	0,239770359	0,810508287	1	protein_codin	dispersed gene family protein 1 (DGF-1)
TcG_10501	166,6059955	-0,056407409	0,137492638	-0,41025767	0,681616942	0,834183363		
TcG_10502	116,276293	0,228485375	0,163071235	1,401138441	0,161172678	0,364502567	protein_codin	retrotransposon hot spot (RHS) protein
TcG_10503	238,1185347	0,282903757	0,115583203	2,447619975	0,014380324	0,065801909	protein_codin	putative retrotransposon hot spot (RHS) protein
TcG_10504	17,55736116	0,963864599	0,439984136	2,190680344	0,028474932	0,109896925	protein_codin	hypothetical protein
TcG_10505	21,87854223	0,575110799	0,381272074	1,508400007	0,131452181	0,322260891	protein_codin	hypothetical protein
TcG_10506	122,0859854	0,375336842	0,170577777	2,200385352	0,027779566	0,107932859	protein_codin	surface protein-2
TcG_10507	89,99447877	0,725663281	0,194656873	3,727909887	0,000193074	0,001939856	protein_codin	hypothetical protein
TcG_10508	147,3282274	0,285254344	0,14858001	1,919870272	0,054874288	0,17679942	protein_codin	hypothetical protein
TcG_10509	67,26737797	-0,071363845	0,210571525	-0,33890549	0,734680931	0,864076059		
TcG_10510	25,99558968	0,42933735	0,352085928	1,219410705	0,222688349	0,448804202		
TcG_10511	2,843317745	-1,74348979	1,121258027	-1,55494074	0,119960183	1	protein_codin	surface protease GP63
TcG_10512	135,6926703	-0,419781966	0,152955479	-2,74447158	0,00606084	0,033280045	protein_codin	mucin-associated surface protein (MASP)
TcG_10513	18,44279771	-0,832015637	0,411389194	-2,02245379	0,043129493	0,149157111		
TcG_10514	27,96247869	-0,098239031	0,326736673	-0,30066729	0,763668213	0,880822291	protein_codin	mucin TcMUCII
TcG_10515	42,03126474	-0,218432664	0,265635559	-0,82230205	0,410905011	0,637576557		
TcG_10516	38,40152345	-0,46590708	0,28826809	-1,61622842	0,106044936	0,280446616		
TcG_10517	6,829935448	-0,715960773	0,749912185	-0,95472615	0,339716202	1	protein_codin	putative surface protease GP63
TcG_10518	11,72726744	0,035449679	0,513365308	0,069053515	0,944947023	1	protein_codin	putative surface protease GP63
TcG_10519	12,24230909	-0,555544881	0,521552732	-1,0651749	0,286796789	1		
TcG_10520	105,1498297	0,162709522	0,181384786	0,897040628	0,369697238	0,601251011	protein_codin	putative trans-sialidase
TcG_10521	74,34108114	0,4610442	0,203006844	2,271077125	0,023142309	0,094206411	protein_codin	hypothetical protein
TcG_10522	113,3819774	0,208464754	0,173874199	1,19894013	0,230551226	0,457783463		
TcG_10523	35,31261281	0,466920378	0,297219202	1,570963028	0,116191237	0,297106967	protein_codin	hypothetical protein
TcG_10524	12,84389306	0,33073306	0,483798055	0,683618002	0,494216414	1	protein_codin	retrotransposon hot spot (RHS) protein
TcG_10525	0,155988004	0,503022807	4,080472857	0,123275616	0,901888849	1	protein_codin	retrotransposon hot spot (RHS) protein
TcG_10526	153,5517869	0,347082167	0,150505548	2,306108791	0,021104554	0,087835603	protein_codin	putative retrotransposon hot spot (RHS) protein
TcG_10527	245,6905468	0,100103631	0,113068084	0,88533941	0,3759736	0,606538655	protein_codin	sister chromatid cohesion protein DCC1
TcG_10528	487,0876182	-0,2999077	0,087104141	-3,44309348	0,0005751	0,004877829	protein_codin	hypothetical protein
TcG_10529	54,59921069	0,148921571	0,239192415	0,622601563	0,533546384	0,734103975	protein_codin	hypothetical protein
TcG_10530	31,398907	-0,276152156	0,318545717	-0,8669153	0,38598842	0,616495979		
TcG_10531	53,99347831	-0,217951476	0,241148049	-0,90380775	0,36609735	0,598251607		
TcG_10532	104,3406764	-0,459294645	0,1784048	-2,57445229	0,010039893	0,049540973	protein_codin	trans-sialidase
TcG_10533	51,9837075	-0,196168939	0,245514929	-0,79901023	0,424284482	0,650097383	protein_codin	trans-sialidase
TcG_10534	50,74525442	-0,409139719	0,244602506	-1,67267182	0,09439192	0,258540137	protein_codin	trans-sialidase
TcG_10535	210,4920805	-0,406089212	0,124255713	-3,26817337	0,00108244	0,008321934		
TcG_10536	32,35926194	-0,771409478	0,315205276	-2,44732413	0,014392134	0,065829952	protein_codin	hypothetical protein
TcG_10537	41,53013321	-0,282708356	0,267342108	-1,05747785	0,290293561	0,523456792	protein_codin	trans-sialidase
TcG_10538	31,01884436	0,232604389	0,334382629	0,695623424	0,486664693	0,698438886	protein_codin	hypothetical protein
TcG_10539	11,14313029	-0,658035512	0,538813457	-1,22126778	0,22198465	1	protein_codin	hypothetical protein
TcG_10540	6,13588619	-1,431841283	0,718512318	-1,99278599	0,046284886	1	protein_codin	hypothetical protein
TcG_10541	36,15866912	-0,443979747	0,28661526	-1,54904434	0,121371061	0,305696764	protein_codin	hypothetical protein
TcG_10542	17,70574599	0,066880512	0,408410483	0,163758069	0,869921602	0,937311604	protein_codin	hypothetical protein
TcG_10543	73,38729561	-0,803014392	0,210594903	-3,81307611	0,000137248	0,001452858	protein_codin	mucin-associated surface protein (MASP)
TcG_10544	92,10153138	-0,282324777	0,180854262	-1,56106234	0,118509043	0,300645011		
TcG_10545	19,93650517	-0,22819122	0,383652839	-0,59478569	0,551986731	0,747373577	protein_codin	putative trans-sialidase

TcG_10546	2,6100924	-0,827813517	1,163375378	-0,71156183	0,476736148	1	protein_codin putative trans-sialidase
TcG_10547	14,67699838	0,030657734	0,482187816	0,063580482	0,949304273	1	protein_codin putative trans-sialidase
TcG_10548	37,16207315	-0,399857057	0,290742941	-1,37529412	0,169040279	0,375479423	protein_codin putative trans-sialidase
TcG_10549	170,549831	-0,007424504	0,138547185	-0,05358827	0,9572632	0,98019014	protein_codin RNA editing complex protein MP90
TcG_10550	164,9369151	-0,139721861	0,143692281	-0,9723686	0,330867209	0,565235831	protein_codin beta galactofuranosyl glycosyltransferase
TcG_10551	65,93493843	0,175394667	0,219499396	0,799066745	0,424251711	0,650097383	
TcG_10552	37,48330552	0,165039814	0,28084259	0,587659492	0,55676087	0,751383977	protein_codin hypothetical protein
TcG_10553	352,7068061	-0,361265131	0,095194754	-3,79501094	0,000147637	0,001552198	protein_codin sialidase-like protein
TcG_10554	28,86245882	-0,134121469	0,325197336	-0,41243102	0,680023531	0,833113316	protein_codin trans-sialidase
TcG_10555	6,439738533	-0,232992645	0,672467378	-0,34647427	0,728986316	1	protein_codin trans-sialidase
TcG_10556	14,78086431	0,382931611	0,489723998	0,781933524	0,434253642	1	protein_codin trans-sialidase
TcG_10557	19,23205789	-0,579388119	0,444017085	-1,30487799	0,191934399	0,408102761	protein_codin putative trans-sialidase
TcG_10558	15,84292318	0,135114193	0,46527853	0,290394213	0,77151467	1	protein_codin hypothetical protein
TcG_10559	755,5274297	0,329934565	0,069819057	4,725566041	2,29475E-06	4,22016E-05	protein_codin putative retrotransposon hot spot (RHS) protein
TcG_10560	6,718663877	-0,017307704	0,671523971	-0,02577377	0,979437784	1	protein_codin retrotransposon hot spot (RHS) protein
TcG_10561	11,69594419	-0,249988928	0,498717198	-0,5012639	0,616185407	1	protein_codin retrotransposon hot spot (RHS) protein
TcG_10562	9,310207085	0,163719601	0,622039013	0,263198285	0,792397753	1	protein_codin hypothetical protein
TcG_10563	10,18648617	0,748007234	0,59230797	1,262868765	0,206636347	1	protein_codin hypothetical protein
TcG_10564	23,97868759	-0,185328373	0,364747623	-0,50810029	0,611383012	0,789796547	protein_codin mucin-associated surface protein (MASP)
TcG_10565	2,699975142	-1,077701816	1,154875063	-0,93317611	0,350729049	1	protein_codin hypothetical protein
TcG_10566	1,002806097	2,083335566	1,93166526	1,078517903	0,280802697	1	protein_codin hypothetical protein
TcG_10567	10,54804298	0,571781134	0,545240259	1,048677393	0,294326622	1	protein_codin surface protease GP63
TcG_10568	5,360609887	0,464699193	0,776774512	0,598242071	0,549678423	1	protein_codin hypothetical protein
TcG_10569	10,9314355	0,324310802	0,536872086	0,604074621	0,545794032	1	
TcG_10570	9,123764019	0,204042706	0,576026131	0,354224739	0,723170465	1	
TcG_10571	1,153102055	-1,933583056	1,777108012	-1,08805038	0,276572865	1	protein_codin putative trans-sialidase
TcG_10572	1,526783483	-1,898148536	1,631003463	-1,16379185	0,244508376	1	protein_codin trans-sialidase
TcG_10573	8,772372954	-0,33415065	0,588407892	-0,56788948	0,570110018	1	protein_codin hypothetical protein
TcG_10574	5,11088109	-0,909623875	0,791531777	-1,14919439	0,250475835	1	protein_codin hypothetical protein
TcG_10575	2,412382129	-0,262511708	1,158516198	-0,22659304	0,820740204	1	protein_codin dispersed gene family protein 1 (DGF-1)
TcG_10576	3,466452539	0,132527965	1,028437856	0,128863367	0,897465764	1	protein_codin dispersed gene family protein 1 (DGF-1)
TcG_10577	68,77609545	0,015786347	0,236241076	0,06682304	0,946722581	0,974824726	protein_codin dispersed gene family protein 1 (DGF-1)
TcG_10578	25,11354748	-0,787407431	0,34867005	-2,2583168	0,023925916	0,096621004	protein_codin dispersed gene family protein 1 (DGF-1)
TcG_10579	53,69161424	-0,121790863	0,242215058	-0,50282119	0,615089997	0,792057258	protein_codin mucin-associated surface protein (MASP)
TcG_10580	5,360240848	0,688173403	0,787624359	0,873733011	0,382263669	1	protein_codin surface protease GP63
TcG_10581	6,502915406	0,302987483	0,706949539	0,428584314	0,668225763	1	protein_codin surface protease GP63
TcG_10582	7,750037968	0,773897869	0,629932595	1,228540759	0,219244039	1	protein_codin hypothetical protein
TcG_10583	42,42998603	-0,001094554	0,262989606	-0,00416197	0,99667924	0,998839568	protein_codin putative mucin-associated surface protein (MASP)
TcG_10584	37,36059068	-0,427069699	0,292082946	-1,46215212	0,143699528	0,34019263	protein_codin mucin TcMUCII
TcG_10585	12,23971744	0,158156503	0,493887562	0,32022775	0,748795689	1	protein_codin mucin TcMUCII
TcG_10586	13,50649013	-0,031584586	0,492852921	-0,06408522	0,948902373	1	protein_codin hypothetical protein
TcG_10587	11,76204485	-0,597786354	0,540502222	-1,10598316	0,268733809	1	
TcG_10588	47,08558291	0,16213883	0,254149412	0,637966573	0,52349544	0,72759335	protein_codin hypothetical protein
TcG_10589	101,8747472	0,213563087	0,177415849	1,203743005	0,228688925	0,455746126	protein_codin hypothetical protein
TcG_10590	166,9386727	-0,106335666	0,13838844	-0,76838547	0,442258209	0,664667154	protein_codin hypothetical protein
TcG_10591	829,2836214	0,129671345	0,072160995	1,796972792	0,072339939	0,216182287	protein_codin hypothetical protein
TcG_10592	110,0455477	0,118328404	0,356781496	0,331655105	0,740149705	0,867561457	protein_codin hypothetical protein
TcG_10593	19,0834516	0,384924971	0,438065825	0,87869208	0,379568254	0,610703762	protein_codin protein ARV1
TcG_10594	24,83712273	0,023479276	0,34269878	0,068512867	0,945377377	0,974691882	protein_codin hypothetical protein
TcG_10595	14,41674507	0,089260913	0,460852495	0,193686513	0,846421353	1	protein_codin putative mitotic centromere-associated kinesin (MCAK)
TcG_10596	40,44845539	-0,220689156	0,281602765	-0,78368959	0,433222272	0,657580669	protein_codin hypothetical protein
TcG_10597	15,26832365	-0,080731788	0,478866754	-0,16858925	0,866119735	1	protein_codin putative kinesin
TcG_10598	0,417901025	0,396008574	2,541866886	0,15579438	0,876195101	1	protein_codin putative kinesin
TcG_10599	21,02561864	0,401147263	0,379007585	1,058414869	0,289866343	0,523276948	protein_codin ATP-dependent DEAD/H RNA helicase
TcG_10600	61,69291914	0,663020461	0,227087315	2,919671937	0,003504	0,021651919	protein_codin trans-sialidase
TcG_10601	26,44435506	0,403872552	0,343393007	1,176123403	0,239545567	0,467943845	protein_codin hypothetical protein
TcG_10602	24,25830736	0,00019815	0,36166068	0,000547889	0,999562848	0,99981271	protein_codin hypothetical protein

TcG_10603	48,68516985	0,338571516	0,264440606	1,280331038	0,200428736	0,419618239	protein_codin retrotransposon hot spot (RHS) protein
TcG_10604	23,18168222	0,28204831	0,386088981	0,730526702	0,465068297	0,682138496	protein_codin putative retrotransposon hot spot (RHS) protein
TcG_10605	434,6275374	0,183045747	0,091583074	1,998685343	0,04564241	0,154925766	protein_codin putative retrotransposon hot spot (RHS) protein
TcG_10606	27,90422196	0,358944347	0,338010248	1,061933326	0,288265954	0,521281309	protein_codin mitotic centromere-associated kinesin (MCAK)
TcG_10607	19,74161996	0,556644511	0,392653412	1,417648475	0,15629342	0,357690793	protein_codin hypothetical protein
TcG_10608	67,1176014	0,029116689	0,221054399	0,131717303	0,895207899	0,948981304	protein_codin hypothetical protein
TcG_10609	26,14467452	0,211969092	0,348932962	0,607477983	0,543533749	0,740868473	
TcG_10610	125,3239528	0,00944141	0,167236277	0,056455514	0,954978934	0,978889315	protein_codin ATP-dependent DEAD/H RNA helicase
TcG_10611	1,732403416	2,007034156	1,482622479	1,353705468	0,175830363	1	protein_codin dispersed gene family protein 1 (DGF-1)
TcG_10612	12,31460478	0,777110014	0,515804004	1,50659942	0,131913366	1	protein_codin dispersed gene family protein 1 (DGF-1)
TcG_10613	87,86761352	0,01648955	0,188191101	0,087621305	0,930177668	0,966240456	protein_codin dispersed gene family protein 1 (DGF-1)
TcG_10614	5,690788123	0,8458761	0,774648192	1,091948717	0,274855643	1	
TcG_10615	20,09265714	0,001020864	0,400253334	0,002550545	0,997964962	0,999325424	protein_codin hypothetical protein
TcG_10616	10,55430538	-0,853623836	0,587697271	-1,45248903	0,146365679	1	protein_codin trans-sialidase
TcG_10617	8,126478805	0,358614383	0,609700097	0,588181607	0,556410402	1	protein_codin Sialidase 85-1.3
TcG_10618	12,69563052	0,77441363	0,50430681	1,535600183	0,124636469	1	protein_codin hypothetical protein
TcG_10619	34,01973554	-0,227265538	0,297200237	-0,76468828	0,444457187	0,666426941	protein_codin mucin-associated surface protein (MASP)
TcG_10620	9,202135437	0,167540288	0,581098438	0,288316535	0,773104451	1	protein_codin hypothetical protein
TcG_10621	15,9264138	0,11083188	0,443727585	0,2497746	0,802761664	1	protein_codin trans-sialidase
TcG_10622	6,766455665	-0,314859755	0,675701733	-0,46597447	0,641233777	1	protein_codin trans-sialidase
TcG_10623	25,85338693	0,312543677	0,347836217	0,898536903	0,368899381	0,600861456	protein_codin putative trans-sialidase
TcG_10624	325,6852002	0,071954753	0,100610511	0,715181271	0,47449704	0,690210007	protein_codin retrotransposon hot spot (RHS) protein
TcG_10625	79,63845917	0,637475823	0,197083932	3,234539808	0,001218389	0,009160454	protein_codin retrotransposon hot spot (RHS) protein
TcG_10626	0					1	protein_codin retrotransposon hot spot (RHS) protein
TcG_10627	8,749303858	-0,328390374	0,580309997	-0,56588785	0,571470024	1	protein_codin hypothetical protein
TcG_10628	10,75044706	0,201187056	0,530100514	0,379526243	0,70429712	1	
TcG_10629	17,5350447	0,745230365	0,418741663	1,779690038	0,075126702	0,221255202	protein_codin trans-sialidase
TcG_10630	8,911181784	-0,032369122	0,584080034	-0,05541898	0,955804672	1	protein_codin trans-sialidase
TcG_10631	7,124747439	0,481815787	0,729162672	0,660779557	0,508753695	1	protein_codin surface glycoprotein Tc-85/20
TcG_10632	71,27901127	0,043294747	0,213534788	0,202752661	0,83932836	0,921926912	protein_codin retrotransposon hot spot (RHS) protein
TcG_10633	93,75870796	0,139942444	0,185197967	0,755637044	0,44986684	0,670632682	protein_codin retrotransposon hot spot (RHS) protein
TcG_10634	0					1	protein_codin dispersed gene family protein 1 (DGF-1)
TcG_10635	36,97780978	0,303657442	0,289009854	1,050681965	0,293404682	0,526465332	protein_codin dispersed gene family protein 1 (DGF-1)
TcG_10636	284,4189413	0,318732305	0,105986807	3,007282835	0,002635944	0,017118177	protein_codin dispersed gene family protein 1 (DGF-1)
TcG_10637	23,79764371	-0,760817009	0,367771787	-2,06872043	0,038572329	0,137676835	protein_codin putative mucin TcMUCII
TcG_10638	78,06829497	-0,124849435	0,19975783	-0,62500396	0,53196846	0,732933197	protein_codin mucin-associated surface protein (MASP)
TcG_10639	3,916396115	-0,363102416	0,8699856	-0,41736601	0,676410715	1	protein_codin surface protease GP63
TcG_10640	3,862327759	0,285371602	0,940886669	0,303300718	0,761660699	1	protein_codin surface protease GP63
TcG_10641	4,955543098	-0,048839355	0,797742585	-0,06122195	0,95118245	1	protein_codin hypothetical protein
TcG_10642	3,27272296	-1,549243739	1,03790683	-1,49266167	0,135525772	1	protein_codin putative syntaxin binding protein
TcG_10643	6,337440835	0,254043086	0,699469401	0,363193995	0,716459973	1	protein_codin putative syntaxin binding protein
TcG_10644	34,78904955	0,027821362	0,294724128	0,09439798	0,924793021	0,963208309	protein_codin hypothetical protein
TcG_10645	16,37191622	-0,685717113	0,444785314	-1,5416811	0,123151106	1	protein_codin mucin TcMUCII
TcG_10646	137,5742466	-0,111619164	0,153151984	-0,72881304	0,466116032	0,682733292	
TcG_10647	270,5477141	-0,142966379	0,109059498	-1,31090259	0,189890675	0,404960997	protein_codin mucin-associated surface protein (MASP)
TcG_10648	186,8043024	0,231898168	0,138728595	1,671596021	0,09460401	0,258998596	protein_codin adenylate cyclase
TcG_10649	107,3672891	0,031475943	0,167610059	0,187792684	0,851039171	0,928053891	protein_codin receptor-type adenylate cyclase
TcG_10650	166,29919	0,095909944	0,160374378	0,598037826	0,549814694	0,745961428	protein_codin trans-sialidase
TcG_10651	91,14395994	-0,140269659	0,192103209	-0,73017864	0,465280999	0,682138496	protein_codin hypothetical protein
TcG_10652	569,8475759	-0,399199102	0,082692459	-4,82751516	1,38247E-06	2,69652E-05	protein_codin putative proteasome regulatory ATPase subunit 2
TcG_10653	336,5246866	0,008860426	0,099606203	0,088954555	0,929118026	0,96557269	protein_codin putative NADH-cytochrome b5 reductase
TcG_10654	1,067721804	-0,098571876	1,871128478	-0,05268044	0,957986522	1	
TcG_10655	262,6834862	0,114137939	0,113307561	1,007328538	0,31377692	0,548080717	protein_codin regulator of sigma E protease
TcG_10656	92,05526018	-0,287517054	0,181085105	-1,58774547	0,112343907	0,29018174	protein_codin mucin-like glycoprotein
TcG_10657	143,8168535	-0,425346286	0,148807255	-2,85837062	0,004258227	0,025396702	protein_codin mucin-associated surface protein (MASP)
TcG_10658	34,93287132	0,268507643	0,303428521	0,884912341	0,376203912	0,606723068	protein_codin trans-sialidase
TcG_10659	364,1153486	0,147906143	0,109349454	1,352600657	0,176183242	0,386017218	protein_codin retrotransposon hot spot protein (RHS)

TcG_10660	6,871990233	-0,276270418	0,683969232	-0,40392229	0,686269865	1	protein_codin retrotransposon hot spot (RHS) protein
TcG_10661	14,20760167	0,148604487	0,463363707	0,320708084	0,74843162	1	protein_codin retrotransposon hot spot (RHS) protein
TcG_10662	8,236205134	0,42234617	0,676344675	0,624454048	0,532329439	1	protein_codin hypothetical protein
TcG_10663	21,18295192	0,425448875	0,377530051	1,126927179	0,259773271	0,492108097	
TcG_10664	18,03451761	-0,503424392	0,404724312	-1,24386991	0,213547447	0,436980474	protein_codin putative ABC transporter
TcG_10665	325,2074496	-0,154926648	0,099994105	-1,54935781	0,121295729	0,305573455	protein_codin hypothetical protein
TcG_10666	145,8214385	0,052101038	0,148089391	0,351821541	0,724972103	0,858172586	protein_codin hypothetical protein
TcG_10667	135,5094563	0,433984142	0,162601971	2,668996806	0,007607817	0,039866202	protein_codin hypothetical protein
TcG_10668	198,8696427	0,041470624	0,126713124	0,32727963	0,743456398	0,86954228	protein_codin D-alanyl-glycyl endopeptidase-like protein
TcG_10669	62,1798771	-0,560673741	0,223581769	-2,50768989	0,012152327	0,05763277	protein_codin D-alanyl-glycyl endopeptidase-like protein
TcG_10670	274,0574946	-0,236422834	0,112654632	-2,0986517	0,035847615	0,1308952	protein_codin D-alanyl-glycyl endopeptidase-like protein
TcG_10671	73,96240435	-0,154209719	0,204127584	-0,75545752	0,449974512	0,670706894	protein_codin dispersed gene family protein 1 (DGF-1)
TcG_10672	85,48742663	-0,058226278	0,218045239	-0,2670376	0,789440208	0,895015489	protein_codin dispersed gene family protein 1 (DGF-1)
TcG_10673	134,9548291	-0,330012566	0,155840449	-2,117631	0,034206331	0,126255033	protein_codin dispersed gene family protein 1 (DGF-1)
TcG_10674	119,4689847	-0,392993951	0,161583455	-2,43214227	0,01500981	0,067745876	protein_codin dispersed gene family protein 1 (DGF-1)
TcG_10675	116,611109	-0,648233694	0,163015497	-3,97651578	6,99324E-05	0,000825888	protein_codin hypothetical protein
TcG_10676	1094,318315	-0,051762251	0,069847747	-0,74107259	0,458649427	0,677438813	protein_codin surface protein-2
TcG_10677	178,523991	0,029765488	0,135454186	0,219745794	0,826069137	0,915112889	protein_codin putative 50S ribosomal protein L17
TcG_10678	70,7245194	-0,165629932	0,217230315	-0,76246233	0,445784118	0,667552641	protein_codin hypothetical protein
TcG_10679	138,3076725	0,099802588	0,14898481	0,669884321	0,502931536	0,711484963	protein_codin hypothetical protein
TcG_10680	469,4898115	-0,182180815	0,085610624	-2,12801645	0,03333572	0,123671998	protein_codin putative eukaryotic translation initiation factor 1A
TcG_10681	175,9906012	-0,23941644	0,135046506	-1,77284438	0,076254497	0,223384224	protein_codin anti-silencing protein a-like protein
TcG_10682	123,8108467	0,316131159	0,160296669	1,972162993	0,048591003	0,162053931	protein_codin trafficking protein particle complex 2
TcG_10683	276,7600918	-0,284768699	0,107819414	-2,64116347	0,008262184	0,042506957	protein_codin putative 6-phosphogluconolactonase
TcG_10684	27,30324585	0,094891377	0,349524002	0,27148744	0,786016158	0,893522685	protein_codin hypothetical protein
TcG_10685	310,8576422	0,107907048	0,102676377	1,050943281	0,29328464	0,52633145	protein_codin subtilisin-like serine peptidase
TcG_10686	23,81394493	-0,072749698	0,365643706	-0,19896335	0,842291411	0,923105402	
TcG_10687	135,3389386	-0,068880513	0,159325628	-0,43232538	0,66550494	0,825042607	protein_codin hypothetical protein
TcG_10688	82,59139519	-0,063477844	0,197373071	-0,3216135	0,747745515	0,872708728	protein_codin retrotransposon hot spot (RHS) protein
TcG_10689	14,51892985	-0,076501724	0,468444775	-0,16331002	0,870274347	1	
TcG_10690	730,42944	0,18605139	0,070120312	2,653316638	0,007970506	0,041392329	protein_codin putative retrotransposon hot spot (RHS) protein
TcG_10691	193,5436163	-0,035382364	0,131372909	-0,26932771	0,787677513	0,894530881	protein_codin sialidase-like protein
TcG_10692	37,66758274	-0,342413427	0,29136007	-1,17522427	0,239905001	0,468488007	protein_codin trans-sialidase
TcG_10693	9,993975879	-0,574132293	0,556219617	-1,03220432	0,30197641	1	protein_codin hypothetical protein
TcG_10694	57,31538348	0,106665744	0,229575935	0,464620755	0,642203067	0,810066503	protein_codin hypothetical protein
TcG_10695	20,22558448	-1,368084548	0,421674177	-3,2444115	0,001176936	0,008918233	protein_codin hypothetical protein
TcG_10696	9,610648232	-0,421220996	0,5751326	-0,73238936	0,463930952	1	protein_codin putative trans-sialidase
TcG_10697	32,15411796	-0,591731803	0,303717264	-1,94829821	0,051379291	0,168684291	protein_codin target of rapamycin (TOR) kinase 1
TcG_10698	43,40592354	-0,366761959	0,265798836	-1,37984787	0,167633489	0,373644017	protein_codin putative profilin
TcG_10699	190,811656	-0,022094172	0,129231619	-0,17096568	0,86425075	0,934985064	protein_codin mucin-associated surface protein (MASP)
TcG_10700	0					1	
TcG_10701	6,887632761	0,607291641	0,668427699	0,908537516	0,363594302	1	protein_codin hypothetical protein
TcG_10702	2,029660636	0,417092374	1,260205044	0,330971833	0,740665762	1	protein_codin structural maintenance of chromosome protein 4
TcG_10703	32,57702166	0,259377048	0,315152659	0,823020338	0,410496428	0,637295575	protein_codin putative trans-sialidase
TcG_10704	7,683655443	-0,194837682	0,619851546	-0,31432959	0,753270727	1	protein_codin hypothetical protein
TcG_10705	3,408530509	-1,095952621	0,935396983	-1,17164438	0,241339861	1	protein_codin hypothetical protein
TcG_10706	0					1	protein_codin hypothetical protein
TcG_10707	2,901723286	1,24078653	1,068228149	1,16153701	0,245423581	1	protein_codin hypothetical protein
TcG_10708	23,62666379	0,758432355	0,367884752	2,061603126	0,039245536	0,139472543	protein_codin putative profilin
TcG_10709	12,31680315	0,367766511	0,490646961	0,749554243	0,453523218	1	protein_codin hypothetical protein
TcG_10710	48,28599888	1,187249485	0,264757019	4,484298426	7,31542E-06	0,000116584	protein_codin hypothetical protein
TcG_10711	153,1540343	0,010679498	0,148057725	0,072130637	0,942497945	0,973191135	protein_codin surface protease GP63
TcG_10712	221,5015395	0,129910621	0,119768367	1,084682245	0,278062414	0,510721486	protein_codin surface protease GP63
TcG_10713	0,701286704	2,70705338	2,054732337	1,317472516	0,187680288	1	protein_codin putative trans-sialidase
TcG_10714	41,22955388	-0,036023575	0,287129887	-0,1254609	0,900158676	0,951486034	protein_codin hypothetical protein
TcG_10715	31,73734274	0,531579043	0,316668494	1,678660976	0,093218135	0,256051521	protein_codin putative trans-sialidase
TcG_10716	18,45512942	-0,096267857	0,450499982	-0,21369114	0,830787917	0,917601778	protein_codin target of rapamycin (TOR) kinase 1

TcG_10717	10,15642317	0,851182727	0,596784992	1,426280383	0,153787425	1	protein_codin hypothetical protein
TcG_10718	66,00110673	-0,159526681	0,214259869	-0,74454764	0,456545228	0,676399932	protein_codin putative protein tyrosine phosphatase-like protein
TcG_10719	402,3608909	-0,047880619	0,092724435	-0,51637543	0,605592226	0,786889311	protein_codin Alpha/beta hydrolase fold protein
TcG_10720	40,11054366	-0,106835672	0,27274551	-0,3917046	0,695276489	0,842340007	protein_codin hypothetical protein
TcG_10721	83,46304467	0,00874646	0,192282607	0,045487525	0,963718718	0,983670608	protein_codin hypothetical protein
TcG_10722	77,27009422	0,010738148	0,204904704	0,052405573	0,958205534	0,98064539	protein_codin hypothetical protein
TcG_10723	17,76887845	0,335194353	0,435111128	0,77036493	0,44108345	0,663546065	protein_codin hypothetical protein
TcG_10724	34,19144192	0,613907032	0,298393955	2,057370872	0,039650565	0,140357911	
TcG_10725	15,8346289	-0,092199067	0,433153899	-0,21285522	0,831439893	1	protein_codin hypothetical protein
TcG_10726	61,93451037	0,526800468	0,227804103	2,312515273	0,020749305	0,086599944	protein_codin hypothetical protein
TcG_10727	51,57369092	0,175950417	0,246530761	0,713705732	0,475409161	0,691032669	protein_codin dispersed gene family protein 1 (DGF-1)
TcG_10728	24,43577683	0,374083148	0,360333154	1,038159112	0,299195985	0,532813508	protein_codin dispersed gene family protein 1 (DGF-1)
TcG_10729	1,005188382	0,099414818	1,667829206	0,059607313	0,952468393	1	protein_codin hypothetical protein
TcG_10730	2,713524462	-1,586854187	1,117493224	-1,42001236	0,155604084	1	protein_codin hypothetical protein
TcG_10731	2,305431873	-2,03158436	1,369645894	-1,48329168	0,137996932	1	protein_codin hypothetical protein
TcG_10732	2,757803563	1,549938951	1,179373674	1,314205146	0,188777177	1	
TcG_10733	12,69428042	-1,941707438	0,540582502	-3,59187993	0,000328301	1	protein_codin mucin TcMUCII
TcG_10734	5,95333191	0,345998623	0,706358345	0,48983441	0,624251079	1	
TcG_10735	172,2935683	-0,200200344	0,138168067	-1,44896247	0,147348063	0,3460723	protein_codin mucin-associated surface protein (MASP)
TcG_10736	433,5350442	-0,101288644	0,093219823	-1,08655692	0,277232673	0,50960142	protein_codin AAA family ATPase-like protein
TcG_10737	427,7188314	0,031429738	0,088822551	0,353848633	0,723452324	0,857397569	protein_codin RING finger protein
TcG_10738	235,329586	0,072243498	0,118180263	0,611299176	0,541001536	0,739766765	protein_codin hypothetical protein
TcG_10739	208,171289	0,109958156	0,134349452	0,818448862	0,413100931	0,639864624	protein_codin hypothetical protein
TcG_10740	52,64941658	0,071703991	0,244208146	0,293618342	0,769049559	0,884364283	protein_codin hypothetical protein
TcG_10741	0					1	protein_codin mitochondrial heat shock
TcG_10742	376,5593081	-0,255260766	0,102039756	-2,5015815	0,012363998	0,058421405	protein_codin Flagellar attachment zone protein 1
TcG_10743	147,5965486	0,21525767	0,151960479	1,416537191	0,156618283	0,358187807	protein_codin hypothetical protein
TcG_10744	1133,730478	0,191282818	0,059589879	3,20998838	0,001327403	0,009833309	protein_codin L1Tc protein
TcG_10745	629,7669968	0,538030477	0,076860226	7,000115731	2,55751E-12	1,80679E-10	protein_codin coiled-coil domain containing 39
TcG_10746	1159,00896	0,496418207	0,060284746	8,234557504	1,80223E-16	2,15264E-14	protein_codin putative ribonuclease inhibitor
TcG_10747	63,94362312	0,533560696	0,22303042	2,392322521	0,016742123	0,073462439	protein_codin hypothetical protein
TcG_10748	0					1	
TcG_10749	5,907975894	-1,557215467	0,755493201	-2,06119058	0,039284862	1	protein_codin trans-sialidase
TcG_10750	12,4462596	-0,433822522	0,500652761	-0,86651379	0,386208467	1	protein_codin trans-sialidase
TcG_10751	8,405504964	-0,138632646	0,593190821	-0,23370666	0,815212716	1	
TcG_10752	5,192207249	-1,388486485	0,830034383	-1,67280599	0,094365496	1	protein_codin dispersed gene family protein 1 (DGF-1)
TcG_10753	20,73386341	0,157714939	0,377698647	0,417568186	0,676262861	0,830261895	protein_codin dispersed gene family protein 1 (DGF-1)
TcG_10754	34,55891058	-0,596909488	0,334047674	-1,7868991	0,073953798	0,219474566	protein_codin mucin TcMUCII
TcG_10755	3,643071939	-0,774209739	0,930387237	-0,8321371	0,405331562	1	protein_codin trans-sialidase
TcG_10756	3,166334251	-0,949811846	1,044950128	-0,90895424	0,363374283	1	protein_codin hypothetical protein
TcG_10757	9,570722025	-0,309709465	0,597304269	-0,51851206	0,60410105	1	protein_codin telomerase reverse transcriptase
TcG_10758	11,91917584	0,40036479	0,517450595	0,773725635	0,439093057	1	protein_codin hypothetical protein
TcG_10759	0,938070566	0,848909746	1,825276375	0,465085593	0,641870163	1	protein_codin putative surface protease GP63
TcG_10760	31,872519	-0,163471578	0,325146907	-0,50276221	0,615131465	0,792057258	protein_codin mucin-associated surface protein (MASP)
TcG_10761	54,28733416	0,287458046	0,236228384	1,216864974	0,223655594	0,450179159	protein_codin mucin-associated surface protein (MASP)
TcG_10762	69,1454665	-0,108161702	0,211504115	-0,5113929	0,609075968	0,788218003	protein_codin mucin TcMUCII
TcG_10763	81,08854453	-0,117841196	0,192285295	-0,61284559	0,539978441	0,738978292	protein_codin putative syntaxin binding protein
TcG_10764	13,90432821	0,403019516	0,465776904	0,865262988	0,386894461	1	protein_codin syntaxin binding protein
TcG_10765	9,967093944	0,936513834	0,575286626	1,627908232	0,103544346	1	protein_codin hypothetical protein
TcG_10766	33,97969747	0,044158466	0,29709914	0,148632088	0,881843952	0,942184067	protein_codin surface protease GP63
TcG_10767	25,38926689	0,734916258	0,380922428	1,929306874	0,053692777	0,173863754	protein_codin surface protease GP63
TcG_10768	32,50285063	0,033425094	0,314003864	0,106448034	0,915226884	0,958753949	protein_codin regulator of sigma E protease
TcG_10769	121,8806676	-0,571508603	0,168819484	-3,38532373	0,000710944	0,005829435	protein_codin cytochrome b5
TcG_10770	477,5557004	-0,413815367	0,08541675	-4,844663	1,26827E-06	2,50326E-05	protein_codin succinate dehydrogenase flavoprotein subunit
TcG_10771	181,262384	-0,229406539	0,135956207	-1,68735613	0,091534894	0,252686034	protein_codin putative ADP-ribosylation factor-like protein 3A
TcG_10772	21,1961487	-0,003503581	0,37275769	-0,00939908	0,992500727	0,997050572	
TcG_10773	65,10934798	0,081261133	0,224257336	0,362356632	0,717085543	0,854924172	protein_codin RNA editing complex protein MP90

TcG_10774	67,45276337	0,129987962	0,217230941	0,59838604	0,549582378	0,745866398	protein_codin target of rapamycin (TOR) kinase 1
TcG_10775	266,3580324	0,067709127	0,111312541	0,608279409	0,543002176	0,74066673	protein_codin beta galactofuranosyl glycosyltransferase
TcG_10776	76,62358276	0,000937611	0,209252152	0,004480773	0,996424872	0,998838776	protein_codin hypothetical protein
TcG_10777	90,2993382	-0,002893372	0,185956852	-0,01555937	0,987585917	0,995144411	protein_codin putative trans-sialidase
TcG_10778	39,66903088	-0,522406519	0,275438305	-1,89663714	0,057875845	0,183611595	protein_codin trans-sialidase
TcG_10779	9,191390642	-0,251410916	0,596993846	-0,42112815	0,673661505	1	protein_codin hypothetical protein
TcG_10780	26,18943153	0,006181086	0,339947106	0,018182493	0,985493269	0,99432608	protein_codin hypothetical protein
TcG_10781	4,492511709	0,163387439	0,840535836	0,194384857	0,845874546	1	protein_codin retrotransposon hot spot (RHS) protein
TcG_10782	623,5850948	0,052851329	0,078578437	0,672593278	0,501206079	0,710072589	protein_codin putative retrotransposon hot spot (RHS) protein
TcG_10783	75,34999419	-0,410139637	0,19891772	-2,06185571	0,039221475	0,139435414	protein_codin c71 surface protein
TcG_10784	39,81468771	0,068406008	0,282075476	0,242509591	0,808385326	0,905185309	protein_codin hypothetical protein
TcG_10785	28,73169316	0,086842863	0,323722428	0,268263352	0,788496614	0,894874711	protein_codin hypothetical protein
TcG_10786	8,255683828	0,176133946	0,624705285	0,281947265	0,777983947	1	protein_codin hypothetical protein
TcG_10787	24,36947318	0,268944979	0,352512739	0,762936906	0,445501025	0,667214952	protein_codin hypothetical protein
TcG_10788	19,29818196	-0,059547072	0,42990456	-0,13851231	0,889835544	0,946535728	
TcG_10789	204,3518697	0,554978766	0,127429929	4,355168134	1,32965E-05	0,000194511	protein_codin putative short chain dehydrogenase
TcG_10790	10,97824202	-0,177345135	0,552976627	-0,32071	0,748430167	1	
TcG_10791	101,3452284	0,644732476	0,17723327	3,637762115	0,000275017	0,00259898	protein_codin hypothetical protein
TcG_10792	172,6367199	0,310401532	0,135124404	2,297153762	0,02161	0,089387168	protein_codin hypothetical protein
TcG_10793	78,42685971	0,256965171	0,209653088	1,225668429	0,220323475	0,445413229	
TcG_10794	126,6366982	-0,051872119	0,15826713	-0,32775042	0,743100376	0,869301389	protein_codin putative UDP-Gal or UDP-GlcNAc-dependent glycosyltransferase
TcG_10795	30,68033335	0,394216516	0,360890929	1,092342546	0,274682566	0,507556451	protein_codin hypothetical protein
TcG_10796	14,08576528	0,305085107	1,094655149	0,278704309	0,780471756	1	protein_codin putative mucin TcMUCII
TcG_10797	10,44843901	0,008664318	0,551557693	0,015708815	0,987466694	1	protein_codin hypothetical protein
TcG_10798	7,64457284	0,438781583	0,629834337	0,696661896	0,48601441	1	protein_codin hypothetical protein
TcG_10799	1,188139541	-0,820597086	1,597694497	-0,51361326	0,607522404	1	protein_codin hypothetical protein
TcG_10800	2,864763405	0,334126016	1,023028424	0,326604822	0,743966796	1	protein_codin hypothetical protein
TcG_10801	13,8457286	0,128433761	0,483284104	0,265752091	0,790430142	1	protein_codin hypothetical protein
TcG_10802	33,23409443	-0,497228663	0,299232846	-1,66167809	0,096577336	0,262848254	protein_codin hypothetical protein
TcG_10803	633,2102346	0,153898026	0,074154969	2,07535689	0,03795347	0,136145608	protein_codin hypothetical protein
TcG_10804	393,3986319	-0,057600994	0,094636243	-0,60865681	0,54275194	0,740499762	protein_codin hypothetical protein
TcG_10805	48,62663251	0,189574972	0,266441503	0,711506916	0,476770167	0,691952794	protein_codin hypothetical protein
TcG_10806	503,810166	-0,14688327	0,087137505	-1,68564926	0,091863376	0,253351363	protein_codin profilin
TcG_10807	347,9053247	-0,125412593	0,102006756	-1,22945379	0,218901712	0,443701056	protein_codin miltefosine transporter beta subunit
TcG_10808	202,5877152	-0,187806985	0,127174805	-1,47676252	0,139739306	0,334291079	protein_codin ras-related rab-4
TcG_10809	18,2741627	0,152604178	0,424442214	0,359540529	0,719190764	0,855882956	
TcG_10810	3,534231857	-0,025422789	0,914926453	-0,0277867	0,977832272	1	protein_codin surface protease GP63
TcG_10811	21,96485738	0,064467496	0,377690895	0,17068851	0,864468698	0,934985064	protein_codin hypothetical protein
TcG_10812	18,33654371	-0,570304896	0,406636452	-1,40249329	0,160767993	0,363729343	
TcG_10813	244,8033234	-0,104802672	0,118357917	-0,88547243	0,375901884	0,606538655	protein_codin dispersed gene family protein 1 (DGF-1)
TcG_10814	235,7587287	-0,072768617	0,119237404	-0,61028347	0,541674042	0,740087861	protein_codin hypothetical protein
TcG_10815	200,1920671	-0,158645636	0,126219858	-1,25689918	0,208790144	0,431432604	protein_codin solute carrier family 50 (sugar transporter)
TcG_10816	56,63901413	0,354108144	0,232079181	1,525807455	0,127057824	0,315290628	protein_codin glyceraldehyde 3-phosphate dehydrogenase
TcG_10817	183,7828612	0,207429673	0,146324814	1,417597382	0,156308345	0,357690793	protein_codin putative dispersed gene family protein 1 (DGF-1)
TcG_10818	6,927507707	0,68362204	0,685556191	0,997178712	0,318677772	1	protein_codin hypothetical protein
TcG_10819	6,222094379	-0,845986813	0,69761227	-1,21268912	0,225248699	1	protein_codin hypothetical protein
TcG_10820	9,114451066	-0,252091692	0,575262156	-0,43822054	0,661226421	1	protein_codin hypothetical protein
TcG_10821	78,12150925	-0,42125681	0,214703005	-1,96204431	0,049757325	0,164993809	protein_codin putative 90 kDa surface protein
TcG_10822	34,54475362	-0,102521022	0,303872455	-0,33738175	0,735829137	0,864944729	protein_codin putative mucin TcMUCII
TcG_10823	9,653797826	0,004820148	0,582743687	0,008271472	0,993400395	1	protein_codin hypothetical protein
TcG_10824	6,539349334	-0,160104515	0,736740239	-0,21731474	0,827963069	1	protein_codin hypothetical protein
TcG_10825	37,00402438	0,35593177	0,295105516	1,206116966	0,227772394	0,454603093	protein_codin hypothetical protein
TcG_10826	2,685606415	0,388638495	1,078178052	0,360458548	0,71850425	1	protein_codin trans-sialidase
TcG_10827	4,277635939	0,074755693	0,824654526	0,090650922	0,927769969	1	protein_codin trans-sialidase
TcG_10828	3,980585542	-0,101560846	0,853952235	-0,11893036	0,905330529	1	protein_codin hypothetical protein
TcG_10829	48,48237329	0,383528737	0,255411477	1,501611211	0,133197546	0,324411766	protein_codin hypothetical protein
TcG_10830	41,34599235	0,229501017	0,273119037	0,840296669	0,400742069	0,631067681	protein_codin putative trans-sialidase

TcG_10831	13,81008975	0,167128233	0,461348795	0,36226004	0,717157717	1	protein_codin hypothetical protein
TcG_10832	57,17472415	-0,277965556	0,232003267	-1,19811052	0,230873993	0,458312605	protein_codin putative mucin TcMUCII
TcG_10833	4,270978222	-1,102864511	0,884595598	-1,24674429	0,212491273	1	protein_codin mucin-associated surface protein (MASP)
TcG_10834	5,040204184	-0,79899811	0,776962856	-1,02836076	0,303780161	1	protein_codin hypothetical protein
TcG_10835	3,81428332	-1,360265994	0,944066817	-1,44085775	0,149624873	1	protein_codin hypothetical protein
TcG_10836	0,626347624	-0,249464434	2,223093324	-0,11221501	0,91065293	1	protein_codin hypothetical protein
TcG_10837	6,822906241	-0,33815665	0,655773117	-0,51566104	0,606091168	1	protein_codin mucin TcMUCII
TcG_10838	2,195392299	3,290417954	1,508731303	2,180917136	0,029189546	1	
TcG_10839	493,3581578	0,158610979	0,089174524	1,778657993	0,07529585	0,221549936	protein_codin putative dihydroorotase
TcG_10840	813,3845683	0,110412129	0,067389762	1,638411023	0,101335981	0,271400526	protein_codin orotidine-5-phosphate decarboxylase/orotate phosphoribosyltransferase
TcG_10841	73,0723486	0,303446864	0,218242703	1,390410126	0,164404373	0,369718375	
TcG_10842	256,5809881	0,110953604	0,122092461	0,908767042	0,363473107	0,595447368	protein_codin putative trans-sialidase
TcG_10843	14,46329785	0,952700314	0,48562296	1,96181069	0,049784529	1	protein_codin hypothetical protein
TcG_10844	18,35867555	-0,085609703	0,443146585	-0,19318597	0,846813327	0,925932352	protein_codin hypothetical protein
TcG_10845	36,65384524	0,379588571	0,295643435	1,283940469	0,199162786	0,418025369	
TcG_10846	142,2237407	-0,296826322	0,147777895	-2,00859758	0,044579828	0,152540426	protein_codin putative mucin-associated surface protein (MASP)
TcG_10847	124,5479294	-0,12870345	0,158092235	-0,81410355	0,41558563	0,64239863	
TcG_10848	10,84394415	-0,04833918	0,520634985	-0,09284658	0,926025442	1	protein_codin hypothetical protein
TcG_10849	11,47418117	-0,275313731	0,512684377	-0,53700433	0,591264622	1	protein_codin hypothetical protein
TcG_10850	5,900242614	0,103405634	0,721630149	0,143294503	0,886057597	1	protein_codin hypothetical protein
TcG_10851	9,367789545	0,183745015	0,570097473	0,32230456	0,747221979	1	protein_codin hypothetical protein
TcG_10852	25,79357415	-0,265631128	0,347142098	-0,76519422	0,4441559	0,666406404	protein_codin hypothetical protein
TcG_10853	24,63261994	-0,725571413	0,351650021	-2,06333391	0,039080914	0,139124749	
TcG_10854	28,70340044	-0,255289199	0,326335775	-0,78228996	0,434044187	0,658558262	protein_codin hypothetical protein
TcG_10855	52,41538408	-0,179501924	0,251932178	-0,71250098	0,476154604	0,691579195	protein_codin hypothetical protein
TcG_10856	26,67077753	0,265381301	0,345452205	0,768214233	0,442359919	0,664667154	
TcG_10857	19,65888076	-0,444823697	0,396557182	-1,12171389	0,26198411	0,494282475	protein_codin hypothetical protein
TcG_10858	11,09106491	-0,568387825	0,522154766	-1,08854283	0,276355541	1	protein_codin N-acetyltransferase complex ARD1 subunit
TcG_10859	42,40903583	0,558413978	0,274651737	2,033171116	0,042035238	0,146245706	protein_codin trans-sialidase
TcG_10860	41,78793634	-0,052582731	0,270194012	-0,19461102	0,845697478	0,925035391	protein_codin putative myosin heavy chain
TcG_10861	43,68808	0,081313233	0,262775772	0,309439613	0,756987141	0,8777475	protein_codin subtilisin-like serine peptidase
TcG_10862	19,767828	0,616338384	0,391593894	1,573922355	0,115505401	0,295838856	protein_codin subtilisin-like serine peptidase
TcG_10863	25,5846115	0,424310209	0,344095314	1,233118242	0,217531652	0,441851635	protein_codin putative kinesin
TcG_10864	25,86593287	0,064763541	0,344545167	0,187968217	0,850901566	0,928053891	protein_codin subtilisin-like serine peptidase
TcG_10865	44,21560562	-0,037899904	0,267895427	-0,14147276	0,887496482	0,94569431	protein_codin dynein
TcG_10866	42,07757558	0,068786639	0,269066343	0,255649362	0,798221599	0,899367446	protein_codin putative myosin heavy chain
TcG_10867	35,33615862	0,398718542	0,30122921	1,323638375	0,185623174	0,399522589	protein_codin hypothetical protein
TcG_10868	45,882752	-0,151816223	0,258444638	-0,58742261	0,556919915	0,75151108	protein_codin trans-sialidase
TcG_10869	20,26077144	-0,067327672	0,378326152	-0,17796198	0,858752833	0,932070224	protein_codin hypothetical protein
TcG_10870	20,94683296	0,291429782	0,415043791	0,702166346	0,482575431	0,696286283	protein_codin hypothetical protein
TcG_10871	25,5264932	-0,420815723	0,355489451	-1,1837643	0,236506376	0,463805496	protein_codin retrotransposon hot spot protein (RHS)
TcG_10872	193,2450552	0,069537995	0,130309568	0,533636912	0,593592765	0,778863106	protein_codin retrotransposon hot spot (RHS) protein
TcG_10873	36,05214793	0,346816381	0,292737339	1,184735717	0,236121956	0,463600927	protein_codin trans-sialidase
TcG_10874	34,20861311	0,077005407	0,304332935	0,253030148	0,800244917	0,900622067	protein_codin putative trans-sialidase
TcG_10875	4,992485638	-0,528681218	0,801937002	-0,6592553	0,509731842	1	
TcG_10876	20,43028636	0,379856869	0,411846713	0,922325847	0,356358636	0,590414866	protein_codin hypothetical protein
TcG_10877	2,054172417	-0,135232202	1,299143801	-0,10409333	0,917095286	1	protein_codin hypothetical protein
TcG_10878	0,534000759	0,261756182	2,520388668	0,103855483	0,917284036	1	protein_codin hypothetical protein
TcG_10879	0,770238516	-1,951474499	2,034869126	-0,9590172	0,337550079	1	protein_codin hypothetical protein
TcG_10880	30,01665455	0,086965365	0,321953257	0,270117986	0,787069485	0,893930698	protein_codin putative trans-sialidase
TcG_10881	41,07951157	-0,148867169	0,279329834	-0,53294404	0,59407232	0,779189213	protein_codin putative kinesin
TcG_10882	14,75118232	-0,100806886	0,447014485	-0,22551145	0,821581417	1	protein_codin putative kinesin
TcG_10883	43,01052257	0,021835185	0,265180168	0,082340943	0,934375597	0,969000687	protein_codin hypothetical protein
TcG_10884	47,195063	0,061906828	0,250807599	0,246829953	0,805039828	0,903850005	protein_codin trans-sialidase
TcG_10885	25,6721612	0,044063614	0,347165417	0,126923973	0,899000573	0,950695567	protein_codin hypothetical protein
TcG_10886	84,39002604	0,040220495	0,194534654	0,206752341	0,836203278	0,920523248	
TcG_10887	29,51967196	-0,128947354	0,329591999	-0,39123327	0,695624822	0,842340007	protein_codin trans-sialidase

TcG_10888	9,602982244	-0,940799747	0,57560758	-1,63444642	0,102165149	1	protein_codin hypothetical protein
TcG_10889	16,14365856	0,412749328	0,429906266	0,960091446	0,337009193	1	protein_codin hypothetical protein
TcG_10890	30,70243421	-0,055506662	0,320148656	-0,17337778	0,862354492	0,934140118	protein_codin beta galactofuranosyl glycosyltransferase
TcG_10891	22,93553453	0,267104272	0,362836173	0,736156679	0,461635353	0,679450496	protein_codin serine/threonine protein phosphatase
TcG_10892	8,879949959	-0,800549459	0,631191553	-1,2683146	0,204685627	1	protein_codin SH3 domain protein
TcG_10893	19,32651123	-0,962082781	0,402273246	-2,39161513	0,016774422	0,073533278	protein_codin mucin TcMUCII
TcG_10894	37,73755458	-0,152568949	0,297522171	-0,51279859	0,60809222	0,788022108	protein_codin hypothetical protein
TcG_10895	16,67650479	0,243045461	0,422481495	0,575280726	0,565101454	1	protein_codin putative syntaxin binding protein
TcG_10896	8,362728627	-0,439415556	0,635373886	-0,6915858	0,489197484	1	protein_codin syntaxin binding protein
TcG_10897	6,543478502	0,446065313	0,681495378	0,654539015	0,5127646	1	protein_codin hypothetical protein
TcG_10898	7,711692488	0,902212919	0,644868368	1,399065242	0,16179342	1	protein_codin hypothetical protein
TcG_10899	4,525083239	-0,72339664	0,806867892	-0,89654905	0,369959597	1	protein_codin surface protease GP63
TcG_10900	18,75359833	-0,176843718	0,406664291	-0,43486414	0,663661043	0,824134711	protein_codin putative elongation factor 1-gamma (EF-1-gamma)
TcG_10901	21,32899696	-0,325266452	0,39471197	-0,82406027	0,409905314	0,636890088	protein_codin hypothetical protein
TcG_10902	373,1971867	-0,205446775	0,09693072	-2,1195218	0,034046394	0,125744825	protein_codin putative dynamin-like protein
TcG_10903	70,68127976	0,01561242	0,216406397	0,072143984	0,942487323	0,973191135	protein_codin hypothetical protein
TcG_10904	0,781802848	0,328756738	1,95376118	0,168268641	0,866371943	1	
TcG_10905	0,145251675	-1,420530545	4,080472857	-0,3481289	0,727743378	1	
TcG_10906	215,6862683	0,95846583	0,124019569	7,72834349	1,08955E-14	1,11713E-12	protein_codin dispersed gene family protein 1 (DGF-1)
TcG_10907	90,46217833	1,076723184	0,21323635	5,049435447	4,43118E-07	9,74372E-06	protein_codin dispersed gene family protein 1 (DGF-1)
TcG_10908	44,33611363	1,098633578	0,277480392	3,959319681	7,51636E-05	0,000874342	protein_codin dispersed gene family protein 1 (DGF-1)
TcG_10909	64,13833385	0,821214947	0,223996417	3,666196794	0,000246185	0,002376225	protein_codin dispersed gene family protein 1 (DGF-1)
TcG_10910	460,7172017	-0,017934251	0,086587735	-0,2071223	0,835914346	0,920523248	protein_codin putative trans-sialidase
TcG_10911	46,22170356	-0,049708996	0,259741288	-0,19137888	0,848228767	0,926605553	protein_codin hypothetical protein
TcG_10912	437,4389609	0,005735295	0,096420286	0,059482241	0,95256801	0,977542335	protein_codin trans-sialidase-like protein
TcG_10913	246,7634456	-0,049301721	0,117137945	-0,420886	0,673838327	0,829023911	protein_codin putative cell cycle sequence binding phosphoprotein (RBP33)
TcG_10914	62,10605459	0,32840671	0,227248405	1,445144184	0,148417391	0,347246342	protein_codin hypothetical protein
TcG_10915	36,42072013	0,334462294	0,289580986	1,154987067	0,248095723	0,477445495	protein_codin hypothetical protein
TcG_10916	6,28317093	-0,613628151	0,687740164	-0,89223835	0,372265189	1	
TcG_10917	29,16716886	-0,508905516	0,34173095	-1,48919937	0,136434876	0,329163749	
TcG_10918	12,1037477	0,450185625	0,509456241	0,883659063	0,376880285	1	protein_codin hypothetical protein
TcG_10919	77,22436779	0,019518067	0,216024309	0,090351253	0,928008093	0,965201068	protein_codin hypothetical protein
TcG_10920	100,5987042	-0,304747014	0,172785317	-1,76373213	0,077777089	0,226280448	protein_codin putative kinesin
TcG_10921	183,1540122	-0,466311798	0,13058525	-3,57093774	0,000355705	0,003234854	protein_codin putative kinesin
TcG_10922	17,99554137	-0,330870298	0,426589861	-0,77561688	0,437975229	0,661461733	protein_codin putative kinesin
TcG_10923	694,2450681	0,59324233	0,078627628	7,54496025	4,52425E-14	4,22726E-12	
TcG_10924	264,2379408	-0,286933957	0,110252426	-2,6025183	0,009254188	0,046435261	protein_codin putative aspartyl aminopeptidase, putative,metallo-peptidase, clan MH, family M20
TcG_10925	0,719407094	0,307042706	2,080804357	0,147559623	0,882690323	1	protein_codin putative trans-sialidase
TcG_10926	1,225911917	1,431829341	1,653469887	0,865954289	0,38651523	1	protein_codin Sialidase 85-1.3
TcG_10927	5,709172999	0,14983635	0,739245984	0,202688081	0,83937884	1	protein_codin hypothetical protein
TcG_10928	46,86471216	-0,436008632	0,254456483	-1,71348997	0,086622436	0,243949331	protein_codin mucin-associated surface protein (MASP)
TcG_10929	7,687810898	0,898428866	0,660518195	1,360187913	0,173770467	1	protein_codin hypothetical protein
TcG_10930	18,41260468	0,369293797	0,420563933	0,878091932	0,379893831	0,610721305	protein_codin hypothetical protein
TcG_10931	2,787595217	0,422108342	1,033887693	0,408272915	0,683073322	1	protein_codin hypothetical protein
TcG_10932	7,057337001	-0,707034025	0,68580091	-1,03096105	0,302559086	1	
TcG_10933	770,6119657	-0,491331263	0,071313676	-6,88972001	5,59023E-12	3,7566E-10	protein_codin viral A-type inclusion protein
TcG_10934	98,08296091	0,314995092	0,187471009	1,680233618	0,092911871	0,255452524	protein_codin putative antigenic protein
TcG_10935	229,7845571	0,380695269	0,12166745	3,128982062	0,00175413	0,012392288	protein_codin putative transmembrane amino acid transporter
TcG_10936	167,7125944	0,342673734	0,146399142	2,340681294	0,019248591	0,081511027	protein_codin hypothetical protein
TcG_10937	1039,166252	0,245641596	0,060975927	4,028501191	5,61336E-05	0,000681723	protein_codin acetyl-CoA C-acetyltransferase
TcG_10938	43,59697816	0,195135949	0,271226863	0,719456571	0,471859652	0,687841712	protein_codin putative dispersed gene family protein 1 (DGF-1)
TcG_10939	34,415979	-0,068591757	0,29994964	-0,22867758	0,819119524	0,910866571	protein_codin dispersed gene family protein 1 (DGF-1)
TcG_10940	18,88761382	-0,098518211	0,399394586	-0,24666887	0,8051645	0,903850005	protein_codin dispersed gene family protein 1 (DGF-1)
TcG_10941	0,116927736	0,503022807	4,080472857	0,123275616	0,901888849	1	protein_codin dispersed gene family protein 1 (DGF-1)
TcG_10942	23,87332003	0,876466298	0,364358034	2,405508365	0,016149982	0,071526641	protein_codin dispersed gene family protein 1 (DGF-1)
TcG_10943	23,74324272	0,673821651	0,368554099	1,828284239	0,067506904	0,206892139	protein_codin dispersed gene family protein 1 (DGF-1)
TcG_10944	3,702079414	0,683009548	0,923873621	0,739288938	0,459731565	1	protein_codin dispersed gene family protein 1 (DGF-1)

TcG_10945	132,3709357	0,011670003	0,159265795	0,073273756	0,941588275	0,972960076	protein_codin dispersed gene family protein 1 (DGF-1)
TcG_10946	10,68388837	0,250984126	0,541052868	0,463880964	0,642733032	1	
TcG_10947	147,9456012	0,165442961	0,1505235	1,099117159	0,271716966	0,50482886	protein_codin surface protein-2
TcG_10948	30,1228615	0,382164631	0,340426407	1,122605716	0,261604987	0,493882251	protein_codin hypothetical protein
TcG_10949	79,90876144	-0,030150702	0,198165028	-0,15214946	0,879069053	0,941484386	protein_codin trans-sialidase
TcG_10950	36,36901327	0,005821667	0,293109551	0,019861744	0,984153663	0,993987719	protein_codin trans-sialidase
TcG_10951	12,69810555	0,391129893	0,532197855	0,734933239	0,462380154	1	protein_codin dispersed protein family protein 1
TcG_10952	41,95433495	0,543645264	0,274190444	1,982728706	0,047397741	0,159181631	protein_codin dispersed gene family protein 1 (DGF-1)
TcG_10953	88,0129896	0,311352981	0,209525324	1,485992127	0,137281204	0,330261633	protein_codin dispersed gene family protein 1 (DGF-1)
TcG_10954	7,781290295	0,413479553	0,623449478	0,663212606	0,507194399	1	protein_codin dispersed gene family protein 1 (DGF-1)
TcG_10955	17,90796516	0,237816706	0,419905764	0,566357328	0,571150897	0,762455846	protein_codin dispersed gene family protein 1 (DGF-1)
TcG_10956	2,291366834	1,783120001	1,257079146	1,418462796	0,156055693	1	protein_codin dispersed gene family protein 1 (DGF-1)
TcG_10957	4,473636207	1,170084922	0,89970477	1,300520973	0,193422473	1	protein_codin dispersed gene family protein 1 (DGF-1)
TcG_10958	4,879020905	-0,080135392	0,771567017	-0,10386057	0,917279997	1	protein_codin hypothetical protein
TcG_10959	1,516681657	0,525586926	1,531371964	0,343213105	0,731438147	1	protein_codin hypothetical protein
TcG_10960	1,44191339	3,77000623	1,680479108	2,243411543	0,024870288	1	protein_codin hypothetical protein
TcG_10961	4,278477266	0,250314221	0,854738774	0,292854646	0,769633257	1	protein_codin hypothetical protein
TcG_10962	0,338239883	-2,234864625	3,050011161	-0,73273982	0,463717133	1	protein_codin hypothetical protein
TcG_10963	2,730148169	1,124057401	1,219038387	0,92208532	0,356484074	1	protein_codin hypothetical protein
TcG_10964	3,820128542	-0,051287314	0,896971147	-0,05717833	0,954403138	1	protein_codin hypothetical protein
TcG_10965	43,65891131	0,108996697	0,276927547	0,39359283	0,693881675	0,84137238	protein_codin mucin-associated surface protein (MASP)
TcG_10966	5,476677849	0,764947431	0,79474904	0,962501863	0,335797568	1	protein_codin hypothetical protein
TcG_10967	75,94281562	-0,24335618	0,200770241	-1,21211281	0,225469202	0,451820114	protein_codin mucin TcMUCI
TcG_10968	78,75131904	-0,28544332	0,205816969	-1,38687943	0,165478532	0,370915599	protein_codin mucin-associated surface protein (MASP)
TcG_10969	21,50201119	-0,190503111	0,379675737	-0,50175213	0,615841889	0,792562564	protein_codin trans-sialidase
TcG_10970	44,41915452	0,282905493	0,270203706	1,047008189	0,295095801	0,527946256	protein_codin amastigote surface protein 4
TcG_10971	102,0917407	-0,391543059	0,171998496	-2,27643304	0,022820104	0,093359366	protein_codin trans-sialidase
TcG_10972	739,7973038	0,107336965	0,069845994	1,536766239	0,124350564	0,310501213	protein_codin trans-sialidase
TcG_10973	78,26715585	-1,226772894	0,208966438	-5,8706695	4,34039E-09	1,49768E-07	protein_codin trans-sialidase-like protein
TcG_10974	34,26581984	-0,561879314	0,300711985	-1,86849658	0,061692887	0,1936007	protein_codin mucin-associated surface protein (MASP)
TcG_10975	61,96898004	-0,380981513	0,244138431	-1,5605143	0,118638394	0,300690664	protein_codin mucin TcMUCII
TcG_10976	20,75793891	-0,411994572	0,380742436	-1,08208209	0,279216052	0,512149969	
TcG_10977	26,67373028	0,122185899	0,362426555	0,337132854	0,736016752	0,864944729	protein_codin hypothetical protein
TcG_10978	29,08924759	-0,023771599	0,354027621	-0,06714617	0,946465338	0,974694377	protein_codin trans-sialidase
TcG_10979	13,69156615	0,204639446	0,471312013	0,434191025	0,66414973	1	protein_codin hypothetical protein
TcG_10980	12,51914892	0,237577844	0,487137868	0,487701449	0,625761325	1	protein_codin hypothetical protein
TcG_10981	5,190921837	-0,635035317	0,821629184	-0,77289771	0,439582919	1	
TcG_10982	16,07635066	-0,741291019	0,446429389	-1,66048884	0,09681615	1	protein_codin trans-sialidase
TcG_10983	12,18188844	-0,31267012	0,508852686	-0,61446098	0,538910751	1	protein_codin hypothetical protein
TcG_10984	24,41700376	0,371431411	0,374329723	0,992257329	0,321072013	0,556034397	protein_codin hypothetical protein
TcG_10985	22,10403313	-0,092424794	0,378041803	-0,244483	0,806856767	0,904614097	
TcG_10986	46,51077472	0,069830854	0,262917475	0,265599897	0,790547365	0,895247949	protein_codin trans-sialidase
TcG_10987	40,23980117	0,273665955	0,298919411	0,91551751	0,359920015	0,592630203	protein_codin hypothetical protein
TcG_10988	23,19810094	-0,028894108	0,364041436	-0,07937038	0,936738025	0,970581896	protein_codin hypothetical protein
TcG_10989	39,02116863	0,3303827	0,27702573	1,192606547	0,2330235	0,460404208	protein_codin target of rapamycin (TOR) kinase 1
TcG_10990	17,1269748	0,25459357	0,430896019	0,590846882	0,554623019	0,749417743	protein_codin putative target of rapamycin (TOR) kinase 1
TcG_10991	4,96729817	-0,247120673	0,846206876	-0,2920334	0,770261086	1	protein_codin hypothetical protein
TcG_10992	19,38426396	-0,205490112	0,394200052	-0,52128383	0,602169065	0,784755061	protein_codin mitochondrial processing peptide beta subunit
TcG_10993	170,7938595	0,229924788	0,1390965	1,652987583	0,098333391	0,265642661	protein_codin putative glycine dehydrogenase, putative,glycine cleavage system P-protein
TcG_10994	1,543644813	-0,40452982	1,412968871	-0,28629776	0,774650075	1	protein_codin hypothetical protein
TcG_10995	1,79187078	4,040520836	1,613473885	2,504236897	0,012271584	1	protein_codin hypothetical protein
TcG_10996	3,883769681	0,104868836	0,867859709	0,120836161	0,903820807	1	protein_codin hypothetical protein
TcG_10997	9,558086923	0,254617492	0,568525693	0,447855735	0,654257317	1	protein_codin hypothetical protein
TcG_10998	19,3647797	0,452393947	0,39614852	1,141980658	0,25346206	0,484454814	
TcG_10999	42,94376095	0,44106648	0,272046731	1,621289398	0,104955588	0,278328093	protein_codin putative trans-sialidase
TcG_11000	204,12794	0,104325132	0,125714748	0,829855957	0,406620229	0,634833846	protein_codin hypothetical protein
TcG_11001	391,5004312	0,027528027	0,104434743	0,263590703	0,792095323	0,896041689	protein_codin putative SNF2 DNA repair protein

TcG_11002	610,5910931	-0,212979419	0,077798277	-2,73758529	0,006189207	0,033840562	protein_codin putative protein kinase-like protein
TcG_11003	56,79216314	-0,02783978	0,239686703	-0,11615071	0,907533102	0,955230917	protein_codin Unc104-like kinesin
TcG_11004	1,491213216	-3,141177727	1,665447416	-1,8860864	0,059283312	1	protein_codin mucin-associated surface protein (MASP)
TcG_11005	2,983254435	-2,655016334	1,185442202	-2,23968434	0,025111423	1	protein_codin hypothetical protein
TcG_11006	3,121692607	-1,658779735	1,920380051	-0,8637768	0,387710515	1	protein_codin hypothetical protein
TcG_11007	0					1	protein_codin hypothetical protein
TcG_11008	3,472287046	-0,854625614	0,924128513	-0,92479087	0,3550747	1	protein_codin hypothetical protein
TcG_11009	6,371186117	0,067122411	0,712225992	0,094243136	0,92491602	1	protein_codin hypothetical protein
TcG_11010	5,395931786	-1,258736133	0,778956938	-1,61592518	0,10611049	1	protein_codin hypothetical protein
TcG_11011	31,90998009	0,073444835	0,307206477	0,239073197	0,811048825	0,906853087	protein_codin putative mucin-associated surface protein (MASP)
TcG_11012	2,614745947	-2,4312683	1,233798445	-1,97055549	0,048774742	1	protein_codin trans-sialidase
TcG_11013	6,651739559	-0,400915894	0,686762436	-0,58377668	0,55937057	1	protein_codin trans-sialidase
TcG_11014	41,78524609	0,035818168	0,296635828	0,120747951	0,903890678	0,953490587	protein_codin trans-sialidase
TcG_11015	1,45756528	0,913576117	1,575473734	0,579873912	0,56199965	1	protein_codin hypothetical protein
TcG_11016	0,116927736	0,503022807	4,080472857	0,123275616	0,901888849	1	protein_codin hypothetical protein
TcG_11017	6,583611438	-0,590445914	0,686822038	-0,85967817	0,38996647	1	protein_codin hypothetical protein
TcG_11018	2,333393334	-0,933551265	1,213901736	-0,76905011	0,441863563	1	protein_codin hypothetical protein
TcG_11019	49,4113618	-0,452331333	0,246206059	-1,83720634	0,06617944	0,20397845	protein_codin mucin-associated surface protein (MASP)
TcG_11020	1,229162527	-2,961037463	1,797361217	-1,64743594	0,099468473	1	protein_codin hypothetical protein
TcG_11021	2,493766598	-2,881733226	1,382519648	-2,08440671	0,037123188	1	protein_codin surface protease GP63
TcG_11022	0,638385169	-2,974621413	2,29321366	-1,29714098	0,194582684	1	protein_codin syntaxin binding protein
TcG_11023	2,010110373	0,10533212	1,21650644	0,086585748	0,931000795	1	protein_codin putative syntaxin binding protein
TcG_11024	5,180977916	-0,593992877	0,803549794	-0,73921104	0,459778859	1	protein_codin hypothetical protein
TcG_11025	4,633896322	-0,43465373	0,796122832	-0,54596315	0,585091268	1	protein_codin hypothetical protein
TcG_11026	574,272402	0,653240734	0,080139733	8,151271604	3,60117E-16	4,21446E-14	protein_codin putative retrotransposon hot spot (RHS) protein
TcG_11027	93,46405582	0,70441665	0,192399655	3,661215759	0,000251021	0,002413554	protein_codin target of rapamycin (TOR) kinase 1
TcG_11028	52,40601787	-0,075188228	0,256651348	-0,29295863	0,76955377	0,884364283	protein_codin putative rab1 small GTP-binding protein
TcG_11029	71,13941095	0,699679304	0,212900419	3,286415813	0,001014711	0,00787282	protein_codin putative mitotic centromere-associated kinesin (MCAK)
TcG_11030	178,1806793	0,479700814	0,137794269	3,481282765	0,000499018	0,004330807	protein_codin hypothetical protein
TcG_11031	194,8493104	0,355161661	0,140626699	2,525563519	0,01155129	0,055304282	protein_codin hypothetical protein
TcG_11032	44,85817996	0,58368091	0,268156683	2,176641298	0,029507341	0,112903585	protein_codin dispersed gene family protein 1 (DGF-1)
TcG_11033	103,1229943	0,680575421	0,180784325	3,764570969	0,000166835	0,001718181	protein_codin dispersed gene family protein 1 (DGF-1)
TcG_11034	31,00893829	0,085526621	0,308934396	0,276843956	0,781899927	0,891730737	protein_codin trans-sialidase
TcG_11035	20,5930944	-0,037573181	0,376936629	-0,09968037	0,920598088	0,96150609	protein_codin hypothetical protein
TcG_11036	23,42136862	0,216501596	0,363925294	0,594906701	0,551905838	0,747373577	protein_codin hypothetical protein
TcG_11037	6,932752317	-1,262333795	0,676967188	-1,86468978	0,062224901	1	protein_codin hypothetical protein
TcG_11038	80,38222455	-0,332913387	0,202238397	-1,64614332	0,099734255	0,268164557	protein_codin putative retrotransposon hot spot (RHS) protein
TcG_11039	29,78076377	0,04743435	0,31591736	0,15014797	0,880647875	0,942069315	protein_codin hypothetical protein
TcG_11040	161,2410426	-0,133507364	0,138326396	-0,96516188	0,334463738	0,568936844	protein_codin mucin-associated surface protein (MASP)
TcG_11041	55,04527363	-0,534696754	0,238548815	-2,24145634	0,024996531	0,099697539	
TcG_11042	16,88601321	-0,158507697	0,416483024	-0,38058621	0,703510314	0,847019692	protein_codin hypothetical protein
TcG_11043	15,2337548	-0,28818115	0,482372156	-0,59742493	0,550223715	1	protein_codin hypothetical protein
TcG_11044	6,583040814	0,073592529	0,663681947	0,110885235	0,911707354	1	
TcG_11045	44,66679484	-0,707423775	0,267169116	-2,64785012	0,008100543	0,041879915	protein_codin mucin-associated surface protein (MASP)
TcG_11046	22,43808464	0,422618759	0,396388782	1,06617235	0,286345739	0,519593067	protein_codin hypothetical protein
TcG_11047	90,92335326	-0,224298045	0,188720134	-1,18852207	0,23462779	0,462469815	protein_codin trans-sialidase
TcG_11048	57,41850623	0,278842098	0,233206863	1,195685643	0,231819256	0,459199504	
TcG_11049	87,84362406	0,105978647	0,197539276	0,536494053	0,591617145	0,777210826	protein_codin mucin-associated surface protein (MASP)
TcG_11050	113,2477815	-0,013327675	0,18613699	-0,07160143	0,942919101	0,973191135	protein_codin mucin TcMUCII
TcG_11051	36,10856733	0,108435785	0,287697072	0,376909588	0,706240796	0,848727637	
TcG_11052	4,406294507	0,598881744	0,833050416	0,718902161	0,472201205	1	protein_codin hypothetical protein
TcG_11053	19,8772005	-0,402593991	0,407662199	-0,98756763	0,323364447	0,557588877	protein_codin putative surface protease GP63
TcG_11054	0,469265227	-2,470320505	2,566008638	-0,96270935	0,335693404	1	
TcG_11055	55,72014483	-0,35621418	0,233047872	-1,52850218	0,126387903	0,31409915	protein_codin mucin-associated surface protein (MASP)
TcG_11056	33,5182594	-0,481405085	0,299650857	-1,60655334	0,108152386	0,283624614	protein_codin serine-alanine-and proline-rich protein
TcG_11057	12,11458227	-0,168727067	0,511561365	-0,32982762	0,741530217	1	protein_codin serine-alanine-and proline-rich protein
TcG_11058	27,70206476	0,479577799	0,344299594	1,392908409	0,163647489	0,368373773	protein_codin hypothetical protein

TcG_11059	43,10967142	-0,107326562	0,261932304	-0,40974924	0,681989907	0,834223169	protein_codin mucin TcMUCII
TcG_11060	3,925045382	0,493737454	0,903796854	0,546292512	0,584864883	1	protein_codin mucin-associated surface protein (MASP)
TcG_11061	3,635829923	0,096276859	0,903143409	0,106601962	0,915104762	1	protein_codin hypothetical protein
TcG_11062	15,58430448	0,253145681	0,481149048	0,526127365	0,598799716	1	protein_codin hypothetical protein
TcG_11063	4,354409015	-7,87795E-05	0,812466784	-9,6963E-05	0,999922634	1	protein_codin hypothetical protein
TcG_11064	28,78564844	-0,243717448	0,323290952	-0,75386412	0,450930818	0,67161389	protein_codin mucin TcMUCII
TcG_11065	155,083134	-0,293796798	0,145662397	-2,01697078	0,043698563	0,150420362	protein_codin mucin-associated surface protein (MASP)
TcG_11066	53,89207783	0,447986062	0,24705418	1,813311002	0,069783867	0,211431979	
TcG_11067	90,2258239	-0,243184661	0,200033454	-1,21571995	0,224091621	0,450750958	protein_codin mucin-associated surface protein (MASP)
TcG_11068	15,47166449	-0,183043954	0,45695554	-0,40057279	0,688734684	1	
TcG_11069	9,209721657	-0,591195111	0,596129531	-0,99172257	0,321332877	1	protein_codin surface protease GP63
TcG_11070	7,85061543	-0,344327072	0,62958017	-0,54691537	0,584436875	1	protein_codin hypothetical protein
TcG_11071	2,669169029	0,119745214	1,114341062	0,107458316	0,914425393	1	protein_codin hypothetical protein
TcG_11072	0,846538379	1,74528242	1,910600667	0,913473155	0,360993746	1	protein_codin hypothetical protein
TcG_11073	19,05123306	-0,212924976	0,404592511	-0,52627019	0,598700494	0,782640632	protein_codin mucin TcMUCII
TcG_11074	3,579917337	-2,057766597	1,012985363	-2,03138828	0,042215622	1	
TcG_11075	70,41911701	-0,116005634	0,210592709	-0,55085304	0,58173442	0,770899576	protein_codin putative trans-sialidase
TcG_11076	215,186752	0,311259209	0,121553363	2,560679543	0,010446767	0,051135561	protein_codin trans-sialidase
TcG_11077	55,7871325	0,283999826	0,242222265	1,172476138	0,241005947	0,469729485	protein_codin trans-sialidase
TcG_11078	142,5050635	0,046942135	0,149378515	0,314249577	0,753331491	0,875527481	protein_codin putative mucin-associated surface protein (MASP)
TcG_11079	20,10239649	-0,044987357	0,398796741	-0,11280773	0,910182986	0,956414305	protein_codin hypothetical protein
TcG_11080	5,931311315	-0,766957059	0,724017536	-1,0593073	0,289459853	1	
TcG_11081	28,75240375	0,403425288	0,359051204	1,123587062	0,261188247	0,493587786	protein_codin hypothetical protein
TcG_11082	25,10361296	0,363038921	0,347548904	1,044569318	0,296222066	0,529282905	protein_codin hypothetical protein
TcG_11083	178,7237836	-0,140314418	0,135960003	-1,03202717	0,302059389	0,535346443	protein_codin calmodulin
TcG_11084	205,7992404	-0,307884546	0,129178328	-2,38340712	0,017153212	0,074758218	protein_codin hypothetical protein
TcG_11085	222,6330616	-0,377348045	0,133044557	-2,83625316	0,004564625	0,026831934	protein_codin NUDIX hydrolase
TcG_11086	180,526584	0,353640572	0,137388648	2,574015949	0,010052564	0,049561279	protein_codin L1Tc protein
TcG_11087	112,6622849	0,49313555	0,170455405	2,893047309	0,003815238	0,023203859	protein_codin hypothetical protein
TcG_11088	84,36575127	0,523334308	0,197588784	2,648603312	0,008082514	0,041819234	protein_codin hypothetical protein
TcG_11089	1006,152272	0,005302163	0,062056285	0,085441197	0,931910643	0,967364315	protein_codin hypothetical protein
TcG_11090	541,549532	0,036390237	0,085435484	0,425938211	0,670152871	0,826966786	protein_codin hypothetical protein
TcG_11091	285,1760946	-0,320364957	0,107242593	-2,98729215	0,002814606	0,018056493	
TcG_11092	560,5096512	-0,352200312	0,07959709	-4,42478877	9,65367E-06	0,000147751	protein_codin ubiquitin-conjugating enzyme E2
TcG_11093	23,47207512	0,208082784	0,353470294	0,58868535	0,556072367	0,750892126	protein_codin dispersed protein family protein 1 (DGF-1)
TcG_11094	5,802749742	0,132432825	0,745013772	0,177758895	0,85891233	1	protein_codin dispersed gene family protein 1 (DGF-1)
TcG_11095	14,94562219	0,209110977	0,449739202	0,464960528	0,641959724	1	protein_codin dispersed gene family protein 1 (DGF-1)
TcG_11096	0,155721614	0,503022807	4,080472857	0,123275616	0,901888849	1	protein_codin dispersed protein family protein 1
TcG_11097	88,27049483	0,08850358	0,191401058	0,462398593	0,643795508	0,810875005	
TcG_11098	621,0404378	0,009503881	0,074813514	0,127034278	0,898913268	0,950695567	protein_codin UDP-Gal or UDP-GlcNAc-dependent glycosyltransferase
TcG_11099	821,4892896	-0,084770053	0,075302598	-1,12572549	0,260281733	0,492617789	
TcG_11100	530,8414145	0,231393017	0,083146435	2,782957785	0,005386581	0,03036931	protein_codin hypothetical protein
TcG_11101	1000,92529	0,115686958	0,06498405	1,780236183	0,075037316	0,221170712	protein_codin putative citrate transporter
TcG_11102	93,04036056	0,017165642	0,186539351	0,092021559	0,926680911	0,964306367	protein_codin ATPase
TcG_11103	26,58659471	-0,241421278	0,336721195	-0,71697678	0,473388425	0,689203102	protein_codin hypothetical protein
TcG_11104	17,93152584	-0,745504995	0,418244356	-1,78246277	0,074673796	0,220594239	protein_codin hypothetical protein
TcG_11105	72,53056975	-0,0800467	0,206935207	-0,38682011	0,698889388	0,844142481	
TcG_11106	57,71145931	-0,402874421	0,235674063	-1,70945591	0,087366531	0,245121514	protein_codin putative mucin TcMUCII
TcG_11107	220,7290552	-0,602056409	0,120127971	-5,01179204	5,39255E-07	1,16781E-05	protein_codin putative mucin-associated surface protein (MASP)
TcG_11108	18,07690654	0,467874375	0,426054222	1,09815688	0,272135992	0,505040461	protein_codin hypothetical protein
TcG_11109	5,691953503	1,167091438	0,766783187	1,522061853	0,127993583	1	protein_codin structural maintenance of chromosome protein 4
TcG_11110	16,13162907	1,259193247	0,463689902	2,715593419	0,006615711	1	protein_codin hypothetical protein
TcG_11111	6,921378043	0,422900073	0,674088216	0,627366067	0,530419306	1	protein_codin target of rapamycin (TOR) kinase 1
TcG_11112	167,1802221	0,262048767	0,146029303	1,794494404	0,072734291	0,21694934	protein_codin target of rapamycin (TOR) kinase 1
TcG_11113	329,1058362	-0,288169294	0,102688154	-2,80625644	0,005012079	0,02883314	
TcG_11114	45,78821577	-0,635461838	0,258257668	-2,46057297	0,013871536	0,06390283	
TcG_11115	88,48551216	0,11309104	0,20795862	0,543815109	0,586568711	0,775089539	protein_codin dispersed gene family protein 1 (DGF-1)

TcG_11116	82,45216578	-0,360836279	0,195600203	-1,84476434	0,065071826	0,201260593	
TcG_11117	20,20873067	-0,112810484	0,412584173	-0,27342417	0,784527177	0,89340789	
TcG_11118	530,6510718	0,159105779	0,08167755	1,947974418	0,051418024	0,168684291	protein_codin putative retrotransposon hot spot (RHS) protein
TcG_11119	8,066272762	-0,106863812	0,625535019	-0,17083586	0,864352832	1	protein_codin hypothetical protein
TcG_11120	0,548227089	-0,494168331	2,319377258	-0,21306078	0,831279554	1	
TcG_11121	8,321563086	-0,636862472	0,615964897	-1,03392657	0,301170493	1	protein_codin hypothetical protein
TcG_11122	5,487399131	0,222682123	0,740446054	0,300740509	0,763612376	1	protein_codin hypothetical protein
TcG_11123	13,10224142	-0,540269707	0,479581382	-1,12654437	0,259935169	1	protein_codin hypothetical protein
TcG_11124	10,01455794	-0,047366616	0,548981157	-0,08628095	0,931243081	1	protein_codin hypothetical protein
TcG_11125	19,44298525	0,113932942	0,400940722	0,284164055	0,77628466	0,888310845	protein_codin trans-sialidase
TcG_11126	39,96933677	0,022782906	0,274817552	0,082901931	0,933929518	0,968711495	protein_codin hypothetical protein
TcG_11127	27,90356186	-0,297597709	0,329967045	-0,90190131	0,367109297	0,599390612	protein_codin hypothetical protein
TcG_11128	35,991361	0,446045835	0,302732409	1,473399682	0,140643296	0,335424707	protein_codin putative trans-sialidase
TcG_11129	21,24076765	0,4749714	0,377416464	1,258480869	0,208217902	0,430787966	protein_codin hypothetical protein
TcG_11130	24,79238769	0,210188683	0,355323833	0,59154119	0,554157865	0,749109966	protein_codin hypothetical protein
TcG_11131	0					1	protein_codin hypothetical protein
TcG_11132	255,4539567	-0,000854428	0,114778157	-0,00744417	0,994060469	0,997677114	protein_codin mucin-associated surface protein (MASP)
TcG_11133	466,8241359	-0,151691979	0,08756373	-1,73236087	0,08320933	0,237045316	
TcG_11134	0,27264935	1,353296468	3,06414982	0,441654798	0,658739024	1	
TcG_11135	274,5523704	-0,011560618	0,11102931	-0,10412222	0,917072361	0,959992806	protein_codin hypothetical protein
TcG_11136	65,74969927	-0,044625886	0,214369637	-0,20817261	0,835094196	0,92007617	protein_codin hypothetical protein
TcG_11137	80,07070257	-0,03048009	0,201276581	-0,15143386	0,879633479	0,941623821	
TcG_11138	174,9962671	0,150605197	0,138184212	1,089887156	0,275762855	0,508108849	protein_codin hypothetical protein
TcG_11139	62,25211534	-0,292468961	0,21795776	-1,34186074	0,179641154	0,390785281	protein_codin hypothetical protein
TcG_11140	119,4138295	0,142158408	0,162439195	0,875148439	0,381493144	0,611592579	protein_codin hypothetical protein
TcG_11141	12,86512385	0,114988991	0,486617646	0,23630255	0,813197908	1	
TcG_11142	12,50203021	-0,283008645	0,487526247	-0,5804993	0,561577959	1	protein_codin hypothetical protein
TcG_11143	8,550505756	-0,132341984	0,615269248	-0,21509605	0,829692434	1	protein_codin hypothetical protein
TcG_11144	0,314371616	-2,1715226	2,910784339	-0,74602662	0,455651335	1	
TcG_11145	78,91527885	-0,267418707	0,20003069	-1,33688839	0,181259071	0,392829705	protein_codin mucin-associated surface protein (MASP)
TcG_11146	43,92325396	0,255549296	0,264164704	0,967386225	0,333350993	0,567804265	protein_codin hypothetical protein
TcG_11147	33,23017235	0,292699885	0,299041959	0,978792027	0,32768275	0,56219937	
TcG_11148	55,37356081	0,195917447	0,24044055	0,81482698	0,415171355	0,642127262	protein_codin helicase-like protein
TcG_11149	29,2919836	0,27595433	0,321750516	0,857665541	0,391077173	0,621027978	protein_codin trans-sialidase
TcG_11150	29,08609046	0,273319021	0,333125066	0,820469696	0,411948398	0,638506239	protein_codin target of rapamycin (TOR) kinase 1
TcG_11151	42,6523304	0,100998081	0,276595417	0,36514734	0,715001425	0,853669261	protein_codin putative target of rapamycin (TOR) kinase 1
TcG_11152	540,754441	0,058624475	0,085605253	0,684823336	0,493455407	0,703987578	protein_codin RNA-binding protein NOB1
TcG_11153	520,133018	-0,069039203	0,082955077	-0,83224807	0,405268934	0,634304216	protein_codin gamma carbonic dehydratase
TcG_11154	16,75416887	-0,494655268	0,419406056	-1,17941852	0,238231566	1	protein_codin hypothetical protein
TcG_11155	20,81598927	0,543321785	0,400437104	1,356821783	0,174837845	0,383940725	protein_codin putative mucin-associated surface protein (MASP)
TcG_11156	2,22458025	-1,664132002	1,205320939	-1,38065468	0,167385161	1	protein_codin hypothetical protein
TcG_11157	1,786596117	0,120829688	1,366902257	0,088396728	0,929561361	1	protein_codin hypothetical protein
TcG_11158	2,19987497	0,409414592	1,162210939	0,35227219	0,724634143	1	protein_codin hypothetical protein
TcG_11159	3,520400639	0,123047513	0,906267554	0,135773937	0,891999996	1	protein_codin hypothetical protein
TcG_11160	9,940651438	-0,102264004	0,592312866	-0,17265201	0,862924971	1	protein_codin hypothetical protein
TcG_11161	2,769869939	1,118744228	1,098170497	1,018734551	0,308329005	1	protein_codin hypothetical protein
TcG_11162	33,64563989	0,28797848	0,307524582	0,936440522	0,349046423	0,58360833	
TcG_11163	3,280011443	-0,551926832	0,967782777	-0,57030033	0,56847402	1	protein_codin hypothetical protein
TcG_11164	31,02912533	-0,985607485	0,320521714	-3,07501003	0,002104955	0,014370825	protein_codin mucin TcMUCII
TcG_11165	11,78474332	-0,054814595	0,507436011	-0,10802267	0,913977705	1	protein_codin surface protease GP63
TcG_11166	12,63566822	0,242135209	0,491729237	0,492415726	0,622425485	1	protein_codin surface protease GP63
TcG_11167	64,40103068	0,140687059	0,219479357	0,641003605	0,521520341	0,725401608	
TcG_11168	5,491575089	0,041371069	0,746094882	0,055450145	0,955779848	1	protein_codin hypothetical protein
TcG_11169	74,72539088	0,154830595	0,201347318	0,768972723	0,441909504	0,664412602	protein_codin receptor-type adenylate cyclase
TcG_11170	172,1541871	0,239550938	0,144933388	1,652834735	0,098364503	0,265652944	protein_codin hypothetical protein
TcG_11171	219,5017487	-0,10697671	0,121892873	-0,87762892	0,380145131	0,610721305	protein_codin esag4
TcG_11172	58,71187166	-0,448742347	0,242775839	-1,8483814	0,064547187	0,200065198	protein_codin trans-sialidase

TcG_11173	16,63940598	-0,228282502	0,436075268	-0,52349335	0,600630979	1	protein_codin	putative trans-sialidase
TcG_11174	31,19161938	-0,292906795	0,313137762	-0,93539276	0,349585928	0,584106942	protein_codin	trans-sialidase
TcG_11175	10,20534186	-0,282208106	0,549510334	-0,51356287	0,607557643	1	protein_codin	surface protease GP63
TcG_11176	33,54023406	-0,160918829	0,313829343	-0,51275903	0,608119894	0,788022108		
TcG_11177	4,648320463	-0,100476489	0,829975398	-0,1210596	0,903643829	1		
TcG_11178	28,66333843	0,186339963	0,322040386	0,578622966	0,562843605	0,756157932	protein_codin	putative trans-sialidase
TcG_11179	48,93110792	-0,033666751	0,254185394	-0,13244959	0,894628694	0,948843651		
TcG_11180	54,16585565	0,151107692	0,238109387	0,634614593	0,525679808	0,728873483	protein_codin	mucin-associated surface protein (MASP)
TcG_11181	196,5260085	0,13462794	0,130958454	1,028020228	0,303940313	0,536835661	protein_codin	UDP-Gal or UDP-GlcNAc-dependent glycosyltransferase
TcG_11182	45,91468578	-0,020432761	0,263650433	-0,07749944	0,938226237	0,971428881	protein_codin	UDP-Gal or UDP-GlcNAc-dependent glycosyltransferase
TcG_11183	11,19527993	-0,044786995	0,524038302	-0,08546512	0,931891626	1	protein_codin	hypothetical protein
TcG_11184	1,381633531	1,638078331	1,580512256	1,036422416	0,300005117	1	protein_codin	hypothetical protein
TcG_11185	17,28088266	0,230733789	0,426598615	0,540868584	0,588598166	0,775648564	protein_codin	hypothetical protein
TcG_11186	6,290805191	1,261071469	0,741318699	1,701119195	0,088920607	1	protein_codin	hypothetical protein
TcG_11187	12,79936161	0,731163423	0,527900586	1,385039992	0,166040239	1	protein_codin	hypothetical protein
TcG_11188	9,344076999	-0,181861034	0,559998128	-0,32475293	0,745368071	1	protein_codin	surface protease GP63
TcG_11189	6,903118216	-0,594317462	0,659098684	-0,90171241	0,36720966	1	protein_codin	surface protease GP63
TcG_11190	84,57722825	0,334354795	0,18971097	1,762443129	0,077994457	0,22670441	protein_codin	hypothetical protein
TcG_11191	106,82271	-0,063850113	0,177183845	-0,36036081	0,718577332	0,8554703	protein_codin	mucin TcMUCI
TcG_11192	43,07522898	0,024046896	0,261642509	0,091907449	0,926771573	0,964306367		
TcG_11193	111,3412986	0,017463469	0,175887908	0,099287488	0,920910013	0,96150609	protein_codin	hypothetical protein
TcG_11194	92,59796073	0,191027839	0,184906032	1,033107665	0,301553516	0,534874318	protein_codin	hypothetical protein
TcG_11195	18,3265413	0,308036908	0,418352463	0,736309537	0,461542344	0,679450496		
TcG_11196	73,26713024	0,312891513	0,225903763	1,385065519	0,166032434	0,371648335	protein_codin	retrotransposon hot spot (RHS) protein
TcG_11197	19,25595335	0,141704001	0,398582822	0,35551959	0,722200371	0,857285556	protein_codin	retrotransposon hot spot (RHS) protein
TcG_11198	182,0594104	0,076399328	0,131848231	0,579449017	0,562286238	0,75575967	protein_codin	putative retrotransposon hot spot (RHS) protein
TcG_11199	10,32099918	-0,180644791	0,604303726	-0,29893046	0,764993105	1	protein_codin	hypothetical protein
TcG_11200	11,85127881	0,337241039	0,51697802	0,652331483	0,514187355	1	protein_codin	hypothetical protein
TcG_11201	5,564526997	0,837927255	0,745705944	1,123669809	0,261153129	1	protein_codin	hypothetical protein
TcG_11202	41,05732164	0,000132309	0,296930587	0,000445588	0,999644472	0,99981271		
TcG_11203	10,46678604	0,076139127	0,539923857	0,141018267	0,887855511	1		
TcG_11204	17,78447651	0,543573876	0,423009518	1,285015711	0,198786794	0,417614468	protein_codin	dispersed gene family protein 1 (DGF-1)
TcG_11205	58,7365481	0,285733734	0,243983359	1,171119765	0,24155064	0,470195853	protein_codin	dispersed gene family protein 1 (DGF-1)
TcG_11206	5,060954875	1,313556254	0,835599591	1,571992457	0,115952301	1	protein_codin	dispersed gene family protein 1 (DGF-1)
TcG_11207	17,84180146	0,84654605	0,443146695	1,910306588	0,05609375	0,179283362	protein_codin	dispersed protein family protein 1 (DGF-1)
TcG_11208	1028,494898	0,401617764	0,063441043	6,330566899	2,44262E-10	1,10118E-08	protein_codin	60S ribosomal protein L34
TcG_11209	290,5707827	0,240097324	0,112075575	2,142280537	0,032170914	0,120469363	protein_codin	hypothetical protein
TcG_11210	429,7595348	0,048185143	0,090928493	0,529923478	0,596164987	0,780668859	protein_codin	hypothetical protein
TcG_11211	279,8914664	-0,077542079	0,1080583	-0,71759485	0,473007135	0,688821099	protein_codin	hypothetical protein
TcG_11212	64,01768865	0,385427671	0,22136511	1,741140103	0,081659023	0,234473716	protein_codin	hypothetical protein
TcG_11213	10,14537653	-0,18502652	0,57250213	-0,32318922	0,746551941	1		
TcG_11214	32,60877975	0,080154088	0,312917713	0,256150688	0,797834484	0,89910615	protein_codin	dynein
TcG_11215	74,46437958	0,114764117	0,199737345	0,574575158	0,565578657	0,757986619	protein_codin	hypothetical protein
TcG_11216	80,20778819	-0,182877871	0,196646426	-0,92998319	0,352379786	0,586675127	protein_codin	putative trans-sialidase
TcG_11217	58,41952585	0,2496748	0,228599365	1,092193757	0,274747946	0,507556451	protein_codin	putative kinesin
TcG_11218	293,4455595	-0,301912794	0,108331149	-2,78694353	0,005320775	0,030056801	protein_codin	putative retrotransposon hot spot (RHS) protein
TcG_11219	475,8541895	0,183877381	0,090221909	2,038056867	0,041544246	0,145125552	protein_codin	oxidoreductase-like protein
TcG_11220	620,2383423	-0,014461723	0,07684222	-0,18820022	0,850719698	0,928053891	protein_codin	hypothetical protein
TcG_11221	194,9552943	0,183125507	0,125775742	1,455968407	0,145401353	0,342611365	protein_codin	hypothetical protein
TcG_11222	19,45214127	-0,250384865	0,409158572	-0,61195068	0,540570392	0,739439027	protein_codin	putative trans-sialidase
TcG_11223	57,01847794	-0,074622798	0,233883832	-0,31905924	0,74968159	0,873736134	protein_codin	hypothetical protein
TcG_11224	196,0209612	-0,018508085	0,133270205	-0,13887639	0,889547831	0,946535728	protein_codin	putative trans-sialidase
TcG_11225	16,13915607	0,343106126	0,427938845	0,801764389	0,422689262	1	protein_codin	trans-sialidase
TcG_11226	1045,84025	0,277108477	0,061910324	4,475965522	7,60668E-06	0,000120485	protein_codin	putative retrotransposon hot spot (RHS) protein
TcG_11227	26,40226091	-0,172100951	0,345449681	-0,49819398	0,618347325	0,793033999	protein_codin	mucin TcMUCII
TcG_11228	9,085649603	-0,388135723	0,595212505	-0,65209605	0,514339212	1	protein_codin	hypothetical protein
TcG_11229	4,980950138	-0,721201371	0,775253607	-0,93027799	0,352227169	1	protein_codin	hypothetical protein

TcG_11230	4,017161802	-0,592375946	0,870098685	-0,68081467	0,495988769	1	protein_codin mucin-associated surface protein (MASP)
TcG_11231	25,44256919	0,584525182	0,35016847	1,669268456	0,095064189	0,259833672	protein_codin dispersed gene family protein 1 (DGF-1)
TcG_11232	240,4285425	-0,098854429	0,114488162	-0,86344673	0,387891897	0,618342807	protein_codin dispersed gene family protein 1 (DGF-1)
TcG_11233	121,226241	-0,133484024	0,167453317	-0,79714171	0,425368732	0,651205356	protein_codin dispersed gene family protein 1 (DGF-1)
TcG_11234	177,3611303	0,003300707	0,146609195	0,02251364	0,982038232	0,993289929	protein_codin trans-sialidase
TcG_11235	11,72587782	-0,077390785	0,52005315	-0,14881322	0,881701017	1	protein_codin hypothetical protein
TcG_11236	111,5264166	-0,10705385	0,175920007	-0,60853709	0,542831312	0,74052085	protein_codin mucin-associated surface protein (MASP)
TcG_11237	123,253397	0,048415187	0,162511128	0,297919213	0,765764824	0,882186661	protein_codin trans-sialidase
TcG_11238	11,110795	-0,472860927	0,527926483	-0,89569465	0,370415868	1	protein_codin trans-sialidase
TcG_11239	50,42055217	-0,249645009	0,248678694	-1,0038858	0,315433662	0,550228005	protein_codin trans-sialidase
TcG_11240	19,36987263	0,578289917	0,428428543	1,349793159	0,17708234	0,387183019	protein_codin hypothetical protein
TcG_11241	53,51449019	-0,791275087	0,24467014	-3,23404844	0,001220488	0,009165584	protein_codin mucin-associated surface protein (MASP)
TcG_11242	30,83257009	-0,184322094	0,329070919	-0,56012878	0,575391599	0,766252913	protein_codin hypothetical protein
TcG_11243	708,7597807	-0,014239005	0,072418149	-0,19662205	0,844123317	0,924394313	protein_codin hypothetical protein
TcG_11244	104,5514892	-0,410908684	0,173378875	-2,37000432	0,017787877	0,076856438	protein_codin hypothetical protein
TcG_11245	6,812653359	0,828898995	0,683045798	1,213533554	0,224925894	1	protein_codin trans-sialidase
TcG_11246	34,75355809	-0,341245603	0,294030004	-1,16058089	0,245812383	0,474324447	protein_codin mucin-associated surface protein (MASP)
TcG_11247	33,8831192	-0,280776352	0,306353015	-0,91651245	0,35939818	0,592630203	protein_codin hypothetical protein
TcG_11248	24,84424188	0,250179392	0,362611968	0,68993694	0,490233844	0,701302546	protein_codin trans-sialidase
TcG_11249	28,66659515	0,112394676	0,324901375	0,345934751	0,729391751	0,860563425	protein_codin trans-sialidase
TcG_11250	3,143662101	0,639441139	0,990468348	0,645594724	0,518541855	1	protein_codin calpain-like cysteine peptidase
TcG_11251	127,62114	-0,424344238	0,160767712	-2,63948671	0,008303168	0,042679904	
TcG_11252	232,4539375	-0,384421593	0,122518057	-3,13767294	0,001702948	0,012089677	protein_codin putative calpain-like cysteine peptidase
TcG_11253	516,4614248	-0,317854927	0,089883525	-3,53629797	0,000405777	0,003621979	protein_codin putative calpain-like cysteine peptidase, putative,cysteine peptidase, Clan CA, family C2
TcG_11254	7,739549134	-0,762851425	0,646463916	-1,18003713	0,237985447	1	protein_codin mucin-associated surface protein (MASP)
TcG_11255	16,85093499	0,50582847	0,432902685	1,168457688	0,242622197	0,471015545	
TcG_11256	24,12401138	0,476427391	0,360634578	1,321080728	0,186474448	0,400387872	protein_codin hypothetical protein
TcG_11257	7,134280889	0,062319201	0,704838296	0,08841631	0,929545799	1	protein_codin hypothetical protein
TcG_11258	14,63212917	0,114318871	0,478105524	0,239108033	0,811021813	1	protein_codin hypothetical protein
TcG_11259	28,05377027	-0,088739503	0,331246876	-0,26789537	0,788779861	0,894874711	protein_codin dispersed gene family protein 1 (DGF-1)
TcG_11260	56,67445721	0,443935286	0,237849111	1,86645762	0,061977368	0,194335531	protein_codin dispersed gene family protein 1 (DGF-1)
TcG_11261	17,89832577	0,011614929	0,443445947	0,026192434	0,97910385	0,99133944	protein_codin dispersed gene family protein 1 (DGF-1)
TcG_11262	44,63323033	0,242405163	0,261632458	0,926510284	0,354180855	0,588321059	protein_codin hypothetical protein
TcG_11263	11,19574886	-0,187578452	0,514251207	-0,36476035	0,715290303	1	protein_codin hypothetical protein
TcG_11264	12,84510713	0,26165888	0,489076818	0,535005689	0,592645923	1	protein_codin dispersed gene family protein 1 (DGF-1)
TcG_11265	19,74691258	0,5383015	0,400309696	1,344712617	0,178718058	0,389799325	protein_codin dispersed gene family protein 1 (DGF-1)
TcG_11266	82,11069663	0,381653516	0,199444696	1,913580677	0,055673756	0,178505081	protein_codin dispersed gene family protein 1 (DGF-1)
TcG_11267	29,66626421	0,202065474	0,320136024	0,631186304	0,527918716	0,730324327	protein_codin mucin TcMUC
TcG_11268	38,68821203	-1,131015196	0,285966437	-3,95506272	7,65146E-05	0,000885612	protein_codin mucin TcMUCII
TcG_11269	3,161010319	-1,133238814	0,991820398	-1,1425847	0,25321106	1	protein_codin trans-sialidase
TcG_11270	108,6608254	0,008371888	0,168616705	0,049650405	0,960400978	0,981885783	protein_codin putative trans-sialidase
TcG_11271	28,36165482	-0,357043913	0,354602014	-1,00688631	0,313989413	0,548203939	protein_codin hypothetical protein
TcG_11272	3,820435255	-1,016733698	0,900625097	-1,12892001	0,258931577	1	protein_codin hypothetical protein
TcG_11273	16,54727659	-0,218347539	0,440341398	-0,49585967	0,619993425	1	protein_codin trans-sialidase
TcG_11274	6,472981771	-0,025372381	0,729219539	-0,03479389	0,972244096	1	protein_codin trans-sialidase
TcG_11275	10,11803892	-0,256313358	0,585373854	-0,43786267	0,661485839	1	protein_codin trans-sialidase
TcG_11276	5,477404238	0,265474061	0,804890747	0,329826205	0,741531285	1	protein_codin trans-sialidase
TcG_11277	33,4585927	-0,568980494	0,303127381	-1,87703431	0,060513392	0,190956093	
TcG_11278	12,65256366	-0,329450758	0,511624965	-0,64393018	0,519620708	1	protein_codin mucin-associated surface protein (MASP)
TcG_11279	12,20386682	0,515676142	0,514337317	1,002603009	0,316052443	1	protein_codin hypothetical protein
TcG_11280	8,613336305	0,134292902	0,604707335	0,222079169	0,824252254	1	protein_codin hypothetical protein
TcG_11281	16,41842191	0,361215775	0,459013174	0,786939887	0,431317056	1	protein_codin hypothetical protein
TcG_11282	6,215033797	-0,013102932	0,707467387	-0,0185209	0,985223305	1	protein_codin hypothetical protein
TcG_11283	1,965053826	-0,125323857	1,250383766	-0,10022831	0,920163068	1	protein_codin hypothetical protein
TcG_11284	18,89597436	0,354259054	0,404379986	0,876054863	0,381000209	0,611048137	protein_codin hypothetical protein
TcG_11285	24,29729654	-0,230362618	0,351557843	-0,65526235	0,512298856	0,718385781	protein_codin helicase-like protein
TcG_11286	23,31410309	-0,400100809	0,371112088	-1,07811312	0,280983279	0,514211384	protein_codin trans-sialidase

TcG_11287	324,8882006	-0,020189972	0,100665401	-0,20056516	0,841038601	0,922466573	protein_codin putative retrotransposon hot spot (RHS) protein
TcG_11288	56,72046575	0,864767986	0,240500897	3,595695472	0,000323526	0,002984374	protein_codin putative retrotransposon hot spot (RHS) protein
TcG_11289	15,12034979	0,557662863	0,450718508	1,23727527	0,215984906	1	protein_codin hypothetical protein
TcG_11290	8,565120222	0,792851528	0,656696086	1,207334024	0,227303532	1	protein_codin hypothetical protein
TcG_11291	3,520400639	0,123047513	0,906267554	0,135773937	0,891999996	1	
TcG_11292	168,8140228	0,385828493	0,150849595	2,557703213	0,010536598	0,051444171	protein_codin RNA-binding protein
TcG_11293	139,3146858	-0,08288986	0,14833029	-0,55881951	0,576284906	0,766706337	protein_codin glutathione-S-transferase/glutaredoxin
TcG_11294	447,6317203	0,273723543	0,088567589	3,09056107	0,001997787	0,013720428	protein_codin trifunctional enzyme alpha subunit, mitochondrial precursor-like protein
TcG_11295	0,459623291	-2,652712423	2,530139326	-1,0484452	0,294433539	1	protein_codin trans-sialidase
TcG_11296	0					1	protein_codin hypothetical protein
TcG_11297	0,311976008	1,510501932	3,146303199	0,480087848	0,631164929	1	protein_codin hypothetical protein
TcG_11298	38,15107207	-0,087484653	0,286881308	-0,30495069	0,760403708	0,879069782	protein_codin trans-sialidase
TcG_11299	16,45083972	-0,635322491	0,466670089	-1,36139535	0,173388784	1	protein_codin trans-sialidase
TcG_11300	35,94180271	-0,14296486	0,301186264	-0,47467258	0,635020368	0,805049347	protein_codin mucin-like glycoprotein
TcG_11301	9,998054491	-1,038943837	0,556085132	-1,86831796	0,061717765	1	protein_codin putative trans-sialidase
TcG_11302	38,87545706	-0,124835014	0,289194919	-0,43166392	0,665985691	0,825196732	protein_codin trans-sialidase
TcG_11303	50,48845255	-0,424516365	0,255843465	-1,65928164	0,097059053	0,263482126	protein_codin hypothetical protein
TcG_11304	9,44612411	-0,416332892	0,591765381	-0,70354385	0,481716893	1	protein_codin trans-sialidase
TcG_11305	10,51189095	0,912546325	0,580926203	1,570847243	0,116218135	1	protein_codin trans-sialidase
TcG_11306	36,57263297	0,039903225	0,284992508	0,140014996	0,888648142	0,94628587	protein_codin hypothetical protein
TcG_11307	144,3256388	0,038404	0,147150886	0,260983814	0,794104989	0,897216926	protein_codin putative retrotransposon hot spot (RHS) protein
TcG_11308	59,09877403	0,071462134	0,226017313	0,316179912	0,751865954	0,87492919	protein_codin retrotransposon hot spot (RHS) protein
TcG_11309	12,91663815	0,912717804	0,498083179	1,832460603	0,066882826	1	protein_codin hypothetical protein
TcG_11310	4,838939136	0,438567748	0,815420808	0,537842233	0,590685973	1	protein_codin trans-sialidase
TcG_11311	291,063412	0,10864056	0,104507204	1,03955092	0,298548589	0,532231897	protein_codin hypothetical protein
TcG_11312	0					1	protein_codin thimet oligopeptidase
TcG_11313	0					1	protein_codin hypothetical protein
TcG_11314	4,509302626	-0,043169867	0,803785337	-0,0537082	0,957167646	1	protein_codin hypothetical protein
TcG_11315	258,9630799	-0,079212636	0,115257907	-0,68726423	0,491916238	0,702635507	
TcG_11316	24,07583861	-0,460438216	0,364879488	-1,2618912	0,206987939	0,429162896	protein_codin putative trans-sialidase
TcG_11317	0					1	protein_codin hypothetical protein
TcG_11318	1,215901867	-2,719168274	1,778653981	-1,52877867	0,126319321	1	protein_codin hypothetical protein
TcG_11319	0,614516902	-2,923786983	2,209719194	-1,32314866	0,185785947	1	protein_codin hypothetical protein
TcG_11320	2,127038109	0,257488977	1,191706657	0,216067415	0,828935199	1	protein_codin hypothetical protein
TcG_11321	26,4795225	-0,401393917	0,336128251	-1,19416894	0,23241189	0,459642541	protein_codin hypothetical protein
TcG_11322	40,28152047	-0,04895627	0,282760156	-0,17313709	0,862543672	0,934140118	protein_codin hypothetical protein
TcG_11323	27,67363541	-0,185380399	0,340156997	-0,54498482	0,58576396	0,77437942	protein_codin hypothetical protein
TcG_11324	16,51725166	-0,097820762	0,419357882	-0,2332632	0,815557032	1	protein_codin mucin TcMUCII
TcG_11325	11,10042247	-0,664115136	0,529176628	-1,2549971	0,209479811	1	protein_codin hypothetical protein
TcG_11326	6,616832915	0,184068198	0,690537472	0,266557871	0,789809596	1	protein_codin hypothetical protein
TcG_11327	0,927587303	-0,166402636	1,898537151	-0,08764782	0,930156596	1	protein_codin hypothetical protein
TcG_11328	12,94159993	-0,66098201	0,475961148	-1,38873102	0,164914558	1	protein_codin hypothetical protein
TcG_11329	14,79699462	-0,506751294	0,461133467	-1,09892543	0,271800593	1	protein_codin hypothetical protein
TcG_11330	8,277974255	-1,004759304	0,611441601	-1,64326291	0,100328549	1	protein_codin hypothetical protein
TcG_11331	175,643804	-0,319839901	0,132350937	-2,41660475	0,015666018	0,069837047	protein_codin mucin-associated surface protein (MASP)
TcG_11332	63,50020279	-0,504852547	0,221480741	-2,27944219	0,022640793	0,092691245	protein_codin mucin TcMUCII
TcG_11333	20,88128244	-0,141263568	0,390309697	-0,36192687	0,717406678	0,855087918	protein_codin hypothetical protein
TcG_11334	0,494227887	-1,278901833	2,548340486	-0,50185673	0,615768302	1	protein_codin hypothetical protein
TcG_11335	36,46028733	0,382458172	0,297564501	1,285295022	0,198689209	0,417560888	protein_codin protein kinase
TcG_11336	37,55943923	0,697384414	0,308617928	2,259701562	0,023839779	0,096407568	protein_codin putative trans-sialidase
TcG_11337	30,94274646	0,056524738	0,312787538	0,180712883	0,856592946	0,930653214	protein_codin trans-sialidase
TcG_11338	24,55860978	0,353296756	0,360582299	0,979795063	0,327187289	0,561765254	protein_codin hypothetical protein
TcG_11339	374,3492411	0,030746559	0,094824739	0,324246181	0,745751662	0,871496958	protein_codin retrotransposon hot spot (RHS) protein
TcG_11340	44,71213713	0,219899418	0,282107006	0,779489389	0,435691486	0,659399643	protein_codin hypothetical protein
TcG_11341	56,99735694	-0,016704635	0,248344561	-0,06726395	0,946371578	0,974694377	
TcG_11342	81,74242555	0,256980697	0,191962493	1,338702643	0,180667495	0,392062146	protein_codin ATP-dependent DEAD/H RNA helicase
TcG_11343	13,97380637	-0,547449383	0,470252965	-1,16415934	0,244359443	1	protein_codin hypothetical protein

TcG_11344	25,83189101	-0,209925831	0,352350278	-0,59578733	0,551317309	0,746821273	protein_codin trans-sialidase
TcG_11345	47,05993008	0,297948052	0,262636254	1,134451349	0,25660527	0,488583182	protein_codin trans-sialidase
TcG_11346	0,756278576	-1,907568997	3,816453778	-0,49982762	0,617196463	1	protein_codin hypothetical protein
TcG_11347	0,469265227	-2,470320505	2,566008638	-0,96270935	0,335693404	1	protein_codin hypothetical protein
TcG_11348	39,64671818	-0,27255822	0,278051151	-0,98024489	0,326965251	0,561467229	protein_codin mucin-associated surface protein (MASP)
TcG_11349	7,624456302	0,122698472	0,652401558	0,18807201	0,850820202	1	protein_codin trans-sialidase
TcG_11350	72,26942455	0,056579816	0,202972173	0,278756516	0,780431687	0,89067206	protein_codin putative trans-sialidase
TcG_11351	3,311714648	-0,910748674	0,955445283	-0,95321908	0,340479081	1	protein_codin surface protease GP63
TcG_11352	4,890627327	1,555001257	0,87074045	1,785837855	0,074125516	1	protein_codin surface protease GP63
TcG_11353	1,535281757	0,946096683	1,452968795	0,651147283	0,514951417	1	protein_codin syntaxin binding protein
TcG_11354	6,087616811	-0,635095988	0,739019852	-0,85937609	0,390133059	1	protein_codin putative syntaxin binding protein
TcG_11355	1,632248611	0,334596411	1,39398018	0,240029533	0,810307362	1	protein_codin hypothetical protein
TcG_11356	5,457563417	0,267857577	0,735073926	0,364395426	0,71556275	1	protein_codin hypothetical protein
TcG_11357	14,75614251	-0,274560997	0,460598455	-0,59609622	0,55111095	1	protein_codin trans-sialidase
TcG_11358	10,4334826	0,200017719	0,546038005	0,366307322	0,714135767	1	
TcG_11359	19,16565323	0,201092091	0,40444777	0,497201631	0,619046871	0,793656861	protein_codin hypothetical protein
TcG_11360	83,99273228	-0,995041151	0,196379579	-5,06692781	4,04288E-07	8,97332E-06	protein_codin mucin-associated surface protein (MASP)
TcG_11361	118,850105	-0,561502546	0,163069074	-3,44334171	0,000574573	0,004876924	protein_codin mucin-associated surface protein (MASP)
TcG_11362	52,57156126	-0,335540593	0,24497708	-1,36968158	0,170786323	0,377476218	protein_codin trans-sialidase
TcG_11363	82,51006461	-0,149996201	0,203482084	-0,73714697	0,461032981	0,6791517	protein_codin trans-sialidase
TcG_11364	1,657197946	0,587019982	1,376925732	0,426326539	0,669869922	1	protein_codin target of rapamycin (TOR) kinase 1
TcG_11365	1,474719999	-0,7496187	1,423416712	-0,52663334	0,598448234	1	protein_codin hypothetical protein
TcG_11366	1,741135511	0,043361943	2,661691924	0,01629112	0,987002142	1	protein_codin surface protease GP63
TcG_11367	2,801808223	0,239215874	1,102689145	0,216938631	0,828256171	1	protein_codin hypothetical protein
TcG_11368	0,756840188	-0,42123777	2,117387077	-0,19894226	0,842307912	1	protein_codin hypothetical protein
TcG_11369	586,8929928	0,30090301	0,082016162	3,668825782	0,000243667	0,002354568	protein_codin ubiquitin hydrolase, putative,cysteine peptidase, Clan CA, family C19
TcG_11370	10,05965298	-0,830824377	0,562978391	-1,47576601	0,140006717	1	protein_codin hypothetical protein
TcG_11371	3,094720505	-0,634030434	0,982143902	-0,64555757	0,518565921	1	protein_codin hypothetical protein
TcG_11372	35,71976337	-0,27961188	0,28760781	-0,9721985	0,330951809	0,565235831	protein_codin retrotransposon hot spot (RHS) protein
TcG_11373	15,73187604	0,094818216	0,440495008	0,215253782	0,829569462	1	protein_codin putative retrotransposon hot spot (RHS) protein
TcG_11374	22,14185265	-0,371875801	0,36496764	-1,01892814	0,308237081	0,541260203	protein_codin retrotransposon hot spot protein (RHS)
TcG_11375	19,83496937	0,094090681	0,4163786	0,225973863	0,821221749	0,911367354	protein_codin retrotransposon hot spot protein (RHS)
TcG_11376	5,802778925	-0,483261452	0,748380258	-0,64574319	0,518445688	1	protein_codin retrotransposon hot spot (RHS) protein
TcG_11377	15,83893232	-0,495434742	0,441168827	-1,12300487	0,261435426	1	protein_codin hypothetical protein
TcG_11378	46,0657645	0,085107683	0,256199325	0,332193237	0,73974335	0,86729518	protein_codin trans-sialidase
TcG_11379	21,87293912	0,16404768	0,374711025	0,437797847	0,661532832	0,822726426	protein_codin ATP-dependent DEAD/H RNA helicase
TcG_11380	7,428875535	0,145802525	0,660786918	0,220649836	0,825365094	1	
TcG_11381	24,91793657	0,012198298	0,348761768	0,034976019	0,972098863	0,987776155	protein_codin putative trans-sialidase
TcG_11382	8,955459896	0,298464419	0,574620023	0,519411797	0,603473606	1	protein_codin hypothetical protein
TcG_11383	14,36548046	-1,467086188	0,474751932	-3,09021636	0,002000107	1	protein_codin subtilisin-like serine peptidase
TcG_11384	4,115278879	-1,467813328	0,888715449	-1,65161226	0,09861362	1	protein_codin subtilisin-like serine peptidase
TcG_11385	74,77057948	0,170200214	0,204888448	0,830696977	0,406144835	0,634690323	
TcG_11386	159,5006932	0,190472517	0,152038097	1,252794663	0,210280444	0,432895627	protein_codin putative mitochondrial carrier protein
TcG_11387	386,3976303	-0,263490777	0,094649414	-2,78386061	0,005371611	0,030314412	protein_codin cell morphogenesis protein Las1
TcG_11388	225,3599366	-0,102071904	0,126647	-0,80595595	0,420268248	0,646815611	protein_codin hypothetical protein
TcG_11389	298,0517642	0,031155592	0,108067386	0,288297819	0,773118777	0,886918879	protein_codin hypothetical protein
TcG_11390	126,9888074	0,037064625	0,159338537	0,232615571	0,816059935	0,90921145	protein_codin trans-sialidase
TcG_11391	26,82274198	-0,004557052	0,375946326	-0,01212155	0,990328639	0,996036512	protein_codin hypothetical protein
TcG_11392	54,29451287	0,05594189	0,244438812	0,228858459	0,818978928	0,910797645	protein_codin hypothetical protein
TcG_11393	288,5635926	-0,293633368	0,109340768	-2,68548843	0,007242388	0,038332713	protein_codin mucin-associated surface protein (MASP)
TcG_11394	53,46790005	0,334363529	0,243314641	1,374202256	0,169378901	0,37606421	protein_codin hypothetical protein
TcG_11395	403,0871275	-0,146612896	0,094934879	-1,54435227	0,122503016	0,307355751	protein_codin putative retrotransposon hot spot (RHS) protein
TcG_11396	16,7034944	0,397128579	0,448577934	0,885305649	0,375991804	1	protein_codin hypothetical protein
TcG_11397	42,6381922	0,343550598	0,266418216	1,28951617	0,197218699	0,415601281	protein_codin trans-sialidase
TcG_11398	42,03712528	0,001912594	0,271215062	0,007051945	0,994373408	0,997731905	protein_codin hypothetical protein
TcG_11399	34,93044037	0,15336985	0,301080113	0,509398807	0,610472713	0,789056132	protein_codin hypothetical protein
TcG_11400	7,213830436	0,465802476	0,666264497	0,699125465	0,484473626	1	protein_codin dispersed gene family protein 1 (DGF-1)

TcG_11401	70,19761249	0,304886614	0,230289497	1,323927572	0,1855271	0,399390001	protein_codin dispersed gene family protein 1 (DGF-1)
TcG_11402	61,85905598	0,066428226	0,22358909	0,297099583	0,766390483	0,882731895	
TcG_11403	29,07448313	-0,282318653	0,339843692	-0,83073089	0,406125672	0,634690323	protein_codin putative trans-sialidase
TcG_11404	11,80812744	0,067025241	0,578998805	0,115760587	0,907842288	1	protein_codin trans-sialidase
TcG_11405	207,811501	0,345154201	0,128106734	2,694270553	0,007054288	0,037520323	protein_codin putative trans-sialidase
TcG_11406	1624,633414	0,184833112	0,061299937	3,015225157	0,002567886	0,016780328	protein_codin L1Tc protein
TcG_11407	41,98601421	0,167201554	0,271448196	0,615961189	0,537920135	0,73738082	protein_codin trans-sialidase
TcG_11408	31,73750481	0,333243822	0,317002751	1,051233218	0,29315149	0,526173999	protein_codin mucin TcMUCII
TcG_11409	459,0405522	0,043171656	0,092530832	0,466565089	0,640811069	0,809291154	protein_codin retrotransposon hot spot (RHS) protein
TcG_11410	12,06244719	0,667109937	0,521000195	1,28044086	0,200390131	1	protein_codin hypothetical protein
TcG_11411	7,376352163	0,744813703	0,644601344	1,155464085	0,247900432	1	protein_codin subtilisin-like serine peptidase
TcG_11412	32,91870937	-0,042678045	0,303162001	-0,14077637	0,888046614	0,945946339	protein_codin subtilisin-like serine peptidase
TcG_11413	39,33930596	0,271086235	0,309439341	0,876056139	0,380999515	0,611048137	protein_codin putative kinesin
TcG_11414	8,452088794	0,043650629	0,59532431	0,073322437	0,941549538	1	protein_codin dispersed gene family protein 1 (DGF-1)
TcG_11415	20,43890007	-0,006865303	0,381918231	-0,01797584	0,985658124	0,99432608	protein_codin dispersed gene family protein 1 (DGF-1)
TcG_11416	30,13634221	0,113064513	0,319108766	0,354313402	0,723104025	0,857335574	protein_codin dispersed gene family protein 1 (DGF-1)
TcG_11417	10,54229568	0,306762243	0,533799859	0,574676515	0,565510093	1	protein_codin dispersed gene family protein 1 (DGF-1)
TcG_11418	107,3130728	-0,723387644	0,168432981	-4,29480995	1,74843E-05	0,00024704	protein_codin mucin TcMUCII
TcG_11419	81,4018163	-0,65785116	0,198493569	-3,31421902	0,000918995	0,007292791	protein_codin putative mucin-associated surface protein (MASP)
TcG_11420	6,784862266	0,241451953	0,694501091	0,347662452	0,728093698	1	protein_codin surface protease GP63
TcG_11421	8,443259353	0,030547114	0,601981717	0,050744255	0,959529311	1	protein_codin surface protease GP63
TcG_11422	3,803027469	-0,385811406	0,916400311	-0,4210075	0,673749604	1	protein_codin hypothetical protein
TcG_11423	11,21757164	0,204922436	0,519724359	0,394290614	0,693366492	1	
TcG_11424	48,84801153	-0,290756746	0,263795409	-1,10220548	0,270372349	0,503380048	protein_codin trans-sialidase
TcG_11425	102,4410659	-0,574301396	0,177034596	-3,24400658	0,00117861	0,008919254	protein_codin mucin-associated surface protein (MASP)
TcG_11426	40,87752699	-0,51917749	0,271549434	-1,91190784	0,055888016	0,178906968	
TcG_11427	5,087689834	-0,708488053	0,811039059	-0,87355602	0,382360085	1	
TcG_11428	16,72553607	0,158129962	0,4370233	0,361834168	0,717475958	1	protein_codin hypothetical protein
TcG_11429	13,92051478	0,156095078	0,497535404	0,313736625	0,753721082	1	protein_codin hypothetical protein
TcG_11430	393,4550196	0,106417969	0,095662958	1,112426071	0,265955	0,498666135	protein_codin trans-sialidase-like protein
TcG_11431	114,365762	0,000570534	0,169426088	0,003367453	0,997313166	0,999104158	protein_codin beta galactofuranosyl glycosyltransferase
TcG_11432	13,42502165	0,583922672	0,499910297	1,1680549	0,242784621	1	protein_codin hypothetical protein
TcG_11433	63,26259008	0,379625095	0,247740552	1,532349433	0,125436229	0,312378851	protein_codin trans-sialidase
TcG_11434	93,10485475	-0,029665892	0,186381243	-0,1591678	0,873536673	0,939322955	protein_codin putative mucin-associated surface protein (MASP)
TcG_11435	147,1765449	-0,191467804	0,147379315	-1,29914977	0,193892535	0,410814106	protein_codin trans-sialidase
TcG_11436	42,78689803	-0,425304195	0,263211778	-1,61582509	0,106132135	0,280549147	protein_codin trans-sialidase
TcG_11437	341,272344	-0,195154111	0,100791428	-1,93621734	0,052841088	0,171681672	
TcG_11438	411,4590707	0,81392351	0,094256181	8,635226871	5,86106E-18	8,48828E-16	protein_codin dispersed gene family protein 1 (DGF-1)
TcG_11439	17,27007895	0,891000199	0,426490853	2,089142574	0,036694891	0,133233158	protein_codin hypothetical protein
TcG_11440	85,05524858	0,875679881	0,202744275	4,319134929	1,56642E-05	0,00022378	protein_codin dispersed gene family protein 1 (DGF-1)
TcG_11441	45,72562796	-0,118475275	0,263407746	-0,44977901	0,652869795	0,816333849	protein_codin hypothetical protein
TcG_11442	17,31218171	0,070981711	0,417757459	0,169911296	0,865079898	0,935010385	protein_codin hypothetical protein
TcG_11443	90,60348713	-0,014451293	0,185022594	-0,07810556	0,937744084	0,97127686	protein_codin retrotransposon hot spot (RHS) protein
TcG_11444	329,1897843	0,080924756	0,098799802	0,819078124	0,412741842	0,6393939	protein_codin putative retrotransposon hot spot (RHS) protein
TcG_11445	68,48717956	0,208159171	0,212560279	0,979294779	0,327434349	0,561939619	protein_codin retrotransposon hot spot (RHS) protein
TcG_11446	33,99866449	-0,110316572	0,30988176	-0,35599569	0,72184379	0,857117481	
TcG_11447	73,58996425	-0,05837566	0,201846566	-0,28920809	0,772422142	0,886418674	protein_codin putative retrotransposon hot spot (RHS) protein
TcG_11448	32,61173171	-0,058226461	0,31658512	-0,1839204	0,854075902	0,929165368	protein_codin trans-sialidase
TcG_11449	31,85102042	0,189680459	0,312981399	0,60604387	0,544485622	0,741642419	protein_codin trans-sialidase
TcG_11450	0,573889029	1,026419886	2,174050325	0,472123333	0,636838758	1	protein_codin retrotransposon hot spot (RHS) protein
TcG_11451	141,458148	0,172334652	0,151938301	1,134241012	0,256693465	0,4886708	protein_codin hypothetical protein
TcG_11452	58,71787219	0,413202886	0,228966245	1,804645419	0,071130186	0,21392986	protein_codin hypothetical protein
TcG_11453	333,616653	-0,010357717	0,100676439	-0,10288124	0,918057224	0,96050307	protein_codin putative trans-sialidase
TcG_11454	238,5169301	-0,133526536	0,119225424	-1,11995019	0,262734988	0,494886615	protein_codin 85 kDa surface glycoprotein
TcG_11455	15,70077782	-0,038004667	0,456126736	-0,08332041	0,933596771	1	protein_codin hypothetical protein
TcG_11456	28,03946168	0,124945798	0,339382477	0,368156303	0,712756691	0,852489876	protein_codin trans-sialidase
TcG_11457	62,22121719	0,012094507	0,220165096	0,054933807	0,956191199	0,979698552	protein_codin trans-sialidase

TcG_11458	49,27339636	0,141167489	0,249177623	0,566533572	0,571031118	0,762440697	protein_codin	hypothetical protein
TcG_11459	48,2060885	0,470297633	0,258788515	1,817304884	0,069170443	0,210398726	protein_codin	hypothetical protein
TcG_11460	46,38956814	0,38351889	0,262126012	1,463108855	0,143437595	0,339919816	protein_codin	mucin-associated surface protein (MASP)
TcG_11461	48,81059101	0,475996517	0,249766898	1,905763016	0,056680959	0,180661787	protein_codin	hypothetical protein
TcG_11462	45,27293119	0,575803075	0,261933857	2,19827662	0,027929399	0,108405368	protein_codin	hypothetical protein
TcG_11463	0					1	protein_codin	hypothetical protein
TcG_11464	15,24748108	0,641430621	0,455369389	1,408594069	0,158955234	1	protein_codin	hypothetical protein
TcG_11465	39,15361462	0,436238183	0,283737136	1,537472988	0,124177525	0,310211809	protein_codin	hypothetical protein
TcG_11466	12,11213808	-0,953761584	0,538199889	-1,77213263	0,076372544	1	protein_codin	hypothetical protein
TcG_11467	51,38203022	0,068760348	0,244565672	0,281152899	0,778593129	0,889535549	protein_codin	hypothetical protein
TcG_11468	24,48340179	-0,977669895	0,37559506	-2,60298923	0,009241486	0,046391618	protein_codin	hypothetical protein
TcG_11469	18,77781524	-0,492797332	0,398125995	-1,2377924	0,215793047	0,439553137	protein_codin	thimet oligopeptidase
TcG_11470	42,42495262	-0,721895624	0,282515764	-2,55524016	0,010611456	0,051787837		
TcG_11471	2,506691713	-1,260271447	1,227179236	-1,02696608	0,304436437	1		
TcG_11472	306,3896014	-0,131774512	0,107428857	-1,22662119	0,219965002	0,445077631	protein_codin	putative retrotransposon hot spot (RHS) protein
TcG_11473	58,15438971	0,042680797	0,231816067	0,184114921	0,853923301	0,929148282	protein_codin	hypothetical protein
TcG_11474	65,65703611	0,10333549	0,213804609	0,483317412	0,628870379	0,801074673	protein_codin	trans-sialidase
TcG_11475	131,1299213	0,106335253	0,163678253	0,64966024	0,515911712	0,721815372	protein_codin	trans-sialidase-like protein
TcG_11476	47,67583251	0,067460396	0,251762982	0,267952008	0,788736261	0,894874711	protein_codin	hypothetical protein
TcG_11477	140,0006584	0,155695666	0,163032235	0,954999269	0,339578067	0,57368788	protein_codin	hypothetical protein
TcG_11478	318,5334464	-0,072759766	0,11116074	-0,65454554	0,512760399	0,718795158	protein_codin	retrotransposon hot spot (RHS) protein
TcG_11479	135,2898263	-0,057392449	0,154008875	-0,37265676	0,709403921	0,850052867	protein_codin	putative retrotransposon hot spot (RHS) protein
TcG_11480	58,2044643	-0,162550301	0,238071149	-0,68278034	0,494745651	0,70453824		
TcG_11481	1,39895475	0,329276146	1,577691943	0,208707503	0,834676582	1	protein_codin	hypothetical protein
TcG_11482	2,830292166	-2,389620952	1,2859069	-1,85831568	0,063124199	1	protein_codin	hypothetical protein
TcG_11483	9,660988389	0,068360966	0,554623325	0,123256565	0,901903935	1		
TcG_11484	11,23738823	0,716695005	0,540954525	1,324871078	0,185213915	1		
TcG_11485	9,513770941	-2,318866099	0,64366784	-3,60258188	0,000315072	1	protein_codin	mucin TcMUCII
TcG_11486	2,055773518	-0,498565891	1,288151296	-0,38703985	0,698726703	1		
TcG_11487	20,18063525	-0,19580434	0,391763245	-0,49980273	0,617213987	0,793033999	protein_codin	mucin-associated surface protein (MASP)
TcG_11488	83,90810139	0,14980952	0,196652017	0,761800071	0,446179338	0,667885505	protein_codin	dispersed gene family protein 1 (DGF-1)
TcG_11489	25,92000123	0,04631987	0,356146954	0,130058309	0,896520295	0,949883084		
TcG_11490	190,9253936	-0,060887851	0,132739215	-0,45870281	0,646447597	0,812019442		
TcG_11491	510,741018	0,02232475	0,082021946	0,272180203	0,785483463	0,893514718	protein_codin	putative retrotransposon hot spot (RHS) protein
TcG_11492	61,31566783	0,23488228	0,226609036	1,036508886	0,299964796	0,533675126	protein_codin	putative trans-sialidase
TcG_11493	125,8396649	0,582588041	0,163050729	3,573047749	0,00035285	0,00321141	protein_codin	dispersed gene family protein 1 (DGF-1)
TcG_11494	283,8489134	0,597088091	0,119489596	4,996988106	5,82327E-07	1,25639E-05	protein_codin	dispersed gene family protein 1 (DGF-1)
TcG_11495	17,31521929	-0,341556184	0,442107514	-0,77256362	0,439780679	0,662760009	protein_codin	hypothetical protein
TcG_11496	7,655669232	-0,429576032	0,629305798	-0,6826189	0,494847687	1	protein_codin	hypothetical protein
TcG_11497	10,58576946	0,162622065	0,53289499	0,305167187	0,760238822	1	protein_codin	mucin-associated surface protein (MASP)
TcG_11498	14,74211348	-0,359327037	0,446898339	-0,80404648	0,421370132	1	protein_codin	hypothetical protein
TcG_11499	6,63654073	0,398145072	0,732409745	0,543609741	0,586710055	1	protein_codin	hypothetical protein
TcG_11500	49,40601757	-0,318264542	0,254742136	-1,24935963	0,211533566	0,43452566		
TcG_11501	62,63562358	0,125574046	0,221078411	0,568006823	0,570030335	0,761925641	protein_codin	mucin TcMUCII
TcG_11502	171,8194996	-0,304731872	0,139087534	-2,1909359	0,028456431	0,109869556	protein_codin	mucin-associated surface protein (MASP)
TcG_11503	95,24977544	0,134710581	0,186837612	0,721003549	0,470907331	0,686925321	protein_codin	trans-sialidase
TcG_11504	2,296872839	0,597938551	2,857636657	0,209242329	0,83425907	1	protein_codin	hypothetical protein
TcG_11505	2,32222405	0,401511375	1,174343508	0,341902835	0,732424016	1	protein_codin	hypothetical protein
TcG_11506	1,904787291	-1,062261353	1,327838075	-0,79999314	0,423714771	1	protein_codin	hypothetical protein
TcG_11507	4,75170133	-1,172498368	0,822051714	-1,42630731	0,153779656	1	protein_codin	hypothetical protein
TcG_11508	17,20239521	0,590794364	0,419993824	1,406673932	0,159524099	0,362110397	protein_codin	mucin TcMUCII
TcG_11509	1,679971819	0,197485257	1,333553881	0,148089447	0,882272178	1		
TcG_11510	40,61870255	0,195603279	0,277664795	0,704458335	0,481147388	0,695361812		
TcG_11511	40,52580868	0,560805885	0,303485152	1,84788574	0,064618874	0,200233825	protein_codin	putative trans-sialidase
TcG_11512	46,2919952	-0,334613844	0,26704192	-1,25303864	0,210191645	0,432895627	protein_codin	trans-sialidase
TcG_11513	72,22769138	-0,400694388	0,212491634	-1,88569489	0,059336082	0,187560733	protein_codin	retrotransposon hot spot protein (RHS)
TcG_11514	37,9945976	-0,140664084	0,280512348	-0,50145416	0,616051532	0,792562564	protein_codin	putative retrotransposon hot spot (RHS) protein

TcG_11515	71,7250035	0,165186492	0,22388127	0,737830776	0,460617289	0,678659097	protein_codin	trans-sialidase
TcG_11516	76,35888214	0,469042646	0,218686051	2,144821971	0,031967084	0,11991016	protein_codin	trans-sialidase
TcG_11517	30,73746224	-0,529893166	0,370416646	-1,43053281	0,152564155	0,352816028		
TcG_11518	23,26676506	0,054132503	0,376933126	0,143613015	0,885806063	0,944588039	protein_codin	hypothetical protein
TcG_11519	5,339247898	0,817051318	0,77556787	1,053487836	0,292117466	1	protein_codin	hypothetical protein
TcG_11520	6,775273911	0,377197479	0,66832621	0,56439127	0,572487879	1	protein_codin	hypothetical protein
TcG_11521	0,324841555	-0,458748506	2,940479636	-0,15601146	0,876023992	1	protein_codin	putative trans-sialidase
TcG_11522	0,807744502	1,687520936	1,945319872	0,867477354	0,385680515	1	protein_codin	helicase-like protein
TcG_11523	0,116927736	0,503022807	4,080472857	0,123275616	0,901888849	1	protein_codin	hypothetical protein
TcG_11524	0,350783209	1,758746969	2,99002236	0,58820529	0,556394507	1	protein_codin	hypothetical protein
TcG_11525	8,766508783	0,812928032	0,612550488	1,327120046	0,184468975	1	protein_codin	hypothetical protein
TcG_11526	6,27067816	0,338683023	0,71036685	0,476771999	0,633524487	1	protein_codin	dispersed gene family protein 1 (DGF-1)
TcG_11527	23,56474341	0,317114633	0,400263992	0,792263705	0,42820693	0,653564154	protein_codin	dispersed gene family protein 1 (DGF-1)
TcG_11528	10,28087766	0,293676782	0,557061691	0,527188975	0,598062364	1	protein_codin	dispersed gene family protein 1 (DGF-1)
TcG_11529	9,244980512	1,118781818	0,599811867	1,865221213	0,062150403	1	protein_codin	hypothetical protein
TcG_11530	12,57817905	-0,244708621	0,539597611	-0,45350205	0,65018727	1	protein_codin	retrotransposon hot spot (RHS) protein
TcG_11531	30,32649776	0,22126257	0,330942575	0,66858297	0,503761539	0,712038696	protein_codin	retrotransposon hot spot (RHS) protein
TcG_11532	29,24019791	0,101440475	0,33038382	0,307038265	0,758814247	0,878108456	protein_codin	hypothetical protein
TcG_11533	7,802868052	-0,260266928	0,644486657	-0,40383602	0,686333305	1		
TcG_11534	58,24011374	0,127129236	0,236093094	0,538470795	0,590252063	0,776238879		
TcG_11535	3,727781677	-0,007444812	0,885999442	-0,00840273	0,993295672	1		
TcG_11536	27,74059817	-0,045098287	0,327258916	-0,13780614	0,890393633	0,94667588	protein_codin	hypothetical protein
TcG_11537	49,70262966	0,064930111	0,266527858	0,243614726	0,807529222	0,904926354	protein_codin	retrotransposon hot spot (RHS) protein
TcG_11538	23,2559068	0,254417503	0,363619395	0,699680783	0,48412668	0,697418541	protein_codin	retrotransposon hot spot (RHS) protein
TcG_11539	26,10643373	-0,077733012	0,359353852	-0,21631328	0,828743555	0,916457611	protein_codin	retrotransposon hot spot (RHS) protein
TcG_11540	41,65140033	0,501380676	0,287302036	1,745134436	0,080961471	0,232932605	protein_codin	hypothetical protein
TcG_11541	19,55322422	-0,538619833	0,408748091	-1,31773052	0,187593875	0,402046363		
TcG_11542	29,54747708	-0,336247822	0,340367483	-0,98789643	0,323203373	0,557573663		
TcG_11543	18,75063252	-0,306059949	0,395728199	-0,7734095	0,439280068	0,662631709	protein_codin	hypothetical protein
TcG_11544	17,88209771	-0,809156571	0,409825737	-1,97439179	0,048337211	0,161570239	protein_codin	hypothetical protein
TcG_11545	12,21698206	-0,429695257	0,502875213	-0,85447691	0,392840798	1	protein_codin	hypothetical protein
TcG_11546	141,549784	-0,015413022	0,147367936	-0,10458871	0,916702175	0,959735311	protein_codin	surface protein-2
TcG_11547	543,432778	0,780663826	0,084283905	9,262312059	2,00053E-20	3,79969E-18	protein_codin	dispersed gene family protein 1 (DGF-1)
TcG_11548	269,533506	0,733632504	0,115334842	6,360892244	2,00585E-10	9,20059E-09	protein_codin	dispersed gene family protein 1 (DGF-1)
TcG_11549	147,1224971	0,245521189	0,16460563	1,491572249	0,135811318	0,328363512	protein_codin	hypothetical protein
TcG_11550	71,25729771	-0,025600167	0,215232937	-0,11894168	0,905321561	0,953903154	protein_codin	hypothetical protein
TcG_11551	347,8695842	0,142977475	0,099700907	1,434063932	0,151554019	0,351799471	protein_codin	putative syntaxin binding protein
TcG_11552	94,77147229	0,17847633	0,180412463	0,989268297	0,322531891	0,557248729	protein_codin	hypothetical protein
TcG_11553	190,3290925	0,026866468	0,127616162	0,21052559	0,833257482	0,919089983	protein_codin	surface protease GP63
TcG_11554	189,0707325	-0,185445938	0,140476258	-1,32012299	0,186793959	0,400784893	protein_codin	mucin-associated surface protein (MASP)
TcG_11555	138,7102426	-0,002558389	0,150750529	-0,01697101	0,986459743	0,994631244	protein_codin	elongation factor 1-gamma (EF-1-gamma)
TcG_11556	49,28713869	-0,321312499	0,251995852	-1,27507059	0,20228426	0,422382768	protein_codin	hypothetical protein
TcG_11557	42,1816695	-0,073396454	0,271260816	-0,27057522	0,786717759	0,893794073	protein_codin	trans-sialidase
TcG_11558	22,25575115	0,489959301	0,366630881	1,336383064	0,181424099	0,392966837	protein_codin	trans-sialidase
TcG_11559	117,5562983	-0,167529361	0,161955831	-1,03441389	0,300942714	0,534199829	protein_codin	putative calpain-like cysteine peptidase
TcG_11560	35,69453103	0,365327235	0,313660744	1,164720934	0,244131973	0,472284694	protein_codin	calpain-like cysteine peptidase, putative,cysteine peptidase, Clan CA, family C2
TcG_11561	3,599220455	0,294305929	0,959210786	0,306820913	0,758979689	1	protein_codin	calpain-like cysteine peptidase
TcG_11562	20,14281699	0,368375311	0,412196054	0,893689563	0,371488011	0,602808656	protein_codin	trans-sialidase
TcG_11563	2,554718739	0,415385103	1,142109724	0,363699822	0,716082176	1	protein_codin	mucin TcMUC
TcG_11564	0,94826079	1,85906385	1,948930856	0,953889074	0,340139795	1	protein_codin	hypothetical protein
TcG_11565	1,170285541	0,784823943	1,707958706	0,459509905	0,645868041	1	protein_codin	hypothetical protein
TcG_11566	0,428370964	1,990401553	2,587616184	0,769202777	0,441772942	1	protein_codin	hypothetical protein
TcG_11567	1,05232298	3,269962262	1,84561937	1,771742493	0,076437313	1		
TcG_11568	44,66815333	0,108201612	0,271641036	0,398325721	0,690390103	0,83922987	protein_codin	hypothetical protein
TcG_11569	287,1500195	0,035823784	0,114956605	0,311628758	0,755322676	0,876519283	protein_codin	regulator of sigma E protease
TcG_11570	167,757995	-0,253904691	0,134494442	-1,88784523	0,059046731	0,186865727	protein_codin	trans-sialidase
TcG_11571	42,96315328	-0,369893192	0,261330957	-1,4154205	0,156945244	0,358581659	protein_codin	mucin-associated surface protein (MASP)

TcG_11572	21,02222634	0,42515422	0,384507201	1,105711983	0,268851202	0,501496548	protein_codin hypothetical protein
TcG_11573	2,465286939	-0,406808424	1,177534919	-0,34547462	0,729737591	1	
TcG_11574	17,88278763	-0,256348848	0,419818069	-0,6106189	0,541451907	0,740033242	protein_codin putative trans-sialidase
TcG_11575	9,590076338	0,774706242	0,593182548	1,306016579	0,19154692	1	protein_codin trans-sialidase
TcG_11576	40,48715747	0,241325507	0,27852567	0,866439015	0,386249456	0,616572913	protein_codin trans-sialidase
TcG_11577	7,571451602	0,218833306	0,656042107	0,333565946	0,738707119	1	protein_codin hypothetical protein
TcG_11578	8,315497876	0,026800606	0,607653825	0,044105056	0,964820663	1	protein_codin hypothetical protein
TcG_11579	132,2547378	0,339268746	0,165478033	2,050234343	0,040341568	0,142022914	protein_codin GP63 group II protein
TcG_11580	421,9847195	-0,109412908	0,089968183	-1,2161289	0,223935823	0,450515792	protein_codin XPA-interacting protein
TcG_11581	329,0515031	-0,142057721	0,100189464	-1,41789082	0,156222642	0,357690793	protein_codin hypothetical protein
TcG_11582	259,503118	0,21807148	0,11393879	1,913935375	0,055628414	0,178477962	protein_codin hypothetical protein
TcG_11583	70,52586547	0,068804866	0,211761462	0,324916842	0,74524401	0,871193331	protein_codin hypothetical protein
TcG_11584	7,691592105	0,187250658	0,616477035	0,30374312	0,761323604	1	protein_codin trans-sialidase
TcG_11585	7,769725964	0,222275117	0,620497208	0,358220979	0,720177949	1	protein_codin putative trans-sialidase
TcG_11586	15,97540913	0,288728015	0,44907036	0,642946053	0,520259105	1	protein_codin hypothetical protein
TcG_11587	120,9871718	0,069004165	0,164803788	0,41870497	0,675431763	0,830121185	protein_codin putative trans-sialidase
TcG_11588	35,15228713	0,235514219	0,298800491	0,788198902	0,430580371	0,655385122	
TcG_11589	24,15172656	0,811039679	0,374089303	2,168037609	0,030155825	0,114929405	protein_codin putative retrotransposon hot spot (RHS) protein
TcG_11590	68,90309236	-0,269670106	0,214698943	-1,25603835	0,209102067	0,431627308	protein_codin retrotransposon hot spot (RHS) protein
TcG_11591	24,12294997	0,362842254	0,351745961	1,031546329	0,302284694	0,535372803	protein_codin retrotransposon hot spot (RHS) protein
TcG_11592	24,01486317	0,181296755	0,35699884	0,507835696	0,611568574	0,789925697	protein_codin retrotransposon hot spot (RHS) protein
TcG_11593	267,4462324	0,144892972	0,109565425	1,322433348	0,18602389	0,399879894	protein_codin retrotransposon hot spot (RHS) protein
TcG_11594	74,90815754	-0,165416884	0,200785786	-0,82384758	0,410026167	0,636890088	protein_codin ATP-dependent DEAD/H RNA helicase
TcG_11595	44,85420919	0,17622153	0,278787171	0,632100569	0,527321164	0,729846255	protein_codin putative kinesin
TcG_11596	95,88275895	0,169321855	0,17707211	0,956231084	0,338955499	0,57332217	protein_codin hypothetical protein
TcG_11597	29,60103422	-0,411067133	0,342382884	-1,20060655	0,22990386	0,456889557	protein_codin mitotic centromere-associated kinesin (MCAK)
TcG_11598	34,89897437	0,277159466	0,296421749	0,935017309	0,349779379	0,584276801	protein_codin hypothetical protein
TcG_11599	56,15382647	-0,368323724	0,248674271	-1,48114931	0,138566791	0,332388167	protein_codin mucin-associated surface protein (MASP)
TcG_11600	7,137231068	0,210790121	0,6690736	0,315047734	0,752725412	1	
TcG_11601	11,14301428	0,437429463	0,528565101	0,827579161	0,407908874	1	protein_codin surface protease GP63
TcG_11602	7,036485107	0,867195046	0,67931523	1,276572359	0,201753266	1	protein_codin putative surface protease GP63
TcG_11603	14,65420179	0,22063424	0,458647875	0,481053662	0,63047836	1	protein_codin retrotransposon hot spot (RHS) protein
TcG_11604	10,8369217	0,05310241	0,533553094	0,099526008	0,920720639	1	protein_codin retrotransposon hot spot (RHS) protein
TcG_11605	12,589806	-0,236262524	0,484435418	-0,48770696	0,625757423	1	protein_codin hypothetical protein
TcG_11606	22,46226454	-0,069134551	0,367294328	-0,18822657	0,850699045	0,928053891	
TcG_11607	109,2478754	0,070185314	0,170937902	0,410589536	0,681373538	0,83397357	
TcG_11608	1,236381856	2,394359416	1,711546619	1,398944902	0,161829506	1	protein_codin hypothetical protein
TcG_11609	1,331863933	-2,075534761	1,652433876	-1,25604709	0,209098898	1	protein_codin hypothetical protein
TcG_11610	1,747664571	0,189667504	1,352609617	0,140223389	0,888483493	1	protein_codin hypothetical protein
TcG_11611	0,857807489	2,992029954	2,048621974	1,460508572	0,144150353	1	protein_codin hypothetical protein
TcG_11612	8,849749158	0,465252898	0,626589864	0,742515838	0,457774859	1	protein_codin trans-sialidase
TcG_11613	15,76210256	-0,219847894	0,462107199	-0,47575085	0,634251887	1	protein_codin trans-sialidase
TcG_11614	24,46699228	-0,24628326	0,360997836	-0,68222919	0,495094042	0,704861092	protein_codin trans-sialidase
TcG_11615	93,97549023	0,015676352	0,181764321	0,086245487	0,931271272	0,966993095	protein_codin mucin-associated surface protein (MASP)
TcG_11616	26,22879999	0,198258947	0,382068759	0,518909076	0,603824148	0,785809669	protein_codin dispersed gene family protein 1 (DGF-1)
TcG_11617	89,43414681	-0,096138854	0,190664526	-0,50423042	0,614099464	0,791403156	protein_codin dispersed gene family protein 1 (DGF-1)
TcG_11618	27,85604628	0,502488555	0,330576909	1,520035252	0,128502116	0,317446805	protein_codin dispersed gene family protein 1 (DGF-1)
TcG_11619	3,126381204	0,071657331	0,998999931	0,071729065	0,942817525	1	protein_codin thimet oligopeptidase
TcG_11620	24,30046673	0,068710527	0,363466636	0,189042184	0,850059754	0,9278184	
TcG_11621	3,31122776	1,246289347	1,025644413	1,2151281	0,224317238	1	protein_codin hypothetical protein
TcG_11622	2,375413301	-1,085260902	1,194974992	-0,90818712	0,363779365	1	protein_codin hypothetical protein
TcG_11623	215,6789033	-0,18167815	0,127486154	-1,42508143	0,154133664	0,355169577	protein_codin putative surface protease GP63
TcG_11624	11,00449131	1,132308023	0,545506459	2,07570049	0,037921661	1	protein_codin hypothetical protein
TcG_11625	15,60290265	-0,262495015	0,437620332	-0,59982363	0,548623784	1	protein_codin hypothetical protein
TcG_11626	23,07544419	0,027574034	0,648369804	0,042528251	0,966077591	0,984950279	protein_codin hypothetical protein
TcG_11627	0					1	protein_codin hypothetical protein
TcG_11628	8,341239527	-0,091258931	0,665005285	-0,13723038	0,890848694	1	protein_codin hypothetical protein

TcG_11629	7,421082752	0,374465898	0,681165552	0,549742858	0,582495757	1	protein_codin dispersed protein family protein 1 (DGF-1)
TcG_11630	36,53617477	0,013585811	0,29352088	0,046285671	0,96308256	0,983194514	protein_codin dispersed gene family protein 1 (DGF-1)
TcG_11631	10,93224368	-0,15940736	0,546165154	-0,29186659	0,770388631	1	protein_codin dispersed protein family protein 1 (DGF-1)
TcG_11632	0,350783209	1,758746969	2,99002236	0,58820529	0,556394507	1	
TcG_11633	472,0553574	-0,250981531	0,092654316	-2,70879481	0,006752809	0,036322045	protein_codin retrotransposon hot spot (RHS) protein
TcG_11634	228,9658918	-0,427479192	0,120948638	-3,53438615	0,000408723	0,00363987	protein_codin retrotransposon hot spot (RHS) protein
TcG_11635	5,798886697	0,553750015	0,776553478	0,713086774	0,475792063	1	protein_codin hypothetical protein
TcG_11636	20,1211731	-0,646678622	0,397157554	-1,62826721	0,103468239	0,275518965	protein_codin hypothetical protein
TcG_11637	19,60021427	0,140646125	0,388768193	0,361773743	0,717521115	0,855087918	protein_codin hypothetical protein
TcG_11638	5,092005654	-0,841760051	0,782337978	-1,07595448	0,281947616	1	protein_codin hypothetical protein
TcG_11639	4,8754714	-1,438492703	0,845536476	-1,70127812	0,088890775	1	protein_codin hypothetical protein
TcG_11640	69,56925044	-0,522397905	0,214827798	-2,43170535	0,015027927	0,067801232	protein_codin mucin-associated surface protein (MASP)
TcG_11641	29,22408493	-0,020003629	0,325129377	-0,06152514	0,950940997	0,97699764	protein_codin 90 kDa surface protein
TcG_11642	11,72443405	-0,191007095	0,540763804	-0,35321723	0,723925589	1	protein_codin hypothetical protein
TcG_11643	1,173085208	-1,451105745	1,872187997	-0,77508549	0,438289147	1	protein_codin prolyl-tRNA synthetase, putative,bifunctional aminoacyl-tRNA synthetase
TcG_11644	1,337413998	-2,059721941	1,647881317	-1,24992129	0,211328302	1	protein_codin ubiquitin-protein ligase-like
TcG_11645	1,293296123	0,758805081	1,582026353	0,47964124	0,631482517	1	protein_codin reverse transcriptase endonuclease
TcG_11646	0,598023686	1,027640128	2,245589121	0,457626072	0,647221109	1	protein_codin reverse transcriptase endonuclease
TcG_11647	0					1	protein_codin hypothetical protein
TcG_11648	0,27291574	1,35431792	3,063093298	0,442140604	0,658387464	1	protein_codin dispersed protein family protein 1
TcG_11649	14,53401511	-0,140679906	0,471130728	-0,29860057	0,765244828	1	protein_codin dispersed gene family protein 1 (DGF-1)
TcG_11650	1,270995463	-1,145780573	1,618724201	-0,7078294	0,479051209	1	protein_codin dispersed protein family protein 1
TcG_11651	0,402975414	0,247428563	2,726002926	0,090766066	0,927678474	1	protein_codin putative dispersed gene family protein 1 (DGF-1)
TcG_11652	0,155988004	0,503022807	4,080472857	0,123275616	0,901888849	1	protein_codin dispersed gene family protein 1 (DGF-1)
TcG_11653	0					1	
TcG_11654	75,50523751	-0,259993528	0,202617495	-1,28317413	0,199431077	0,418209675	protein_codin mucin TcMUCII
TcG_11655	68,91617715	0,138362369	0,230223058	0,600992663	0,547844873	0,744642269	protein_codin mucin-associated surface protein (MASP)
TcG_11656	11,71263648	0,168759592	0,504325139	0,334624589	0,737908294	1	protein_codin hypothetical protein
TcG_11657	12,74693345	-0,698971251	0,485683288	-1,4391503	0,150107943	1	protein_codin hypothetical protein
TcG_11658	6,321509582	0,099444559	0,692451836	0,143612239	0,885806675	1	
TcG_11659	94,62502109	-0,56561976	0,182113628	-3,1058618	0,001897254	0,013186311	protein_codin mucin-associated surface protein (MASP)
TcG_11660	34,3878179	-0,287626865	0,302865808	-0,94968417	0,342272753	0,576576323	protein_codin hypothetical protein
TcG_11661	10,33980793	-0,018964366	0,530121157	-0,03577365	0,971462847	1	protein_codin hypothetical protein
TcG_11662	20,7199211	0,434642312	0,384187944	1,131327308	0,257917355	0,490195288	protein_codin hypothetical protein
TcG_11663	43,68494929	0,285175703	0,280425754	1,016938348	0,309182759	0,542345412	protein_codin putative trans-sialidase
TcG_11664	3,981284823	0,002871564	0,869479559	0,003302624	0,997364892	1	protein_codin structural maintenance of chromosome protein 4
TcG_11665	13,43134634	0,411547373	0,483436429	0,851295741	0,39460509	1	protein_codin hypothetical protein
TcG_11666	7,539562937	-0,548000348	0,640020258	-0,85622344	0,391874199	1	protein_codin hypothetical protein
TcG_11667	3,930788946	-0,33541728	0,872469309	-0,38444594	0,70064796	1	protein_codin hypothetical protein
TcG_11668	0,300145286	-1,420535481	3,227725303	-0,44010421	0,659861636	1	protein_codin hypothetical protein
TcG_11669	0,652611499	-3,135370342	2,250818913	-1,39299093	0,163622532	1	protein_codin hypothetical protein
TcG_11670	3,716539566	-1,379415283	1,001766224	-1,37698322	0,168517435	1	protein_codin hypothetical protein
TcG_11671	6,194558121	-0,009389498	0,680768409	-0,0137925	0,988995526	1	protein_codin hypothetical protein
TcG_11672	6,055205379	-0,392453	0,722427526	-0,54324204	0,586963167	1	protein_codin hypothetical protein
TcG_11673	30,71746717	-0,420006148	0,323958491	-1,29648137	0,194809695	0,412174055	protein_codin mucin TcMUCII
TcG_11674	58,78049394	-0,164973663	0,24773652	-0,66592388	0,505459759	0,712526678	protein_codin mucin-associated surface protein (MASP)
TcG_11675	53,67306321	0,452446676	0,243313743	1,859519607	0,062953523	0,196492326	
TcG_11676	14,89767255	0,267988867	0,452748141	0,591915997	0,553906841	1	protein_codin hypothetical protein
TcG_11677	208,4405484	0,025327138	0,122392318	0,206934046	0,836061367	0,920523248	protein_codin Alpha-(1,3)-fucosyltransferase, family GT10
TcG_11678	943,2706963	-0,091587328	0,068143797	-1,3440303	0,17893859	0,389799325	protein_codin MGT2 magnesium transporter
TcG_11679	77,7724227	0,036522105	0,201878493	0,180911321	0,856437182	0,930571246	
TcG_11680	124,575857	0,099923167	0,169886129	0,588177314	0,556413283	0,75122326	protein_codin mucin-associated surface protein (MASP)
TcG_11681	19,35583711	0,216978972	0,409449161	0,529928969	0,59616118	0,780668859	protein_codin hypothetical protein
TcG_11682	27,74340804	0,060559994	0,332940198	0,181894511	0,855665512	0,930168946	protein_codin hypothetical protein
TcG_11683	21,7570422	-0,185635161	0,371866901	-0,49919786	0,617640002	0,793033999	protein_codin trans-sialidase
TcG_11684	59,46443888	0,003893228	0,228176089	0,017062385	0,986386847	0,994631244	protein_codin putative trans-sialidase
TcG_11685	43,1743701	-0,200823462	0,262240094	-0,76579999	0,443795317	0,666037895	protein_codin putative retrotransposon hot spot (RHS) protein

TcG_11686	52,34332676	-0,36721731	0,243334193	-1,5091069	0,131271466	0,322090471	
TcG_11687	9,411037262	-0,362877987	0,577874838	-0,62795256	0,530035015	1	protein_codin hypothetical protein
TcG_11688	2,178135921	-0,416460897	1,18254335	-0,35217389	0,724707854	1	protein_codin hypothetical protein
TcG_11689	1,372731198	-0,84402288	1,54699324	-0,54558925	0,585348316	1	protein_codin hypothetical protein
TcG_11690	6,283423932	0,091566515	0,76100762	0,120322731	0,904227498	1	protein_codin hypothetical protein
TcG_11691	227,8670832	-0,221507905	0,123682269	-1,79094309	0,073302428	0,218100137	protein_codin mucin-associated surface protein (MASP)
TcG_11692	34,4104159	-0,053934961	0,295777794	-0,1823496	0,855308379	0,929867963	protein_codin hypothetical protein
TcG_11693	12,0389743	0,165704304	0,522461447	0,317160826	0,751121573	1	protein_codin mucin-associated surface protein (MASP)
TcG_11694	4,988602358	0,63186134	0,837377992	0,754571228	0,450506293	1	protein_codin mucin TcMUCII
TcG_11695	5,091068175	0,281054147	0,78965544	0,355919978	0,721900494	1	
TcG_11696	2,433868111	0,647631852	1,219588453	0,531024913	0,595401516	1	protein_codin hypothetical protein
TcG_11697	2,560424525	-1,066877494	1,086801189	-0,98166758	0,326263642	1	
TcG_11698	6,117749035	1,556351773	0,762262961	2,041751802	0,041176157	1	protein_codin mucin-associated surface protein (MASP)
TcG_11699	266,1327386	0,270268296	0,111309345	2,428082697	0,015178882	0,06821665	protein_codin retrotransposon hot spot (RHS) protein
TcG_11700	2,954981053	0,895342489	1,058553111	0,845817258	0,397654697	1	
TcG_11701	168,4760793	-0,224850858	0,135683412	-1,65717279	0,097484546	0,264138434	protein_codin retrotransposon hot spot (RHS) protein
TcG_11702	7,257135816	-0,050022238	0,672461536	-0,07438676	0,94070264	1	protein_codin hypothetical protein
TcG_11703	4,974213905	0,650759018	0,796245671	0,817284215	0,413766026	1	protein_codin hypothetical protein
TcG_11704	8,483832321	-0,502904145	0,60329144	-0,83360066	0,404506051	1	
TcG_11705	236,266054	-0,286254367	0,120497468	-2,37560483	0,017520215	0,075940595	protein_codin putative mucin-associated surface protein (MASP)
TcG_11706	39,9472765	-0,136499035	0,283123931	-0,48211762	0,629722394	0,801688869	protein_codin elongation factor 1-alpha (EF-1-alpha)
TcG_11707	58,91508489	-0,195038298	0,229356516	-0,85037173	0,395118446	0,624620319	
TcG_11708	117,5233293	0,213601738	0,166493324	1,282944765	0,199511428	0,418302462	protein_codin mucin TcMUCII
TcG_11709	23,68824596	-0,287774532	0,353395907	-0,81431201	0,41546623	0,64239863	
TcG_11710	0					1	protein_codin thimet oligopeptidase
TcG_11711	7,756397141	-0,554190372	0,628607582	-0,88161579	0,377984612	1	
TcG_11712	1,945778901	-0,678011498	1,258202679	-0,53887304	0,589974463	1	
TcG_11713	9,560640084	0,138911057	0,558253973	0,248831291	0,803491285	1	protein_codin trans-sialidase
TcG_11714	43,50511786	0,284769552	0,26277019	1,083720922	0,278488556	0,511342062	protein_codin trans-sialidase
TcG_11715	14,77698199	-0,352437286	0,452731172	-0,77846923	0,436292441	1	protein_codin trans-sialidase
TcG_11716	55,91367042	0,122125591	0,251264554	0,48604385	0,626936076	0,799788744	protein_codin dispersed gene family protein 1 (DGF-1)
TcG_11717	74,92105977	0,42951454	0,215468089	1,993402095	0,046217435	0,156434472	protein_codin dispersed gene family protein 1 (DGF-1)
TcG_11718	114,4708585	0,307655584	0,178887664	1,719825599	0,085464148	0,24133259	protein_codin stage-specific surface protein GP82
TcG_11719	191,0716294	0,109419756	0,127258179	0,859824938	0,38988555	0,620165295	protein_codin trans-sialidase
TcG_11720	470,7151897	0,053365435	0,09470585	0,563486154	0,573103885	0,764270444	
TcG_11721	297,230676	-0,023425606	0,113528282	-0,20634159	0,836524096	0,920635641	protein_codin retrotransposon hot spot (RHS) protein
TcG_11722	102,7034157	0,293577001	0,179970673	1,631249112	0,102837766	0,274218265	protein_codin hypothetical protein
TcG_11723	202,3754356	0,09091632	0,126212755	0,720341776	0,471314589	0,687306586	protein_codin putative retrotransposon hot spot (RHS) protein
TcG_11724	94,5412365	-0,235745166	0,180997119	-1,30248021	0,192752278	0,409315964	protein_codin retrotransposon hot spot (RHS) protein
TcG_11725	39,29729969	-0,222061576	0,28679995	-0,77427341	0,438769123	0,662126125	protein_codin hypothetical protein
TcG_11726	90,57638028	-0,091394628	0,188710357	-0,48431167	0,628164694	0,800692914	protein_codin hypothetical protein
TcG_11727	298,5170312	0,124913303	0,105629413	1,182561744	0,236982881	0,464589014	protein_codin putative UDP-Gal or UDP-GlcNAc-dependent glycosyltransferase
TcG_11728	37,6804285	0,227490694	0,291122227	0,781426743	0,434551547	0,658735343	
TcG_11729	31,74612401	-0,092735733	0,306140824	-0,30291855	0,761951937	0,879778971	
TcG_11730	26,09720169	0,063736031	0,341246311	0,186774271	0,851837621	0,928229751	protein_codin hypothetical protein
TcG_11731	47,53159213	-0,080156915	0,263559216	-0,30413247	0,761026972	0,879398329	protein_codin retrotransposon hot spot (RHS) protein
TcG_11732	280,3985675	0,043494485	0,12019593	0,361863212	0,717454252	0,855087918	protein_codin retrotransposon hot spot (RHS) protein
TcG_11733	18,14577461	0,148246041	0,408212688	0,363158826	0,716486243	0,854413373	protein_codin retrotransposon hot spot (RHS) protein
TcG_11734	4,538540783	0,631848879	0,883517831	0,715151247	0,47451559	1	protein_codin putative retrotransposon hot spot (RHS) protein
TcG_11735	11,37755536	0,887205813	0,530563943	1,672193945	0,094486084	1	protein_codin retrotransposon hot spot protein (RHS)
TcG_11736	19,69187481	0,32954229	0,401407092	0,820967783	0,411664619	0,638151764	protein_codin retrotransposon hot spot (RHS) protein
TcG_11737	7,571045645	0,296697406	0,63690868	0,465839791	0,641330181	1	protein_codin retrotransposon hot spot (RHS) protein
TcG_11738	11,37599551	0,177511716	0,509443735	0,348442239	0,727508086	1	protein_codin retrotransposon hot spot protein (RHS)
TcG_11739	119,4190774	0,107392302	0,159353139	0,673926493	0,500358048	0,709261227	protein_codin putative retrotransposon hot spot (RHS) protein
TcG_11740	85,61377785	-0,010771894	0,188085431	-0,05727128	0,954329097	0,978742756	protein_codin putative retrotransposon hot spot (RHS) protein
TcG_11741	36,02483471	0,184872377	0,295745173	0,625106995	0,531900837	0,732933197	protein_codin retrotransposon hot spot (RHS) protein
TcG_11742	50,44413587	-0,043973926	0,253775332	-0,17327896	0,862432159	0,934140118	protein_codin hypothetical protein

TcG_11743	22,35165054	0,475326684	0,382381957	1,243067763	0,213842864	0,437459217	protein_codin	hypothetical protein
TcG_11744	50,42178485	-0,127536991	0,260093011	-0,49035147	0,62388521	0,797066211	protein_codin	trans-sialidase
TcG_11745	42,74470583	0,256968747	0,268374938	0,957499047	0,338315427	0,572891338	protein_codin	ATP-dependent DEAD/H RNA helicase
TcG_11746	9,982042602	-0,359309027	0,562512555	-0,63875735	0,5229808	1		
TcG_11747	35,9046889	0,319107778	0,293093398	1,088757987	0,276260623	0,508736401	protein_codin	dispersed gene family protein 1 (DGF-1)
TcG_11748	79,55976997	-0,236662941	0,202824484	-1,16683615	0,243276544	0,471574709	protein_codin	dispersed gene family protein 1 (DGF-1)
TcG_11749	44,8676664	-0,317902768	0,262932698	-1,20906518	0,226637807	0,453275614	protein_codin	trans-sialidase
TcG_11750	54,21962152	0,082330384	0,249420139	0,330087156	0,741334108	0,868278957	protein_codin	putative mucin-associated surface protein (MASP)
TcG_11751	266,6869549	0,102628233	0,127236782	0,806592491	0,419901302	0,646561352	protein_codin	structural maintenance of chromosome protein 4
TcG_11752	32,5978902	0,396128064	0,306376267	1,292946312	0,196029628	0,413999139	protein_codin	hypothetical protein
TcG_11753	2,44474867	1,292537927	1,200630354	1,076549433	0,281681605	1	protein_codin	hypothetical protein
TcG_11754	1,431430128	2,612905746	1,654976522	1,57881741	0,114377944	1	protein_codin	hypothetical protein
TcG_11755	4,865205194	0,136807776	0,829853121	0,164857819	0,869055893	1	protein_codin	hypothetical protein
TcG_11756	4,394504108	-0,557321679	0,83553113	-0,66702683	0,504754998	1		
TcG_11757	0,155988004	0,503022807	4,080472857	0,123275616	0,901888849	1		
TcG_11758	59,60293898	-0,32744792	0,234056806	-1,39901046	0,161809846	0,365515476	protein_codin	hypothetical protein
TcG_11759	17,92334326	0,423570693	0,413603329	1,024098851	0,305788587	0,538836287	protein_codin	hypothetical protein
TcG_11760	40,93669807	-0,369892941	0,282379858	-1,30991262	0,190225397	0,405510846	protein_codin	hypothetical protein
TcG_11761	14,16645714	-0,38563125	0,499710347	-0,77170956	0,440286469	1	protein_codin	hypothetical protein
TcG_11762	0,874862318	0,728287021	1,859475349	0,391662638	0,695307499	1	protein_codin	trans-sialidase
TcG_11763	4,849422749	-0,154965234	0,819934719	-0,18899704	0,850095137	1	protein_codin	hypothetical protein
TcG_11764	3,110334553	-0,301688578	1,095766877	-0,27532186	0,783068969	1	protein_codin	hypothetical protein
TcG_11765	1,204439259	-0,005291937	1,690723872	-0,00312998	0,997502639	1	protein_codin	hypothetical protein
TcG_11766	36,13278629	0,14938009	0,291089442	0,513175912	0,607828274	0,788022108	protein_codin	mucin-associated surface protein (MASP)
TcG_11767	32,9633378	-0,956646357	0,308299234	-3,10298	0,001915826	0,013291472	protein_codin	mucin TcMUCII
TcG_11768	0,783636844	-3,302230925	2,034954294	-1,62275435	0,104641929	1	protein_codin	hypothetical protein
TcG_11769	1,399248139	-3,05906022	1,691805256	-1,80816333	0,070581085	1	protein_codin	hypothetical protein
TcG_11770	5,973188951	-0,923772689	0,761223567	-1,21353664	0,224924715	1	protein_codin	hypothetical protein
TcG_11771	45,15253804	0,441769048	0,259256777	1,703982642	0,08838433	0,247049662	protein_codin	surface protease GP63
TcG_11772	13,40387522	0,216156203	0,490009093	0,441126921	0,659121115	1	protein_codin	hypothetical protein
TcG_11773	7,682764847	0,212356485	0,635276345	0,334274189	0,738172666	1	protein_codin	putative syntaxin binding protein
TcG_11774	18,83774687	-0,53071713	0,39491304	-1,34388353	0,178986053	0,389799325	protein_codin	putative syntaxin binding protein
TcG_11775	17,67777028	0,280537529	0,410415208	0,683545646	0,494262117	0,70453824	protein_codin	hypothetical protein
TcG_11776	57,8921318	-0,311483717	0,226458973	-1,37545319	0,168990989	0,375441916	protein_codin	trans-sialidase
TcG_11777	25,2013386	0,670762192	0,350547533	1,913470011	0,055687909	0,178505081	protein_codin	nucleoside deoxyribosyltransferase
TcG_11778	7,969083227	0,82174055	0,651465168	1,261372965	0,207174504	1	protein_codin	hypothetical protein
TcG_11779	38,50028063	0,013555832	0,280670571	0,048298016	0,961478735	0,98216299	protein_codin	trans-sialidase
TcG_11780	109,0897724	0,286977316	0,178044899	1,611825539	0,106999909	0,282082846	protein_codin	trans-sialidase
TcG_11781	4,969979988	0,49276346	0,784890853	0,627811444	0,530127468	1	protein_codin	trans-sialidase
TcG_11782	16,85697455	-0,424375029	0,428392571	-0,99062182	0,321870278	0,556678465	protein_codin	structural maintenance of chromosome protein 4
TcG_11783	4,207727403	0,703952516	0,864393389	0,814389056	0,415422106	1		
TcG_11784	875,3700087	-0,44488547	0,065883546	-6,75260362	1,45215E-11	8,90193E-10	protein_codin	hypothetical protein
TcG_11785	181,8579619	-0,541133678	0,132038436	-4,09830421	4,16188E-05	0,000525267	protein_codin	type IB topoisomerase small subunit
TcG_11786	20,14274316	-0,607660855	0,389083885	-1,56177338	0,118341384	0,300482857	protein_codin	hypothetical protein
TcG_11787	1848,301497	0,274339347	0,06581942	4,168060835	3,07202E-05	0,000403542	protein_codin	L1Tc protein
TcG_11788	7,024681384	0,08292207	0,658333381	0,125957565	0,899765515	1	protein_codin	hypothetical protein
TcG_11789	18,41599938	0,67222413	0,422554541	1,590857663	0,111641612	0,289304344	protein_codin	elongation factor 1-gamma (EF-1-gamma)
TcG_11790	57,65064303	-0,128102095	0,236090208	-0,54259809	0,587406557	0,775648564	protein_codin	putative mucin-associated surface protein (MASP)
TcG_11791	20,26312016	-0,027408936	0,387491574	-0,07073428	0,943609242	0,973371575	protein_codin	mucin TcMUCII
TcG_11792	0					1	protein_codin	hypothetical protein
TcG_11793	30,7789874	0,413716875	0,348204168	1,188144524	0,234776476	0,462684172	protein_codin	hypothetical protein
TcG_11794	11,47553606	-0,199011793	0,584655938	-0,3403913	0,733561872	1	protein_codin	target of rapamycin (TOR) kinase 1
TcG_11795	5,330598632	0,115852149	0,750595088	0,154347065	0,877336089	1	protein_codin	hypothetical protein
TcG_11796	34,9090982	0,365906359	0,29838785	1,226277676	0,220094199	0,44520638	protein_codin	selenocysteine-tRNA-specific elongation factor
TcG_11797	0,753745299	1,464639247	1,998080553	0,733023123	0,463544326	1	protein_codin	retrotransposon hot spot (RHS) protein
TcG_11798	7,518067593	0,435843608	0,660395104	0,659974014	0,509270506	1	protein_codin	retrotransposon hot spot (RHS) protein
TcG_11799	17,06947198	0,257886985	0,424770734	0,607120417	0,543771002	0,740930357	protein_codin	retrotransposon hot spot (RHS) protein

TcG_11800	3,025497991	-1,30247146	1,128649542	-1,15400876	0,248496577	1	protein_codin	hypothetical protein
TcG_11801	2,010909544	0,114243885	1,270419793	0,08992609	0,928345948	1	protein_codin	hypothetical protein
TcG_11802	1,631536006	-0,013507203	1,337509584	-0,01009877	0,991942483	1	protein_codin	trans-sialidase
TcG_11803	250,6988076	0,293068125	0,115997783	2,526497637	0,011520616	0,055224601	protein_codin	hypothetical protein
TcG_11804	316,3635343	0,565578216	0,104899944	5,391596934	6,98343E-08	1,87726E-06	protein_codin	hypothetical protein
TcG_11805	45,92075209	0,8644604	0,274469996	3,149562475	0,001635151	0,011698514		
TcG_11806	3,430298389	-0,341929743	0,924474046	-0,36986408	0,711483766	1	protein_codin	hypothetical protein
TcG_11807	0,155721614	0,503022807	4,080472857	0,123275616	0,901888849	1	protein_codin	hypothetical protein
TcG_11808	14,73764915	-0,245861544	0,463045151	-0,53096667	0,595441873	1	protein_codin	hypothetical protein
TcG_11809	35,04194889	0,311550298	0,304987746	1,021517429	0,307009356	0,540086607	protein_codin	elongation factor 1-gamma (EF-1-gamma)
TcG_11810	45,14345305	0,419505847	0,261660588	1,603244305	0,108880727	0,284889817	protein_codin	mucin-associated surface protein (MASP)
TcG_11811	119,6864059	-0,365078718	0,162127281	-2,25180312	0,024334716	0,09782343		
TcG_11812	68,94309242	-0,047367731	0,225176709	-0,21035804	0,833388239	0,91912273	protein_codin	putative trans-sialidase
TcG_11813	1086,667202	0,136423793	0,059689953	2,285540283	0,022281167	0,091412749	protein_codin	putative retrotransposon hot spot (RHS) protein
TcG_11814	16,04511726	0,527948045	0,440742669	1,197860071	0,230971496	1	protein_codin	mucin TcMUCII
TcG_11815	5,522132448	0,404383724	0,745216029	0,542639595	0,587377971	1	protein_codin	hypothetical protein
TcG_11816	0,300973289	-0,458748581	3,014498415	-0,15218073	0,879044389	1	protein_codin	hypothetical protein
TcG_11817	0,507359824	-2,754314599	2,63041628	-1,04710217	0,295052458	1	protein_codin	hypothetical protein
TcG_11818	1,046644258	-1,603153737	1,763365284	-0,90914444	0,363273889	1	protein_codin	hypothetical protein
TcG_11819	423,8562649	0,047058596	0,090519029	0,519875171	0,603150582	0,785225212	protein_codin	trans-sialidase
TcG_11820	40,59354646	0,201671853	0,275550278	0,731887678	0,464237128	0,681704862	protein_codin	hypothetical protein
TcG_11821	40,04102898	-0,177585285	0,274353394	-0,64728664	0,51744645	0,722827895	protein_codin	hypothetical protein
TcG_11822	4,762796761	1,078586638	0,823086902	1,310416476	0,190054983	1		
TcG_11823	22,71610847	-0,195861783	0,370353212	-0,52885131	0,596908598	0,780914975	protein_codin	putative surface protease GP63
TcG_11824	13,4048127	-0,220030668	0,491949121	-0,44726306	0,654685137	1	protein_codin	hypothetical protein
TcG_11825	244,5216196	0,11789698	0,120787402	0,976070171	0,329029692	0,563425659	protein_codin	putative ferric reductase
TcG_11826	230,6524921	0,076387723	0,123999534	0,616032343	0,537873174	0,73738082	protein_codin	ferric reductase
TcG_11827	98,83242984	-0,231644275	0,17512954	-1,32270247	0,185934342	0,399879894	protein_codin	amastigote surface protein 4
TcG_11828	6,896731108	-1,002104353	0,688244239	-1,45603014	0,145384289	1	protein_codin	trans-sialidase
TcG_11829	5,747642888	-0,862264072	0,720491236	-1,19677246	0,231395254	1	protein_codin	hypothetical protein
TcG_11830	6,01174463	0,13468282	0,705179519	0,190990827	0,848532779	1	protein_codin	mucin-associated surface protein (MASP)
TcG_11831	16,35935584	0,135836688	0,431932353	0,314486023	0,75315193	1	protein_codin	trans-sialidase
TcG_11832	91,63607124	-0,100637352	0,196673357	-0,51169794	0,608862427	0,788218003	protein_codin	trans-sialidase
TcG_11833	148,6390539	-0,052765966	0,143681235	-0,36724326	0,71343757	0,852784519	protein_codin	trans-sialidase
TcG_11834	12,11423129	-0,341718813	0,504439053	-0,67742339	0,498137363	1		
TcG_11835	179,3186581	-0,231882981	0,143218738	-1,6190827	0,105429471	0,279201337	protein_codin	hypothetical protein
TcG_11836	187,209992	0,205230719	0,135347919	1,516319721	0,129438527	0,319107199	protein_codin	target of rapamycin (TOR) kinase 1
TcG_11837	306,5270753	0,192662577	0,107880129	1,785894938	0,074116271	0,219844115	protein_codin	SH3 domain protein
TcG_11838	248,6701898	0,148769797	0,115985764	1,282655671	0,199612737	0,418439148	protein_codin	structural maintenance of chromosome protein 4
TcG_11839	106,0014196	0,331472837	0,186797043	1,774507942	0,07597917	0,222972305	protein_codin	hypothetical protein
TcG_11840	10,45171372	-0,072817337	0,542301767	-0,13427457	0,893185461	1	protein_codin	putative amino acid transporter
TcG_11841	3,447857167	-1,071258598	0,942396494	-1,13673873	0,255647522	1	protein_codin	syntaxin binding protein
TcG_11842	5,319134256	-1,087868759	0,781850142	-1,39140316	0,164103207	1	protein_codin	hypothetical protein
TcG_11843	2,740966401	0,709331333	1,108681237	0,639797364	0,522304346	1	protein_codin	putative syntaxin binding protein
TcG_11844	3,546269401	-0,505931459	0,928470417	-0,54490854	0,585816423	1	protein_codin	surface protease GP63
TcG_11845	3,135854161	-0,220507721	0,976024384	-0,2259244	0,821260222	1	protein_codin	hypothetical protein
TcG_11846	7,286394761	-0,846963438	0,679241576	-1,2469252	0,212424925	1	protein_codin	hypothetical protein
TcG_11847	9,011474258	0,241037073	0,585753137	0,411499414	0,680706371	1	protein_codin	hypothetical protein
TcG_11848	25,59478722	-0,658054297	0,360013621	-1,82785944	0,067570649	0,20694516	protein_codin	trans-sialidase
TcG_11849	65,46205976	-0,685551273	0,223165341	-3,07194329	0,002126701	0,014468562	protein_codin	putative trans-sialidase
TcG_11850	53,05686531	-0,178022098	0,241617678	-0,73679252	0,461248532	0,679291481	protein_codin	putative trans-sialidase
TcG_11851	31,24108662	-0,16035813	0,314603989	-0,50971423	0,610251683	0,789016404	protein_codin	trans-sialidase
TcG_11852	6,638565467	-0,387625056	0,675294687	-0,57400875	0,565961883	1	protein_codin	surface protease GP63
TcG_11853	24,34487731	0,384801862	0,355628675	1,082032718	0,27923799	0,512149969	protein_codin	mucin-like glycoprotein
TcG_11854	17,42999766	-0,016587177	0,440161333	-0,03768431	0,969939383	0,987033994	protein_codin	hypothetical protein
TcG_11855	14,53282304	0,336649091	0,466458184	0,721713332	0,470470744	1	protein_codin	trans-sialidase
TcG_11856	13,22818216	0,027621649	0,472875254	0,058412126	0,953420356	1	protein_codin	Sialidase 85-1.3

TcG_11857	23,78213311	0,209630891	0,358950679	0,584010292	0,559213388	0,753815743	
TcG_11858	28,11896853	0,47384726	0,358652392	1,321188065	0,186438665	0,400387872	protein_codin hypothetical protein
TcG_11859	26,52170707	0,436209145	0,359767247	1,212475982	0,225330231	0,451820114	protein_codin hypothetical protein
TcG_11860	49,90049109	0,458549233	0,250064696	1,833722396	0,066695212	0,205108511	protein_codin hypothetical protein
TcG_11861	45,95655711	0,618072152	0,261523983	2,36334789	0,01811066	0,077801299	protein_codin hypothetical protein
TcG_11862	50,57390068	0,324048965	0,267323568	1,212197514	0,225436783	0,451820114	protein_codin trans-sialidase
TcG_11863	18,42546288	-0,743609625	0,41954998	-1,77239819	0,076328482	0,223487944	protein_codin hypothetical protein
TcG_11864	5,087407936	-0,812932369	0,761733519	-1,0672136	0,285875394	1	protein_codin trans-sialidase
TcG_11865	10,77605844	-0,48062296	0,535669536	-0,89723781	0,369592034	1	protein_codin putative trans-sialidase
TcG_11866	4,387688838	-2,403863207	0,949206575	-2,53249743	0,01132532	1	
TcG_11867	18,80331718	-0,160741876	0,399833211	-0,40202232	0,687667598	0,837783048	protein_codin hypothetical protein
TcG_11868	8,518338285	0,298991491	0,617238859	0,4844016	0,628100883	1	protein_codin mucin-associated surface protein (MASP)
TcG_11869	46,628073	-0,476994308	0,265821781	-1,79441393	0,072747125	0,21694934	protein_codin hypothetical protein
TcG_11870	2,903695016	-1,214438831	1,105087806	-1,09895234	0,271788855	1	protein_codin hypothetical protein
TcG_11871	11,03765828	-0,030329859	0,525494711	-0,05771677	0,953974232	1	protein_codin trans-sialidase
TcG_11872	3,956422054	-0,25085572	0,868993433	-0,2886739	0,772830941	1	protein_codin hypothetical protein
TcG_11873	24,61273078	0,522502501	0,352765572	1,481160697	0,138563757	0,332388167	protein_codin hypothetical protein
TcG_11874	123,5104589	0,048707118	0,158527427	0,307247264	0,758655173	0,878015178	protein_codin retrotransposon hot spot (RHS) protein
TcG_11875	27,61489167	0,273208139	0,351427369	0,777424194	0,436908543	0,660407355	protein_codin putative retrotransposon hot spot (RHS) protein
TcG_11876	196,4229137	0,1368205	0,129432235	1,05708211	0,290474115	0,523456792	protein_codin retrotransposon hot spot (RHS) protein
TcG_11877	525,4137824	-0,029426301	0,087270526	-0,33718486	0,735977546	0,864944729	protein_codin protein kinase
TcG_11878	543,826191	0,275319777	0,080247478	3,430883868	0,000601618	0,00505322	protein_codin glycine dehydrogenase
TcG_11879	615,938107	0,136009182	0,084559255	1,608448204	0,107737052	0,282727561	protein_codin glycine dehydrogenase
TcG_11880	147,0974169	0,191677176	0,145968848	1,313137559	0,1891366	0,403784161	protein_codin glycine dehydrogenase, putative,glycine cleavage system P-protein
TcG_11881	1419,306825	0,05345898	0,055387157	0,965187283	0,334451015	0,568936844	protein_codin putative glycine dehydrogenase, putative,glycine cleavage system P-protein
TcG_11882	1,966594433	0,456441531	1,298053021	0,351635506	0,725111633	1	protein_codin putative GDP-mannose 4,6 dehydratase
TcG_11883	185,0969903	-0,150569136	0,13184086	-1,14205214	0,253432348	0,484454814	protein_codin hypothetical protein
TcG_11884	180,1519629	-0,443703117	0,132081835	-3,3593046	0,000781389	0,006338608	protein_codin putative SNARE protein
TcG_11885	38,63177588	0,146720758	0,284807118	0,515158325	0,606442389	0,787342169	protein_codin retrotransposon hot spot (RHS) protein
TcG_11886	13,77132937	0,291395196	0,467450471	0,623371274	0,533040571	1	protein_codin retrotransposon hot spot (RHS) protein
TcG_11887	23,0035086	0,330127447	0,37243221	0,886409496	0,375396904	0,60592763	protein_codin retrotransposon hot spot (RHS) protein
TcG_11888	34,23624736	-0,076848263	0,295942671	-0,2596728	0,795116172	0,897705774	protein_codin trans-sialidase
TcG_11889	5,468437479	-0,439070164	0,726751712	-0,60415429	0,545741068	1	protein_codin trans-sialidase
TcG_11890	4,962070325	-0,887781761	0,787645786	-1,12713326	0,259686144	1	protein_codin trans-sialidase
TcG_11891	15,24823221	-0,601047516	0,455077797	-1,32075773	0,18658216	1	protein_codin Sialidase 85-1.3
TcG_11892	30,56753925	-0,746120806	0,357165384	-2,08900649	0,036707139	0,133235874	protein_codin putative elongation factor 1-gamma (EF-1-gamma)
TcG_11893	110,8283771	-0,50656884	0,168265145	-3,01053935	0,002607842	0,016974411	protein_codin putative mucin-associated surface protein (MASP)
TcG_11894	79,50414119	0,076619283	0,203326121	0,376829513	0,706300307	0,848727637	protein_codin dispersed gene family protein 1 (DGF-1)
TcG_11895	31,96048618	0,278950186	0,325722212	0,85640517	0,391773706	0,621398293	protein_codin dispersed protein family protein 1 (DGF-1)
TcG_11896	18,83934792	0,802737666	0,412295531	1,946995798	0,051535238	0,168764068	protein_codin hypothetical protein
TcG_11897	32,72410575	-0,219872668	0,308460519	-0,71280652	0,475965493	0,691391199	protein_codin trans-sialidase
TcG_11898	5,142535026	-0,236642184	0,825511098	-0,28666142	0,77437158	1	protein_codin hypothetical protein
TcG_11899	2,534101082	-1,797222286	1,17301584	-1,53213812	0,125488354	1	protein_codin hypothetical protein
TcG_11900	94,66883299	-0,248626336	0,179489354	-1,38518709	0,165995267	0,37163694	protein_codin retrotransposon hot spot (RHS) protein
TcG_11901	280,284424	-0,207827512	0,106812069	-1,9457306	0,051687109	0,169103777	protein_codin retrotransposon hot spot (RHS) protein
TcG_11902	38,4410133	-0,220215132	0,282535521	-0,77942459	0,435729645	0,659399643	protein_codin hypothetical protein
TcG_11903	27,62842011	-0,673507421	0,341309865	-1,97330195	0,048461171	0,161760625	protein_codin retrotransposon hot spot (RHS) protein
TcG_11904	220,3038287	-0,015425071	0,120586345	-0,12791723	0,898214476	0,950297956	protein_codin retrotransposon hot spot (RHS) protein
TcG_11905	0,467431231	2,090445105	2,518488183	0,830039672	0,406516354	1	protein_codin dispersed gene family protein 1 (DGF-1)
TcG_11906	0					1	protein_codin hypothetical protein
TcG_11907	0					1	protein_codin trans-sialidase
TcG_11908	0					1	protein_codin putative trans-sialidase
TcG_11909	59,56071776	-0,612733945	0,224290185	-2,73188033	0,006297401	0,034302627	protein_codin mucin TcMUCII
TcG_11910	3,818499185	-0,309894614	0,926562862	-0,33445611	0,738035402	1	protein_codin hypothetical protein
TcG_11911	10,80935725	-0,045222037	0,579340067	-0,07805784	0,93778204	1	
TcG_11912	6,039407131	-0,798168613	0,714740269	-1,1167254	0,264111754	1	protein_codin hypothetical protein
TcG_11913	5,127102708	0,004023847	0,760635282	0,005290113	0,99577912	1	protein_codin hypothetical protein

TcG_11914	2,836572823	-1,462150619	1,117673708	-1,30820883	0,190802487	1	protein_codin	hypothetical protein
TcG_11915	3,141588713	0,255261391	0,973857942	0,262113579	0,793233878	1	protein_codin	hypothetical protein
TcG_11916	2,365513922	-1,656857661	1,207518742	-1,37211755	0,170026846	1	protein_codin	hypothetical protein
TcG_11917	8,066045704	0,677227471	0,635089842	1,066349083	0,286265869	1	protein_codin	mucin TcMUCII
TcG_11918	2,410561457	0,950844156	1,175187122	0,809100218	0,418457503	1	protein_codin	hypothetical protein
TcG_11919	2,750102555	-0,560591225	1,087847247	-0,51532164	0,606328281	1	protein_codin	hypothetical protein
TcG_11920	26,67925219	0,13279587	0,335541188	0,395766227	0,692277493	0,84069156	protein_codin	putative mucin TcMUCII
TcG_11921	16,94506278	0,486565058	0,432946422	1,123845893	0,261078408	0,493587786	protein_codin	hypothetical protein
TcG_11922	87,37879147	-0,263505374	0,198487605	-1,32756589	0,18432156	0,397237647	protein_codin	mucin-associated surface protein (MASP)
TcG_11923	30,47176916	-0,003479713	0,33217129	-0,01047566	0,991641787	0,996781867	protein_codin	putative trans-sialidase
TcG_11924	995,3278746	-0,064249374	0,06462046	-0,99425746	0,320097539	0,555187139	protein_codin	L1Tc protein
TcG_11925	1647,268381	0,154977901	0,060955861	2,542461037	0,011007487	0,053205151	protein_codin	L1Tc protein
TcG_11926	236,9608382	0,135446574	0,121837635	1,111697337	0,266268305	0,498946237	protein_codin	membrane associated protein
TcG_11927	11,80559606	0,24142519	0,526223955	0,458787914	0,646386475	1	protein_codin	complement regulatory protein
TcG_11928	184,8320448	-0,356360663	0,134701089	-2,64556631	0,00815543	0,042107314	protein_codin	sialidase-like protein
TcG_11929	8,366400062	-0,339013426	0,601925117	-0,56321529	0,573288296	1	protein_codin	putative elongation factor 1-gamma (EF-1-gamma)
TcG_11930	14,79583297	-0,347110953	0,443850309	-0,78204509	0,434188077	1	protein_codin	putative mucin TcMUCII
TcG_11931	24,54331644	-0,434230167	0,347725752	-1,24877196	0,211748494	0,434677188	protein_codin	hypothetical protein
TcG_11932	23,33028333	-0,377875153	0,363214163	-1,04036459	0,298170547	0,531804796	protein_codin	trans-sialidase
TcG_11933	72,88658847	-0,304238092	0,212610727	-1,43096304	0,152440809	0,352671568	protein_codin	mucin-associated surface protein (MASP)
TcG_11934	18,88080109	-0,627138408	0,396535365	-1,58154471	0,113753545	0,29274735	protein_codin	mucin TcMUCII
TcG_11935	14,42420978	-0,585245933	0,459086764	-1,27480463	0,2023784	1	protein_codin	mucin TcMUCII
TcG_11936	11,22699652	-0,519494788	0,51753068	-1,00379515	0,315477361	1	protein_codin	hypothetical protein
TcG_11937	11,394167	0,053429932	0,529047773	0,100992642	0,919556301	1	protein_codin	hypothetical protein
TcG_11938	14,19532902	-0,110561446	0,476633245	-0,23196335	0,816566478	1	protein_codin	hypothetical protein
TcG_11939	29,9256508	0,3151812	0,323847094	0,973240785	0,330433645	0,565078112	protein_codin	hypothetical protein
TcG_11940	32,69690434	0,159172528	0,306910281	0,518628857	0,604019581	0,785958094	protein_codin	trans-sialidase
TcG_11941	45,66851668	0,445633462	0,265167985	1,680570385	0,092846392	0,255333088	protein_codin	hypothetical protein
TcG_11942	25,56362989	-0,014164725	0,354723912	-0,03993169	0,968147588	0,986366334	protein_codin	putative amino acid transporter
TcG_11943	529,2018689	0,281485751	0,086069195	3,27045872	0,001073732	0,00827145	protein_codin	hypothetical protein
TcG_11944	242,8331133	0,610812711	0,116518145	5,242211093	1,58664E-07	3,95017E-06	protein_codin	hypothetical protein
TcG_11945	486,0998096	0,319041075	0,087245928	3,656801909	0,000255382	0,002443312	protein_codin	terbinafine resistance locus protein (yip1)
TcG_11946	377,9447651	0,567286849	0,095784718	5,922519393	3,17046E-09	1,13724E-07	protein_codin	hypothetical protein
TcG_11947	331,1482636	0,695323579	0,101669734	6,839042001	7,97245E-12	5,1316E-10	protein_codin	hypothetical protein
TcG_11948	53,68782216	-0,139556002	0,235241295	-0,59324619	0,553016405	0,748298441	protein_codin	putative trans-sialidase
TcG_11949	144,3458187	-0,078742542	0,149133561	-0,52800014	0,597499237	0,781334781	protein_codin	amastigote surface protein 4
TcG_11950	30,09902947	0,187737689	0,321355615	0,584205413	0,55908212	0,753726489	protein_codin	hypothetical protein
TcG_11951	44,39562319	0,128615802	0,270161244	0,476070515	0,634024142	0,804197117	protein_codin	hypothetical protein
TcG_11952	0					1	protein_codin	hypothetical protein
TcG_11953	39,87162009	-0,119634145	0,286988869	-0,41685988	0,676780902	0,830633848	protein_codin	hypothetical protein
TcG_11954	56,15828726	0,126750853	0,230315074	0,550336765	0,582088412	0,771194386	protein_codin	hypothetical protein
TcG_11955	198,3450297	-0,393056073	0,130913788	-3,00240394	0,002678565	0,017338417	protein_codin	mucin-associated surface protein (MASP)
TcG_11956	7,749904998	-0,480386891	0,638653379	-0,75218719	0,451938495	1	protein_codin	surface protease GP63
TcG_11957	13,96265936	-0,361782235	0,495788648	-0,72971061	0,465567096	1	protein_codin	surface protease GP63
TcG_11958	7,35188978	-0,903815789	0,640216642	-1,41173429	0,158028212	1	protein_codin	syntaxin binding protein
TcG_11959	4,99061132	0,741768506	0,823791066	0,900432812	0,367889966	1	protein_codin	putative syntaxin binding protein
TcG_11960	2,055451297	0,968325021	1,239621668	0,781145607	0,434716861	1	protein_codin	hypothetical protein
TcG_11961	0,545831481	2,353994432	2,357183978	0,998646883	0,31796578	1	protein_codin	hypothetical protein
TcG_11962	0,951189179	-0,226581371	1,794715655	-0,12624918	0,899534687	1	protein_codin	hypothetical protein
TcG_11963	26,97418238	0,043295025	0,335771384	0,128941974	0,897403564	0,950181186	protein_codin	mucin TcMUCII
TcG_11964	38,99828717	-0,039982955	0,276126338	-0,1447995	0,884869178	0,944023415		
TcG_11965	23,2342257	0,260359645	0,360831795	0,721554056	0,470568695	0,686737486	protein_codin	putative target of rapamycin (TOR) kinase 1
TcG_11966	36,8434693	-0,216167438	0,299373196	-0,72206678	0,470253423	0,686450317	protein_codin	hypothetical protein
TcG_11967	28,21470173	0,616931196	0,341828047	1,804799815	0,071106013	0,21392986	protein_codin	hypothetical protein
TcG_11968	18,89003047	1,026714746	0,42403575	2,42129289	0,015465411	0,069297684	protein_codin	amino acid transporter
TcG_11969	62,0230257	0,34943008	0,232037363	1,505921613	0,132087298	0,322861483	protein_codin	hypothetical protein
TcG_11970	13,64535691	0,290214368	0,465709975	0,623165454	0,533175801	1	protein_codin	trans-sialidase

TcG_11971	2,305391551	-0,023622143	1,260277365	-0,01874361	0,985045641	1	
TcG_11972	0,6499495	-0,306228688	2,200246378	-0,13917927	0,889308488	1	protein_codin trans-sialidase
TcG_11973	1,81283116	-1,574011129	1,409736854	-1,11652833	0,264196053	1	protein_codin hypothetical protein
TcG_11974	2,030024437	-0,182602287	1,297894629	-0,14069115	0,888113938	1	
TcG_11975	0,689722372	0,936118941	2,175680909	0,430264814	0,667003019	1	
TcG_11976	43,68638591	-0,456609875	0,276394428	-1,65202272	0,098529919	0,265975684	protein_codin mucin-associated surface protein (MASP)
TcG_11977	23,06939268	0,427401927	0,367998668	1,161422485	0,24547013	0,474081834	protein_codin dispersed gene family protein 1 (DGF-1)
TcG_11978	12,72587432	1,092370874	0,520986069	2,096737204	0,036016845	1	protein_codin dispersed gene family protein 1 (DGF-1)
TcG_11979	9,674588265	0,825101173	0,568555506	1,451223608	0,146717605	1	protein_codin hypothetical protein
TcG_11980	4,828957853	0,751372795	0,856183457	0,877583875	0,380169583	1	protein_codin hypothetical protein
TcG_11981	4,654949414	-0,096203243	0,801476388	-0,12003254	0,904457375	1	
TcG_11982	4,301808374	0,130219046	0,850410451	0,153124936	0,878299752	1	protein_codin hypothetical protein
TcG_11983	1019,346026	0,018963223	0,062270303	0,304530771	0,760723553	0,879264075	protein_codin retrotransposon hot spot protein (RHS)
TcG_11984	36,75625431	-0,414704294	0,299626819	-1,38406934	0,166337224	0,371899474	protein_codin hypothetical protein
TcG_11985	41,38711283	-0,319420265	0,273235699	-1,1690283	0,242392229	0,470898628	protein_codin hypothetical protein
TcG_11986	10,72674101	0,387328723	0,548403169	0,706284619	0,480011156	1	protein_codin hypothetical protein
TcG_11987	4,391267173	-0,329101551	0,837858923	-0,39278874	0,694475521	1	protein_codin hypothetical protein
TcG_11988	7,831240068	0,589696896	0,623053754	0,94646231	0,343912837	1	protein_codin syntaxin binding protein
TcG_11989	6,892046343	-0,306315556	0,667763158	-0,45871886	0,646436068	1	protein_codin hypothetical protein
TcG_11990	0,910335238	-1,510920849	3,7967832	-0,39794762	0,690668795	1	protein_codin syntaxin binding protein
TcG_11991	3,719904583	0,035404621	0,911093861	0,038859466	0,969002434	1	protein_codin hypothetical protein
TcG_11992	3,487622861	2,04129422	1,092238936	1,868908124	0,061635598	1	protein_codin surface protease GP63
TcG_11993	100,5011892	-0,294280904	0,17435724	-1,68780433	0,091448797	0,252568714	protein_codin retrotransposon hot spot (RHS) protein
TcG_11994	17,17380275	0,3567821	0,443719841	0,804070649	0,421356175	0,647488005	protein_codin hypothetical protein
TcG_11995	49,56206398	0,123738874	0,245425223	0,504181571	0,61413379	0,791403156	protein_codin trans-sialidase
TcG_11996	18,08324337	0,173876353	0,410963401	0,423094496	0,672226318	0,828290346	protein_codin putative mucin TcMUCII
TcG_11997	2,006776092	-0,995362907	1,260001748	-0,78996947	0,429545601	1	protein_codin hypothetical protein
TcG_11998	5,584634559	0,11501689	0,747245375	0,153921181	0,877671883	1	protein_codin hypothetical protein
TcG_11999	1,307995666	-2,05315139	1,657500177	-1,23870357	0,215455295	1	
TcG_12000	97,00188279	-0,867912594	0,180628192	-4,80496751	1,54777E-06	2,97387E-05	protein_codin mucin-associated surface protein (MASP)
TcG_12001	206,0351781	0,040697845	0,122872044	0,331221357	0,740477289	0,867634494	protein_codin mucin-associated surface protein (MASP)
TcG_12002	116,3255078	-0,03977373	0,163164714	-0,24376429	0,80741338	0,90488406	protein_codin trans-sialidase
TcG_12003	24,2818338	0,017885416	0,349427433	0,051184923	0,959178166	0,981173255	protein_codin trans-sialidase
TcG_12004	103,4847802	0,098265876	0,181372719	0,541789727	0,587963369	0,775648564	protein_codin trans-sialidase
TcG_12005	124,5126949	0,048820033	0,157709727	0,309556257	0,756898425	0,877732474	protein_codin trans-sialidase
TcG_12006	199,7829844	-0,163413985	0,128745598	-1,26927824	0,204341845	0,425466046	protein_codin trans-sialidase
TcG_12007	11,73474238	0,199105262	0,511914388	0,388942501	0,697318682	1	protein_codin hypothetical protein
TcG_12008	11,7111182	-0,34762694	0,506235117	-0,68669069	0,492277667	1	protein_codin syntaxin binding protein
TcG_12009	4,946749667	-2,065554446	0,849076422	-2,43270734	0,014986408	1	protein_codin hypothetical protein
TcG_12010	6,234486713	-0,462981512	0,681035768	-0,67981967	0,49661865	1	protein_codin hypothetical protein
TcG_12011	16,46318768	0,091790503	0,429601762	0,213664169	0,830808955	1	
TcG_12012	91,45987033	-0,743942052	0,184428455	-4,03377045	5,48889E-05	0,000669711	protein_codin mucin-associated surface protein (MASP)
TcG_12013	41,66792803	-0,695682618	0,270787854	-2,56910569	0,010196135	0,050119824	protein_codin trans-sialidase
TcG_12014	35,24400385	-0,055926153	0,301965821	-0,1852069	0,85306676	0,928875734	
TcG_12015	98,62956563	0,073459786	0,180062539	0,407968182	0,683297035	0,835357122	protein_codin L1Tc protein
TcG_12016	55,33735293	0,181103997	0,257406818	0,703571095	0,481699918	0,695708707	protein_codin retrotransposon hot spot protein (RHS)
TcG_12017	24,28283082	-0,375140678	0,349420594	-1,07360781	0,282998496	0,516187118	protein_codin hypothetical protein
TcG_12018	26,51821781	-0,340229963	0,349932543	-0,972273	0,330914753	0,565235831	protein_codin glutamine synthetase
TcG_12019	5,64856014	-0,358217302	0,720119212	-0,49744167	0,618877628	1	protein_codin hypothetical protein
TcG_12020	7,138305225	0,730148217	0,716033858	1,01971186	0,307865135	1	protein_codin hypothetical protein
TcG_12021	5,824839842	0,02556646	0,714174116	0,035798637	0,97144292	1	protein_codin hypothetical protein
TcG_12022	7,003958563	1,143170681	0,690777276	1,654904873	0,097943796	1	protein_codin hypothetical protein
TcG_12023	0,615344905	-1,716476007	2,233536369	-0,76850148	0,442189311	1	protein_codin hypothetical protein
TcG_12024	414,5860828	0,740676802	0,092598629	7,998787962	1,2565E-15	1,44137E-13	protein_codin hypothetical protein
TcG_12025	265,381775	0,299222278	0,114259738	2,618790172	0,008824221	0,04478205	protein_codin hypothetical protein
TcG_12026	0,747731033	-0,555671401	3,735918973	-0,14873754	0,881760737	1	protein_codin hypothetical protein
TcG_12027	0,442035682	0,397306084	2,502129891	0,158787154	0,87383657	1	protein_codin hypothetical protein

TcG_12028	7,587534227	0,696191449	0,662415787	1,050988612	0,29326382	1	protein_codin surface protease GP63
TcG_12029	14,26142159	-0,417110298	0,460913339	-0,90496469	0,365484092	1	protein_codin putative target of rapamycin (TOR) kinase 1
TcG_12030	26,69526994	0,323190741	0,352677973	0,916390492	0,35946212	0,592630203	protein_codin hypothetical protein
TcG_12031	3,666355907	1,783148954	1,035575172	1,721892338	0,085089023	1	protein_codin structural maintenance of chromosome protein 4
TcG_12032	14,34336204	0,388613855	0,48160887	0,806907596	0,419719722	1	protein_codin dispersed gene family protein 1 (DGF-1)
TcG_12033	3,207698352	0,850817698	1,001175673	0,84981859	0,395425953	1	protein_codin amastigote surface protein
TcG_12034	39,11737657	0,339849682	0,280677222	1,210820315	0,225964277	0,452435657	protein_codin putative dispersed gene family protein 1 (DGF-1)
TcG_12035	24,67930313	0,240556505	0,363754129	0,66131622	0,508409542	0,715118726	
TcG_12036	31,05181286	-0,112349219	0,322767351	-0,34808111	0,727779267	0,859799184	
TcG_12037	91,07557253	-0,150774006	0,185573897	-0,81247421	0,416519576	0,643439442	protein_codin trans-sialidase-like protein
TcG_12038	30,27628736	-0,563321966	0,314323977	-1,79216989	0,073105757	0,217799278	protein_codin dispersed gene family protein 1 (DGF-1)
TcG_12039	53,81530721	-0,393275723	0,243055644	-1,61804811	0,105652231	0,27966341	protein_codin putative dispersed gene family protein 1 (DGF-1)
TcG_12040	10,27998578	-0,572652551	0,53845795	-1,06350468	0,287553143	1	
TcG_12041	58,05047484	0,375704737	0,237706966	1,580537348	0,113983861	0,293209816	protein_codin dispersed gene family protein 1 (DGF-1)
TcG_12042	42,34472307	0,044833774	0,267711624	0,167470404	0,866999933	0,93578156	protein_codin phosphatidylinositol 3-related kinase
TcG_12043	43,30363096	0,421850364	0,273408156	1,542932623	0,122847124	0,307742007	protein_codin putative target of rapamycin (TOR) kinase 1
TcG_12044	58,07399788	-0,048976438	0,226688739	-0,21605148	0,828947617	0,91651752	protein_codin mucin-associated surface protein (MASP)
TcG_12045	51,17371072	0,056855029	0,24557722	0,231515889	0,816914043	0,909580769	protein_codin trans-sialidase
TcG_12046	56,48050205	0,231720141	0,239002624	0,969529695	0,332280971	0,566340797	protein_codin hypothetical protein
TcG_12047	64,14630444	0,233615522	0,222745215	1,048801529	0,294269473	0,527256689	protein_codin hypothetical protein
TcG_12048	20,58885426	0,473113488	0,455285719	1,039157323	0,298731575	0,532231897	
TcG_12049	64,79446064	-0,132387968	0,216721175	-0,61086771	0,541287162	0,739895358	protein_codin mucin-associated surface protein (MASP)
TcG_12050	33,70695296	-0,064444892	0,309055175	-0,20852229	0,834821183	0,920026465	protein_codin mucin-associated surface protein (MASP)
TcG_12051	20,37493297	-0,192477493	0,383767382	-0,5015473	0,615986001	0,792562564	protein_codin regulator of sigma E protease
TcG_12052	8,853348089	0,439300926	0,600285185	0,73182037	0,464278214	1	protein_codin trans-sialidase
TcG_12053	56,92607616	-0,107742649	0,232622602	-0,46316501	0,643246097	0,810687402	protein_codin trans-sialidase
TcG_12054	5,627622031	0,952339397	0,753776882	1,263423461	0,206437037	1	protein_codin dispersed gene family protein 1 (DGF-1)
TcG_12055	17,00036735	0,196643642	0,418798731	0,469542115	0,638682189	0,807731988	protein_codin dispersed gene family protein 1 (DGF-1)
TcG_12056	0,911300909	-1,12574557	1,896603933	-0,5935586	0,55280738	1	protein_codin hypothetical protein
TcG_12057	2,928124829	0,516673819	1,03365589	0,499850892	0,617180073	1	protein_codin hypothetical protein
TcG_12058	7,942414358	-0,998899756	0,638680877	-1,56400448	0,117816515	1	protein_codin mucin TcMUCII
TcG_12059	5,473468023	0,406025337	0,745395012	0,544711637	0,58595186	1	protein_codin hypothetical protein
TcG_12060	65,07368094	0,02767771	0,213521174	0,129625131	0,896863019	0,950082741	protein_codin trans-sialidase
TcG_12061	56,33889433	-0,005032439	0,230873884	-0,02179735	0,982609608	0,99358657	protein_codin trans-sialidase
TcG_12062	21,43100333	0,133112118	0,372380514	0,35746263	0,720745497	0,85670516	protein_codin hypothetical protein
TcG_12063	26,62377677	-0,161951424	0,35064415	-0,46186832	0,644175754	0,811082798	protein_codin dispersed gene family protein 1 (DGF-1)
TcG_12064	29,45511306	0,703201899	0,34518264	2,037187901	0,041631216	0,145326685	protein_codin dispersed gene family protein 1 (DGF-1)
TcG_12065	6,182892066	-0,983217852	0,720620953	-1,36440364	0,172440563	1	protein_codin hypothetical protein
TcG_12066	125,2365074	0,105451116	0,173158224	0,60898705	0,542533023	0,740452532	protein_codin mucin-associated surface protein (MASP)
TcG_12067	62,18688695	0,144423146	0,235317225	0,613738099	0,539388408	0,738607031	protein_codin putative trans-sialidase
TcG_12068	82,15076132	0,183753481	0,198163969	0,927279982	0,353781185	0,58808501	protein_codin putative retrotransposon hot spot (RHS) protein
TcG_12069	22,42393242	0,273839048	0,364902666	0,750444088	0,452987286	0,673551167	protein_codin hypothetical protein
TcG_12070	24,91626589	0,531172048	0,38907473	1,365218577	0,172184361	0,379841586	protein_codin hypothetical protein
TcG_12071	12,1049357	0,274897772	0,499757883	0,550061904	0,582276915	1	protein_codin hypothetical protein
TcG_12072	38,16045297	0,119408258	0,293281572	0,40714545	0,683901163	0,835947285	protein_codin trans-sialidase
TcG_12073	39,66193769	-0,279850665	0,279529788	-1,00114792	0,316755303	0,551702786	protein_codin putative kinesin
TcG_12074	258,2914214	0,052698711	0,114830082	0,45892775	0,64628605	0,812019442	protein_codin retrotransposon hot spot (RHS) protein
TcG_12075	289,1967621	0,024712302	0,106032636	0,23306317	0,815712353	0,909172037	protein_codin retrotransposon hot spot (RHS) protein
TcG_12076	18,02281986	-0,033724579	0,406547339	-0,08295363	0,933888408	0,968711495	protein_codin retrotransposon hot spot protein (RHS)
TcG_12077	7,347679996	0,446447792	0,658455865	0,678022349	0,497757523	1	protein_codin hypothetical protein
TcG_12078	15,42423348	0,329043861	0,444168124	0,740809263	0,458809098	1	
TcG_12079	13,54886517	0,266655088	0,488225181	0,546172337	0,58494748	1	protein_codin retrotransposon hot spot protein (RHS)
TcG_12080	167,8463242	-0,059979169	0,14361528	-0,4176378	0,676211958	0,830261895	protein_codin putative retrotransposon hot spot (RHS) protein
TcG_12081	11,36584779	-0,178423813	0,50801365	-0,35121854	0,725424401	1	protein_codin hypothetical protein
TcG_12082	21,38667932	-0,245492351	0,390996061	-0,627864	0,530093036	0,731981763	protein_codin hypothetical protein
TcG_12083	3,014517571	0,5839229	1,830566089	0,318984878	0,74973798	1	protein_codin hypothetical protein
TcG_12084	17,35712147	-0,267861407	0,426839679	-0,6275457	0,530301587	0,731981763	protein_codin hypothetical protein

TcG_12085	4,317615634	-0,509095679	0,85161083	-0,5978032	0,549971255	1	protein_codin hypothetical protein
TcG_12086	35,74663247	0,321542261	0,30382767	1,058304732	0,289916536	0,523286024	
TcG_12087	1,04451504	0,170764434	1,724828725	0,099003705	0,921135328	1	protein_codin hypothetical protein
TcG_12088	4,237481279	-0,084857931	0,820766294	-0,10338866	0,917654508	1	protein_codin mucin TcMUCII
TcG_12089	5,750728765	-0,240020102	0,705360642	-0,34027998	0,733645696	1	protein_codin hypothetical protein
TcG_12090	1,068116916	0,120305675	1,643116993	0,073217961	0,941632674	1	
TcG_12091	10,87967879	-0,331292811	0,530212992	-0,62482967	0,532082853	1	protein_codin complement regulatory protein
TcG_12092	17,6897861	-0,236612266	0,416272458	-0,56840721	0,569758496	0,761822026	
TcG_12093	23,0730766	-0,154822476	0,35948912	-0,43067361	0,666705713	0,825438095	protein_codin hypothetical protein
TcG_12094	29,99899991	0,416242966	0,339700709	1,225322632	0,220453685	0,445465872	protein_codin retrotransposon hot spot (RHS) protein
TcG_12095	7,738700694	-0,191880402	0,67283387	-0,28518244	0,775504376	1	
TcG_12096	2560,473855	0,285679275	0,052841839	5,406308353	6,4337E-08	1,73351E-06	protein_codin L1Tc protein
TcG_12097	72,35143191	-0,068792328	0,223901485	-0,30724373	0,758657859	0,878015178	protein_codin L1Tc protein
TcG_12098	34,51295734	-0,216422625	0,291683785	-0,74197688	0,458101343	0,677244118	protein_codin putative trans-sialidase
TcG_12099	174,418656	-0,148743242	0,132962729	-1,11868373	0,263275089	0,495482515	protein_codin gp85-like protein
TcG_12100	62,57860132	0,389402747	0,220298153	1,767616941	0,077124964	0,225023881	protein_codin surface protease GP63
TcG_12101	366,9557013	-0,277362062	0,095192271	-2,91370358	0,003571689	0,021918213	protein_codin hypothetical protein
TcG_12102	489,5580946	-0,315007871	0,087068799	-3,61791912	0,000296981	0,002783838	protein_codin vacuolar type H+ ATPase subunit
TcG_12103	49,14259506	-0,194750994	0,287555335	-0,67726441	0,498238208	0,707424985	protein_codin complement regulatory protein
TcG_12104	9,275897934	0,44271784	0,588694971	0,752032651	0,452031424	1	protein_codin hypothetical protein
TcG_12105	23,0926325	0,126542219	0,36140301	0,350141575	0,726232451	0,85867223	protein_codin trans-sialidase
TcG_12106	32,08637209	0,290051293	0,308924224	0,938907571	0,347778202	0,582277203	protein_codin hypothetical protein
TcG_12107	56,97161532	0,363092224	0,248508434	1,461086119	0,143991809	0,340606186	protein_codin hypothetical protein
TcG_12108	166,3553351	-0,042480427	0,152740757	-0,2781211	0,780919399	0,890875557	protein_codin hypothetical protein
TcG_12109	35,3327504	-0,237133774	0,29986902	-0,79079117	0,429065862	0,653987669	protein_codin putative RNA-binding protein
TcG_12110	16,77732418	-0,279230884	0,418506506	-0,66720799	0,504639289	0,71221932	protein_codin zinc finger protein ZFP1
TcG_12111	2,48691715	0,739371386	1,130948724	0,653762077	0,513265103	1	protein_codin hypothetical protein
TcG_12112	3,008893688	1,224934736	1,081250306	1,132887297	0,257261585	1	protein_codin hypothetical protein
TcG_12113	7,300545333	0,733740101	0,672647747	1,090823695	0,275350468	1	protein_codin hypothetical protein
TcG_12114	2,86488775	0,238386248	1,065498473	0,223732135	0,82296574	1	
TcG_12115	39,32199621	0,095485893	0,287521127	0,33210044	0,739813418	0,86729518	protein_codin hypothetical protein
TcG_12116	25,61032278	0,127412829	0,339337194	0,375475578	0,707306806	0,849060062	protein_codin hypothetical protein
TcG_12117	62,27144716	-0,017509911	0,227128325	-0,07709259	0,938549888	0,971677152	protein_codin trans-sialidase
TcG_12118	4,830640856	0,17128935	0,814761495	0,210232505	0,833486211	1	protein_codin hypothetical protein
TcG_12119	1,444295674	1,632165986	1,522654534	1,071921404	0,283755335	1	protein_codin hypothetical protein
TcG_12120	2,474791206	-0,165514506	1,128344416	-0,14668793	0,883378346	1	protein_codin surface protease GP63
TcG_12121	4,552640575	0,604081965	0,844648685	0,715187244	0,47449335	1	protein_codin hypothetical protein
TcG_12122	51,03895654	0,272875548	0,243795306	1,119281383	0,263020115	0,495182167	protein_codin mucin-associated surface protein (MASP)
TcG_12123	33,99685353	0,518172885	0,330272277	1,56892637	0,116665097	0,2979716	protein_codin hypothetical protein
TcG_12124	27,73928879	0,739173774	0,333813164	2,214333803	0,026805825	0,105243066	protein_codin hypothetical protein
TcG_12125	2,397792063	-1,749627287	1,244052836	-1,40639307	0,159607437	1	protein_codin putative trans-sialidase
TcG_12126	0,652611499	-3,135370342	2,250818913	-1,39299093	0,163622532	1	
TcG_12127	663,1993536	0,441380776	0,077421376	5,701019519	1,19093E-08	3,70917E-07	protein_codin ATP-binding cassette protein subfamily C, member 1
TcG_12128	19,50322936	0,368128957	0,402687463	0,914180328	0,360622099	0,593321165	protein_codin putative dispersed gene family protein 1 (DGF-1)
TcG_12129	55,02407046	0,558537117	0,239262028	2,334416043	0,019573948	0,082617033	protein_codin dispersed gene family protein 1 (DGF-1)
TcG_12130	189,9167131	0,494219823	0,138591451	3,566019544	0,000362444	0,003285822	protein_codin helicase-like protein
TcG_12131	14,39986105	0,606696272	0,457569973	1,325909279	0,184869749	1	protein_codin hypothetical protein
TcG_12132	36,42996873	0,599004112	0,290083772	2,064934924	0,038929158	0,138779454	protein_codin hypothetical protein
TcG_12133	20,73444977	0,221005059	0,389617841	0,56723547	0,570554212	0,762194568	protein_codin putative trans-sialidase
TcG_12134	91,2898069	0,351528978	0,190036905	1,849793215	0,064343359	0,199593617	protein_codin target of rapamycin (TOR) kinase 1
TcG_12135	66,6969262	-0,227634876	0,212228476	-1,07259346	0,28345356	0,516854414	protein_codin putative retrotransposon hot spot (RHS) protein
TcG_12136	10,9612177	-0,224790949	0,549443476	-0,40912479	0,682448081	1	protein_codin retrotransposon hot spot (RHS) protein
TcG_12137	64,34021312	0,218955584	0,214790779	1,019390054	0,308017826	0,541121233	protein_codin retrotransposon hot spot (RHS) protein
TcG_12138	16,38707635	-0,350648861	0,482605601	-0,72657437	0,467486733	1	protein_codin hypothetical protein
TcG_12139	3,444202499	0,42728352	0,940625663	0,454254584	0,6496456	1	protein_codin putative syntaxin binding protein
TcG_12140	1,340499875	0,676716533	1,503178201	0,450190491	0,652573092	1	protein_codin hypothetical protein
TcG_12141	3,362914183	-0,122690126	0,94904659	-0,12927724	0,897138278	1	protein_codin surface protease GP63

TcG_12142	3,026062082	-0,812131705	0,989510633	-0,82074076	0,411793951	1	protein_codin	hypothetical protein
TcG_12143	4,480965364	0,111764329	0,815489534	0,137051825	0,890989831	1	protein_codin	surface protease GP63
TcG_12144	68,34930778	-0,250505807	0,220088782	-1,13820343	0,255035545	0,486388031	protein_codin	putative complement regulatory protein
TcG_12145	12,79719177	-0,535587906	0,491178526	-1,09041393	0,275530849	1	protein_codin	hypothetical protein
TcG_12146	14,66309678	-0,895694569	0,459704817	-1,94841241	0,051365636	1	protein_codin	hypothetical protein
TcG_12147	6,328019396	-0,917361571	0,694382763	-1,32111801	0,18646202	1	protein_codin	hypothetical protein
TcG_12148	26,37180081	0,10643198	0,363584096	0,29273002	0,769728522	0,884364283	protein_codin	retrotransposon hot spot protein (RHS)
TcG_12149	792,927901	-0,22715264	0,067972112	-3,34185058	0,000832218	0,006700544	protein_codin	putative retrotransposon hot spot (RHS) protein
TcG_12150	243,2056556	0,113526187	0,115974044	0,978893061	0,327632821	0,562196959	protein_codin	hypothetical protein
TcG_12151	30,24105281	0,33208895	0,323372942	1,026953427	0,304442395	0,537227191	protein_codin	hypothetical protein
TcG_12152	19,79172365	-0,348135129	0,40696891	-0,85543421	0,392310811	0,621708802	protein_codin	mitotic centromere-associated kinesin (MCAK)
TcG_12153	3,675855862	0,236486961	0,887616615	0,266429173	0,7899087	1	protein_codin	trans-sialidase
TcG_12154	13,61339323	0,850106835	0,511325309	1,662555754	0,096401394	1	protein_codin	cytochrome P450
TcG_12155	20,63543067	0,507576976	0,390694422	1,29916617	0,193886909	0,410814106	protein_codin	trans-sialidase
TcG_12156	36,56746842	0,157144375	0,296792875	0,529474893	0,596476057	0,780668859	protein_codin	trans-sialidase
TcG_12157	56,85031085	0,000409507	0,2355657	0,001738396	0,998612961	0,999561967	protein_codin	mucin-associated surface protein (MASP)
TcG_12158	771,4578476	0,020474725	0,067790674	0,30202864	0,76263023	0,880133662	protein_codin	retrotransposon hot spot (RHS) protein
TcG_12159	338,7296886	0,050518424	0,098459956	0,51308599	0,607891171	0,788022108	protein_codin	retrotransposon hot spot (RHS) protein
TcG_12160	36,81154261	0,378967933	0,288241776	1,314757141	0,188591536	0,402917487	protein_codin	trans-sialidase
TcG_12161	40,35104919	0,346873155	0,281363862	1,232827671	0,217640065	0,441916894	protein_codin	dispersed gene family protein 1 (DGF-1)
TcG_12162	76,99089741	0,399728377	0,1988674	2,010024654	0,04442858	0,152264203	protein_codin	dispersed gene family protein 1 (DGF-1)
TcG_12163	4,33166214	-0,700195575	0,828243195	-0,84539852	0,397888368	1	protein_codin	hypothetical protein
TcG_12164	2,357013197	-0,453021535	1,177933252	-0,38459016	0,700541087	1	protein_codin	hypothetical protein
TcG_12165	0					1	protein_codin	trans-sialidase
TcG_12166	3,881984954	-0,330446555	0,958223601	-0,34485328	0,730204673	1	protein_codin	hypothetical protein
TcG_12167	6,797470369	0,041762611	0,666763955	0,062634777	0,950057336	1	protein_codin	hypothetical protein
TcG_12168	34,16239768	0,16443271	0,3031444	0,54242371	0,58752665	0,775648564	protein_codin	hypothetical protein
TcG_12169	8,115618066	0,898782069	0,624035317	1,440274364	0,149789791	1	protein_codin	hypothetical protein
TcG_12170	24,97407107	0,52849782	0,363532473	1,453784348	0,146006104	0,343477508	protein_codin	trans-sialidase
TcG_12171	13,538397	0,927071757	0,50512847	1,83531876	0,066458473	1	protein_codin	dispersed gene family protein 1 (DGF-1)
TcG_12172	31,1512251	1,012372629	0,366016123	2,76592359	0,005676183	0,031602524	protein_codin	dispersed gene family protein 1 (DGF-1)
TcG_12173	2,055731012	1,016541084	1,287830965	0,789343564	0,429911231	1	protein_codin	trans-sialidase
TcG_12174	2,970361889	-0,543932525	1,064134791	-0,51115003	0,609246009	1	protein_codin	trans-sialidase
TcG_12175	3,659375969	0,935577728	0,92459275	1,011880883	0,311595011	1	protein_codin	putative trans-sialidase
TcG_12176	24,2790462	0,772318293	0,367945557	2,099001546	0,035816764	0,130823781	protein_codin	hypothetical protein
TcG_12177	7,945428316	-0,248991574	0,677270834	-0,36763959	0,713141988	1	protein_codin	hypothetical protein
TcG_12178	2,357361426	0,813893593	1,25735405	0,647306614	0,517433522	1		
TcG_12179	5,346323414	0,136361748	0,756841475	0,18017214	0,85701743	1	protein_codin	hypothetical protein
TcG_12180	14,19629104	-0,355611854	0,474028429	-0,75019098	0,453139688	1		
TcG_12181	1,600297002	1,858791288	1,514355721	1,227446935	0,219654655	1		
TcG_12182	41,59991848	0,646242998	0,278496643	2,320469617	0,020315487	0,085126665	protein_codin	putative kinesin
TcG_12183	49,27797783	0,526210146	0,253041328	2,079542302	0,037567534	0,1350473	protein_codin	putative trans-sialidase
TcG_12184	108,9274554	0,415683777	0,18110236	2,295297406	0,021716086	0,089633977	protein_codin	hypothetical protein
TcG_12185	19,22450747	0,683288297	0,430886808	1,585772144	0,112791007	0,291046014	protein_codin	putative kinesin
TcG_12186	21,54351949	-0,210641763	0,391897022	-0,53749263	0,590927371	0,776685709	protein_codin	mitotic centromere-associated kinesin (MCAK)
TcG_12187	25,52714271	0,008203521	0,36981715	0,02218264	0,982302266	0,99336249	protein_codin	subtilisin-like serine peptidase
TcG_12188	12,71409511	0,285127428	0,494849392	0,576190317	0,564486549	1	protein_codin	kinesin
TcG_12189	53,6696709	0,017489769	0,242545891	0,072109114	0,942515073	0,973191135	protein_codin	putative kinesin
TcG_12190	30,24415206	0,306903549	0,323283109	0,949333697	0,34245092	0,576607522		
TcG_12191	720,9945997	0,436710108	0,074603589	5,85374126	4,80636E-09	1,63651E-07	protein_codin	hypothetical protein
TcG_12192	36,61566192	0,666503093	0,302879766	2,200553382	0,027767657	0,107932859	protein_codin	hypothetical protein
TcG_12193	6,21936506	0,665447799	0,710890817	0,936075953	0,349234083	1	protein_codin	hypothetical protein
TcG_12194	1,370069199	0,499899786	1,507992789	0,331500117	0,740266753	1	protein_codin	hypothetical protein
TcG_12195	8,703007146	1,251608437	0,632102512	1,980071925	0,047695447	1	protein_codin	hypothetical protein
TcG_12196	4,577386113	-0,122928246	0,805666942	-0,15257948	0,878729905	1	protein_codin	hypothetical protein
TcG_12197	5,768918245	-0,276267166	0,755042562	-0,36589615	0,71444257	1	protein_codin	hypothetical protein
TcG_12198	183,1269522	0,217453726	0,145662973	1,492855197	0,135475095	0,327754114	protein_codin	retrotransposon hot spot (RHS) protein

TcG_12199	309,1865743	0,004233032	0,108851553	0,038888115	0,968979592	0,986693405	protein_codin retrotransposon hot spot (RHS) protein
TcG_12200	11,13499537	0,712399584	0,526137426	1,354018072	0,175730612	1	protein_codin hypothetical protein
TcG_12201	21,09884462	0,338770833	0,39513847	0,85734713	0,391253069	0,621127152	protein_codin hypothetical protein
TcG_12202	67,48336951	0,114135946	0,210261464	0,542828645	0,587247789	0,775633024	protein_codin L1Tc protein
TcG_12203	39,69820164	0,881790579	0,293696686	3,002385182	0,00267873	0,017338417	protein_codin hypothetical protein
TcG_12204	213,6542388	0,158233511	0,13193755	1,199306114	0,230408939	0,457579356	protein_codin putative mucin-associated surface protein (MASP)
TcG_12205	33,45477111	-0,161696998	0,296414023	-0,54551062	0,585402378	0,774078059	protein_codin hypothetical protein
TcG_12206	90,32678291	0,115409019	0,191664929	0,602139468	0,547081304	0,74395352	protein_codin dispersed gene family protein 1 (DGF-1)
TcG_12207	53,85099432	0,401550723	0,239726248	1,675038621	0,093926654	0,25750928	protein_codin dispersed gene family protein 1 (DGF-1)
TcG_12208	0,29050335	-2,096028338	3,222358999	-0,65046394	0,515392586	1	protein_codin putative dolichyl-P-Man:GDP-Man7GlcNAc2-PP-dolichyl alpha-1,6-mannosyltransferase
TcG_12209	322,69577	0,165443933	0,104424456	1,584340867	0,113116171	0,291598993	protein_codin putative ribosomal protein L11
TcG_12210	129,7045609	0,007426658	0,152738515	0,048623349	0,961219462	0,981984718	protein_codin hypothetical protein
TcG_12211	236,5144162	-0,086483177	0,121861718	-0,7096829	0,477900798	0,69263931	protein_codin putative esterase
TcG_12212	11,59187734	-0,456946002	0,513232406	-0,8903296	0,373288932	1	protein_codin surface protease GP63
TcG_12213	49,6442161	0,238749715	0,250171252	0,954345125	0,339908973	0,573995826	protein_codin surface protease GP63
TcG_12214	77,33805524	-0,051172403	0,199351874	-0,25669386	0,797415108	0,898983307	
TcG_12215	135,6959246	-0,182405742	0,151027939	-1,20776158	0,227138984	0,453964511	
TcG_12216	106,6050359	-0,282820742	0,174241288	-1,62315572	0,104556122	0,277522847	protein_codin hypothetical protein
TcG_12217	44,80081929	-0,185011662	0,261293402	-0,70806098	0,47890739	0,693404277	
TcG_12218	21,94897083	-0,11794975	0,368723714	-0,31988653	0,749054347	0,873397335	protein_codin mucin TcMUCII
TcG_12219	2,478547598	-0,661557748	1,087615945	-0,60826411	0,543012319	1	protein_codin hypothetical protein
TcG_12220	2,293453897	0,516435088	1,211899245	0,426136983	0,670008033	1	
TcG_12221	6,365435725	1,559372451	0,740259713	2,106520756	0,035159142	1	protein_codin hypothetical protein
TcG_12222	13,77894162	0,756155207	0,527456728	1,433587187	0,151690102	1	protein_codin hypothetical protein
TcG_12223	12,85470405	1,512213651	0,530112809	2,852626128	0,00433596	1	
TcG_12224	14,85054162	0,853986847	0,473261816	1,804470208	0,071157626	1	protein_codin trans-sialidase
TcG_12225	28,68586692	0,727425386	0,329685598	2,206421485	0,027354502	0,106782096	
TcG_12226	27,37396343	-0,193824562	0,332586033	-0,58278022	0,560041262	0,754054394	protein_codin hypothetical protein
TcG_12227	27,07163582	-0,267486447	0,330697152	-0,80885622	0,418597855	0,645187541	protein_codin retrotransposon hot spot (RHS) protein
TcG_12228	112,5006382	0,101780172	0,167180132	0,60880543	0,542653414	0,740452532	protein_codin putative retrotransposon hot spot (RHS) protein
TcG_12229	22,23567621	-0,189884502	0,374667009	-0,5068087	0,612289048	0,790679994	protein_codin trans-sialidase
TcG_12230	51,98378004	-0,050790292	0,239036507	-0,21247923	0,831733182	0,918195393	protein_codin putative trans-sialidase
TcG_12231	136,5636843	0,009405344	0,156114575	0,060246417	0,951959377	0,977350584	
TcG_12232	1,766317742	-0,735401488	1,352383726	-0,54378168	0,58659172	1	protein_codin trans-sialidase
TcG_12233	0,389577086	1,883588912	2,667528218	0,706117708	0,48011494	1	protein_codin hypothetical protein
TcG_12234	6,780764408	-0,10573823	0,65894326	-0,16046636	0,872513715	1	protein_codin hypothetical protein
TcG_12235	0,573889029	1,026419886	2,174050325	0,472123333	0,636838758	1	
TcG_12236	60,84453001	-0,512922119	0,227939671	-2,25025383	0,024432837	0,098120917	protein_codin sialidase-like protein
TcG_12237	1,28049973	-0,676364055	1,54583357	-0,43754002	0,661719757	1	protein_codin trans-sialidase
TcG_12238	39,95143003	-0,144595264	0,273741539	-0,5282182	0,597347896	0,78122505	protein_codin trans-sialidase
TcG_12239	0,927320913	-0,18393331	1,808007194	-0,10173262	0,918968909	1	protein_codin hypothetical protein
TcG_12240	0,155721614	0,503022807	4,080472857	0,123275616	0,901888849	1	protein_codin hypothetical protein
TcG_12241	2,546069473	-1,197400426	1,165828839	-1,02708081	0,304382415	1	protein_codin surface protease GP63
TcG_12242	2,96337294	0,385652076	1,022257186	0,377255432	0,70598379	1	protein_codin hypothetical protein
TcG_12243	3,750164241	0,19157414	0,934021575	0,205106761	0,837488715	1	protein_codin hypothetical protein
TcG_12244	246,9365751	-0,00049378	0,117415366	-0,00420541	0,996644579	0,998839568	protein_codin L1Tc protein
TcG_12245	19,10280351	-0,40620721	0,396041395	-1,02566857	0,305047835	0,537951182	
TcG_12246	41,20986885	-0,185949213	0,288136701	-0,64535067	0,518699963	0,723619237	protein_codin hypothetical protein
TcG_12247	17,18628725	-0,853504618	0,438353459	-1,94706943	0,051526412	0,168764068	protein_codin trans-sialidase
TcG_12248	16,91534752	-0,354259723	0,417448447	-0,84863107	0,396086611	0,625894637	protein_codin SH3 domain protein
TcG_12249	91,18643239	0,306620706	0,19972756	1,535194773	0,124735992	0,311108715	protein_codin retrotransposon hot spot (RHS) protein
TcG_12250	34,41291034	-0,410791747	0,337115407	-1,21854931	0,223015298	0,449216975	protein_codin hypothetical protein
TcG_12251	59,43644137	-0,018390007	0,235245598	-0,07817365	0,937689923	0,97127686	
TcG_12252	28,35881683	0,314271945	0,328257353	0,95739499	0,338367926	0,572896506	protein_codin cytochrome P450
TcG_12253	37,45095101	-0,263700914	0,300618296	-0,87719516	0,380380645	0,610738657	protein_codin hypothetical protein
TcG_12254	16,33191821	0,088330114	0,424779136	0,207943626	0,835272985	1	protein_codin hypothetical protein
TcG_12255	11,39946442	-0,711965706	0,511306373	-1,39244442	0,163787861	1	protein_codin putative retrotransposon hot spot (RHS) protein

TcG_12256	0,389843477	1,884304201	2,666932449	0,706543655	0,479850114	1	protein_codin retrotransposon hot spot protein (RHS)
TcG_12257	12,86162208	0,677989827	0,49866381	1,359613056	0,173952404	1	protein_codin putative retrotransposon hot spot (RHS) protein
TcG_12258	252,1937044	-0,030389428	0,114230491	-0,26603604	0,79021145	0,895042512	protein_codin exo-alpha-sialidase
TcG_12259	28,43943283	0,003870286	0,356960096	0,010842348	0,991349228	0,996682762	
TcG_12260	22,86409899	0,26183913	0,37452926	0,699115283	0,484479988	0,697636732	protein_codin putative retrotransposon hot spot (RHS) protein
TcG_12261	38,35256011	0,71828358	0,293234361	2,449520505	0,014304656	0,065604944	protein_codin helicase-like protein
TcG_12262	41,75104816	0,490551139	0,275823546	1,77849624	0,075322389	0,221549936	protein_codin trans-sialidase
TcG_12263	74,00478433	0,223970715	0,216664628	1,033720716	0,301266746	0,534447484	protein_codin target of rapamycin (TOR) kinase 1
TcG_12264	58,79601979	-0,038253778	0,234284995	-0,16327882	0,870298907	0,937398327	protein_codin putative trans-sialidase
TcG_12265	19,08017287	0,177166823	0,402092548	0,440612052	0,659493876	0,821160242	protein_codin serine/threonine protein phosphatase
TcG_12266	12,18044048	0,689578091	0,513335125	1,343329254	0,179165384	1	protein_codin hypothetical protein
TcG_12267	29,60929841	0,558456798	0,337306344	1,655636803	0,097795392	0,264733041	protein_codin putative trans-sialidase
TcG_12268	7,535531209	-0,301530527	0,624951115	-0,48248658	0,629460328	1	protein_codin structural maintenance of chromosome protein 4
TcG_12269	1,111379789	-0,344676894	1,652744023	-0,20854826	0,834800903	1	protein_codin hypothetical protein
TcG_12270	0,624986841	-1,473189537	2,279661729	-0,64623164	0,518129352	1	protein_codin hypothetical protein
TcG_12271	0,301239679	-0,45874858	3,013621183	-0,15222503	0,879009452	1	protein_codin hypothetical protein
TcG_12272	2,852863529	0,890589411	1,06386184	0,837128824	0,40252016	1	protein_codin hypothetical protein
TcG_12273	19,30053508	0,255692769	0,409332766	0,624657468	0,532195893	0,732933197	protein_codin trans-sialidase
TcG_12274	128,8222896	-0,184995166	0,28085475	-0,65868626	0,510097255	0,716970375	
TcG_12275	8,088686609	-0,680779378	0,63411804	-1,07358463	0,283008891	1	protein_codin hypothetical protein
TcG_12276	15,86913558	-0,180435442	0,444821326	-0,40563577	0,68501024	1	protein_codin surface protease GP63
TcG_12277	1,618610891	-1,528040853	1,427557706	-1,07038815	0,28444463	1	protein_codin hypothetical protein
TcG_12278	1,301972388	0,634511042	1,510543521	0,42005479	0,674445429	1	protein_codin hypothetical protein
TcG_12279	1,572492521	2,745833125	1,64155574	1,672701729	0,094386029	1	protein_codin hypothetical protein
TcG_12280	4,70396911	0,053481637	0,827951303	0,064595148	0,948496348	1	protein_codin hypothetical protein
TcG_12281	52,59380407	0,313052376	0,240855305	1,299752882	0,193685679	0,410802512	protein_codin hypothetical protein
TcG_12282	231,0061926	0,093963884	0,118113883	0,795536321	0,426301592	0,651783849	protein_codin rab1 small GTP-binding protein
TcG_12283	443,0160423	-0,028080014	0,088459849	-0,3174323	0,7509156	0,87456599	protein_codin hypothetical protein
TcG_12284	3,833034412	-1,291025184	0,925858633	-1,39440854	0,163194271	1	protein_codin hypothetical protein
TcG_12285	22,03235756	-0,017320233	0,379617126	-0,04562553	0,96360872	0,983644989	protein_codin putative trans-sialidase
TcG_12286	413,5807692	-0,267318158	0,110810069	-2,41239952	0,015847905	0,070593552	protein_codin calpain-like cysteine peptidase
TcG_12287	87,44324946	-0,431395806	0,206409108	-2,09000373	0,036617465	0,133035418	protein_codin calpain-like cysteine peptidase
TcG_12288	26,99477614	0,188126765	0,3385862	0,555624431	0,578467627	0,768240018	protein_codin trans-sialidase
TcG_12289	20,57097978	0,215423173	0,394571828	0,545966939	0,585088663	0,773751541	protein_codin putative trans-sialidase
TcG_12290	21,14501655	-0,679865963	0,375181086	-1,81210085	0,069970616	0,211886971	protein_codin mucin TcMUCII
TcG_12291	4,563577231	-1,099943241	0,861590905	-1,27664212	0,201728624	1	protein_codin hypothetical protein
TcG_12292	6,384056392	-0,590736499	0,694522445	-0,85056502	0,395011026	1	protein_codin hypothetical protein
TcG_12293	44,20017761	0,109514364	0,27409577	0,399547806	0,689489606	0,838680499	protein_codin NUP-1 protein
TcG_12294	103,4836431	-0,16682296	0,187593412	-0,88927942	0,37385293	0,604496256	protein_codin NUP-1 protein
TcG_12295	556,5774129	-0,234065563	0,079836217	-2,93182181	0,0033698	0,020911895	protein_codin NUP-1 protein
TcG_12296	48,56162923	0,012461563	0,251263384	0,049595618	0,960444638	0,981885783	protein_codin hypothetical protein
TcG_12297	78,69959616	-0,137785596	0,197611305	-0,69725564	0,485642826	0,698269767	protein_codin mucin-associated surface protein (MASP)
TcG_12298	61,44978536	0,107387316	0,224378063	0,478599887	0,632223299	0,802906018	protein_codin hypothetical protein
TcG_12299	75,22315485	0,204934936	0,202333801	1,012855666	0,311129107	0,54476981	protein_codin hypothetical protein
TcG_12300	75,44225789	-0,047150303	0,217414745	-0,21686801	0,828311209	0,916179436	protein_codin hypothetical protein
TcG_12301	11,54563922	0,5754869	0,533288027	1,079129608	0,280529954	1	protein_codin hypothetical protein
TcG_12302	6,156528366	-1,364514757	0,74175172	-1,83958422	0,065829303	1	protein_codin hypothetical protein
TcG_12303	3,223643631	-1,399410097	1,016936764	-1,37610336	0,168789636	1	protein_codin hypothetical protein
TcG_12304	3,774699218	-1,752995192	0,949135985	-1,84693787	0,064756146	1	protein_codin surface protease GP63
TcG_12305	25,70298222	-1,226622149	0,388051382	-3,16097869	0,0015724	0,01131542	protein_codin mucin-associated surface protein (MASP)
TcG_12306	30,06487627	-0,585620088	0,328383871	-1,78333999	0,074530976	0,220451886	protein_codin mucin TcMUCII
TcG_12307	34,30640254	-0,445621727	0,296736712	-1,50174113	0,133163976	0,324411766	
TcG_12308	7,659778165	0,470271297	0,627566568	0,749356836	0,45364216	1	
TcG_12309	8,348210517	0,222125593	0,618183405	0,359319889	0,719355797	1	protein_codin hypothetical protein
TcG_12310	8,440877419	-0,206906317	0,58861878	-0,35151158	0,725204586	1	protein_codin hypothetical protein
TcG_12311	14,90834748	-0,184144128	0,458806046	-0,40135506	0,688158731	1	protein_codin hypothetical protein
TcG_12312	14,08198801	0,740103481	0,500596972	1,478441784	0,139289569	1	protein_codin hypothetical protein

TcG_12313	35,20923164	-0,033727244	0,303718875	-0,11104757	0,911578622	0,956922163	protein_codin retrotransposon hot spot (RHS) protein
TcG_12314	18,11758215	0,165246571	0,41935363	0,394050651	0,693543644	0,841257963	protein_codin retrotransposon hot spot (RHS) protein
TcG_12315	13,81914343	0,123040338	0,471526756	0,26094031	0,794138538	1	protein_codin retrotransposon hot spot (RHS) protein
TcG_12316	55,78969069	-0,117125981	0,235854387	-0,49660294	0,619469083	0,793990967	protein_codin retrotransposon hot spot (RHS) protein
TcG_12317	47,14149077	-0,278633696	0,263443902	-1,05765855	0,290211139	0,523456792	protein_codin hypothetical protein
TcG_12318	61,67497004	-0,600890225	0,222347369	-2,70248408	0,006882348	0,036847913	protein_codin mucin TcMUCII
TcG_12319	12,01311731	-0,135027253	0,492827078	-0,27398505	0,784096109	1	
TcG_12320	8,677670228	-0,39862552	0,611169394	-0,6522341	0,514250164	1	protein_codin putative surface protease GP63
TcG_12321	6,253905775	-0,571877148	0,730264946	-0,78310913	0,433563029	1	protein_codin surface protease GP63
TcG_12322	63,08094929	0,705183644	0,232773714	3,029481429	0,00244974	0,016200162	protein_codin putative retrotransposon hot spot (RHS) protein
TcG_12323	40,41877219	0,767677881	0,291439657	2,634088607	0,008436347	0,043268488	protein_codin hypothetical protein
TcG_12324	17,13800854	0,348877689	0,422540205	0,825667439	0,408992768	0,636605346	protein_codin putative amino acid transporter
TcG_12325	33,14875467	0,408904139	0,311930494	1,310882224	0,189897557	0,404960997	protein_codin putative GAG protein
TcG_12326	36,95161358	0,04944046	0,286450544	0,172596844	0,862968333	0,934425337	protein_codin trans-sialidase
TcG_12327	19,01017594	0,109679488	0,412086045	0,266156763	0,790118481	0,895024708	protein_codin putative protein tyrosine phosphatase
TcG_12328	36,2544683	0,441552863	0,296405541	1,489691662	0,136305328	0,329163749	protein_codin target of rapamycin (TOR) kinase 1
TcG_12329	12,76721359	0,132993362	0,504335061	0,263700409	0,79201078	1	protein_codin serine/threonine protein phosphatase
TcG_12330	72,05075092	0,395907709	0,223213661	1,773671503	0,076117503	0,223104838	protein_codin trans-sialidase
TcG_12331	126,5960905	-0,162840386	0,154290468	-1,05541443	0,291235827	0,523709187	protein_codin sialidase
TcG_12332	57,96333378	0,527463301	0,240364631	2,194429767	0,028204528	0,109180642	protein_codin rab1 small GTP-binding protein
TcG_12333	76,31274309	0,18944366	0,201680713	0,939324627	0,347564098	0,582002839	protein_codin trans-sialidase
TcG_12334	36,27626338	0,367098842	0,309902748	1,184561428	0,236190895	0,463600927	protein_codin hypothetical protein
TcG_12335	40,83444687	0,800121948	0,275815763	2,900929013	0,003720582	0,022665484	protein_codin structural maintenance of chromosome protein 4
TcG_12336	65,06659384	0,736519222	0,224520401	3,280411122	0,001036559	0,00803316	protein_codin hypothetical protein
TcG_12337	175,6896146	0,094734899	0,135549489	0,698895285	0,484617474	0,697747987	protein_codin putative retrotransposon hot spot (RHS) protein
TcG_12338	66,39387479	0,075415636	0,229395531	0,328758089	0,742338544	0,86893659	protein_codin retrotransposon hot spot (RHS) protein
TcG_12339	26,25322325	-0,025540404	0,341621585	-0,07476227	0,940403865	0,972380114	protein_codin putative retrotransposon hot spot (RHS) protein
TcG_12340	17,55378019	0,043653398	0,441589887	0,098855068	0,921253345	0,961663127	protein_codin retrotransposon hot spot (RHS) protein
TcG_12341	13,7047864	0,25949437	0,506898676	0,511925522	0,608703135	1	protein_codin hypothetical protein
TcG_12342	12,9511565	0,190491325	0,502962089	0,378738932	0,70488174	1	protein_codin retrotransposon hot spot (RHS) protein
TcG_12343	33,54366456	-0,427709458	0,306225694	-1,39671317	0,162499843	0,366431137	protein_codin retrotransposon hot spot (RHS) protein
TcG_12344	29,62673853	-0,195738469	0,336462194	-0,58175472	0,56073191	0,754426375	protein_codin trans-sialidase
TcG_12345	31,41074641	-0,033937334	0,308416412	-0,11003738	0,91237973	0,957238745	
TcG_12346	28,21366286	0,305164596	0,340142524	0,897166849	0,369629892	0,601225878	protein_codin mucin-associated surface protein (MASP)
TcG_12347	17,92860714	0,46617581	0,422567235	1,103199139	0,269940695	0,502775797	protein_codin hypothetical protein
TcG_12348	49,46604926	0,087751084	0,257974655	0,340153894	0,733740637	0,863266603	protein_codin putative trans-sialidase
TcG_12349	21,85906228	-0,440037878	0,385275701	-1,14213763	0,253396815	0,484454814	protein_codin dispersed gene family protein 1 (DGF-1)
TcG_12350	32,28669759	-0,685733622	0,303782653	-2,25731659	0,023988299	0,09677174	protein_codin putative dispersed gene family protein 1 (DGF-1)
TcG_12351	22,89248276	0,083514608	0,380465216	0,219506554	0,826255475	0,915112889	
TcG_12352	4,681845248	1,133100131	0,879222427	1,288752534	0,197484132	1	protein_codin putative elongation factor 1-gamma (EF-1-gamma)
TcG_12353	58,74031969	-0,351386157	0,225886604	-1,55558653	0,119806445	0,302917264	protein_codin mucin-associated surface protein (MASP)
TcG_12354	28,01643194	0,604408294	0,338165927	1,787312812	0,073886944	0,219332344	protein_codin dispersed gene family protein 1 (DGF-1)
TcG_12355	23,56348139	-0,263094712	0,376911053	-0,69802865	0,48515928	0,698140808	protein_codin dispersed protein family protein 1 (DGF-1)
TcG_12356	49,5046317	0,199667426	0,26946113	0,740987859	0,458700802	0,677438813	protein_codin dispersed gene family protein 1 (DGF-1)
TcG_12357	76,0174514	0,058685099	0,19997759	0,293458378	0,769171809	0,884364283	protein_codin hypothetical protein
TcG_12358	311,2946216	0,243687495	0,10732189	2,270622462	0,023169843	0,094257653	protein_codin GP63 group I member a protein
TcG_12359	50,36466002	0,26659235	0,249842787	1,067040411	0,285953588	0,519187736	protein_codin serine/threonine protein phosphatase
TcG_12360	151,6120866	0,136800151	0,148690885	0,920030508	0,357556817	0,59135536	protein_codin putative rab1 small GTP-binding protein
TcG_12361	12,39230207	-0,112664117	0,507417116	-0,22203452	0,82428701	1	protein_codin hypothetical protein
TcG_12362	50,41000258	0,015376859	0,283958229	0,054151834	0,956814195	0,980060942	protein_codin structural maintenance of chromosome protein 4
TcG_12363	229,998417	0,398494567	0,121713081	3,274048811	0,001060183	0,008183398	protein_codin Tbingi protein
TcG_12364	326,2830306	0,072010165	0,101308954	0,710797634	0,477209647	0,692243767	protein_codin UDP-Gal or UDP-GlcNAc-dependent glycosyltransferase
TcG_12365	33,69105837	0,403696847	0,303341988	1,330830757	0,183244705	0,395505431	protein_codin hypothetical protein
TcG_12366	231,4480187	0,741018323	0,124223255	5,965214172	2,44313E-09	8,95764E-08	protein_codin Tbingi protein
TcG_12367	216,5268814	-0,535704029	0,128633336	-4,16458165	3,11924E-05	0,000407895	protein_codin putative complement regulatory protein
TcG_12368	211,938631	-0,056707863	0,126347918	-0,44882309	0,653559279	0,81657908	protein_codin hypothetical protein
TcG_12369	879,7043039	-0,112742083	0,065301789	-1,7264777	0,084261508	0,239095707	protein_codin putative cytochrome-b5 reductase

TcG_12370	24,36857969	-0,152596543	0,356266062	-0,42832186	0,668416805	0,826255403	protein_codin hypothetical protein
TcG_12371	36,99955706	-0,282218517	0,286108111	-0,98640516	0,323934333	0,558191541	protein_codin subtilisin-like serine peptidase
TcG_12372	13,48922944	-0,889941312	0,498177462	-1,78639417	0,07403546	1	protein_codin subtilisin-like serine peptidase
TcG_12373	78,86516955	-0,265030436	0,210655135	-1,25812473	0,20834665	0,430977377	protein_codin subtilisin-like serine peptidase
TcG_12374	31,15651013	0,026330231	0,311886243	0,084422546	0,932720482	0,967890686	protein_codin putative kinesin
TcG_12375	0					1	protein_codin hypothetical protein
TcG_12376	5,140221256	0,749601517	0,841994046	0,890269379	0,373321259	1	protein_codin hypothetical protein
TcG_12377	0,300973289	-0,458748581	3,014498415	-0,15218073	0,879044389	1	protein_codin hypothetical protein
TcG_12378	1,994090369	-0,25447292	1,242925289	-0,2047371	0,837777532	1	protein_codin trans-sialidase
TcG_12379	4,129349489	-0,592279752	0,891121855	-0,66464507	0,506277532	1	protein_codin hypothetical protein
TcG_12380	9,362616956	-0,342019504	0,577075539	-0,59267718	0,553397215	1	protein_codin hypothetical protein
TcG_12381	10,5481103	-0,126195739	0,538784299	-0,23422312	0,814811768	1	protein_codin surface protease GP63
TcG_12382	12,37204217	-0,413540737	0,492780601	-0,83919849	0,401357934	1	protein_codin putative mucin-associated surface protein (MASP)
TcG_12383	1,2131022	-0,268819298	1,535684871	-0,17504848	0,861041542	1	
TcG_12384	63,38441844	0,031408274	0,468167195	0,067087729	0,946511862	0,974694377	protein_codin hypothetical protein
TcG_12385	74,33364995	0,080905279	0,209225211	0,386689914	0,698985784	0,844142481	protein_codin retrotransposon hot spot protein (RHS)
TcG_12386	90,71963227	-0,029264459	0,183447722	-0,15952479	0,873255432	0,939244099	protein_codin putative retrotransposon hot spot (RHS) protein
TcG_12387	92,26283234	-0,185653805	0,186149949	-0,99733471	0,318602072	0,553576194	protein_codin retrotransposon hot spot (RHS) protein
TcG_12388	247,5583999	-0,058759287	0,114767093	-0,51198724	0,608659943	0,788174776	protein_codin retrotransposon hot spot (RHS) protein
TcG_12389	7,248759796	-0,327499292	0,693133452	-0,47249096	0,636576391	1	protein_codin hypothetical protein
TcG_12390	10,49863769	0,356274178	0,546571517	0,651834512	0,514507935	1	protein_codin syntaxin binding protein
TcG_12391	0,428370964	1,990401553	2,587616184	0,769202777	0,441772942	1	protein_codin putative syntaxin binding protein
TcG_12392	0					1	protein_codin putative syntaxin binding protein
TcG_12393	7,375963907	0,89639944	0,714117858	1,2552542	0,209386496	1	protein_codin putative syntaxin binding protein
TcG_12394	25,00177374	-0,154191129	0,344209108	-0,44795773	0,654183706	0,817094887	protein_codin dispersed gene family protein 1 (DGF-1)
TcG_12395	52,54996899	0,170493573	0,251691188	0,677391902	0,498157335	0,707424985	protein_codin dispersed gene family protein 1 (DGF-1)
TcG_12396	15,44677212	-0,095640151	0,465447689	-0,20547991	0,83719719	1	protein_codin hypothetical protein
TcG_12397	1,164804055	-0,787737267	1,670117072	-0,4716659	0,637165282	1	protein_codin thimet oligopeptidase
TcG_12398	0,300145286	-1,420535481	3,227725303	-0,44010421	0,659861636	1	protein_codin hypothetical protein
TcG_12399	8,488347146	0,849993583	0,633240617	1,342291635	0,179501455	1	protein_codin putative elongation factor 1-gamma (EF-1-gamma)
TcG_12400	3,25542156	0,786011844	0,977084561	0,804446079	0,421139398	1	protein_codin surface protease GP63
TcG_12401	3,406245636	-0,752308016	1,016098779	-0,74038866	0,459064196	1	protein_codin surface protease GP63
TcG_12402	256,9068755	-0,011122457	0,113533535	-0,09796627	0,921959079	0,962091476	protein_codin putative nuclear receptor binding factor
TcG_12403	21,61815353	-0,242742986	0,401034881	-0,60529145	0,544985359	0,741887014	
TcG_12404	315,7759391	-0,078763554	0,106686027	-0,73827433	0,460347765	0,678401068	protein_codin kinetoplast DNA-associated protein
TcG_12405	134,8764223	-0,265580548	0,151986421	-1,74739654	0,080568577	0,232148104	protein_codin mucin-associated surface protein (MASP)
TcG_12406	69,08466905	0,336124148	0,213981181	1,570811727	0,116226387	0,297131272	protein_codin hypothetical protein
TcG_12407	37,05423344	0,581553485	0,296400712	1,962051581	0,049756479	0,164993809	protein_codin hypothetical protein
TcG_12408	3,943842718	1,484492968	0,935843004	1,586262827	0,112679701	1	protein_codin mucin TcMUCII
TcG_12409	7,874962831	0,090368166	0,617235958	0,146407812	0,88359946	1	protein_codin hypothetical protein
TcG_12410	7,948832332	-0,244998115	0,644891437	-0,37990598	0,704015207	1	protein_codin hypothetical protein
TcG_12411	5,585944815	-0,026123336	0,754919019	-0,03460416	0,972395387	1	protein_codin hypothetical protein
TcG_12412	13,53933673	0,106619641	0,476390446	0,223807262	0,822907279	1	protein_codin hypothetical protein
TcG_12413	64,47419489	-0,513291752	0,240043013	-2,1383324	0,032489774	0,121506303	protein_codin mucin-associated surface protein (MASP)
TcG_12414	44,47551067	0,14577364	0,279553018	0,521452574	0,602051536	0,784755061	protein_codin putative trans-sialidase
TcG_12415	32,27352875	-0,004053334	0,303701321	-0,01334645	0,989351392	0,995541618	protein_codin putative profilin
TcG_12416	37,1836887	0,044333075	0,280803704	0,157879237	0,874551959	0,939591895	protein_codin rab1 small GTP-binding protein
TcG_12417	22,77452769	-0,006493199	0,373314374	-0,01739338	0,986122791	0,994614548	protein_codin hypothetical protein
TcG_12418	8,543252867	-0,505508203	0,591614815	-0,85445494	0,392852965	1	protein_codin selenocysteine-tRNA-specific elongation factor
TcG_12419	10,54563714	-0,375785238	0,54513631	-0,68934179	0,490608208	1	protein_codin putative trans-sialidase
TcG_12420	112,1730773	0,276412112	0,169959373	1,626342264	0,103876866	0,27628957	
TcG_12421	100,7976614	-0,038794397	0,18412462	-0,21069642	0,833124172	0,919089983	protein_codin trans-sialidase
TcG_12422	226,1133887	-0,176026433	0,135326434	-1,30075424	0,193342589	0,410268724	protein_codin putative complement regulatory protein
TcG_12423	22,47675948	0,229078022	0,374436111	0,611794683	0,540673608	0,739492908	protein_codin retrotransposon hot spot (RHS) protein
TcG_12424	27,5961205	0,260347018	0,333724511	0,780125548	0,43531698	0,659399643	
TcG_12425	9,458788951	0,112176663	0,567593215	0,197635666	0,843330128	1	protein_codin hypothetical protein
TcG_12426	84,76269171	-0,019763155	0,190820319	-0,10356945	0,917511033	0,960175645	protein_codin retrotransposon hot spot (RHS) protein

TcG_12427	24,70226289	0,21236493	0,348285272	0,609744216	0,542031259	0,740196534	protein_codin putative retrotransposon hot spot (RHS) protein
TcG_12428	300,2751525	0,187537115	0,121464741	1,543963403	0,122597199	0,307514861	protein_codin helicase-like protein
TcG_12429	18,66944338	0,002740607	0,411098159	0,00666655	0,994680902	0,997783662	protein_codin hypothetical protein
TcG_12430	7,992891352	0,557953939	0,642777494	0,868035897	0,385374681	1	protein_codin trans-sialidase
TcG_12431	6,243950658	0,175788683	0,694852793	0,252986941	0,800278304	1	protein_codin hypothetical protein
TcG_12432	19,50636901	-0,107328615	0,400338784	-0,26809447	0,788626601	0,894874711	protein_codin retrotransposon hot spot (RHS) protein
TcG_12433	2,540736529	0,587791799	1,092694469	0,537928777	0,590626221	1	protein_codin retrotransposon hot spot (RHS) protein
TcG_12434	5,461379922	-0,866152266	0,834716887	-1,03765993	0,299428407	1	protein_codin hypothetical protein
TcG_12435	8,686987493	0,696159968	0,59407394	1,17184061	0,241261055	1	protein_codin hypothetical protein
TcG_12436	7,536114546	0,797003618	0,65960586	1,208302815	0,226930806	1	protein_codin hypothetical protein
TcG_12437	23,91349456	0,762853306	0,376669656	2,025258193	0,042840859	0,148298235	protein_codin surface protein-2
TcG_12438	49,20670043	-0,454987083	0,251030528	-1,8124771	0,06991251	0,211766363	protein_codin trans-sialidase-like protein
TcG_12439	36,59155185	0,397894473	0,287702558	1,383006378	0,16666291	0,372555755	protein_codin dispersed gene family protein 1 (DGF-1)
TcG_12440	8,17231994	-0,264078507	0,606320575	-0,43554271	0,663168544	1	
TcG_12441	23,50760378	-0,103203905	0,363009991	-0,28430045	0,776180139	0,888310845	protein_codin hypothetical protein
TcG_12442	58,33609117	-0,072609456	0,228746064	-0,31742385	0,750922015	0,87456599	protein_codin hypothetical protein
TcG_12443	1,619310172	-1,335565474	1,514349892	-0,88193982	0,377809351	1	
TcG_12444	57,96401779	-0,164558148	0,232666594	-0,7072702	0,479398581	0,693855335	protein_codin putative mucin-associated surface protein (MASP)
TcG_12445	5,517838964	-0,313724667	0,728672207	-0,43054293	0,666800746	1	
TcG_12446	11,10412271	0,475580682	0,543987128	0,874249882	0,381982186	1	protein_codin hypothetical protein
TcG_12447	7,654618964	-0,715122796	0,657902207	-1,08697431	0,277048164	1	protein_codin retrotransposon hot spot protein (RHS)
TcG_12448	4,157622326	-0,832279799	0,884764527	-0,94067944	0,346869159	1	protein_codin retrotransposon hot spot (RHS) protein
TcG_12449	13,39443334	0,015805837	0,472138228	0,033477138	0,973294097	1	protein_codin retrotransposon hot spot (RHS) protein
TcG_12450	0,469265227	-2,470320505	2,566008638	-0,96270935	0,335693404	1	protein_codin hypothetical protein
TcG_12451	3,870549344	-0,685445198	0,909267506	-0,75384328	0,450943333	1	protein_codin putative syntaxin binding protein
TcG_12452	1,963104432	1,195522899	1,335111691	0,895447854	0,370547729	1	protein_codin putative syntaxin binding protein
TcG_12453	2,44725318	-1,807872815	1,242478598	-1,45505348	0,145654455	1	protein_codin hypothetical protein
TcG_12454	4,053422403	-0,029261634	0,849644521	-0,03443985	0,972526403	1	protein_codin surface protease GP63
TcG_12455	1,968690443	-1,665599866	3,847466968	-0,43290817	0,665081488	1	protein_codin surface protease GP63
TcG_12456	29,2076321	-0,210427606	0,339688068	-0,61947306	0,535604765	0,735555719	protein_codin hypothetical protein
TcG_12457	33,0050481	-0,464727912	0,303480185	-1,53132868	0,12568818	0,312897132	protein_codin hypothetical protein
TcG_12458	7,43608415	-0,213783819	0,641830933	-0,33308432	0,739070636	1	protein_codin trans-sialidase
TcG_12459	5,505226483	-0,1654656	0,808452046	-0,20466965	0,837830231	1	protein_codin hypothetical protein
TcG_12460	8,983343883	-0,555073312	0,578012687	-0,96031337	0,336897522	1	protein_codin hypothetical protein
TcG_12461	57,28274151	0,328130627	0,239305861	1,371176727	0,170319871	0,376919082	protein_codin retrotransposon hot spot (RHS) protein
TcG_12462	261,0494273	0,205489778	0,11410777	1,80083948	0,071728184	0,215117279	protein_codin retrotransposon hot spot (RHS) protein
TcG_12463	62,8069445	0,409806134	0,220219717	1,860896654	0,062758772	0,196042906	protein_codin putative kinesin
TcG_12464	91,32919698	0,205805586	0,181648509	1,13298803	0,25721928	0,489028976	protein_codin subtilisin-like serine peptidase
TcG_12465	43,13143366	-0,191801756	0,262821288	-0,72978014	0,465524587	0,682138496	protein_codin mucin TcMUCII
TcG_12466	8,90322411	0,274111868	0,631939124	0,433763091	0,664460487	1	protein_codin hypothetical protein
TcG_12467	11,76997469	0,097690895	0,517384272	0,188816901	0,850236325	1	
TcG_12468	119,8015402	0,226621357	0,163424913	1,386700188	0,165533203	0,370960869	protein_codin hypothetical protein
TcG_12469	25,36295146	-0,057509825	0,357325167	-0,16094535	0,872136441	0,938476159	protein_codin helicase-like protein
TcG_12470	60,86066349	-0,617310186	0,221911139	-2,78179	0,005406001	0,030449162	
TcG_12471	300,3813401	-0,577033825	0,109364447	-5,27624691	1,31856E-07	3,35019E-06	protein_codin putative UDP-Gal or UDP-GlcNAc-dependent glycosyltransferase
TcG_12472	0					1	protein_codin hypothetical protein
TcG_12473	137,0186824	0,267244275	0,151357343	1,765651209	0,077454383	0,225757606	protein_codin dispersed gene family protein 1 (DGF-1)
TcG_12474	42,35412642	0,513855858	0,296349752	1,733950692	0,082926832	0,236811141	protein_codin retrotransposon hot spot (RHS) protein
TcG_12475	48,06268533	0,197709952	0,26894351	0,735135612	0,462256908	0,679744706	
TcG_12476	11,40133996	0,22646719	0,513874916	0,440704892	0,659426654	1	protein_codin hypothetical protein
TcG_12477	7,075888098	-0,96079579	0,662793919	-1,44961467	0,147166002	1	protein_codin trans-sialidase
TcG_12478	1,695177145	0,349483648	1,313653824	0,266039379	0,790208881	1	
TcG_12479	23,02795025	0,106957611	0,372138959	0,287413097	0,773796034	0,886918879	protein_codin hypothetical protein
TcG_12480	421,7812136	0,162887051	0,089667939	1,816558437	0,069284752	0,210580572	protein_codin hypothetical protein
TcG_12481	189,526393	-0,101398683	0,132719559	-0,76400708	0,444863025	0,666776586	protein_codin hypothetical protein
TcG_12482	252,4121072	-0,04078837	0,114962967	-0,35479573	0,72274263	0,857335574	protein_codin hypothetical protein
TcG_12483	156,8976586	0,734090783	0,148236455	4,952160934	7,33939E-07	1,54327E-05	protein_codin dispersed protein family protein 1

TcG_12484	720,9548785	0,203154271	0,071812351	2,828960048	0,004669953	0,027233751	protein_codin cytochrome c oxidase subunit 10
TcG_12485	2,327232323	3,369180977	1,492034906	2,258111364	0,023938717	1	protein_codin hypothetical protein
TcG_12486	5,11741015	-0,797685493	0,785397445	-1,01564564	0,309798161	1	protein_codin hypothetical protein
TcG_12487	2,530040522	-1,157820416	1,114212082	-1,03913827	0,298740435	1	protein_codin hypothetical protein
TcG_12488	5,188482168	-0,825285486	0,803179894	-1,02752259	0,304174453	1	protein_codin trans-sialidase
TcG_12489	19,8288726	-0,195535435	0,407938454	-0,47932582	0,631706854	0,802906018	protein_codin dispersed gene family protein 1 (DGF-1)
TcG_12490	21,72401296	0,164052295	0,373855501	0,438812039	0,660797737	0,822243957	protein_codin dispersed gene family protein 1 (DGF-1)
TcG_12491	9,249368085	-0,249466892	0,593112327	-0,42060649	0,674042456	1	protein_codin putative dispersed gene family protein 1 (DGF-1)
TcG_12492	3,604686498	-0,620940938	0,923974089	-0,67203285	0,501562786	1	protein_codin hypothetical protein
TcG_12493	2,589911945	-0,53549244	1,141498779	-0,46911346	0,63898854	1	protein_codin hypothetical protein
TcG_12494	2,094944455	1,339563545	1,282115951	1,044806863	0,296112242	1	
TcG_12495	2,484530553	-0,553786755	1,164249889	-0,4756597	0,634316835	1	
TcG_12496	151,6217893	-0,148310503	0,143635264	-1,03254938	0,301814827	0,535010187	protein_codin putative mucin-associated surface protein (MASP)
TcG_12497	53,84709742	0,093169446	0,242925486	0,383530966	0,701326115	0,845322279	protein_codin trans-sialidase
TcG_12498	9,790292952	0,272630415	0,54833021	0,497201157	0,619047205	1	protein_codin trans-sialidase
TcG_12499	14,3731786	0,042371639	0,46701582	0,090728488	0,927708334	1	protein_codin hypothetical protein
TcG_12500	16,40312542	0,018534991	0,427531594	0,043353501	0,965419744	1	protein_codin hypothetical protein
TcG_12501	26,66488816	-0,098324529	0,340332637	-0,28890714	0,772652439	0,886595143	protein_codin trans-sialidase
TcG_12502	163,2327475	0,197605321	0,14007653	1,410695431	0,158334439	0,360533035	protein_codin glycine dehydrogenase
TcG_12503	268,3931726	0,015874742	0,115353398	0,137618329	0,890542069	0,94667588	protein_codin putative retrotransposon hot spot (RHS) protein
TcG_12504	32,81137423	0,213047801	0,300633353	0,708663223	0,478533492	0,69303613	protein_codin dispersed gene family protein 1 (DGF-1)
TcG_12505	21,47690627	-0,461255044	0,38771552	-1,18967392	0,234174579	0,461890819	protein_codin putative dispersed protein family protein 1 (DGF-1)
TcG_12506	62,9921243	0,613117245	0,247004289	2,482212949	0,013056922	0,061048224	protein_codin putative trans-sialidase
TcG_12507	57,48674686	0,295515543	0,236949883	1,247164754	0,212337094	0,435576765	protein_codin hypothetical protein
TcG_12508	32,03905804	0,267410715	0,319557634	0,836815291	0,402696401	0,632851796	protein_codin putative dispersed gene family protein 1 (DGF-1)
TcG_12509	26,876603	-0,100410054	0,343098878	-0,29265632	0,769784862	0,884364283	
TcG_12510	11,66193809	0,749298002	0,5200846	1,4407233	0,14966287	1	protein_codin hypothetical protein
TcG_12511	19,25634374	0,331264355	0,395224171	0,83816826	0,40193621	0,63220648	protein_codin hypothetical protein
TcG_12512	68,53352532	-0,126355995	0,215774599	-0,58559254	0,558149363	0,752819386	protein_codin retrotransposon hot spot (RHS) protein
TcG_12513	98,71039923	0,264984579	0,192409919	1,377187723	0,168454217	0,374941548	protein_codin retrotransposon hot spot protein (RHS)
TcG_12514	85,539649	-0,007899845	0,192500325	-0,04103809	0,967265534	0,985814433	protein_codin hypothetical protein
TcG_12515	88,36115564	0,248057928	0,196281686	1,263785399	0,206307063	0,428287697	
TcG_12516	91,52605662	0,019333885	0,191536335	0,100941081	0,919597231	0,961308205	
TcG_12517	105,1389756	0,11215598	0,17367121	0,645794888	0,518412199	0,723391996	protein_codin putative kinesin
TcG_12518	57,72780177	0,400551975	0,242648534	1,650749618	0,098789712	0,266366674	protein_codin subtilisin-like serine peptidase
TcG_12519	127,3930612	0,118690713	0,161584071	0,734544636	0,462616866	0,680187692	protein_codin putative kinesin
TcG_12520	44,14022658	-0,194335691	0,26372434	-0,73688948	0,461189565	0,679291481	protein_codin amastigote surface protein 4
TcG_12521	44,8300914	0,263425136	0,286638318	0,919015776	0,358087321	0,591973602	protein_codin target of rapamycin (TOR) kinase 1
TcG_12522	46,95482892	0,201946075	0,256372576	0,787705448	0,430869018	0,655640721	protein_codin target of rapamycin (TOR) kinase 1
TcG_12523	99,84697889	0,423987409	0,18009199	2,354282433	0,018558504	0,079167463	protein_codin hypothetical protein
TcG_12524	36,29281069	0,744101702	0,300293532	2,477914513	0,01321528	0,061614583	protein_codin hypothetical protein
TcG_12525	63,61740408	0,358883803	0,218194511	1,644788415	0,100013452	0,268790502	protein_codin structural maintenance of chromosome protein 4
TcG_12526	25,87869824	0,110387358	0,356955838	0,309246541	0,757133993	0,877829926	protein_codin trans-sialidase
TcG_12527	21,48429422	0,049740247	0,38271453	0,129966969	0,89659256	0,949883084	protein_codin trans-sialidase
TcG_12528	123,318759	-0,128810397	0,162918545	-0,79064294	0,429152382	0,653987669	protein_codin amastigote surface protein 4
TcG_12529	390,9578545	-0,409724382	0,099421507	-4,121084	3,77094E-05	0,000481699	protein_codin hypothetical protein
TcG_12530	34,34007448	0,636737081	0,309348803	2,058314355	0,039559967	0,14012283	protein_codin dispersed gene family protein 1 (DGF-1)
TcG_12531	86,01311647	-0,150495762	0,195301634	-0,77058117	0,440955224	0,663546065	protein_codin hypothetical protein
TcG_12532	534,5113988	-0,294776483	0,080583043	-3,65804607	0,000254145	0,002435507	protein_codin hypothetical protein
TcG_12533	51,71368421	-0,165673	0,241015717	-0,687395	0,491833845	0,702635507	protein_codin trans-sialidase
TcG_12534	2,319704097	1,329479437	1,211329647	1,097537272	0,272406597	1	protein_codin hypothetical protein
TcG_12535	12,99608223	0,337981204	0,488769474	0,691494093	0,489255092	1	protein_codin hypothetical protein
TcG_12536	23,03846889	0,015300572	0,379088691	0,040361457	0,967804958	0,98610397	protein_codin hypothetical protein
TcG_12537	15,43432884	0,215069288	0,450496694	0,477404808	0,633073891	1	protein_codin retrotransposon hot spot (RHS) protein
TcG_12538	140,9828179	0,08106634	0,153699558	0,527433788	0,597892385	0,781760656	
TcG_12539	81,77212871	0,152002814	0,196265675	0,774474772	0,438650082	0,66208961	protein_codin putative retrotransposon hot spot (RHS) protein
TcG_12540	66,7417721	-0,302448662	0,218901231	-1,38166725	0,167073894	0,373072109	protein_codin mucin-associated surface protein (MASP)

TcG_12541	8,236960309	-0,477511826	0,617814751	-0,77290454	0,439578878	1	protein_codin hypothetical protein
TcG_12542	48,5175316	0,024634188	0,251763441	0,097846567	0,92205413	0,962091476	protein_codin putative retrotransposon hot spot (RHS) protein
TcG_12543	198,4963128	-0,042251398	0,135389009	-0,31207406	0,754984237	0,876363937	protein_codin mucin-associated surface protein (MASP)
TcG_12544	6,866282329	-0,441808505	0,674539917	-0,65497755	0,512482206	1	protein_codin hypothetical protein
TcG_12545	3,561869839	-0,329362797	0,914985476	-0,35996506	0,718873266	1	protein_codin hypothetical protein
TcG_12546	64,46824762	0,193850968	0,225446803	0,85985237	0,389870427	0,620165295	protein_codin hypothetical protein
TcG_12547	39,90926313	0,199163859	0,280453817	0,710148505	0,47761205	0,692608846	protein_codin trans-sialidase
TcG_12548	30,59755306	-0,064026115	0,313567535	-0,20418605	0,838208115	0,92144011	
TcG_12549	10,41459847	-0,378818737	0,535789521	-0,70702901	0,479548448	1	protein_codin hypothetical protein
TcG_12550	3,900234066	-0,718406721	0,889303562	-0,8078307	0,419188048	1	protein_codin thimet oligopeptidase
TcG_12551	2,903093081	-0,251663433	1,034960884	-0,24316227	0,807879697	1	
TcG_12552	67,27801246	0,337771843	0,211028007	1,600601967	0,109465106	0,286031286	protein_codin putative retrotransposon hot spot (RHS) protein
TcG_12553	30,45407946	-0,103777824	0,337305108	-0,30766751	0,758335341	0,878015178	protein_codin mucin-associated surface protein (MASP)
TcG_12554	147,218085	0,112049942	0,149301739	0,750493215	0,452957709	0,673551167	protein_codin mucin-associated surface protein (MASP)
TcG_12555	349,0143643	0,521721137	0,099386935	5,249393571	1,52601E-07	3,81864E-06	protein_codin putative retrotransposon hot spot (RHS) protein
TcG_12556	193,9624122	0,552354296	0,130560584	4,230635921	2,33032E-05	0,000316148	
TcG_12557	17,04720644	0,285885917	0,428610247	0,667006726	0,504767837	0,71221932	protein_codin dispersed gene family protein 1 (DGF-1)
TcG_12558	16,76272256	0,063083981	0,425833216	0,148142462	0,882230339	1	protein_codin dispersed gene family protein 1 (DGF-1)
TcG_12559	2,20014136	0,408830624	1,179648143	0,346569972	0,728914408	1	protein_codin dispersed protein family protein 1
TcG_12560	327,7381747	0,305220314	0,101552266	3,005549009	0,002651019	0,017187858	protein_codin surface protease GP63
TcG_12561	141,7561263	0,290704099	0,15722455	1,848973964	0,064461572	0,199906792	
TcG_12562	162,9818521	0,159257738	0,141805306	1,123073195	0,261406409	0,493587786	
TcG_12563	354,6235147	0,257405482	0,096429931	2,669352559	0,007599763	0,039860052	protein_codin surface protease GP63
TcG_12564	65,09125948	0,150491377	0,217022451	0,693436905	0,488035409	0,699453024	protein_codin hypothetical protein
TcG_12565	150,4608302	0,205072069	0,1483691	1,382175059	0,166917956	0,372910035	protein_codin retrotransposon hot spot (RHS) protein
TcG_12566	17,91016514	0,047481773	0,410872292	0,115563336	0,907998622	0,95541477	protein_codin putative trans-sialidase
TcG_12567	12,38246529	-0,4979846	0,497910493	-1,00014884	0,317238486	1	protein_codin putative trans-sialidase
TcG_12568	20,35765639	-0,025283916	0,384048881	-0,06583515	0,94750907	0,975394476	protein_codin hypothetical protein
TcG_12569	39,90088822	-0,074084251	0,283604099	-0,26122419	0,793919624	0,897137693	protein_codin surface protease GP63
TcG_12570	154,2714561	0,08103268	0,143516637	0,564622202	0,572330761	0,763503074	protein_codin complement regulatory protein
TcG_12571	25,85530538	-0,002708131	0,336071217	-0,00805821	0,993570551	0,997617506	protein_codin hypothetical protein
TcG_12572	17,75589828	-0,324382955	0,426942173	-0,75978195	0,447384936	0,668546514	protein_codin hypothetical protein
TcG_12573	66,41118714	-0,252172255	0,214238732	-1,17706193	0,239170789	0,467367696	protein_codin ATPase
TcG_12574	14,81386245	-0,01187851	0,452374946	-0,02625811	0,979051468	1	protein_codin retrotransposon hot spot (RHS) protein
TcG_12575	22,69418613	0,093773999	0,370700771	0,252964133	0,80029593	0,900622067	protein_codin hypothetical protein
TcG_12576	9,522664262	-1,058561065	0,582706822	-1,81662721	0,069274215	1	protein_codin hypothetical protein
TcG_12577	14,2754681	-0,476562316	0,46042934	-1,03503898	0,300650705	1	protein_codin trans-sialidase
TcG_12578	29,58253439	-0,037912165	0,315623805	-0,1201182	0,904389511	0,953695494	protein_codin trans-sialidase
TcG_12579	96,03307684	0,410157163	0,186087114	2,204113729	0,027516347	0,107233231	protein_codin L1Tc protein
TcG_12580	69,85175416	0,323095993	0,220700656	1,463955741	0,143206042	0,339673155	protein_codin L1Tc protein
TcG_12581	2706,790555	0,163986783	0,054588614	3,004047373	0,002664138	0,017263259	protein_codin L1Tc protein
TcG_12582	1081,482729	0,232903364	0,075034992	3,103930039	0,001909685	0,013256806	protein_codin L1Tc protein
TcG_12583	16,07502241	0,385115342	0,435324445	0,884662798	0,376338526	1	protein_codin hypothetical protein
TcG_12584	8,406738793	1,081249463	0,649680278	1,664279339	0,096056619	1	protein_codin trans-sialidase
TcG_12585	11,48239966	0,428954681	0,512828565	0,836448494	0,402902641	1	protein_codin trans-sialidase
TcG_12586	69,43588824	0,189074954	0,210440781	0,898471072	0,368934461	0,600861456	protein_codin putative trans-sialidase
TcG_12587	52,82314385	-0,238148318	0,249205806	-0,95562909	0,339259658	0,573397354	protein_codin putative protein kinase, putative,casein kinase I
TcG_12588	485,8140209	-0,032367443	0,092087837	-0,35148445	0,725224936	0,858172586	protein_codin putative myosin heavy chain
TcG_12589	592,7064521	0,129052436	0,079880283	1,615573096	0,106186645	0,28057088	protein_codin retrotransposon hot spot (RHS) protein
TcG_12590	9,100441857	0,224058174	0,566186401	0,395732171	0,69230262	1	protein_codin hypothetical protein
TcG_12591	11,10717347	0,553585684	0,550618617	1,005388607	0,314709764	1	protein_codin trans-sialidase
TcG_12592	556,7543453	-0,011011934	0,083058526	-0,13258041	0,894525225	0,948820769	protein_codin retrotransposon hot spot (RHS) protein
TcG_12593	12,04052607	0,864130659	0,519159266	1,664480855	0,096016374	1	protein_codin hypothetical protein
TcG_12594	123,8442676	0,173630841	0,168109151	1,032845864	0,301676036	0,535009729	protein_codin retrotransposon hot spot (RHS) protein
TcG_12595	20,78633448	-0,557837964	0,381167733	-1,46349734	0,143331341	0,339782167	protein_codin putative trans-sialidase
TcG_12596	13,423397	-0,246595408	0,470396838	-0,52422846	0,600119652	1	protein_codin putative trans-sialidase
TcG_12597	15,41725383	0,237392591	0,457078218	0,51936973	0,603502936	1	protein_codin hypothetical protein

TcG_12598	1,131647384	-2,750683068	3,959339801	-0,69473276	0,487222794	1	protein_codin hypothetical protein
TcG_12599	45,56238644	0,508124935	0,281449536	1,805385582	0,071014366	0,213819698	protein_codin putative mucin-associated surface protein (MASP)
TcG_12600	35,66754597	0,291170729	0,288378334	1,009683097	0,312647143	0,546766762	protein_codin dispersed gene family protein 1 (DGF-1)
TcG_12601	35,32887735	0,23924187	0,297346718	0,804588906	0,421056946	0,647488005	protein_codin dispersed gene family protein 1 (DGF-1)
TcG_12602	703,5252655	-0,169746577	0,071881815	-2,3614676	0,018202762	0,078028056	protein_codin retrotransposon hot spot (RHS) protein
TcG_12603	24,53766488	0,402802813	0,359306211	1,121057195	0,262263517	0,494320012	protein_codin hypothetical protein
TcG_12604	100,9770893	0,107091315	0,177670364	0,602752832	0,54667313	0,743573008	
TcG_12605	159,6597769	0,225385691	0,143194992	1,573977466	0,115492659	0,295838856	protein_codin L1Tc protein
TcG_12606	0					1	protein_codin L1Tc protein
TcG_12607	302,855773	0,396873668	0,104034727	3,814819131	0,000136283	0,001444622	protein_codin L1Tc protein
TcG_12608	14,60359891	0,633268573	0,454650204	1,392869877	0,163659143	1	protein_codin dispersed gene family protein 1 (DGF-1)
TcG_12609	52,15334163	-0,030027114	0,243850156	-0,12313756	0,901998168	0,952705039	protein_codin trans-sialidase
TcG_12610	21,55203823	0,049956181	0,376687229	0,132619788	0,894494082	0,948820769	
TcG_12611	5,281794705	-0,764207922	0,779006598	-0,98100314	0,326591191	1	protein_codin hypothetical protein
TcG_12612	11,91882323	0,112634686	0,531042413	0,212101112	0,832028152	1	protein_codin dispersed gene family protein 1 (DGF-1)
TcG_12613	37,64122103	-0,034067705	0,309739882	-0,10998811	0,912418804	0,957238745	protein_codin dispersed gene family protein 1 (DGF-1)
TcG_12614	17,62512381	-0,354028005	0,413545855	-0,8560792	0,391953973	0,621398293	protein_codin mucin-associated surface protein (MASP)
TcG_12615	41,49468912	-0,758719543	0,2859711	-2,65313363	0,007974829	0,041396224	protein_codin mucin TcMUCII
TcG_12616	5,364572526	-0,15396625	0,75360579	-0,20430609	0,838114314	1	protein_codin putative trans-sialidase
TcG_12617	2,70857759	-0,432667471	1,091505683	-0,39639507	0,691813603	1	protein_codin hypothetical protein
TcG_12618	28,84581811	-0,745370828	0,335685771	-2,22044213	0,02638877	0,103887287	protein_codin hypothetical protein
TcG_12619	61,96777738	-0,396690023	0,221516904	-1,79078894	0,07332717	0,218117739	protein_codin hypothetical protein
TcG_12620	33,64929813	-0,612740398	0,300343998	-2,04012866	0,041337513	0,144693784	protein_codin mitotic centromere-associated kinesin (MCAK)
TcG_12621	52,56822008	0,276553185	0,259521607	1,065626823	0,286592369	0,519715008	protein_codin hypothetical protein
TcG_12622	39,22378697	0,694764171	0,314846918	2,206672928	0,027336918	0,106749421	protein_codin dispersed protein family protein 1
TcG_12623	18,23139193	0,603752355	0,438279359	1,377551426	0,168341827	0,374862273	protein_codin dispersed gene family protein 1 (DGF-1)
TcG_12624	1,360160873	0,283683357	1,596215085	0,177722514	0,858940903	1	protein_codin dispersed gene family protein 1 (DGF-1)
TcG_12625	2,54008835	0,611396707	1,165526296	0,524567064	0,599884188	1	protein_codin dispersed gene family protein 1 (DGF-1)
TcG_12626	2,041224967	1,257677982	1,293032619	0,972657583	0,330723514	1	protein_codin hypothetical protein
TcG_12627	6,814012237	0,627001977	0,754610985	0,830894315	0,406033335	1	protein_codin dispersed gene family protein 1 (DGF-1)
TcG_12628	5,767747584	0,97696391	0,758941444	1,287271788	0,197999573	1	protein_codin hypothetical protein
TcG_12629	7,369283826	-0,067745647	0,634994702	-0,10668695	0,915037337	1	protein_codin hypothetical protein
TcG_12630	5,808317794	0,334120802	0,745264662	0,44832503	0,653918639	1	protein_codin hypothetical protein
TcG_12631	13,10031742	0,911161466	0,495361607	1,839386526	0,065858354	1	protein_codin retrotransposon hot spot (RHS) protein
TcG_12632	40,16680302	0,08874232	0,289675408	0,306350894	0,759337493	0,878478701	protein_codin trans-sialidase
TcG_12633	24,75091589	0,635854713	0,371221717	1,712870458	0,086736374	0,244092211	protein_codin trans-sialidase
TcG_12634	522,0460654	0,742869374	0,08433943	8,808090962	1,27295E-18	2,01642E-16	protein_codin dispersed protein family protein 1 (DGF-1)
TcG_12635	158,7985809	0,485309927	0,145572683	3,33379805	0,000856688	0,006868921	protein_codin dispersed gene family protein 1 (DGF-1)
TcG_12636	20,49522412	-0,005927079	0,391107622	-0,0151546	0,987908843	0,995145161	protein_codin hypothetical protein
TcG_12637	10,65399599	-0,756561128	0,551143945	-1,37271059	0,169842336	1	protein_codin hypothetical protein
TcG_12638	46,75122624	0,170333612	0,254473883	0,669355968	0,503268432	0,71168901	protein_codin hypothetical protein
TcG_12639	48,22148542	-0,241664292	0,256978639	-0,94040615	0,34700927	0,5815781	protein_codin hypothetical protein
TcG_12640	4652,084563	0,444646292	0,041277154	10,772213	4,65672E-27	1,79842E-24	protein_codin putative hexose transporter
TcG_12641	5,528146715	0,72931619	0,789579601	0,923676586	0,355654726	1	protein_codin retrotransposon hot spot protein (RHS)
TcG_12642	9,279785138	0,148939627	0,642228791	0,231910542	0,816607497	1	
TcG_12643	7,949311531	-0,374633971	0,606227123	-0,61797626	0,536590987	1	protein_codin dispersed protein family protein 1
TcG_12644	30,52064061	-0,161848436	0,316533415	-0,51131548	0,609130165	0,788218003	protein_codin hypothetical protein
TcG_12645	10,9285252	-0,873605186	0,551828679	-1,58310943	0,113396524	1	protein_codin hypothetical protein
TcG_12646	5,813055365	0,273054515	0,717057114	0,380798837	0,703352522	1	protein_codin hypothetical protein
TcG_12647	5,557616564	-0,813069744	0,763327331	-1,06516524	0,286801158	1	
TcG_12648	43,37227208	-0,122596678	0,304612054	-0,40246824	0,687339457	0,837648015	protein_codin dispersed gene family protein 1 (DGF-1)
TcG_12649	50,47774519	0,007367778	0,245346549	0,030030085	0,97604306	0,989788723	protein_codin dispersed gene family protein 1 (DGF-1)
TcG_12650	4,64947188	1,725505377	0,967830743	1,782858613	0,074609322	1	protein_codin hypothetical protein
TcG_12651	6,646093693	0,019831011	0,705433862	0,028111793	0,977572988	1	protein_codin putative leucine-rich repeat protein
TcG_12652	4,803255655	0,23543903	0,796821014	0,295472918	0,767632638	1	protein_codin mucin-associated surface protein (MASP)
TcG_12653	5,413648052	-0,645224882	0,772981954	-0,83472179	0,403874369	1	protein_codin hypothetical protein
TcG_12654	0,863297986	-1,045201145	1,92159747	-0,54392304	0,586494431	1	protein_codin mucin TcMUCII

TcG_12655	3,716878848	-0,35518552	0,882454223	-0,40249739	0,68731801	1	protein_codin hypothetical protein
TcG_12656	3,38368756	0,273544784	0,975928116	0,280291939	0,779253535	1	protein_codin hypothetical protein
TcG_12657	3,437558041	0,321082464	0,942728292	0,340588552	0,733413349	1	protein_codin hypothetical protein
TcG_12658	42,56167561	0,333737524	0,303214876	1,100663424	0,271043171	0,50430483	protein_codin kinesin
TcG_12659	30,12438336	0,81249364	0,333550888	2,435891103	0,014855156	0,067322838	protein_codin subtilisin-like serine peptidase
TcG_12660	16,9158923	0,890148373	0,429676788	2,07166968	0,038296257	0,136971017	protein_codin retrotransposon hot spot (RHS) protein
TcG_12661	34,80691668	1,046962684	0,318436574	3,287821718	0,001009658	0,00784041	protein_codin SH3 domain protein
TcG_12662	44,44004012	1,041116015	0,272087659	3,826399247	0,000130031	0,001388519	protein_codin surface protease GP63
TcG_12663	17,13216113	0,040564622	0,417345597	0,097196717	0,922570175	0,96227026	protein_codin hypothetical protein
TcG_12664	28,80711392	0,147457687	0,319252303	0,46188449	0,644164157	0,811082798	protein_codin trans-sialidase
TcG_12665	112,1251986	0,721137856	0,17166197	4,200917986	2,65835E-05	0,000355244	protein_codin dispersed gene family protein 1 (DGF-1)
TcG_12666	127,1839566	0,80807928	0,168168964	4,805162981	1,54625E-06	2,97387E-05	protein_codin dispersed gene family protein 1 (DGF-1)
TcG_12667	1,460356001	-0,357719607	1,408708261	-0,25393449	0,799546172	1	protein_codin hypothetical protein
TcG_12668	0,350783209	1,758746969	2,99002236	0,58820529	0,556394507	1	protein_codin trans-sialidase
TcG_12669	0,4558669	-0,458748245	2,666213369	-0,17205984	0,863390485	1	protein_codin hypothetical protein
TcG_12670	5,365183408	-0,847943059	0,781044774	-1,08565231	0,277632849	1	protein_codin hypothetical protein
TcG_12671	2,934552166	-0,820278824	1,077721619	-0,7611231	0,446583542	1	protein_codin hypothetical protein
TcG_12672	5,426881713	-0,043341875	0,748956002	-0,05786972	0,953852401	1	
TcG_12673	97,31646707	-0,111495371	0,17967612	-0,62053528	0,534905437	0,735099868	protein_codin putative trans-sialidase
TcG_12674	111,0053441	-0,410978649	0,167877143	-2,44809176	0,014361508	0,065767758	protein_codin hypothetical protein
TcG_12675	26,18128036	-0,59190491	0,341516174	-1,73316802	0,08306581	0,236811141	protein_codin mucin TcMUCII
TcG_12676	31,12828659	-0,154683993	0,3434014	-0,4504466	0,652388452	0,8161724	
TcG_12677	34,28003458	0,655997803	0,303138897	2,16401725	0,030463026	0,115605149	protein_codin SH3 domain protein
TcG_12678	26,35672877	-0,009845291	0,362788979	-0,02713779	0,978349834	0,990851444	protein_codin hypothetical protein
TcG_12679	32,70135071	0,266444589	0,311234284	0,856090099	0,391947945	0,621398293	protein_codin putative GAG protein
TcG_12680	40,91203058	0,513556062	0,287799455	1,784423332	0,074354903	0,220159333	protein_codin putative amino acid transporter
TcG_12681	38,61303897	0,12521544	0,284005189	0,440891382	0,659291633	0,820996653	protein_codin hypothetical protein
TcG_12682	20,95447845	0,083973241	0,384580949	0,218349975	0,827156439	0,915585603	protein_codin putative retrotransposon hot spot (RHS) protein
TcG_12683	25,02596013	-0,361243307	0,363318867	-0,99428722	0,320083054	0,555187139	protein_codin retrotransposon hot spot (RHS) protein
TcG_12684	1,312875217	2,262617112	1,731597862	1,306664302	0,191326748	1	
TcG_12685	8,799017691	0,045342573	0,595644366	0,076123566	0,939320792	1	protein_codin target of rapamycin (TOR) kinase 1
TcG_12686	15,63580876	0,354555241	0,462512823	0,766584672	0,443328492	1	protein_codin putative trans-sialidase
TcG_12687	13,8688299	-0,656396154	0,474302045	-1,38392014	0,166382908	1	protein_codin helicase-like protein
TcG_12688	4,584241706	0,438672361	0,820292148	0,534775765	0,592804924	1	protein_codin hypothetical protein
TcG_12689	3,433415003	1,521561575	1,055356882	1,441750749	0,149372702	1	protein_codin hypothetical protein
TcG_12690	2,113271668	-1,315955307	1,280914441	-1,02735613	0,304252799	1	protein_codin hypothetical protein
TcG_12691	41,98587966	0,05214204	0,278612712	0,187148819	0,851543953	0,928214153	protein_codin hypothetical protein
TcG_12692	29,36515788	0,023594219	0,329319104	0,07164546	0,942884061	0,973191135	protein_codin trans-sialidase
TcG_12693	2,177087773	0,664187737	1,285356493	0,516734261	0,605341676	1	protein_codin hypothetical protein
TcG_12694	32,06278642	0,058739389	0,318505268	0,184422033	0,853682386	0,929098927	protein_codin dispersed gene family protein 1 (DGF-1)
TcG_12695	213,5008164	-0,024954287	0,123740679	-0,201666	0,840177846	0,922245432	protein_codin sialidase-like protein
TcG_12696	59,2591252	0,563603462	0,233029523	2,418592523	0,015580683	0,069642191	protein_codin trans-sialidase-like protein
TcG_12697	50,05319456	0,081731798	0,270770642	0,301848818	0,762767314	0,880133662	protein_codin putative kinesin
TcG_12698	63,32271775	-0,087515163	0,21753427	-0,40230518	0,687459444	0,837683625	protein_codin subtilisin-like serine peptidase
TcG_12699	51,61601214	0,039202444	0,25153062	0,155855554	0,87614688	0,940607649	protein_codin putative kinesin
TcG_12700	769,4864688	-0,034125921	0,073349032	-0,46525387	0,641749664	0,809837619	protein_codin putative glutamine synthetase
TcG_12701	5,40861033	0,292991079	0,793569757	0,369206458	0,711973842	1	protein_codin hypothetical protein
TcG_12702	3,501048187	0,495590597	0,9451321	0,524361194	0,600027342	1	protein_codin hypothetical protein
TcG_12703	4,537642073	-0,124418555	0,8315811	-0,14961686	0,881066904	1	protein_codin trans-sialidase
TcG_12704	38,98807207	0,163321222	0,278816862	0,585765225	0,558033296	0,752750467	protein_codin retrotransposon hot spot (RHS) protein
TcG_12705	9,550652334	0,379306657	0,605332054	0,626609238	0,530915411	1	protein_codin hypothetical protein
TcG_12706	3,660102249	0,036728519	0,904957873	0,040585888	0,967626035	1	protein_codin trans-sialidase
TcG_12707	29,249465	0,231923792	0,32661005	0,710093862	0,477645932	0,692608846	protein_codin trans-sialidase
TcG_12708	39,04088587	0,015956137	0,284992291	0,05598796	0,955351398	0,979184475	protein_codin dispersed gene family protein 1 (DGF-1)
TcG_12709	2,258523343	-1,473995237	1,242621683	-1,18619791	0,235544153	1	protein_codin hypothetical protein
TcG_12710	104,0721311	-1,155348184	0,176786697	-6,53526652	6,34964E-11	3,29896E-09	
TcG_12711	5,851476465	-0,617322911	0,745544557	-0,82801612	0,407661371	1	protein_codin mucin TcMUCII

TcG_12712	24,59876096	-0,121693151	0,355775551	-0,34205035	0,732313002	0,862516869	protein_codin mucin TcMUCII
TcG_12713	41,98266294	-0,152932911	0,26582501	-0,57531423	0,565078799	0,757837672	protein_codin mucin TcMUCII
TcG_12714	71,24218924	0,035732852	0,216287417	0,165210035	0,868778667	0,936741226	protein_codin trans-sialidase-like protein
TcG_12715	24,1796245	0,302375276	0,349086423	0,866190307	0,386385808	0,616705603	protein_codin putative kinesin
TcG_12716	9,754183343	0,058130726	0,561727182	0,103485692	0,917577505	1	protein_codin putative trans-sialidase
TcG_12717	46,00091781	0,277480788	0,260376432	1,065690877	0,286563403	0,519715008	protein_codin hypothetical protein
TcG_12718	21,82983777	0,650071634	0,397118607	1,636970976	0,101636534	0,271946299	protein_codin putative kinesin
TcG_12719	91,32548156	-0,319025353	0,198848323	-1,60436532	0,108633551	0,28454709	protein_codin putative trans-sialidase
TcG_12720	12,17491958	-0,135687709	0,510946914	-0,26556127	0,79057712	1	protein_codin hypothetical protein
TcG_12721	1,18464954	0,302518536	1,720804698	0,175800622	0,860450581	1	
TcG_12722	79,58622177	0,045307392	0,218526692	0,20733116	0,835751241	0,920523248	protein_codin trans-sialidase
TcG_12723	51,4604724	-0,002353818	0,256989209	-0,00915921	0,99269211	0,997081126	protein_codin hypothetical protein
TcG_12724	442,0402247	-0,104556758	0,087412763	-1,19612691	0,231647037	0,458936829	
TcG_12725	21,89086602	0,23145829	0,369963512	0,625624644	0,53156117	0,732689576	protein_codin trans-sialidase
TcG_12726	5,82340997	-0,357404733	0,71316651	-0,50115187	0,616264241	1	protein_codin hypothetical protein
TcG_12727	15,45103262	0,260971532	0,443769106	0,588079541	0,556478905	1	protein_codin retrotransposon hot spot (RHS) protein
TcG_12728	155,3075757	0,314648602	0,142036639	2,215263633	0,026741974	0,105027972	protein_codin putative dispersed gene family protein 1 (DGF-1)
TcG_12729	132,4489334	-0,1300937	0,15419307	-0,84370653	0,398833419	0,628777247	protein_codin dispersed gene family protein 1 (DGF-1)
TcG_12730	2,469871333	-0,602061194	1,098205516	-0,5482227	0,583538997	1	protein_codin hypothetical protein
TcG_12731	163,3739217	-0,247306389	0,136732679	-1,80868533	0,070499904	0,212933234	protein_codin trans-sialidase-like protein
TcG_12732	35,65179863	0,344217204	0,301790199	1,140584436	0,254042897	0,485059493	protein_codin hypothetical protein
TcG_12733	35,17996879	0,201865977	0,307001664	0,657540337	0,510833543	0,71748302	protein_codin hypothetical protein
TcG_12734	19,6692948	-0,018152667	0,385920353	-0,04703734	0,962483466	0,982669496	protein_codin hypothetical protein
TcG_12735	10,76197107	0,68813338	0,531941915	1,293625036	0,19579497	1	protein_codin hypothetical protein
TcG_12736	1,213368591	-0,268484184	1,535611058	-0,17483866	0,861206408	1	
TcG_12737	7,255106488	0,137449699	0,642408922	0,213959823	0,830578388	1	protein_codin putative trans-sialidase
TcG_12738	16,08988669	-0,375403923	0,428985887	-0,87509621	0,38152156	1	protein_codin trans-sialidase
TcG_12739	13,78606492	-0,597133314	0,463655487	-1,28788148	0,197787224	1	protein_codin trans-sialidase
TcG_12740	8,522769438	0,754938945	0,602949105	1,252077395	0,210541664	1	protein_codin putative trans-sialidase
TcG_12741	13,88624757	0,34630601	0,4663607	0,742571169	0,457741349	1	protein_codin putative GAG protein
TcG_12742	39,7151218	0,486167184	0,283988314	1,711926721	0,086910174	0,244343914	protein_codin hypothetical protein
TcG_12743	38,12923321	0,311348459	0,291833478	1,066870263	0,286030425	0,519187736	protein_codin target of rapamycin (TOR) kinase 1
TcG_12744	3,170978213	-0,004743017	0,993455607	-0,00477426	0,996190704	1	protein_codin hypothetical protein
TcG_12745	2,348616646	-0,321158477	1,117496803	-0,28739096	0,773812984	1	protein_codin hypothetical protein
TcG_12746	6,13632812	-0,984732519	0,750950198	-1,31131535	0,189751245	1	protein_codin hypothetical protein
TcG_12747	6,691674074	0,151831993	0,67157012	0,226085093	0,821135239	1	
TcG_12748	0,155721614	0,503022807	4,080472857	0,123275616	0,901888849	1	
TcG_12749	110,16893	0,210969288	0,175279723	1,203614911	0,228738454	0,455746126	protein_codin hypothetical protein
TcG_12750	74,32523582	-0,046784275	0,206073425	-0,22702721	0,820402585	0,911143056	protein_codin retrotransposon hot spot (RHS) protein
TcG_12751	74,3781024	0,080006693	0,207845029	0,38493436	0,700286048	0,844381264	protein_codin hypothetical protein
TcG_12752	53,82053199	-0,031402496	0,237310052	-0,13232687	0,894725757	0,948859736	protein_codin hypothetical protein
TcG_12753	56,71555982	-0,221589441	0,237884464	-0,93150026	0,351594849	0,586017576	protein_codin mucin-associated surface protein (MASP)
TcG_12754	31,86232226	0,686429897	0,316473628	2,168995572	0,03008302	0,11472741	protein_codin mucin TcMUCII
TcG_12755	7,770825565	-0,575797536	0,644335934	-0,8936294	0,371520209	1	
TcG_12756	135,6531976	-0,030878461	0,151886618	-0,20329942	0,838901011	0,921717128	protein_codin retrotransposon hot spot (RHS) protein
TcG_12757	32,06984159	0,485653925	0,318883576	1,522981933	0,127763226	0,316545406	protein_codin putative retrotransposon hot spot (RHS) protein
TcG_12758	89,07725923	0,122707917	0,186891899	0,65657162	0,511456402	0,717683589	
TcG_12759	17,85880525	0,415174788	0,419237963	0,990308188	0,322023505	0,556777247	protein_codin retrotransposon hot spot (RHS) protein
TcG_12760	31,67008066	-0,003233176	0,320703288	-0,01008152	0,991956248	0,996781867	protein_codin kinesin
TcG_12761	53,03991115	-0,291576134	0,2562001	-1,13807971	0,255087202	0,486388031	protein_codin subtilisin-like serine peptidase
TcG_12762	25,67545557	-0,06499814	0,347185224	-0,18721459	0,851492385	0,928214153	protein_codin putative trans-sialidase
TcG_12763	27,70509642	0,00560781	0,324252619	0,017294572	0,986201616	0,994614548	protein_codin mitotic centromere-associated kinesin (MCAK)
TcG_12764	17,31465021	0,151726595	0,417034868	0,363822326	0,71599069	0,854234181	
TcG_12765	21,64872122	0,114466722	0,382270354	0,299439182	0,76460497	0,881292267	protein_codin hypothetical protein
TcG_12766	30,70674195	-0,08868823	0,30885548	-0,28715123	0,77399653	0,886918879	protein_codin hypothetical protein
TcG_12767	1,081386522	-1,191968622	1,878352968	-0,63458181	0,525701196	1	protein_codin hypothetical protein
TcG_12768	160,785276	0,035218958	0,139464187	0,25253048	0,800631058	0,90076825	protein_codin trans-sialidase

TcG_12769	85,48823537	0,086047545	0,201192807	0,427686984	0,668879027	0,826307698	protein_codin trans-sialidase
TcG_12770	37,36757087	-0,107239813	0,290445669	-0,36922504	0,711959995	0,851969479	protein_codin mucin TcMUCII
TcG_12771	13,75679884	-0,185842501	0,467863319	-0,39721537	0,691208649	1	protein_codin hypothetical protein
TcG_12772	41,81653526	0,014360282	0,266172972	0,05395094	0,956974252	0,980067505	protein_codin hypothetical protein
TcG_12773	40,56598506	-0,283878679	0,27680121	-1,02556878	0,305094891	0,537951182	protein_codin hypothetical protein
TcG_12774	18,36686683	0,392123923	0,41197977	0,95180383	0,341196479	0,575582762	protein_codin hypothetical protein
TcG_12775	6,696115229	-1,559532084	0,737620096	-2,11427548	0,034491745	1	
TcG_12776	5,813601469	0,298032671	0,717836342	0,415181921	0,678008732	1	protein_codin hypothetical protein
TcG_12777	17,11356195	-0,300737424	0,4327414	-0,69495875	0,487081151	0,698603888	protein_codin hypothetical protein
TcG_12778	13,57564911	-0,827209395	0,469851972	-1,76057449	0,07831045	1	protein_codin mucin TcMUCII
TcG_12779	6,468379964	-0,314753336	0,686396653	-0,45855896	0,646550913	1	protein_codin RNaseH
TcG_12780	21,03480229	-0,388269205	0,376418192	-1,03148363	0,302314079	0,535372803	protein_codin L1Tc protein
TcG_12781	8,096761513	-0,470012679	0,616063214	-0,7629293	0,445505559	1	protein_codin hypothetical protein
TcG_12782	50,41992305	-0,505605715	0,251386082	-2,01127171	0,044296765	0,151975814	protein_codin mucin TcMUCI
TcG_12783	166,9457144	0,083565961	0,137732203	0,606727837	0,544031545	0,741198199	protein_codin dispersed gene family protein 1 (DGF-1)
TcG_12784	6,070102158	-0,485562887	0,703745087	-0,68996984	0,490213152	1	
TcG_12785	28,68570271	-0,596435756	0,32007088	-1,86344898	0,062399124	0,195182572	protein_codin putative trans-sialidase
TcG_12786	7,720506487	0,241243523	0,649580387	0,371383631	0,710351815	1	protein_codin hypothetical protein
TcG_12787	4,618859051	0,887026542	0,935085789	0,948604452	0,342821826	1	protein_codin hypothetical protein
TcG_12788	11,40950755	0,588353603	0,5268997	1,116633018	0,264151268	1	protein_codin hypothetical protein
TcG_12789	221,3100507	-0,386335554	0,12038753	-3,20909942	0,001331514	0,009848091	protein_codin cAMP phosphodiesterase
TcG_12790	177,2456237	-0,417037372	0,147419478	-2,82891634	0,004670591	0,027233751	protein_codin putative cAMP specific phosphodiesterase
TcG_12791	17,04234841	1,22690736	0,443665218	2,765390002	0,005685478	0,031638782	protein_codin retrotransposon hot spot (RHS) protein
TcG_12792	23,17872699	0,900333031	0,380142427	2,36840975	0,017864738	0,07704558	protein_codin retrotransposon hot spot (RHS) protein
TcG_12793	18,95555555	-0,117050519	0,418811295	-0,27948272	0,7798744	0,890390004	protein_codin hypothetical protein
TcG_12794	80,22322544	0,005911302	0,204225269	0,028945006	0,976908451	0,990177192	protein_codin putative retrotransposon hot spot (RHS) protein
TcG_12795	10,03318038	-0,481644766	0,556677665	-0,86521302	0,386921878	1	protein_codin retrotransposon hot spot (RHS) protein
TcG_12796	7,786503558	0,084323007	0,647530134	0,130222522	0,896390376	1	
TcG_12797	89,9236845	0,277549472	0,19289517	1,438861696	0,150189712	0,350190784	protein_codin hypothetical protein
TcG_12798	161,8204654	0,225300049	0,140382575	1,604900391	0,108515727	0,284384351	protein_codin hypothetical protein
TcG_12799	55,94327035	-0,09777727	0,235559331	-0,41508553	0,67807929	0,831522719	
TcG_12800	24,06531322	-0,251007165	0,364458293	-0,688713	0,491003894	0,701878188	
TcG_12801	18,55566695	0,985232309	0,415721229	2,369935042	0,017791211	0,076856438	protein_codin hypothetical protein
TcG_12802	5,759335876	0,243661358	0,718200566	0,339266452	0,734409013	1	protein_codin putative GAG protein
TcG_12803	26,86219736	0,836933128	0,341362418	2,45174361	0,014216592	0,065284753	protein_codin putative trans-sialidase
TcG_12804	704,8550778	0,415421771	0,077080977	5,389420168	7,06854E-08	1,89574E-06	protein_codin putative mannosyl-oligosaccharide 1,2-alpha-mannosidase IB
TcG_12805	1103,856724	0,38516735	0,063354949	6,079514832	1,20547E-09	4,71843E-08	protein_codin putative mannosyl-oligosaccharide 1,2-alpha-mannosidase IB
TcG_12806	59,58177494	-0,546750319	0,231742552	-2,3593005	0,018309422	0,078335661	protein_codin mucin-associated surface protein (MASP)
TcG_12807	281,3443332	0,206239821	0,108099479	1,907870622	0,05640794	0,180039227	protein_codin hypothetical protein
TcG_12808	20,66924334	-0,113048963	0,393272183	-0,28745731	0,773762187	0,886918879	
TcG_12809	20,06609592	-0,176374794	0,384960976	-0,45816279	0,646835492	0,812383308	protein_codin trans-sialidase
TcG_12810	58,98388722	-0,265113891	0,229392757	-1,15572041	0,247795537	0,477220593	protein_codin putative trans-sialidase
TcG_12811	49,08617417	-0,272637983	0,263567465	-1,03441441	0,300942472	0,534199829	
TcG_12812	1643,829484	0,239401793	0,064201132	3,728934155	0,000192291	0,001933931	protein_codin L1Tc protein
TcG_12813	7,22000937	0,175069424	0,650367632	0,269185328	0,78778707	1	protein_codin hypothetical protein
TcG_12814	4,021484118	0,567208406	0,886344365	0,639941346	0,522210733	1	
TcG_12815	144,3376838	-0,113539847	0,147694796	-0,76874643	0,442043859	0,66452837	protein_codin putative trans-sialidase
TcG_12816	15,72280029	0,056417289	0,465051964	0,121313947	0,903442374	1	protein_codin hypothetical protein
TcG_12817	43,99296788	-0,007519257	0,26750901	-0,02810843	0,977575674	0,990224843	protein_codin trans-sialidase
TcG_12818	7,705949276	0,843638758	0,664791798	1,269027024	0,204431426	1	protein_codin surface protease GP63
TcG_12819	6,876982647	0,958365517	0,685432756	1,398190426	0,162055891	1	protein_codin hypothetical protein
TcG_12820	24,83264257	-0,217156457	0,356211287	-0,60962823	0,542108106	0,740196534	protein_codin trans-sialidase
TcG_12821	26,93075947	0,003574309	0,335520968	0,010653012	0,991500287	0,996748141	protein_codin hypothetical protein
TcG_12822	27,63256122	0,375557854	0,346175097	1,084878309	0,277975556	0,510651984	protein_codin target of rapamycin (TOR) kinase 1
TcG_12823	0					1	protein_codin hypothetical protein
TcG_12824	0,701553094	2,707439836	2,05454854	1,317778472	0,187577816	1	protein_codin trans-sialidase

TcG_12825	0,76867091	1,535796318	2,067383313	0,74286965	0,457560602	1	protein_codin trans-sialidase
TcG_12826	296,5154378	-0,300708364	0,106968722	-2,81118029	0,004936012	0,028523009	protein_codin putative trans-sialidase
TcG_12827	68,47382968	-0,426457331	0,217622317	-1,95962131	0,050040068	0,165599609	protein_codin mucin-associated surface protein (MASP)
TcG_12828	2,50170326	-0,92124696	1,12361419	-0,81989616	0,412275306	1	protein_codin hypothetical protein
TcG_12829	701,2759114	0,013261271	0,071817318	0,184652835	0,853501342	0,929047136	
TcG_12830	4080,448349	0,572103634	0,041127388	13,91052673	5,46753E-44	7,91835E-41	protein_codin tyrosine aminotransferase
TcG_12831	348,2038596	0,214278621	0,097613854	2,195166073	0,028151687	0,109061055	protein_codin putative retrotransposon hot spot (RHS) protein
TcG_12832	34,34382836	-0,256120587	0,307652834	-0,83249871	0,405127506	0,634304216	protein_codin mucin-associated surface protein (MASP)
TcG_12833	66,87757032	0,306648378	0,212038213	1,446193936	0,148122814	0,346907403	protein_codin mucin TcMUCII
TcG_12834	15,87185517	0,071448769	0,435670203	0,163997374	0,869733211	1	
TcG_12835	24,76766299	-0,465018714	0,366624198	-1,26837976	0,204662365	0,425941828	protein_codin retrotransposon hot spot (RHS) protein
TcG_12836	33,92717516	0,109534531	0,321098908	0,341123961	0,733010264	0,862784475	protein_codin trans-sialidase
TcG_12837	145,6789624	-0,084866667	0,160609765	-0,52840291	0,597219719	0,781145594	protein_codin putative retrotransposon hot spot (RHS) protein
TcG_12838	160,7853792	-0,213930232	0,143717271	-1,48854922	0,136606112	0,329389889	protein_codin putative retrotransposon hot spot (RHS) protein
TcG_12839	11,14169566	0,447436535	0,518852409	0,862358017	0,388490536	1	protein_codin serine/threonine protein phosphatase
TcG_12840	18,04173877	0,065378959	0,414986731	0,157544698	0,874815584	0,939787979	protein_codin hypothetical protein
TcG_12841	18,12151744	0,401846271	0,41431322	0,969909362	0,332091673	0,566340797	protein_codin putative trans-sialidase
TcG_12842	38,65769963	0,476605547	0,286371786	1,664289465	0,096054597	0,262041101	protein_codin retrotransposon hot spot (RHS) protein
TcG_12843	48,07803167	-0,008778367	0,264835402	-0,0331465	0,973557761	0,988345381	protein_codin putative retrotransposon hot spot (RHS) protein
TcG_12844	7,050305545	0,188046398	0,642627527	0,292621137	0,769811755	1	protein_codin putative trans-sialidase
TcG_12845	4,356779744	1,968956541	1,048926003	1,877116723	0,060502098	1	protein_codin putative trans-sialidase
TcG_12846	3,627438036	0,504958363	0,986060531	0,51209672	0,60858332	1	protein_codin hypothetical protein
TcG_12847	6,197952544	0,69833447	0,7301574	0,956416343	0,338861931	1	
TcG_12848	25,76565616	-0,249239671	0,35381317	-0,70443865	0,481159645	0,695361812	protein_codin hypothetical protein
TcG_12849	141,2051592	0,11218748	0,158647325	0,707150154	0,479473171	0,693876612	protein_codin retrotransposon hot spot (RHS) protein
TcG_12850	28,39900096	0,161260461	0,337008348	0,478505836	0,632290221	0,802906018	protein_codin trans-sialidase
TcG_12851	36,11282171	-0,322026918	0,307389452	-1,04761863	0,294814349	0,527931847	protein_codin trans-sialidase
TcG_12852	5,239841162	0,217996984	0,767449676	0,284053784	0,776369164	1	protein_codin mucin TcMUCII
TcG_12853	1,124778117	-1,132218905	1,722363904	-0,65736335	0,510947312	1	protein_codin hypothetical protein
TcG_12854	15,36515566	-0,459717227	0,437854606	-1,04993124	0,29374973	1	
TcG_12855	52,37418279	-0,166780427	0,240742559	-0,692775	0,488450762	0,699788614	protein_codin trans-sialidase
TcG_12856	50,51325399	-0,024349597	0,244856983	-0,09944416	0,920785621	0,96150609	protein_codin trans-sialidase
TcG_12857	23,29359865	-0,621074327	0,364951482	-1,70179971	0,08879292	0,247952465	protein_codin hypothetical protein
TcG_12858	0,441769291	0,396475077	2,50258318	0,158426333	0,874120865	1	protein_codin hypothetical protein
TcG_12859	2,584252469	0,316121531	1,124827852	0,281039921	0,77867978	1	protein_codin hypothetical protein
TcG_12860	1,888993355	0,327709614	1,244898048	0,26324213	0,792363961	1	protein_codin hypothetical protein
TcG_12861	2,934234674	-0,880348259	1,05135893	-0,83734321	0,402399679	1	protein_codin hypothetical protein
TcG_12862	5,149370799	-0,87225536	0,769127812	-1,13408376	0,256759416	1	protein_codin hypothetical protein
TcG_12863	23,27191985	0,978311654	0,383605665	2,550305544	0,010762855	0,052341661	
TcG_12864	154,413736	-0,37740383	0,144726259	-2,60770804	0,009115066	0,045896201	protein_codin trans-sialidase
TcG_12865	81,93901465	-0,142697704	0,200093537	-0,71315499	0,475749856	0,691164619	protein_codin trans-sialidase
TcG_12866	227,268494	-0,508192487	0,130787394	-3,88563816	0,000102061	0,001136955	protein_codin proton motive P-type ATPase 1
TcG_12867	8,760672212	0,142944247	0,61455152	0,23259929	0,816072579	1	protein_codin hypothetical protein
TcG_12868	7,155655626	-0,000350956	0,641723234	-0,0005469	0,999563641	1	protein_codin hypothetical protein
TcG_12869	22,71407631	0,012985412	0,36929826	0,035162396	0,971950247	0,987776155	protein_codin trans-sialidase
TcG_12870	40,70938389	0,192937847	0,272744143	0,707395013	0,479321035	0,693829774	protein_codin rab1 small GTP-binding protein
TcG_12871	10,35501503	0,910867275	0,560631701	1,624715965	0,104223094	1	protein_codin hypothetical protein
TcG_12872	35,08484326	0,109824803	0,305832757	0,35910085	0,719519646	0,855976447	protein_codin hypothetical protein
TcG_12873	11,5068652	0,062814295	0,515825464	0,121774321	0,903077752	1	protein_codin hypothetical protein
TcG_12874	10,8123851	0,536130797	0,531771791	1,008197136	0,313359832	1	protein_codin hypothetical protein
TcG_12875	43,33664704	0,730339487	0,277473483	2,63210552	0,00848575	0,043425752	protein_codin multidrug resistance-associated protein
TcG_12876	27,3633175	1,257106196	0,360725759	3,484936035	0,000492255	0,004277704	protein_codin multidrug resistance-associated protein
TcG_12877	206,3173695	0,376866051	0,124660015	3,023151007	0,002501574	0,016443652	protein_codin mucin-associated surface protein (MASP)
TcG_12878	71,97077605	0,451045482	0,211785222	2,129730665	0,033193856	0,123294526	
TcG_12879	13,67306008	-0,691084147	0,470285198	-1,4695001	0,141697194	1	protein_codin trans-sialidase
TcG_12880	7,262026922	-0,591810294	0,677705023	-0,87325647	0,382523303	1	protein_codin trans-sialidase
TcG_12881	45,91946827	-0,025942809	0,262669457	-0,09876599	0,921324073	0,961663127	protein_codin putative retrotransposon hot spot (RHS) protein

TcG_12882	30,09764857	-0,037172793	0,316036588	-0,1176218	0,90636733	0,954710879	protein_codin retrotransposon hot spot (RHS) protein
TcG_12883	102,8658016	-0,0950208	0,174337706	-0,54503872	0,585726888	0,77437942	protein_codin retrotransposon hot spot (RHS) protein
TcG_12884	35,03610045	-0,435328995	0,305776116	-1,42368541	0,15453756	0,355746507	protein_codin mucin-associated surface protein (MASP)
TcG_12885	3,073868963	1,64891126	1,10325884	1,494582414	0,135023459	1	protein_codin hypothetical protein
TcG_12886	4,663032756	-0,564989044	0,800930279	-0,70541601	0,480551382	1	protein_codin hypothetical protein
TcG_12887	99,5534428	0,039704931	0,194743631	0,20388308	0,838444867	0,92147811	protein_codin proton motive ATPase
TcG_12888	826,7365048	-0,302659847	0,067290599	-4,49780283	6,86593E-06	0,000110483	protein_codin proton motive ATPase
TcG_12889	5,886090101	-0,142274251	0,726932978	-0,19571853	0,844830483	1	protein_codin hypothetical protein
TcG_12890	5,568093265	-0,861932763	0,81706473	-1,05491368	0,291464804	1	protein_codin hypothetical protein
TcG_12891	1,20250354	-1,476588472	1,731302038	-0,85287745	0,393727266	1	protein_codin hypothetical protein
TcG_12892	3,927694057	0,014445748	0,878319647	0,016447029	0,986877761	1	protein_codin hypothetical protein
TcG_12893	22,13037144	0,232485948	0,371594637	0,625643981	0,531548485	0,732689576	
TcG_12894	0,987600772	1,979939579	1,830332716	1,081737523	0,279369175	1	
TcG_12895	109,6809627	-0,599339021	0,177786823	-3,37111048	0,000748658	0,006112724	protein_codin mucin TcMUCII
TcG_12896	0,97939772	-1,359815763	1,94279564	-0,69992733	0,483972689	1	
TcG_12897	4,872119068	-1,306188556	0,805565224	-1,62145599	0,104919882	1	protein_codin hypothetical protein
TcG_12898	5,716699041	0,584651396	0,761928165	0,767331387	0,442884512	1	protein_codin hypothetical protein
TcG_12899	39,04560794	-0,005839959	0,284844774	-0,02050225	0,983642717	0,99376391	protein_codin trans-sialidase
TcG_12900	183,4581462	-0,100225692	0,13531624	-0,74067748	0,458889015	0,677544046	protein_codin sialidase
TcG_12901	20,83777891	0,468649449	0,377947054	1,239987039	0,214980188	0,438870501	
TcG_12902	39,33883707	-0,474321183	0,277882162	-1,70691483	0,087837885	0,245988886	protein_codin putative mucin TcMUCII
TcG_12903	1,911460515	2,115717721	1,435114449	1,47425017	0,140414246	1	protein_codin hypothetical protein
TcG_12904	0,481095949	0,532926224	2,572744524	0,20714308	0,835898119	1	protein_codin hypothetical protein
TcG_12905	1,288840451	1,428846426	1,585674046	0,901097189	0,367536647	1	protein_codin hypothetical protein
TcG_12906	23,07409463	0,117346373	0,372586816	0,314950416	0,752799301	0,875344511	protein_codin dispersed gene family protein 1 (DGF-1)
TcG_12907	4,47942659	0,820260171	0,846790279	0,968669801	0,332709965	1	protein_codin dispersed gene family protein 1 (DGF-1)
TcG_12908	19,24861679	-0,392430583	0,391532887	-1,00229277	0,316202212	0,551153727	protein_codin mucin-associated surface protein (MASP)
TcG_12909	22,60287087	-0,488635765	0,385160621	-1,26865452	0,204564309	0,425814243	protein_codin hypothetical protein
TcG_12910	34,32659003	-0,540327432	0,297001078	-1,81927768	0,068869078	0,209702269	protein_codin surface protease GP63
TcG_12911	5,500362089	-0,174788234	0,748198015	-0,23361227	0,815286004	1	protein_codin mucin TcMUCII
TcG_12912	0,884770644	1,463065956	1,937279582	0,755216733	0,45011895	1	protein_codin hypothetical protein
TcG_12913	2,142394778	-1,855279794	1,348142044	-1,37617531	0,168767365	1	protein_codin trans-sialidase
TcG_12914	6,828694439	-0,628629032	0,681884599	-0,92189944	0,35658103	1	
TcG_12915	51,01283505	-0,178490183	0,250683906	-0,71201293	0,476456767	0,691757908	protein_codin trans-sialidase
TcG_12916	17,34866661	-0,117706439	0,409832909	-0,28720592	0,77395465	0,886918879	
TcG_12917	2,294488722	-0,633365366	1,147929132	-0,55174605	0,581122354	1	protein_codin hypothetical protein
TcG_12918	10,55199287	0,13720008	0,532323814	0,257738009	0,79660911	1	protein_codin hypothetical protein
TcG_12919	1271,031726	0,175360399	0,059556407	2,944442218	0,003235374	0,020263719	protein_codin kinesin-like protein
TcG_12920	17,17875549	0,875827762	0,43046773	2,034595628	0,041891578	0,145883925	protein_codin kinesin-like protein
TcG_12921	85,40987375	-0,007110453	0,204320277	-0,03480053	0,972238801	0,987776155	protein_codin putative trans-sialidase
TcG_12922	21,82399908	0,246892136	0,36856659	0,669871179	0,502939914	0,711484963	
TcG_12923	15,4117772	-0,054828253	0,467154454	-0,11736644	0,906569681	1	protein_codin hypothetical protein
TcG_12924	15,36179431	-0,071332657	0,445259102	-0,16020483	0,872719728	1	protein_codin hypothetical protein
TcG_12925	10,48829683	-0,116359177	0,560642752	-0,20754603	0,835583449	1	protein_codin trans-sialidase
TcG_12926	10,70805721	-1,023517266	0,544871464	-1,87845636	0,060318762	1	protein_codin mucin-associated surface protein (MASP)
TcG_12927	1,47762139	0,485643037	1,553821373	0,312547533	0,754624445	1	
TcG_12928	8,041635711	0,68880248	0,625043417	1,102007415	0,270458448	1	protein_codin hypothetical protein
TcG_12929	5,22082171	0,650397223	0,824255431	0,789072415	0,430069684	1	protein_codin hypothetical protein
TcG_12930	7,405839649	0,295851771	0,633699035	0,466864797	0,640596613	1	protein_codin hypothetical protein
TcG_12931	10,21456824	0,19009647	0,551497148	0,344691665	0,730326185	1	protein_codin putative ATP-dependent DEAD/H RNA helicase
TcG_12932	136,584387	0,171816252	0,154163518	1,114506556	0,265061933	0,497508899	protein_codin retrotransposon hot spot (RHS) protein
TcG_12933	153,6186824	0,265261529	0,142324198	1,863783762	0,062352077	0,195088081	protein_codin retrotransposon hot spot protein (RHS)
TcG_12934	32,19014342	-0,335323955	0,307588334	-1,09017124	0,275637719	0,50795906	protein_codin hypothetical protein
TcG_12935	22,17390811	-0,076054743	0,379340697	-0,20049191	0,841095881	0,922466573	protein_codin hypothetical protein
TcG_12936	7,196745222	-0,195586884	0,658788512	-0,29688873	0,766551461	1	protein_codin hypothetical protein
TcG_12937	47,78448595	0,788430048	0,258016814	3,055731268	0,002245124	0,015114471	protein_codin hypothetical protein
TcG_12938	36,73352089	0,122138698	0,289748132	0,421534032	0,673365166	0,828900214	protein_codin putative target of rapamycin (TOR) kinase 1

TcG_12939	28,13367315	0,461877856	0,344608982	1,340295464	0,180149306	0,391485489	protein_codin target of rapamycin (TOR) kinase 1
TcG_12940	20,36238536	-0,216192502	0,400479164	-0,53983458	0,589311116	0,775648564	
TcG_12941	44,13042799	0,402524646	0,278362439	1,446045117	0,148164548	0,346935014	protein_codin retrotransposon hot spot (RHS) protein
TcG_12942	55,94492021	-0,24555859	0,235016593	-1,0448564	0,296089346	0,52923344	protein_codin retrotransposon hot spot protein (RHS)
TcG_12943	71,93789915	0,362023974	0,215585467	1,67925964	0,093101453	0,255852333	protein_codin putative retrotransposon hot spot (RHS) protein
TcG_12944	2,413338854	0,108828121	1,114923874	0,097610361	0,922241697	1	protein_codin hypothetical protein
TcG_12945	7,228441931	-1,200070648	0,665994475	-1,80192283	0,071557548	1	protein_codin hypothetical protein
TcG_12946	4,057229896	-0,245585448	0,87146574	-0,28180735	0,778091237	1	protein_codin hypothetical protein
TcG_12947	90,13957791	-0,005321285	0,186004586	-0,02860835	0,977176949	0,990177192	protein_codin protein kinase, putative,serine/threonine protein kinase
TcG_12948	30,62971227	0,370536622	0,329326878	1,125133255	0,260532571	0,492901759	protein_codin hypothetical protein
TcG_12949	53,75074838	0,428280985	0,244657811	1,750530599	0,080026799	0,231180714	protein_codin helicase-like protein
TcG_12950	7,553800141	0,241919956	0,664931865	0,363826685	0,715987435	1	protein_codin hypothetical protein
TcG_12951	1,790618899	-0,579702448	1,283892546	-0,45151944	0,651615214	1	
TcG_12952	29,29787414	8,22048E-05	0,318691444	0,000257945	0,99979419	0,99981271	protein_codin putative trans-sialidase
TcG_12953	18,80417614	0,165842512	0,398672338	0,415987005	0,677419512	0,830962833	protein_codin putative GAG protein
TcG_12954	28,56793972	-0,141835277	0,323032516	-0,4390743	0,660607699	0,822193662	protein_codin trans-sialidase
TcG_12955	25,94642878	0,113006269	0,339815756	0,332551588	0,739472792	0,867087269	protein_codin putative trans-sialidase
TcG_12956	32,56826598	-0,21564053	0,319452871	-0,67503081	0,499656192	0,708743467	protein_codin trans-sialidase
TcG_12957	17,68364221	0,318016214	0,417991798	0,760819268	0,446765024	0,668244199	protein_codin mitotic centromere-associated kinesin (MCAK)
TcG_12958	6,708291349	-0,296764037	0,727170642	-0,40810784	0,683194508	1	protein_codin hypothetical protein
TcG_12959	0					1	protein_codin structural maintenance of chromosome protein 4
TcG_12960	15,58768993	0,126539395	0,450530382	0,280867617	0,778811939	1	protein_codin trans-sialidase
TcG_12961	28,49402588	-0,092520602	0,322215952	-0,28713849	0,774006282	0,886918879	protein_codin trans-sialidase
TcG_12962	4,888813833	-0,036728522	0,810798029	-0,04529922	0,963868805	1	protein_codin hypothetical protein
TcG_12963	4,655244571	1,131760436	0,879703351	1,286525093	0,198259867	1	protein_codin hypothetical protein
TcG_12964	3,692142257	0,42789037	0,908618914	0,470923909	0,637695074	1	protein_codin hypothetical protein
TcG_12965	22,83588148	0,11216271	0,39351388	0,2850286	0,77562223	0,888230528	protein_codin putative retrotransposon hot spot (RHS) protein
TcG_12966	49,94638352	0,294683896	0,262593666	1,122204888	0,261775335	0,494123335	protein_codin retrotransposon hot spot (RHS) protein
TcG_12967	10,09849924	0,467245111	0,550206654	0,849217484	0,395760286	1	protein_codin hypothetical protein
TcG_12968	37,03745569	-0,167249226	0,301360422	-0,55498073	0,578907844	0,768472306	protein_codin hypothetical protein
TcG_12969	6,966883348	0,011426068	0,661913748	0,01726217	0,986227465	1	protein_codin trans-sialidase
TcG_12970	713,5688021	-0,019904026	0,070641446	-0,28176131	0,77812654	0,889256383	protein_codin host cell signaling surface protein
TcG_12971	21,69459936	0,012830886	0,373491603	0,034353881	0,972594959	0,987858399	protein_codin putative dispersed gene family protein 1 (DGF-1)
TcG_12972	20,67868572	0,18349229	0,385262209	0,476278974	0,633875643	0,804197117	protein_codin dispersed gene family protein 1 (DGF-1)
TcG_12973	8,093906016	0,185002114	0,604585149	0,305998442	0,759605832	1	protein_codin structural maintenance of chromosome protein 4
TcG_12974	11,80671958	0,390514809	0,516735308	0,755734712	0,449808268	1	protein_codin putative mucin-associated surface protein (MASP)
TcG_12975	81,56430348	0,650365399	0,198528672	3,275926803	0,001053159	0,008150898	protein_codin dispersed gene family protein 1 (DGF-1)
TcG_12976	7,477848507	0,262023596	0,627384904	0,417644089	0,676207357	1	protein_codin hypothetical protein
TcG_12977	7,738569214	-0,137995833	0,642028887	-0,21493711	0,829816352	1	protein_codin hypothetical protein
TcG_12978	11,39001064	0,387445612	0,532744513	0,727263448	0,467064585	1	protein_codin hypothetical protein
TcG_12979	12,31962509	0,192160124	0,506149788	0,379650705	0,704204717	1	protein_codin putative leucine-rich repeat protein
TcG_12980	104,9337255	0,281894746	0,180195639	1,564381623	0,117727974	0,299247687	protein_codin retrotransposon hot spot (RHS) protein
TcG_12981	47,09107989	0,107737711	0,273498969	0,393923644	0,693637413	0,841257963	protein_codin retrotransposon hot spot protein (RHS)
TcG_12982	7,083398014	0,781828534	0,688450118	1,135635704	0,25610906	1	protein_codin hypothetical protein
TcG_12983	53,08560166	0,111640598	0,238660034	0,467780866	0,639941305	0,808545252	protein_codin hypothetical protein
TcG_12984	3,989794588	-0,137513199	0,854435674	-0,16094038	0,872140361	1	protein_codin hypothetical protein
TcG_12985	122,4526533	0,12069516	0,163957102	0,73613865	0,461646323	0,679450496	protein_codin putative trans-sialidase
TcG_12986	79,36012214	0,011230011	0,213389772	0,052626755	0,958029299	0,98064539	protein_codin trans-sialidase-like protein
TcG_12987	24,35005756	-0,346838569	0,349757134	-0,99165545	0,321365628	0,556054685	protein_codin trans-sialidase
TcG_12988	210,8791412	0,080986374	0,132740499	0,610110509	0,541788605	0,740087861	protein_codin hypothetical protein
TcG_12989	116,8766654	-0,058077757	0,170252104	-0,34112799	0,733007233	0,862784475	protein_codin complement regulatory protein
TcG_12990	50,93129883	0,167643469	0,246549247	0,679959362	0,496530193	0,706384923	protein_codin putative trans-sialidase
TcG_12991	126,1018636	-0,486400537	0,165675742	-2,93585851	0,00332626	0,020697126	protein_codin sialidase
TcG_12992	66,76322927	-0,180317099	0,219284856	-0,82229618	0,410908351	0,637576557	protein_codin trans-sialidase
TcG_12993	4,233733899	-1,028019828	0,855792635	-1,20124874	0,229654726	1	
TcG_12994	204,5878204	0,144388828	0,125449528	1,150971471	0,249743973	0,478982564	protein_codin hypothetical protein
TcG_12995	114,1294699	-0,039209667	0,178545638	-0,21960585	0,826178135	0,915112889	protein_codin hypothetical protein

TcG_12996	4,291085369	-0,056152311	0,852999366	-0,06582925	0,947513766	1	protein_codin hypothetical protein
TcG_12997	8,509435952	-0,102766213	0,608907642	-0,16877143	0,865976432	1	protein_codin hypothetical protein
TcG_12998	13,58400064	0,191460809	0,477119775	0,401284581	0,688210614	1	
TcG_12999	16,59054397	1,365656641	0,475218433	2,873745097	0,004056362	1	protein_codin retrotransposon hot spot (RHS) protein
TcG_13000	22,66297293	0,553020582	0,387953683	1,425480944	0,154018225	0,355044797	protein_codin retrotransposon hot spot (RHS) protein
TcG_13001	36,32181811	0,110440614	0,286483804	0,385503866	0,699864143	0,844367272	protein_codin trans-sialidase
TcG_13002	23,70056724	-0,147471256	0,362516199	-0,40679908	0,684155562	0,835965655	protein_codin N-acetyltransferase complex ARD1 subunit
TcG_13003	35,3246647	-0,176962847	0,296591737	-0,59665468	0,550737958	0,746546058	protein_codin hypothetical protein
TcG_13004	0,913094583	0,185694647	1,881143835	0,098713689	0,9213656	1	protein_codin hypothetical protein
TcG_13005	0,145251675	-1,420530545	4,080472857	-0,3481289	0,727743378	1	protein_codin hypothetical protein
TcG_13006	0					1	protein_codin hypothetical protein
TcG_13007	0					1	protein_codin hypothetical protein
TcG_13008	8,967829661	-0,218921682	0,572872052	-0,3821476	0,702351884	1	protein_codin dispersed protein family protein 1
TcG_13009	15,58195376	-0,054625816	0,433032025	-0,12614729	0,899615336	1	protein_codin hypothetical protein
TcG_13010	21,67087777	-0,227152644	0,376122616	-0,60393243	0,545888569	0,742854706	protein_codin hypothetical protein
TcG_13011	34,2263353	-0,270770225	0,297497994	-0,91015815	0,36273911	0,595082383	protein_codin retrotransposon hot spot (RHS) protein
TcG_13012	36,26751591	0,180067066	0,320722946	0,561441169	0,574496825	0,76562855	protein_codin hypothetical protein
TcG_13013	39,4685246	-0,205692565	0,285675244	-0,72002237	0,471511225	0,687506802	protein_codin retrotransposon hot spot (RHS) protein
TcG_13014	23,99450717	0,122007434	0,357602257	0,341181945	0,732966615	0,862784475	protein_codin hypothetical protein
TcG_13015	7,611673583	-0,635322251	0,622396895	-1,02076706	0,307364812	1	protein_codin mucin-associated surface protein (MASP)
TcG_13016	2,476280711	0,172977383	1,094097263	0,158100554	0,874377564	1	protein_codin hypothetical protein
TcG_13017	2,01264365	-0,956079645	1,258502657	-0,75969617	0,44743622	1	protein_codin hypothetical protein
TcG_13018	3,325756426	-0,09388737	0,997610964	-0,09411221	0,925020023	1	protein_codin hypothetical protein
TcG_13019	4,423953176	-0,015701776	0,906750666	-0,01731653	0,986184098	1	protein_codin mucin-associated surface protein (MASP)
TcG_13020	10,5335268	-0,308907344	0,557011831	-0,5545795	0,579182313	1	protein_codin hypothetical protein
TcG_13021	6,362501831	-0,626915618	0,72636785	-0,86308283	0,388091929	1	protein_codin trans-sialidase
TcG_13022	7,247018769	0,38934191	0,657909582	0,591786351	0,553993664	1	protein_codin trans-sialidase
TcG_13023	89,49017059	-0,030070751	0,189601198	-0,15860001	0,873984024	0,939417284	
TcG_13024	27,08959367	-0,006320974	0,336332086	-0,01879385	0,985005562	0,994129886	protein_codin putative trans-sialidase
TcG_13025	5,001485382	-0,049185618	0,832686956	-0,05906856	0,952897501	1	protein_codin hypothetical protein
TcG_13026	4,455749638	0,010535299	0,838217849	0,012568688	0,989971902	1	protein_codin hypothetical protein
TcG_13027	20,43918233	-0,180625833	0,38463043	-0,4696088	0,63863454	0,807731988	protein_codin hypothetical protein
TcG_13028	5,858167742	-0,175718245	0,729332657	-0,24093017	0,809609235	1	protein_codin hypothetical protein
TcG_13029	12,67901173	-0,370127226	0,486647316	-0,76056564	0,44691655	1	protein_codin putative elongation factor 1-gamma (EF-1-gamma)
TcG_13030	11,12375107	0,238840308	0,550408955	0,433932453	0,664337493	1	protein_codin trans-sialidase
TcG_13031	6,971749576	0,680442137	0,669149322	1,016876376	0,309212243	1	protein_codin trans-sialidase
TcG_13032	2,930464607	0,968828152	1,053122161	0,919957995	0,35759471	1	protein_codin hypothetical protein
TcG_13033	52,55599015	0,012416223	0,245416241	0,050592509	0,959650232	0,981466419	protein_codin putative trans-sialidase
TcG_13034	84,95278014	0,104755532	0,227584211	0,460293497	0,645305568	0,811781793	protein_codin trans-sialidase
TcG_13035	23,31384594	-0,424780376	0,648329939	-0,65519167	0,512344356	0,718385781	protein_codin hypothetical protein
TcG_13036	1,709137084	0,146436551	1,306518693	0,112081482	0,9107588	1	protein_codin mucin-associated surface protein (MASP)
TcG_13037	1,214936196	-2,863964133	1,7557499	-1,6311914	0,10284994	1	protein_codin hypothetical protein
TcG_13038	1,751838346	-0,511482485	1,41044979	-0,36263785	0,716875429	1	protein_codin hypothetical protein
TcG_13039	1,274669951	-0,810979684	1,708724468	-0,47461115	0,635064158	1	
TcG_13040	0,701020314	2,706632999	2,17304287	1,245549748	0,212929741	1	protein_codin mucin-associated surface protein (MASP)
TcG_13041	8,530682542	-0,31107896	0,592683717	-0,52486503	0,599677018	1	
TcG_13042	1,993249043	-0,716809443	1,242082338	-0,577103	0,563869878	1	protein_codin hypothetical protein
TcG_13043	38,81581161	-0,133158382	0,288942028	-0,46084809	0,644907597	0,811369249	protein_codin surface protease GP63
TcG_13044	8,984781842	-0,283322985	0,602904582	-0,46993006	0,638404988	1	protein_codin putative dispersed gene family protein 1 (DGF-1)
TcG_13045	6,137013726	-0,268611989	0,751428077	-0,35746866	0,720740983	1	protein_codin putative dispersed gene family protein 1 (DGF-1)
TcG_13046	6,945655096	-0,133363456	0,712673734	-0,18713115	0,851557804	1	protein_codin dispersed gene family protein 1 (DGF-1)
TcG_13047	3,688974476	-0,06875094	0,928582492	-0,07403859	0,940979678	1	
TcG_13048	53,38806095	0,50271977	0,240723603	2,088369251	0,036764539	0,133355843	protein_codin hypothetical protein
TcG_13049	23,39634501	-1,033518888	0,363542451	-2,84291115	0,004470353	0,02639873	protein_codin mucin-associated surface protein (MASP)
TcG_13050	2,916366999	1,123264913	1,053614013	1,066106658	0,28637543	1	
TcG_13051	4,221598944	-0,336952349	0,875891305	-0,38469653	0,700462263	1	protein_codin hypothetical protein
TcG_13052	48,48574089	0,163658632	0,253232101	0,646279171	0,518098575	0,723215673	protein_codin dispersed gene family protein 1 (DGF-1)

TcG_13053	28,70566256	0,185150044	0,32607394	0,567816136	0,570159822	0,762010808	protein_codin retrotransposon hot spot (RHS) protein
TcG_13054	10,70701578	0,035318545	0,549136203	0,064316549	0,948718177	1	protein_codin hypothetical protein
TcG_13055	0,846271989	1,744895683	1,997141606	0,873696526	0,382283543	1	
TcG_13056	37,02820283	0,023431478	0,294180236	0,079650075	0,936515568	0,970438187	
TcG_13057	5,628555132	-0,273791094	0,77891025	-0,35150532	0,725209285	1	protein_codin hypothetical protein
TcG_13058	12,92316633	-0,675140417	0,478727053	-1,41028257	0,158456264	1	protein_codin hypothetical protein
TcG_13059	21,04665403	0,453117386	0,389485372	1,16337459	0,244677554	0,472864744	protein_codin hypothetical protein
TcG_13060	74,05248303	-0,597714447	0,210591226	-2,83826852	0,004535901	0,02669017	protein_codin mucin-associated surface protein (MASP)
TcG_13061	39,64634122	0,479503557	0,280334432	1,710469719	0,087179048	0,244802825	protein_codin dispersed gene family protein 1 (DGF-1)
TcG_13062	10,12631611	-1,363143609	0,591135828	-2,30597359	0,021112108	1	protein_codin hypothetical protein
TcG_13063	7,316363214	-0,143124695	0,702098928	-0,2038526	0,838468683	1	protein_codin hypothetical protein
TcG_13064	9,184232714	-0,05639064	0,579587258	-0,09729448	0,922492541	1	protein_codin hypothetical protein
TcG_13065	45,30100621	0,033101598	0,265521133	0,124666529	0,900787557	0,951748911	protein_codin trans-sialidase
TcG_13066	132,1776138	-0,317449154	0,154715192	-2,05182924	0,040186261	0,141691423	protein_codin putative NADH-dependent fumarate reductase
TcG_13067	43,25564806	0,275501659	0,267822376	1,028673044	0,303633341	0,536498717	protein_codin hypothetical protein
TcG_13068	164,4147175	-0,096941674	0,149647704	-0,64779927	0,517114785	0,722625968	protein_codin mucin-associated surface protein (MASP)
TcG_13069	316,8881708	0,334574765	0,104181913	3,211447708	0,00132068	0,00979603	protein_codin hypothetical protein
TcG_13070	343,9653191	0,103510728	0,099404593	1,041307291	0,297732954	0,531106082	protein_codin putative retrotransposon hot spot (RHS) protein
TcG_13071	542,0911533	-0,05343663	0,078867086	-0,677553	0,498055154	0,707424985	protein_codin putative mitotubule-associated protein Gb4
TcG_13072	60,16218263	-0,053630111	0,22803494	-0,23518375	0,814066121	0,908210889	protein_codin hypothetical protein
TcG_13073	3,927758834	-0,956498139	0,897054207	-1,06626571	0,286303547	1	
TcG_13074	4,606311987	-0,985164164	0,810085042	-1,21612437	0,223937547	1	
TcG_13075	16,0751679	-0,591132782	0,44400896	-1,33135327	0,183072797	1	protein_codin trans-sialidase
TcG_13076	4,769279928	-0,389995359	0,804000044	-0,48506883	0,627627523	1	protein_codin hypothetical protein
TcG_13077	13,06152171	0,567721588	0,484936925	1,170712227	0,241714469	1	
TcG_13078	199,4984273	1,13242442	0,133379615	8,490236103	2,06218E-17	2,91371E-15	protein_codin phospholipase A1
TcG_13079	135,6090242	0,056024077	0,157800596	0,355030834	0,722566492	0,857335574	protein_codin retrotransposon hot spot (RHS) protein
TcG_13080	37,98447026	0,317783701	0,287231534	1,10636773	0,268567387	0,501174141	protein_codin putative retrotransposon hot spot (RHS) protein
TcG_13081	22,70792686	-0,017676108	0,373620429	-0,04731034	0,96226589	0,98256823	protein_codin retrotransposon hot spot (RHS) protein
TcG_13082	69,91674663	-0,104682953	0,212669758	-0,49223244	0,622555038	0,796665292	protein_codin hypothetical protein
TcG_13083	38,82825457	0,020480013	0,276246797	0,074136652	0,940901651	0,972634416	protein_codin hypothetical protein
TcG_13084	52,34505321	0,058917856	0,259848394	0,226739349	0,820626427	0,911143056	protein_codin hypothetical protein
TcG_13085	126,7505793	-0,081966096	0,155849207	-0,52593207	0,598935406	0,782859388	protein_codin putative retrotransposon hot spot (RHS) protein
TcG_13086	615,8546905	0,050376349	0,076410256	0,659287792	0,50971098	0,716514367	protein_codin trans-sialidase-like protein
TcG_13087	1112,072228	0,065348228	0,063285181	1,032599207	0,3017915	0,535010187	protein_codin putative amino acid transporter PAT1
TcG_13088	292,9619464	0,679616434	0,111421895	6,099487286	1,06409E-09	4,2077E-08	protein_codin hypothetical protein
TcG_13089	1013,503828	0,021437273	0,064721564	0,331223033	0,740476023	0,867634494	protein_codin amino acid transporter
TcG_13090	614,5737746	0,276523566	0,091126087	3,034515956	0,00240922	0,015959534	protein_codin structural maintenance of chromosome protein 4
TcG_13091	25,39605075	-0,019574204	0,374396458	-0,05228202	0,958303979	0,980649169	protein_codin hypothetical protein
TcG_13092	8,802428333	-0,300839783	0,623593897	-0,482429	0,629501221	1	protein_codin putative target of rapamycin (TOR) kinase 1
TcG_13093	12,05957489	0,047072485	0,50498146	0,093216264	0,925731753	1	protein_codin putative target of rapamycin (TOR) kinase 1
TcG_13094	48,35904369	-0,089802194	0,256683841	-0,34985527	0,726447318	0,858752908	protein_codin dispersed gene family protein 1 (DGF-1)
TcG_13095	13,27298503	1,07562589	0,509397679	2,111564175	0,034723848	1	protein_codin mucin TcMUCII
TcG_13096	10,15659898	0,441546108	0,567275651	0,778362524	0,436355326	1	protein_codin hypothetical protein
TcG_13097	1025,549081	0,178357048	0,06199558	2,876931654	0,004015626	0,024131244	protein_codin trans-sialidase-like protein
TcG_13098	1283,500685	-0,299175554	0,056306311	-5,31335739	1,07624E-07	2,77095E-06	protein_codin putative P-type H+-ATPase
TcG_13099	4256,306814	0,015660118	0,040651537	0,38522819	0,70006836	0,844367272	protein_codin stress-70 protein, mitochondrial
TcG_13100	85,82926149	0,035200635	0,188957249	0,186288882	0,852218226	0,928425046	protein_codin hypothetical protein
TcG_13101	21,01073338	-0,310352949	0,401647576	-0,77269967	0,439700143	0,662749321	protein_codin dispersed gene family protein 1 (DGF-1)
TcG_13102	79,50069516	0,598137803	0,203108436	2,944918555	0,003230397	0,020263619	protein_codin rab1 small GTP-binding protein
TcG_13103	22,39767501	0,31026199	0,374546497	0,828367085	0,407462641	0,635720733	protein_codin hypothetical protein
TcG_13104	13,10047784	-0,145940624	0,481036687	-0,30338772	0,761594402	1	protein_codin helicase-like protein
TcG_13105	30,01305874	-0,075309989	0,323028272	-0,23313745	0,815654673	0,909172037	protein_codin trans-sialidase
TcG_13106	53,60762717	0,061780049	0,240908032	0,256446613	0,797605999	0,898993352	protein_codin surface protein-2
TcG_13107	96,43942543	0,017259963	0,177473603	0,097253692	0,92252493	0,96227026	protein_codin Sialidase 85-1.3
TcG_13108	16,50172411	0,07311846	0,419988008	0,174096542	0,861789591	1	
TcG_13109	33,25192008	0,528766823	0,310819771	1,701200735	0,0889053	0,248146665	protein_codin dispersed gene family protein 1 (DGF-1)

TcG_13110	48,61248608	0,082576108	0,257744888	0,320379228	0,74868087	0,873184674	protein_codin dispersed gene family protein 1 (DGF-1)
TcG_13111	73,66296513	-0,352113951	0,206277102	-1,70699485	0,087823009	0,245988886	protein_codin hypothetical protein
TcG_13112	86,18229164	0,096489877	0,201551308	0,47873605	0,632126416	0,802906018	protein_codin dispersed gene family protein 1 (DGF-1)
TcG_13113	43,18531628	0,078790108	0,26379845	0,298675402	0,765187727	0,881784862	protein_codin putative trans-sialidase
TcG_13114	30,86916807	-0,060706836	0,311934832	-0,19461384	0,845695266	0,925035391	
TcG_13115	40,70793661	0,132929944	0,273380753	0,486244706	0,626793678	0,799695139	protein_codin trans-sialidase
TcG_13116	4,990592074	-0,679652554	0,79477432	-0,85515163	0,392467211	1	protein_codin hypothetical protein
TcG_13117	12,37573899	-0,785306588	0,512514489	-1,53226222	0,12545774	1	protein_codin hypothetical protein
TcG_13118	450,4590803	-0,080649217	0,085412688	-0,94422995	0,345052156	0,579219686	protein_codin trans-sialidase
TcG_13119	256,8643166	-0,03382489	0,114470577	-0,29548982	0,767619728	0,883509739	protein_codin trans-sialidase
TcG_13120	47,33403612	-0,029426263	0,27212955	-0,10813329	0,913889963	0,957873088	protein_codin hypothetical protein
TcG_13121	88,41018526	0,306710189	0,189200942	1,621081721	0,105000114	0,278382452	protein_codin putative beta galactofuranosyl glycosyltransferase
TcG_13122	47,09453053	0,195032924	0,257194518	0,758309024	0,448266006	0,669107182	protein_codin putative beta galactofuranosyl glycosyltransferase
TcG_13123	259,856742	0,063991065	0,112641436	0,568095257	0,569970288	0,761925641	protein_codin dispersed gene family protein 1 (DGF-1)
TcG_13124	15,44903131	-1,284079577	0,458820412	-2,79865399	0,005131609	1	protein_codin hypothetical protein
TcG_13125	40,68393547	0,191854107	0,275973745	0,695189708	0,486936421	0,698594881	protein_codin dispersed gene family protein 1 (DGF-1)
TcG_13126	19,42362322	0,487294782	0,407597578	1,19552914	0,231880358	0,459225958	protein_codin hypothetical protein
TcG_13127	10,8272527	0,81727659	0,543896687	1,502632045	0,132933955	1	
TcG_13128	19,3176759	0,189975107	0,404589983	0,469549704	0,638676766	0,807731988	protein_codin putative trans-sialidase
TcG_13129	23,24189357	0,24583727	0,363873369	0,675612152	0,499286924	0,70839313	protein_codin putative trans-sialidase
TcG_13130	9,279088337	0,198698373	0,570681611	0,348177283	0,727707047	1	protein_codin hypothetical protein
TcG_13131	94,47268899	-0,344621265	0,183846392	-1,87450654	0,060860641	0,19150771	protein_codin mucin TcMUCII
TcG_13132	3,766560046	-0,113991416	0,890573932	-0,1279977	0,898150792	1	protein_codin mucin TcMUCII
TcG_13133	2,409733519	-1,205487415	1,222606792	-0,98599764	0,32413427	1	protein_codin hypothetical protein
TcG_13134	5,632522149	-0,714043074	0,737533612	-0,96814987	0,332969524	1	protein_codin hypothetical protein
TcG_13135	173,7590824	0,136582696	0,137768447	0,991393157	0,321493641	0,556110081	
TcG_13136	199,6214135	0,319310094	0,126528277	2,523626358	0,011615131	0,05556272	protein_codin surface protease GP63
TcG_13137	54,38391345	0,102244954	0,242018	0,422468387	0,672683171	0,828677003	protein_codin trans-sialidase
TcG_13138	213,3254934	-0,000974107	0,125610427	-0,00775499	0,993812476	0,997677114	
TcG_13139	5,615749218	-0,324152384	0,728592718	-0,44490204	0,65639055	1	protein_codin putative trans-sialidase
TcG_13140	5,270993599	-0,202881178	0,737626643	-0,27504589	0,78328098	1	protein_codin putative trans-sialidase
TcG_13141	8,066791868	0,361705057	0,606614363	0,596268534	0,550995848	1	protein_codin hypothetical protein
TcG_13142	21,0620895	-0,396877058	0,383616133	-1,03456821	0,300870605	0,534199829	protein_codin hypothetical protein
TcG_13143	19,48049771	0,190779543	0,398065916	0,47926621	0,631749258	0,802906018	protein_codin dispersed gene family protein 1 (DGF-1)
TcG_13144	24,85972003	0,683742697	0,359585727	1,901473409	0,057240032	0,182193136	protein_codin hypothetical protein
TcG_13145	23,39424077	0,159136474	0,362609926	0,438864087	0,66076002	0,822243957	protein_codin syntaxin binding protein
TcG_13146	26,24271412	-0,186816603	0,346327839	-0,53942127	0,589596208	0,775816202	protein_codin hypothetical protein
TcG_13147	43,33833757	0,304261353	0,275501159	1,104392282	0,269423007	0,50225824	protein_codin surface protease GP63
TcG_13148	67,21617107	0,562921359	0,212974427	2,643140622	0,008214091	0,042353565	protein_codin hypothetical protein
TcG_13149	8,986700434	-0,342948077	0,604203481	-0,56760361	0,570304155	1	protein_codin hypothetical protein
TcG_13150	61,7227286	0,120883021	0,234823824	0,514781759	0,606705534	0,787507317	protein_codin putative trans-sialidase
TcG_13151	63,2350185	0,095702725	0,228925618	0,418051619	0,675909377	0,83017833	protein_codin putative trans-sialidase
TcG_13152	103,4995252	0,147314275	0,172727303	0,852871971	0,393730307	0,623531894	protein_codin hypothetical protein
TcG_13153	123,911547	0,151403404	0,178667298	0,847404117	0,396769911	0,626624319	protein_codin retrotransposon hot spot (RHS) protein
TcG_13154	182,696291	0,223851418	0,140176502	1,596925418	0,110282333	0,287130587	protein_codin retrotransposon hot spot (RHS) protein
TcG_13155	62,9993256	0,030641198	0,221533358	0,138314151	0,889992142	0,946535728	protein_codin receptor-type adenylate cyclase
TcG_13156	26,75908421	0,216965397	0,344653411	0,629517624	0,529010234	0,731076654	protein_codin hypothetical protein
TcG_13157	172,1648777	-0,285896175	0,136976883	-2,08718559	0,036871362	0,133423365	protein_codin trans-sialidase-like protein
TcG_13158	124,8914122	-0,094595227	0,157335816	-0,60123136	0,5476859	0,744600896	
TcG_13159	56,74226277	0,642830056	0,245754211	2,615743806	0,008903333	0,045124243	protein_codin target of rapamycin (TOR) kinase 1
TcG_13160	89,53454008	0,480784941	0,187757122	2,560674856	0,010446908	0,051135561	protein_codin target of rapamycin (TOR) kinase 1
TcG_13161	53,44617953	0,523596359	0,250354571	2,091419209	0,036490502	0,132730628	protein_codin trans-sialidase
TcG_13162	2,231100299	0,810200723	1,189046843	0,681386716	0,495626826	1	protein_codin hypothetical protein
TcG_13163	3,268957557	0,466117791	0,966046298	0,482500468	0,629450466	1	protein_codin mucin TcMUC
TcG_13164	3,79449325	-0,224578199	0,963114468	-0,23317913	0,815622307	1	protein_codin dispersed protein family protein 1
TcG_13165	102,7640258	0,301239877	0,18106956	1,663669348	0,096178525	0,262317418	protein_codin putative dispersed gene family protein 1 (DGF-1)
TcG_13166	73,58841266	-0,096516086	0,22269141	-0,43340732	0,664718886	0,824768789	protein_codin hypothetical protein

TcG_13167	44,28351326	0,19646824	0,262875562	0,747381149	0,454833521	0,674756913	protein_codin hypothetical protein
TcG_13168	4,444906377	0,402100514	0,862381621	0,466267491	0,641024045	1	protein_codin hypothetical protein
TcG_13169	28,43615116	0,480507344	0,360034424	1,334615003	0,182002385	0,393924833	protein_codin retrotransposon hot spot (RHS) protein
TcG_13170	18,57827526	-0,38113089	0,440727589	-0,86477657	0,387161436	0,617348252	
TcG_13171	24,38890805	0,296352023	0,350123698	0,846420922	0,397317971	0,626985292	protein_codin putative trans-sialidase
TcG_13172	505,8416649	-0,031266197	0,084600078	-0,36957646	0,711698093	0,851832036	
TcG_13173	5,665435145	-0,752452507	0,76440488	-0,98436382	0,324936661	1	protein_codin hypothetical protein
TcG_13174	75,46281374	0,471912144	0,218887817	2,155954367	0,031087229	0,117359606	protein_codin hypothetical protein
TcG_13175	7,498069283	-0,311897938	0,641975178	-0,48584112	0,627079819	1	protein_codin hypothetical protein
TcG_13176	8,579622305	-0,354499358	0,598157759	-0,59265194	0,55341411	1	protein_codin hypothetical protein
TcG_13177	11,10232684	0,377143666	0,518940582	0,726756934	0,467374869	1	protein_codin retrotransposon hot spot (RHS) protein
TcG_13178	10,65320477	0,12351603	0,558476156	0,221166166	0,824963053	1	
TcG_13179	80,81249324	0,067728406	0,195061304	0,347216003	0,728429049	0,860128308	protein_codin retrotransposon hot spot protein (RHS)
TcG_13180	101,5683826	0,12529745	0,179413609	0,698372049	0,484944552	0,697958705	protein_codin retrotransposon hot spot (RHS) protein
TcG_13181	81,69256593	0,139828107	0,193626857	0,722152439	0,470200761	0,686450317	protein_codin hypothetical protein
TcG_13182	59,3140669	0,067877653	0,231940221	0,2926515	0,769788545	0,884364283	protein_codin dispersed gene family protein 1 (DGF-1)
TcG_13183	94,1470328	-0,204867417	0,180402044	-1,13561583	0,25611738	0,488055258	protein_codin dispersed gene family protein 1 (DGF-1)
TcG_13184	19,61711994	0,270979778	0,401606729	0,674739138	0,499841514	0,708798748	protein_codin hypothetical protein
TcG_13185	37,27644583	0,16325866	0,282751906	0,577391898	0,563674746	0,756774978	
TcG_13186	52,41374781	0,263510214	0,242131585	1,088293434	0,276465588	0,508918066	protein_codin dispersed gene family protein 1 (DGF-1)
TcG_13187	165,1256053	0,215967009	0,137968207	1,56533895	0,117503455	0,29894489	protein_codin putative trans-sialidase
TcG_13188	25,21488888	-0,036016856	0,355650419	-0,10127039	0,919335823	0,961200581	
TcG_13189	53,95159807	-0,077634803	0,245854597	-0,31577527	0,752173088	0,87492919	protein_codin trans-sialidase
TcG_13190	107,9126359	0,568236732	0,195350086	2,908812297	0,003628046	0,022228738	protein_codin hypothetical protein
TcG_13191	149,2654602	-0,228388288	0,152402217	-1,49858902	0,133980283	0,32557556	
TcG_13192	4,069755616	0,651417679	0,982828607	0,662798858	0,507459385	1	protein_codin dispersed protein family protein 1 (DGF-1)
TcG_13193	464,4685293	0,15431599	0,085063928	1,814117848	0,069659584	0,211276425	protein_codin methyltransferase
TcG_13194	3,028150978	-0,229304072	1,028611458	-0,22292584	0,823593226	1	protein_codin hypothetical protein
TcG_13195	10,73439111	0,500882668	0,54214467	0,923891161	0,355542986	1	protein_codin trans-sialidase
TcG_13196	2,06017336	0,557614503	1,206632219	0,462124659	0,643991928	1	protein_codin trans-sialidase
TcG_13197	53,52953399	-0,085539985	0,239129616	-0,35771389	0,720557437	0,85670516	protein_codin mucin-associated surface protein (MASP)
TcG_13198	10,8425405	0,601531333	0,574688187	1,046709063	0,295233783	1	protein_codin elongation factor 1-alpha (EF-1-alpha)
TcG_13199	38,28113055	0,196258069	0,282373772	0,695029387	0,487036885	0,698603888	protein_codin subtilisin-like serine peptidase
TcG_13200	29,45232936	0,18502357	0,329518166	0,561497328	0,574458552	0,76562855	protein_codin subtilisin-like serine peptidase
TcG_13201	26,4328065	0,129980713	0,341168268	0,380987113	0,703212811	0,846749494	protein_codin putative kinesin
TcG_13202	468,7205804	-0,580617294	0,083993889	-6,91261351	4,75805E-12	3,24275E-10	protein_codin hypothetical protein
TcG_13203	4,979608601	0,542883704	0,810565789	0,669758965	0,503011456	1	protein_codin hypothetical protein
TcG_13204	6,60664269	0,066831357	0,668675306	0,099945903	0,920387274	1	protein_codin hypothetical protein
TcG_13205	2,328799928	1,5721751	1,229957675	1,278235122	0,201166536	1	
TcG_13206	45,34172008	-0,009235088	0,263700005	-0,03502119	0,972062844	0,987776155	protein_codin hypothetical protein
TcG_13207	41,96083417	0,207874082	0,274238288	0,758005323	0,448447796	0,669292305	protein_codin hypothetical protein
TcG_13208	251,8412912	0,384267345	0,119838911	3,206532337	0,001343452	0,009907852	protein_codin rab1 small GTP-binding protein
TcG_13209	10,68691234	-0,432737861	0,543489024	-0,7962219	0,425903073	1	protein_codin trans-sialidase
TcG_13210	8,33346473	0,280887604	0,611293412	0,459497189	0,64587717	1	protein_codin hypothetical protein
TcG_13211	27,92519453	-0,416536068	0,324772275	-1,28254811	0,199650439	0,418442472	protein_codin hypothetical protein
TcG_13212	21,24711595	-0,295405861	0,376867136	-0,78384617	0,433130379	0,657527329	protein_codin hypothetical protein
TcG_13213	33,07781458	-0,218725708	0,313679307	-0,69729084	0,485620799	0,698269767	protein_codin hypothetical protein
TcG_13214	264,1568008	0,159935024	0,110555439	1,446649982	0,14799498	0,346888496	protein_codin putative retrotransposon hot spot (RHS) protein
TcG_13215	92,52052079	0,29265528	0,183381345	1,595883595	0,110514785	0,287412861	protein_codin putative retrotransposon hot spot (RHS) protein
TcG_13216	36,4998907	-0,313861235	0,291194259	-1,07784142	0,28110453	0,514255504	protein_codin dispersed gene family protein 1 (DGF-1)
TcG_13217	12,39152517	-0,14321476	0,48619164	-0,29456442	0,768326642	1	protein_codin hypothetical protein
TcG_13218	23,79478715	-0,022831834	0,382755781	-0,05965118	0,952433456	0,977496481	protein_codin hypothetical protein
TcG_13219	17,1729536	-0,076941565	0,459438118	-0,16746883	0,86700117	0,93578156	
TcG_13220	27,17292303	0,0467703	0,352025578	0,132860517	0,894303693	0,948820769	protein_codin trans-sialidase
TcG_13221	12,37801829	0,045668641	0,513596897	0,08891923	0,929146101	1	protein_codin trans-sialidase
TcG_13222	10,97466059	0,234366478	0,522656065	0,448414347	0,65385419	1	protein_codin hypothetical protein
TcG_13223	94,03111811	-0,548346111	0,183397167	-2,98993774	0,002790343	0,017910757	protein_codin mucin-associated surface protein (MASP)

TcG_13224	59,23512952	0,166067647	0,228726984	0,726051838	0,467806993	0,684344927	protein_codin hypothetical protein
TcG_13225	0,233855473	1,175456718	3,508398891	0,335040785	0,737594321	1	
TcG_13226	29,78797971	-0,103164444	0,319774377	-0,32261635	0,746985806	0,872173491	protein_codin hypothetical protein
TcG_13227	21,62796068	0,208241146	0,385433765	0,540277383	0,589005753	0,775648564	protein_codin 90 kDa surface protein
TcG_13228	33,83646003	0,039618546	0,322221762	0,122954284	0,902143299	0,952714635	protein_codin hypothetical protein
TcG_13229	13,74143599	0,22280698	0,486485447	0,457993104	0,646957396	1	protein_codin hypothetical protein
TcG_13230	37,71075844	-0,010946063	0,288002414	-0,03800684	0,969682226	0,986975162	protein_codin trans-sialidase
TcG_13231	30,78222432	0,311329112	0,325485762	0,956506087	0,338816611	0,573276734	protein_codin hypothetical protein
TcG_13232	21,60744722	0,198477118	0,383891297	0,517013852	0,605146489	0,786698766	protein_codin surface protease GP63
TcG_13233	15,49218896	-0,229162577	0,43904628	-0,5219554	0,601701384	1	
TcG_13234	44,91194594	-0,177311757	0,26556613	-0,66767459	0,504341332	0,712076368	protein_codin hypothetical protein
TcG_13235	48,68725916	0,193446313	0,252930943	0,764818689	0,444379517	0,666426941	protein_codin hypothetical protein
TcG_13236	37,65181931	0,144236908	0,2879014	0,500994119	0,616375262	0,792657426	protein_codin hypothetical protein
TcG_13237	40,71447753	0,303923626	0,29476908	1,031056672	0,302514245	0,535430803	
TcG_13238	51,23094259	-0,277170533	0,2481159	-1,11710105	0,26395112	0,496193848	protein_codin mucin TcMUCII
TcG_13239	79,63152847	-0,422475409	0,195677983	-2,15903395	0,030847533	0,116644751	protein_codin mucin-associated surface protein (MASP)
TcG_13240	10,61666883	-0,041658747	0,532986967	-0,07816091	0,937700056	1	protein_codin hypothetical protein
TcG_13241	17,91162024	-0,530113193	0,405012591	-1,30888077	0,190574741	0,405956783	protein_codin hypothetical protein
TcG_13242	24,42610903	-0,943111203	0,373144378	-2,52746995	0,011488765	0,055140363	protein_codin hypothetical protein
TcG_13243	27,07530533	0,105759746	0,343636628	0,307766219	0,758260228	0,878015178	protein_codin putative trans-sialidase
TcG_13244	2,814926486	1,265498015	1,106644257	1,143545459	0,252812192	1	protein_codin hypothetical protein
TcG_13245	12,67839429	-0,178322913	0,498478252	-0,35773459	0,720541944	1	protein_codin hypothetical protein
TcG_13246	16,93711224	0,770829923	0,445094875	1,731832841	0,083303329	0,237229207	
TcG_13247	78,4838538	-0,077789283	0,199249384	-0,39041166	0,696232167	0,842635108	protein_codin trans-sialidase
TcG_13248	181,5324443	-0,059007913	0,13256952	-0,4451092	0,656240838	0,818341013	protein_codin putative surface protease GP63, putative,metallopeptidase
TcG_13249	27,76628364	0,176729698	0,331644034	0,532889726	0,594109918	0,779189213	protein_codin dispersed gene family protein 1 (DGF-1)
TcG_13250	28,9422553	0,142991813	0,352721549	0,40539574	0,685186642	0,836557076	protein_codin dispersed protein family protein 1
TcG_13251	19,27777463	-1,742186882	0,431266948	-4,03969488	5,35208E-05	0,000656877	protein_codin mucin-associated surface protein (MASP)
TcG_13252	7,213986572	-1,158428601	0,735391194	-1,57525493	0,115197614	1	protein_codin putative mucin TcMUCII
TcG_13253	128,9540048	-0,371517596	0,157984375	-2,35160975	0,018692374	0,0795939	protein_codin putative trans-sialidase
TcG_13254	18,99361385	0,300642942	0,40889622	0,735254882	0,46218428	0,679744706	protein_codin hypothetical protein
TcG_13255	172,7999791	-0,503308744	0,137296803	-3,6658446	0,000246524	0,002376225	protein_codin putative trans-sialidase
TcG_13256	103,0105115	0,22115134	0,173805158	1,27240953	0,20322765	0,423412255	protein_codin putative retrotransposon hot spot (RHS) protein
TcG_13257	29,62689549	0,242821426	0,333835927	0,727367566	0,467000818	0,683597154	
TcG_13258	70,46277977	-0,16265513	0,210610495	-0,77230306	0,439934951	0,662906274	protein_codin trans-sialidase
TcG_13259	57,17311602	0,02866011	0,230156971	0,124524188	0,90090025	0,951748911	
TcG_13260	81,38886517	-0,275643417	0,19393367	-1,42132832	0,155221335	0,356612014	protein_codin sialidase
TcG_13261	135,5837401	-0,298053675	0,153312425	-1,94409341	0,051884188	0,169534522	protein_codin hypothetical protein
TcG_13262	52,41981721	-0,471397898	0,277257796	-1,70021512	0,089090468	0,248483911	protein_codin hypothetical protein
TcG_13263	5,366715003	0,410986591	0,770672824	0,533282839	0,593837805	1	protein_codin hypothetical protein
TcG_13264	8,305374038	-0,33180129	0,646002317	-0,51362245	0,607515984	1	protein_codin hypothetical protein
TcG_13265	9,515033121	-0,461238979	0,576286097	-0,80036458	0,423499598	1	protein_codin hypothetical protein
TcG_13266	48,68392707	0,152256583	0,254546023	0,598149524	0,549740168	0,745961428	protein_codin hypothetical protein
TcG_13267	831,2031741	0,189386709	0,067459807	2,807400703	0,004994307	0,028745179	protein_codin L1Tc protein
TcG_13268	52,12190841	0,241974541	0,257882075	0,938314696	0,348082709	0,582618646	
TcG_13269	18,5686796	0,143074372	0,402468503	0,355492097	0,722220964	0,857285556	protein_codin trans-sialidase
TcG_13270	4,194227417	-1,205794245	0,856727574	-1,40744185	0,159296408	1	protein_codin peptide hydrolase
TcG_13271	15,3340959	-0,166808036	0,444973525	-0,37487182	0,707755796	1	protein_codin surface protease GP63
TcG_13272	23,02191211	-0,01302253	0,378605406	-0,03439605	0,972561335	0,987858399	protein_codin hypothetical protein
TcG_13273	39,99159674	0,103240536	0,310344672	0,332664116	0,739387839	0,867087269	protein_codin hypothetical protein
TcG_13274	119,5047991	0,158173408	0,159890875	0,989258506	0,32253668	0,557248729	protein_codin dispersed gene family protein 1 (DGF-1)
TcG_13275	1,853698425	-0,646231476	1,376916832	-0,46933225	0,638832166	1	protein_codin hypothetical protein
TcG_13276	42,37443721	0,080697665	0,266894775	0,302357606	0,762379469	0,880036717	
TcG_13277	51,15759448	0,13285974	0,253568048	0,523960889	0,600305743	0,783502945	protein_codin putative retrotransposon hot spot (RHS) protein
TcG_13278	21,35946171	0,908390221	0,414397168	2,192076325	0,028373996	0,109726674	protein_codin structural maintenance of chromosome protein 4
TcG_13279	57,64266525	0,396527584	0,251904382	1,574119439	0,115459839	0,295838856	protein_codin hypothetical protein
TcG_13280	33,34894241	0,524598537	0,306415113	1,71205177	0,086887128	0,244343914	protein_codin hypothetical protein

TcG_13281	11,78582439	-0,014331031	0,50879296	-0,02816673	0,977529176	1	protein_codin putative trans-sialidase
TcG_13282	3,609703717	0,615747579	0,934462812	0,658932138	0,509939347	1	protein_codin trans-sialidase
TcG_13283	1,119187729	2,222337238	1,769738912	1,255742993	0,209209166	1	protein_codin hypothetical protein
TcG_13284	3,63871799	0,541080708	0,921588705	0,587117339	0,557124903	1	protein_codin hypothetical protein
TcG_13285	5,480782995	-0,360634102	0,78989782	-0,45655792	0,647988834	1	protein_codin hypothetical protein
TcG_13286	90,64784413	0,165870408	0,184804975	0,897542982	0,369429249	0,601128826	
TcG_13287	28,50109113	-0,235550647	0,340412072	-0,69195739	0,488964091	0,700177723	protein_codin hypothetical protein
TcG_13288	8,323724874	-0,014852165	0,650769067	-0,02282248	0,981791875	1	protein_codin hypothetical protein
TcG_13289	6,926104483	0,354012286	0,666744751	0,53095624	0,595449104	1	protein_codin hypothetical protein
TcG_13290	4,953242717	0,019142237	0,816662206	0,023439601	0,981299616	1	
TcG_13291	9,618710495	-2,130211123	0,633949079	-3,36022434	0,000778792	1	protein_codin mucin TcMUCII
TcG_13292	22,27867466	-0,331793346	0,366058223	-0,90639501	0,364726813	0,596685239	protein_codin mucin-associated surface protein (MASP)
TcG_13293	45,86865182	0,18504675	0,260954322	0,709115483	0,478252816	0,692834318	protein_codin hypothetical protein
TcG_13294	34,90232342	-0,22422534	0,32213848	-0,69605264	0,486395863	0,698294875	protein_codin trans-sialidase
TcG_13295	640,2624514	-0,267294739	0,07451531	-3,58711168	0,000334361	0,003067227	protein_codin UDP-Gal or UDP-GlcNAc-dependent glycosyltransferase
TcG_13296	120,167003	-0,36071773	0,159716469	-2,25848801	0,023915251	0,096611612	
TcG_13297	2,176479916	-1,124932816	1,258692091	-0,89373154	0,371465548	1	protein_codin hypothetical protein
TcG_13298	5,735845661	-0,852204965	0,764946053	-1,11407198	0,265248307	1	protein_codin hypothetical protein
TcG_13299	112,3516908	-0,232544228	0,167137409	-1,3913356	0,164123682	0,369158801	protein_codin dispersed gene family protein 1 (DGF-1)
TcG_13300	7,365178632	-0,144097345	0,651500763	-0,22117755	0,824954186	1	protein_codin hypothetical protein
TcG_13301	8,760841191	0,285579143	0,580212504	0,492197499	0,622579732	1	protein_codin hypothetical protein
TcG_13302	8,788192963	-0,485857738	0,598122238	-0,81230509	0,416616591	1	protein_codin hypothetical protein
TcG_13303	82,93633736	0,320380636	0,193231646	1,658013287	0,097314784	0,263987144	protein_codin hypothetical protein
TcG_13304	154,6297338	0,13413809	0,14708378	0,911984243	0,361777012	0,59446156	protein_codin hypothetical protein
TcG_13305	31,03138099	0,174380418	0,312967559	0,557183686	0,577401935	0,767704707	protein_codin hypothetical protein
TcG_13306	18,81191598	-0,273187283	0,424872566	-0,6429864	0,520232923	0,724773499	protein_codin hypothetical protein
TcG_13307	50,66605594	-0,088465323	0,244836819	-0,36132361	0,717857545	0,855087918	protein_codin trans-sialidase
TcG_13308	17,66783495	0,472056969	0,418325813	1,128443319	0,259132742	0,491636632	protein_codin hypothetical protein
TcG_13309	129,689281	-0,455544249	0,159147667	-2,86239979	0,004204462	0,02510974	protein_codin putative mucin-associated surface protein (MASP)
TcG_13310	754,9451704	-0,002520195	0,069972724	-0,03601683	0,971268943	0,98762955	protein_codin retrotransposon hot spot (RHS) protein
TcG_13311	41,82144976	0,207966051	0,279064167	0,74522664	0,456134721	0,676062028	protein_codin trans-sialidase
TcG_13312	100,0773783	-0,048032369	0,190954295	-0,25153856	0,801397751	0,901455761	protein_codin trans-sialidase
TcG_13313	41,01731771	0,144488783	0,283977421	0,508803774	0,610889774	0,78940095	protein_codin trans-sialidase
TcG_13314	33,72603268	-0,045182767	0,296049442	-0,15261899	0,878698748	0,941484386	protein_codin trans-sialidase
TcG_13315	27,62009902	0,715858606	0,344876743	2,075694057	0,037922256	0,136111294	protein_codin hypothetical protein
TcG_13316	1,953586841	0,920018746	1,353037693	0,679965348	0,496526402	1	protein_codin hypothetical protein
TcG_13317	1,420693798	1,672303167	1,566630176	1,067452417	0,28576759	1	protein_codin hypothetical protein
TcG_13318	6,994374972	-0,973072831	0,680115597	-1,43074624	0,152502956	1	protein_codin hypothetical protein
TcG_13319	11,58235073	-0,085393948	0,50010811	-0,17075098	0,864419577	1	protein_codin hypothetical protein
TcG_13320	23,13577427	0,037002209	0,387539074	0,095479944	0,92393362	0,962825591	protein_codin hypothetical protein
TcG_13321	39,5862928	0,350794748	0,292980735	1,197330424	0,23117779	0,458517724	protein_codin trans-sialidase
TcG_13322	12,08143835	-0,219944294	0,500873526	-0,43912142	0,66057356	1	protein_codin trans-sialidase
TcG_13323	33,47760983	-0,42697268	0,298015164	-1,43272132	0,151937493	0,35209328	protein_codin trans-sialidase
TcG_13324	11,12340877	0,56501859	0,523791862	1,078708224	0,280717818	1	
TcG_13325	22,10523903	-0,245807349	0,389026808	-0,63185195	0,527483621	0,729914268	protein_codin mucin-associated surface protein (MASP)
TcG_13326	171,212429	-0,630755836	0,141049184	-4,47188574	7,75329E-06	0,000122551	protein_codin mucin TcMUCI
TcG_13327	22,07077149	0,287722603	0,391302215	0,735295103	0,46215979	0,679744706	protein_codin hypothetical protein
TcG_13328	25,2035519	-0,325074959	0,34986537	-0,929143	0,352814983	0,587146567	
TcG_13329	7,959672633	-0,139857316	0,640020905	-0,21851992	0,827024041	1	protein_codin hypothetical protein
TcG_13330	29,41168194	-0,710191344	0,328369976	-2,16277795	0,030558262	0,115853412	protein_codin mucin TcMUCII
TcG_13331	291,312581	-0,2169763	0,113872414	-1,90543339	0,056723758	0,180748477	
TcG_13332	96,44137051	-0,476716926	0,183462541	-2,59844284	0,009364763	0,046848078	protein_codin putative trans-sialidase
TcG_13333	4,810680039	-1,033824675	0,80455909	-1,28495804	0,198806946	1	
TcG_13334	4,436869825	-0,163149108	0,830343079	-0,19648397	0,844231375	1	
TcG_13335	0,892716318	-1,109608292	1,964105391	-0,56494336	0,572112292	1	protein_codin trans-sialidase
TcG_13336	25,94733961	-0,122442602	0,338621268	-0,36159159	0,717657253	0,855087918	protein_codin hypothetical protein
TcG_13337	7,516638072	-0,431669696	0,63642085	-0,67827711	0,497596009	1	protein_codin hypothetical protein

TcG_13338	13,41192995	0,238728758	0,507020074	0,470846758	0,637750171	1	protein_codin	dispersed gene family protein 1 (DGF-1)
TcG_13339	165,5666502	-0,294573442	0,138926732	-2,12035105	0,033976451	0,12556656	protein_codin	hypothetical protein
TcG_13340	36,43331768	0,271291864	0,293362024	0,924768178	0,355086507	0,588980997	protein_codin	hypothetical protein
TcG_13341	41,94431727	0,734809243	0,28284853	2,597889556	0,009379866	0,046903377	protein_codin	dispersed gene family protein 1 (DGF-1)
TcG_13342	3,451464335	1,252305522	1,014031611	1,234976808	0,216839136	1	protein_codin	hypothetical protein
TcG_13343	4,379675843	0,199491236	0,837609412	0,238167376	0,811751279	1	protein_codin	hypothetical protein
TcG_13344	3,637916986	-0,64177615	0,899160893	-0,71375007	0,475381739	1	protein_codin	hypothetical protein
TcG_13345	2,582077007	-0,113917978	1,069347715	-0,10653034	0,915161582	1	protein_codin	hypothetical protein
TcG_13346	37,92462422	0,14119178	0,284929014	0,49553318	0,620223807	0,794638176	protein_codin	90 kDa surface protein
TcG_13347	34,58636499	0,585072786	0,322638642	1,813399602	0,069770211	0,211431979	protein_codin	protein kinase, putative,serine/threonine protein kinase
TcG_13348	27,79710661	-0,407435298	0,338916314	-1,2021708	0,229297358	0,456467215	protein_codin	mucin TcMUCII
TcG_13349	52,83060852	-0,245789234	0,249820872	-0,98386188	0,325183428	0,559651692	protein_codin	mucin-associated surface protein (MASP)
TcG_13350	20,61045762	-0,053855211	0,865513815	-0,0622234	0,950384929	0,976946126	protein_codin	dispersed gene family protein 1 (DGF-1)
TcG_13351	62,81698209	-0,205462929	0,228991035	-0,89725316	0,369583845	0,601225878	protein_codin	putative trans-sialidase
TcG_13352	71,09067881	0,005874885	0,207025692	0,028377564	0,977361018	0,990224843	protein_codin	trans-sialidase
TcG_13353	203,8297236	0,086195304	0,13097097	0,65812526	0,510457646	0,717215897	protein_codin	putative retrotransposon hot spot (RHS) protein
TcG_13354	135,062878	0,076998917	0,153831035	0,500542152	0,616693384	0,792744873	protein_codin	retrotransposon hot spot (RHS) protein
TcG_13355	48,32246594	-0,268275535	0,253798296	-1,0570423	0,290492282	0,523456792		
TcG_13356	3,031654238	0,988631769	1,047741888	0,94358332	0,345382619	1	protein_codin	putative D-isomer specific 2-hydroxyacid dehydrogenase-protein
TcG_13357	109,8004809	-0,235920839	0,174669996	-1,35066608	0,176802423	0,387081042	protein_codin	putative D-isomer specific 2-hydroxyacid dehydrogenase-protein
TcG_13358	12,3052384	1,007269579	0,523700531	1,923369407	0,054433674	1	protein_codin	dispersed gene family protein 1 (DGF-1)
TcG_13359	26,83930082	-0,058626879	0,333870964	-0,17559742	0,860610231	0,933293722	protein_codin	trans-sialidase
TcG_13360	266,5879745	-0,016731982	0,111769159	-0,14970124	0,881000327	0,942069315	protein_codin	trans-sialidase
TcG_13361	24,29254079	-0,112266659	0,367682165	-0,30533616	0,76011014	0,878818091		
TcG_13362	69,18536835	0,045535849	0,21627791	0,210543226	0,833243718	0,919089983	protein_codin	putative dispersed gene family protein 1 (DGF-1)
TcG_13363	6,272412266	0,012206273	0,685040527	0,017818322	0,985783788	1	protein_codin	dispersed protein family protein 1
TcG_13364	82,11530136	-0,12468765	0,191226291	-0,6520424	0,51437382	0,720229102	protein_codin	trans-sialidase
TcG_13365	149,1025164	-0,413064393	0,151463093	-2,727162	0,006388167	0,034748028	protein_codin	trans-sialidase
TcG_13366	17,63465472	-0,301966861	0,406801713	-0,74229496	0,457908645	0,677211722	protein_codin	mucin-associated surface protein (MASP)
TcG_13367	306,8301613	0,264477262	0,104989988	2,519071254	0,011766484	0,056108134	protein_codin	putative retrotransposon hot spot (RHS) protein
TcG_13368	13,30179775	0,390257483	0,478533608	0,815527847	0,414770231	1	protein_codin	hypothetical protein
TcG_13369	9,014768281	0,362699176	0,594607561	0,609980767	0,541874548	1	protein_codin	hypothetical protein
TcG_13370	14,34497373	0,875903785	0,492049629	1,780112681	0,075057522	1	protein_codin	trans-sialidase
TcG_13371	8,833829109	-0,208887096	0,576630368	-0,36225476	0,717161661	1	protein_codin	putative mitotic centromere-associated kinesin (MCAK)
TcG_13372	19,59593353	-0,001603237	0,40206516	-0,00398751	0,996818439	0,9988876	protein_codin	subtilisin-like serine peptidase
TcG_13373	69,01215098	0,236533296	0,216894563	1,090545071	0,275473111	0,507736472	protein_codin	hypothetical protein
TcG_13374	11,57346391	-0,341385521	0,509132633	-0,67052375	0,502523973	1	protein_codin	trans-sialidase
TcG_13375	10,04079736	-0,233192079	0,546778907	-0,42648331	0,669755706	1		
TcG_13376	10,03740477	-0,227583832	0,555751688	-0,40950633	0,682168124	1		
TcG_13377	10,19320265	-0,999228337	0,549837483	-1,81731579	0,069168774	1	protein_codin	trans-sialidase
TcG_13378	7,319658393	0,010702211	0,653942155	0,016365684	0,986942656	1	protein_codin	trans-sialidase
TcG_13379	1,987234776	-1,772471914	1,328852518	-1,33383644	0,182257465	1	protein_codin	putative trans-sialidase
TcG_13380	11,21481661	0,52287895	0,555695041	0,940945863	0,346732603	1		
TcG_13381	80,26013517	0,010786904	0,208907834	0,051634752	0,958819728	0,980917031	protein_codin	hypothetical protein
TcG_13382	51,03767145	0,133024865	0,244378505	0,544339467	0,586207893	0,774789487	protein_codin	rab1 small GTP-binding protein
TcG_13383	109,4471444	0,162425146	0,180122613	0,901747666	0,367190926	0,599390612	protein_codin	protein kinase
TcG_13384	192,9978631	-0,425102719	0,131793081	-3,22553137	0,001257391	0,009404859	protein_codin	putative trans-sialidase
TcG_13385	8,749198759	0,475654859	0,590753275	0,805166691	0,420723495	1	protein_codin	hypothetical protein
TcG_13386	5,846776192	0,053276319	0,738398157	0,072151207	0,942481575	1	protein_codin	hypothetical protein
TcG_13387	8,988272768	-1,037260213	0,595483962	-1,74187766	0,081529853	1	protein_codin	hypothetical protein
TcG_13388	16,99813048	-0,318541788	0,424998993	-0,74951186	0,45354875	0,674035553	protein_codin	hypothetical protein
TcG_13389	21,3488557	-0,058065663	0,380698157	-0,15252415	0,878773543	0,941484386	protein_codin	rab1 small GTP-binding protein
TcG_13390	7,189109648	1,072451761	0,704307307	1,522704295	0,127832703	1	protein_codin	dispersed gene family protein 1 (DGF-1)
TcG_13391	28,59979216	0,256477646	0,336864697	0,761366949	0,446437923	0,668040475	protein_codin	dispersed gene family protein 1 (DGF-1)
TcG_13392	53,47410018	-0,070113329	0,240295348	-0,2917798	0,770454988	0,884686967	protein_codin	dispersed gene family protein 1 (DGF-1)
TcG_13393	0,600419294	-1,762951393	2,247341994	-0,78446066	0,432769854	1		
TcG_13394	3,596598779	-0,213342063	0,947074388	-0,22526431	0,821773659	1	protein_codin	hypothetical protein

TcG_13395	13,46846075	-0,39971848	0,477298808	-0,83745962	0,402334264	1	protein_codin putative mucin-associated surface protein (MASP)
TcG_13396	183,8834475	0,187504883	0,137103712	1,367613466	0,171433099	0,378544671	protein_codin putative retrotransposon hot spot (RHS) protein
TcG_13397	3,931761113	1,1948089	0,973325808	1,22755288	0,219614859	1	protein_codin hypothetical protein
TcG_13398	23,37573506	-0,531590326	0,361495356	-1,47053155	0,141417847	0,3366698	protein_codin dispersed gene family protein 1 (DGF-1)
TcG_13399	46,88881679	-0,202078772	0,261972555	-0,77137382	0,440485386	0,663390574	protein_codin hypothetical protein
TcG_13400	81,71693198	0,192104837	0,197335261	0,973494731	0,330307478	0,565029151	protein_codin dispersed gene family protein 1 (DGF-1)
TcG_13401	14,97827083	0,376157403	0,496627304	0,757423928	0,448795925	1	protein_codin hypothetical protein
TcG_13402	4,43199677	0,029000008	0,805770561	0,035990404	0,971290011	1	
TcG_13403	54,24408629	-0,156503219	0,247697305	-0,63183255	0,527496303	0,729914268	protein_codin retrotransposon hot spot (RHS) protein
TcG_13404	2,986886417	-0,904948889	1,071333019	-0,8446943	0,398281545	1	protein_codin putative retrotransposon hot spot (RHS) protein
TcG_13405	16,89213815	0,158528089	0,423208307	0,374586431	0,707968066	0,849437494	protein_codin retrotransposon hot spot (RHS) protein
TcG_13406	3,12192122	-1,448723638	1,03069759	-1,40557585	0,159850112	1	protein_codin hypothetical protein
TcG_13407	2,749629342	0,610079618	1,035254925	0,589303758	0,555657524	1	protein_codin hypothetical protein
TcG_13408	9,36364277	-0,077294829	0,559625448	-0,13811886	0,890146481	1	
TcG_13409	3,490292038	-1,46071061	0,982596146	-1,48658288	0,137125012	1	protein_codin hypothetical protein
TcG_13410	5,229982105	-0,617584479	0,815474731	-0,75733123	0,448851447	1	
TcG_13411	77,76214422	1,07681548	0,220317769	4,887556212	1,02095E-06	2,06436E-05	protein_codin putative kinesin
TcG_13412	76,23693271	0,1394853	0,205642467	0,678290345	0,497587619	0,707281334	protein_codin trans-sialidase
TcG_13413	11,4084594	0,809452031	0,521350459	1,552606345	0,120517209	1	protein_codin surface protein-2
TcG_13414	30,05708716	-0,171765682	0,330410806	-0,51985492	0,603164699	0,785225212	protein_codin trans-sialidase
TcG_13415	27,71884149	-0,301986825	0,331689034	-0,91045164	0,362584374	0,595028691	protein_codin hypothetical protein
TcG_13416	29,07526338	-0,150334447	0,329226299	-0,45662952	0,647937357	0,812603825	protein_codin trans-sialidase
TcG_13417	16,96247028	0,289952687	0,434057201	0,668005707	0,504129949	0,712076368	protein_codin hypothetical protein
TcG_13418	16,59836626	0,156420829	0,443561211	0,352647674	0,724352592	1	protein_codin hypothetical protein
TcG_13419	3,409766883	0,579713969	0,975196867	0,5944584	0,552205558	1	
TcG_13420	46,1967833	0,212120977	0,259791496	0,816504699	0,414211537	0,641133904	protein_codin protein kinase
TcG_13421	41,92981782	-0,283059936	0,270992414	-1,04453085	0,296239854	0,529282905	protein_codin beta galactofuranosyl glycosyltransferase
TcG_13422	0,428637354	1,990244214	2,439304538	0,815906412	0,414553664	1	protein_codin hypothetical protein
TcG_13423	2,096038848	1,604734614	1,308355702	1,226527779	0,220000128	1	
TcG_13424	20,82897061	-0,242782386	0,389610369	-0,62314149	0,533191547	0,733938133	protein_codin retrotransposon hot spot (RHS) protein
TcG_13425	12,71036318	0,39890216	0,538675467	0,74052409	0,45898205	1	protein_codin retrotransposon hot spot protein (RHS)
TcG_13426	36,82113921	0,56151453	0,311109902	1,804875148	0,071094222	0,21392986	protein_codin retrotransposon hot spot (RHS) protein
TcG_13427	37,56759485	0,416899216	0,293380199	1,421020453	0,155310814	0,356746845	protein_codin retrotransposon hot spot (RHS) protein
TcG_13428	30,35308696	0,458905759	0,320014174	1,434016976	0,151567419	0,351799471	protein_codin retrotransposon hot spot (RHS) protein
TcG_13429	136,0342246	0,128171859	0,164186308	0,780648892	0,435009028	0,659170101	protein_codin retrotransposon hot spot (RHS) protein
TcG_13430	42,80158985	-0,098871438	0,268564265	-0,36814815	0,712762768	0,852489876	protein_codin putative elongation factor 1-gamma (EF-1-gamma)
TcG_13431	235,451921	-0,387298421	0,116245702	-3,33172249	0,000863103	0,006910787	protein_codin mucin-associated surface protein (MASP)
TcG_13432	234,8574175	0,539570814	0,118957193	4,535840167	5,73746E-06	9,44236E-05	protein_codin ceramide synthase
TcG_13433	131,3106136	0,660690752	0,159287591	4,147785442	3,35707E-05	0,000434581	protein_codin dispersed gene family protein 1 (DGF-1)
TcG_13434	66,61787368	-0,382497364	0,220870987	-1,73176826	0,083314832	0,237229207	protein_codin hypothetical protein
TcG_13435	57,14543238	-0,475119694	0,238136366	-1,99515807	0,046025642	0,155921956	protein_codin mucin-associated surface protein (MASP)
TcG_13436	6,535956772	0,882783588	0,694325954	1,271425304	0,203577385	1	protein_codin hypothetical protein
TcG_13437	3,30931904	-0,309409131	0,953961954	-0,32434116	0,745679758	1	protein_codin hypothetical protein
TcG_13438	19,25505485	-0,14281846	0,424649704	-0,33632064	0,736629087	0,865313252	protein_codin hypothetical protein
TcG_13439	18,11416073	0,128446932	0,406627294	0,315883694	0,752090789	0,87492919	protein_codin hypothetical protein
TcG_13440	61,39532858	-0,327396308	0,22908894	-1,42912315	0,15296884	0,353328743	protein_codin trans-sialidase
TcG_13441	73,66994832	-0,21516428	0,212335777	-1,0133209	0,310906908	0,544463034	protein_codin hypothetical protein
TcG_13442	14,10558683	-0,759477456	0,467673514	-1,62394798	0,104386912	1	protein_codin putative mucin TcMUCII
TcG_13443	17,52238948	-0,556626833	0,416078014	-1,33779439	0,180963471	0,392335849	protein_codin hypothetical protein
TcG_13444	240,0137346	0,240373962	0,11723846	2,05029955	0,040335208	0,142022914	protein_codin trans-sialidase-like protein
TcG_13445	0,27291574	1,35431792	3,063093298	0,442140604	0,658387464	1	protein_codin hypothetical protein
TcG_13446	2,416014176	-0,533294628	1,146452493	-0,46516941	0,641810144	1	protein_codin hypothetical protein
TcG_13447	5,308959474	-0,312603568	0,733013794	-0,42646342	0,669770199	1	
TcG_13448	1,33182361	0,802102879	1,573842521	0,509646212	0,610299342	1	protein_codin hypothetical protein
TcG_13449	1,373279486	-0,47024359	1,545678873	-0,3042311	0,76095183	1	protein_codin mucin-associated surface protein (MASP)
TcG_13450	2,711787812	0,756994269	1,151654878	0,657310001	0,510981607	1	protein_codin hypothetical protein
TcG_13451	281,4778911	0,196880344	0,11864976	1,65934043	0,097047213	0,263482126	protein_codin trans-sialidase

TcG_13452	121,0427361	0,205042836	0,162646104	1,260668598	0,207428272	0,429626894	protein_codin dispersed gene family protein 1 (DGF-1)
TcG_13453	22,8934234	0,140240218	0,414653114	0,338210938	0,735204234	0,864516011	protein_codin trans-sialidase
TcG_13454	29,13594961	-0,05271655	0,343879902	-0,15329931	0,878162248	0,941423575	protein_codin trans-sialidase
TcG_13455	4,390090017	0,051194352	0,849567773	0,060259292	0,951949123	1	
TcG_13456	36,77835456	0,418831483	0,286334667	1,462734105	0,14354015	0,340008225	protein_codin trans-sialidase
TcG_13457	12,7479936	0,748168493	0,495262544	1,510650265	0,13087758	1	protein_codin putative trans-sialidase
TcG_13458	15,88933466	-0,492198324	0,43483048	-1,13193152	0,257663228	1	protein_codin hypothetical protein
TcG_13459	3,970257585	0,962103484	0,896761881	1,072863939	0,283332168	1	
TcG_13460	47,33011206	0,369152296	0,256641032	1,438399362	0,150320774	0,350359751	protein_codin hypothetical protein
TcG_13461	42,27024784	0,132533478	0,267996504	0,494534355	0,62092885	0,795365578	protein_codin hypothetical protein
TcG_13462	228,0354582	0,495981698	0,125847483	3,941133231	8,10976E-05	0,000928455	protein_codin hypothetical protein
TcG_13463	573,9867194	-0,040059419	0,082706139	-0,48435847	0,628131489	0,800692914	protein_codin protein kinase
TcG_13464	1368,579194	0,124620703	0,057585017	2,164116816	0,030455385	0,115605149	protein_codin hypothetical protein
TcG_13465	2294,471873	0,439535672	0,05019239	8,757018115	2,00483E-18	3,05632E-16	protein_codin 40S ribosomal protein S8
TcG_13466	493,6988985	0,068955219	0,088933809	0,775354385	0,438130281	0,661477384	protein_codin fibrillarin
TcG_13467	92,50648026	-0,137553509	0,18125748	-0,75888459	0,447921596	0,668937821	protein_codin hypothetical protein
TcG_13468	481,0606611	-0,362236511	0,088406216	-4,09740998	4,17799E-05	0,000526726	protein_codin hypothetical protein
TcG_13469	7,166501432	-0,800776404	0,674360049	-1,18746122	0,235045746	1	
TcG_13470	131,4783819	-0,173570142	0,153539076	-1,13046233	0,258281461	0,490645845	protein_codin putative ABC transporter
TcG_13471	3049,413219	0,311916431	0,046833513	6,660111813	2,73619E-11	1,55805E-09	protein_codin putative 40S ribosomal protein S23
TcG_13472	133,649359	-0,001191049	0,152366177	-0,00781701	0,993762988	0,997677114	protein_codin hypothetical protein
TcG_13473	421,607906	-0,076349469	0,0924591	-0,82576479	0,408937532	0,636605346	protein_codin hypothetical protein
TcG_13474	120,2150572	0,019966535	0,164062025	0,121701135	0,903135714	0,953154526	protein_codin hypothetical protein
TcG_13475	41,75877445	0,199455213	0,283892693	0,70257255	0,482322174	0,696260243	protein_codin mucin-associated surface protein (MASP)
TcG_13476	290,6452623	-0,518145663	0,10610368	-4,88339014	1,04277E-06	2,09749E-05	protein_codin hypothetical protein
TcG_13477	390,241258	0,22134496	0,092164448	2,401630609	0,016322181	0,072178928	protein_codin hypothetical protein
TcG_13478	24,77541582	-0,353323826	0,368799114	-0,95803871	0,338043241	0,572681532	protein_codin mucin-associated surface protein (MASP)
TcG_13479	253,7310929	0,395350017	0,116890521	3,38222478	0,000719013	0,005891431	protein_codin hypothetical protein
TcG_13480	4,567044897	-0,270942274	0,790889592	-0,34257914	0,731915099	1	protein_codin mucin-associated surface protein (MASP)
TcG_13481	318,0860795	-0,569845195	0,104080031	-5,47506751	4,37345E-08	1,22098E-06	protein_codin rab1 small GTP-binding protein
TcG_13482	135,6978819	-0,013570772	0,151699881	-0,08945803	0,92871791	0,965428814	protein_codin hypothetical protein
TcG_13483	330,0816881	-0,133511475	0,102964687	-1,29667247	0,194743904	0,412174055	protein_codin putative ADP-ribosylation factor 3
TcG_13484	127,4571366	-0,420216646	0,157621037	-2,66599341	0,00767612	0,0400791	protein_codin hypothetical protein
TcG_13485	217,8222116	0,264408492	0,122762273	2,153825317	0,031253873	0,117835138	protein_codin hypothetical protein
TcG_13486	1208,965073	0,1619201	0,062452532	2,592690705	0,009522837	0,047556719	protein_codin basic transcription factor 3a
TcG_13487	75,15110897	-0,273163014	0,203703593	-1,3409828	0,179926037	0,391184662	protein_codin hypothetical protein
TcG_13488	449,6668778	-0,129142534	0,091288387	-1,41466553	0,157166586	0,358945805	protein_codin hypothetical protein
TcG_13489	8,603901191	-0,268842799	0,591775374	-0,45429873	0,649613832	1	protein_codin mucin-associated surface protein (MASP)
TcG_13490	271,9318631	-0,227532798	0,109130557	-2,08495955	0,037072973	0,133850876	protein_codin ATP binding protein-like protein
TcG_13491	152,9864134	-0,306551235	0,149374843	-2,052228	0,04014751	0,141653138	protein_codin hypothetical protein
TcG_13492	134,2401958	-0,376842777	0,156897894	-2,40183451	0,016313087	0,072166255	protein_codin uncharacterized protein
TcG_13493	4,561330164	0,592987942	0,842637048	0,70372878	0,481601694	1	protein_codin hypothetical protein
TcG_13494	2,422599001	0,225699007	1,161492722	0,194318055	0,845926849	1	protein_codin mucin-associated surface protein (MASP)
TcG_13495	646,523959	-0,232828447	0,075677235	-3,07659824	0,002093773	0,014311772	protein_codin hypothetical protein
TcG_13496	8,739784083	-0,460223631	0,632303303	-0,72785264	0,466703794	1	protein_codin mucin-associated surface protein (MASP)
TcG_13497	0,445396961	-2,390354933	2,606559457	-0,91705368	0,359114513	1	
TcG_13498	0,155988004	0,503022807	4,080472857	0,123275616	0,901888849	1	protein_codin hypothetical protein
TcG_13499	0,233855473	1,175456718	3,508398891	0,335040785	0,737594321	1	
TcG_13500	28,42210343	-0,092433393	0,381832664	-0,24207828	0,80871951	0,905265886	protein_codin calpain-like cysteine peptidase
TcG_13501	67,81475192	-0,425807205	0,21706121	-1,96169184	0,049798372	0,164997303	protein_codin glycerol uptake protein
TcG_13502	4,11929265	-0,428406453	0,867902469	-0,49361128	0,62158073	1	protein_codin mucin-associated surface protein (MASP)
TcG_13503	0					1	
